# Synthesis and Preclinical Validation of Novel Indole Derivatives as a GPR17 Agonist for Glioblastoma Treatment

**DOI:** 10.1021/acs.jmedchem.1c00277

**Published:** 2021-07-26

**Authors:** Phung Nguyen, Phuong Doan, Tatu Rimpilainen, Saravanan Konda Mani, Akshaya Murugesan, Olli Yli-Harja, Nuno R. Candeias, Meenakshisundaram Kandhavelu

**Affiliations:** † Molecular Signaling Lab, Faculty of Medicine and Health Technology, Tampere University, 33720 Tampere, Finland; ‡ BioMeditech and Tays Cancer Center, Tampere University, Hospital, P.O. Box 553, 33101 Tampere, Finland; § Faculty of Engineering and Natural Sciences, Tampere University, 33101 Tampere, Finland; ∥ Scigen Research and Innovation Pvt Ltd, Periyar Technology Business Incubator, Thanjavur, Tamil Nadu 613403, India; ⊥ Department of Biotechnology, Lady Doak College, Thallakulam, 625002 Madurai, India; # Computational Systems Biology Group, Faculty of Medicine and Health Technology, Tampere University, P.O. Box 553, 33101 Tampere, Finland; ¶ Institute for Systems Biology, 1441N 34th Street, Seattle, Washington 98103-8904, United States; ∇ LAQV-REQUIMTE, Department of Chemistry, University of Aveiro, 3810-193 Aveiro, Portugal

## Abstract

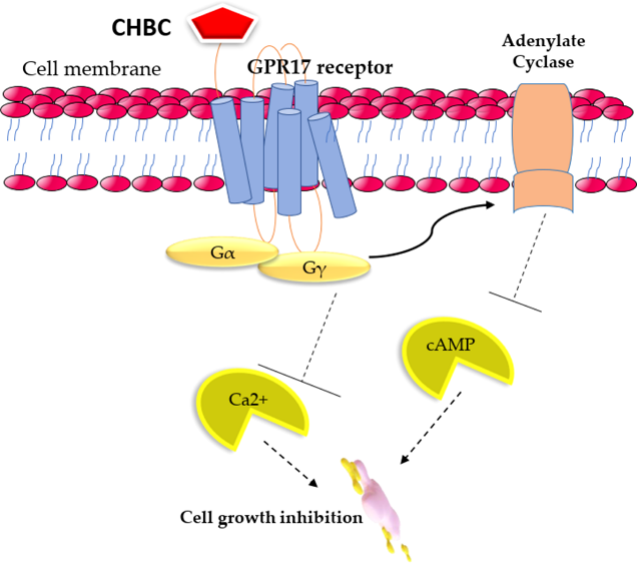

The discovery of a potential ligand-targeting G protein-coupled receptor 17 (GPR17) is important for developing chemotherapeutic agents against glioblastoma multiforme (GBM). We used the integration of ligand- and structure-based cheminformatics and experimental approaches for identifying the potential GPR17 ligand for GBM treatment. Here, we identified a novel indoline-derived phenolic Mannich base as an activator of GPR17 using molecular docking of over 6000 indoline derivatives. One of the top 10 hit molecules, **CHBC**, with a glide score of −8.390 was synthesized through a multicomponent Petasis borono–Mannich reaction. The **CHBC**–GPR17 interaction leads to a rapid decrease of cAMP and Ca^2+^. **CHBC** exhibits the cytotoxicity effect on GBM cells in a dose-dependent manner with an IC_50_ of 85 μM, whereas the known agonist MDL29,951 showed a negligible effect. Our findings suggest that the phenolic Mannich base could be a better GPR17 agonist than MDL29,951, and further uncovering their pharmacological properties could potentiate an inventive GBM treatment.

## Introduction

Human glioblastoma multiforme (GBM), a stage IV glioma, is one of the most common intrinsic and aggressive brain tumors in adults with a median survival of 14.6 months.^1,2^ GBM is among the most difficult tumors to treat since they tend to recur and resist to the current therapy approach despite the combination treatment of surgery, radiation, and chemotherapy with DNA alkylating agents, such as temozolomide (TMZ).^1,3^ In the effort to facilitate the development of more effective target therapies, G protein-coupled receptor (GPCR) has been revealed as a promising candidate that plays a prominent role in the cell signaling process. In fact, approximately 60% of drugs available in the market are targeted to GPCR.^4^ G protein-coupled receptor 17 (GPR17) is an orphan GPCR physiologically located between uracil nucleotides and cysteinyl leukotrienes (CysLTs) that transmits signals through the Gα_i_ protein, leading to adenylyl cyclase inhibition.^5,6^ Many neuronal cancer cells have been proven to express GPR17 at different levels, namely, the level of neuronal GPR17 expression is dramatically increased after undergoing ischemia.^7−13^ Previous studies based on the next-generation sequencing technique revealed high expression of GPR17 in various samples of GBM.^14,15^ Hence, GPR17 is considered a notable target for GBM treatment.

**Figure 1:**
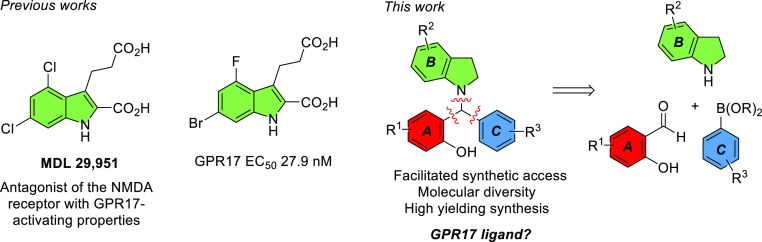
Indole-derived GPR17 ligands.

The number of small-molecule agonists targeted to GPR17 discovered has recently increased due to the aid of computational modeling.^16^ The vast majority of such molecules belong to classes of nucleotides, nucleotide sugars, or cysteinyl leukotrienes. They have been explored to successfully activate GPR17, but none of them show significant outcomes in the phases of clinical trail.^17−21^ Therefore, the finding of outstanding endogenous GPR17 ligands remains an urge. The synthesized indole agonist 3-(2-carboxyethyl)-4,6-dichloro-1*H*-indole-2-carboxylic acid (MDL29,951) is the most notable ligand that has been reported to activate GPR17 (Figure 1). More recently, Baqi and co-workers improved the potency of MDL29,951 indole derivatives by introducing different substituents at 4- and 6-positions of the indole moiety. While substituents at the 6-position of the indole can be large and lipophilic, the 4-position only allows the presence of smaller substituents before decreasing or losing the potency.^22^ Inspired by such previous works and the structural similarity of MDL29,951 with a promising antitumour indoline-derived aminoalkyl phenol reported by us,^23^ we have decided to run docking studies of a library of indoline derivatives. Considering the multicomponent characteristic of the Petasis borono–Mannich (PBM) reaction used for the preparation of the indoline-derived phenolic Mannich bases, a putative ligand identified through its least binding energy could be readily synthesized and tested in vitro (Figure 1).^24,25^ In our previous work, we built a theoretical 3D structural model of human GPR17 by comparative modeling.^16^ Here, we used this advanced model for predicting new ligands, and the lead compound was synthesized for further evaluation. The chain length of the protein is 339 amino acid residues. The template used to model the protein is 5DHG_A, and it shares 30% sequence identity with the GPCR protein. The protein is modeled for the region from 38 to 305 residues, and the model is mainly made up of helices. We used the Sitemap program to find the binding site of the protein and we found 10 pockets or cavities.^26^ Pocket number 67 is considered for our study and has a volume of 436 Å̂3 and a van der Waals (VD) value of 1639 Å̂3. The probe radius used to find the pockets is 2 Å. The downstream signaling pathways including calcium and cAMP mobilization of the lead compound are investigated to validate the activating GPR17 ability of the synthesized compound. The ability to inhibit the GBM cell growth of the top compound is also investigated to elucidate its cytotoxicity effect against GBM proliferation. The findings of this study might open a new opportunity to develop a novel pharmaceutical strategy for brain cancer treatment.

## Results and Discussion

### Design of New GPR17 Ligands

The computational simulations of the GPR17 receptor structure enable “structure-based” ligand design and discovery. A series of small molecules were designed and considered as a GPR17 binder. The GPR17 binding site is sandwiched between the lobes where ligands establish critical hydrogen bonding interactions.^1^ The target protein GPR17 is bound to a known GPR17 activator, and hence, the interactions formed have been taken as ref (2).

**Figure 2:**
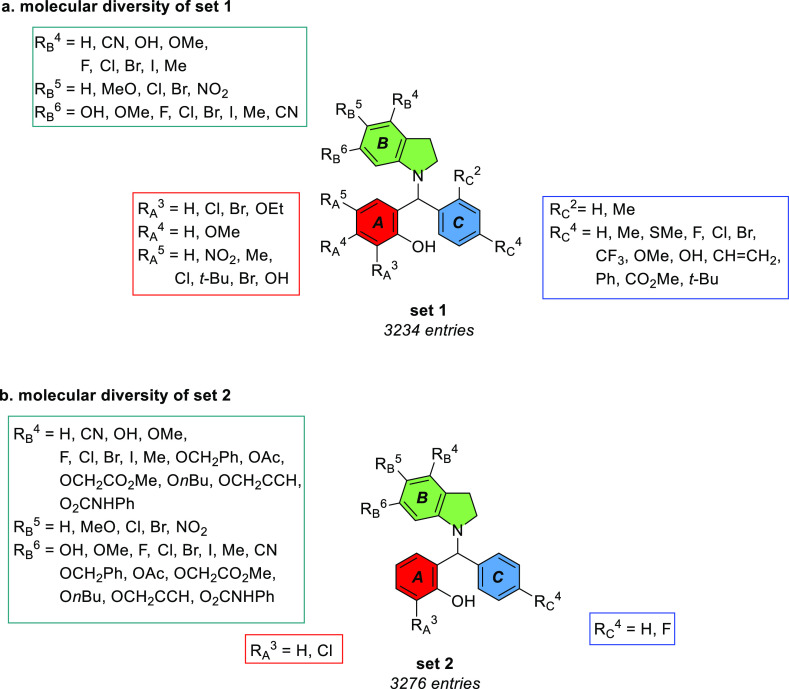
Library of indoline-derived phenolic Schiff bases used in docking.

Two sets of indoline-derived phenolic Mannich bases were built for the docking studies. Envisioning the use of a multicomponent PBM reaction for condensing indoline with phenol and aryl moieties (see below for synthetic details), from substituted salicylaldehydes and boronic acids, respectively, the first set built considered only commercially available substituted components (Figure 2a). Having in mind the reported importance of indole substituents in surrendering agonist activity to the heterocyclic core, non-commercial substituted indolines were considered for the elaboration of a second set of ligands (Figure 2b).^22^ Initially, 3234 ligand entries were modeled and docked with GPR17 target through the virtual screening workflow using glide.^3^ The glide score (gscore) value was used as the criterion to choose the best-docked compounds among the studied compounds from the dataset. Three top compounds out of 3234 showing the highest docking score were picked to build up another library involving 3276 entries. Overall, we obtained 19 best docked protein–ligand complexes with a glide score value greater than −8.0, and the detailed output including the number of hydrogen bonds and glide parameters such as VD forces and electrostatic energies are presented in Supporting Information S1.

**Figure 3:**
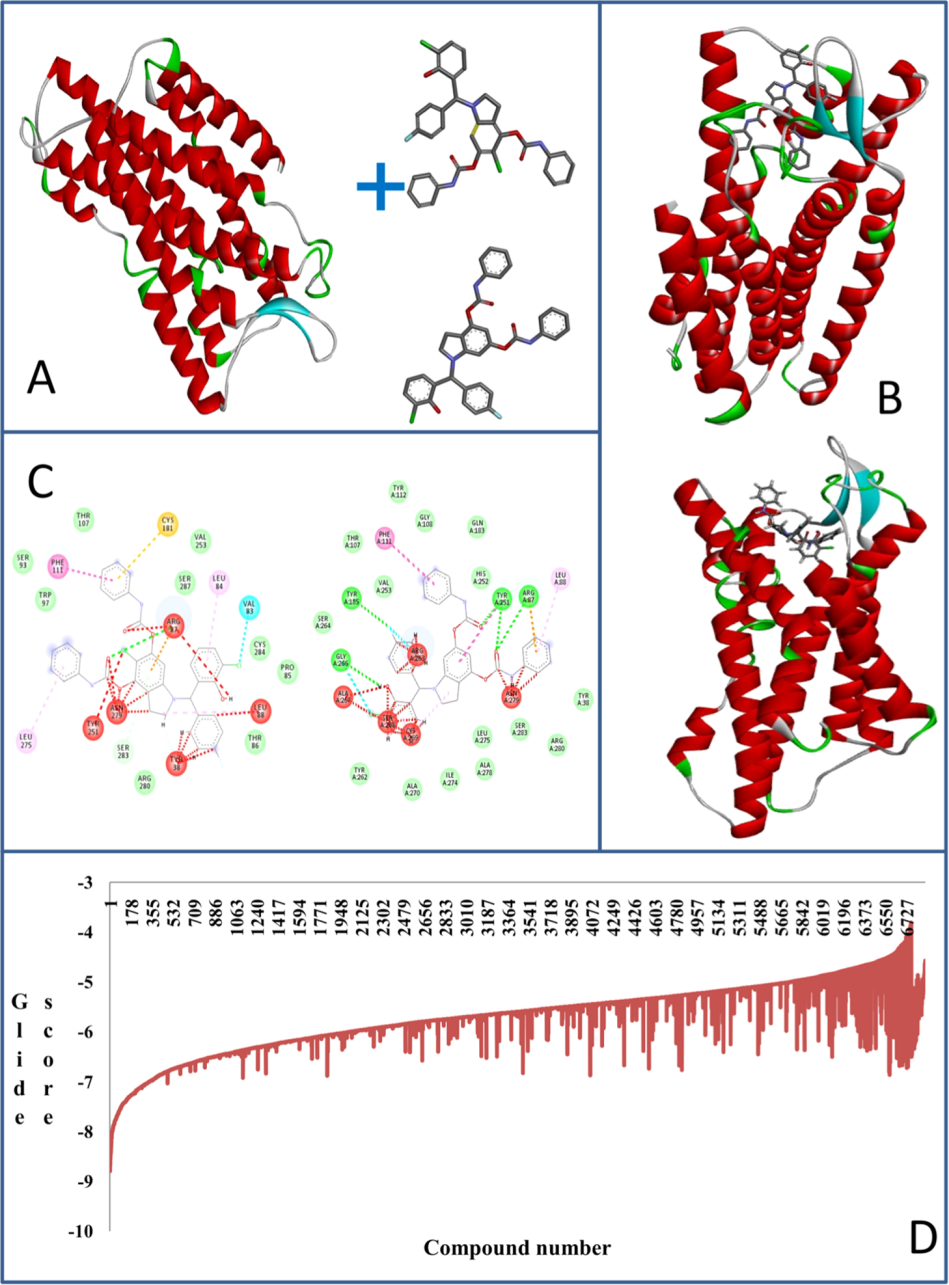
Theoretical model of the GPR17 receptor (template Protein Data Bank identifier): (A) 5DHG_A and structure of the top hit Osma-1792 (top) and the synthesized compound **CHBC** (bottom) and three-dimensional coordinates. (B) The best docked conformation of GPR17-Osma 1792 (top of B) and GPR17–**CHBC** (bottom of B) generated by the glide program is shown. (C) The amino acid residues in the GPR17 receptor interaction with ligand atoms (Osma-1792 in the left and **CHBC** in the right) were presented as a two-dimensional interaction diagram. (D) Glide docking scores of 3276 compounds (each compound has top two or three poses) are presented, and the mean glide score of compounds screened against the GPR17 receptor is −6.2.

From our results, we observed that the compound Osma-1792 is the best GPR17-binding compound with a docking score of −8.79. Due to the synthetic hurdle in the preparation of Osma-1792 (46 atoms), we synthesized a similar compound Osma-1344 (45 atoms; ninth rank, hereafter called as **CHBC**) with a docking score of −8.39. The similarity between the two compounds was computed by fixing Osma-1792 as a reference and superimposing **CHBC** in three dimension by using the LigRMSD tool. Interestingly, properties such as chirality, surface area, and VD force of the compounds are almost similar between the two compounds which was determined as 98%. Another powerful alternate to assess the compound similarity is to compute the Tanimoto coefficient. The Tanimoto coefficient of the two compounds is 0.97, which corroborates the similarity of the compounds. Both compounds have 9 numbers of non-rotatable bonds. The log *P* (octanal/water partition coefficient) of Osma-1792 is 8.34, whereas 7.79 for **CHBC**. The theoretical model of the receptor (A) and its binder **CHBC** (B) is shown in Figure 3. The synthesized inhibitor **CHBC** and the top hit Osma-1792 forms a strong hydrogen bond contact with the residue Arg87 of the protein (Figure 3C). Both the compounds were found to form hydrogen bond interactions with the backbone atoms of Arg87 and the residue Leu88 to form interactions. These residues form interactions with the target protein. Residues Val83, Leu84, Thr86, Phe111, Cys181, Tyr251, Asn279, Arg280, and Ser 287 interact with the compound **CHBC**, which indicates that the synthesized compound also form key interactions with the GPR17 receptor. The receptor forms a couple of π–π stacking interactions and a disulfide salt bridge interaction with the ligand as presented in Figure 3C. The glide score of all the compounds is presented in Figure 3D, which indicates that the ligands in the dataset have a mean glide score of −6.2.

### Synthesis of New GPR17 Ligands

**Figure 4:**
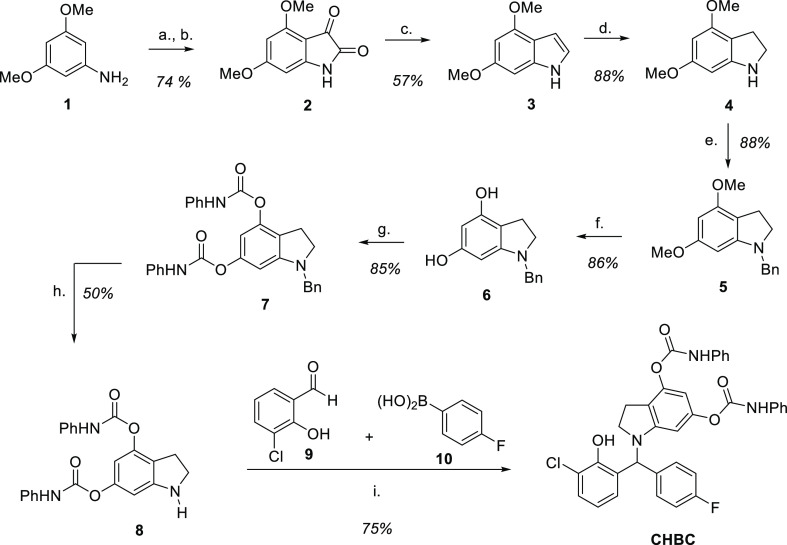
Synthesis scheme for the preparation of the GPR17 ligand **CHBC**. (a) TMSCl (1.5 equiv), MeOH, 0 °C to r.t., and 30 min; (b) (ClCO)_2_ (3.7 equiv), 165 °C, and 3.5 h; (c) LiAlH_4_ (10 equiv), Et_2_O, 0 °C to r.t., and 3 h; (d) NaBH_3_CN (2 equiv), AcOH, 17 °C to r.t., and 30 min; (e) *n*BuLi (1.1 equiv, 2.5 M in hexane), BnCl (1.2 equiv), THF, −78 °C to r.t., and 2 h; (f) BBr_3_ (6 equiv, 1 M in CH_2_Cl_2_), CH_2_Cl_2_, −78 °C to r.t.; (g) PhNCO (2.1 equiv), Et_3_N (2.1 equiv), MeCN, 0 °C to r.t., and 30 min; (h) Pd/C 10%, MeOH/CH_2_Cl_2_ (4:1), r.t., and 3 days; and (i) **9** (1 equiv), **10** (1 equiv), C_2_H_4_Cl_2_, 50 °C, and 1.5 h.

The synthesis scheme of the new GPR17 ligand is shown in Figure 4. In broad lines, the projected preparation of the indoline derivatives required for the multicomponent PBM comprised the synthesis of corresponding indole derivatives, followed by reduction of the heterocyclic ring. Despite several attempts to prepare Osma-1792, the presence of chlorine in the indoline core raised several difficulties, and instead, **CHBC** was prepared according to the scheme provided in Figure 4.

3,5-Dimethoxyaniline **1** was efficiently converted into the corresponding isatin **2** in 74% yield through an uncatalyzed Stolle reaction after its conversion into the hydrochloride salt.^27^ Reduction of isatin with 10 equivalents of lithium aluminum hydride delivered the indole derivative **3**, which was further reduced with NaBH_3_CN in acetic acid to indoline **4**. To ensure the installation of the phenylcarbamate unit without the formation of urea, indoline **4** was *N*-benzylated. The *O*-demethylation of **5** with boron tribromide was achieved in 86% yield, followed by reaction with 2.1 equiv of phenylisocyanate to afford the *N*-benzylated indoline **7** in 85% yield. The *N*-deprotection of **7** was achieved by hydrogenation at atmospheric pressure in only 50% yield, despite the long reaction times. Indoline derivative **8** was condensed with benzaldehyde **9** and aryl boronic acid **10** to provide the target phenolic Mannich base **CHBC** in 75% yield after 1.5 h at 50 °C in dichloroethane.

Preliminary attempts to prepare 5-chloro-4,6-dimethoxy-1*H*-indole, needed for the synthesis of Osma-1792, through a Sugasawa indole synthesis proved problematic in the chloroacetylation step with chloroacetonitrile.^28^ Attempts to install the chloroacetyl unit in the trisubstituted aniline resulted in very low yields of the product despite the stoichiometric use of TiCl_4_ or AlCl_3_. Alternatively, the same trisubstituted indoline was used for the Stolle reaction to prepare the trisubstituted isatin, which could be further reduced to 5-chloro-4,6-dimethoxy-1*H*-indole, with considerable amounts of the dechlorinated side product. After quantitative reduction to the corresponding indoline, attempts to cleave the methyl ether groups with boron tribromide, or with triethylsilane and catalytic B(C_6_F_5_)_3_, proved unproductive.

### CHBC-Mediated GPR17 Ligand Signaling Activation Affects the Ca^2+^ and cAMP Levels

Previously, Ca^2+^ was reported to be a secondary messenger that is mobilized in GPR17 signaling pathway through the activation of the subfamily Gαq protein.^29^ When GPR17 is activated, the β form of phospholipase C hydrolyzes PIP2 into IP3 and DAG in which IP3 serves as an intracellular ligand to open a Ca^2+^ ion channel to release Ca^2+^ into the cytoplasm that binds to DAG, thereby activating the enzyme protein kinase C (PKC).^30^ Besides activation of Gαq signaling process, Gα_i/o_ is also reported to inhibit adenylyl cyclase upon GPR17 activation and thereby reduces the intracellular cAMP levels.^31^

**Figure 5:**
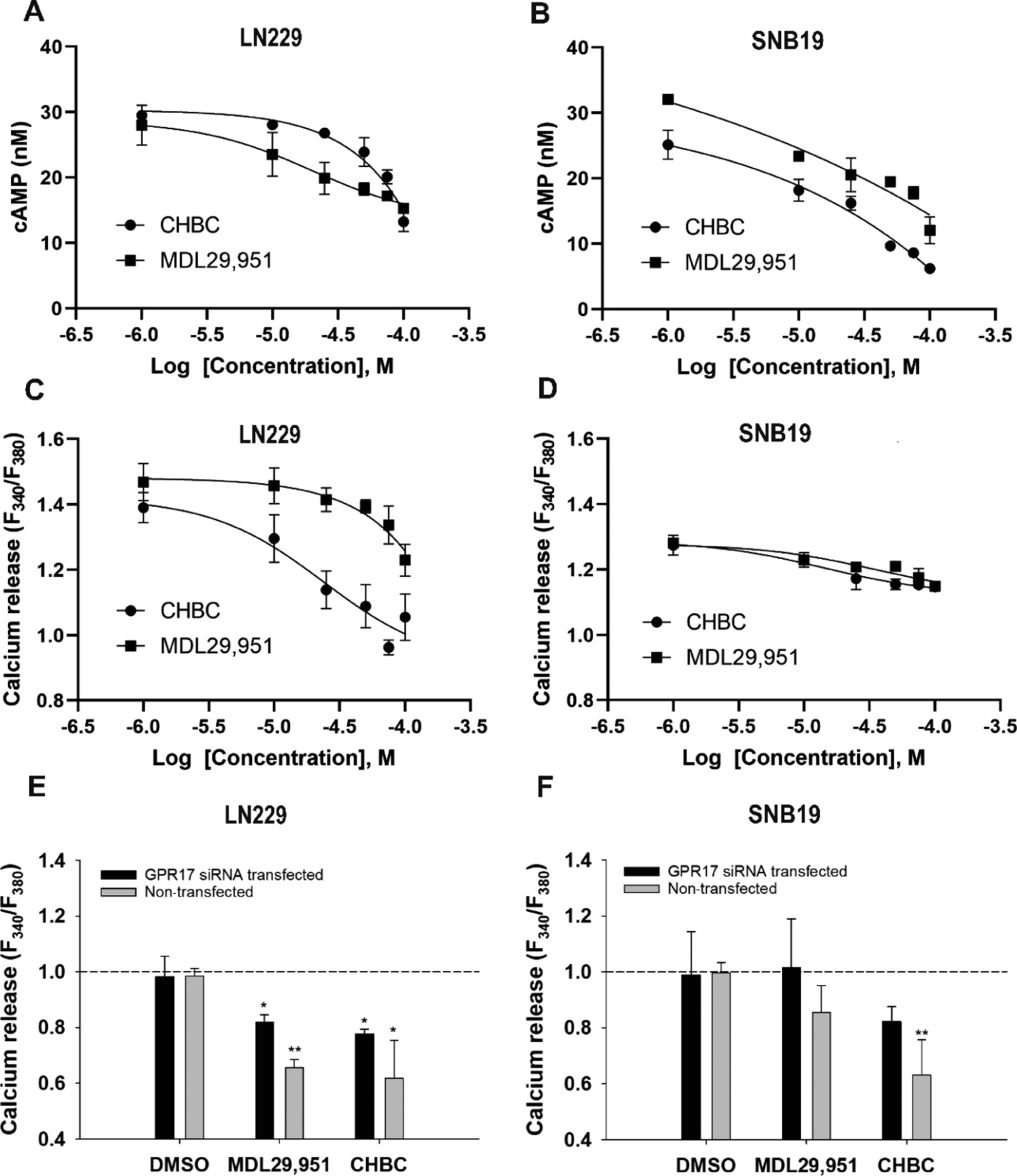
Calcium released and the cAMP level measured in LN229 and SNB19 upon the activation with **CHBC** and MDL29,951. cAMP level (nM) on (A) LN229 and (B) SNB19. Ca^2+^ release was measured as the 340/380 nm ratio in Fura-2 AM-loaded (C) LN229 cells and (D) SNB19. GPR17 silencing by siRNA and its effect on Ca^2+^ signaling activation by **CHBC** and MDL29,951 in (E) LN229 and (F) SNB19 cells. Data are the mean ± SD of three independent experiments. Significant data are denoted by asterisks: *p < 0.05 and **p < 0.01. Kolmogorov–Smirnov test (A–D): *p*-value < 0.05 (B and C).

To determine whether **CHBC** has the agonism activity, we measured the change on downstream effectors, cAMP and Ca^2+^ accumulative levels in LN229 and SNB19 cell lines. As shown in Figure 5, the intracellular Ca^2+^ and cAMP levels decreased in both cell lines when exposed to **CHBC** in a dose-dependent manner. Specifically, **CHBC** rapidly mobilized the cAMP level in LN229 with an EC_50_ of 59.65 μM and SNB19 with an EC_50_ of 19.22 μM. The treated cells with 1 μM tested compound resulted in 2.5-fold and 4-fold higher cAMP level than those treated with a higher compound concentration of 100 μM in LN229 and SNB19, respectively (Figure 5A,B). The known agonist MDL29,951 also showed inverse agonism for the cAMP level in GBM cells with an EC_50_ of 16.62 μM in LN229 and 17.73 μM in SNB19. Simultaneously, the Ca^2+^ level slightly decreased from nearly 1.4 to 1.1 ratio in LN229 treated with either **CHBC** or MDL29,951 as the concentration increases from 1 to 100 μM (Figure 5C). EC_50_ values of 26.94 and 41.93 μM were observed in LN229 when they were exposed to **CHBC** and MDL29,951, respectively. A similar trend was observed in SNB19 in which the Ca^2+^ level significantly decreased from approximately 1.3 to 1.1 ratio as the compound concentration increased from 1 to 100 μM when the cells were treated with either **CHBC** or MDL29,951 (Figure 5D). The EC_50_ values of SNB19 when they were exposed to **CHBC** and MDL29,951 are 7.67 and 26.33 μM, respectively. Gene silencing experiments using GPR17 siRNA were accomplished to validate the targeted binding of the study compound to GPR17. As shown in Figure 5E,F, without GPR17 siRNA transfection in both LN229 and SNB19 cells, there was a 1.6-fold lower level of Ca^2+^ upon the activation of GPR17 receptor by **CHBC**, whereas in cells transfected with GPR17 siRNA, no significant change (MDL29,951) or only 1.1-fold decrease (**CHBC**) in the Ca^2+^ level was observed. These results suggest that **CHBC** can act as an agonist activating endogenous GPR17 in GBM cells and resulting in the involvement of both Gαi and Gαq signaling pathways.

### *In Vitro* Growth Inhibition Effect of **CHBC** on LN229, SNB19, and Non-cancer Cell MEF

**Figure 6:**
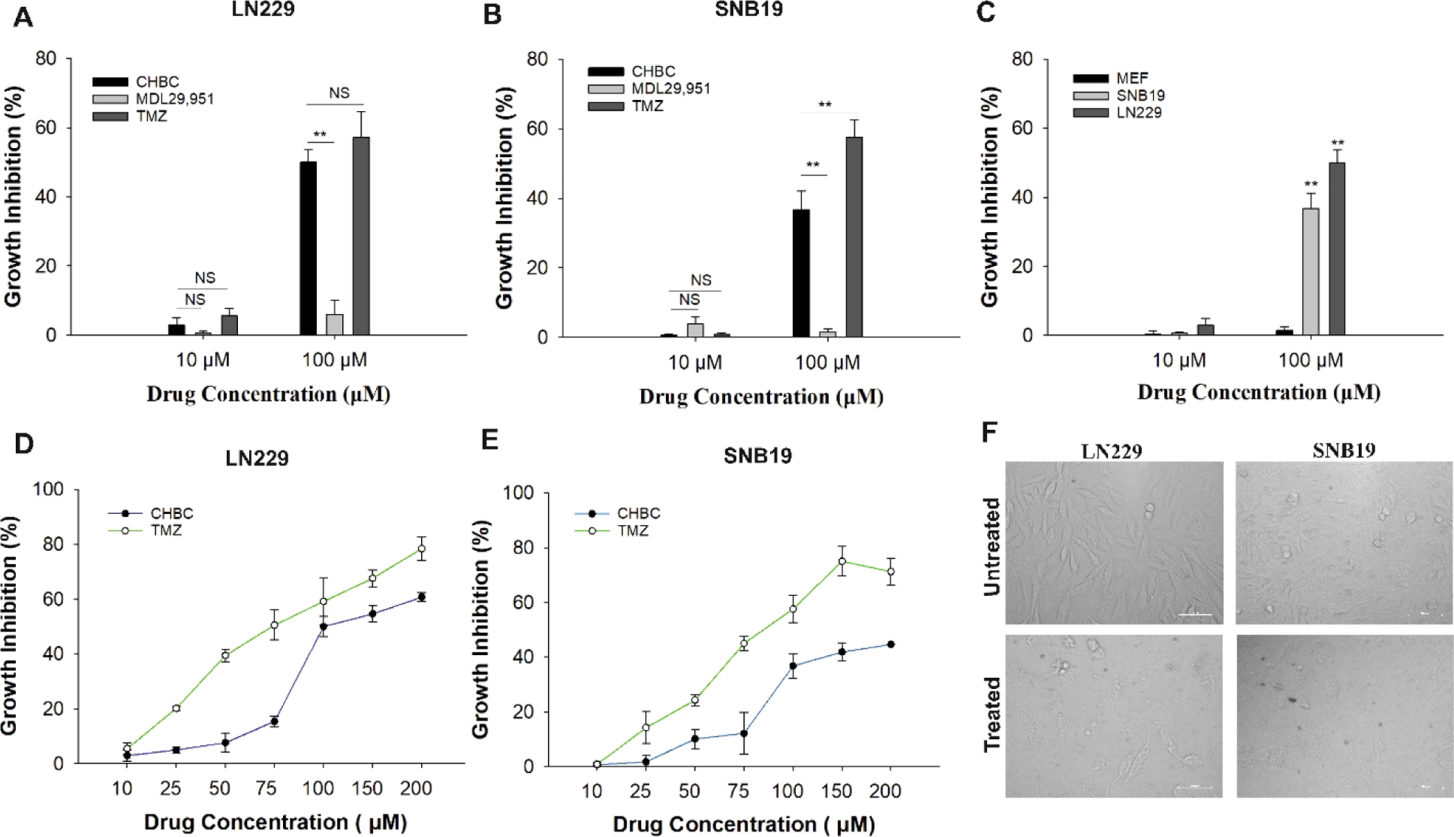
Effect of **CHBC** on GBM cells. Percentage of cell growth inhibition at 10 and 100 μM concentrations of **CHBC**, MDL 29,951, and TMZ in (A) LN229 and (B) SNB19. (C) Percentage of cell growth inhibition of **CHBC** at 10 and 100 μM on SNB19, LN229, and non-tumor cells (MEF). Dynamic cytotoxicity of **CHBC** at 10, 25, 50, 75,100, 150, and 200 μM at 48 h post-treatment on (D) LN229 and (E) SNB19 cell lines. (F) Demonstrated images of morphological changes in GBM cells at 48 h after treatment. All experiments were performed with three biological repeats and two technical repeats. Non-significant data are denoted by NS and significant data by asterisks: **p < 0.01 and *p < 0.05.

The cell growth inhibition assay is a preliminary method to study the biological effect of novel ligands in cancer research. In the present study, **CHBC** was identified as an agonist that binds and interacts with GPR17 as a small agonist. The cell growth inhibition was studied using the Trypan Blue exclusion assay to validate the ability of first-time synthesized **CHBC** in inducing cell death. GBM cell lines were treated with **CHBC**, known agonist MDL29,951 of GPR17, and a chemotherapeutic agent, TMZ, at 10 and 100 μM concentrations. Figure 6A,B shows that **CHBC** has significantly increased the cell death at 100 μM compared to MDL29,951 in both cell lines, and a higher growth inhibition effect was found for LN229. The cytotoxicity study on GBM cells and non-cancerous MEF cells has revealed that **CHBC** induced GBM cell death significantly compared to MEF cells at 100 μM concentration (Figure 6C). Indeed, at a 100 μM treatment concentration, nearly 37 and 50% growth inhibition were recorded in SNB19 and LN229, respectively, whereas less than 5% growth inhibition was observed in MEF at 100 μM. This result suggests that the GPR17 agonist can induce specific cytotoxicity effect on GBM cells. Further study on the dynamic cytotoxicity effect of **CHBC** is shown in Figure 6D,E for LN229 and SNB19, respectively. The percentage of cell death was increased in a dose-dependent manner when the cells were exposed to **CHBC** or TMZ. The IC_50_ values for LN229 treated with **CHBC** and TMZ were observed to be 85.33 ± 2.72 and 67.81 ± 4.51 μM, respectively. For SNB19 treated with **CHBC** and TMZ, the IC_50_ values were 85.54 ± 5.20 and 69.87 ± 4.64 μM, respectively.

## Conclusions

In the present work, we report the molecular and pharmacological characterization of GPR17 for the treatment of GBM. A molecular docking experiment of GPR17 with a library of 6510 indole derivatives was accomplished. A chlorinated derivative of **CHBC** was found to be the best docked compound. Nevertheless, **CHBC** was also a potential agonist and was efficiently synthesized by first preparing the indoline derivative, followed by its use in the multicomponent Petasis borono–Mannich reaction. *In vitro* validation of the GPR17–**CHBC** interaction provides evidence that ligand **CHBC** inhibits the cAMP and calcium levels in LN229 and SNB19 cells in a dose dependent manner. The result reveals that this agonist targets GPR17 through the activation of Gα_i_ and Gα_q_ protein, which is consistent with previous studies.^32−34^ The result from GPR17-silencing further confirms the high selectivity and specificity of this novel GPR17 agonist binding. These results indicate that the activity of **CHBC** is consistent with the virtual screening results. Further cell viability assay shows the potential growth inhibition effect of the lead compound on GBM cells but not on non-cancerous MEF cells. Interestingly, the known agonist MDL29,951 has an insignificant cytotoxicity effect on both GBM cell lines. This suggests that the novel agonist **CHBC** is a better agonist for GBM treatment since inhibiting cancer cell proliferation is a prerequisite in developing potential drug-targeted compounds for cancer treatment. In summary, the synthesized indole derivative **CHBC** can act as a potential GPR17 agonist, thus contributing to finding new strategies for GBM treatment.

## Experimental Section

### Library of Ligands

The two-dimensional structures of indolinobenzylated phenols were drawn using Java Molecular Editor (JME) and translated to the structure data file. A first library of 3234 entries was built, comprising variations at single positions of each of the rings A, B, and C according to Figure 2a. After a preliminary docking screening in which three derivatives were identified as the best ones regarding the glide gscore, namely, Ahma-700 (R_B_^4^ = OH, R_C_^4^ = F, R_A_^3^ = R_A_^4^ = R_A_^5^ = R_B_^5^ = R_B_^6^ = R_C_^2^ = H; gscore = −7.774), Ahma-876 (R_A_^3^ = Cl, R_B_^6^ = OMe, R_C_^4^ = F, R_A_^4^ = R_A_^5^ = R_B_^4^ = R_B_^5^ = R_C_^2^ = H; gscore = −7.639), and Ahma-174 (R_A_^3^ = Cl, R_B_^4^ = CN, R_A_^4^ = R_A_^5^ = R_B_^5^ = R_B_^6^ = R_C_^2^ = R_C_^4^ = H; gscore = −7.624), a new library allowing diversification of substituents in B ring was built. The new library, comprising 3276 entries, allowed multiple diversifications of R_B_^4^–R_B_^6^ (considering replacement of 1, 2, or 3 substituents as described in Figure 2b), while considering R_A_^3^ being Cl or H and R_C_^4^ being F or H. Full disclosure of the structures considered can be found in Supporting Information S2.

All ligands were prepared using the ligand preparation application, LigPrep (Schrödinger). For a given input structure, Ligprep produces various ionization states, tautomers, stereochemistries, and ring conformations to filter molecules using various criteria such as molecular weight, functional groups, and so forth to provide a successfully processed input structure with correct chirality. The MMFF94s force field is used for optimization to produce the low energy conformer of the ligand.^35^ The hydrogen atoms were added to the ligand molecules; their bond orders were fixed. The ionization states of the ligands were generated in the pH range of 5.0–9.0 using Epik 2.3^36^ and optimized by means of OPLS_2005 force field.

### Docking of Ligands on GPCR

The ligands were prepared and incorporated into Maestro. They were subjected to hydrogen additions, removal of salt, ionization, and generation of low-energy ring conformations using LigPrep. The stereochemistry of the original compounds was preserved. Finally, the low-energy 3D structures of all compounds were produced. The virtual screening workflow (VSW) in Maestro was used to dock and to score the compounds. In the first step, glide was run in the high-throughput virtual screen mode.^37^ All the glide protocols were run using default parameters. An extensive search was carried out for generating all possible conformations. Then, such conformations were considered for docking. During the docking process, the glide score was used to select the best conformation for each ligand. Twenty ligand poses were retained for protein structural refinements. A constrained energy minimization was carried out on the protein structure using the OPLS-2005 force field with an implicit solvation model until default criteria were met. All compounds possessing best glide score values were tabulated.

### Preparation of 1-((3-Chloro-2-hydroxyphenyl)(4-fluorophenyl)methyl)indoline-4,6-diyl Bis(phenylcarbamate) (**CHBC**)

#### Preparation of 4,6-Dimethoxyisatin (**2**)

Trimethylsilyl chloride (11.4 mL, 90 mmol, 1.5 equiv) was added slowly over 10 min to a stirred solution of 3,5-dimethoxyaniline (**1**) (9.19 g, 60 mmol) in methanol (90 mL) at 0 °C. The reaction was brought to ambient temperature and left to stir for 30 min. The solvent was evaporated under reduced pressure to yield the hydrochloride salt as white crystals, which is used in the next reaction without further purification. The 3,5-dimethoxyaniline hydrochloride salt (3.99 g, 21.0 mmol) was suspended in oxalyl chloride (6.70 mL, 78.0 mmol, 3.7 equiv) and the mixture was heated to 165 °C for 3.5 h. Oxalyl chloride was evaporated under reduced pressure, and methanol (16 mL) was added to the residue. The resulting suspension was heated to 90 °C, stirred for 1 h, and then filtered while still hot, followed by washing with methanol and dried under vacuum to yield **2** (3.24 g, 15.6 mmol, 74% yield) as a yellow solid. ^1^H NMR (500 MHz, DMSO-*d*_6_): δ 10.93 (s, 1H), 6.18 (d, *J* = 1.7 Hz, 1H), 6.00 (d, *J* = 2.3 Hz, 1H), 3.88 (s, 3H), 3.86 (s, 3H). ^13^C NMR (126 MHz, DMSO-*d*_6_): δ 177.7, 169.8, 160.9, 160.4, 153.2, 100.5, 92.3, 91.3, 56.4, 56.1. Purity is >95%, as validated by ^1^H NMR.

#### Preparation of 4,6-Dimethoxy-1*H*-indole (**3**)

4,6-Dimethoxyisatin (1.64 g, 7.96 mmol) was added in small portions over 10 min to a stirred suspension of lithium aluminum hydride (3.02 g, 79.6 mmol, 10 equiv) in dry diethyl ether (80 mL) at 0 °C. After the addition, the reaction was allowed to reach room temperature (RT) and left to stir for 3 h. The mixture was cooled to 0 °C, and water (3 mL) was added, followed by aqueous NaOH (4 M, 3 mL) and more water (9 mL). The resulting suspension was filtered through Celite, and the solids were extracted with ethyl acetate (4 × 25 mL). Organic filtrates were combined, washed with brine, dried over Na_2_SO_4_, filtered, and evaporated under reduced pressure. The residue was purified by column chromatography, with CH_2_Cl_2_ as an eluent to yield **3** (0.808 g, 4.58 mmol, 57% yield) as a white solid, with similar spectral characterizations to previously described.^38^^1^H NMR (500 MHz, CDCl_3_): δ 8.09 (s, 1H), 6.97–6.98 (m, 1H), 6.55–6.56 (m, 1H), 6.46 (s, 1H), 6.23 (d, *J* = 1.7 Hz, 1H), 3.91 (s, 3H), 3.81 (s, 3H). Purity is >95%, as validated by ^1^H NMR.

#### Preparation of 4,6-Dimethoxy-indoline (**4**)

Sodium cyanoborohydride (557 mg, 8.86 mmol, 2 equiv) was added in small portions over 5 min to a stirred solution of **3** (785 mg, 4.43 mmol) in acetic acid (8.9 mL) at 17 °C. After the addition, the reaction was stirred for 5 min, brought to ambient temperature, and left to stir for an additional 30 min. After evaporation of the bulk of acetic acid under reduced pressure, the oily residue obtained was diluted with CH_2_Cl_2_ (15 mL) and washed with sat. aq. Na_2_CO_3_(15 mL). The solvent layers were separated and the aqueous layer was further extracted with CH_2_Cl_2_ (2 × 15 mL). The organic extracts were combined, dried over Na_2_SO_4_, filtered, and evaporated under reduced pressure. The residue was purified by column chromatography (gradient from CH_2_Cl_2_ to 5% MeCN in CH_2_Cl_2_) to yield **4** (0.701 g, 3.91 mmol, 88% yield) as a white solid with a similar spectral characterization to previously described.^39^^1^HNMR (500 MHz, CDCl_3_): δ 5.92 (d, *J* = 2.3 Hz, 1H), 5.89 (d, *J* = 2.3 Hz, 1H), 3.78 (s, 3H), 3.75 (s, 3H), 3.55 (t, *J* = 8.6 Hz, 2H), 2.92 (t, *J* = 8.3 Hz, 2H). Purity is >95%, as validated by ^1^H NMR.

#### Preparation of 1-Benzyl-4,6-dimethoxyindoline (**5**)

*n*-Butyl lithium (0.46 mL of 2.5 M solution in hexane, 1.14 mmol, 1.1 equiv) was added dropwise to a stirred solution of **4** (186 mg, 1.04 mmol) in dry THF (5 mL) at −78 °C. The reaction was stirred for 10 min, and benzyl chloride (143 μL, 1.24 mmol, 1.2 equiv) was added dropwise. The reaction was then brought to ambient temperature and left to stir for 2 h. The mixture was cooled to 0 °C and sat. aq. NH_4_Cl (5 mL) was added. The mixture was diluted with ethyl acetate (10 mL) and the solvent layers were separated. The aqueous layer was further extracted with ethyl acetate (2 × 10 mL). Organic layers were combined, dried over MgSO_4_, filtered, and evaporated under reduced pressure. The residue was purified by column chromatography (CH_2_Cl_2_/hexane 8:2) to yield **5** (0.246 g, 0.914 mmol, 88% yield) as a white solid. ^1^H NMR (500 MHz, CDCl_3_): δ 7.30–7.35 (m, 4H), 7.23–7.27 (m, 1H), 5.88 (d, *J* = 1.7 Hz, 1H), 5.83 (d, *J* = 2.0 Hz, 1H), 4.23 (s, 2H), 3.79 (s, 3H), 3.74 (s, 3H), 3.32 (t, *J* = 8.6 Hz, 2H), 2.86 (t, *J* = 8.3 Hz, 2H). ^13^C NMR (126 MHz, CDCl_3_): δ 161.5, 156.3, 154.6, 138.4, 128.4, 127.8, 127.1, 108.0, 88.1, 87.3, 55.4, 55.3, 53.8, 53.3, 24.9. Purity is >95%, as validated by ^1^H NMR.

#### Preparation of 1-Benzyl-4,6-dihydroxyindoline (**6**)

Boron tribromide (3.2 mL of 1 M solution in CH_2_Cl_2_, 3.2 mmol, 6 equiv) was added dropwise to a stirred solution of **5** (0.143 g, 0.530 mmol) in dry DCM (2.1 mL) at −78 °C. The mixture was stirred for 10 min and brought to ambient temperature. After 2 h, the reaction was cooled to 0 °C and sat. aq. NaHCO_3_ (6 mL) was added slowly. The resulting mixture was diluted with water (4 mL) and extracted with ethyl acetate (15 mL). The solvent layers were separated, and the aqueous layer was extracted with ethyl acetate (2 × 10 mL). The organic layers were combined, dried over MgSO_4_, filtered, and evaporated under reduced pressure. The residue was purified by column chromatography (hexane/ethyl acetate 1:1) to yield **6** (0.110 g, 0.455 mmol, 86% yield) as an off-white solid. ^1^H NMR (500 MHz, CDCl_3_ + CD_3_OD): δ 7.16–7.35 (m, 7H), 4.18 (s, 2H), 3.29 (t, *J* = 8.3 Hz, 2H), 2.83 (t, *J* = 8.3 Hz, 2H). ^13^C NMR (126 MHz, CDCl_3_ + CD_3_OD): δ 157.5, 154.9, 152.8, 138.3, 129.0, 128.3, 127.9, 127.6, 127.0, 106.0, 92.7, 88.5, 53.8, 53.4, 24.5. Purity is >95%, as validated by ^1^H NMR.

#### Preparation of 1-Benzylindoline-4,6-diyl bis(phenylcarbamate) (**7**)

Phenyl isocyanate (103 μL, 0.949 mmol, 2.1 equiv) was added dropwise to a stirred solution of **6** (0.109 g, 0.452 mmol) and triethylamine (132 μL, 0.949 mmol, 2.1 equiv) in dry acetonitrile (2.3 mL) at 0 °C. The reaction was stirred for 10 min and brought to ambient temperature. After 30 min, volatiles were evaporated under reduced pressure. The residue was purified by column chromatography (gradient from 1 to 1.5% of MeCN in CH_2_Cl_2_) to yield **7** (0.184 g, 0.384 mmol, 85% yield) as a white solid. ^1^H NMR (500 MHz, CDCl_3_): δ 8.48 (s, 1H), 8.37 (s, 1H), 7.44–7.45 (m, 4H), 7.27–7.34 (m, 9H), 7.05–7.09 (m, 2H), 6.34 (s, 1H), 6.21 (s, 1H), 4.22 (s, 2H), 3.37 (t, *J* = 8.3 Hz, 2H), 2.92 (t, *J* = 8.3 Hz, 2H). ^13^C NMR (126 MHz, CDCl_3_ + CD_3_OD): δ 154.4, 151.1, 146.5, 137.7, 137.5, 128.8, 128.8, 128.4, 127.6, 127.10, 123.4, 118.8, 118.6, 104.4, 98.4, 53.2, 52.7, 25.2. Purity is >95%, as validated by ^1^H NMR.

#### Preparation of Indoline-4,6-diyl bis(phenylcarbamate) (**8**)

**7** (0.149 g, 0.310 mmol) in a dry, degassed MeOH/DCM mixture (7.6 mL, 4:1) was added to 10% Pd/C (8.2 mg) under argon at ambient temperature. The reaction flask was then evacuated and filled with hydrogen five times and then left to stir under a hydrogen balloon. After 3 days, the hydrogen balloon was removed and argon was bubbled into the stirring mixture for 15 min. The reaction mixture was filtered through Celite, and solids were washed with DCM (3 × 10 mL). The filtrates were combined and evaporated under reduced pressure. The residue was purified by column chromatography (gradient from hexane/ethyl acetate 3:2 to 1:1) to yield **8** (61 mg, 0.156 mmol, 50% yield) as a white solid. ^1^H NMR (500 MHz, CD_3_OD): δ 7.46–7.49 (m, 4H), 7.27–7.31 (m, 4H), 7.03–7.07 (m, 2H), 6.36 (d, *J* = 1.7 Hz, 1H), 6.31 (d, *J* = 2.3 Hz, 1H), 3.56 (t, *J* = 8.6 Hz, 2H), 2.96 (t, *J* = 8.3 Hz, 2H). ^13^C NMR (126 MHz, CD_3_OD): δ 154.5, 152.8, 151.9, 151.2, 147.1, 138.4, 128.59, 128.57, 123.1, 118.8, 118.6, 104.9, 100.7, 26.4. Purity is >95%, as validated by ^1^H NMR.

#### Preparation of 1-((3-Chloro-2-hydroxyphenyl)(4-fluorophenyl)methyl)indoline-4,6-diylbis(phenylcarbamate) (**CHBC**)

A solution of **9** (23.6 mg, 0.151 mmol), **10** (21.1 mg, 0.151 mmol), and **8** (58.8 mg, 0.151 mmol) in 1,2-dichloroethane (1.5 mL) was stirred while heated to 50 °C. After 1.5 h, the solvent was evaporated under reduced pressure. The residue was purified by column chromatography (CH_2_Cl_2_/MeCN 98:2) to yield **CHBC** (71 mg, 0.113 mmol, 75% yield) as a white solid. ^1^H NMR (500 MHz, CDCl_3_): δ 7.51 (s, 1H), 7.40 (dd, *J* = 24.3, 7.7 Hz, 4H), 7.24–7.33 (m, 8H), 7.05–7.10 (m, 3H), 6.97–7.03 (m, 4H), 6.79 (t, *J* = 8.0 Hz, 1H), 6.47 (d, *J* = 2.3 Hz, 1H), 5.97 (d, *J* = 2.3 Hz, 1H), 5.65 (s, 1H), 3.11–3.22 (m, 2H), 2.83–2.90 (m, 2H). ^13^C NMR (126 MHz, CDCl_3_): δ 163.2, 161.2, 153.2, 151.4, 150.7, 150.6, 150.0, 146.2, 137.3, 134.94, 134.91, 130.2, 130.1, 129.11, 129.05, 128.6, 127.7, 127.5, 123.9, 123.8, 120.9, 120.8, 119.9, 118.6, 115.6, 115.4, 106.3, 100.9, 62.8, 52.2, 25.2. HRMS (ESI/TOF): *m*/*z* calcd for C_35_H_27_ClFN_3_O_5_Na^+^ [M + Na]^+^, 646,1515; found, 646.1520. Purity is >95%, as validated by ^1^H NMR.

### Cell Culture

Human glioblastoma cell lines LN229 and SNB19 (obtained as a gift from Dr. Kirsi Granberg, Faculty of Medicine and Health Technology, Tampere. Finland) and the mouse embryonic fibroblast cell line (MEF) were used to test the efficacy of a novel ligand targeted to the GPR17 receptor protein. Originally, LN229 was derived from a right frontal parieto-occipital glioblastoma patient with the mutation on p52, p16, and p14ARF tumor suppressor genes while SNB19 was obtained from a left parieto-occipital glioblastoma patient. These cell lines were cultured and maintained in Dulbecco’s modified Eagle’s medium (DMEM) (Sigma-Aldrich, USA) supplemented with 10% FBS (Biowest, France), 0.1 mg/mL streptomycin (Sigma-Aldrich, USA), 100 U/mL penicillin (Sigma-Aldrich, USA), and 0.025 mg/mL amphotericin B (Sigma-Aldrich, USA). The culture atmosphere was kept at 37 °C humidified with 5% CO_2_ (v/v).

### Calcium Fura 2 Dynamic Assay

To perform the Calcium Fura 2 assay, LN229 and SNB19 were plated in a black, clear-bottom, 96-well plate (CLS4580-10EA, Corning Sigma-Aldrich) with a density of 5 × 10^4^ cells/well. The used cell concentration is an appropriate quantity to multiply and grow in the given area. After overnight incubation, the cells were washed with pre-warmed 1× phosphate-buffered saline (PBS). The selected compounds were dissolved in dimethyl sulfoxide (DMSO, Sigma-Aldrich, St. Louis, MO, United States) to obtain a stock of 100 mM, from which an intermediate dilution was prepared. The cells were incubated with **CHBC** and the known agonist MDL at different concentrations of 100, 75, 50, 25, 10, and 1 μM for 2 h at 37 °C. The cells were further incubated with 5 μM Fura 2 (47989-1MG-F, Sigma-Aldrich, St. Louis, MO, USA) and 0.1% Pluronic F-127 (P2443-250G, Sigma-Aldrich, St Louis, MO, USA) in 30 min darkness at RT to maximize the activity of the loading dye. The level of fluorescence was recorded using a microplate reader (Spark, Tecan) at dual excitation/emission wavelengths 340/510 and 380/510. The 340/380 ratio was calculated using the following formula 1.
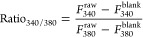
1where *F*_340_^raw^ is the fluorescent intensity of the treated sample measured at 340/510 nm and *F*_380_^raw^ is the fluorescent intensity of the treated sample measured at 380/510 nm. *F*_340_^blank^ and *F*_380_^blank^ are the fluorescent intensities of the untreated sample measured at 340/510 nm and 380/510 nm, respectively. To validate the specificity of the ligand binding to GPR17, the siRNA assay was conducted using the predesigned siRNA against human GPR17 (cat no. AM16704; Thermo Fisher Scientific, USA). LN229 and SNB19 cells with the confluence of 60–70% were transfected with 20 nM of siRNA by Lipofectamine RNAiMAX Transfection Reagents (cat no. 13778030; Thermo Fisher Scientific, USA). After 48 h of transfection, the cells were measured to quantify the changes in the intracellular calcium level.

### cAMP Glo Dynamic Assay

LN229 and SNB19 cells were seeded in a white 96-well plate (136101, NuclonThermoFisher Scientific, USA) with an initial density of 5 × 10^4^ cells/well. After overnight incubation, the cells were washed with PBS prior to incubation with 10 μM Forskolin (F6886-10MG, Sigma-Aldrich, USA) at 37 °C for 15 min. **CHBC** with varying concentrations of 100, 75, 50, 25, 10, and 1 μM were added to the cells for 2 h The cAMP-Glo Assay (V1501, Promega, USA) was conducted following the manufacturer’s instructions. Briefly, the cells were loaded with 20 μL of cAMP Glo lysis buffer, followed by a shaking step of 30 min at RT. Then, the cells were incubated with the cAMP detection solution in 20 min, followed by incubation with the Kinase-Glo Reagent for 10 min at RT. The standard curve was generated for each experiment according to the manufacturer’s protocol. The cAMP level was calculated based on the linear equation generated from a standard curve. The change in the luminescence (ΔRLU) of each sample was measured to calculate the appropriate cAMP concentration. The ΔRLU of each treated sample was calculated using formula 2.
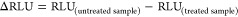
2

### Cell Viability Evaluation

LN229, SNB19, and MEF cells were seeded with a density of 1 × 10^5^ cells/well in a 12-well plate. The used cell concentration is an appropriate quantity to multiply and grow in the given area. When the cells reached 60–70% confluency, they were treated with 10 μM and 100 μM of **CHBC**, MDL29,951, and TMZ for 48 h. The Trypan Blue assay was performed according to the manufacture instructor to determine the cell growth inhibitory effect of **CHBC**. Briefly, cells were collected by the centrifugation method prior to dilution with Trypan Blue (Thermo Fisher Scientific) with a 1:1 ratio. The number of live and dead cells was determined using a Countess II FL Automated Cell Counter (Thermo Fisher Scientific). The percentage of cell growth inhibition was calculated using formula 3
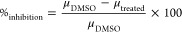
3where μ_DMSO_ is the mean number of untreated cells (control) and μ_treated_ is the mean number of treated cells. DMSO (2%) was used as the negative control.

### Dose–Response Curve for Cell Viability Assessment

LN229 and SNB19 cells were seeded with a density of 1 × 10^5^ cells/well in a 12-well plate. When cells reached 60–70% confluency, they were treated with **CHBC** at 10, 25, 50, 75, 100, 150, and 200 μM. TMZ was used as a positive control. At 48 h post-treatment, the Trypan Blue assay was performed as described above to measure the half-maximal inhibitory concentration (IC_50_) values of **CHBC** and TMZ for each cell line. GraphPad ver 8.0 was used to calculate the IC_50_ values. DMSO (2%) was used as the negative control.

### Statistical Analysis

All the experiments were performed with biological and technical triplicates. The data is presented as the mean ± SD. Statistical analysis was conducted using one-way ANOVA and Kolmogorov–Smirnov test by SPSSver.16.0. The statistical significance was considered with the *P*-values <0.05 and <0.01.

## Supporting Information Available

Two-dimensional structures of indolinobenzylated phenols (set 1: 3234 derivatives; set 2: 3276 derivatives) (PDF)
NMR spectra of the indolinobenzylated phenol derivatives (number of hydrogen bonds and glide parameters such as VD forces and electrostatic energies) (PDF)
Molecular formula strings (CSV)

## Abbreviations

**CHBC**: 1-((3-chloro-2-hydroxyphenyl)(4-fluorophenyl)methyl)indoline-4,6-diyl bis(phenylcarbamate)
CysLTs: cysteinyl leukotrienes
DMEM: Dulbecco’s modified Eagle’s medium
GBM: glioblastoma multiforme
GPR17: G-protein-coupled receptor 17
PKC: protein kinase C
TMZ: temozolomide
VD: Van der Waals
