# Supplementary material for: Synthesis and Preclinical Validation of Novel Indole Derivatives as a GPR17 Agonist for Glioblastoma Treatment
Source: J Med Chem. 2021 Jul 26;64(15):10908–18. doi: 10.1021/acs.jmedchem.1c00277 (PMC8389915; doi:10.1021/acs.jmedchem.1c00277)

Supporting Information

Synthesis and preclinical validation of novel indole derivatives as a GPR17 agonist for glioblastoma treatment

Phung Nguyen[1,2], Phuong Doan[1,2], Tatu Rimpilainen[3], Saravanan Konda Mani[4], Akshaya Murugesan[1,5], Olli Yli-Harja[6,7], Nuno R. Candeias[3,8]** and Meenakshisundaram Kandhavelu[1,2]*

[1]Molecular Signaling Lab, Faculty of Medicine and Health Technology, Tampere University, 33720, Tampere, Finland

[2]BioMeditech and Tays Cancer Center, Tampere University Hospital, P.O. Box 553, 33101 Tampere, Finland

[3]Faculty of Engineering and Natural Sciences, Tampere University, 33101 Tampere, Finland

[4]Scigen Research and Innovation Pvt Ltd, Periyar Technology Business Incubator, Thanjavur - 613403, Tamil Nadu, India

[5]Department of Biotechnology, Lady Doak College, Thallakulam, Madurai, 625002, India

[6]Computational Systems Biology Group, Faculty of Medicine and Health Technology, Tampere University, P.O. Box 553, 33101 Tampere, Finland

[7]Institute for Systems Biology, 1441N 34th Street, Seattle, WA 98103-8904, USA.

[8]LAQV-REQUIMTE, Department of Chemistry, University of Aveiro, 3810-193 Aveiro, Portugal

*Author for correspondence: meenakshisundaram.kandhavelu@tuni.fi

**Author for correspondence: ncandeias@ua.pt

Table of Contents

4,6-dimethoxyisatin (**2**) ^{1}H NMR (500 MHz, DMSO-d_6)……S3
4,6-dimethoxyisatin (**2**) ^{1}H NMR (126 MHz, DMSO-d_6)……S4
4,6-dimethoxy-1*H*-indole (**3**) ^{1}H NMR (500 MHz, $CDCl_3$)……S5
4,6-dimethoxy-1*H*-indoline (**4**) ^{1}H NMR (500 MHz, $CDCl_3$)……S6
1-benzyl-4,6-dimethoxy-1*H*-indoline (**5**) ^{1}H NMR (500 MHz, $CDCl_3$)……S6
1-benzyl-4,6-dimethoxy-1*H*-indoline (**5**) ^{13}C NMR (126 MHz, $CDCl_3$)……S7
1-benzyl-4,6-dihydroxyindoline (**6**) ^{1}H NMR (500 MHz, $CDCl_3$ + drop of Methanol-d_4)……S7
1-benzyl-4,6-dihydroxyindoline (**6**) ^{13}C NMR (126 MHz, $CDCl_3$ + drop of Methanol-d_4)……S8
1-benzylindoline-4,6-diyl bis(phenylcarbamate) (**7**) ^{1}H NMR (500 MHz, $CDCl_3$ + drop of Methanol-d_4)……S8
1-benzylindoline-4,6-diyl bis(phenylcarbamate) (**7**) ^{13}C NMR (126 MHz, $CDCl_3$ + drop of Methanol-d_4)……S8
indoline-4,6-diyl bis(phenylcarbamate) (**8**) ^{1}H NMR (500 MHz, CD_3OD)……S9
indoline-4,6-diyl bis(phenylcarbamate) (**8**) ^{13}C NMR (126 MHz, CD_3OD)……S9
1-((3-chloro-2-hydroxyphenyl)(4-fluorophenyl)methyl)indoline-4,6-diyl bis(phenylcarbamate) (**CHBC**) ^{1}H NMR (500 MHz, $CDCl_3$)……S10
1-((3-chloro-2-hydroxyphenyl)(4-fluorophenyl)methyl)indoline-4,6-diyl bis(phenylcarbamate) (**CHBC**) ^{13}C NMR (126 MHz, $CDCl_3$)……S10

4,6-dimethoxyisatin (**2**) ^{1}H NMR (500 MHz, DMSO-d$_6$)

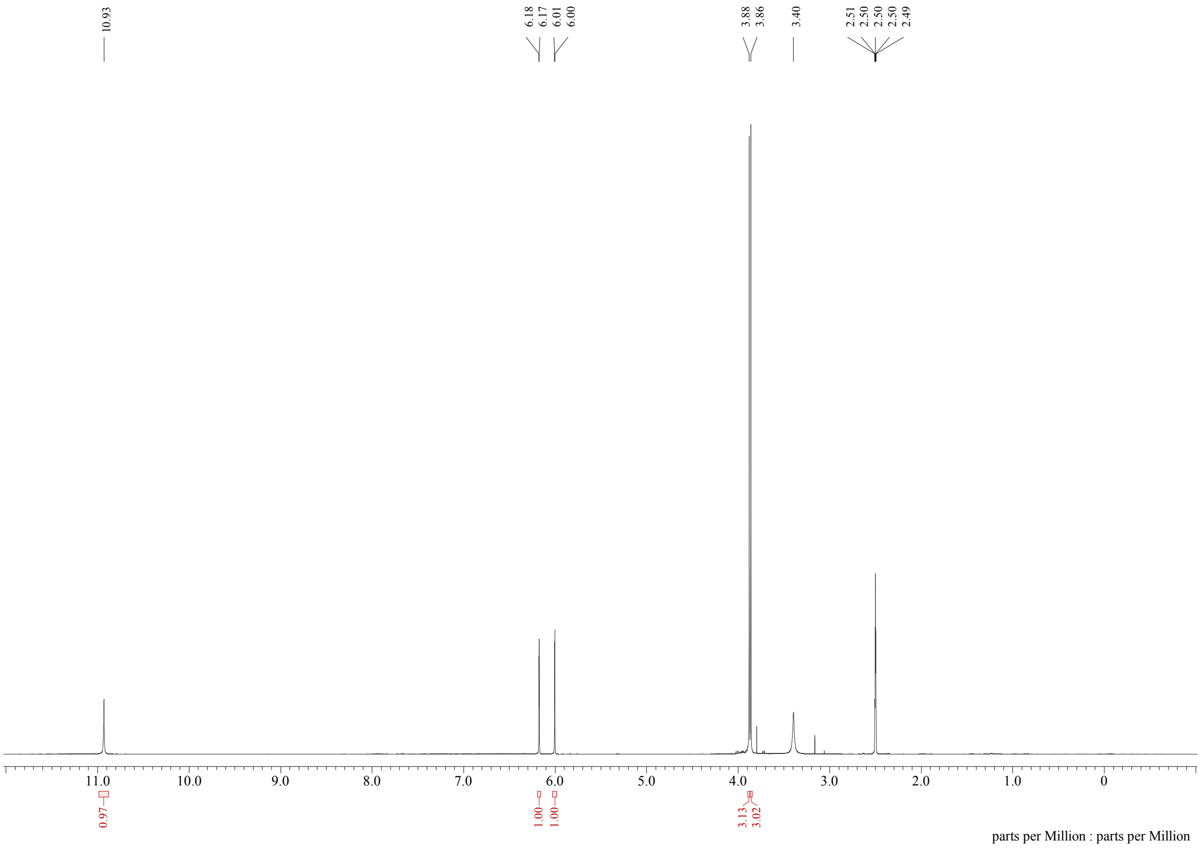

4,6-dimethoxyisatin (**2**) ^{1}H NMR (126 MHz, DMSO-d$_6$)

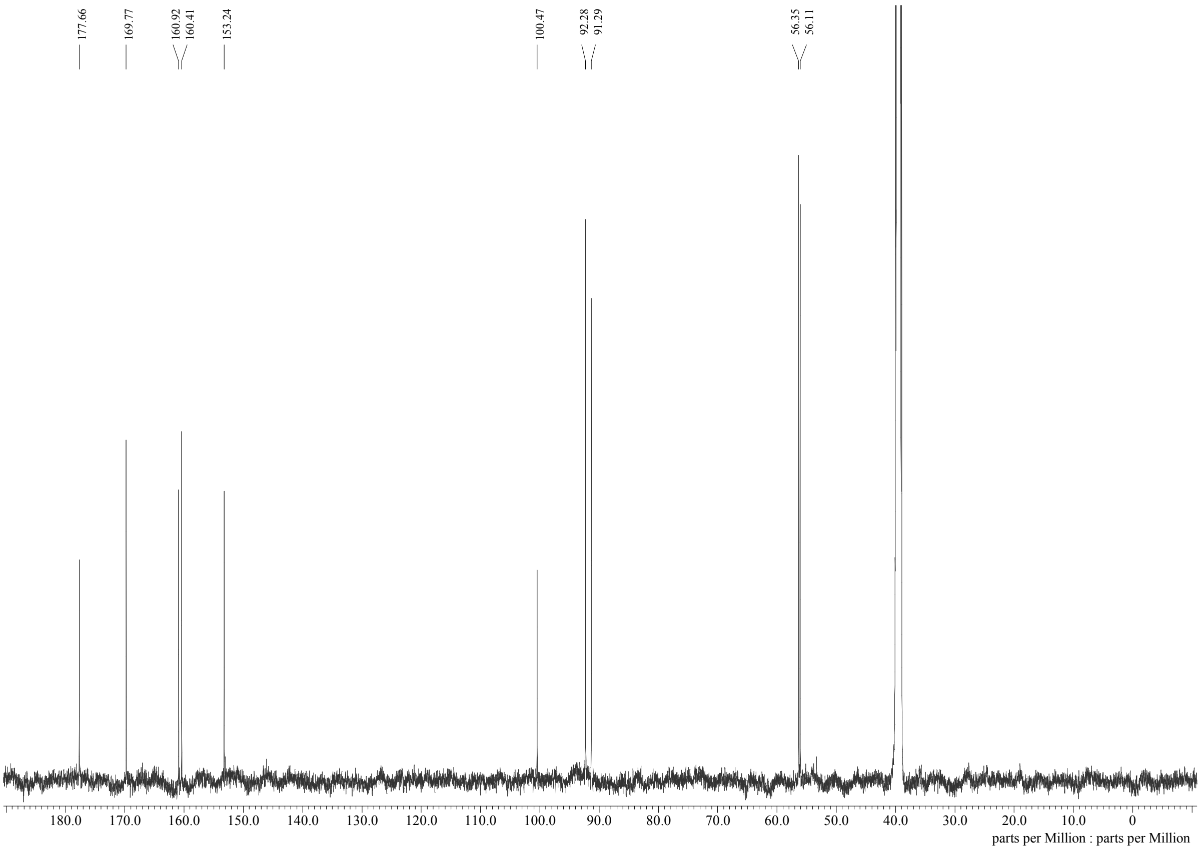

4,6-dimethoxy-1*H*-indole (**3**) ^{1}H NMR (500 MHz, $CDCl_3$)

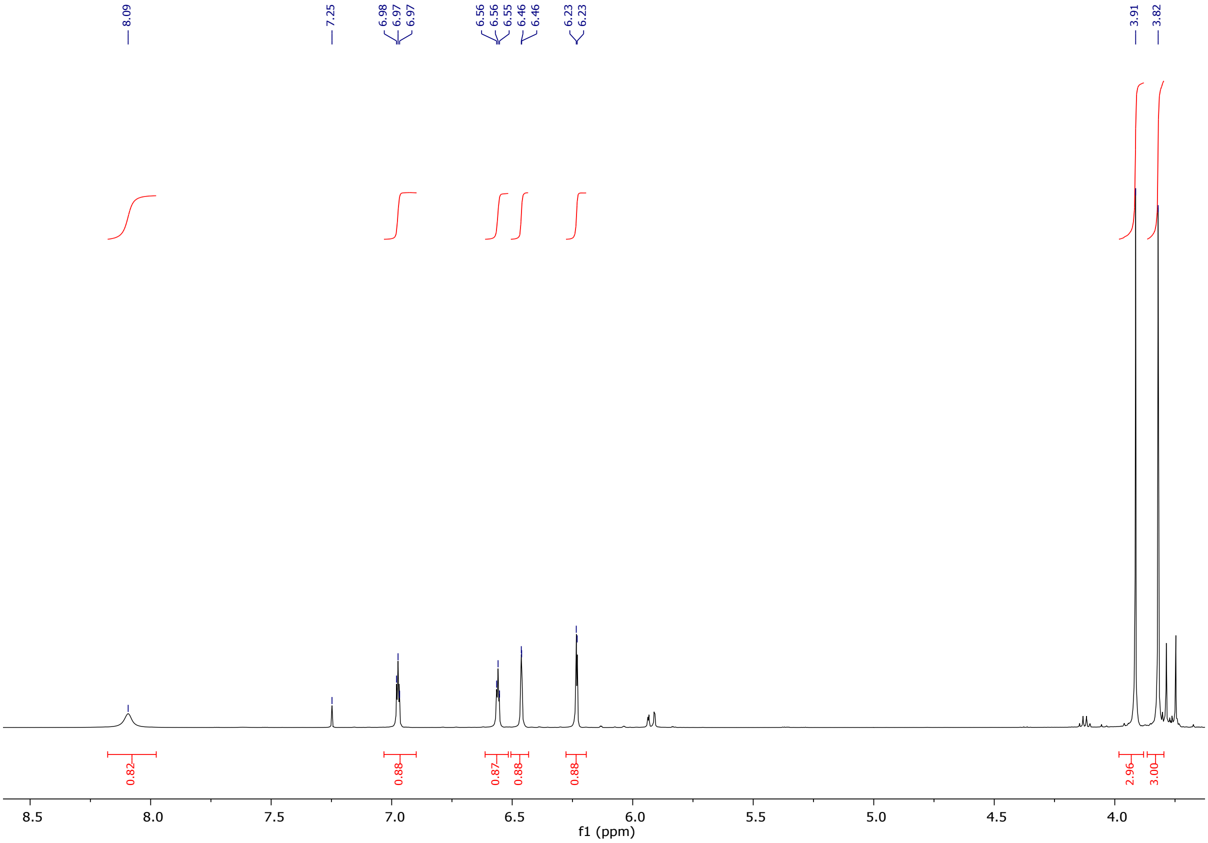

4,6-dimethoxy-1*H*-indoline (**4**) ^{1}H NMR (500 MHz, $CDCl_3$)

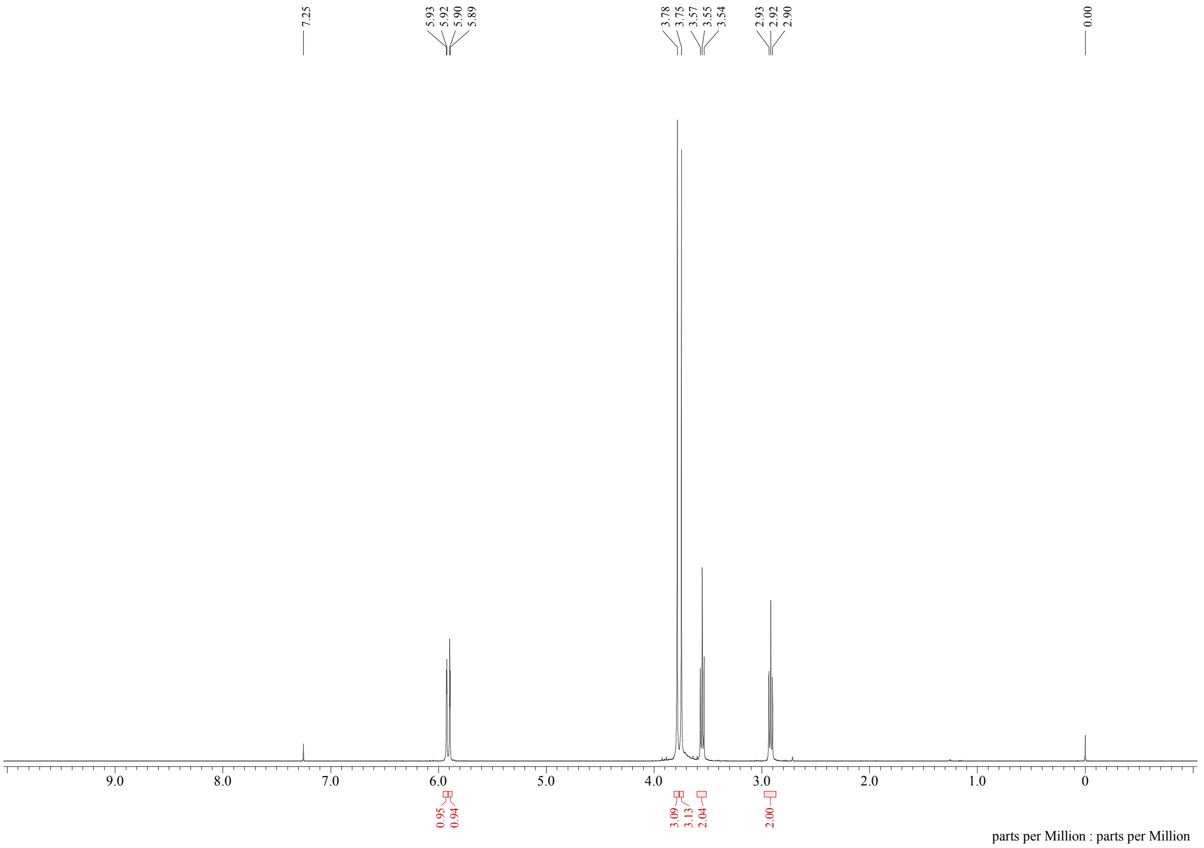

1-benzyl-4,6-dimethoxy-1*H*-indoline (**5**) ^{1}H NMR (500 MHz, $CDCl_3$)

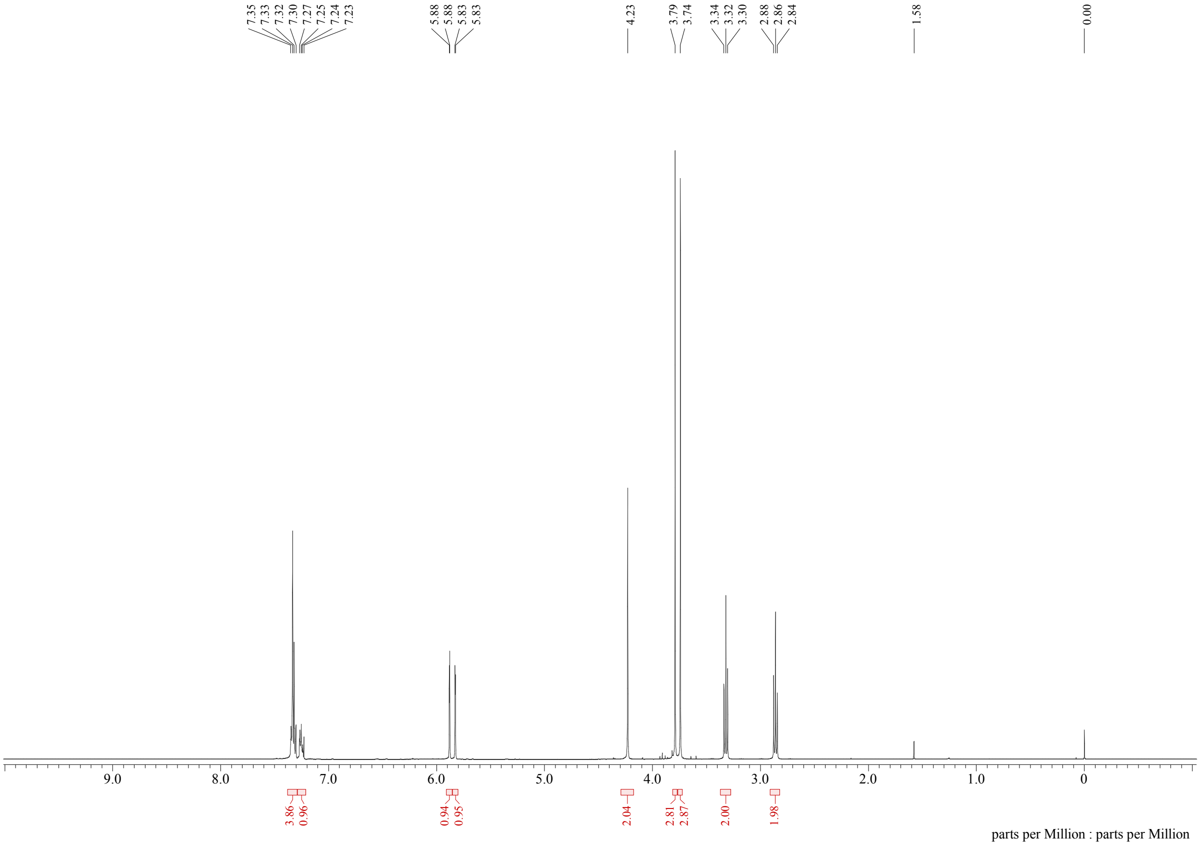

1-benzyl-4,6-dimethoxy-1*H*-indoline (**5**) ^{13}C NMR (126 MHz, $CDCl_3$)

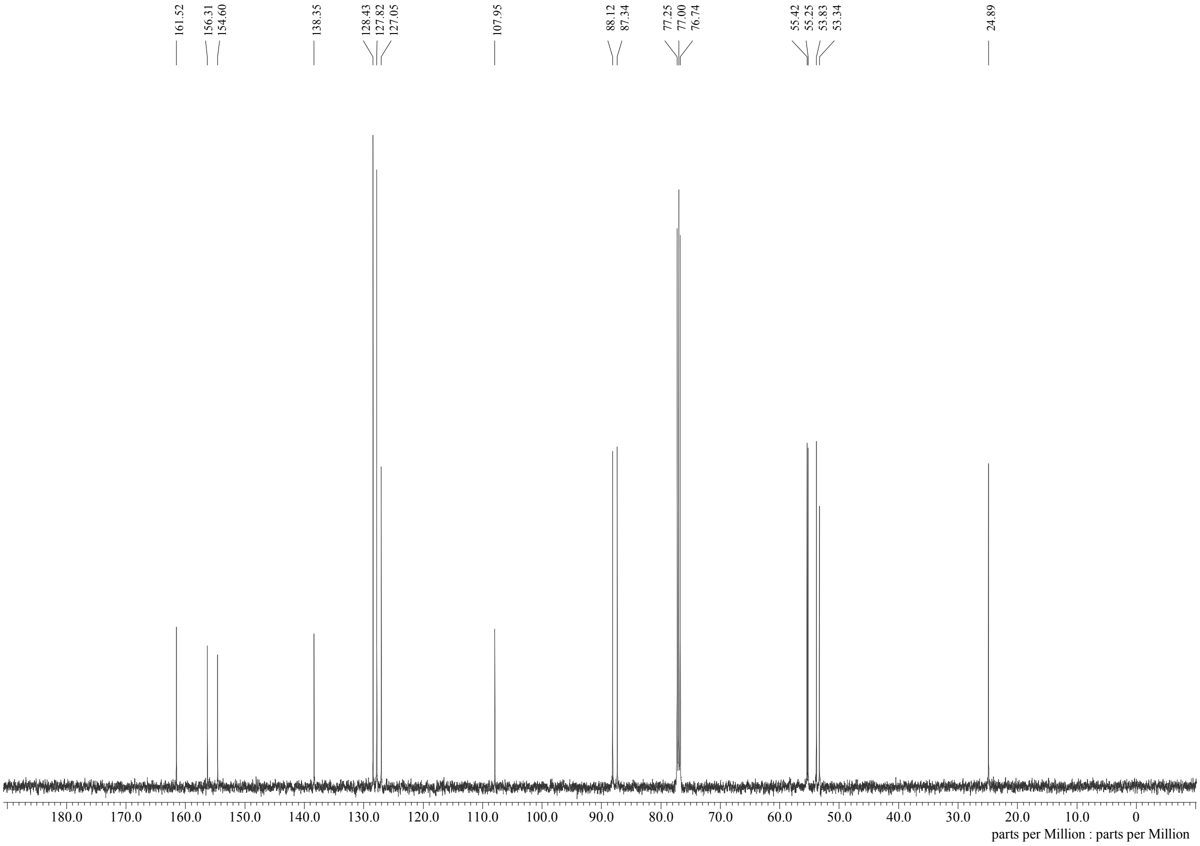

1-benzyl-4,6-dihydroxyindoline (**6**) ^{1}H NMR (500 MHz, $CDCl_3$ + drop of Methanol-d_4)

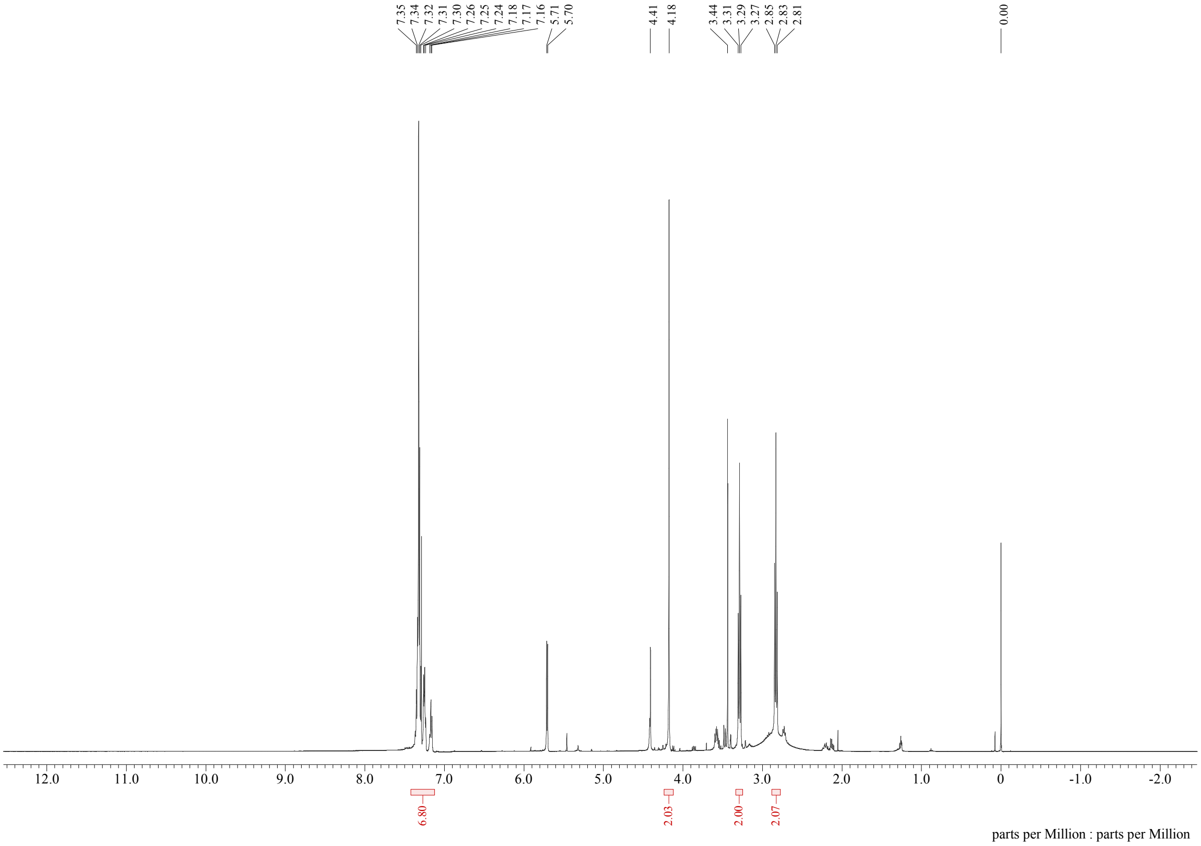

1-benzyl-4,6-dihydroxyindoline (**6**) ^{13}C NMR (126 MHz, $CDCl_3$ + drop of Methanol-d_4)

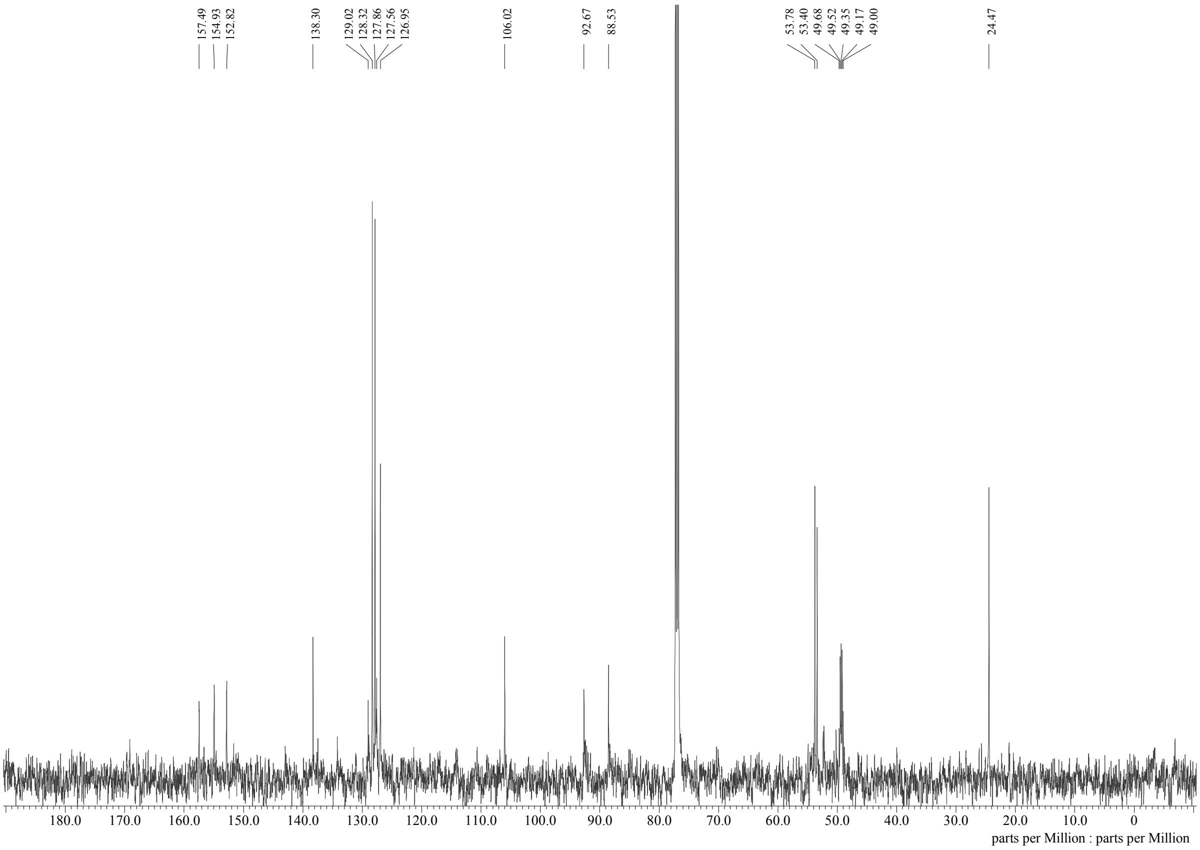

1-benzylindoline-4,6-diyl bis(phenylcarbamate) (**7**) ^{1}H NMR (500 MHz, $CDCl_3$ + drop of Methanol-d_4)

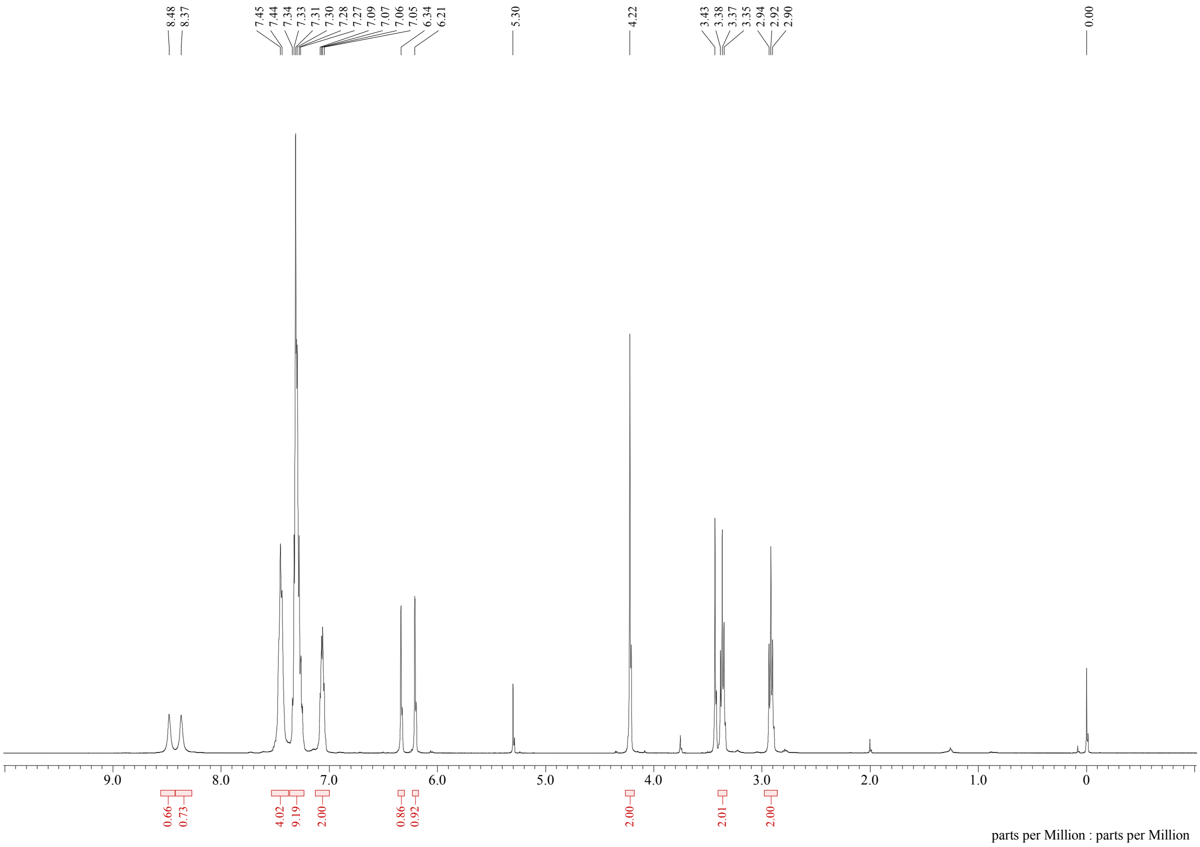

1-benzylindoline-4,6-diyl bis(phenylcarbamate) (**7**) ^{13}C NMR (126 MHz, $CDCl_3$ + drop of Methanol-d_4)

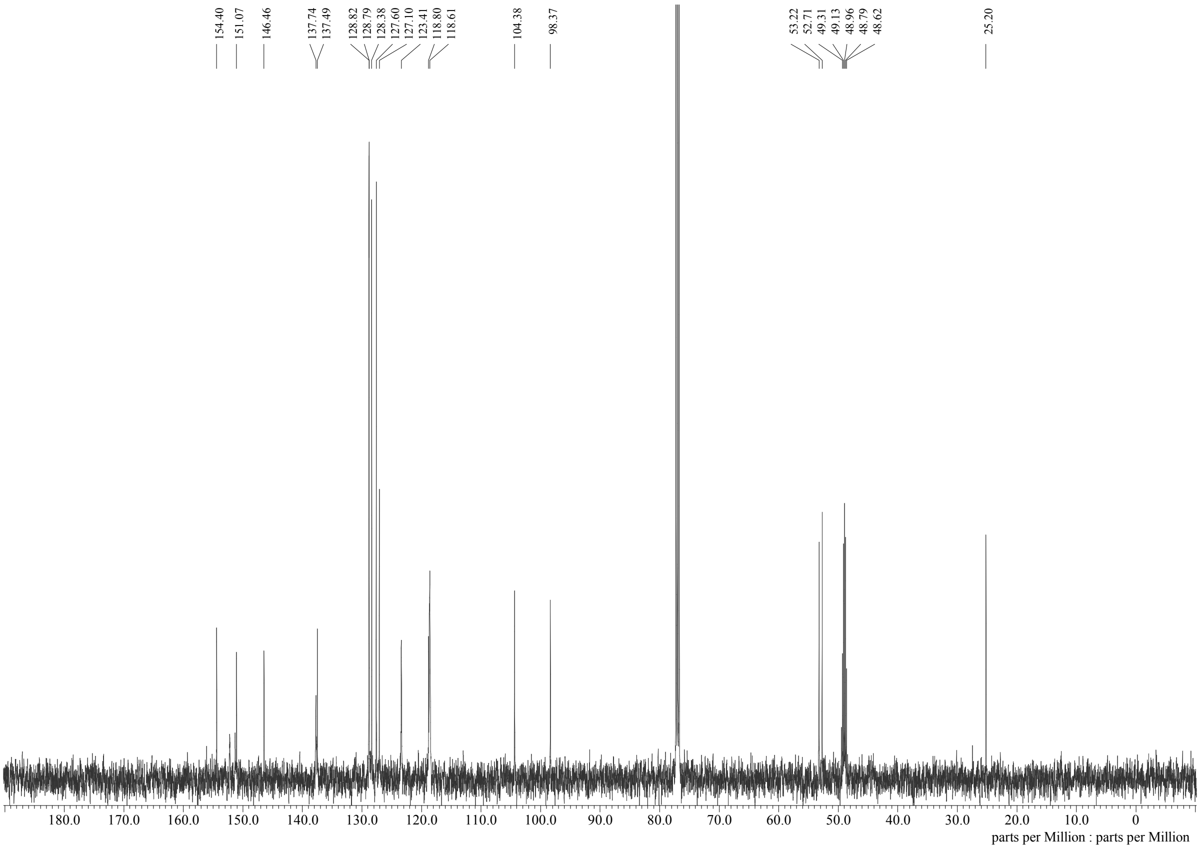

indoline-4,6-diyl bis(phenylcarbamate) (**8**) ^{1}H NMR (500 MHz, CD_3OD)

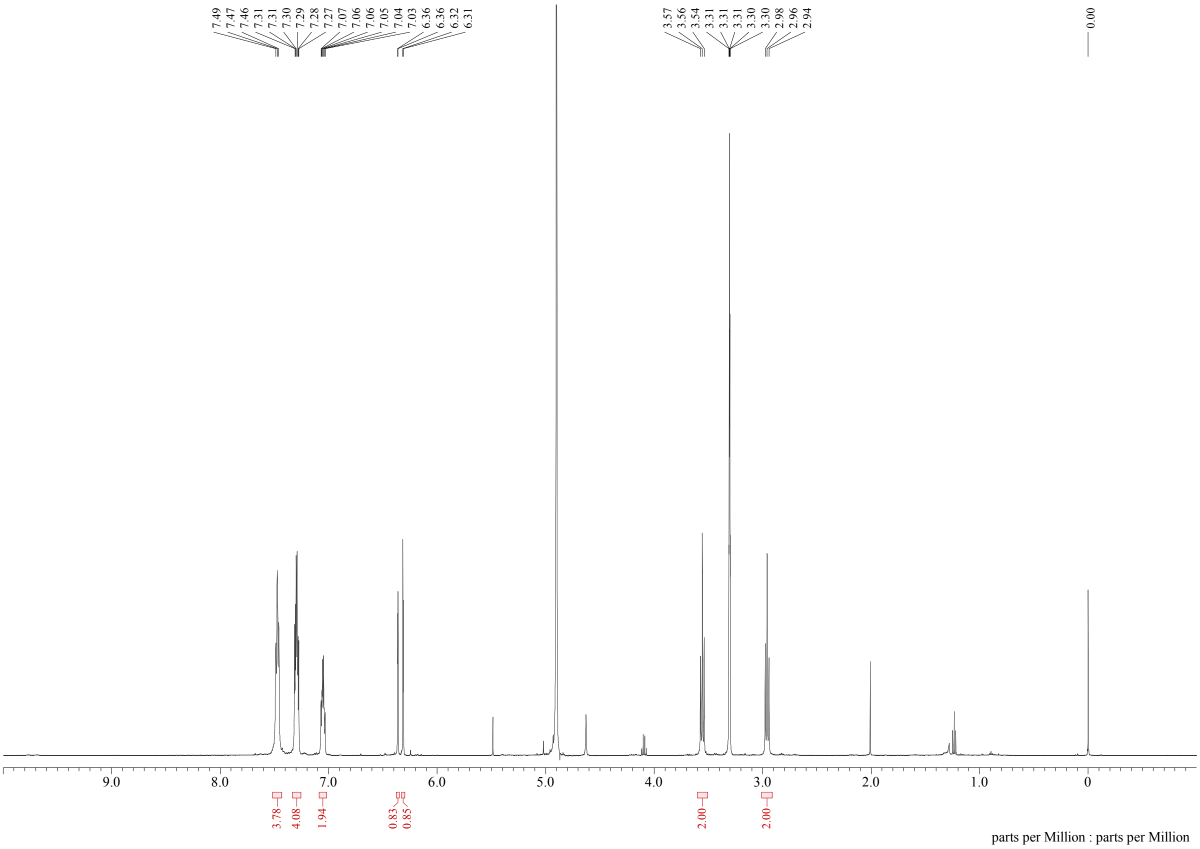

indoline-4,6-diyl bis(phenylcarbamate) (**8**) ^{13}C NMR (126 MHz, CD_3OD)

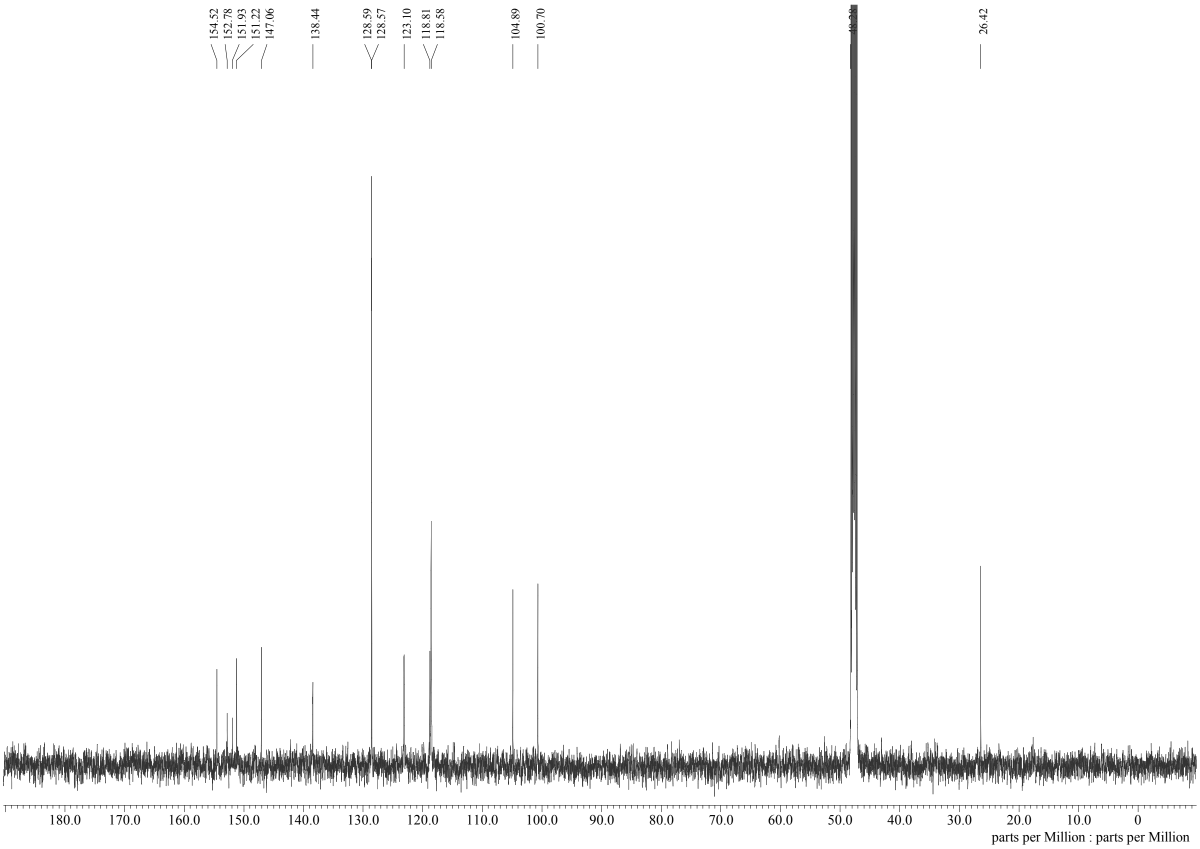

1-((3-chloro-2-hydroxyphenyl)(4-fluorophenyl)methyl)indoline-4,6-diyl bis(phenylcarbamate) (**CHBC**)
^{1}H NMR (500 MHz, $CDCl_3$)

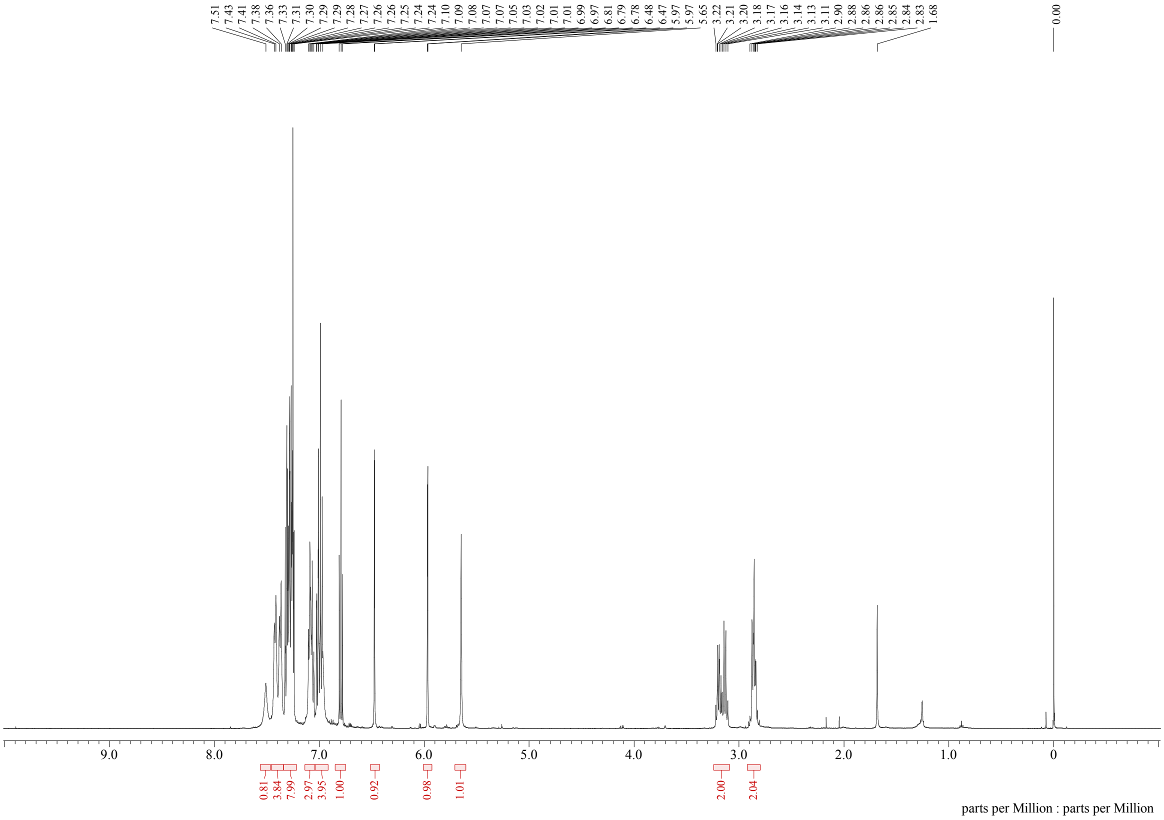

1-((3-chloro-2-hydroxyphenyl)(4-fluorophenyl)methyl)indoline-4,6-diyl bis(phenylcarbamate) (**CHBC**)
^{13}C NMR (126 MHz, $CDCl_3$)

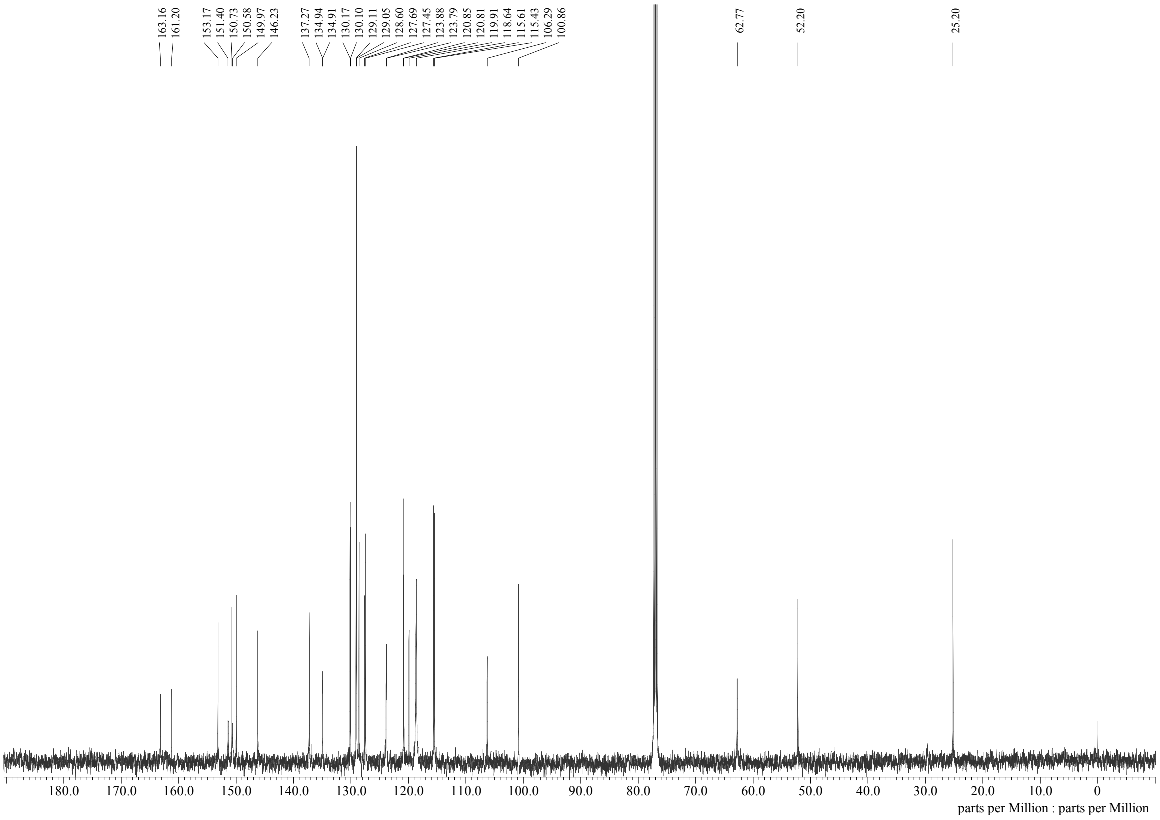

Supplement: Supplementary file 1 — jm1c00277_si_001.pdf [file jm1c00277_si_001.pdf]
