# Supplementary material for: Synthesis and Preclinical Validation of Novel Indole Derivatives as a GPR17 Agonist for Glioblastoma Treatment
Source: J Med Chem. 2021 Jul 26;64(15):10908–18. doi: 10.1021/acs.jmedchem.1c00277 (PMC8389915; doi:10.1021/acs.jmedchem.1c00277)
Supplement: Supplementary file 2 — jm1c00277_si_002.pdf [file jm1c00277_si_002.pdf]

Set 1

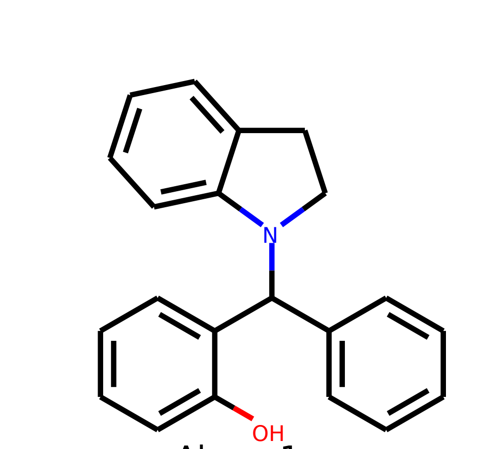
Ahma-1
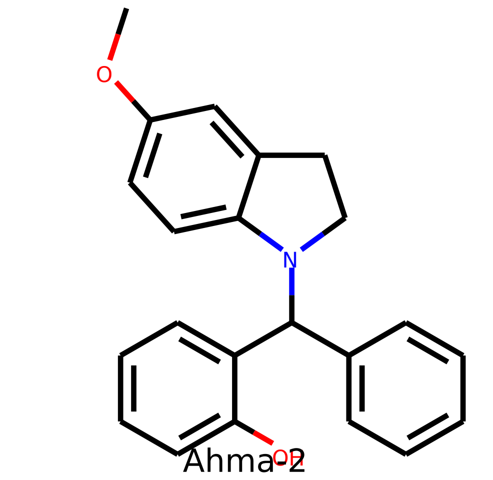
Ahma-2
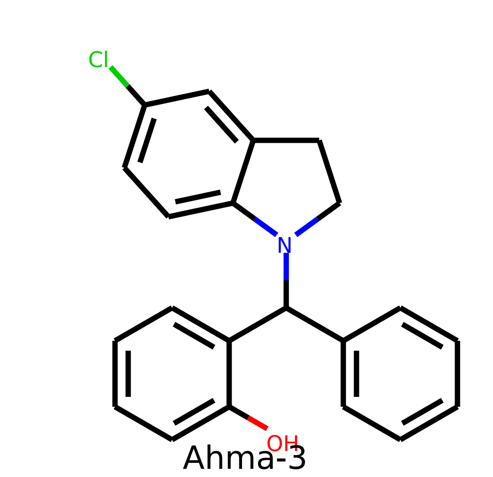
Ahma-3
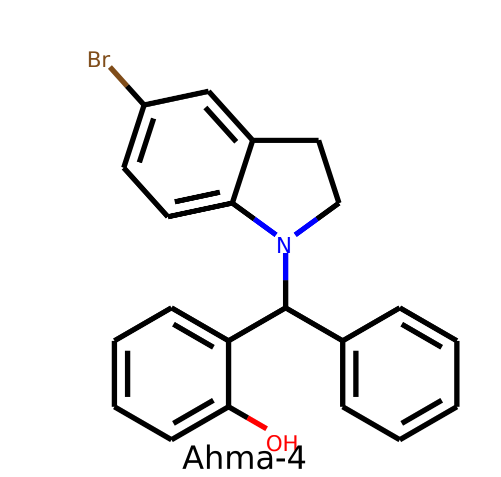
Ahma-4
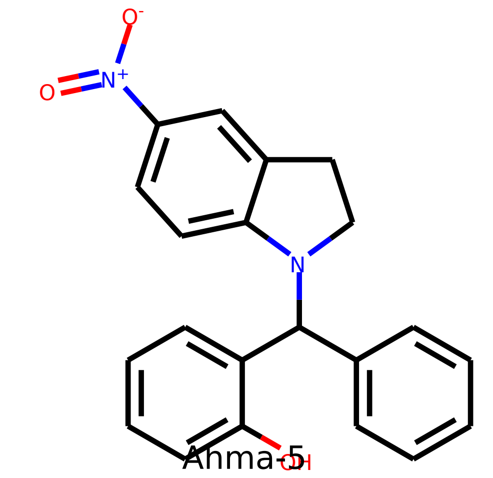
Ahma-5
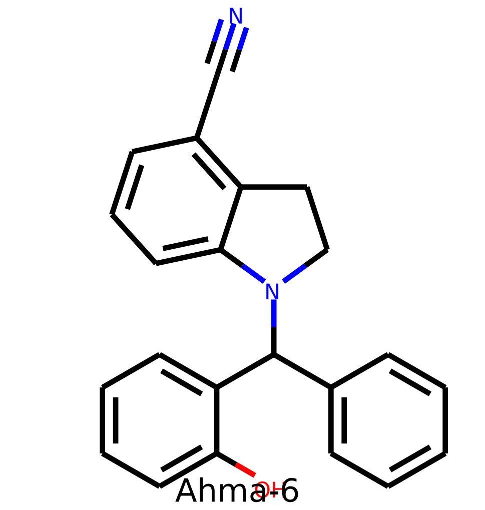
Ahma-6
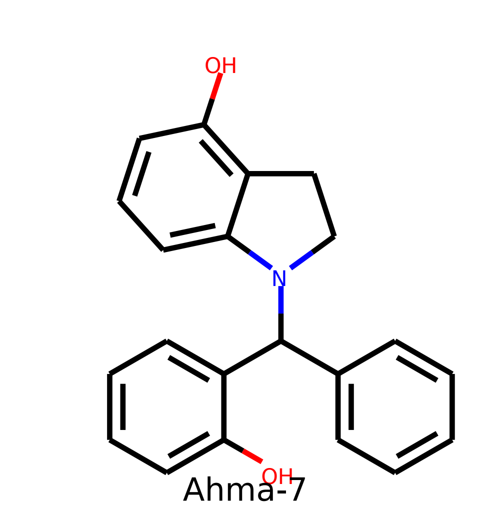
Ahma-7
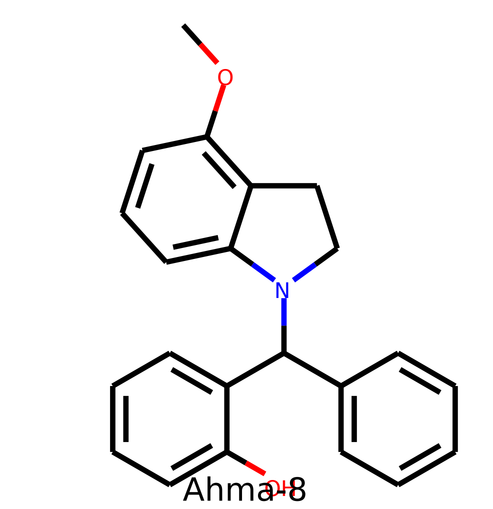
Ahma-8
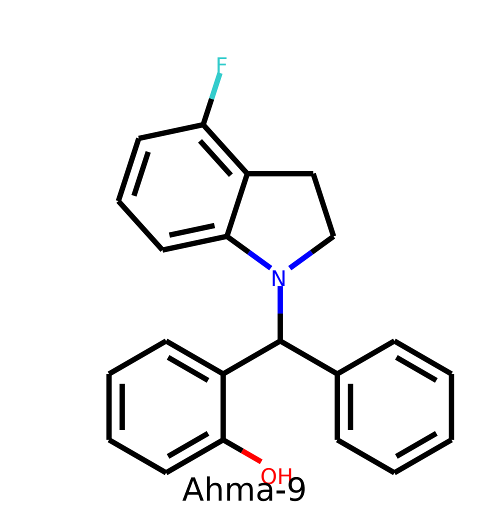
Ahma-9
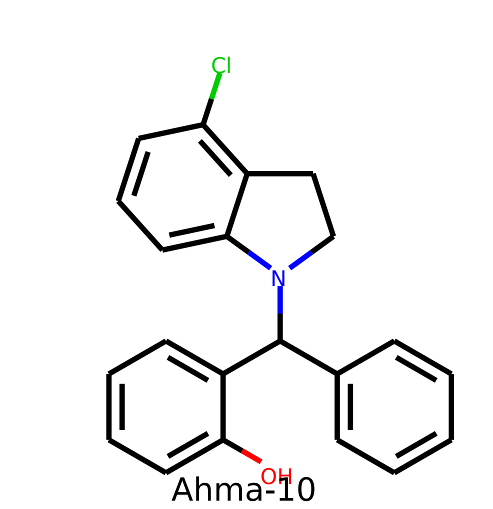
Ahma-10
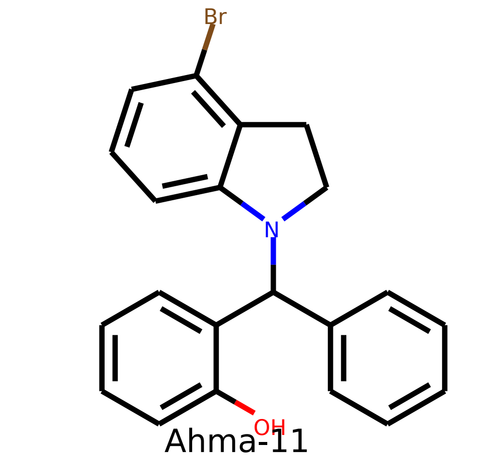
Ahma-11
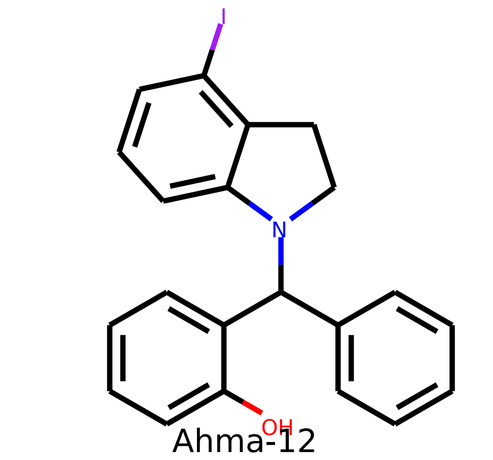
Ahma-12
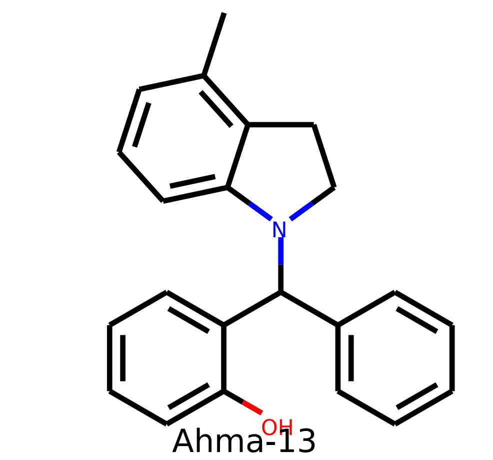
Ahma-13
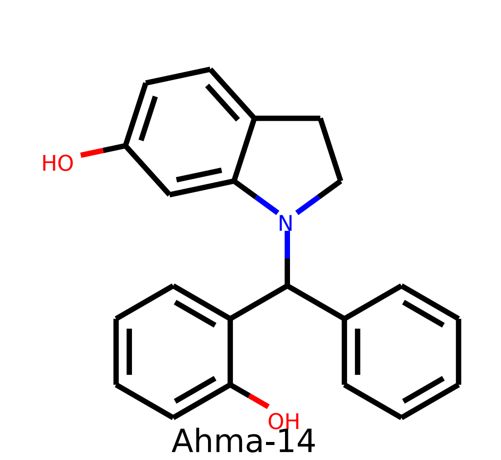
Ahma-14
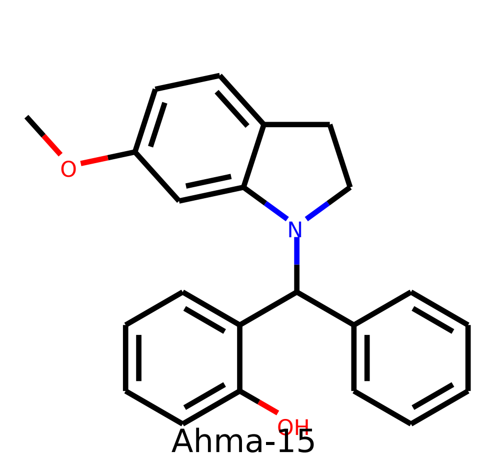
Ahma-15
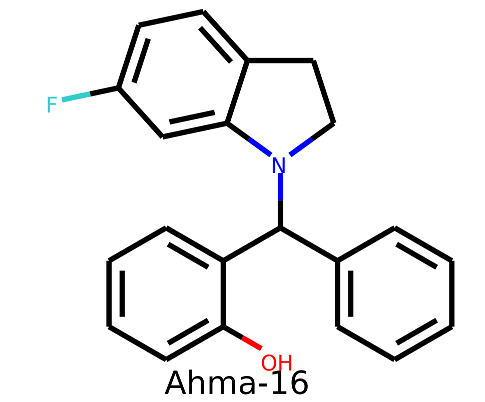
Ahma-16
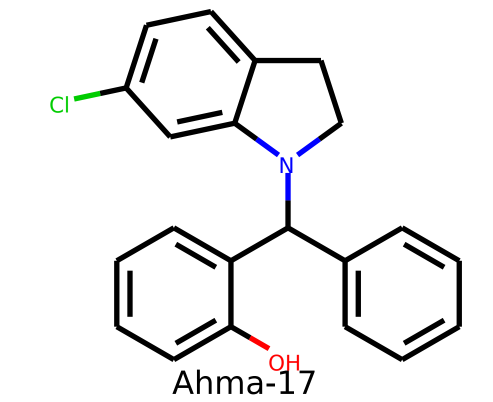
Ahma-17
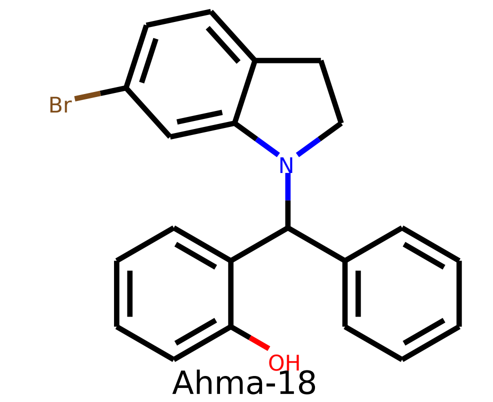
Ahma-18
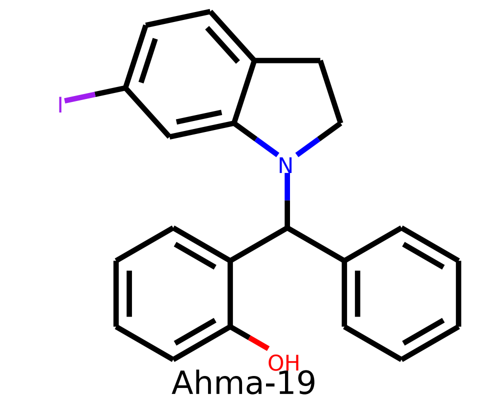
Ahma-19
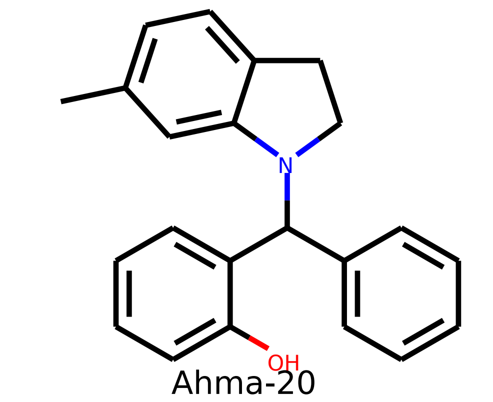
Ahma-20
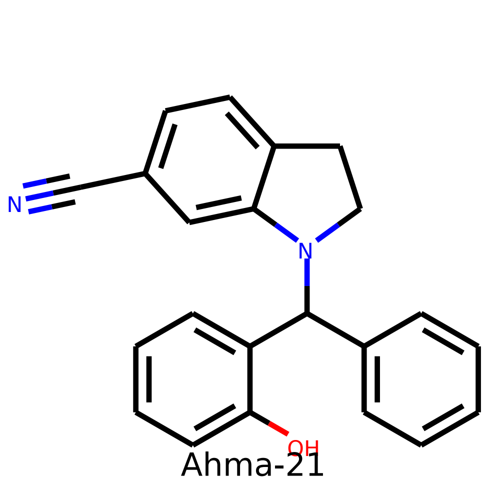
Ahma-21
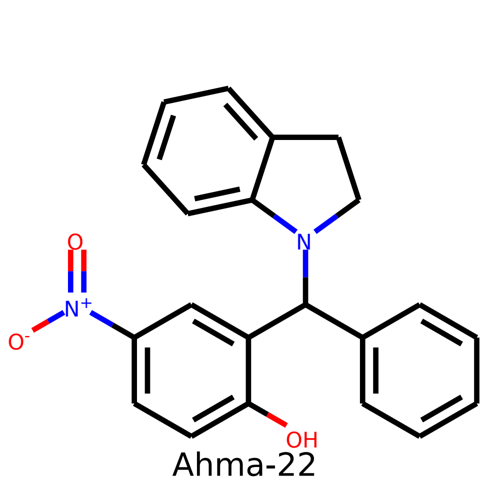
Ahma-22
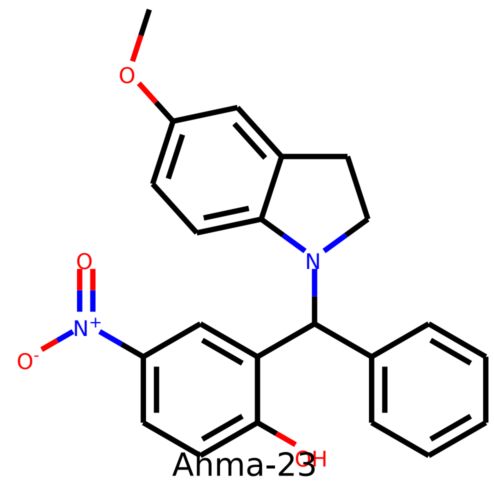
Ahma-23
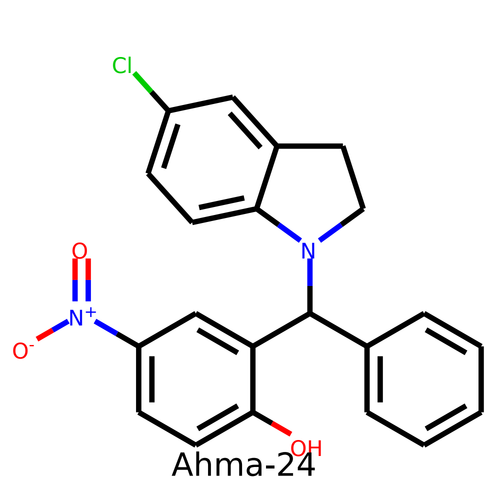
Ahma-24
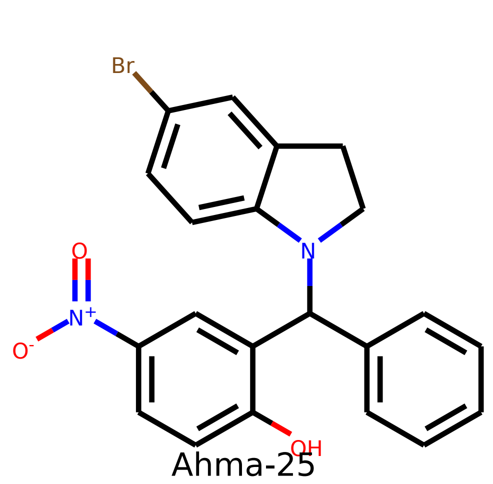
Ahma-25
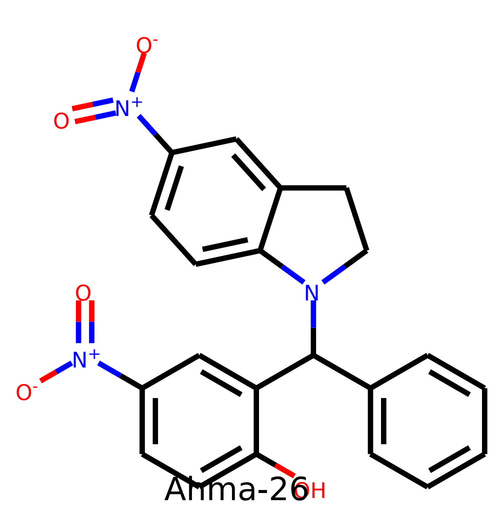
Ahma-26
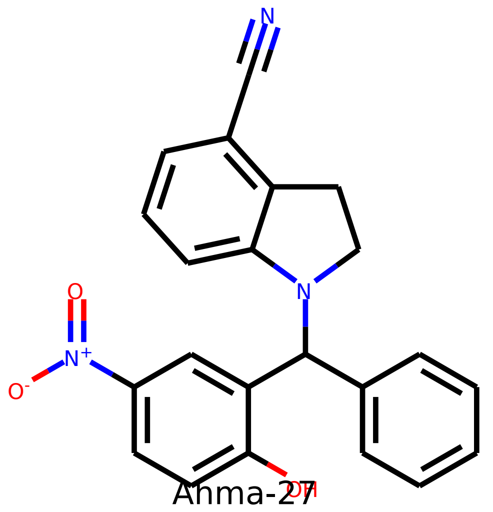
Ahma-27
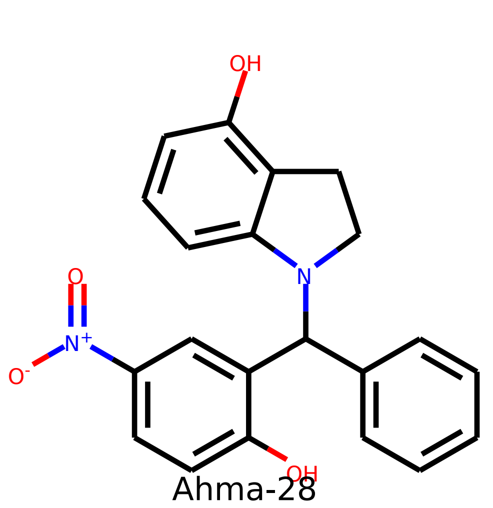
Ahma-28
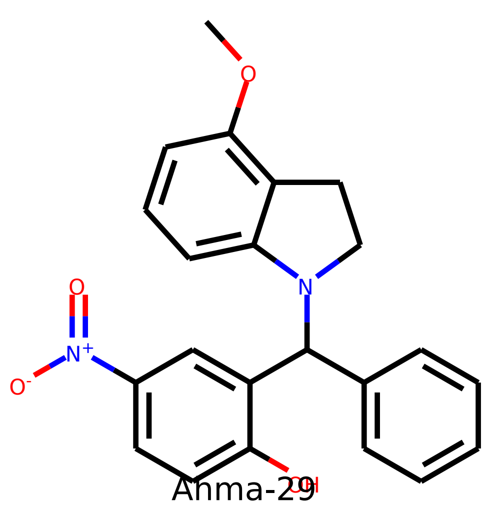
Ahma-29
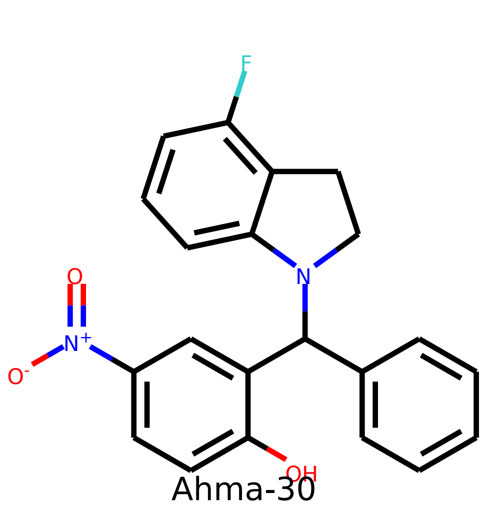
Ahma-30
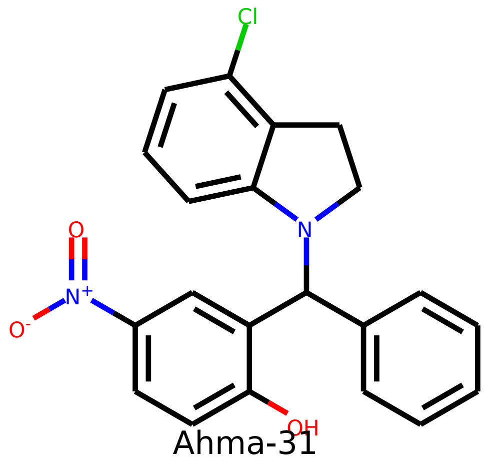
Ahma-31
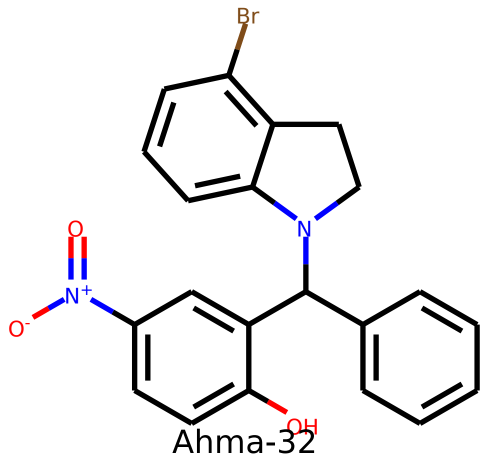
Ahma-32
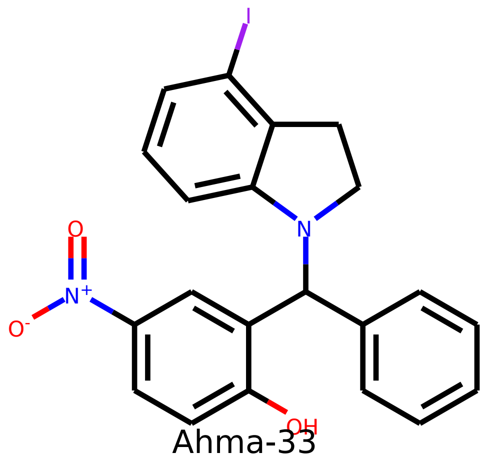
Ahma-33
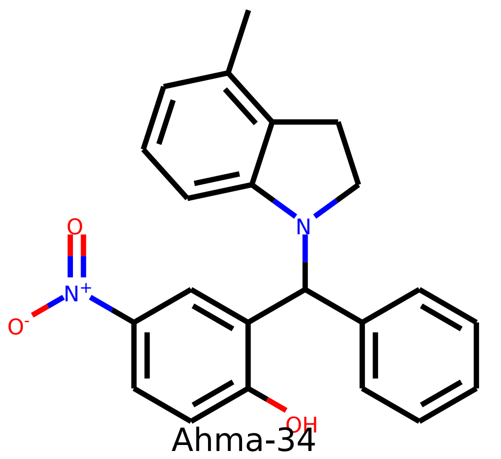
Ahma-34
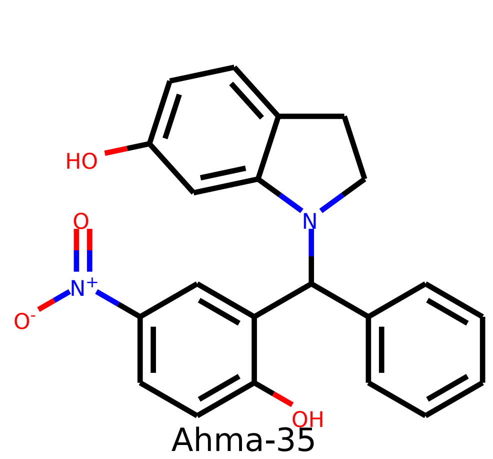
Ahma-35

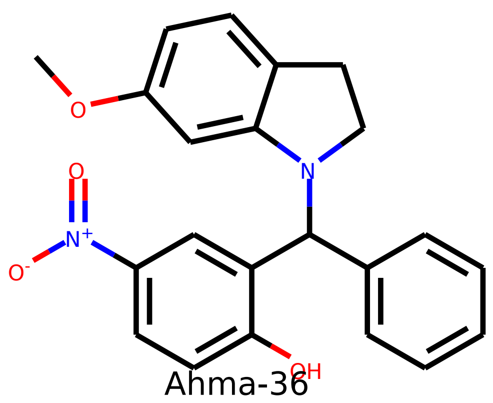
Ahma-36
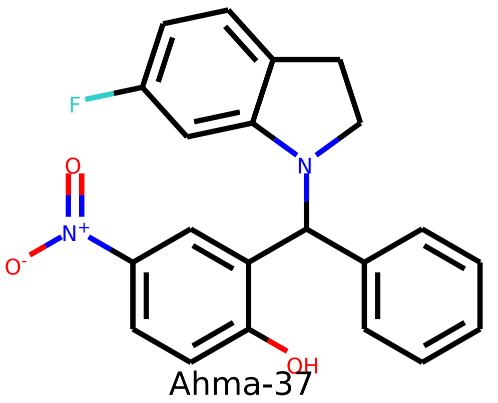
Ahma-37
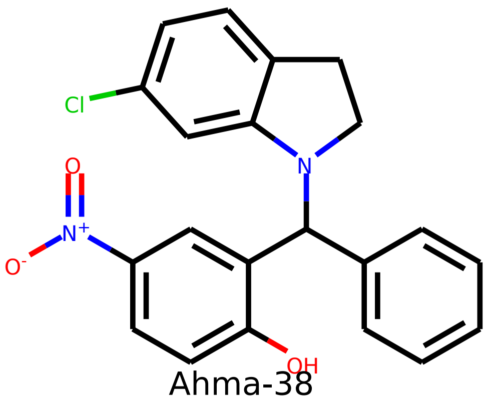
Ahma-38
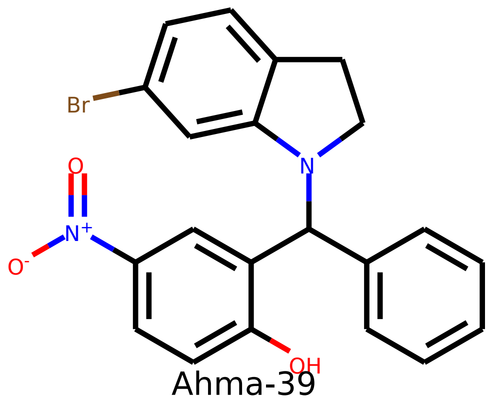
Ahma-39
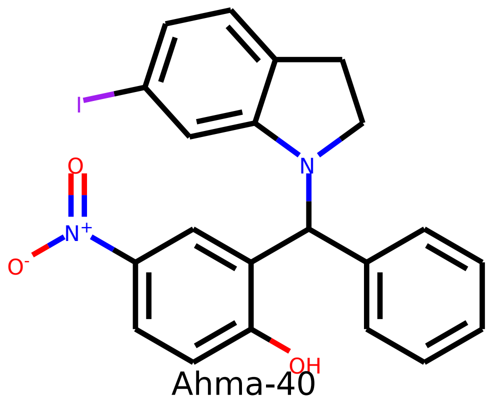
Ahma-40
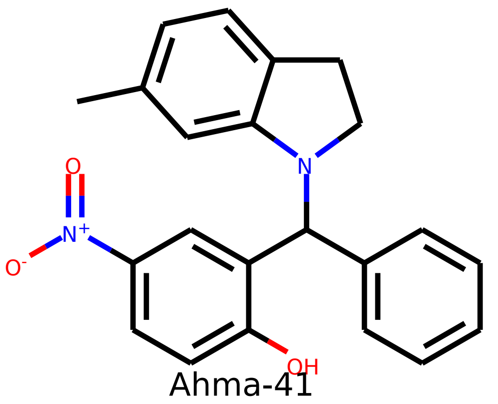
Ahma-41
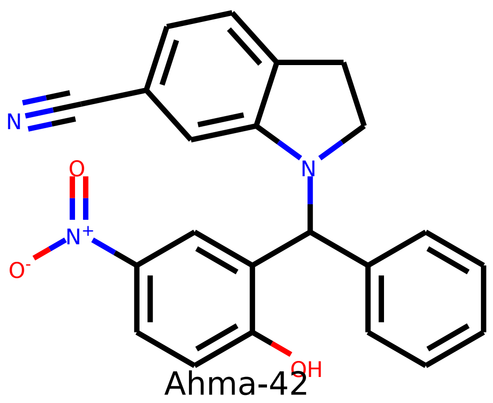
Ahma-42
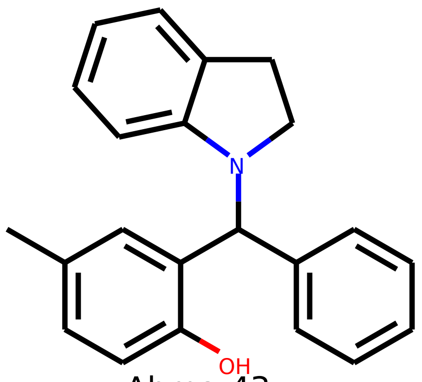
Ahma-43
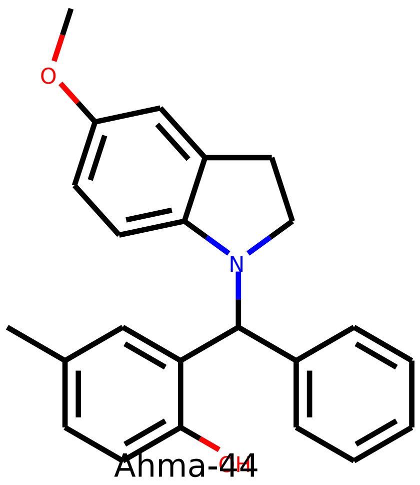
Ahma-44
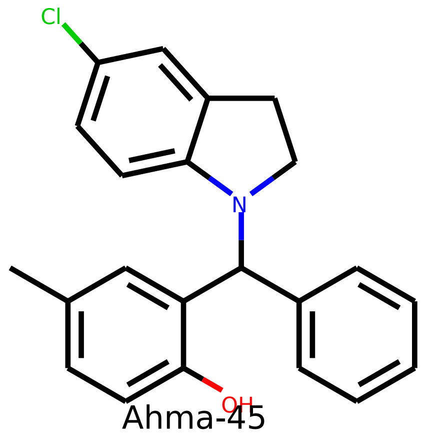
Ahma-45
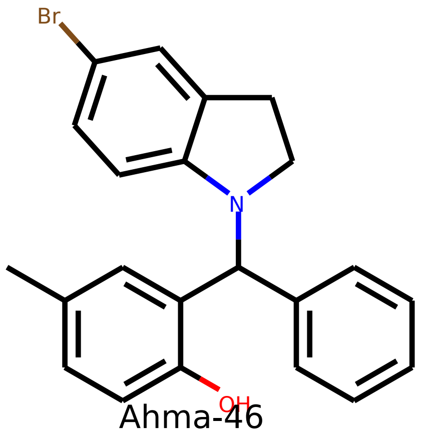
Ahma-46
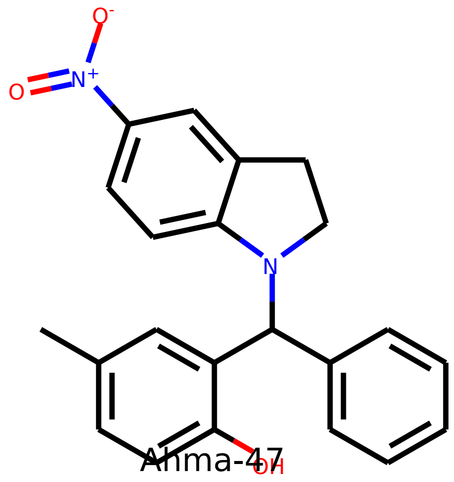
Ahma-47
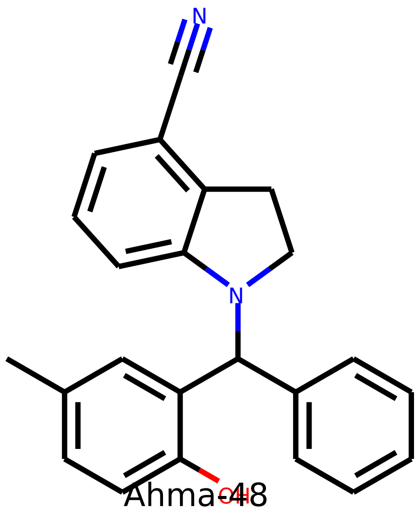
Ahma-48
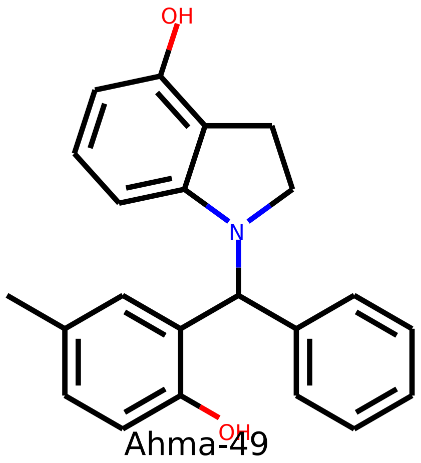
Ahma-49
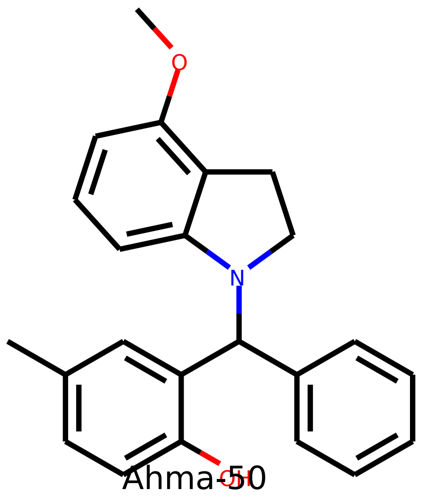
Ahma-50
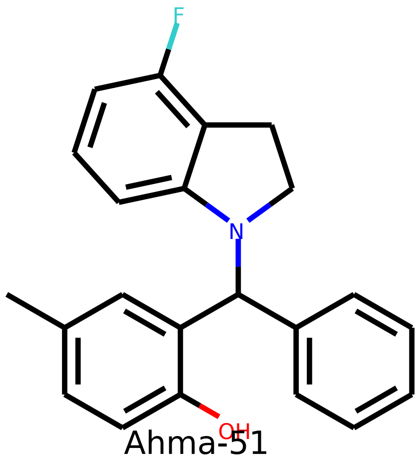
Ahma-51
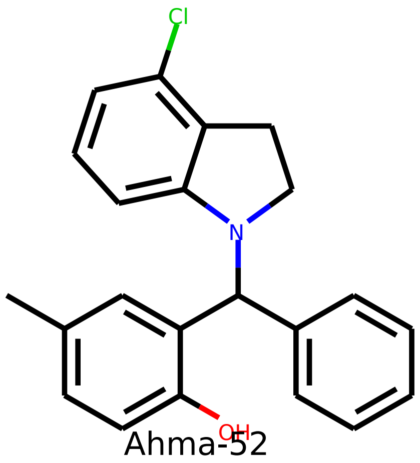
Ahma-52
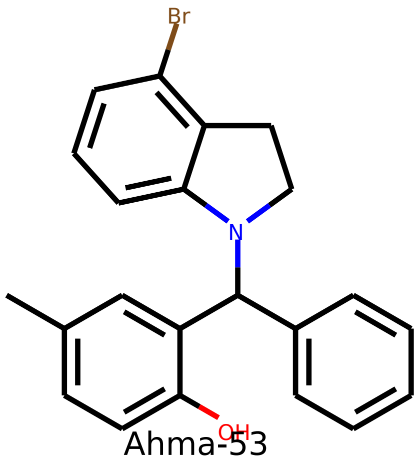
Ahma-53
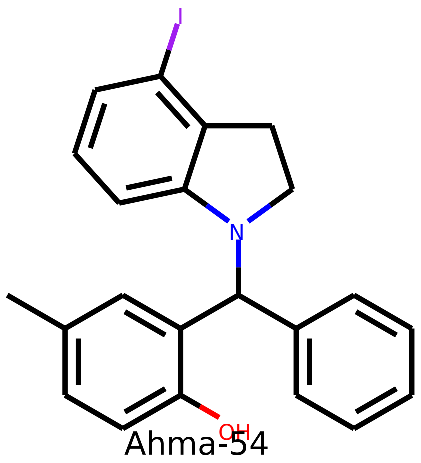
Ahma-54
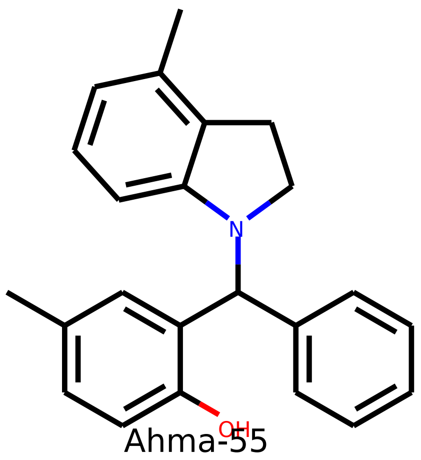
Ahma-55
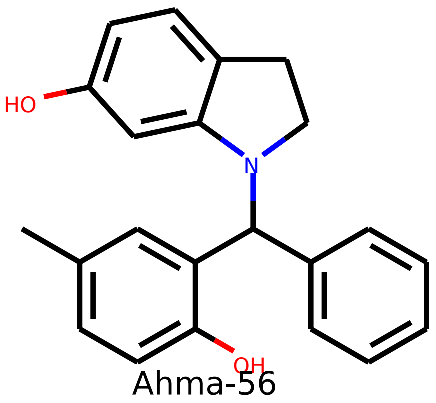
Ahma-56
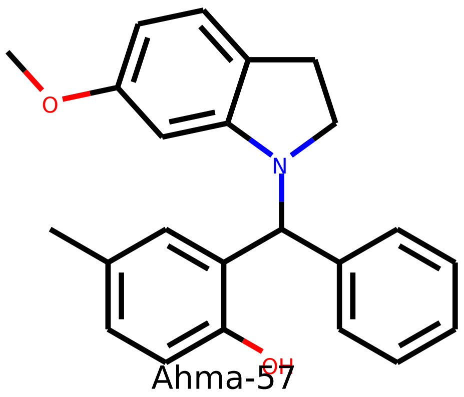
Ahma-57
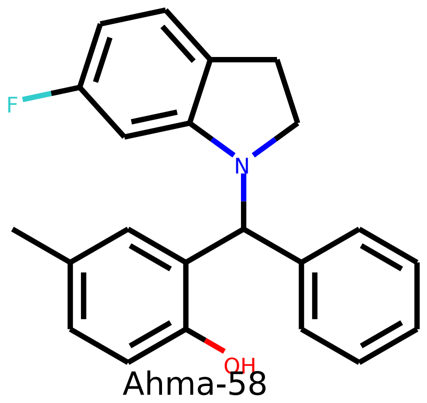
Ahma-58
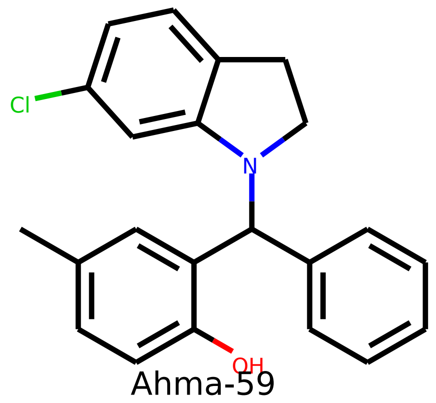
Ahma-59
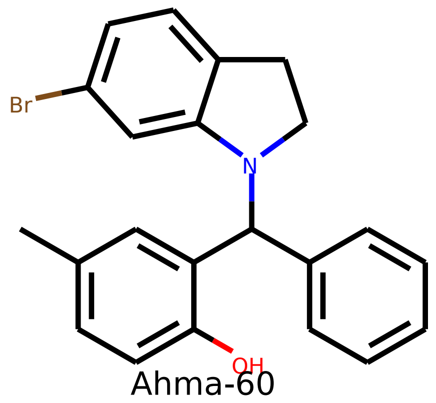
Ahma-60
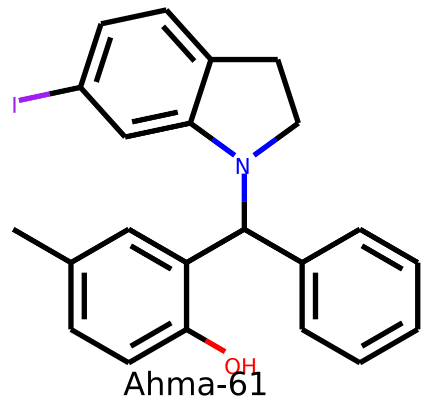
Ahma-61
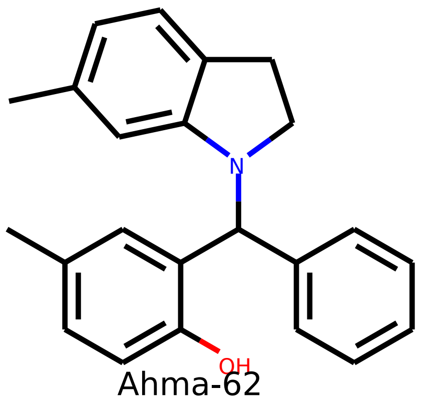
Ahma-62
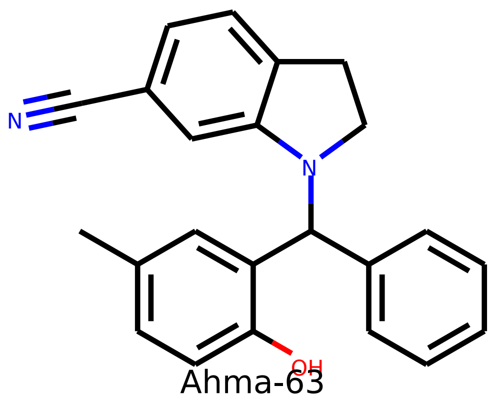
Ahma-63
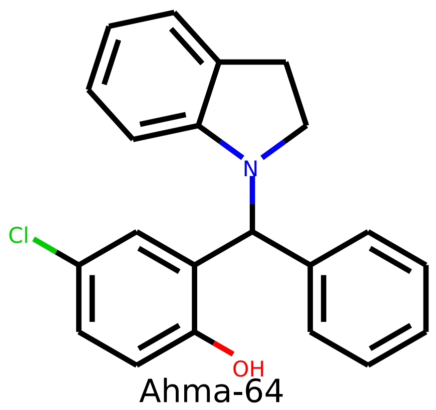
Ahma-64
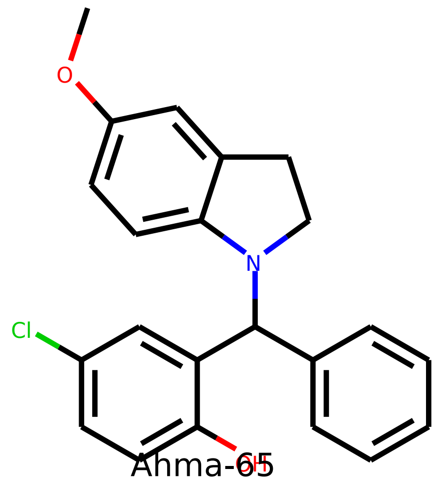
Ahma-65
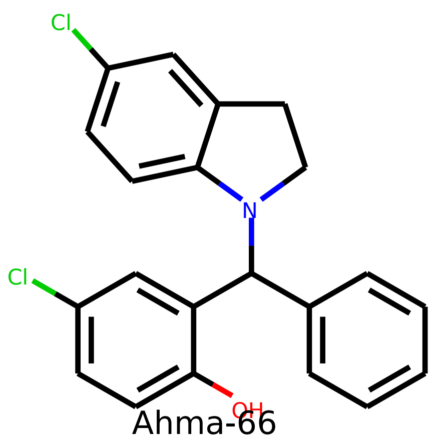
Ahma-66
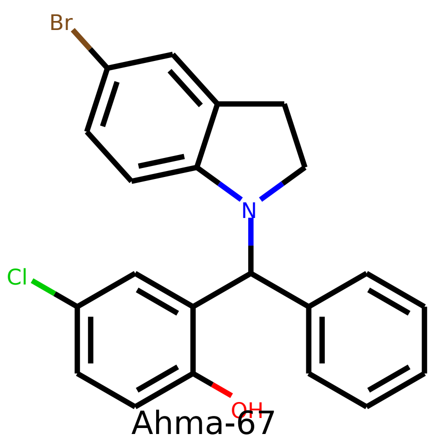
Ahma-67
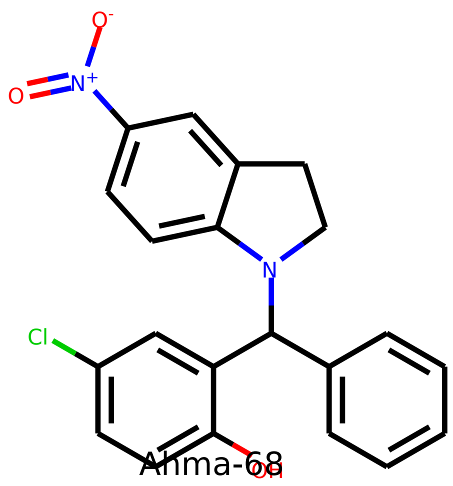
Ahma-68
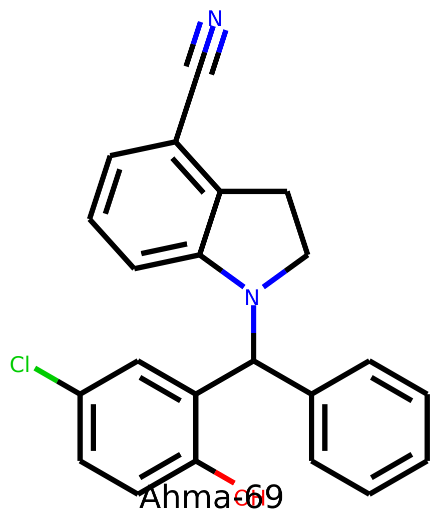
Ahma-69
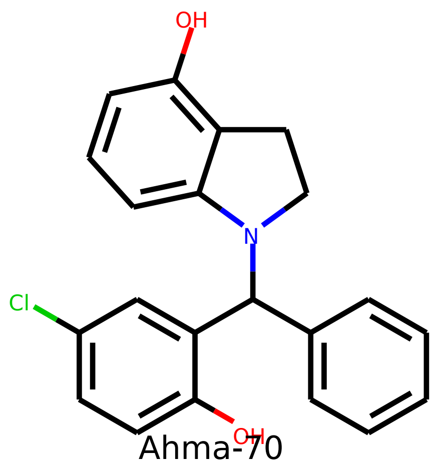
Ahma-70

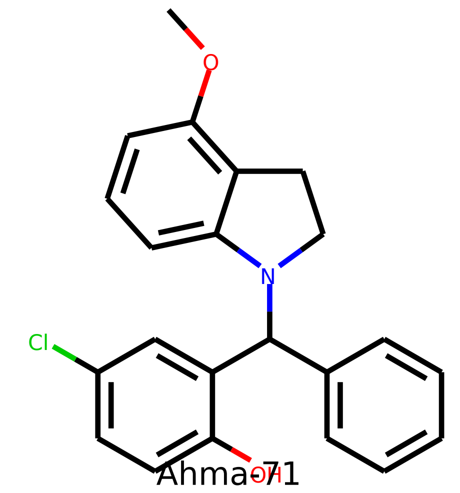
Ahma-71

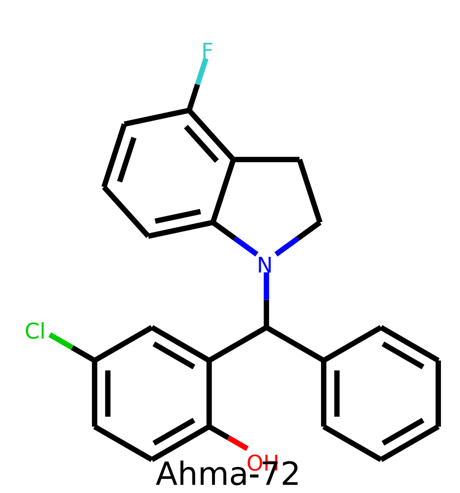
Ahma-72

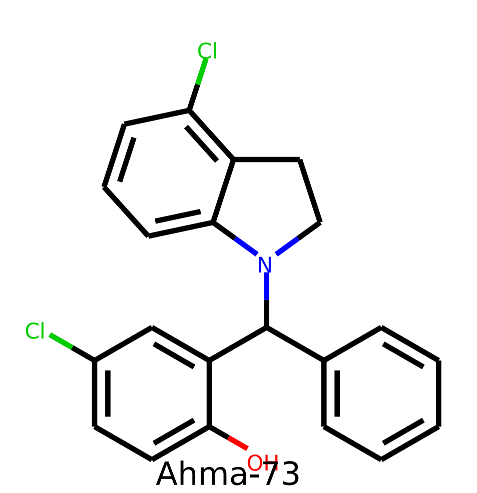
Ahma-73

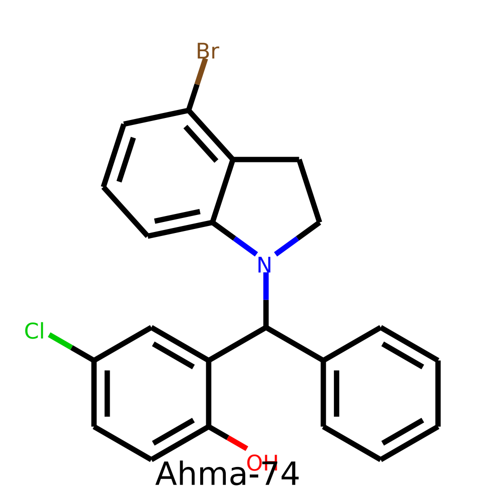
Ahma-74

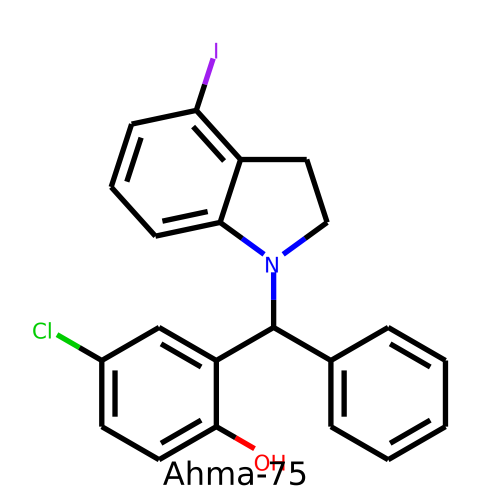
Ahma-75

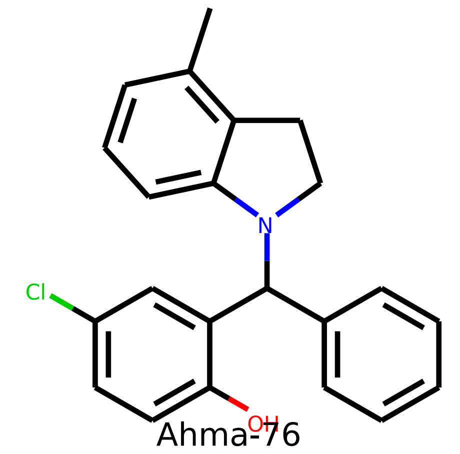
Ahma-76

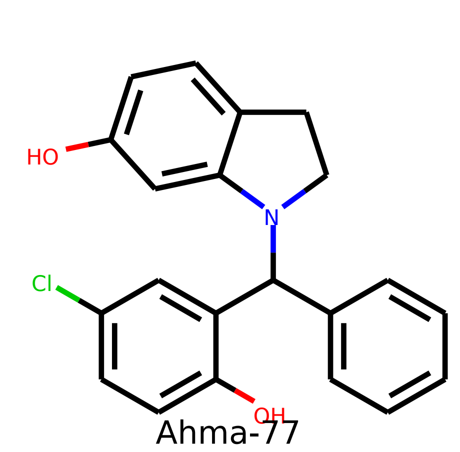
Ahma-77

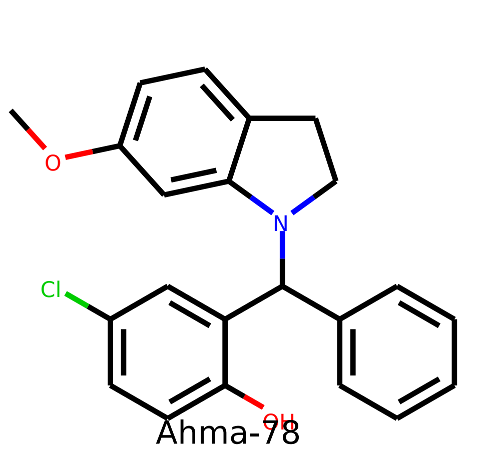
Ahma-78

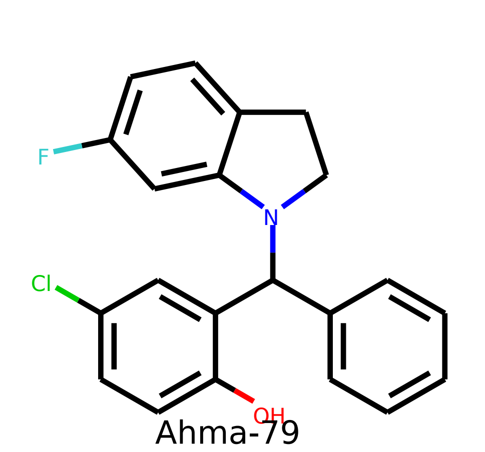
Ahma-79

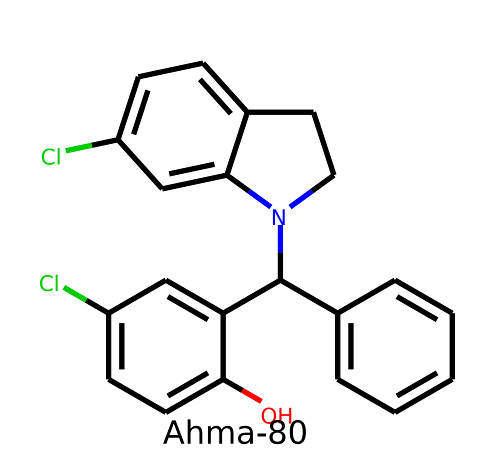
Ahma-80

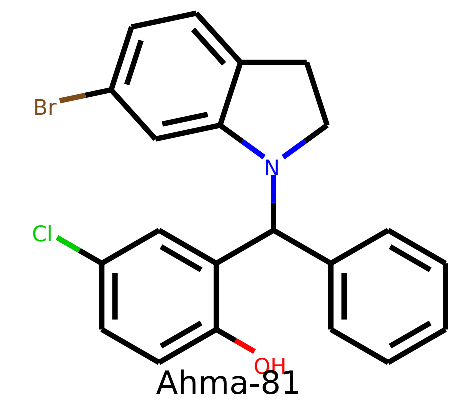
Ahma-81

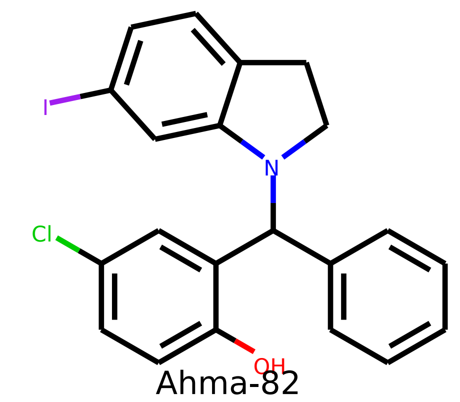
Ahma-82

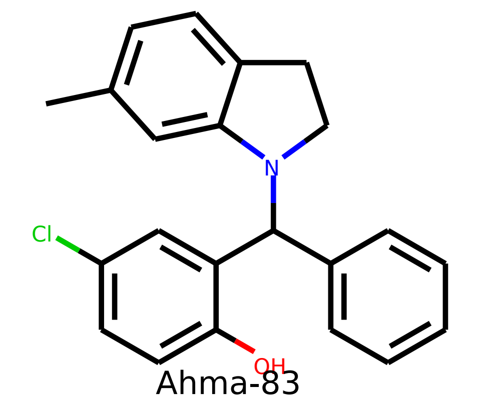
Ahma-83

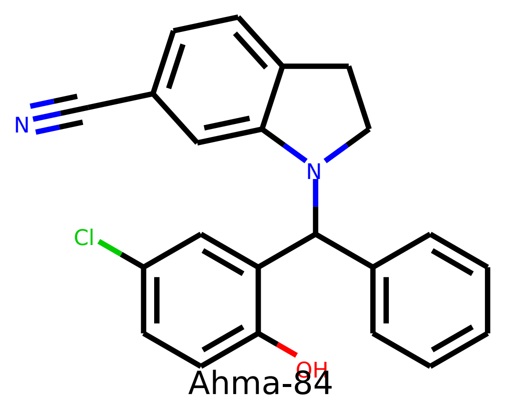
Ahma-84

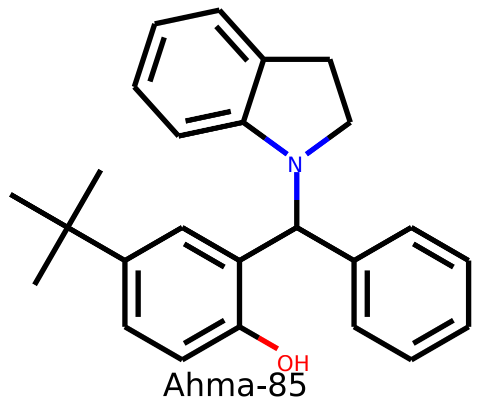
Ahma-85

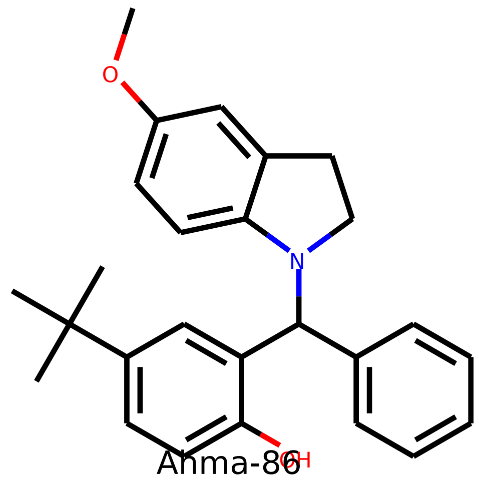
Ahma-86

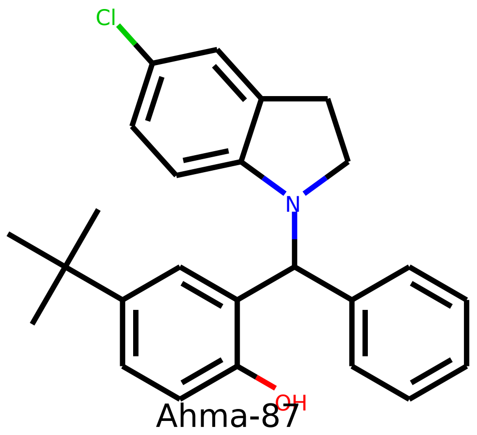
Ahma-87

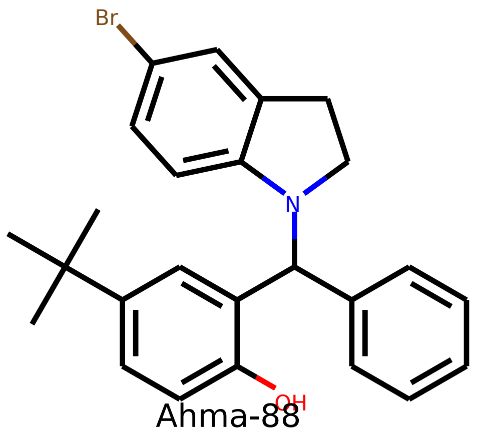
Ahma-88

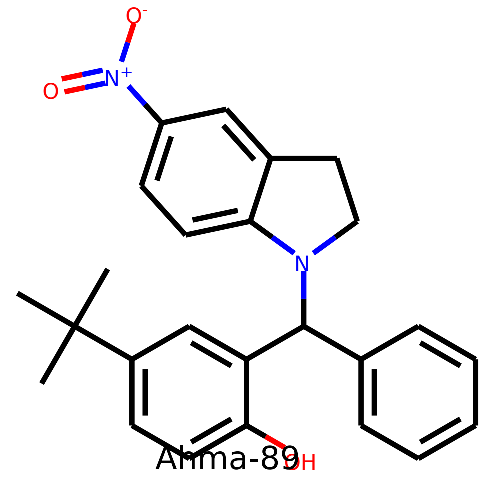
Ahma-89

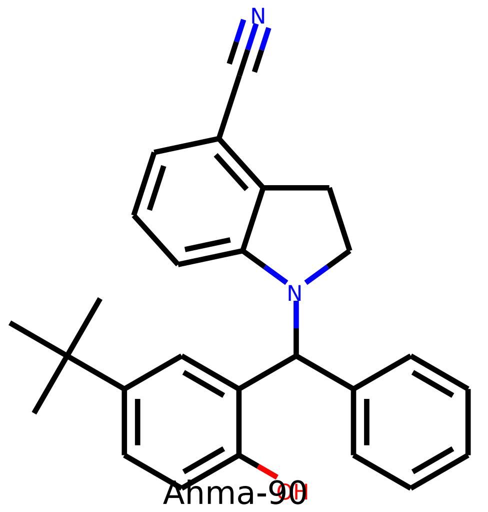
Ahma-90

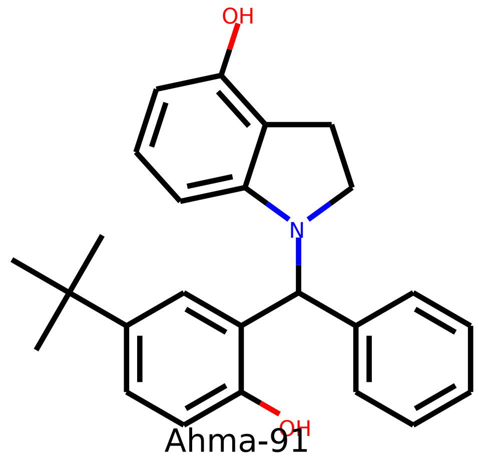
Ahma-91

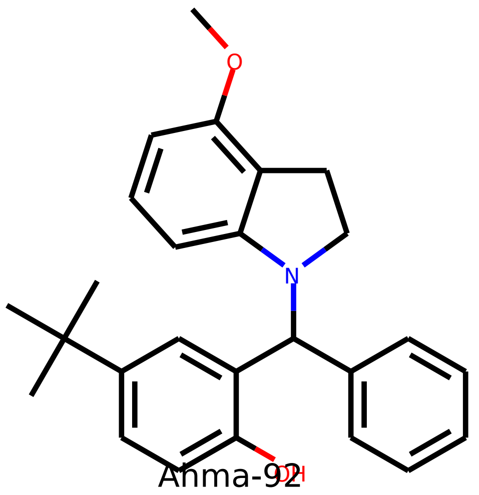
Ahma-92

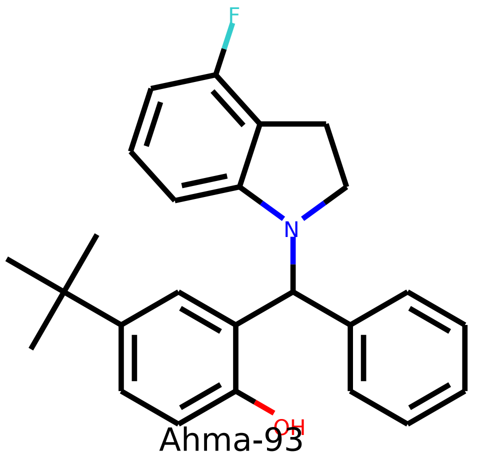
Ahma-93

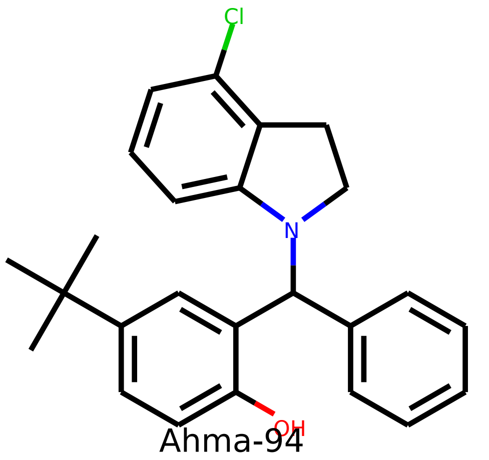
Ahma-94

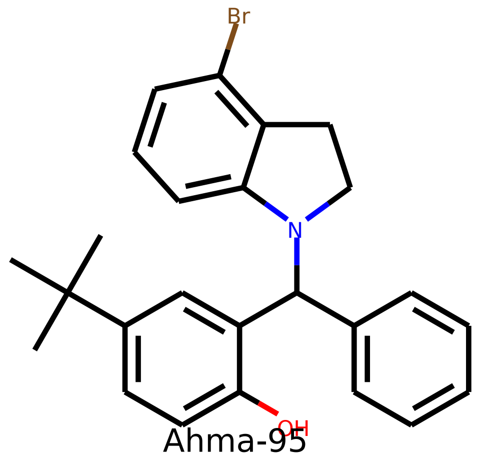
Ahma-95

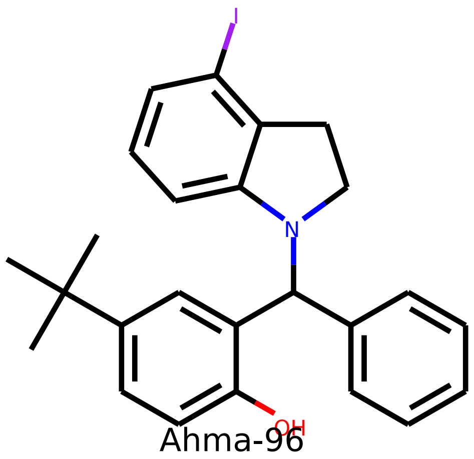
Ahma-96

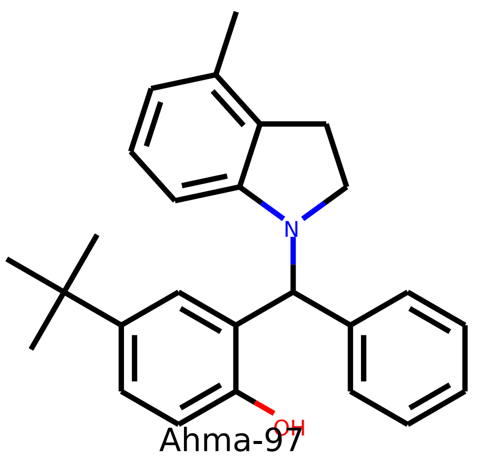
Ahma-97

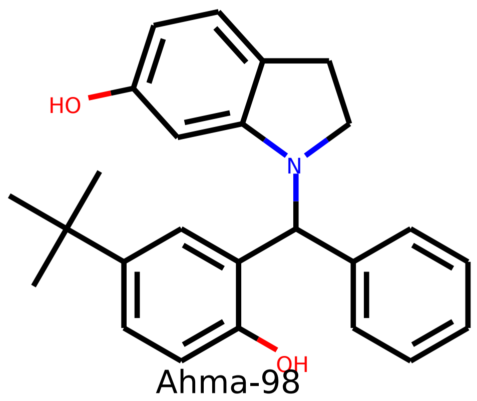
Ahma-98

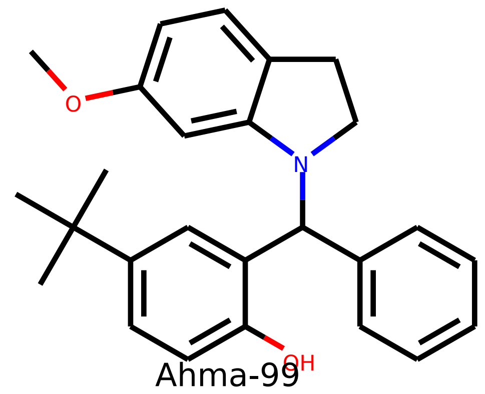
Ahma-99

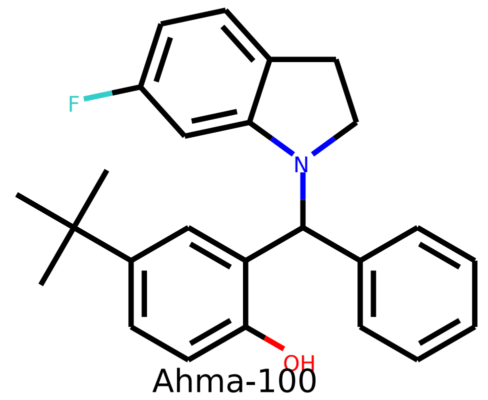
Ahma-100

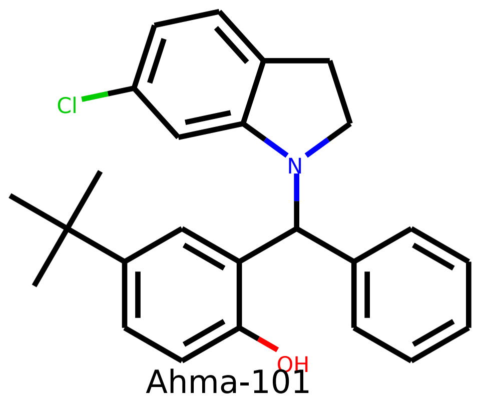
Ahma-101

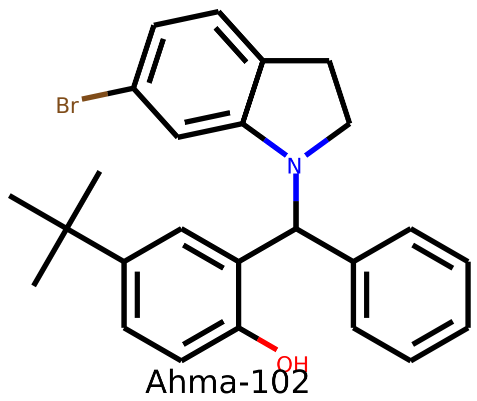
Ahma-102

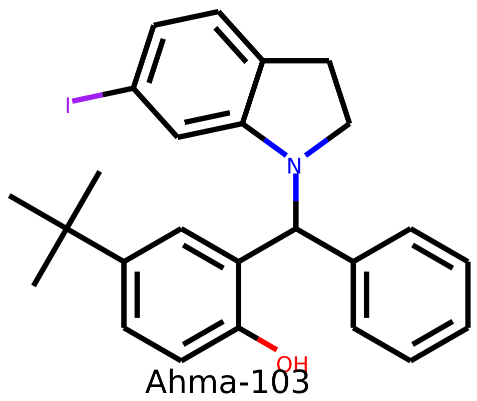
Ahma-103

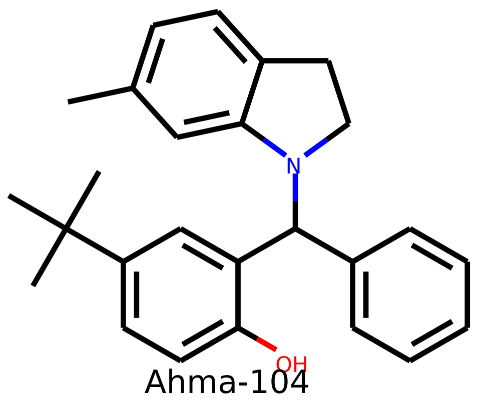
Ahma-104

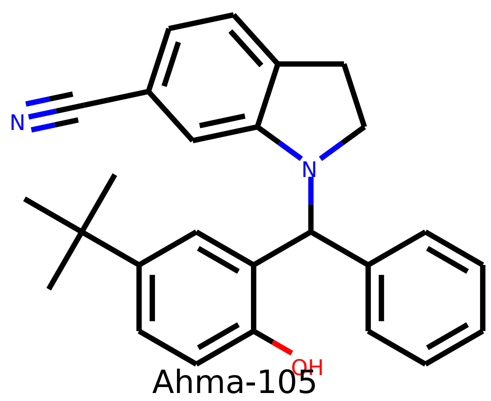
Ahma-105

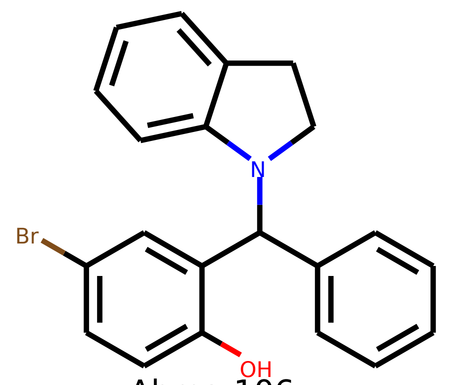
Ahma-106
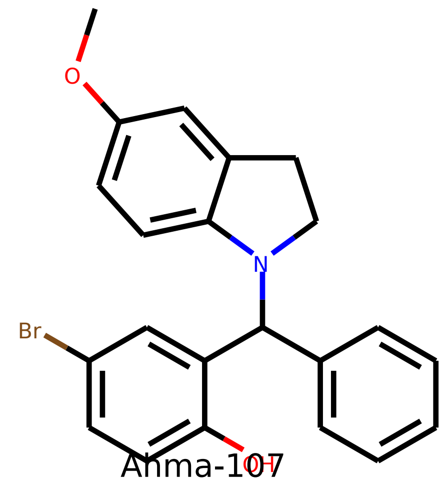
Ahma-107
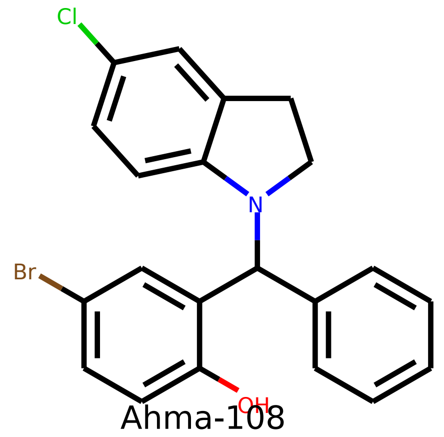
Ahma-108
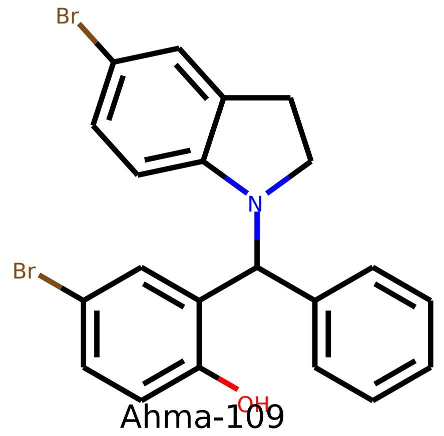
Ahma-109
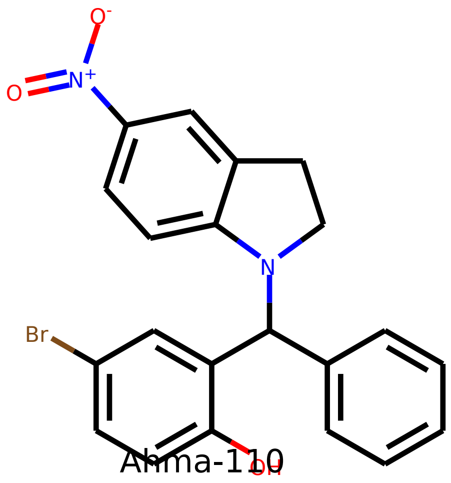
Ahma-110
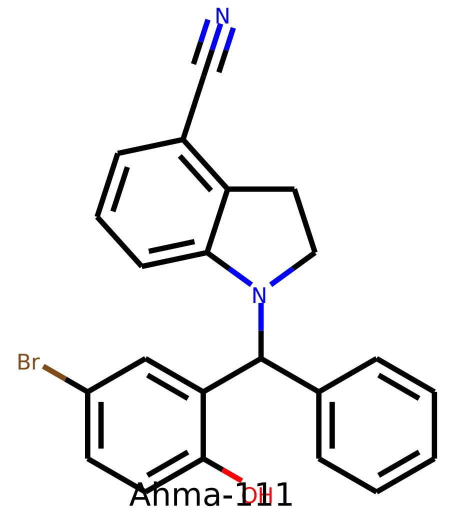
Ahma-111
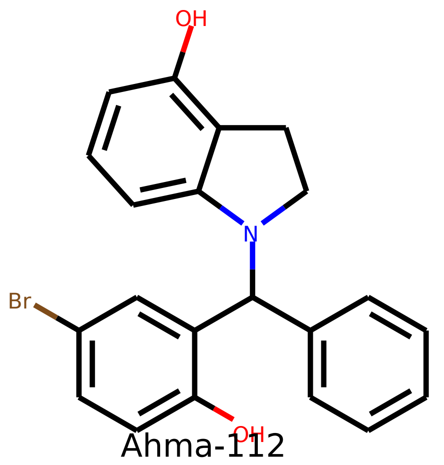
Ahma-112
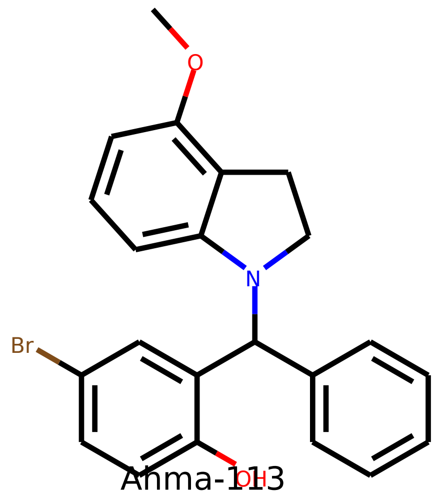
Ahma-113
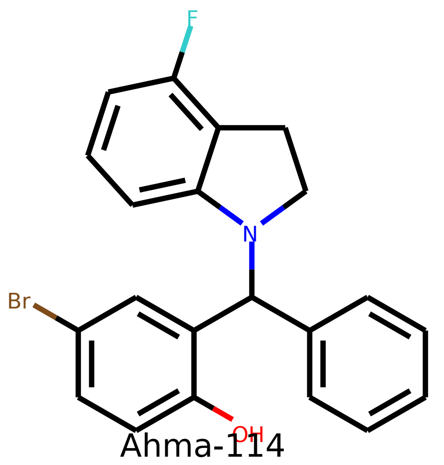
Ahma-114
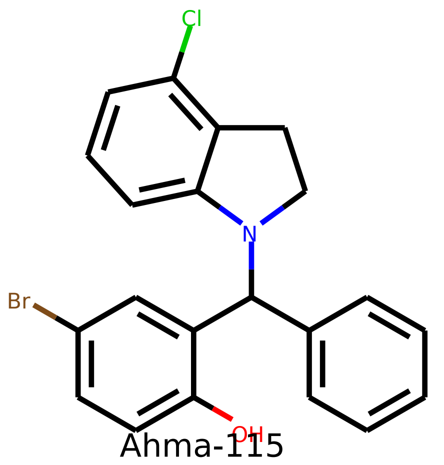
Ahma-115
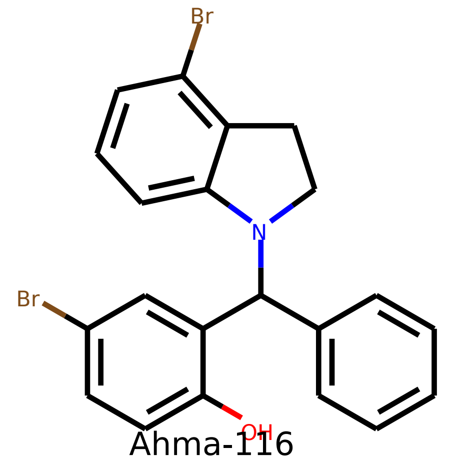
Ahma-116
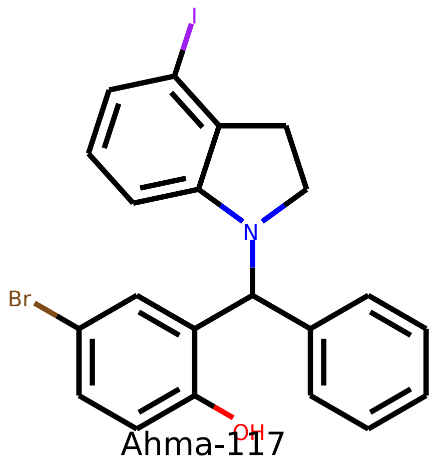
Ahma-117
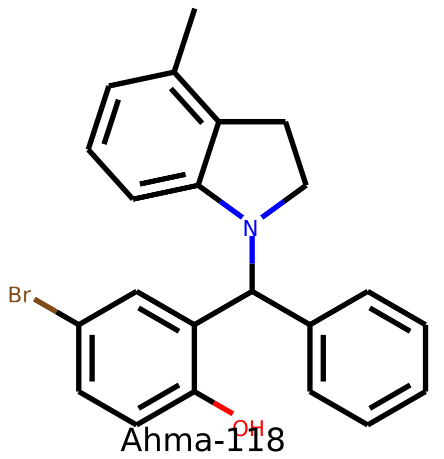
Ahma-118
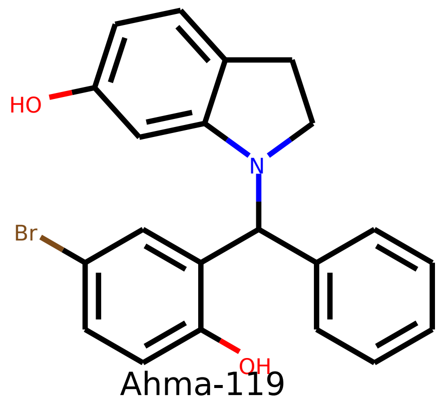
Ahma-119
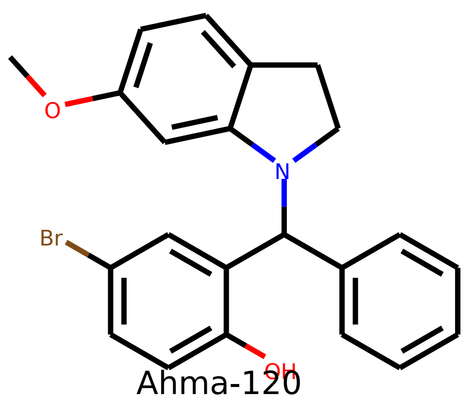
Ahma-120
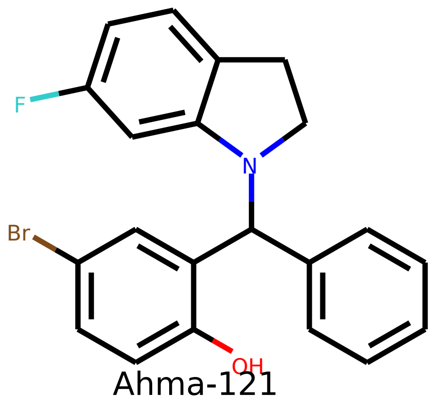
Ahma-121
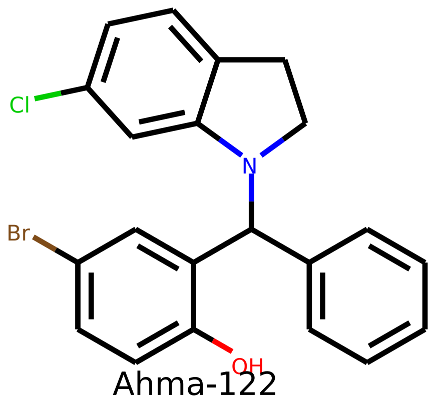
Ahma-122
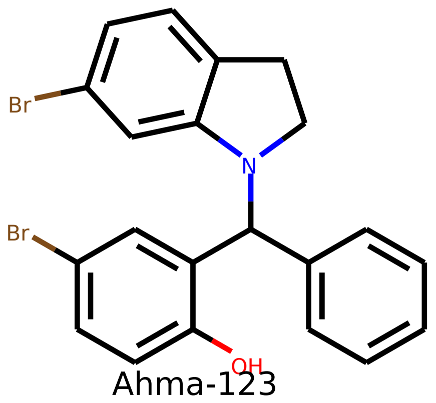
Ahma-123
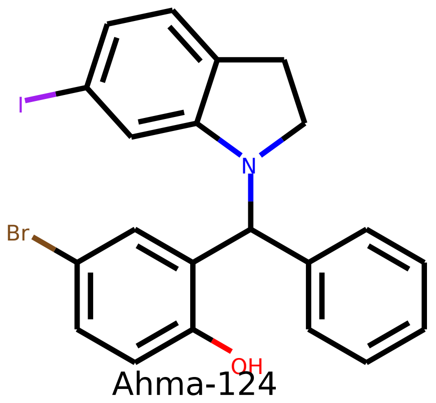
Ahma-124
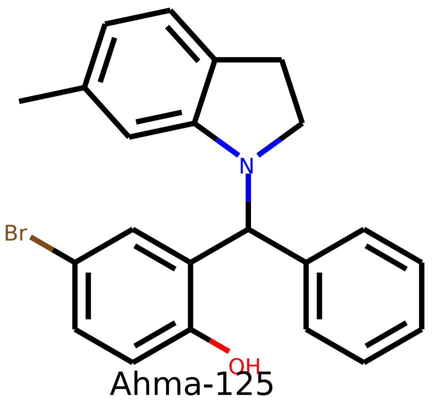
Ahma-125
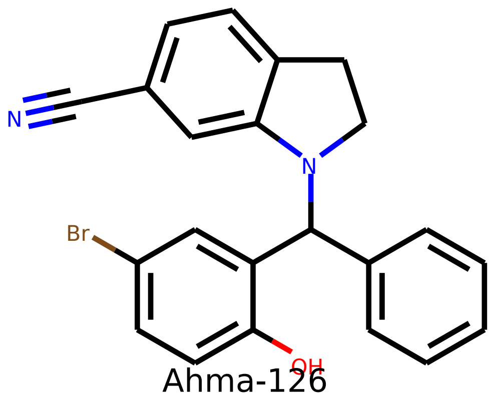
Ahma-126
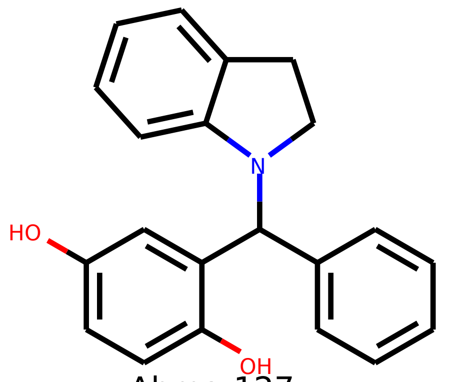
Ahma-127
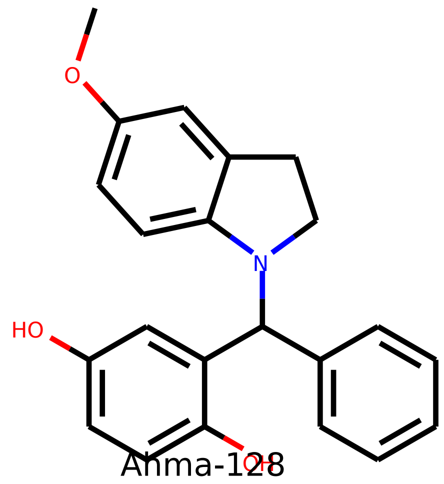
Ahma-128
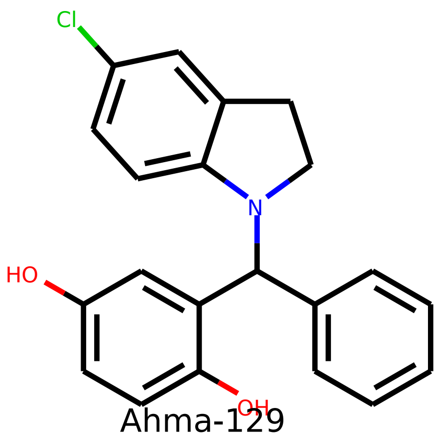
Ahma-129
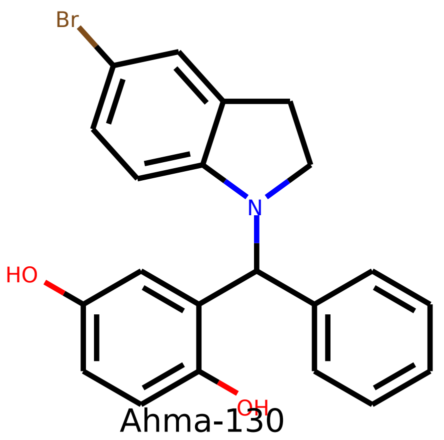
Ahma-130
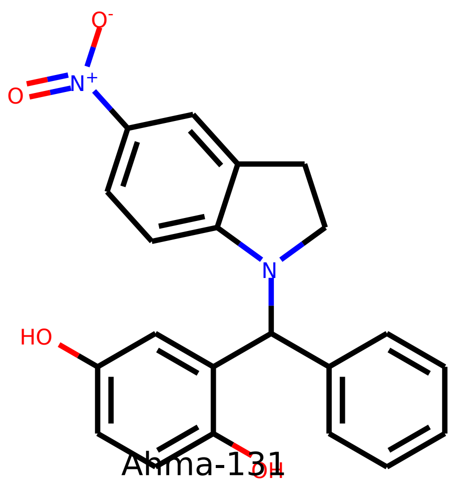
Ahma-131
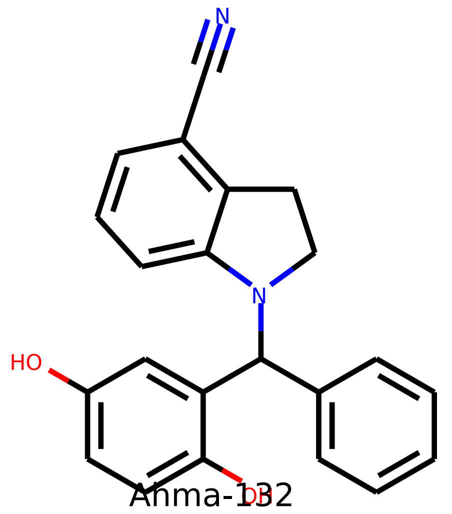
Ahma-132
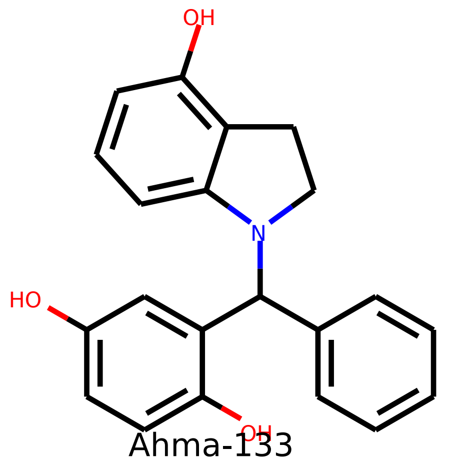
Ahma-133
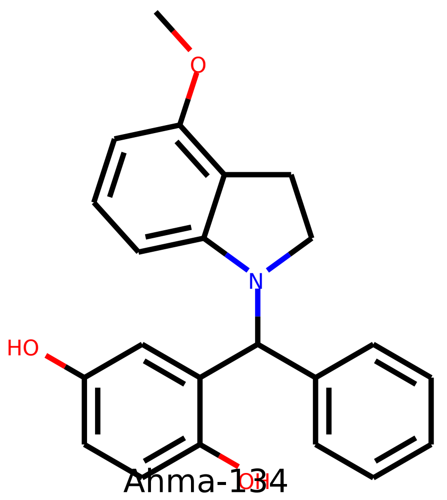
Ahma-134
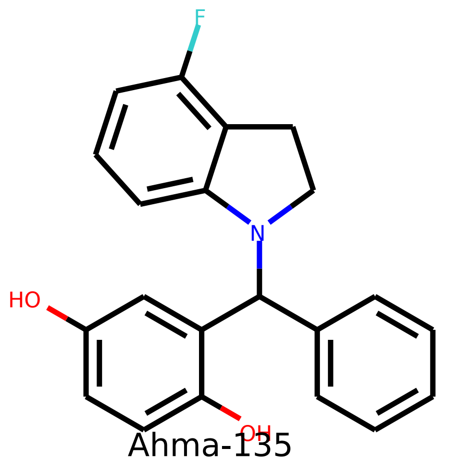
Ahma-135
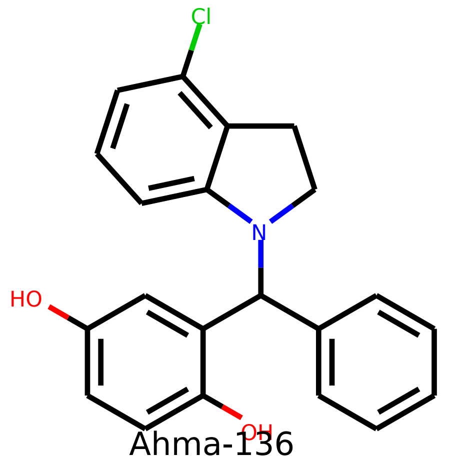
Ahma-136
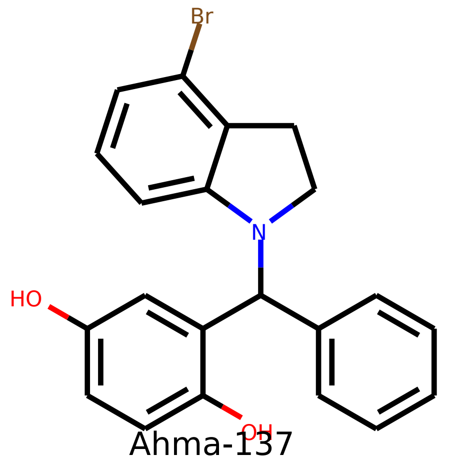
Ahma-137
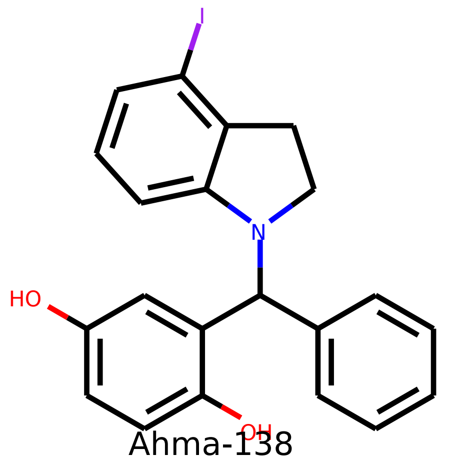
Ahma-138
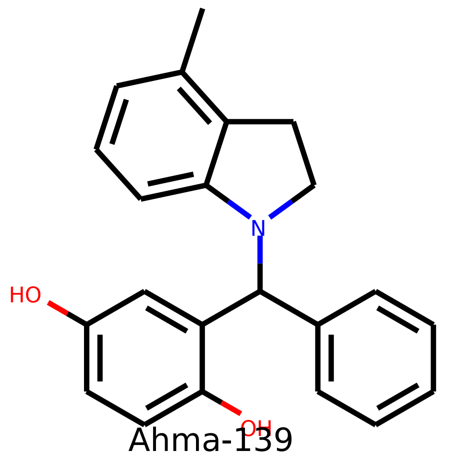
Ahma-139
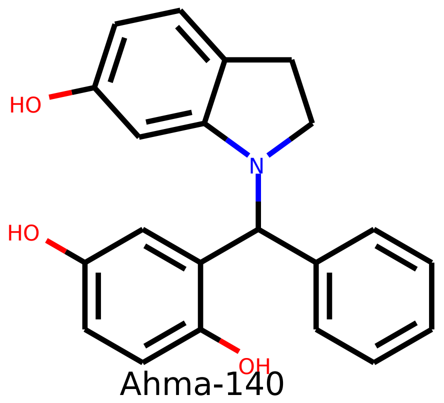
Ahma-140

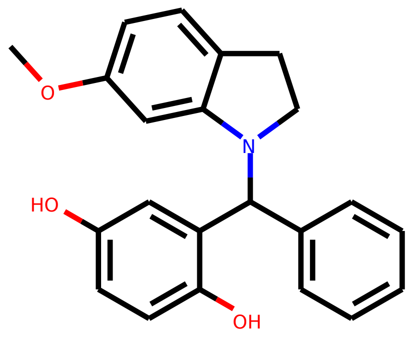
Ahma-141
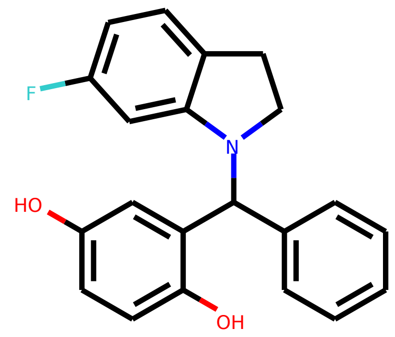
Ahma-142
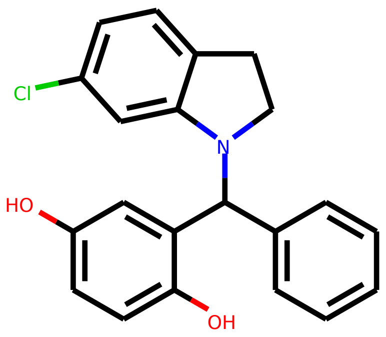
Ahma-143
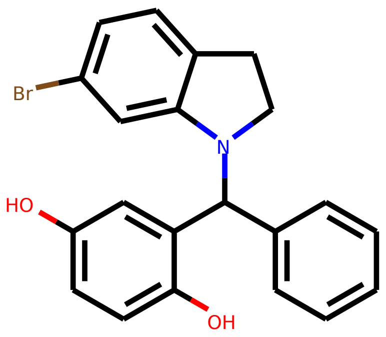
Ahma-144
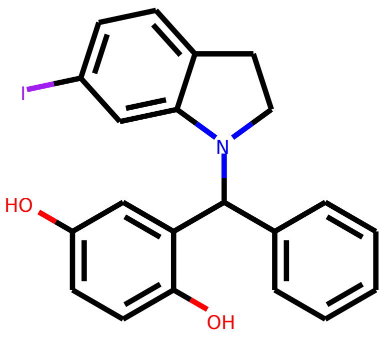
Ahma-145
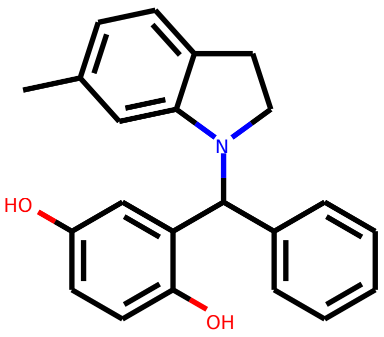
Ahma-146
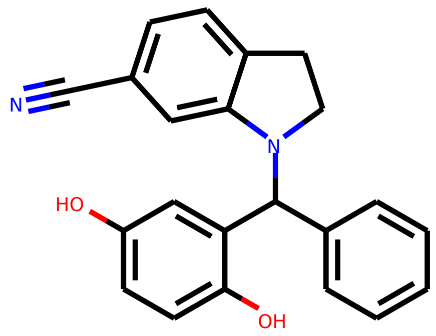
Ahma-147
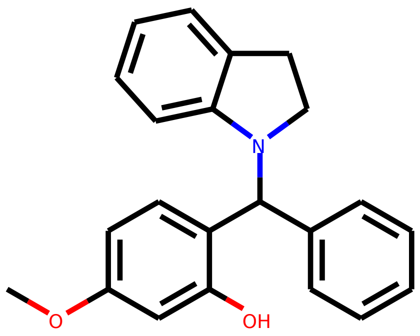
Ahma-148
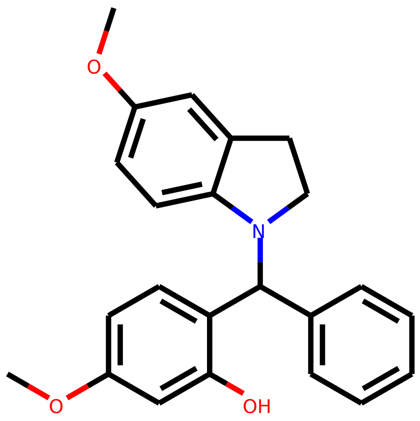
Ahma-149
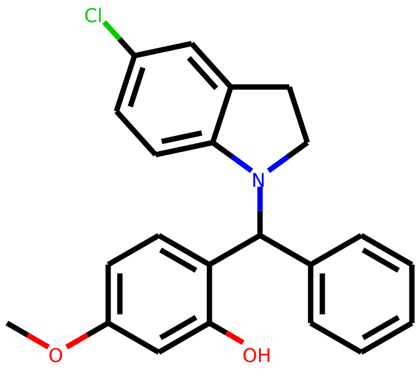
Ahma-150
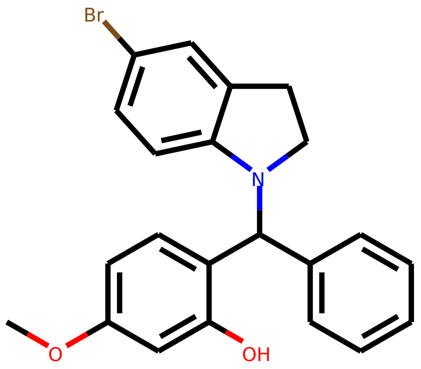
Ahma-151
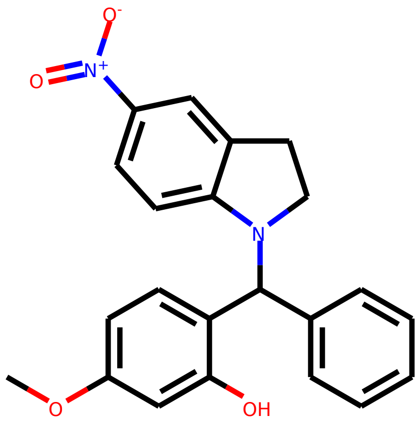
Ahma-152
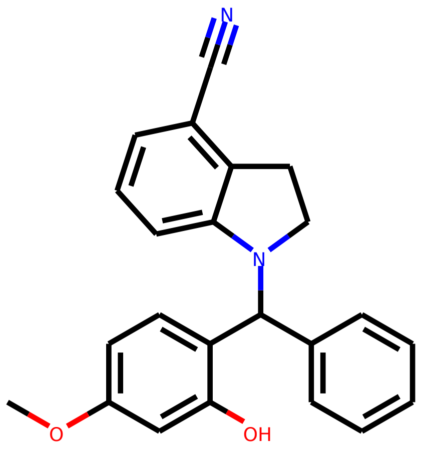
Ahma-153
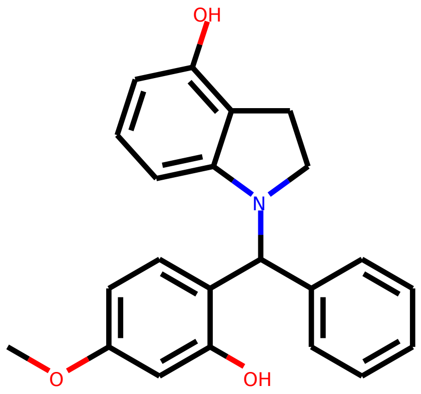
Ahma-154
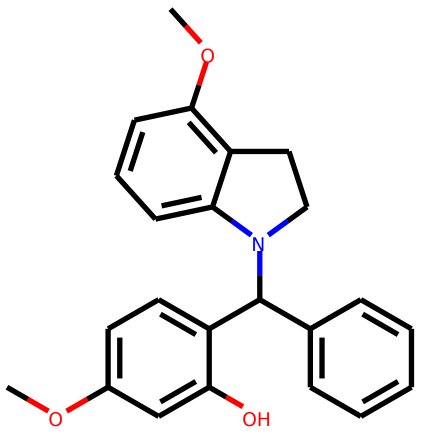
Ahma-155
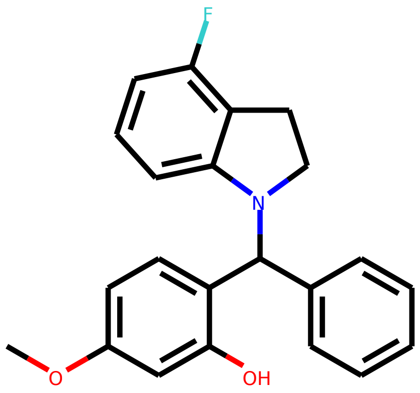
Ahma-156
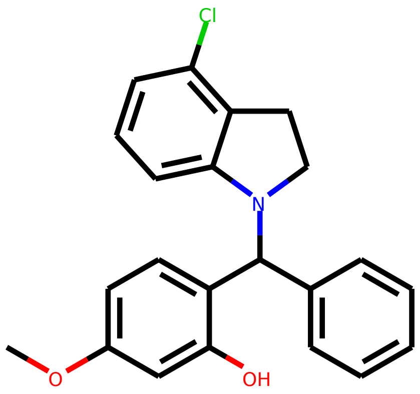
Ahma-157
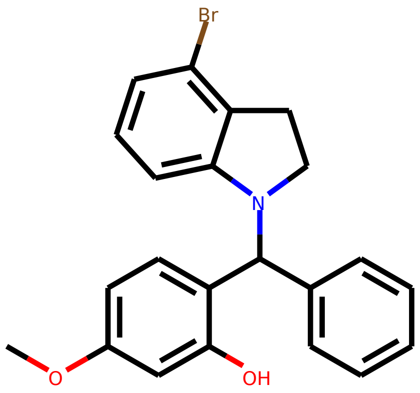
Ahma-158
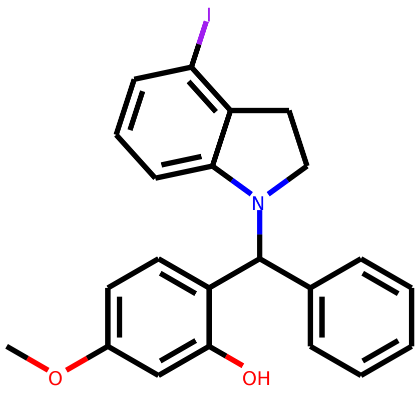
Ahma-159
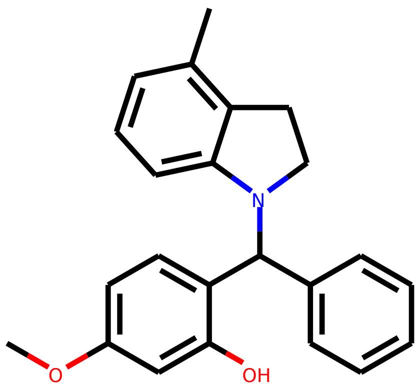
Ahma-160
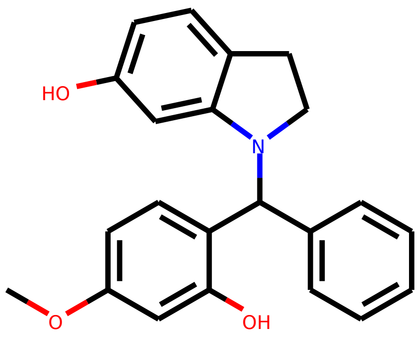
Ahma-161
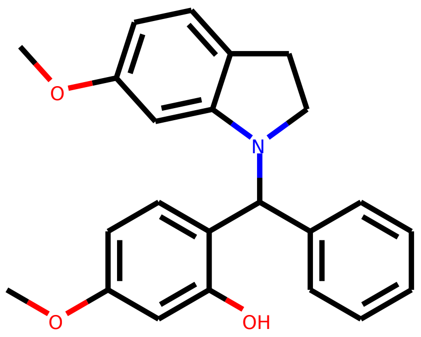
Ahma-162
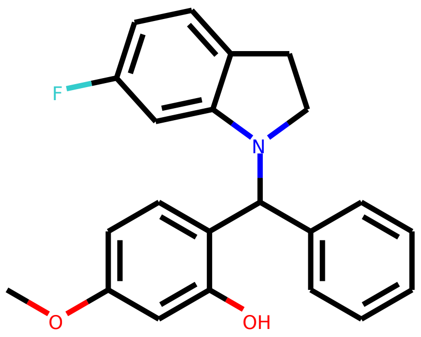
Ahma-163
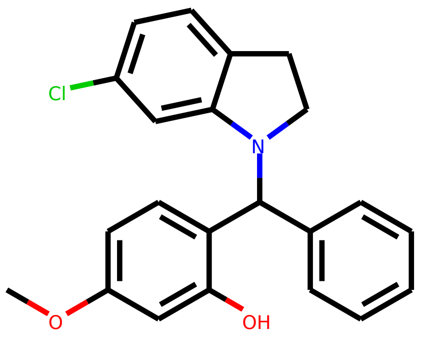
Ahma-164
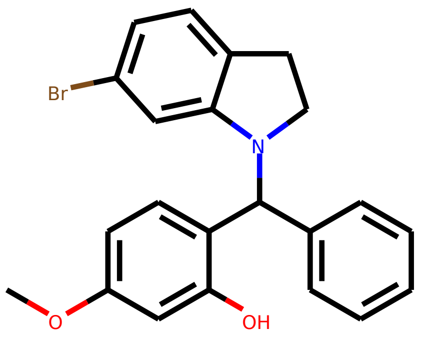
Ahma-165
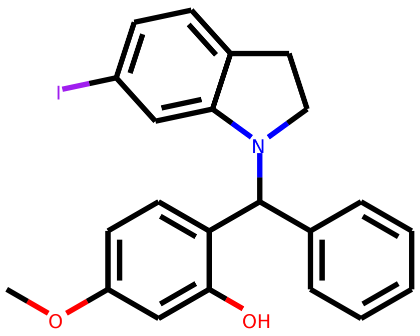
Ahma-166
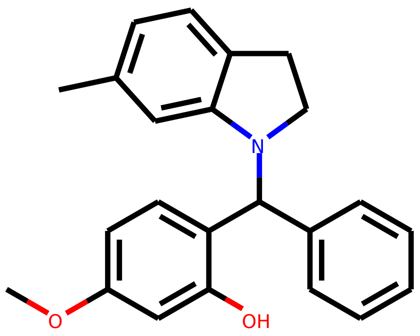
Ahma-167
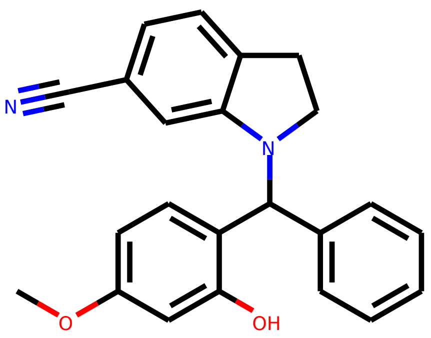
Ahma-168
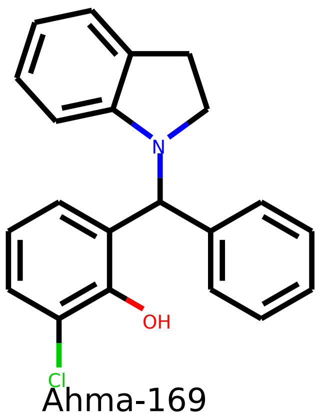
Ahma-169
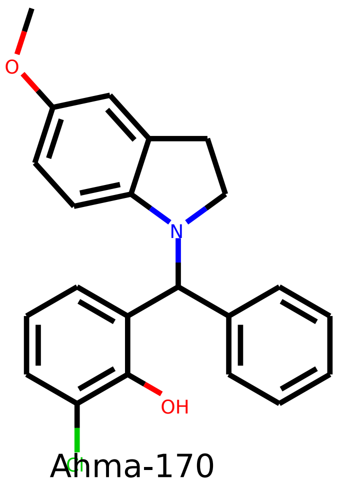
Ahma-170
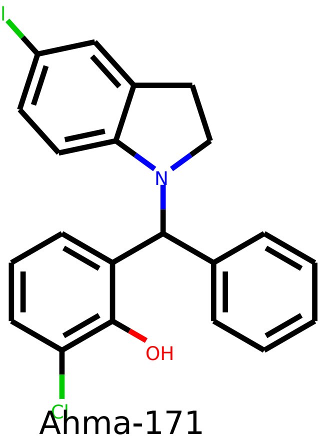
Ahma-171
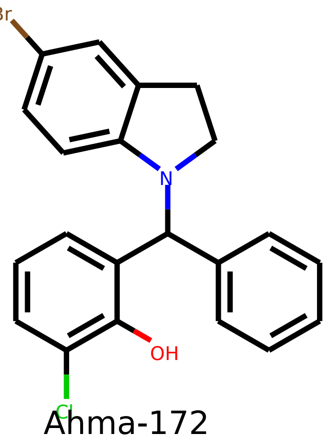
Ahma-172
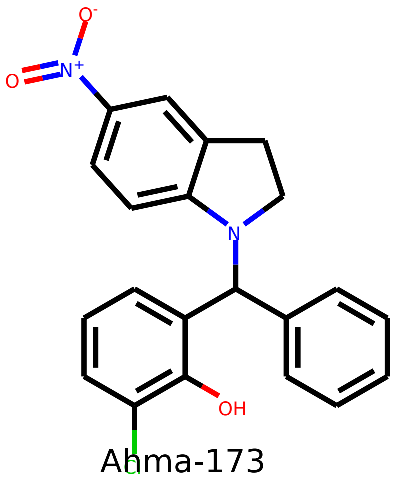
Ahma-173
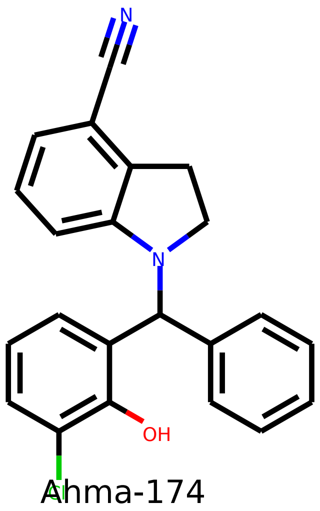
Ahma-174
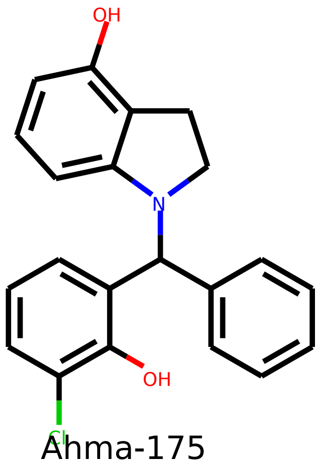
Ahma-175

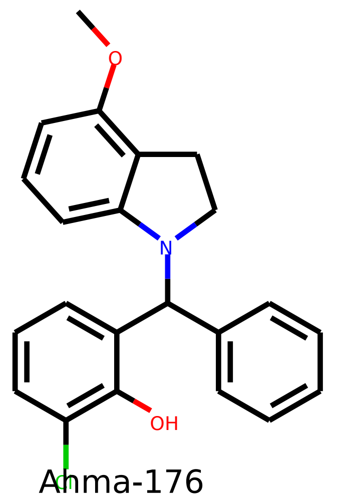
Ahma-176
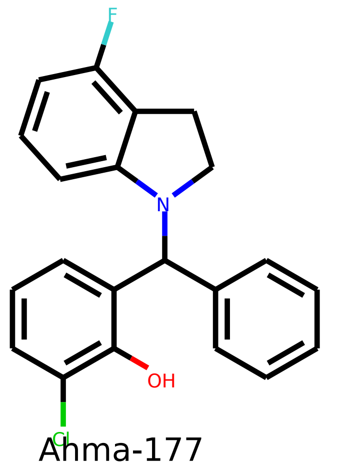
Ahma-177
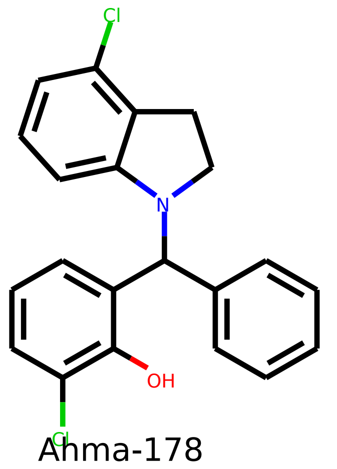
Ahma-178
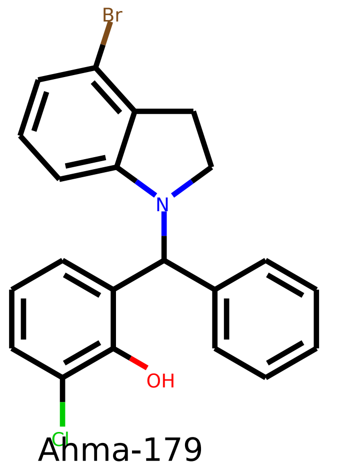
Ahma-179
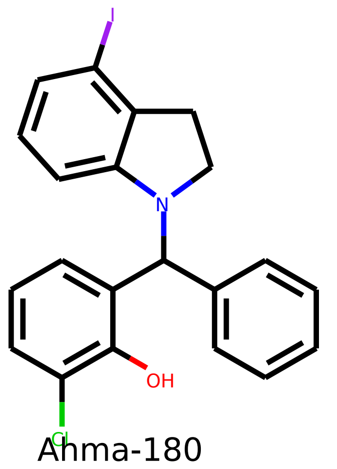
Ahma-180
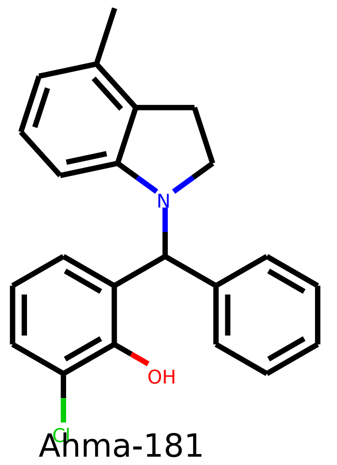
Ahma-181
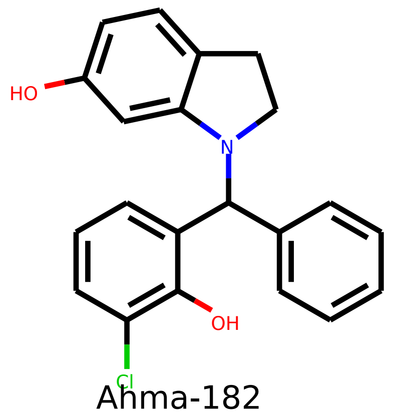
Ahma-182
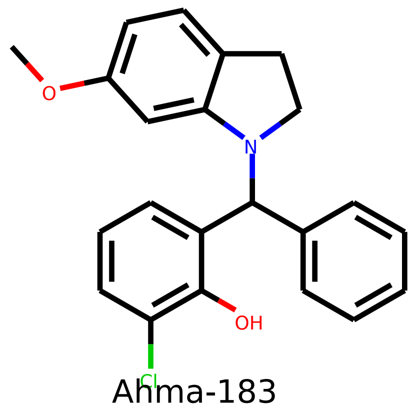
Ahma-183
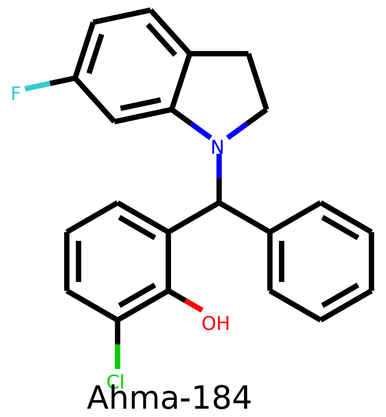
Ahma-184
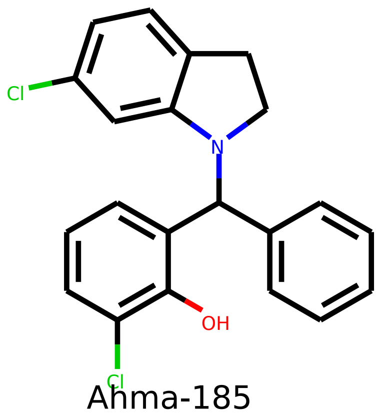
Ahma-185
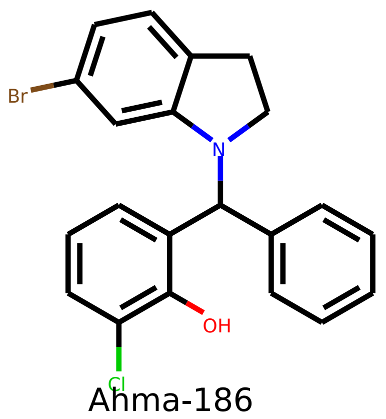
Ahma-186
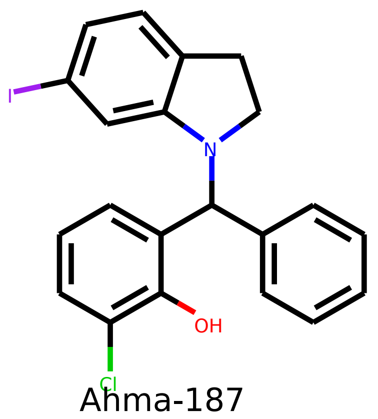
Ahma-187
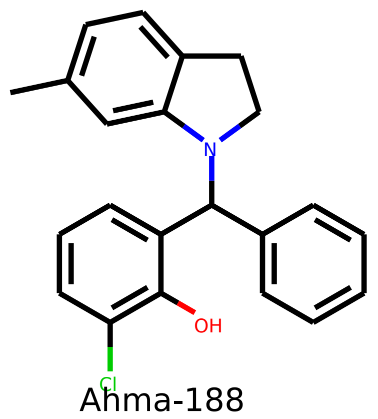
Ahma-188
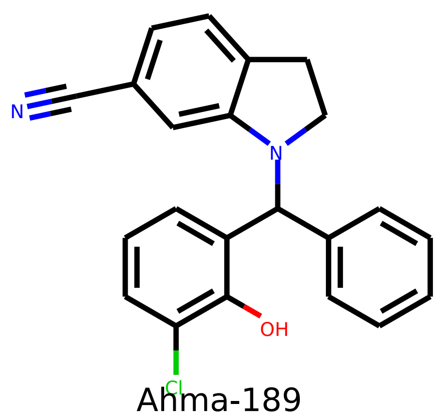
Ahma-189
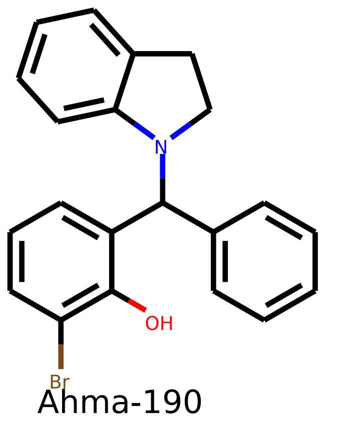
Ahma-190
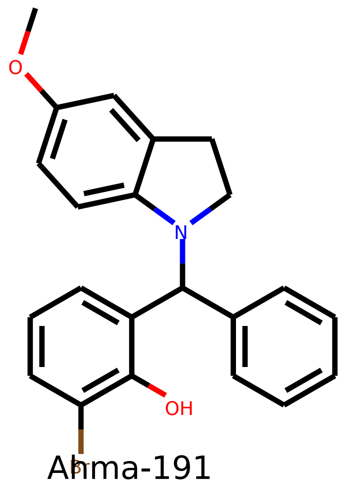
Ahma-191
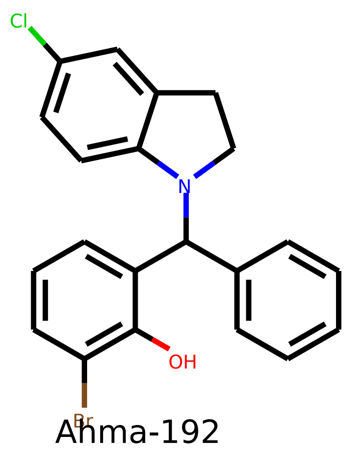
Ahma-192
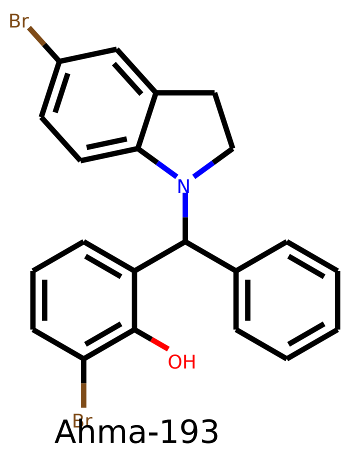
Ahma-193
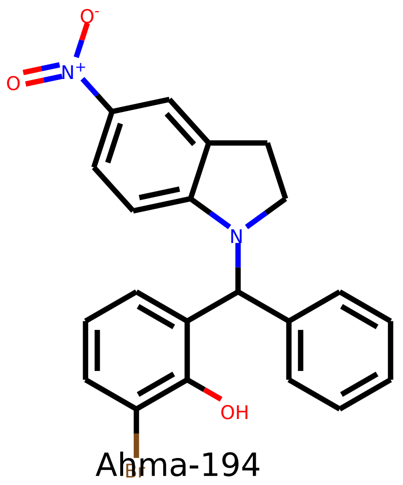
Ahma-194
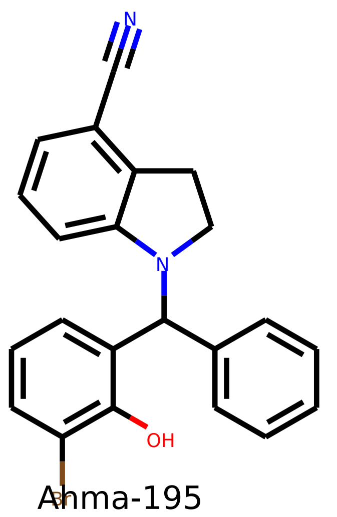
Ahma-195
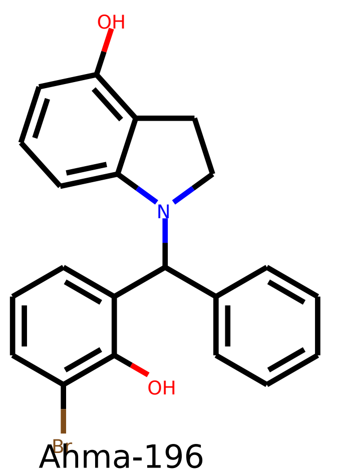
Ahma-196
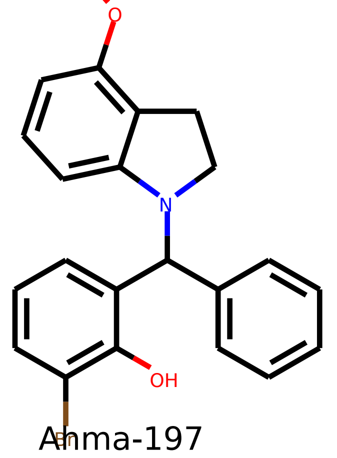
Ahma-197
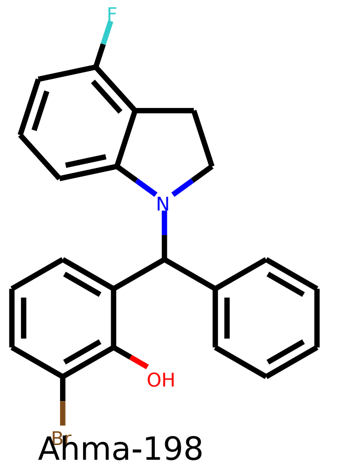
Ahma-198
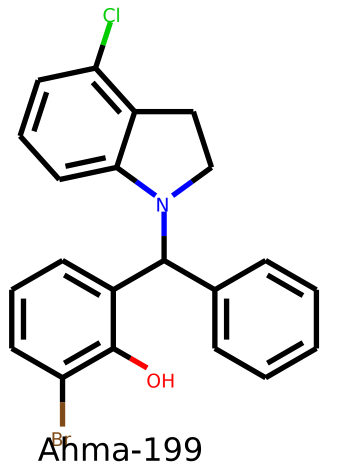
Ahma-199
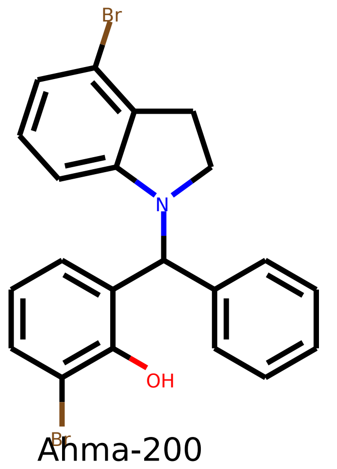
Ahma-200
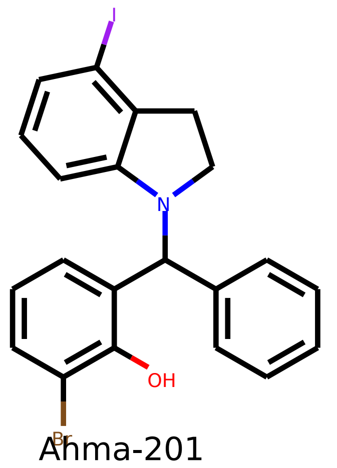
Ahma-201
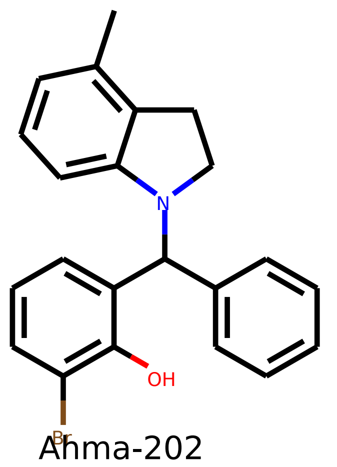
Ahma-202
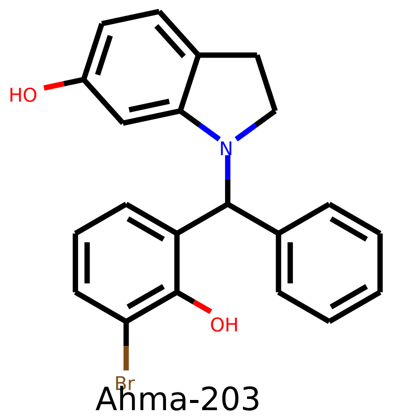
Ahma-203
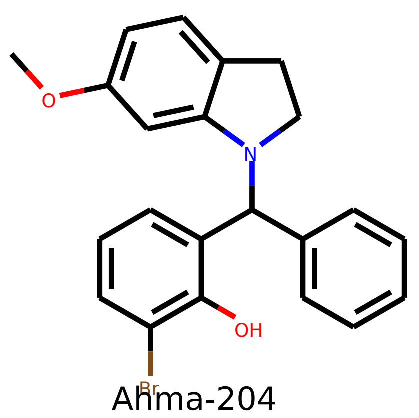
Ahma-204
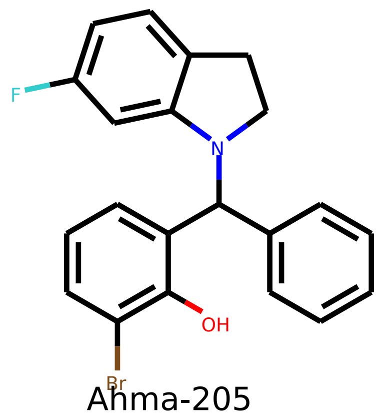
Ahma-205
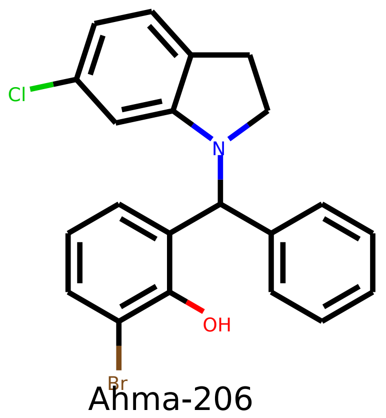
Ahma-206
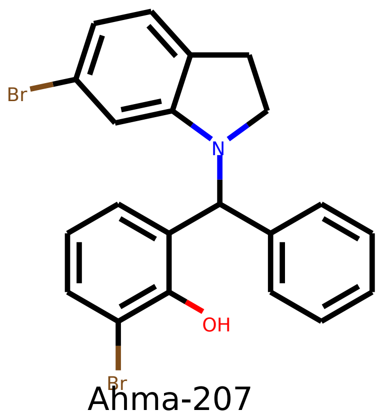
Ahma-207
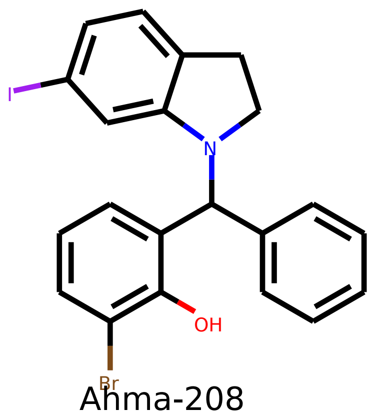
Ahma-208
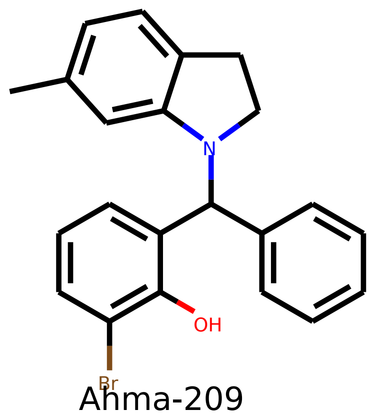
Ahma-209
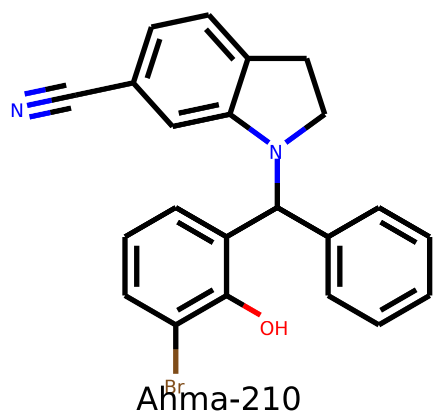
Ahma-210

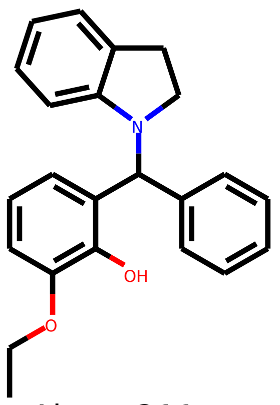
Ahma-211
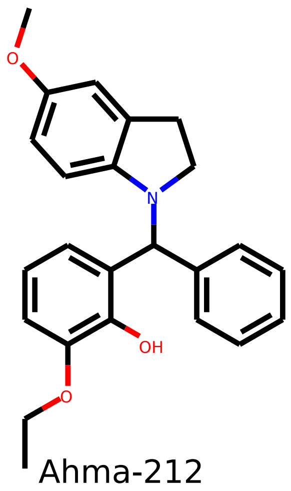
Ahma-212
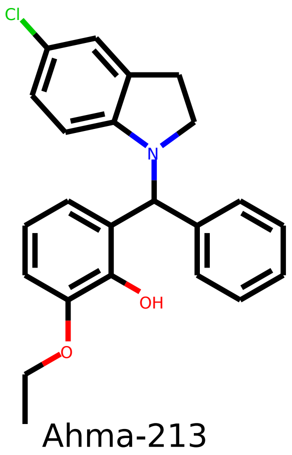
Ahma-213
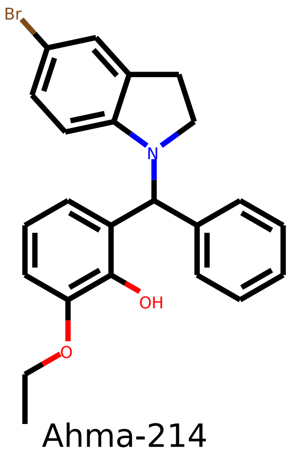
Ahma-214
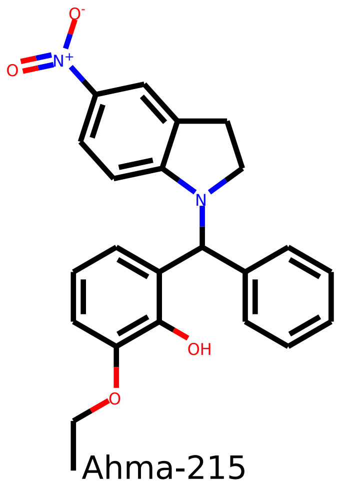
Ahma-215
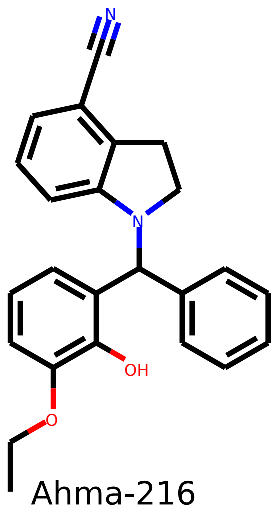
Ahma-216
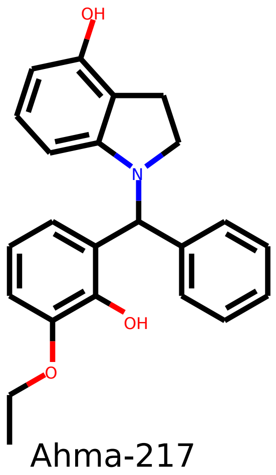
Ahma-217
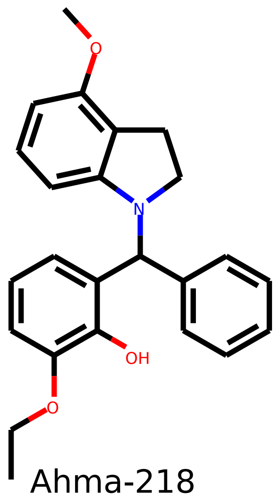
Ahma-218
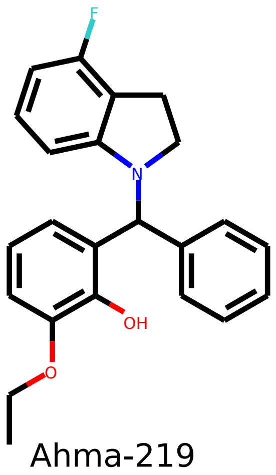
Ahma-219
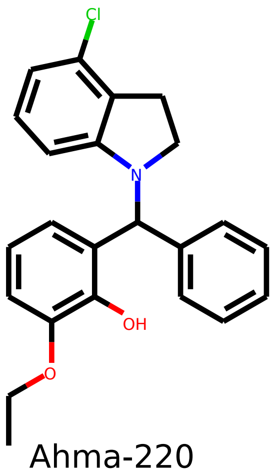
Ahma-220
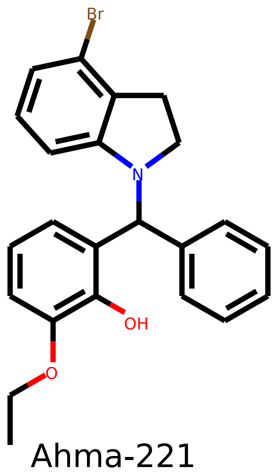
Ahma-221
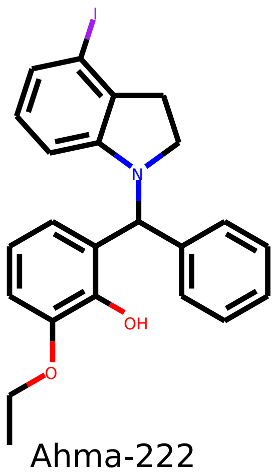
Ahma-222
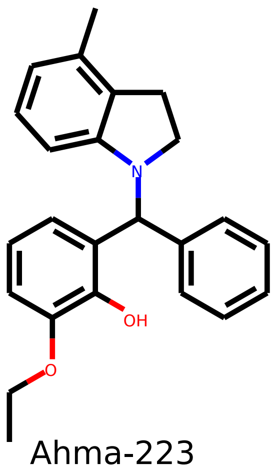
Ahma-223
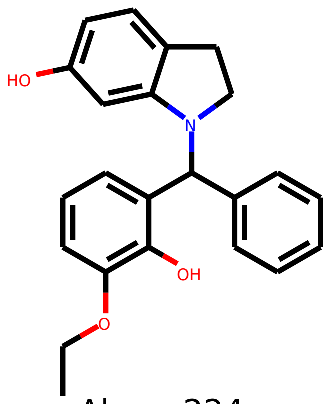
Ahma-224
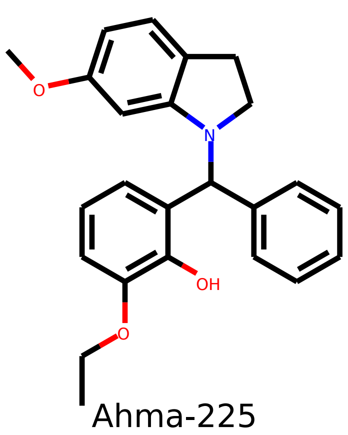
Ahma-225
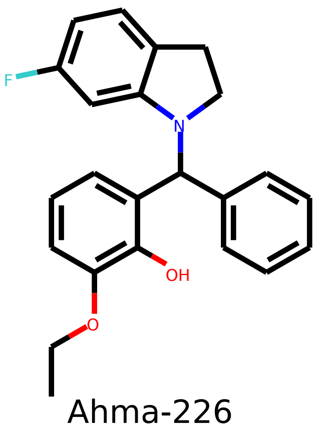
Ahma-226
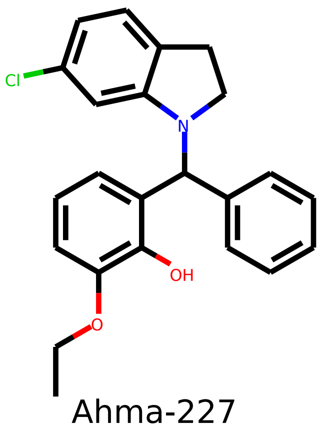
Ahma-227
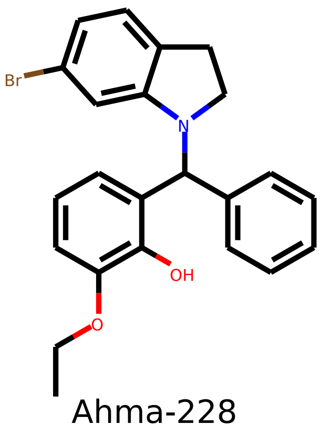
Ahma-228
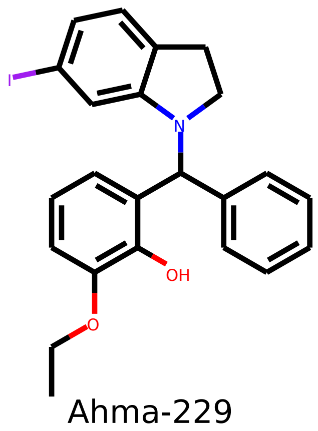
Ahma-229
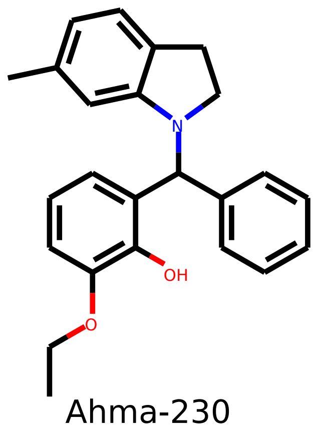
Ahma-230
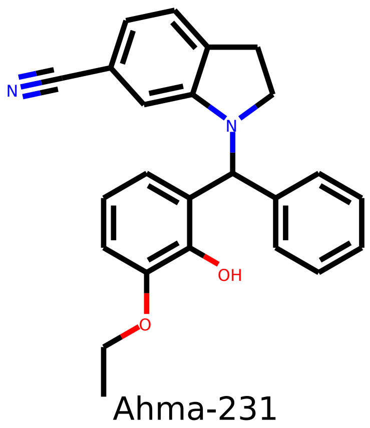
Ahma-231
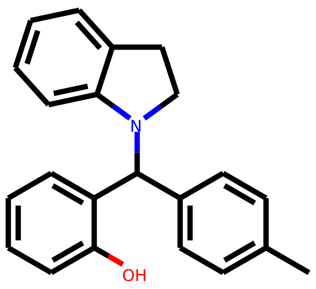
Ahma-232
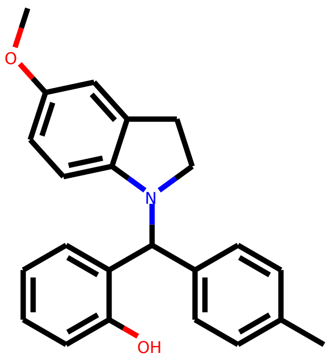
Ahma-233
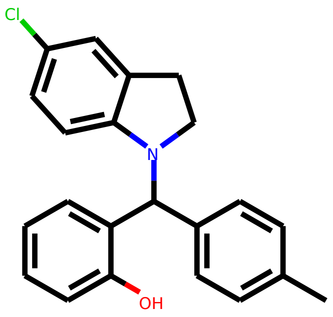
Ahma-234
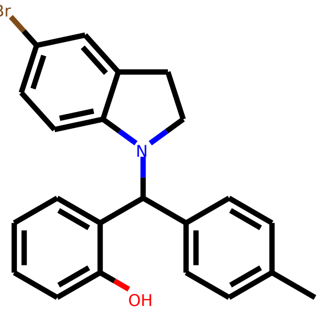
Ahma-235
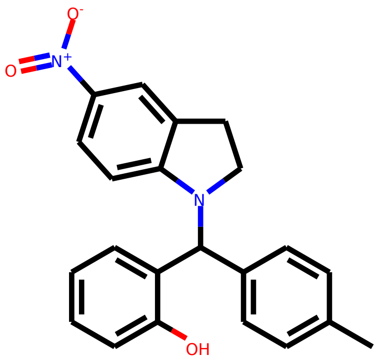
Ahma-236
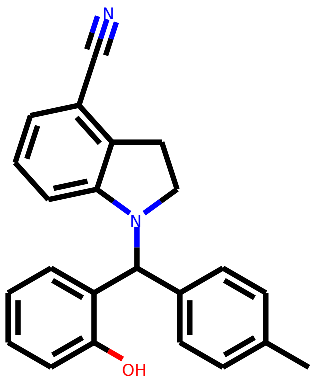
Ahma-237
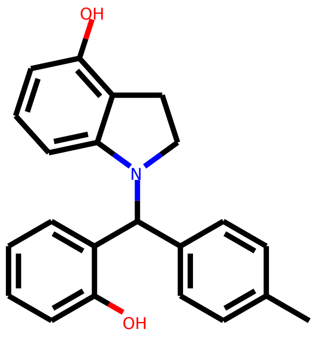
Ahma-238
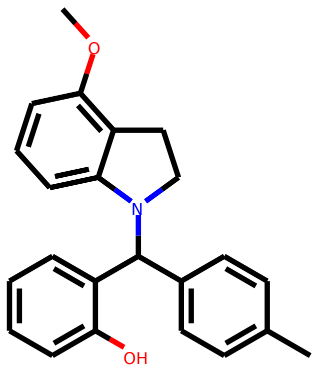
Ahma-239
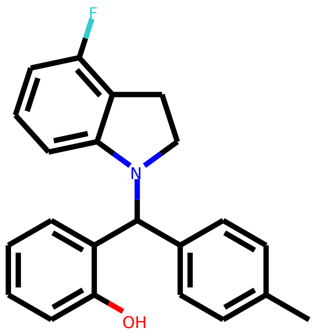
Ahma-240
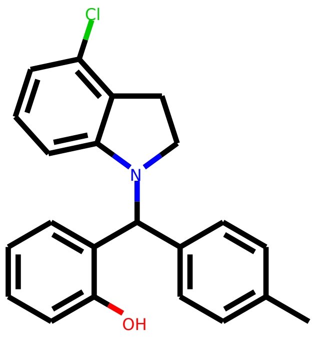
Ahma-241
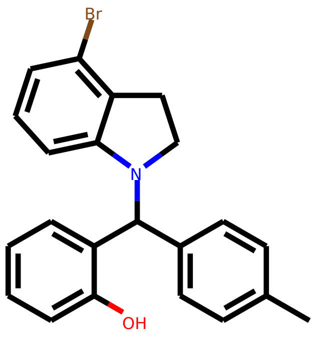
Ahma-242
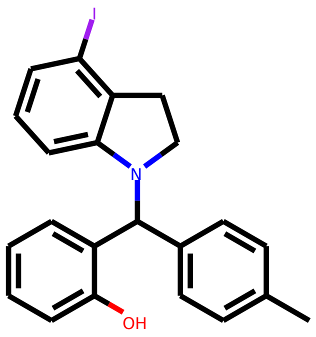
Ahma-243
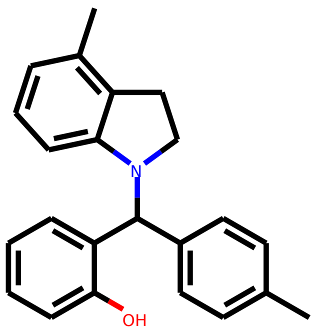
Ahma-244
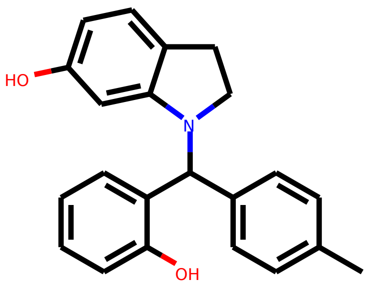
Ahma-245

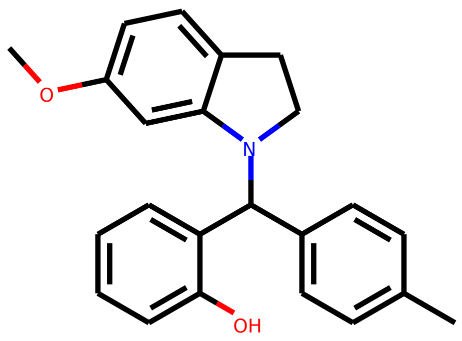
Ahma-246
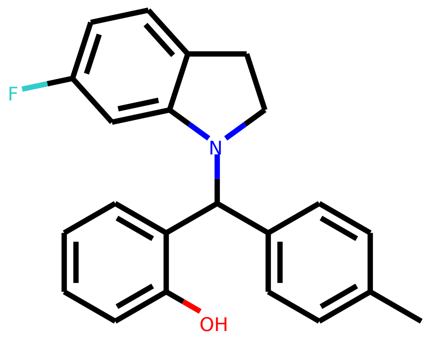
Ahma-247
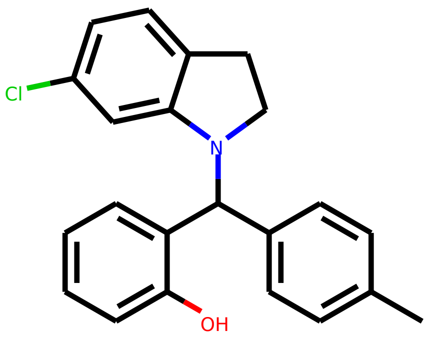
Ahma-248
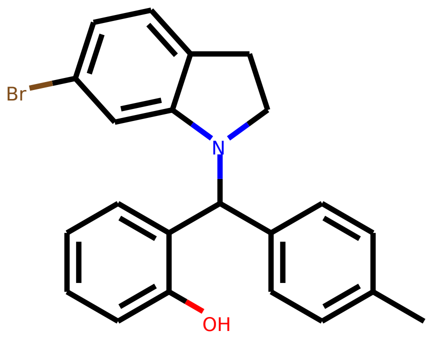
Ahma-249
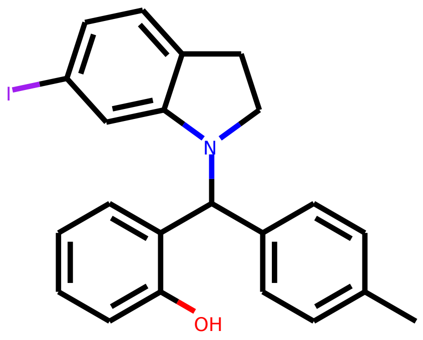
Ahma-250
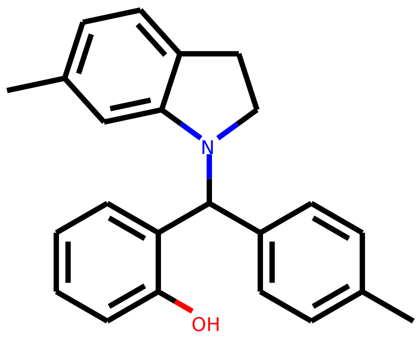
Ahma-251
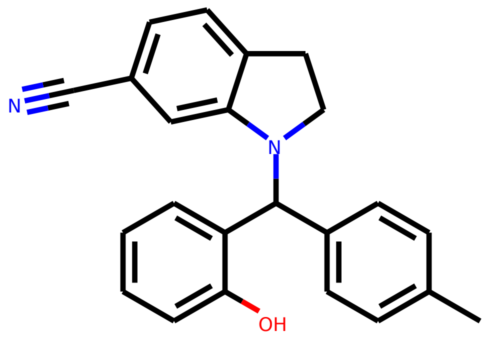
Ahma-252
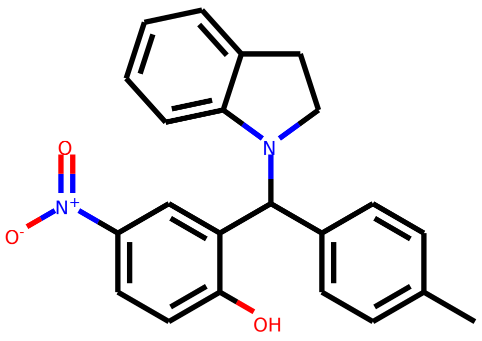
Ahma-253
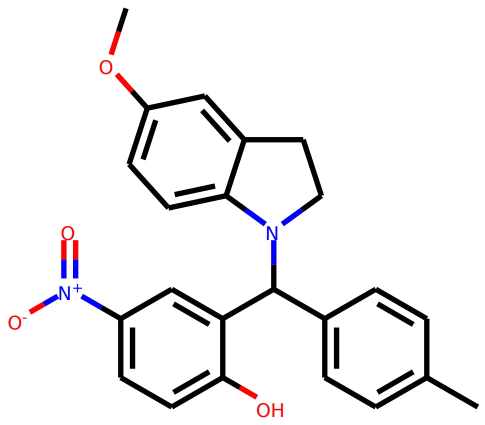
Ahma-254
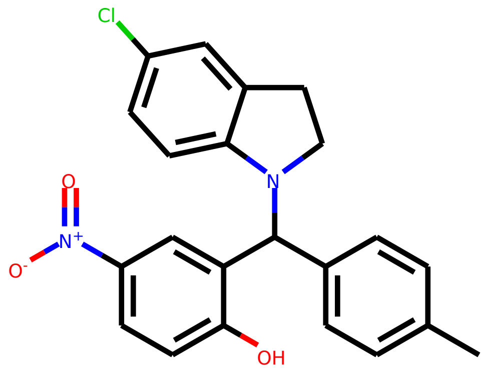
Ahma-255
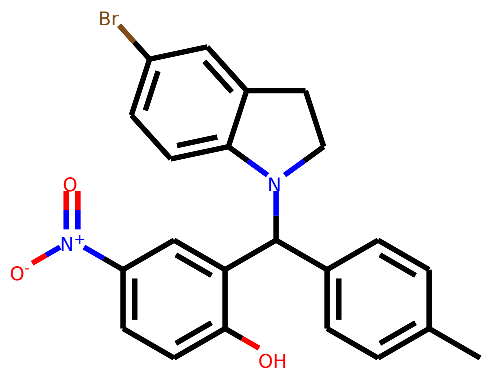
Ahma-256
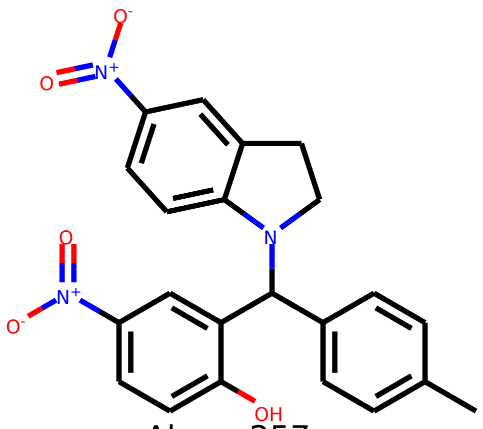
Ahma-257
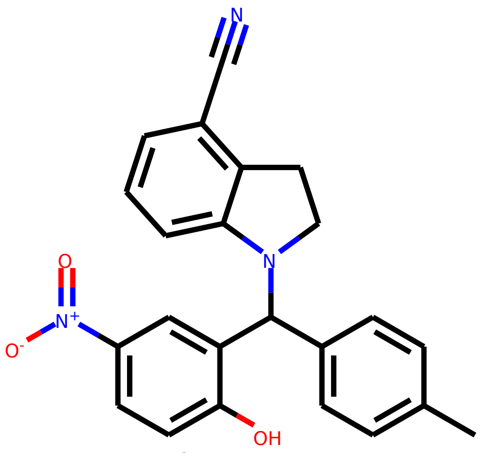
Ahma-258
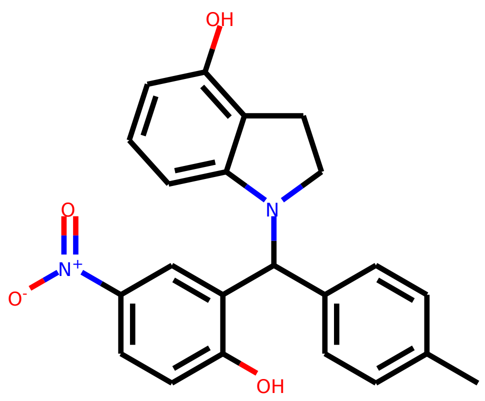
Ahma-259
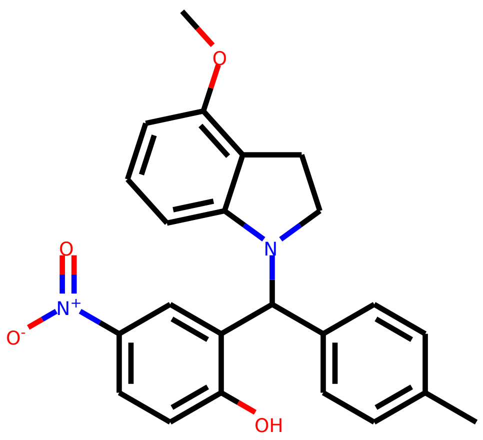
Ahma-260
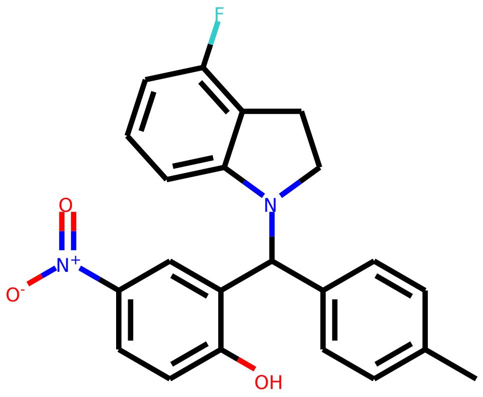
Ahma-261
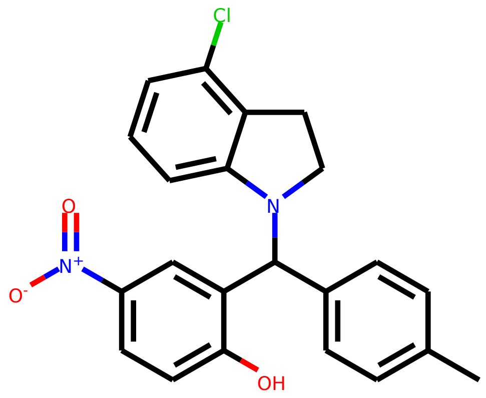
Ahma-262
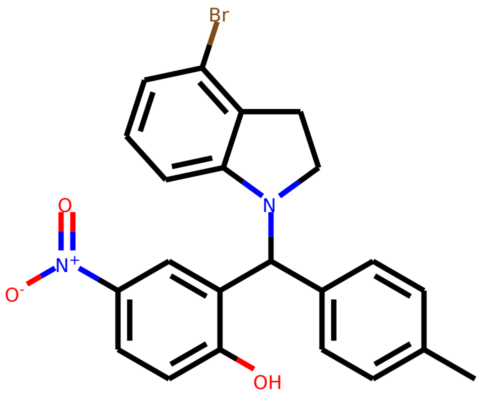
Ahma-263
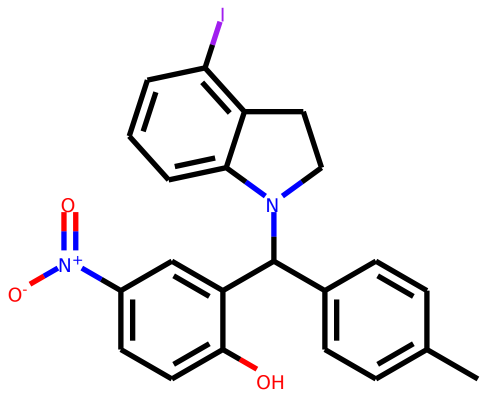
Ahma-264
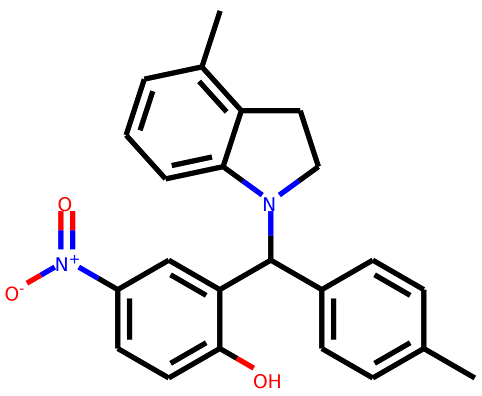
Ahma-265
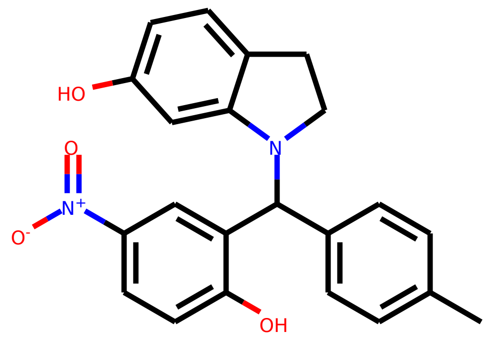
Ahma-266
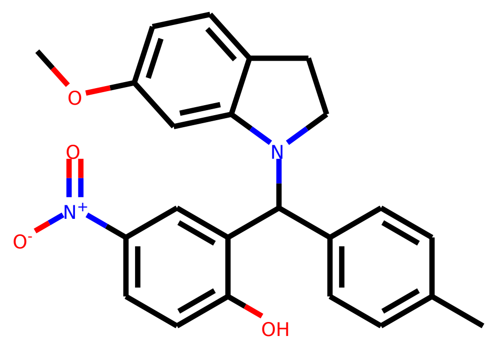
Ahma-267
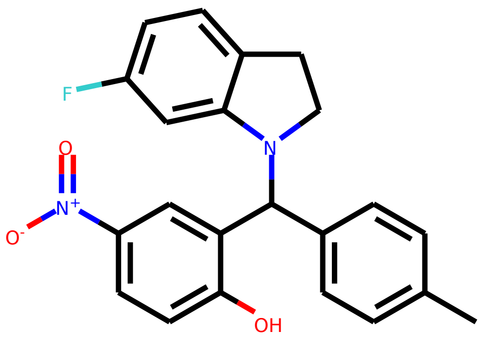
Ahma-268
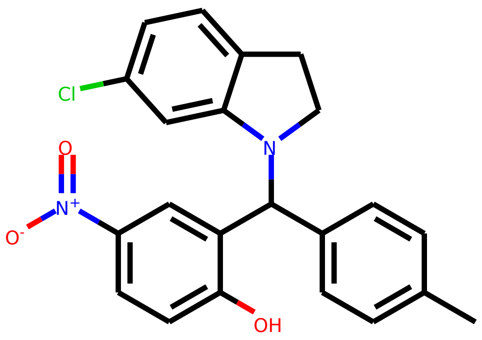
Ahma-269
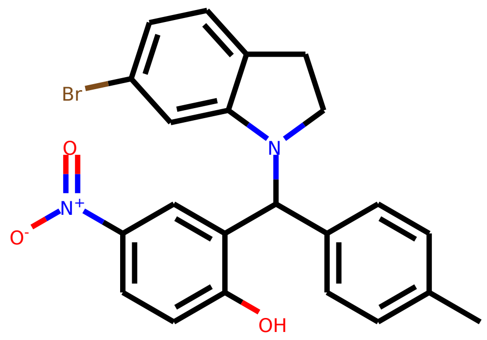
Ahma-270
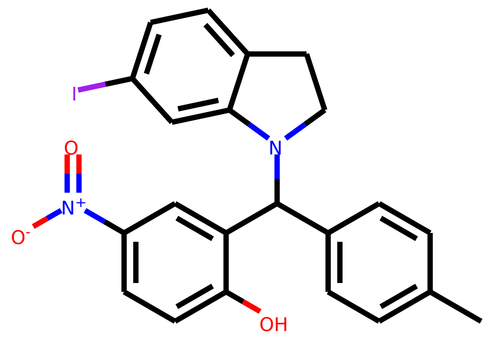
Ahma-271
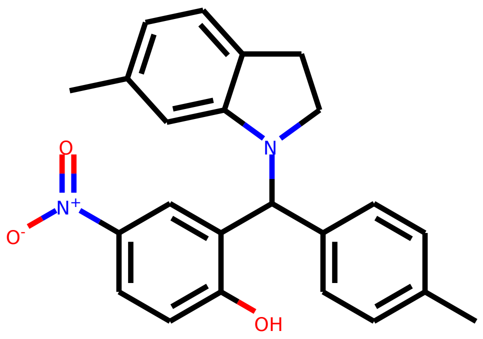
Ahma-272
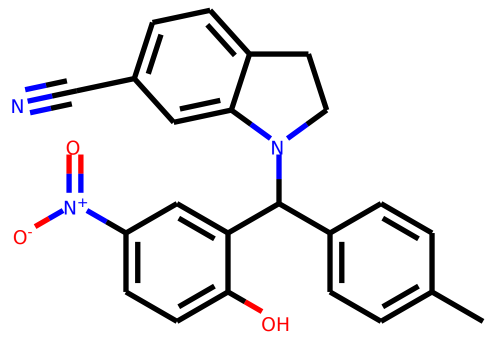
Ahma-273
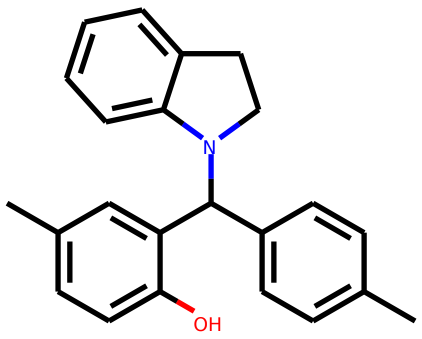
Ahma-274
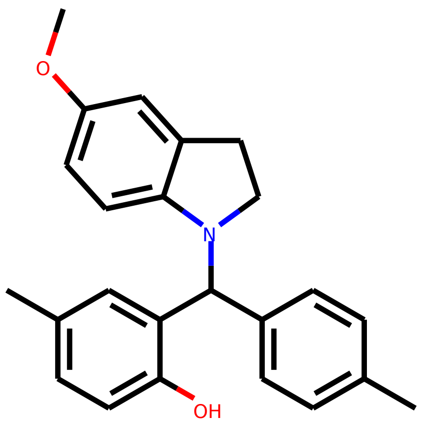
Ahma-275
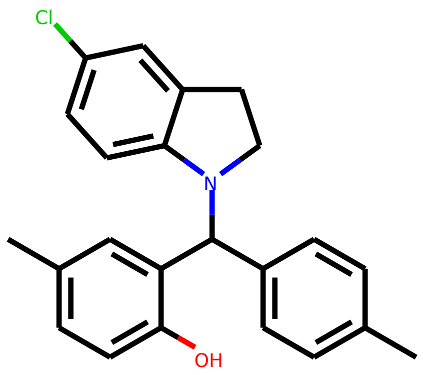
Ahma-276
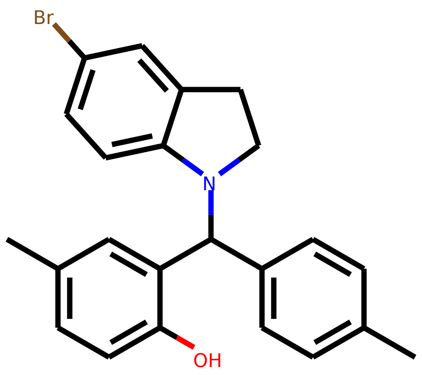
Ahma-277
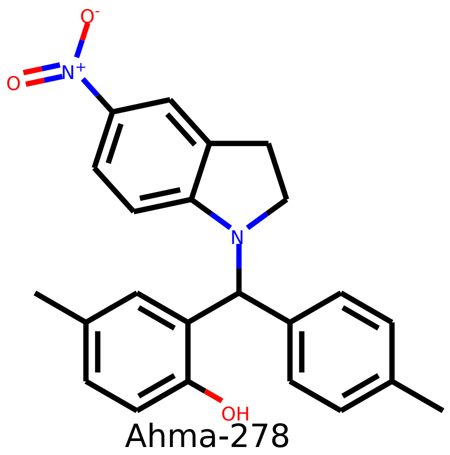
Ahma-278
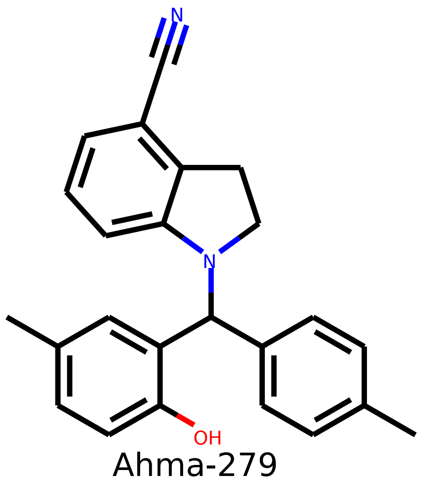
Ahma-279
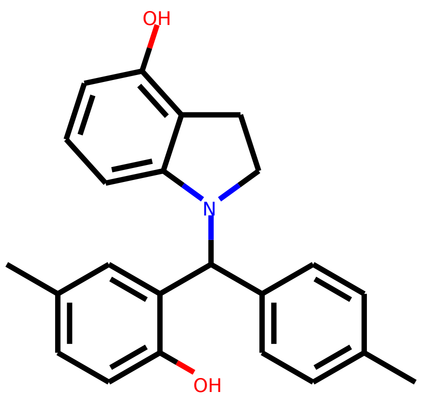
Ahma-280

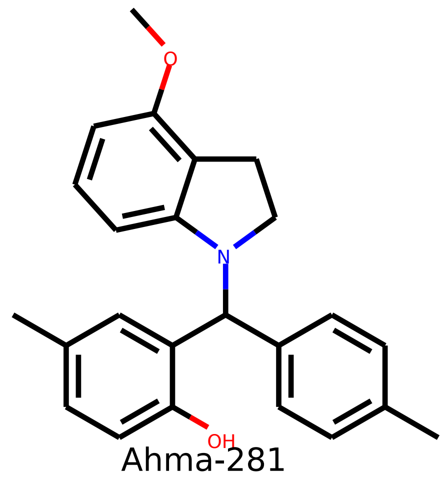
Ahma-281
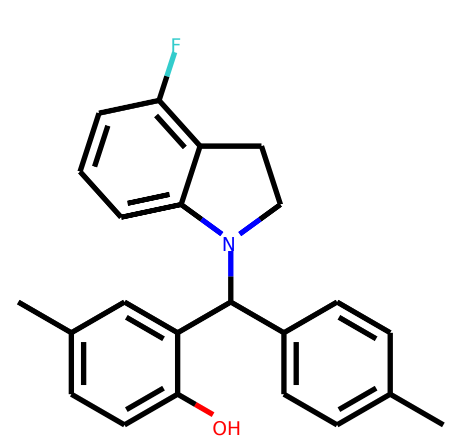
Ahma-282
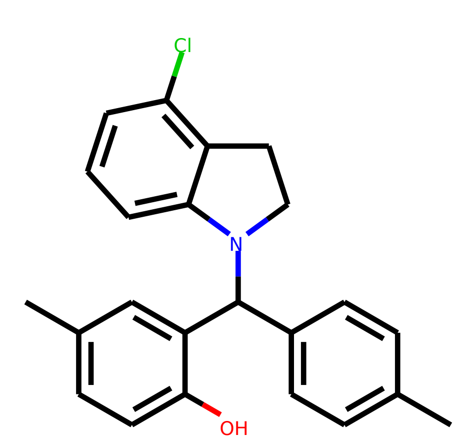
Ahma-283
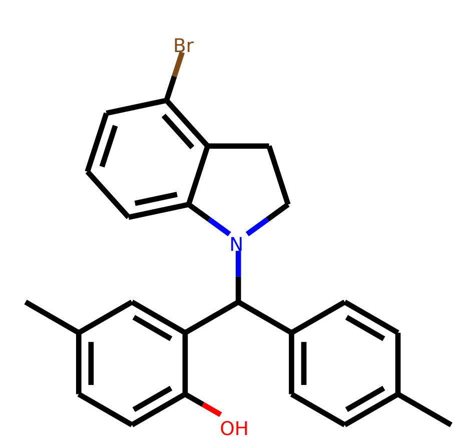
Ahma-284
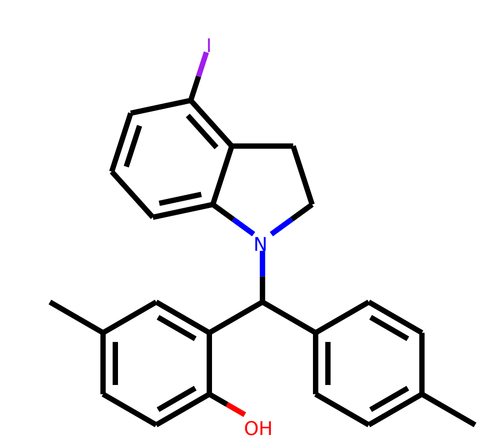
Ahma-285
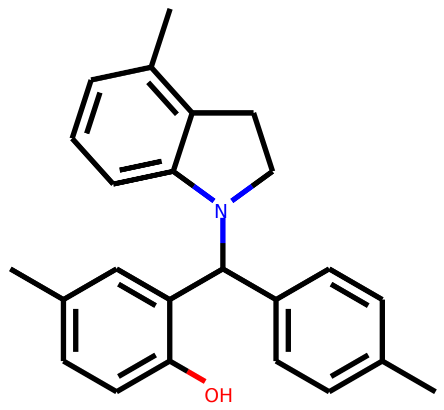
Ahma-286
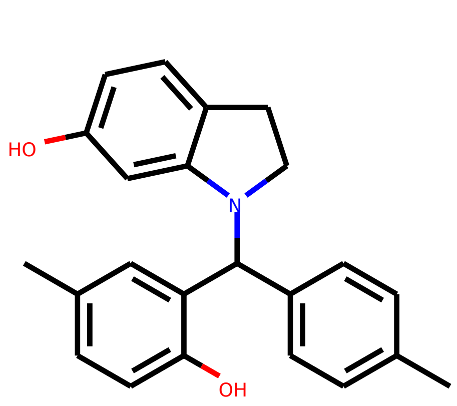
Ahma-287
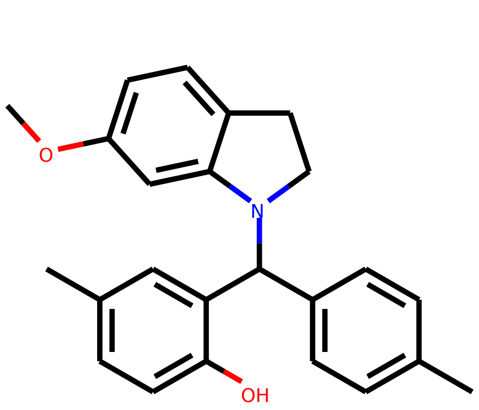
Ahma-288
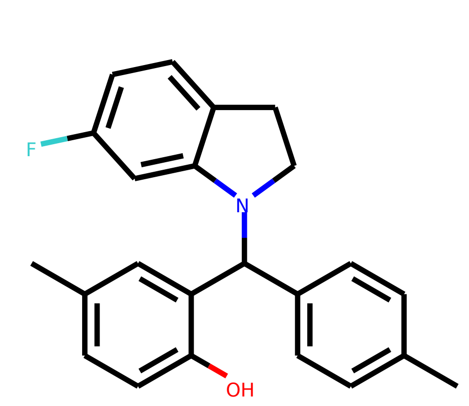
Ahma-289
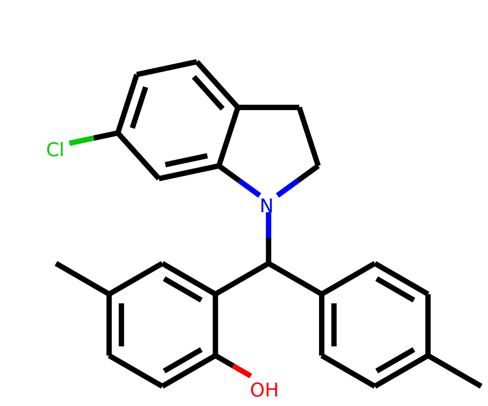
Ahma-290
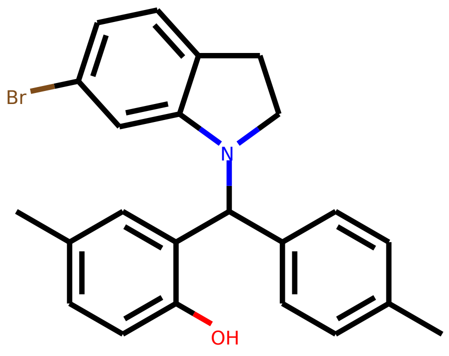
Ahma-291
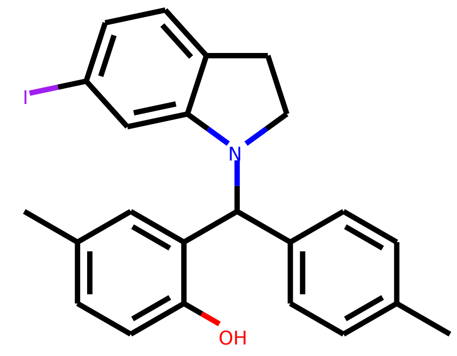
Ahma-292
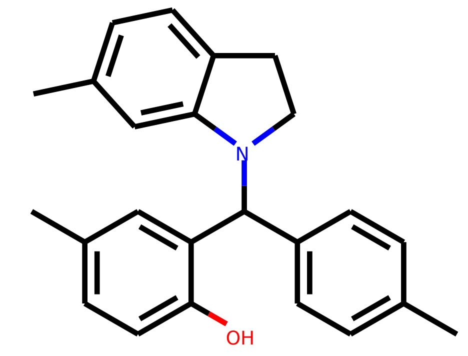
Ahma-293
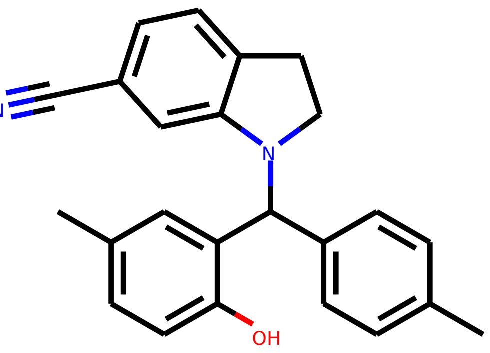
Ahma-294
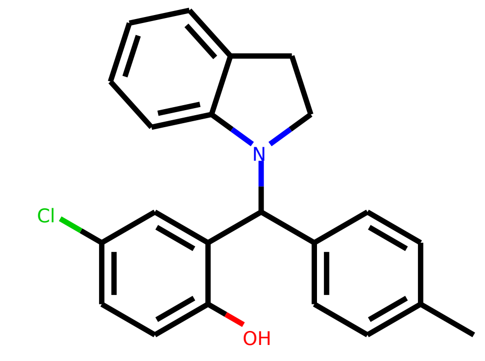
Ahma-295
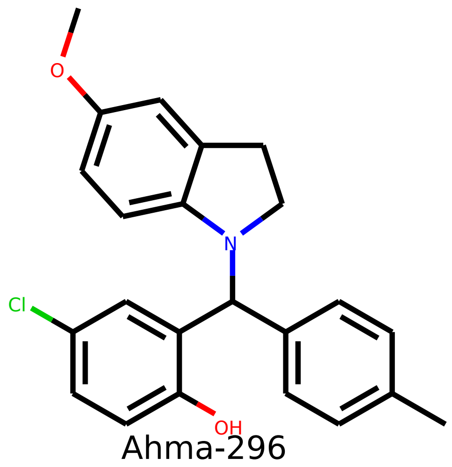
Ahma-296
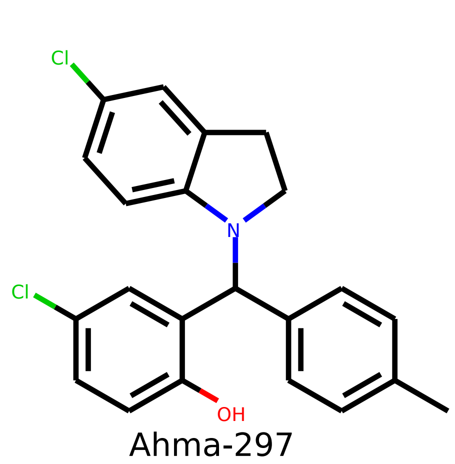
Ahma-297
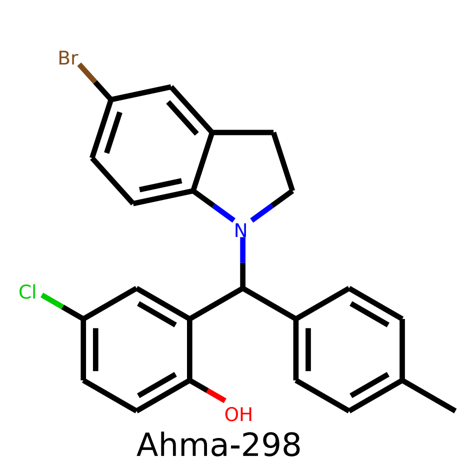
Ahma-298
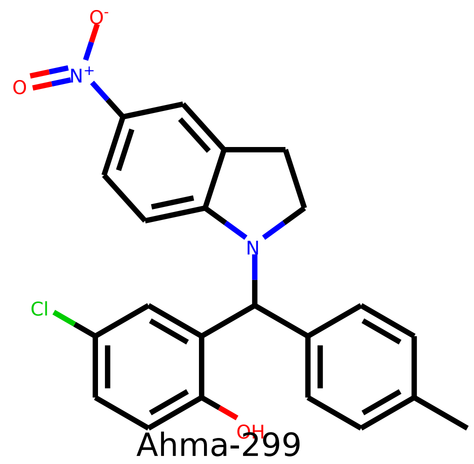
Ahma-299
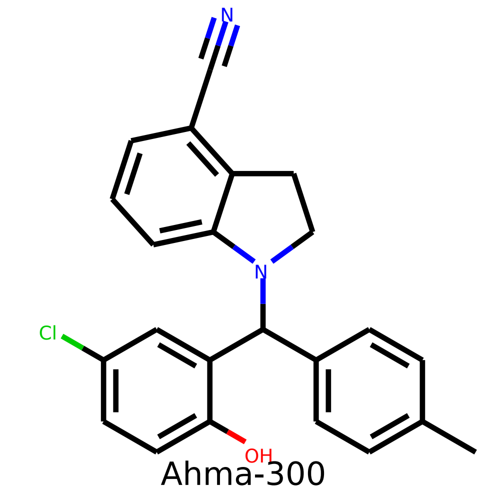
Ahma-300
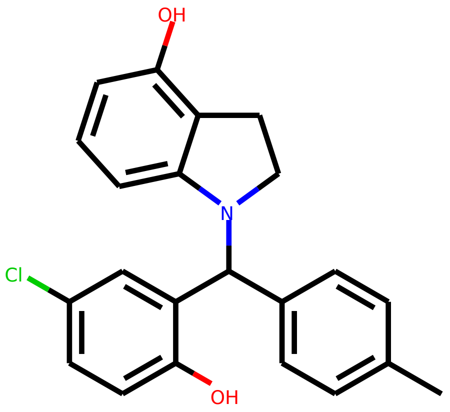
Ahma-301
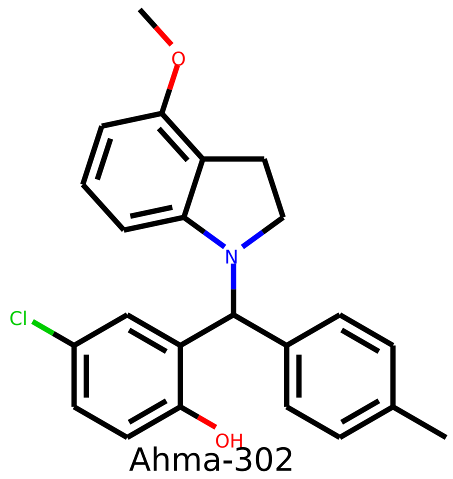
Ahma-302
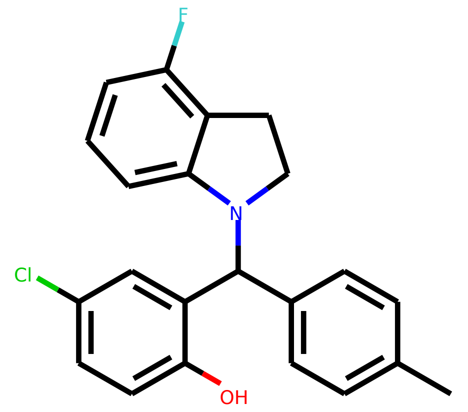
Ahma-303
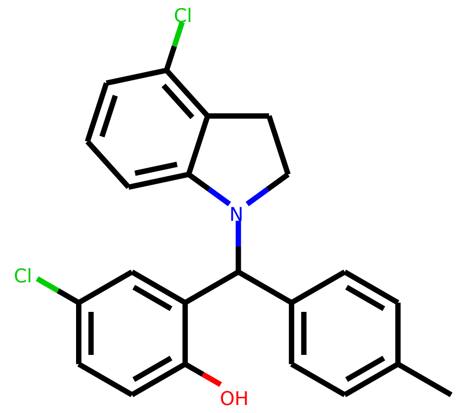
Ahma-304
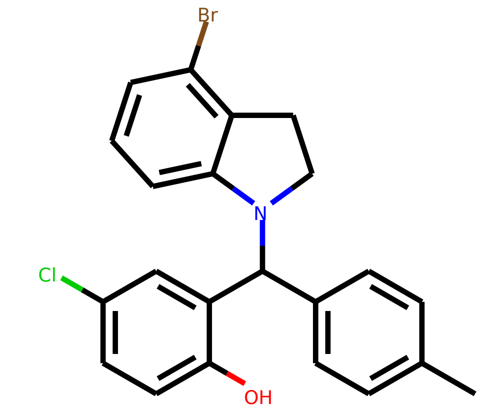
Ahma-305
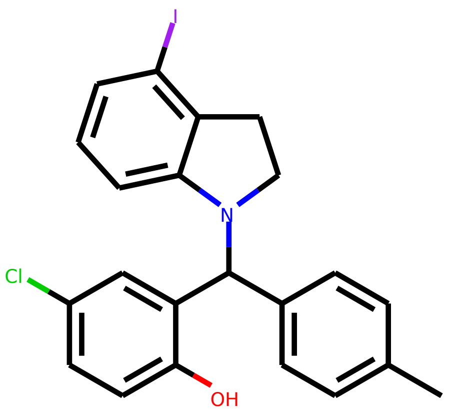
Ahma-306
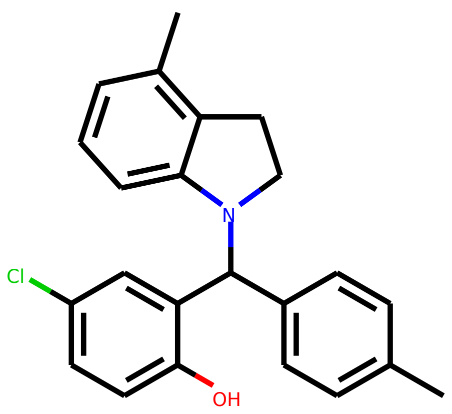
Ahma-307
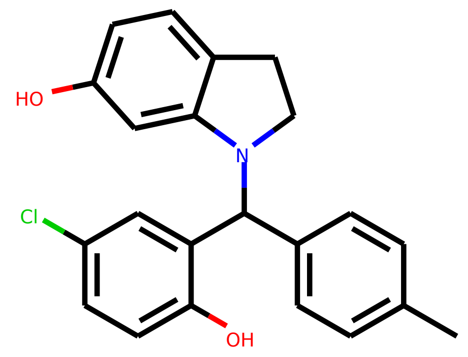
Ahma-308
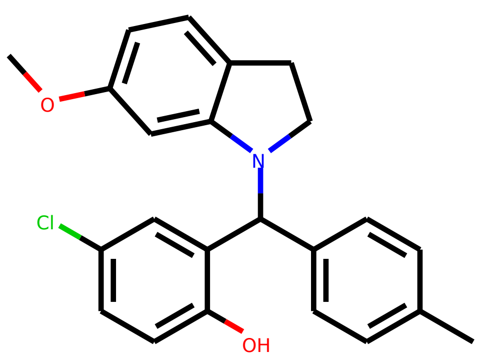
Ahma-309
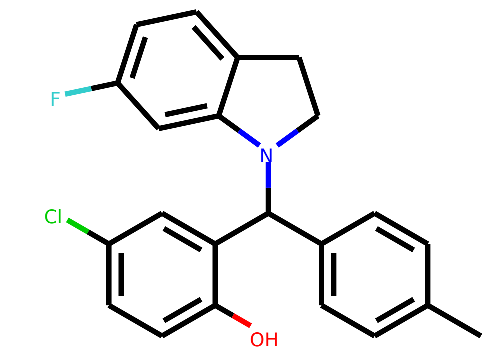
Ahma-310
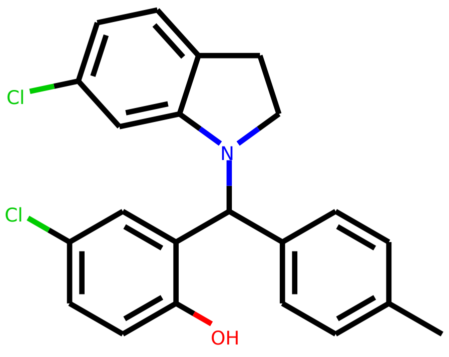
Ahma-311
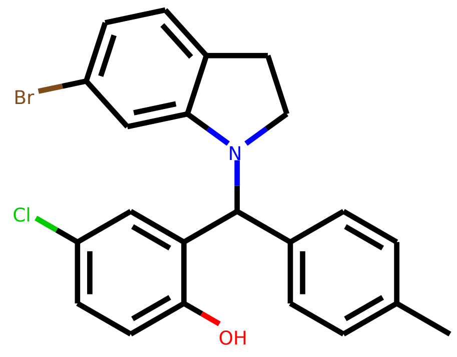
Ahma-312
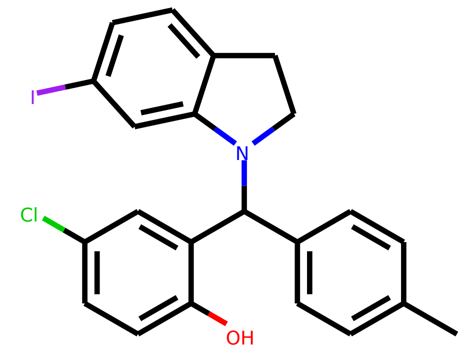
Ahma-313
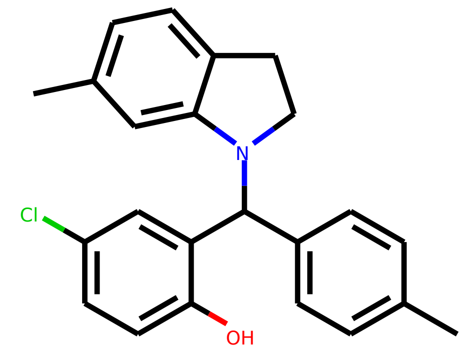
Ahma-314
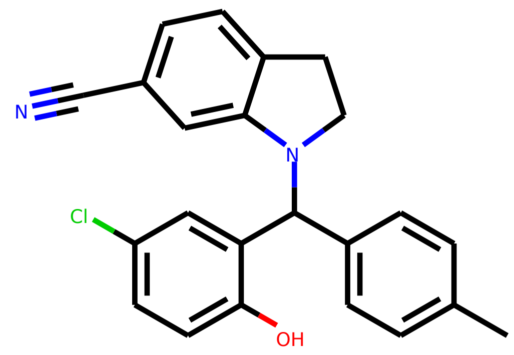
Ahma-315

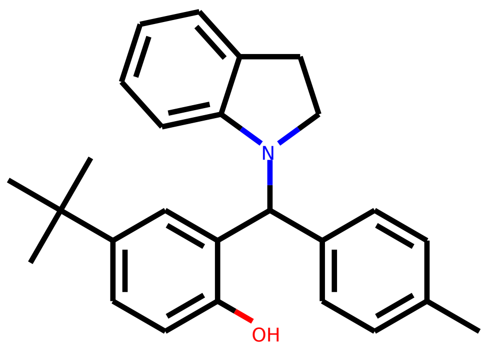
Ahma-316
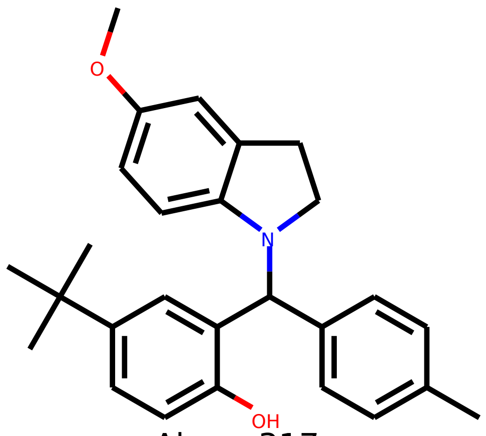
Ahma-317
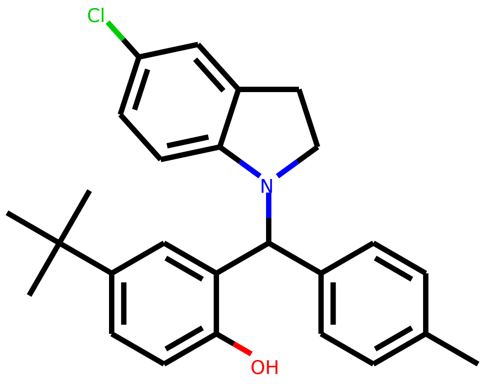
Ahma-318
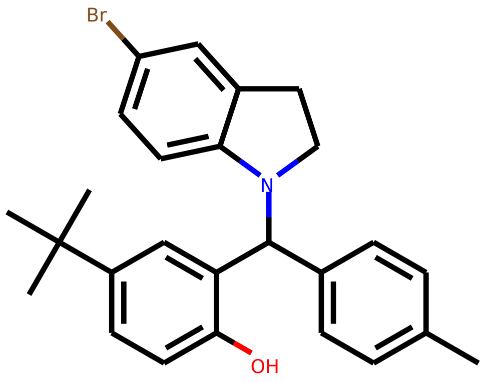
Ahma-319
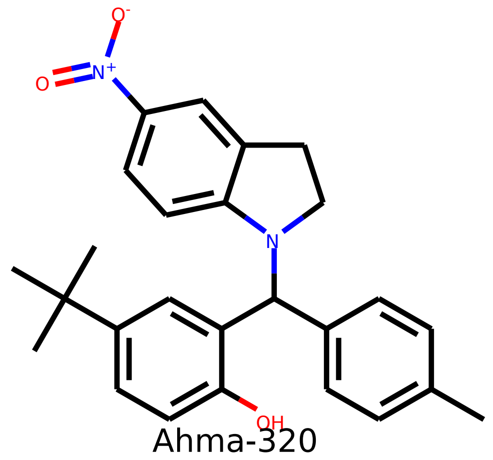
Ahma-320
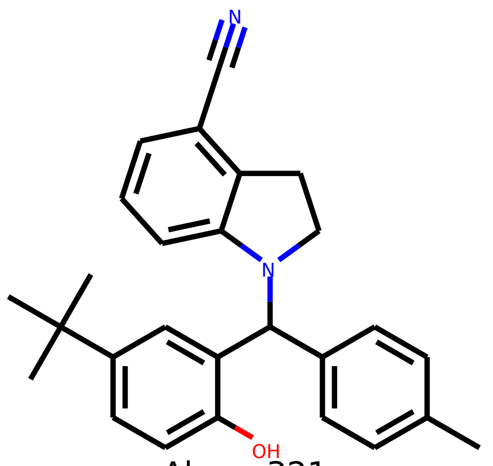
Ahma-321
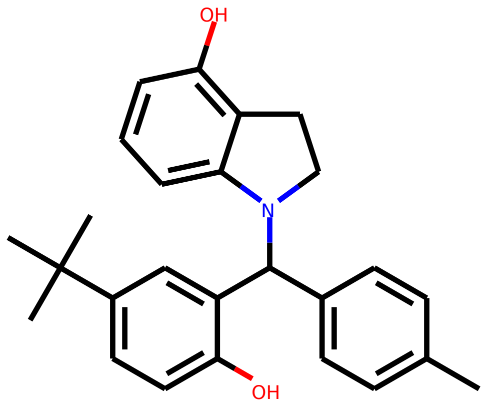
Ahma-322
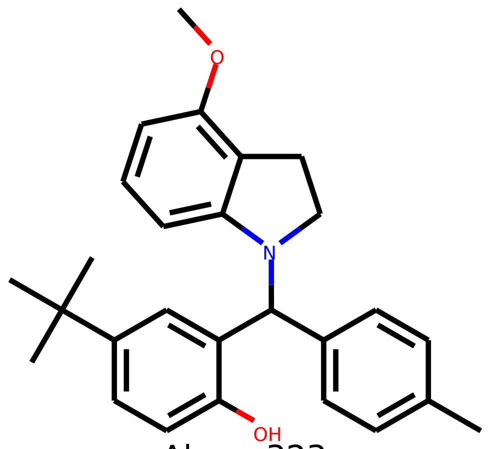
Ahma-323
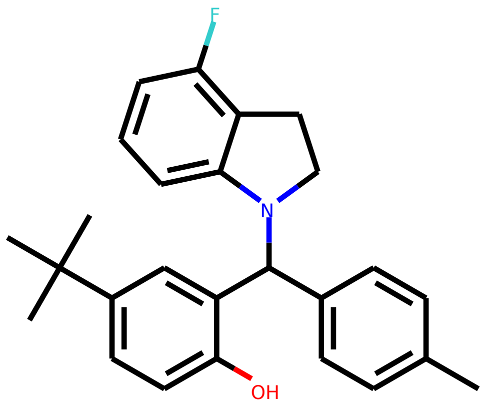
Ahma-324
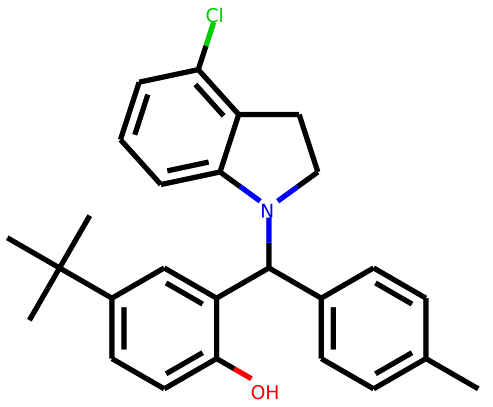
Ahma-325
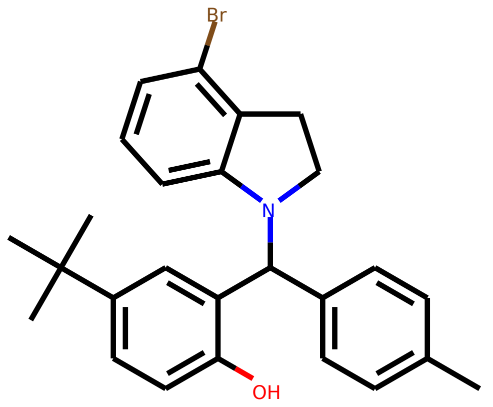
Ahma-326
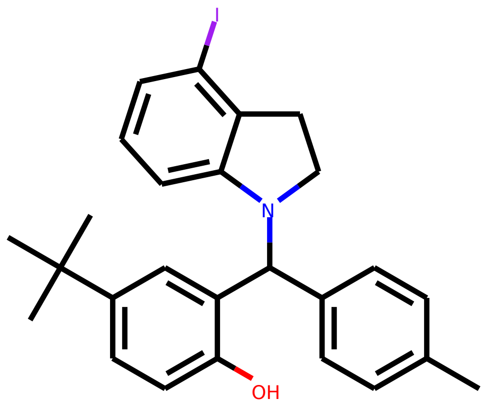
Ahma-327
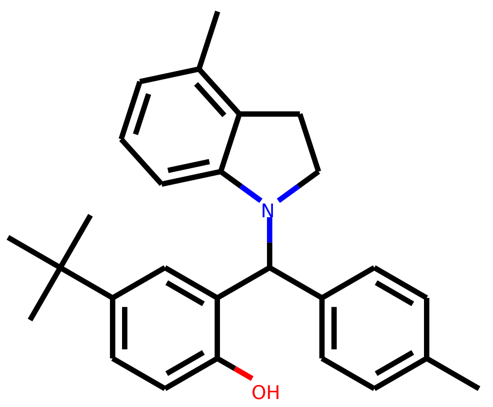
Ahma-328
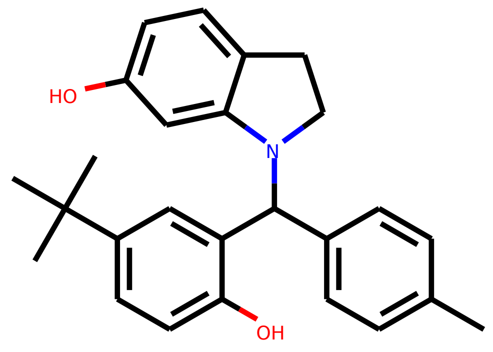
Ahma-329
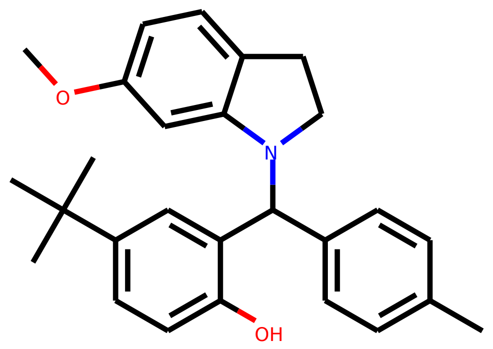
Ahma-330
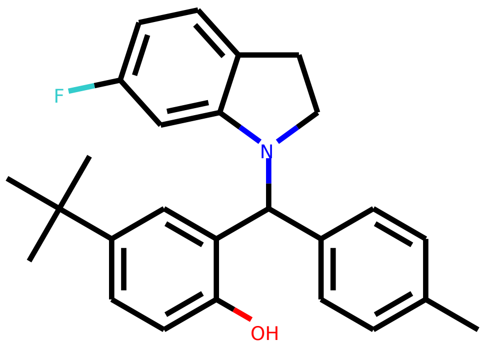
Ahma-331
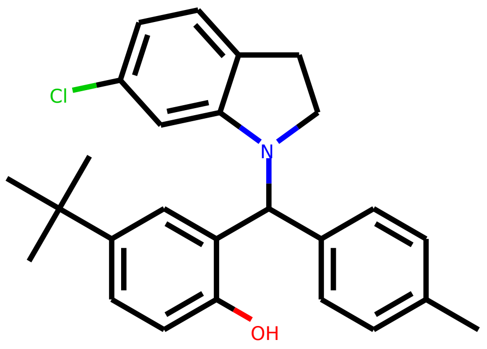
Ahma-332
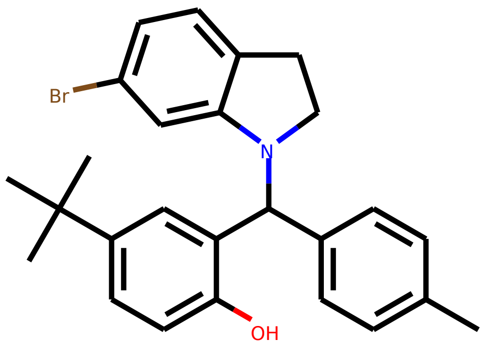
Ahma-333
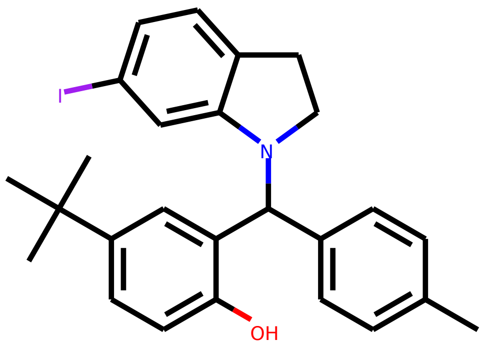
Ahma-334
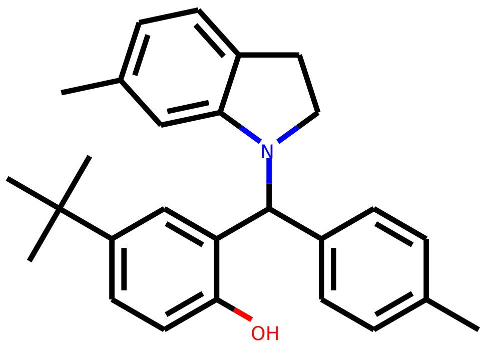
Ahma-335
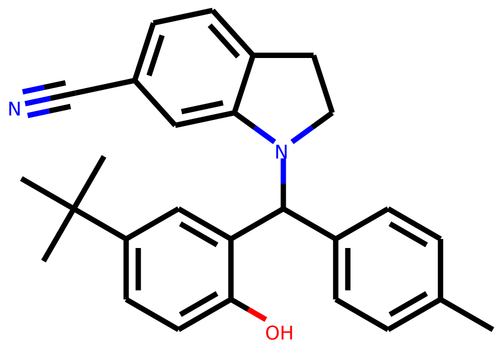
Ahma-336
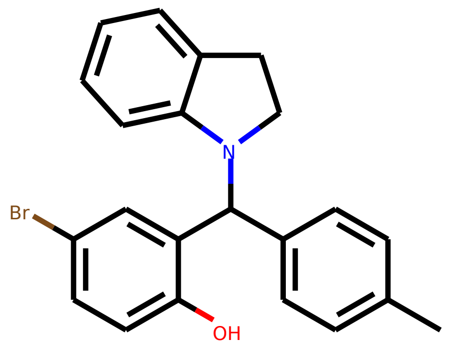
Ahma-337
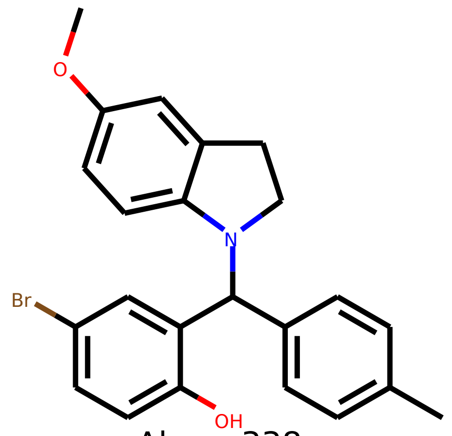
Ahma-338
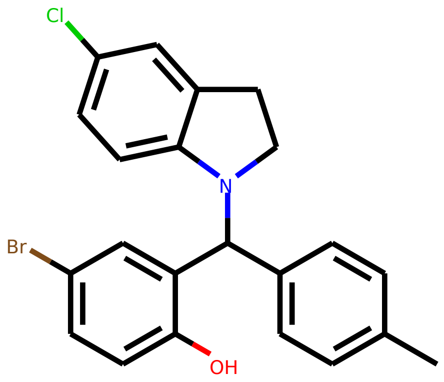
Ahma-339
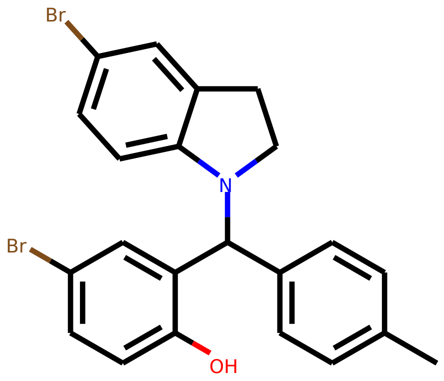
Ahma-340
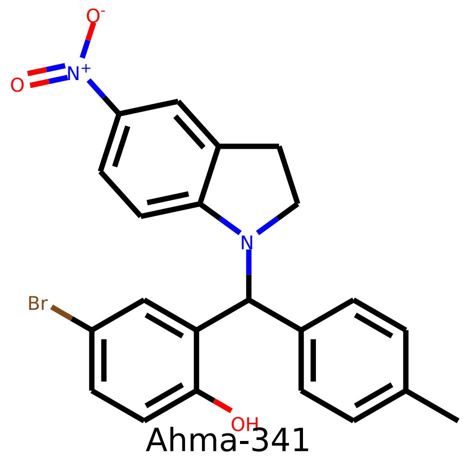
Ahma-341
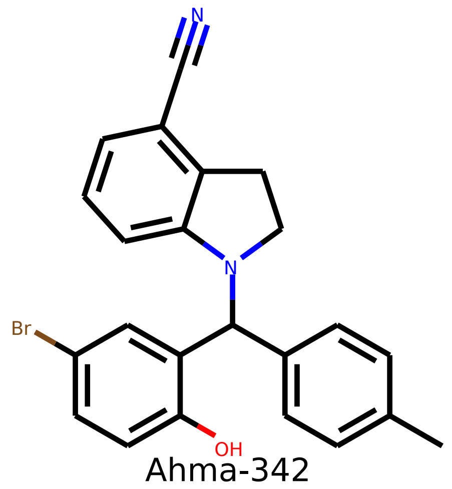
Ahma-342
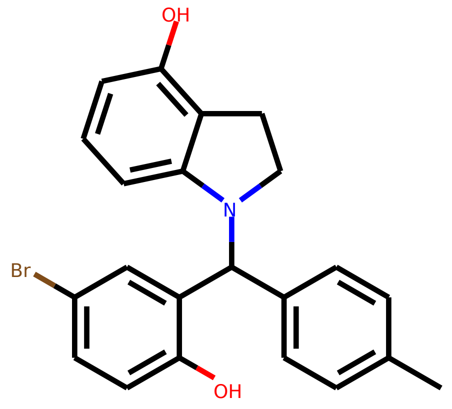
Ahma-343
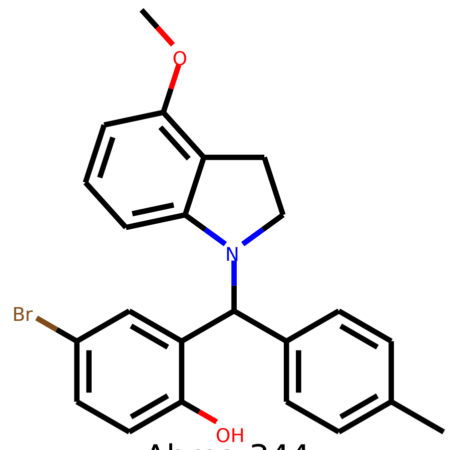
Ahma-344
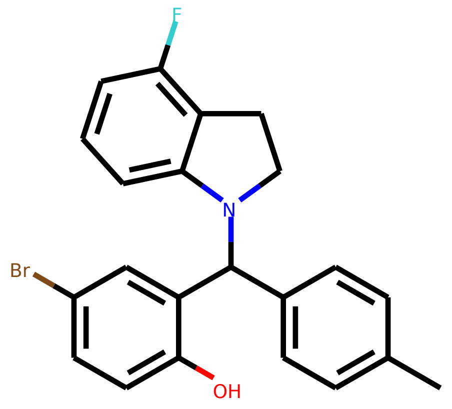
Ahma-345
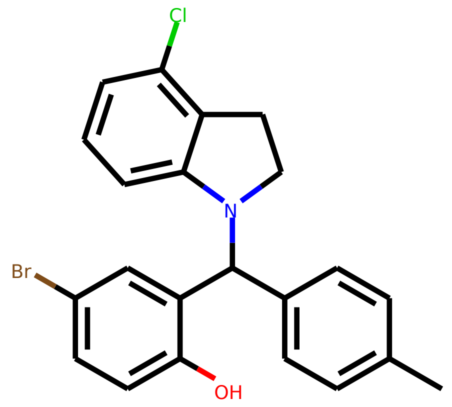
Ahma-346
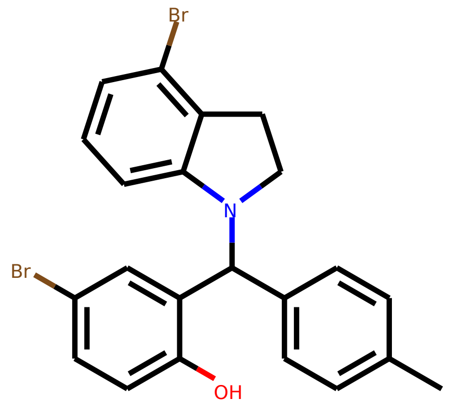
Ahma-347
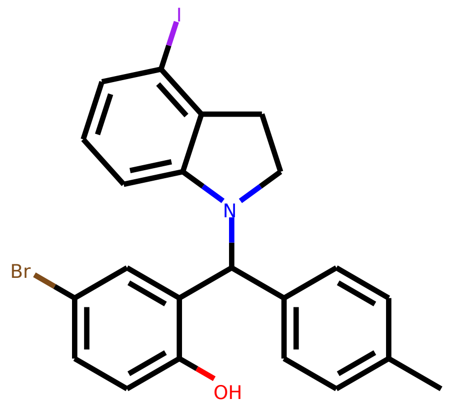
Ahma-348
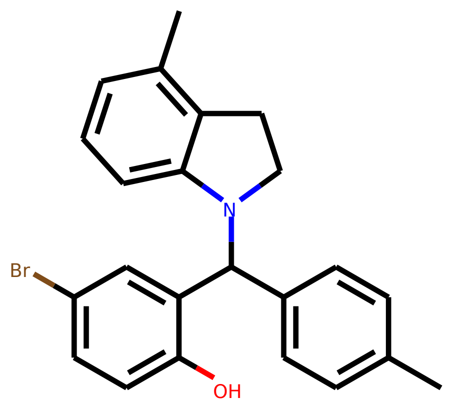
Ahma-349
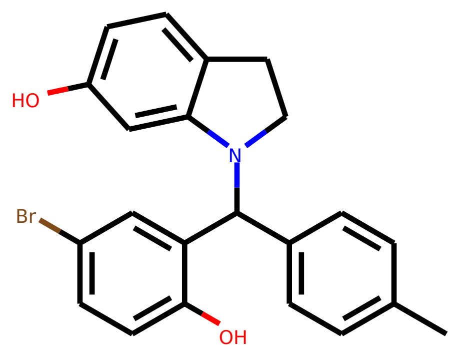
Ahma-350

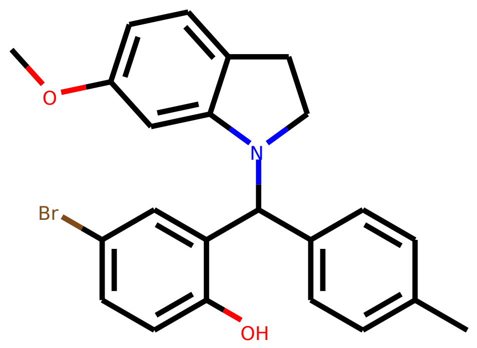
Ahma-351
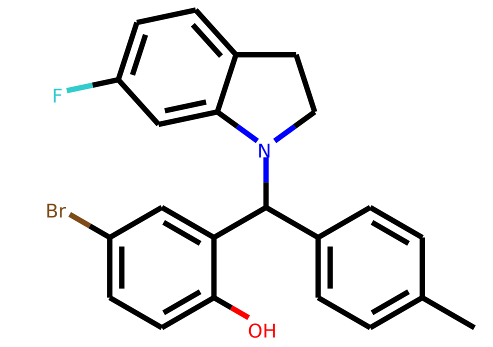
Ahma-352
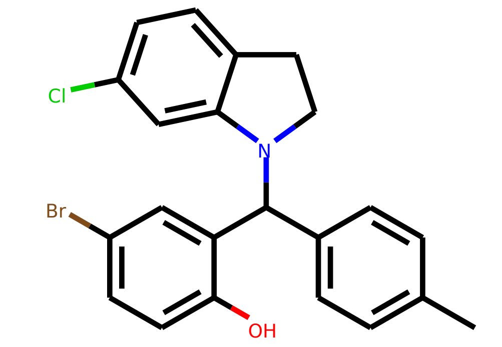
Ahma-353
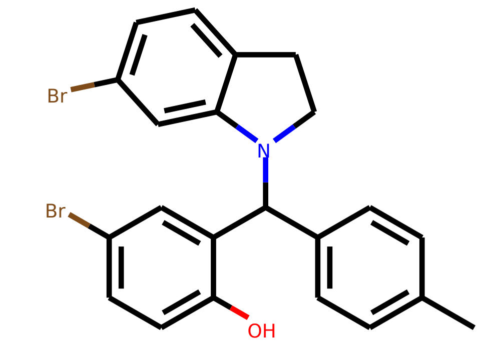
Ahma-354
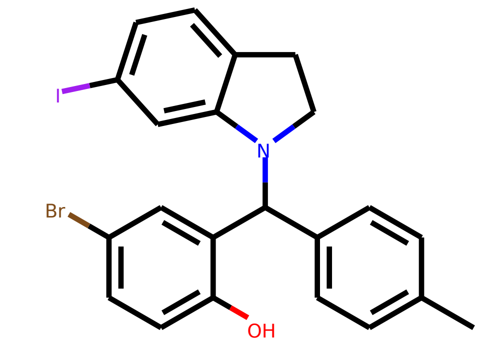
Ahma-355
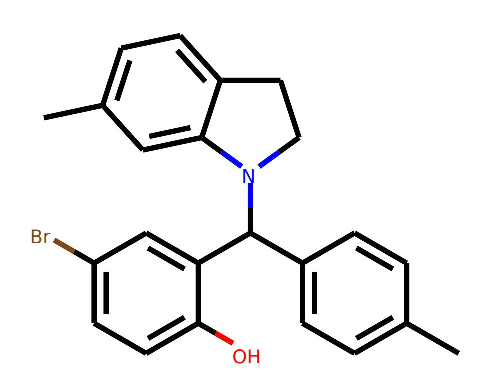
Ahma-356
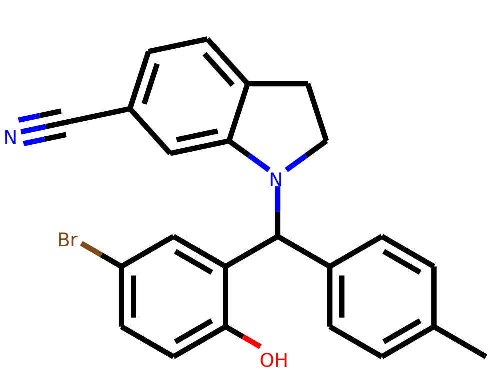
Ahma-357
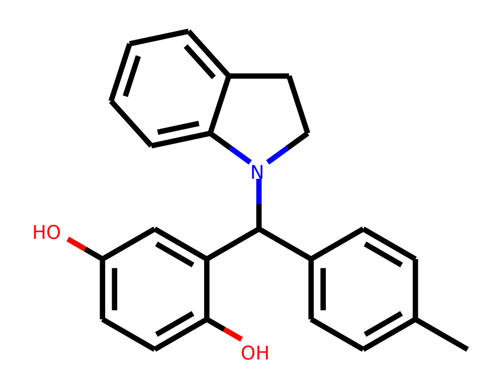
Ahma-358
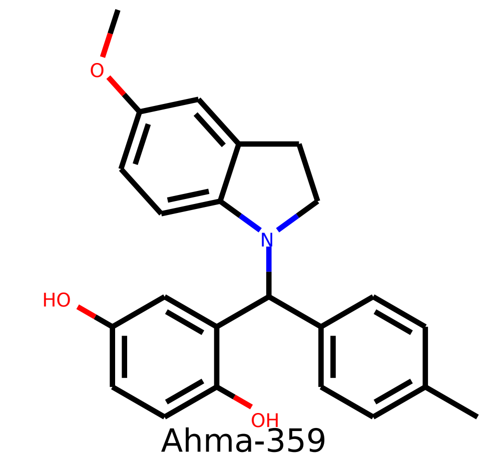
Ahma-359
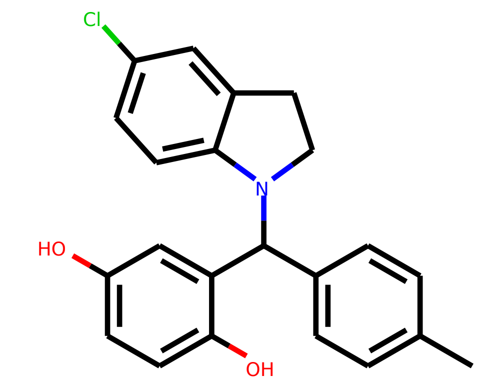
Ahma-360
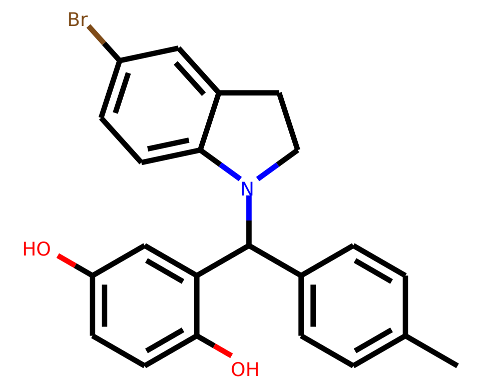
Ahma-361
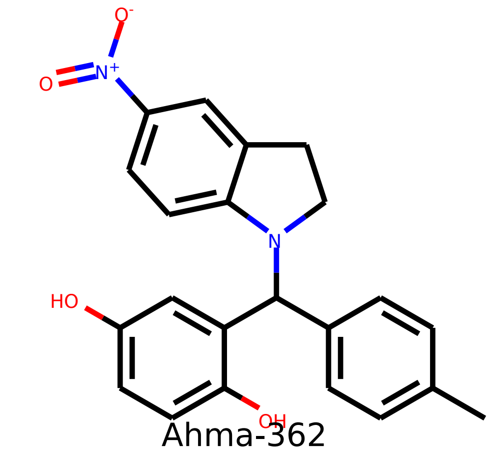
Ahma-362
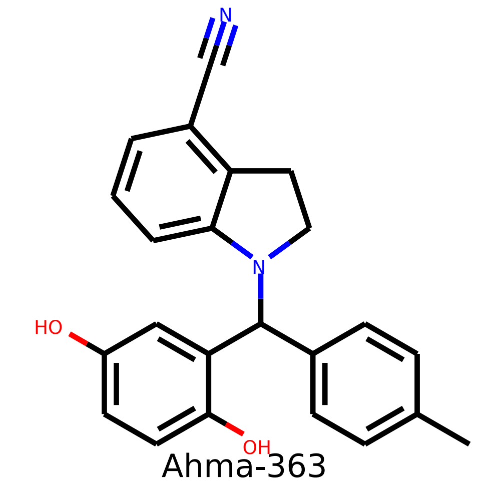
Ahma-363
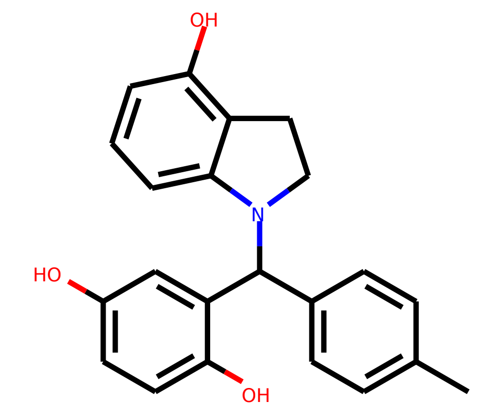
Ahma-364
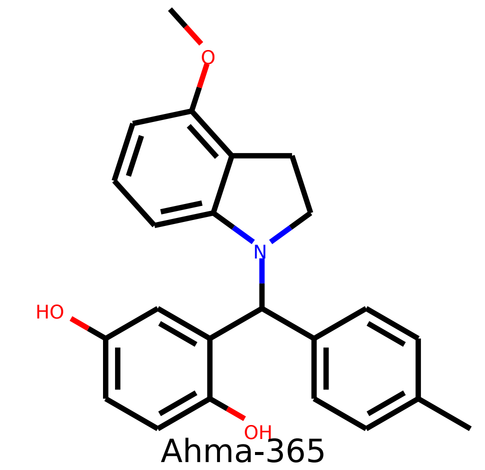
Ahma-365
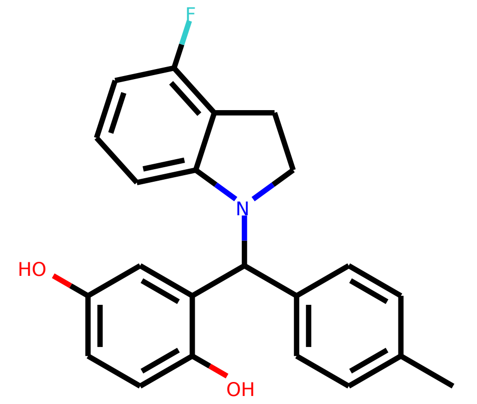
Ahma-366
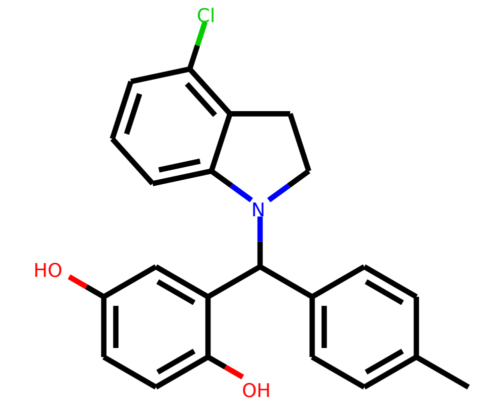
Ahma-367
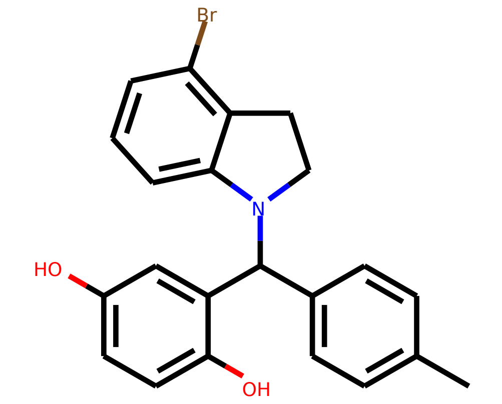
Ahma-368
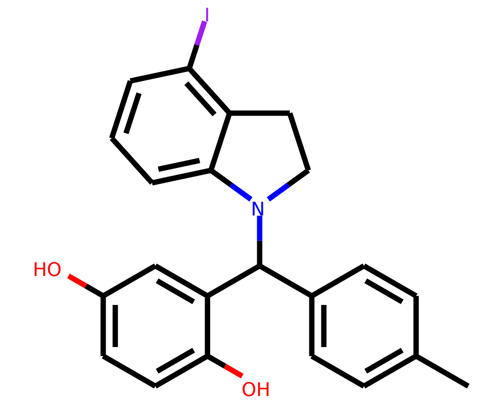
Ahma-369
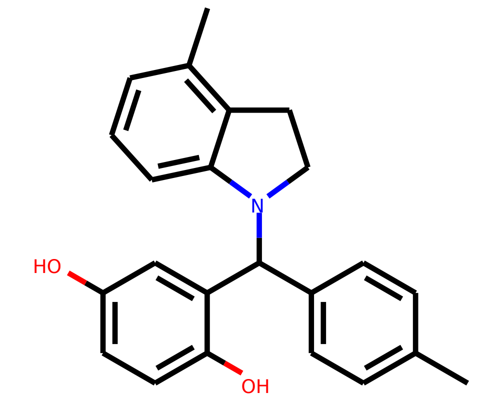
Ahma-370
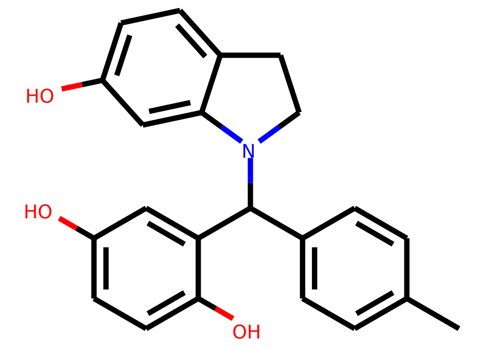
Ahma-371
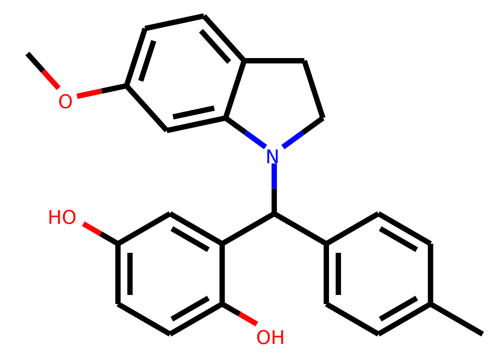
Ahma-372
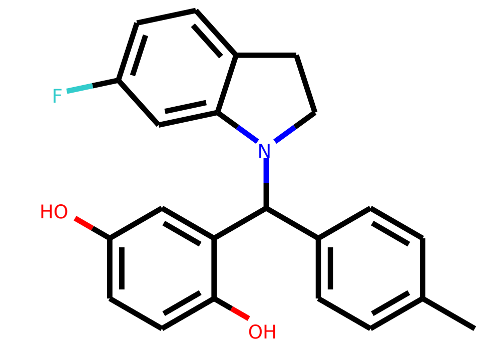
Ahma-373
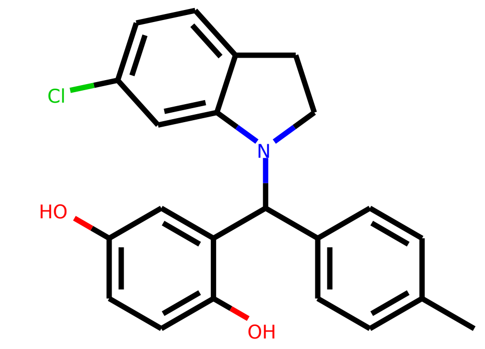
Ahma-374
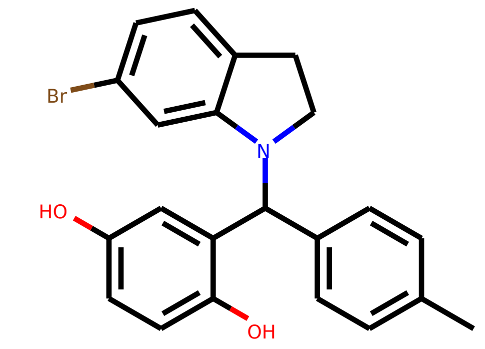
Ahma-375
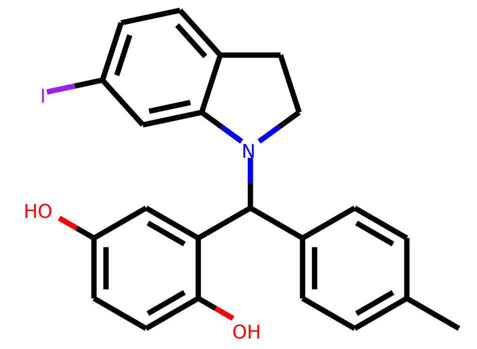
Ahma-376
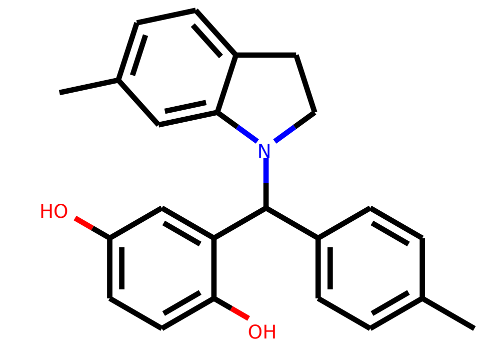
Ahma-377
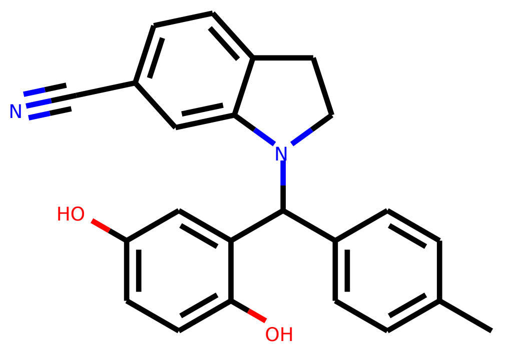
Ahma-378
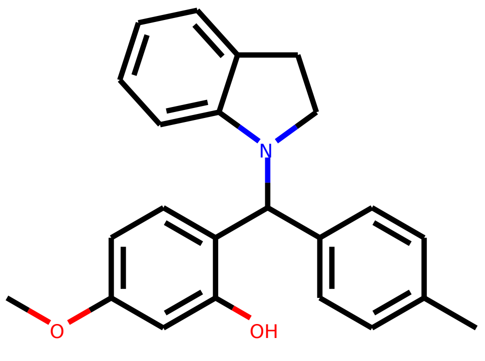
Ahma-379
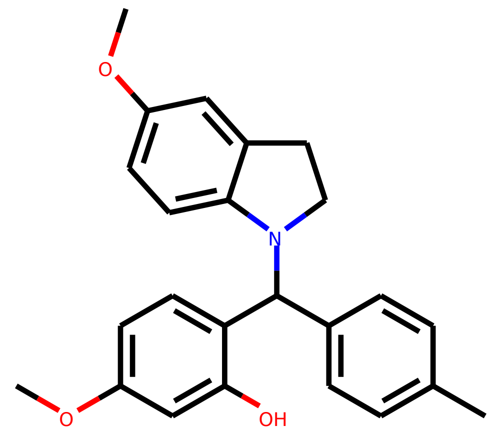
Ahma-380
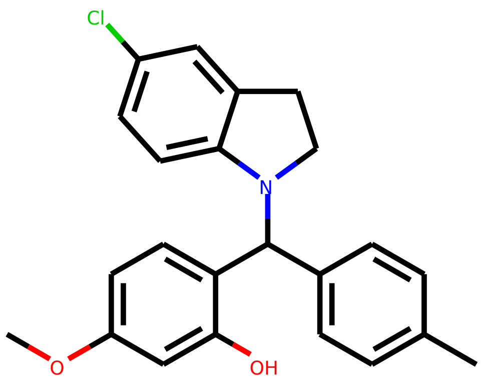
Ahma-381
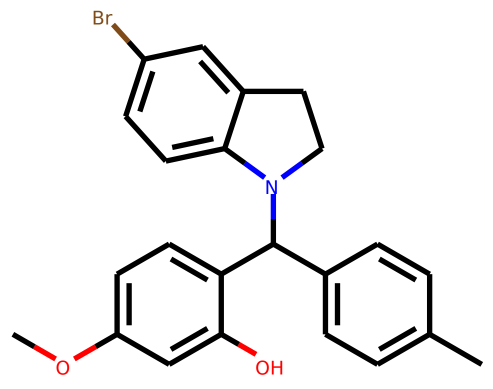
Ahma-382
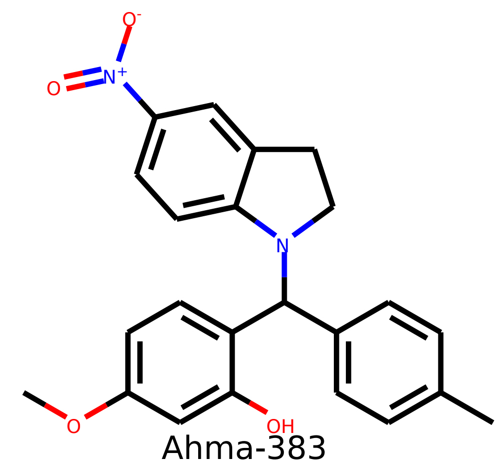
Ahma-383
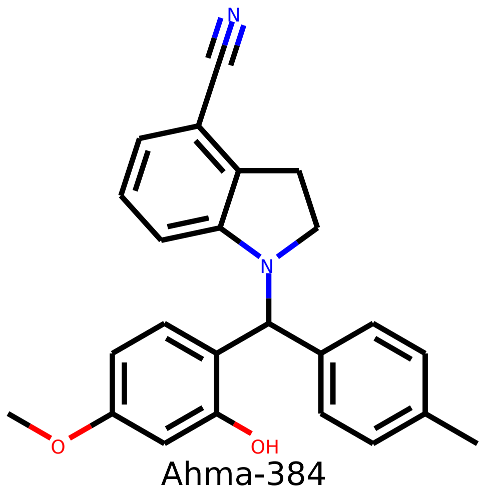
Ahma-384
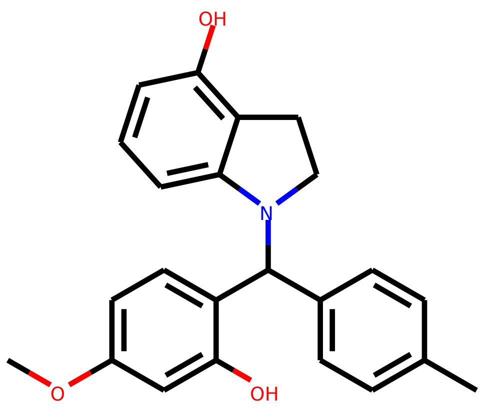
Ahma-385

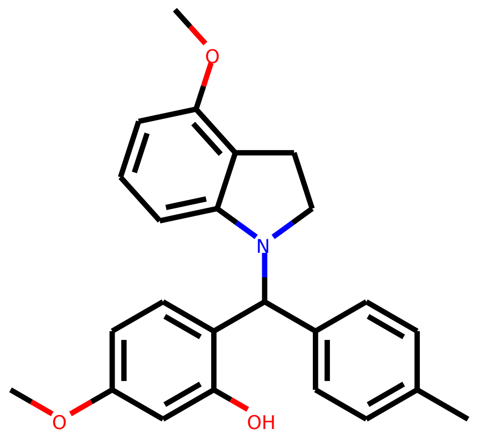
Ahma-386
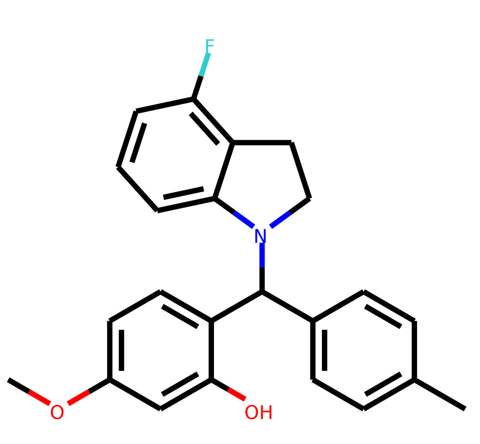
Ahma-387
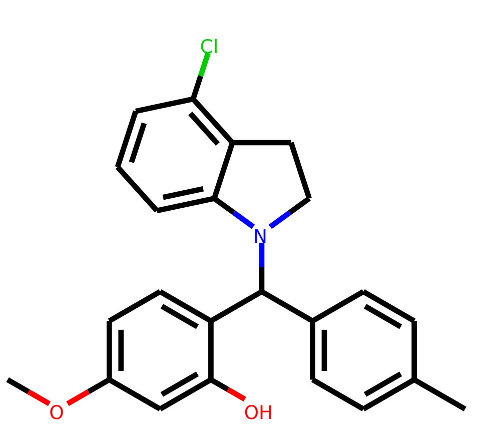
Ahma-388
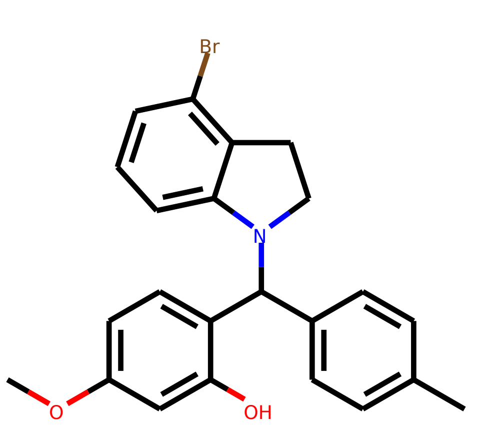
Ahma-389
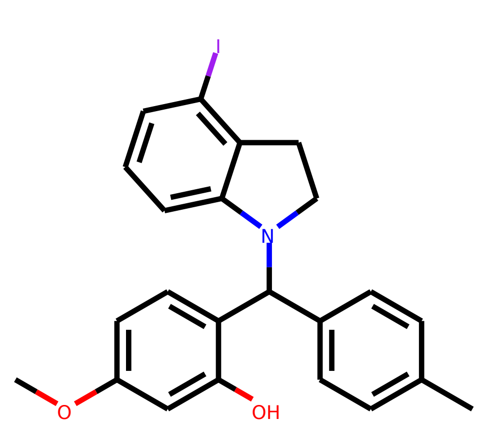
Ahma-390
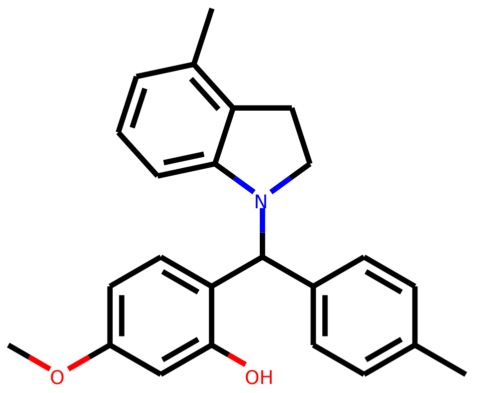
Ahma-391
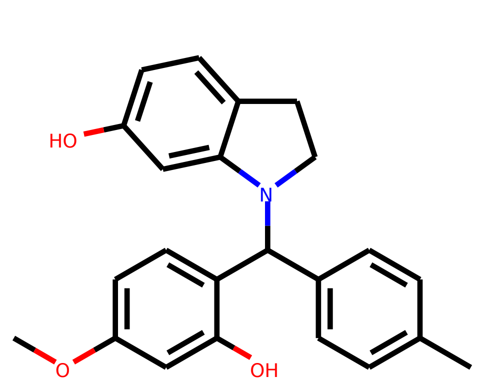
Ahma-392
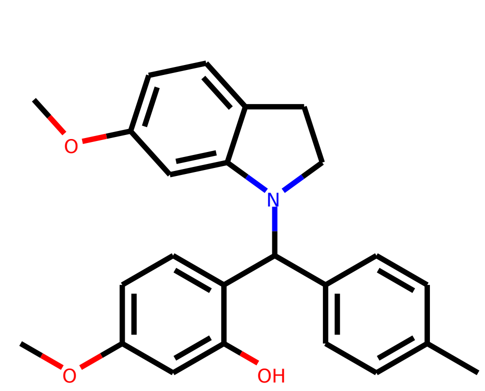
Ahma-393
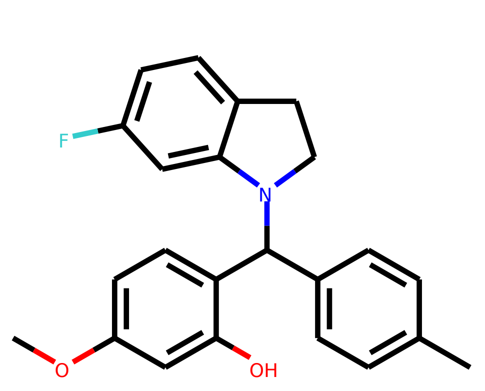
Ahma-394
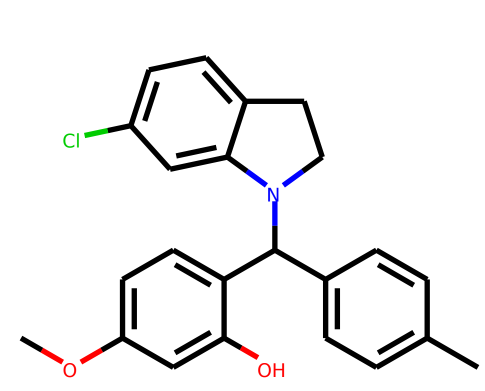
Ahma-395
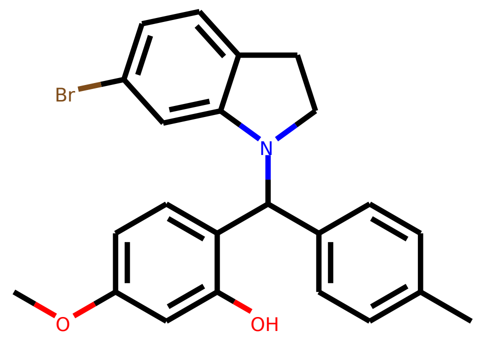
Ahma-396
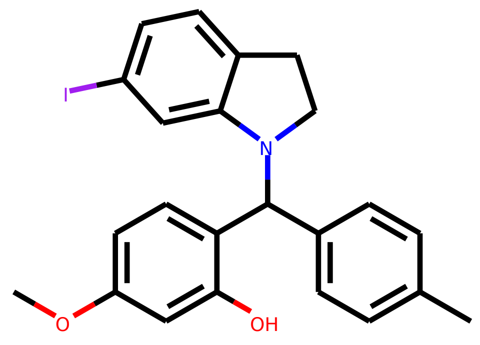
Ahma-397
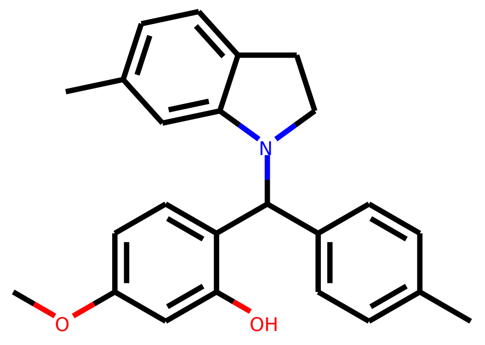
Ahma-398
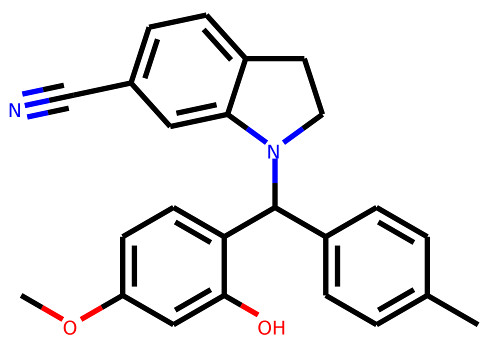
Ahma-399
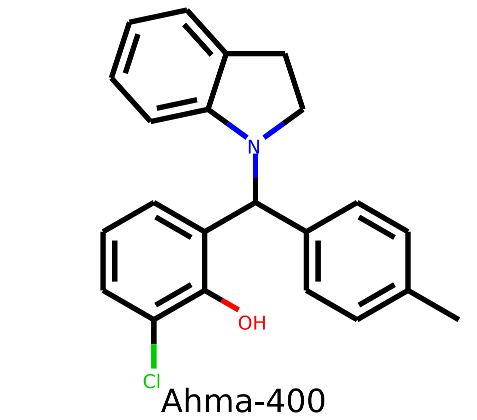
Ahma-400
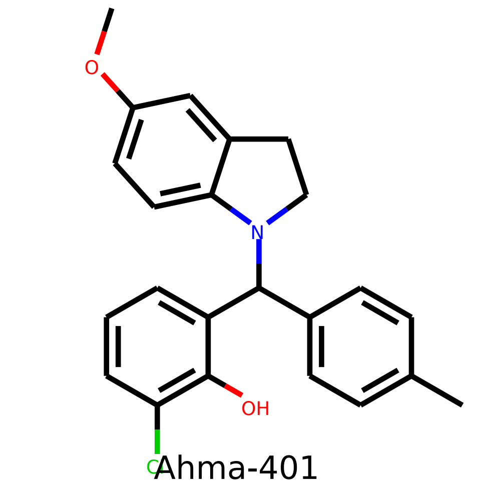
Ahma-401
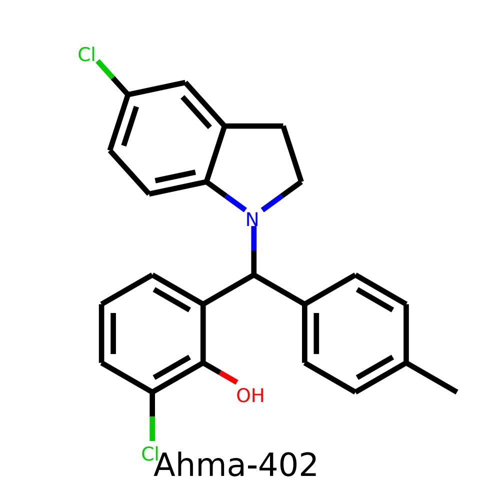
Ahma-402
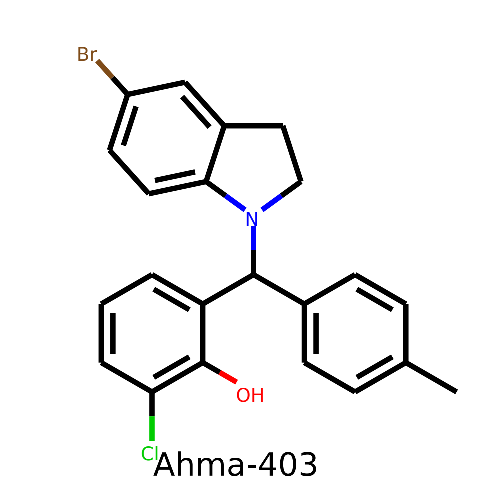
Ahma-403
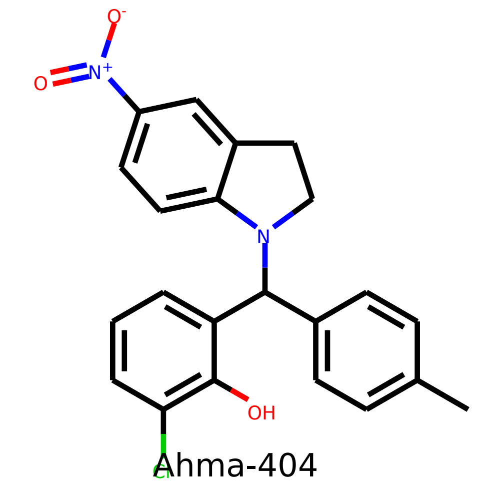
Ahma-404
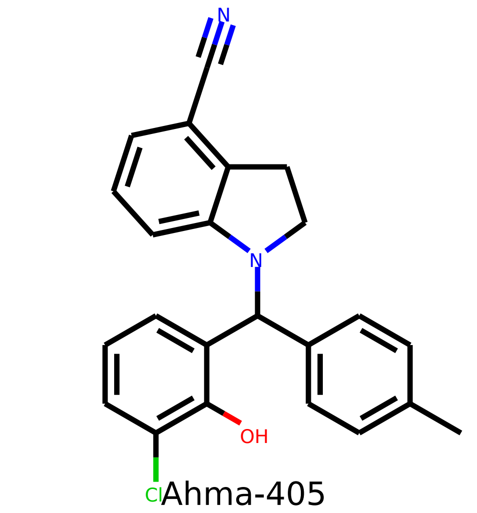
Ahma-405
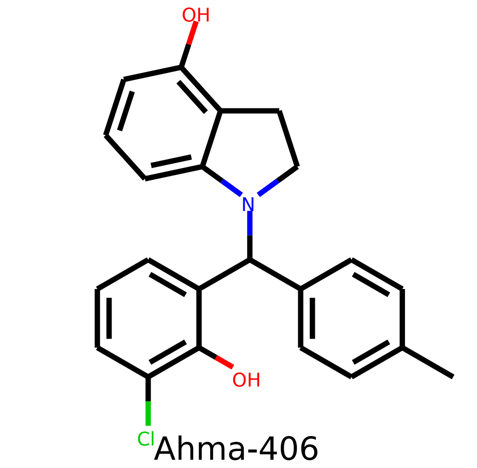
Ahma-406
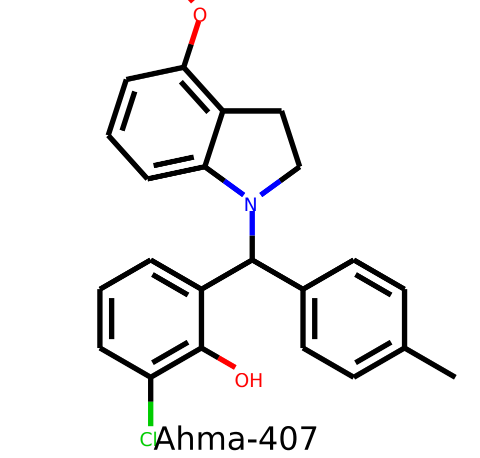
Ahma-407
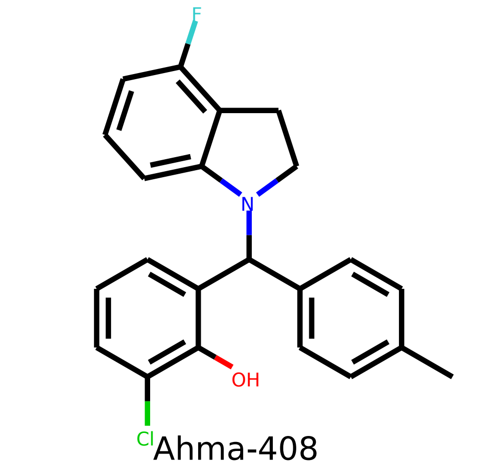
Ahma-408
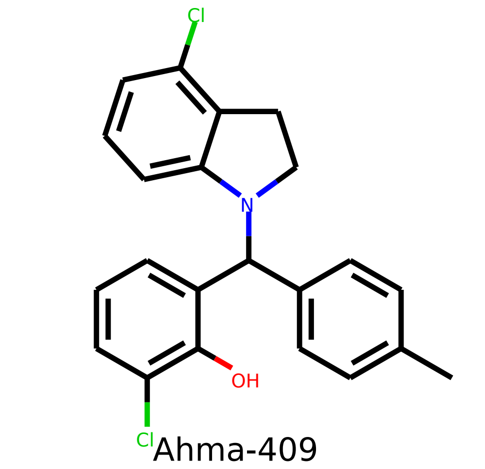
Ahma-409
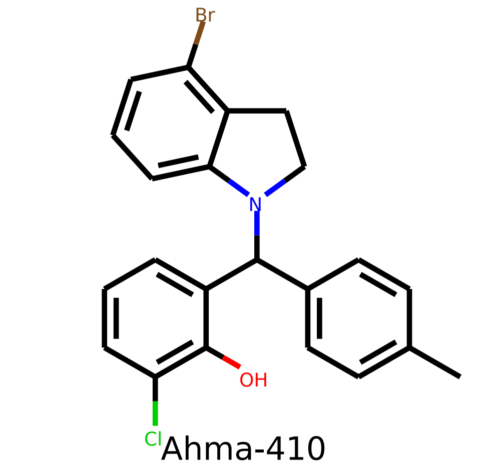
Ahma-410
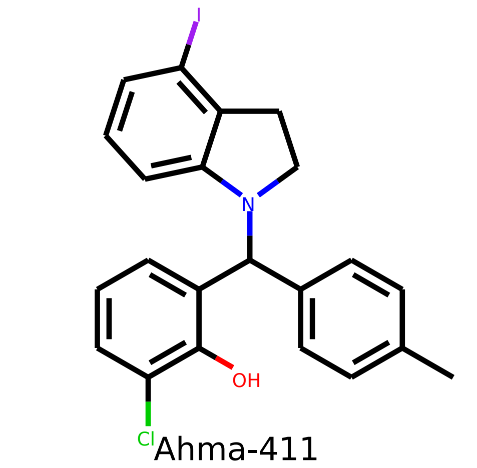
Ahma-411
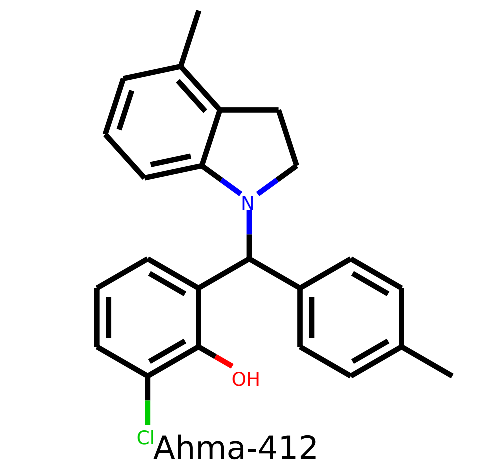
Ahma-412
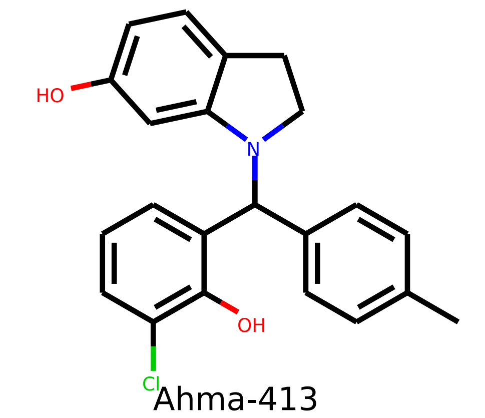
Ahma-413
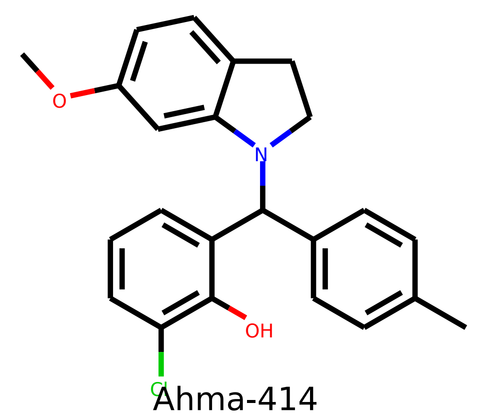
Ahma-414
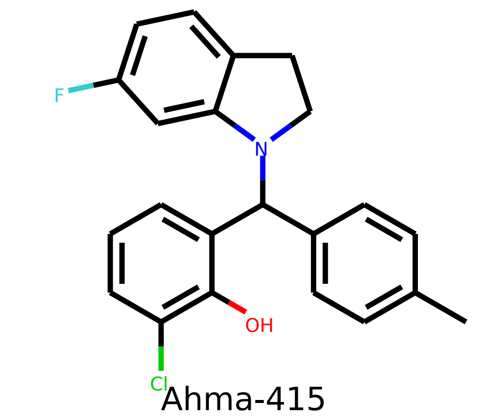
Ahma-415
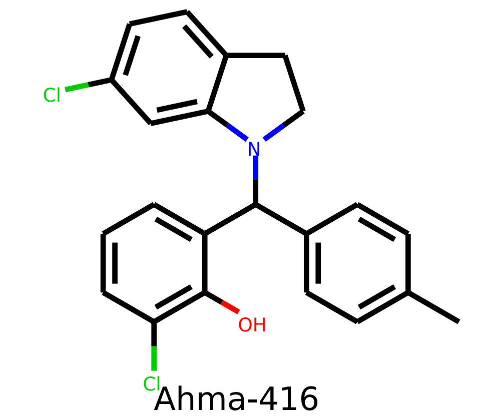
Ahma-416
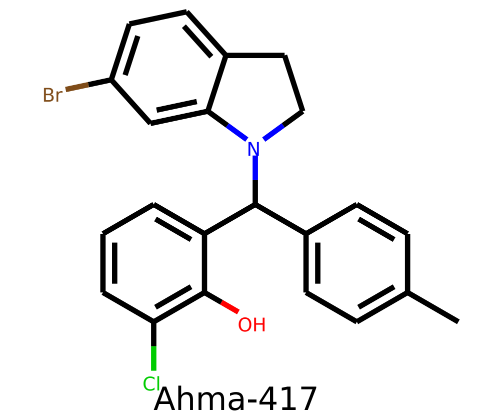
Ahma-417
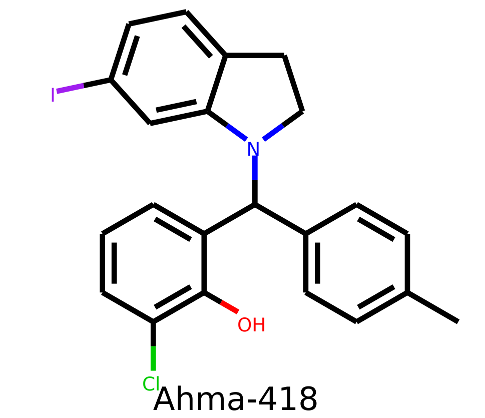
Ahma-418
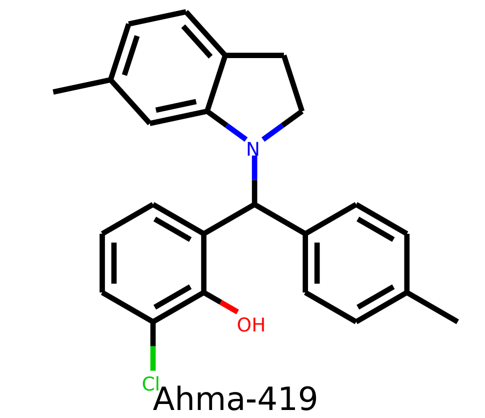
Ahma-419
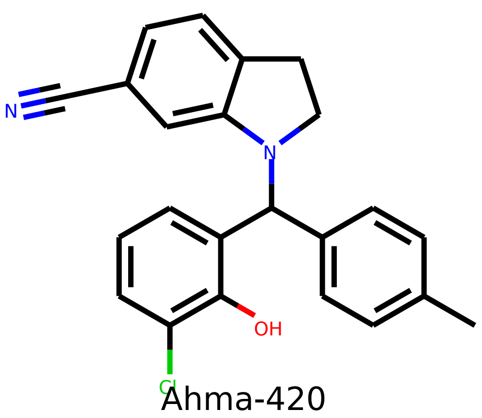
Ahma-420

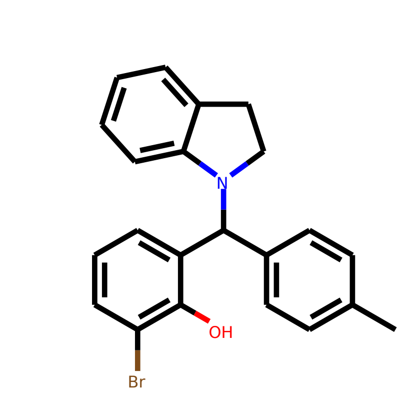
Ahma-421
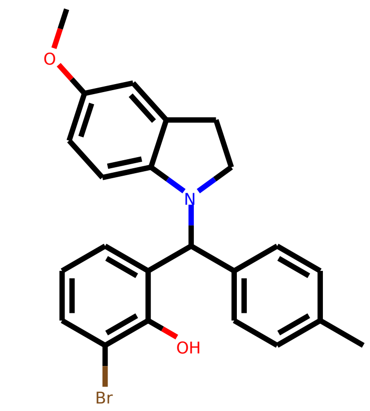
Ahma-422
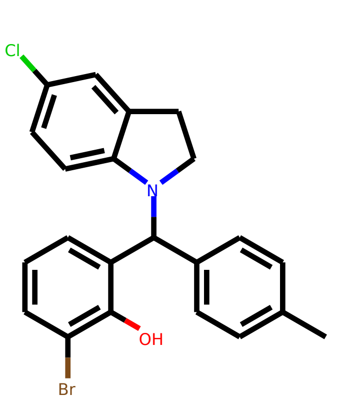
Ahma-423
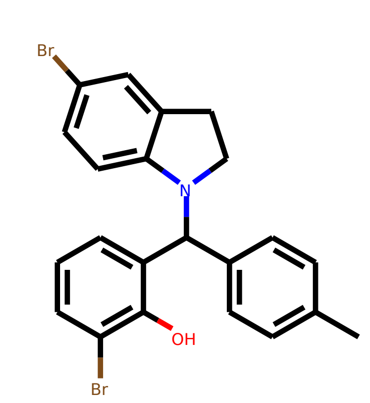
Ahma-424
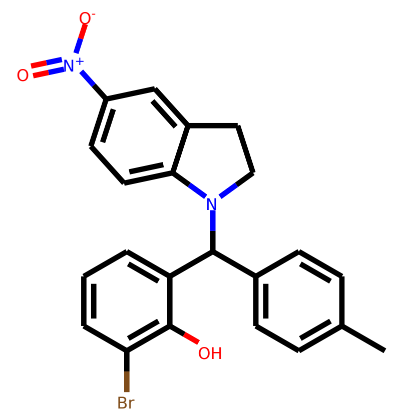
Ahma-425
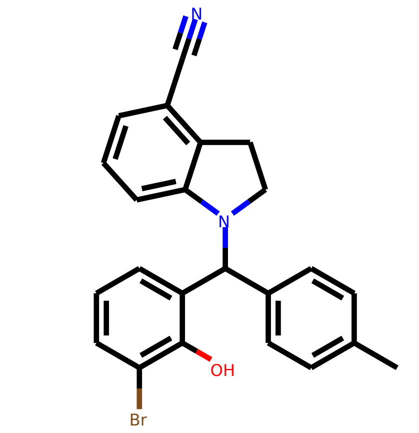
Ahma-426
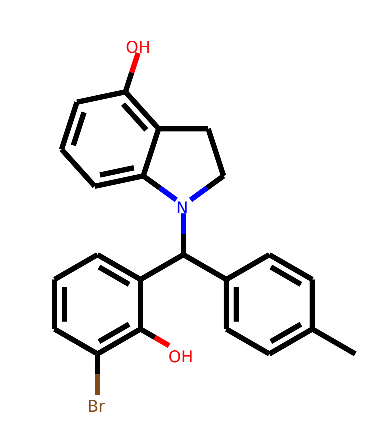
Ahma-427
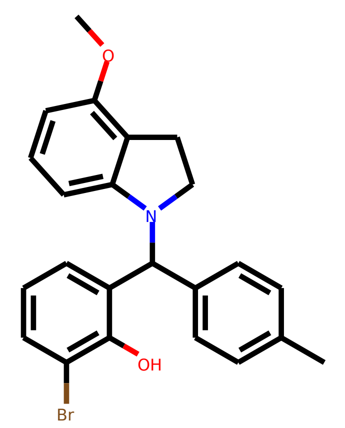
Ahma-428
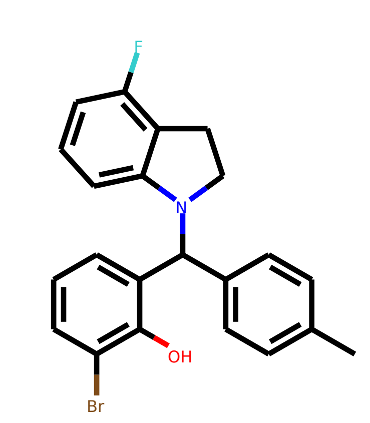
Ahma-429
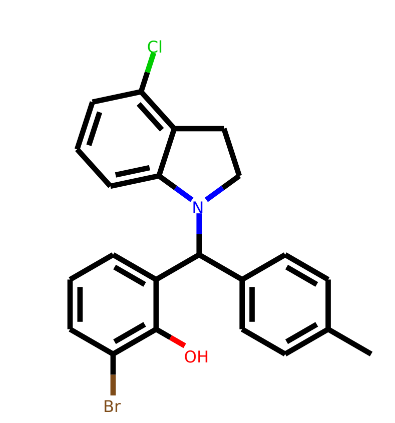
Ahma-430
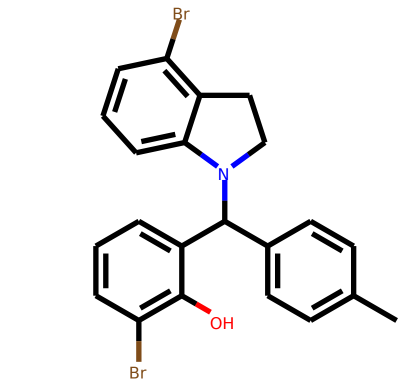
Ahma-431
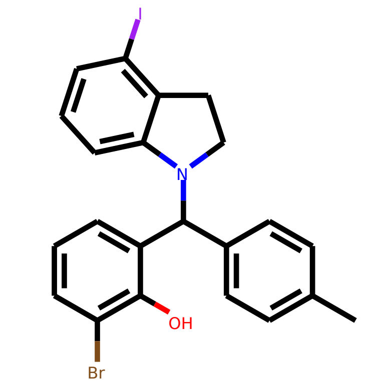
Ahma-432
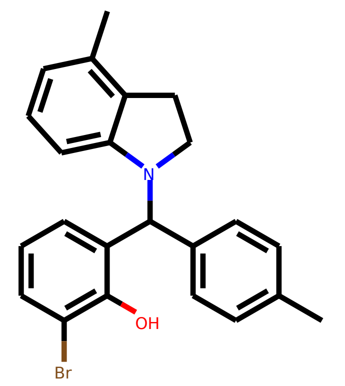
Ahma-433
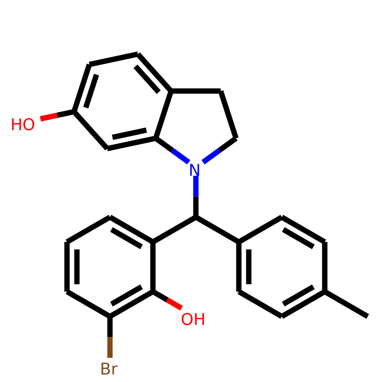
Ahma-434
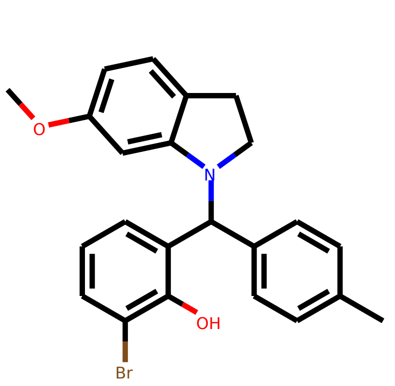
Ahma-435
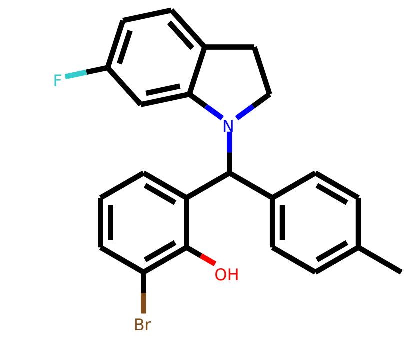
Ahma-436
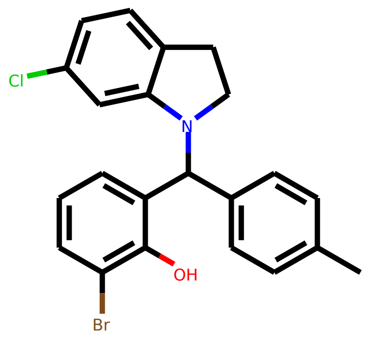
Ahma-437
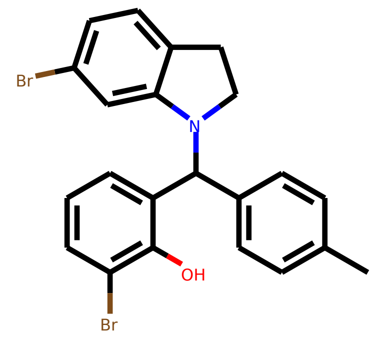
Ahma-438
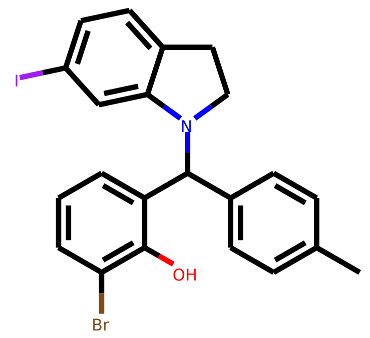
Ahma-439
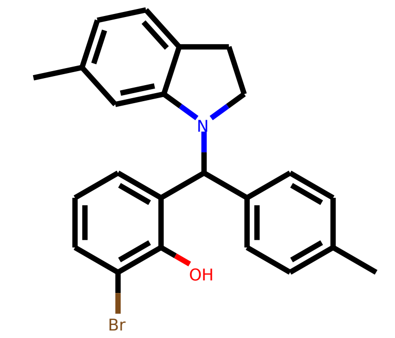
Ahma-440
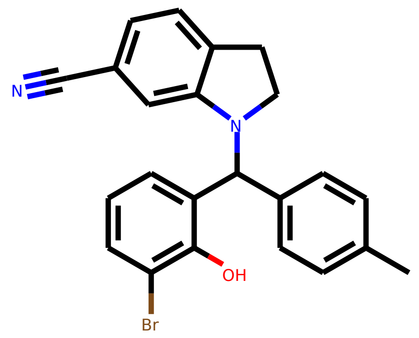
Ahma-441
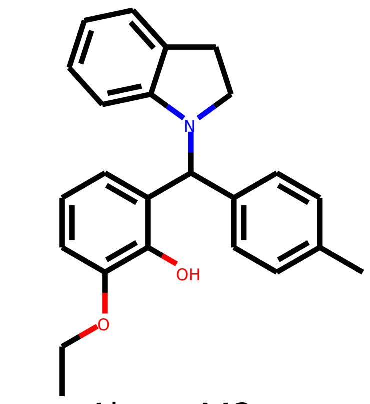
Ahma-442
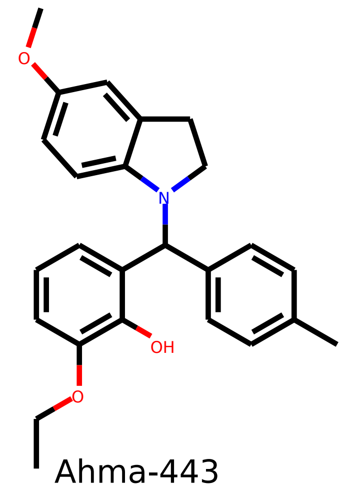
Ahma-443
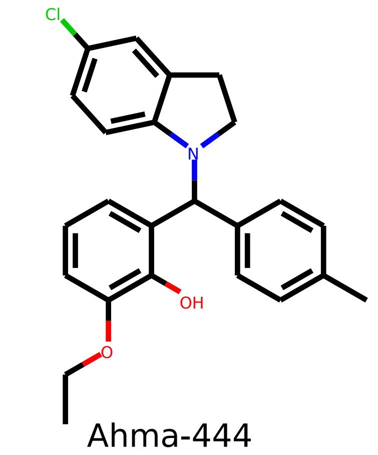
Ahma-444
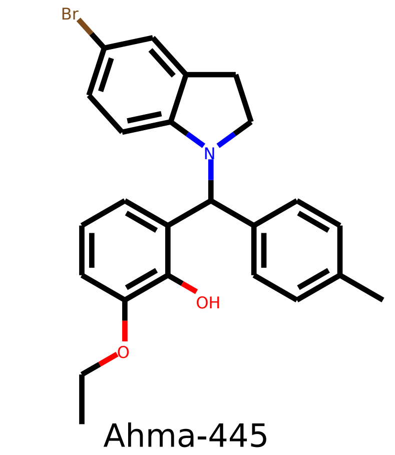
Ahma-445
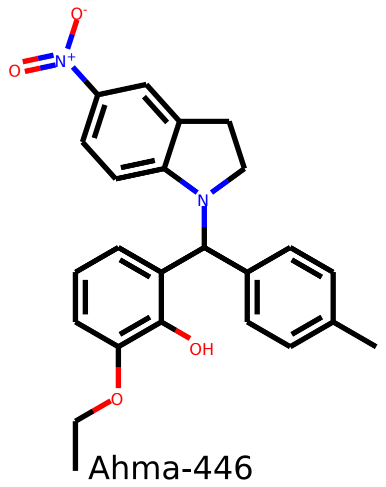
Ahma-446
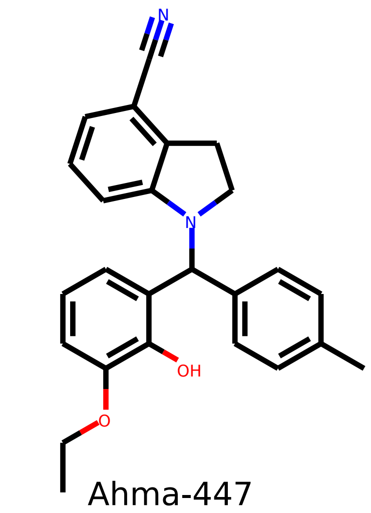
Ahma-447
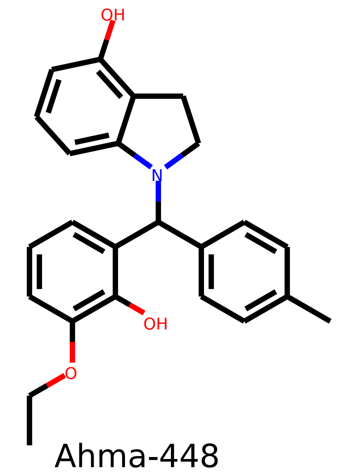
Ahma-448
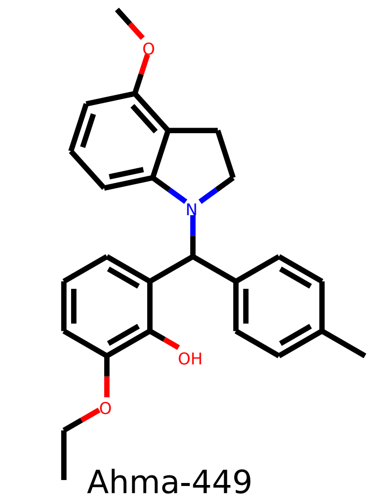
Ahma-449
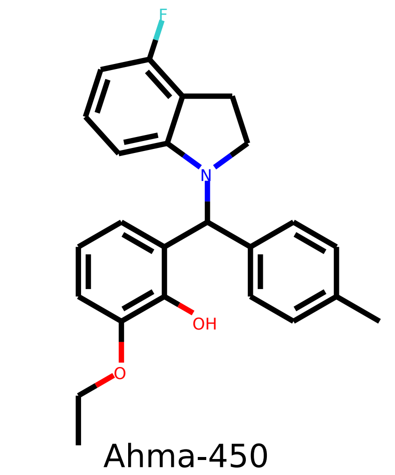
Ahma-450
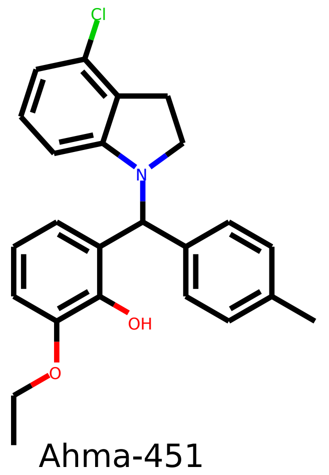
Ahma-451
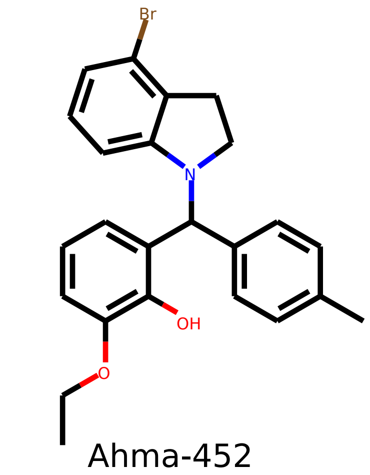
Ahma-452
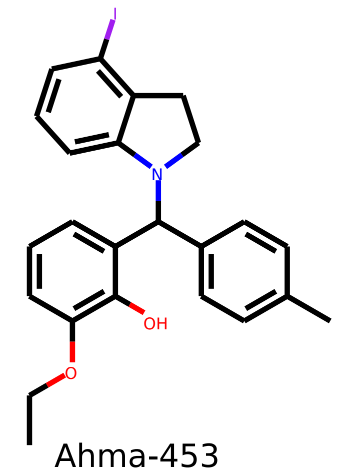
Ahma-453
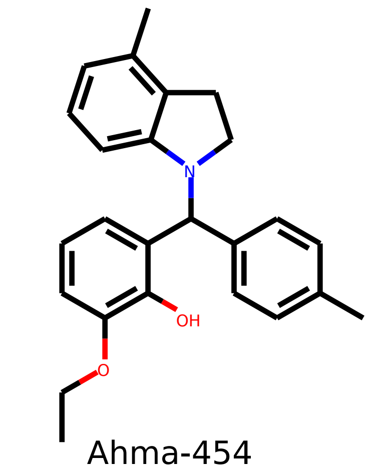
Ahma-454
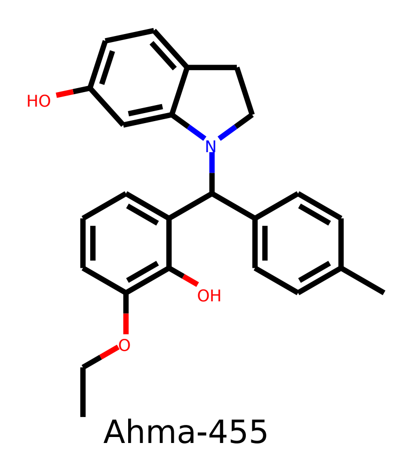
Ahma-455

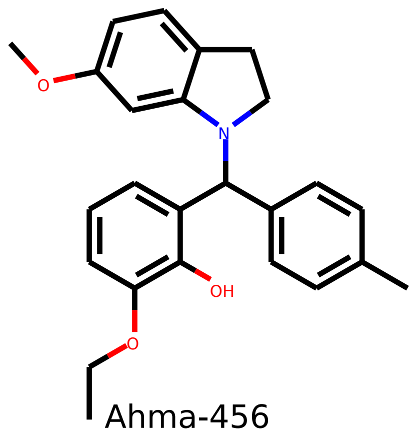

Ahma-456

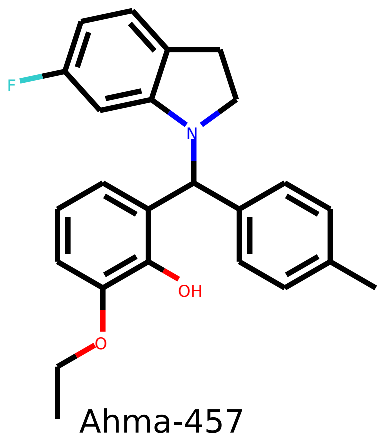

Ahma-457

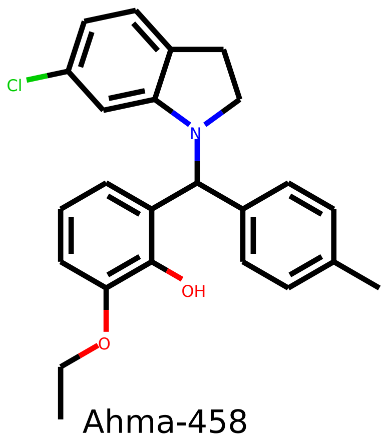

Ahma-458

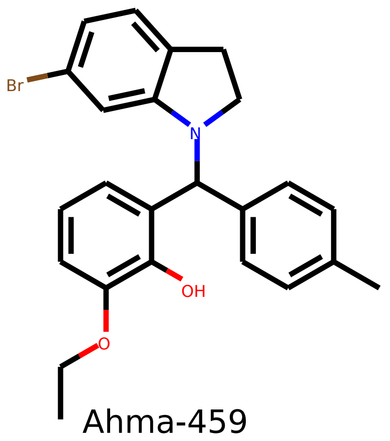

Ahma-459

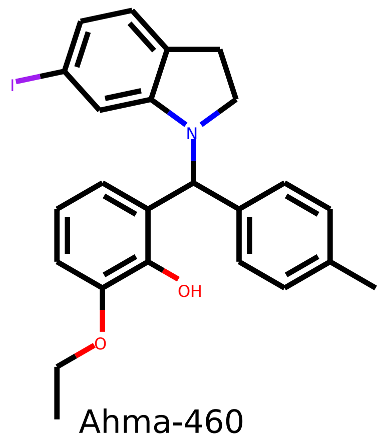

Ahma-460

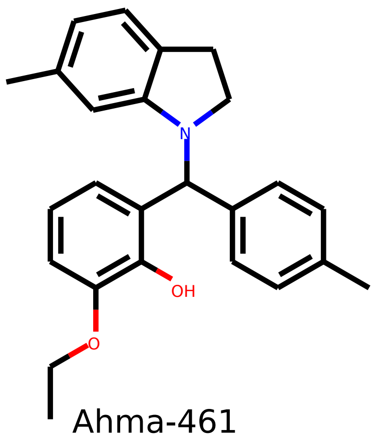

Ahma-461

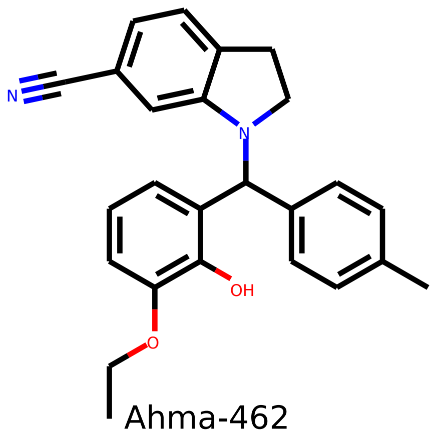

Ahma-462

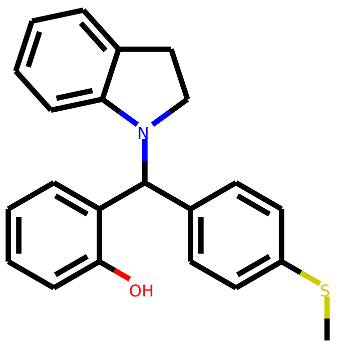

Ahma-463

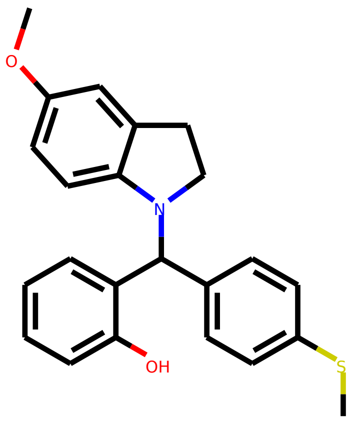

Ahma-464

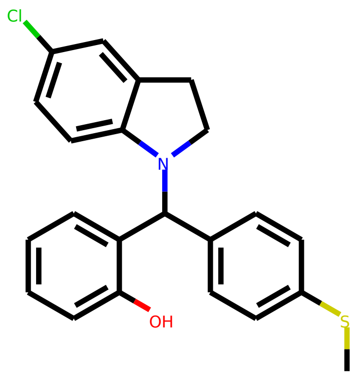

Ahma-465

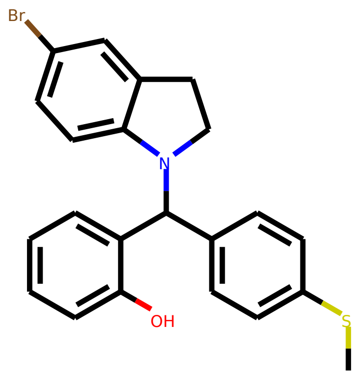

Ahma-466

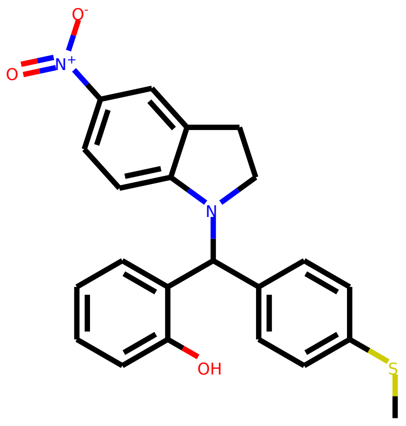

Ahma-467

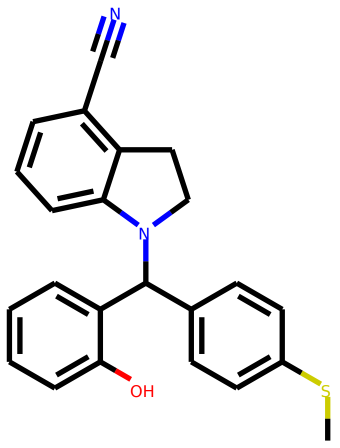

Ahma-468

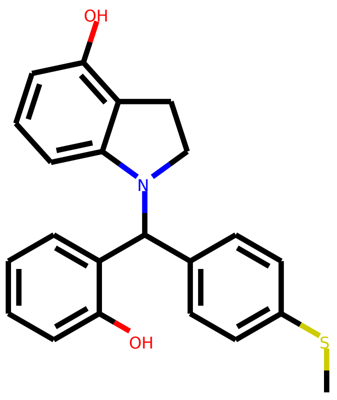

Ahma-469

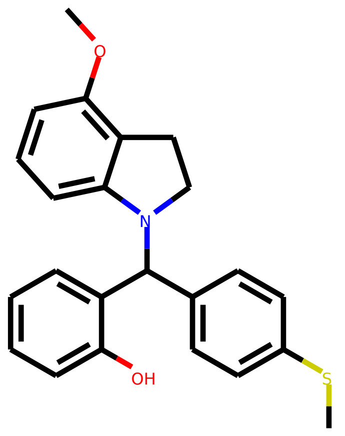

Ahma-470

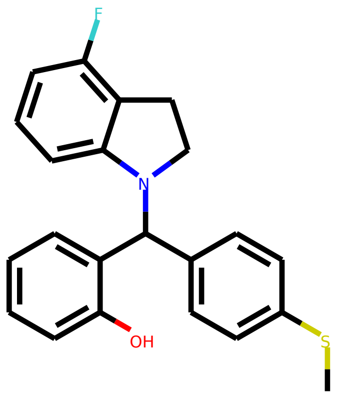

Ahma-471

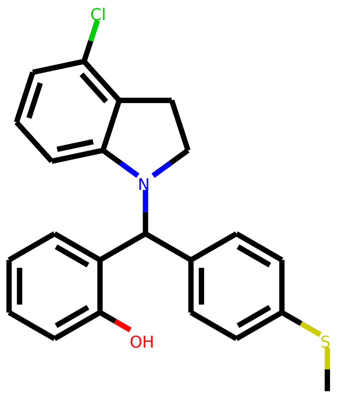

Ahma-472

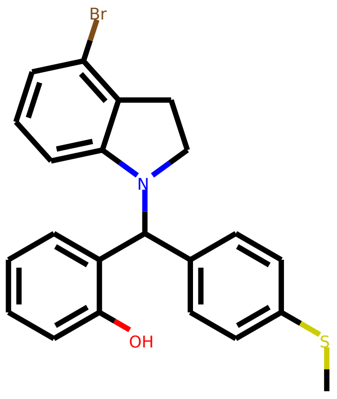

Ahma-473

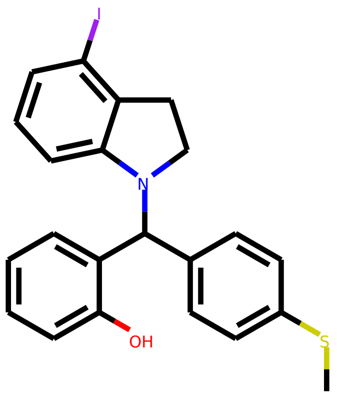

Ahma-474

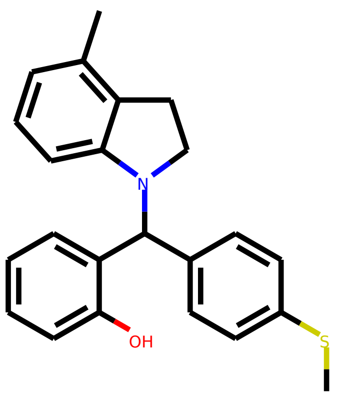

Ahma-475

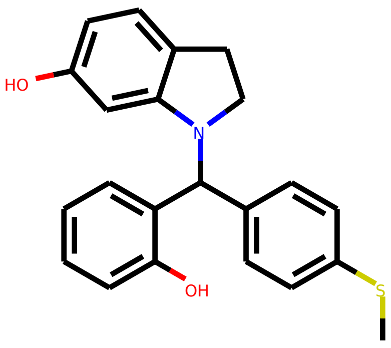

Ahma-476

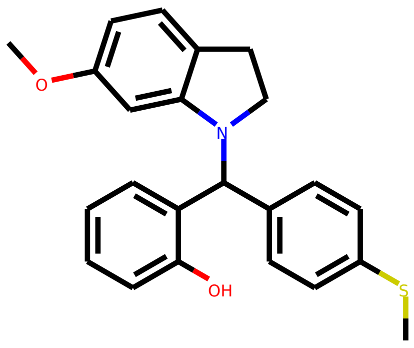

Ahma-477

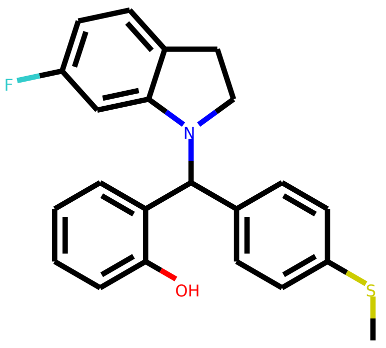

Ahma-478

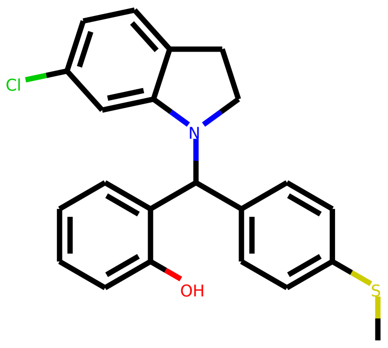

Ahma-479

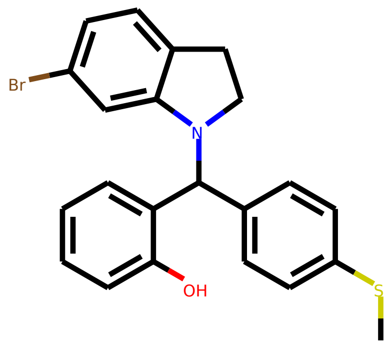

Ahma-480

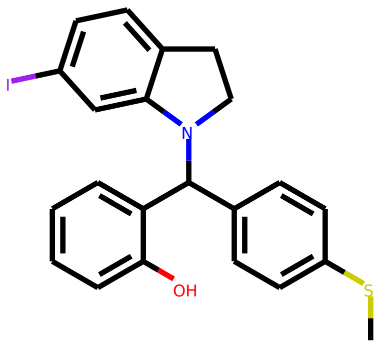

Ahma-481

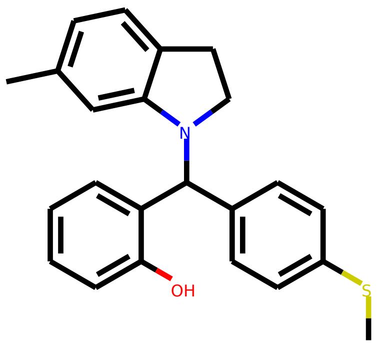

Ahma-482

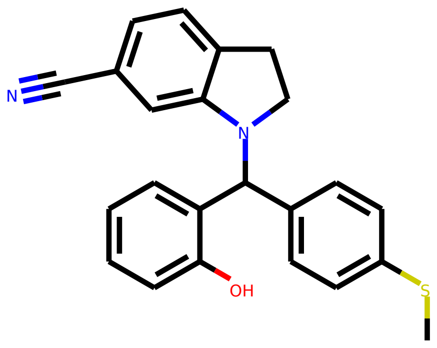

Ahma-483

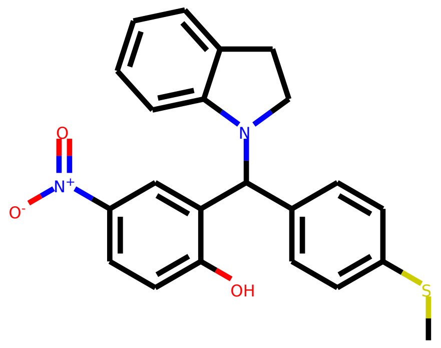

Ahma-484

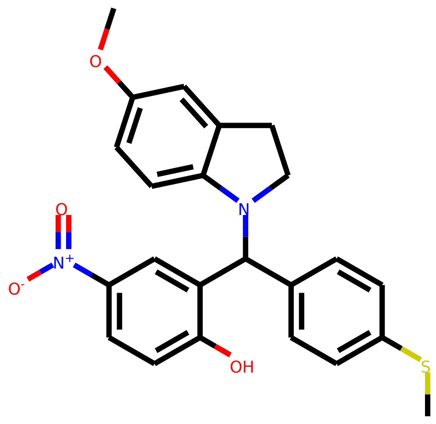

Ahma-485

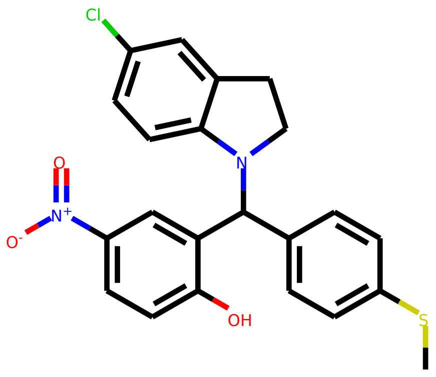

Ahma-486

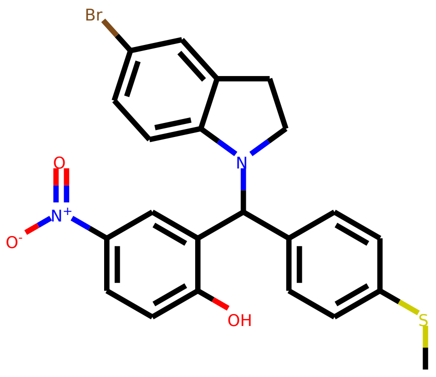

Ahma-487

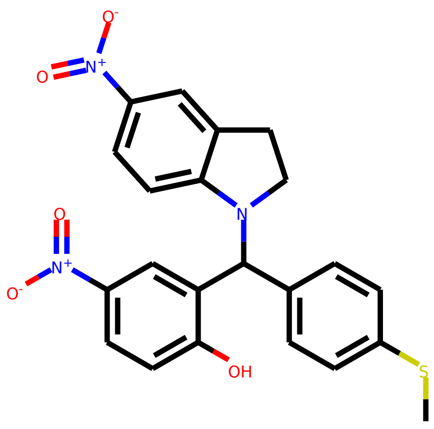

Ahma-488

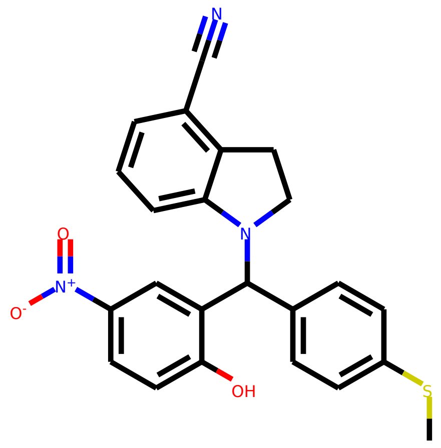

Ahma-489

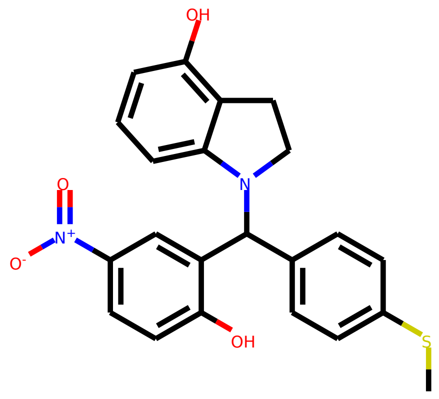

Ahma-490

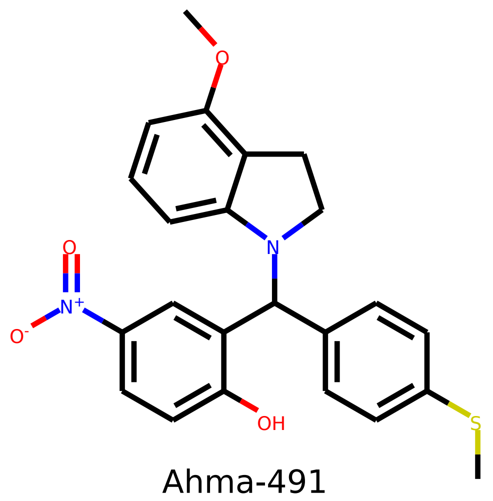
Ahma-491

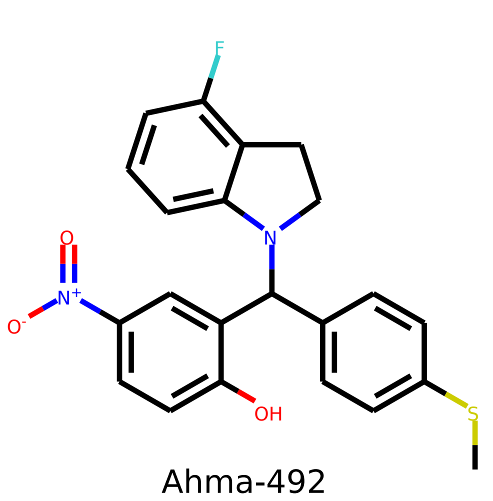
Ahma-492

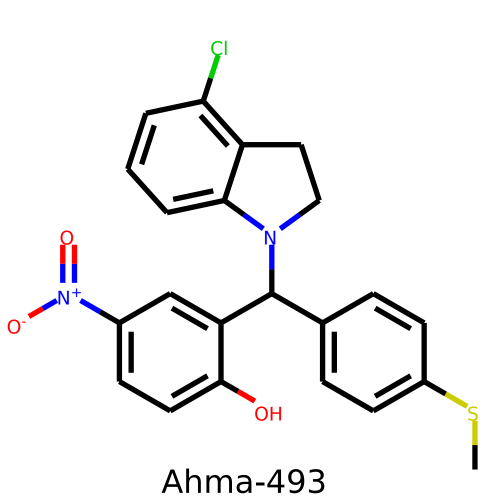
Ahma-493

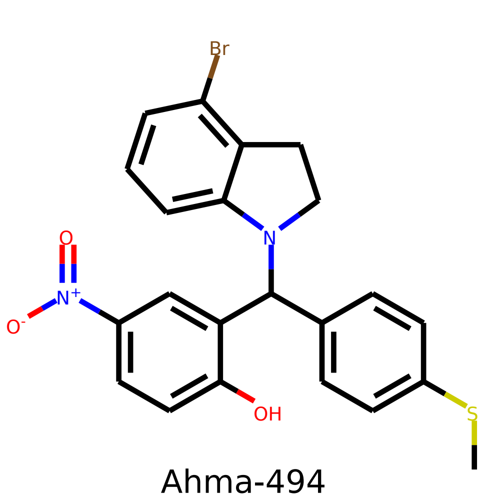
Ahma-494

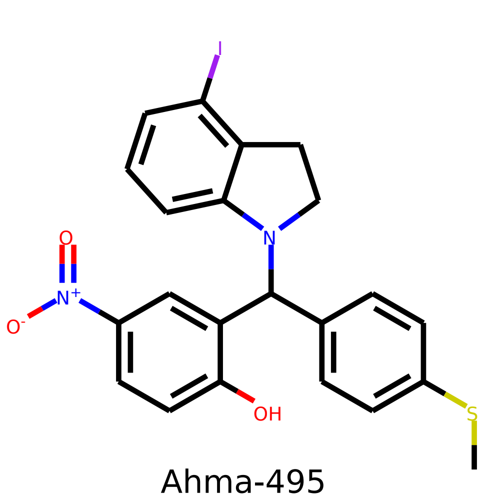
Ahma-495

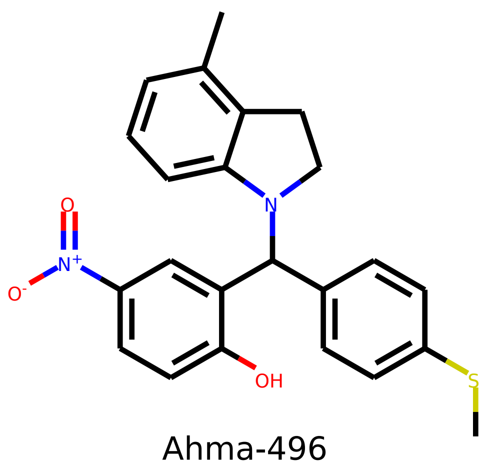
Ahma-496

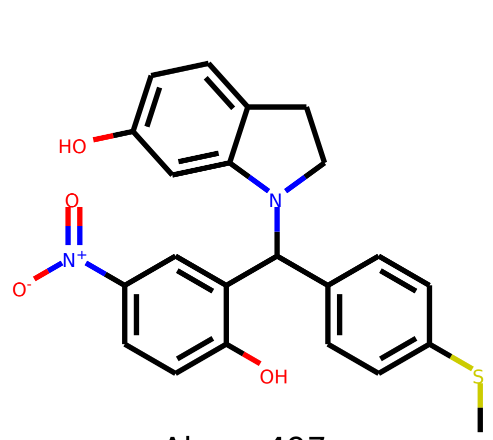
Ahma-497

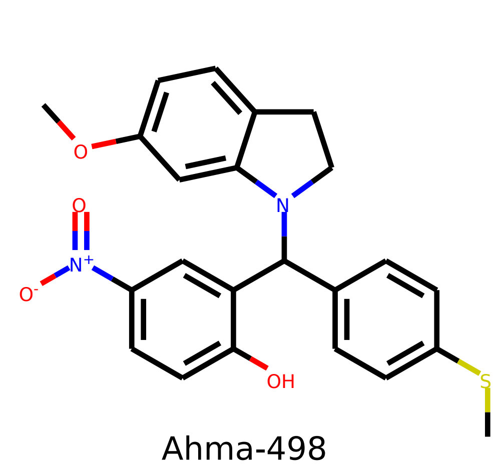
Ahma-498

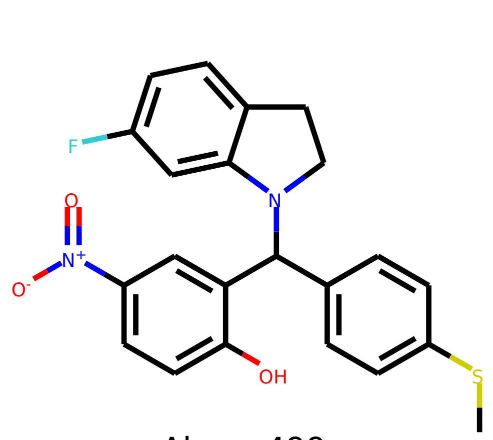
Ahma-499

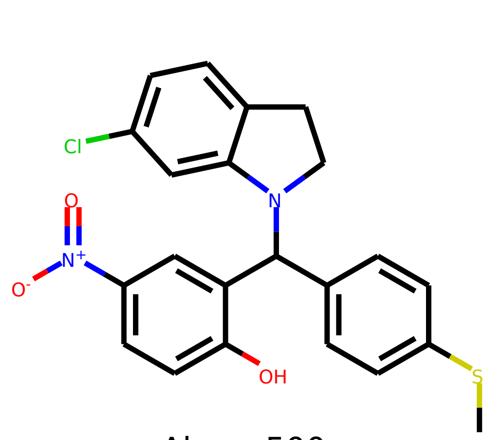
Ahma-500

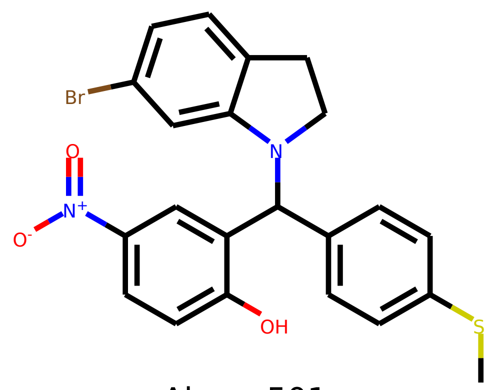
Ahma-501

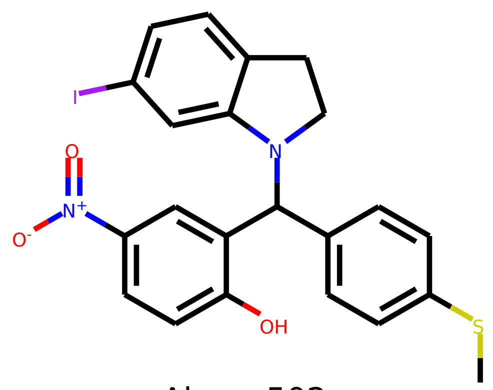
Ahma-502

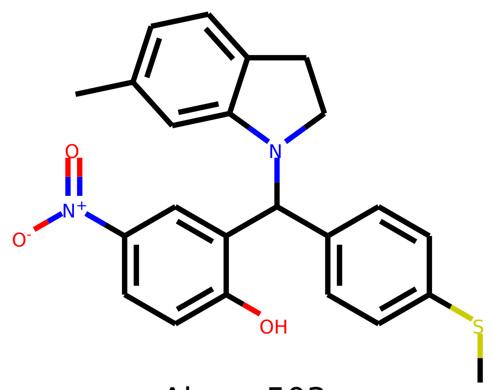
Ahma-503

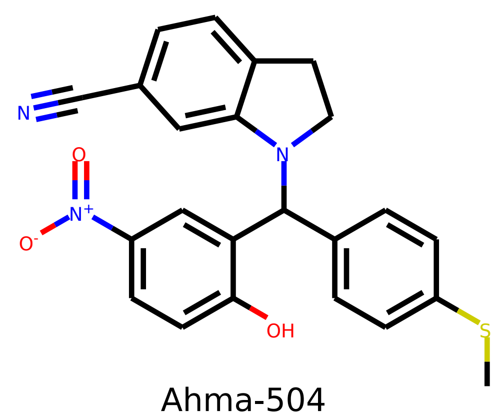
Ahma-504

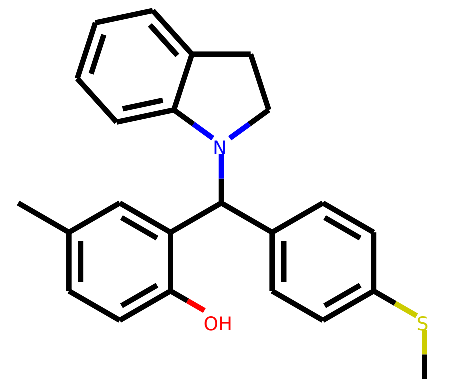
Ahma-505

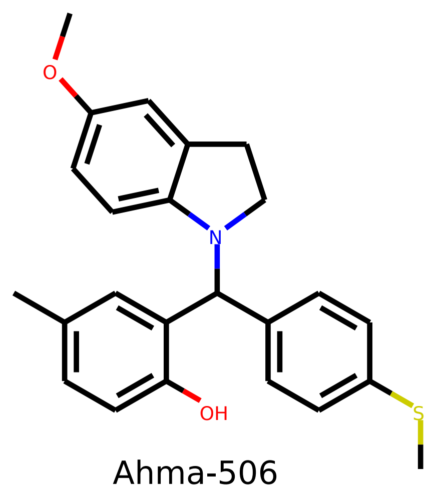
Ahma-506

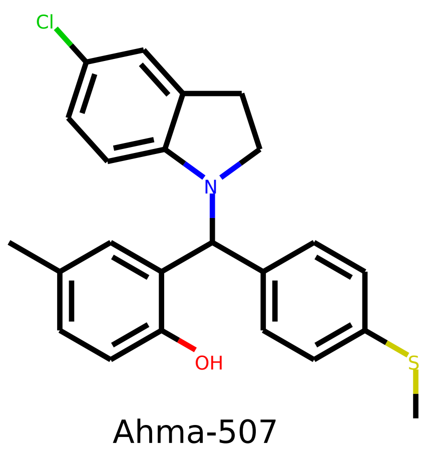
Ahma-507

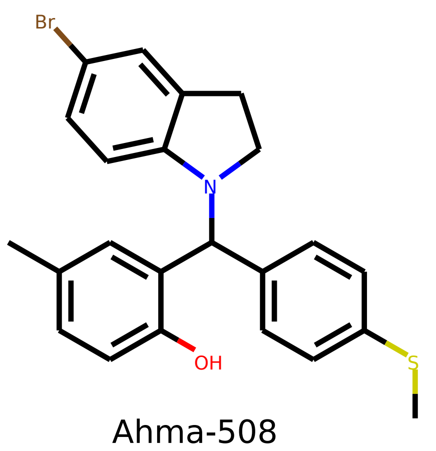
Ahma-508

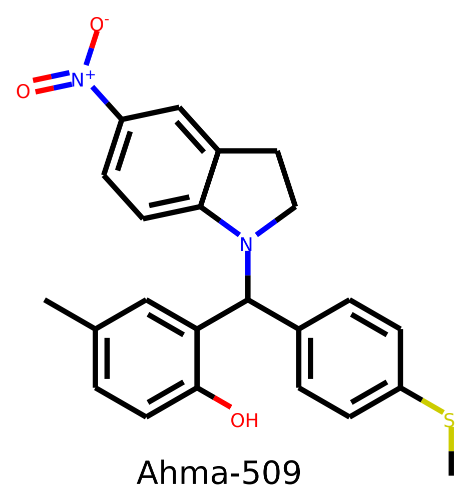
Ahma-509

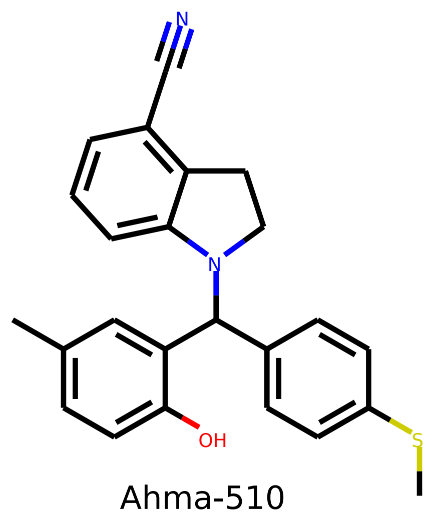
Ahma-510

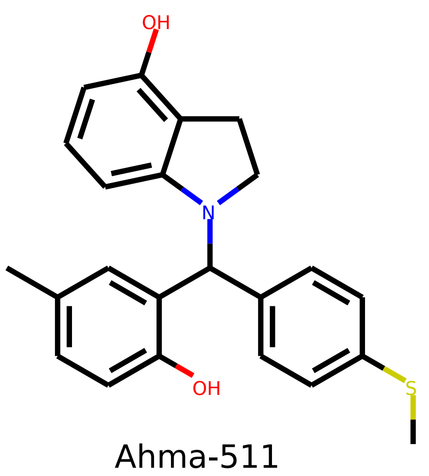
Ahma-511

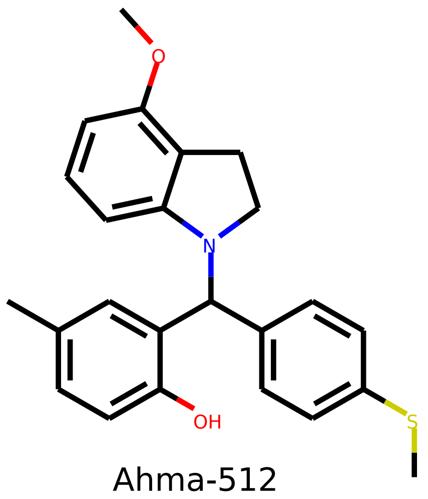
Ahma-512

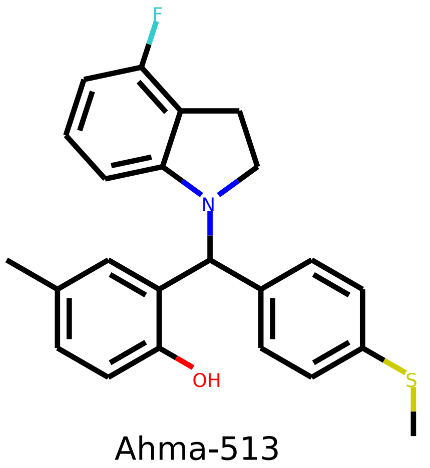
Ahma-513

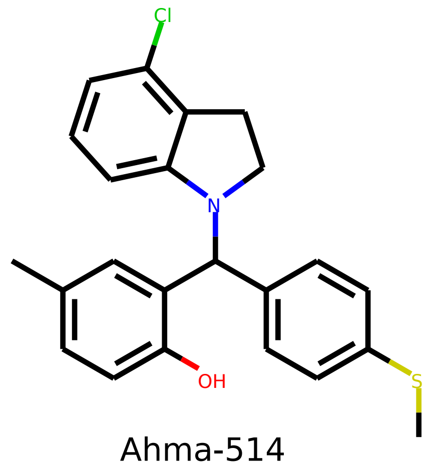
Ahma-514

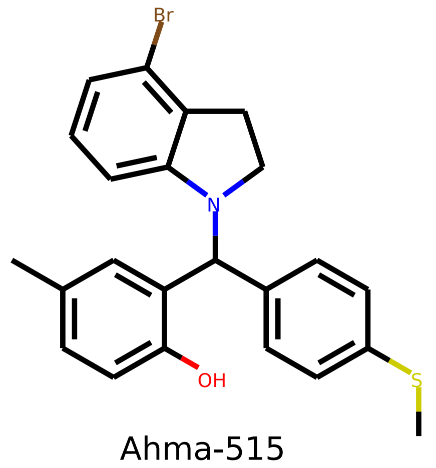
Ahma-515

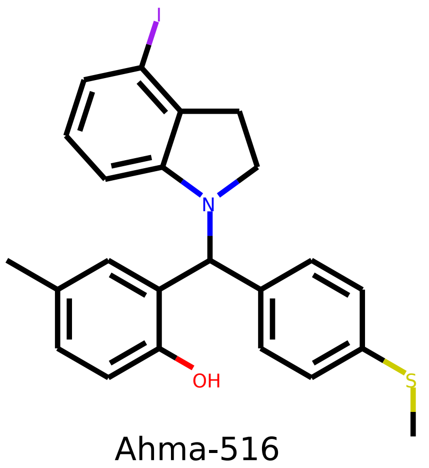
Ahma-516

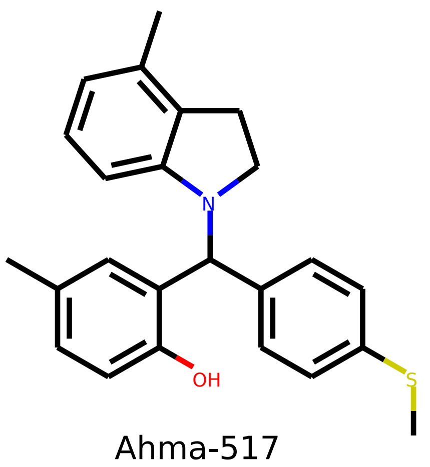
Ahma-517

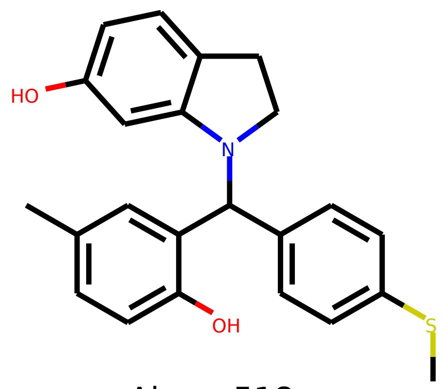
Ahma-518

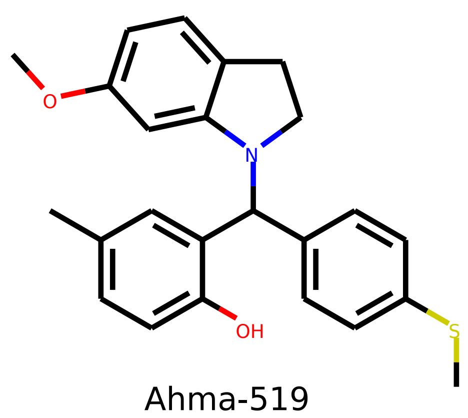
Ahma-519

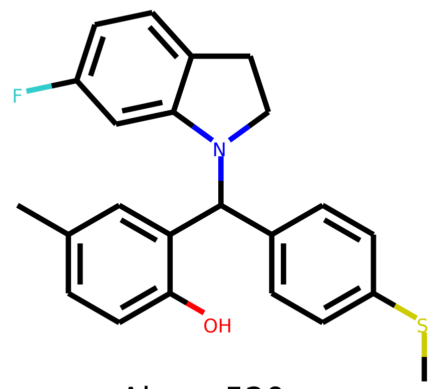
Ahma-520

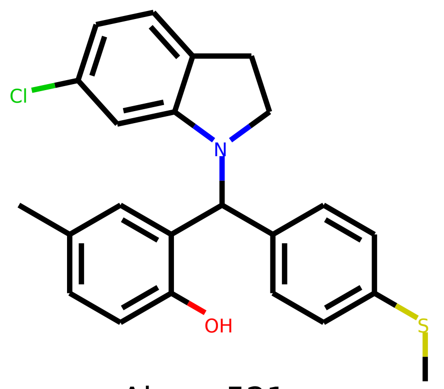
Ahma-521

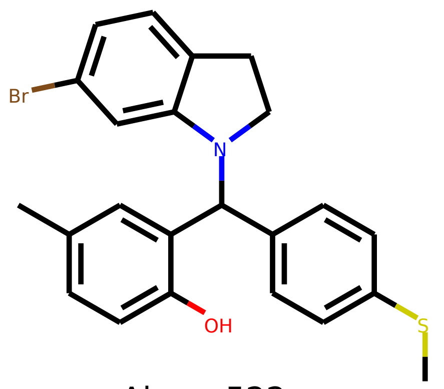
Ahma-522

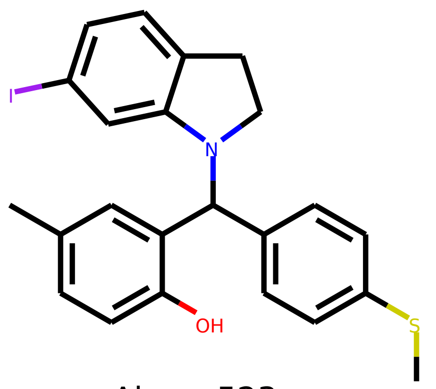
Ahma-523

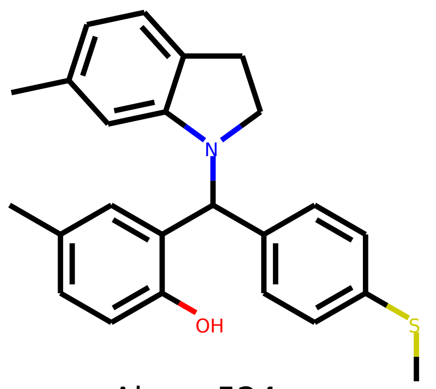
Ahma-524

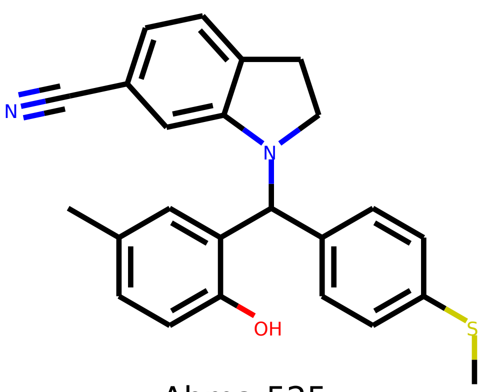
Ahma-525

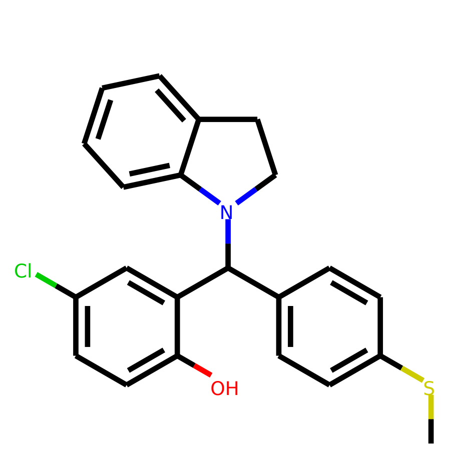
Ahma-526

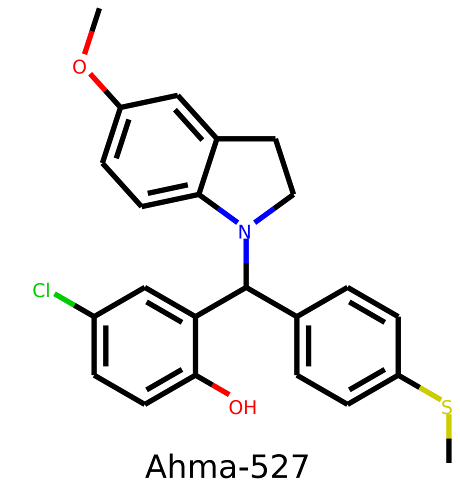
Ahma-527

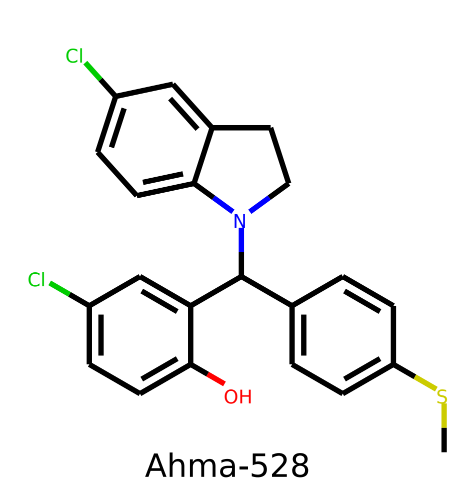
Ahma-528

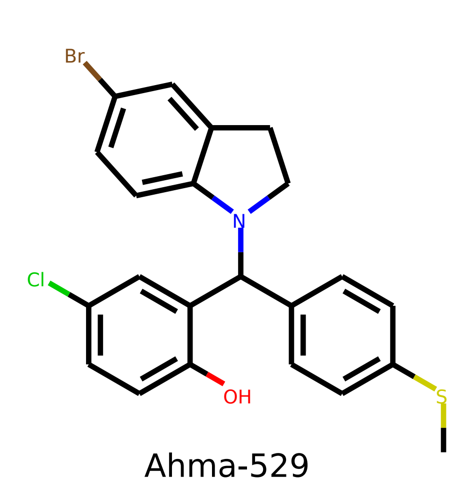
Ahma-529

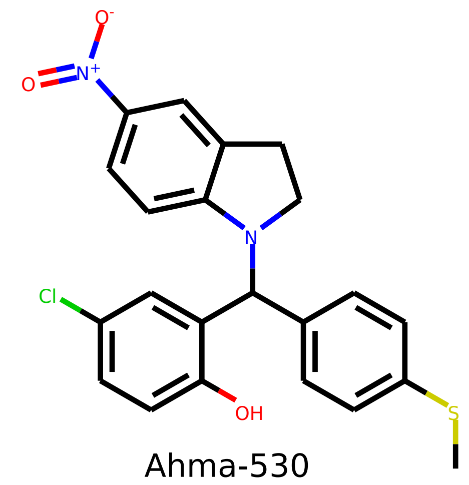
Ahma-530

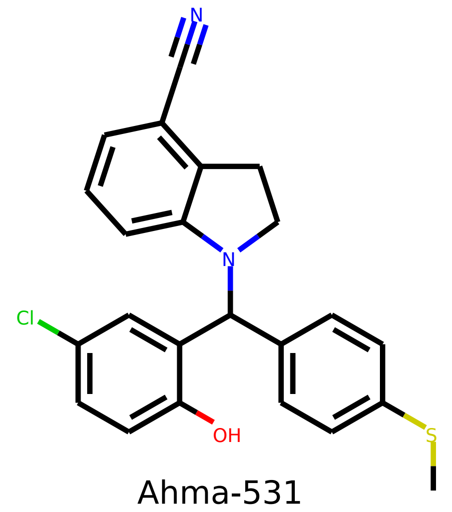
Ahma-531

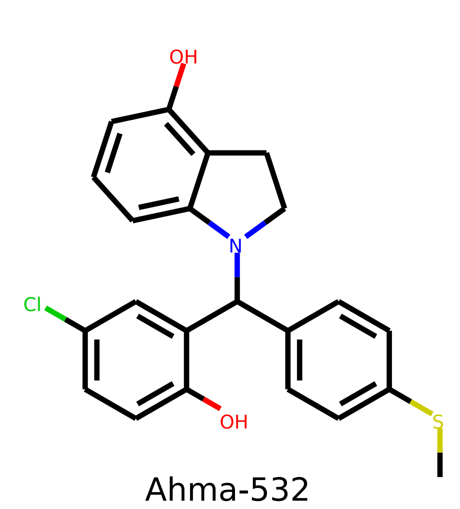
Ahma-532

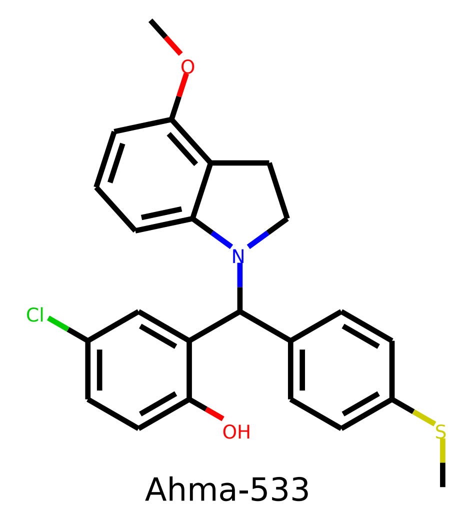
Ahma-533

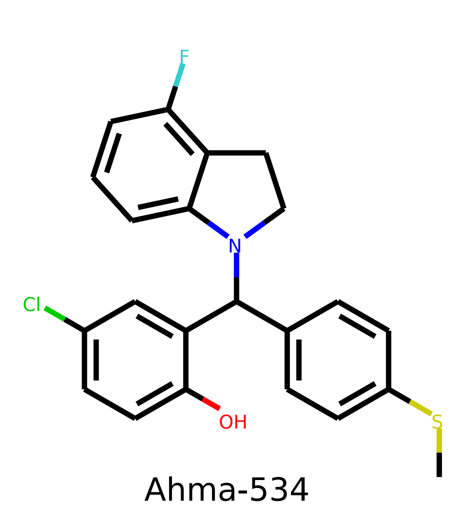
Ahma-534

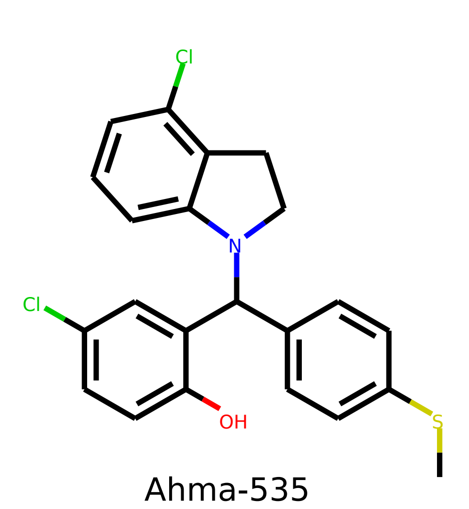
Ahma-535

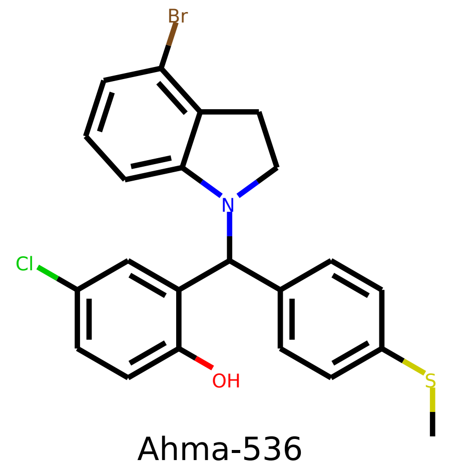
Ahma-536

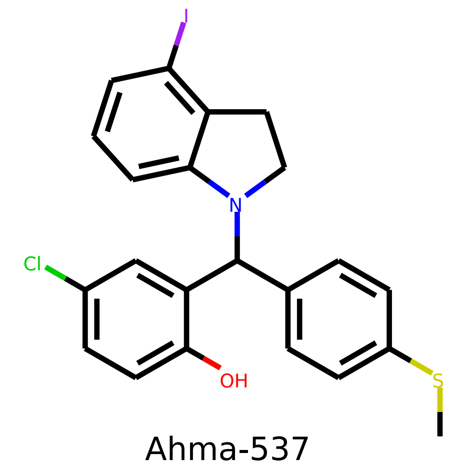
Ahma-537

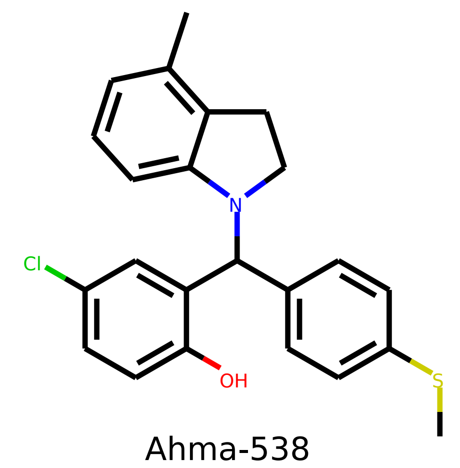
Ahma-538

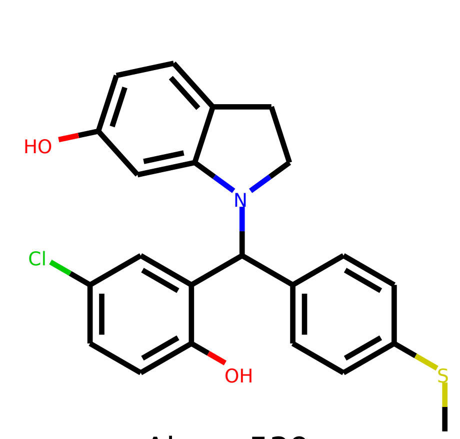
Ahma-539

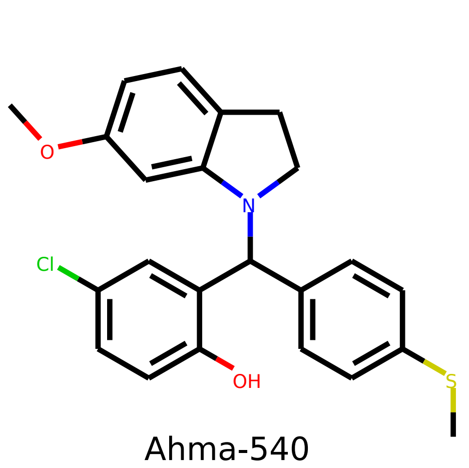
Ahma-540

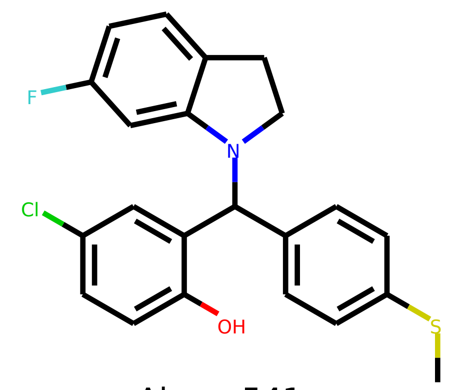
Ahma-541

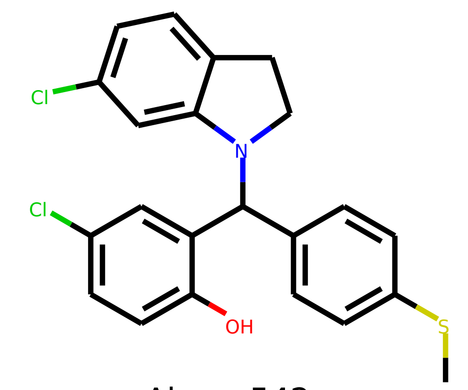
Ahma-542

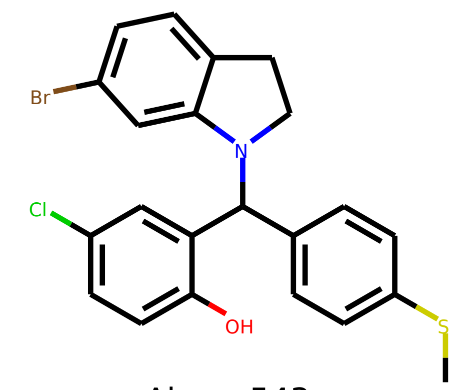
Ahma-543

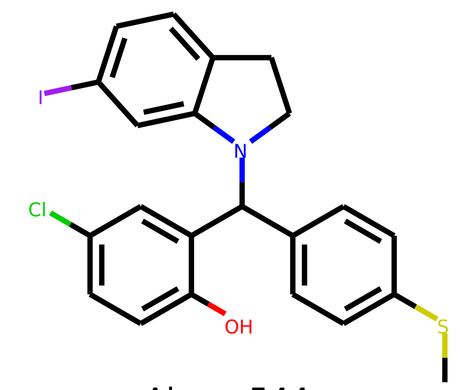
Ahma-544

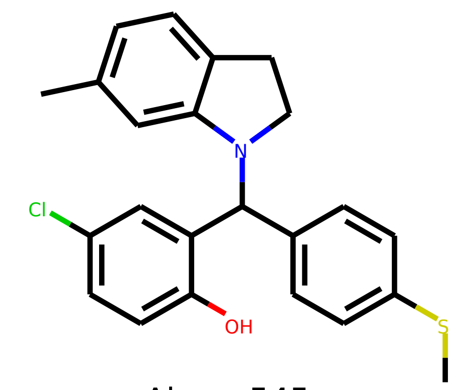
Ahma-545

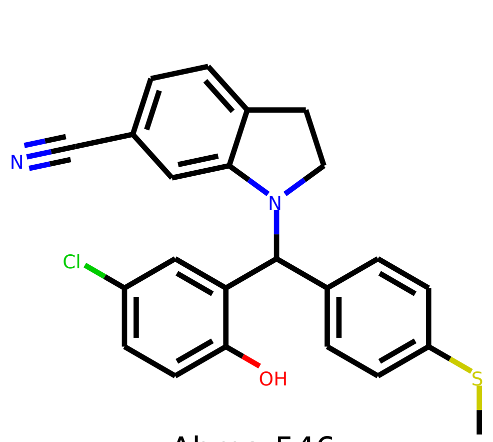
Ahma-546

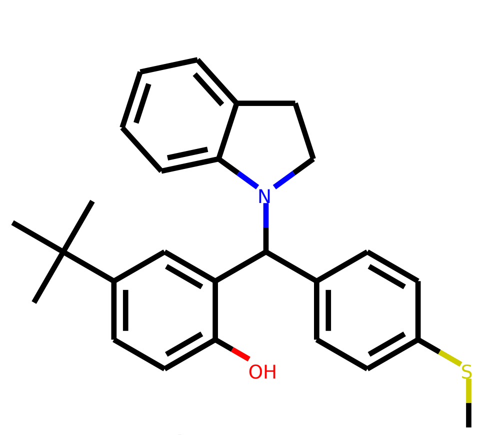
Ahma-547

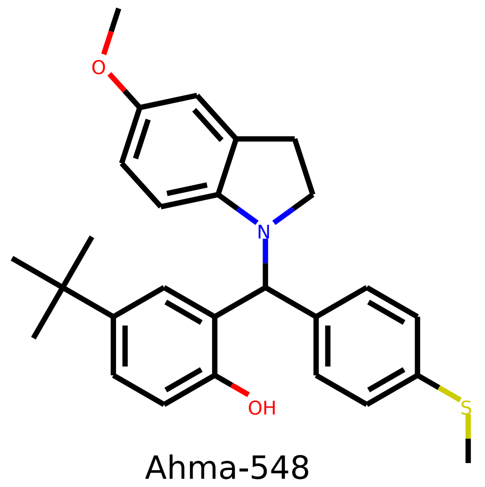
Ahma-548

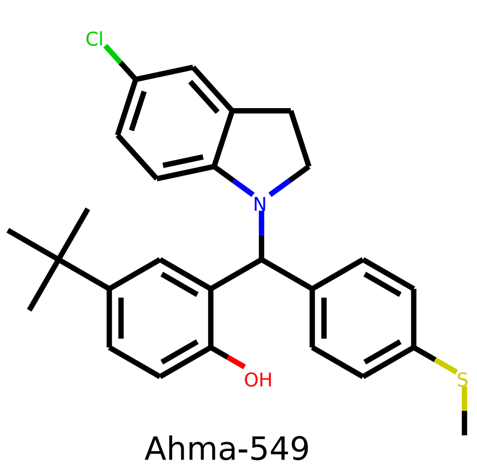
Ahma-549

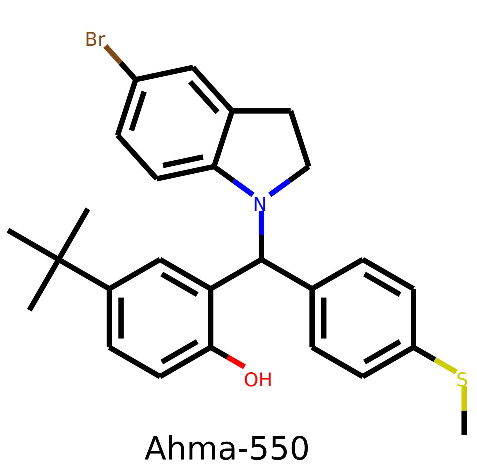
Ahma-550

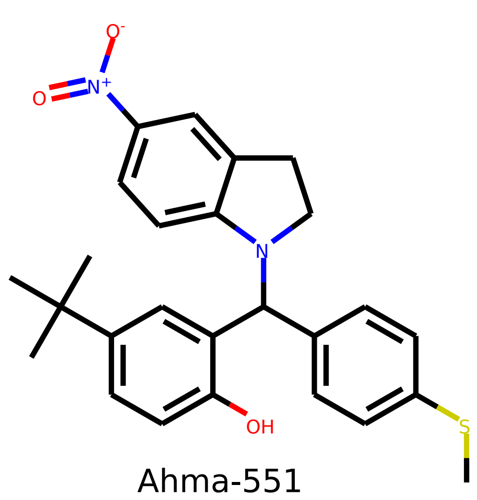
Ahma-551

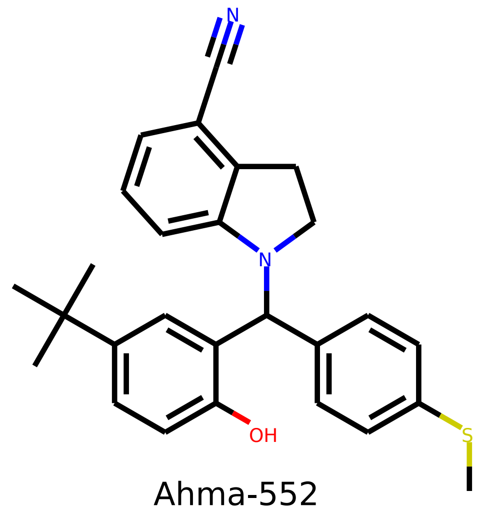
Ahma-552

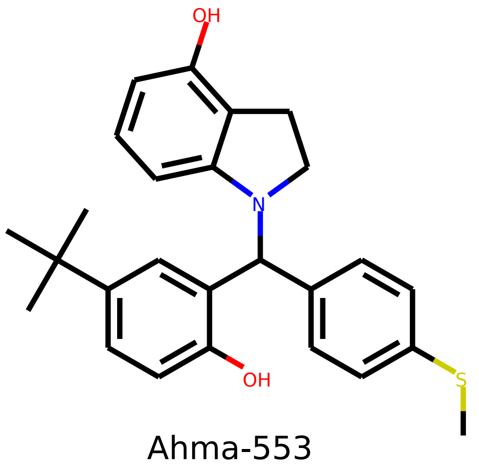
Ahma-553

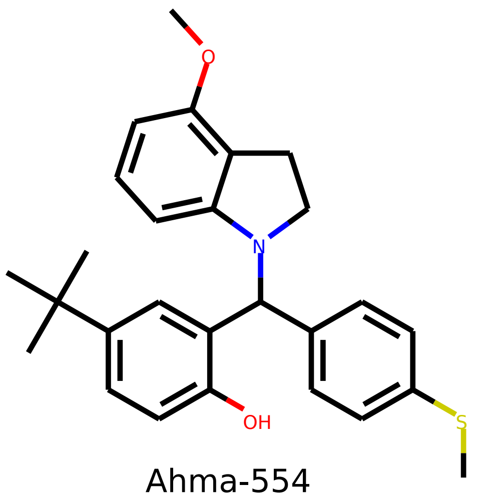
Ahma-554

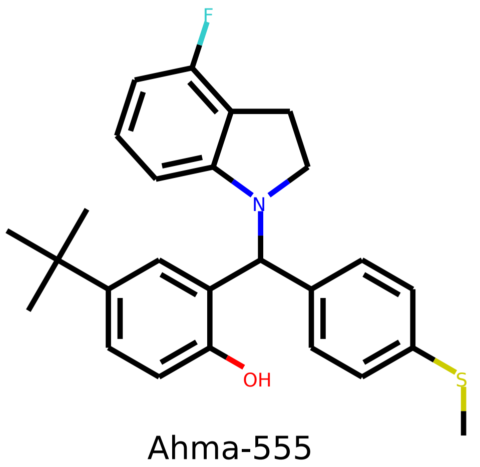
Ahma-555

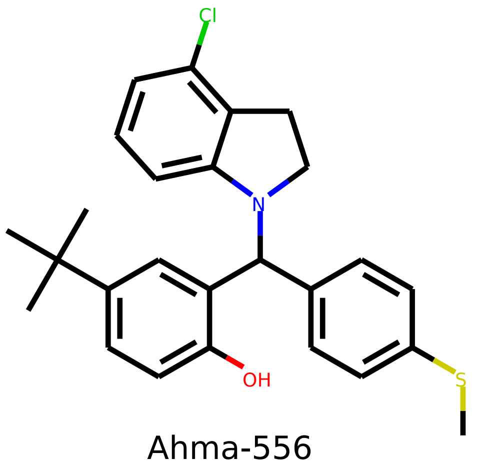
Ahma-556

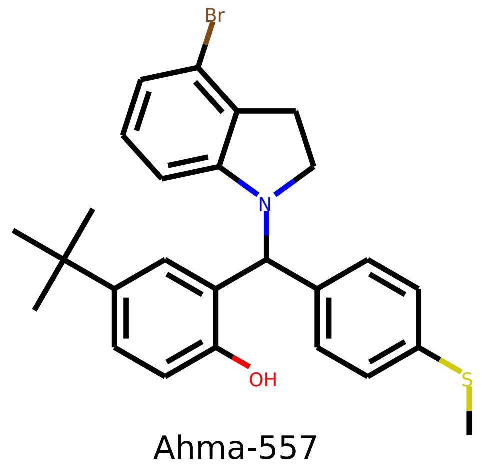
Ahma-557

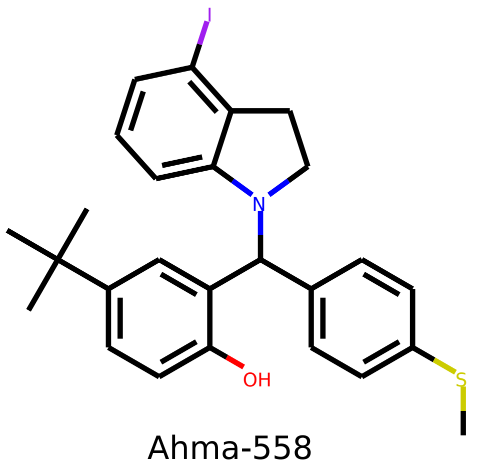
Ahma-558

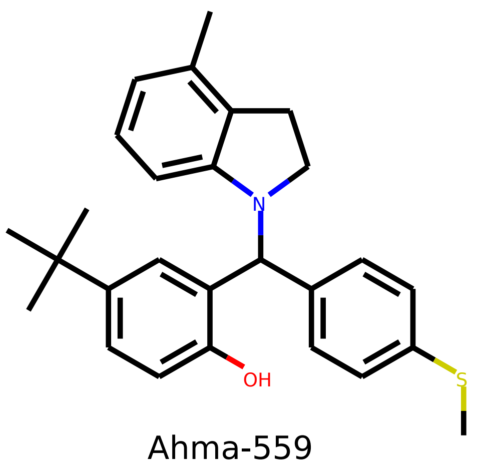
Ahma-559

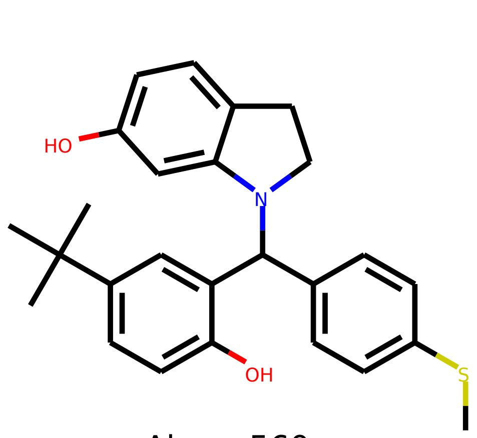
Ahma-560

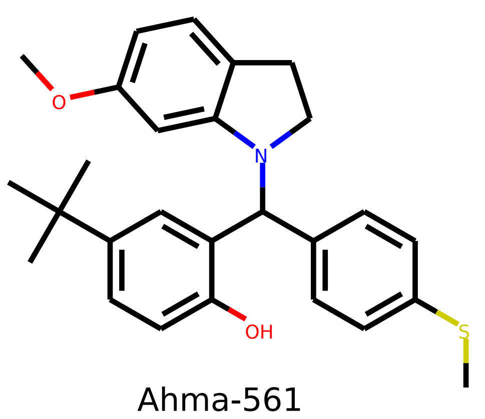

Ahma-561

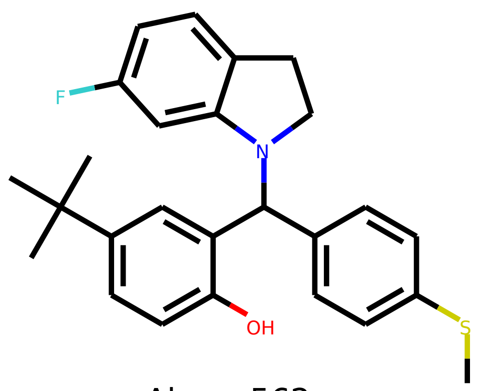

Ahma-562

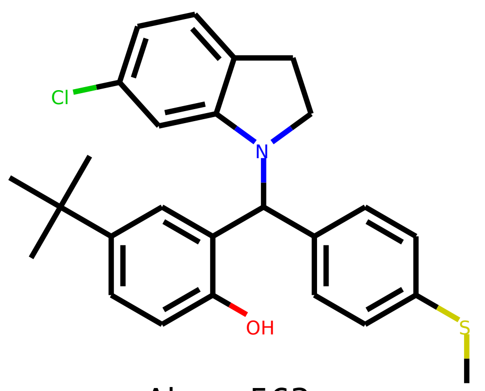

Ahma-563

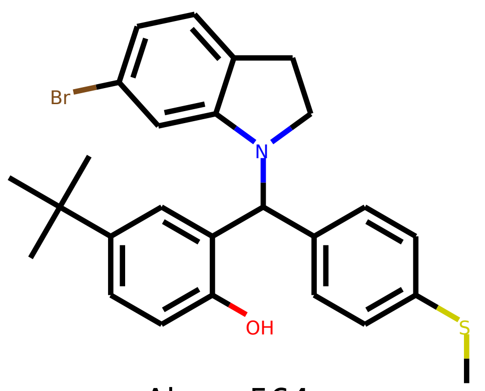

Ahma-564

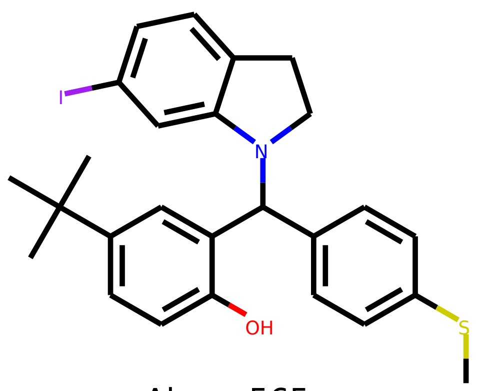

Ahma-565

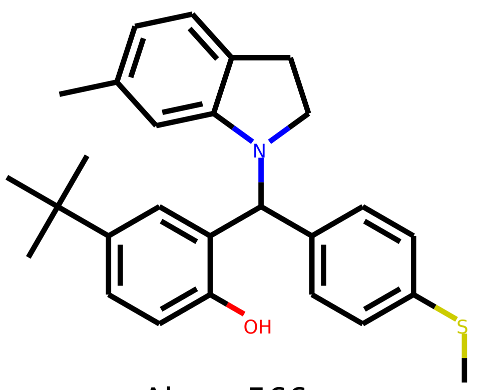

Ahma-566

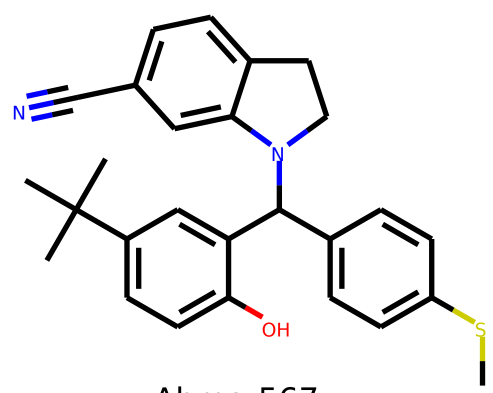

Ahma-567

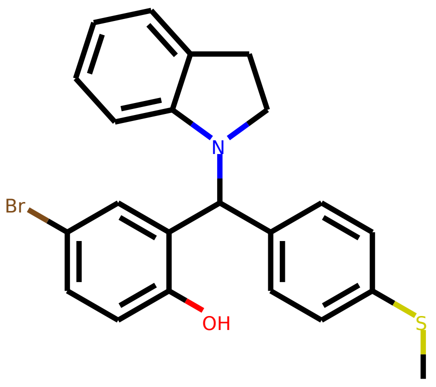

Ahma-568

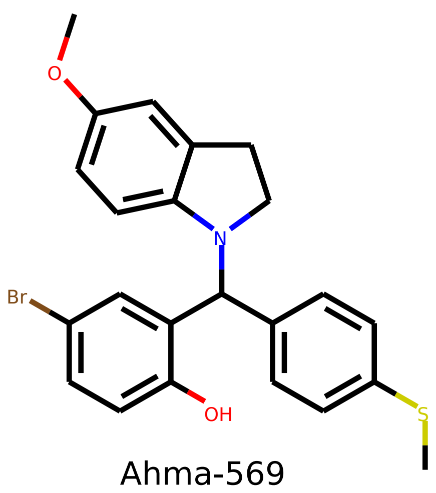

Ahma-569

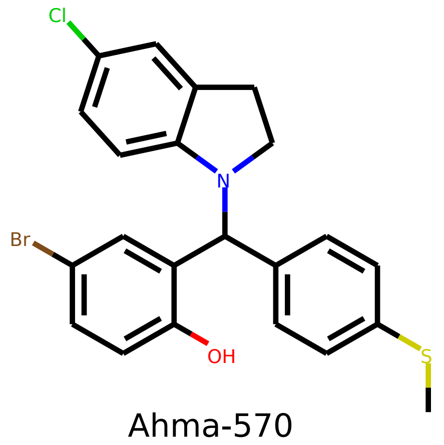

Ahma-570

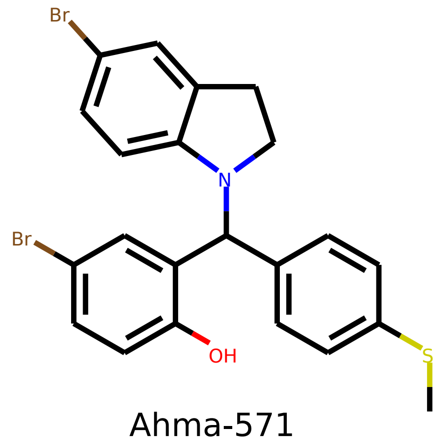

Ahma-571

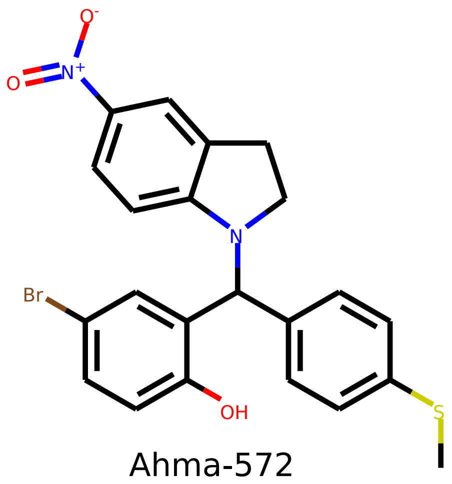

Ahma-572

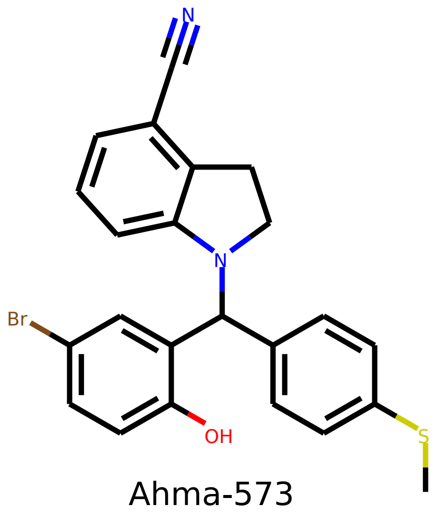

Ahma-573

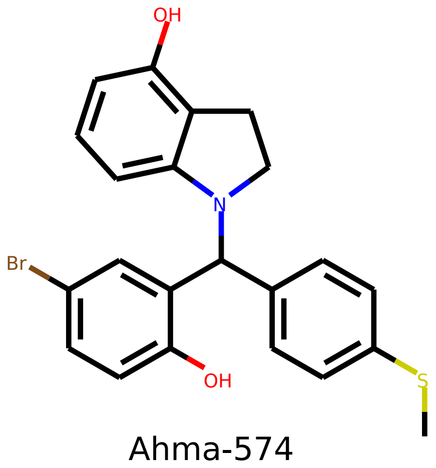

Ahma-574

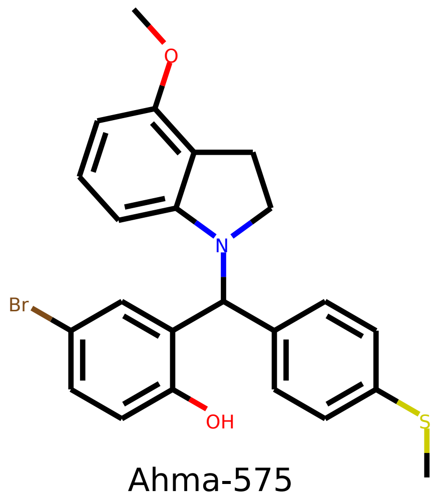

Ahma-575

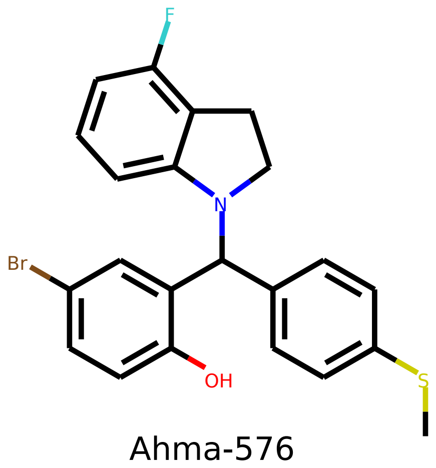

Ahma-576

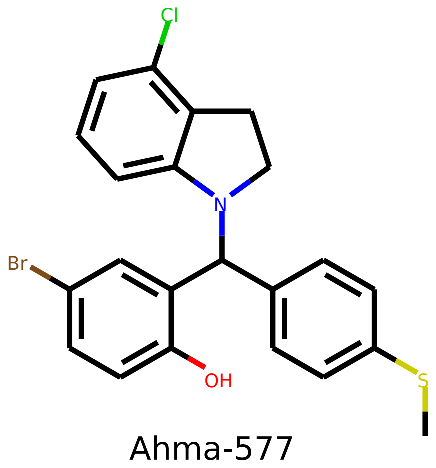

Ahma-577

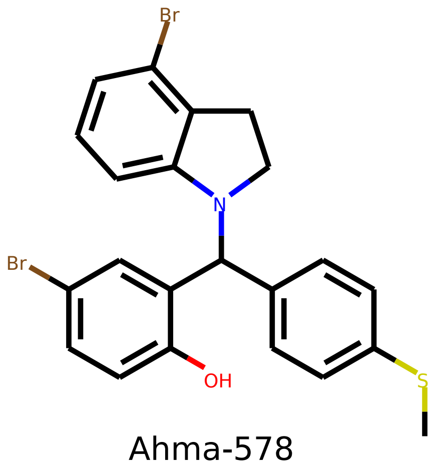

Ahma-578

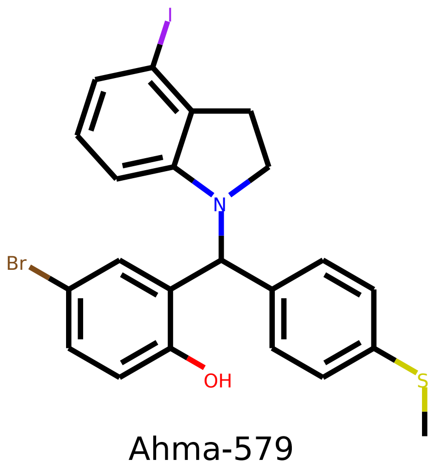

Ahma-579

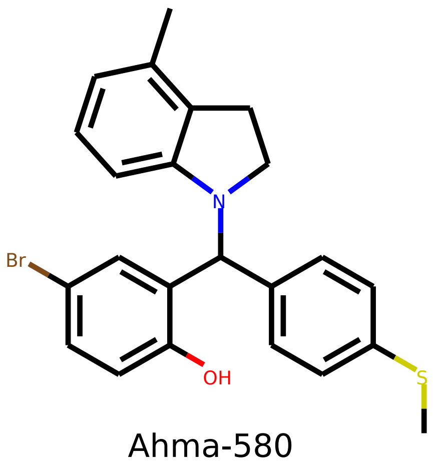

Ahma-580

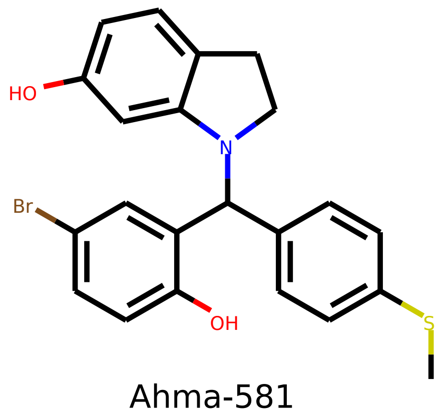

Ahma-581

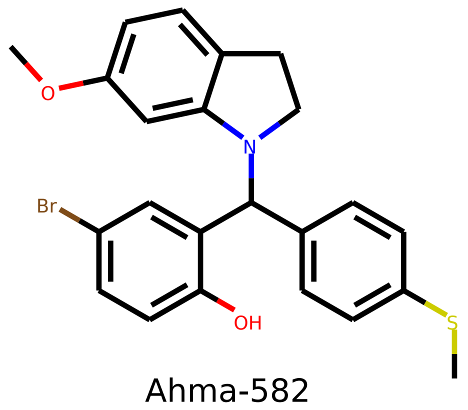

Ahma-582

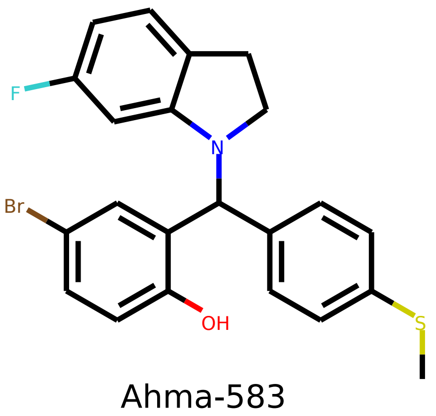

Ahma-583

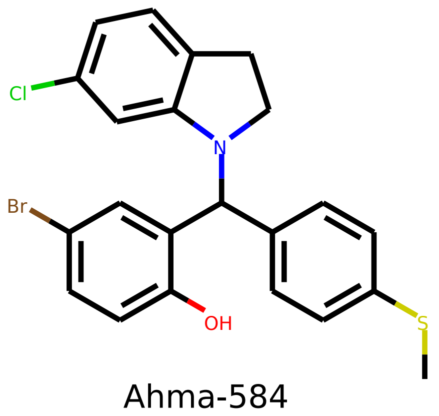

Ahma-584

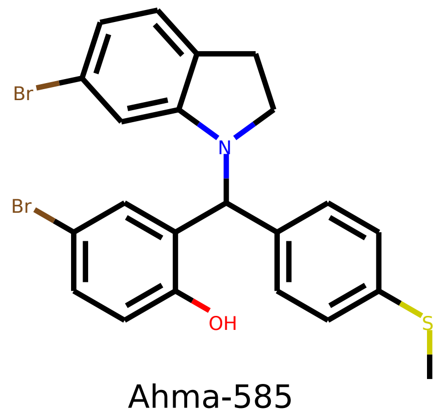

Ahma-585

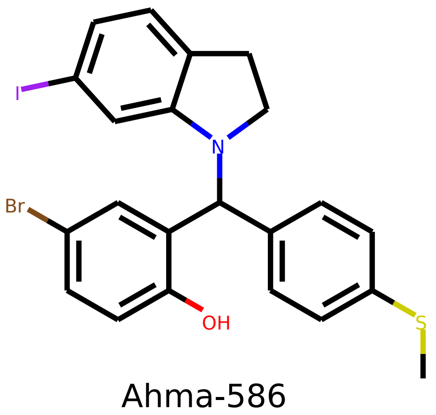

Ahma-586

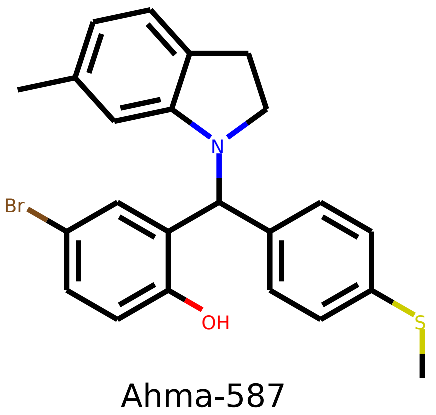

Ahma-587

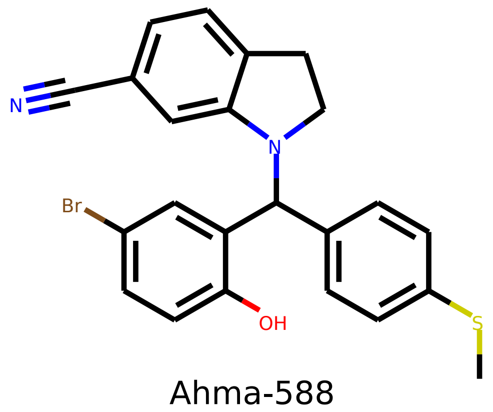

Ahma-588

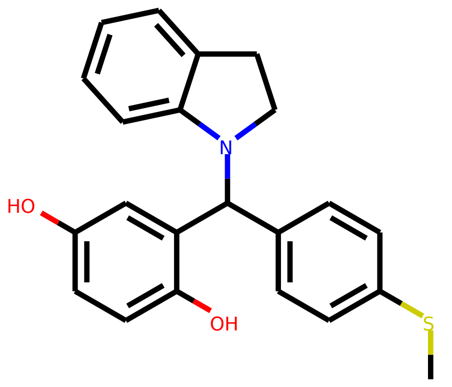

Ahma-589

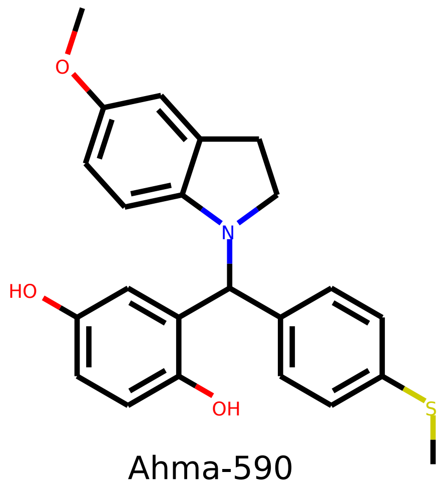

Ahma-590

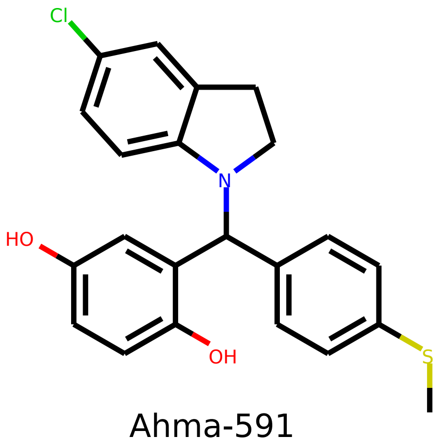

Ahma-591

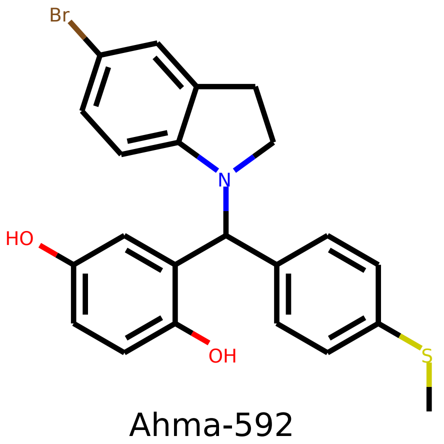

Ahma-592

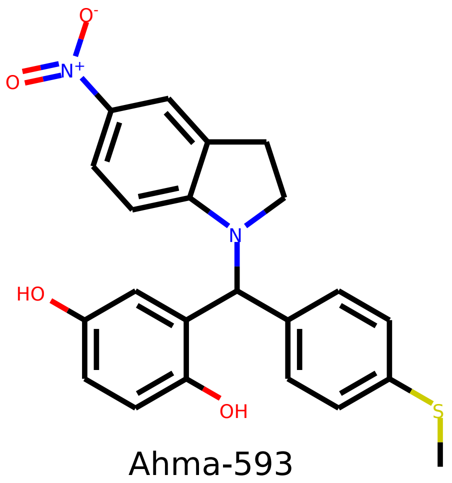

Ahma-593

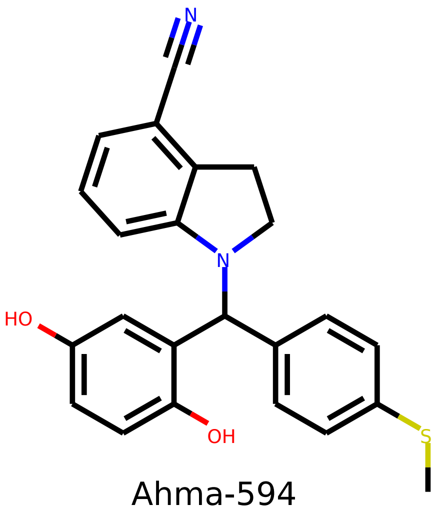

Ahma-594

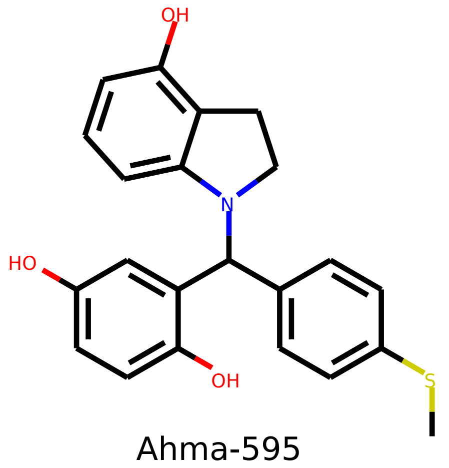

Ahma-595

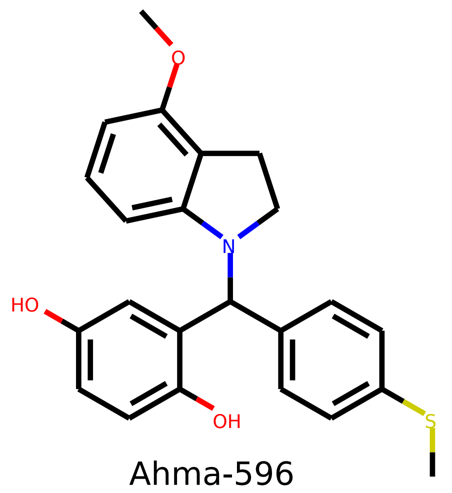
Ahma-596

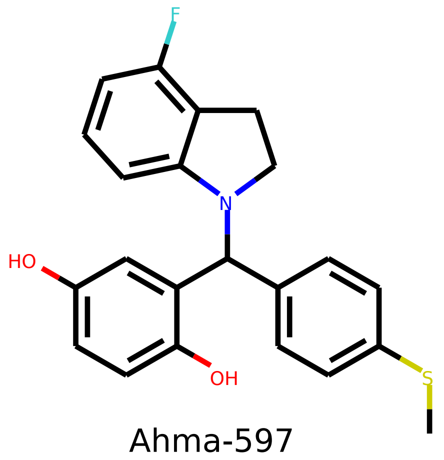
Ahma-597

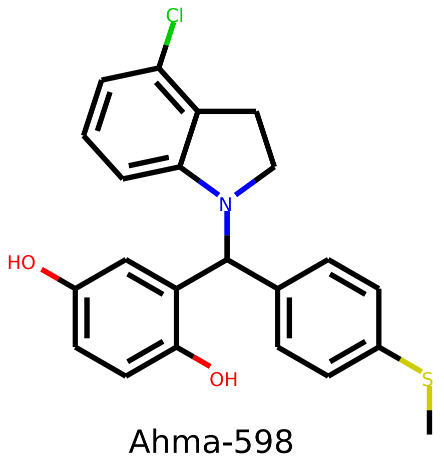
Ahma-598

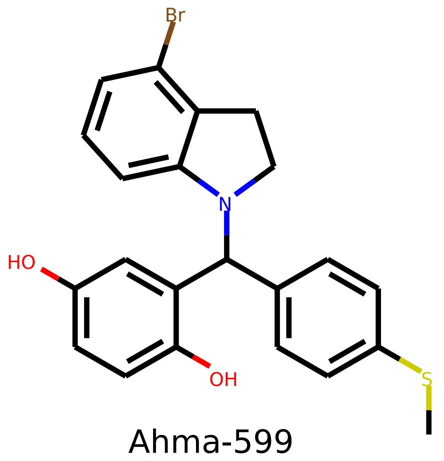
Ahma-599

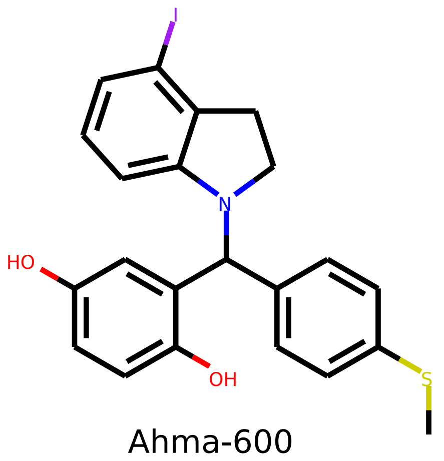
Ahma-600

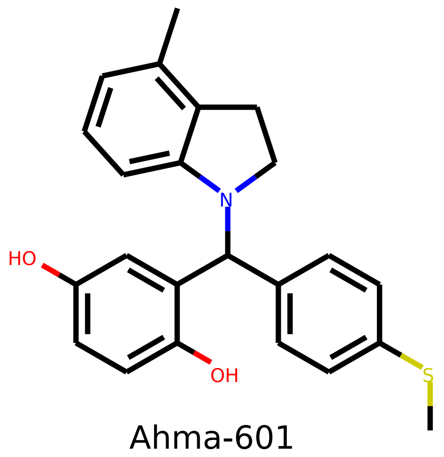
Ahma-601

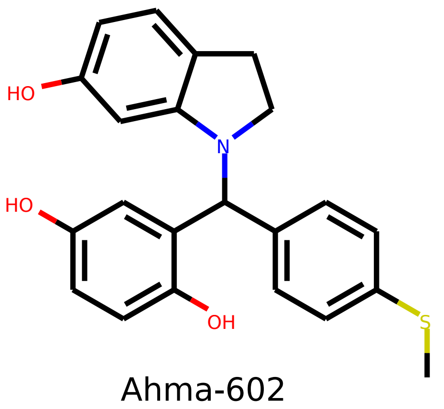
Ahma-602

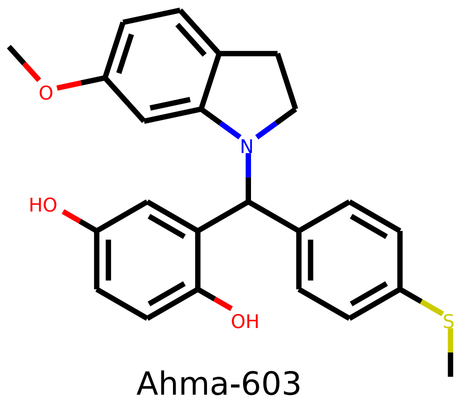
Ahma-603

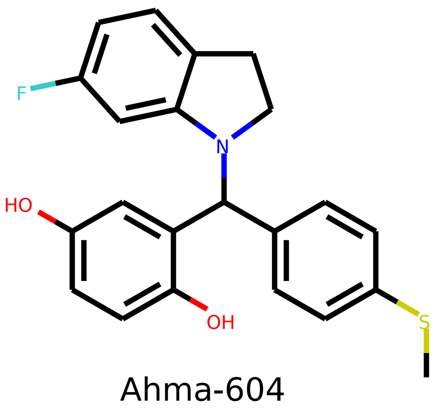
Ahma-604

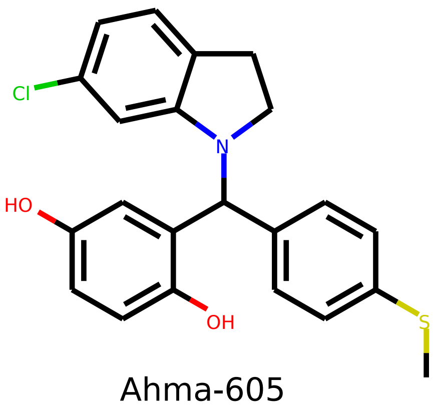
Ahma-605

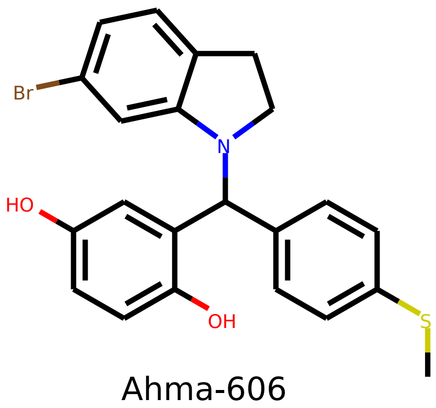
Ahma-606

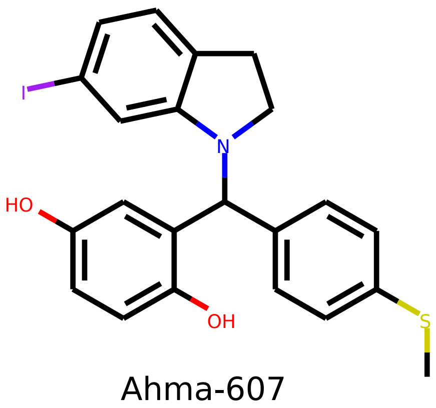
Ahma-607

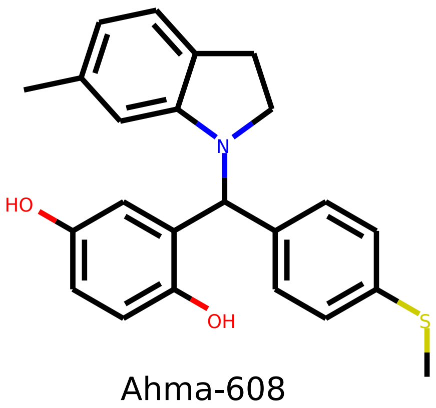
Ahma-608

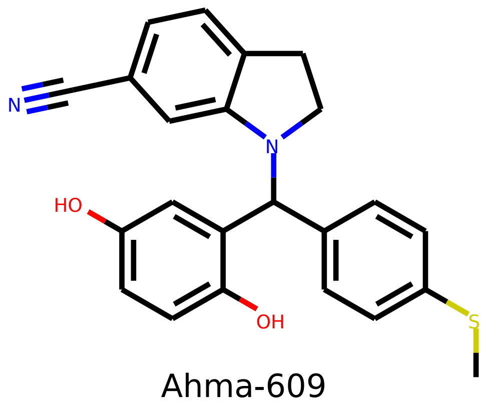
Ahma-609

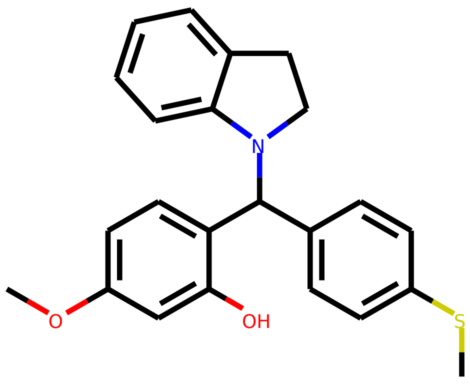
Ahma-610

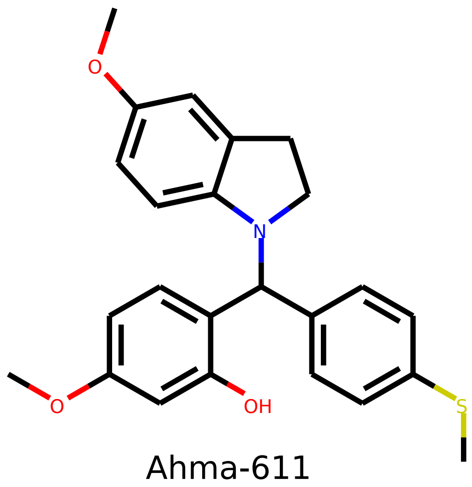
Ahma-611

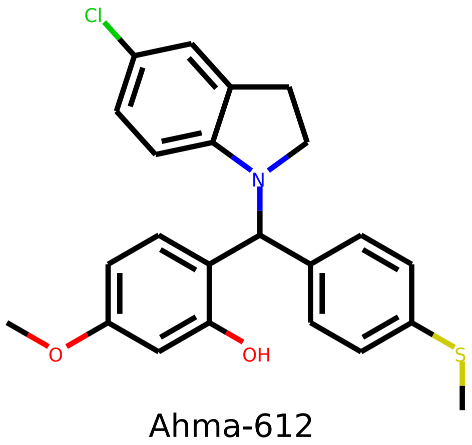
Ahma-612

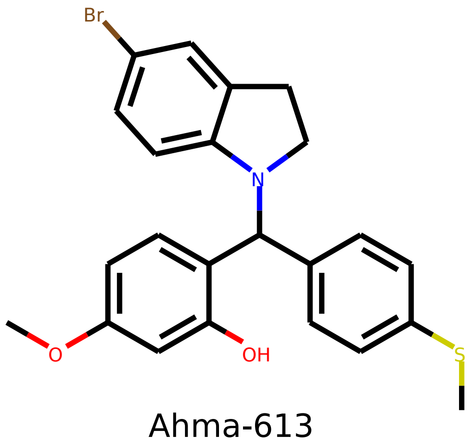
Ahma-613

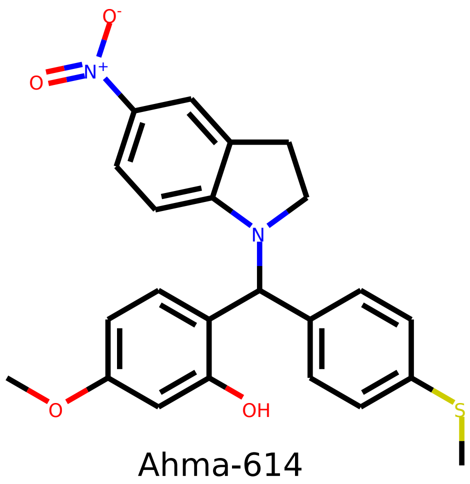
Ahma-614

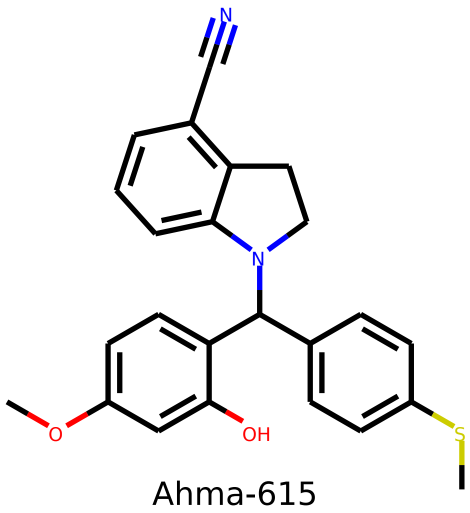
Ahma-615

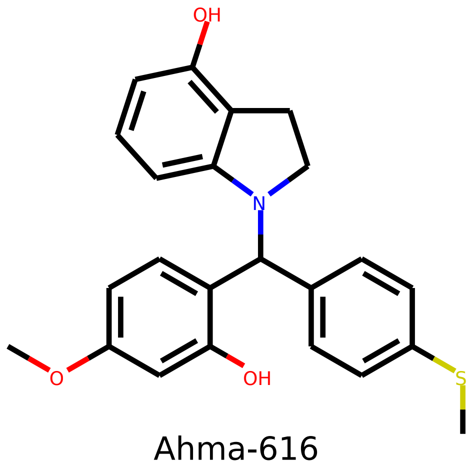
Ahma-616

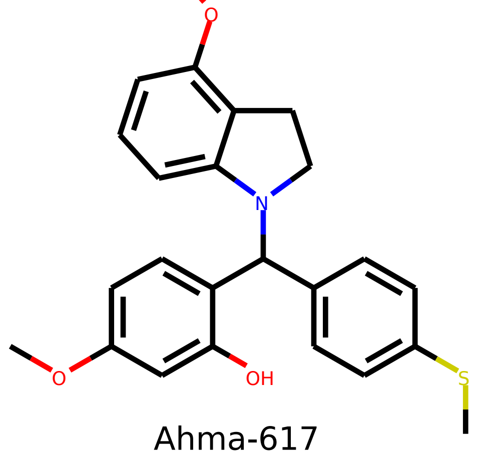
Ahma-617

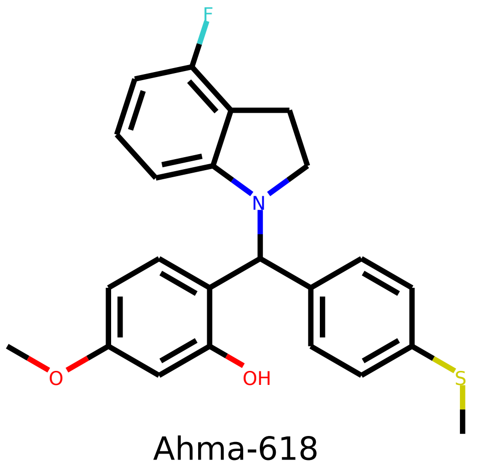
Ahma-618

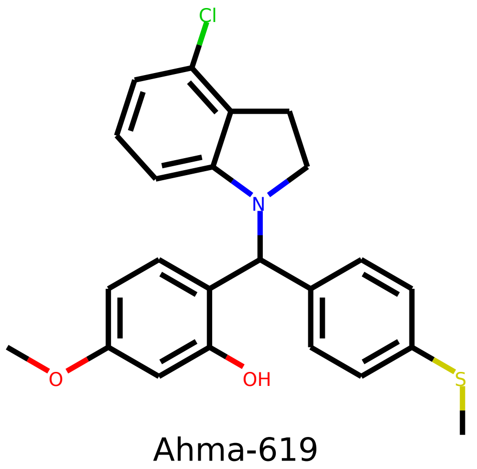
Ahma-619

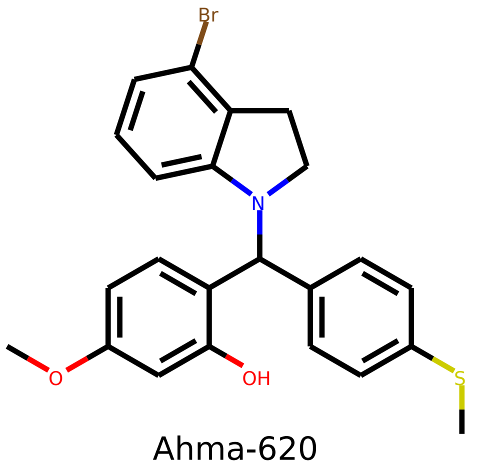
Ahma-620

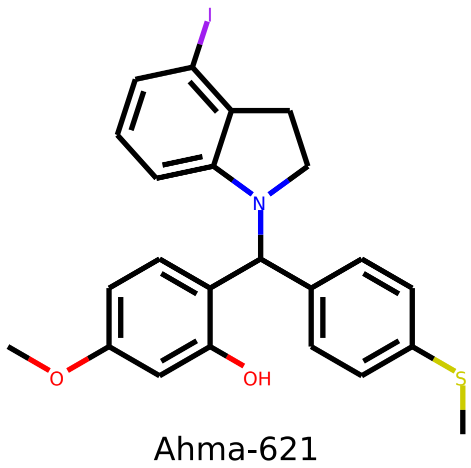
Ahma-621

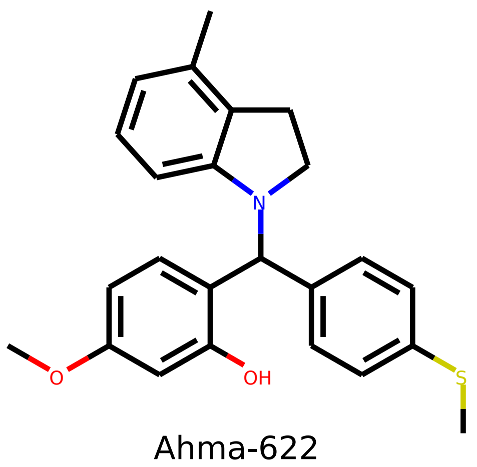
Ahma-622

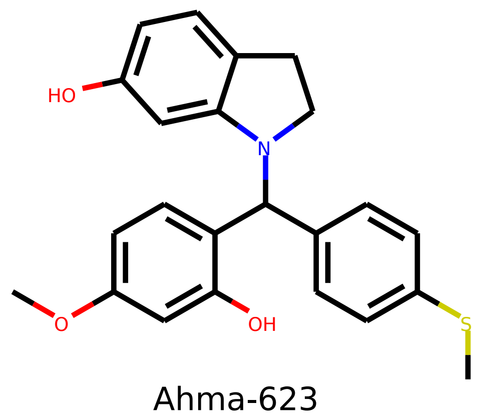
Ahma-623

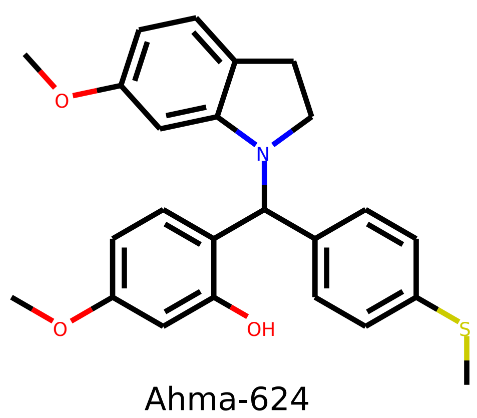
Ahma-624

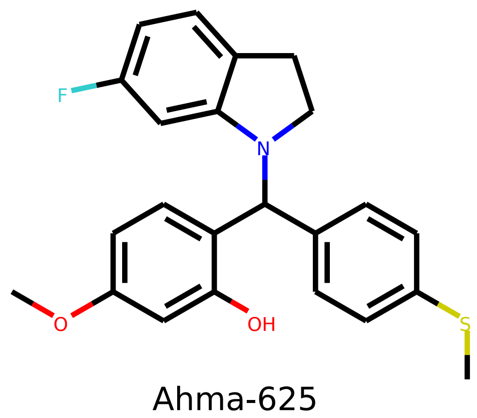
Ahma-625

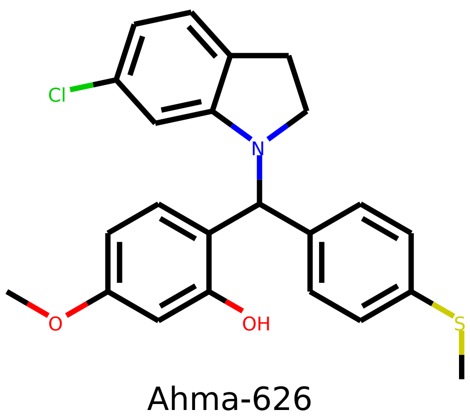
Ahma-626

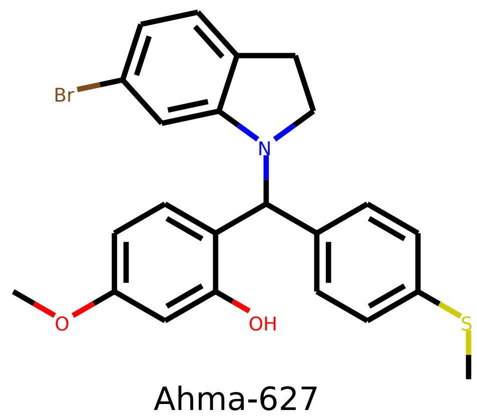
Ahma-627

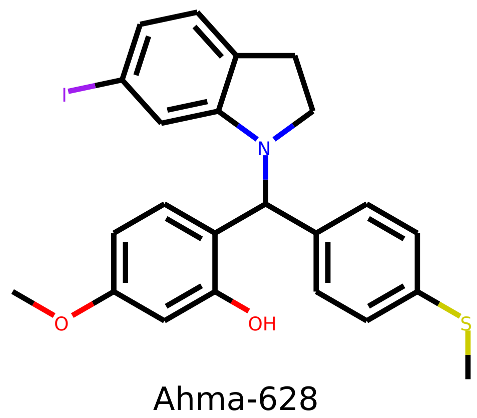
Ahma-628

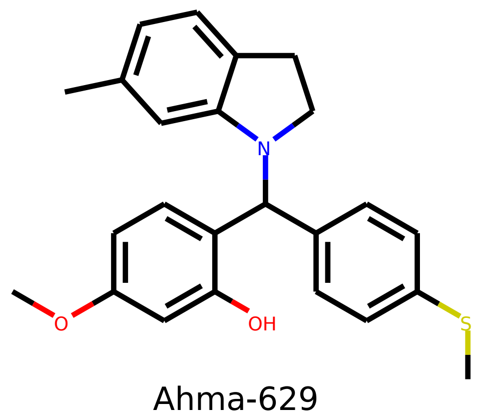
Ahma-629

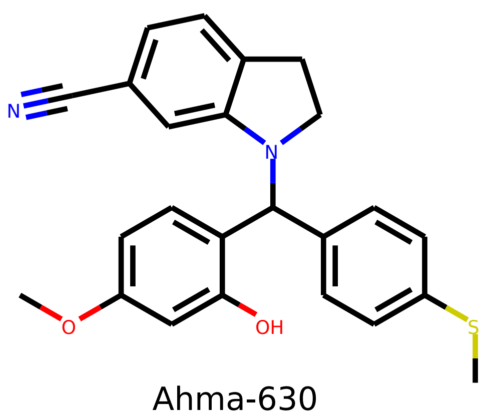
Ahma-630

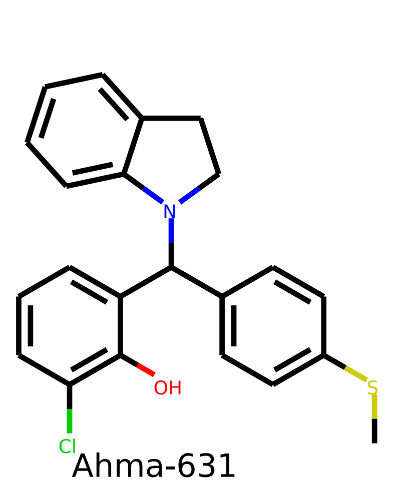
Ahma-631

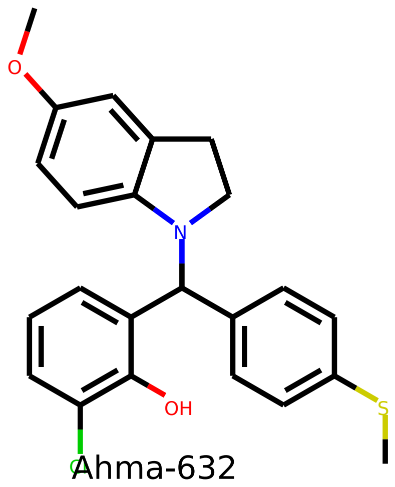
Ahma-632

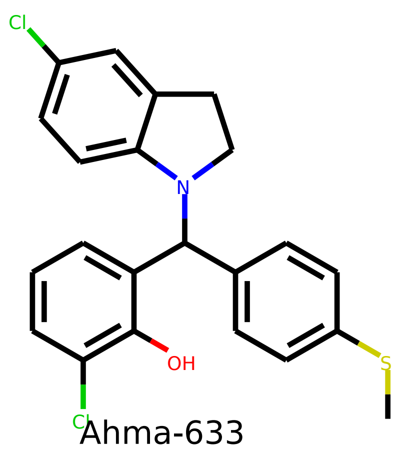
Ahma-633

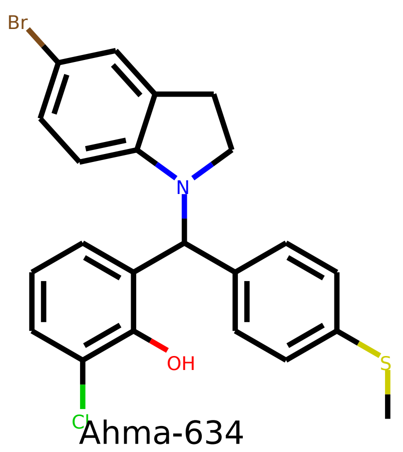
Ahma-634

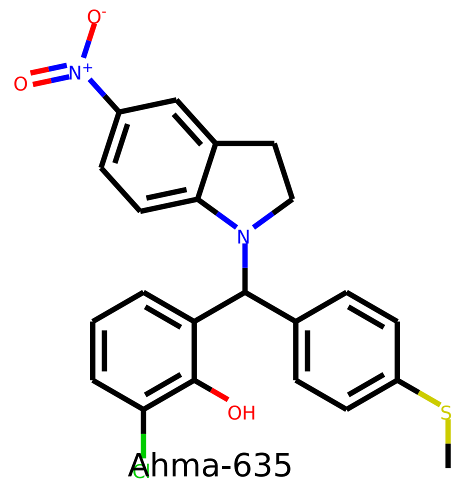
Ahma-635

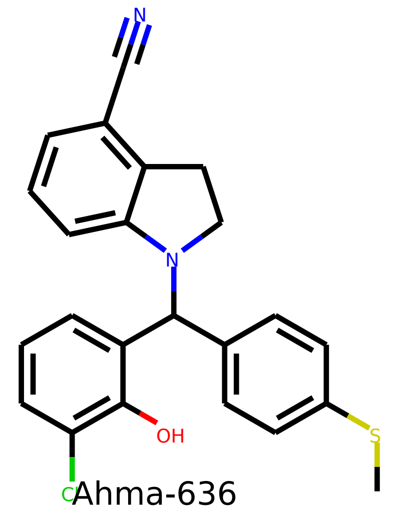
Ahma-636

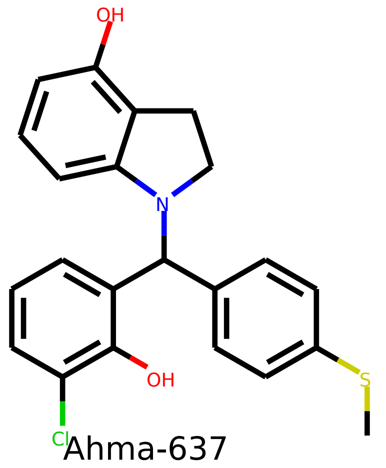
Ahma-637

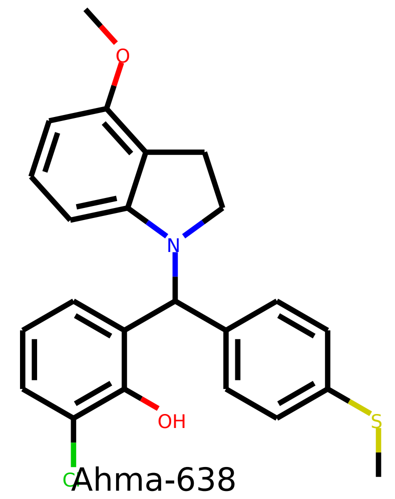
Ahma-638

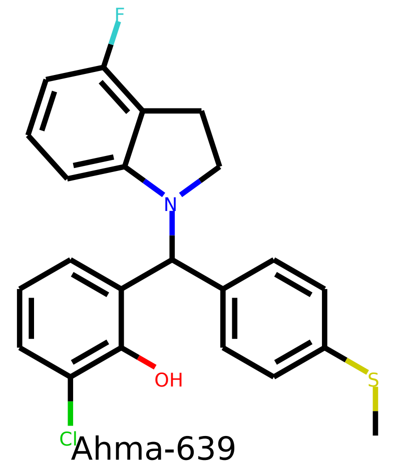
Ahma-639

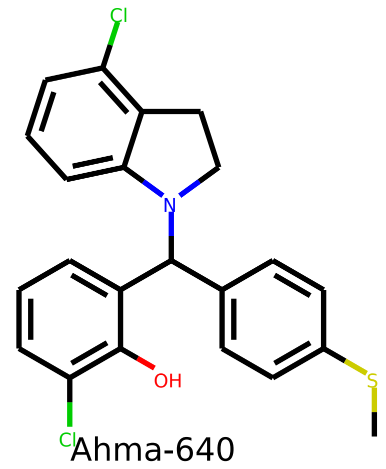
Ahma-640

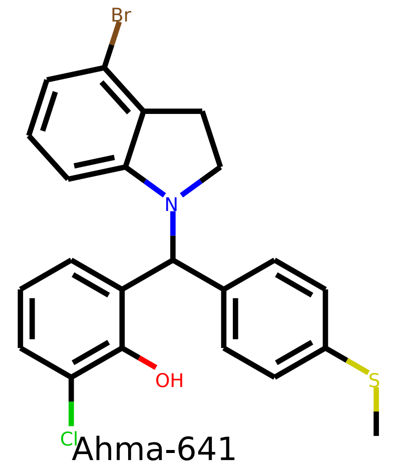
Ahma-641

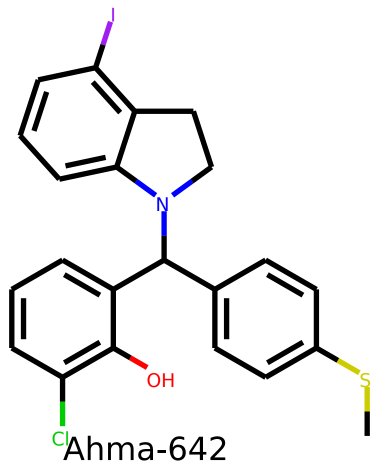
Ahma-642

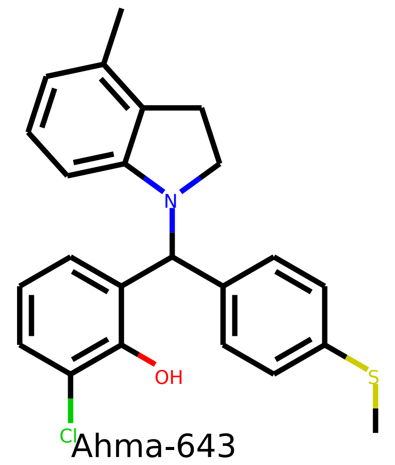
Ahma-643

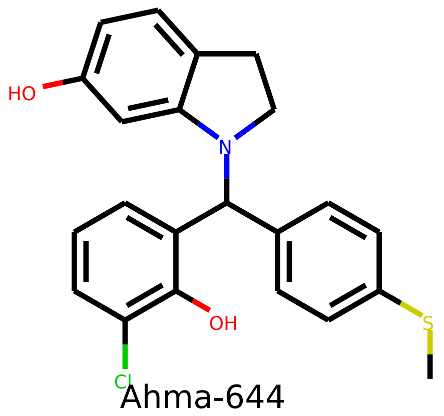
Ahma-644

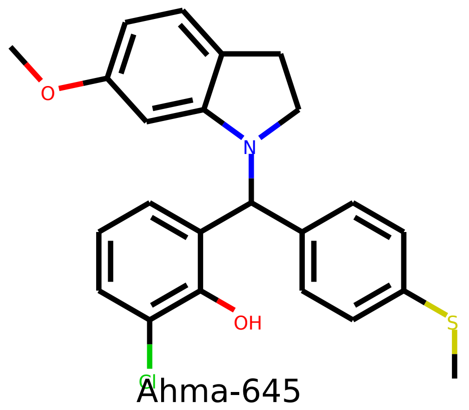
Ahma-645

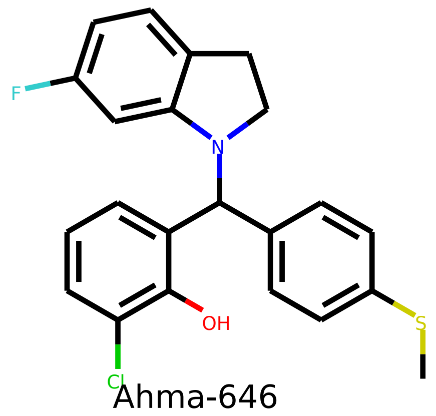
Ahma-646

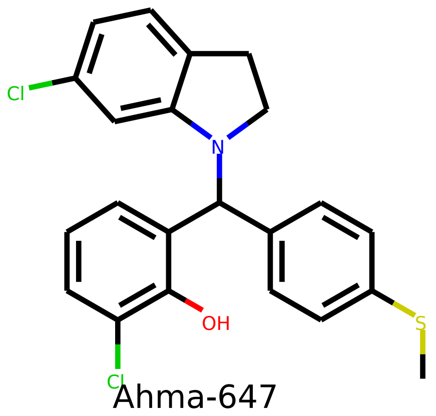
Ahma-647

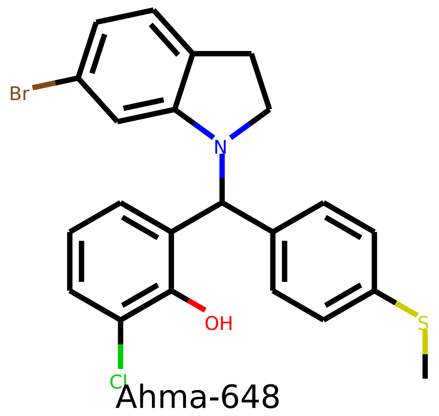
Ahma-648

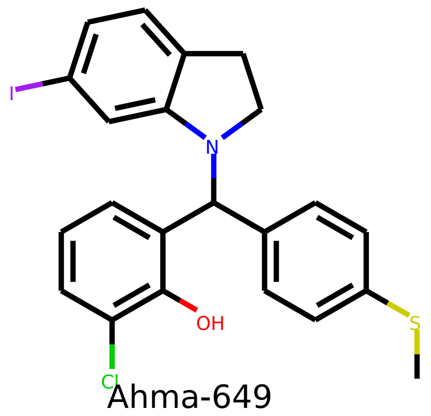
Ahma-649

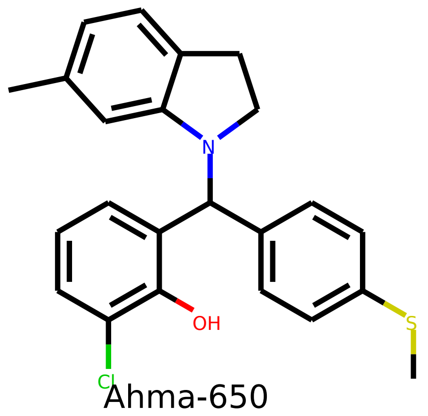
Ahma-650

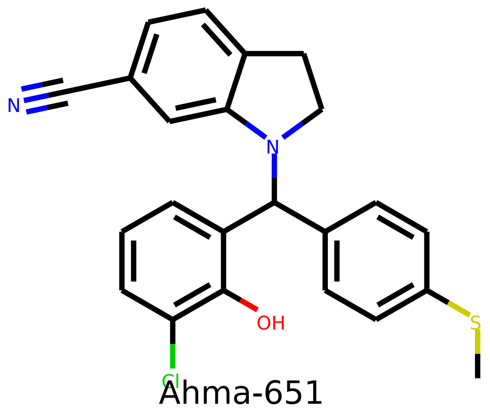
Ahma-651

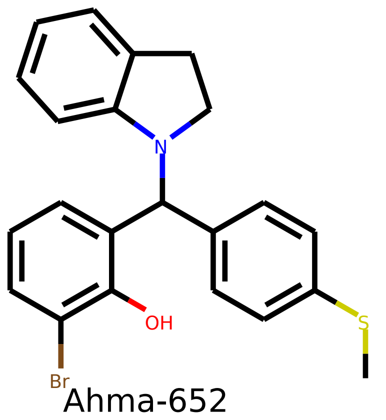
Ahma-652

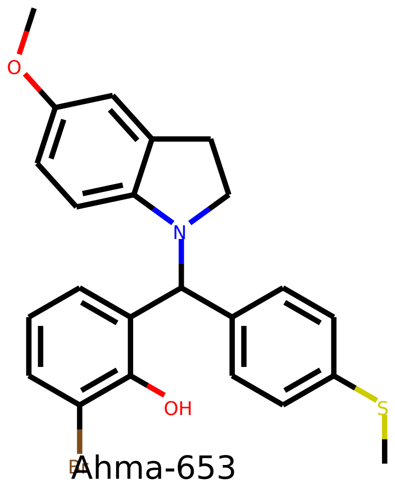
Ahma-653

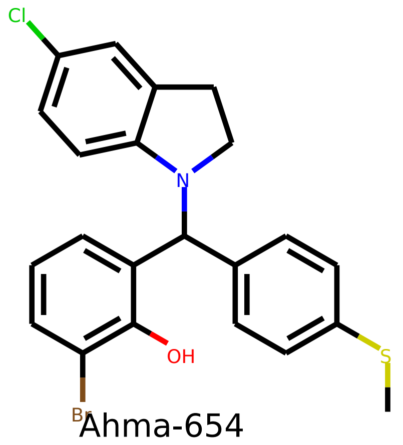
Ahma-654

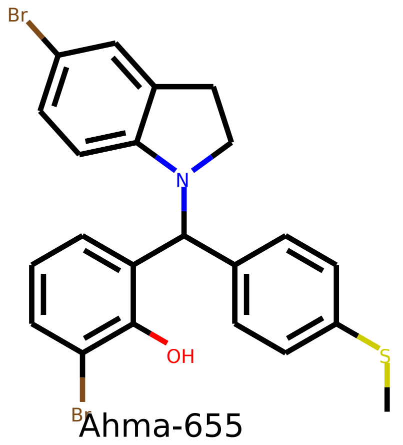
Ahma-655

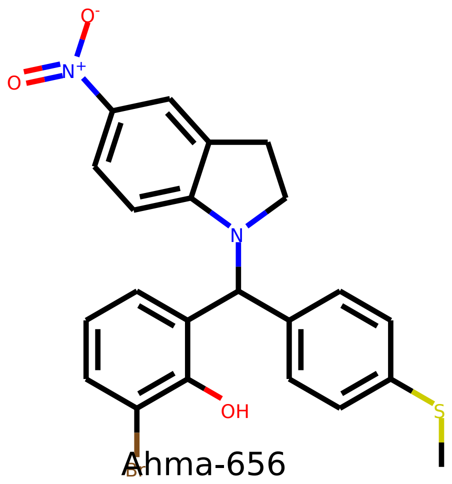
Ahma-656

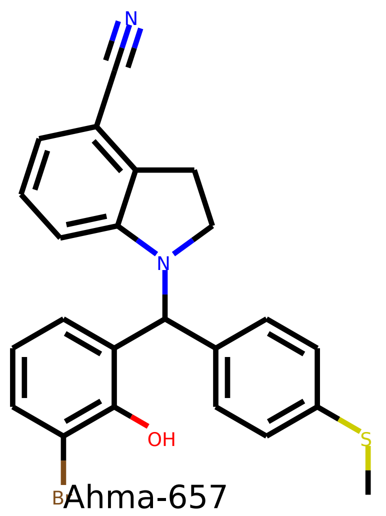
Ahma-657

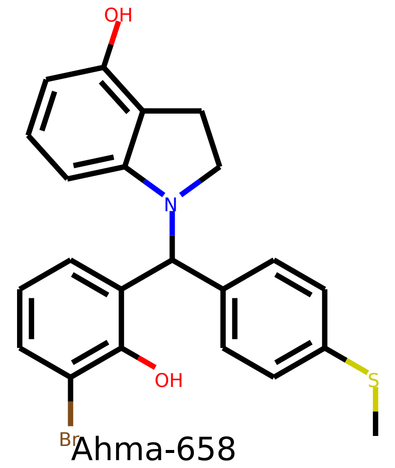
Ahma-658

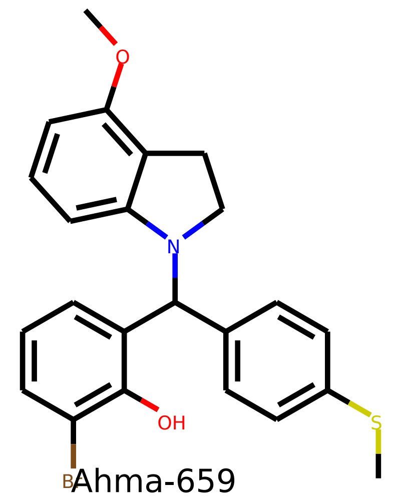
Ahma-659

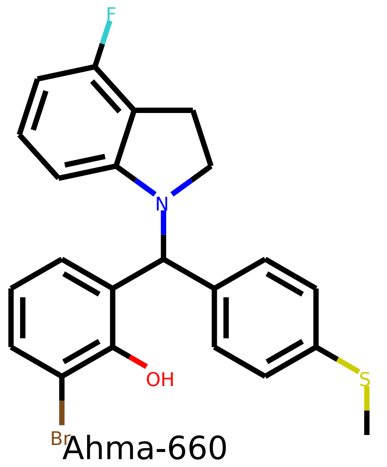
Ahma-660

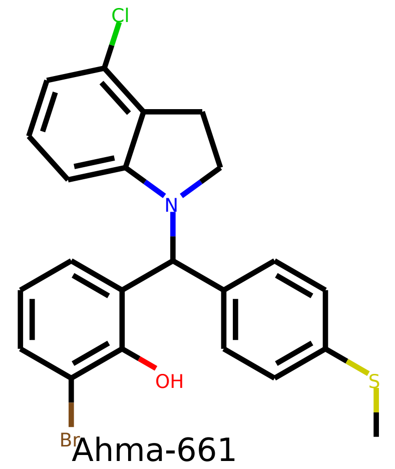
Ahma-661

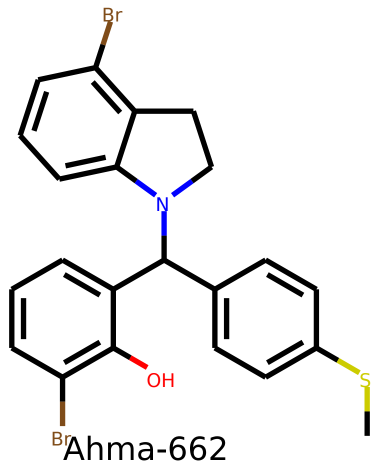
Ahma-662

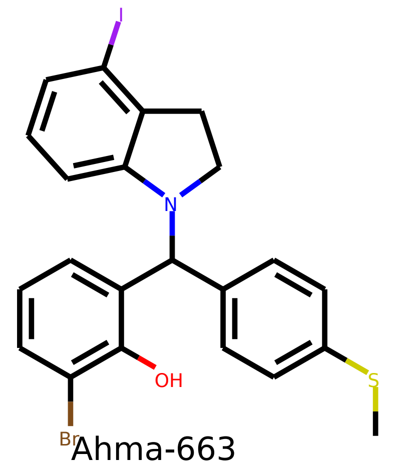
Ahma-663

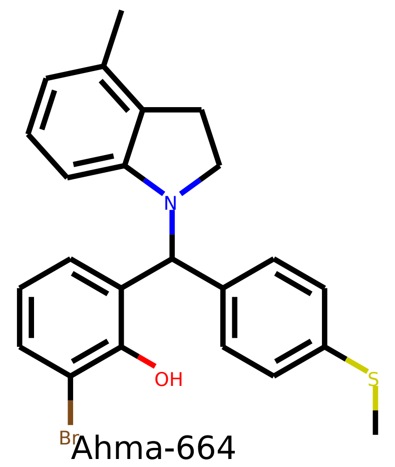
Ahma-664

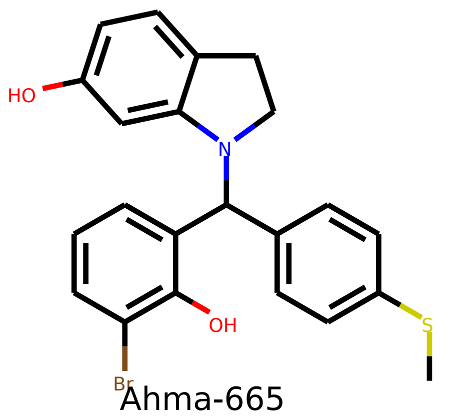
Ahma-665

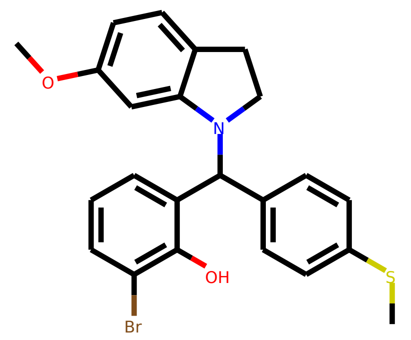
Ahma-666
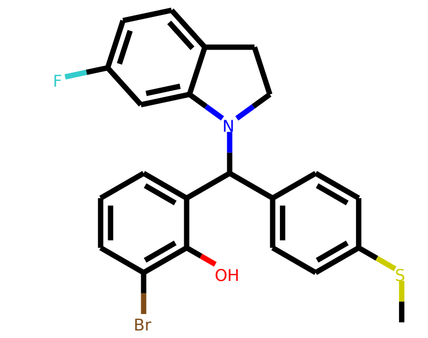
Ahma-667
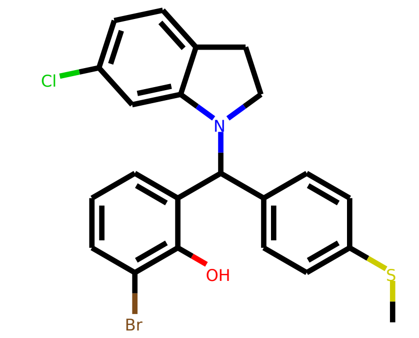
Ahma-668
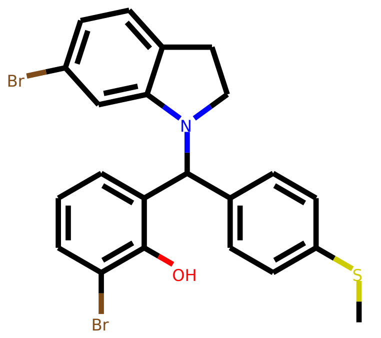
Ahma-669
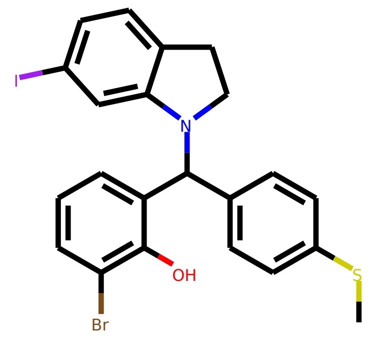
Ahma-670
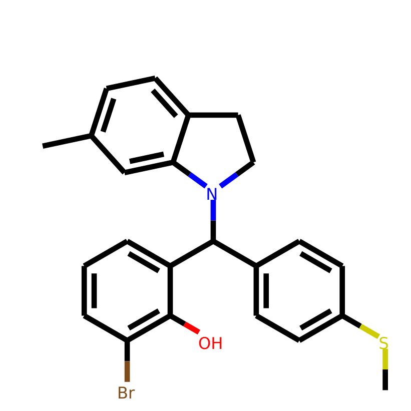
Ahma-671
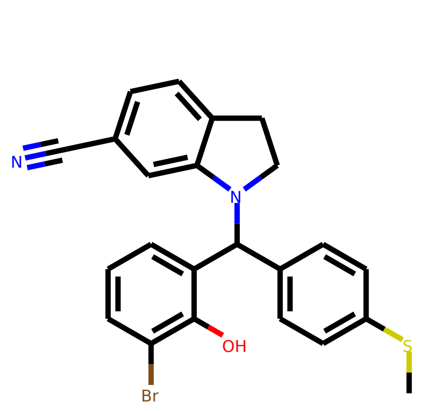
Ahma-672
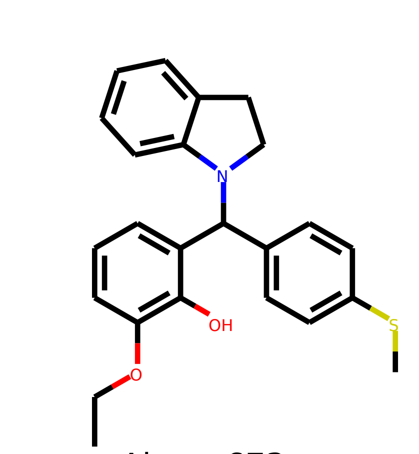
Ahma-673
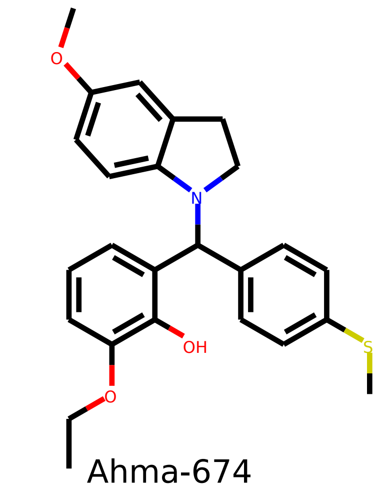
Ahma-674
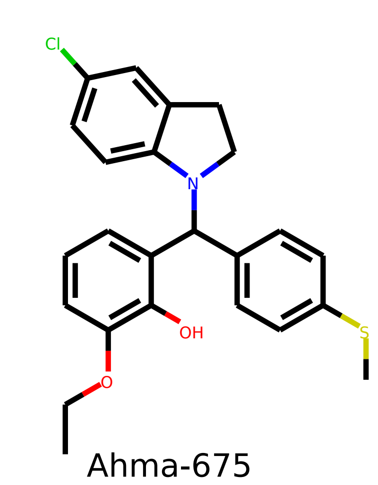
Ahma-675
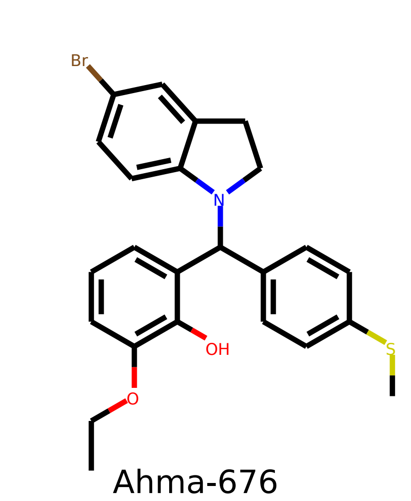
Ahma-676
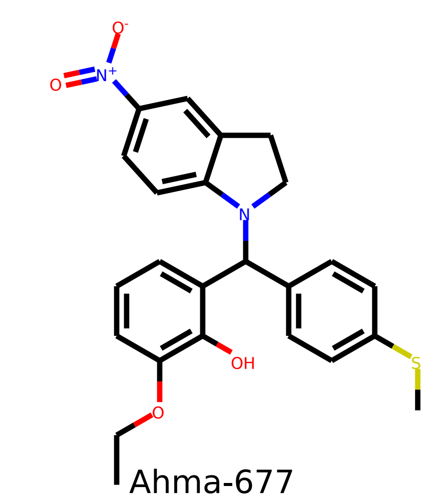
Ahma-677
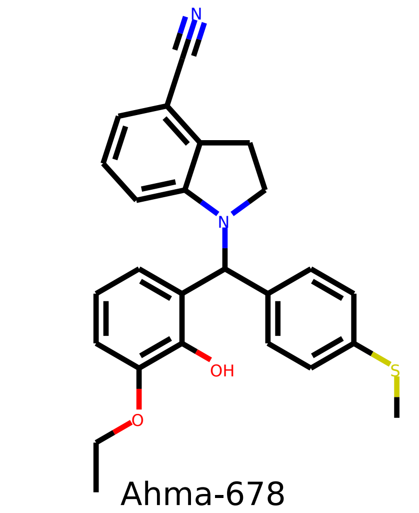
Ahma-678
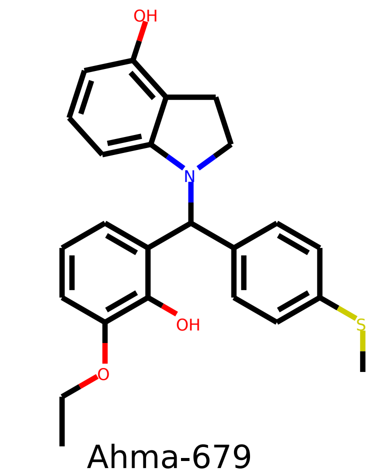
Ahma-679
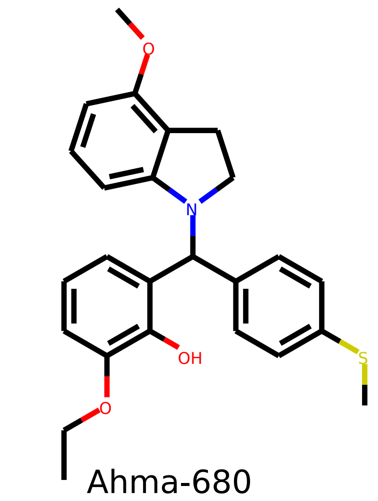
Ahma-680
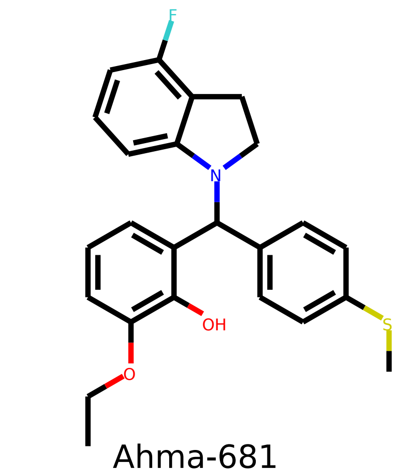
Ahma-681
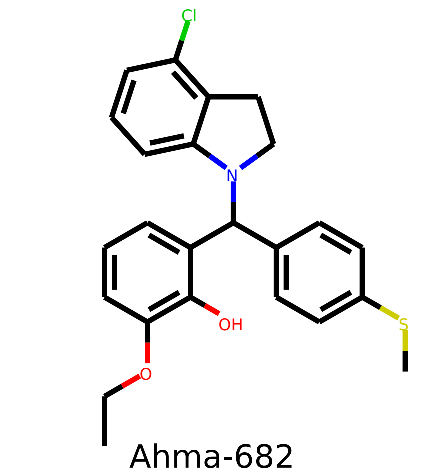
Ahma-682
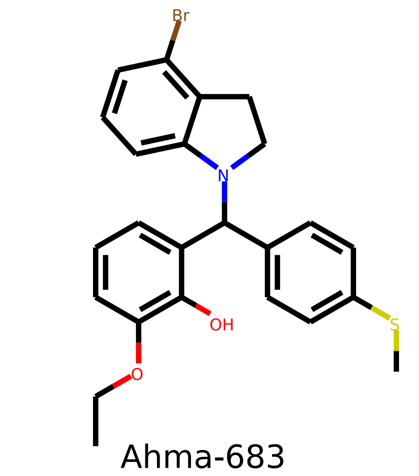
Ahma-683
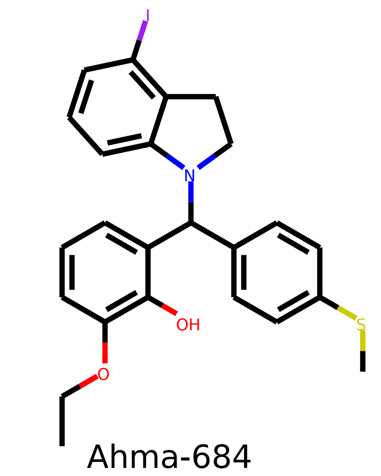
Ahma-684
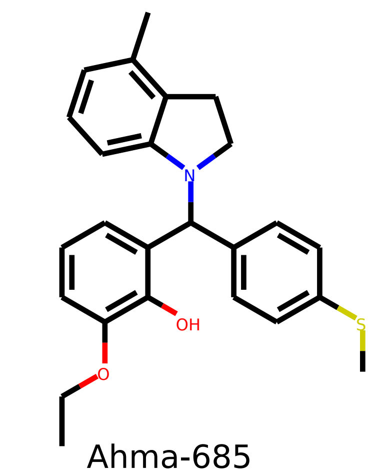
Ahma-685
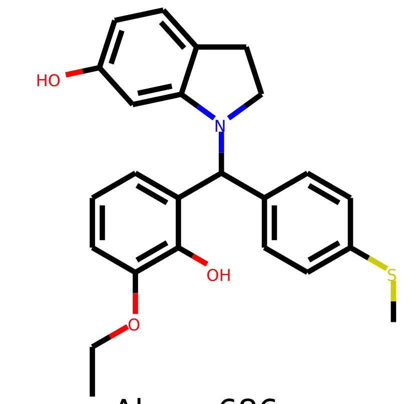
Ahma-686
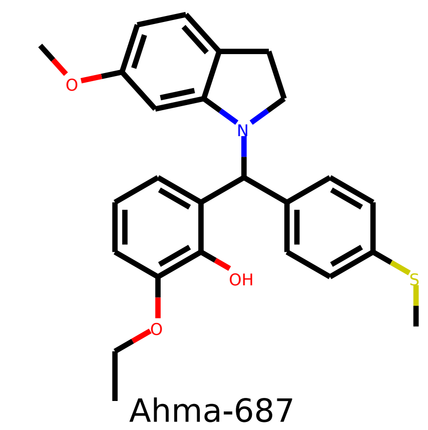
Ahma-687
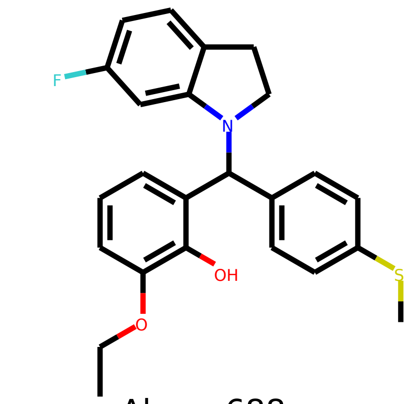
Ahma-688
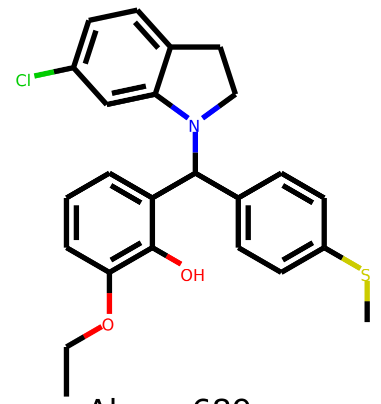
Ahma-689
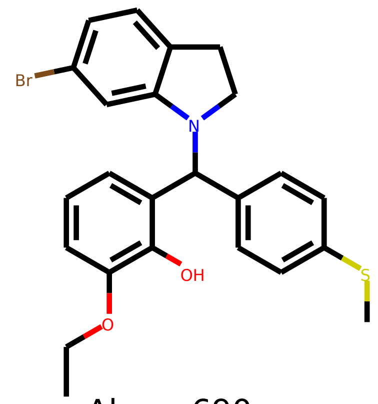
Ahma-690
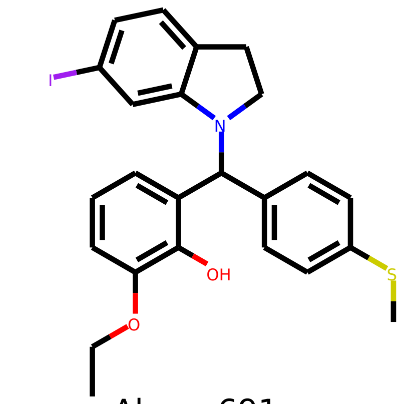
Ahma-691
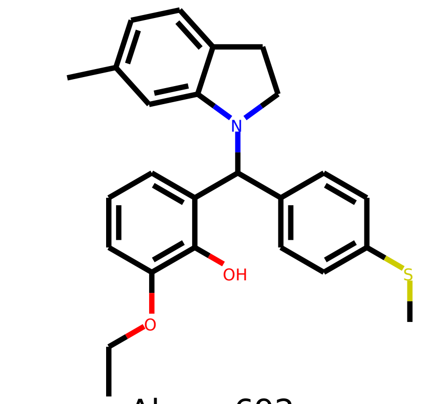
Ahma-692
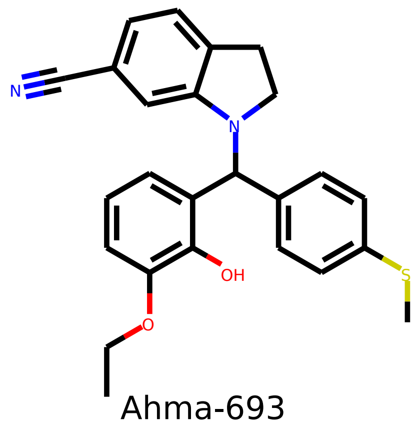
Ahma-693
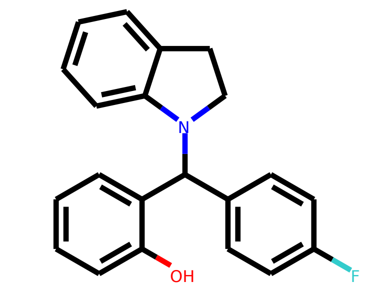
Ahma-694
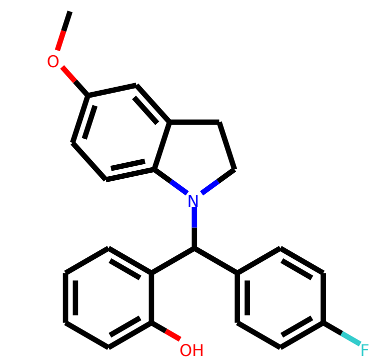
Ahma-695
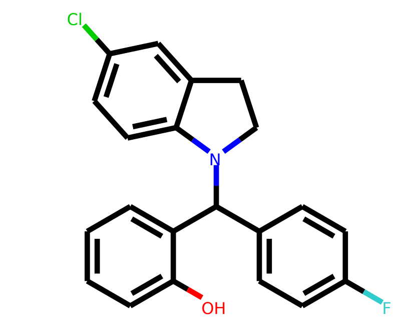
Ahma-696
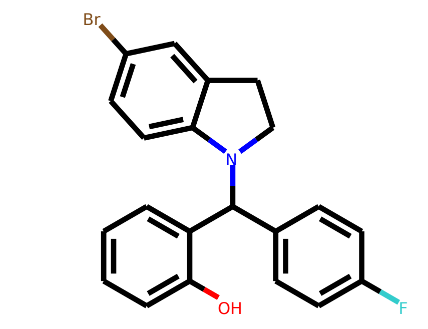
Ahma-697
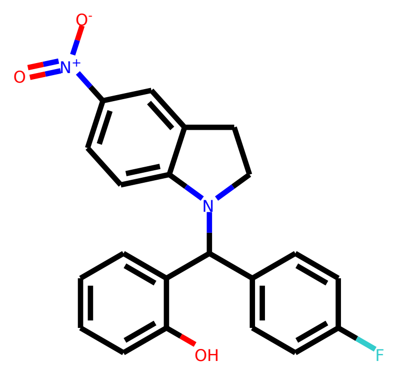
Ahma-698
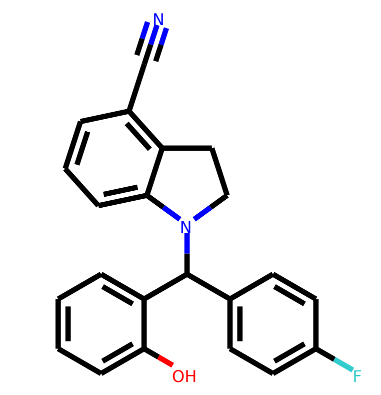
Ahma-699
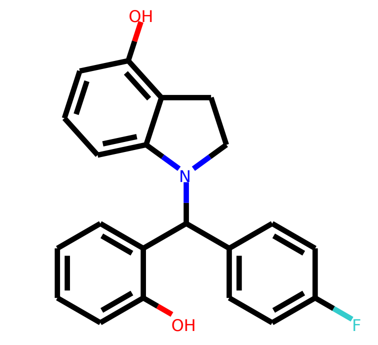
Ahma-700

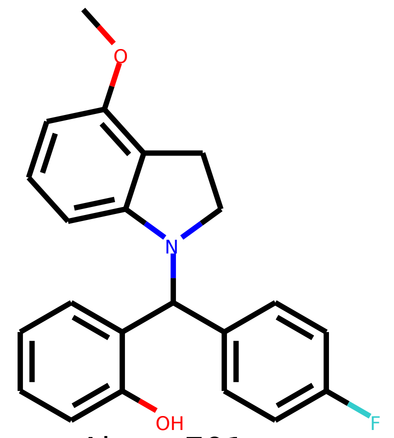
Ahma-701
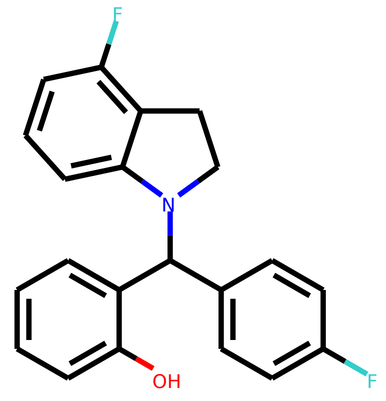
Ahma-702
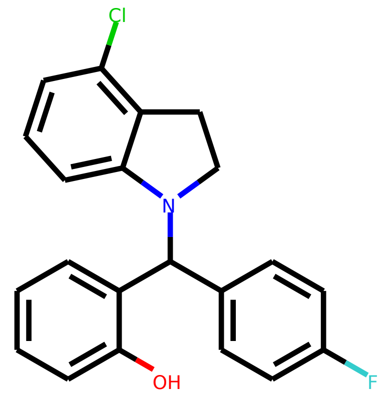
Ahma-703
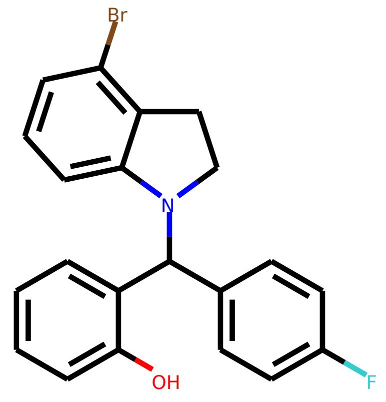
Ahma-704
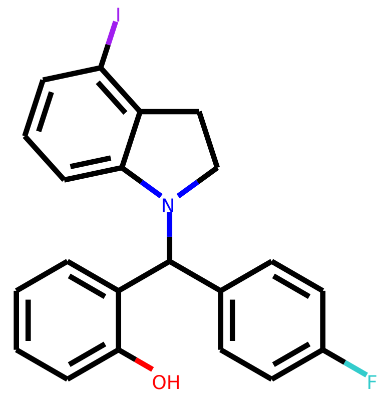
Ahma-705
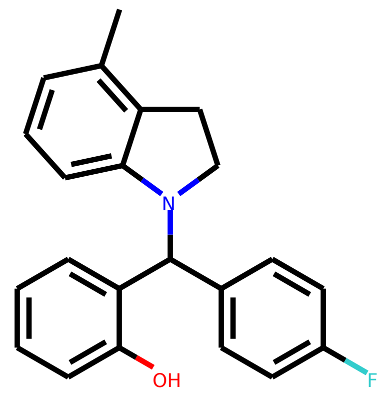
Ahma-706
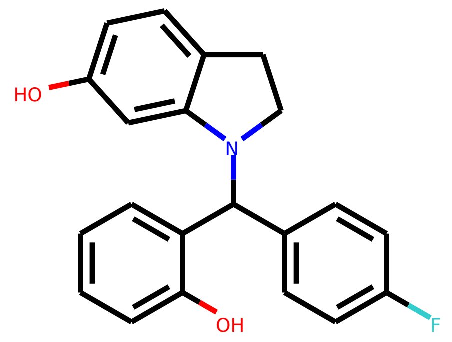
Ahma-707
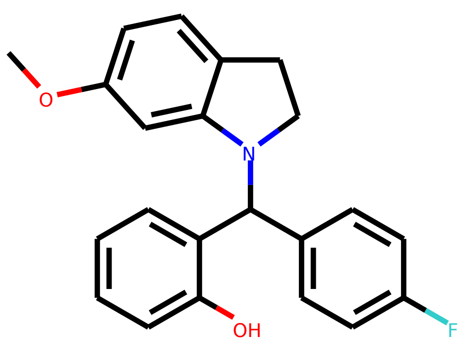
Ahma-708
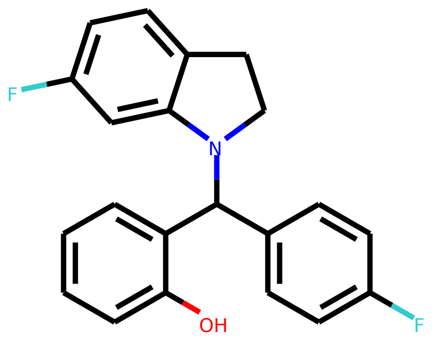
Ahma-709
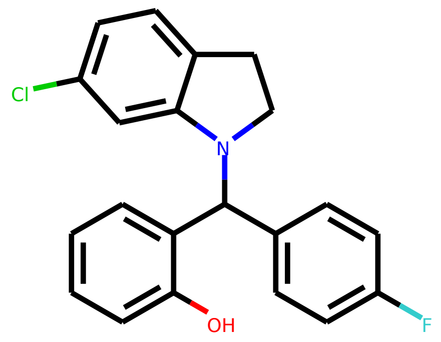
Ahma-710
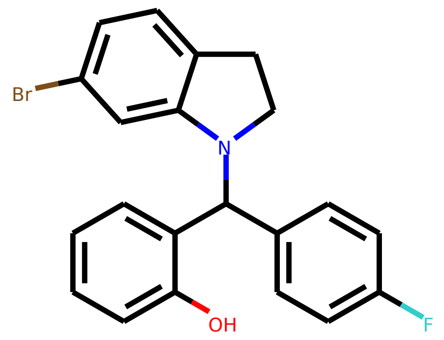
Ahma-711
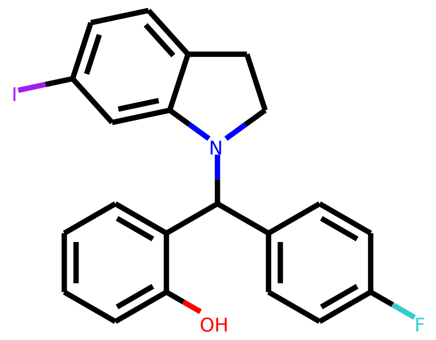
Ahma-712
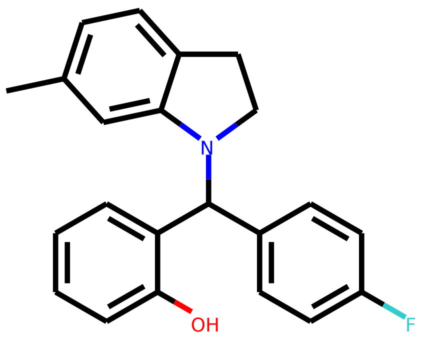
Ahma-713
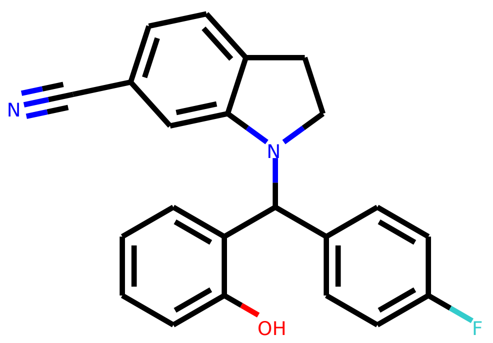
Ahma-714
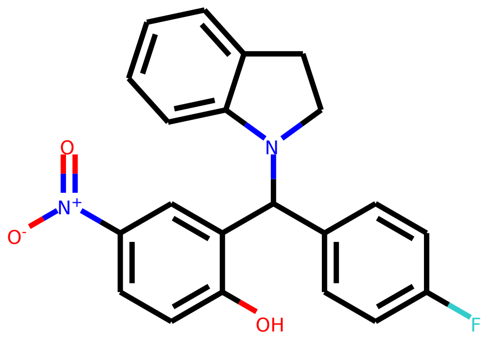
Ahma-715
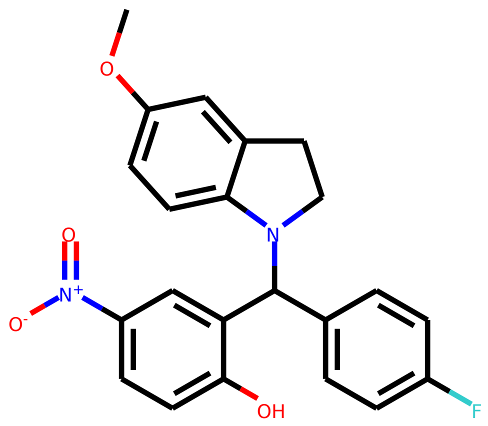
Ahma-716
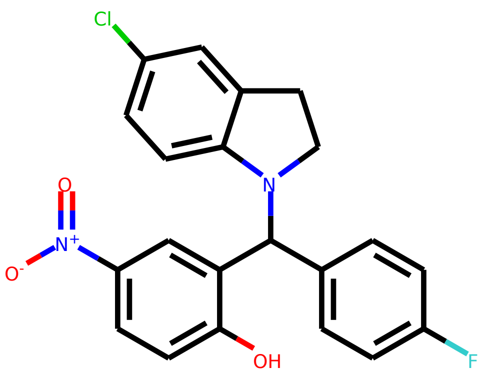
Ahma-717
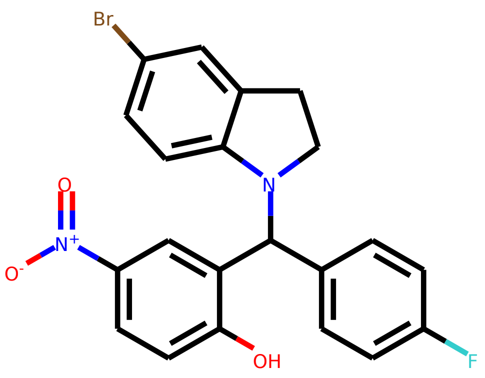
Ahma-718
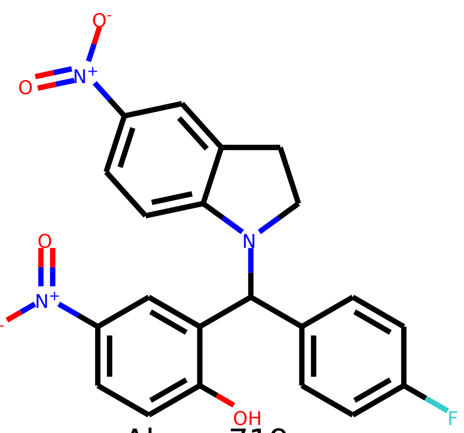
Ahma-719
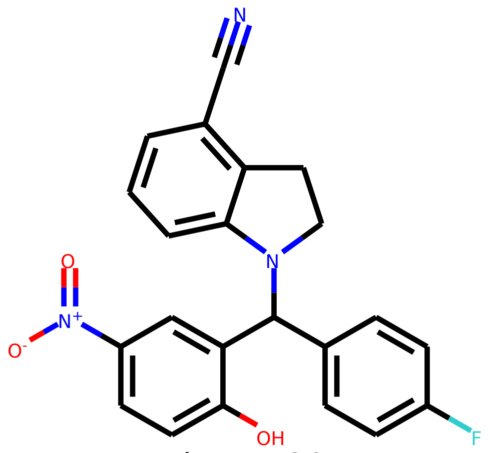
Ahma-720
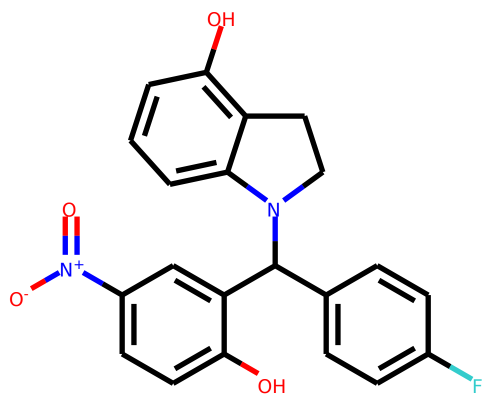
Ahma-721
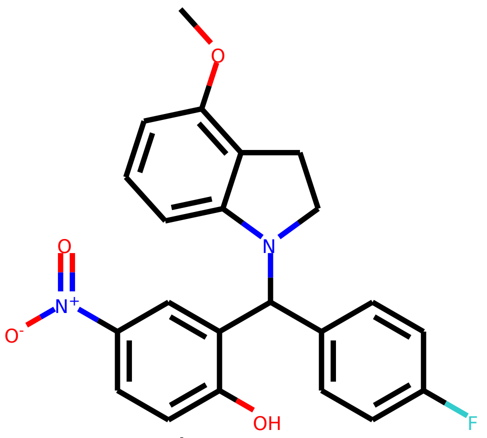
Ahma-722
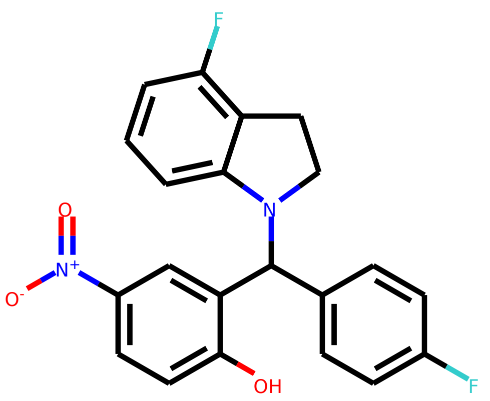
Ahma-723
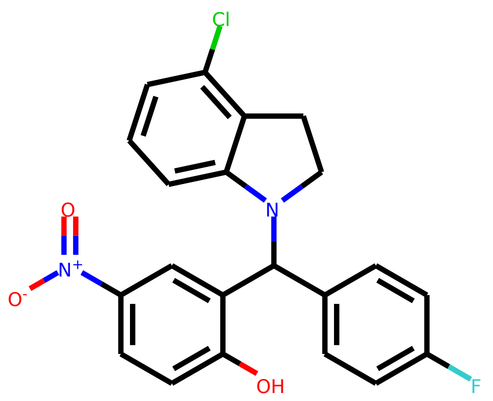
Ahma-724
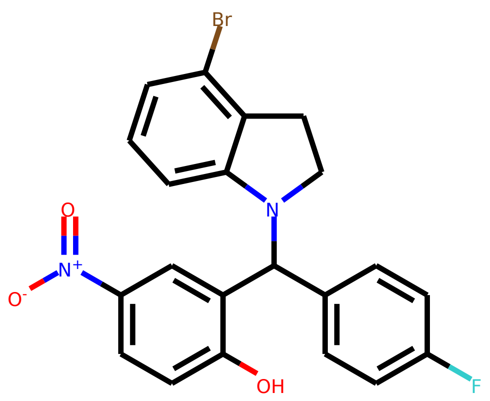
Ahma-725
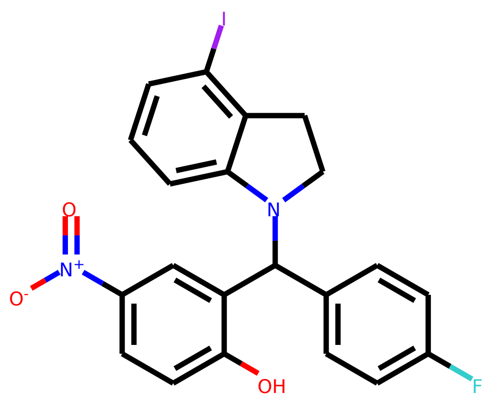
Ahma-726
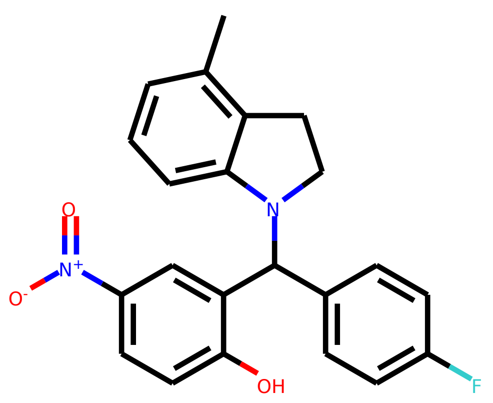
Ahma-727
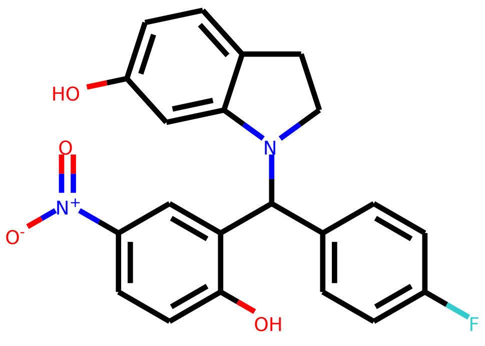
Ahma-728
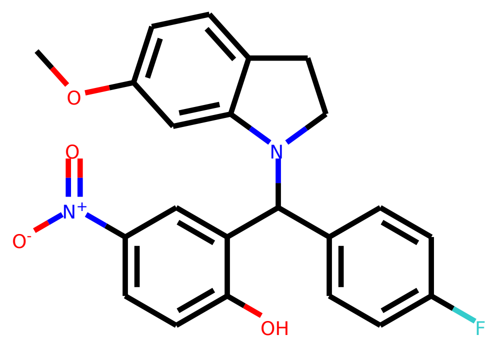
Ahma-729
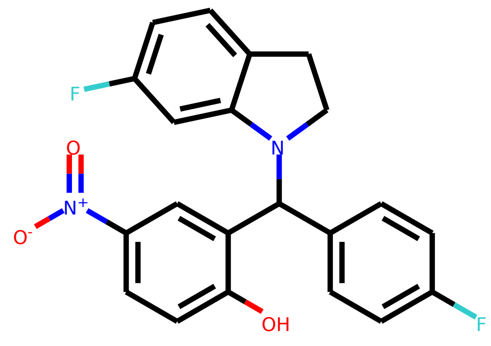
Ahma-730
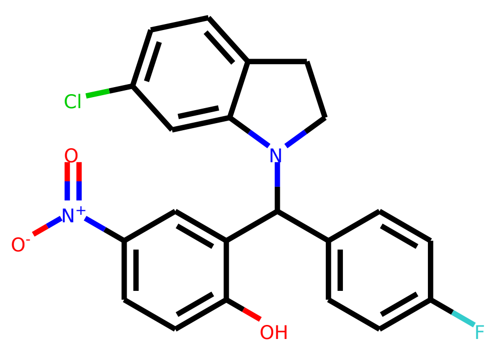
Ahma-731
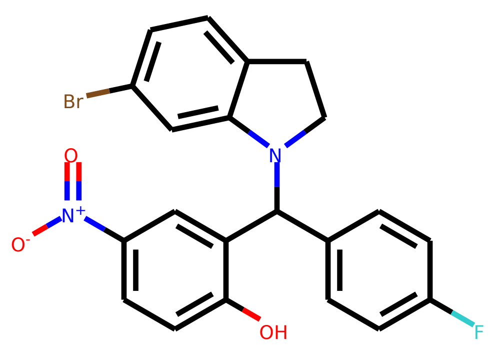
Ahma-732
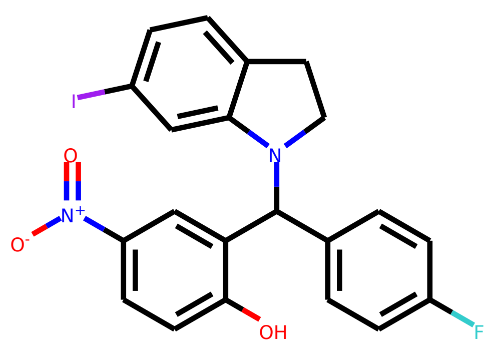
Ahma-733
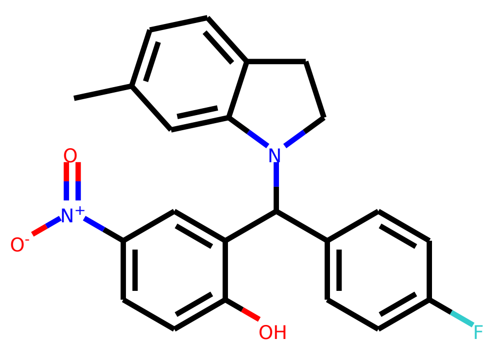
Ahma-734
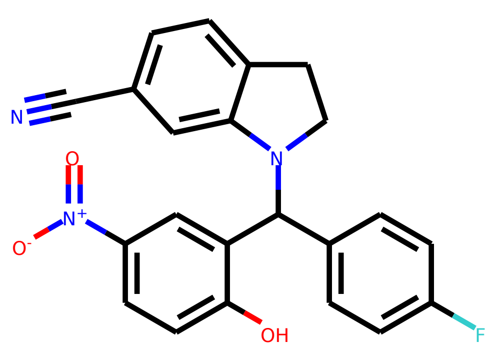
Ahma-735

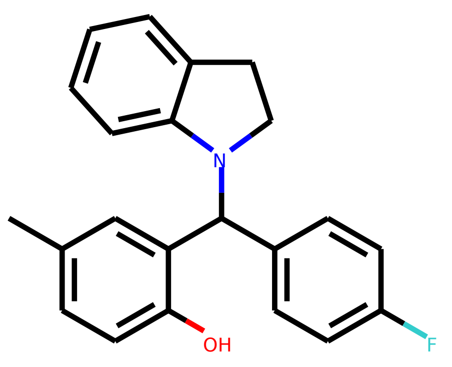
Ahma-736

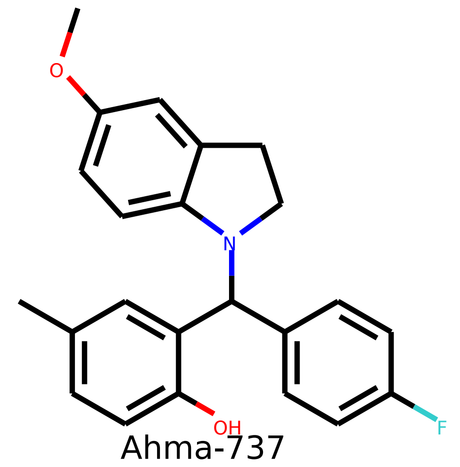
Ahma-737

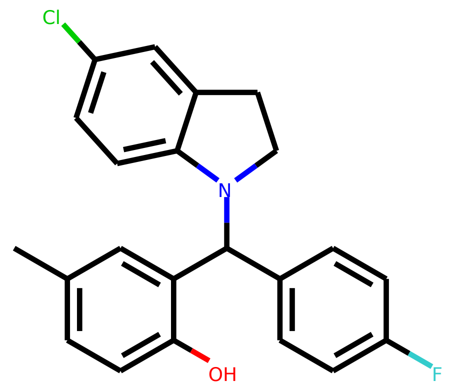
Ahma-738

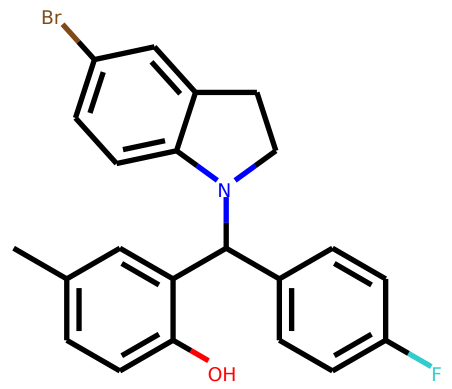
Ahma-739

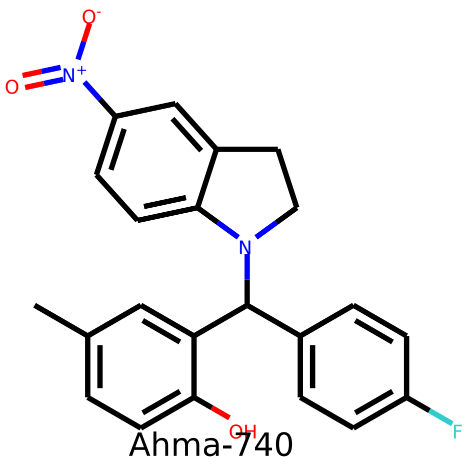
Ahma-740

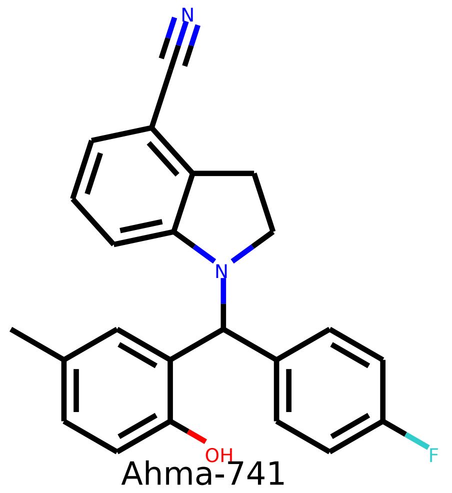
Ahma-741

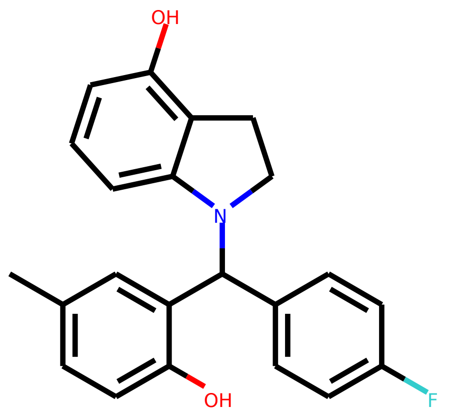
Ahma-742

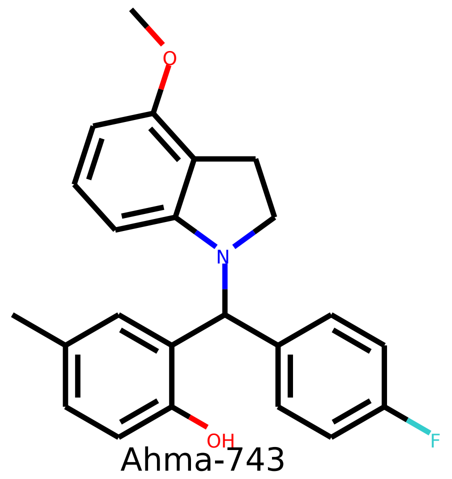
Ahma-743

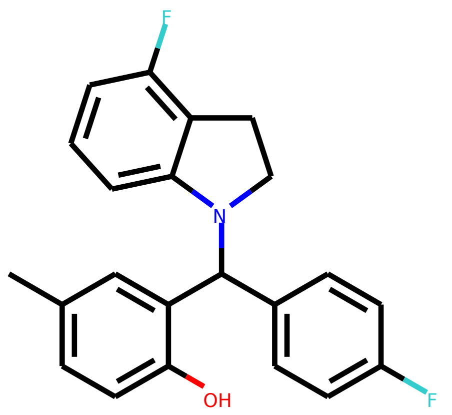
Ahma-744

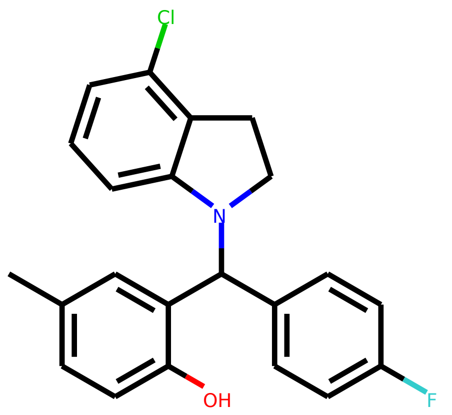
Ahma-745

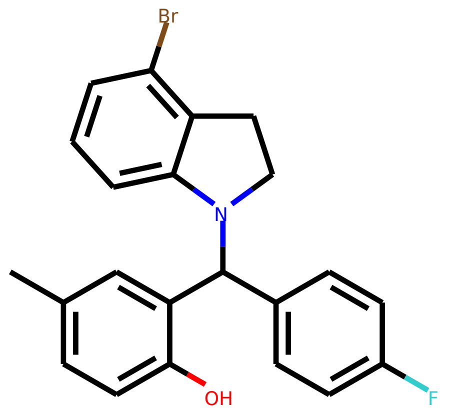
Ahma-746

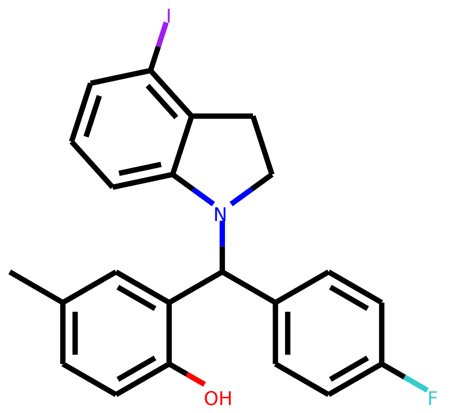
Ahma-747

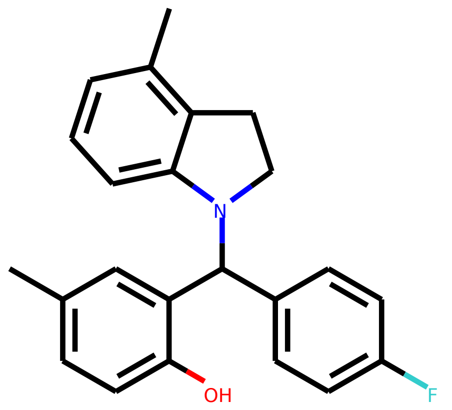
Ahma-748

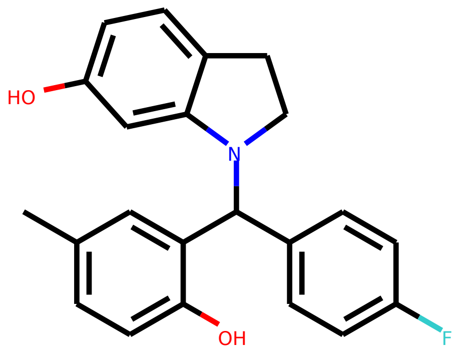
Ahma-749

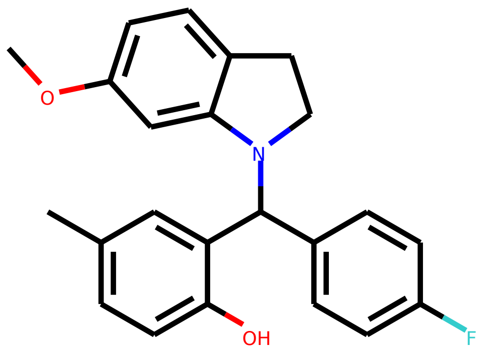
Ahma-750

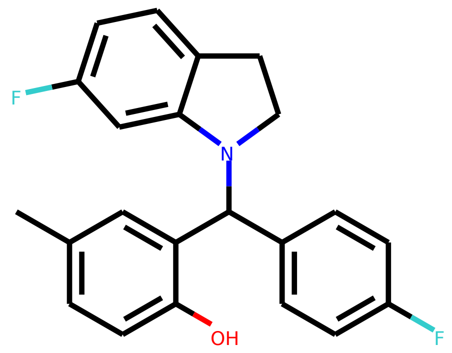
Ahma-751

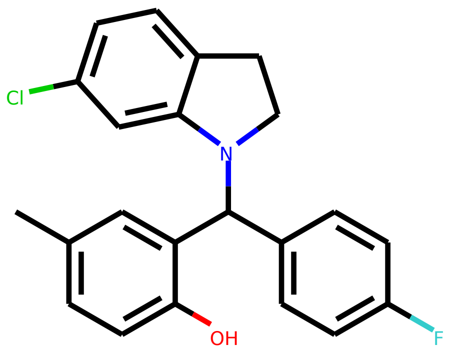
Ahma-752

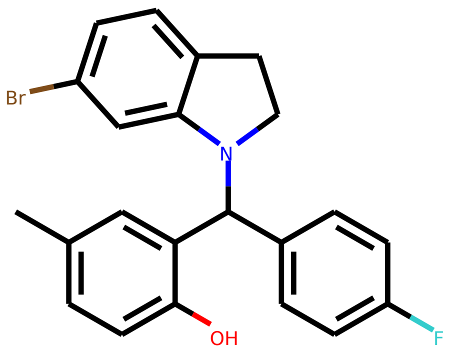
Ahma-753

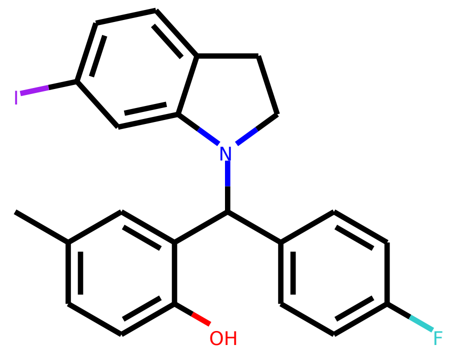
Ahma-754

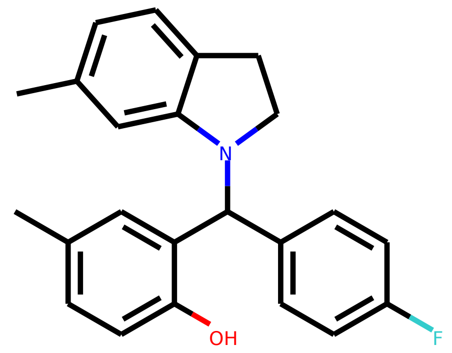
Ahma-755

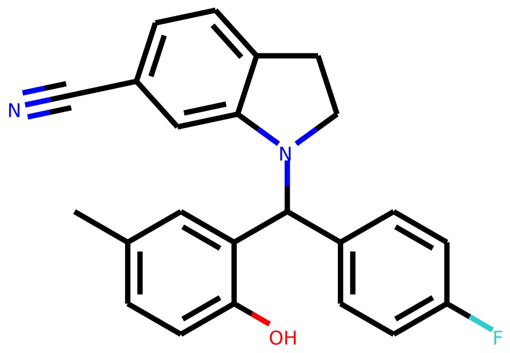
Ahma-756

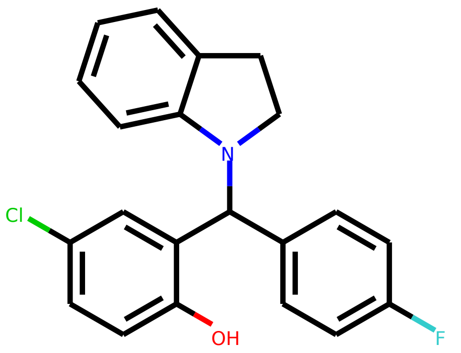
Ahma-757

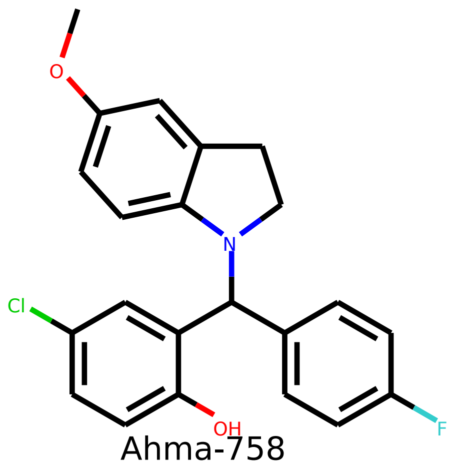
Ahma-758

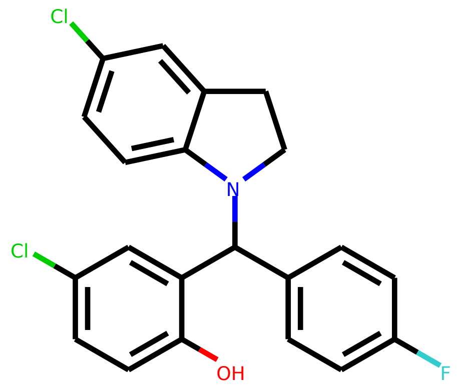
Ahma-759

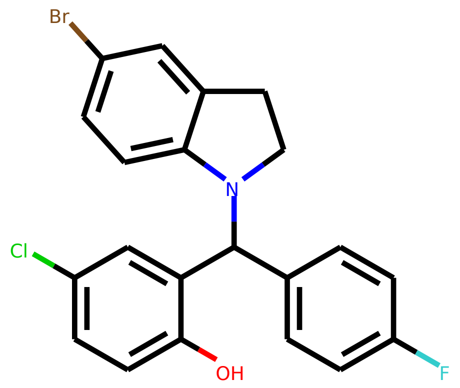
Ahma-760

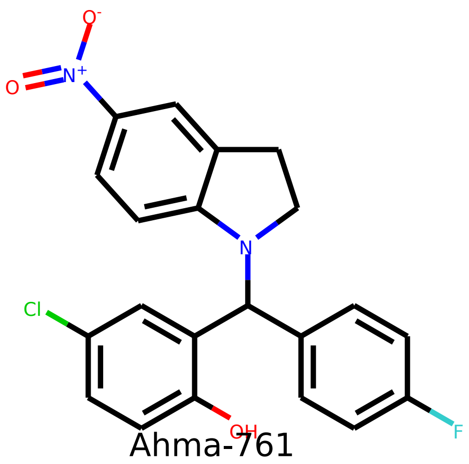
Ahma-761

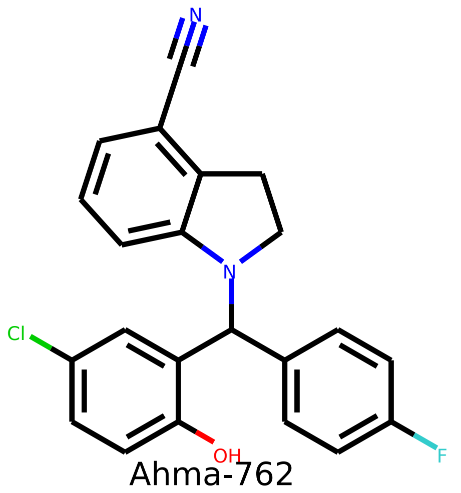
Ahma-762

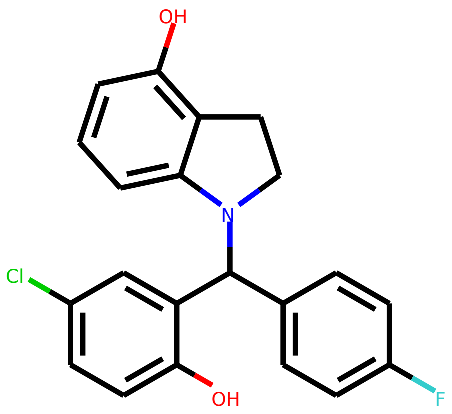
Ahma-763

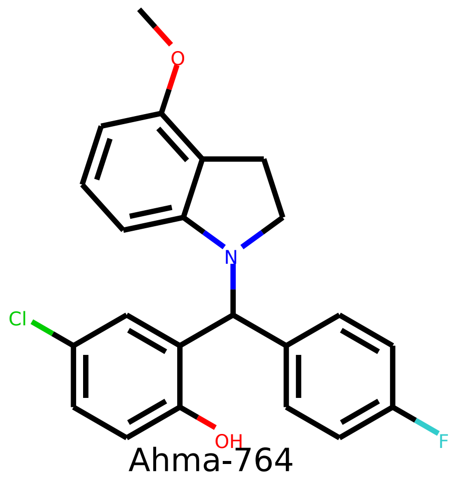
Ahma-764

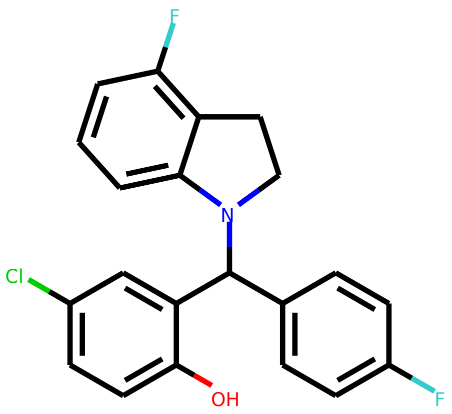
Ahma-765

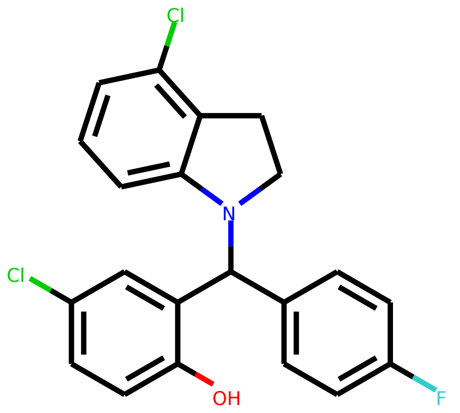
Ahma-766

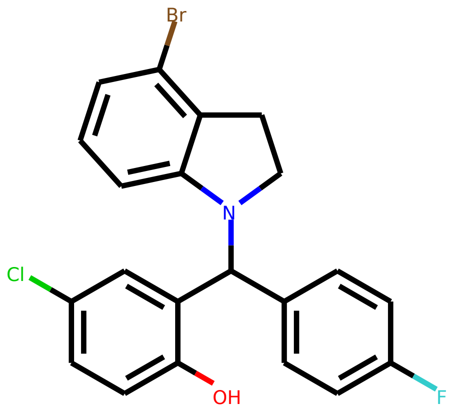
Ahma-767

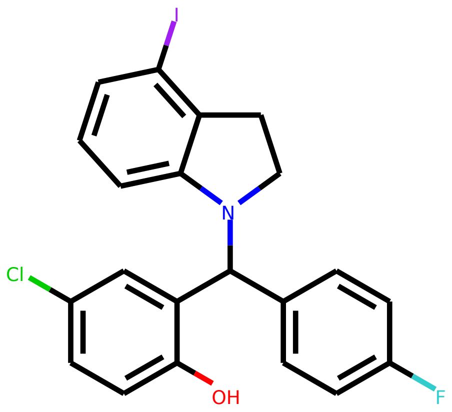
Ahma-768

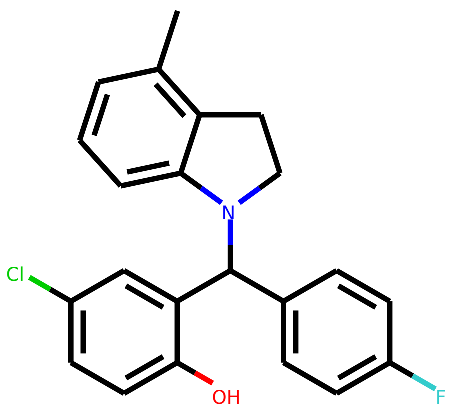
Ahma-769

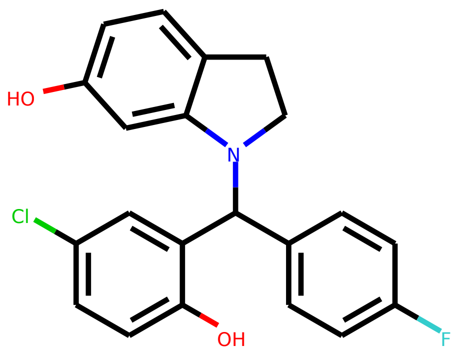
Ahma-770

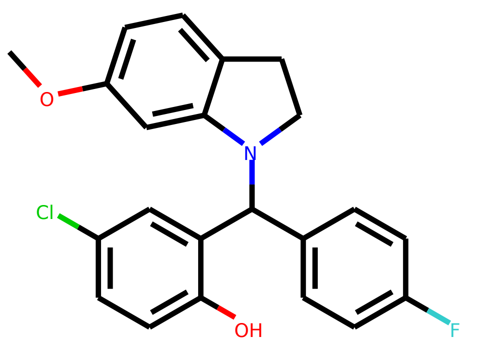
Ahma-771
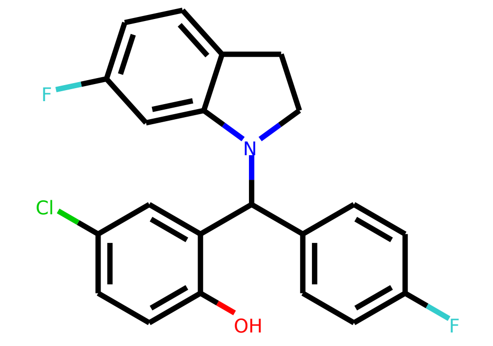
Ahma-772
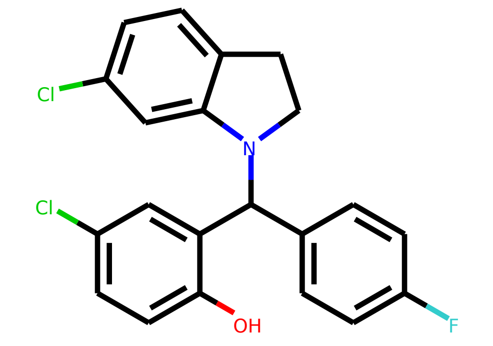
Ahma-773
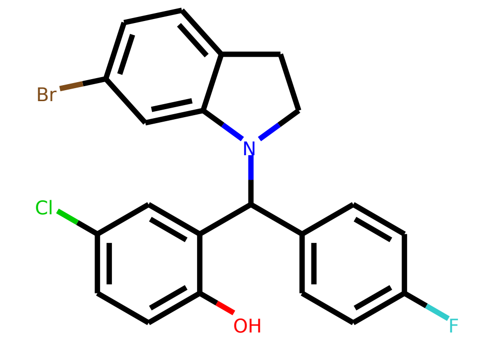
Ahma-774
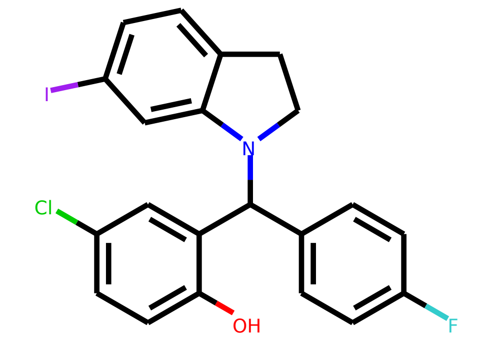
Ahma-775
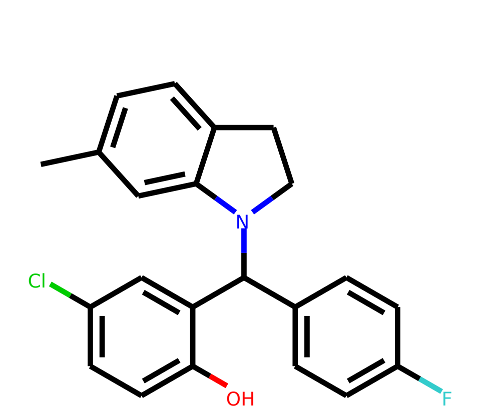
Ahma-776
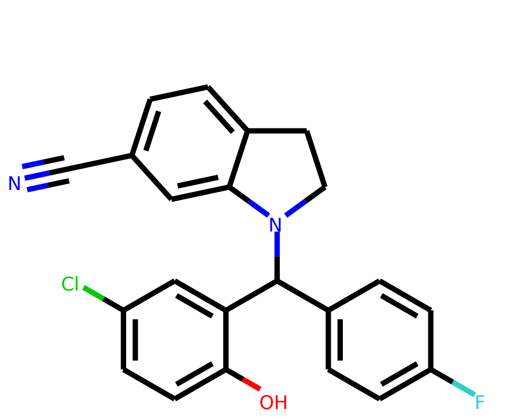
Ahma-777
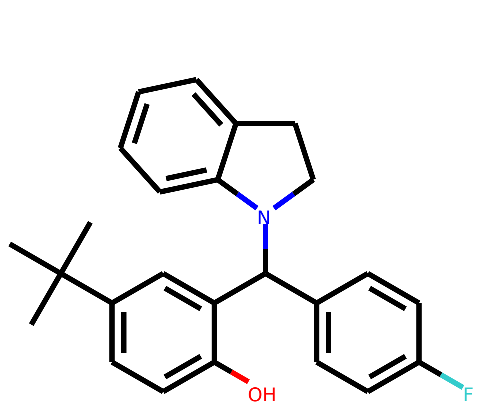
Ahma-778
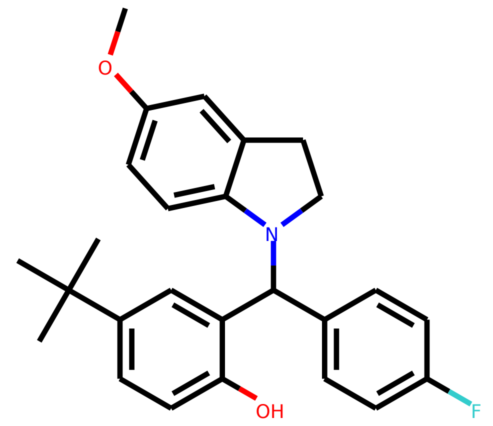
Ahma-779
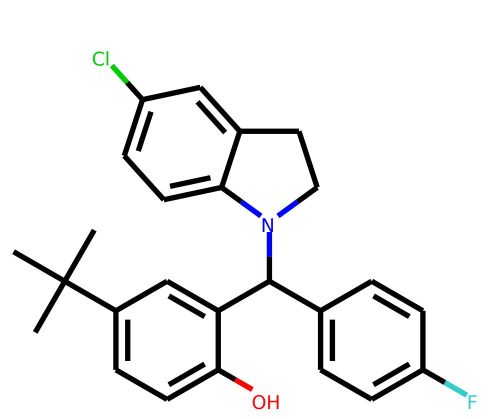
Ahma-780
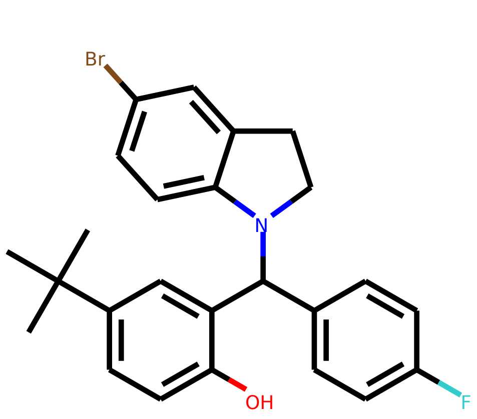
Ahma-781
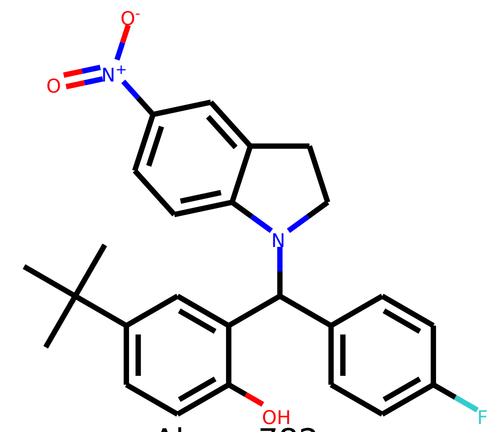
Ahma-782
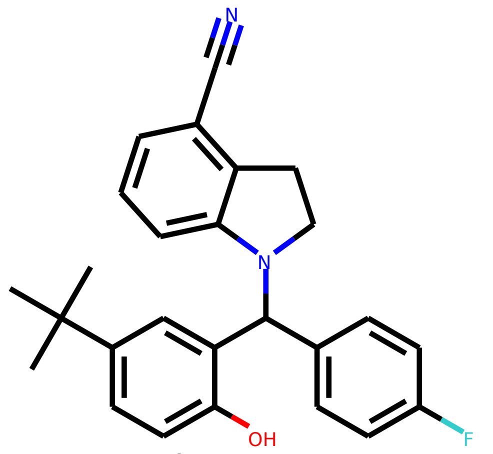
Ahma-783
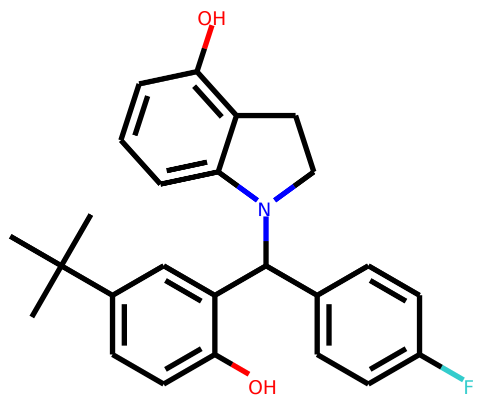
Ahma-784
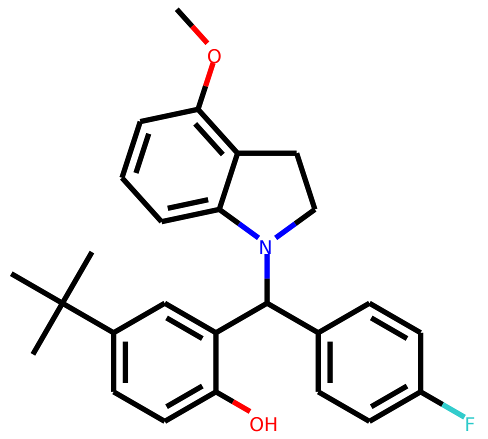
Ahma-785
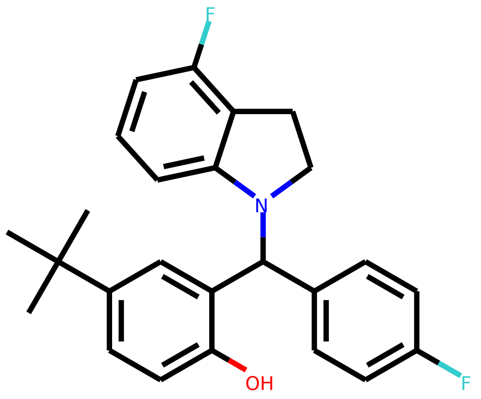
Ahma-786
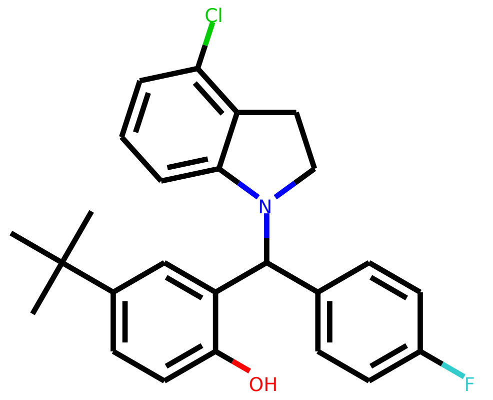
Ahma-787
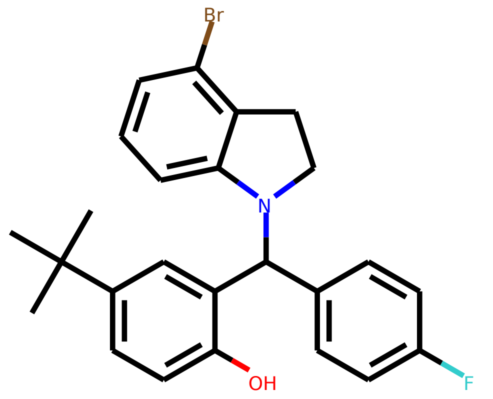
Ahma-788
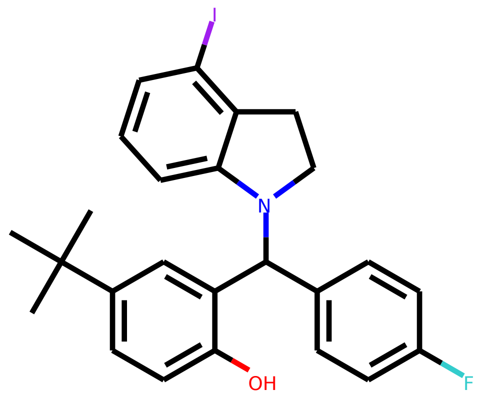
Ahma-789
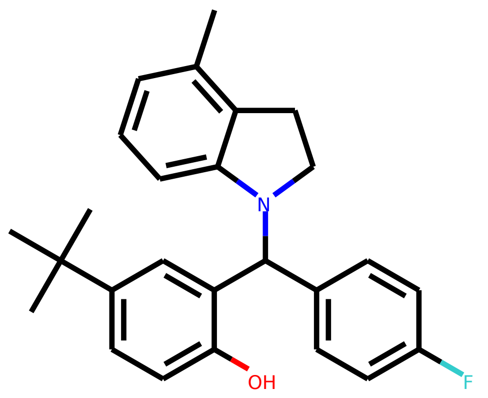
Ahma-790
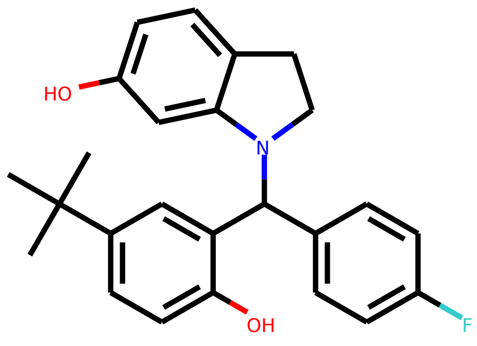
Ahma-791
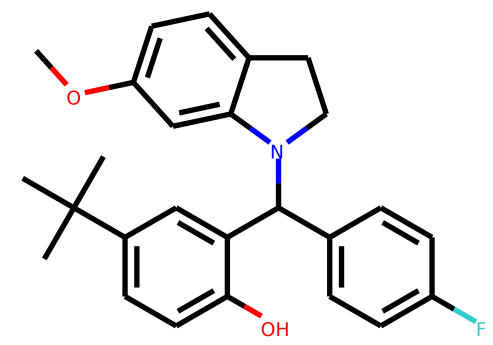
Ahma-792
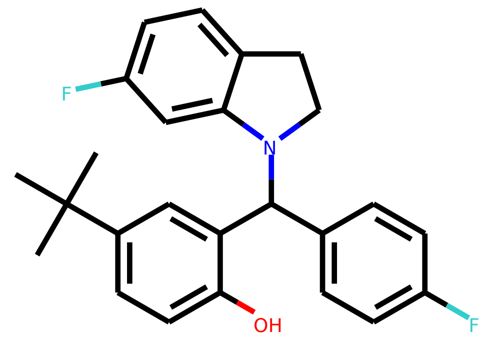
Ahma-793
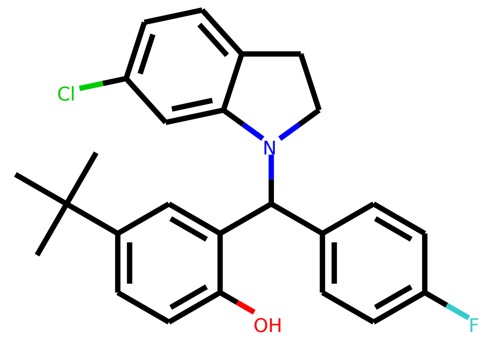
Ahma-794
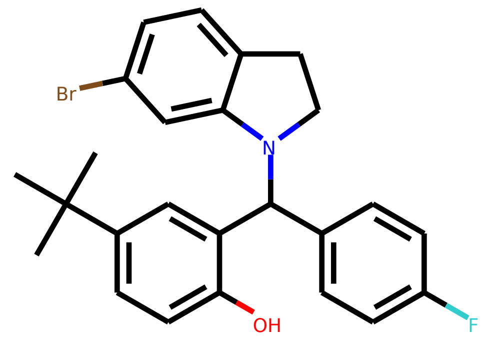
Ahma-795
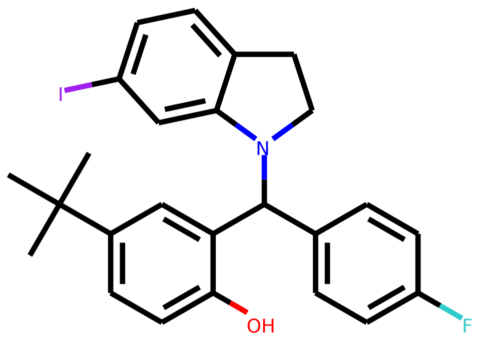
Ahma-796
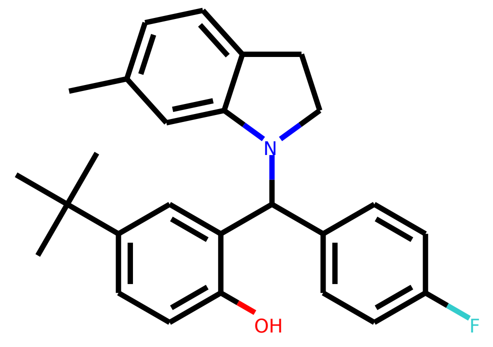
Ahma-797
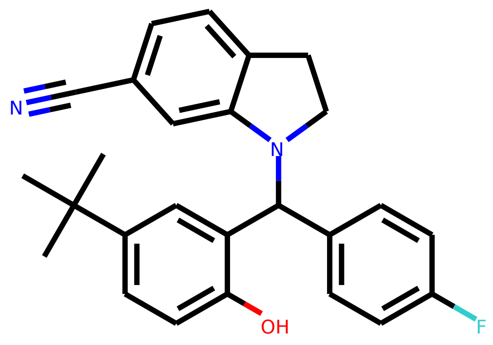
Ahma-798
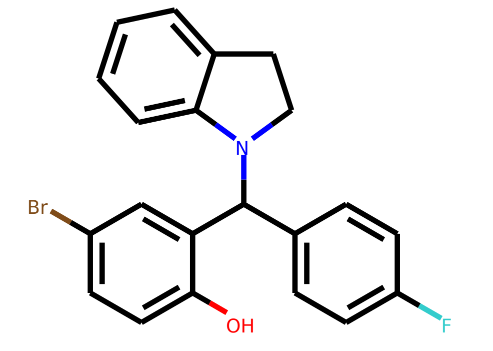
Ahma-799
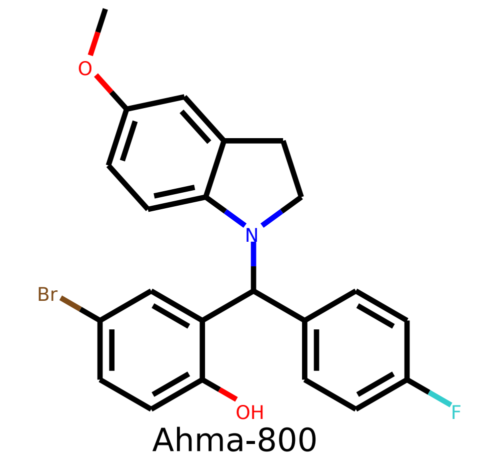
Ahma-800
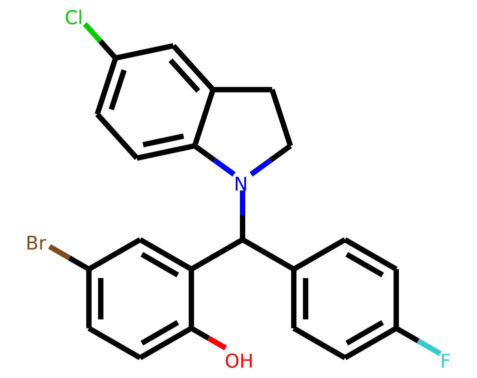
Ahma-801
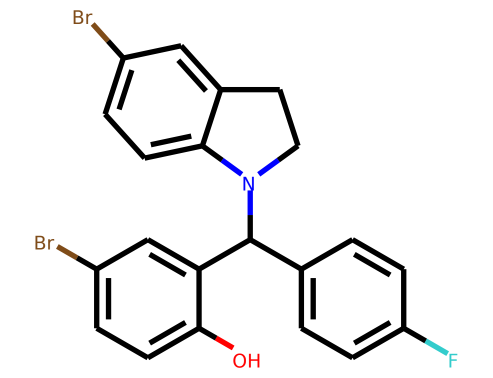
Ahma-802
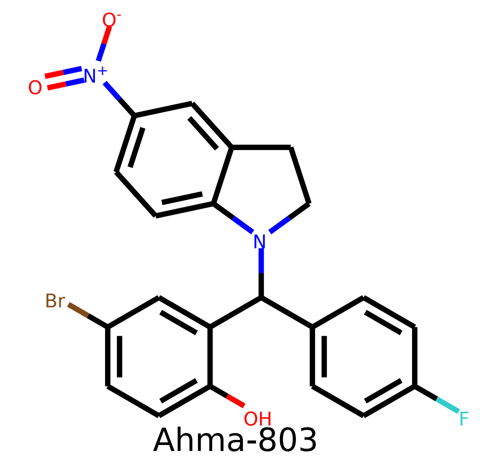
Ahma-803
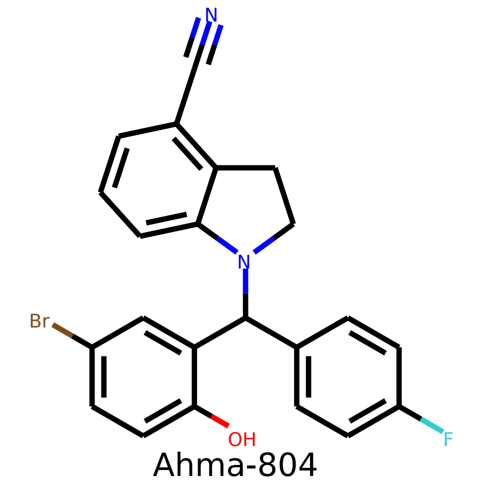
Ahma-804
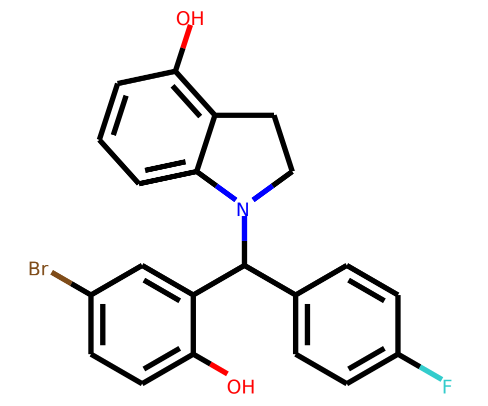
Ahma-805

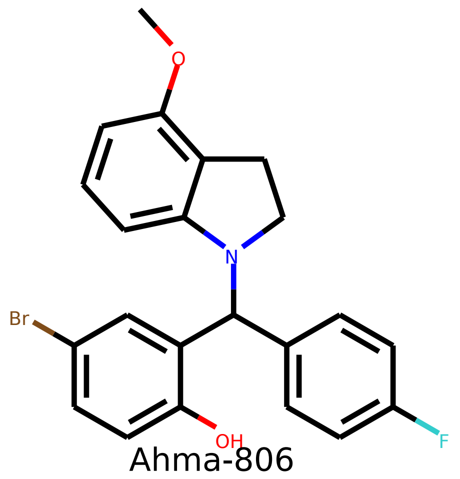
Ahma-806

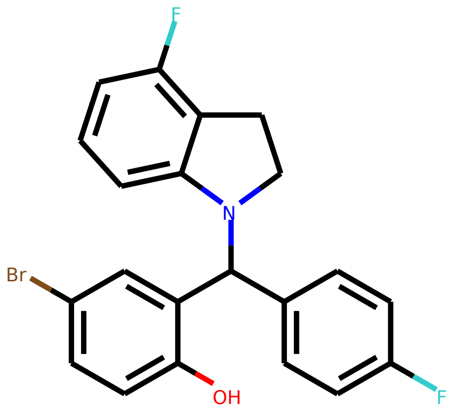
Ahma-807

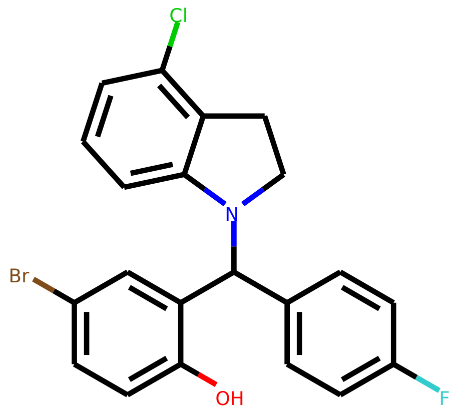
Ahma-808

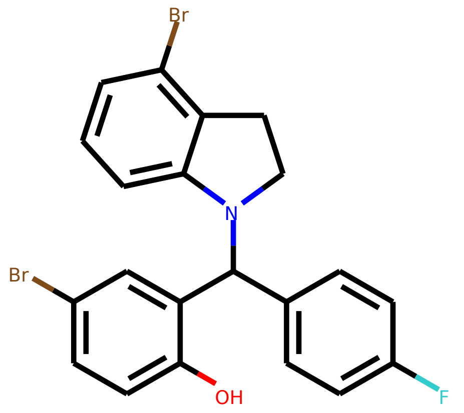
Ahma-809

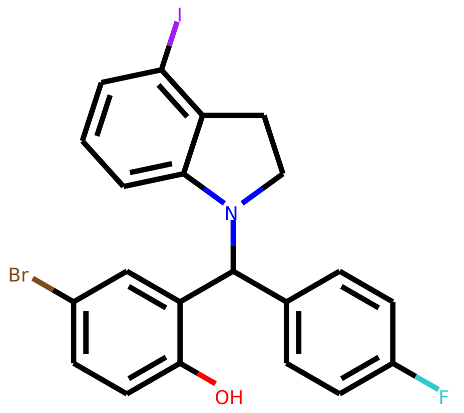
Ahma-810

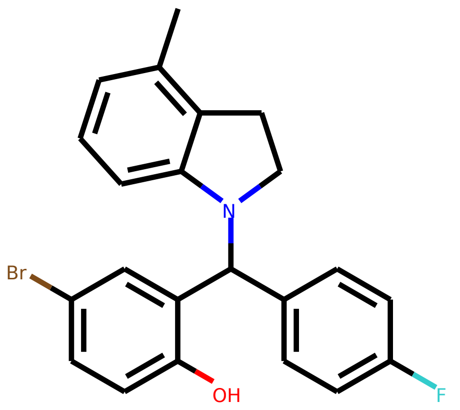
Ahma-811

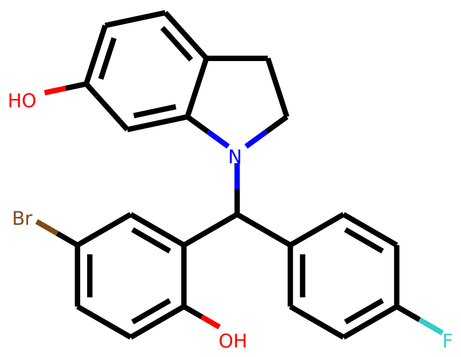
Ahma-812

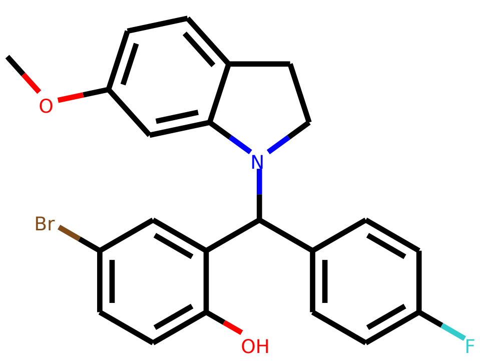
Ahma-813

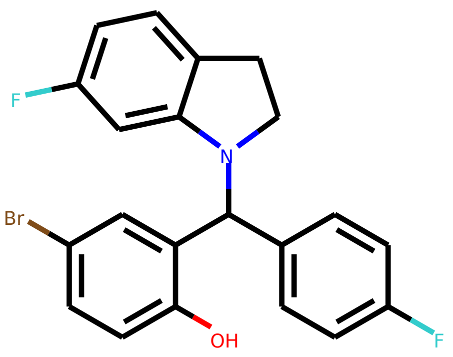
Ahma-814

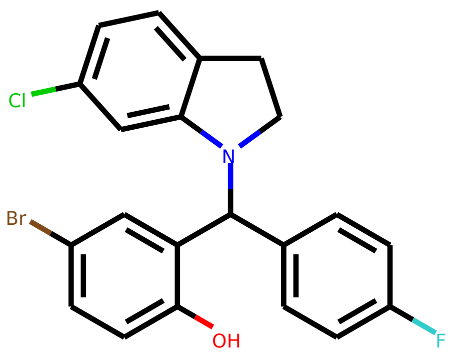
Ahma-815

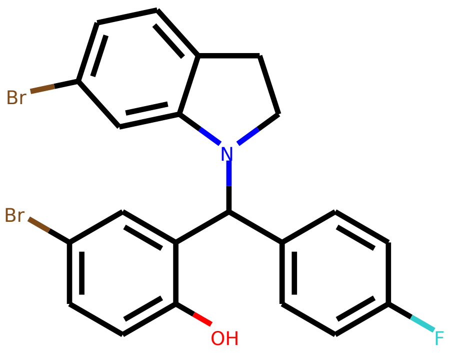
Ahma-816

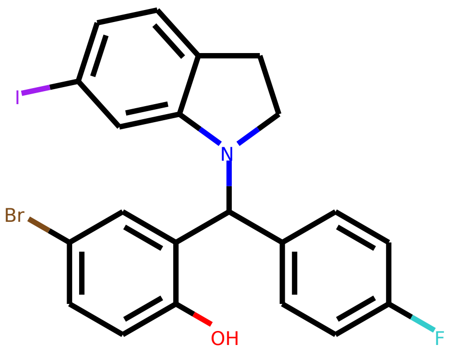
Ahma-817

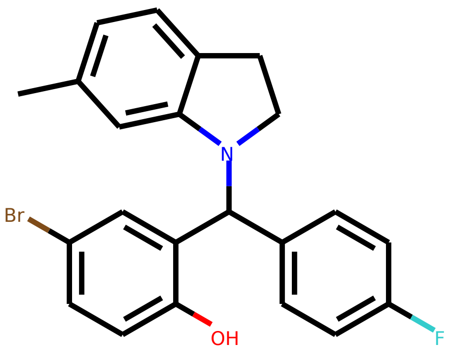
Ahma-818

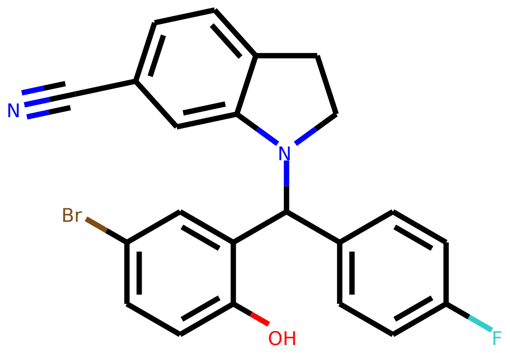
Ahma-819

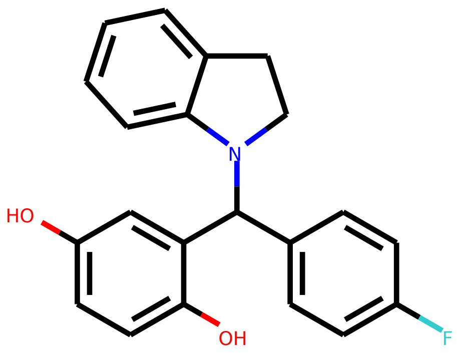
Ahma-820

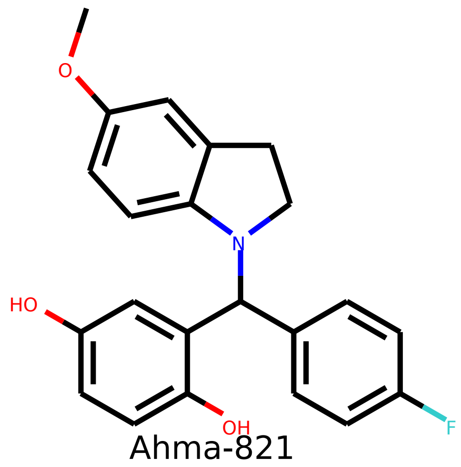
Ahma-821

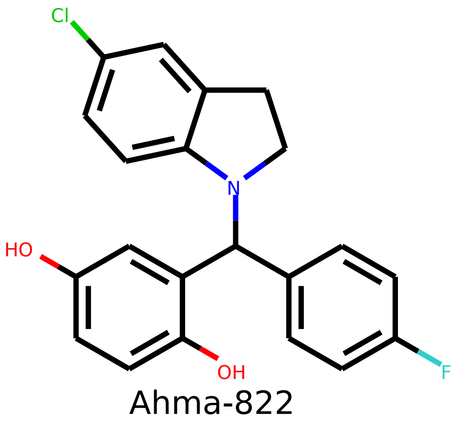
Ahma-822

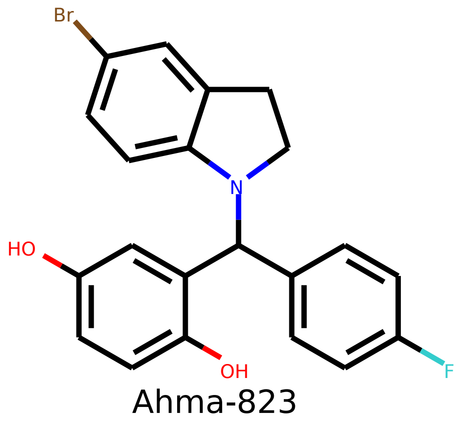
Ahma-823

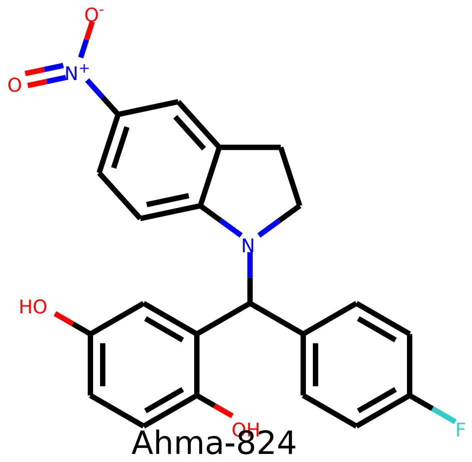
Ahma-824

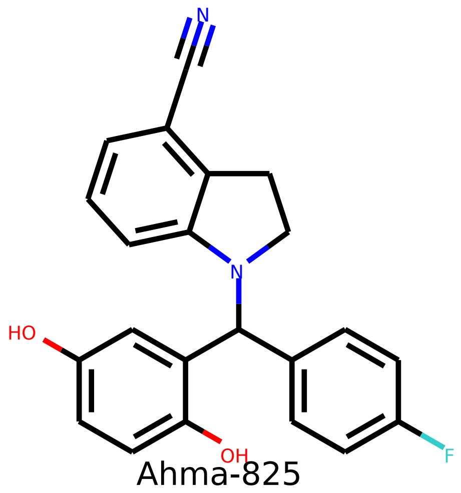
Ahma-825

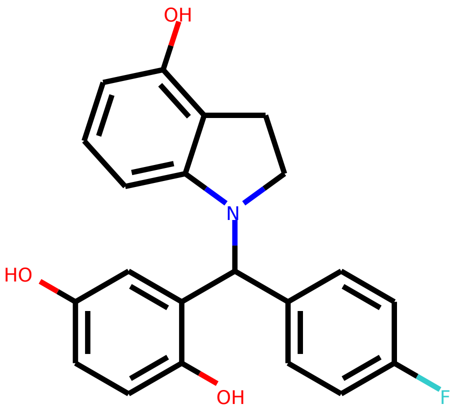
Ahma-826

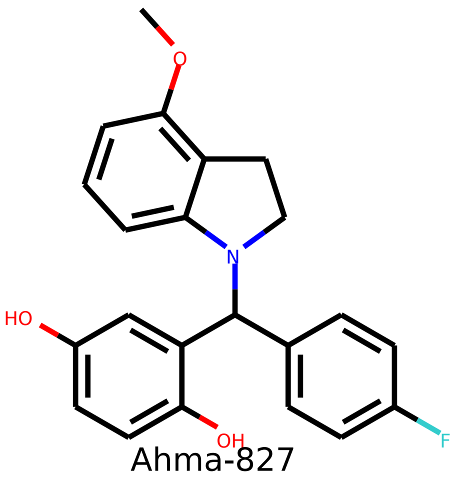
Ahma-827

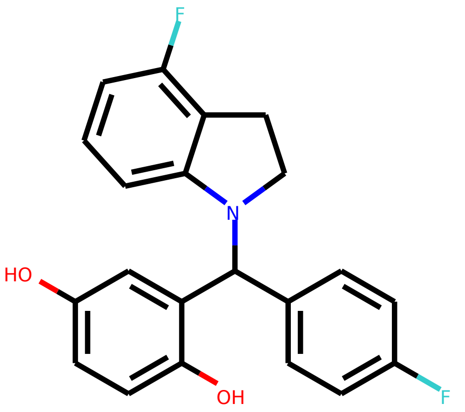
Ahma-828

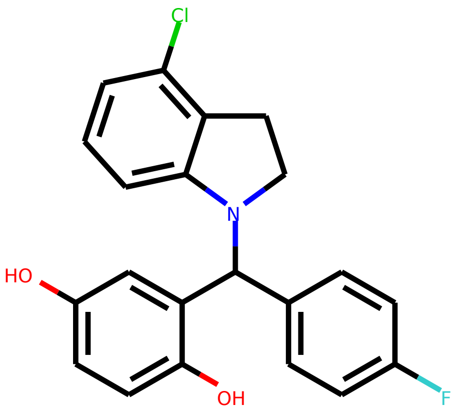
Ahma-829

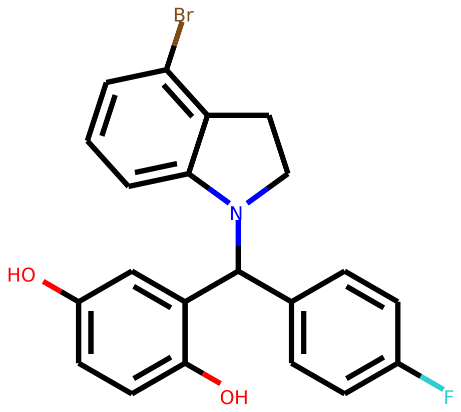
Ahma-830

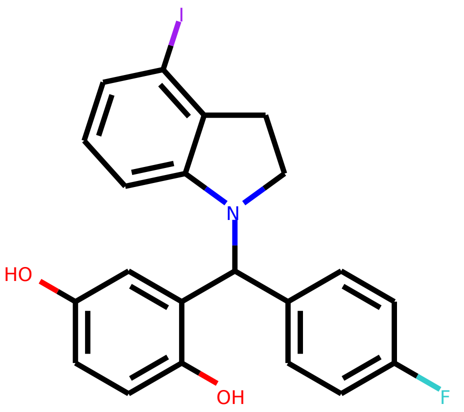
Ahma-831

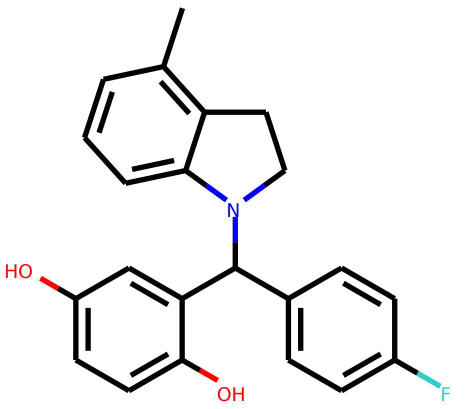
Ahma-832

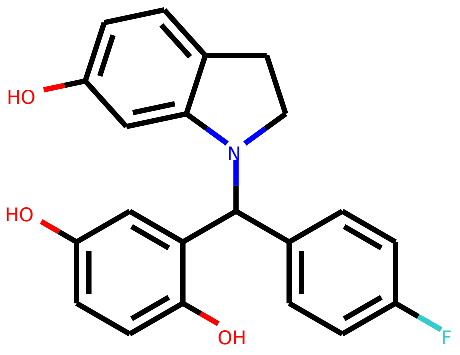
Ahma-833

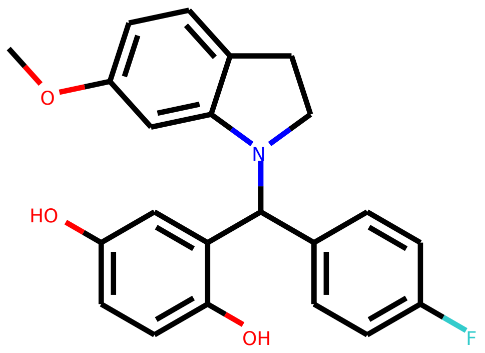
Ahma-834

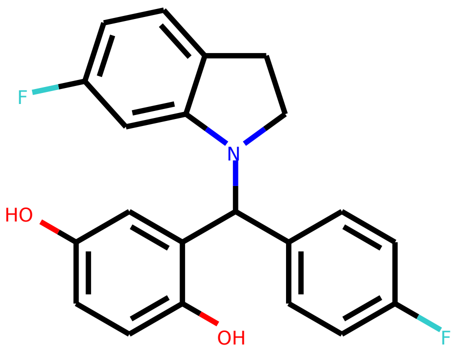
Ahma-835

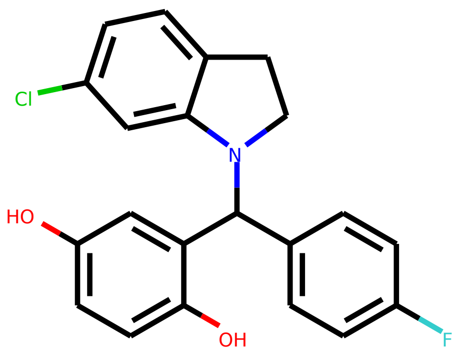
Ahma-836

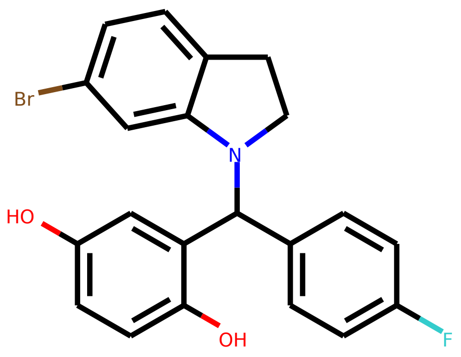
Ahma-837

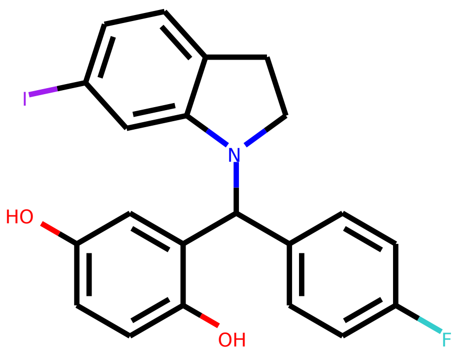
Ahma-838

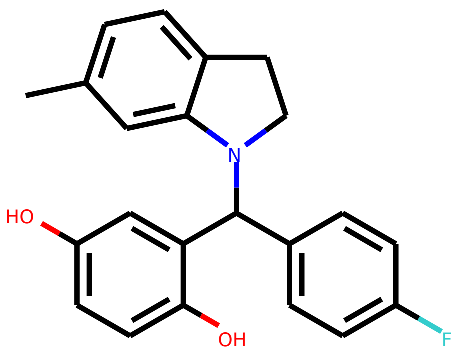
Ahma-839

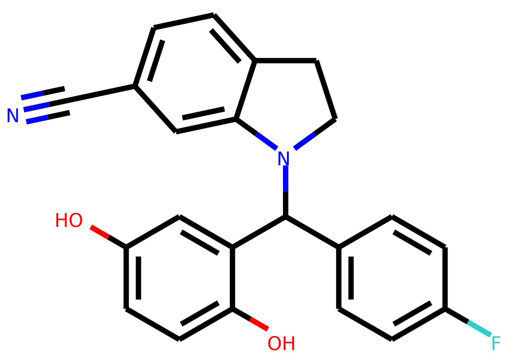
Ahma-840

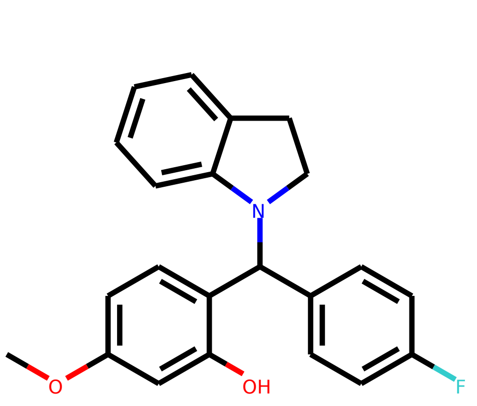
Ahma-841
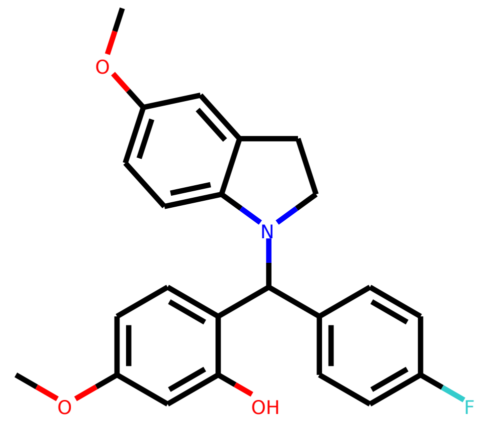
Ahma-842
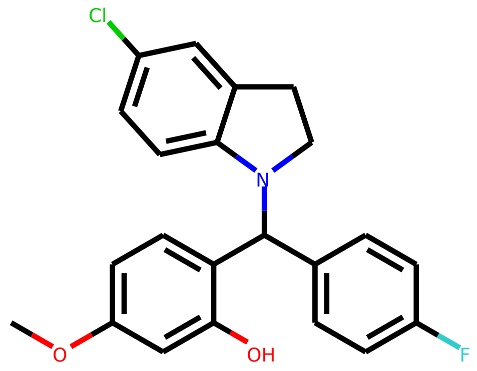
Ahma-843
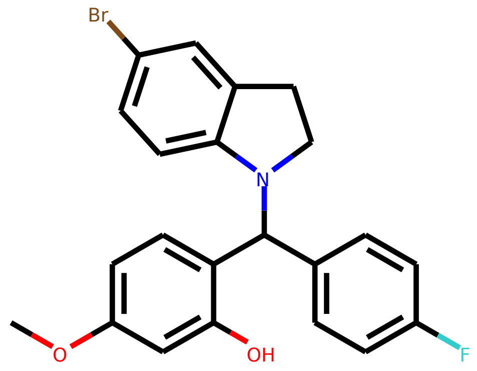
Ahma-844
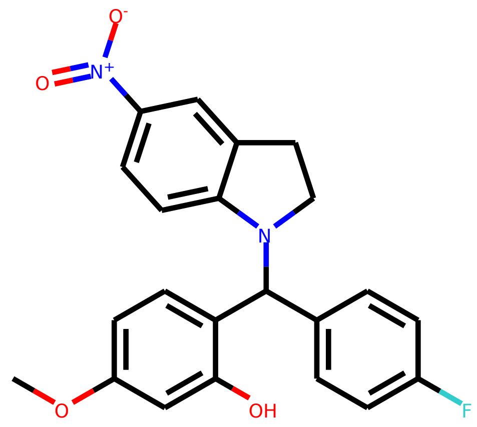
Ahma-845
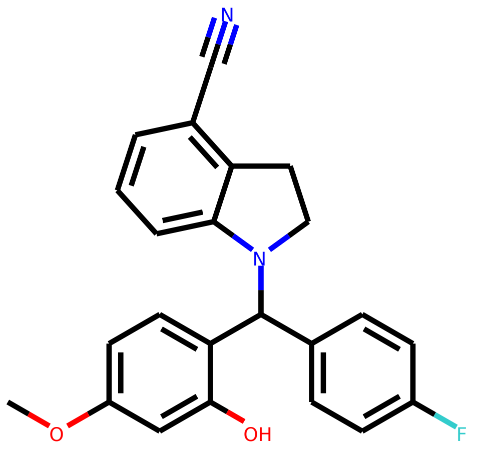
Ahma-846
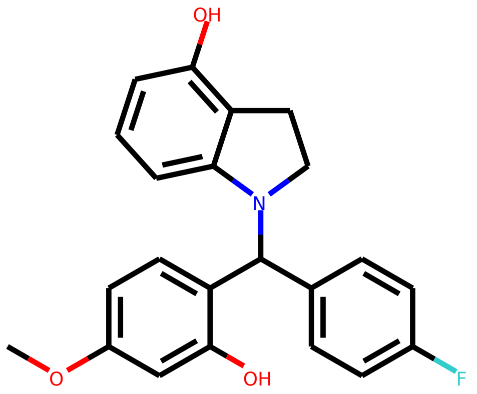
Ahma-847
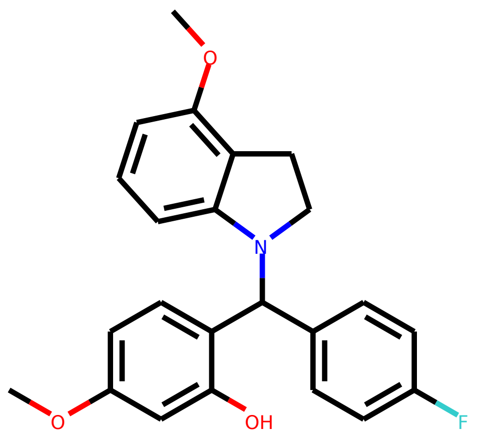
Ahma-848
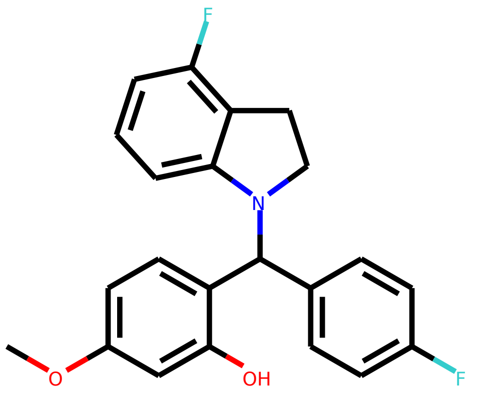
Ahma-849
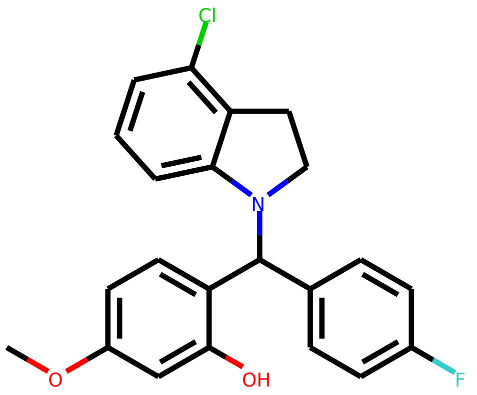
Ahma-850
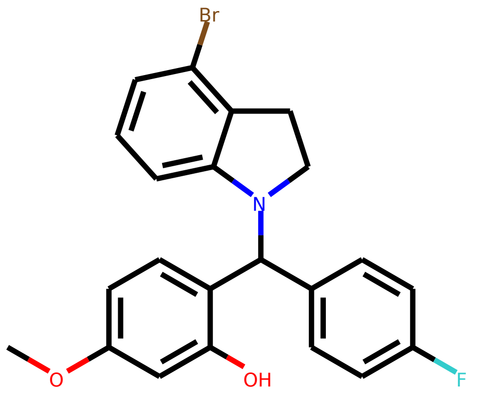
Ahma-851
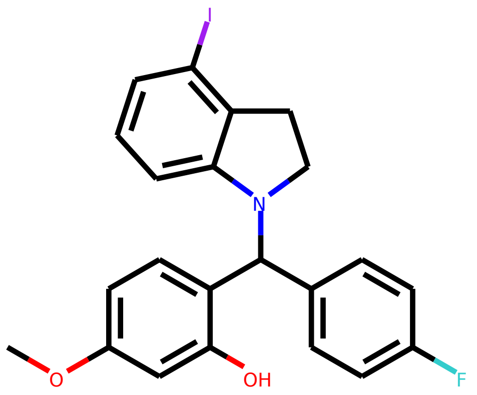
Ahma-852
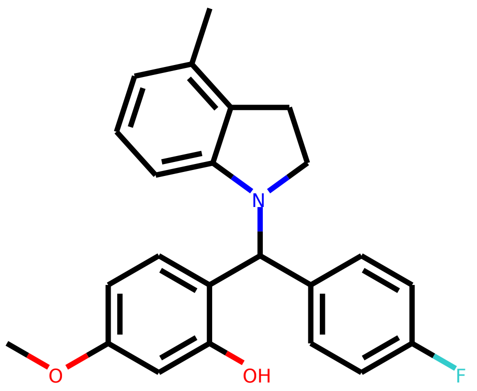
Ahma-853
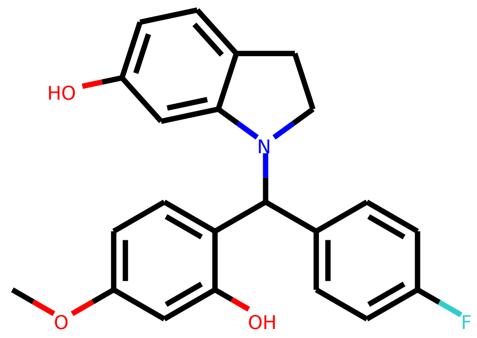
Ahma-854
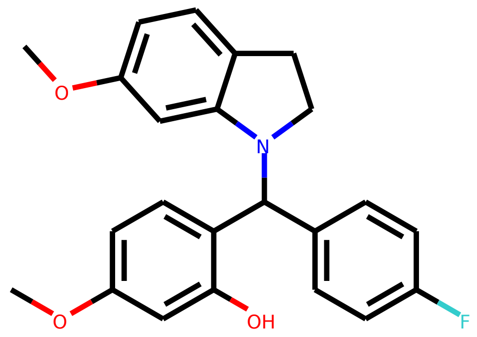
Ahma-855
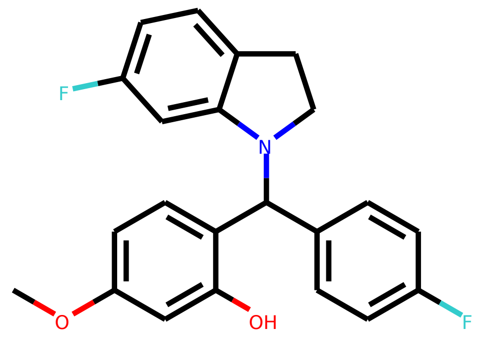
Ahma-856
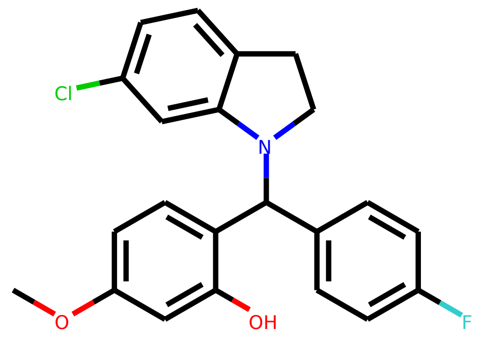
Ahma-857
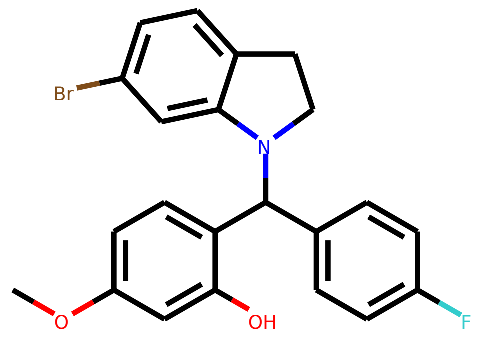
Ahma-858
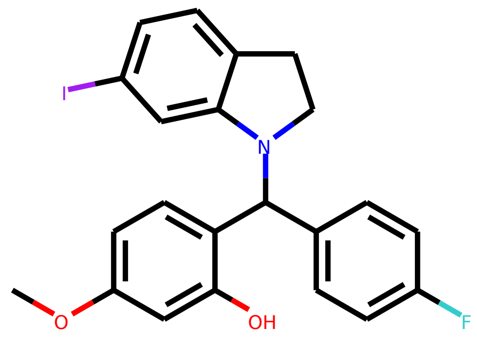
Ahma-859
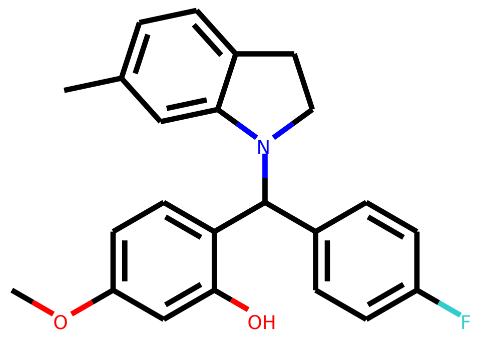
Ahma-860
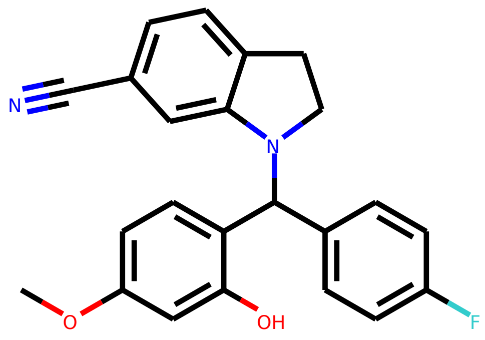
Ahma-861
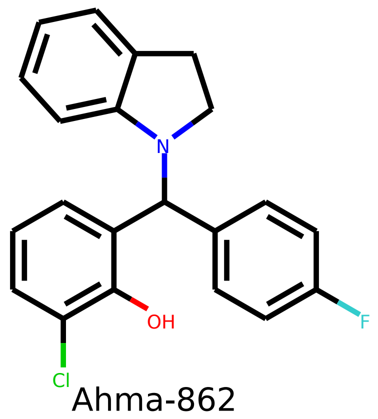
Ahma-862
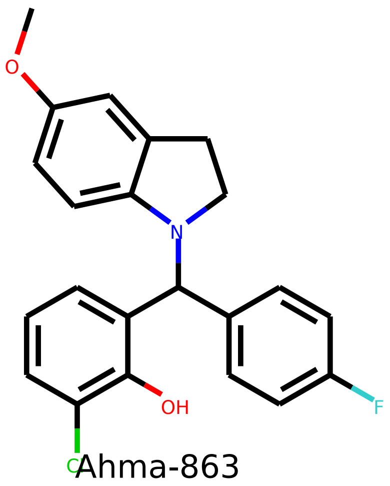
Ahma-863
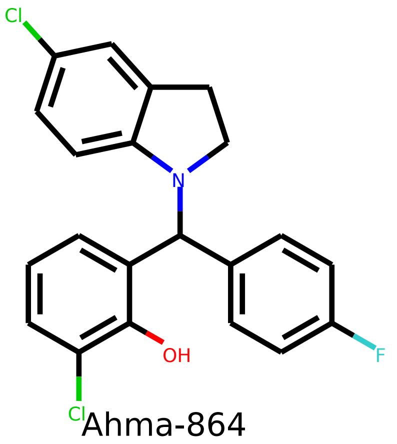
Ahma-864
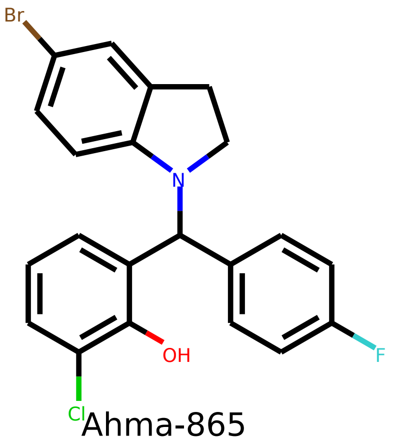
Ahma-865
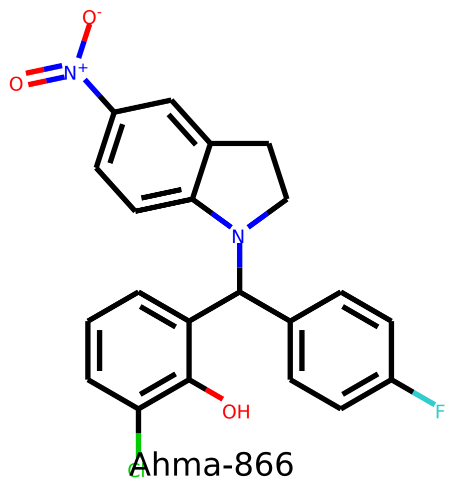
Ahma-866
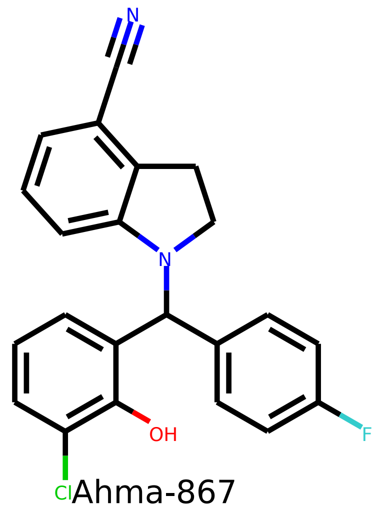
Ahma-867
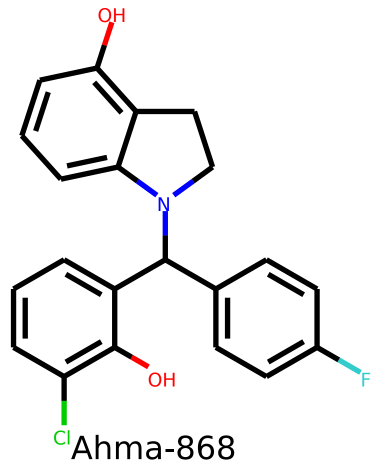
Ahma-868
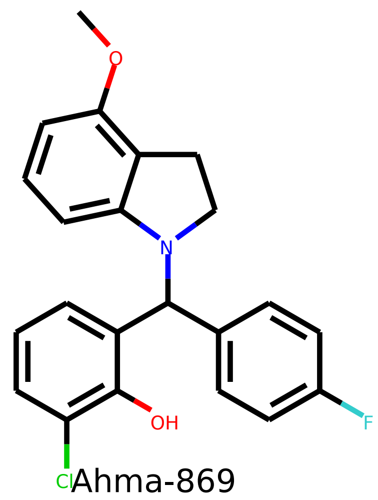
Ahma-869
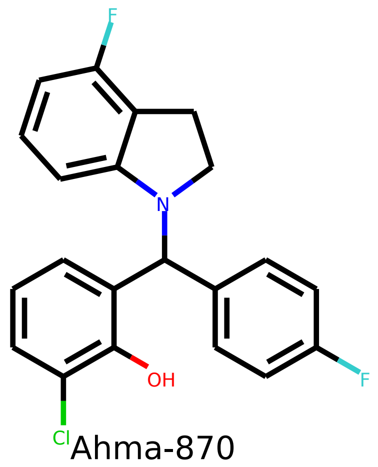
Ahma-870
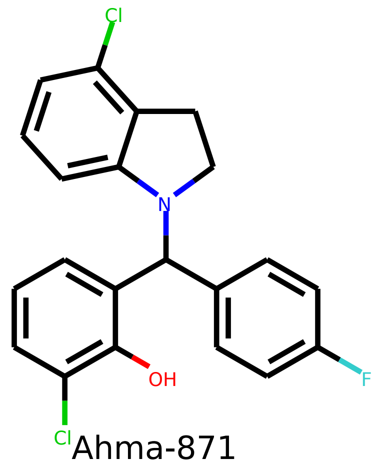
Ahma-871
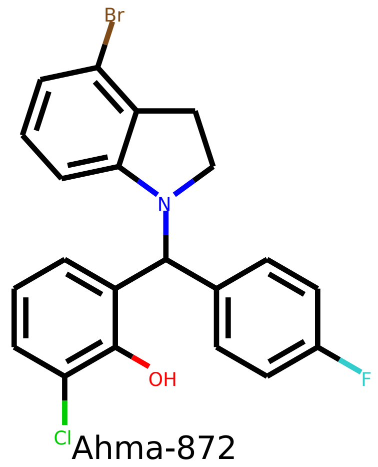
Ahma-872
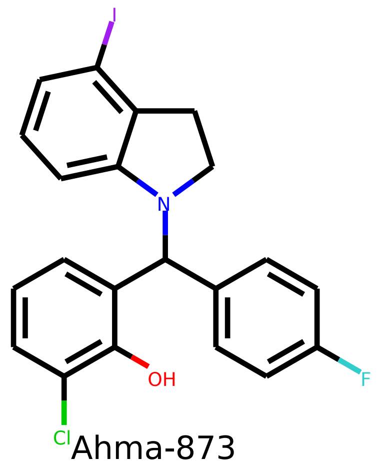
Ahma-873
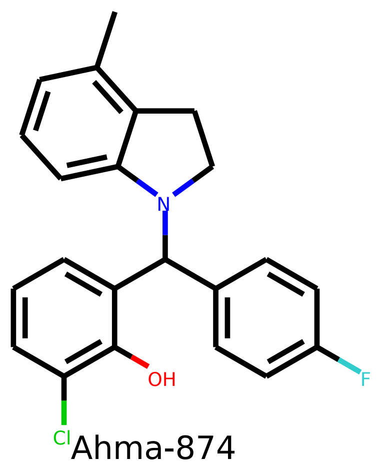
Ahma-874
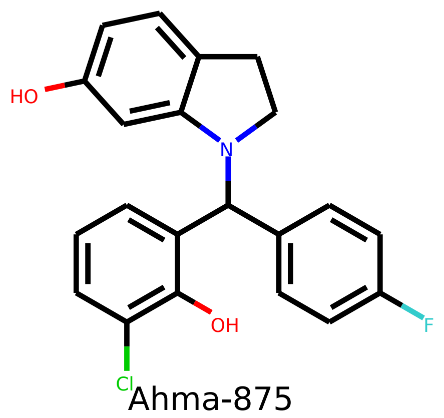
Ahma-875

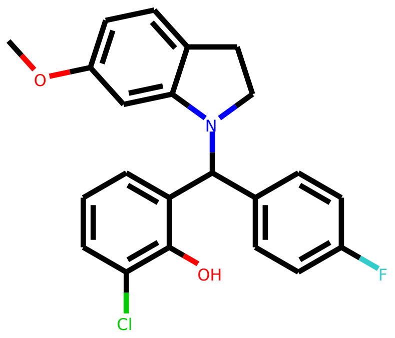
Ahma-876
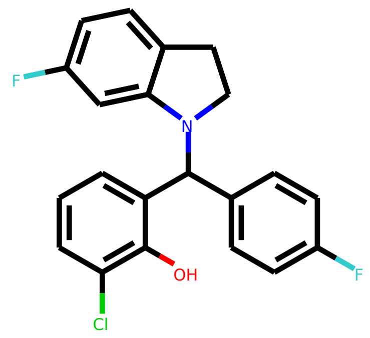
Ahma-877
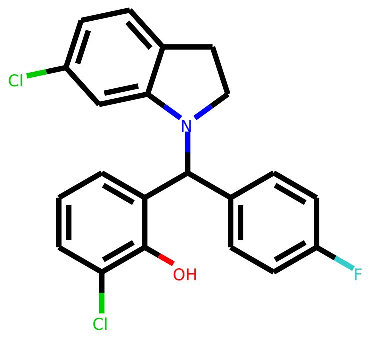
Ahma-878
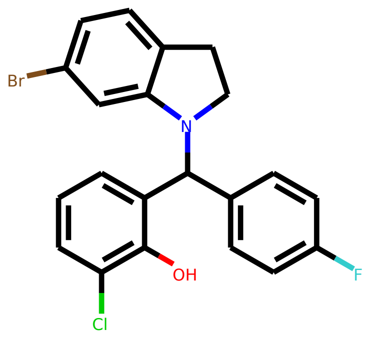
Ahma-879
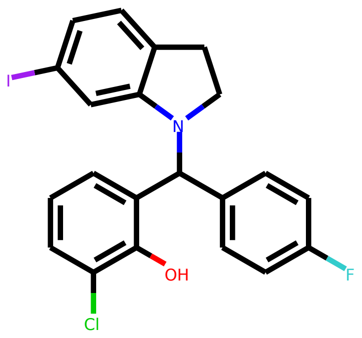
Ahma-880
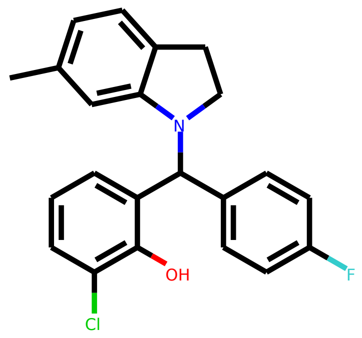
Ahma-881
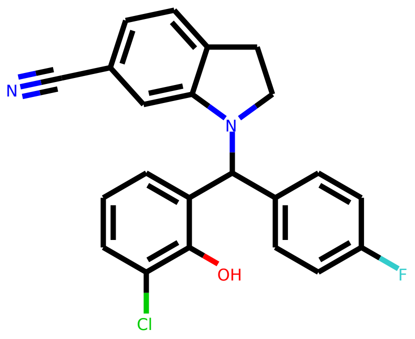
Ahma-882
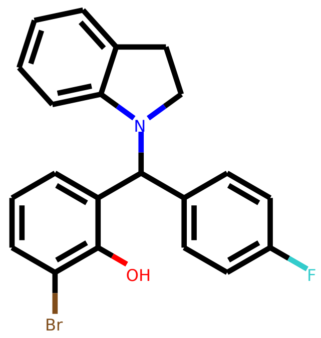
Ahma-883
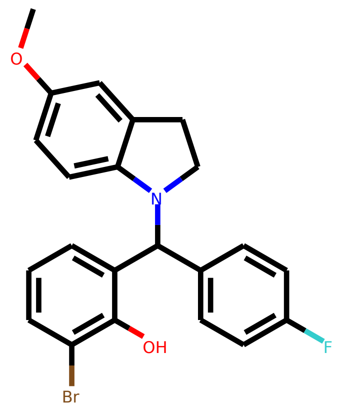
Ahma-884
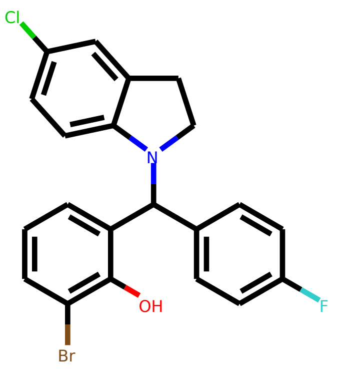
Ahma-885
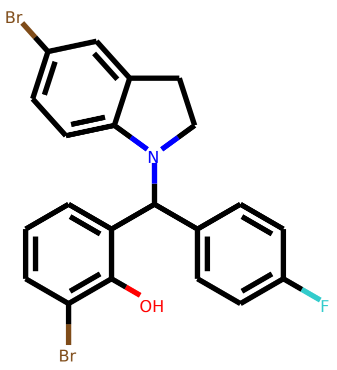
Ahma-886
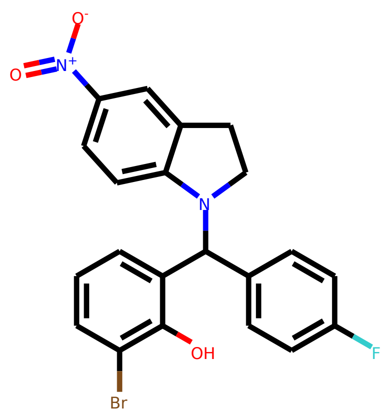
Ahma-887
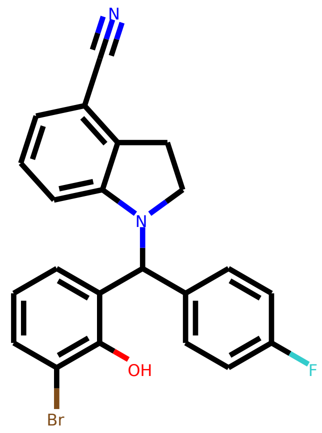
Ahma-888
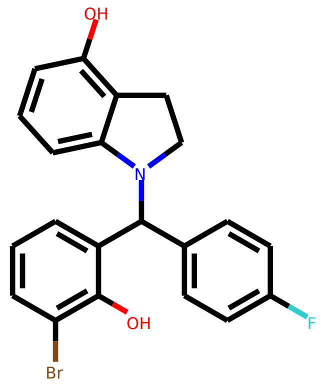
Ahma-889
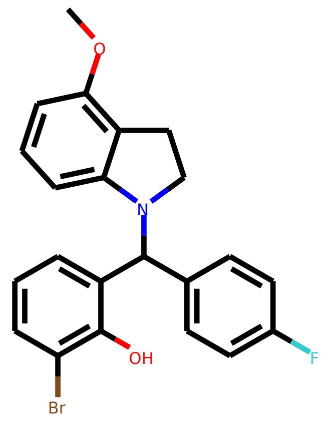
Ahma-890
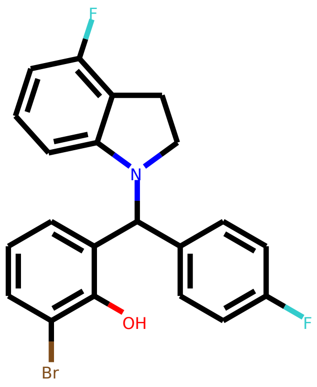
Ahma-891
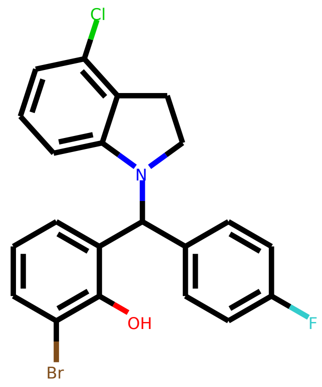
Ahma-892
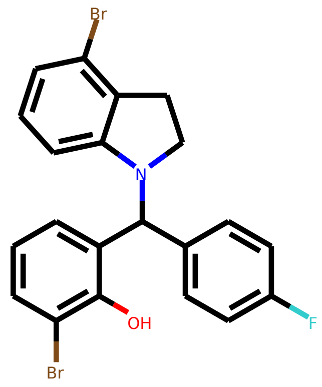
Ahma-893
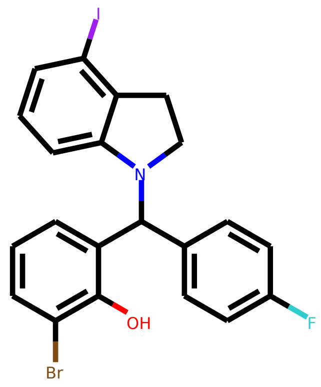
Ahma-894
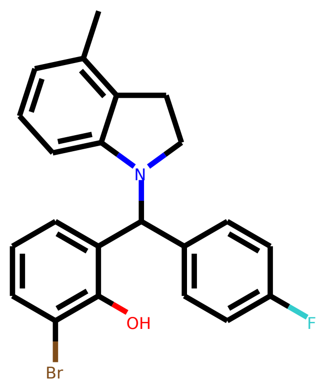
Ahma-895
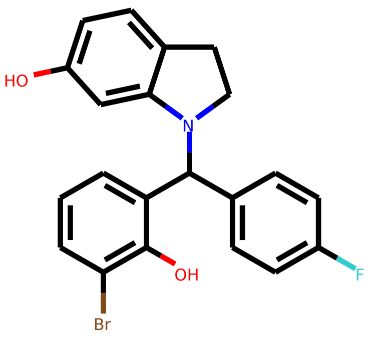
Ahma-896
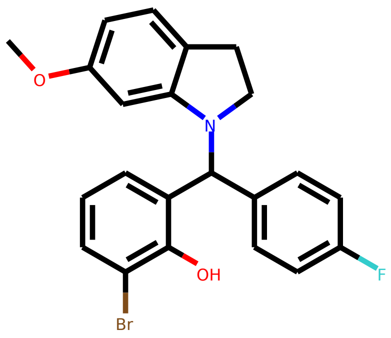
Ahma-897
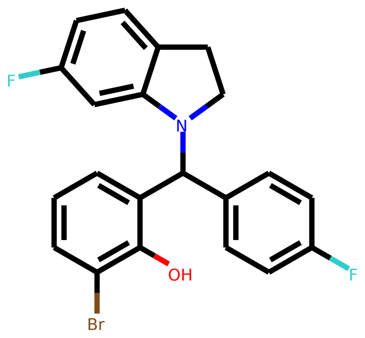
Ahma-898
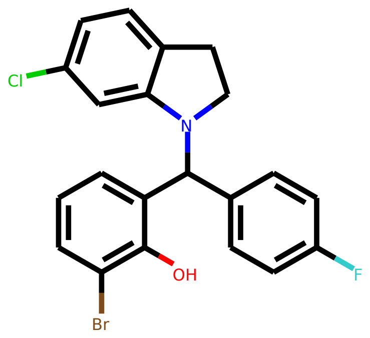
Ahma-899
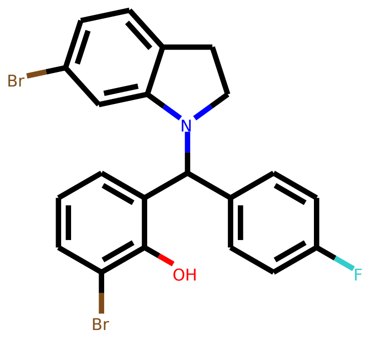
Ahma-900
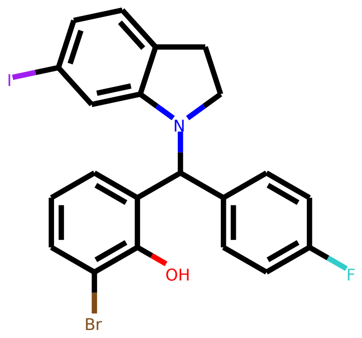
Ahma-901
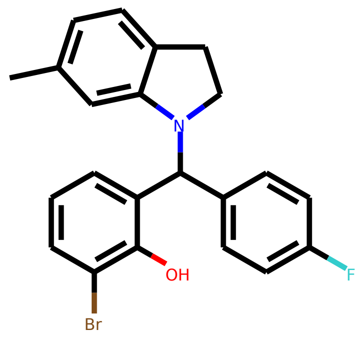
Ahma-902
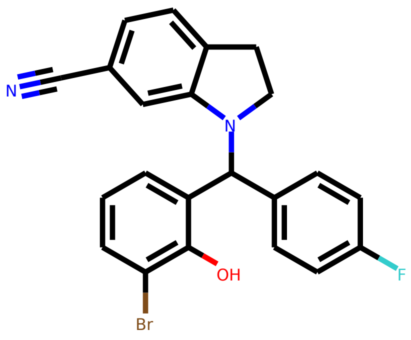
Ahma-903
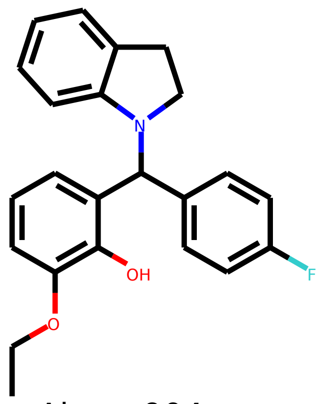
Ahma-904
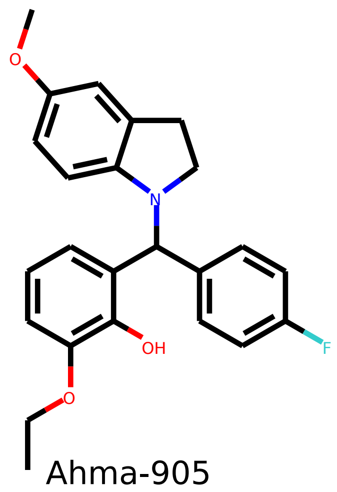
Ahma-905
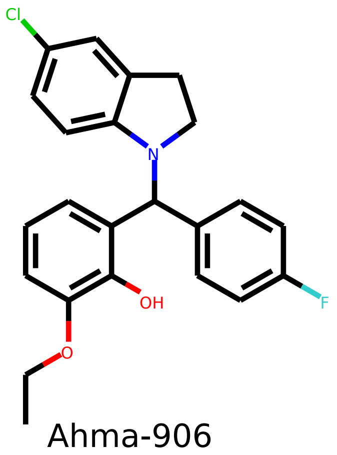
Ahma-906
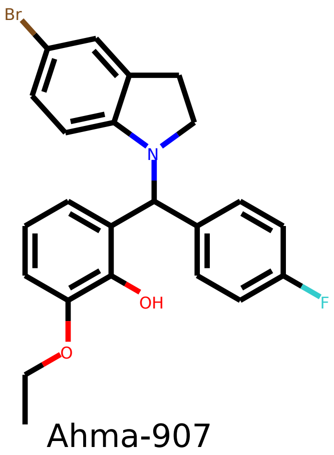
Ahma-907
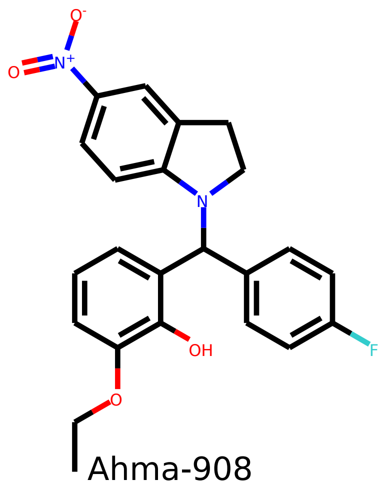
Ahma-908
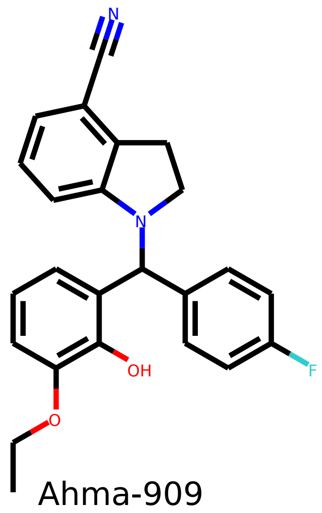
Ahma-909
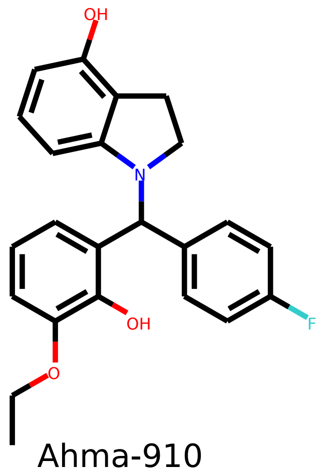
Ahma-910

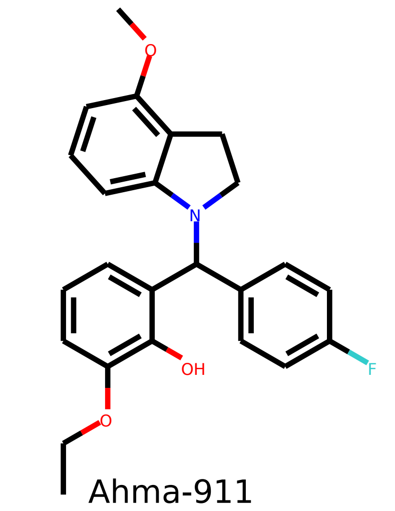
Ahma-911
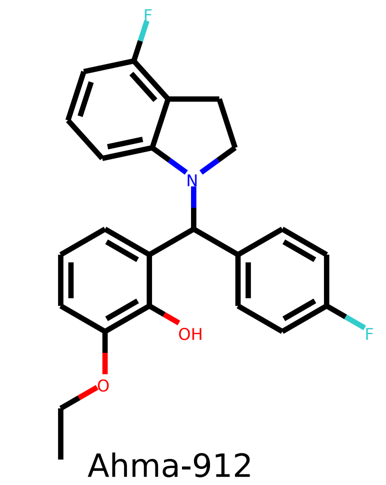
Ahma-912
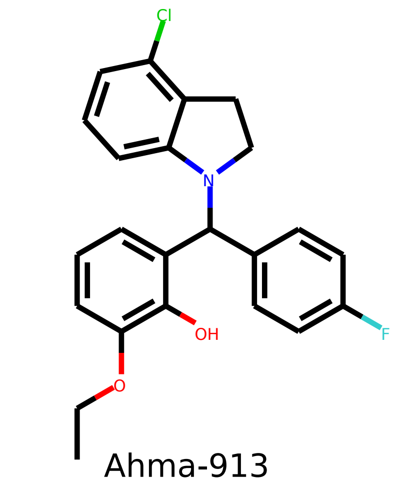
Ahma-913
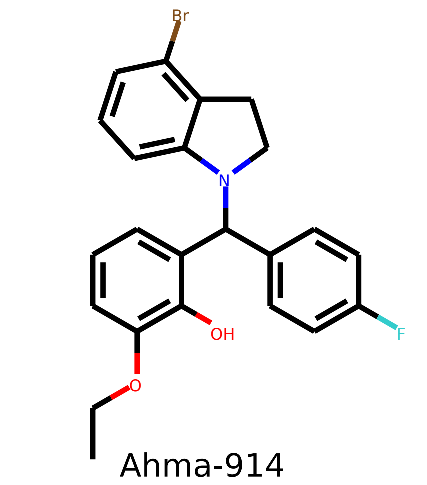
Ahma-914
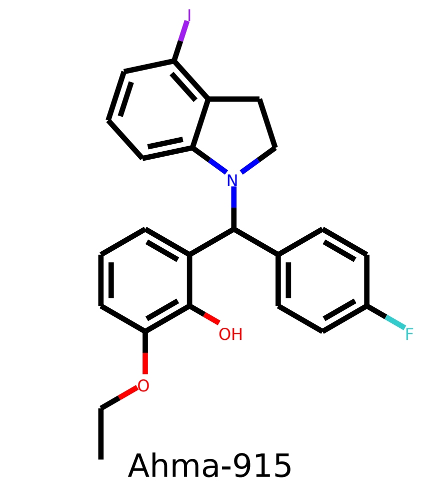
Ahma-915
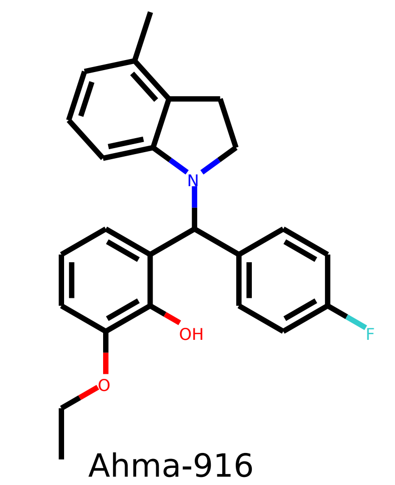
Ahma-916
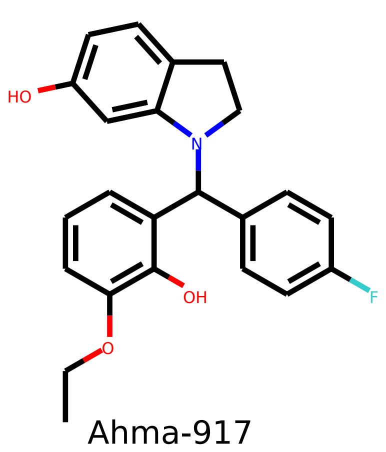
Ahma-917
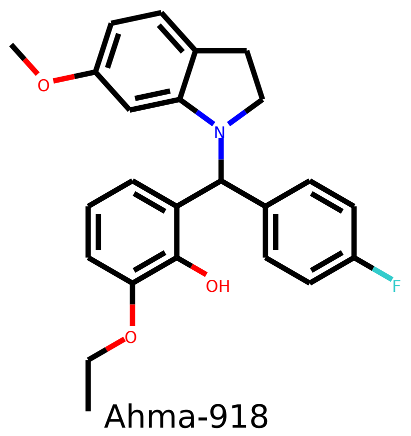
Ahma-918
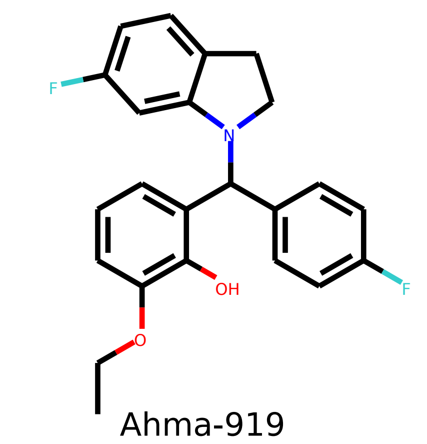
Ahma-919
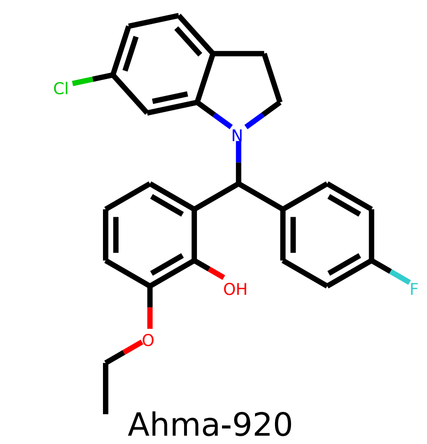
Ahma-920
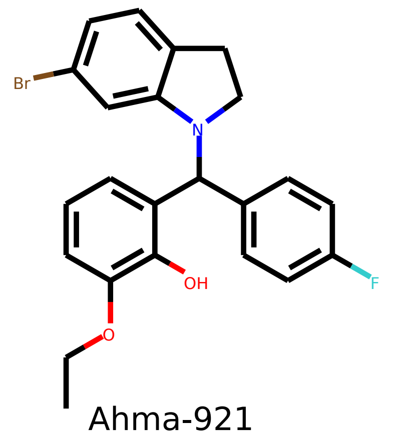
Ahma-921
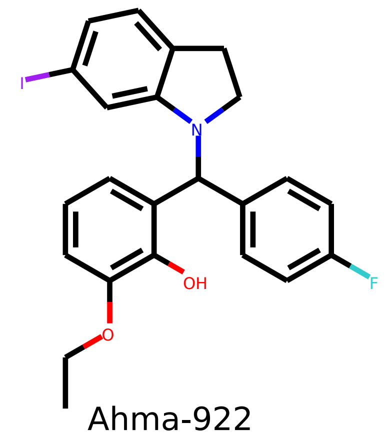
Ahma-922
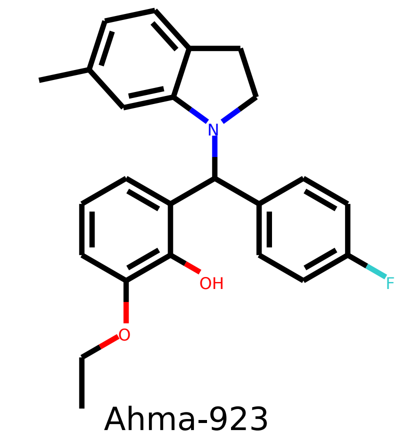
Ahma-923
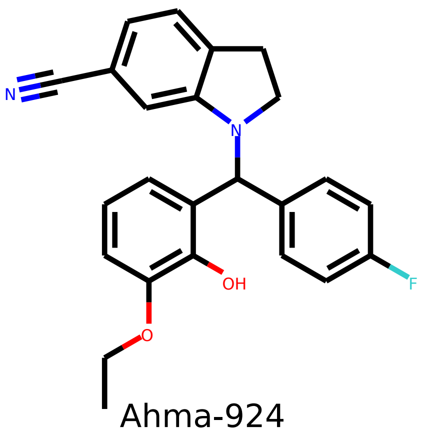
Ahma-924
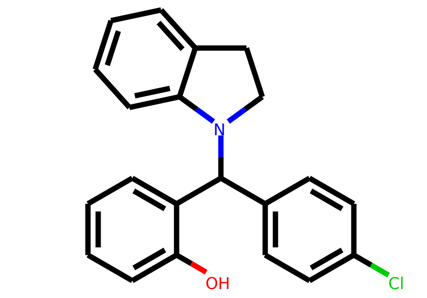
Ahma-925
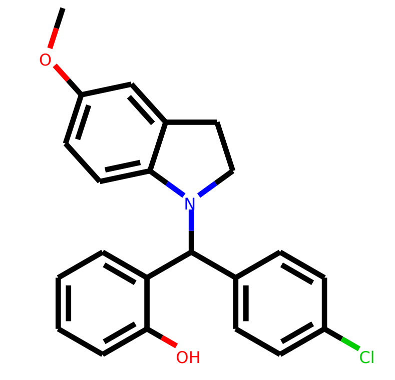
Ahma-926
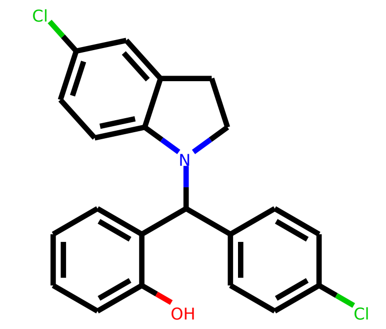
Ahma-927
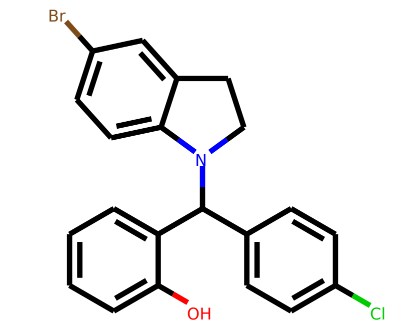
Ahma-928
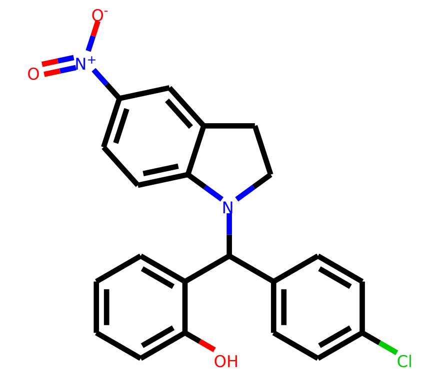
Ahma-929
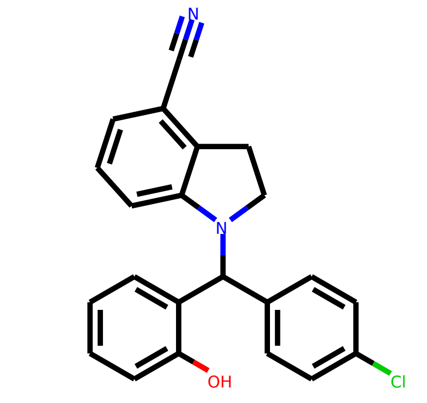
Ahma-930
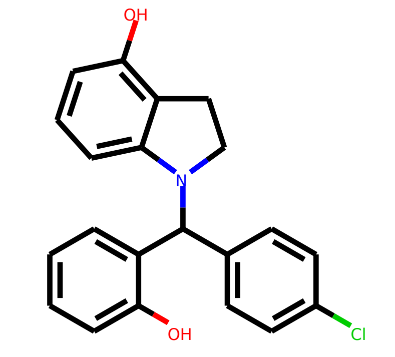
Ahma-931
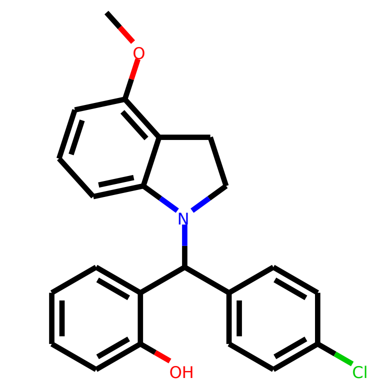
Ahma-932
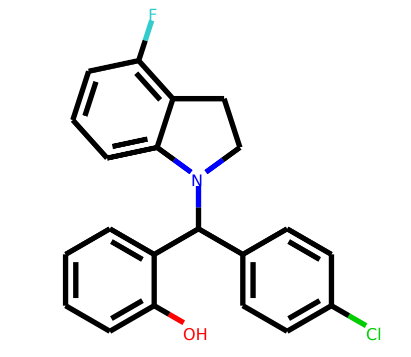
Ahma-933
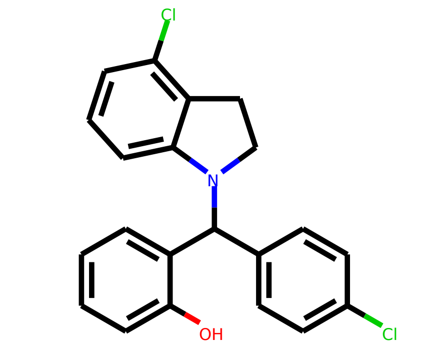
Ahma-934
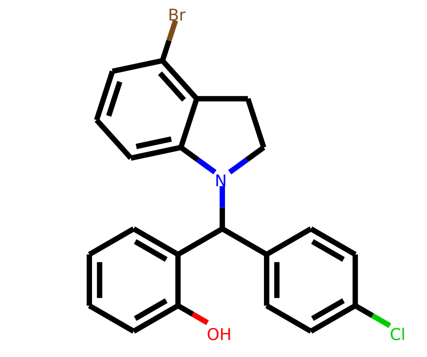
Ahma-935
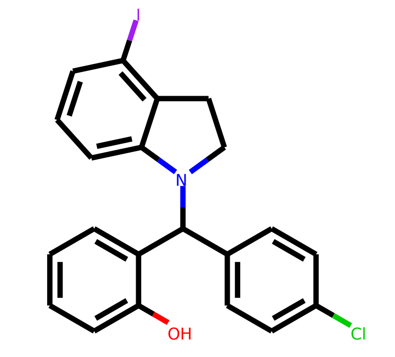
Ahma-936
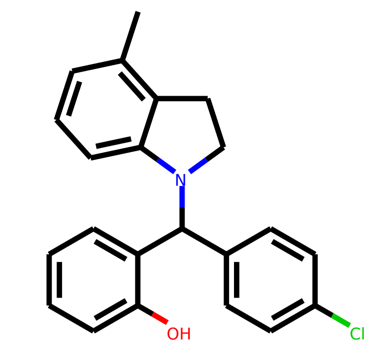
Ahma-937
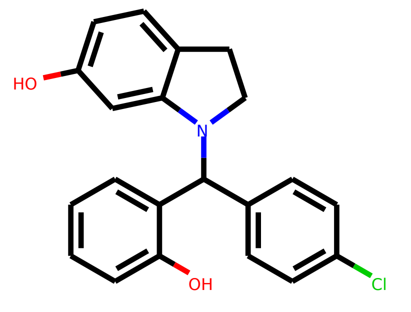
Ahma-938
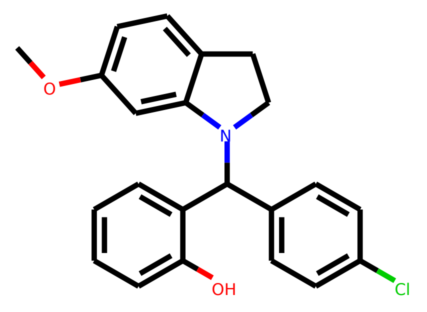
Ahma-939
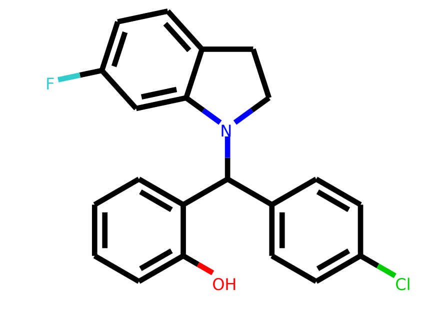
Ahma-940
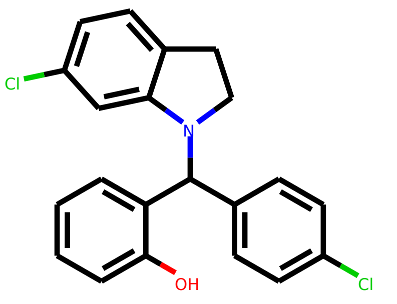
Ahma-941
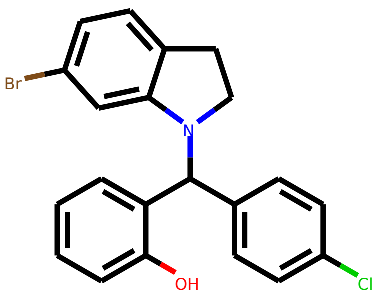
Ahma-942
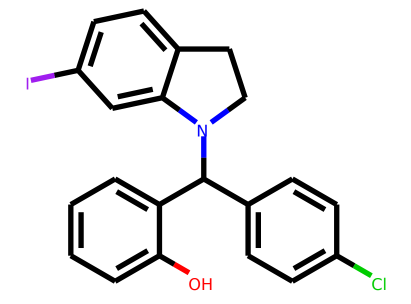
Ahma-943
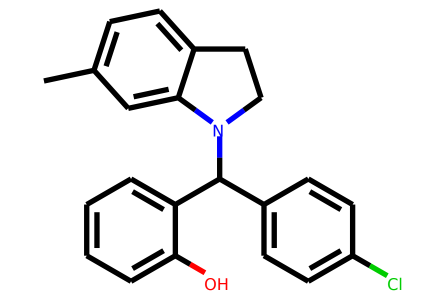
Ahma-944
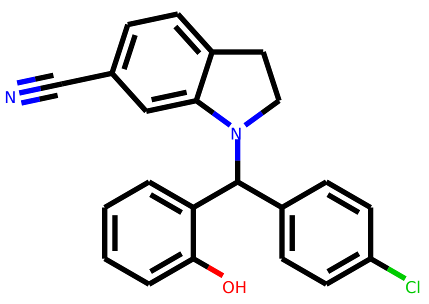
Ahma-945

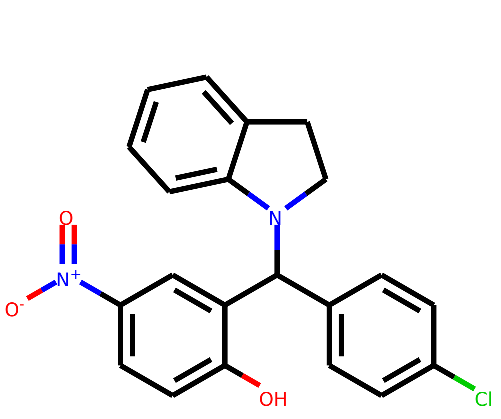
Ahma-946
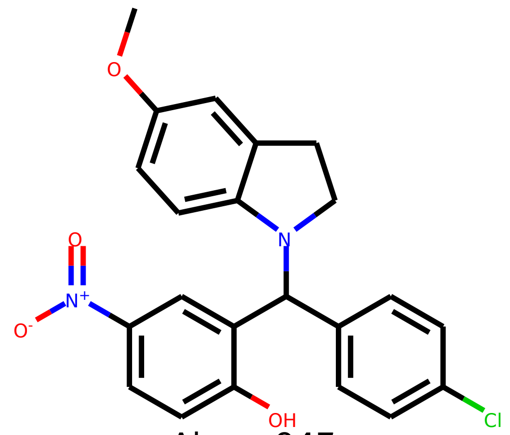
Ahma-947
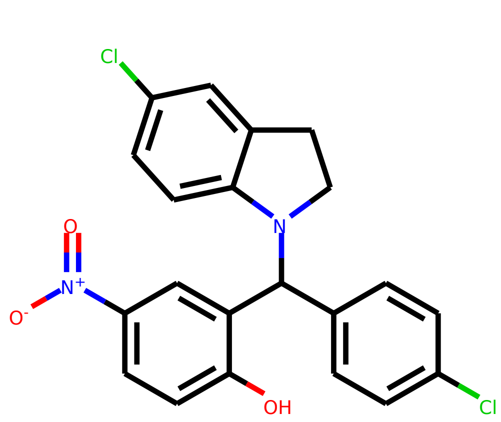
Ahma-948
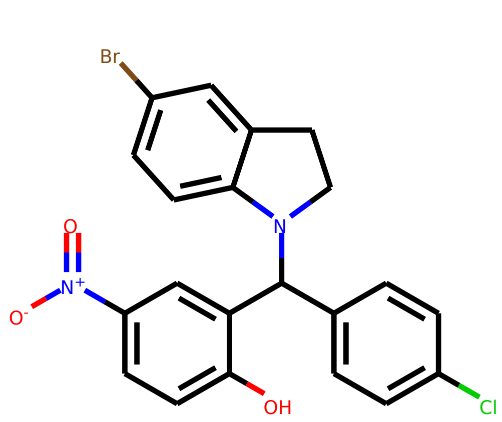
Ahma-949
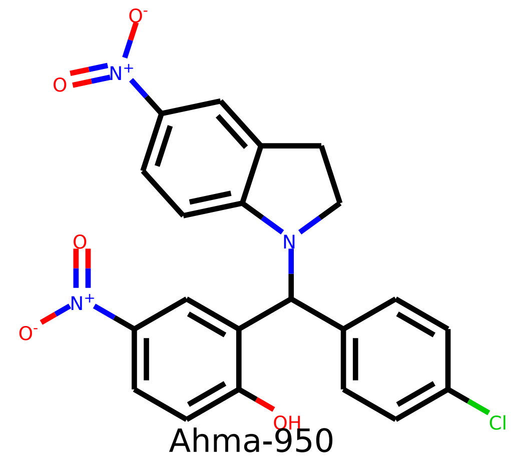
Ahma-950
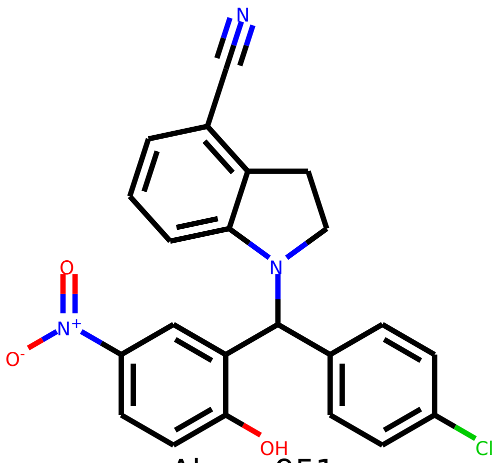
Ahma-951
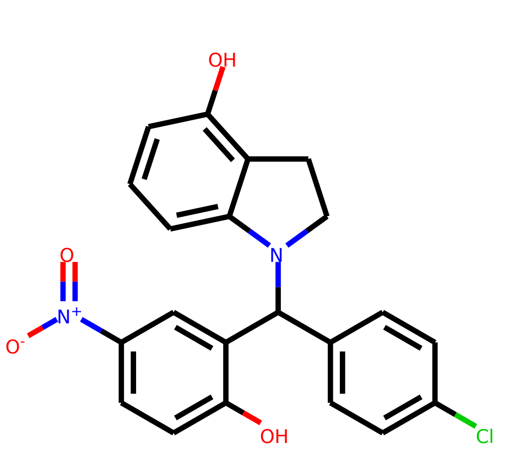
Ahma-952
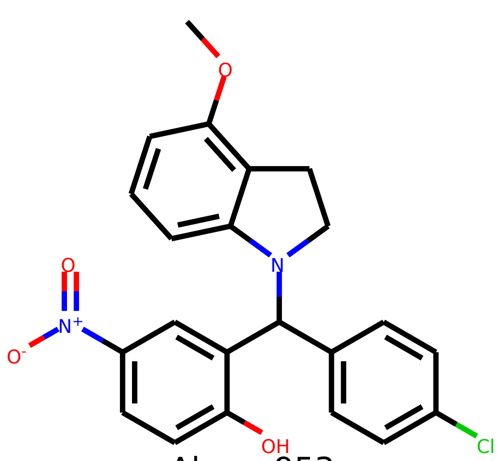
Ahma-953
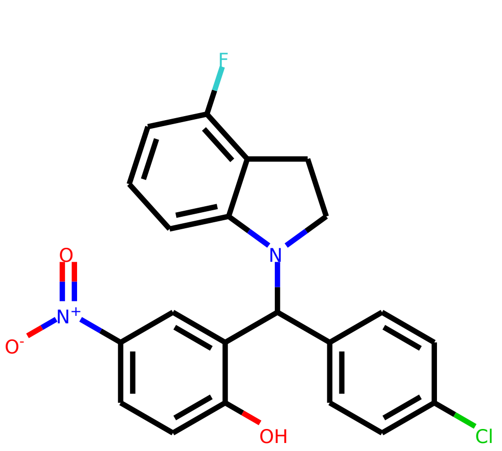
Ahma-954
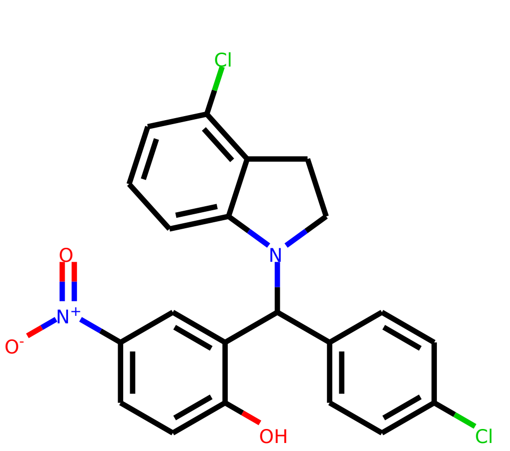
Ahma-955
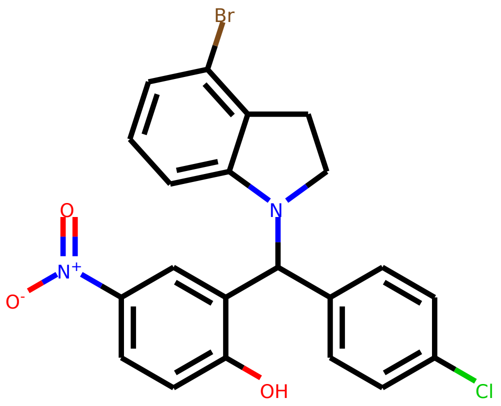
Ahma-956
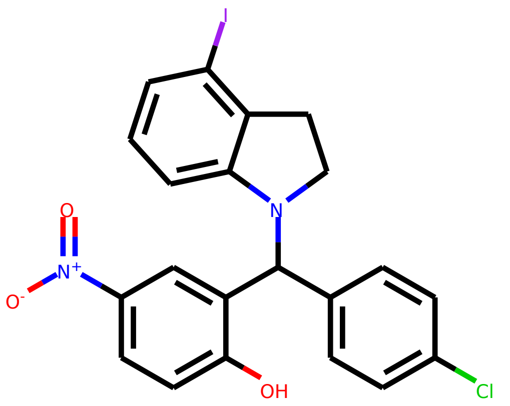
Ahma-957
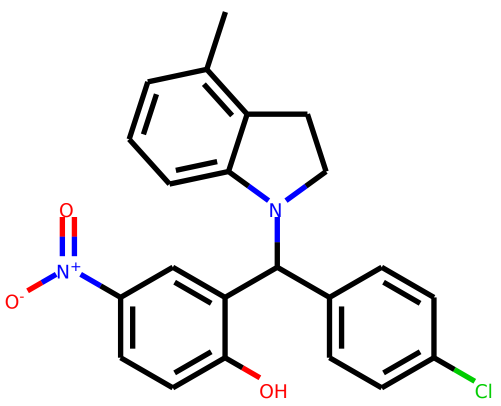
Ahma-958
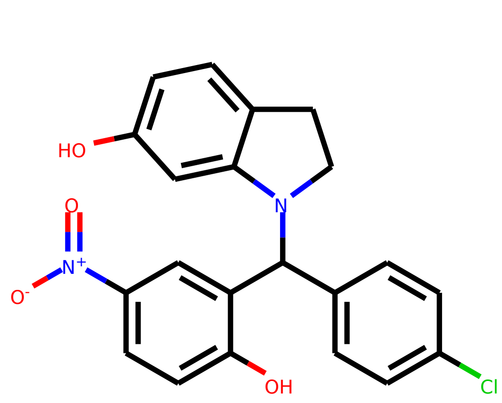
Ahma-959
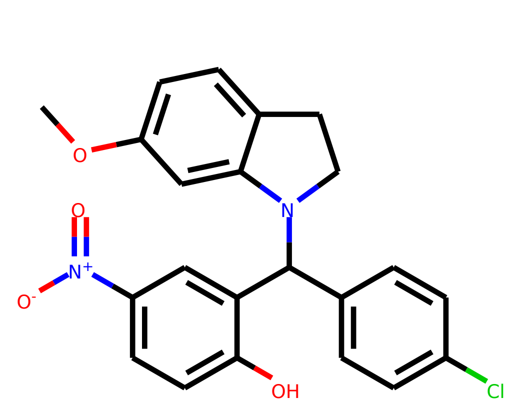
Ahma-960
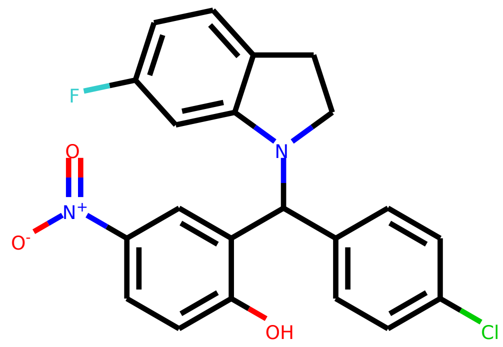
Ahma-961
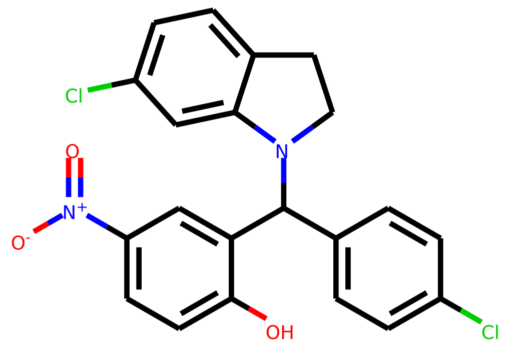
Ahma-962
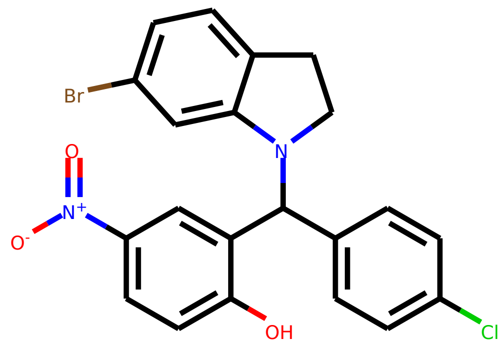
Ahma-963
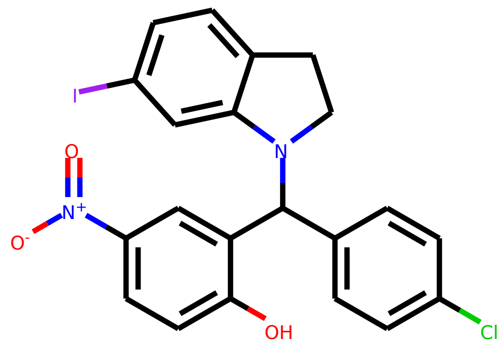
Ahma-964
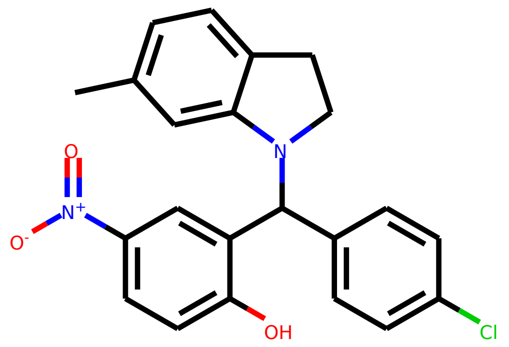
Ahma-965
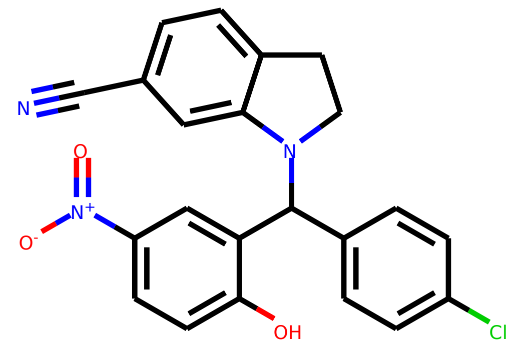
Ahma-966
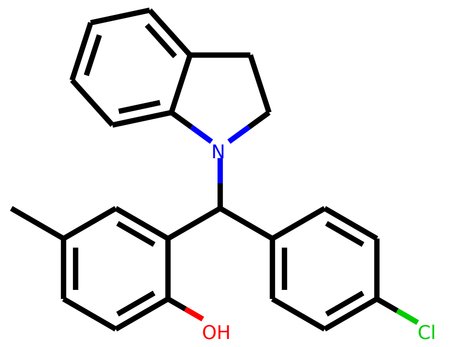
Ahma-967
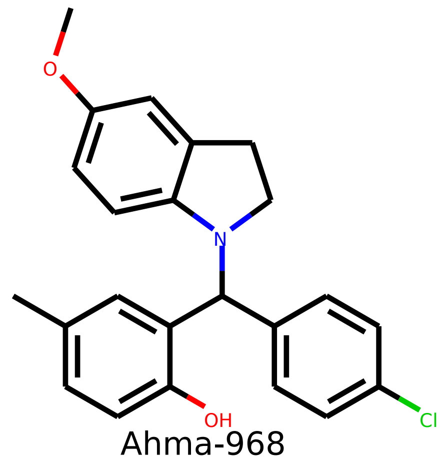
Ahma-968
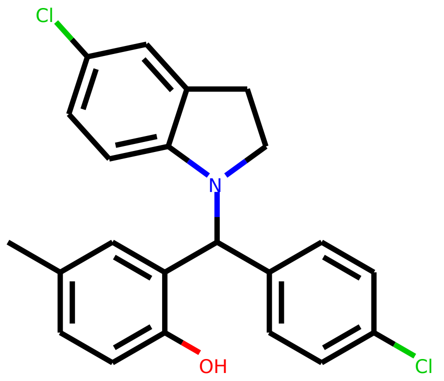
Ahma-969
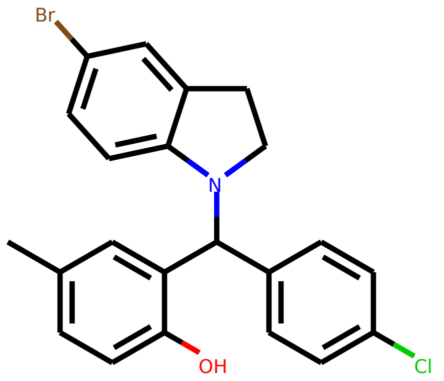
Ahma-970
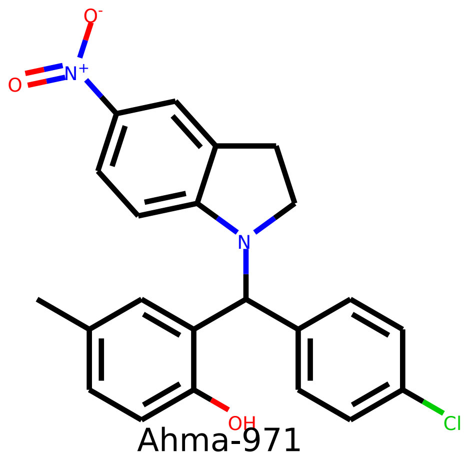
Ahma-971
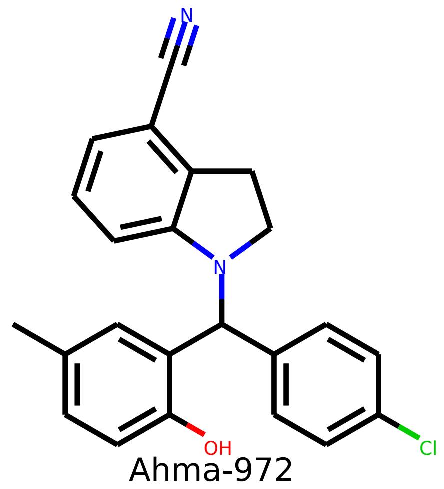
Ahma-972
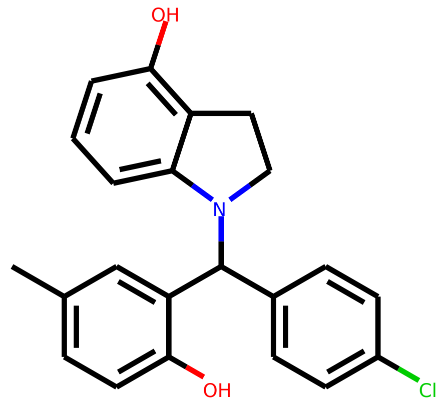
Ahma-973
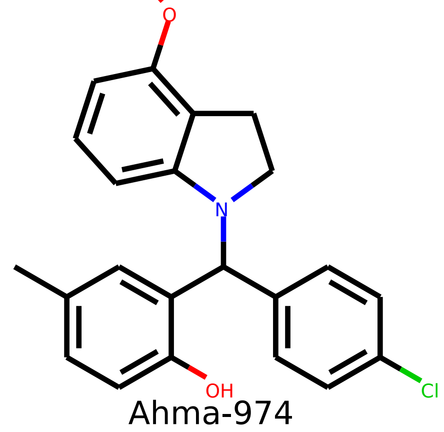
Ahma-974
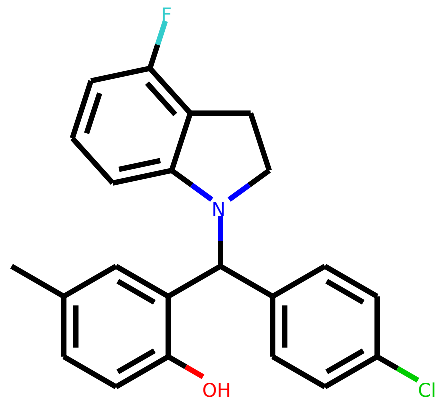
Ahma-975
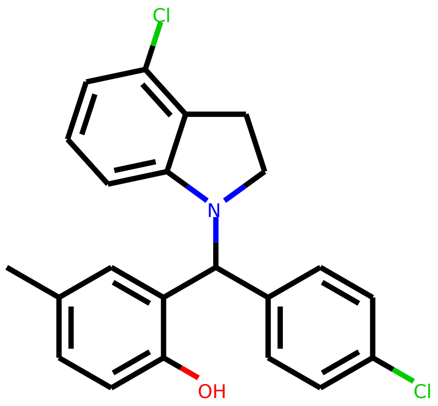
Ahma-976
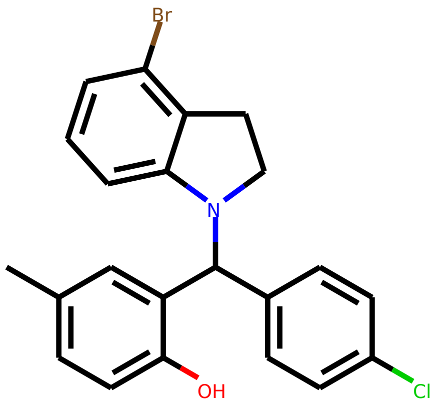
Ahma-977
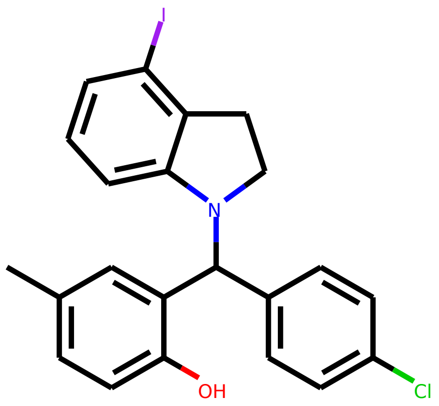
Ahma-978
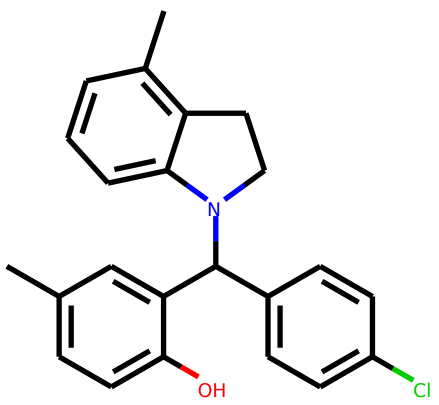
Ahma-979
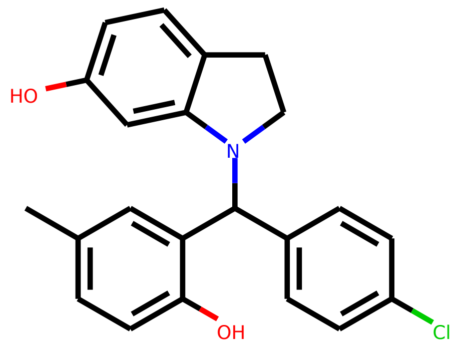
Ahma-980

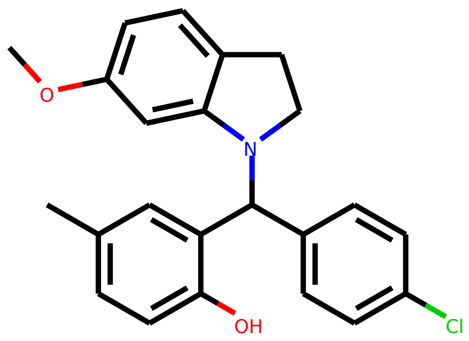
Ahma-981
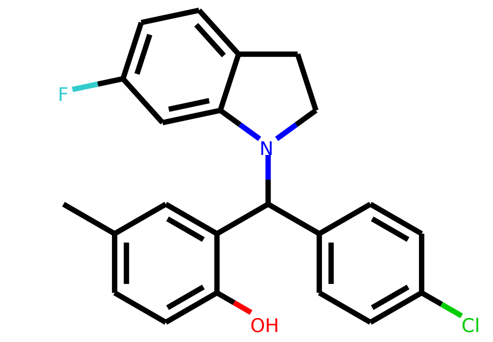
Ahma-982
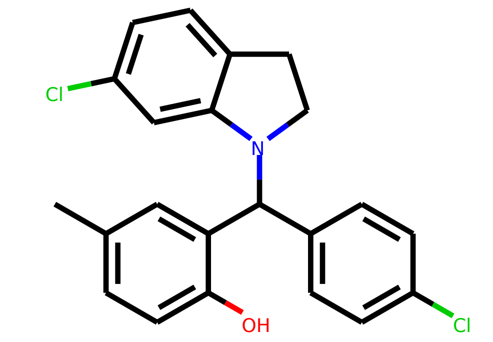
Ahma-983
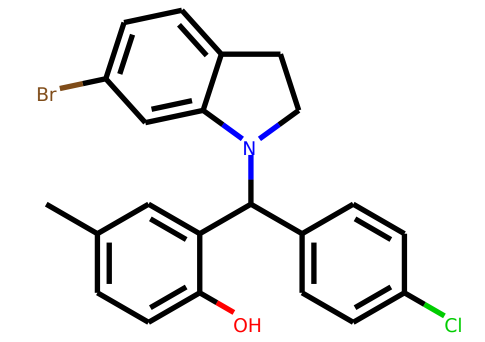
Ahma-984
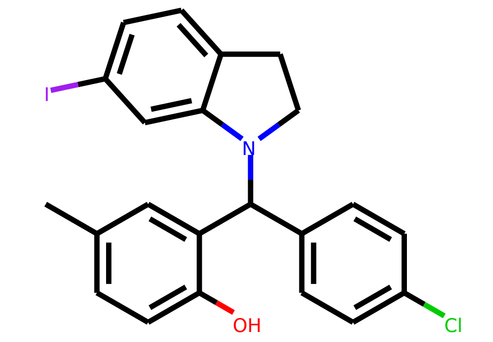
Ahma-985
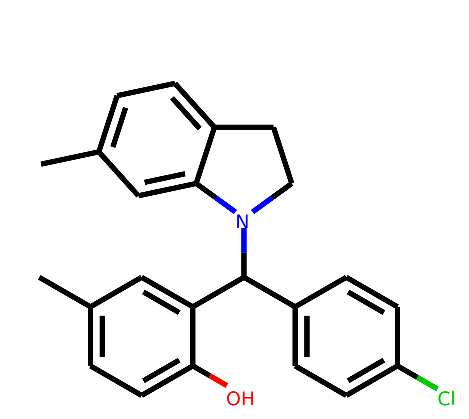
Ahma-986
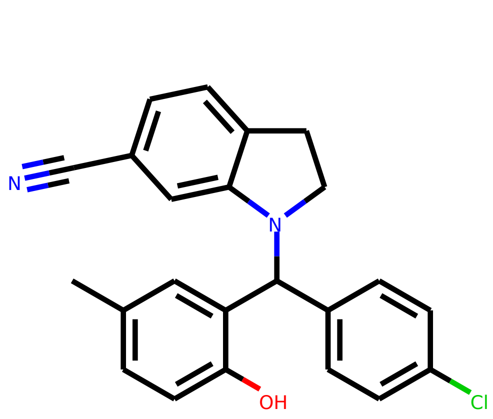
Ahma-987
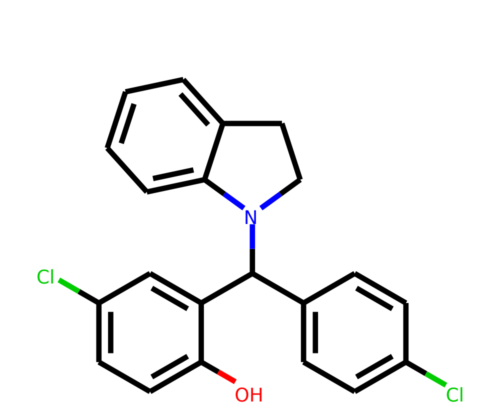
Ahma-988
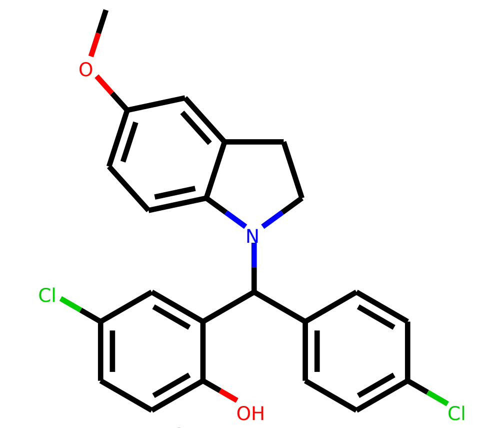
Ahma-989
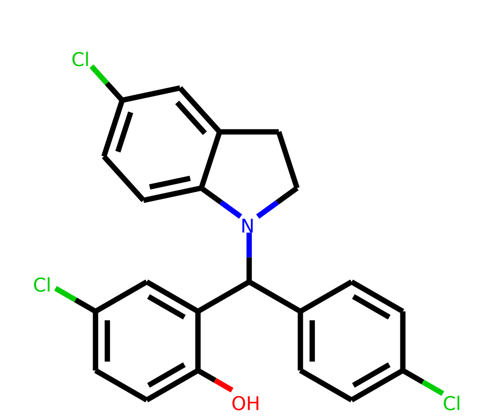
Ahma-990
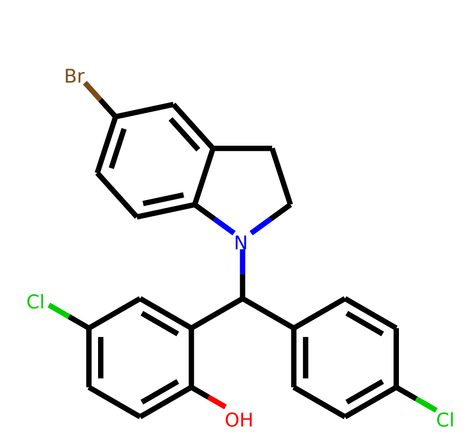
Ahma-991
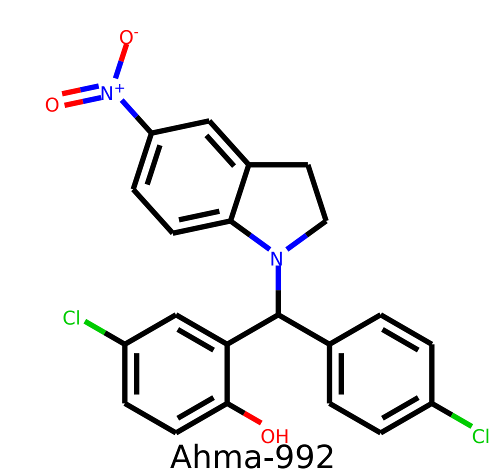
Ahma-992
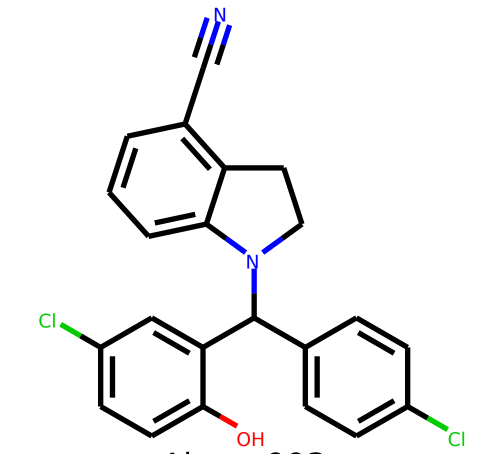
Ahma-993
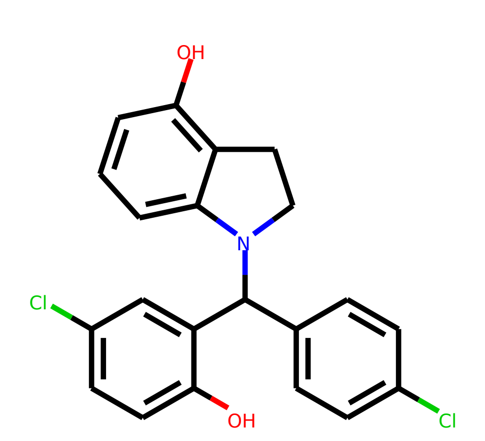
Ahma-994
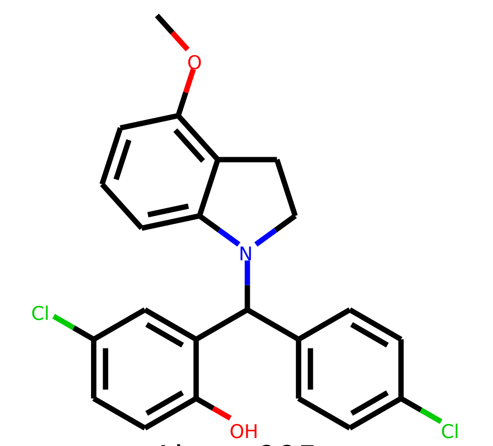
Ahma-995
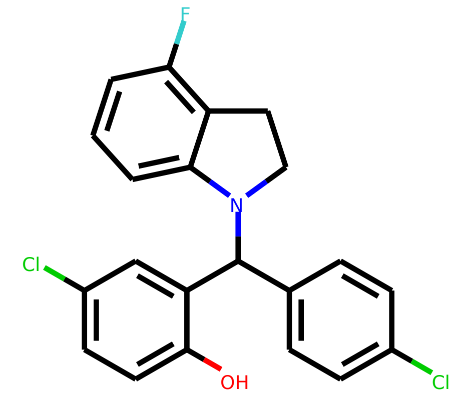
Ahma-996
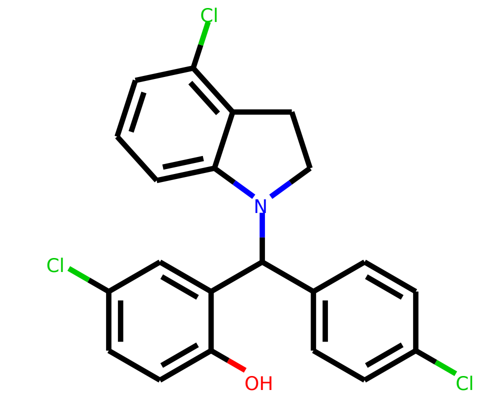
Ahma-997
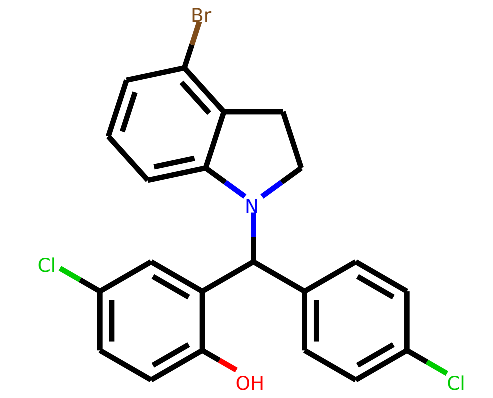
Ahma-998
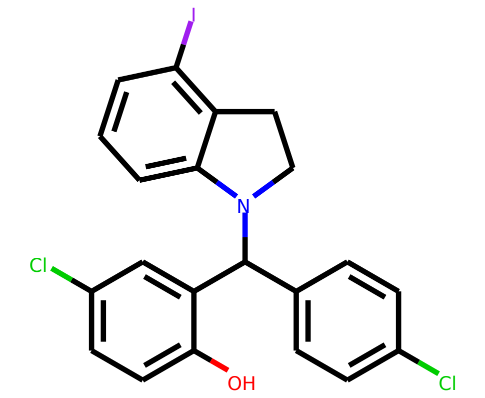
Ahma-999
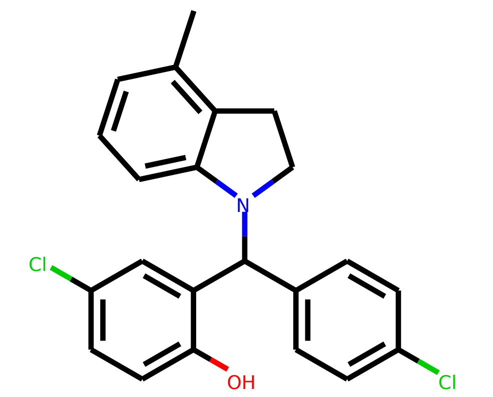
Ahma-1000
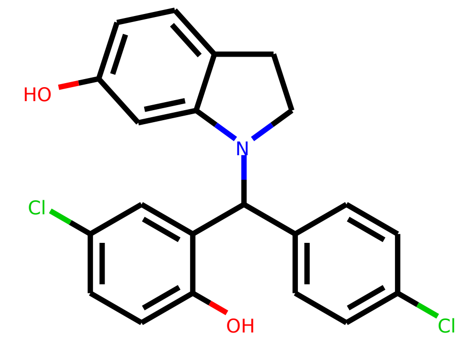
Ahma-1001
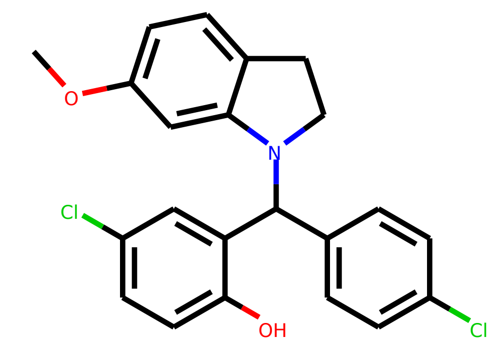
Ahma-1002
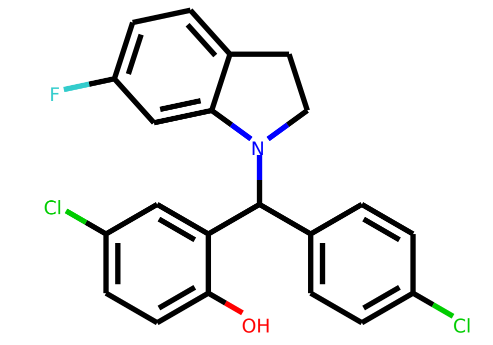
Ahma-1003
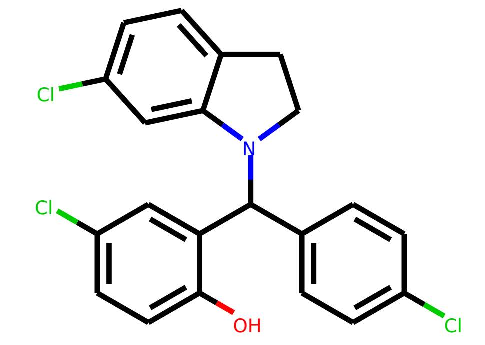
Ahma-1004
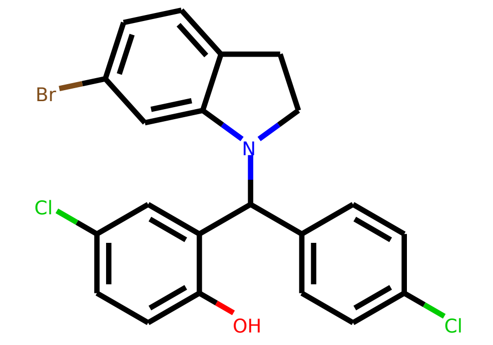
Ahma-1005
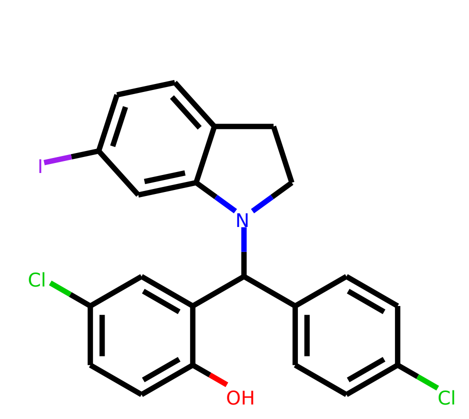
Ahma-1006
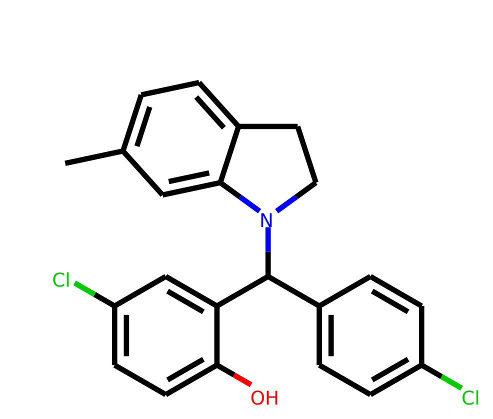
Ahma-1007
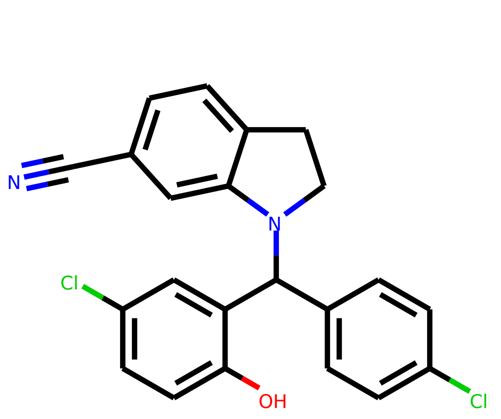
Ahma-1008
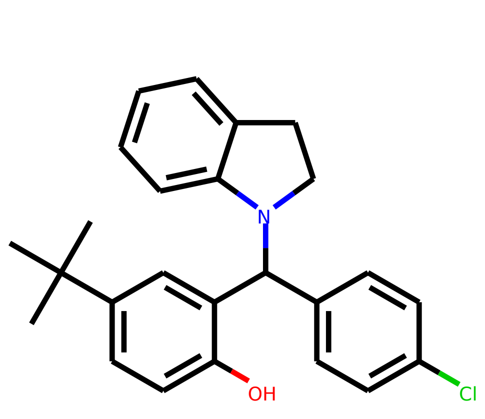
Ahma-1009
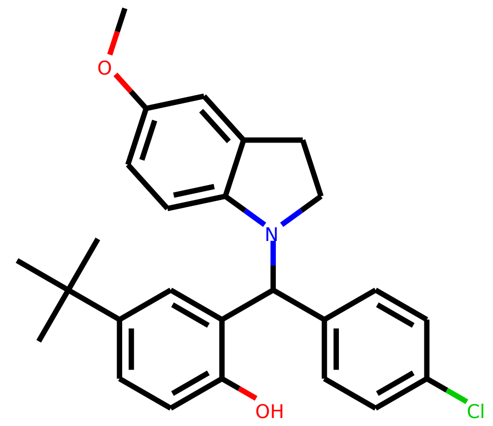
Ahma-1010
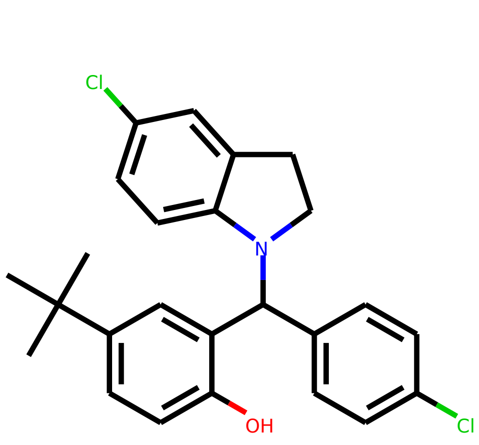
Ahma-1011
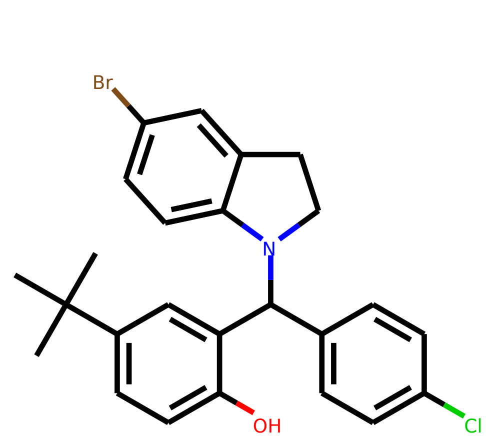
Ahma-1012
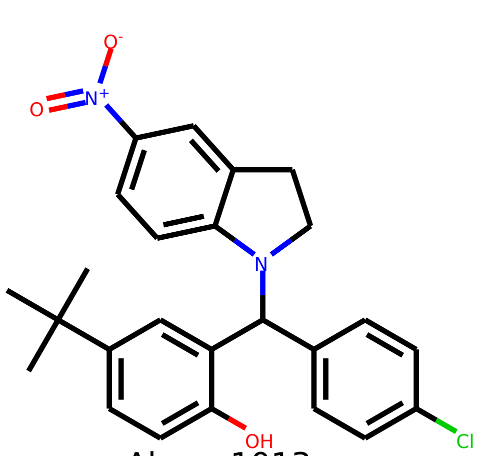
Ahma-1013
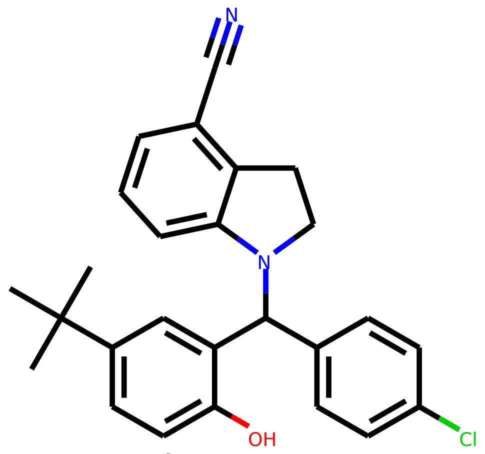
Ahma-1014
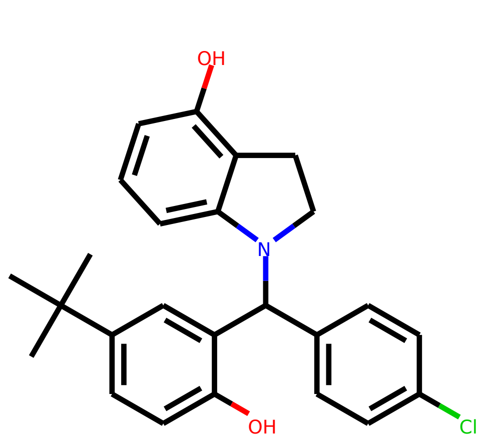
Ahma-1015

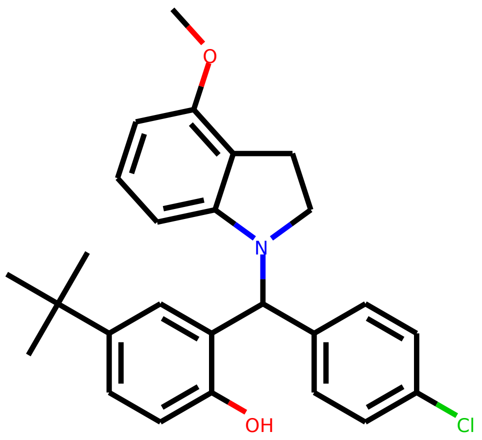
Ahma-1016
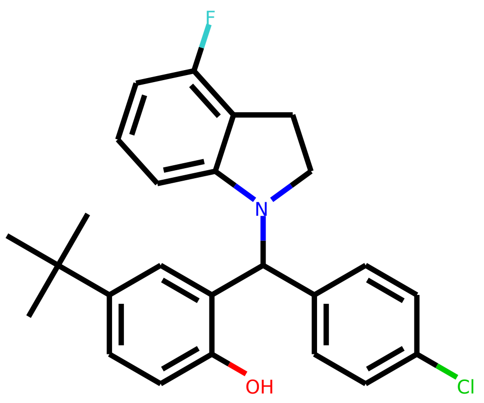
Ahma-1017
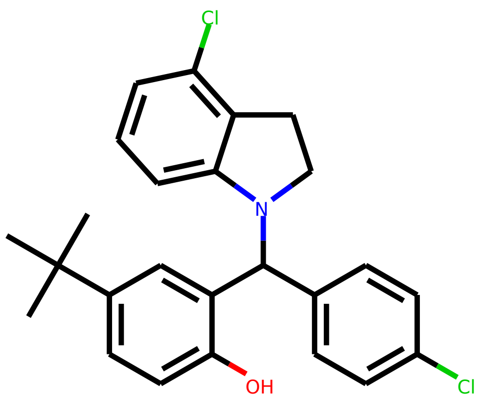
Ahma-1018
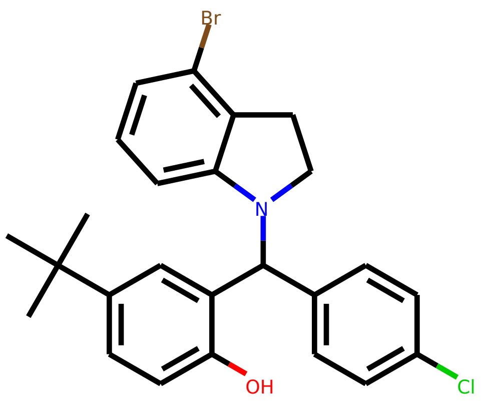
Ahma-1019
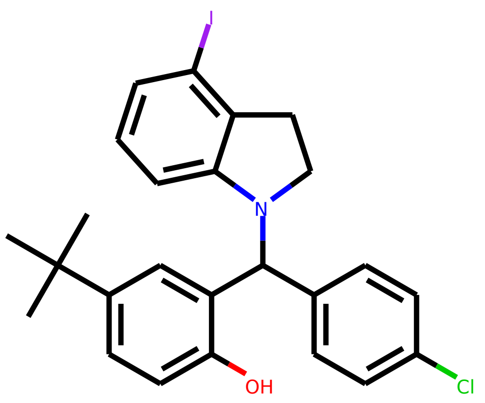
Ahma-1020
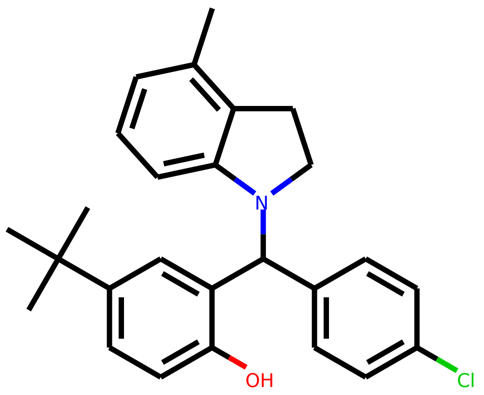
Ahma-1021
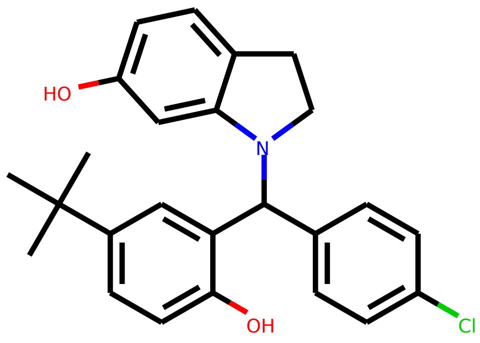
Ahma-1022
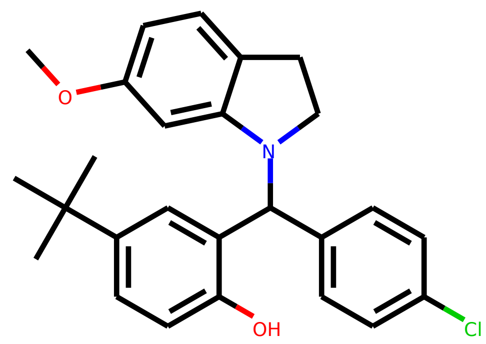
Ahma-1023
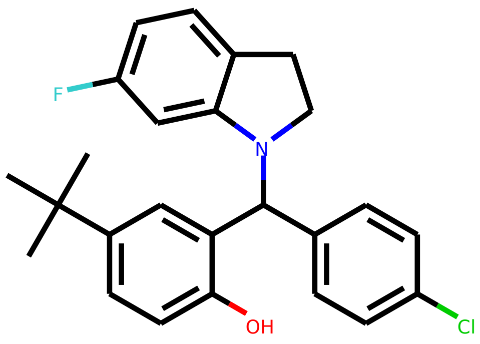
Ahma-1024
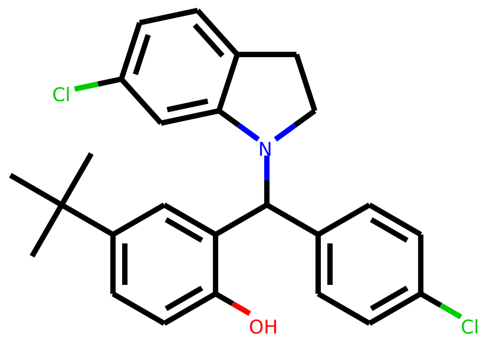
Ahma-1025
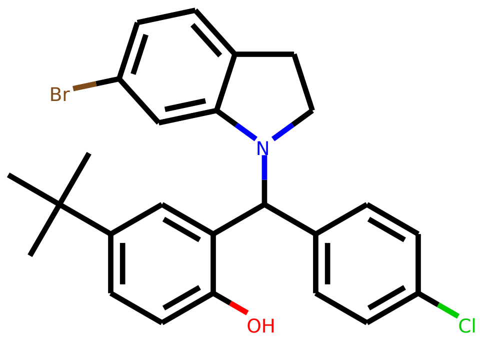
Ahma-1026
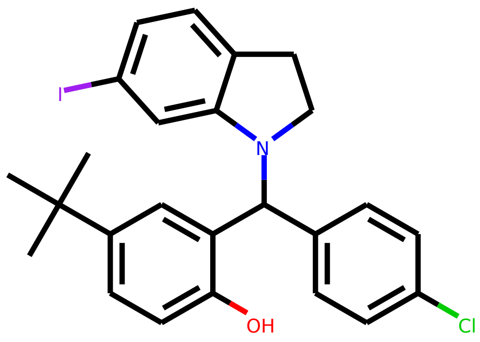
Ahma-1027
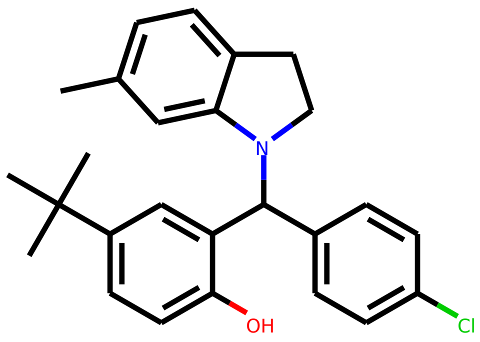
Ahma-1028
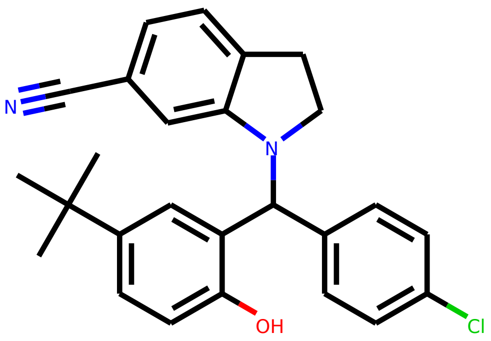
Ahma-1029
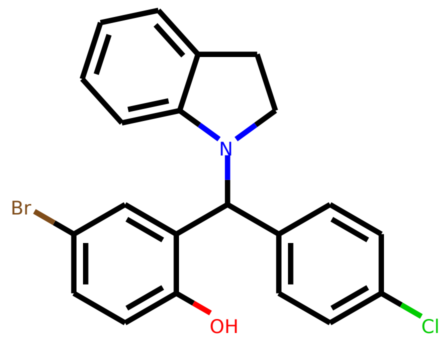
Ahma-1030
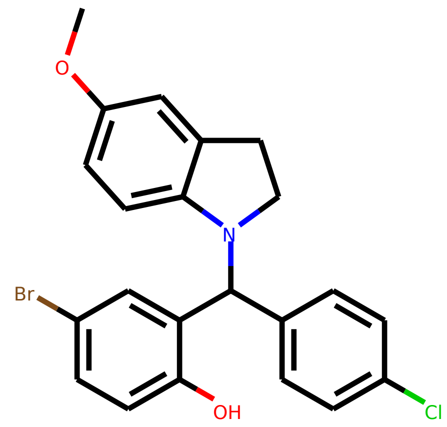
Ahma-1031
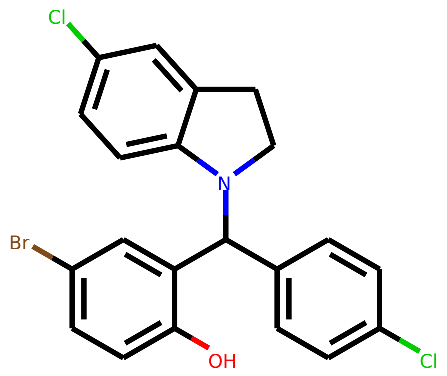
Ahma-1032
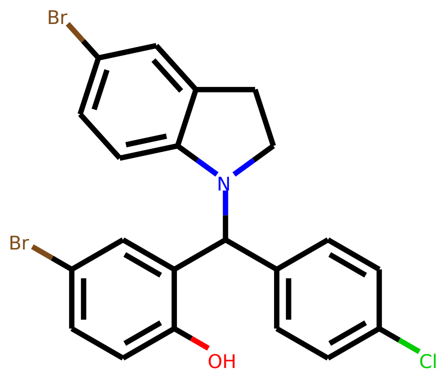
Ahma-1033
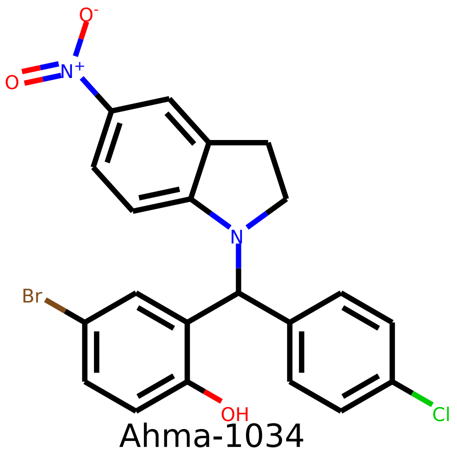
Ahma-1034
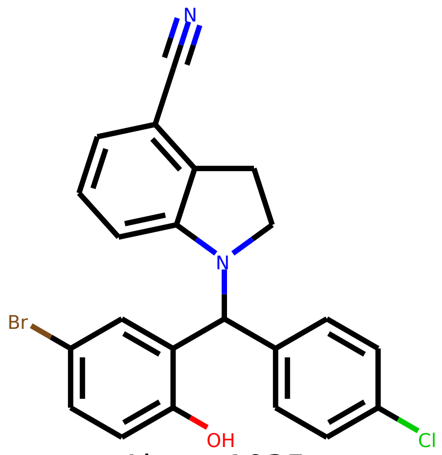
Ahma-1035
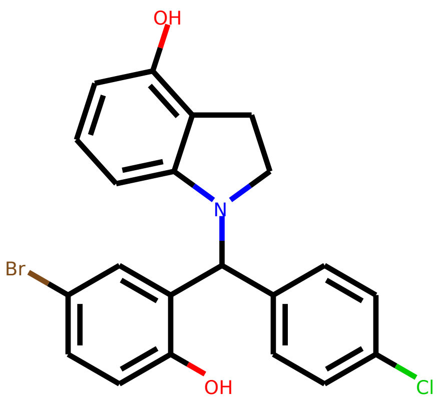
Ahma-1036
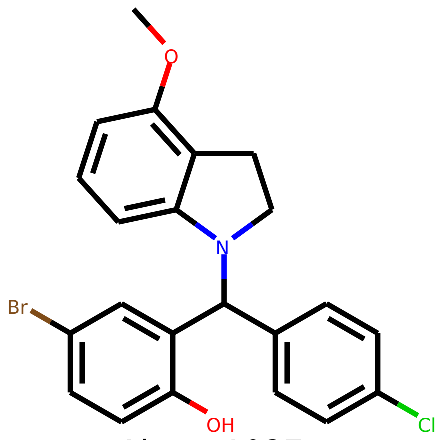
Ahma-1037
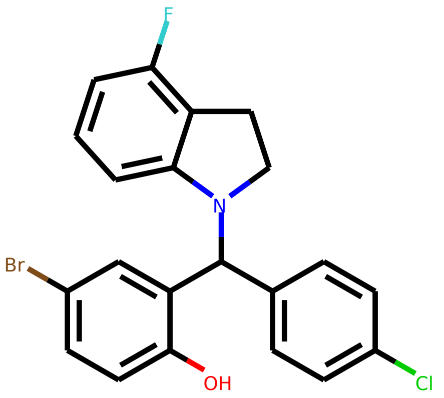
Ahma-1038
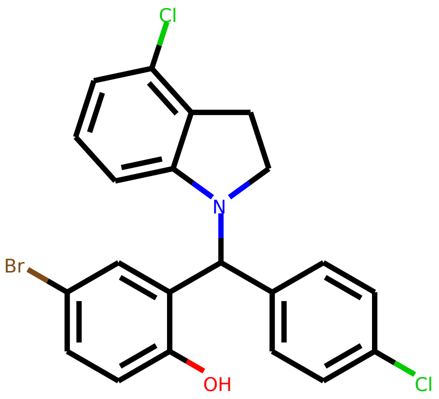
Ahma-1039
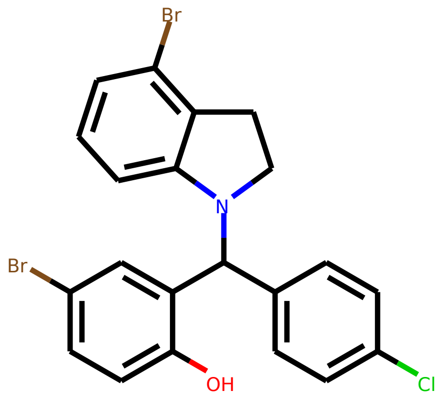
Ahma-1040
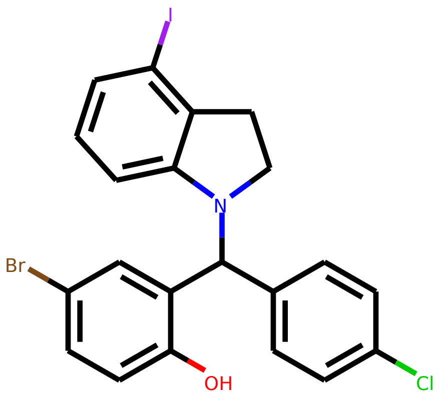
Ahma-1041
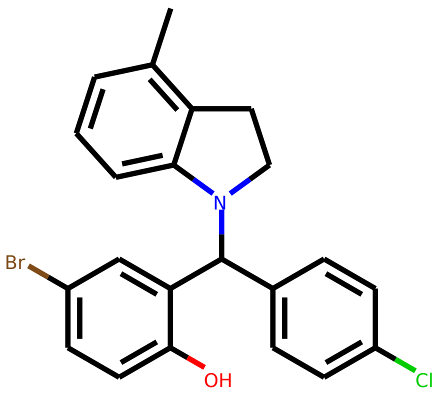
Ahma-1042
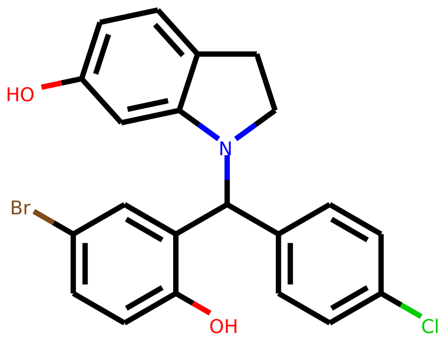
Ahma-1043
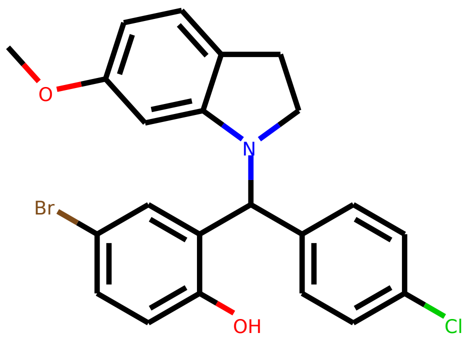
Ahma-1044
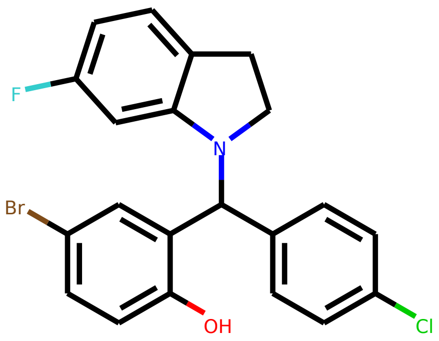
Ahma-1045
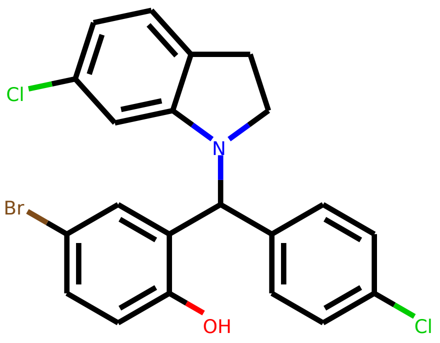
Ahma-1046
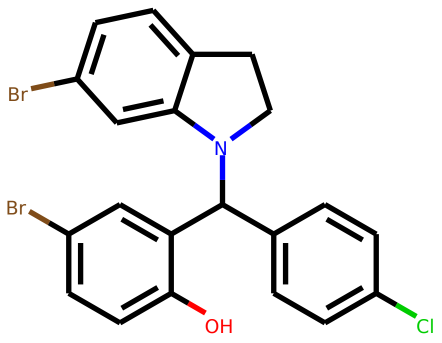
Ahma-1047
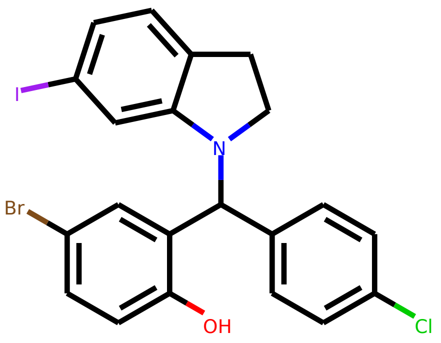
Ahma-1048
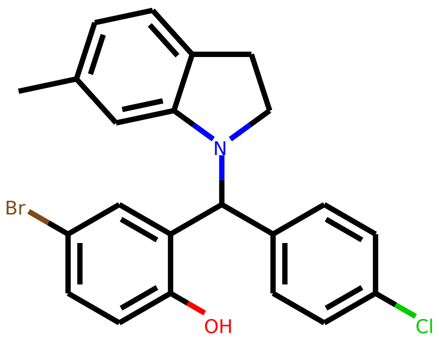
Ahma-1049
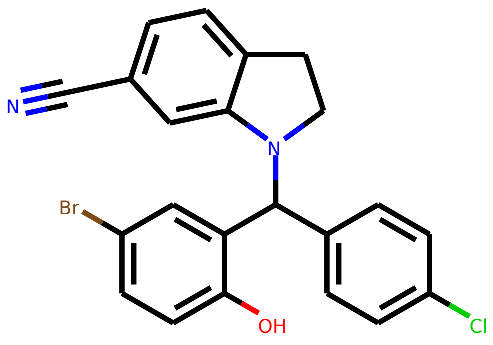
Ahma-1050

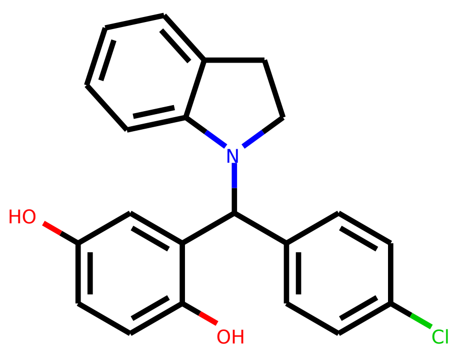
Ahma-1051

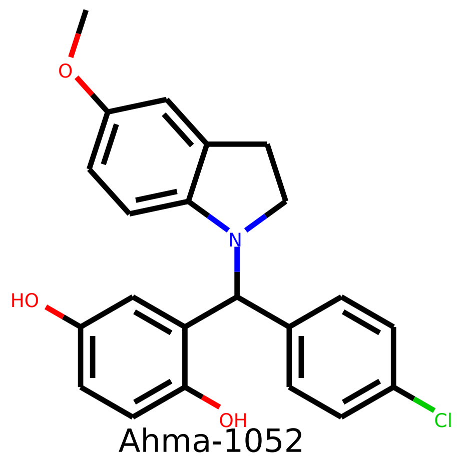
Ahma-1052

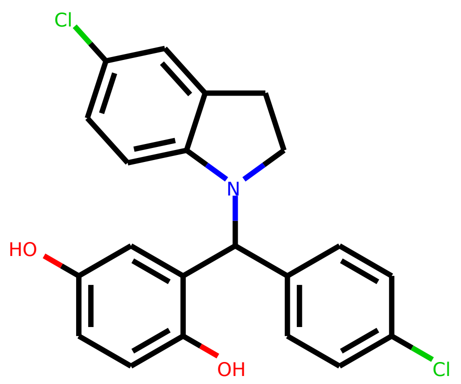
Ahma-1053

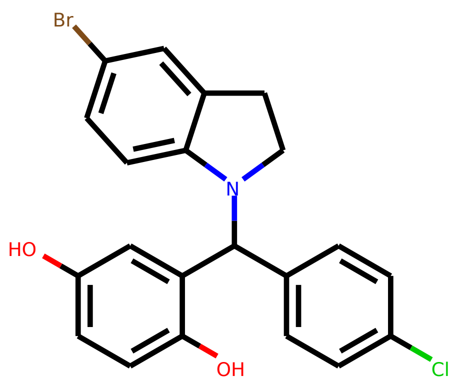
Ahma-1054

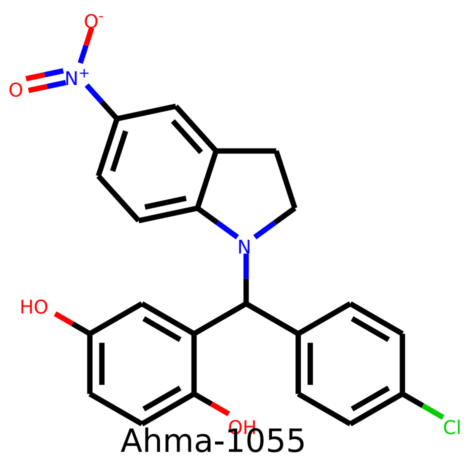
Ahma-1055

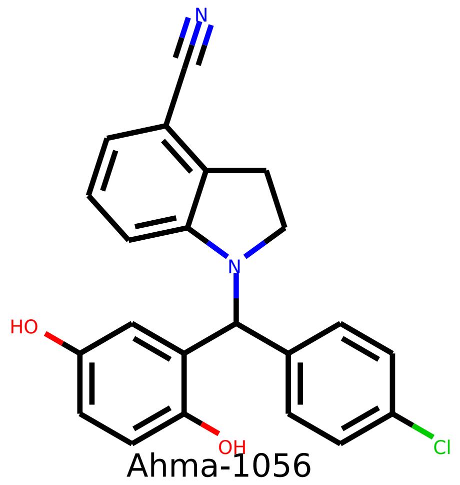
Ahma-1056

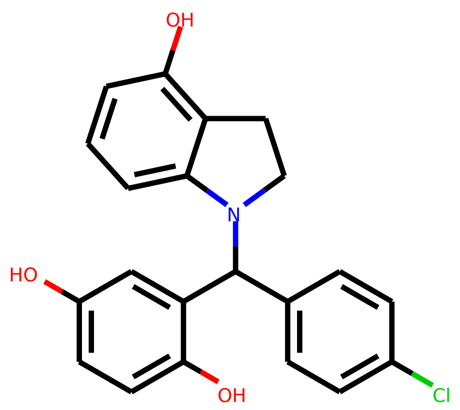
Ahma-1057

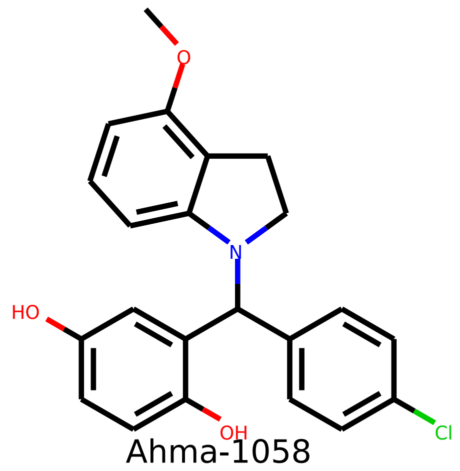
Ahma-1058

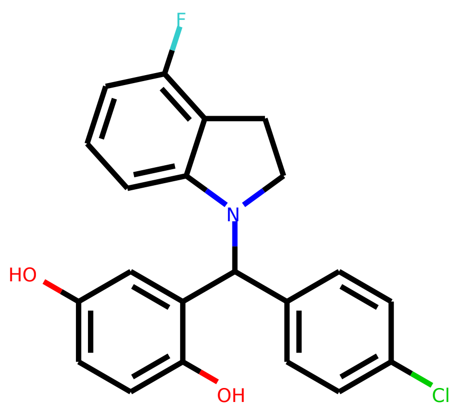
Ahma-1059

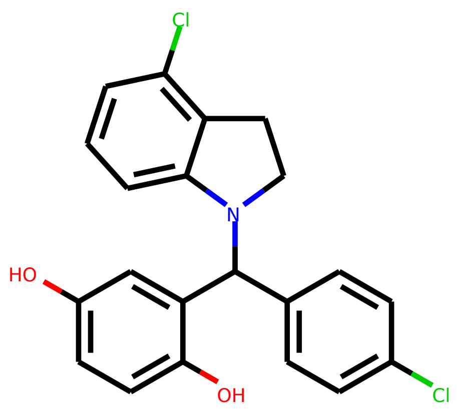
Ahma-1060

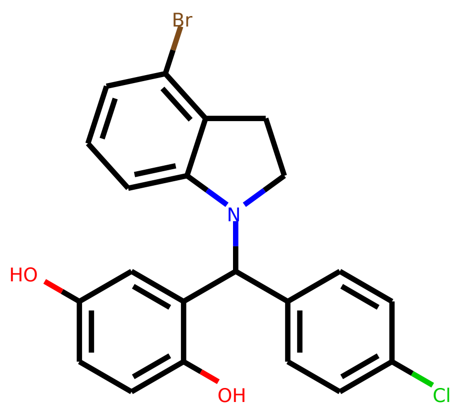
Ahma-1061

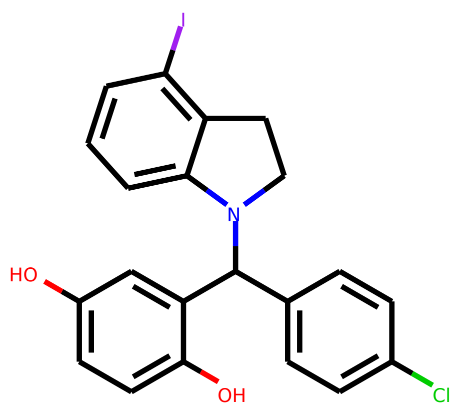
Ahma-1062

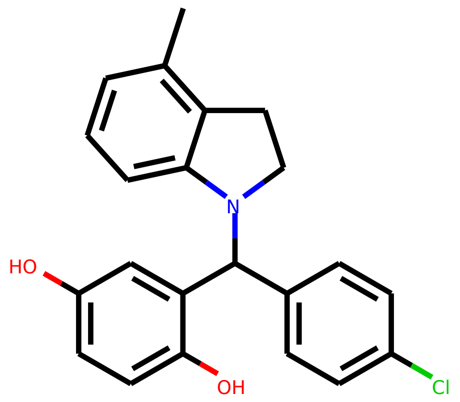
Ahma-1063

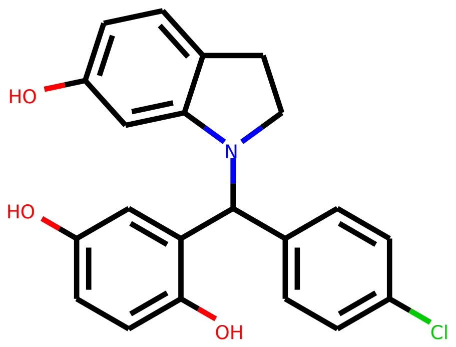
Ahma-1064

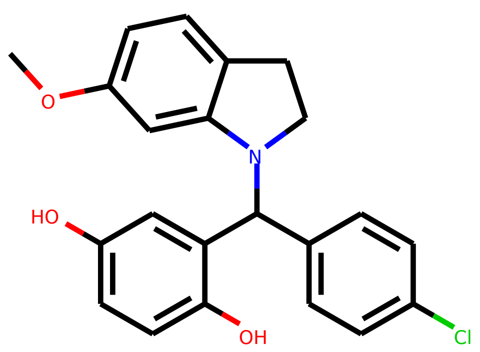
Ahma-1065

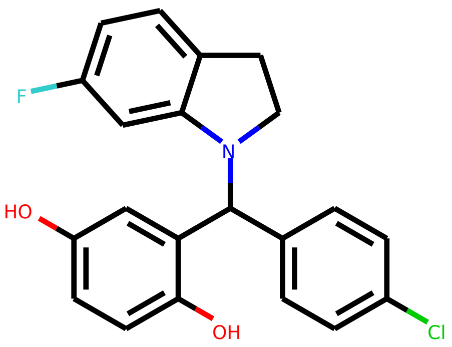
Ahma-1066

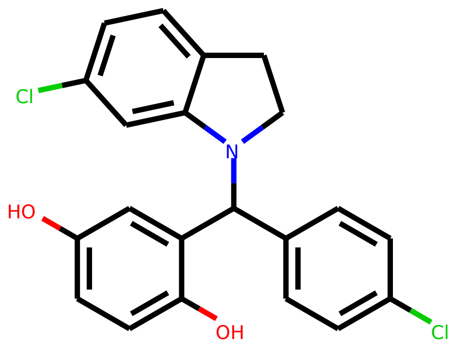
Ahma-1067

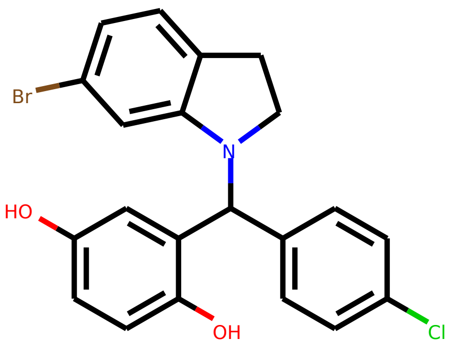
Ahma-1068

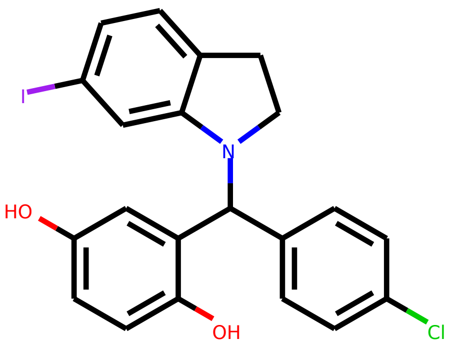
Ahma-1069

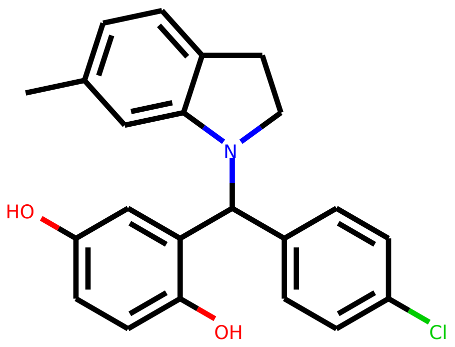
Ahma-1070

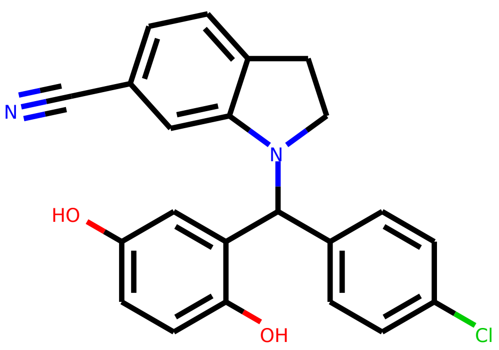
Ahma-1071

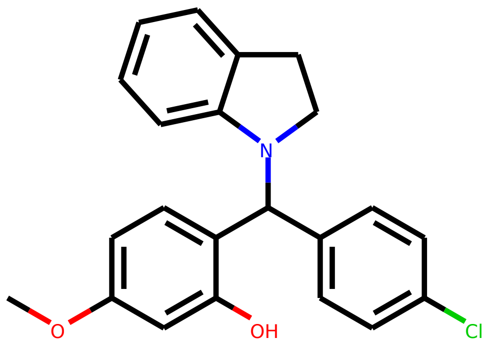
Ahma-1072

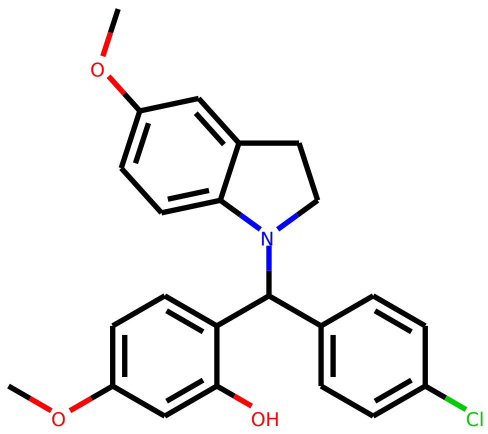
Ahma-1073

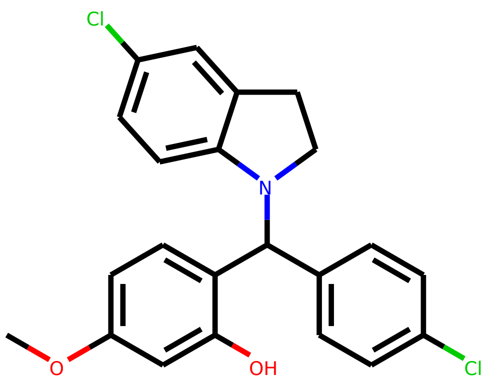
Ahma-1074

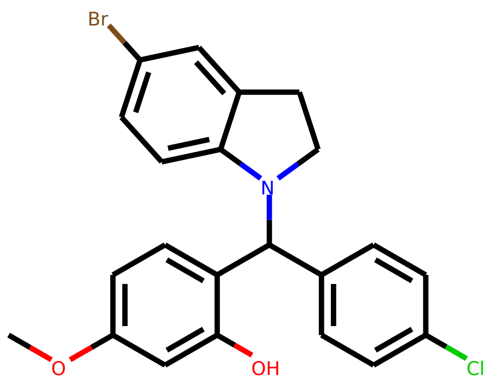
Ahma-1075

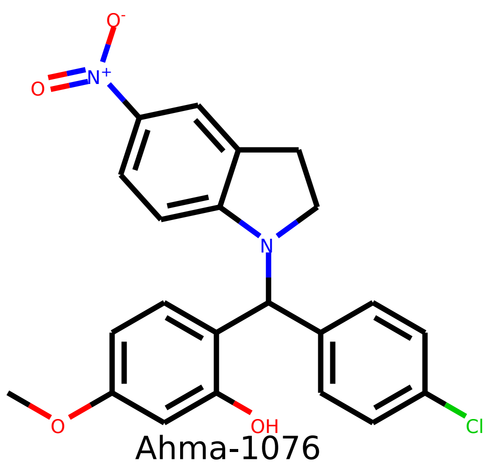
Ahma-1076

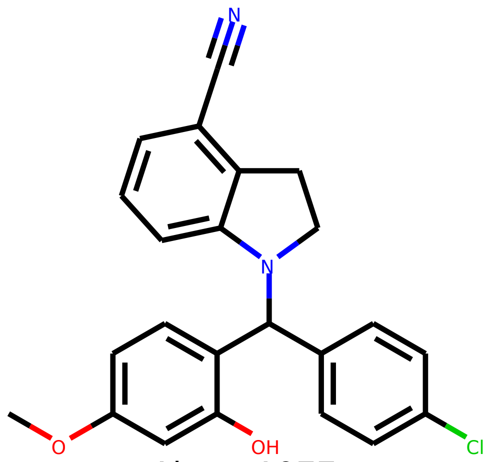
Ahma-1077

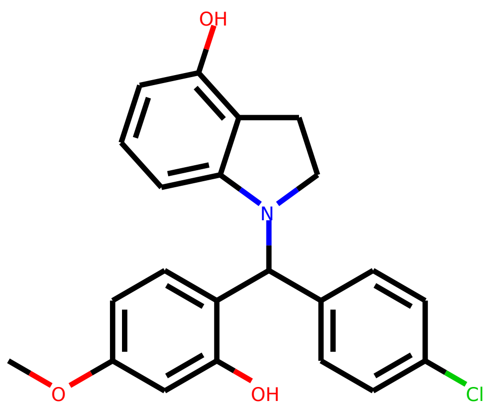
Ahma-1078

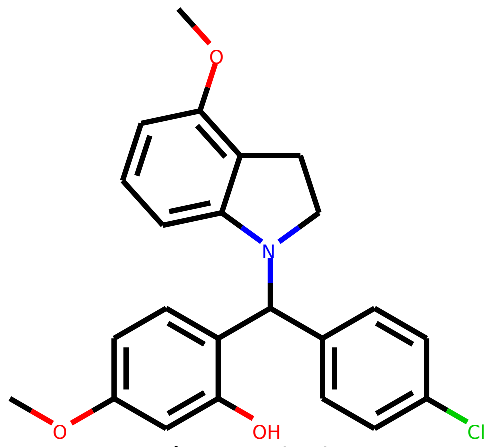
Ahma-1079

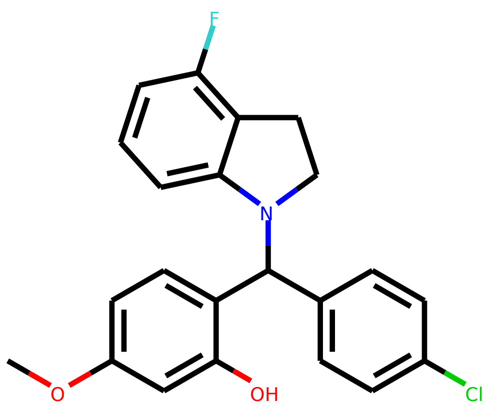
Ahma-1080

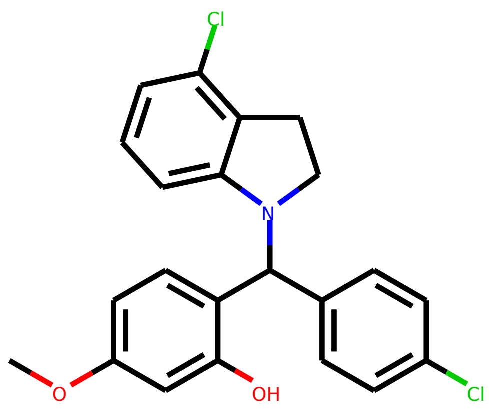
Ahma-1081

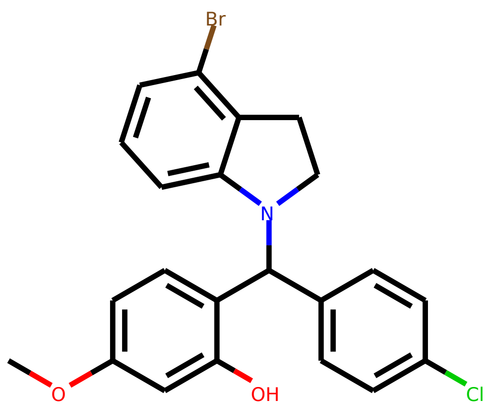
Ahma-1082

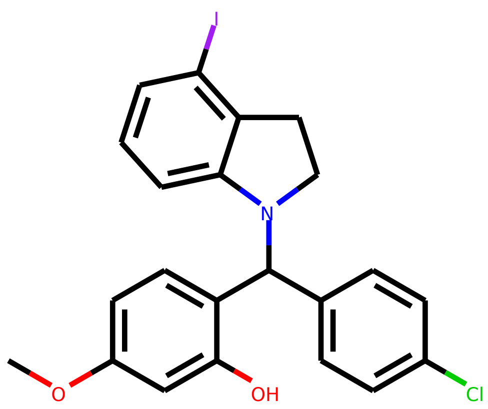
Ahma-1083

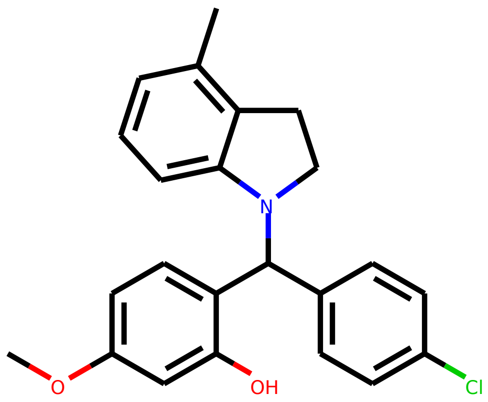
Ahma-1084

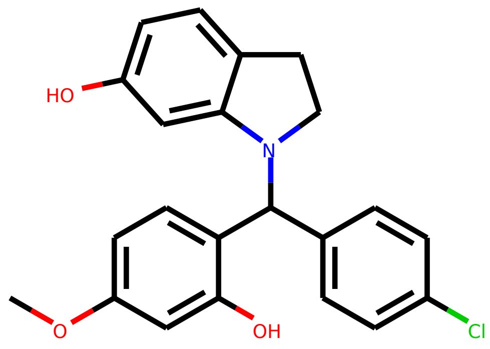
Ahma-1085

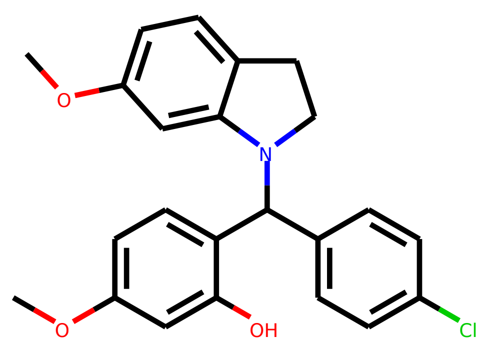
Ahma-1086
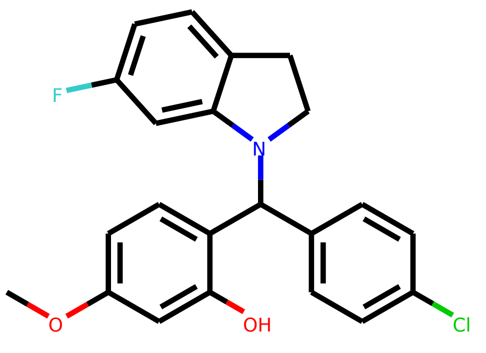
Ahma-1087
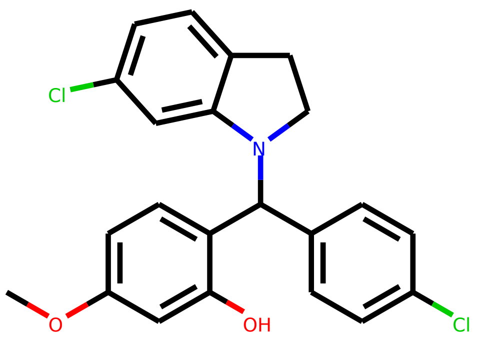
Ahma-1088
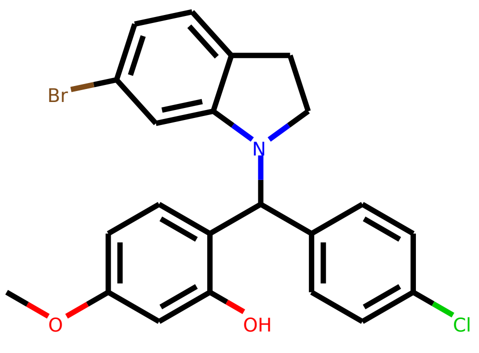
Ahma-1089
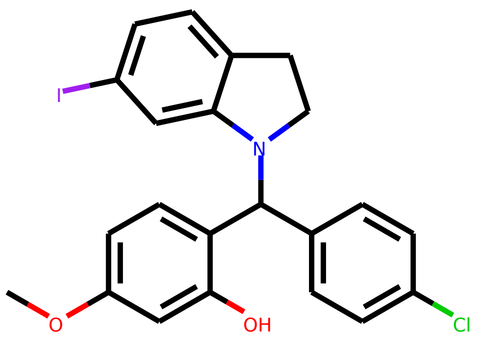
Ahma-1090
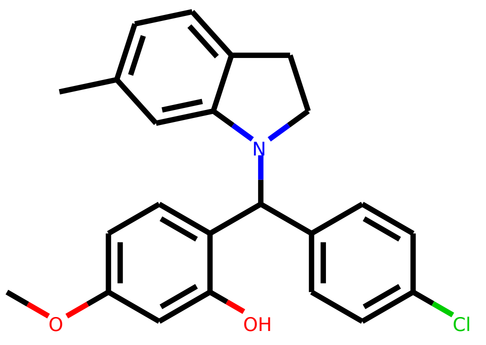
Ahma-1091
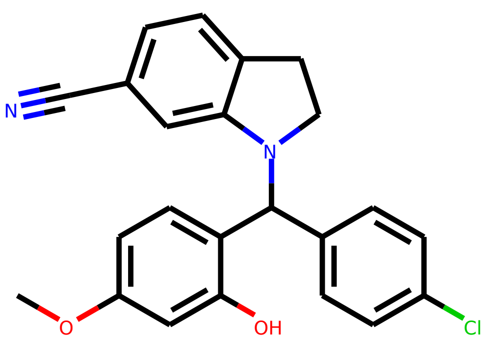
Ahma-1092
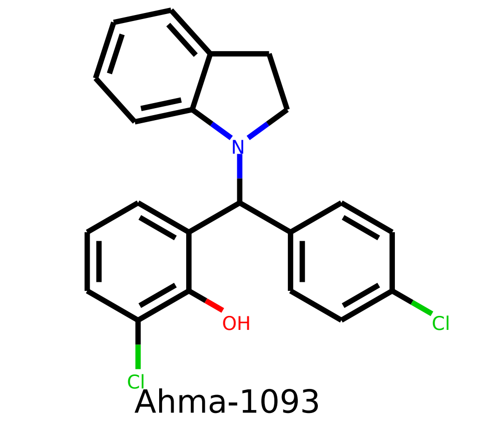
Ahma-1093
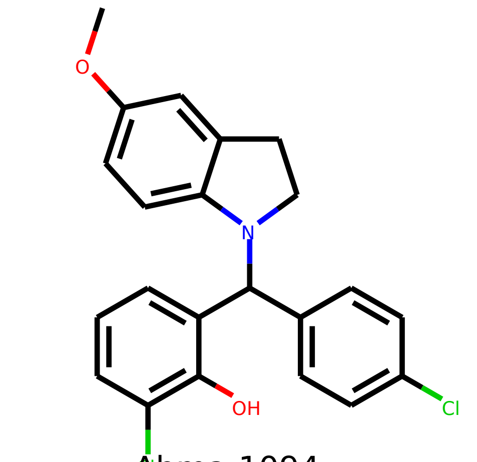
Ahma-1094
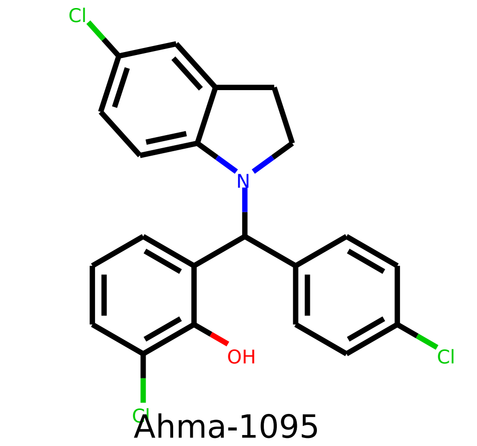
Ahma-1095
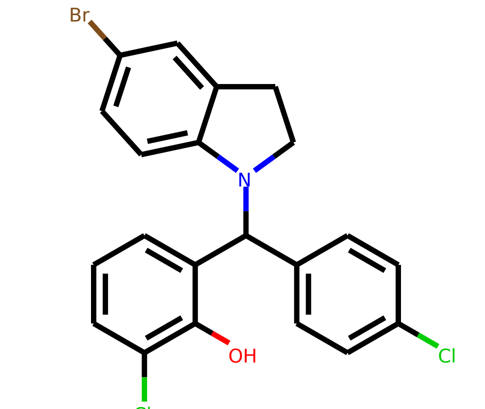
Ahma-1096
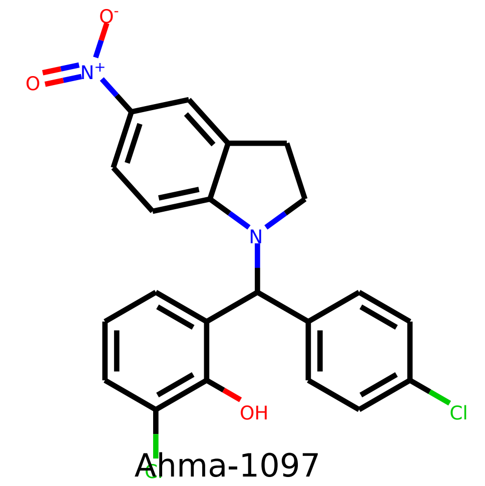
Ahma-1097
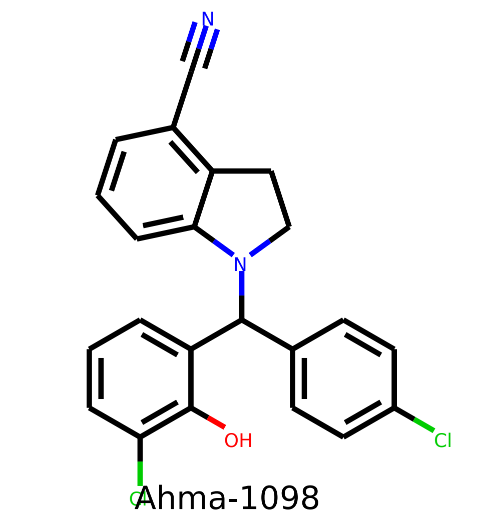
Ahma-1098
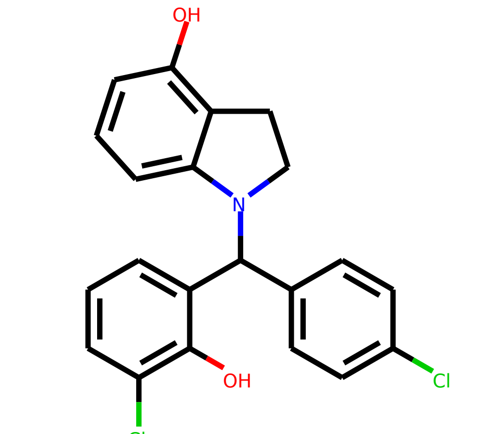
Ahma-1099
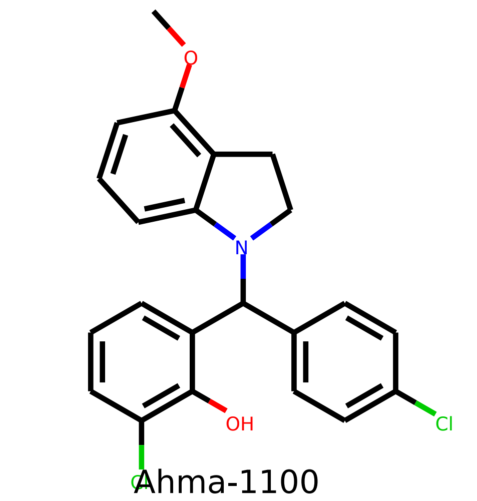
Ahma-1100
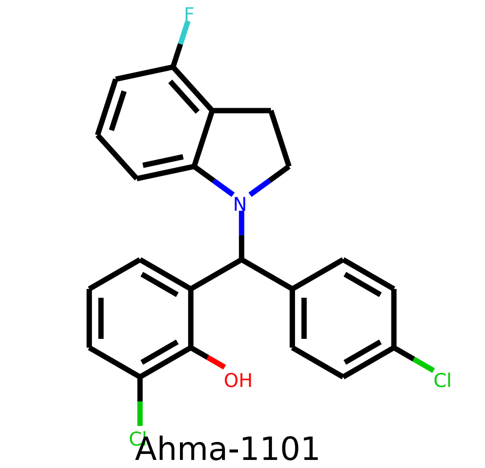
Ahma-1101
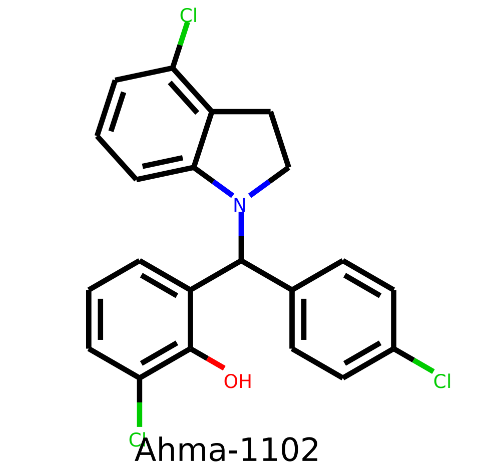
Ahma-1102
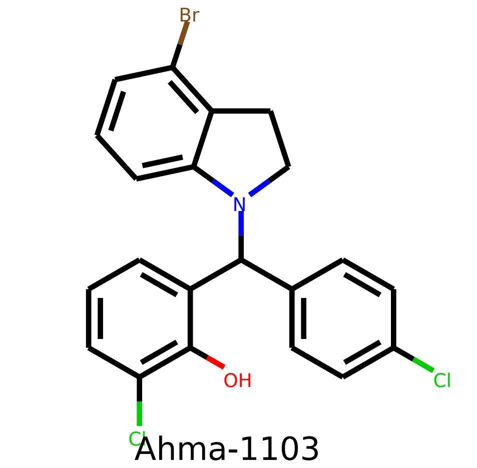
Ahma-1103
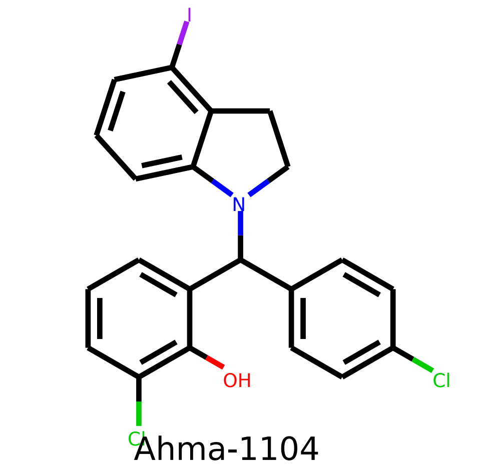
Ahma-1104
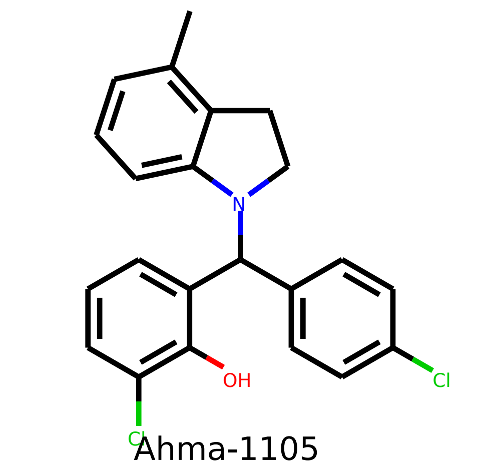
Ahma-1105
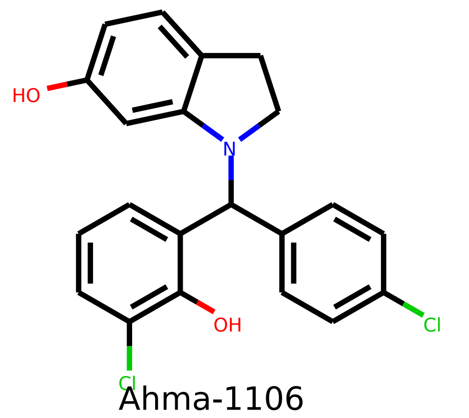
Ahma-1106
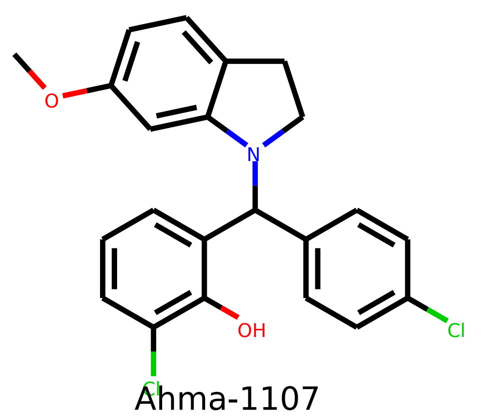
Ahma-1107
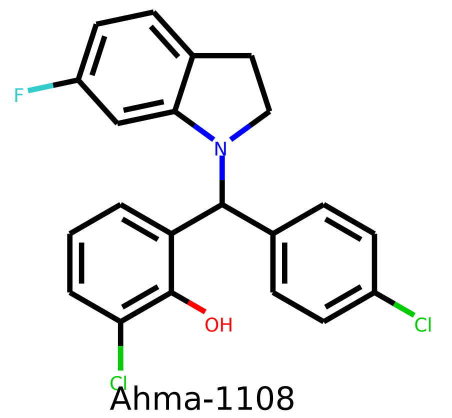
Ahma-1108
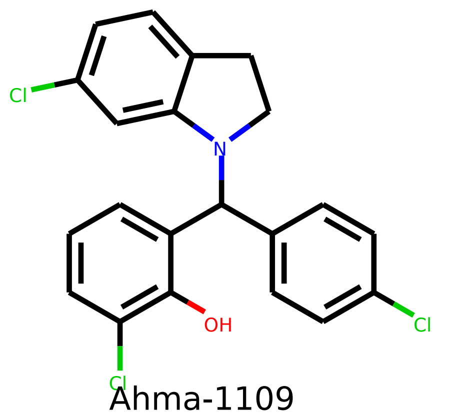
Ahma-1109
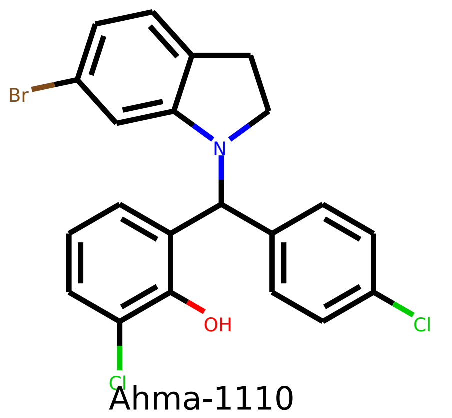
Ahma-1110
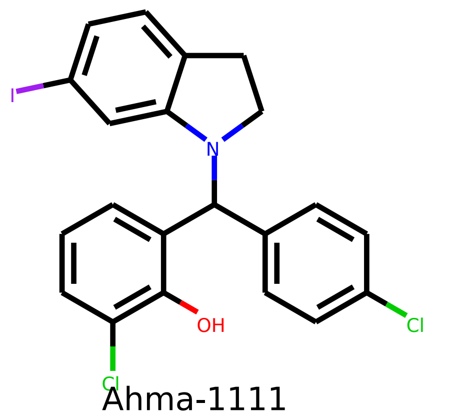
Ahma-1111
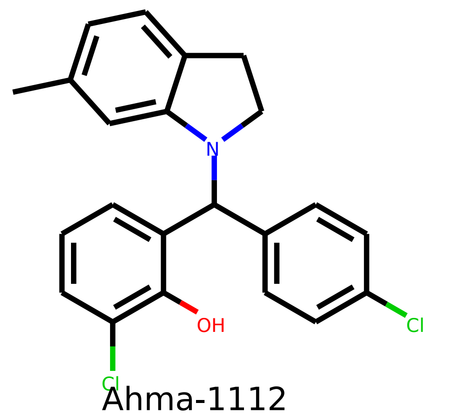
Ahma-1112
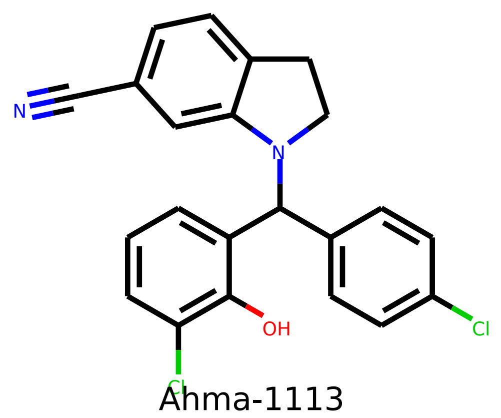
Ahma-1113
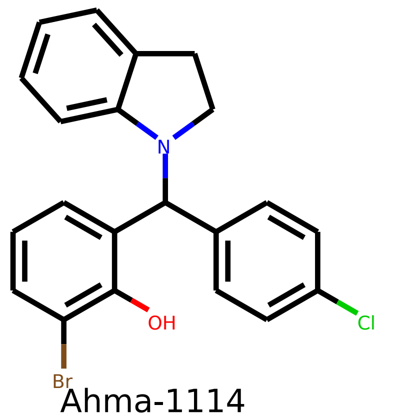
Ahma-1114
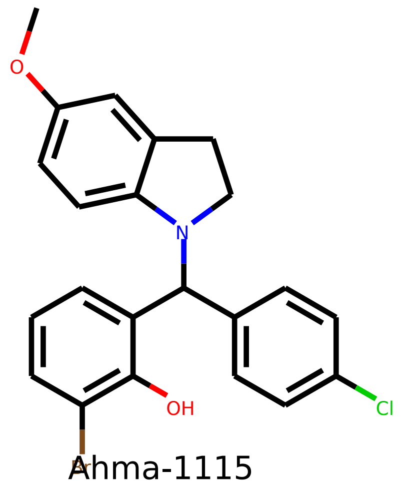
Ahma-1115
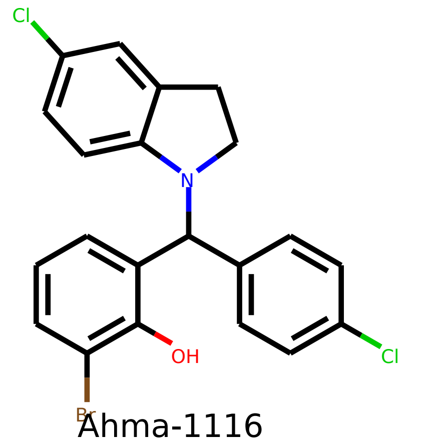
Ahma-1116
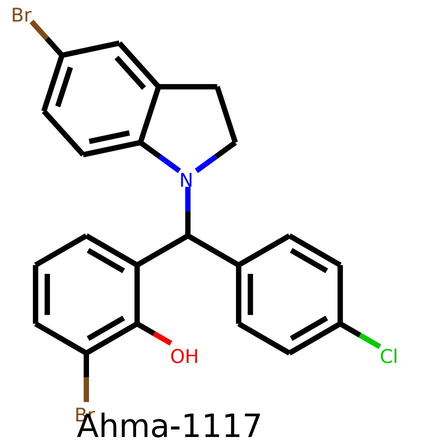
Ahma-1117
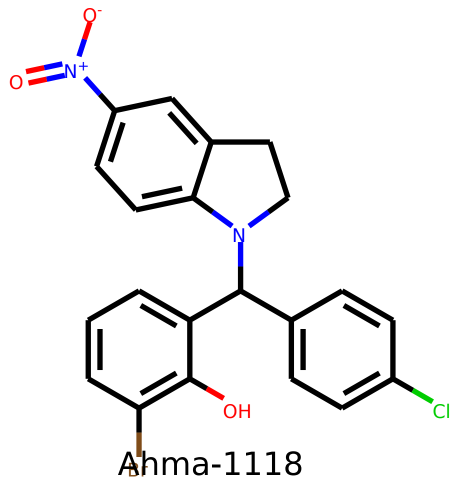
Ahma-1118
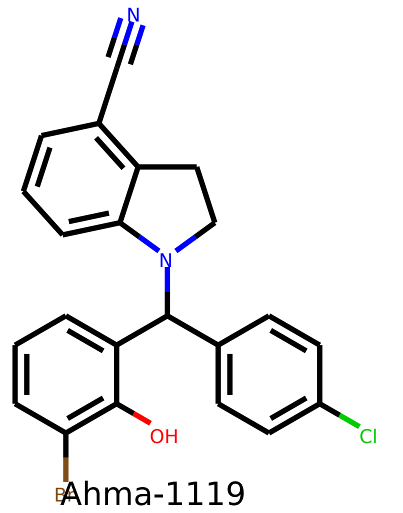
Ahma-1119
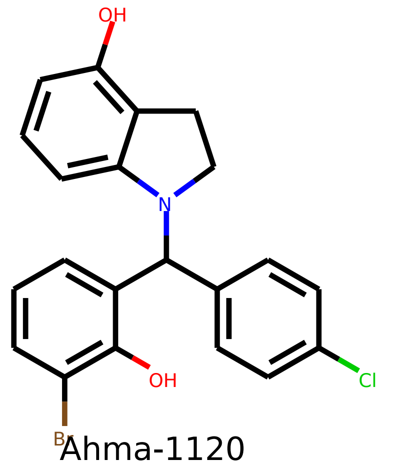
Ahma-1120

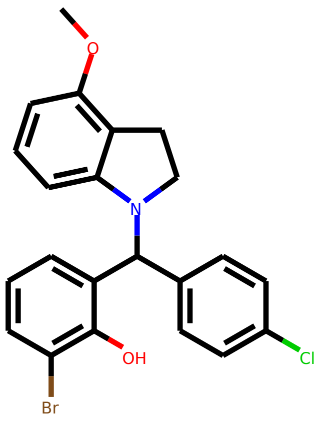
Ahma-1121
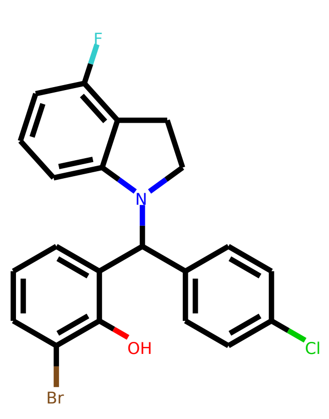
Ahma-1122
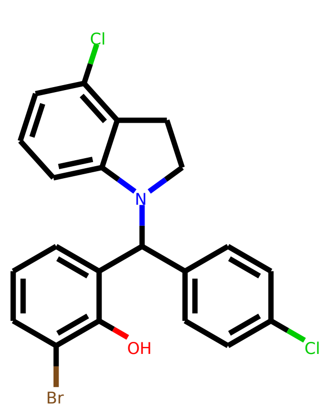
Ahma-1123
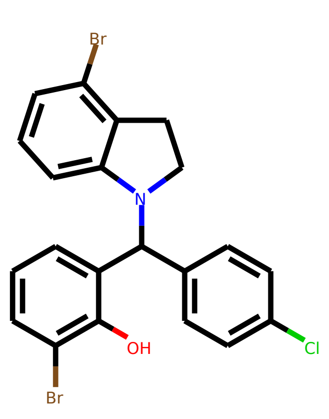
Ahma-1124
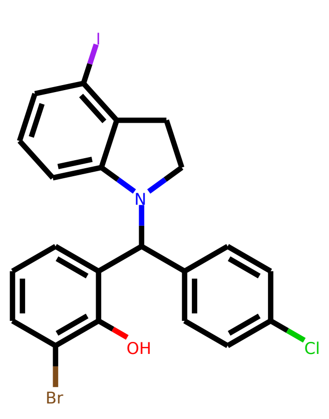
Ahma-1125
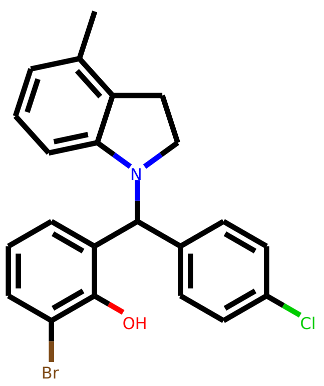
Ahma-1126
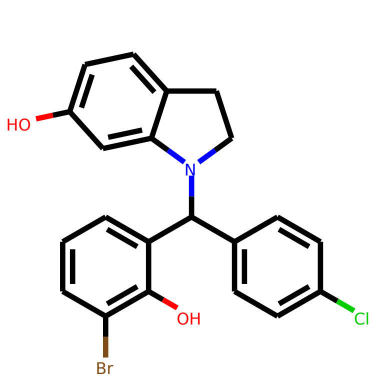
Ahma-1127
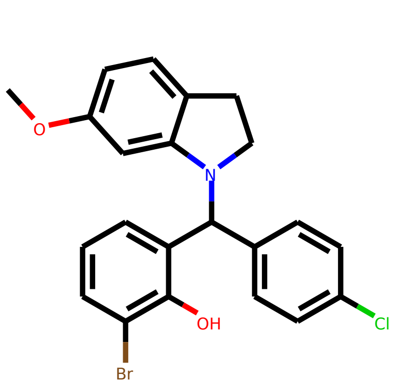
Ahma-1128
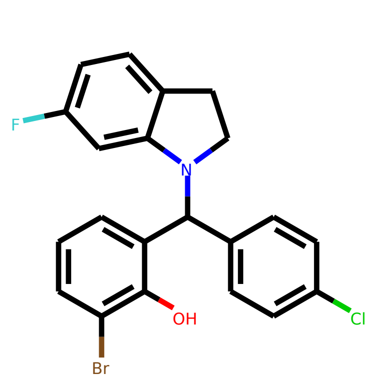
Ahma-1129
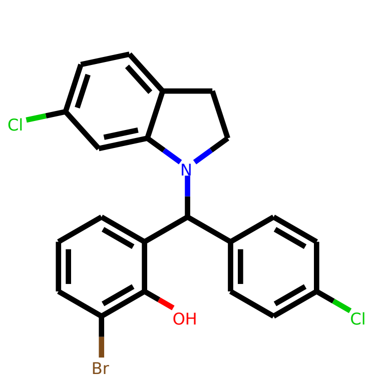
Ahma-1130
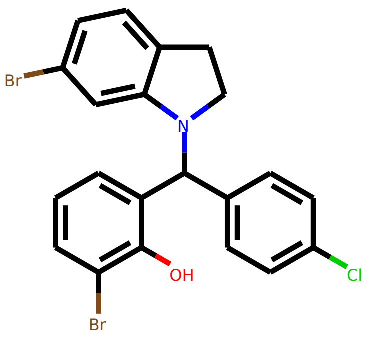
Ahma-1131
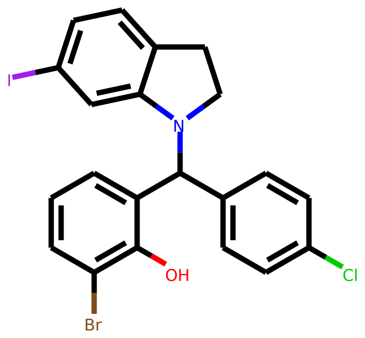
Ahma-1132
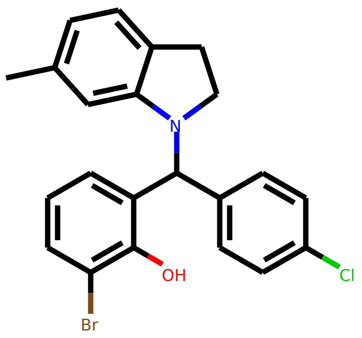
Ahma-1133
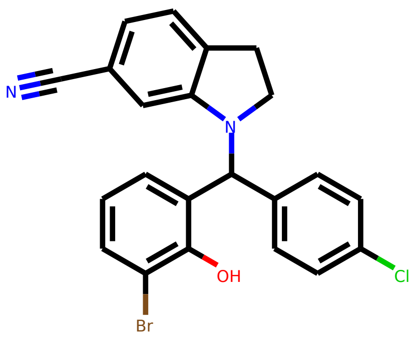
Ahma-1134
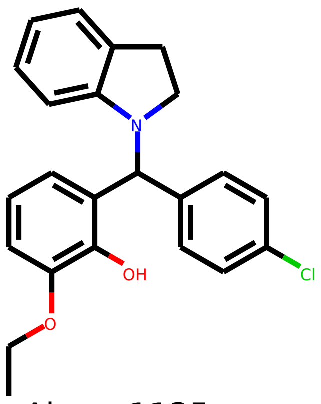
Ahma-1135
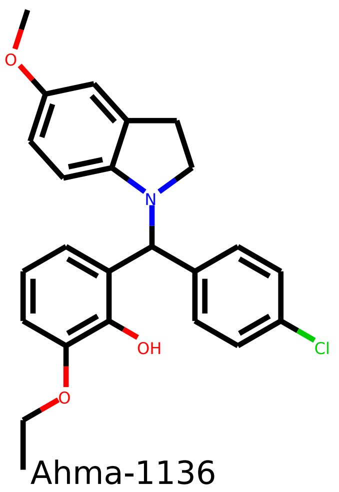
Ahma-1136
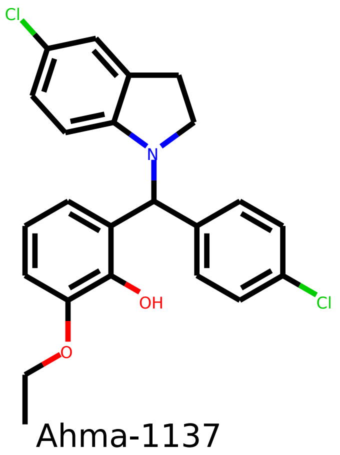
Ahma-1137
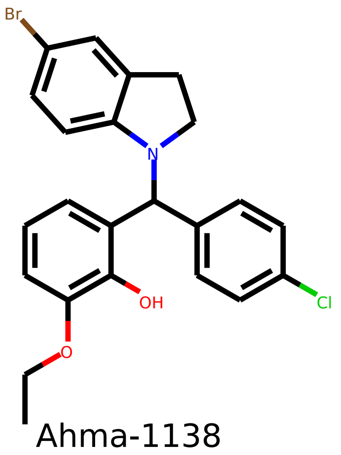
Ahma-1138
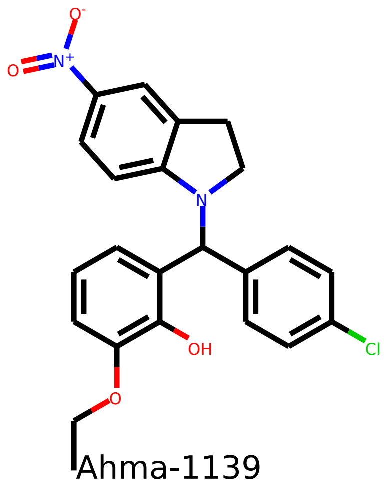
Ahma-1139
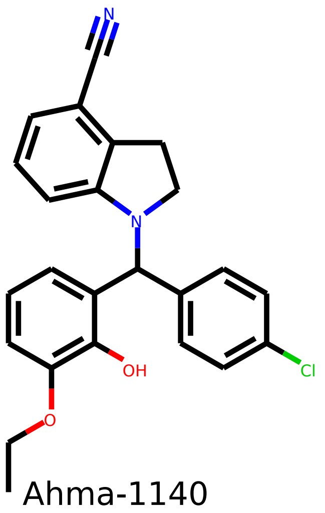
Ahma-1140
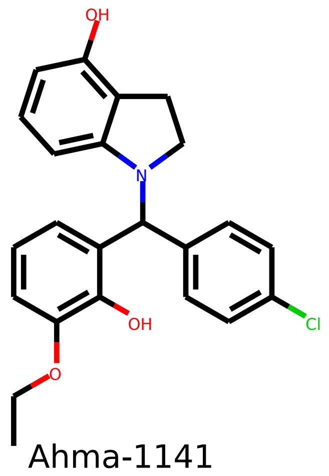
Ahma-1141
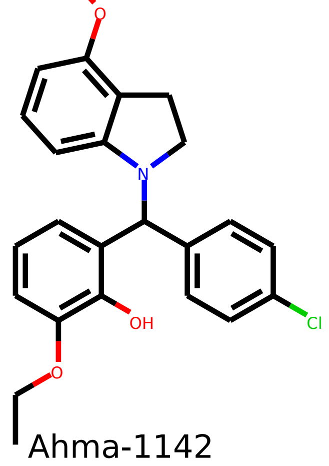
Ahma-1142
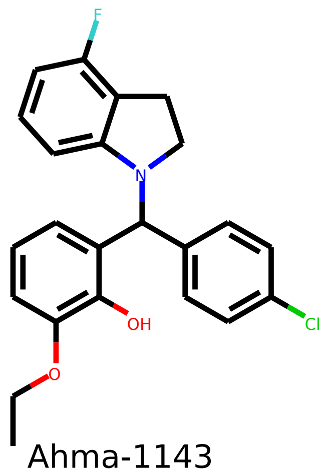
Ahma-1143
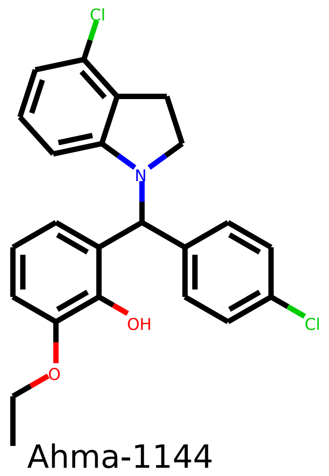
Ahma-1144
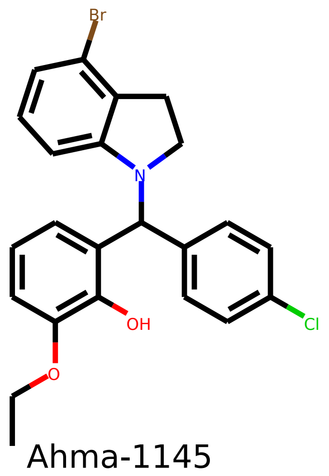
Ahma-1145
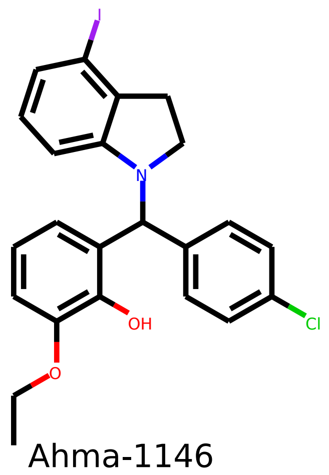
Ahma-1146
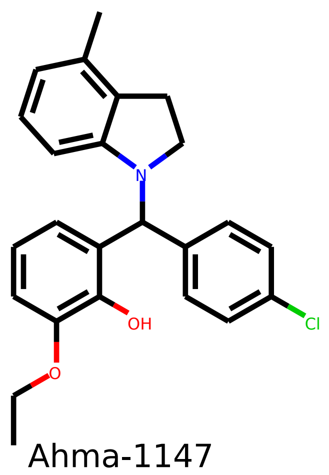
Ahma-1147
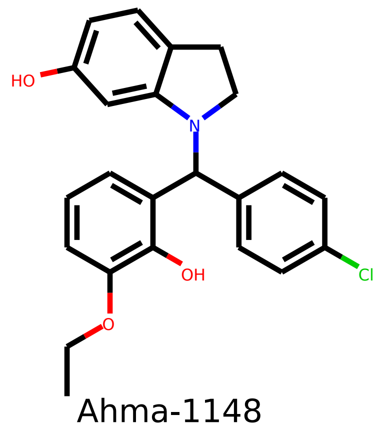
Ahma-1148
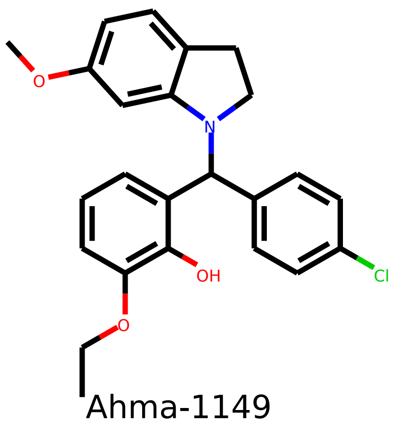
Ahma-1149
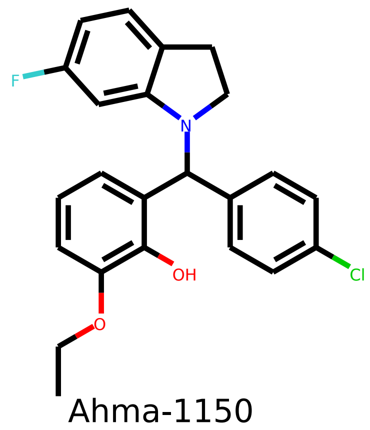
Ahma-1150
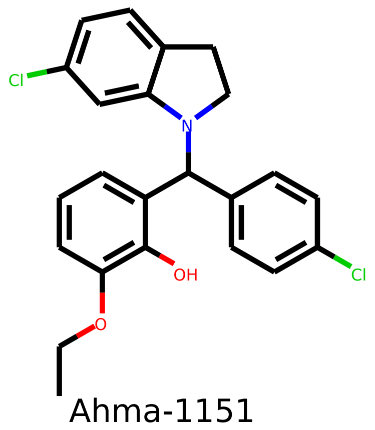
Ahma-1151
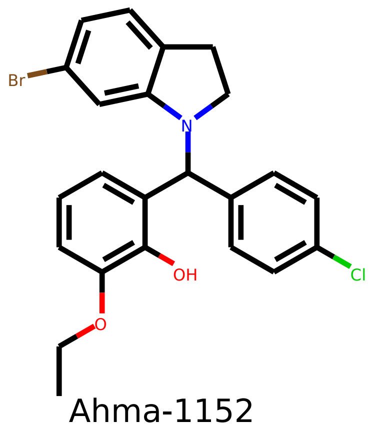
Ahma-1152
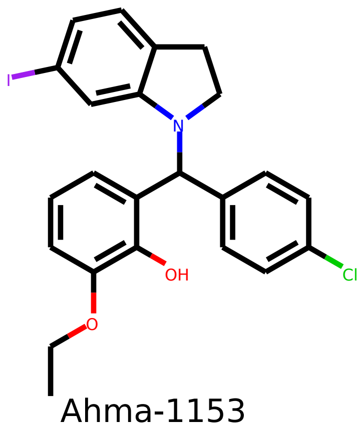
Ahma-1153
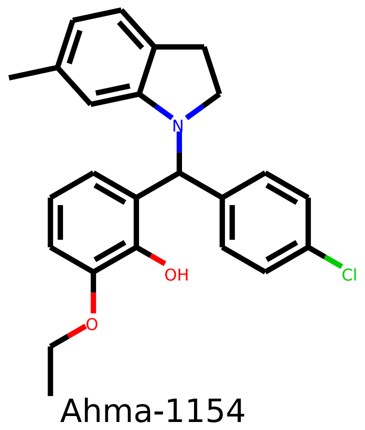
Ahma-1154
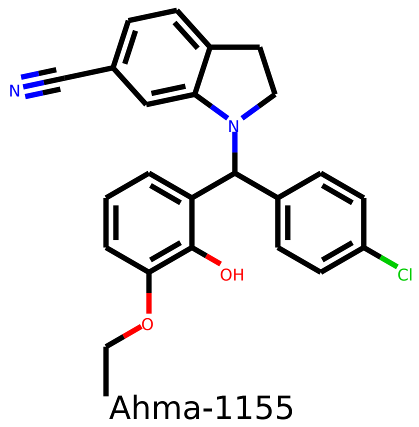
Ahma-1155

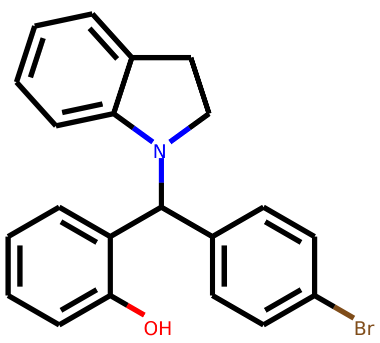

Ahma-1156

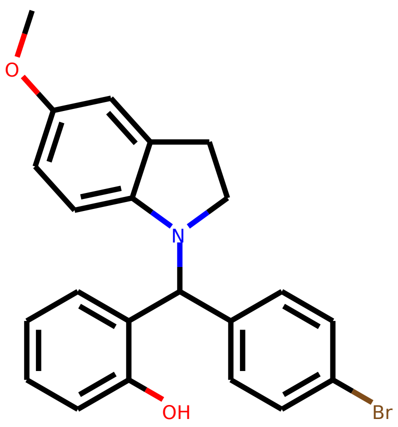

Ahma-1157

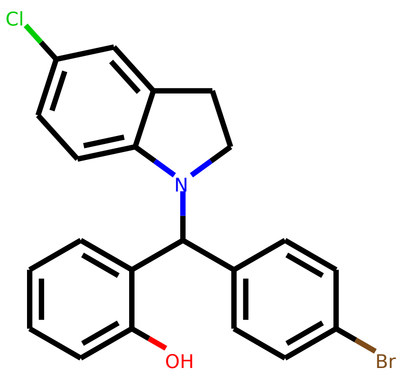

Ahma-1158

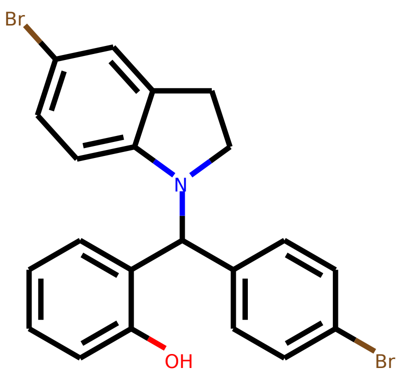

Ahma-1159

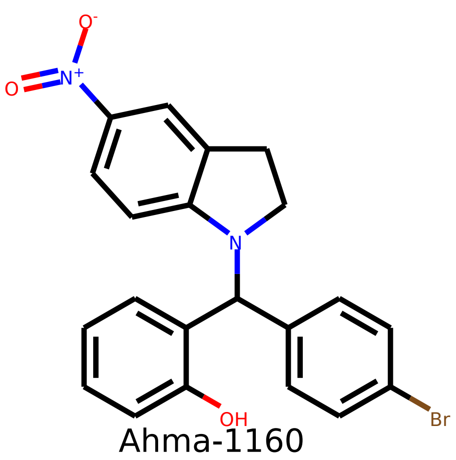

Ahma-1160

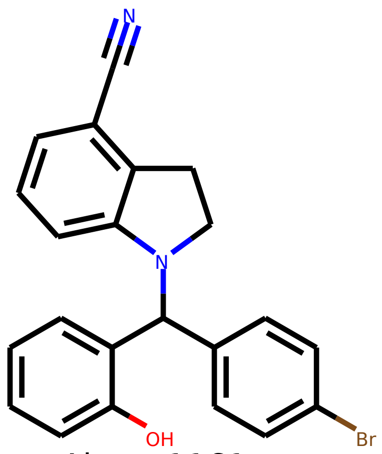

Ahma-1161

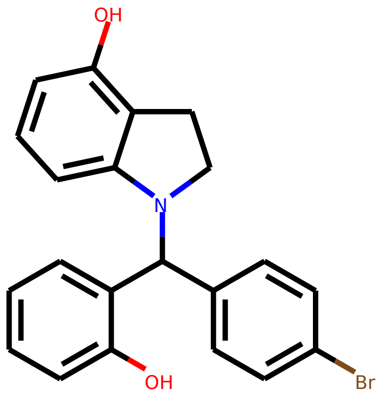

Ahma-1162

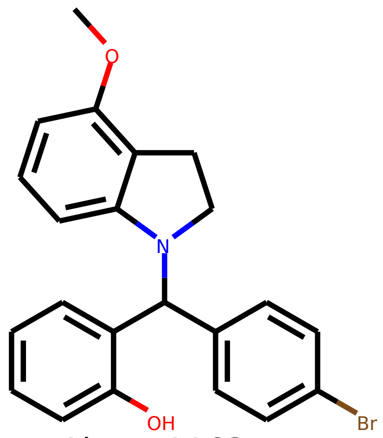

Ahma-1163

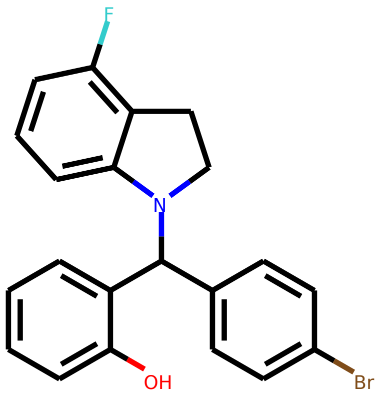

Ahma-1164

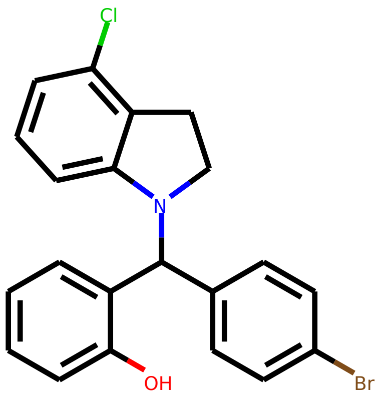

Ahma-1165

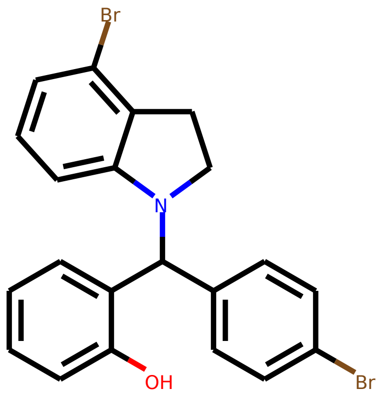

Ahma-1166

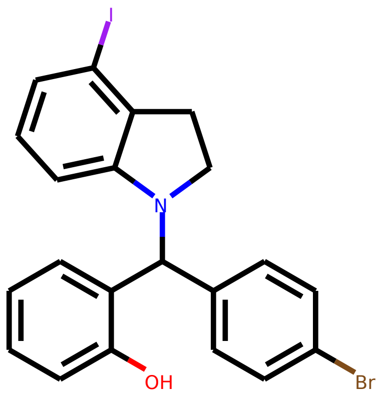

Ahma-1167

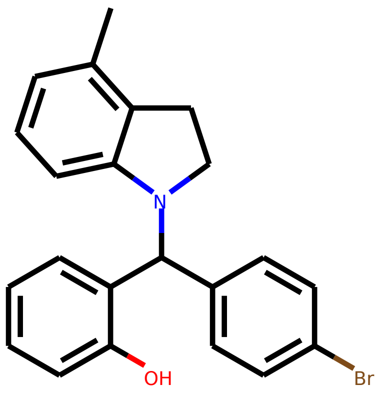

Ahma-1168

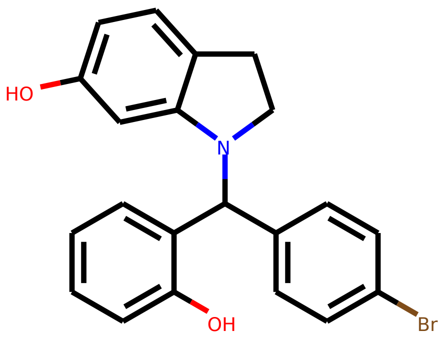

Ahma-1169

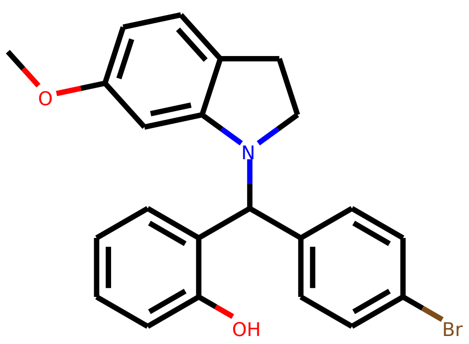

Ahma-1170

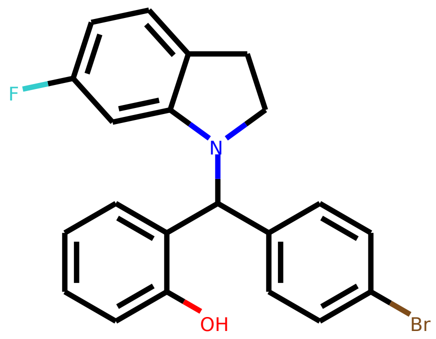

Ahma-1171

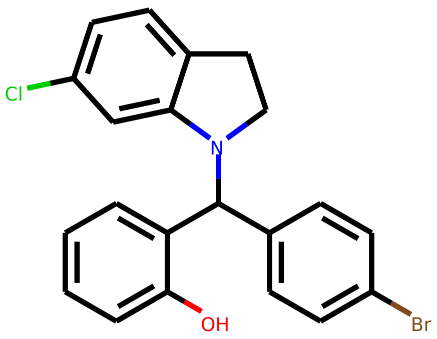

Ahma-1172

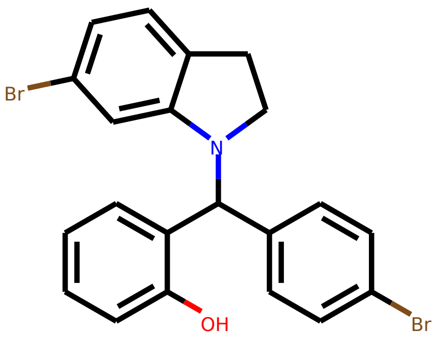

Ahma-1173

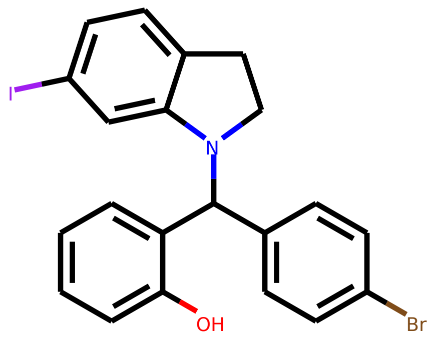

Ahma-1174

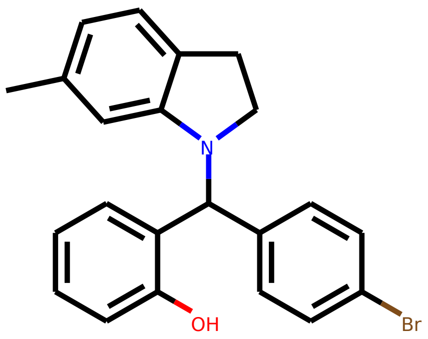

Ahma-1175

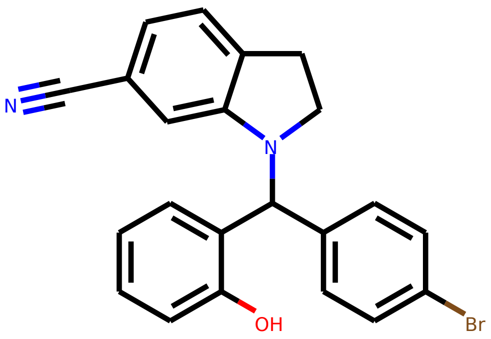

Ahma-1176

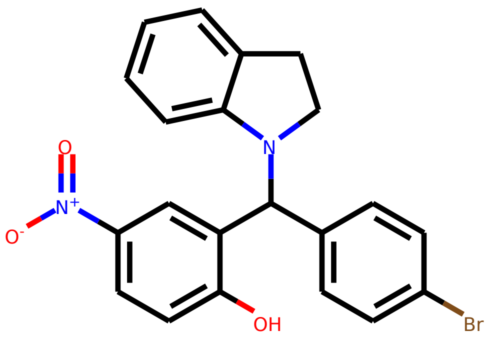

Ahma-1177

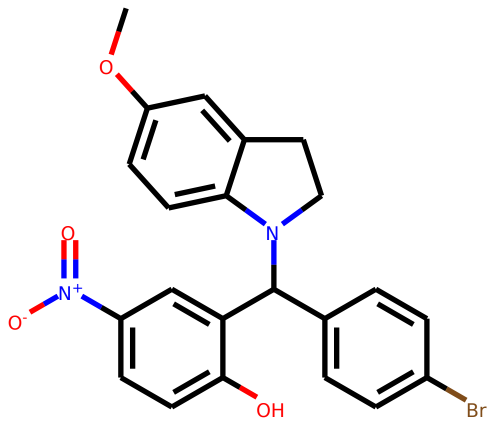

Ahma-1178

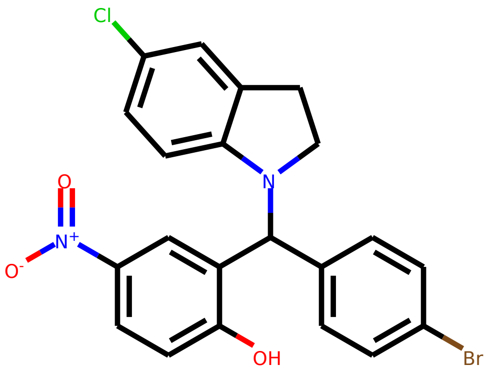

Ahma-1179

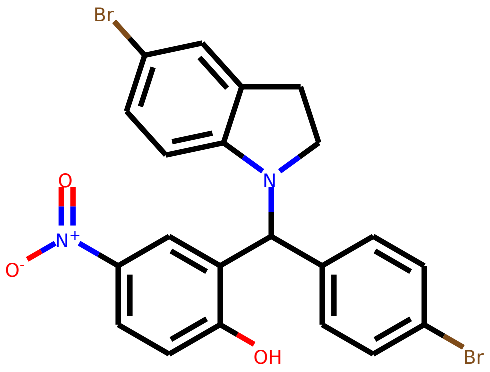

Ahma-1180

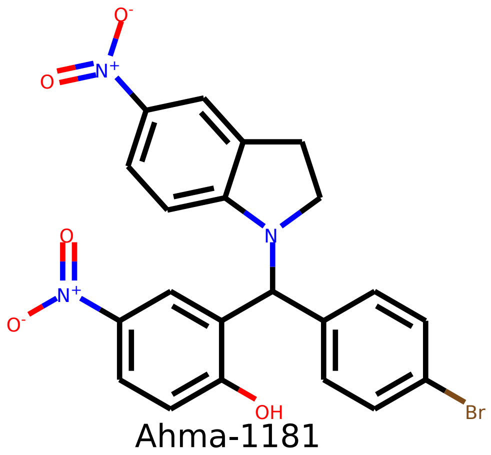

Ahma-1181

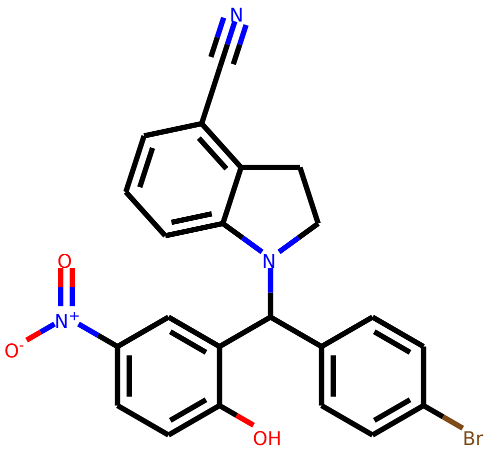

Ahma-1182

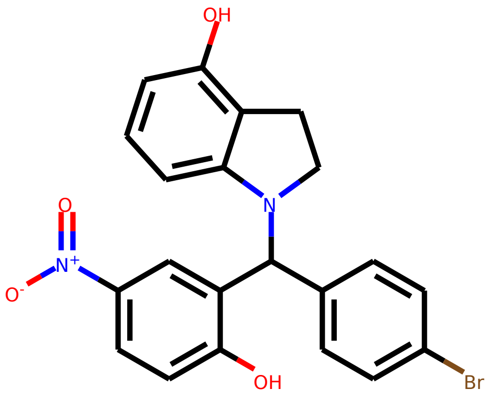

Ahma-1183

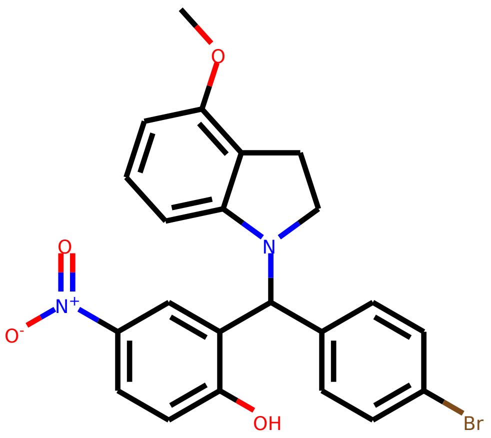

Ahma-1184

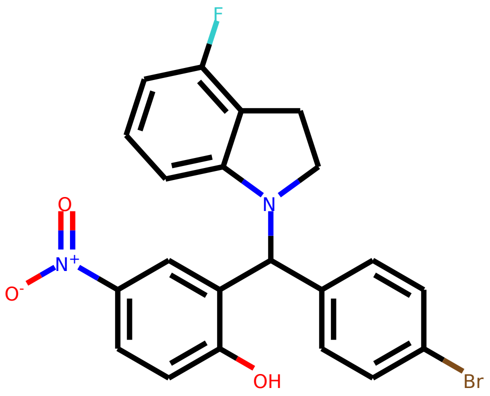

Ahma-1185

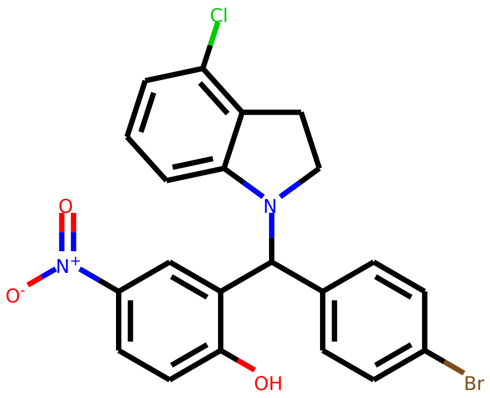

Ahma-1186

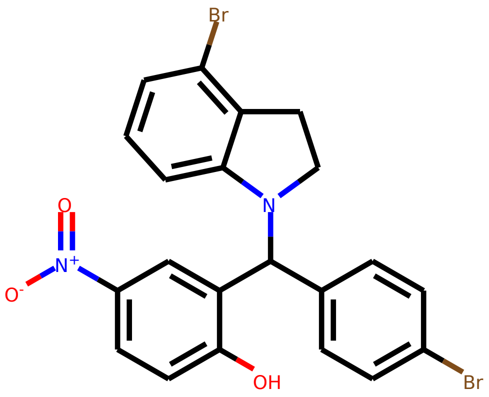

Ahma-1187

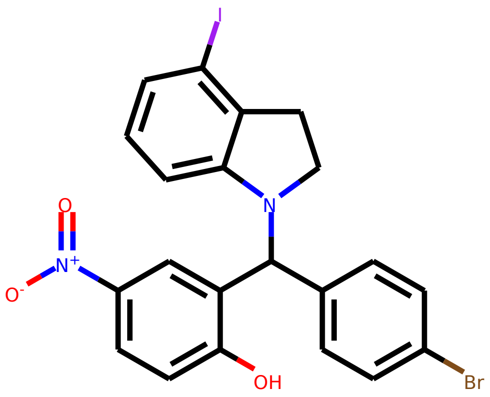

Ahma-1188

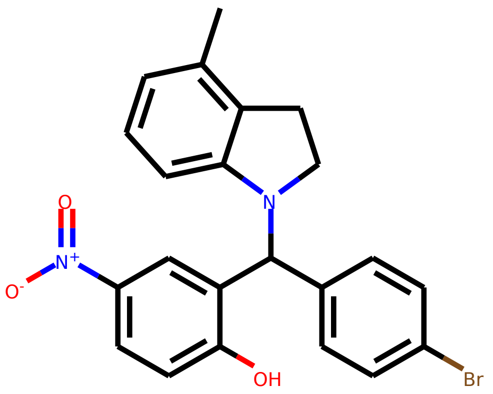

Ahma-1189

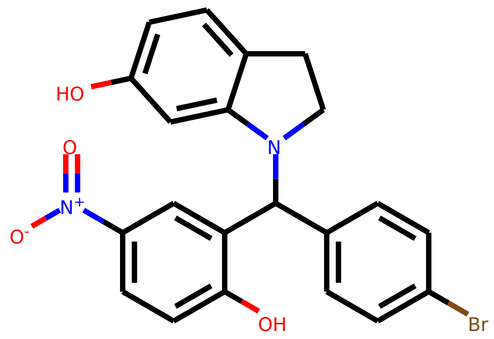

Ahma-1190

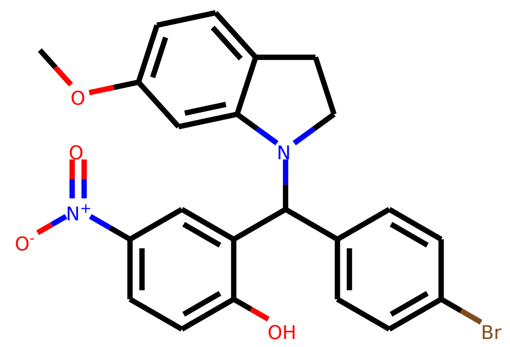
Ahma-1191

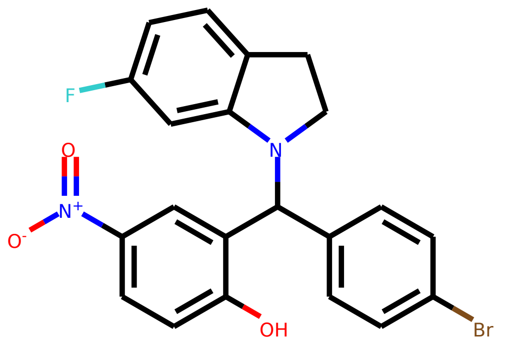
Ahma-1192

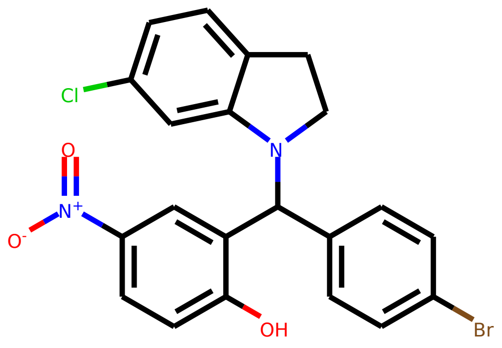
Ahma-1193

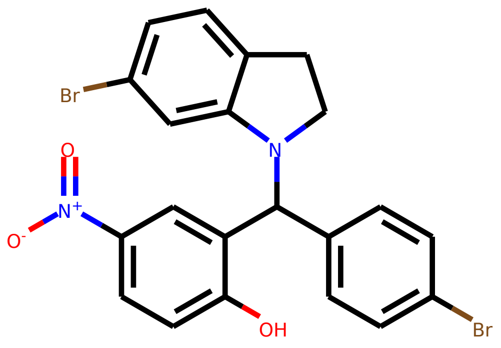
Ahma-1194

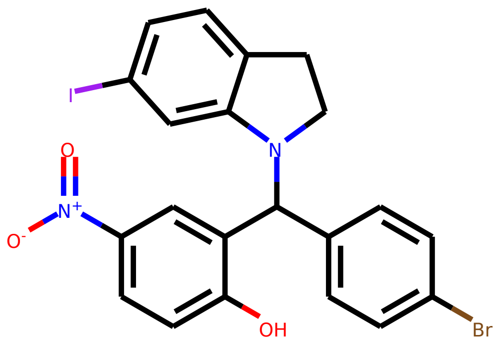
Ahma-1195

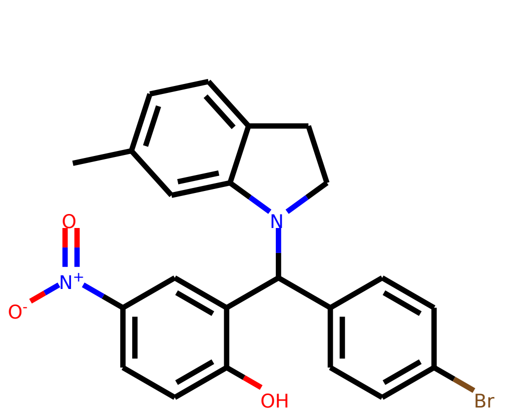
Ahma-1196

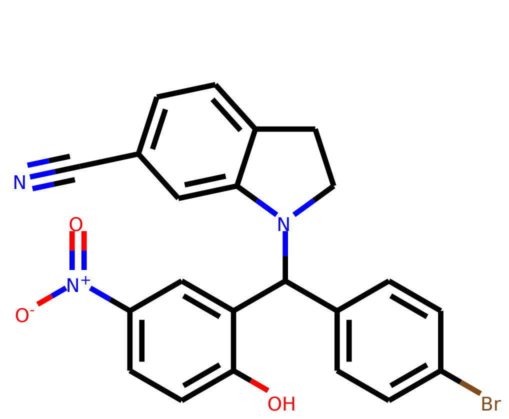
Ahma-1197

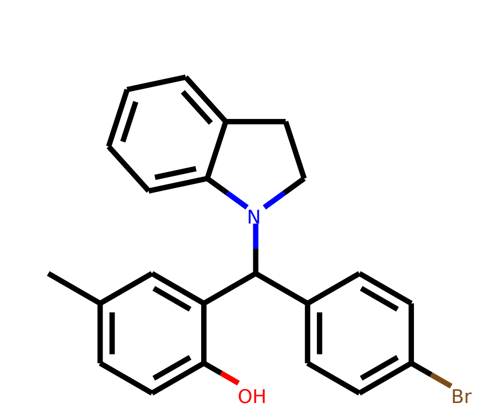
Ahma-1198

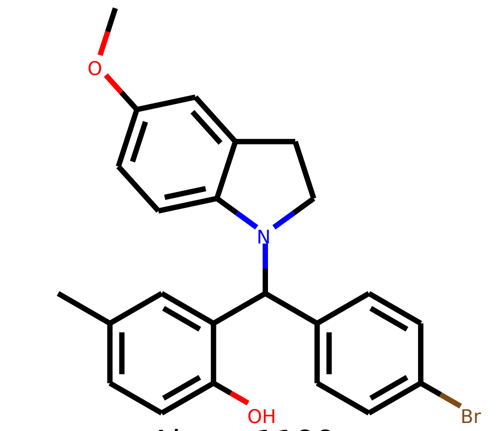
Ahma-1199

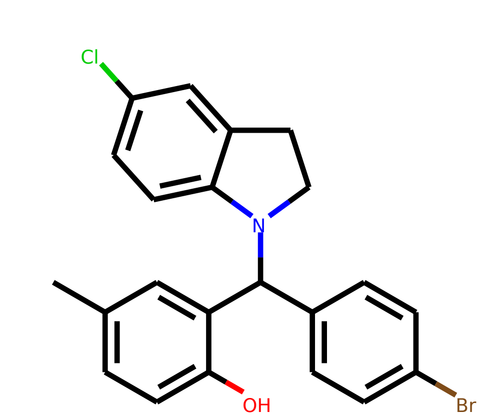
Ahma-1200

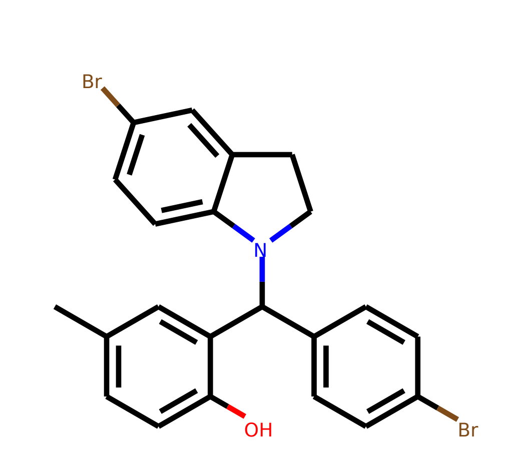
Ahma-1201

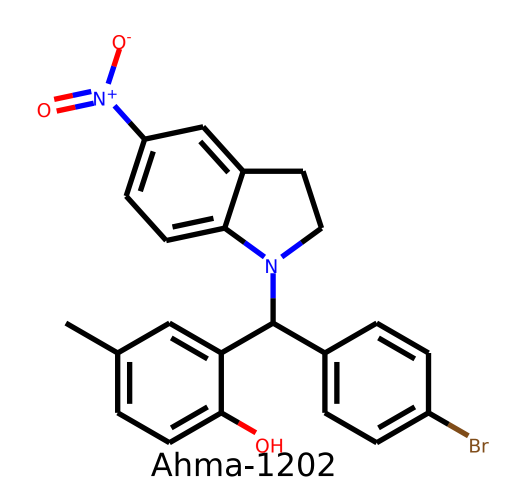
Ahma-1202

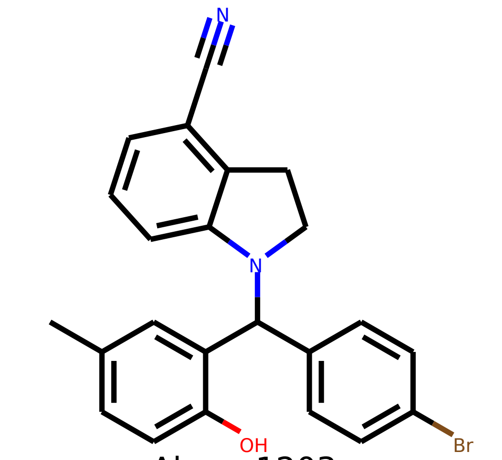
Ahma-1203

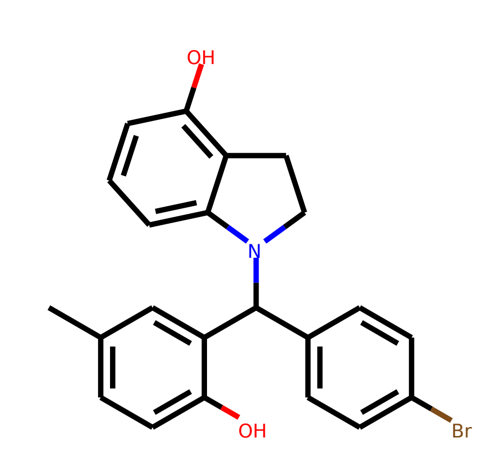
Ahma-1204

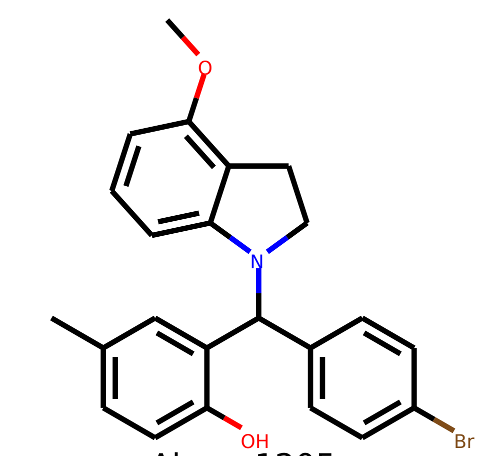
Ahma-1205

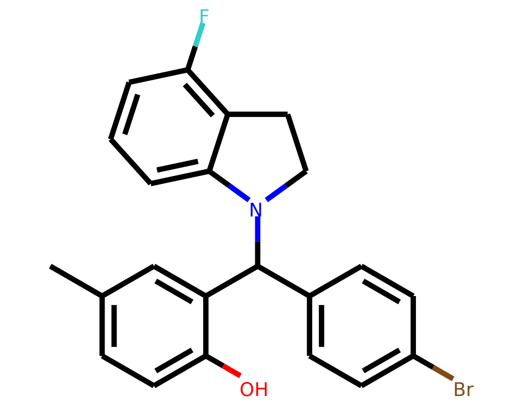
Ahma-1206

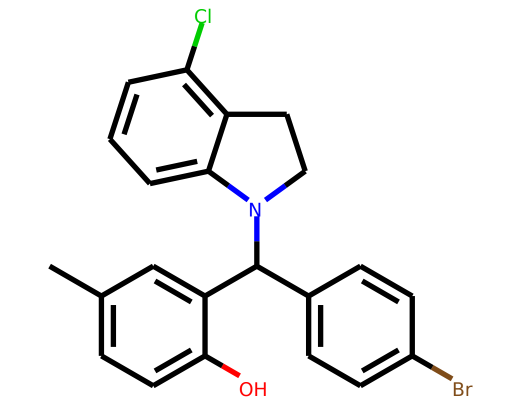
Ahma-1207

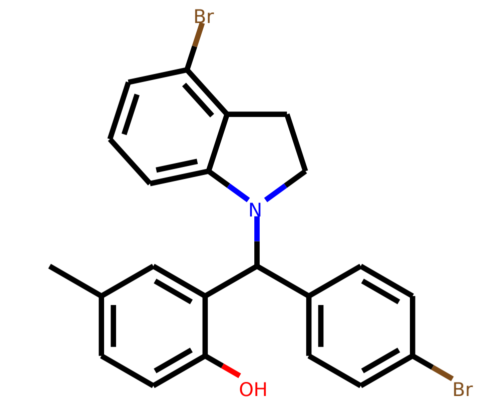
Ahma-1208

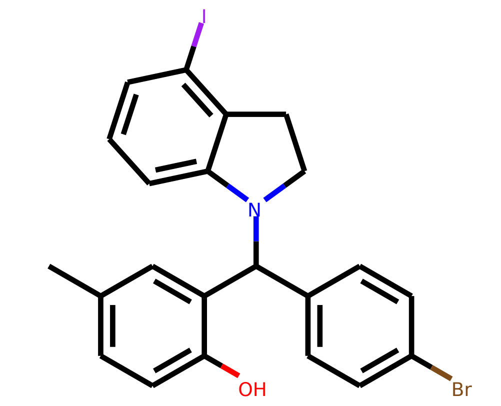
Ahma-1209

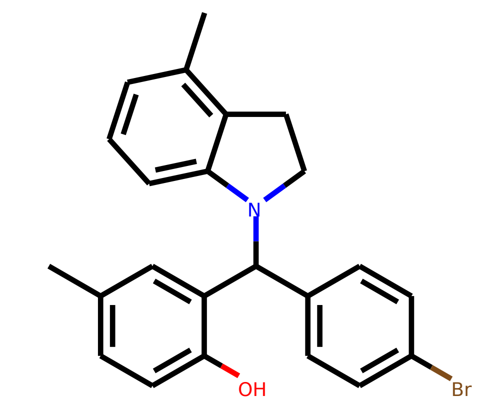
Ahma-1210

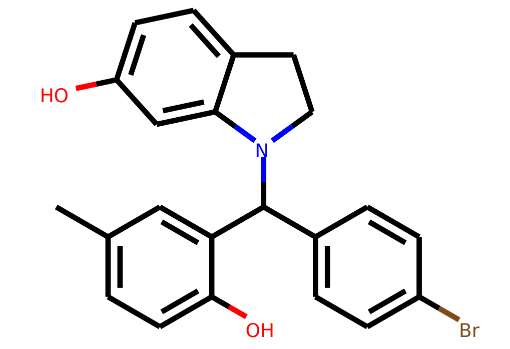
Ahma-1211

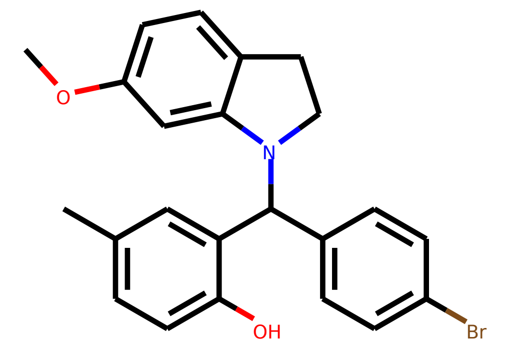
Ahma-1212

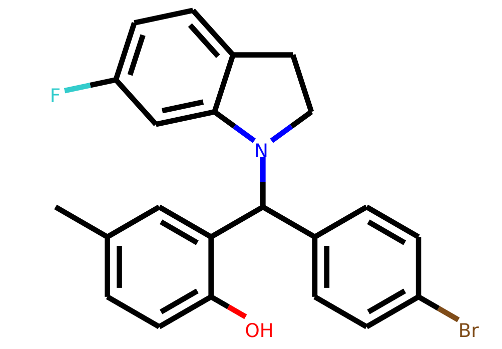
Ahma-1213

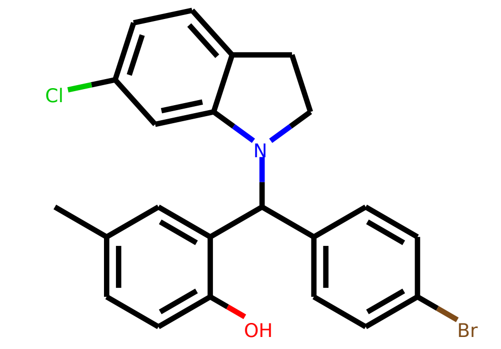
Ahma-1214

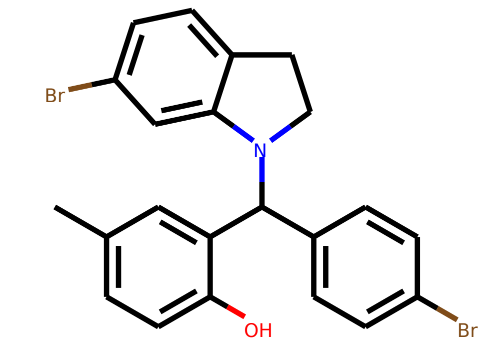
Ahma-1215

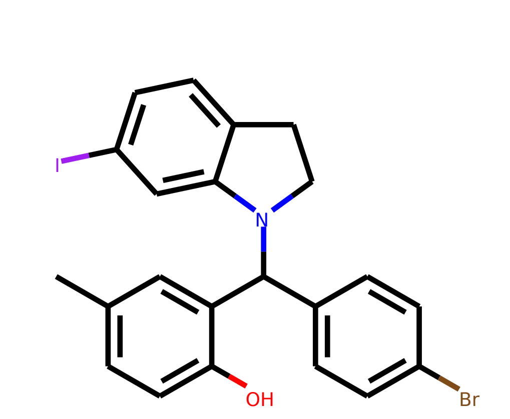
Ahma-1216

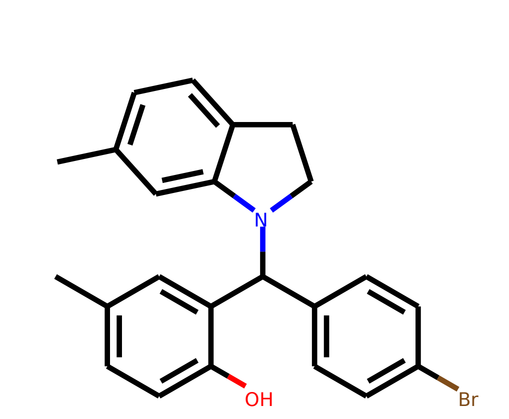
Ahma-1217

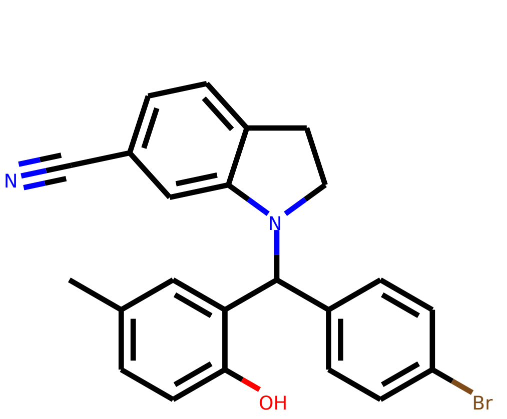
Ahma-1218

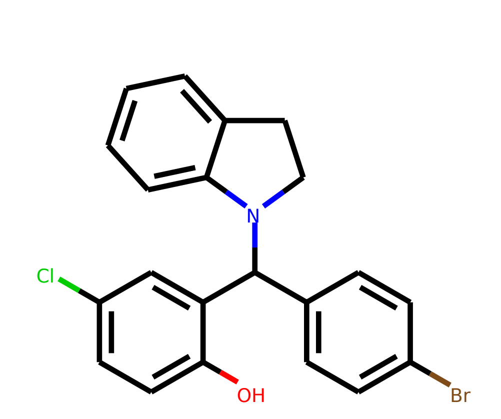
Ahma-1219

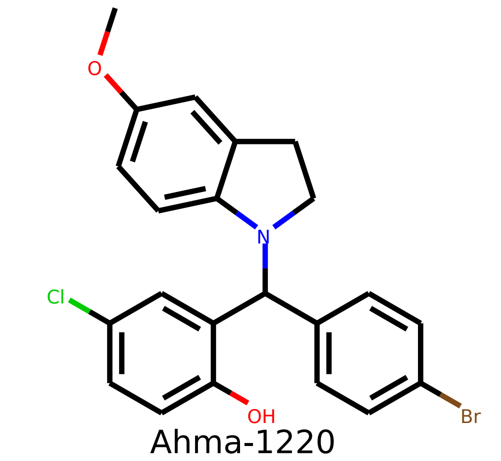
Ahma-1220

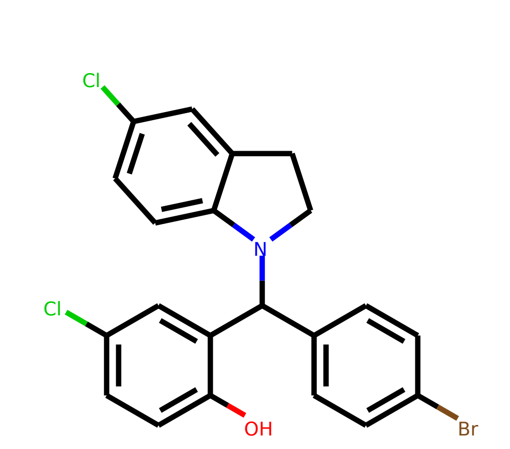
Ahma-1221

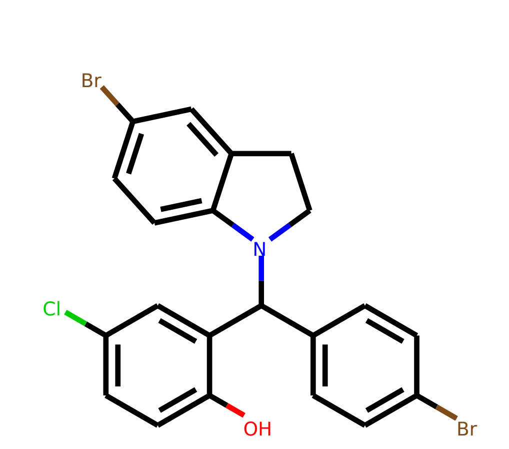
Ahma-1222

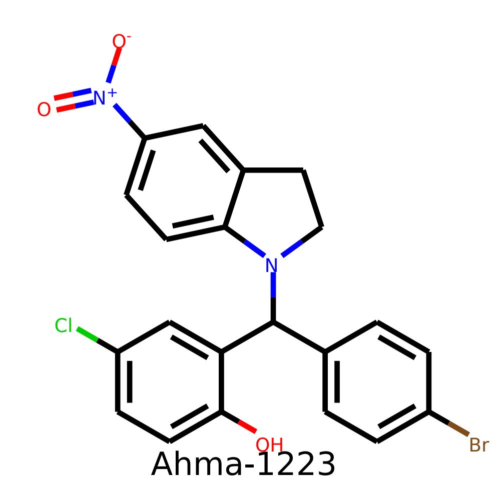
Ahma-1223

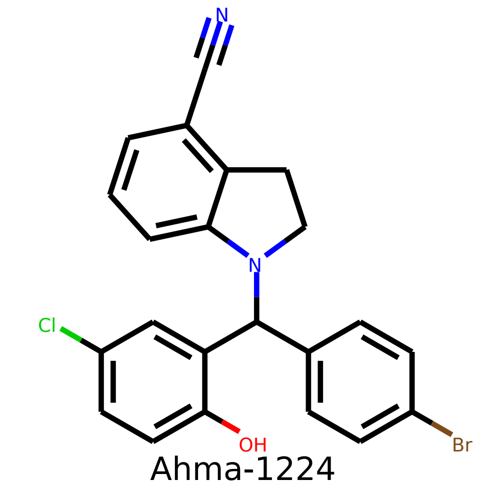
Ahma-1224

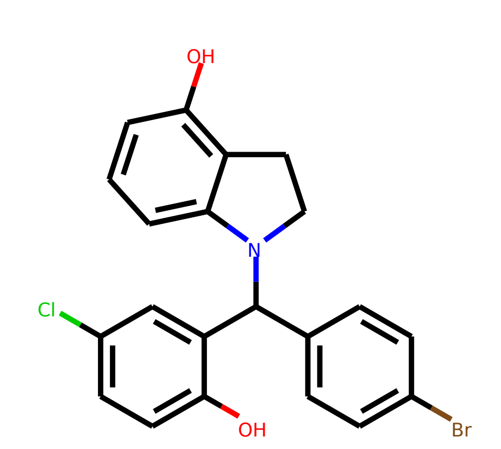
Ahma-1225

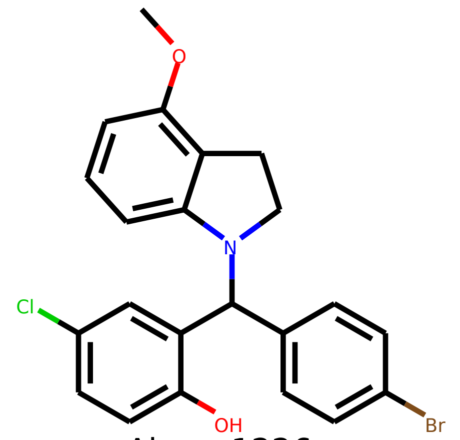

Ahma-1226

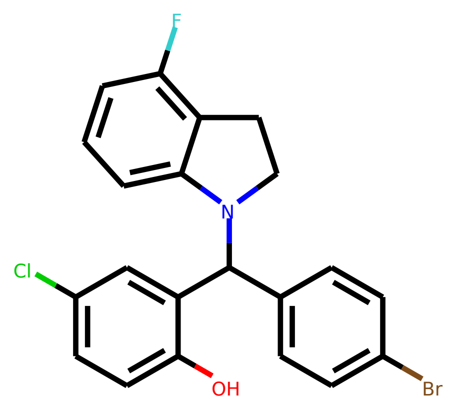

Ahma-1227

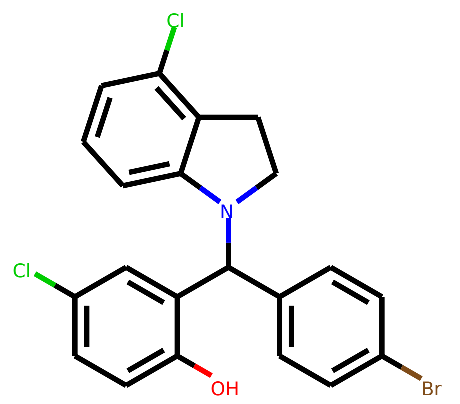

Ahma-1228

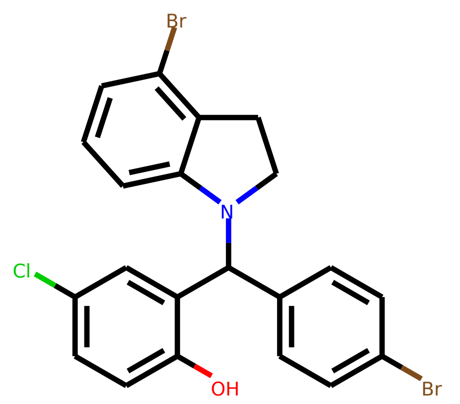

Ahma-1229

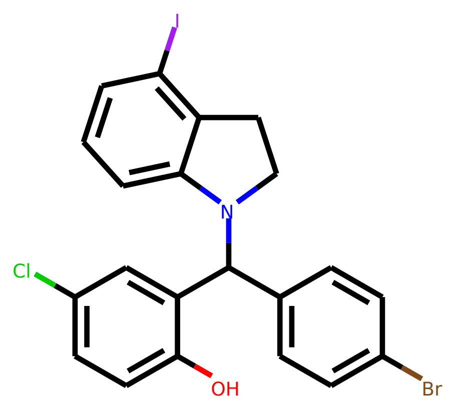

Ahma-1230

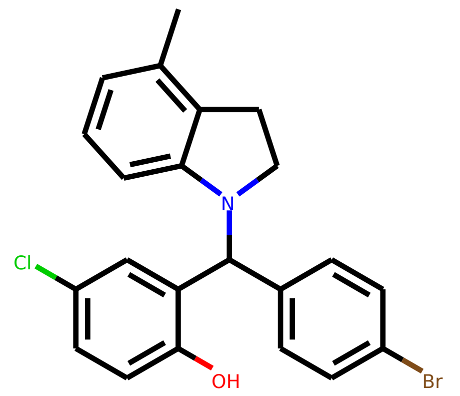

Ahma-1231

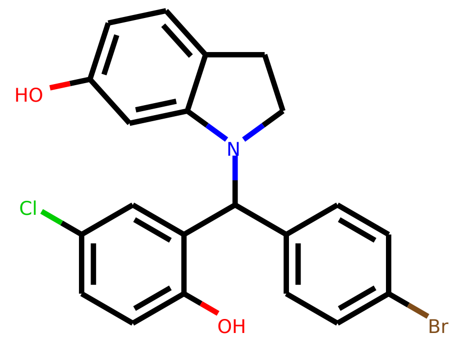

Ahma-1232

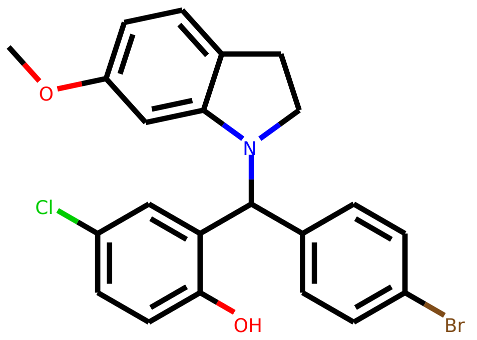

Ahma-1233

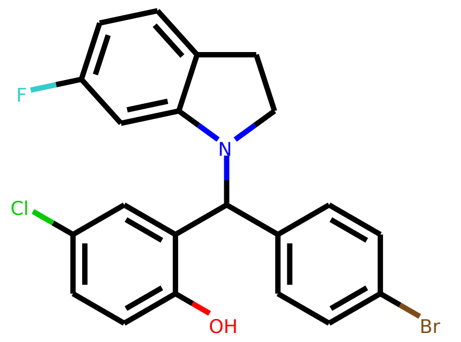

Ahma-1234

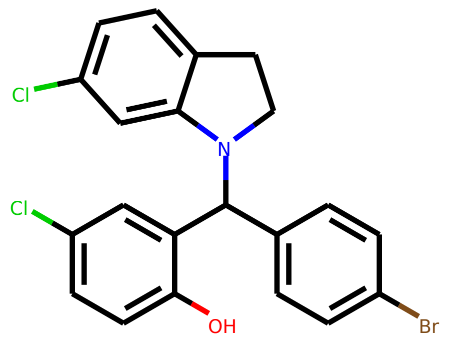

Ahma-1235

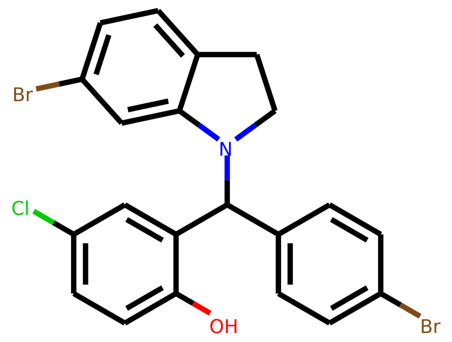

Ahma-1236

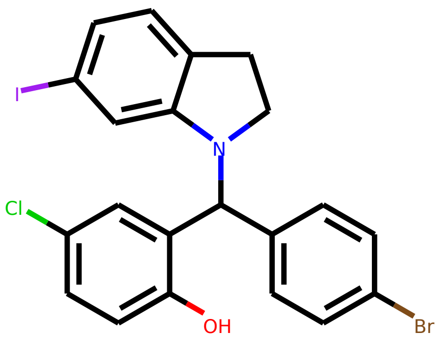

Ahma-1237

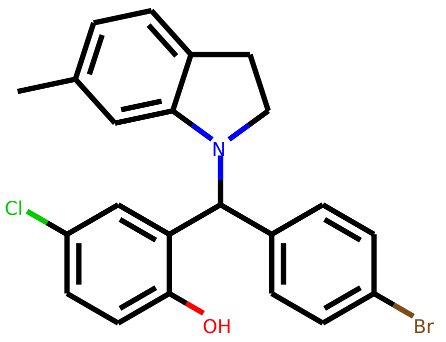

Ahma-1238

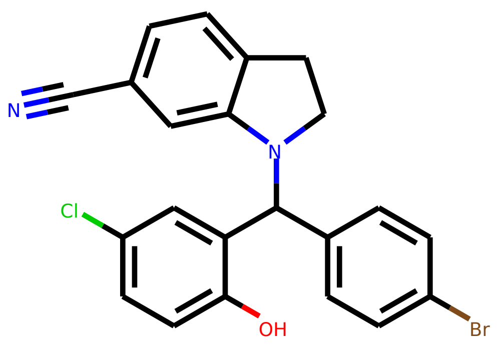

Ahma-1239

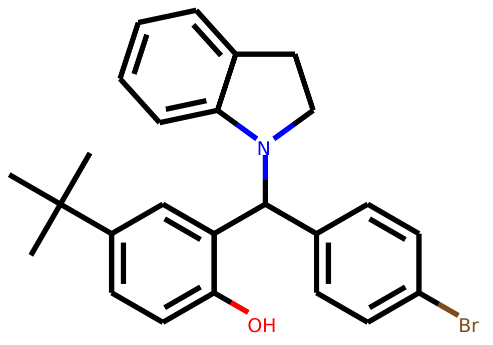

Ahma-1240

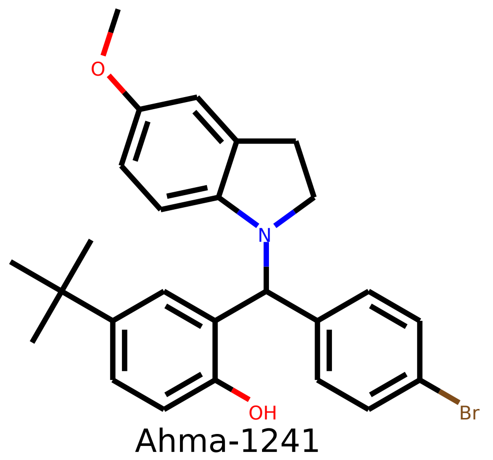

Ahma-1241

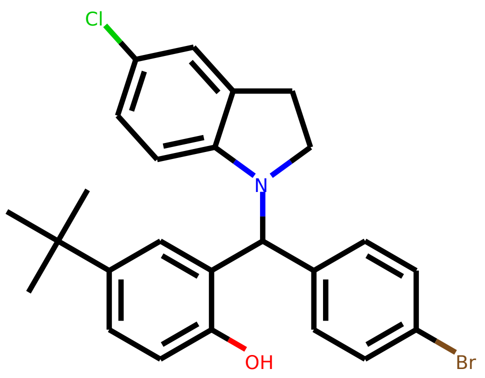

Ahma-1242

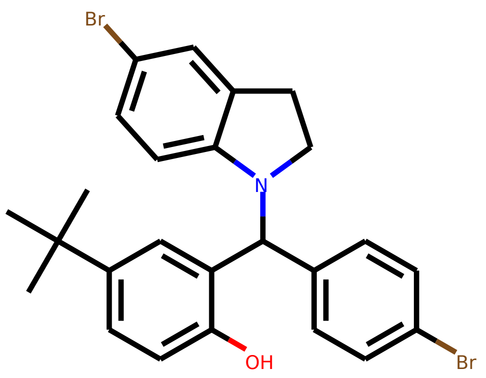

Ahma-1243

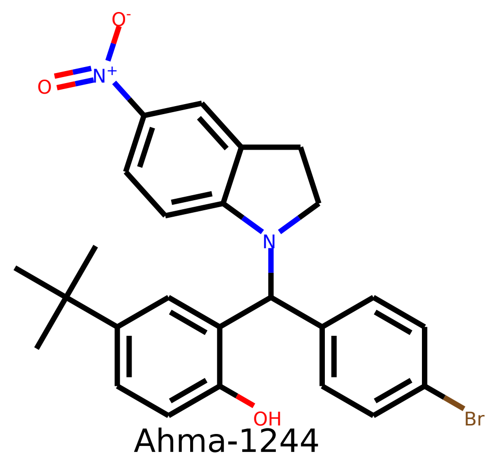

Ahma-1244

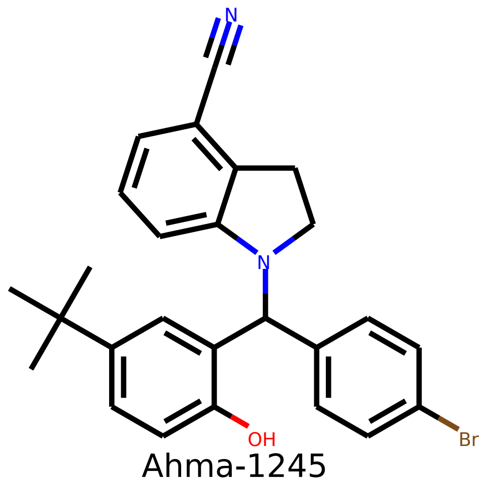

Ahma-1245

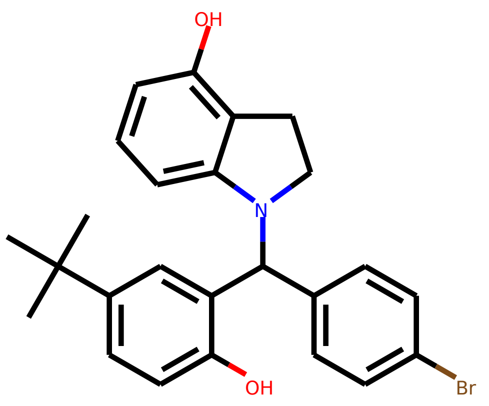

Ahma-1246

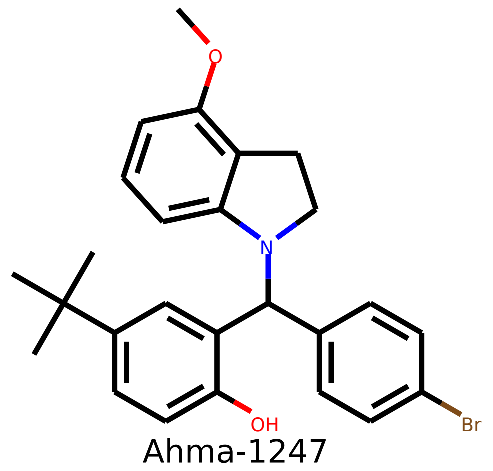

Ahma-1247

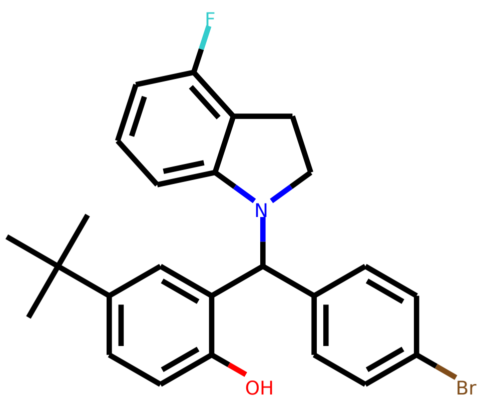

Ahma-1248

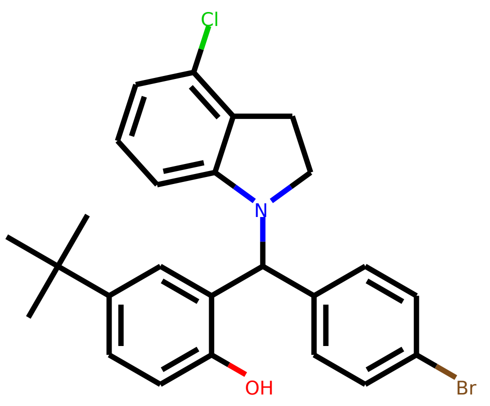

Ahma-1249

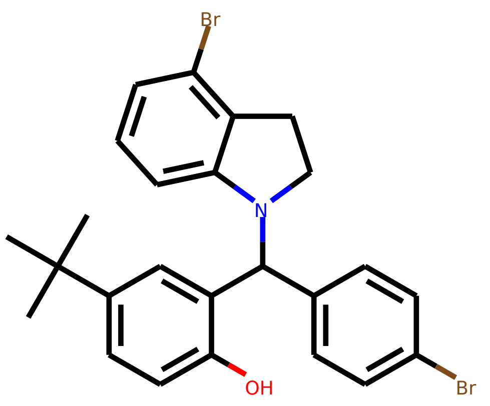

Ahma-1250

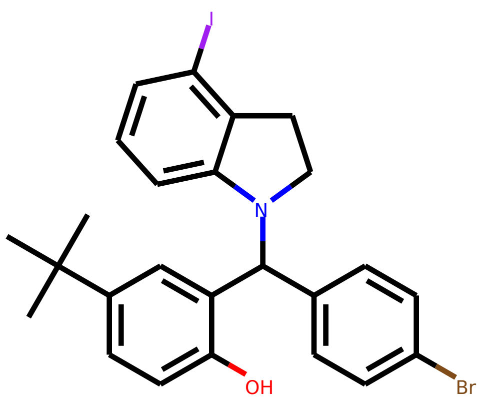

Ahma-1251

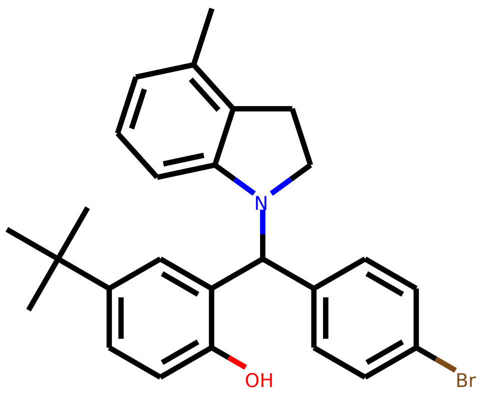

Ahma-1252

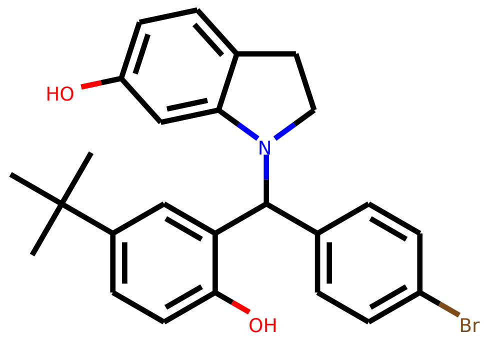

Ahma-1253

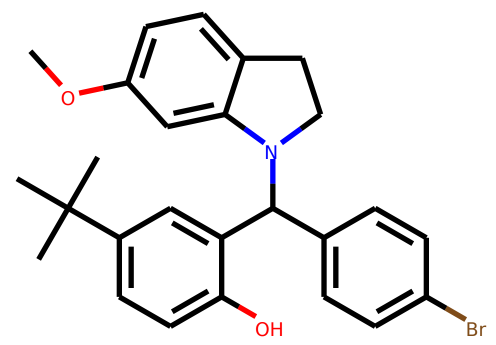

Ahma-1254

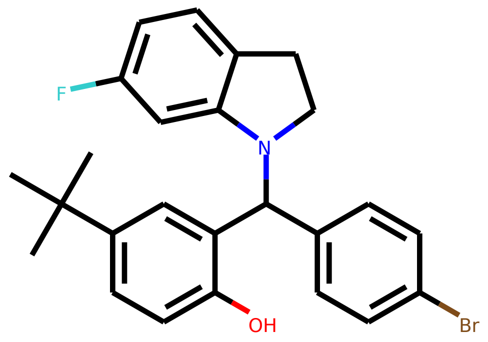

Ahma-1255

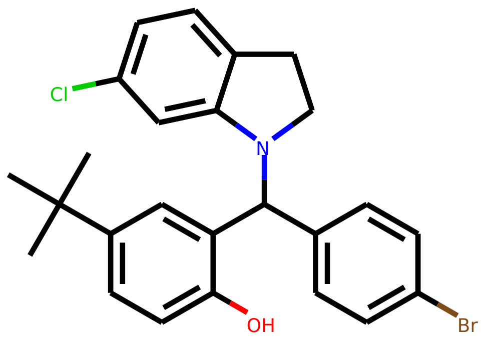

Ahma-1256

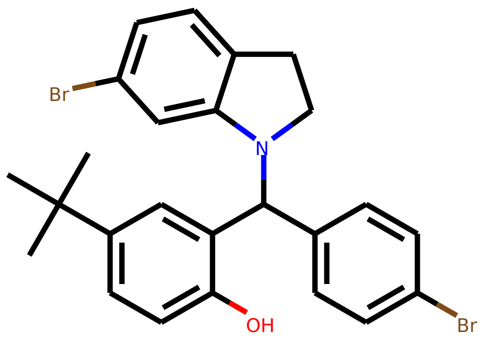

Ahma-1257

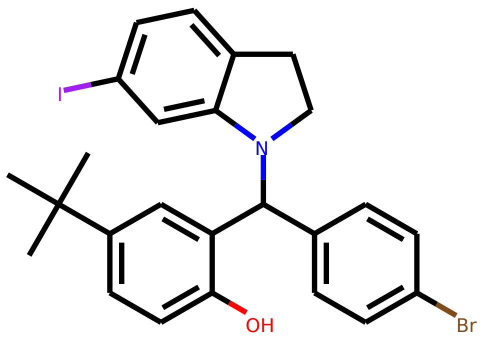

Ahma-1258

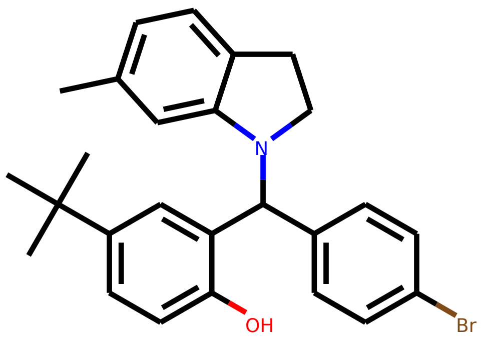

Ahma-1259

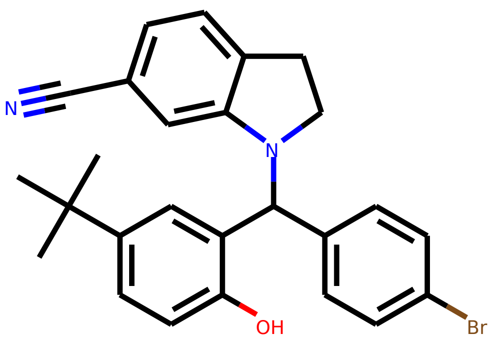

Ahma-1260

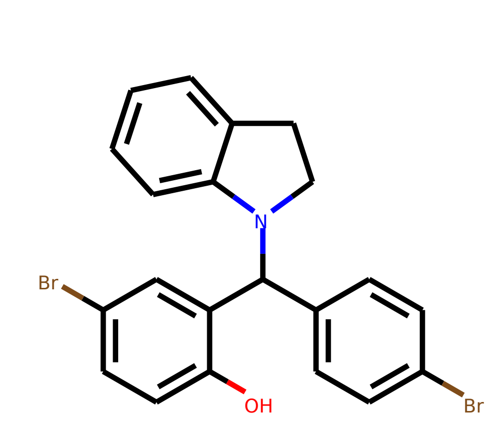
Ahma-1261
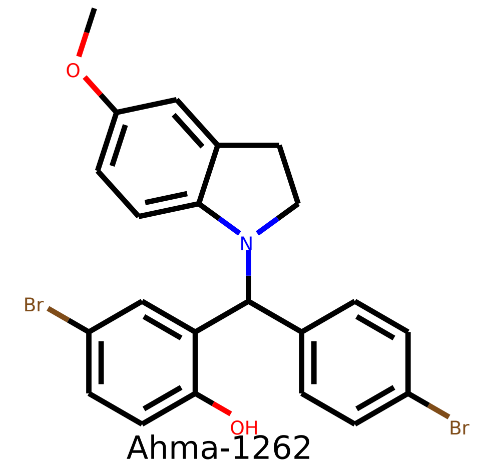
Ahma-1262
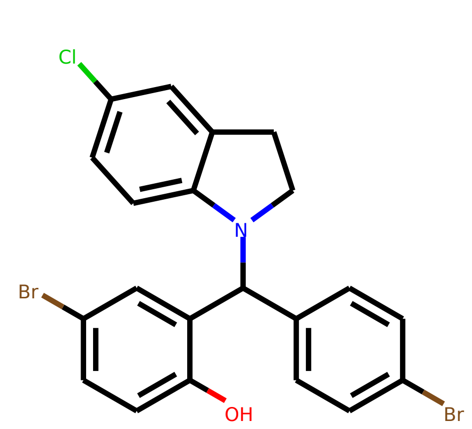
Ahma-1263
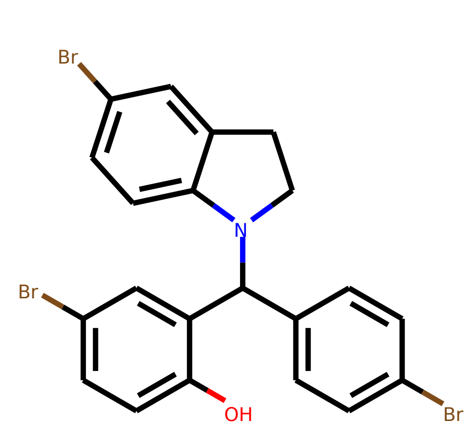
Ahma-1264
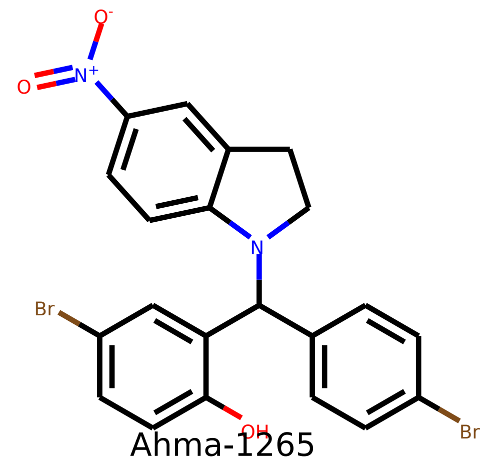
Ahma-1265
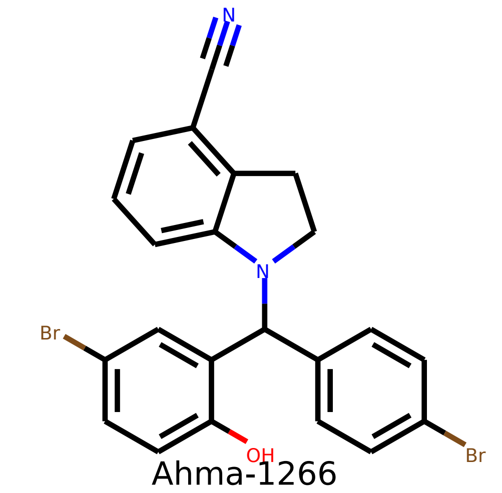
Ahma-1266
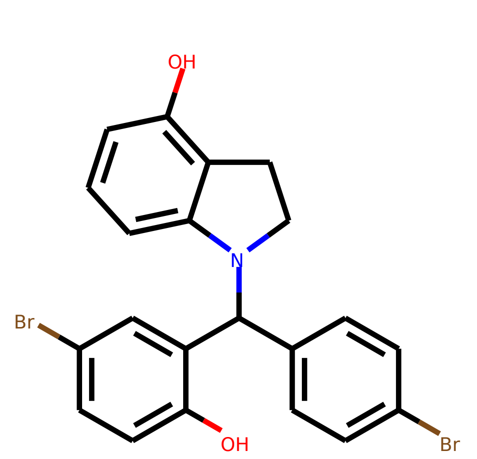
Ahma-1267
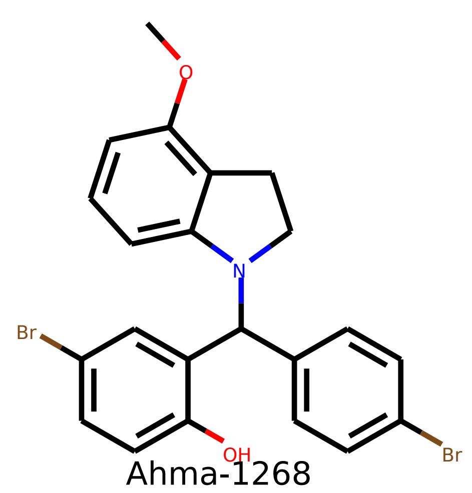
Ahma-1268
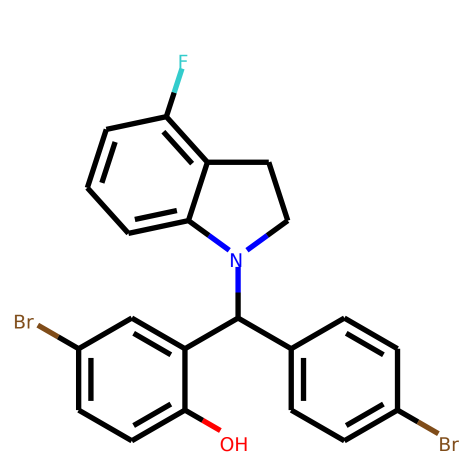
Ahma-1269
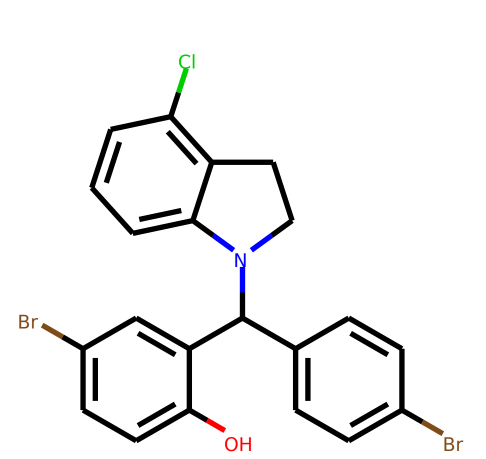
Ahma-1270
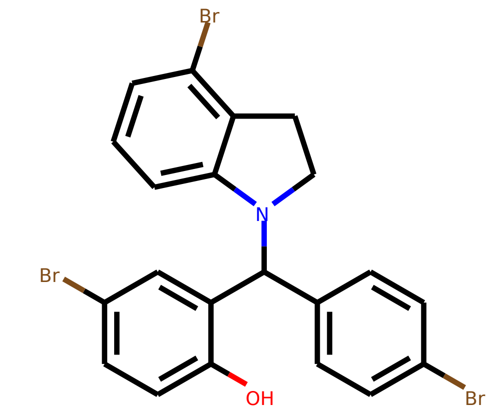
Ahma-1271
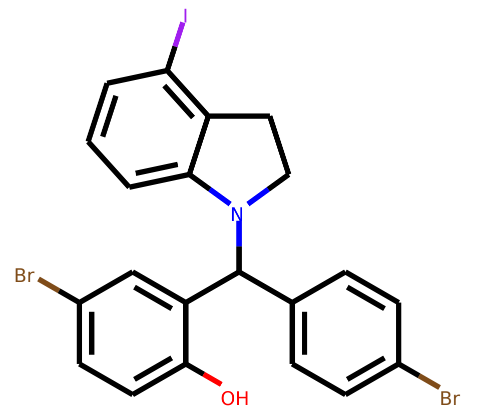
Ahma-1272
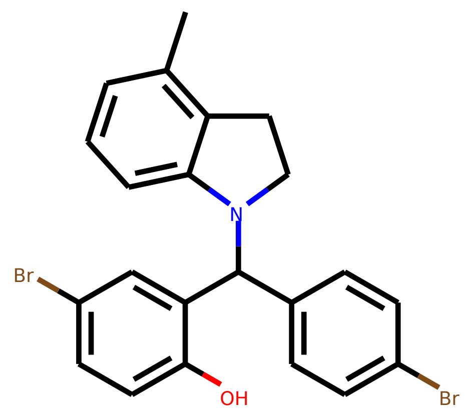
Ahma-1273
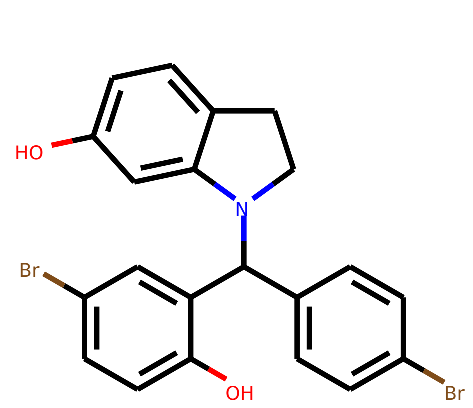
Ahma-1274
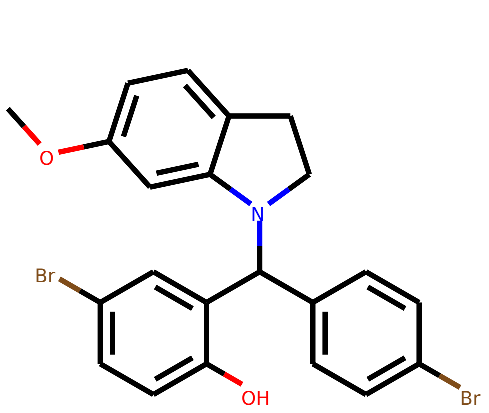
Ahma-1275
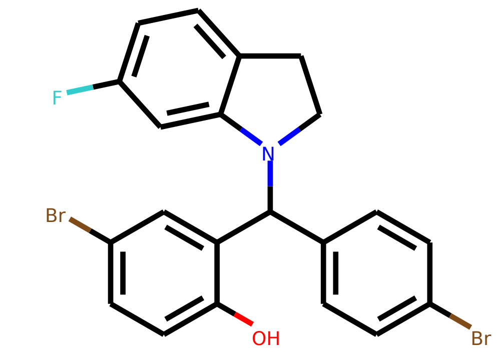
Ahma-1276
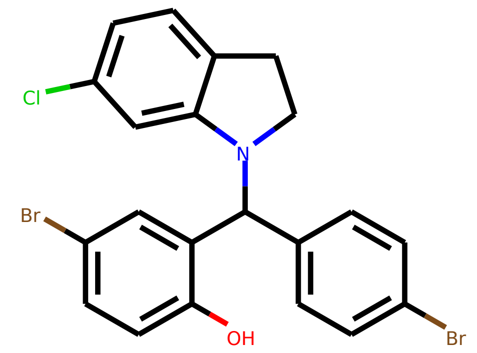
Ahma-1277
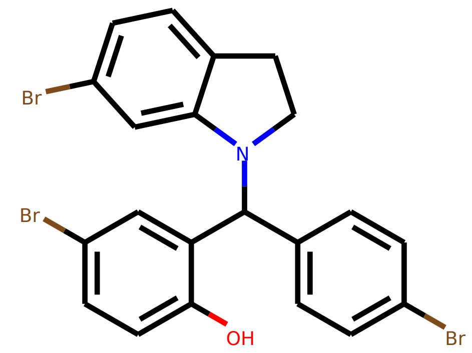
Ahma-1278
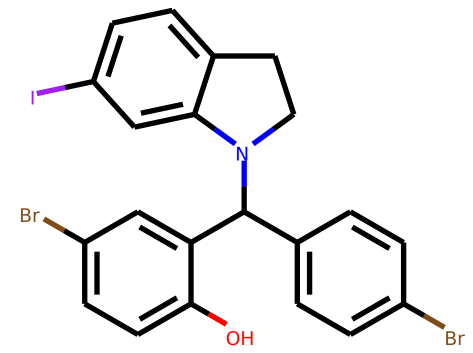
Ahma-1279
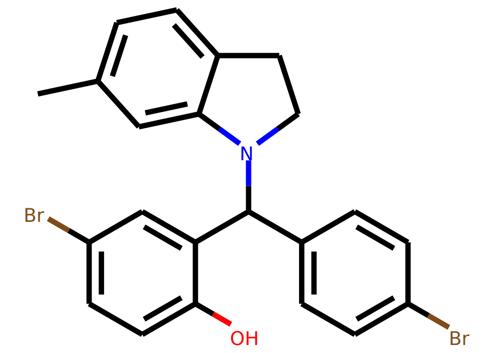
Ahma-1280
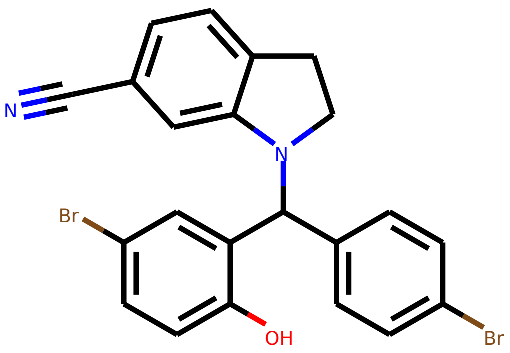
Ahma-1281
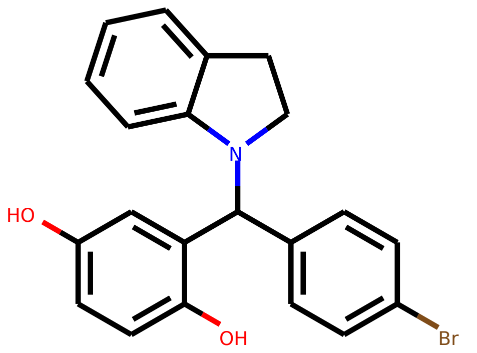
Ahma-1282
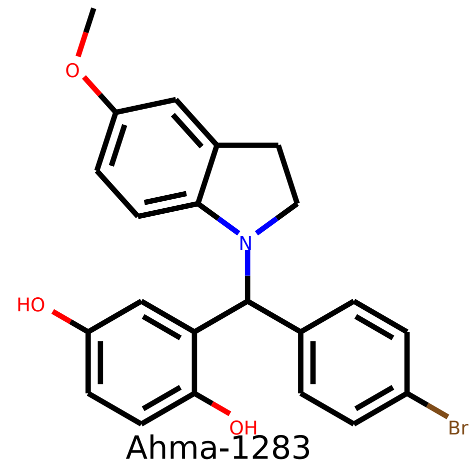
Ahma-1283
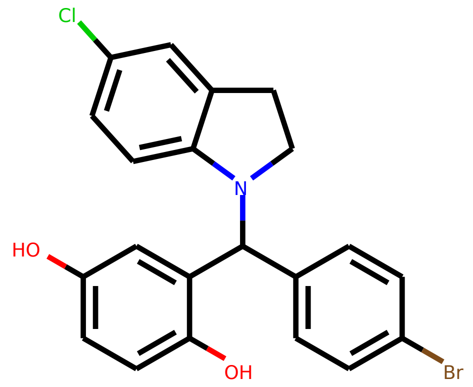
Ahma-1284
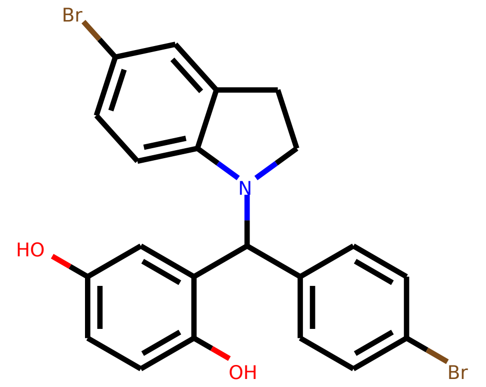
Ahma-1285
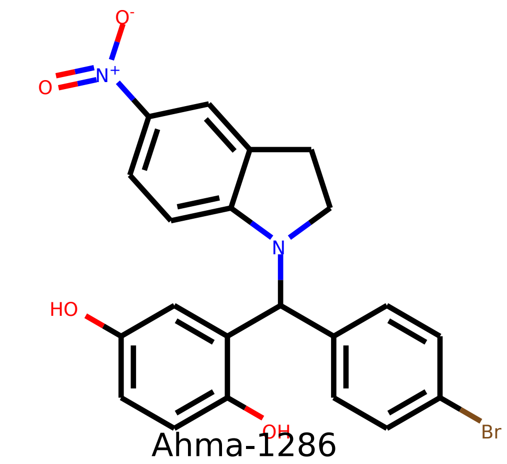
Ahma-1286
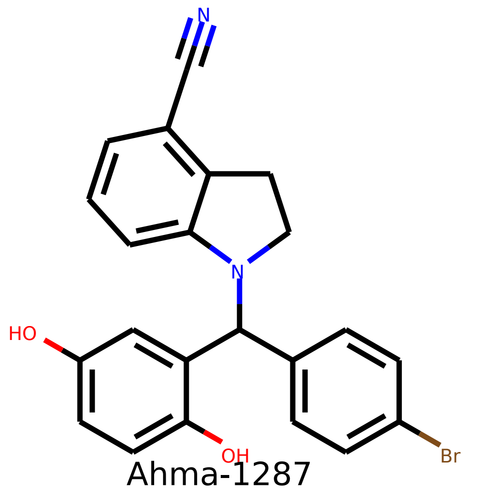
Ahma-1287
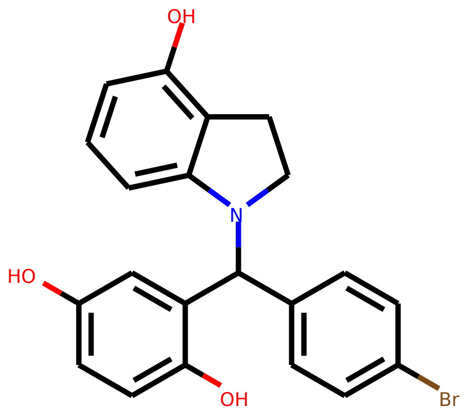
Ahma-1288
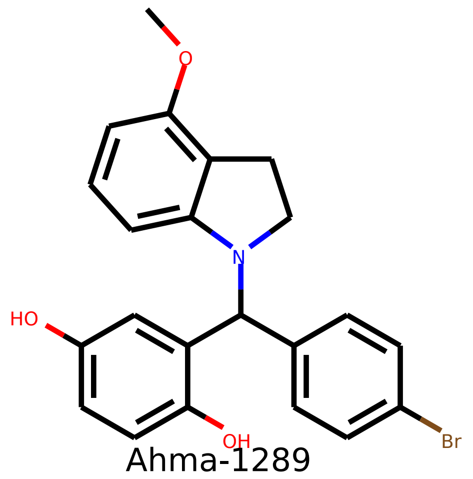
Ahma-1289
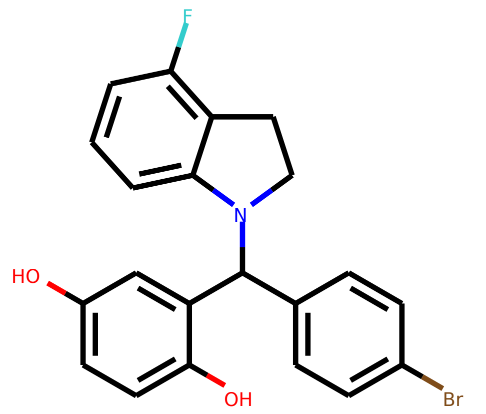
Ahma-1290
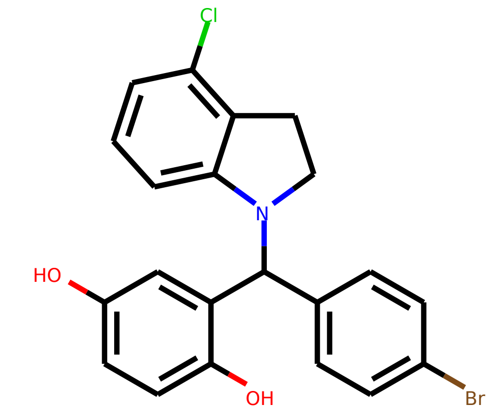
Ahma-1291
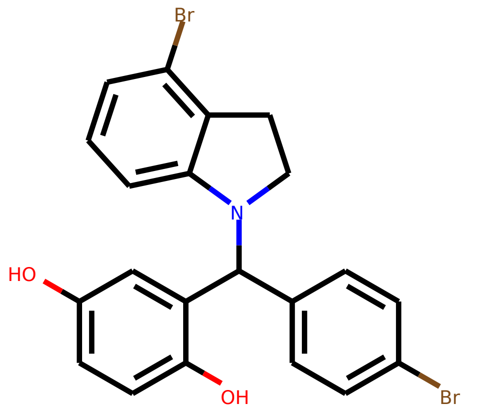
Ahma-1292
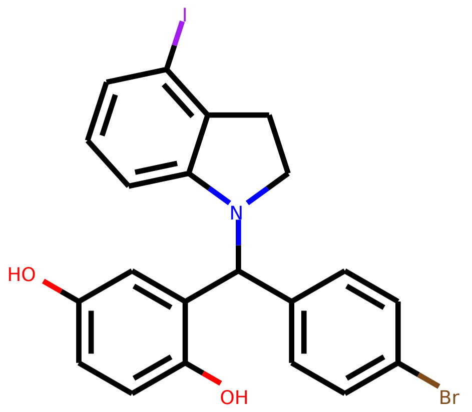
Ahma-1293
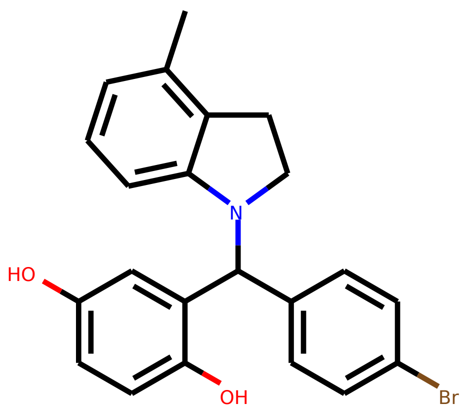
Ahma-1294
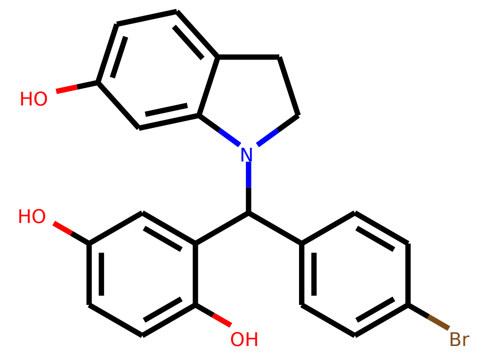
Ahma-1295

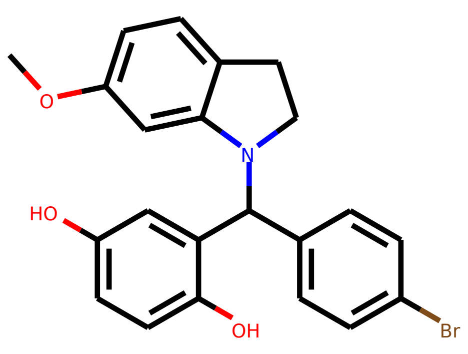
Ahma-1296

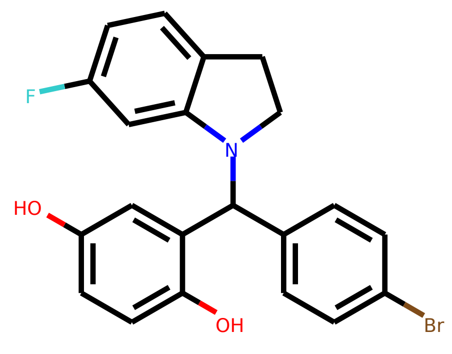
Ahma-1297

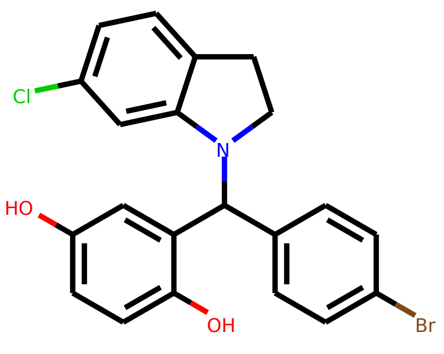
Ahma-1298

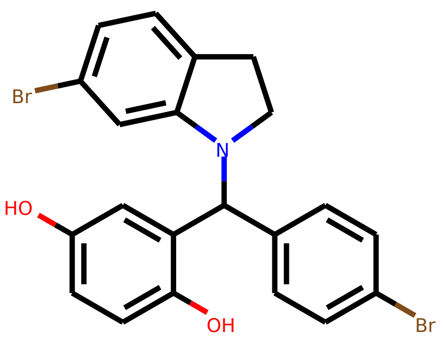
Ahma-1299

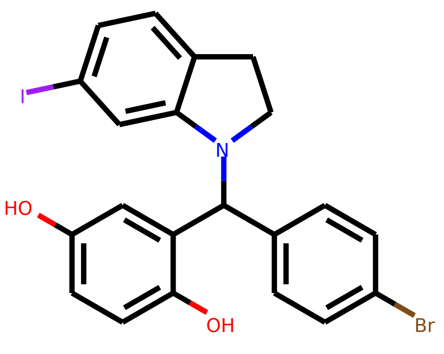
Ahma-1300

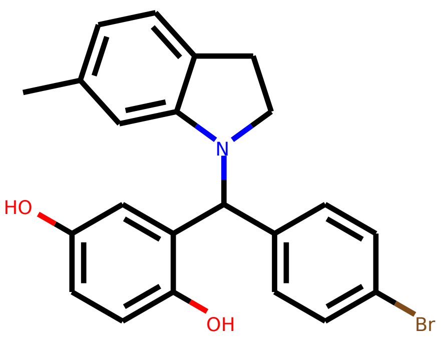
Ahma-1301

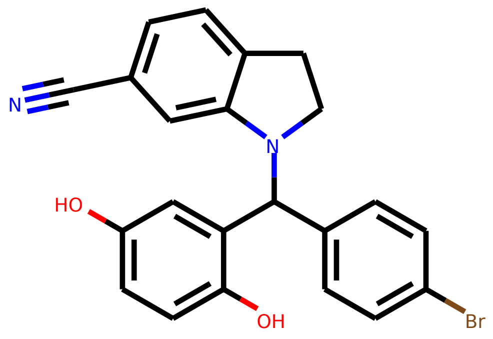
Ahma-1302

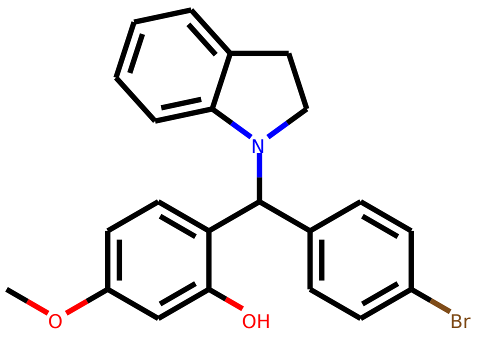
Ahma-1303

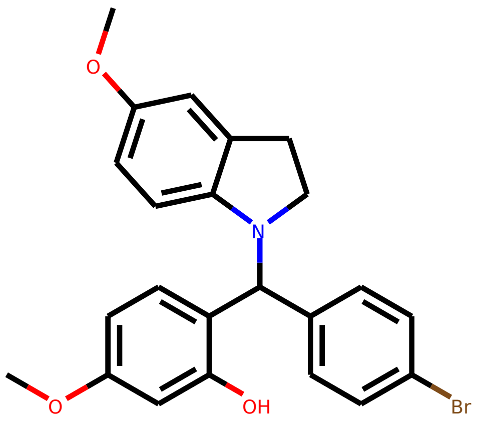
Ahma-1304

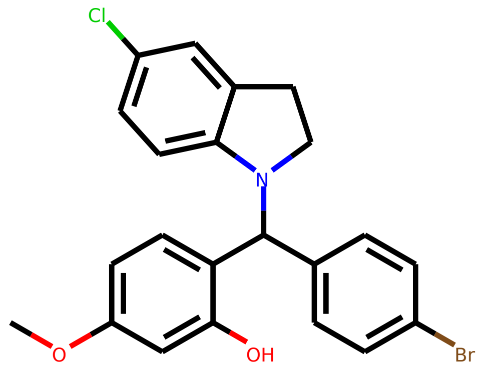
Ahma-1305

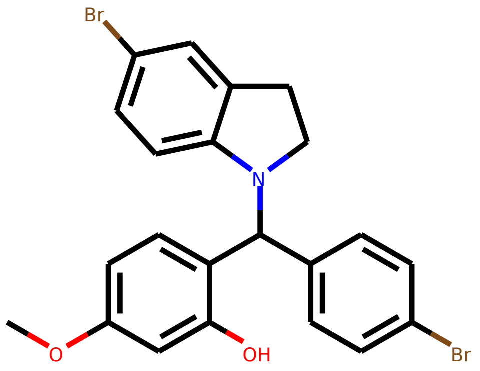
Ahma-1306

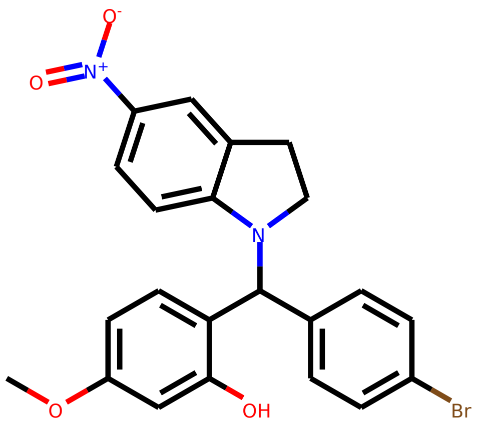
Ahma-1307

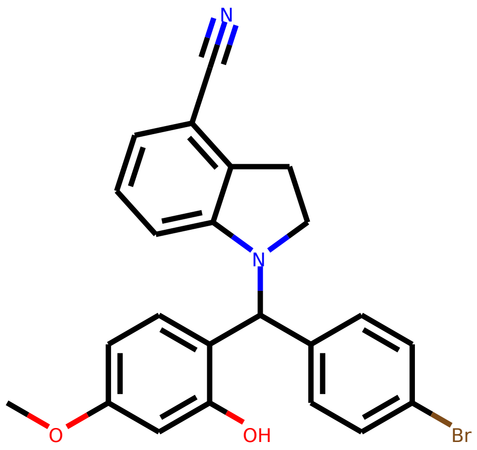
Ahma-1308

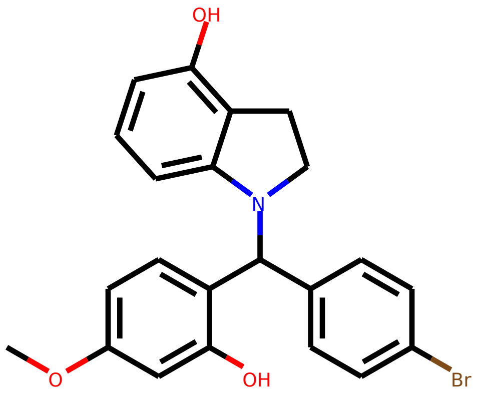
Ahma-1309

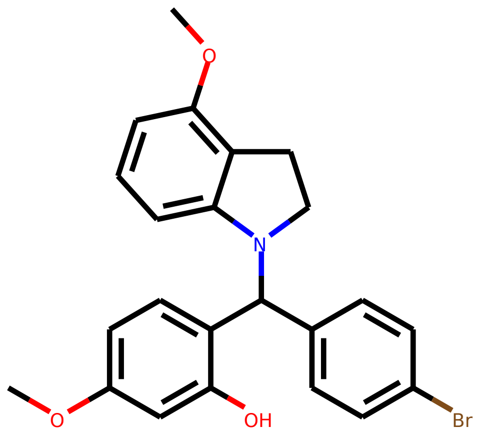
Ahma-1310

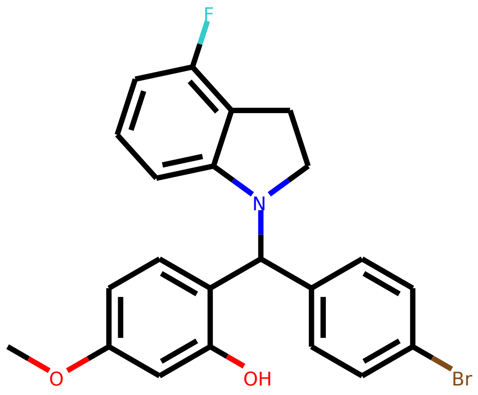
Ahma-1311

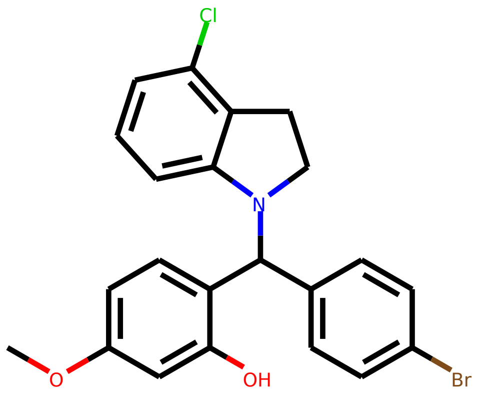
Ahma-1312

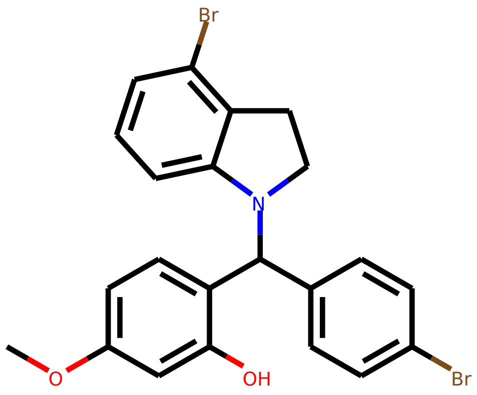
Ahma-1313

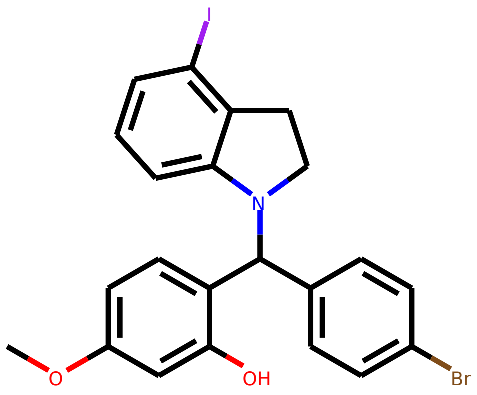
Ahma-1314

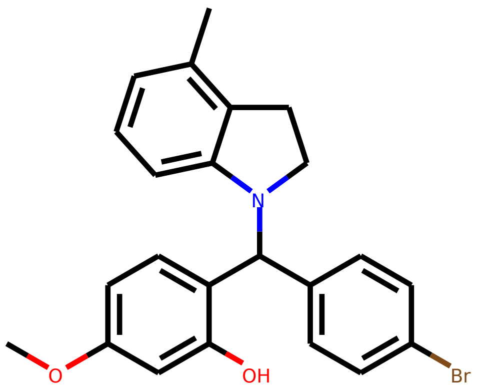
Ahma-1315

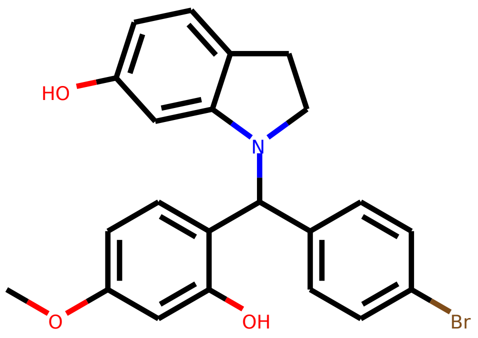
Ahma-1316

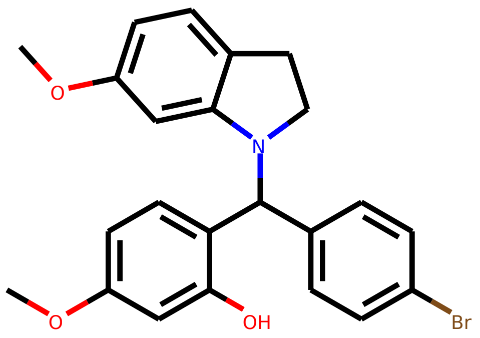
Ahma-1317

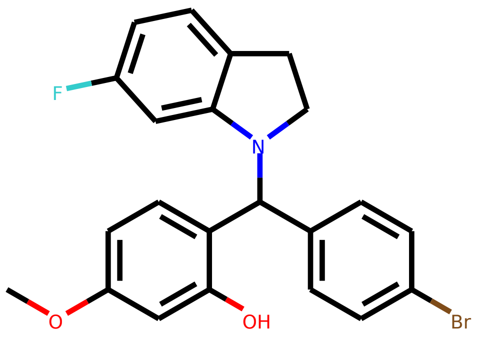
Ahma-1318

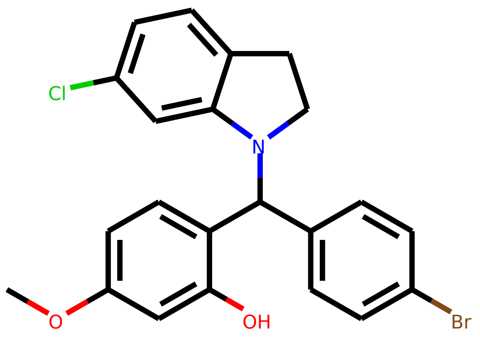
Ahma-1319

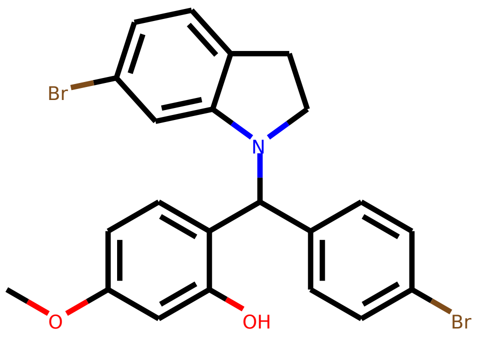
Ahma-1320

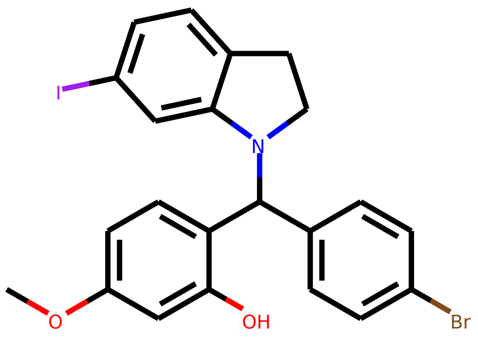
Ahma-1321

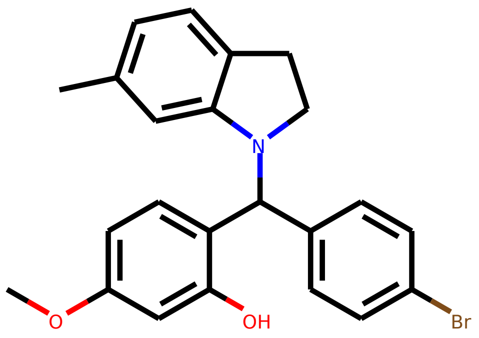
Ahma-1322

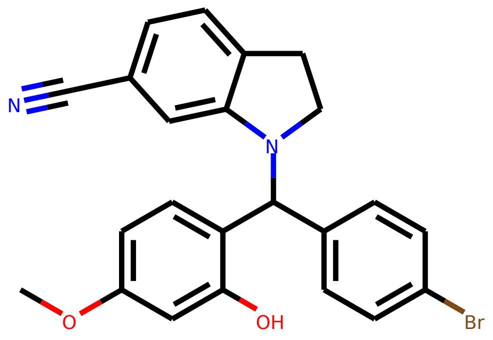
Ahma-1323

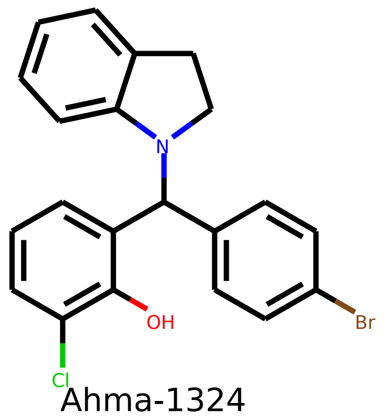
Ahma-1324

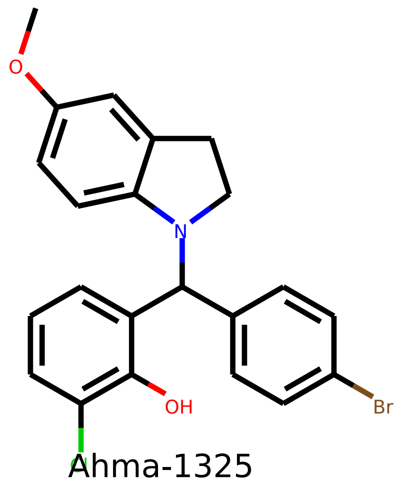
Ahma-1325

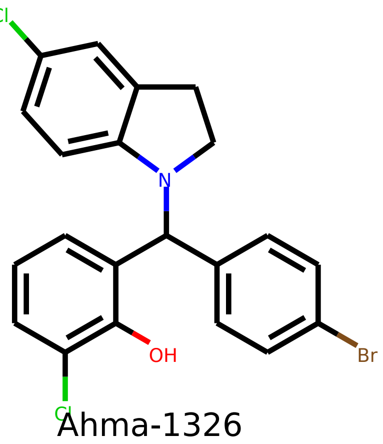
Ahma-1326

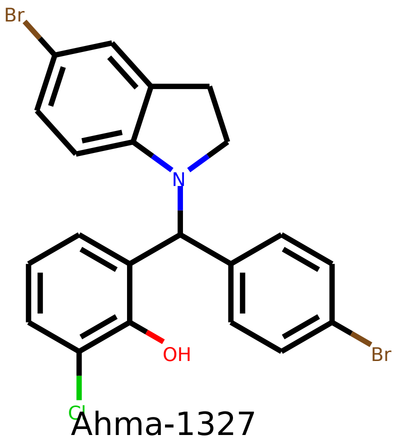
Ahma-1327

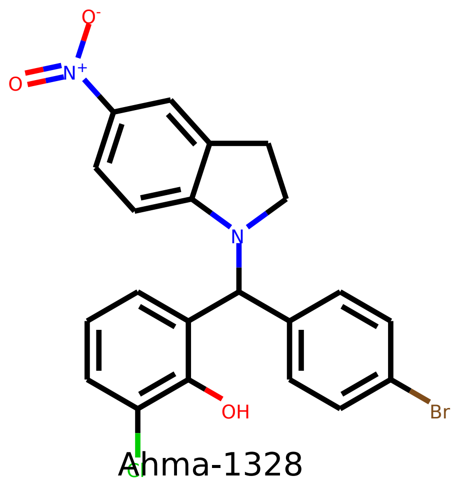
Ahma-1328

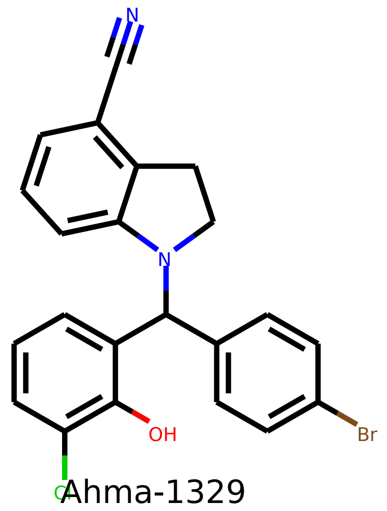
Ahma-1329

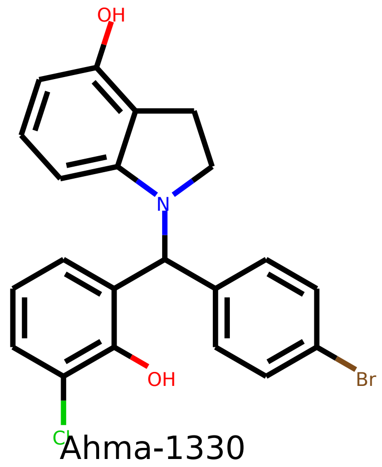
Ahma-1330

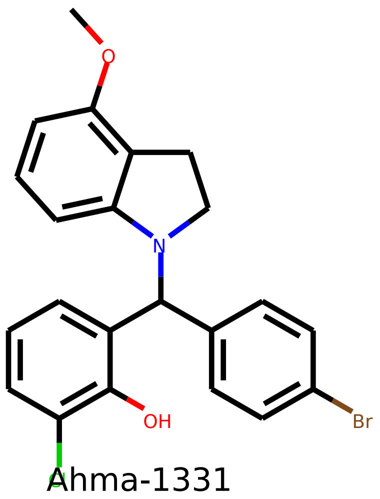
Ahma-1331

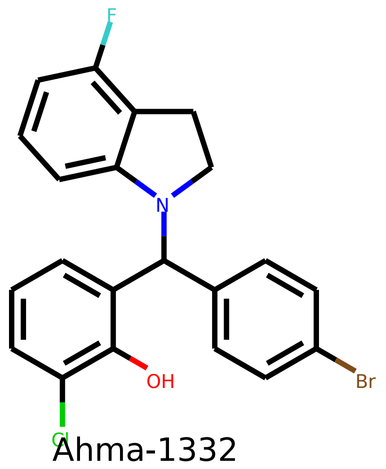
Ahma-1332

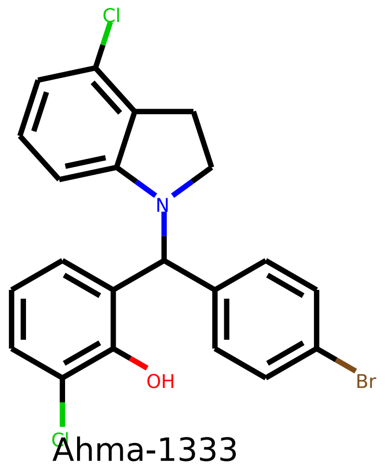
Ahma-1333

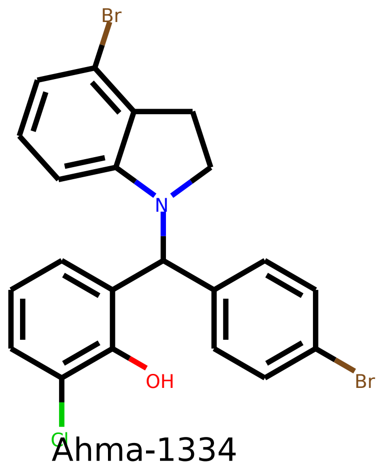
Ahma-1334

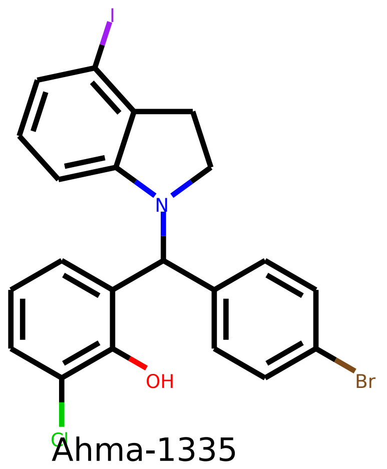
Ahma-1335

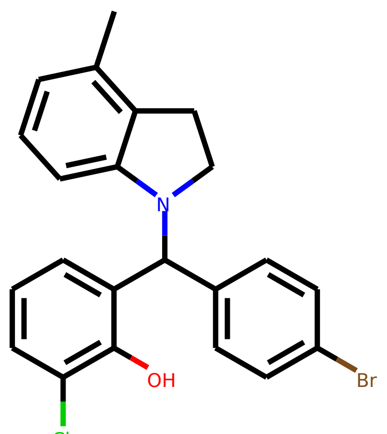
Ahma-1336

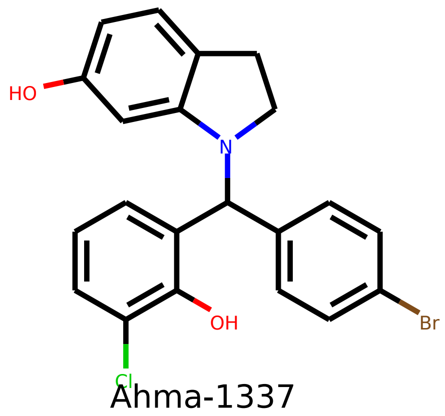
Ahma-1337

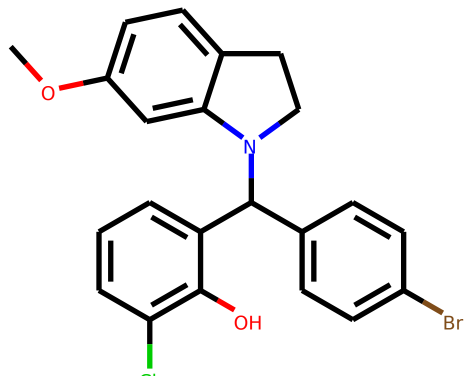
Ahma-1338

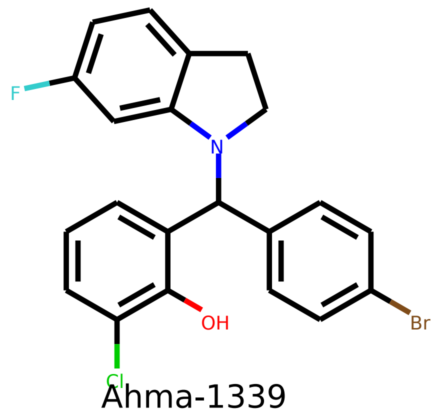
Ahma-1339

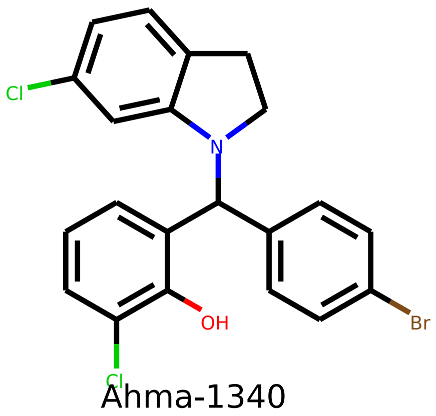
Ahma-1340

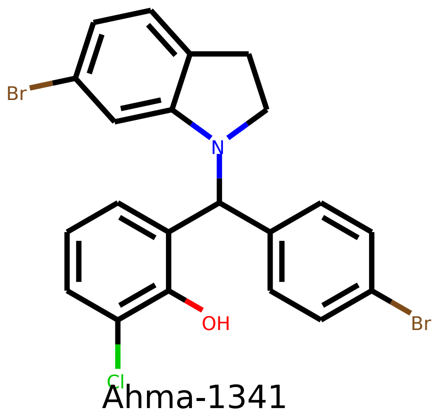
Ahma-1341

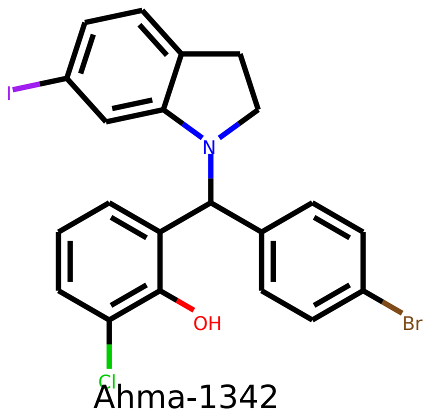
Ahma-1342

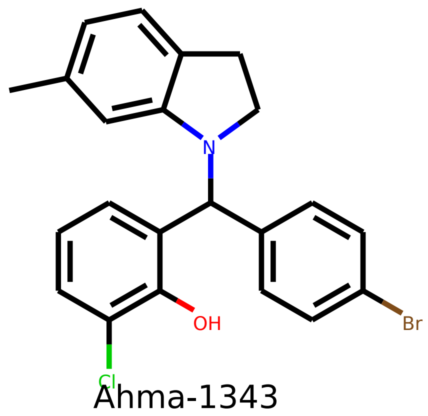
Ahma-1343

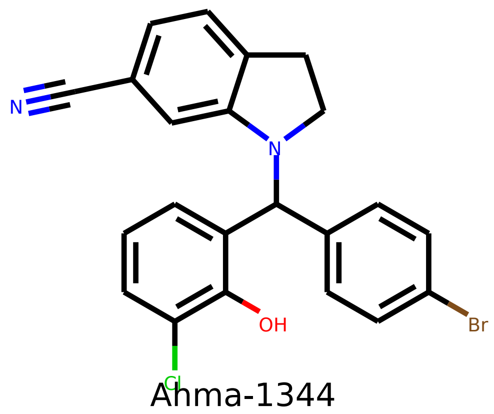
Ahma-1344

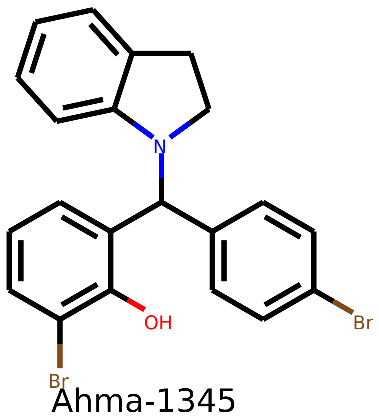
Ahma-1345

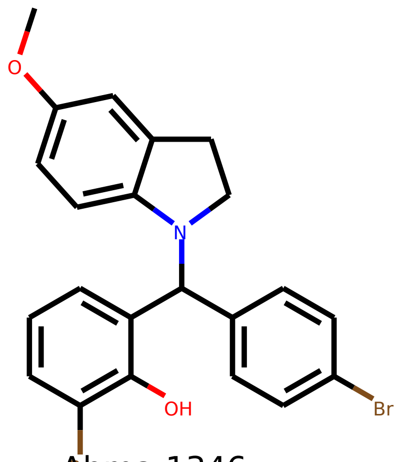
Ahma-1346

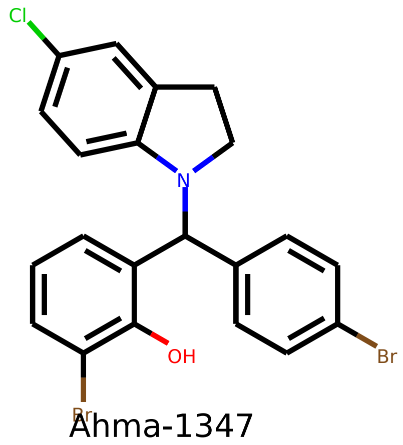
Ahma-1347

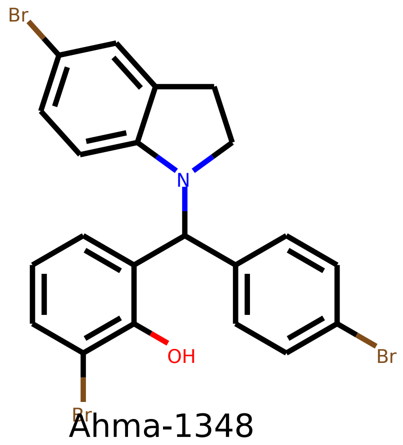
Ahma-1348

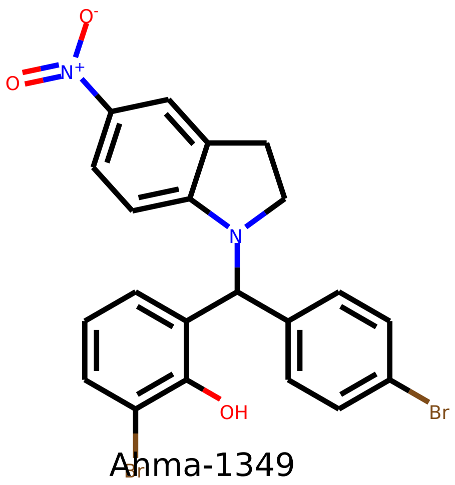
Ahma-1349

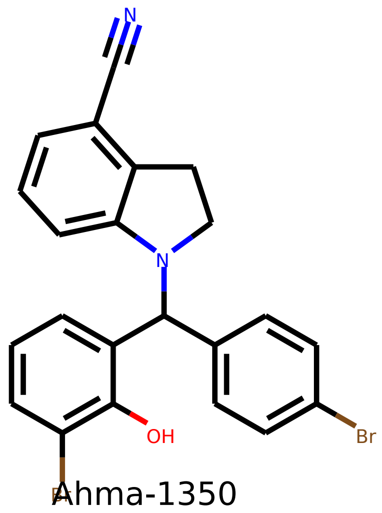
Ahma-1350

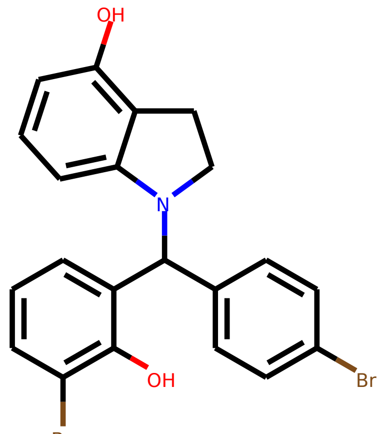
Ahma-1351

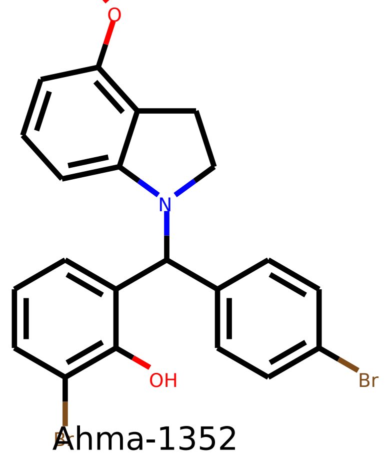
Ahma-1352

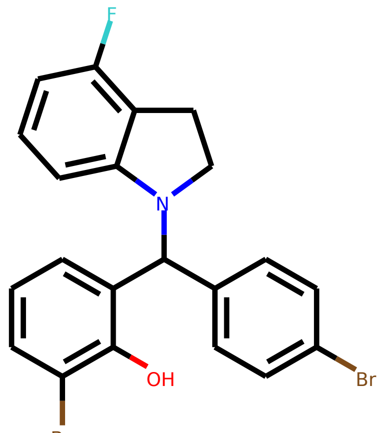
Ahma-1353

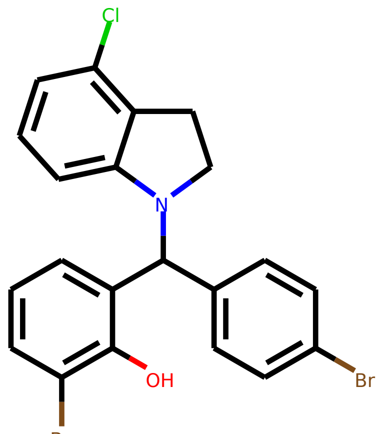
Ahma-1354

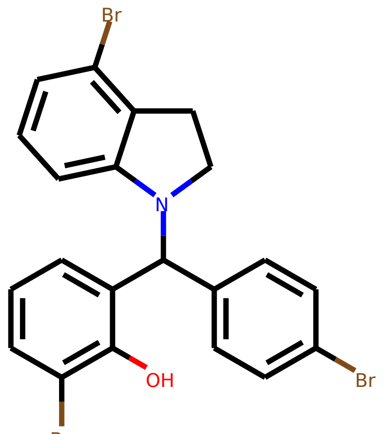
Ahma-1355

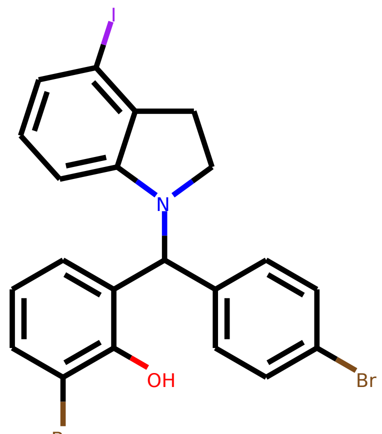
Ahma-1356

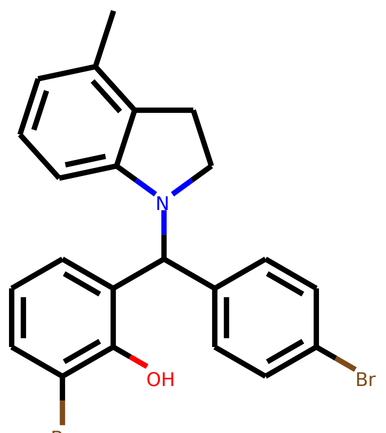
Ahma-1357

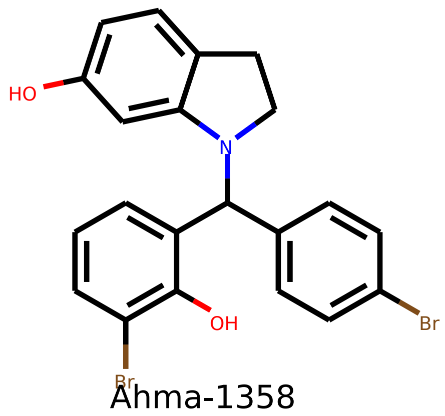
Ahma-1358

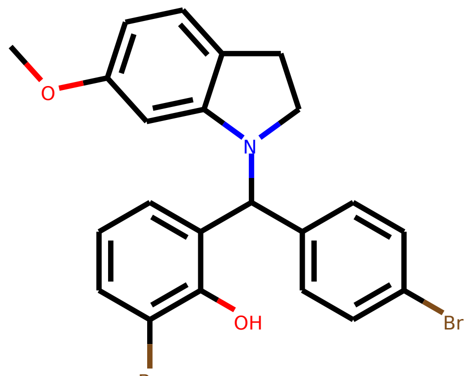
Ahma-1359

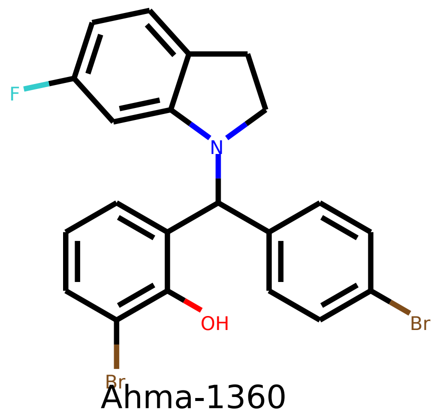
Ahma-1360

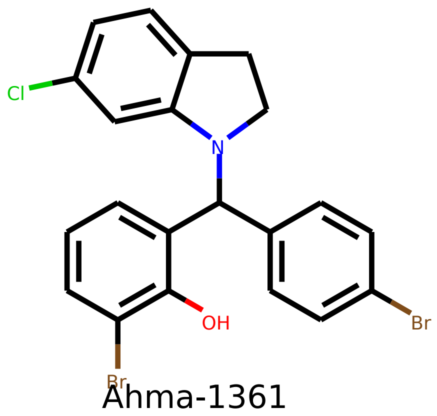
Ahma-1361

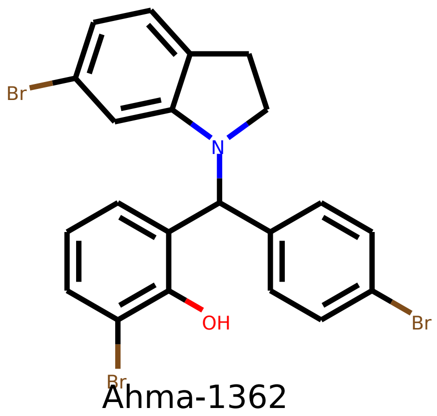
Ahma-1362

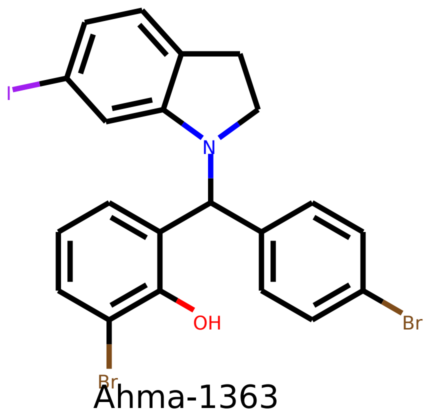
Ahma-1363

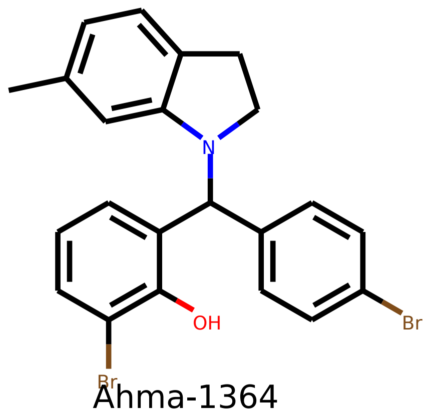
Ahma-1364

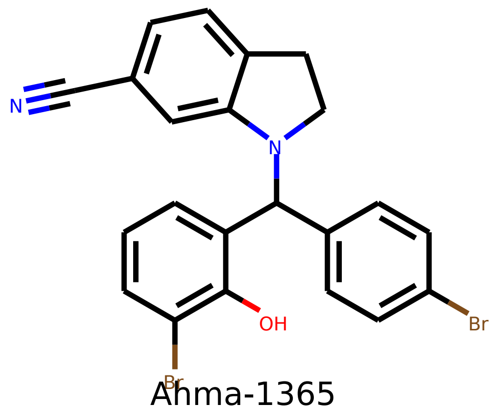
Ahma-1365

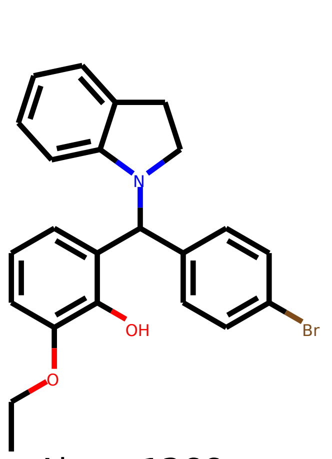
Ahma-1366
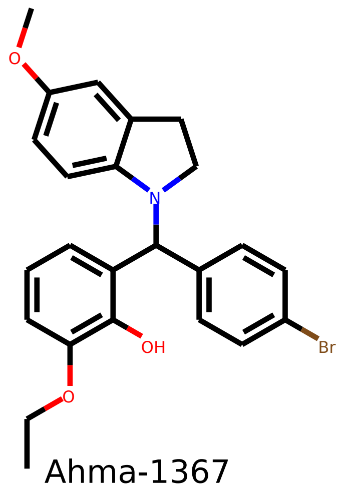
Ahma-1367
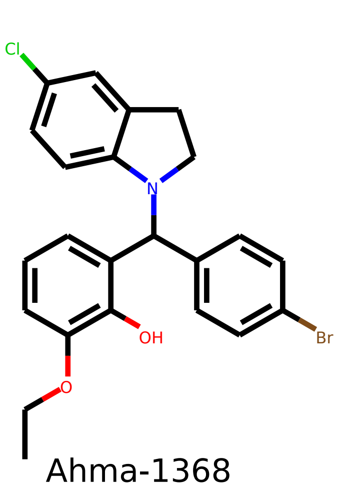
Ahma-1368
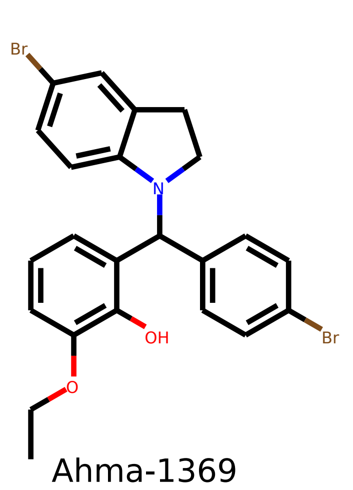
Ahma-1369
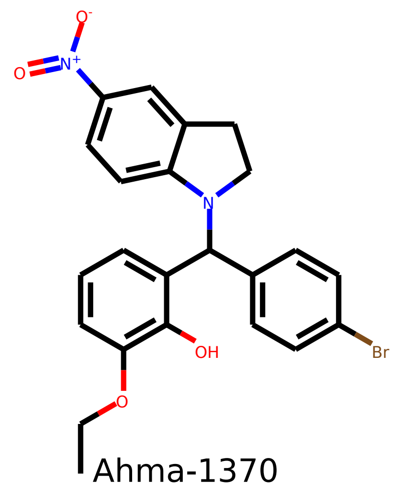
Ahma-1370
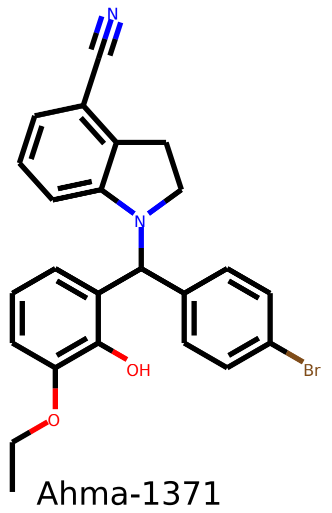
Ahma-1371
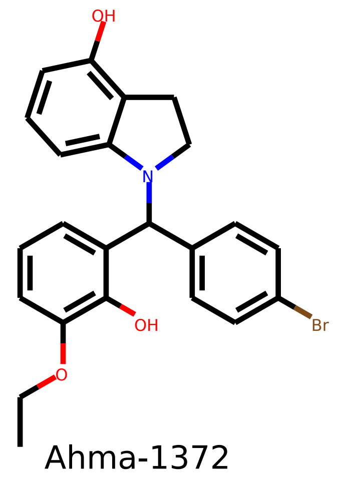
Ahma-1372
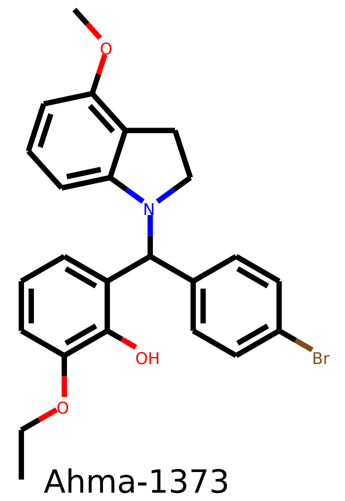
Ahma-1373
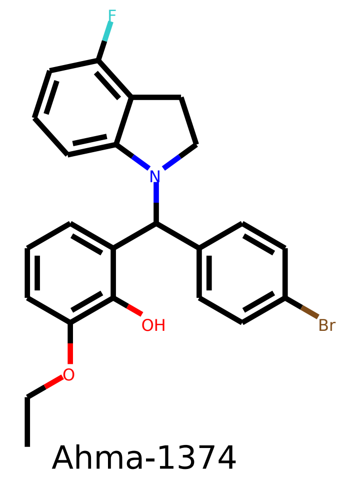
Ahma-1374
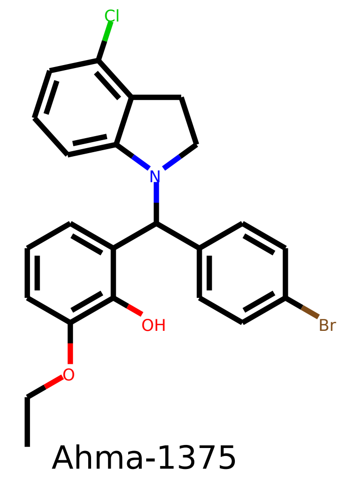
Ahma-1375
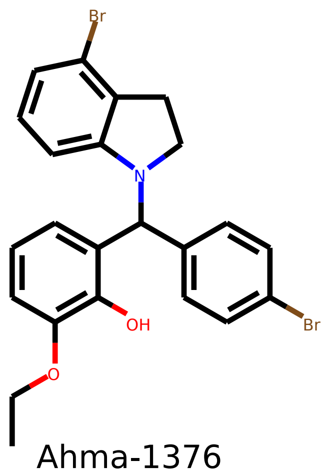
Ahma-1376
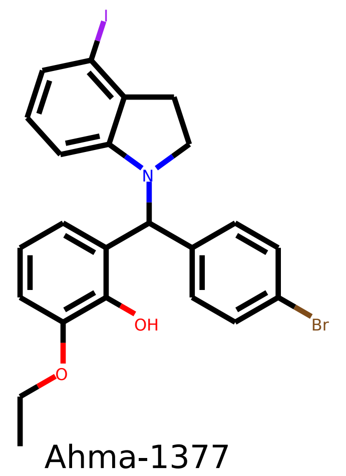
Ahma-1377
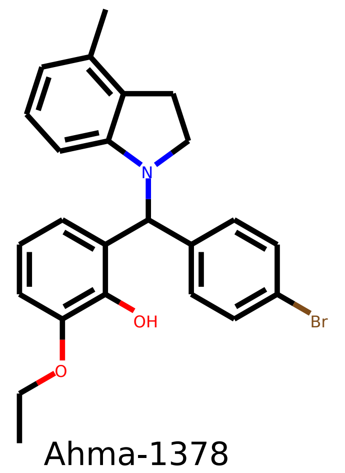
Ahma-1378
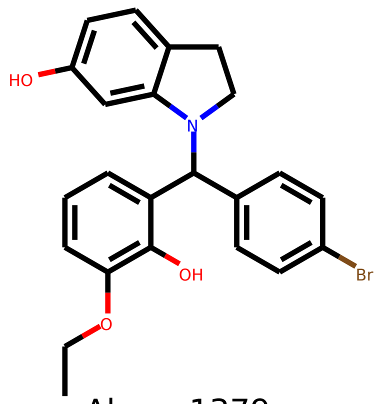
Ahma-1379
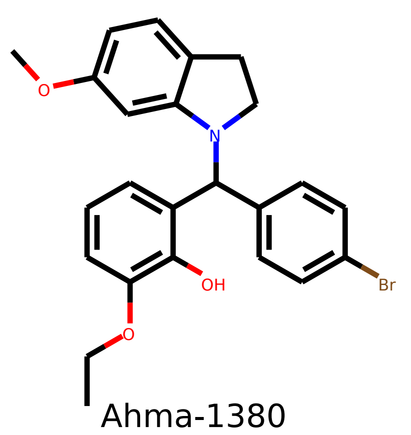
Ahma-1380
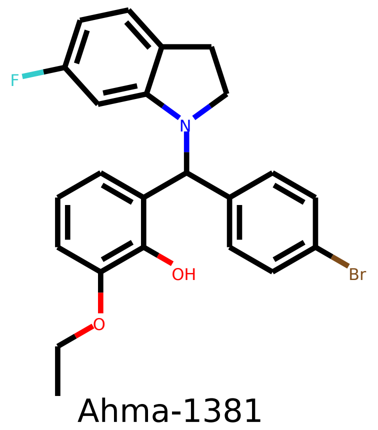
Ahma-1381
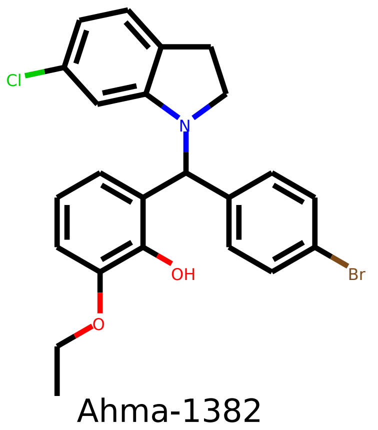
Ahma-1382
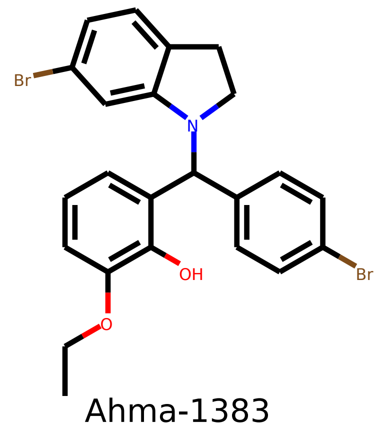
Ahma-1383
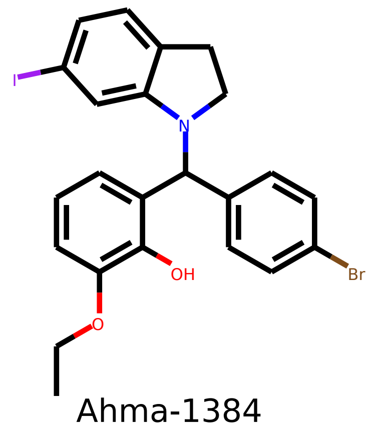
Ahma-1384
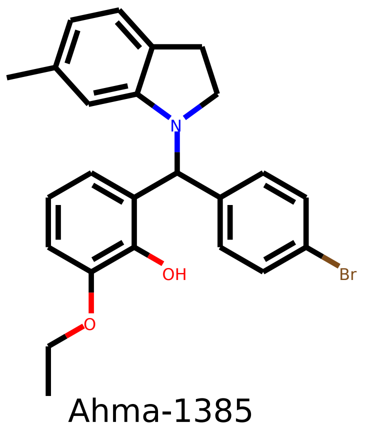
Ahma-1385
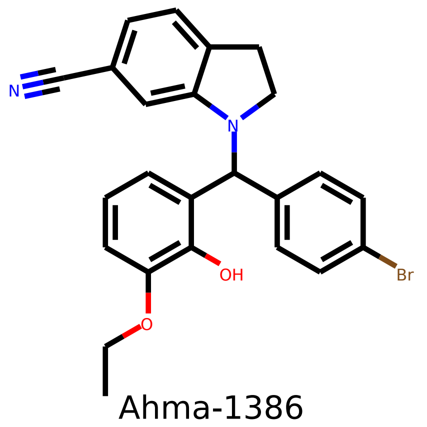
Ahma-1386
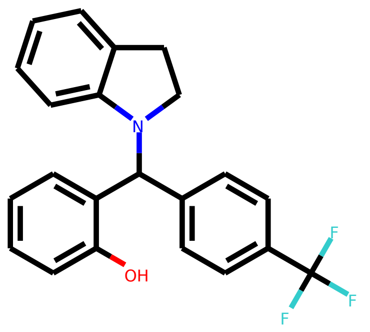
Ahma-1387
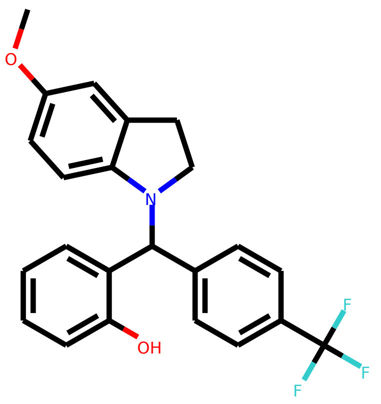
Ahma-1388
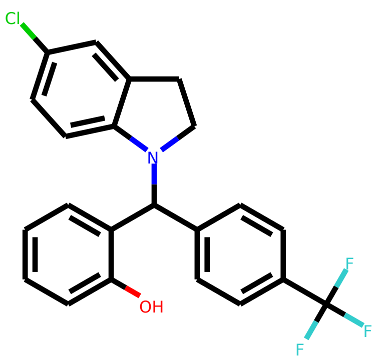
Ahma-1389
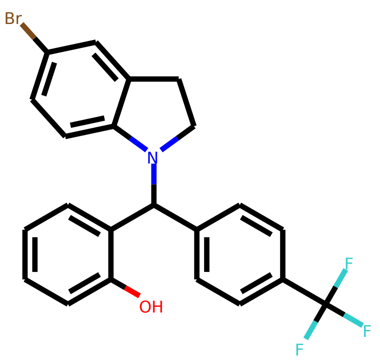
Ahma-1390
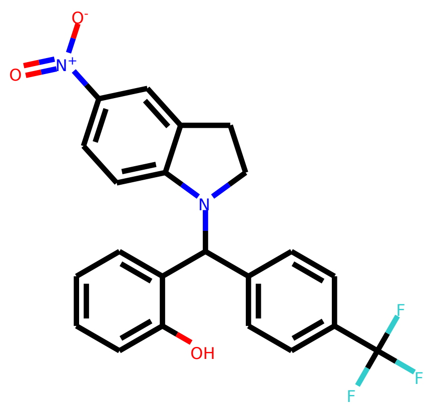
Ahma-1391
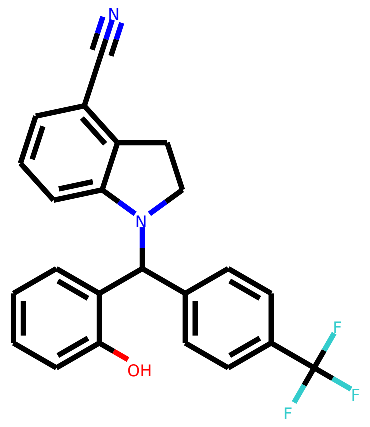
Ahma-1392
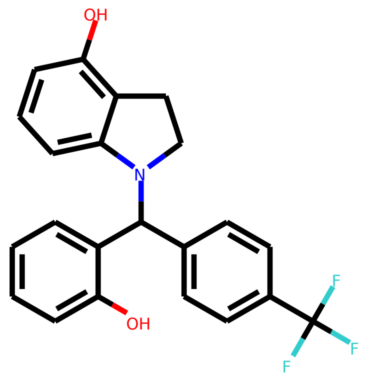
Ahma-1393
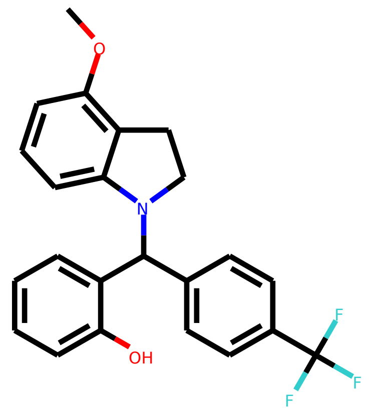
Ahma-1394
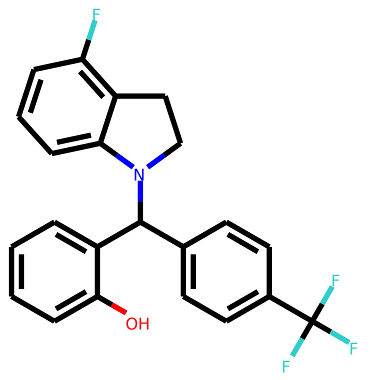
Ahma-1395
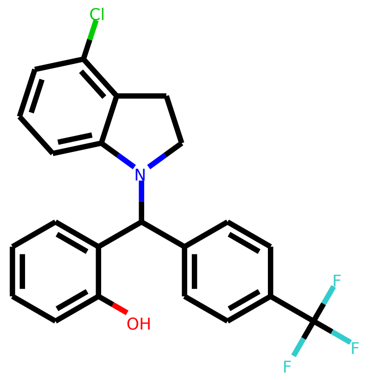
Ahma-1396
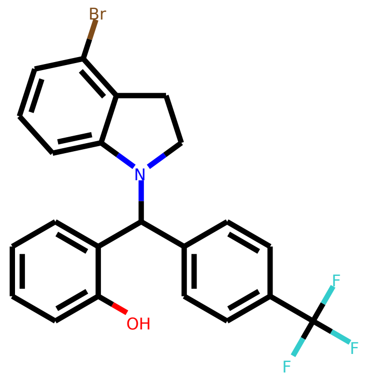
Ahma-1397
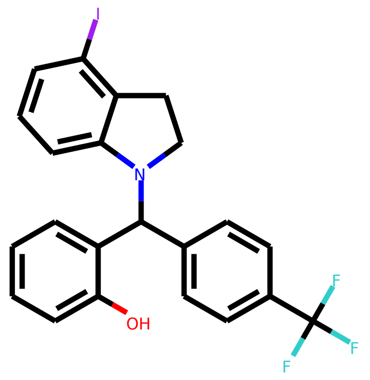
Ahma-1398
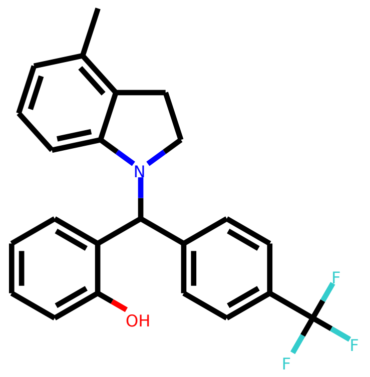
Ahma-1399
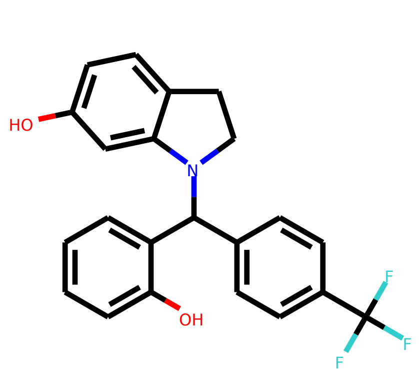
Ahma-1400

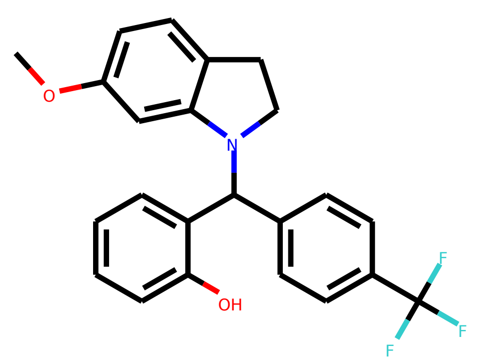
Ahma-1401
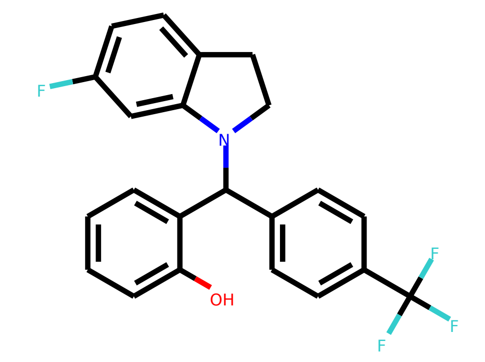
Ahma-1402
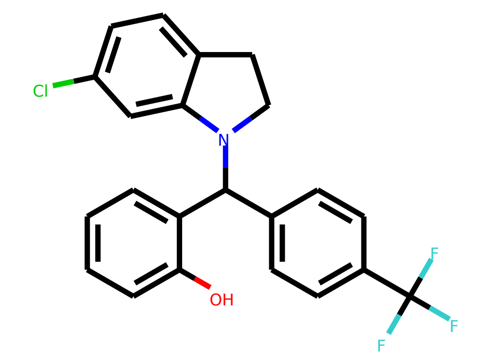
Ahma-1403
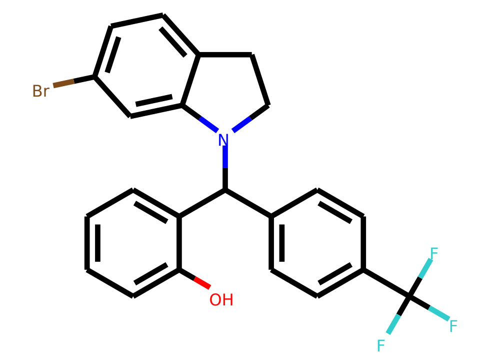
Ahma-1404
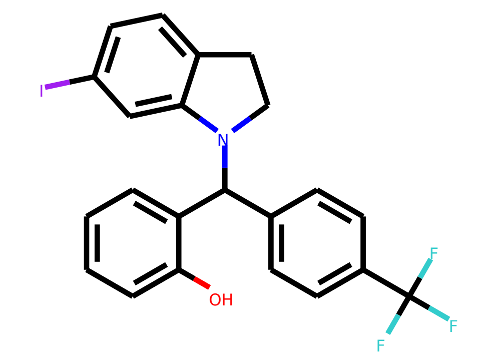
Ahma-1405
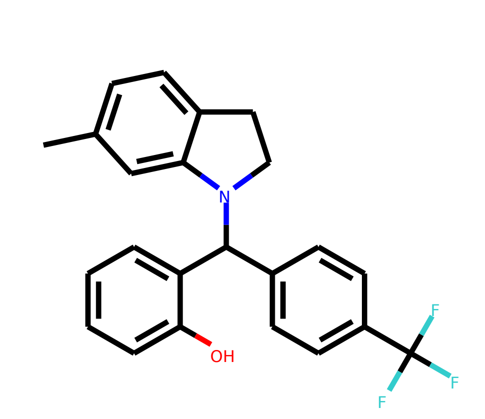
Ahma-1406
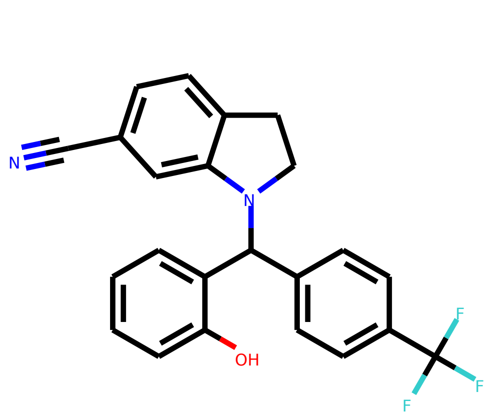
Ahma-1407
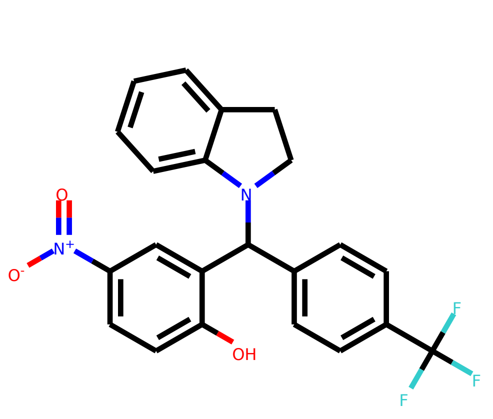
Ahma-1408
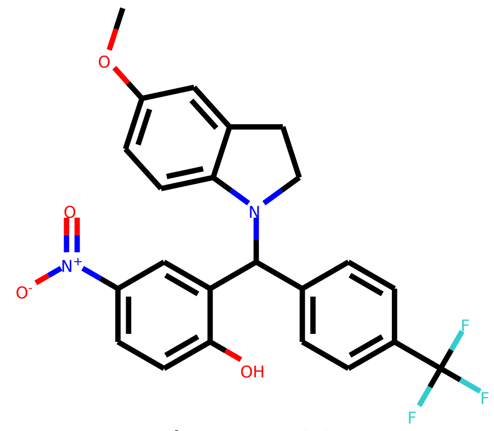
Ahma-1409
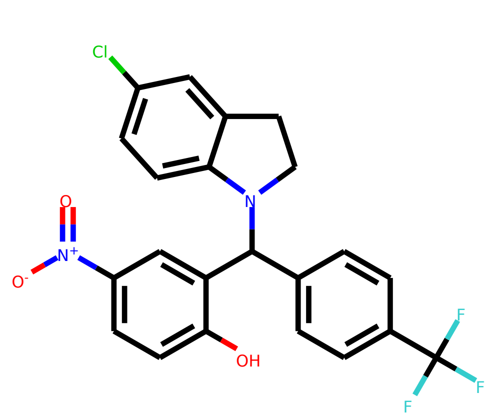
Ahma-1410
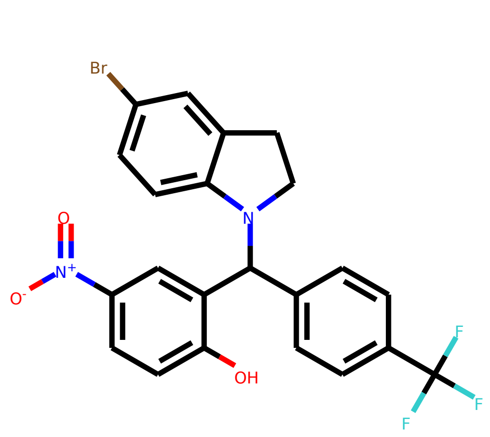
Ahma-1411
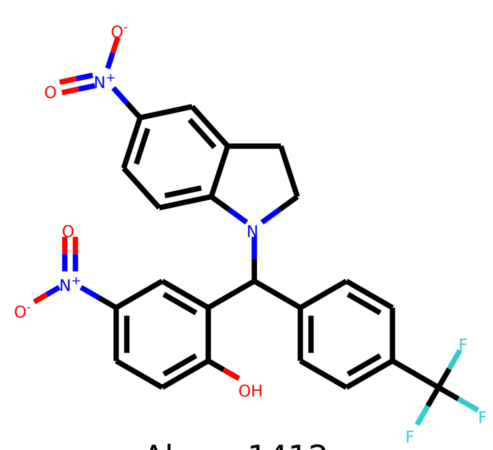
Ahma-1412
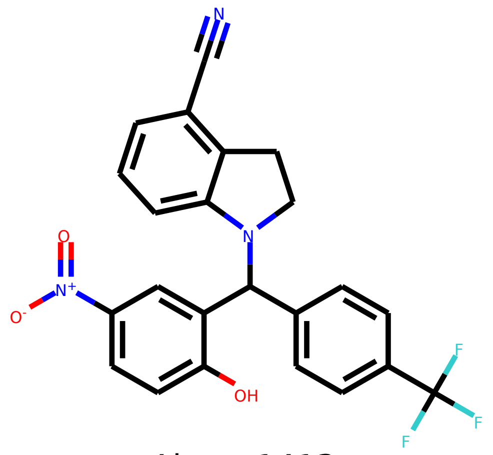
Ahma-1413
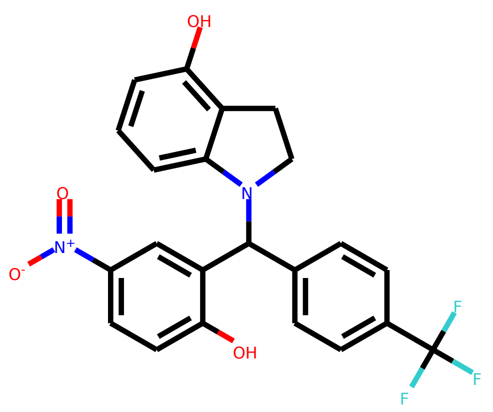
Ahma-1414
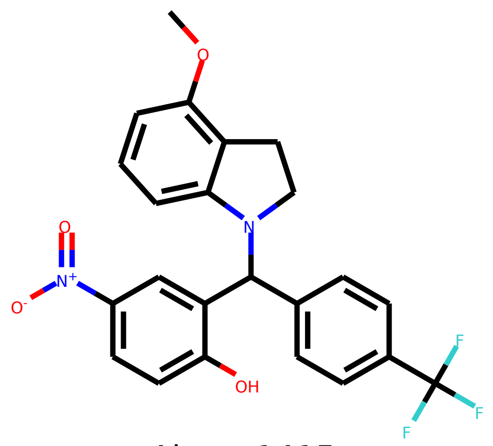
Ahma-1415
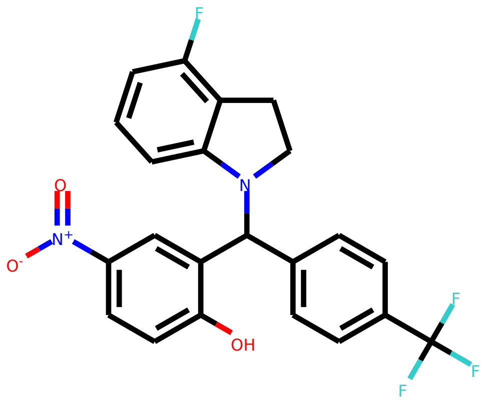
Ahma-1416
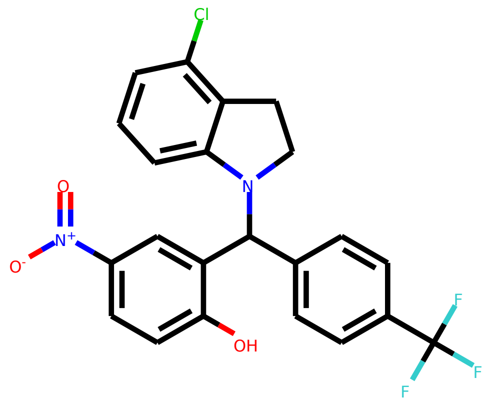
Ahma-1417
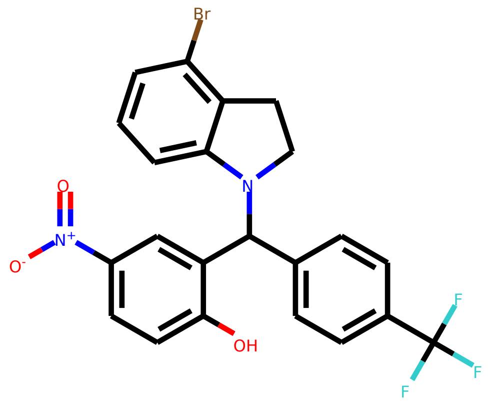
Ahma-1418
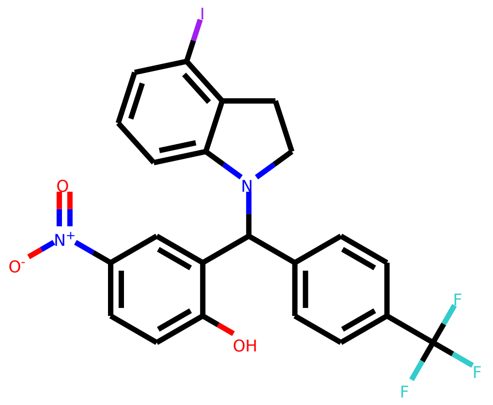
Ahma-1419
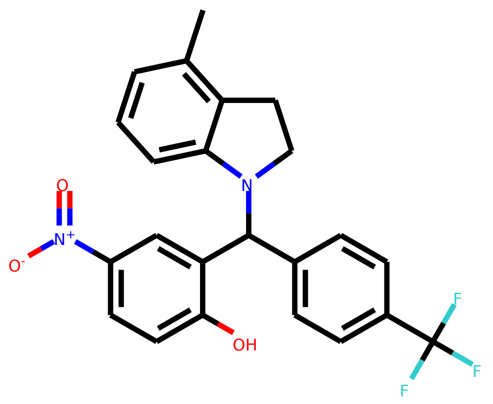
Ahma-1420
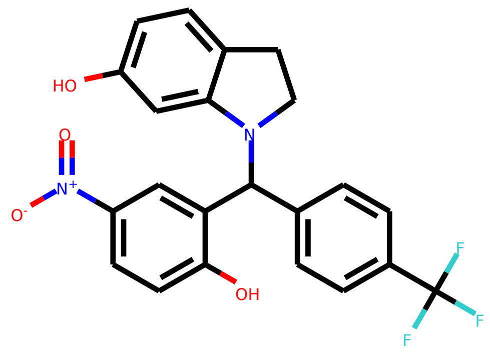
Ahma-1421
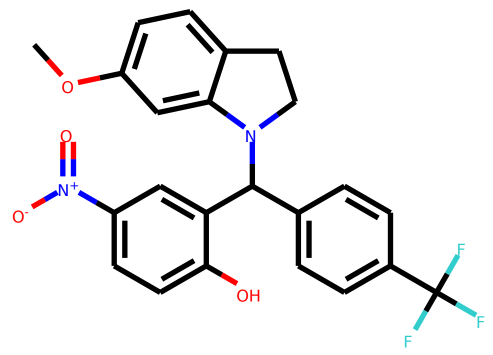
Ahma-1422
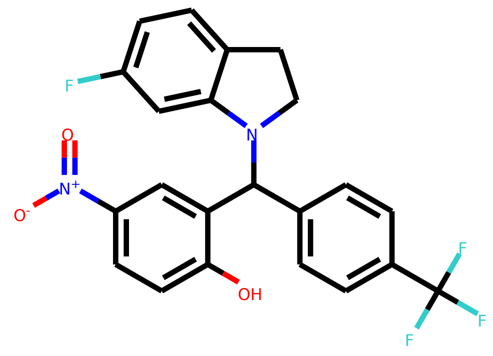
Ahma-1423
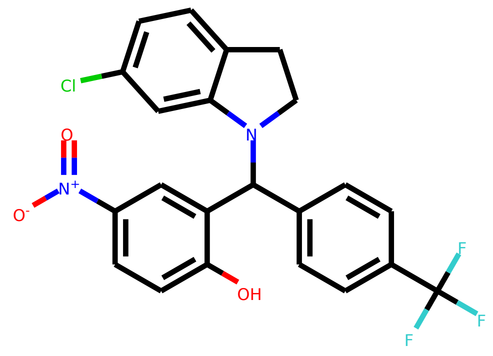
Ahma-1424
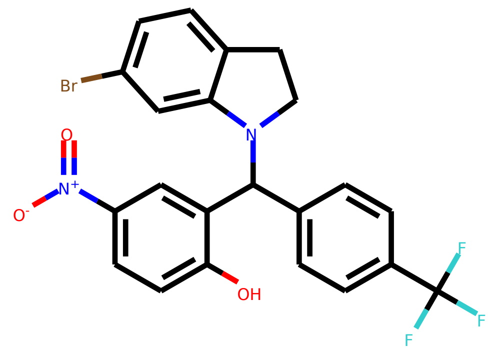
Ahma-1425
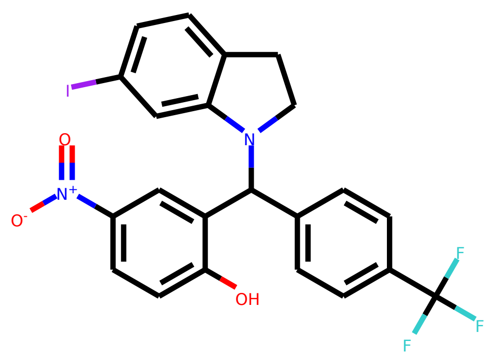
Ahma-1426
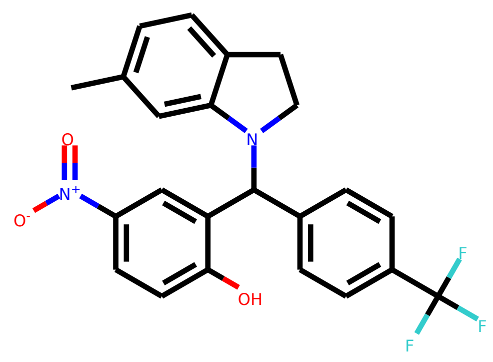
Ahma-1427
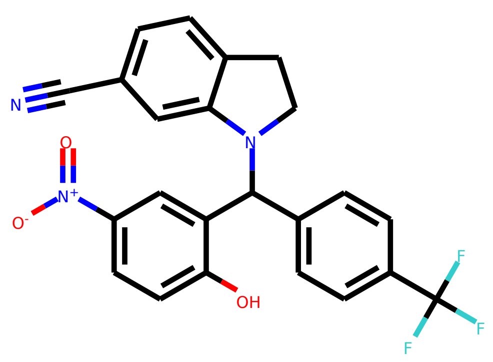
Ahma-1428
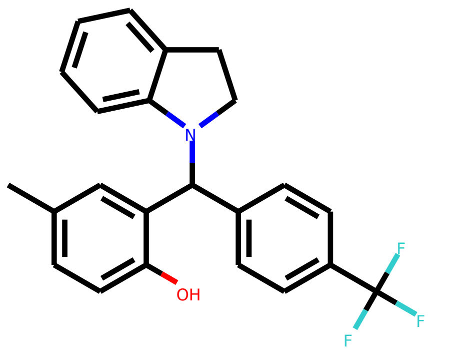
Ahma-1429
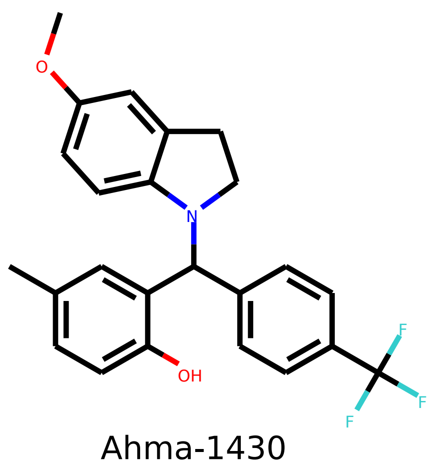
Ahma-1430
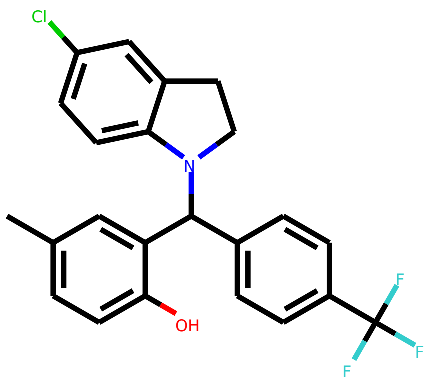
Ahma-1431
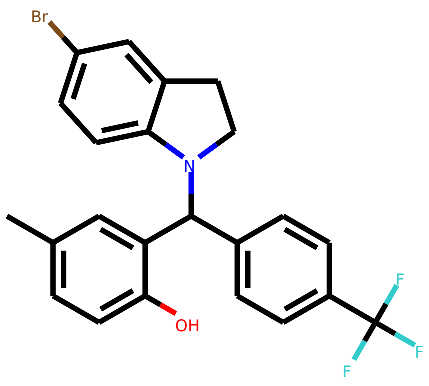
Ahma-1432
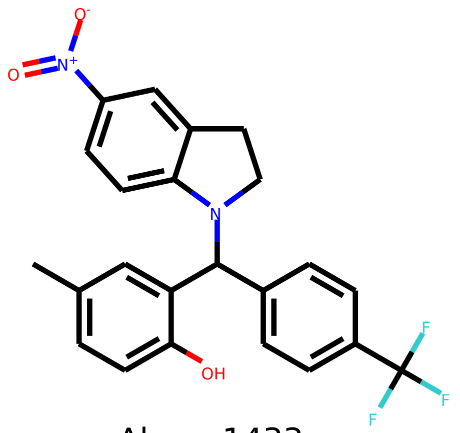
Ahma-1433
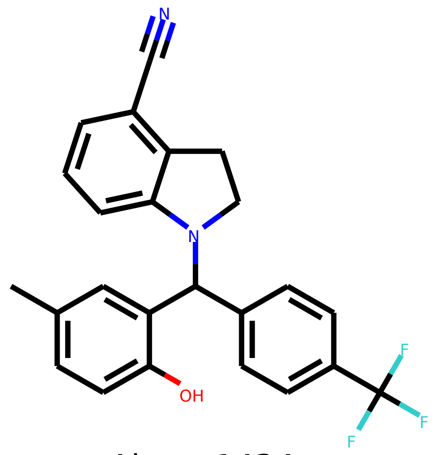
Ahma-1434
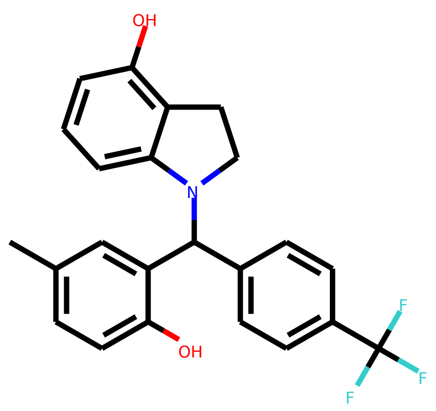
Ahma-1435

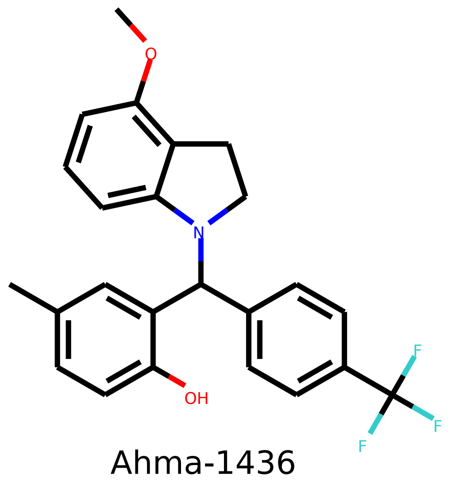

Ahma-1436

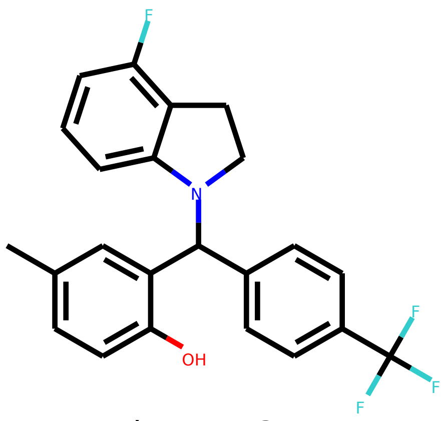

Ahma-1437

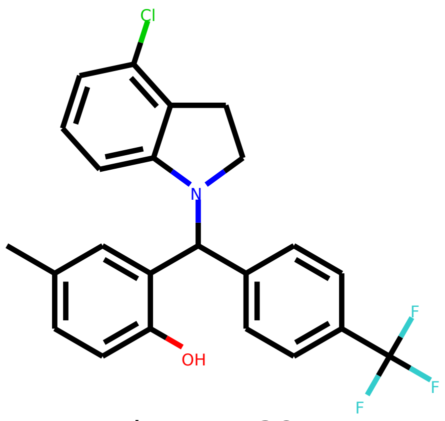

Ahma-1438

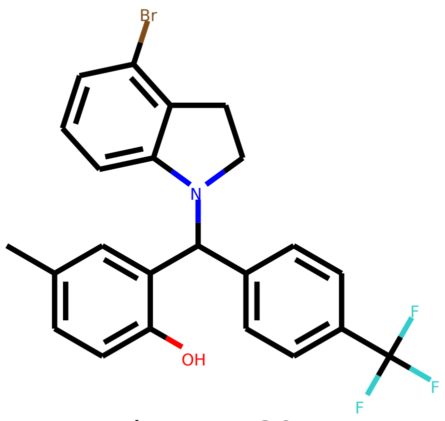

Ahma-1439

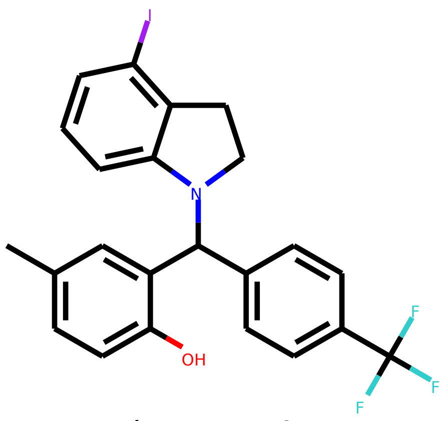

Ahma-1440

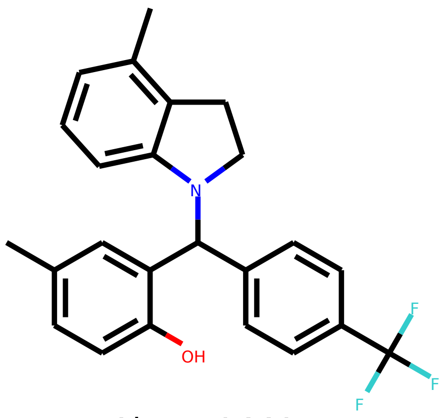

Ahma-1441

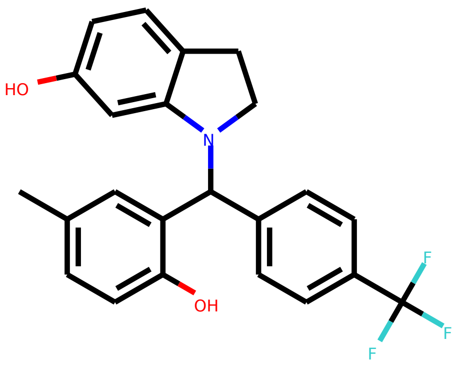

Ahma-1442

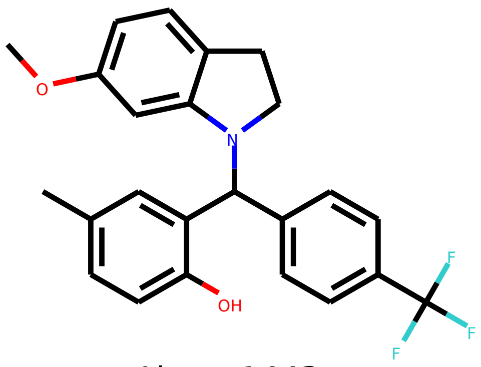

Ahma-1443

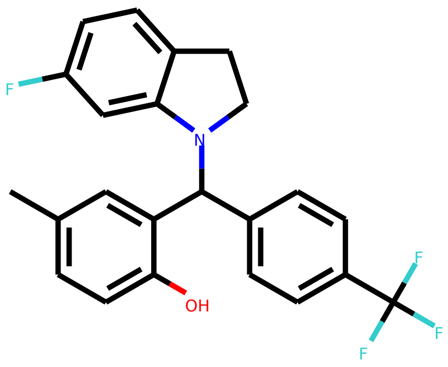

Ahma-1444

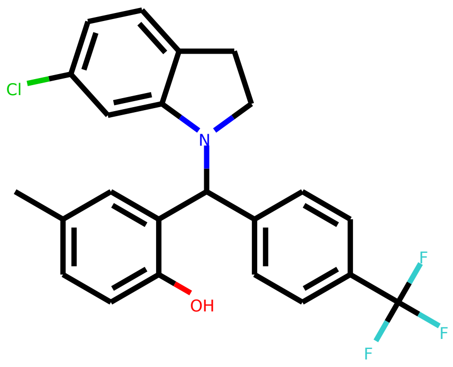

Ahma-1445

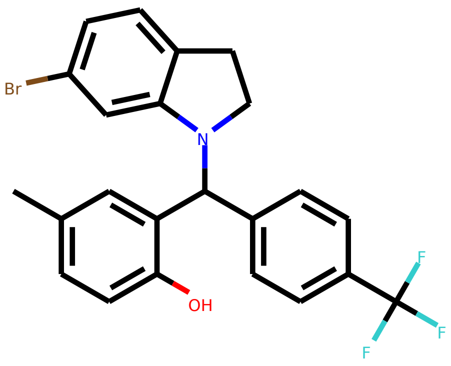

Ahma-1446

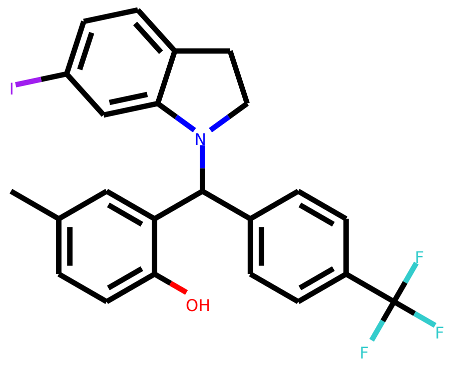

Ahma-1447

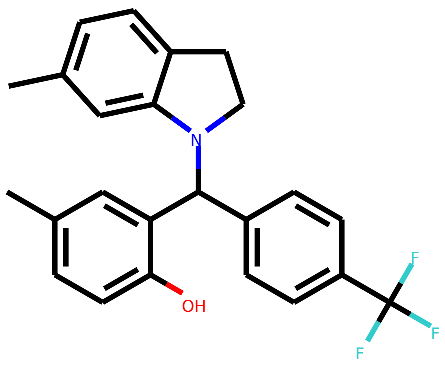

Ahma-1448

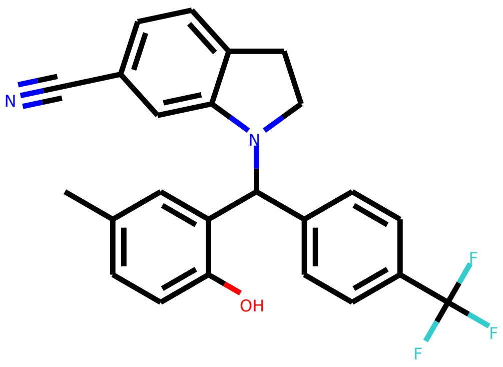

Ahma-1449

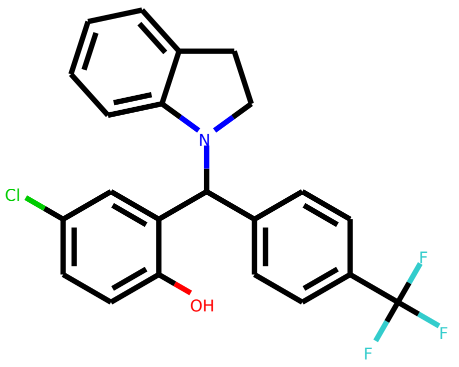

Ahma-1450

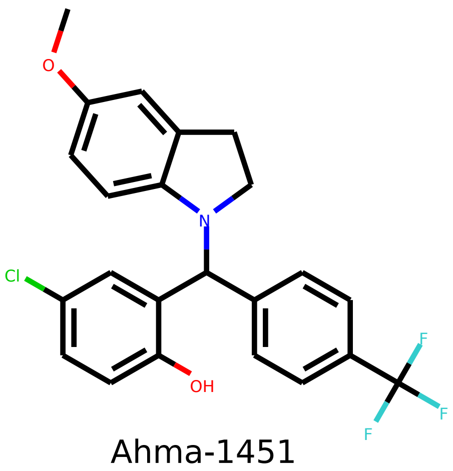

Ahma-1451

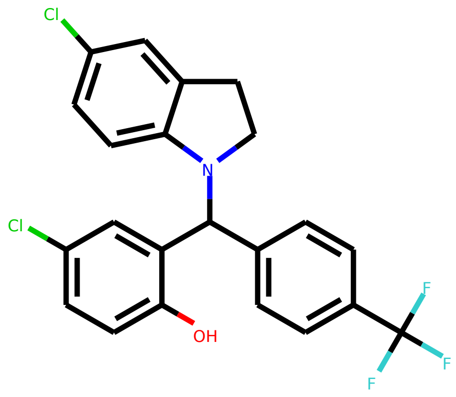

Ahma-1452

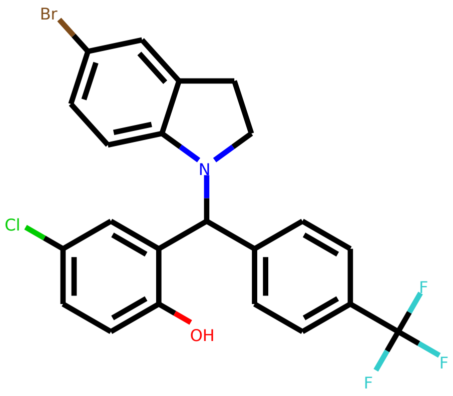

Ahma-1453

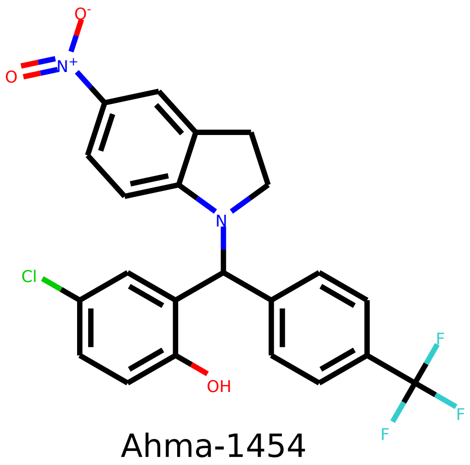

Ahma-1454

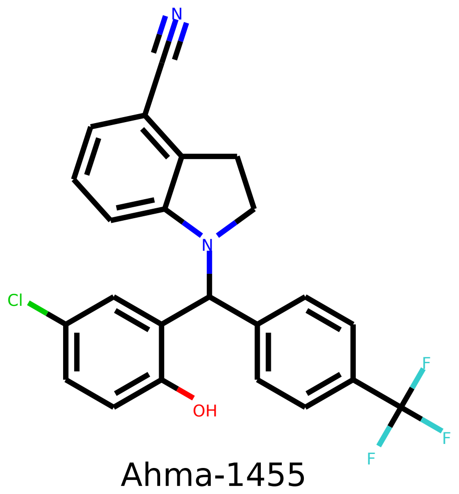

Ahma-1455

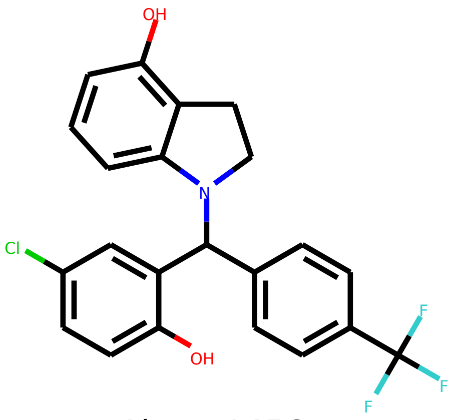

Ahma-1456

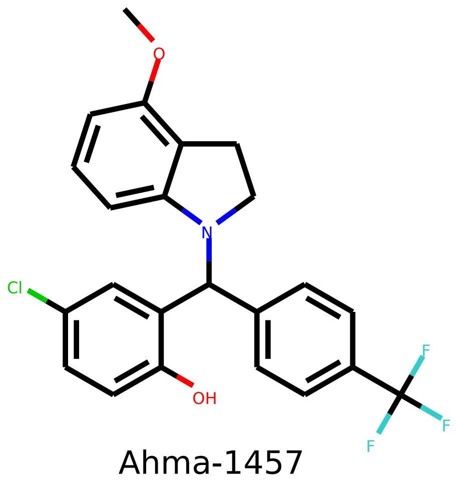

Ahma-1457

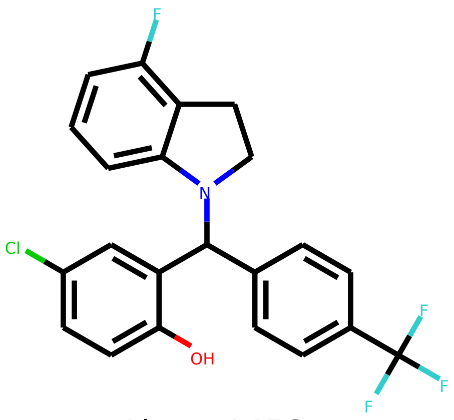

Ahma-1458

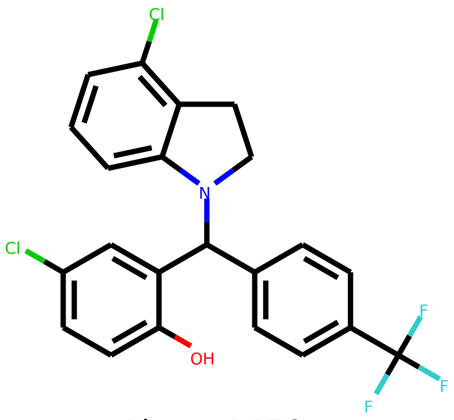

Ahma-1459

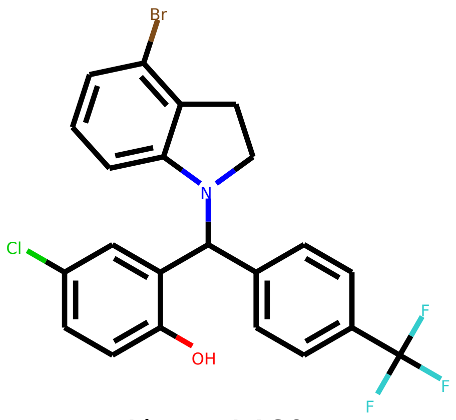

Ahma-1460

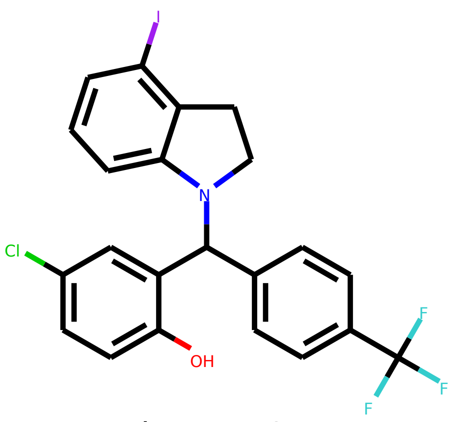

Ahma-1461

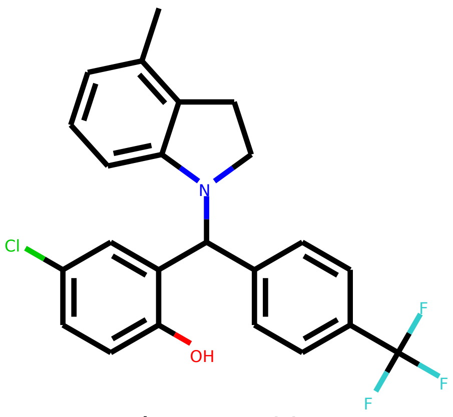

Ahma-1462

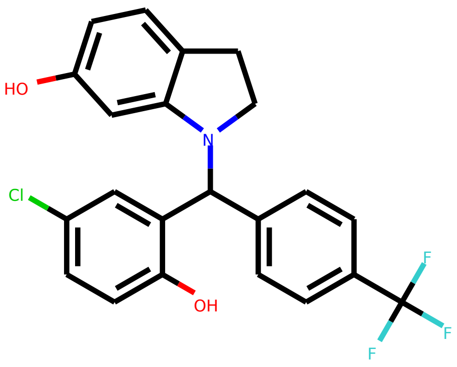

Ahma-1463

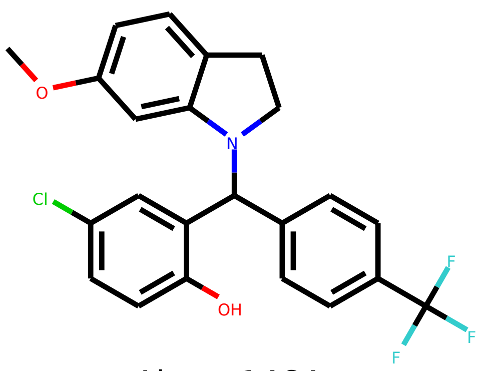

Ahma-1464

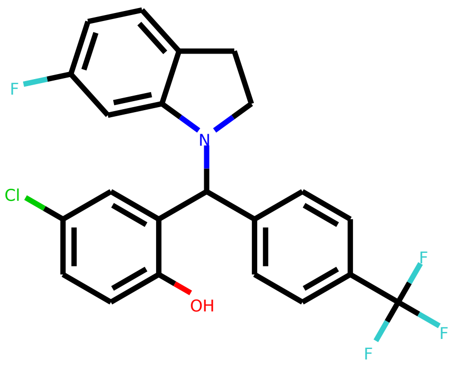

Ahma-1465

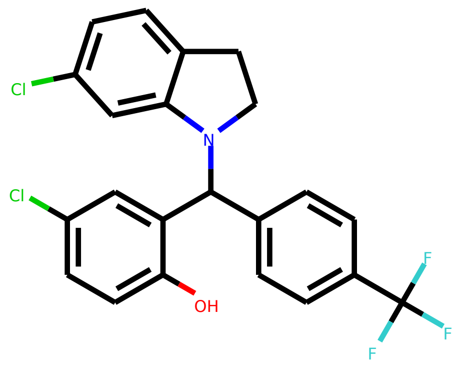

Ahma-1466

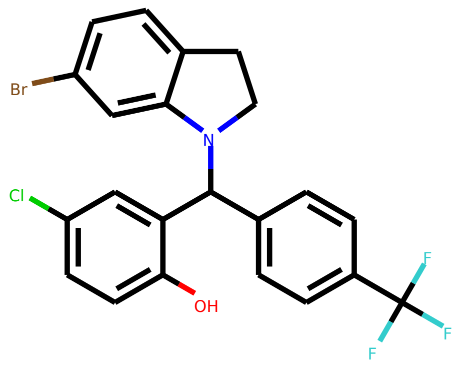

Ahma-1467

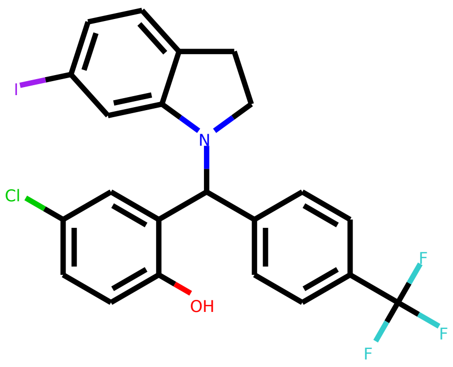

Ahma-1468

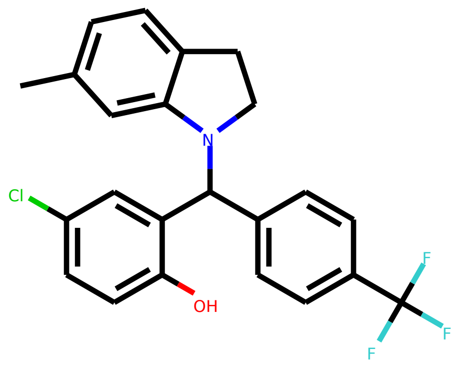

Ahma-1469

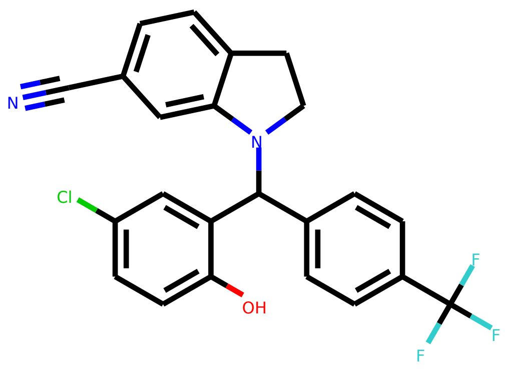

Ahma-1470

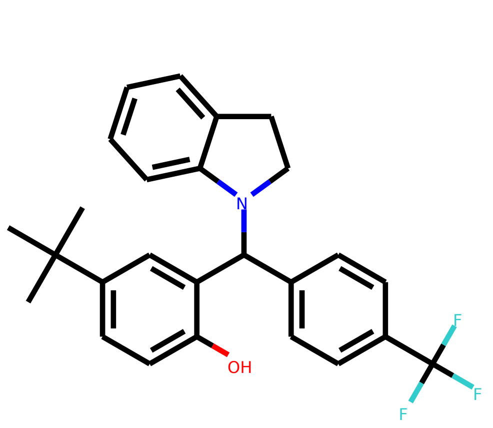

Ahma-1471

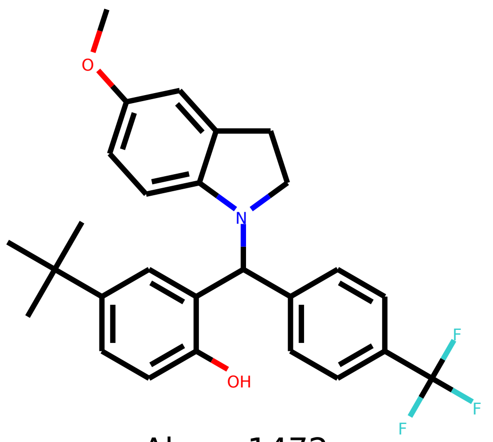

Ahma-1472

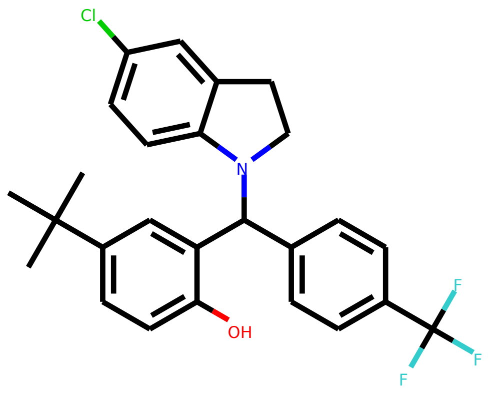

Ahma-1473

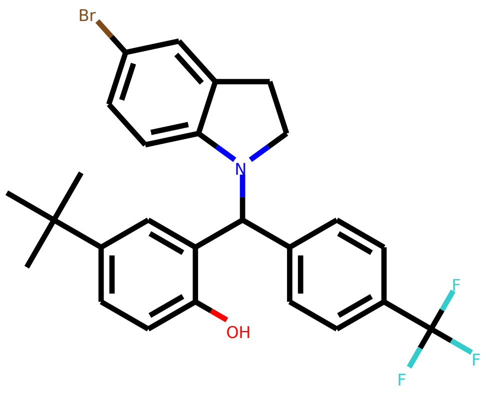

Ahma-1474

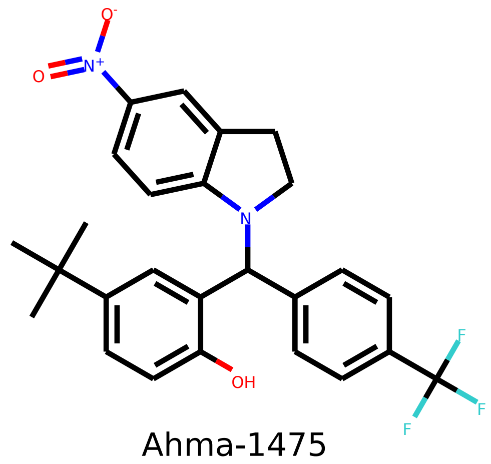

Ahma-1475

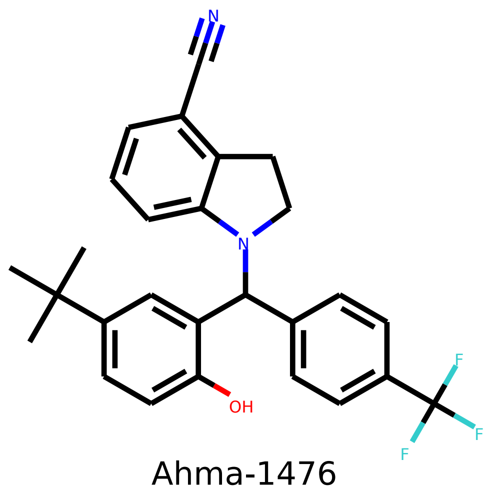

Ahma-1476

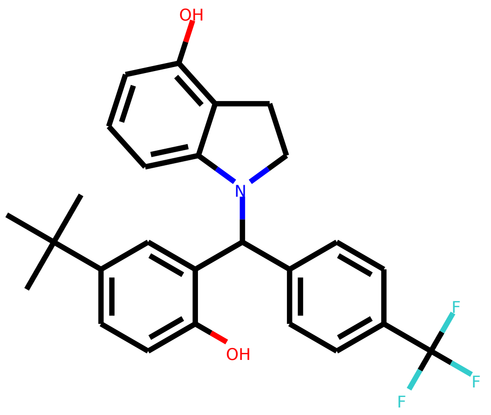

Ahma-1477

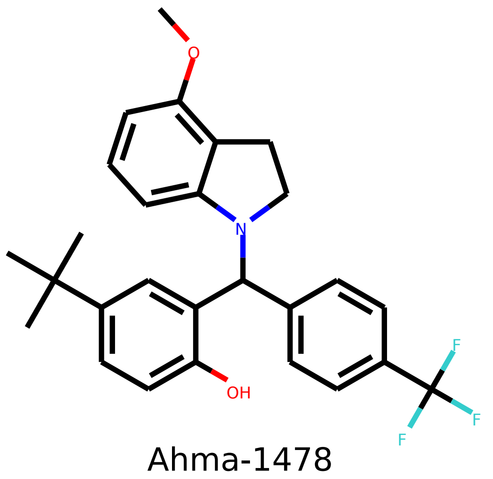

Ahma-1478

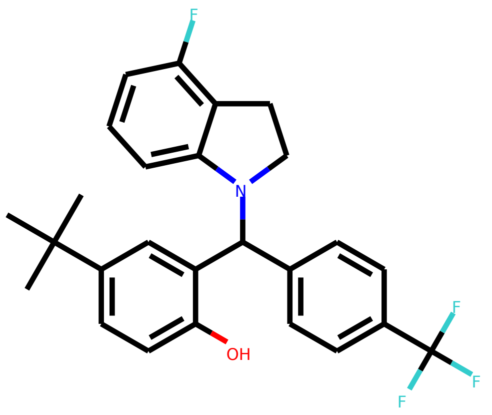

Ahma-1479

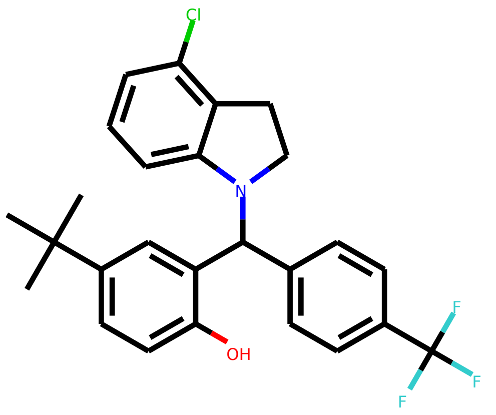

Ahma-1480

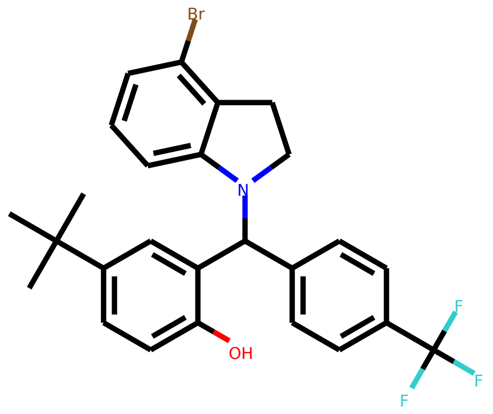

Ahma-1481

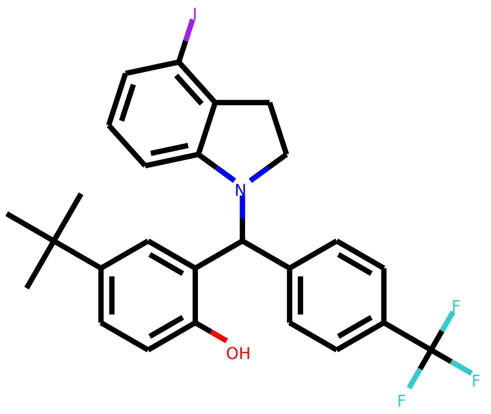

Ahma-1482

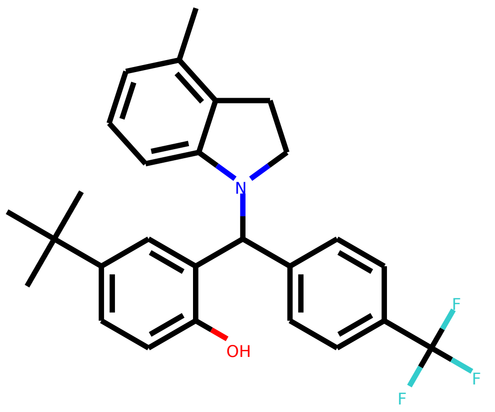

Ahma-1483

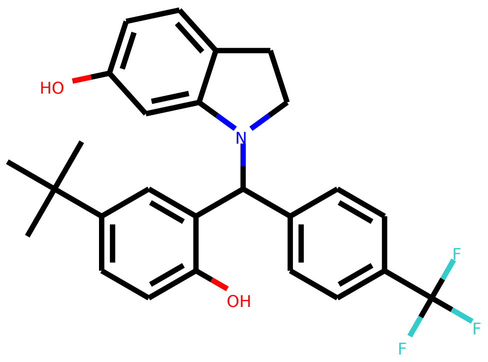

Ahma-1484

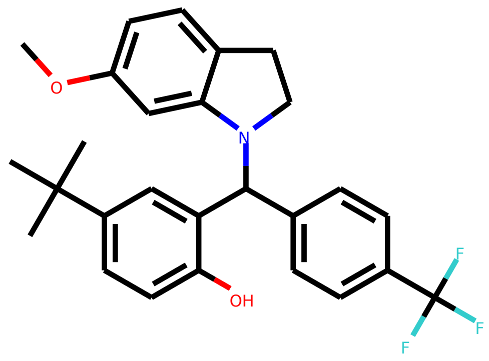

Ahma-1485

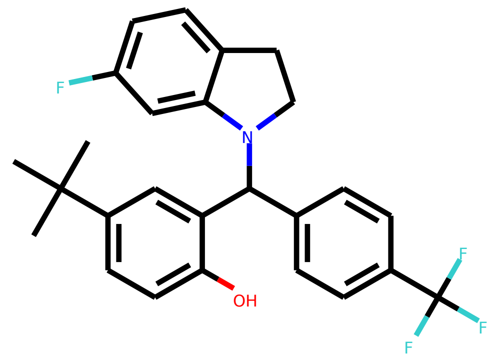

Ahma-1486

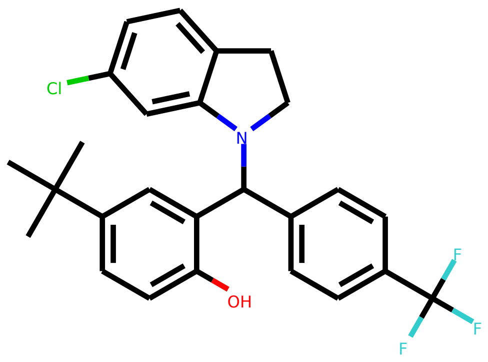

Ahma-1487

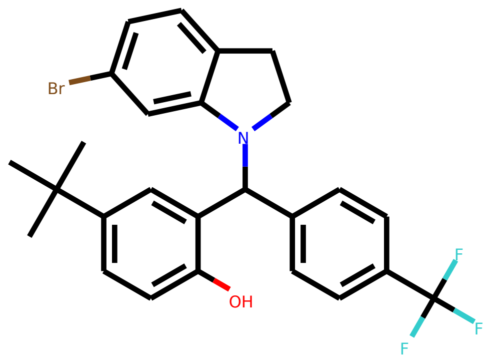

Ahma-1488

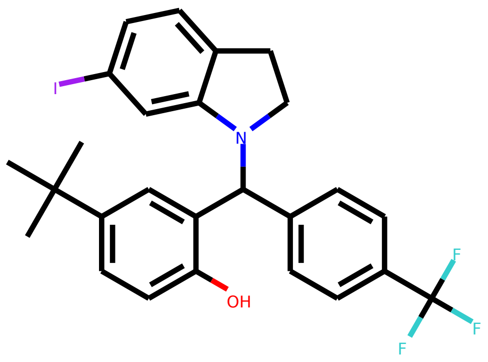

Ahma-1489

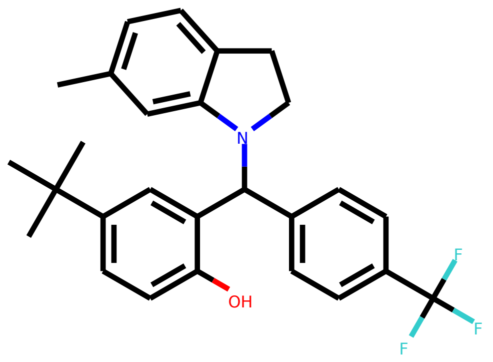

Ahma-1490

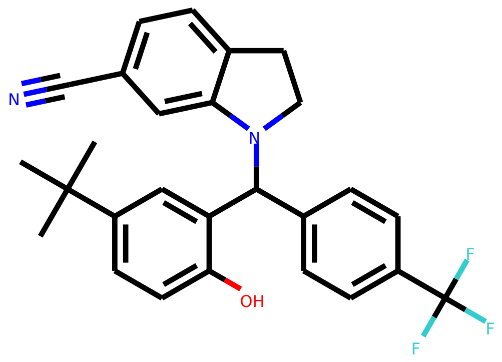

Ahma-1491

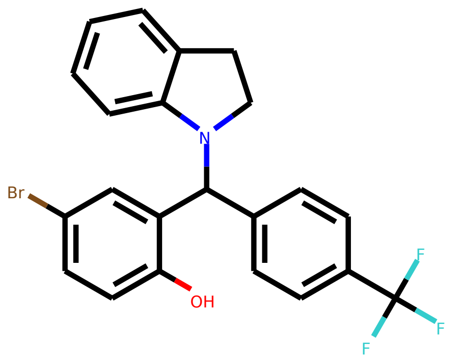

Ahma-1492

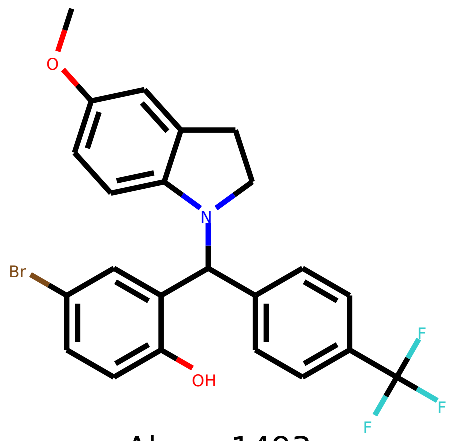

Ahma-1493

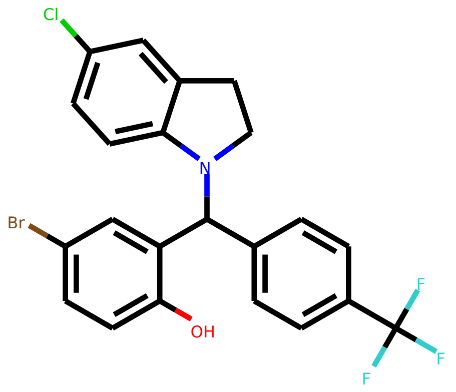

Ahma-1494

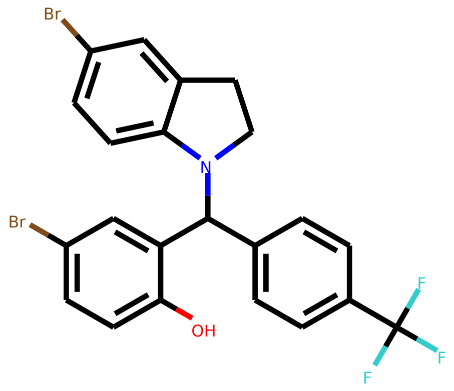

Ahma-1495

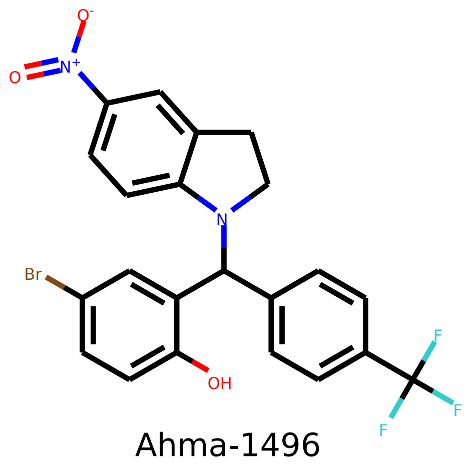

Ahma-1496

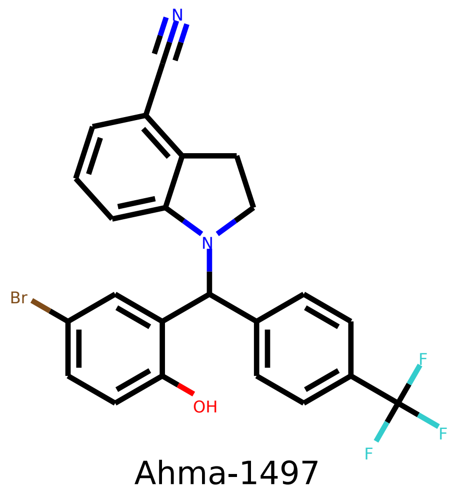

Ahma-1497

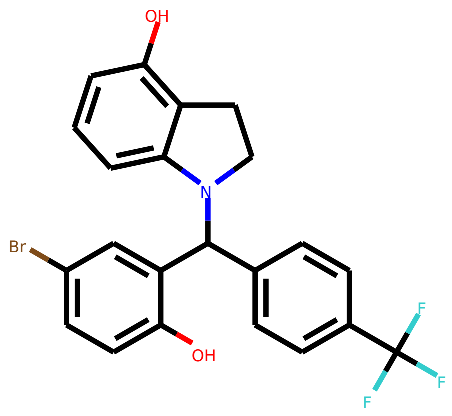

Ahma-1498

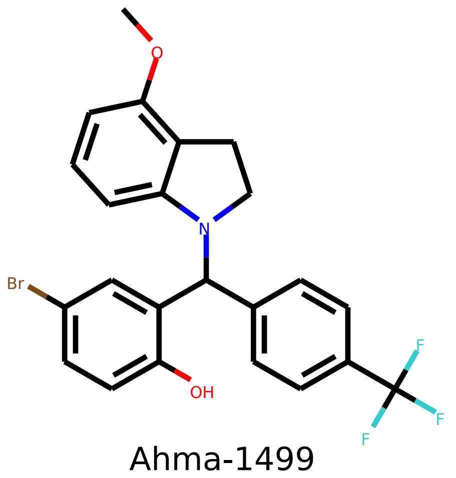

Ahma-1499

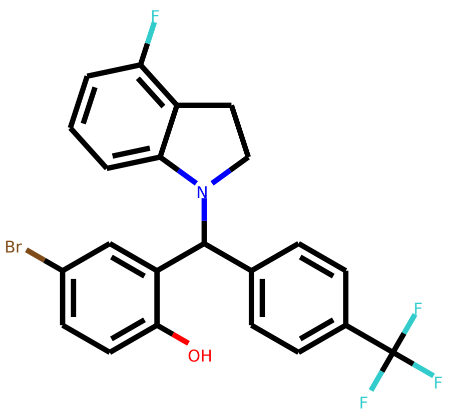

Ahma-1500

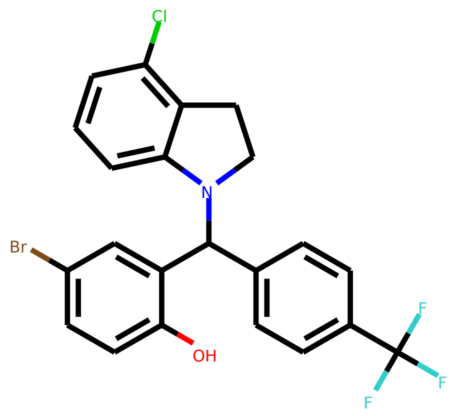

Ahma-1501

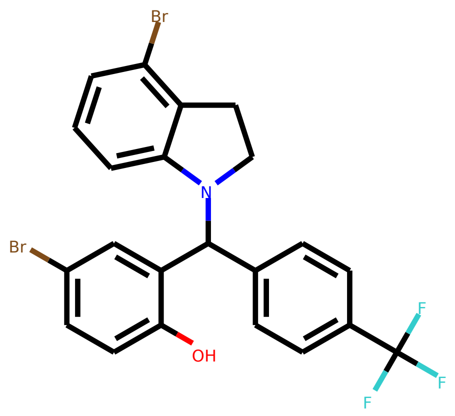

Ahma-1502

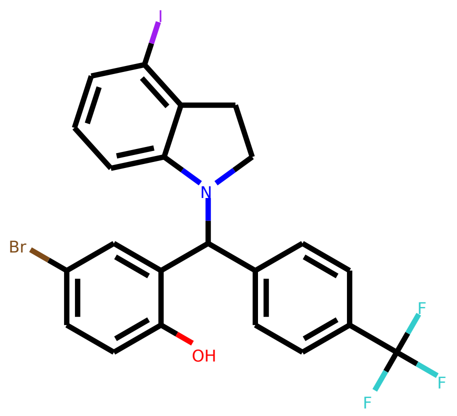

Ahma-1503

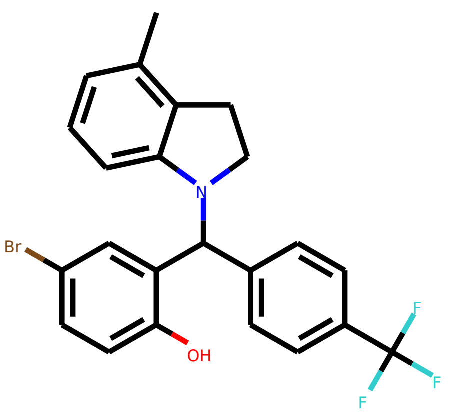

Ahma-1504

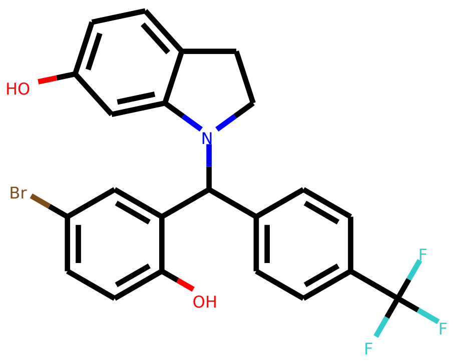

Ahma-1505

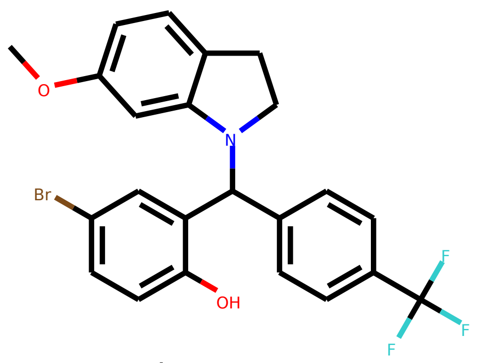
Ahma-1506
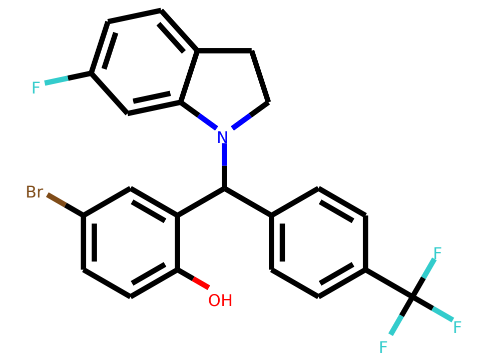
Ahma-1507
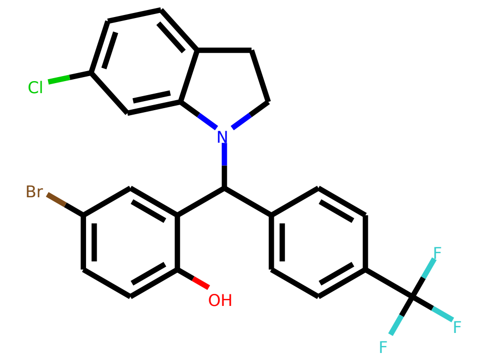
Ahma-1508
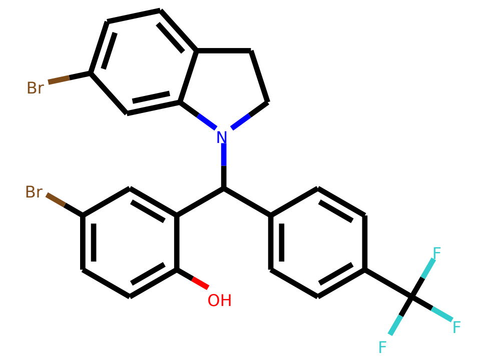
Ahma-1509
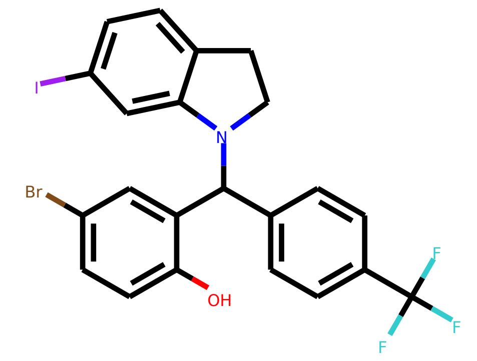
Ahma-1510
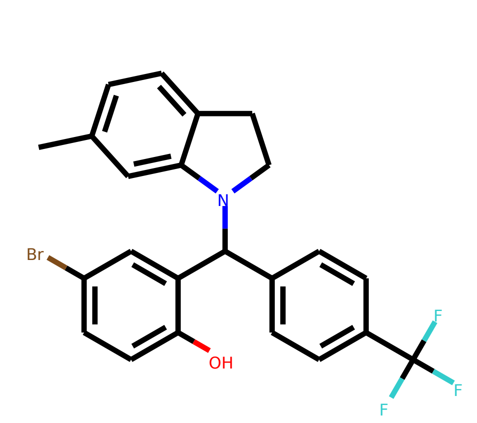
Ahma-1511
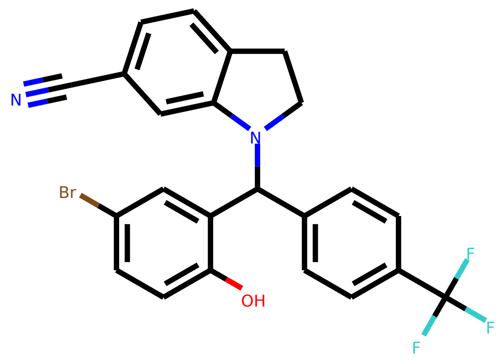
Ahma-1512
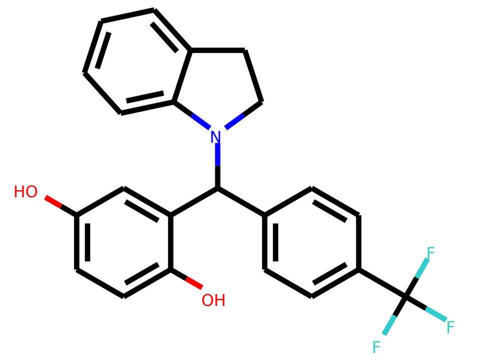
Ahma-1513
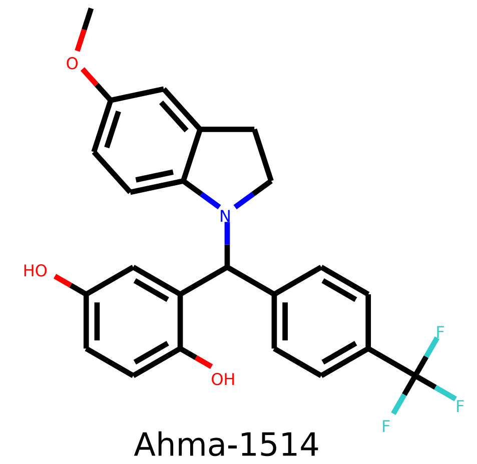
Ahma-1514
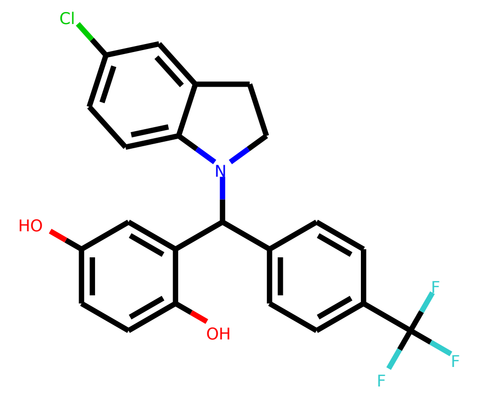
Ahma-1515
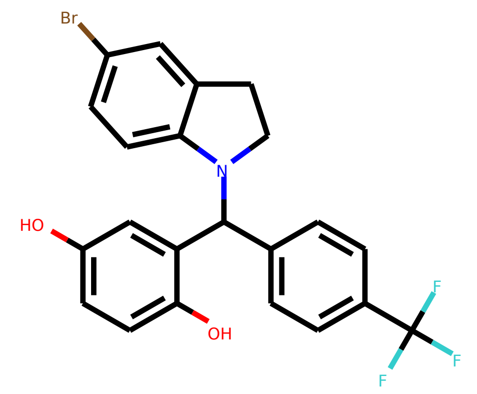
Ahma-1516
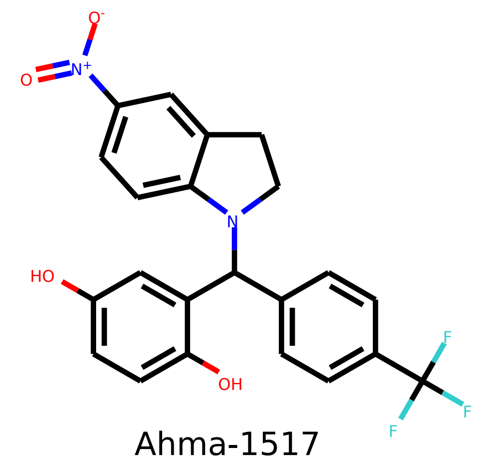
Ahma-1517
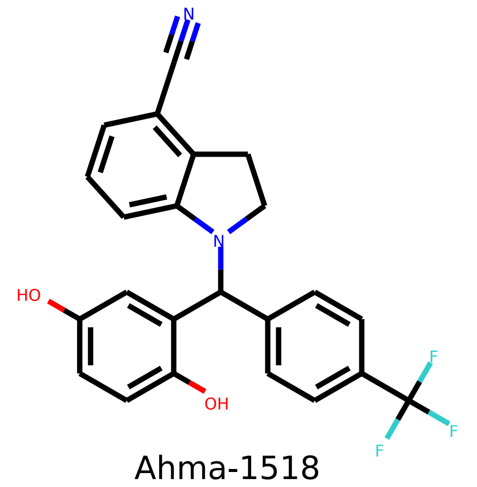
Ahma-1518
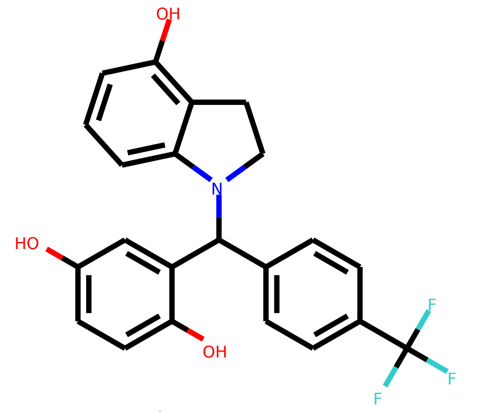
Ahma-1519
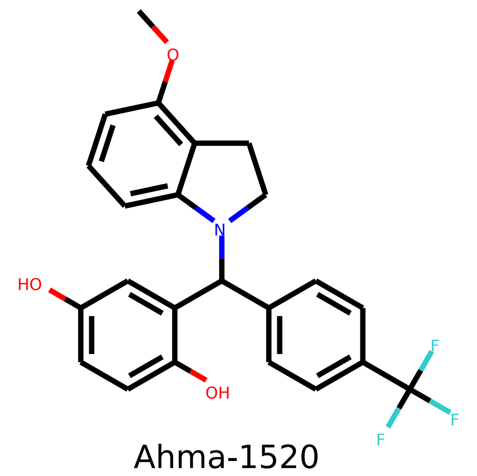
Ahma-1520
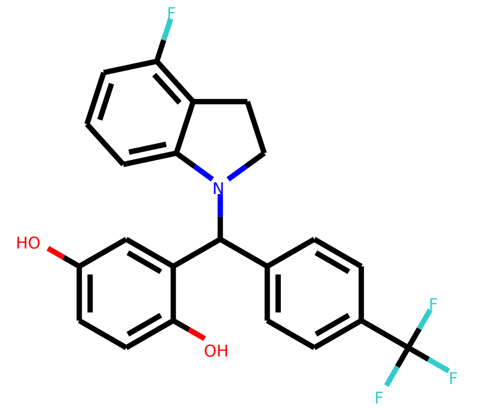
Ahma-1521
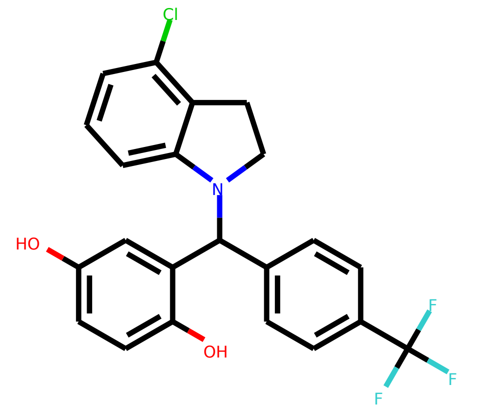
Ahma-1522
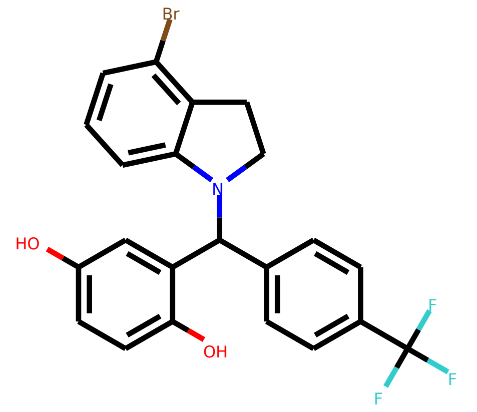
Ahma-1523
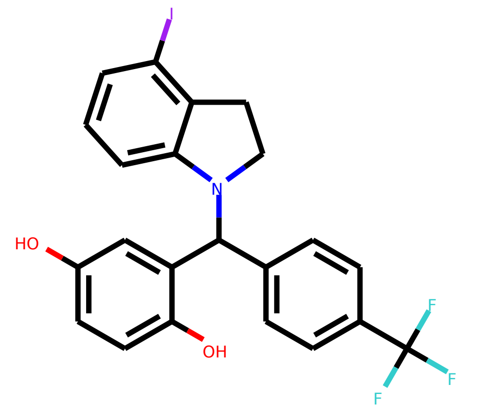
Ahma-1524
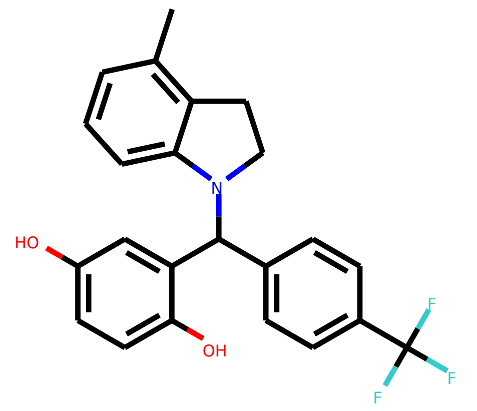
Ahma-1525
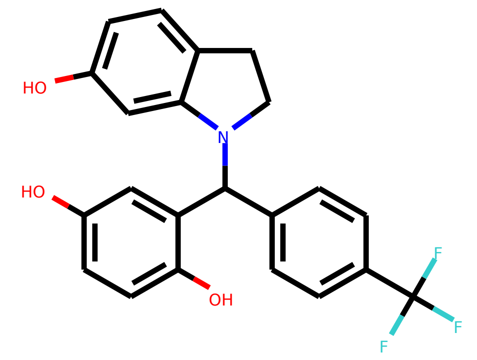
Ahma-1526
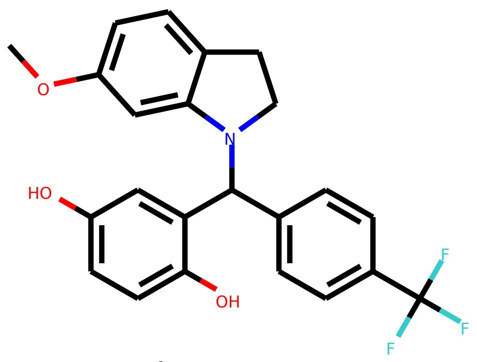
Ahma-1527
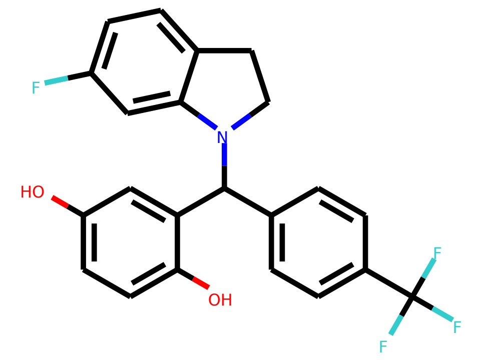
Ahma-1528
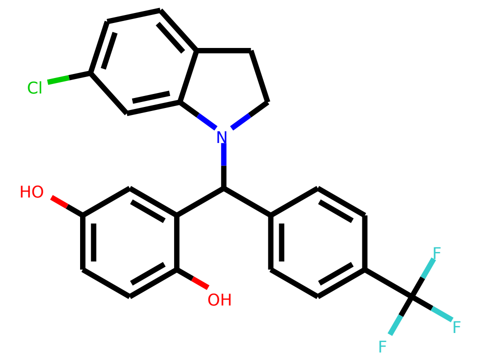
Ahma-1529
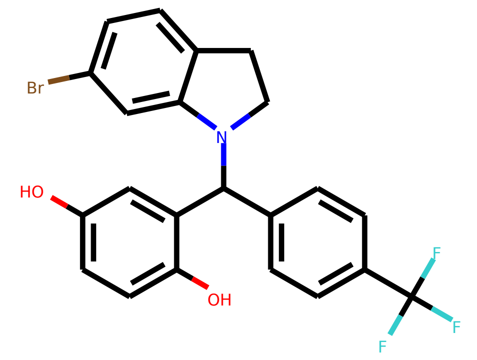
Ahma-1530
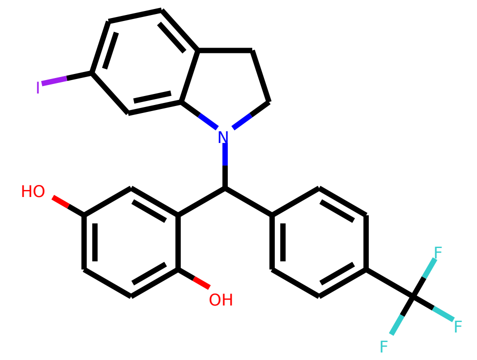
Ahma-1531
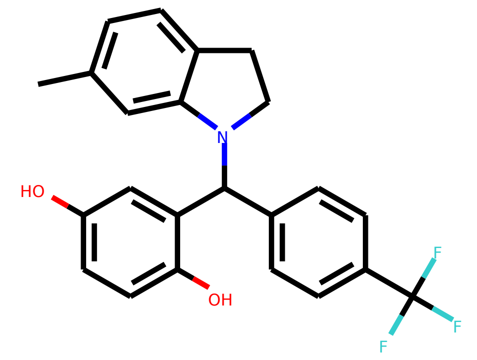
Ahma-1532
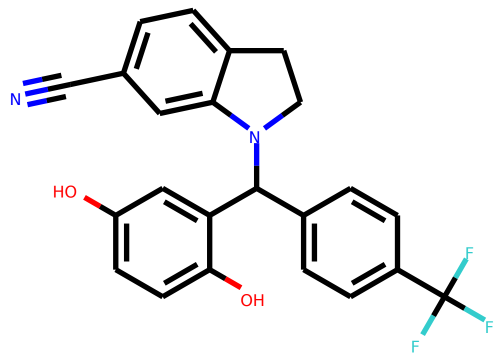
Ahma-1533
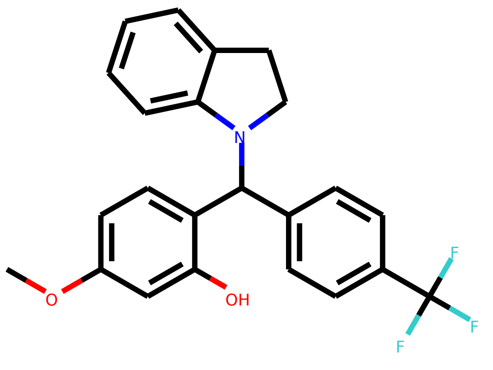
Ahma-1534
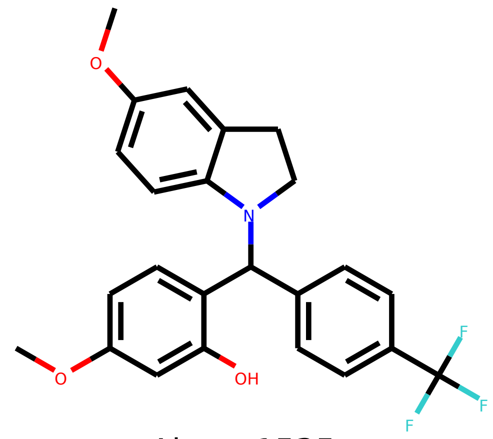
Ahma-1535
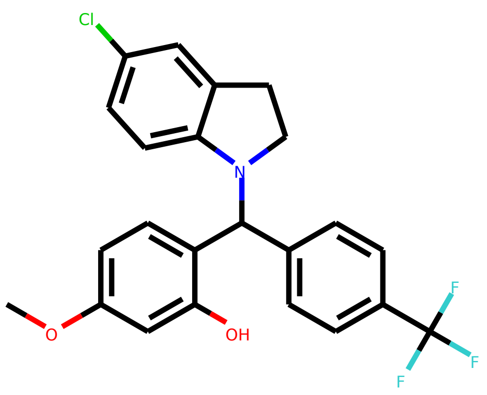
Ahma-1536
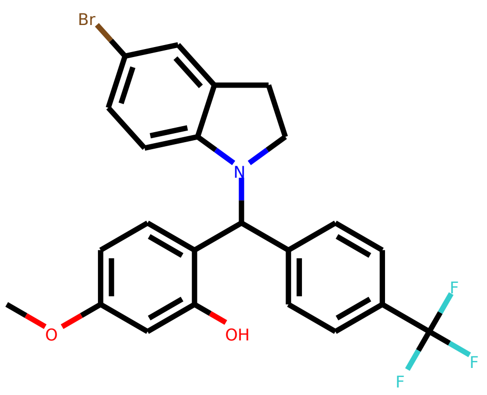
Ahma-1537
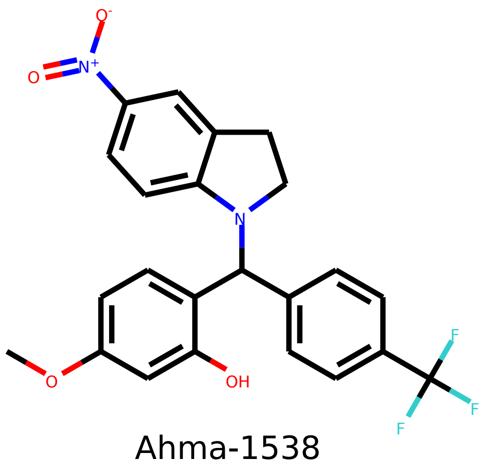
Ahma-1538
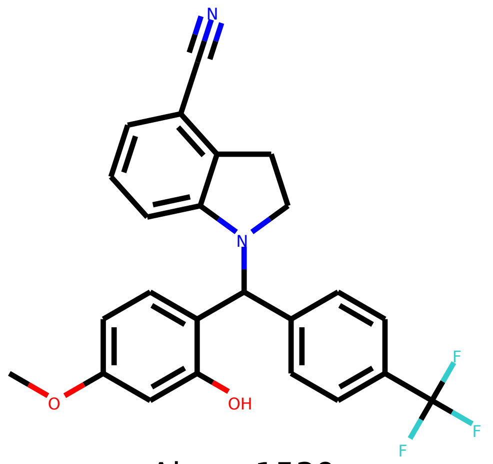
Ahma-1539
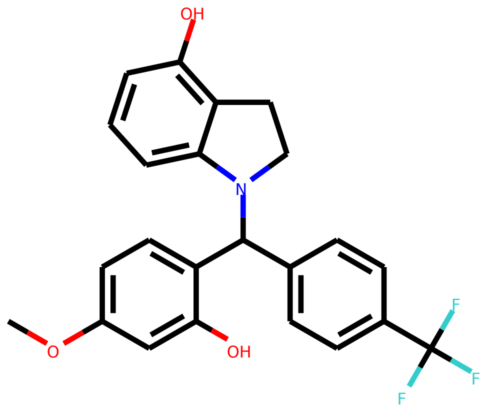
Ahma-1540

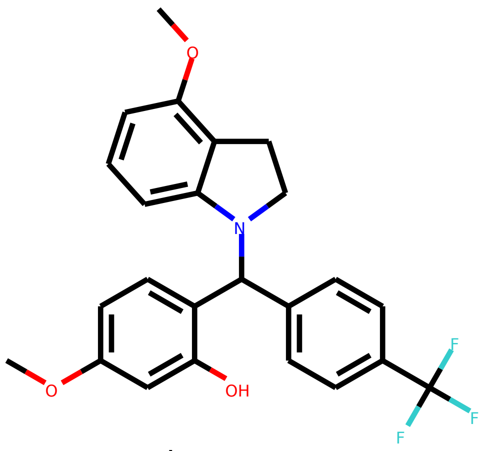
Ahma-1541
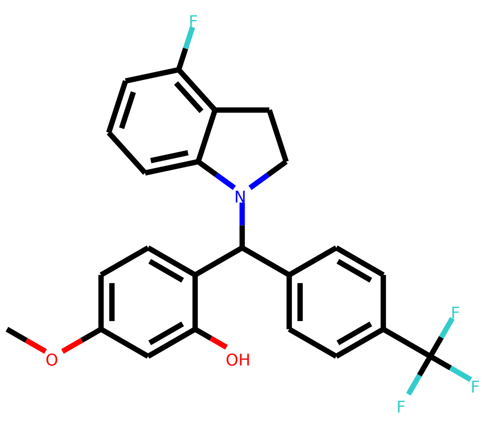
Ahma-1542
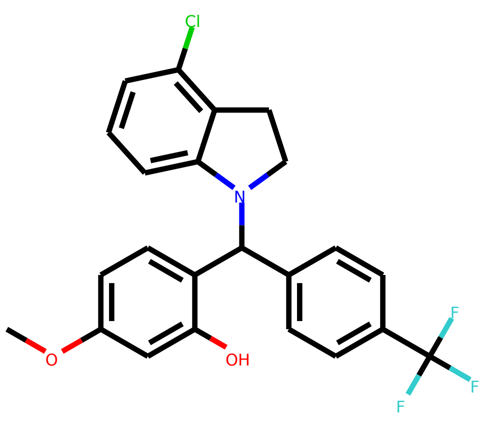
Ahma-1543
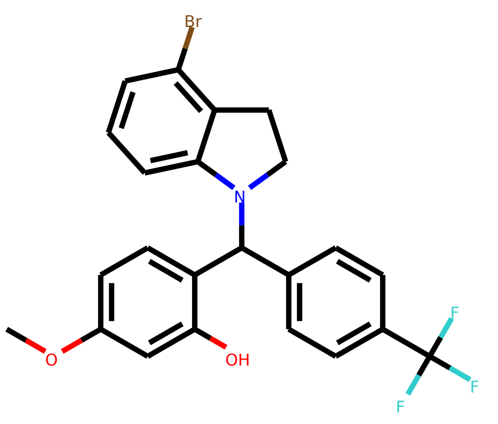
Ahma-1544
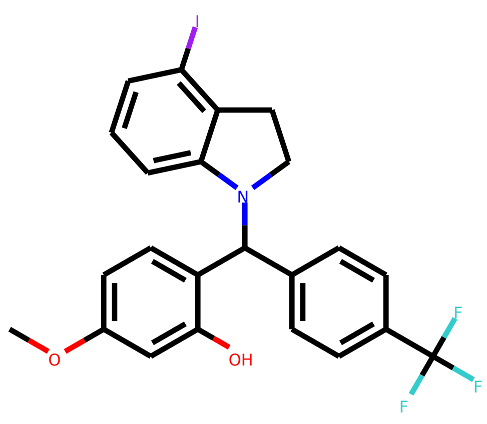
Ahma-1545
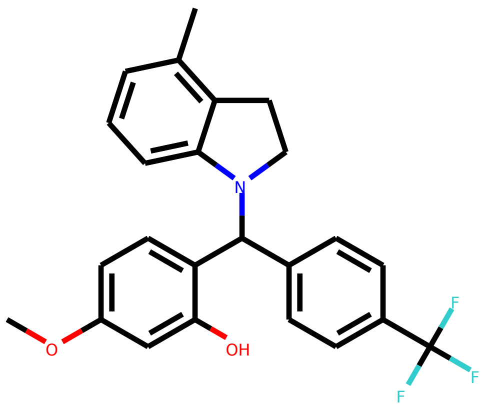
Ahma-1546
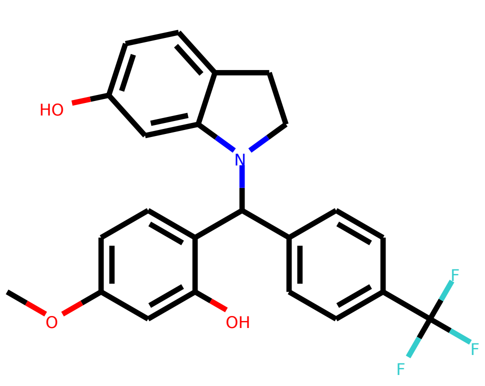
Ahma-1547
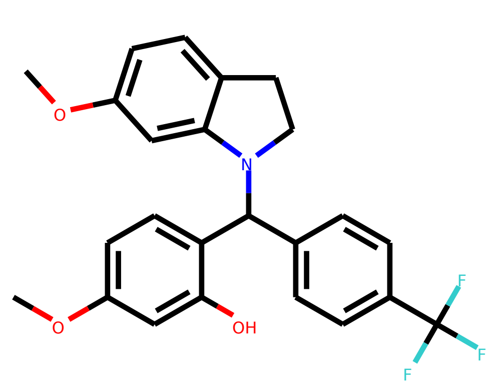
Ahma-1548
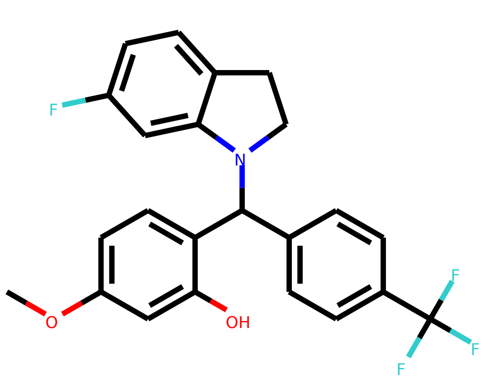
Ahma-1549
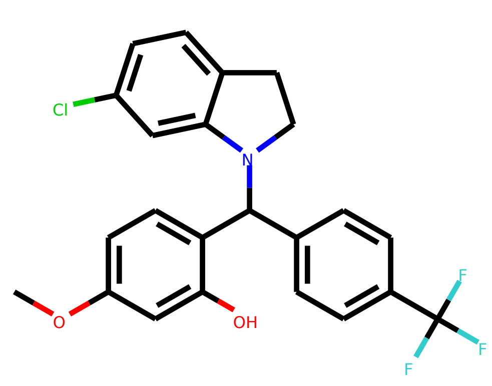
Ahma-1550
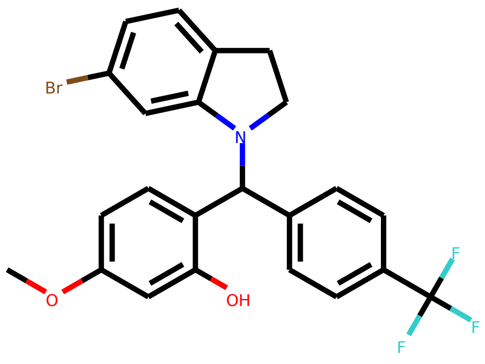
Ahma-1551
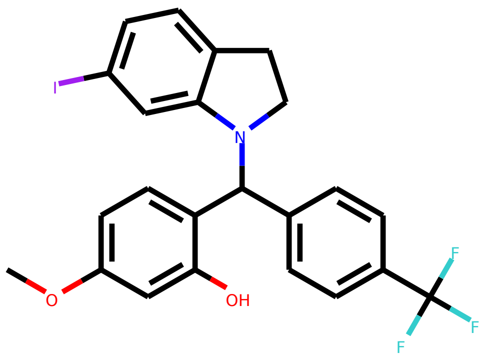
Ahma-1552
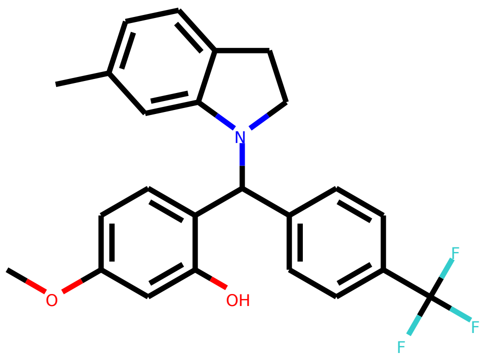
Ahma-1553
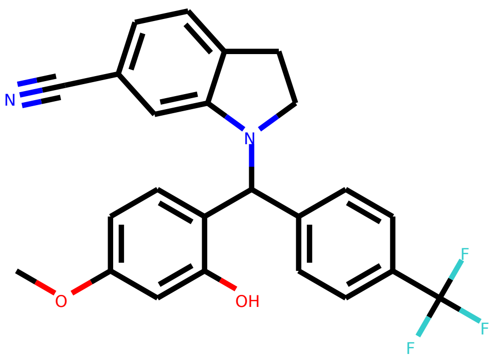
Ahma-1554
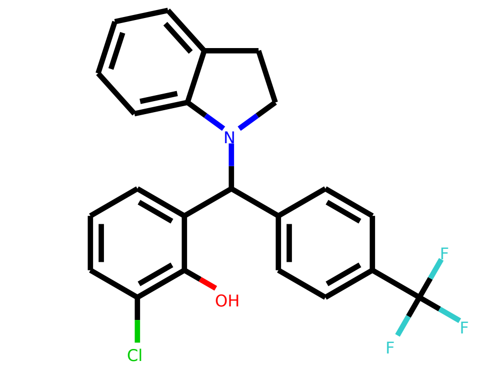
Ahma-1555
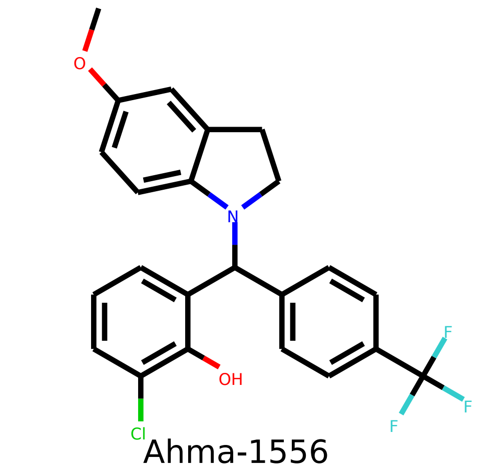
Ahma-1556
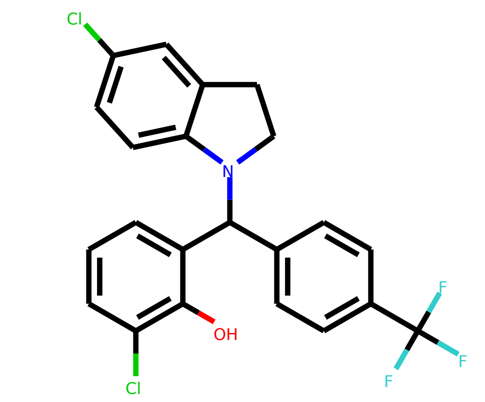
Ahma-1557
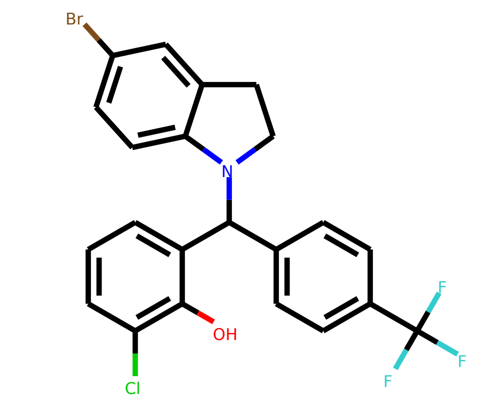
Ahma-1558
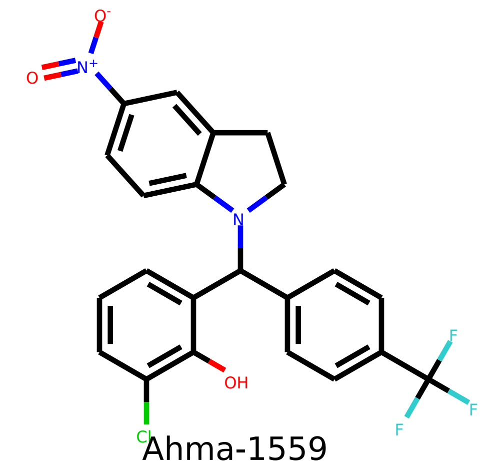
Ahma-1559
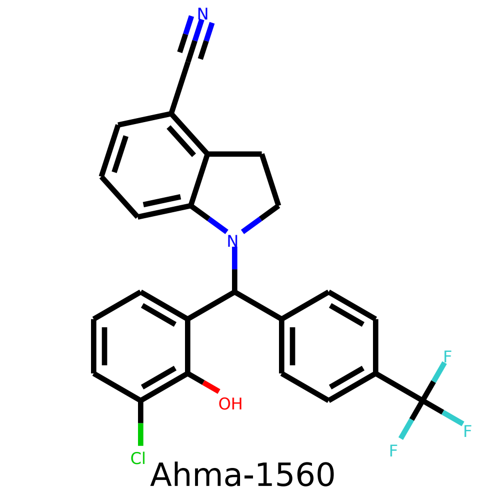
Ahma-1560
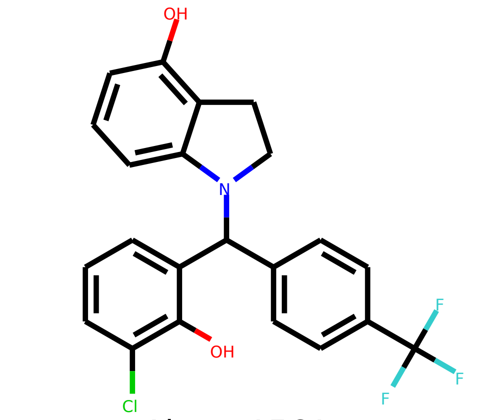
Ahma-1561
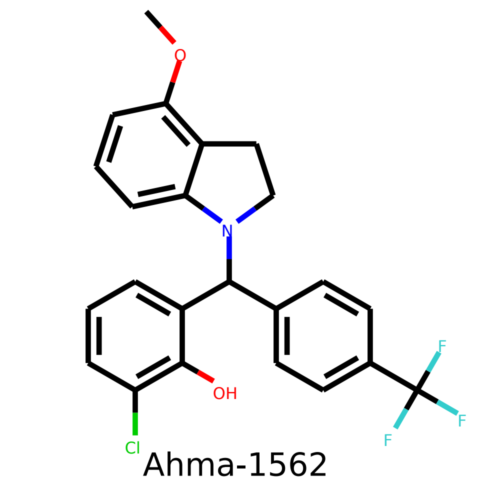
Ahma-1562
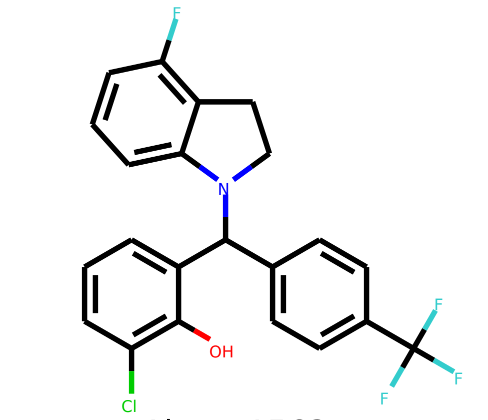
Ahma-1563
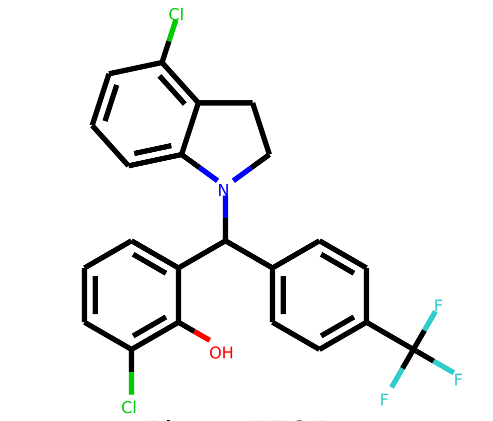
Ahma-1564
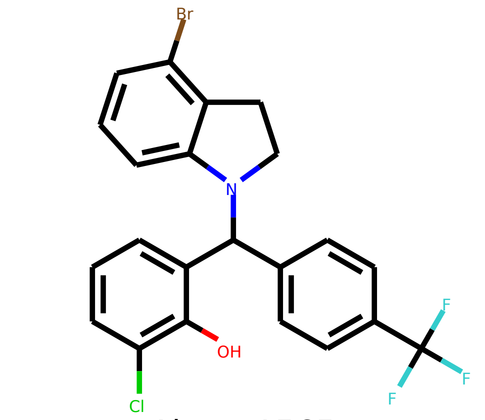
Ahma-1565
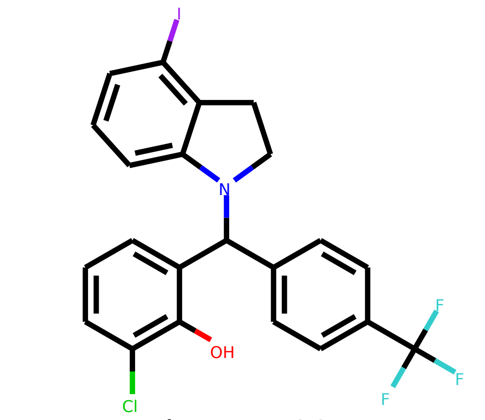
Ahma-1566
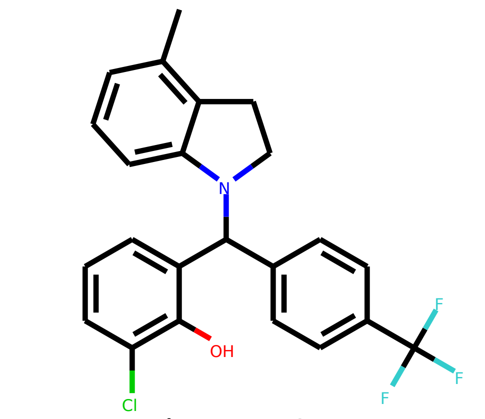
Ahma-1567
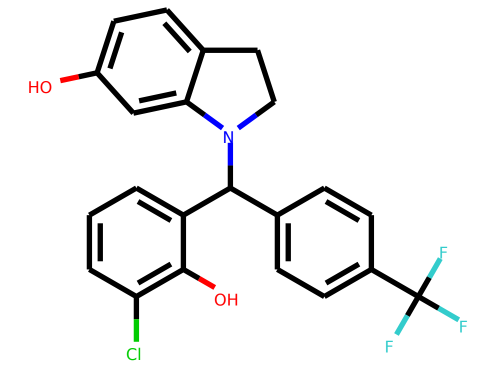
Ahma-1568
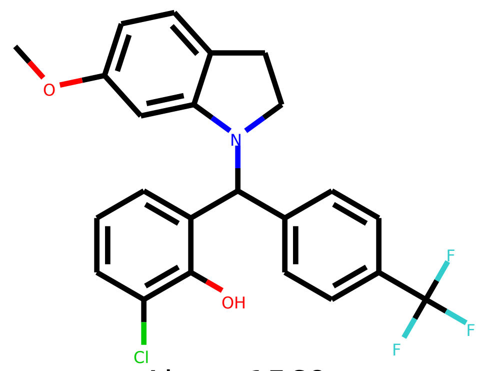
Ahma-1569
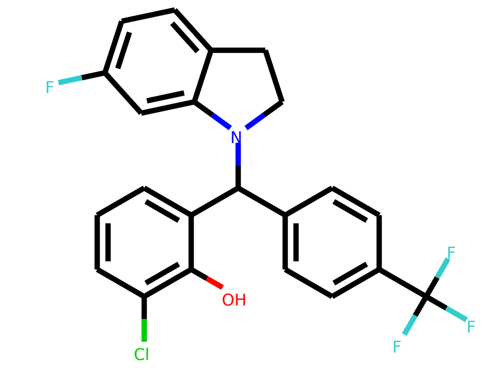
Ahma-1570
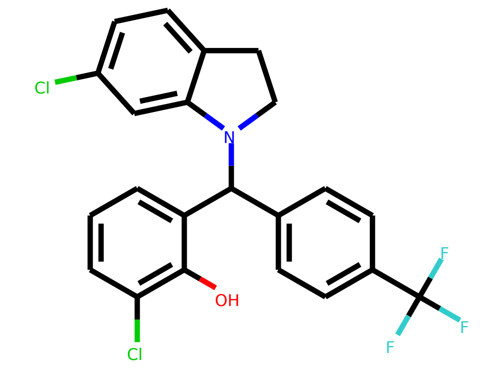
Ahma-1571
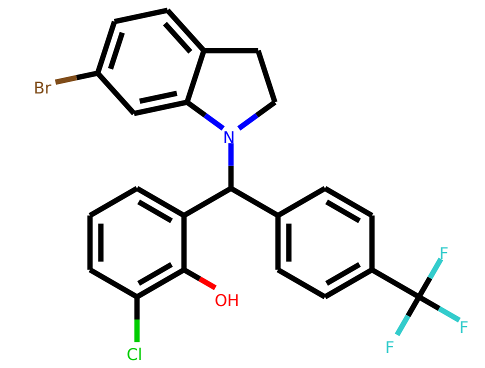
Ahma-1572
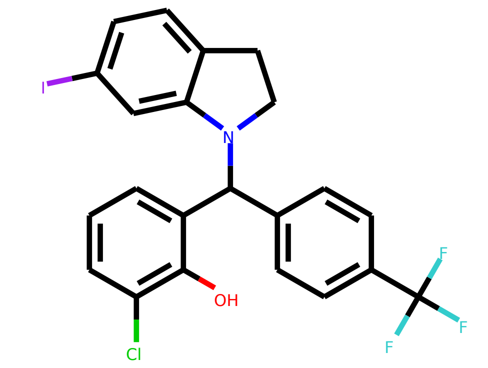
Ahma-1573
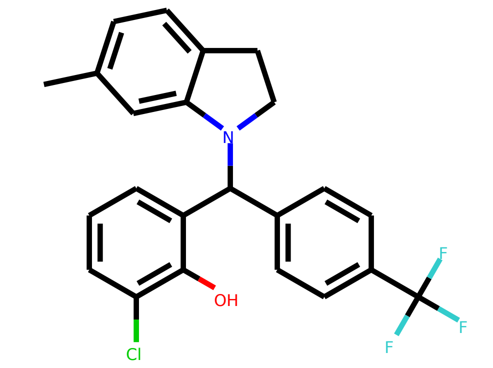
Ahma-1574
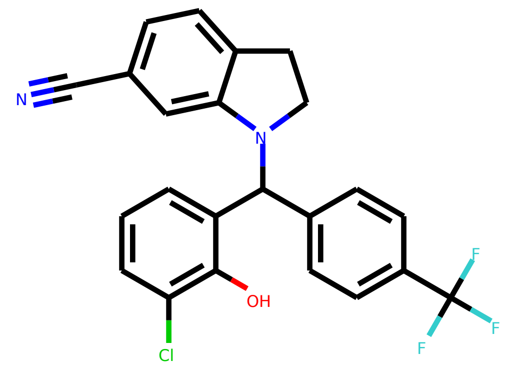
Ahma-1575

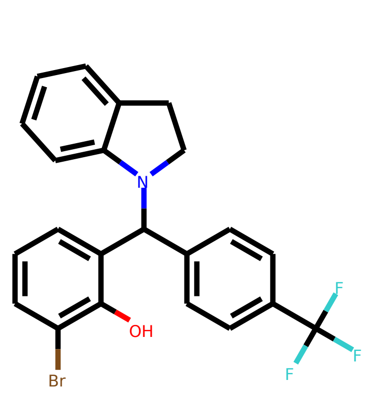
Ahma-1576

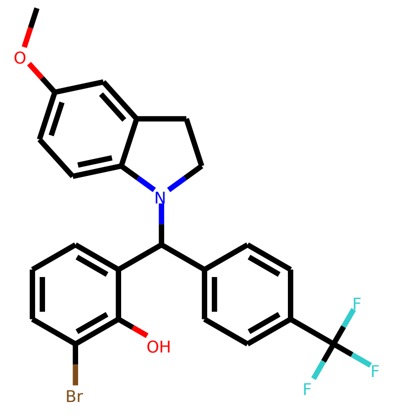
Ahma-1577

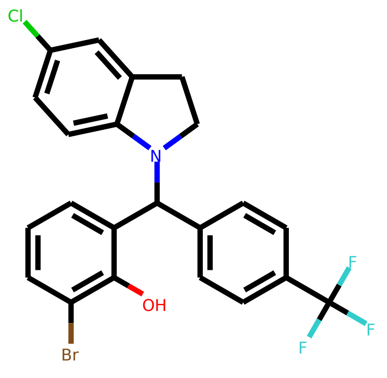
Ahma-1578

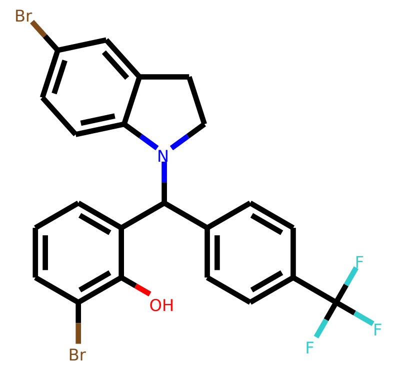
Ahma-1579

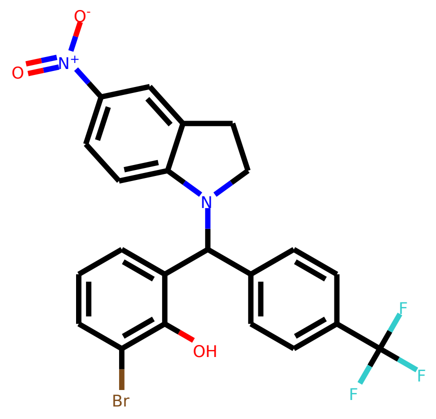
Ahma-1580

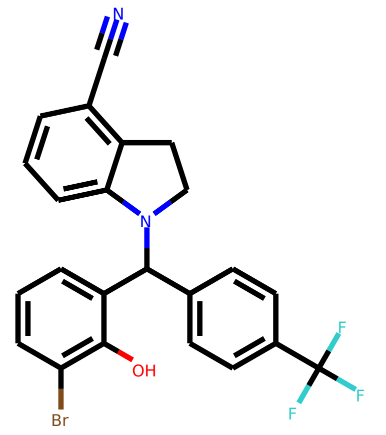
Ahma-1581

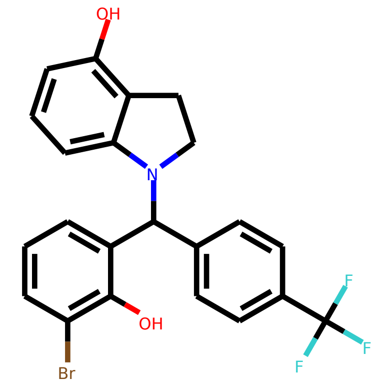
Ahma-1582

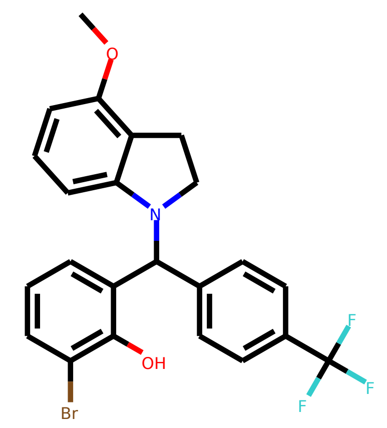
Ahma-1583

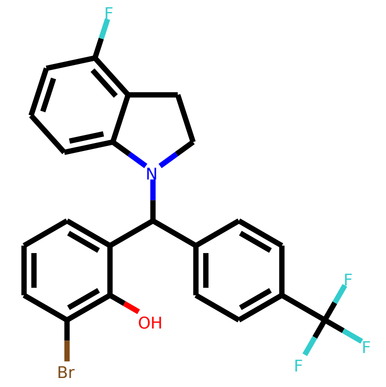
Ahma-1584

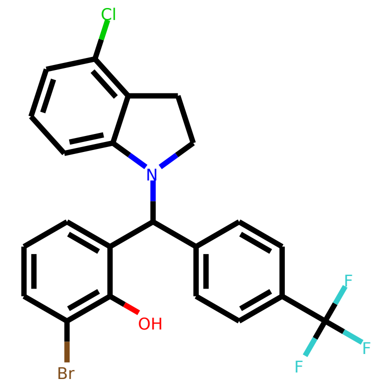
Ahma-1585

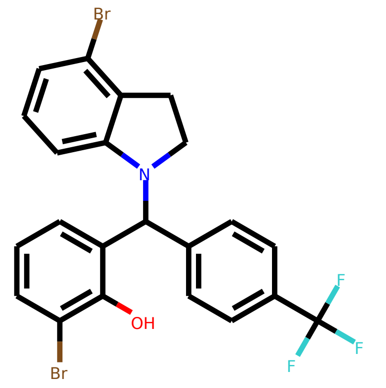
Ahma-1586

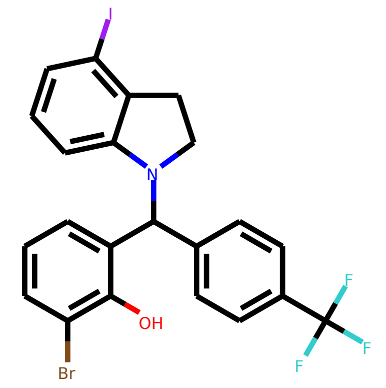
Ahma-1587

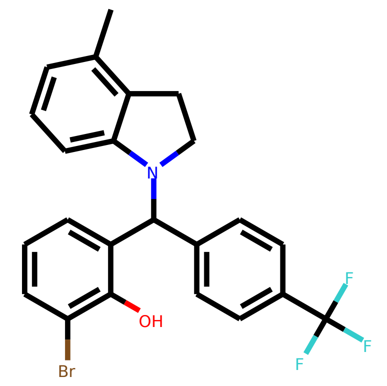
Ahma-1588

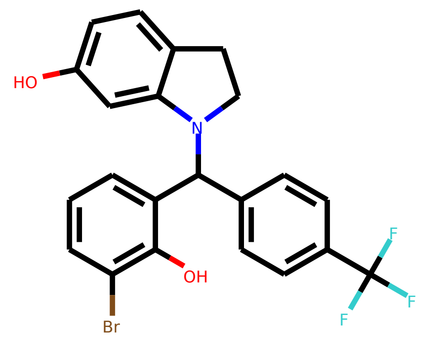
Ahma-1589

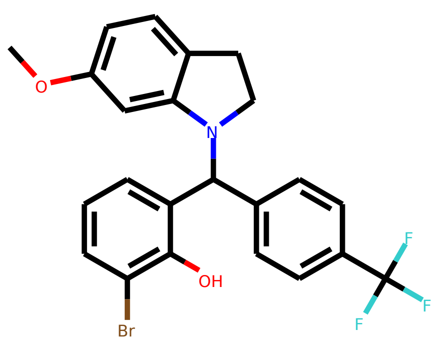
Ahma-1590

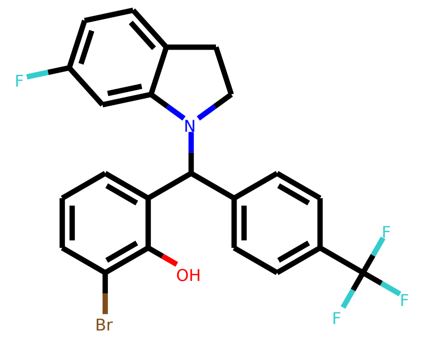
Ahma-1591

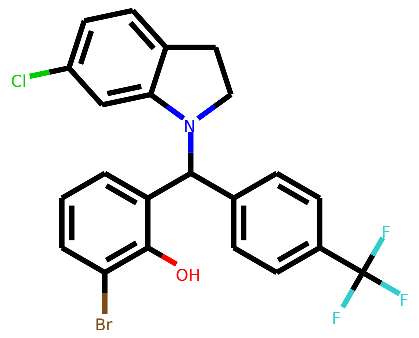
Ahma-1592

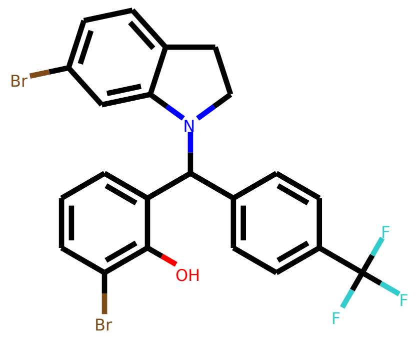
Ahma-1593

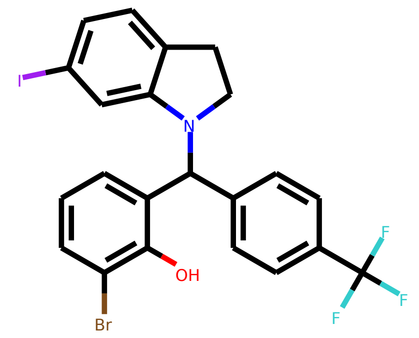
Ahma-1594

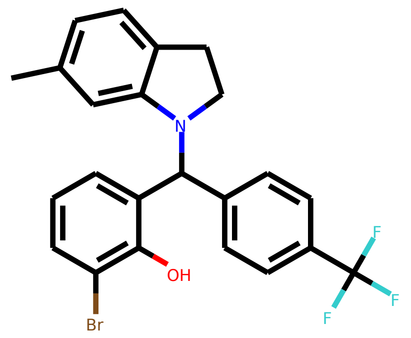
Ahma-1595

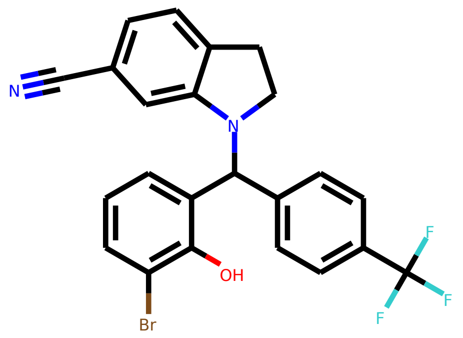
Ahma-1596

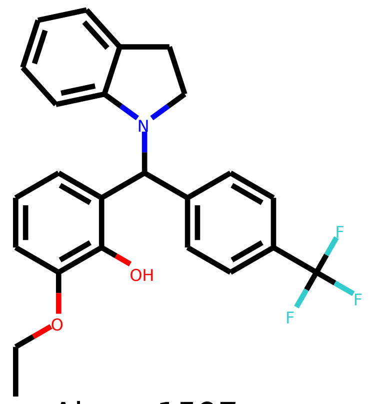
Ahma-1597

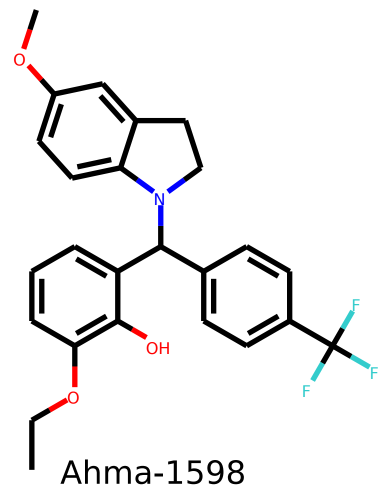
Ahma-1598

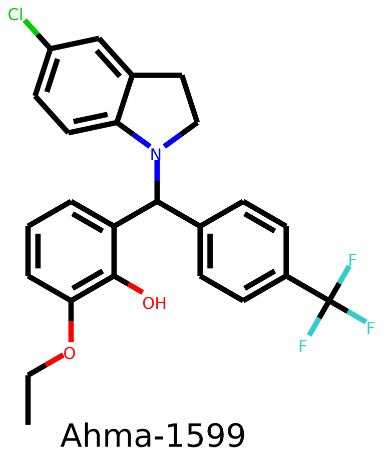
Ahma-1599

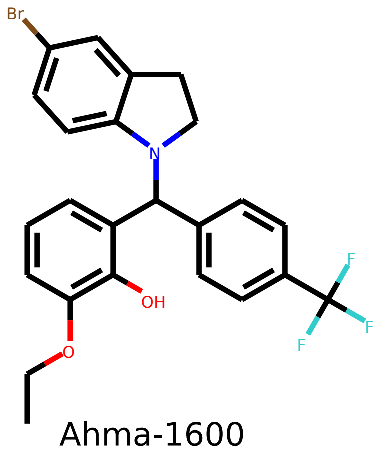
Ahma-1600

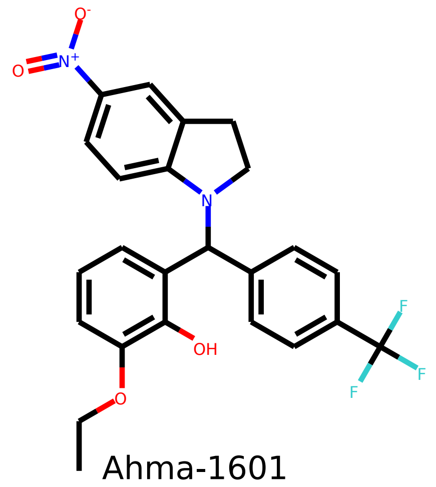
Ahma-1601

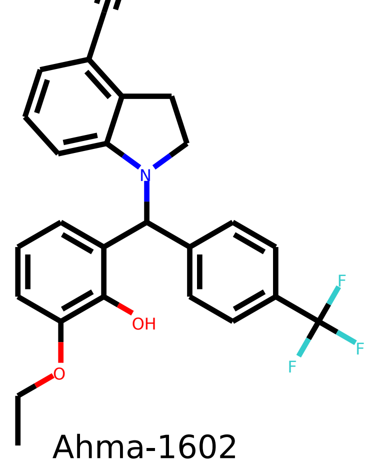
Ahma-1602

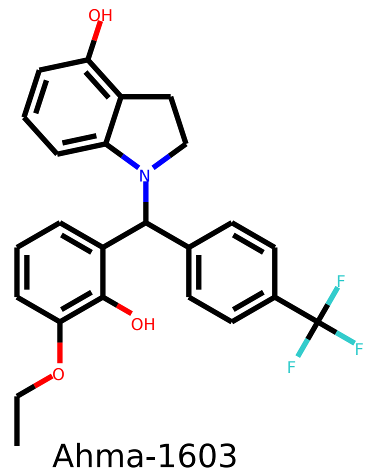
Ahma-1603

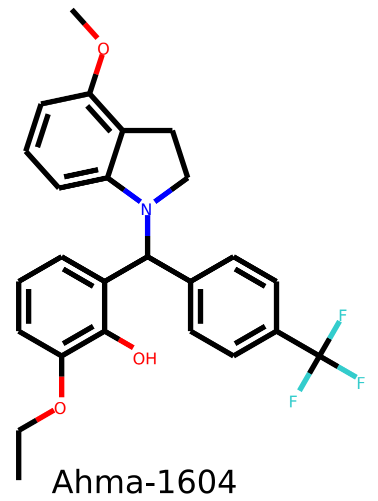
Ahma-1604

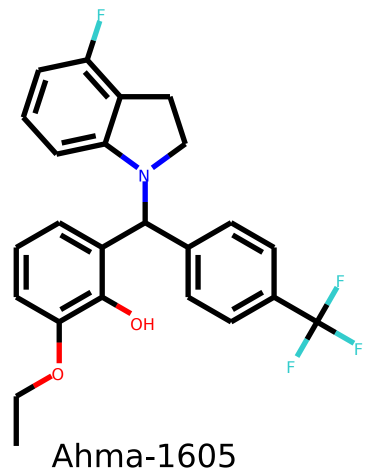
Ahma-1605

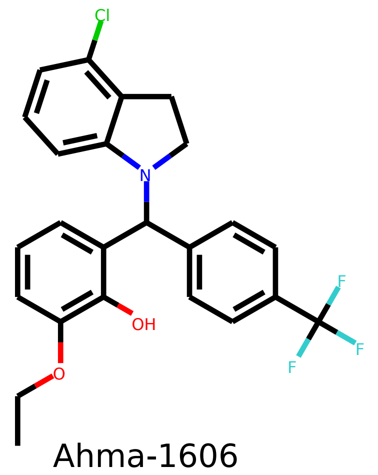
Ahma-1606

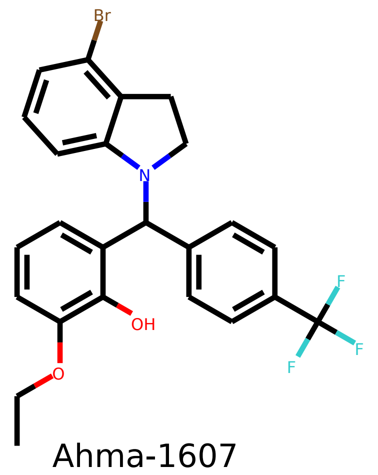
Ahma-1607

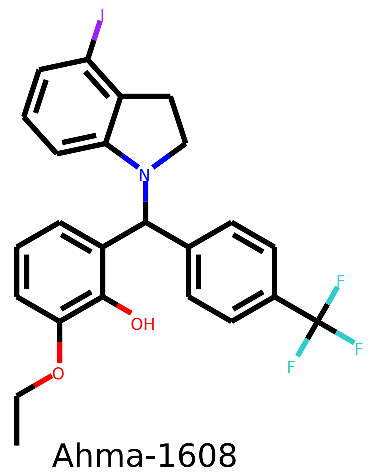
Ahma-1608

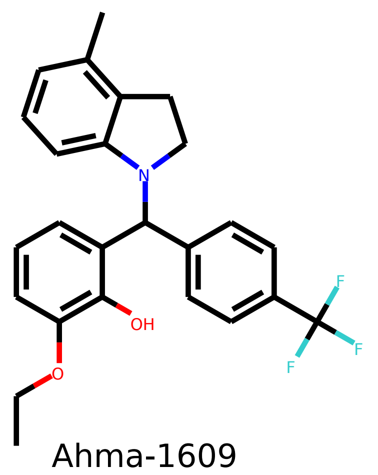
Ahma-1609

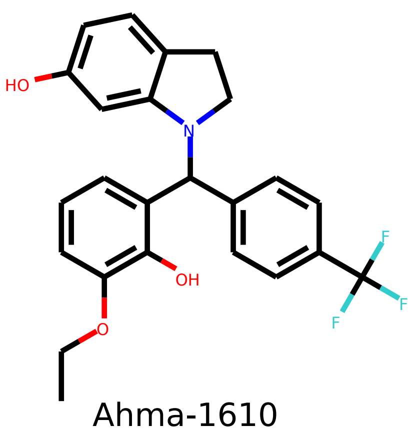
Ahma-1610

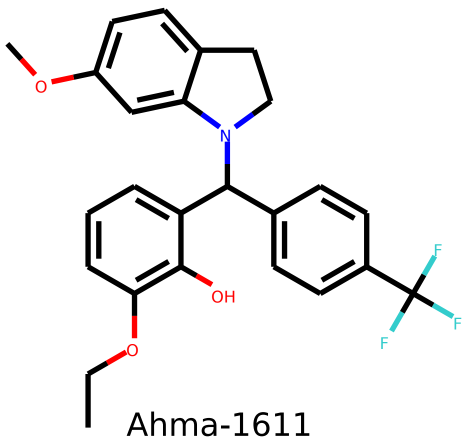
Ahma-1611
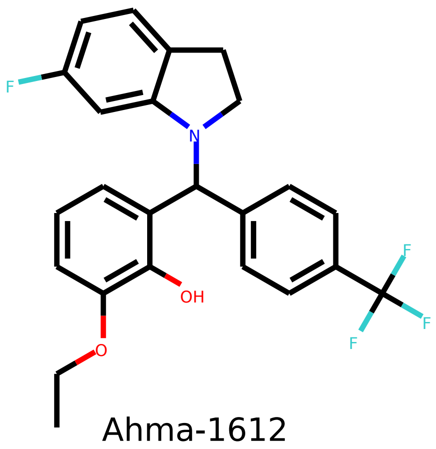
Ahma-1612
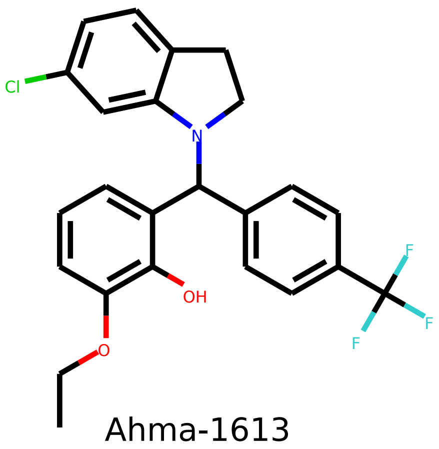
Ahma-1613
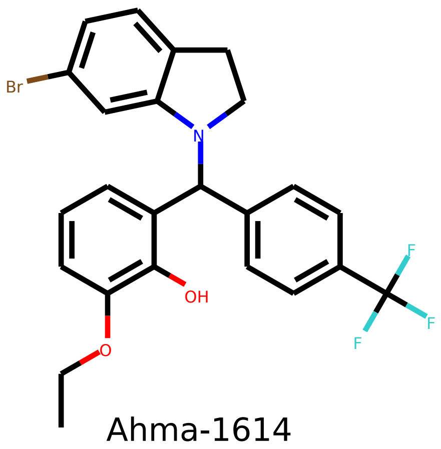
Ahma-1614
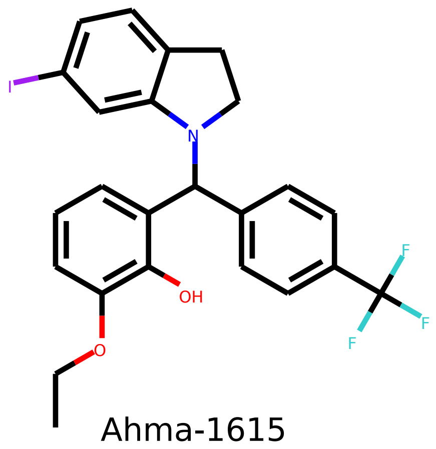
Ahma-1615
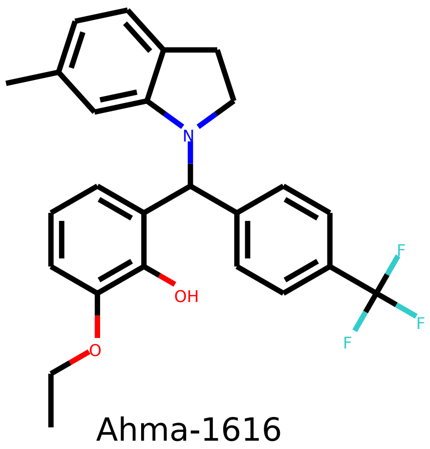
Ahma-1616
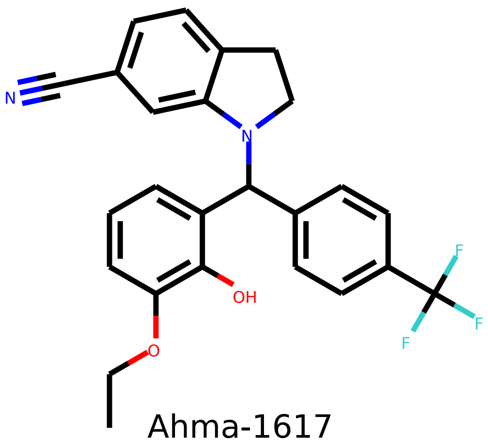
Ahma-1617
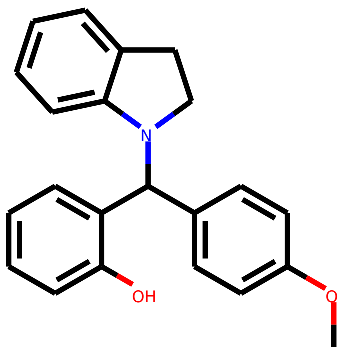
Ahma-1618
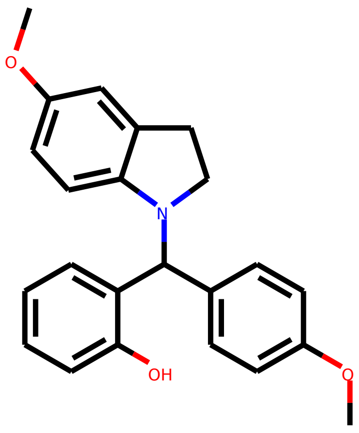
Ahma-1619
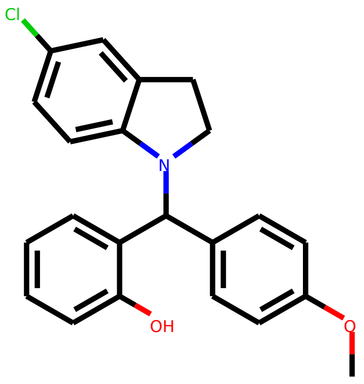
Ahma-1620
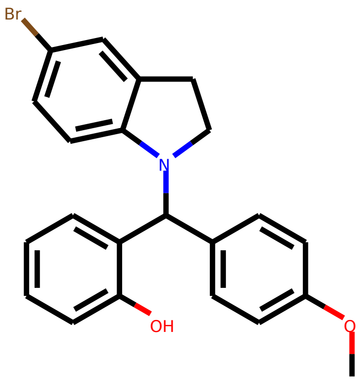
Ahma-1621
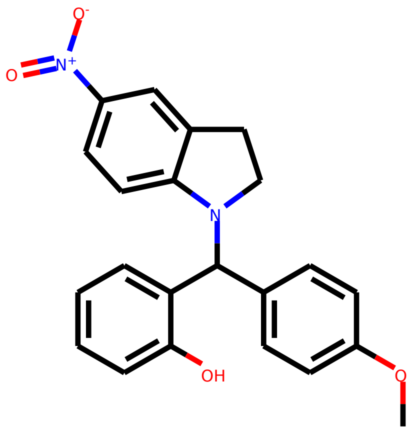
Ahma-1622
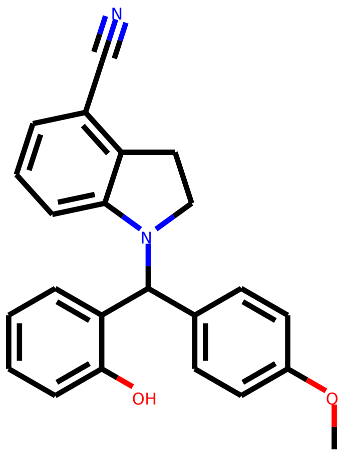
Ahma-1623
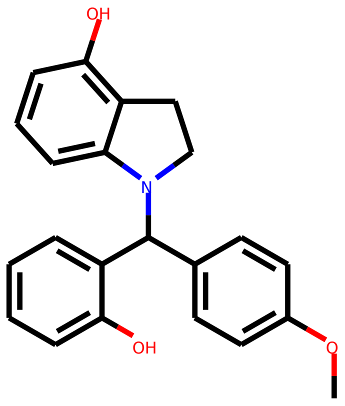
Ahma-1624
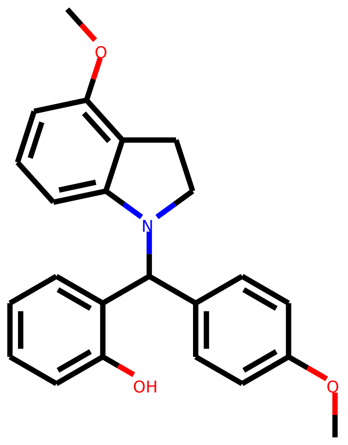
Ahma-1625
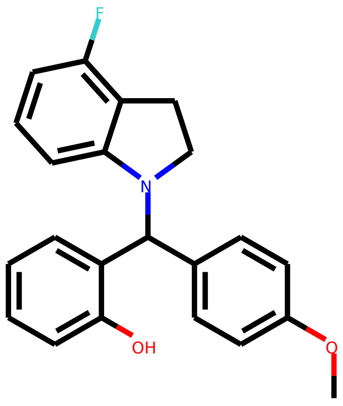
Ahma-1626
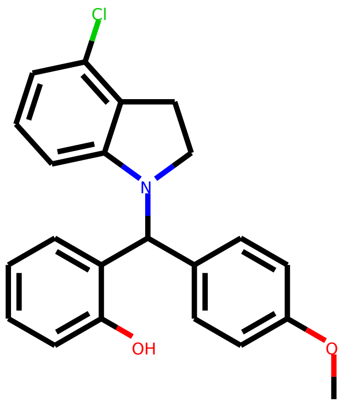
Ahma-1627
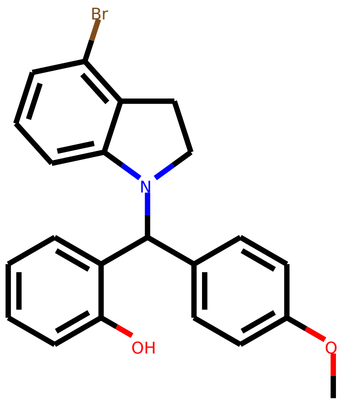
Ahma-1628
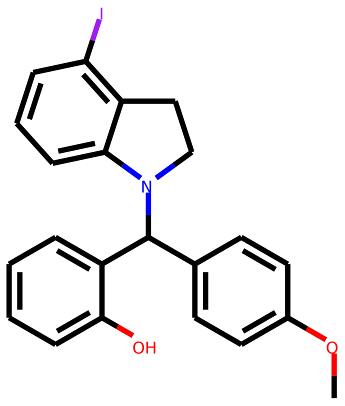
Ahma-1629
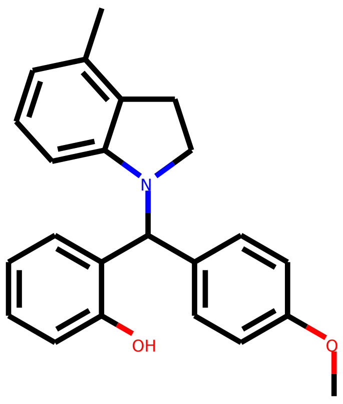
Ahma-1630
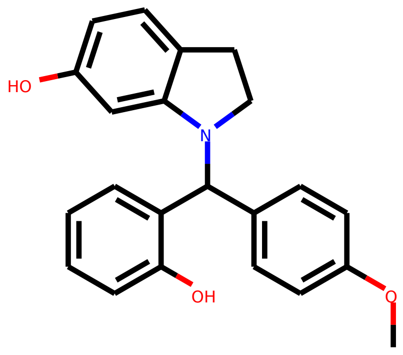
Ahma-1631
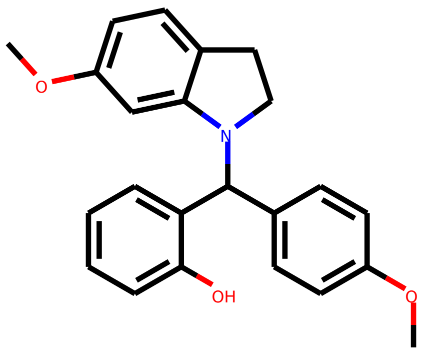
Ahma-1632
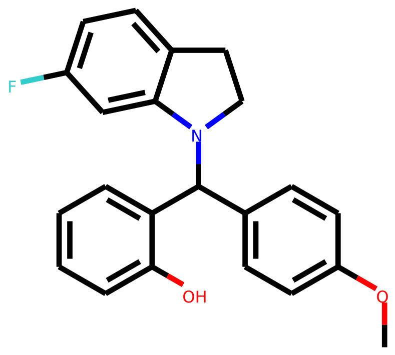
Ahma-1633
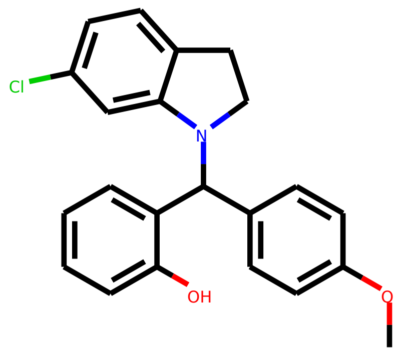
Ahma-1634
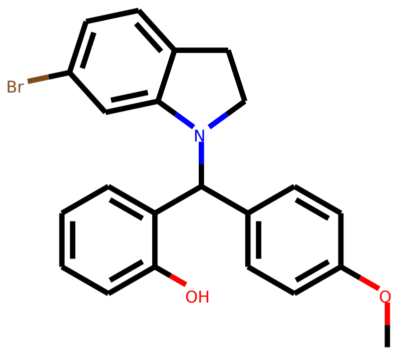
Ahma-1635
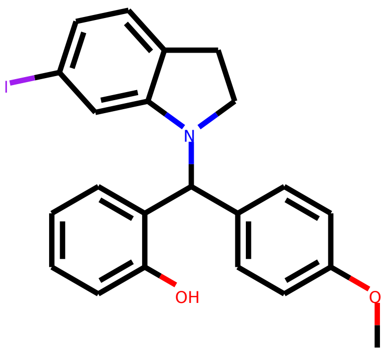
Ahma-1636
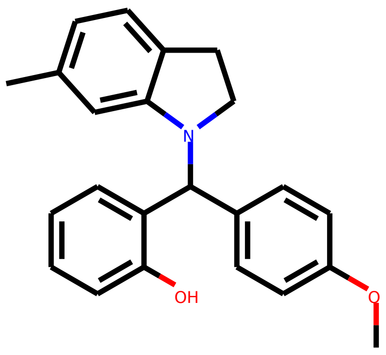
Ahma-1637
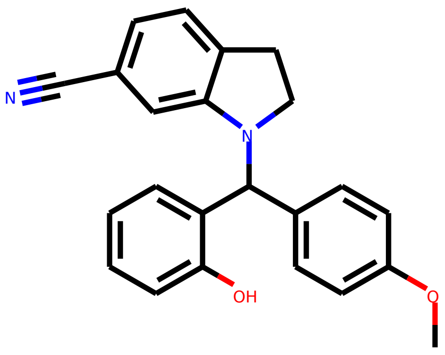
Ahma-1638
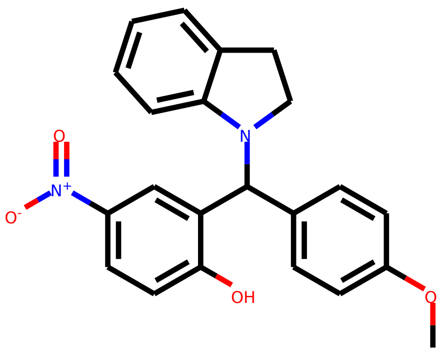
Ahma-1639
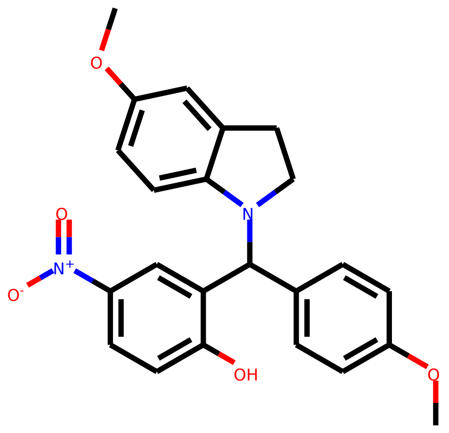
Ahma-1640
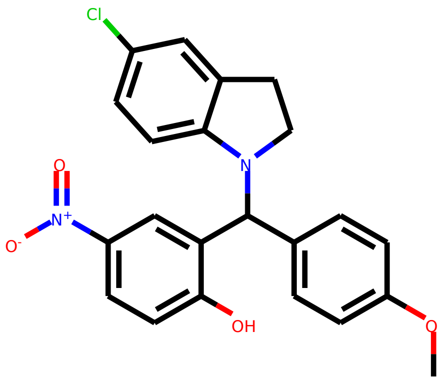
Ahma-1641
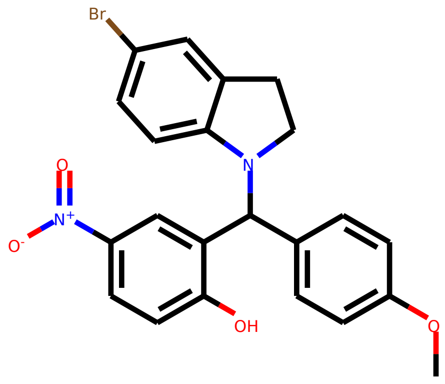
Ahma-1642
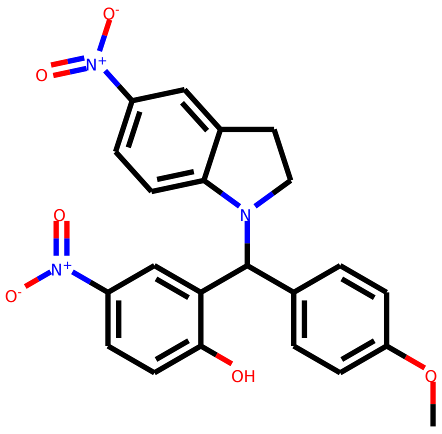
Ahma-1643
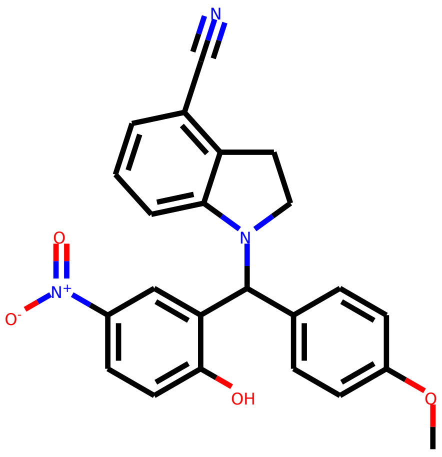
Ahma-1644
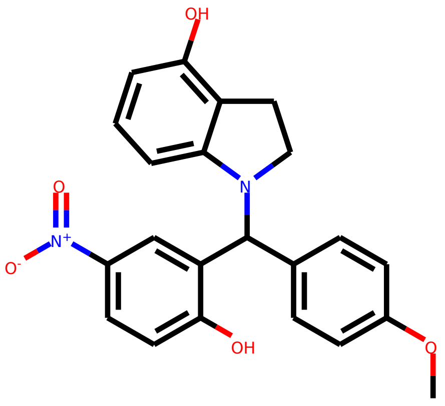
Ahma-1645

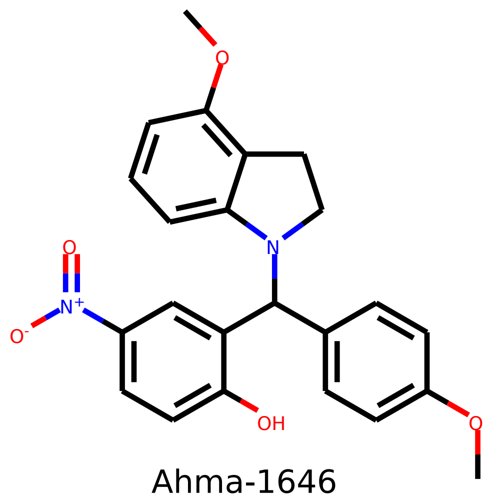
Ahma-1646
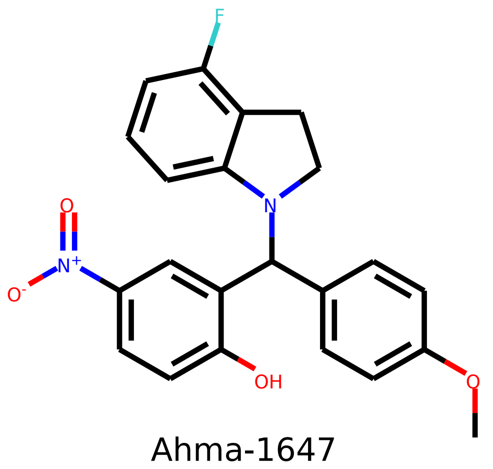
Ahma-1647
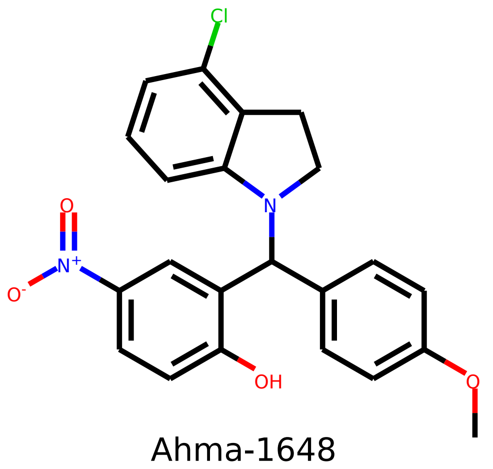
Ahma-1648
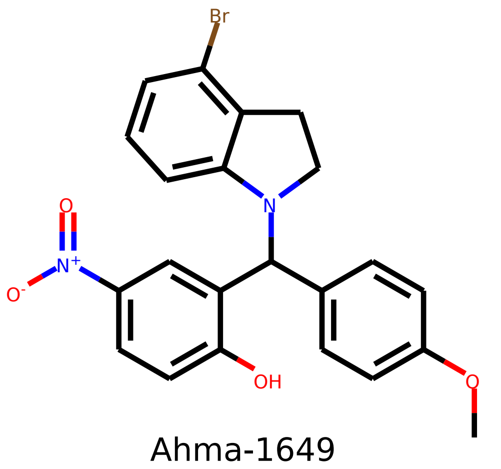
Ahma-1649
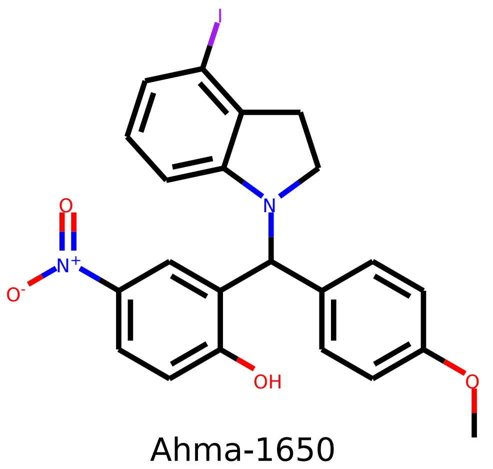
Ahma-1650
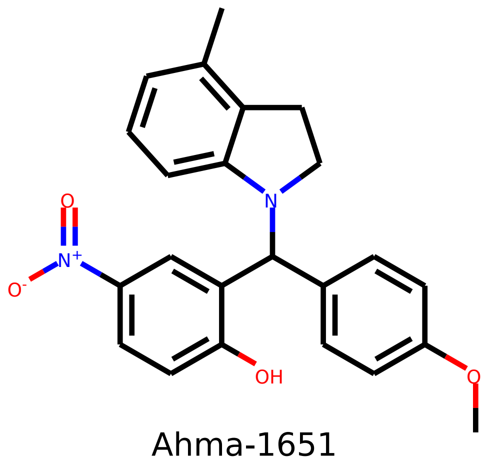
Ahma-1651
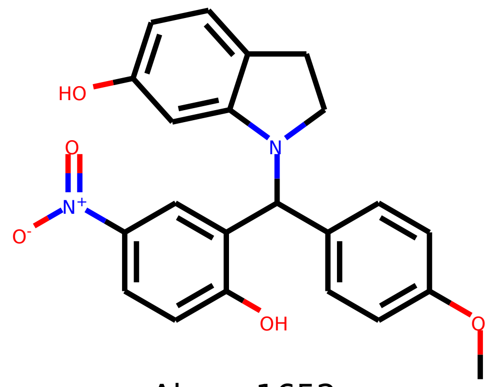
Ahma-1652
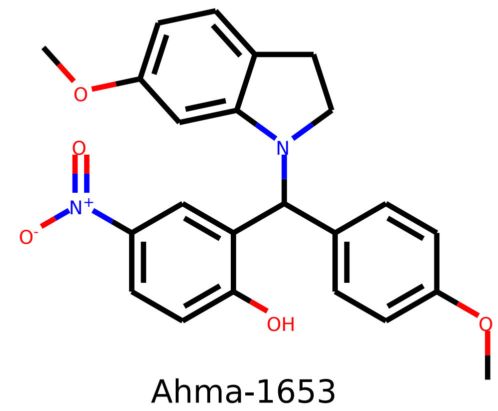
Ahma-1653
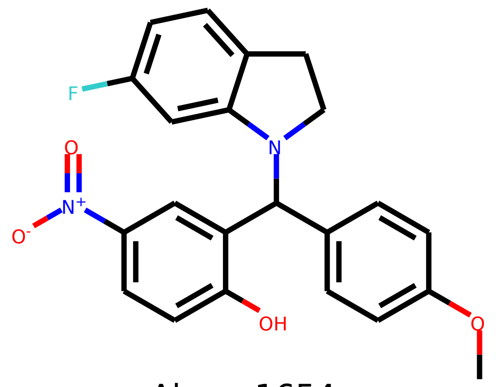
Ahma-1654
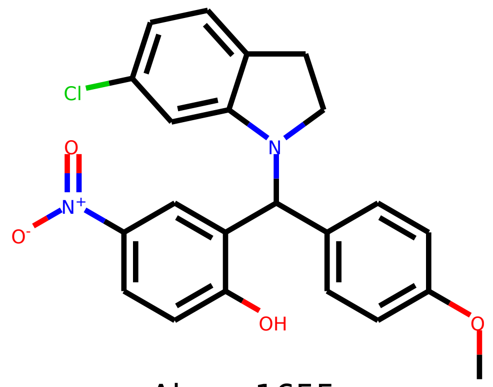
Ahma-1655
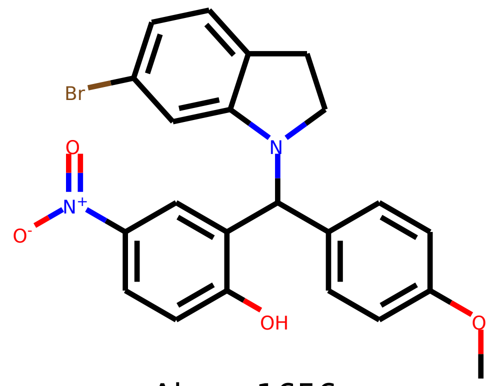
Ahma-1656
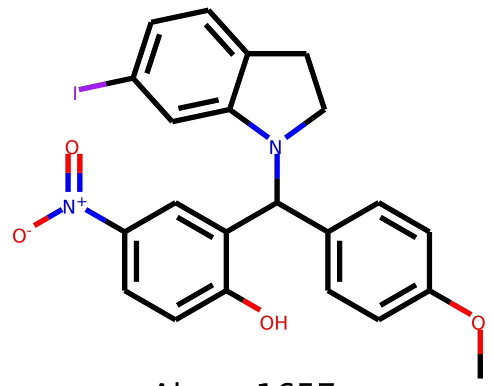
Ahma-1657
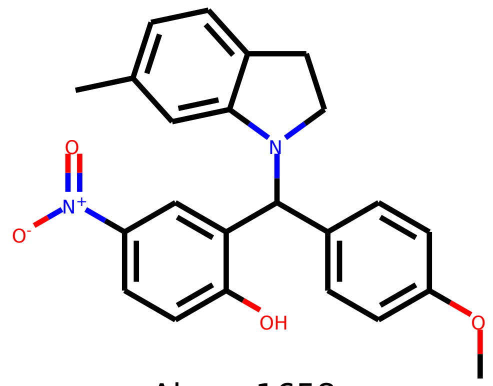
Ahma-1658
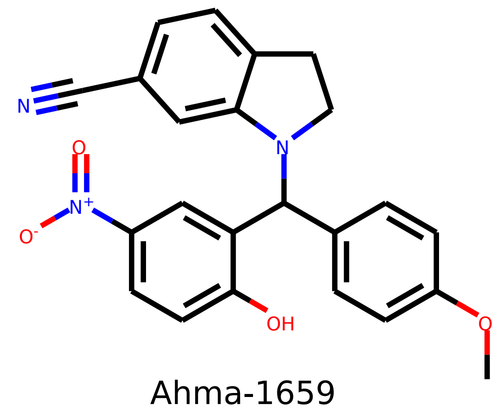
Ahma-1659
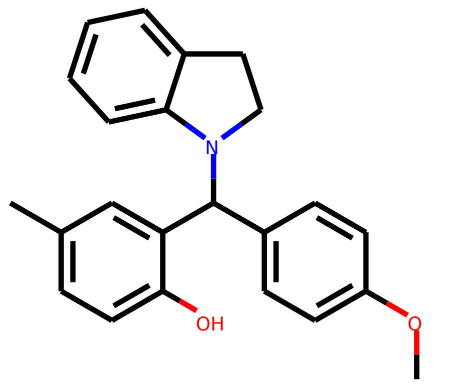
Ahma-1660
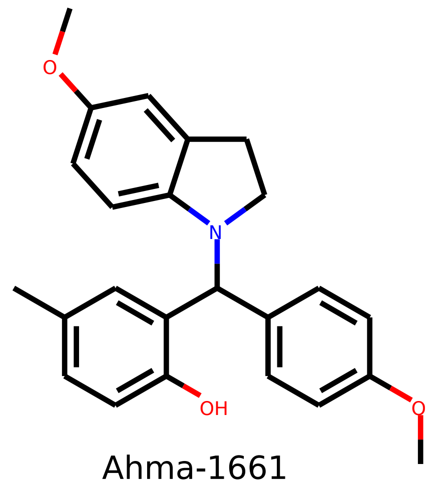
Ahma-1661
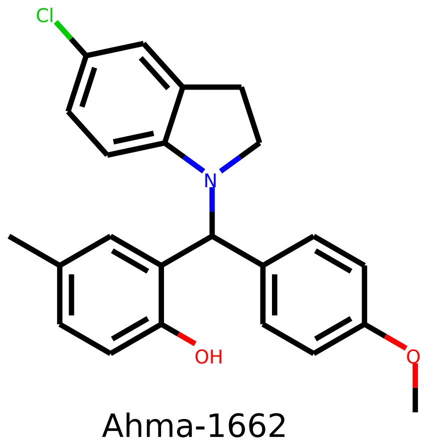
Ahma-1662
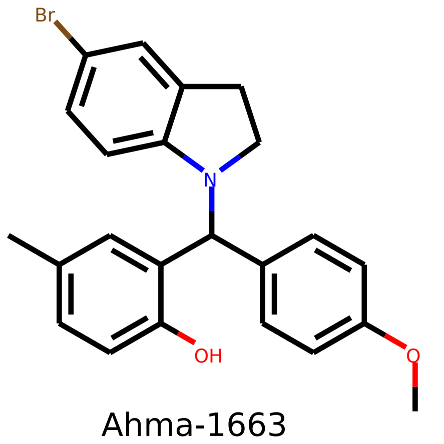
Ahma-1663
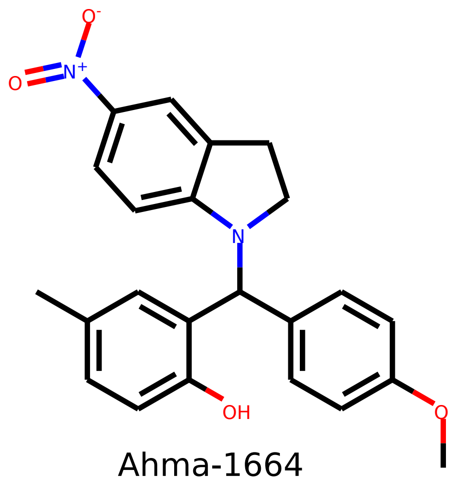
Ahma-1664
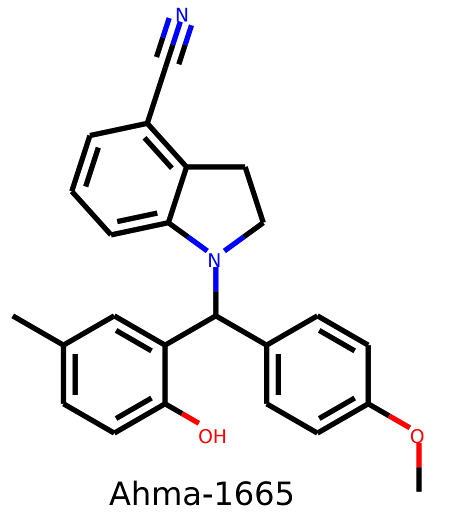
Ahma-1665
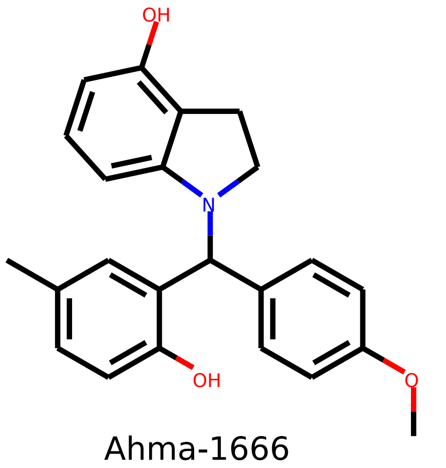
Ahma-1666
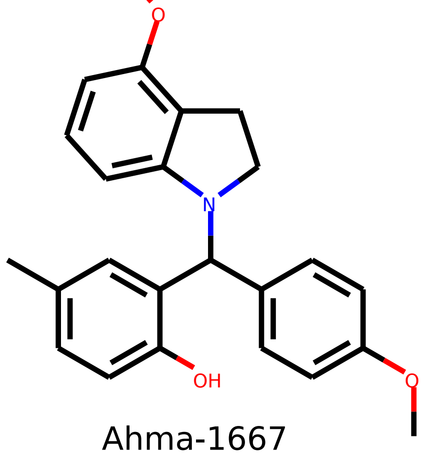
Ahma-1667
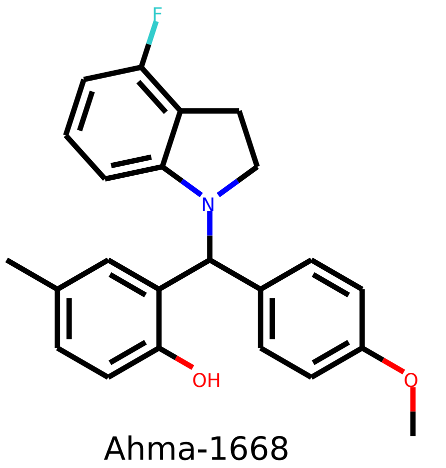
Ahma-1668
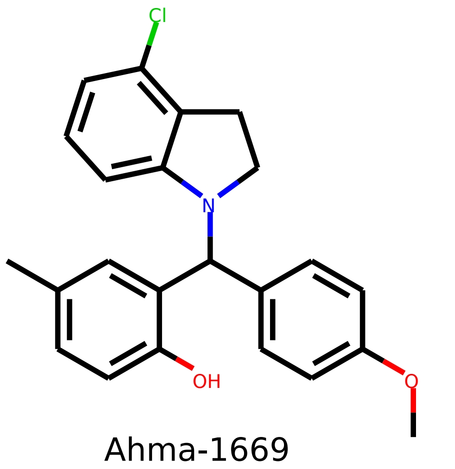
Ahma-1669
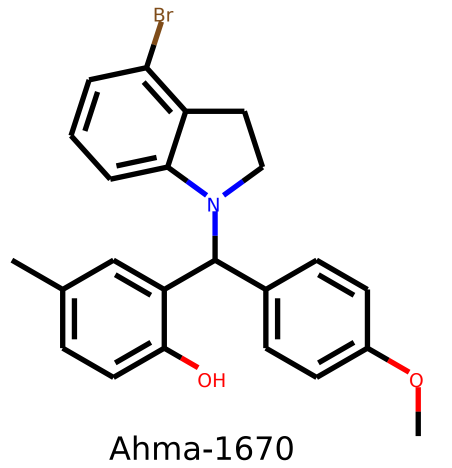
Ahma-1670
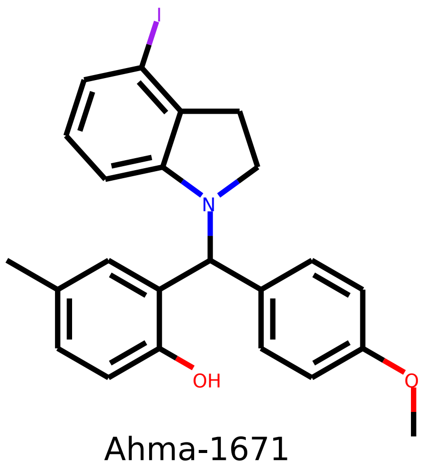
Ahma-1671
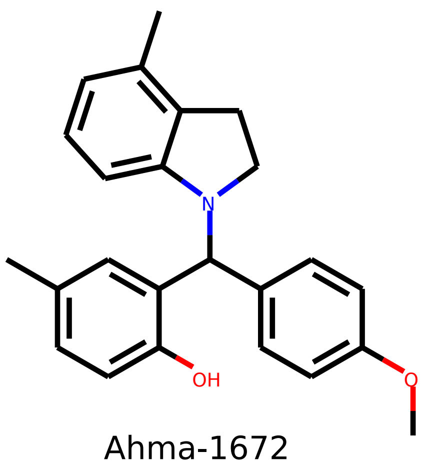
Ahma-1672
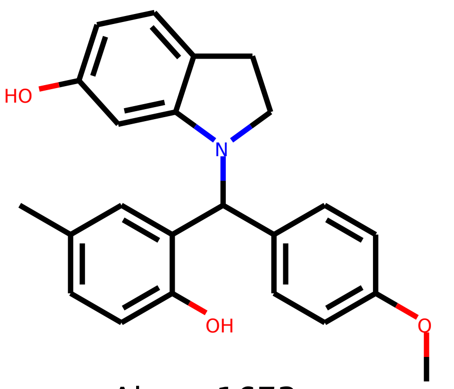
Ahma-1673
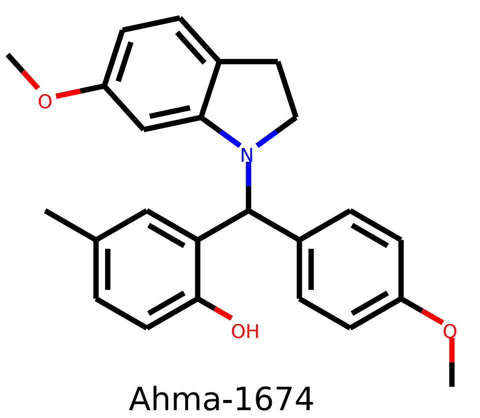
Ahma-1674
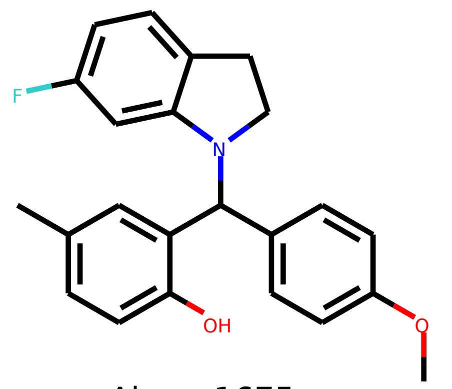
Ahma-1675
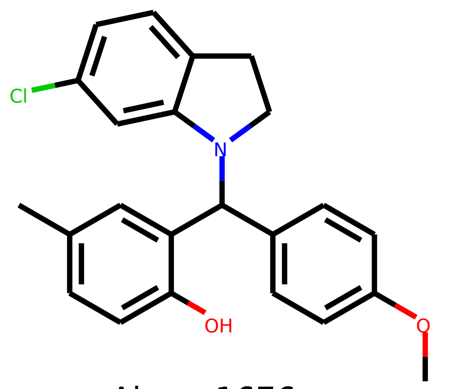
Ahma-1676
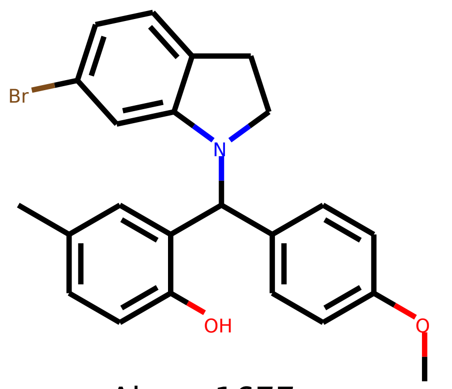
Ahma-1677
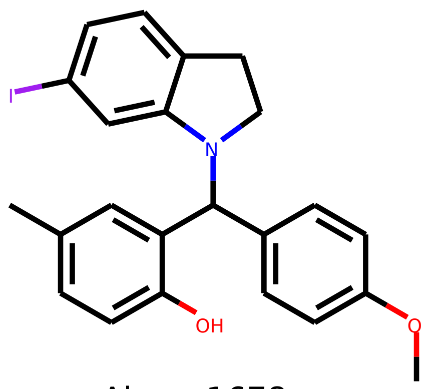
Ahma-1678
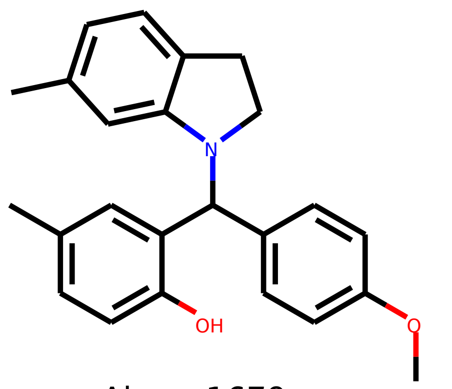
Ahma-1679
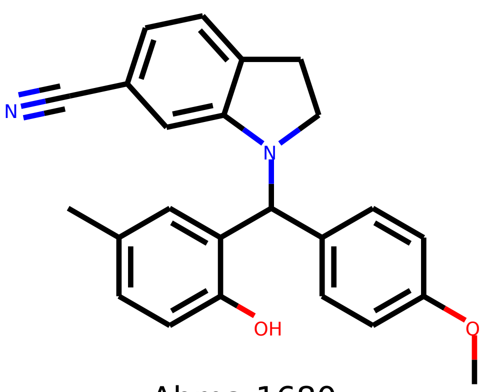
Ahma-1680

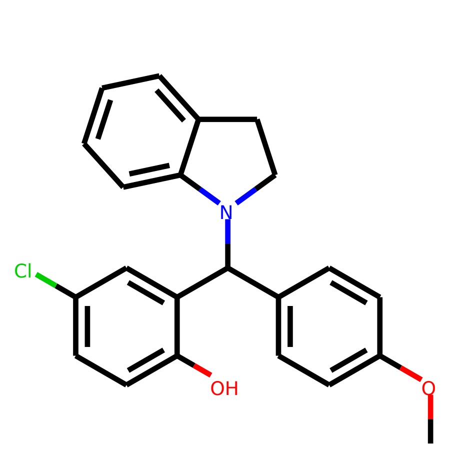
Ahma-1681
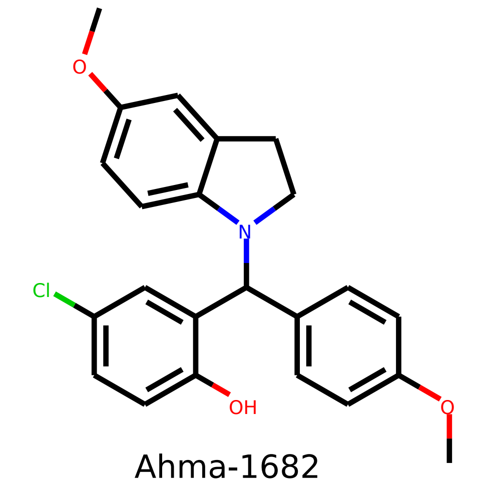
Ahma-1682
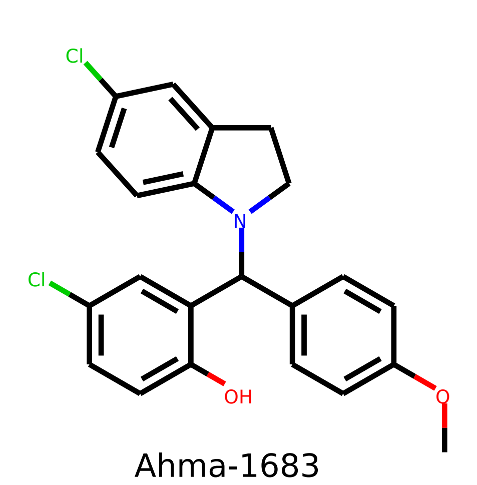
Ahma-1683
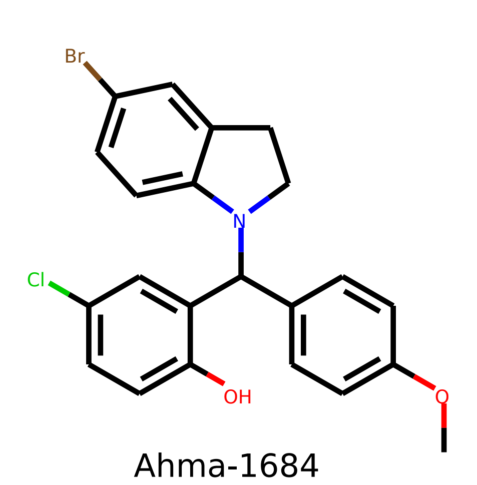
Ahma-1684
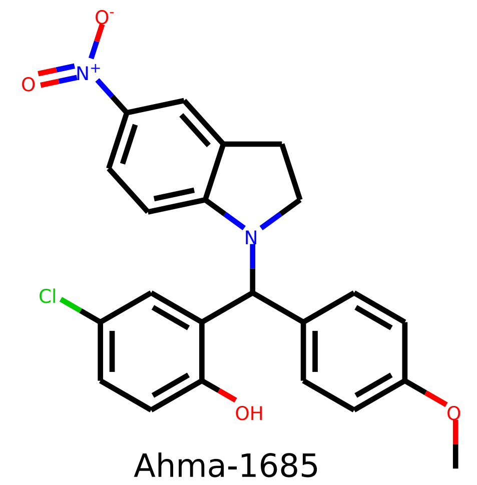
Ahma-1685
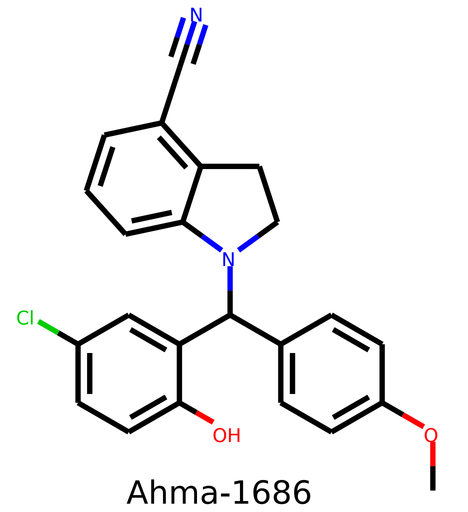
Ahma-1686
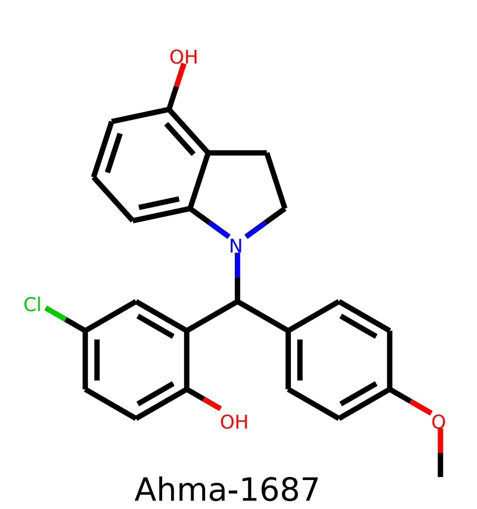
Ahma-1687
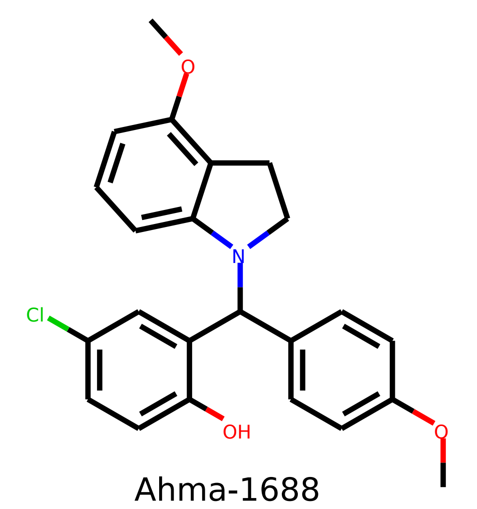
Ahma-1688
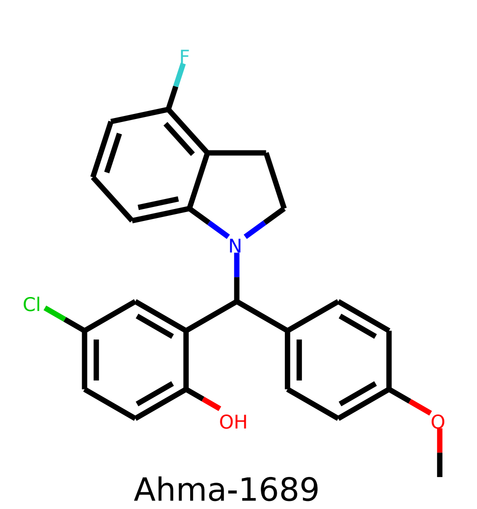
Ahma-1689
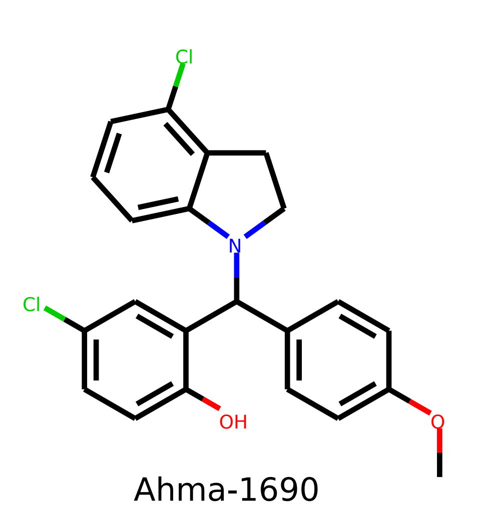
Ahma-1690
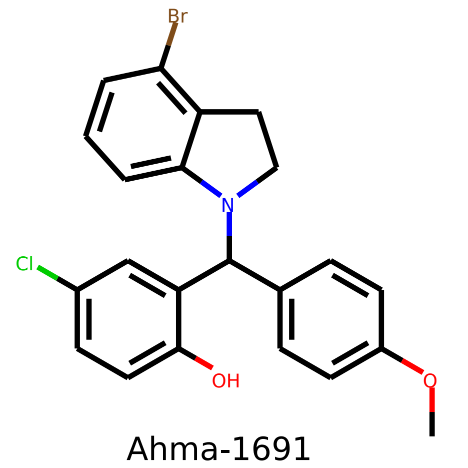
Ahma-1691
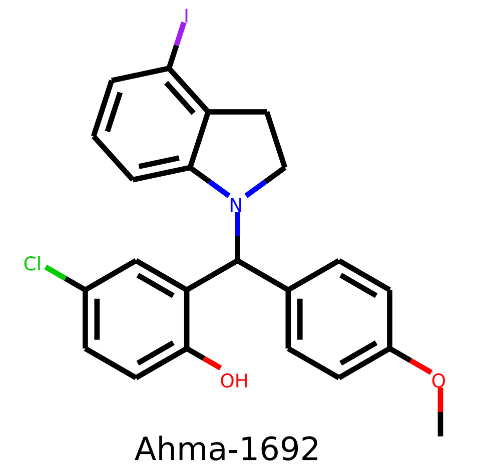
Ahma-1692
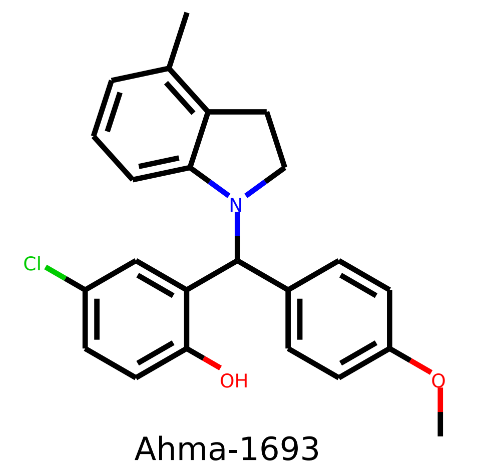
Ahma-1693
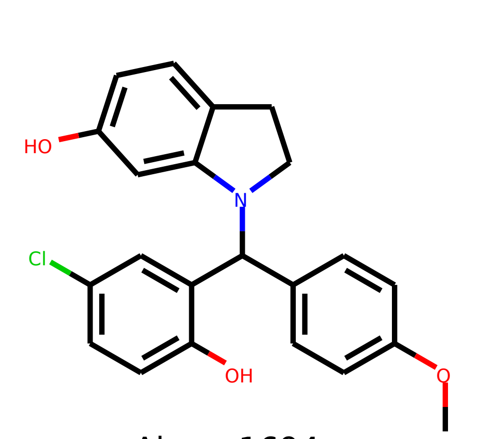
Ahma-1694
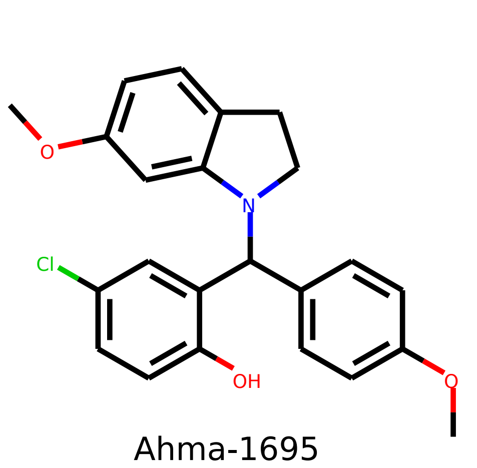
Ahma-1695
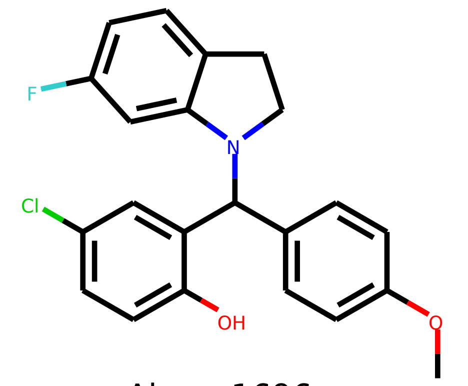
Ahma-1696
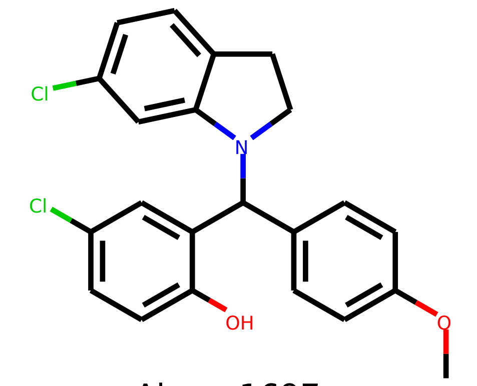
Ahma-1697
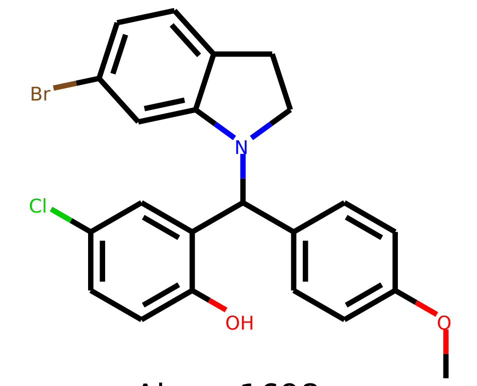
Ahma-1698
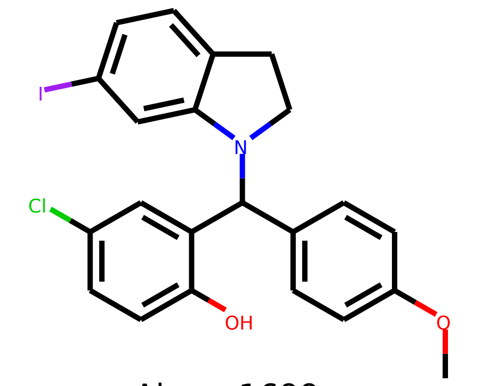
Ahma-1699
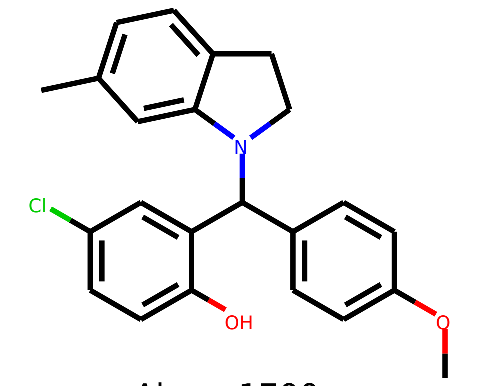
Ahma-1700
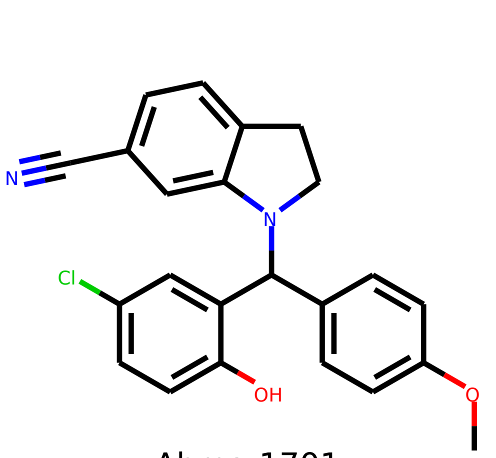
Ahma-1701
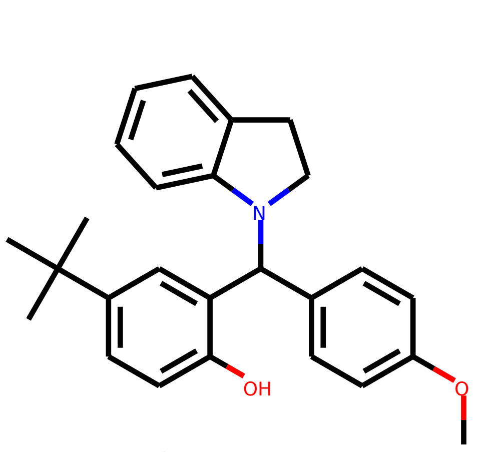
Ahma-1702
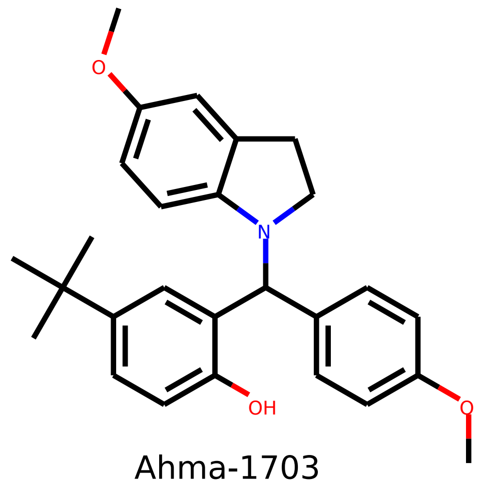
Ahma-1703
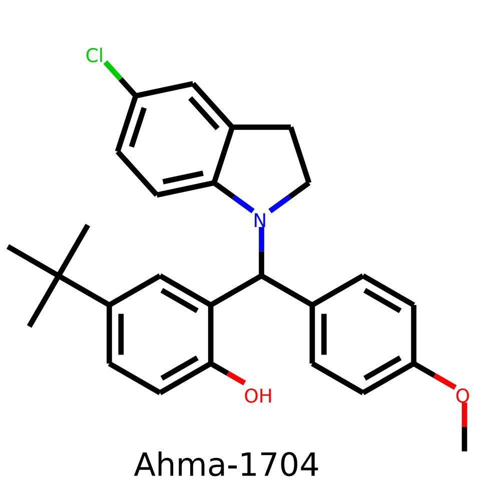
Ahma-1704
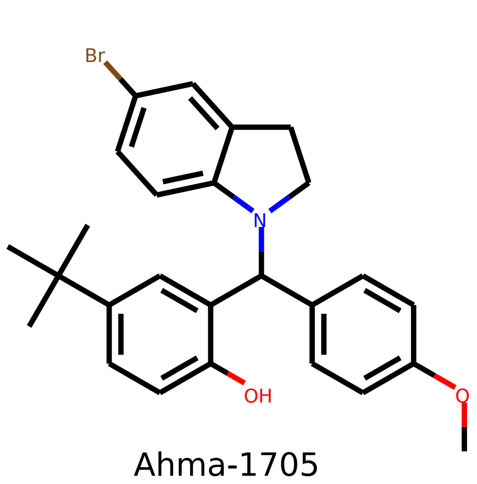
Ahma-1705
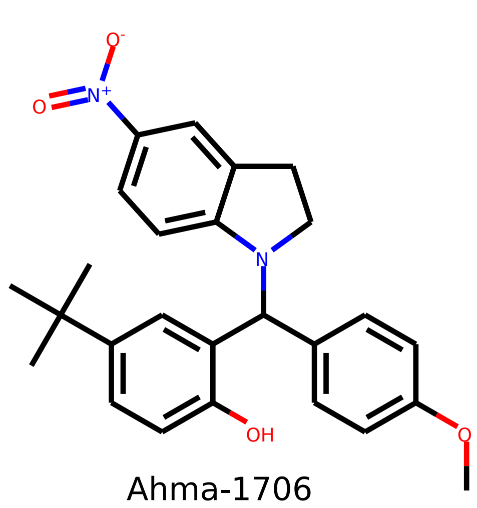
Ahma-1706
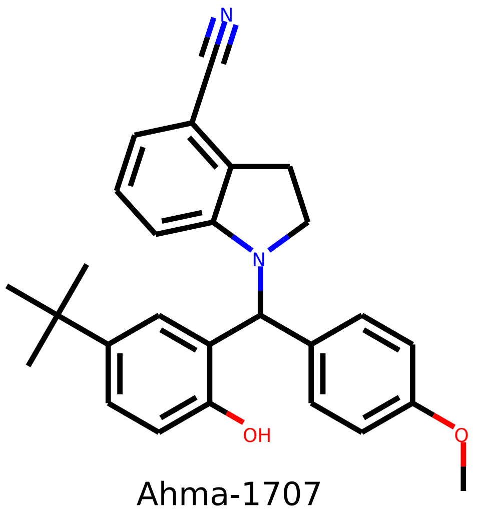
Ahma-1707
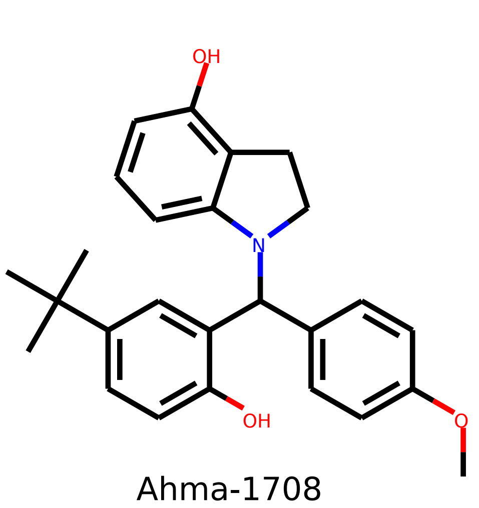
Ahma-1708
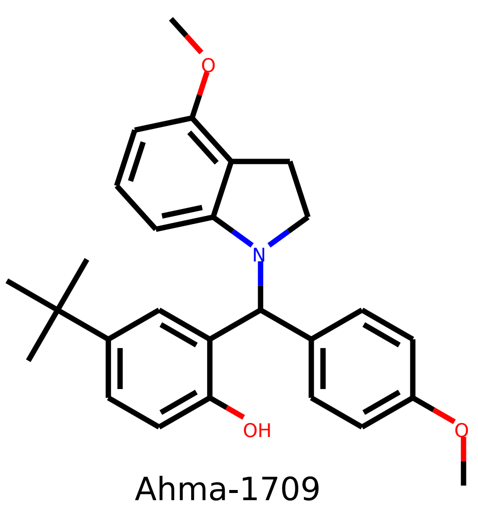
Ahma-1709
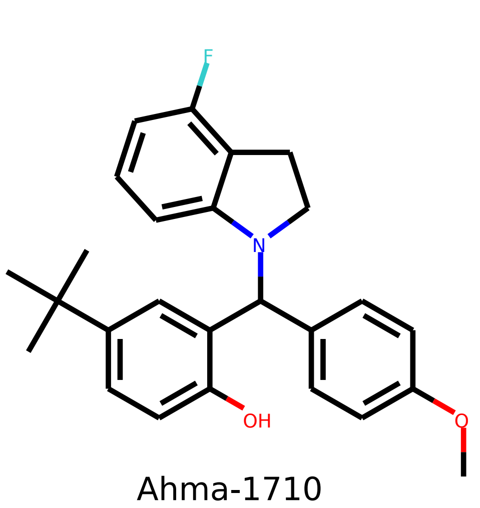
Ahma-1710
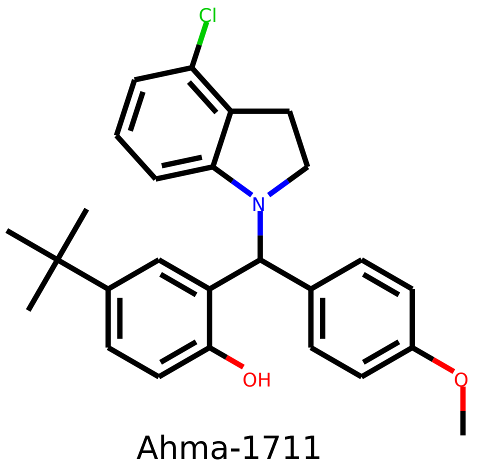
Ahma-1711
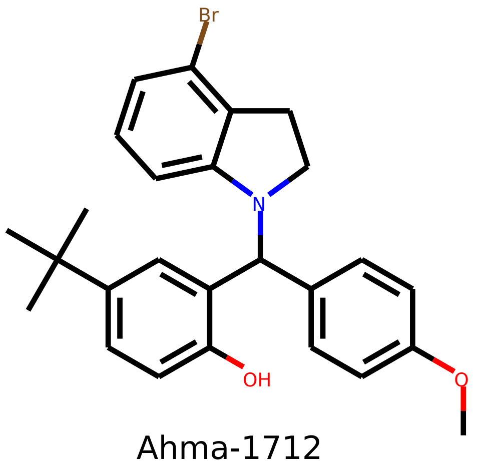
Ahma-1712
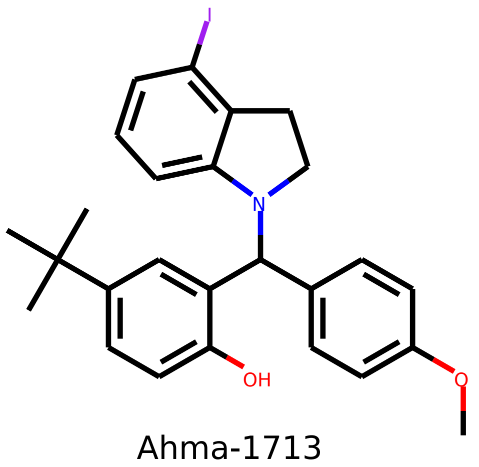
Ahma-1713
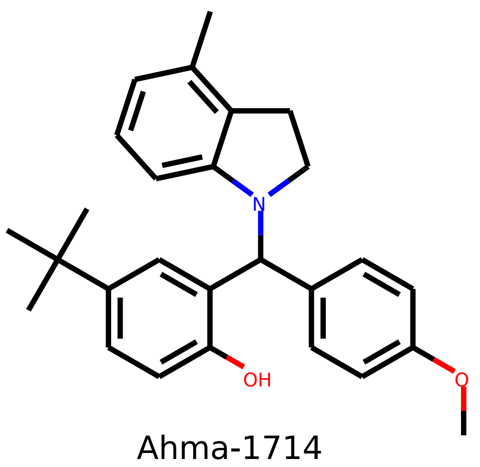
Ahma-1714
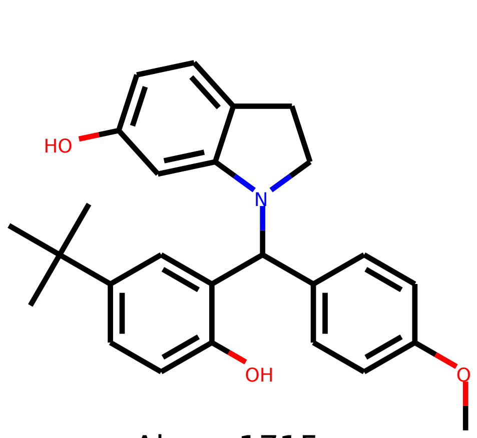
Ahma-1715

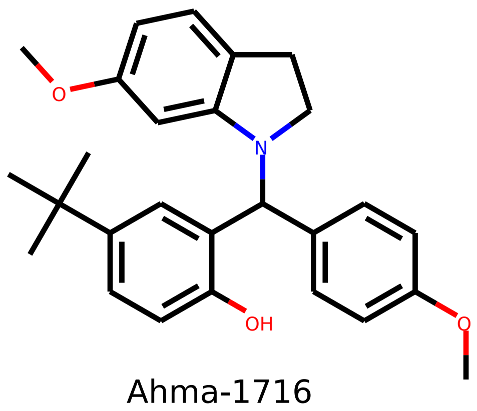
Ahma-1716

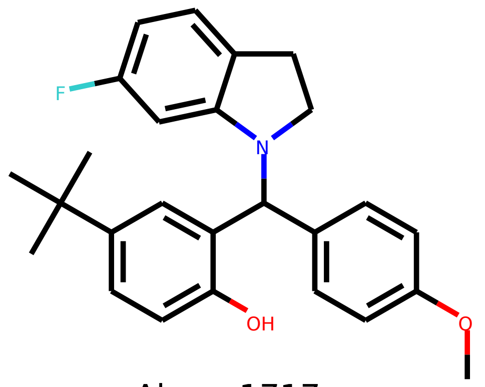
Ahma-1717

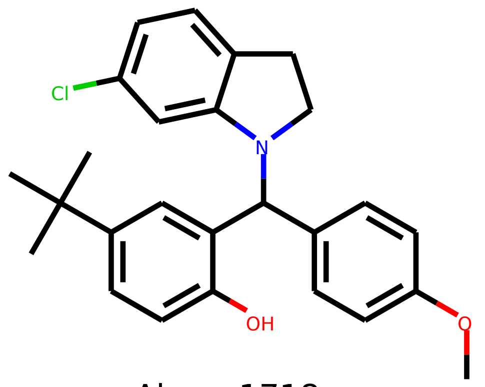
Ahma-1718

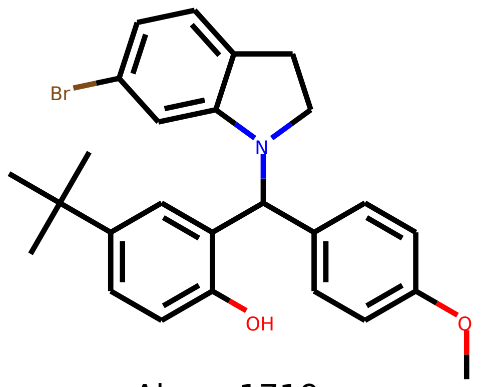
Ahma-1719

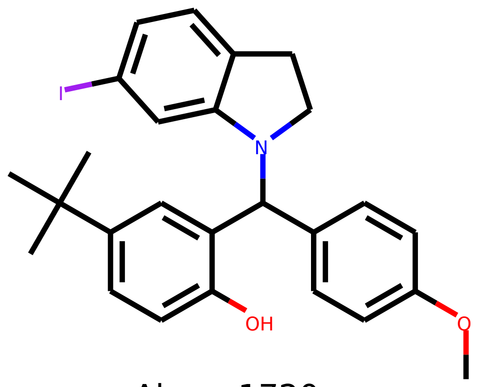
Ahma-1720

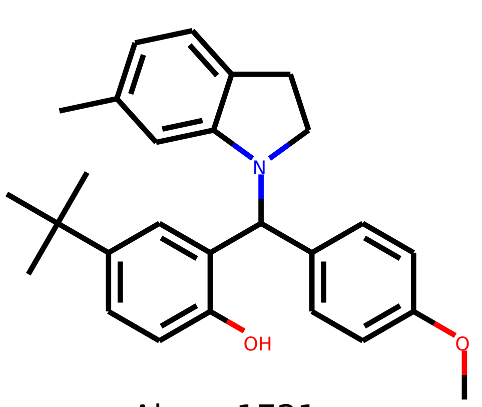
Ahma-1721

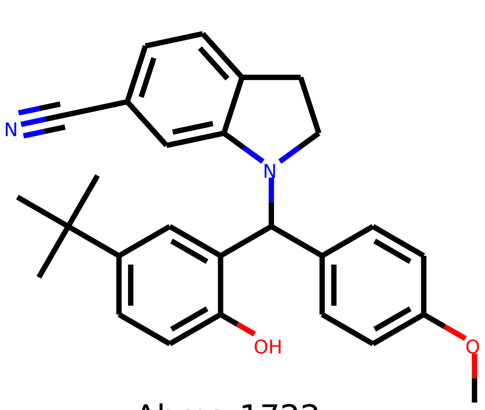
Ahma-1722

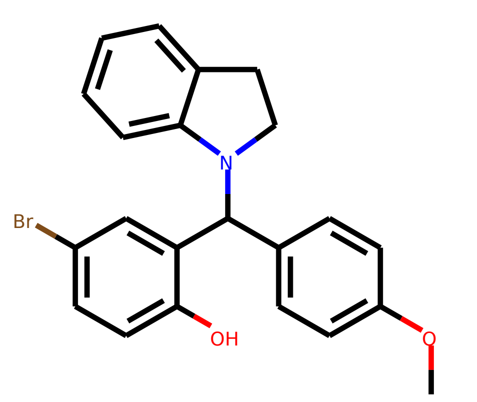
Ahma-1723

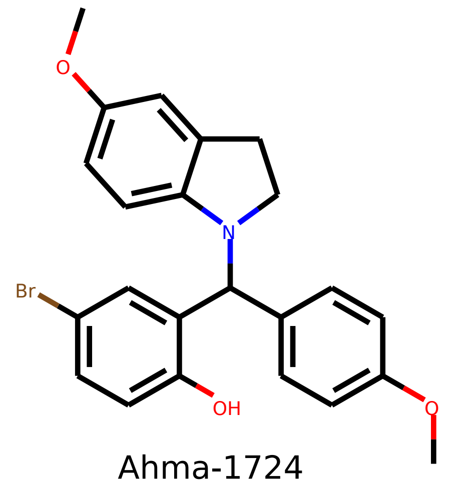
Ahma-1724

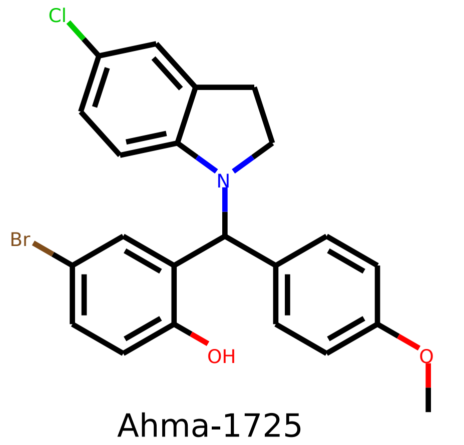
Ahma-1725

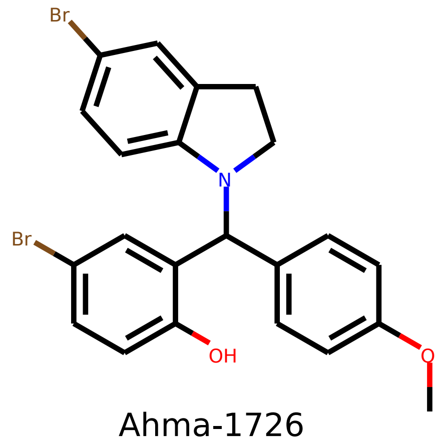
Ahma-1726

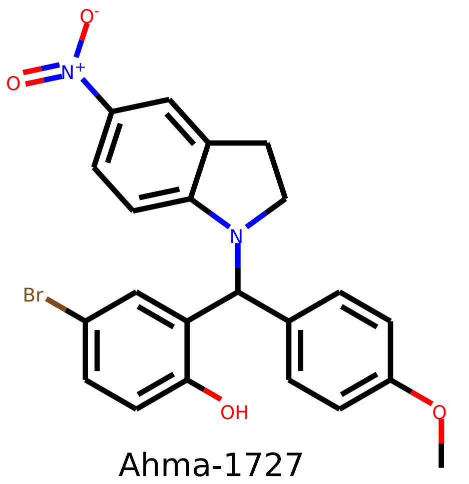
Ahma-1727

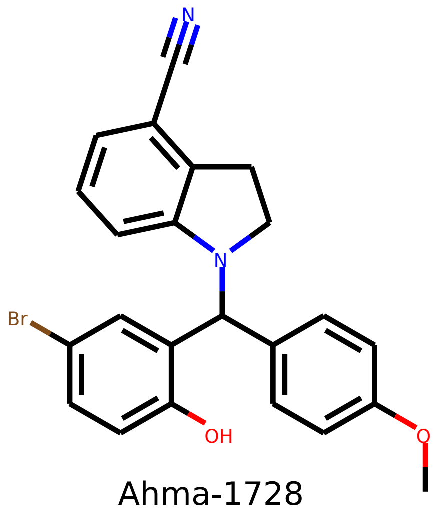
Ahma-1728

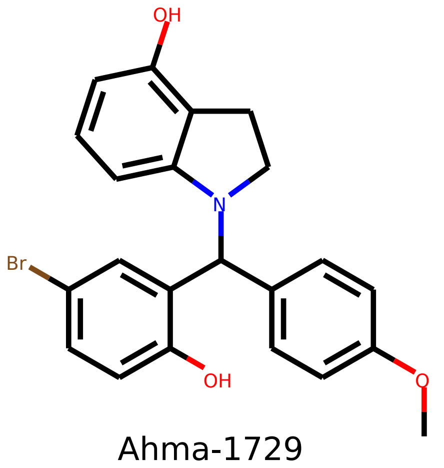
Ahma-1729

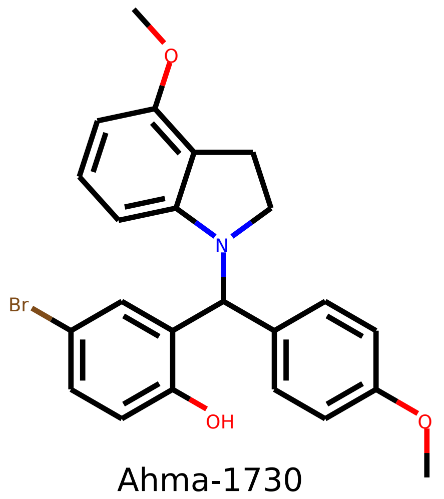
Ahma-1730

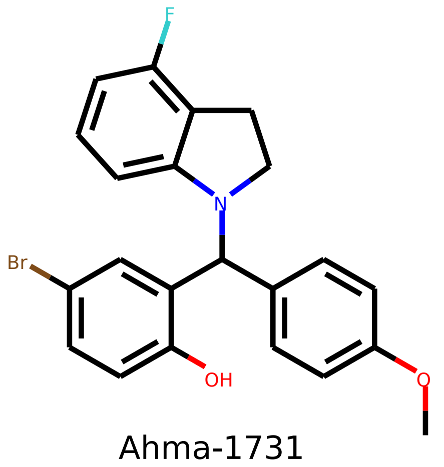
Ahma-1731

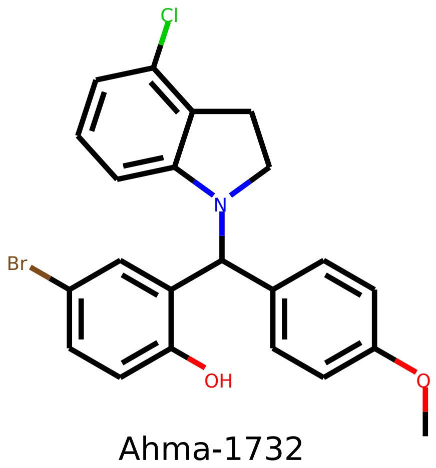
Ahma-1732

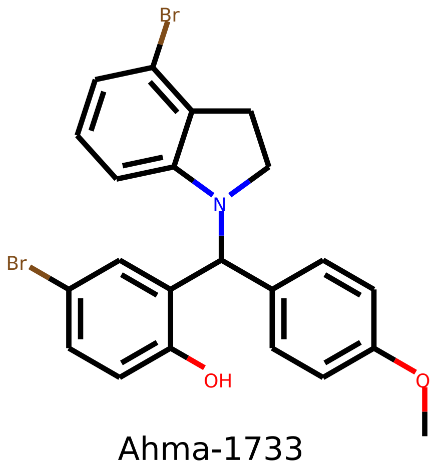
Ahma-1733

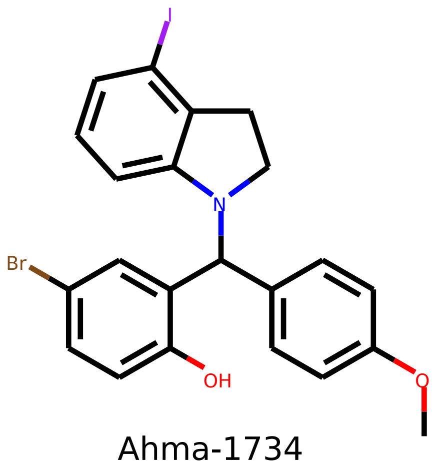
Ahma-1734

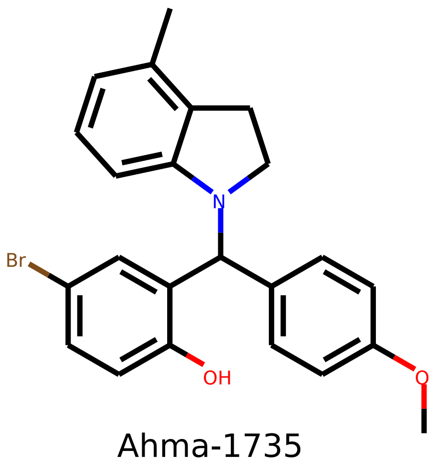
Ahma-1735

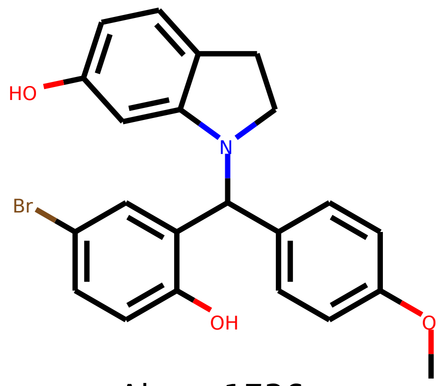
Ahma-1736

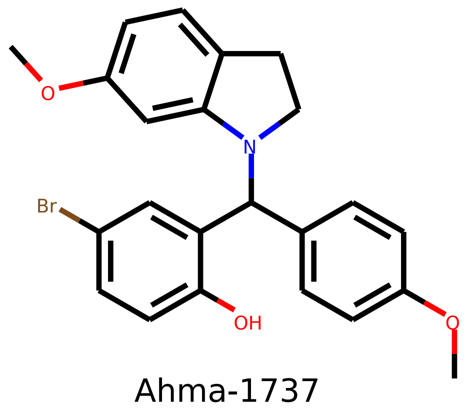
Ahma-1737

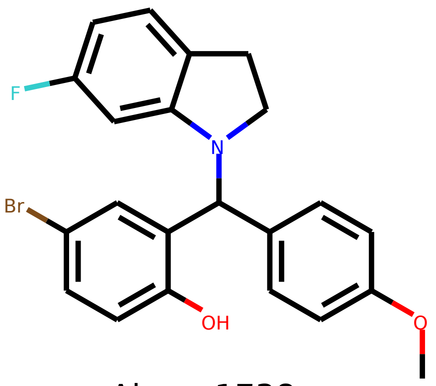
Ahma-1738

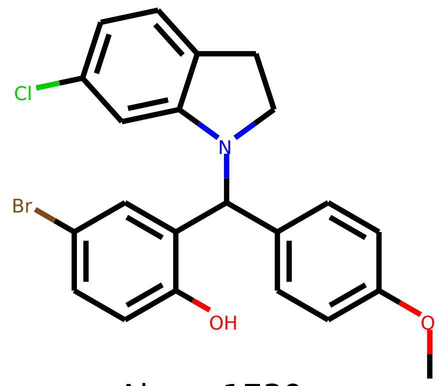
Ahma-1739

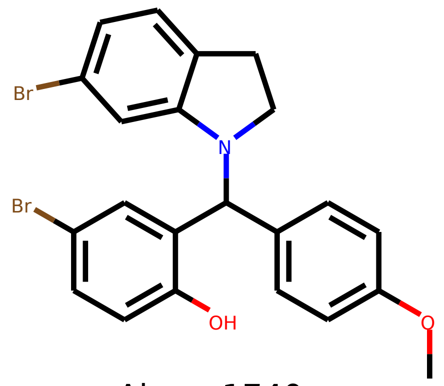
Ahma-1740

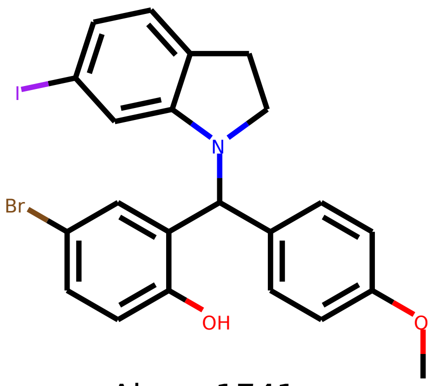
Ahma-1741

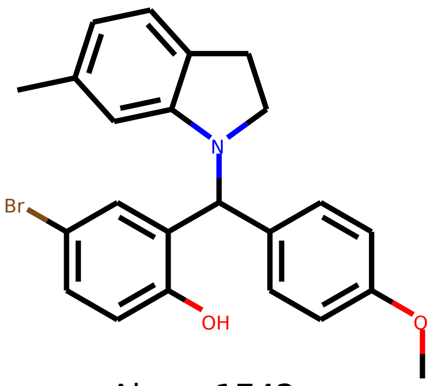
Ahma-1742

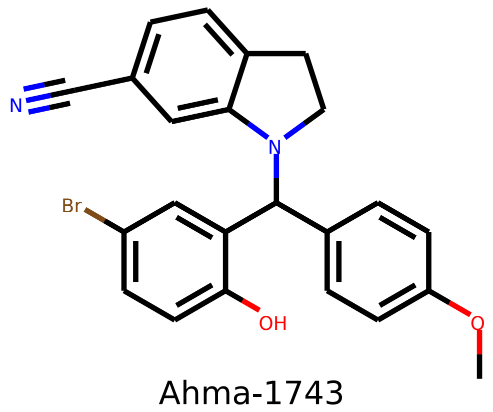
Ahma-1743

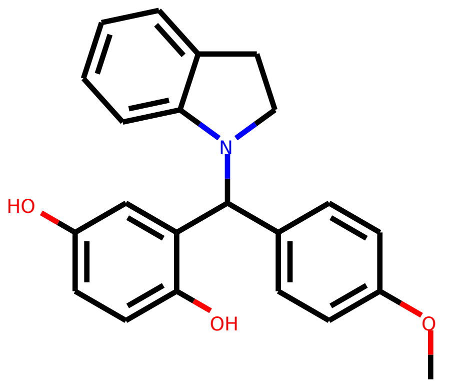
Ahma-1744

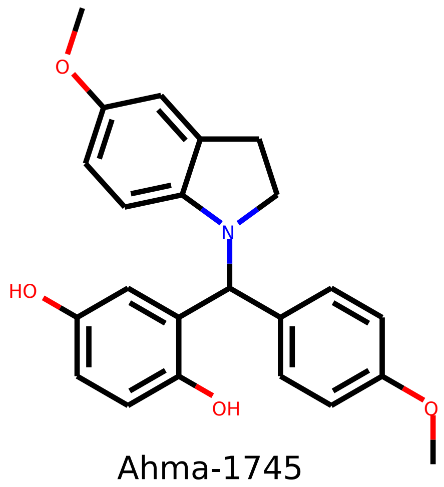
Ahma-1745

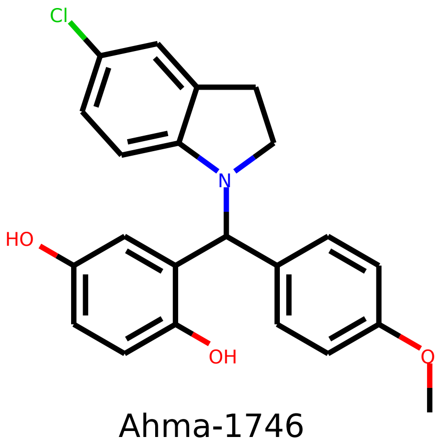
Ahma-1746

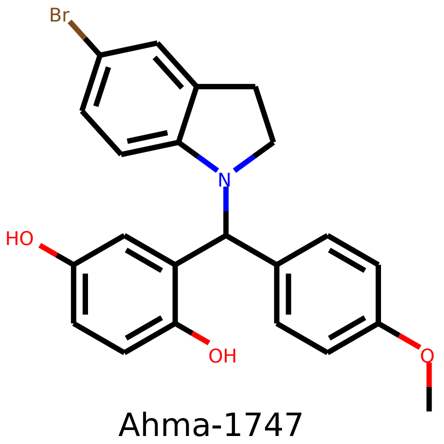
Ahma-1747

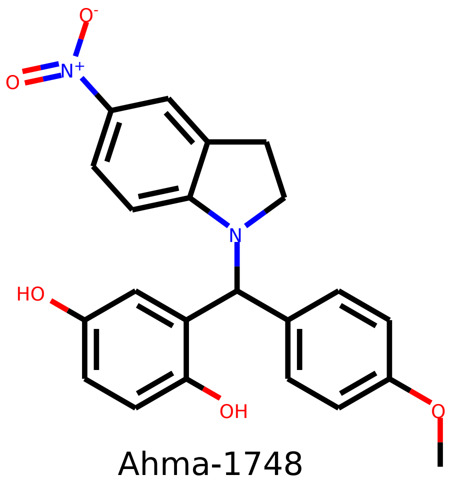
Ahma-1748

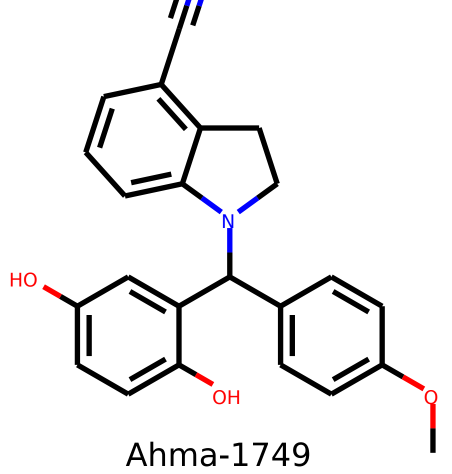
Ahma-1749

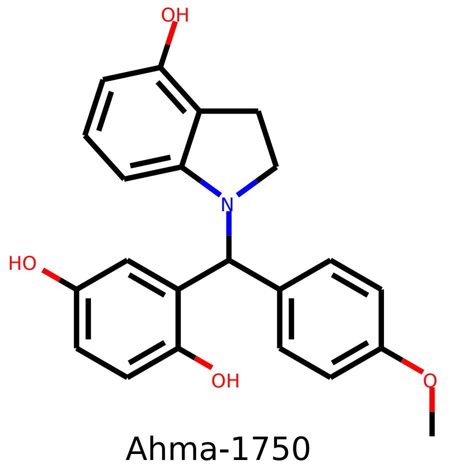
Ahma-1750

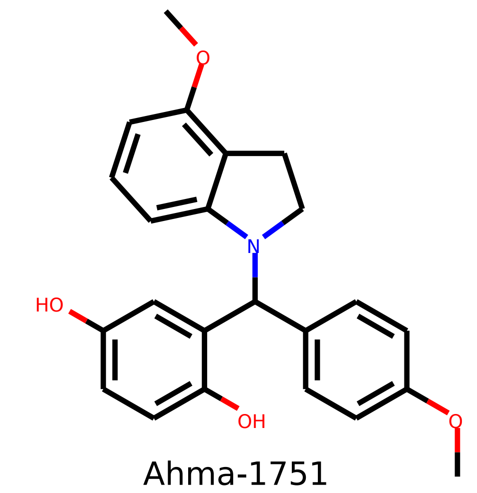
Ahma-1751
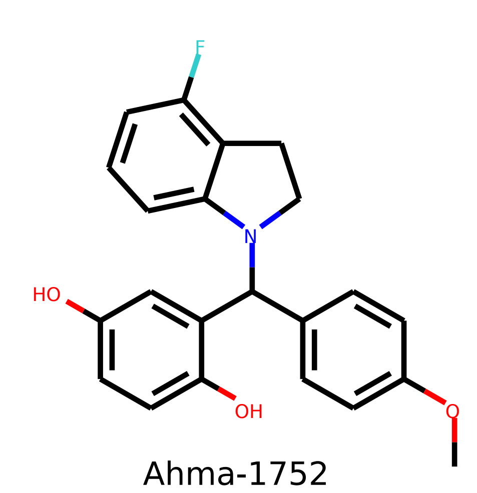
Ahma-1752
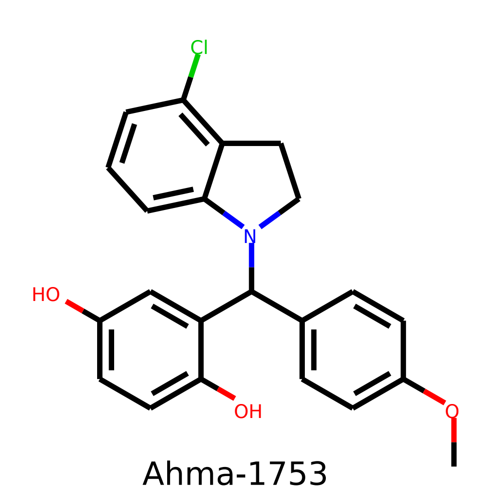
Ahma-1753
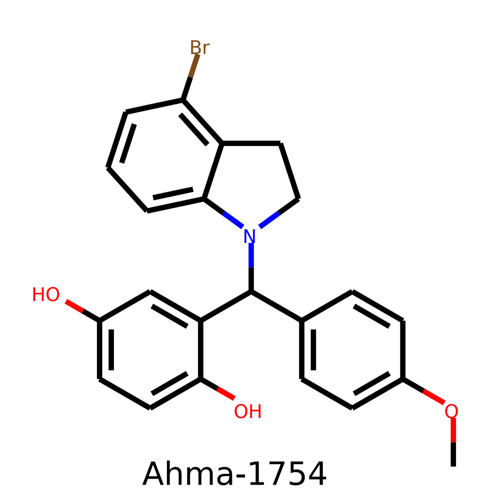
Ahma-1754
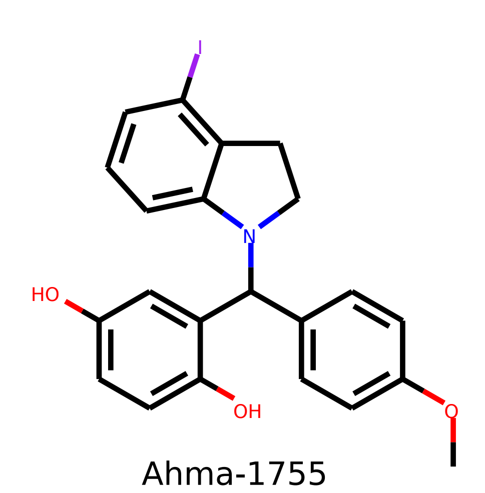
Ahma-1755
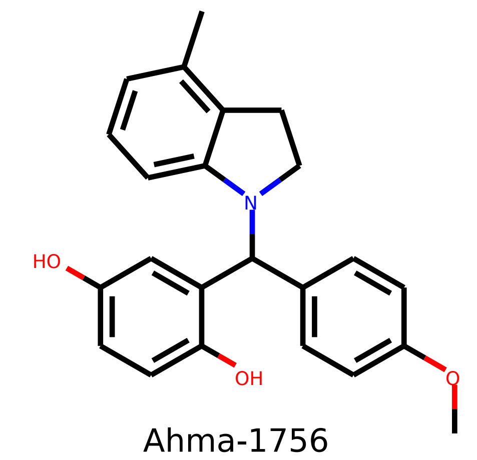
Ahma-1756
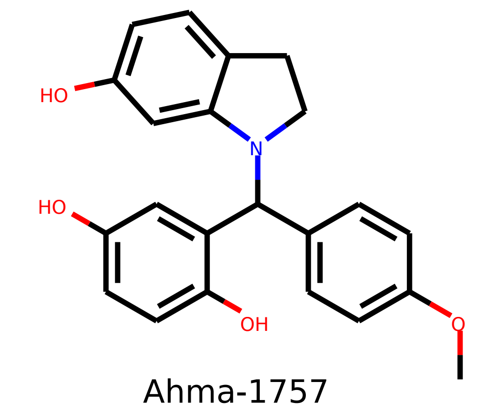
Ahma-1757
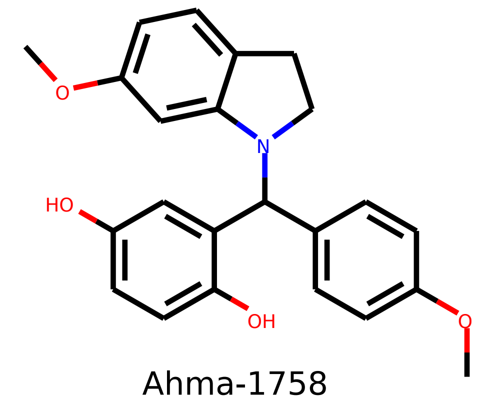
Ahma-1758
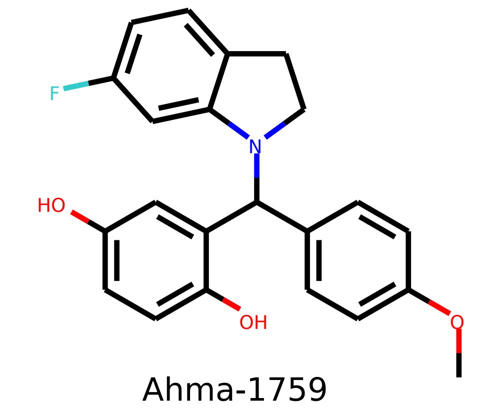
Ahma-1759
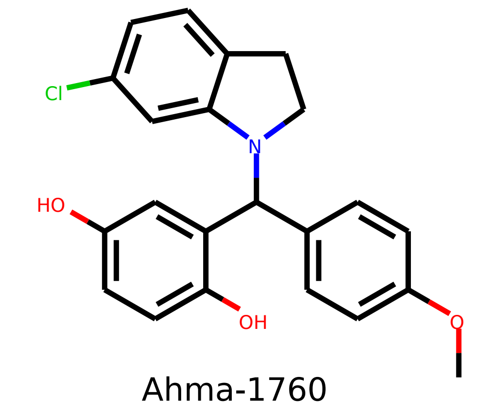
Ahma-1760
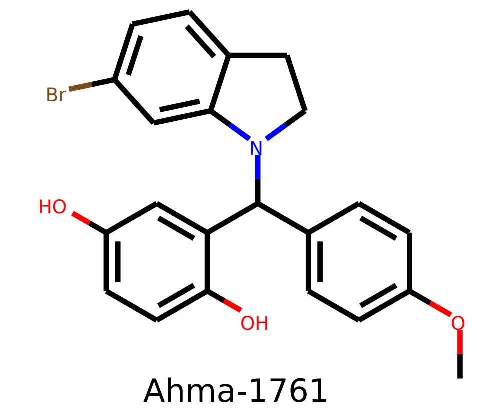
Ahma-1761
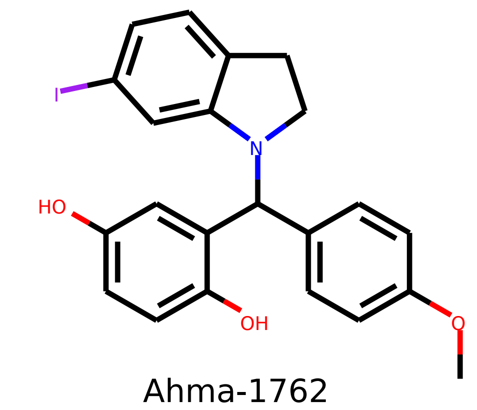
Ahma-1762
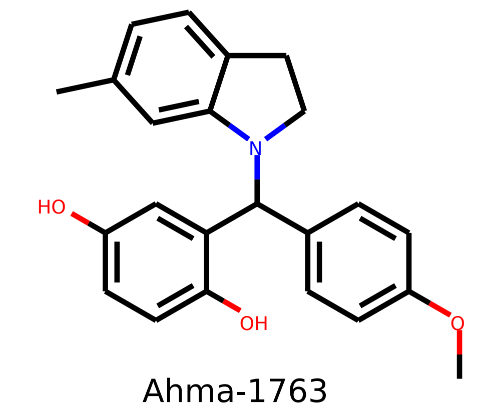
Ahma-1763
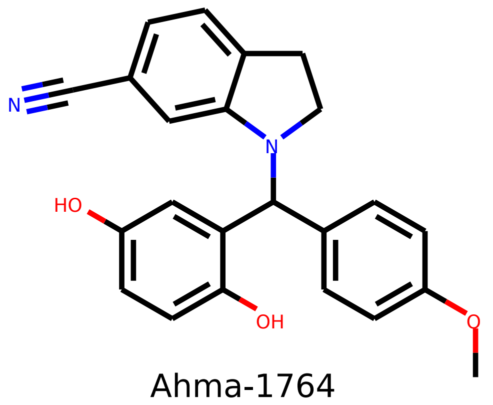
Ahma-1764
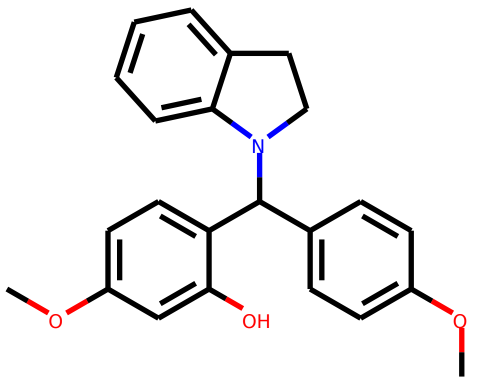
Ahma-1765
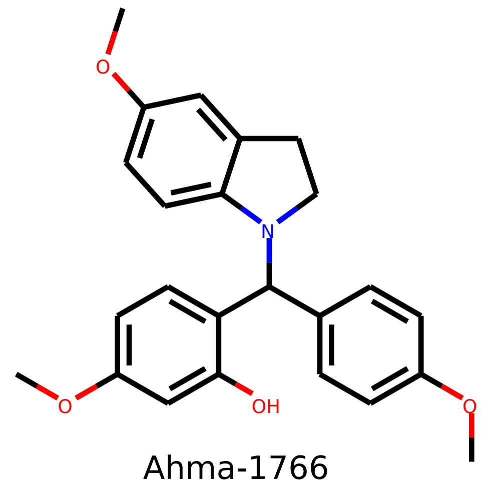
Ahma-1766
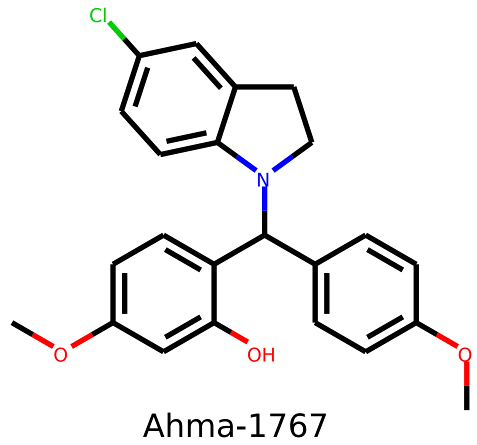
Ahma-1767
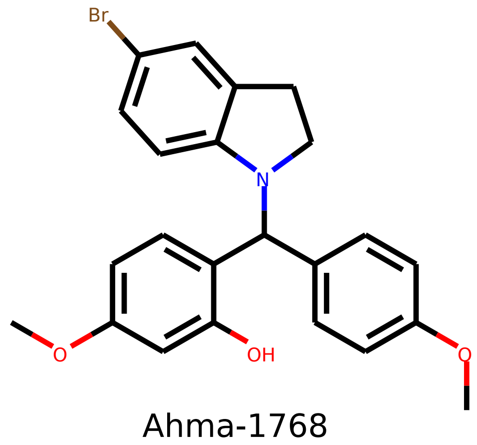
Ahma-1768
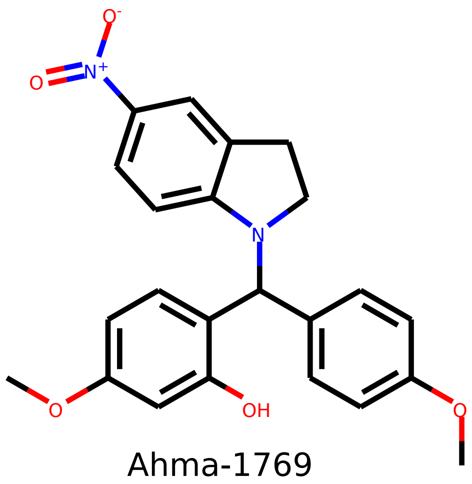
Ahma-1769
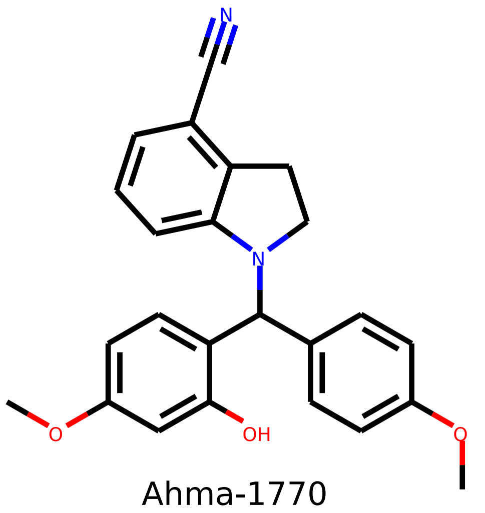
Ahma-1770
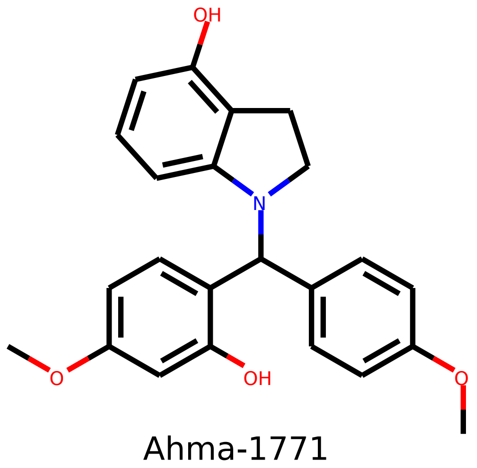
Ahma-1771
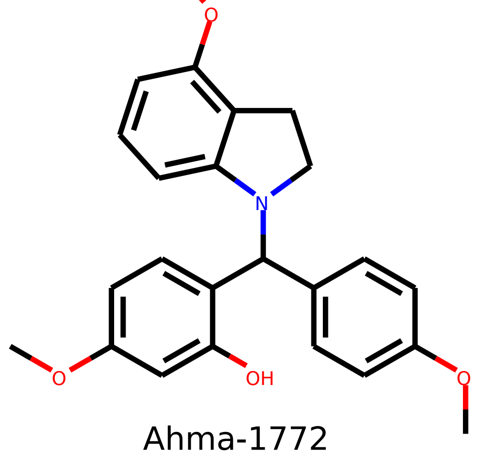
Ahma-1772
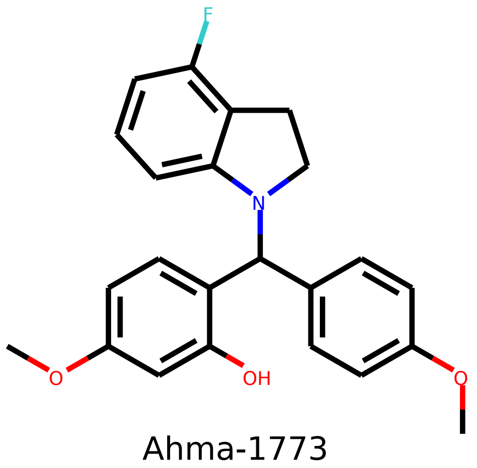
Ahma-1773
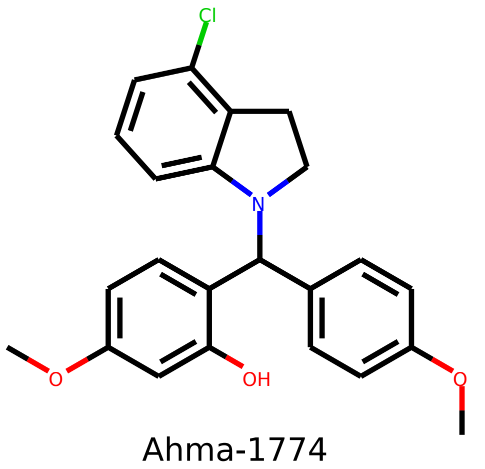
Ahma-1774
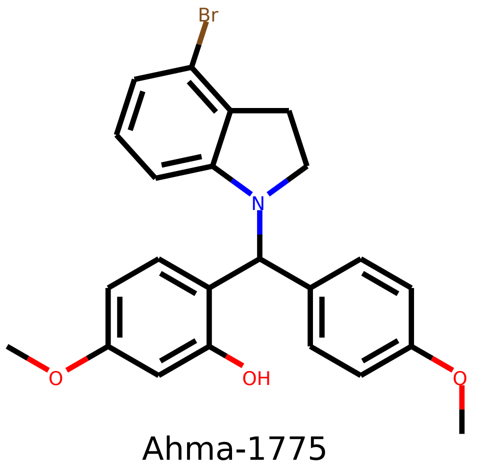
Ahma-1775
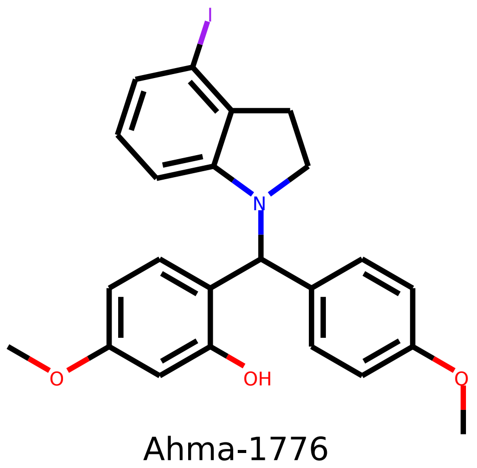
Ahma-1776
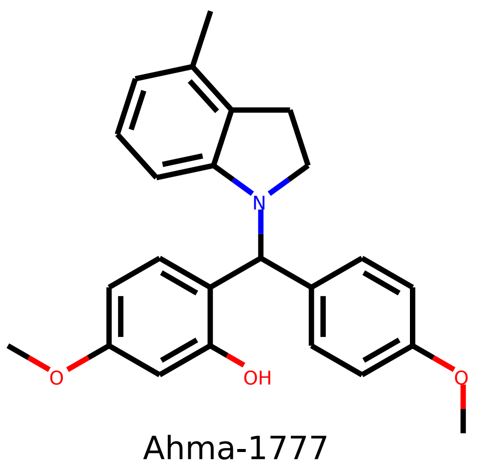
Ahma-1777
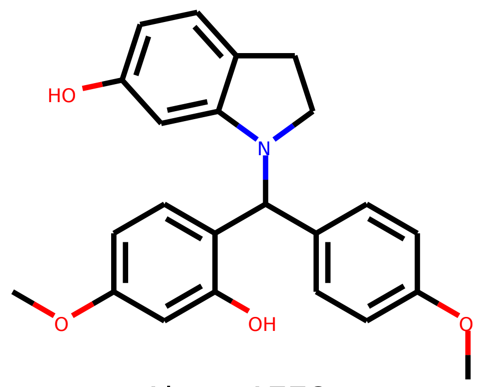
Ahma-1778
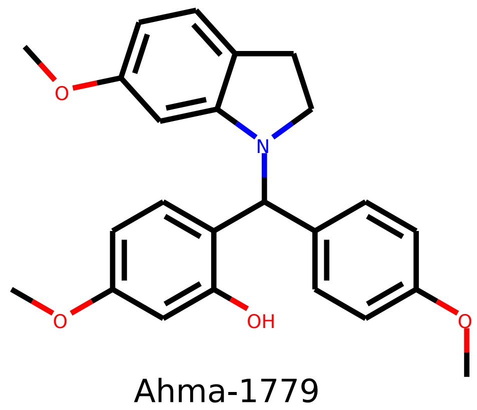
Ahma-1779
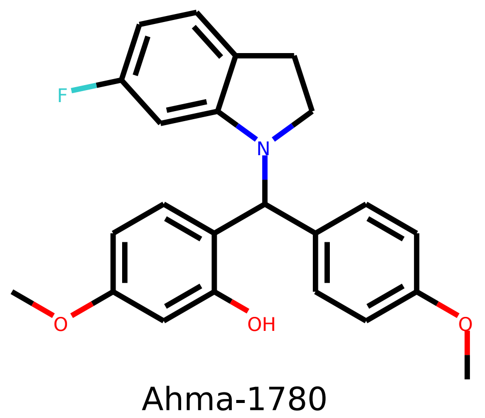
Ahma-1780
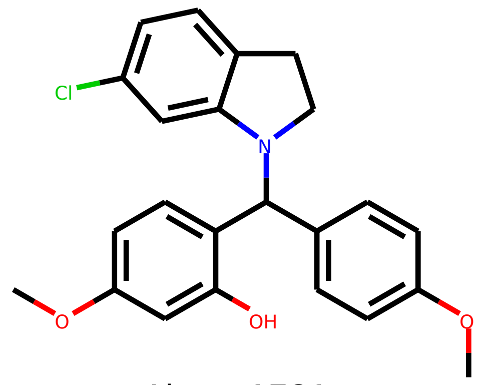
Ahma-1781
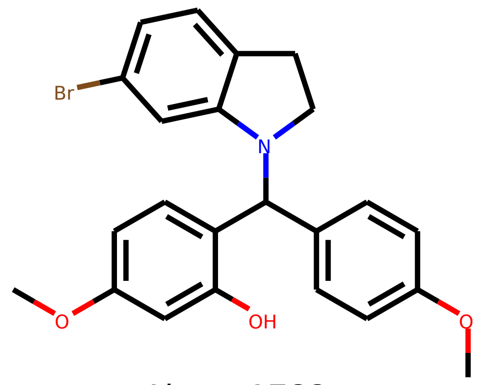
Ahma-1782
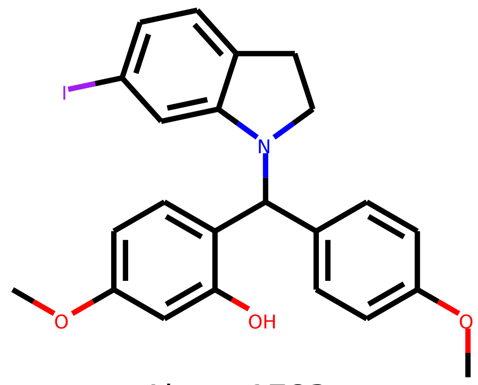
Ahma-1783
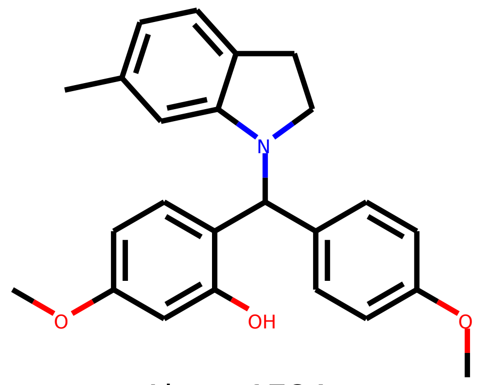
Ahma-1784
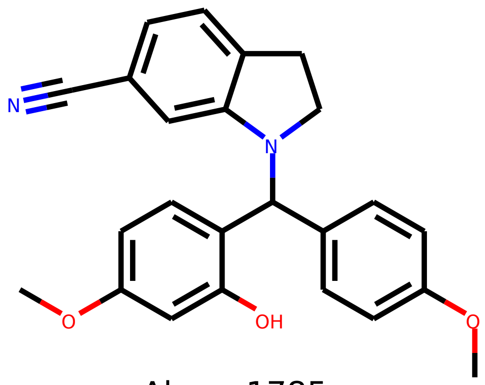
Ahma-1785

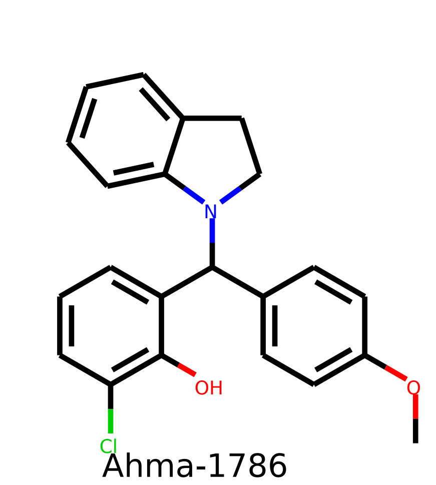
Ahma-1786

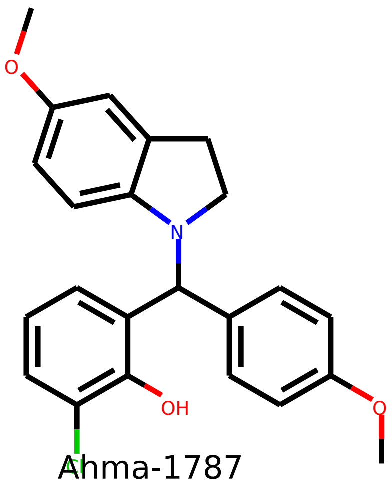
Ahma-1787

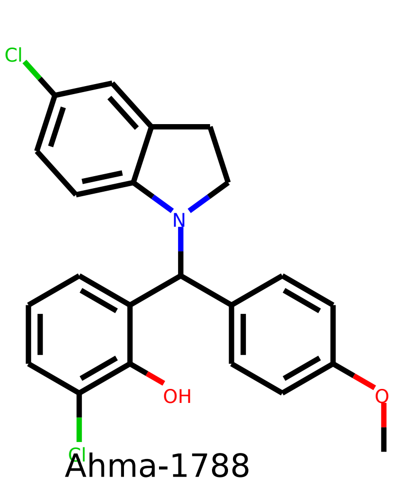
Ahma-1788

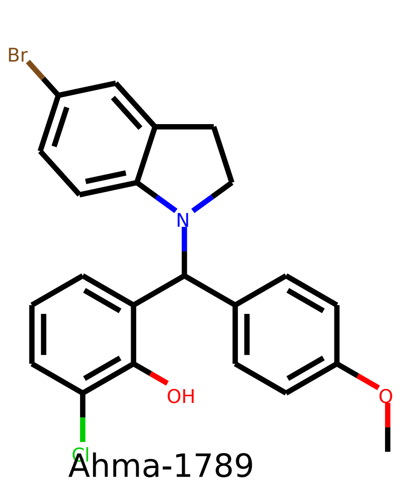
Ahma-1789

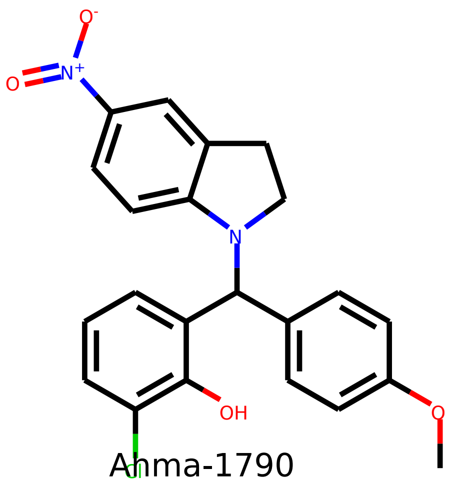
Ahma-1790

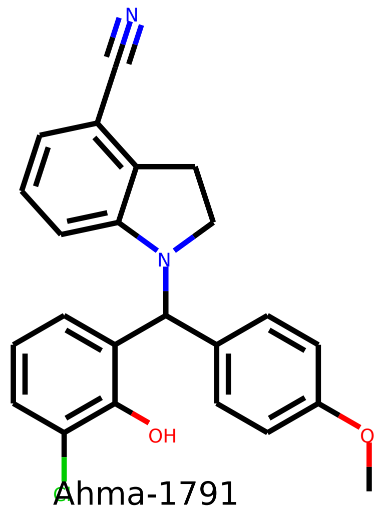
Ahma-1791

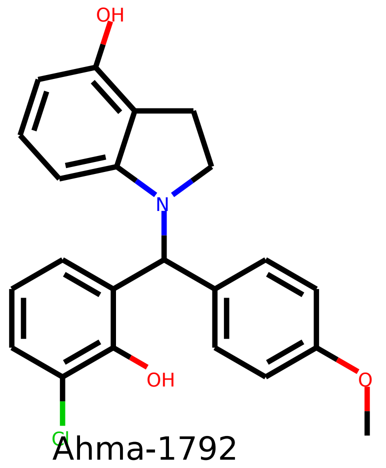
Ahma-1792

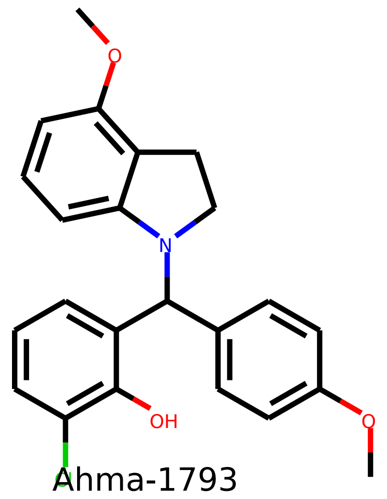
Ahma-1793

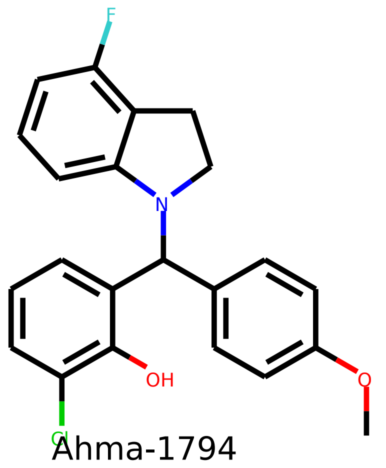
Ahma-1794

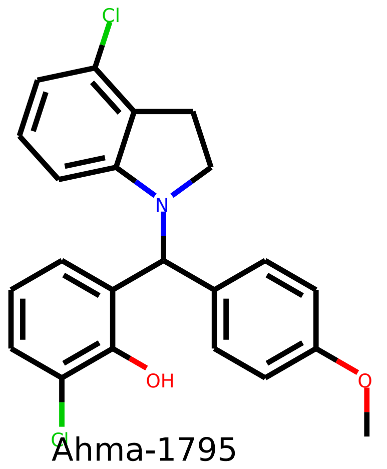
Ahma-1795

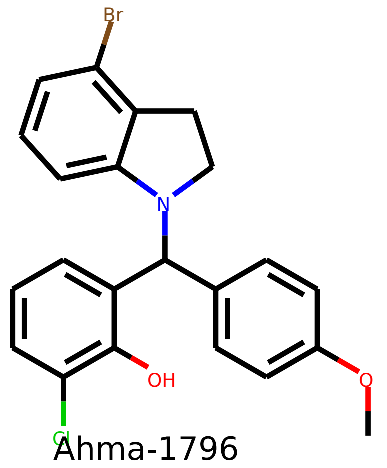
Ahma-1796

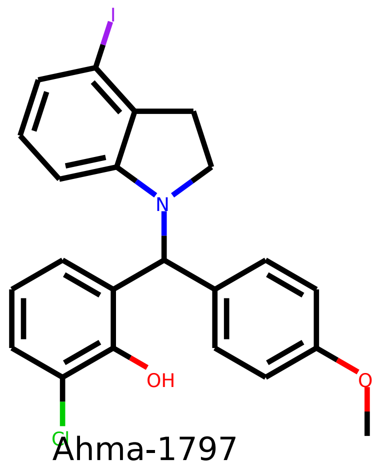
Ahma-1797

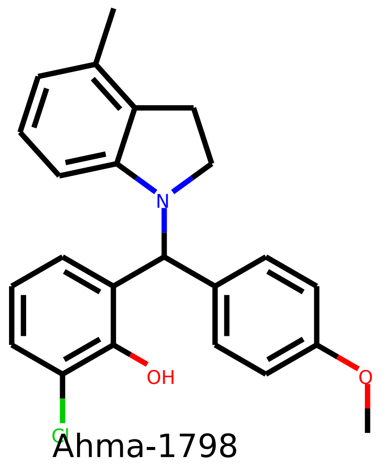
Ahma-1798

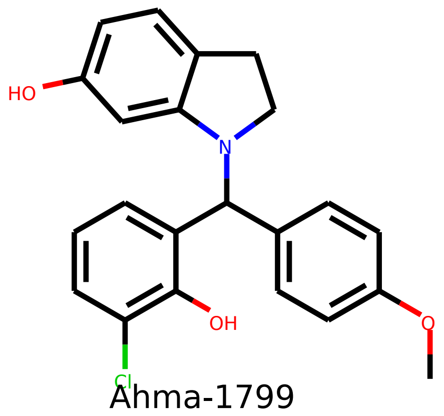
Ahma-1799

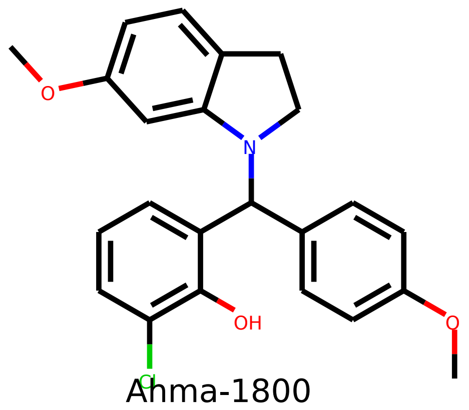
Ahma-1800

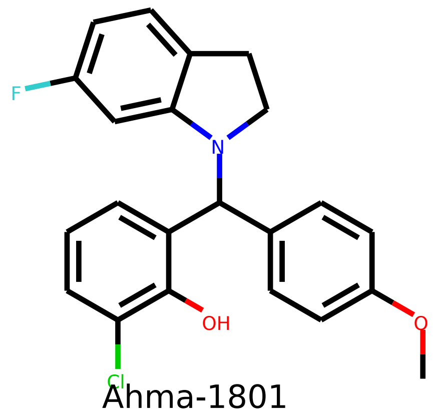
Ahma-1801

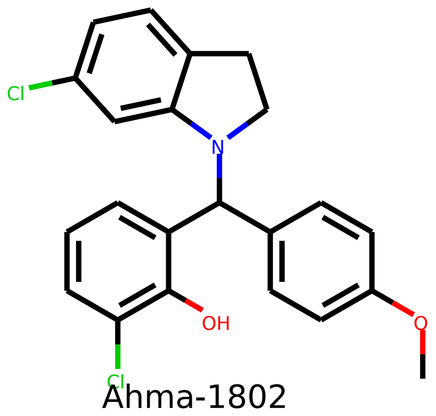
Ahma-1802

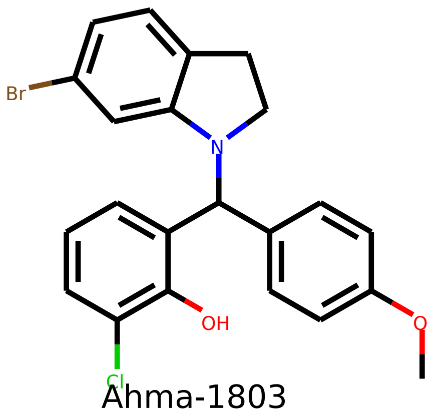
Ahma-1803

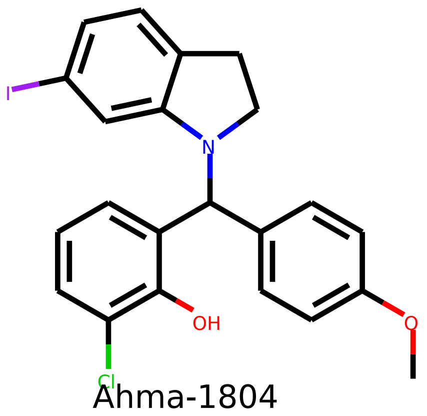
Ahma-1804

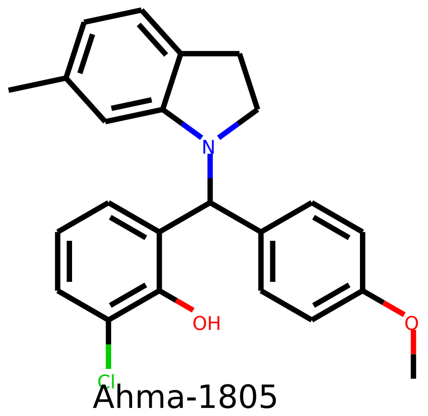
Ahma-1805

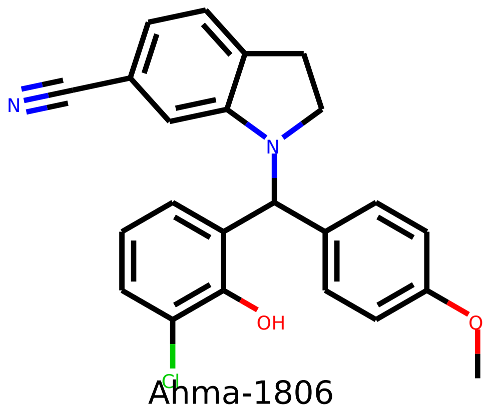
Ahma-1806

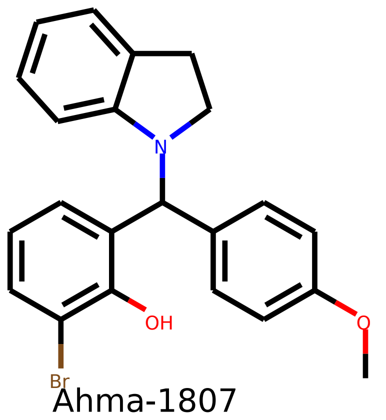
Ahma-1807

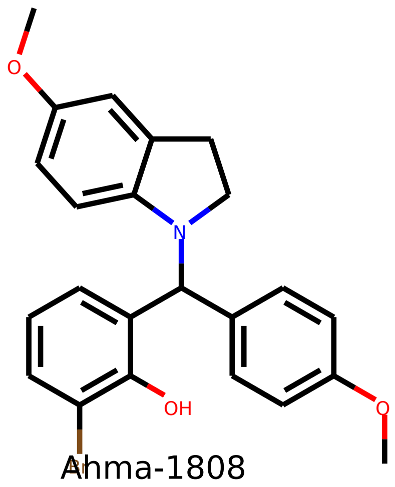
Ahma-1808

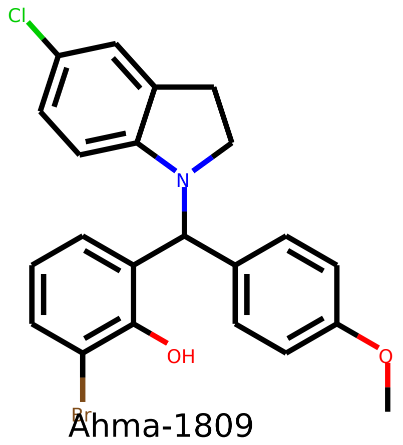
Ahma-1809

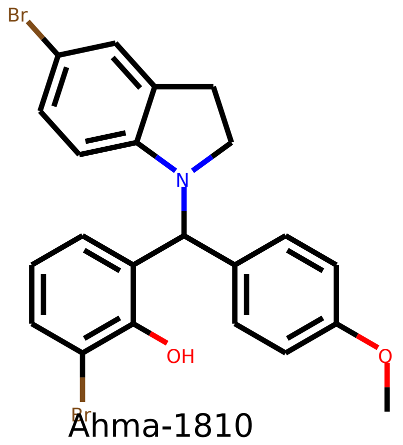
Ahma-1810

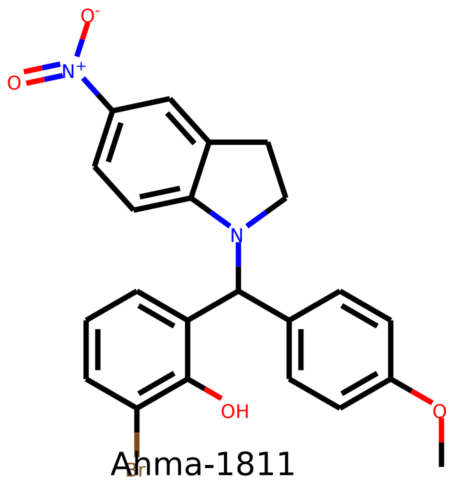
Ahma-1811

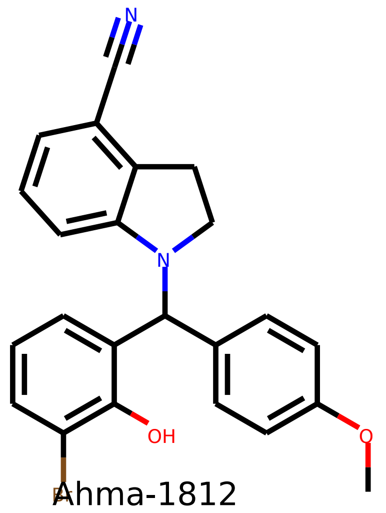
Ahma-1812

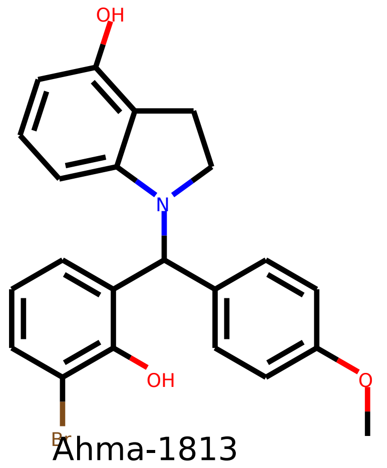
Ahma-1813

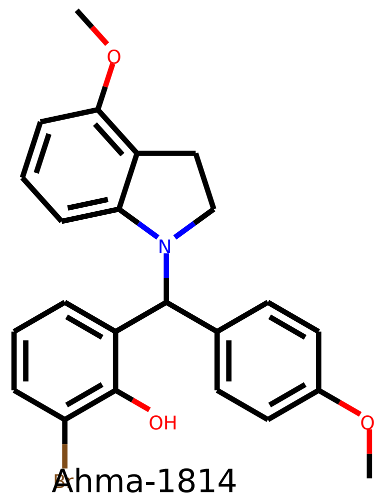
Ahma-1814

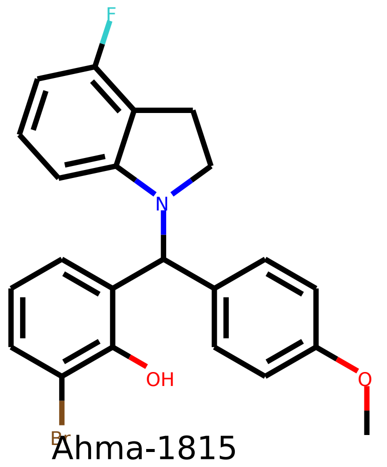
Ahma-1815

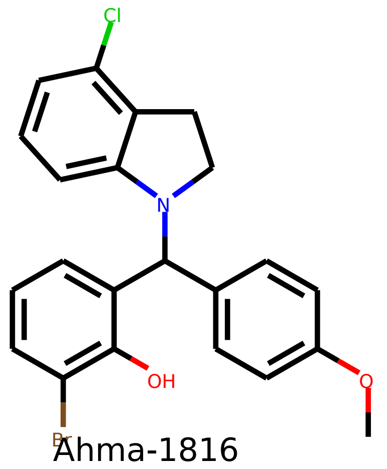
Ahma-1816

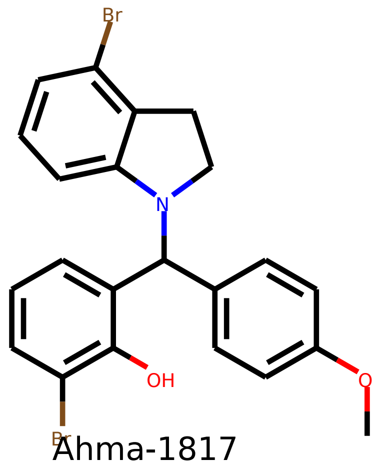
Ahma-1817

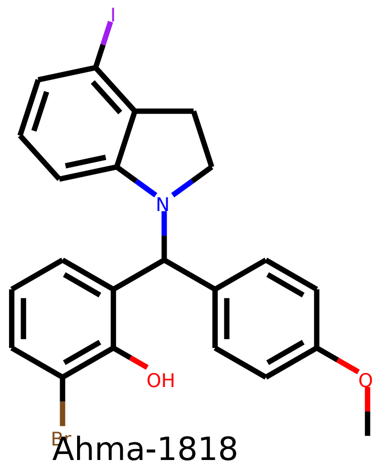
Ahma-1818

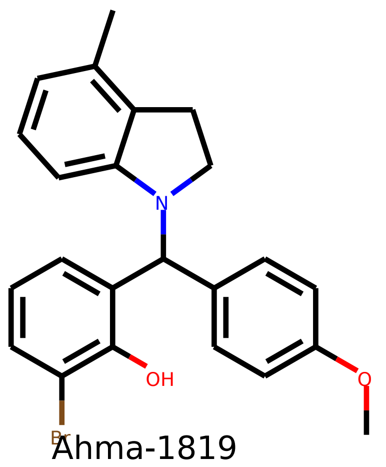
Ahma-1819

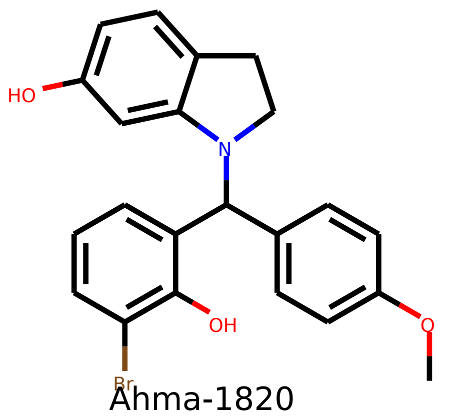
Ahma-1820

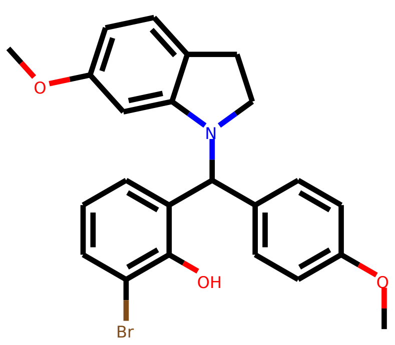
Ahma-1821
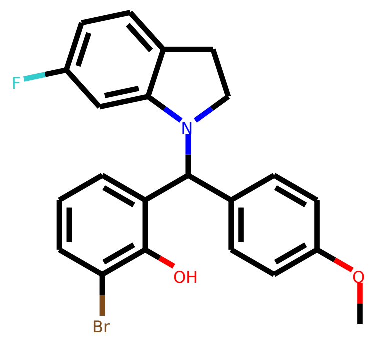
Ahma-1822
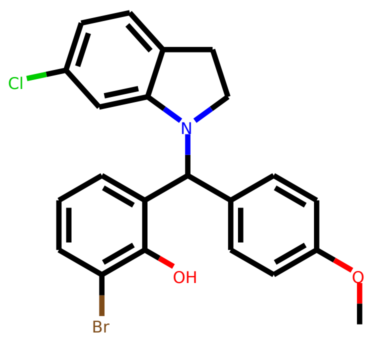
Ahma-1823
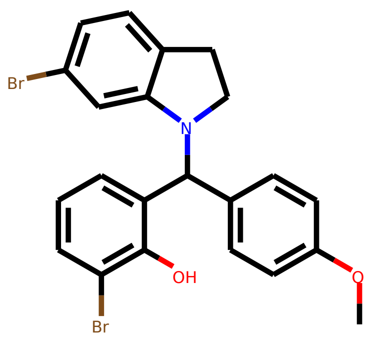
Ahma-1824
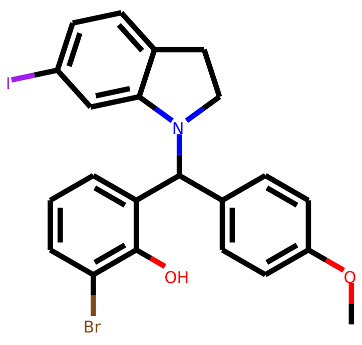
Ahma-1825
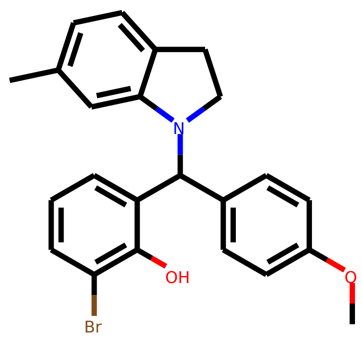
Ahma-1826
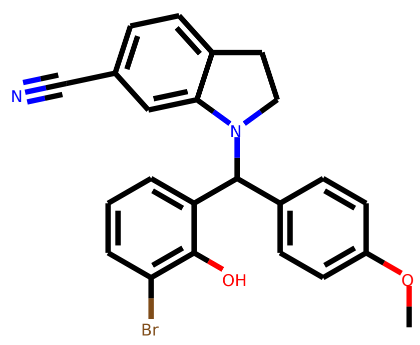
Ahma-1827
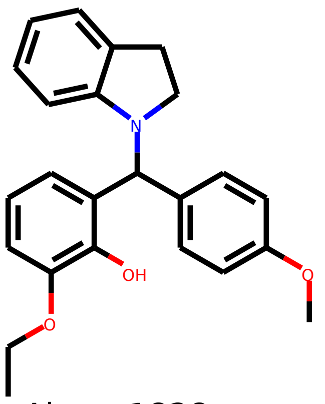
Ahma-1828
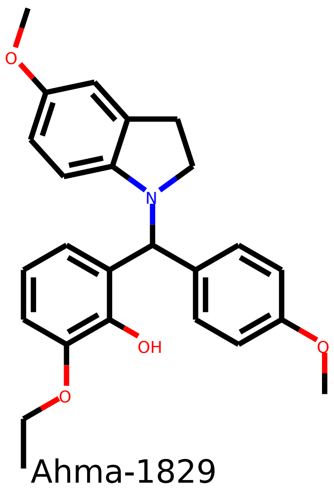
Ahma-1829
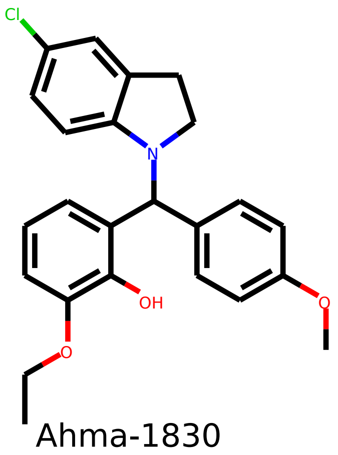
Ahma-1830
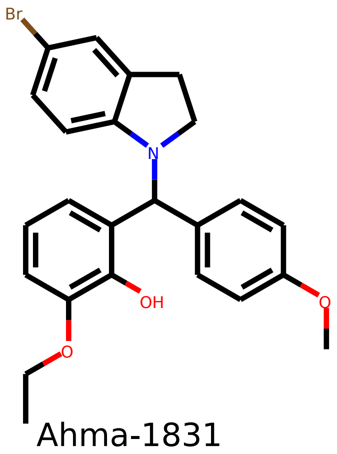
Ahma-1831
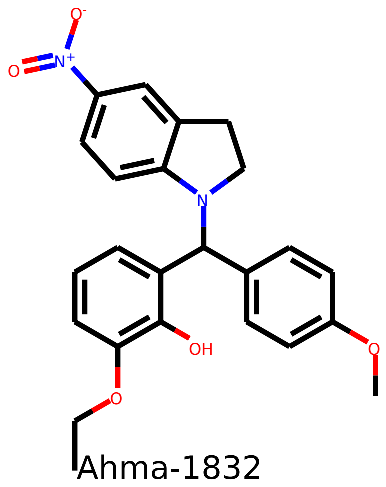
Ahma-1832
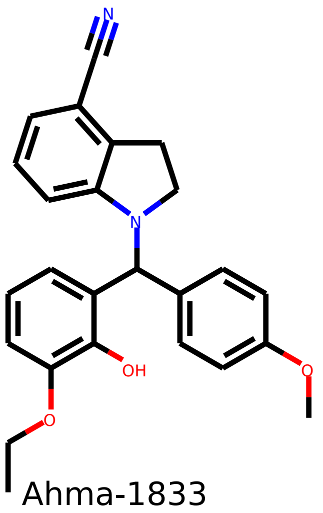
Ahma-1833
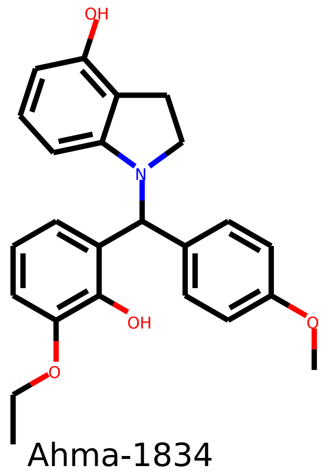
Ahma-1834
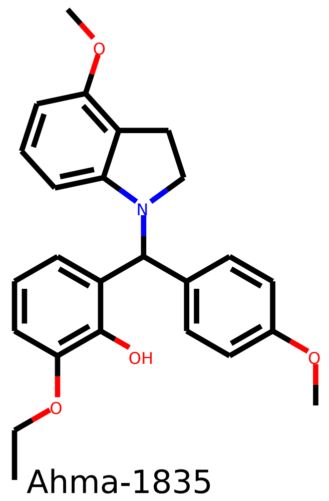
Ahma-1835
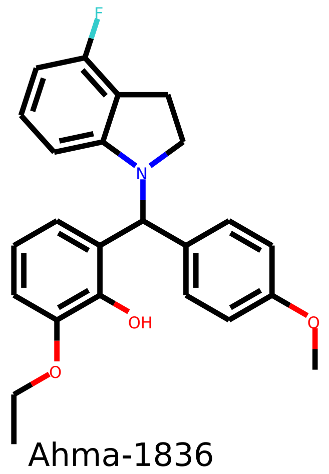
Ahma-1836
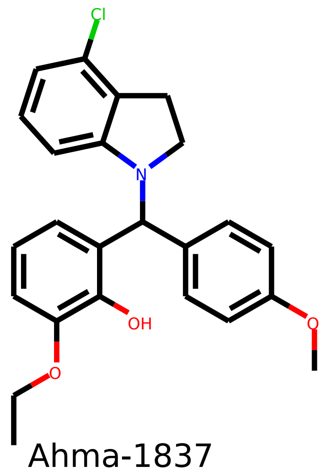
Ahma-1837
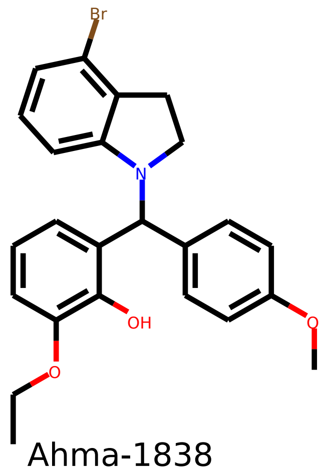
Ahma-1838
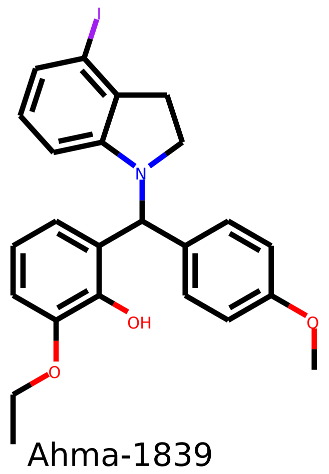
Ahma-1839
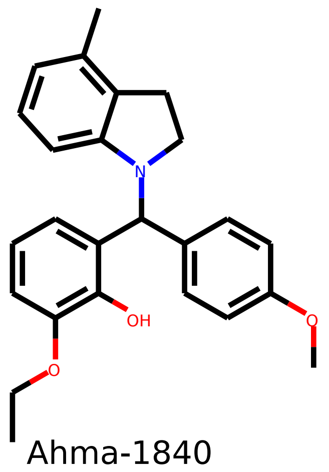
Ahma-1840
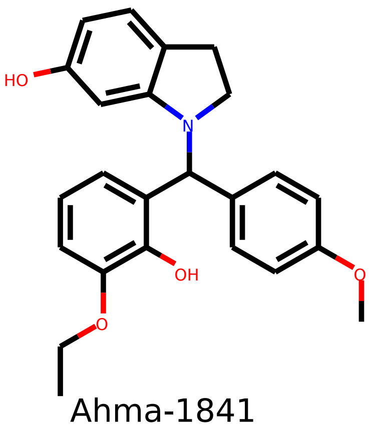
Ahma-1841
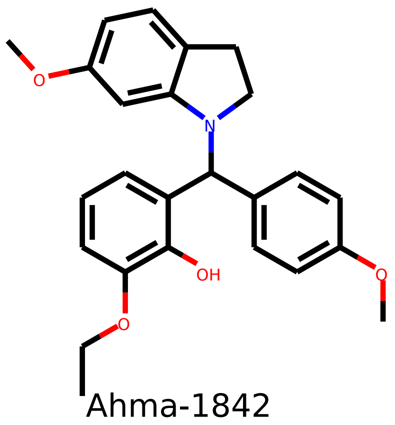
Ahma-1842
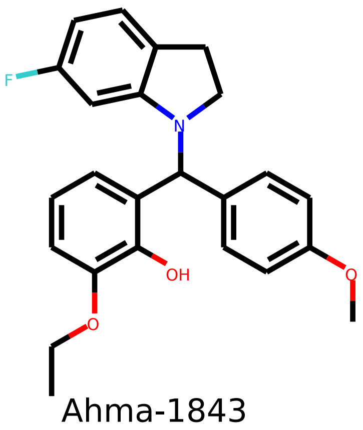
Ahma-1843
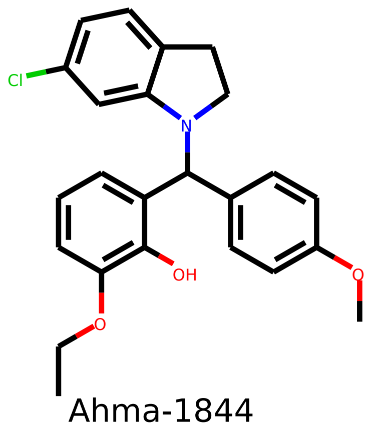
Ahma-1844
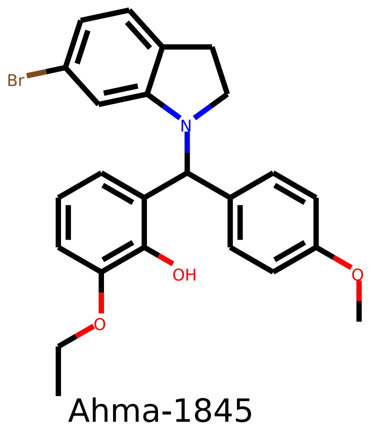
Ahma-1845
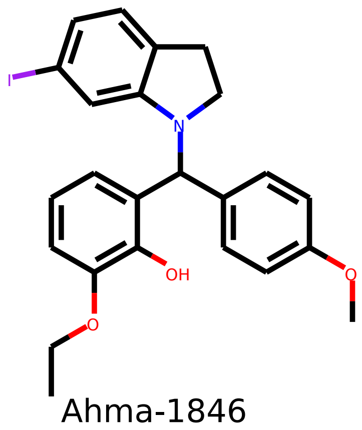
Ahma-1846
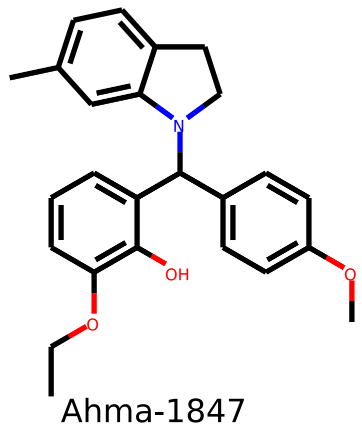
Ahma-1847
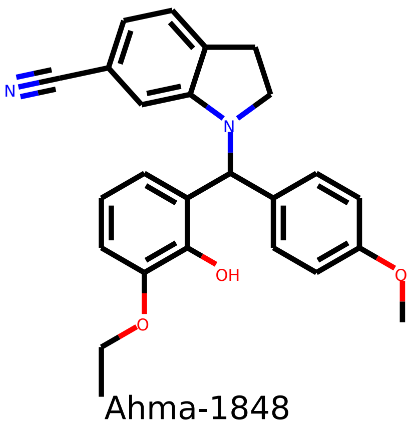
Ahma-1848
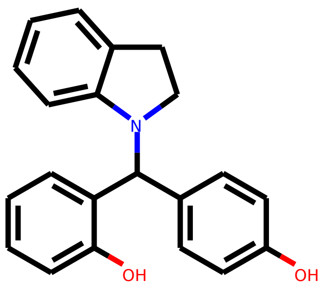
Ahma-1849
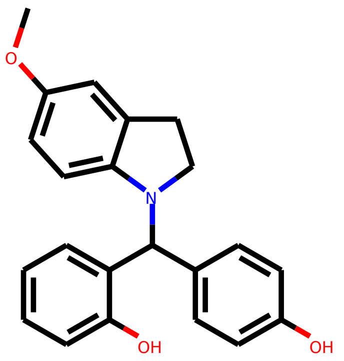
Ahma-1850
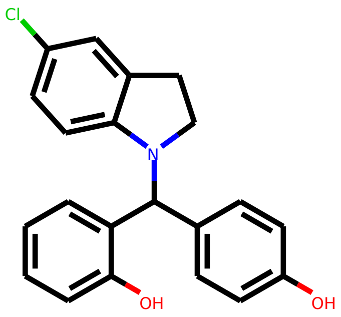
Ahma-1851
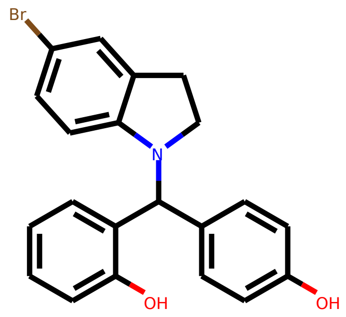
Ahma-1852
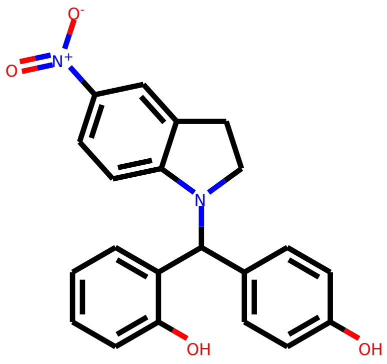
Ahma-1853
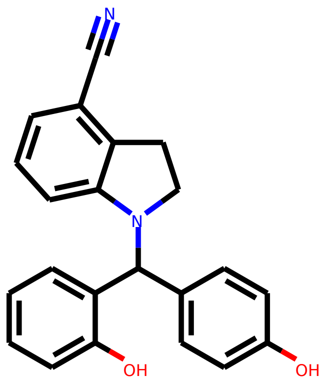
Ahma-1854
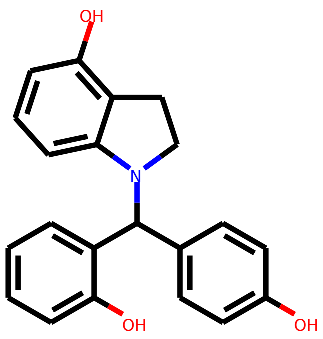
Ahma-1855

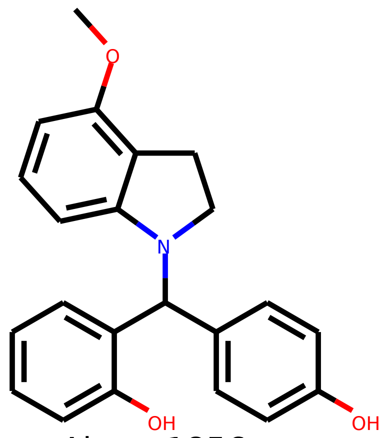
Ahma-1856

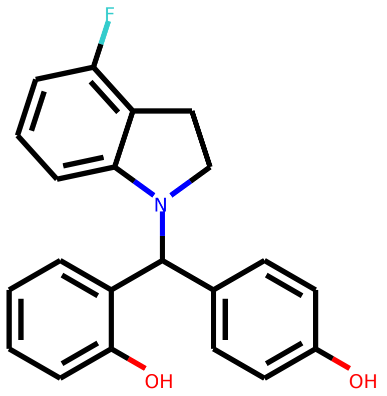
Ahma-1857

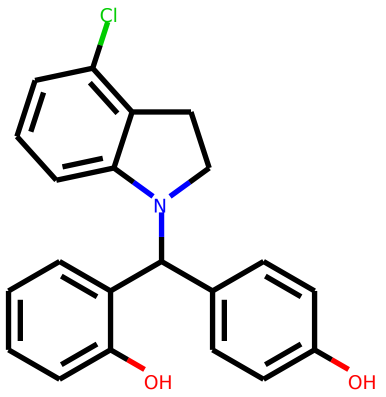
Ahma-1858

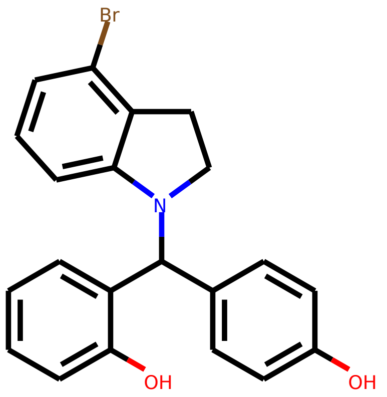
Ahma-1859

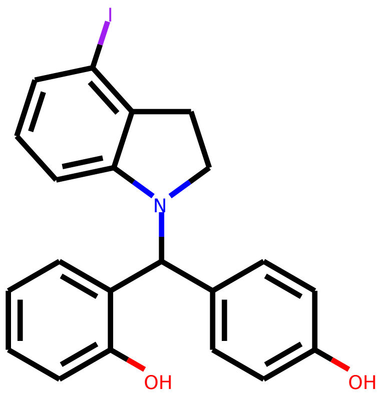
Ahma-1860

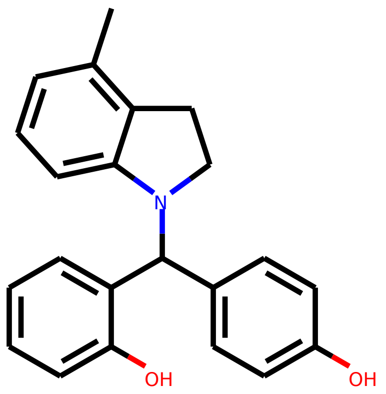
Ahma-1861

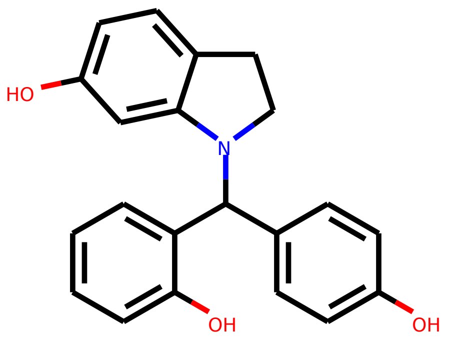
Ahma-1862

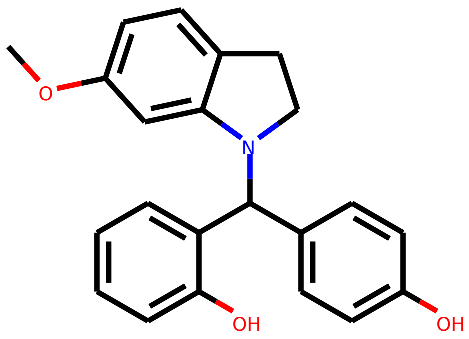
Ahma-1863

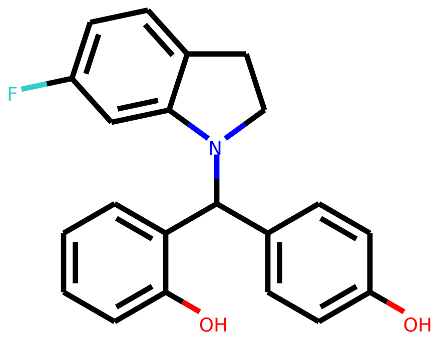
Ahma-1864

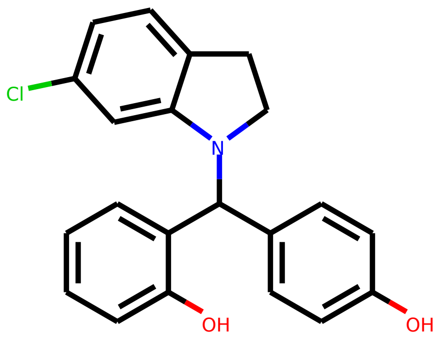
Ahma-1865

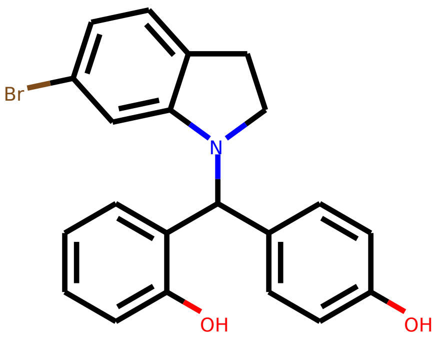
Ahma-1866

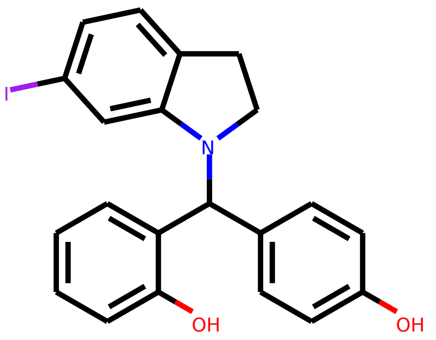
Ahma-1867

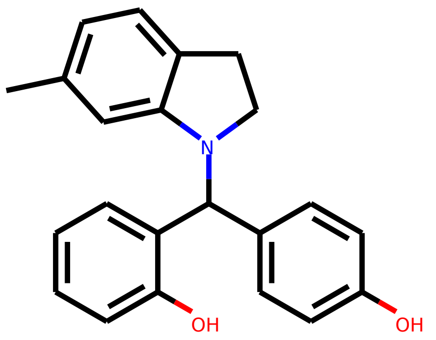
Ahma-1868

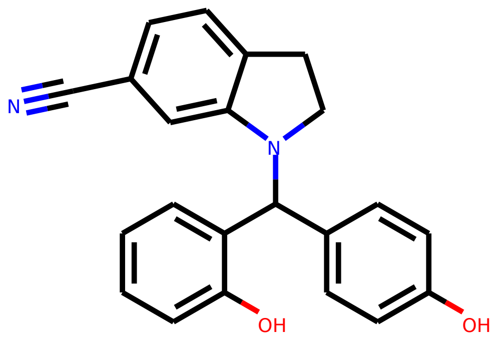
Ahma-1869

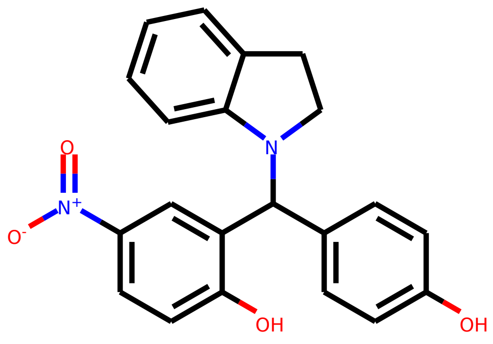
Ahma-1870

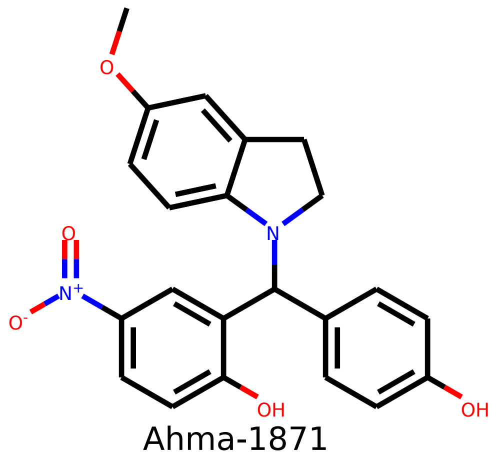
Ahma-1871

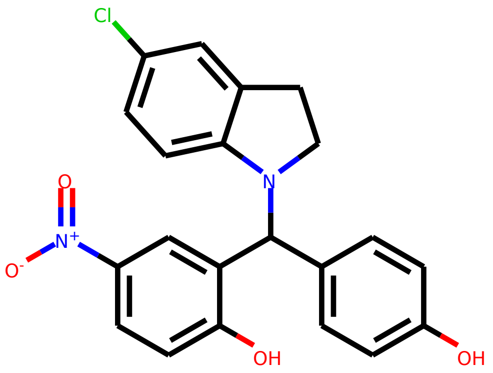
Ahma-1872

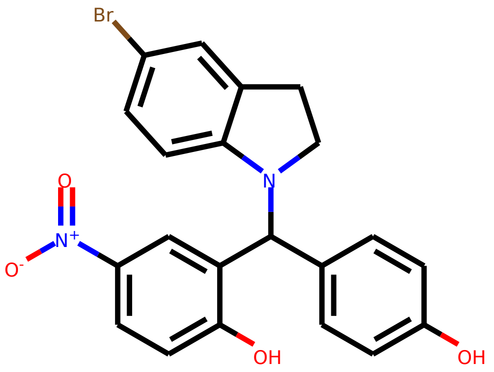
Ahma-1873

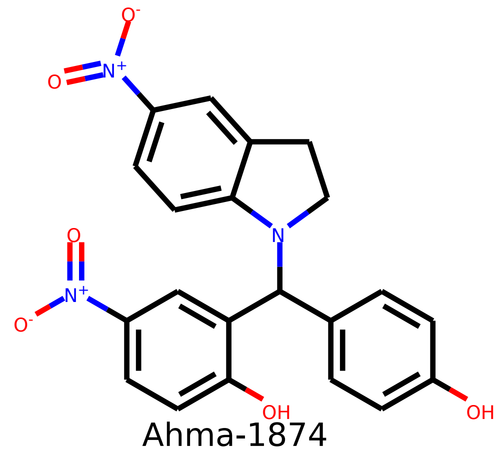
Ahma-1874

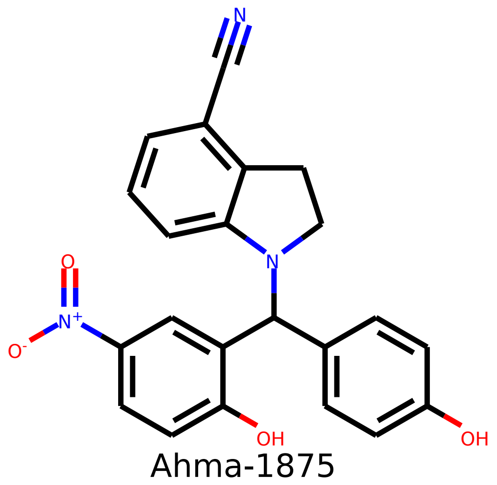
Ahma-1875

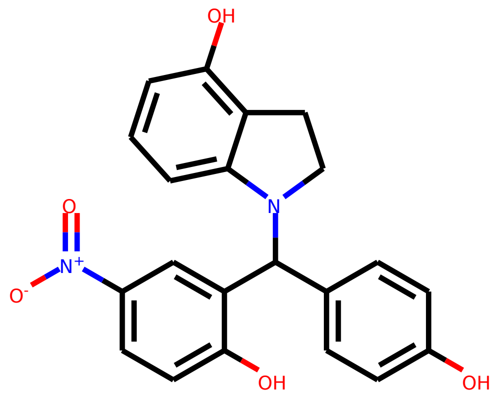
Ahma-1876

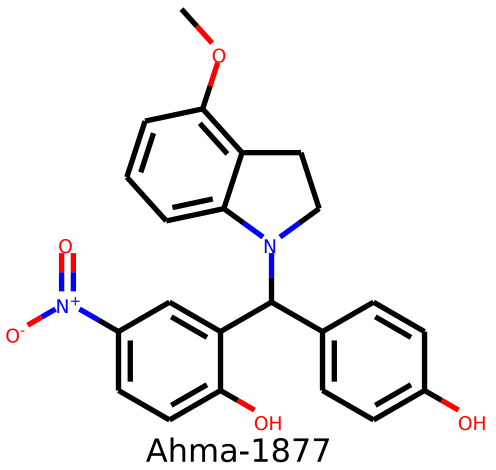
Ahma-1877

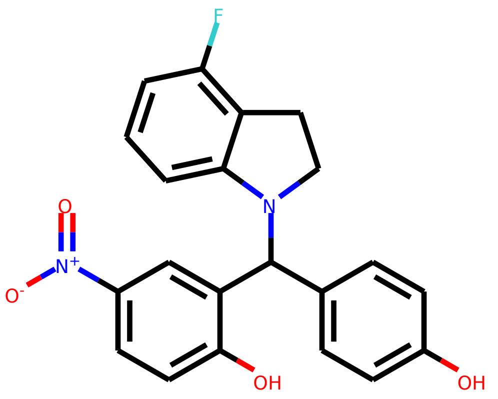
Ahma-1878

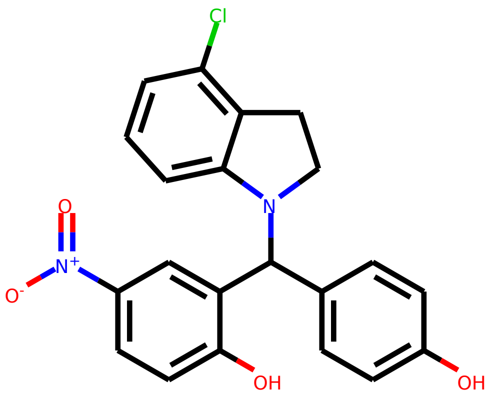
Ahma-1879

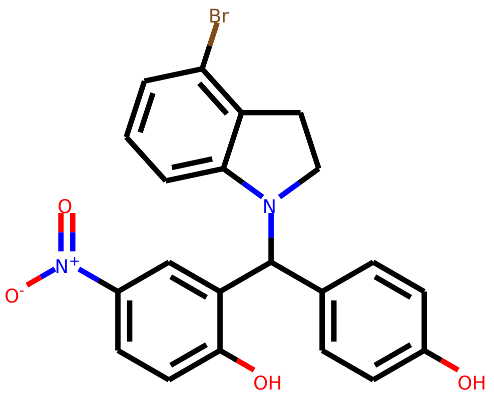
Ahma-1880

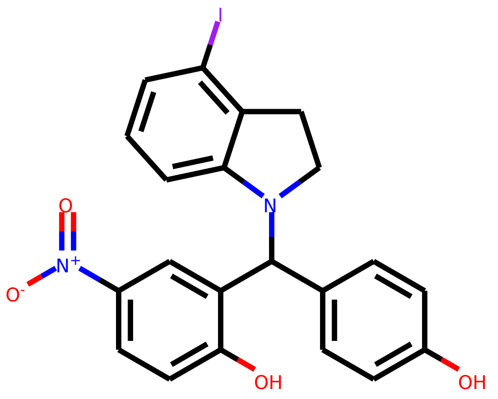
Ahma-1881

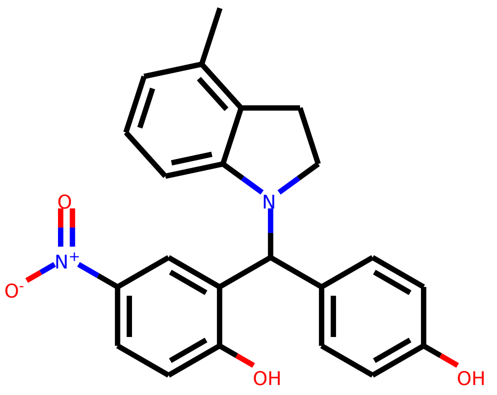
Ahma-1882

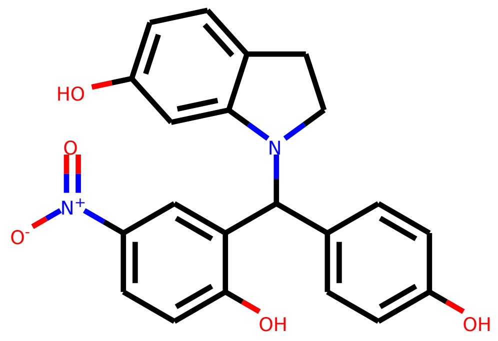
Ahma-1883

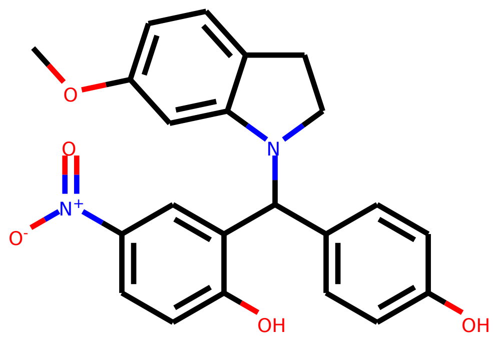
Ahma-1884

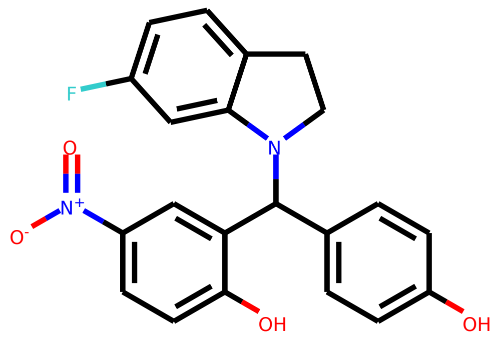
Ahma-1885

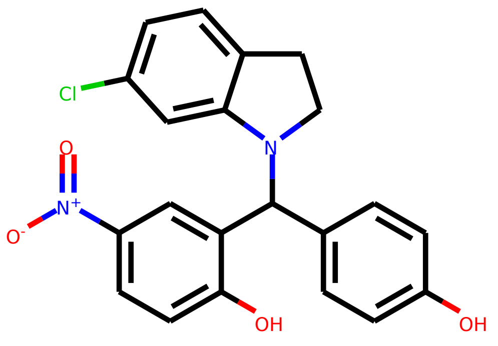
Ahma-1886

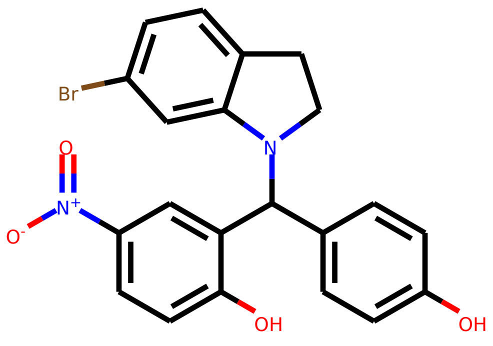
Ahma-1887

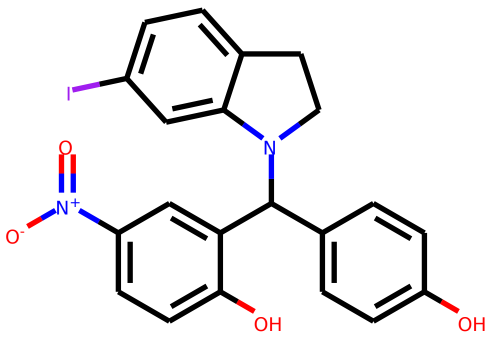
Ahma-1888

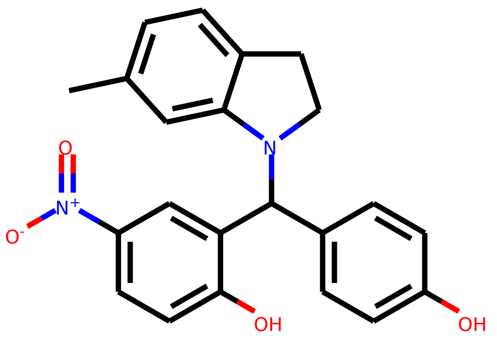
Ahma-1889

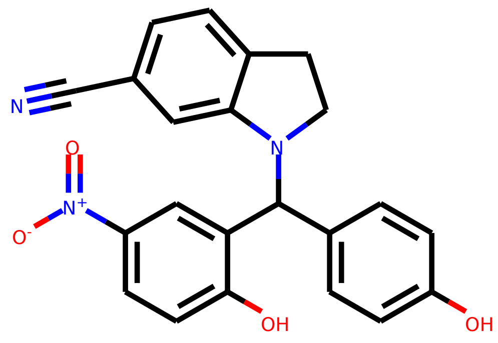
Ahma-1890

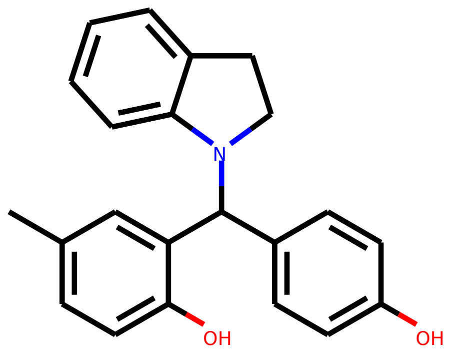
Ahma-1891
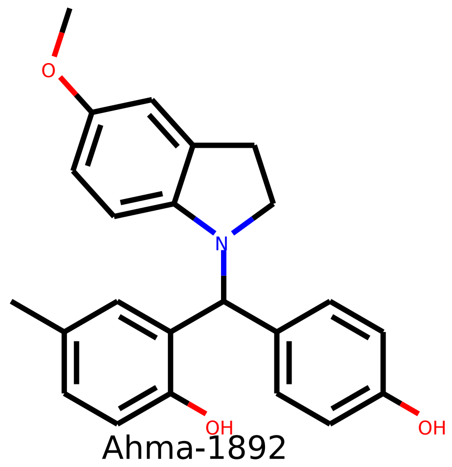
Ahma-1892
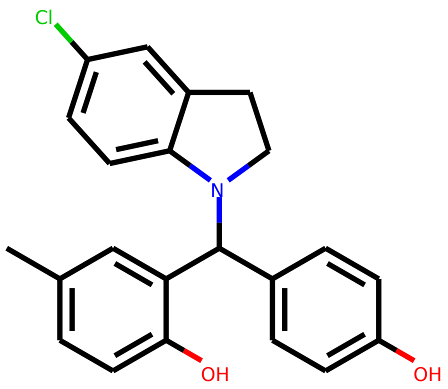
Ahma-1893
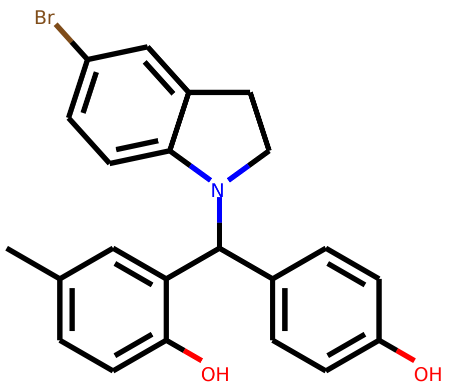
Ahma-1894
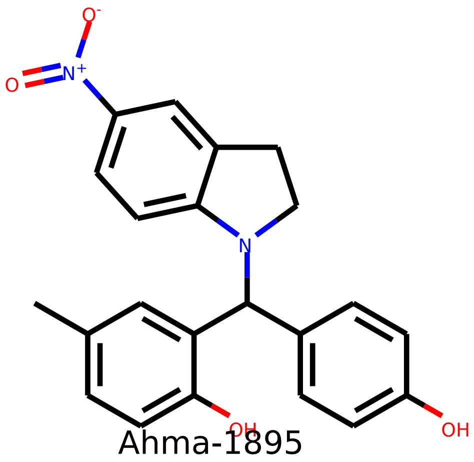
Ahma-1895
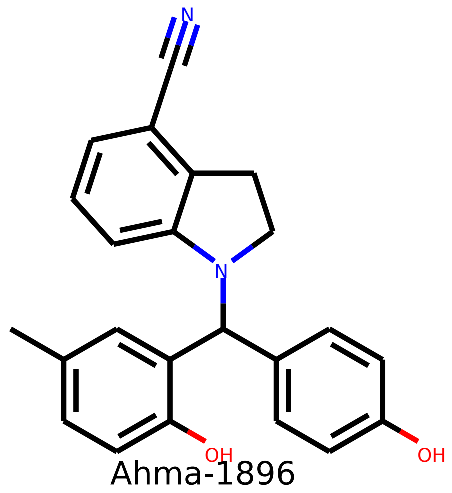
Ahma-1896
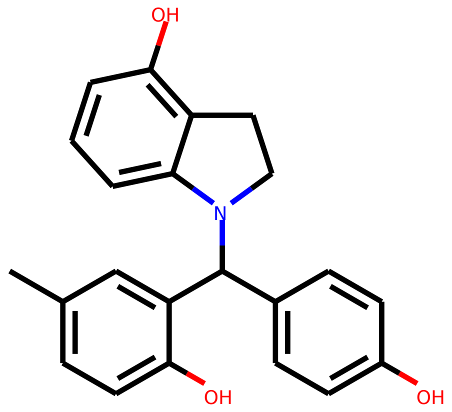
Ahma-1897
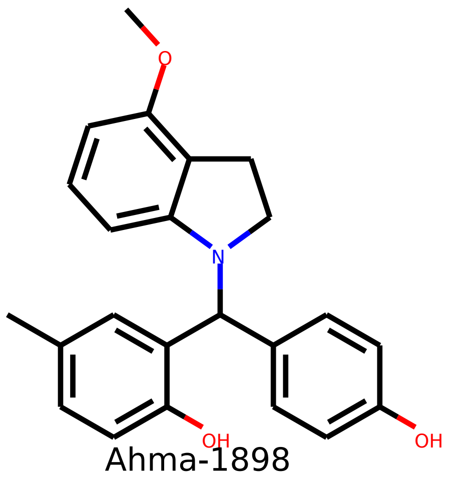
Ahma-1898
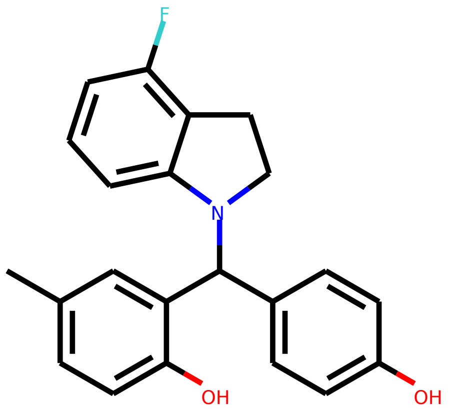
Ahma-1899
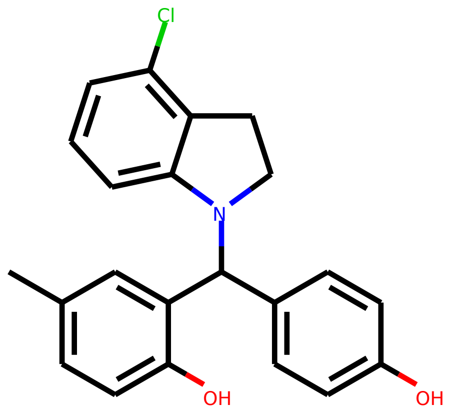
Ahma-1900
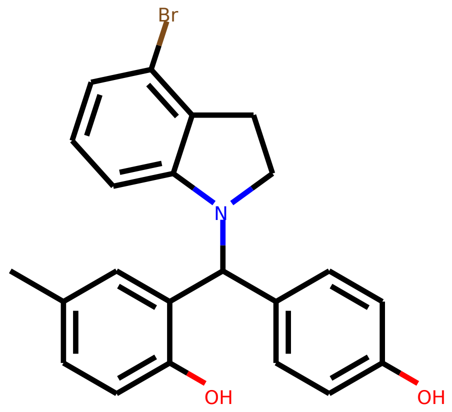
Ahma-1901
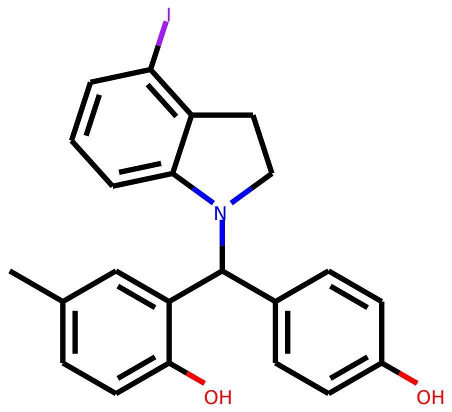
Ahma-1902
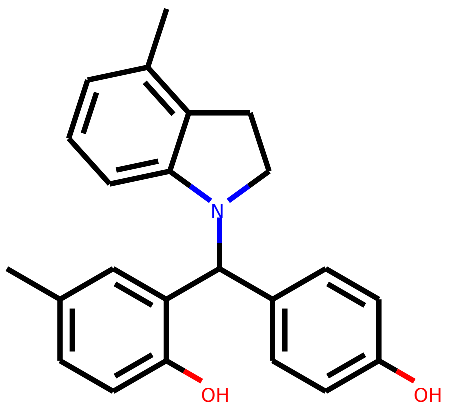
Ahma-1903
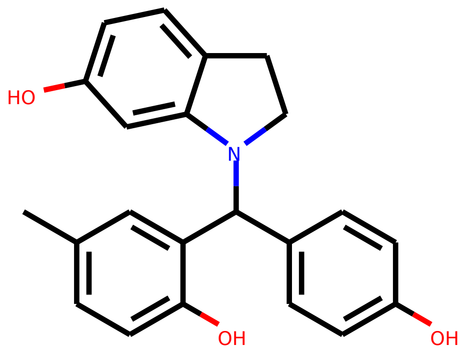
Ahma-1904
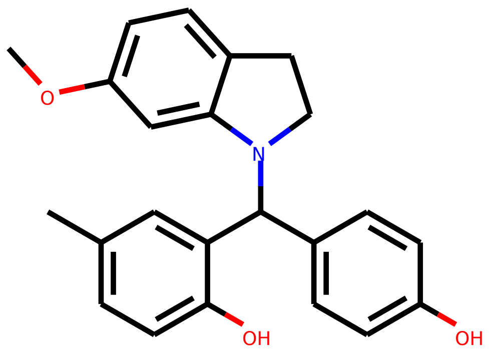
Ahma-1905
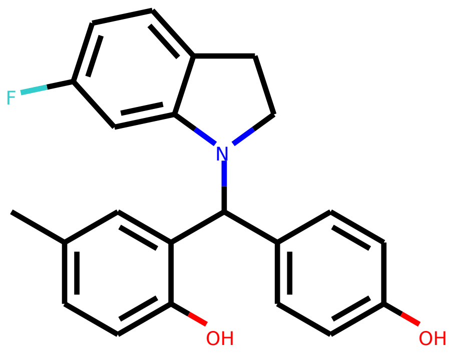
Ahma-1906
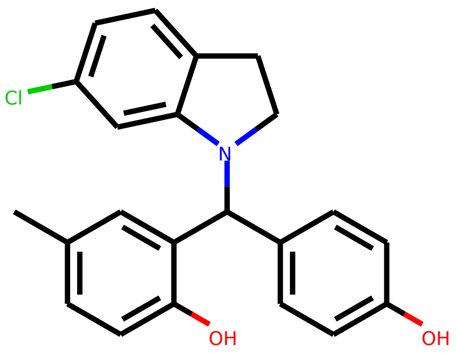
Ahma-1907
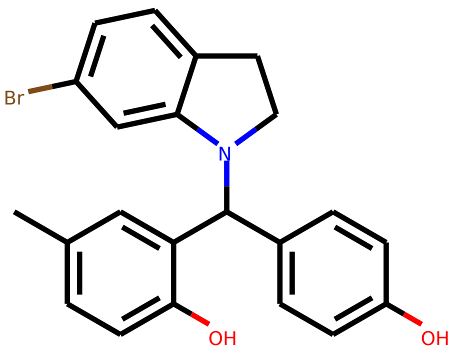
Ahma-1908
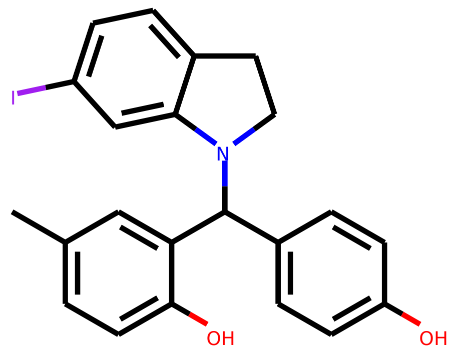
Ahma-1909
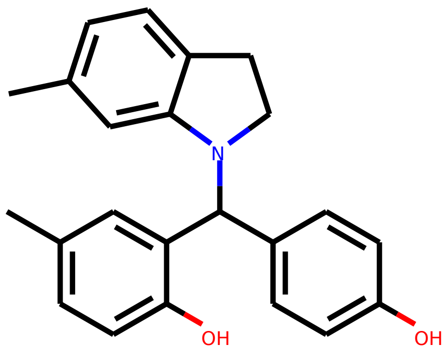
Ahma-1910
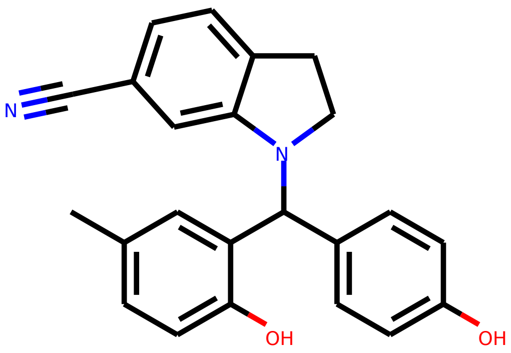
Ahma-1911
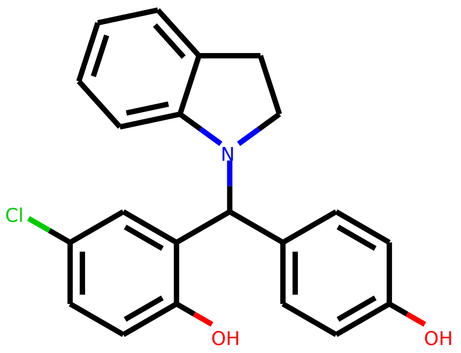
Ahma-1912
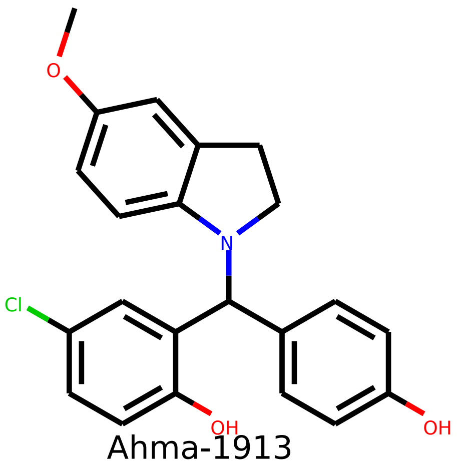
Ahma-1913
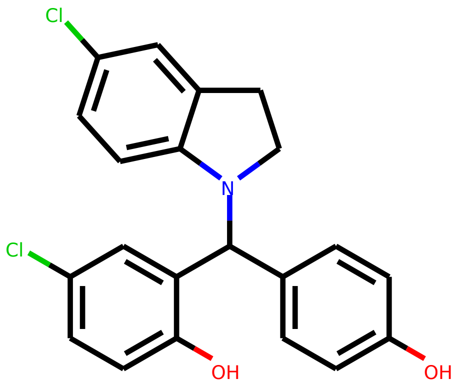
Ahma-1914
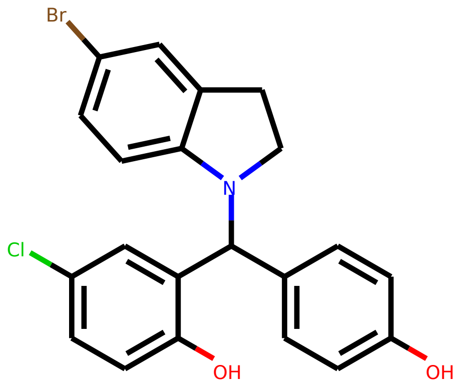
Ahma-1915
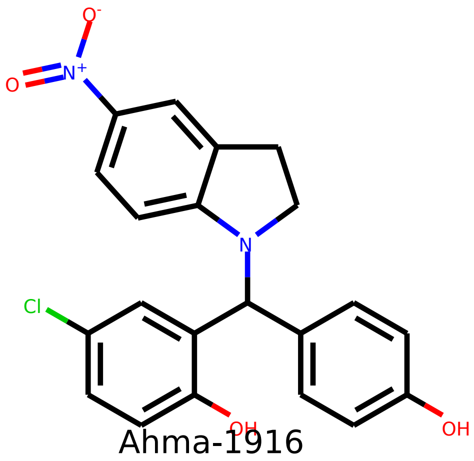
Ahma-1916
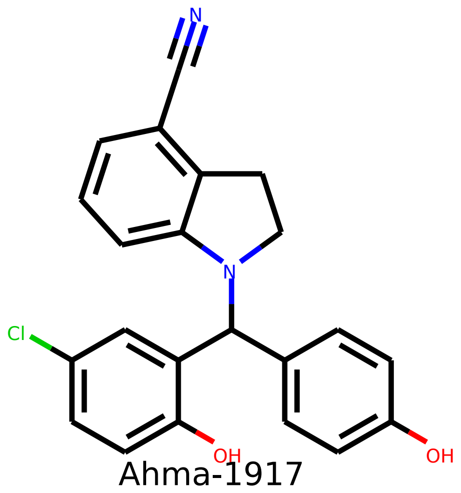
Ahma-1917
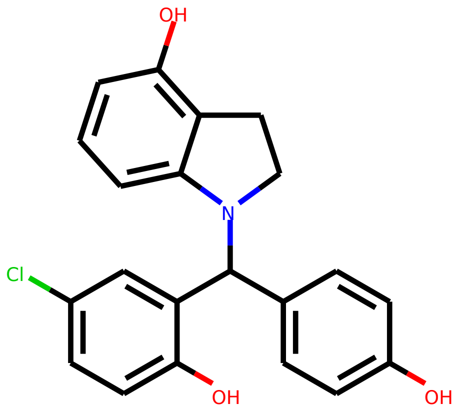
Ahma-1918
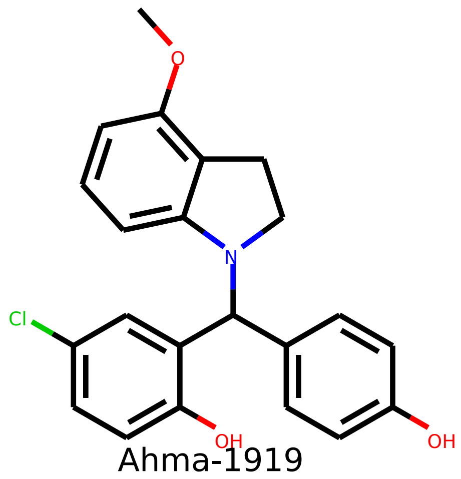
Ahma-1919
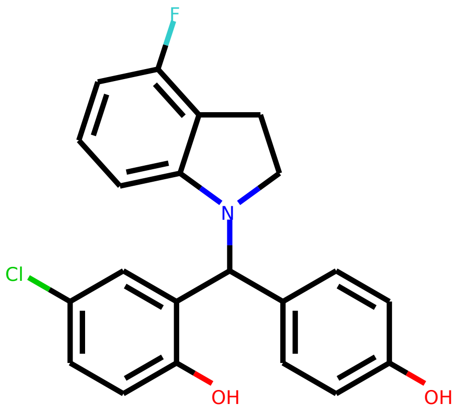
Ahma-1920
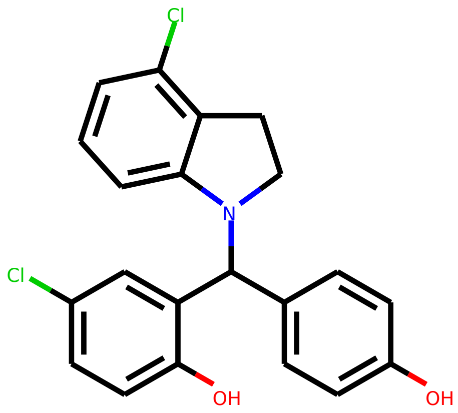
Ahma-1921
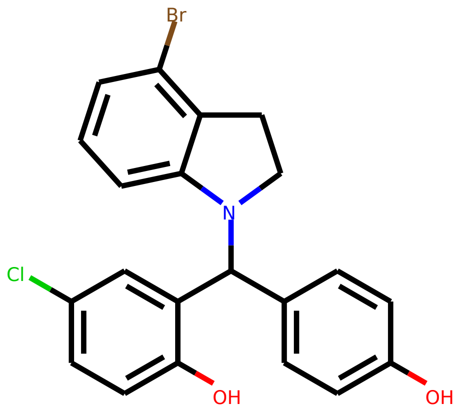
Ahma-1922
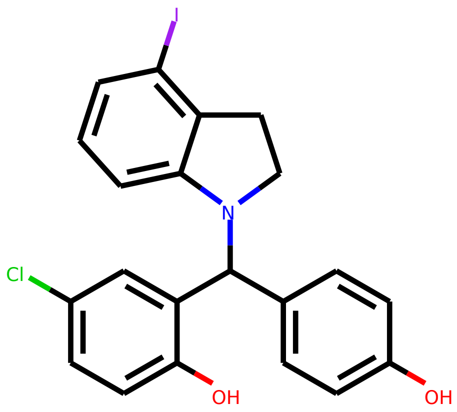
Ahma-1923
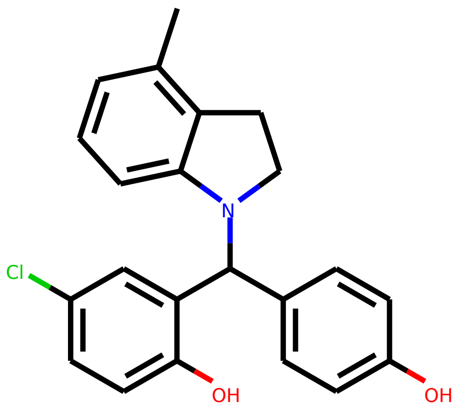
Ahma-1924
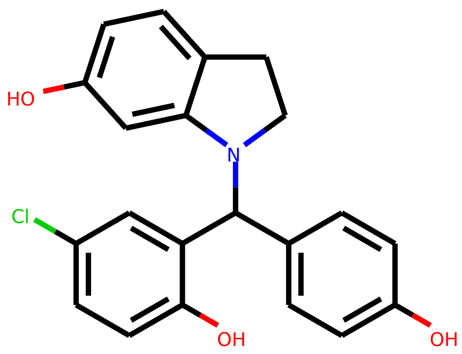
Ahma-1925

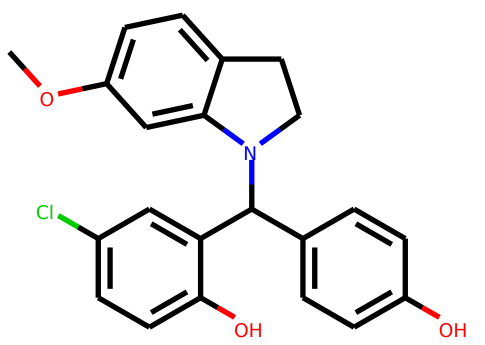
Ahma-1926

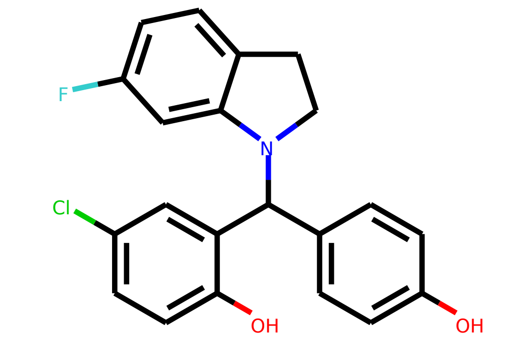
Ahma-1927

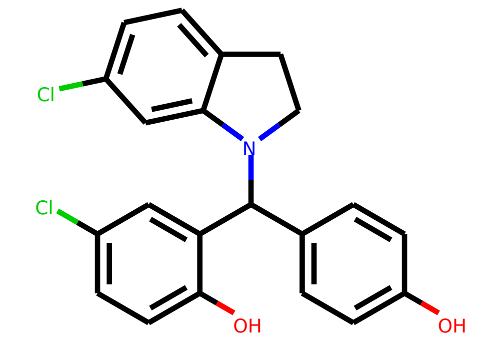
Ahma-1928

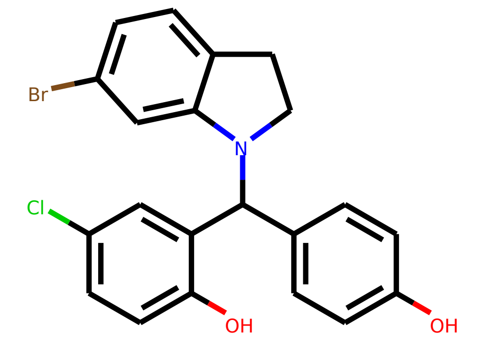
Ahma-1929

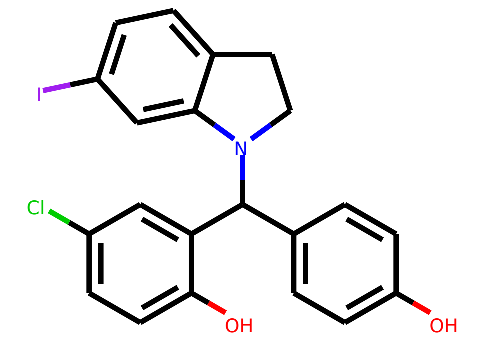
Ahma-1930

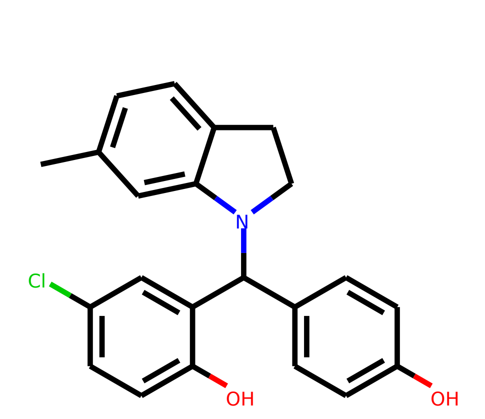
Ahma-1931

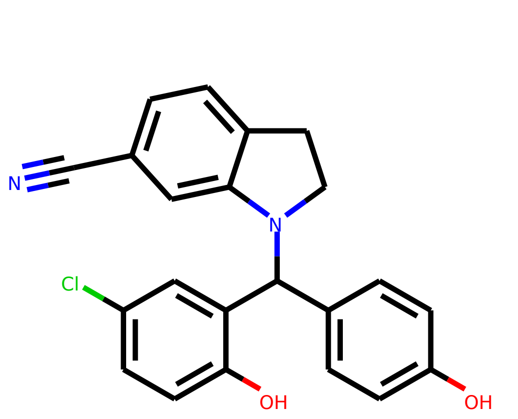
Ahma-1932

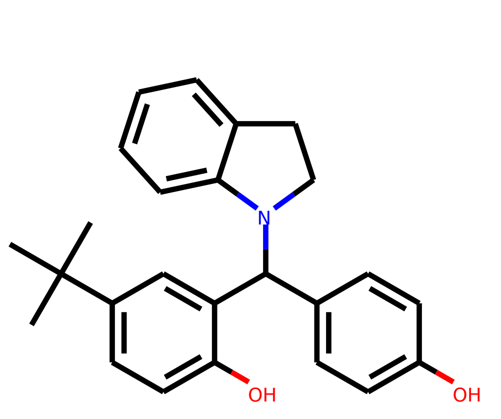
Ahma-1933

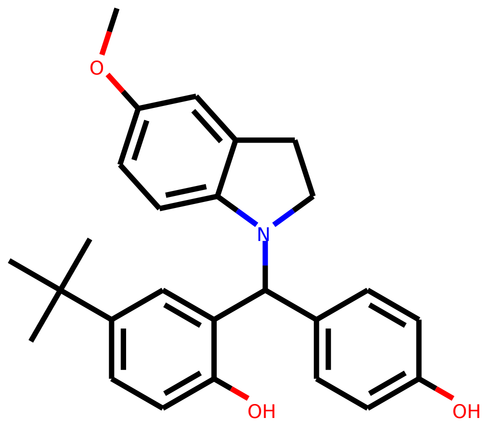
Ahma-1934

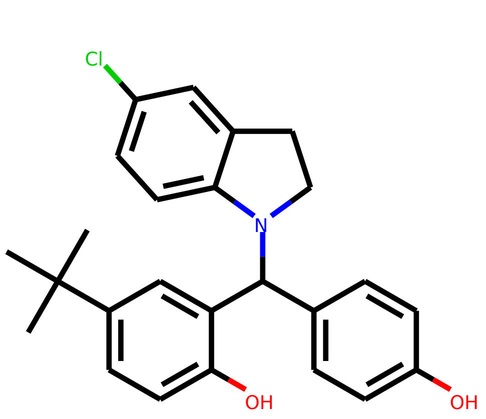
Ahma-1935

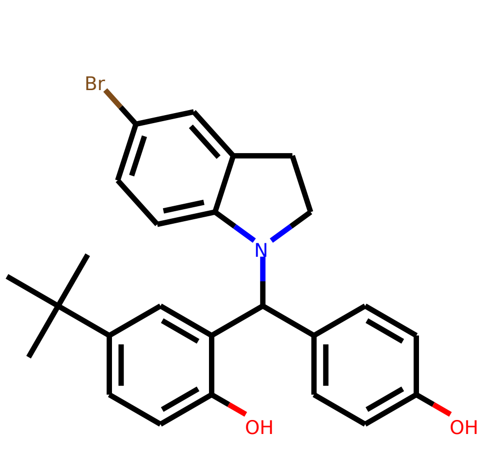
Ahma-1936

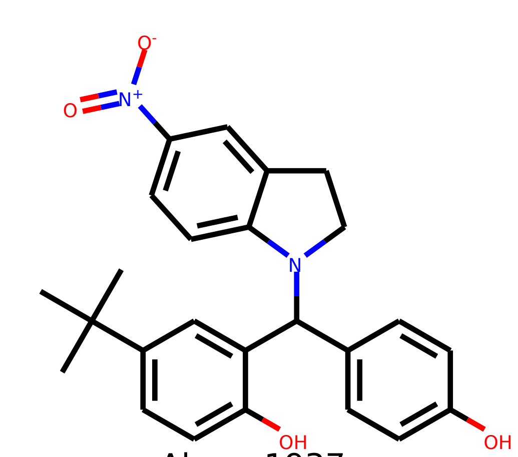
Ahma-1937

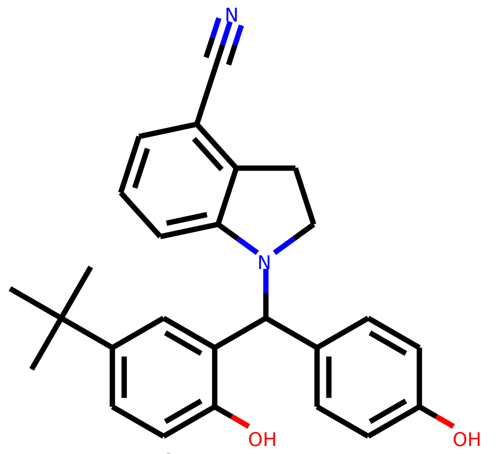
Ahma-1938

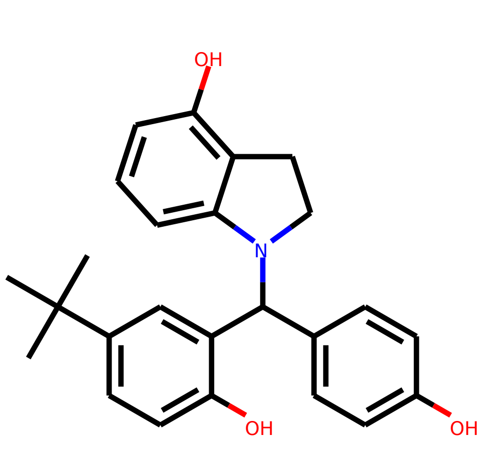
Ahma-1939

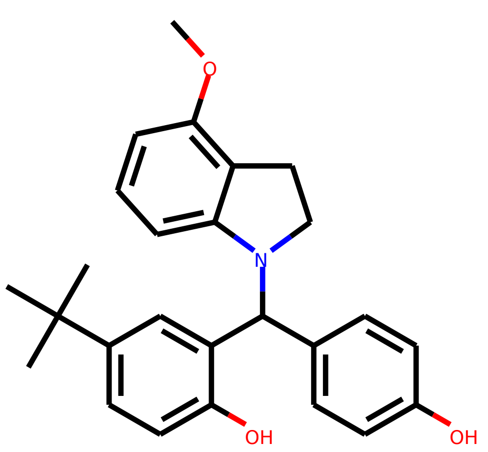
Ahma-1940

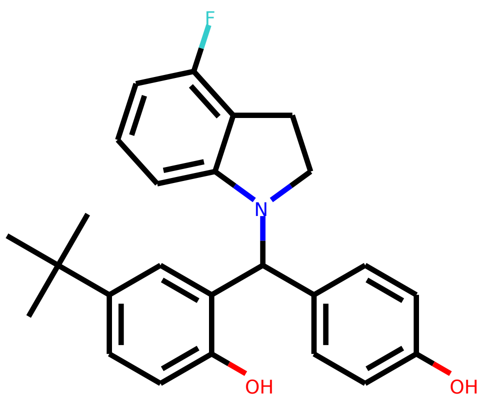
Ahma-1941

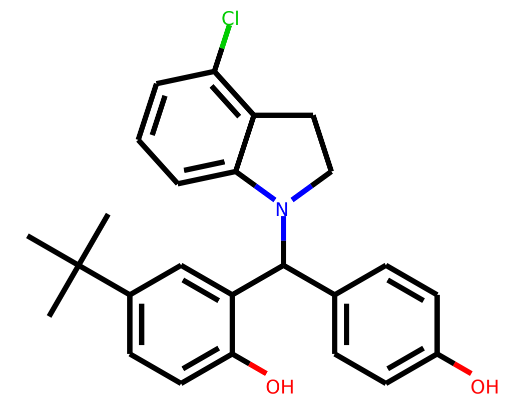
Ahma-1942

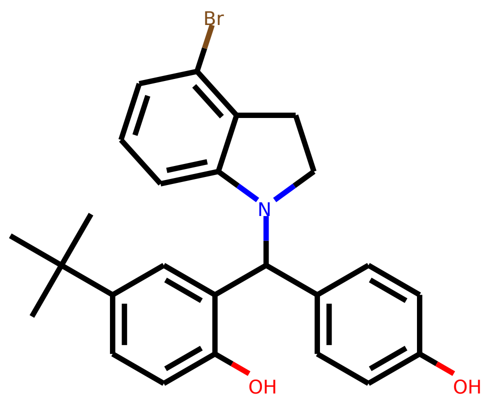
Ahma-1943

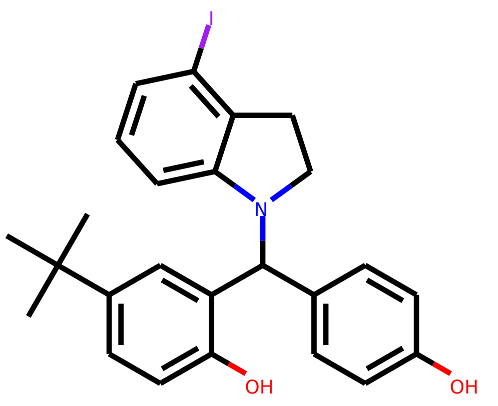
Ahma-1944

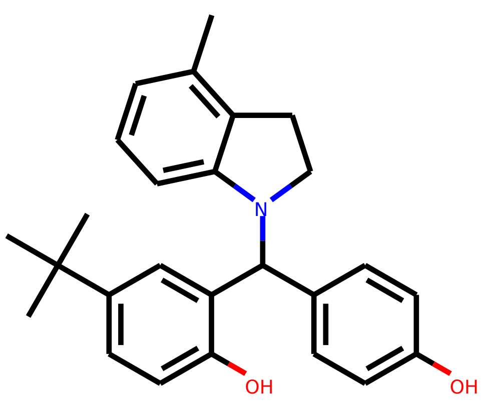
Ahma-1945

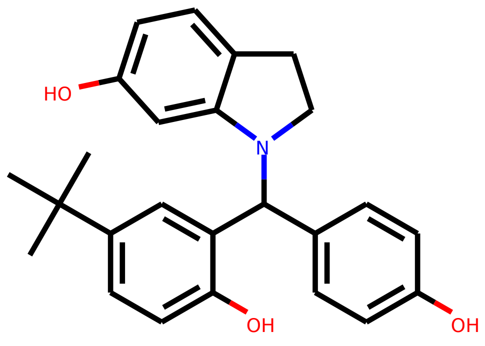
Ahma-1946

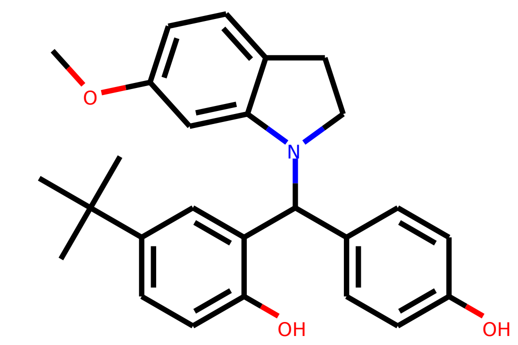
Ahma-1947

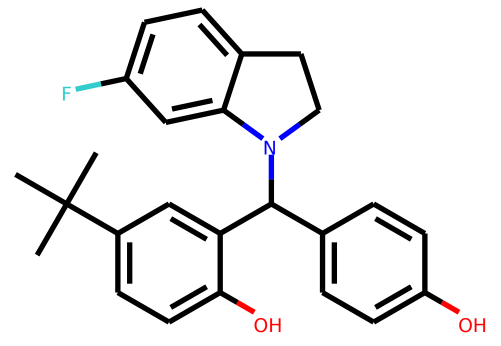
Ahma-1948

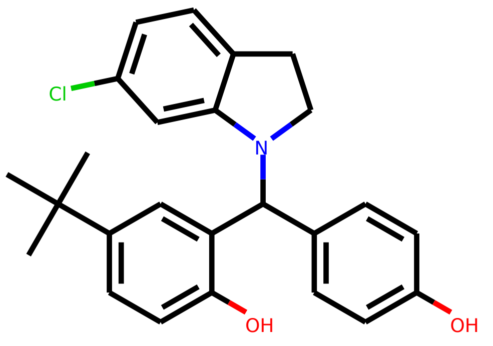
Ahma-1949

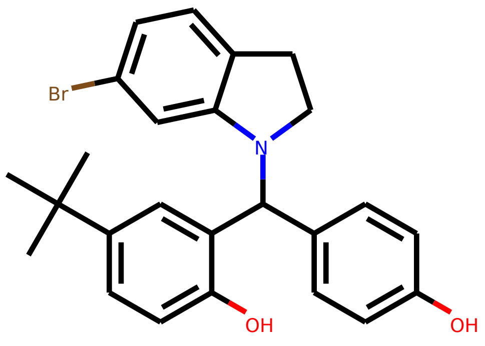
Ahma-1950

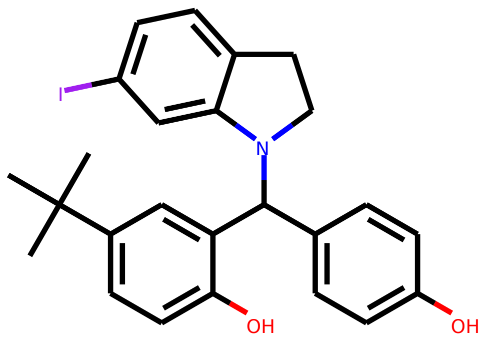
Ahma-1951

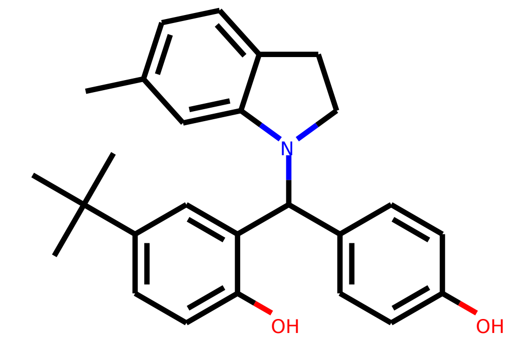
Ahma-1952

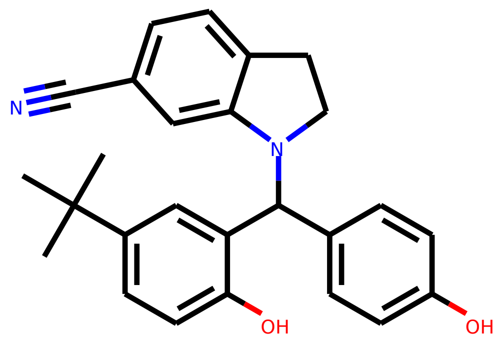
Ahma-1953

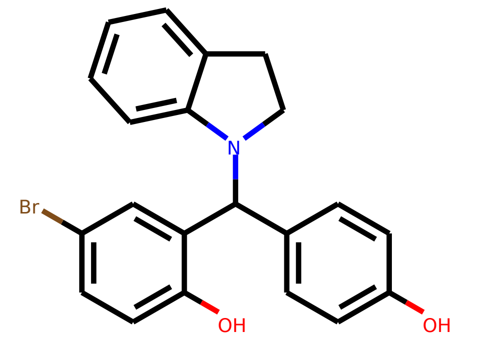
Ahma-1954

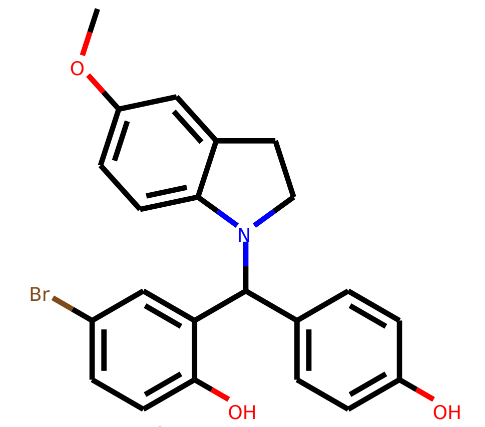
Ahma-1955

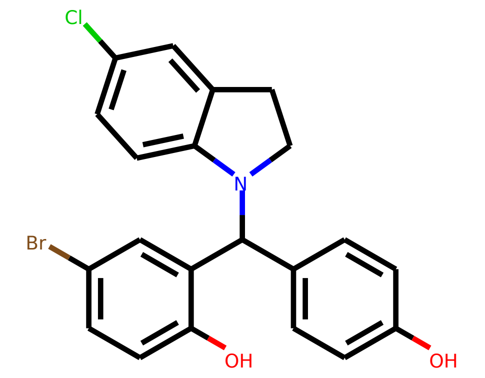
Ahma-1956

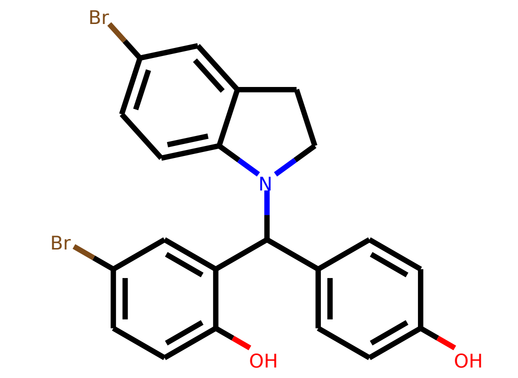
Ahma-1957

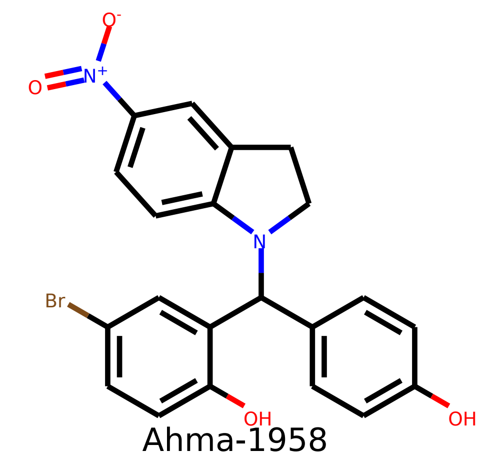
Ahma-1958

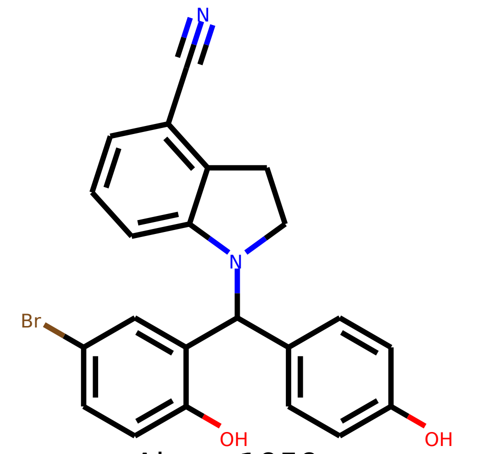
Ahma-1959

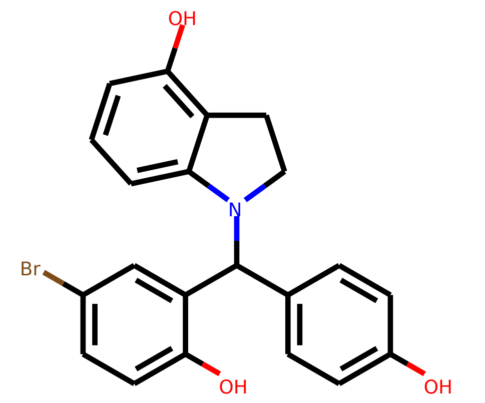
Ahma-1960

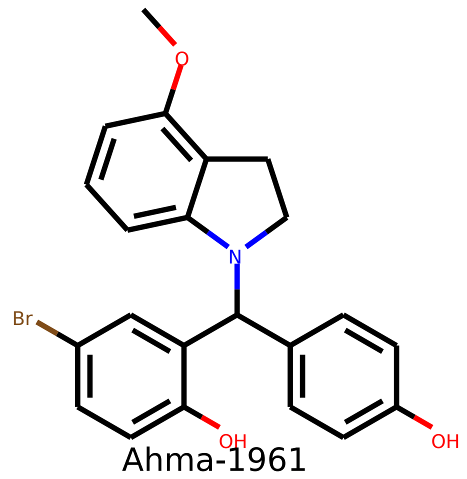
Ahma-1961

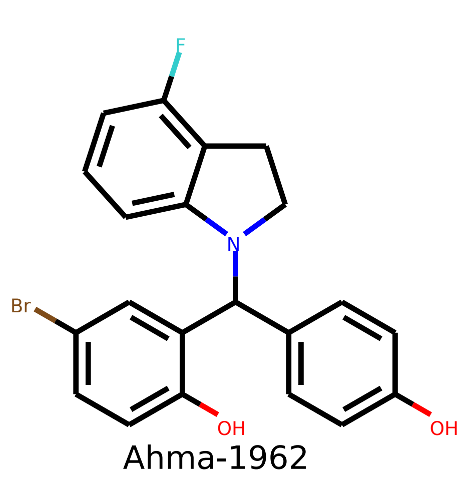
Ahma-1962

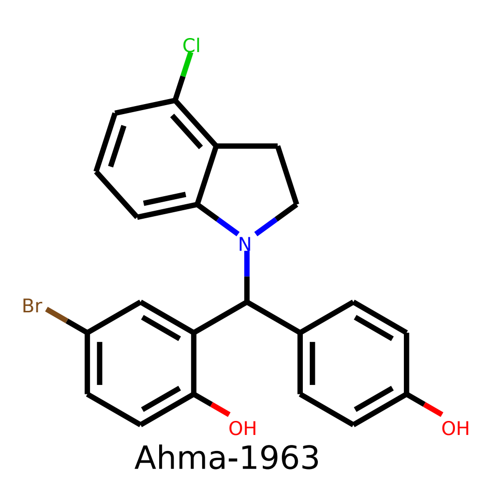
Ahma-1963

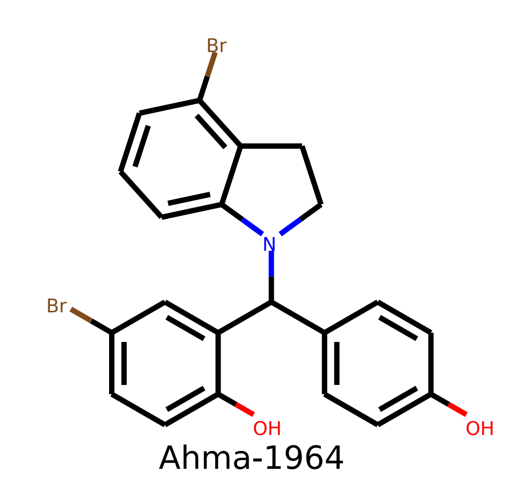
Ahma-1964

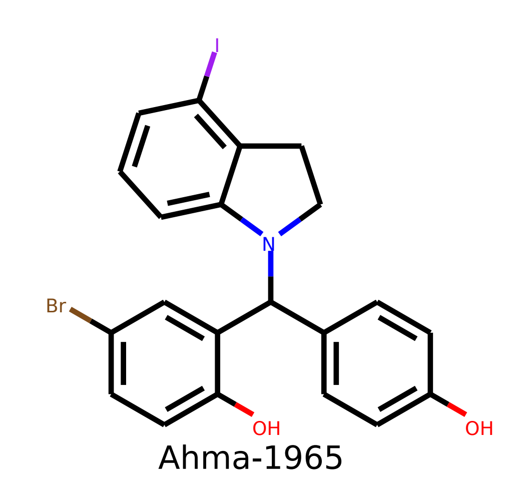
Ahma-1965

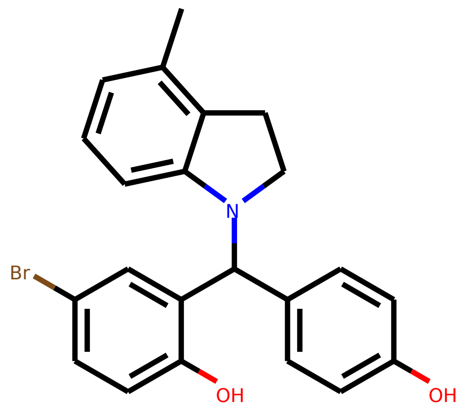
Ahma-1966

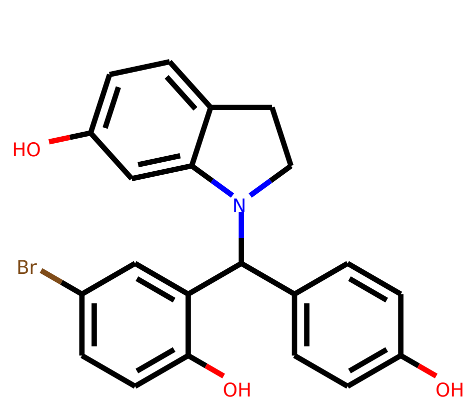
Ahma-1967

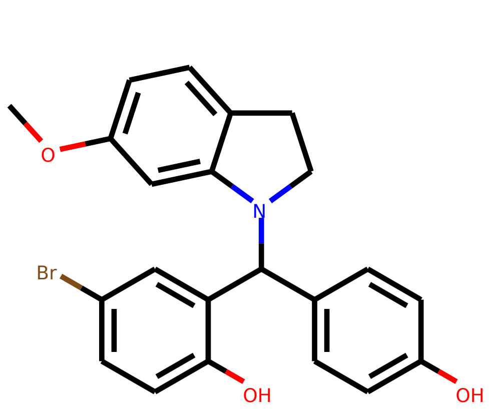
Ahma-1968

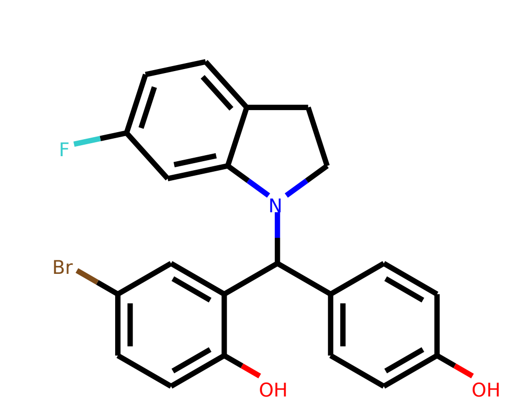
Ahma-1969

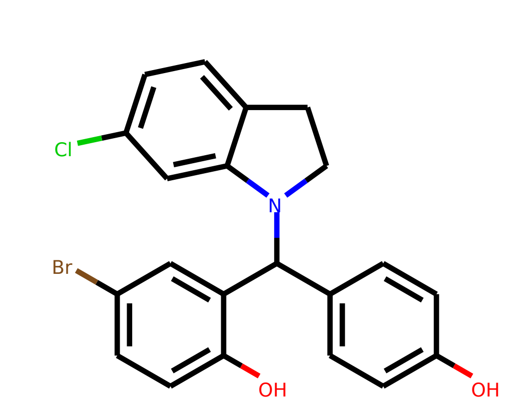
Ahma-1970

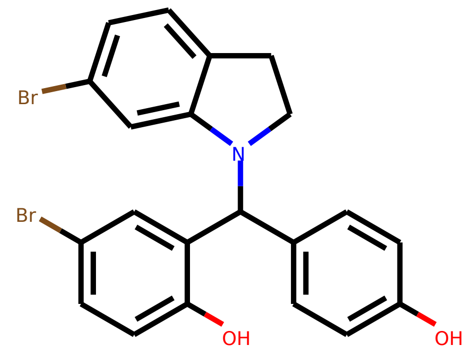
Ahma-1971

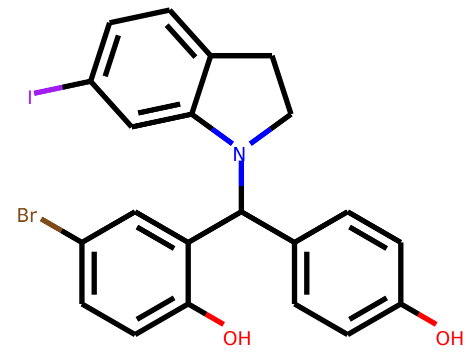
Ahma-1972

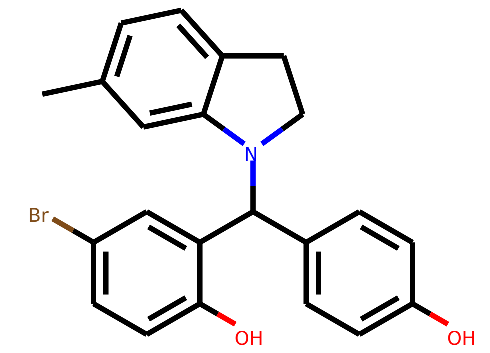
Ahma-1973

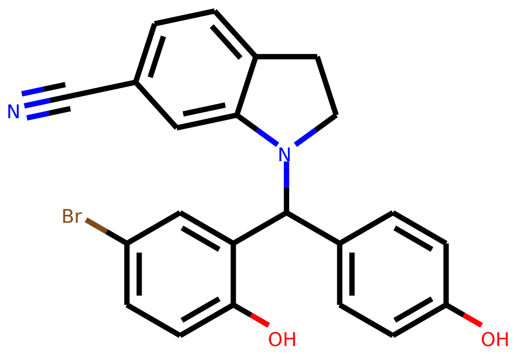
Ahma-1974

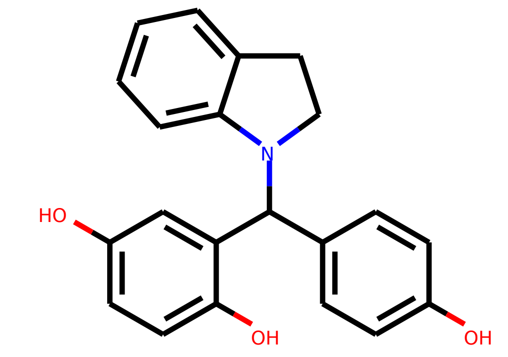
Ahma-1975

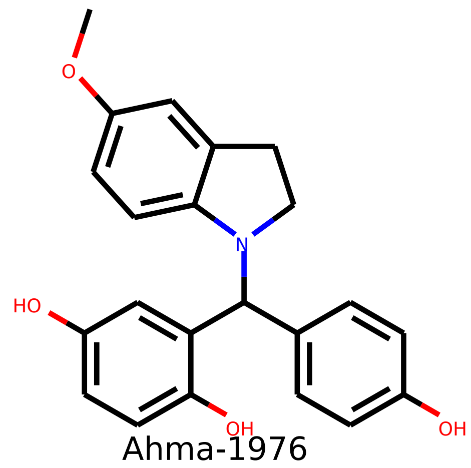
Ahma-1976

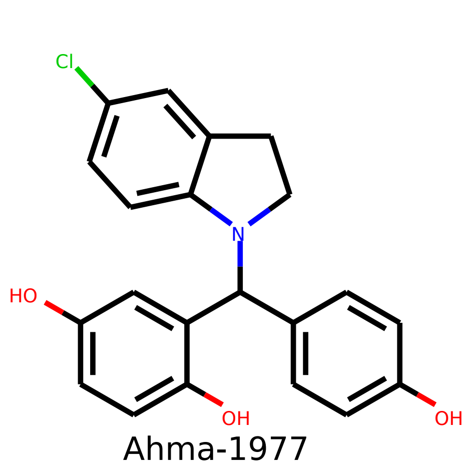
Ahma-1977

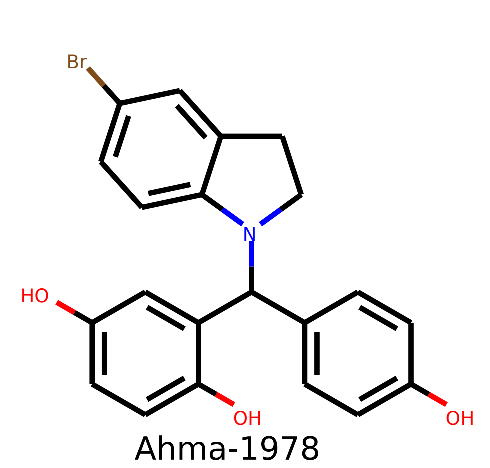
Ahma-1978

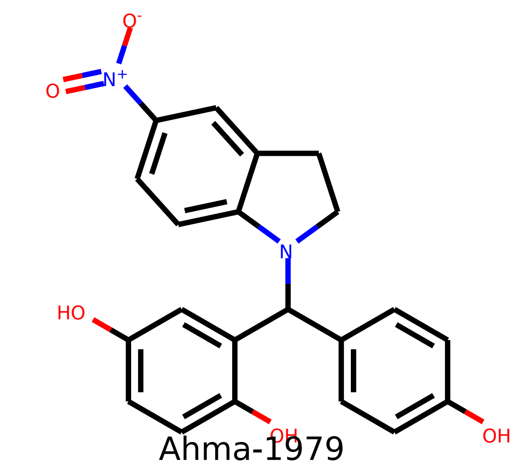
Ahma-1979

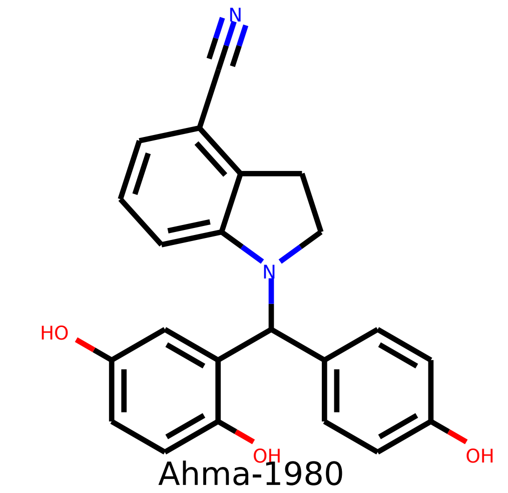
Ahma-1980

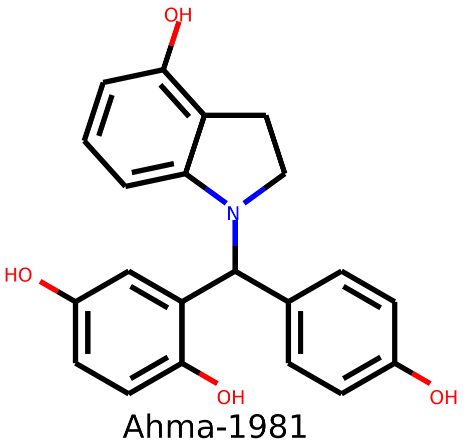
Ahma-1981

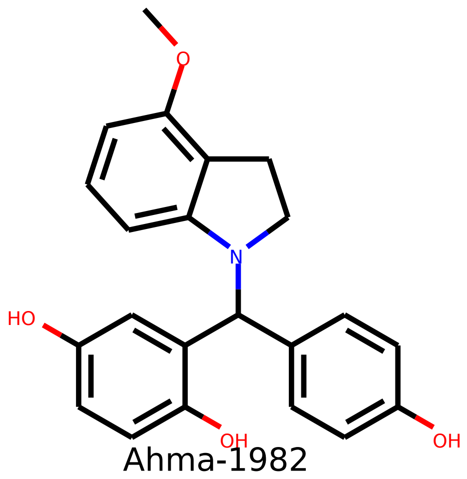
Ahma-1982

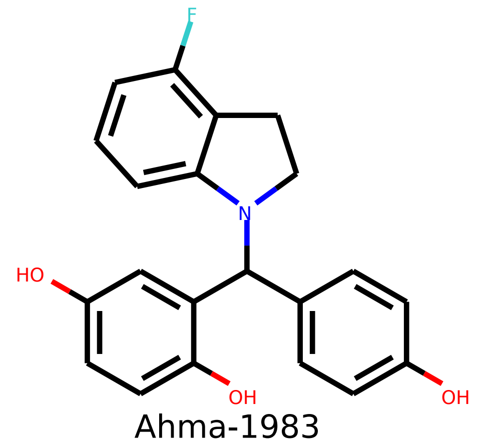
Ahma-1983

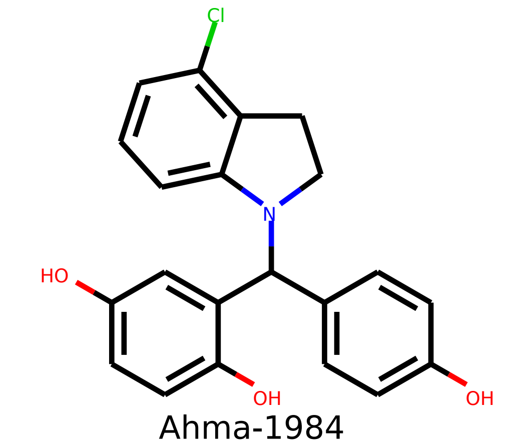
Ahma-1984

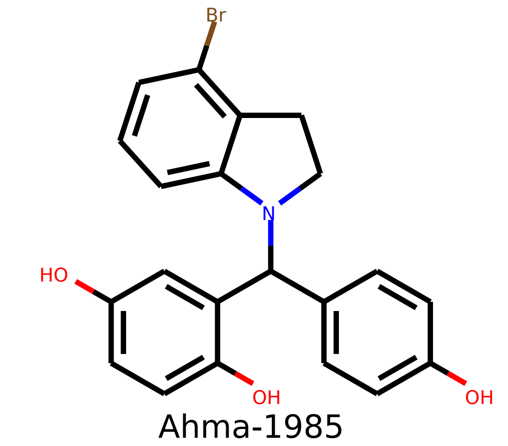
Ahma-1985

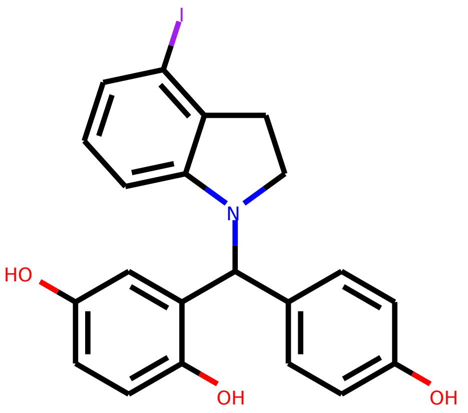
Ahma-1986

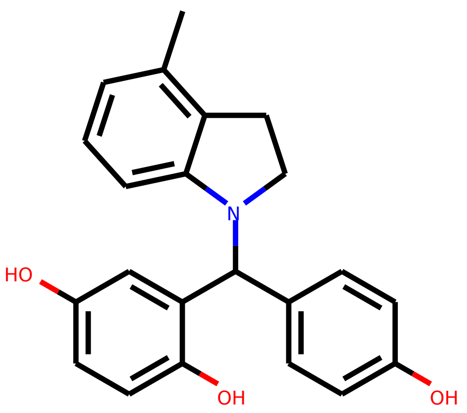
Ahma-1987

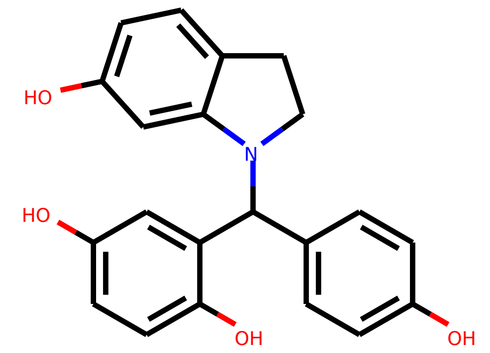
Ahma-1988

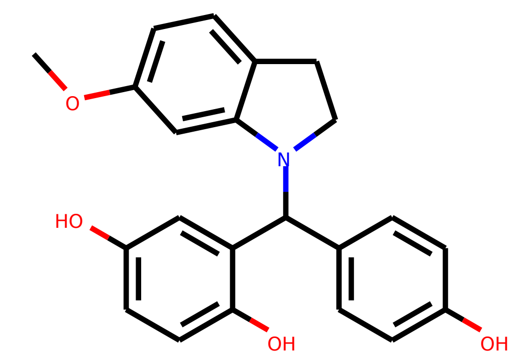
Ahma-1989

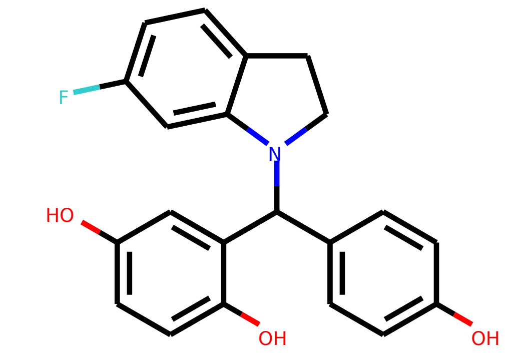
Ahma-1990

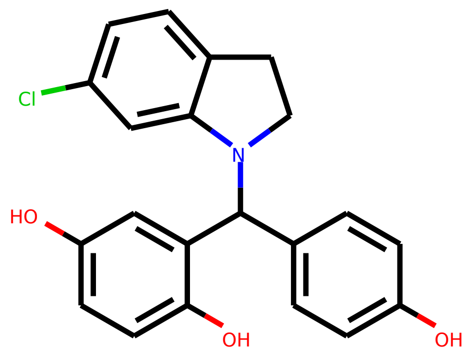
Ahma-1991

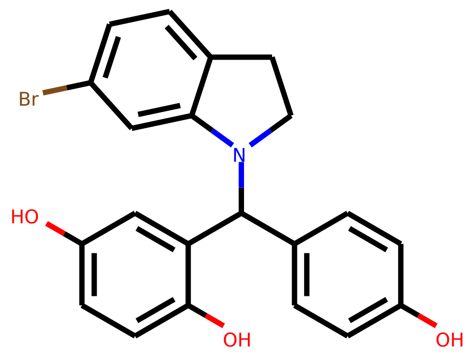
Ahma-1992

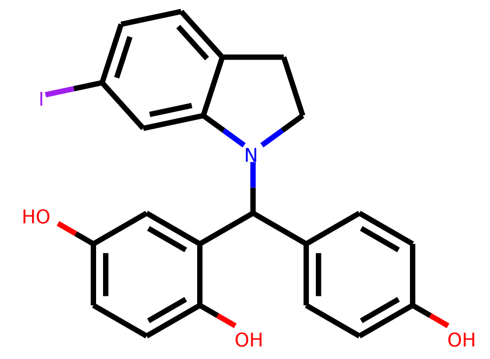
Ahma-1993

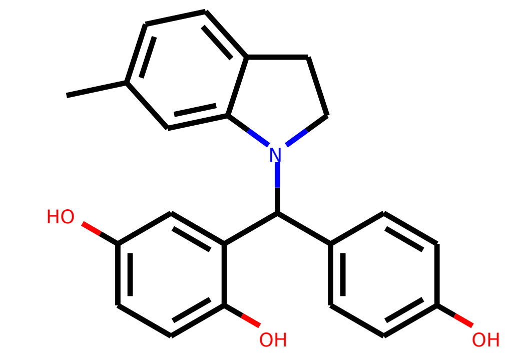
Ahma-1994

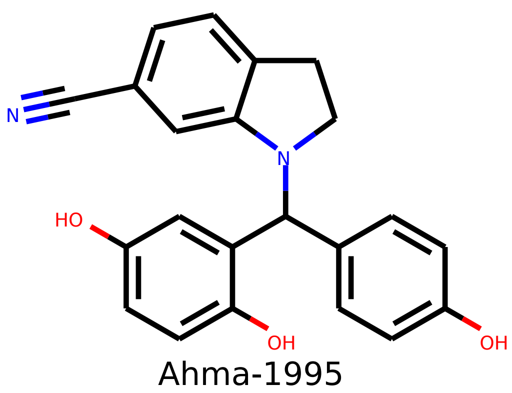
Ahma-1995

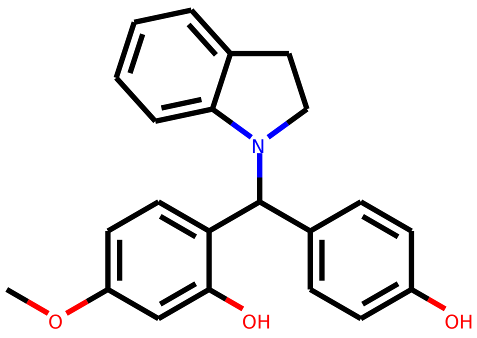
Ahma-1996
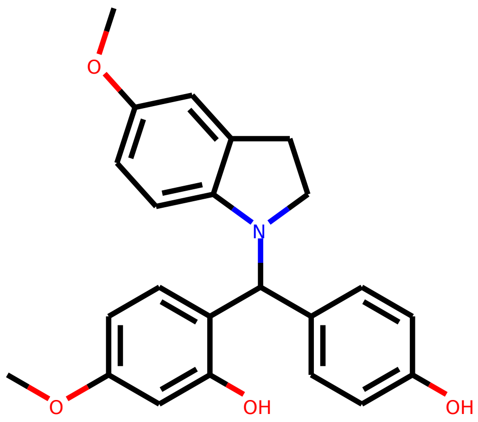
Ahma-1997
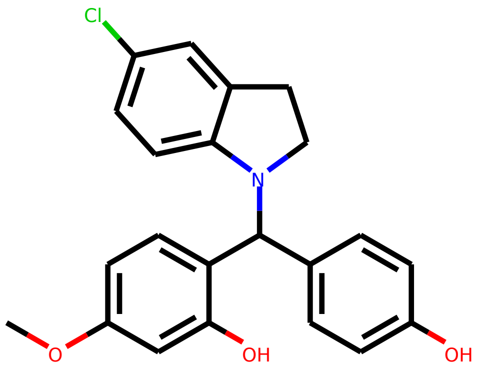
Ahma-1998
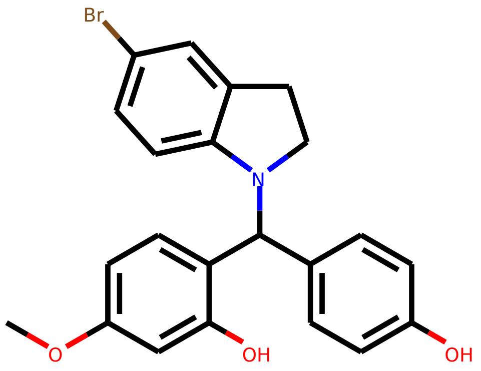
Ahma-1999
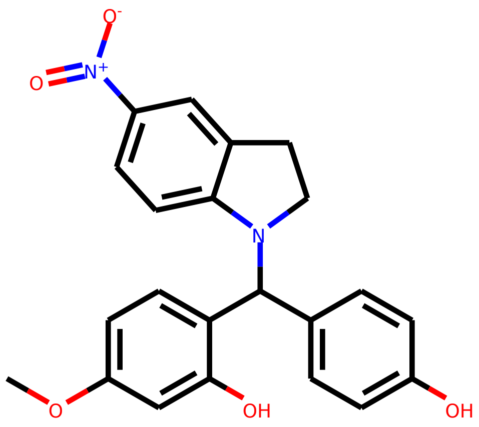
Ahma-2000
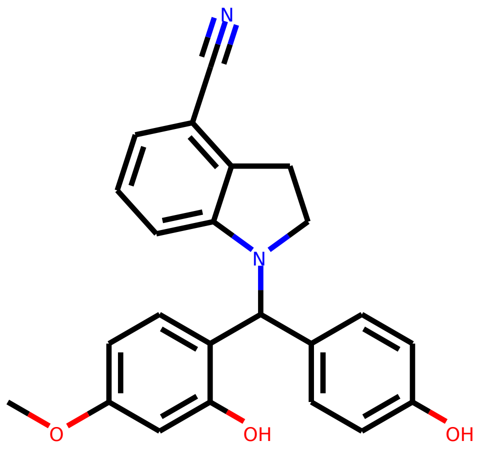
Ahma-2001
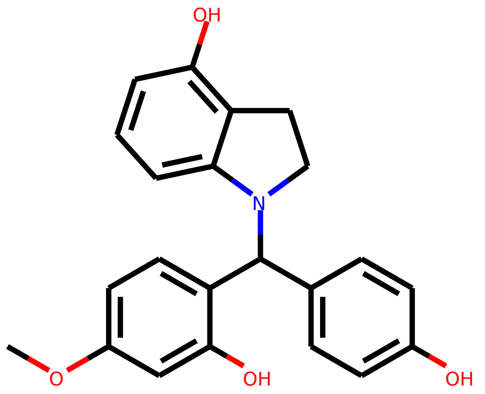
Ahma-2002
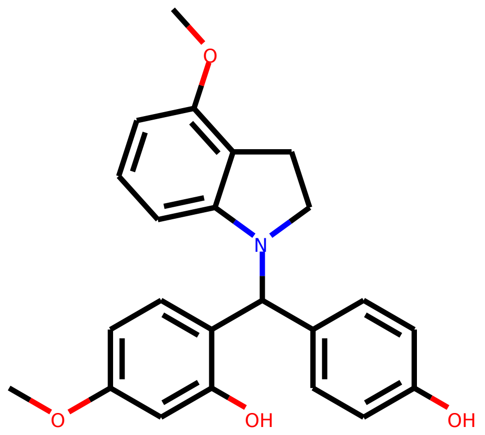
Ahma-2003
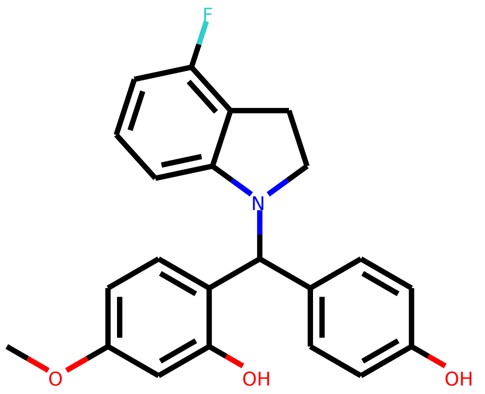
Ahma-2004
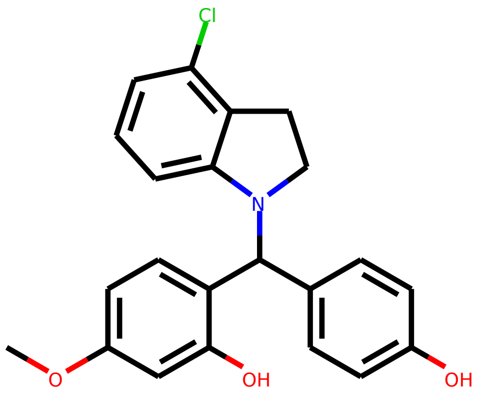
Ahma-2005
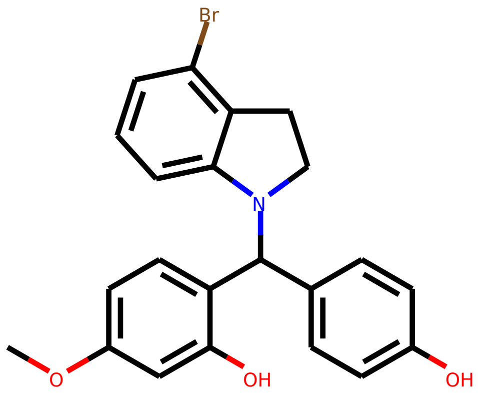
Ahma-2006
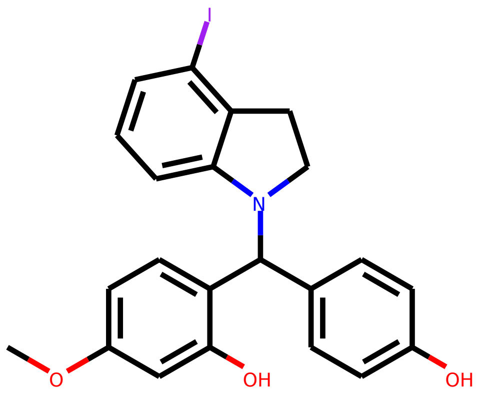
Ahma-2007
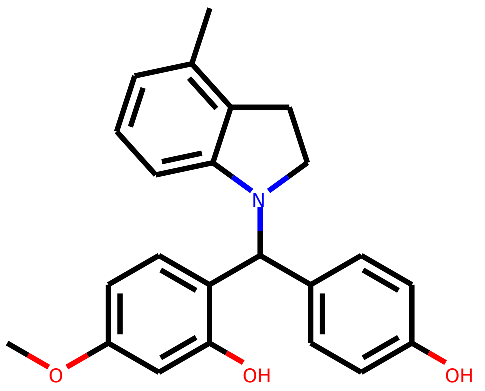
Ahma-2008
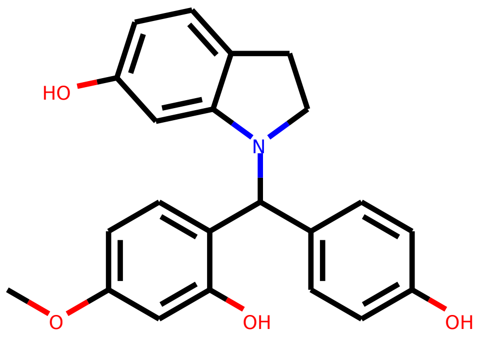
Ahma-2009
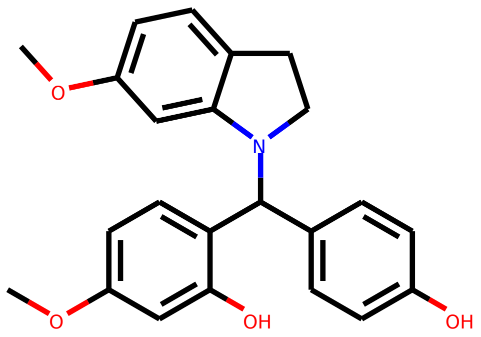
Ahma-2010
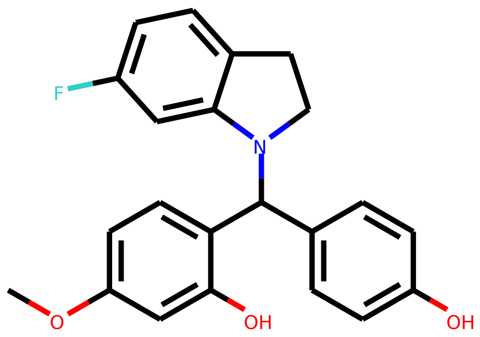
Ahma-2011
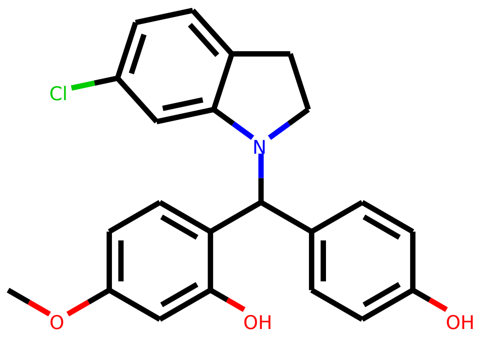
Ahma-2012
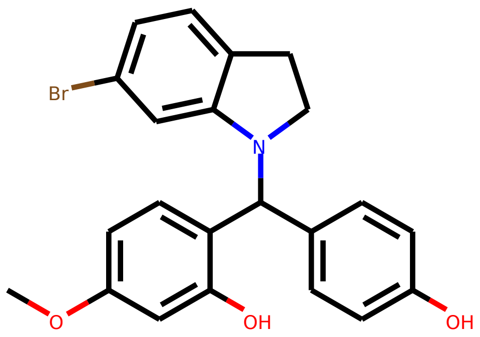
Ahma-2013
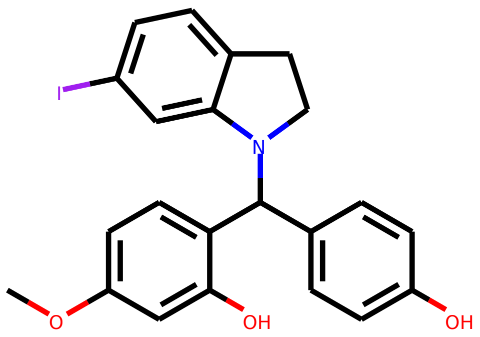
Ahma-2014
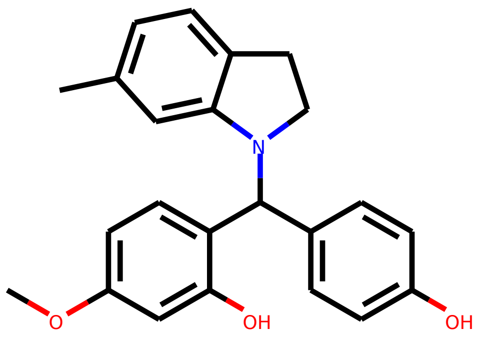
Ahma-2015
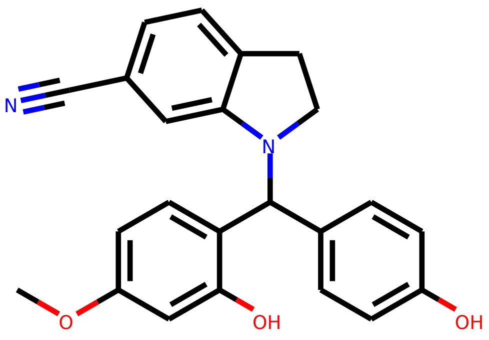
Ahma-2016
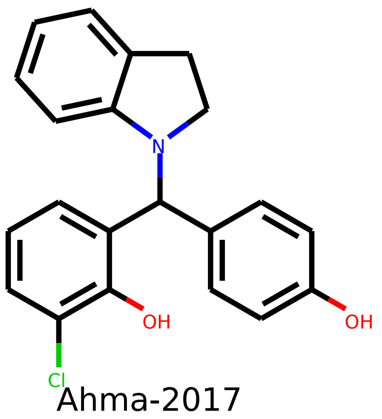
Ahma-2017
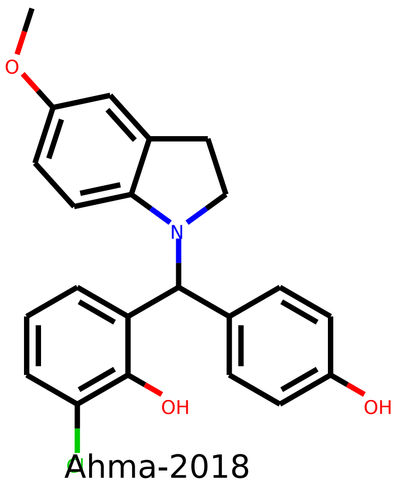
Ahma-2018
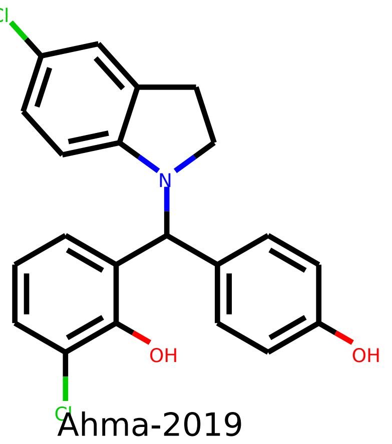
Ahma-2019
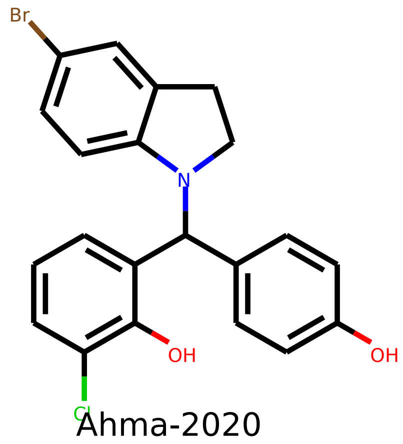
Ahma-2020
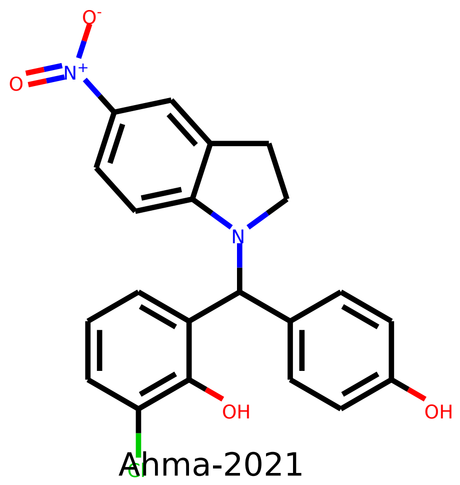
Ahma-2021
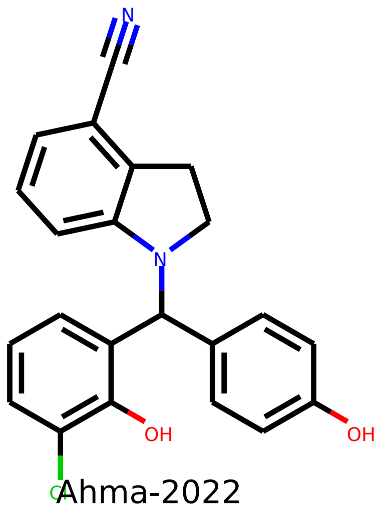
Ahma-2022
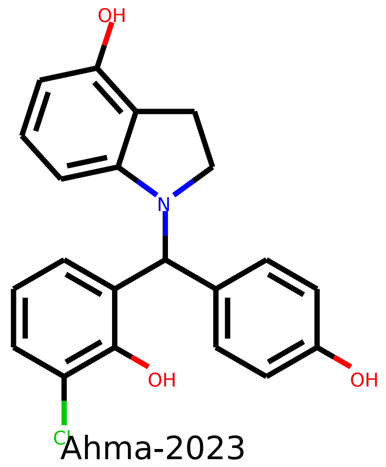
Ahma-2023
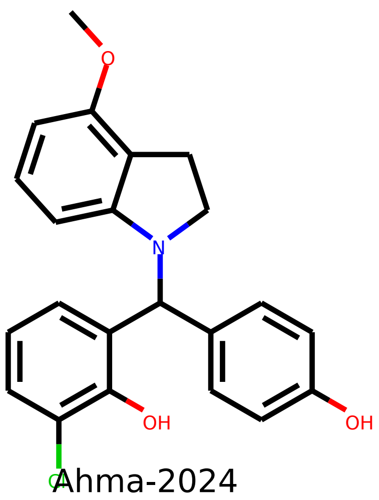
Ahma-2024
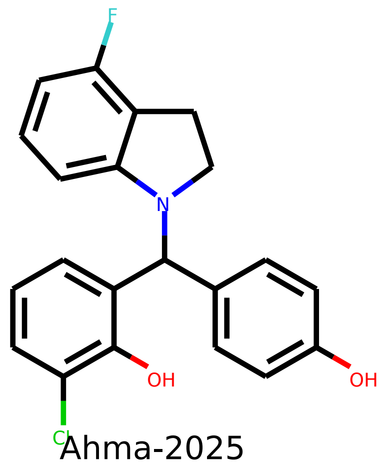
Ahma-2025
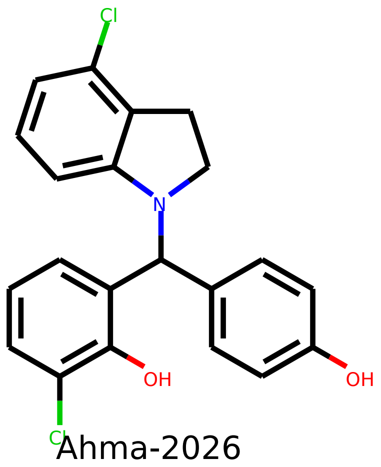
Ahma-2026
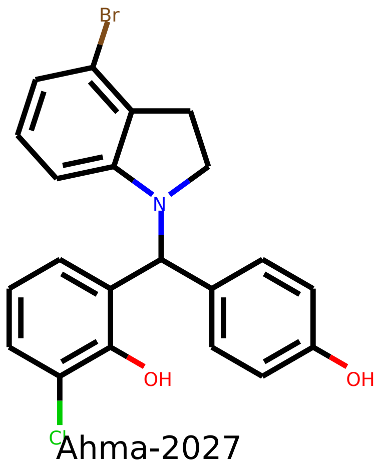
Ahma-2027
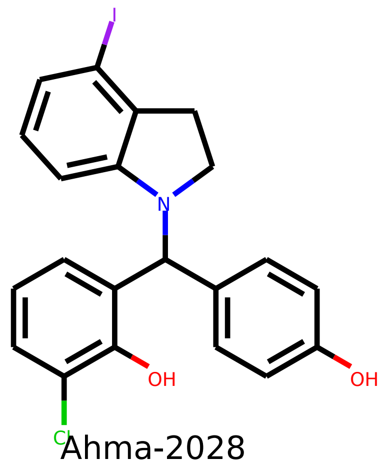
Ahma-2028
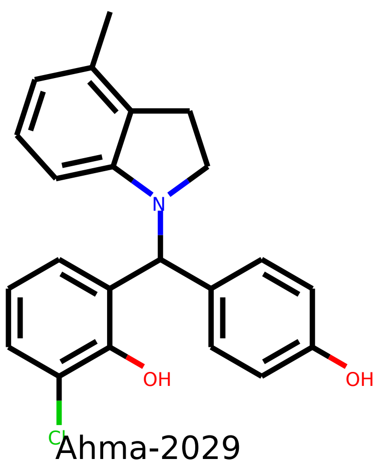
Ahma-2029
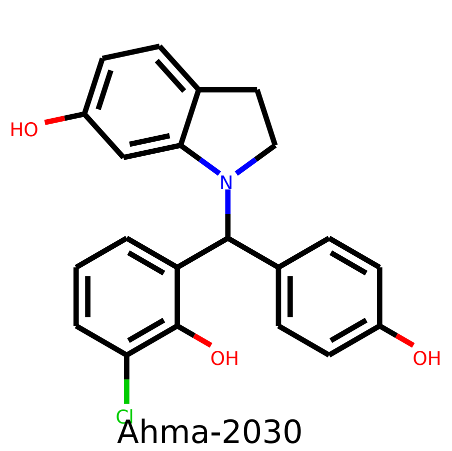
Ahma-2030

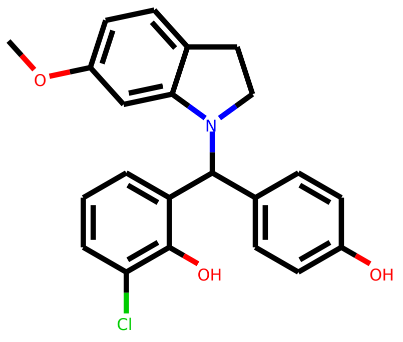
Ahma-2031

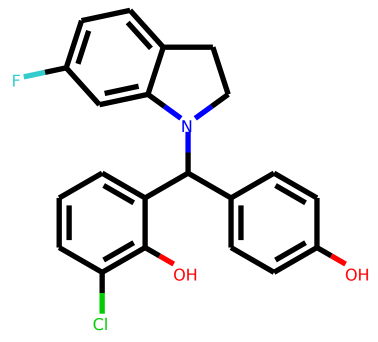
Ahma-2032

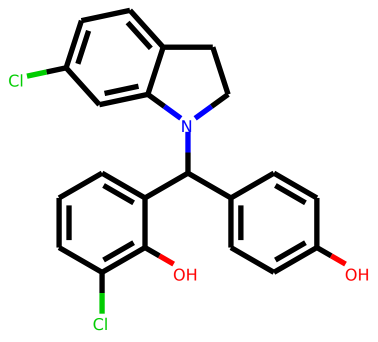
Ahma-2033

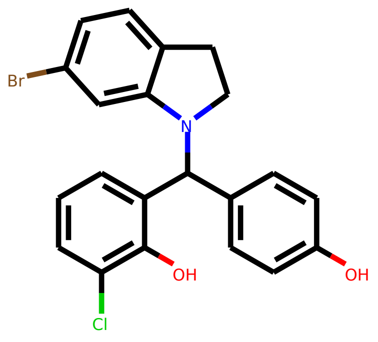
Ahma-2034

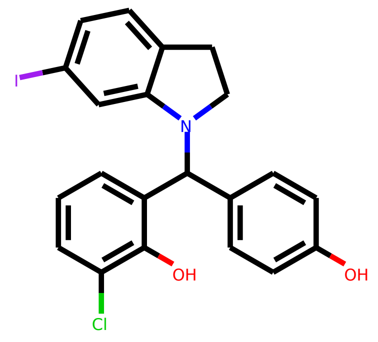
Ahma-2035

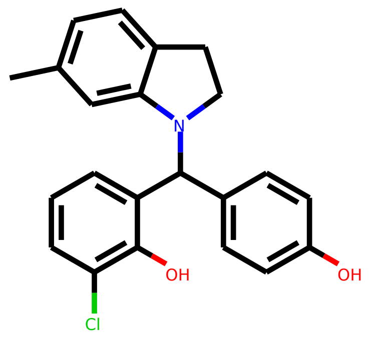
Ahma-2036

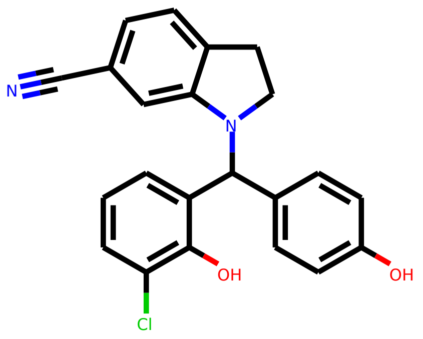
Ahma-2037

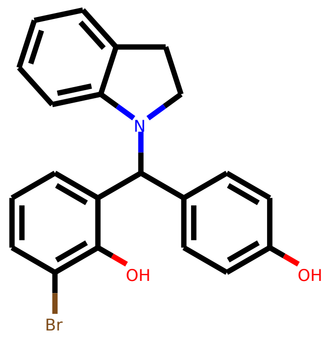
Ahma-2038

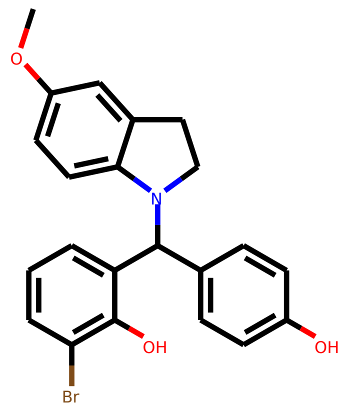
Ahma-2039

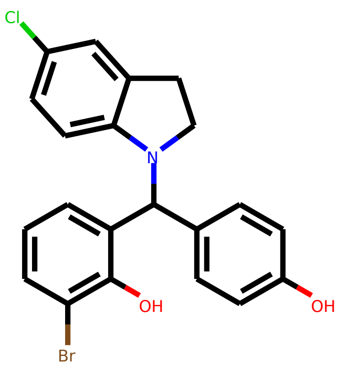
Ahma-2040

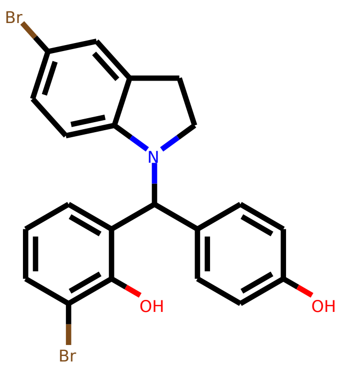
Ahma-2041

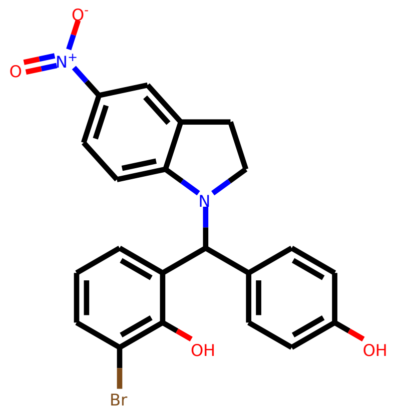
Ahma-2042

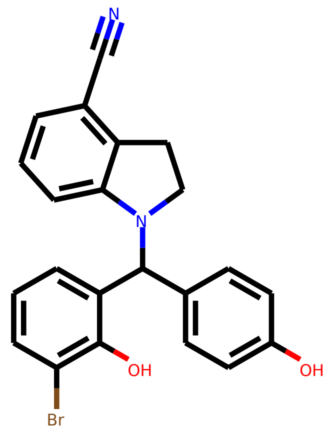
Ahma-2043

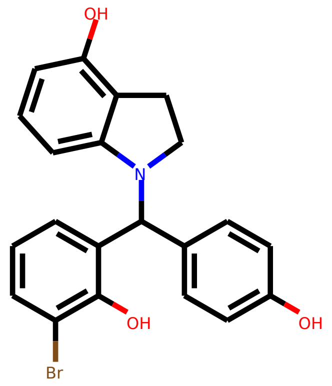
Ahma-2044

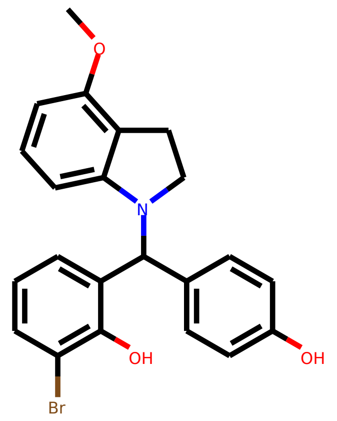
Ahma-2045

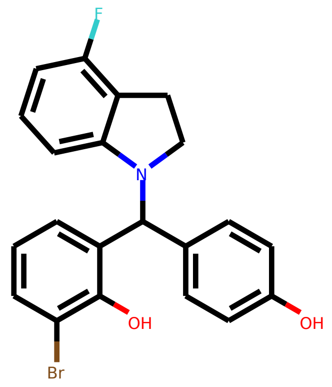
Ahma-2046

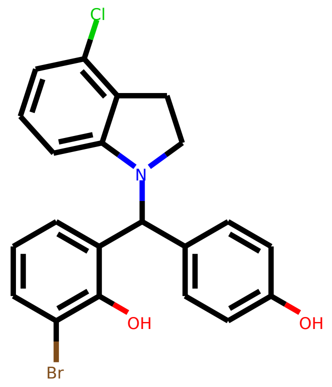
Ahma-2047

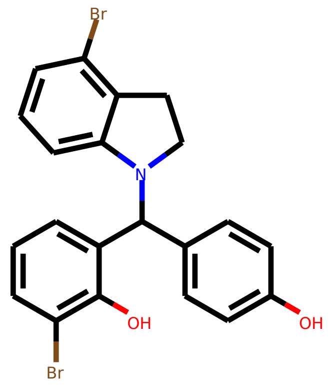
Ahma-2048

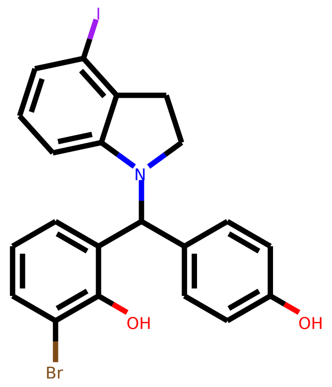
Ahma-2049

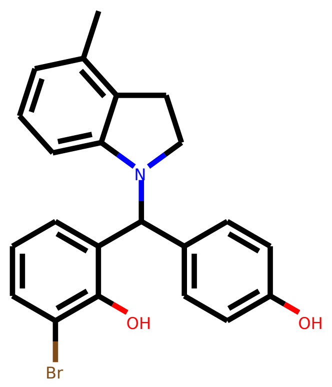
Ahma-2050

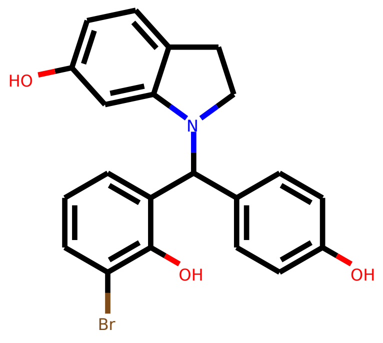
Ahma-2051

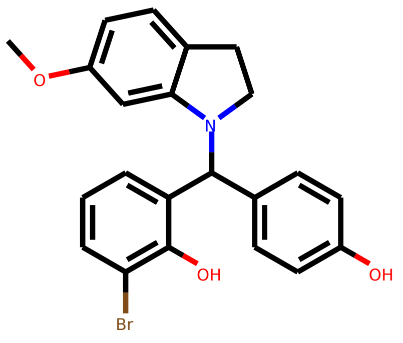
Ahma-2052

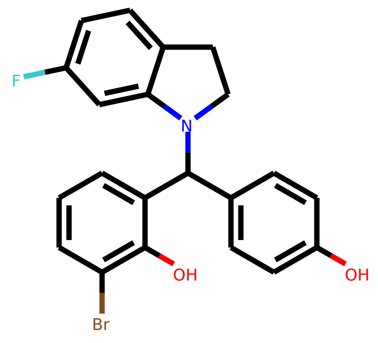
Ahma-2053

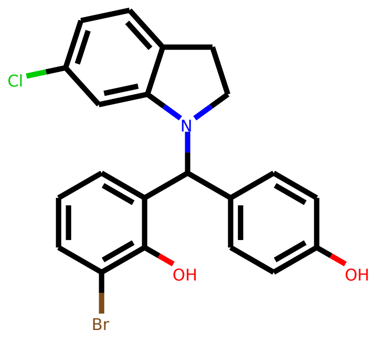
Ahma-2054

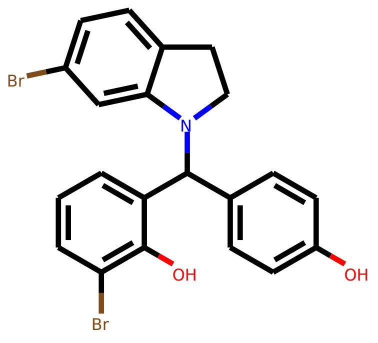
Ahma-2055

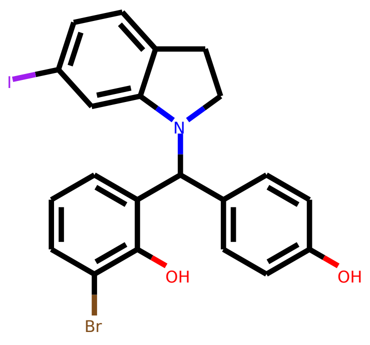
Ahma-2056

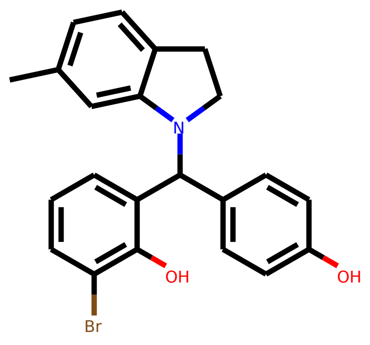
Ahma-2057

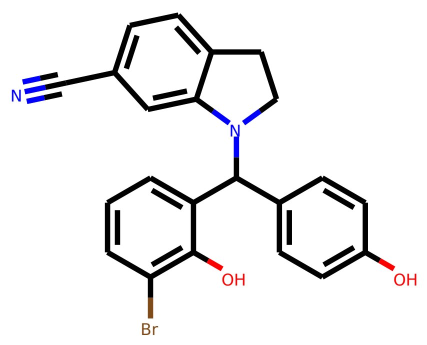
Ahma-2058

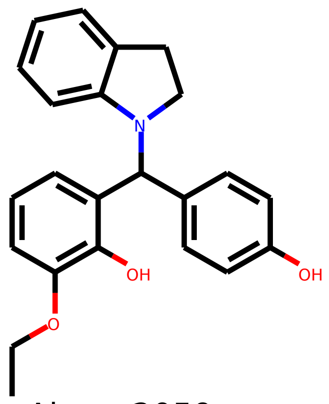
Ahma-2059

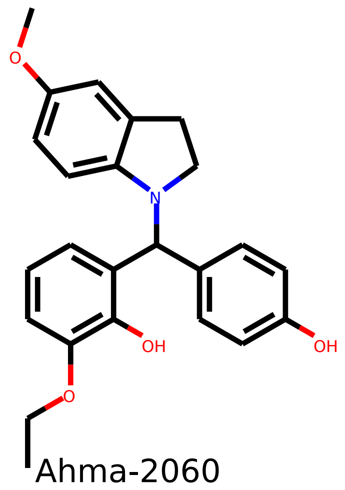
Ahma-2060

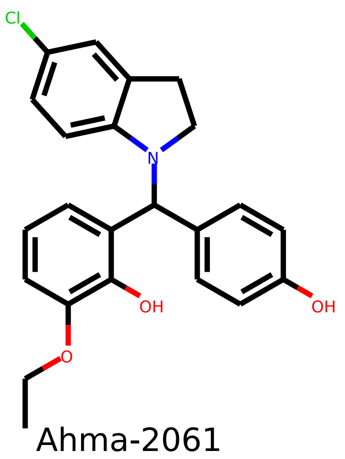
Ahma-2061

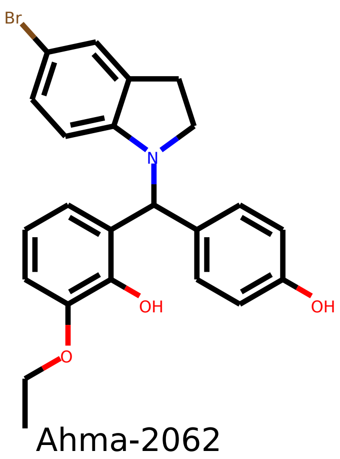
Ahma-2062

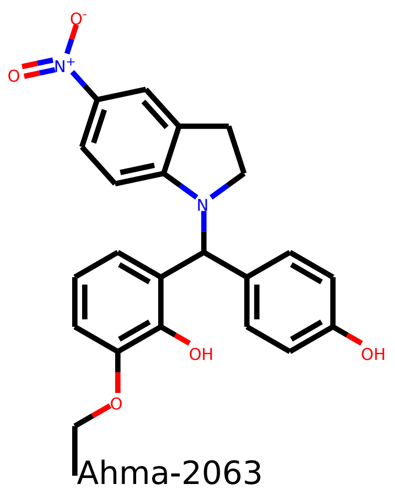
Ahma-2063

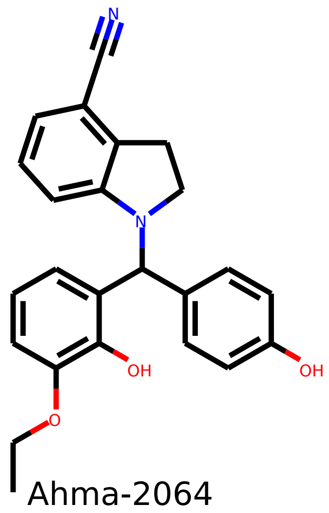
Ahma-2064

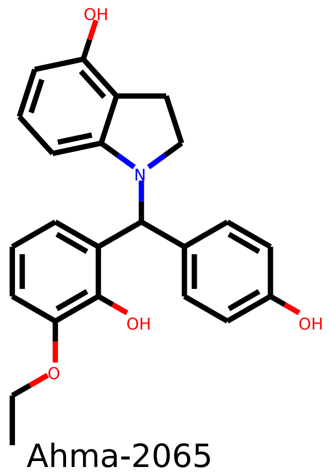
Ahma-2065

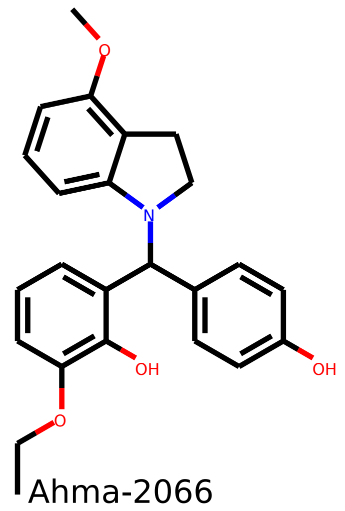
Ahma-2066
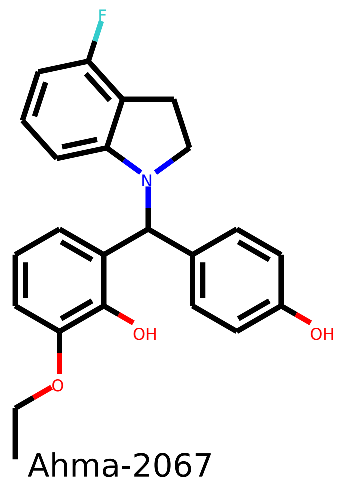
Ahma-2067
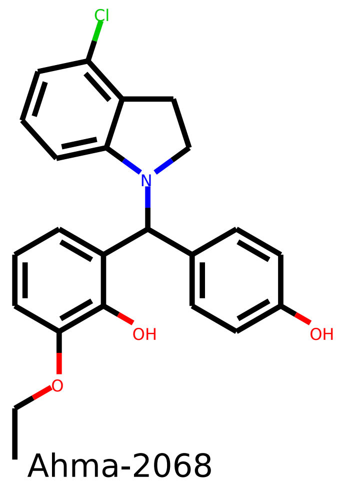
Ahma-2068
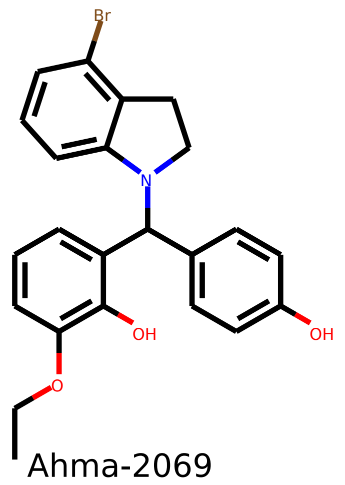
Ahma-2069
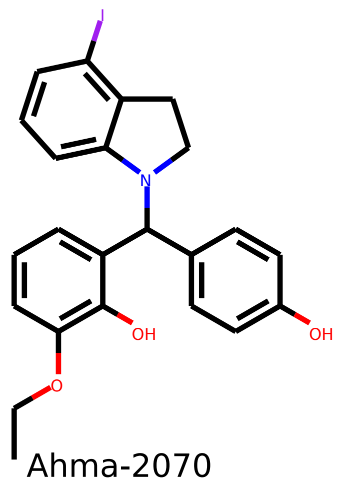
Ahma-2070
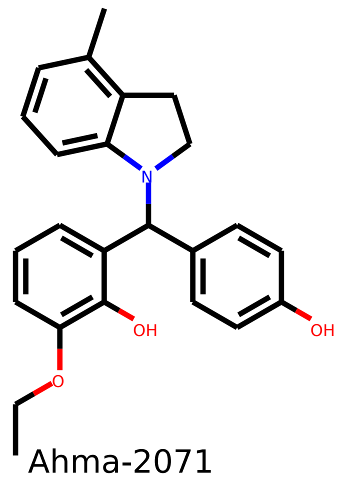
Ahma-2071
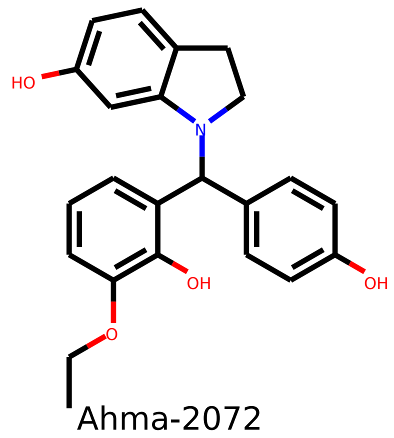
Ahma-2072
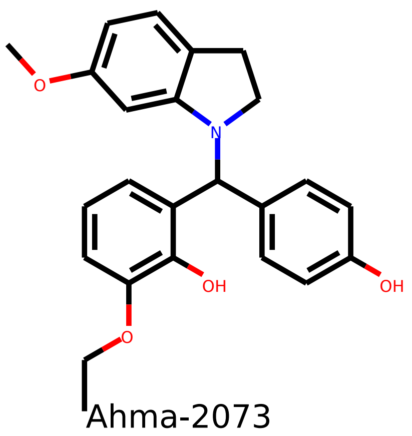
Ahma-2073
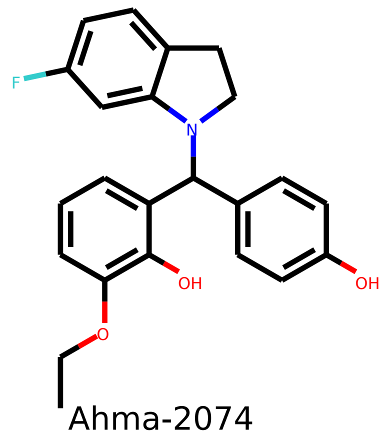
Ahma-2074
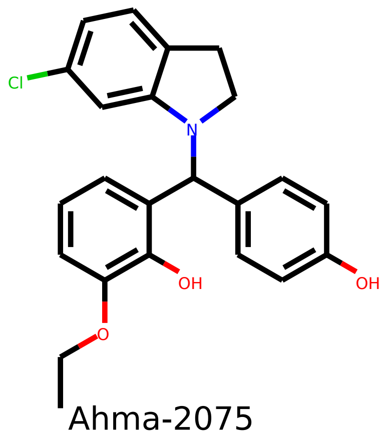
Ahma-2075
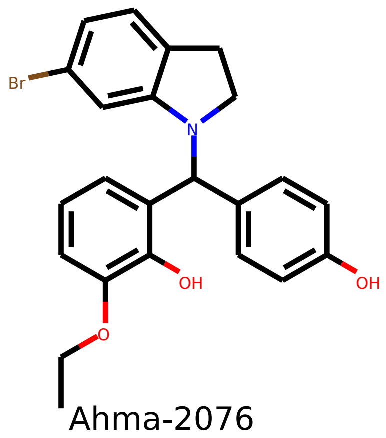
Ahma-2076
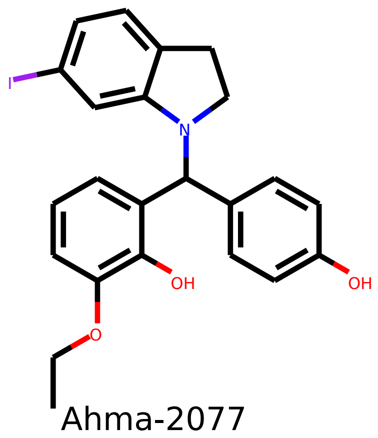
Ahma-2077
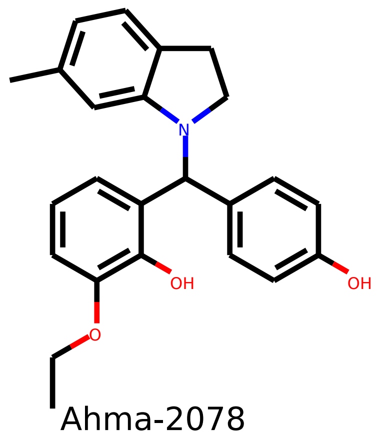
Ahma-2078
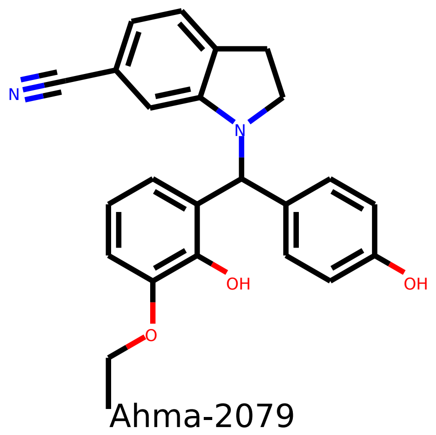
Ahma-2079
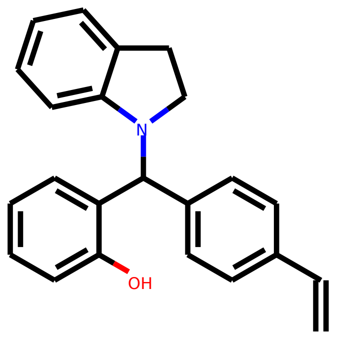
Ahma-2080
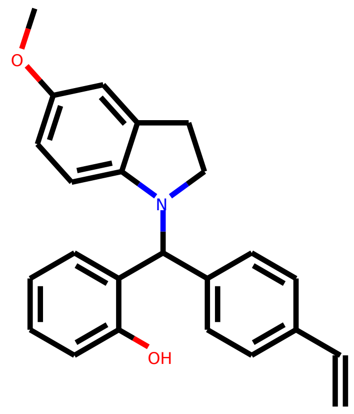
Ahma-2081
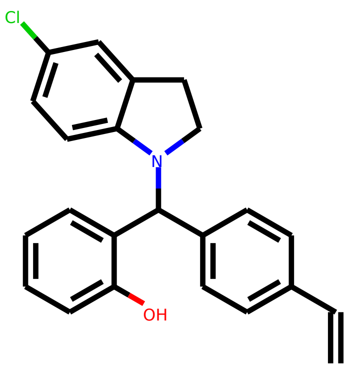
Ahma-2082
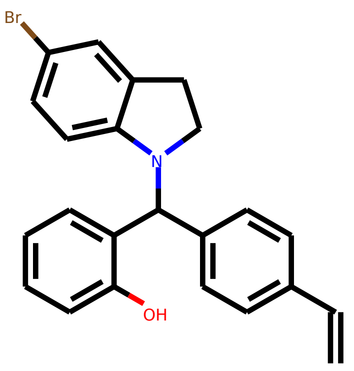
Ahma-2083
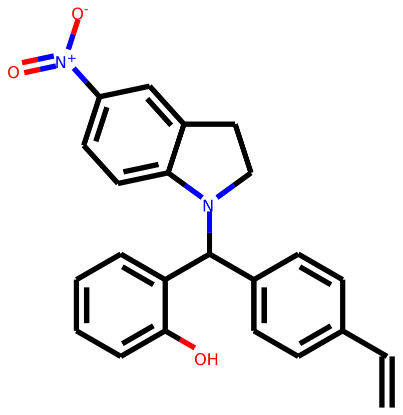
Ahma-2084
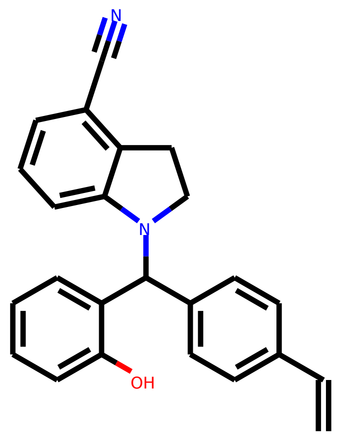
Ahma-2085
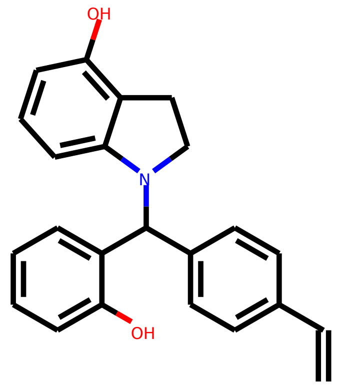
Ahma-2086
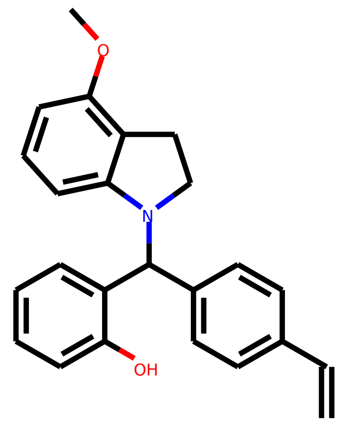
Ahma-2087
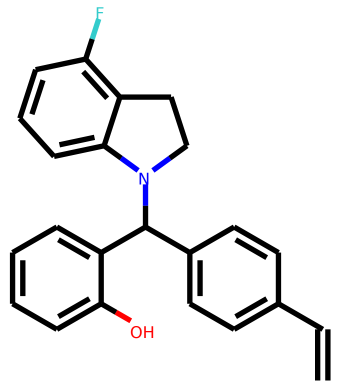
Ahma-2088
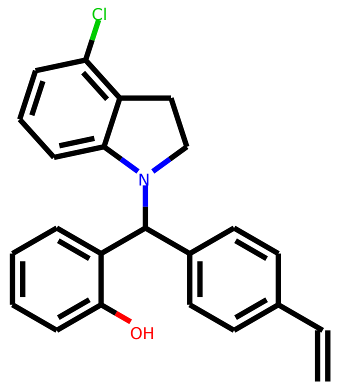
Ahma-2089
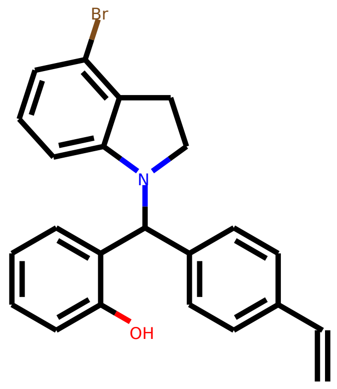
Ahma-2090
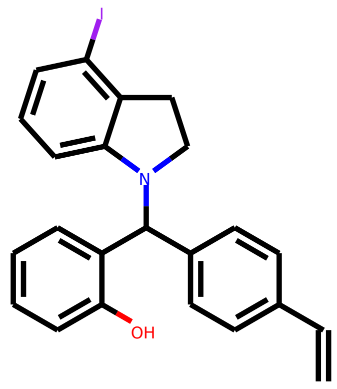
Ahma-2091
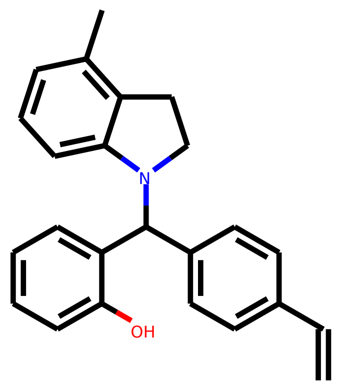
Ahma-2092
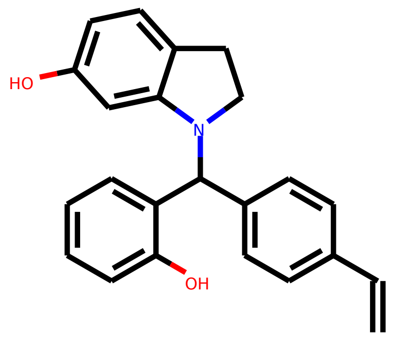
Ahma-2093
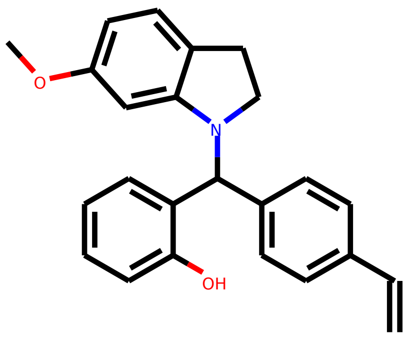
Ahma-2094
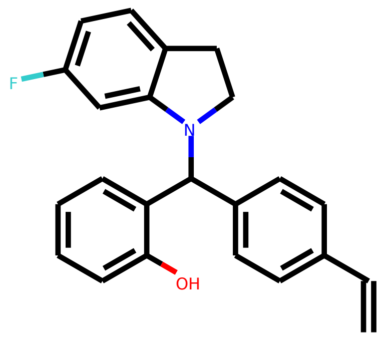
Ahma-2095
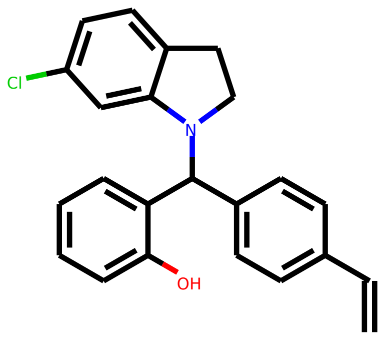
Ahma-2096
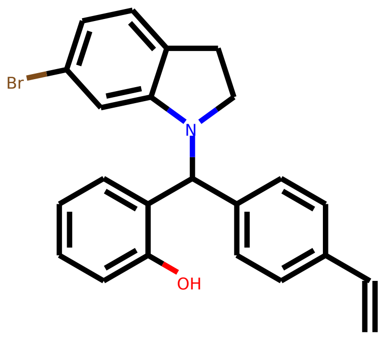
Ahma-2097
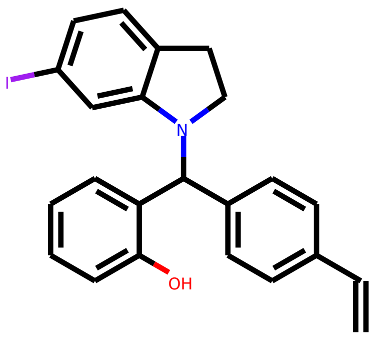
Ahma-2098
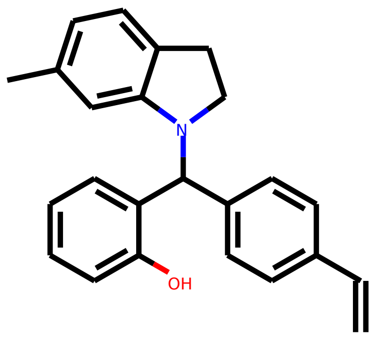
Ahma-2099
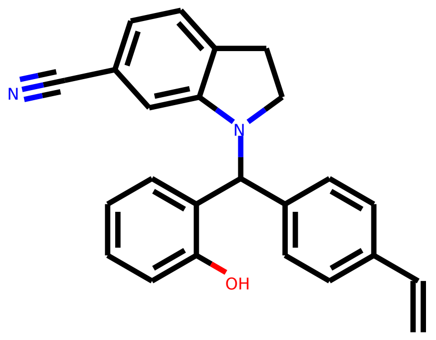
Ahma-2100

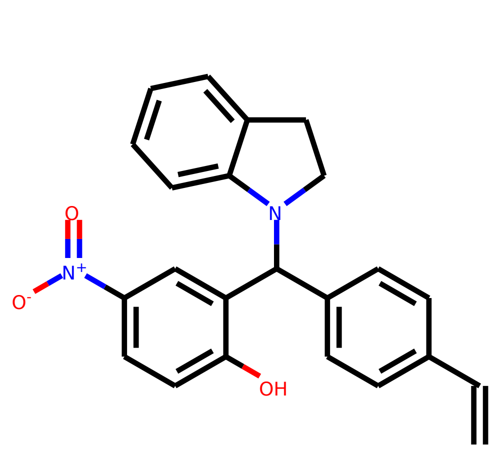
Ahma-2101
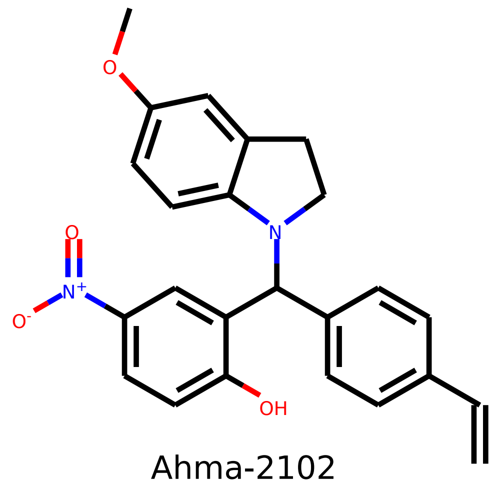
Ahma-2102
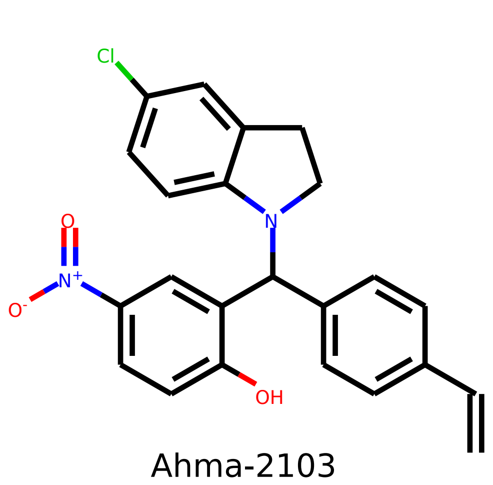
Ahma-2103
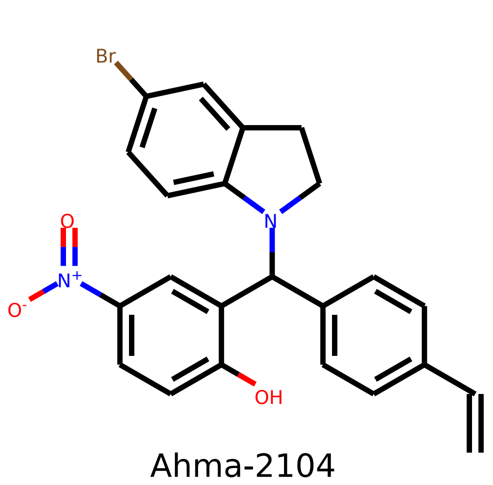
Ahma-2104
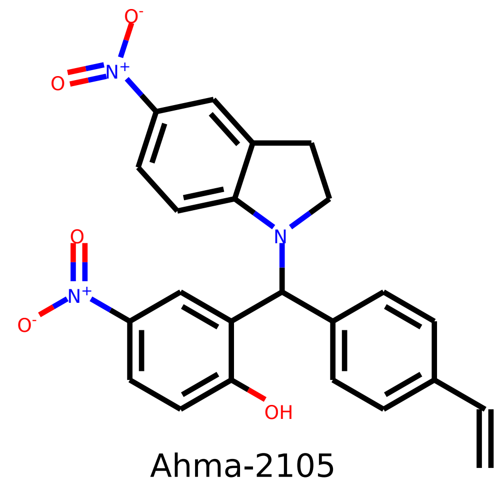
Ahma-2105
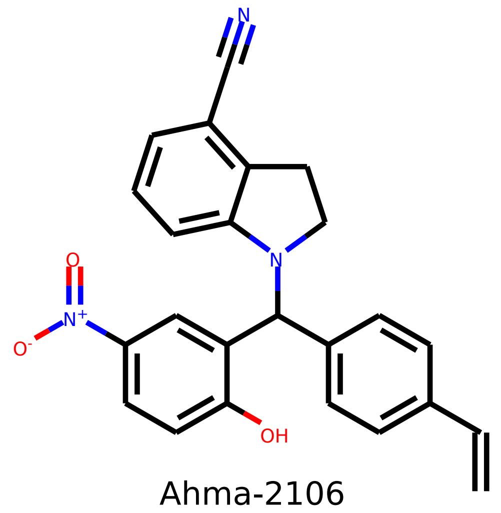
Ahma-2106
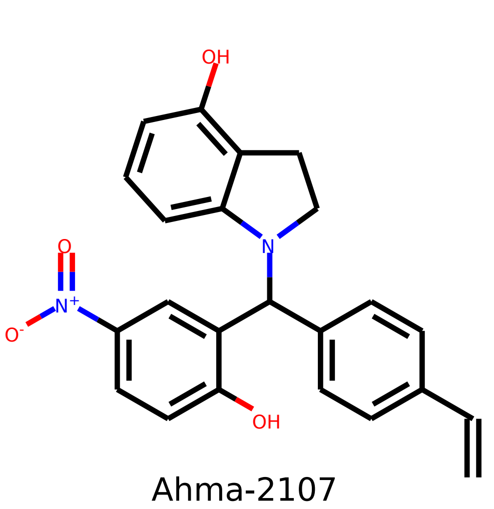
Ahma-2107
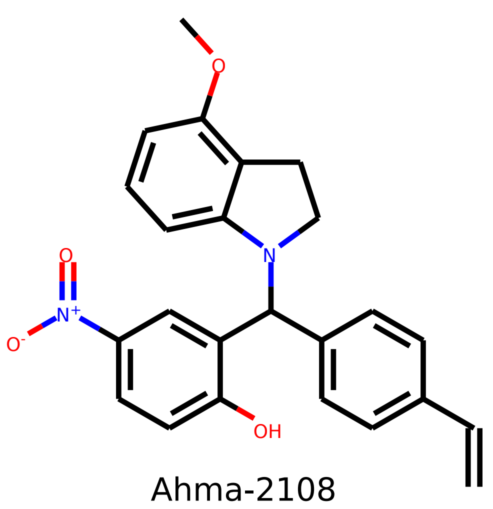
Ahma-2108
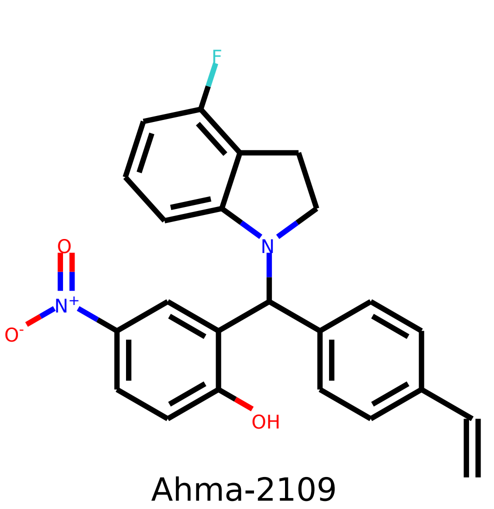
Ahma-2109
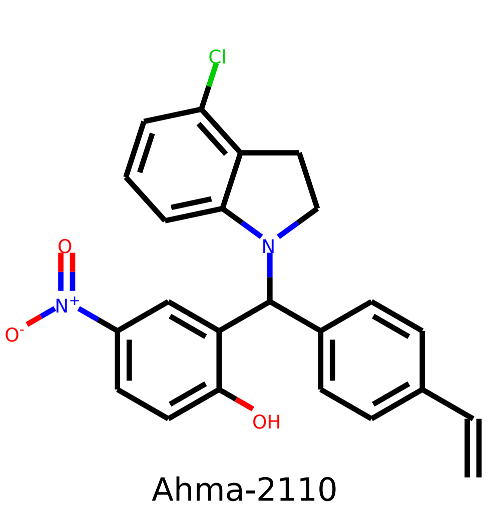
Ahma-2110
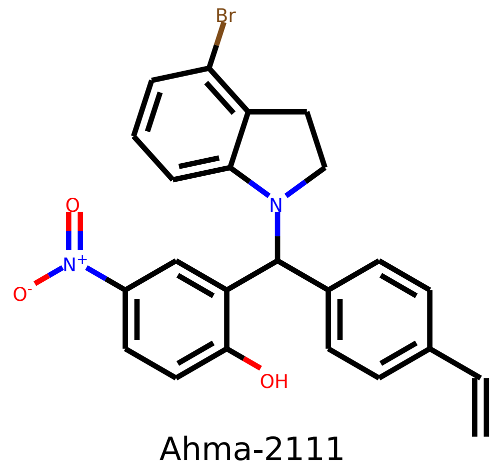
Ahma-2111
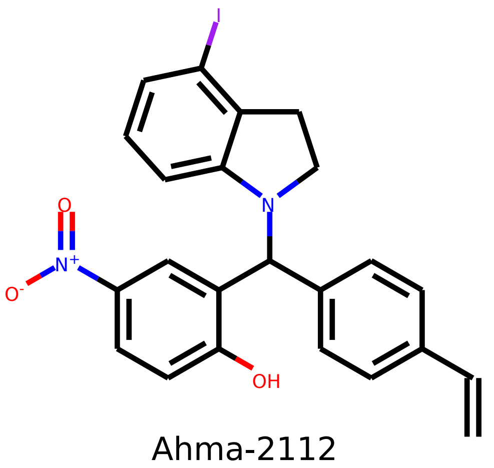
Ahma-2112
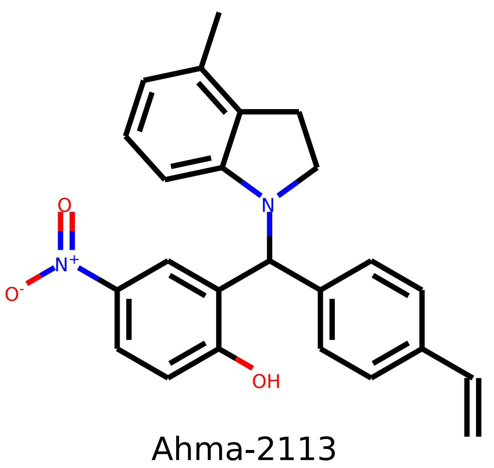
Ahma-2113
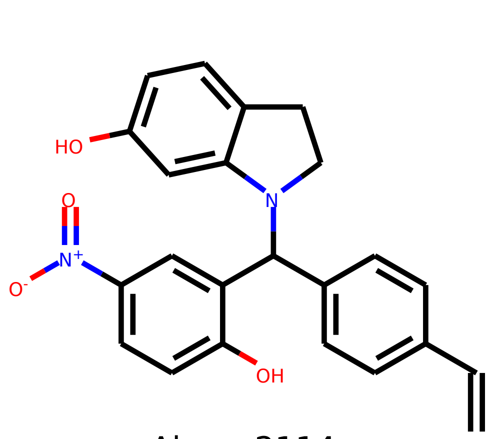
Ahma-2114
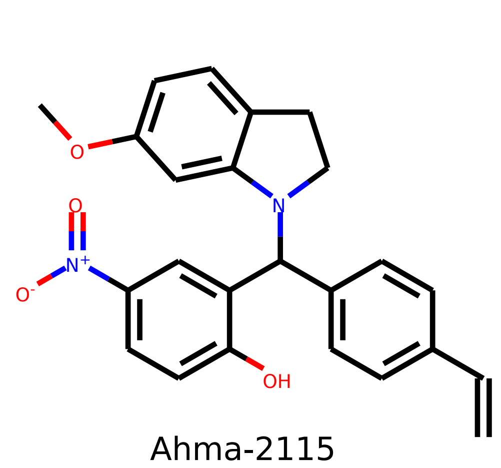
Ahma-2115
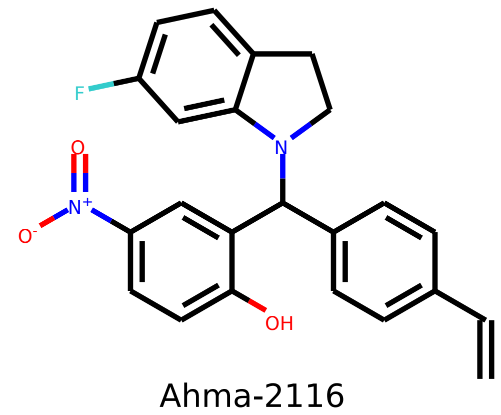
Ahma-2116
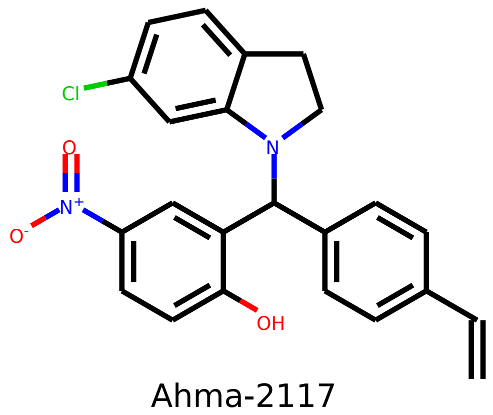
Ahma-2117
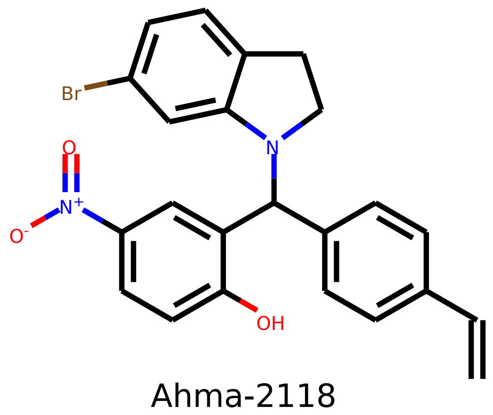
Ahma-2118
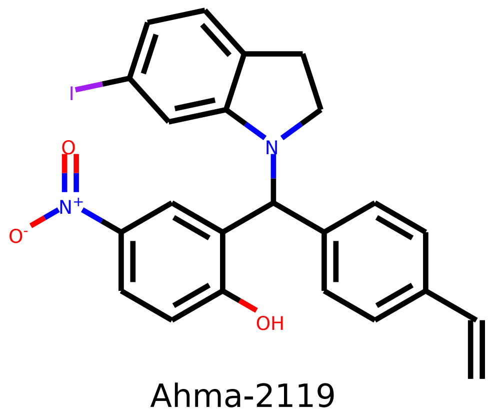
Ahma-2119
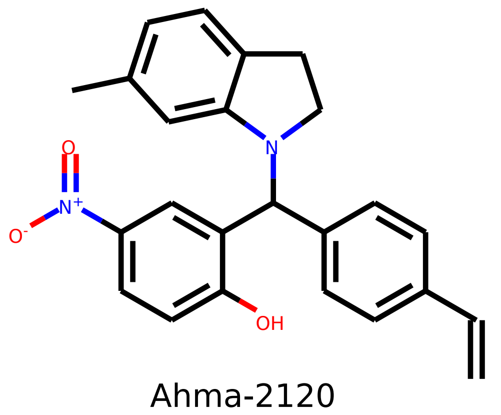
Ahma-2120
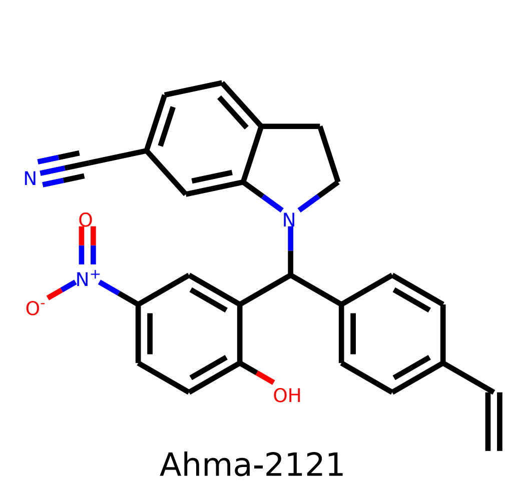
Ahma-2121
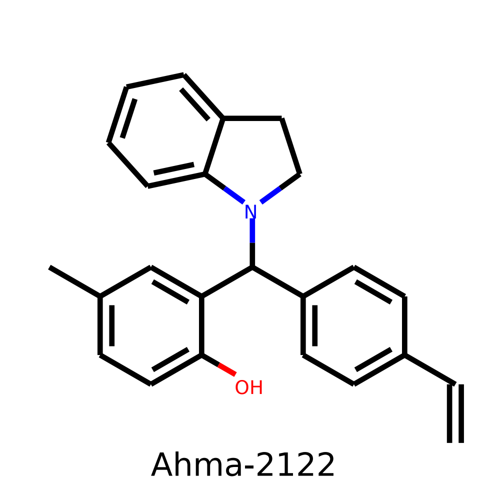
Ahma-2122
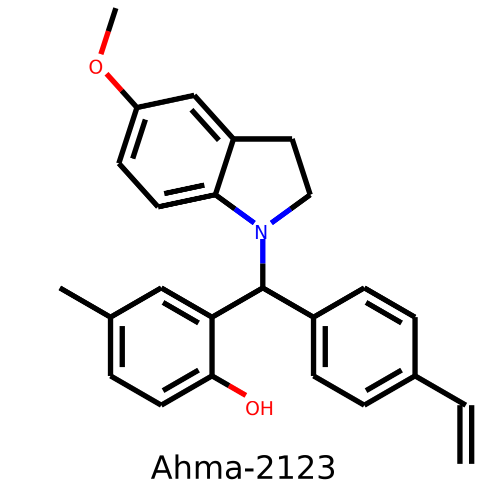
Ahma-2123
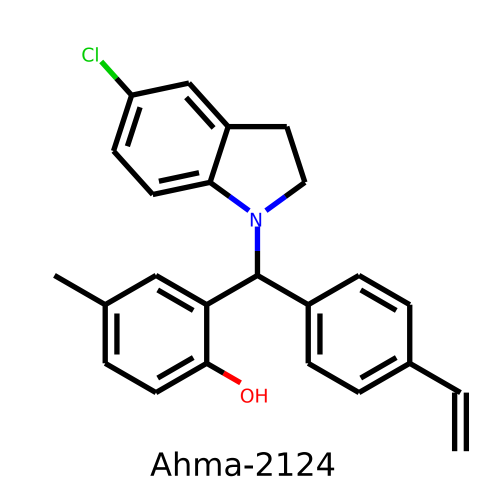
Ahma-2124
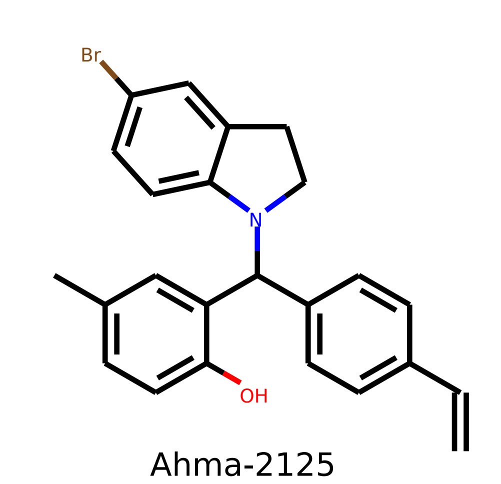
Ahma-2125
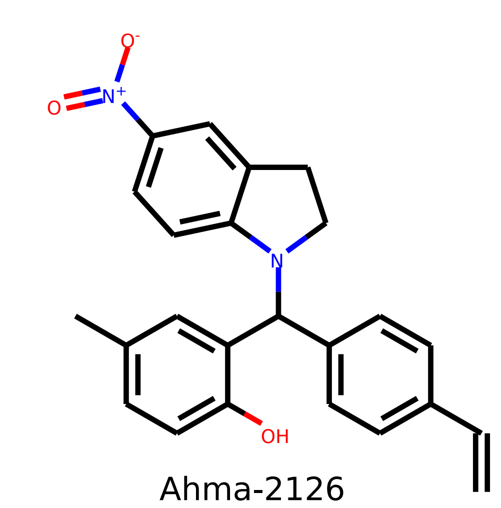
Ahma-2126
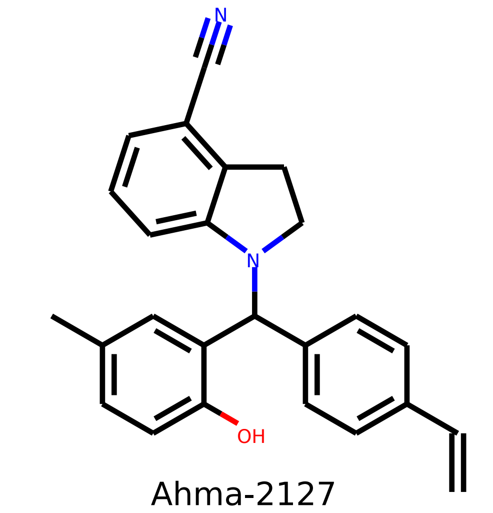
Ahma-2127
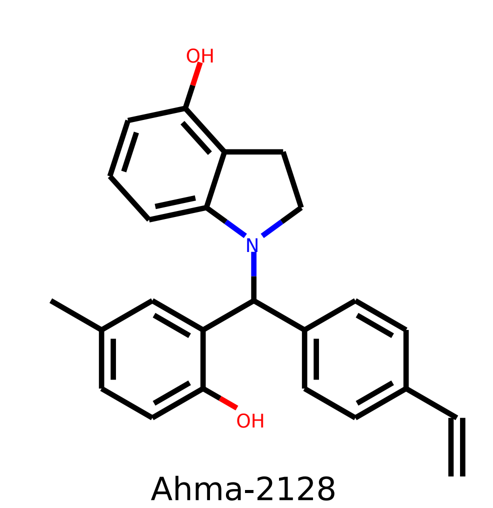
Ahma-2128
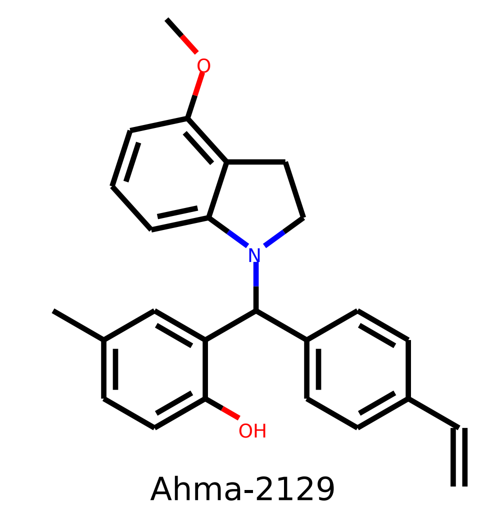
Ahma-2129
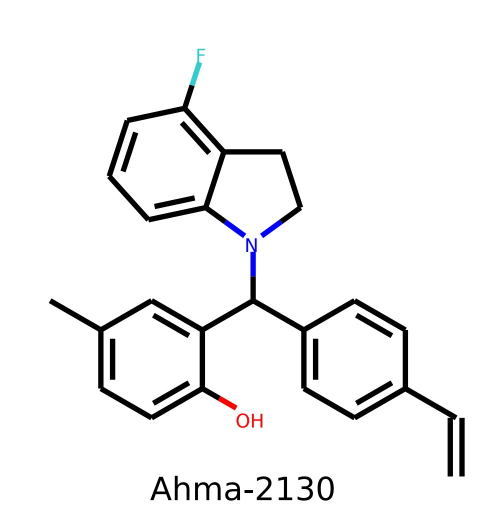
Ahma-2130
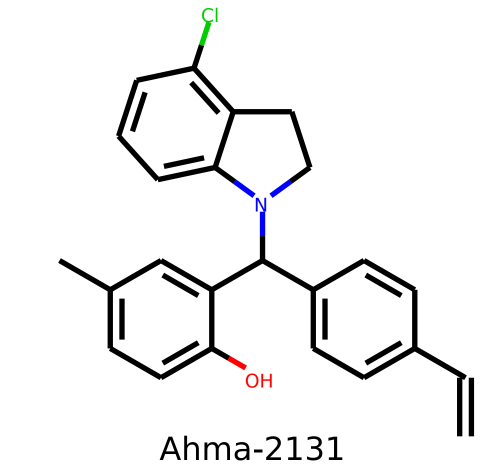
Ahma-2131
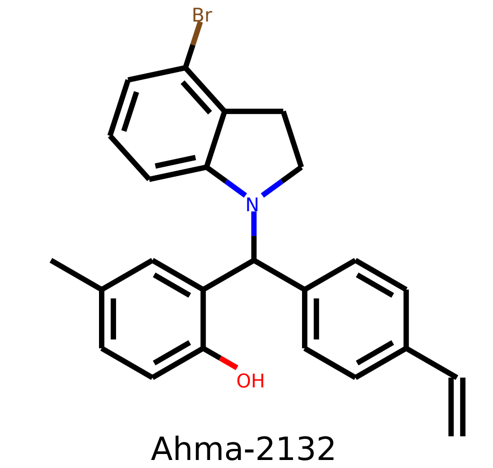
Ahma-2132
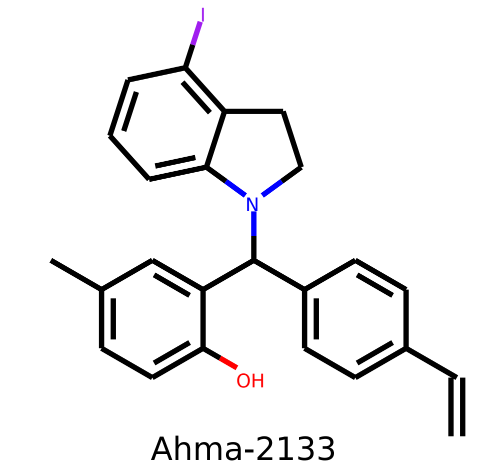
Ahma-2133
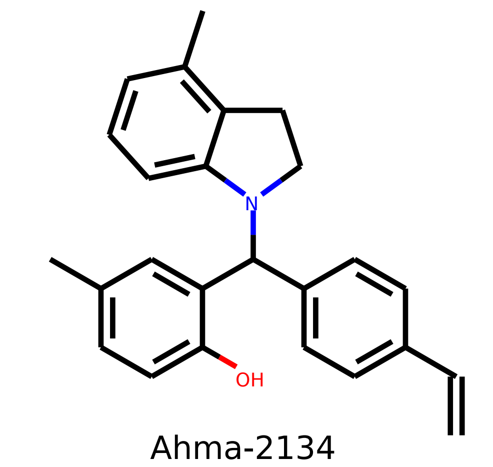
Ahma-2134
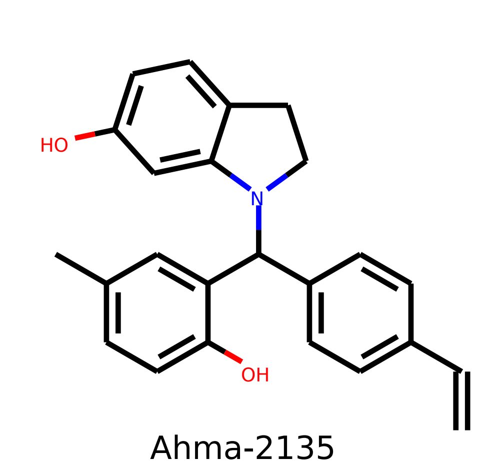
Ahma-2135

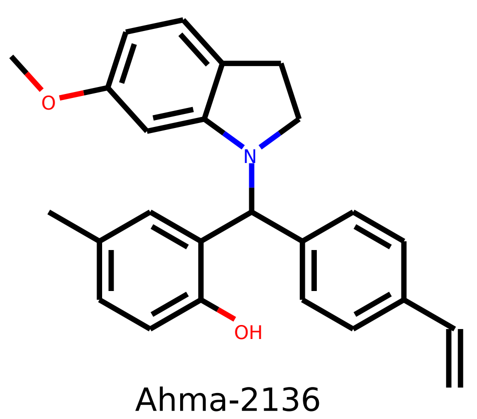
Ahma-2136
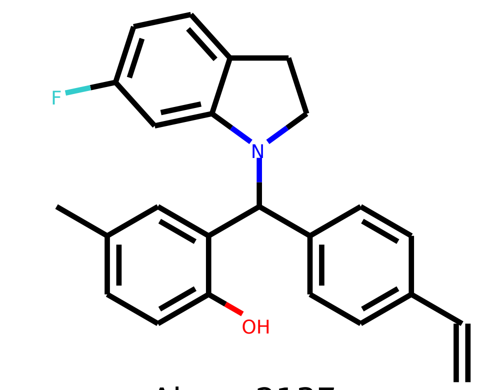
Ahma-2137
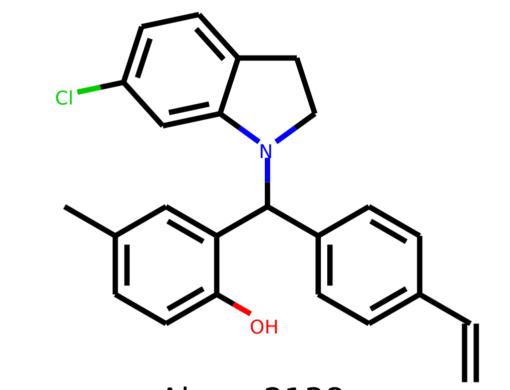
Ahma-2138
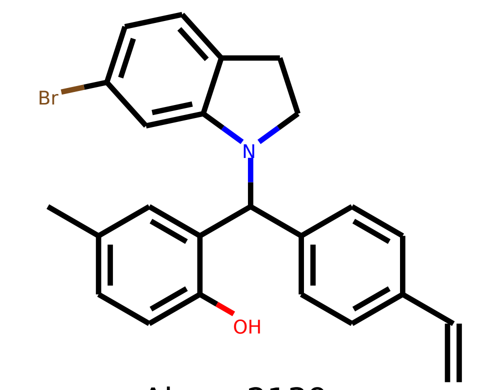
Ahma-2139
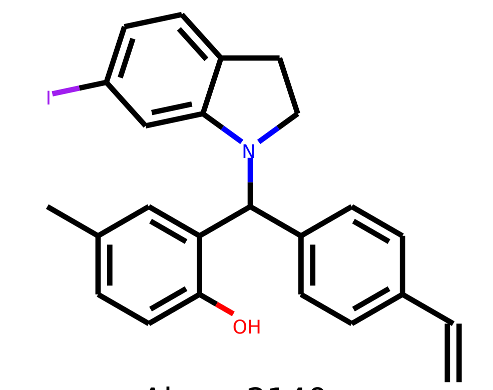
Ahma-2140
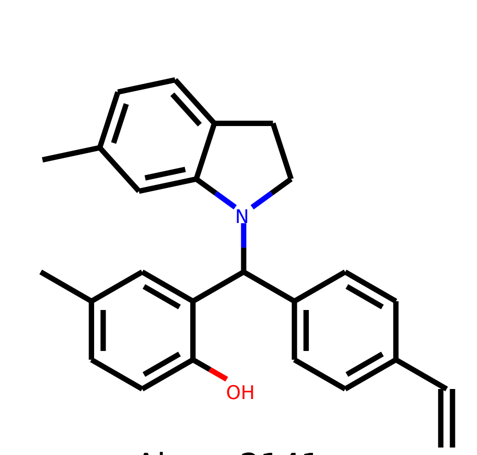
Ahma-2141
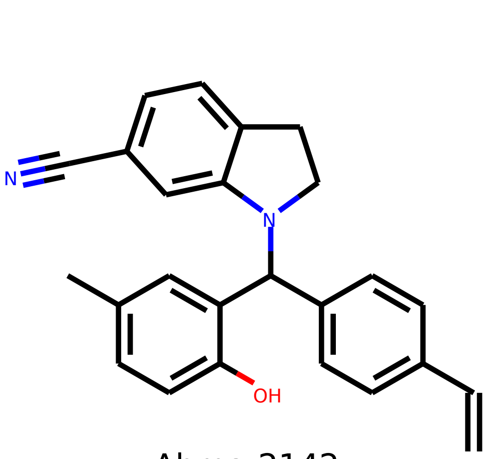
Ahma-2142
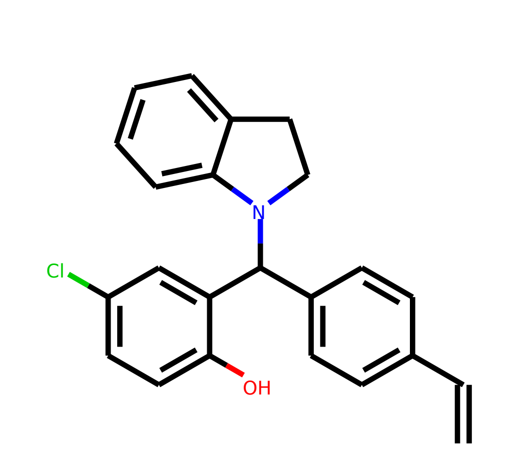
Ahma-2143
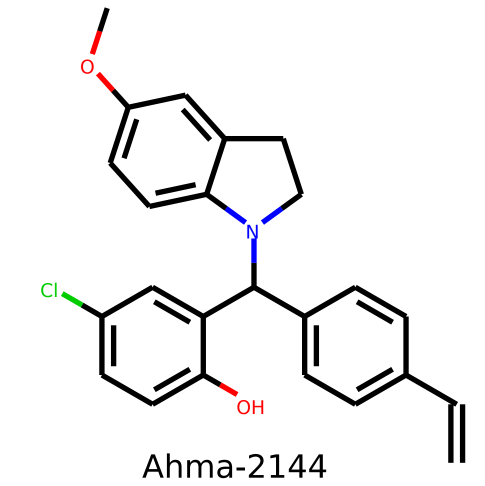
Ahma-2144
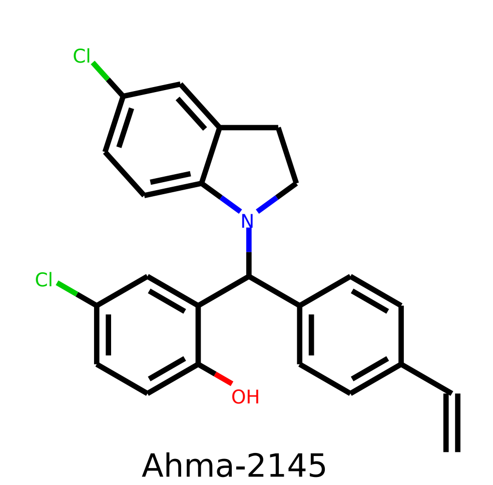
Ahma-2145
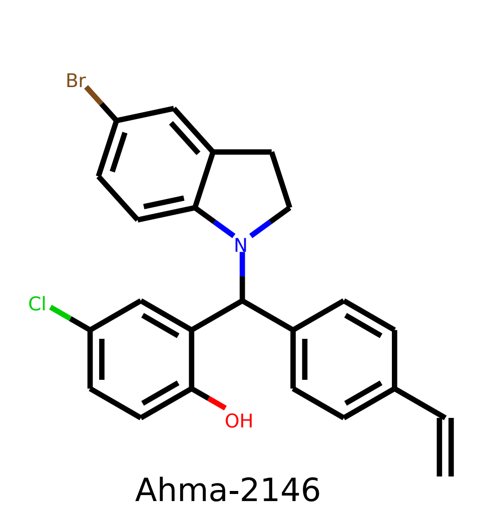
Ahma-2146
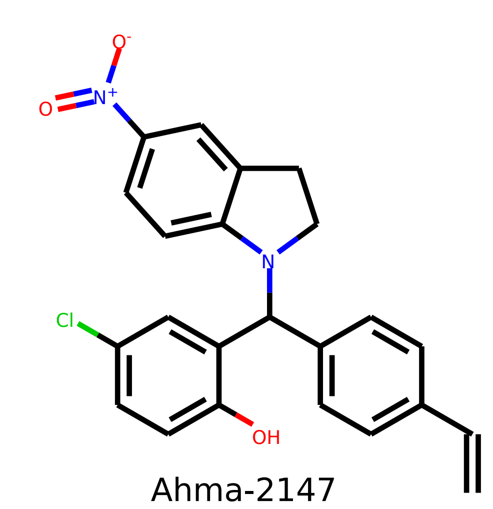
Ahma-2147
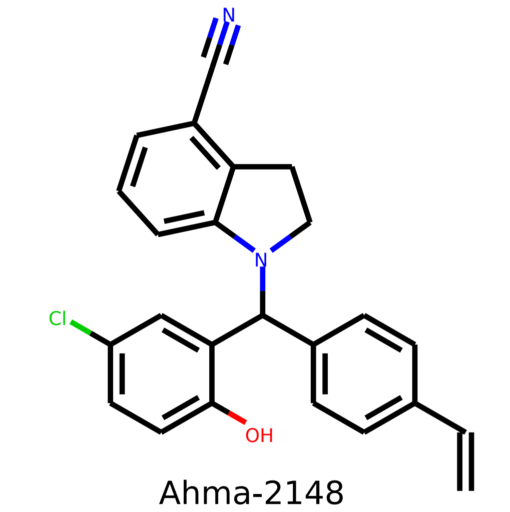
Ahma-2148
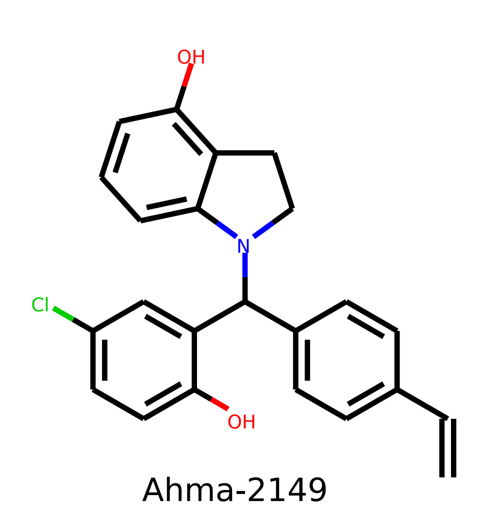
Ahma-2149
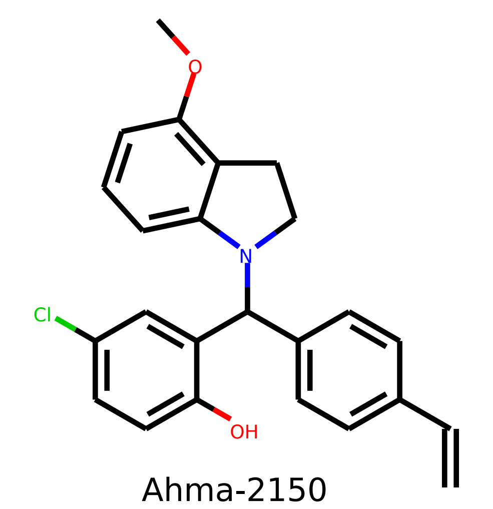
Ahma-2150
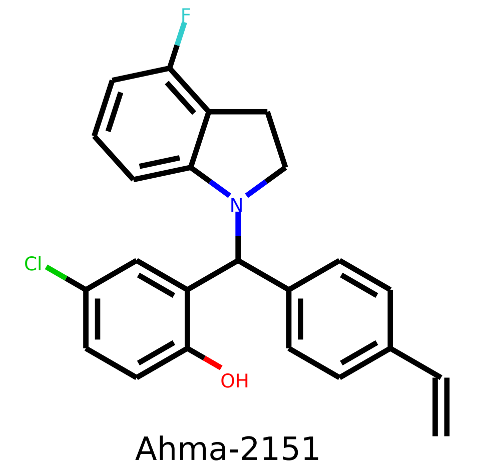
Ahma-2151
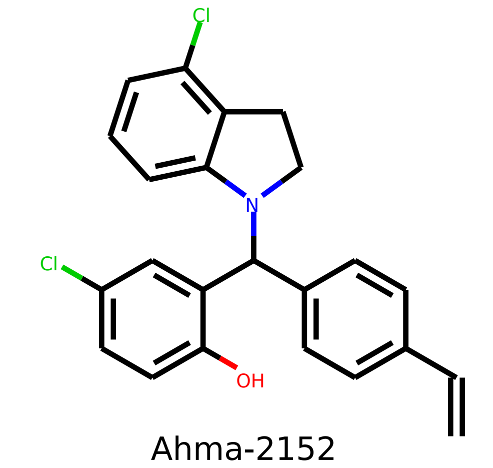
Ahma-2152
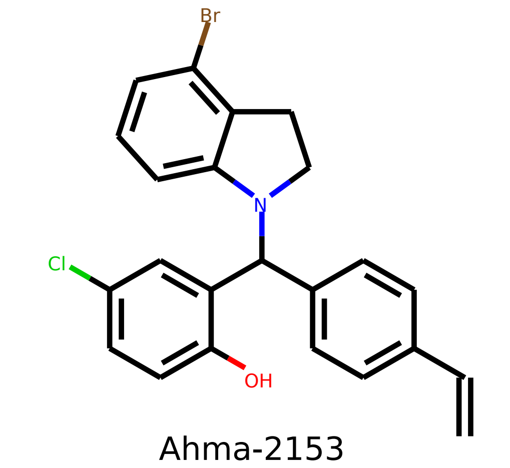
Ahma-2153
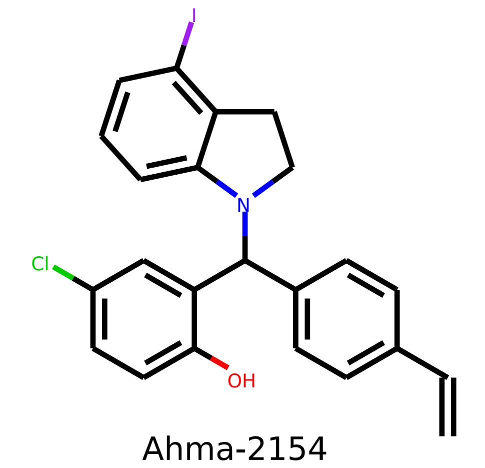
Ahma-2154
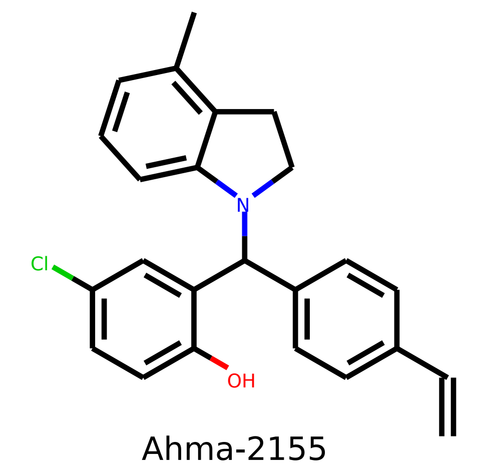
Ahma-2155
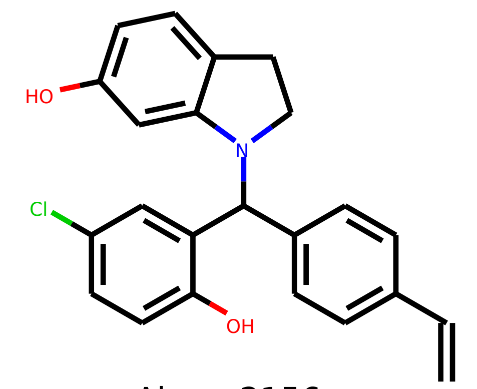
Ahma-2156
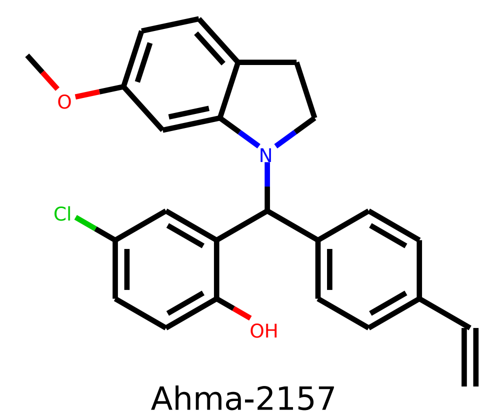
Ahma-2157
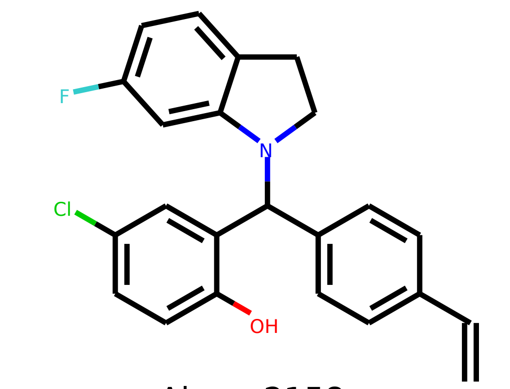
Ahma-2158
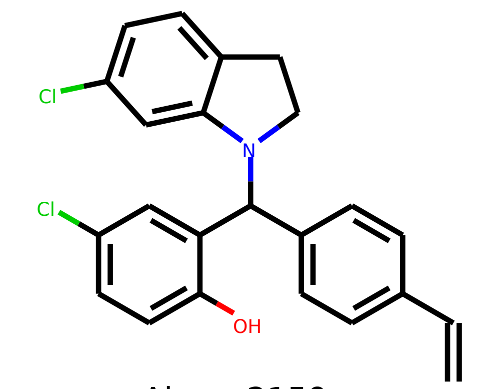
Ahma-2159
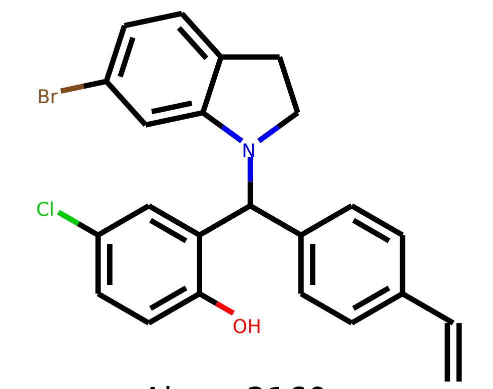
Ahma-2160
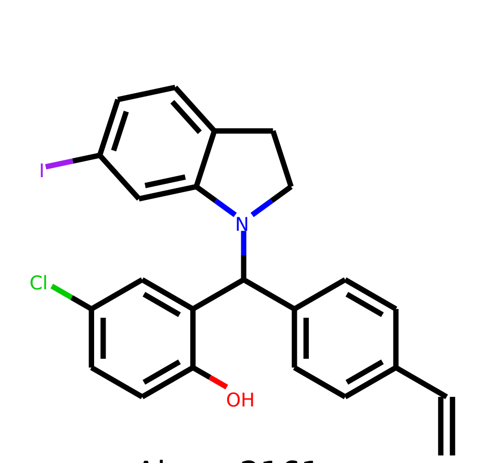
Ahma-2161
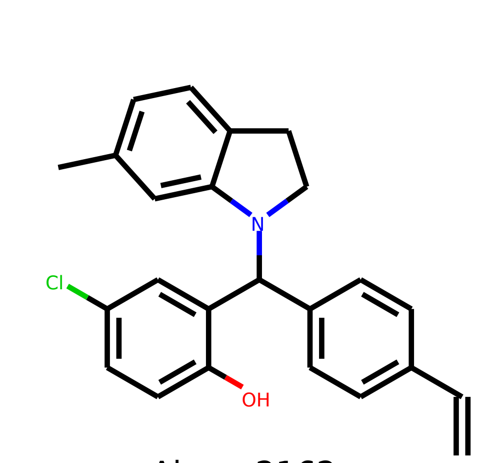
Ahma-2162
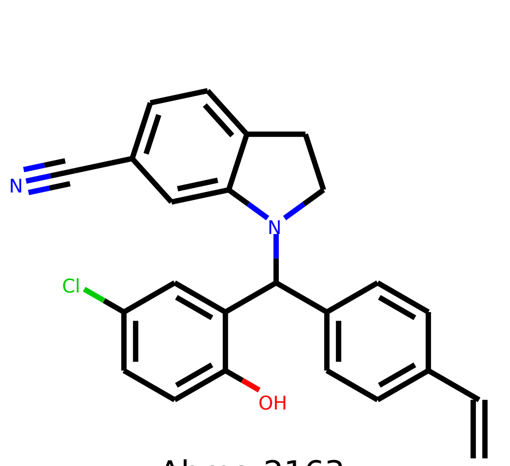
Ahma-2163
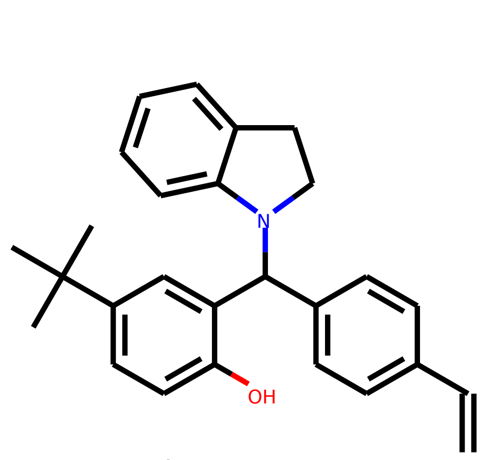
Ahma-2164
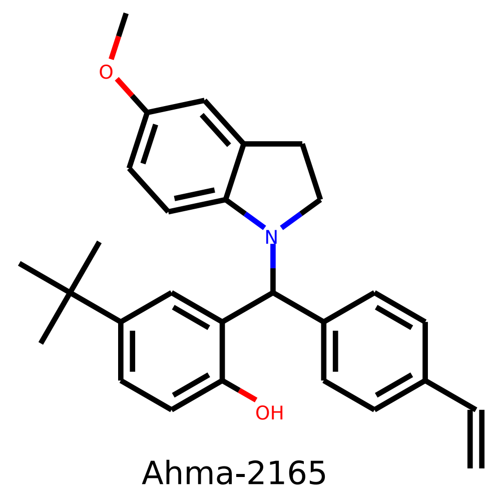
Ahma-2165
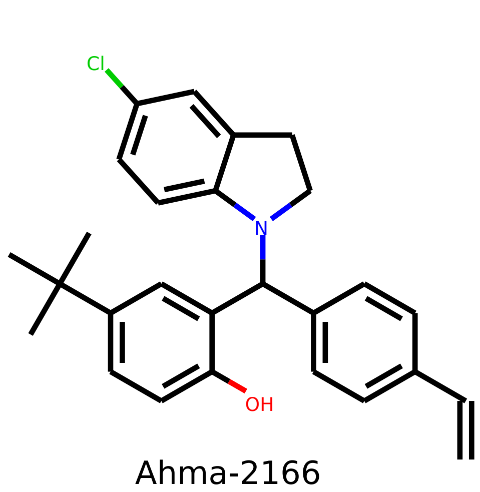
Ahma-2166
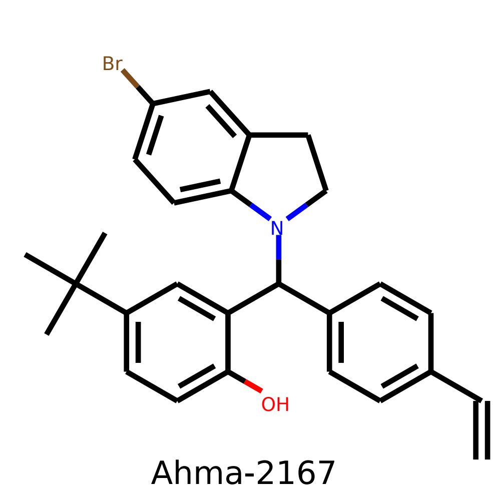
Ahma-2167
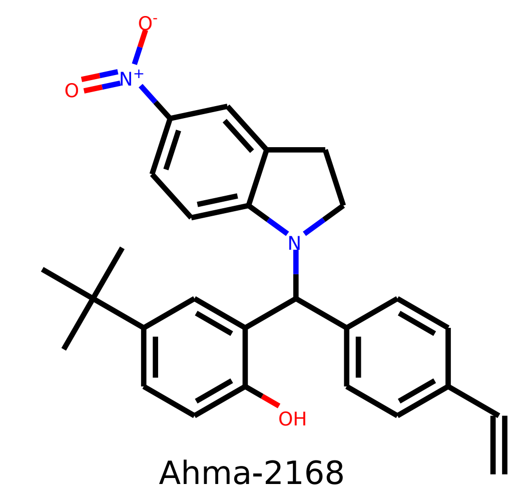
Ahma-2168
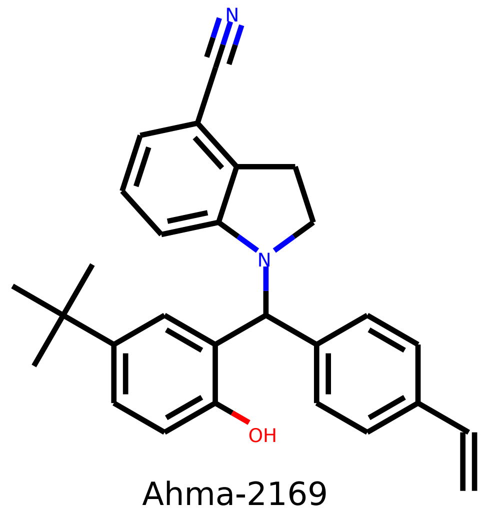
Ahma-2169
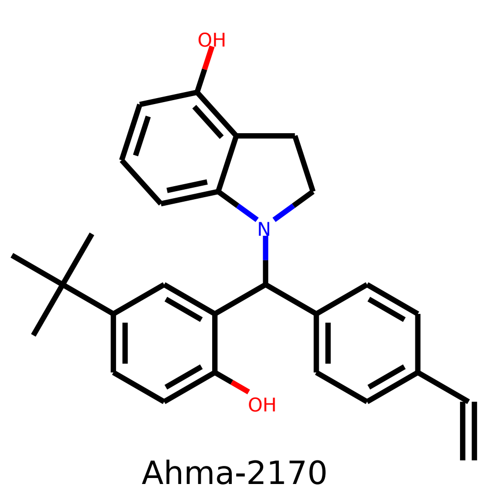
Ahma-2170

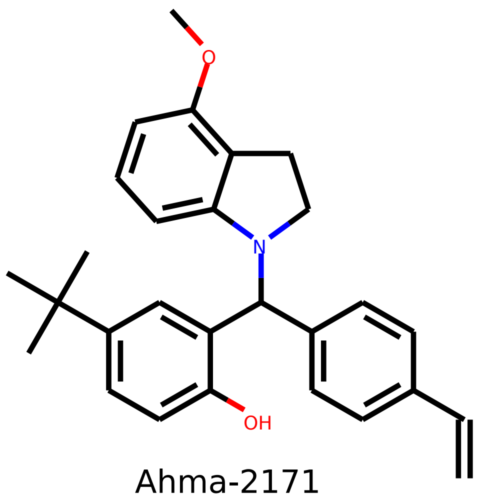
Ahma-2171

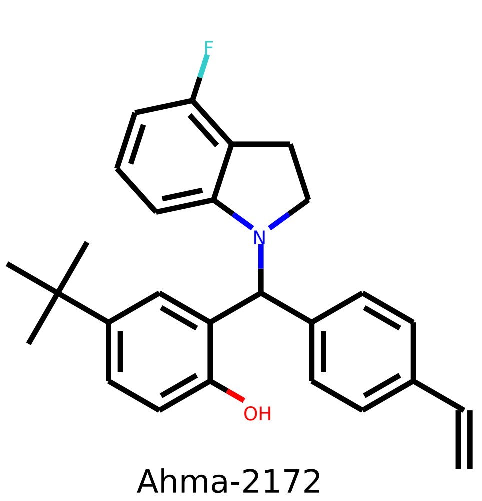
Ahma-2172

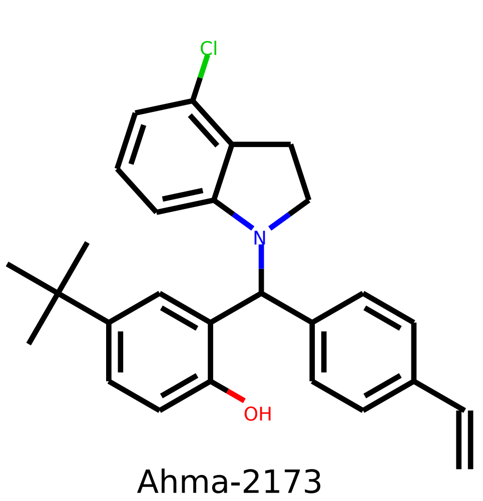
Ahma-2173

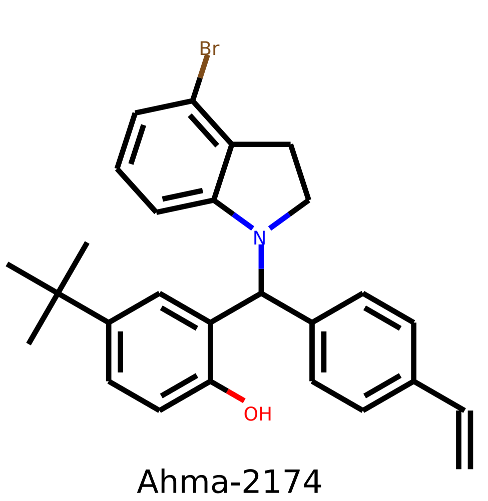
Ahma-2174

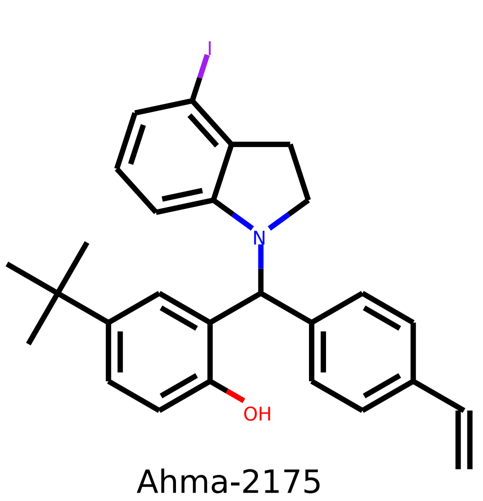
Ahma-2175

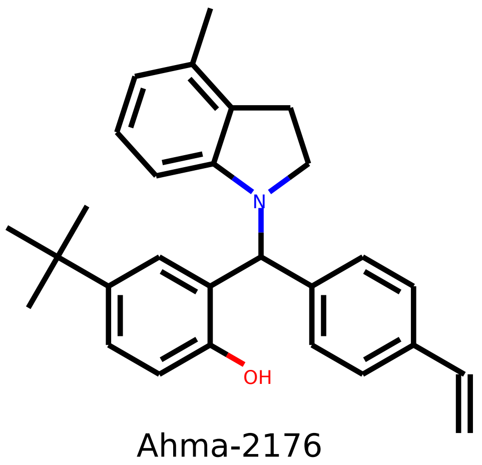
Ahma-2176

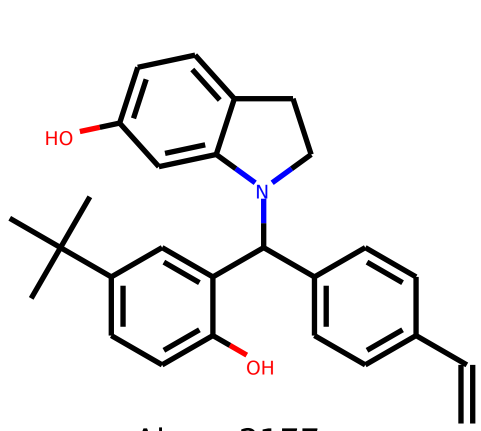
Ahma-2177

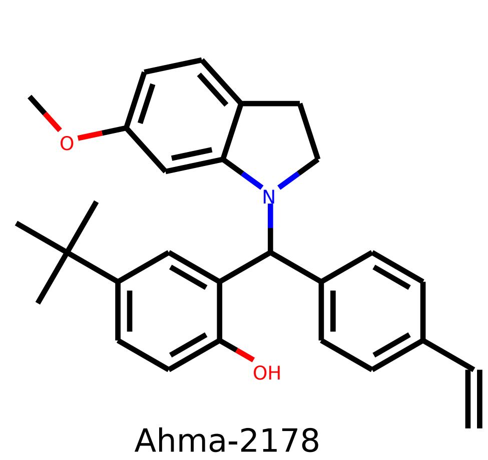
Ahma-2178

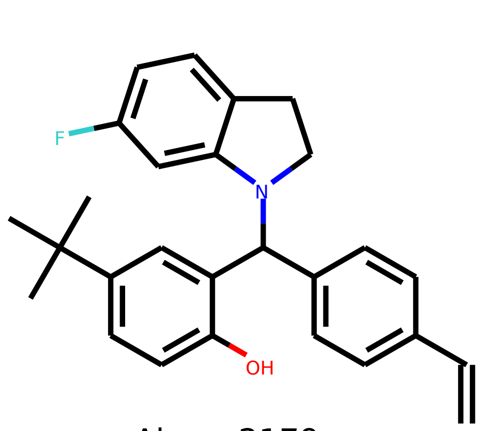
Ahma-2179

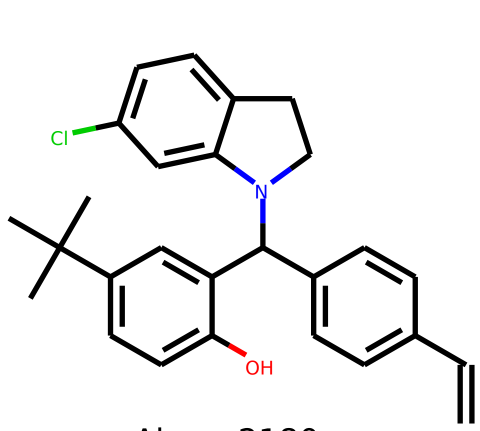
Ahma-2180

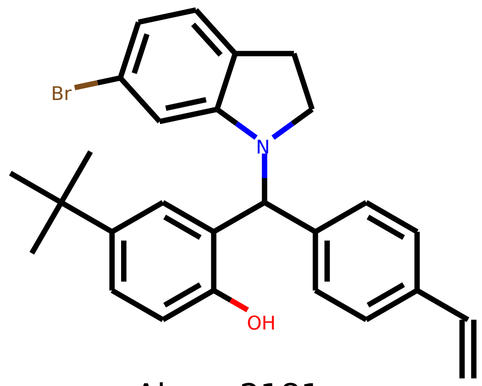
Ahma-2181

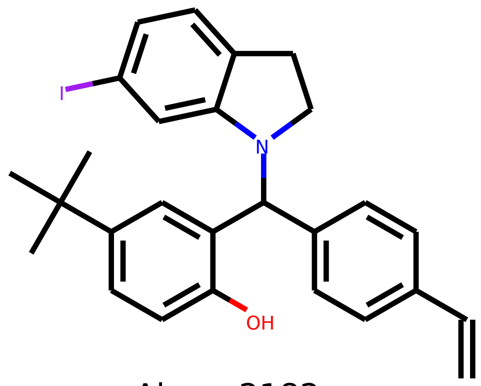
Ahma-2182

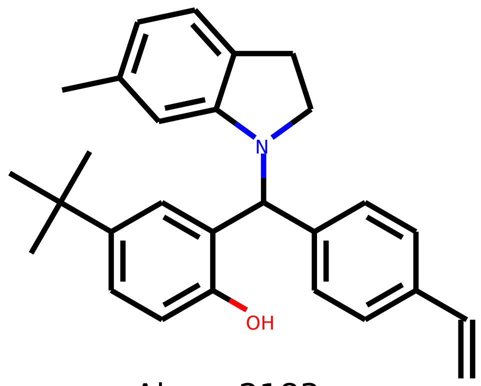
Ahma-2183

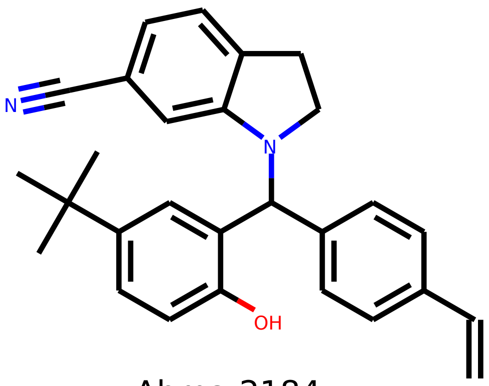
Ahma-2184

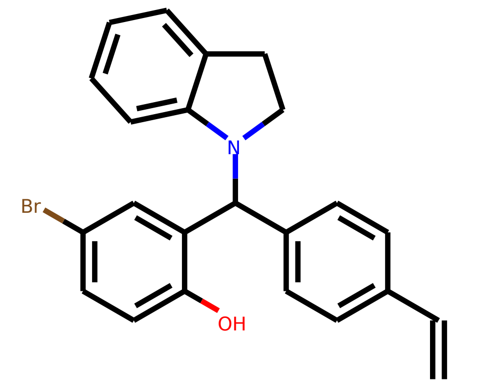
Ahma-2185

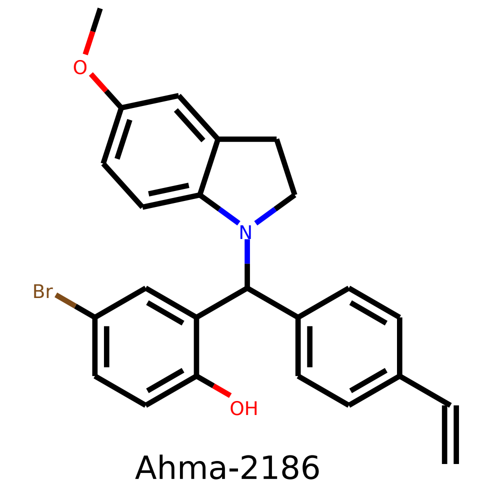
Ahma-2186

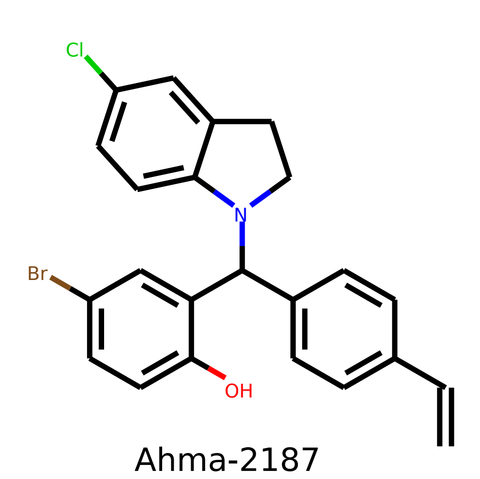
Ahma-2187

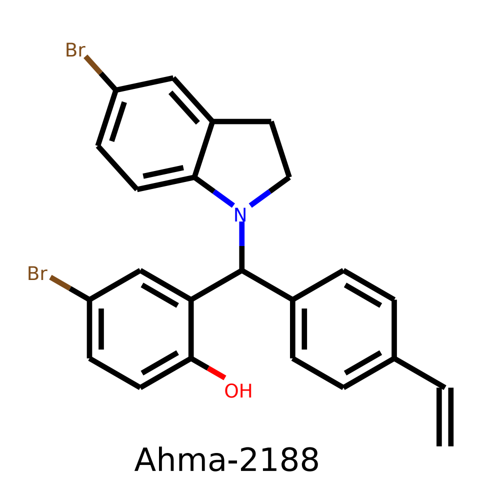
Ahma-2188

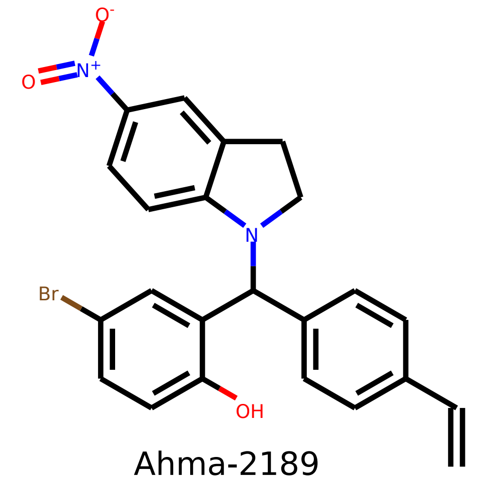
Ahma-2189

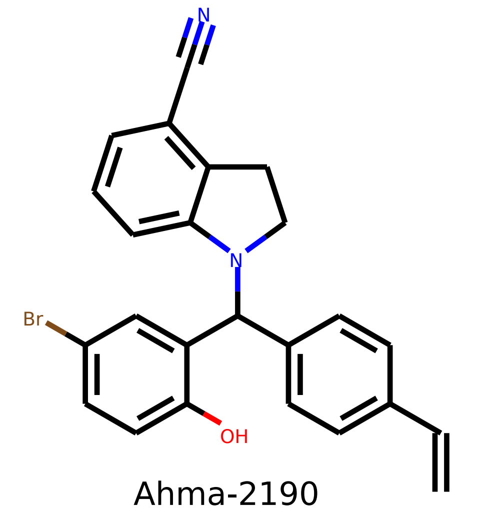
Ahma-2190

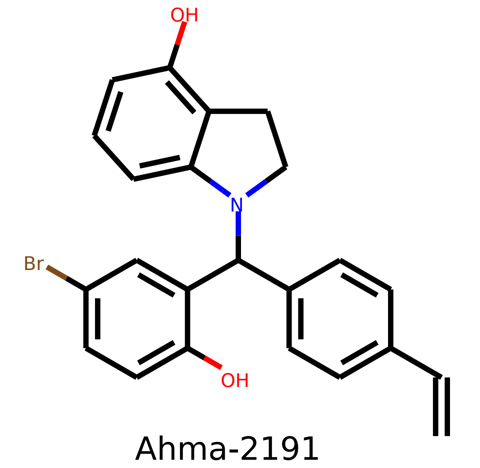
Ahma-2191

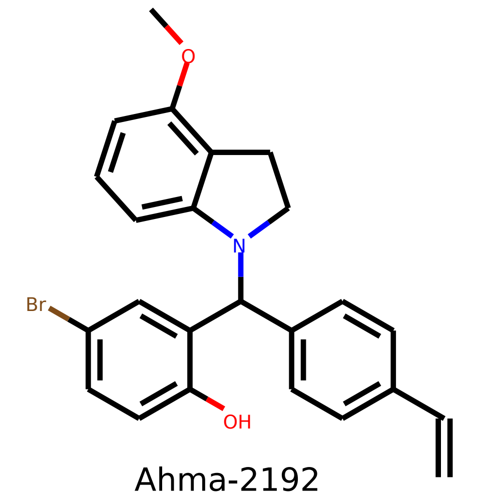
Ahma-2192

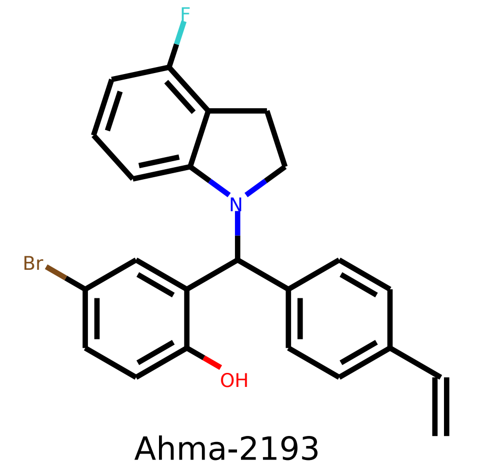
Ahma-2193

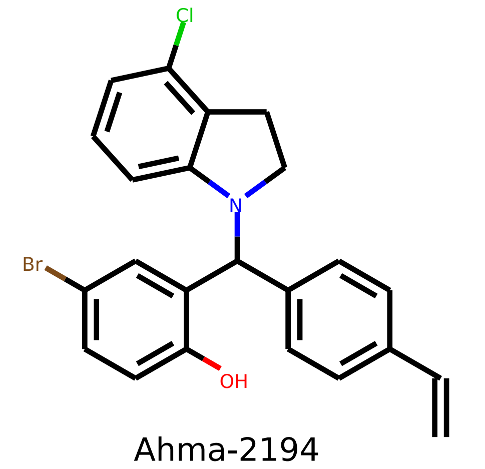
Ahma-2194

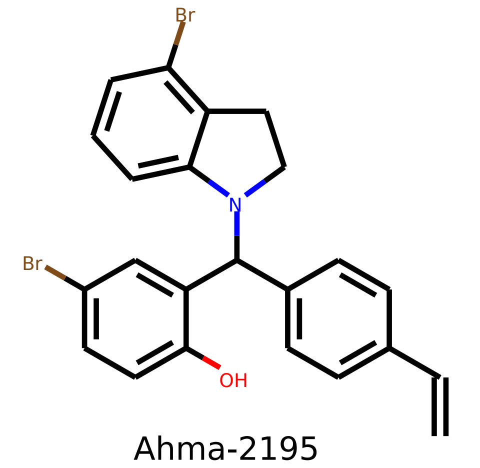
Ahma-2195

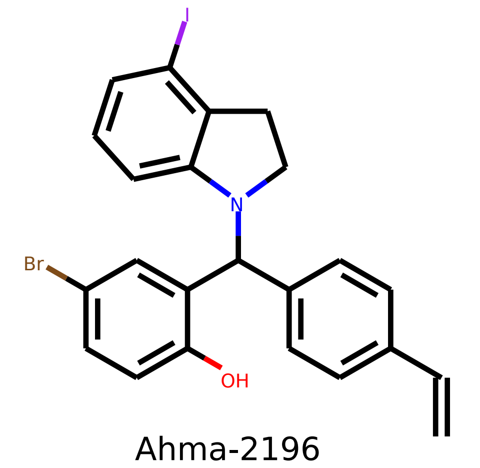
Ahma-2196

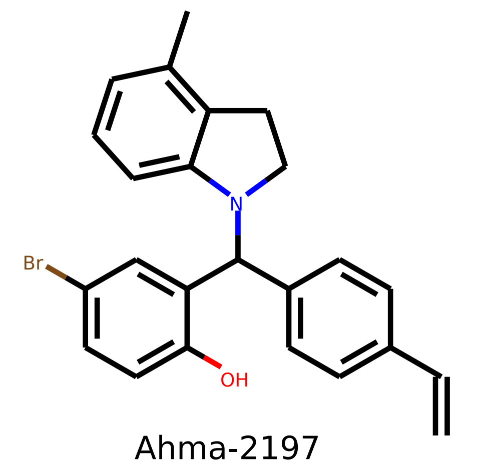
Ahma-2197

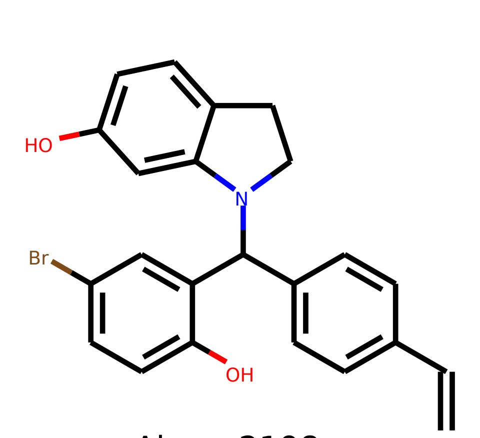
Ahma-2198

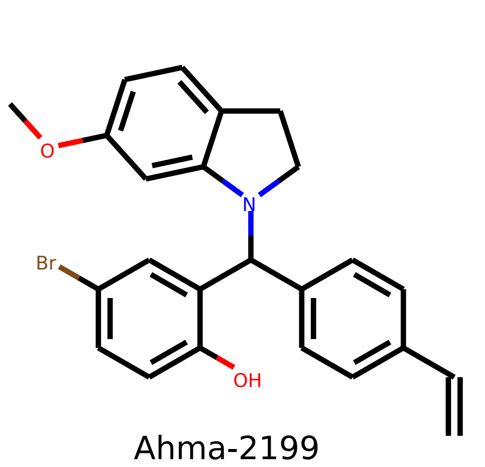
Ahma-2199

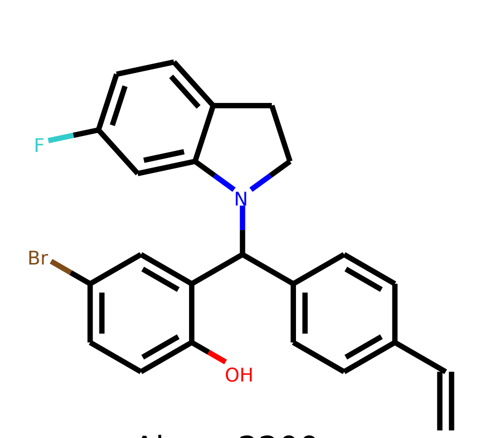
Ahma-2200

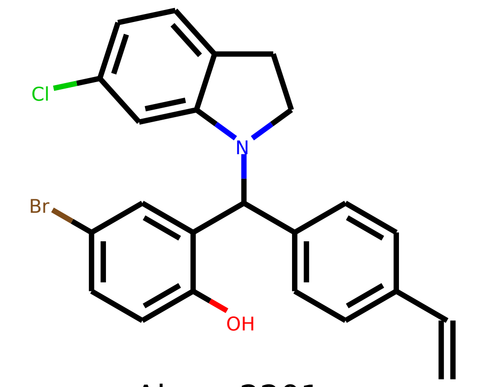
Ahma-2201

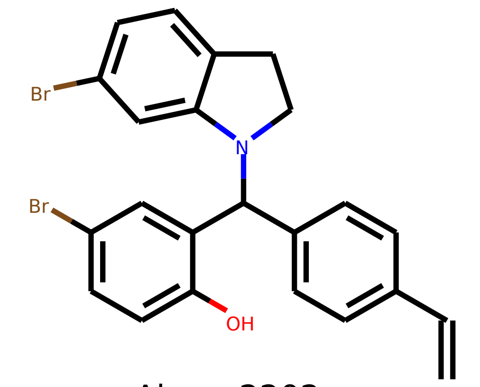
Ahma-2202

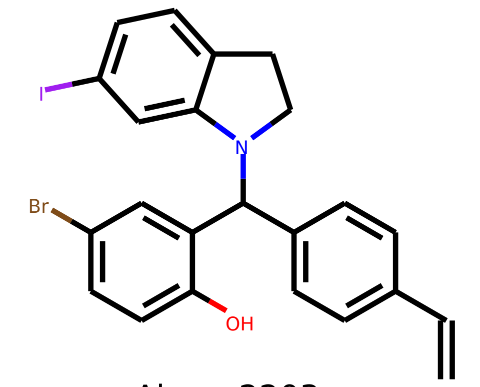
Ahma-2203

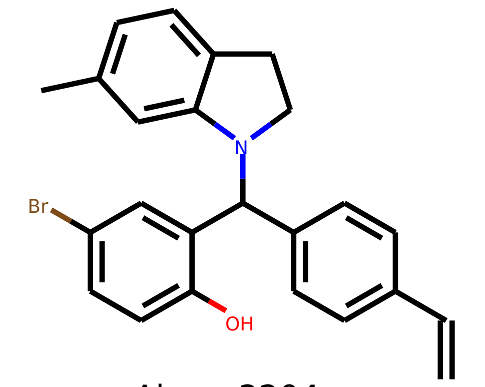
Ahma-2204

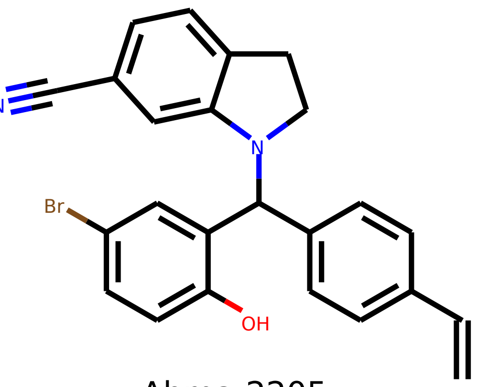
Ahma-2205

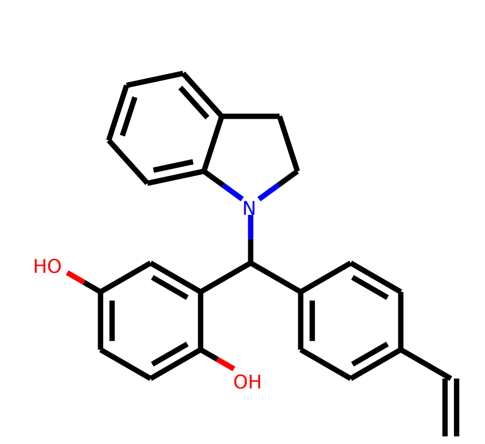
Ahma-2206

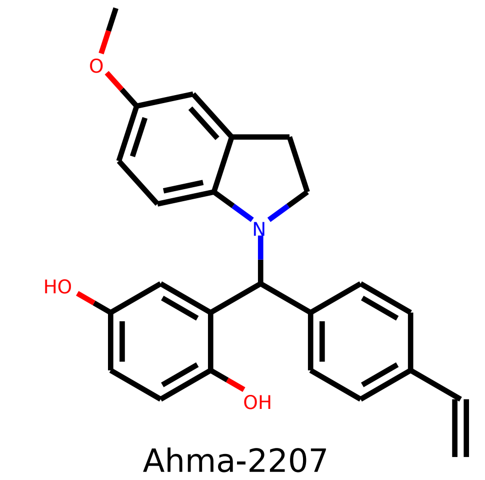
Ahma-2207

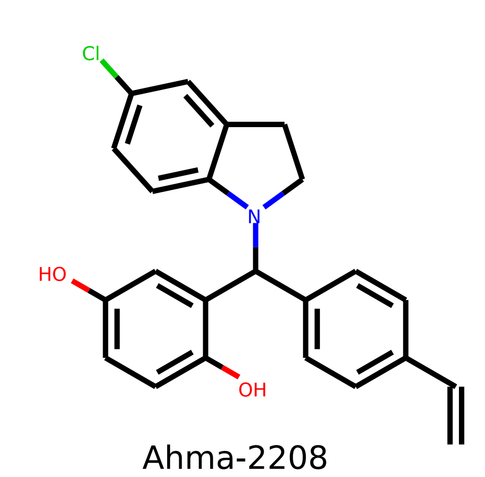
Ahma-2208

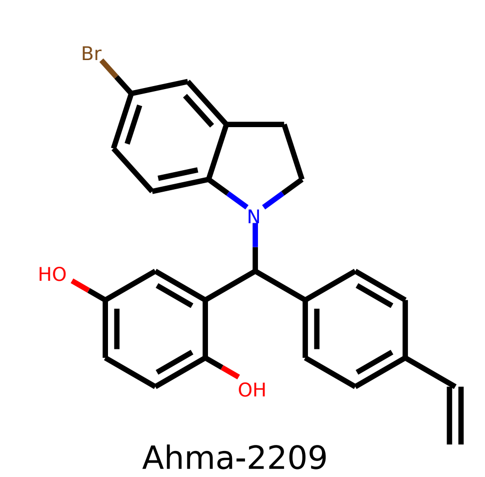
Ahma-2209

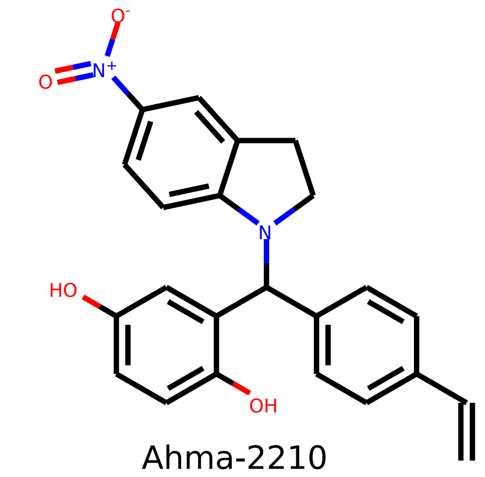
Ahma-2210

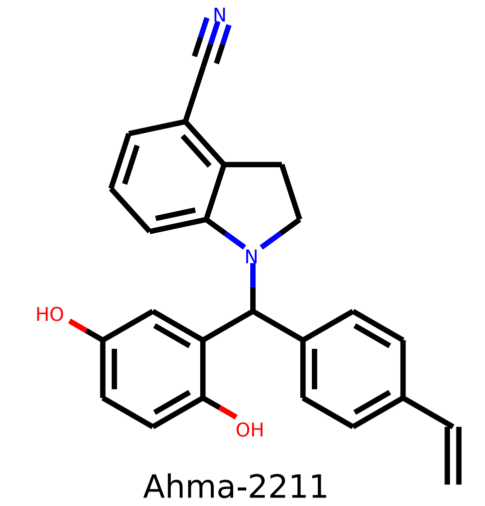
Ahma-2211

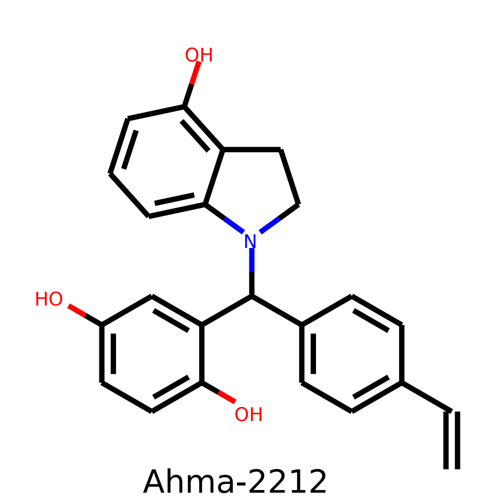
Ahma-2212

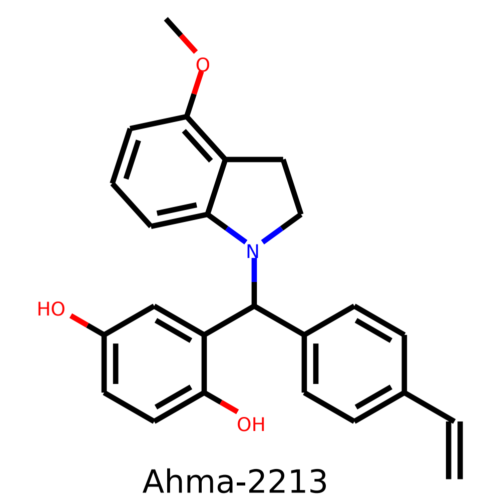
Ahma-2213

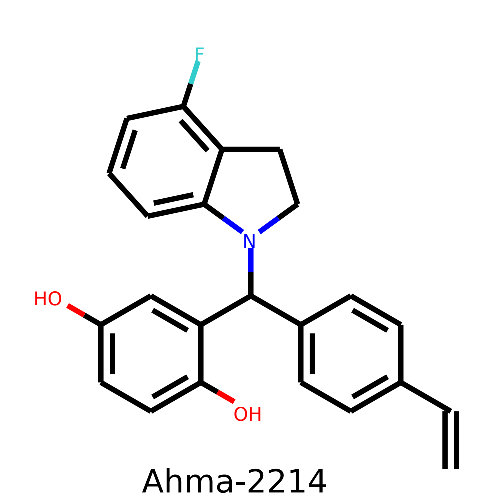
Ahma-2214

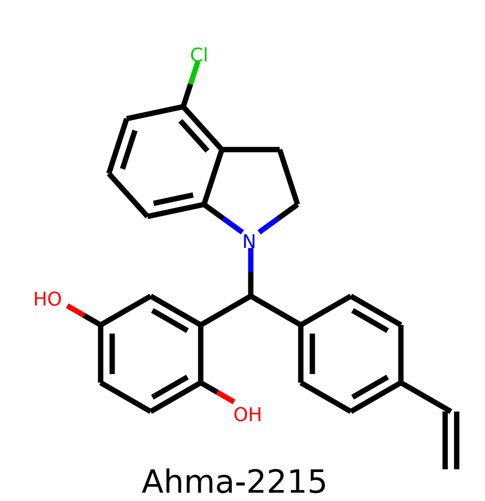
Ahma-2215

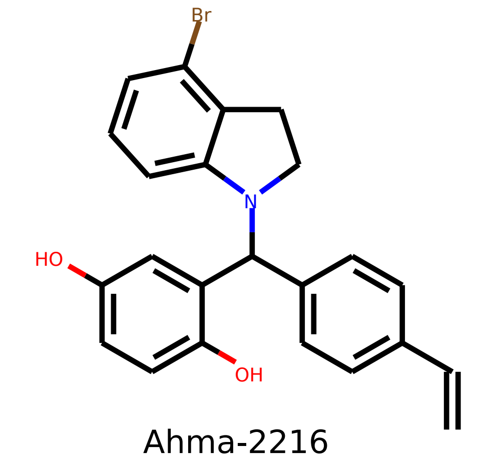
Ahma-2216

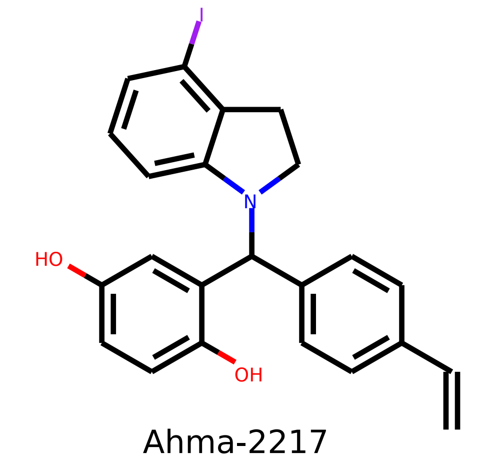
Ahma-2217

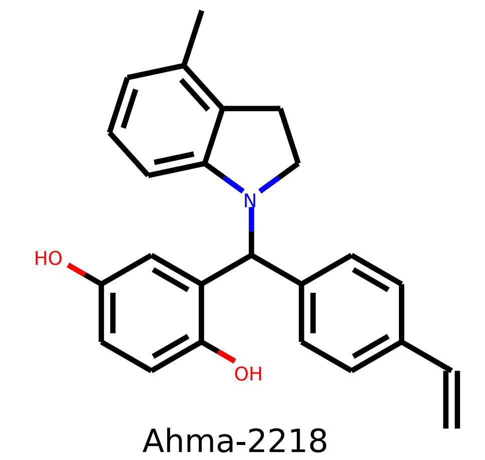
Ahma-2218

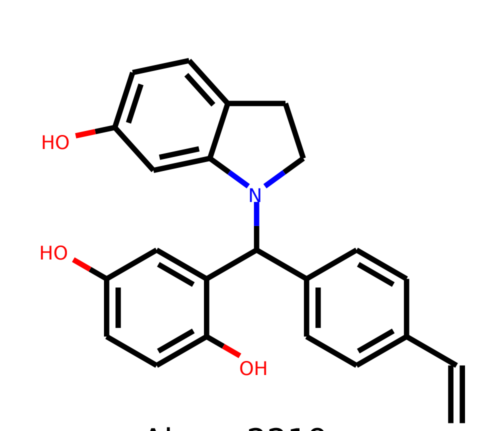
Ahma-2219

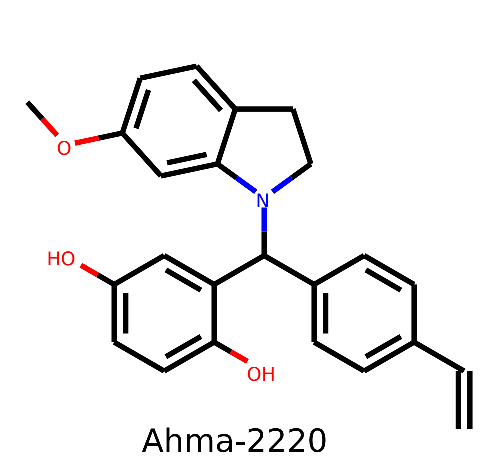
Ahma-2220

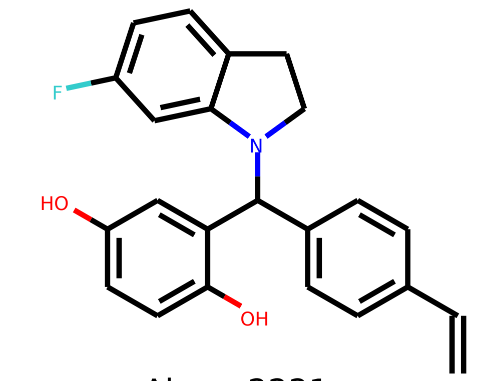
Ahma-2221

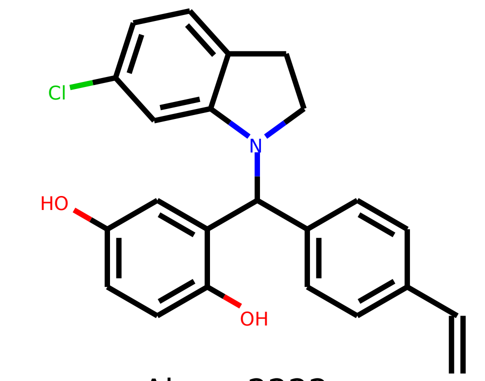
Ahma-2222

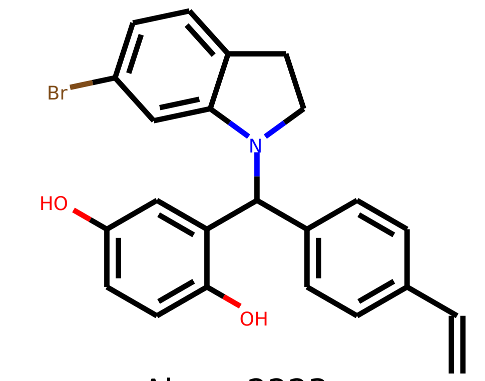
Ahma-2223

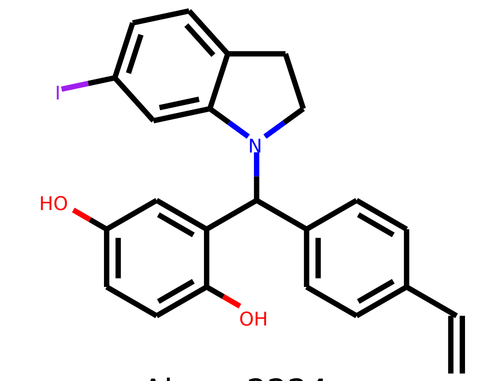
Ahma-2224

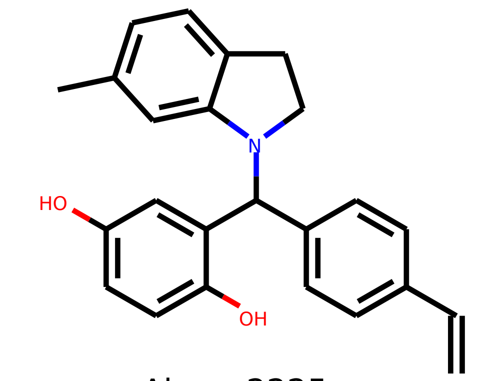
Ahma-2225

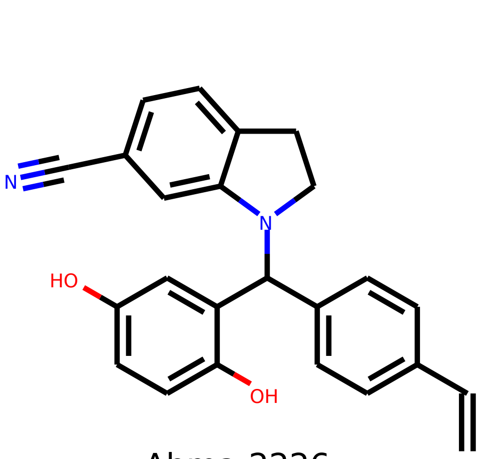
Ahma-2226

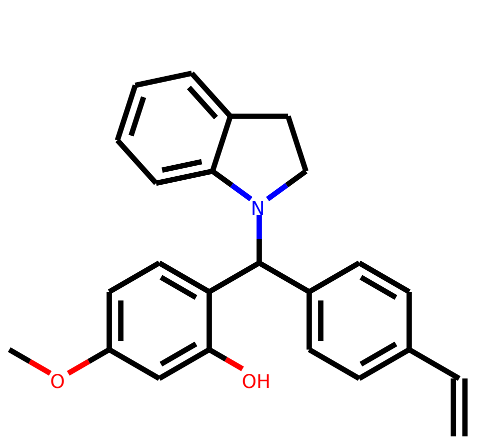
Ahma-2227

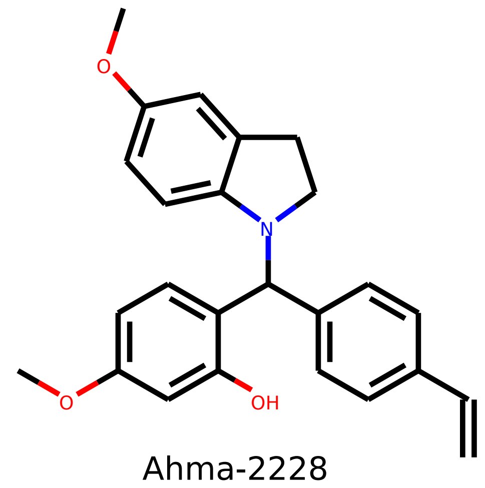
Ahma-2228

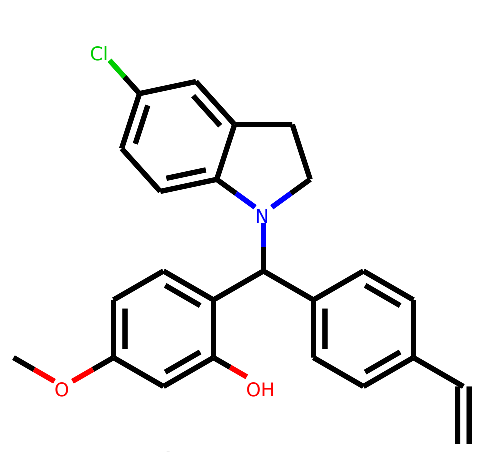
Ahma-2229

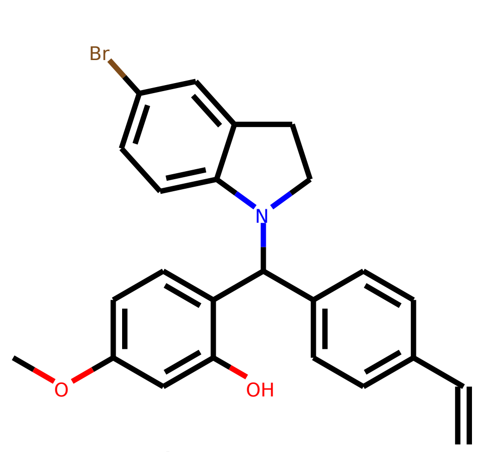
Ahma-2230

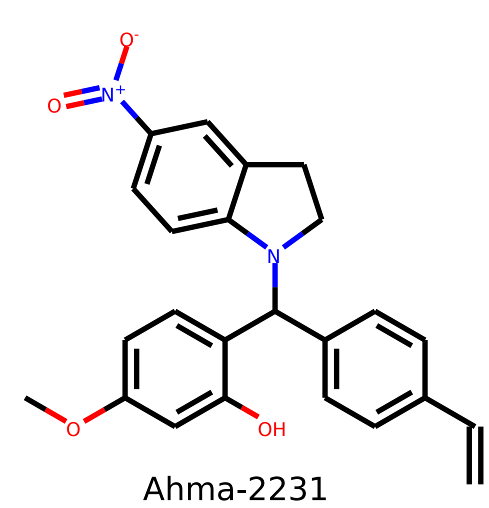
Ahma-2231

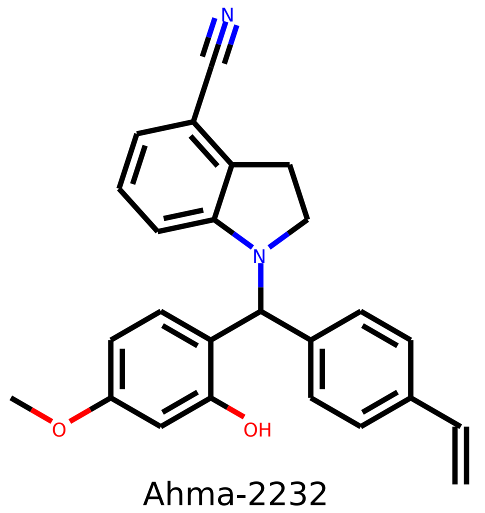
Ahma-2232

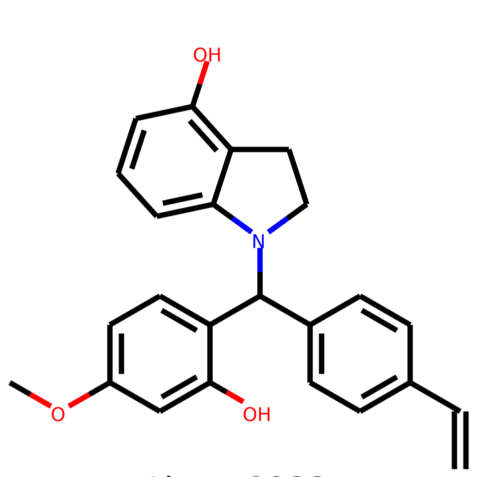
Ahma-2233

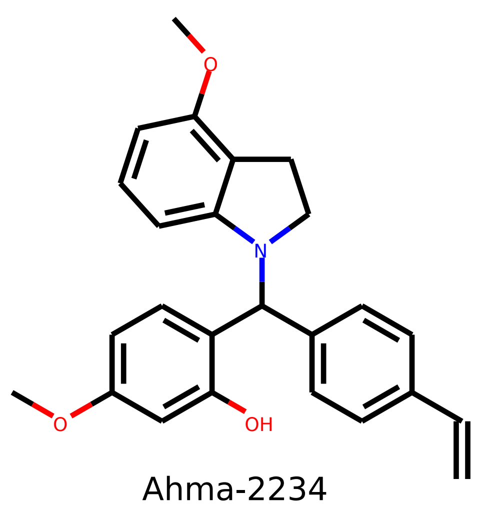
Ahma-2234

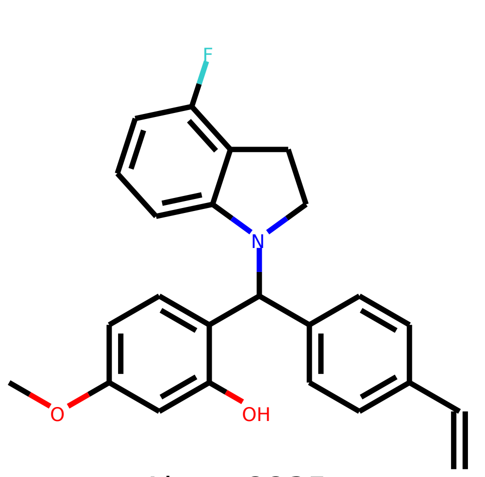
Ahma-2235

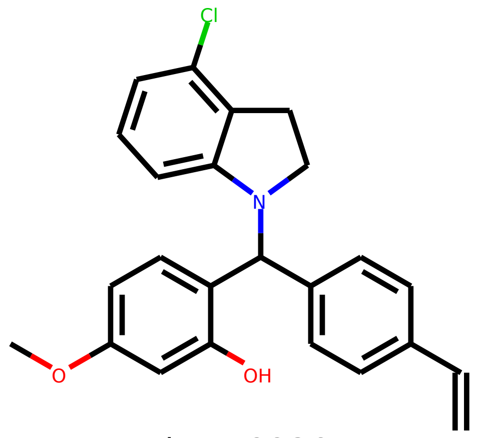
Ahma-2236

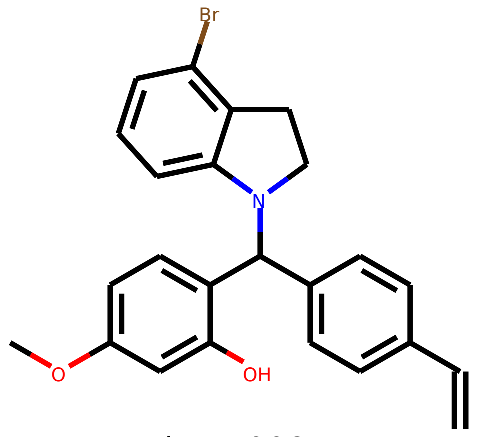
Ahma-2237

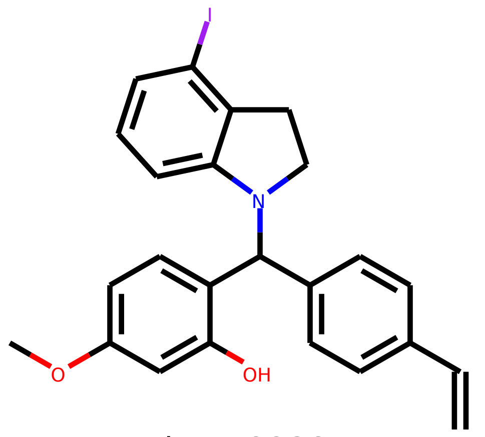
Ahma-2238

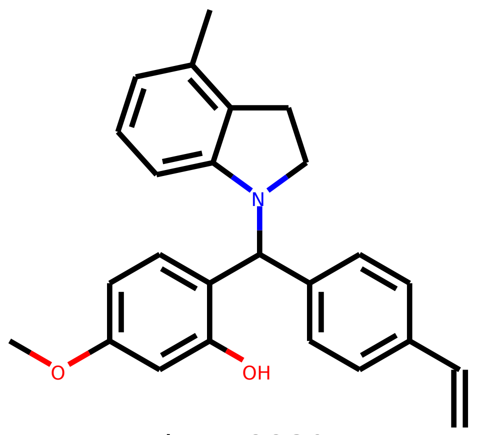
Ahma-2239

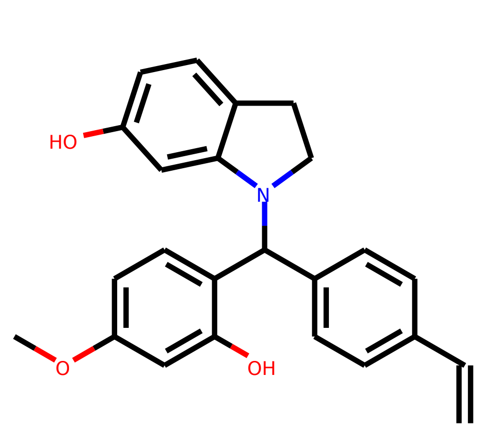
Ahma-2240

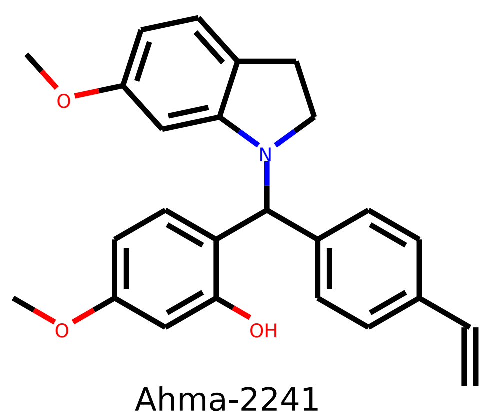
Ahma-2241

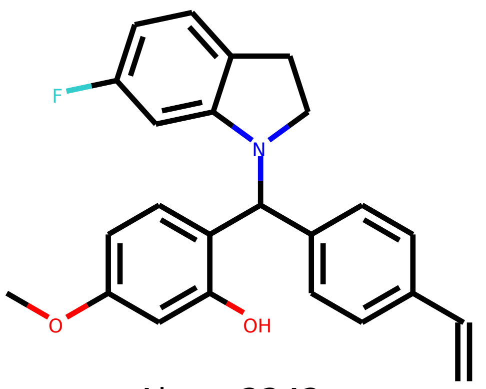
Ahma-2242

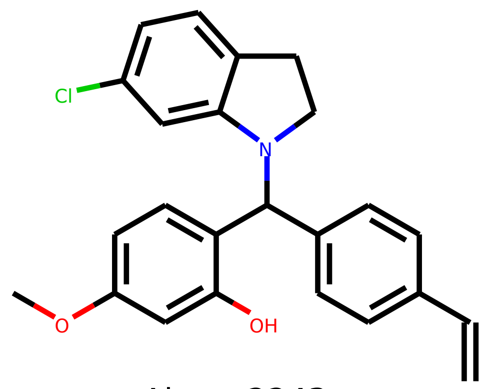
Ahma-2243

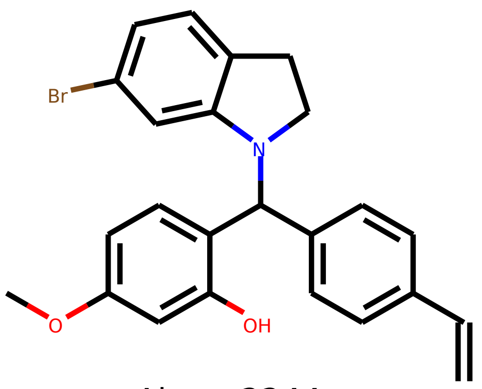
Ahma-2244

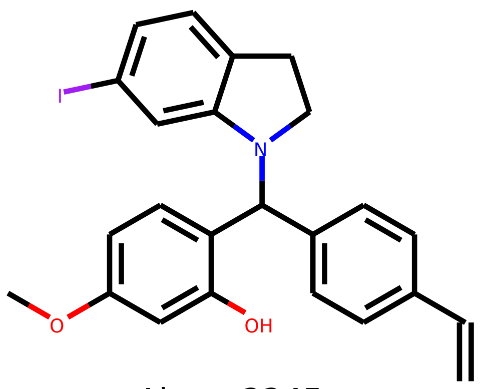
Ahma-2245

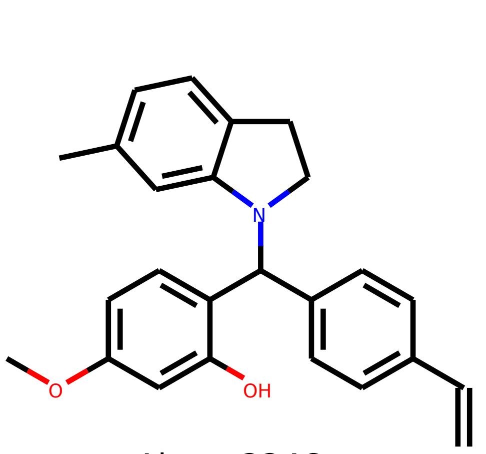
Ahma-2246

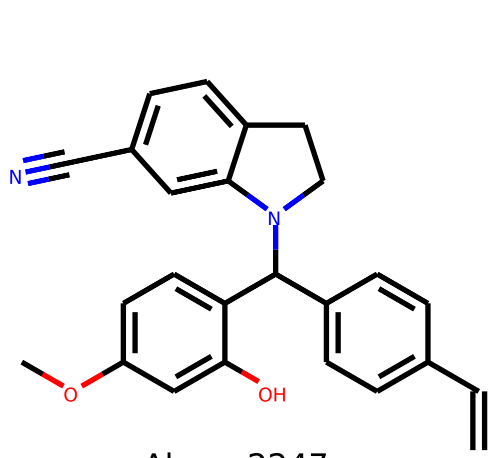
Ahma-2247

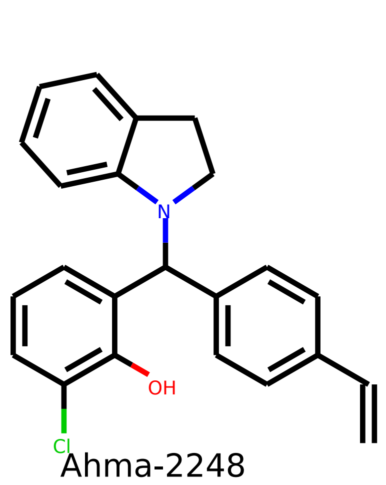
Ahma-2248

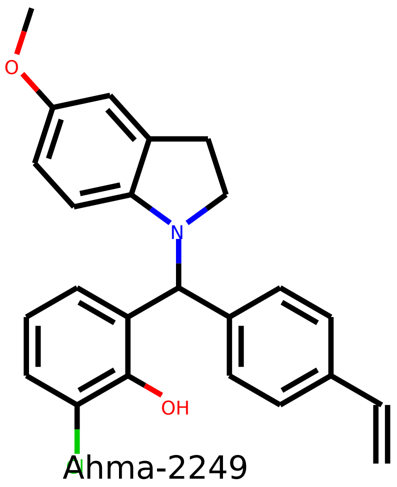
Ahma-2249

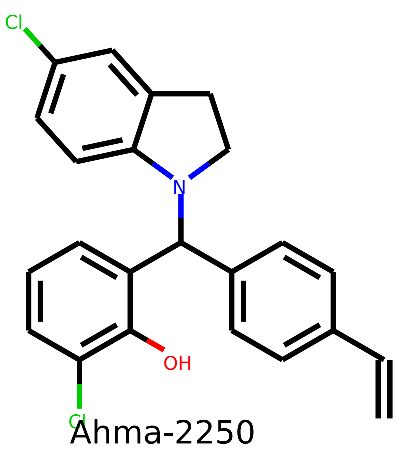
Ahma-2250

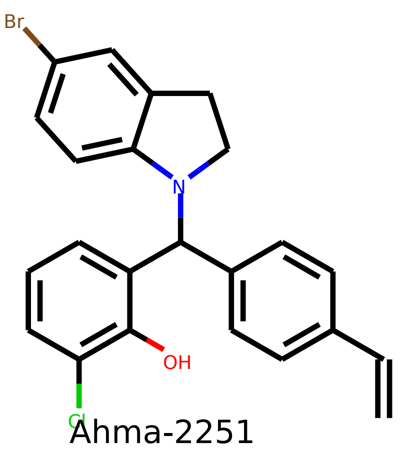
Ahma-2251

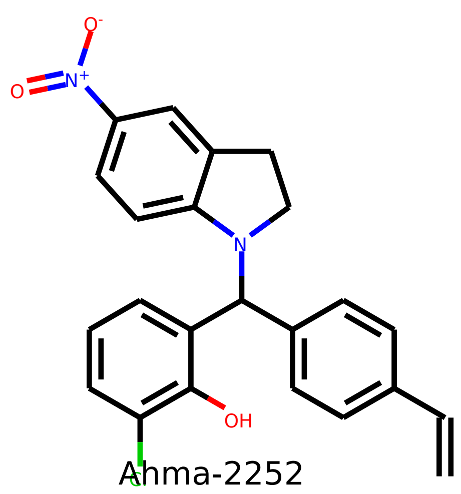
Ahma-2252

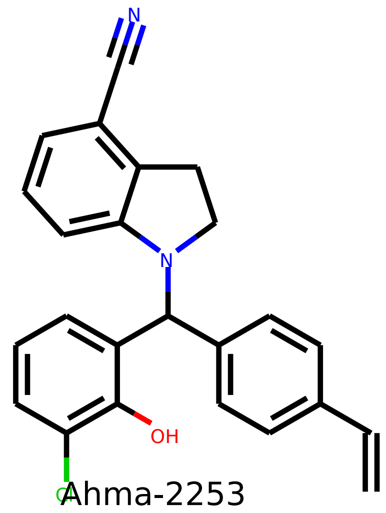
Ahma-2253

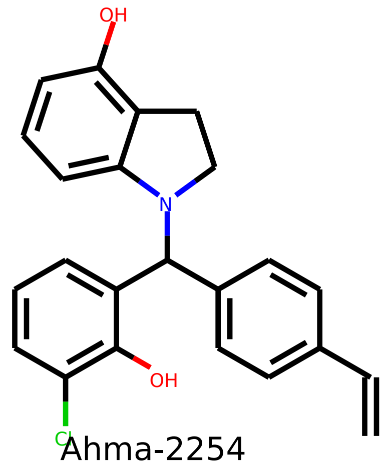
Ahma-2254

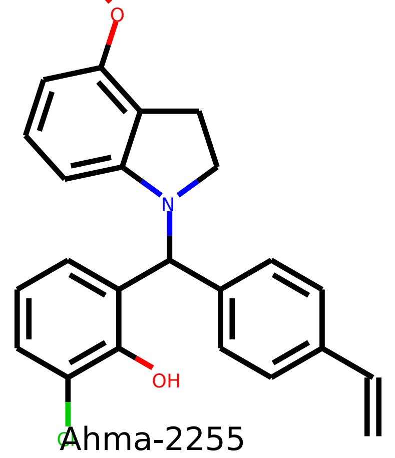
Ahma-2255

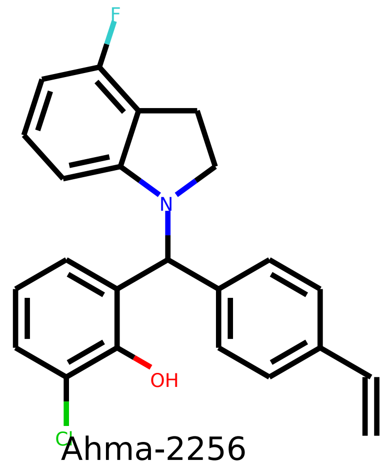
Ahma-2256

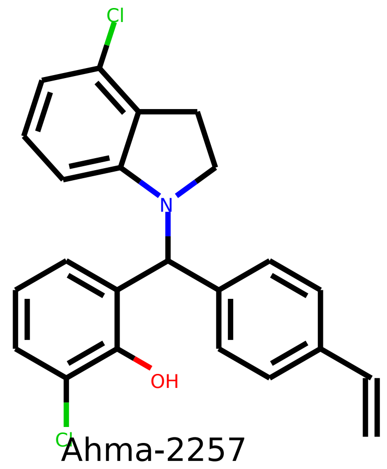
Ahma-2257

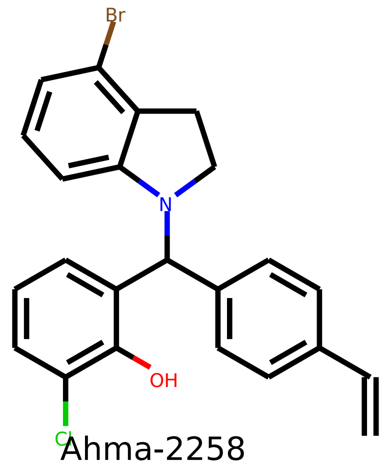
Ahma-2258

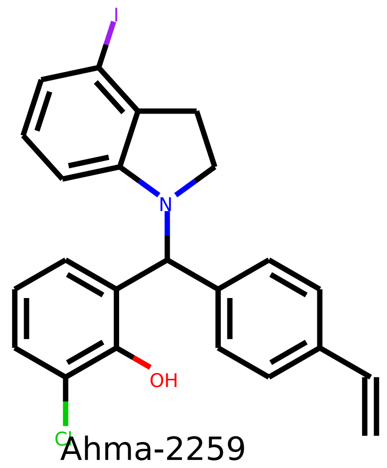
Ahma-2259

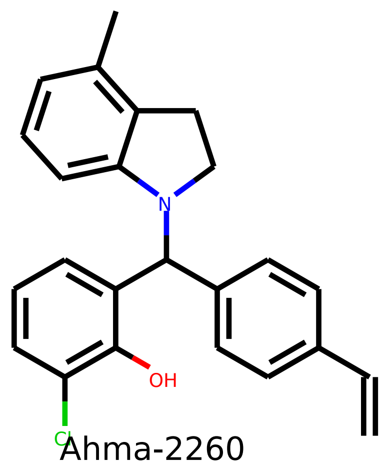
Ahma-2260

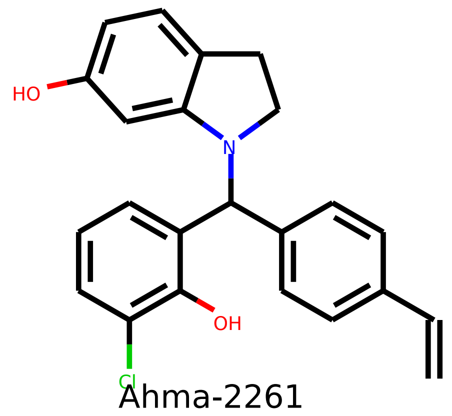
Ahma-2261

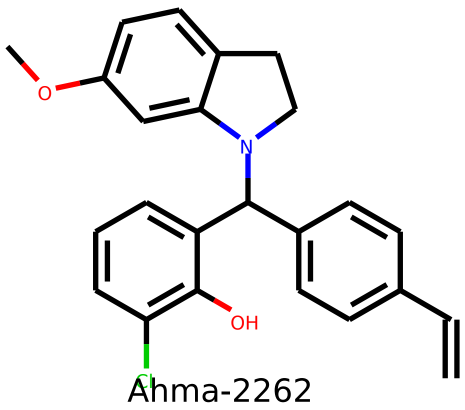
Ahma-2262

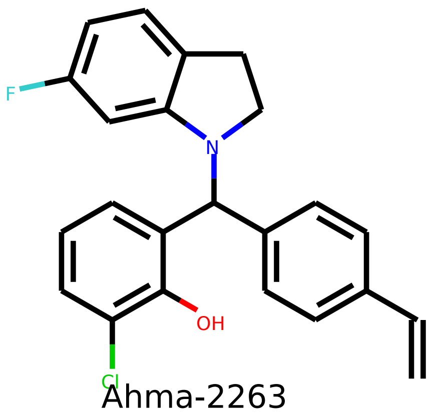
Ahma-2263

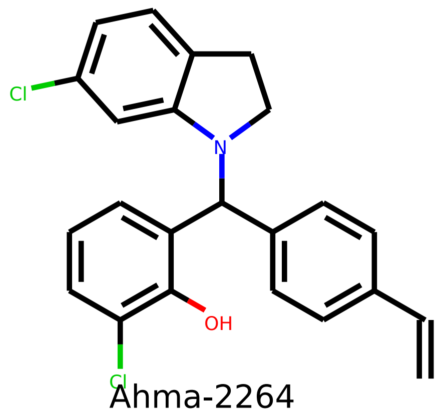
Ahma-2264

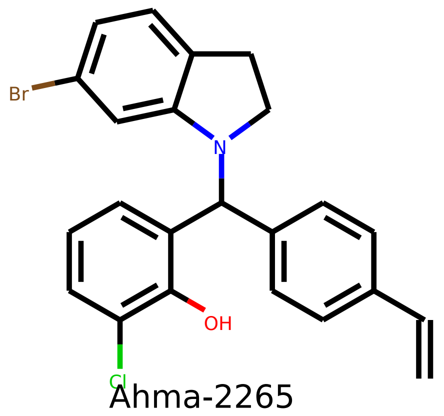
Ahma-2265

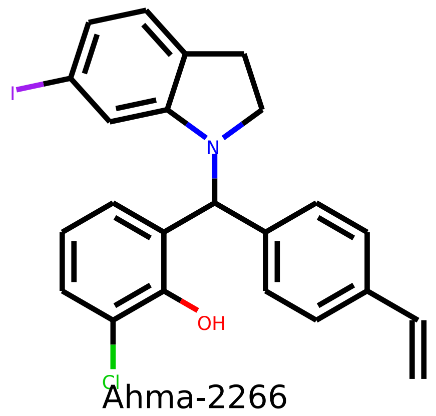
Ahma-2266

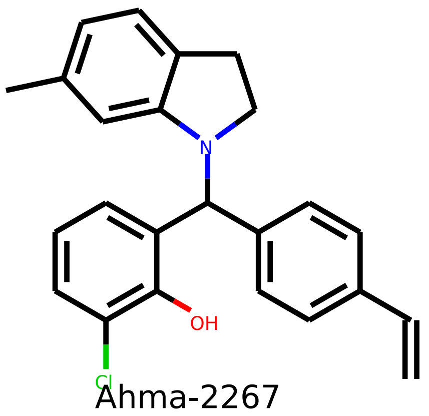
Ahma-2267

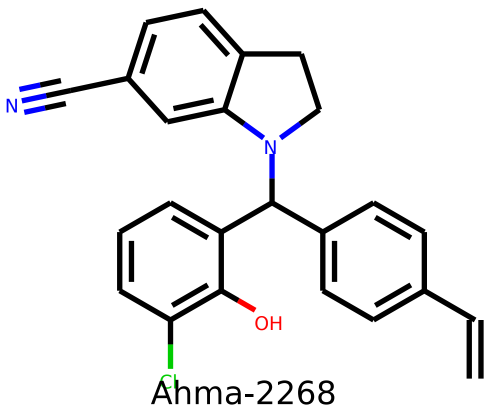
Ahma-2268

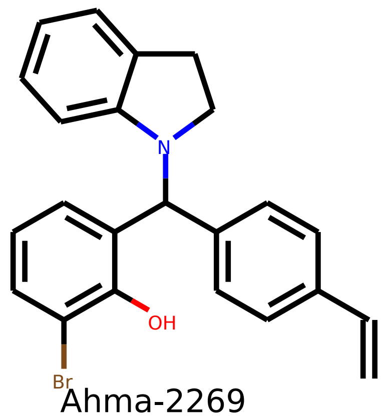
Ahma-2269

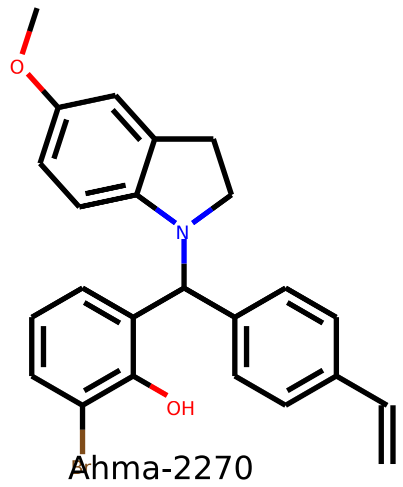
Ahma-2270

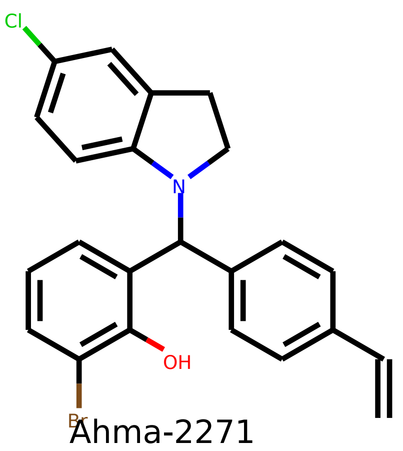
Ahma-2271

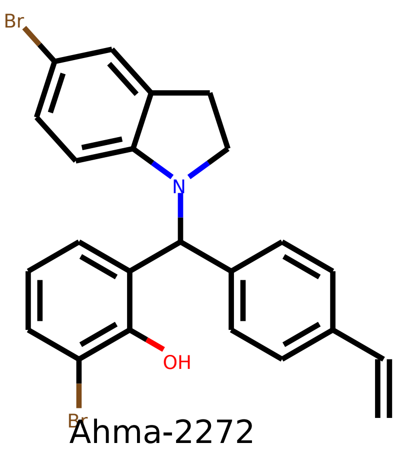
Ahma-2272

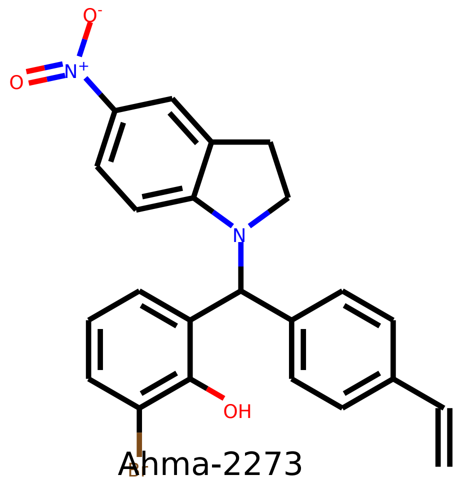
Ahma-2273

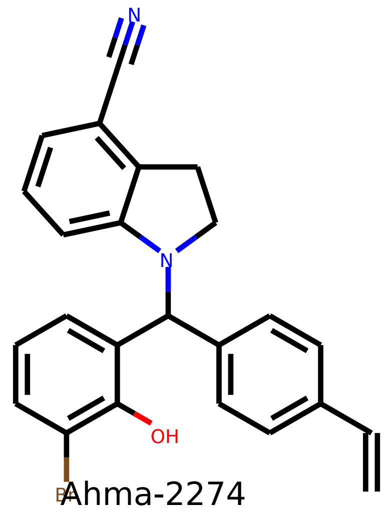
Ahma-2274

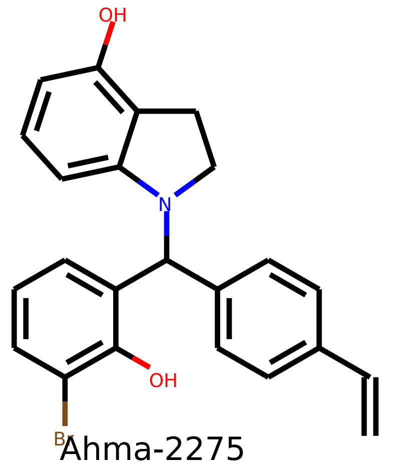
Ahma-2275

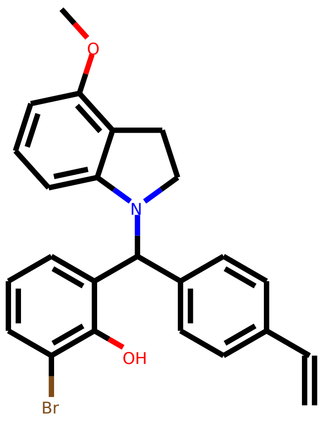
Ahma-2276

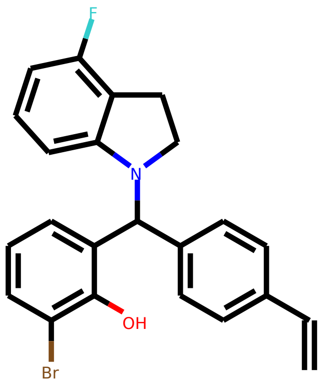
Ahma-2277

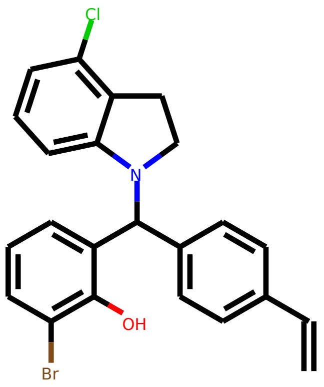
Ahma-2278

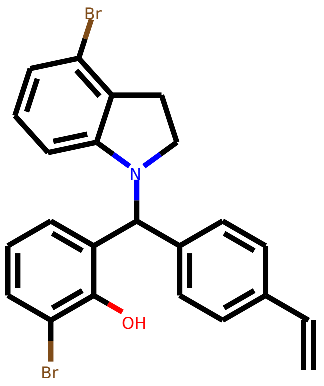
Ahma-2279

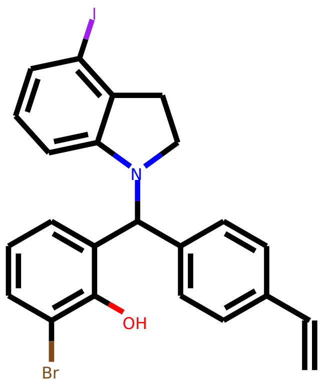
Ahma-2280

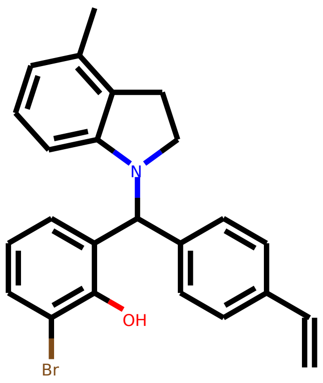
Ahma-2281

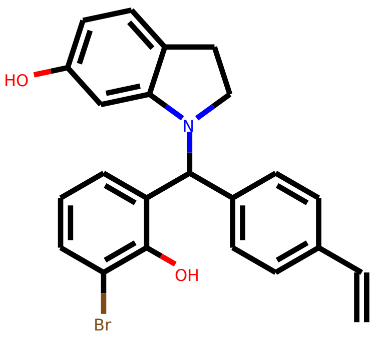
Ahma-2282

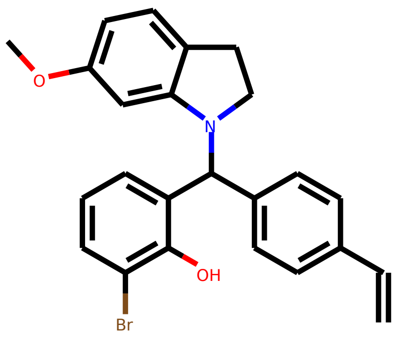
Ahma-2283

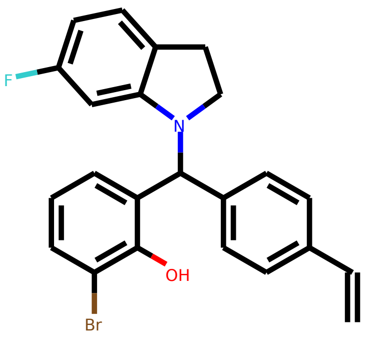
Ahma-2284

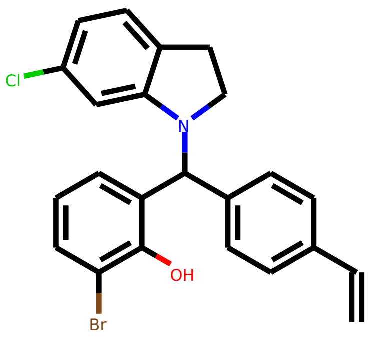
Ahma-2285

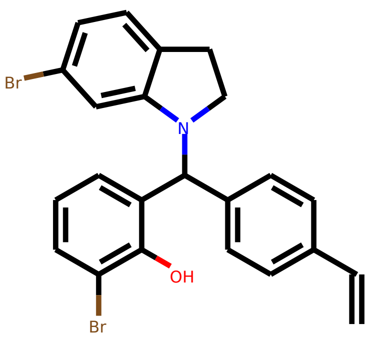
Ahma-2286

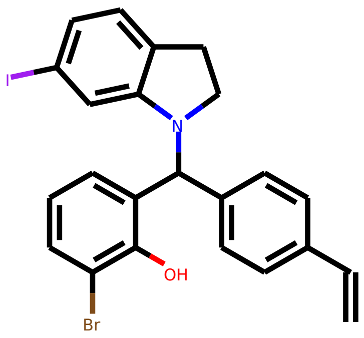
Ahma-2287

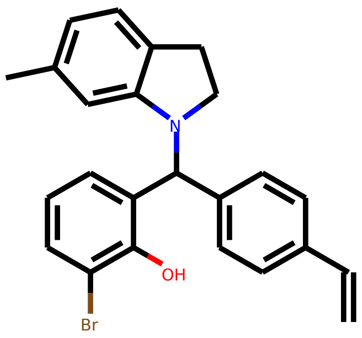
Ahma-2288

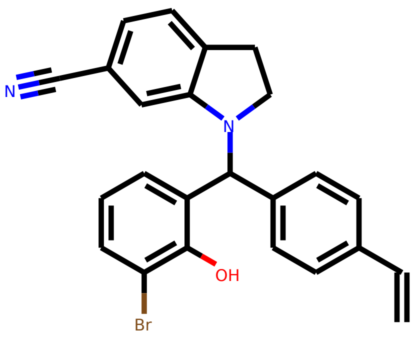
Ahma-2289

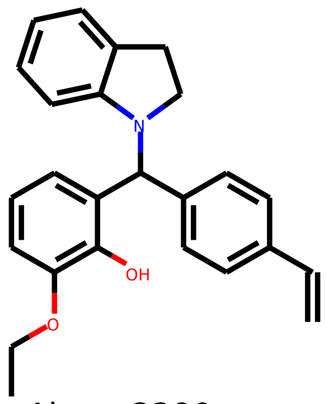
Ahma-2290

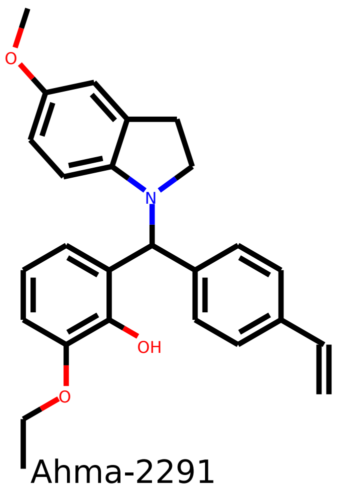
Ahma-2291

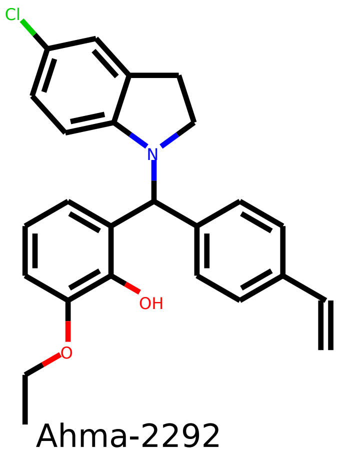
Ahma-2292

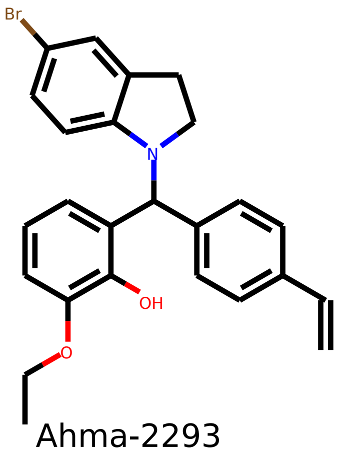
Ahma-2293

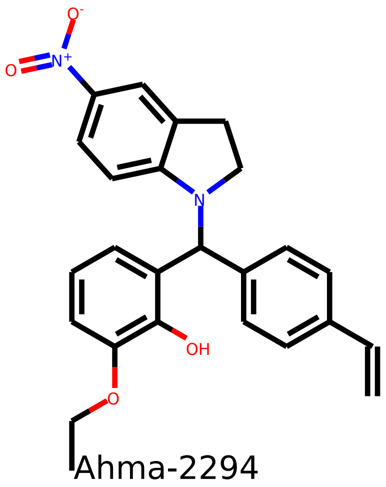
Ahma-2294

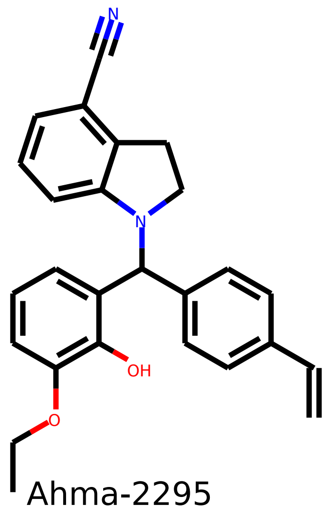
Ahma-2295

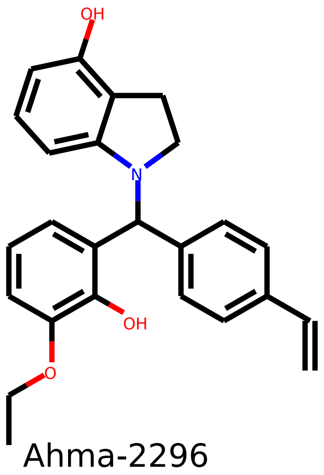
Ahma-2296

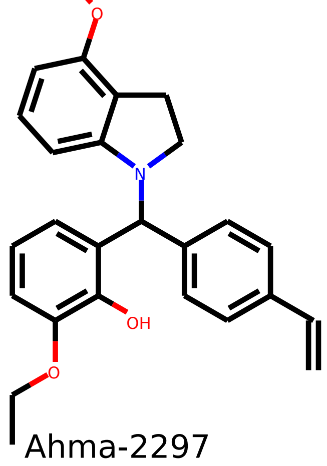
Ahma-2297

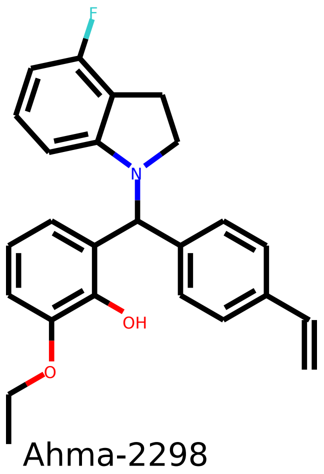
Ahma-2298

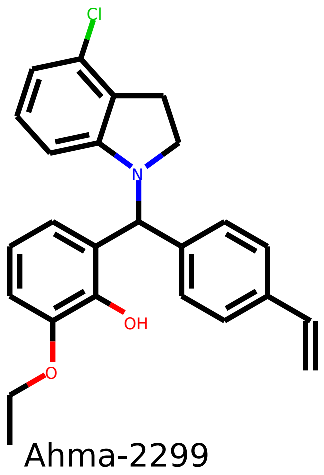
Ahma-2299

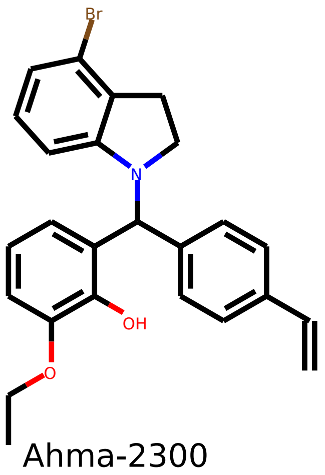
Ahma-2300

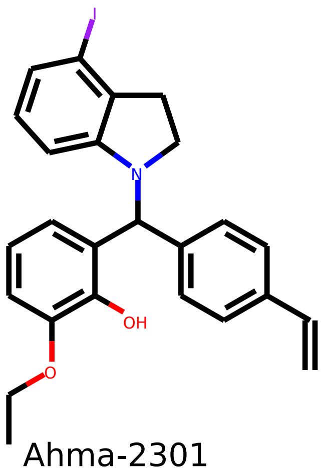
Ahma-2301

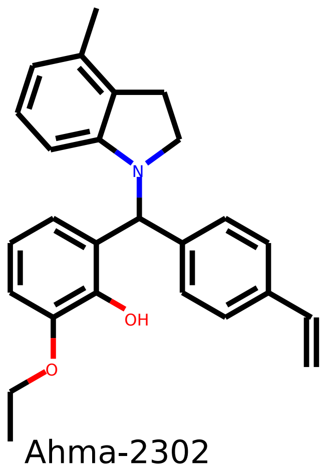
Ahma-2302

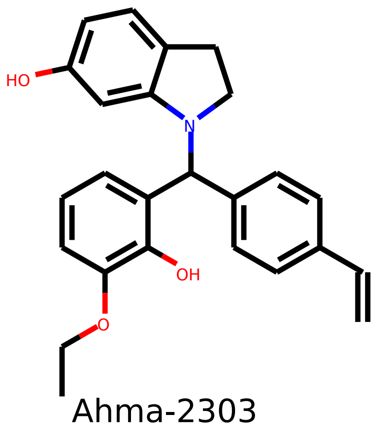
Ahma-2303

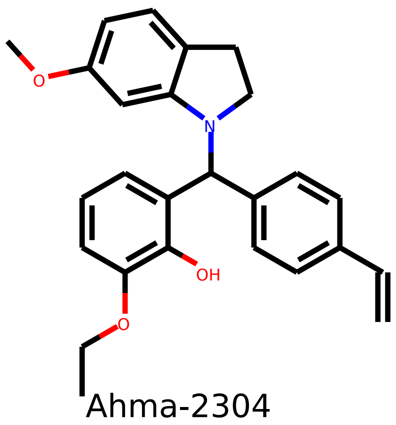
Ahma-2304

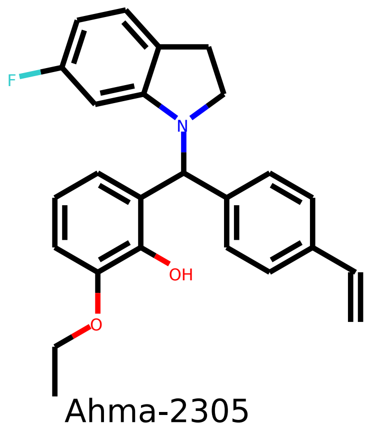
Ahma-2305

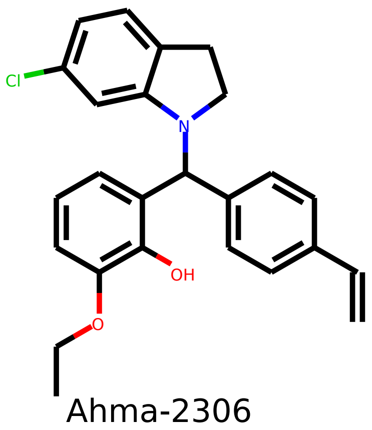
Ahma-2306

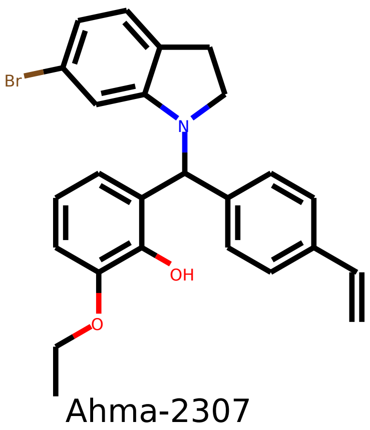
Ahma-2307

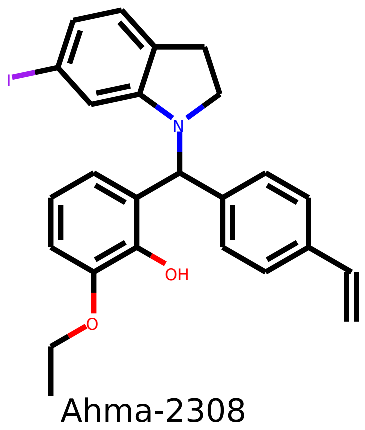
Ahma-2308

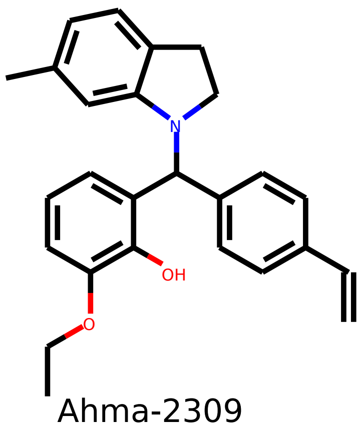
Ahma-2309

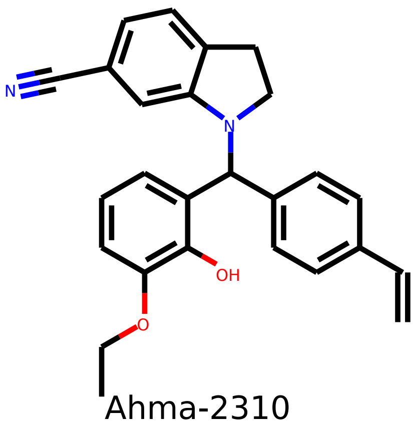
Ahma-2310

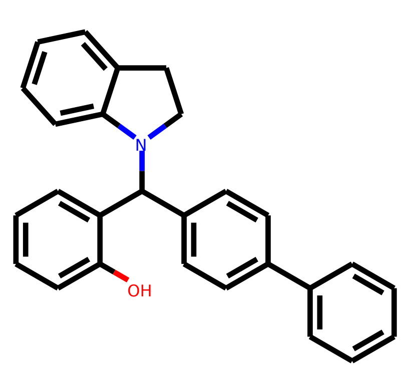
Ahma-2311

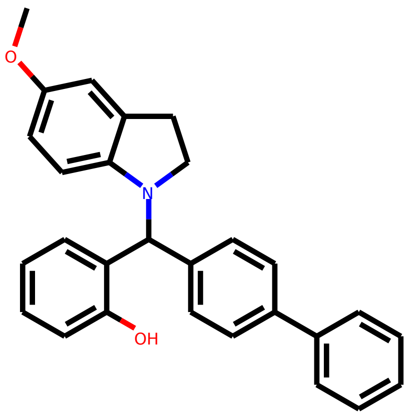
Ahma-2312

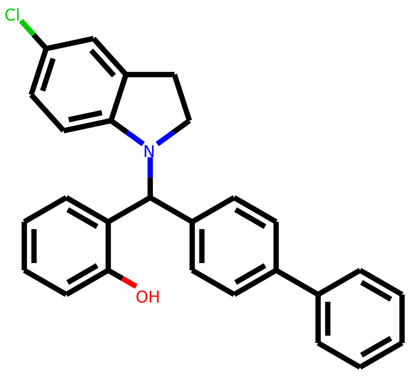
Ahma-2313

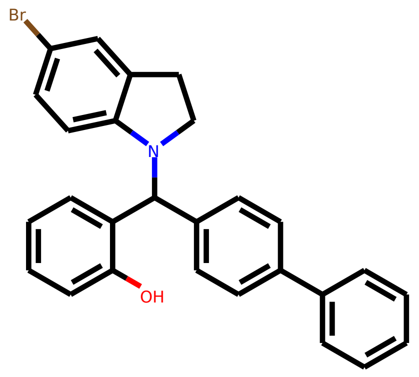
Ahma-2314

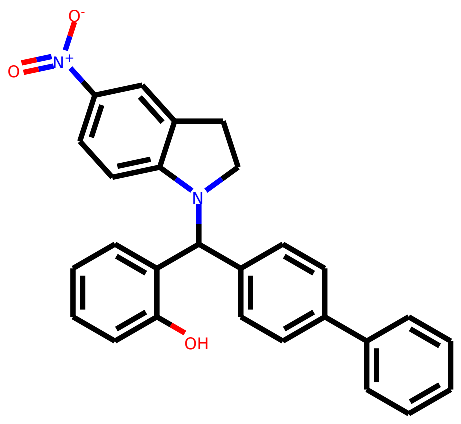
Ahma-2315

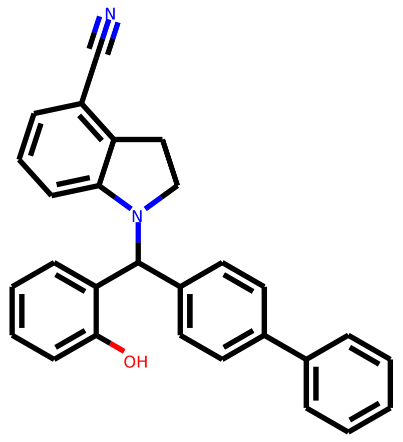
Ahma-2316

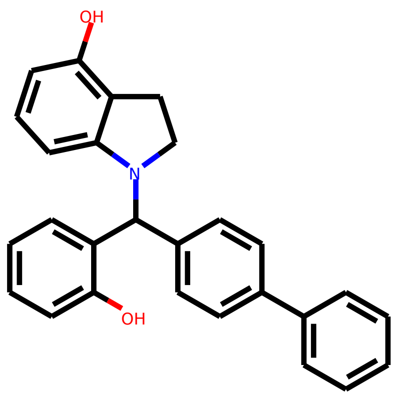
Ahma-2317

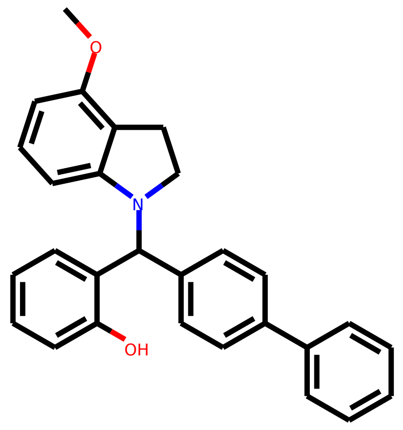
Ahma-2318

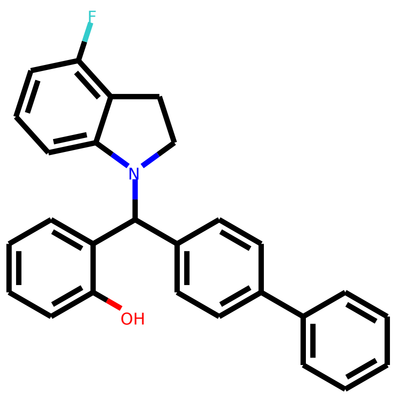
Ahma-2319

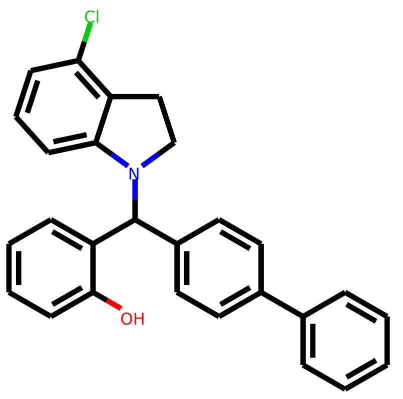
Ahma-2320

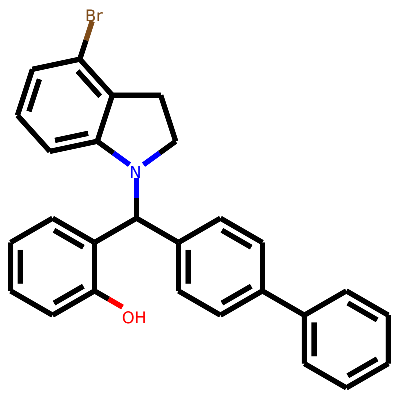
Ahma-2321

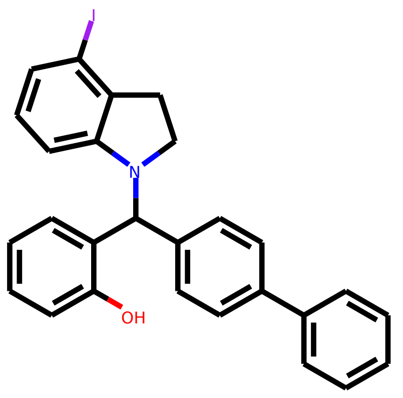
Ahma-2322

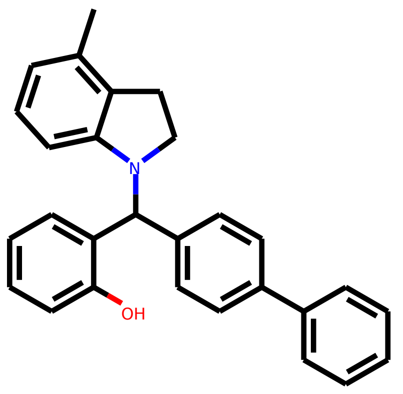
Ahma-2323

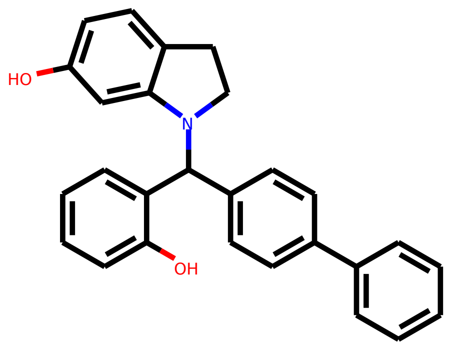
Ahma-2324

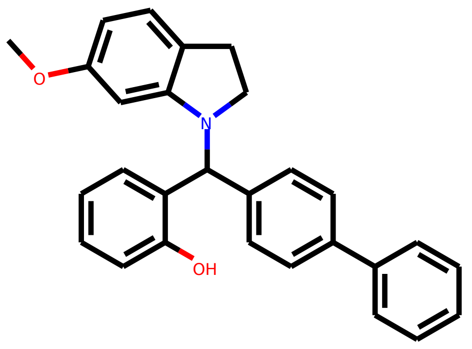
Ahma-2325

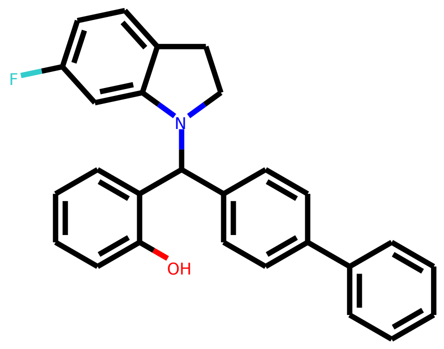
Ahma-2326

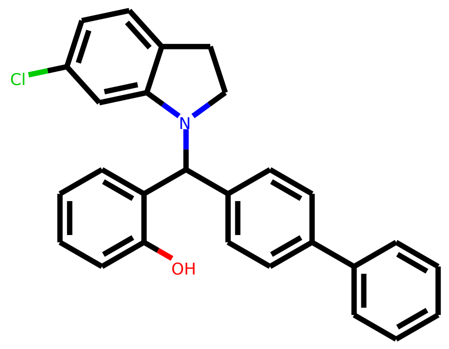
Ahma-2327

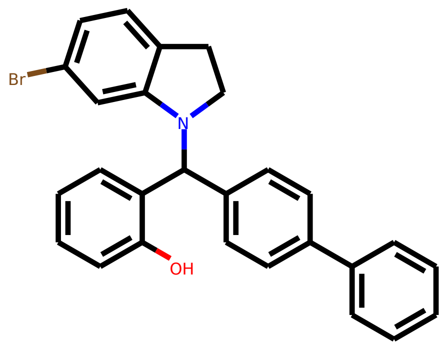
Ahma-2328

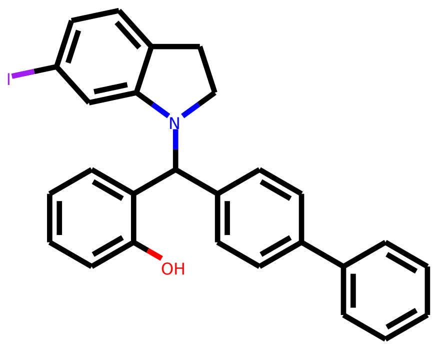
Ahma-2329

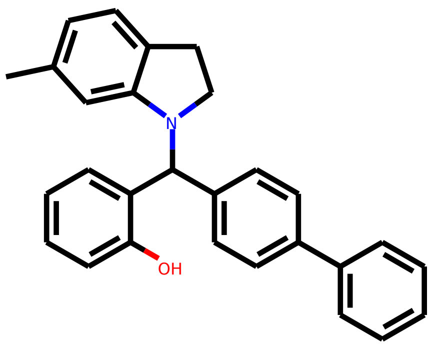
Ahma-2330

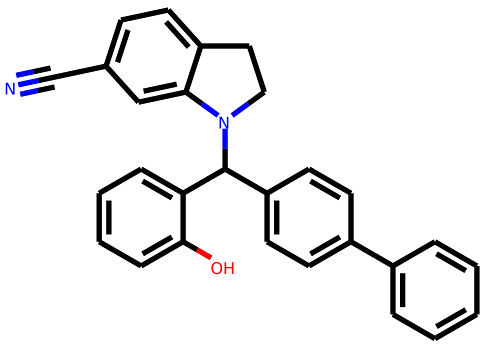
Ahma-2331

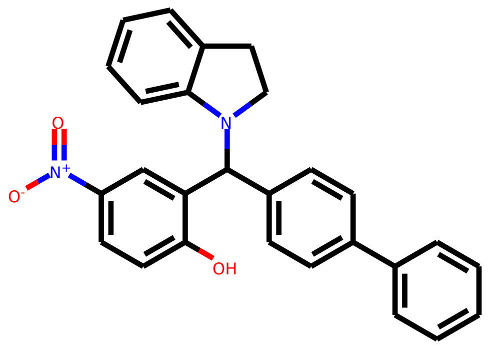
Ahma-2332

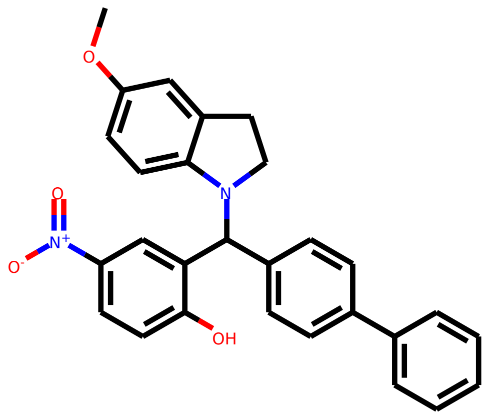
Ahma-2333

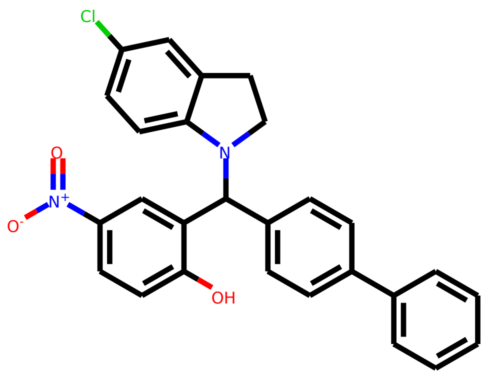
Ahma-2334

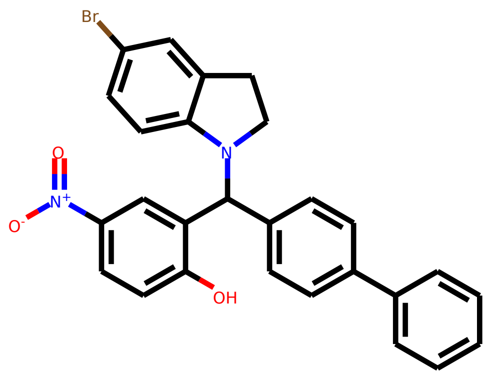
Ahma-2335

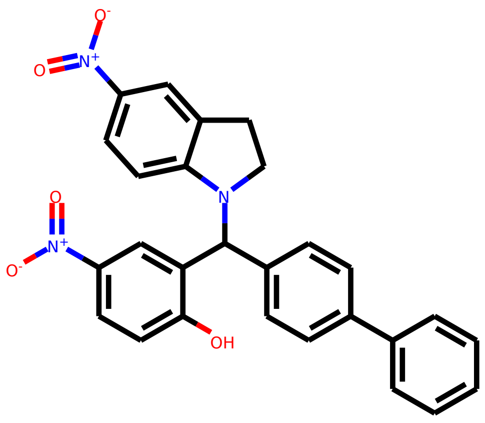
Ahma-2336

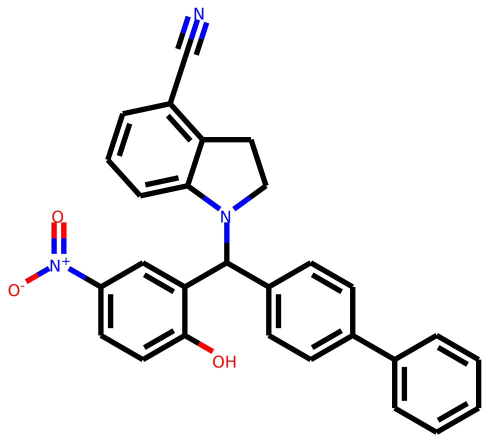
Ahma-2337

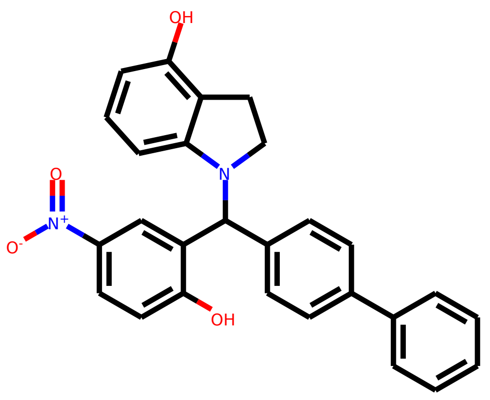
Ahma-2338

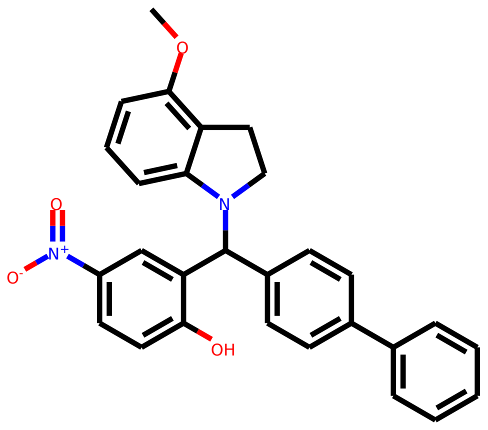
Ahma-2339

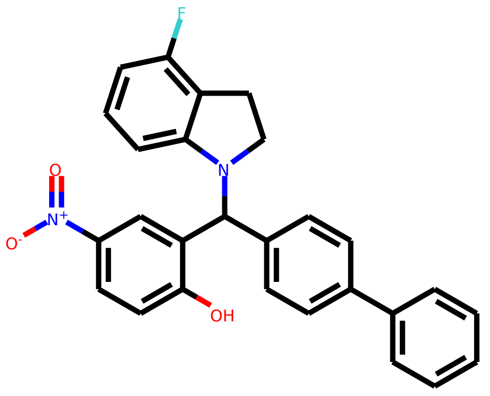
Ahma-2340

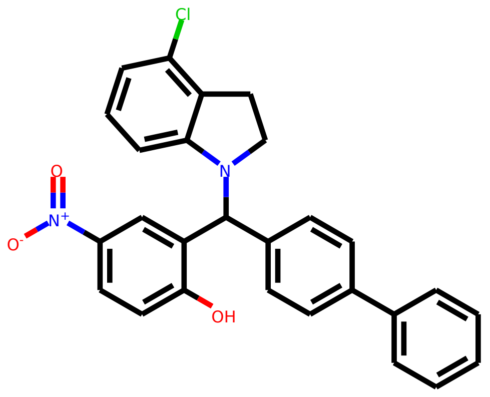
Ahma-2341

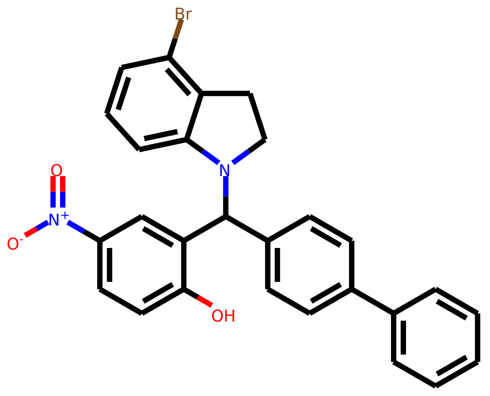
Ahma-2342

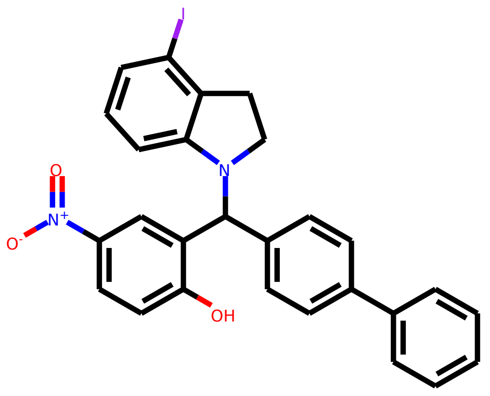
Ahma-2343

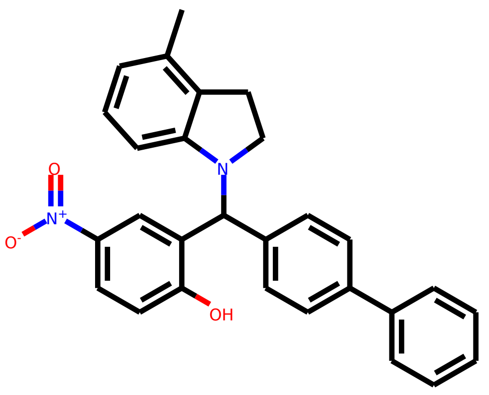
Ahma-2344

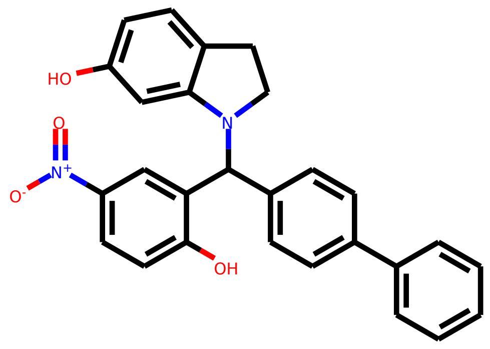
Ahma-2345

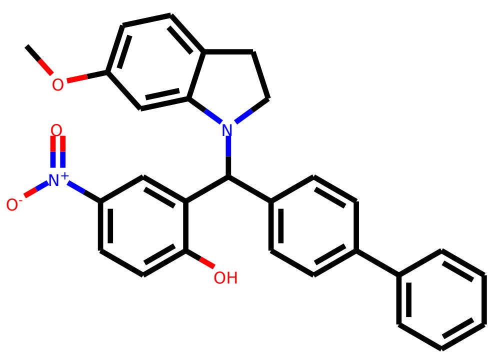
Ahma-2346
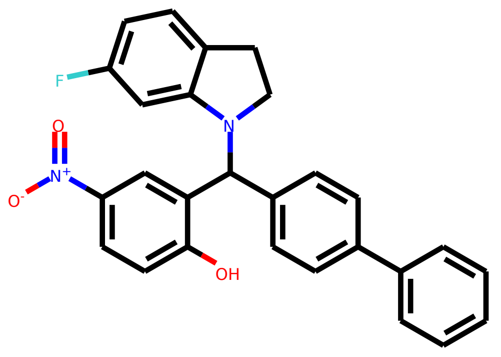
Ahma-2347
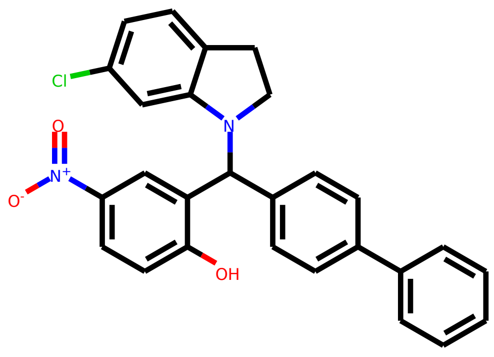
Ahma-2348
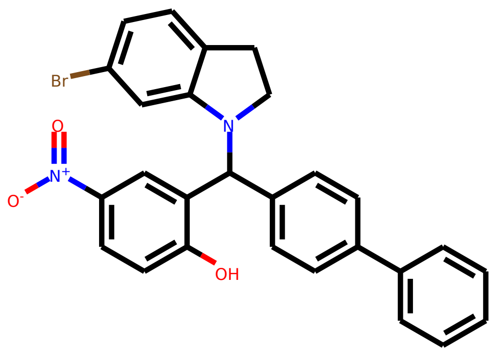
Ahma-2349
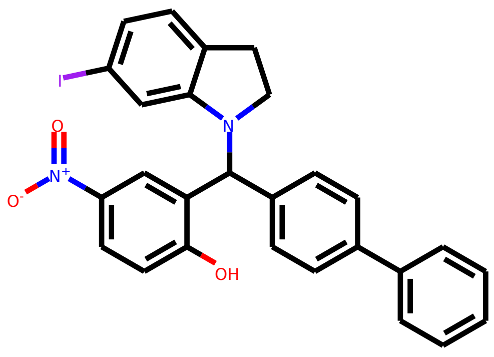
Ahma-2350
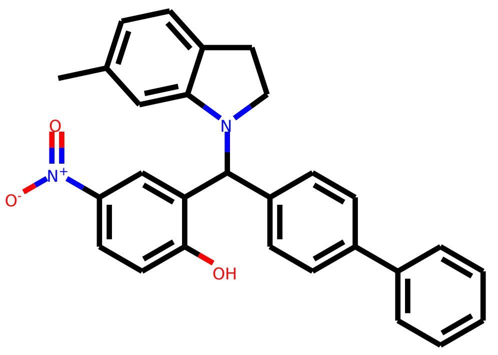
Ahma-2351
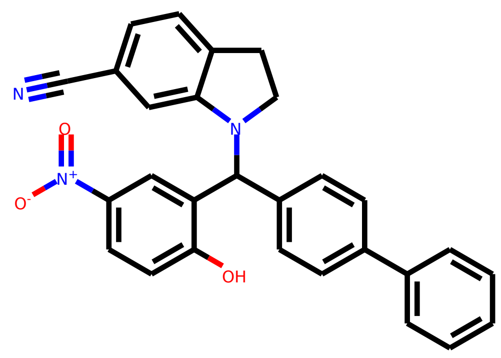
Ahma-2352
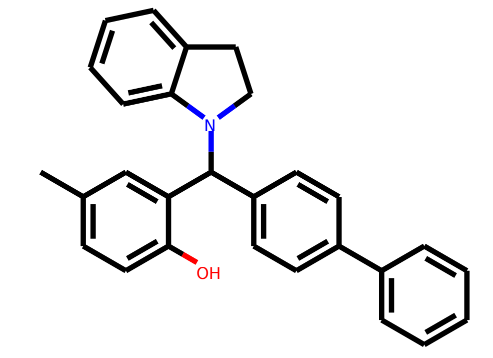
Ahma-2353
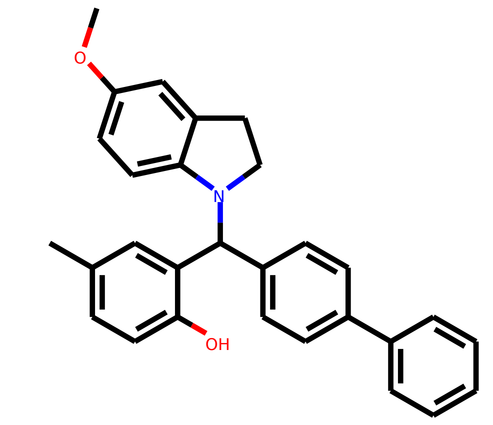
Ahma-2354
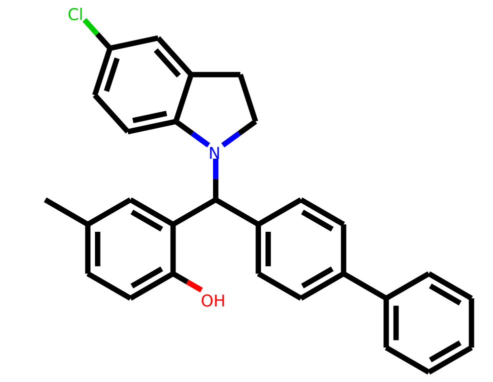
Ahma-2355
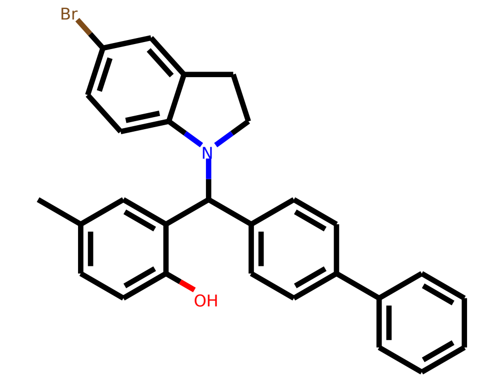
Ahma-2356
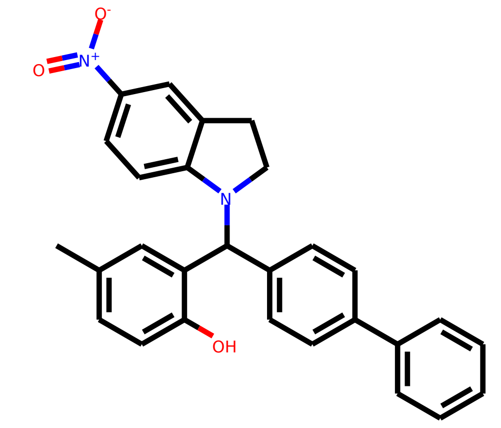
Ahma-2357
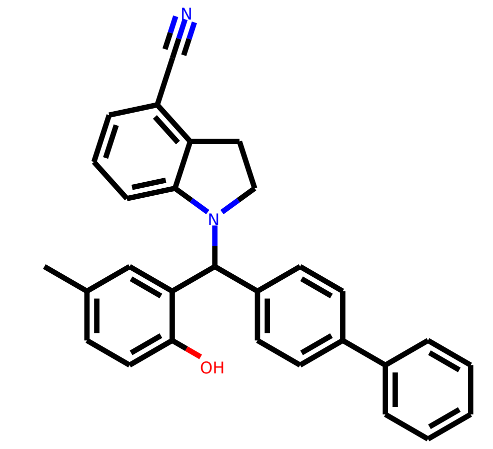
Ahma-2358
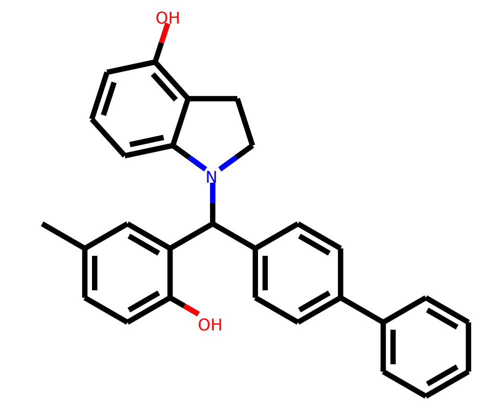
Ahma-2359
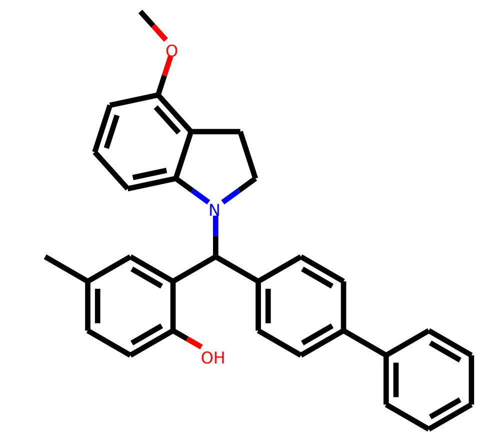
Ahma-2360
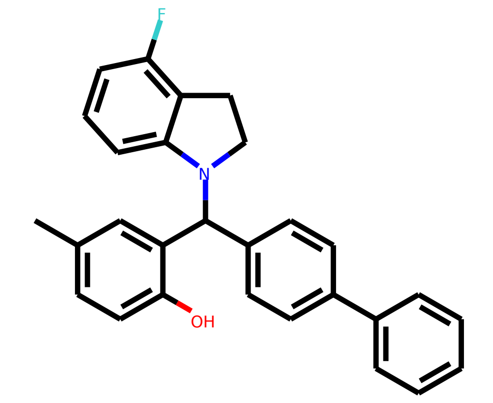
Ahma-2361
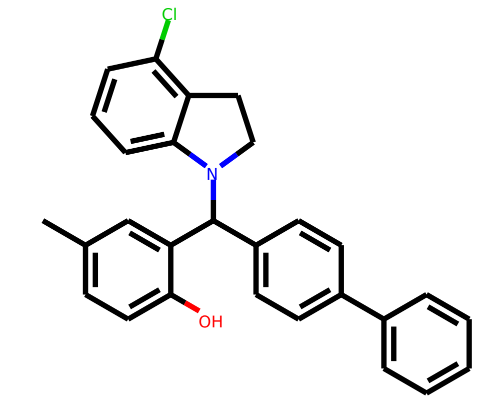
Ahma-2362
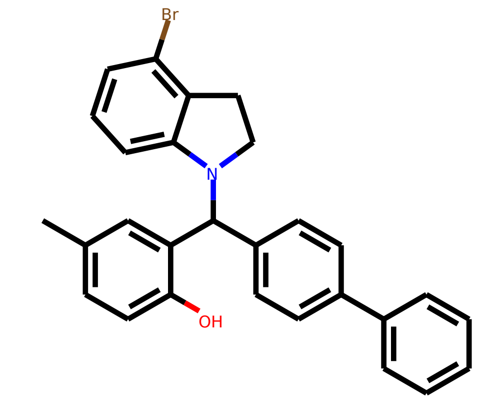
Ahma-2363
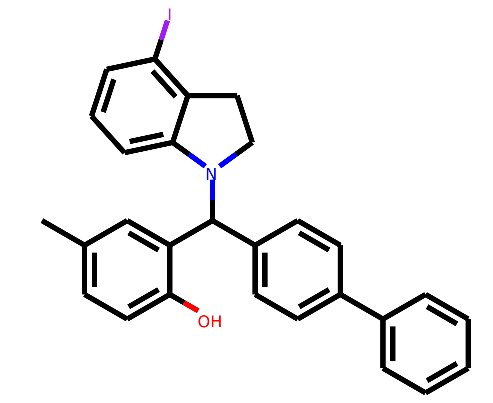
Ahma-2364
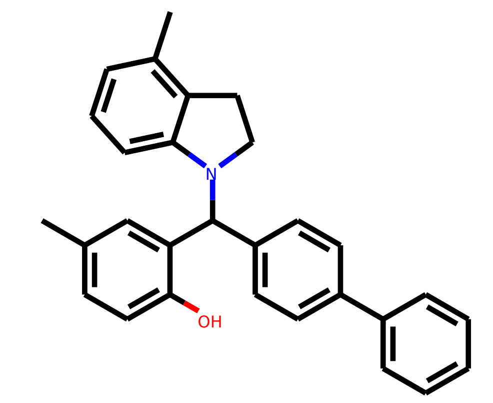
Ahma-2365
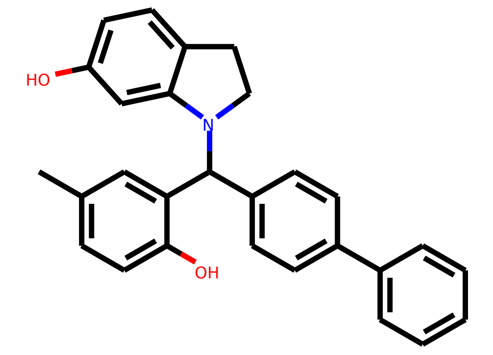
Ahma-2366
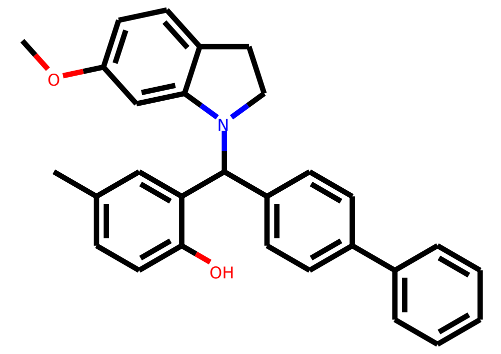
Ahma-2367
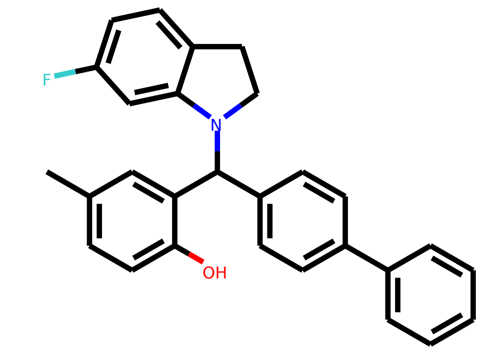
Ahma-2368
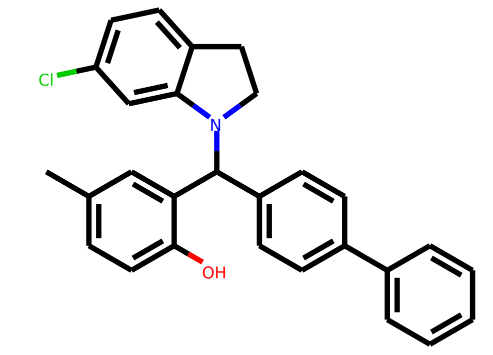
Ahma-2369
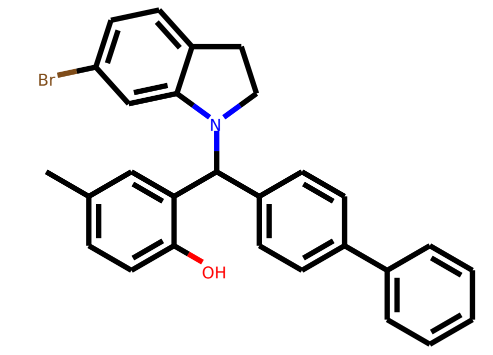
Ahma-2370
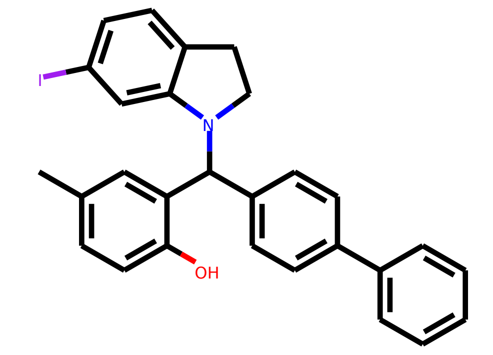
Ahma-2371
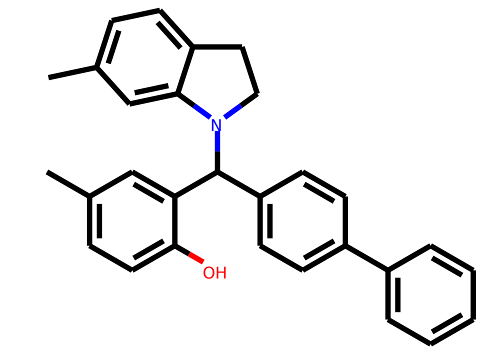
Ahma-2372
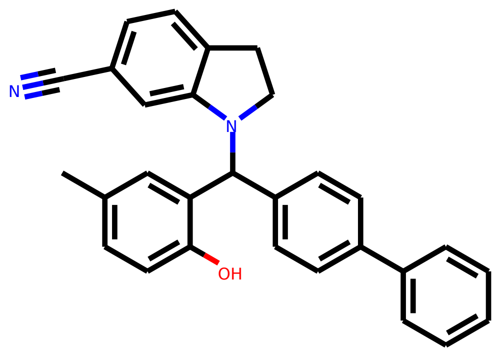
Ahma-2373
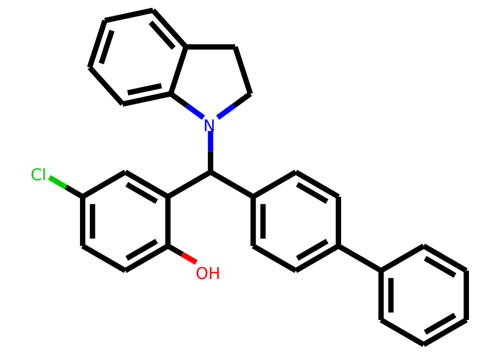
Ahma-2374
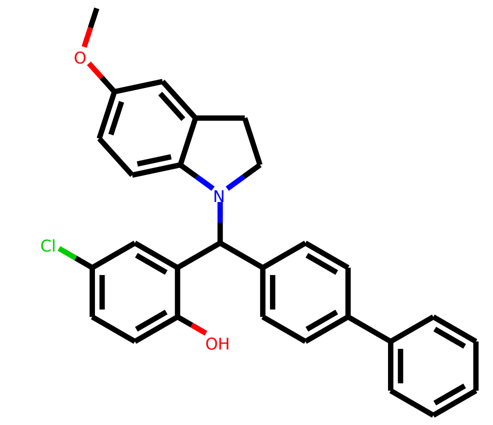
Ahma-2375
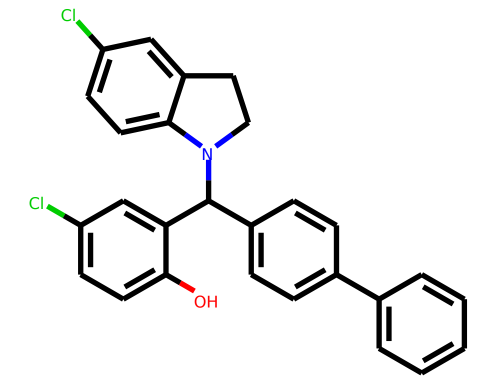
Ahma-2376
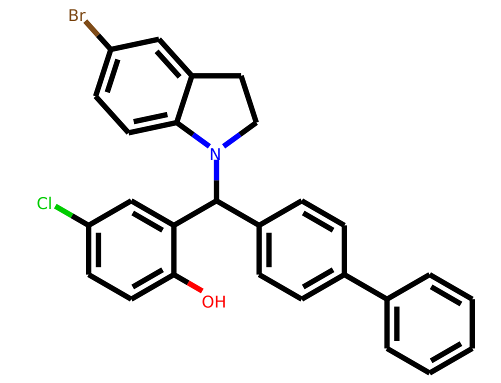
Ahma-2377
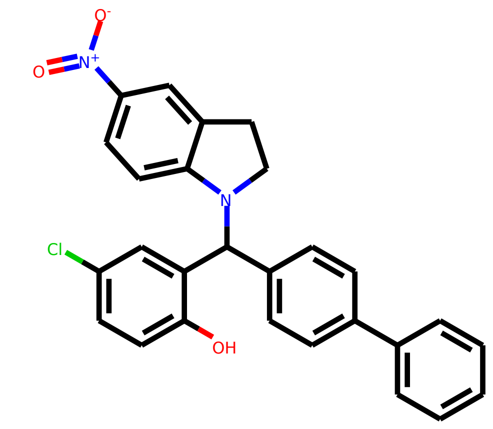
Ahma-2378
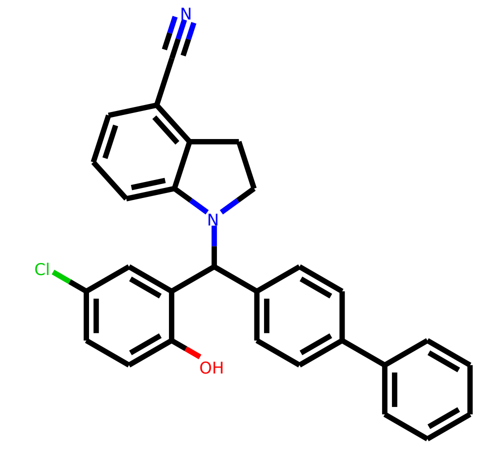
Ahma-2379
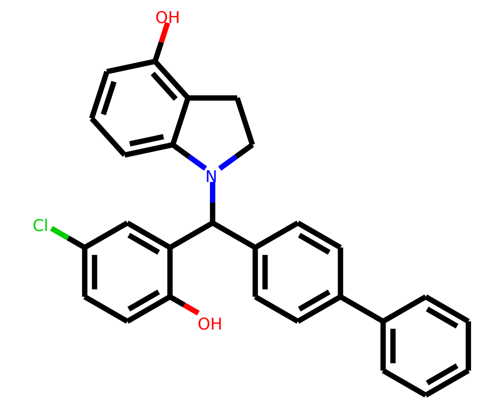
Ahma-2380

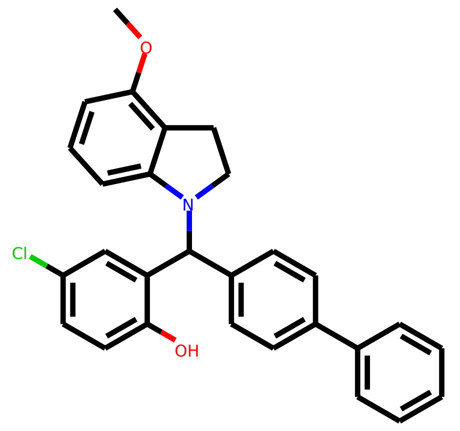
Ahma-2381
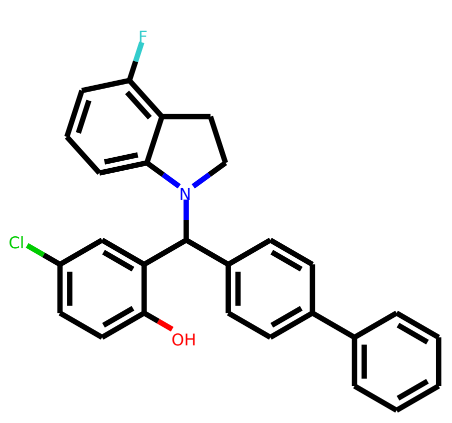
Ahma-2382
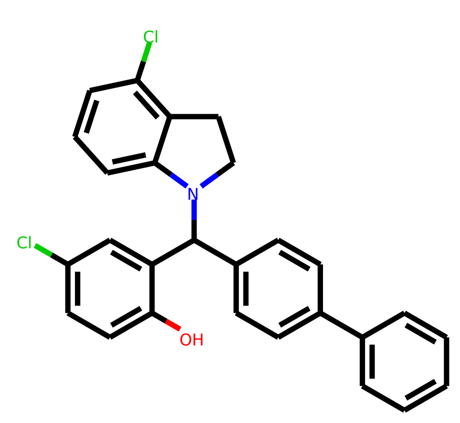
Ahma-2383
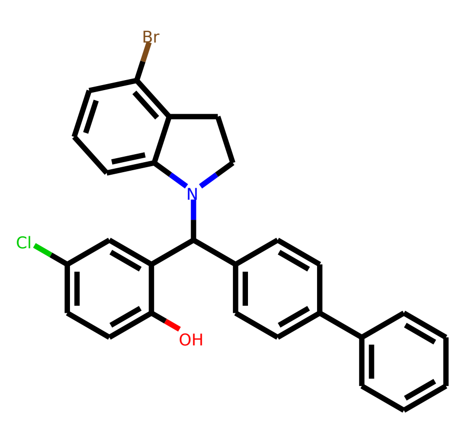
Ahma-2384
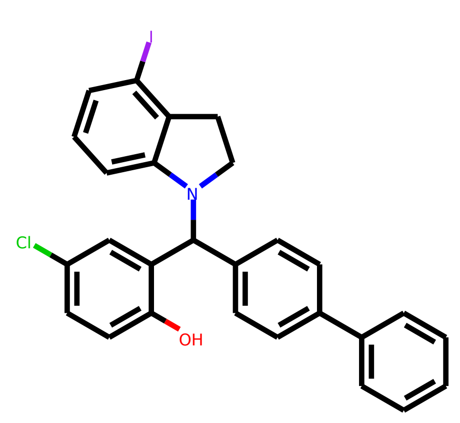
Ahma-2385
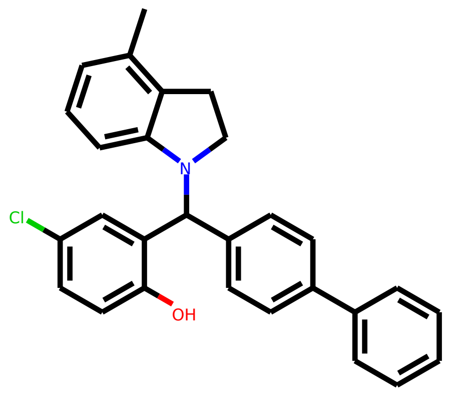
Ahma-2386
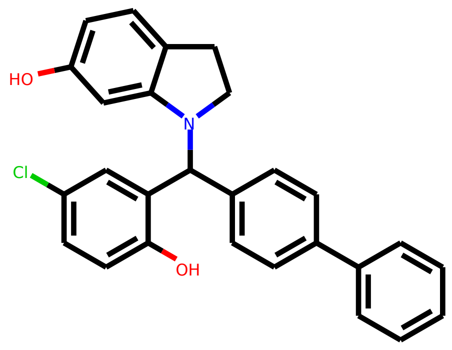
Ahma-2387
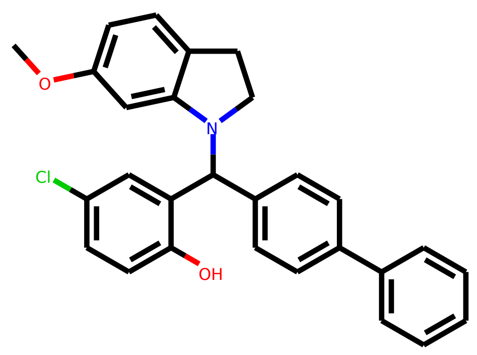
Ahma-2388
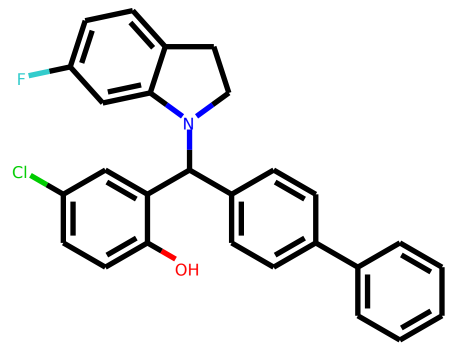
Ahma-2389
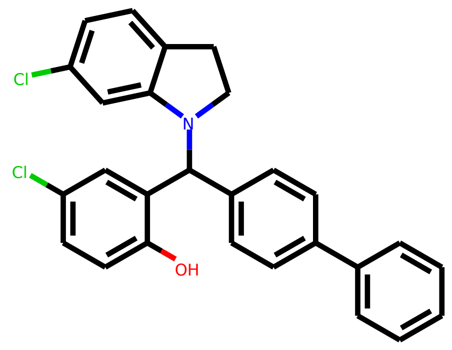
Ahma-2390
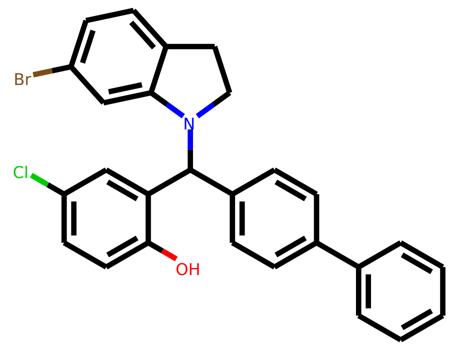
Ahma-2391
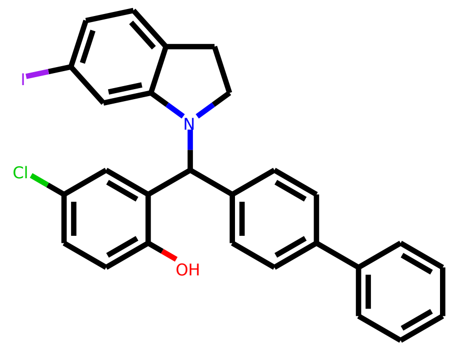
Ahma-2392
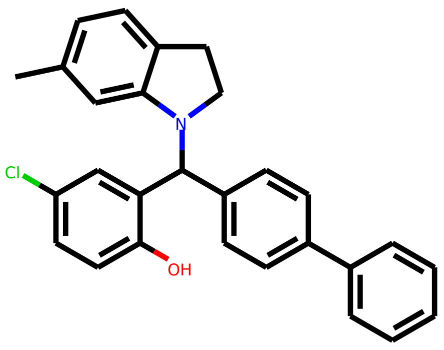
Ahma-2393
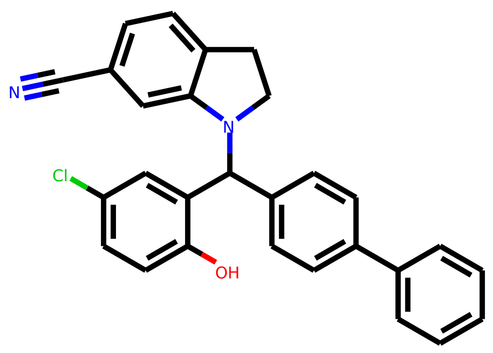
Ahma-2394
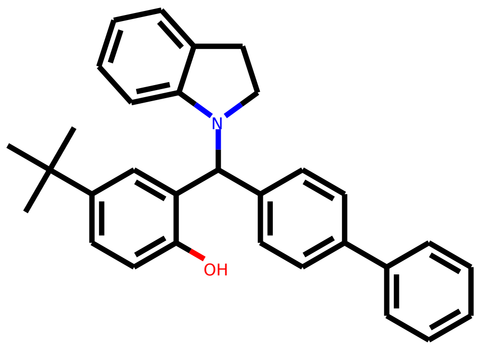
Ahma-2395
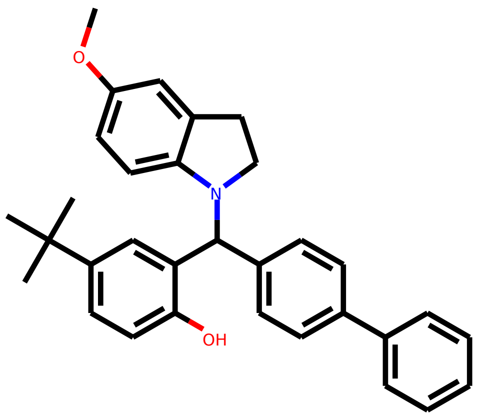
Ahma-2396
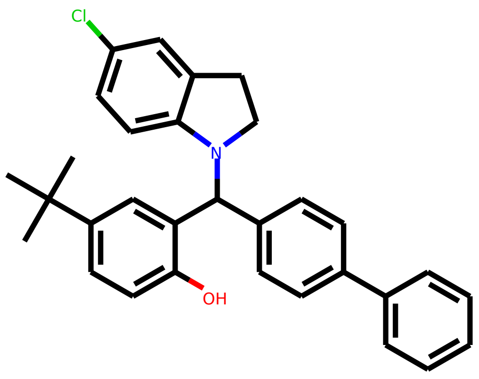
Ahma-2397
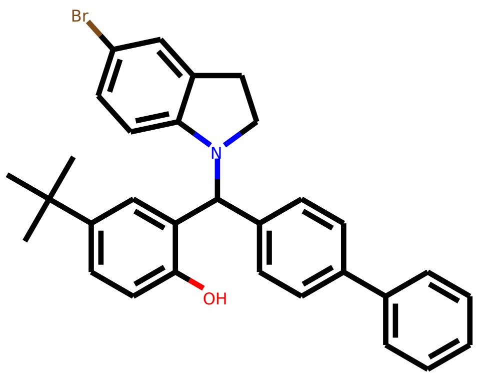
Ahma-2398
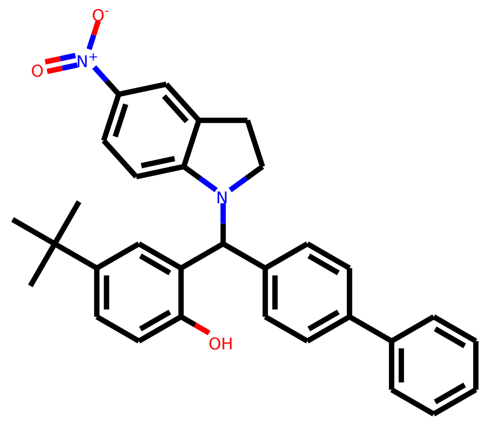
Ahma-2399
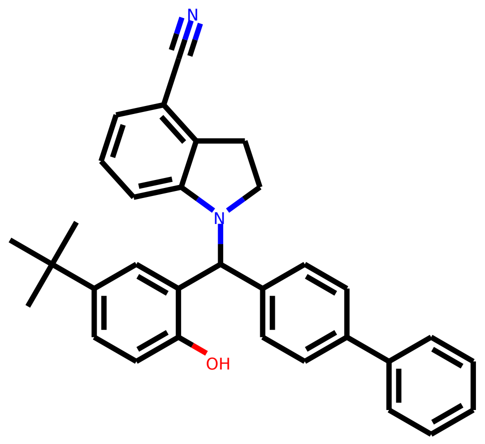
Ahma-2400
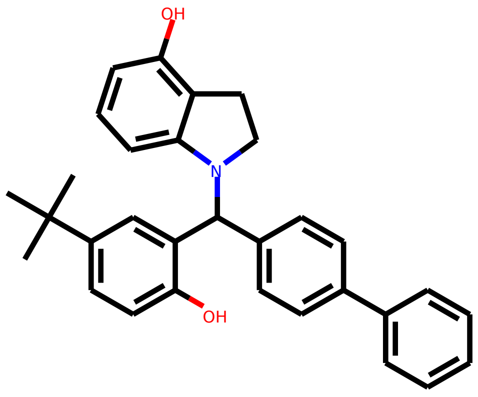
Ahma-2401
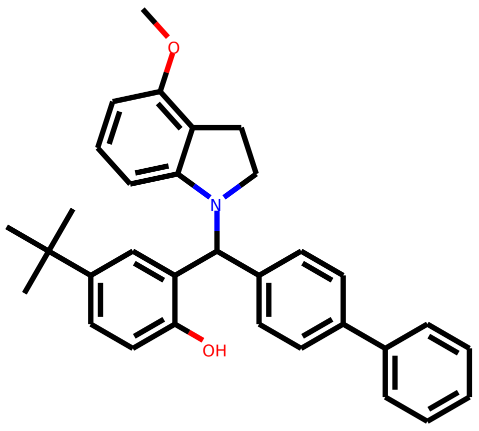
Ahma-2402
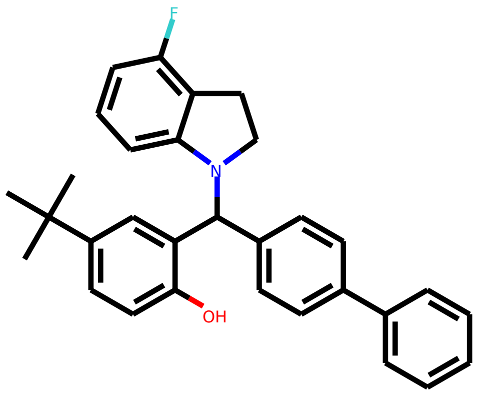
Ahma-2403
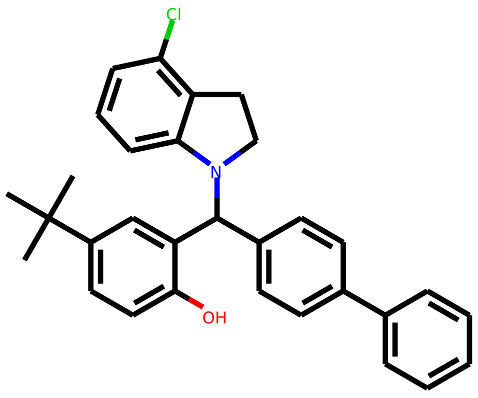
Ahma-2404
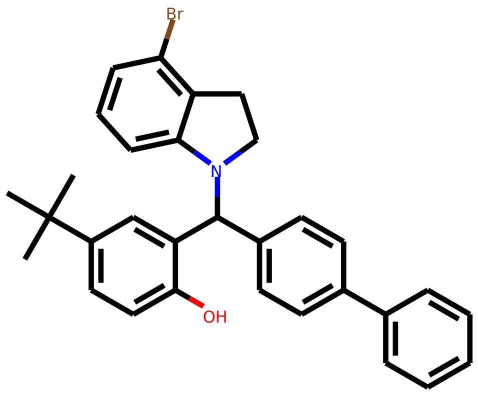
Ahma-2405
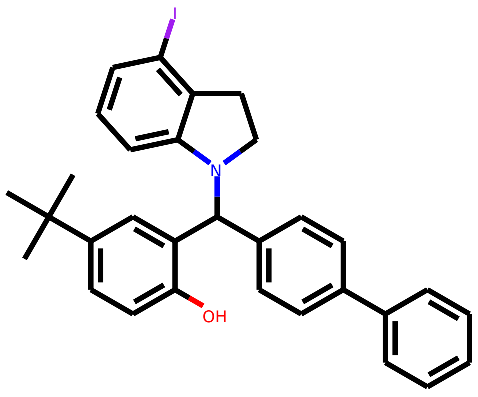
Ahma-2406
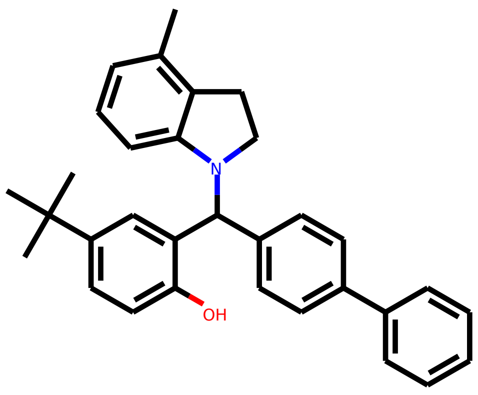
Ahma-2407
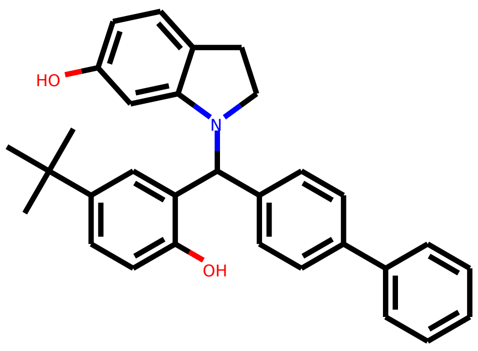
Ahma-2408
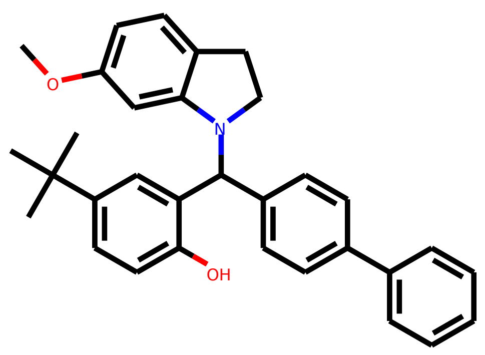
Ahma-2409
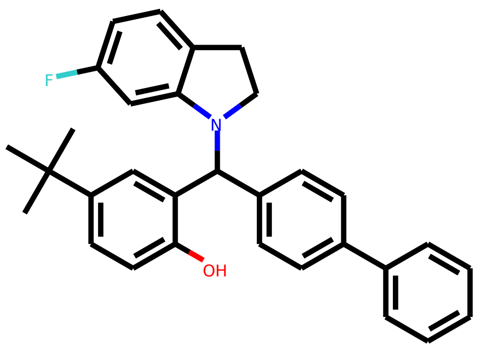
Ahma-2410
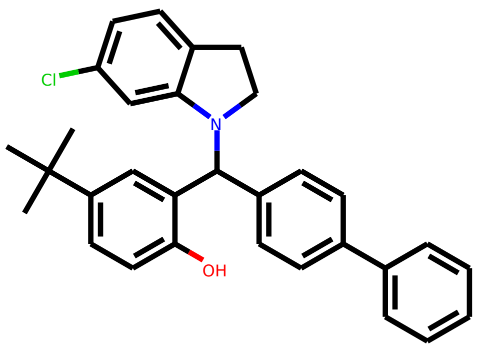
Ahma-2411
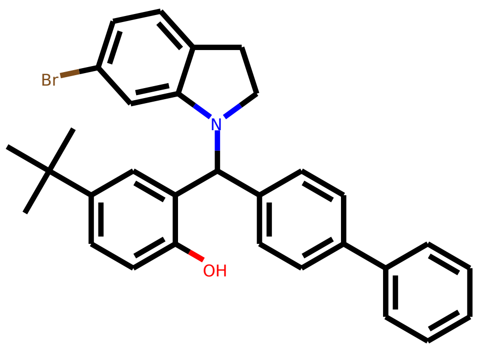
Ahma-2412
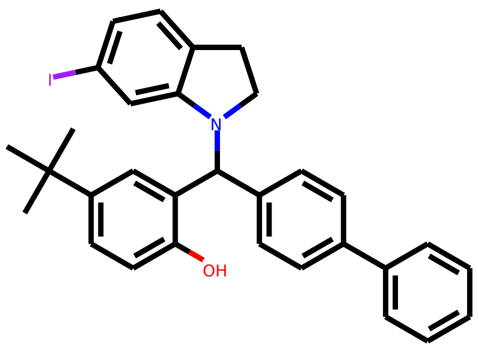
Ahma-2413
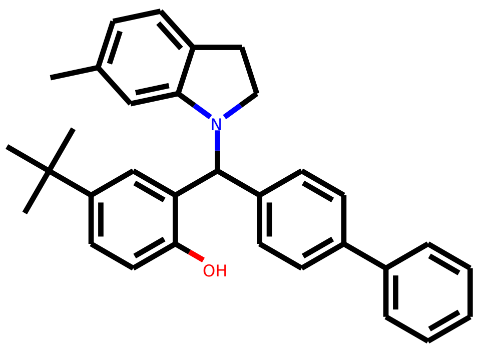
Ahma-2414
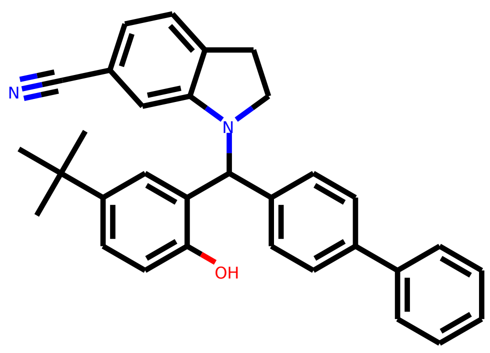
Ahma-2415

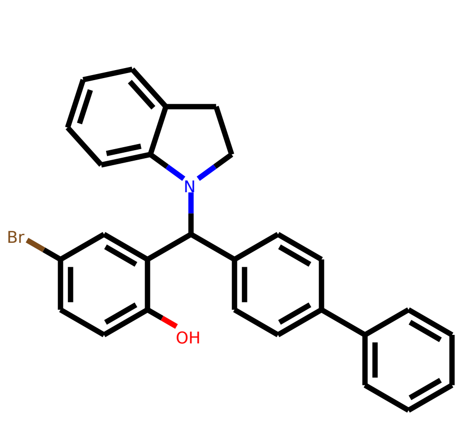
Ahma-2416

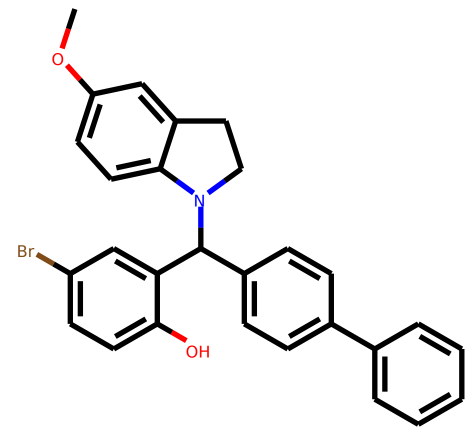
Ahma-2417

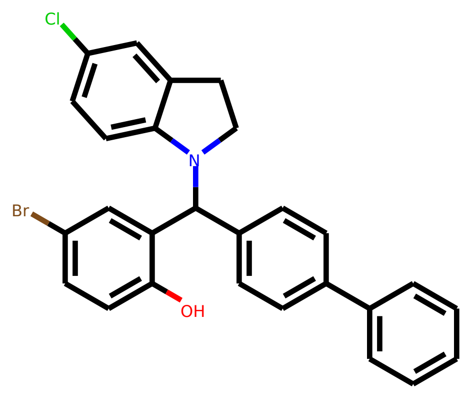
Ahma-2418

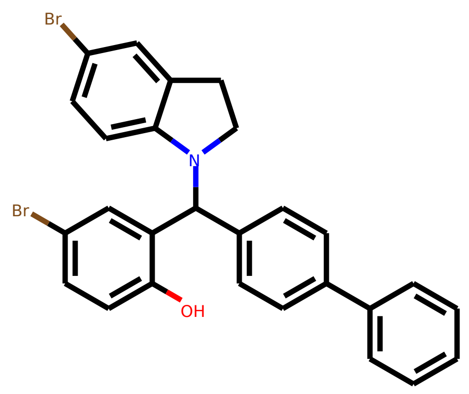
Ahma-2419

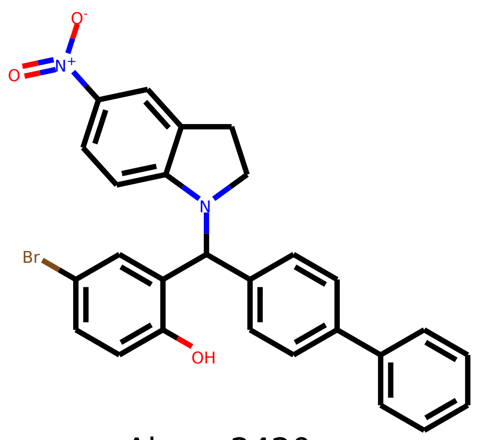
Ahma-2420

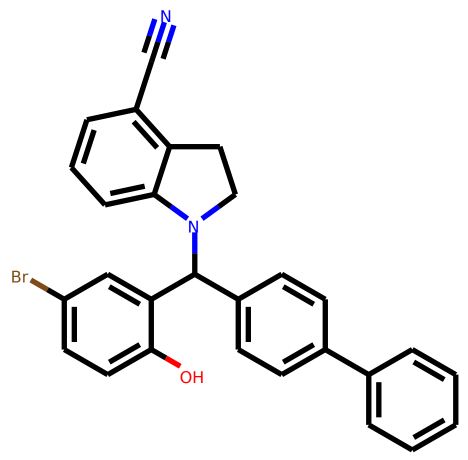
Ahma-2421

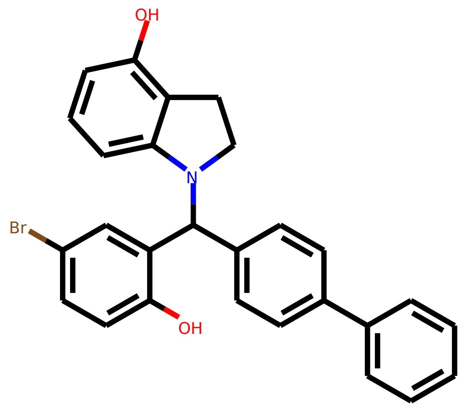
Ahma-2422

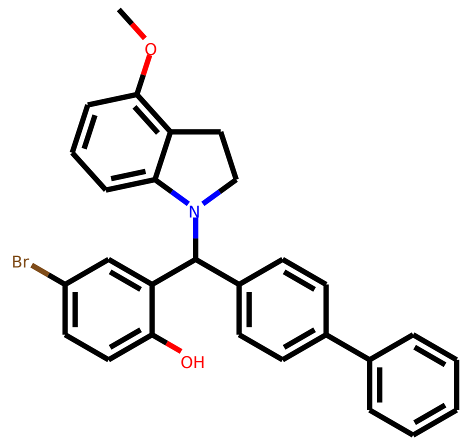
Ahma-2423

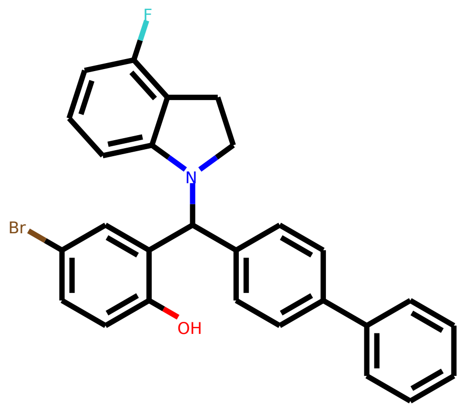
Ahma-2424

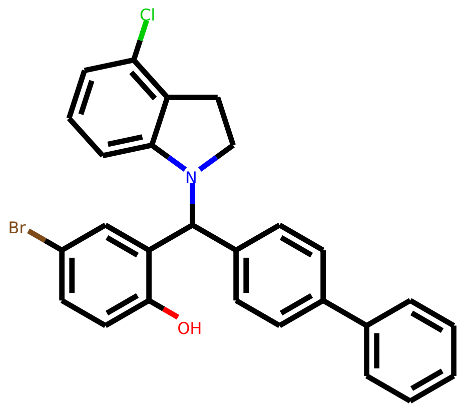
Ahma-2425

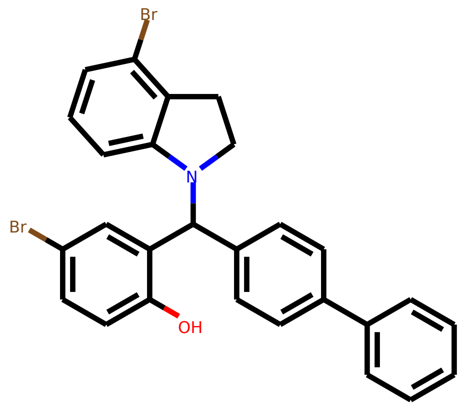
Ahma-2426

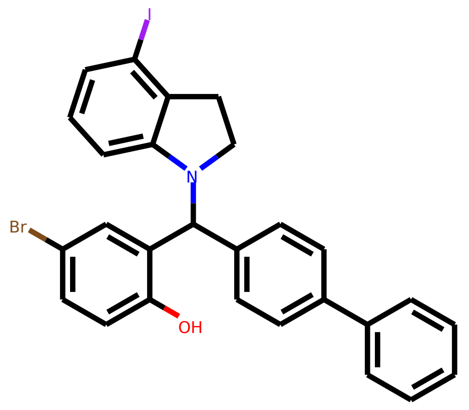
Ahma-2427

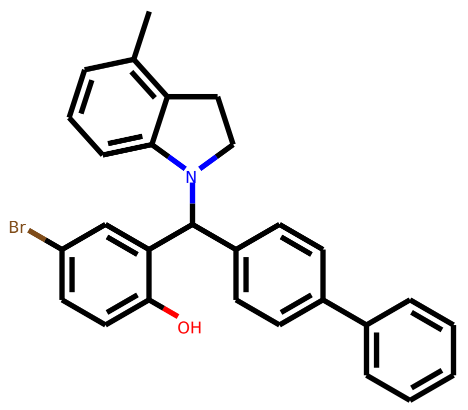
Ahma-2428

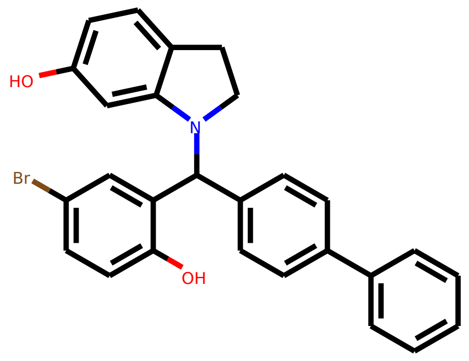
Ahma-2429

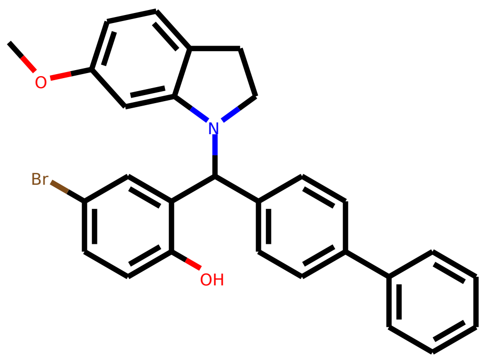
Ahma-2430

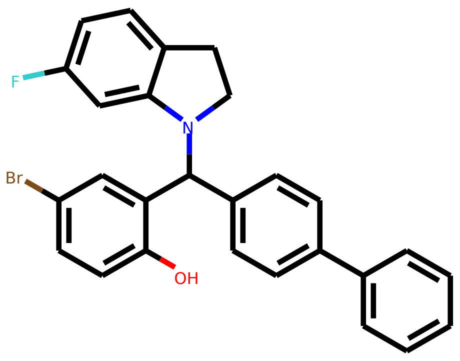
Ahma-2431

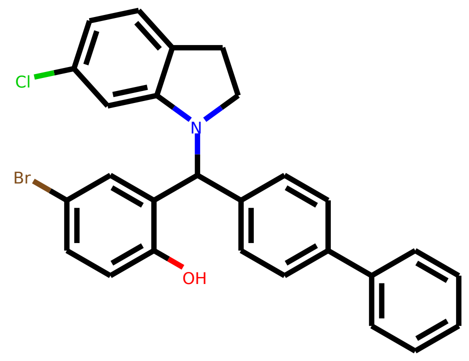
Ahma-2432

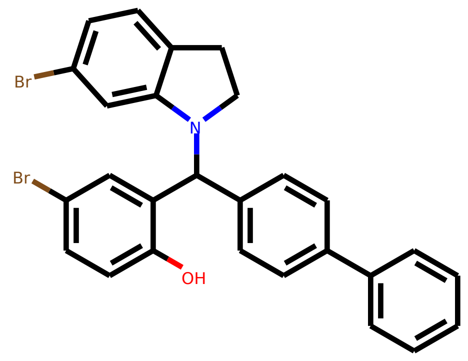
Ahma-2433

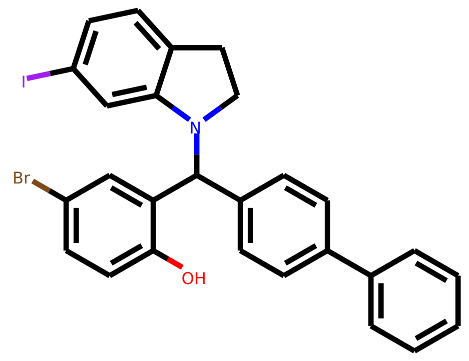
Ahma-2434

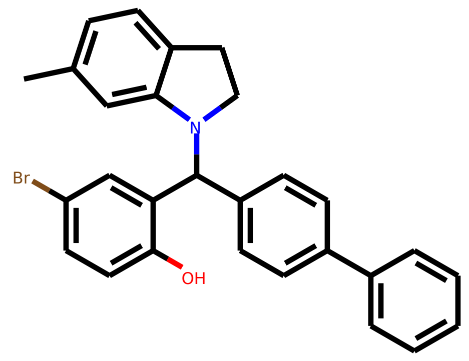
Ahma-2435

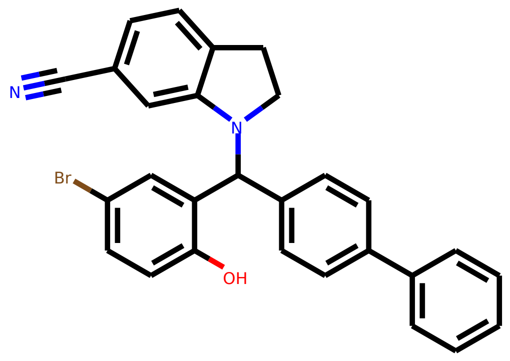
Ahma-2436

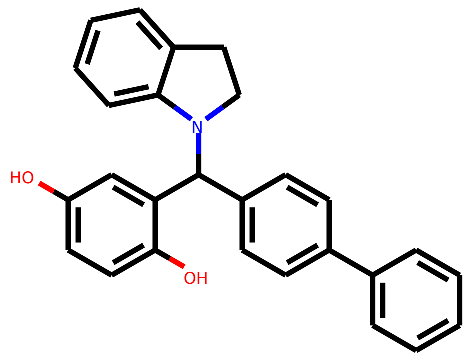
Ahma-2437

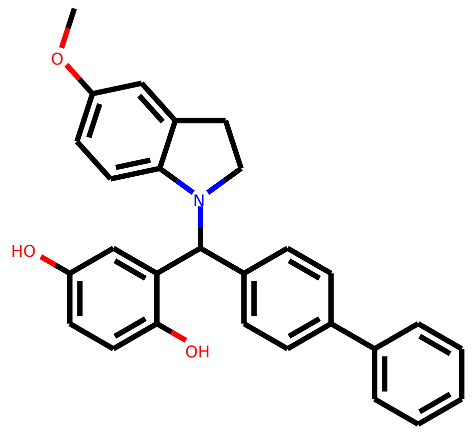
Ahma-2438

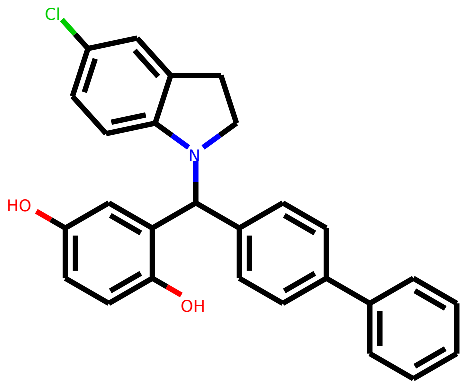
Ahma-2439

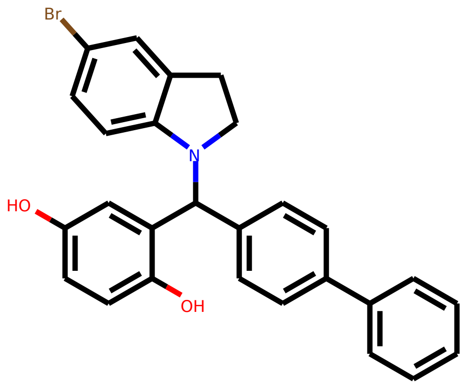
Ahma-2440

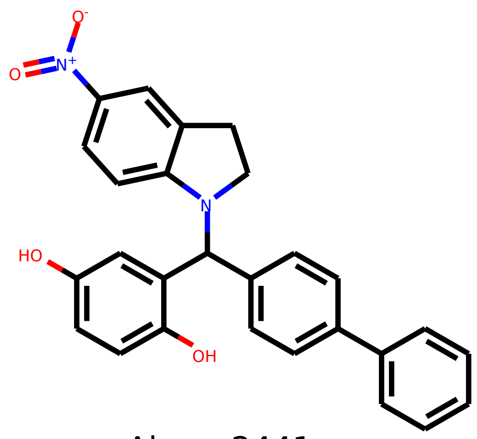
Ahma-2441

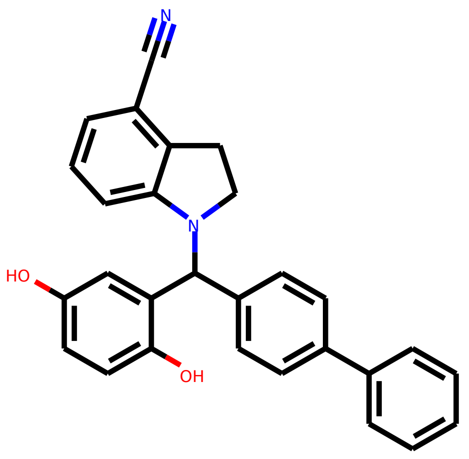
Ahma-2442

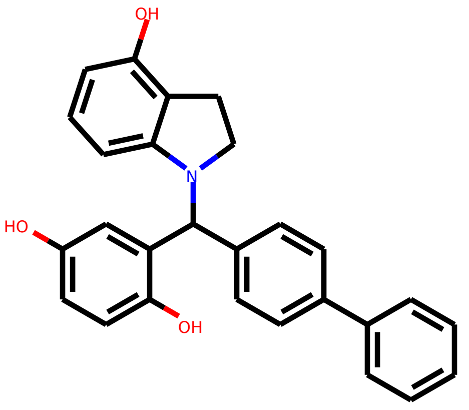
Ahma-2443

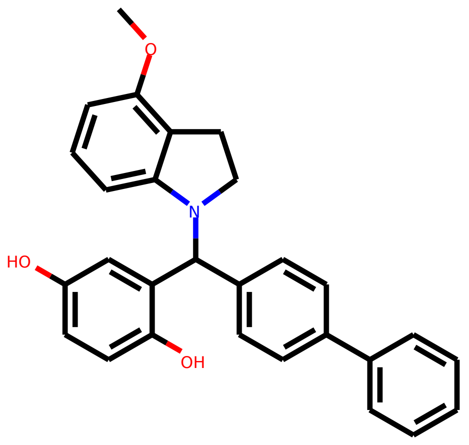
Ahma-2444

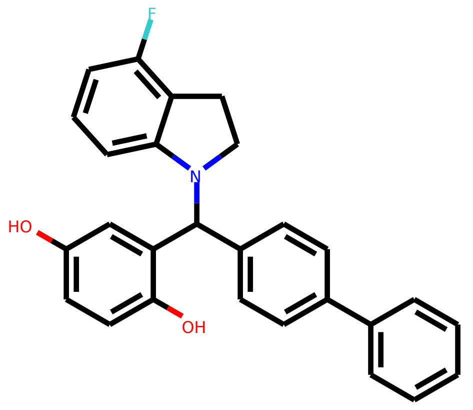
Ahma-2445

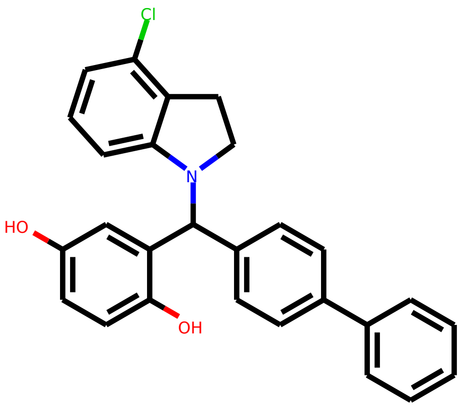
Ahma-2446

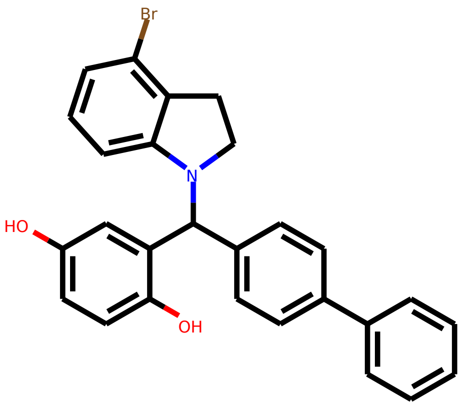
Ahma-2447

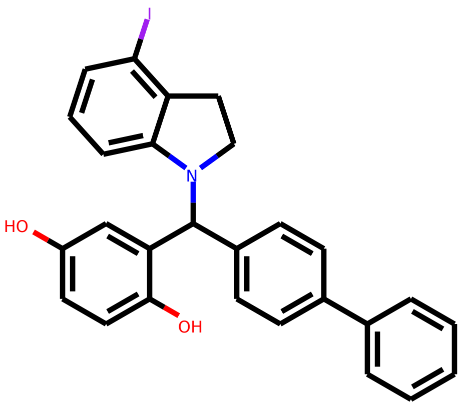
Ahma-2448

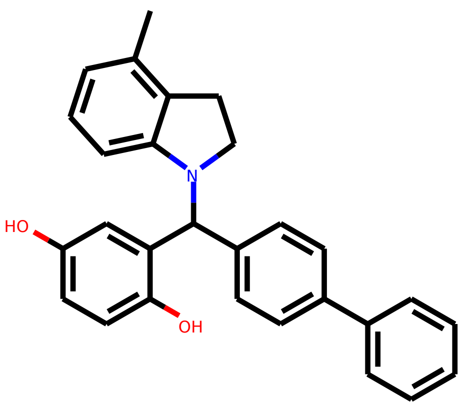
Ahma-2449

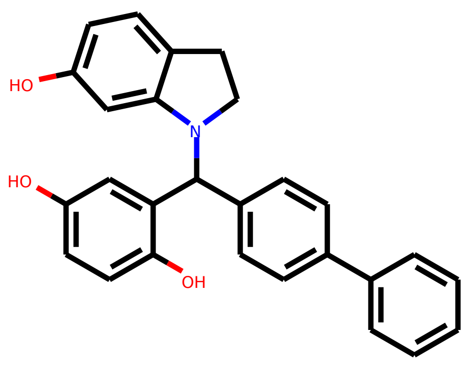
Ahma-2450

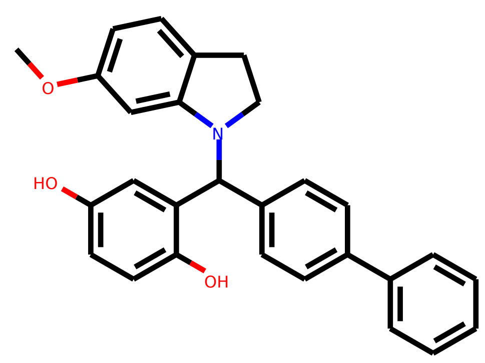
Ahma-2451

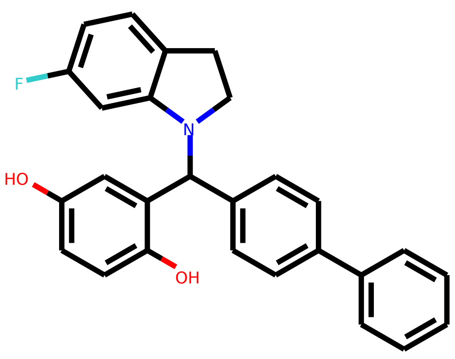
Ahma-2452

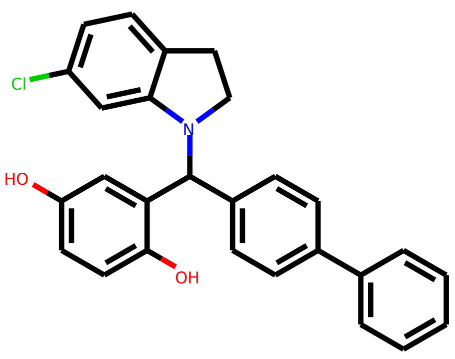
Ahma-2453

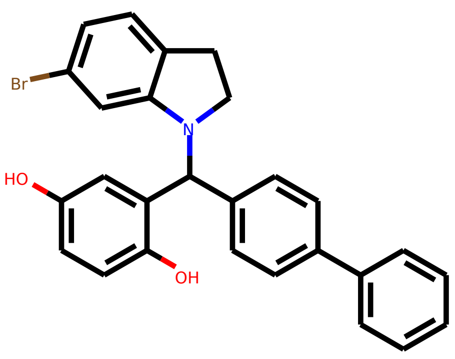
Ahma-2454

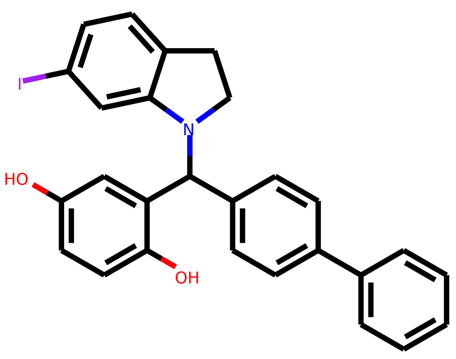
Ahma-2455

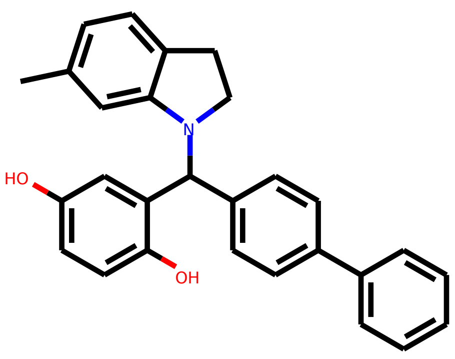
Ahma-2456

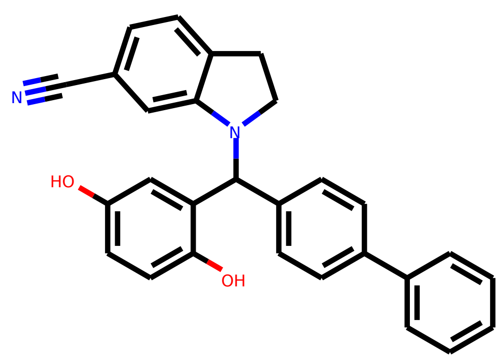
Ahma-2457

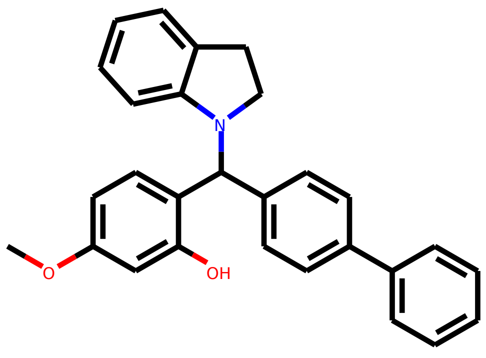
Ahma-2458

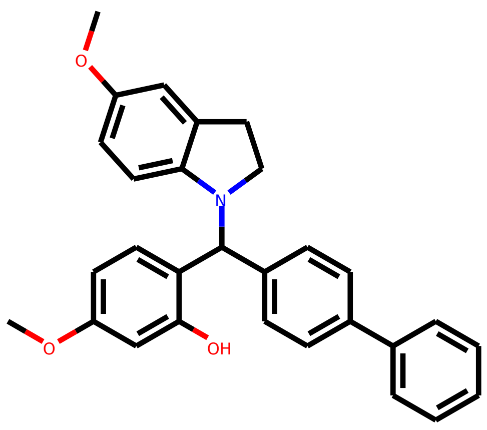
Ahma-2459

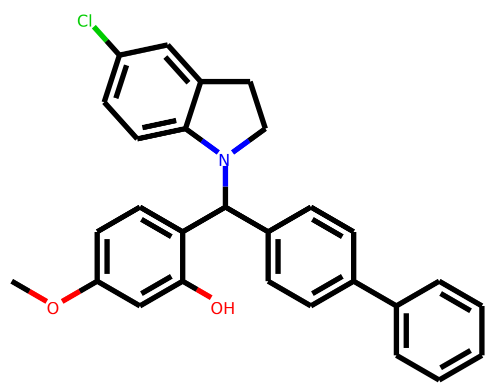
Ahma-2460

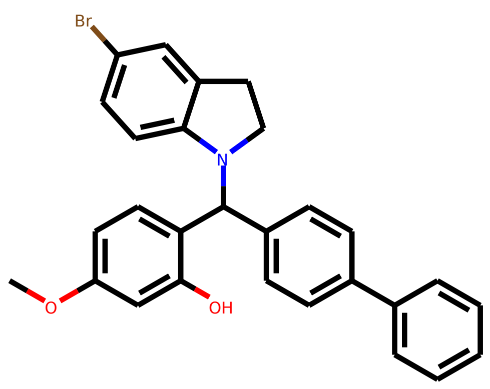
Ahma-2461

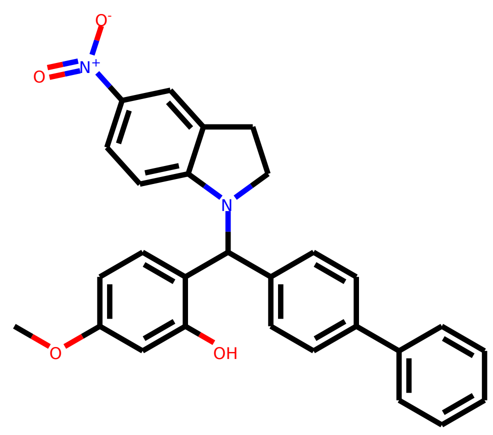
Ahma-2462

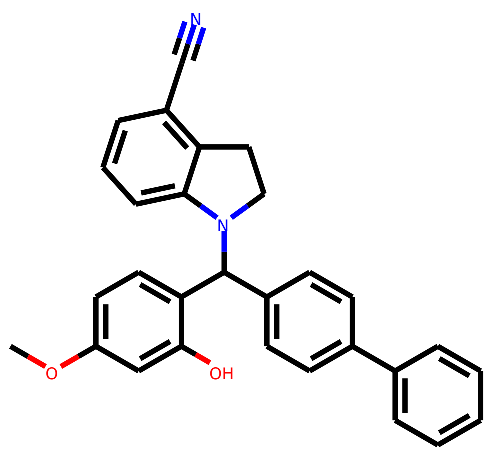
Ahma-2463

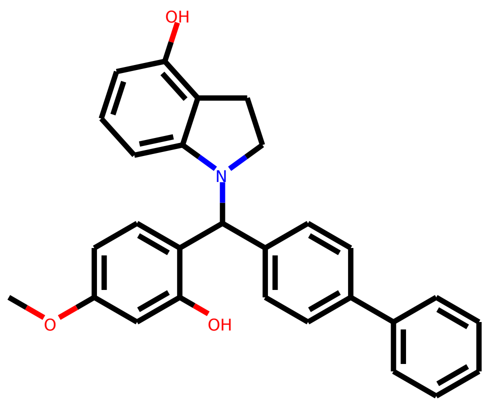
Ahma-2464

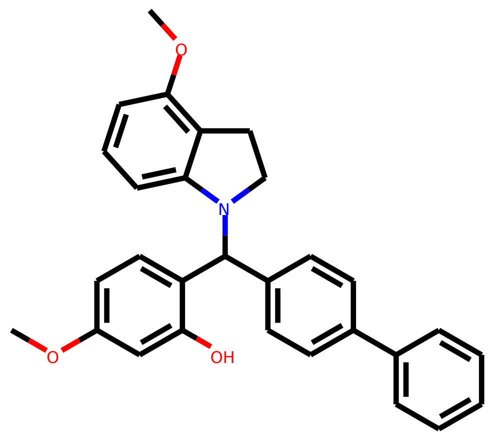
Ahma-2465

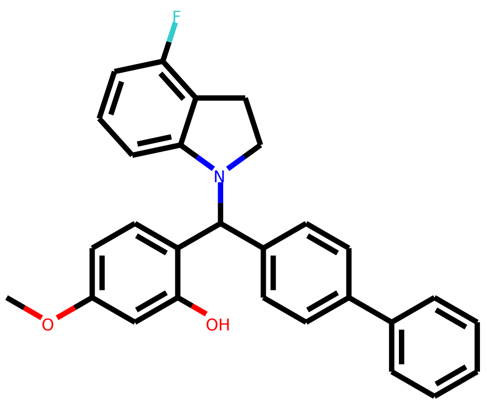
Ahma-2466

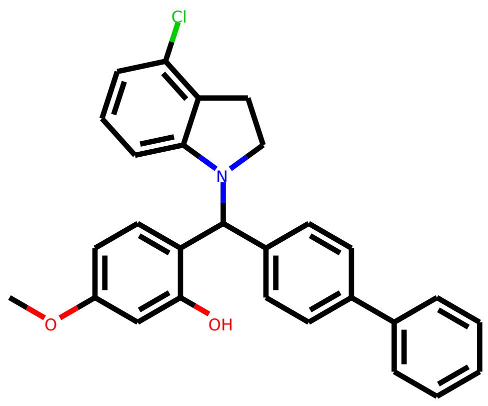
Ahma-2467

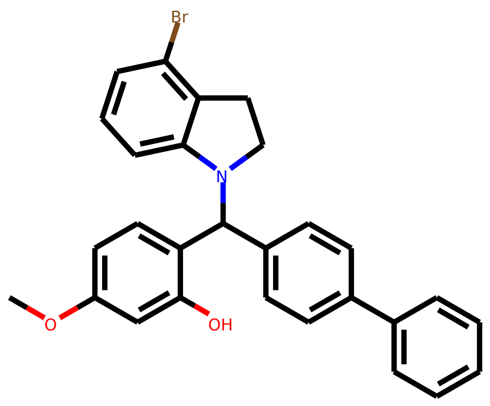
Ahma-2468

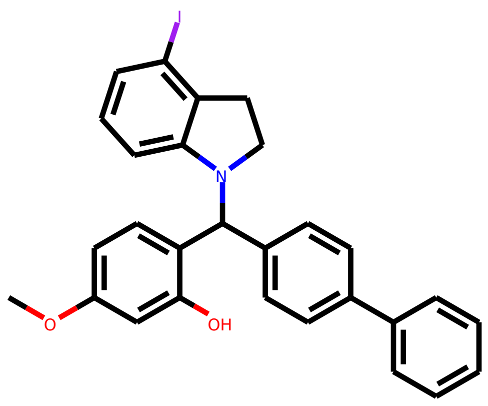
Ahma-2469

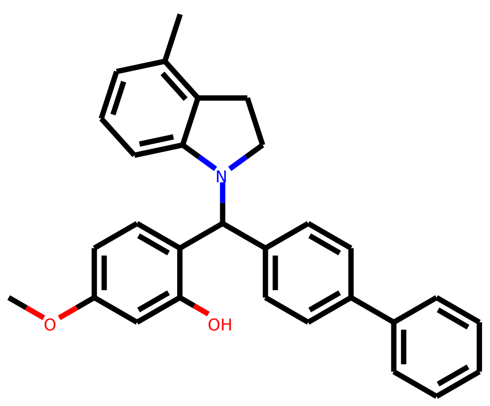
Ahma-2470

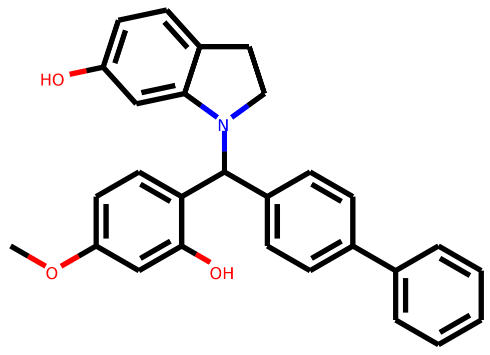
Ahma-2471

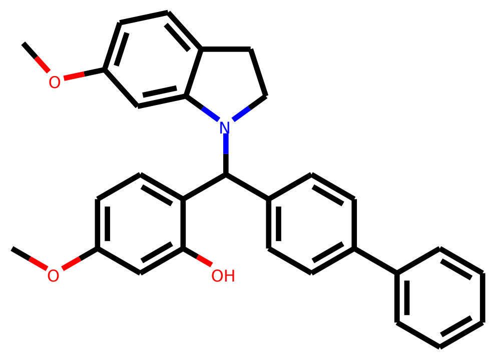
Ahma-2472

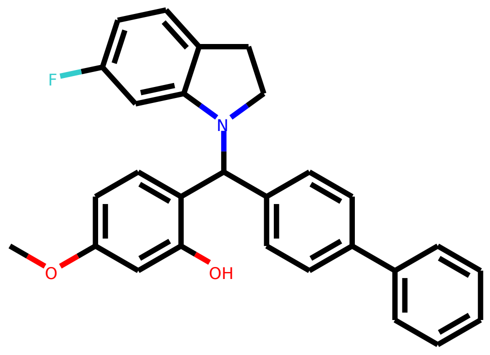
Ahma-2473

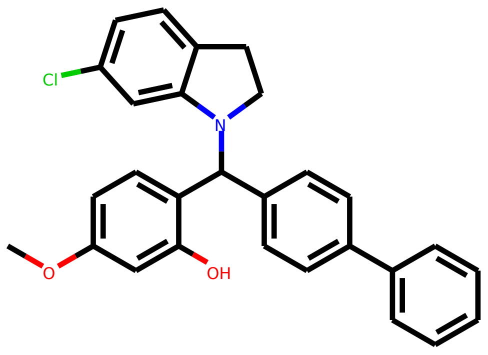
Ahma-2474

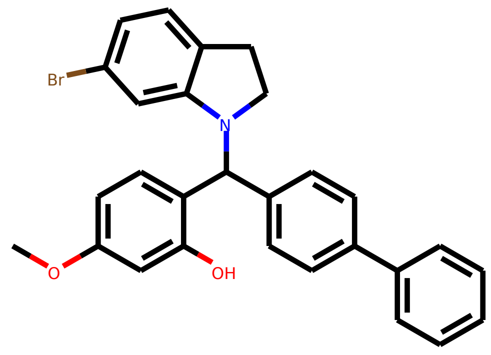
Ahma-2475

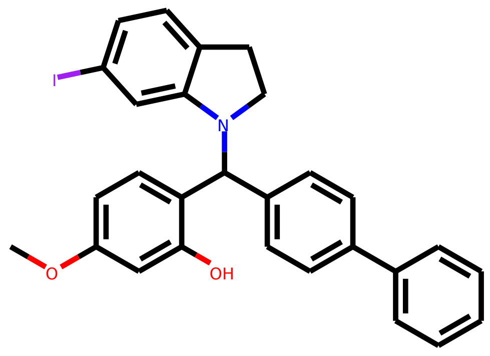
Ahma-2476

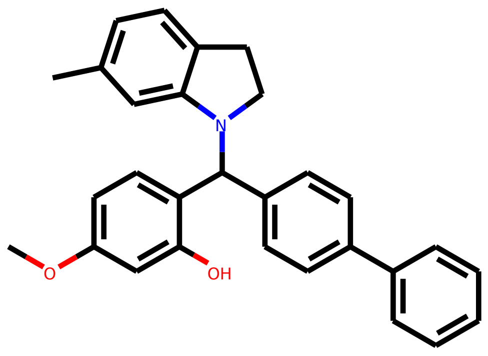
Ahma-2477

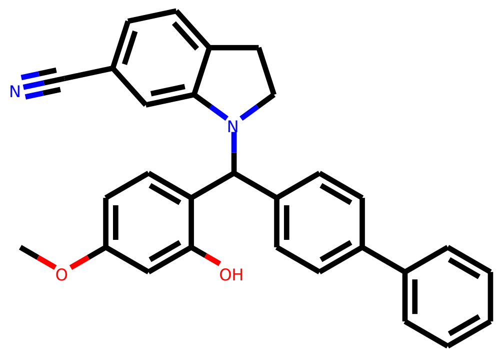
Ahma-2478

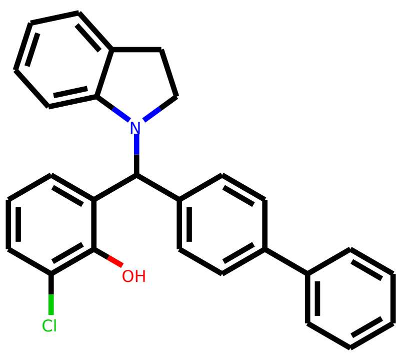
Ahma-2479

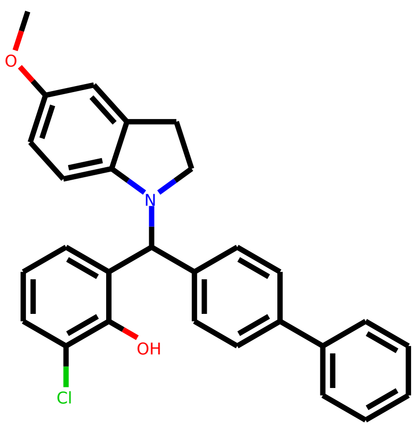
Ahma-2480

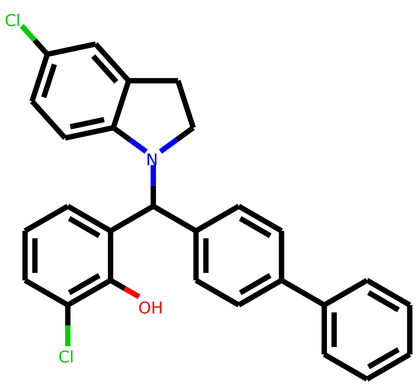
Ahma-2481

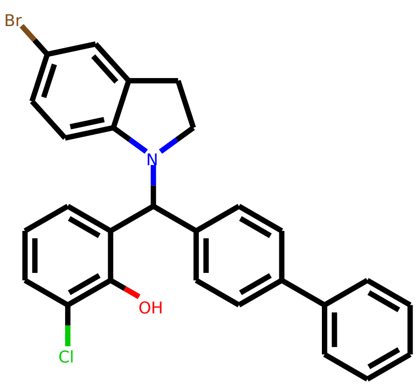
Ahma-2482

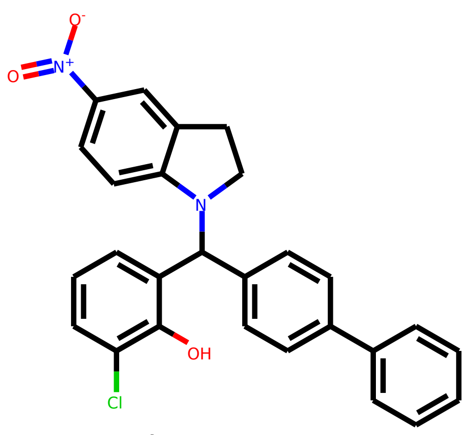
Ahma-2483

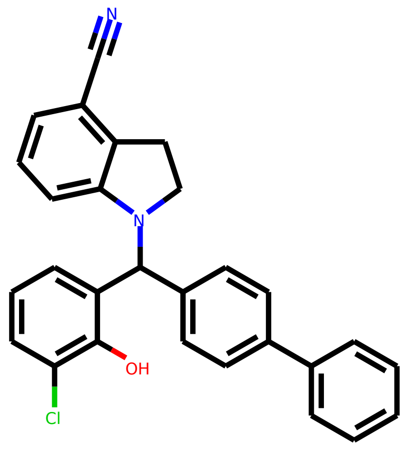
Ahma-2484

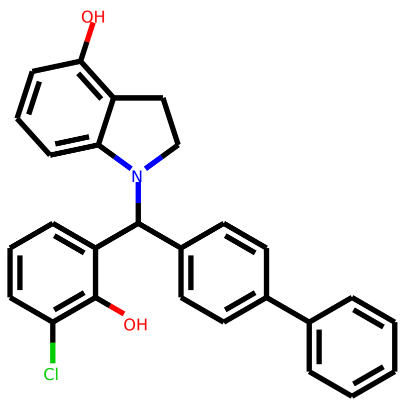
Ahma-2485

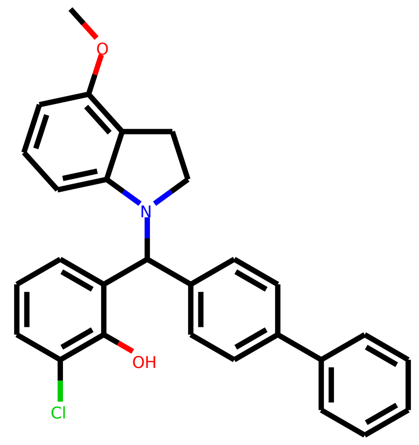
Ahma-2486

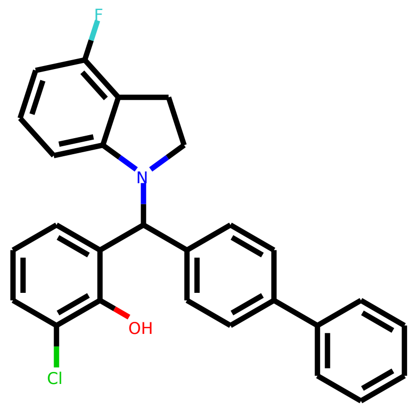
Ahma-2487

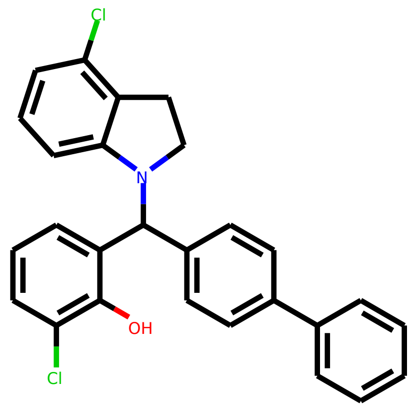
Ahma-2488

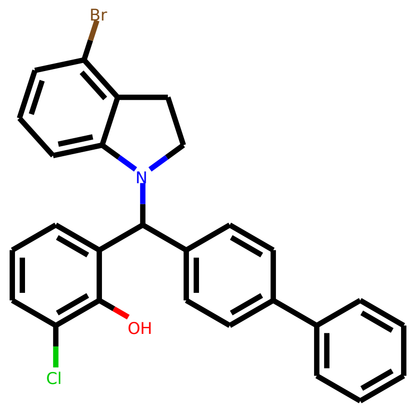
Ahma-2489

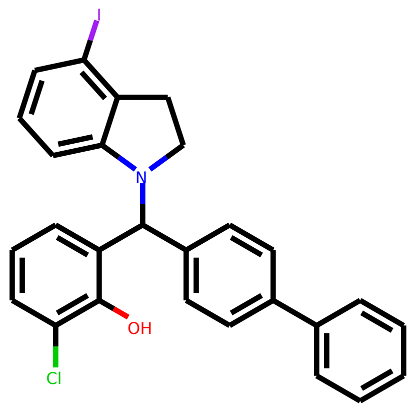
Ahma-2490

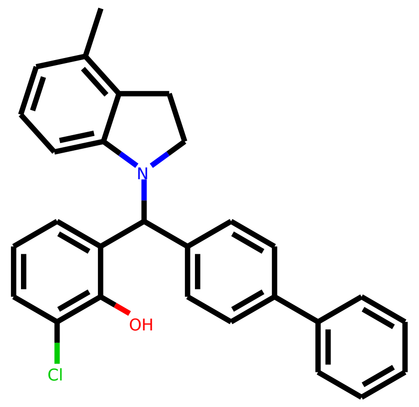
Ahma-2491

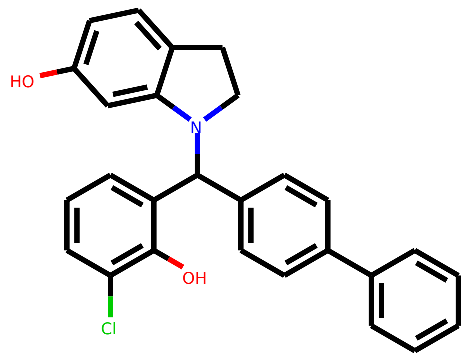
Ahma-2492

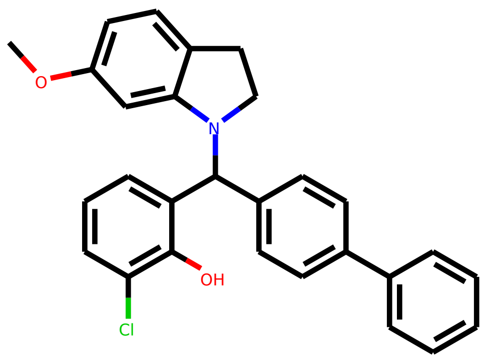
Ahma-2493

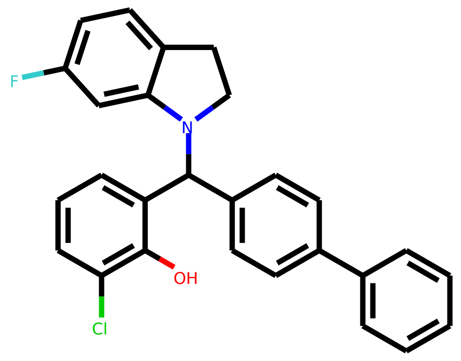
Ahma-2494

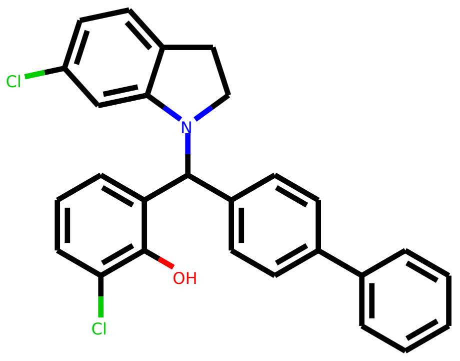
Ahma-2495

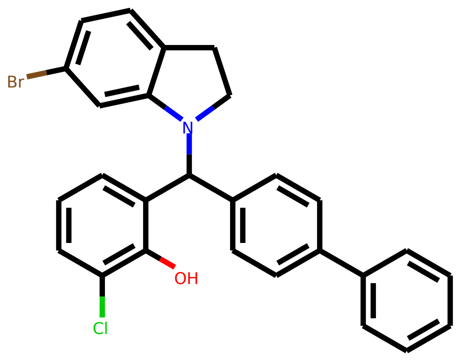
Ahma-2496

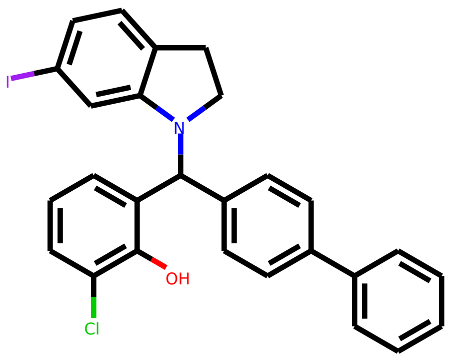
Ahma-2497

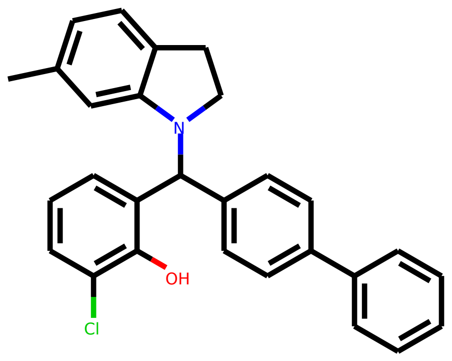
Ahma-2498

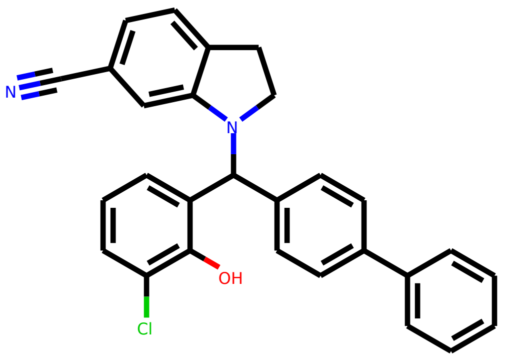
Ahma-2499

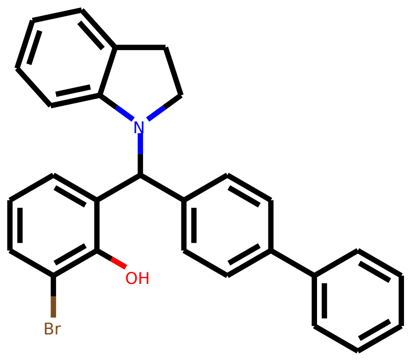
Ahma-2500

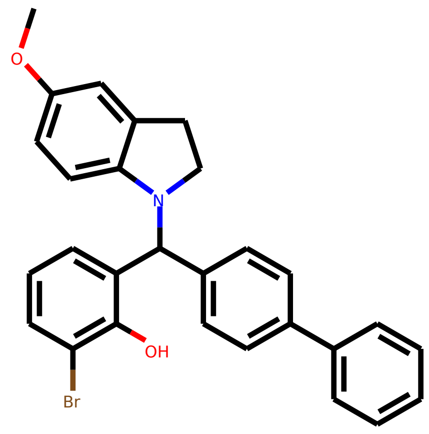
Ahma-2501

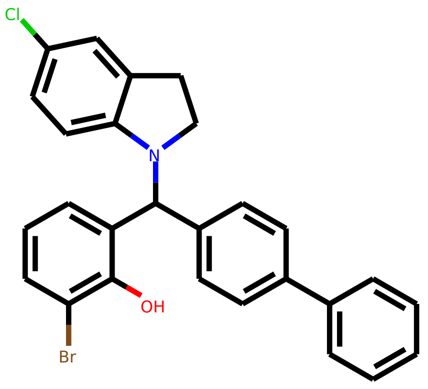
Ahma-2502

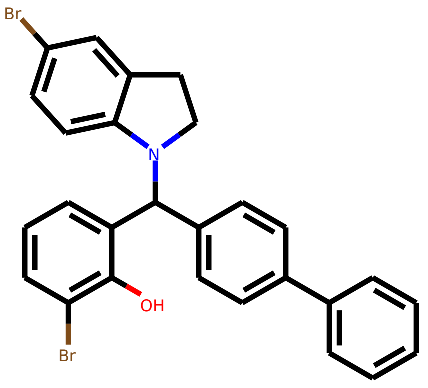
Ahma-2503

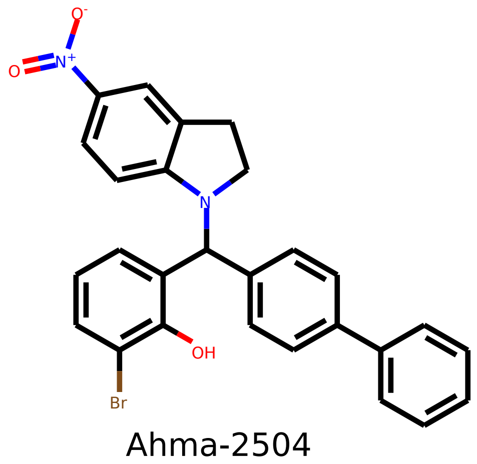
Ahma-2504

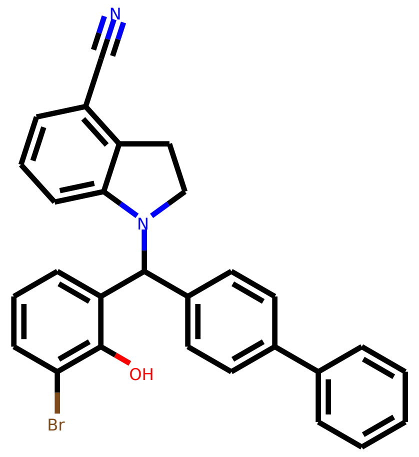
Ahma-2505

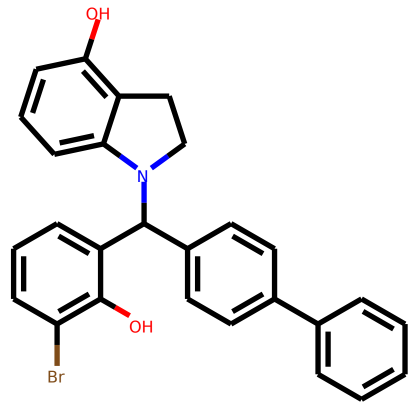
Ahma-2506

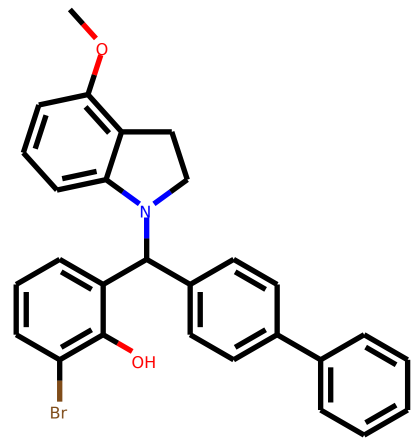
Ahma-2507

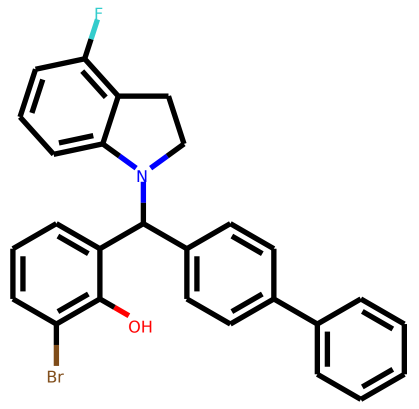
Ahma-2508

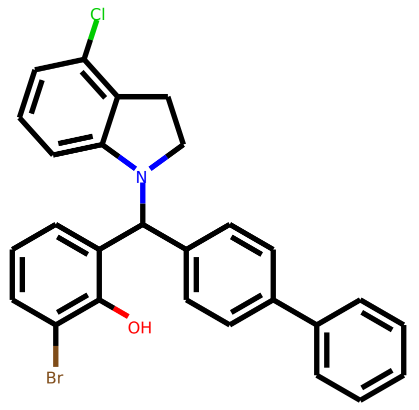
Ahma-2509

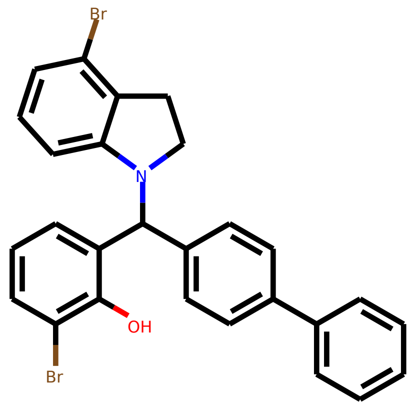
Ahma-2510

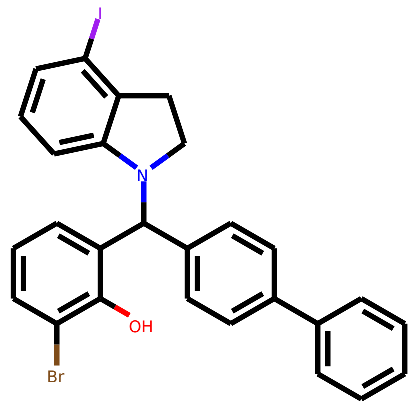
Ahma-2511

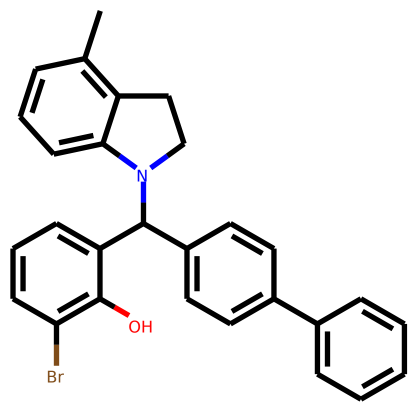
Ahma-2512

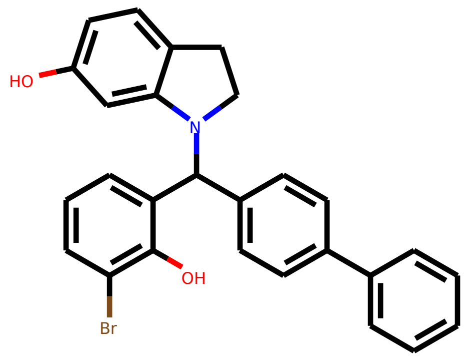
Ahma-2513

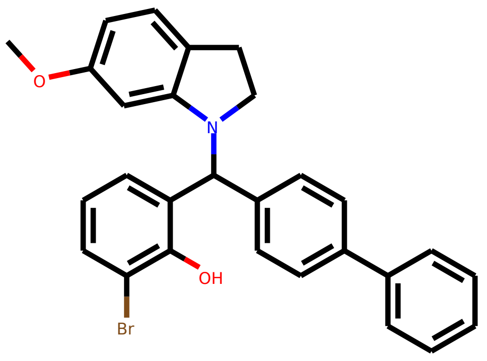
Ahma-2514

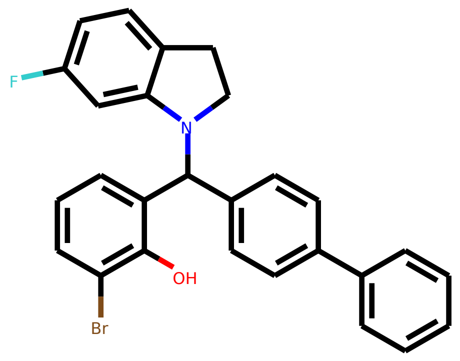
Ahma-2515

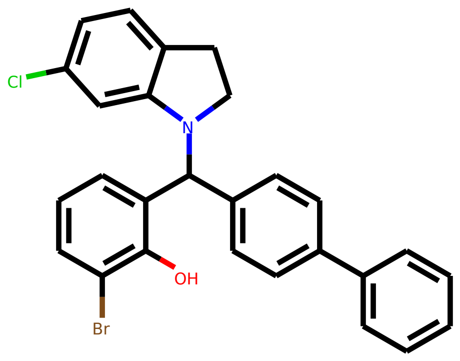
Ahma-2516

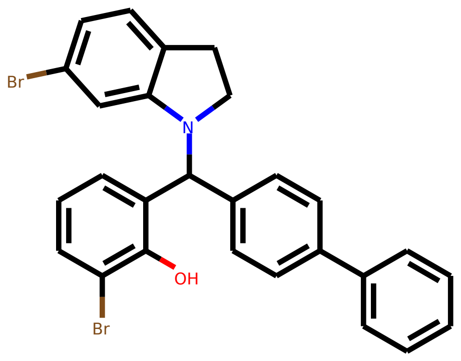
Ahma-2517

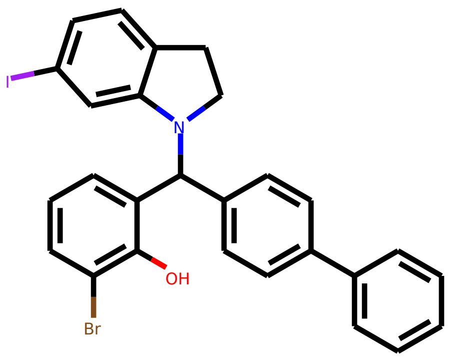
Ahma-2518

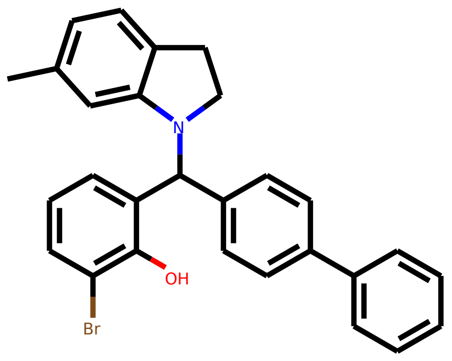
Ahma-2519

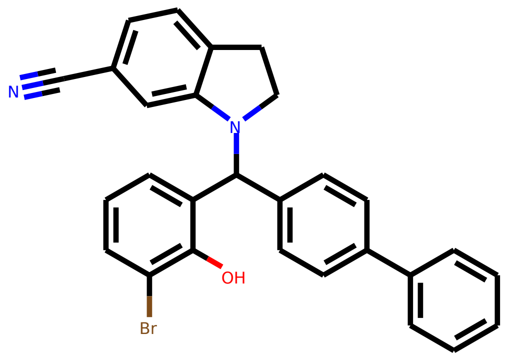
Ahma-2520

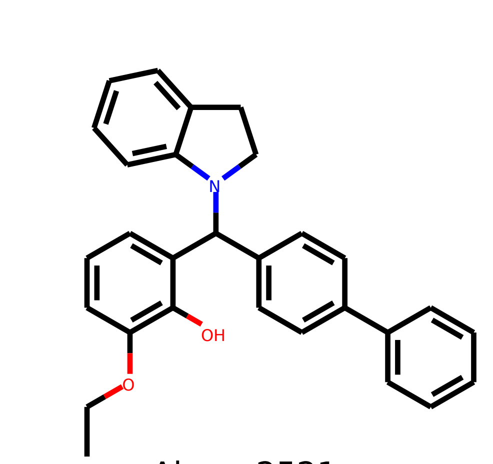
Ahma-2521
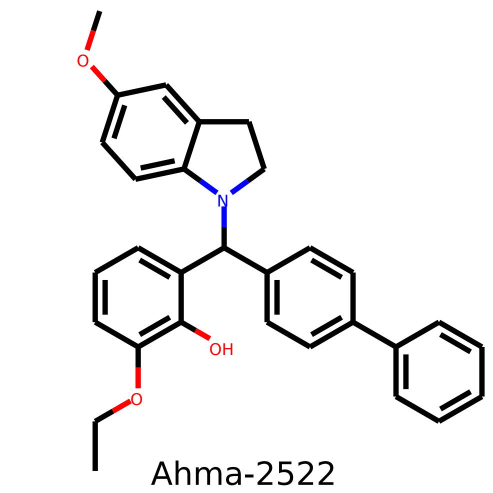
Ahma-2522
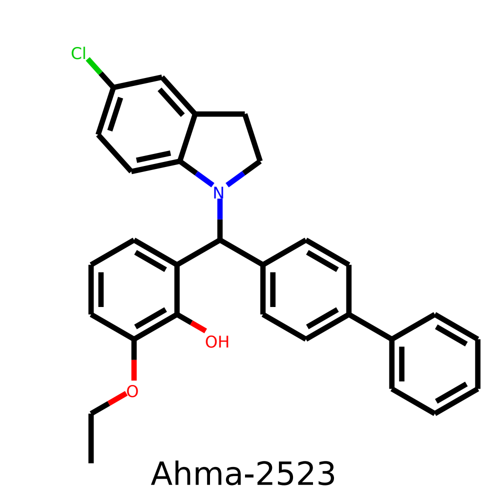
Ahma-2523
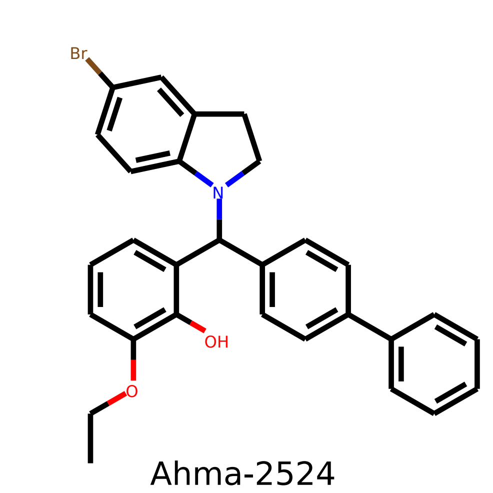
Ahma-2524
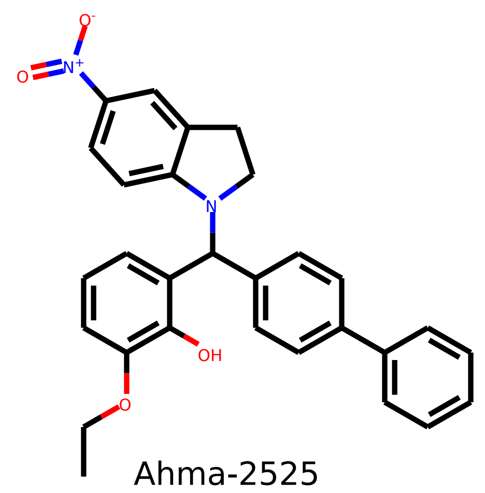
Ahma-2525
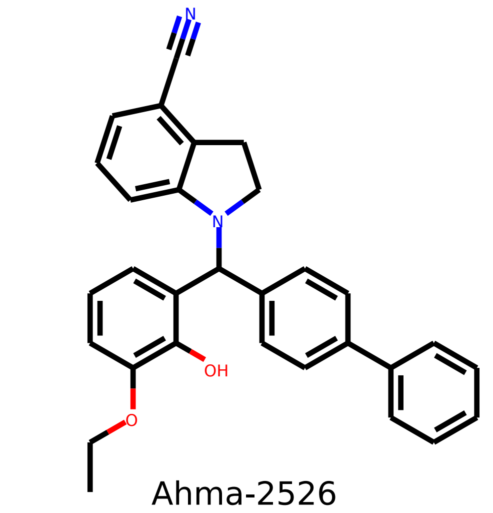
Ahma-2526
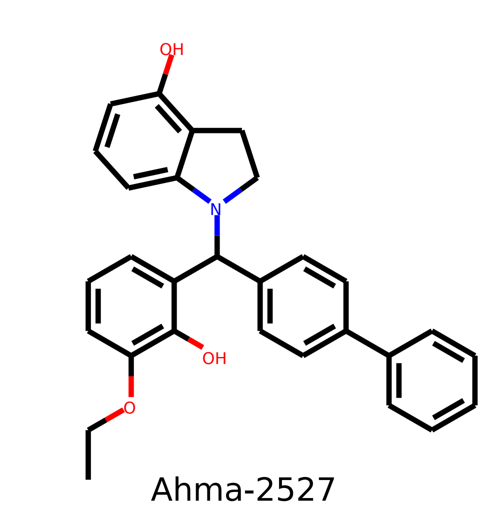
Ahma-2527
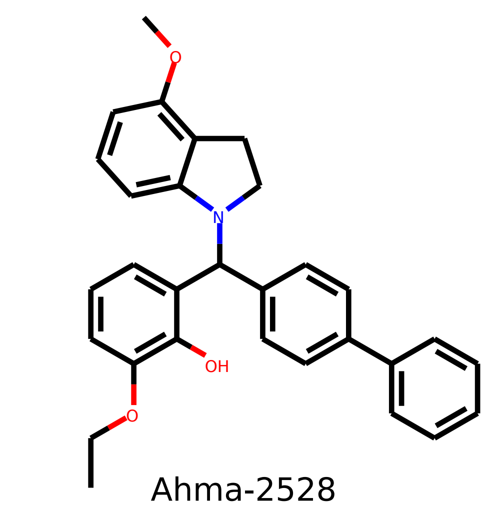
Ahma-2528
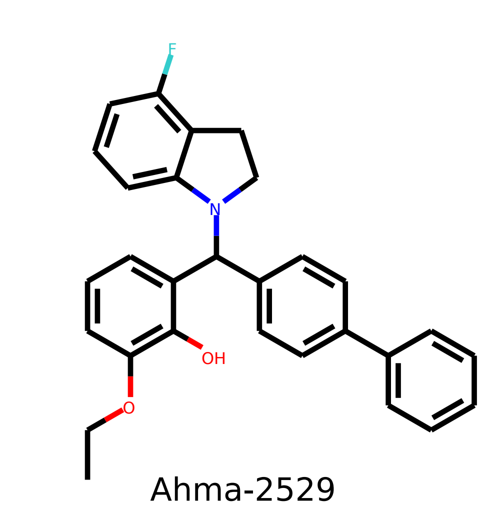
Ahma-2529
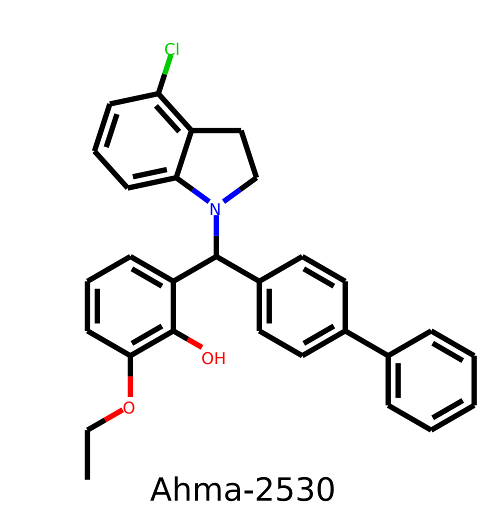
Ahma-2530
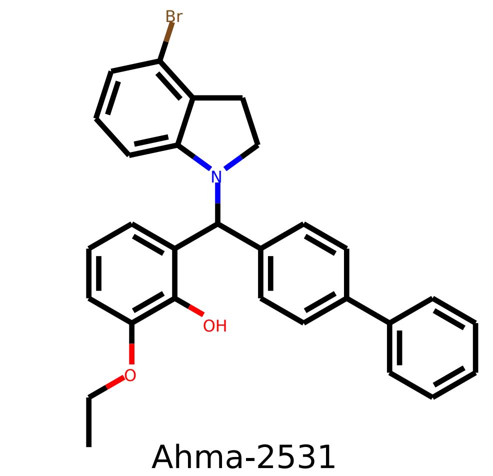
Ahma-2531
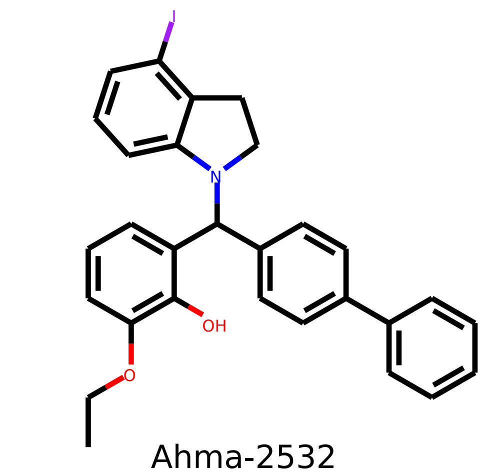
Ahma-2532
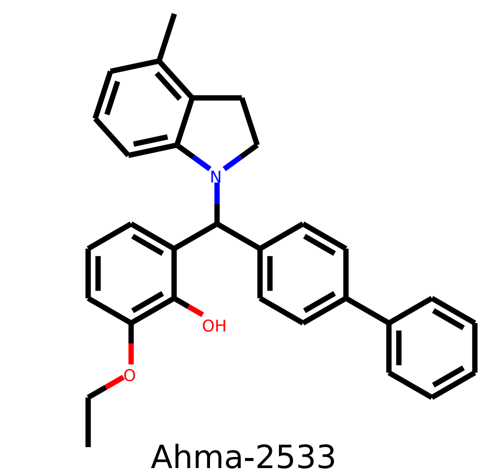
Ahma-2533
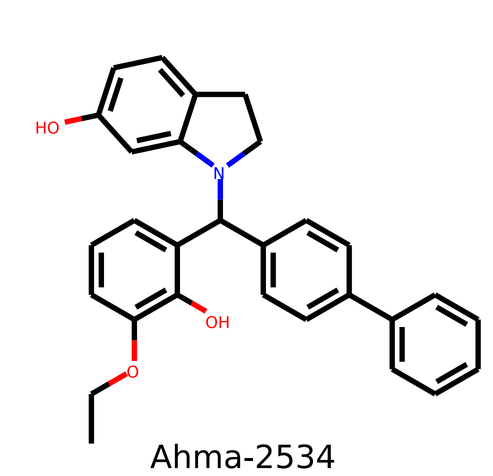
Ahma-2534
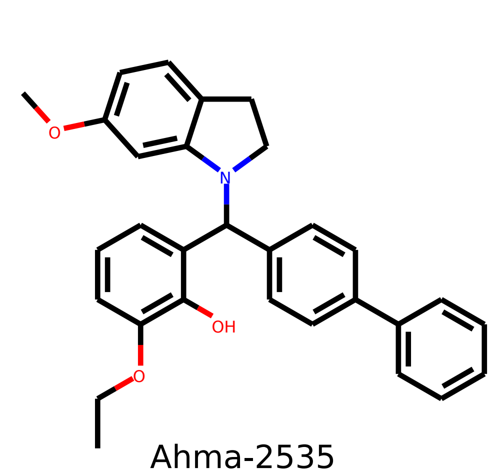
Ahma-2535
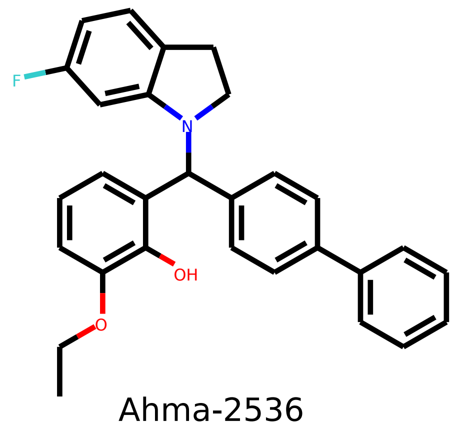
Ahma-2536
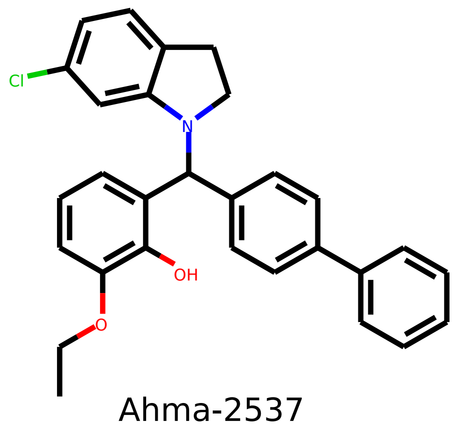
Ahma-2537
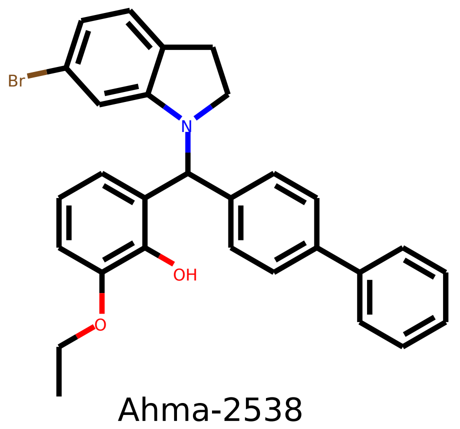
Ahma-2538
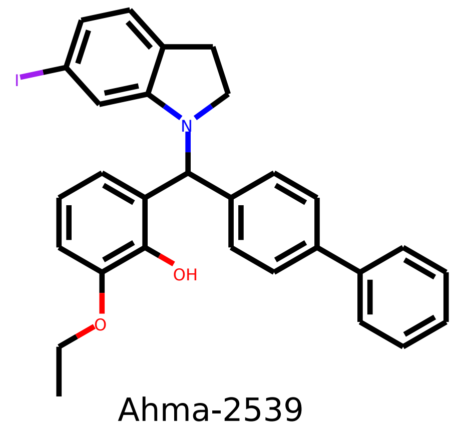
Ahma-2539
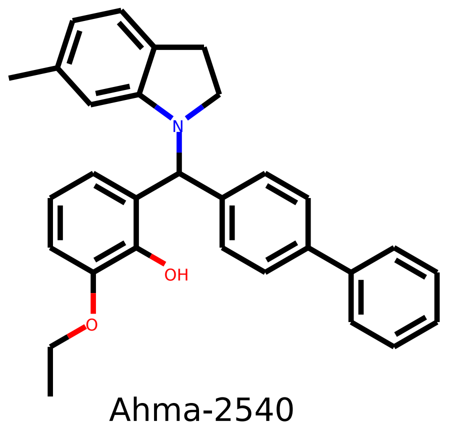
Ahma-2540
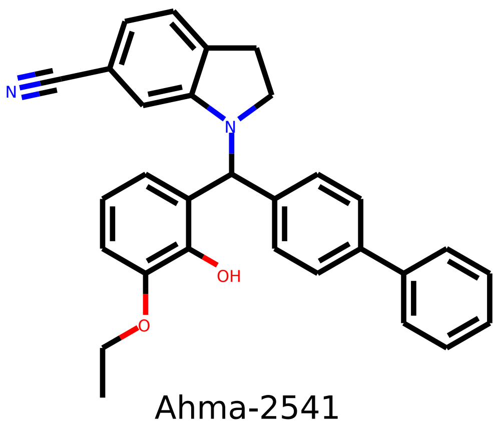
Ahma-2541
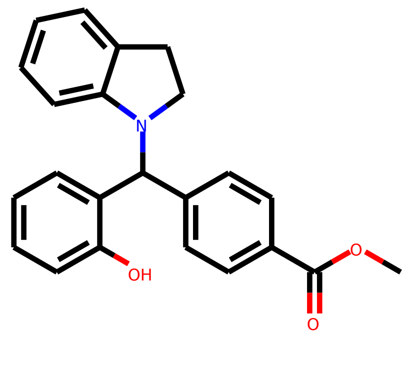
Ahma-2542
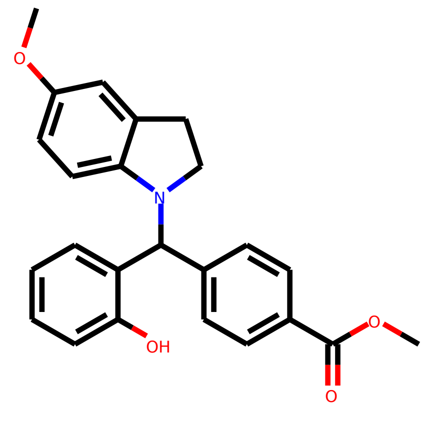
Ahma-2543
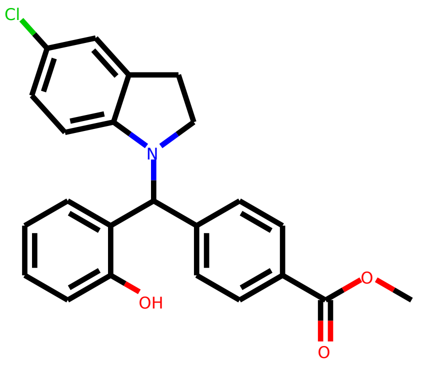
Ahma-2544
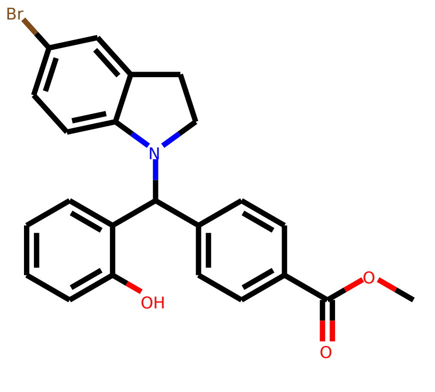
Ahma-2545
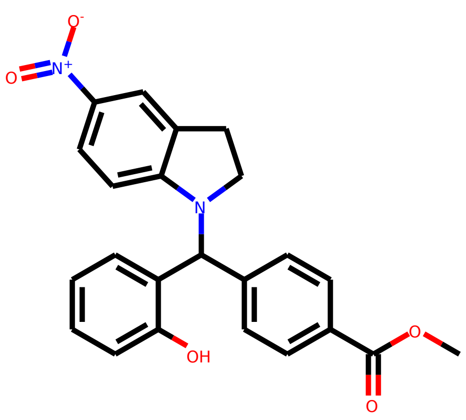
Ahma-2546
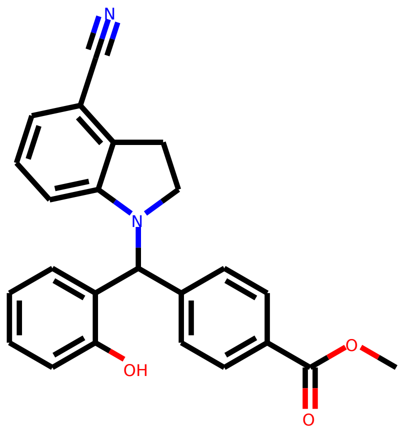
Ahma-2547
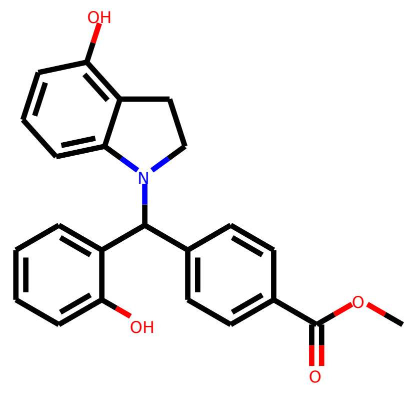
Ahma-2548
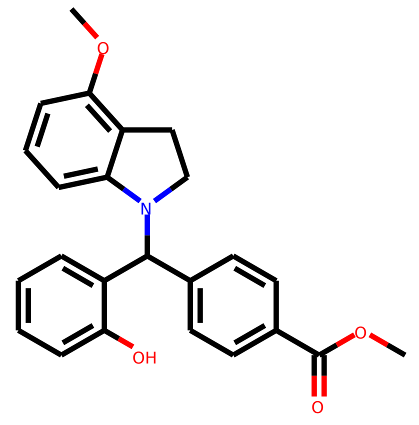
Ahma-2549
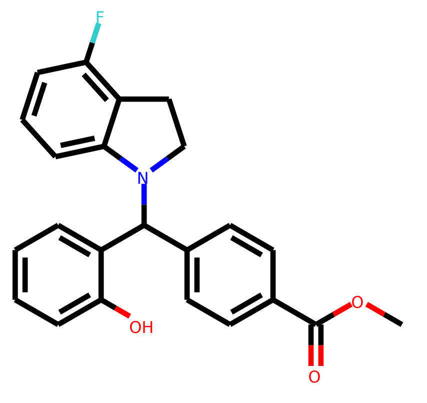
Ahma-2550
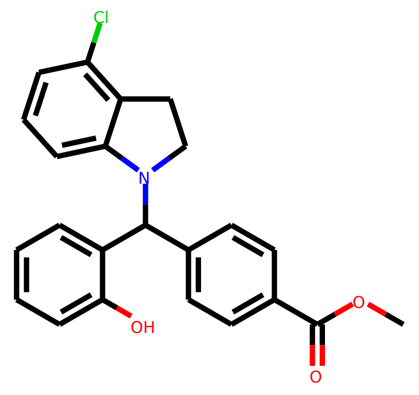
Ahma-2551
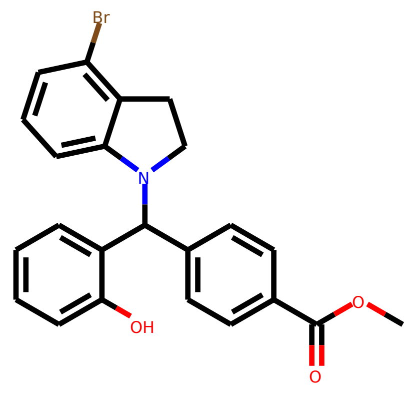
Ahma-2552
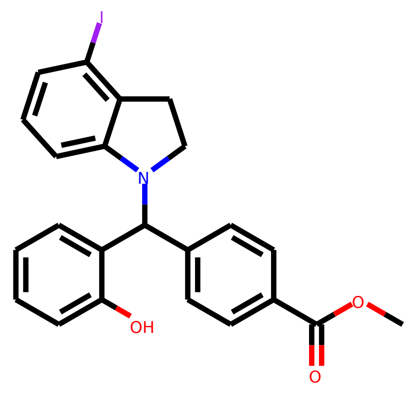
Ahma-2553
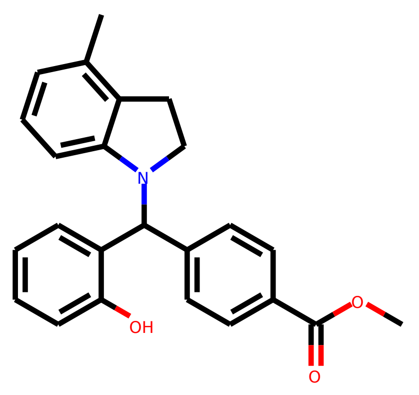
Ahma-2554
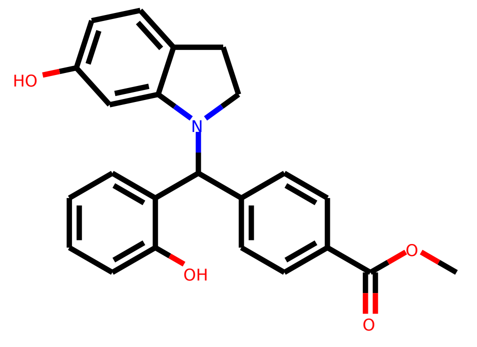
Ahma-2555

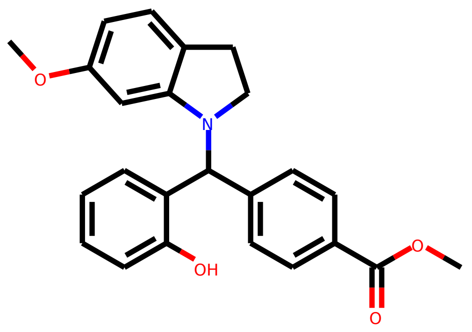
Ahma-2556

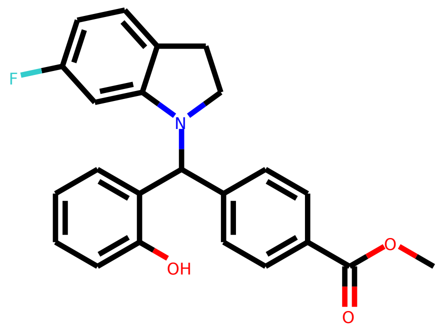
Ahma-2557

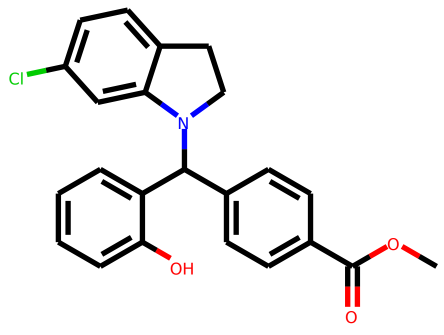
Ahma-2558

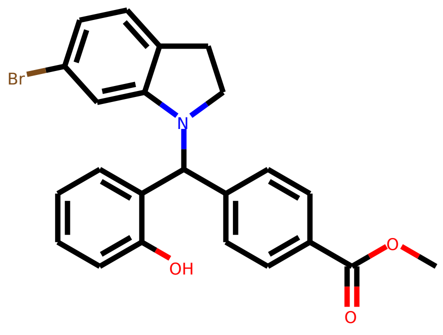
Ahma-2559

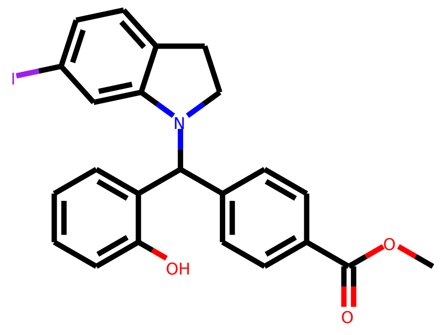
Ahma-2560

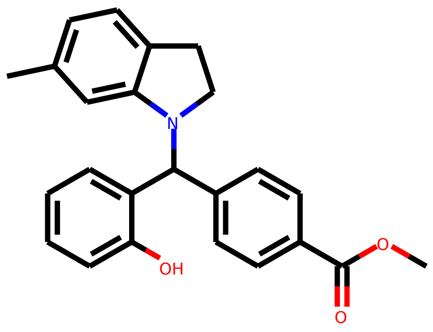
Ahma-2561

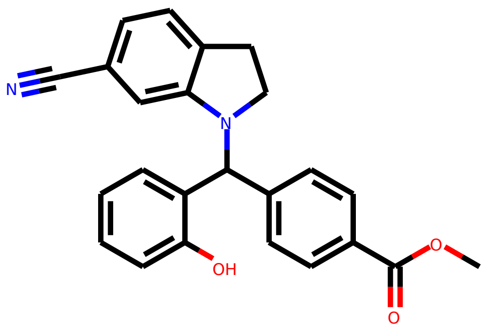
Ahma-2562

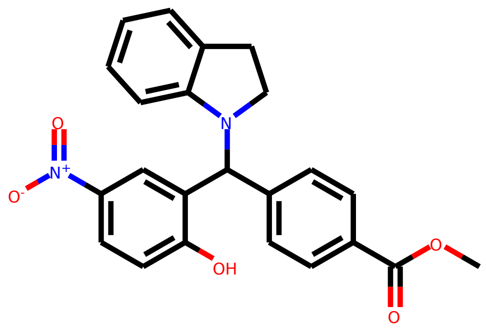
Ahma-2563

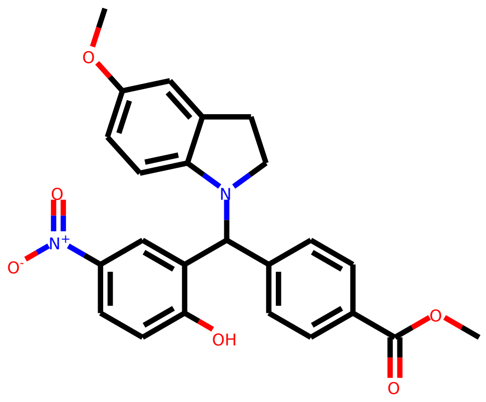
Ahma-2564

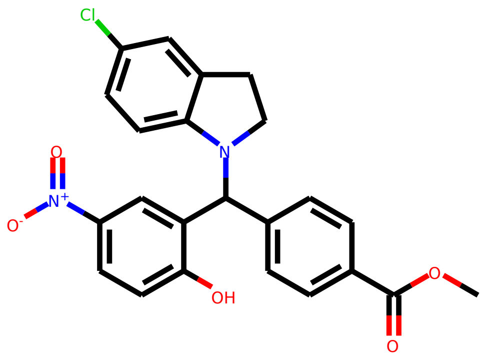
Ahma-2565

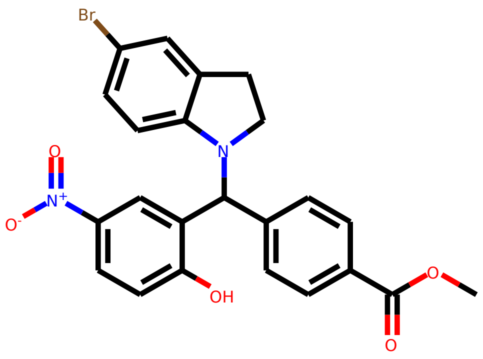
Ahma-2566

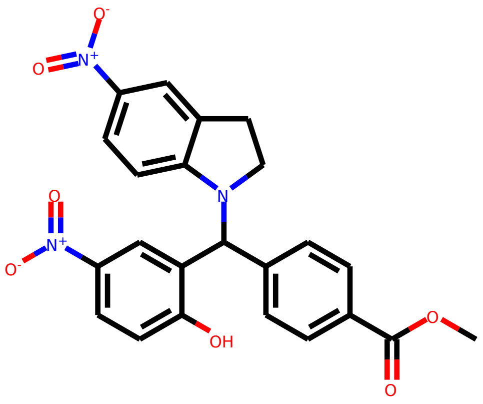
Ahma-2567

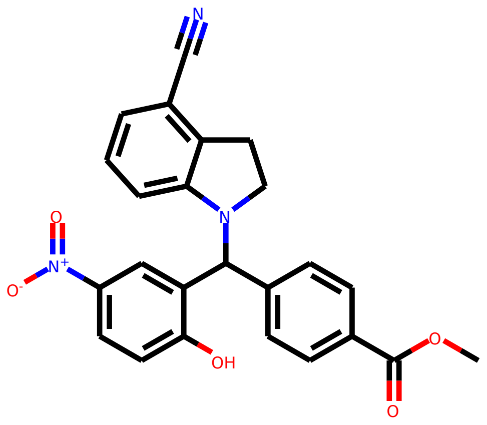
Ahma-2568

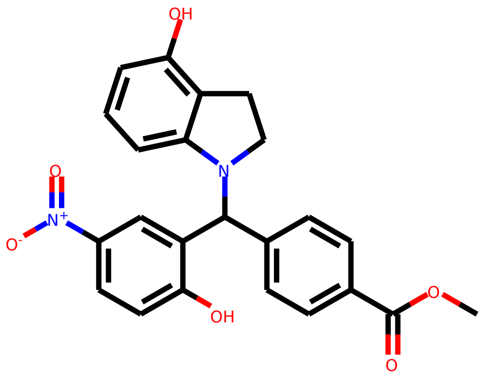
Ahma-2569

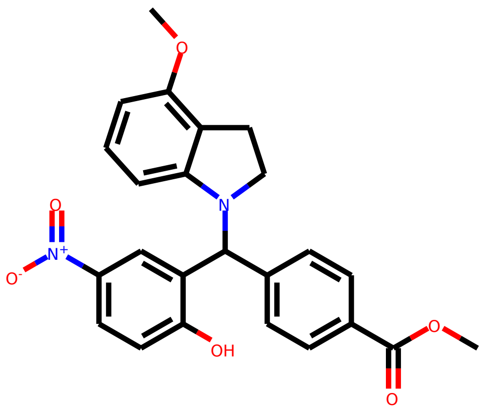
Ahma-2570

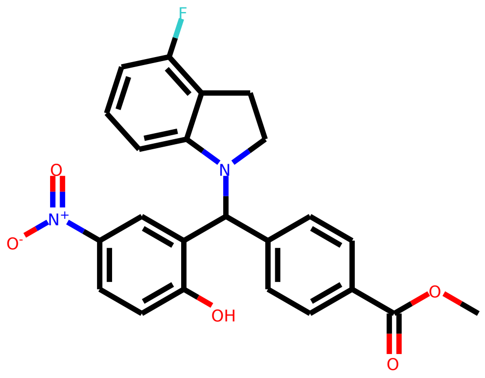
Ahma-2571

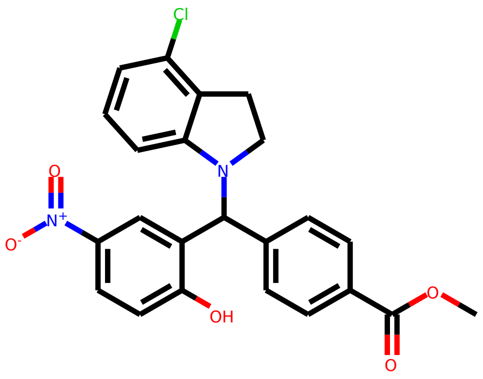
Ahma-2572

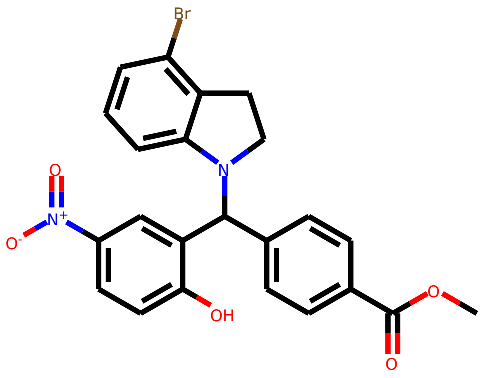
Ahma-2573

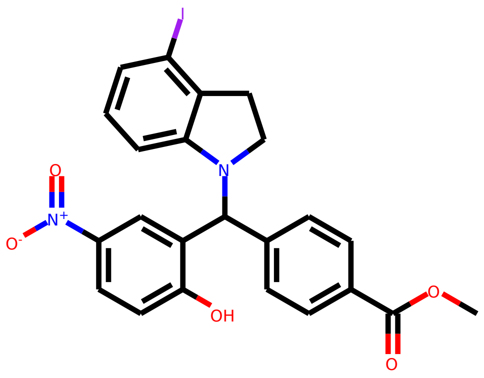
Ahma-2574

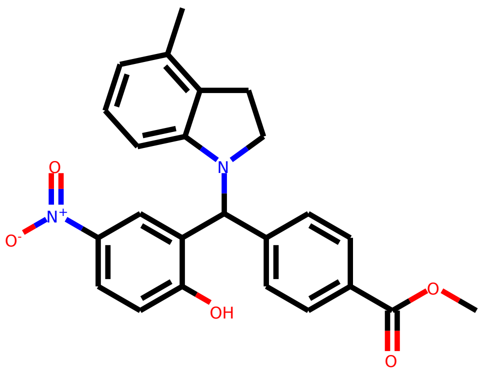
Ahma-2575

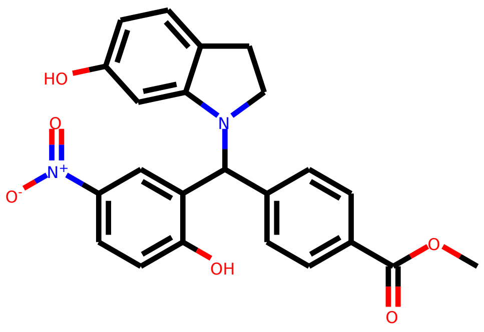
Ahma-2576

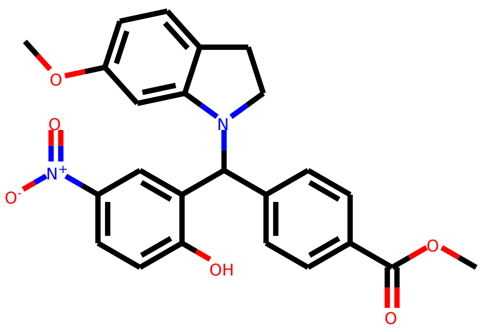
Ahma-2577

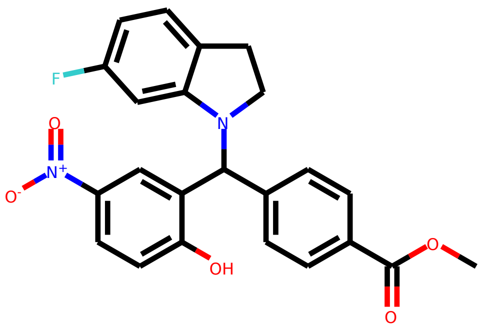
Ahma-2578

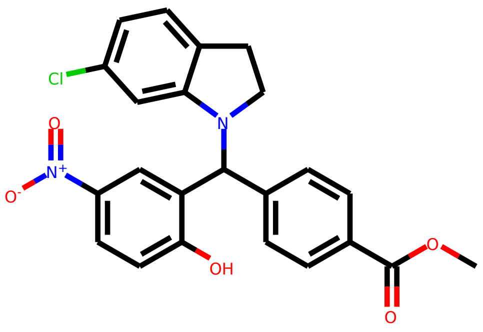
Ahma-2579

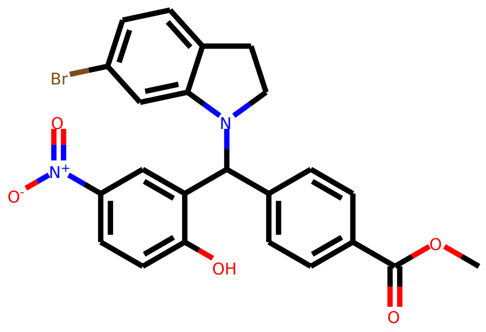
Ahma-2580

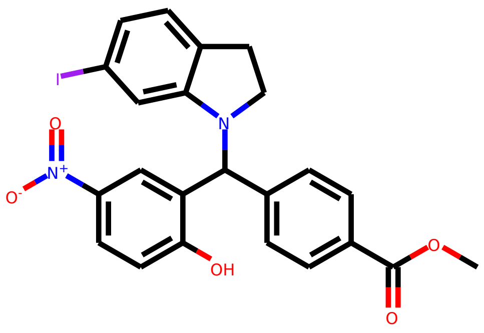
Ahma-2581

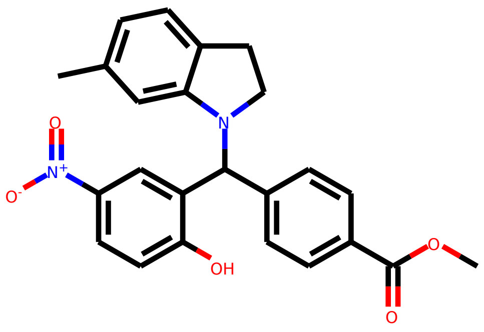
Ahma-2582

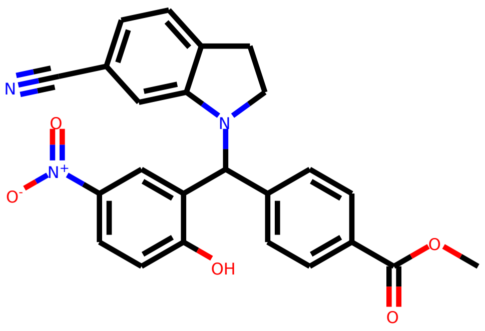
Ahma-2583

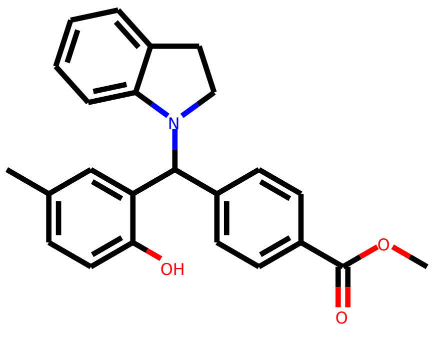
Ahma-2584

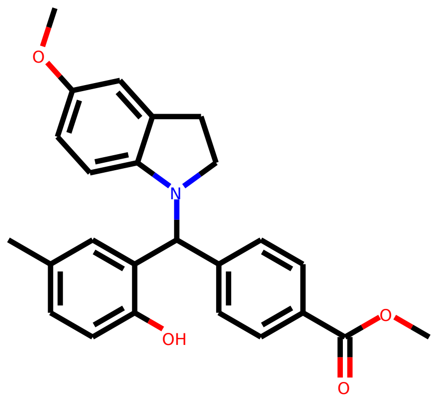
Ahma-2585

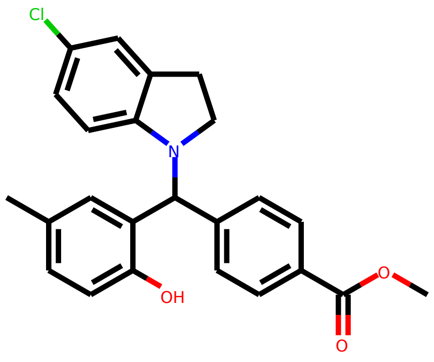
Ahma-2586

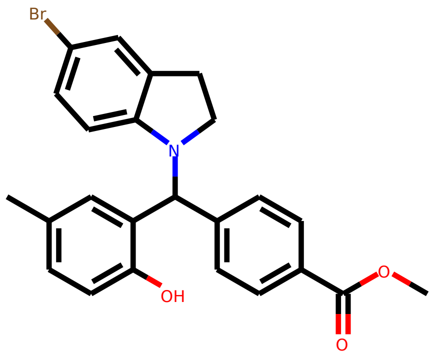
Ahma-2587

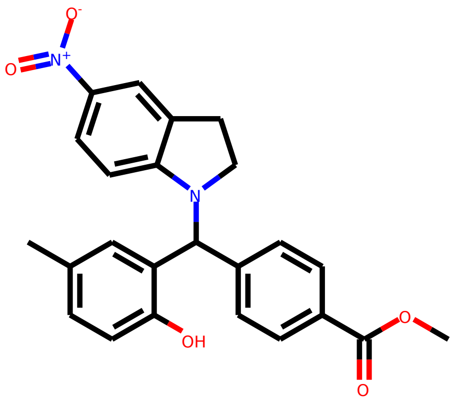
Ahma-2588

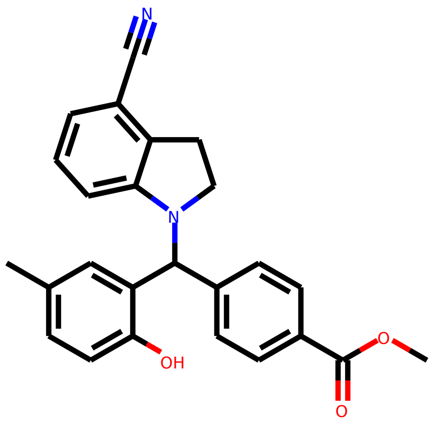
Ahma-2589

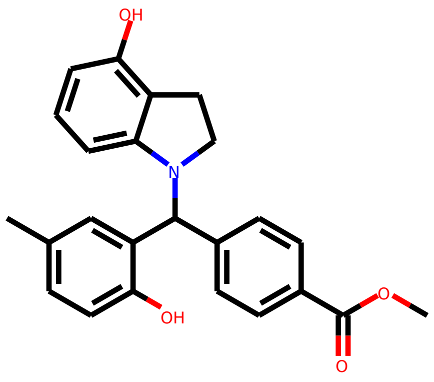
Ahma-2590

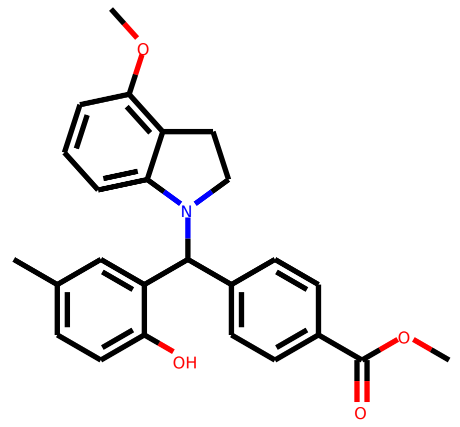
Ahma-2591
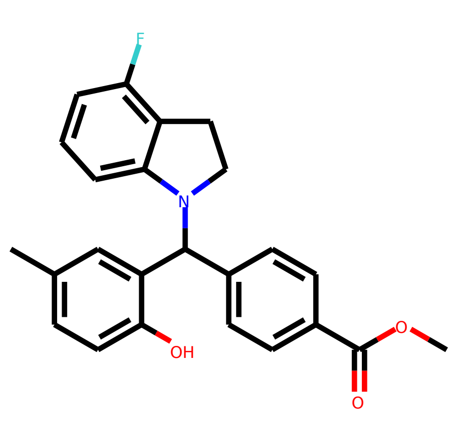
Ahma-2592
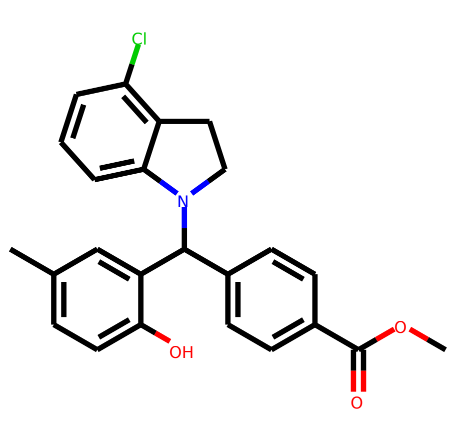
Ahma-2593
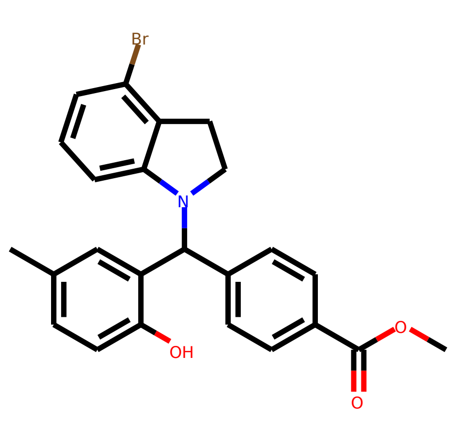
Ahma-2594
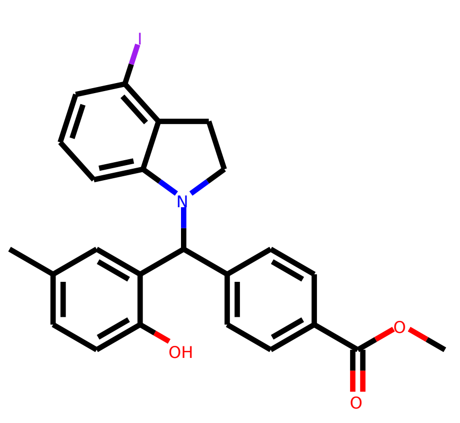
Ahma-2595
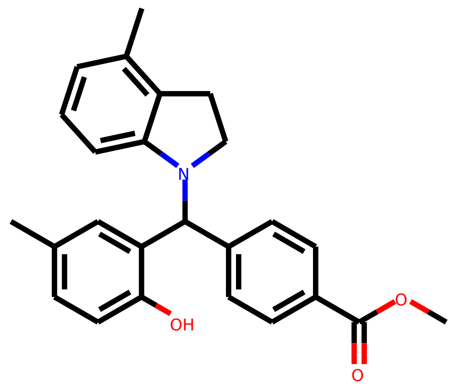
Ahma-2596
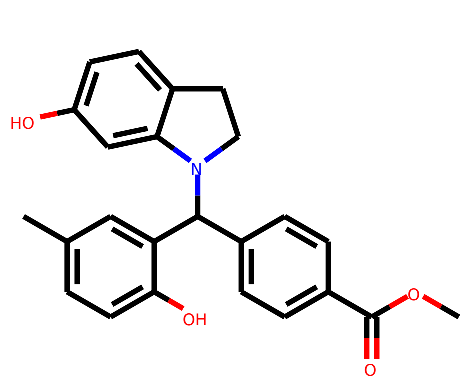
Ahma-2597
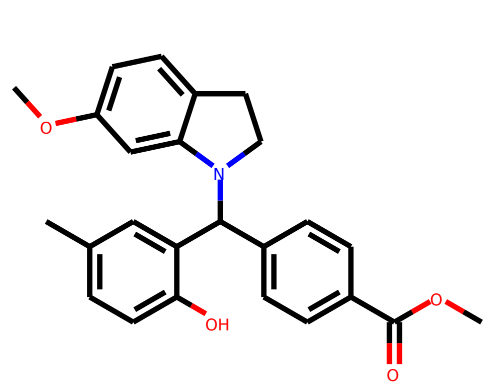
Ahma-2598
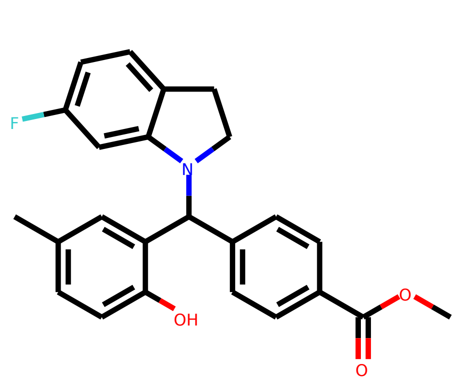
Ahma-2599
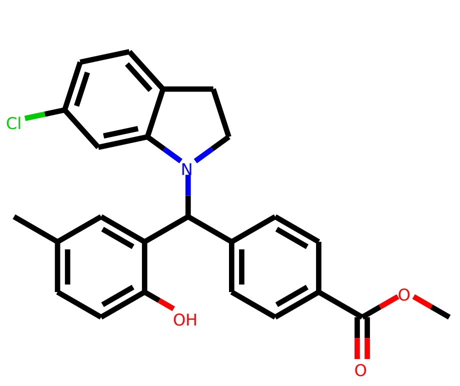
Ahma-2600
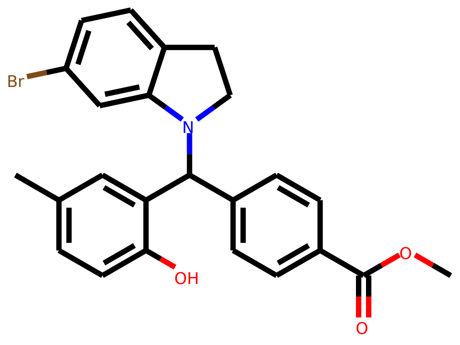
Ahma-2601
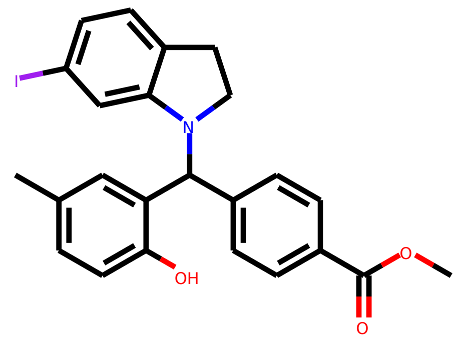
Ahma-2602
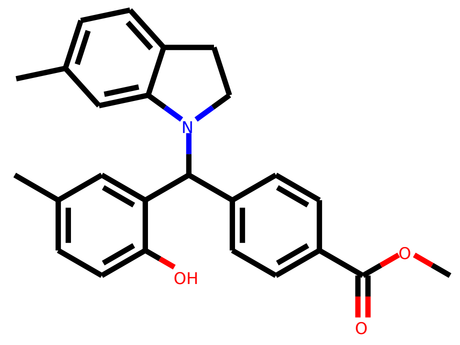
Ahma-2603
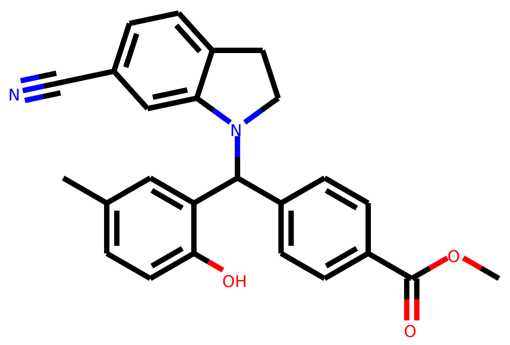
Ahma-2604
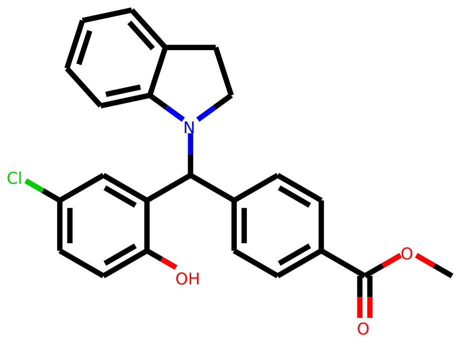
Ahma-2605
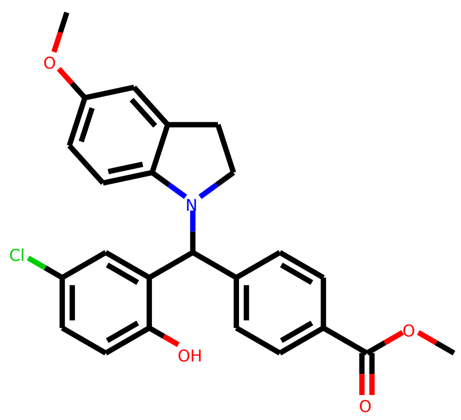
Ahma-2606
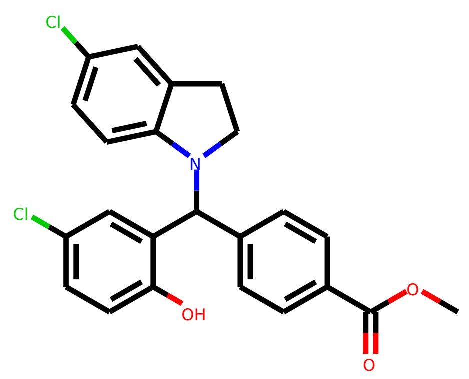
Ahma-2607
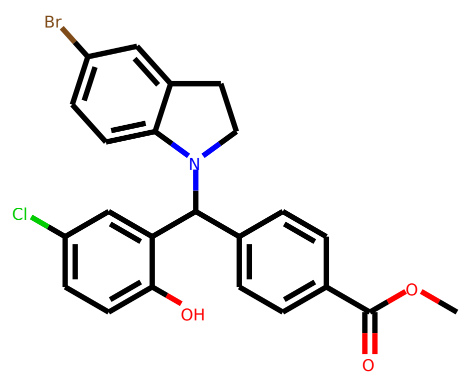
Ahma-2608
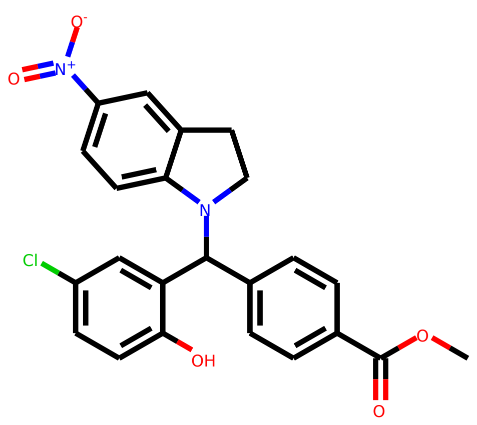
Ahma-2609
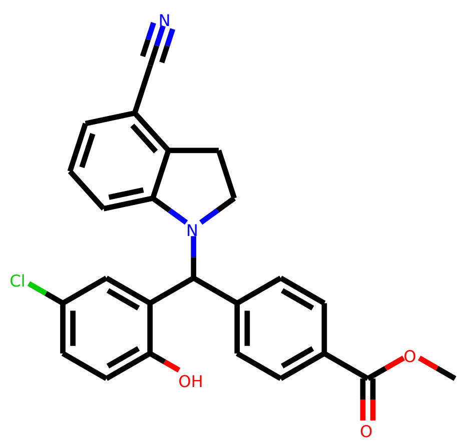
Ahma-2610
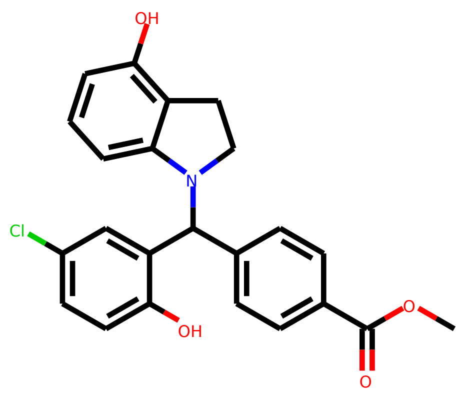
Ahma-2611
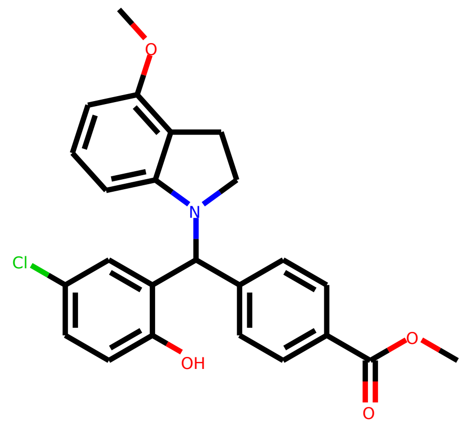
Ahma-2612
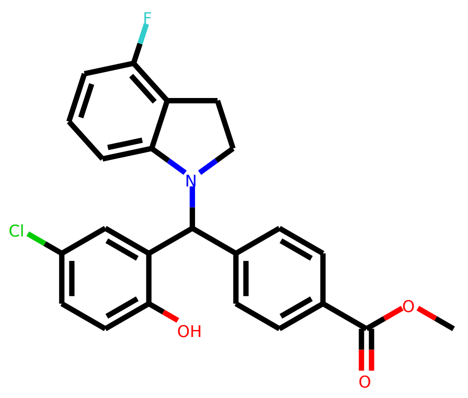
Ahma-2613
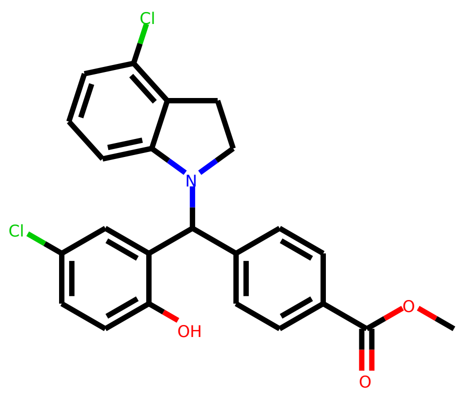
Ahma-2614
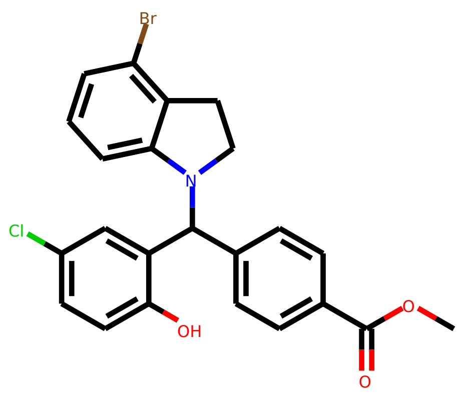
Ahma-2615
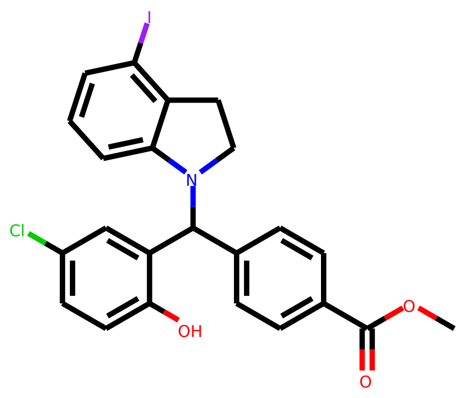
Ahma-2616
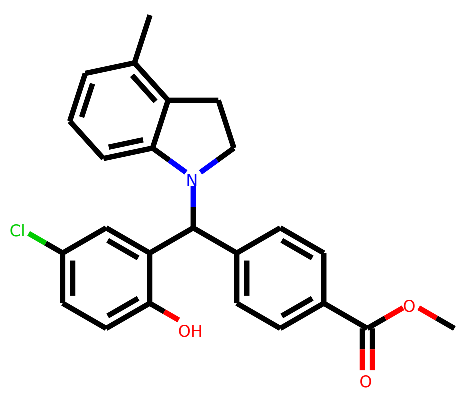
Ahma-2617
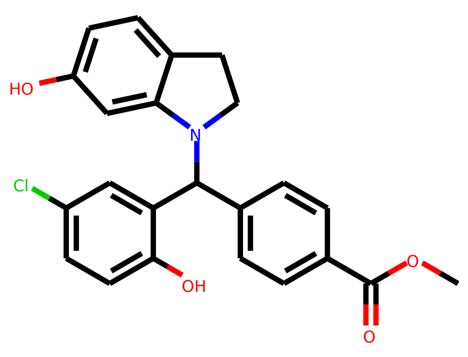
Ahma-2618
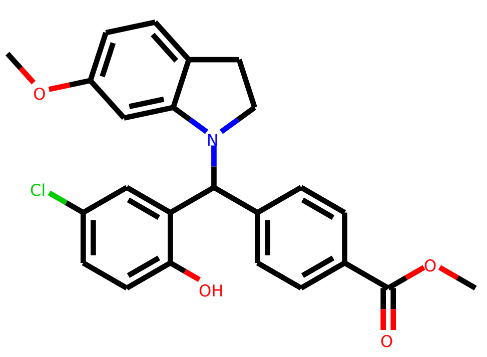
Ahma-2619
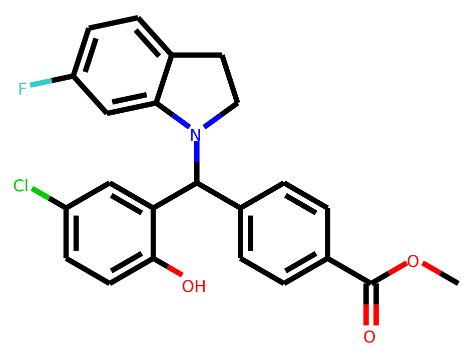
Ahma-2620
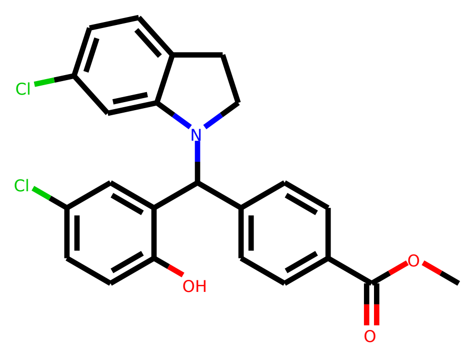
Ahma-2621
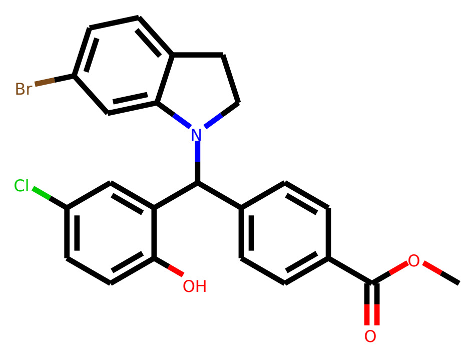
Ahma-2622
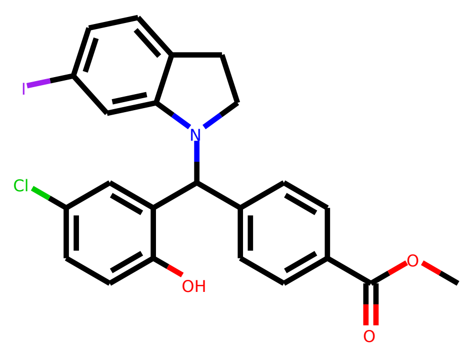
Ahma-2623
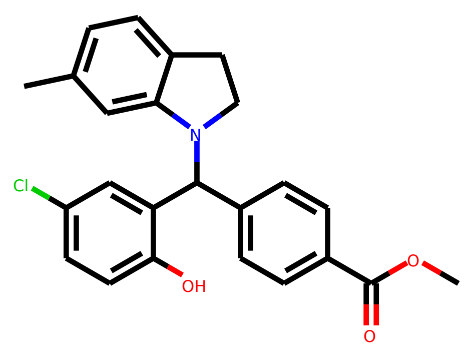
Ahma-2624
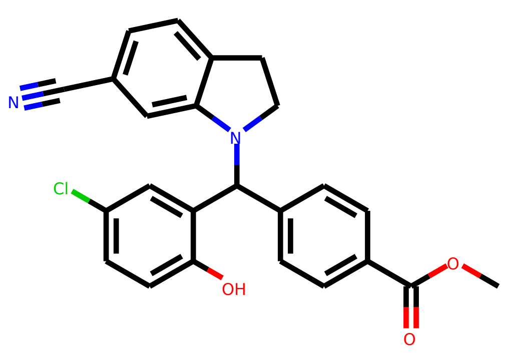
Ahma-2625

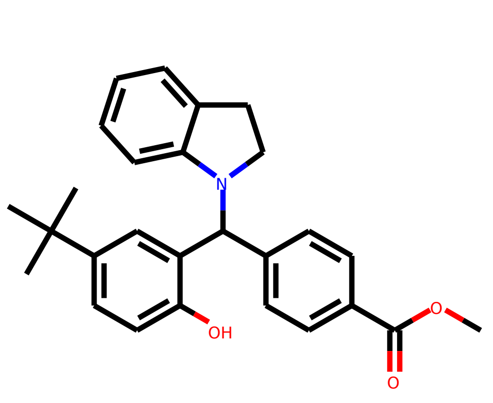
Ahma-2626
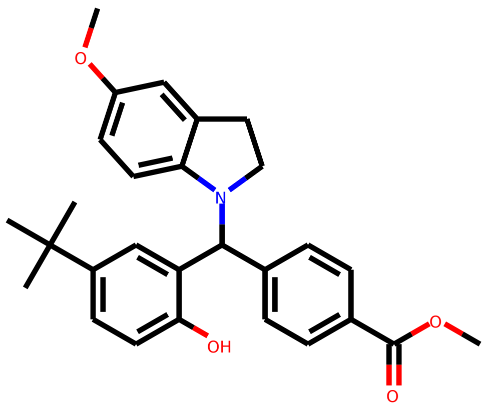
Ahma-2627
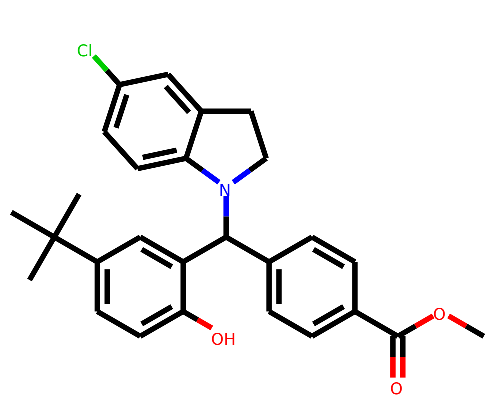
Ahma-2628
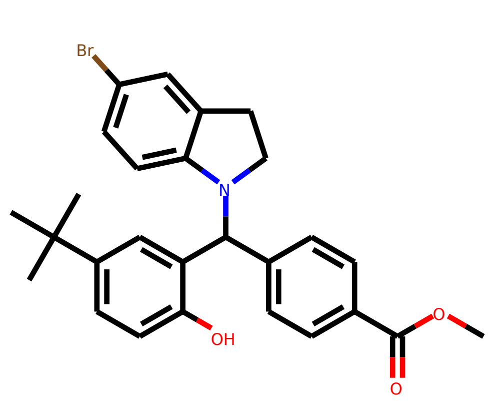
Ahma-2629
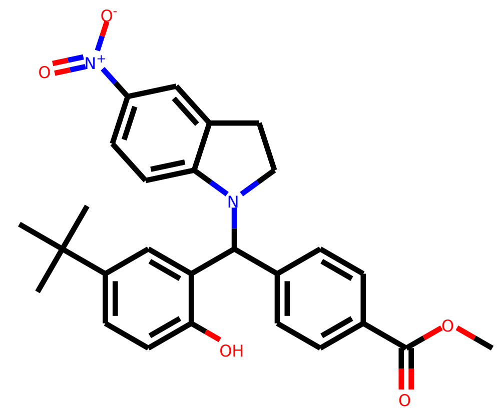
Ahma-2630
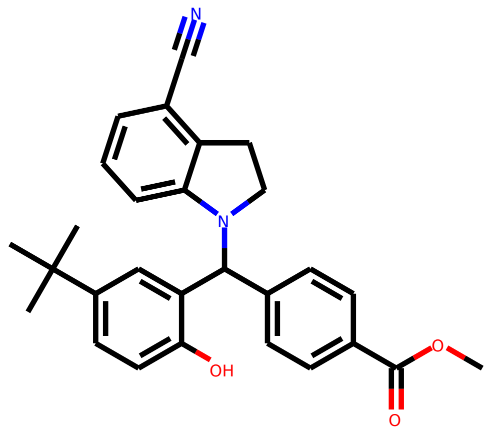
Ahma-2631
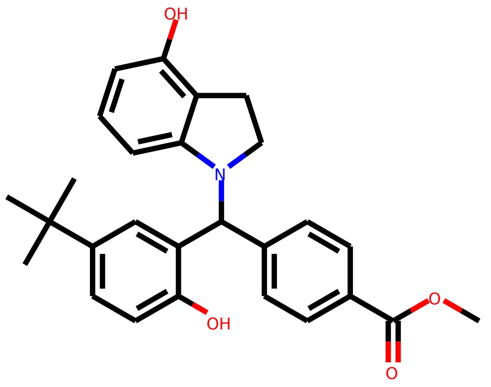
Ahma-2632
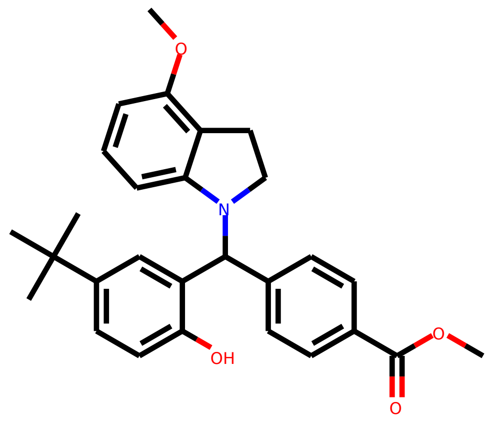
Ahma-2633
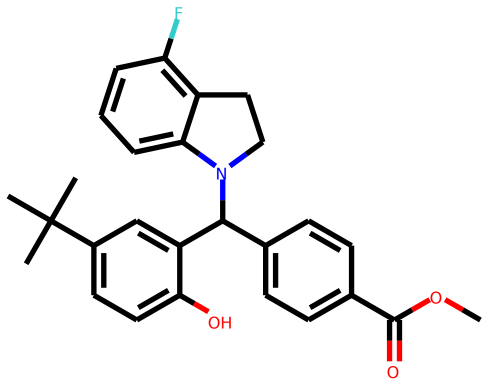
Ahma-2634
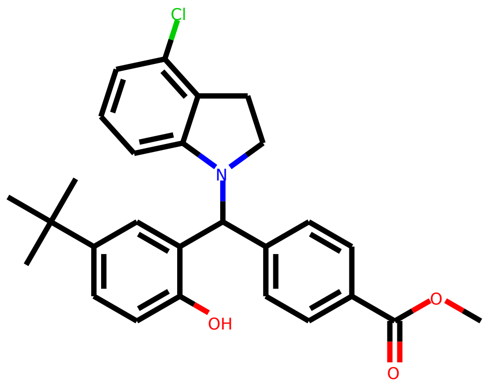
Ahma-2635
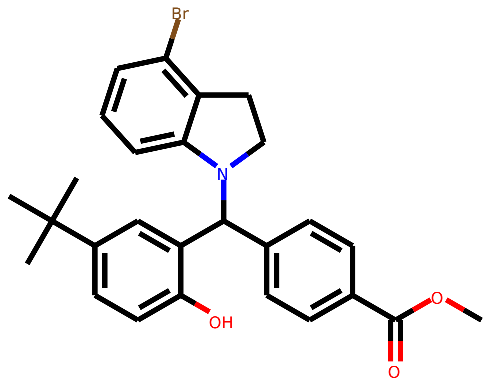
Ahma-2636
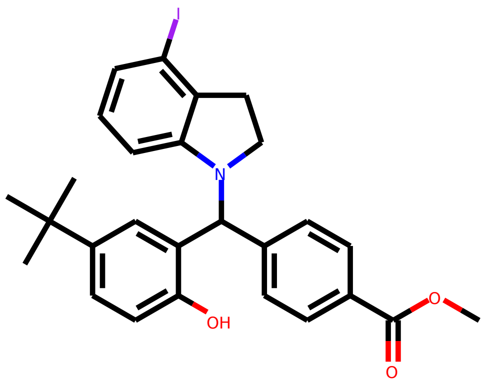
Ahma-2637
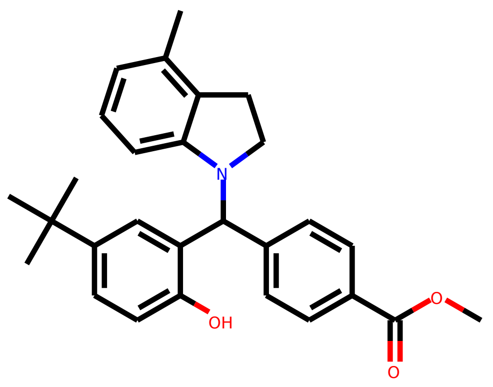
Ahma-2638
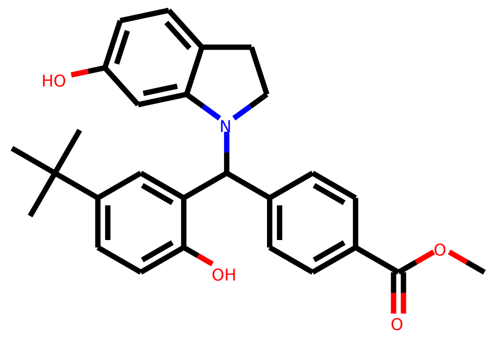
Ahma-2639
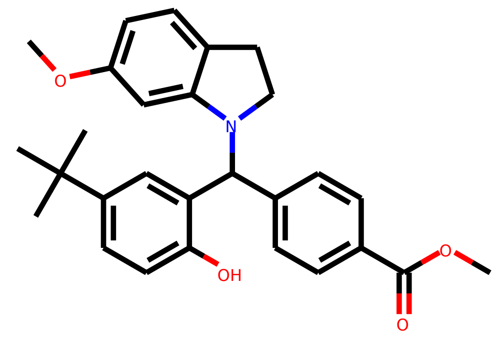
Ahma-2640
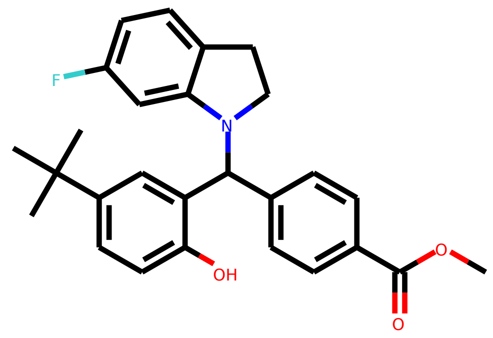
Ahma-2641
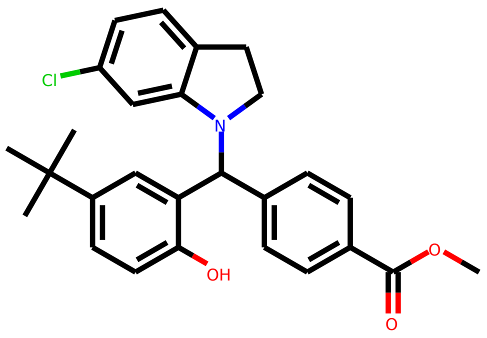
Ahma-2642
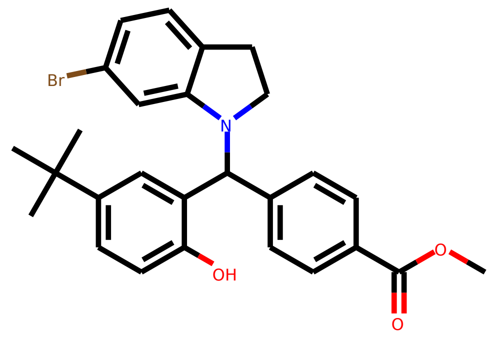
Ahma-2643
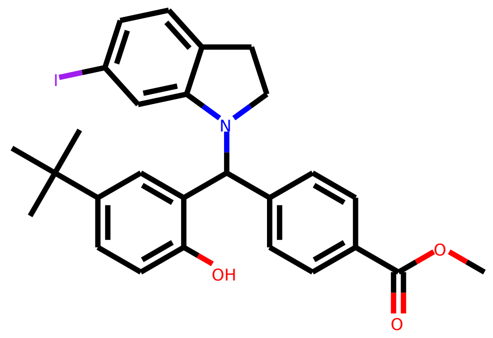
Ahma-2644
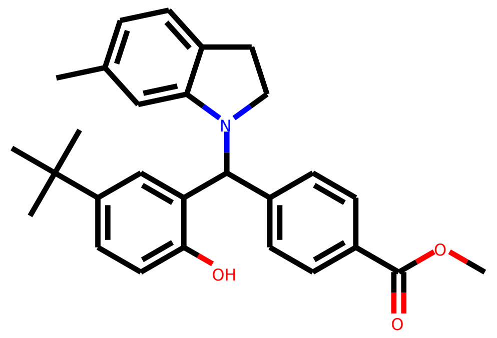
Ahma-2645
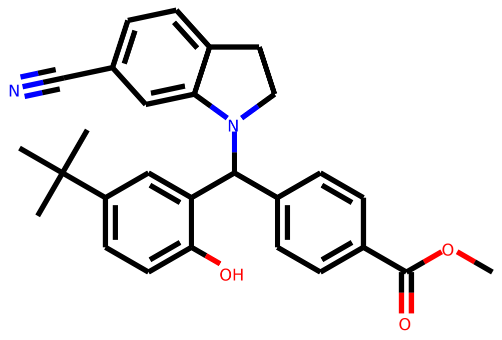
Ahma-2646
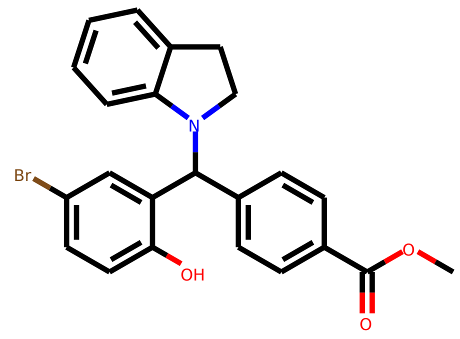
Ahma-2647
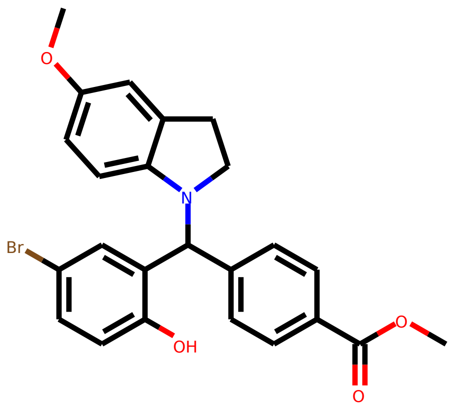
Ahma-2648
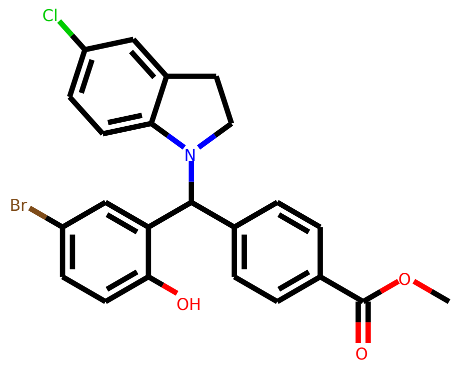
Ahma-2649
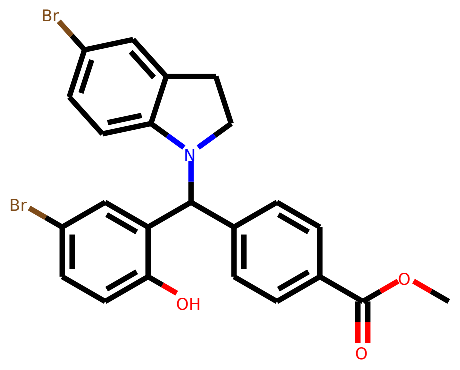
Ahma-2650
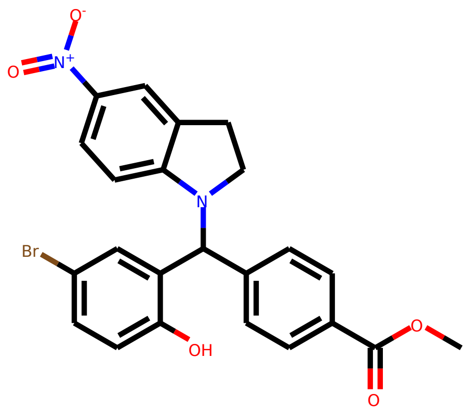
Ahma-2651
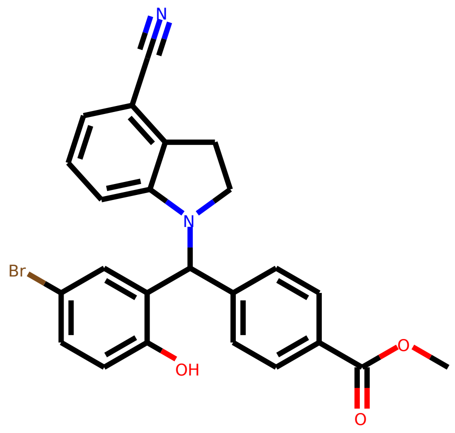
Ahma-2652
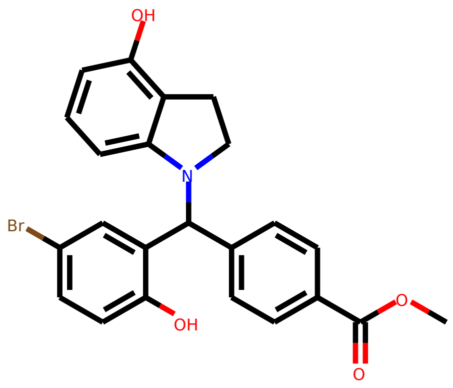
Ahma-2653
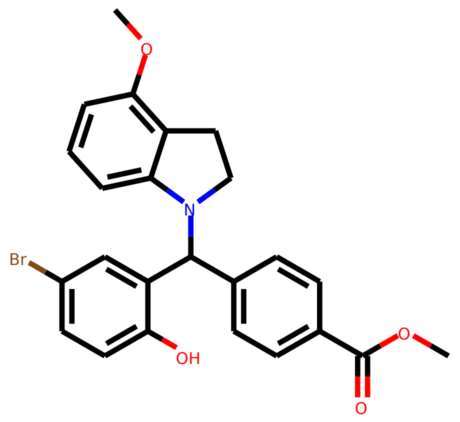
Ahma-2654
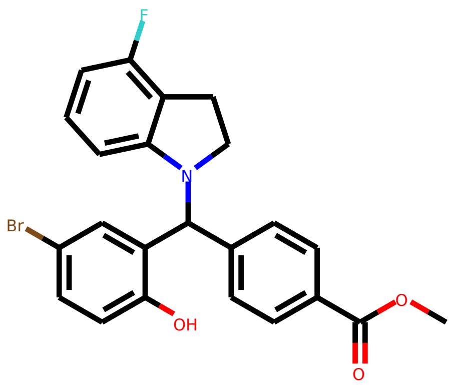
Ahma-2655
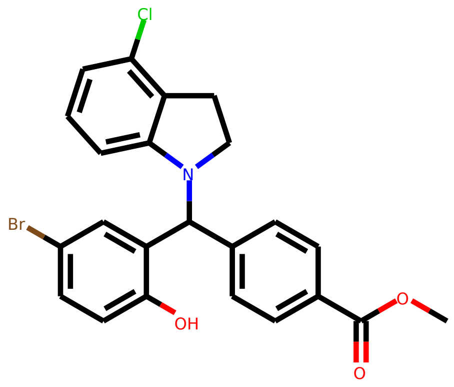
Ahma-2656
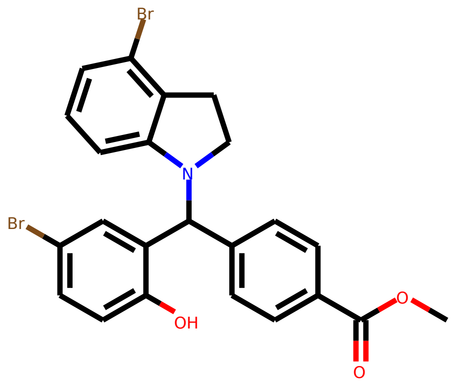
Ahma-2657
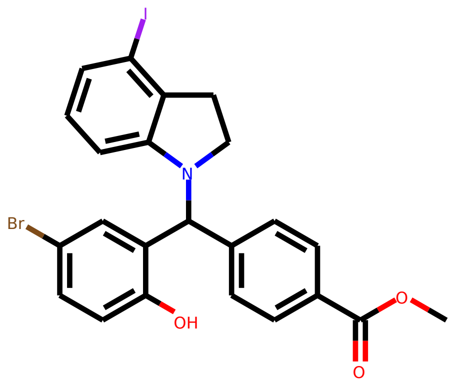
Ahma-2658
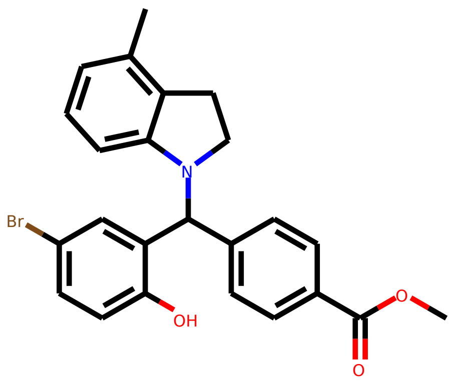
Ahma-2659
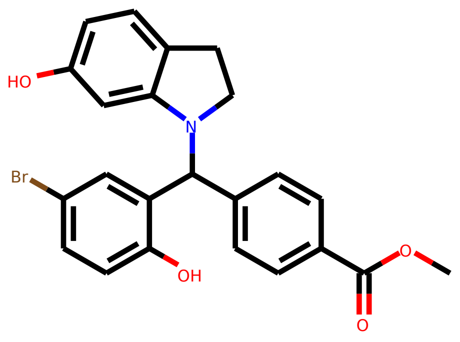
Ahma-2660

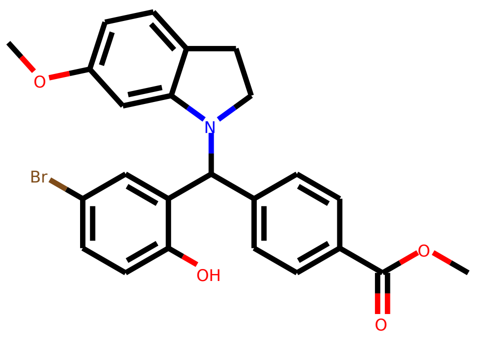
Ahma-2661
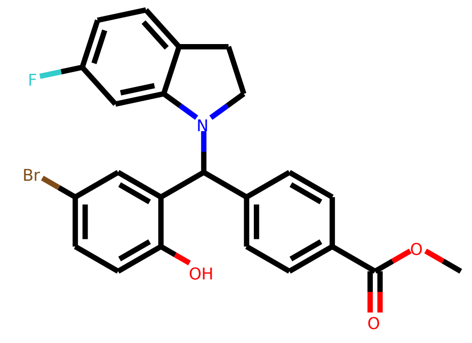
Ahma-2662
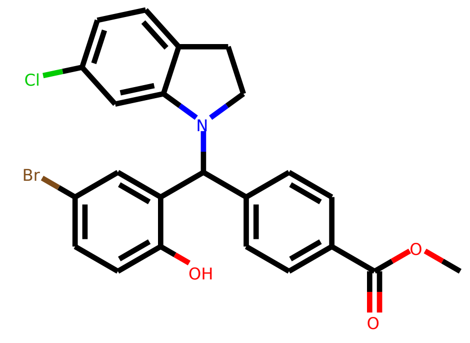
Ahma-2663
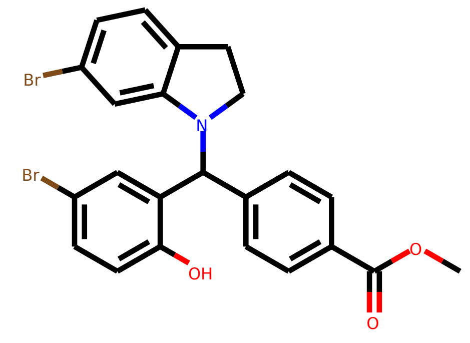
Ahma-2664
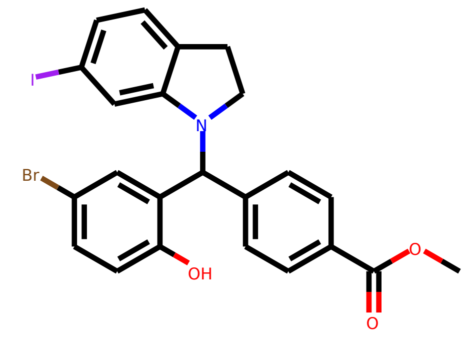
Ahma-2665
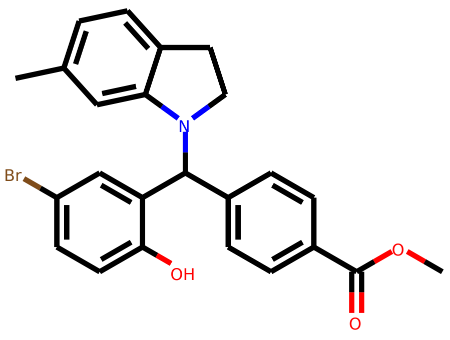
Ahma-2666
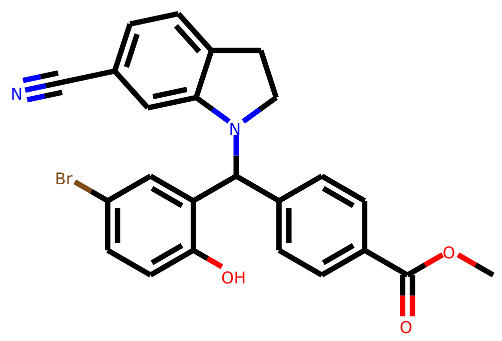
Ahma-2667
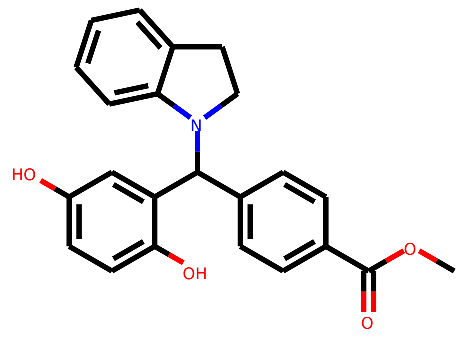
Ahma-2668
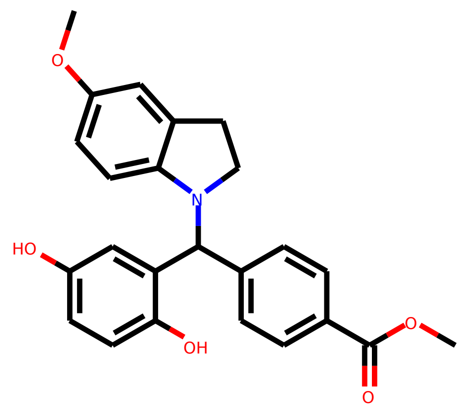
Ahma-2669
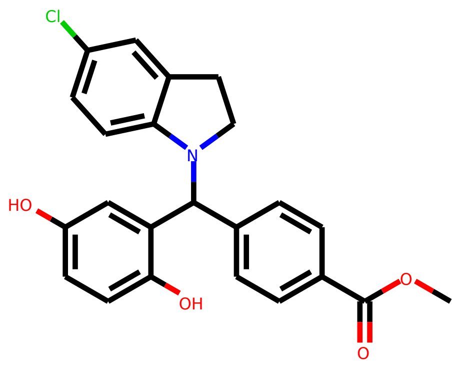
Ahma-2670
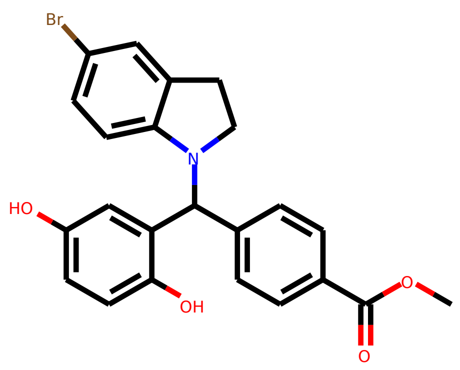
Ahma-2671
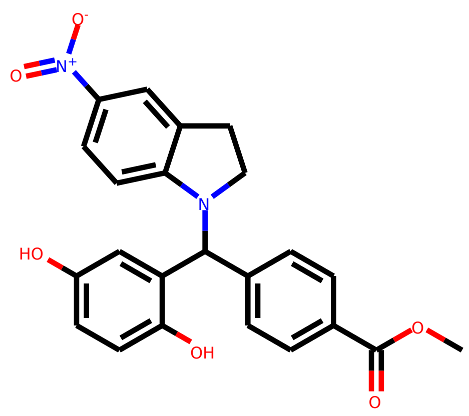
Ahma-2672
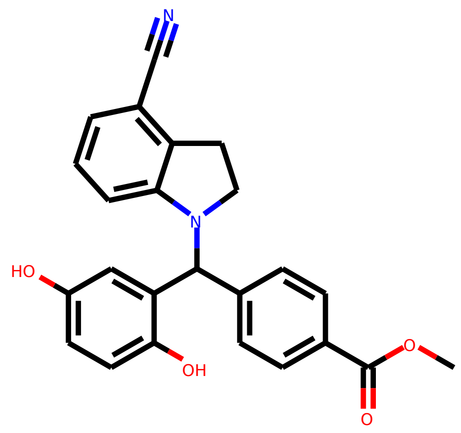
Ahma-2673
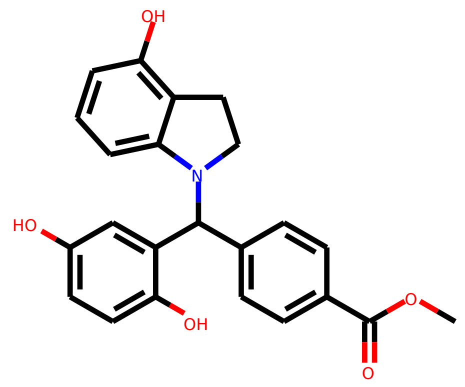
Ahma-2674
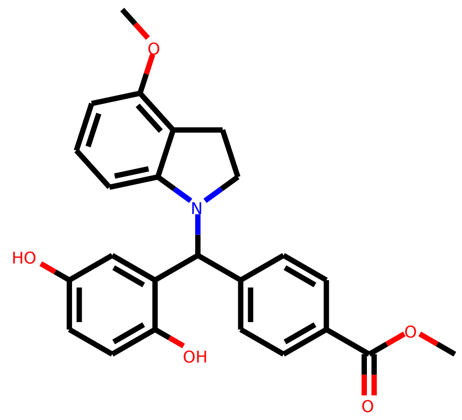
Ahma-2675
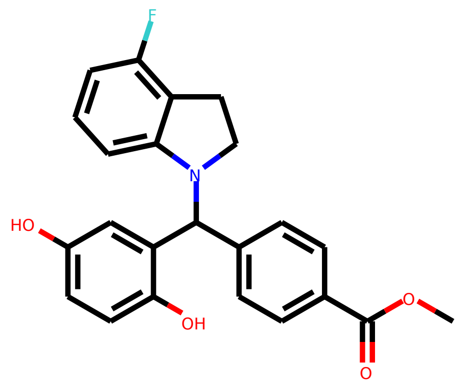
Ahma-2676
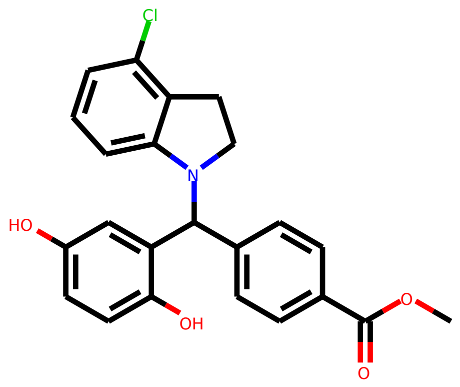
Ahma-2677
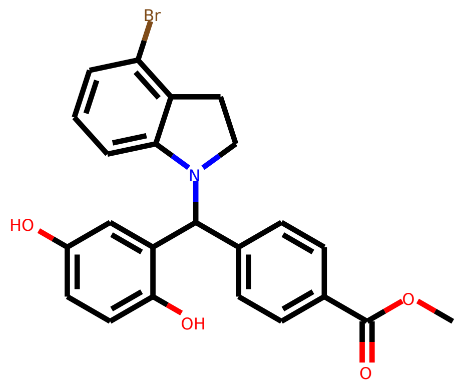
Ahma-2678
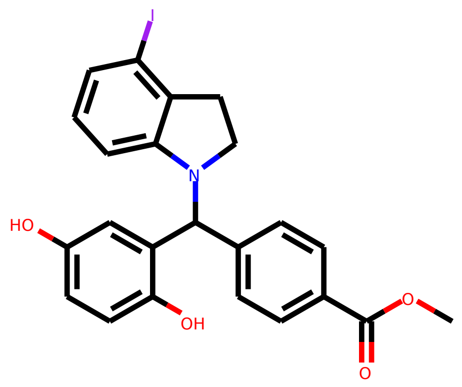
Ahma-2679
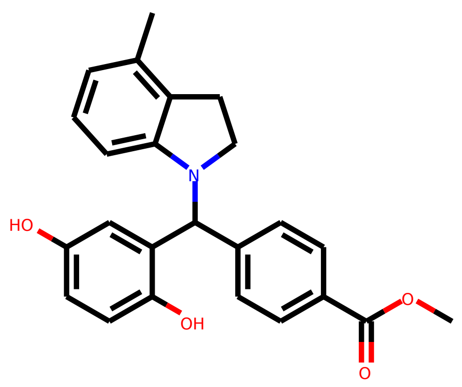
Ahma-2680
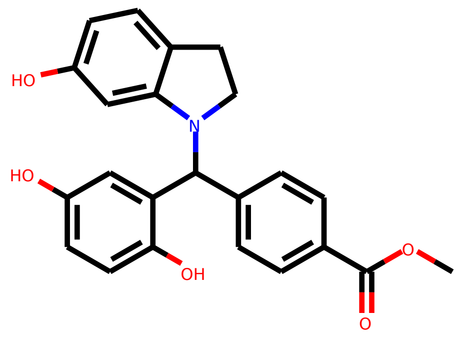
Ahma-2681
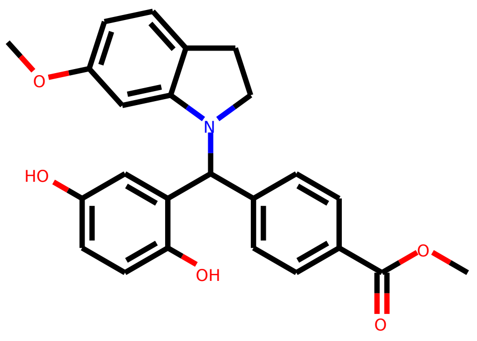
Ahma-2682
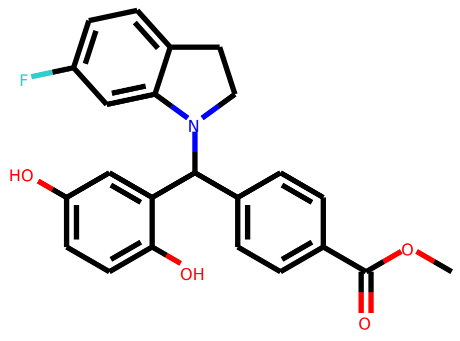
Ahma-2683
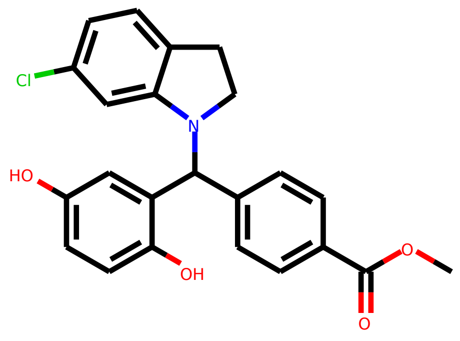
Ahma-2684
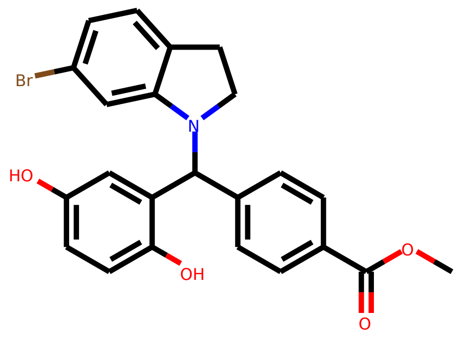
Ahma-2685
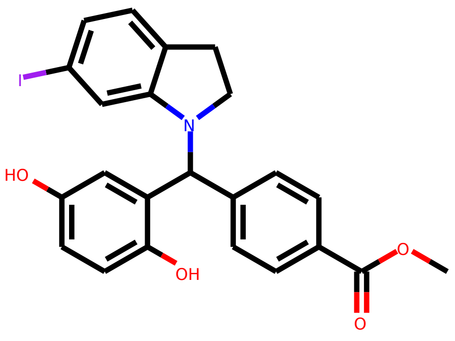
Ahma-2686
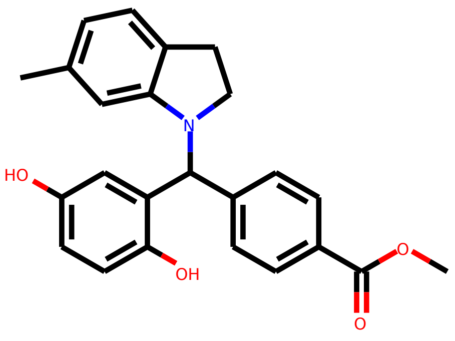
Ahma-2687
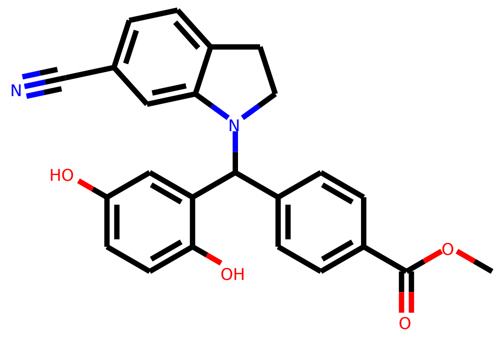
Ahma-2688
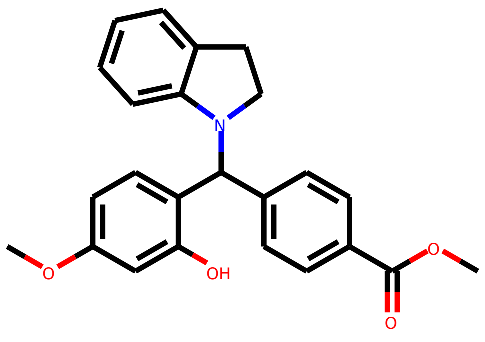
Ahma-2689
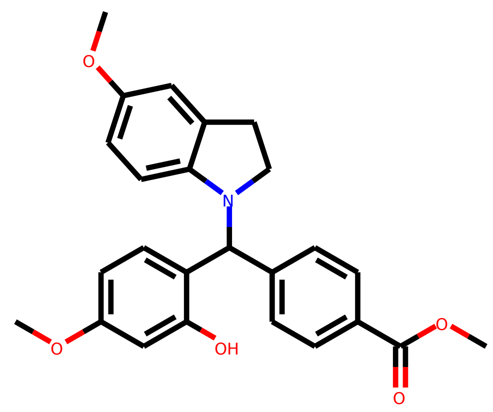
Ahma-2690
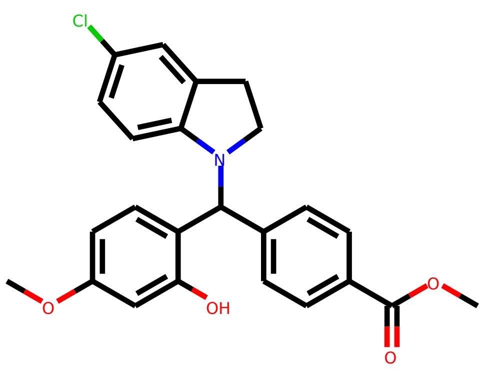
Ahma-2691
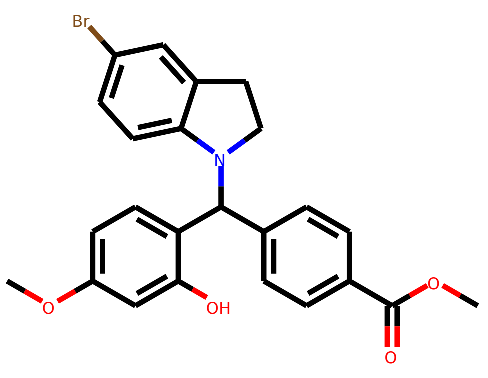
Ahma-2692
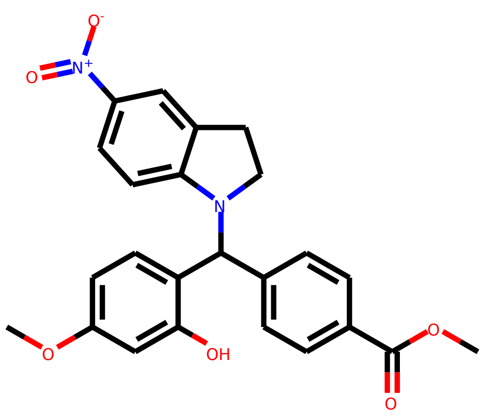
Ahma-2693
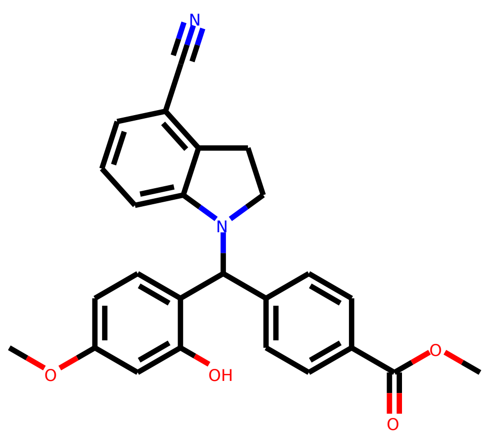
Ahma-2694
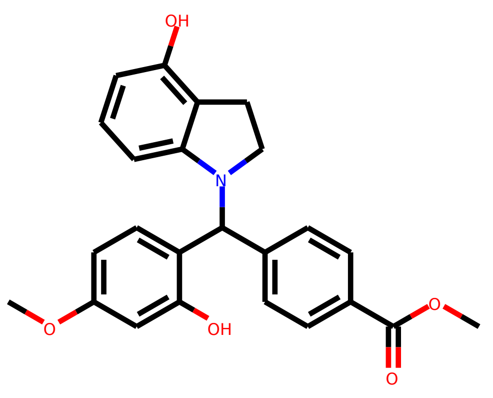
Ahma-2695

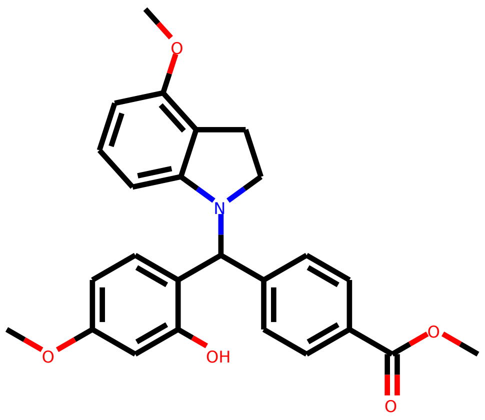
Ahma-2696

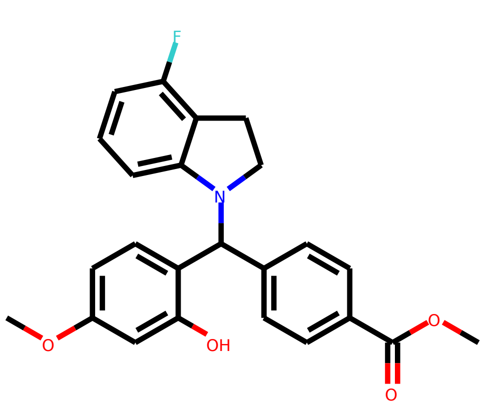
Ahma-2697

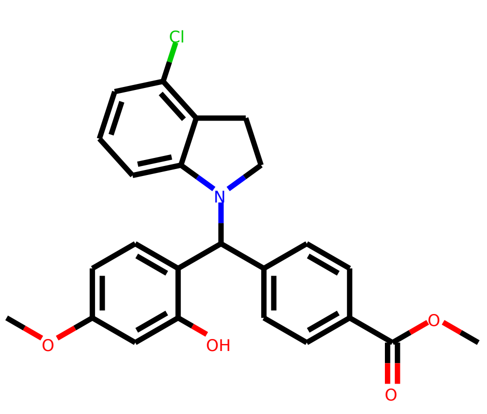
Ahma-2698

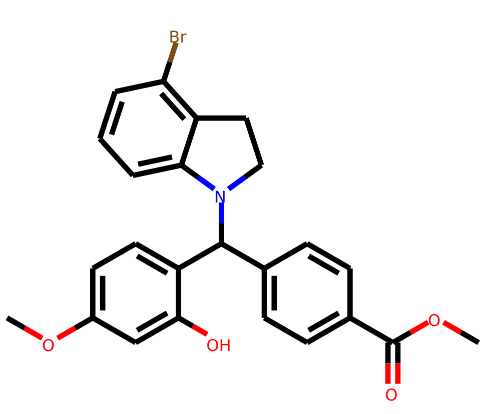
Ahma-2699

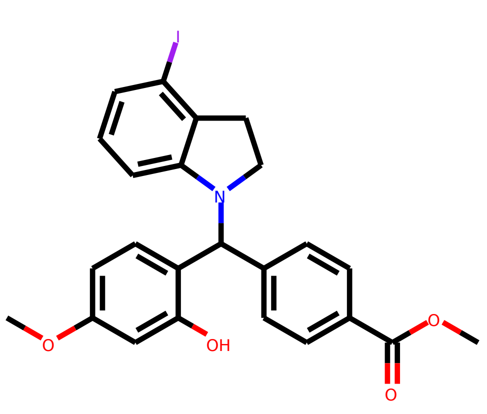
Ahma-2700

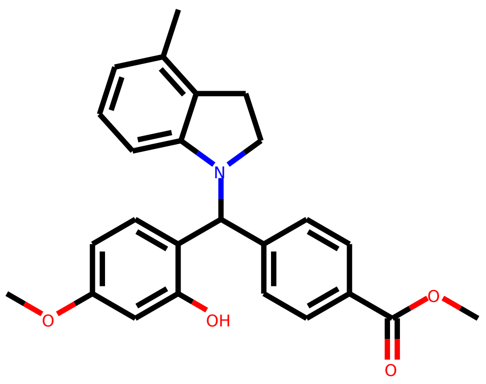
Ahma-2701

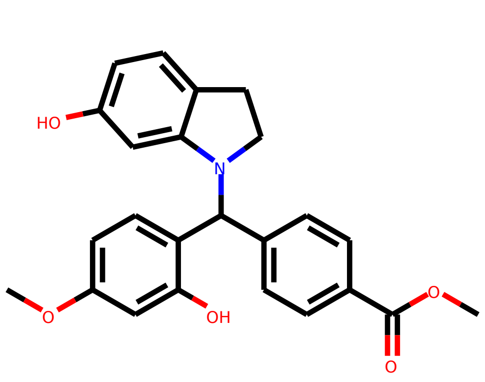
Ahma-2702

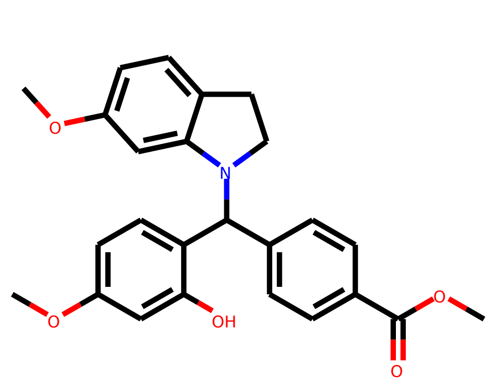
Ahma-2703

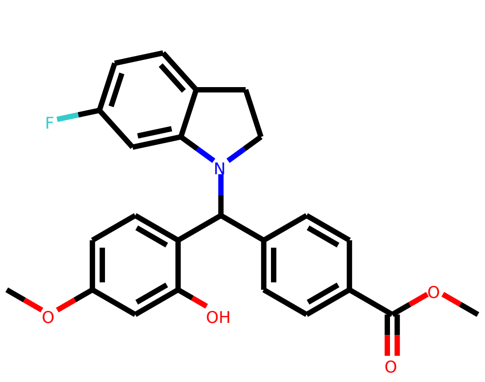
Ahma-2704

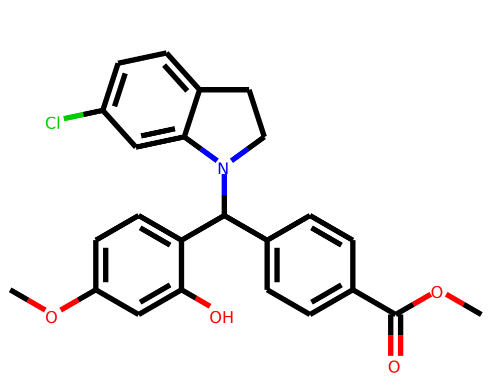
Ahma-2705

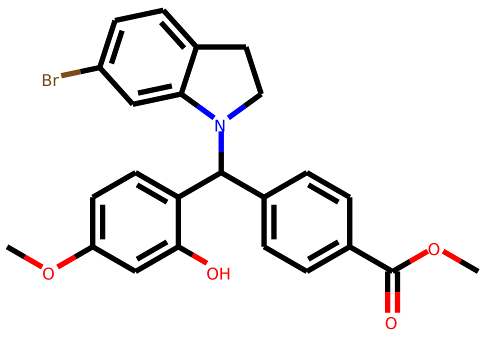
Ahma-2706

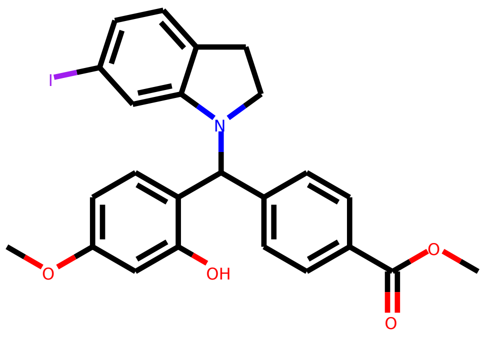
Ahma-2707

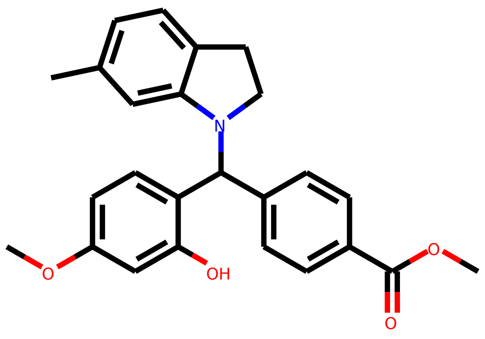
Ahma-2708

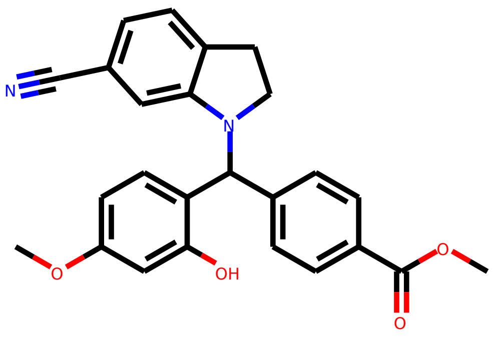
Ahma-2709

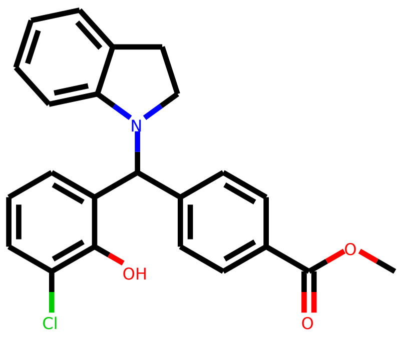
Ahma-2710

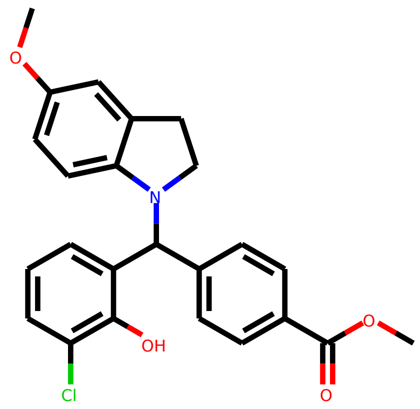
Ahma-2711

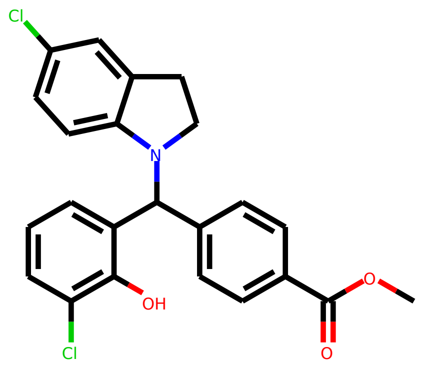
Ahma-2712

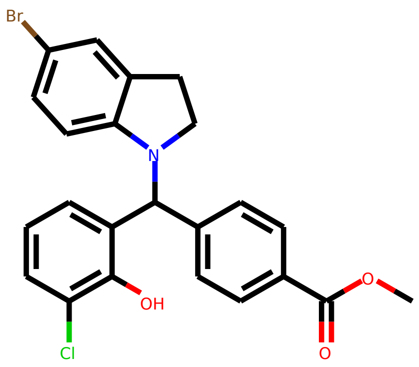
Ahma-2713

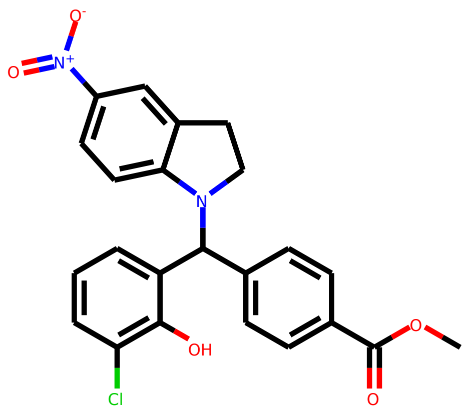
Ahma-2714

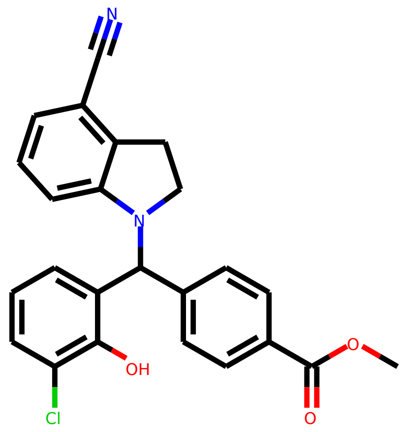
Ahma-2715

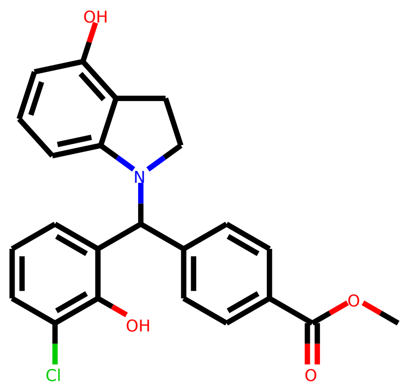
Ahma-2716

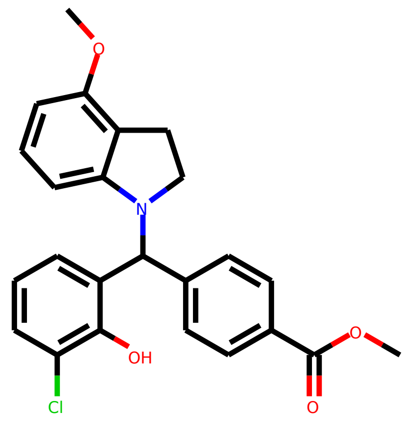
Ahma-2717

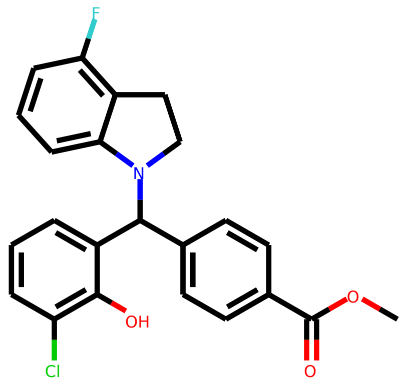
Ahma-2718

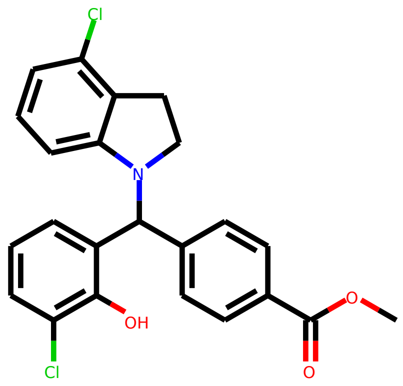
Ahma-2719

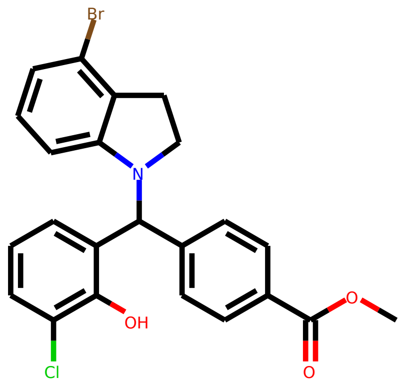
Ahma-2720

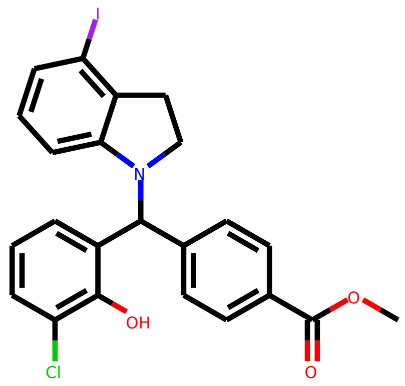
Ahma-2721

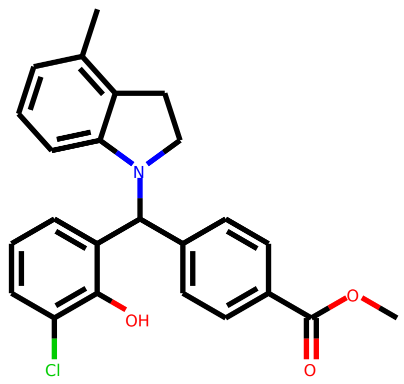
Ahma-2722

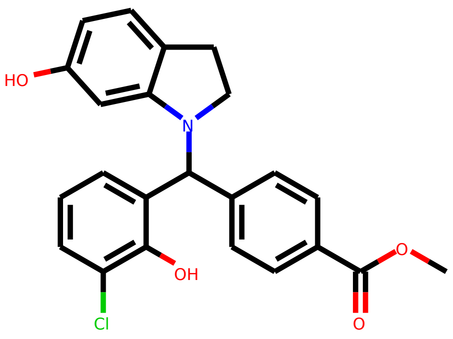
Ahma-2723

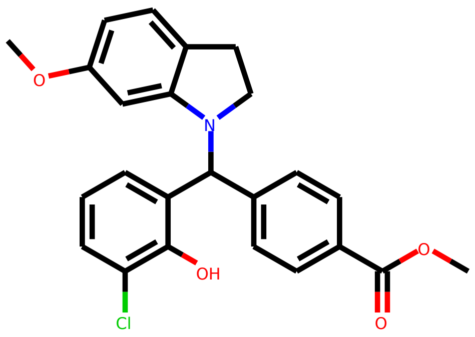
Ahma-2724

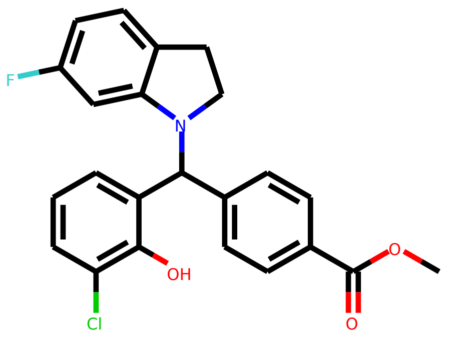
Ahma-2725

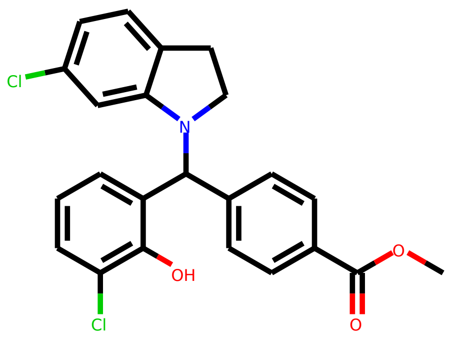
Ahma-2726

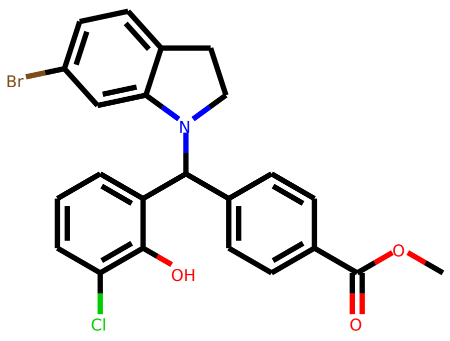
Ahma-2727

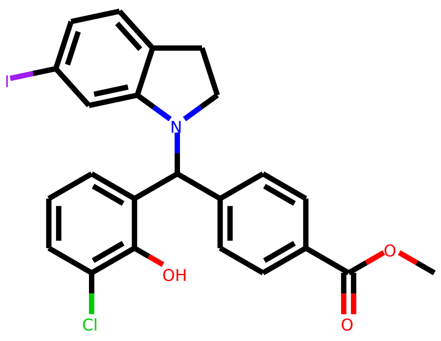
Ahma-2728

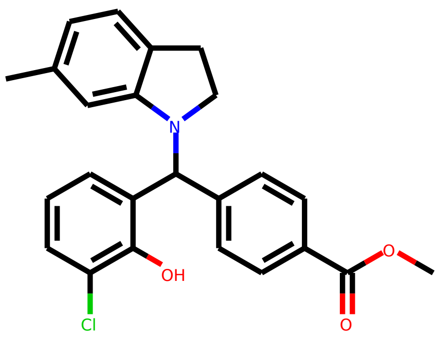
Ahma-2729

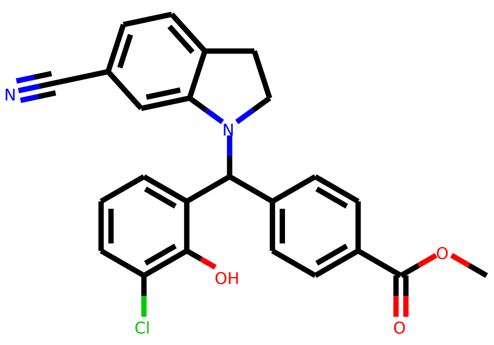
Ahma-2730

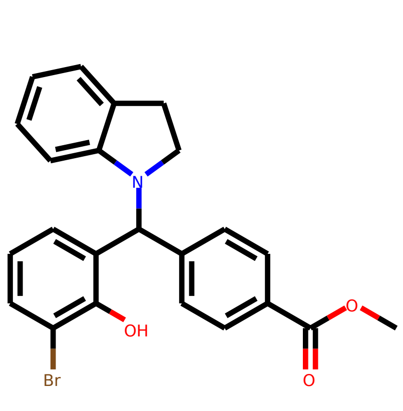
Ahma-2731
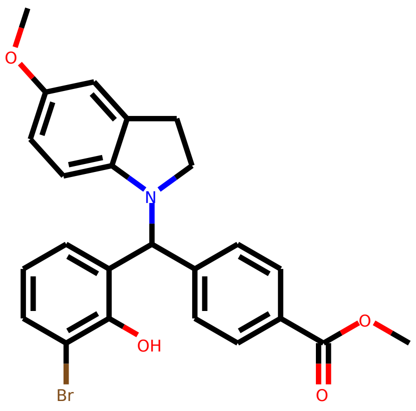
Ahma-2732
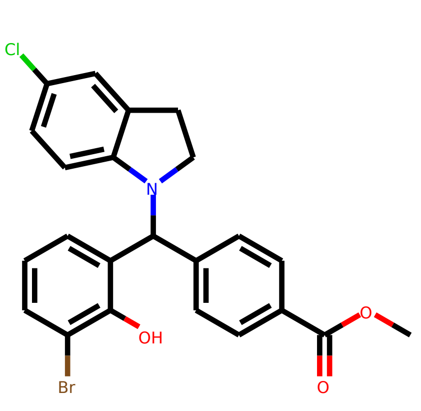
Ahma-2733
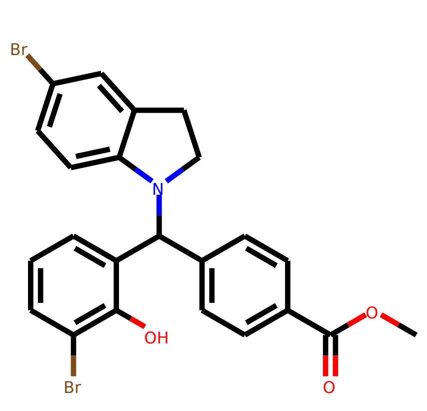
Ahma-2734
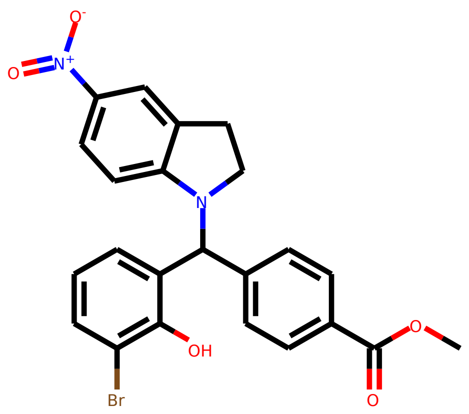
Ahma-2735
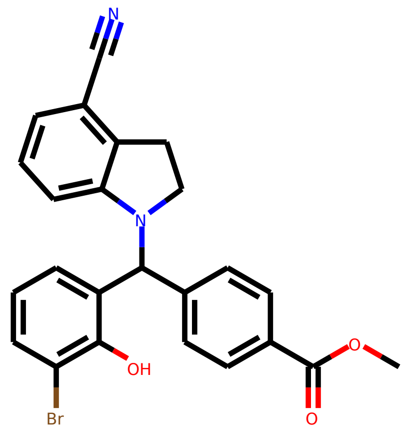
Ahma-2736
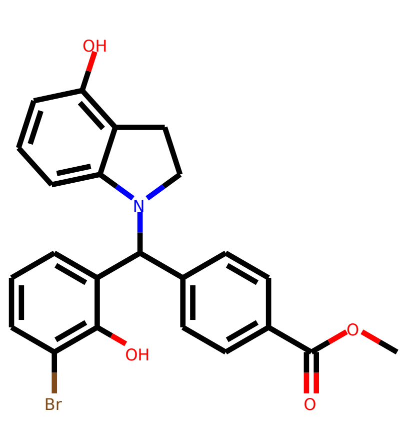
Ahma-2737
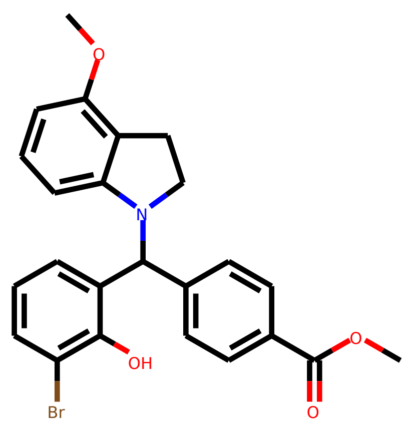
Ahma-2738
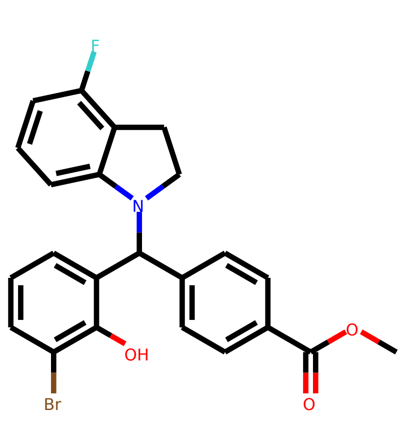
Ahma-2739
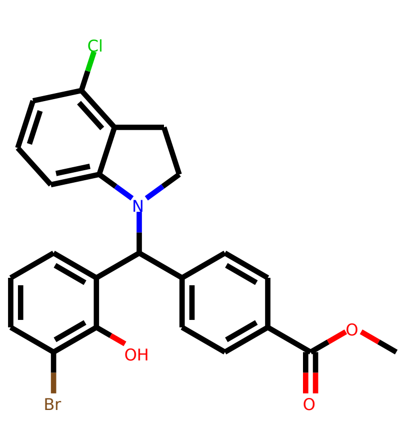
Ahma-2740
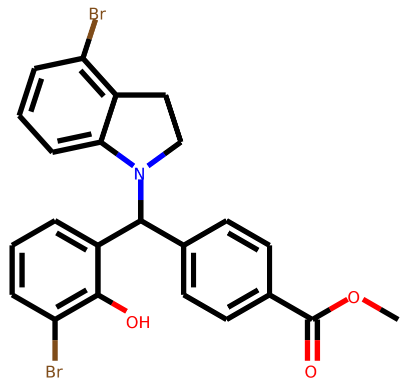
Ahma-2741
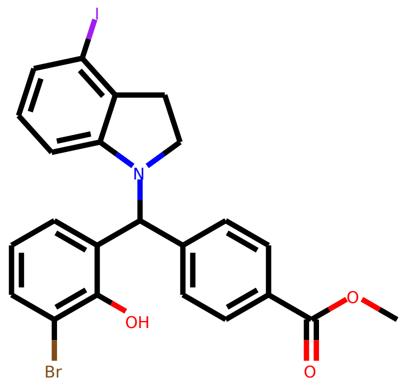
Ahma-2742
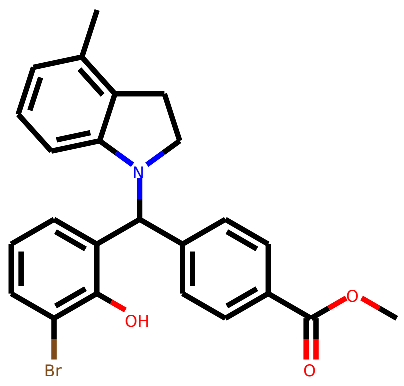
Ahma-2743
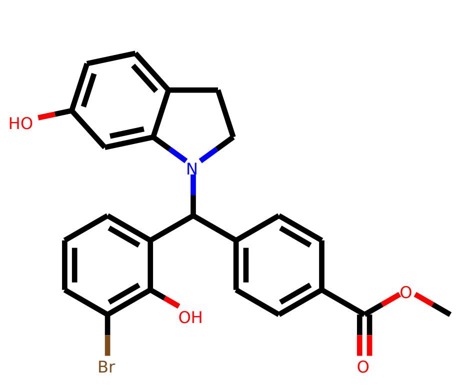
Ahma-2744
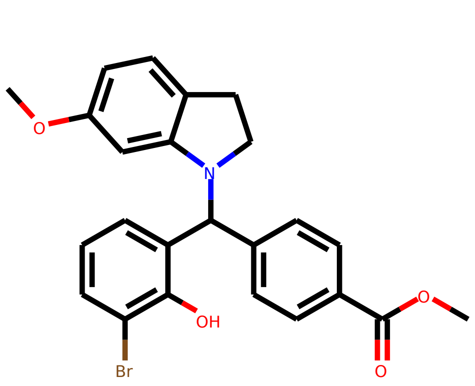
Ahma-2745
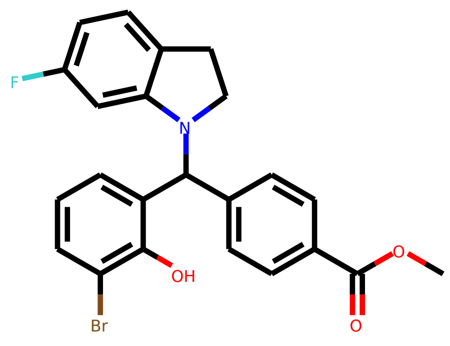
Ahma-2746
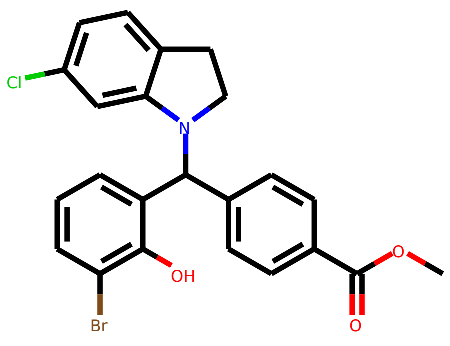
Ahma-2747
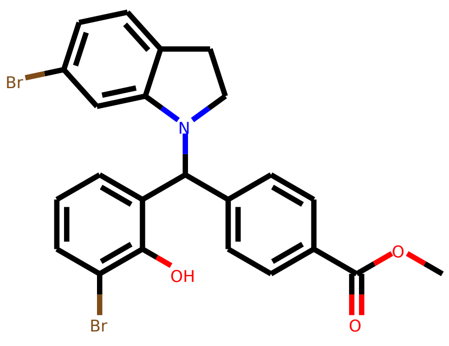
Ahma-2748
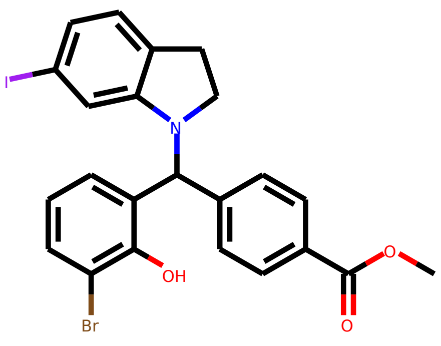
Ahma-2749
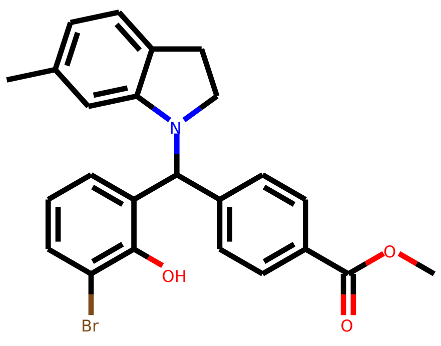
Ahma-2750
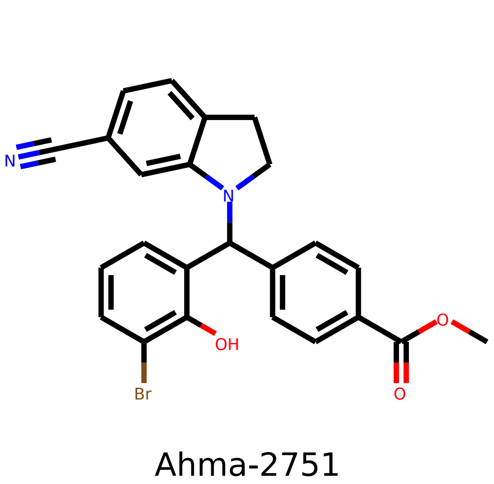
Ahma-2751
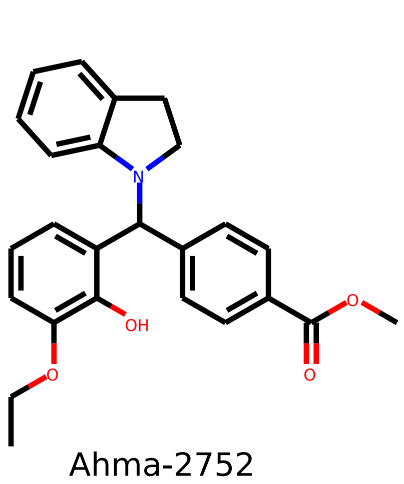
Ahma-2752
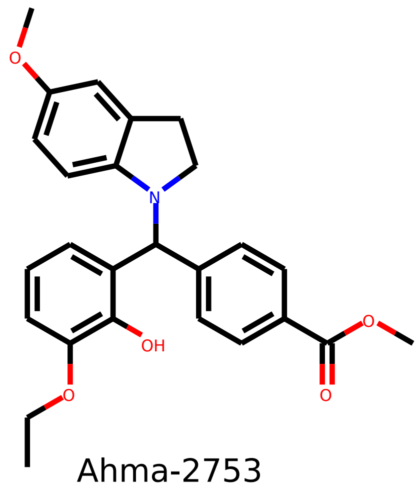
Ahma-2753
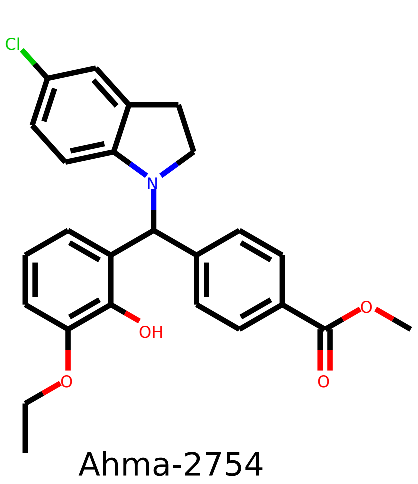
Ahma-2754
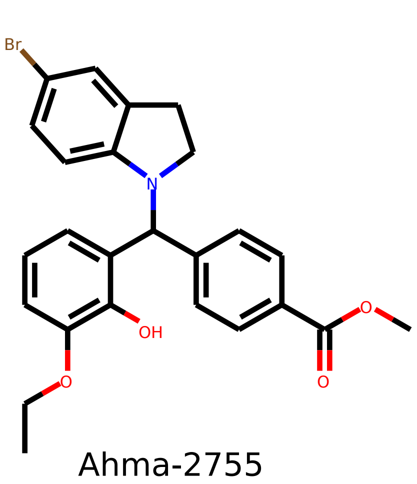
Ahma-2755
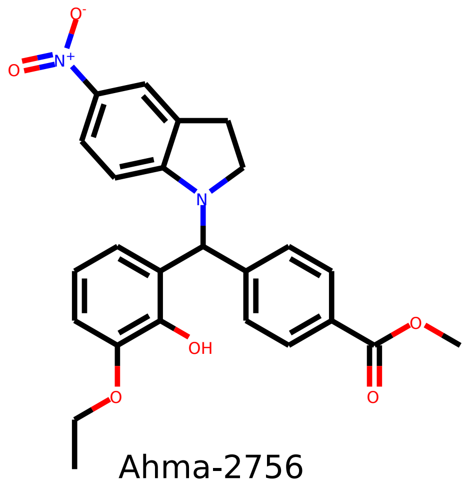
Ahma-2756
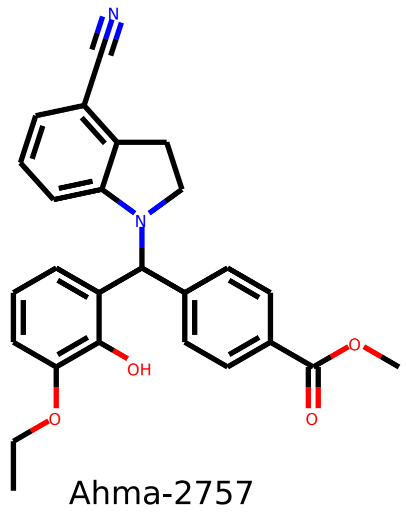
Ahma-2757
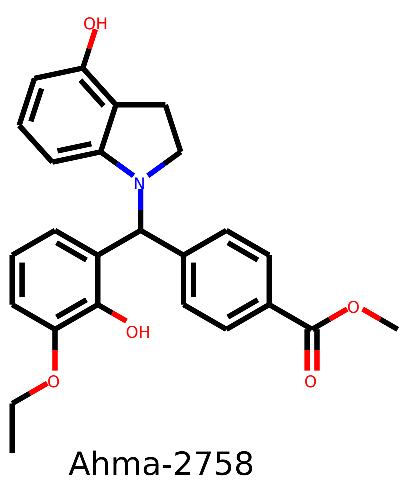
Ahma-2758
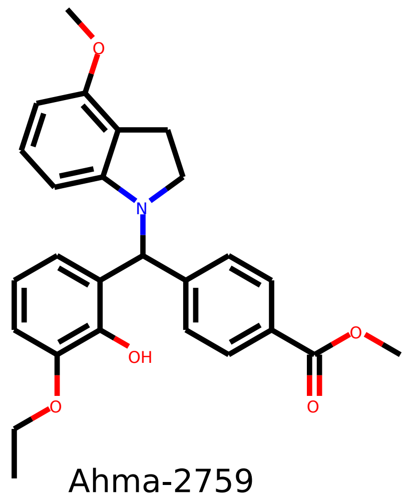
Ahma-2759
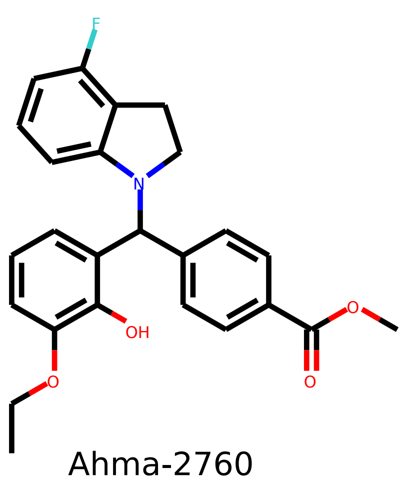
Ahma-2760
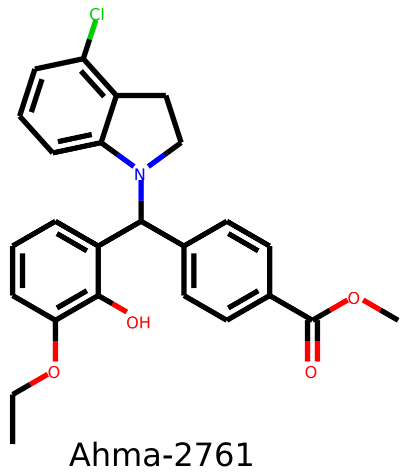
Ahma-2761
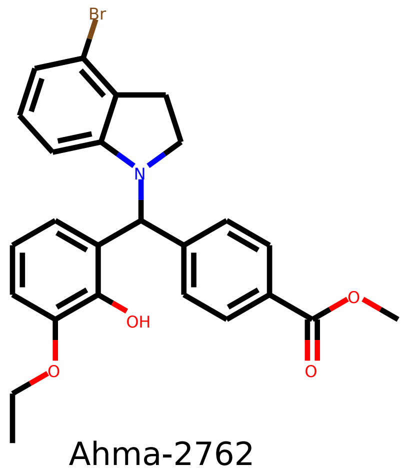
Ahma-2762
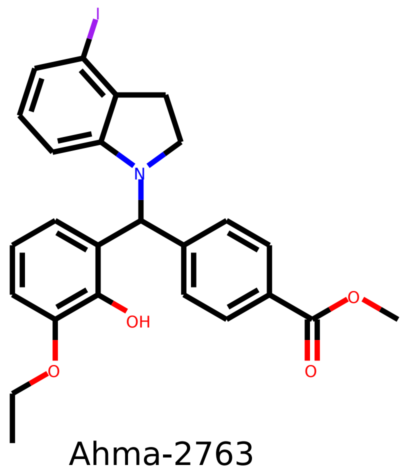
Ahma-2763
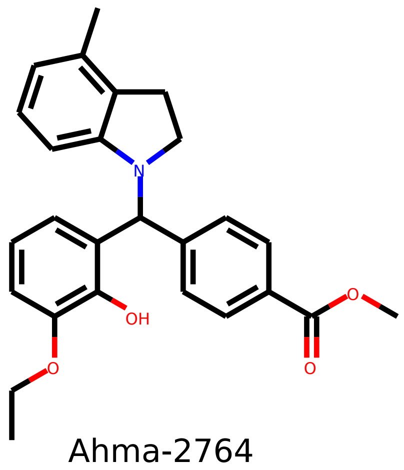
Ahma-2764
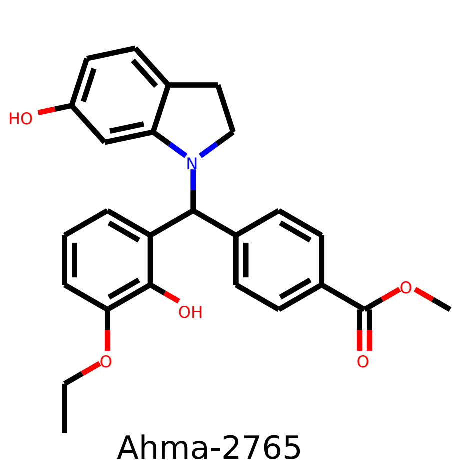
Ahma-2765

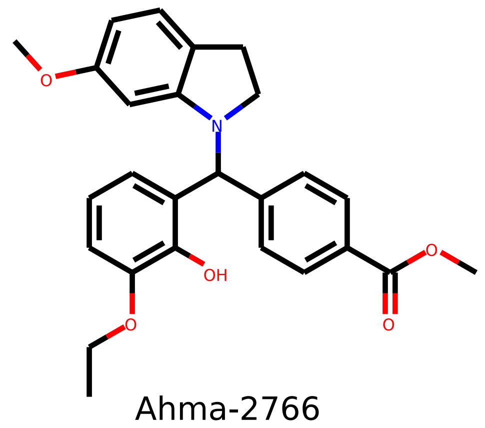

Ahma-2766

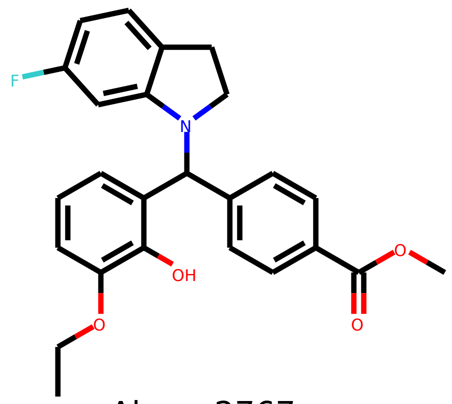

Ahma-2767

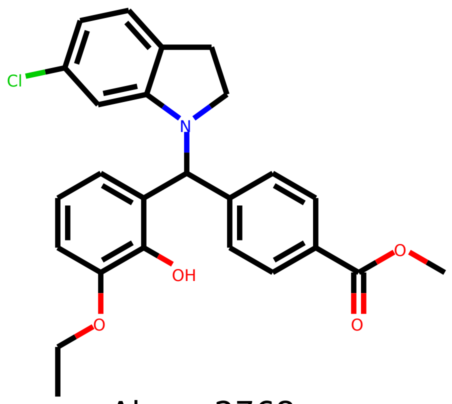

Ahma-2768

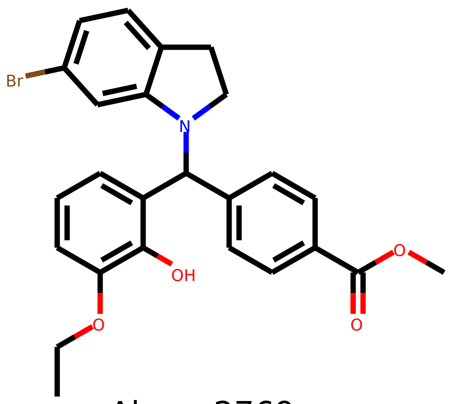

Ahma-2769

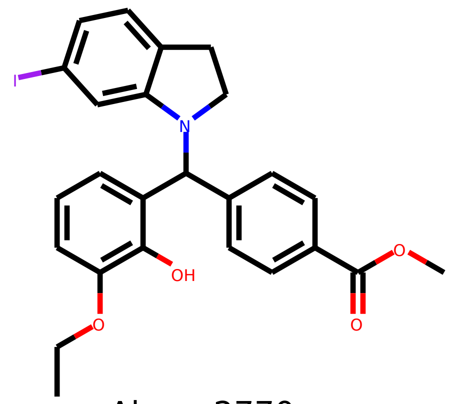

Ahma-2770

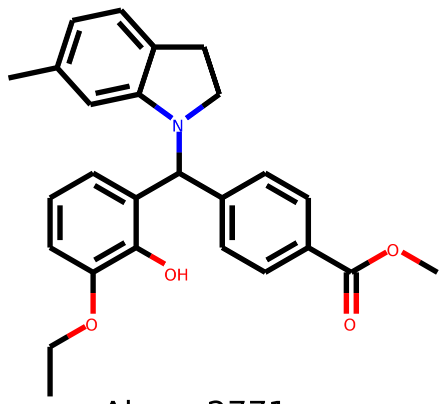

Ahma-2771

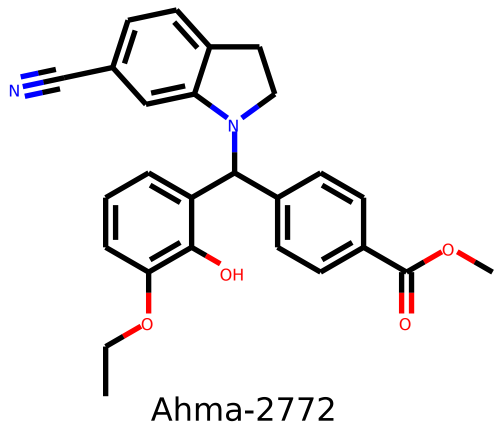

Ahma-2772

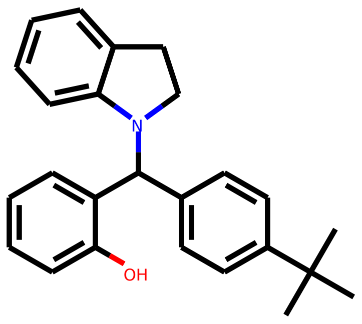

Ahma-2773

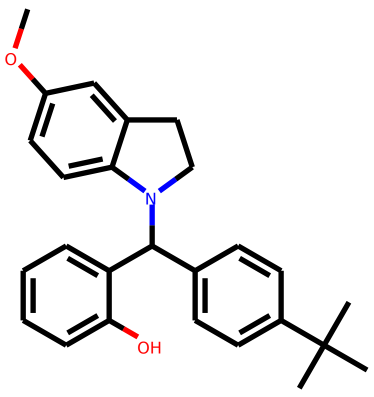

Ahma-2774

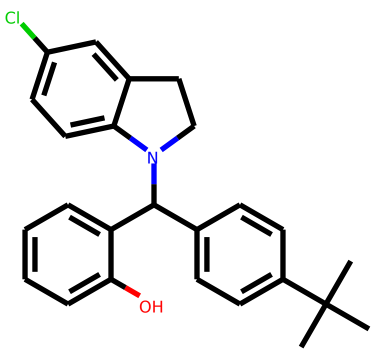

Ahma-2775

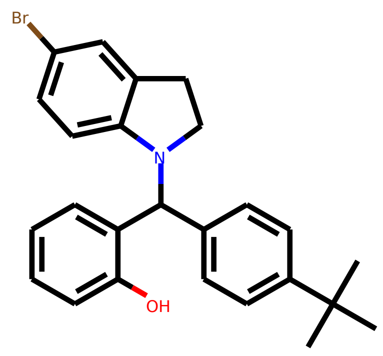

Ahma-2776

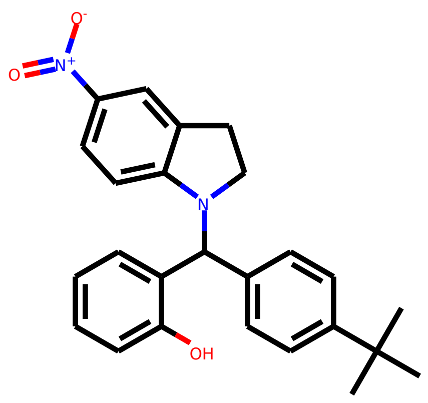

Ahma-2777

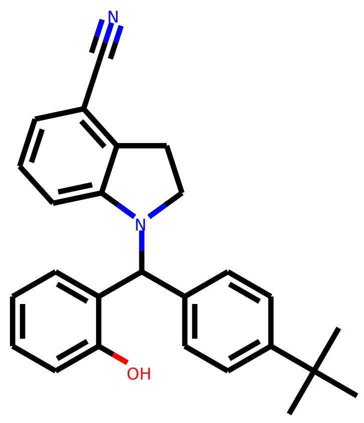

Ahma-2778

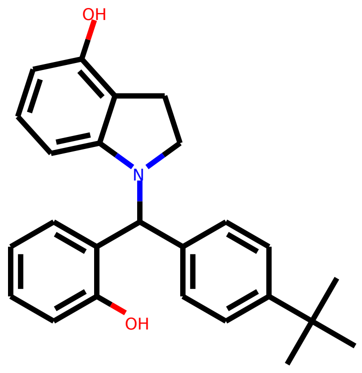

Ahma-2779

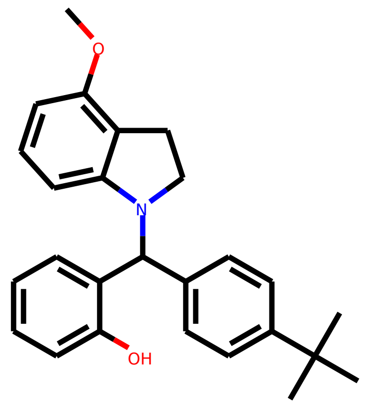

Ahma-2780

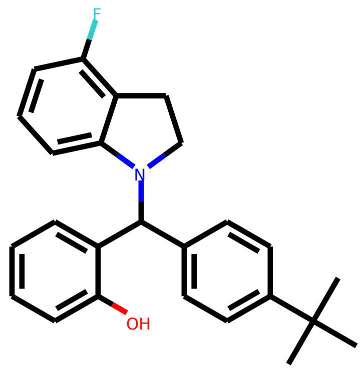

Ahma-2781

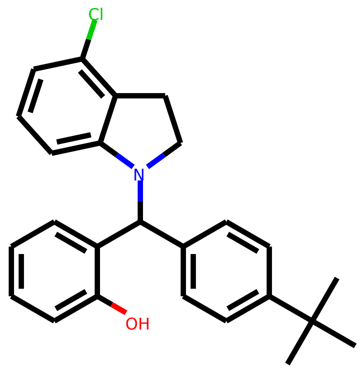

Ahma-2782

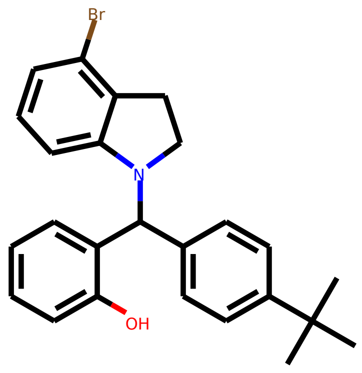

Ahma-2783

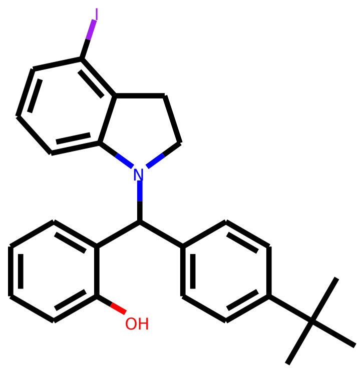

Ahma-2784

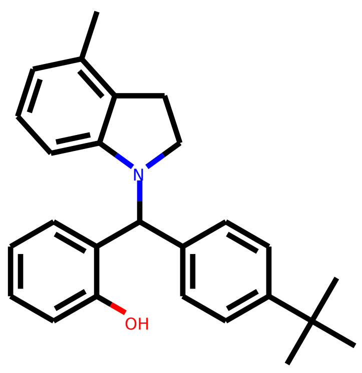

Ahma-2785

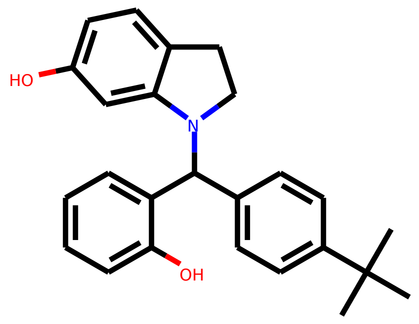

Ahma-2786

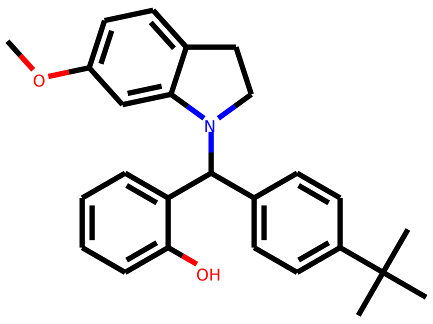

Ahma-2787

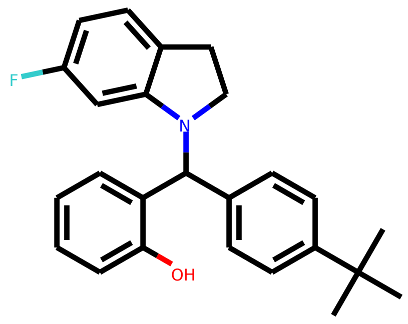

Ahma-2788

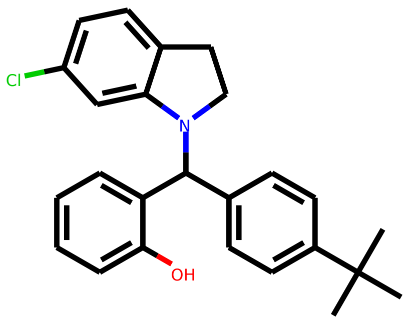

Ahma-2789

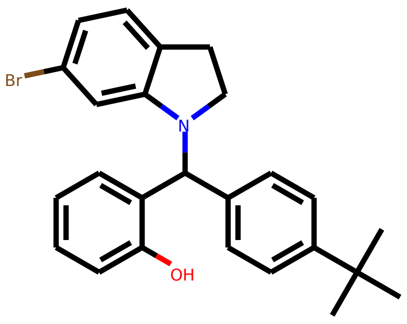

Ahma-2790

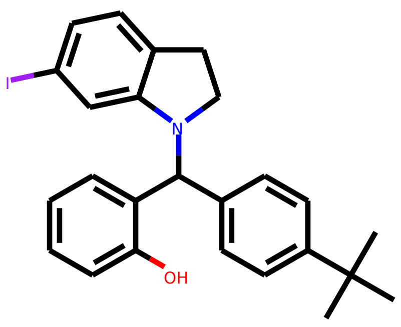

Ahma-2791

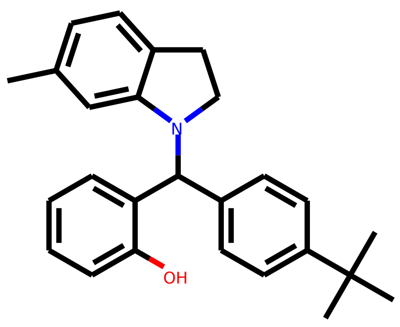

Ahma-2792

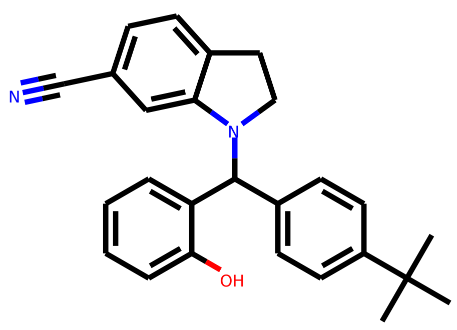

Ahma-2793

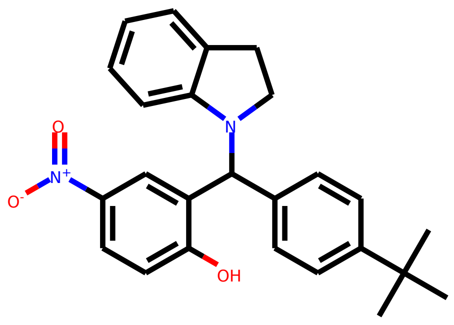

Ahma-2794

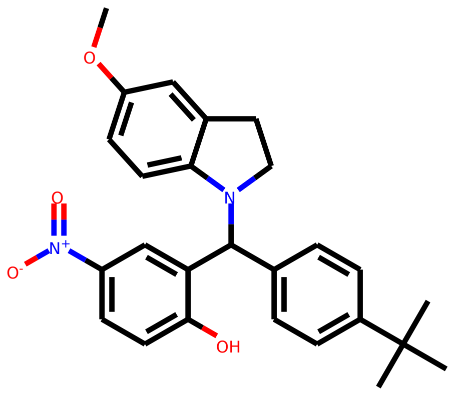

Ahma-2795

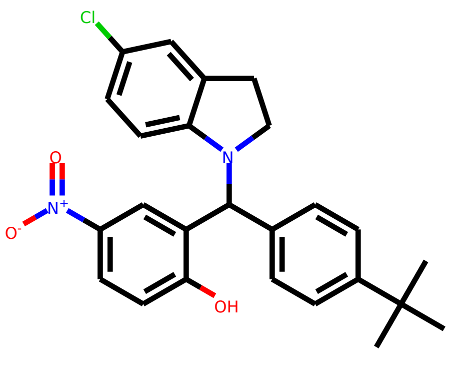

Ahma-2796

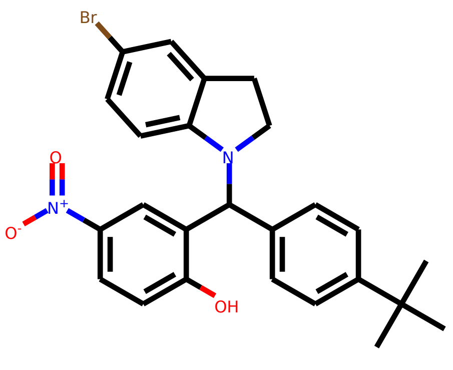

Ahma-2797

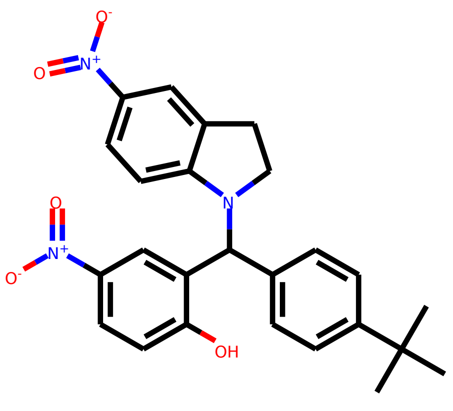

Ahma-2798

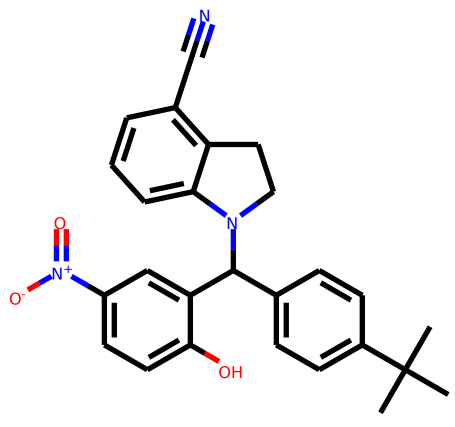

Ahma-2799

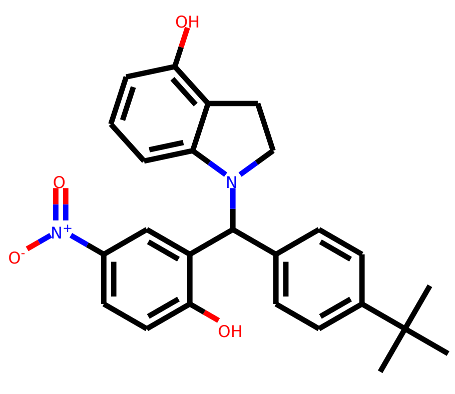

Ahma-2800

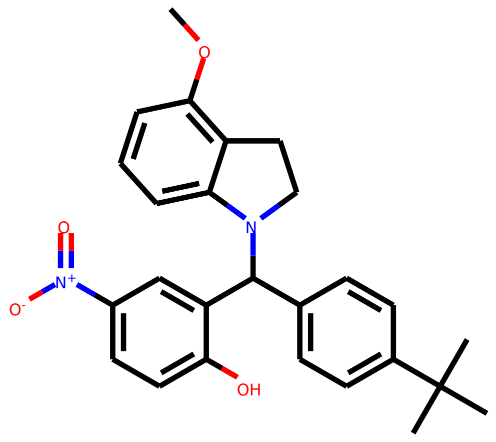
Ahma-2801

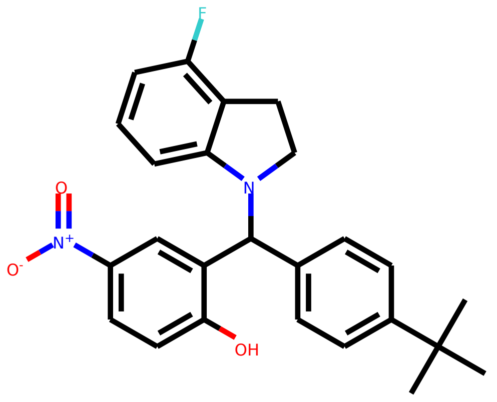
Ahma-2802

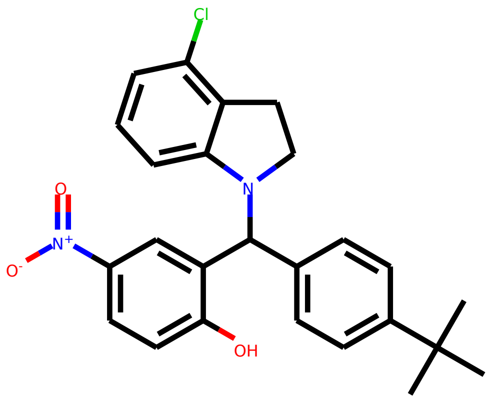
Ahma-2803

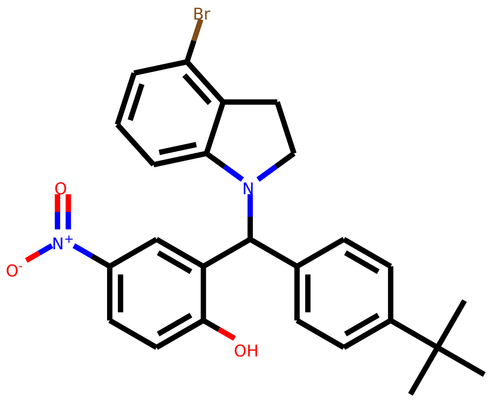
Ahma-2804

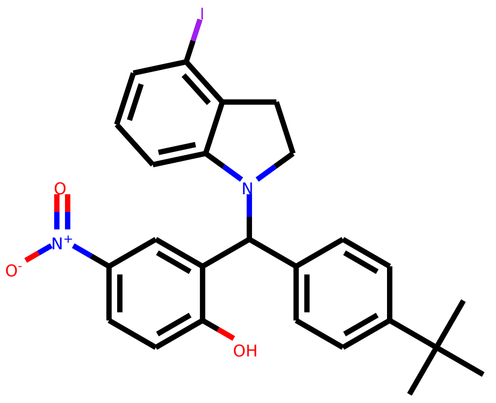
Ahma-2805

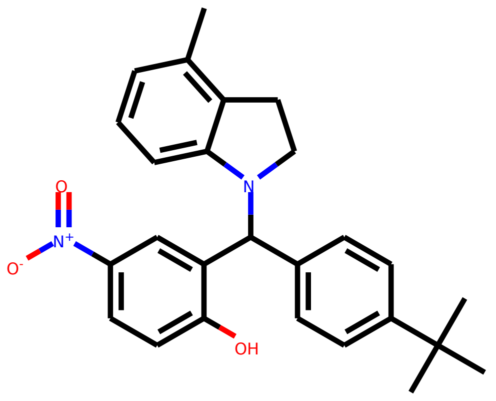
Ahma-2806

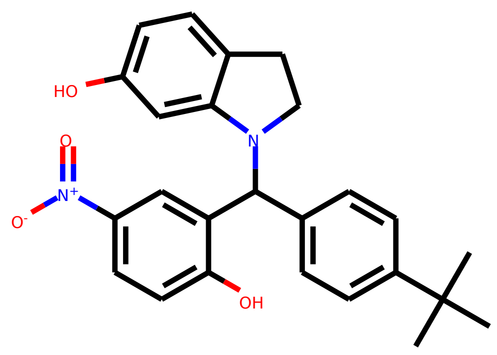
Ahma-2807

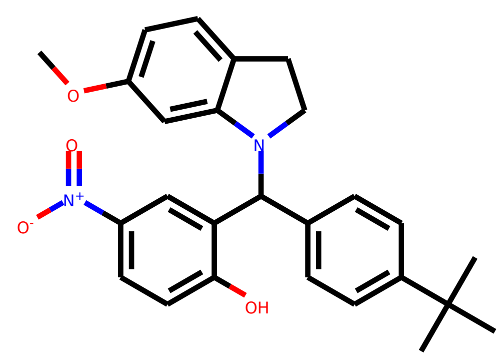
Ahma-2808

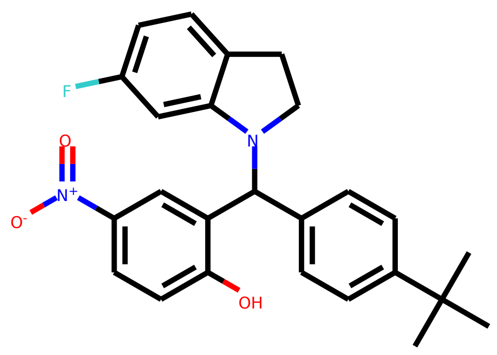
Ahma-2809

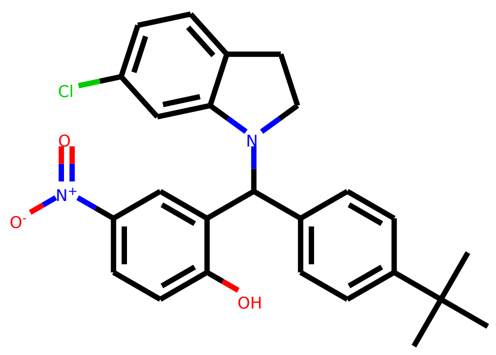
Ahma-2810

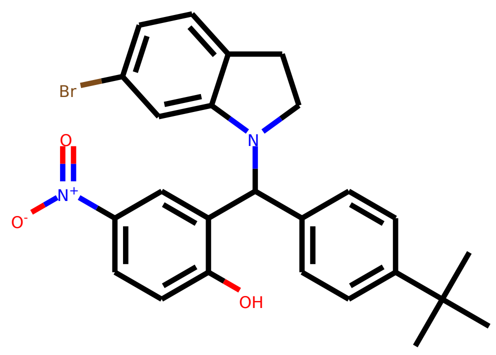
Ahma-2811

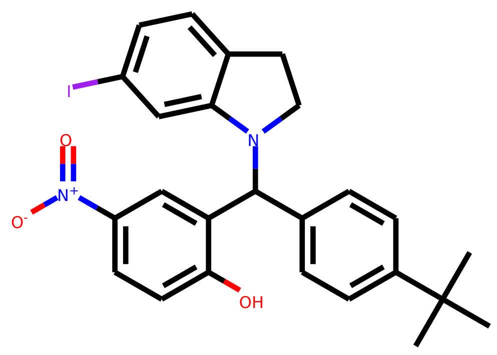
Ahma-2812

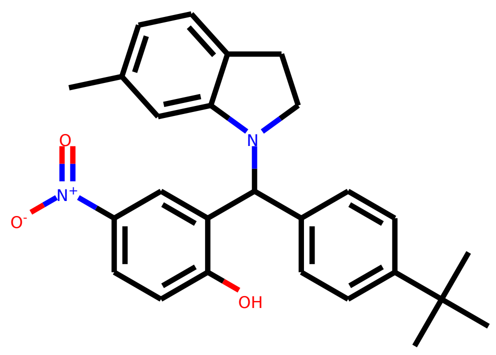
Ahma-2813

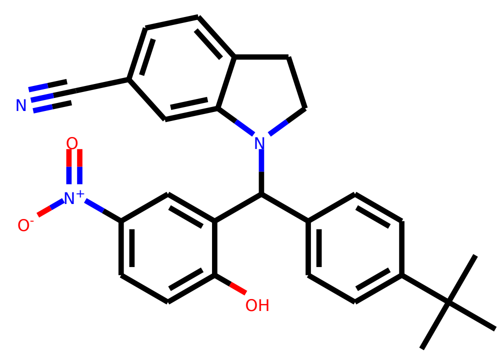
Ahma-2814

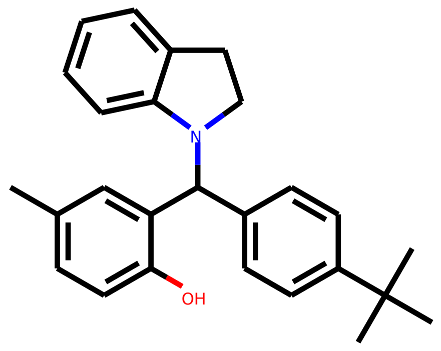
Ahma-2815

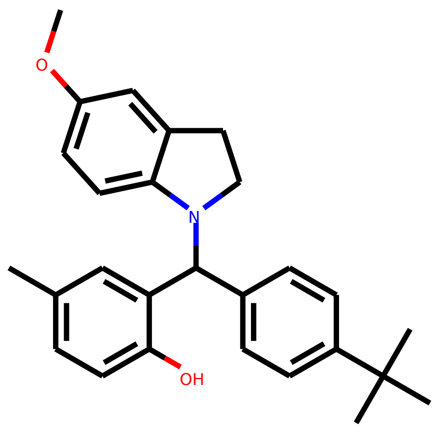
Ahma-2816

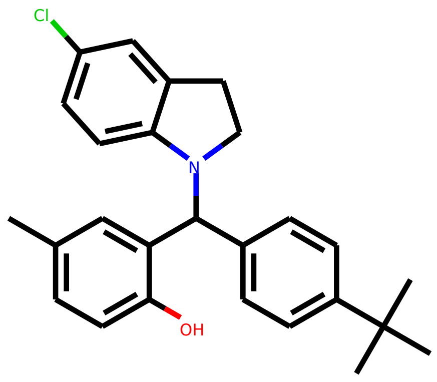
Ahma-2817

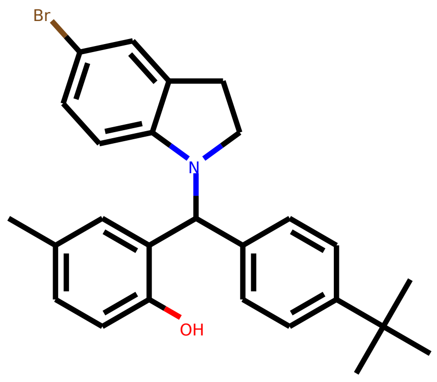
Ahma-2818

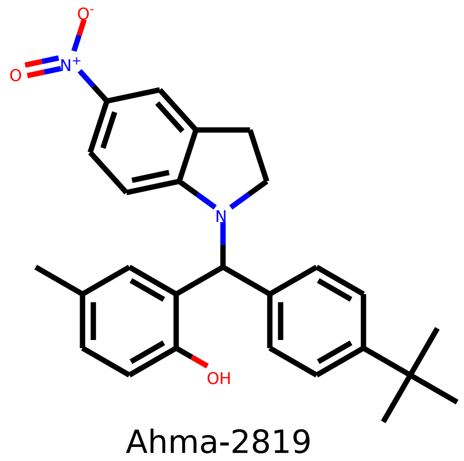
Ahma-2819

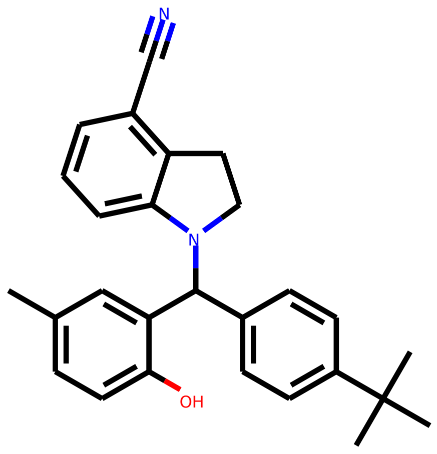
Ahma-2820

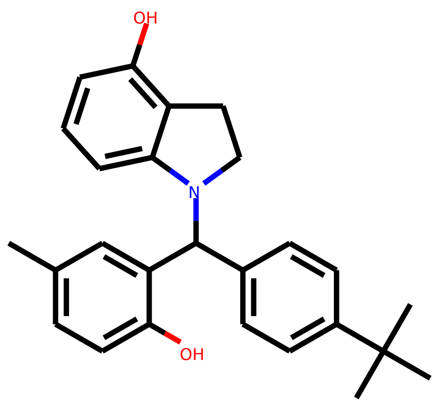
Ahma-2821

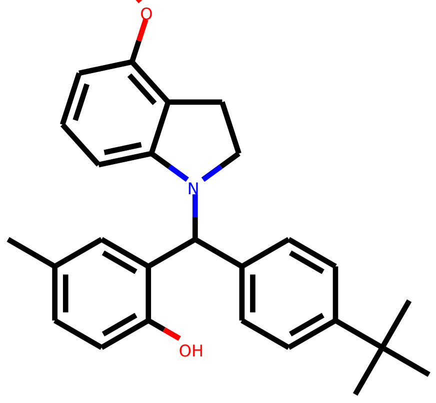
Ahma-2822

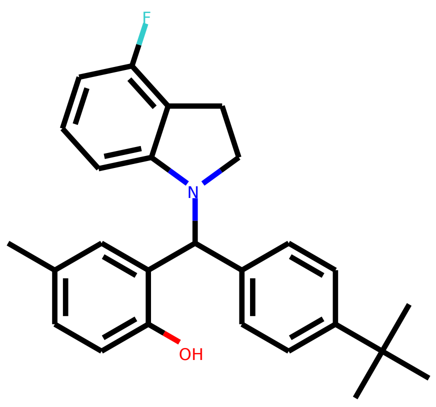
Ahma-2823

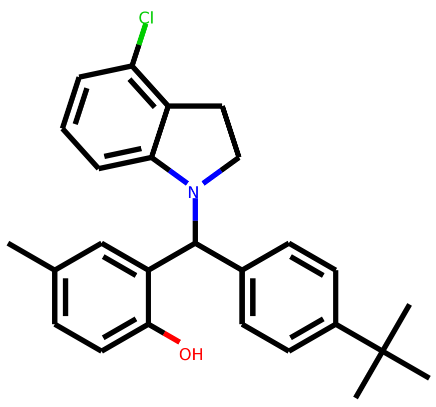
Ahma-2824

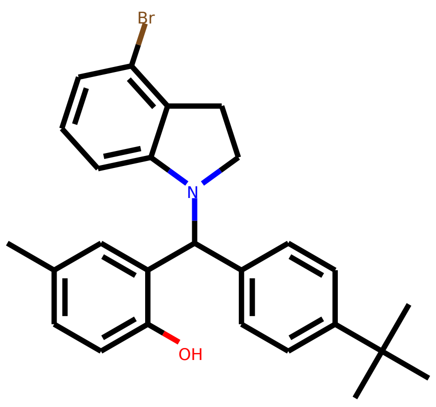
Ahma-2825

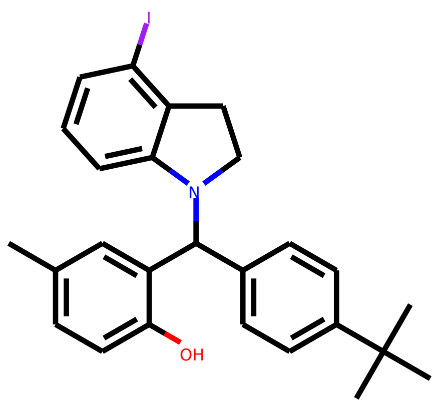
Ahma-2826

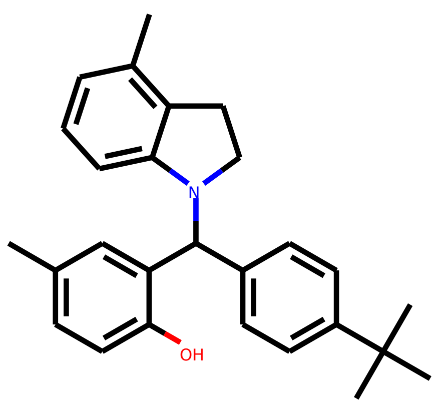
Ahma-2827

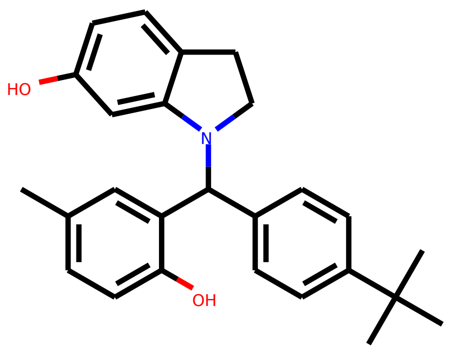
Ahma-2828

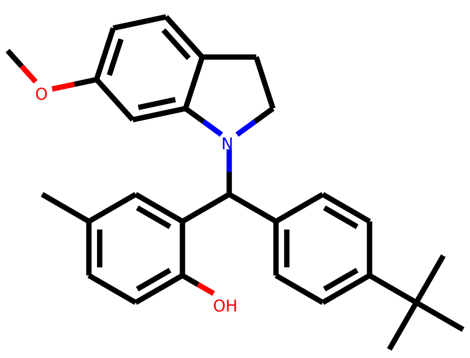
Ahma-2829

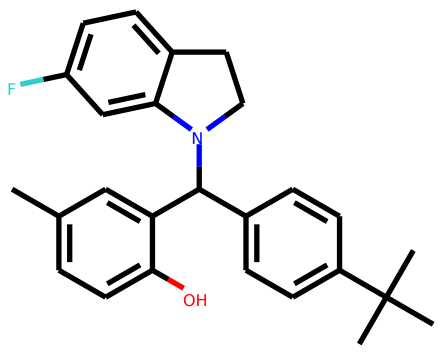
Ahma-2830

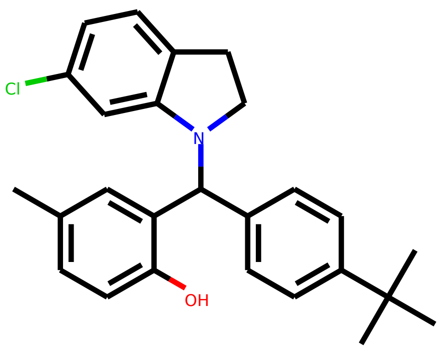
Ahma-2831

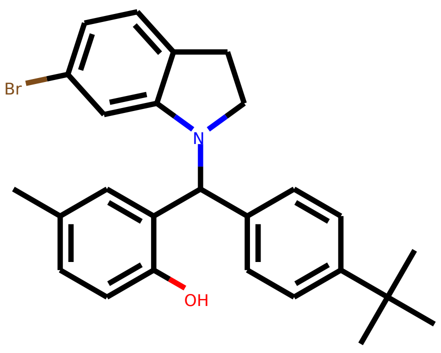
Ahma-2832

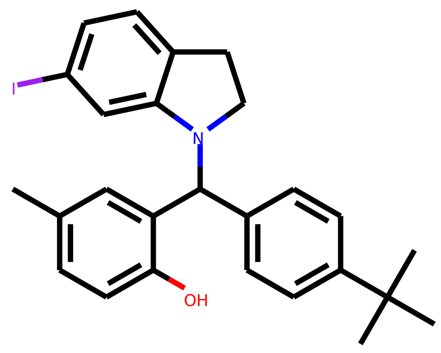
Ahma-2833

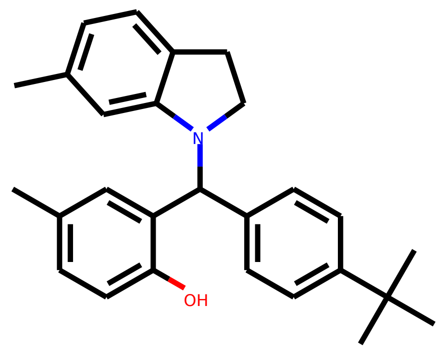
Ahma-2834

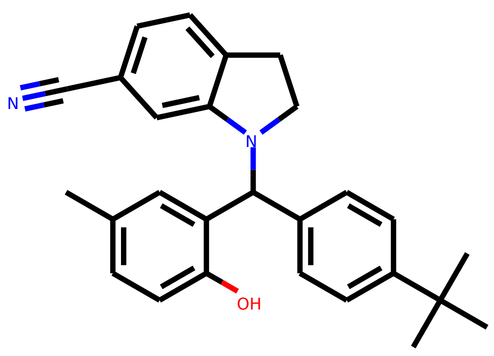
Ahma-2835

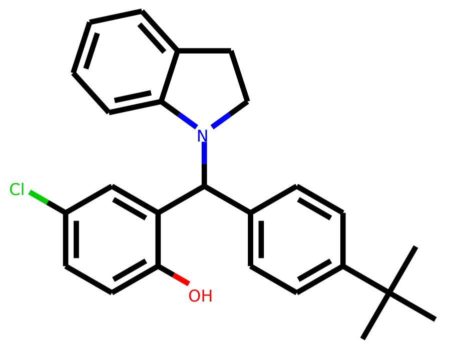

Ahma-2836

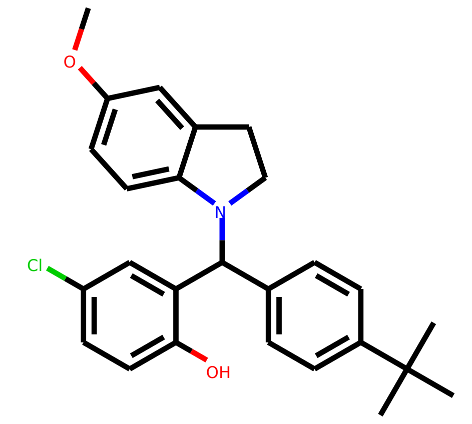

Ahma-2837

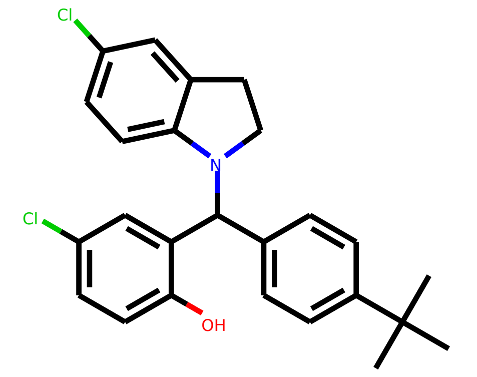

Ahma-2838

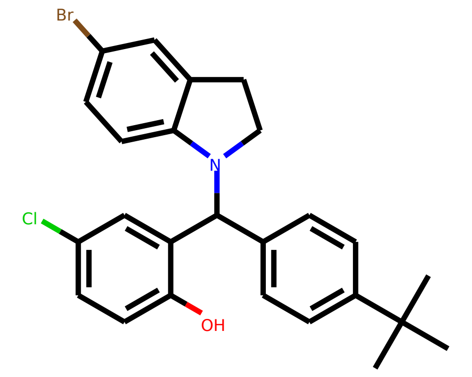

Ahma-2839

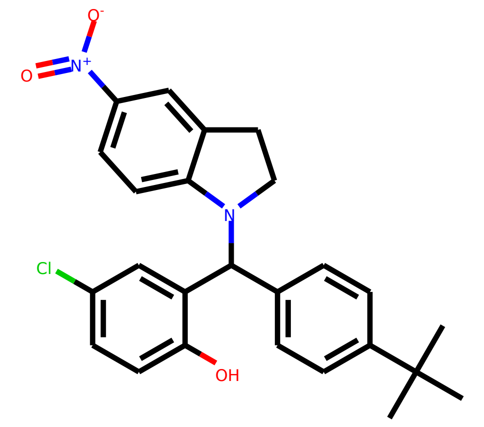

Ahma-2840

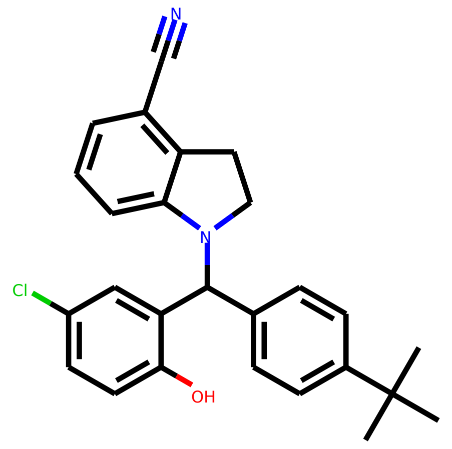

Ahma-2841

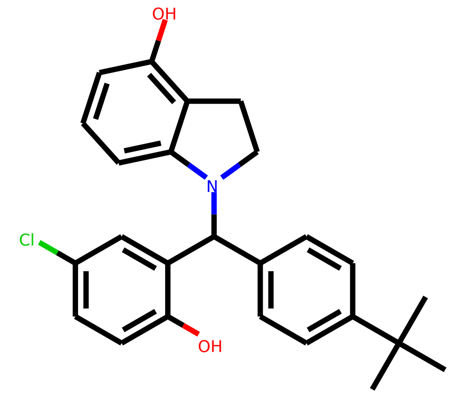

Ahma-2842

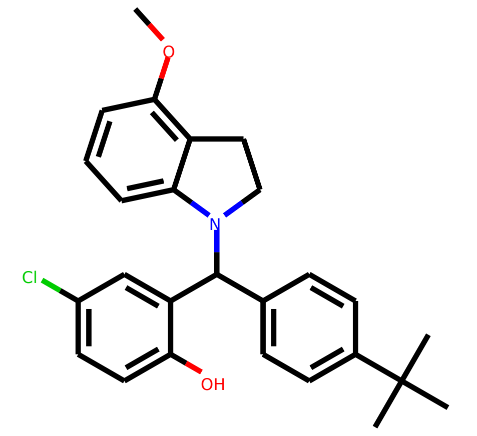

Ahma-2843

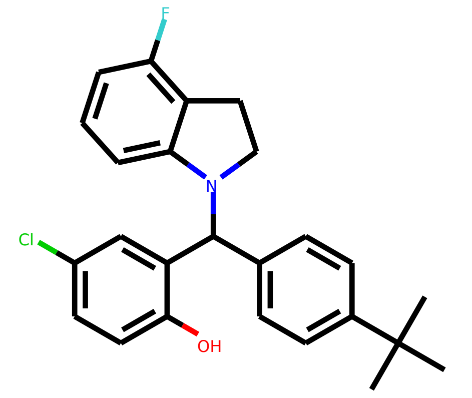

Ahma-2844

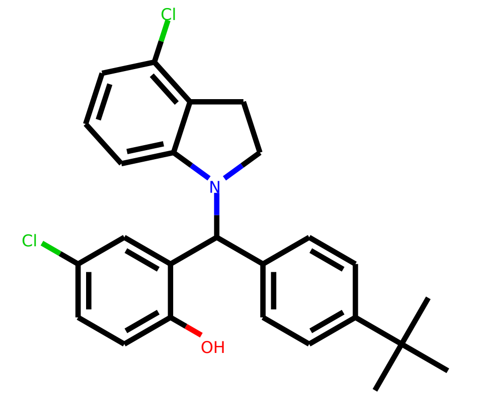

Ahma-2845

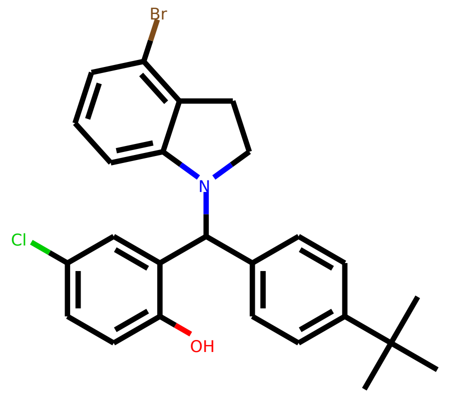

Ahma-2846

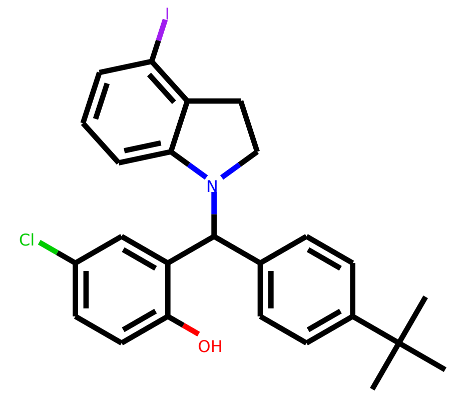

Ahma-2847

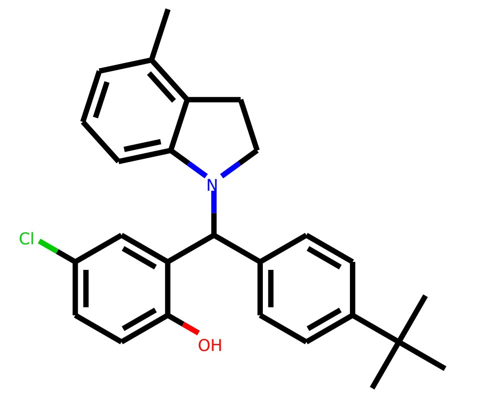

Ahma-2848

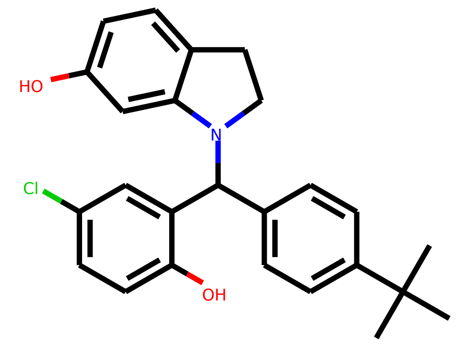

Ahma-2849

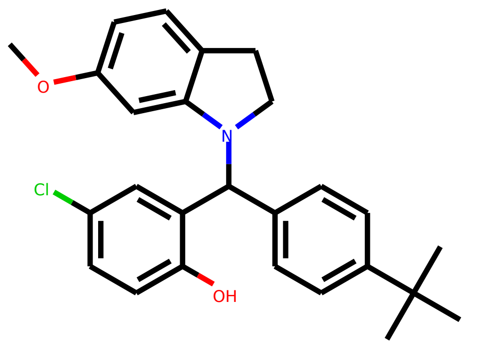

Ahma-2850

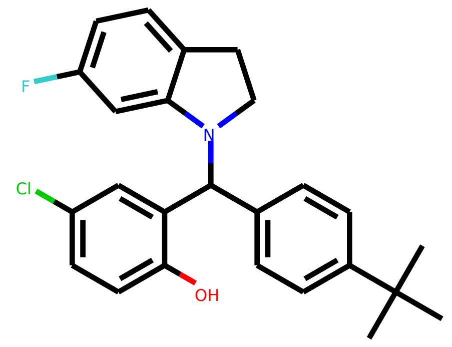

Ahma-2851

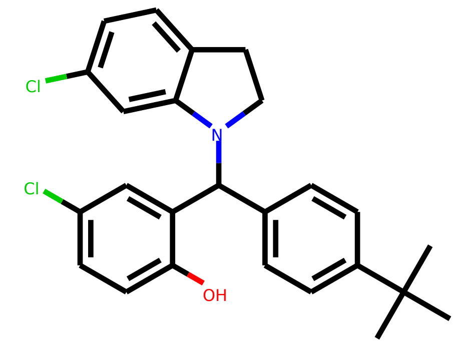

Ahma-2852

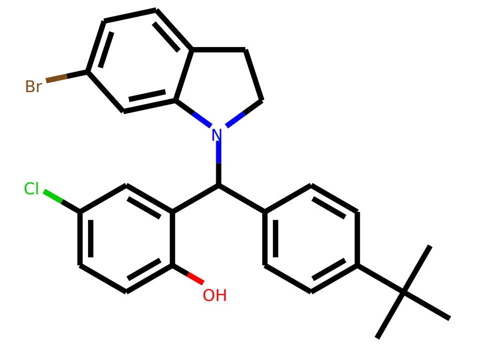

Ahma-2853

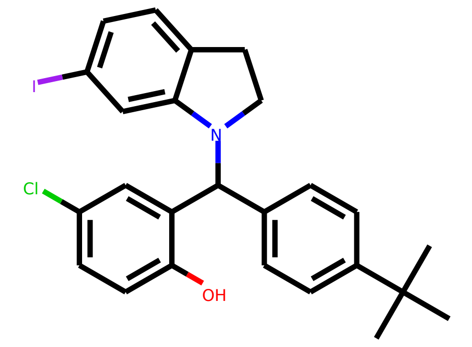

Ahma-2854

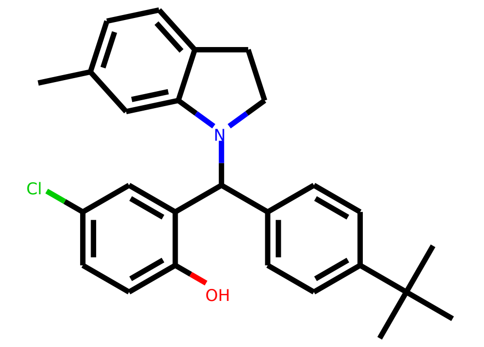

Ahma-2855

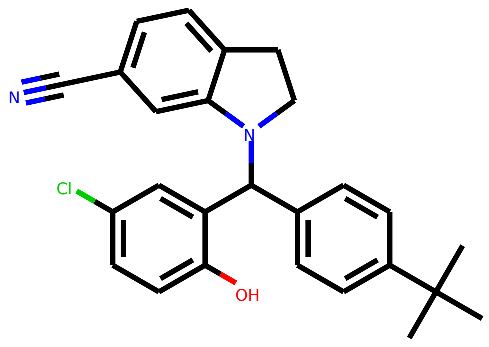

Ahma-2856

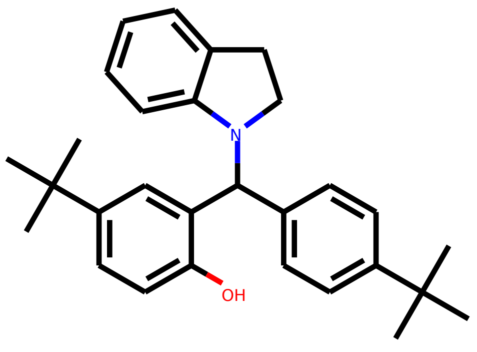

Ahma-2857

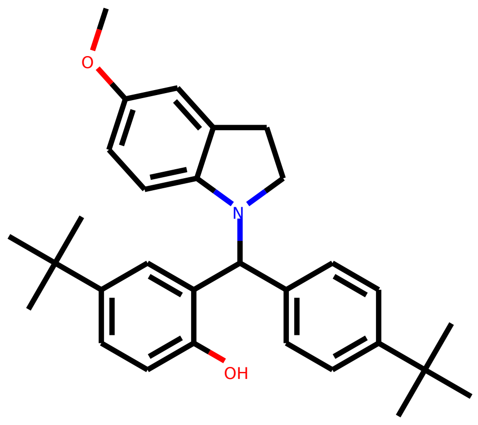

Ahma-2858

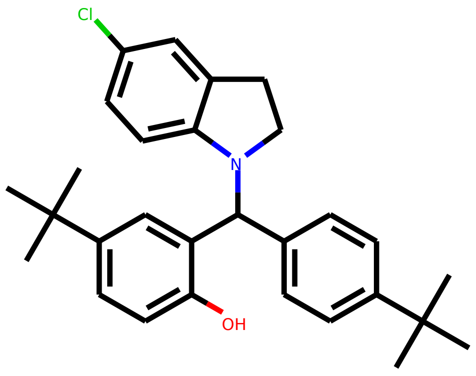

Ahma-2859

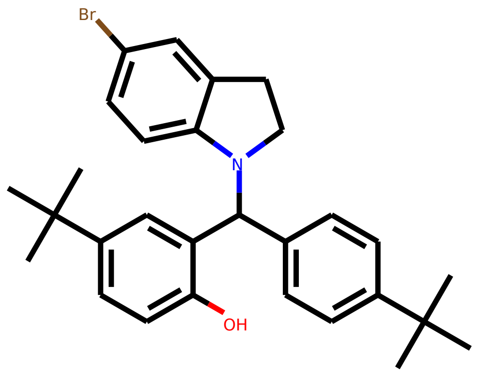

Ahma-2860

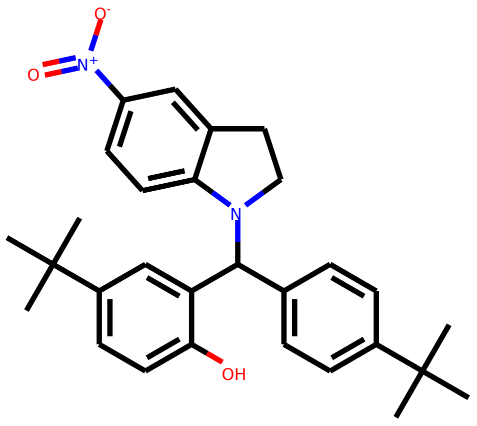

Ahma-2861

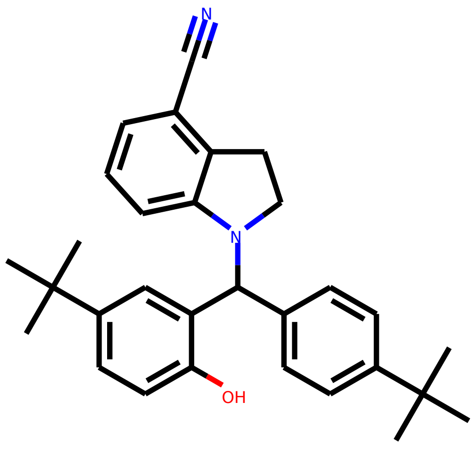

Ahma-2862

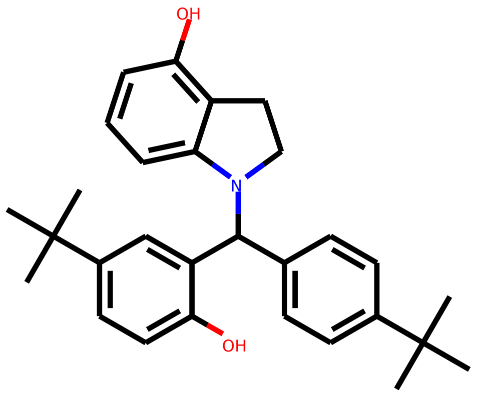

Ahma-2863

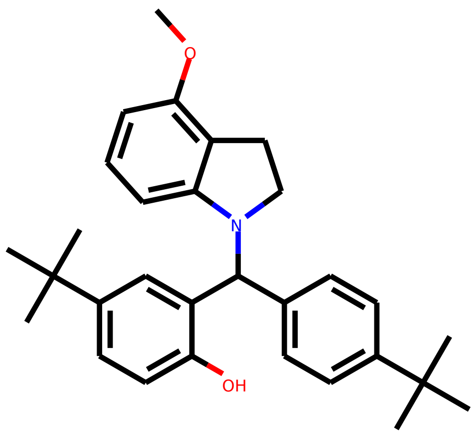

Ahma-2864

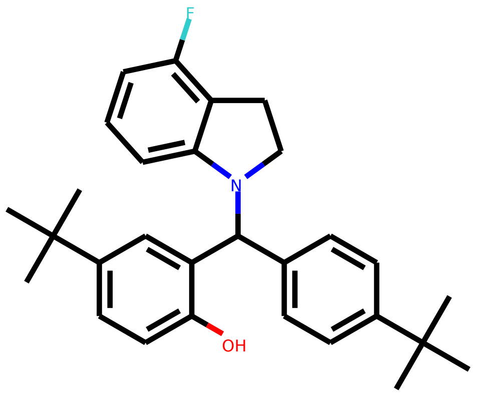

Ahma-2865

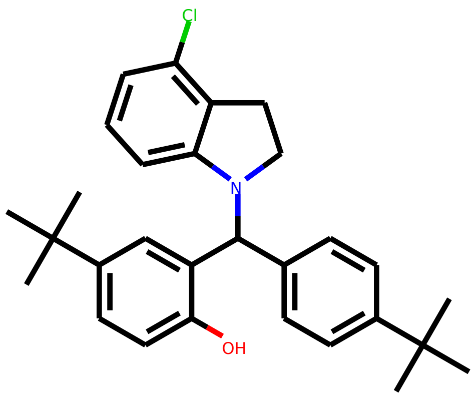

Ahma-2866

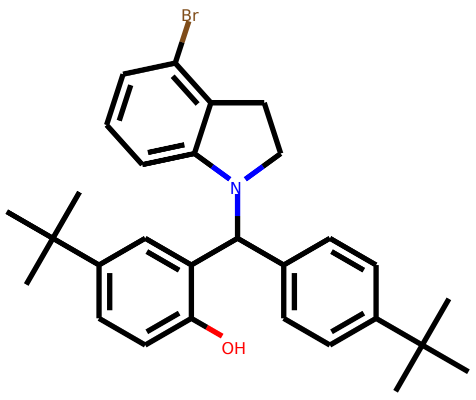

Ahma-2867

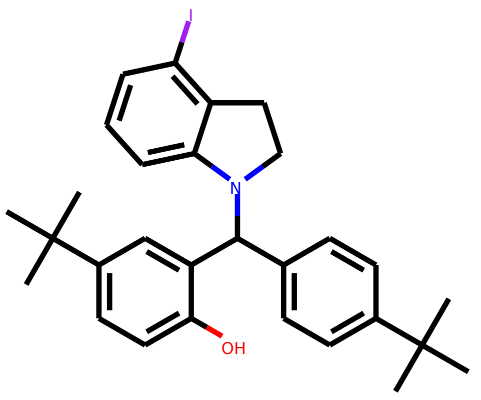

Ahma-2868

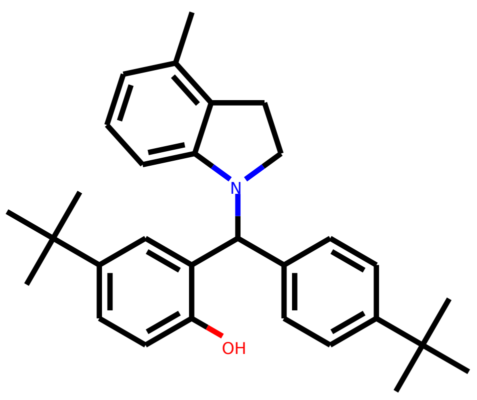

Ahma-2869

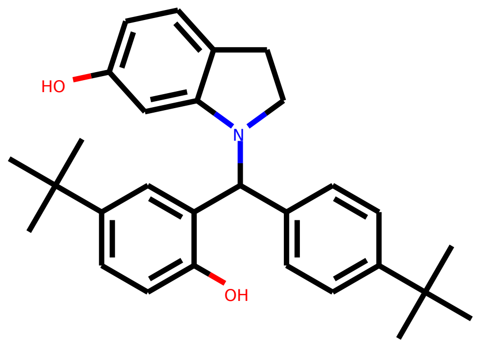

Ahma-2870

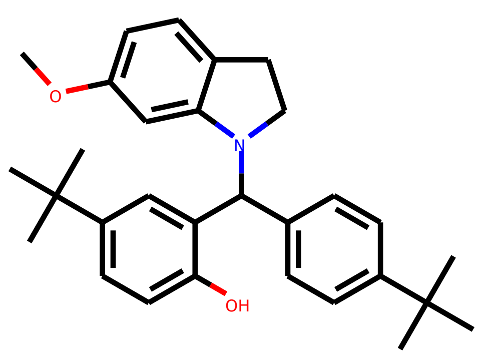
Ahma-2871
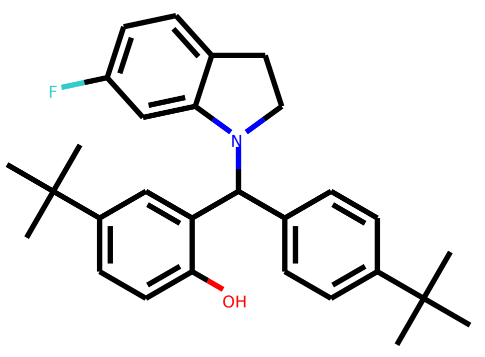
Ahma-2872
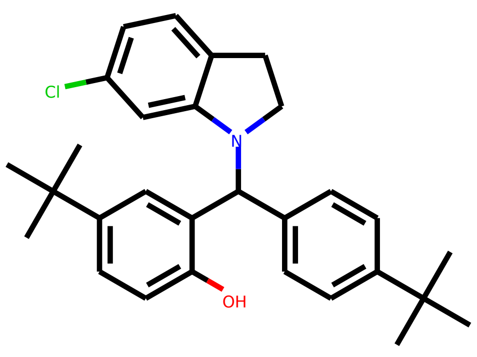
Ahma-2873
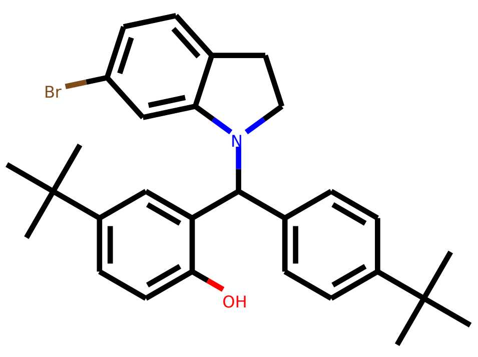
Ahma-2874
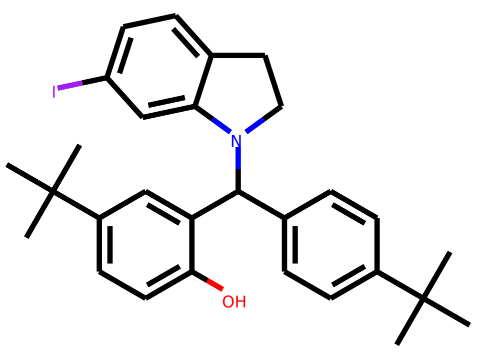
Ahma-2875
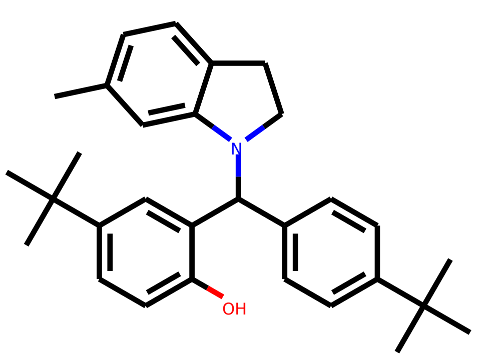
Ahma-2876
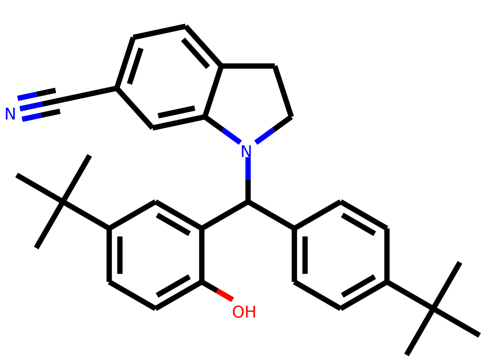
Ahma-2877
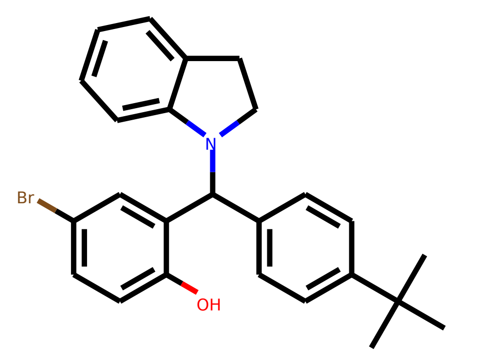
Ahma-2878
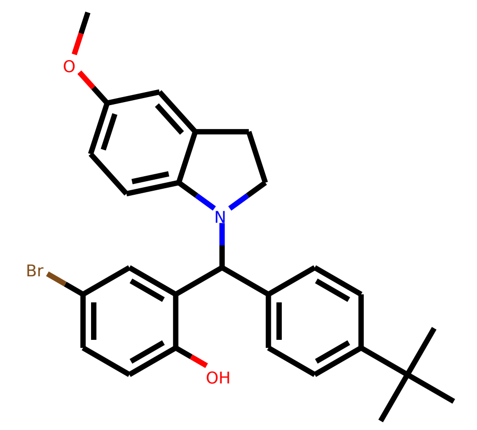
Ahma-2879
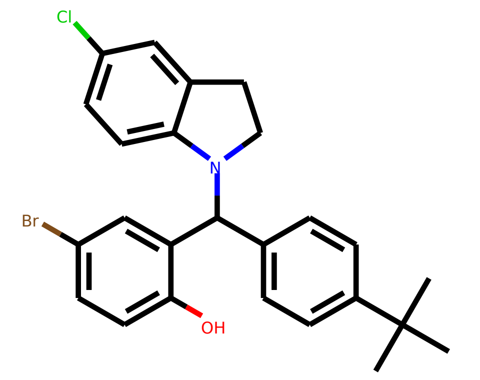
Ahma-2880
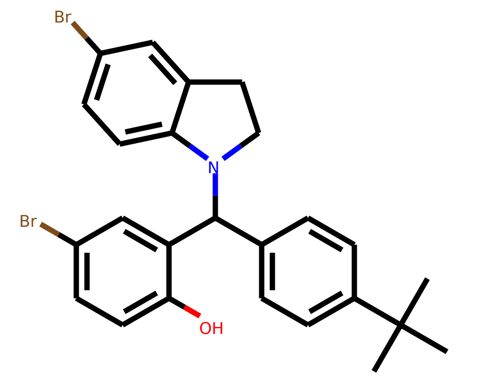
Ahma-2881
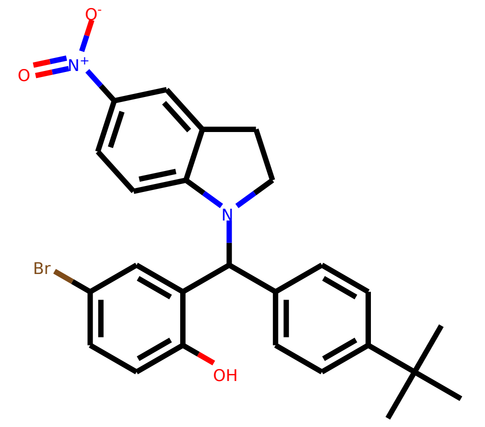
Ahma-2882
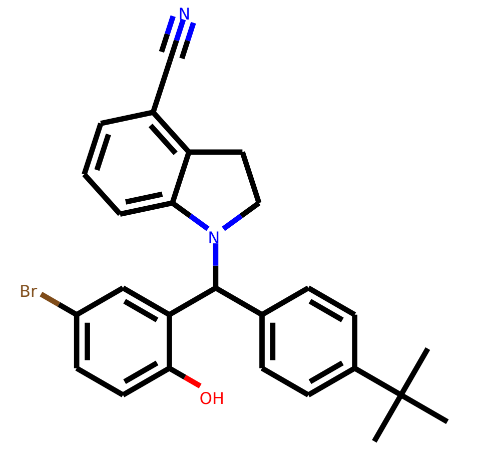
Ahma-2883
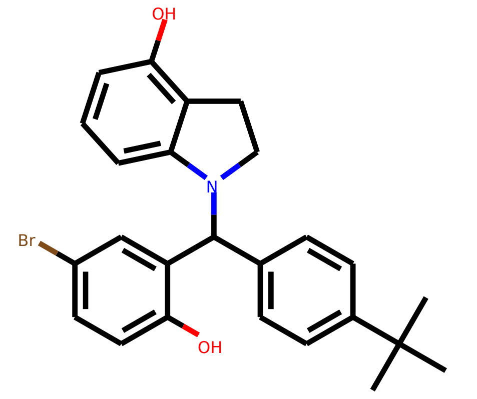
Ahma-2884
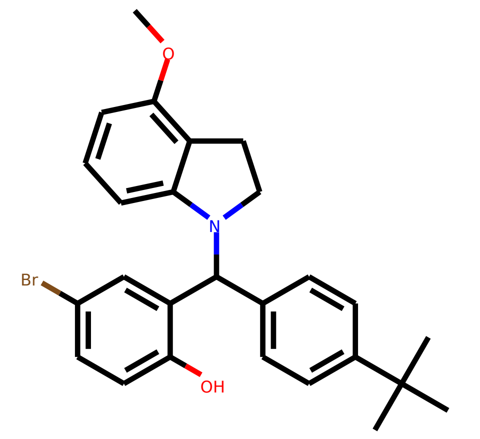
Ahma-2885
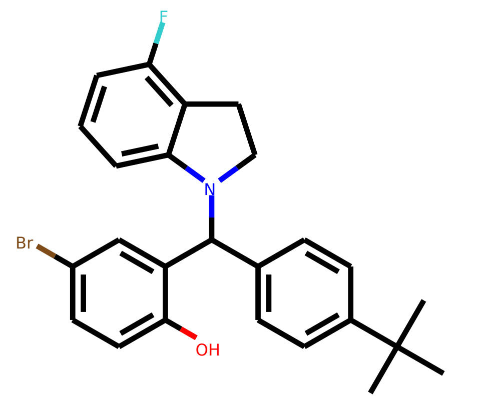
Ahma-2886
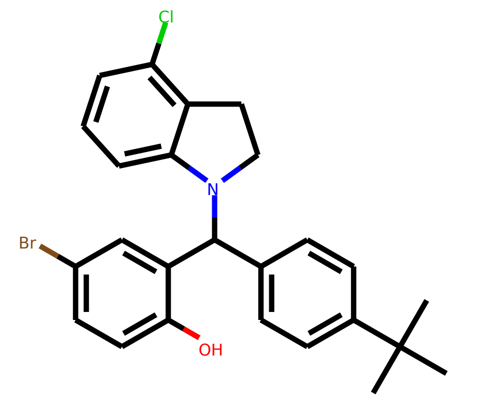
Ahma-2887
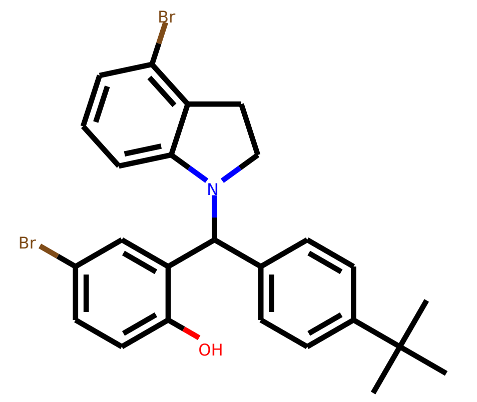
Ahma-2888
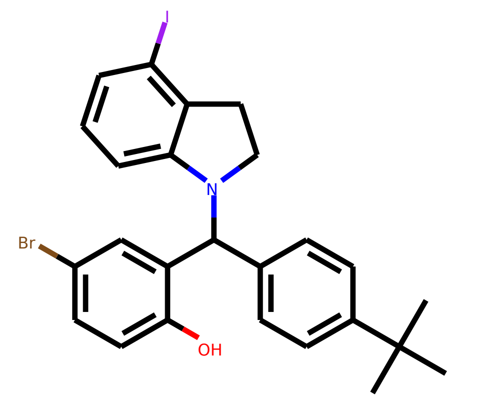
Ahma-2889
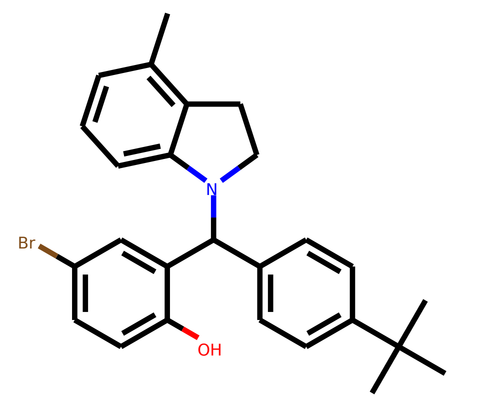
Ahma-2890
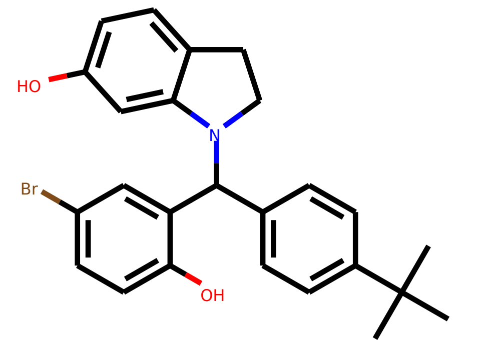
Ahma-2891
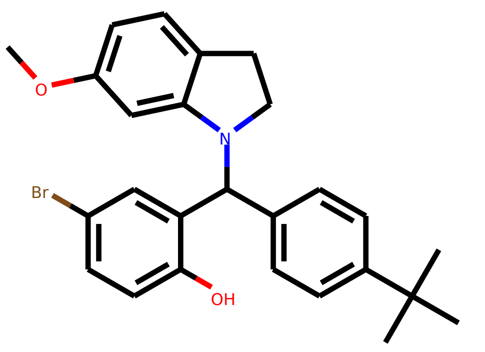
Ahma-2892
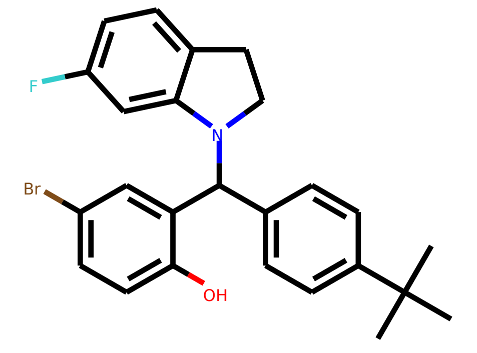
Ahma-2893
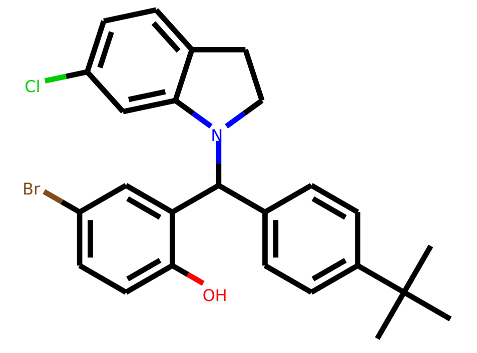
Ahma-2894
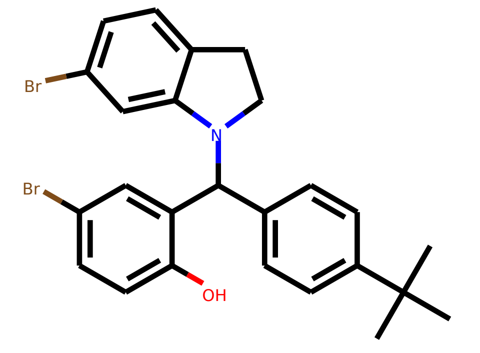
Ahma-2895
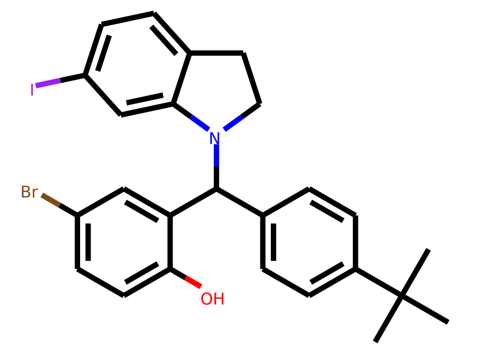
Ahma-2896
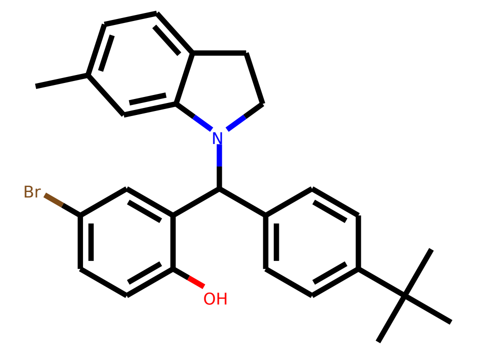
Ahma-2897
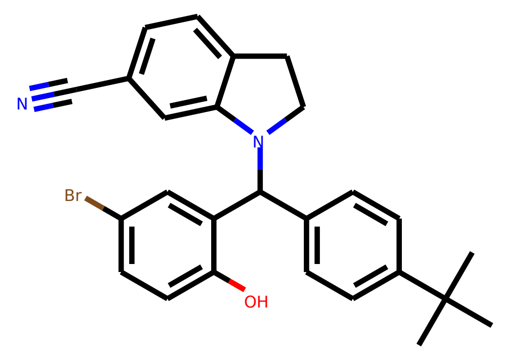
Ahma-2898
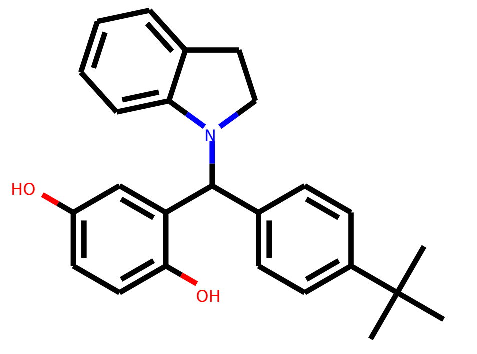
Ahma-2899
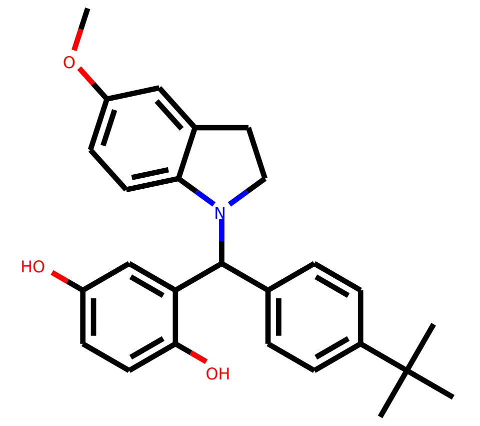
Ahma-2900
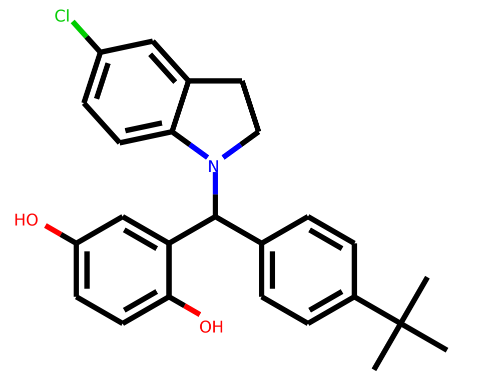
Ahma-2901
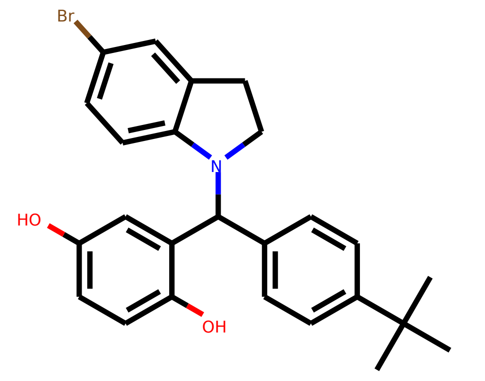
Ahma-2902
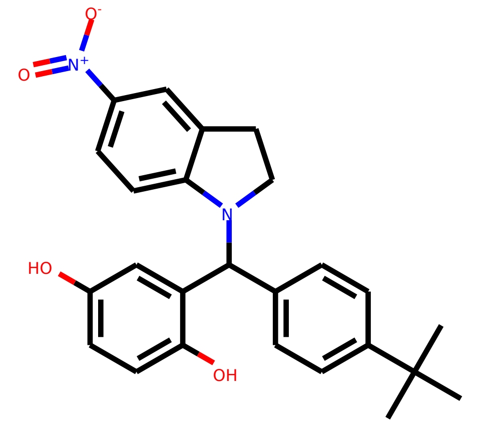
Ahma-2903
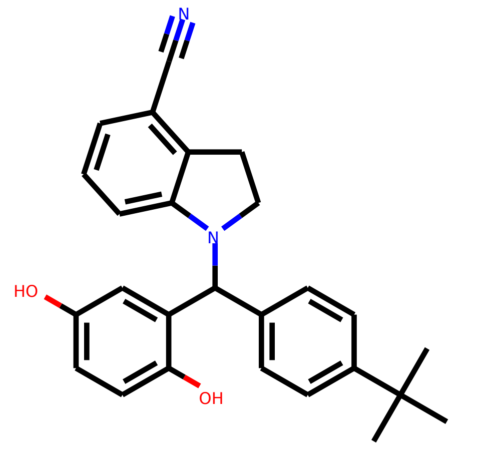
Ahma-2904
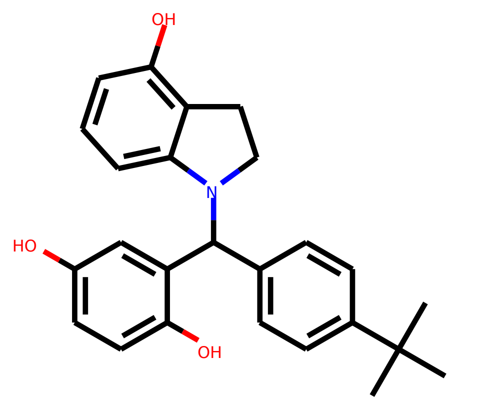
Ahma-2905

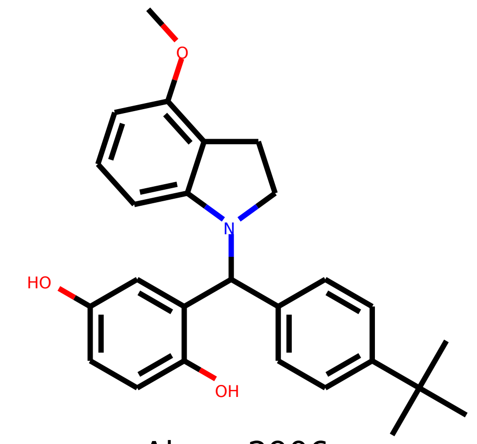
Ahma-2906
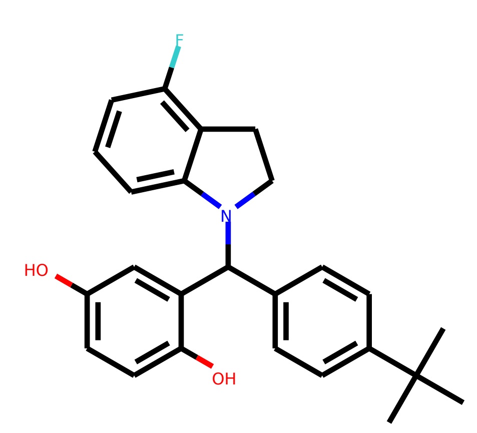
Ahma-2907
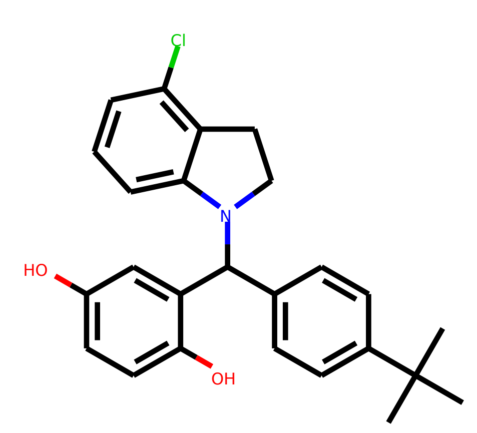
Ahma-2908
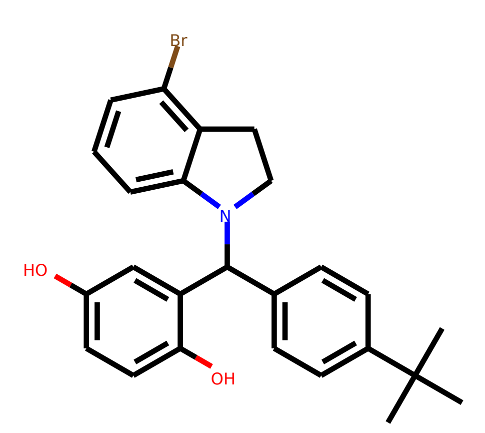
Ahma-2909
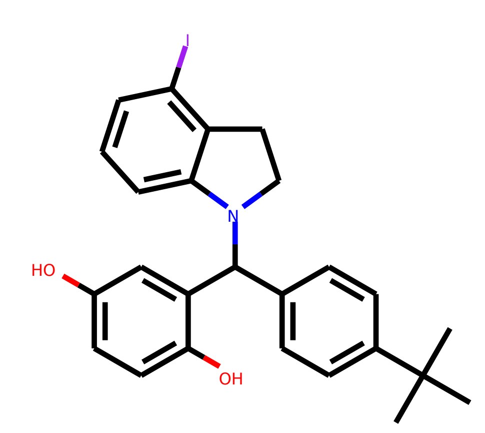
Ahma-2910
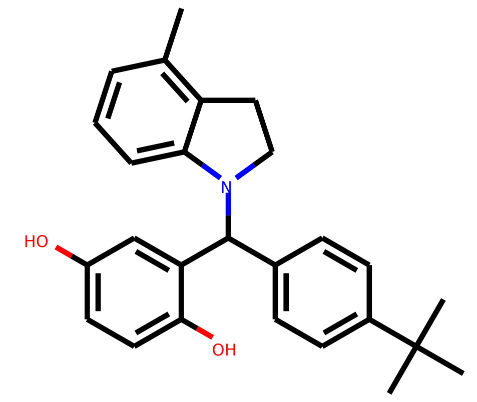
Ahma-2911
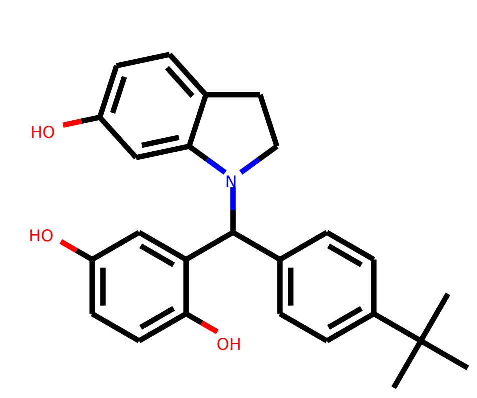
Ahma-2912
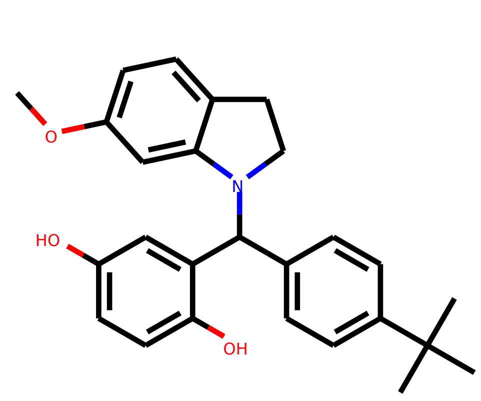
Ahma-2913
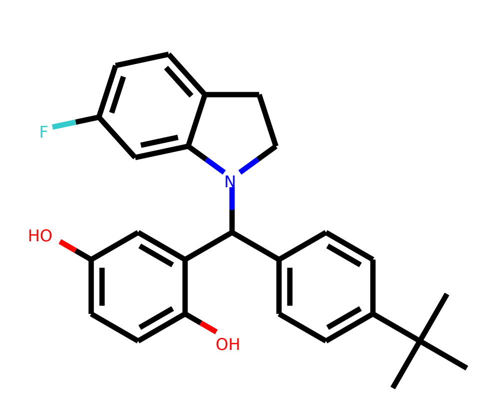
Ahma-2914
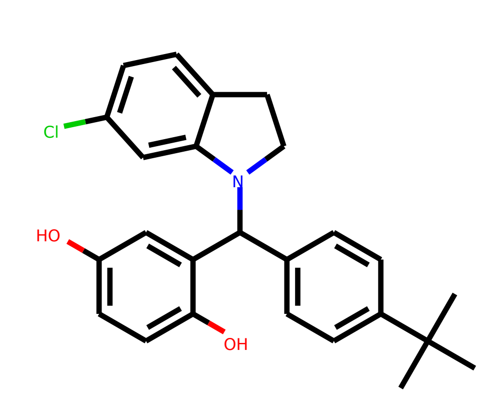
Ahma-2915
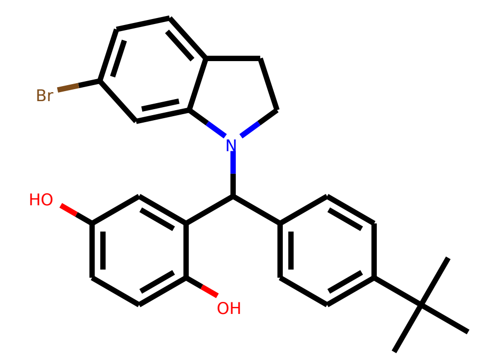
Ahma-2916
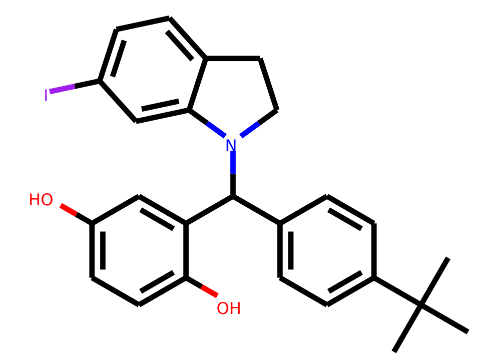
Ahma-2917
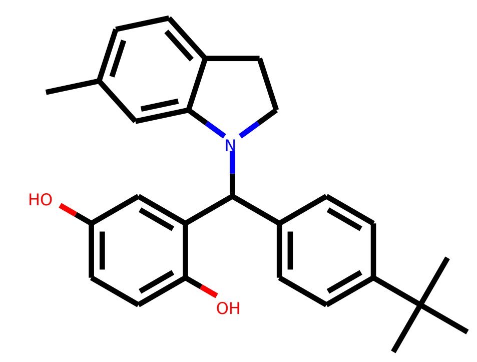
Ahma-2918
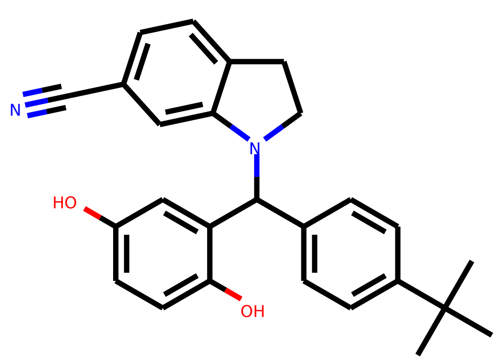
Ahma-2919
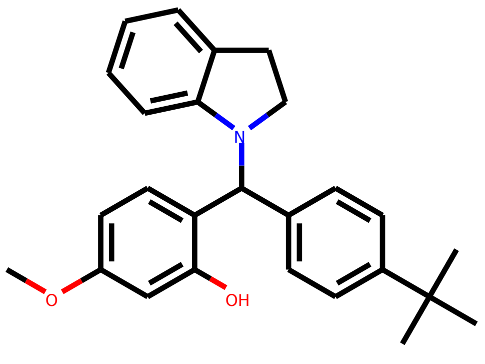
Ahma-2920
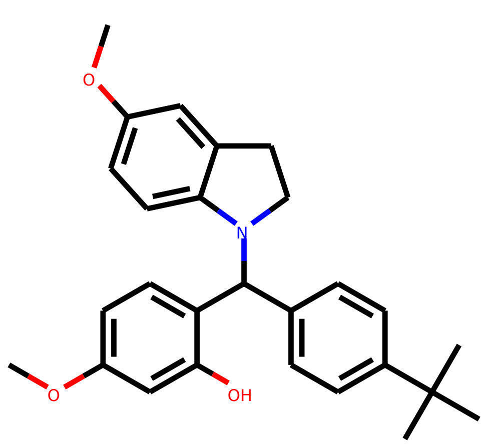
Ahma-2921
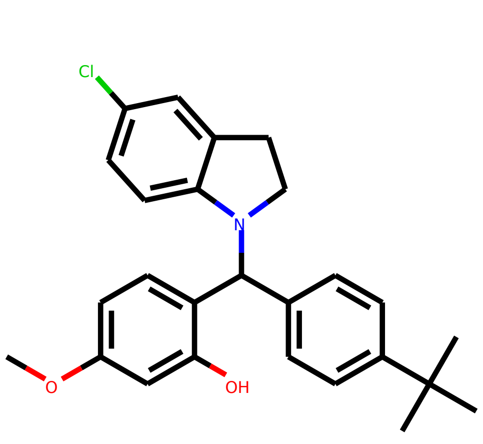
Ahma-2922
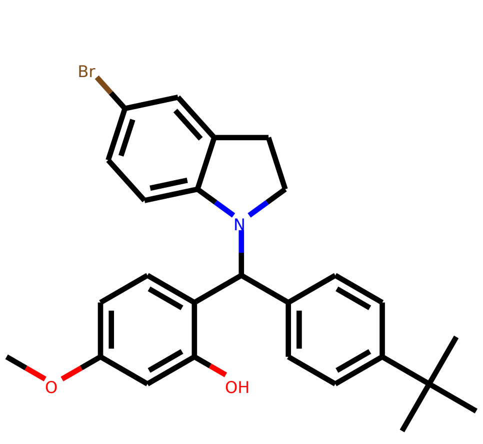
Ahma-2923
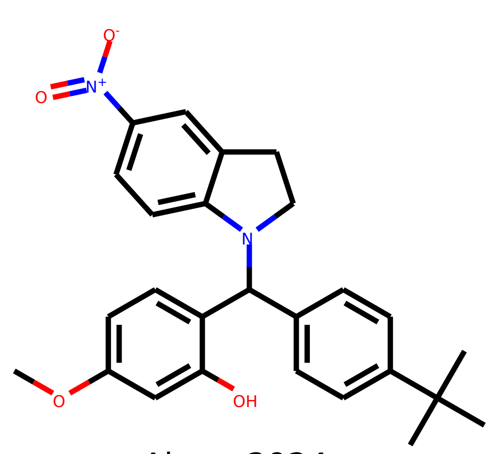
Ahma-2924
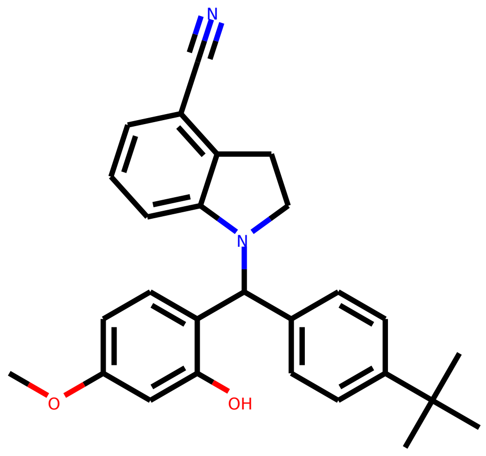
Ahma-2925
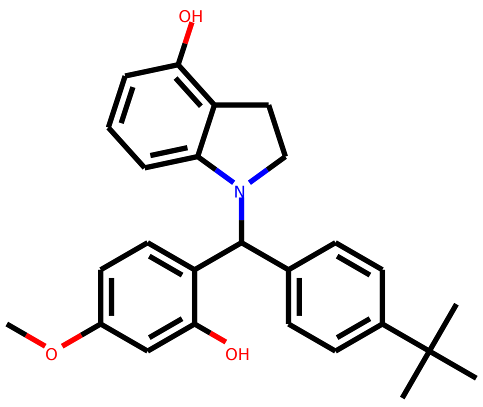
Ahma-2926
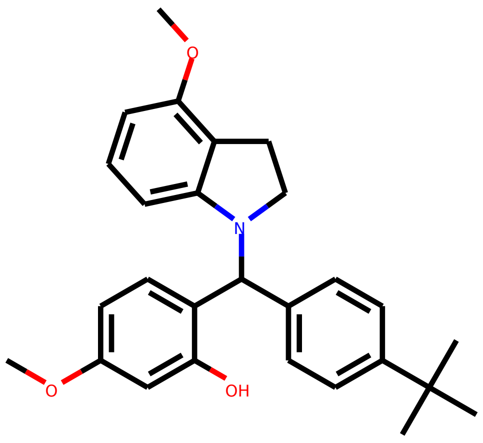
Ahma-2927
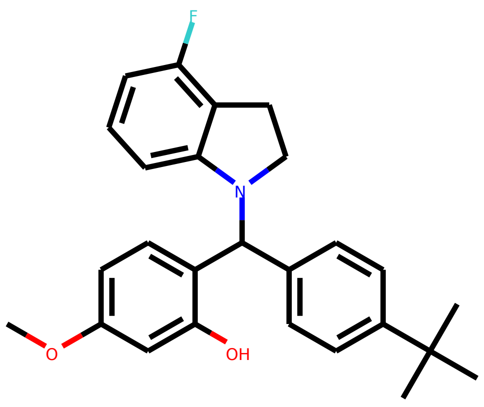
Ahma-2928
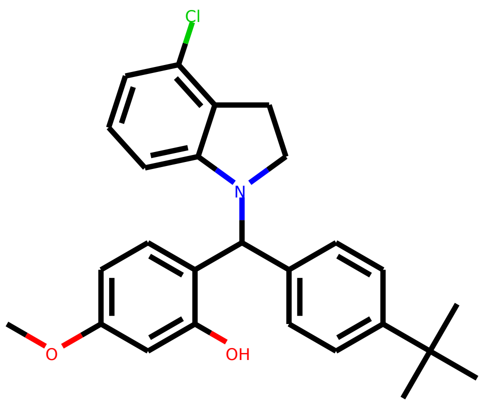
Ahma-2929
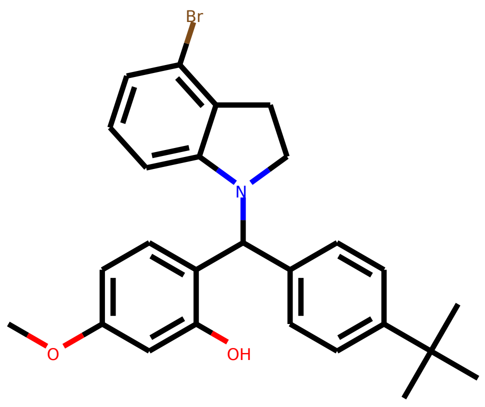
Ahma-2930
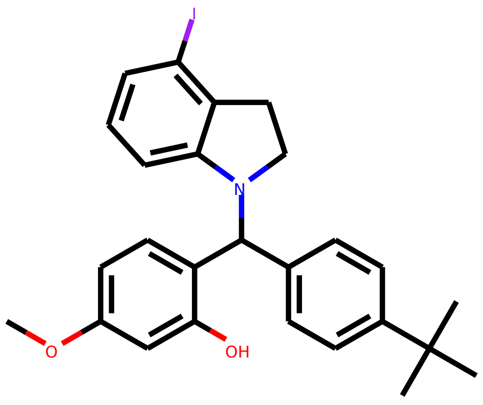
Ahma-2931
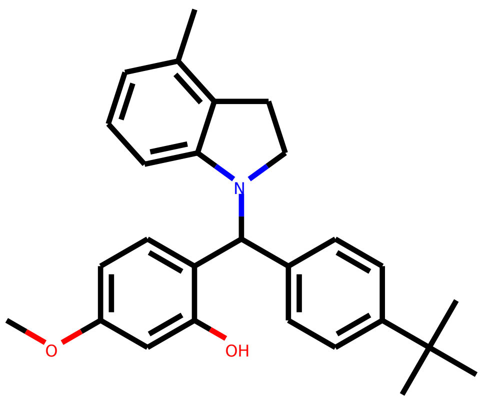
Ahma-2932
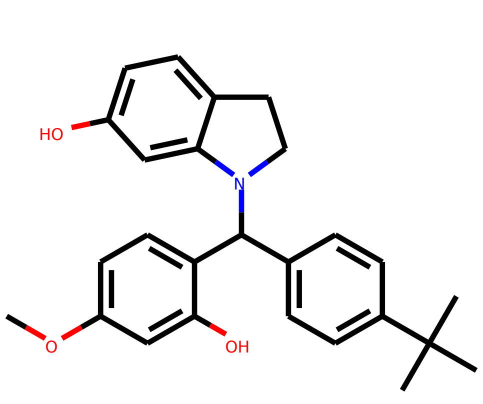
Ahma-2933
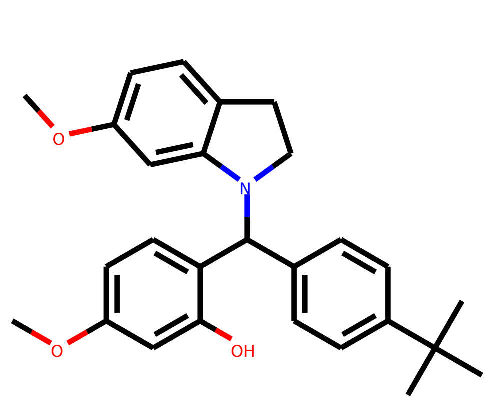
Ahma-2934
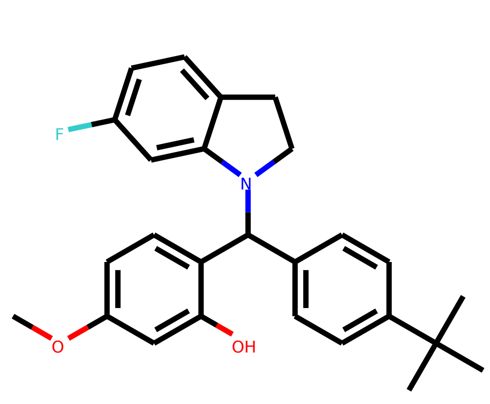
Ahma-2935
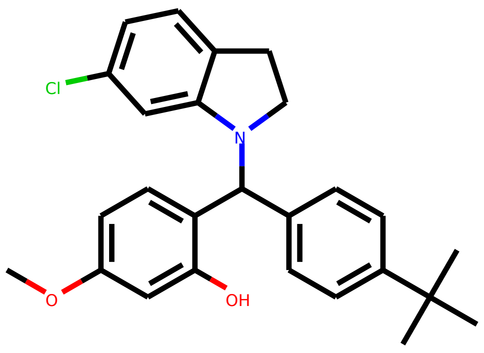
Ahma-2936
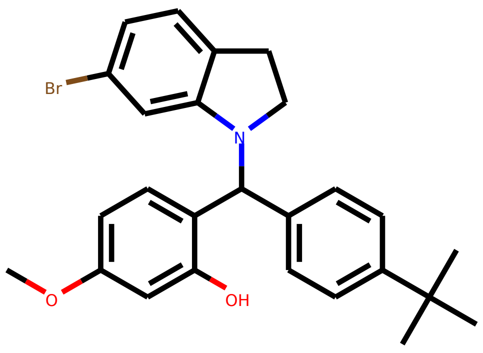
Ahma-2937
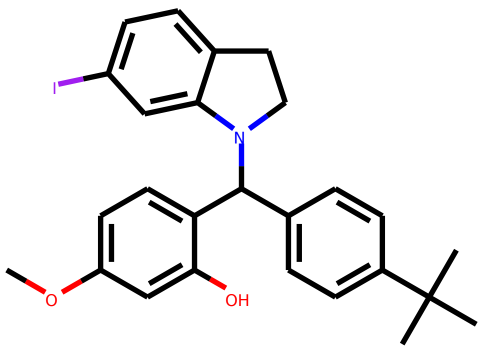
Ahma-2938
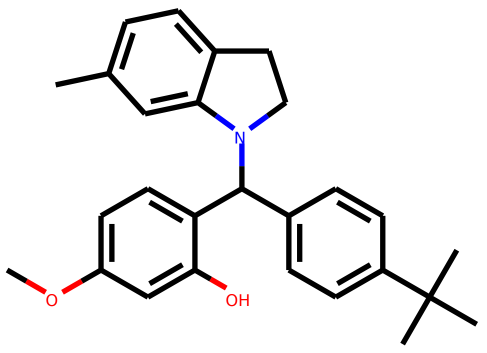
Ahma-2939
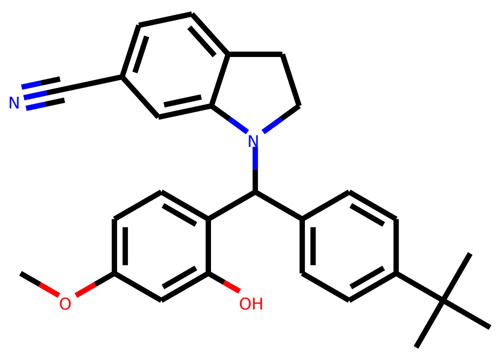
Ahma-2940

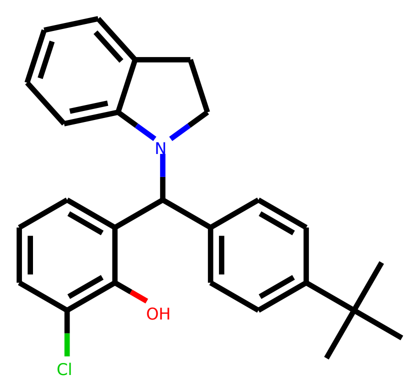
Ahma-2941
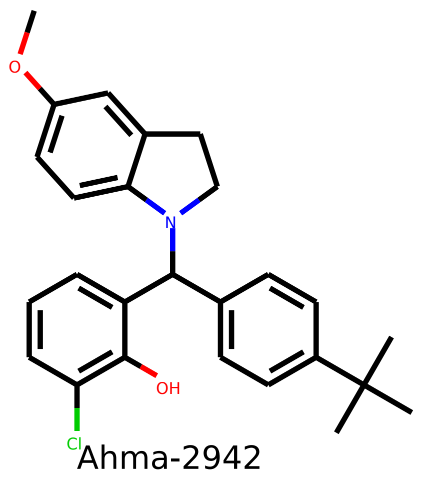
Ahma-2942
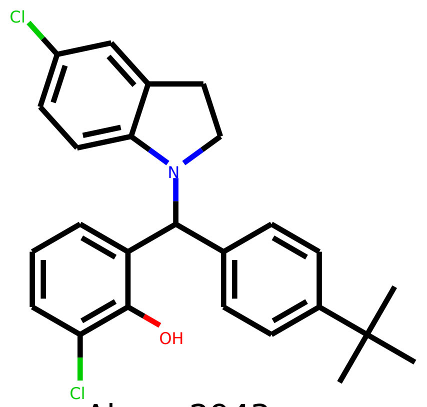
Ahma-2943
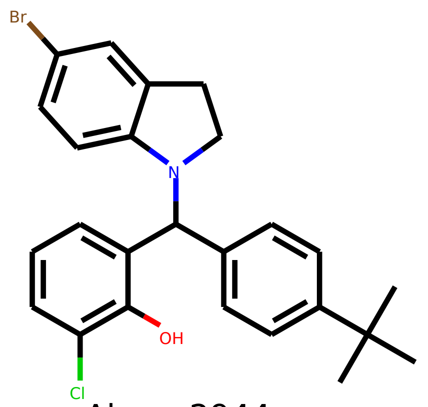
Ahma-2944
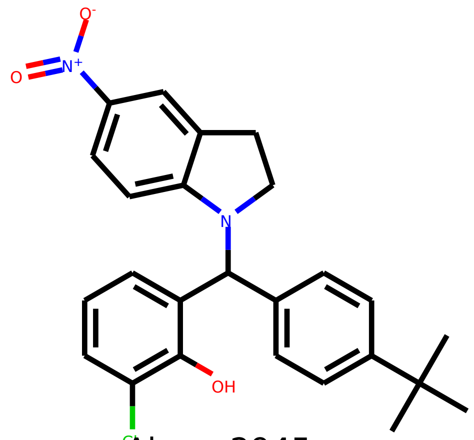
Ahma-2945
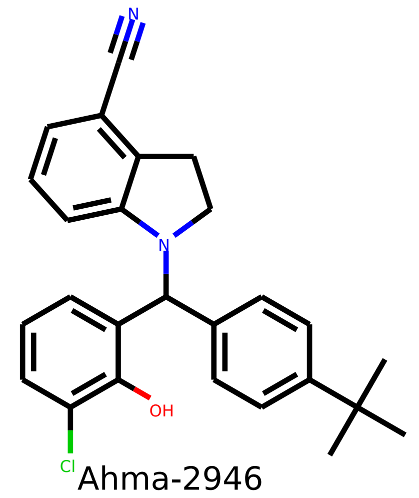
Ahma-2946
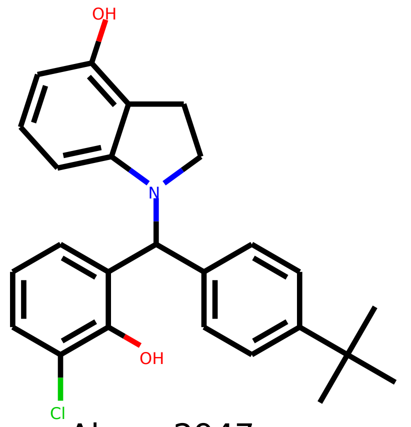
Ahma-2947
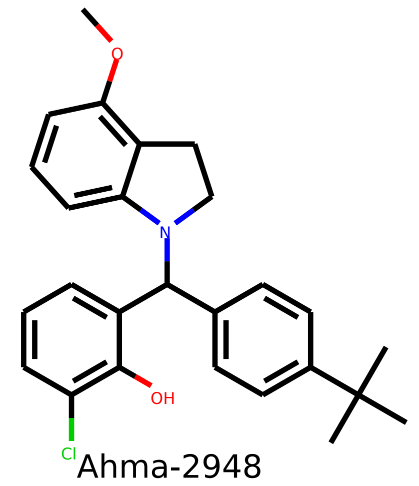
Ahma-2948
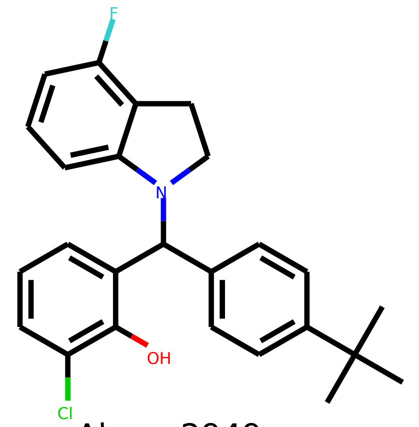
Ahma-2949
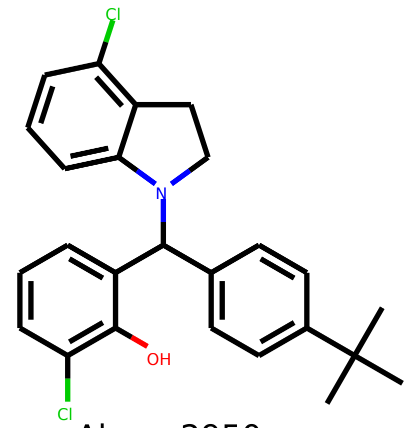
Ahma-2950
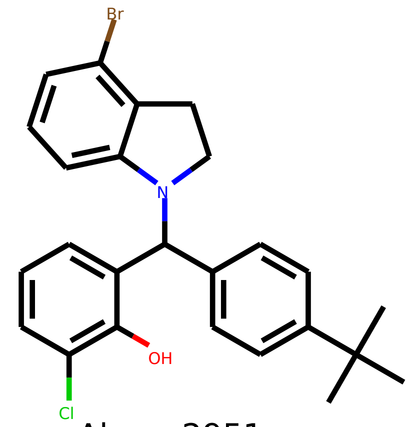
Ahma-2951
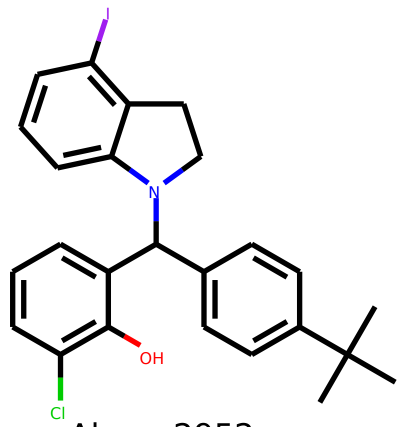
Ahma-2952
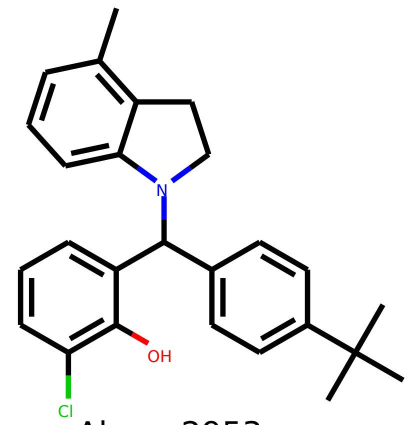
Ahma-2953
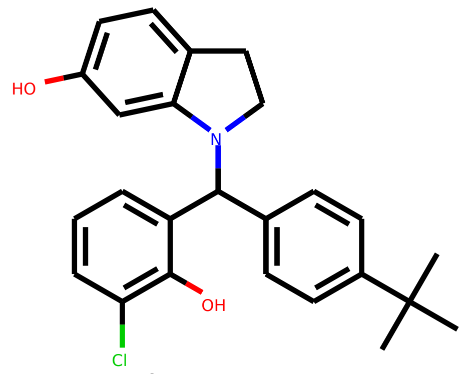
Ahma-2954
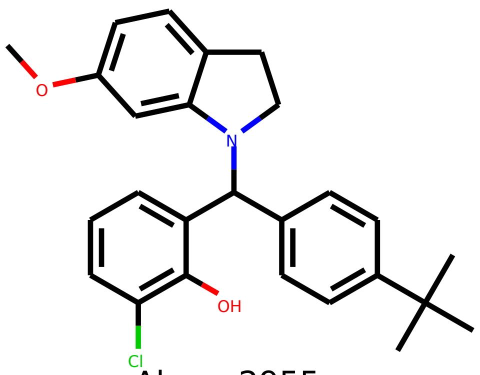
Ahma-2955
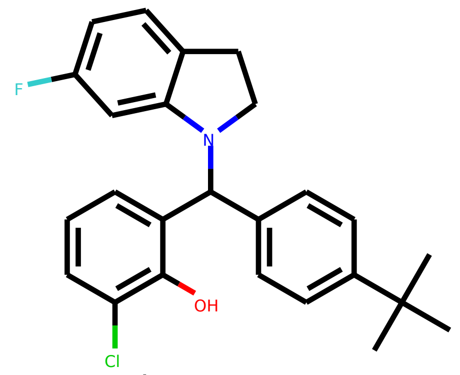
Ahma-2956
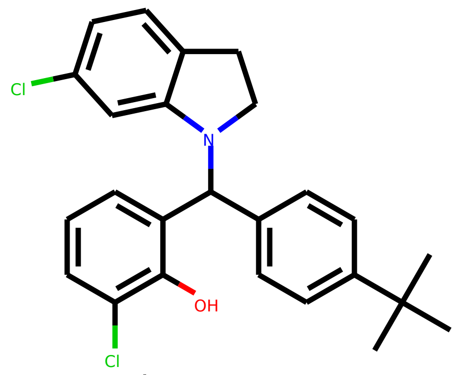
Ahma-2957
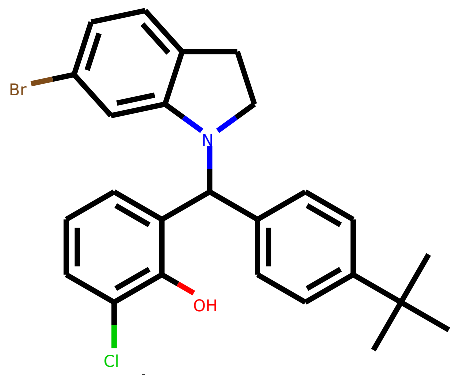
Ahma-2958
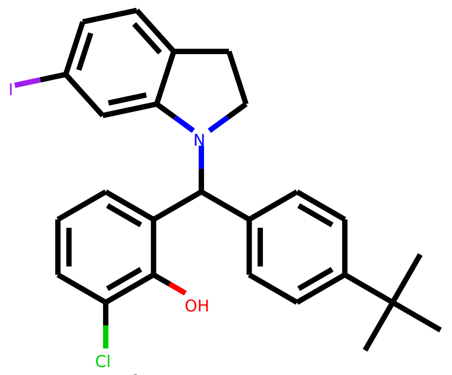
Ahma-2959
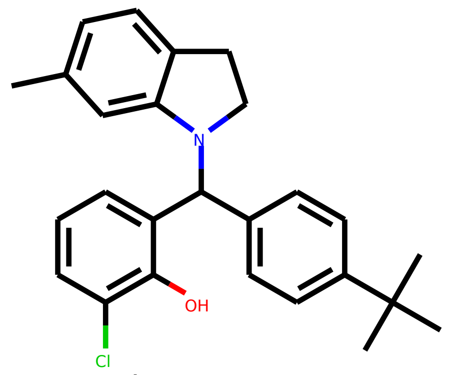
Ahma-2960
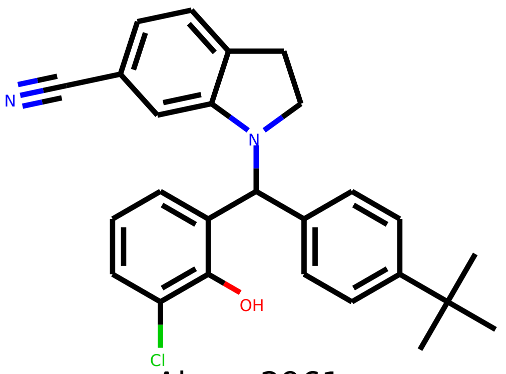
Ahma-2961
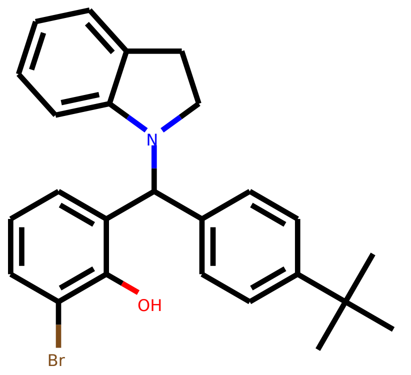
Ahma-2962
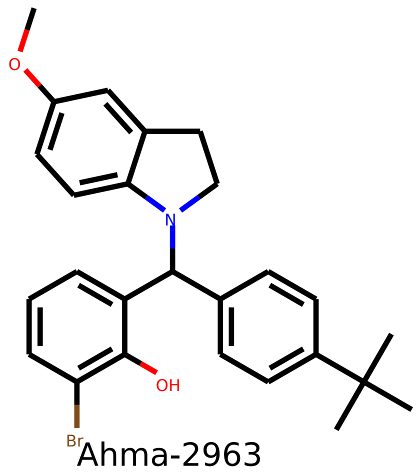
Ahma-2963
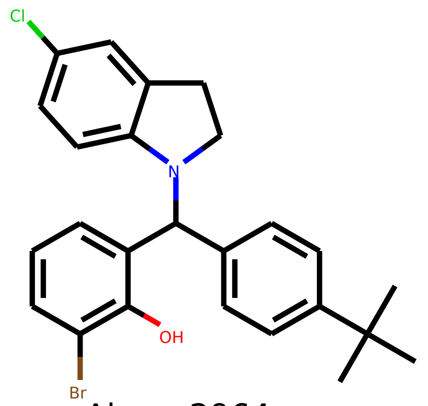
Ahma-2964
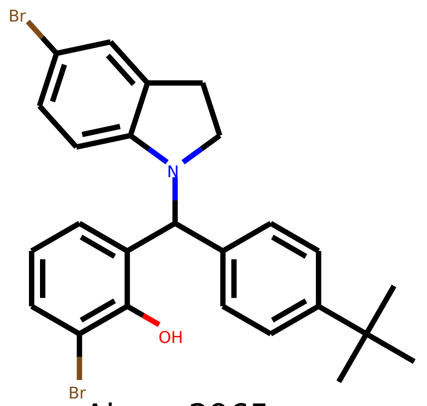
Ahma-2965
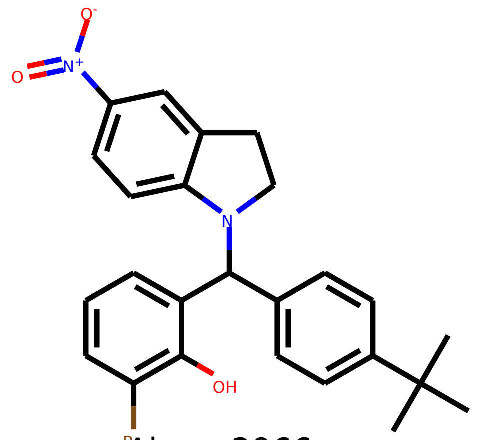
Ahma-2966
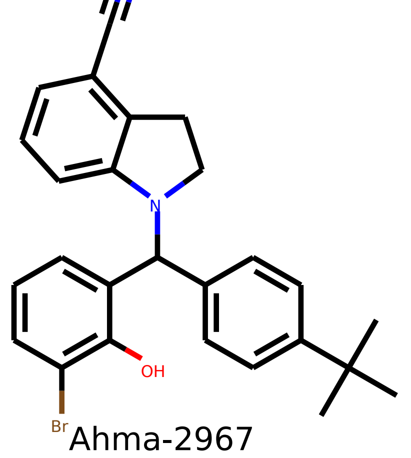
Ahma-2967
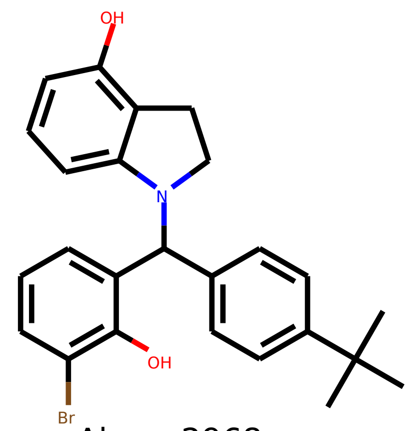
Ahma-2968
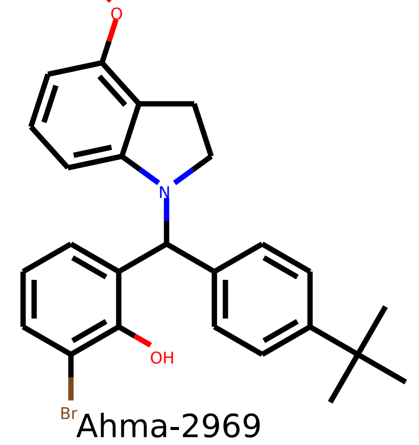
Ahma-2969
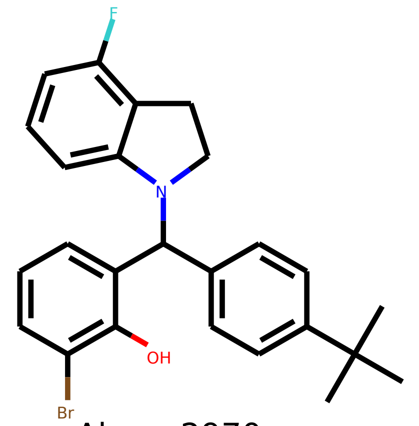
Ahma-2970
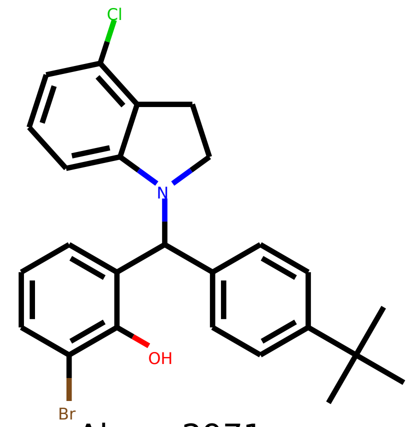
Ahma-2971
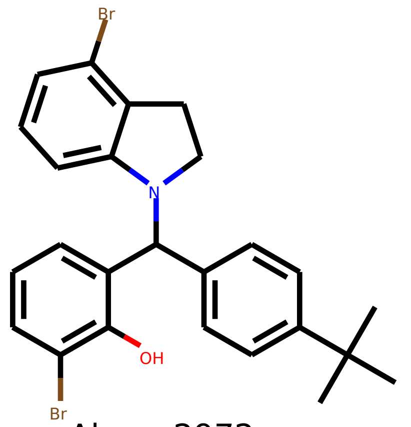
Ahma-2972
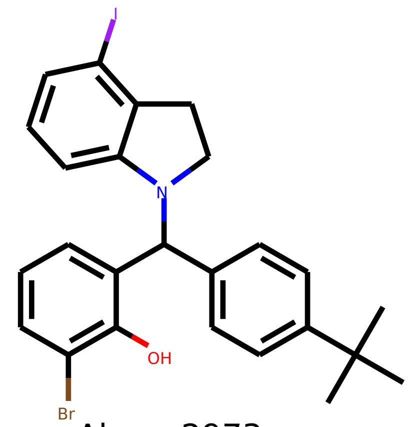
Ahma-2973
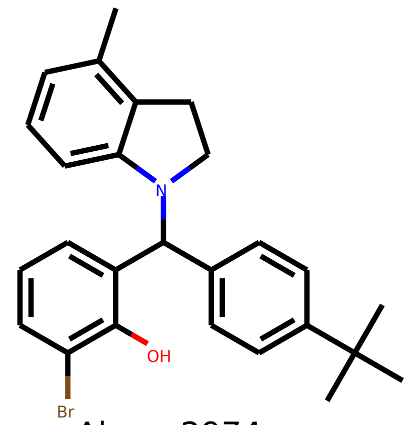
Ahma-2974
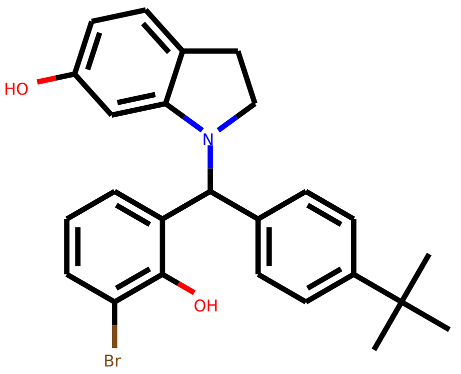
Ahma-2975

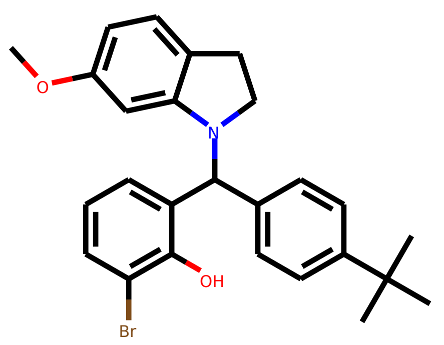
Ahma-2976
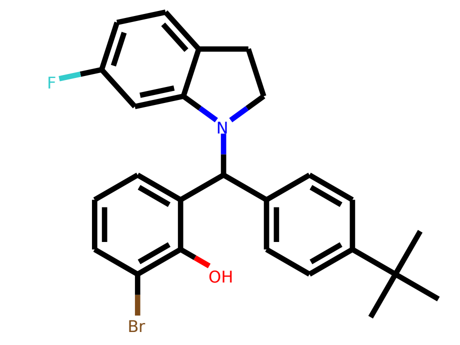
Ahma-2977
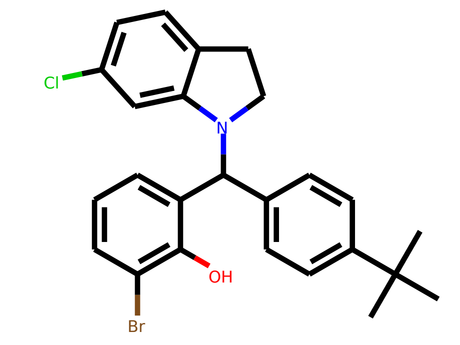
Ahma-2978
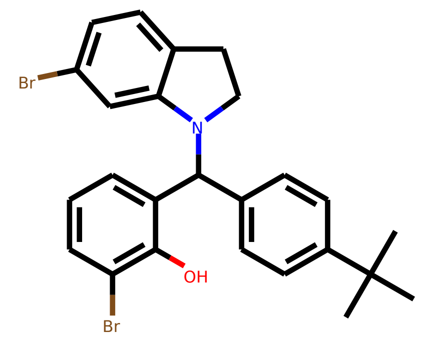
Ahma-2979
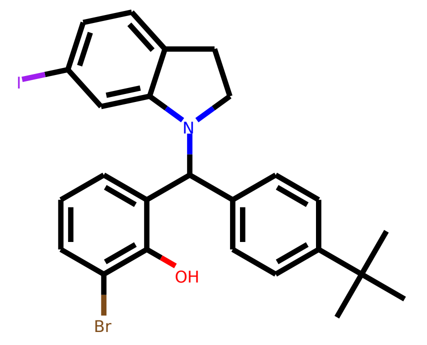
Ahma-2980
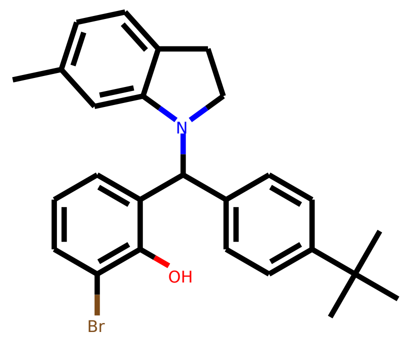
Ahma-2981
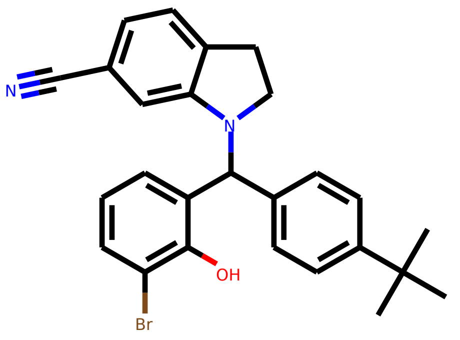
Ahma-2982
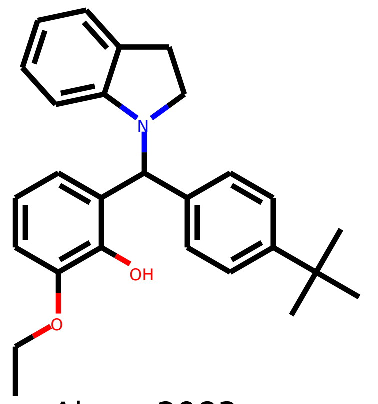
Ahma-2983
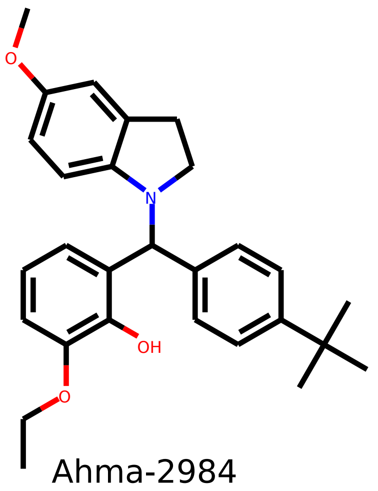
Ahma-2984
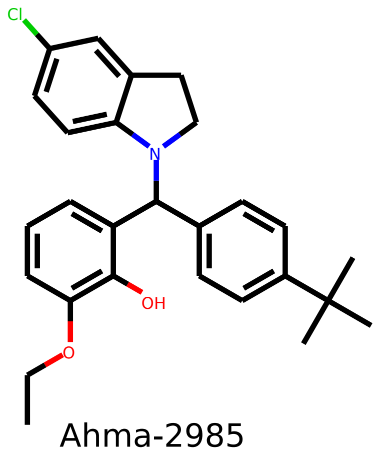
Ahma-2985
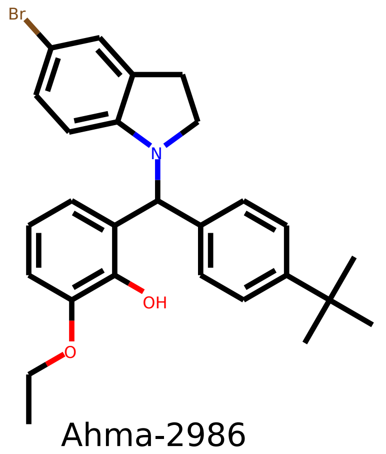
Ahma-2986
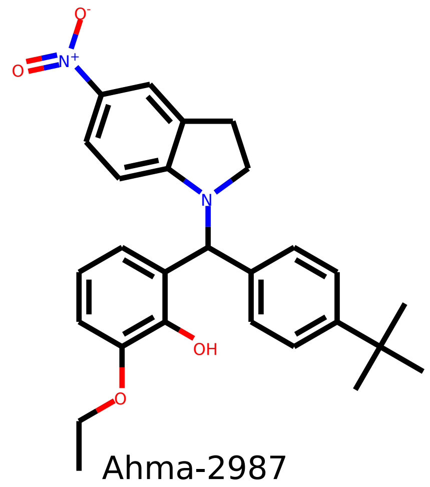
Ahma-2987
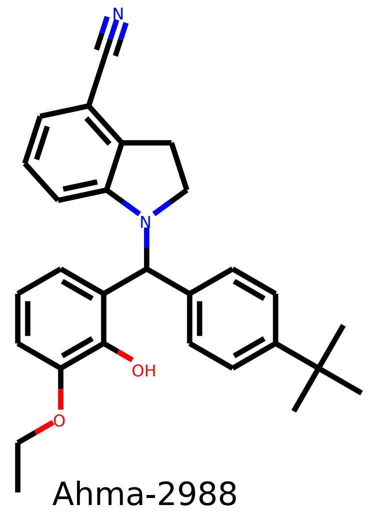
Ahma-2988
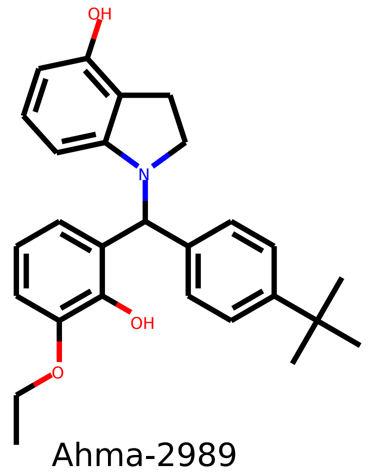
Ahma-2989
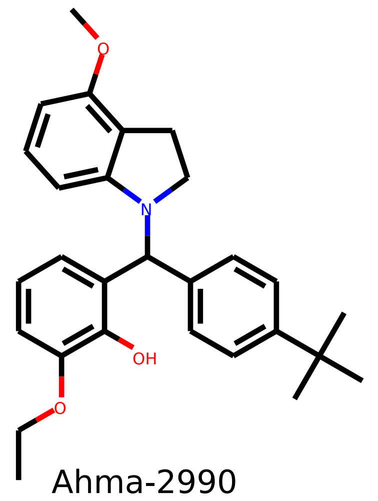
Ahma-2990
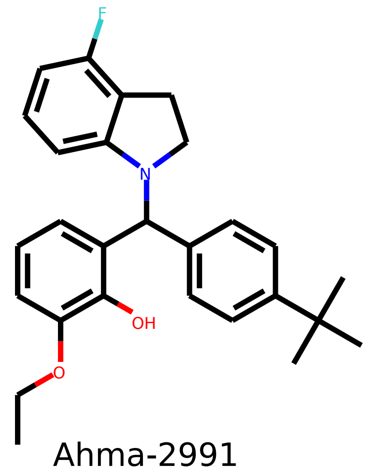
Ahma-2991
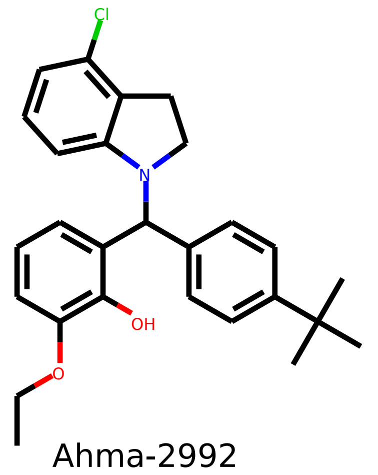
Ahma-2992
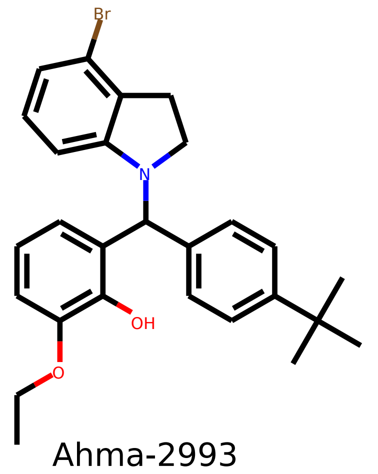
Ahma-2993
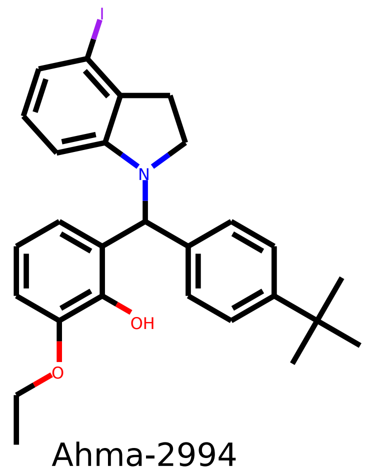
Ahma-2994
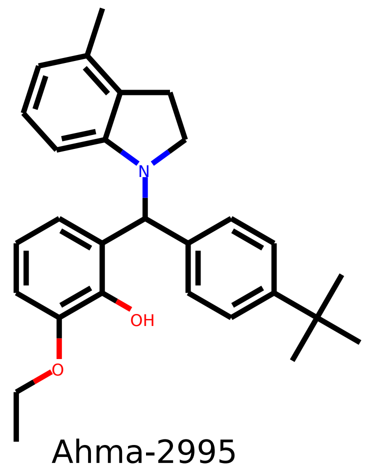
Ahma-2995
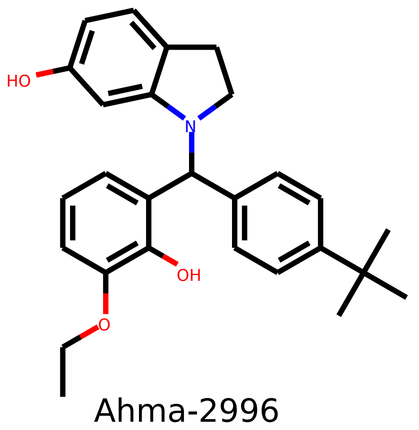
Ahma-2996
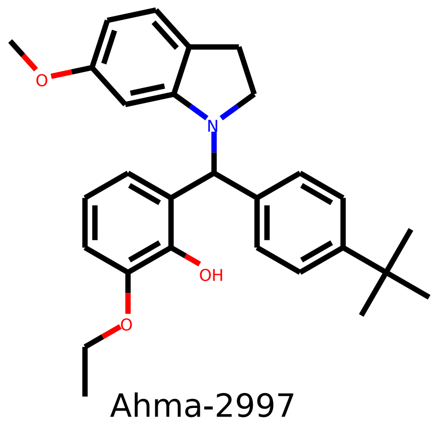
Ahma-2997
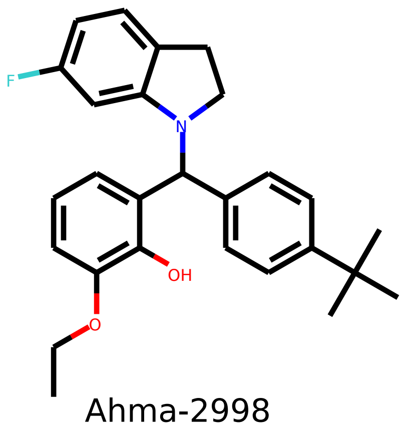
Ahma-2998
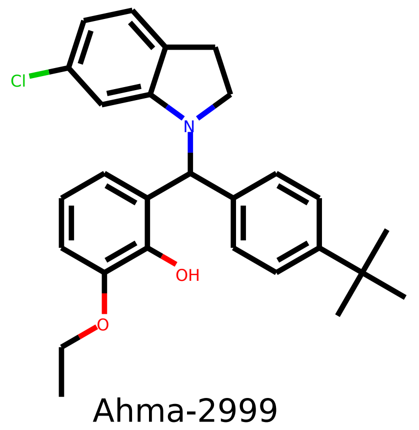
Ahma-2999
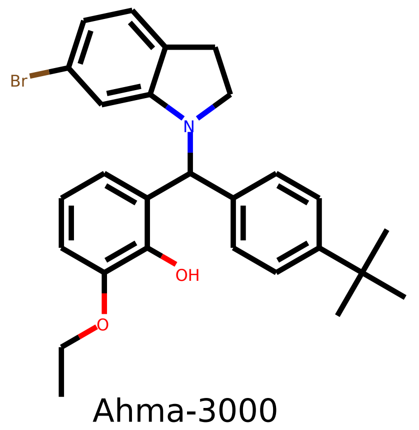
Ahma-3000
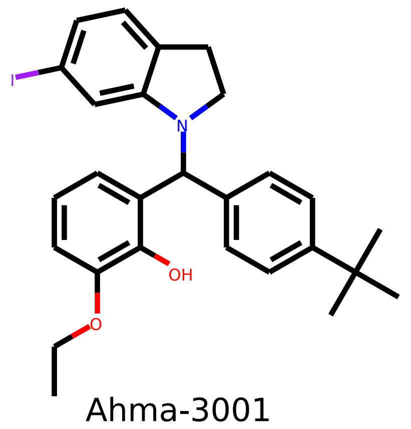
Ahma-3001
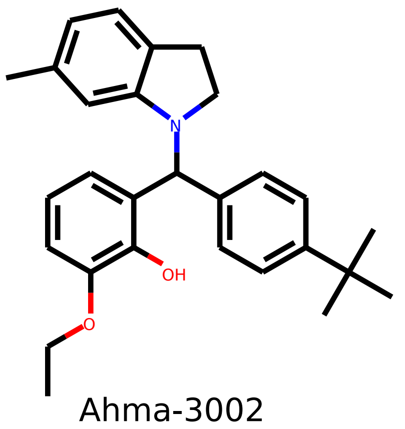
Ahma-3002
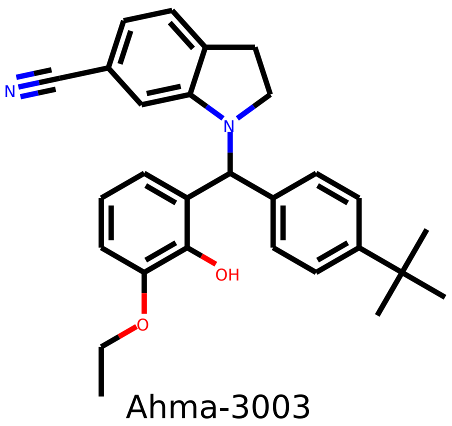
Ahma-3003
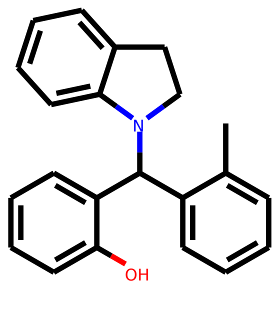
Ahma-3004
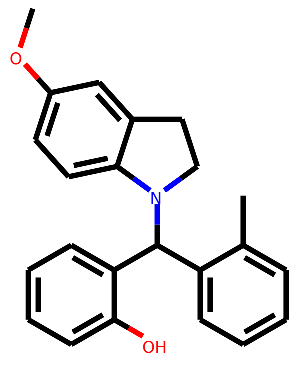
Ahma-3005
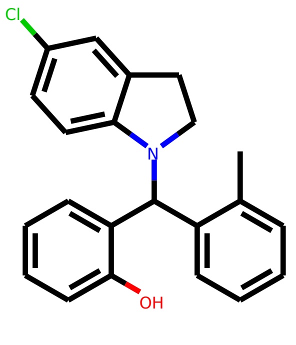
Ahma-3006
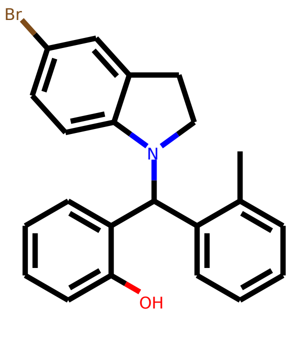
Ahma-3007
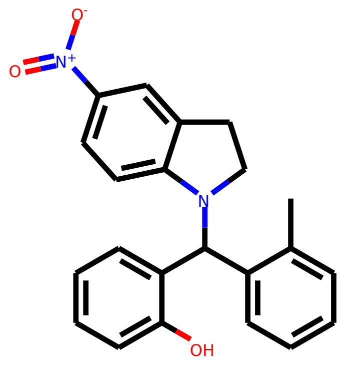
Ahma-3008
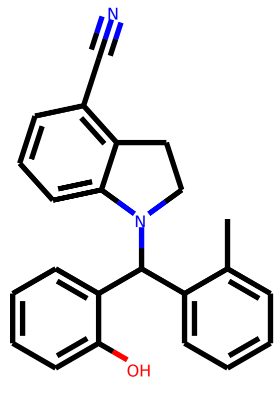
Ahma-3009
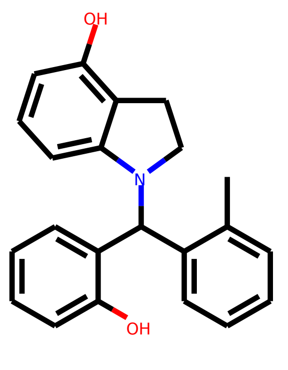
Ahma-3010

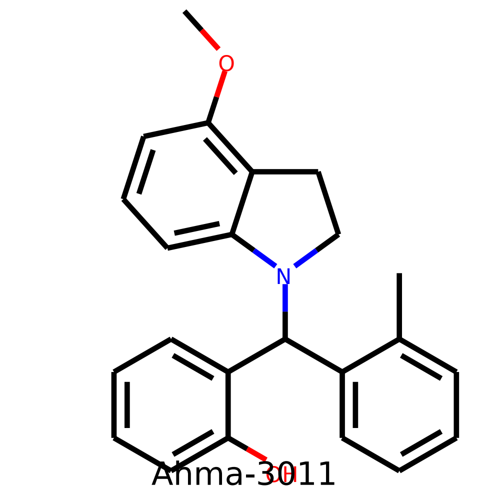
Ahma-3011
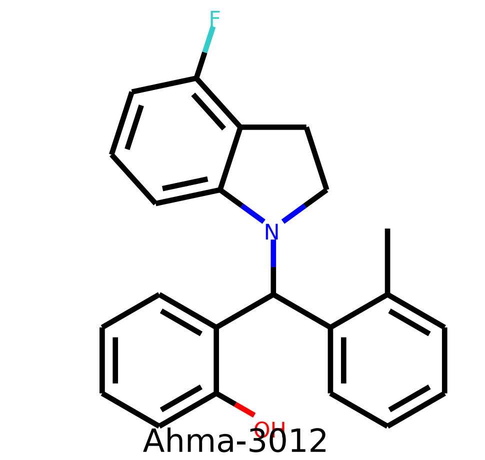
Ahma-3012
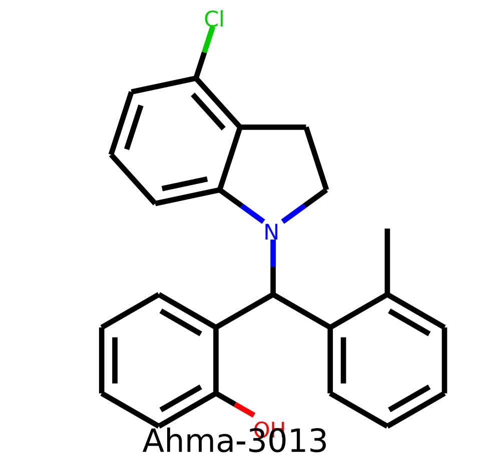
Ahma-3013
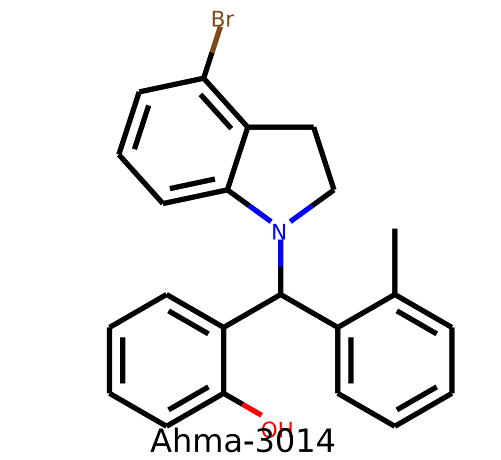
Ahma-3014
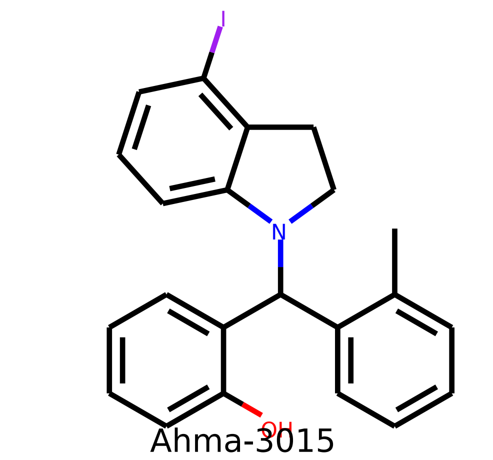
Ahma-3015
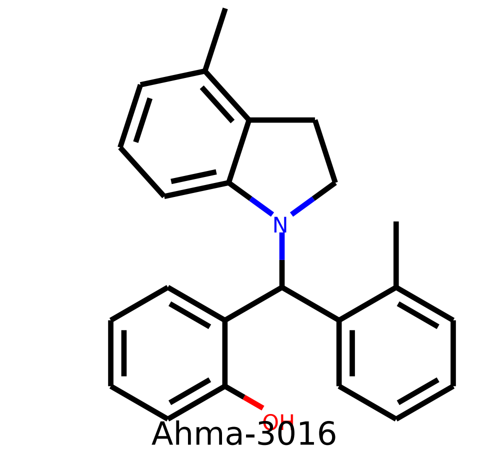
Ahma-3016
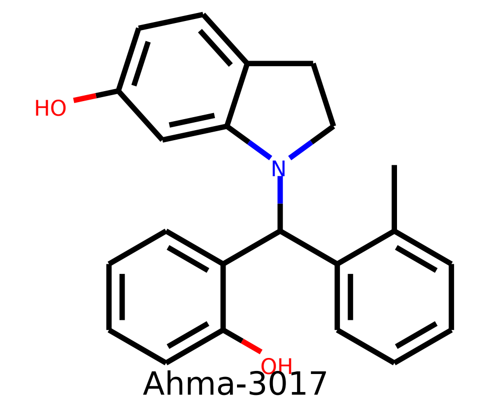
Ahma-3017
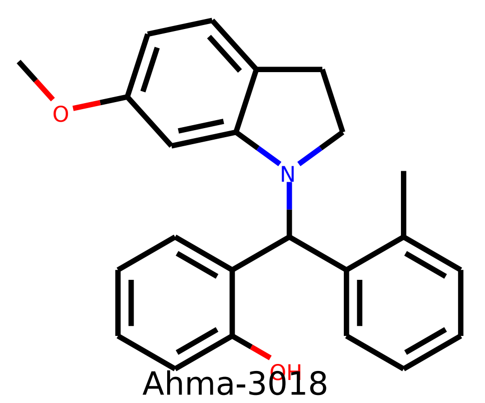
Ahma-3018
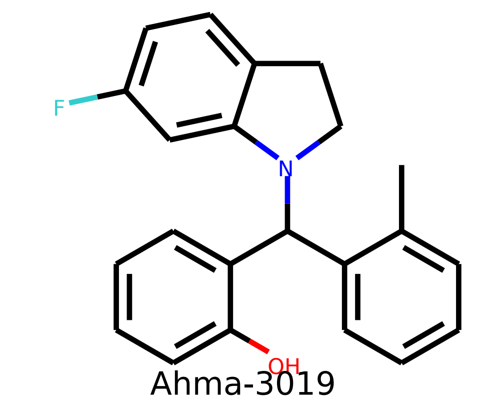
Ahma-3019
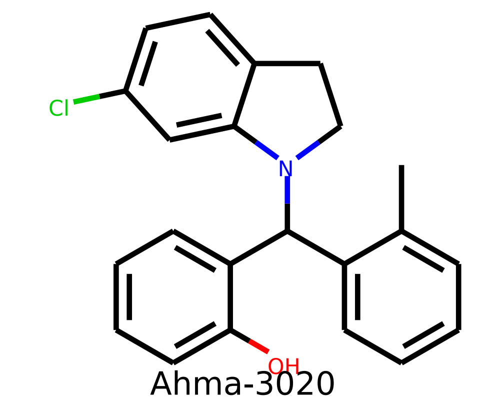
Ahma-3020
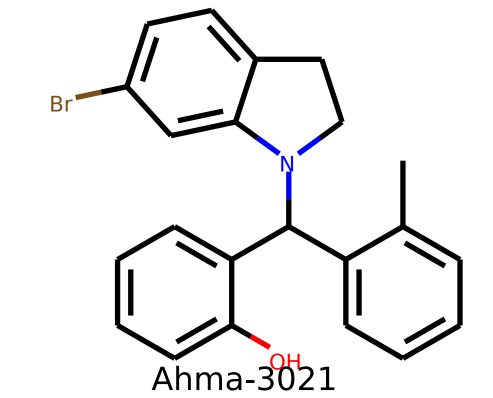
Ahma-3021
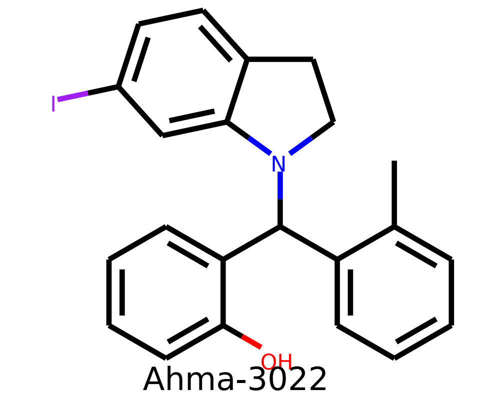
Ahma-3022
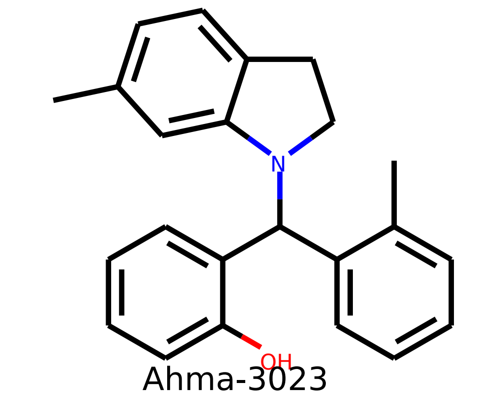
Ahma-3023
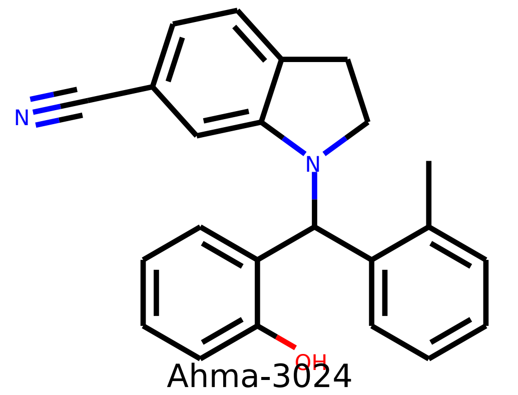
Ahma-3024
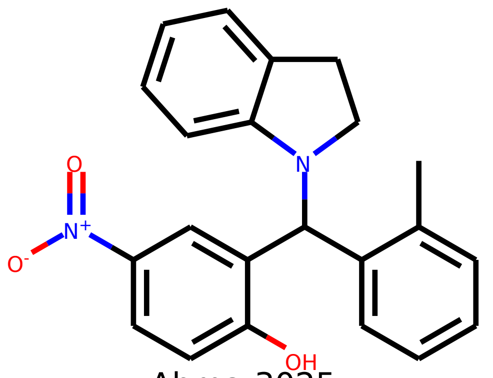
Ahma-3025
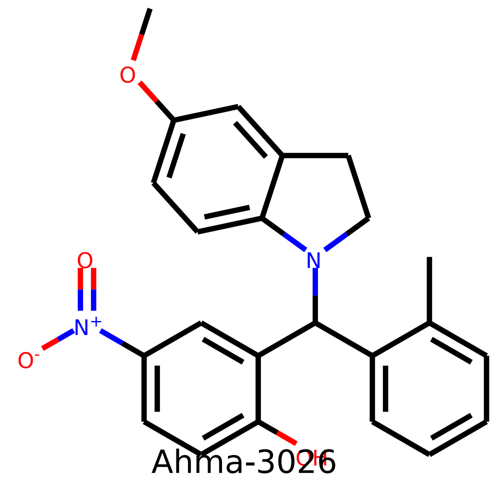
Ahma-3026
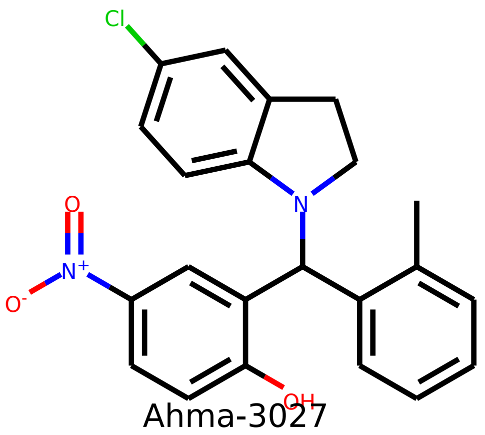
Ahma-3027
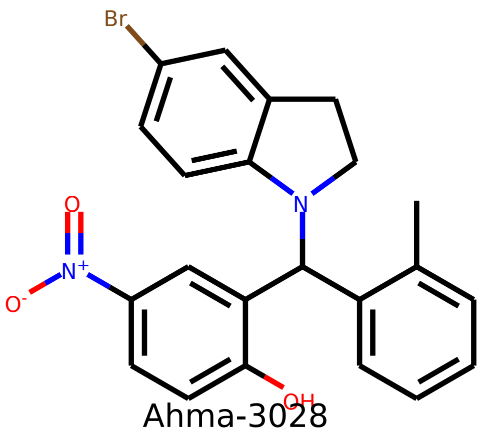
Ahma-3028
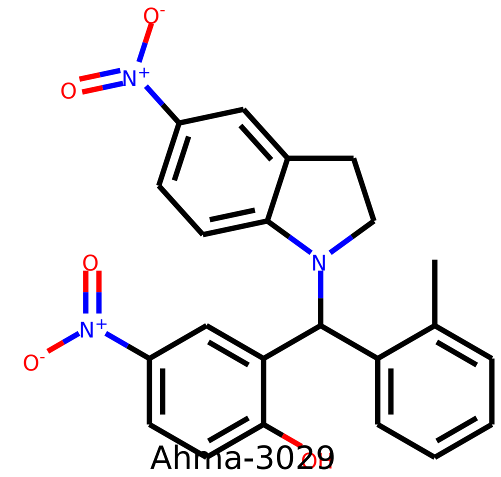
Ahma-3029
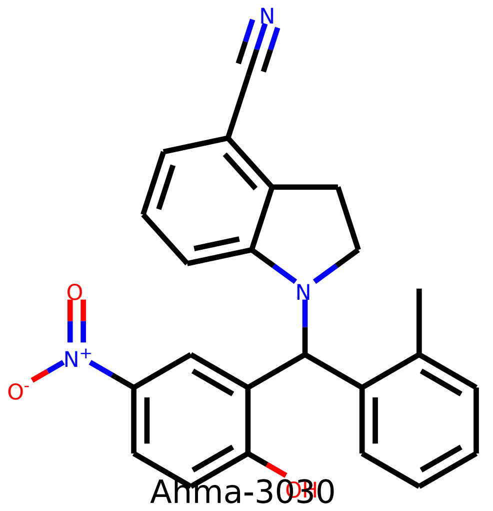
Ahma-3030
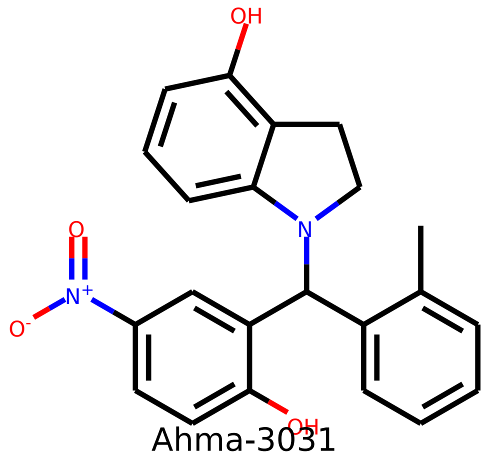
Ahma-3031
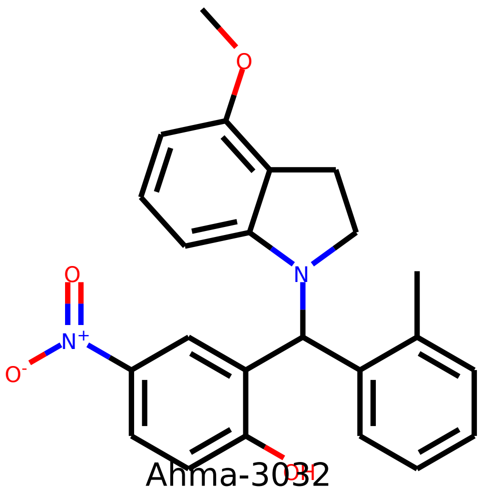
Ahma-3032
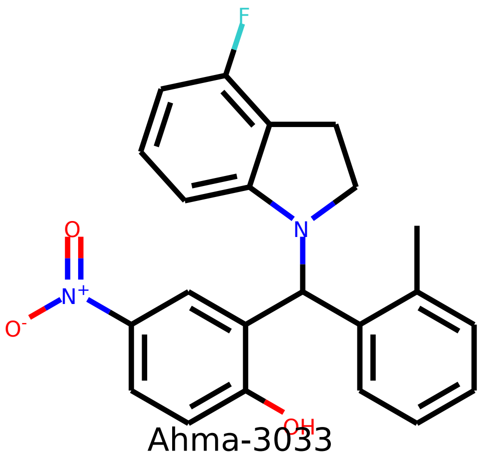
Ahma-3033
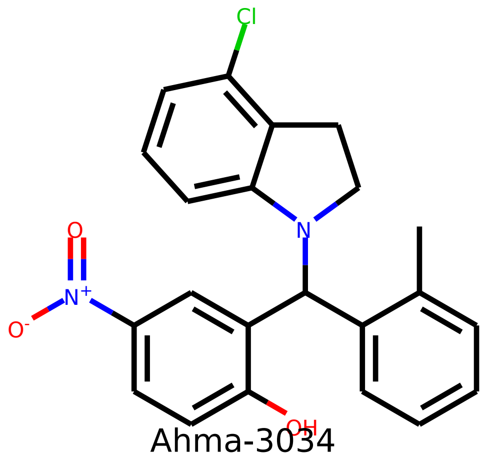
Ahma-3034
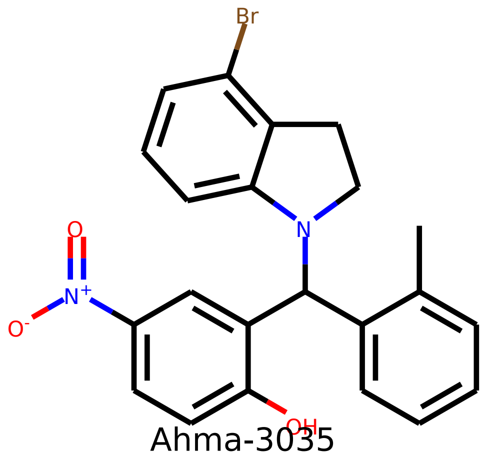
Ahma-3035
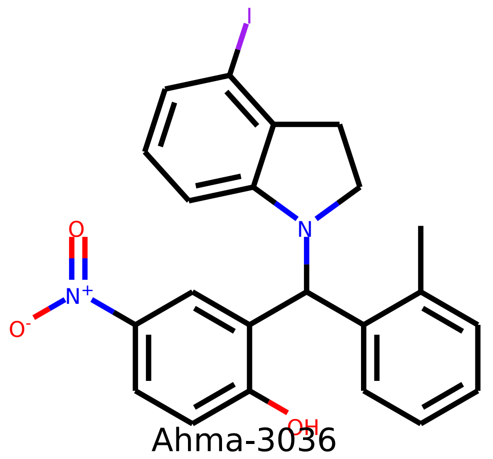
Ahma-3036
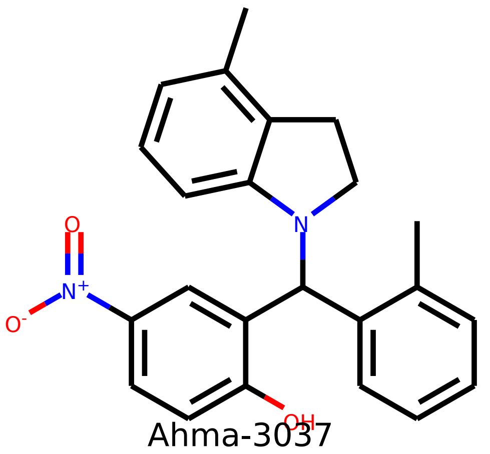
Ahma-3037
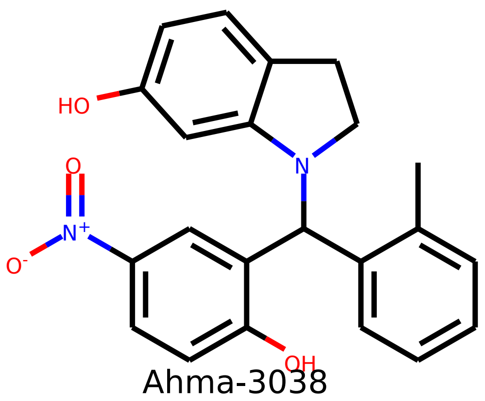
Ahma-3038
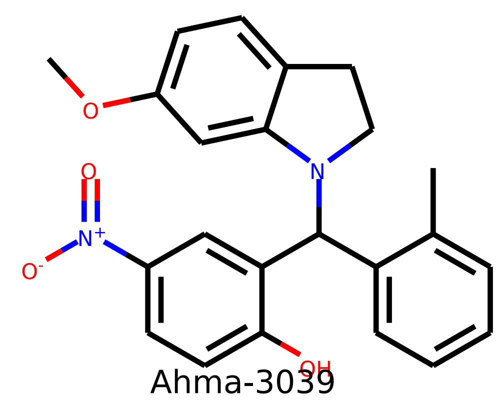
Ahma-3039
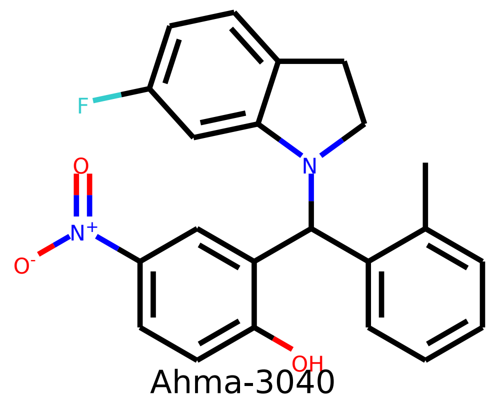
Ahma-3040
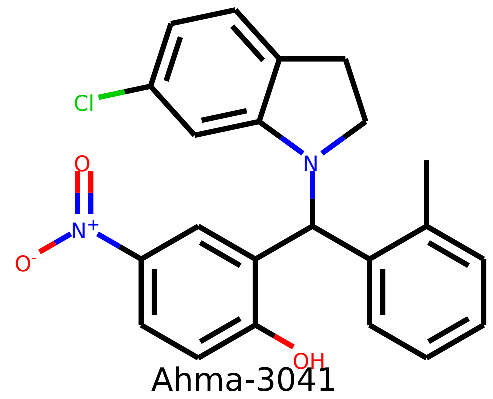
Ahma-3041
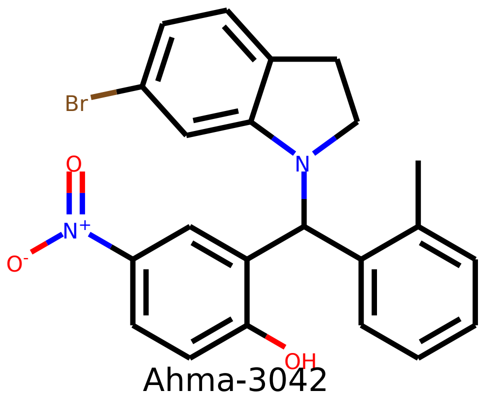
Ahma-3042
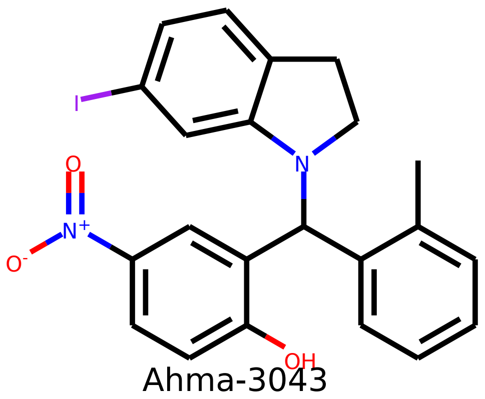
Ahma-3043
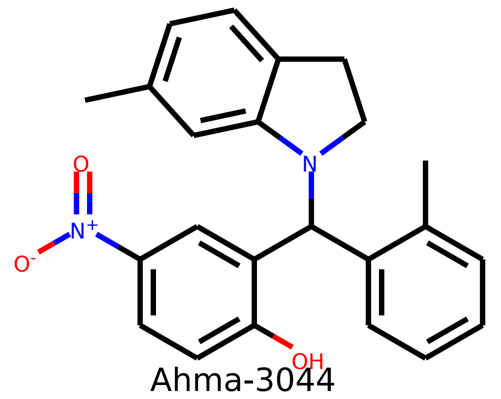
Ahma-3044
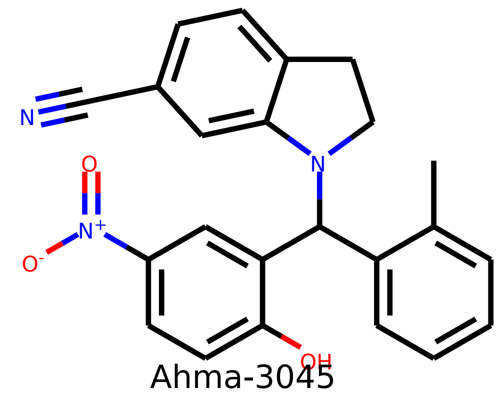
Ahma-3045

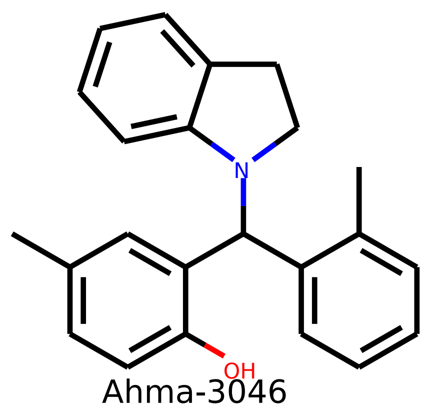
Ahma-3046
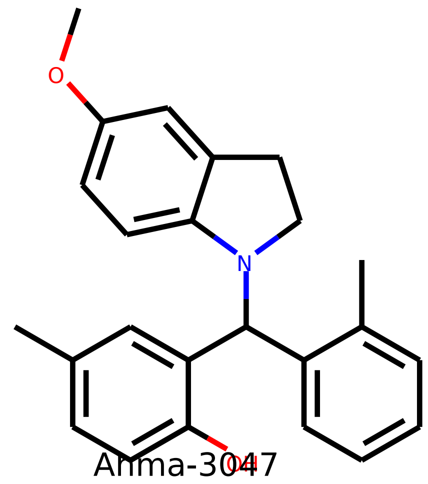
Ahma-3047
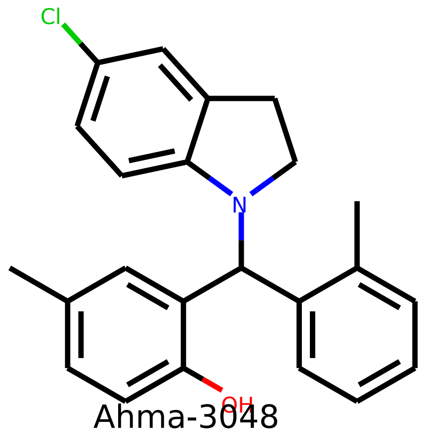
Ahma-3048
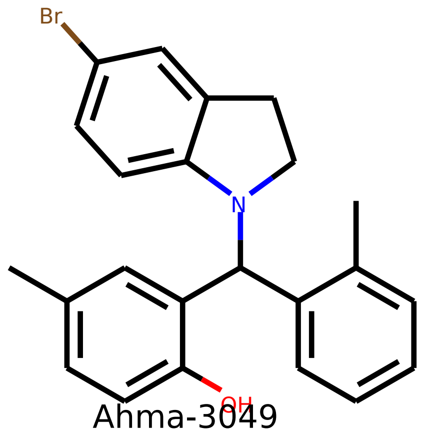
Ahma-3049
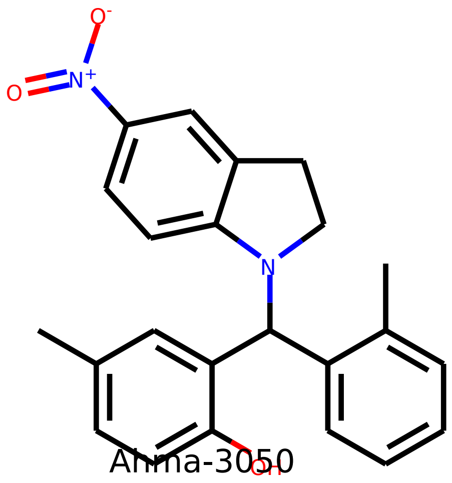
Ahma-3050
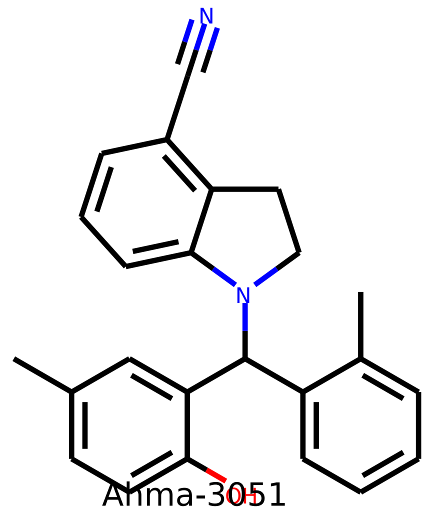
Ahma-3051
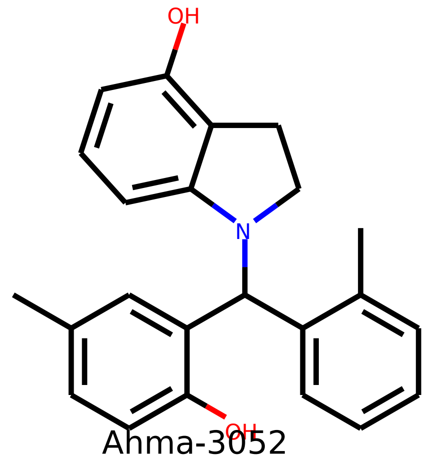
Ahma-3052
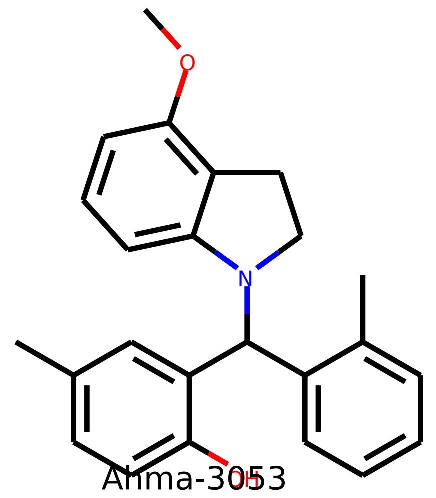
Ahma-3053
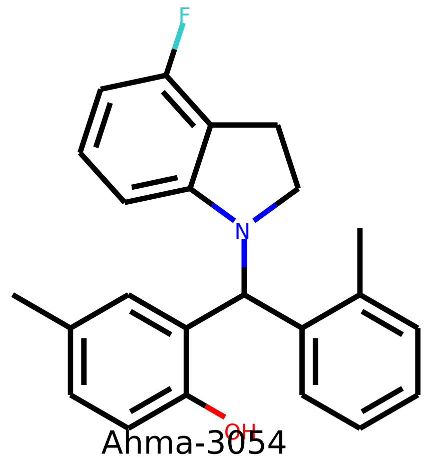
Ahma-3054
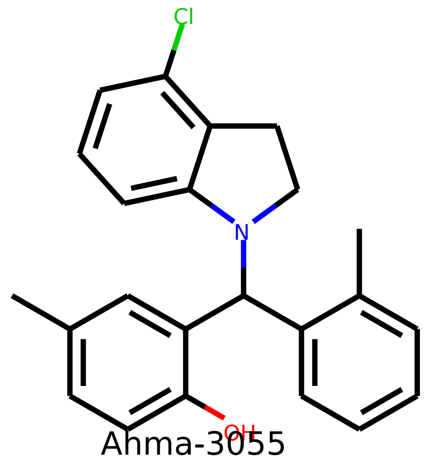
Ahma-3055
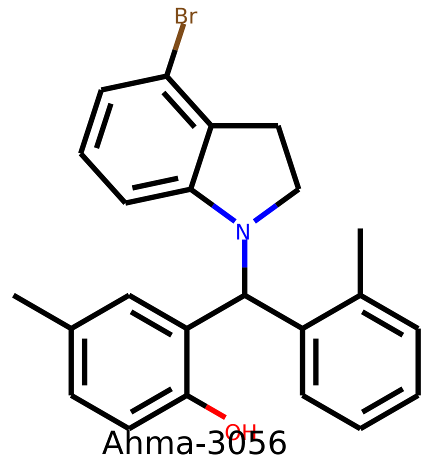
Ahma-3056
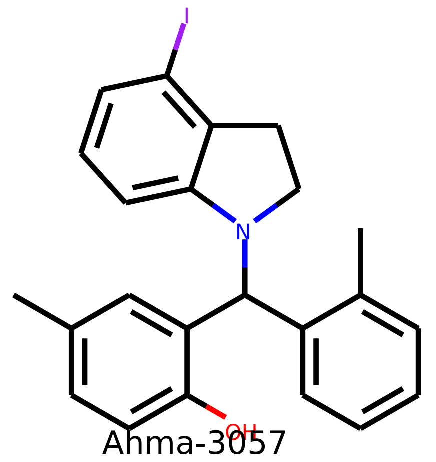
Ahma-3057
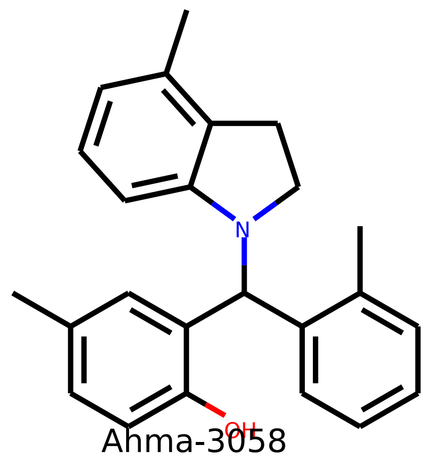
Ahma-3058
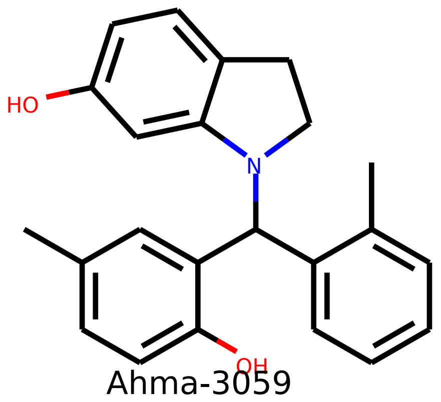
Ahma-3059
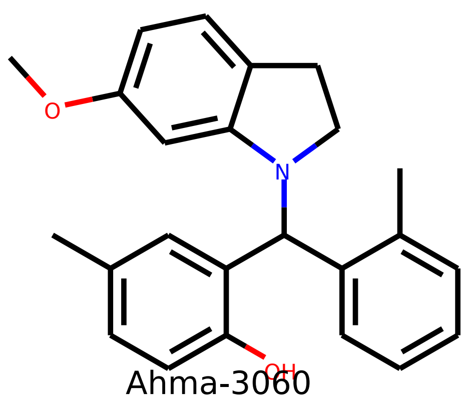
Ahma-3060
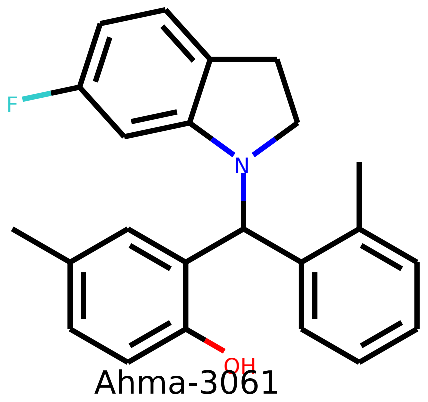
Ahma-3061
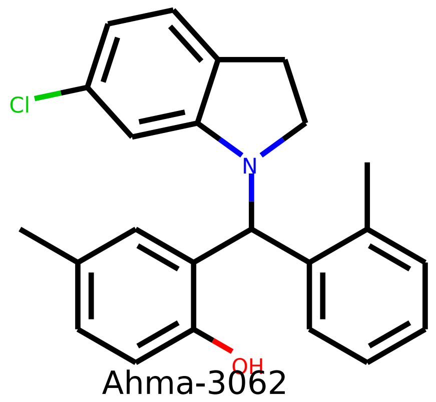
Ahma-3062
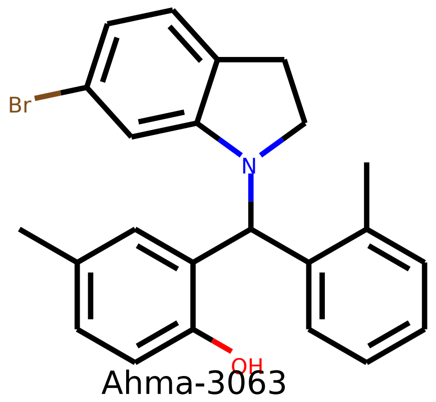
Ahma-3063
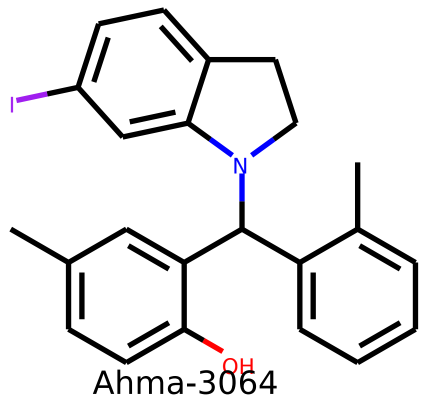
Ahma-3064
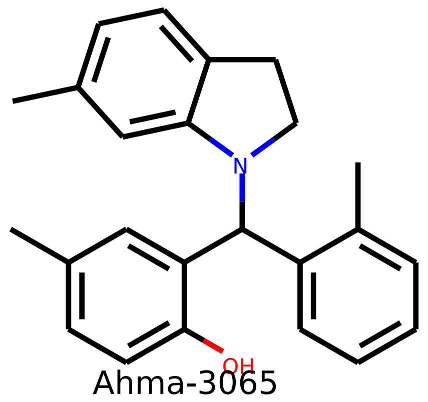
Ahma-3065
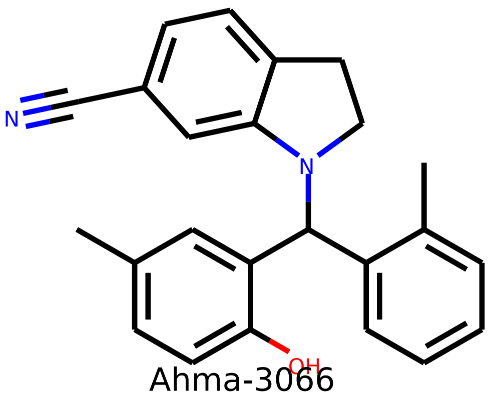
Ahma-3066
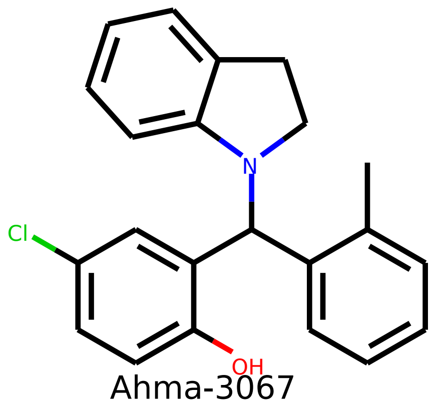
Ahma-3067
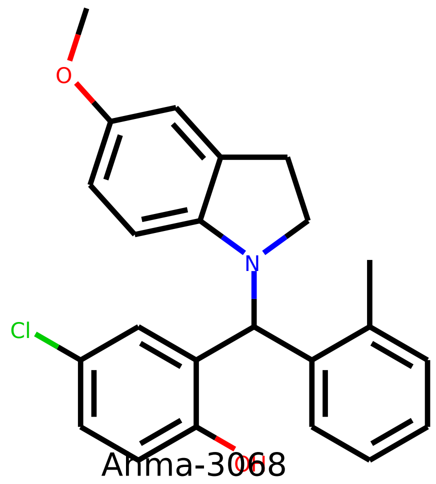
Ahma-3068
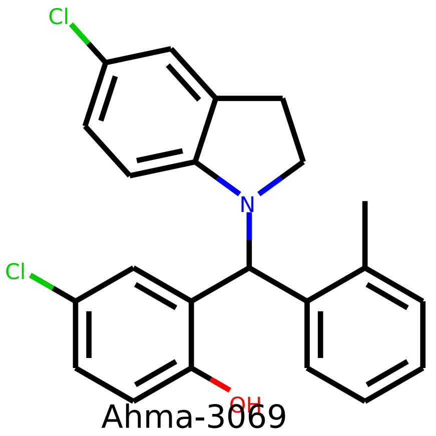
Ahma-3069
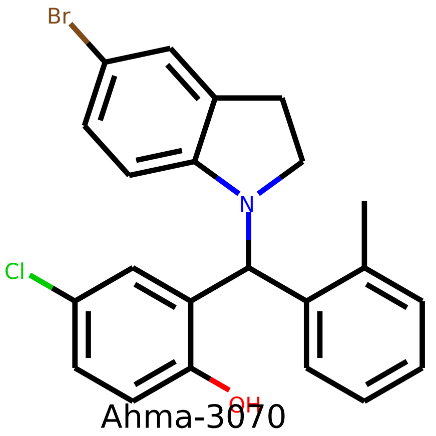
Ahma-3070
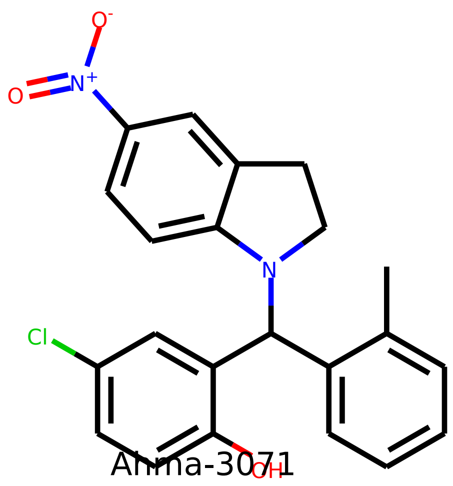
Ahma-3071
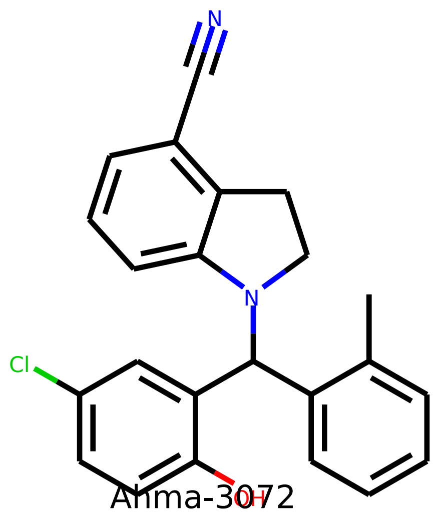
Ahma-3072
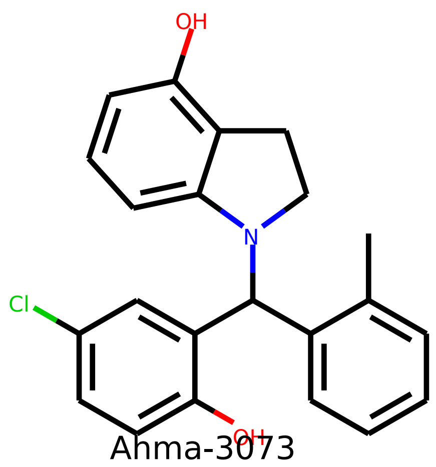
Ahma-3073
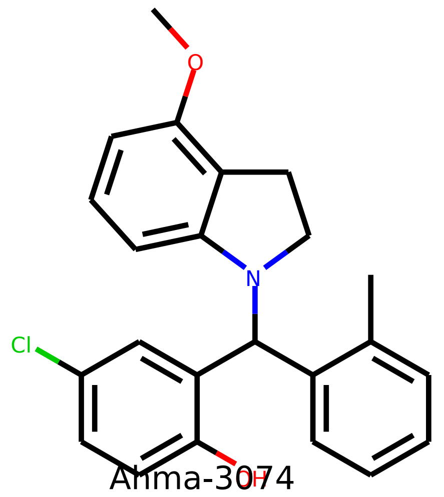
Ahma-3074
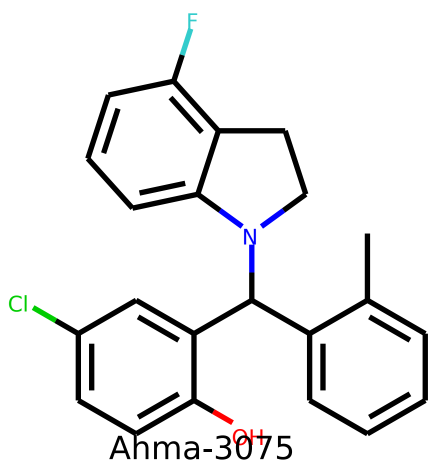
Ahma-3075
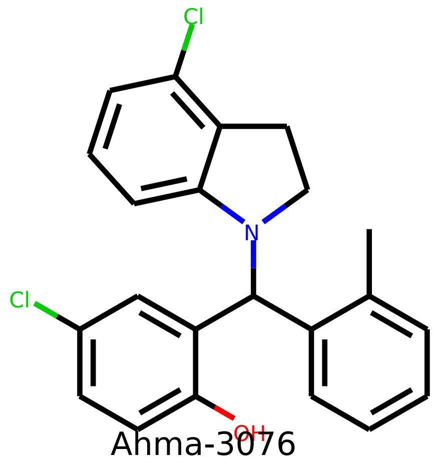
Ahma-3076
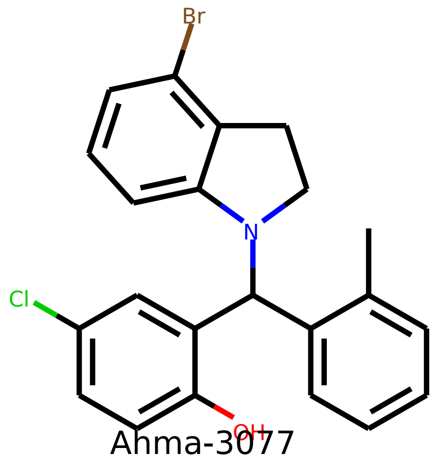
Ahma-3077
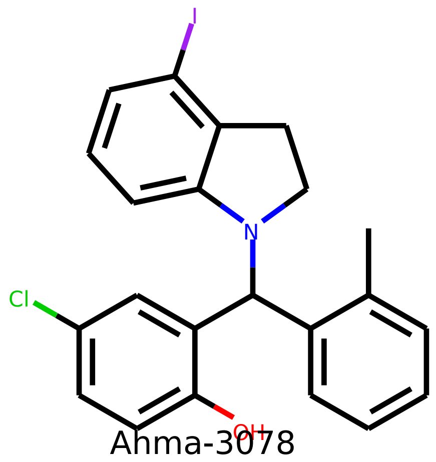
Ahma-3078
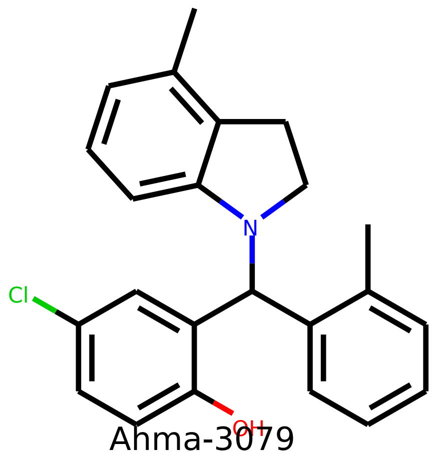
Ahma-3079
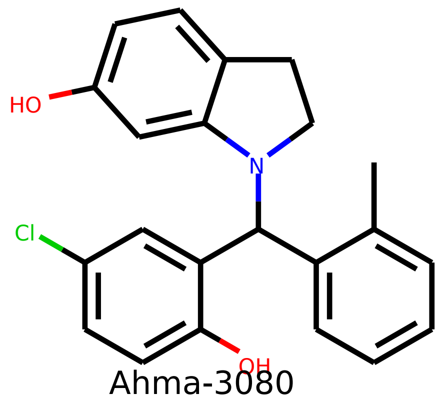
Ahma-3080

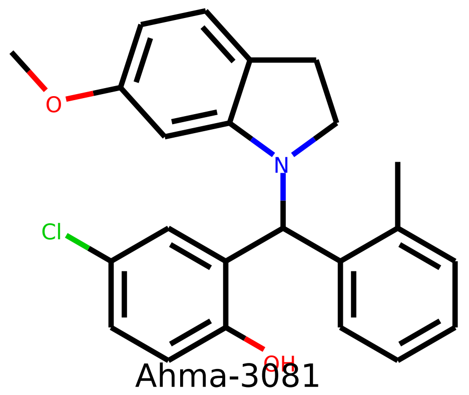
Ahma-3081
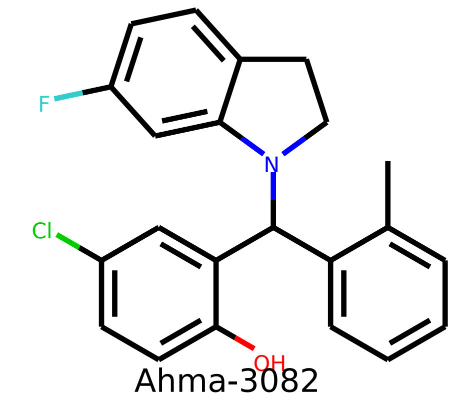
Ahma-3082
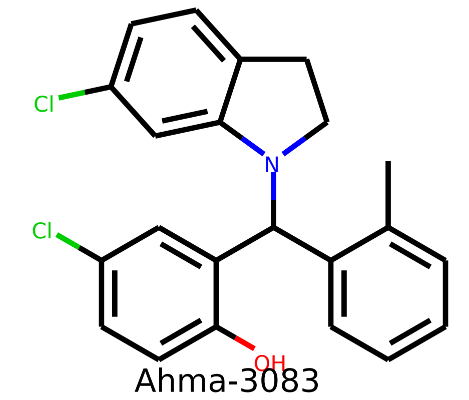
Ahma-3083
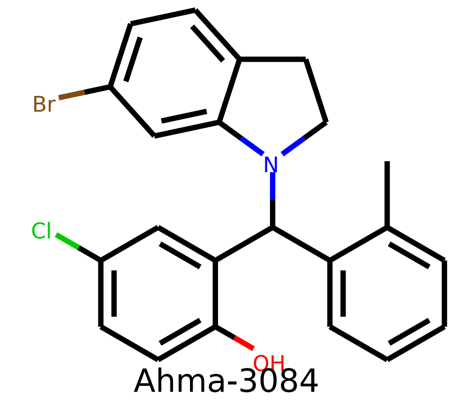
Ahma-3084
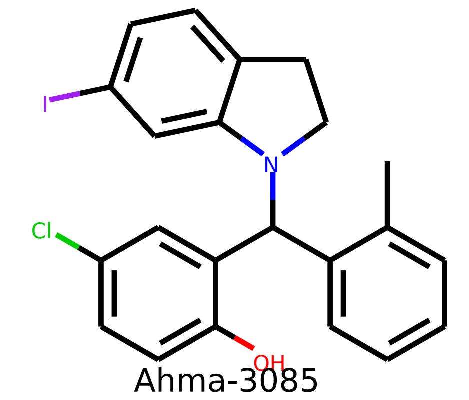
Ahma-3085
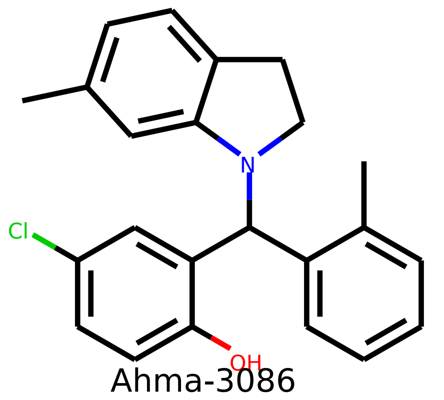
Ahma-3086
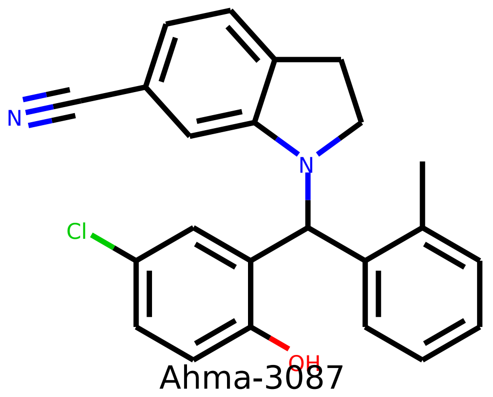
Ahma-3087
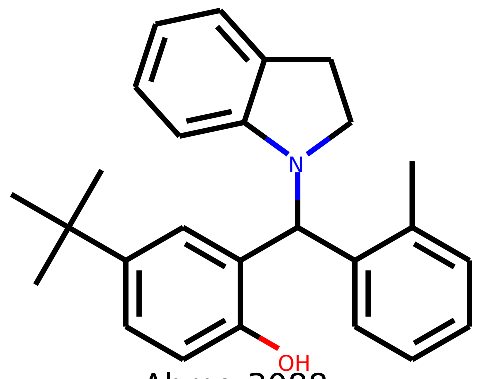
Ahma-3088
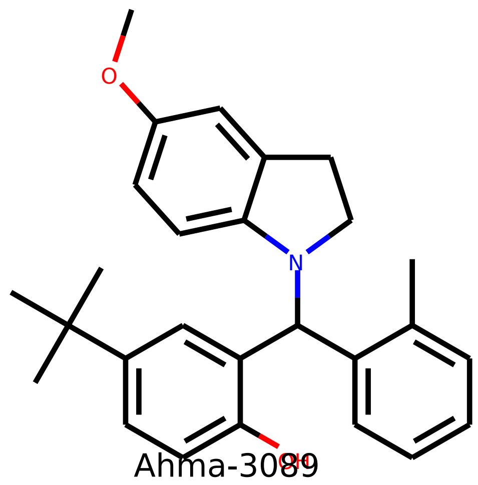
Ahma-3089
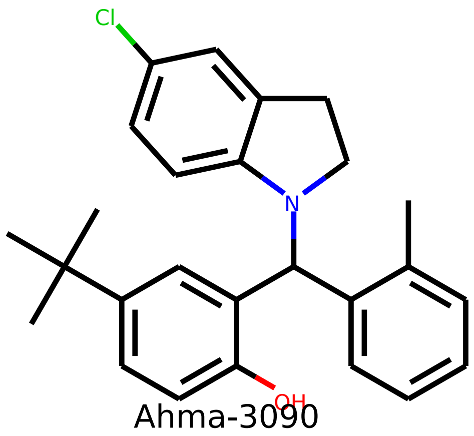
Ahma-3090
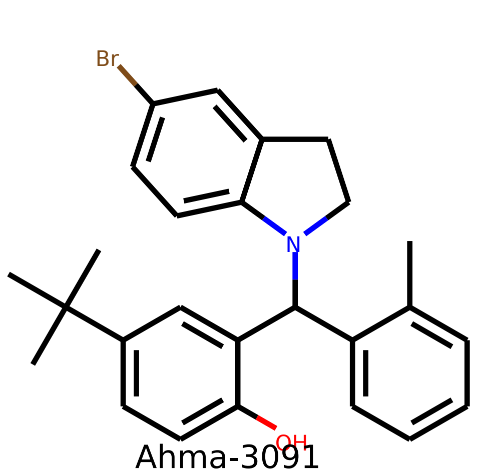
Ahma-3091
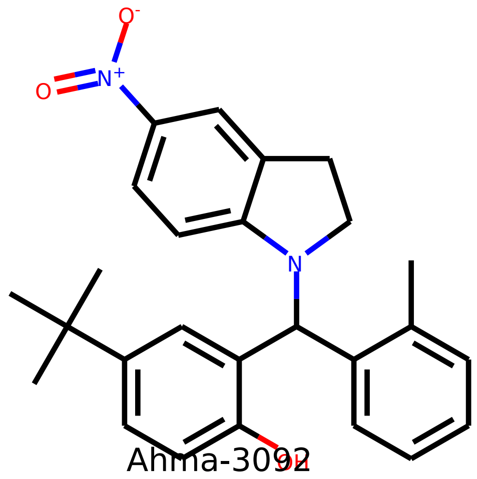
Ahma-3092
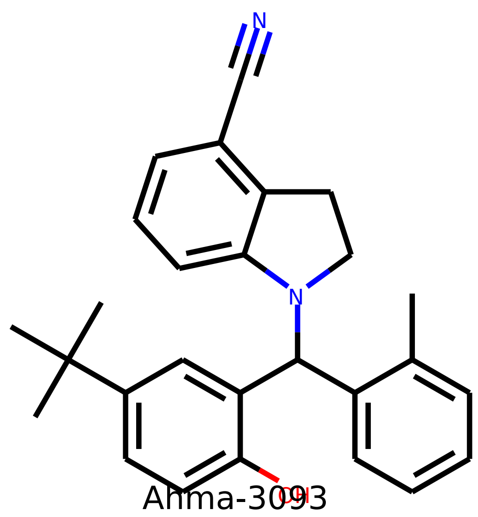
Ahma-3093
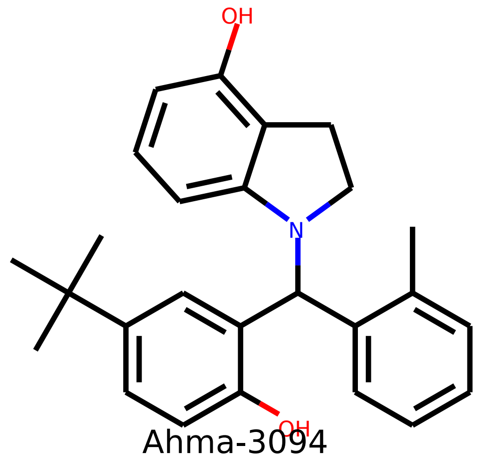
Ahma-3094
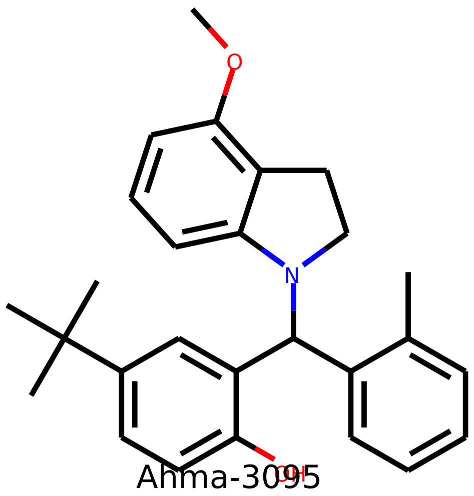
Ahma-3095
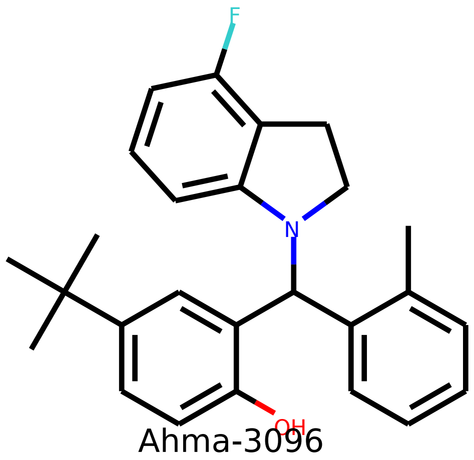
Ahma-3096
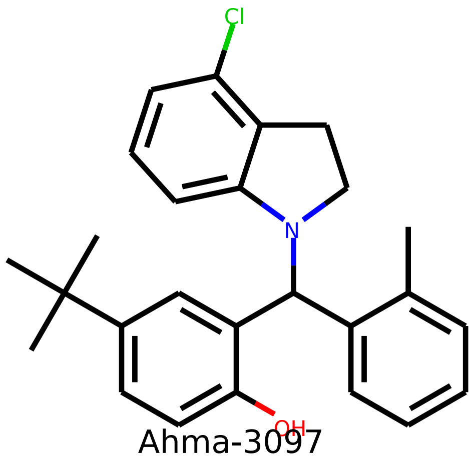
Ahma-3097
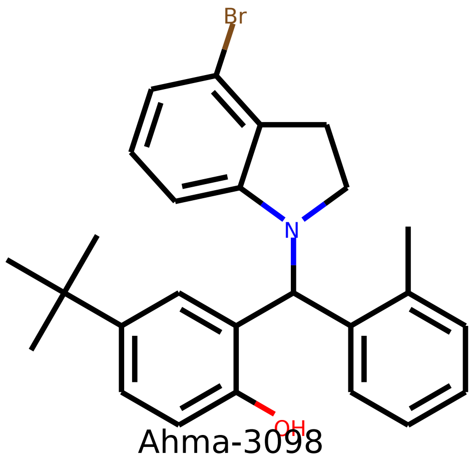
Ahma-3098
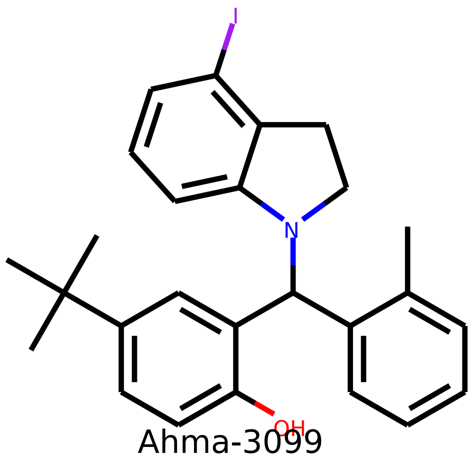
Ahma-3099
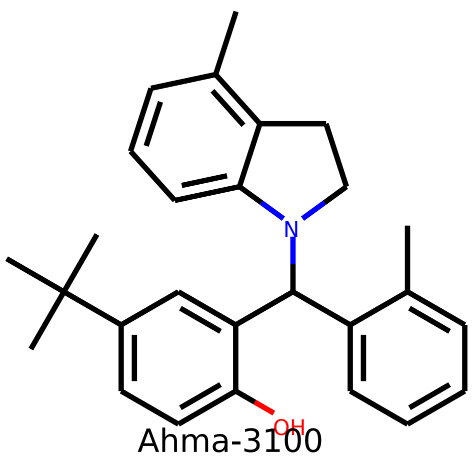
Ahma-3100
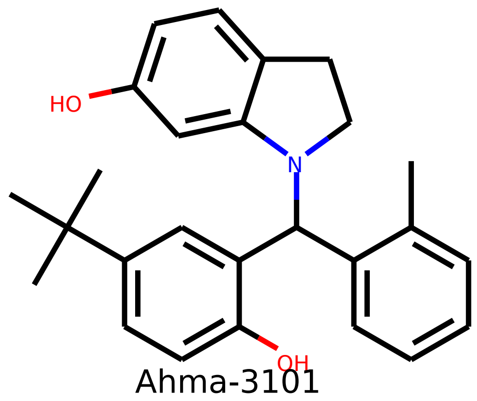
Ahma-3101
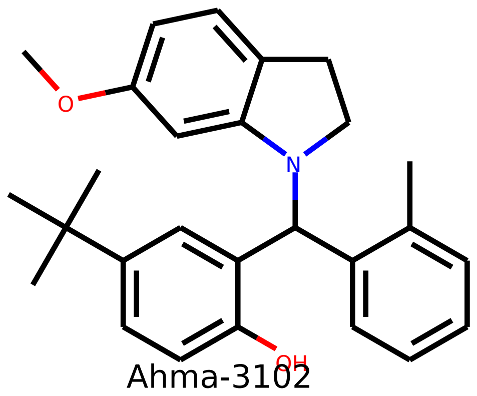
Ahma-3102
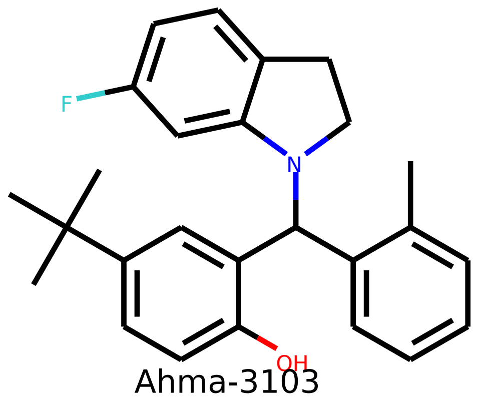
Ahma-3103
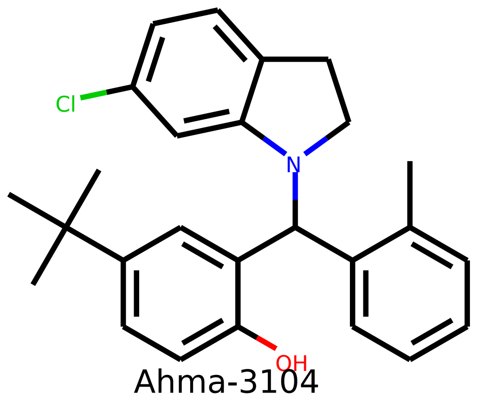
Ahma-3104
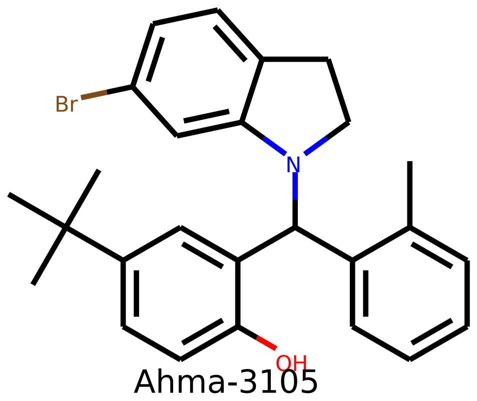
Ahma-3105
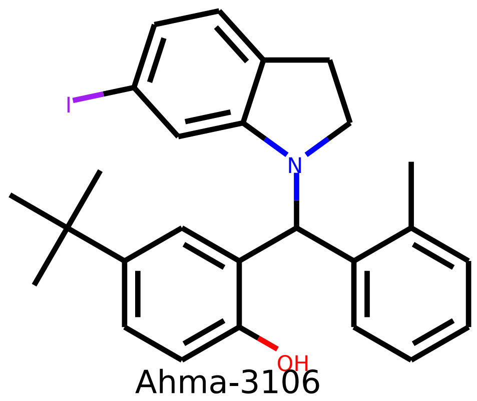
Ahma-3106
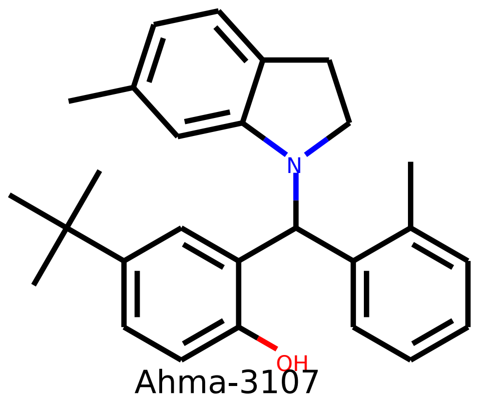
Ahma-3107
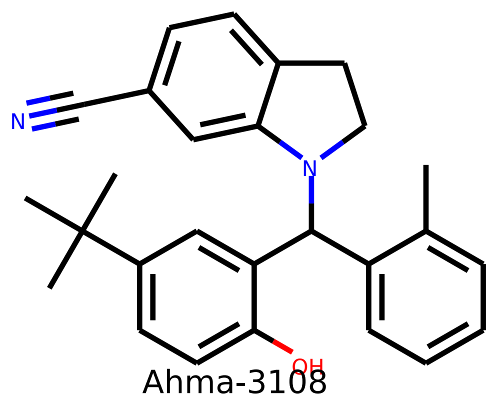
Ahma-3108
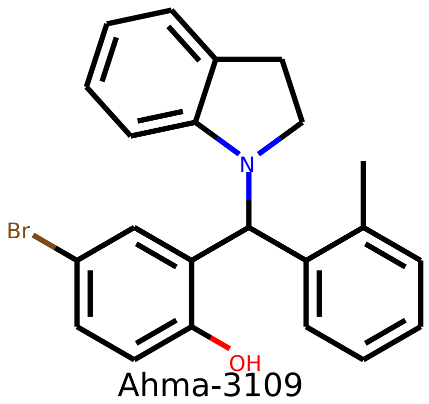
Ahma-3109
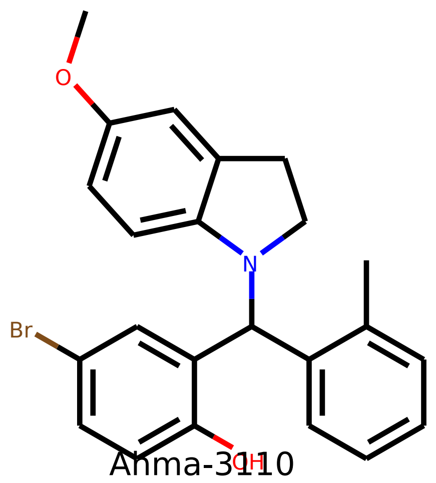
Ahma-3110
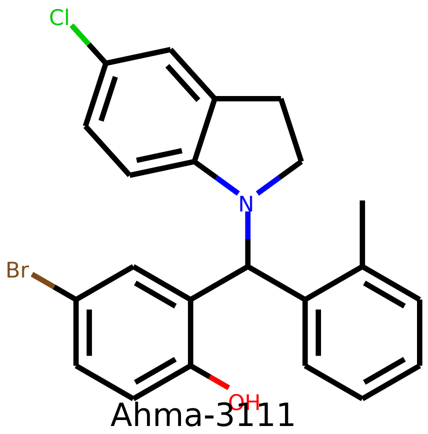
Ahma-3111
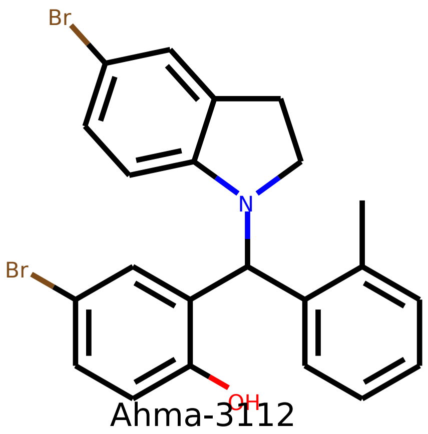
Ahma-3112
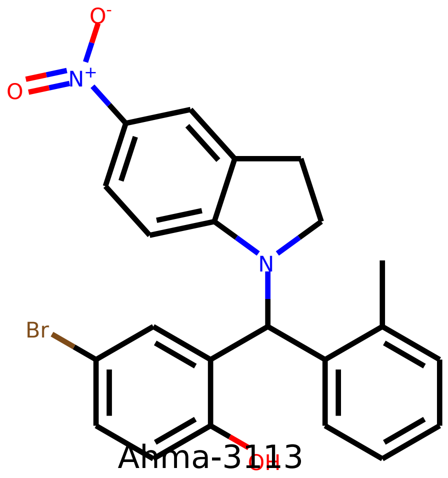
Ahma-3113
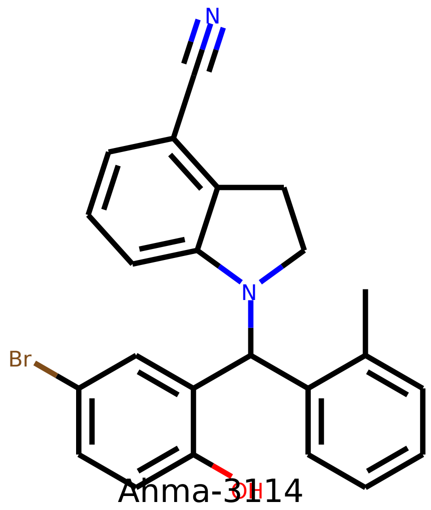
Ahma-3114
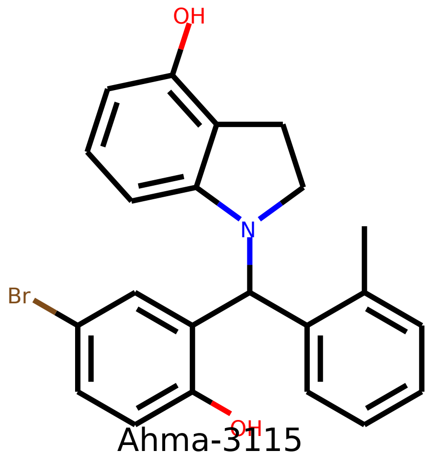
Ahma-3115

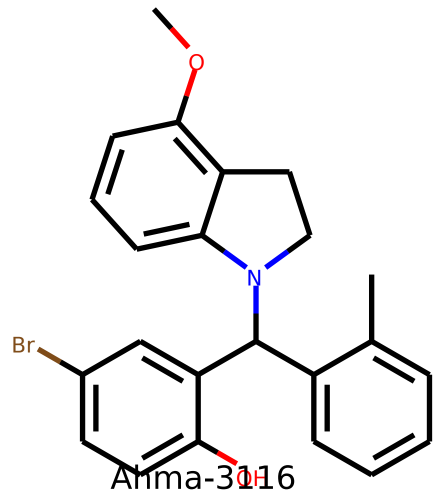
Ahma-3116

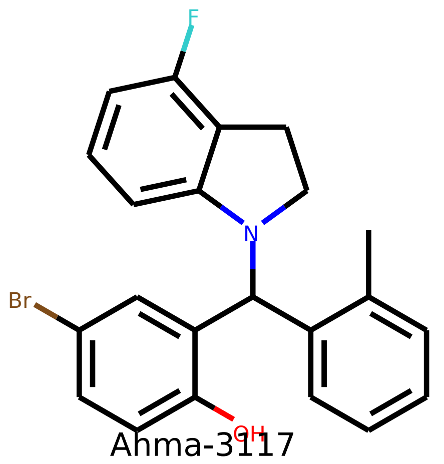
Ahma-3117

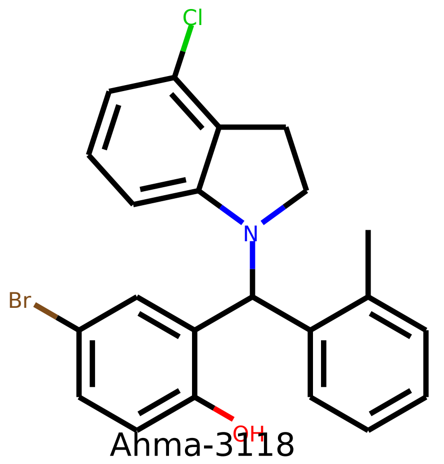
Ahma-3118

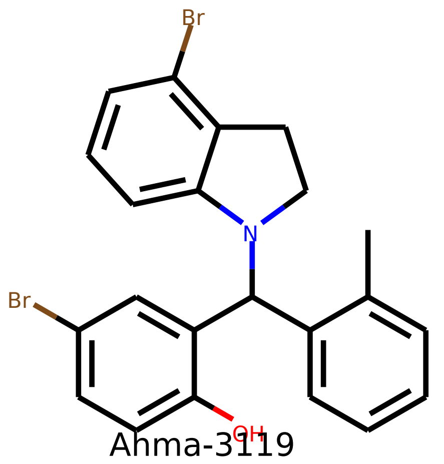
Ahma-3119

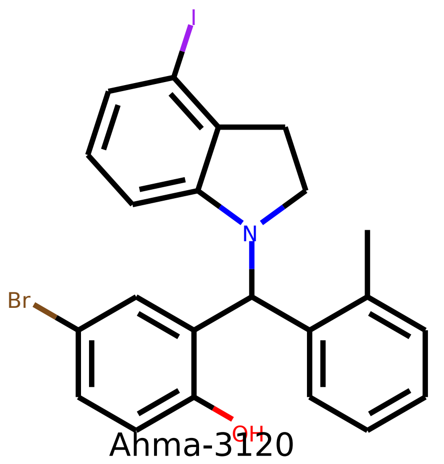
Ahma-3120

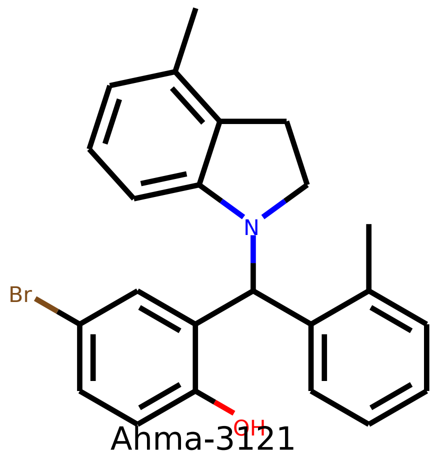
Ahma-3121

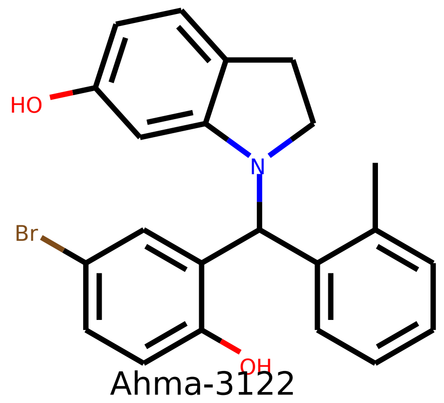
Ahma-3122

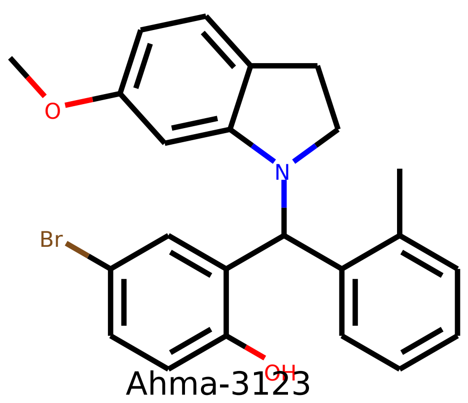
Ahma-3123

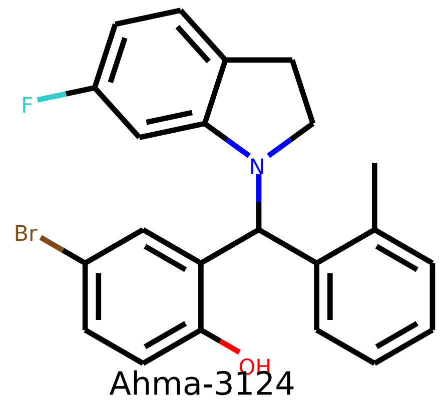
Ahma-3124

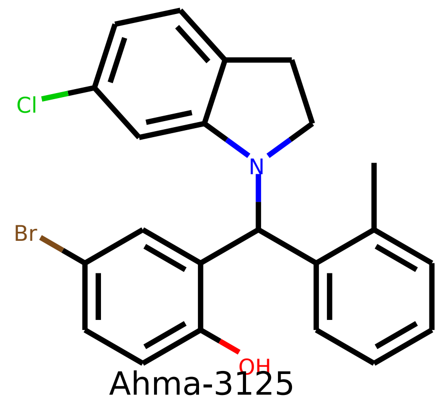
Ahma-3125

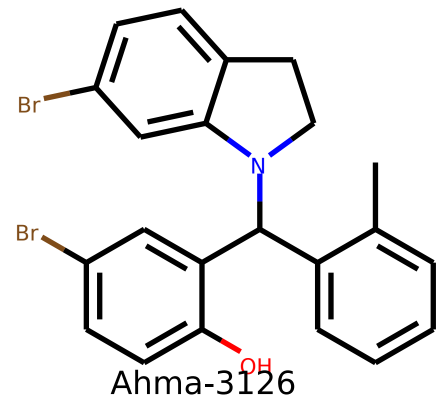
Ahma-3126

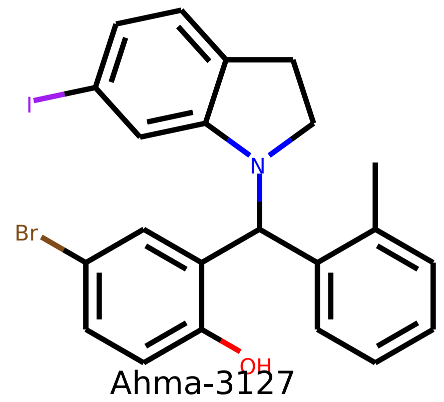
Ahma-3127

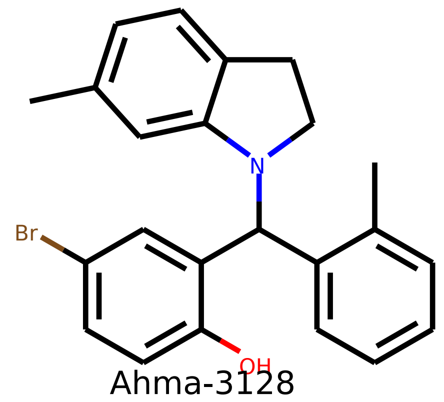
Ahma-3128

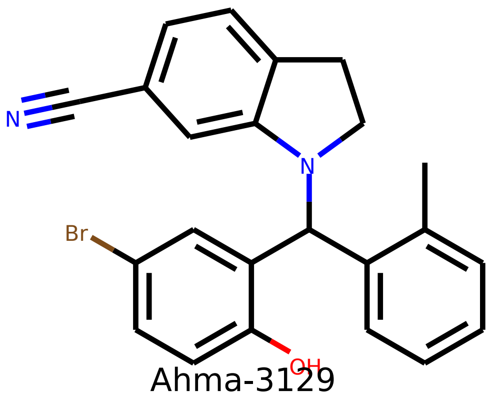
Ahma-3129

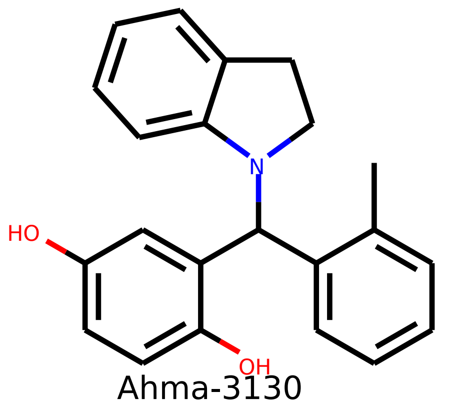
Ahma-3130

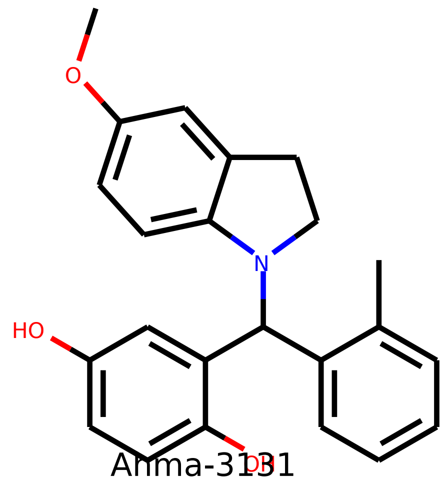
Ahma-3131

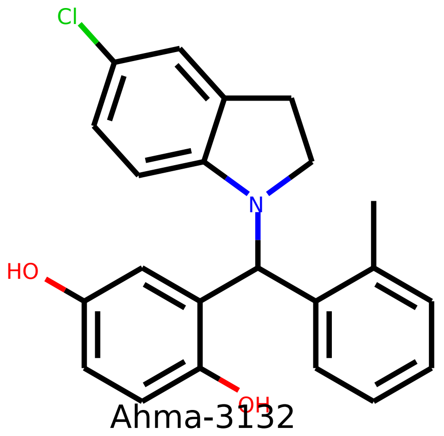
Ahma-3132

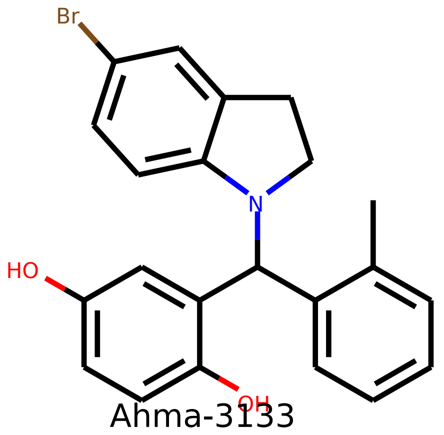
Ahma-3133

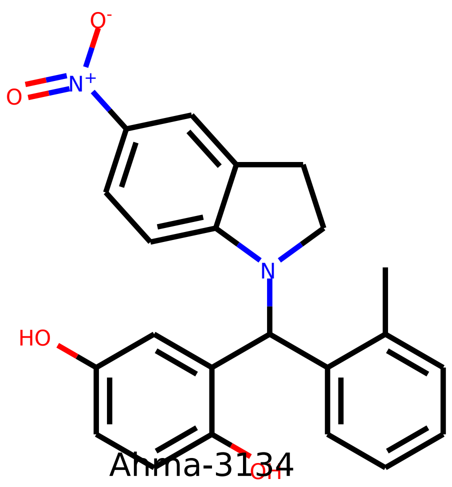
Ahma-3134

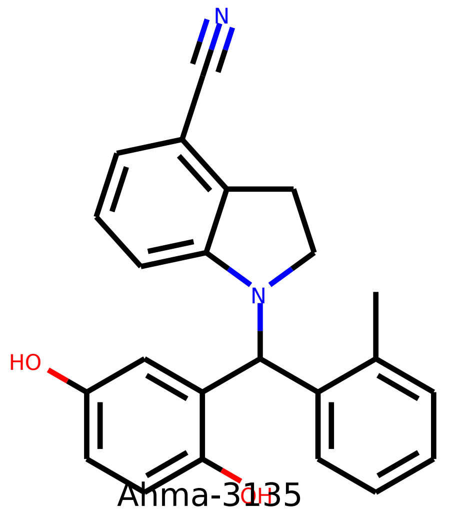
Ahma-3135

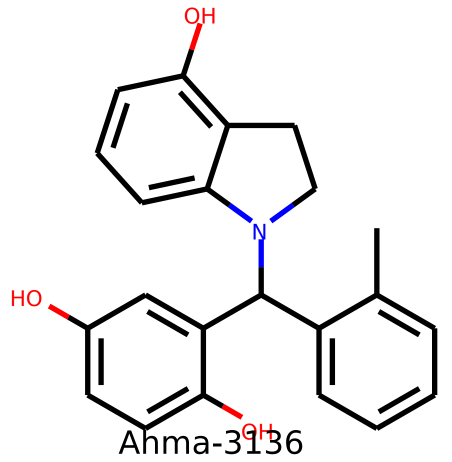
Ahma-3136

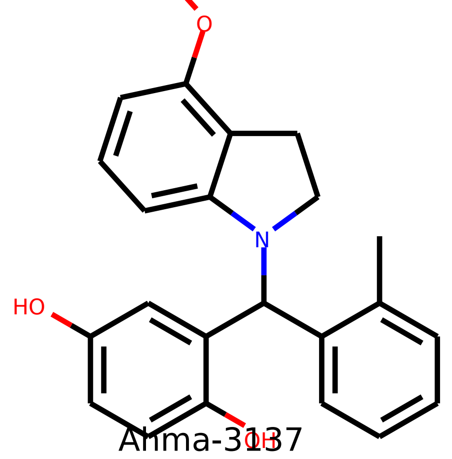
Ahma-3137

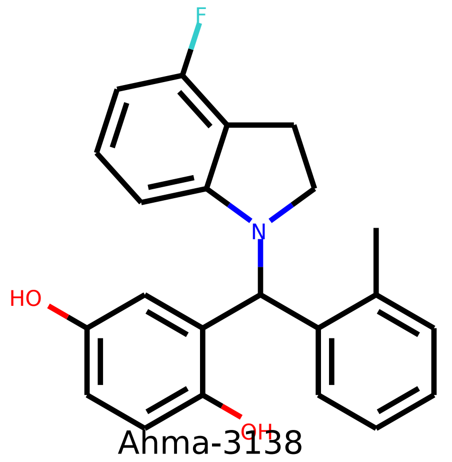
Ahma-3138

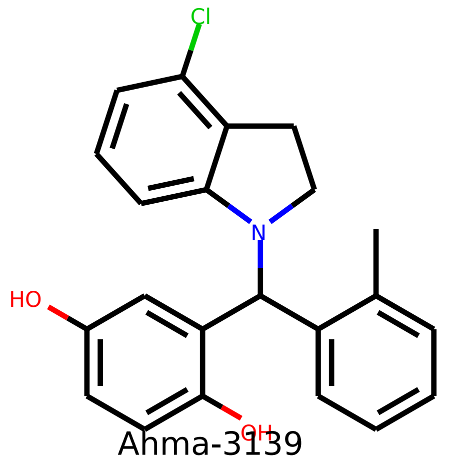
Ahma-3139

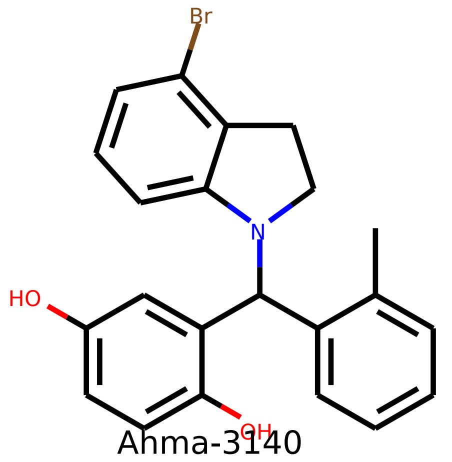
Ahma-3140

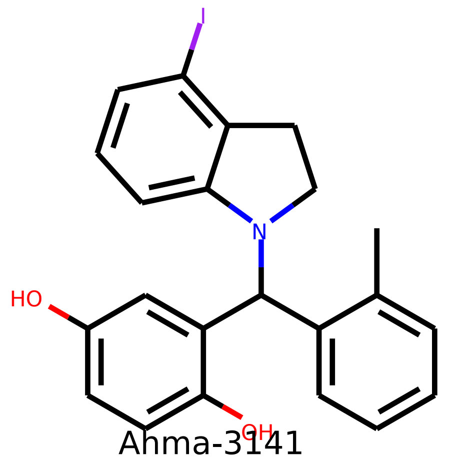
Ahma-3141

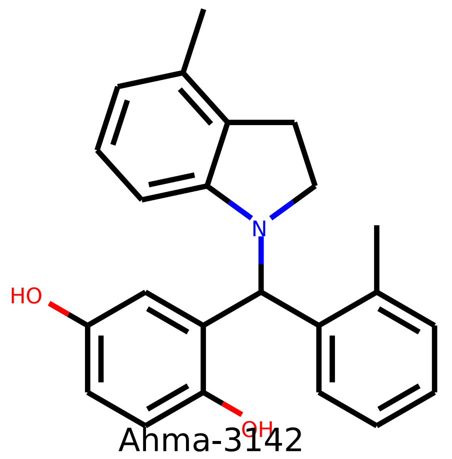
Ahma-3142

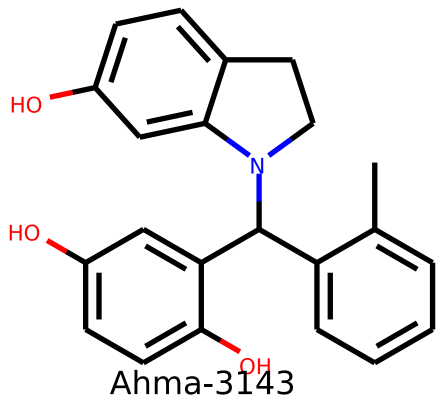
Ahma-3143

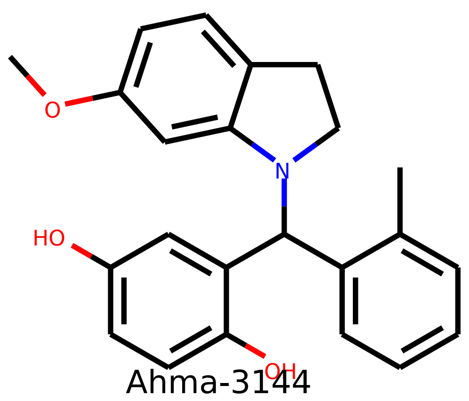
Ahma-3144

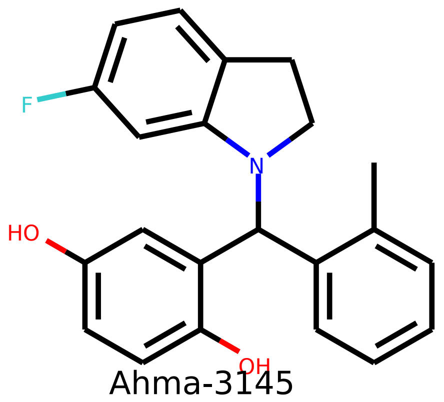
Ahma-3145

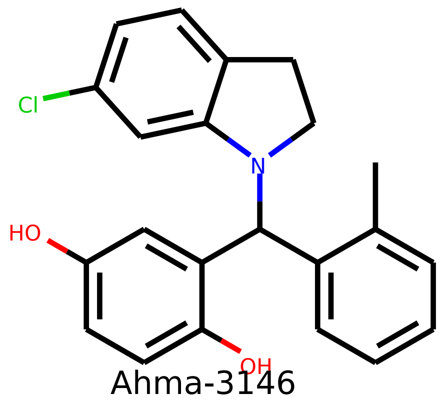
Ahma-3146

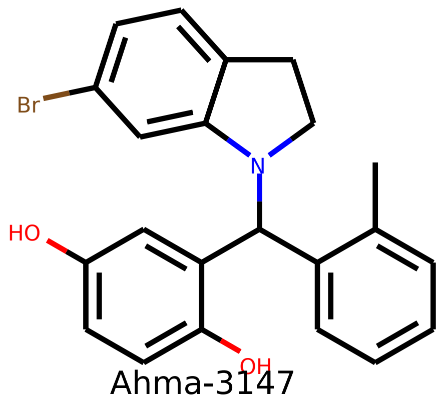
Ahma-3147

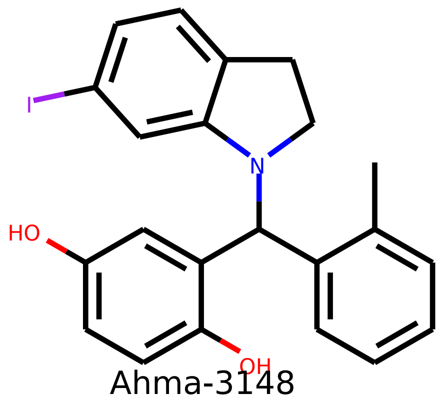
Ahma-3148

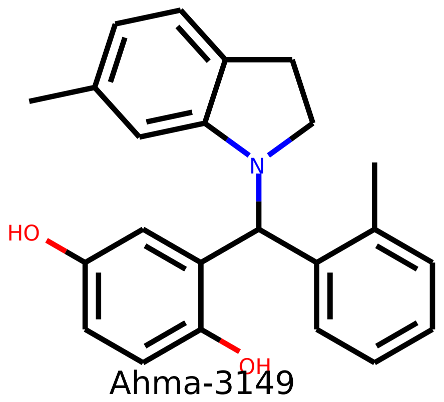
Ahma-3149

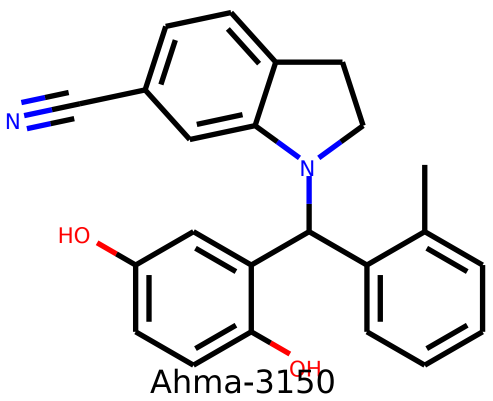
Ahma-3150

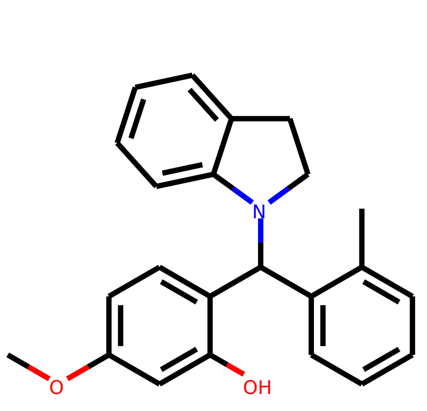

Ahma-3151

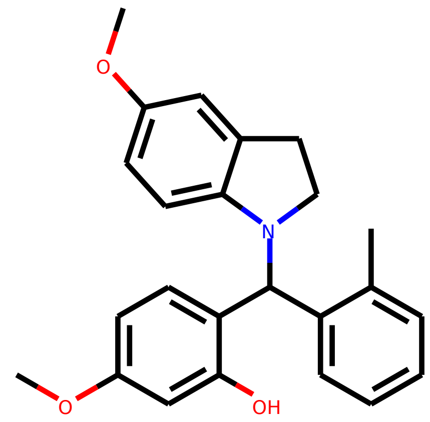

Ahma-3152

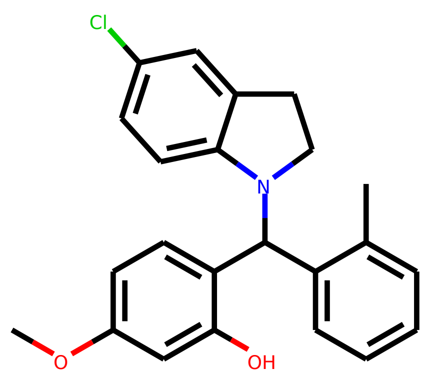

Ahma-3153

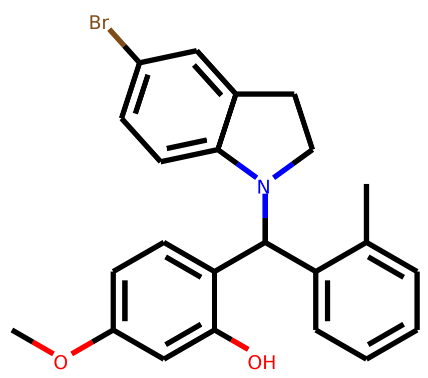

Ahma-3154

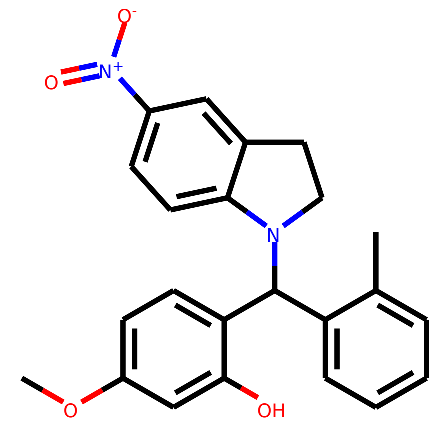

Ahma-3155

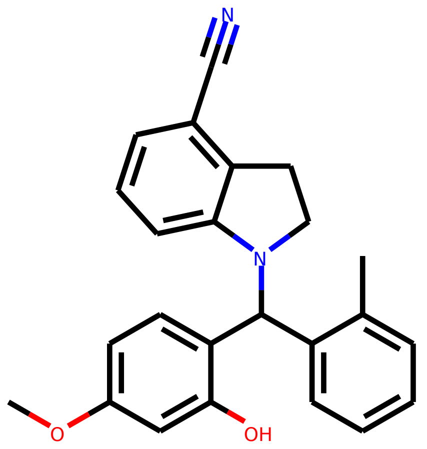

Ahma-3156

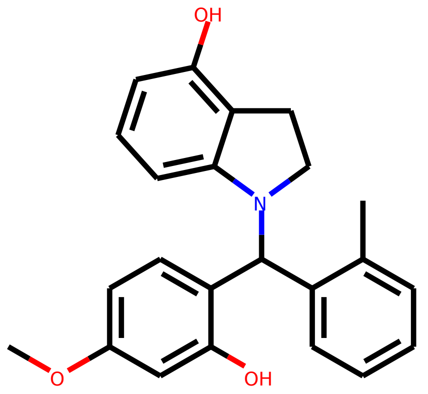

Ahma-3157

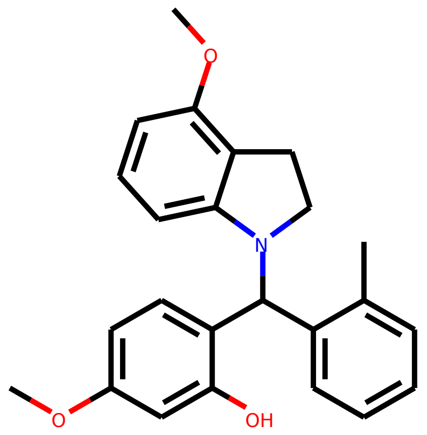

Ahma-3158

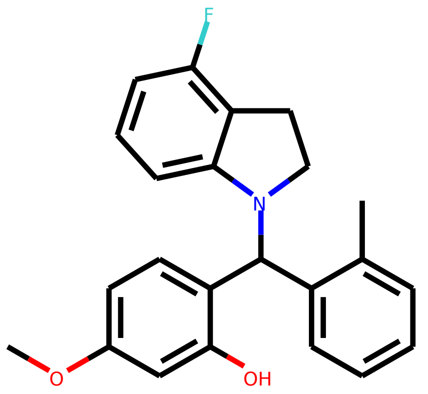

Ahma-3159

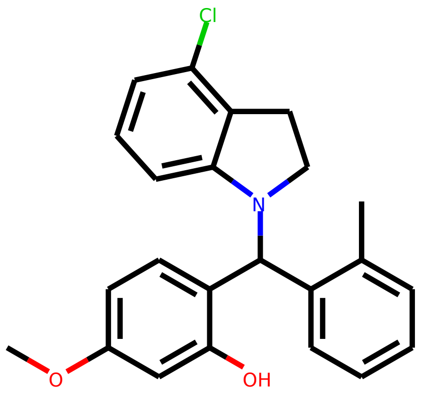

Ahma-3160

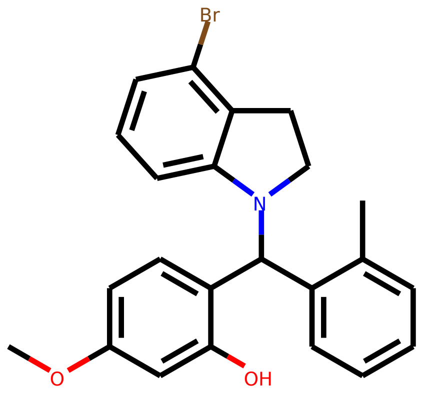

Ahma-3161

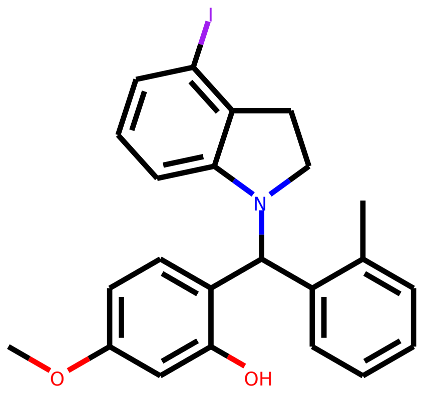

Ahma-3162

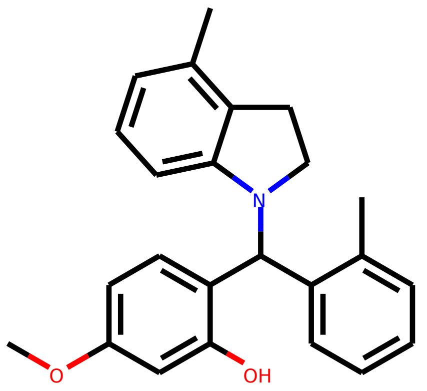

Ahma-3163

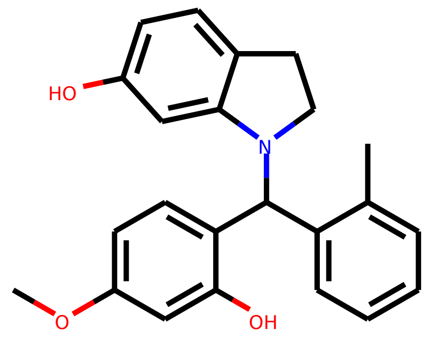

Ahma-3164

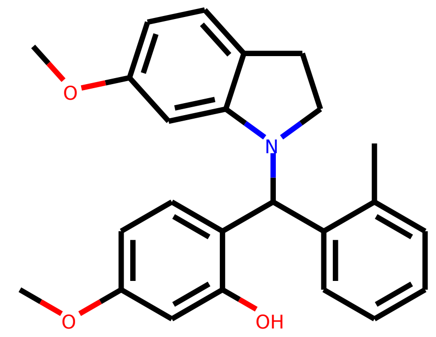

Ahma-3165

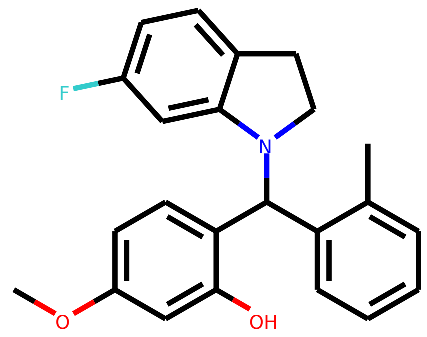

Ahma-3166

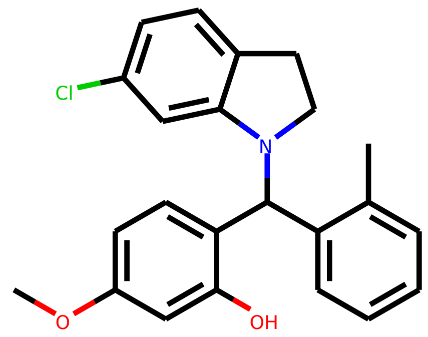

Ahma-3167

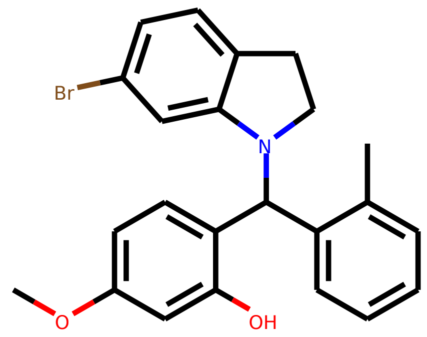

Ahma-3168

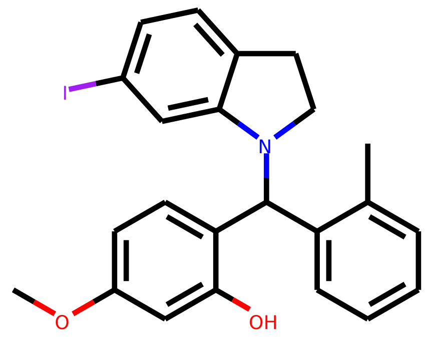

Ahma-3169

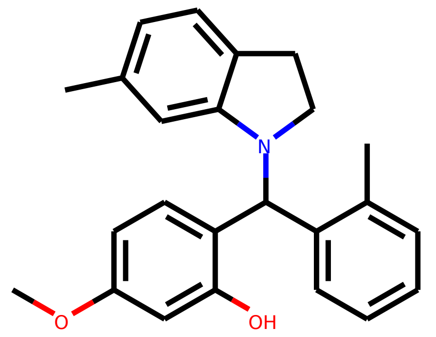

Ahma-3170

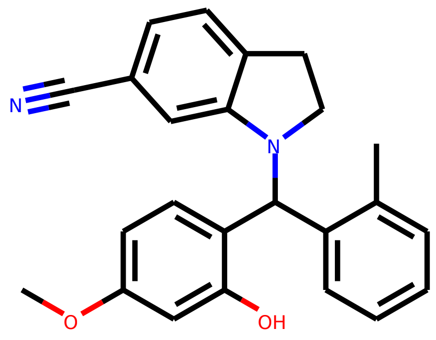

Ahma-3171

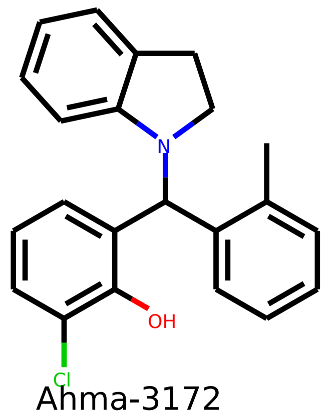

Ahma-3172

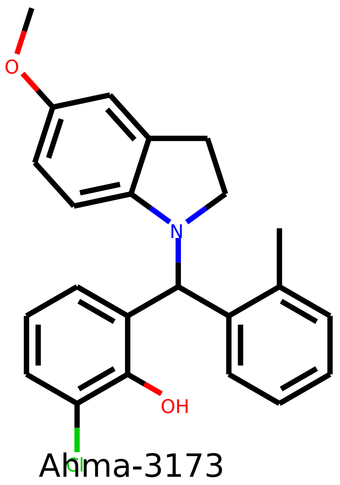

Ahma-3173

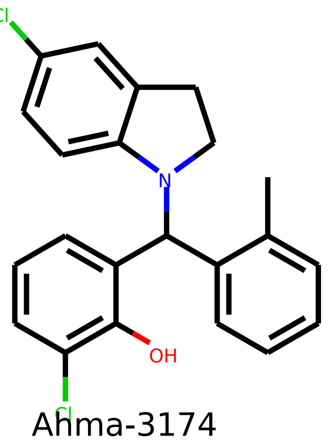

Ahma-3174

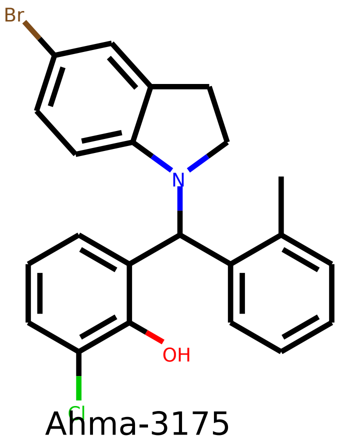

Ahma-3175

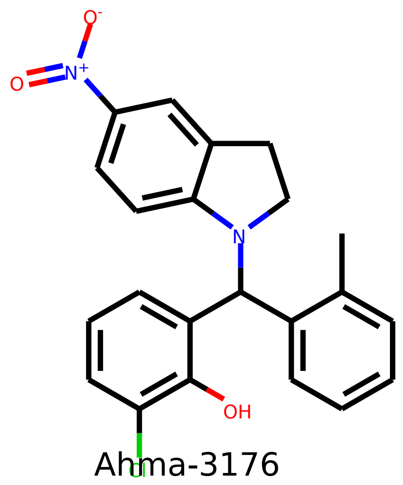

Ahma-3176

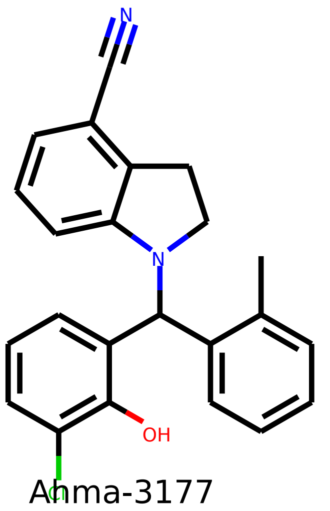

Ahma-3177

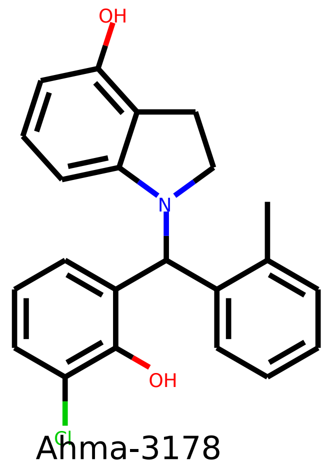

Ahma-3178

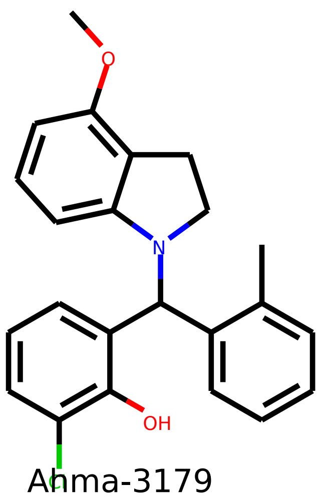

Ahma-3179

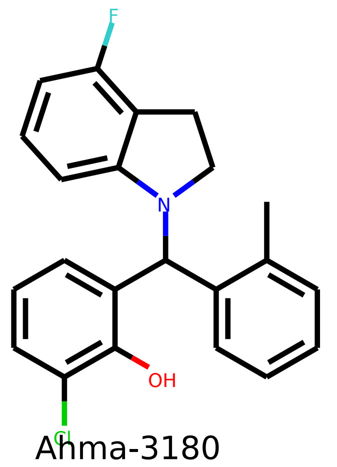

Ahma-3180

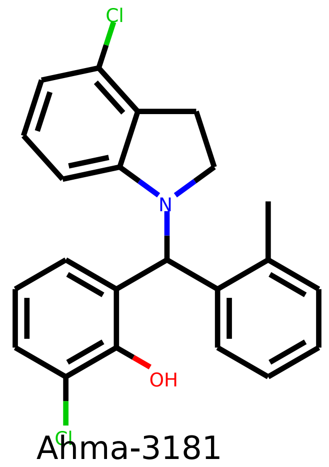

Ahma-3181

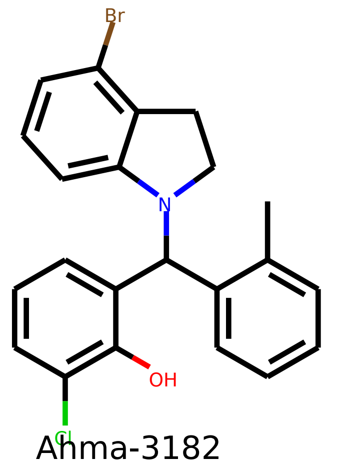

Ahma-3182

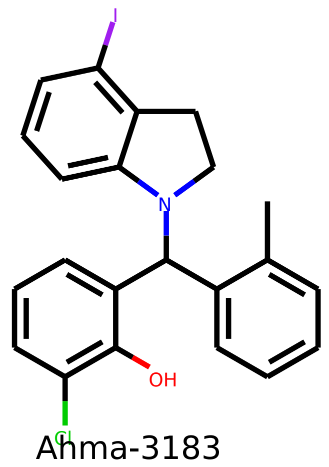

Ahma-3183

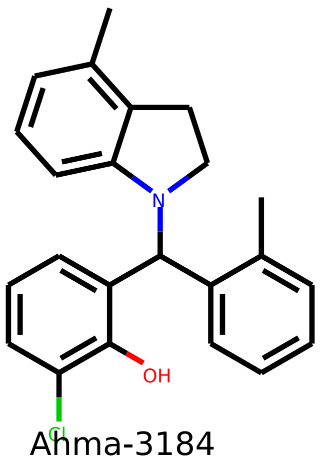

Ahma-3184

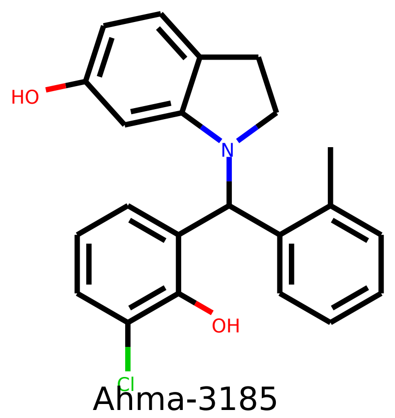

Ahma-3185

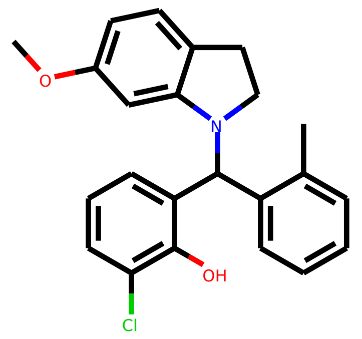

Ahma-3186

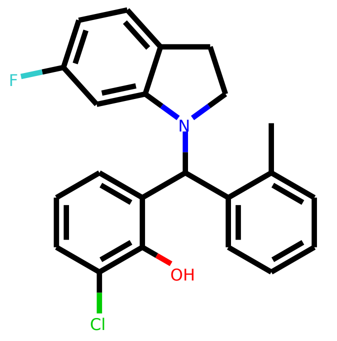

Ahma-3187

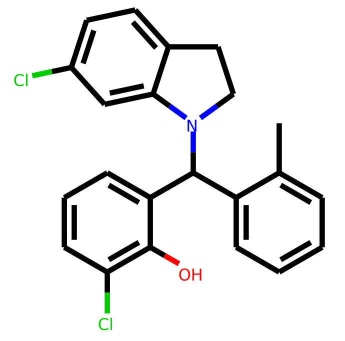

Ahma-3188

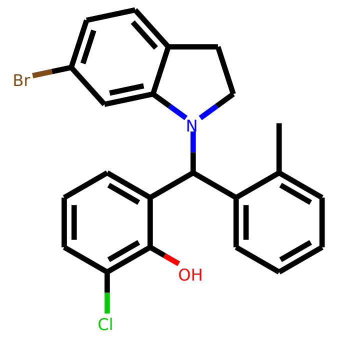

Ahma-3189

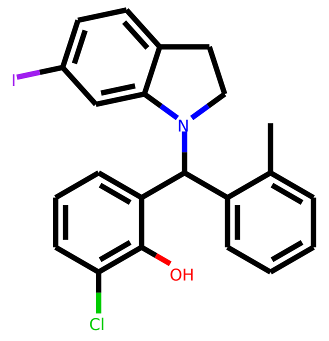

Ahma-3190

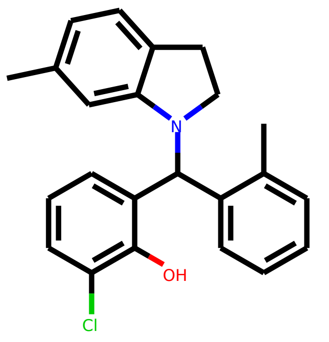

Ahma-3191

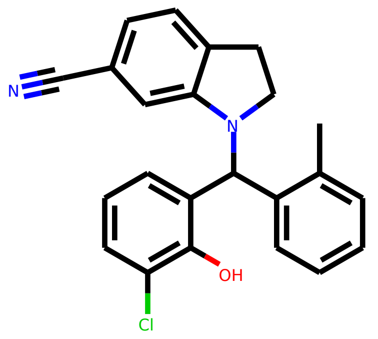

Ahma-3192

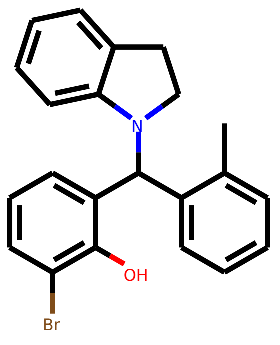

Ahma-3193

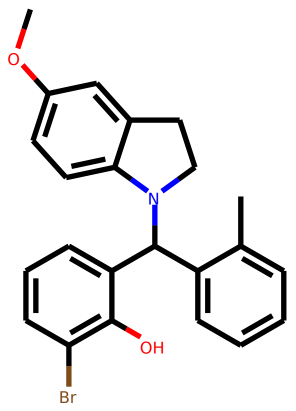

Ahma-3194

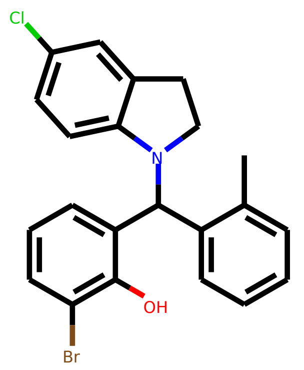

Ahma-3195

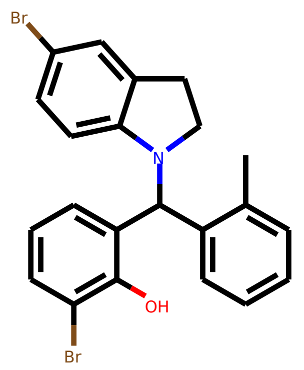

Ahma-3196

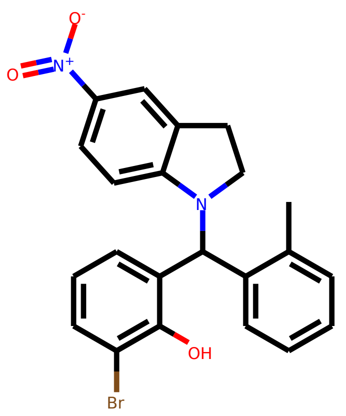

Ahma-3197

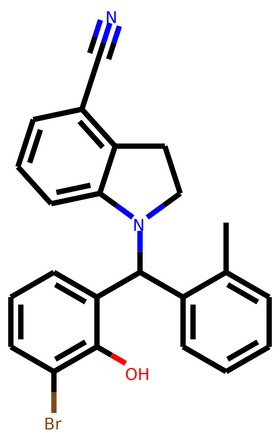

Ahma-3198

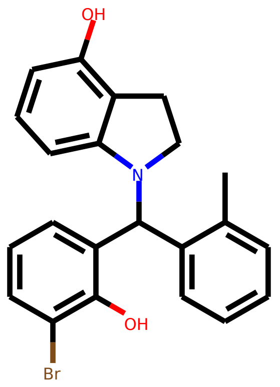

Ahma-3199

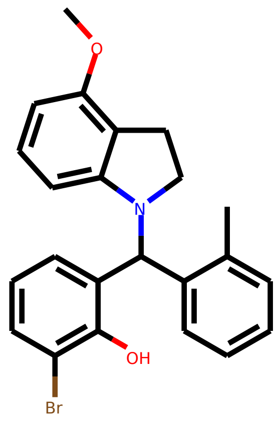

Ahma-3200

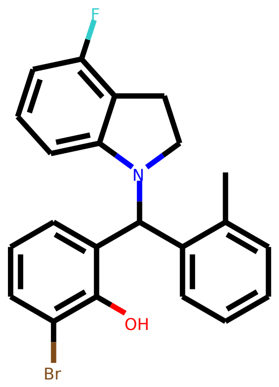

Ahma-3201

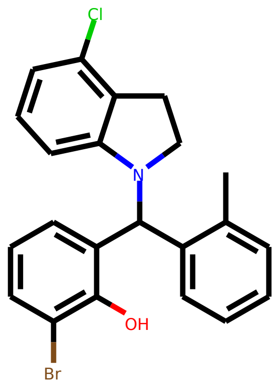

Ahma-3202

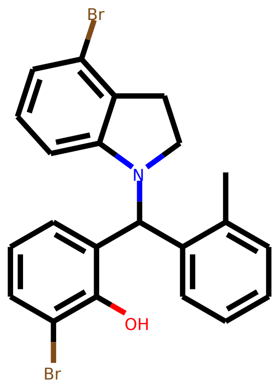

Ahma-3203

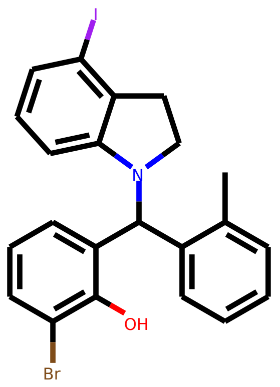

Ahma-3204

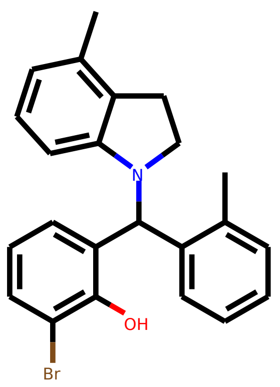

Ahma-3205

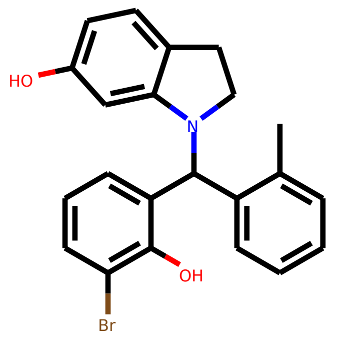

Ahma-3206

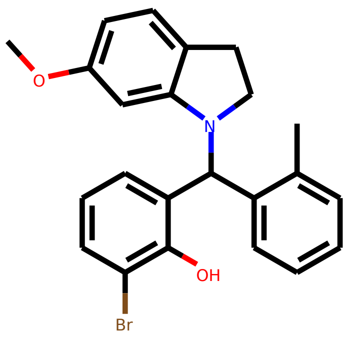

Ahma-3207

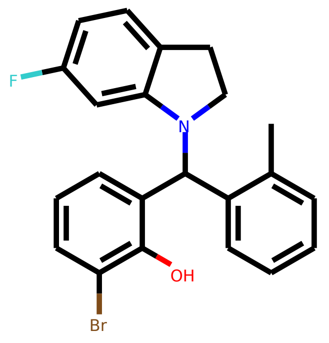

Ahma-3208

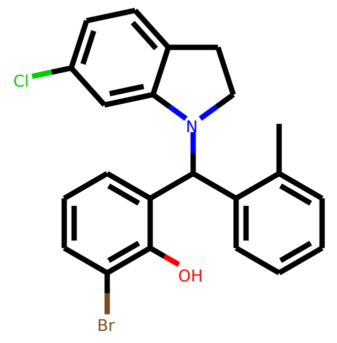

Ahma-3209

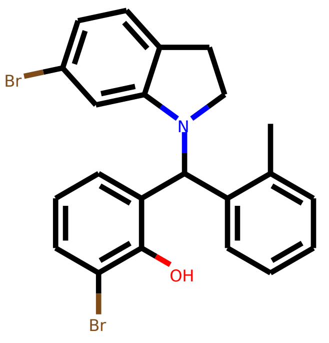

Ahma-3210

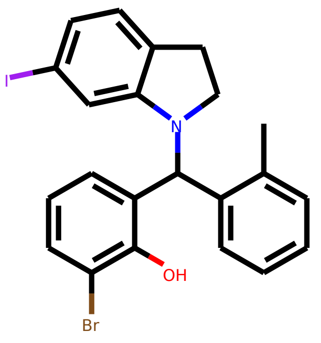

Ahma-3211

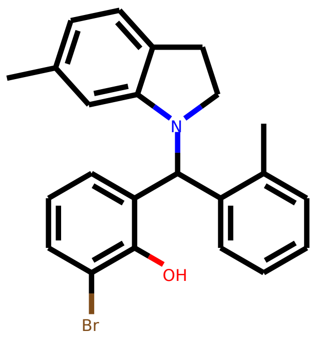

Ahma-3212

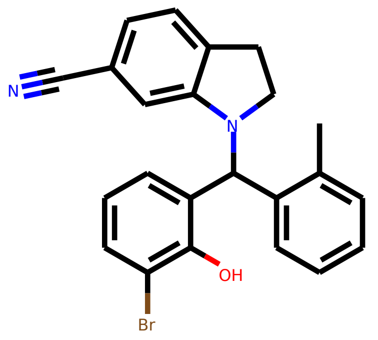

Ahma-3213

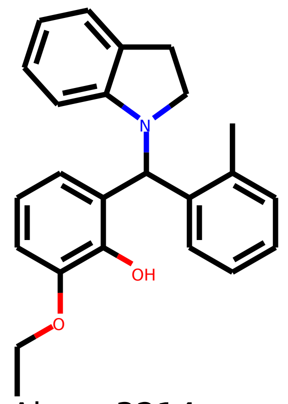

Ahma-3214

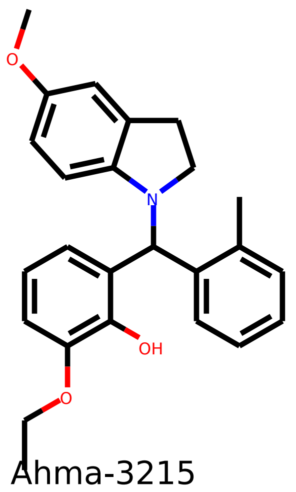

Ahma-3215

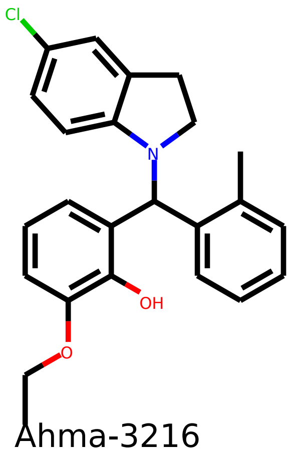

Ahma-3216

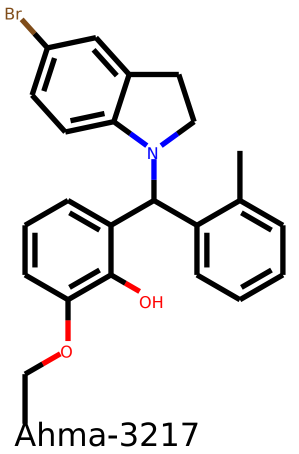

Ahma-3217

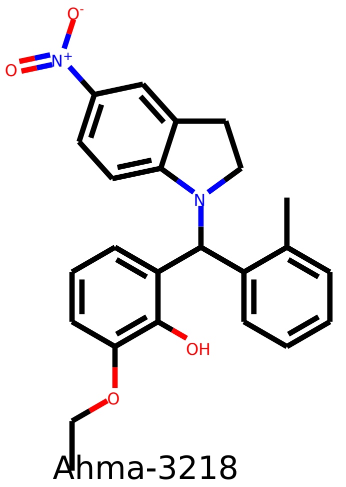

Ahma-3218

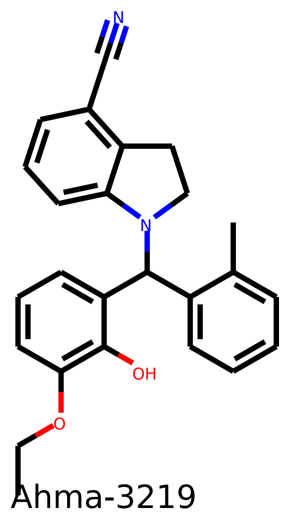

Ahma-3219

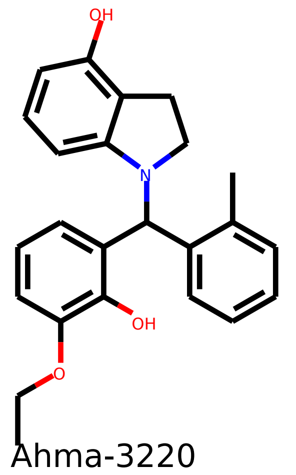

Ahma-3220

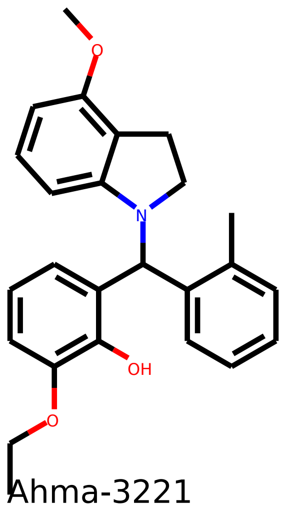
Ahma-3221
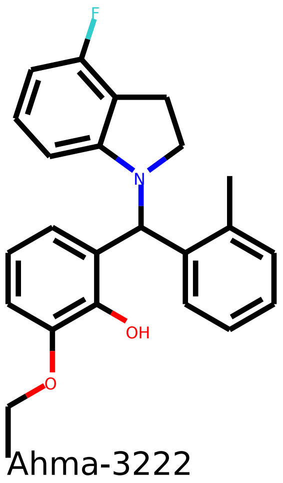
Ahma-3222
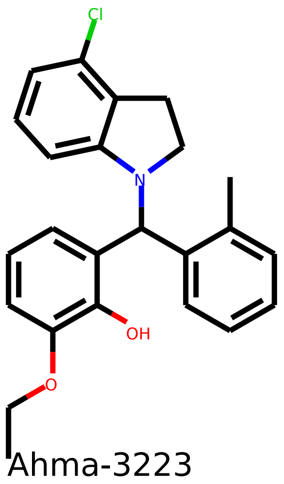
Ahma-3223
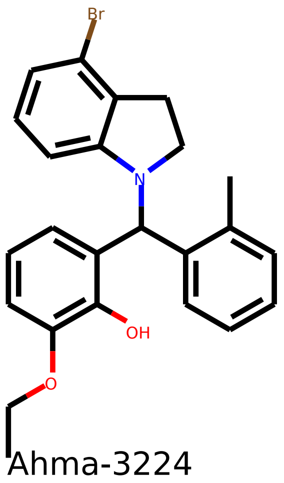
Ahma-3224
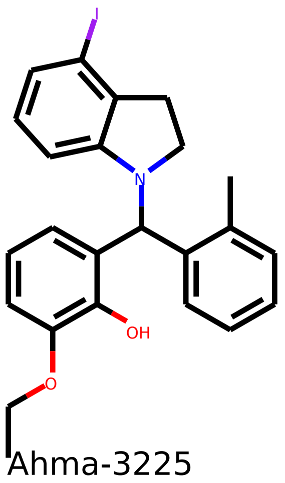
Ahma-3225
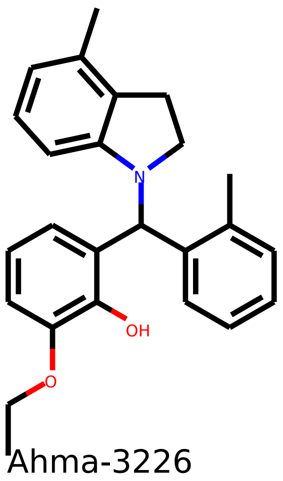
Ahma-3226
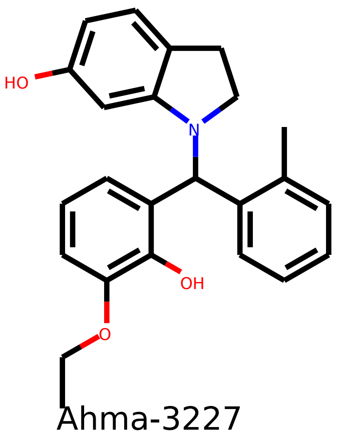
Ahma-3227
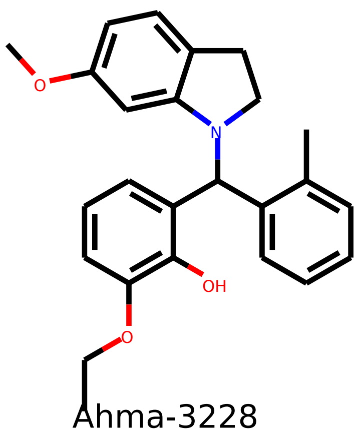
Ahma-3228
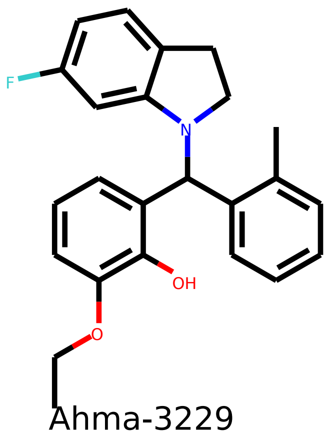
Ahma-3229
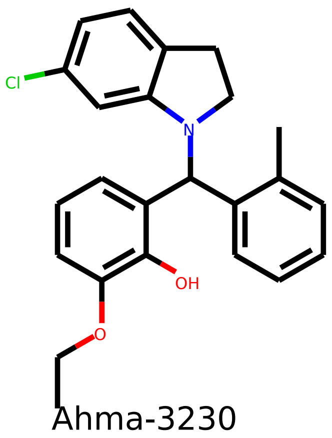
Ahma-3230
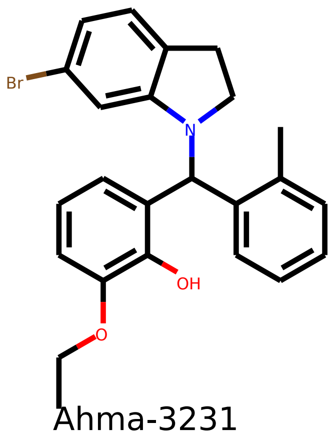
Ahma-3231
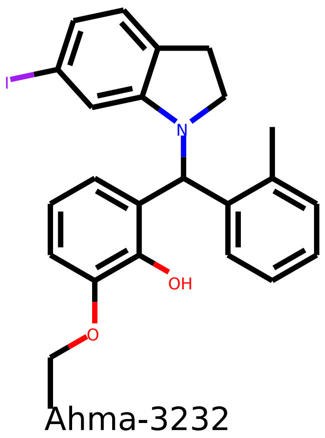
Ahma-3232
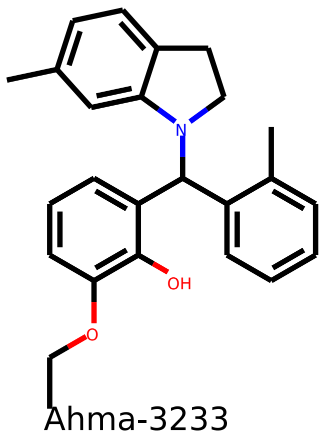
Ahma-3233
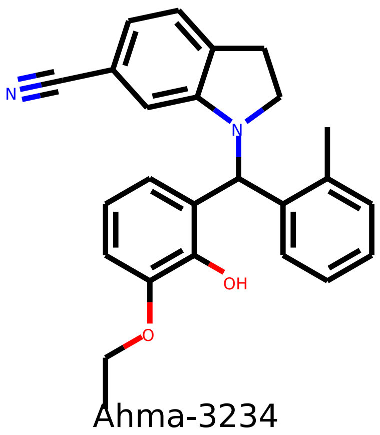
Ahma-3234

Set 2

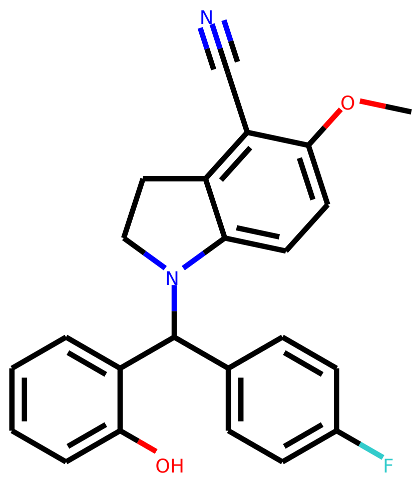
Osma-1
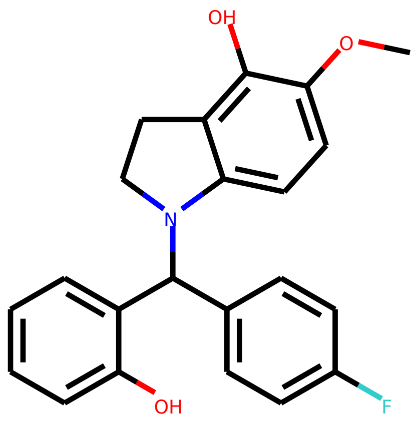
Osma-2
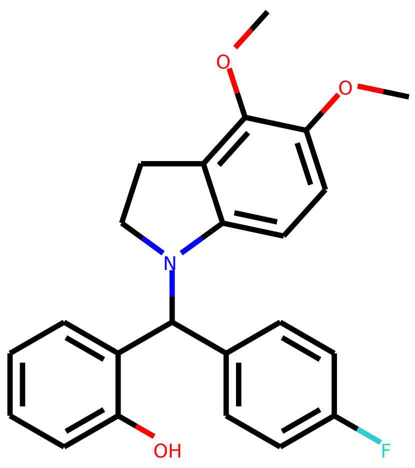
Osma-3
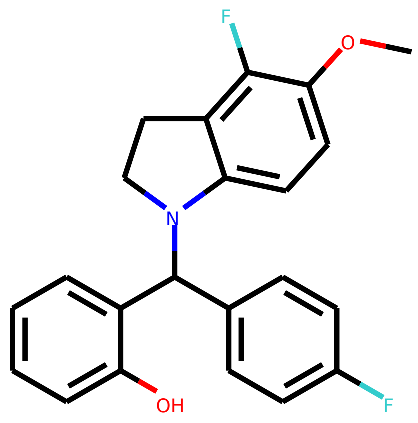
Osma-4
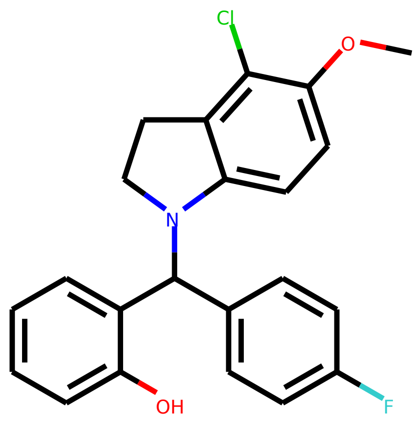
Osma-5
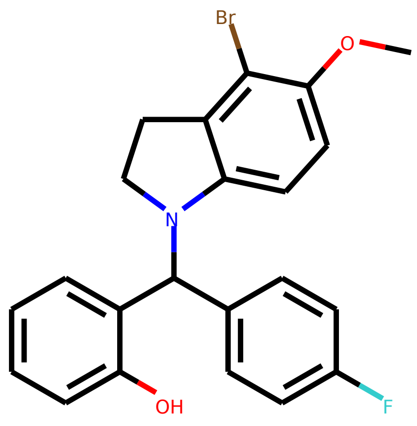
Osma-6
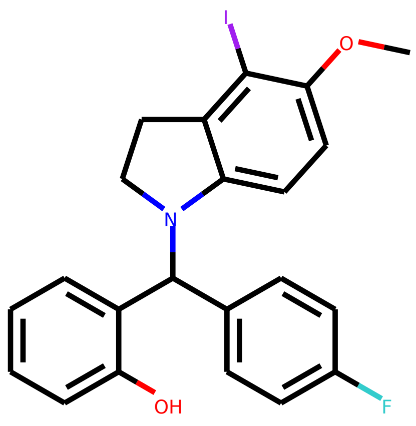
Osma-7
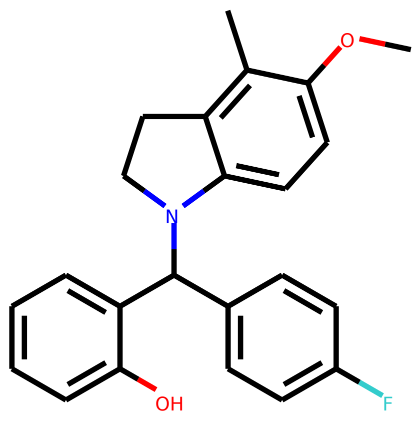
Osma-8
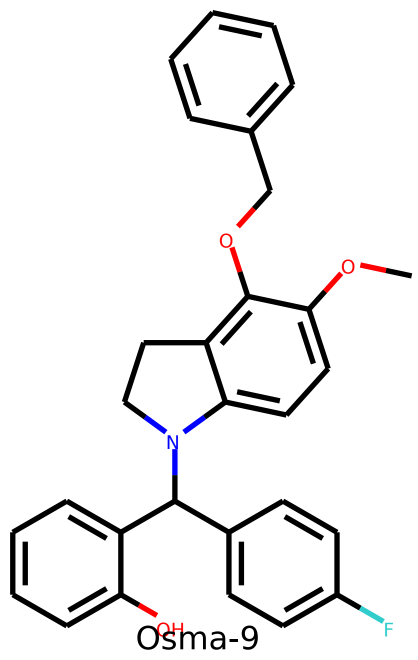
Osma-9
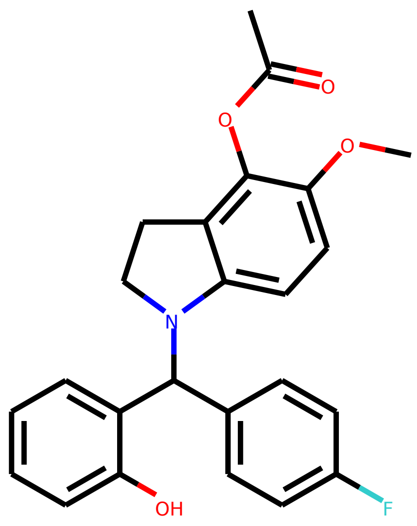
Osma-10
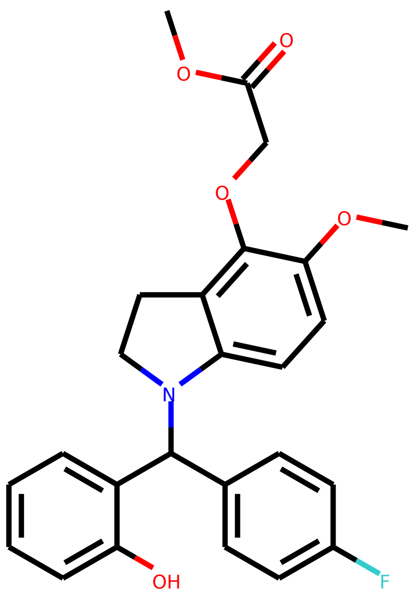
Osma-11
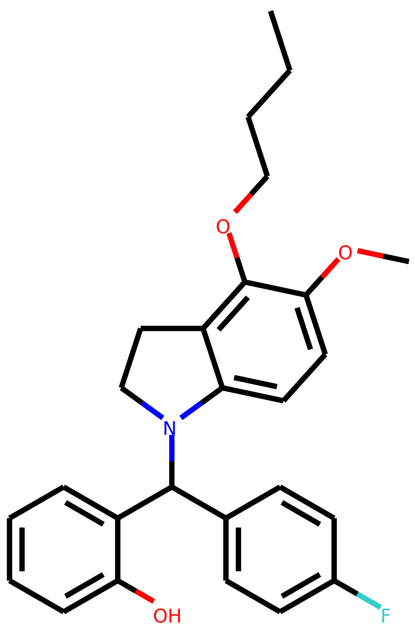
Osma-12
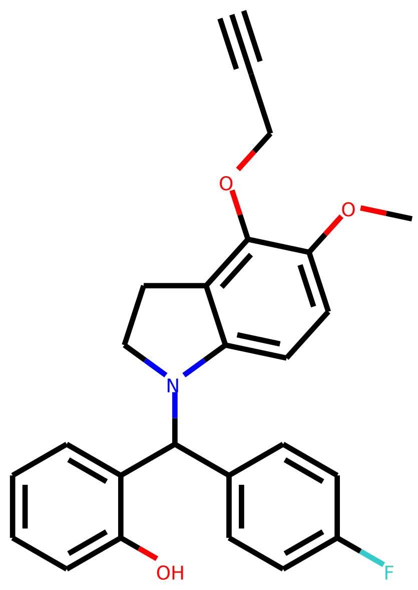
Osma-13
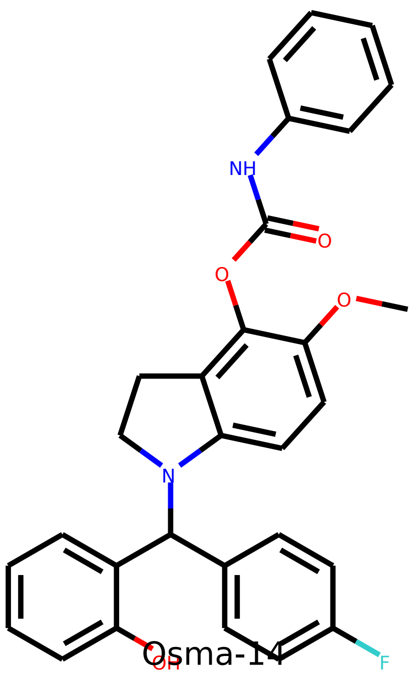
Osma-14
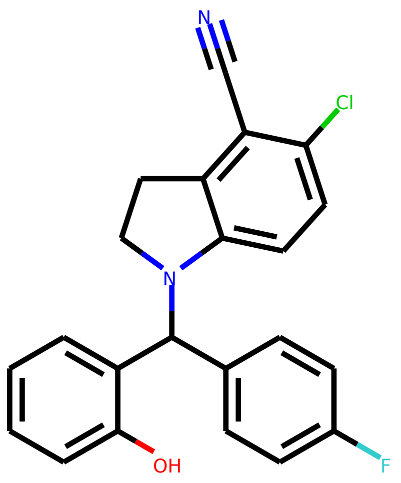
Osma-15
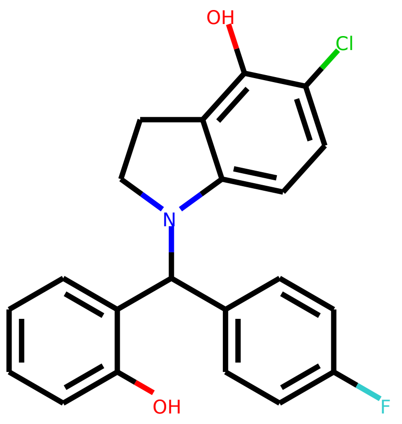
Osma-16
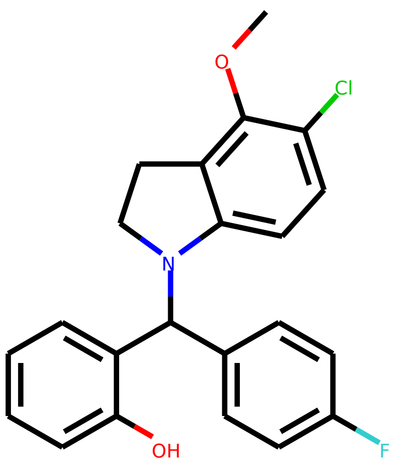
Osma-17
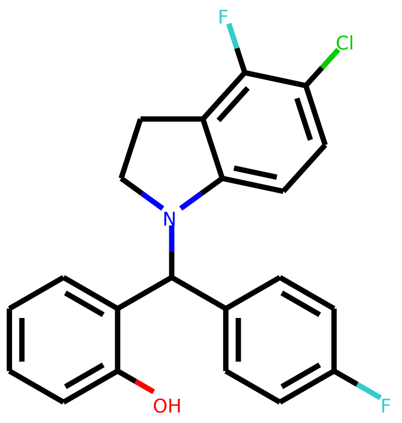
Osma-18
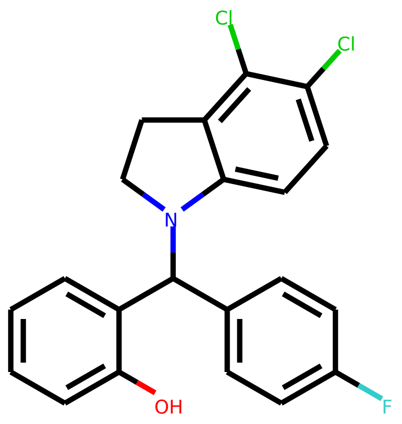
Osma-19
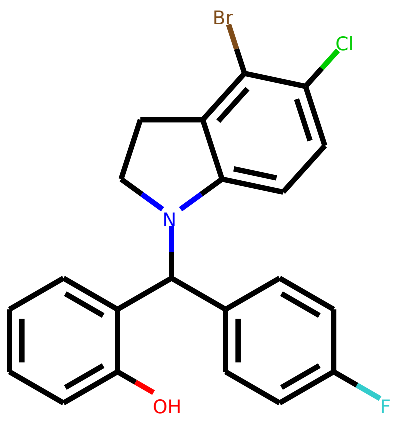
Osma-20
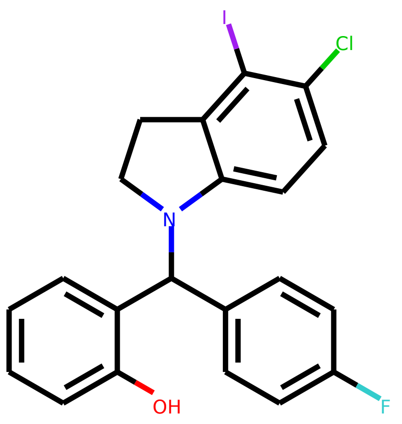
Osma-21
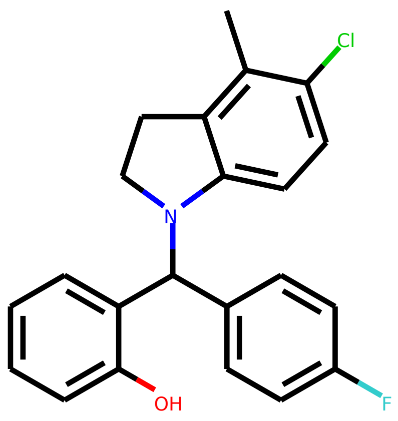
Osma-22
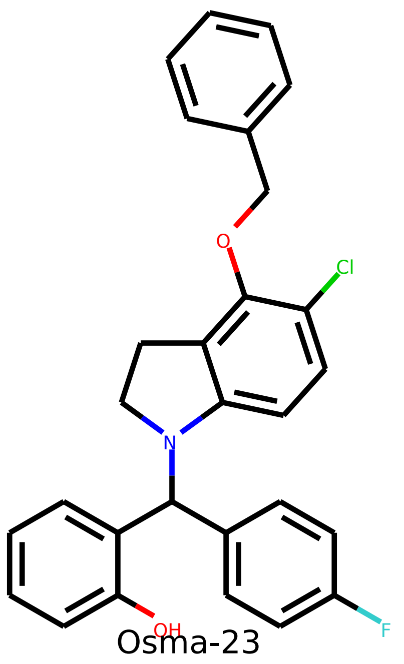
Osma-23
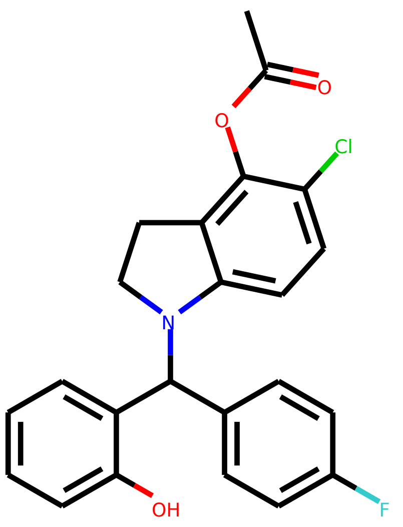
Osma-24
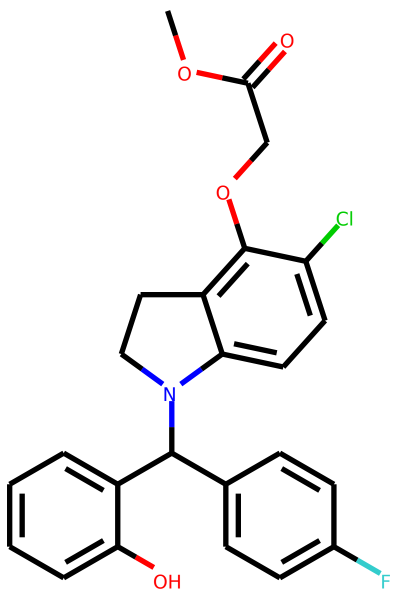
Osma-25
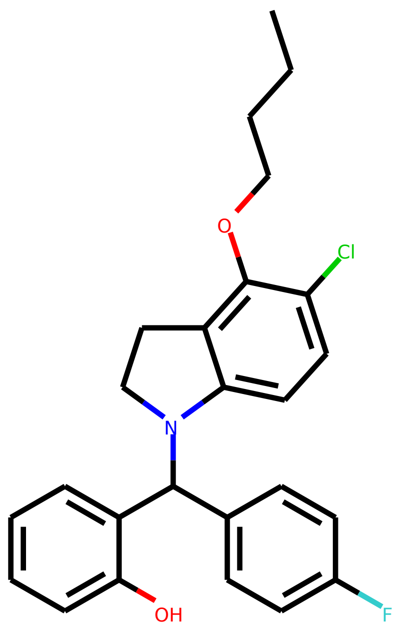
Osma-26
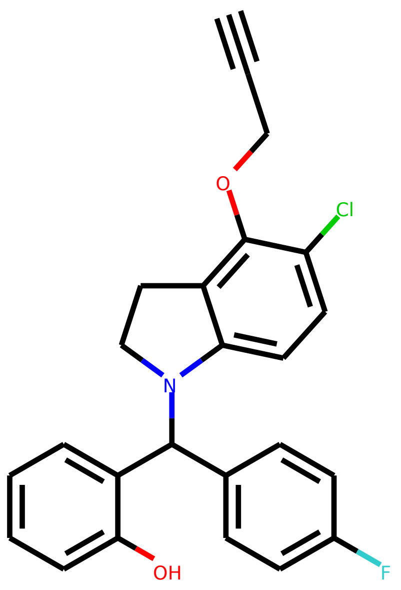
Osma-27
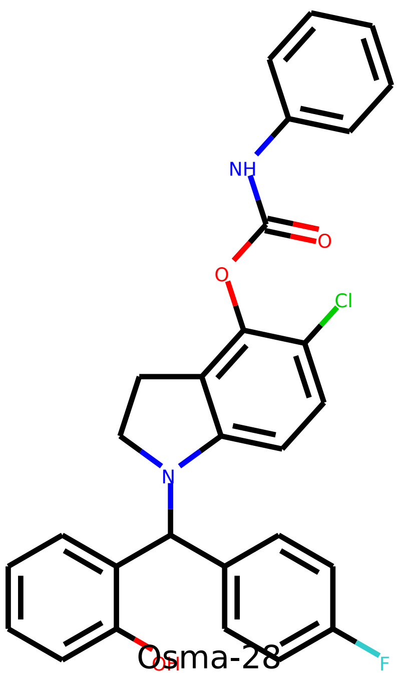
Osma-28
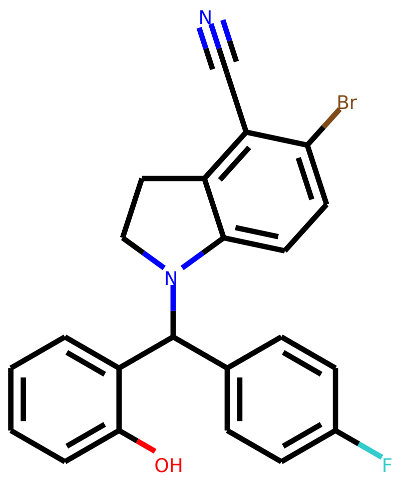
Osma-29
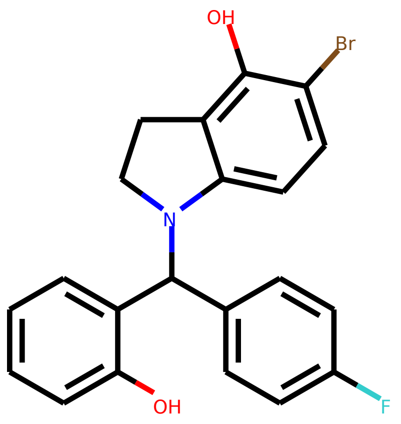
Osma-30
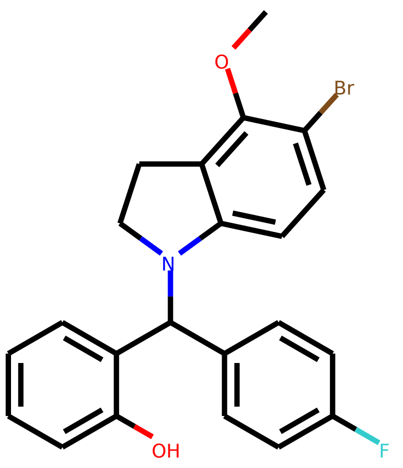
Osma-31
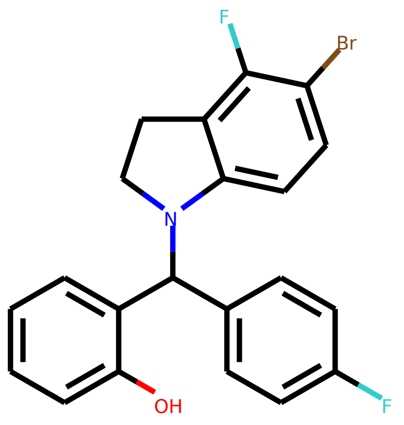
Osma-32
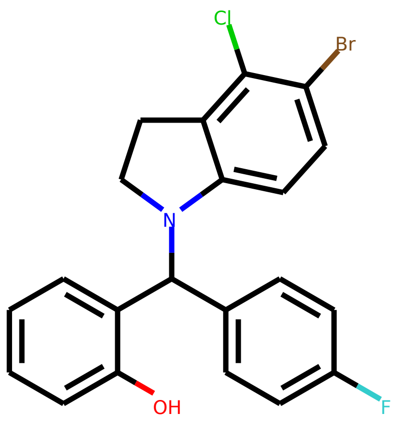
Osma-33
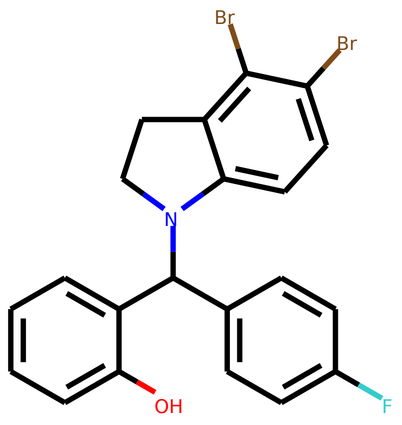
Osma-34
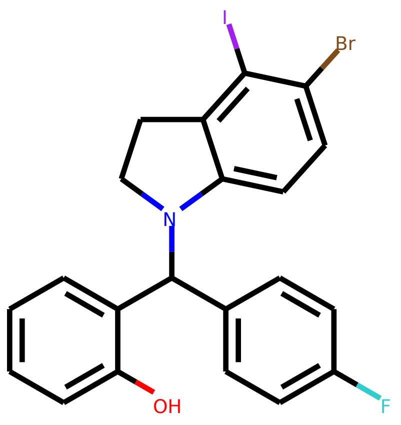
Osma-35

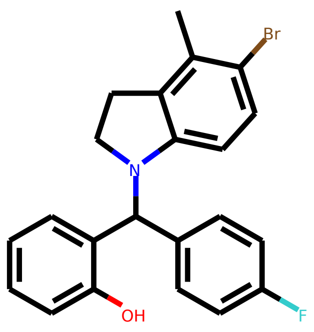
Osma-36

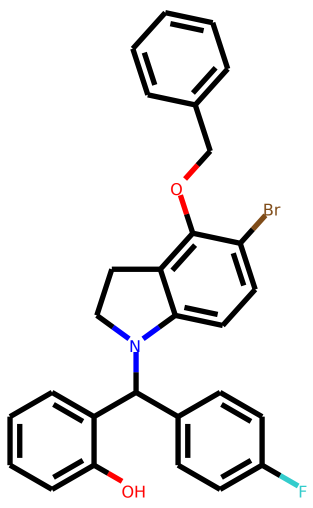
Osma-37

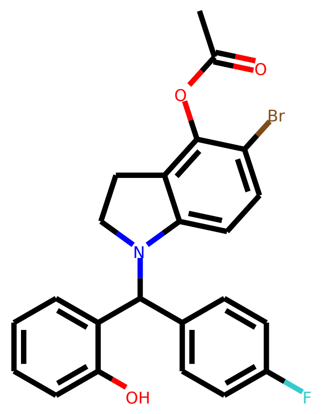
Osma-38

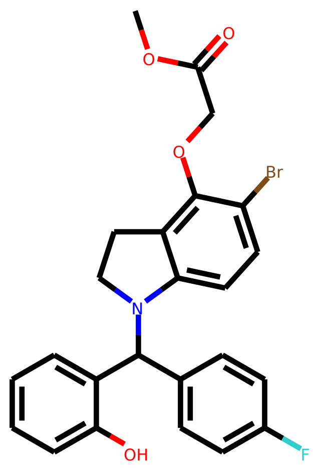
Osma-39

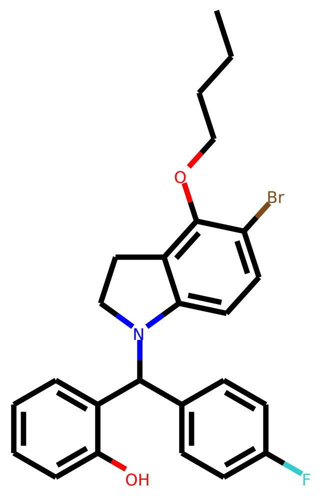
Osma-40

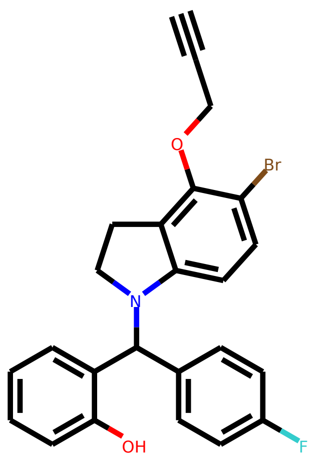
Osma-41

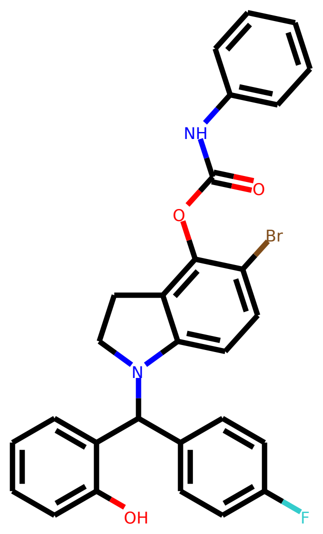
Osma-42

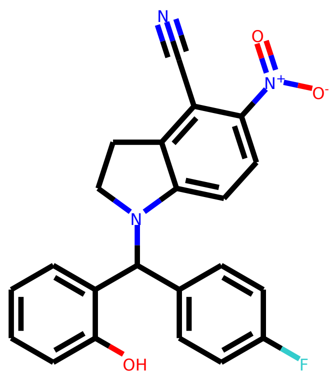
Osma-43

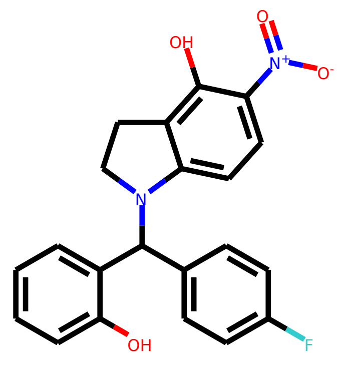
Osma-44

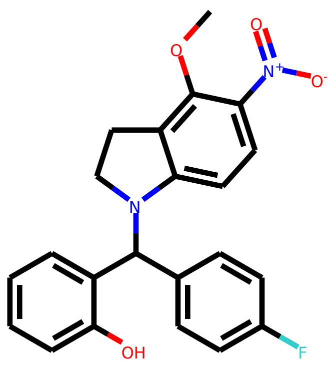
Osma-45

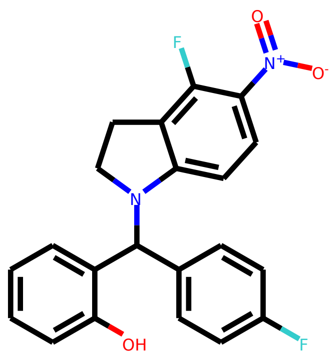
Osma-46

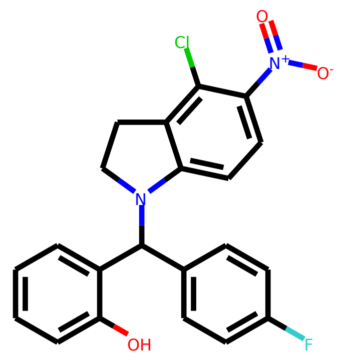
Osma-47

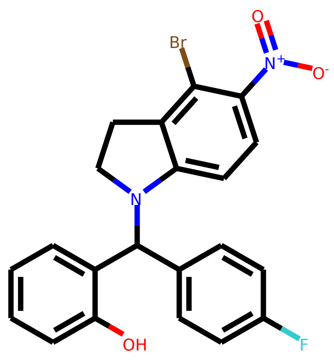
Osma-48

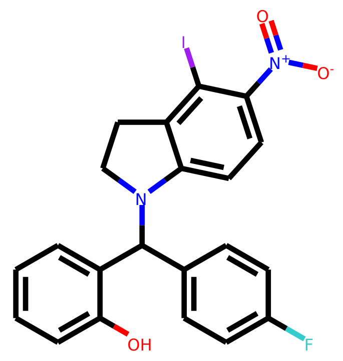
Osma-49

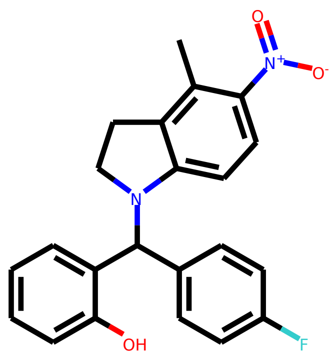
Osma-50

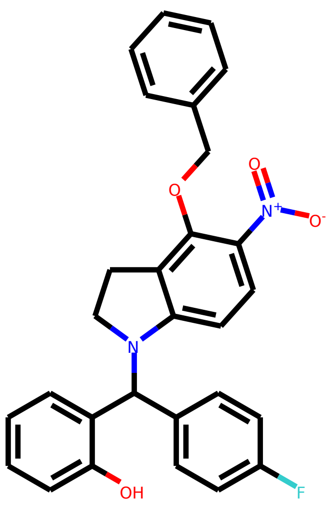
Osma-51

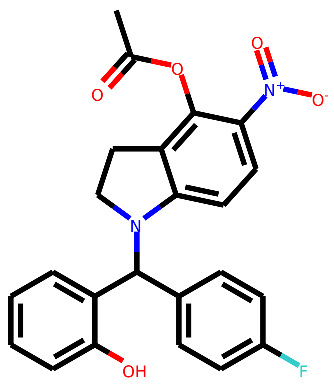
Osma-52

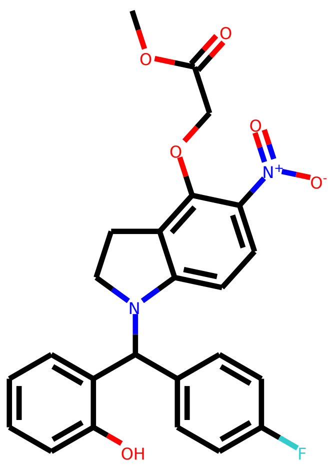
Osma-53

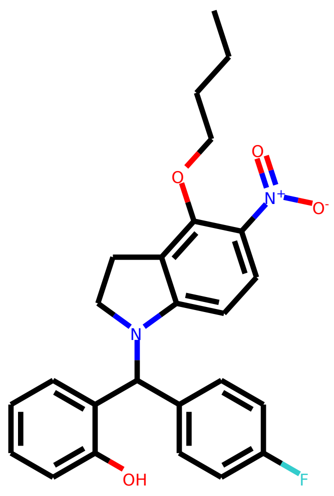
Osma-54

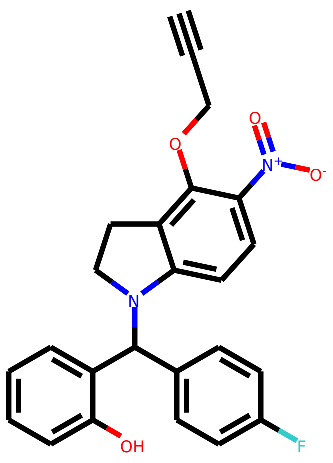
Osma-55

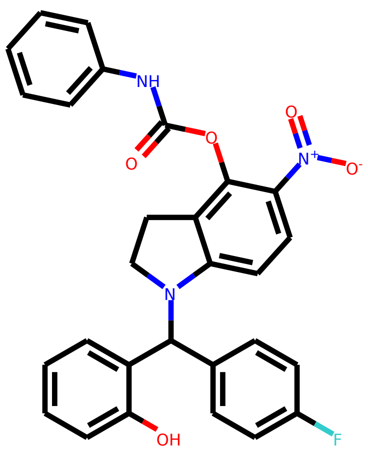
Osma-56

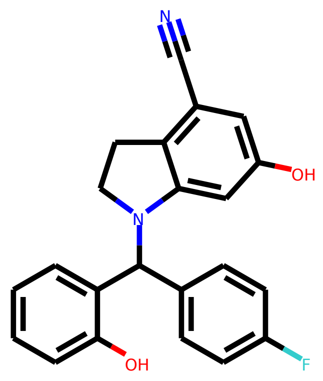
Osma-57

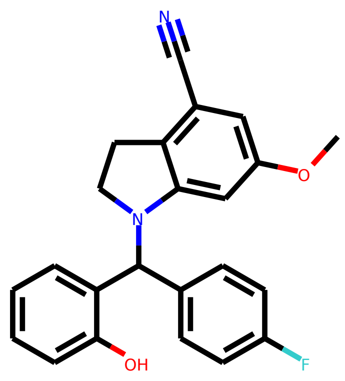
Osma-58

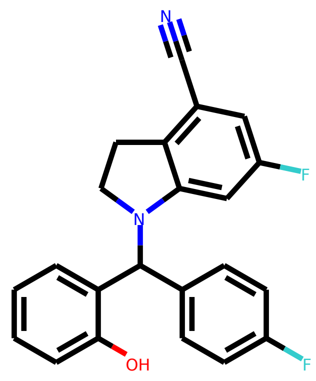
Osma-59

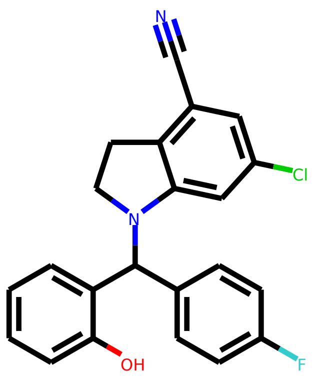
Osma-60

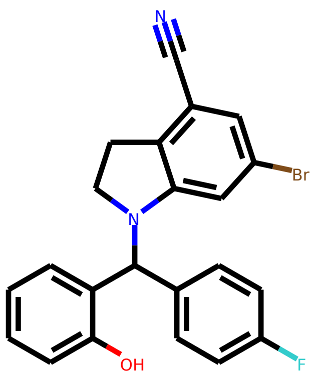
Osma-61

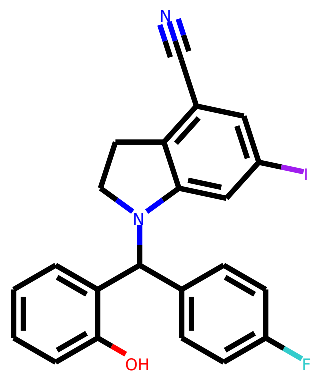
Osma-62

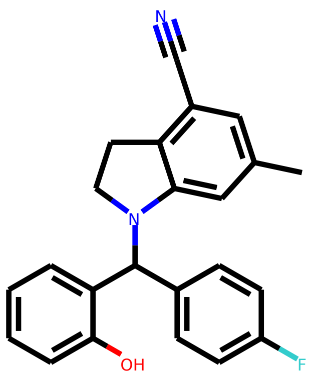
Osma-63

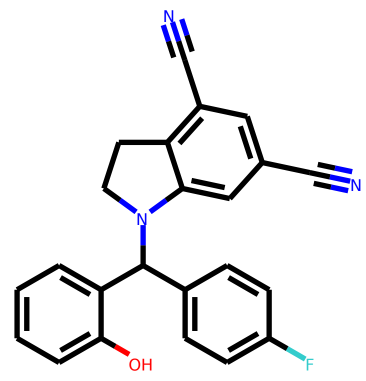
Osma-64

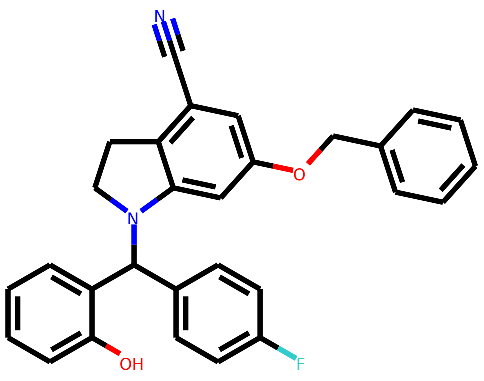
Osma-65

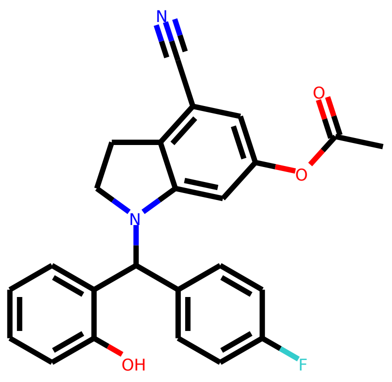
Osma-66

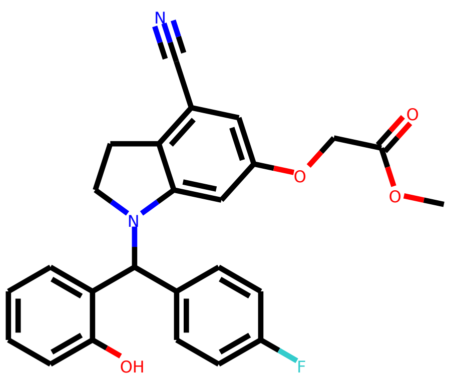
Osma-67

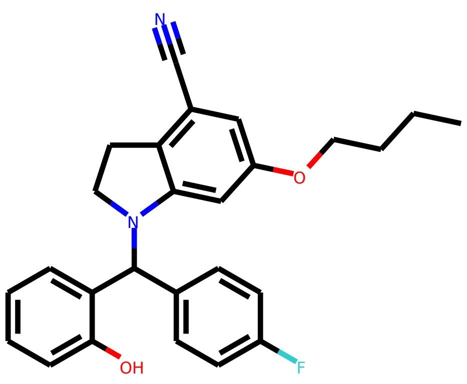
Osma-68

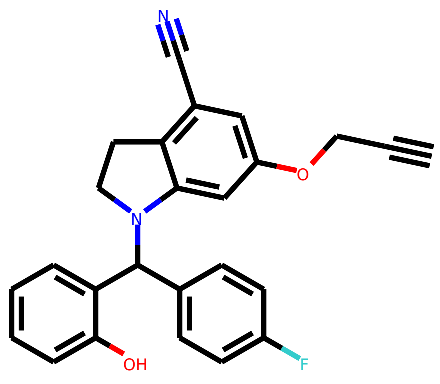
Osma-69

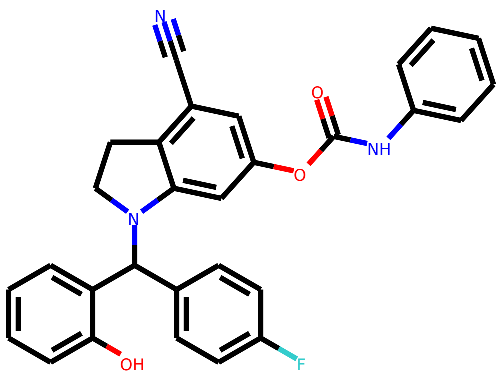
Osma-70

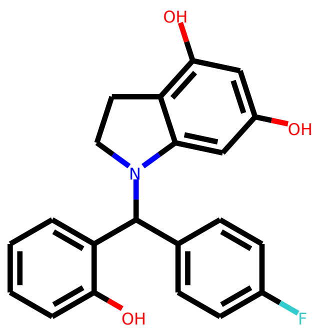
Osma-71

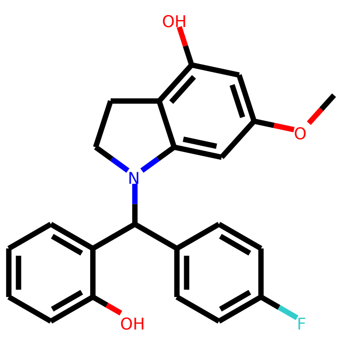
Osma-72

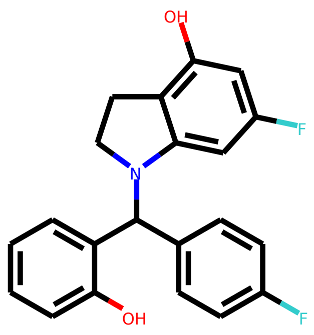
Osma-73

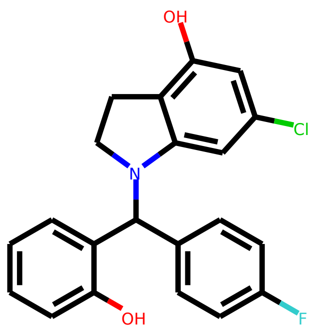
Osma-74

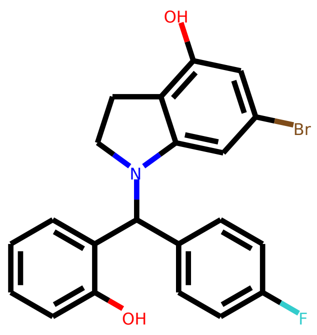
Osma-75

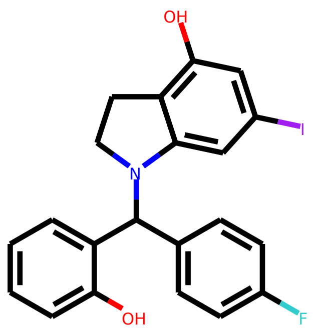
Osma-76

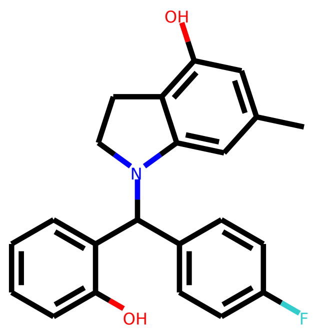
Osma-77

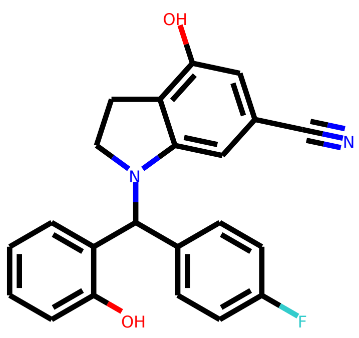
Osma-78

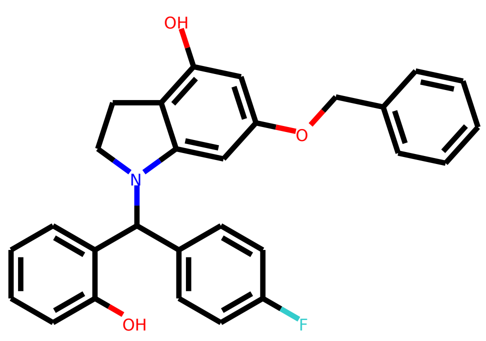
Osma-79

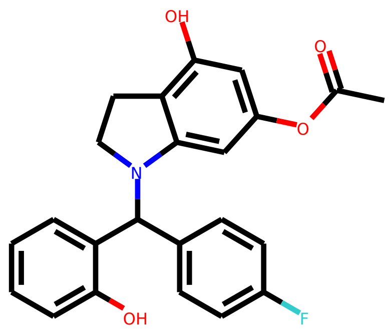
Osma-80

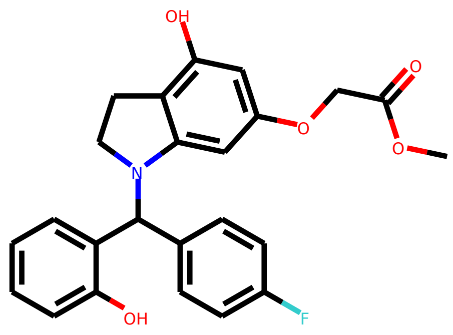
Osma-81

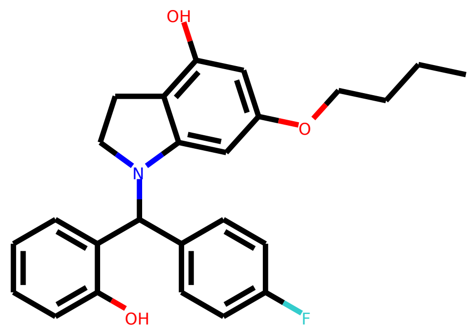
Osma-82

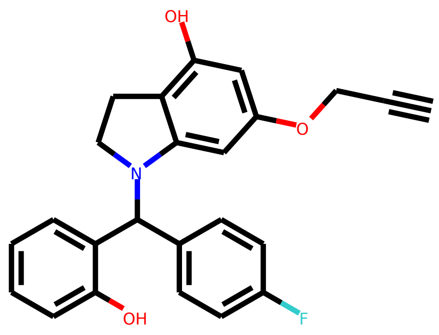
Osma-83

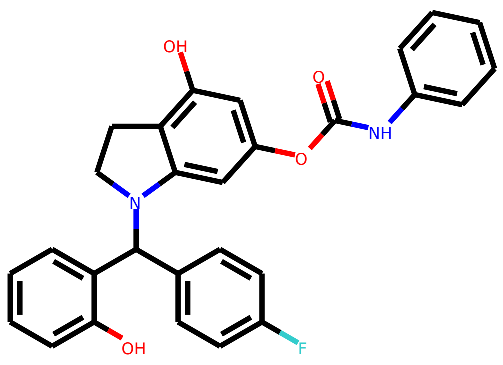
Osma-84

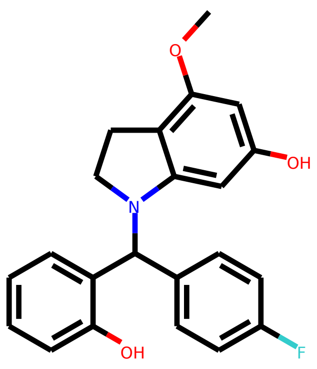
Osma-85

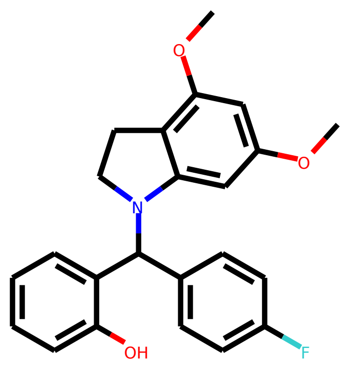
Osma-86

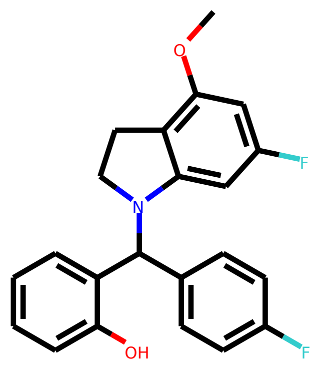
Osma-87

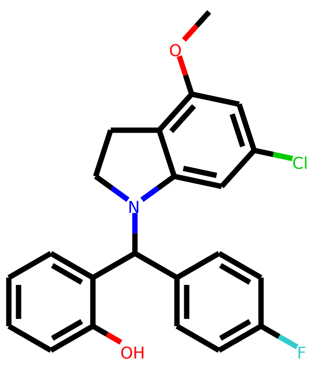
Osma-88

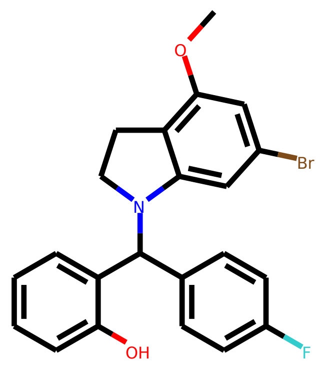
Osma-89

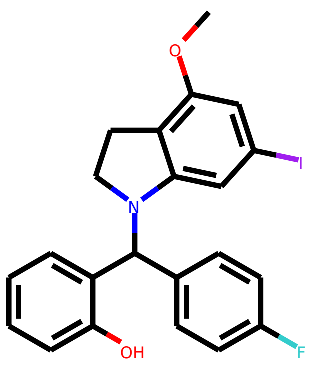
Osma-90

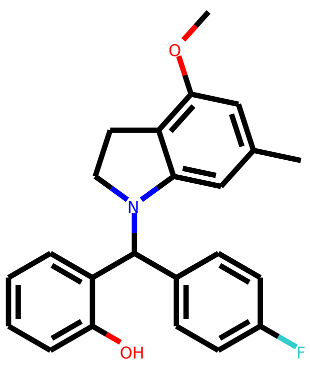
Osma-91

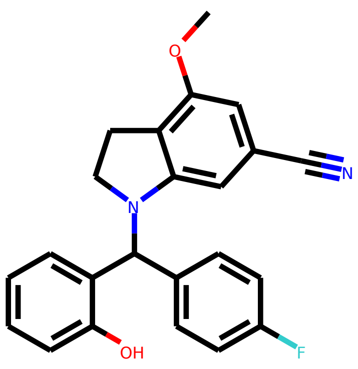
Osma-92

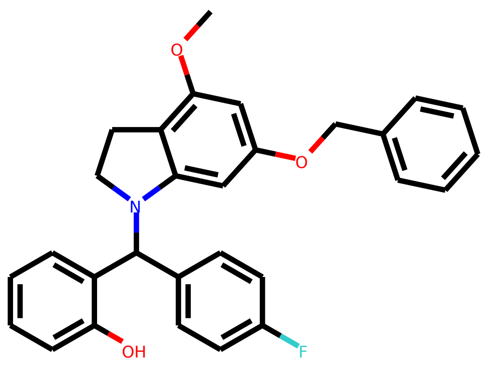
Osma-93

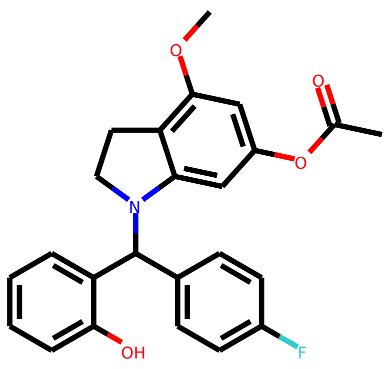
Osma-94

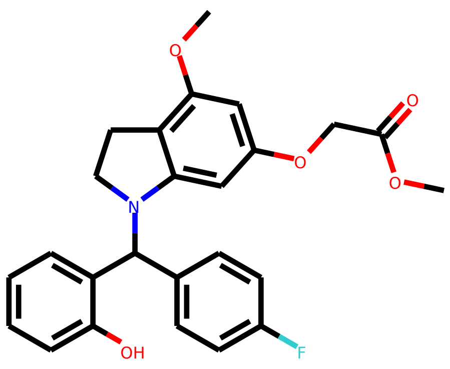
Osma-95

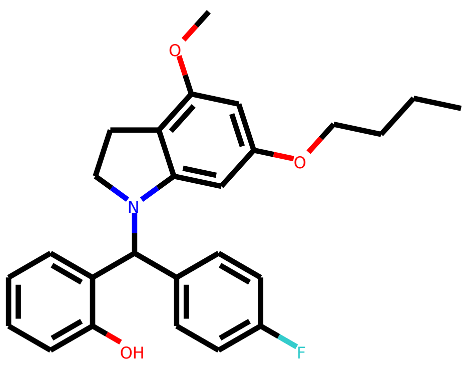
Osma-96

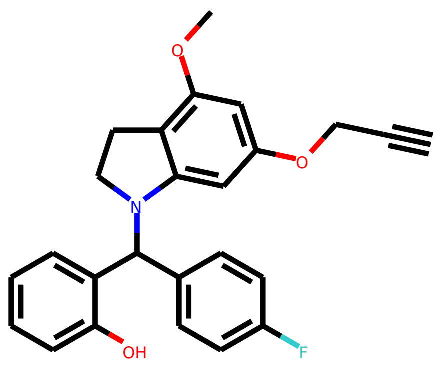
Osma-97

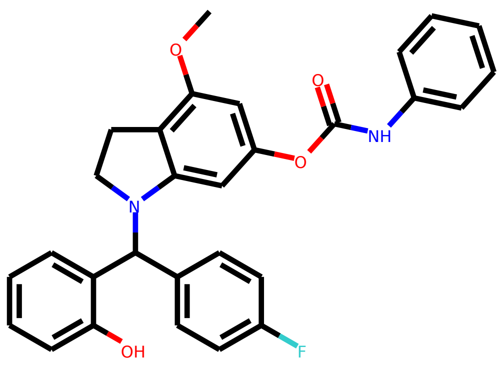
Osma-98

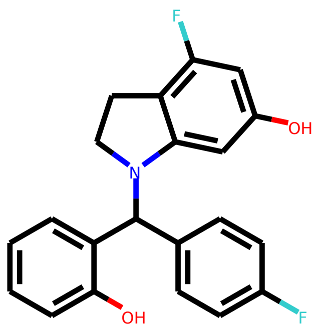
Osma-99

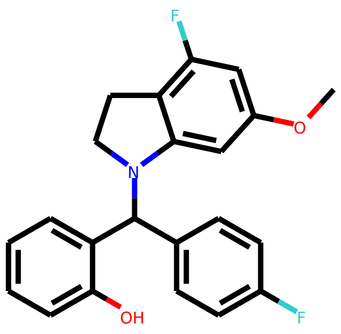
Osma-100

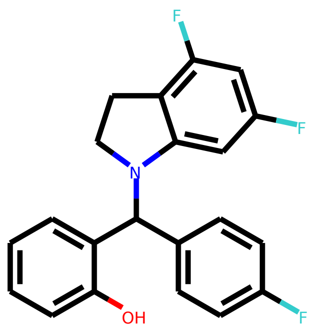
Osma-101

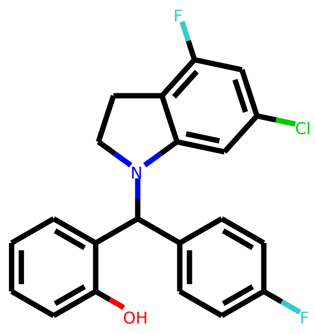
Osma-102

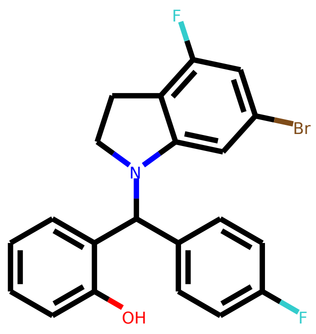
Osma-103

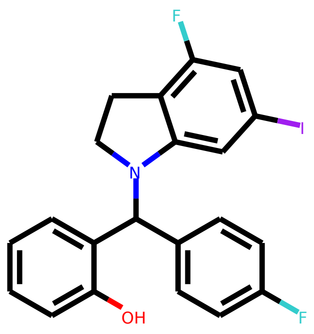
Osma-104

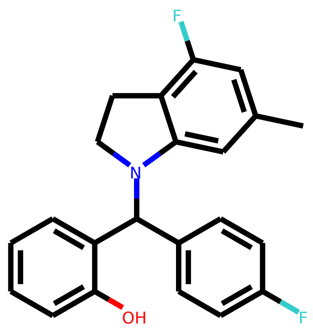
Osma-105

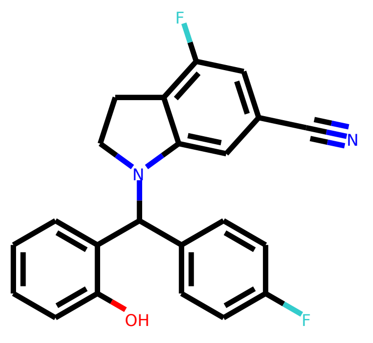
Osma-106
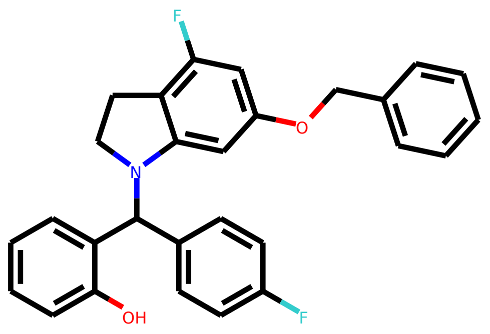
Osma-107
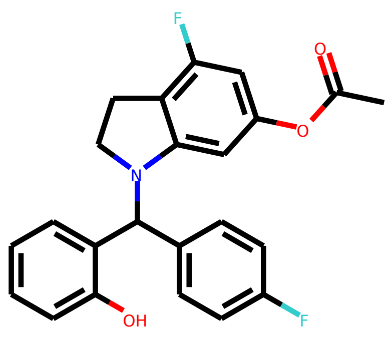
Osma-108
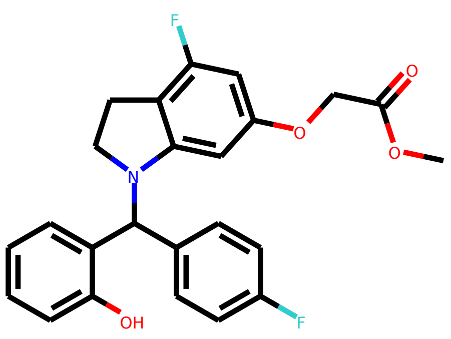
Osma-109
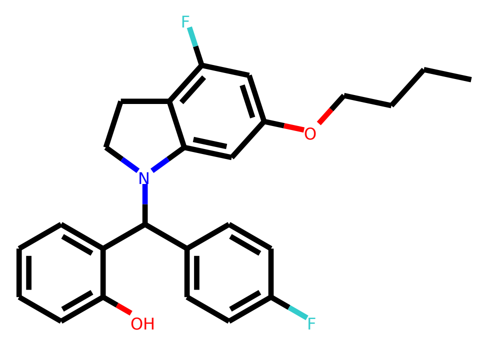
Osma-110
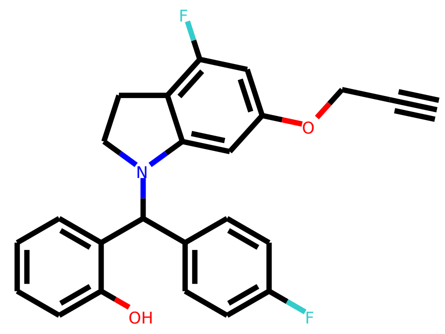
Osma-111
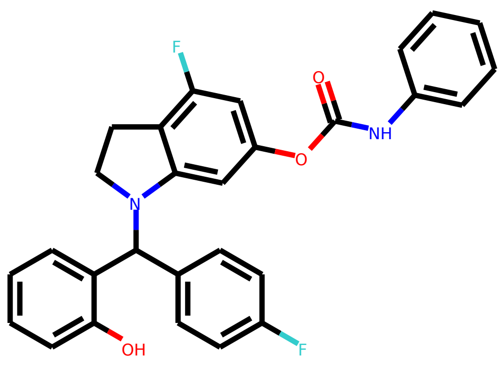
Osma-112
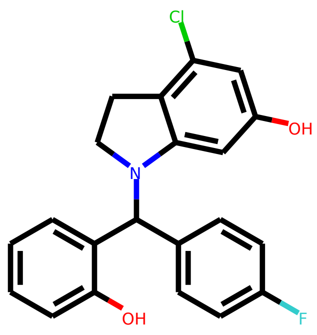
Osma-113
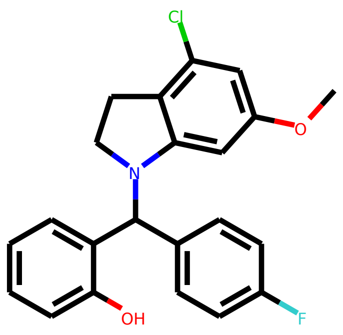
Osma-114
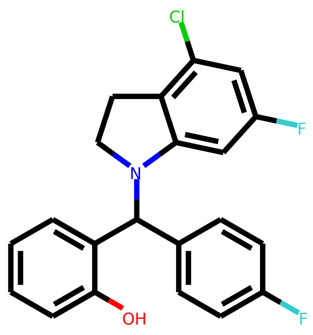
Osma-115
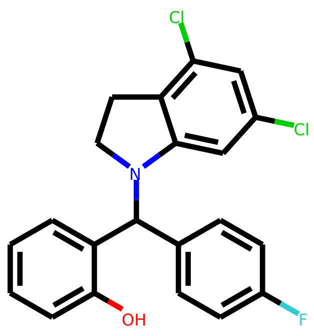
Osma-116
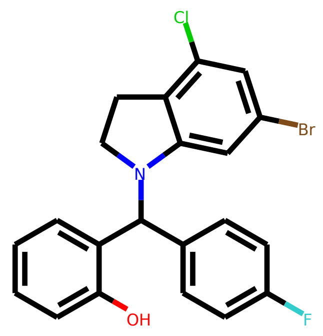
Osma-117
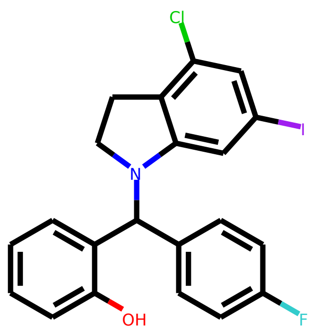
Osma-118
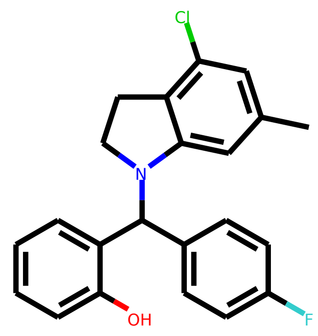
Osma-119
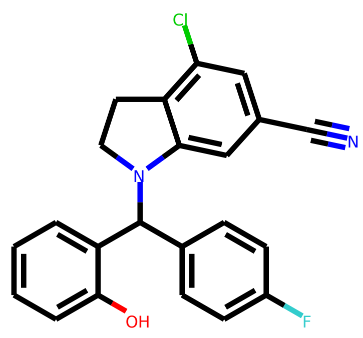
Osma-120
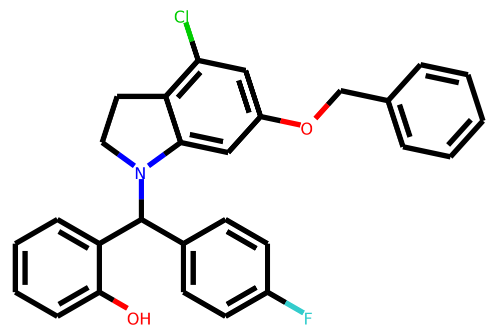
Osma-121
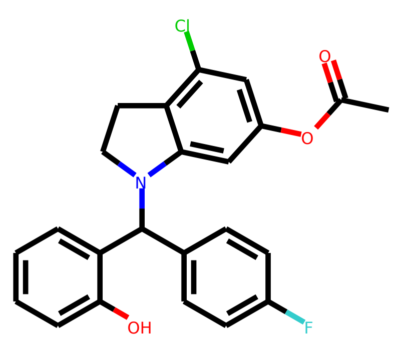
Osma-122
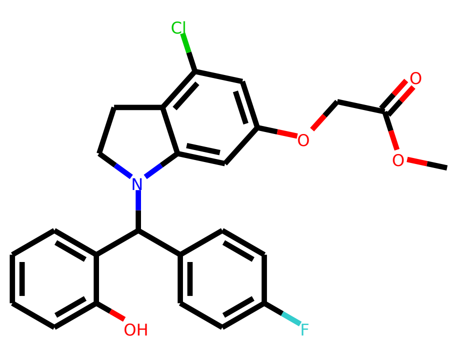
Osma-123
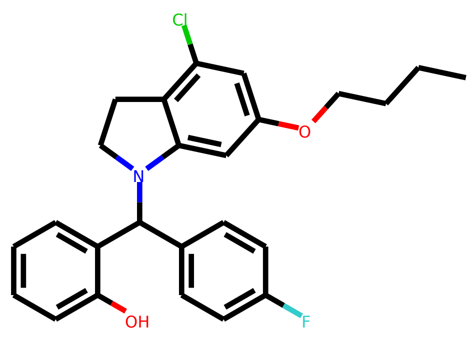
Osma-124
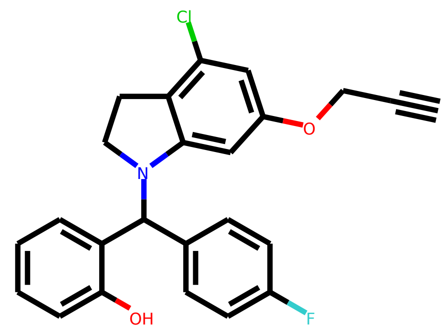
Osma-125
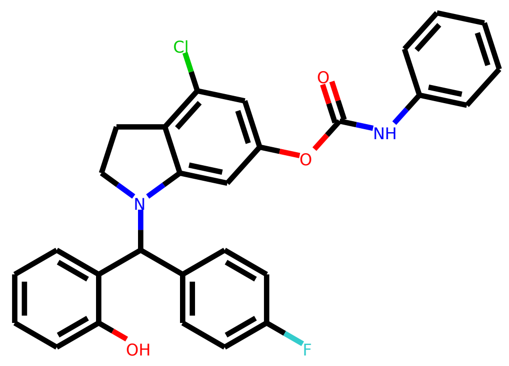
Osma-126
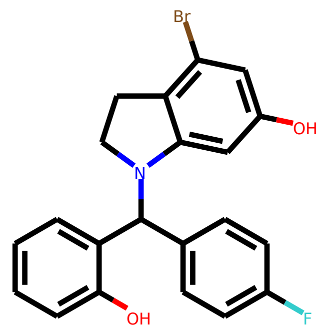
Osma-127
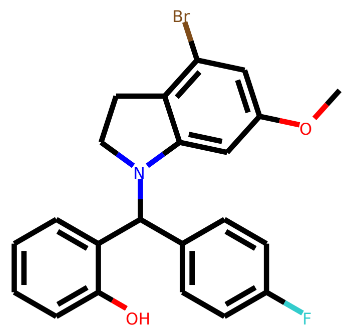
Osma-128
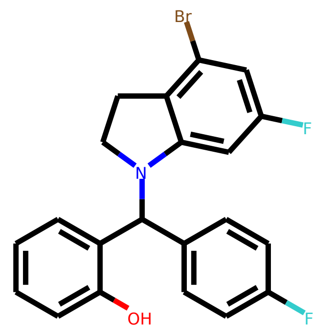
Osma-129
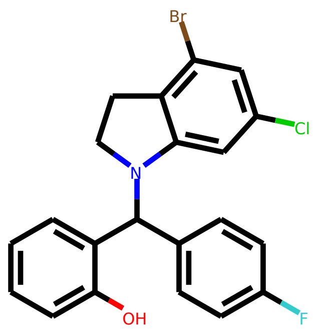
Osma-130
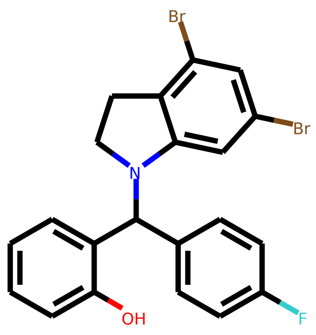
Osma-131
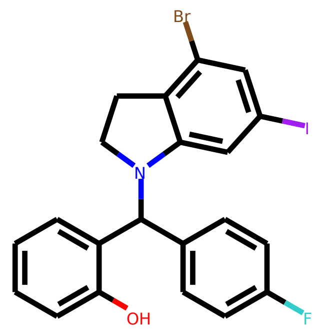
Osma-132
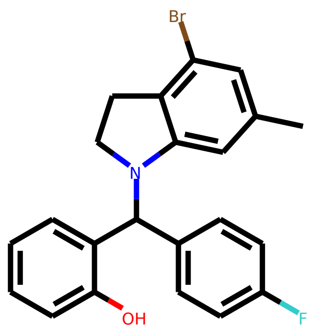
Osma-133
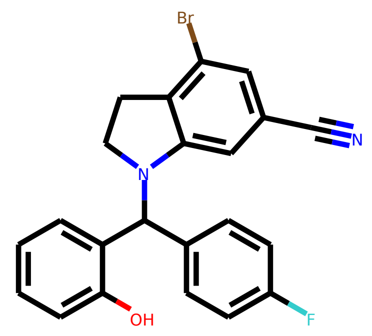
Osma-134
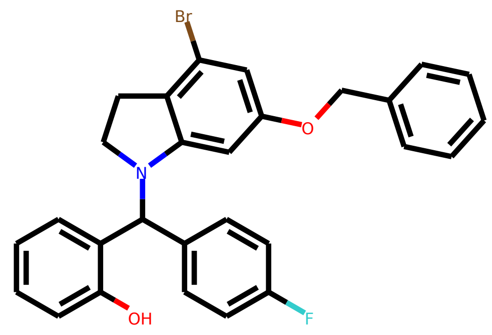
Osma-135
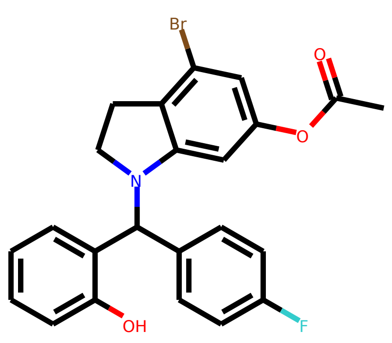
Osma-136
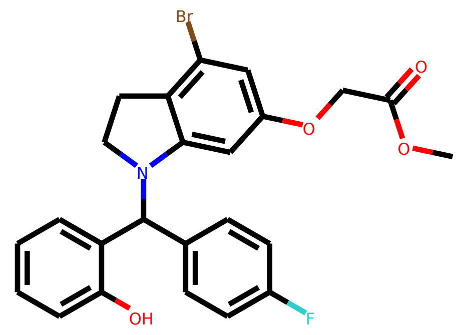
Osma-137
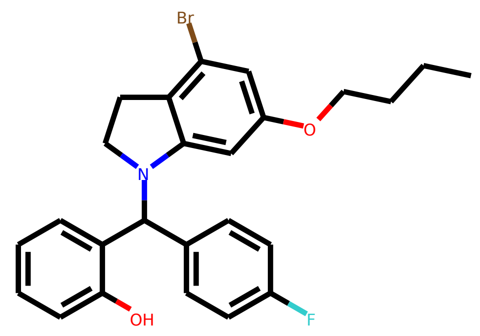
Osma-138
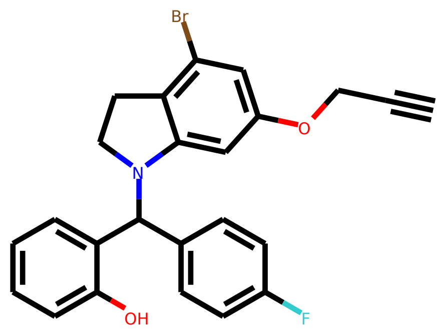
Osma-139
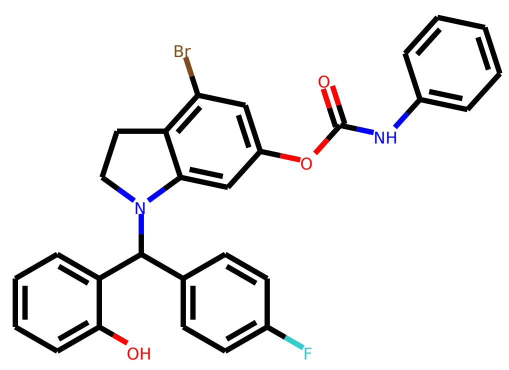
Osma-140

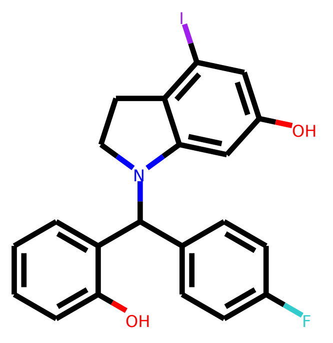

Osma-141

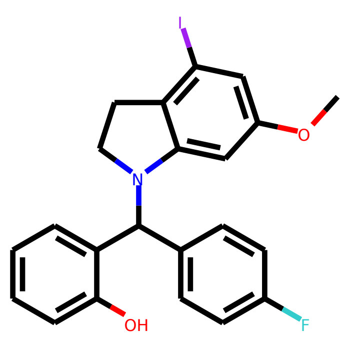

Osma-142

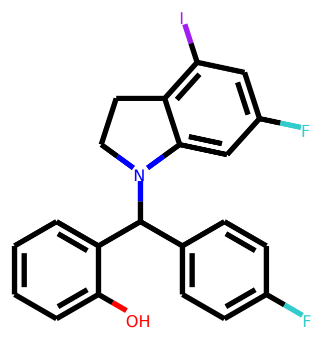

Osma-143

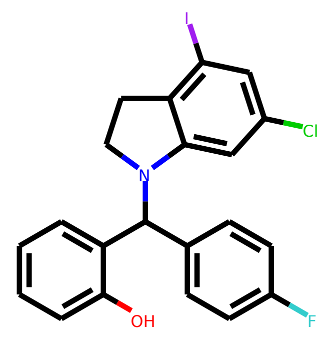

Osma-144

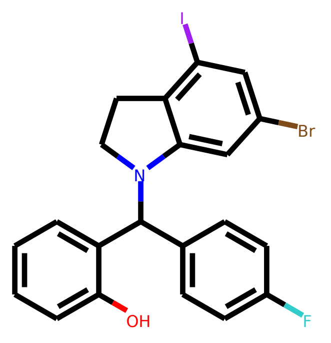

Osma-145

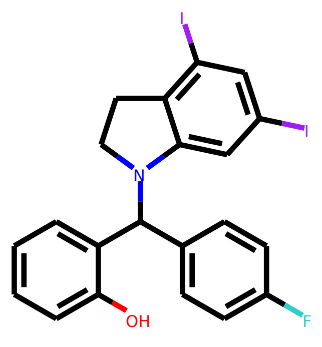

Osma-146

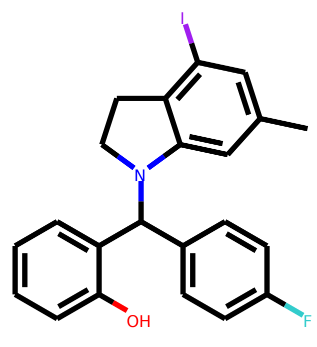

Osma-147

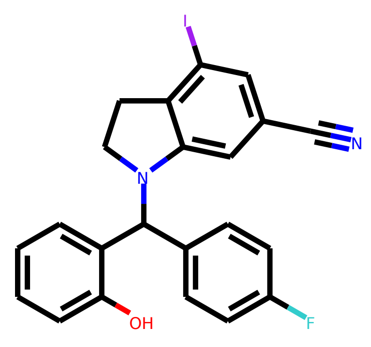

Osma-148

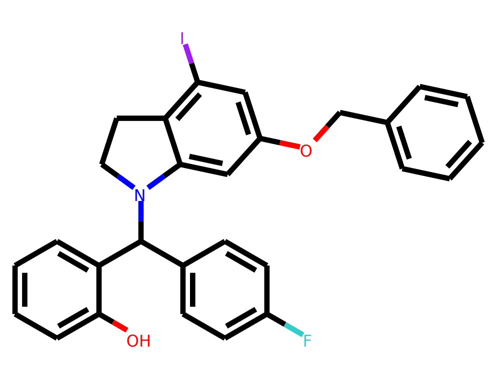

Osma-149

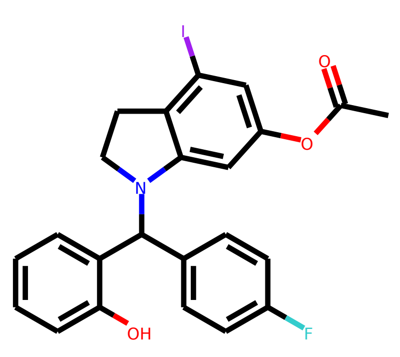

Osma-150

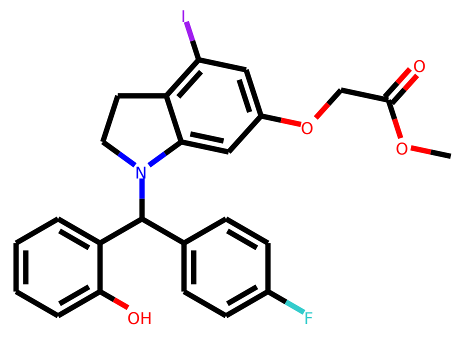

Osma-151

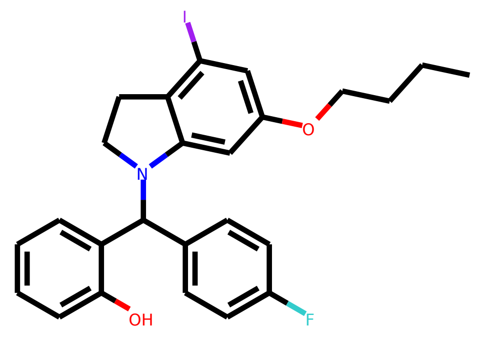

Osma-152

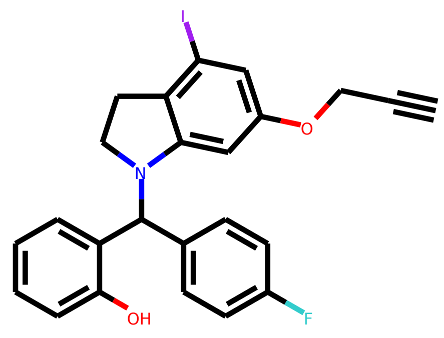

Osma-153

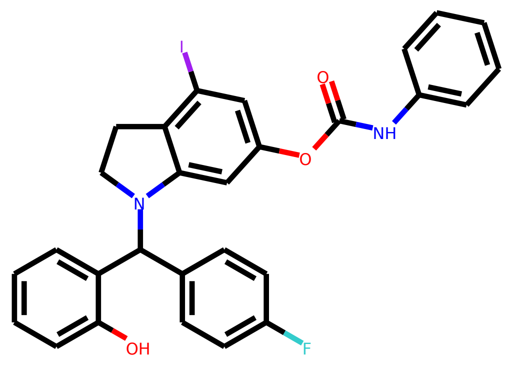

Osma-154

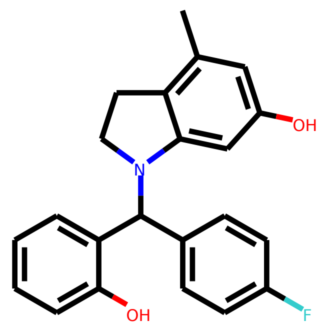

Osma-155

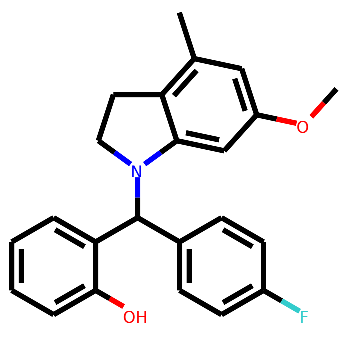

Osma-156

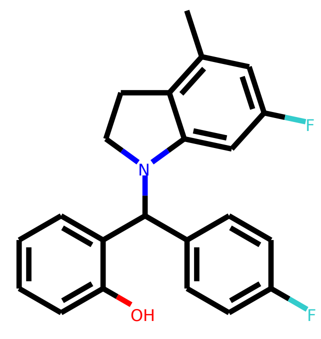

Osma-157

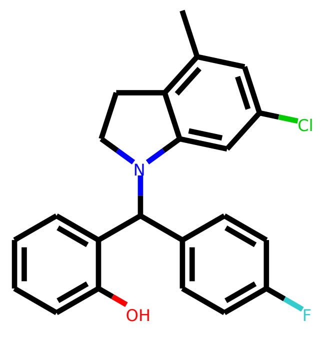

Osma-158

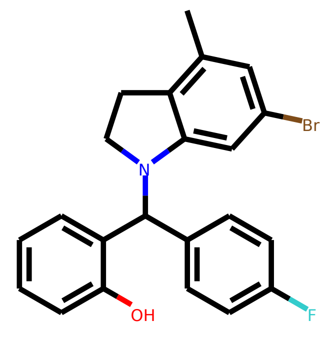

Osma-159

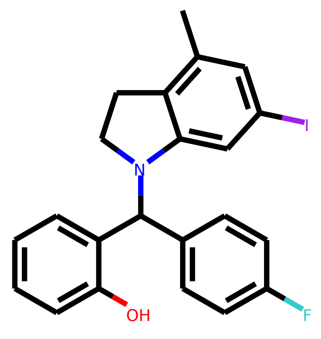

Osma-160

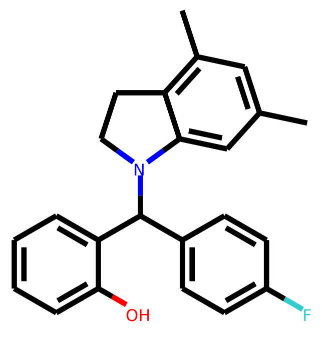

Osma-161

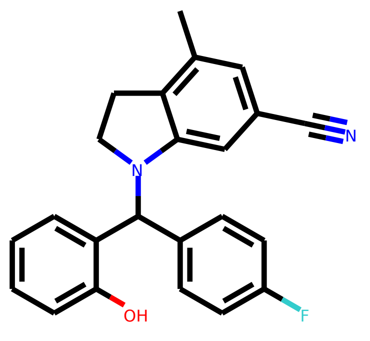

Osma-162

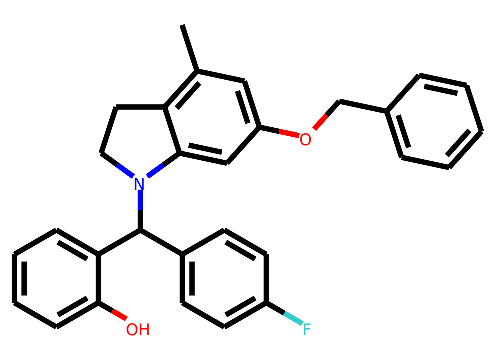

Osma-163

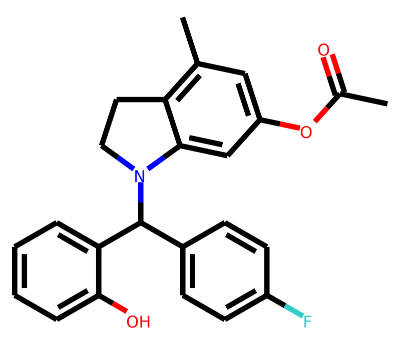

Osma-164

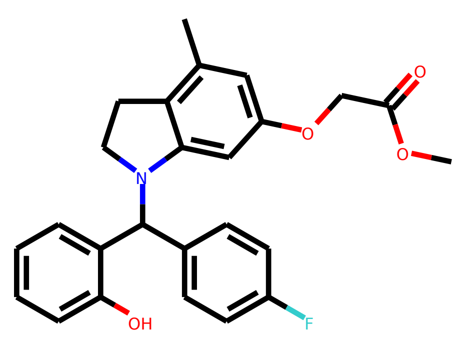

Osma-165

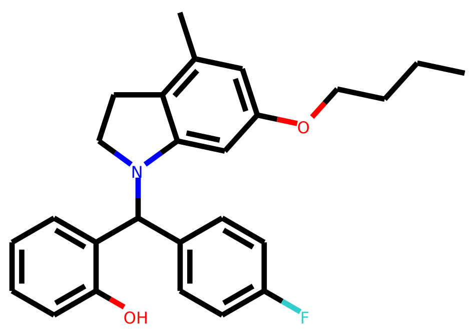

Osma-166

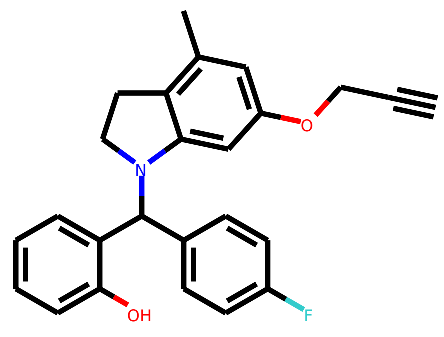

Osma-167

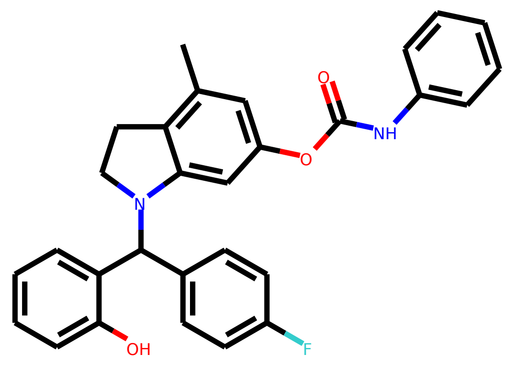

Osma-168

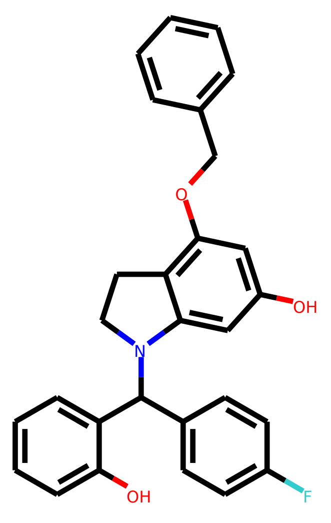

Osma-169

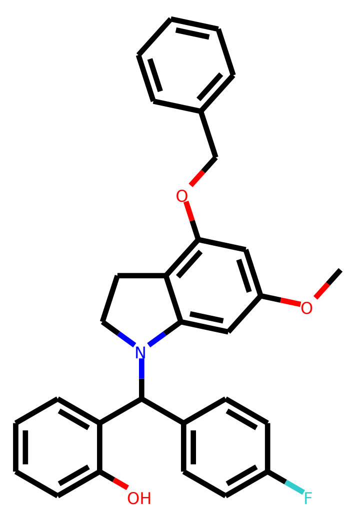

Osma-170

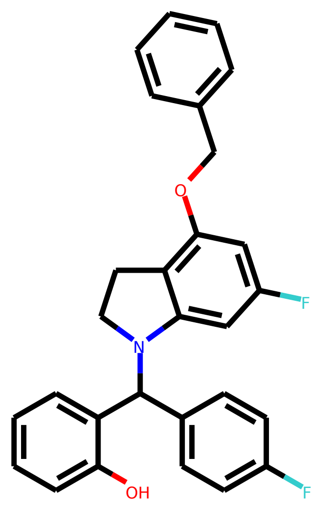

Osma-171

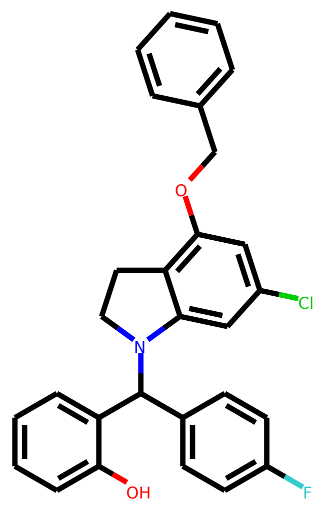

Osma-172

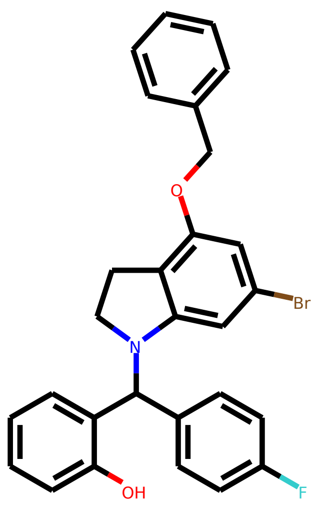

Osma-173

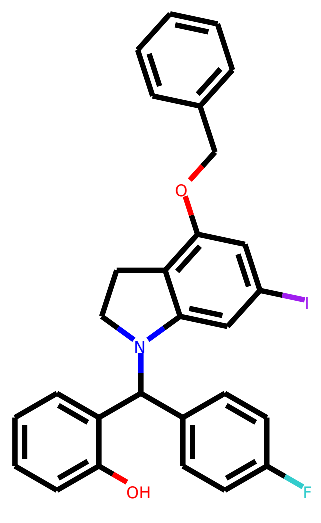

Osma-174

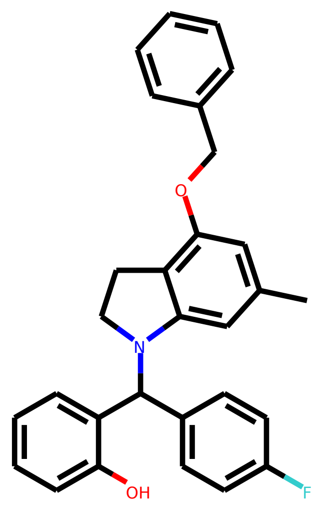

Osma-175

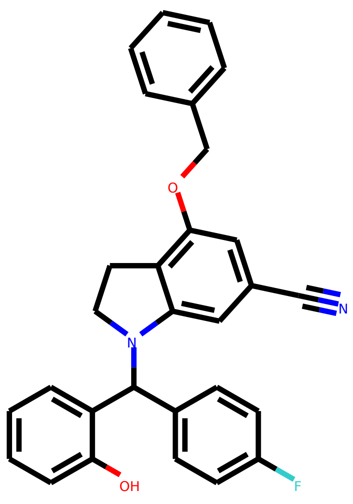
Osma-176

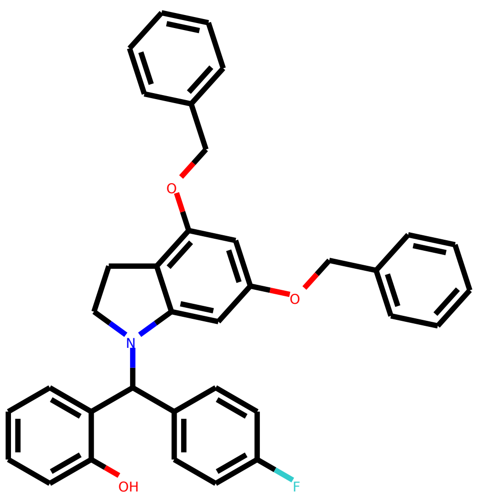
Osma-177

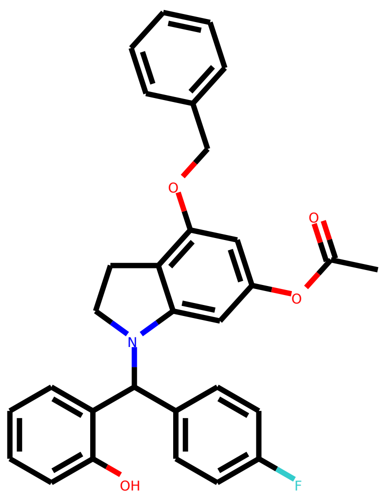
Osma-178

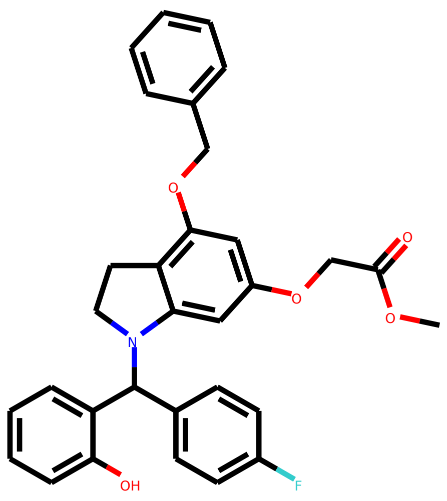
Osma-179

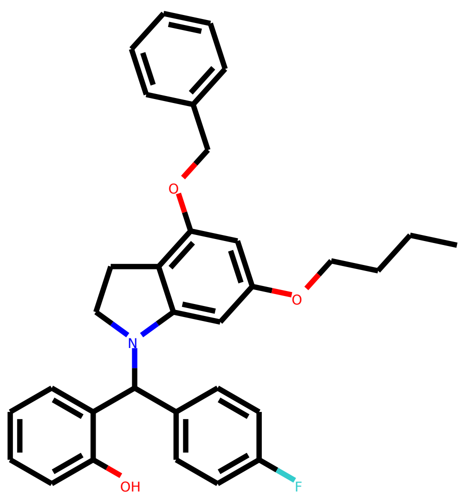
Osma-180

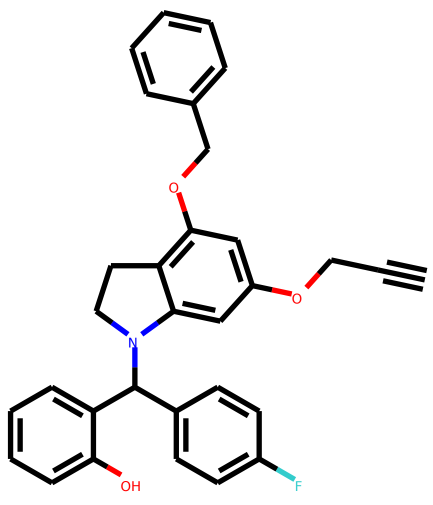
Osma-181

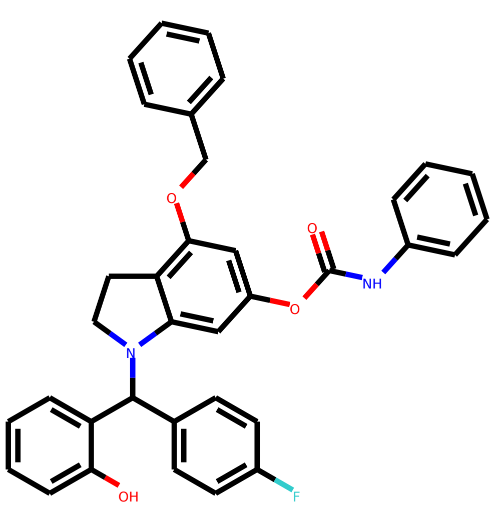
Osma-182

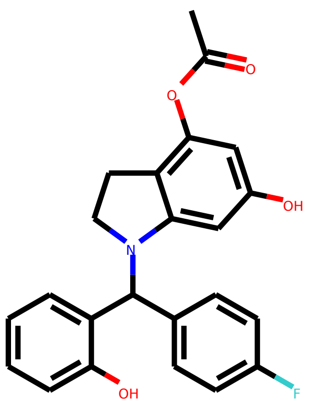
Osma-183

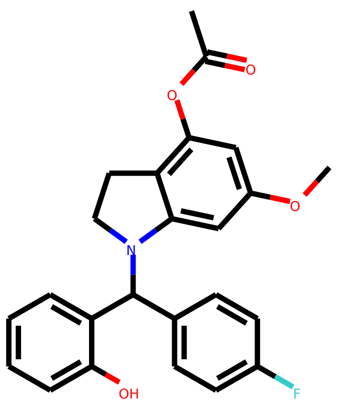
Osma-184

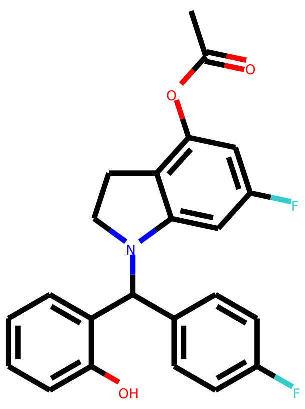
Osma-185

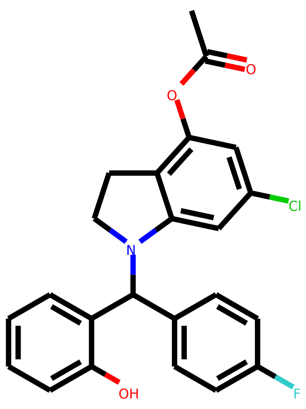
Osma-186

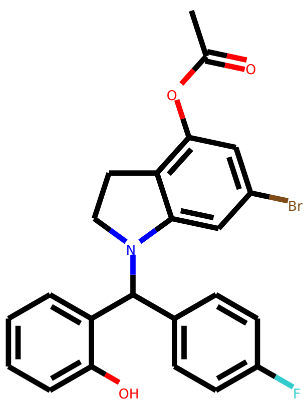
Osma-187

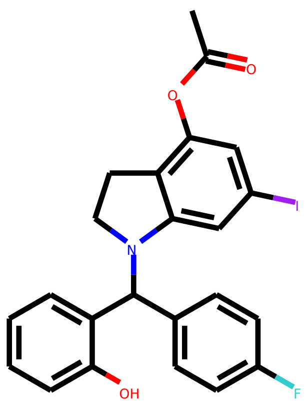
Osma-188

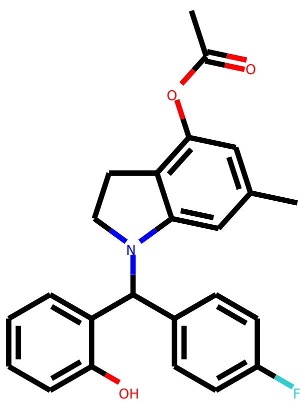
Osma-189

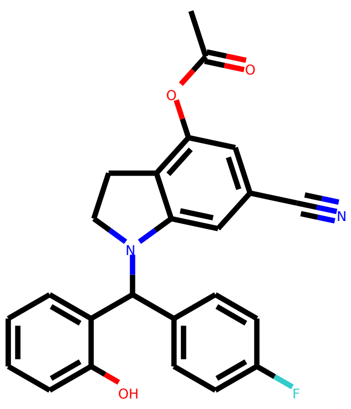
Osma-190

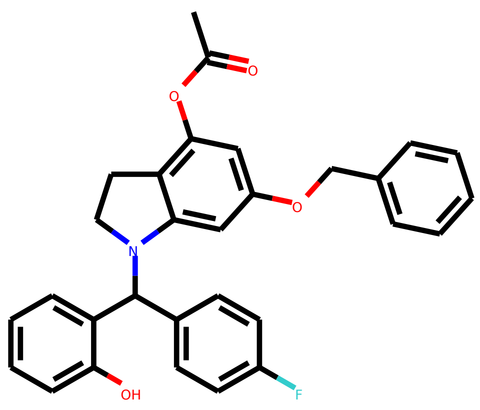
Osma-191

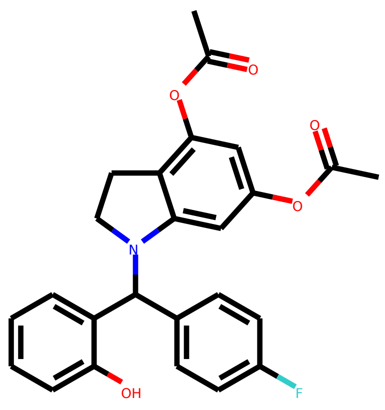
Osma-192

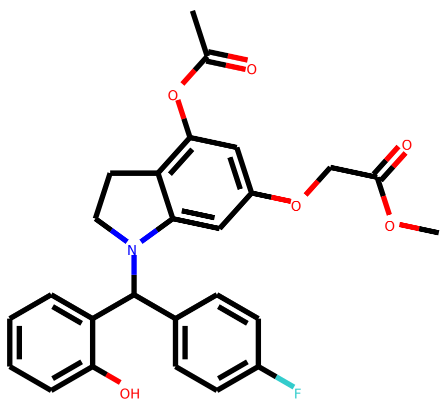
Osma-193

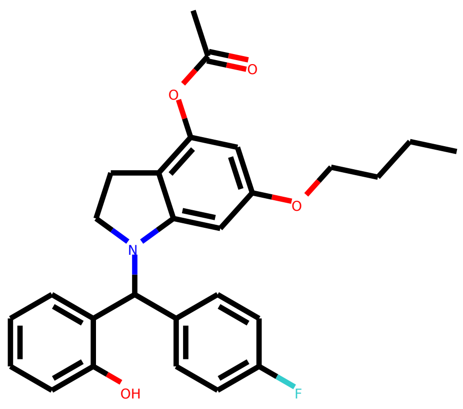
Osma-194

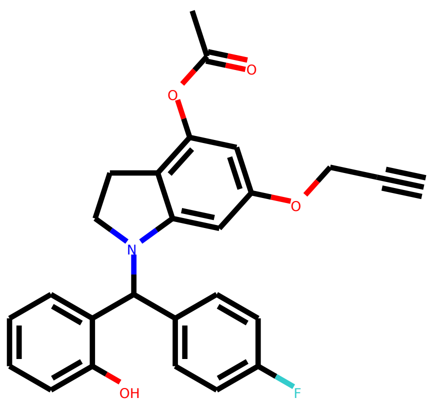
Osma-195

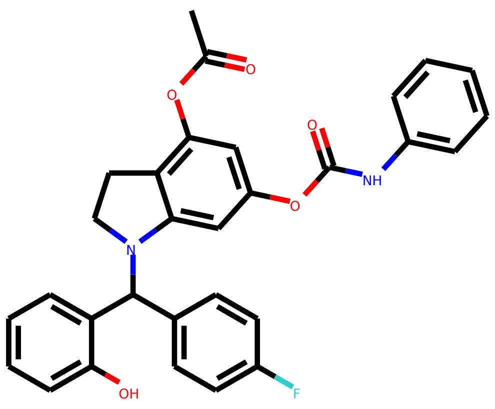
Osma-196

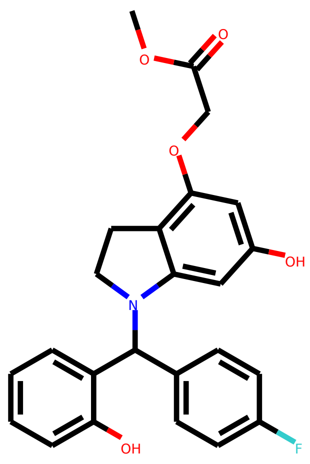
Osma-197

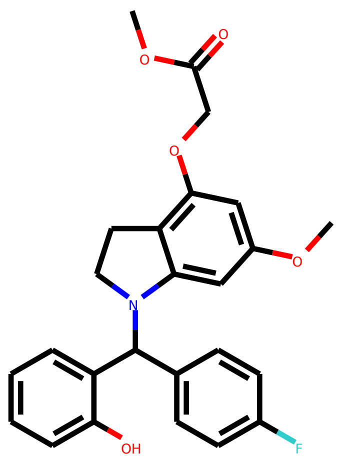
Osma-198

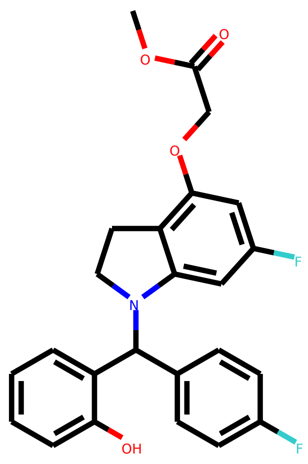
Osma-199

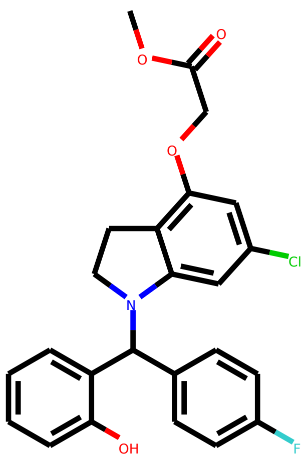
Osma-200

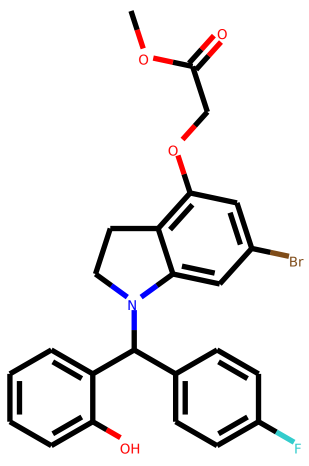
Osma-201

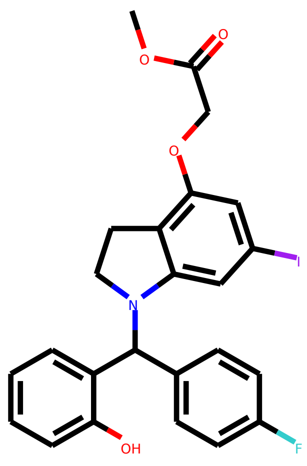
Osma-202

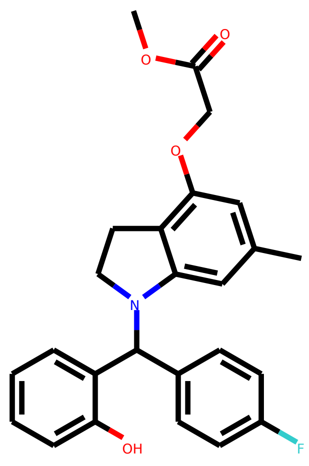
Osma-203

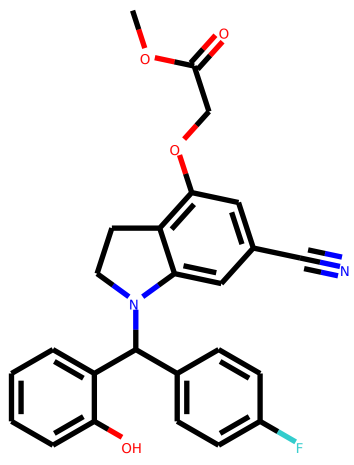
Osma-204

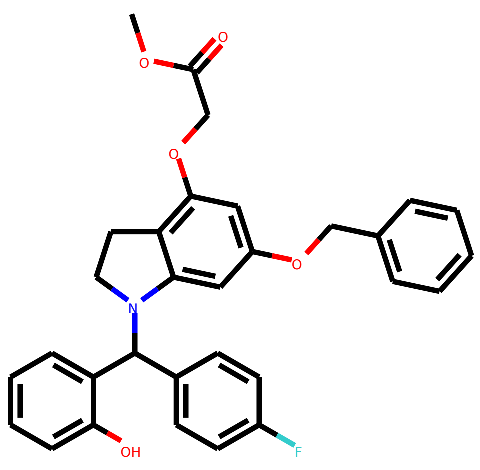
Osma-205

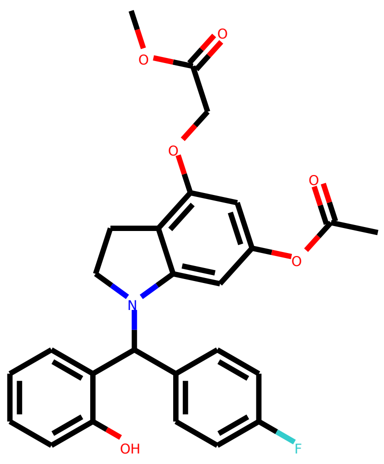
Osma-206

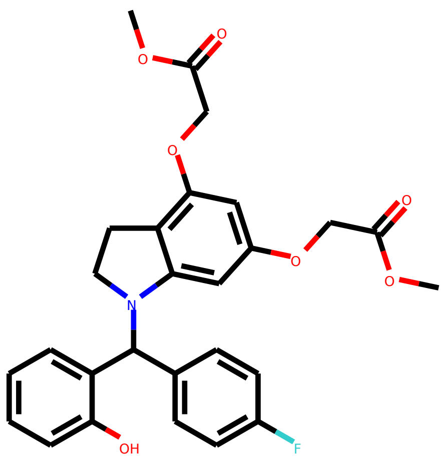
Osma-207

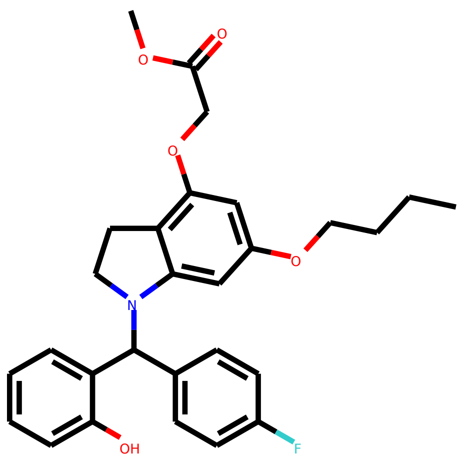
Osma-208

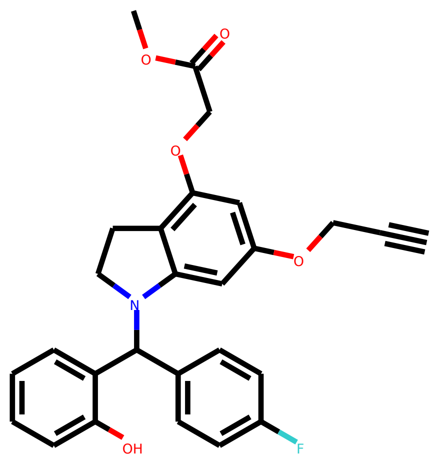
Osma-209

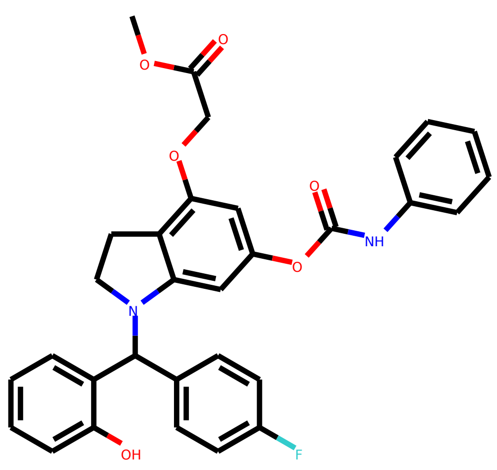
Osma-210

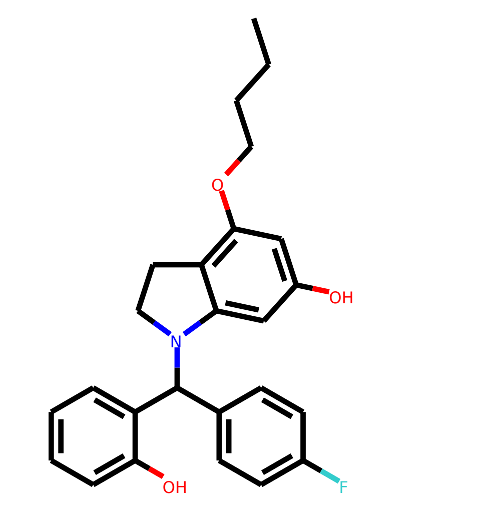

Osma-211

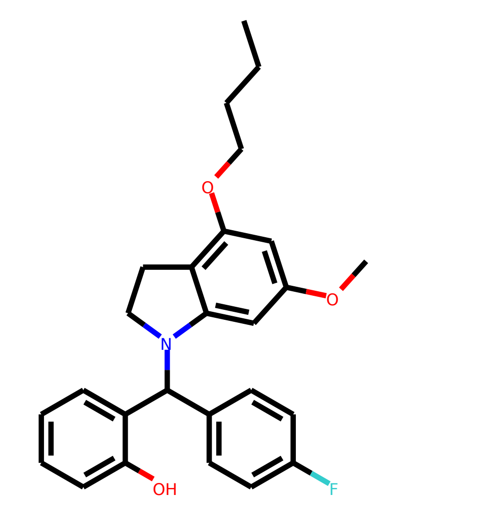

Osma-212

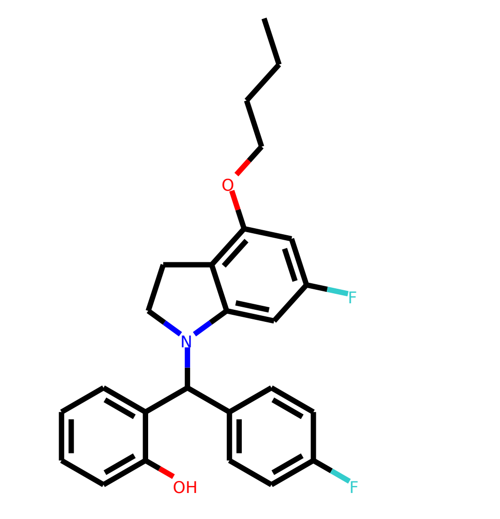

Osma-213

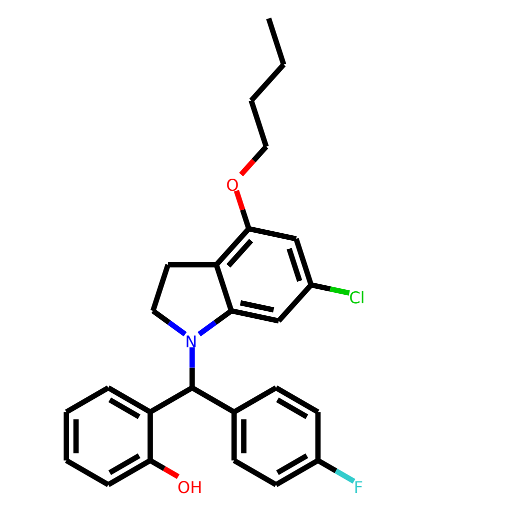

Osma-214

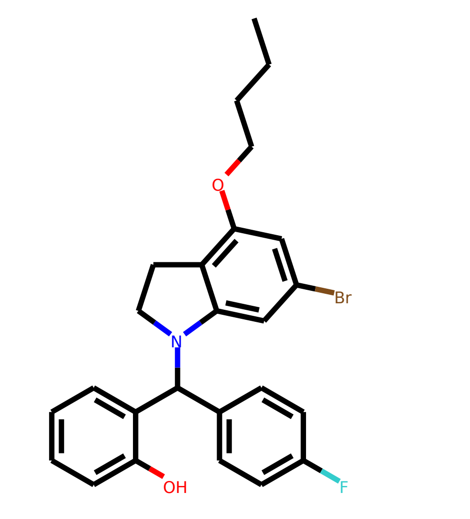

Osma-215

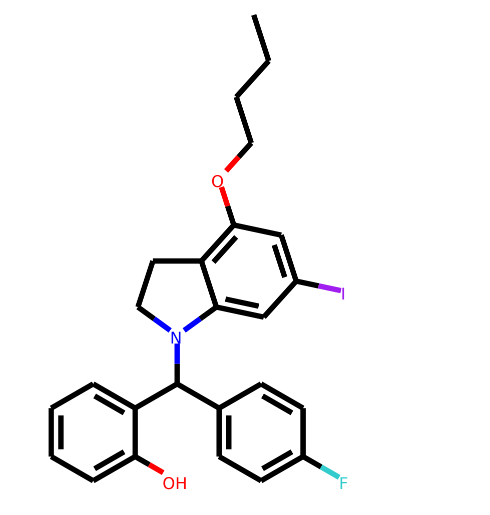

Osma-216

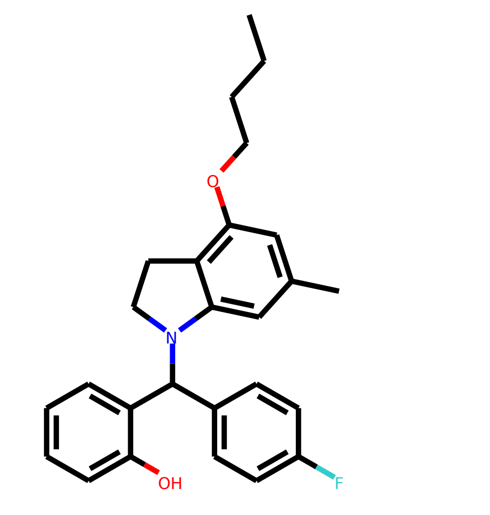

Osma-217

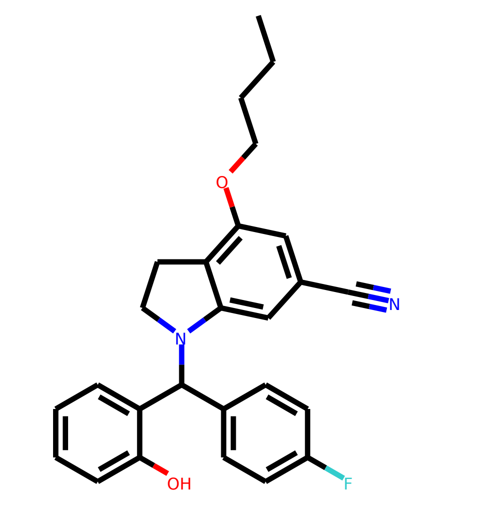

Osma-218

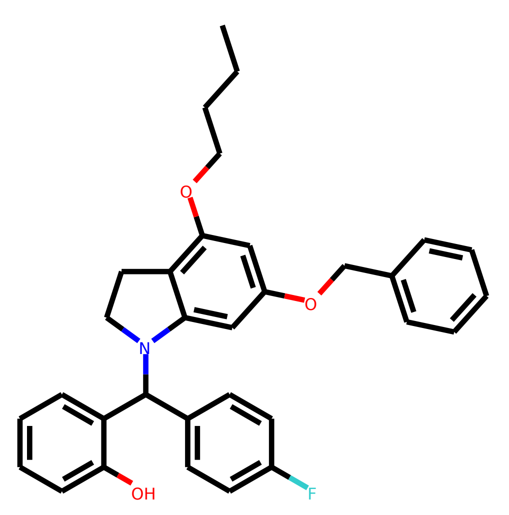

Osma-219

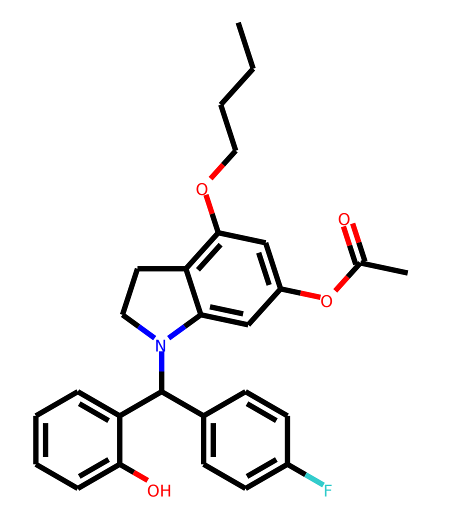

Osma-220

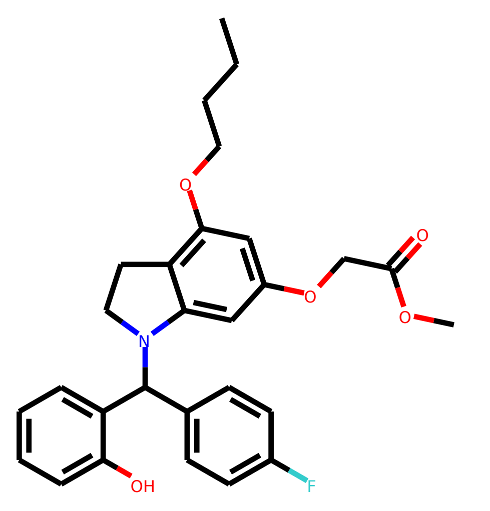

Osma-221

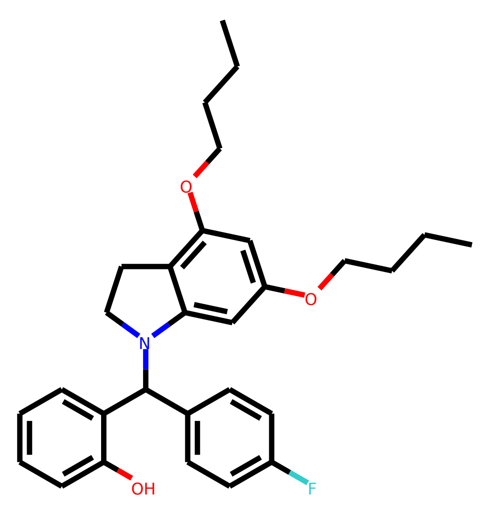

Osma-222

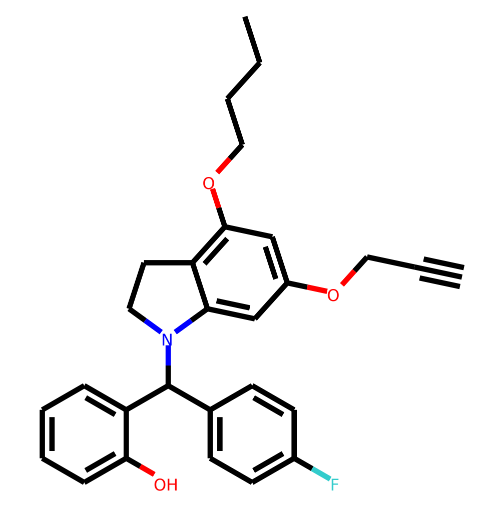

Osma-223

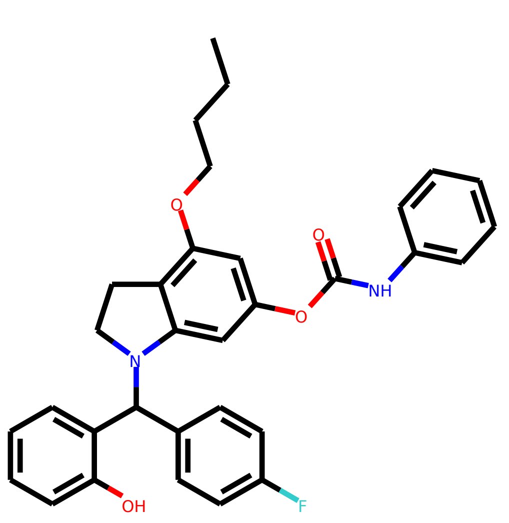

Osma-224

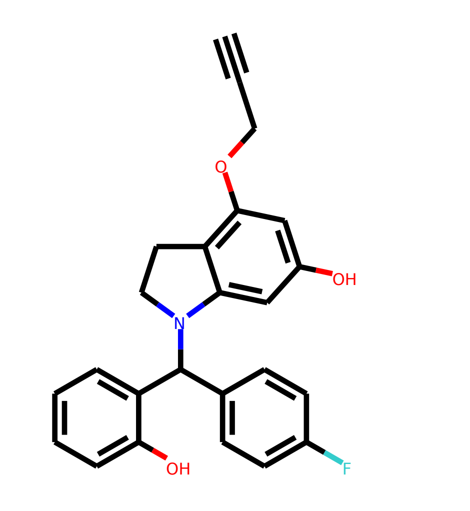

Osma-225

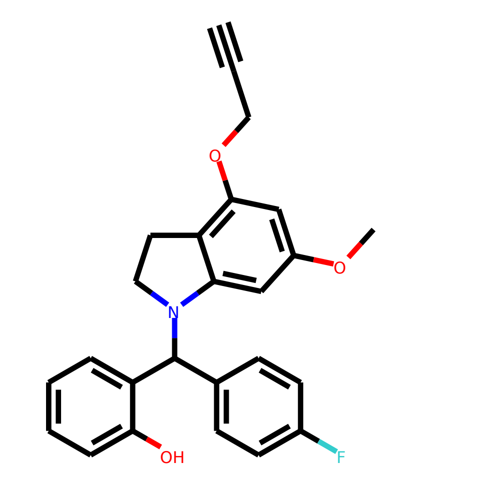

Osma-226

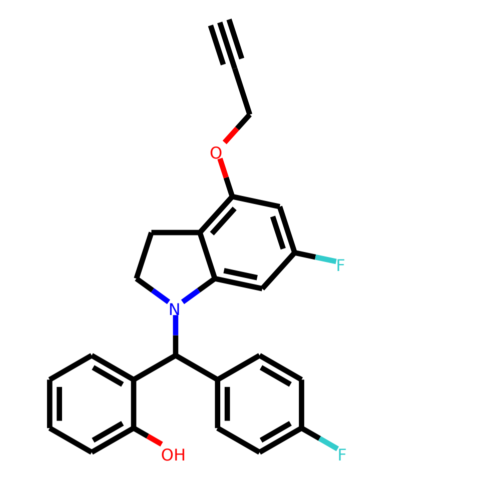

Osma-227

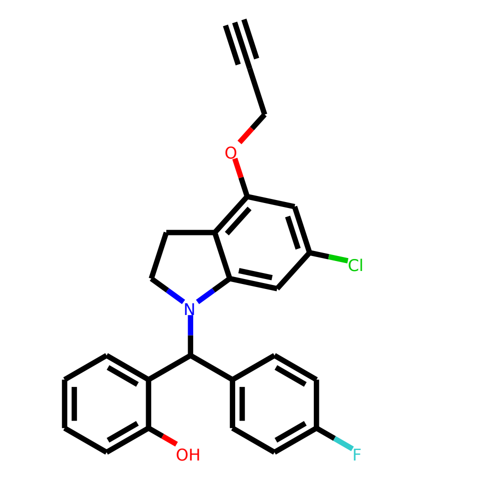

Osma-228

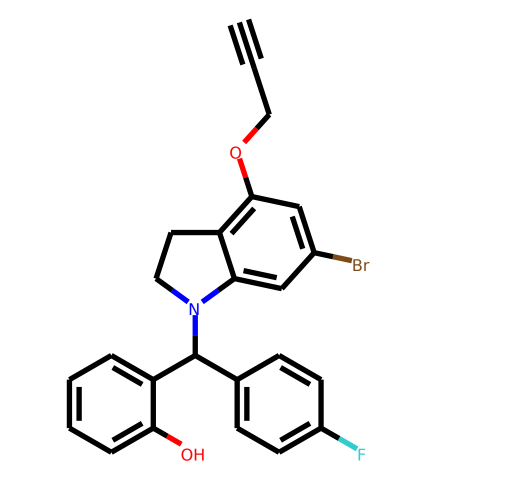

Osma-229

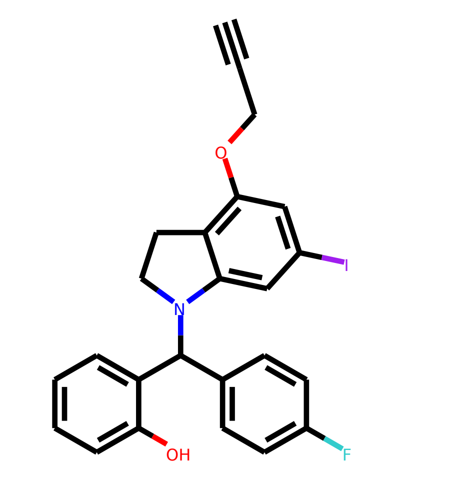

Osma-230

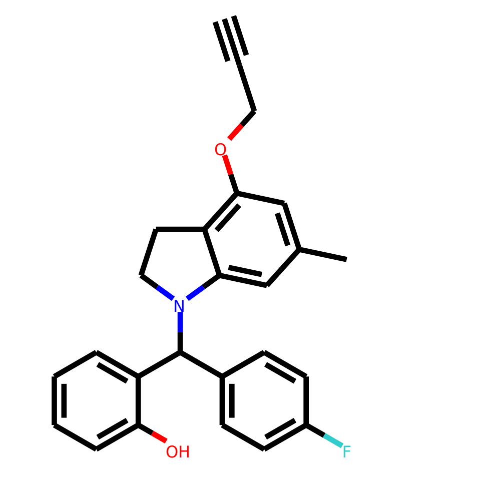

Osma-231

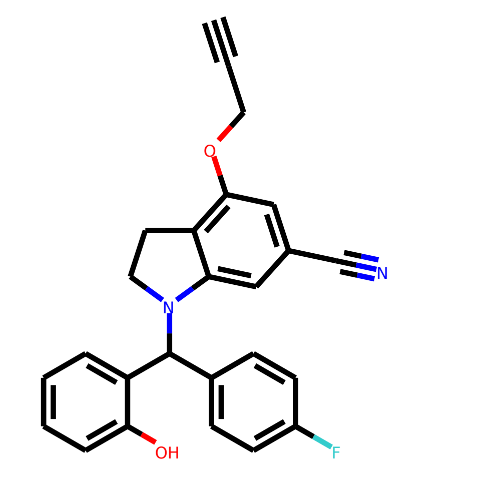

Osma-232

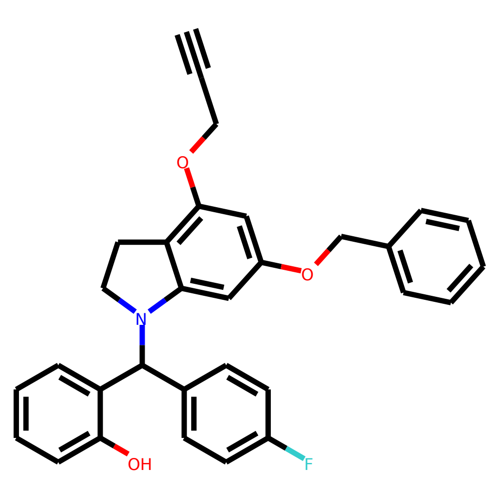

Osma-233

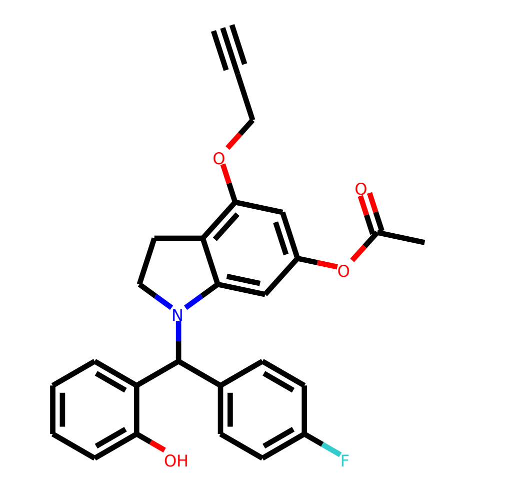

Osma-234

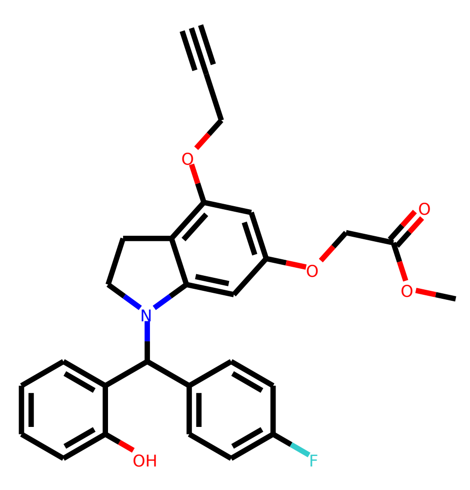

Osma-235

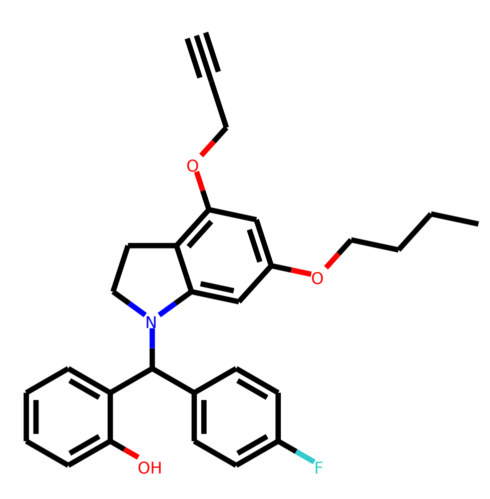

Osma-236

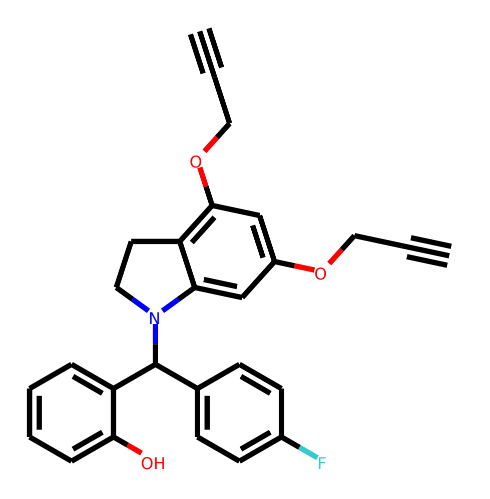

Osma-237

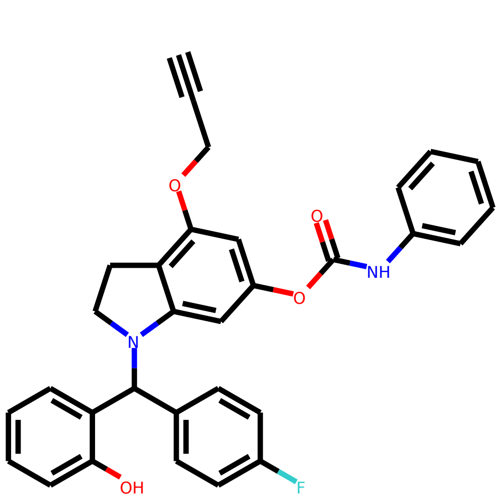

Osma-238

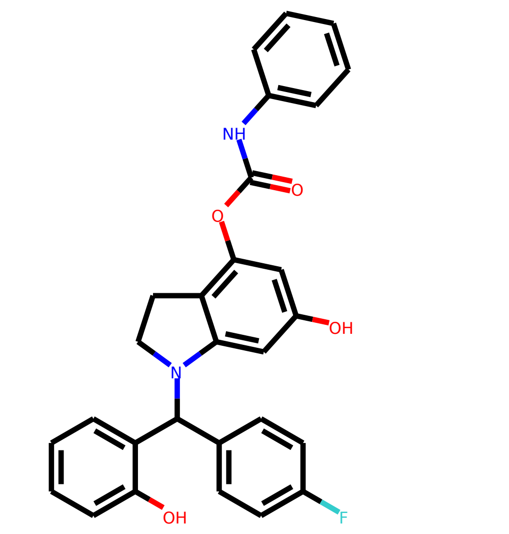

Osma-239

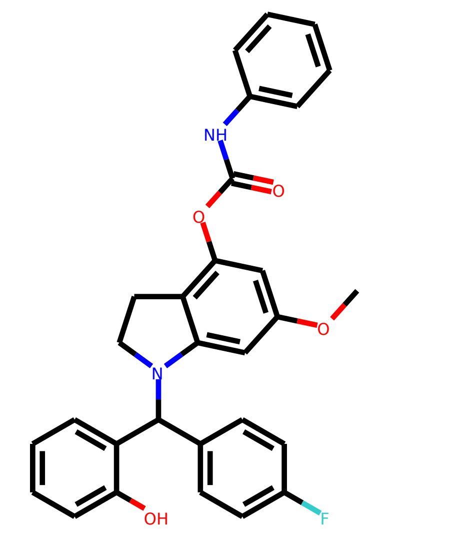

Osma-240

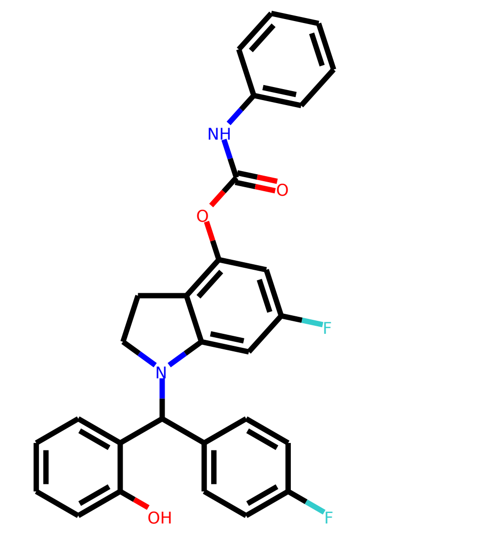

Osma-241

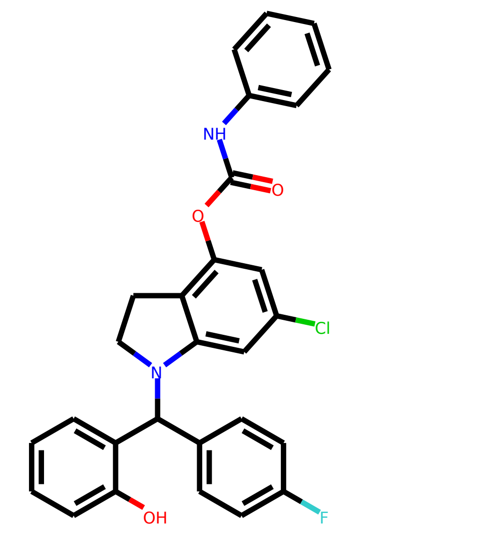

Osma-242

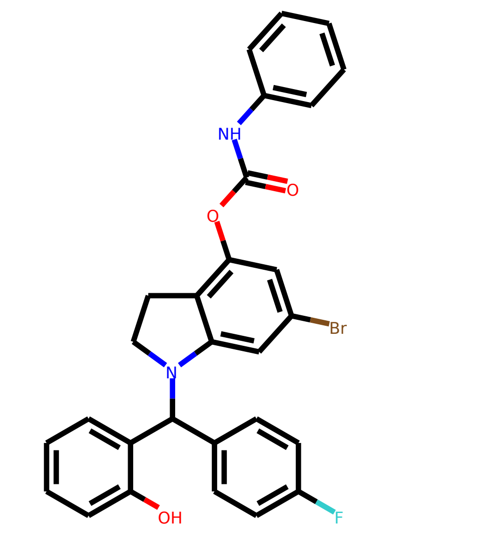

Osma-243

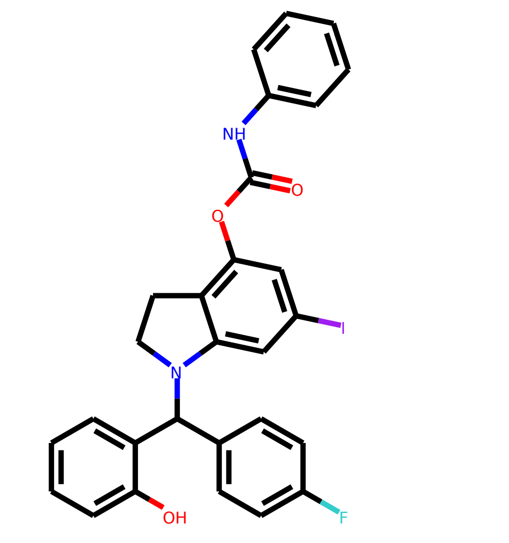

Osma-244

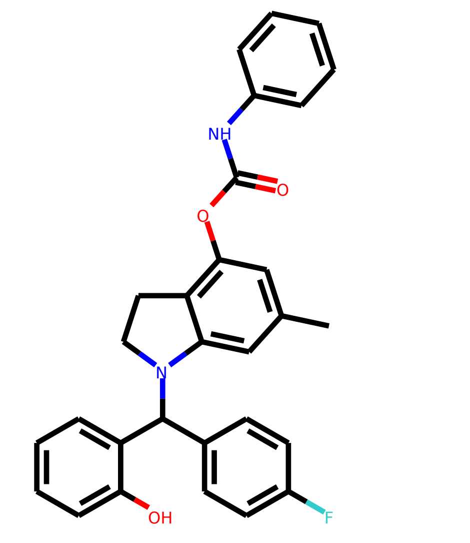

Osma-245

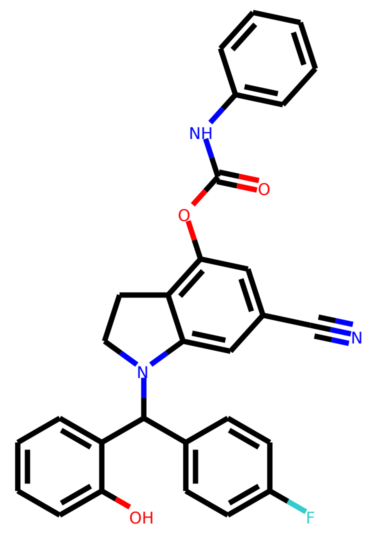

Osma-246

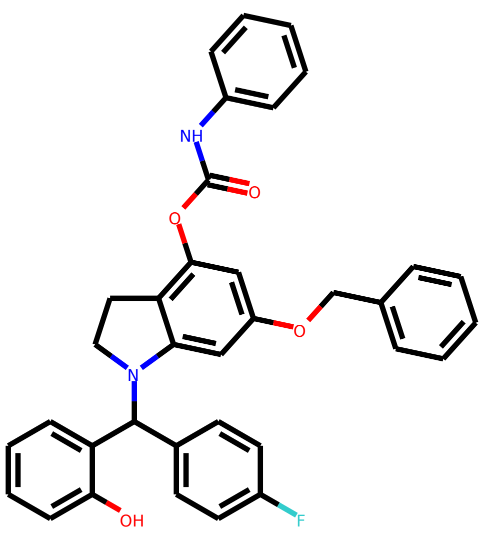

Osma-247

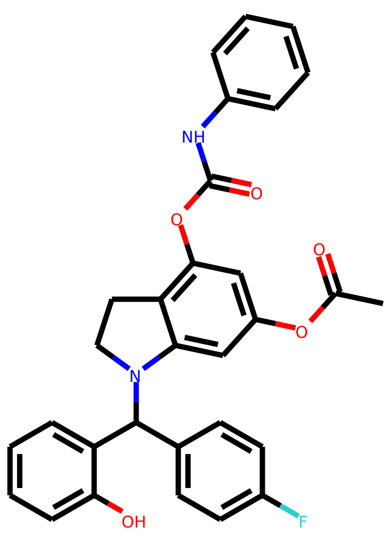

Osma-248

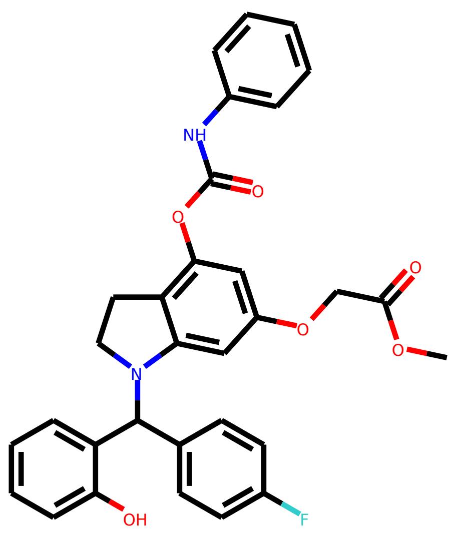

Osma-249

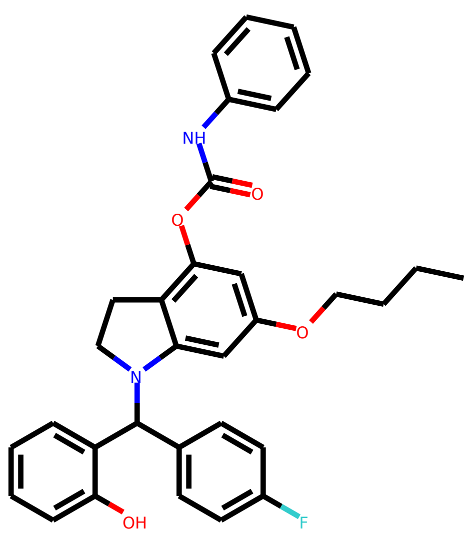

Osma-250

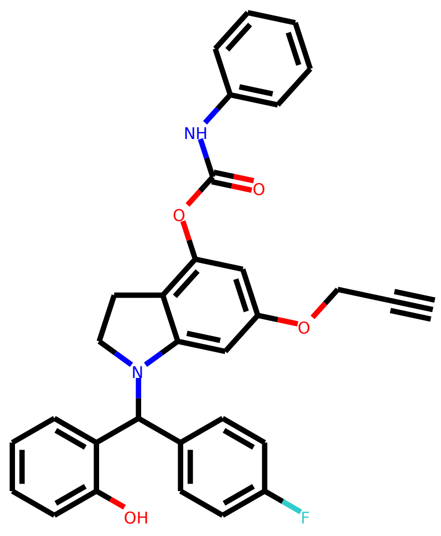

Osma-251

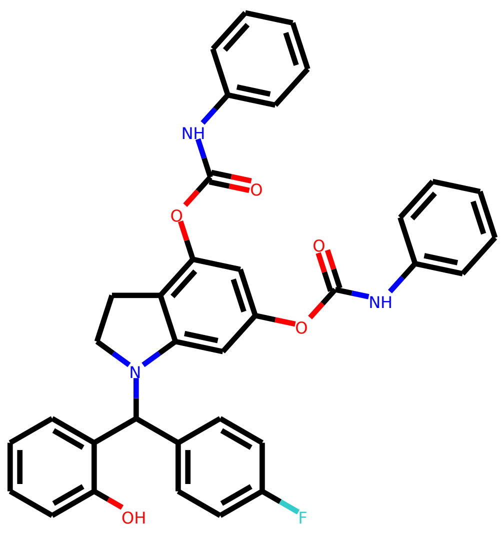

Osma-252

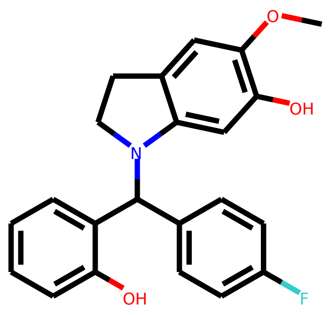

Osma-253

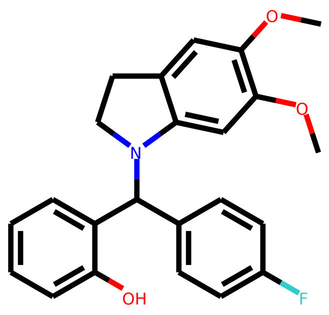

Osma-254

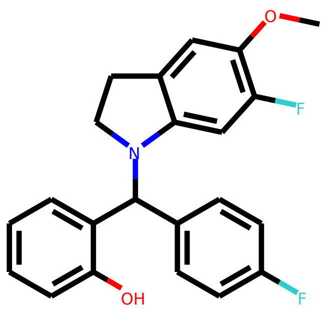

Osma-255

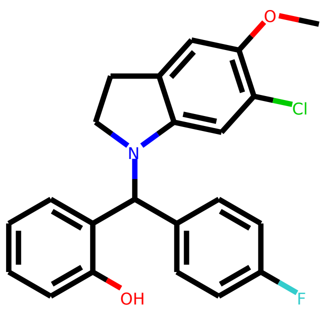

Osma-256

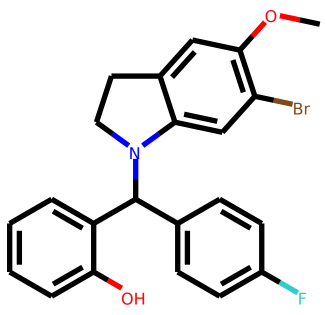

Osma-257

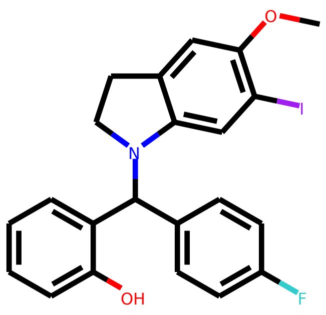

Osma-258

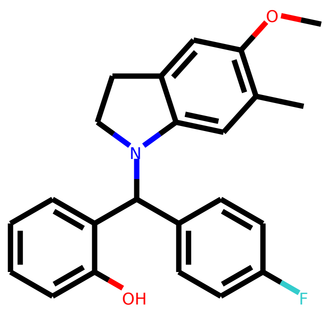

Osma-259

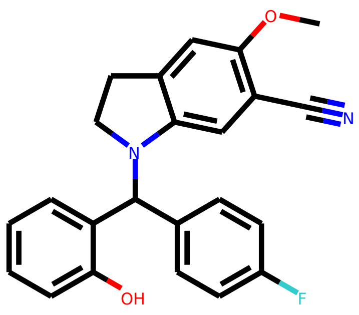

Osma-260

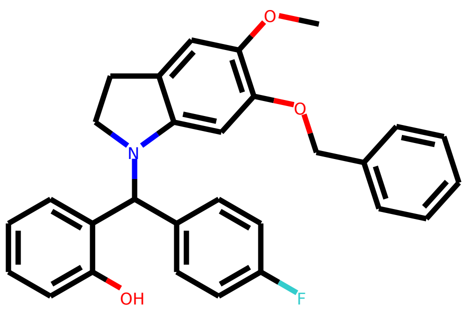

Osma-261

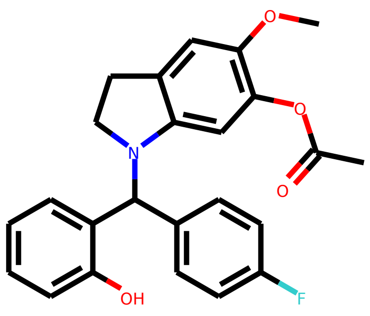

Osma-262

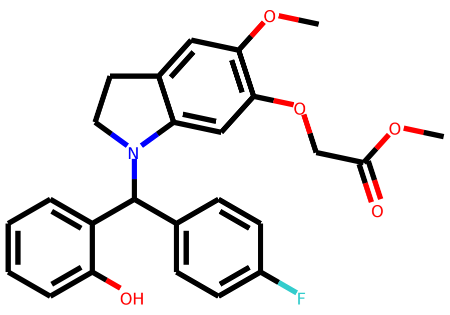

Osma-263

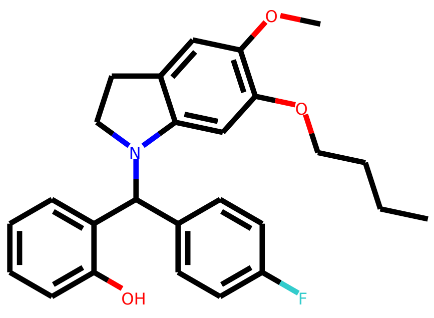

Osma-264

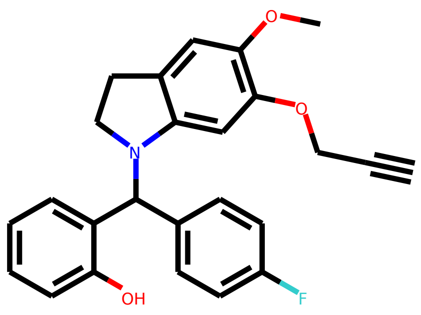

Osma-265

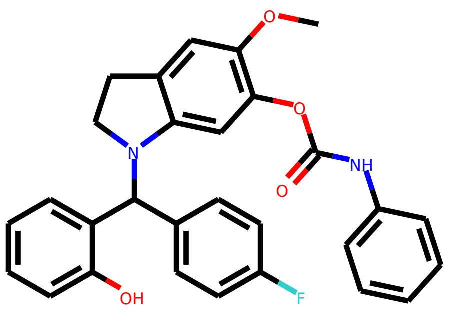

Osma-266

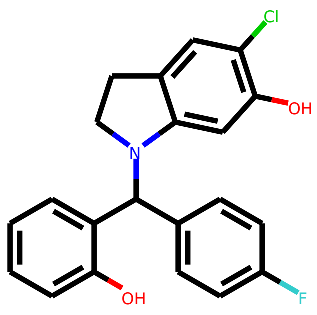

Osma-267

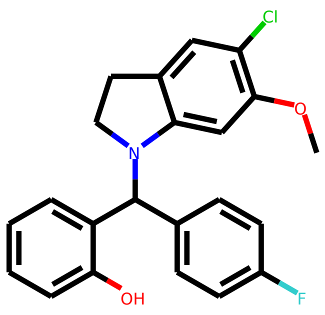

Osma-268

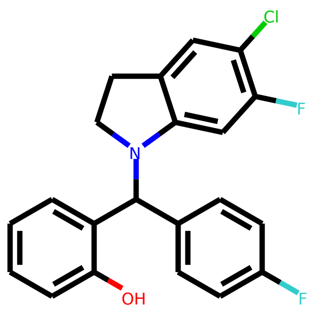

Osma-269

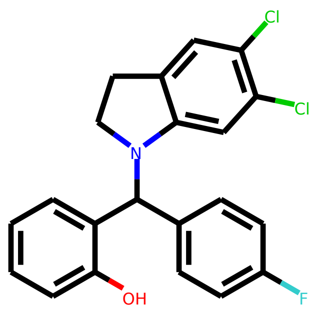

Osma-270

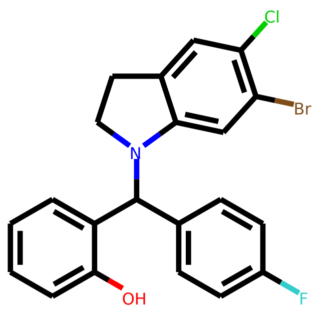

Osma-271

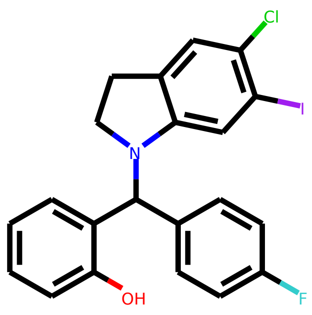

Osma-272

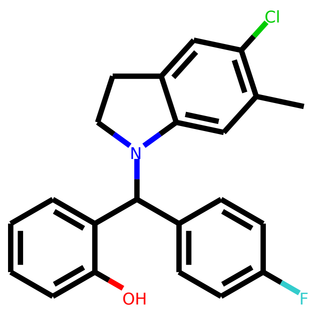

Osma-273

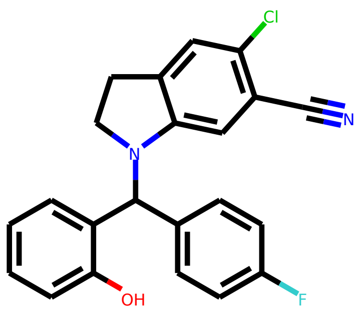

Osma-274

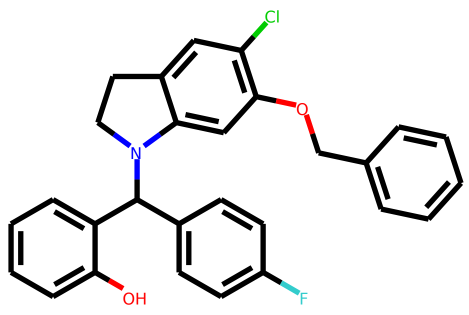

Osma-275

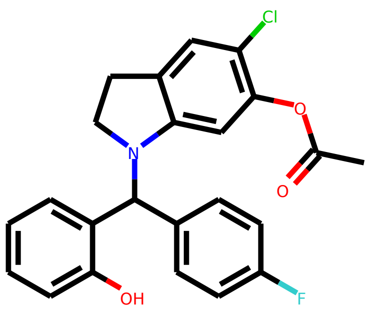

Osma-276

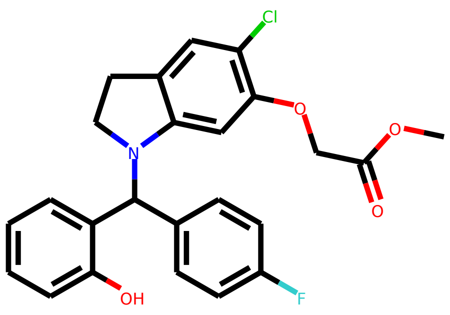

Osma-277

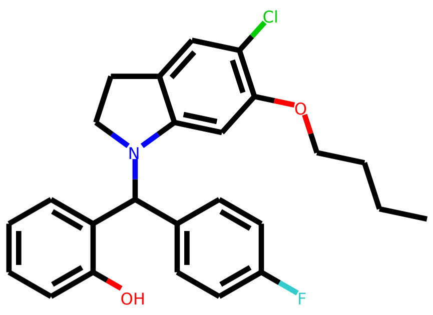

Osma-278

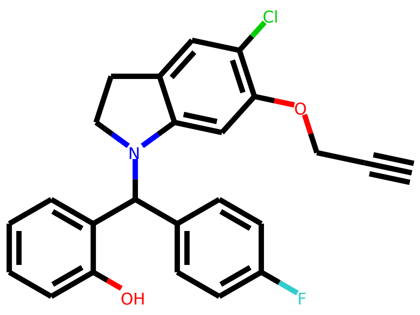

Osma-279

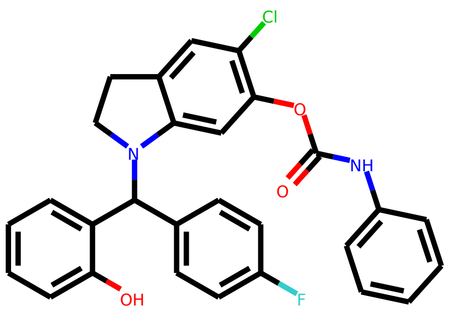

Osma-280

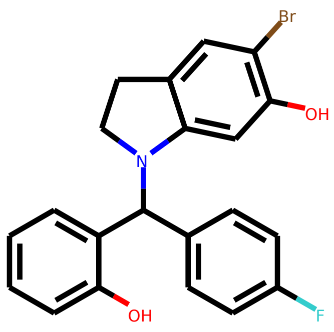

Osma-281

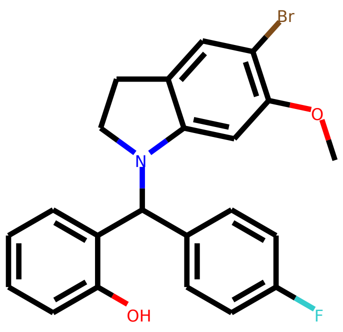

Osma-282

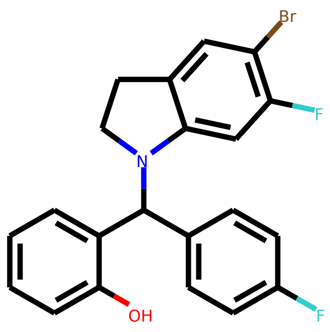

Osma-283

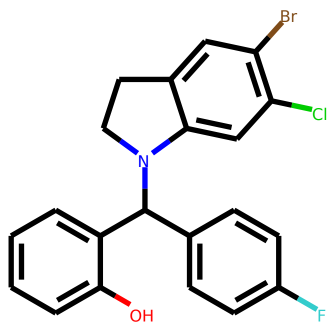

Osma-284

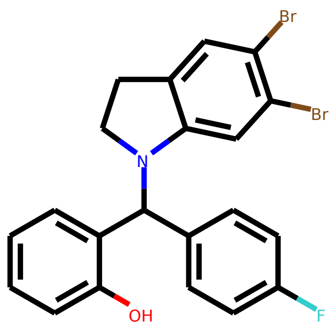

Osma-285

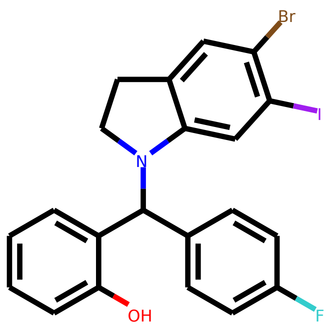

Osma-286

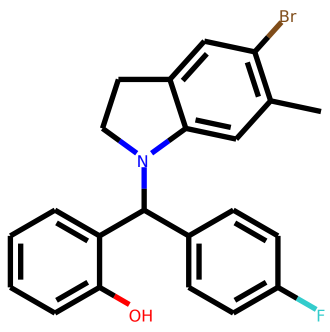

Osma-287

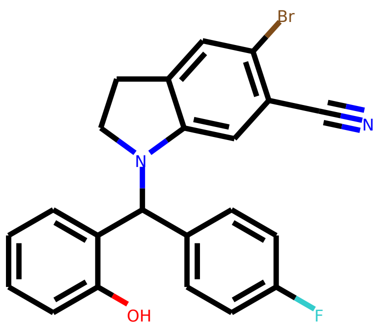

Osma-288

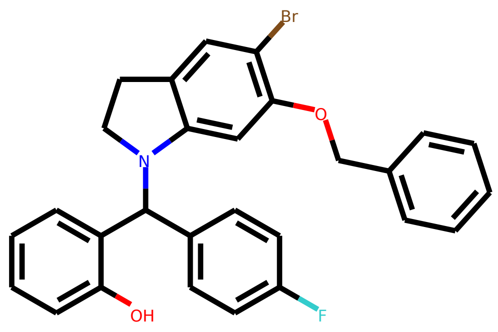

Osma-289

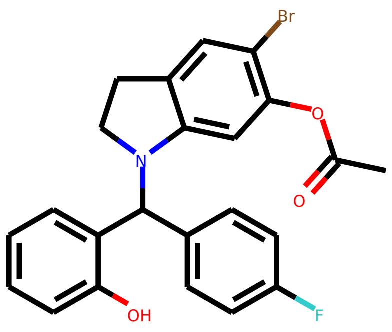

Osma-290

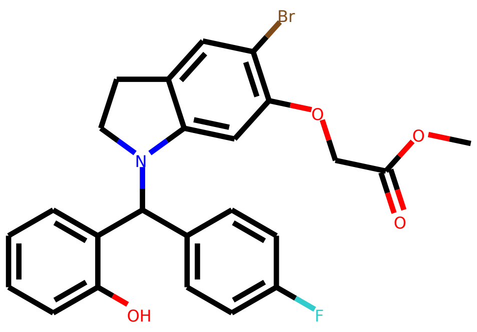

Osma-291

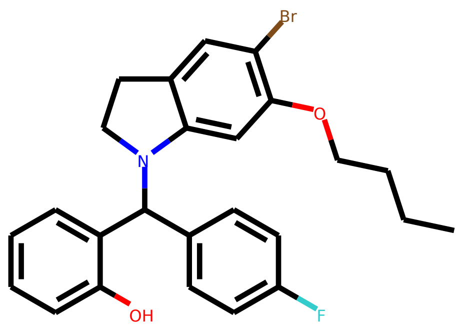

Osma-292

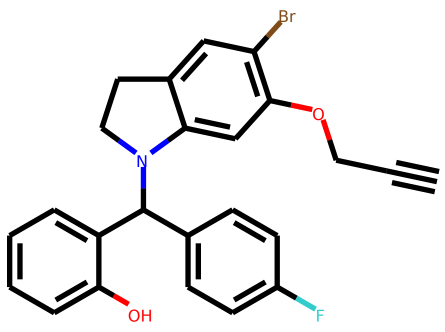

Osma-293

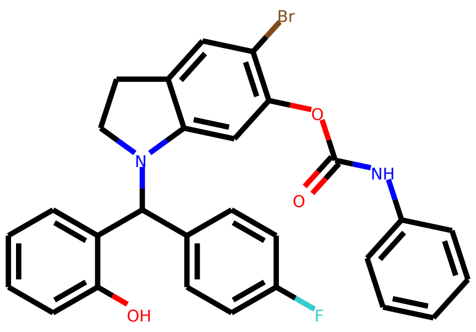

Osma-294

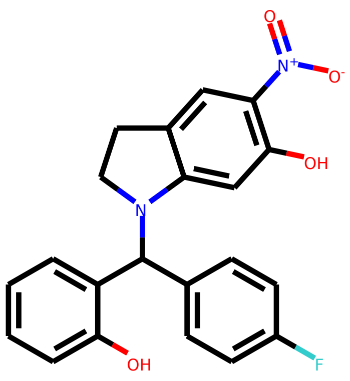

Osma-295

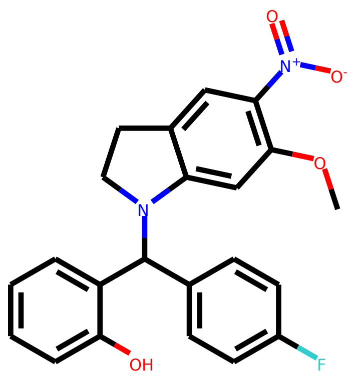

Osma-296

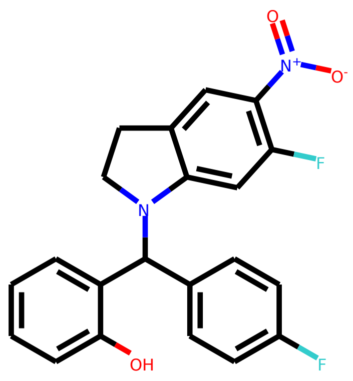

Osma-297

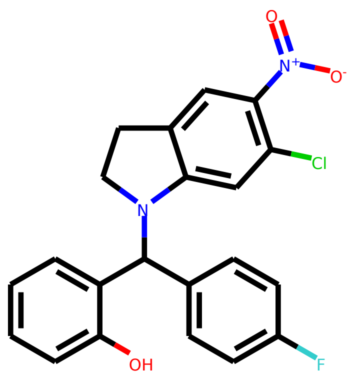

Osma-298

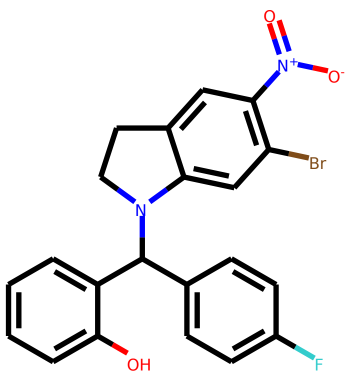

Osma-299

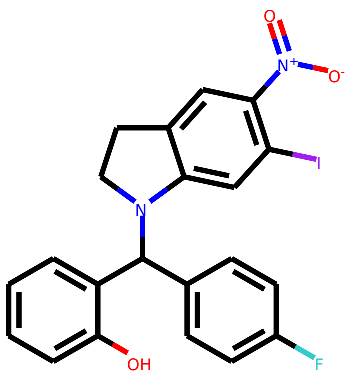

Osma-300

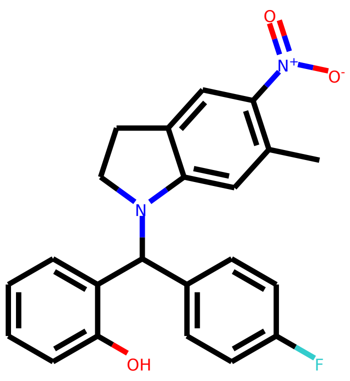

Osma-301

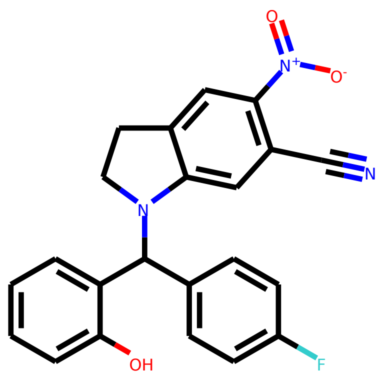

Osma-302

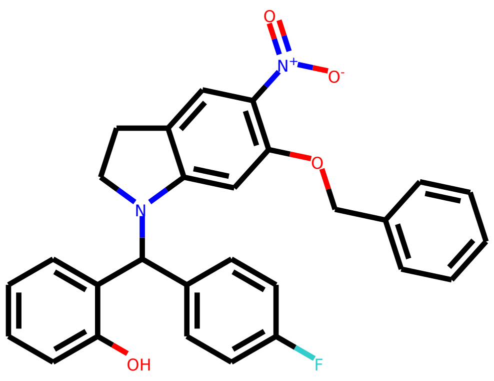

Osma-303

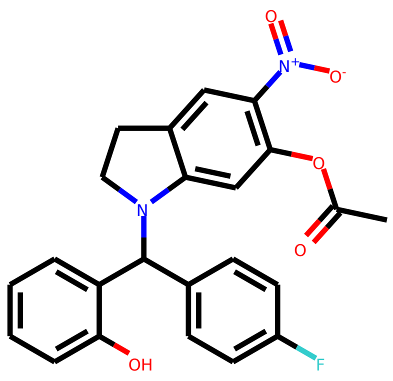

Osma-304

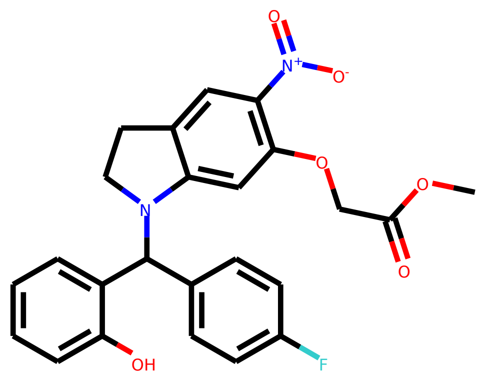

Osma-305

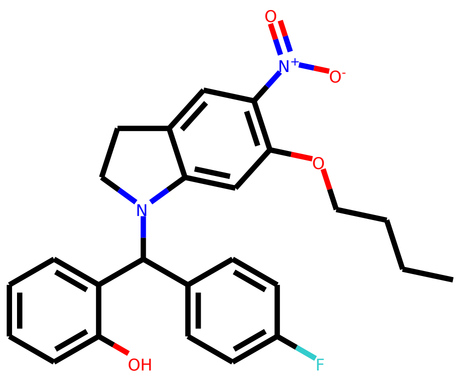

Osma-306

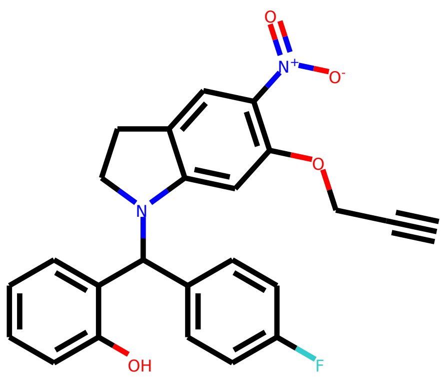

Osma-307

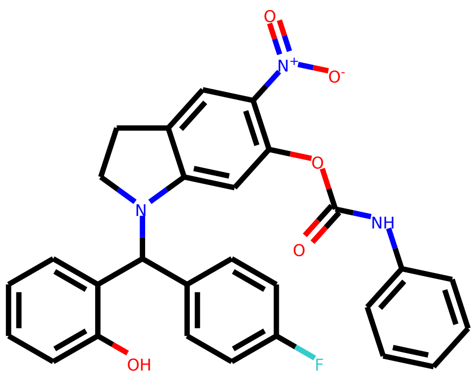

Osma-308

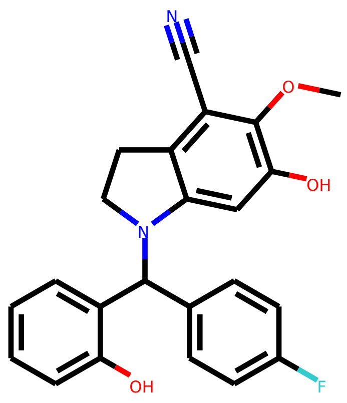

Osma-309

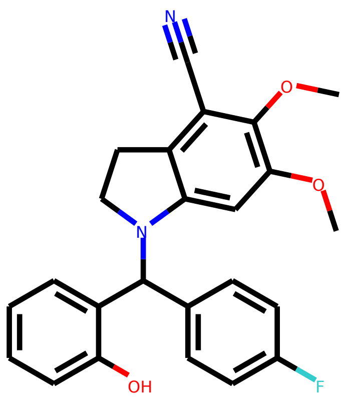

Osma-310

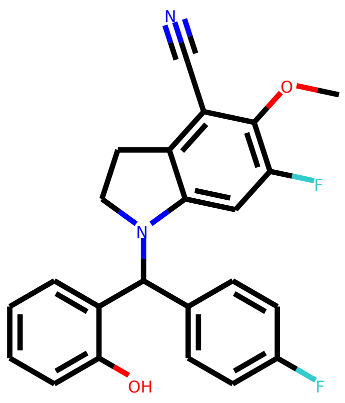

Osma-311

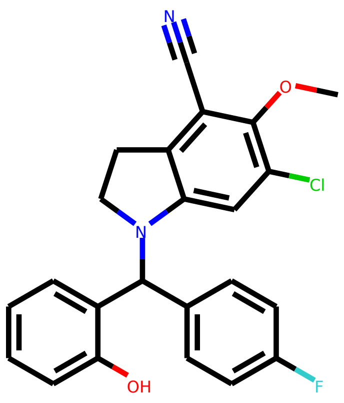

Osma-312

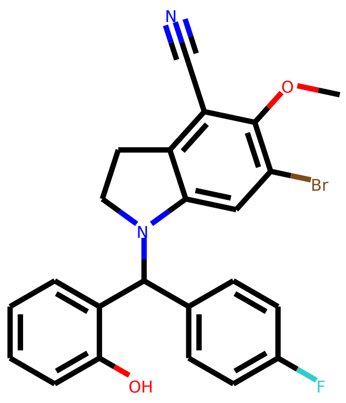

Osma-313

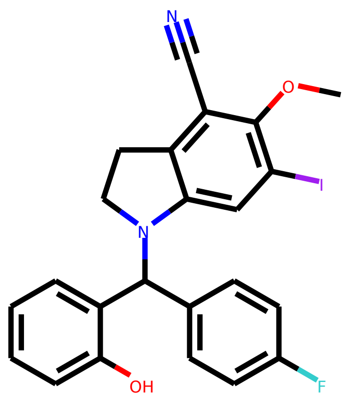

Osma-314

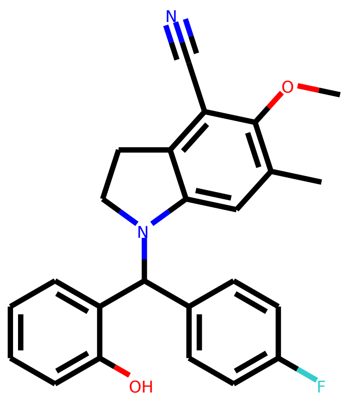

Osma-315

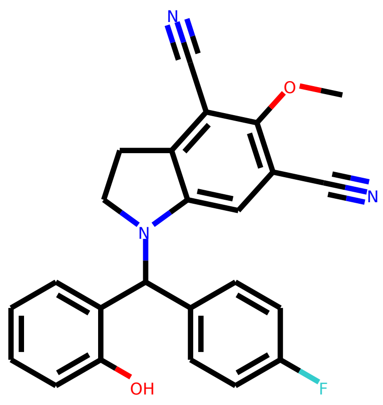

Osma-316

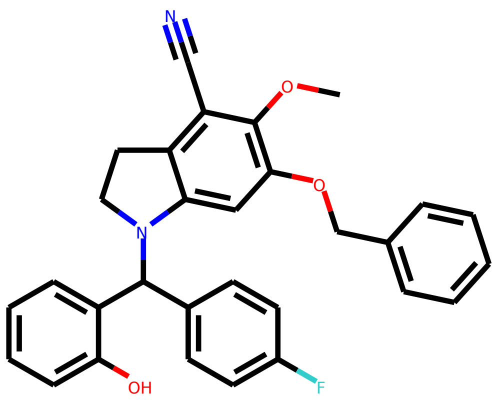

Osma-317

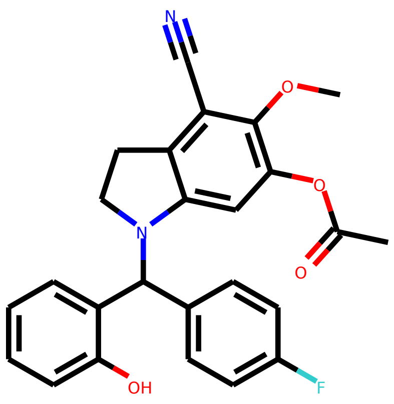

Osma-318

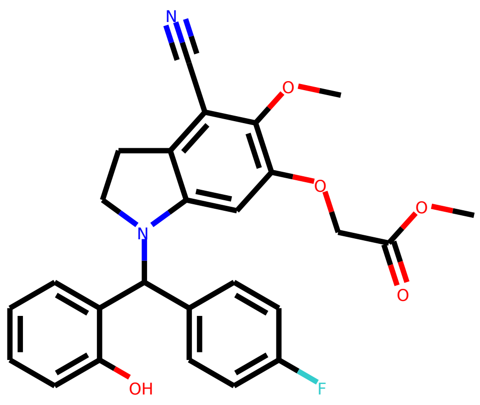

Osma-319

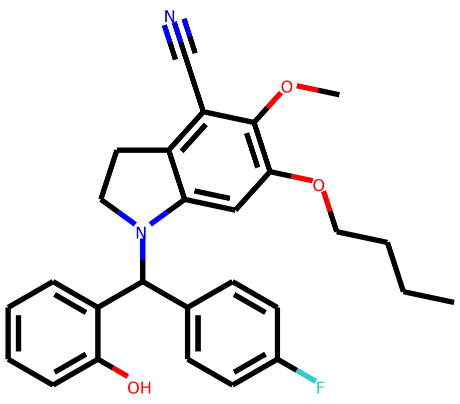

Osma-320

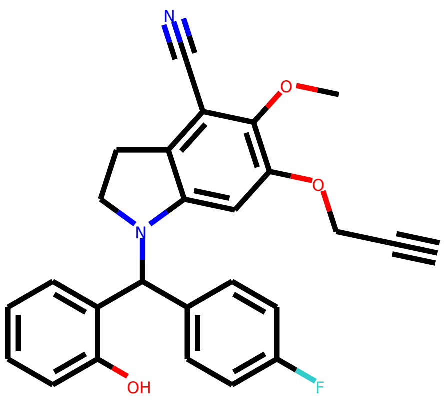

Osma-321

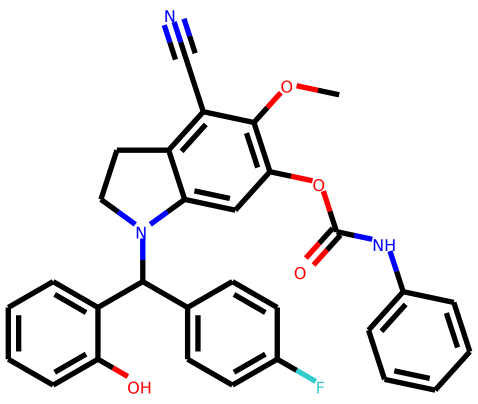

Osma-322

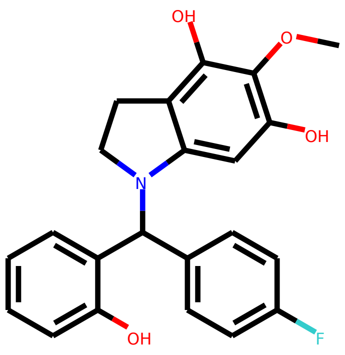

Osma-323

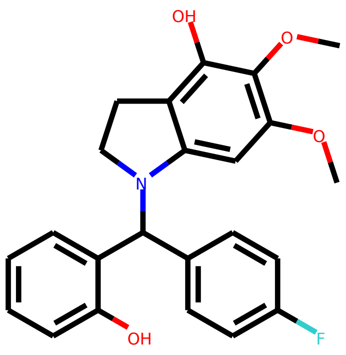

Osma-324

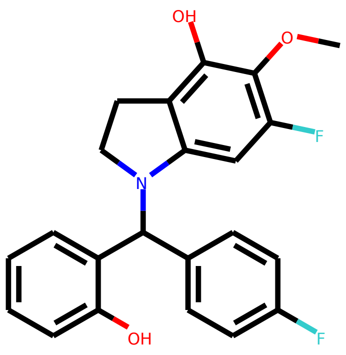

Osma-325

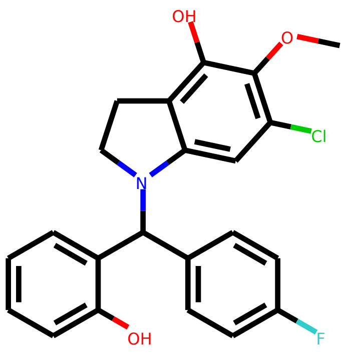

Osma-326

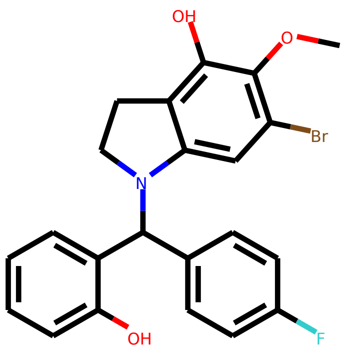

Osma-327

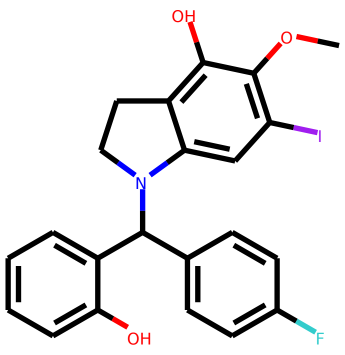

Osma-328

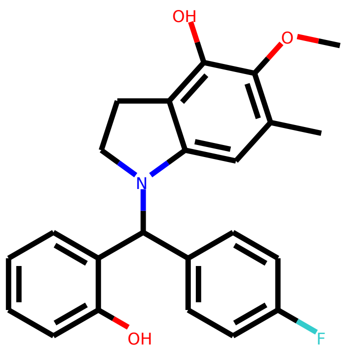

Osma-329

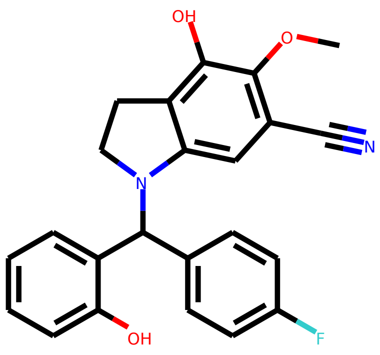

Osma-330

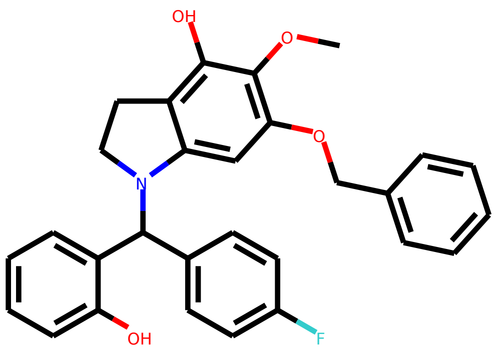

Osma-331

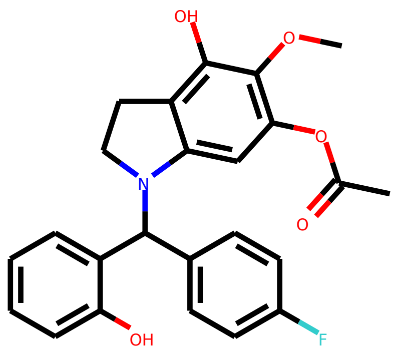

Osma-332

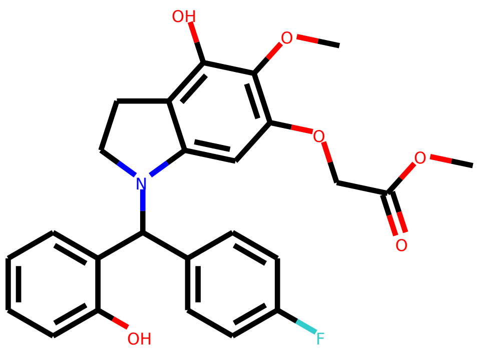

Osma-333

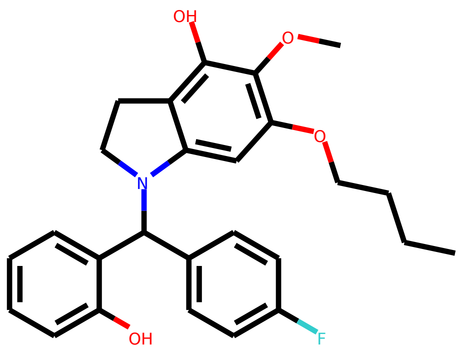

Osma-334

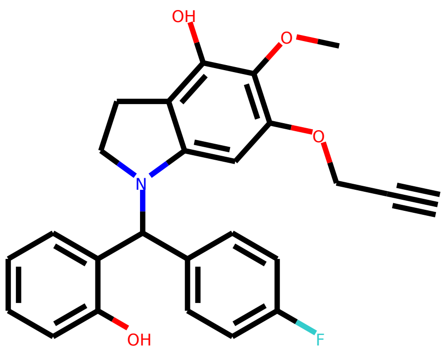

Osma-335

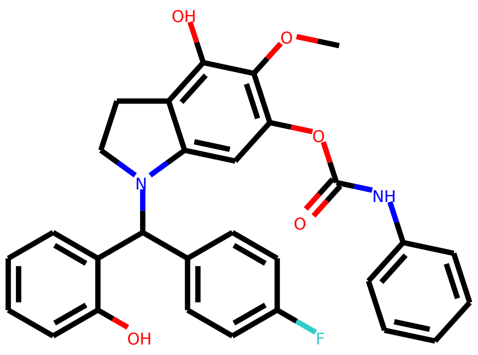

Osma-336

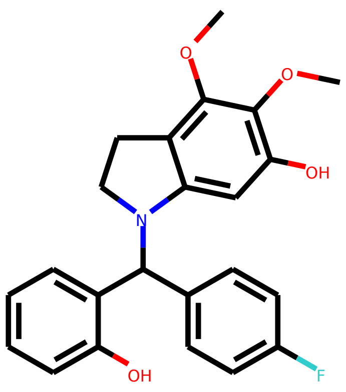

Osma-337

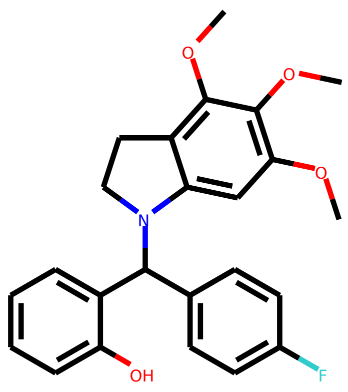

Osma-338

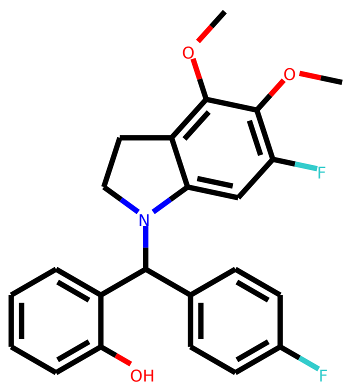

Osma-339

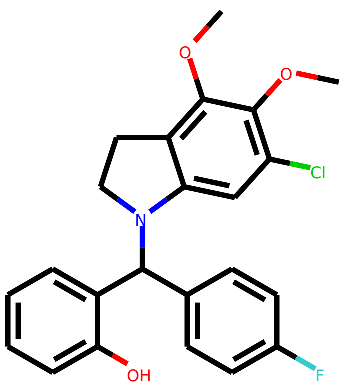

Osma-340

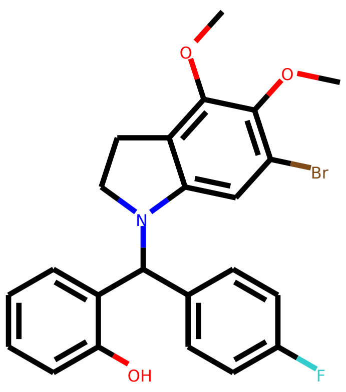

Osma-341

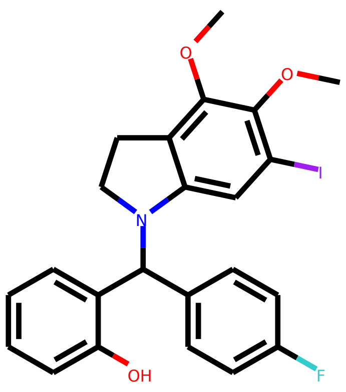

Osma-342

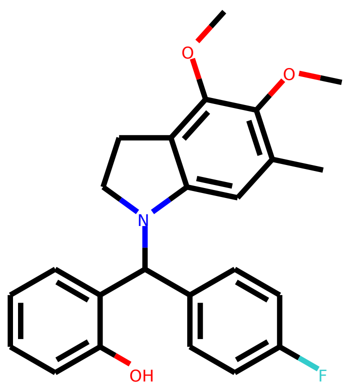

Osma-343

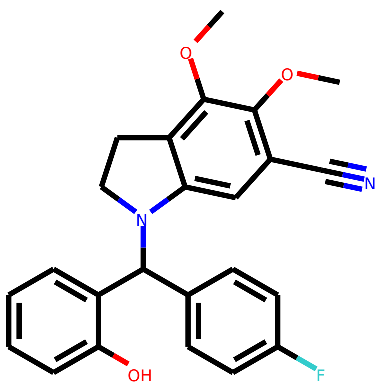

Osma-344

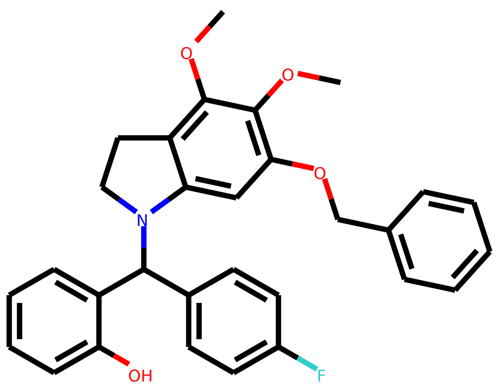

Osma-345

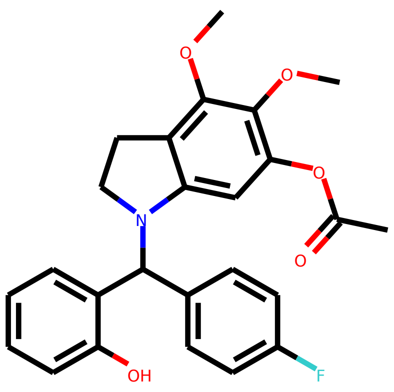

Osma-346

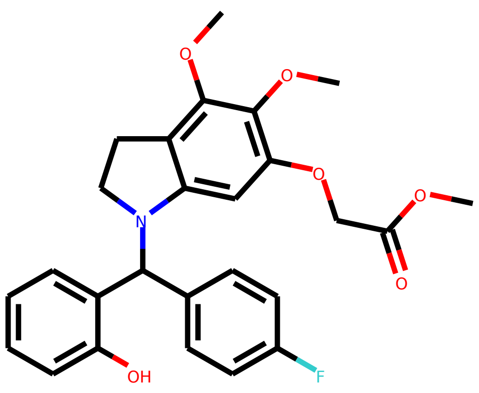

Osma-347

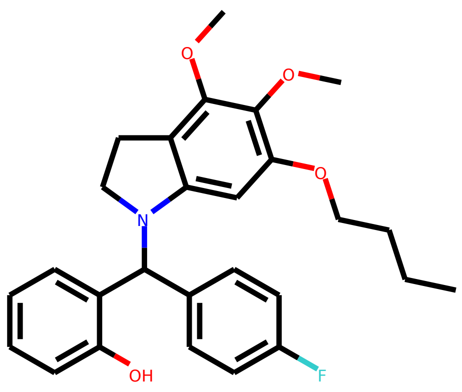

Osma-348

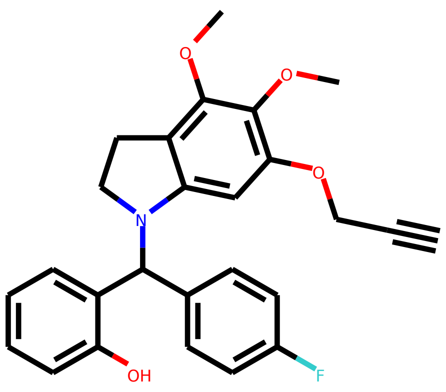

Osma-349

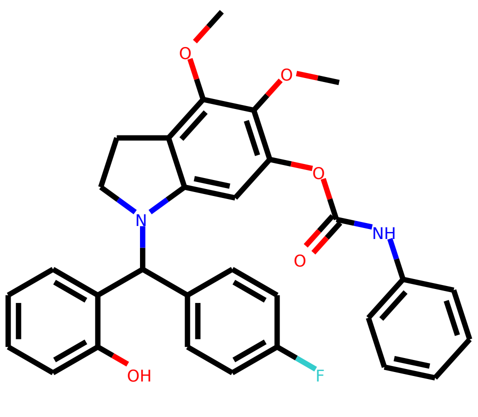

Osma-350

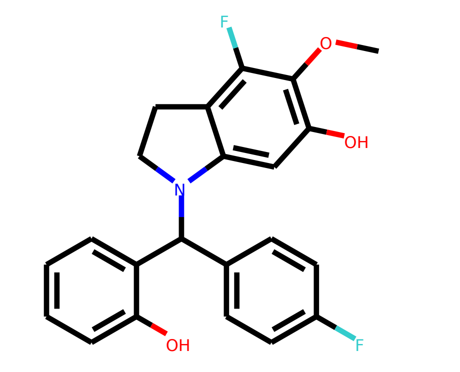
Osma-351
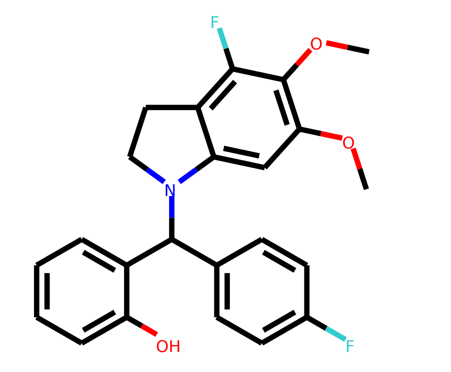
Osma-352
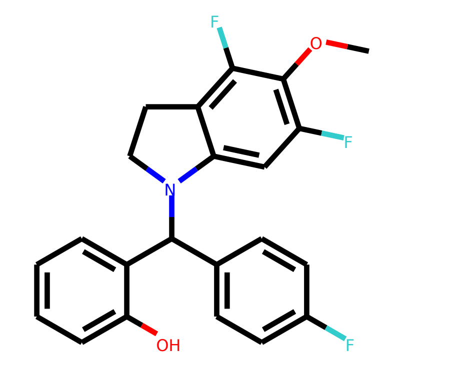
Osma-353
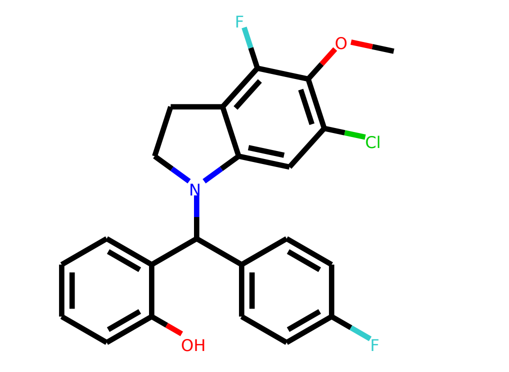
Osma-354
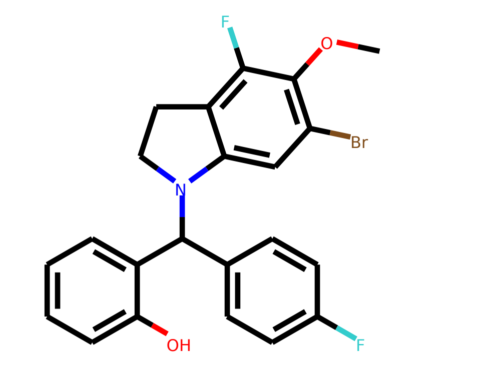
Osma-355
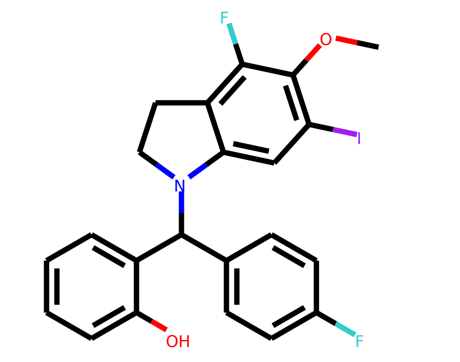
Osma-356
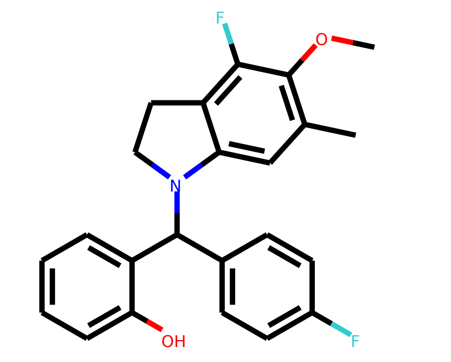
Osma-357
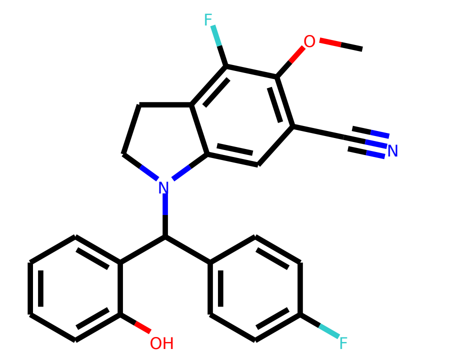
Osma-358
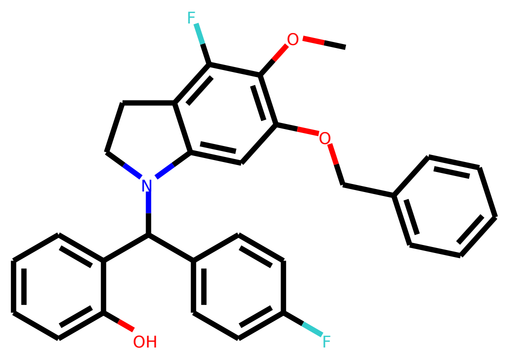
Osma-359
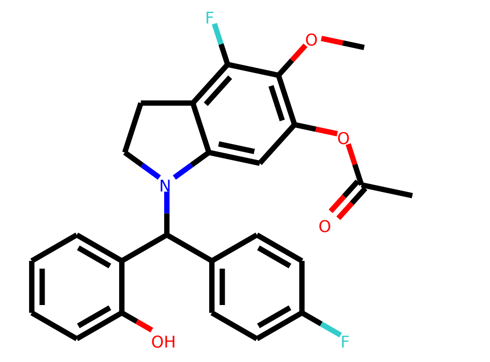
Osma-360
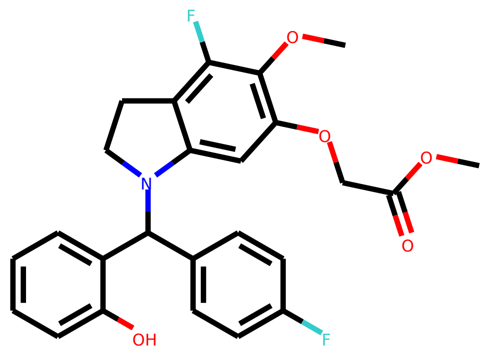
Osma-361
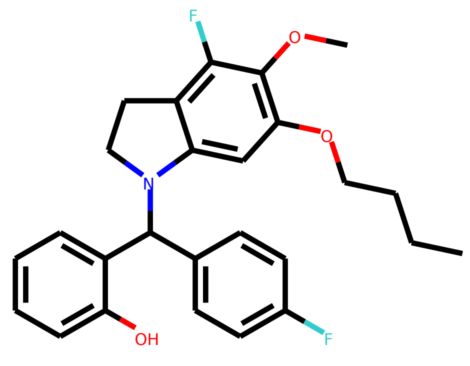
Osma-362
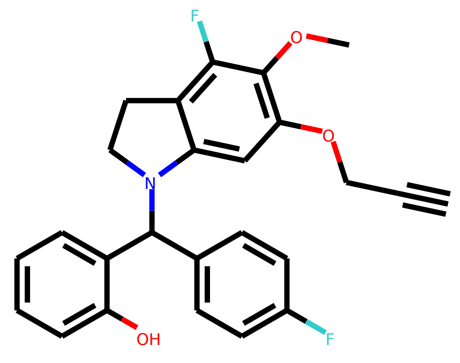
Osma-363
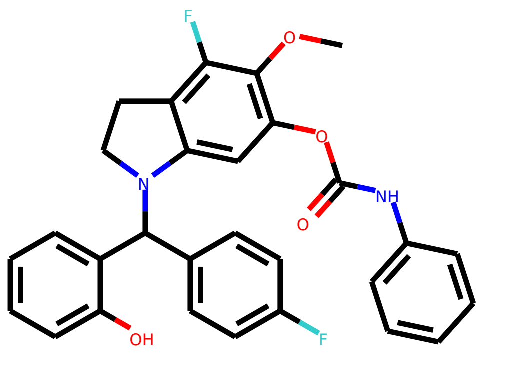
Osma-364
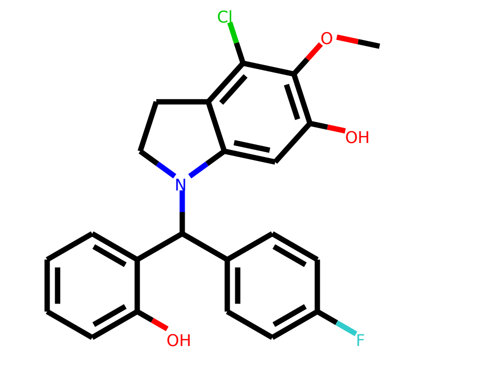
Osma-365
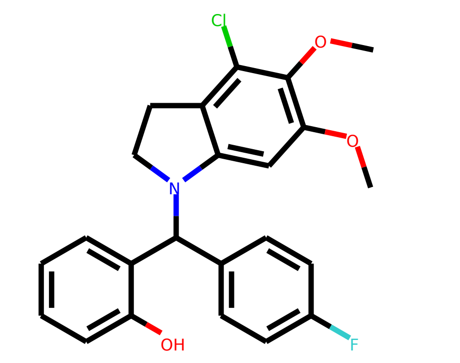
Osma-366
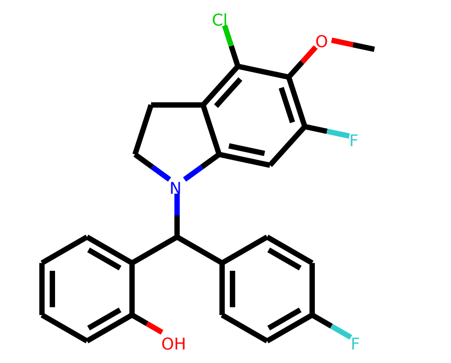
Osma-367
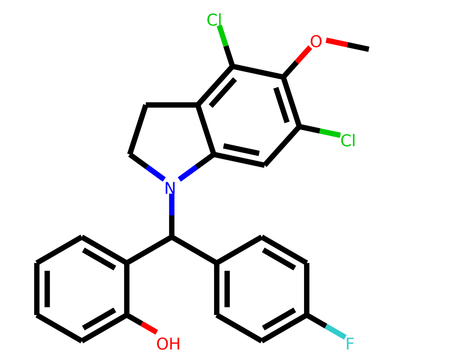
Osma-368
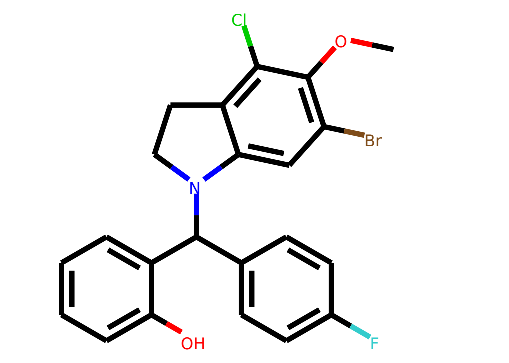
Osma-369
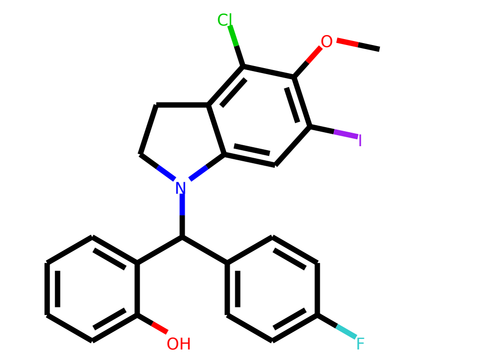
Osma-370
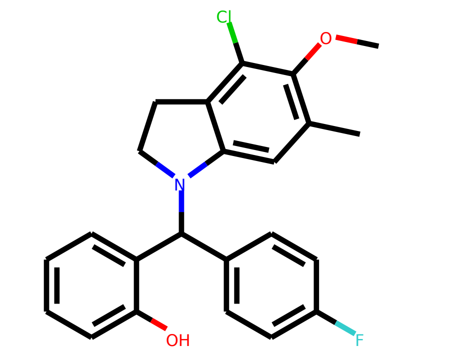
Osma-371
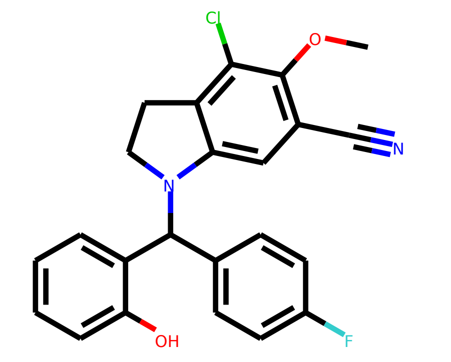
Osma-372
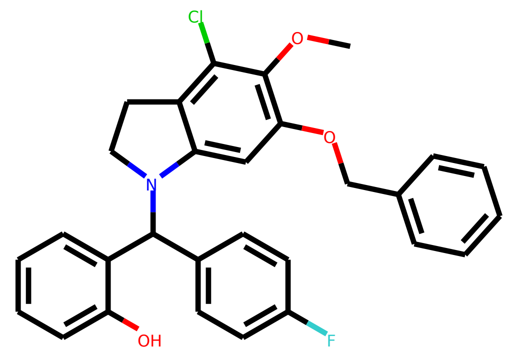
Osma-373
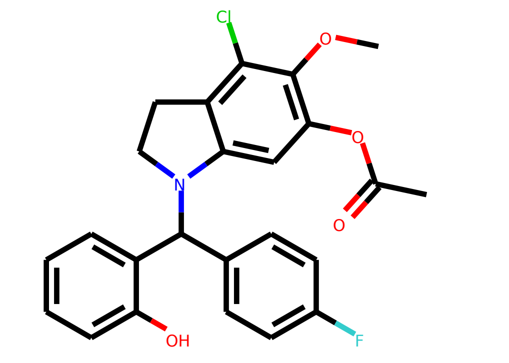
Osma-374
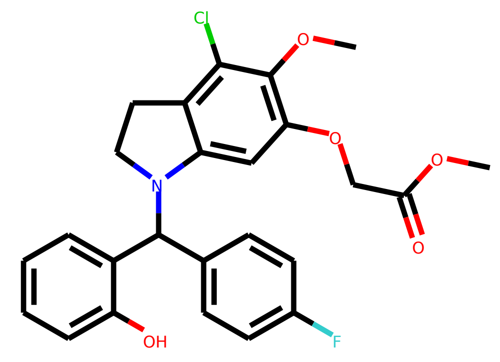
Osma-375
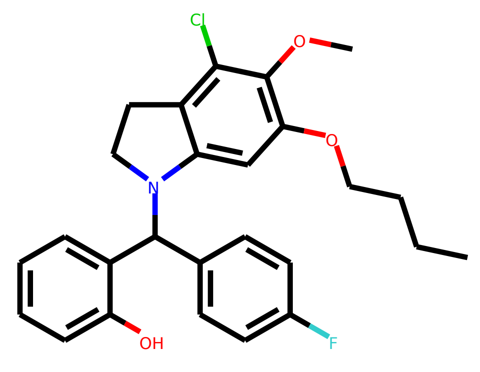
Osma-376
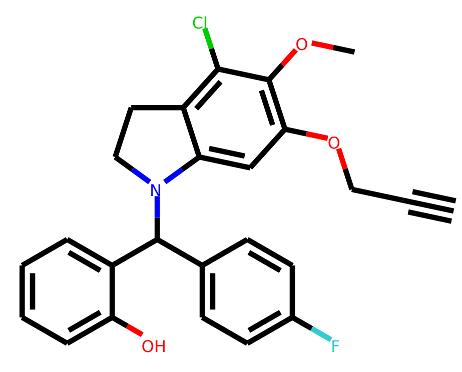
Osma-377
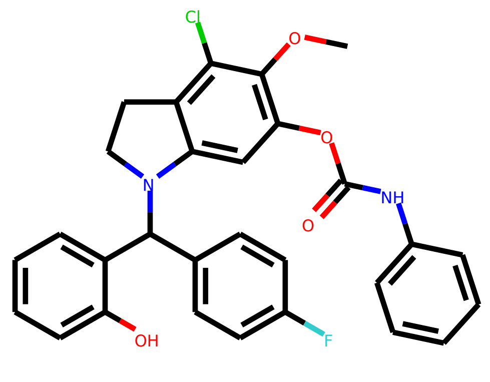
Osma-378
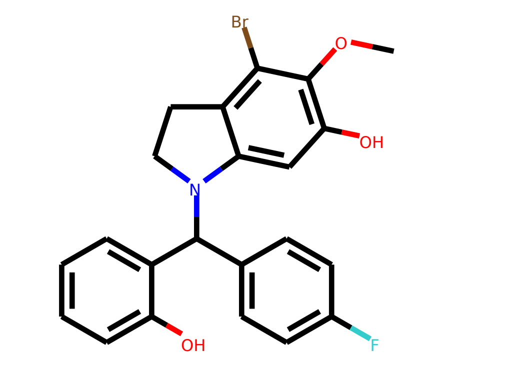
Osma-379
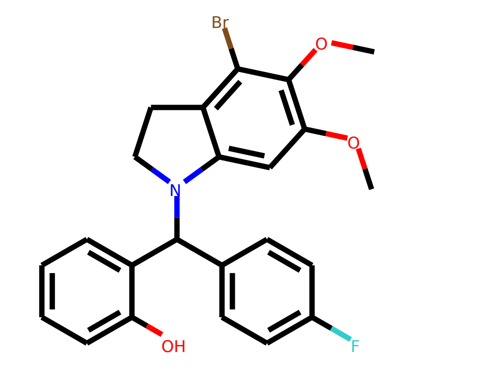
Osma-380
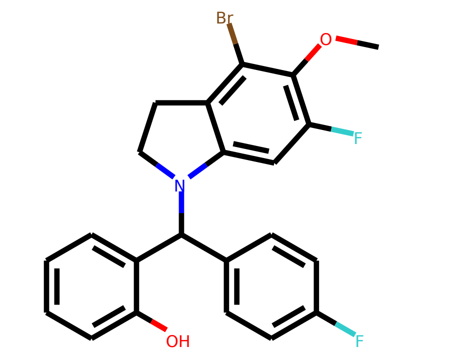
Osma-381
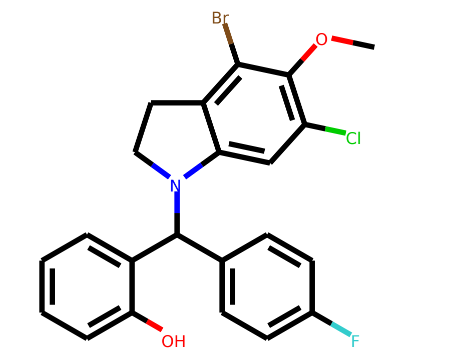
Osma-382
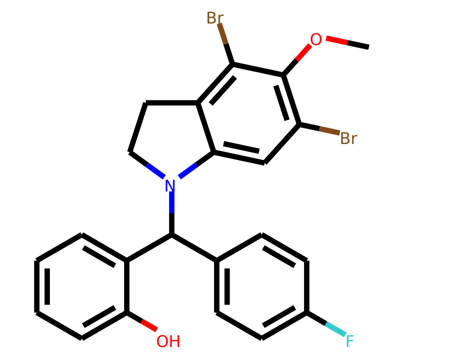
Osma-383
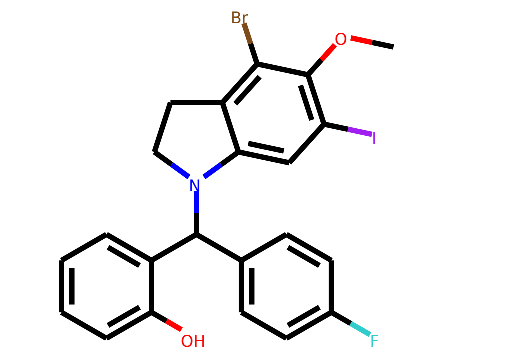
Osma-384
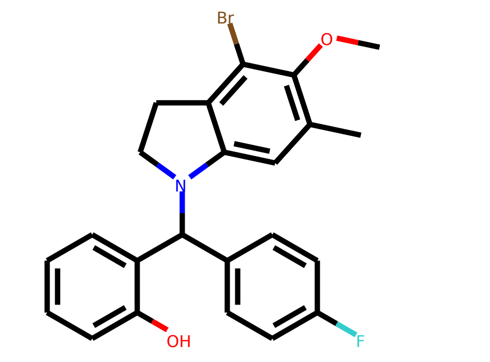
Osma-385

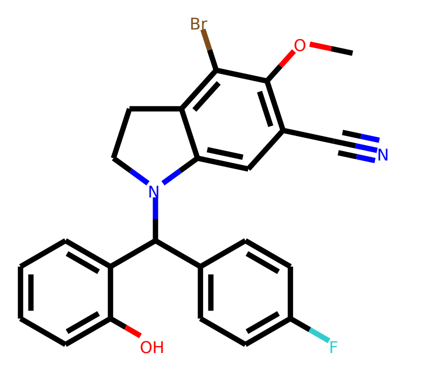

Osma-386

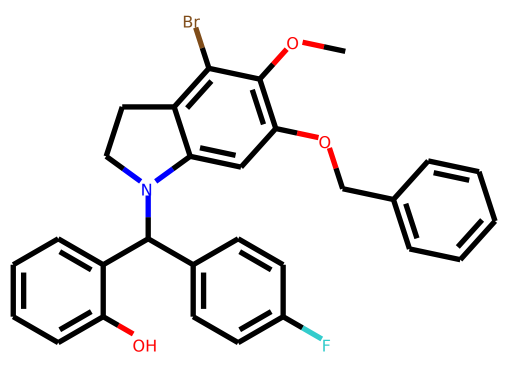

Osma-387

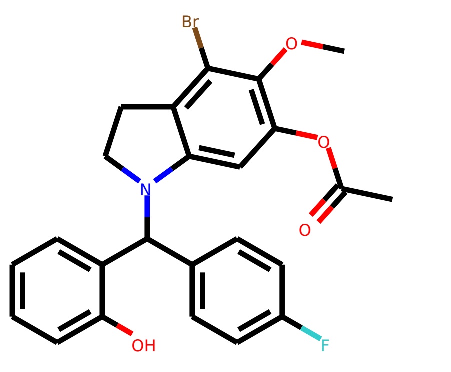

Osma-388

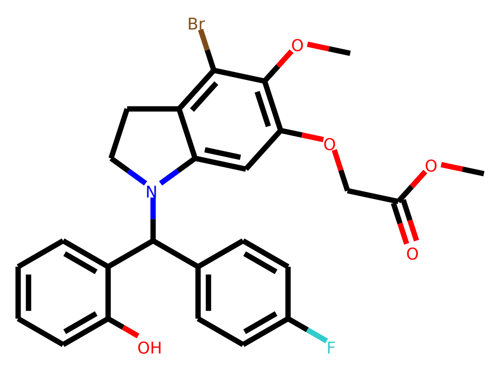

Osma-389

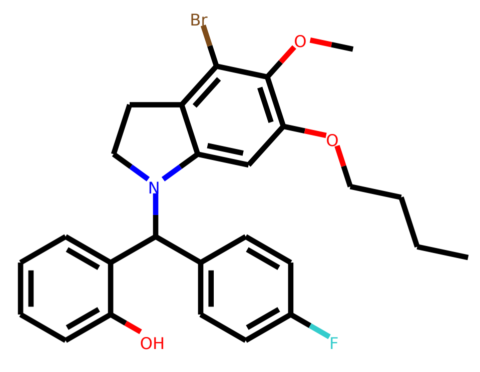

Osma-390

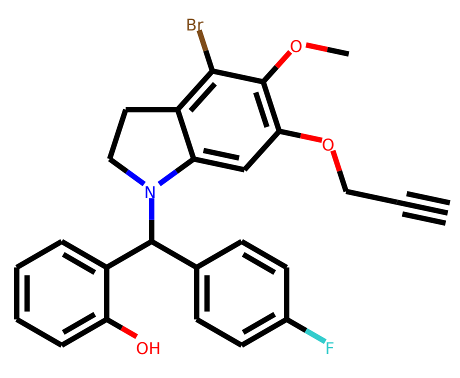

Osma-391

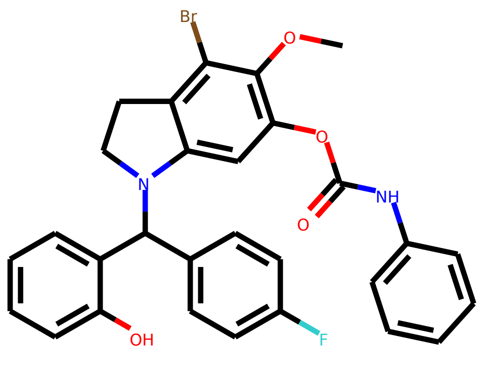

Osma-392

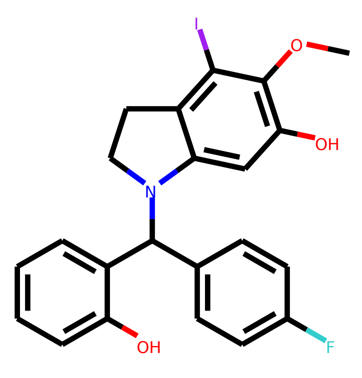

Osma-393

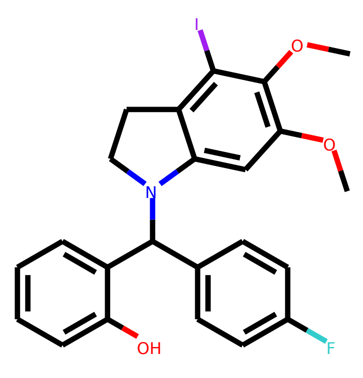

Osma-394

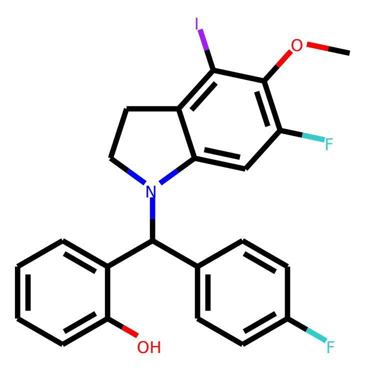

Osma-395

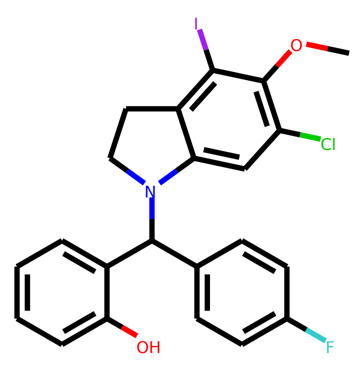

Osma-396

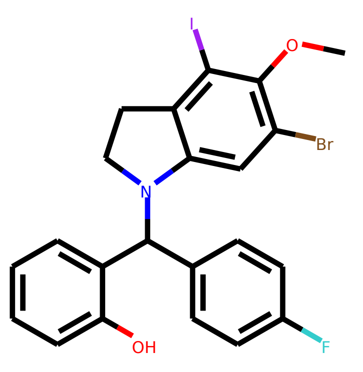

Osma-397

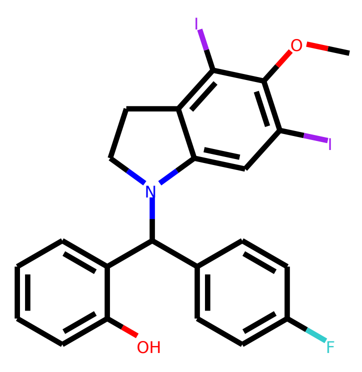

Osma-398

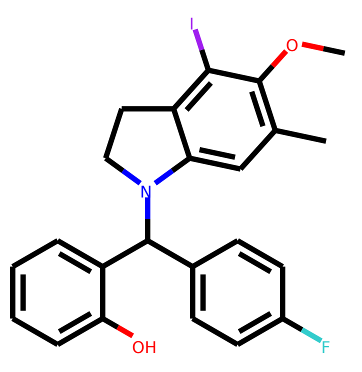

Osma-399

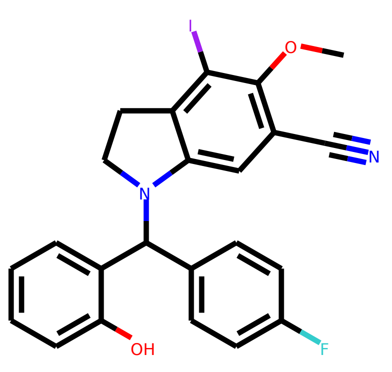

Osma-400

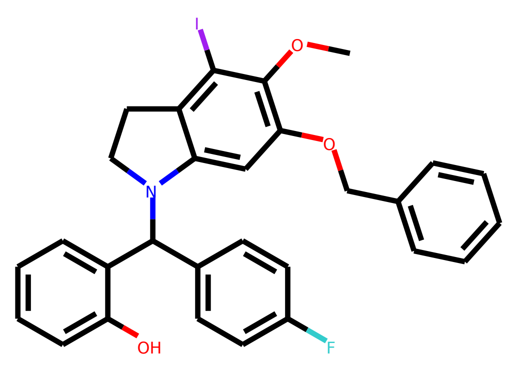

Osma-401

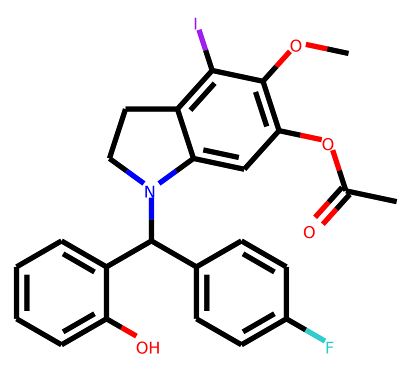

Osma-402

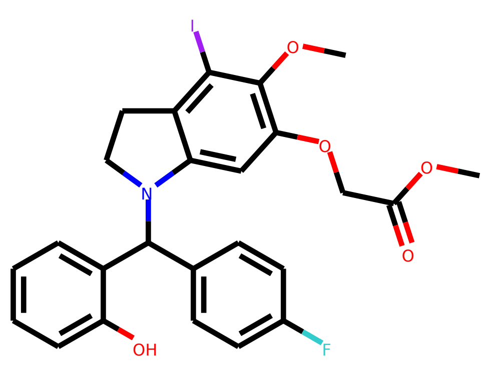

Osma-403

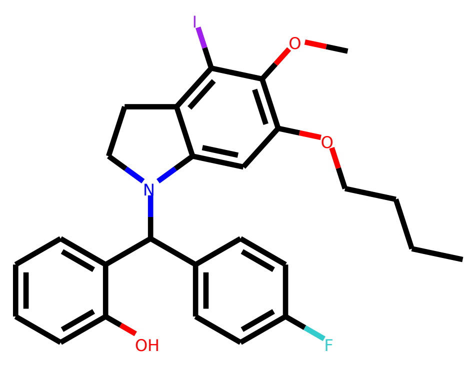

Osma-404

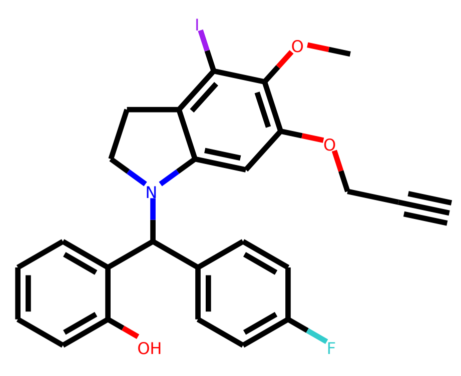

Osma-405

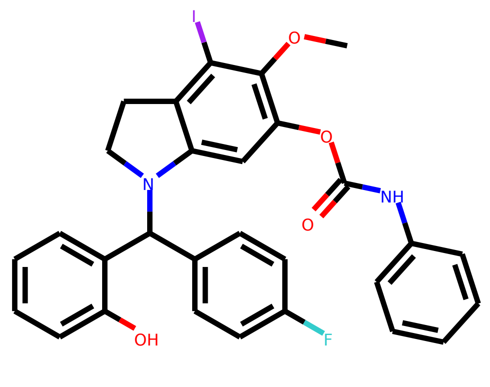

Osma-406

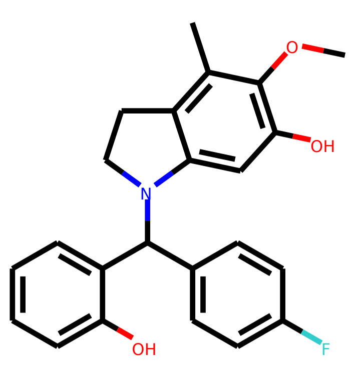

Osma-407

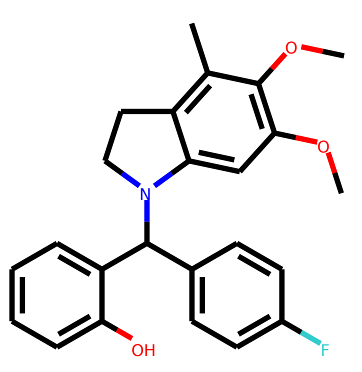

Osma-408

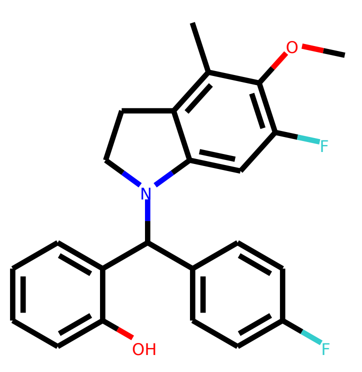

Osma-409

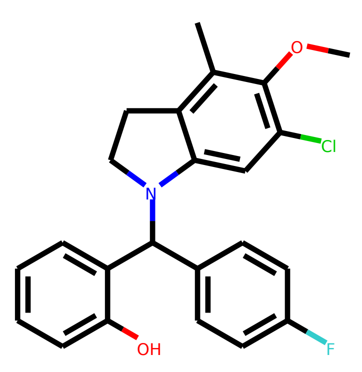

Osma-410

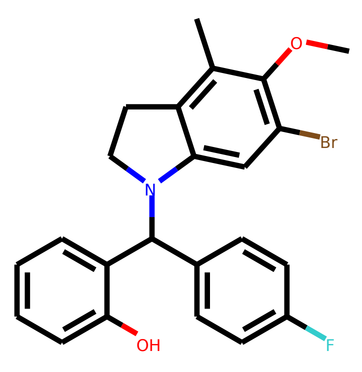

Osma-411

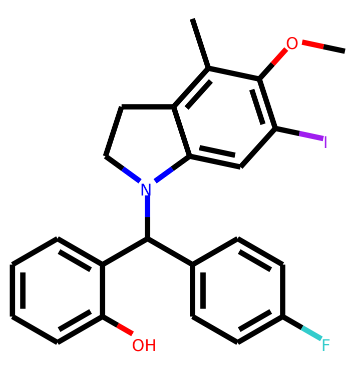

Osma-412

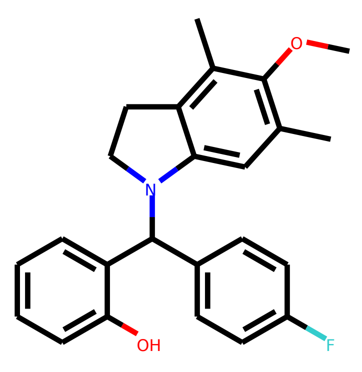

Osma-413

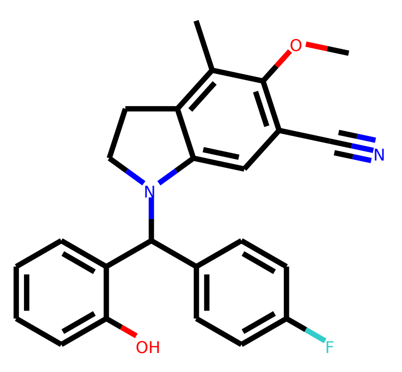

Osma-414

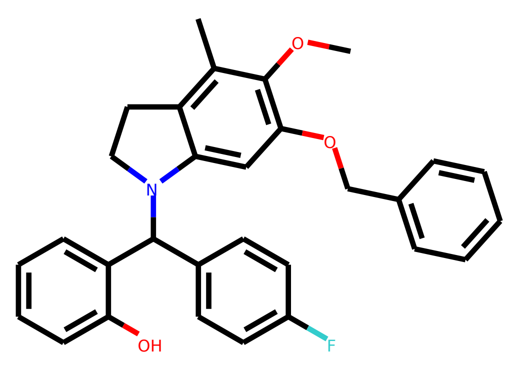

Osma-415

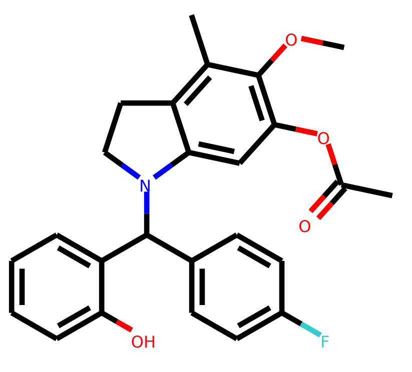

Osma-416

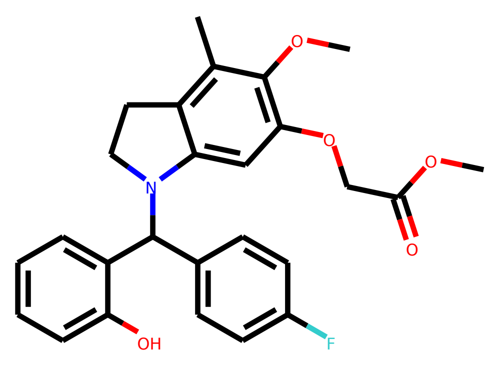

Osma-417

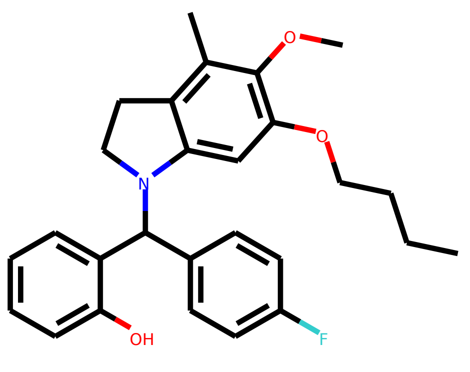

Osma-418

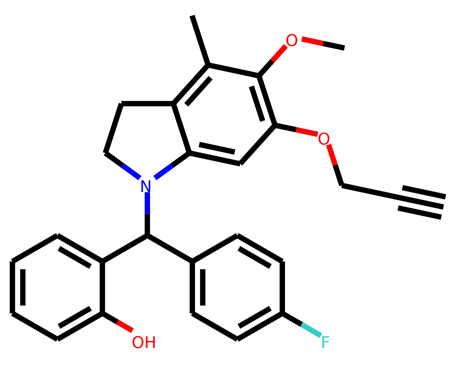

Osma-419

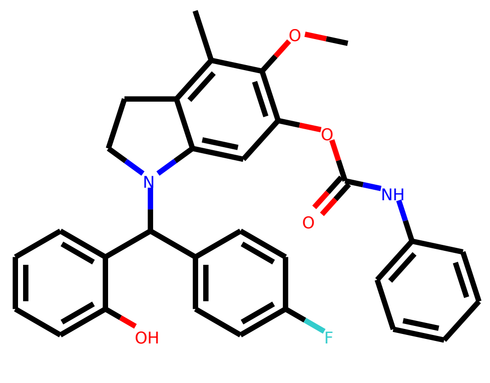

Osma-420

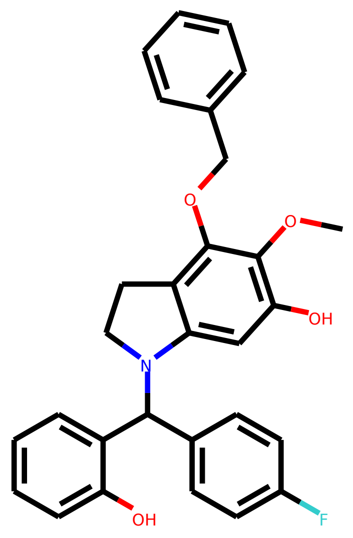
Osma-421
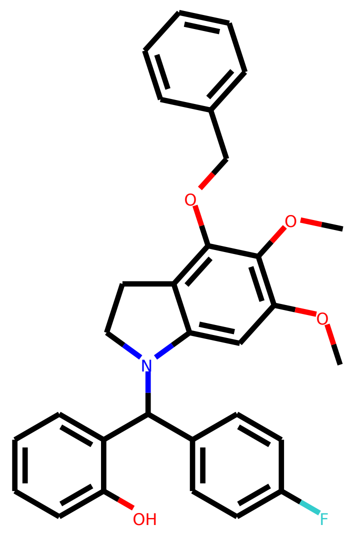
Osma-422
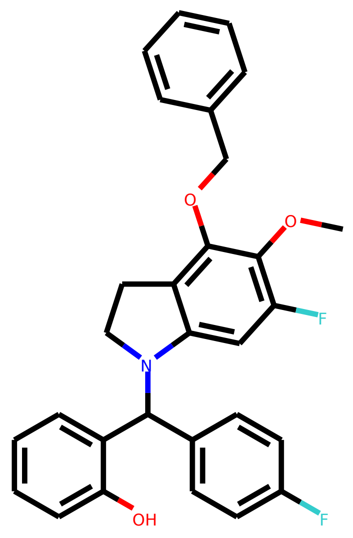
Osma-423
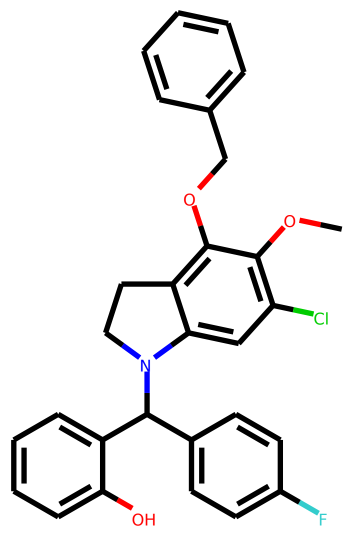
Osma-424
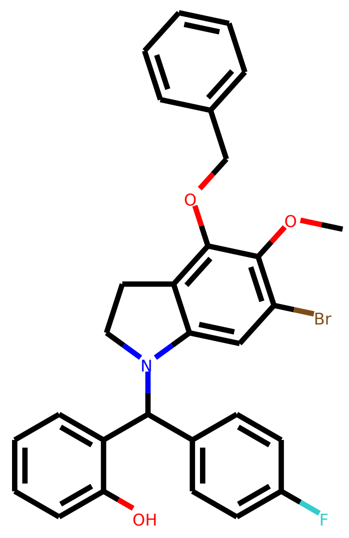
Osma-425
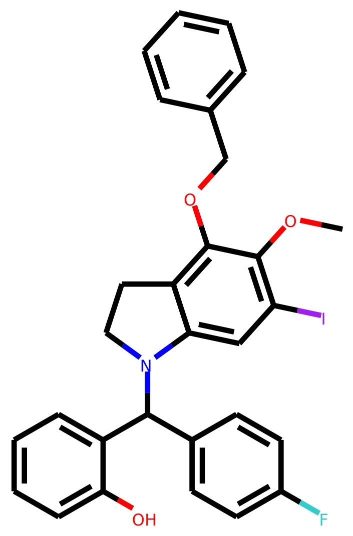
Osma-426
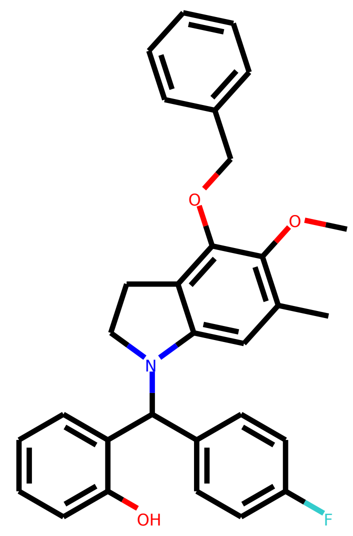
Osma-427
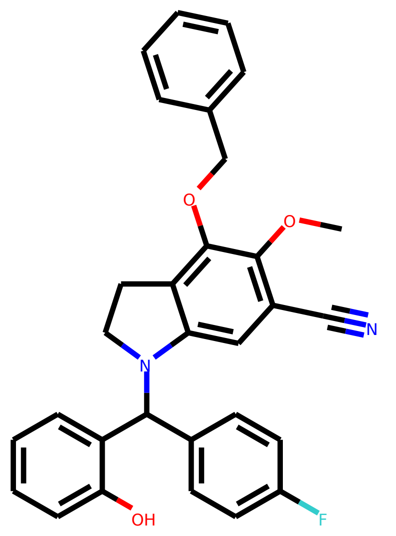
Osma-428
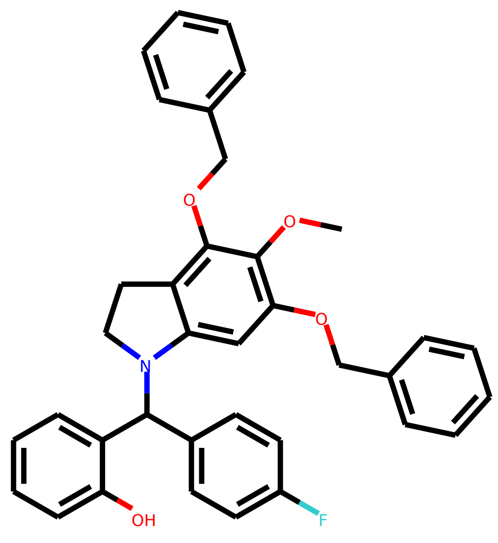
Osma-429
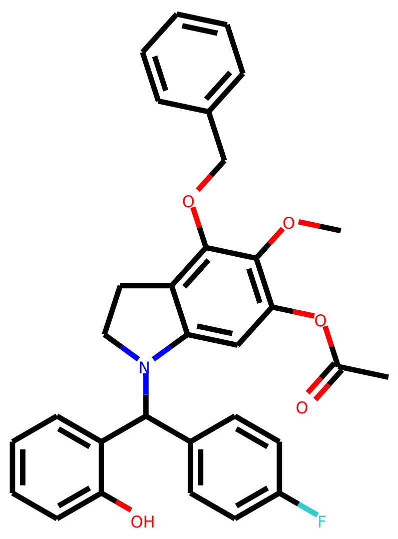
Osma-430
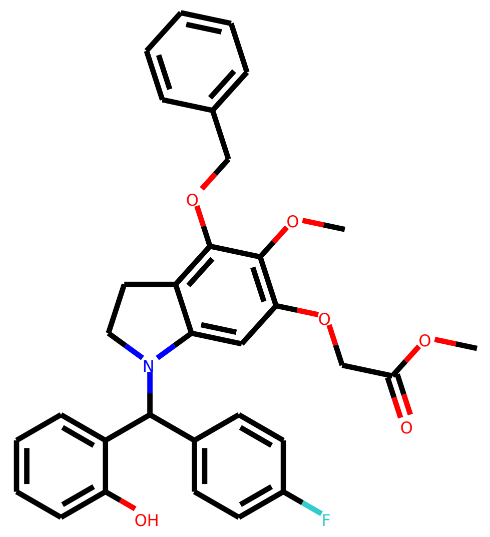
Osma-431
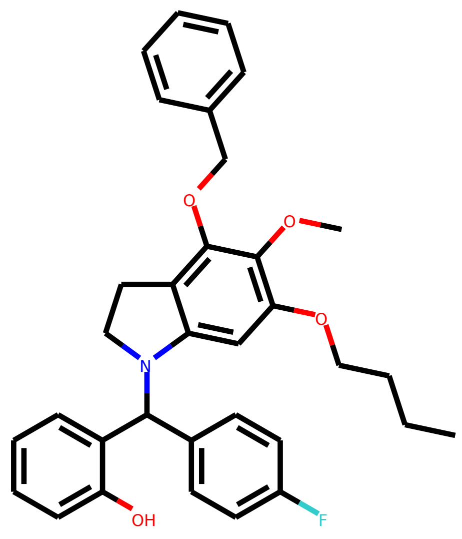
Osma-432
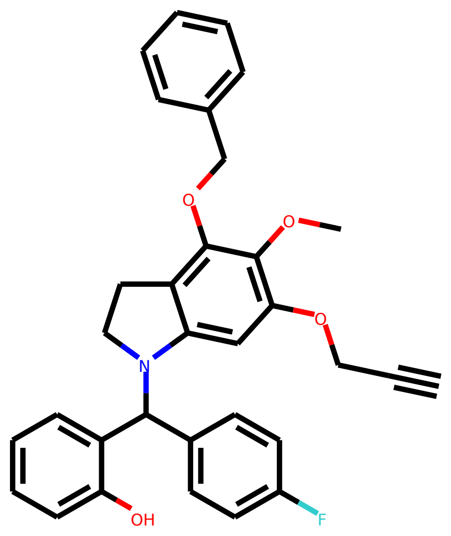
Osma-433
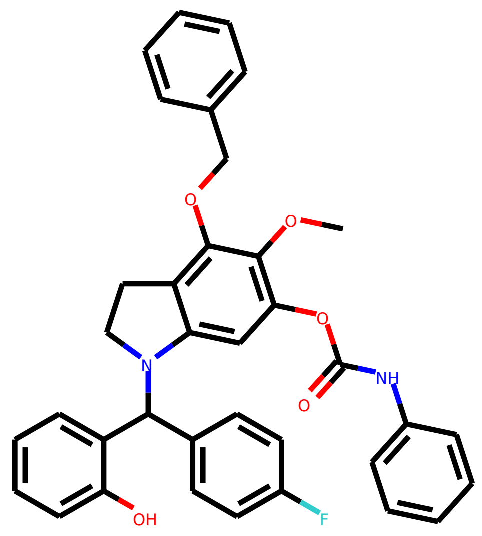
Osma-434
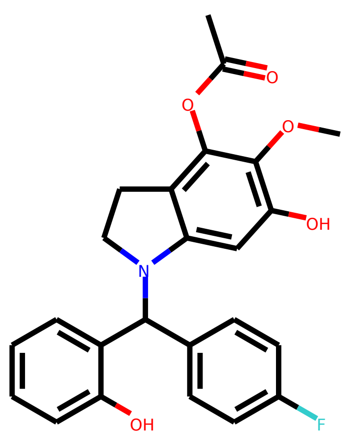
Osma-435
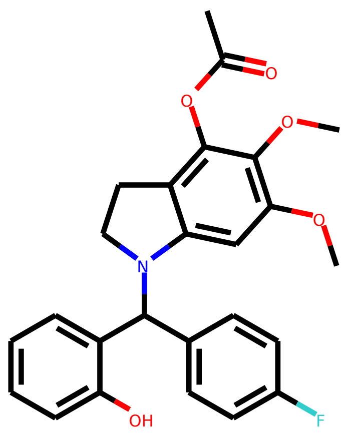
Osma-436
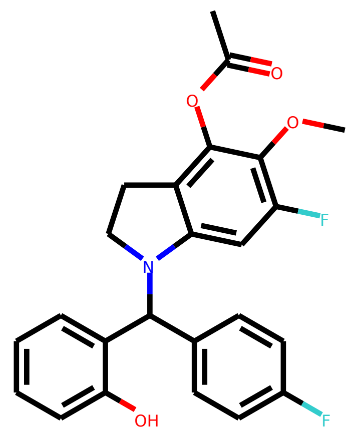
Osma-437
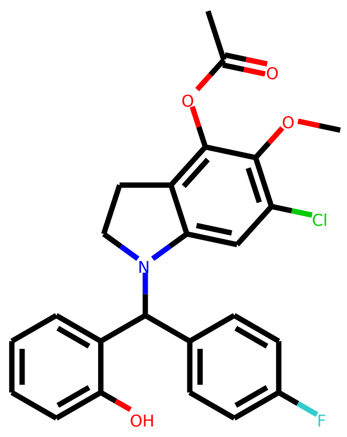
Osma-438
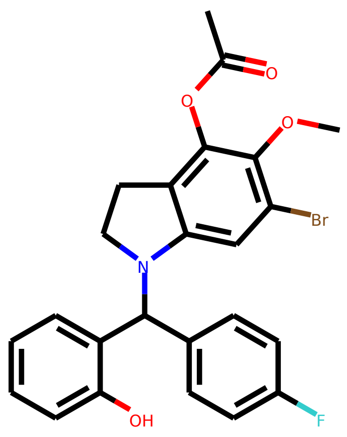
Osma-439
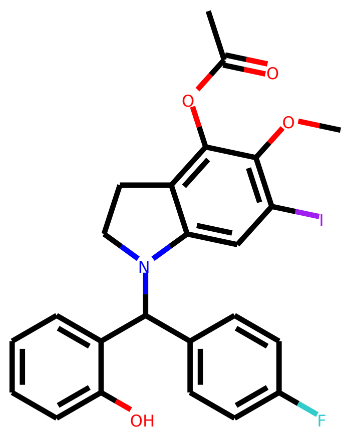
Osma-440
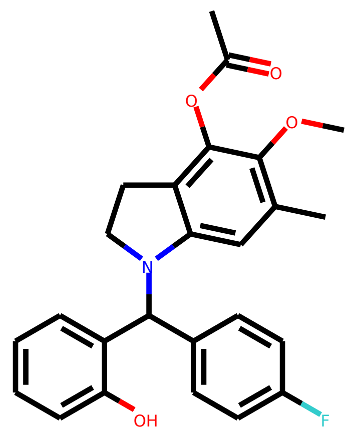
Osma-441
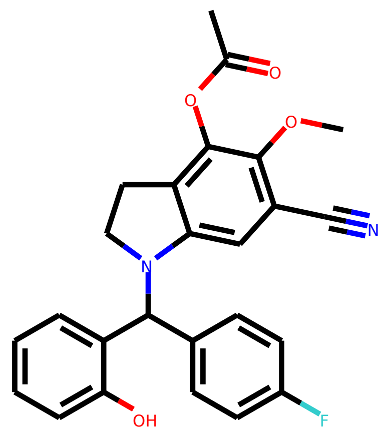
Osma-442
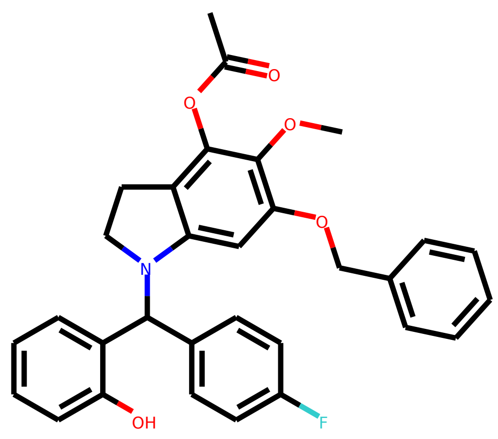
Osma-443
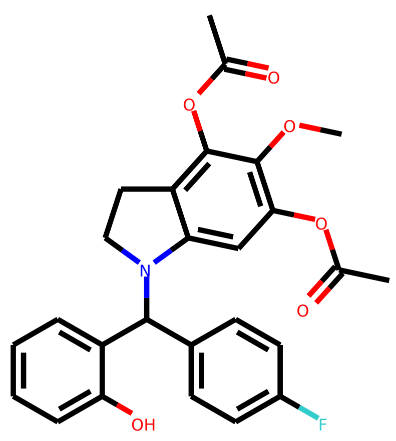
Osma-444
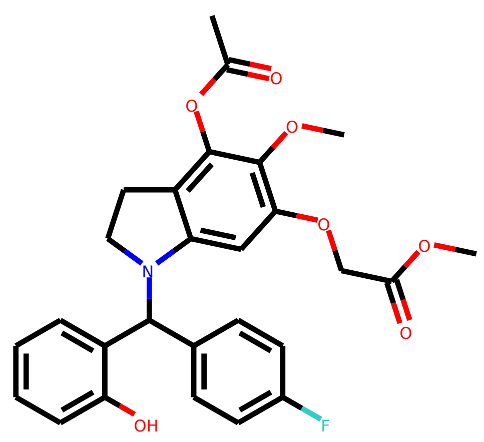
Osma-445
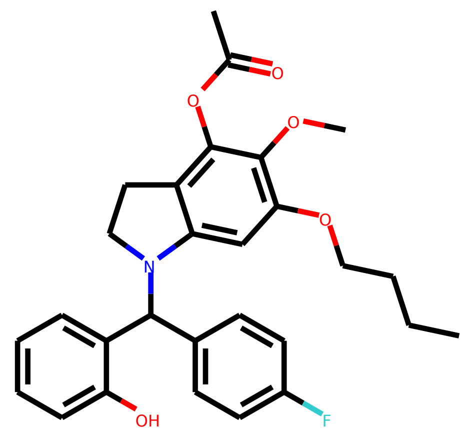
Osma-446
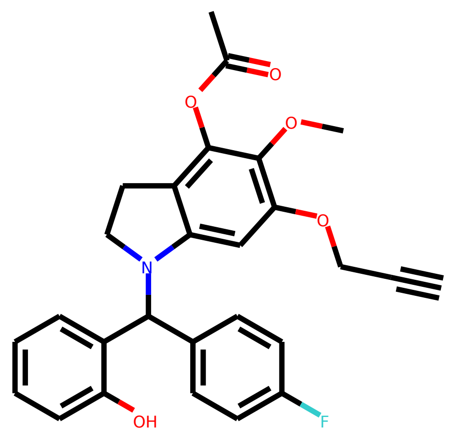
Osma-447
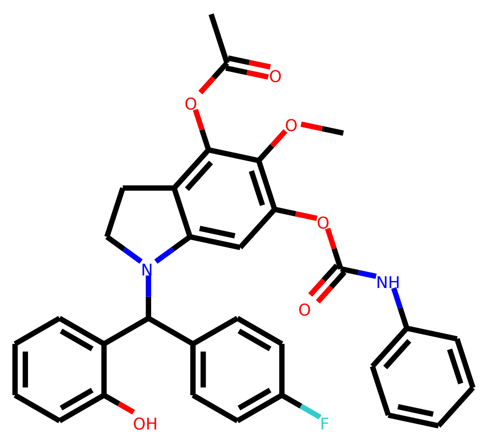
Osma-448
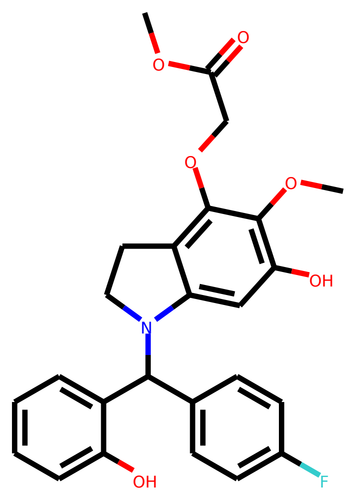
Osma-449
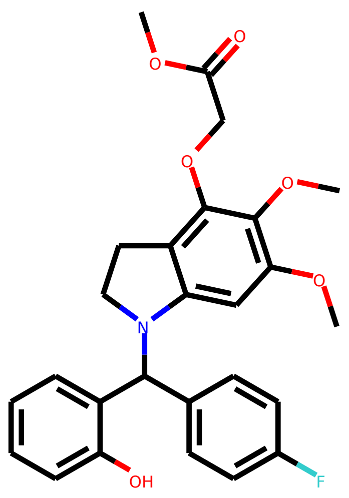
Osma-450
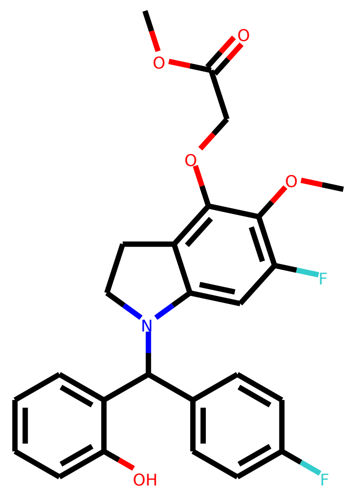
Osma-451
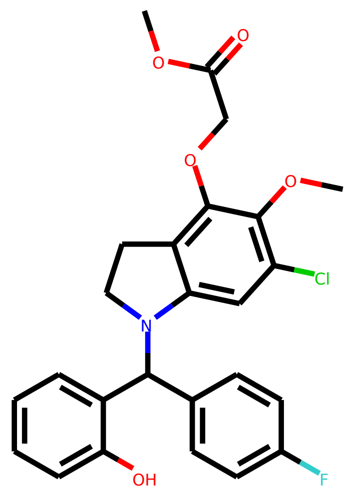
Osma-452
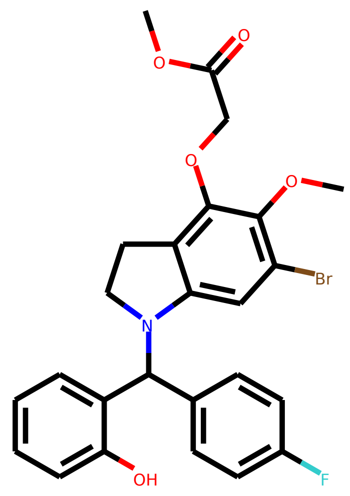
Osma-453
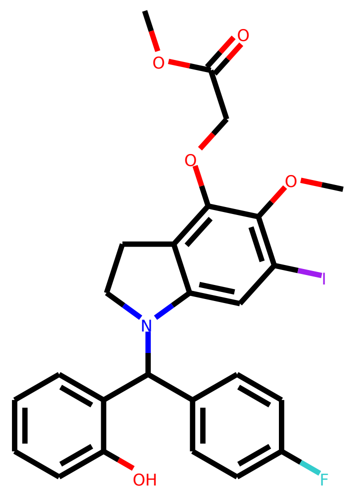
Osma-454
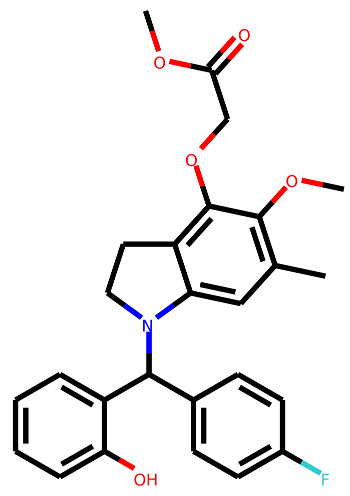
Osma-455

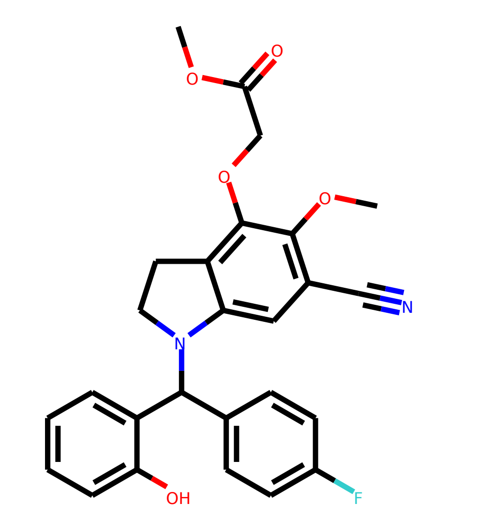
Osma-456
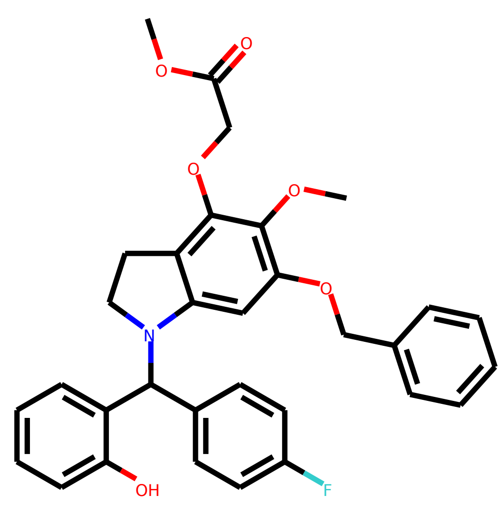
Osma-457
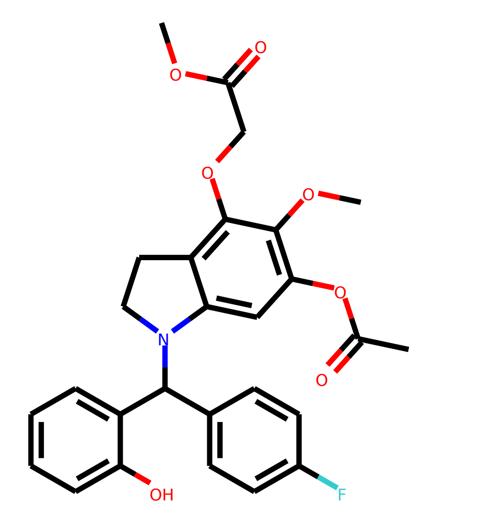
Osma-458
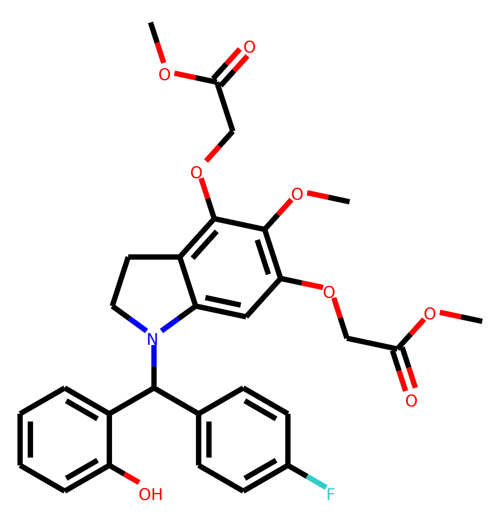
Osma-459
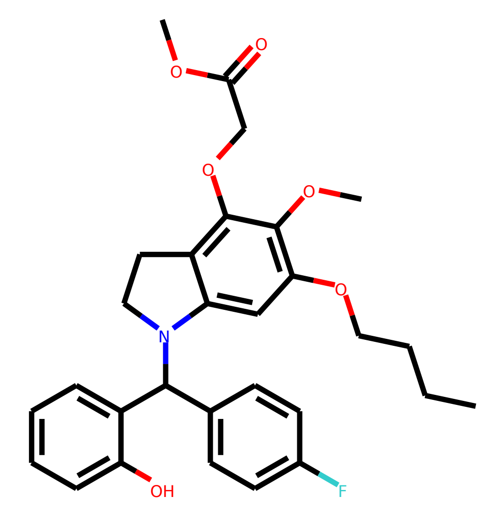
Osma-460
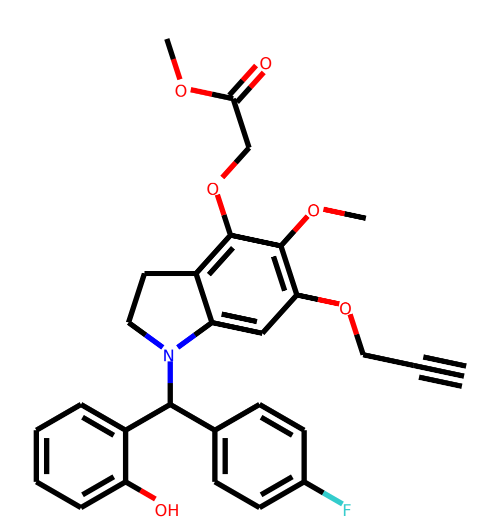
Osma-461
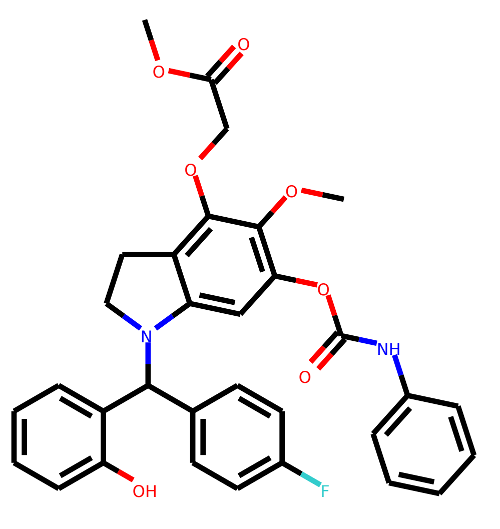
Osma-462
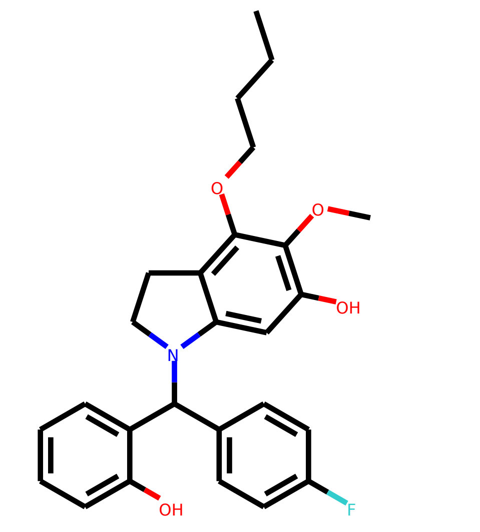
Osma-463
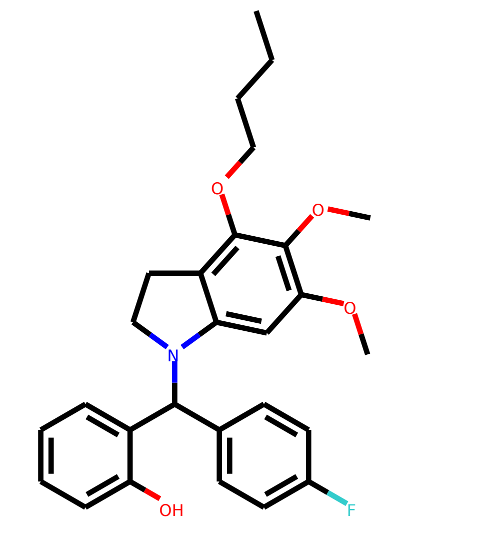
Osma-464
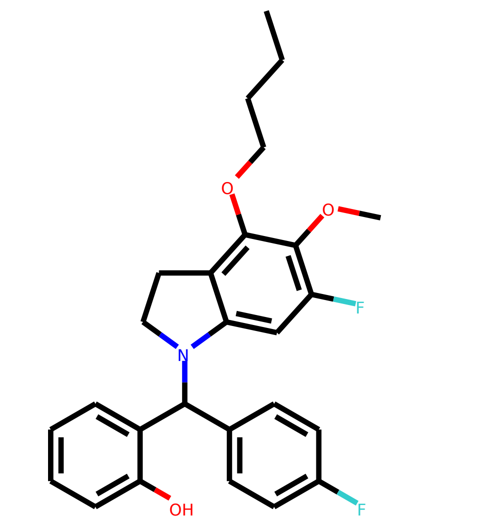
Osma-465
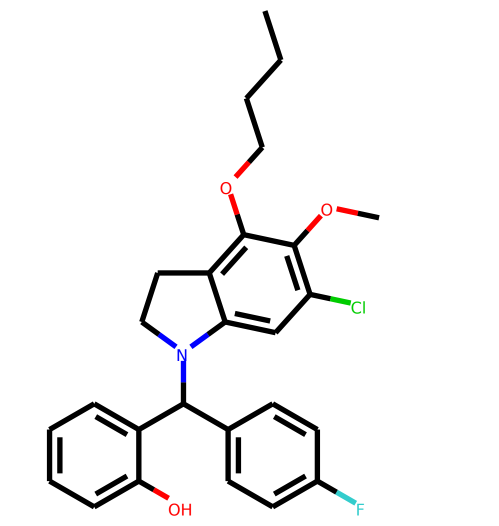
Osma-466
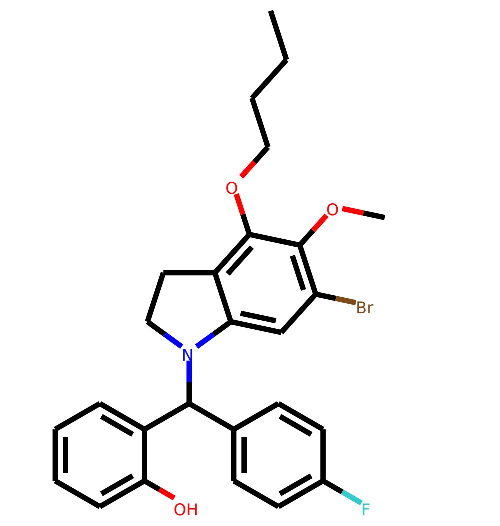
Osma-467
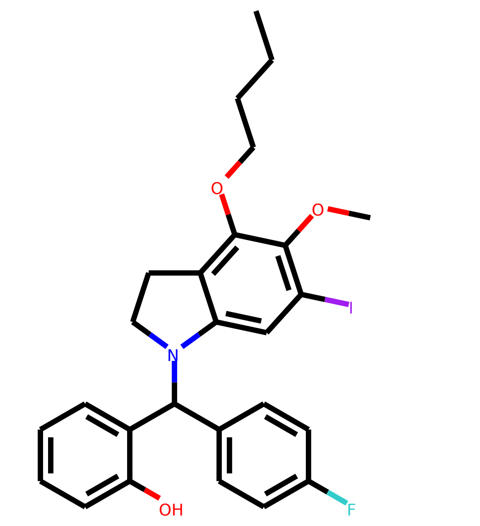
Osma-468
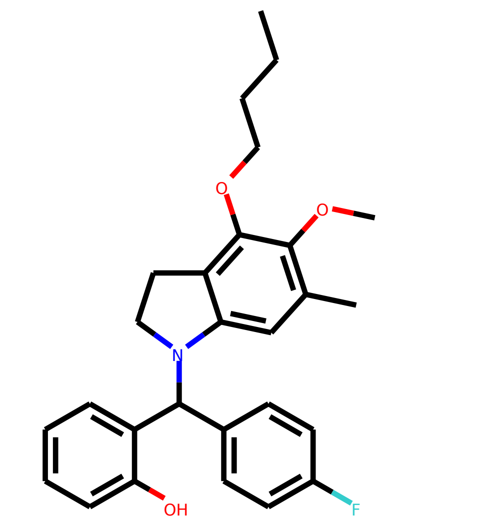
Osma-469
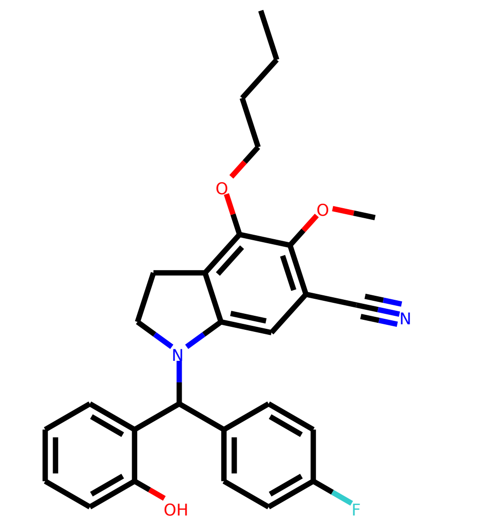
Osma-470
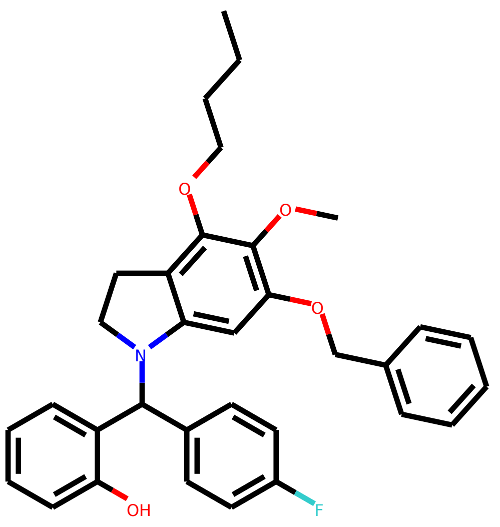
Osma-471
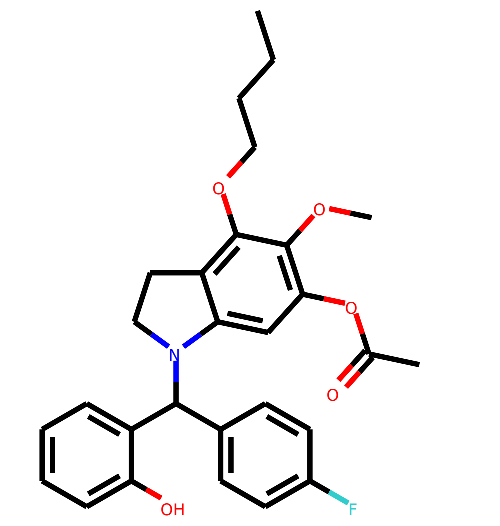
Osma-472
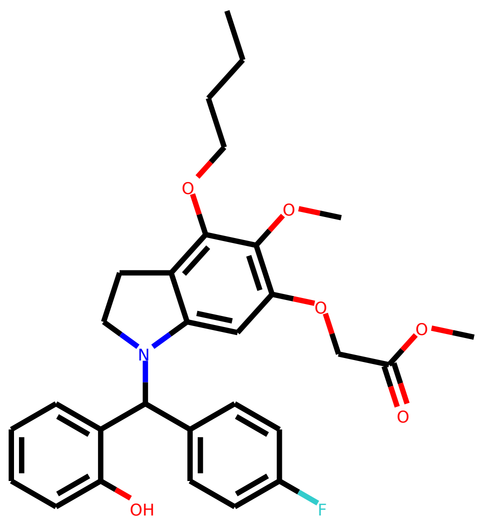
Osma-473
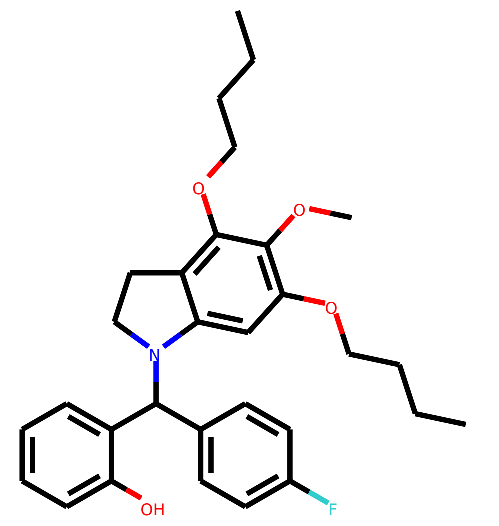
Osma-474
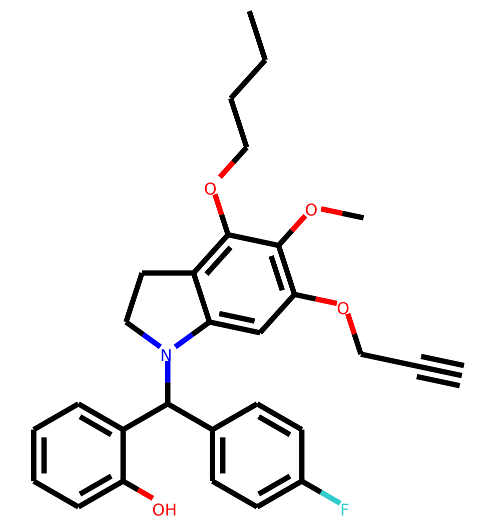
Osma-475
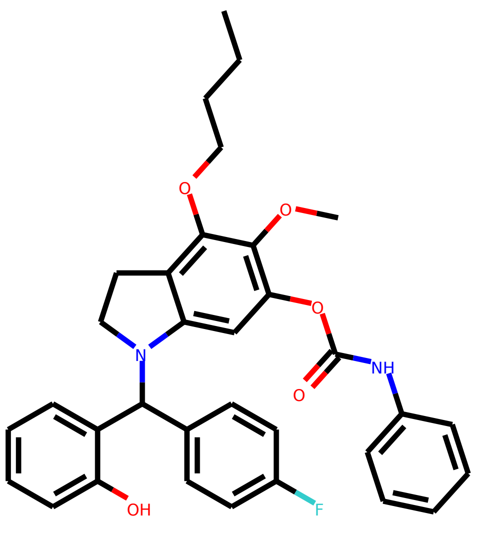
Osma-476
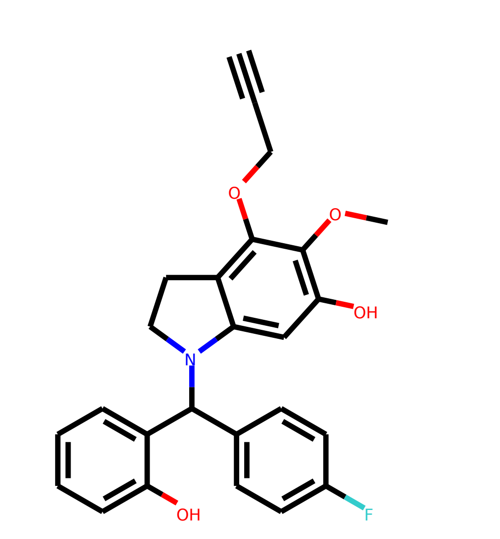
Osma-477
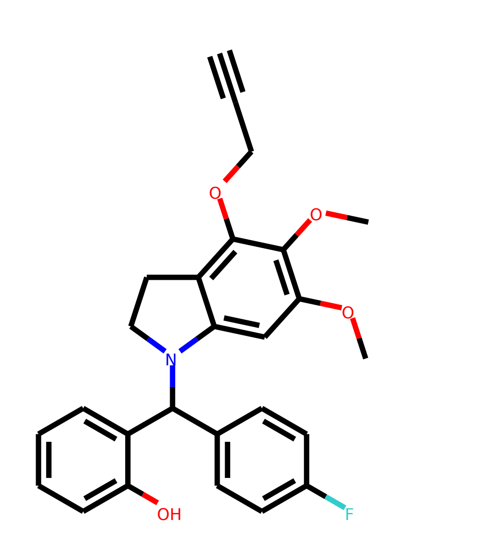
Osma-478
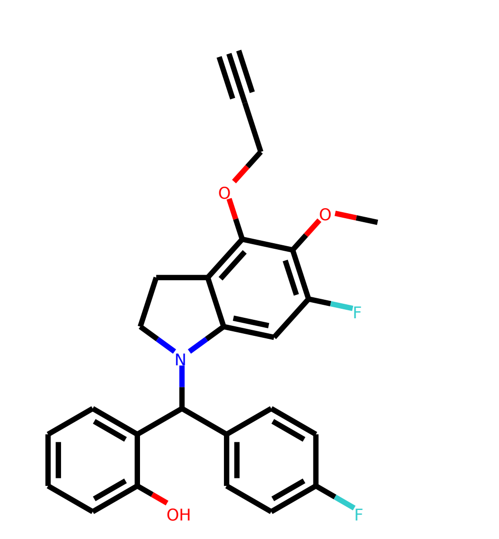
Osma-479
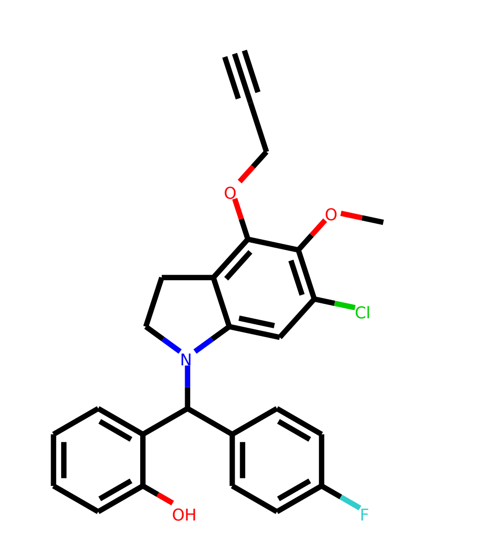
Osma-480
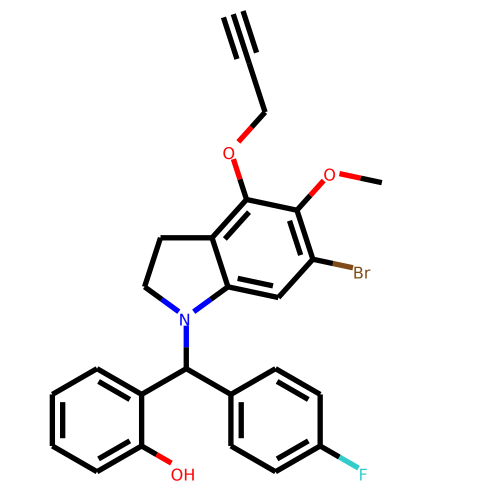
Osma-481
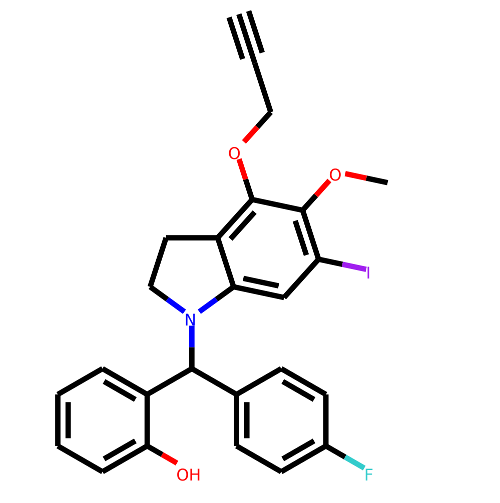
Osma-482
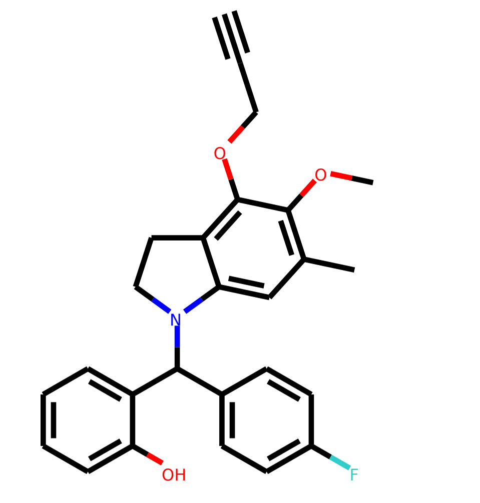
Osma-483
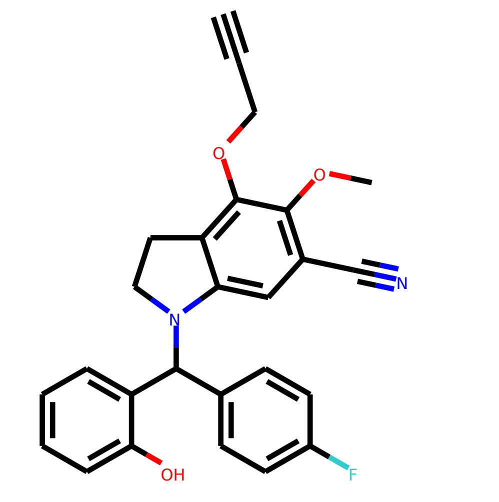
Osma-484
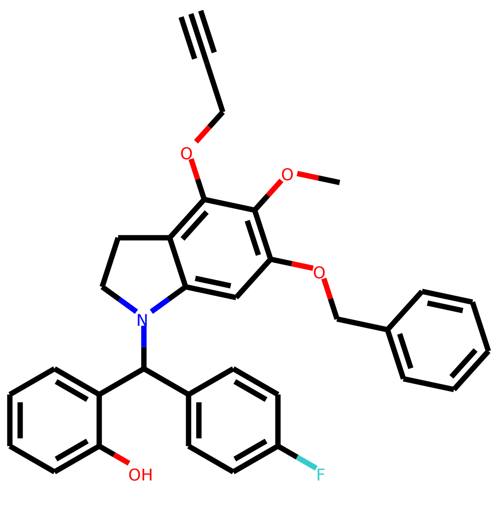
Osma-485
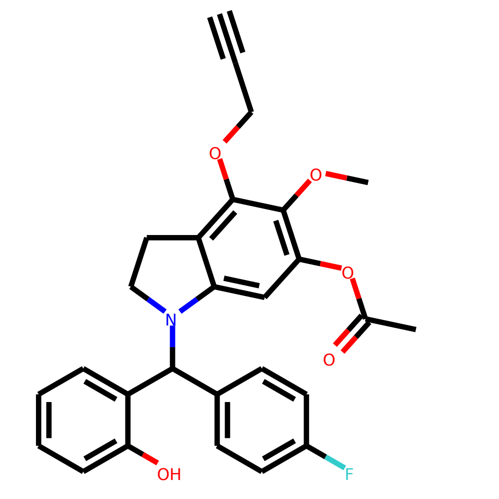
Osma-486
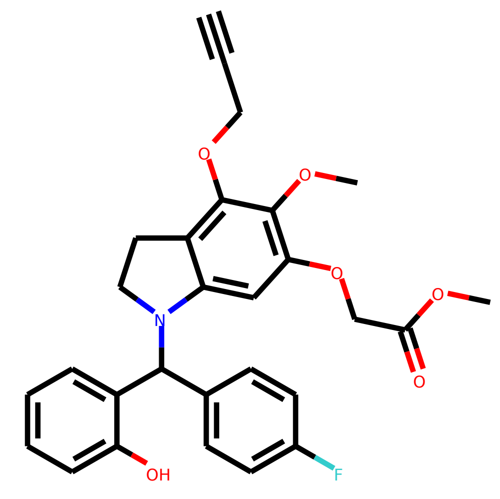
Osma-487
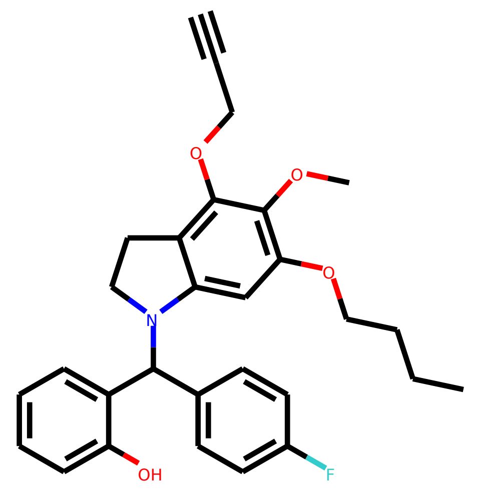
Osma-488
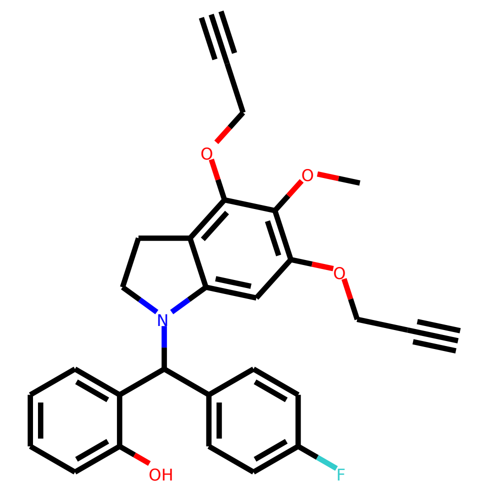
Osma-489
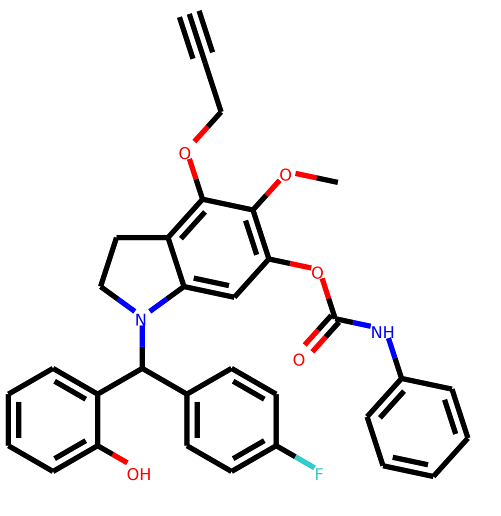
Osma-490

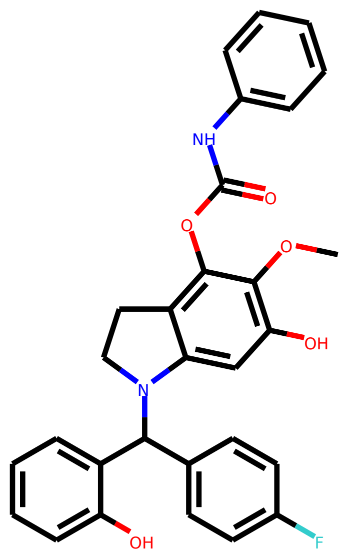
Osma-491
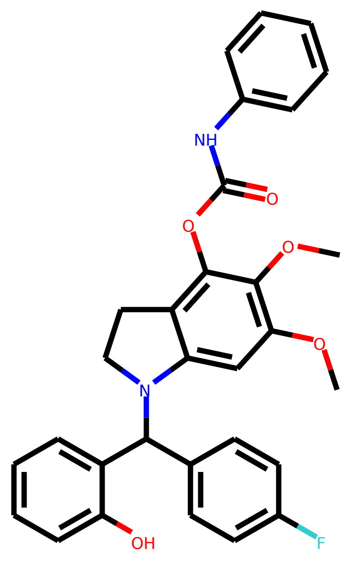
Osma-492
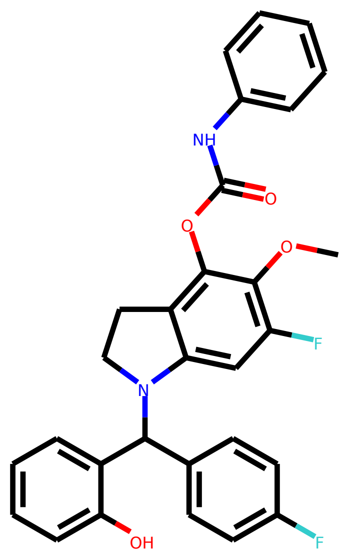
Osma-493
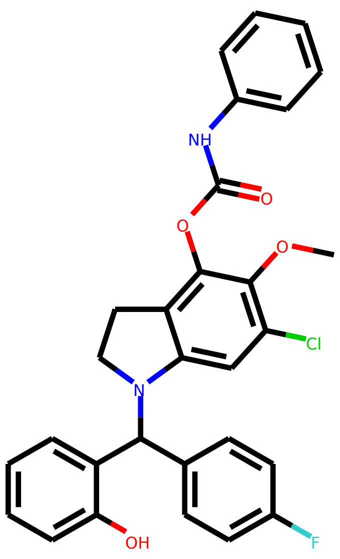
Osma-494
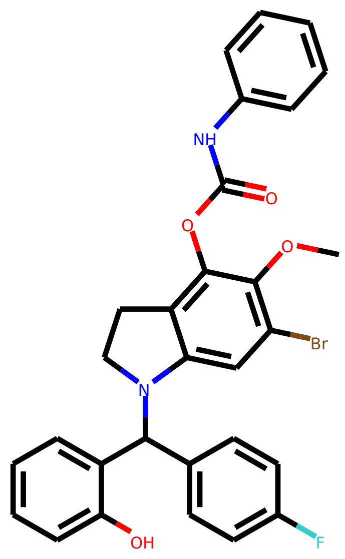
Osma-495
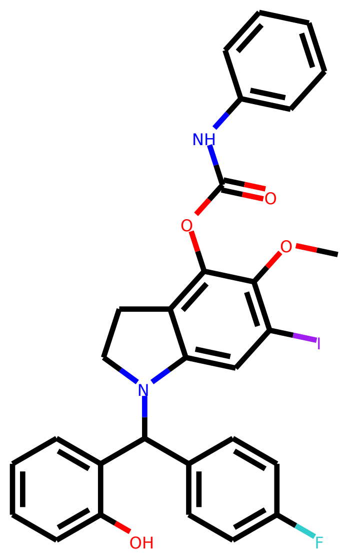
Osma-496
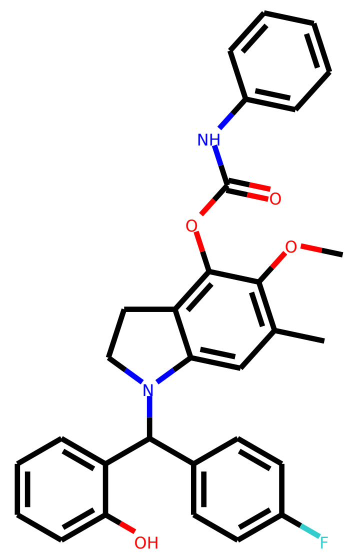
Osma-497
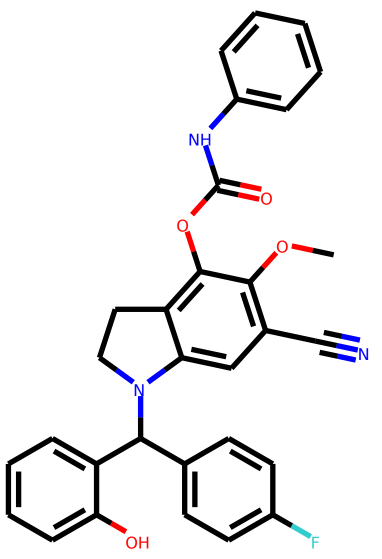
Osma-498
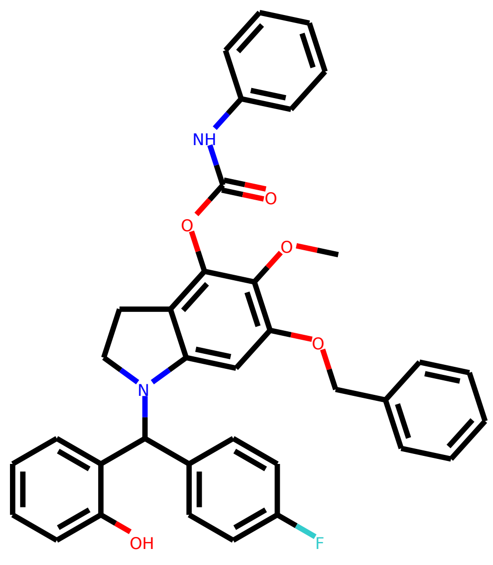
Osma-499
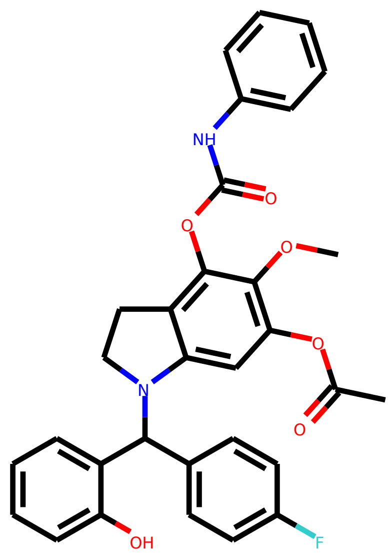
Osma-500
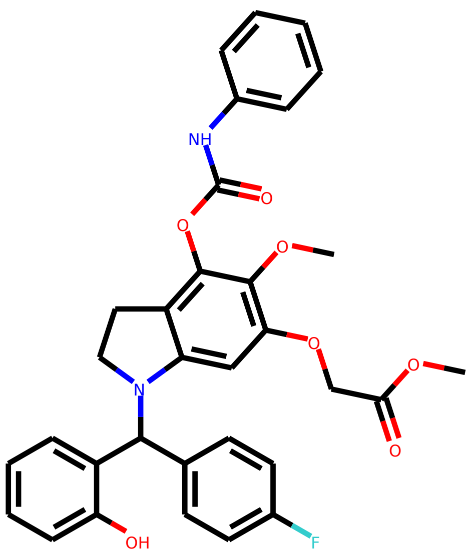
Osma-501
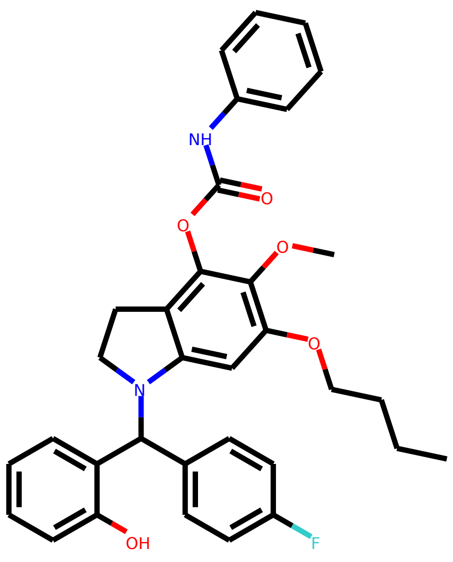
Osma-502
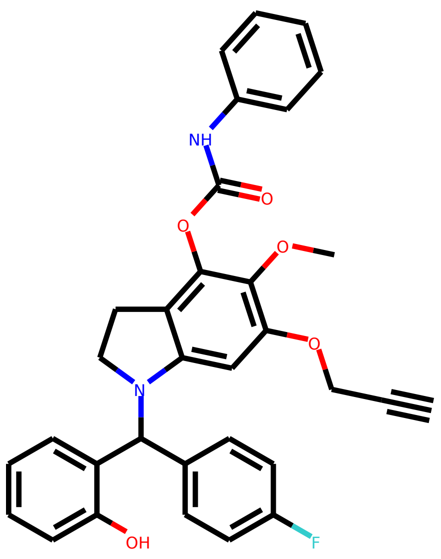
Osma-503
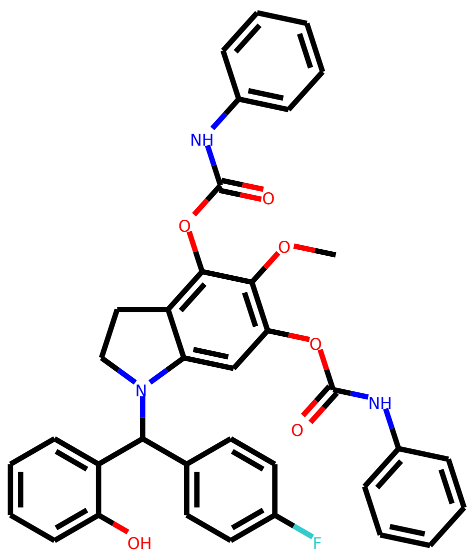
Osma-504
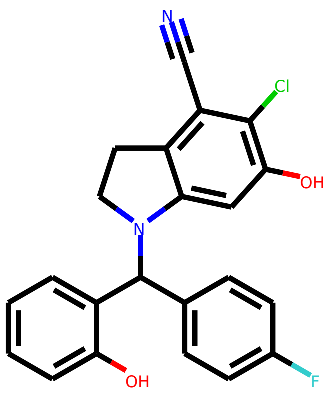
Osma-505
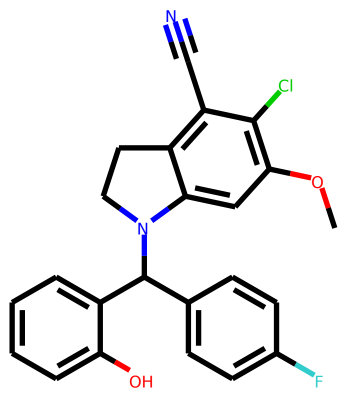
Osma-506
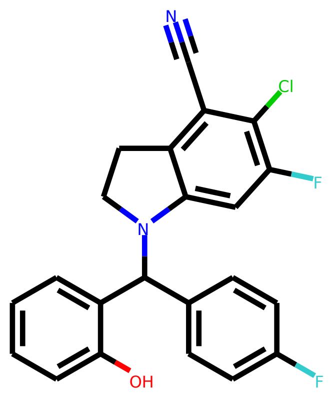
Osma-507
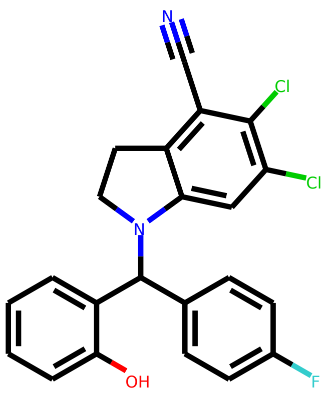
Osma-508
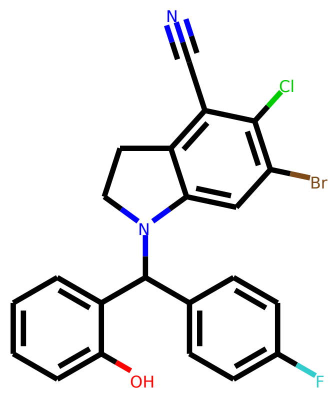
Osma-509
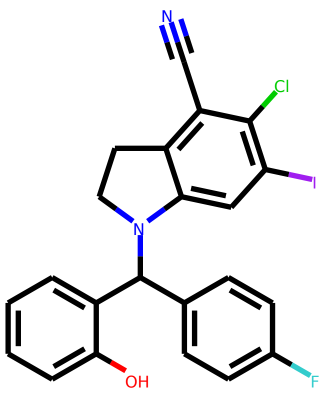
Osma-510
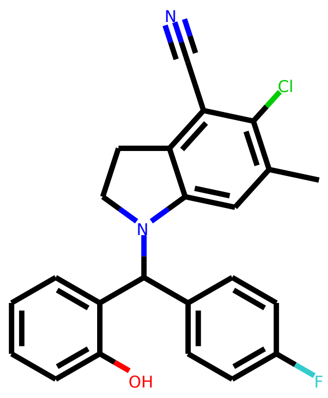
Osma-511
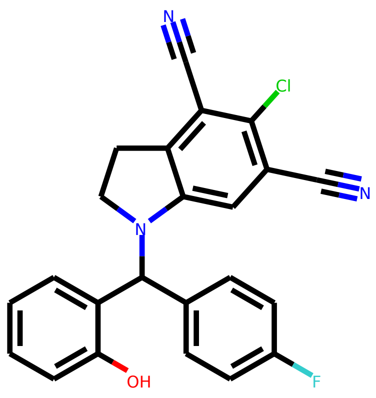
Osma-512
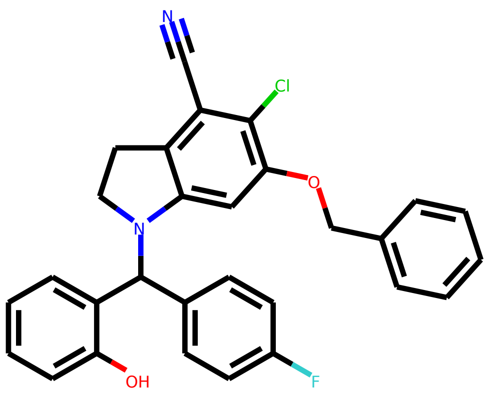
Osma-513
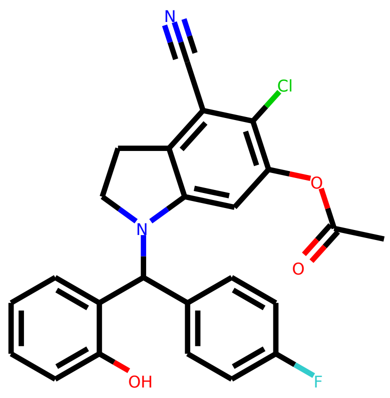
Osma-514
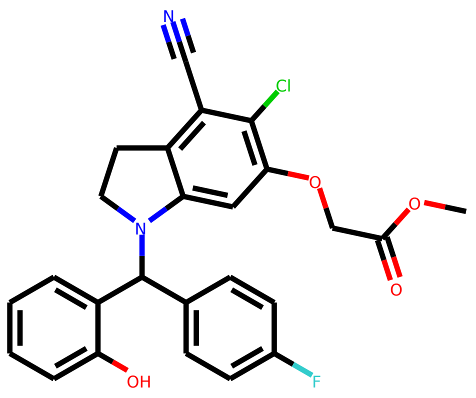
Osma-515
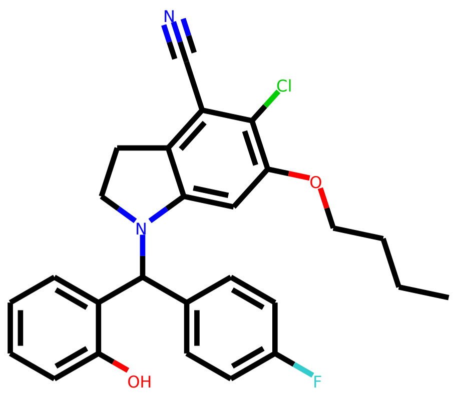
Osma-516
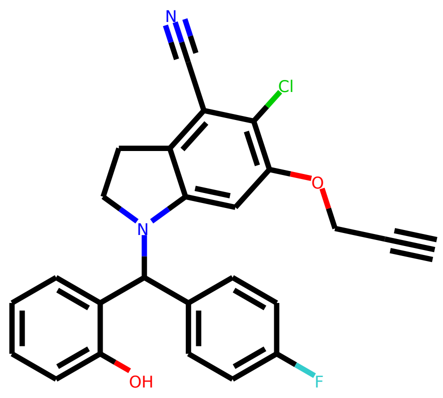
Osma-517
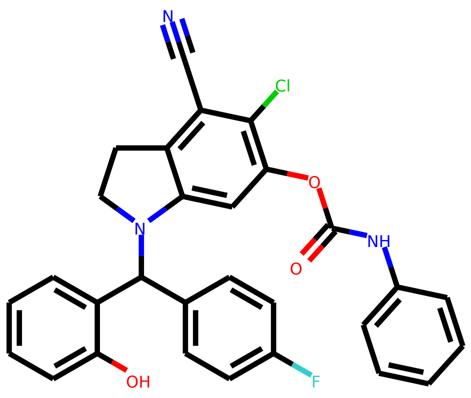
Osma-518
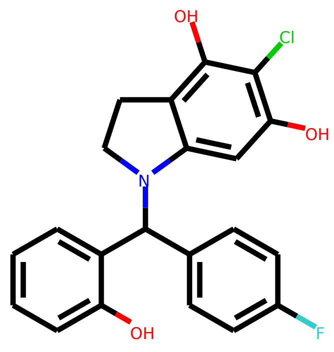
Osma-519
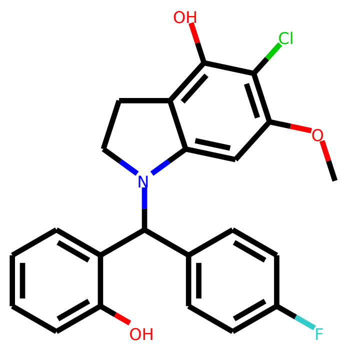
Osma-520
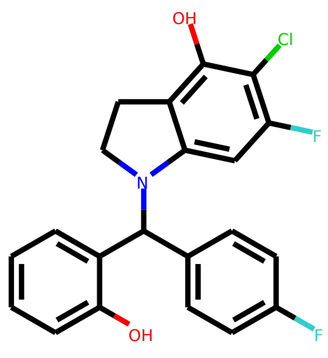
Osma-521
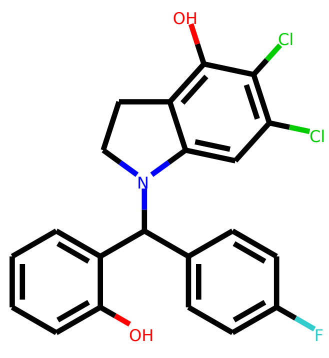
Osma-522
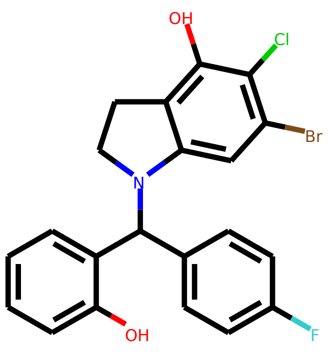
Osma-523
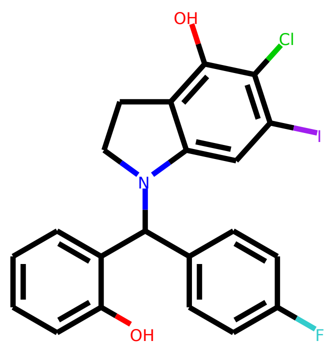
Osma-524
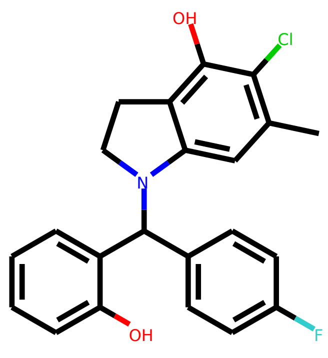
Osma-525

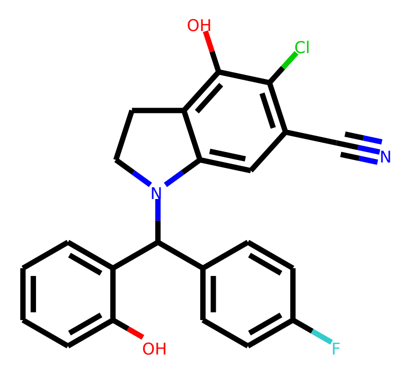
Osma-526

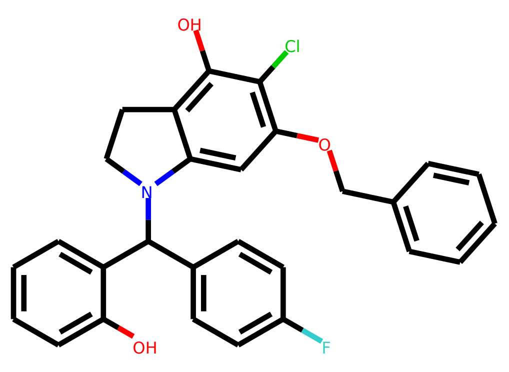
Osma-527

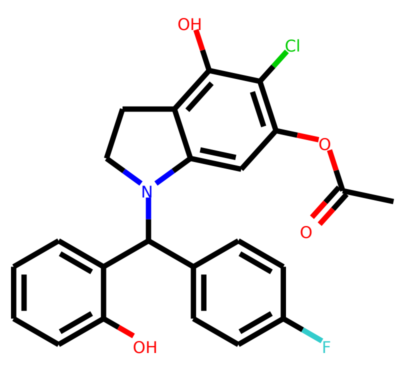
Osma-528

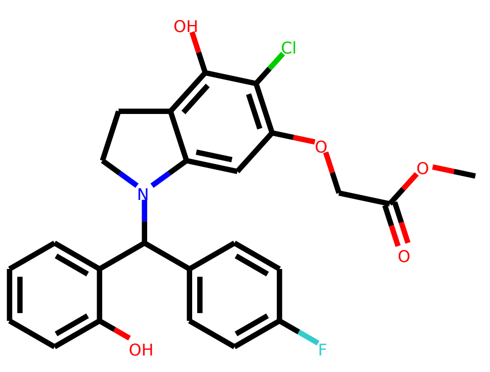
Osma-529

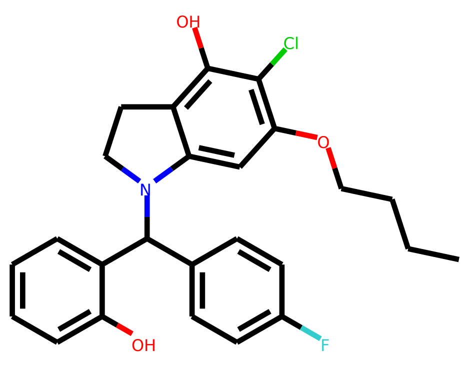
Osma-530

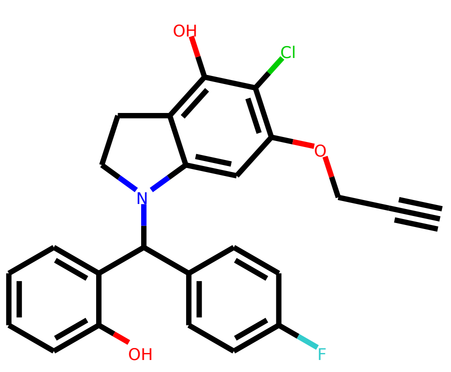
Osma-531

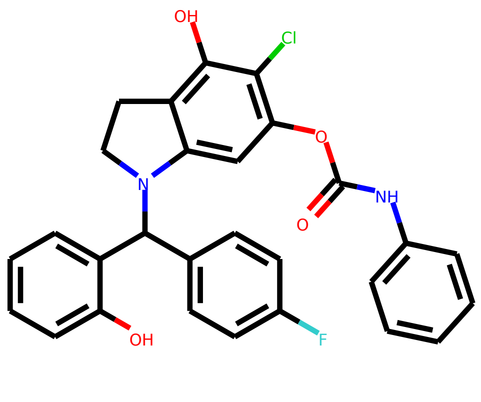
Osma-532

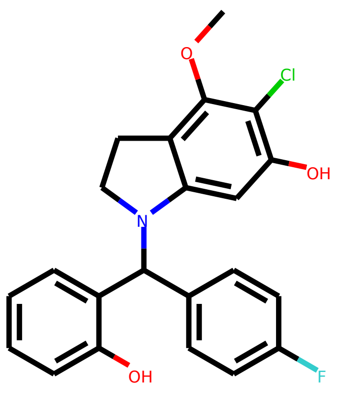
Osma-533

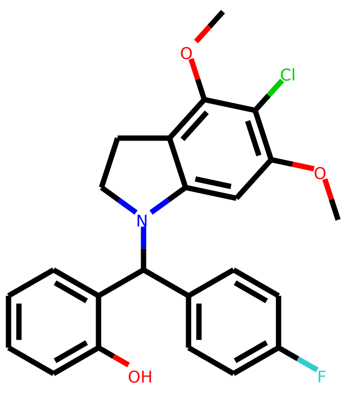
Osma-534

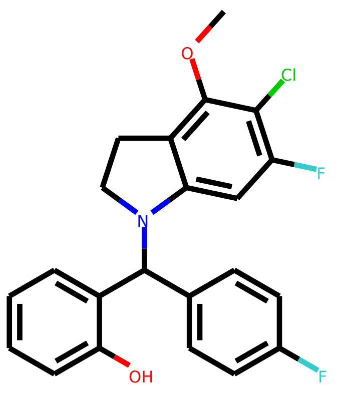
Osma-535

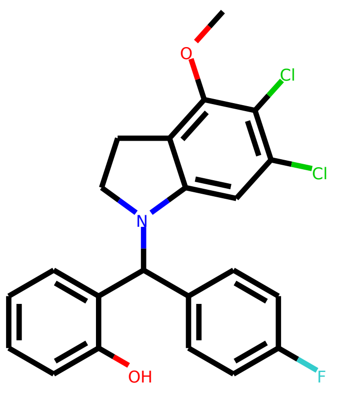
Osma-536

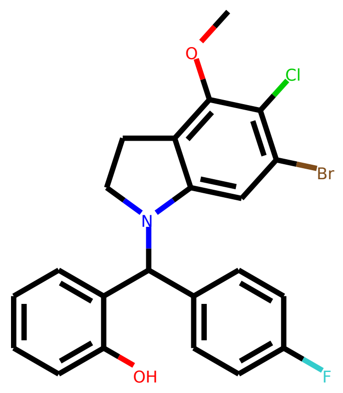
Osma-537

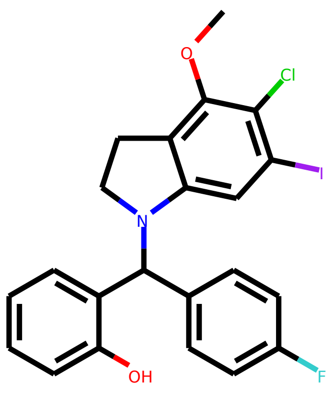
Osma-538

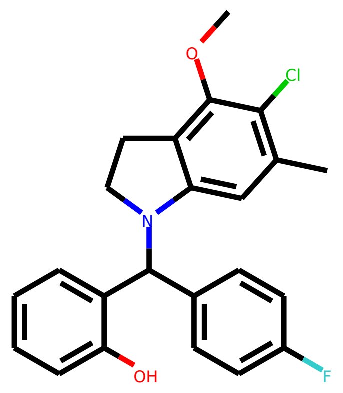
Osma-539

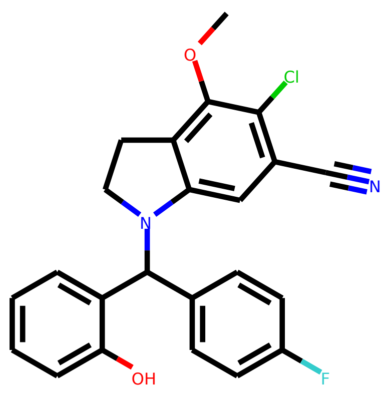
Osma-540

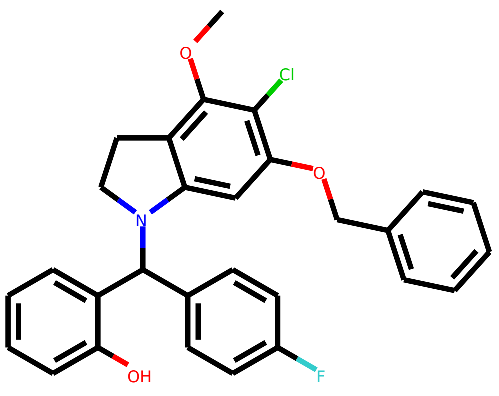
Osma-541

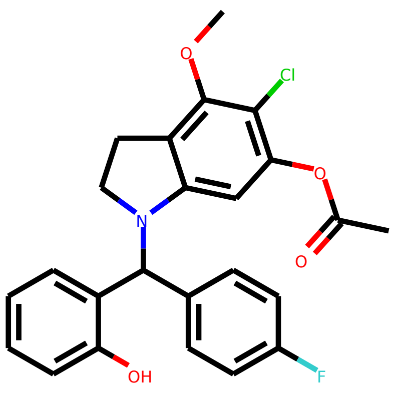
Osma-542

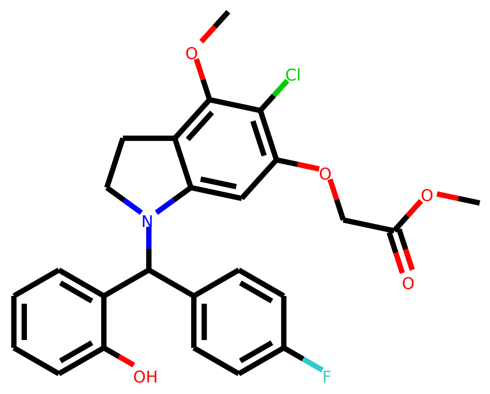
Osma-543

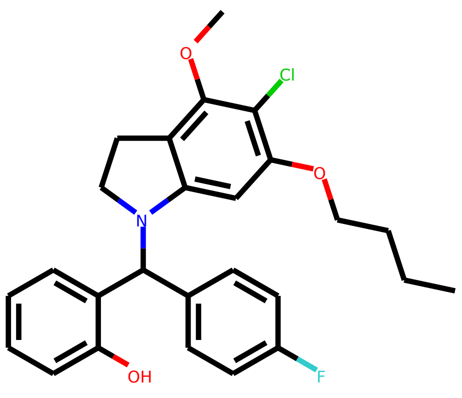
Osma-544

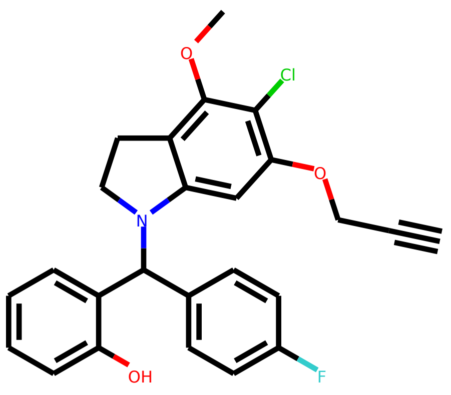
Osma-545

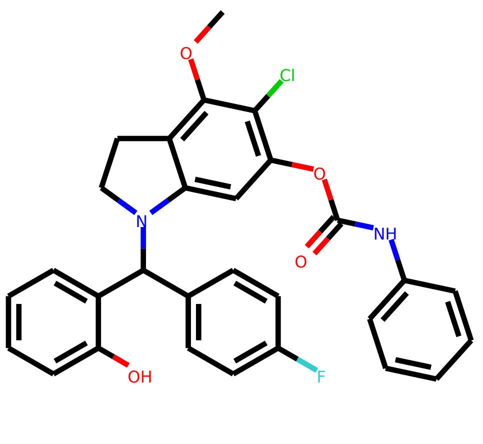
Osma-546

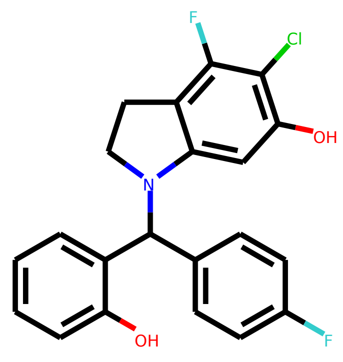
Osma-547

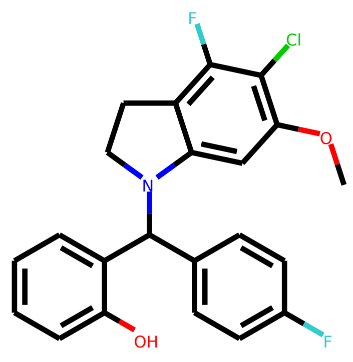
Osma-548

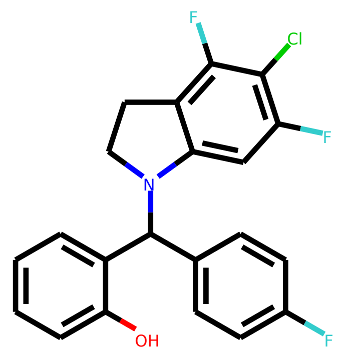
Osma-549

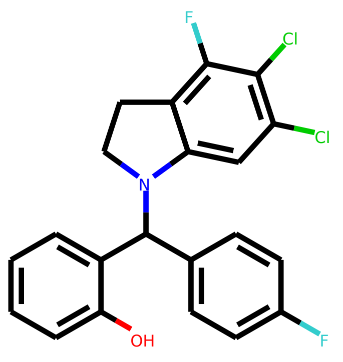
Osma-550

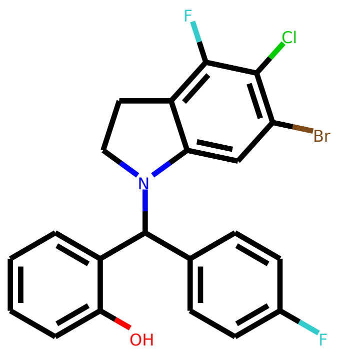
Osma-551

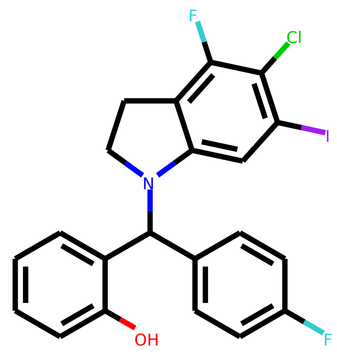
Osma-552

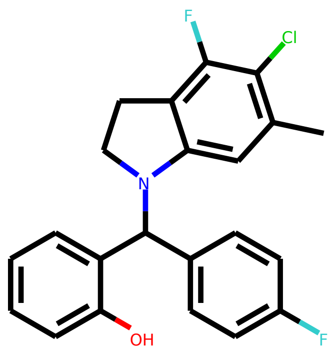
Osma-553

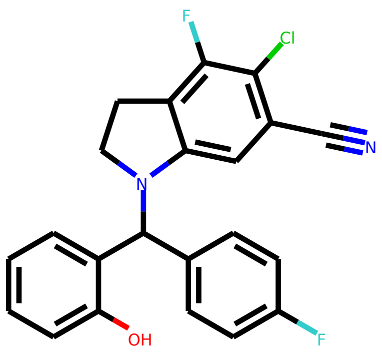
Osma-554

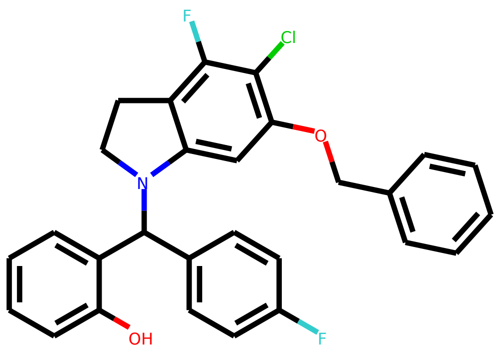
Osma-555

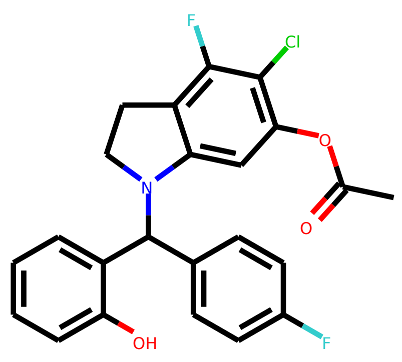
Osma-556

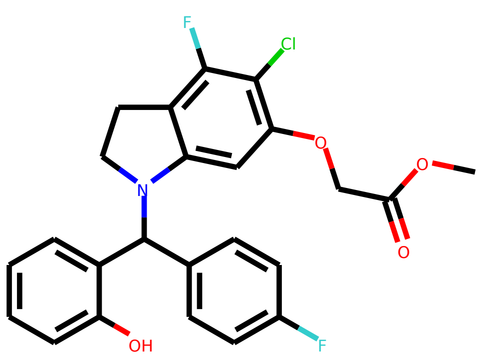
Osma-557

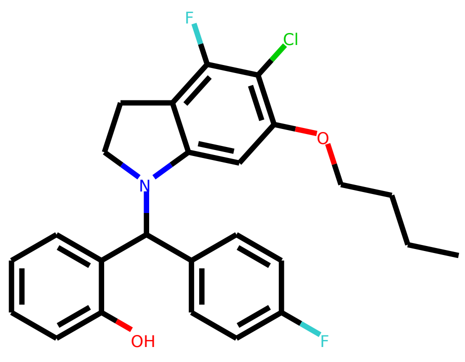
Osma-558

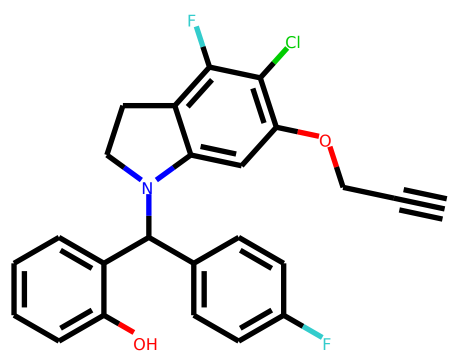
Osma-559

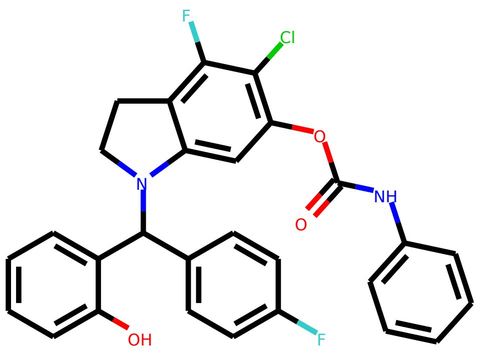
Osma-560

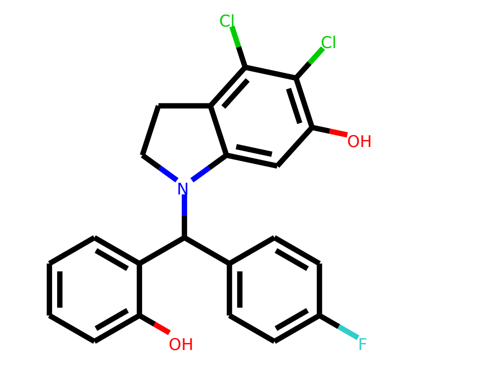

Osma-561

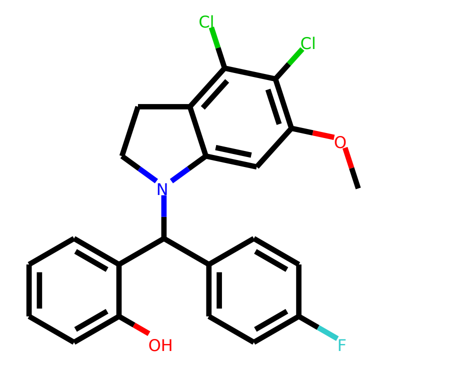

Osma-562

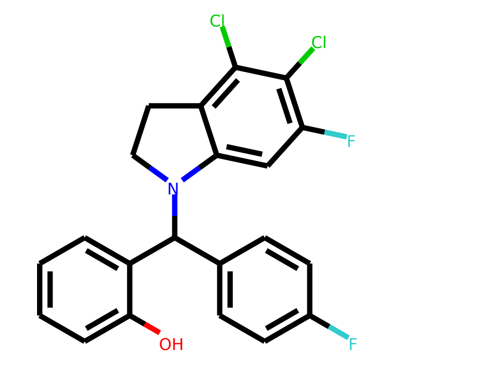

Osma-563

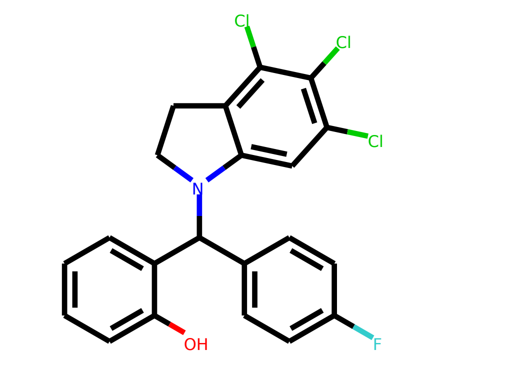

Osma-564

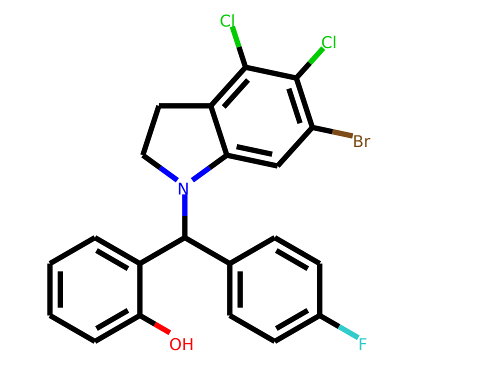

Osma-565

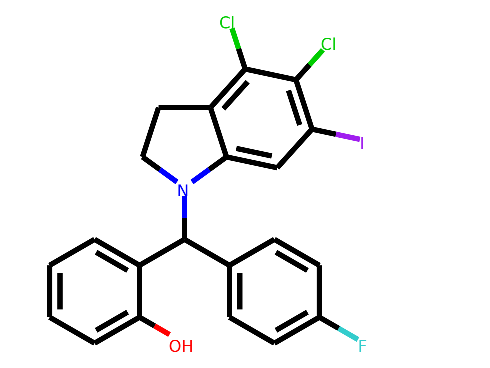

Osma-566

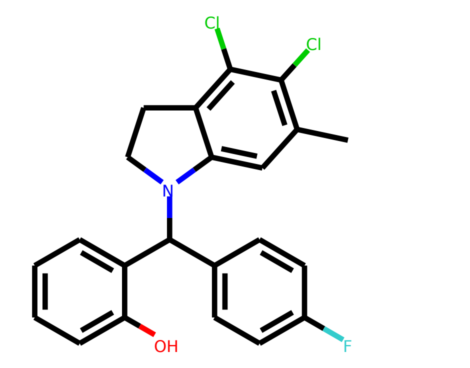

Osma-567

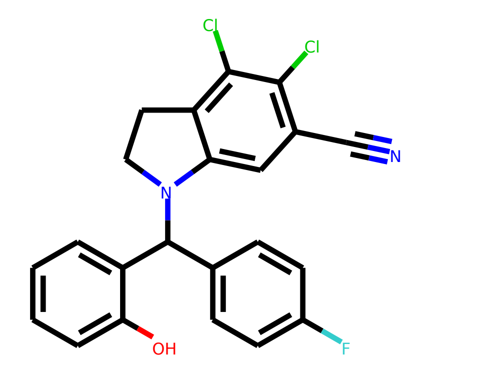

Osma-568

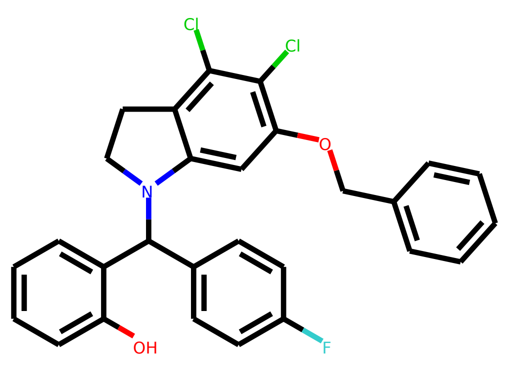

Osma-569

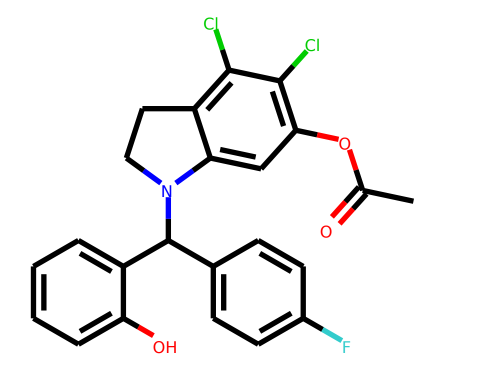

Osma-570

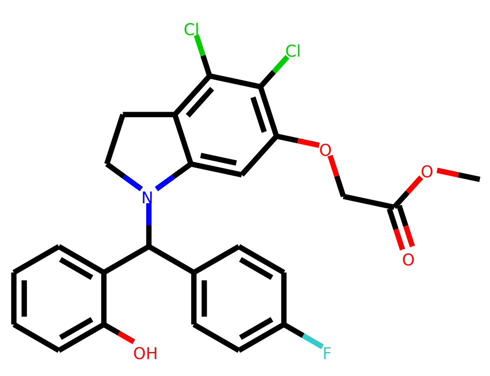

Osma-571

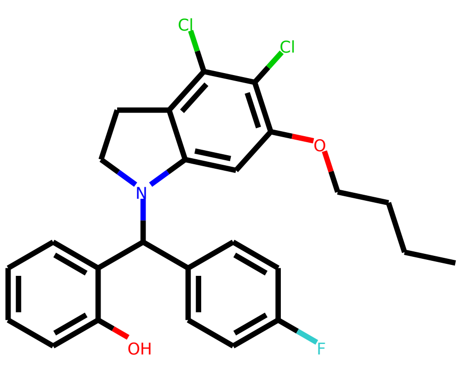

Osma-572

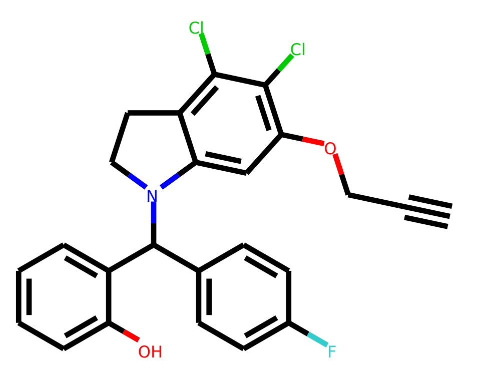

Osma-573

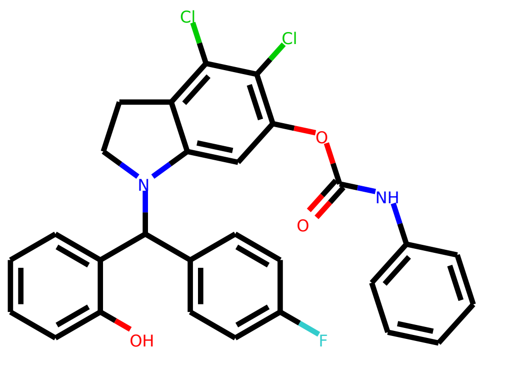

Osma-574

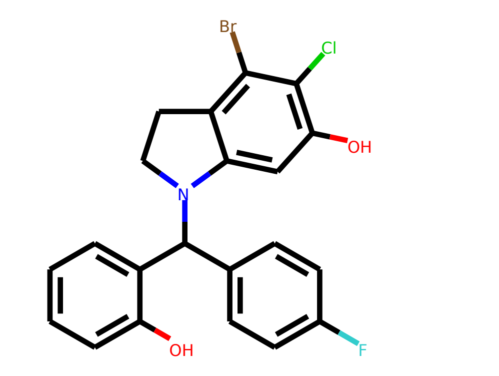

Osma-575

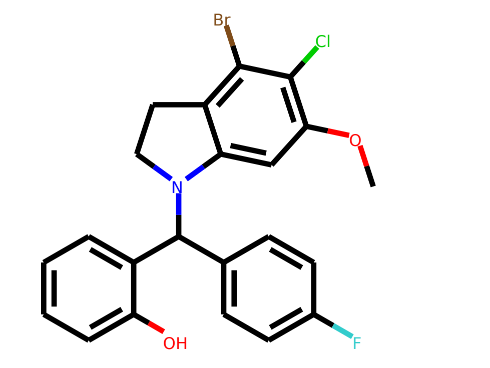

Osma-576

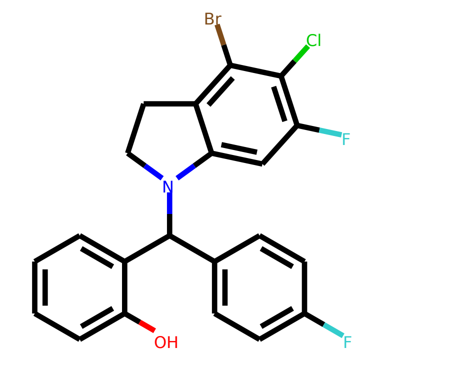

Osma-577

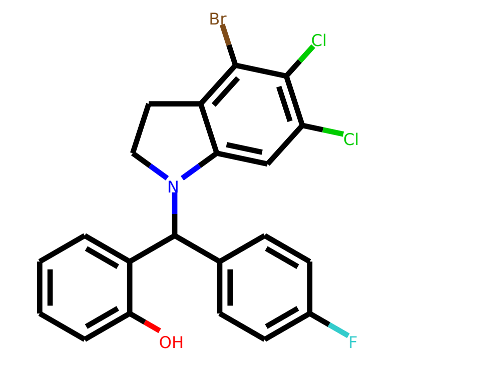

Osma-578

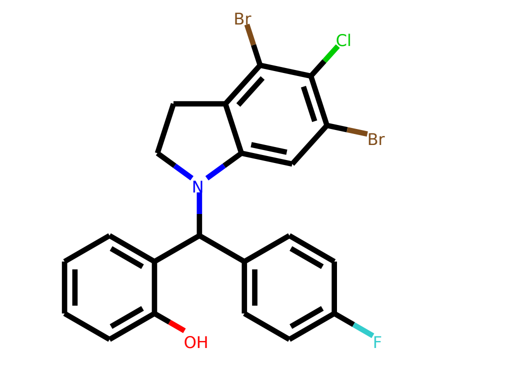

Osma-579

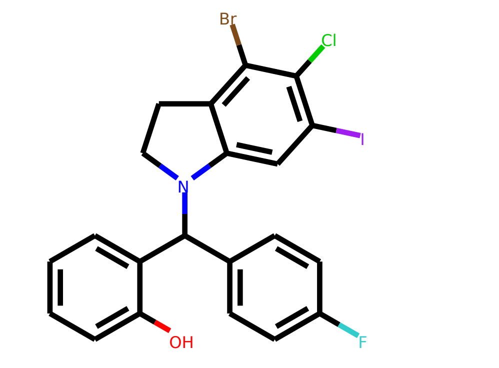

Osma-580

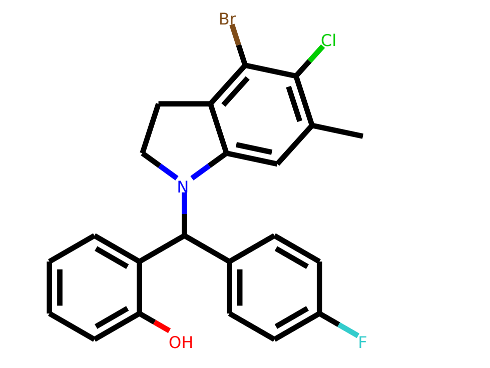

Osma-581

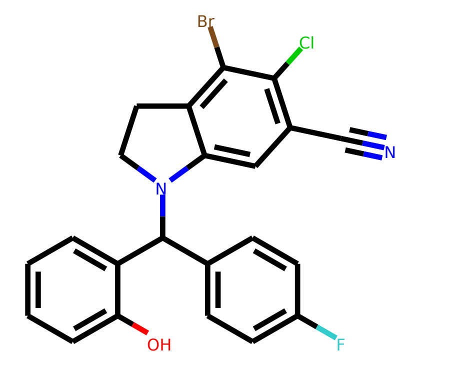

Osma-582

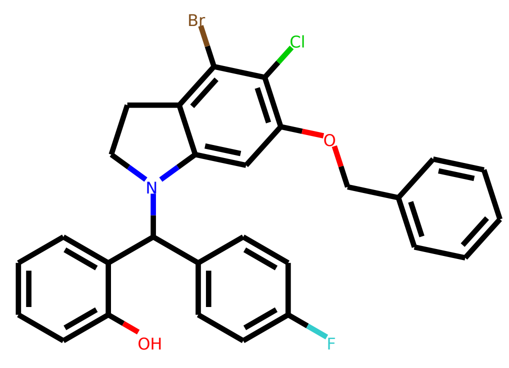

Osma-583

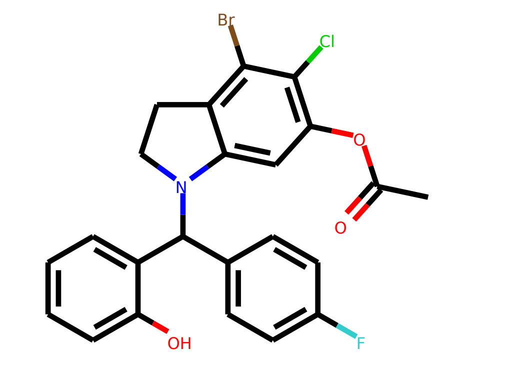

Osma-584

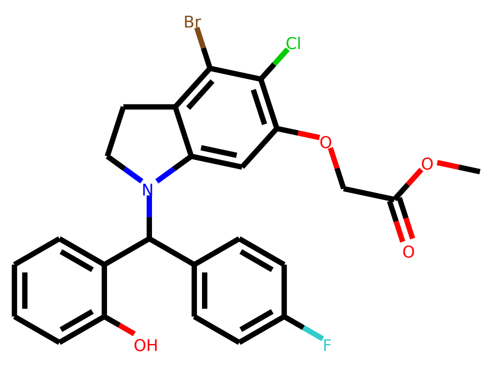

Osma-585

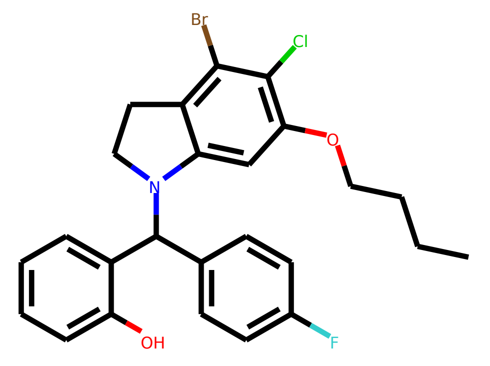

Osma-586

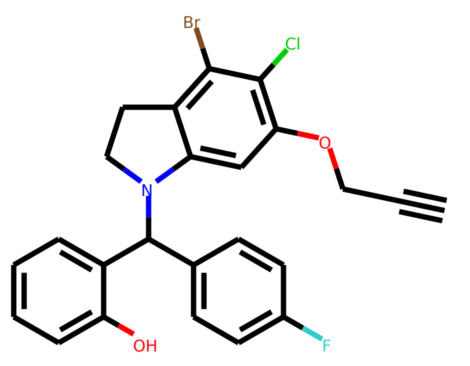

Osma-587

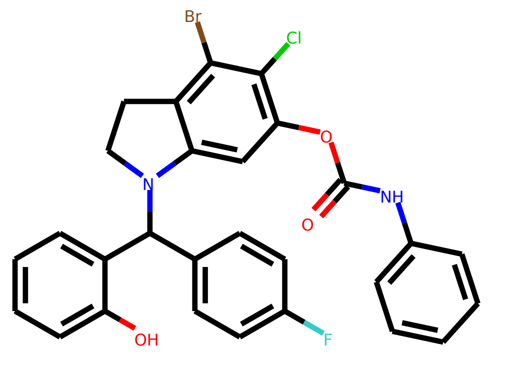

Osma-588

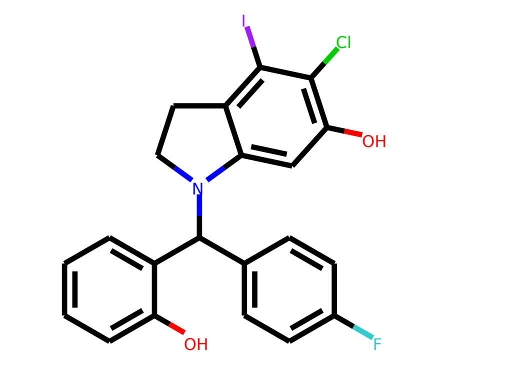

Osma-589

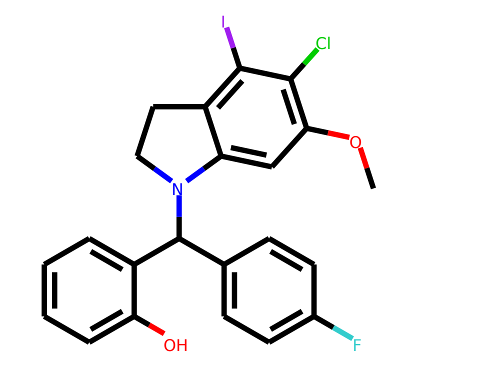

Osma-590

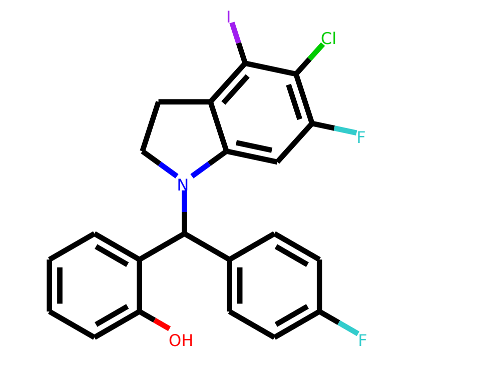

Osma-591

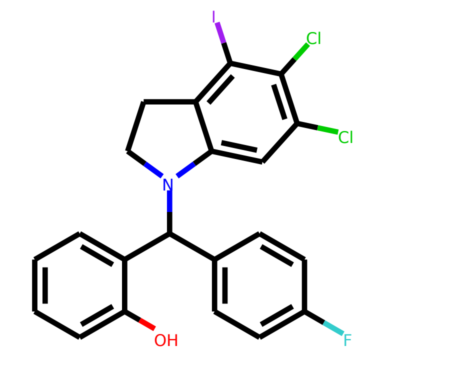

Osma-592

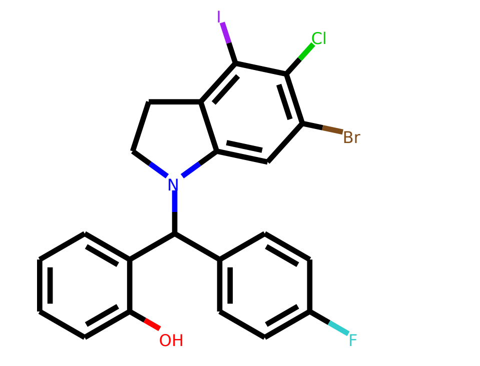

Osma-593

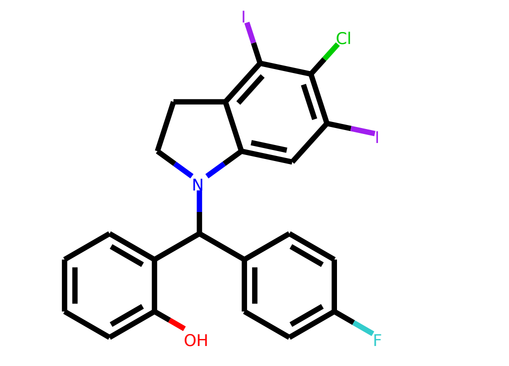

Osma-594

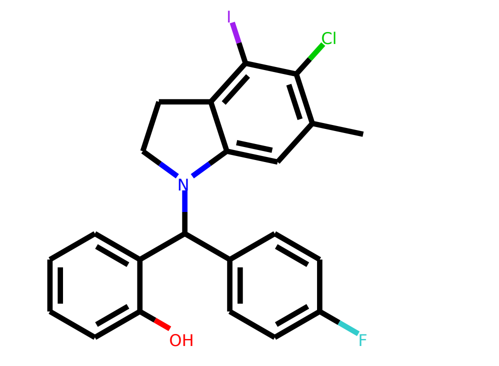

Osma-595

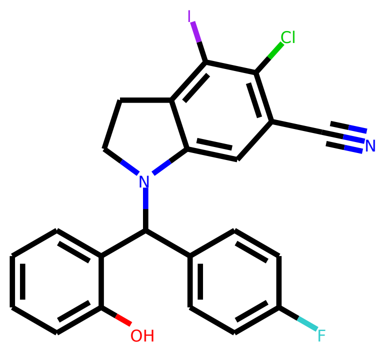
Osma-596
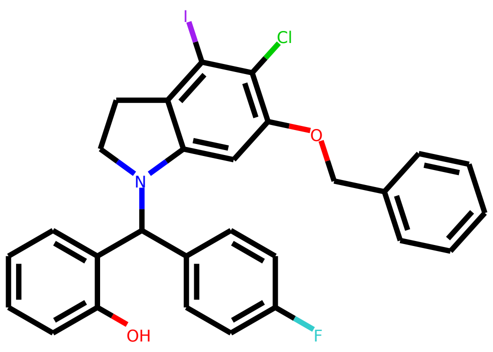
Osma-597
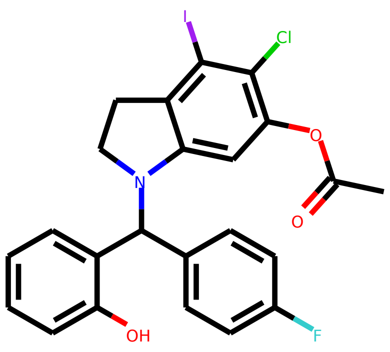
Osma-598
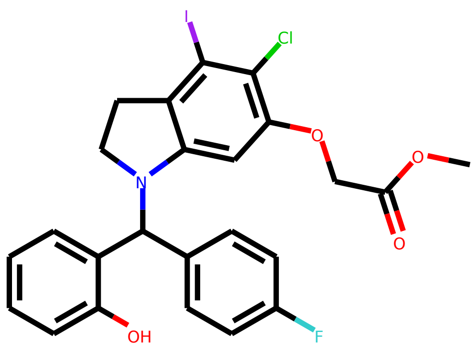
Osma-599
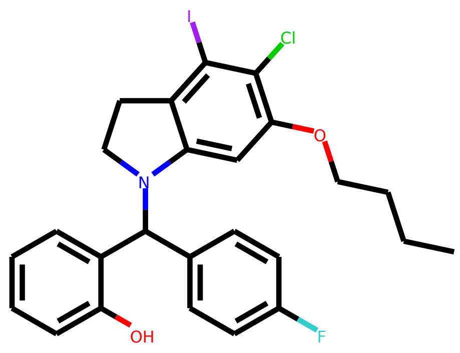
Osma-600
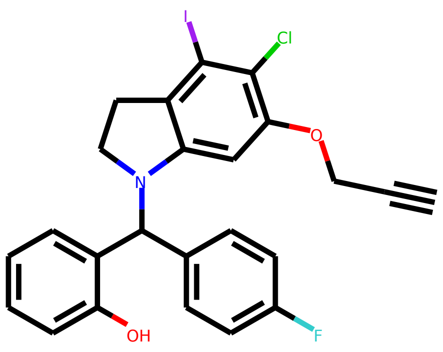
Osma-601
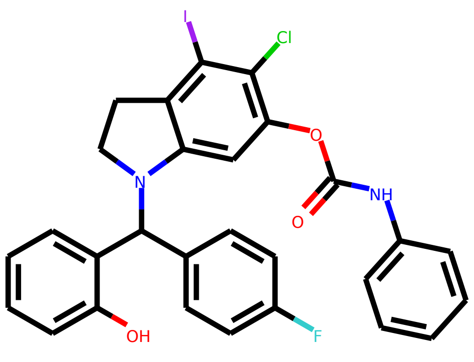
Osma-602
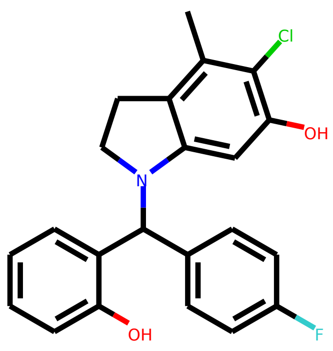
Osma-603
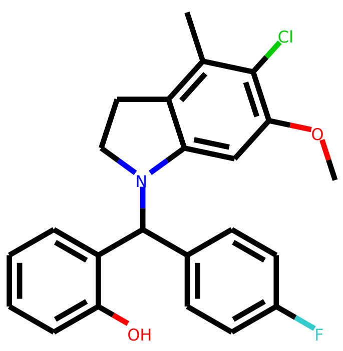
Osma-604
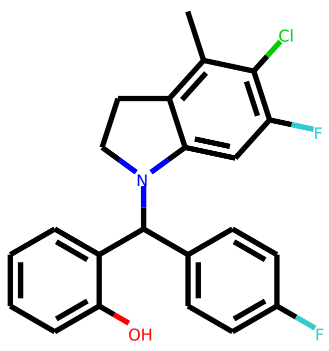
Osma-605
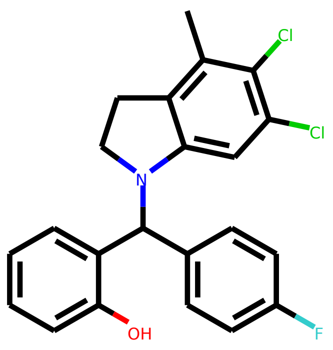
Osma-606
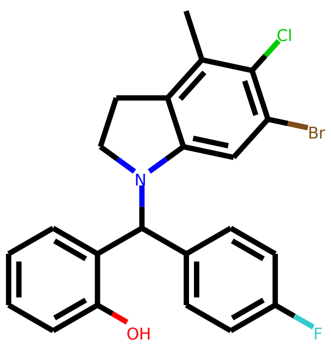
Osma-607
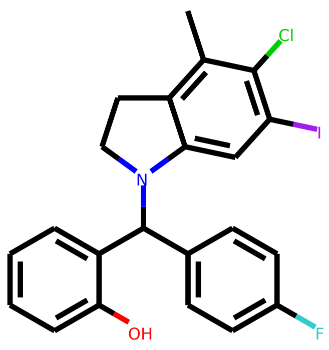
Osma-608
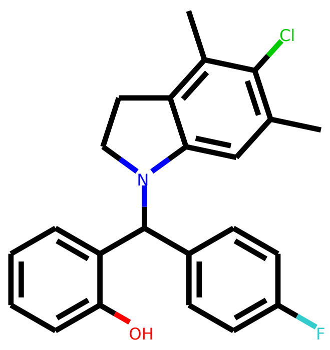
Osma-609
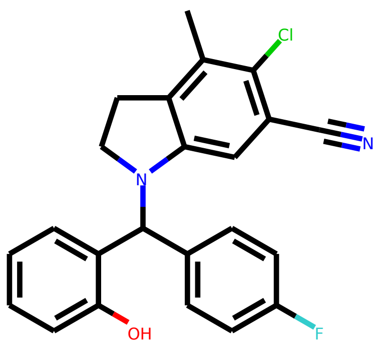
Osma-610
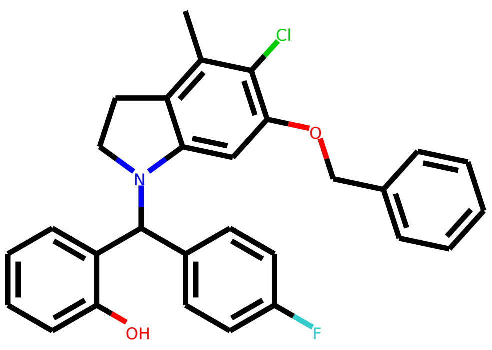
Osma-611
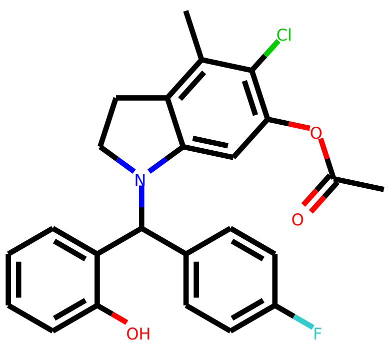
Osma-612
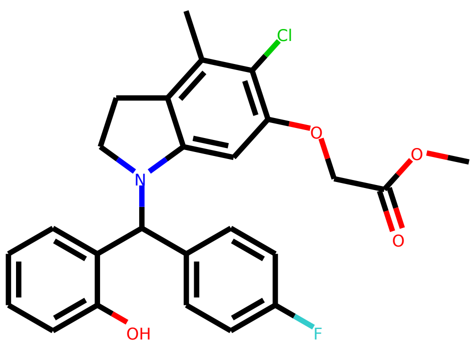
Osma-613
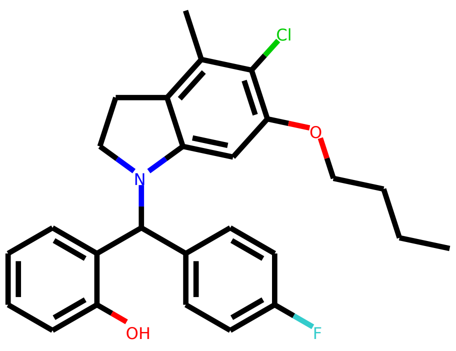
Osma-614
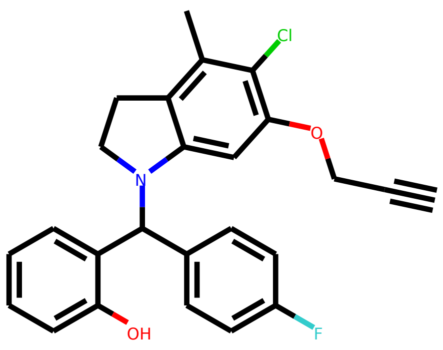
Osma-615
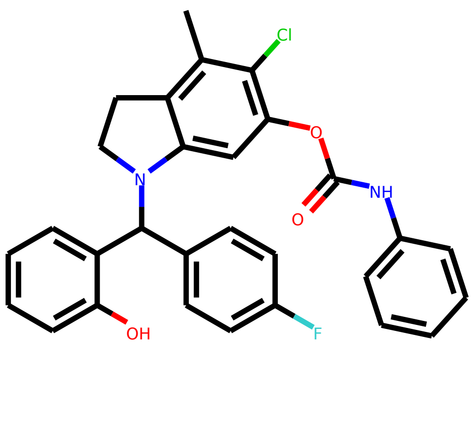
Osma-616
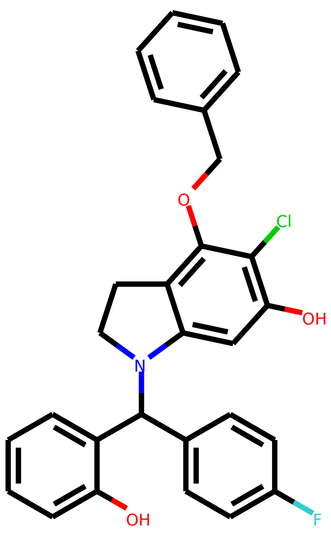
Osma-617
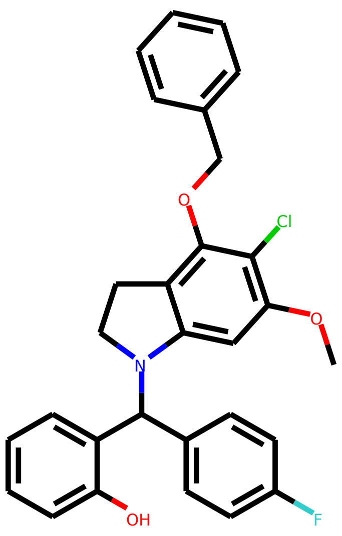
Osma-618
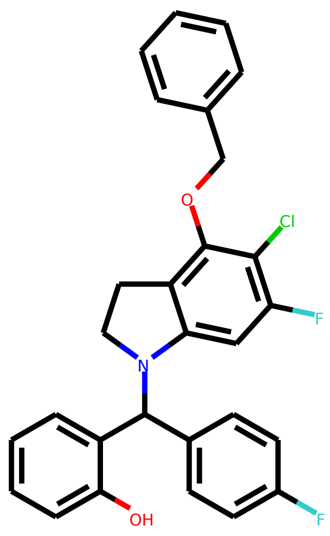
Osma-619
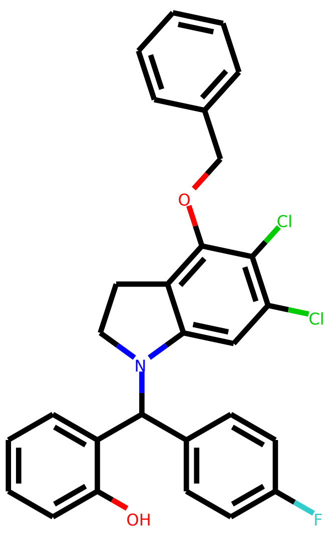
Osma-620
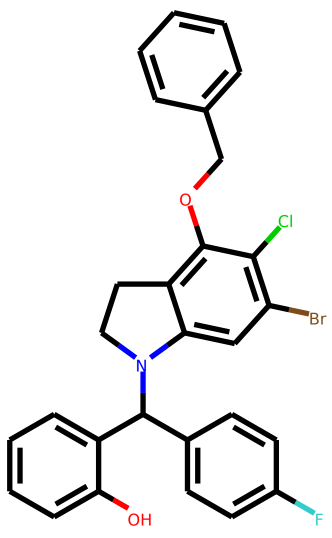
Osma-621
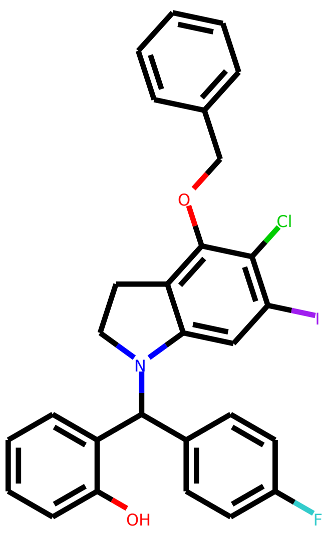
Osma-622
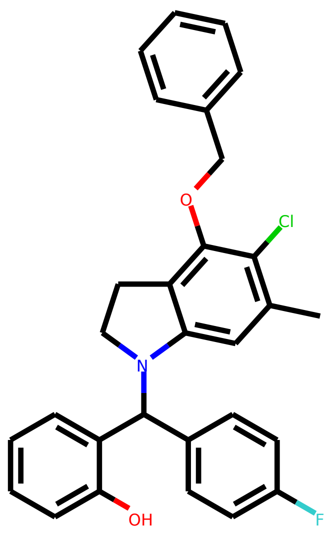
Osma-623
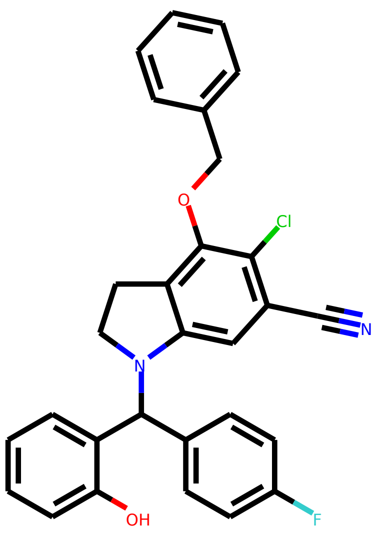
Osma-624
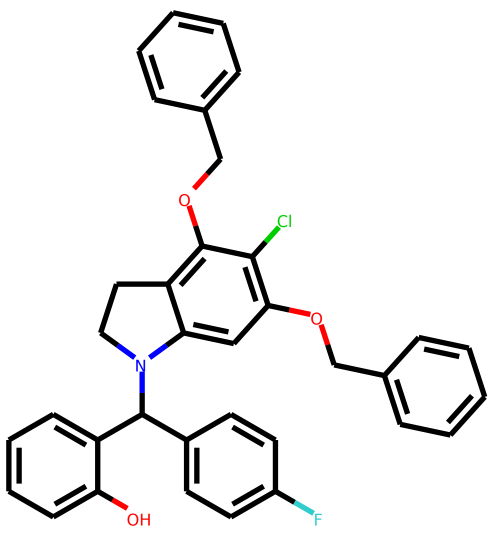
Osma-625
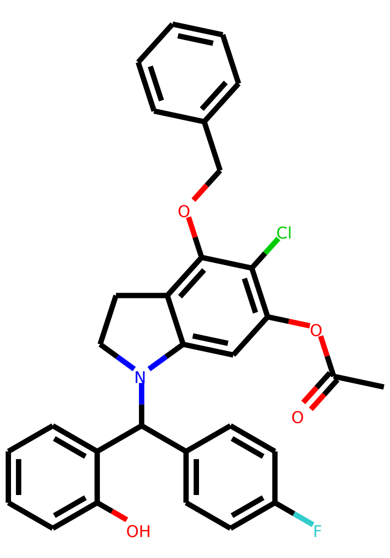
Osma-626
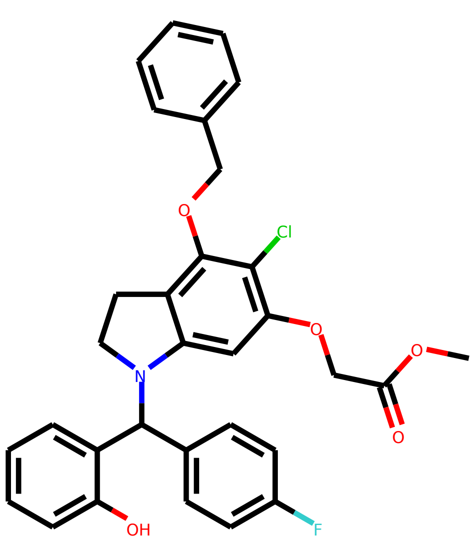
Osma-627
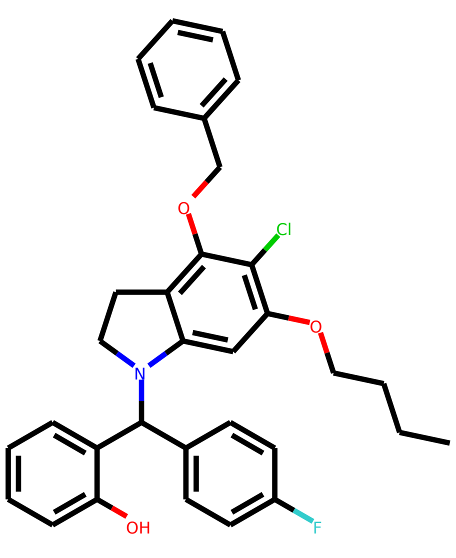
Osma-628
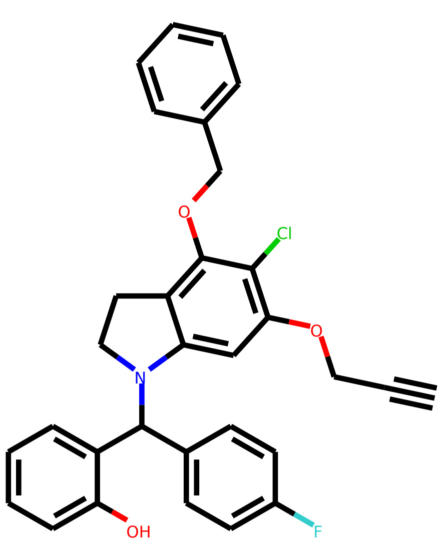
Osma-629
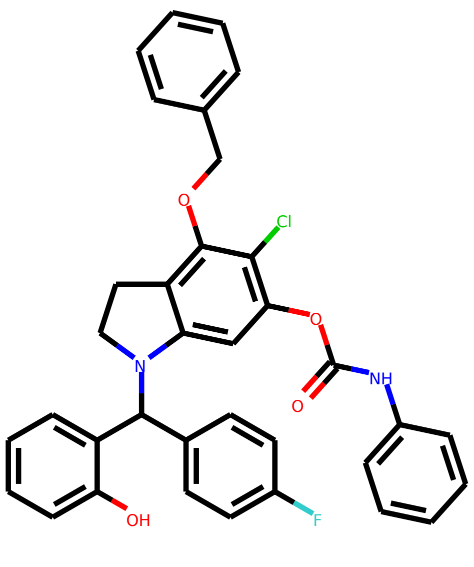
Osma-630

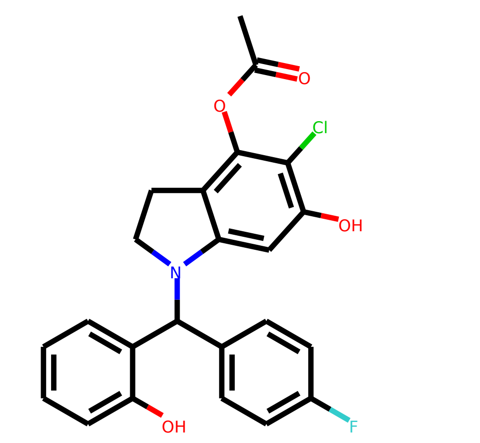

Osma-631

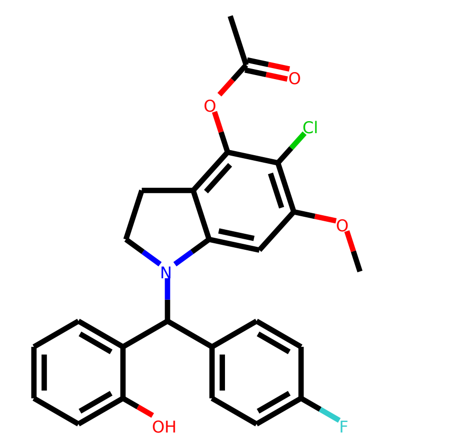

Osma-632

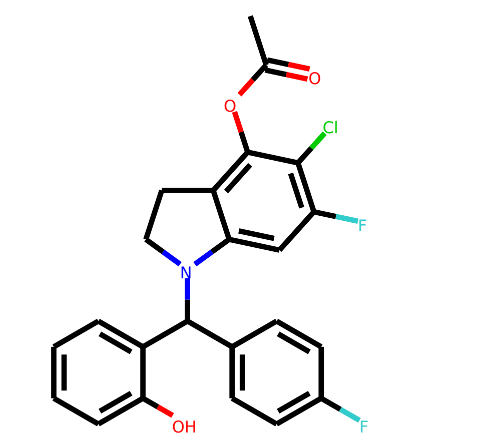

Osma-633

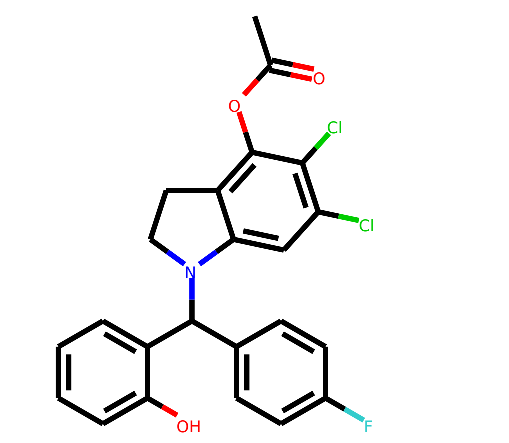

Osma-634

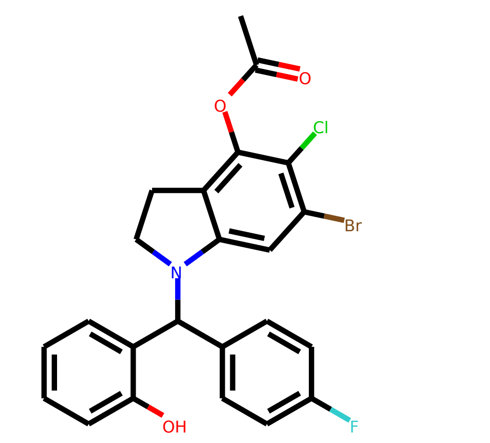

Osma-635

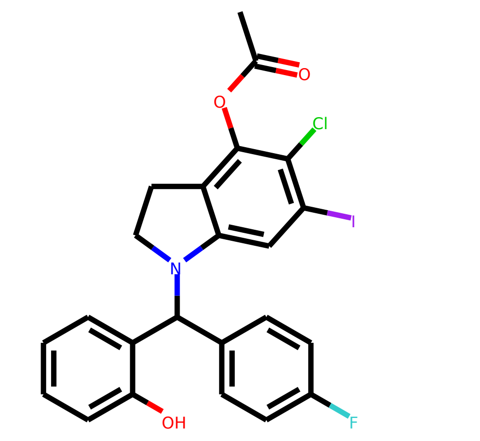

Osma-636

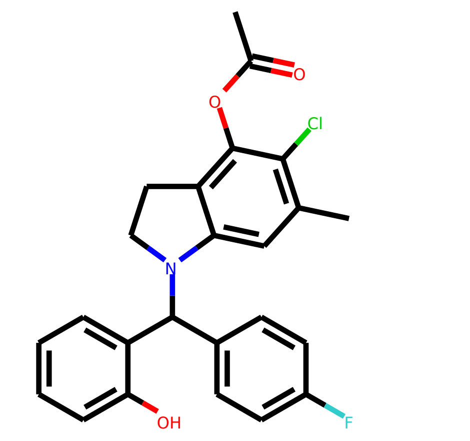

Osma-637

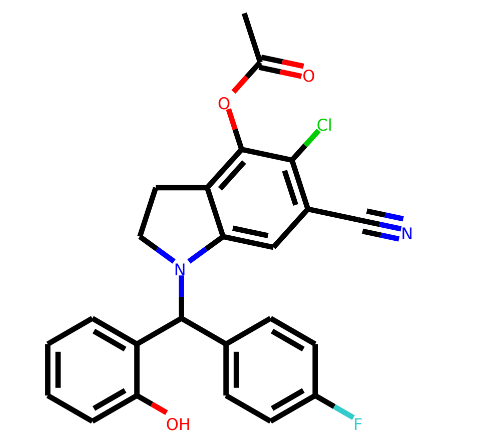

Osma-638

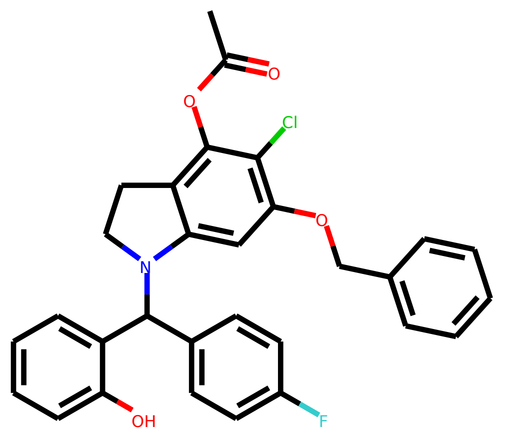

Osma-639

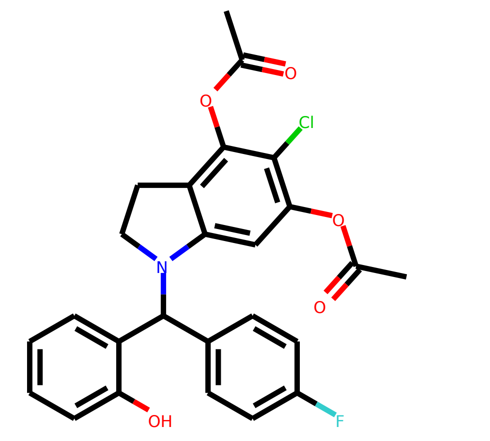

Osma-640

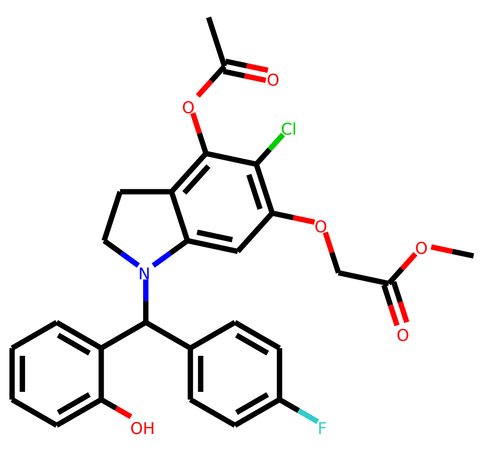

Osma-641

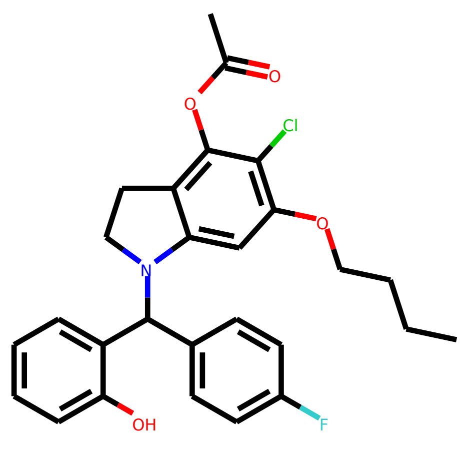

Osma-642

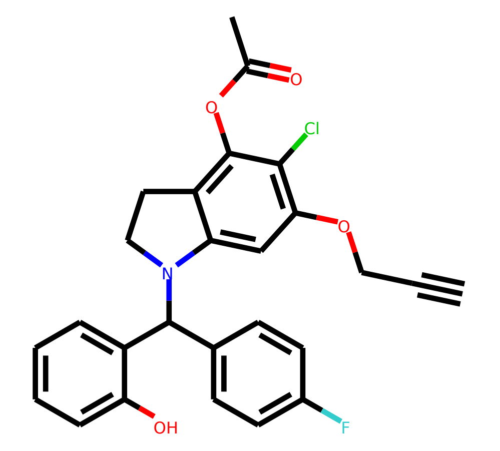

Osma-643

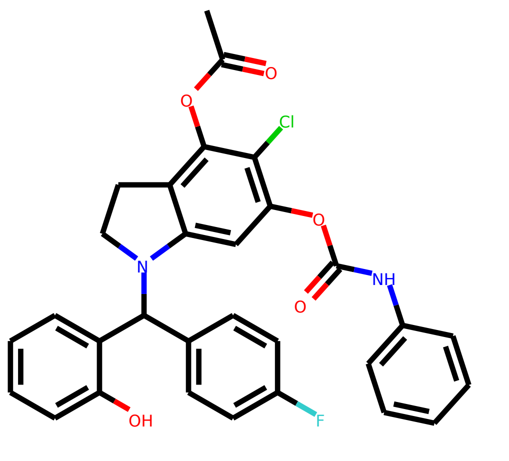

Osma-644

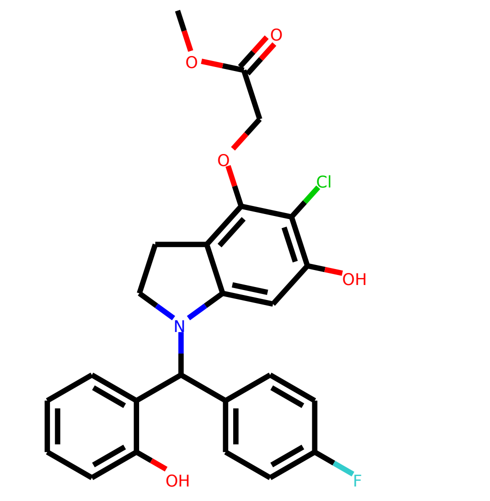

Osma-645

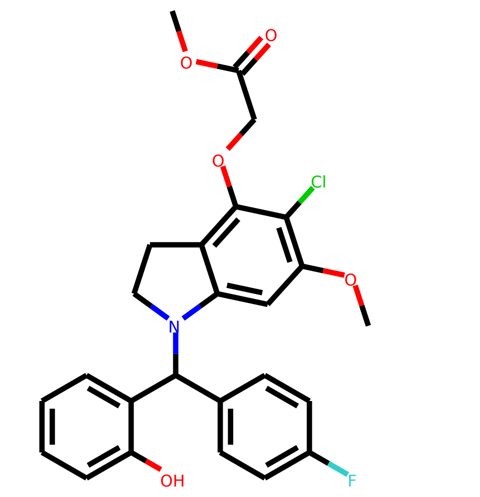

Osma-646

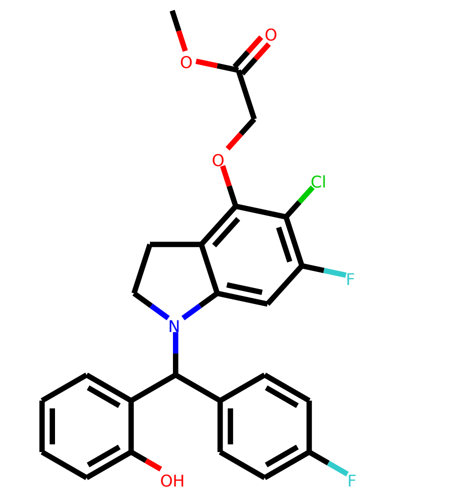

Osma-647

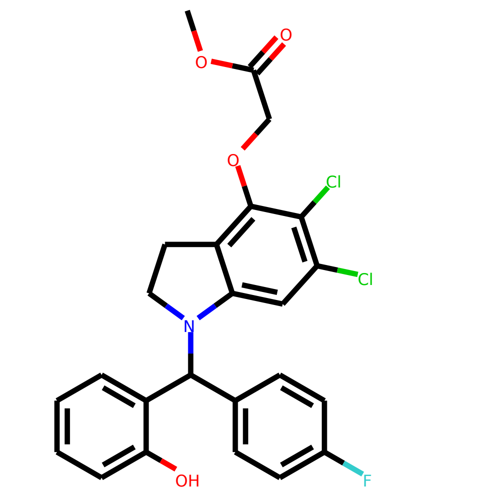

Osma-648

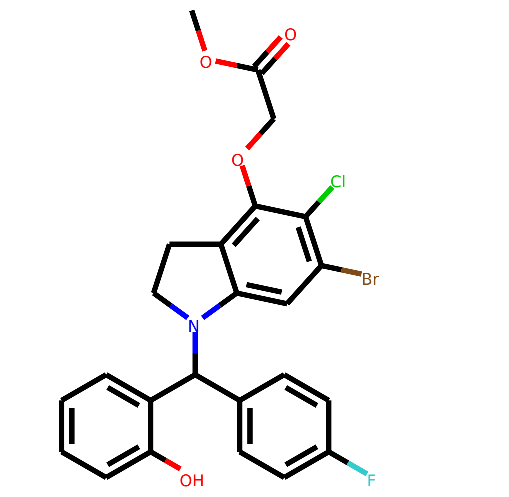

Osma-649

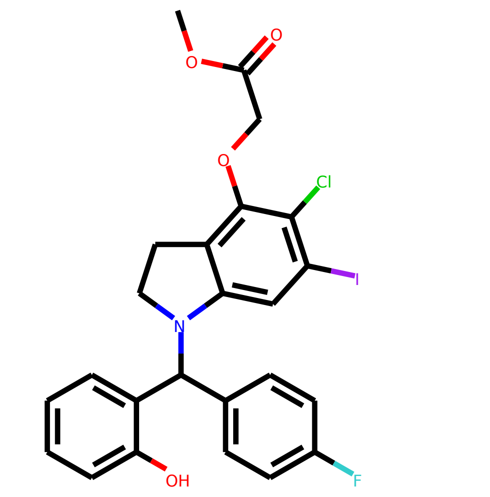

Osma-650

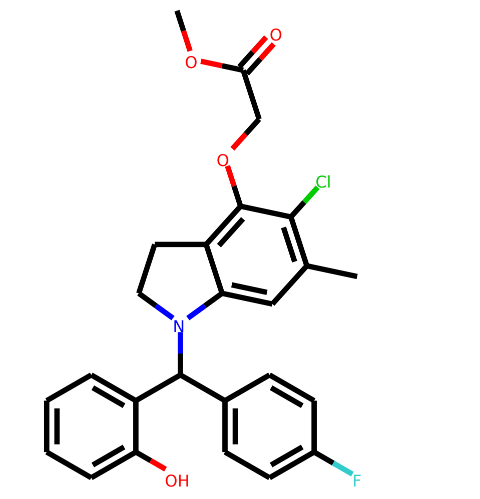

Osma-651

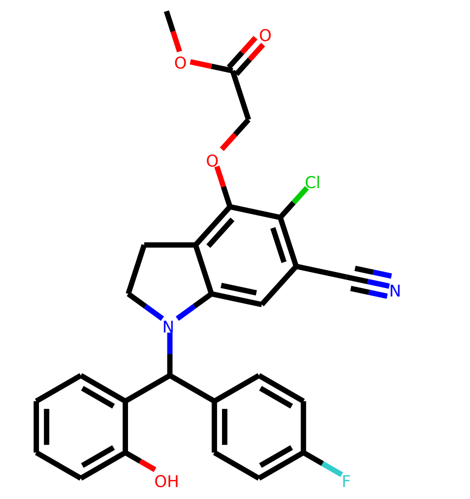

Osma-652

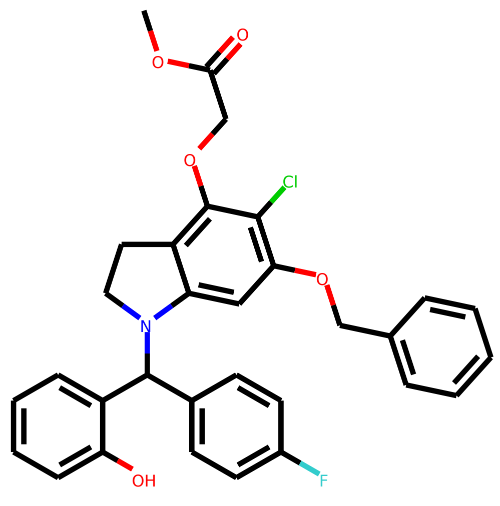

Osma-653

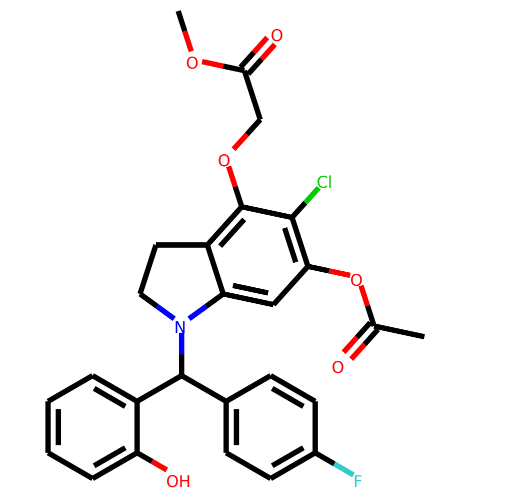

Osma-654

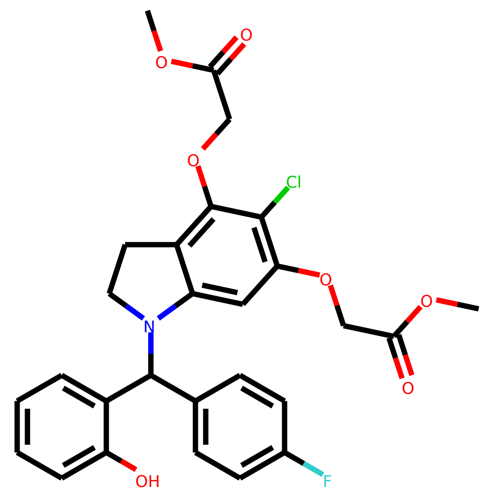

Osma-655

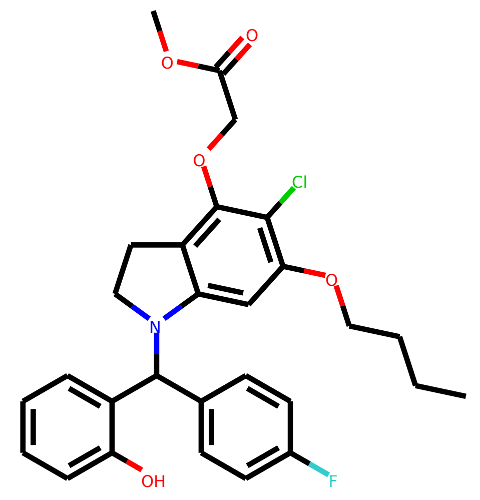

Osma-656

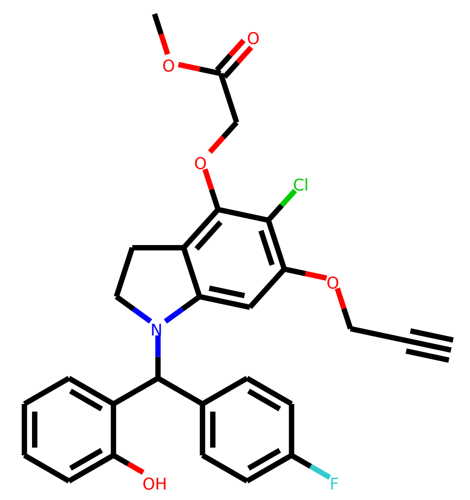

Osma-657

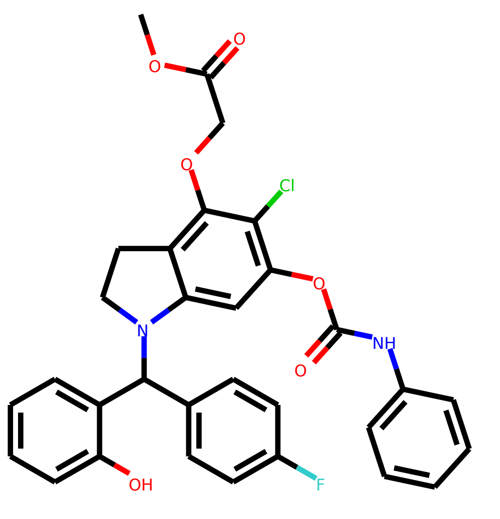

Osma-658

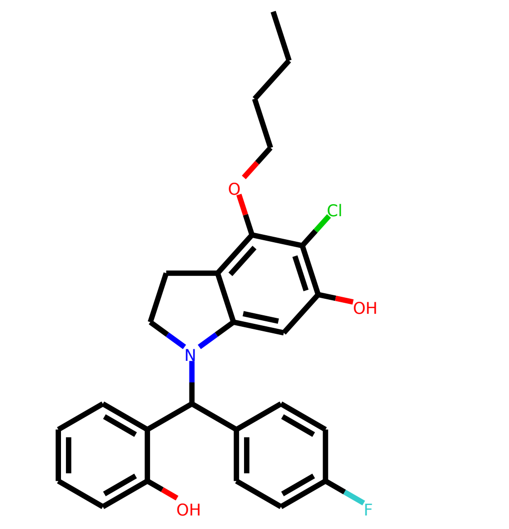

Osma-659

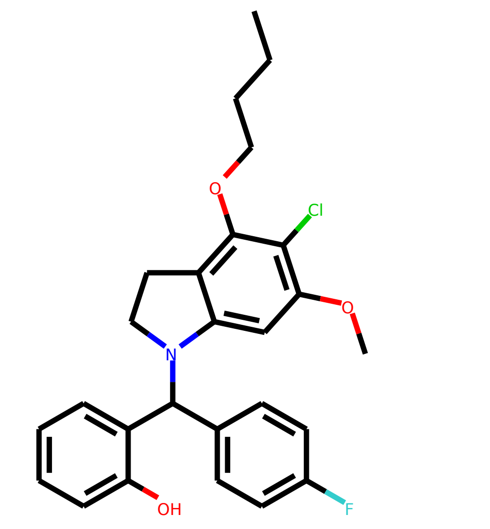

Osma-660

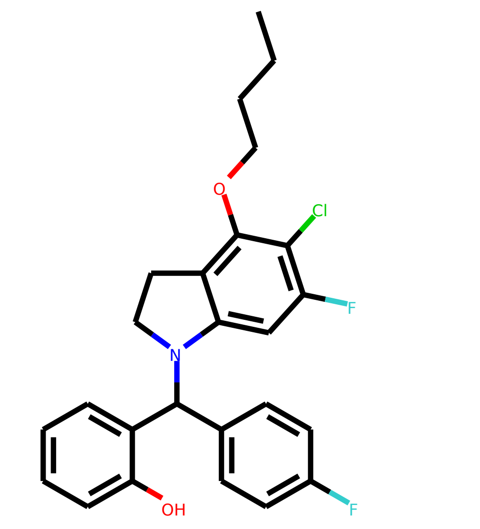

Osma-661

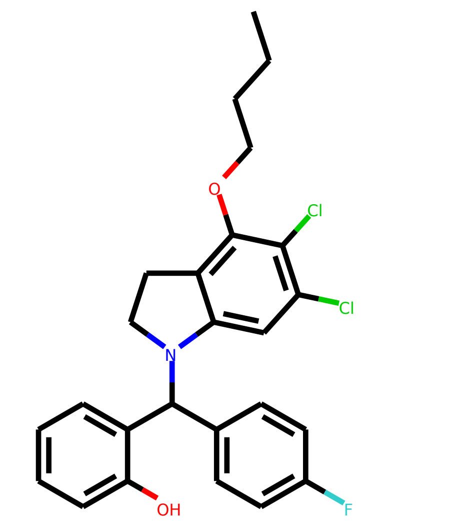

Osma-662

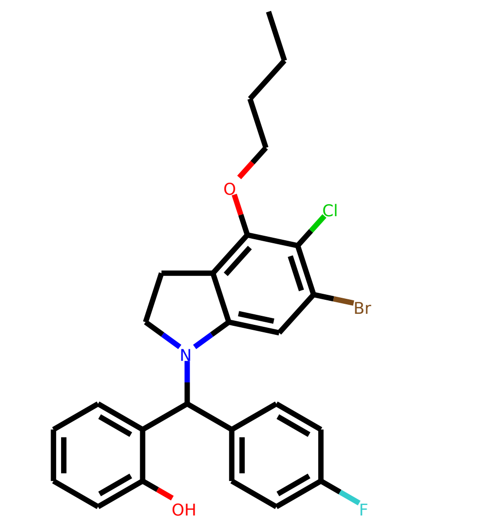

Osma-663

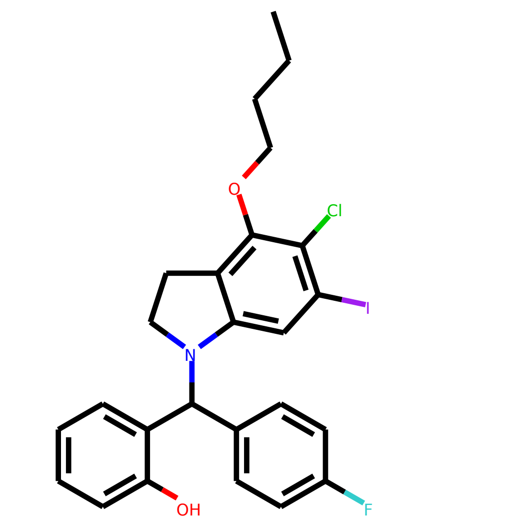

Osma-664

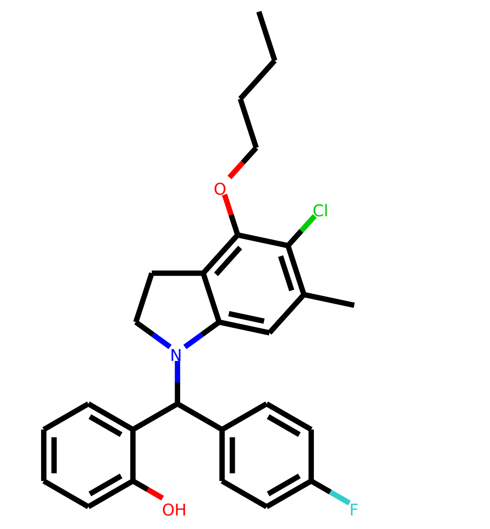

Osma-665

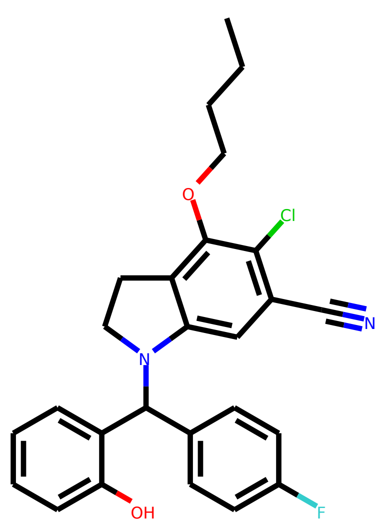

Osma-666

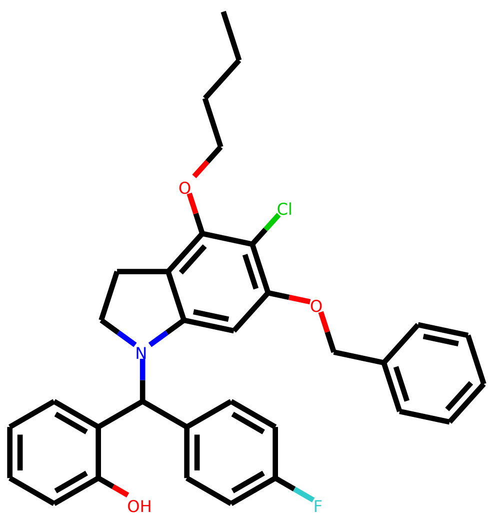

Osma-667

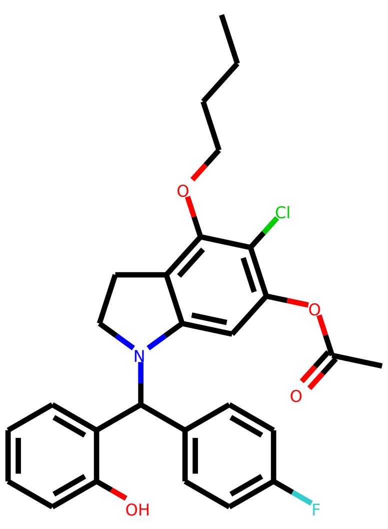

Osma-668

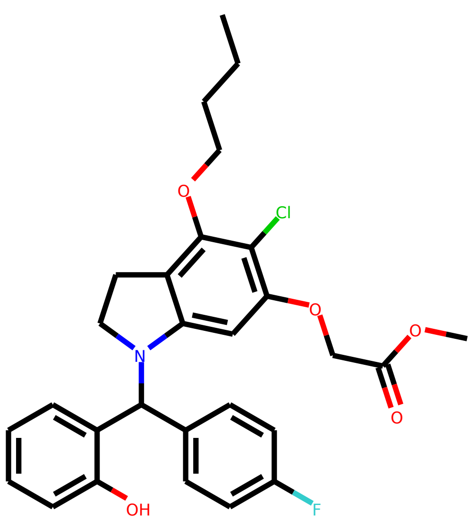

Osma-669

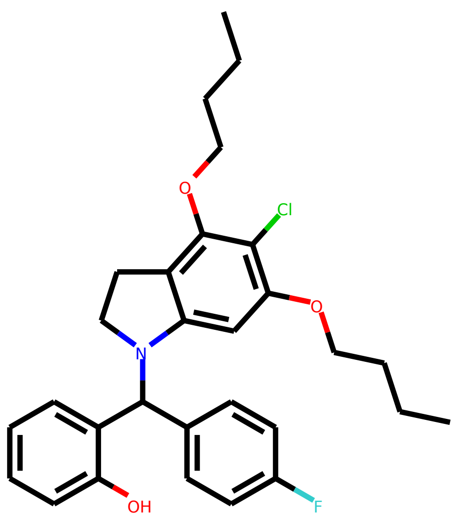

Osma-670

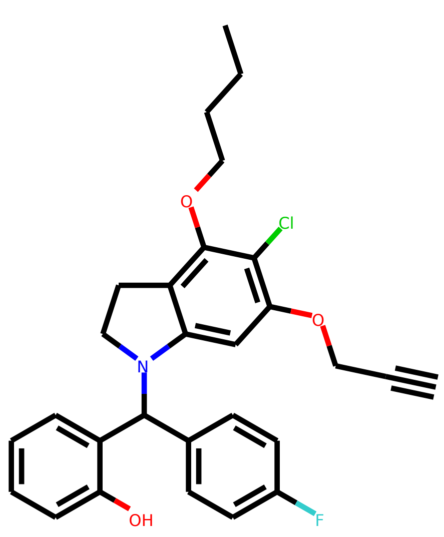

Osma-671

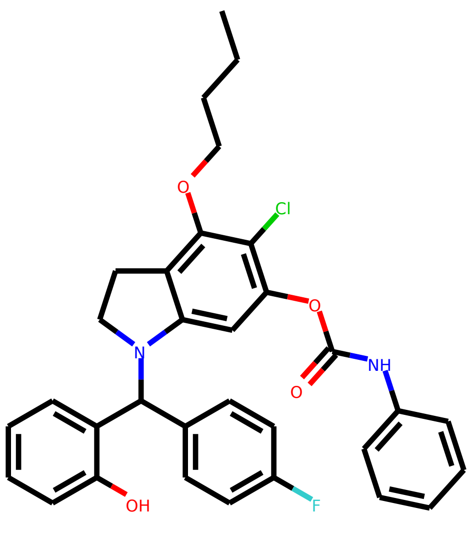

Osma-672

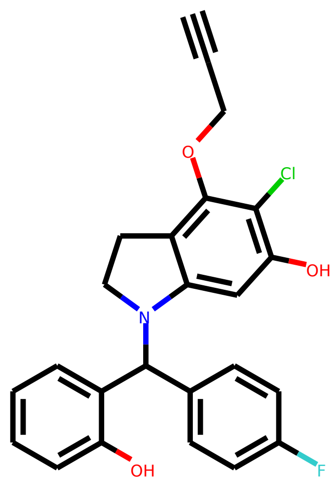

Osma-673

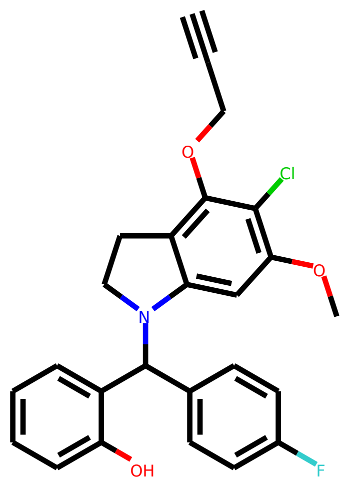

Osma-674

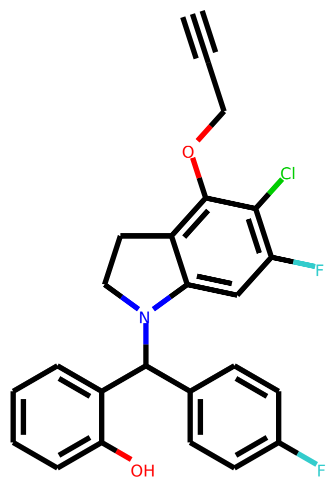

Osma-675

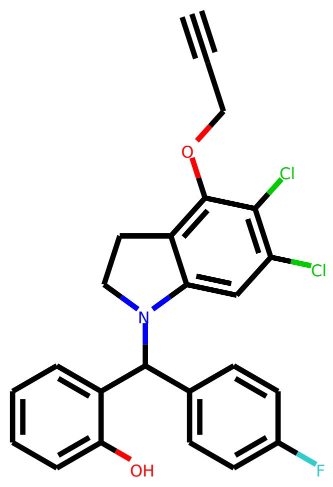

Osma-676

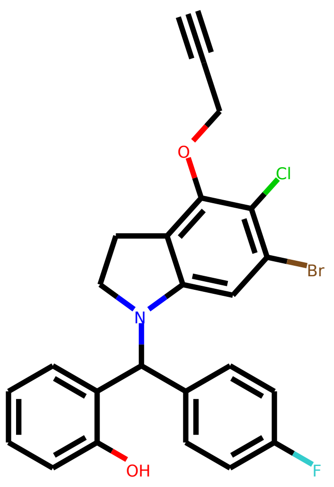

Osma-677

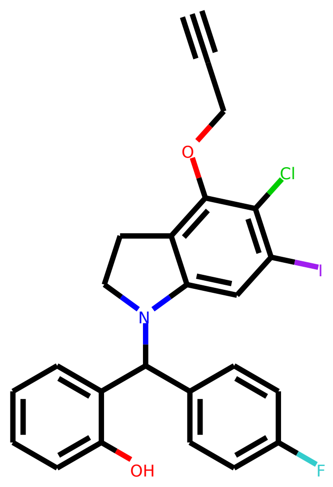

Osma-678

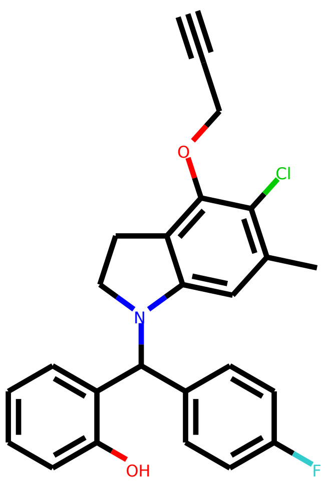

Osma-679

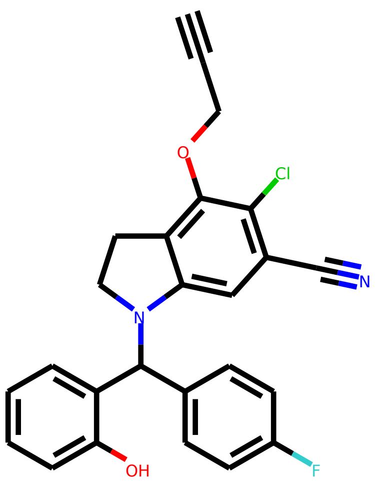

Osma-680

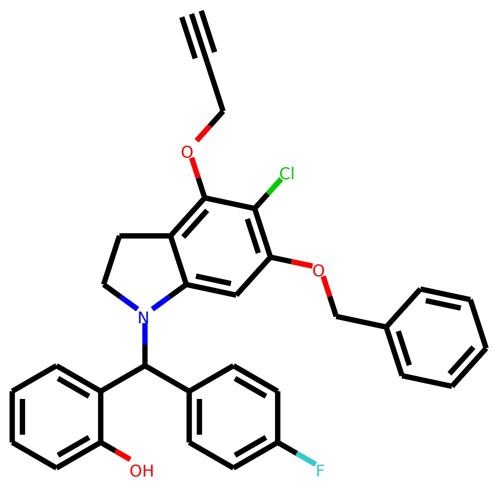

Osma-681

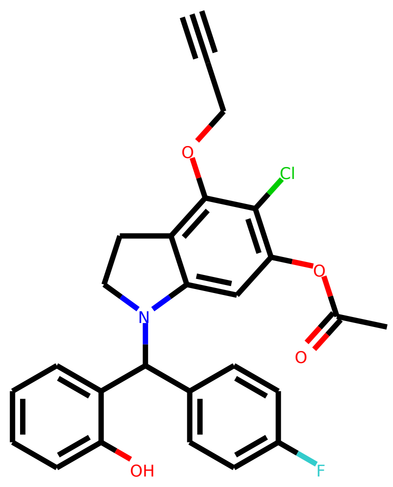

Osma-682

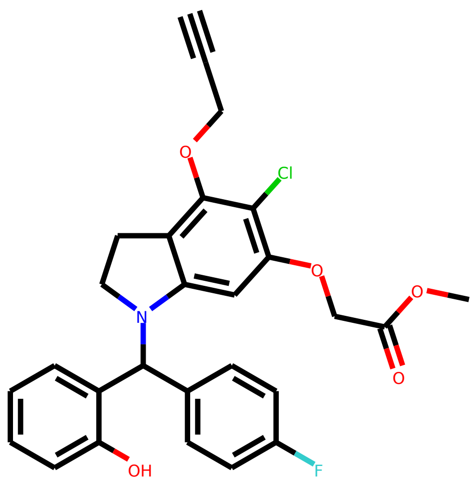

Osma-683

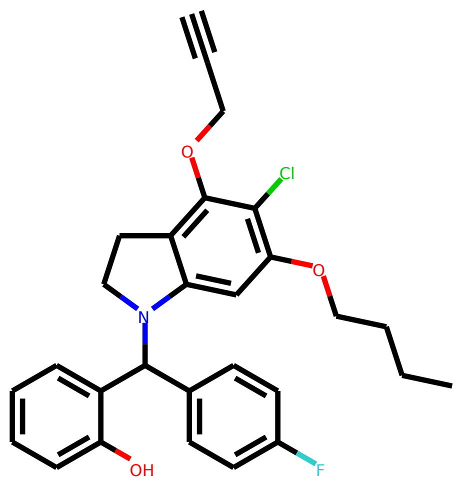

Osma-684

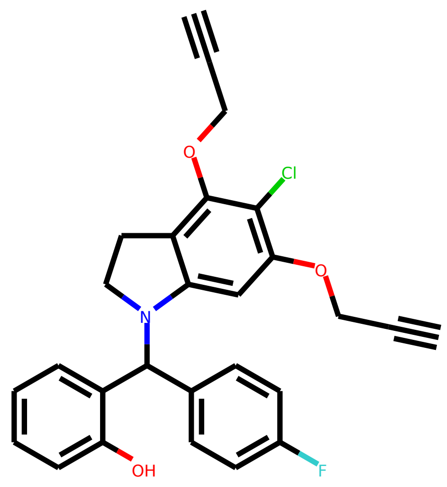

Osma-685

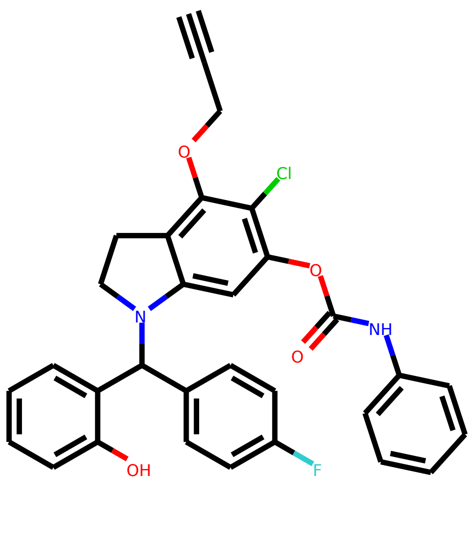

Osma-686

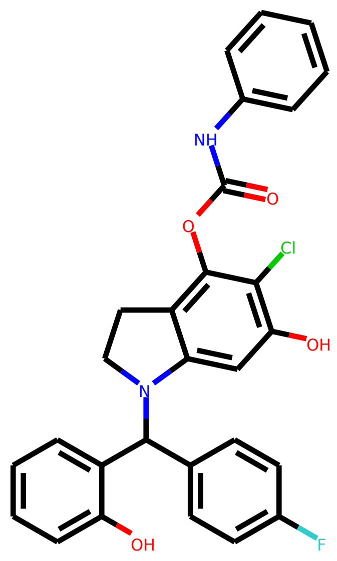

Osma-687

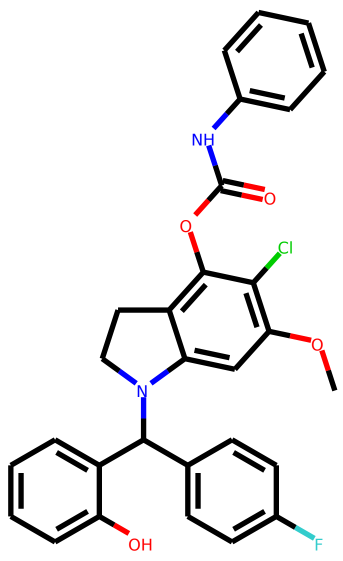

Osma-688

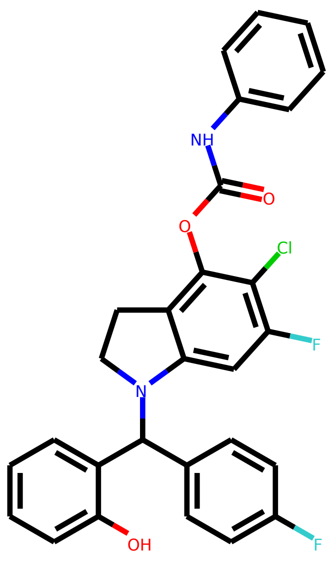

Osma-689

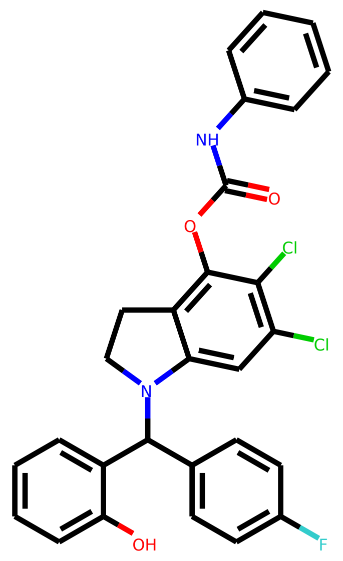

Osma-690

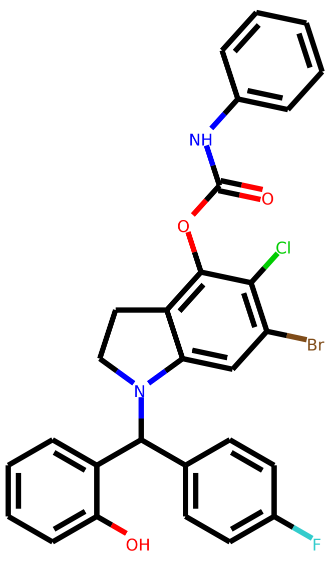

Osma-691

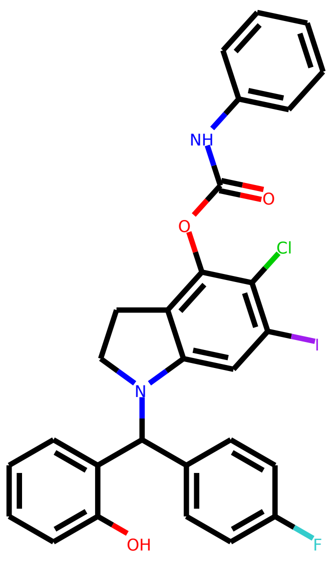

Osma-692

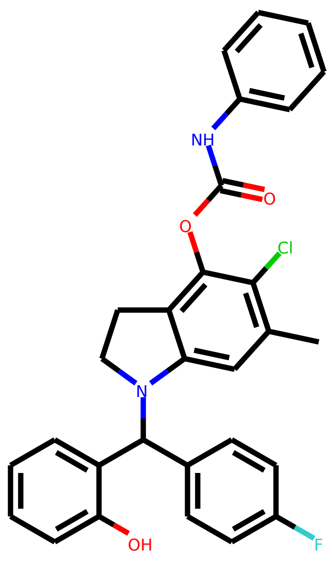

Osma-693

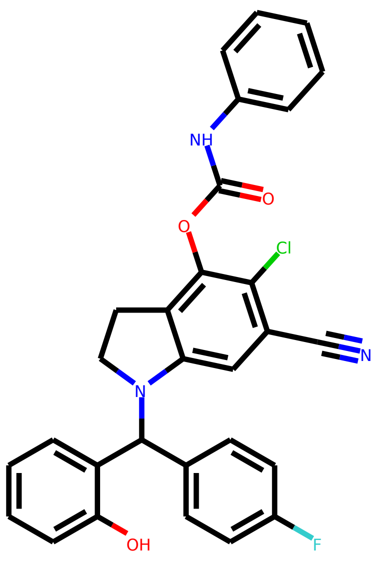

Osma-694

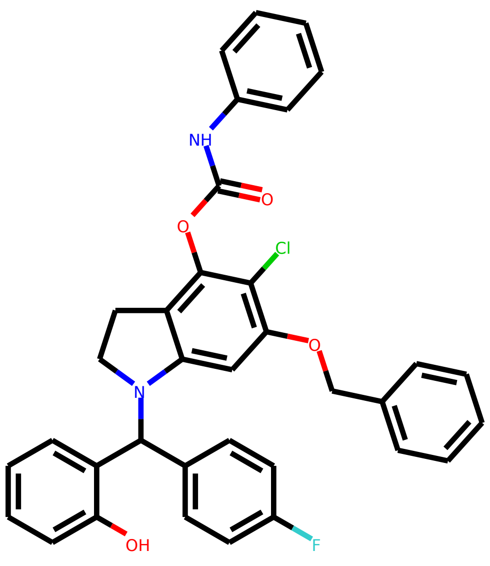

Osma-695

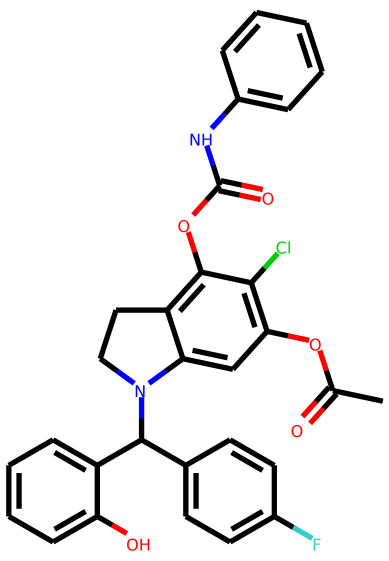

Osma-696

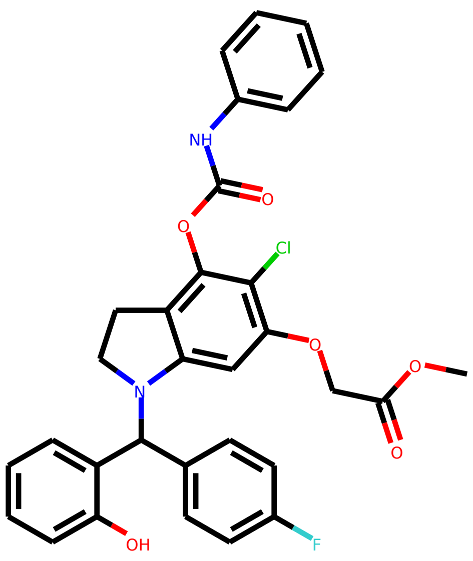

Osma-697

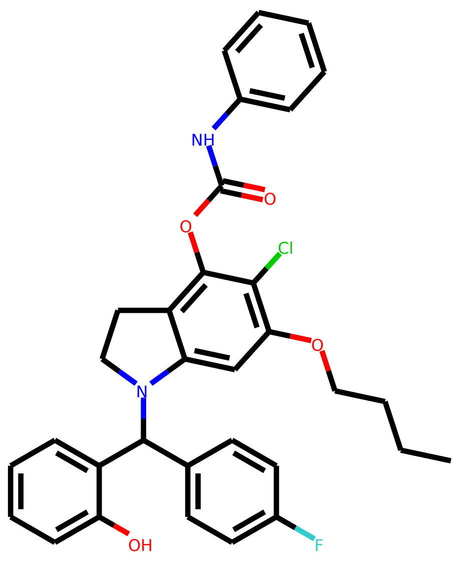

Osma-698

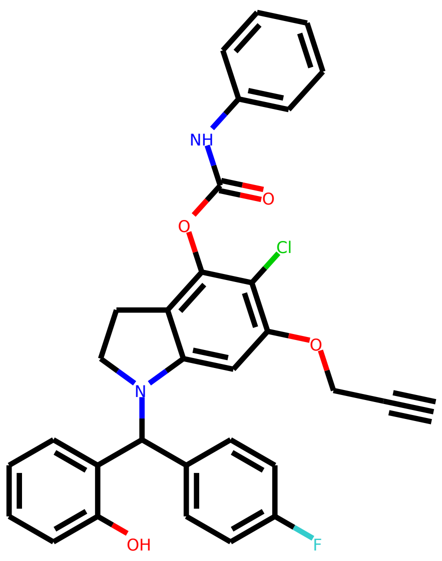

Osma-699

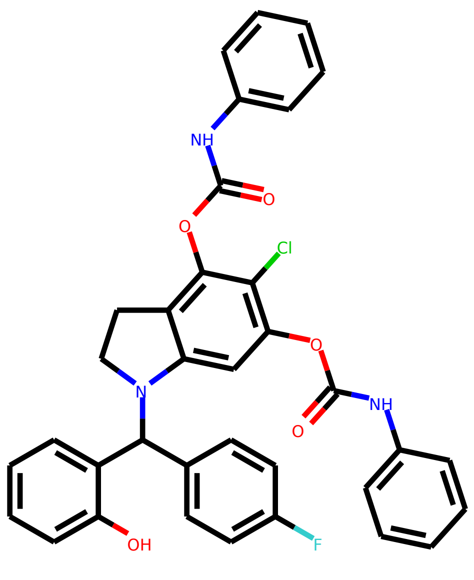

Osma-700

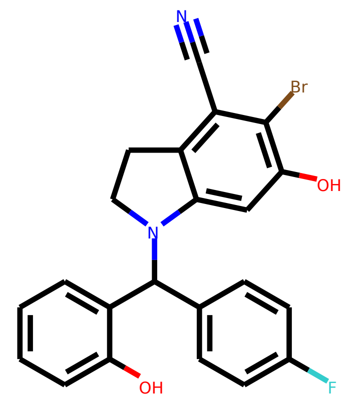

Osma-701

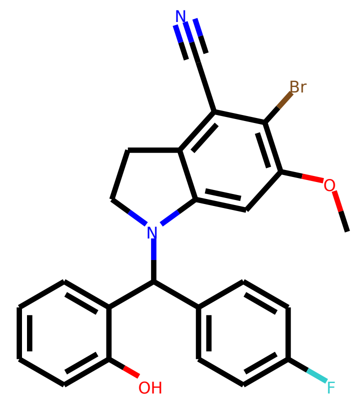

Osma-702

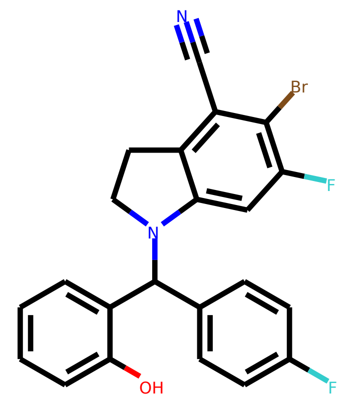

Osma-703

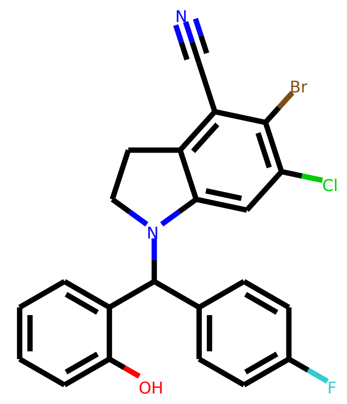

Osma-704

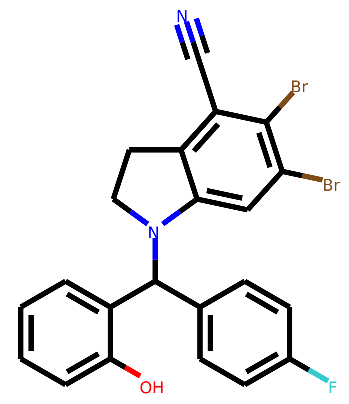

Osma-705

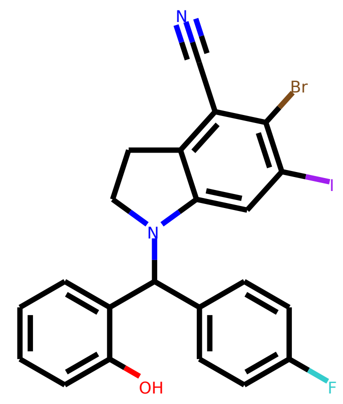

Osma-706

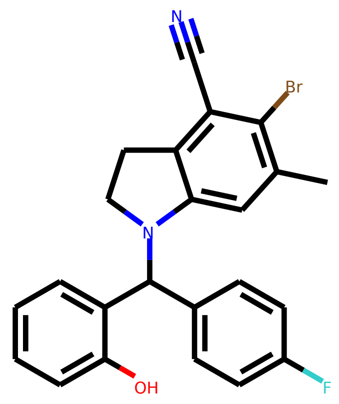

Osma-707

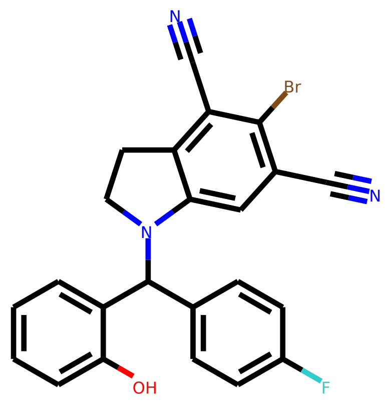

Osma-708

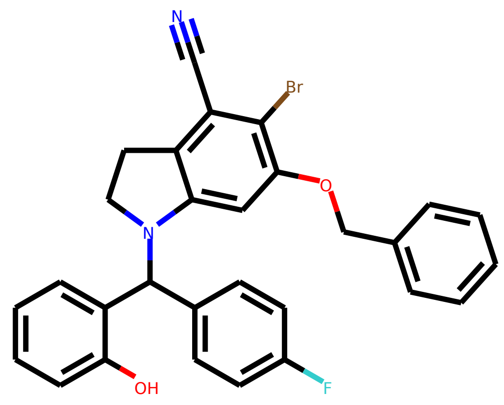

Osma-709

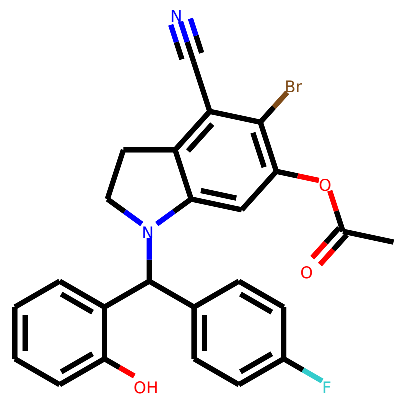

Osma-710

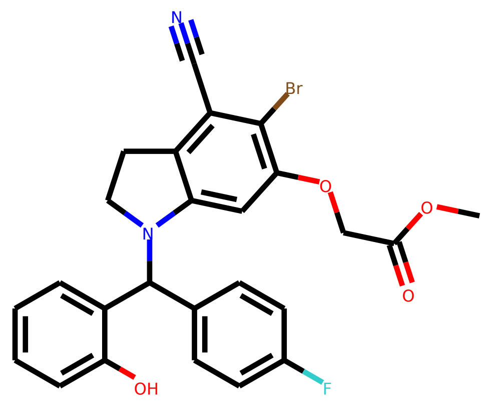

Osma-711

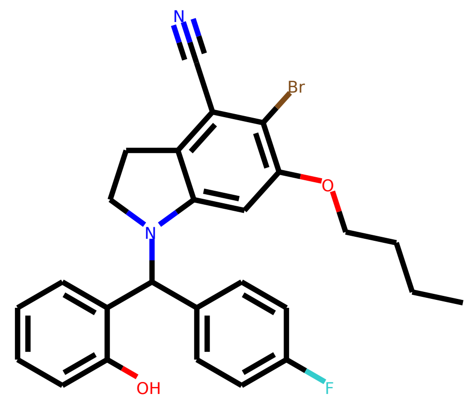

Osma-712

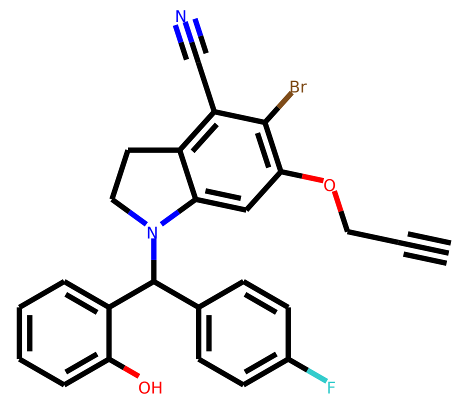

Osma-713

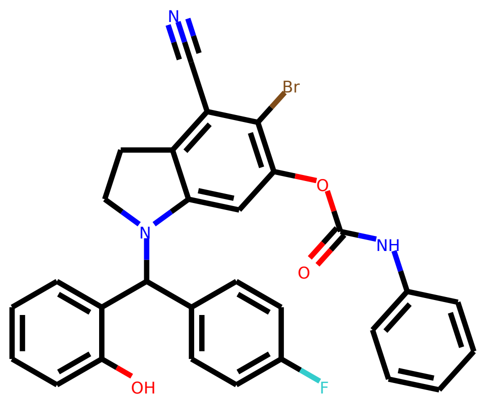

Osma-714

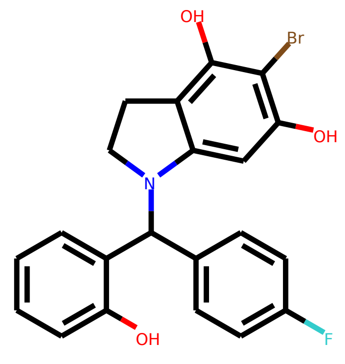

Osma-715

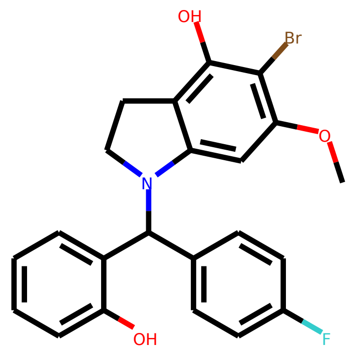

Osma-716

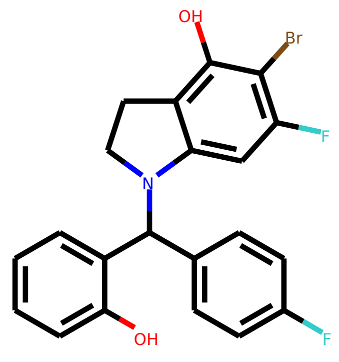

Osma-717

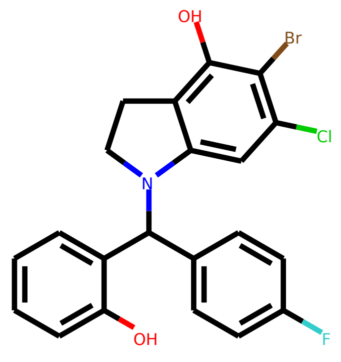

Osma-718

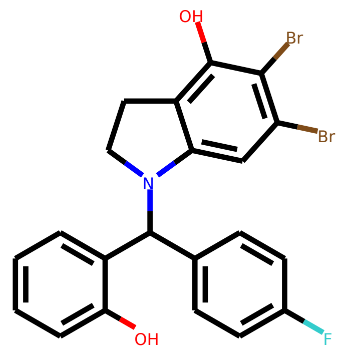

Osma-719

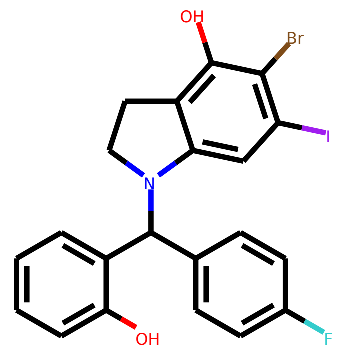

Osma-720

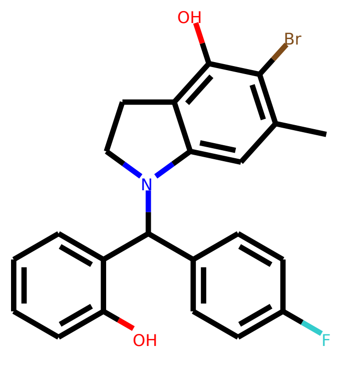

Osma-721

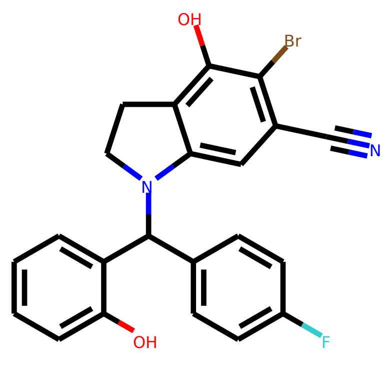

Osma-722

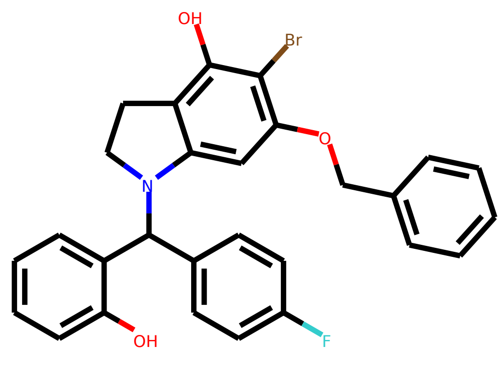

Osma-723

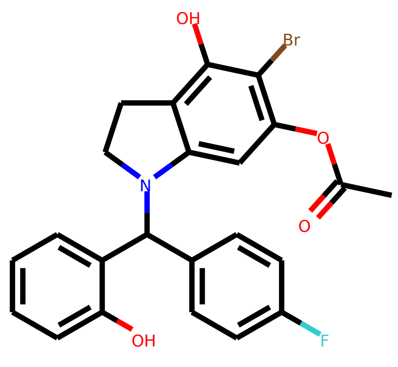

Osma-724

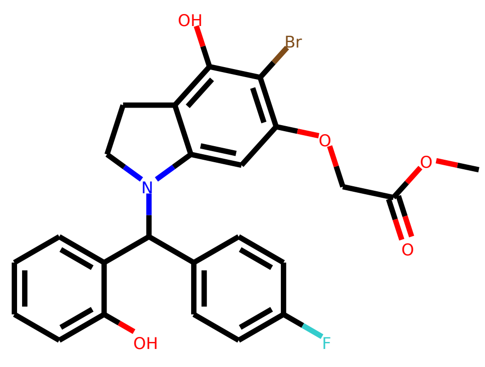

Osma-725

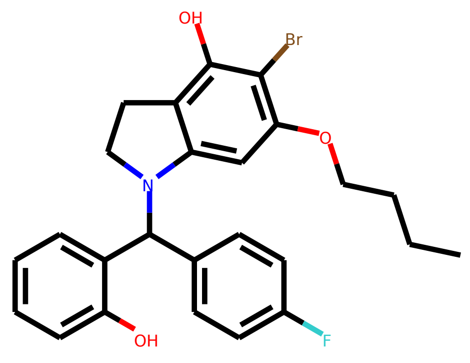

Osma-726

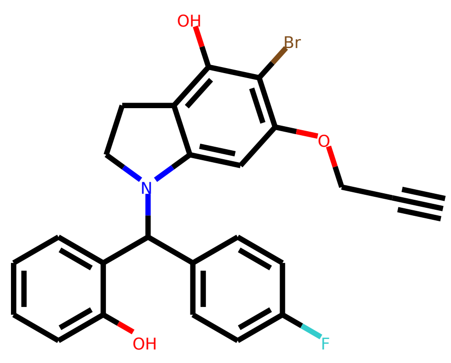

Osma-727

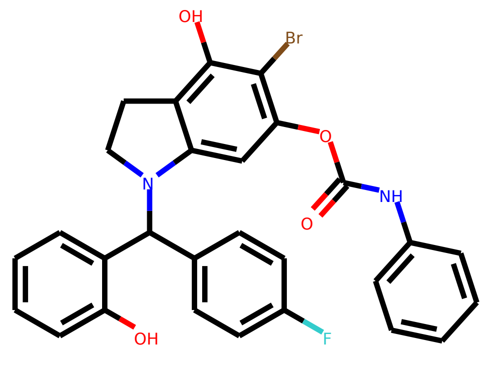

Osma-728

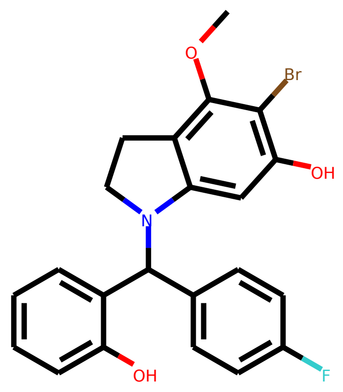

Osma-729

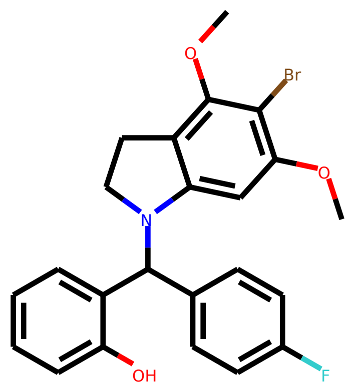

Osma-730

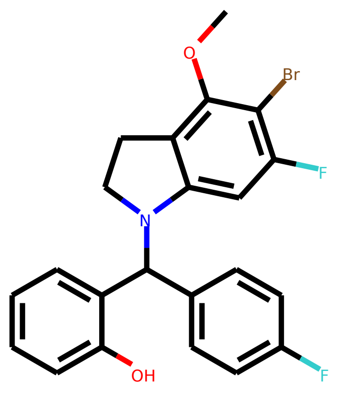

Osma-731

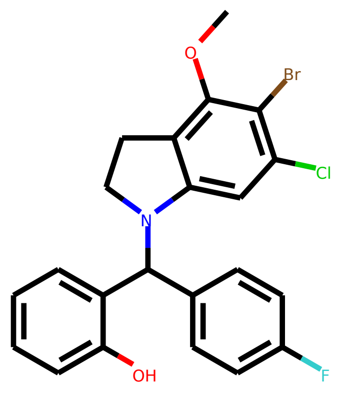

Osma-732

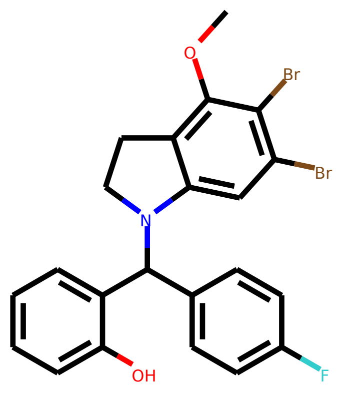

Osma-733

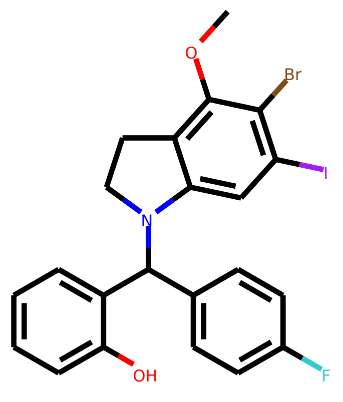

Osma-734

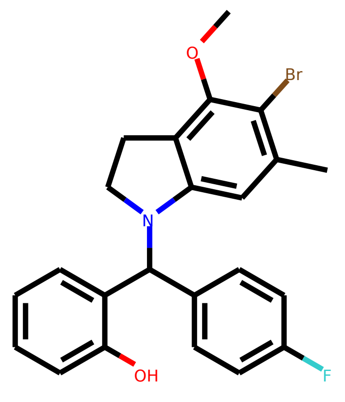

Osma-735

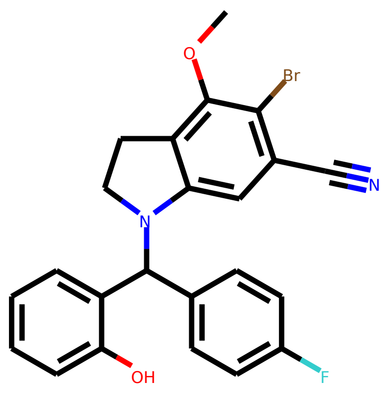

Osma-736

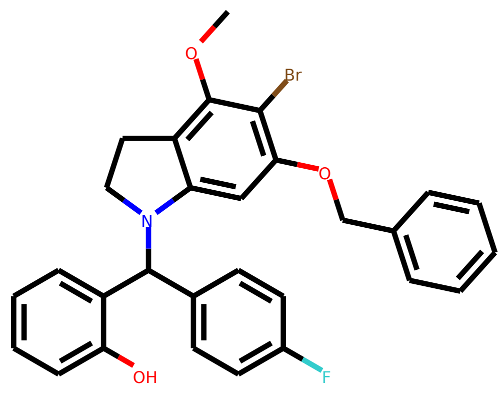

Osma-737

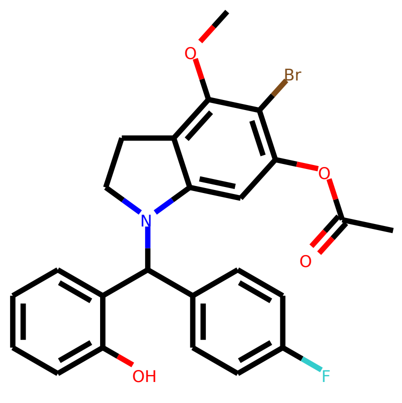

Osma-738

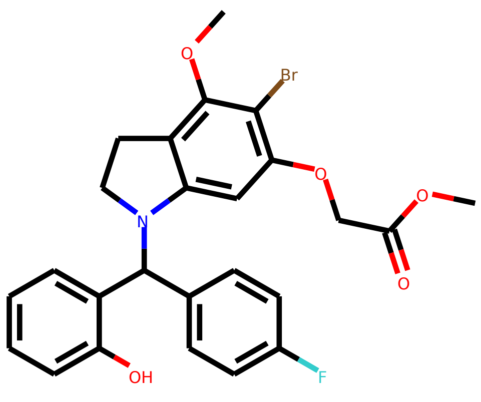

Osma-739

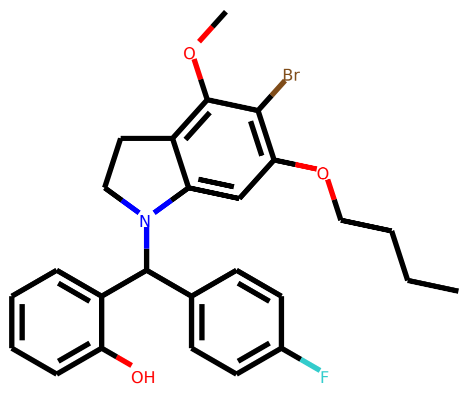

Osma-740

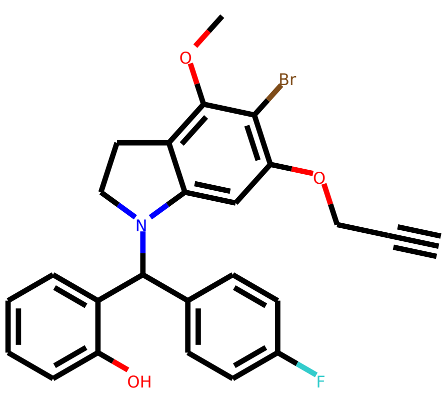

Osma-741

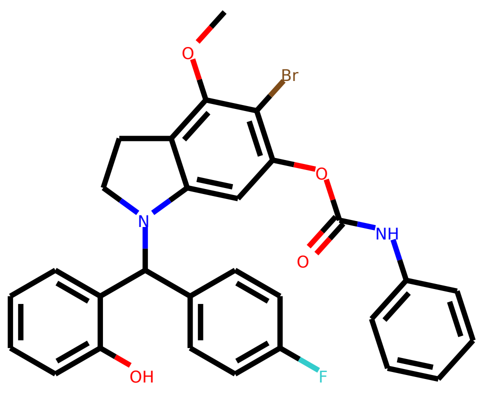

Osma-742

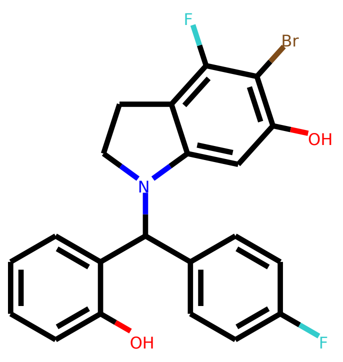

Osma-743

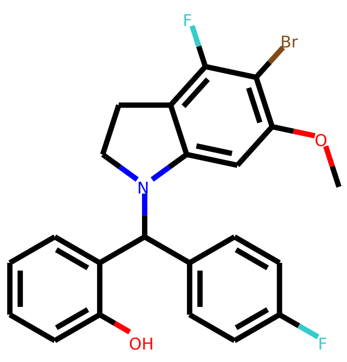

Osma-744

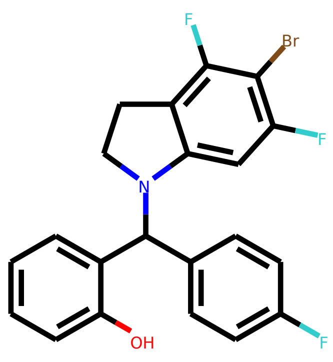

Osma-745

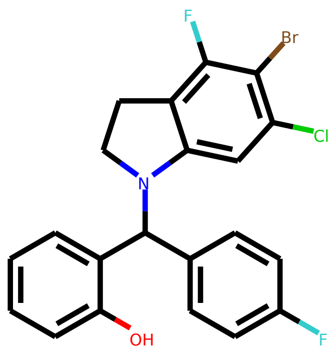

Osma-746

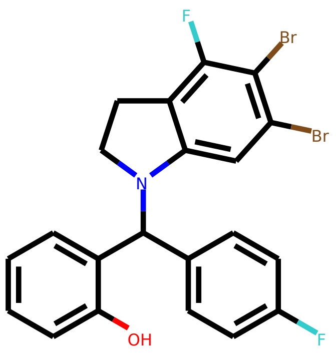

Osma-747

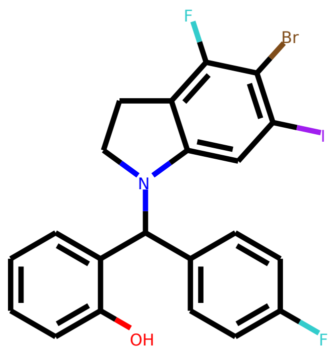

Osma-748

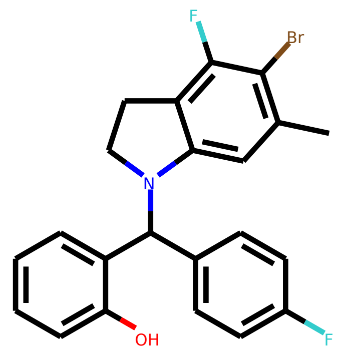

Osma-749

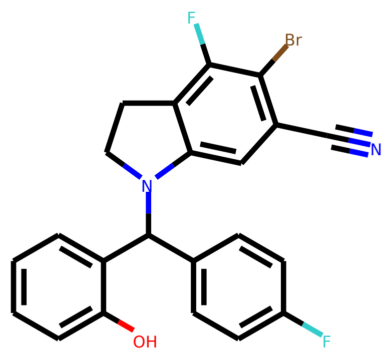

Osma-750

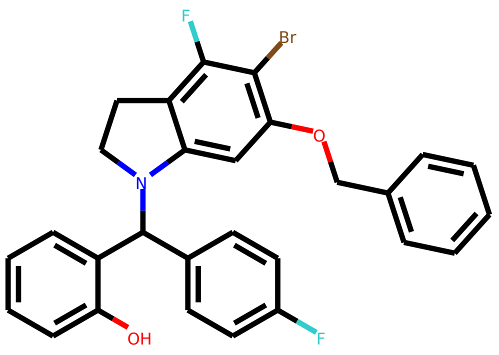

Osma-751

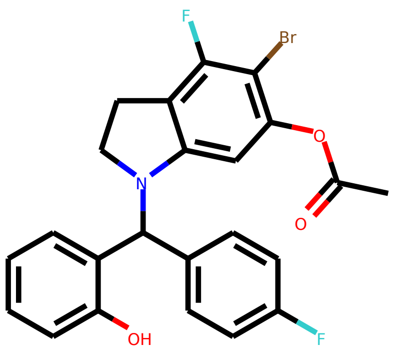

Osma-752

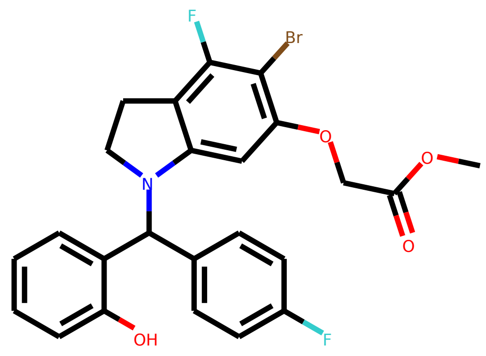

Osma-753

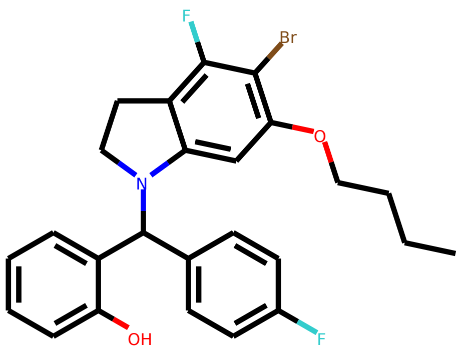

Osma-754

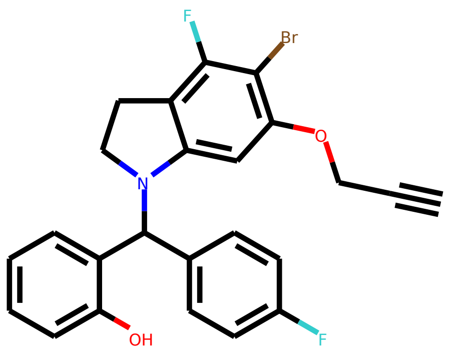

Osma-755

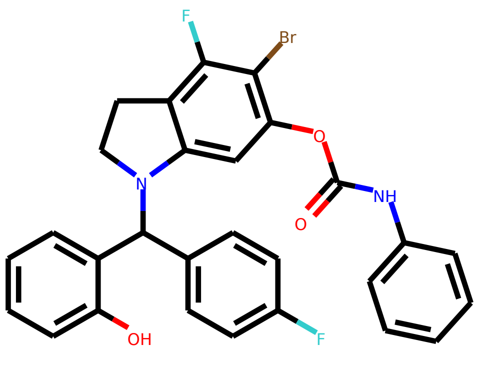

Osma-756

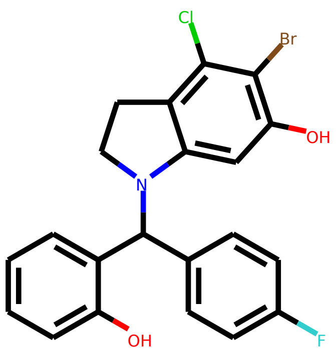

Osma-757

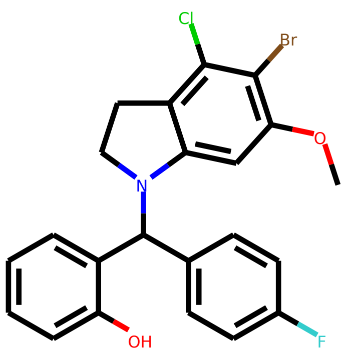

Osma-758

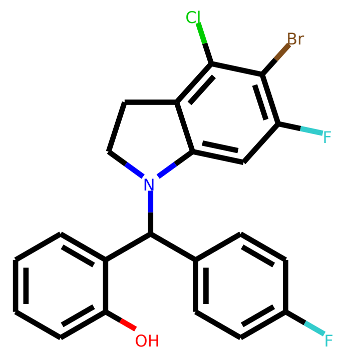

Osma-759

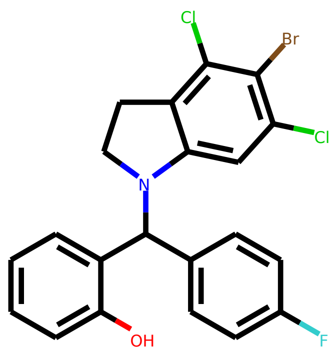

Osma-760

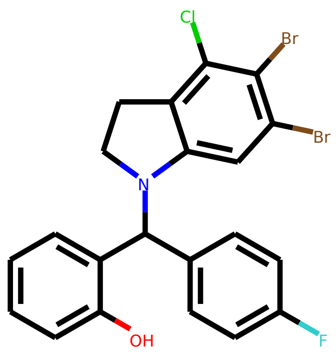

Osma-761

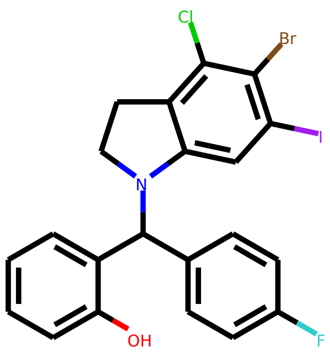

Osma-762

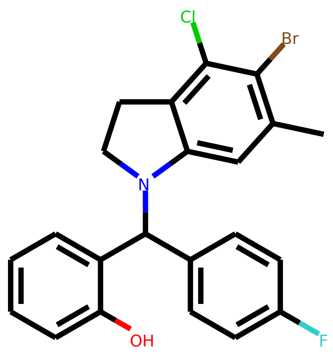

Osma-763

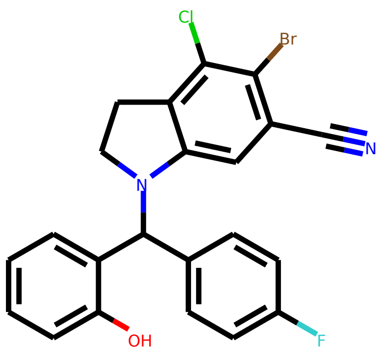

Osma-764

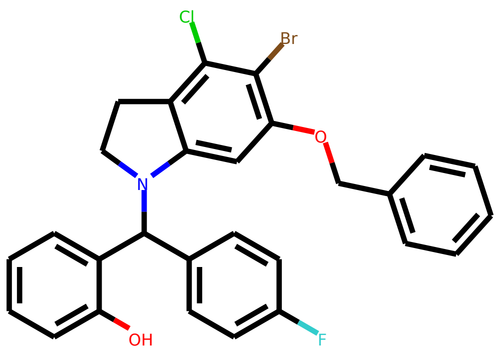

Osma-765

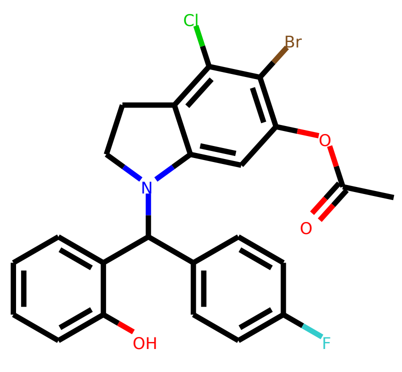

Osma-766

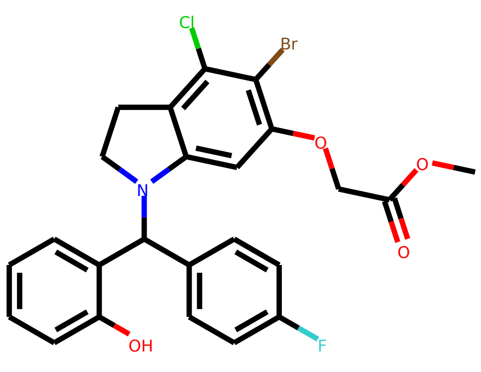

Osma-767

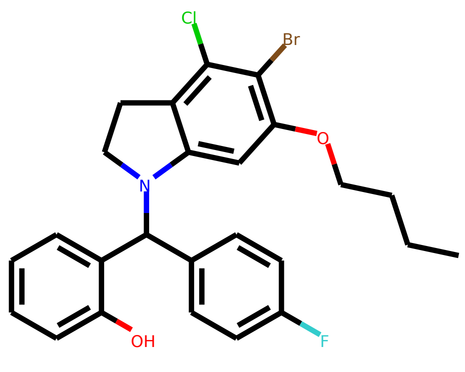

Osma-768

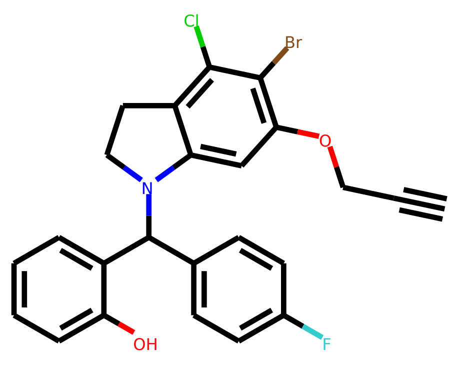

Osma-769

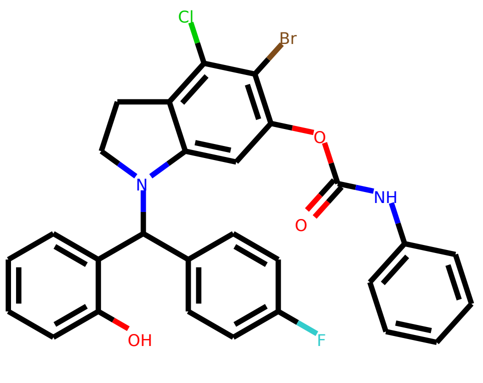

Osma-770

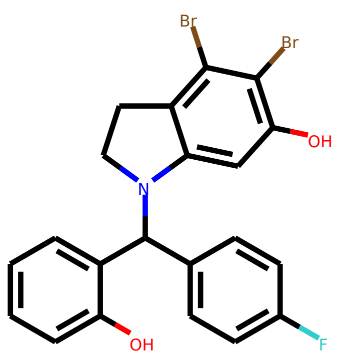

Osma-771

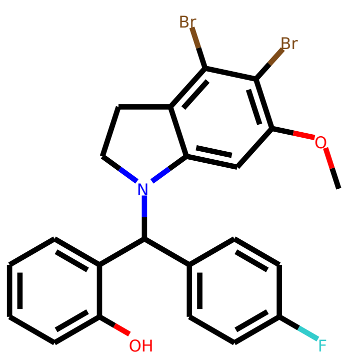

Osma-772

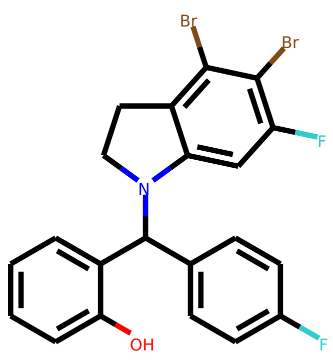

Osma-773

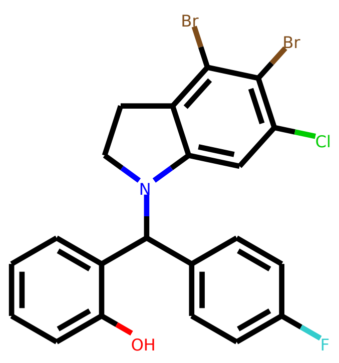

Osma-774

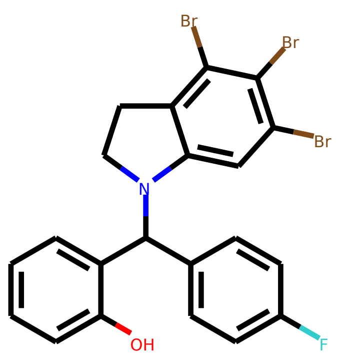

Osma-775

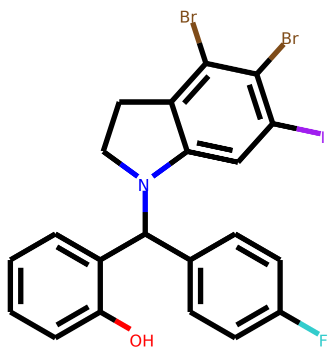

Osma-776

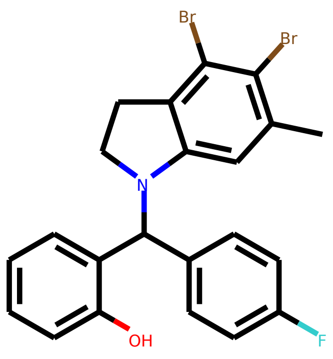

Osma-777

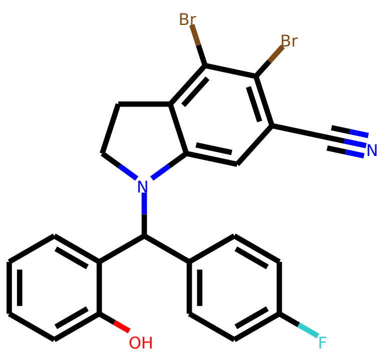

Osma-778

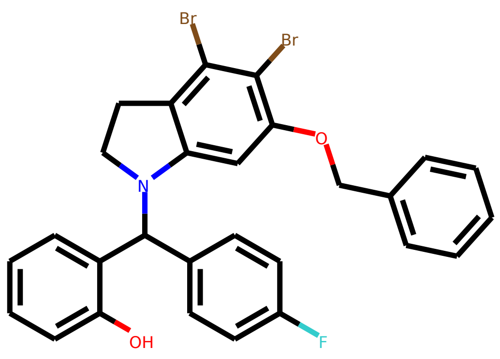

Osma-779

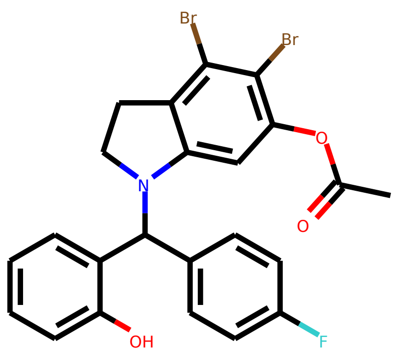

Osma-780

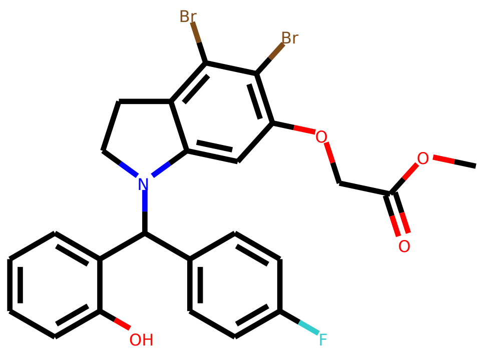

Osma-781

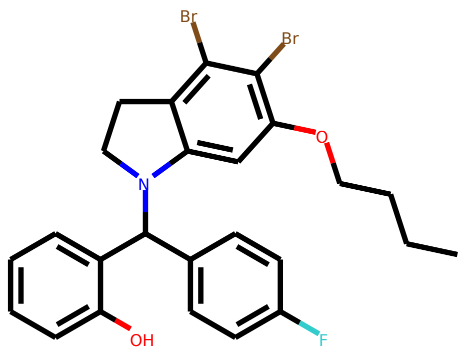

Osma-782

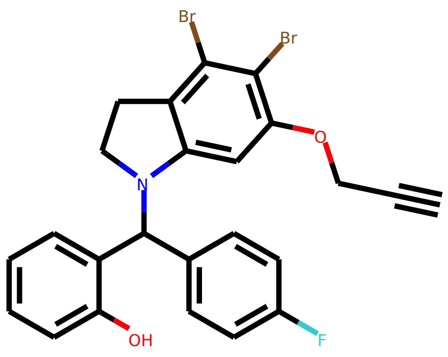

Osma-783

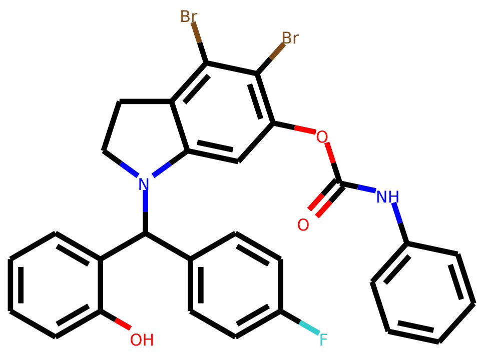

Osma-784

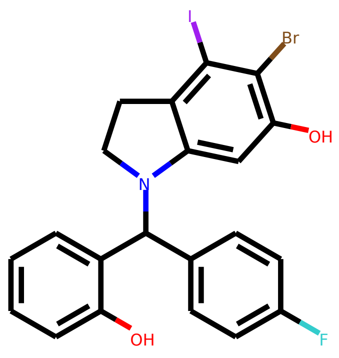

Osma-785

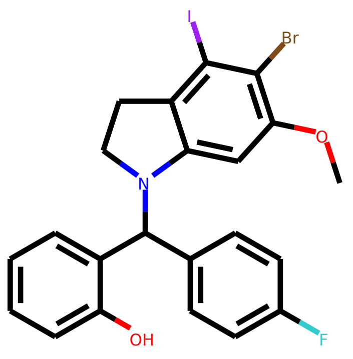

Osma-786

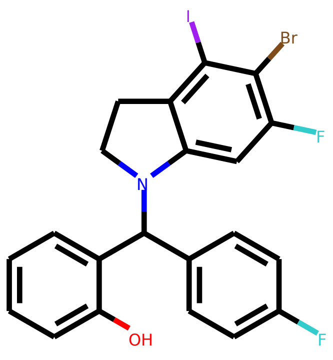

Osma-787

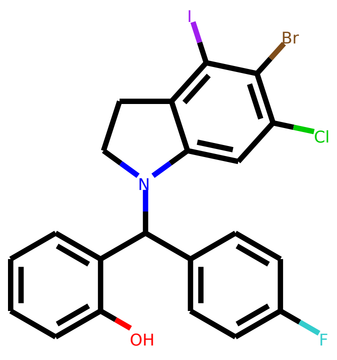

Osma-788

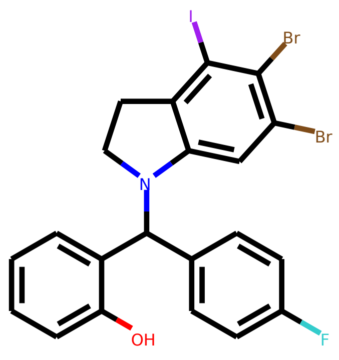

Osma-789

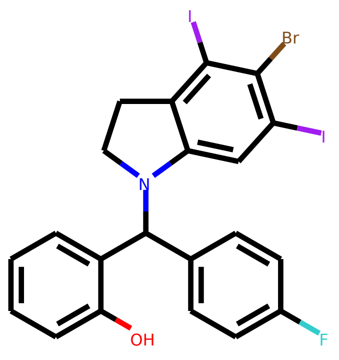

Osma-790

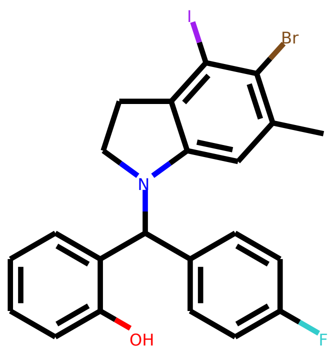

Osma-791

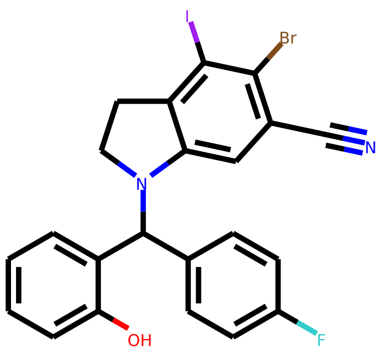

Osma-792

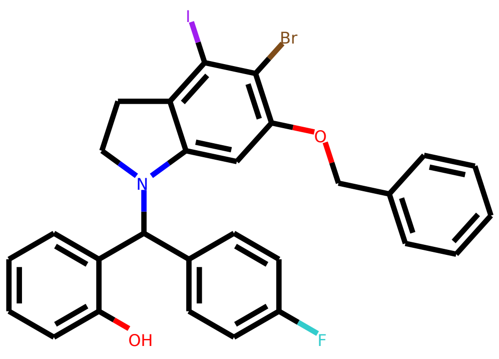

Osma-793

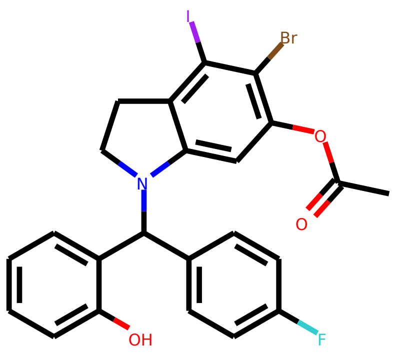

Osma-794

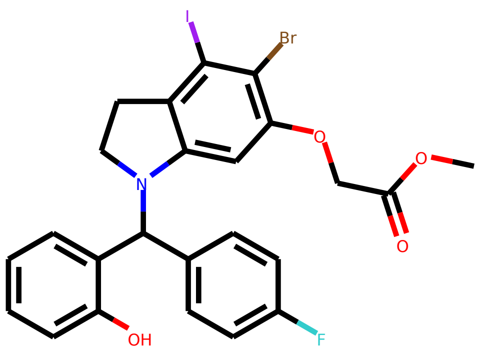

Osma-795

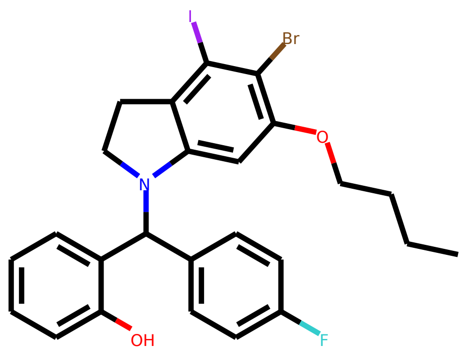

Osma-796

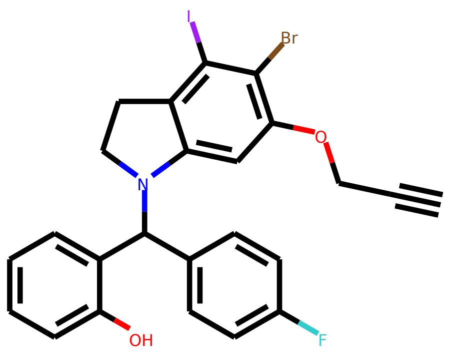

Osma-797

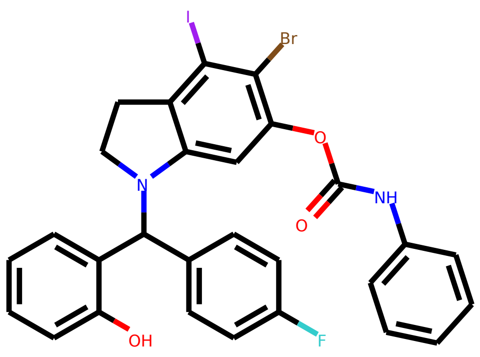

Osma-798

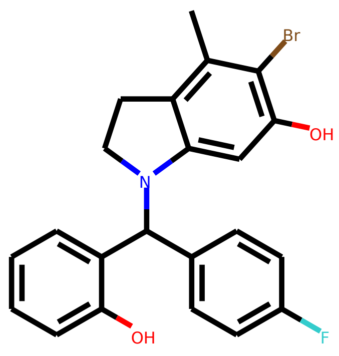

Osma-799

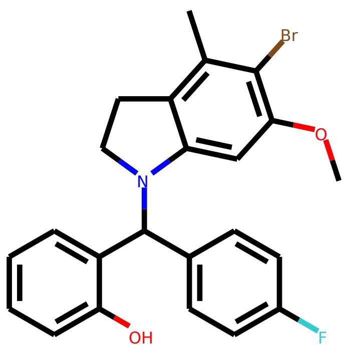

Osma-800

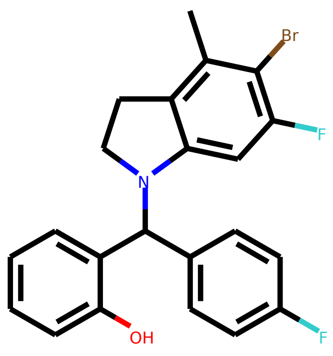

Osma-801

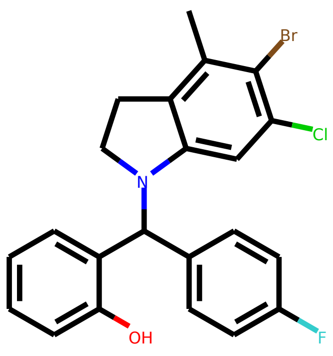

Osma-802

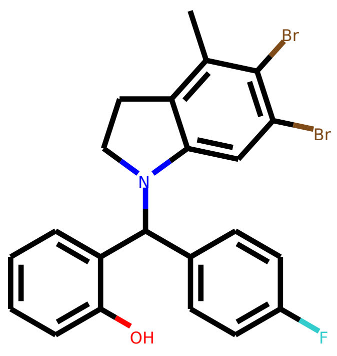

Osma-803

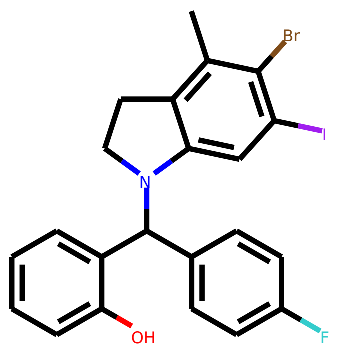

Osma-804

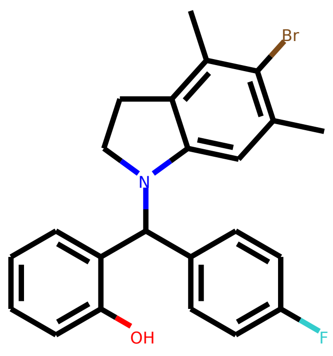

Osma-805

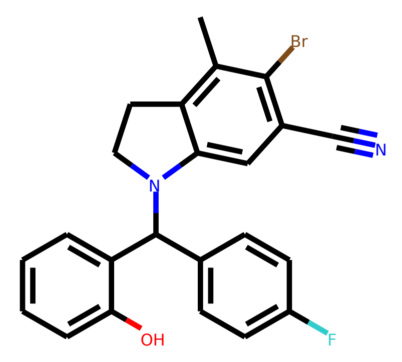

Osma-806

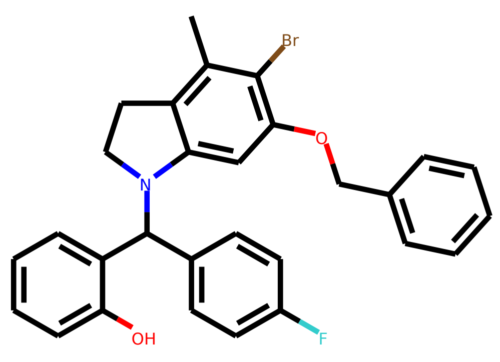

Osma-807

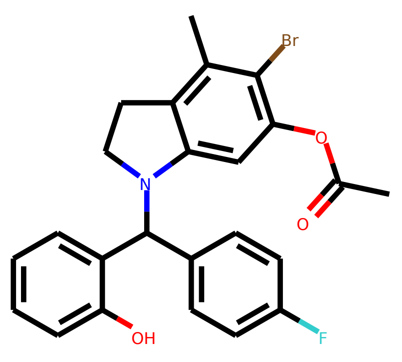

Osma-808

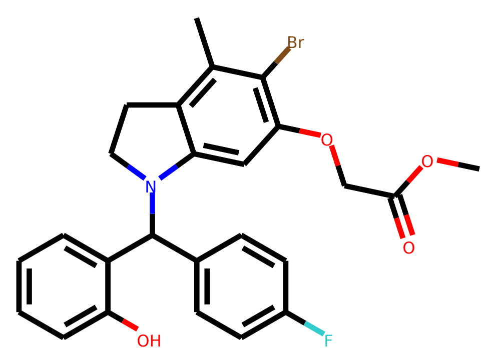

Osma-809

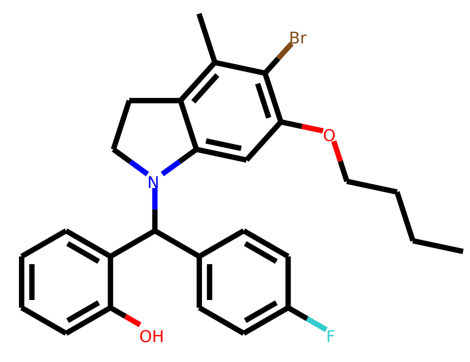

Osma-810

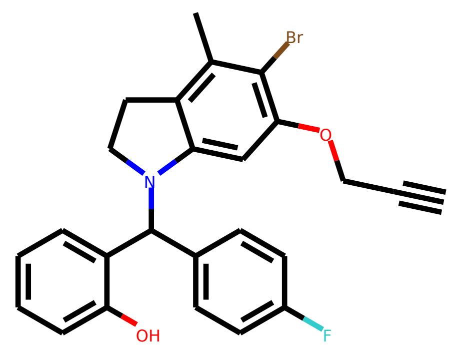

Osma-811

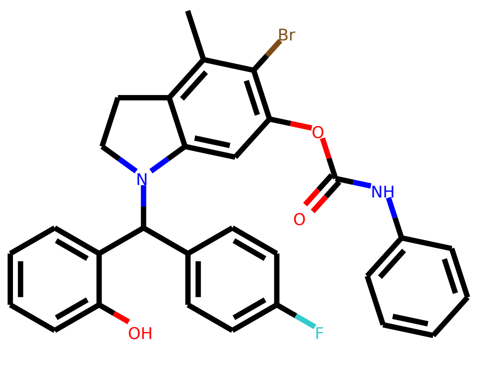

Osma-812

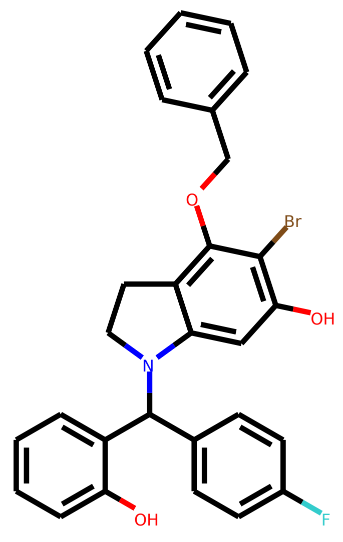

Osma-813

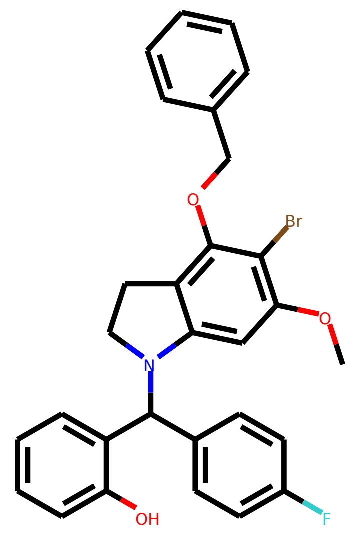

Osma-814

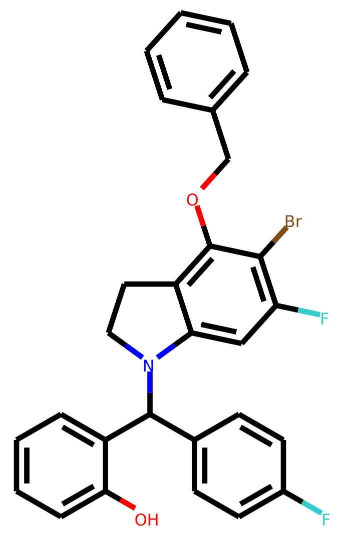

Osma-815

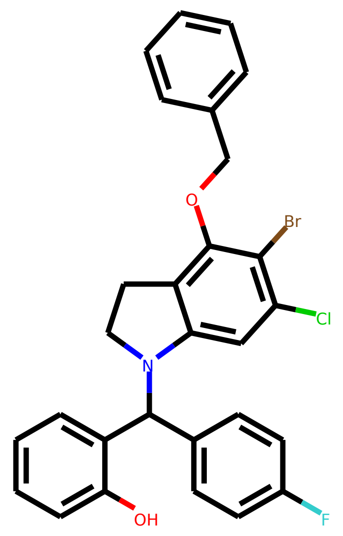

Osma-816

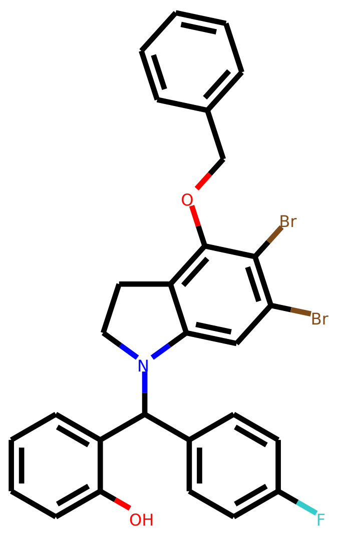

Osma-817

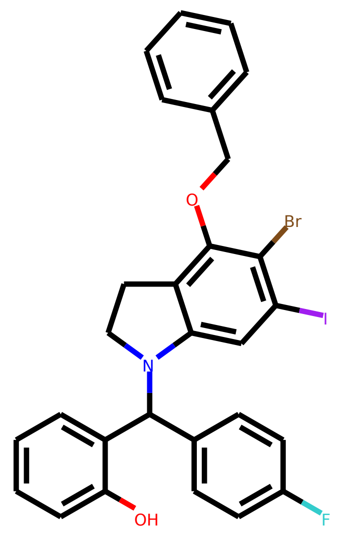

Osma-818

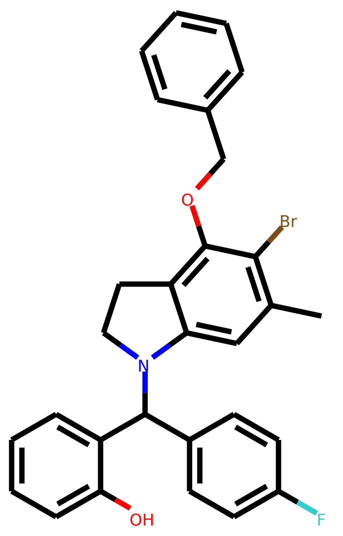

Osma-819

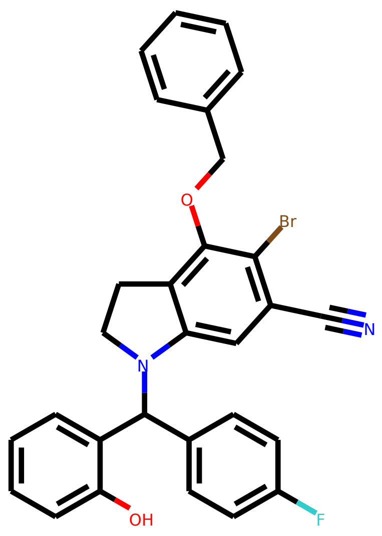

Osma-820

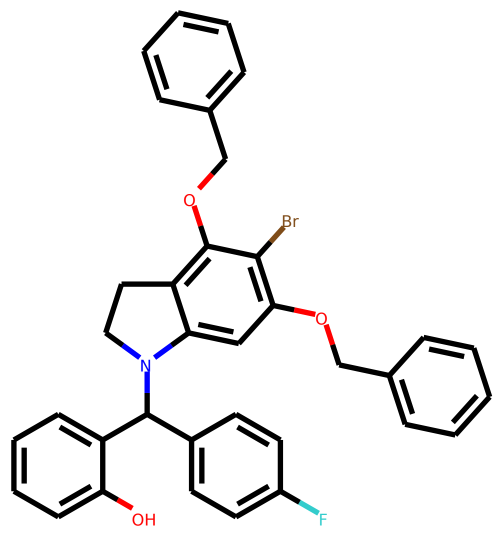

Osma-821

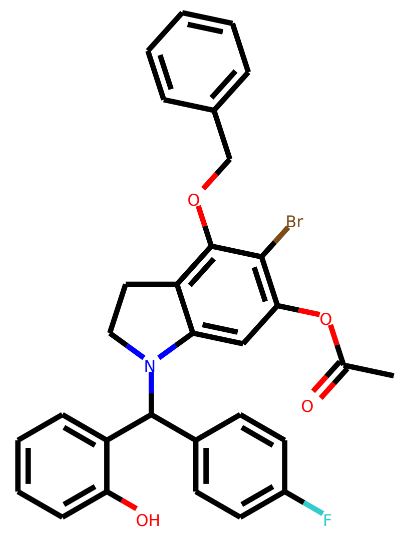

Osma-822

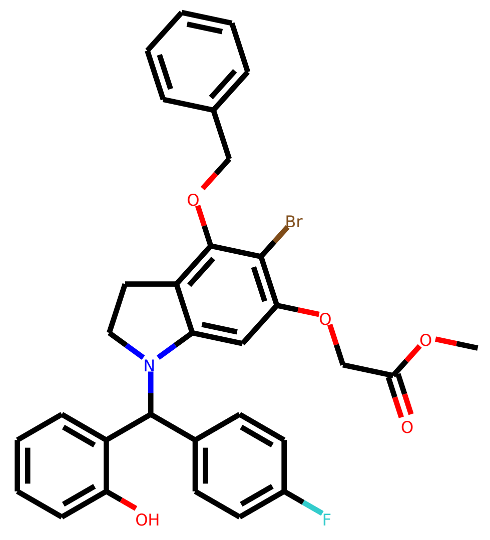

Osma-823

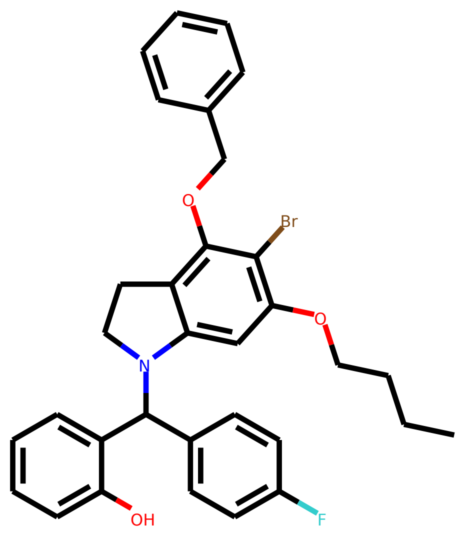

Osma-824

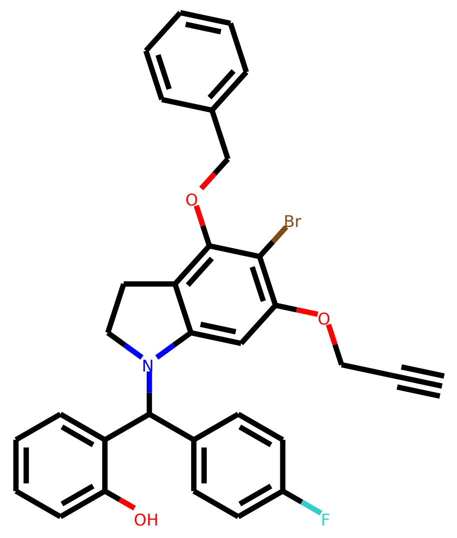

Osma-825

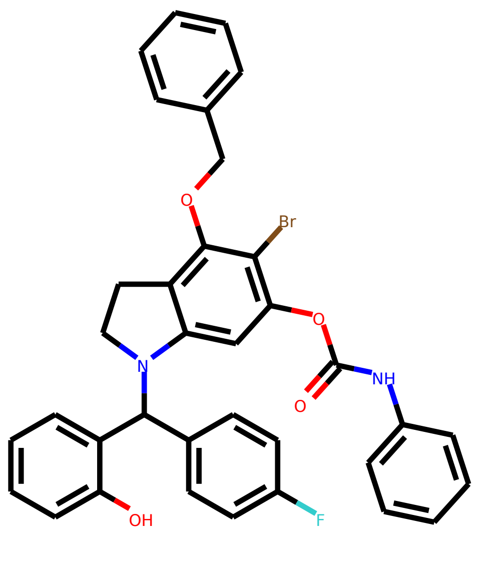

Osma-826

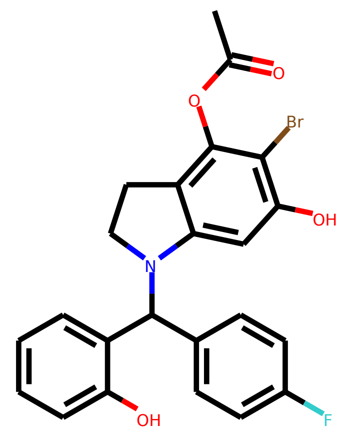

Osma-827

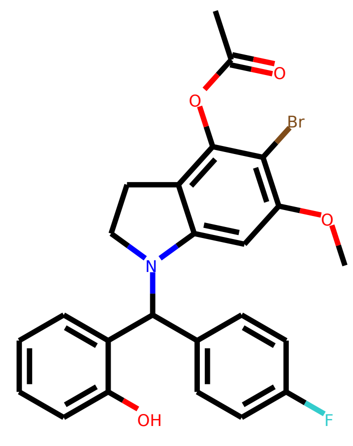

Osma-828

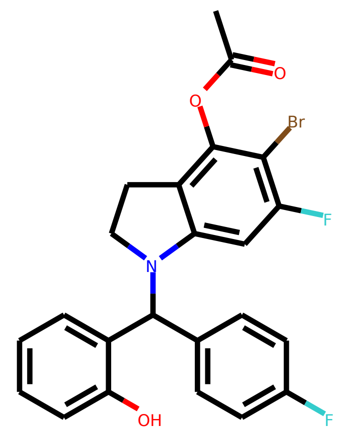

Osma-829

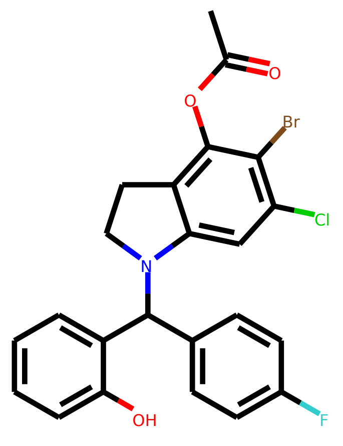

Osma-830

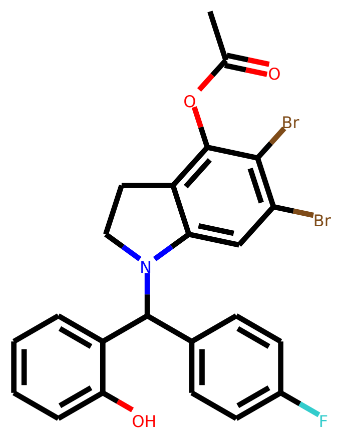

Osma-831

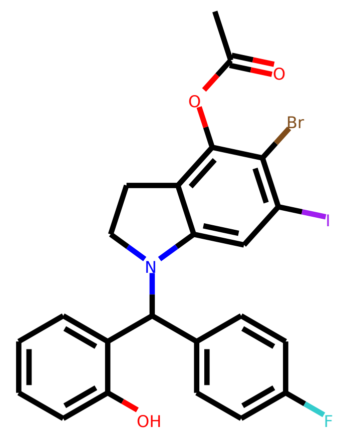

Osma-832

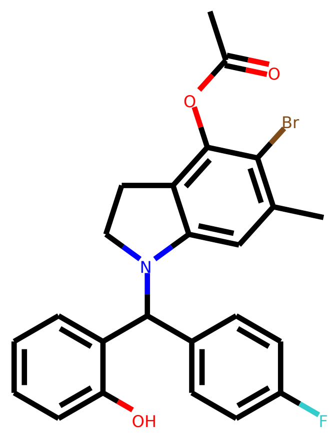

Osma-833

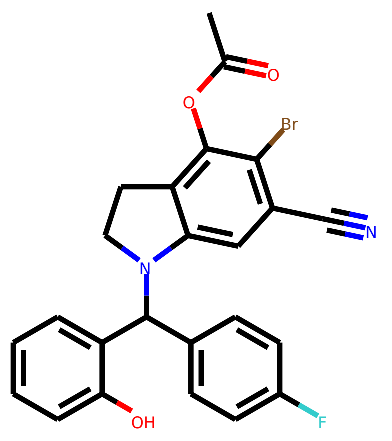

Osma-834

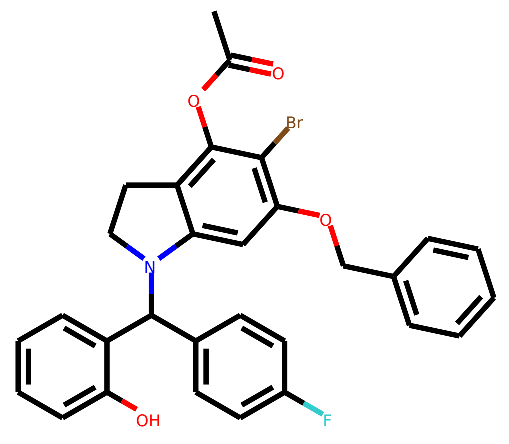

Osma-835

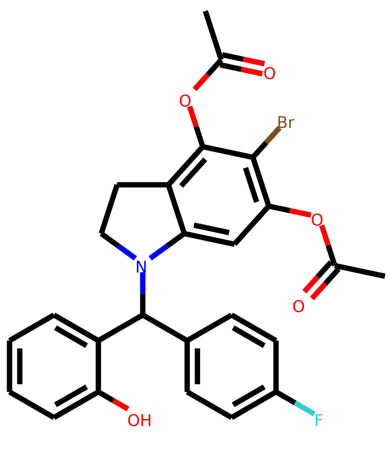

Osma-836

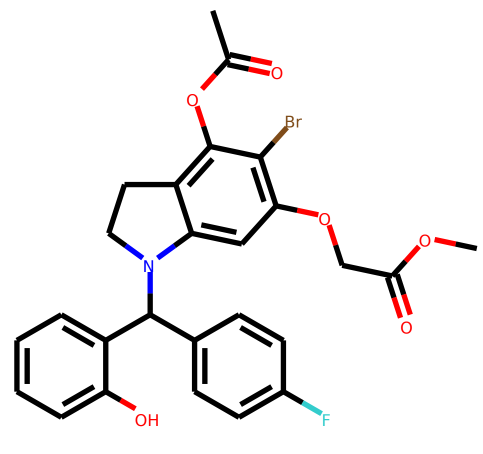

Osma-837

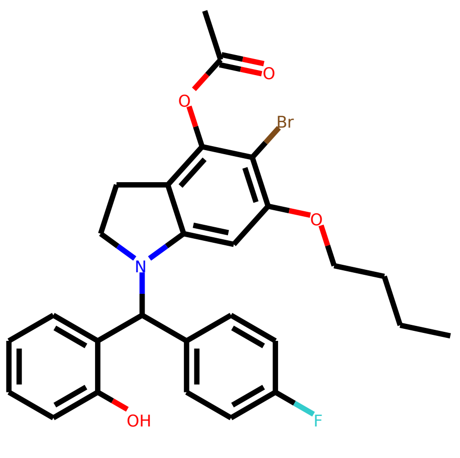

Osma-838

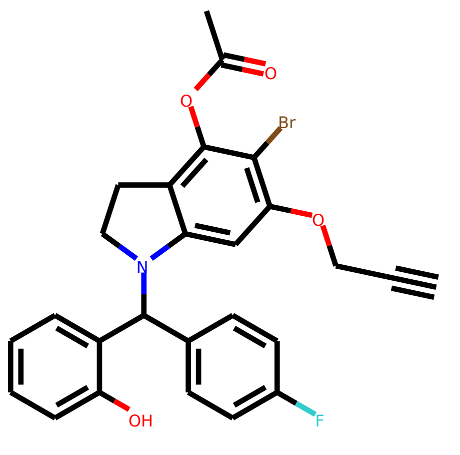

Osma-839

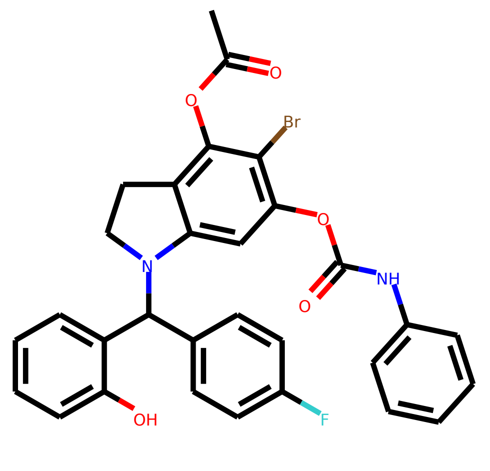

Osma-840

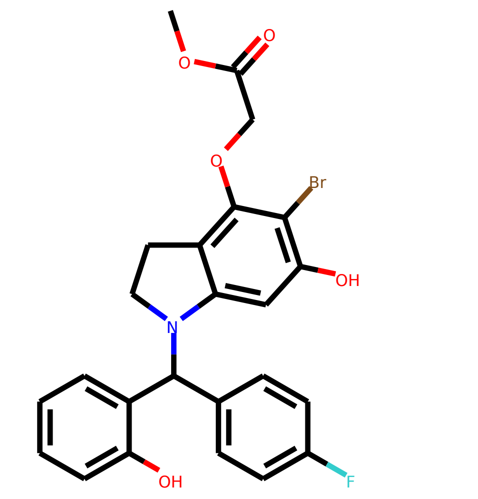
Osma-841

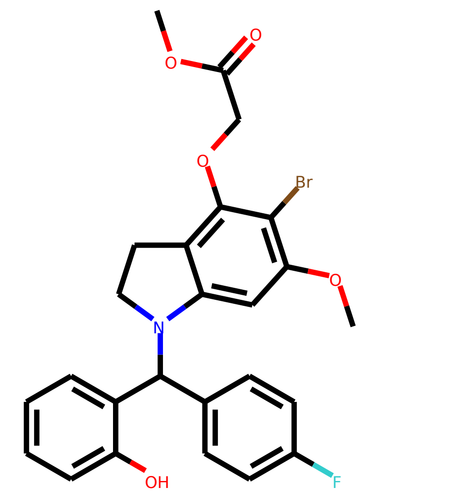
Osma-842

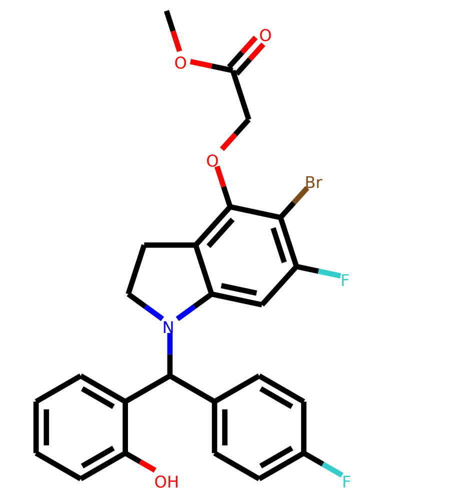
Osma-843

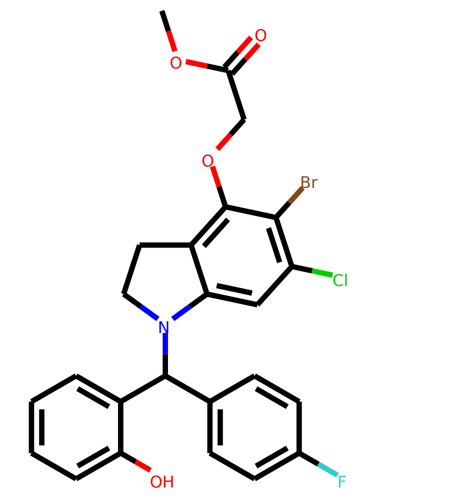
Osma-844

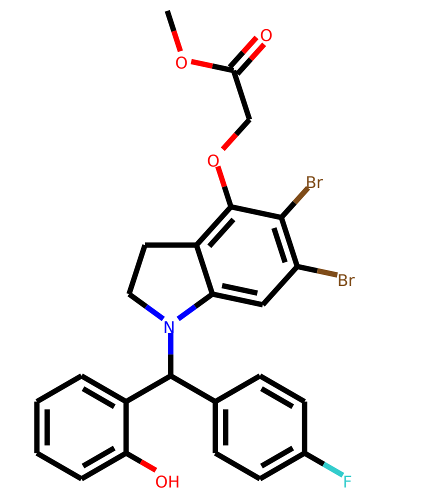
Osma-845

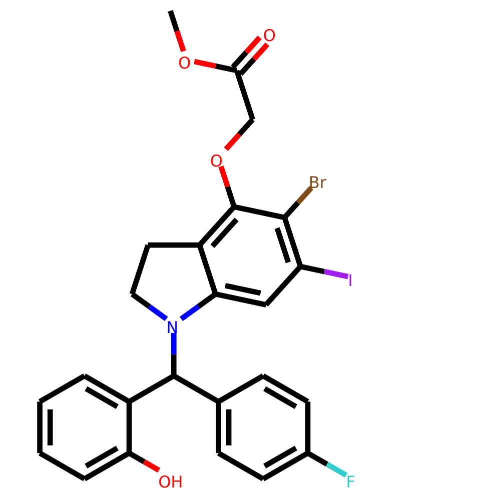
Osma-846

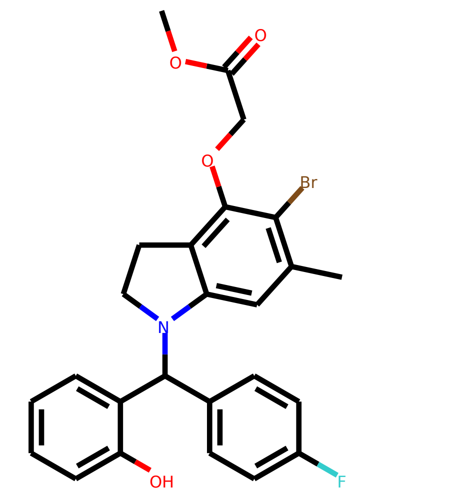
Osma-847

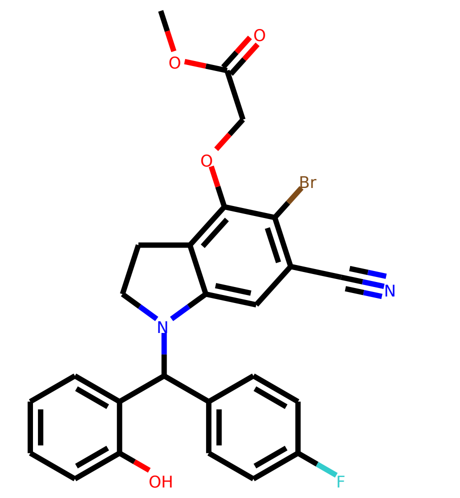
Osma-848

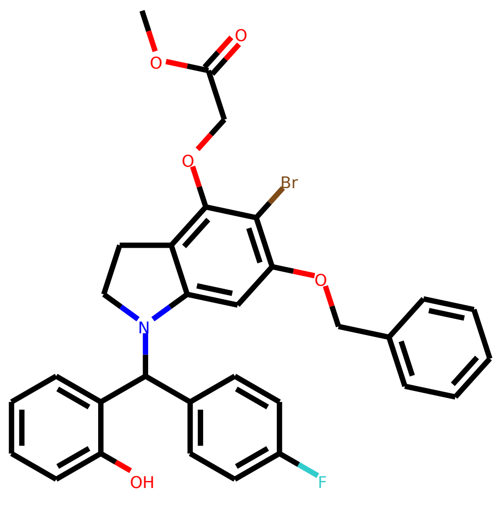
Osma-849

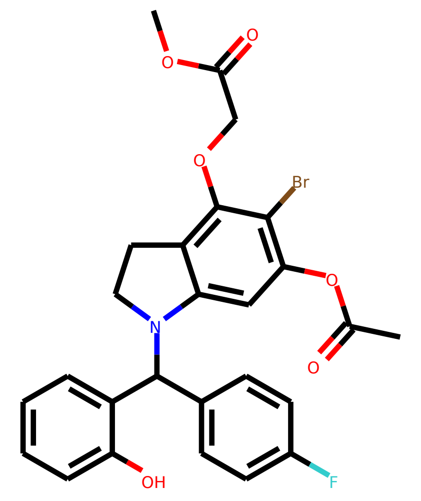
Osma-850

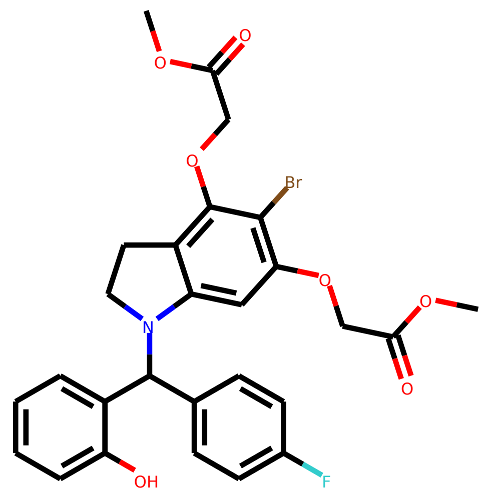
Osma-851

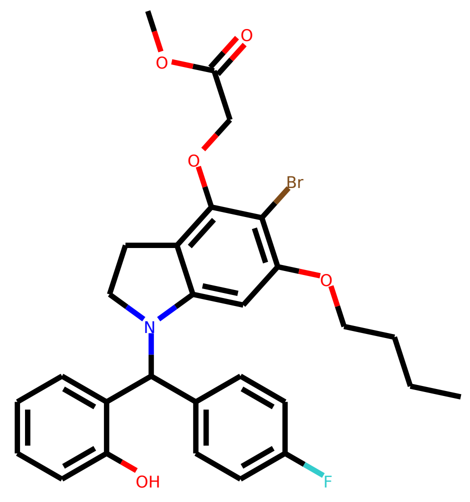
Osma-852

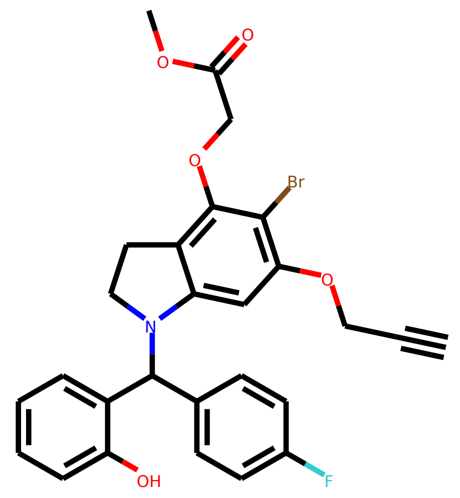
Osma-853

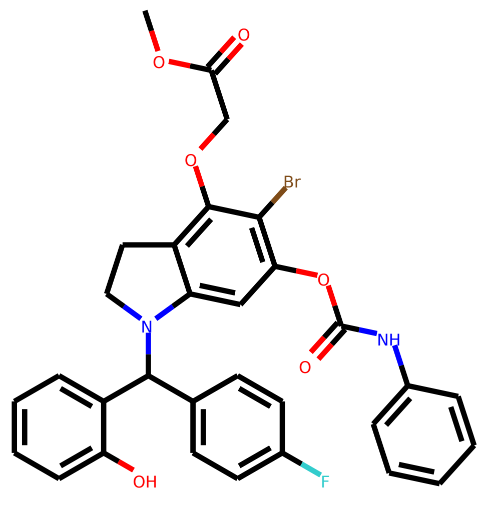
Osma-854

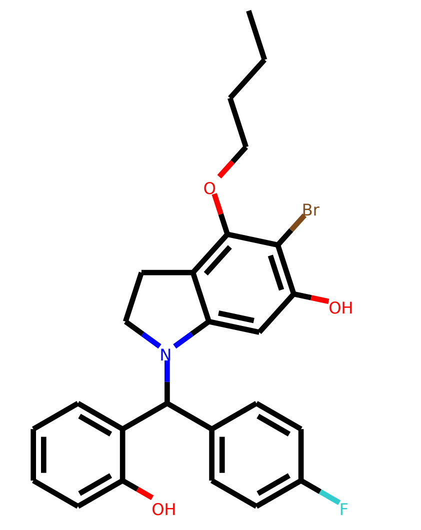
Osma-855

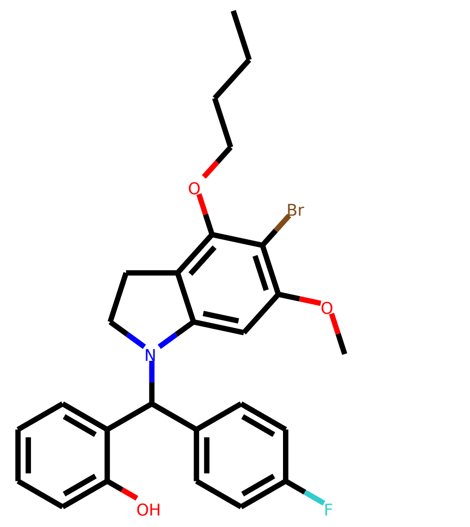
Osma-856

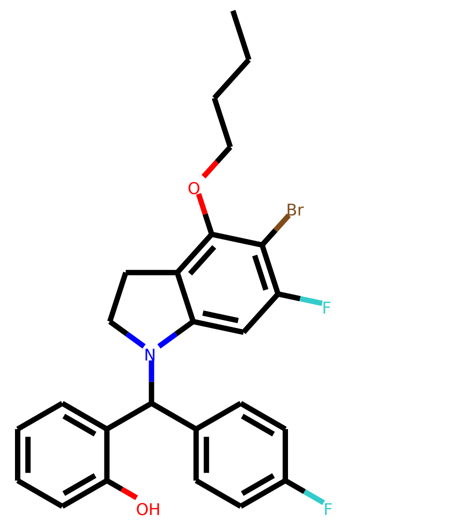
Osma-857

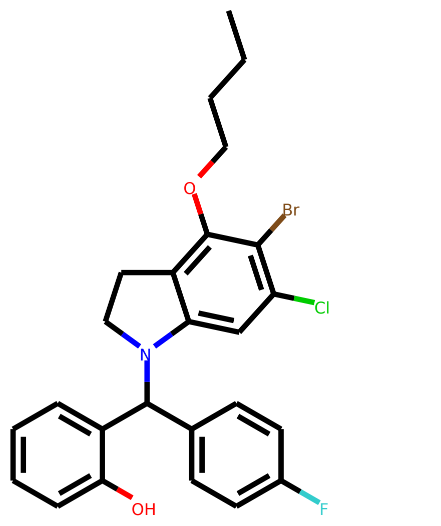
Osma-858

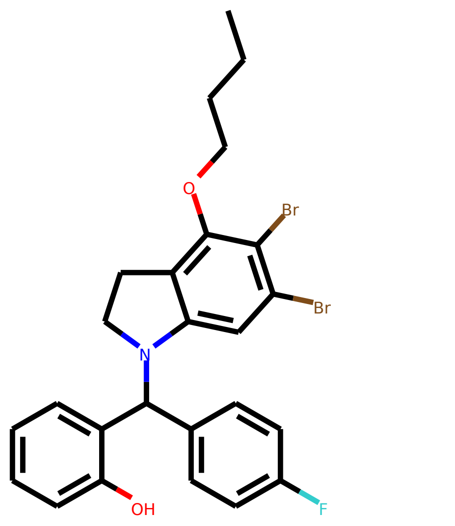
Osma-859

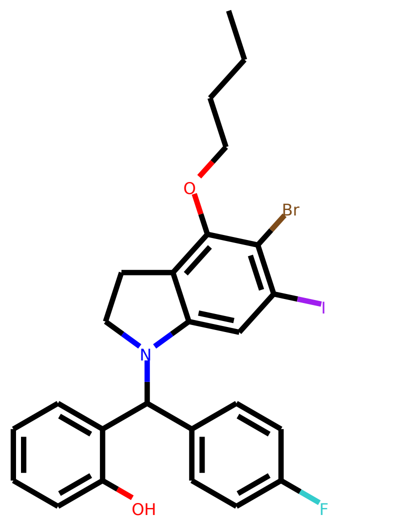
Osma-860

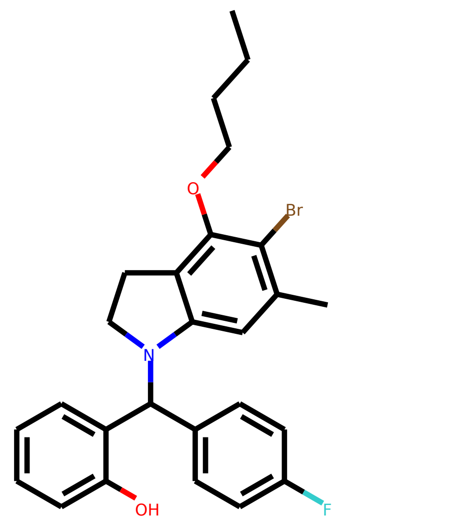
Osma-861

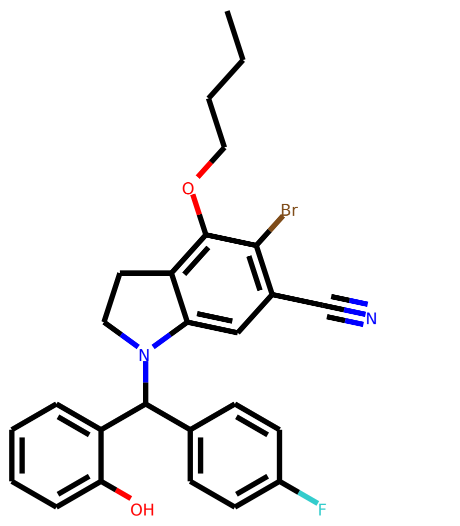
Osma-862

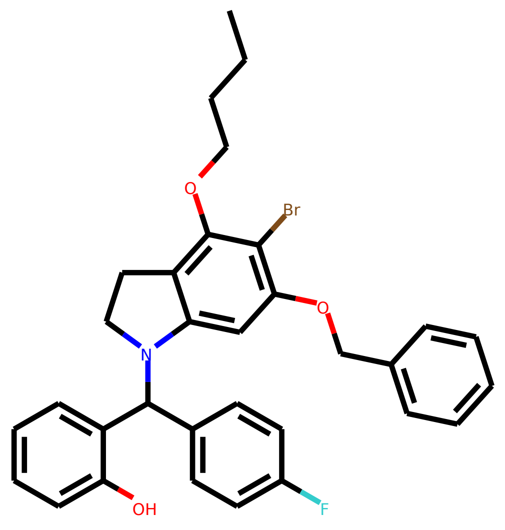
Osma-863

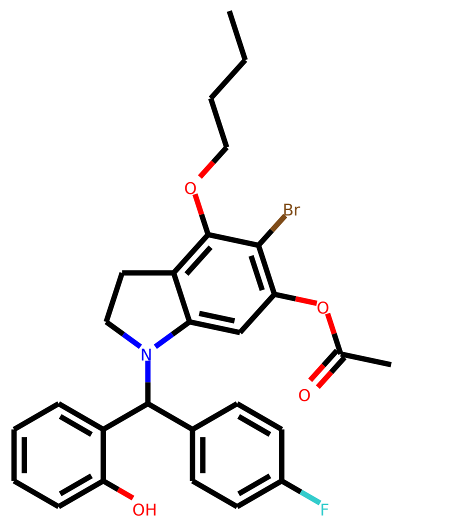
Osma-864

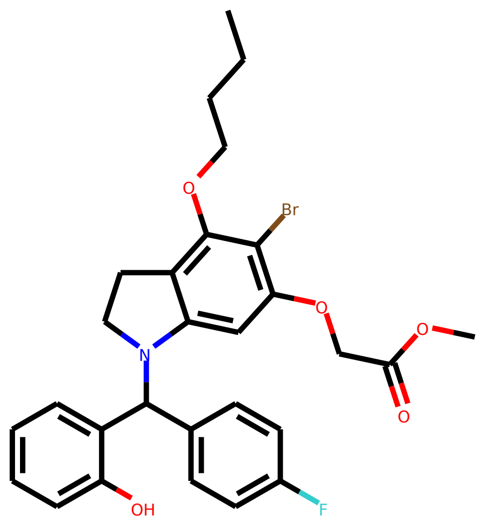
Osma-865

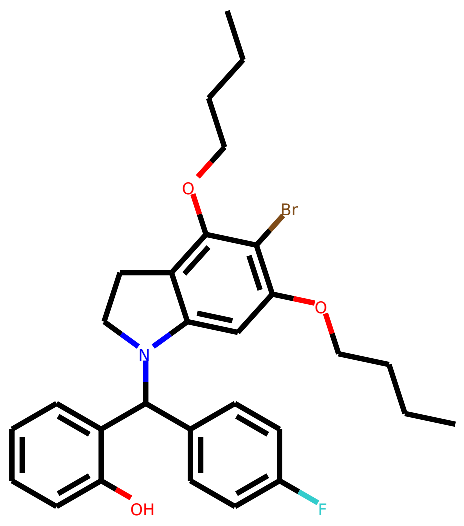
Osma-866

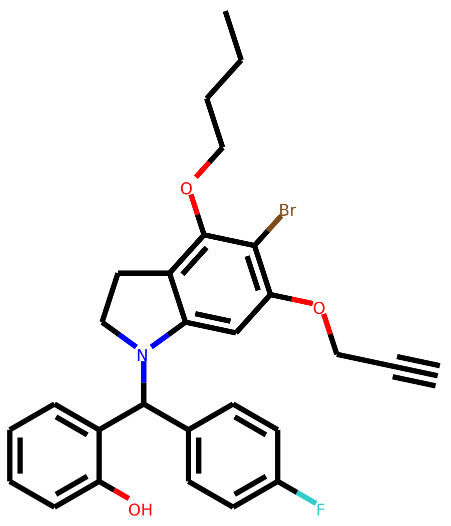
Osma-867

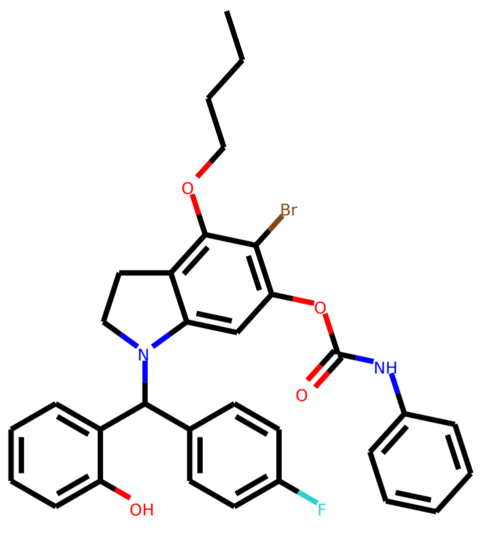
Osma-868

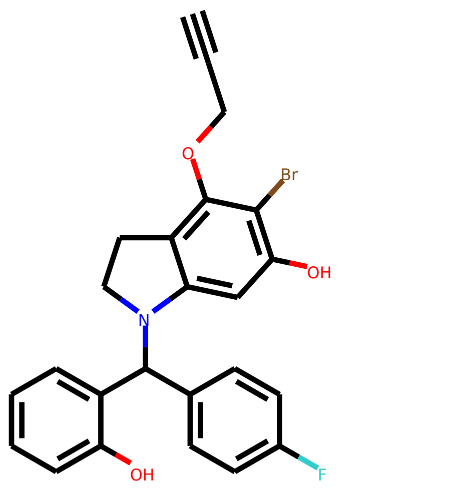
Osma-869

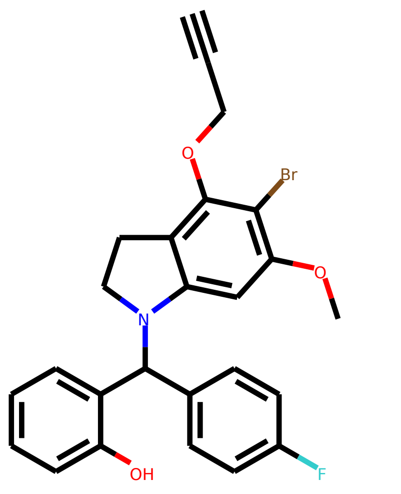
Osma-870

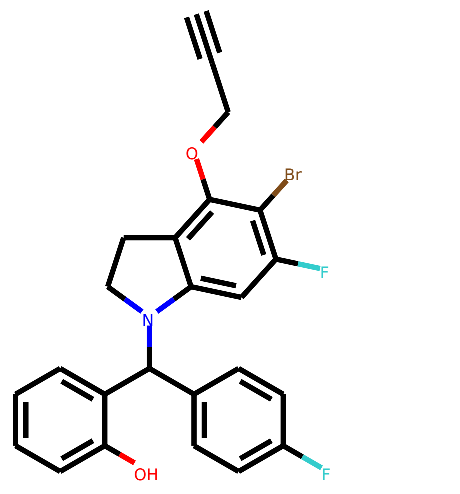
Osma-871

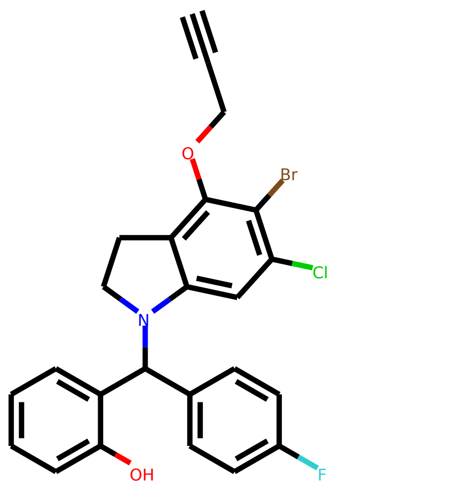
Osma-872

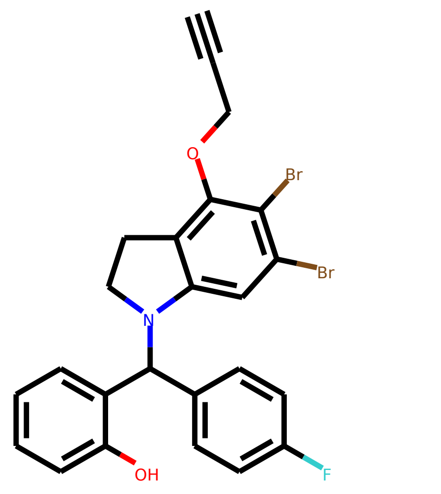
Osma-873

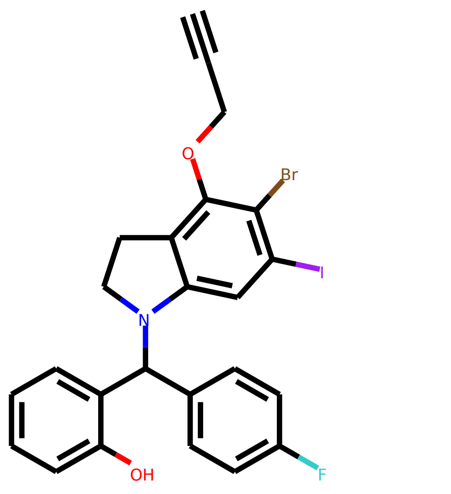
Osma-874

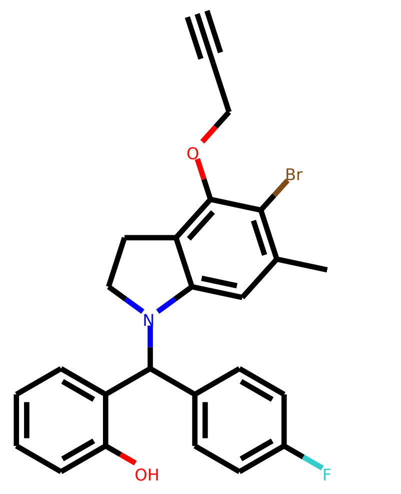
Osma-875

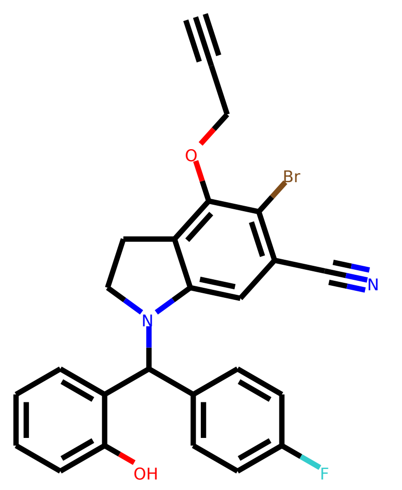
Osma-876
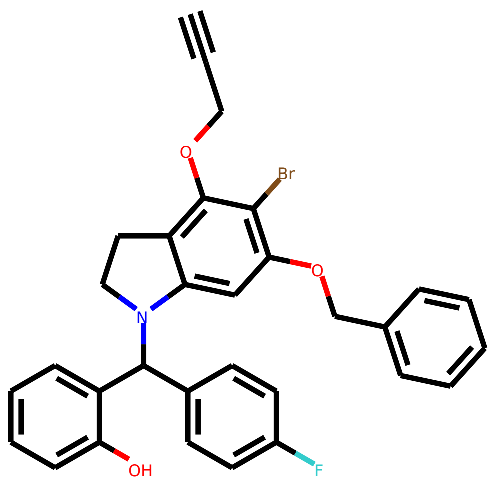
Osma-877
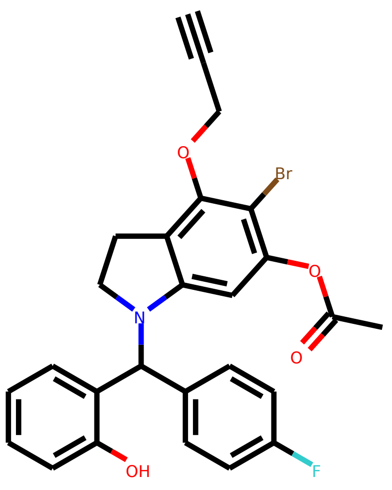
Osma-878
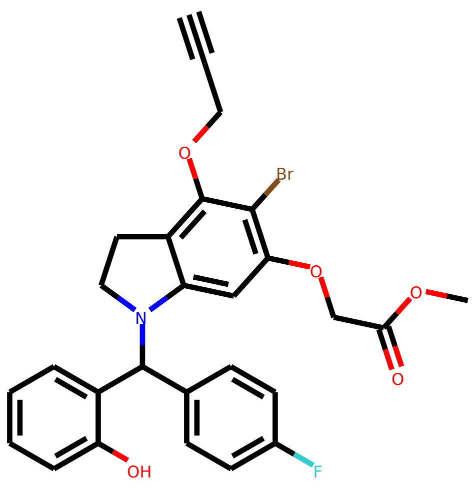
Osma-879
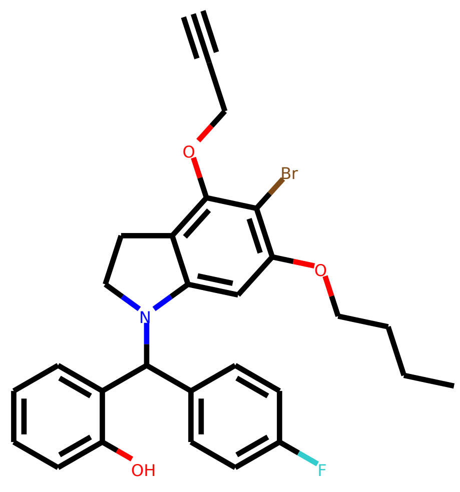
Osma-880
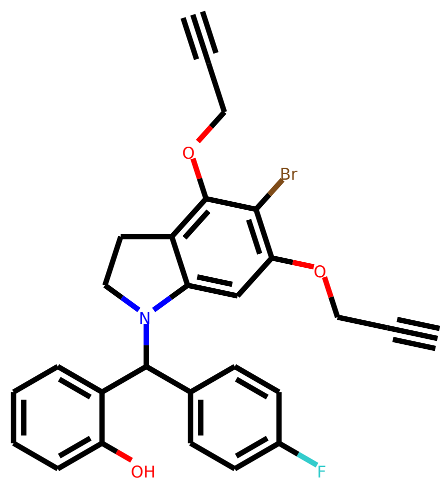
Osma-881
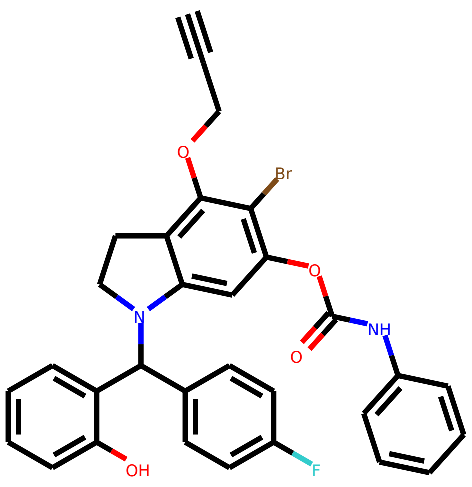
Osma-882
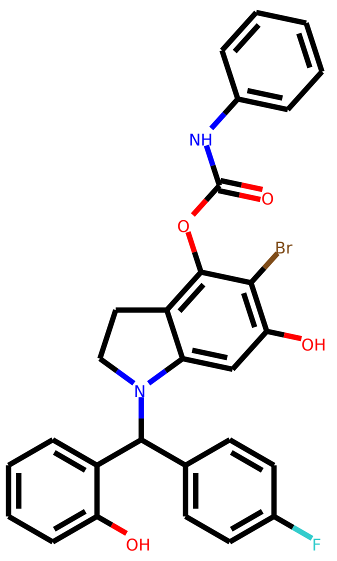
Osma-883
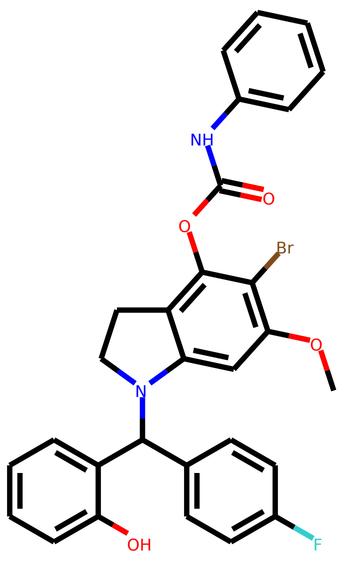
Osma-884
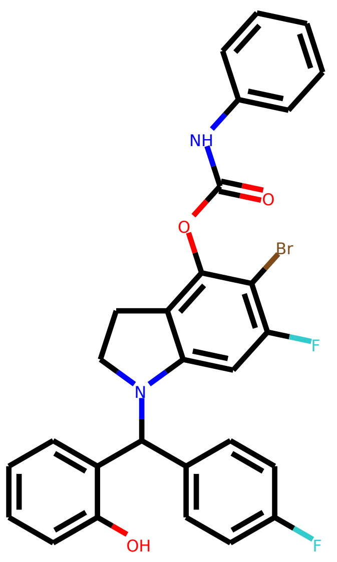
Osma-885
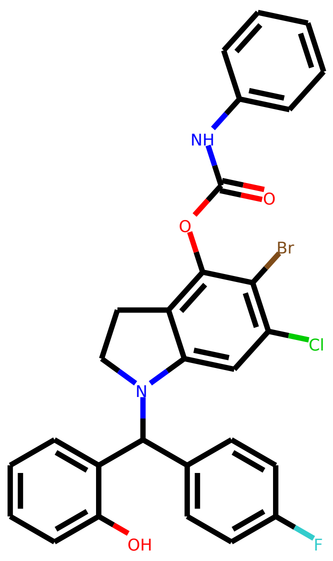
Osma-886
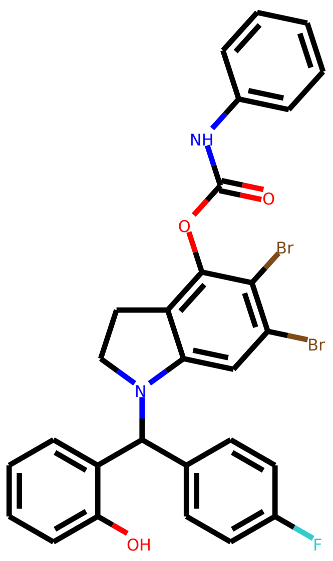
Osma-887
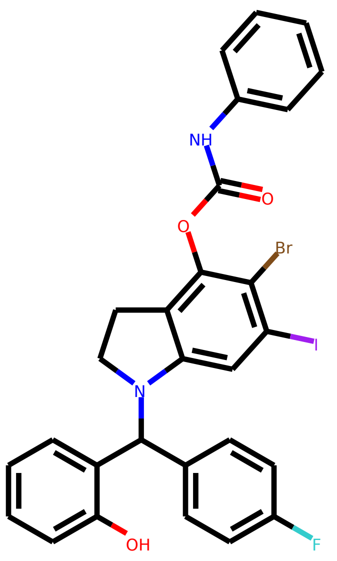
Osma-888
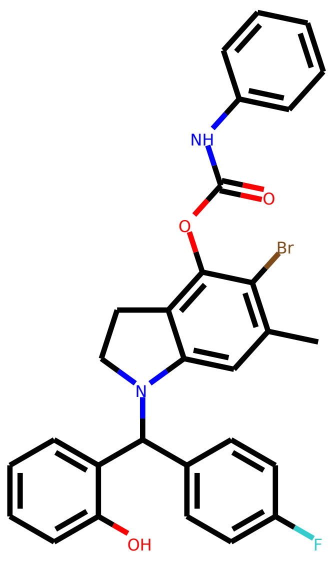
Osma-889
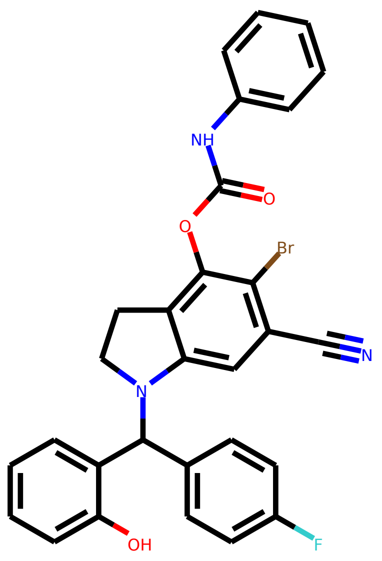
Osma-890
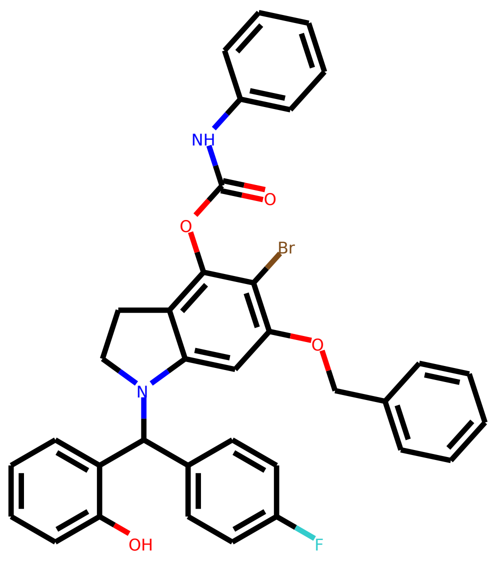
Osma-891
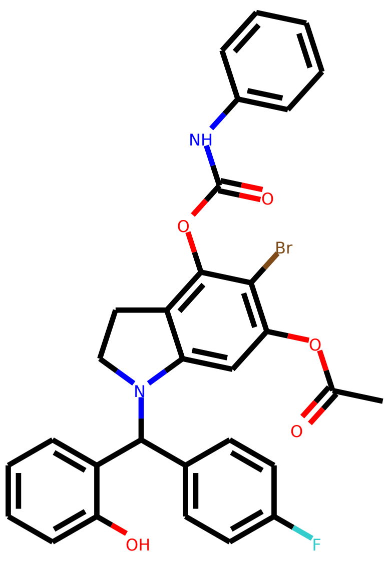
Osma-892
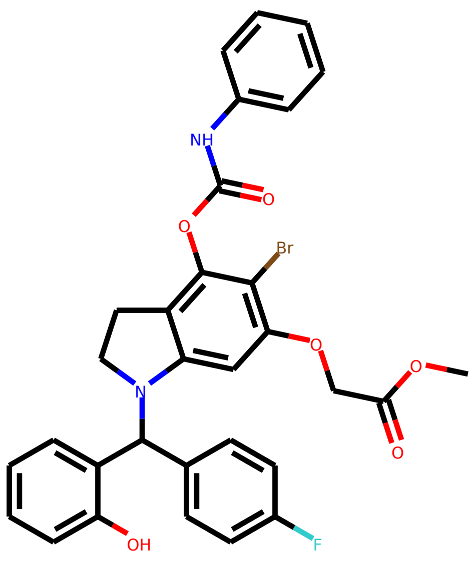
Osma-893
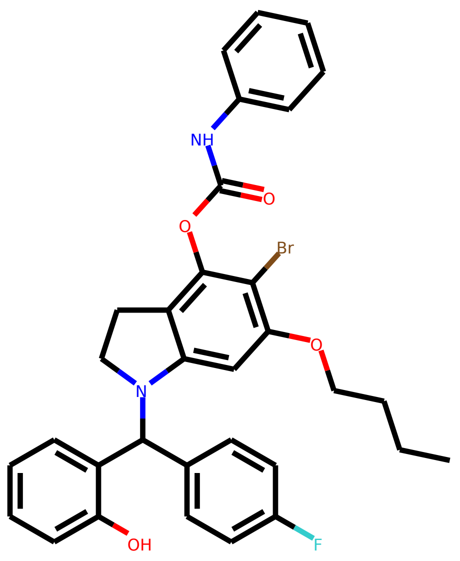
Osma-894
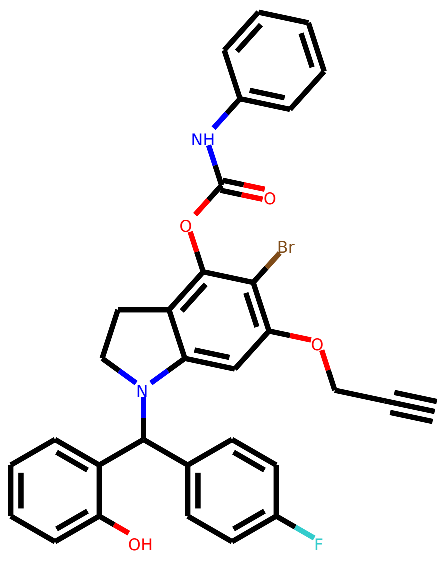
Osma-895
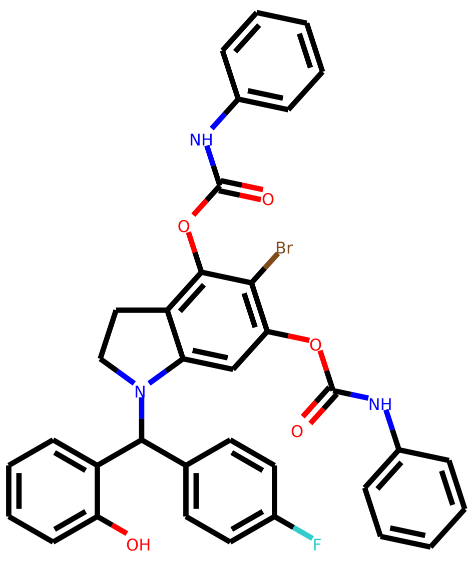
Osma-896
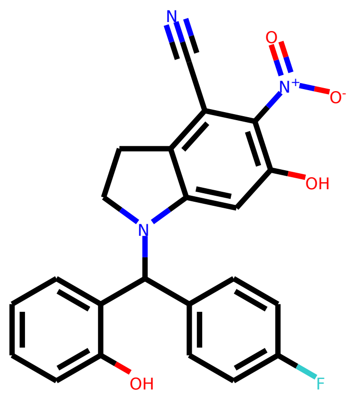
Osma-897
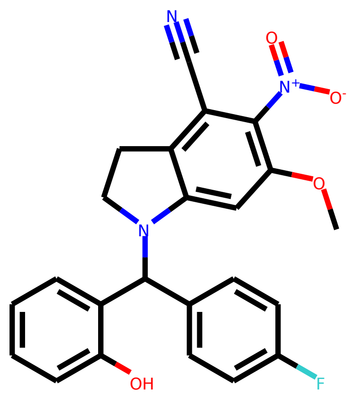
Osma-898
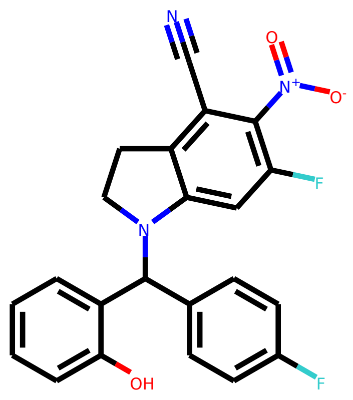
Osma-899
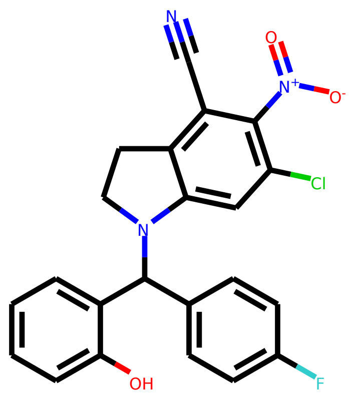
Osma-900
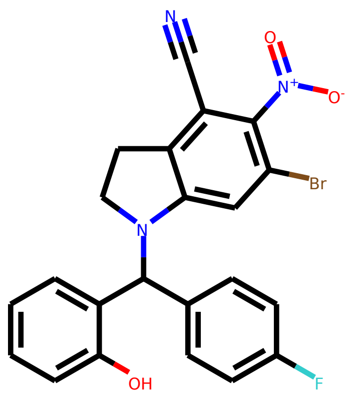
Osma-901
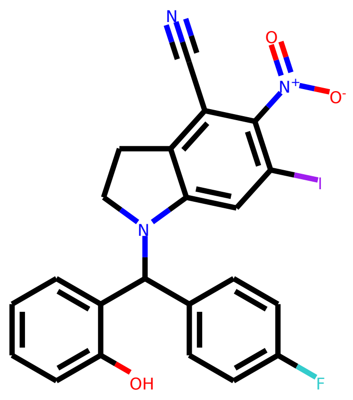
Osma-902
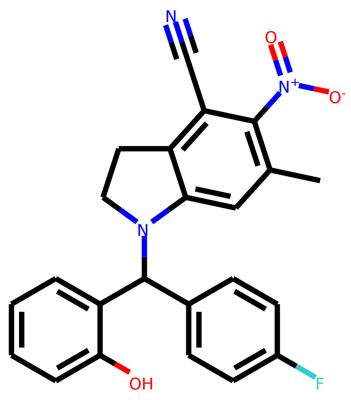
Osma-903
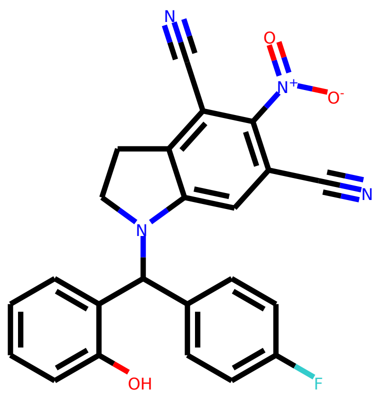
Osma-904
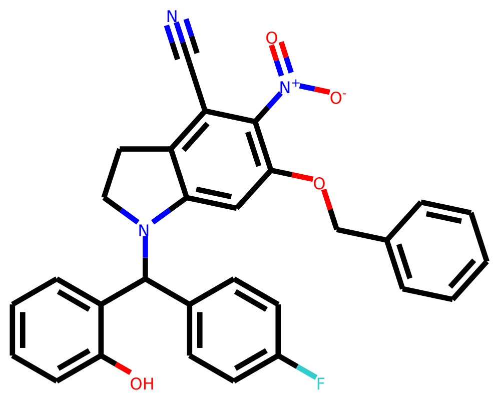
Osma-905
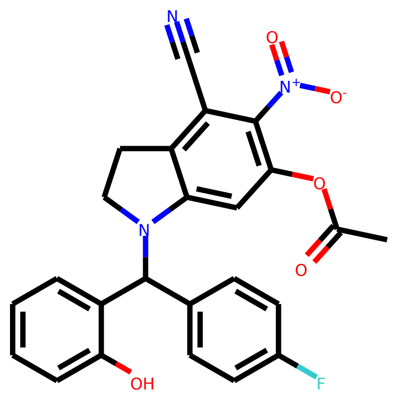
Osma-906
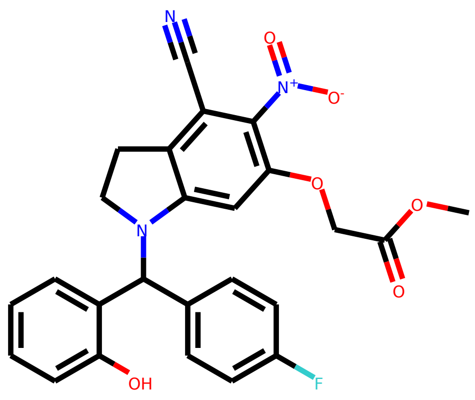
Osma-907
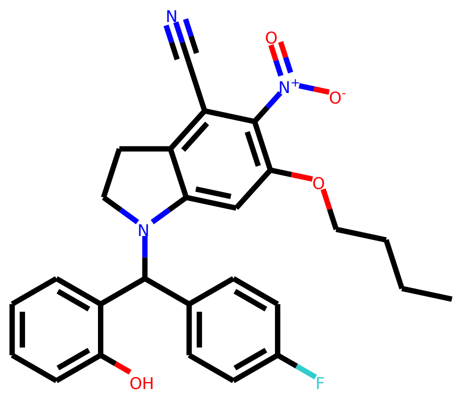
Osma-908
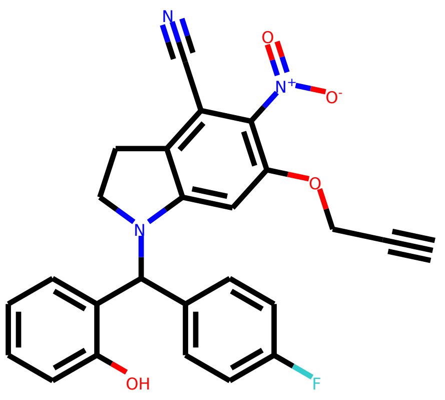
Osma-909
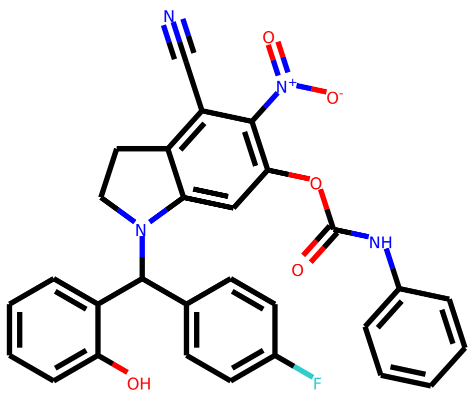
Osma-910

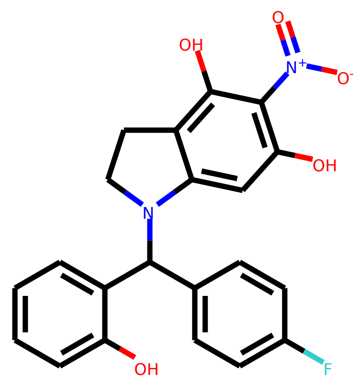
Osma-911
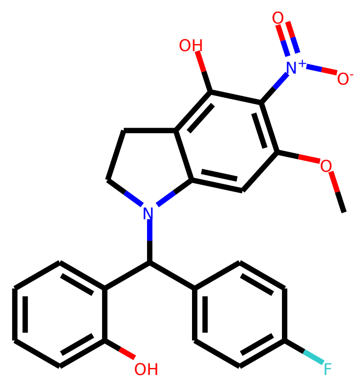
Osma-912
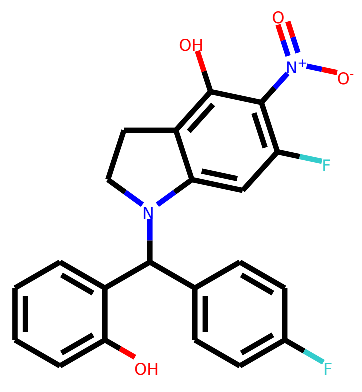
Osma-913
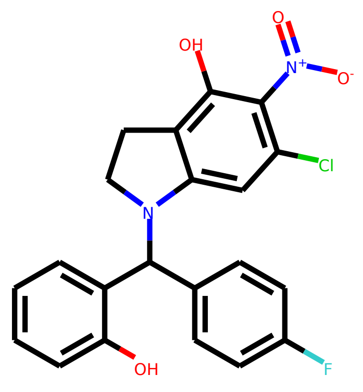
Osma-914
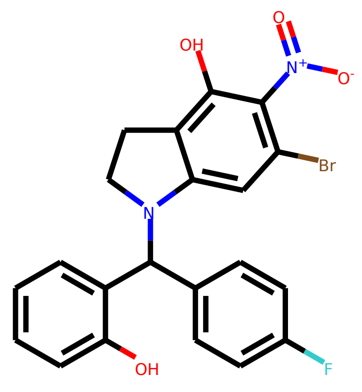
Osma-915
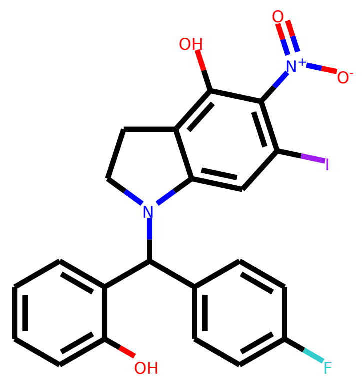
Osma-916
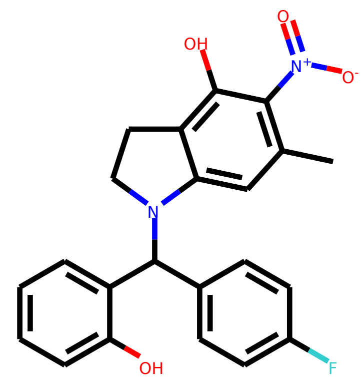
Osma-917
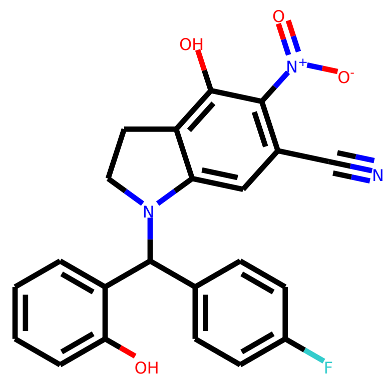
Osma-918
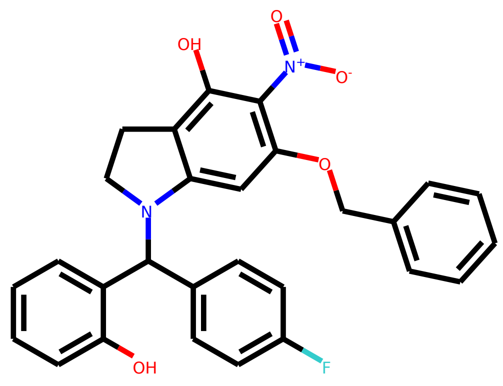
Osma-919
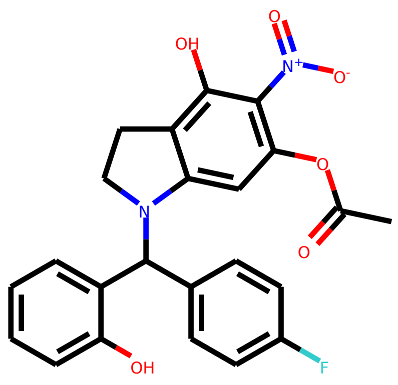
Osma-920
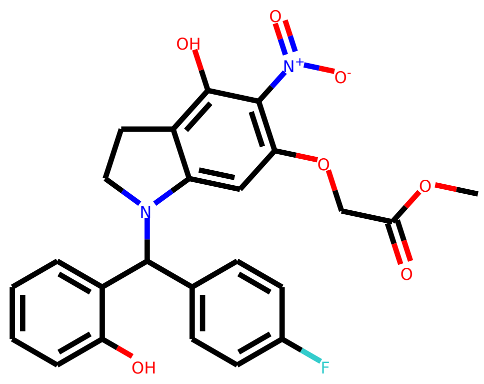
Osma-921
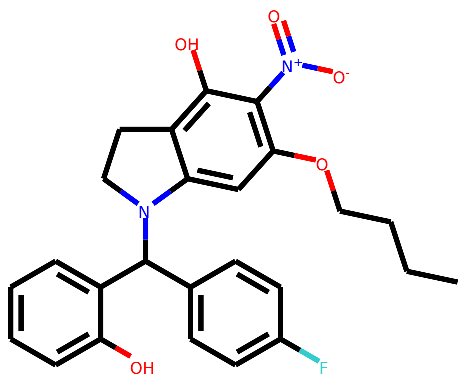
Osma-922
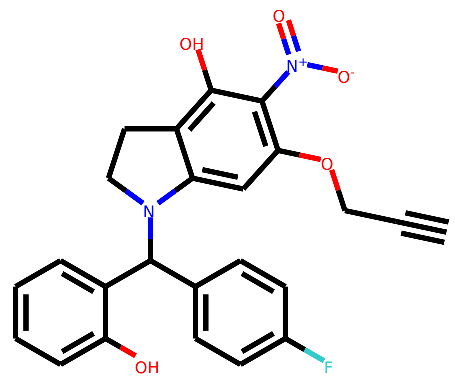
Osma-923
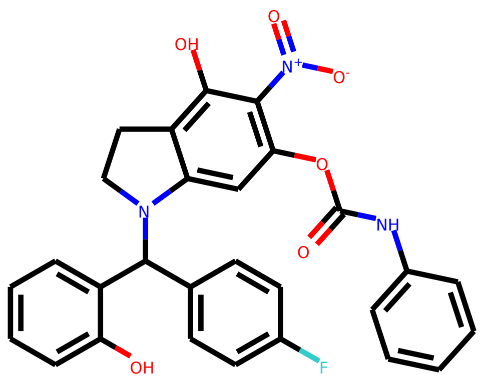
Osma-924
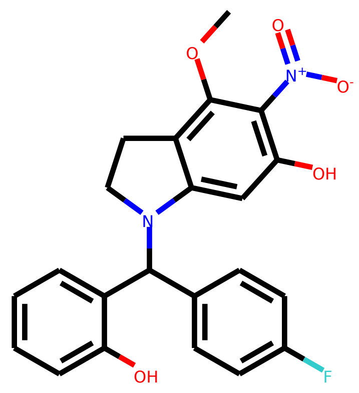
Osma-925
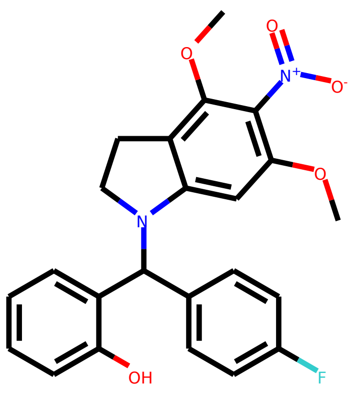
Osma-926
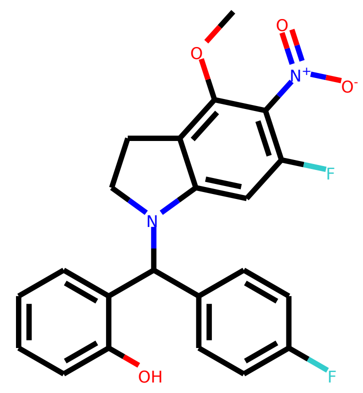
Osma-927
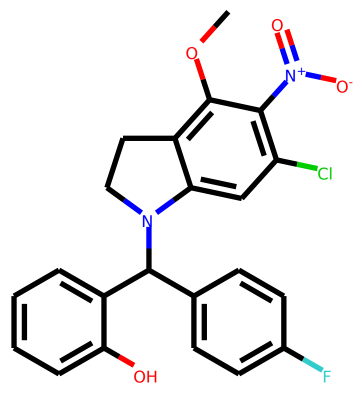
Osma-928
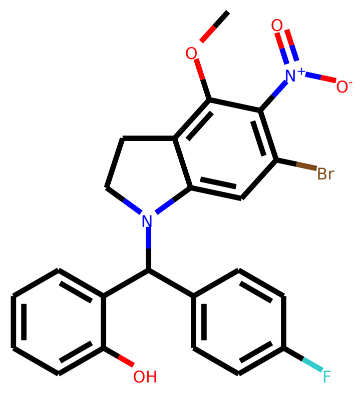
Osma-929
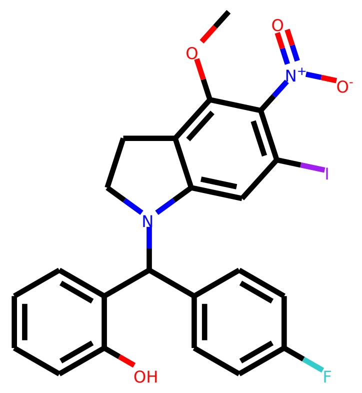
Osma-930
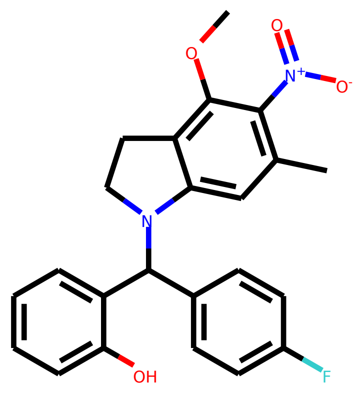
Osma-931
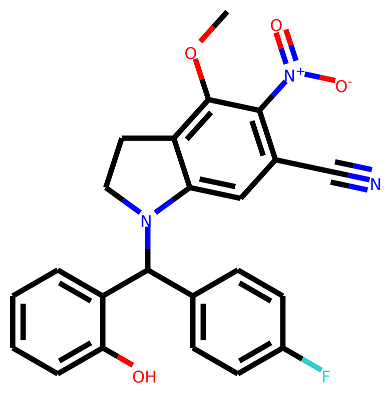
Osma-932
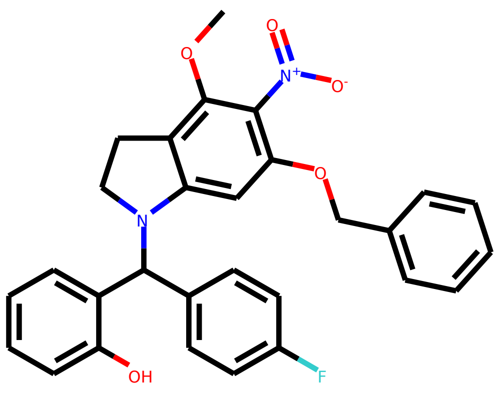
Osma-933
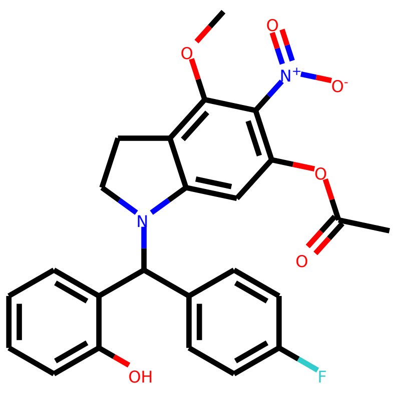
Osma-934
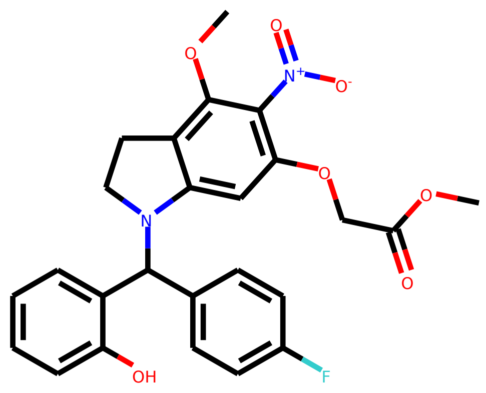
Osma-935
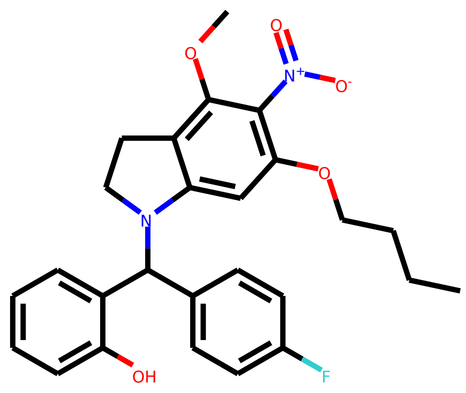
Osma-936
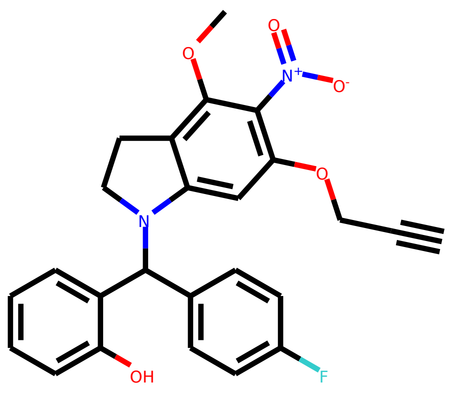
Osma-937
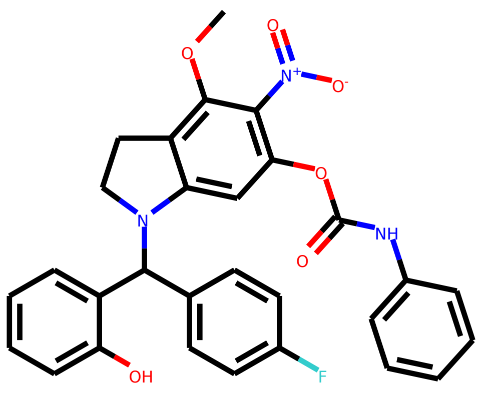
Osma-938
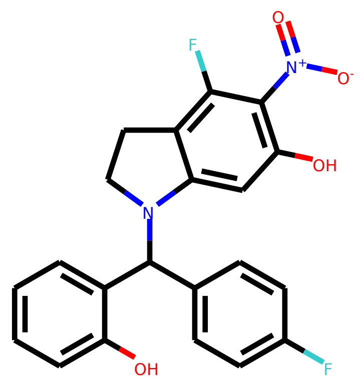
Osma-939
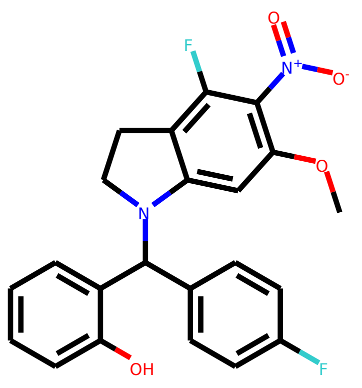
Osma-940
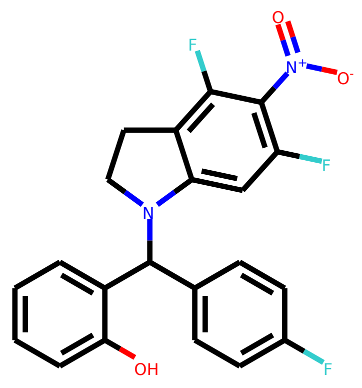
Osma-941
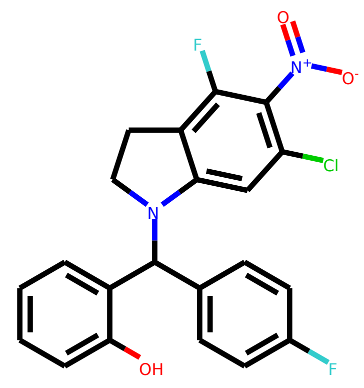
Osma-942
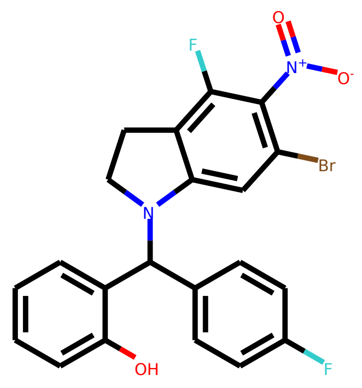
Osma-943
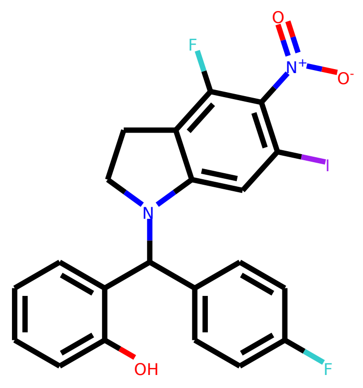
Osma-944
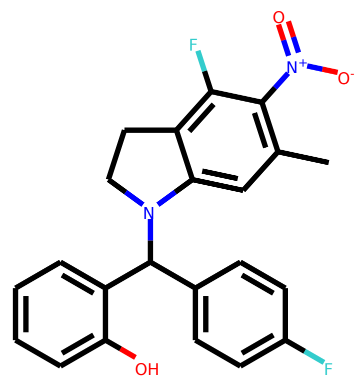
Osma-945

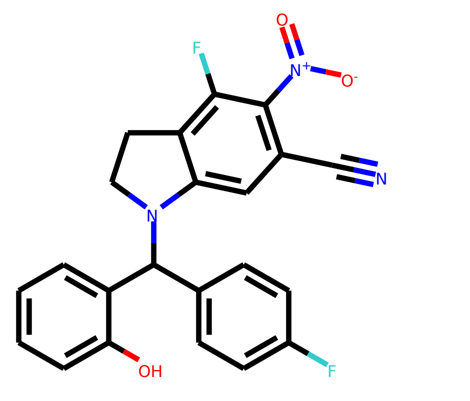

Osma-946

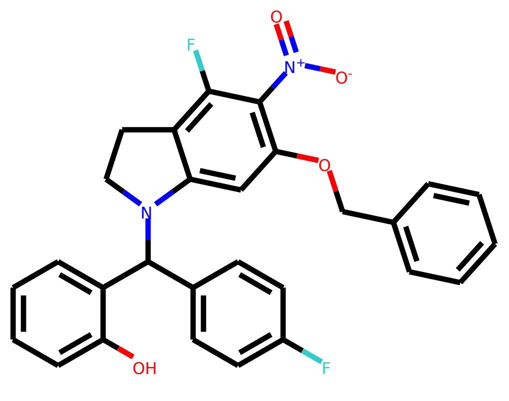

Osma-947

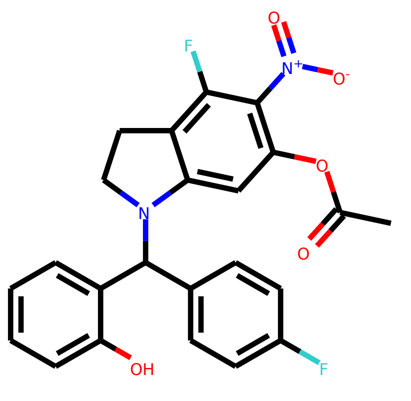

Osma-948

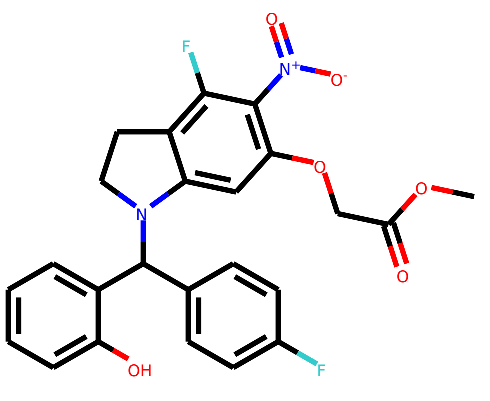

Osma-949

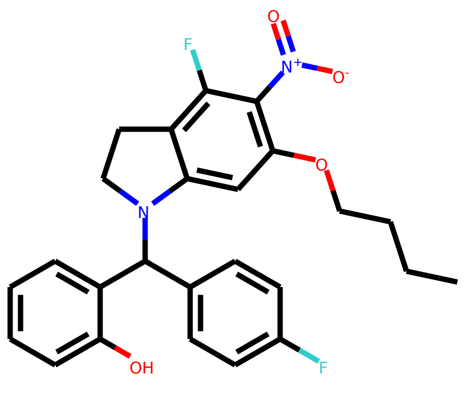

Osma-950

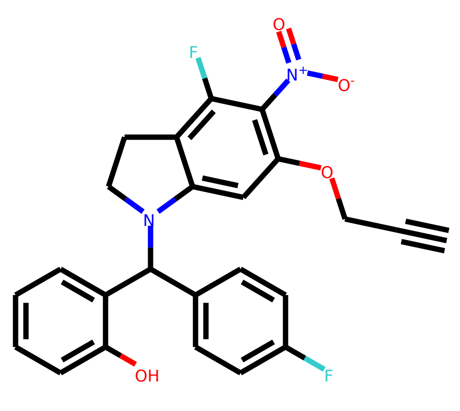

Osma-951

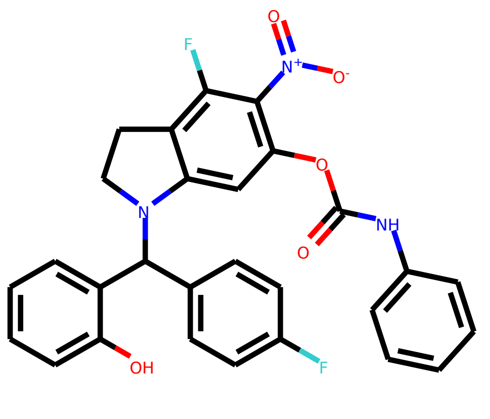

Osma-952

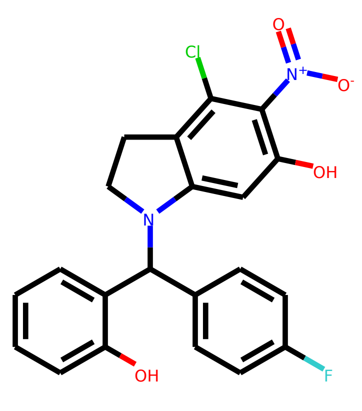

Osma-953

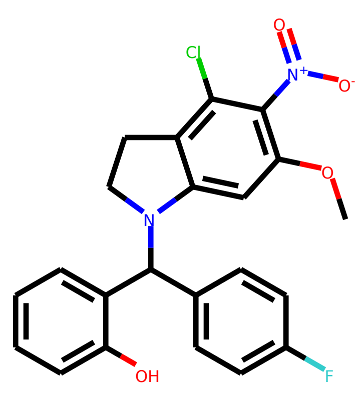

Osma-954

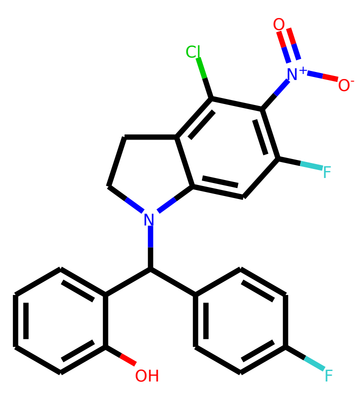

Osma-955

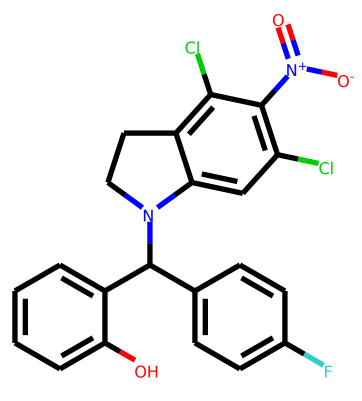

Osma-956

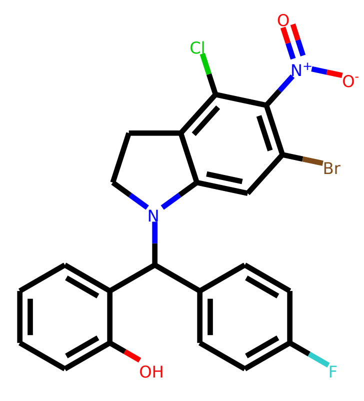

Osma-957

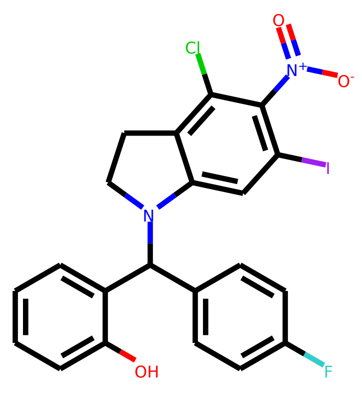

Osma-958

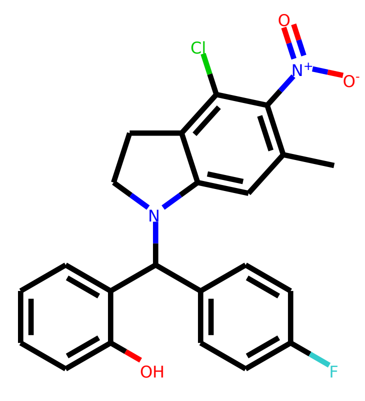

Osma-959

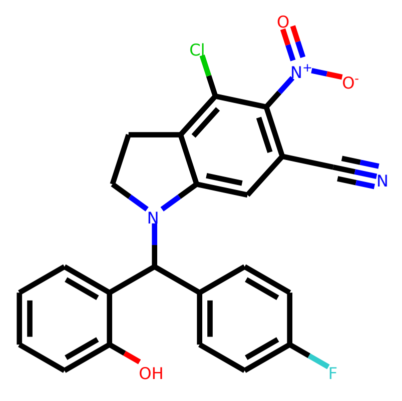

Osma-960

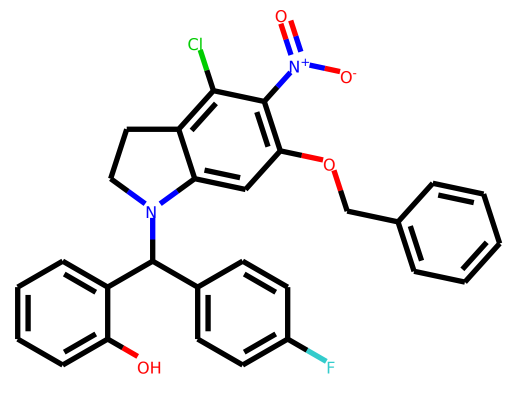

Osma-961

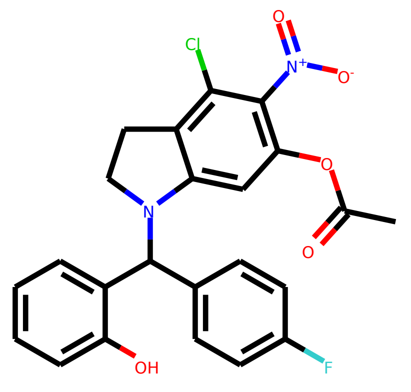

Osma-962

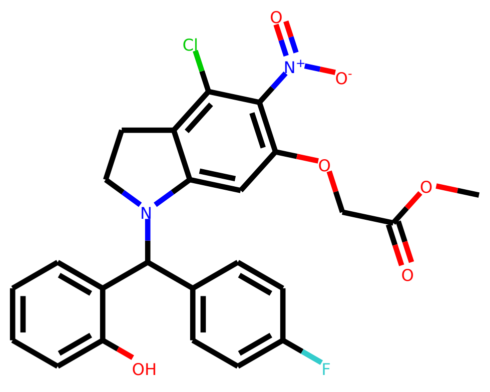

Osma-963

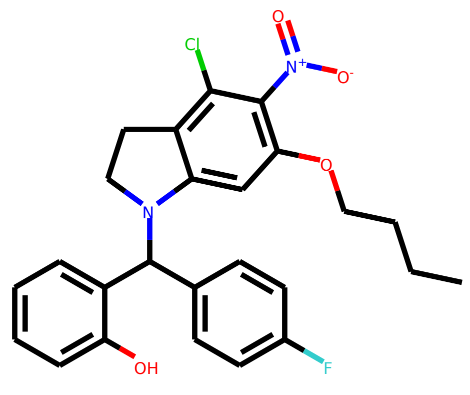

Osma-964

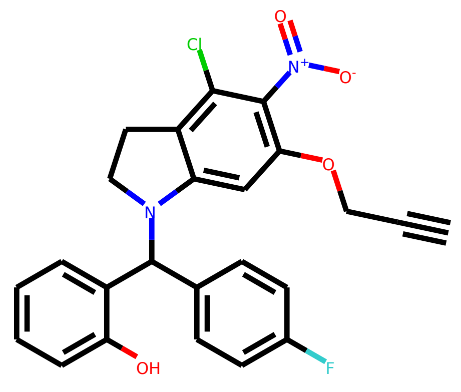

Osma-965

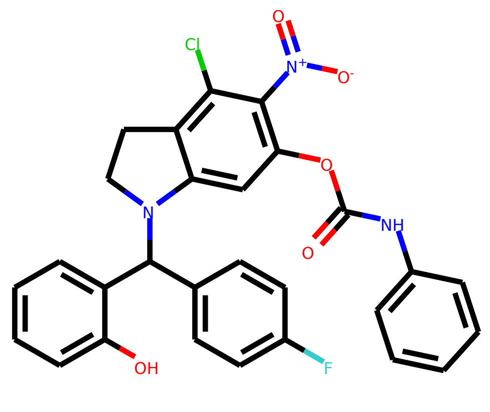

Osma-966

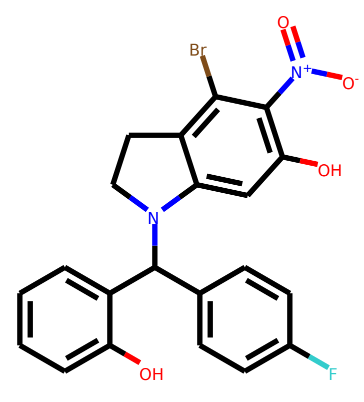

Osma-967

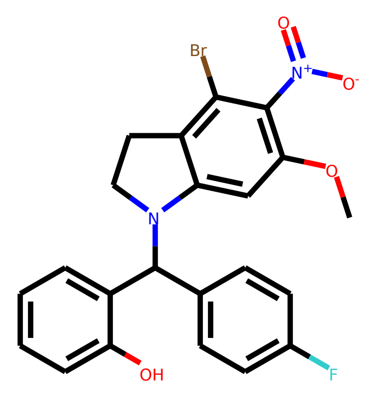

Osma-968

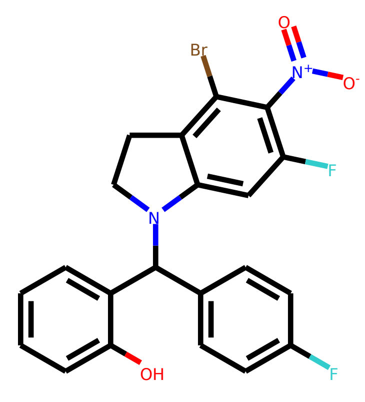

Osma-969

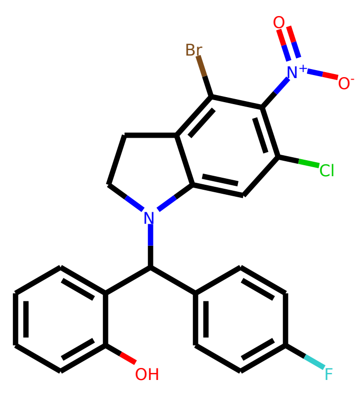

Osma-970

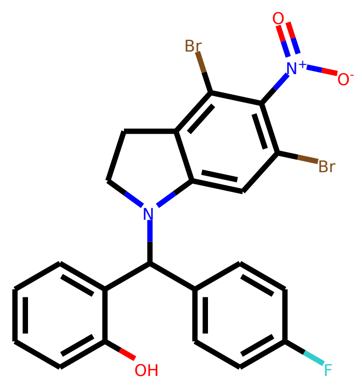

Osma-971

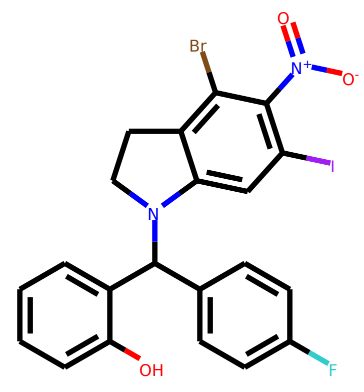

Osma-972

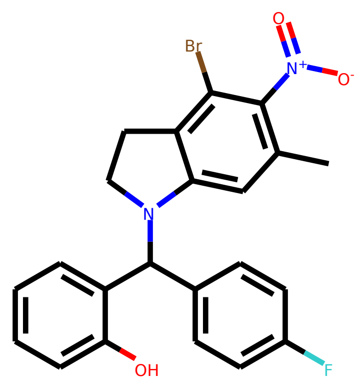

Osma-973

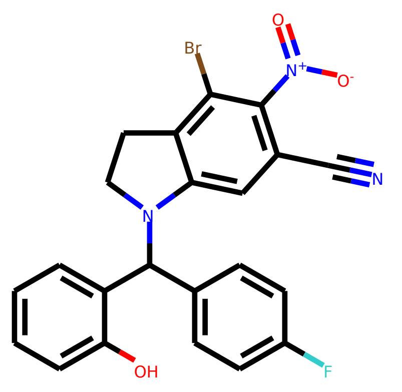

Osma-974

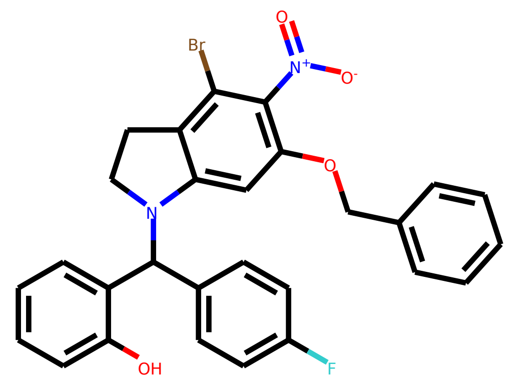

Osma-975

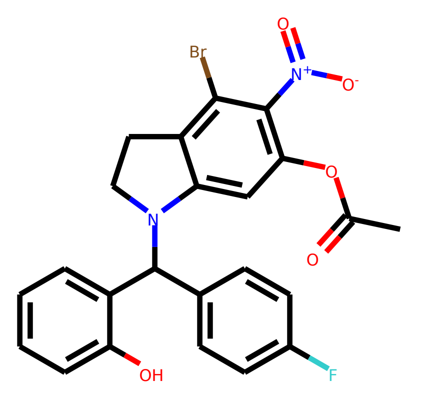

Osma-976

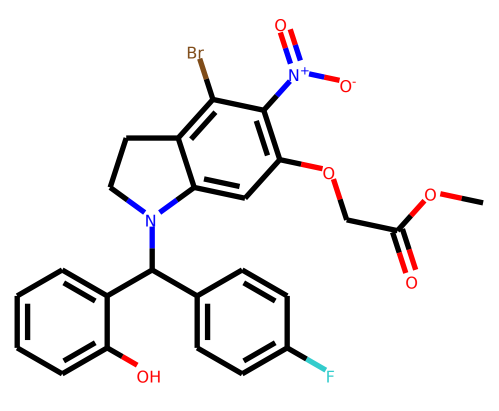

Osma-977

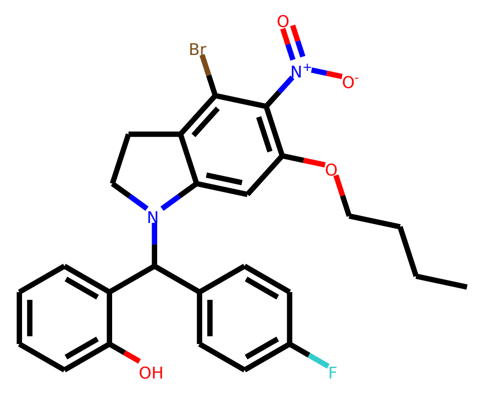

Osma-978

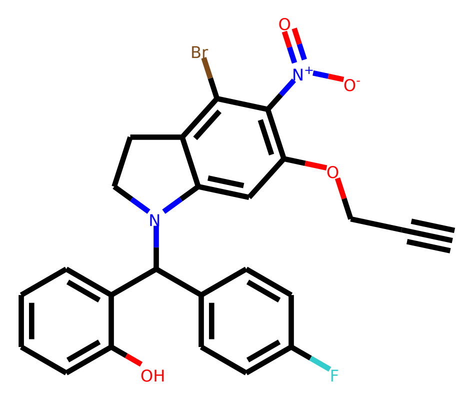

Osma-979

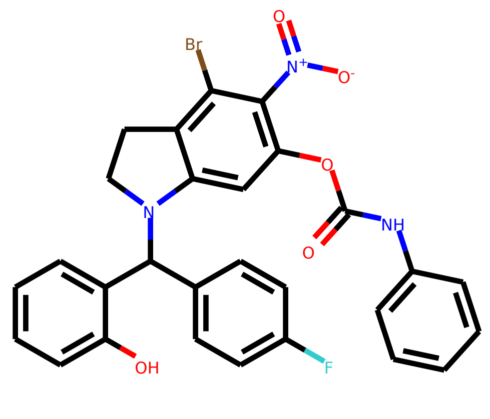

Osma-980

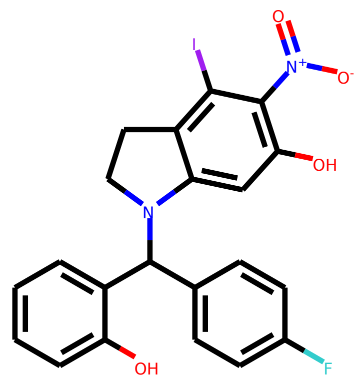
Osma-981

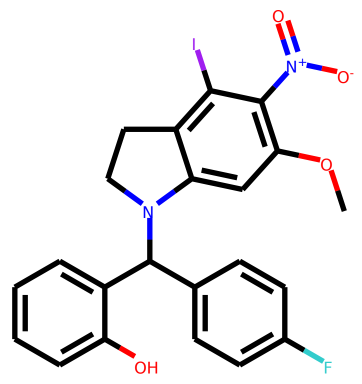
Osma-982

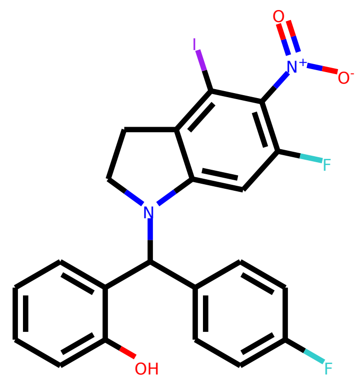
Osma-983

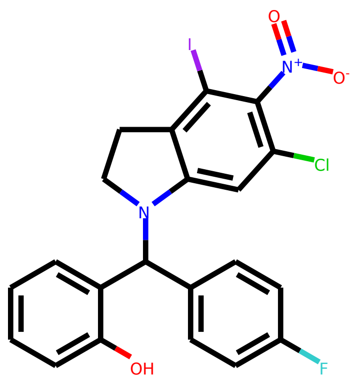
Osma-984

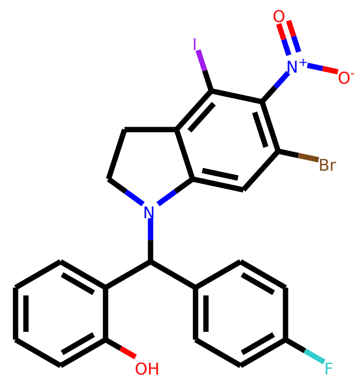
Osma-985

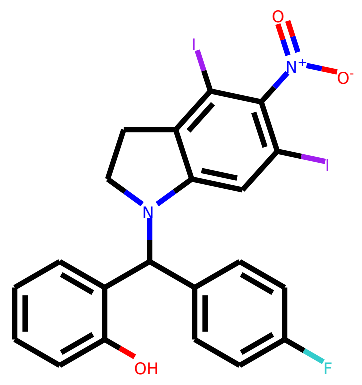
Osma-986

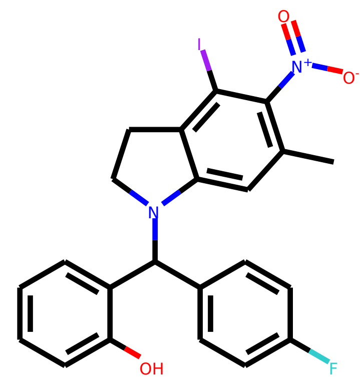
Osma-987

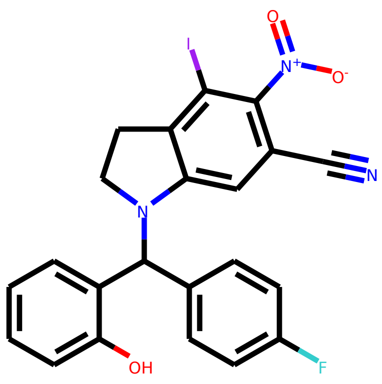
Osma-988

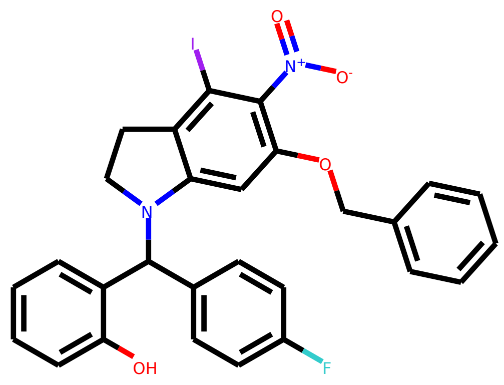
Osma-989

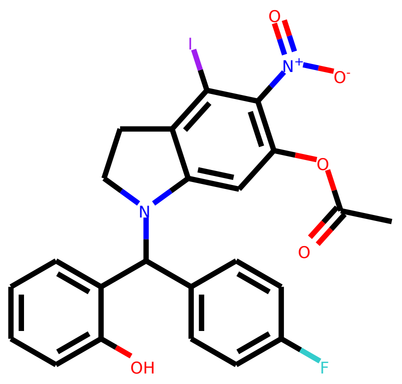
Osma-990

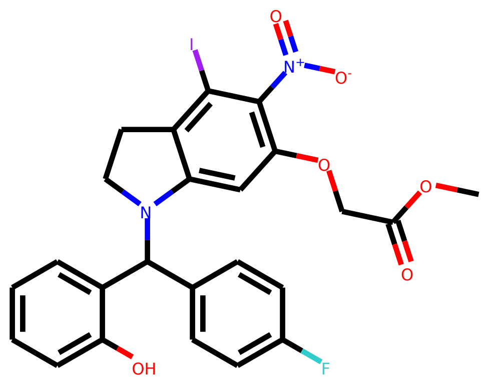
Osma-991

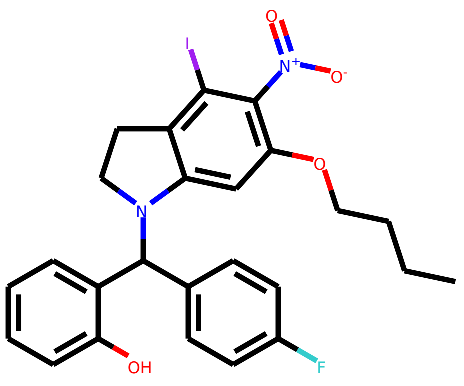
Osma-992

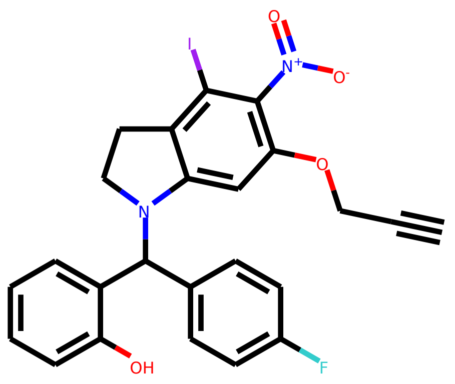
Osma-993

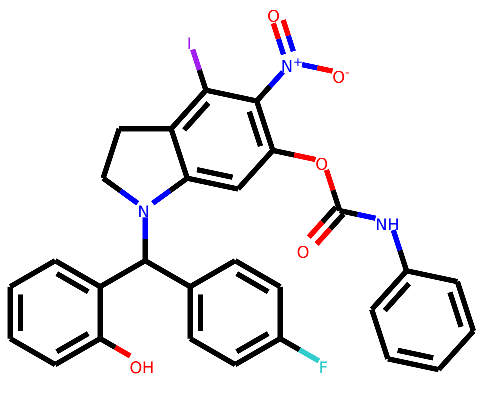
Osma-994

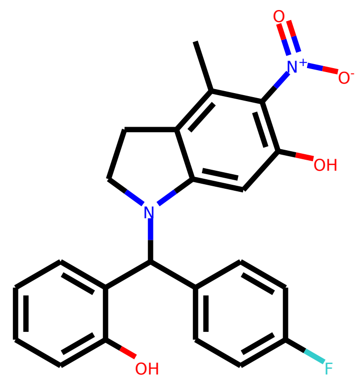
Osma-995

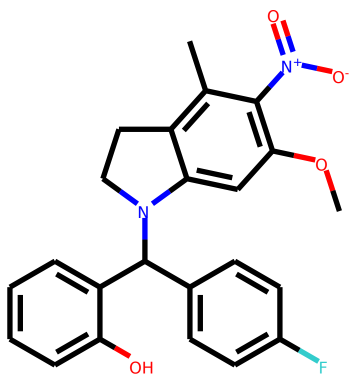
Osma-996

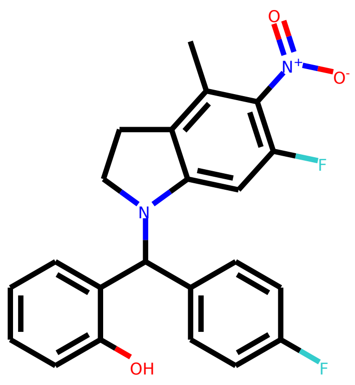
Osma-997

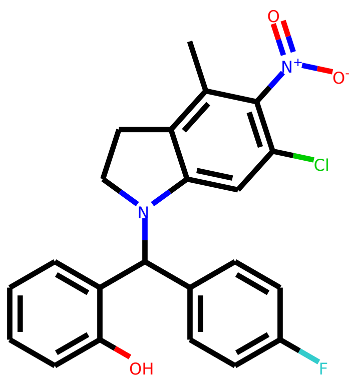
Osma-998

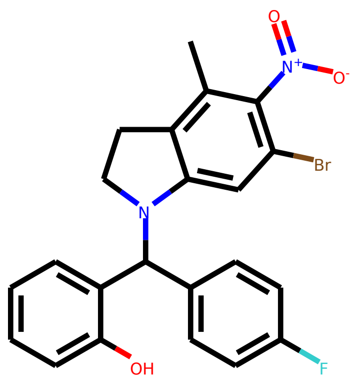
Osma-999

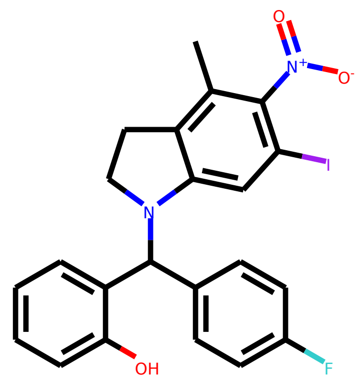
Osma-1000

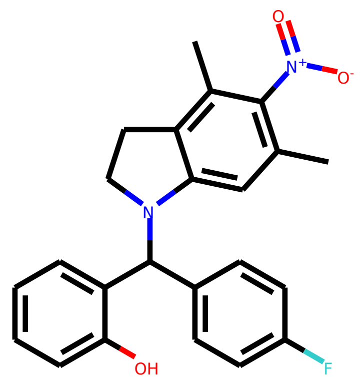
Osma-1001

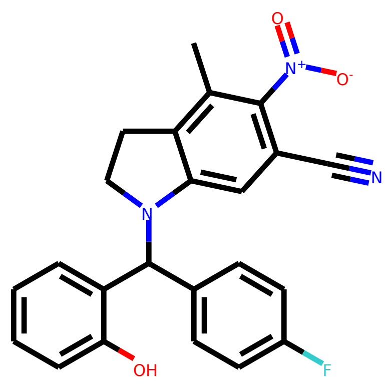
Osma-1002

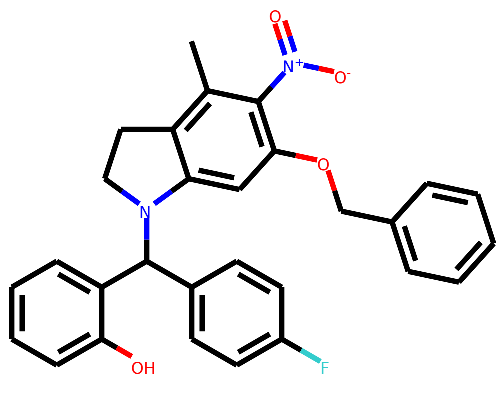
Osma-1003

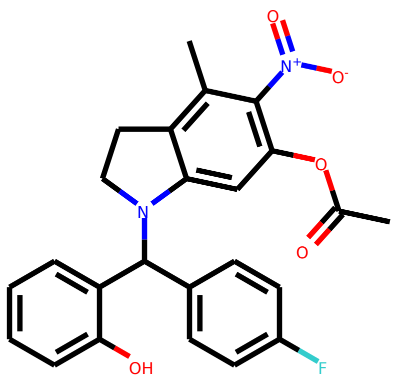
Osma-1004

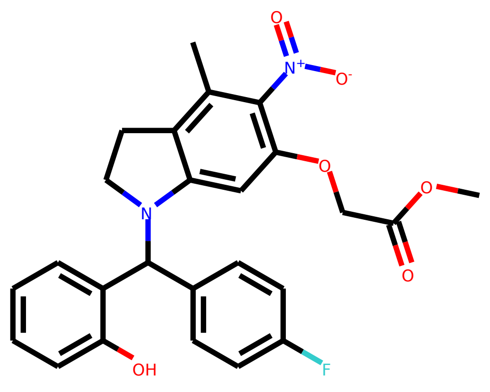
Osma-1005

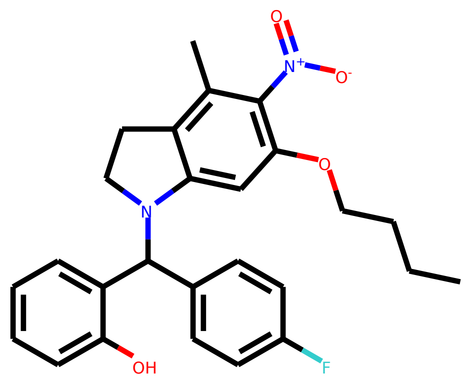
Osma-1006

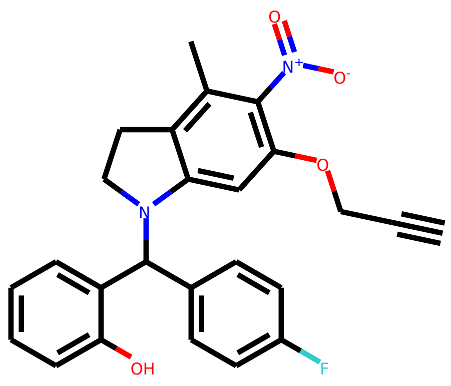
Osma-1007

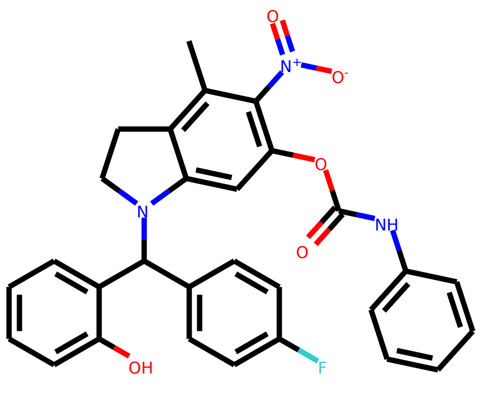
Osma-1008

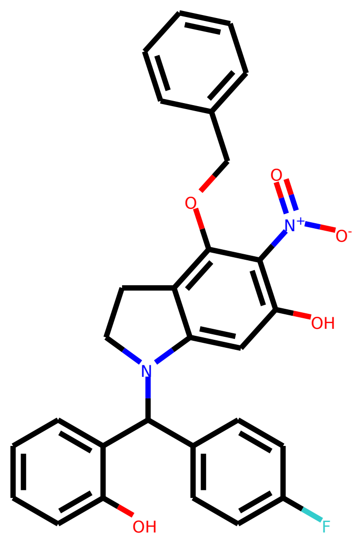
Osma-1009

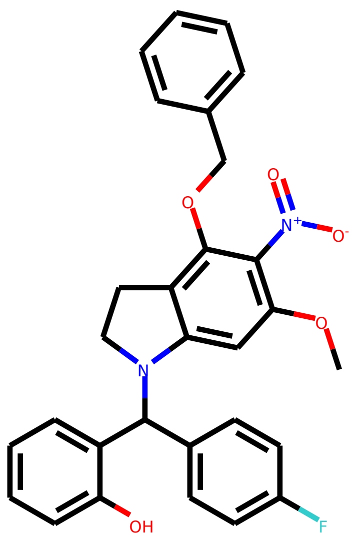
Osma-1010

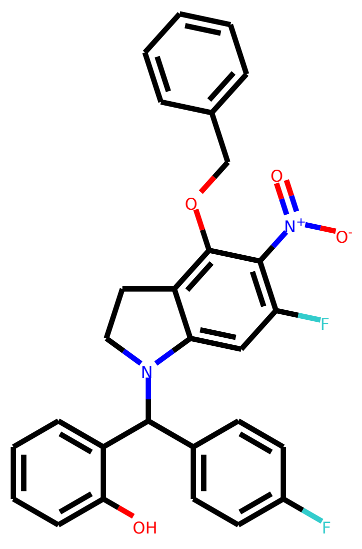
Osma-1011

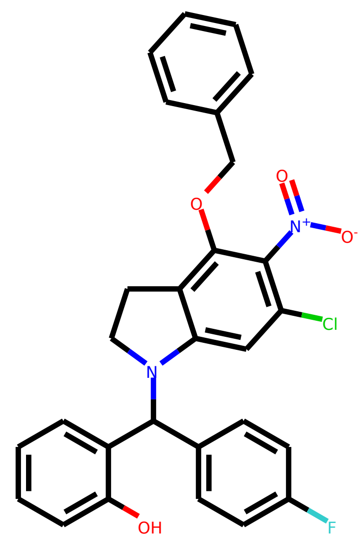
Osma-1012

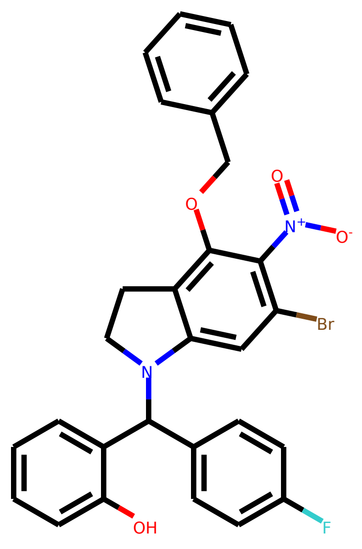
Osma-1013

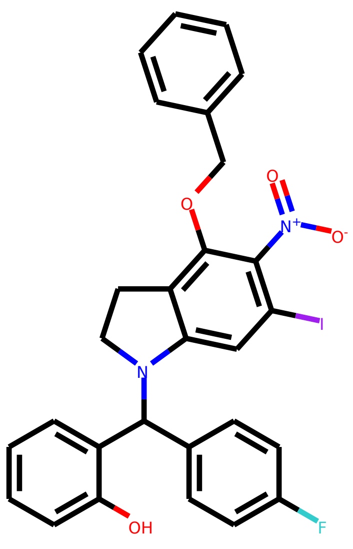
Osma-1014

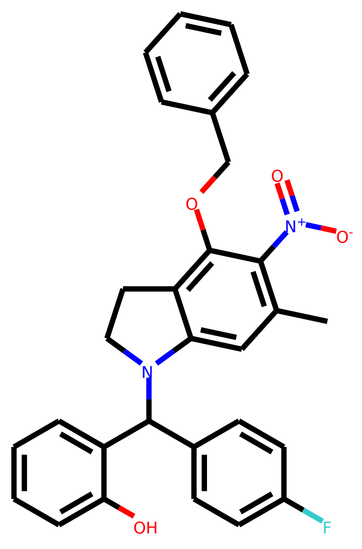
Osma-1015

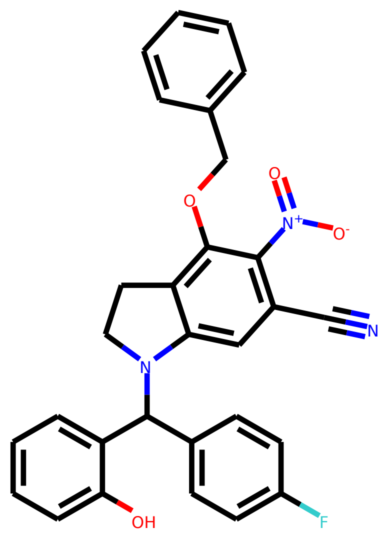
Osma-1016
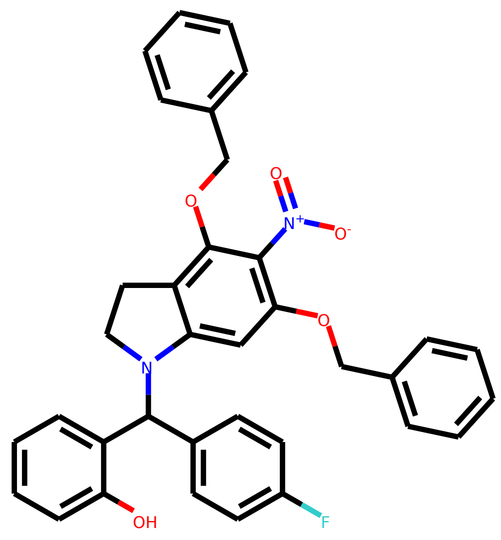
Osma-1017
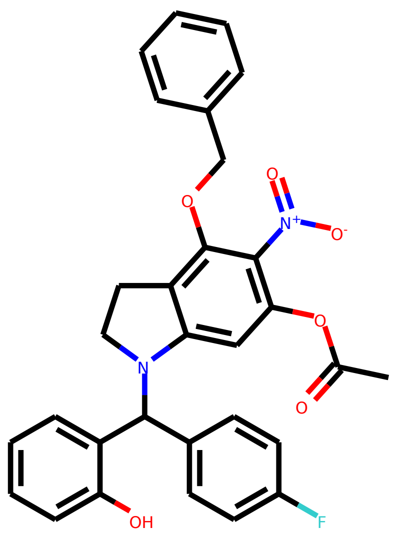
Osma-1018
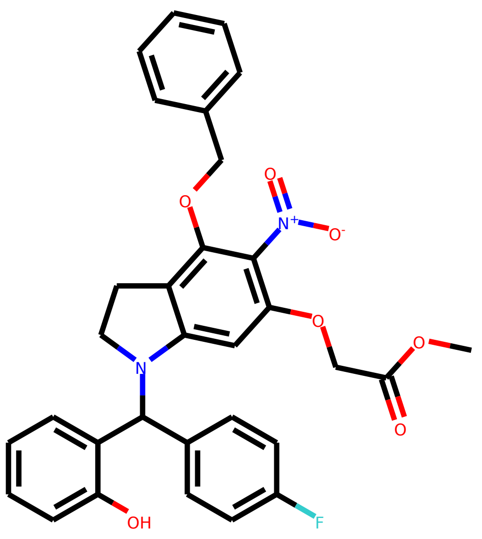
Osma-1019
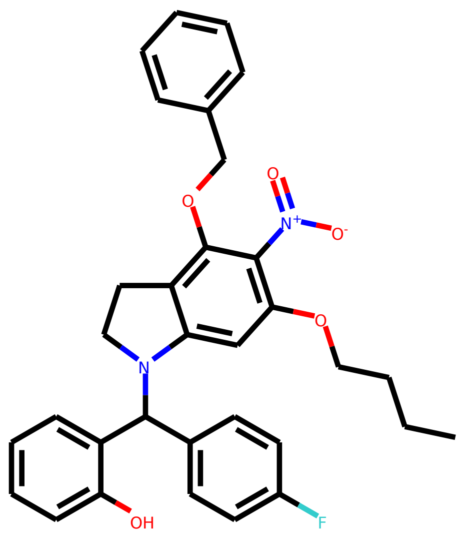
Osma-1020
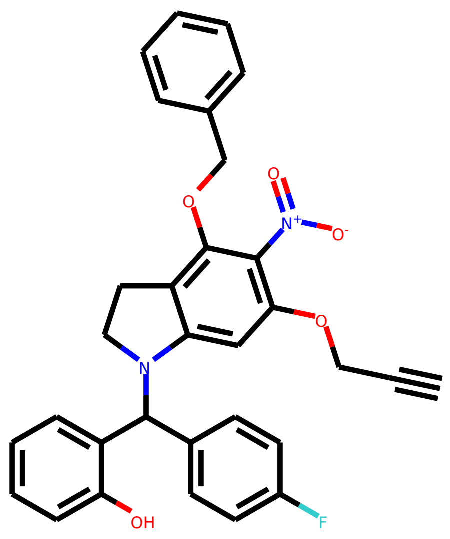
Osma-1021
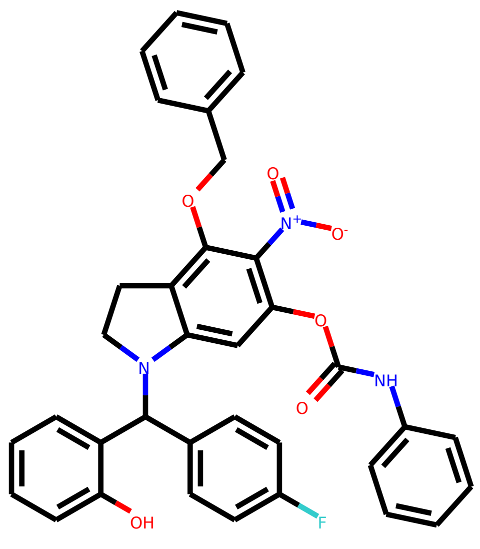
Osma-1022
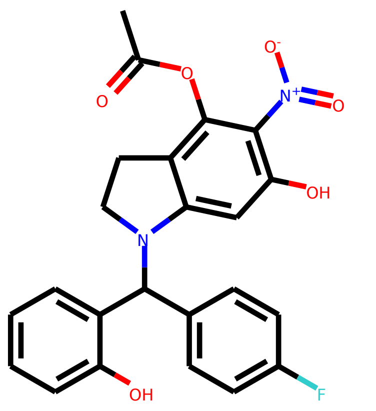
Osma-1023
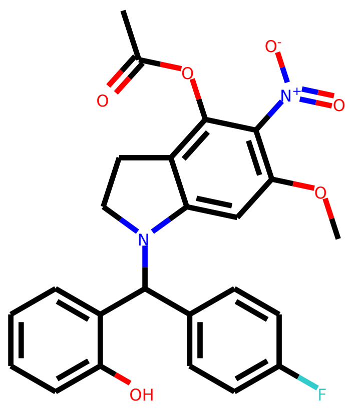
Osma-1024
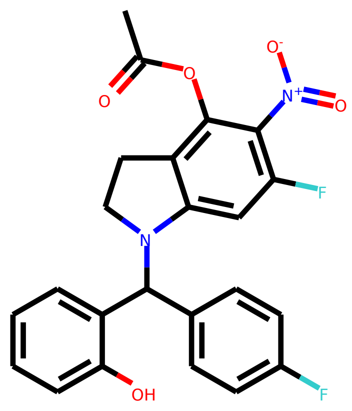
Osma-1025
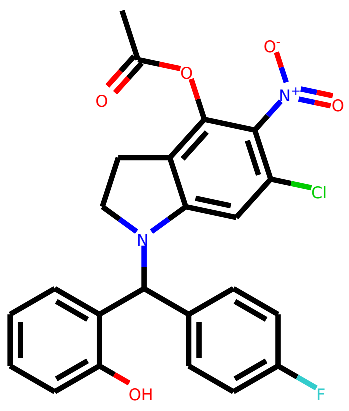
Osma-1026
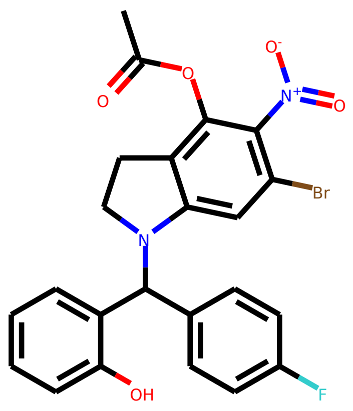
Osma-1027
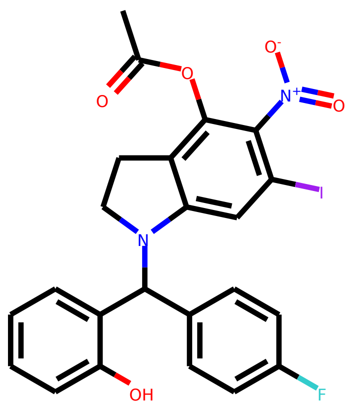
Osma-1028
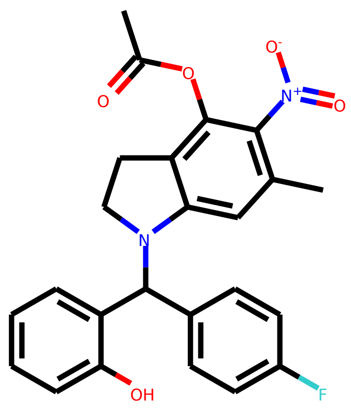
Osma-1029
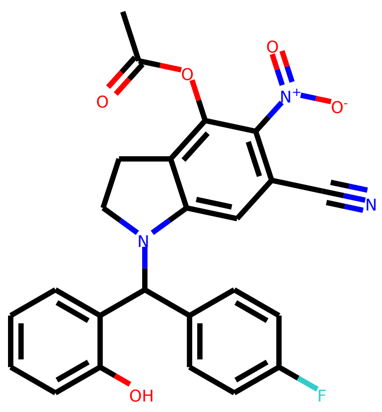
Osma-1030
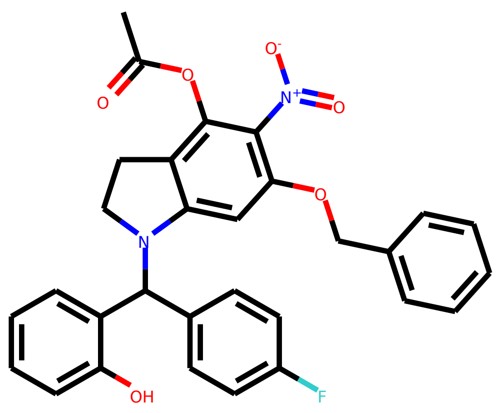
Osma-1031
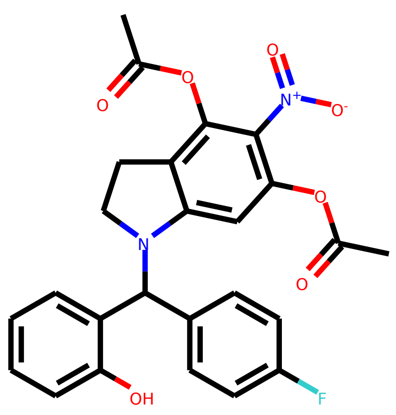
Osma-1032
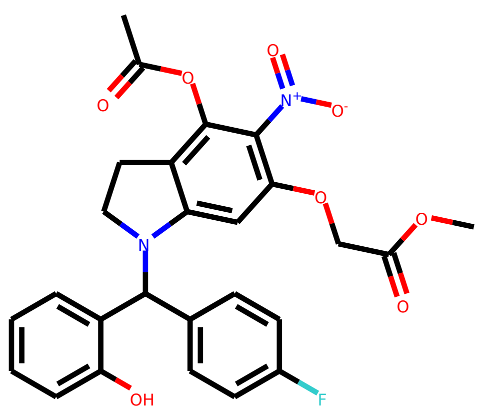
Osma-1033
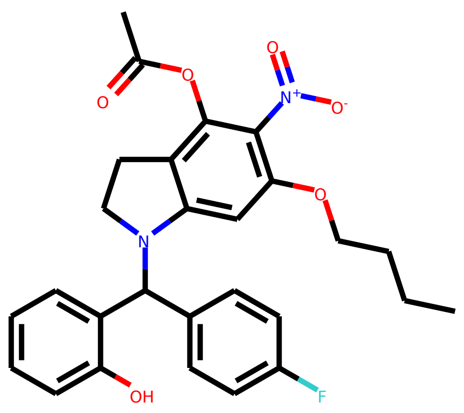
Osma-1034
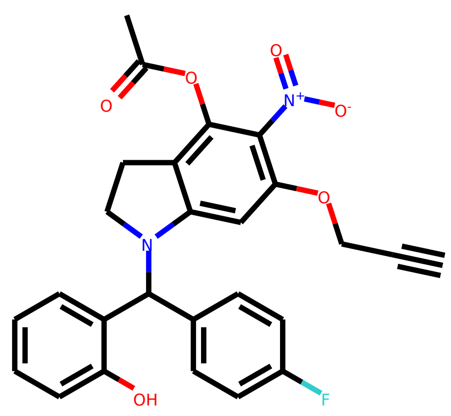
Osma-1035
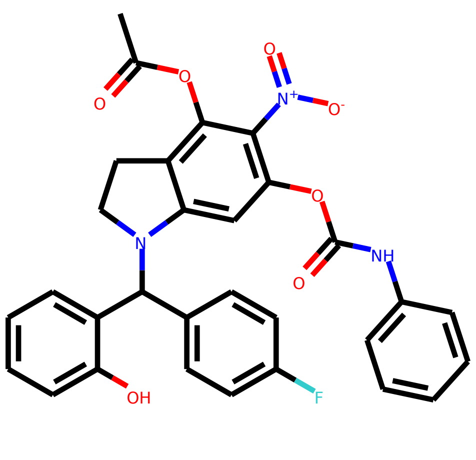
Osma-1036
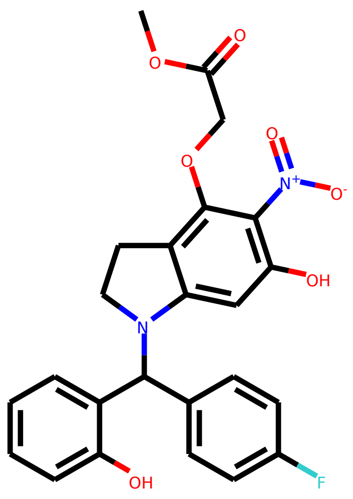
Osma-1037
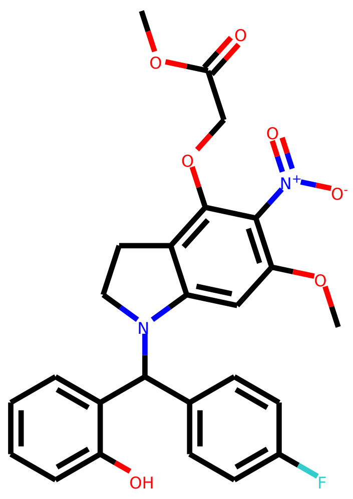
Osma-1038
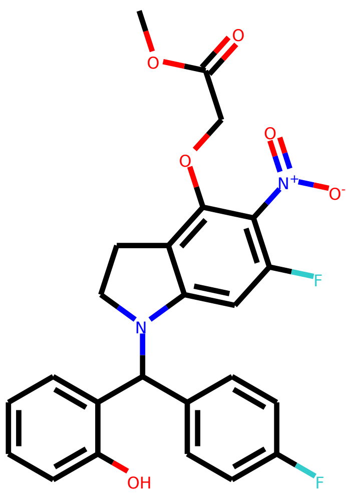
Osma-1039
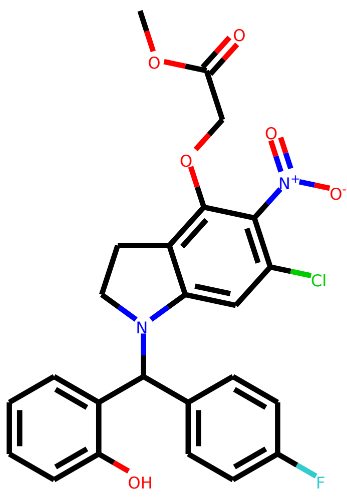
Osma-1040
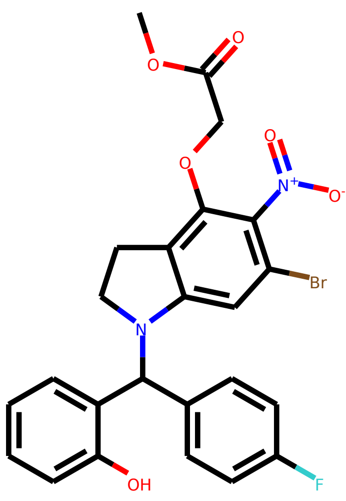
Osma-1041
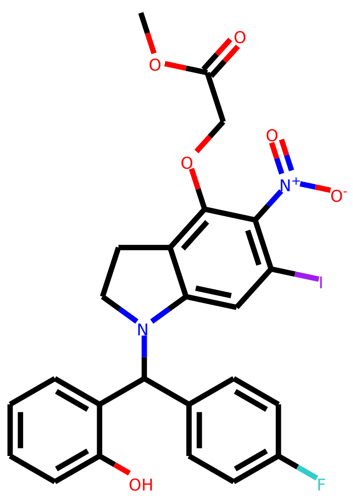
Osma-1042
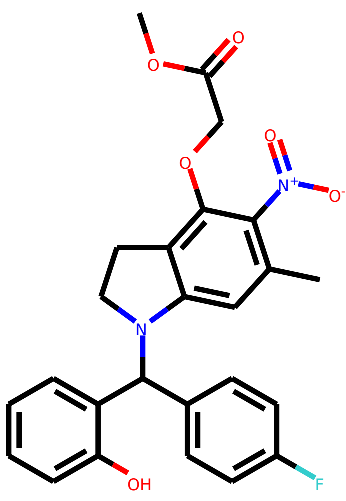
Osma-1043
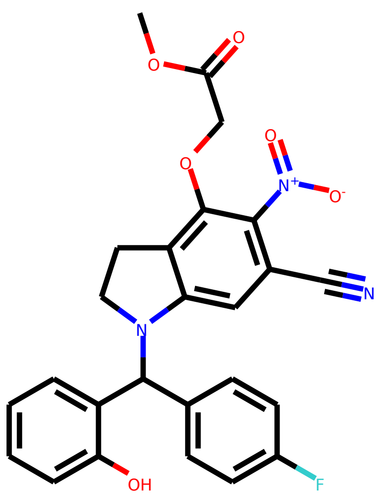
Osma-1044
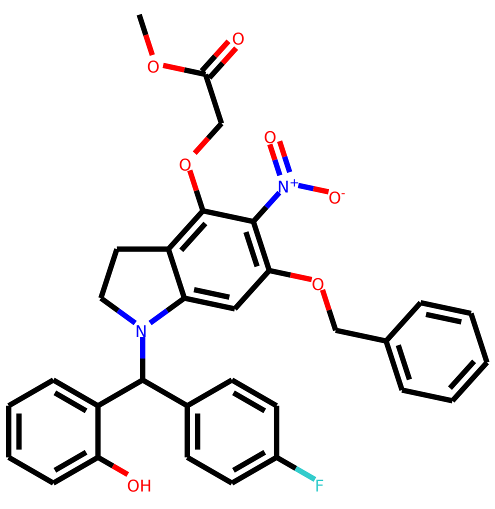
Osma-1045
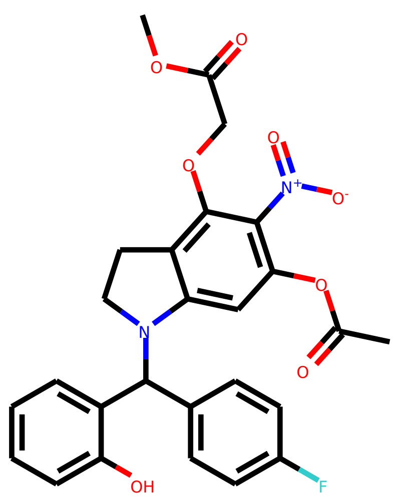
Osma-1046
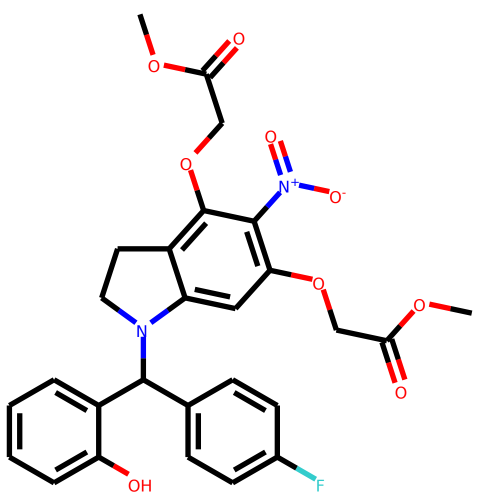
Osma-1047
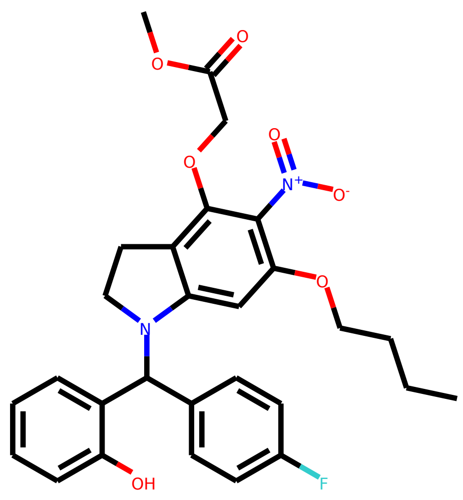
Osma-1048
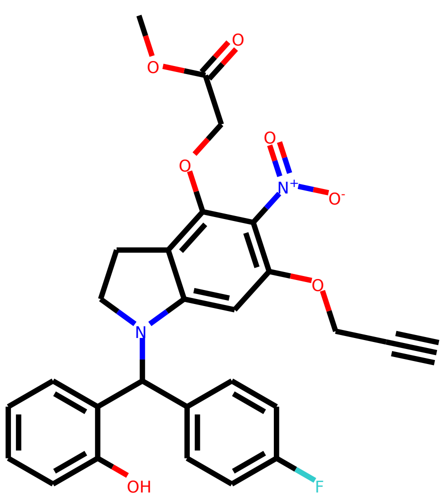
Osma-1049
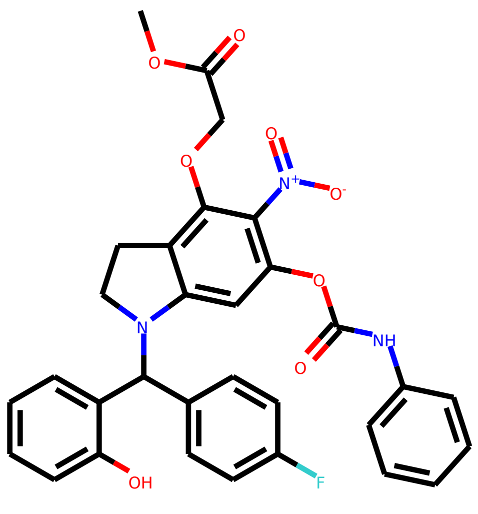
Osma-1050

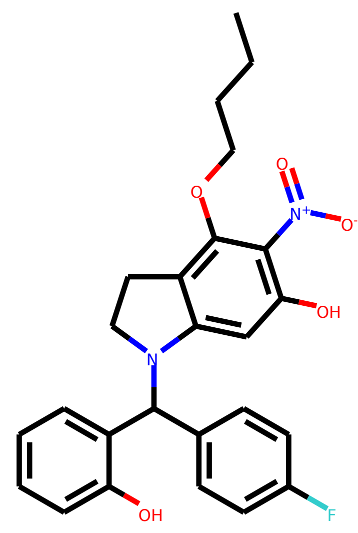
Osma-1051
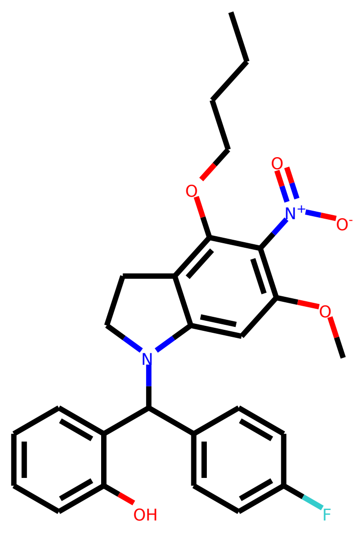
Osma-1052
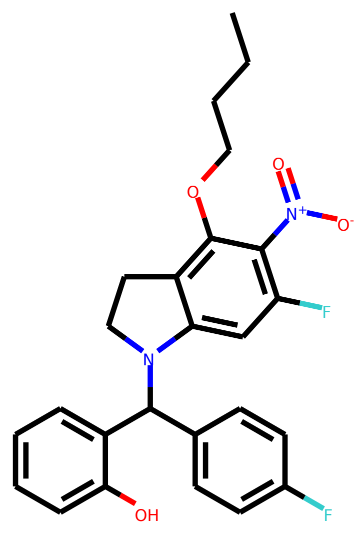
Osma-1053
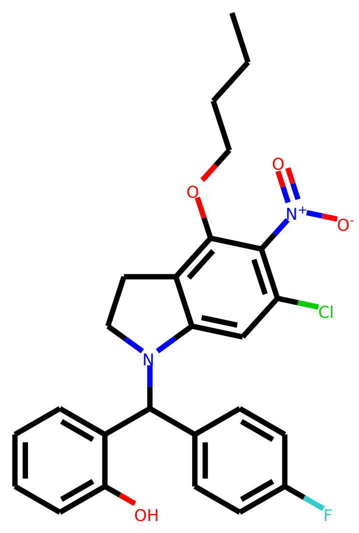
Osma-1054
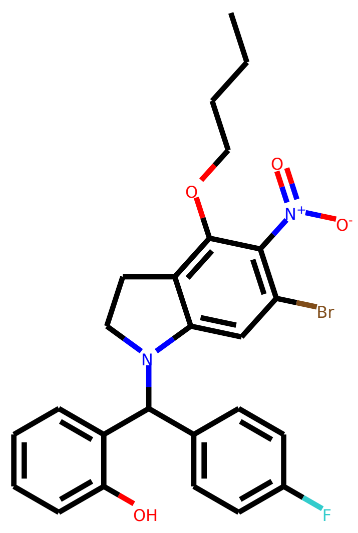
Osma-1055
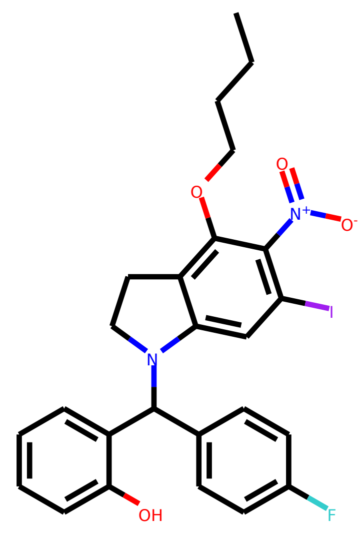
Osma-1056
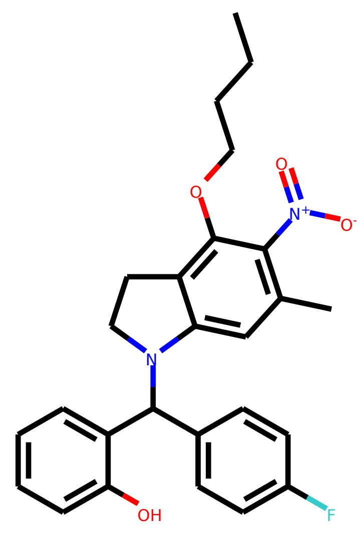
Osma-1057
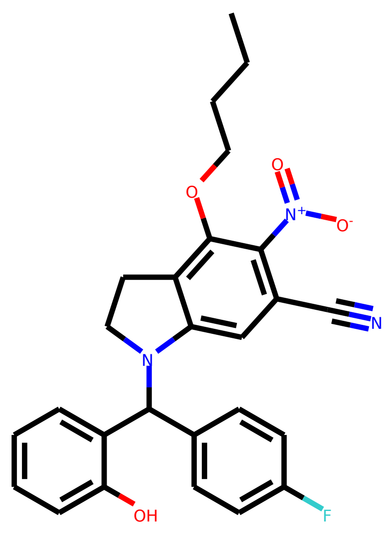
Osma-1058
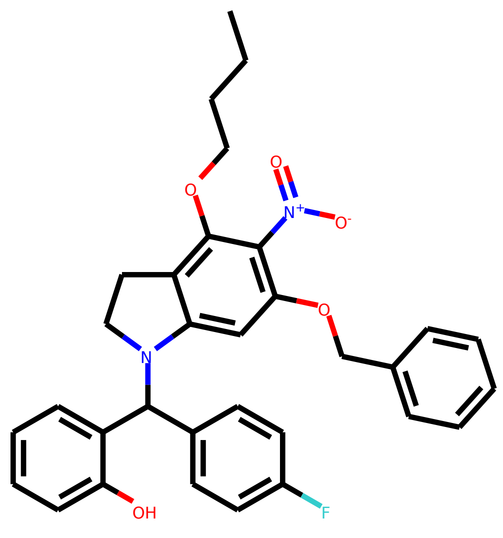
Osma-1059
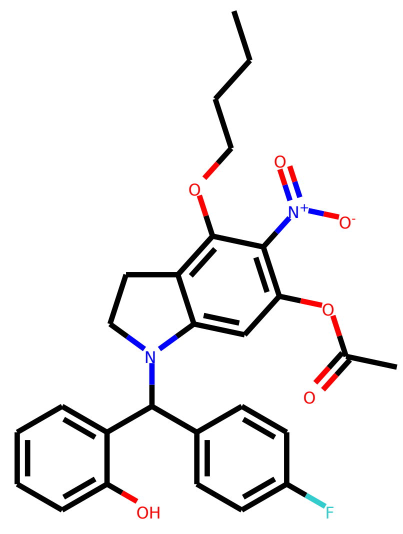
Osma-1060
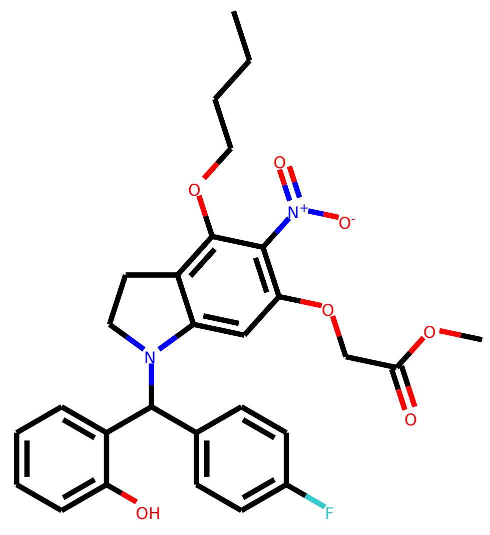
Osma-1061
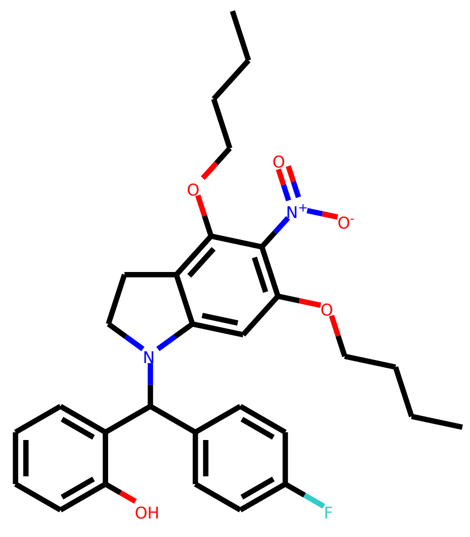
Osma-1062
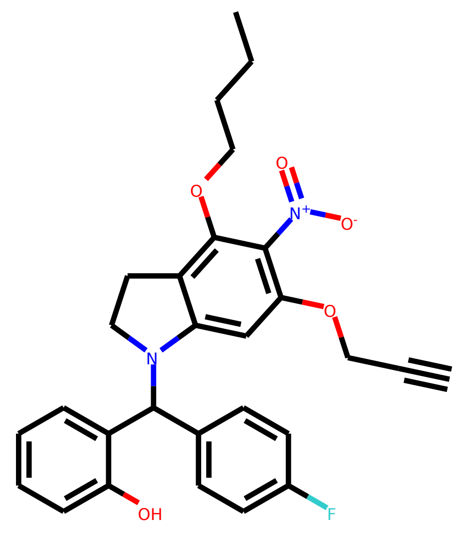
Osma-1063
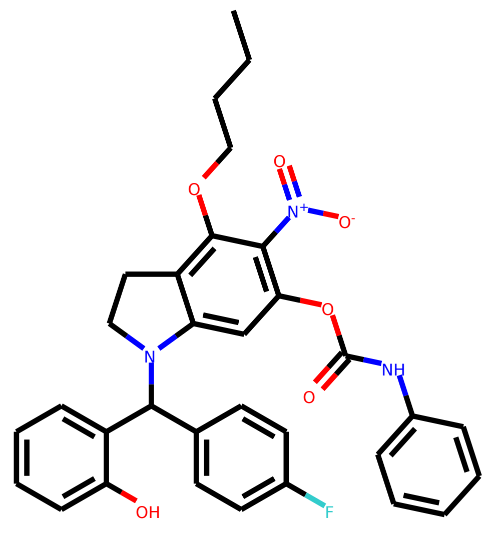
Osma-1064
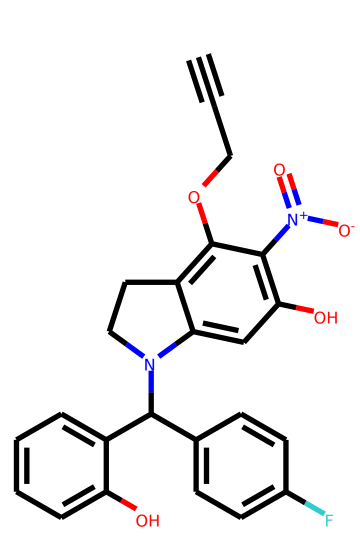
Osma-1065
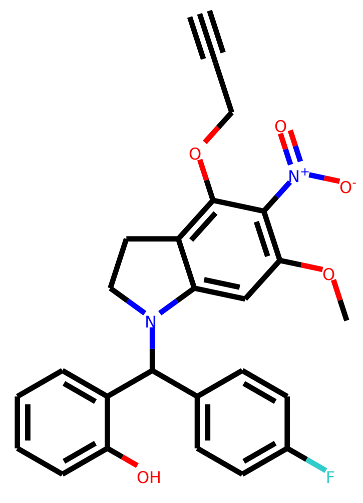
Osma-1066
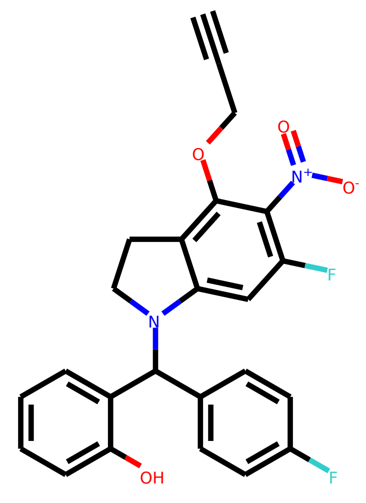
Osma-1067
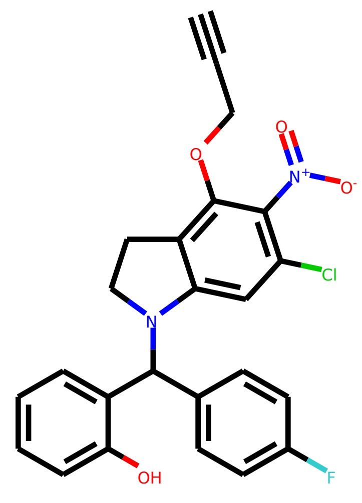
Osma-1068
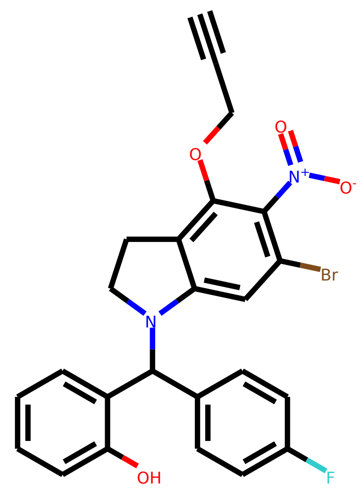
Osma-1069
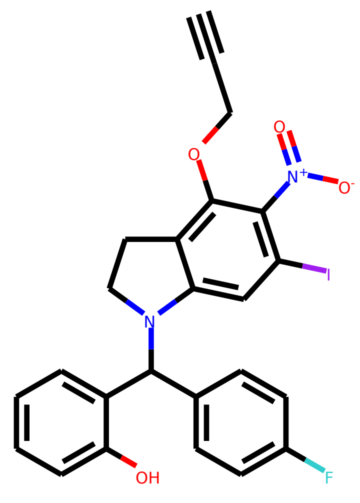
Osma-1070
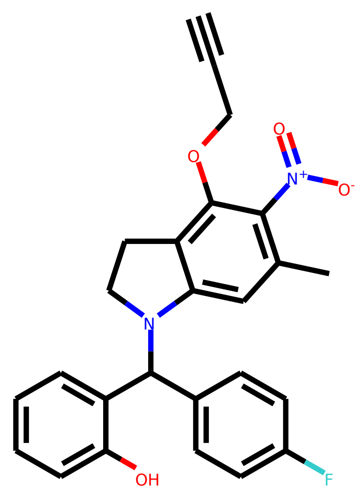
Osma-1071
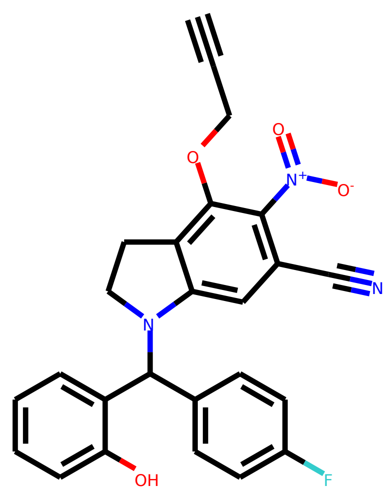
Osma-1072
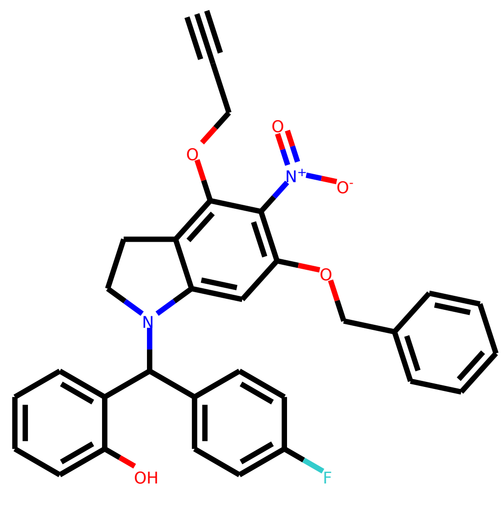
Osma-1073
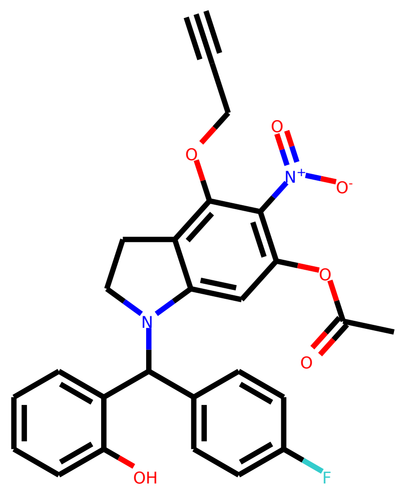
Osma-1074
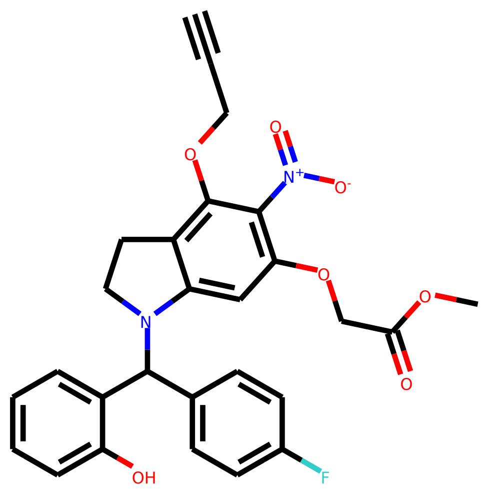
Osma-1075
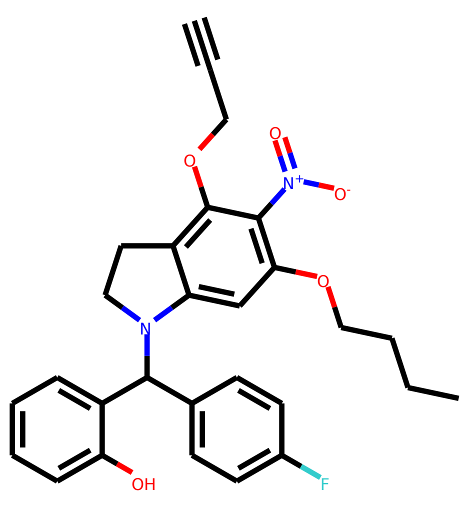
Osma-1076
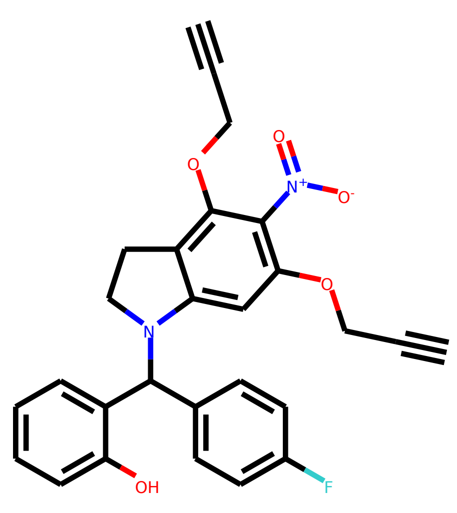
Osma-1077
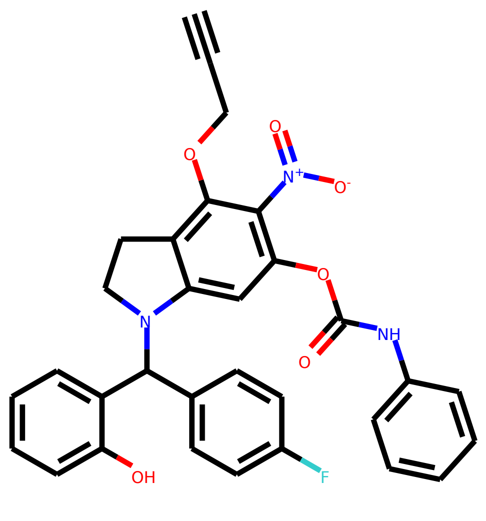
Osma-1078
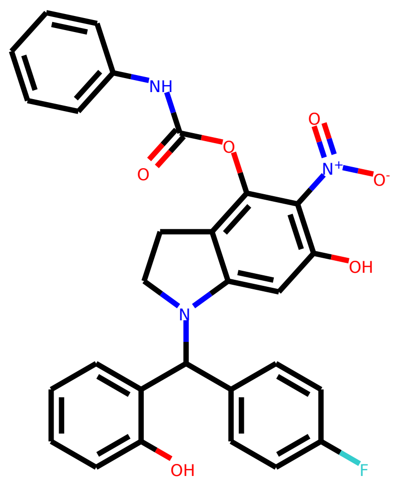
Osma-1079
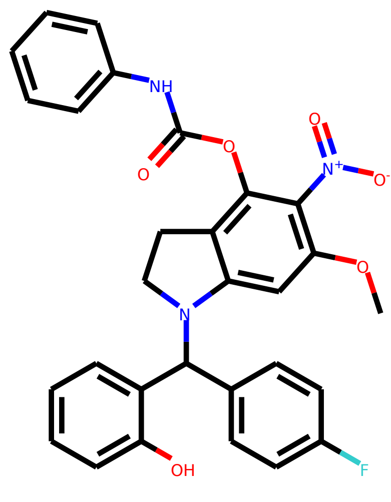
Osma-1080
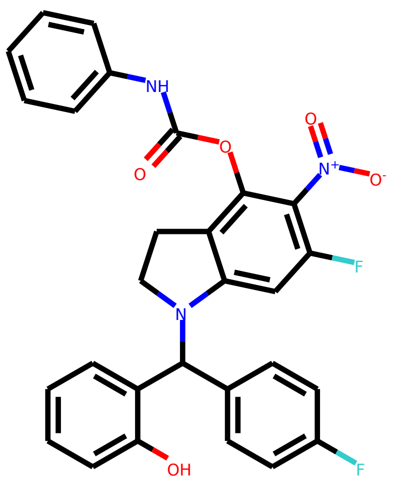
Osma-1081
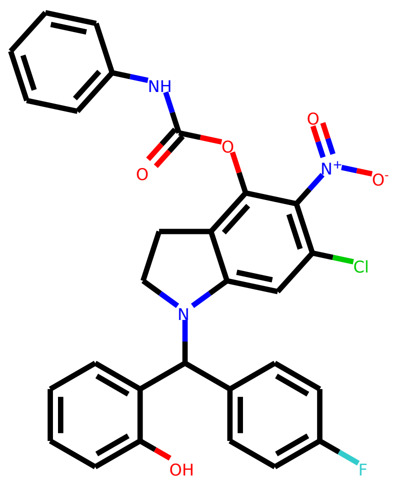
Osma-1082
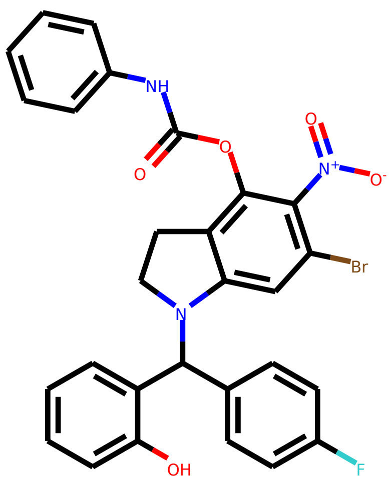
Osma-1083
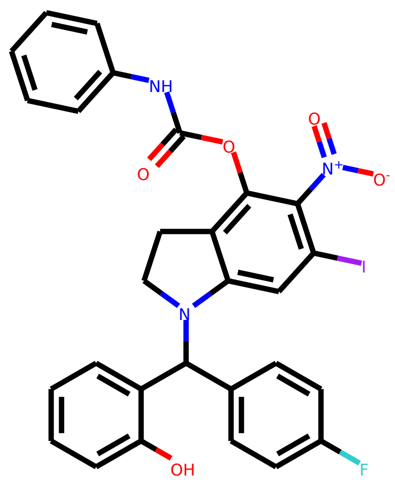
Osma-1084
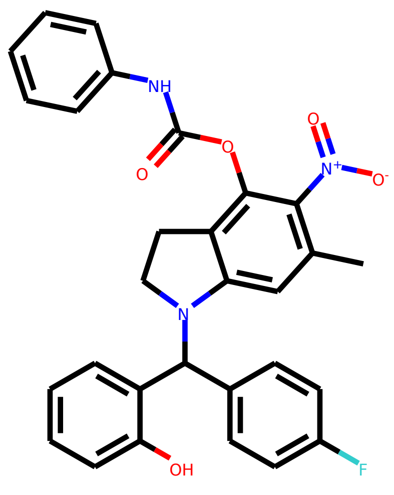
Osma-1085

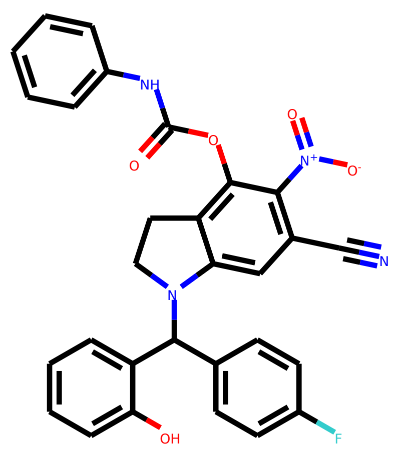

Osma-1086

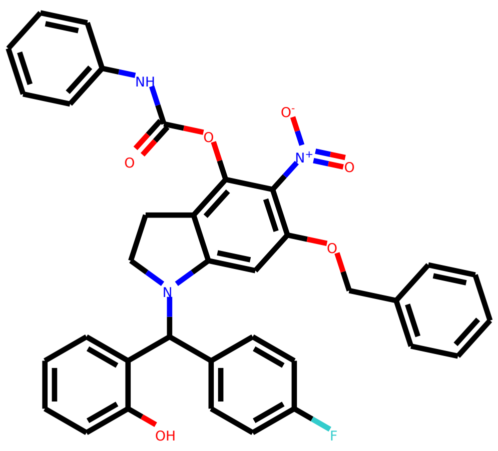

Osma-1087

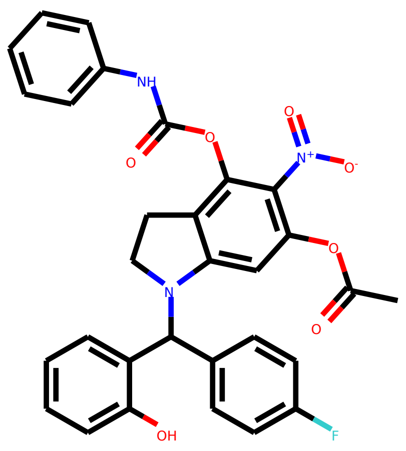

Osma-1088

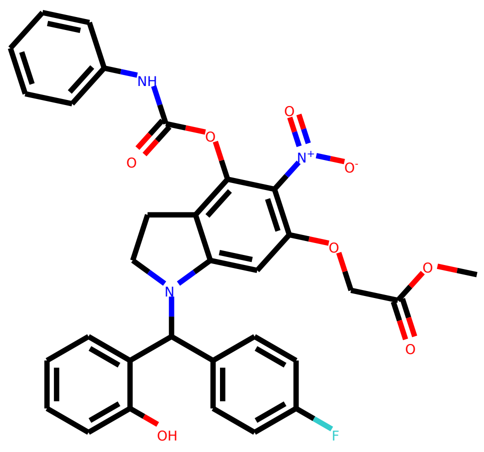

Osma-1089

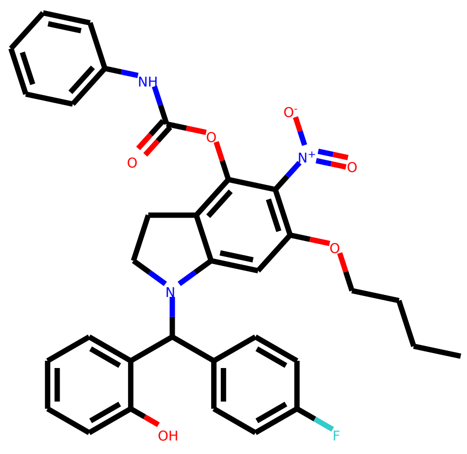

Osma-1090

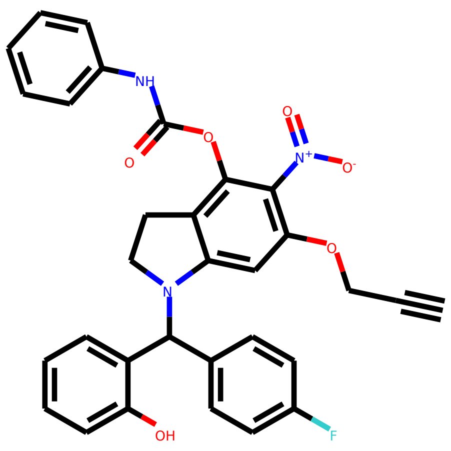

Osma-1091

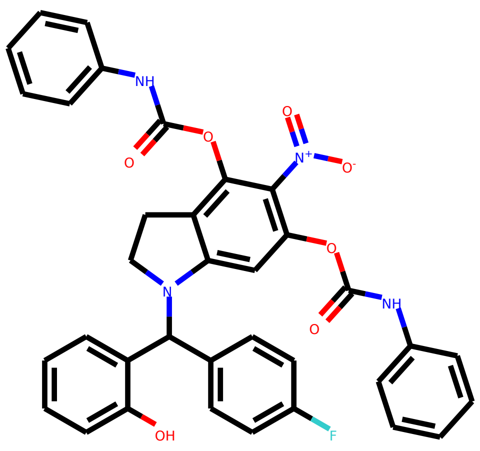

Osma-1092

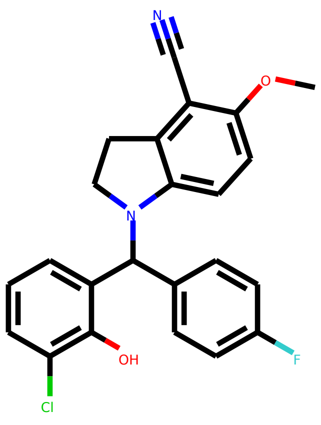

Osma-1093

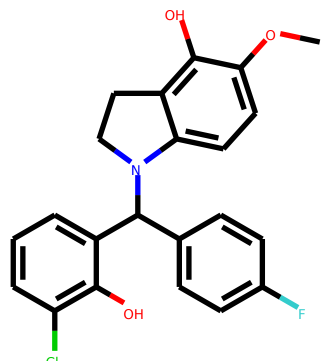

Osma-1094

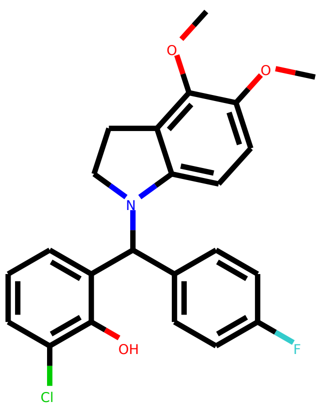

Osma-1095

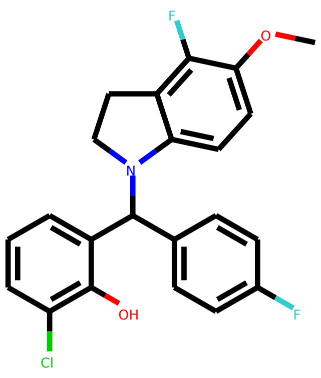

Osma-1096

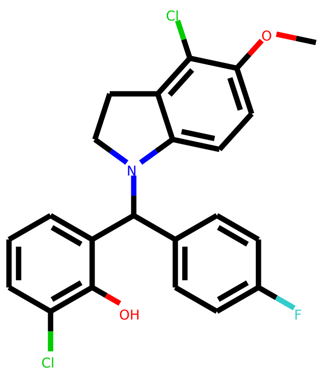

Osma-1097

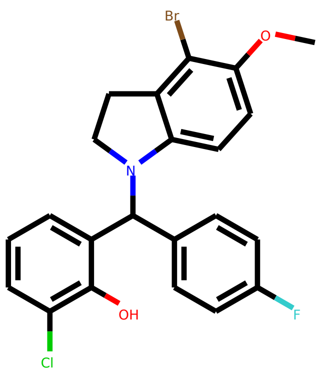

Osma-1098

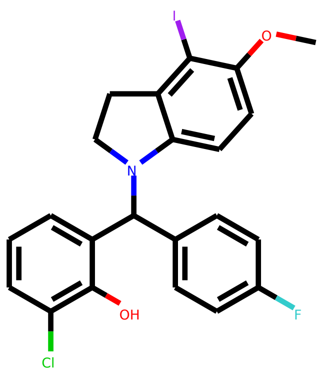

Osma-1099

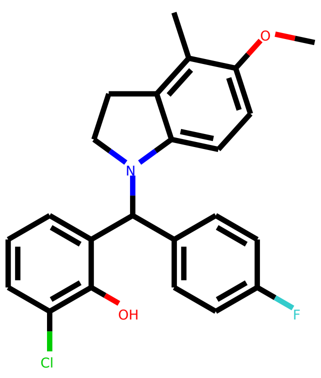

Osma-1100

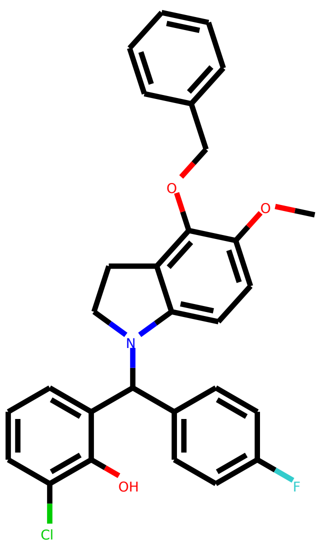

Osma-1101

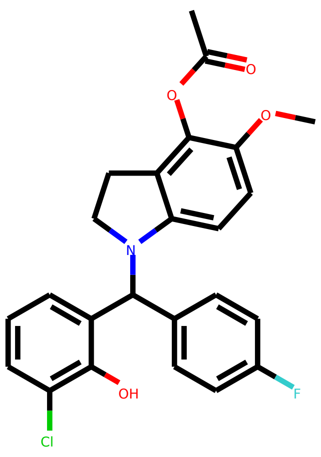

Osma-1102

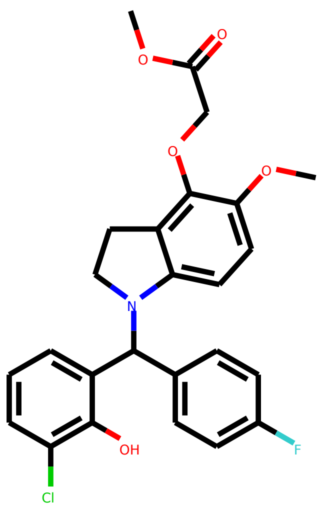

Osma-1103

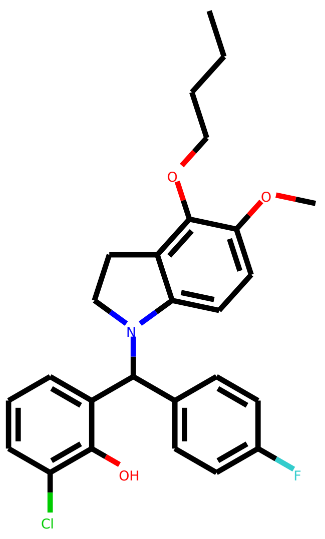

Osma-1104

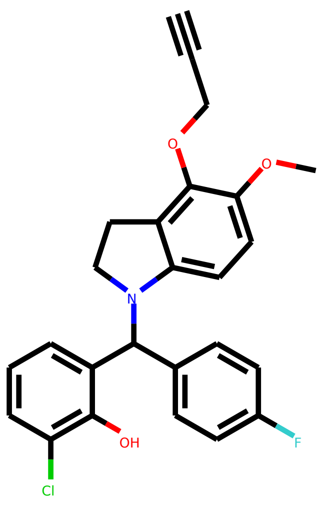

Osma-1105

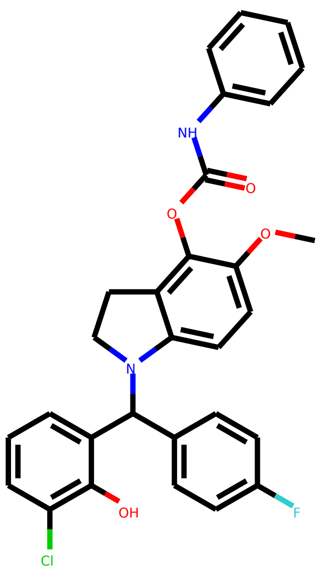

Osma-1106

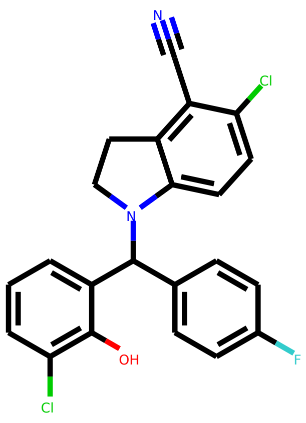

Osma-1107

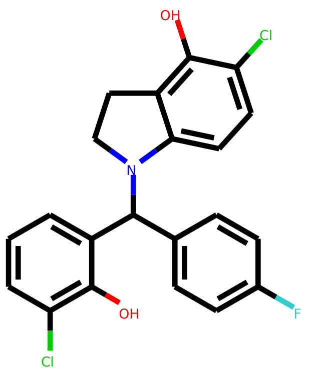

Osma-1108

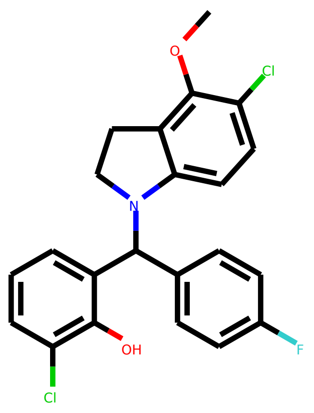

Osma-1109

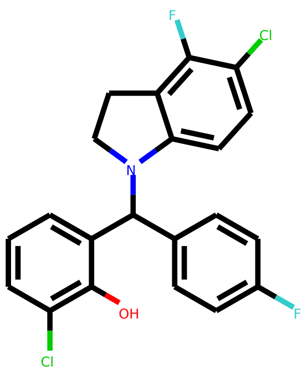

Osma-1110

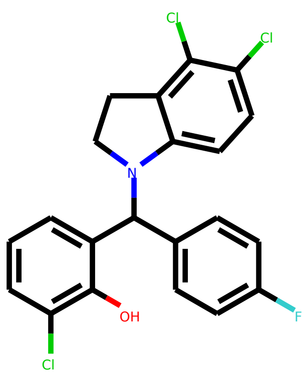

Osma-1111

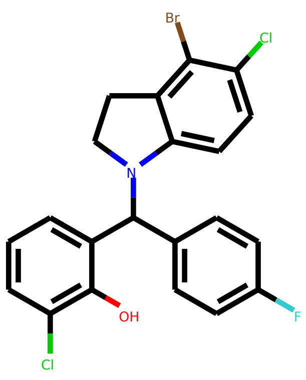

Osma-1112

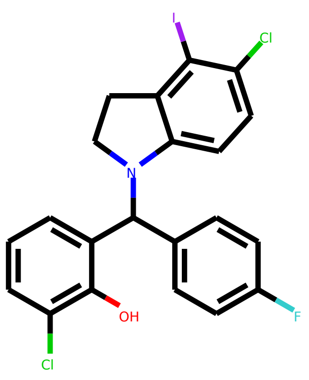

Osma-1113

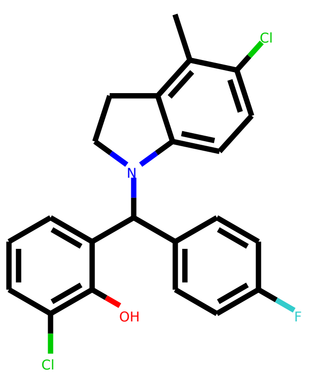

Osma-1114

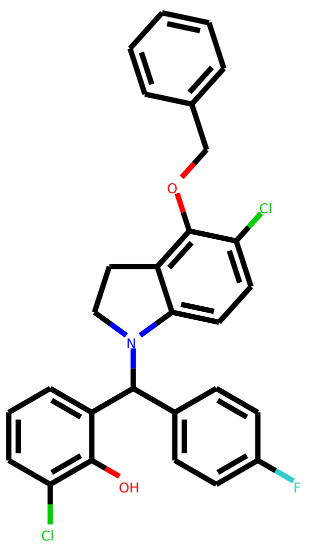

Osma-1115

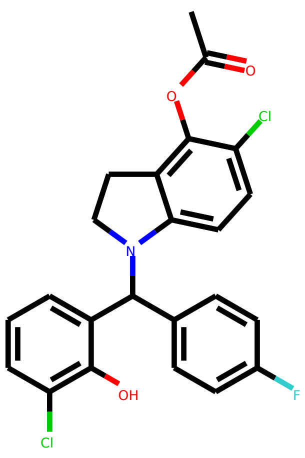

Osma-1116

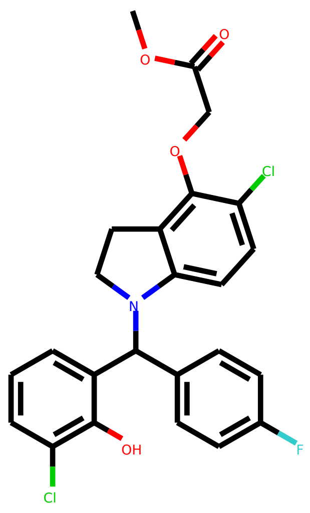

Osma-1117

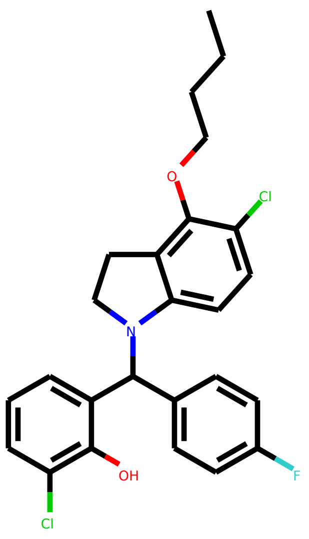

Osma-1118

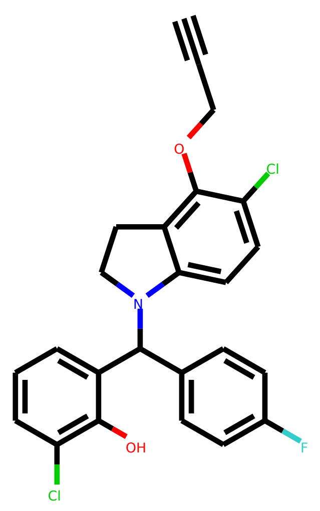

Osma-1119

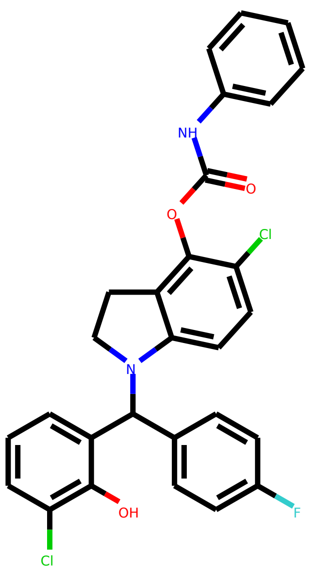

Osma-1120

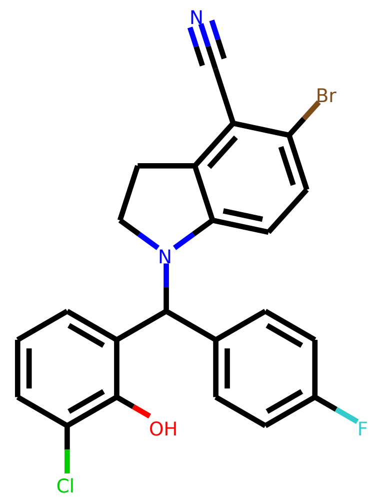
Osma-1121
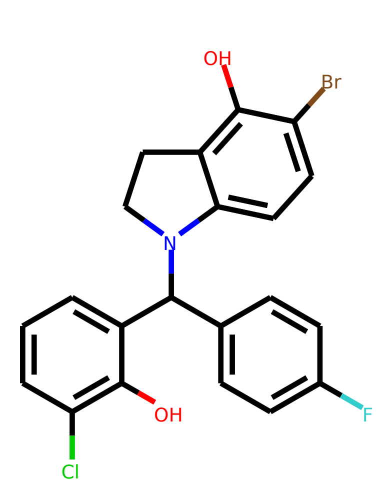
Osma-1122
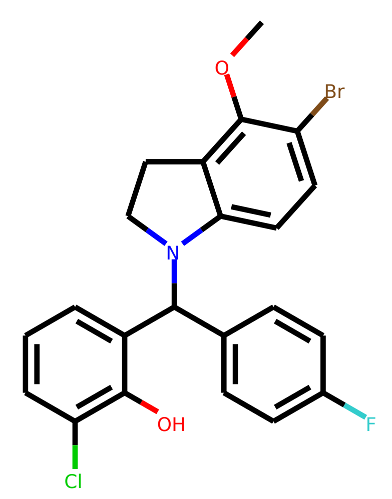
Osma-1123
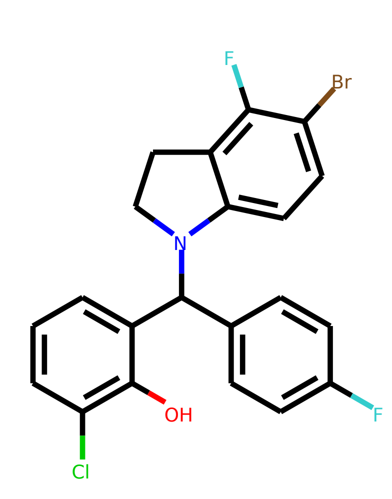
Osma-1124
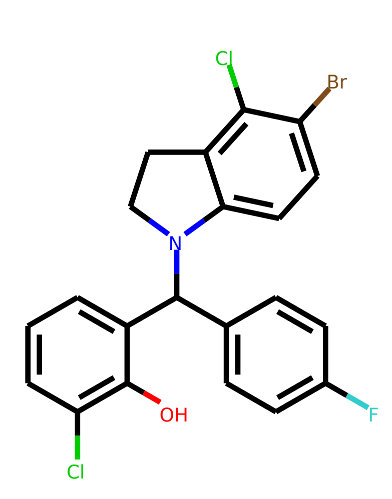
Osma-1125
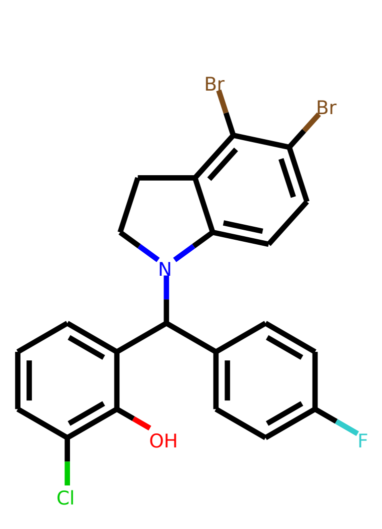
Osma-1126
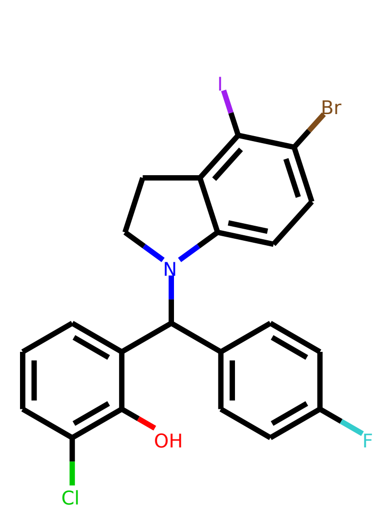
Osma-1127
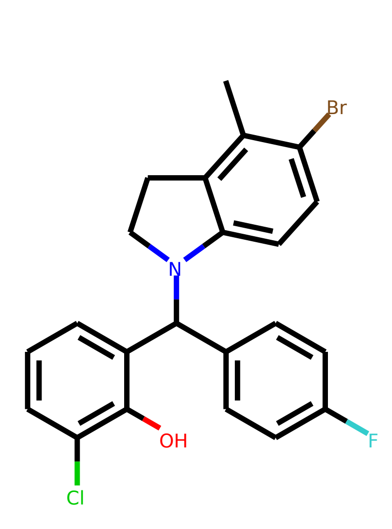
Osma-1128
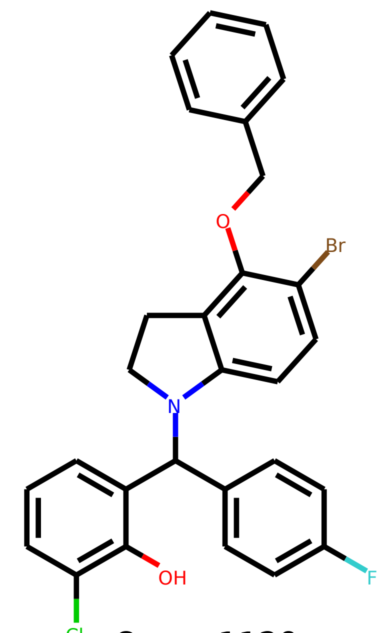
Osma-1129
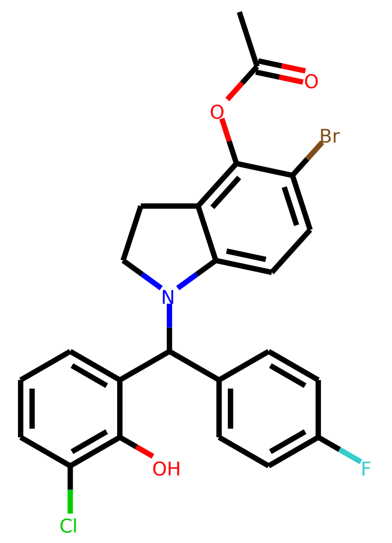
Osma-1130
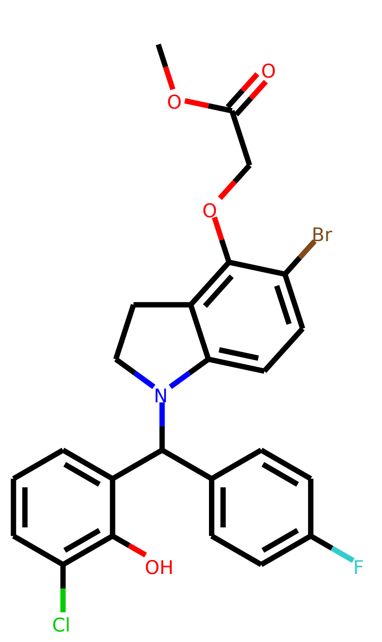
Osma-1131
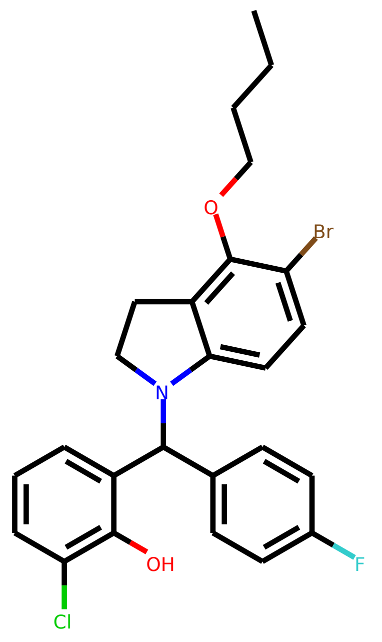
Osma-1132
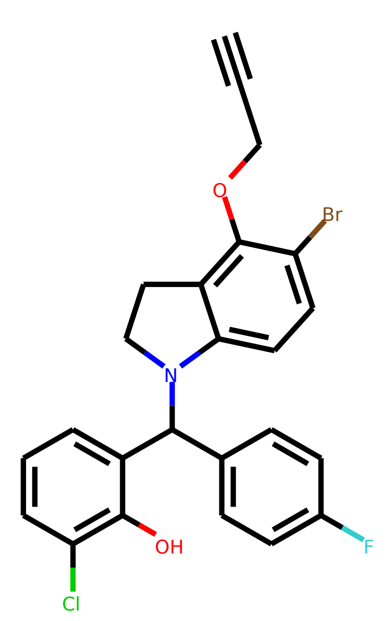
Osma-1133
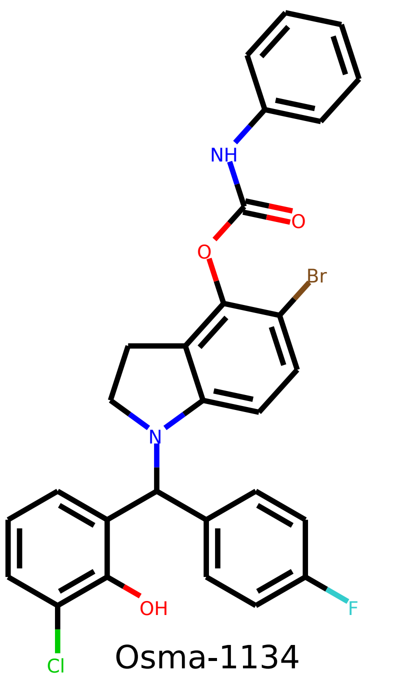
Osma-1134
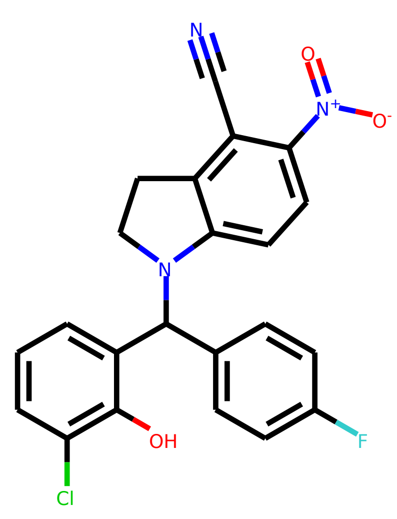
Osma-1135
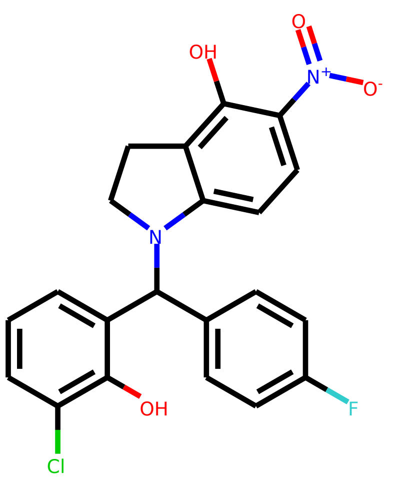
Osma-1136
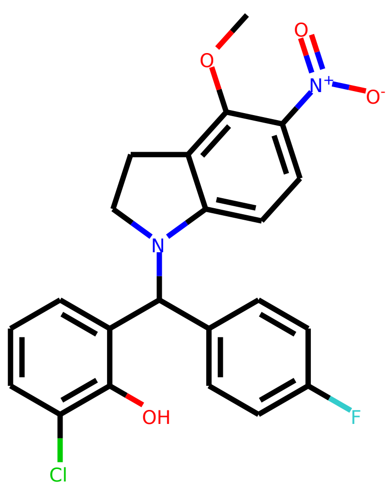
Osma-1137
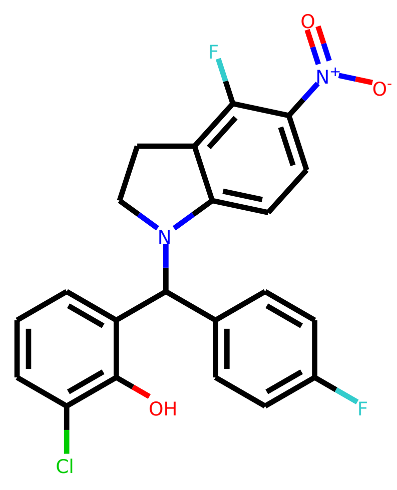
Osma-1138
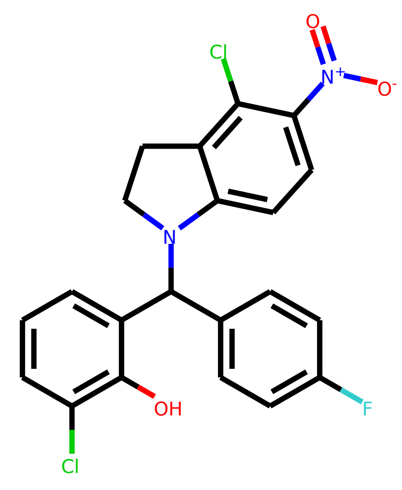
Osma-1139
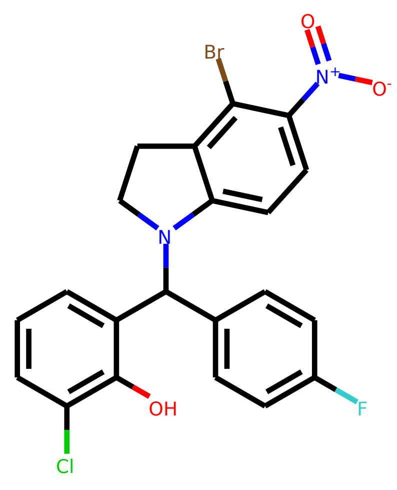
Osma-1140
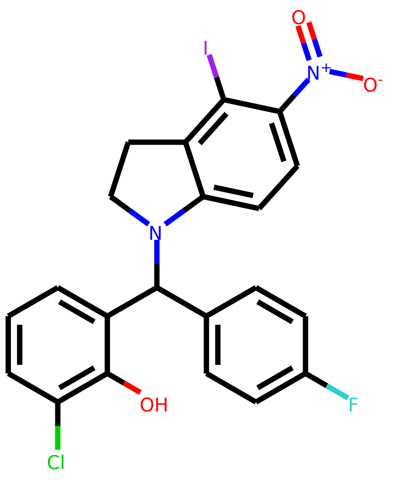
Osma-1141
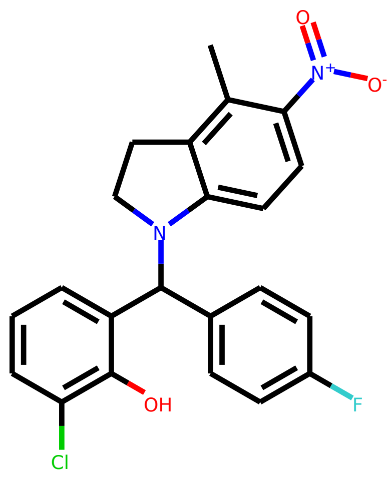
Osma-1142
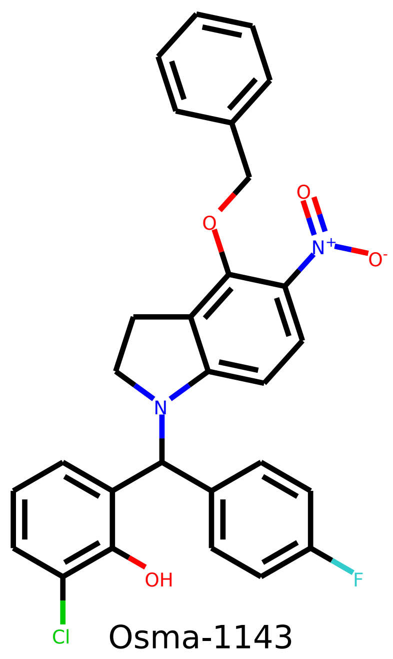
Osma-1143
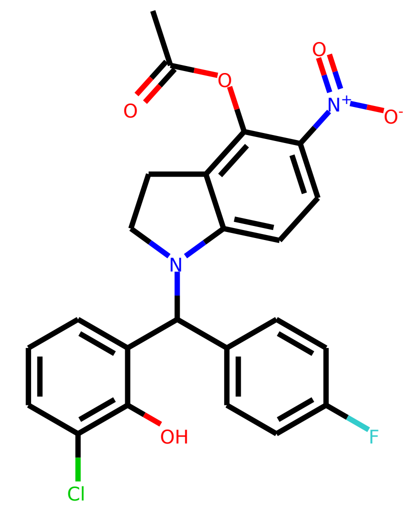
Osma-1144
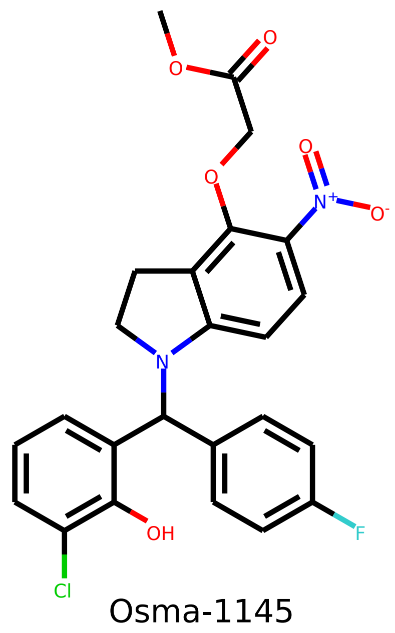
Osma-1145
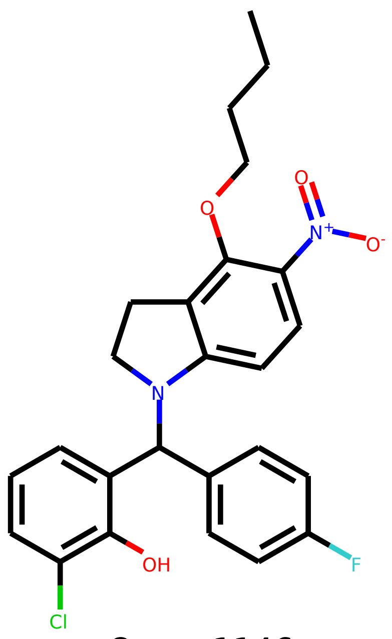
Osma-1146
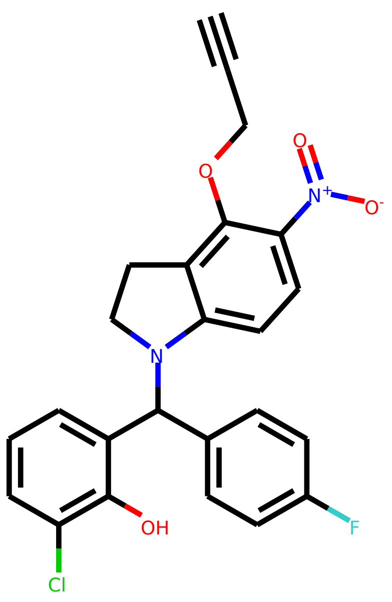
Osma-1147
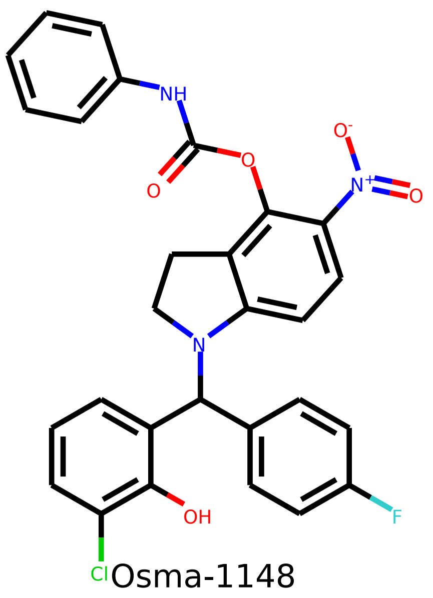
Osma-1148
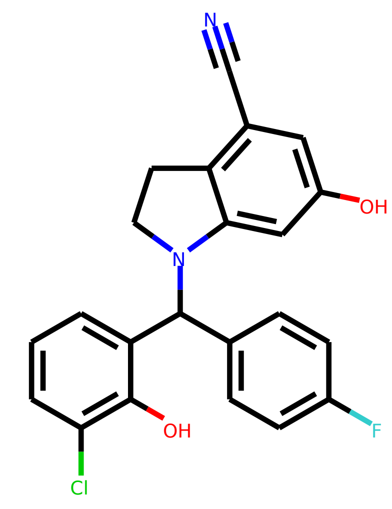
Osma-1149
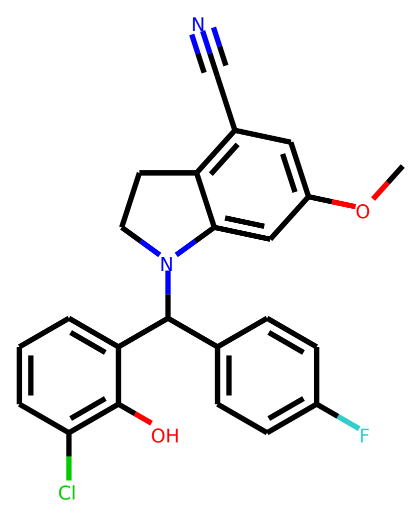
Osma-1150
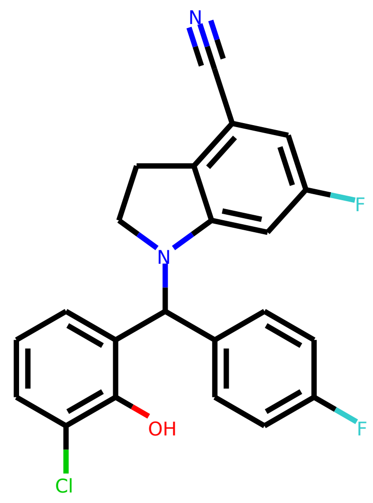
Osma-1151
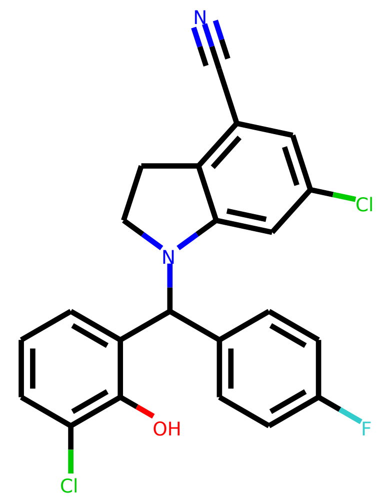
Osma-1152
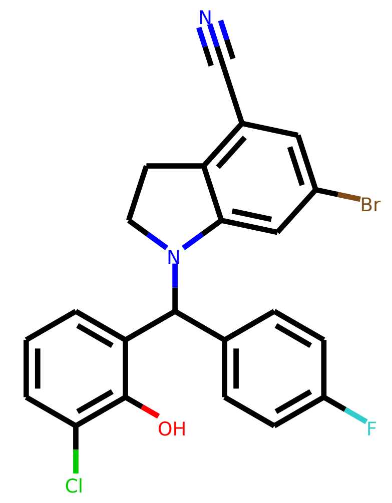
Osma-1153
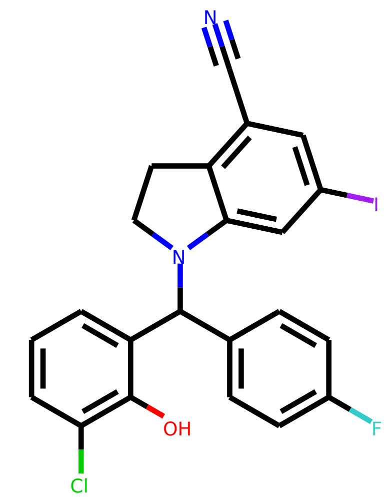
Osma-1154
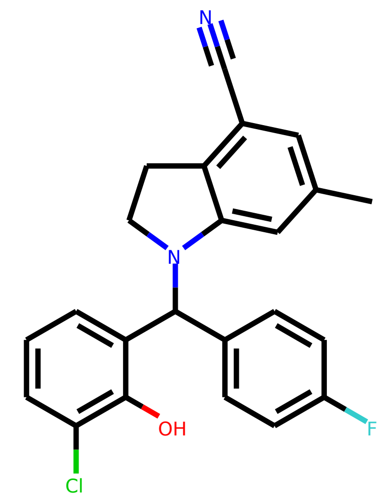
Osma-1155

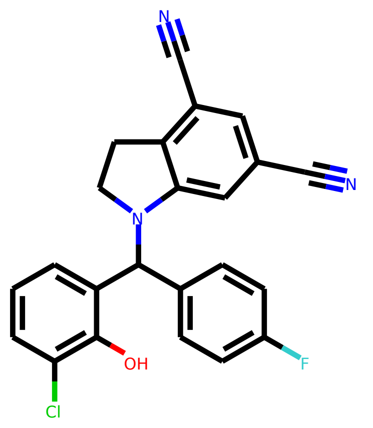
Osma-1156

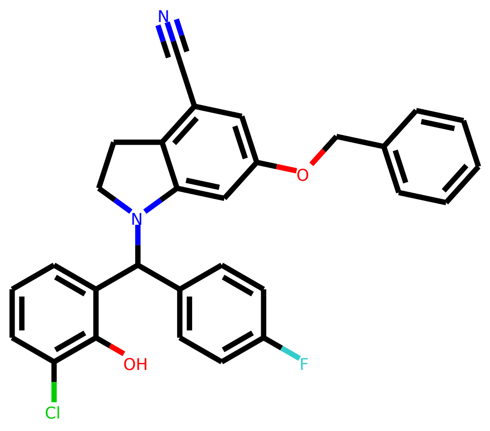
Osma-1157

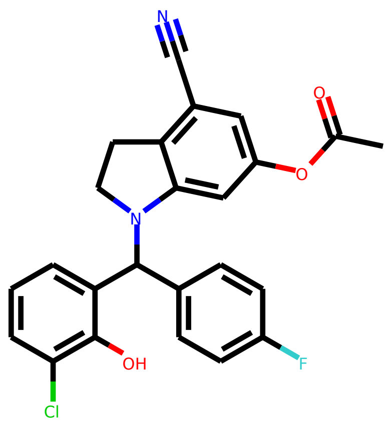
Osma-1158

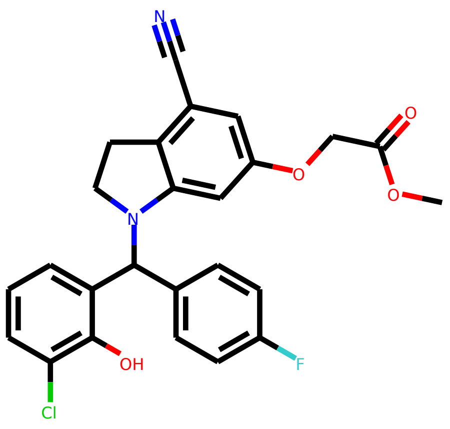
Osma-1159

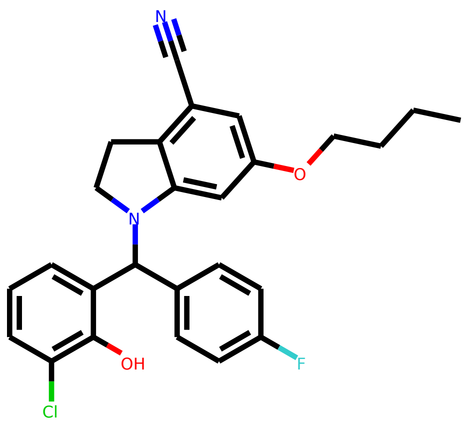
Osma-1160

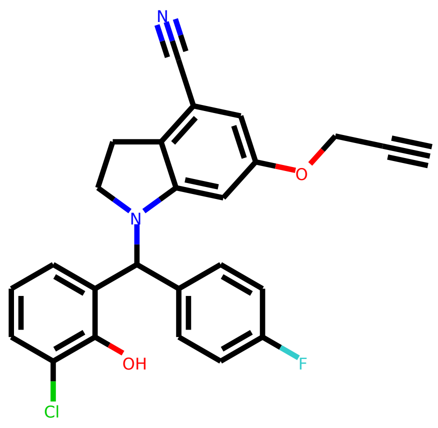
Osma-1161

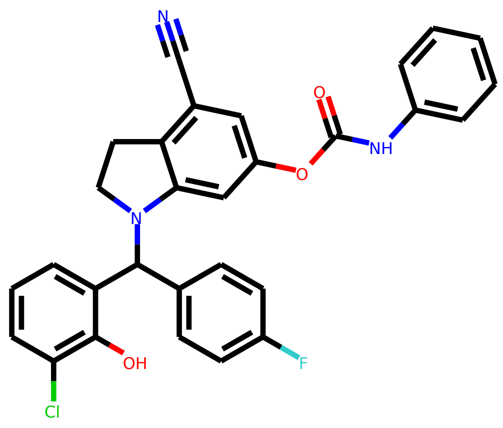
Osma-1162

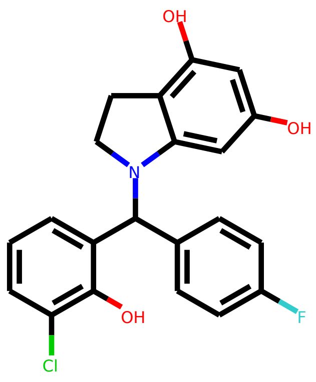
Osma-1163

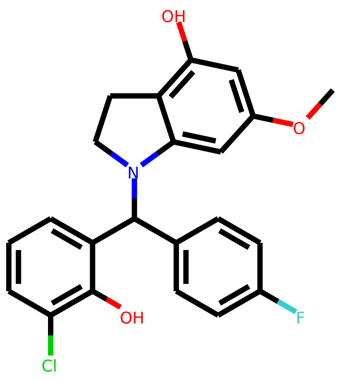
Osma-1164

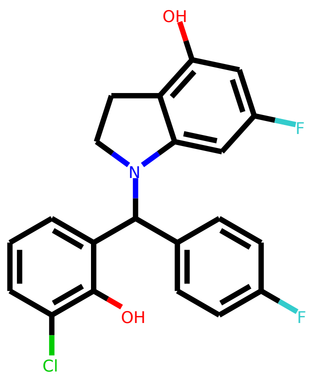
Osma-1165

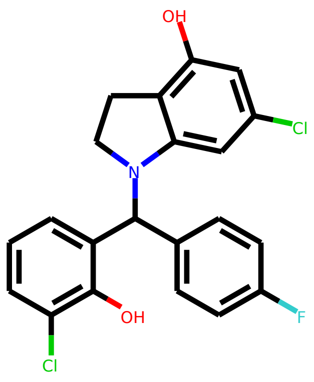
Osma-1166

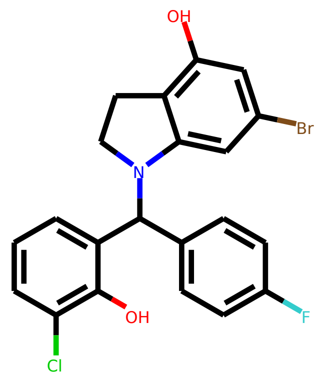
Osma-1167

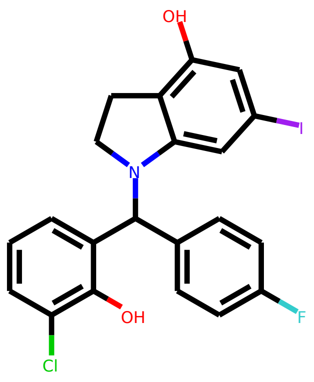
Osma-1168

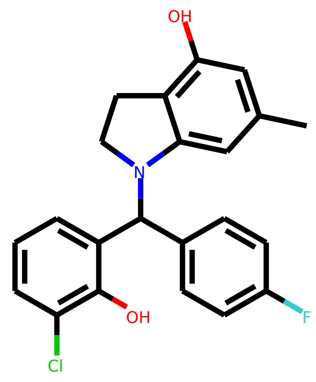
Osma-1169

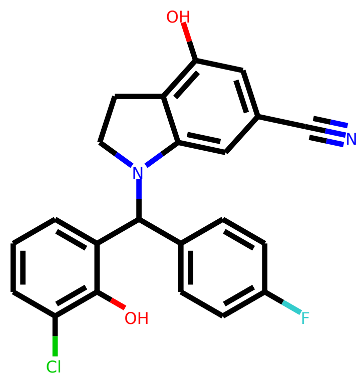
Osma-1170

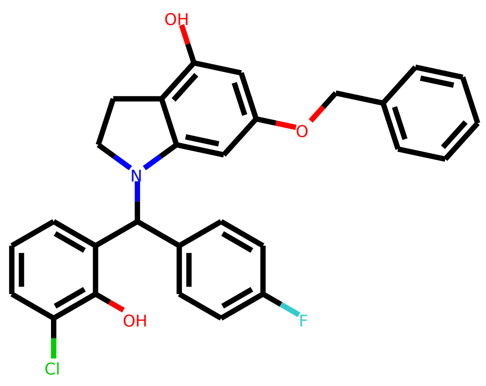
Osma-1171

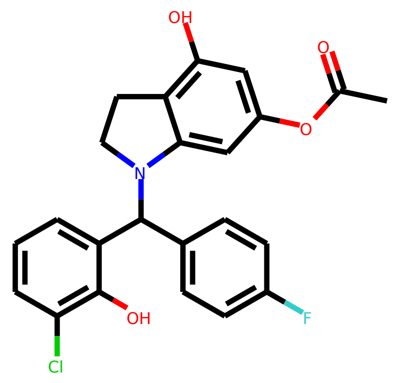
Osma-1172

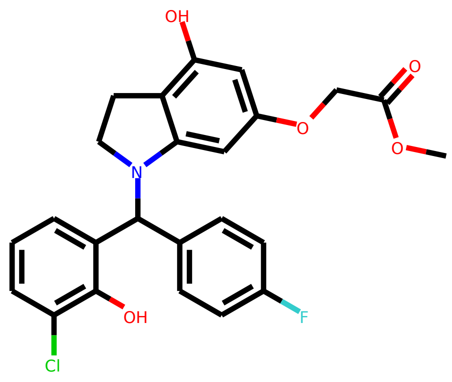
Osma-1173

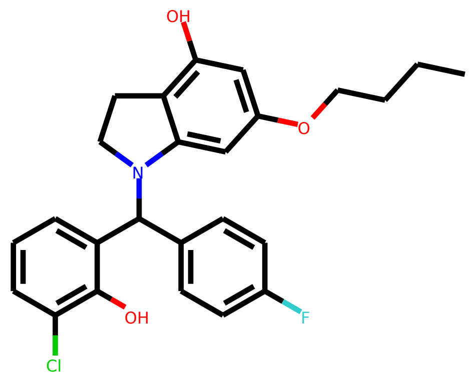
Osma-1174

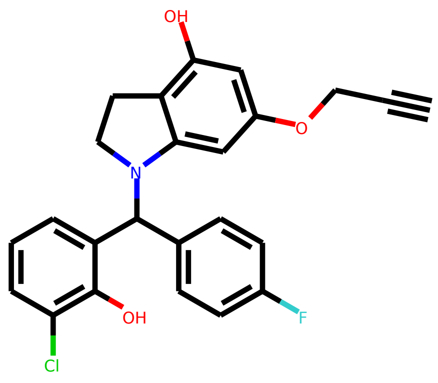
Osma-1175

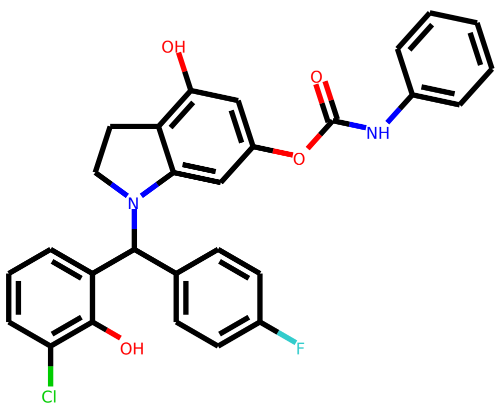
Osma-1176

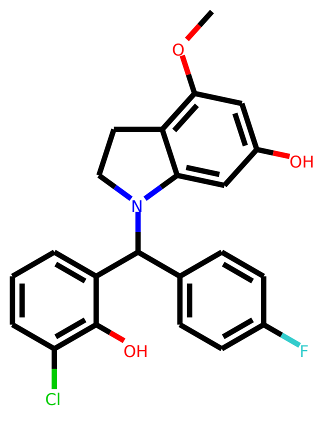
Osma-1177

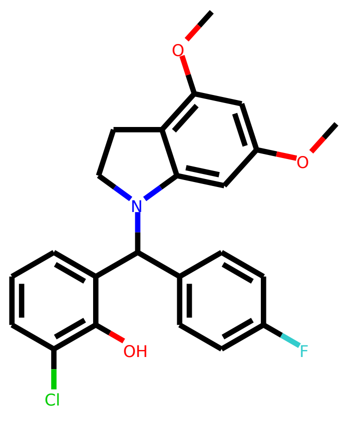
Osma-1178

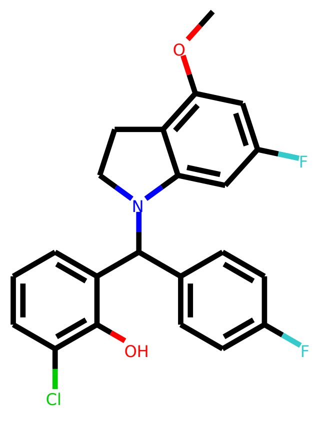
Osma-1179

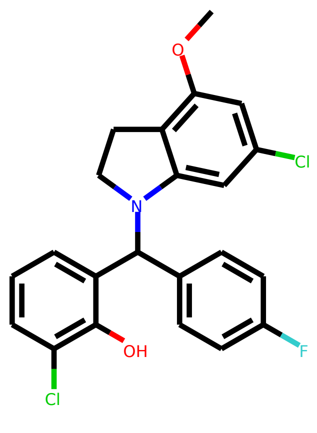
Osma-1180

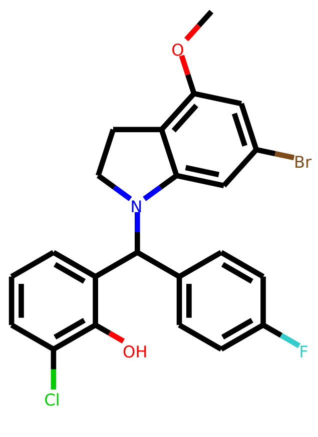
Osma-1181

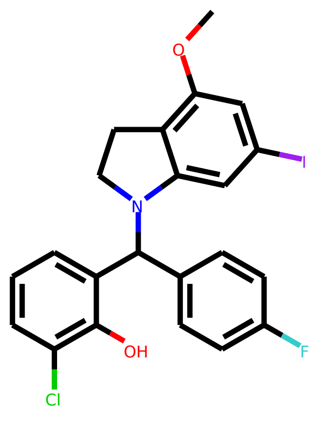
Osma-1182

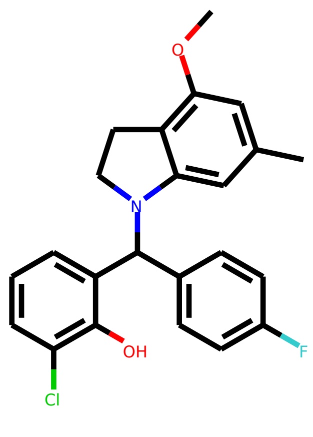
Osma-1183

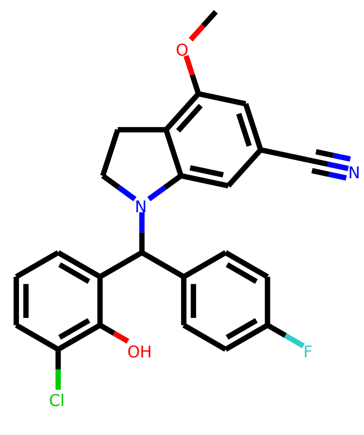
Osma-1184

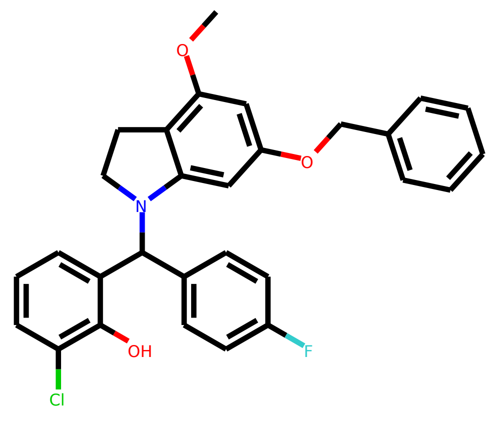
Osma-1185

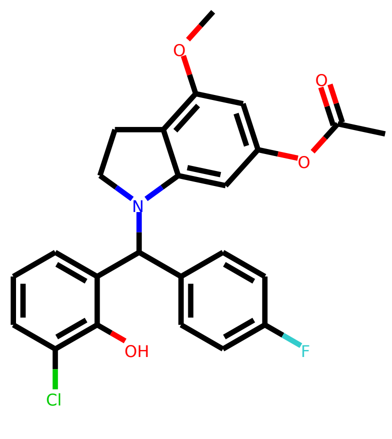
Osma-1186

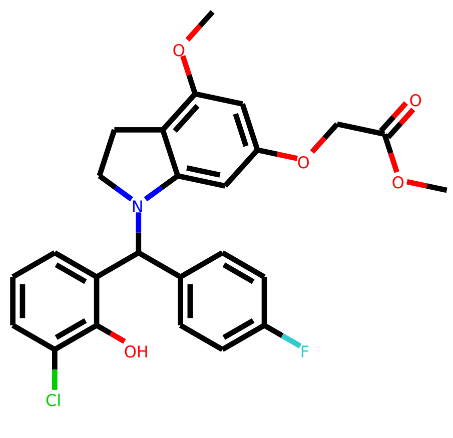
Osma-1187

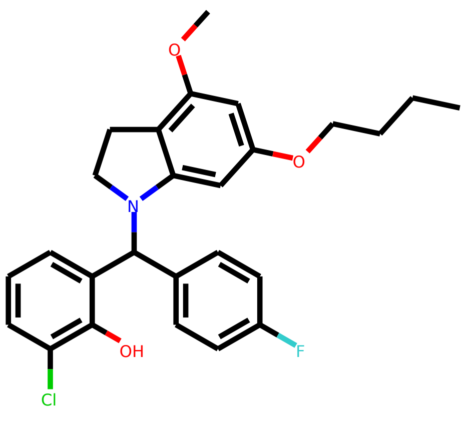
Osma-1188

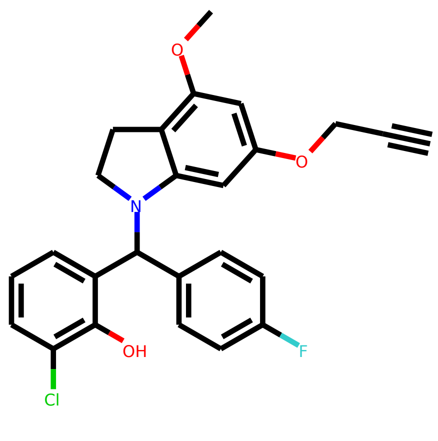
Osma-1189

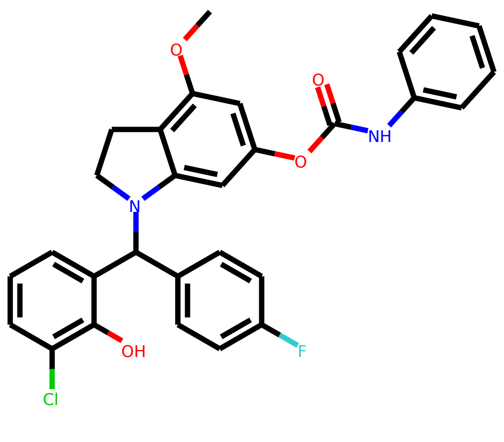
Osma-1190

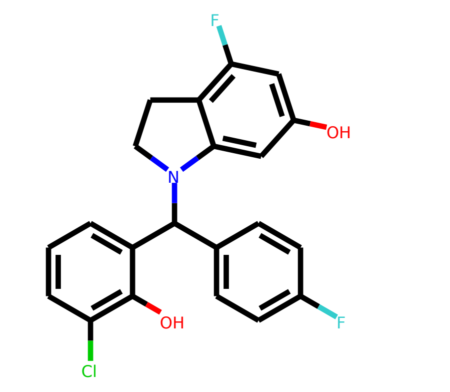

Osma-1191

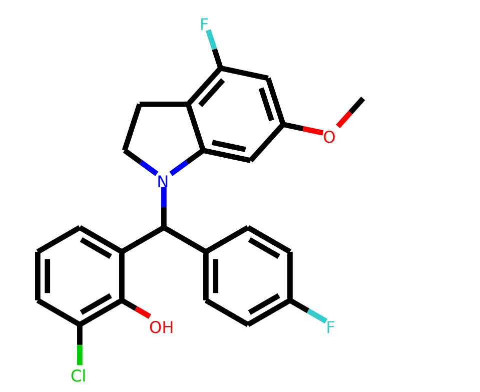

Osma-1192

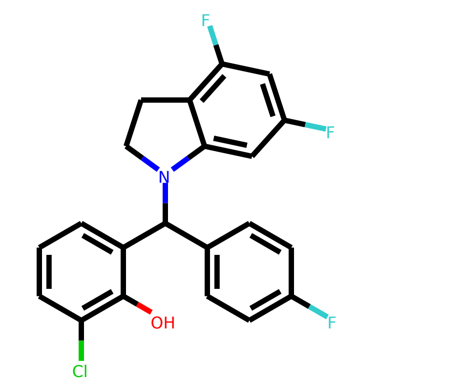

Osma-1193

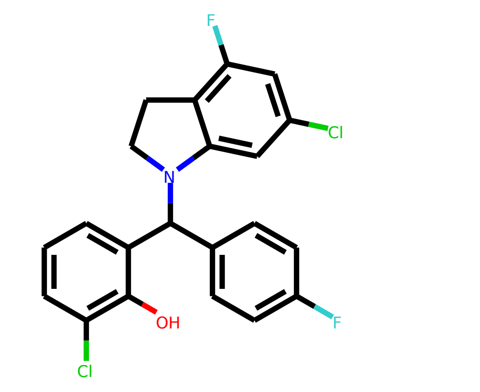

Osma-1194

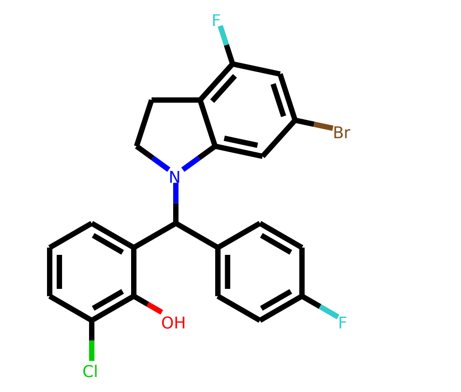

Osma-1195

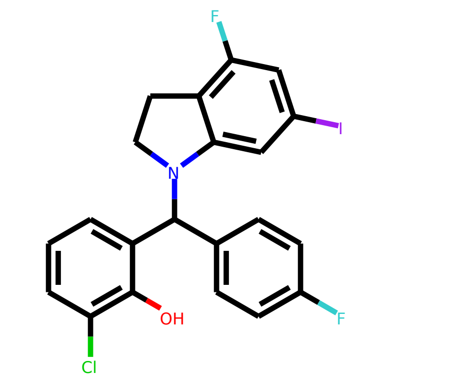

Osma-1196

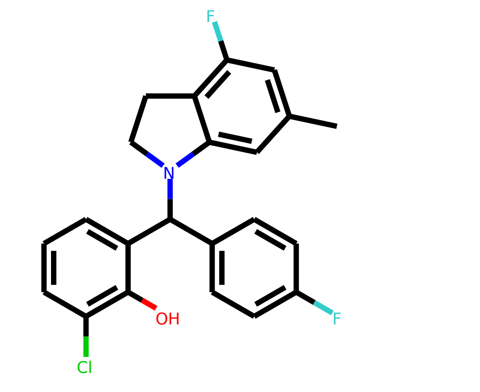

Osma-1197

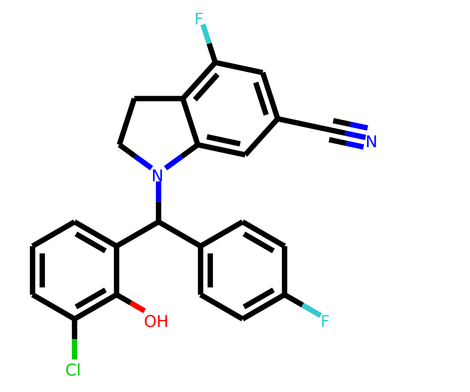

Osma-1198

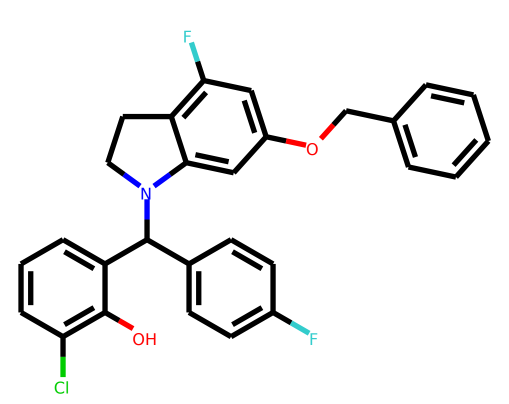

Osma-1199

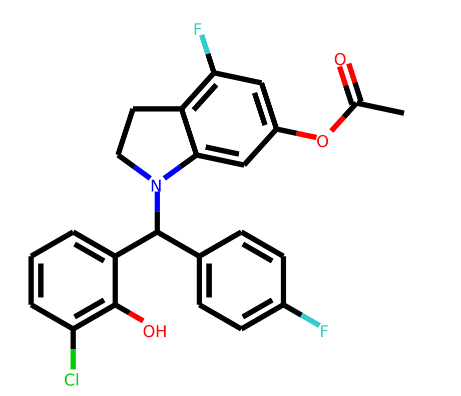

Osma-1200

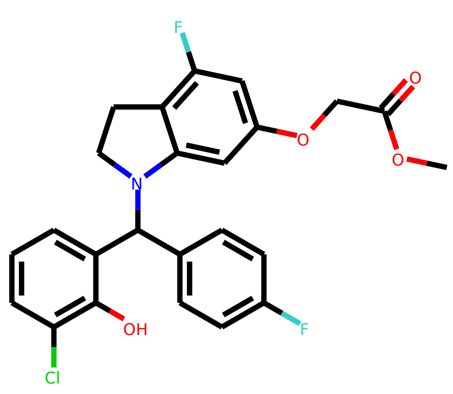

Osma-1201

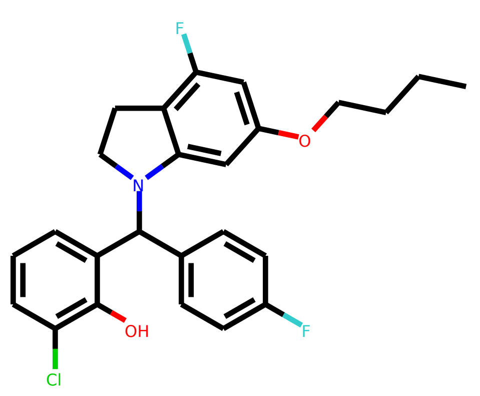

Osma-1202

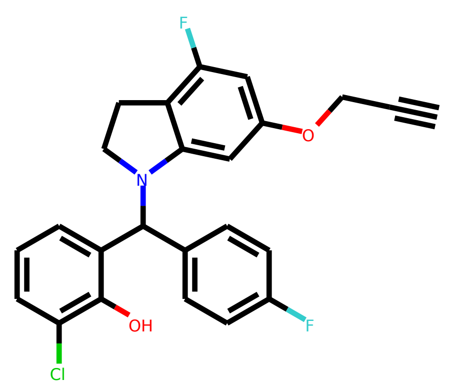

Osma-1203

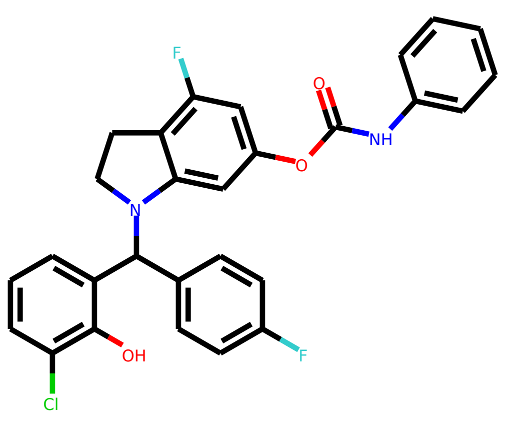

Osma-1204

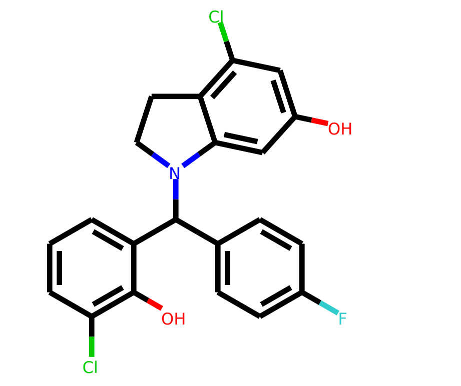

Osma-1205

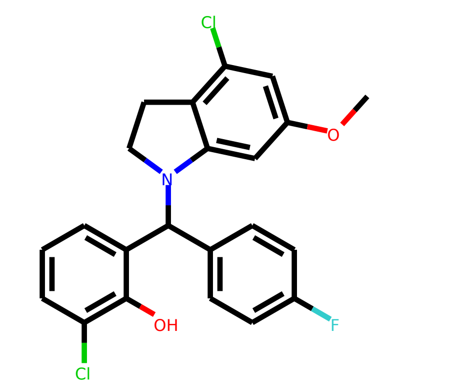

Osma-1206

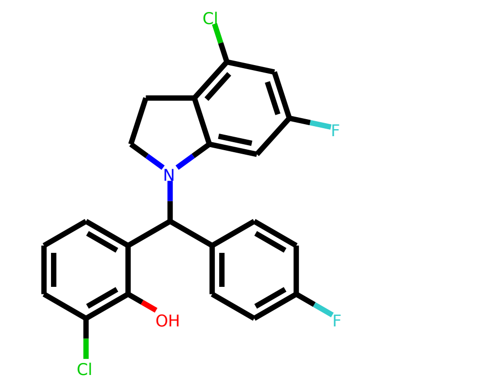

Osma-1207

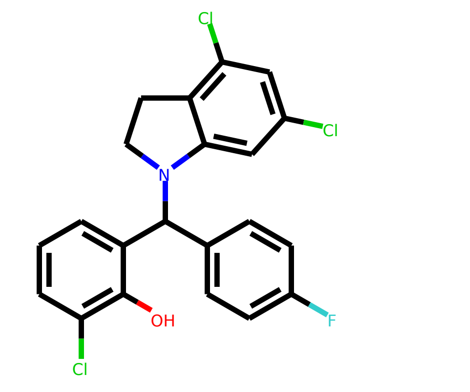

Osma-1208

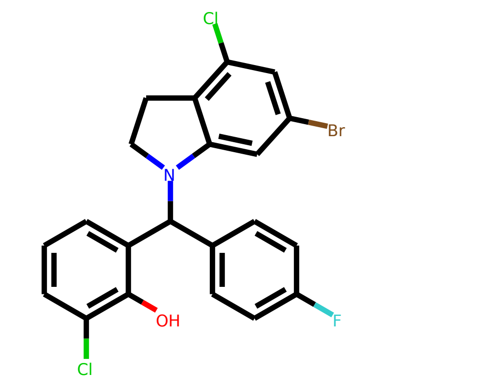

Osma-1209

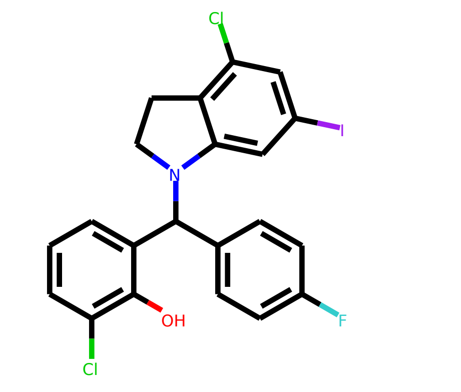

Osma-1210

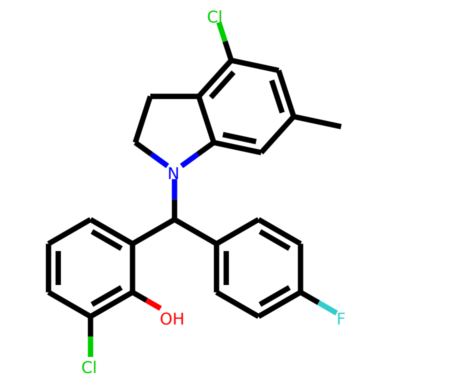

Osma-1211

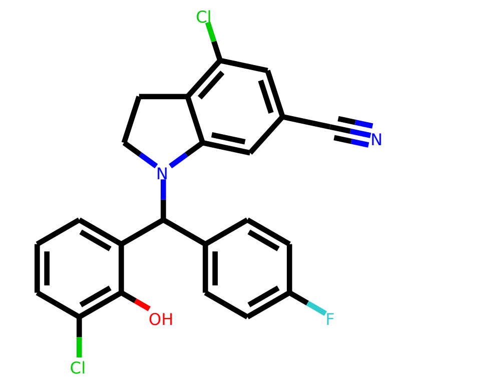

Osma-1212

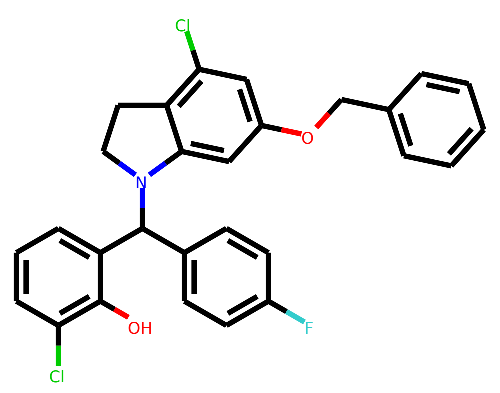

Osma-1213

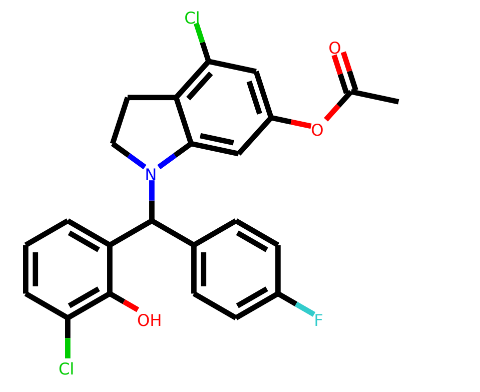

Osma-1214

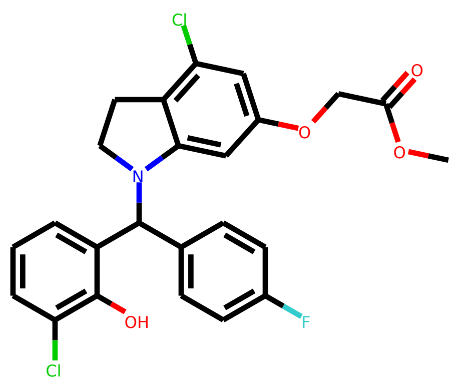

Osma-1215

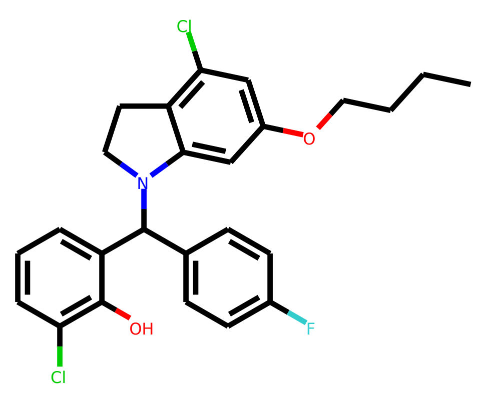

Osma-1216

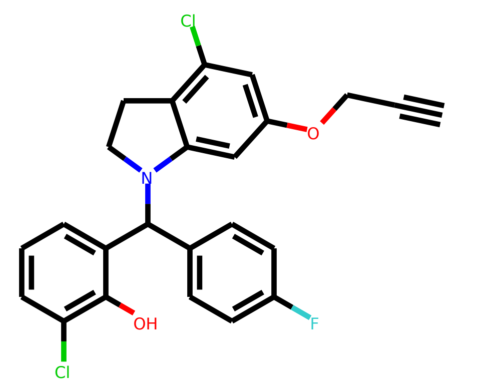

Osma-1217

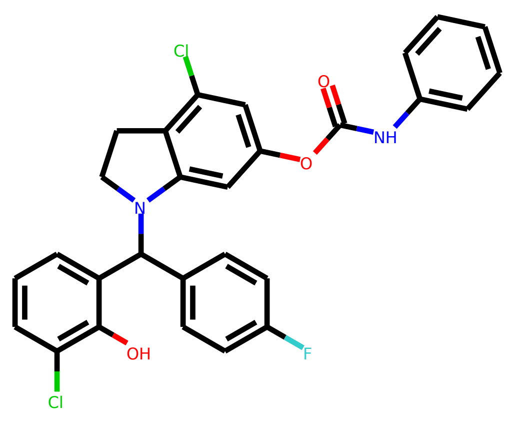

Osma-1218

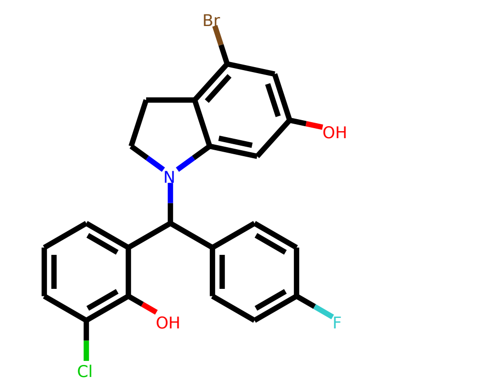

Osma-1219

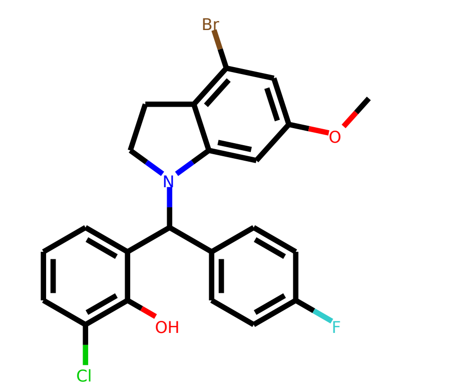

Osma-1220

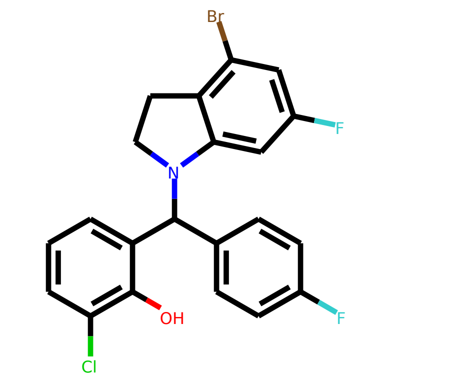

Osma-1221

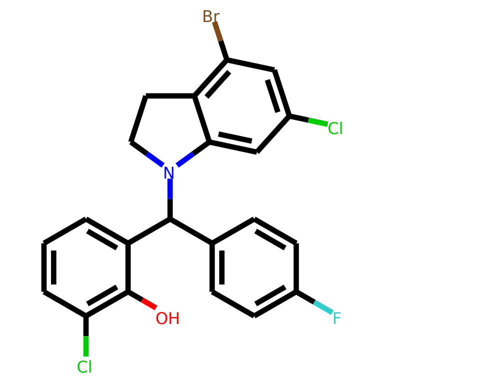

Osma-1222

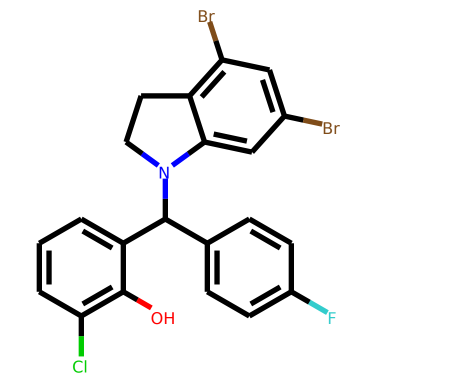

Osma-1223

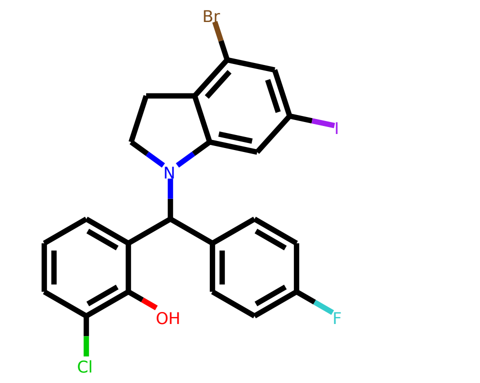

Osma-1224

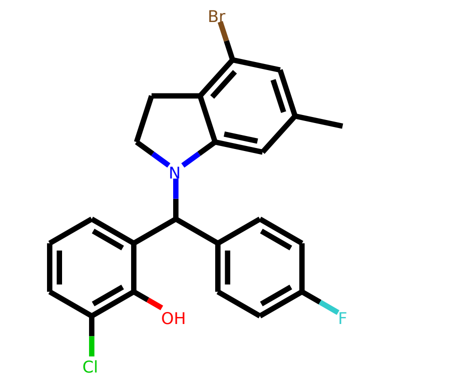

Osma-1225

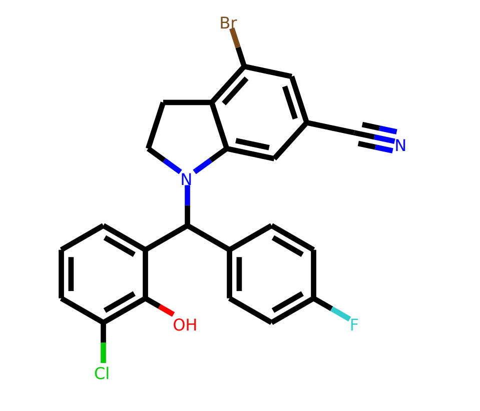

Osma-1226

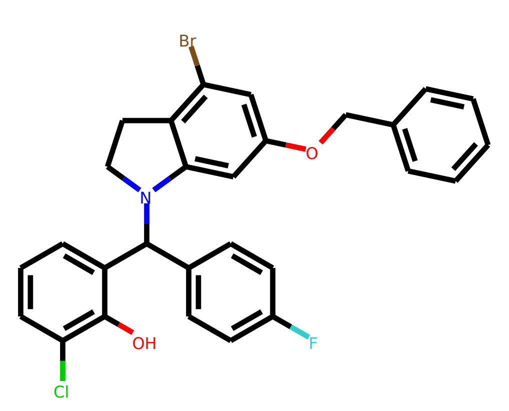

Osma-1227

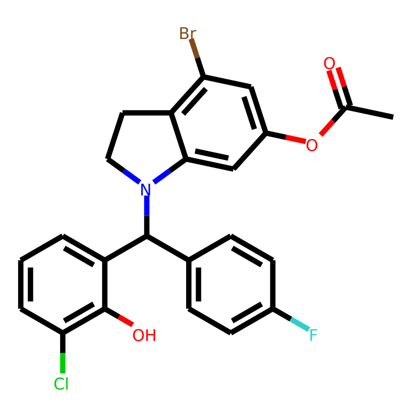

Osma-1228

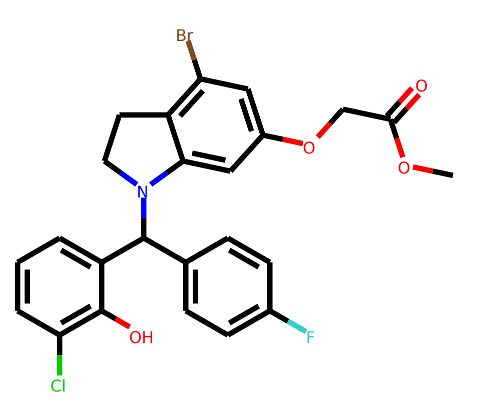

Osma-1229

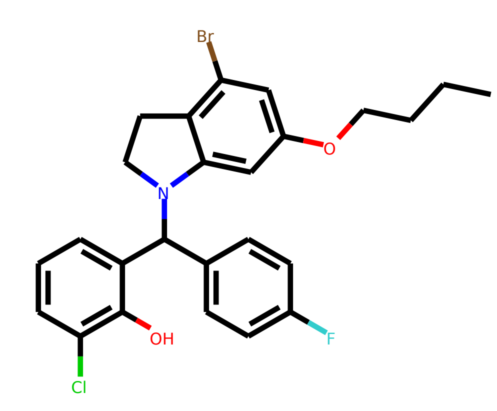

Osma-1230

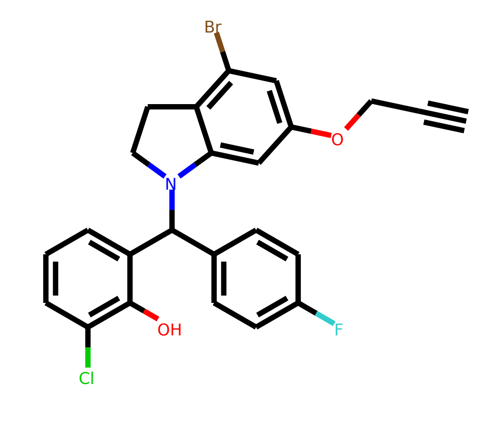

Osma-1231

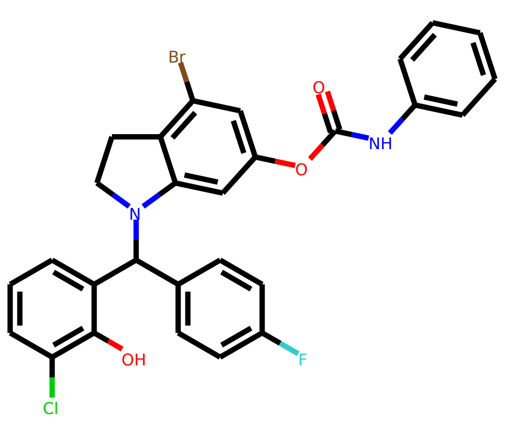

Osma-1232

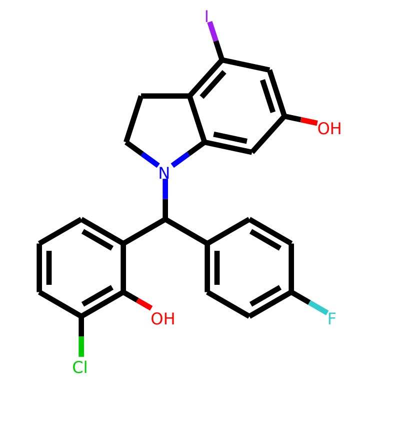

Osma-1233

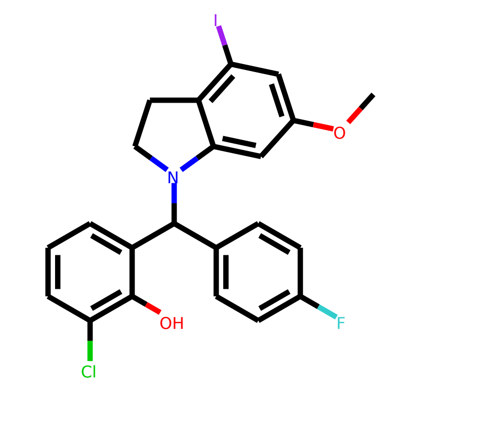

Osma-1234

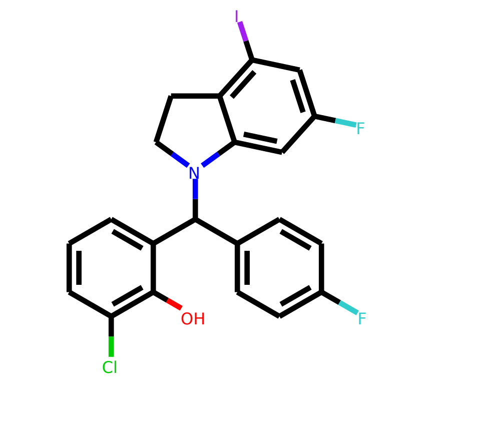

Osma-1235

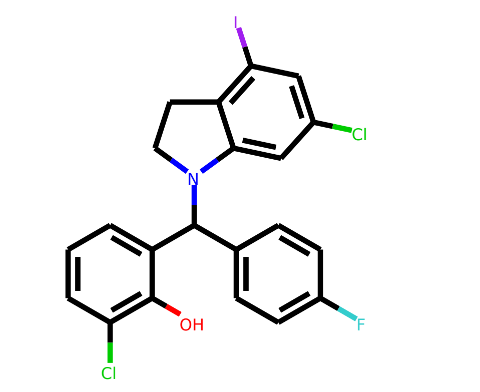

Osma-1236

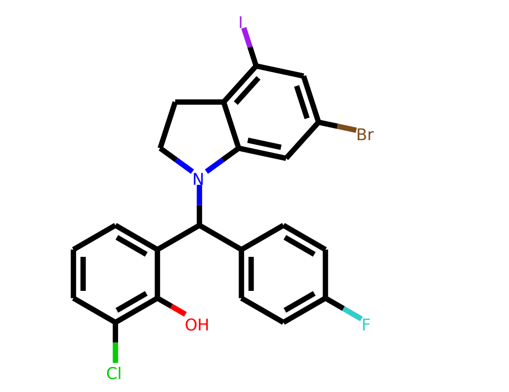

Osma-1237

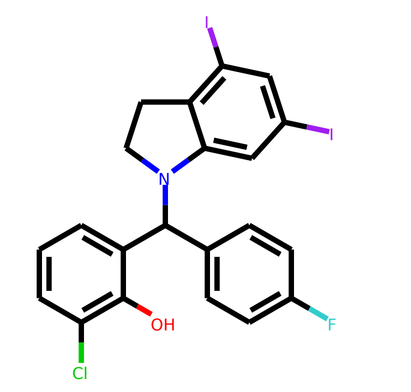

Osma-1238

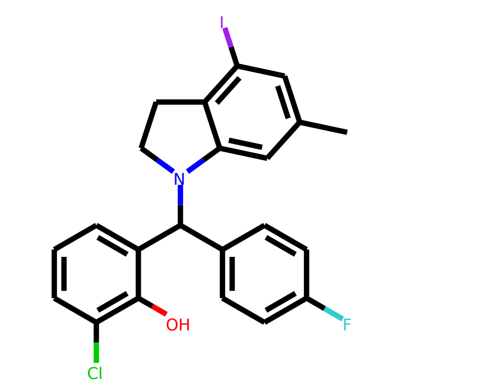

Osma-1239

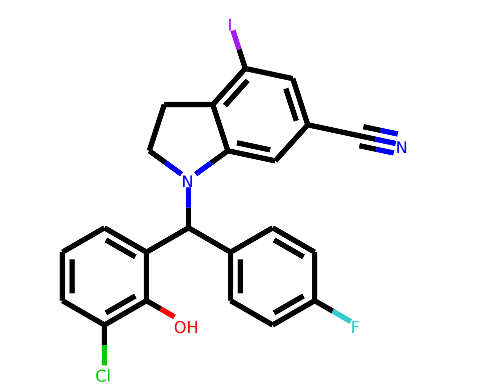

Osma-1240

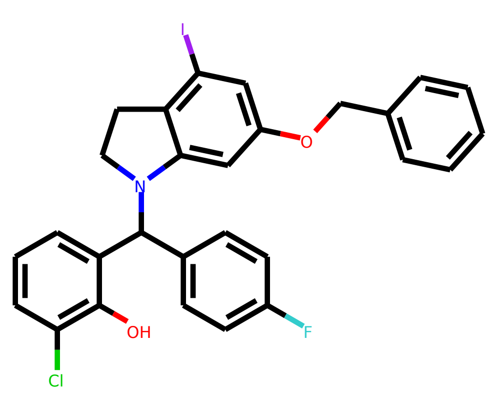

Osma-1241

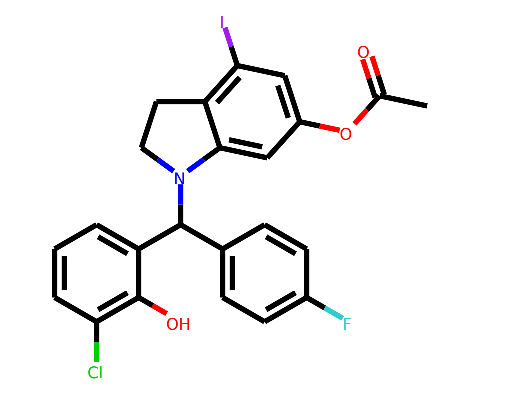

Osma-1242

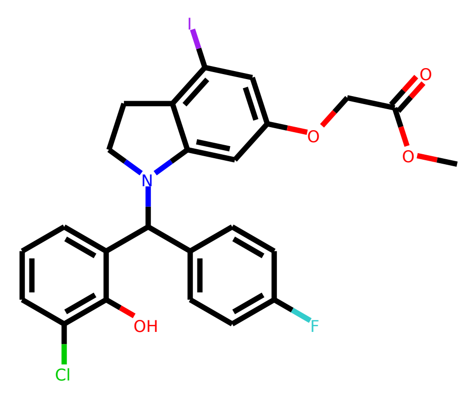

Osma-1243

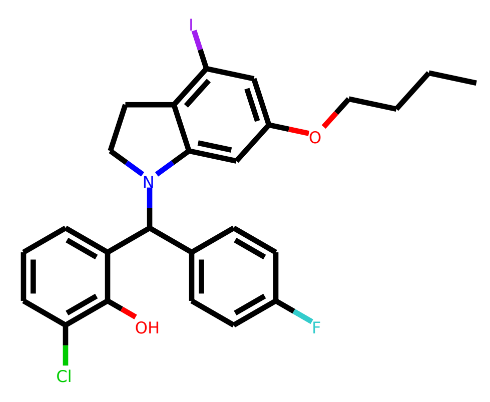

Osma-1244

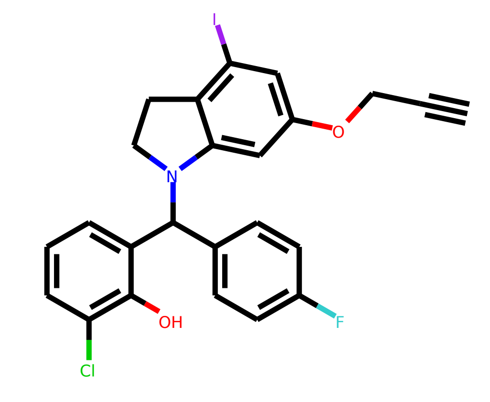

Osma-1245

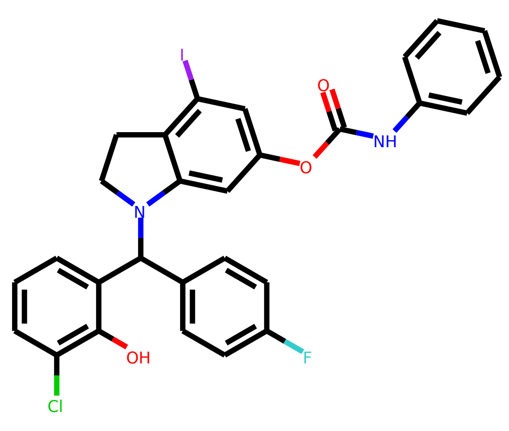

Osma-1246

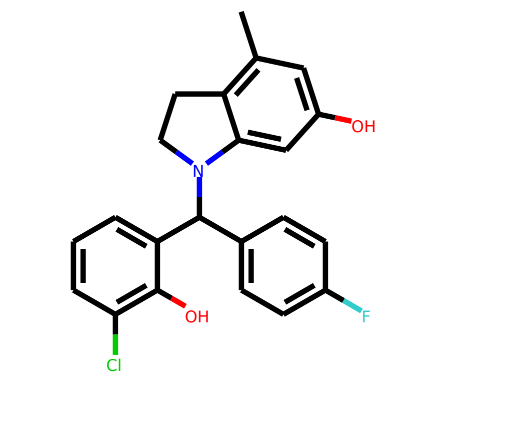

Osma-1247

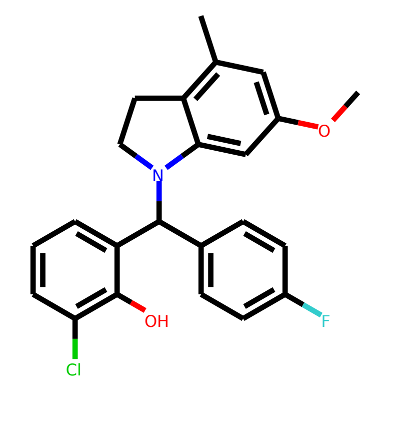

Osma-1248

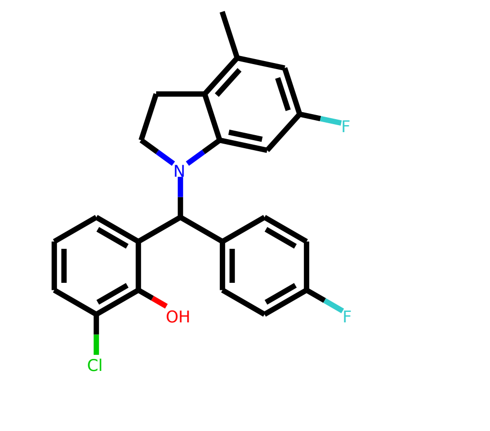

Osma-1249

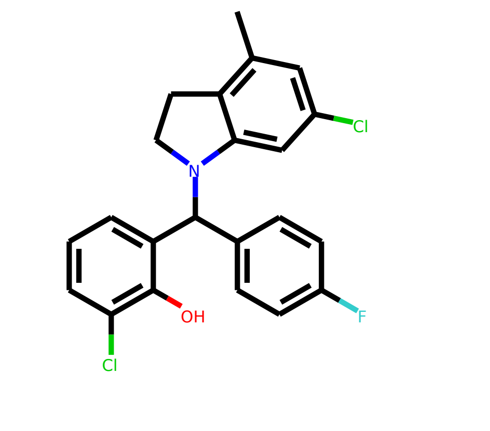

Osma-1250

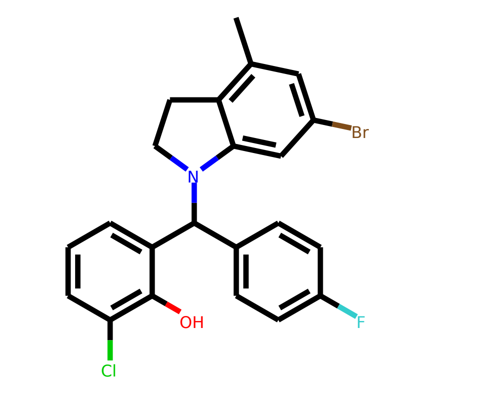

Osma-1251

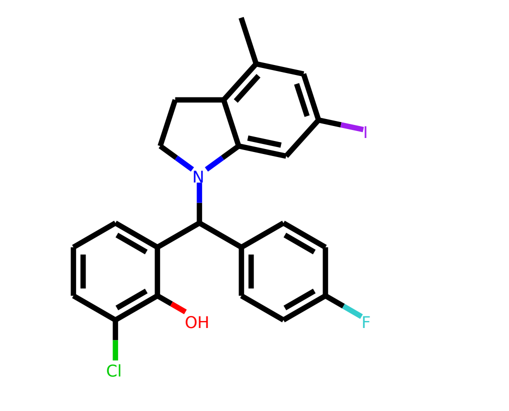

Osma-1252

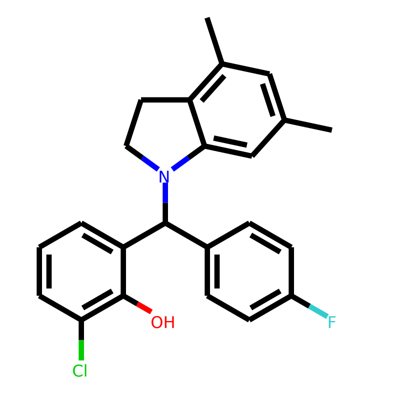

Osma-1253

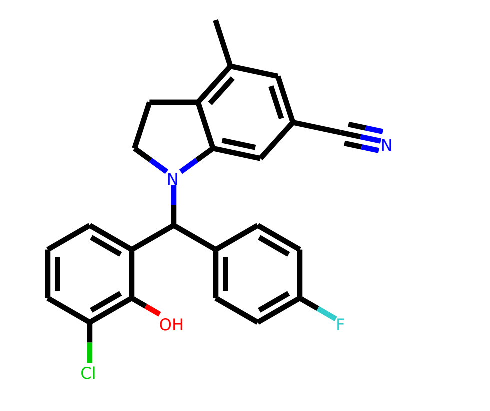

Osma-1254

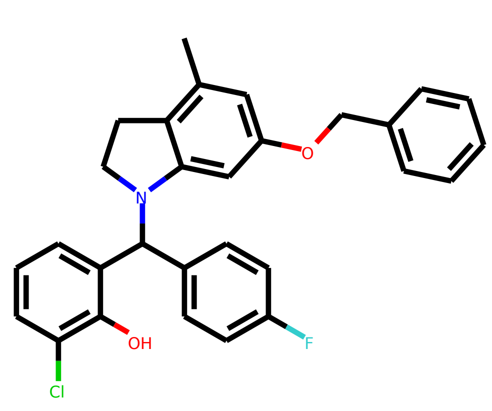

Osma-1255

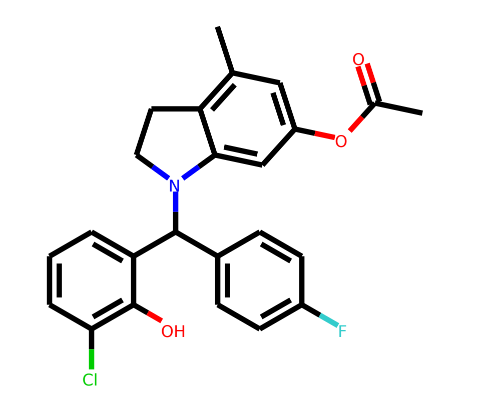

Osma-1256

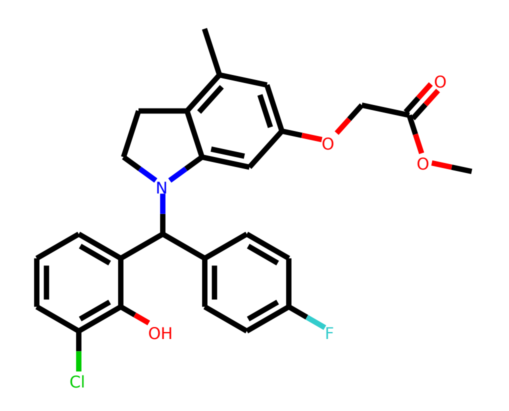

Osma-1257

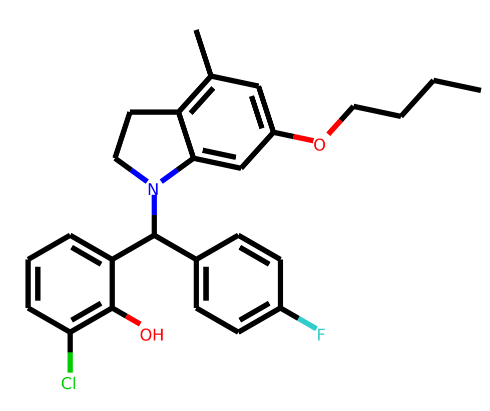

Osma-1258

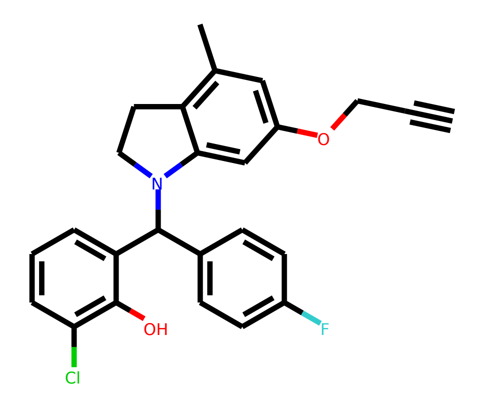

Osma-1259

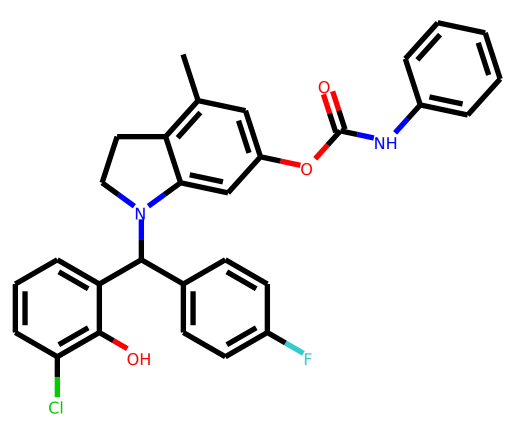

Osma-1260

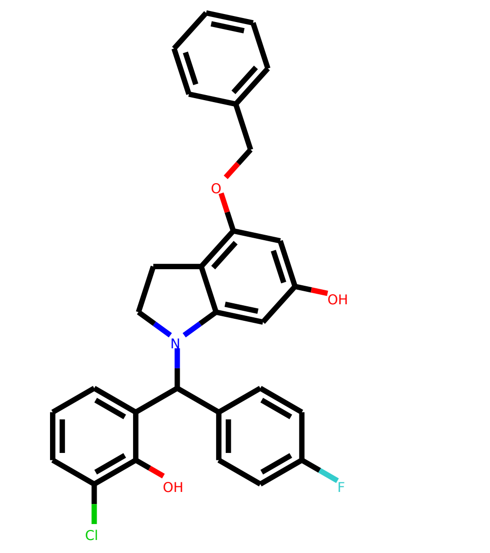
Osma-1261
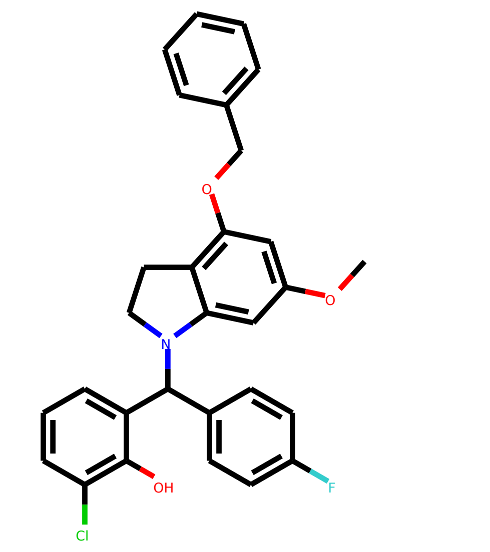
Osma-1262
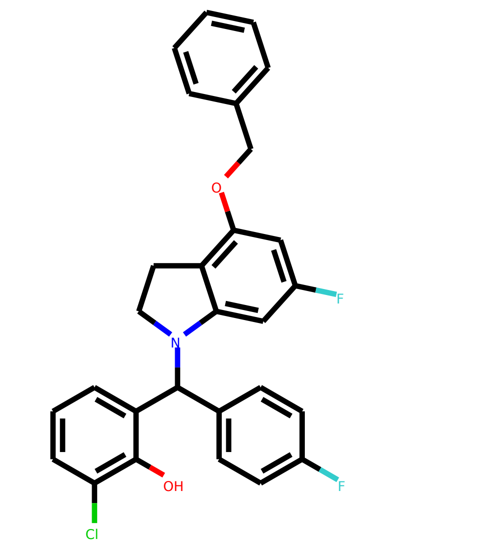
Osma-1263
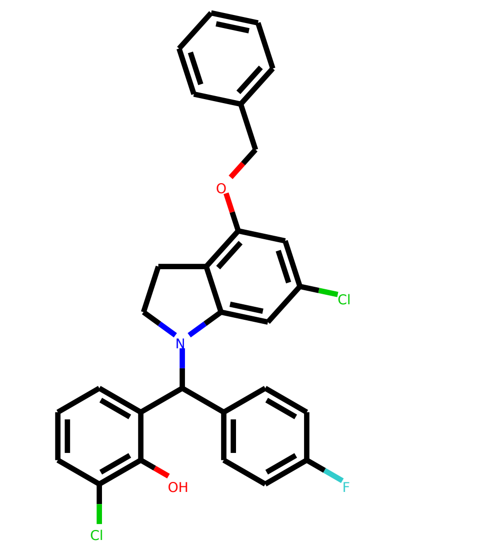
Osma-1264
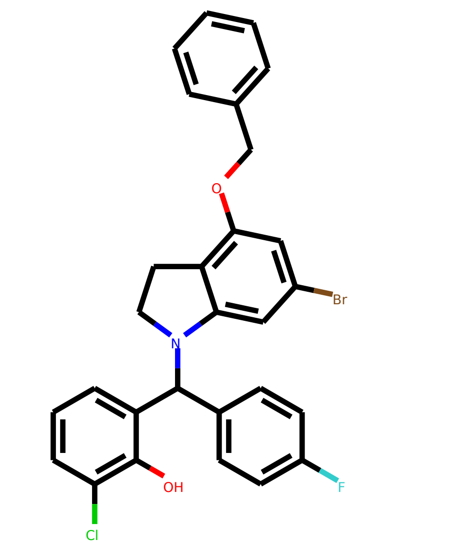
Osma-1265
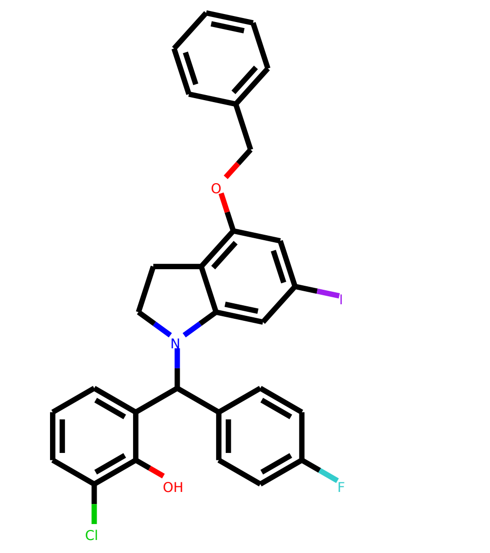
Osma-1266
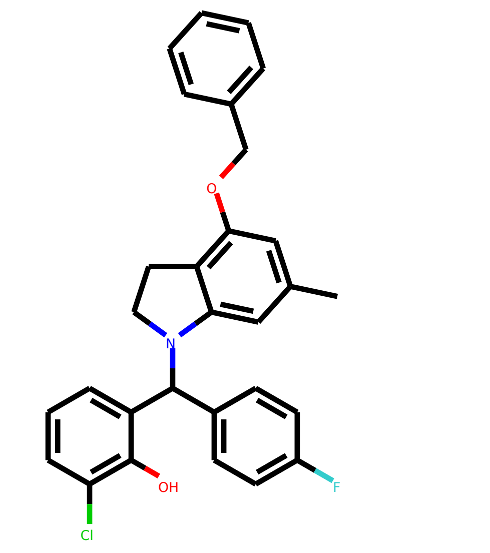
Osma-1267
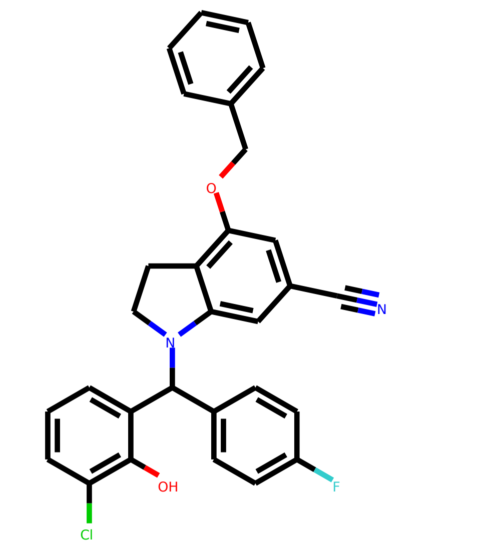
Osma-1268
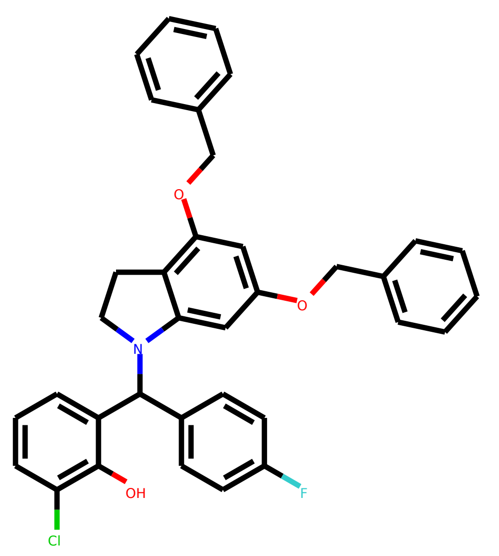
Osma-1269
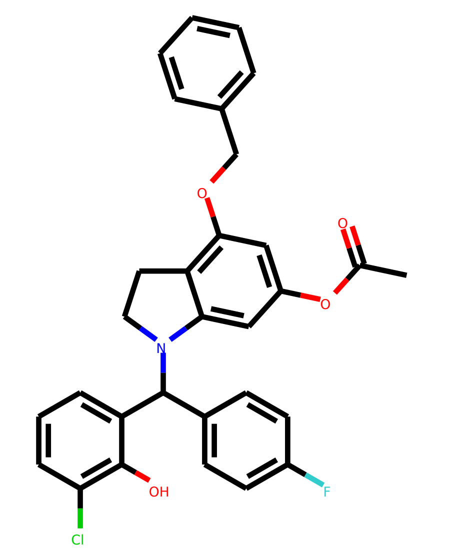
Osma-1270
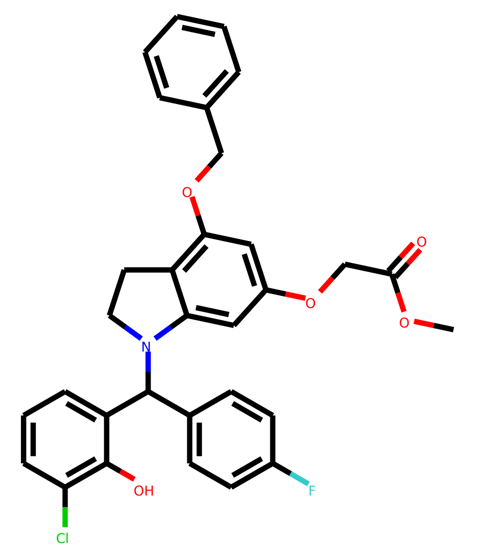
Osma-1271
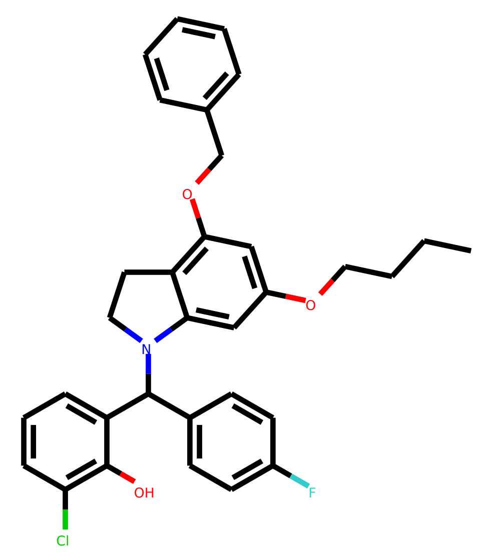
Osma-1272
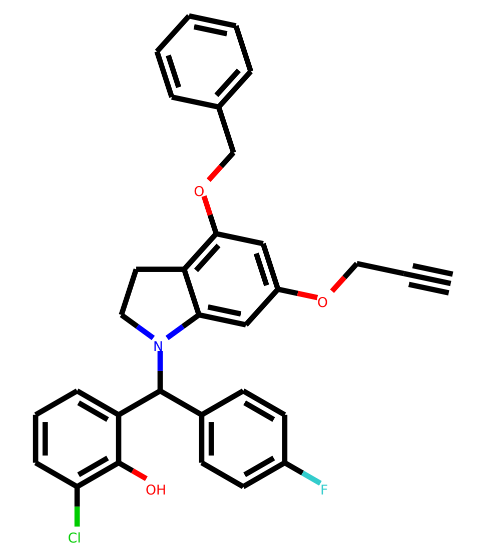
Osma-1273
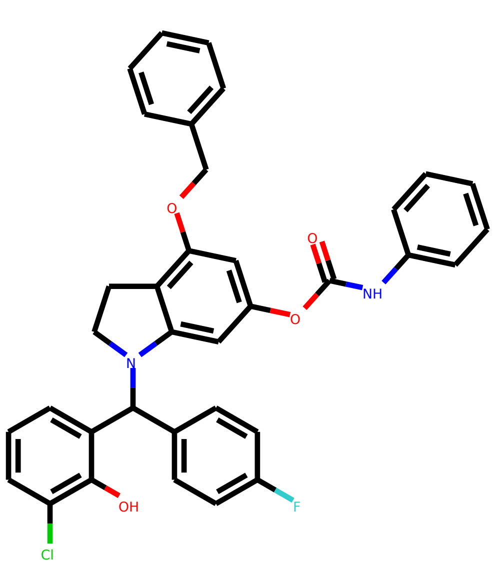
Osma-1274
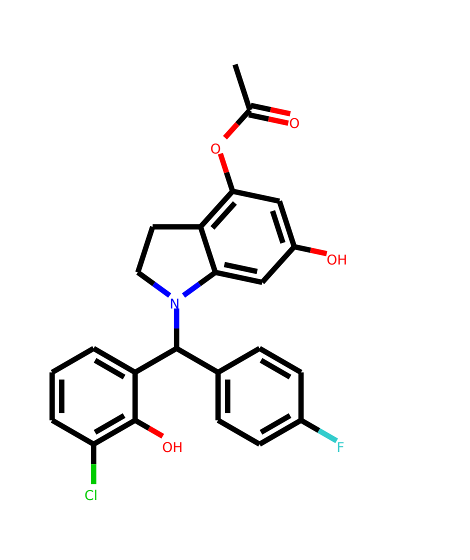
Osma-1275
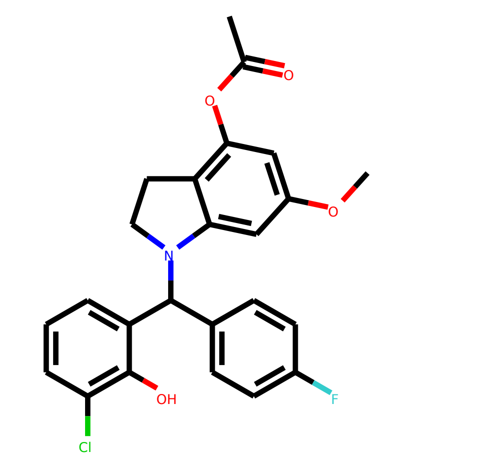
Osma-1276
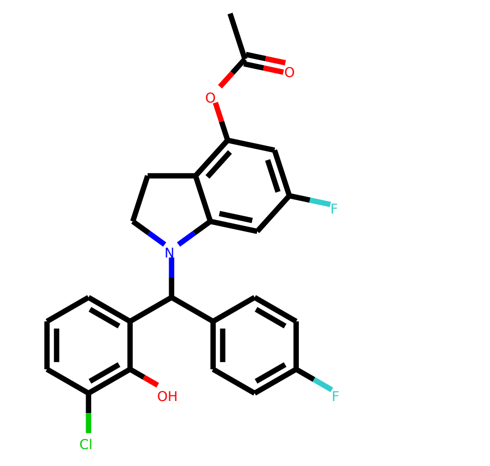
Osma-1277
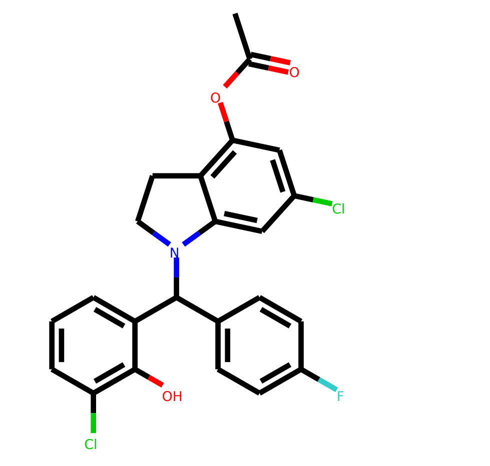
Osma-1278
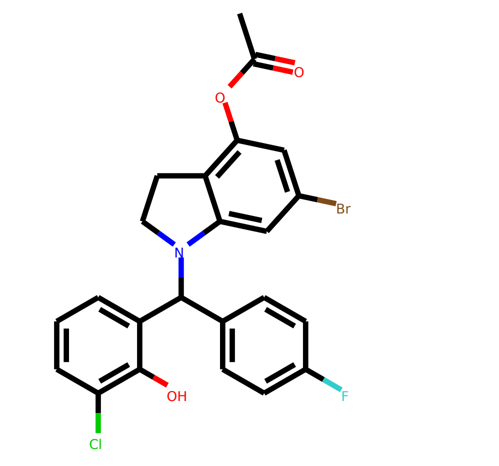
Osma-1279
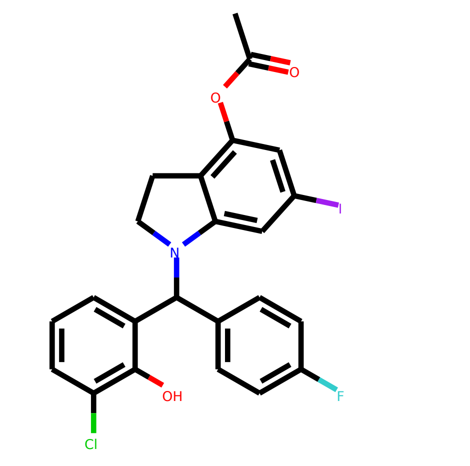
Osma-1280
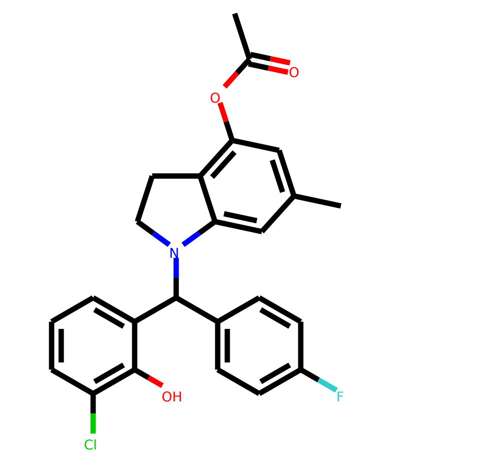
Osma-1281
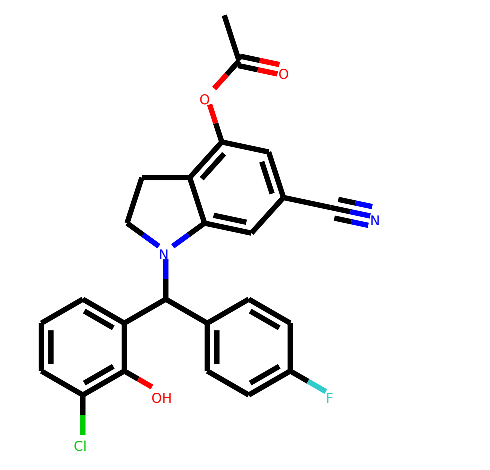
Osma-1282
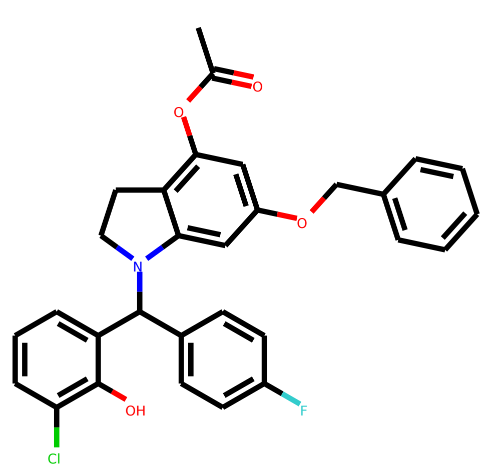
Osma-1283
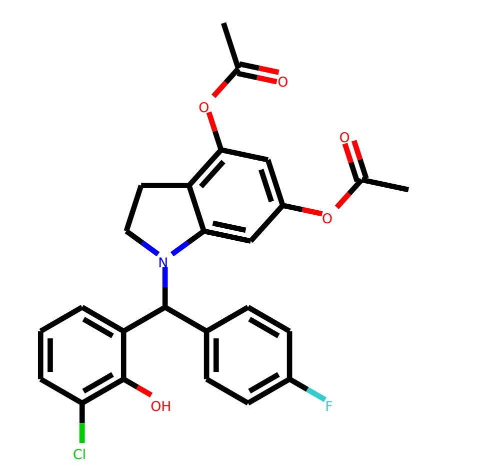
Osma-1284
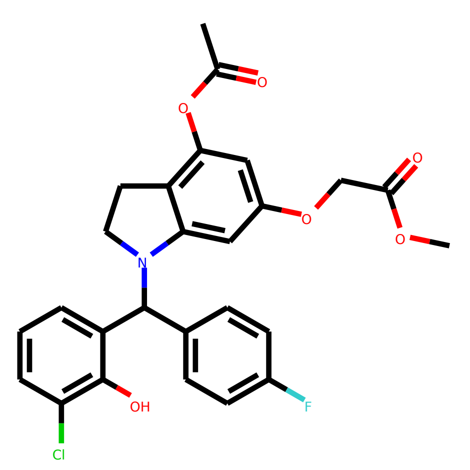
Osma-1285
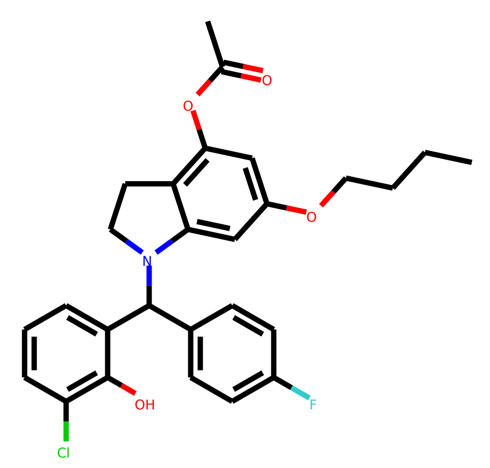
Osma-1286
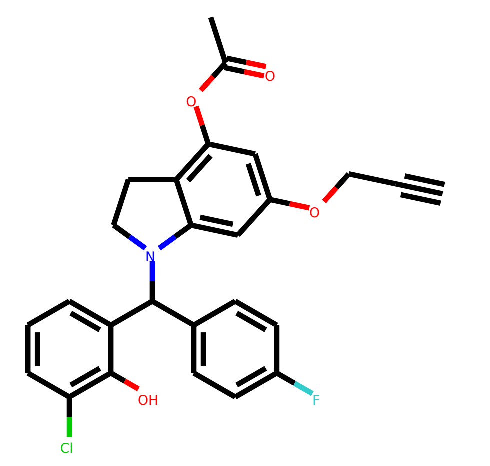
Osma-1287
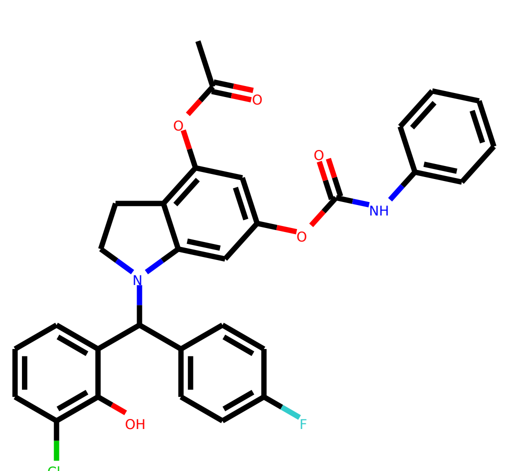
Osma-1288
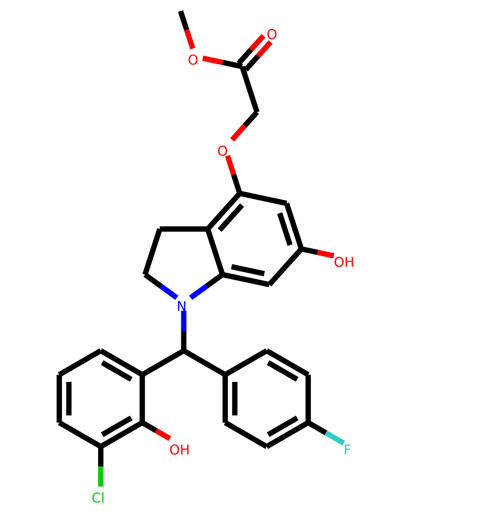
Osma-1289
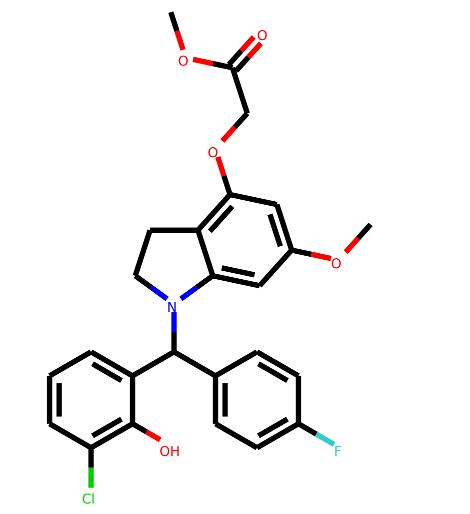
Osma-1290
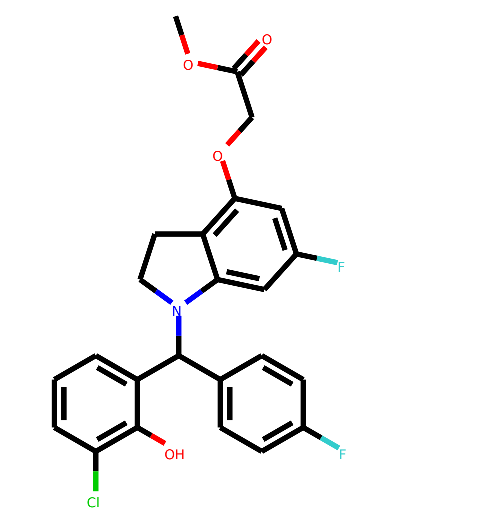
Osma-1291
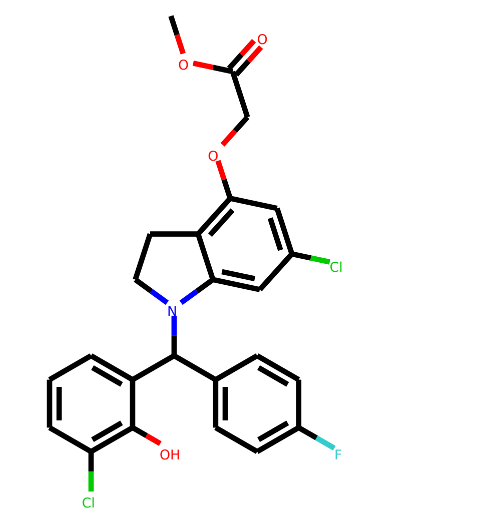
Osma-1292
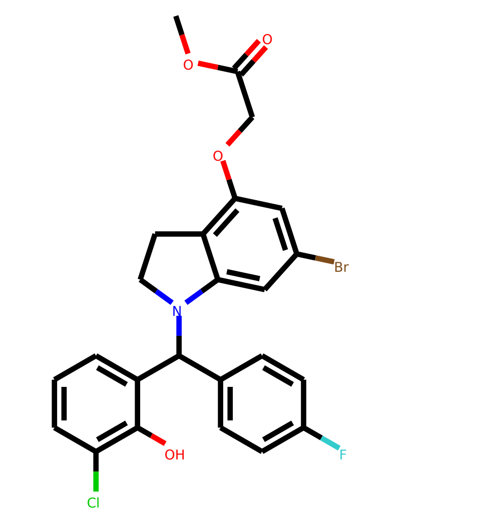
Osma-1293
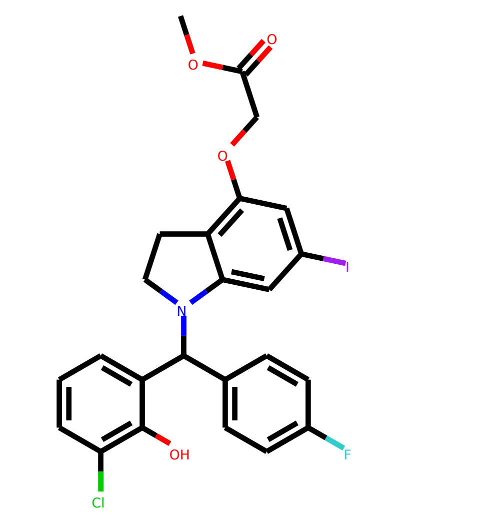
Osma-1294
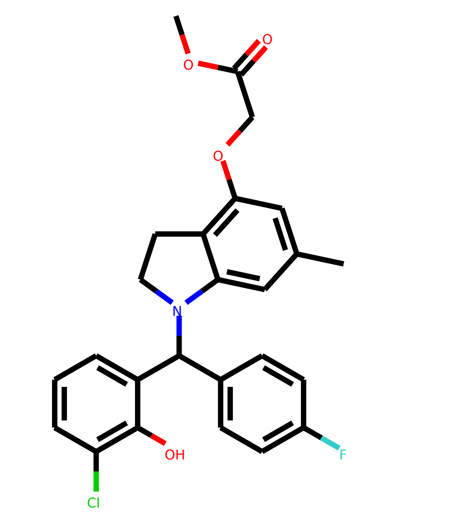
Osma-1295

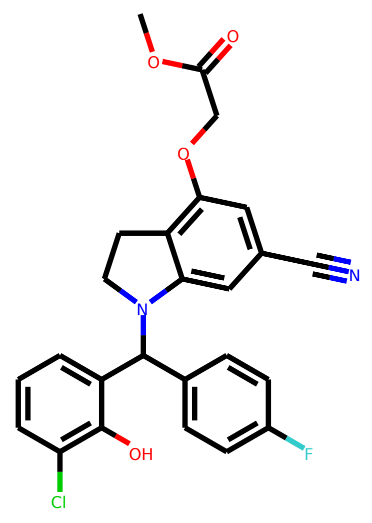
Osma-1296

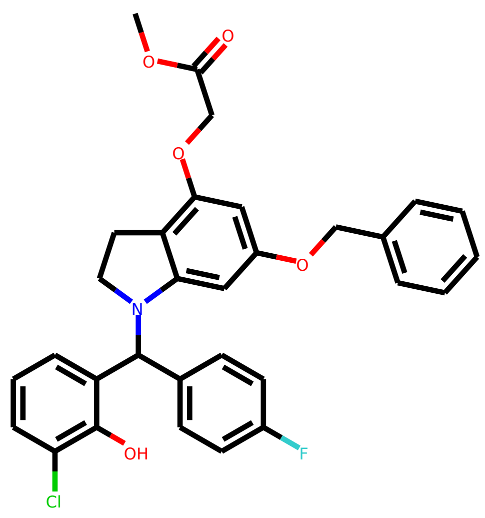
Osma-1297

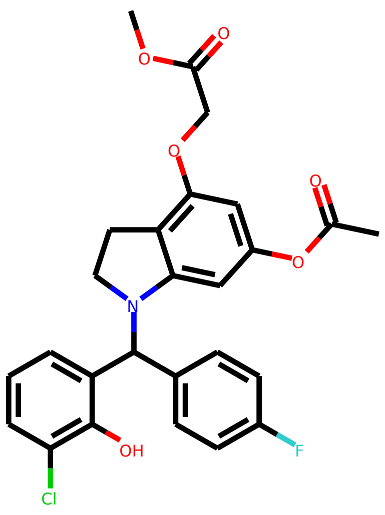
Osma-1298

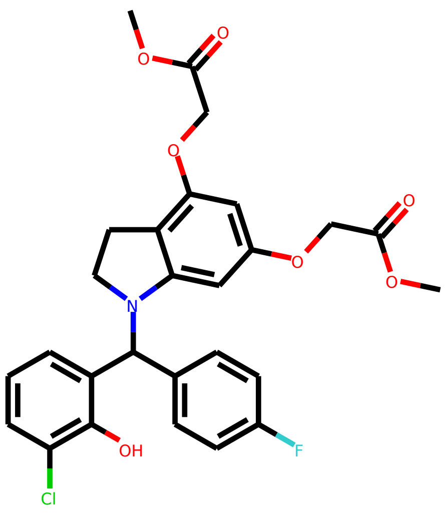
Osma-1299

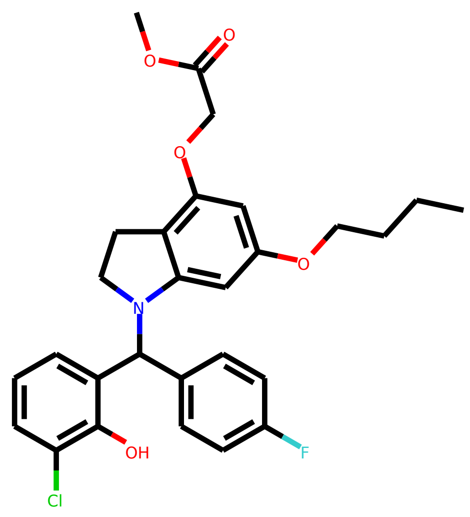
Osma-1300

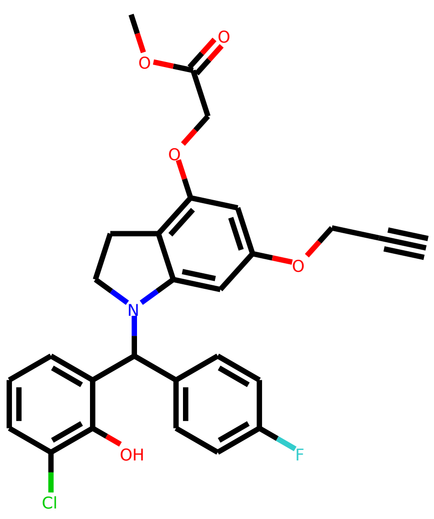
Osma-1301

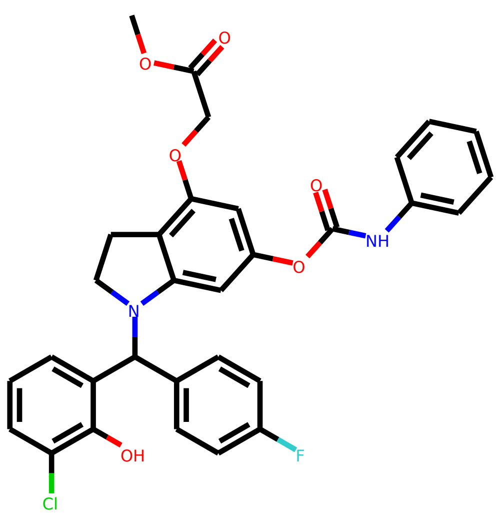
Osma-1302

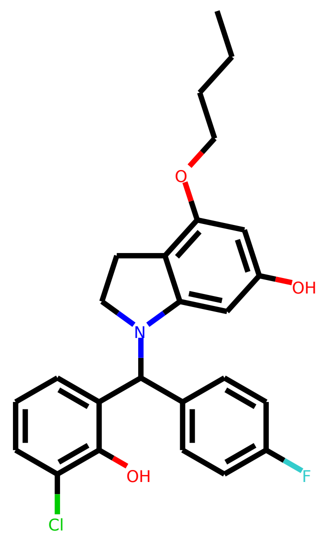
Osma-1303

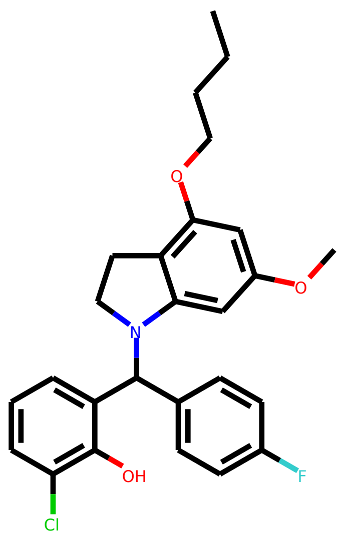
Osma-1304

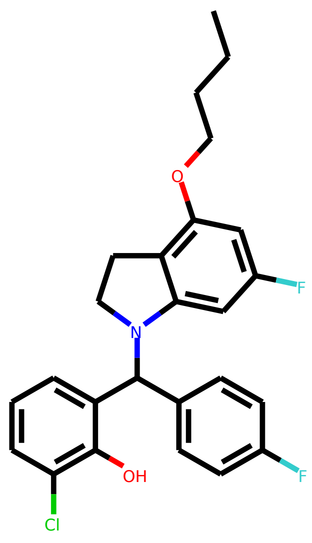
Osma-1305

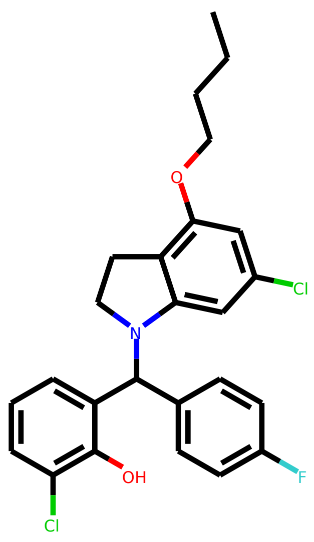
Osma-1306

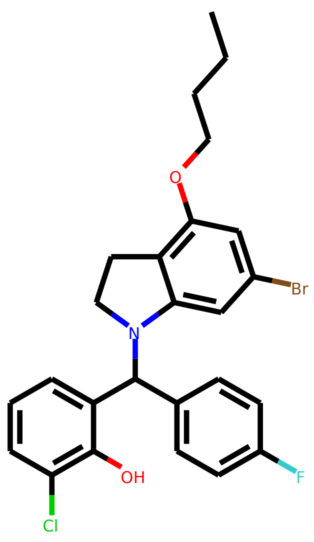
Osma-1307

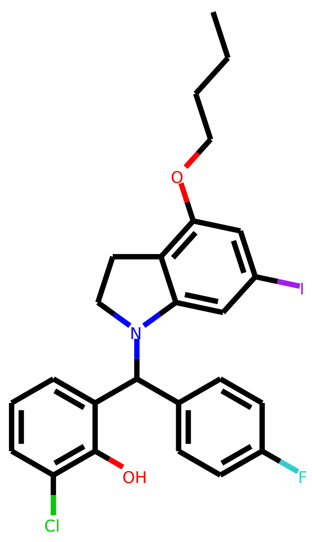
Osma-1308

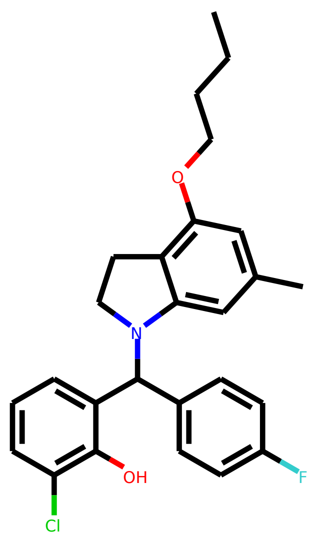
Osma-1309

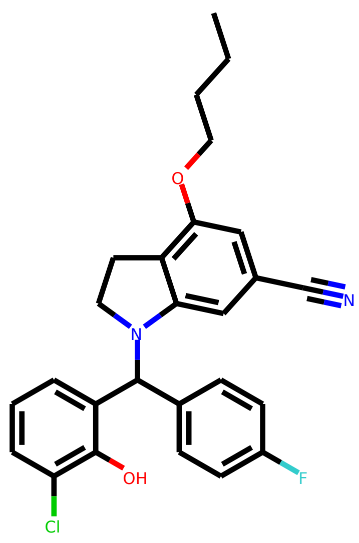
Osma-1310

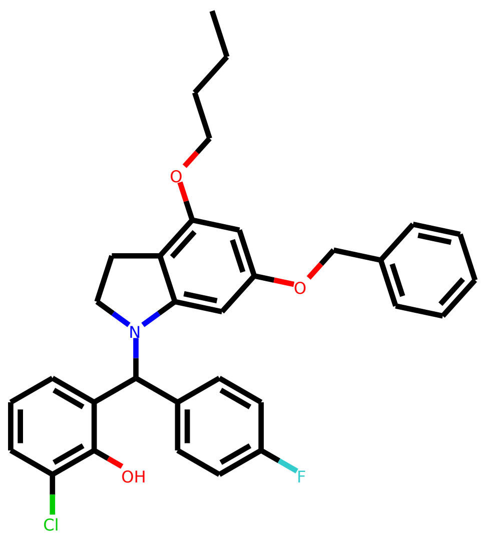
Osma-1311

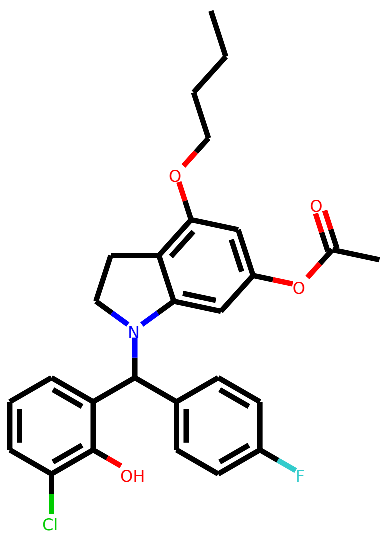
Osma-1312

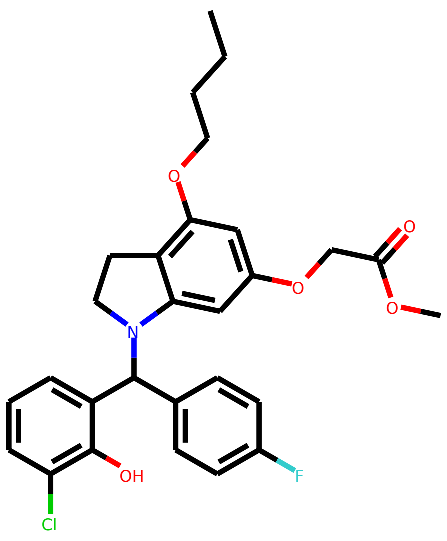
Osma-1313

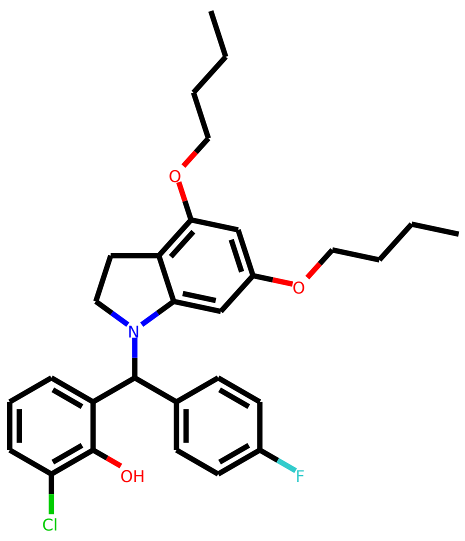
Osma-1314

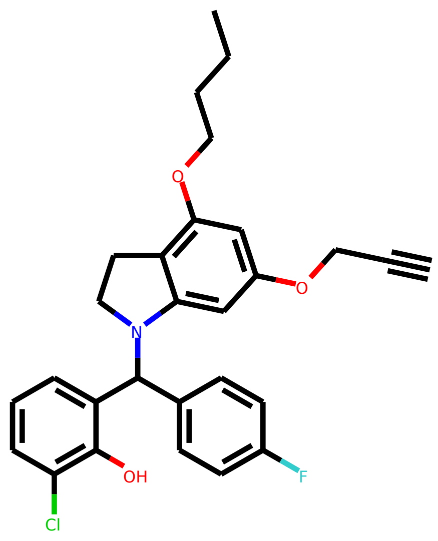
Osma-1315

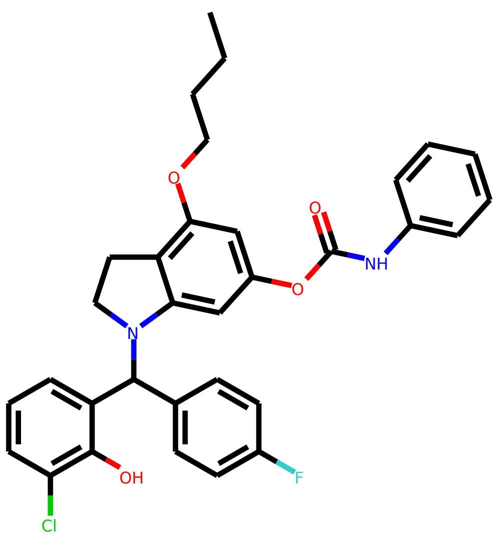
Osma-1316

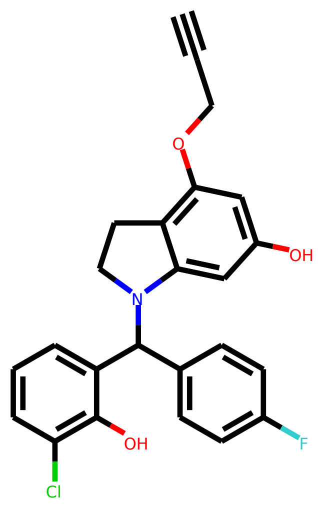
Osma-1317

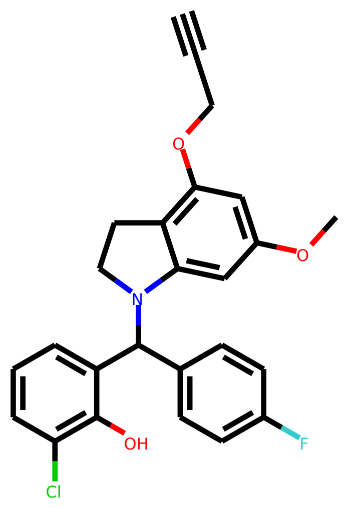
Osma-1318

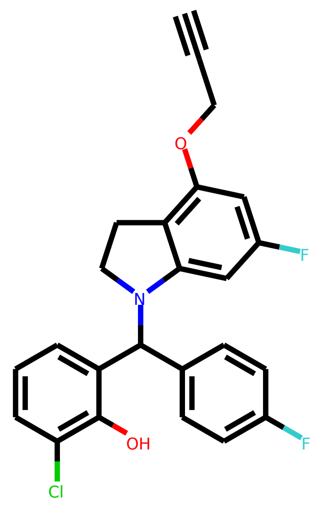
Osma-1319

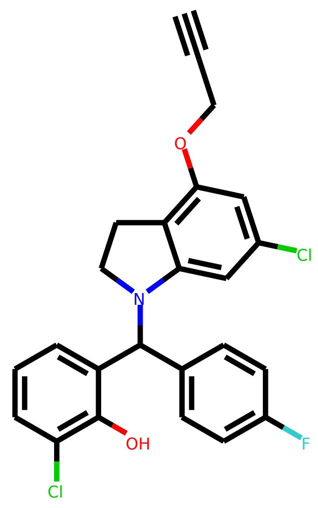
Osma-1320

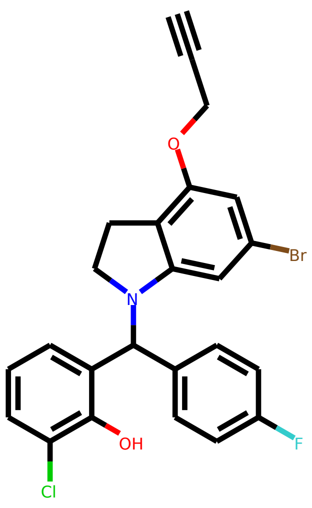
Osma-1321

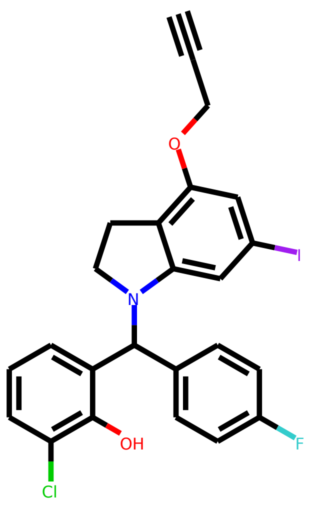
Osma-1322

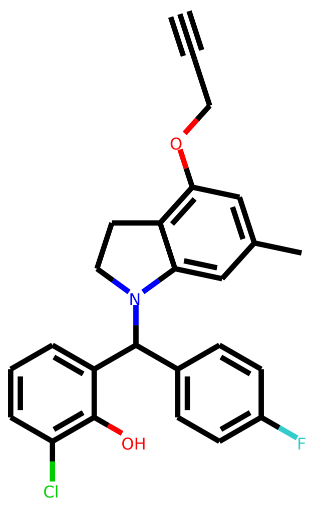
Osma-1323

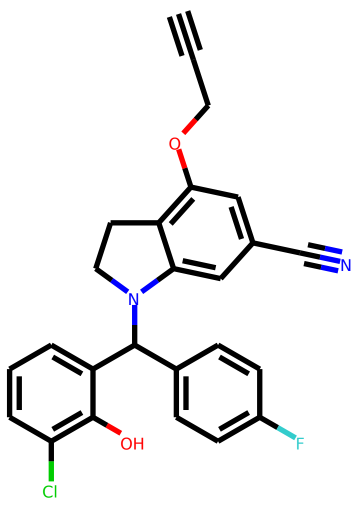
Osma-1324

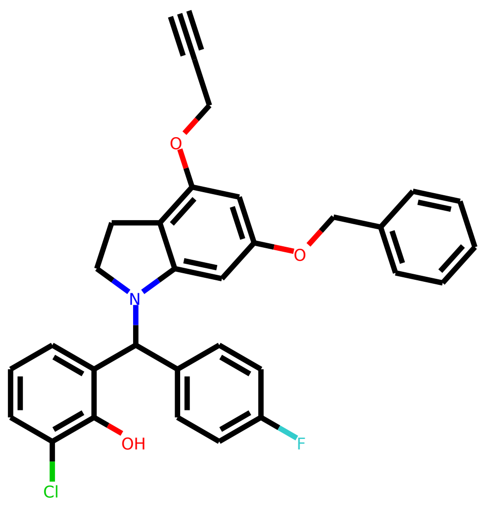
Osma-1325

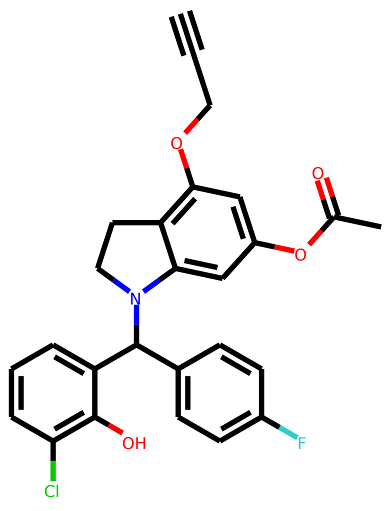
Osma-1326

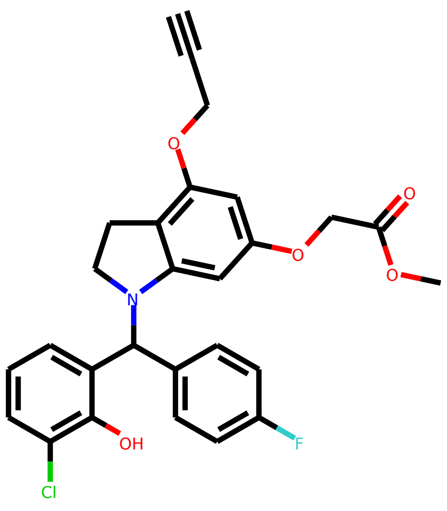
Osma-1327

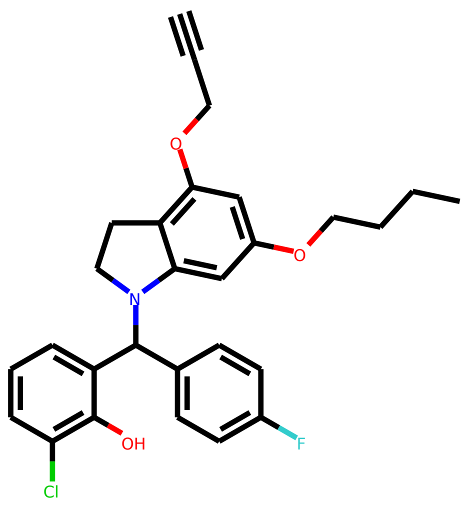
Osma-1328

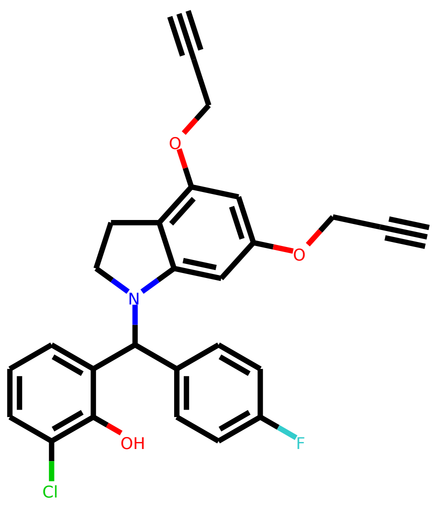
Osma-1329

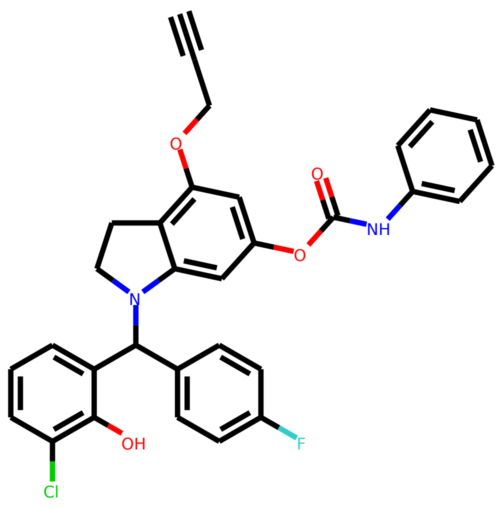
Osma-1330

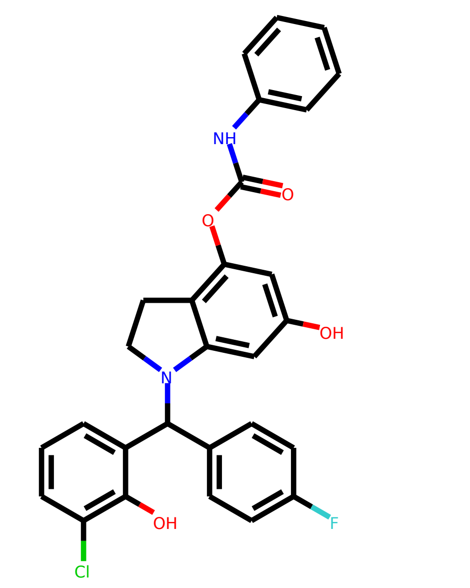
Osma-1331
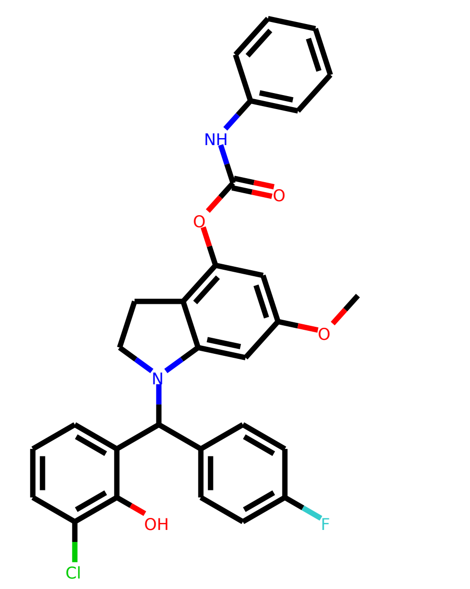
Osma-1332
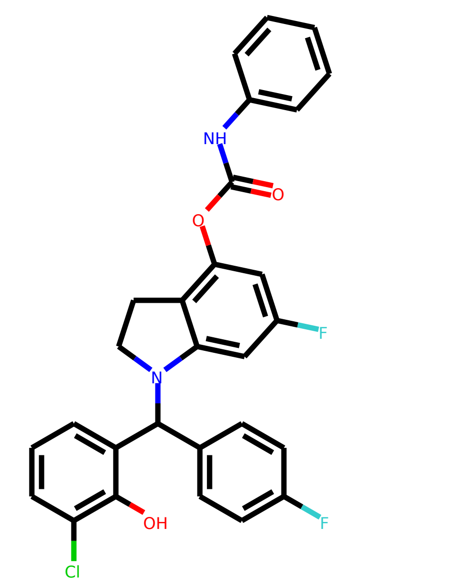
Osma-1333
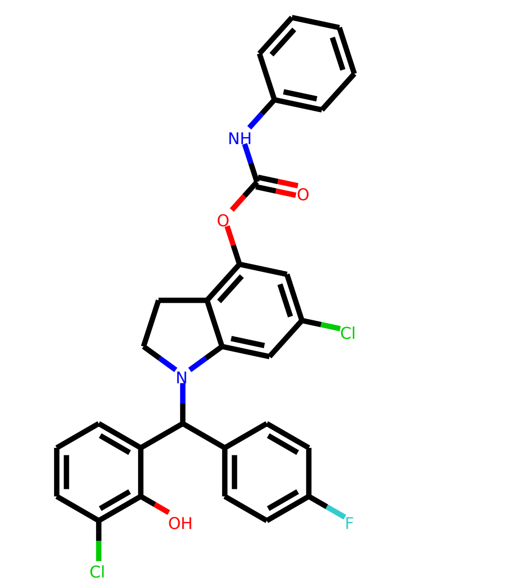
Osma-1334
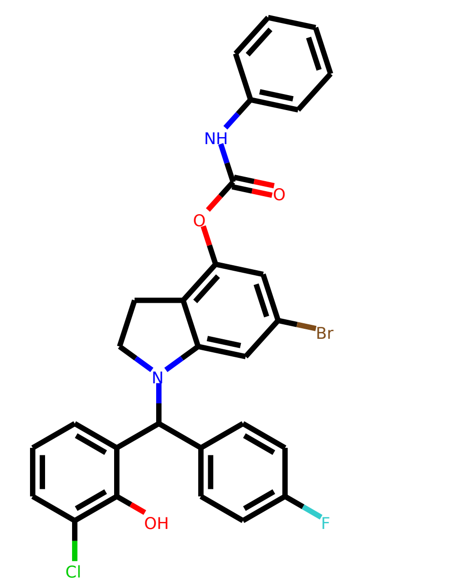
Osma-1335
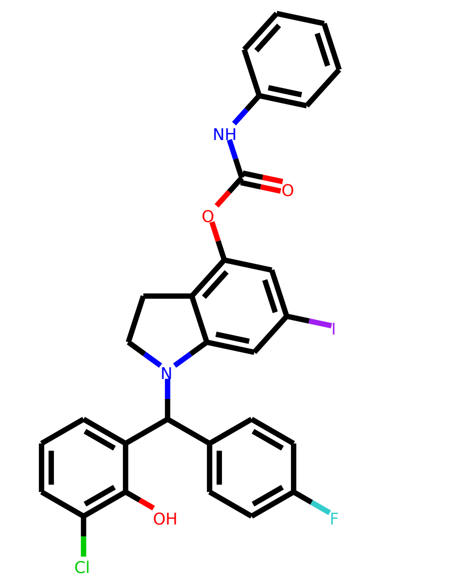
Osma-1336
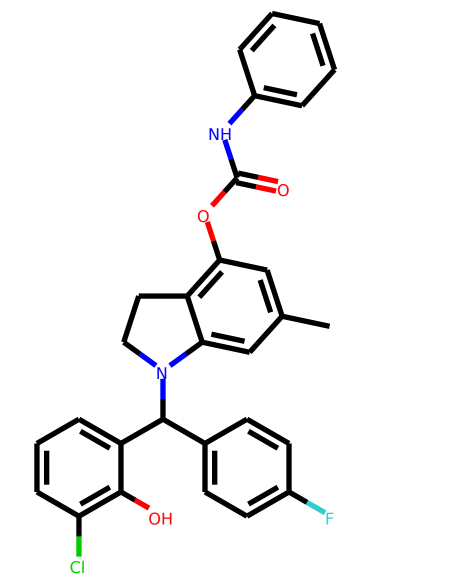
Osma-1337
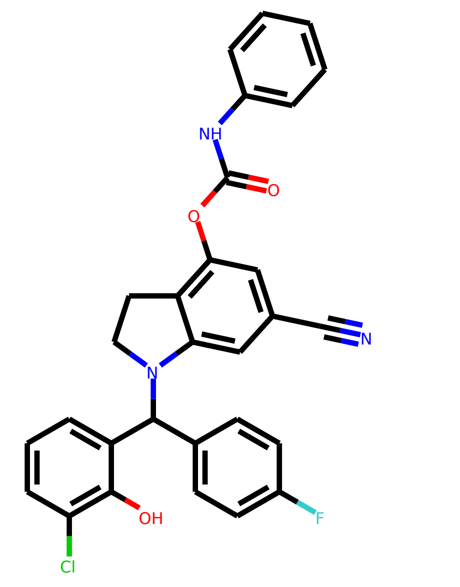
Osma-1338
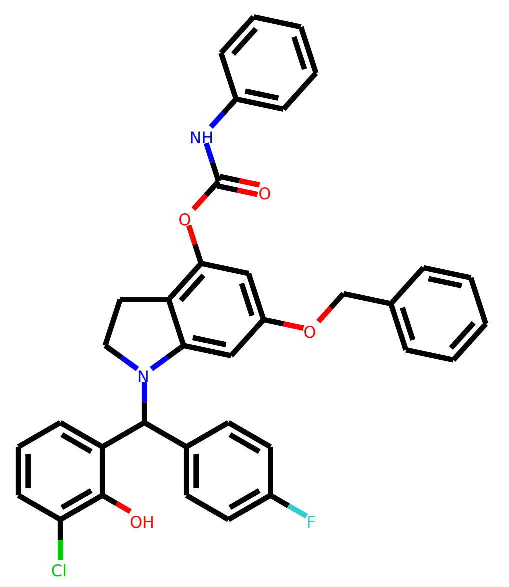
Osma-1339
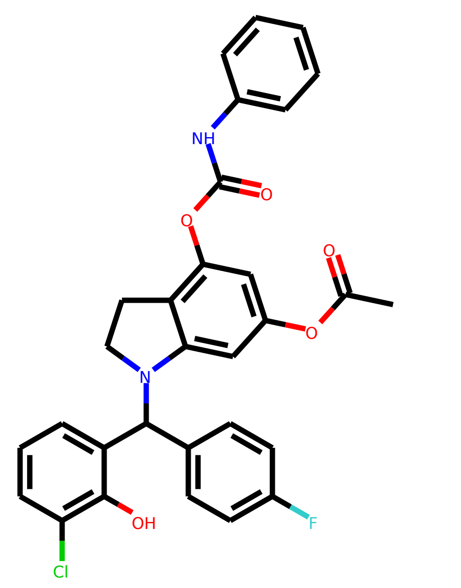
Osma-1340
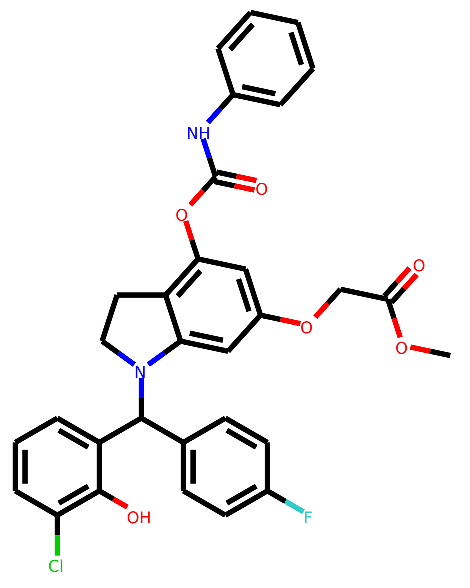
Osma-1341
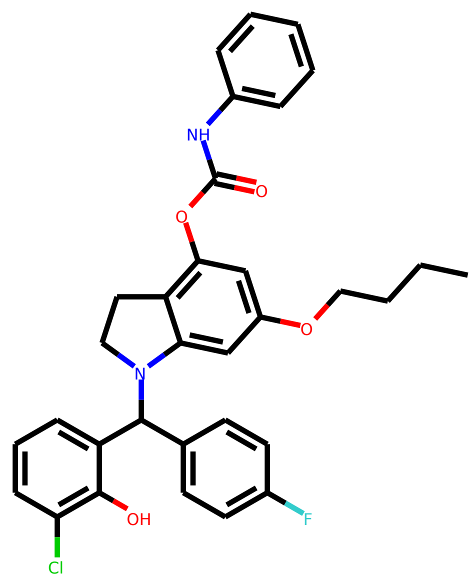
Osma-1342
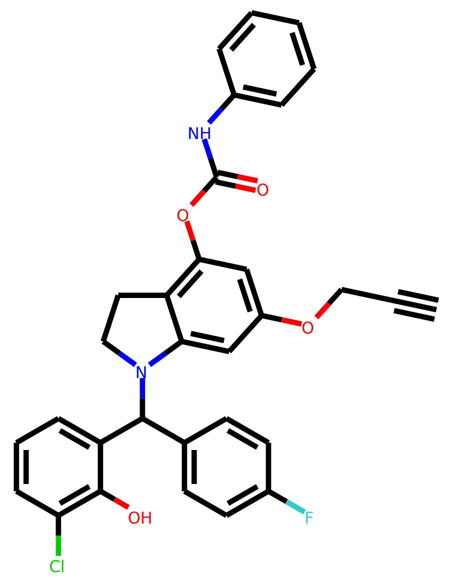
Osma-1343
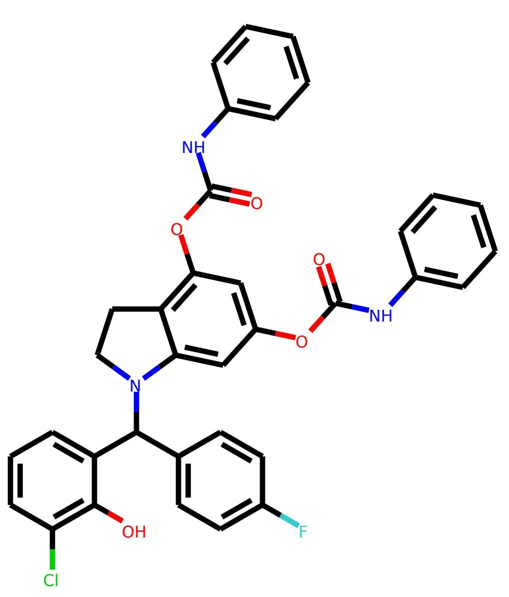
Osma-1344
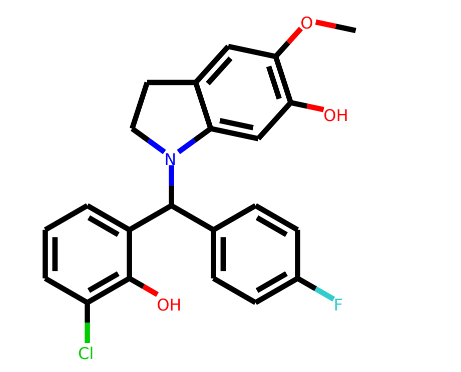
Osma-1345
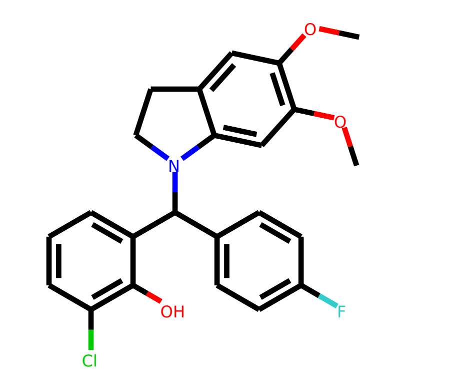
Osma-1346
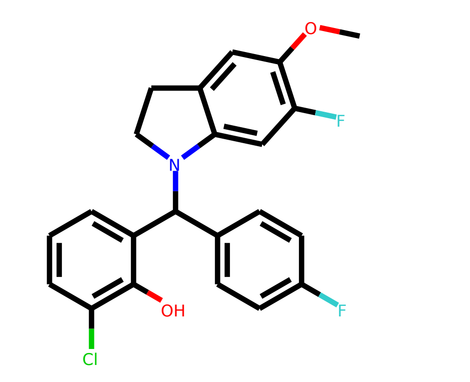
Osma-1347
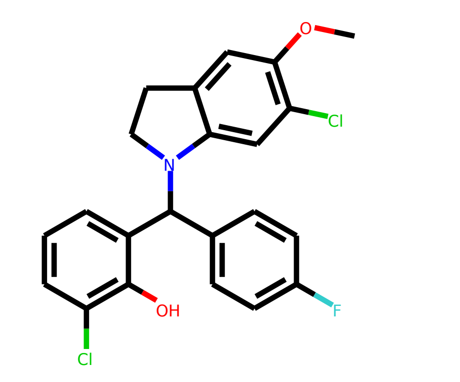
Osma-1348
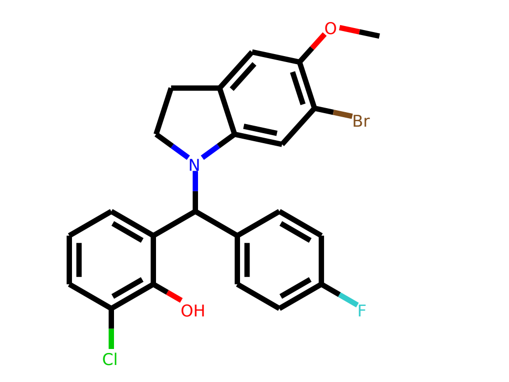
Osma-1349
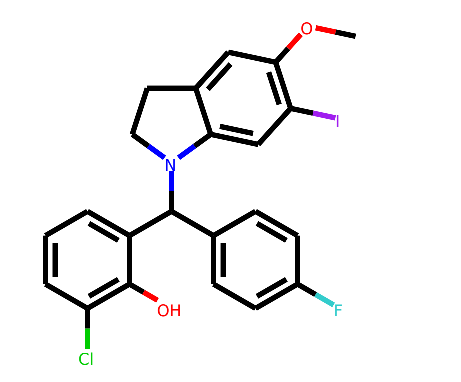
Osma-1350
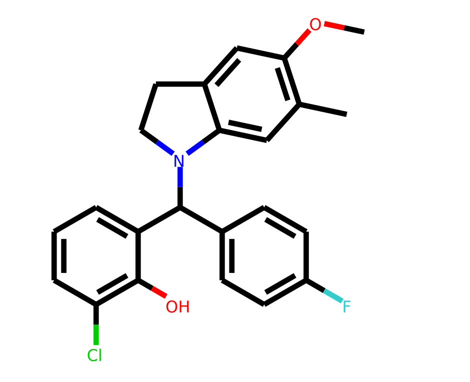
Osma-1351
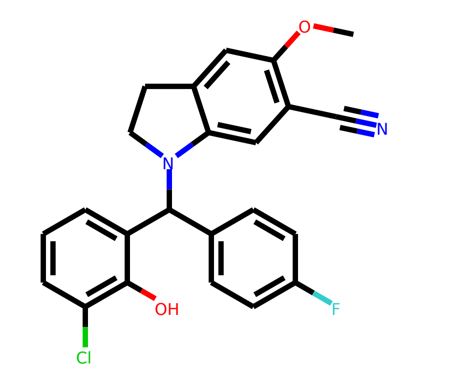
Osma-1352
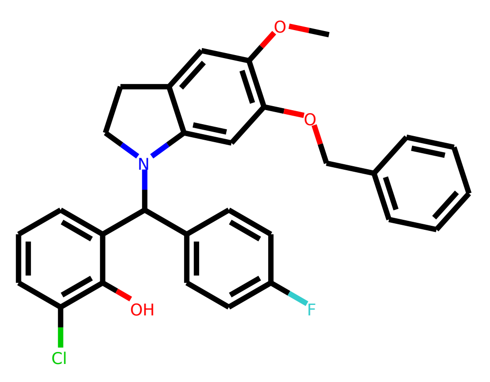
Osma-1353
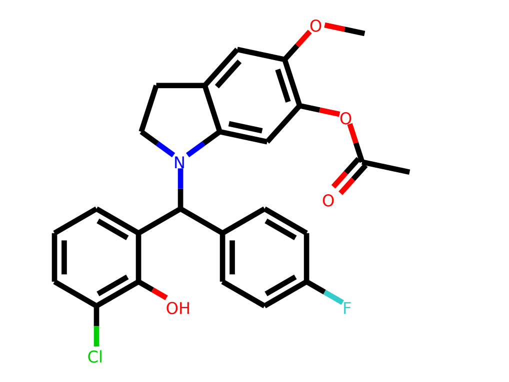
Osma-1354
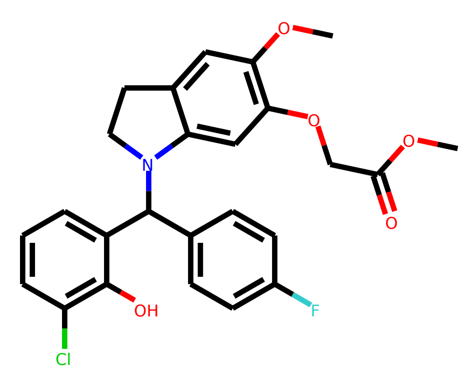
Osma-1355
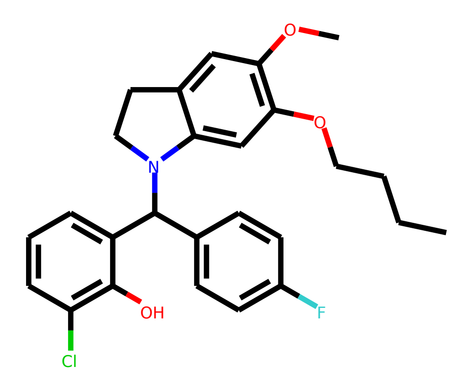
Osma-1356
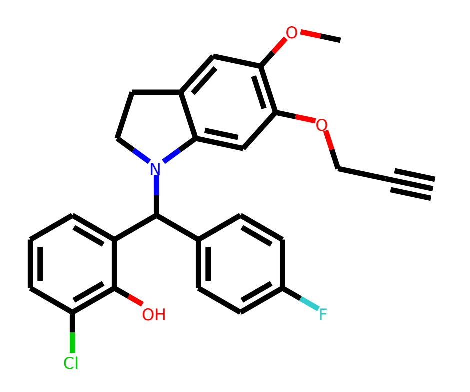
Osma-1357
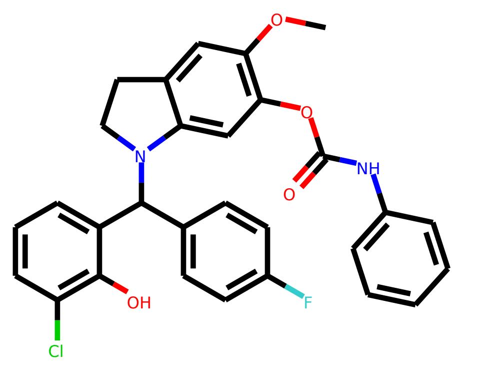
Osma-1358
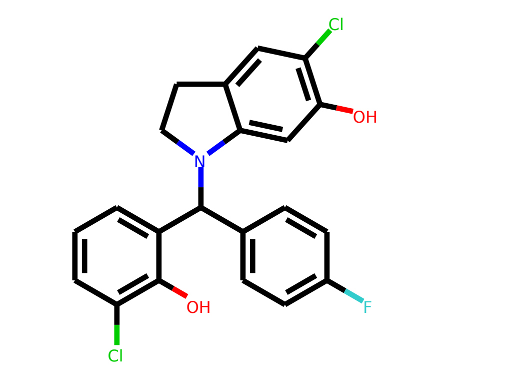
Osma-1359
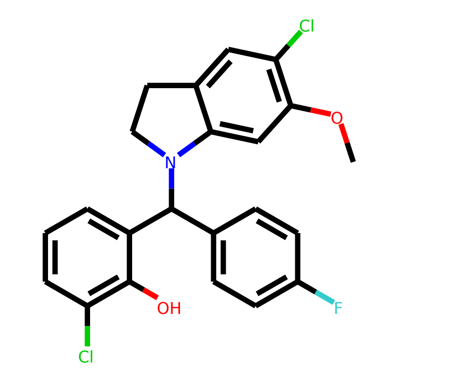
Osma-1360
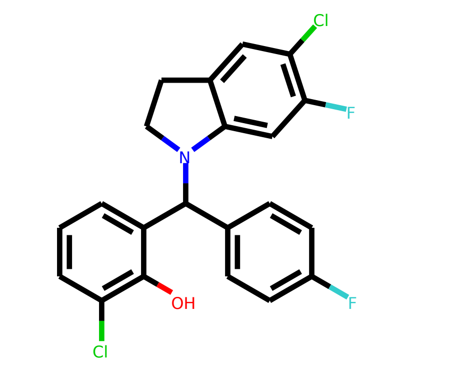
Osma-1361
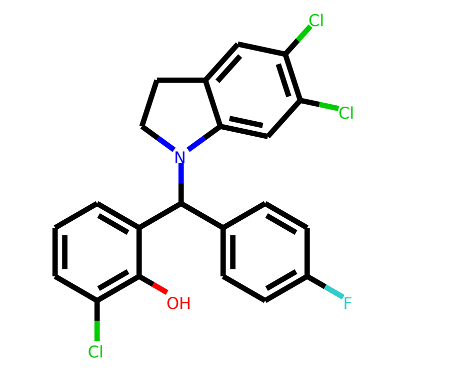
Osma-1362
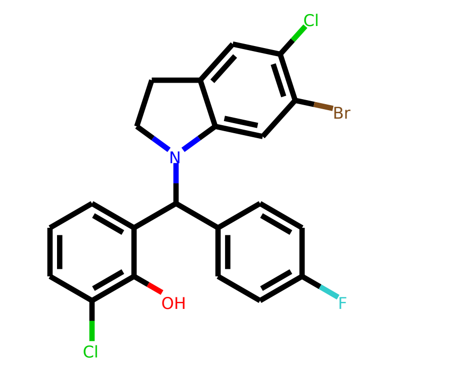
Osma-1363
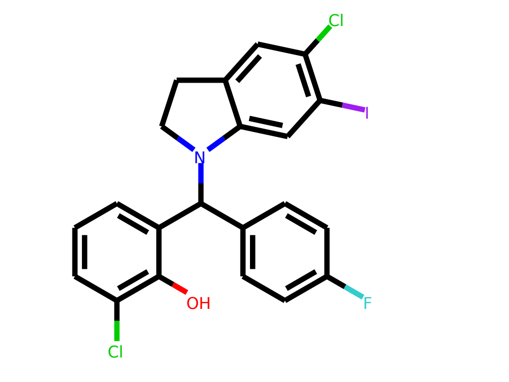
Osma-1364
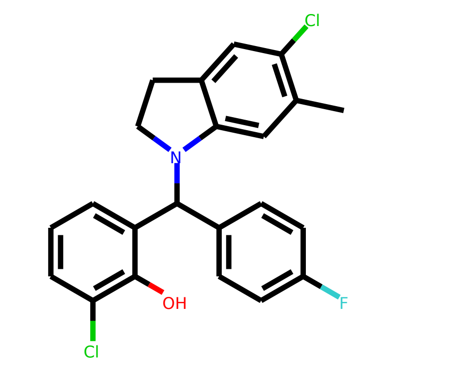
Osma-1365

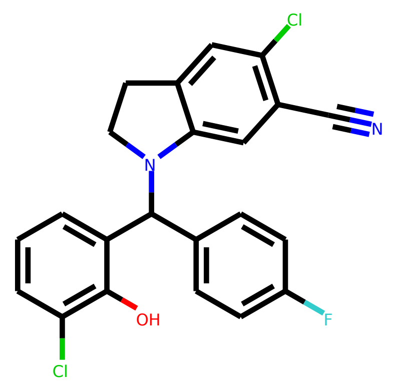

Osma-1366

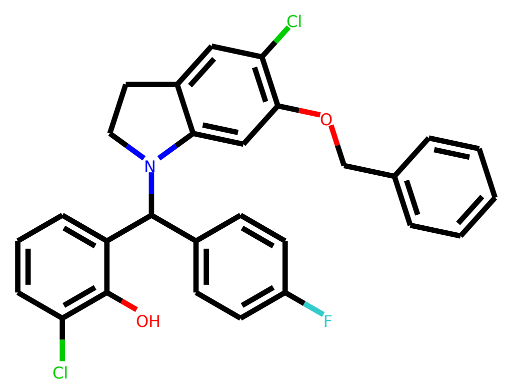

Osma-1367

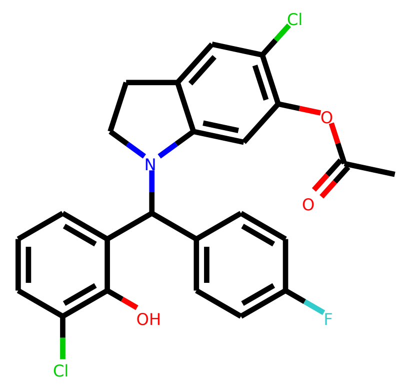

Osma-1368

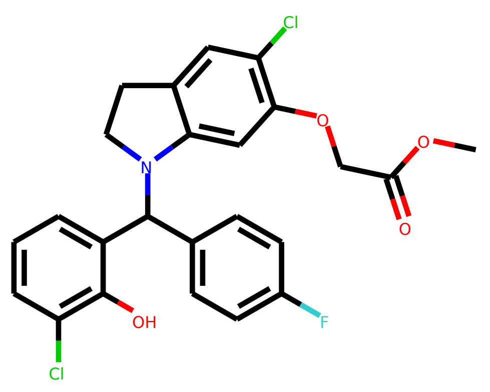

Osma-1369

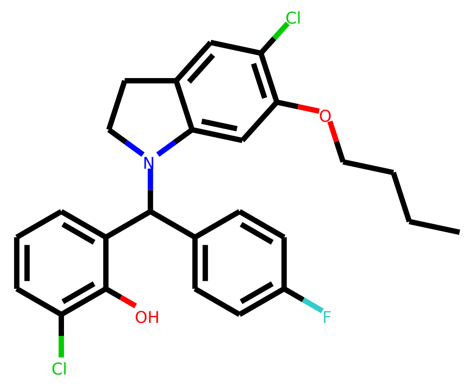

Osma-1370

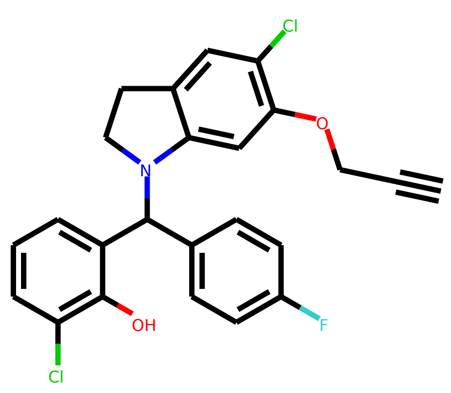

Osma-1371

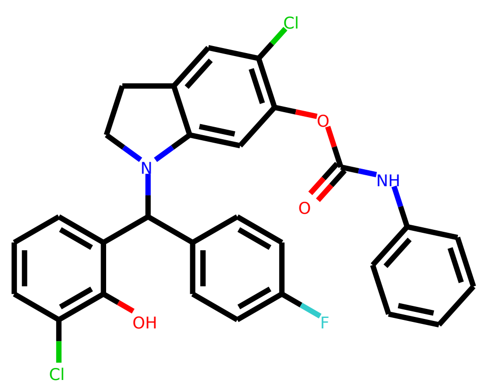

Osma-1372

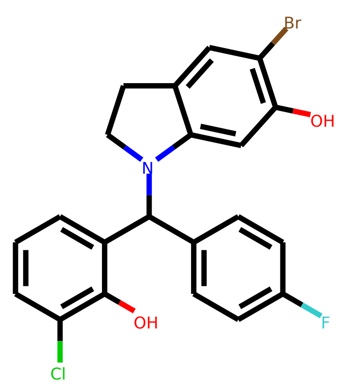

Osma-1373

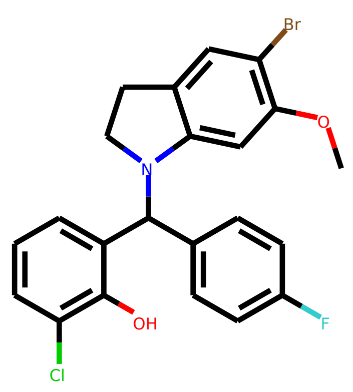

Osma-1374

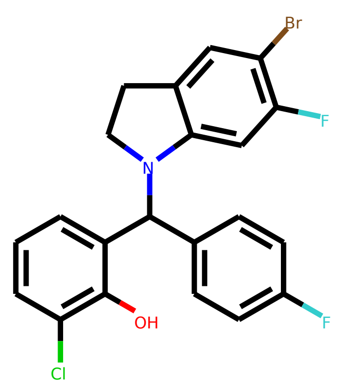

Osma-1375

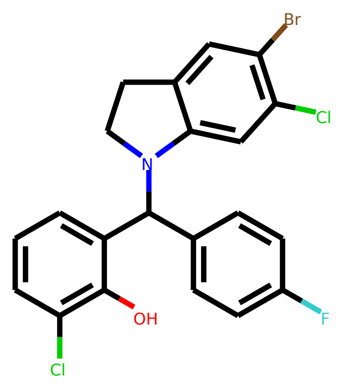

Osma-1376

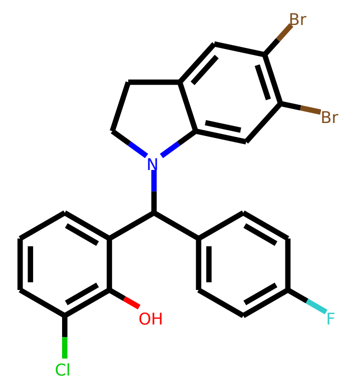

Osma-1377

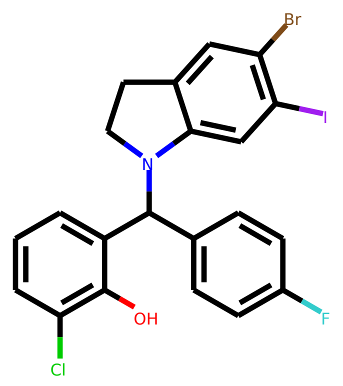

Osma-1378

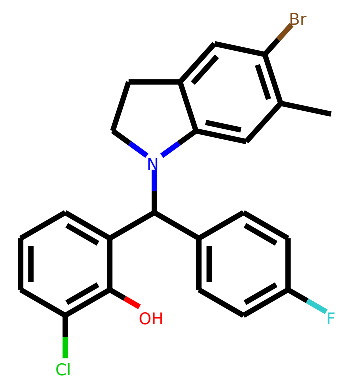

Osma-1379

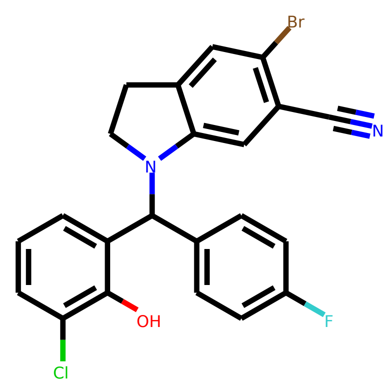

Osma-1380

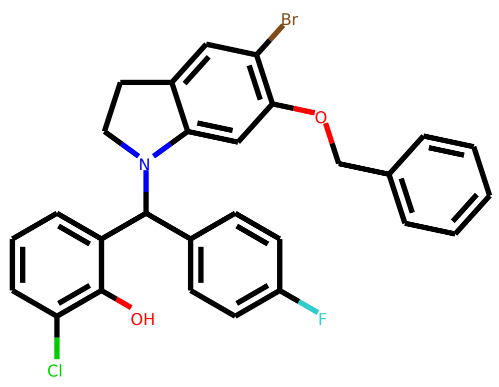

Osma-1381

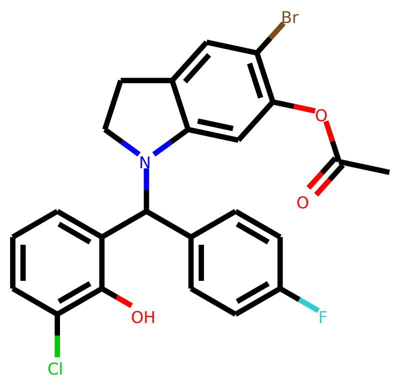

Osma-1382

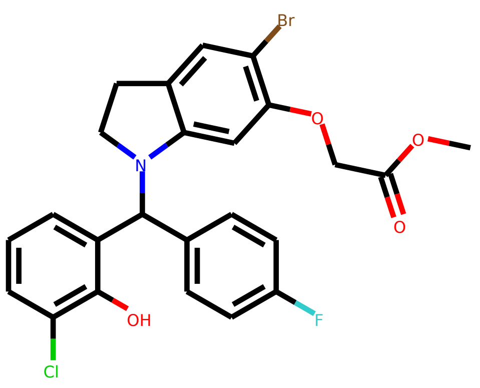

Osma-1383

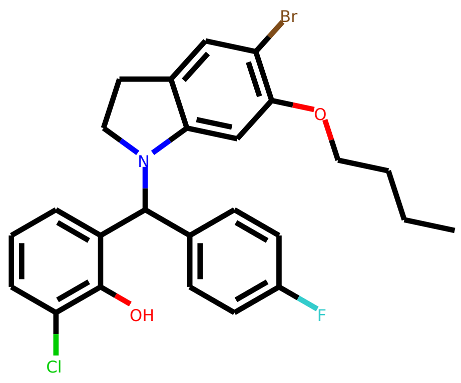

Osma-1384

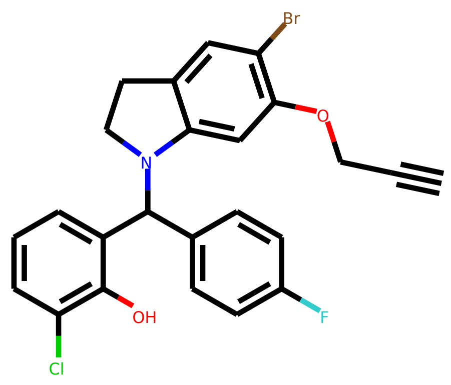

Osma-1385

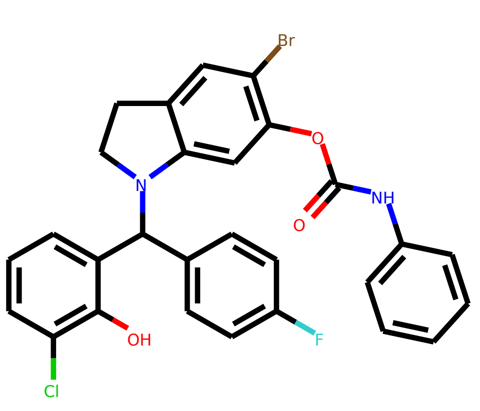

Osma-1386

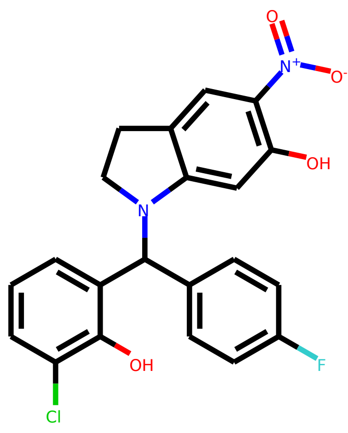

Osma-1387

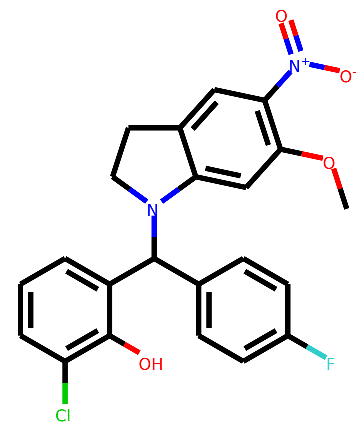

Osma-1388

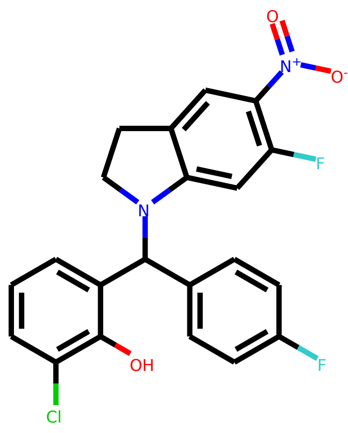

Osma-1389

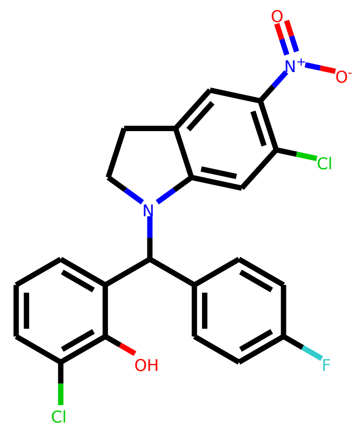

Osma-1390

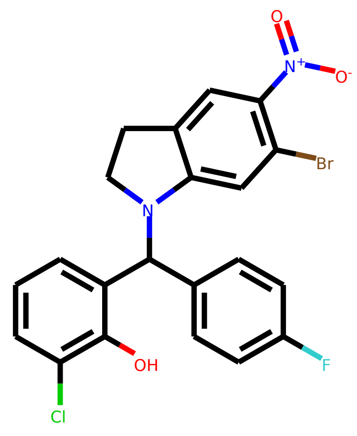

Osma-1391

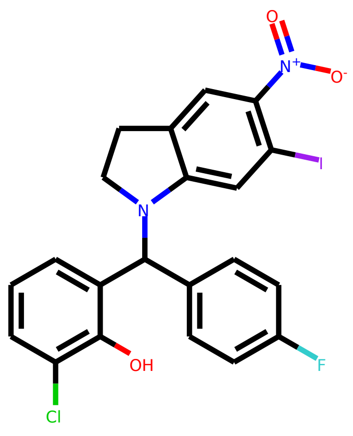

Osma-1392

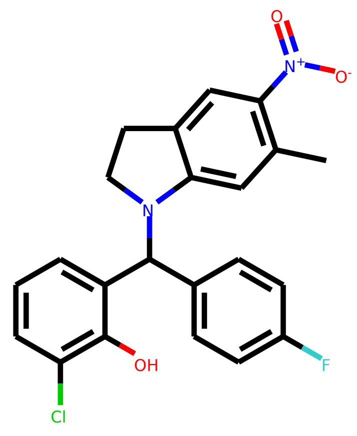

Osma-1393

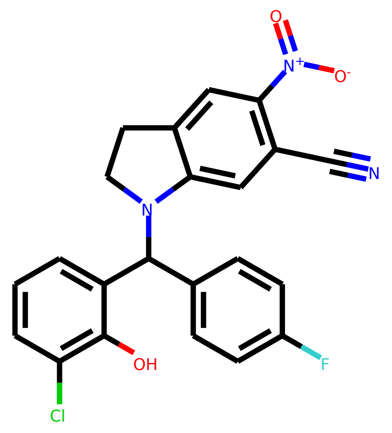

Osma-1394

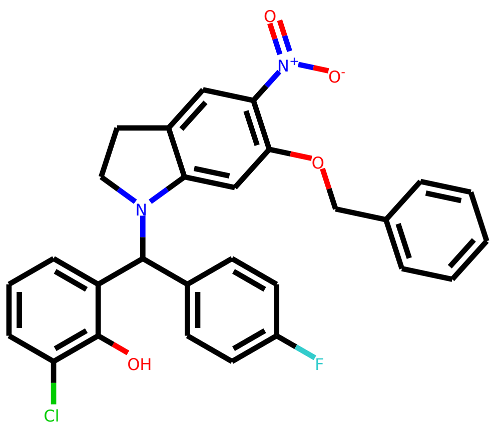

Osma-1395

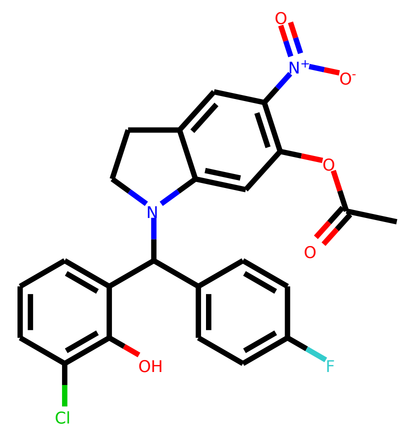

Osma-1396

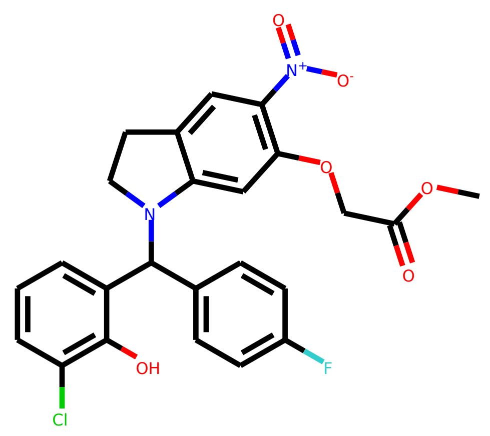

Osma-1397

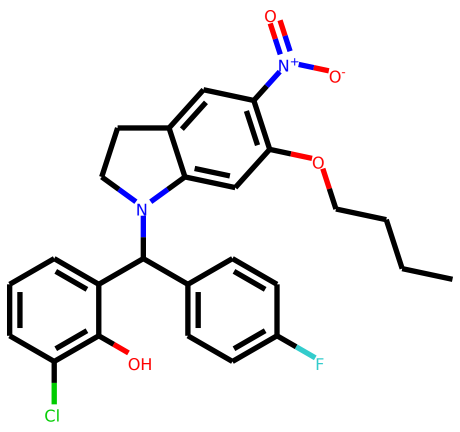

Osma-1398

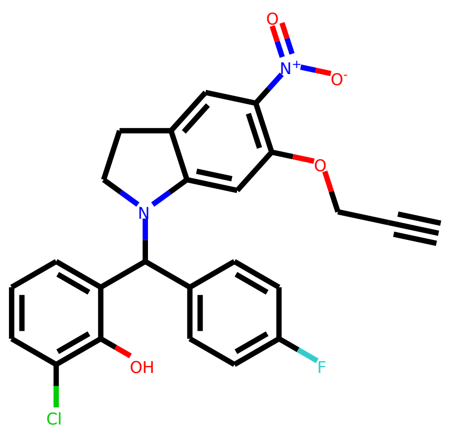

Osma-1399

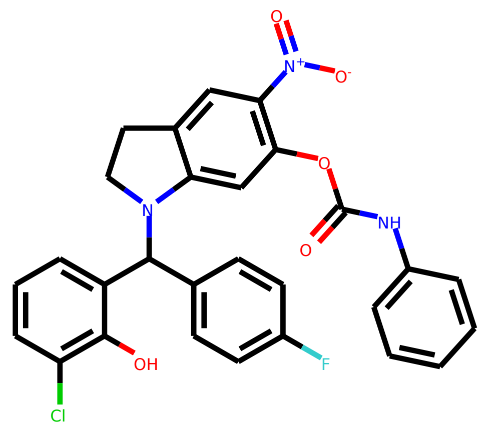

Osma-1400

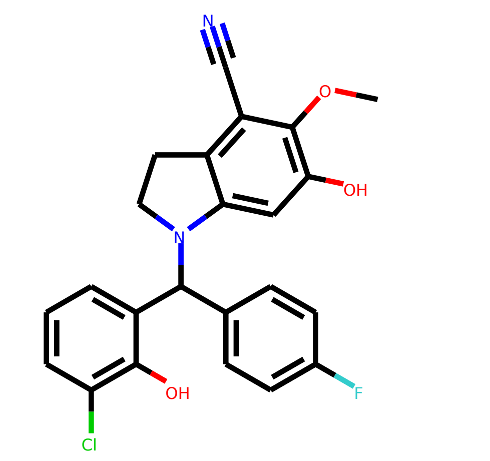

Osma-1401

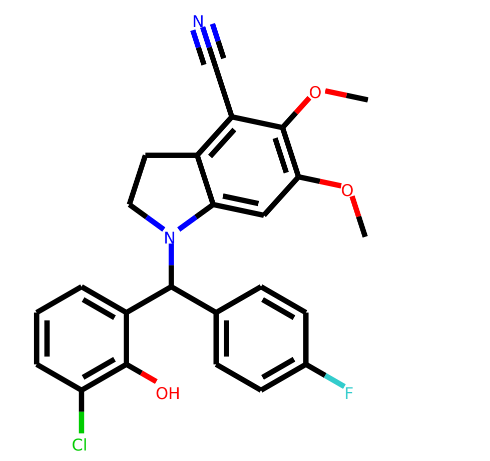

Osma-1402

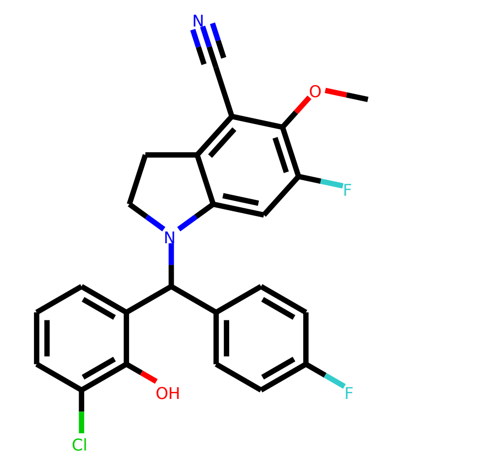

Osma-1403

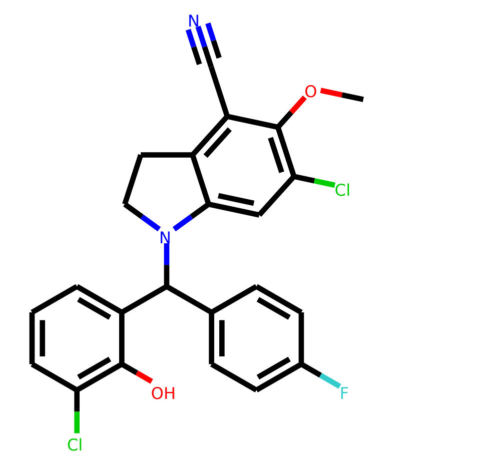

Osma-1404

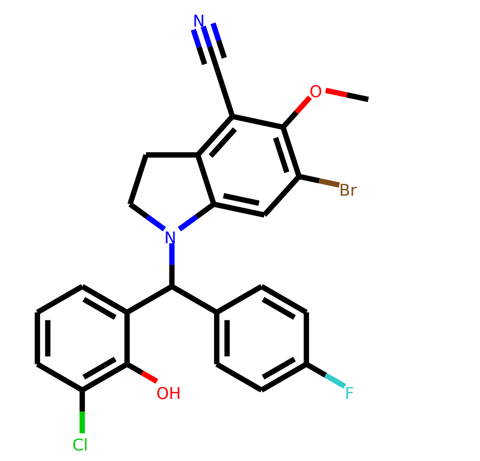

Osma-1405

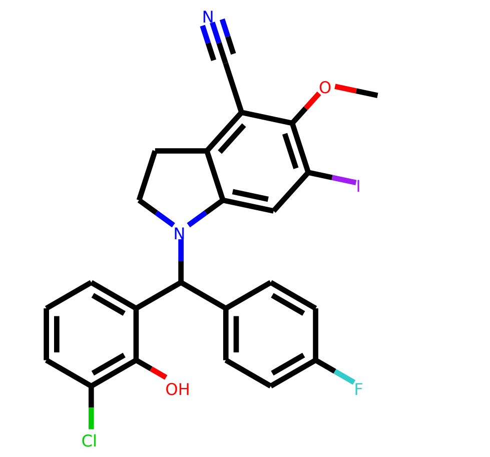

Osma-1406

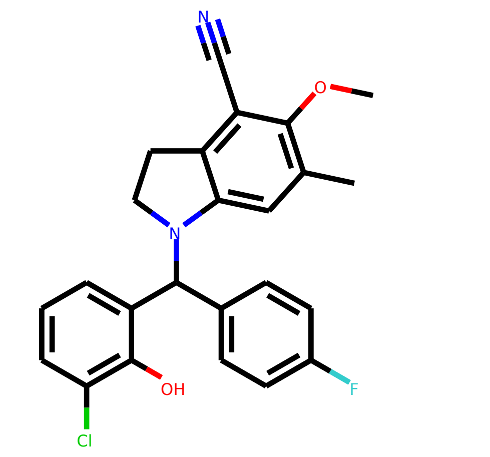

Osma-1407

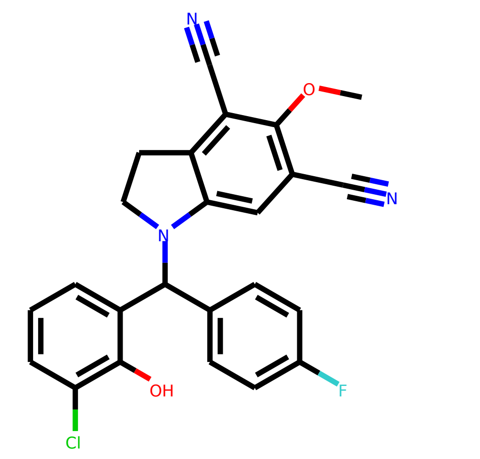

Osma-1408

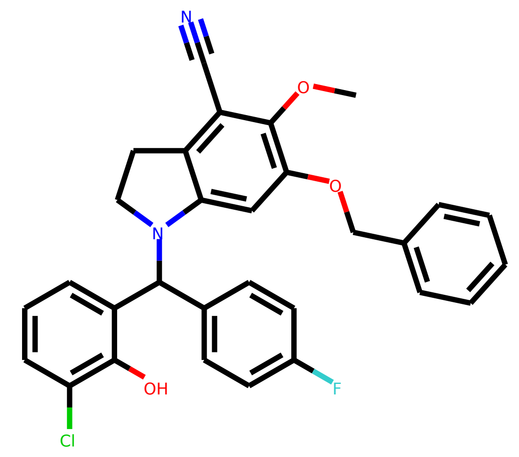

Osma-1409

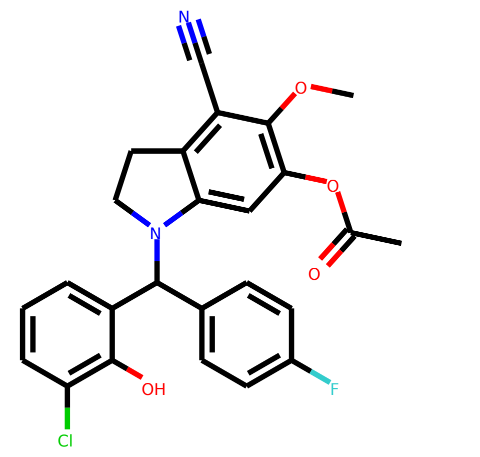

Osma-1410

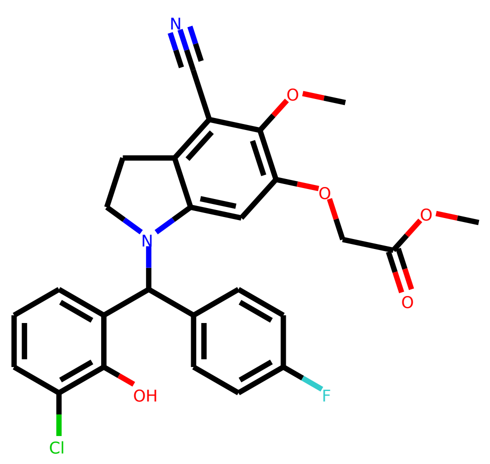

Osma-1411

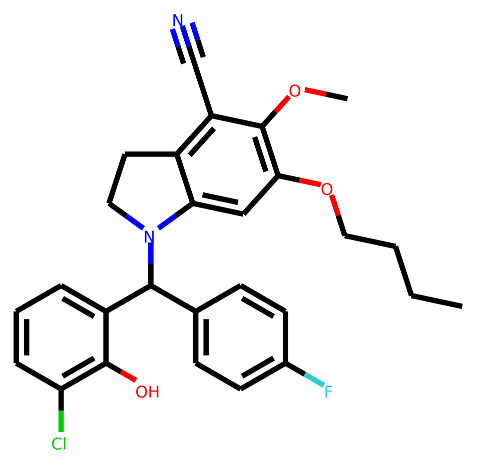

Osma-1412

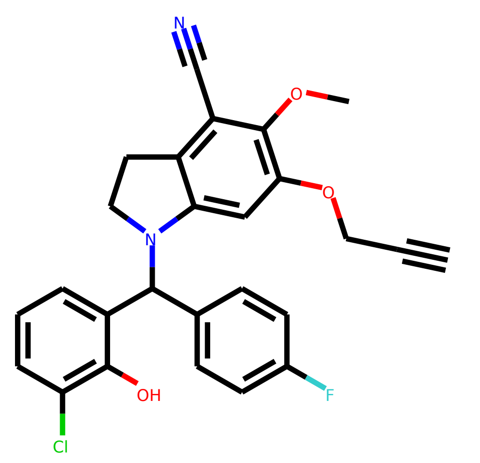

Osma-1413

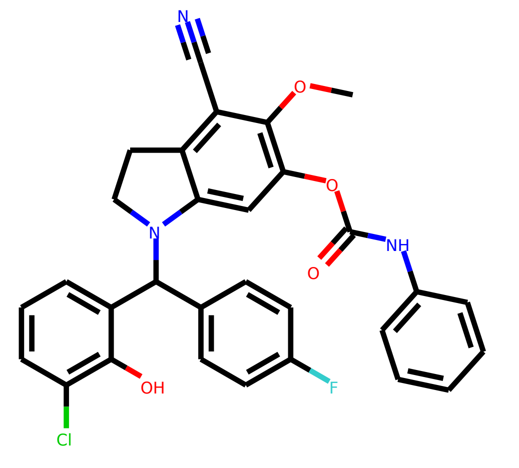

Osma-1414

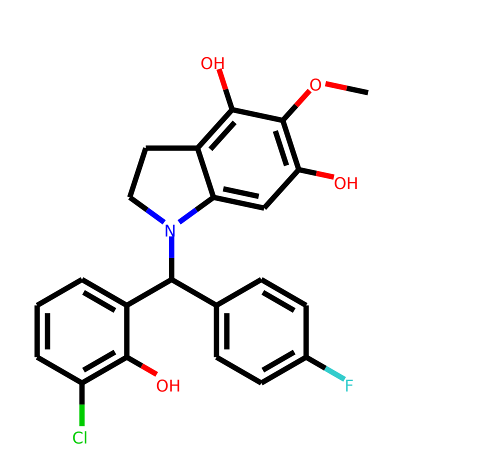

Osma-1415

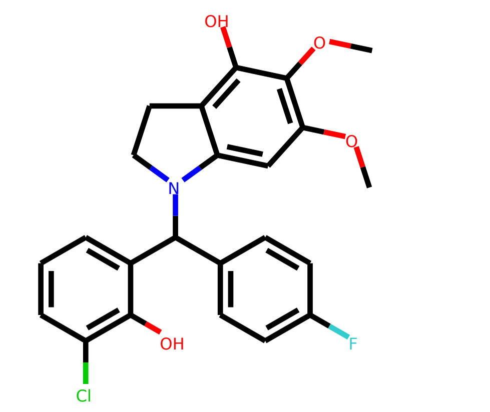

Osma-1416

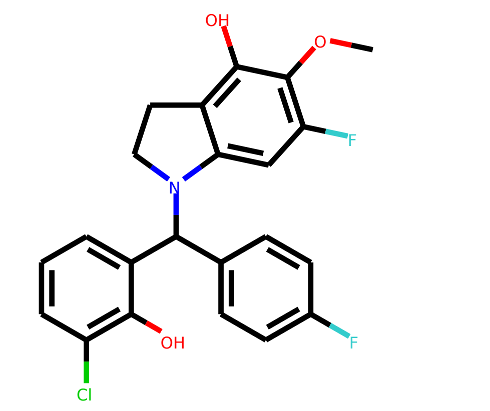

Osma-1417

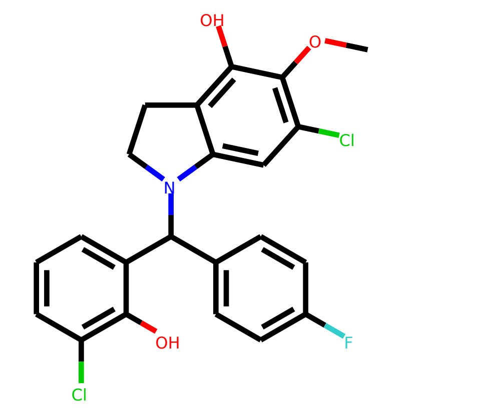

Osma-1418

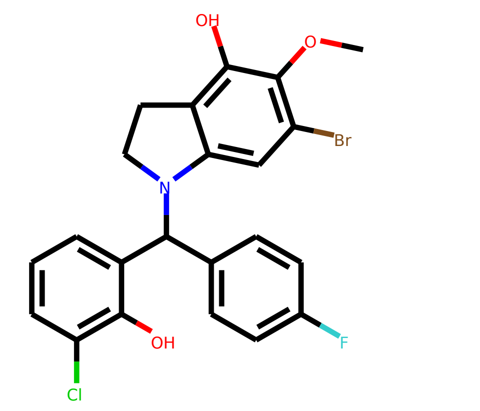

Osma-1419

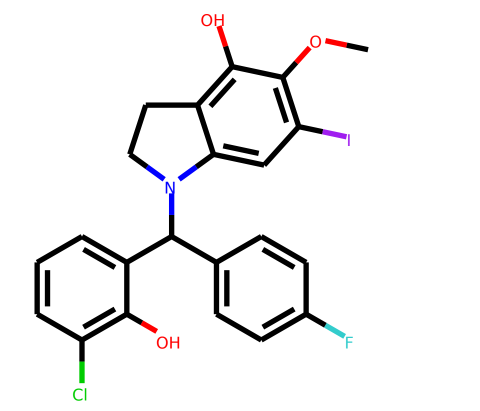

Osma-1420

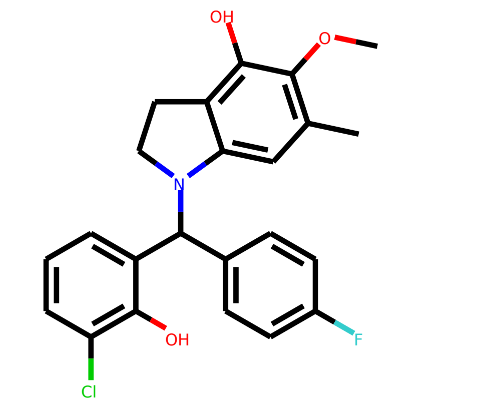

Osma-1421

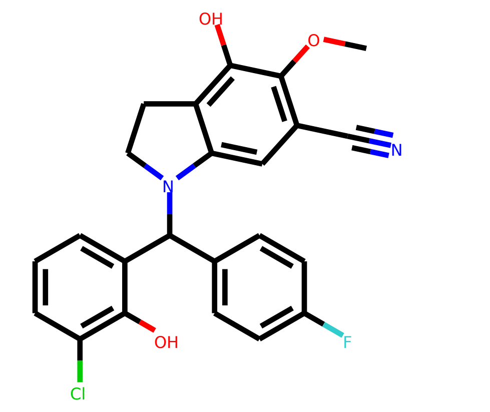

Osma-1422

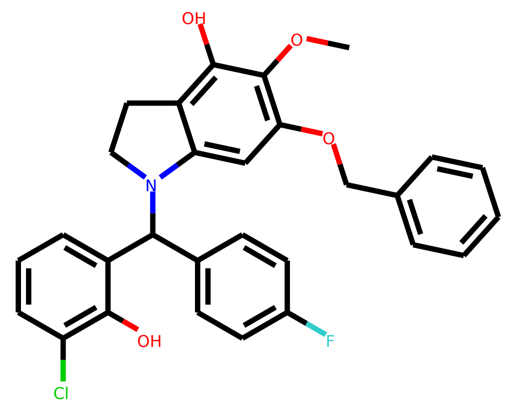

Osma-1423

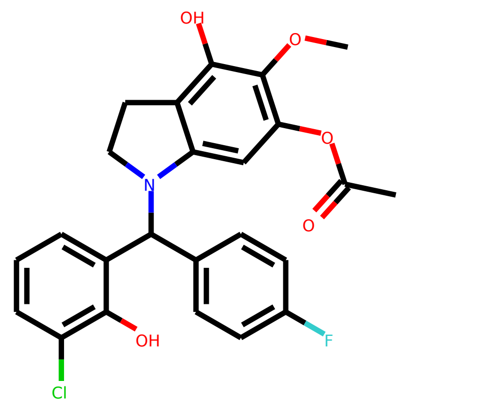

Osma-1424

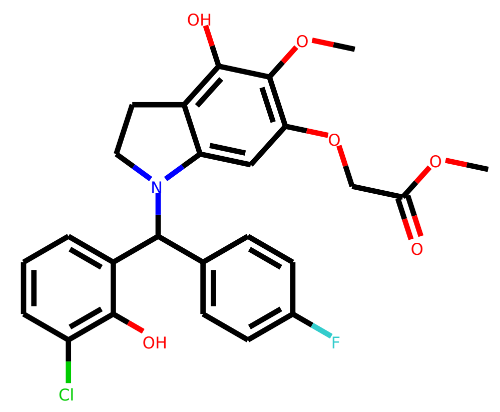

Osma-1425

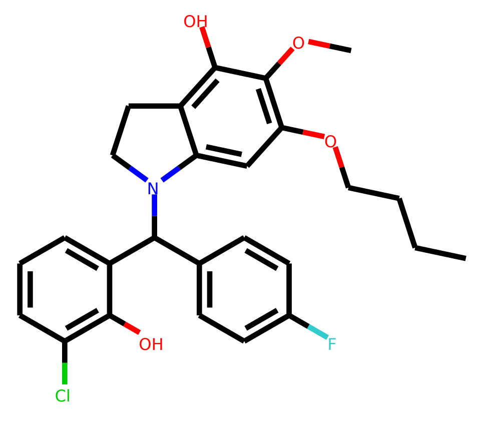

Osma-1426

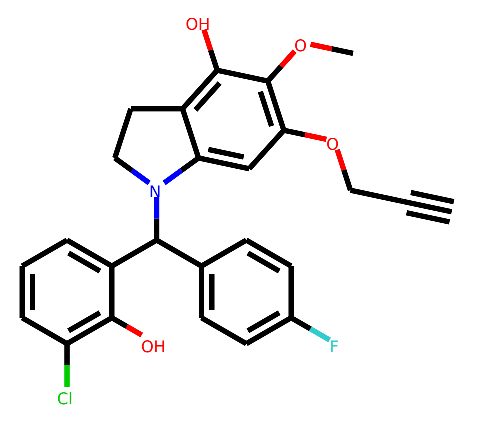

Osma-1427

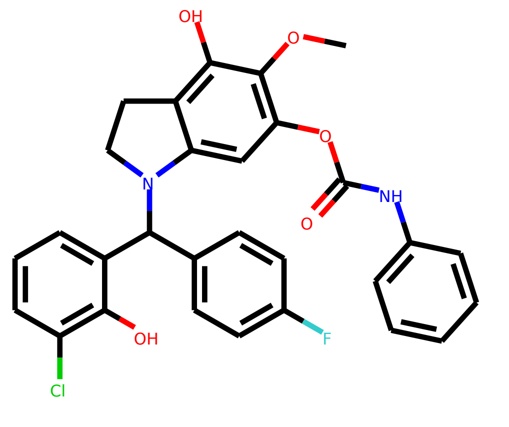

Osma-1428

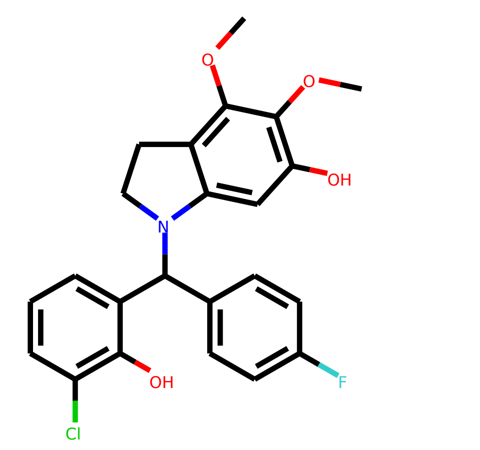

Osma-1429

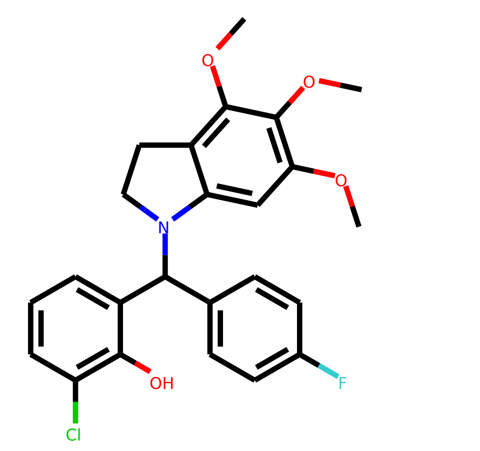

Osma-1430

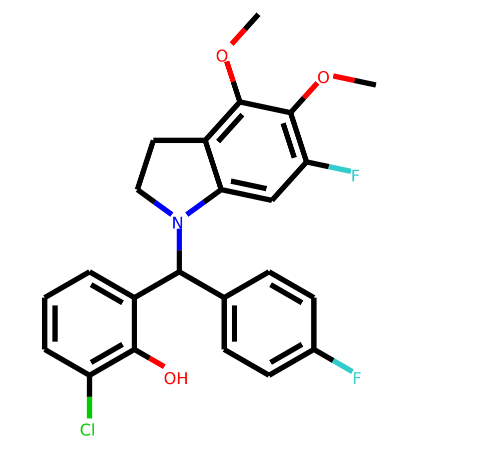

Osma-1431

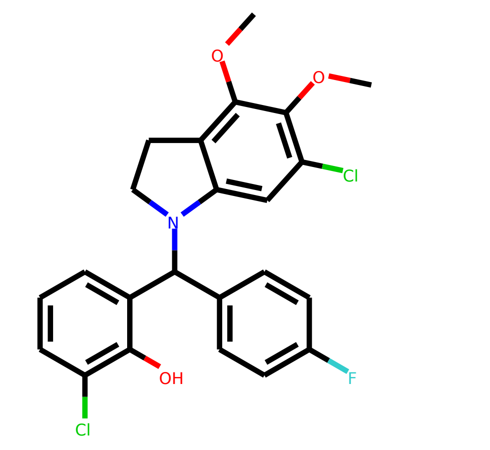

Osma-1432

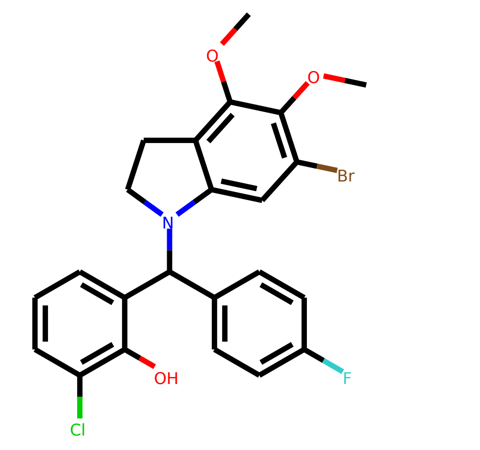

Osma-1433

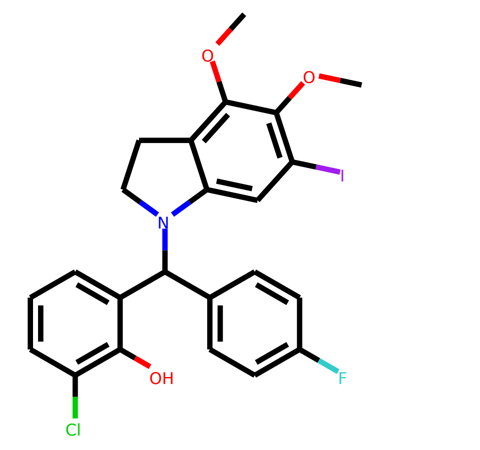

Osma-1434

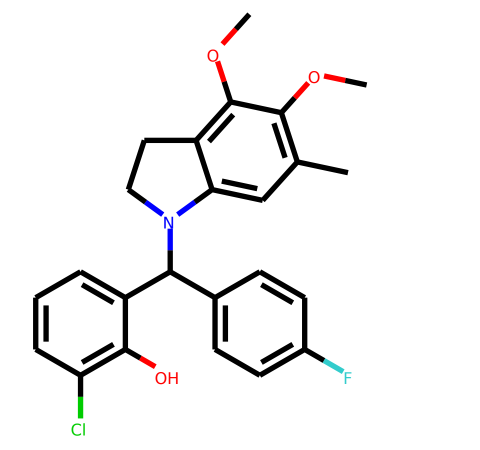

Osma-1435

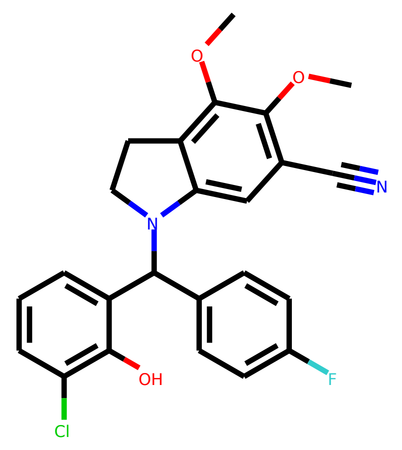

Osma-1436

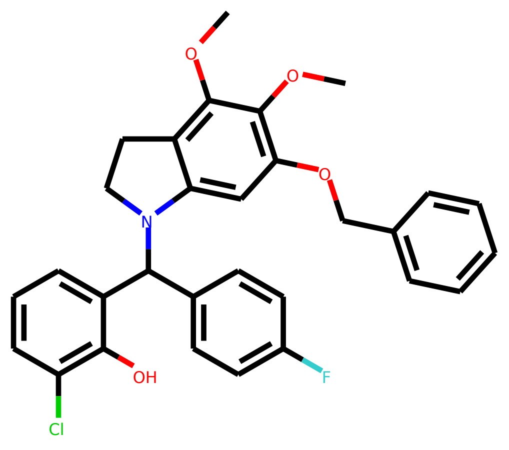

Osma-1437

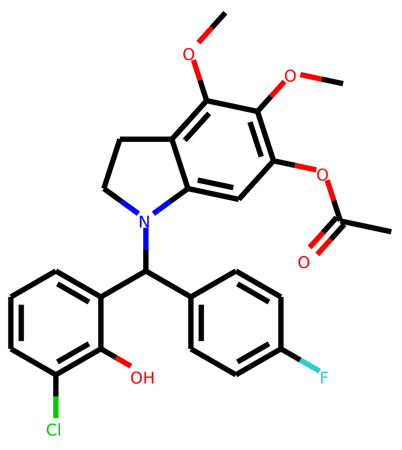

Osma-1438

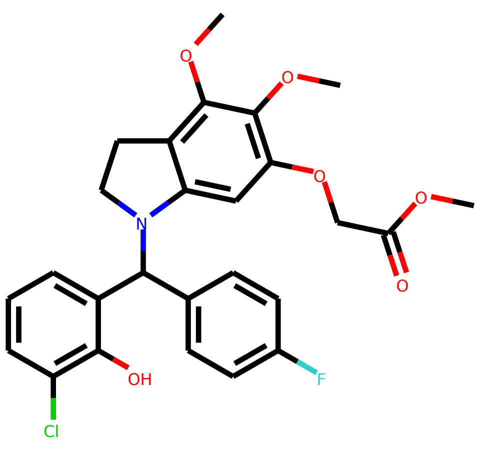

Osma-1439

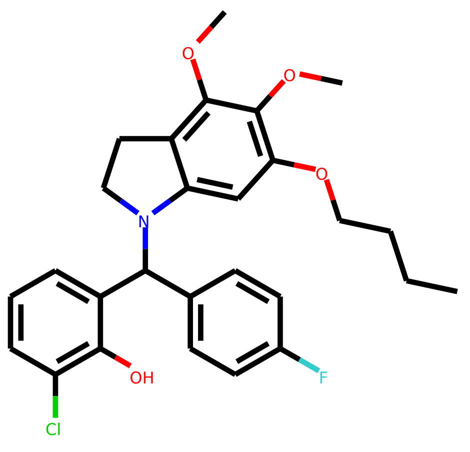

Osma-1440

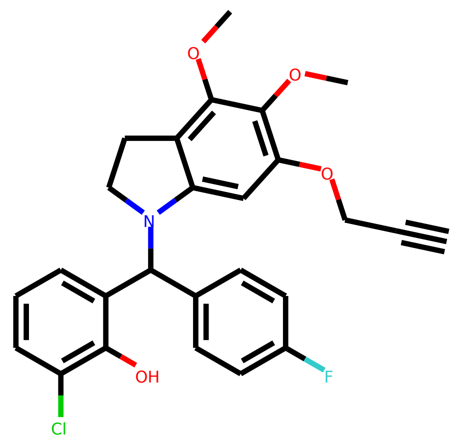

Osma-1441

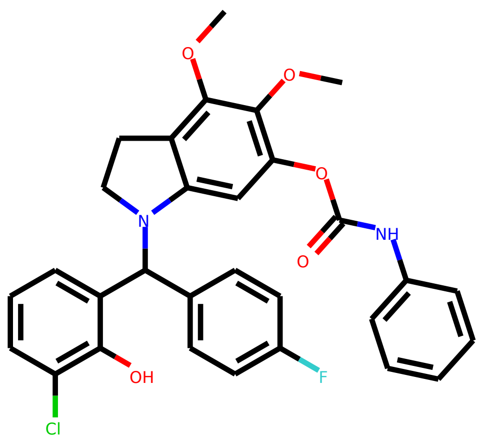

Osma-1442

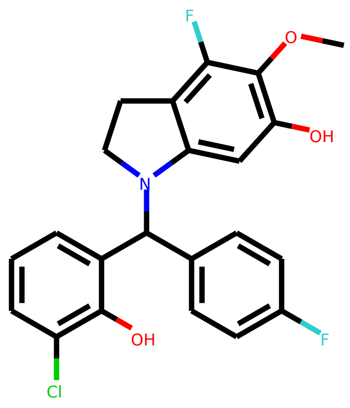

Osma-1443

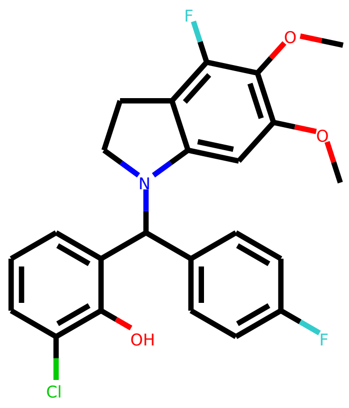

Osma-1444

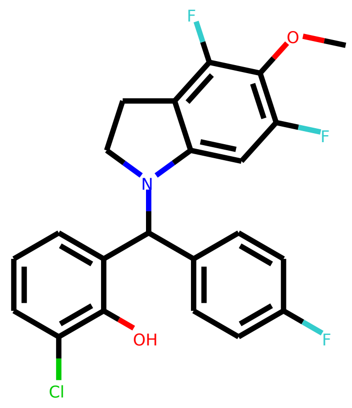

Osma-1445

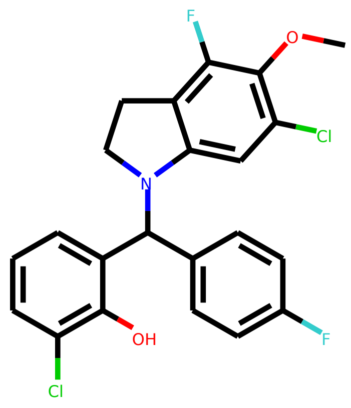

Osma-1446

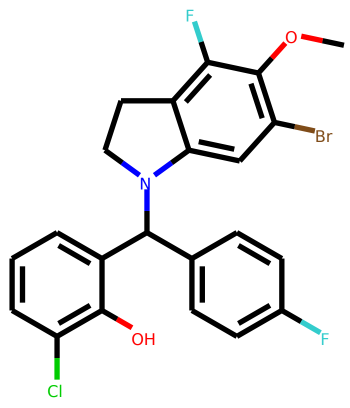

Osma-1447

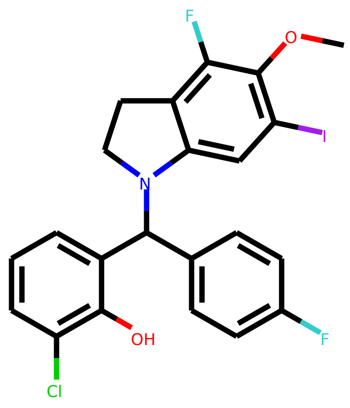

Osma-1448

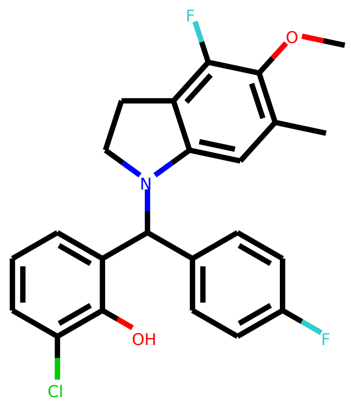

Osma-1449

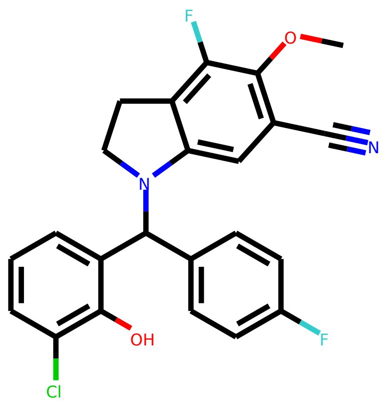

Osma-1450

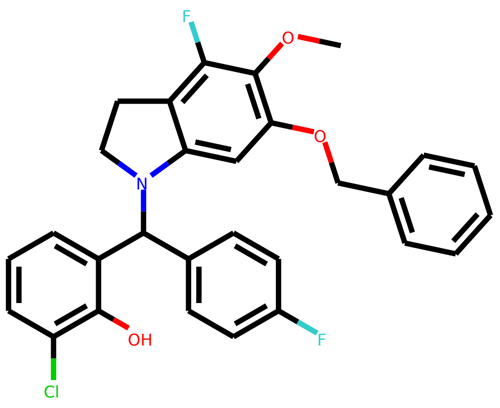

Osma-1451

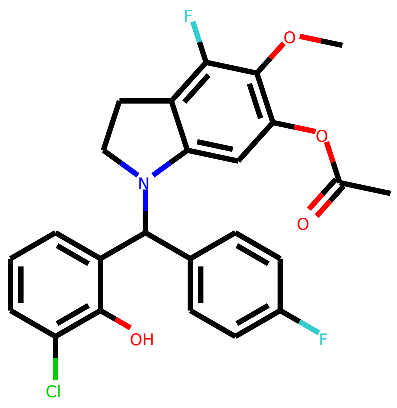

Osma-1452

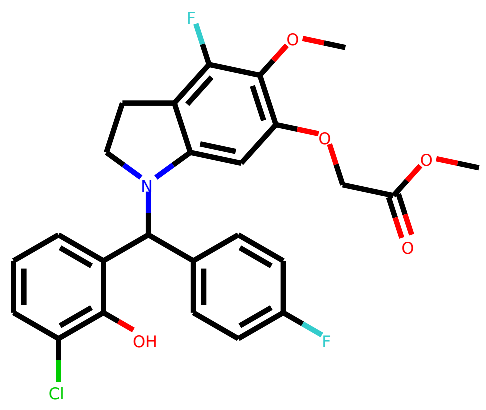

Osma-1453

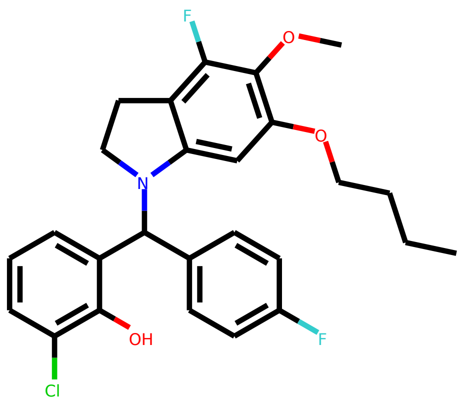

Osma-1454

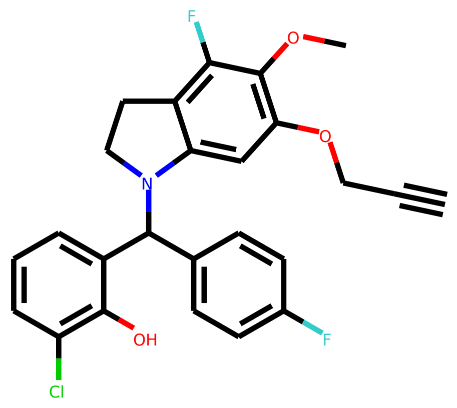

Osma-1455

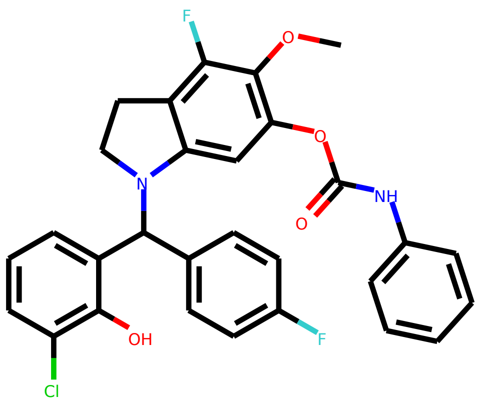

Osma-1456

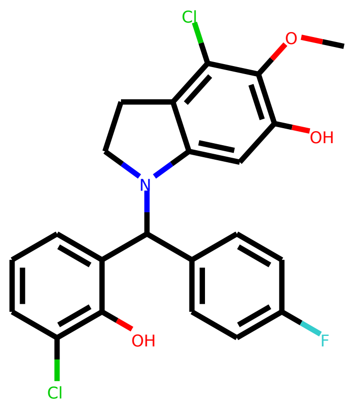

Osma-1457

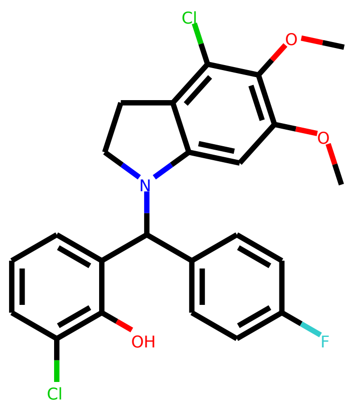

Osma-1458

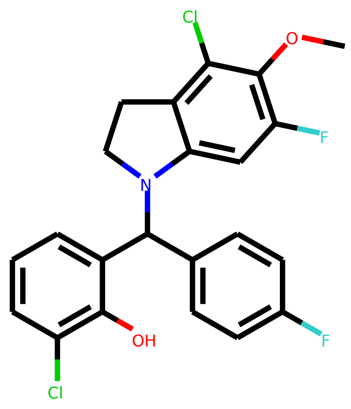

Osma-1459

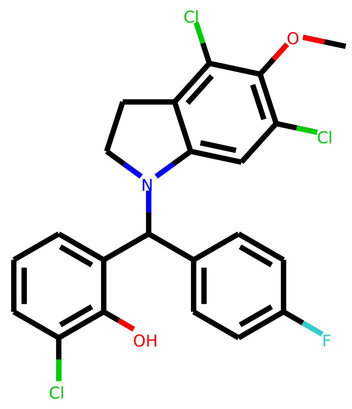

Osma-1460

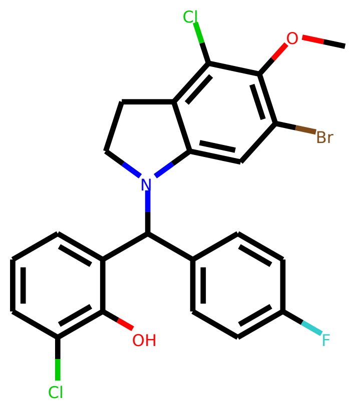

Osma-1461

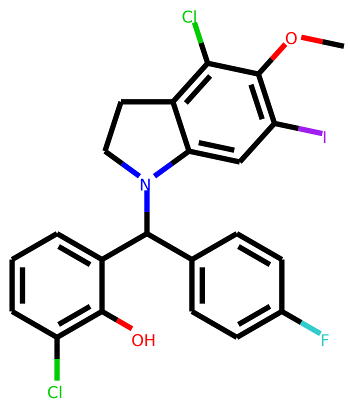

Osma-1462

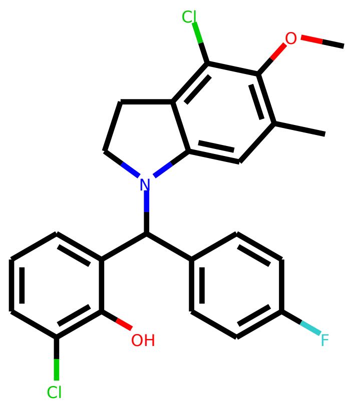

Osma-1463

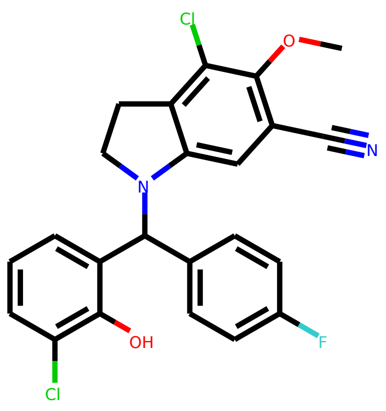

Osma-1464

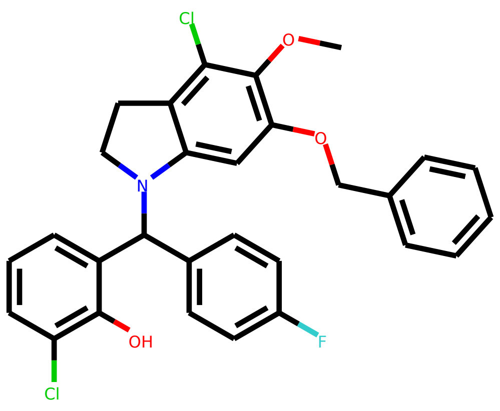

Osma-1465

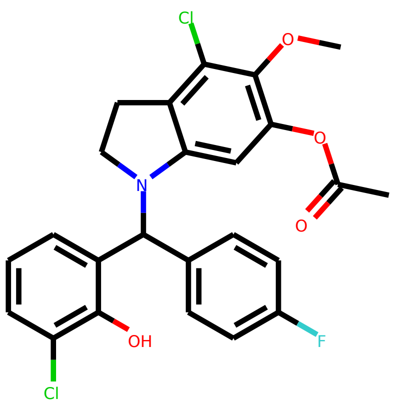

Osma-1466

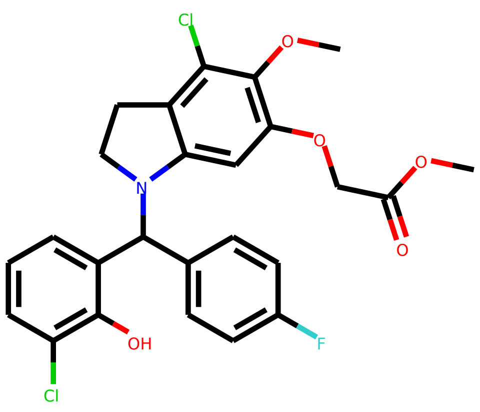

Osma-1467

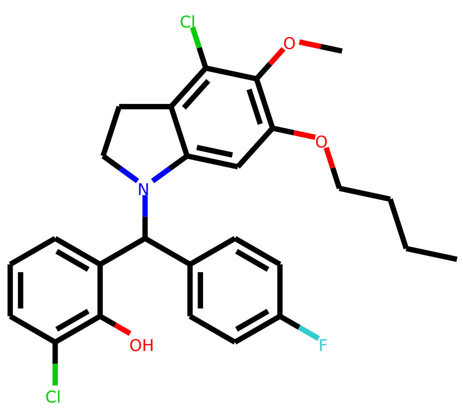

Osma-1468

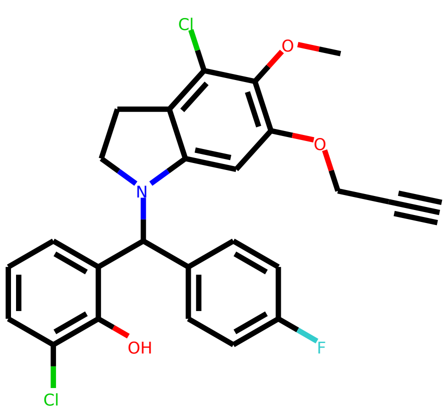

Osma-1469

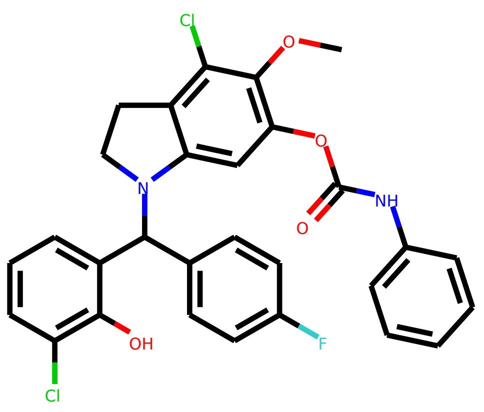

Osma-1470

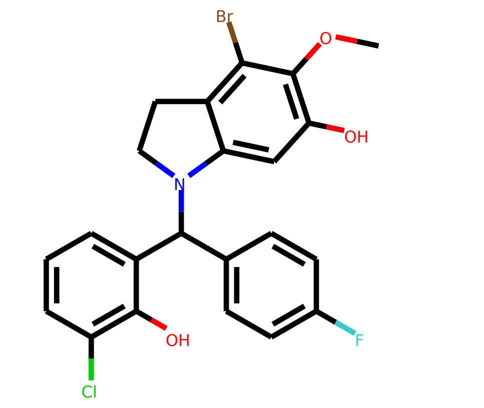
Osma-1471
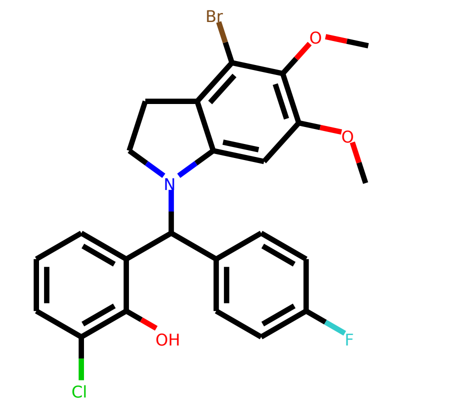
Osma-1472
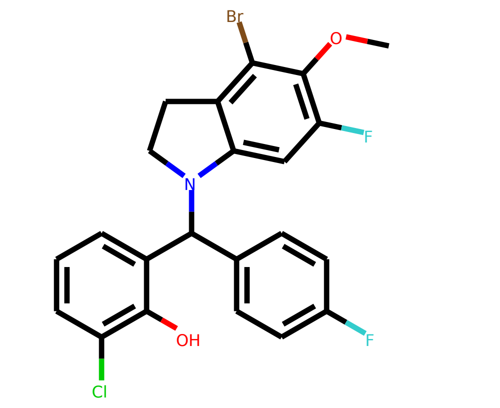
Osma-1473
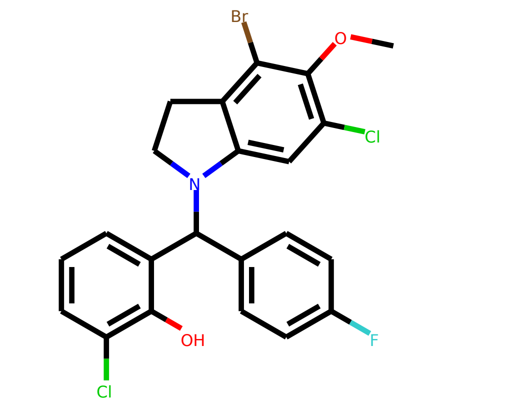
Osma-1474
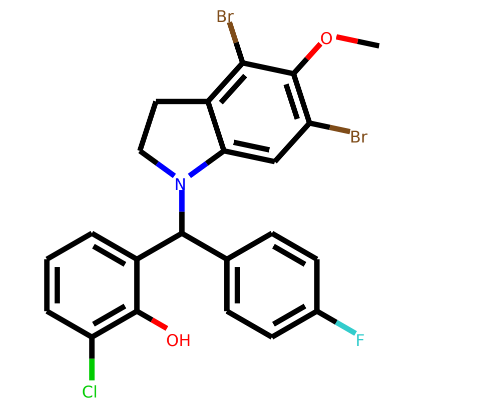
Osma-1475
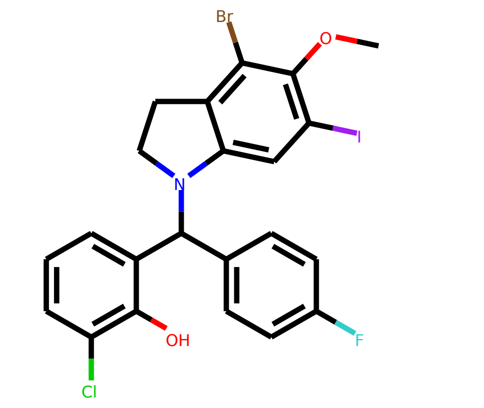
Osma-1476
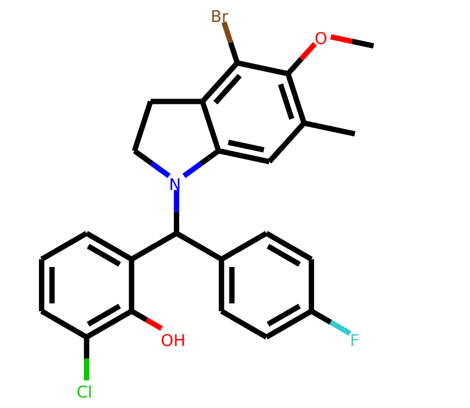
Osma-1477
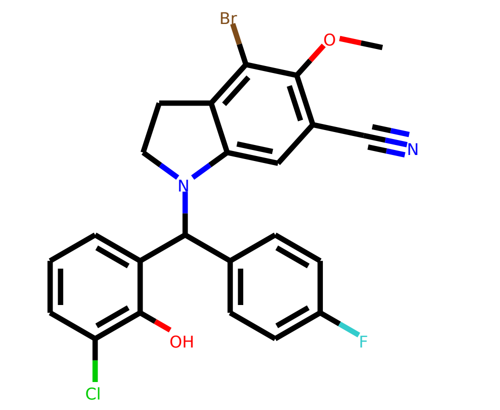
Osma-1478
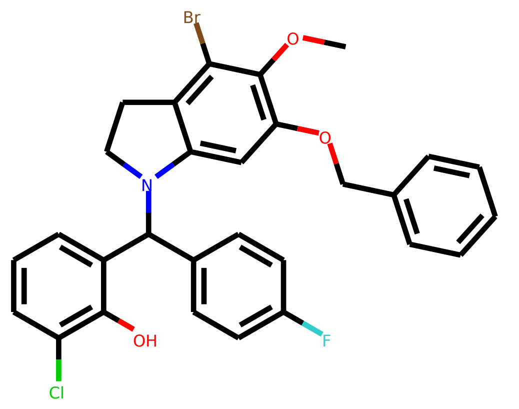
Osma-1479
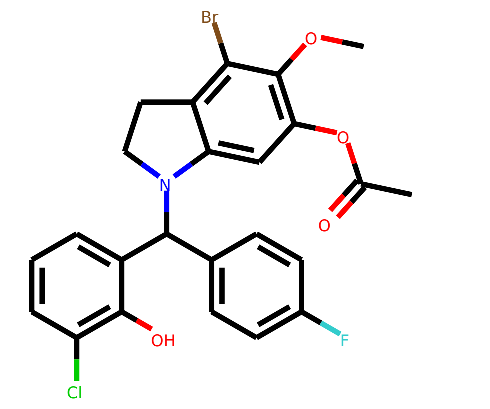
Osma-1480
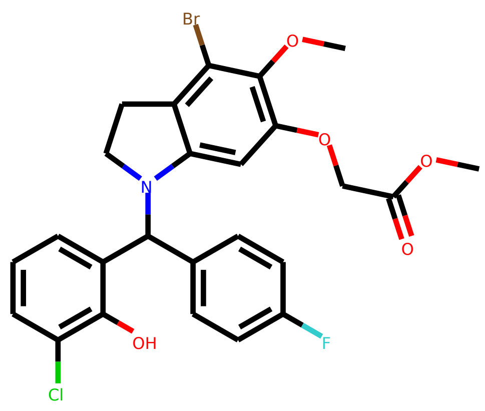
Osma-1481
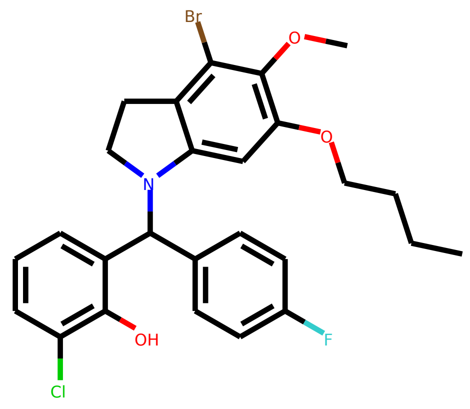
Osma-1482
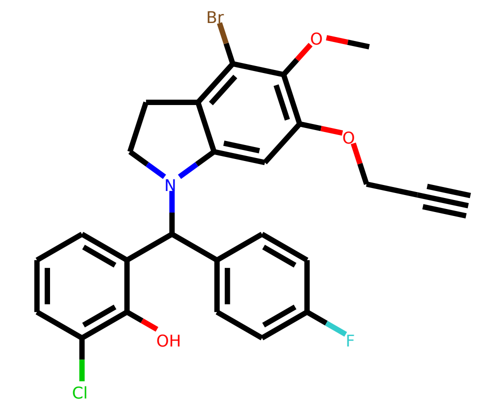
Osma-1483
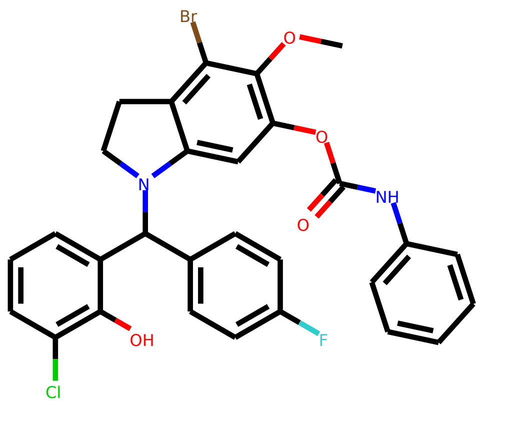
Osma-1484
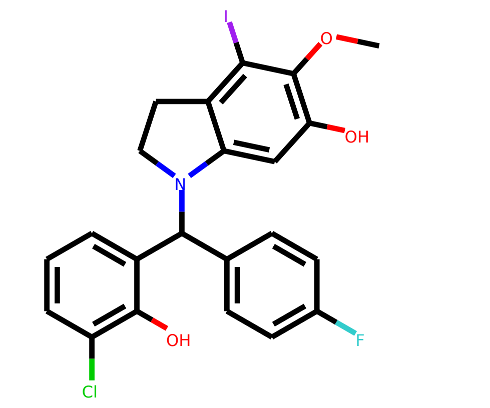
Osma-1485
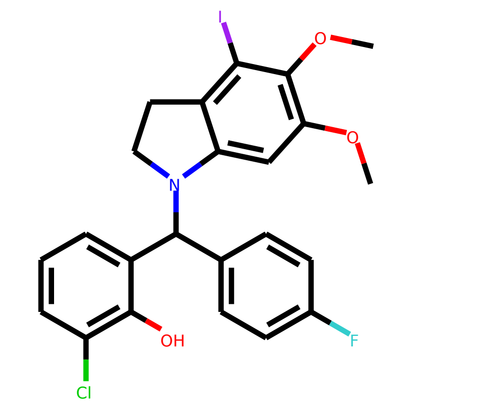
Osma-1486
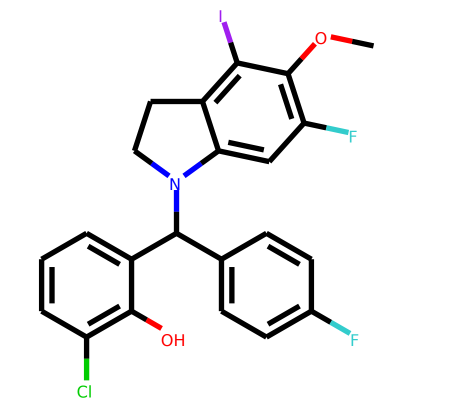
Osma-1487
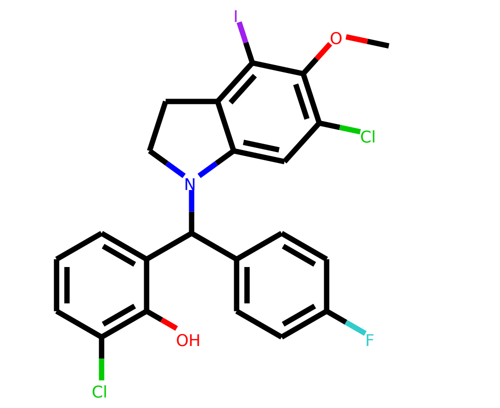
Osma-1488
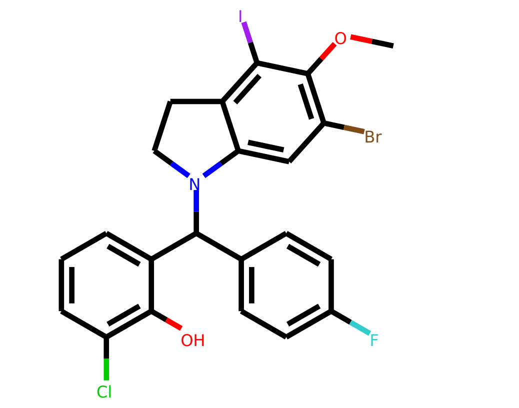
Osma-1489
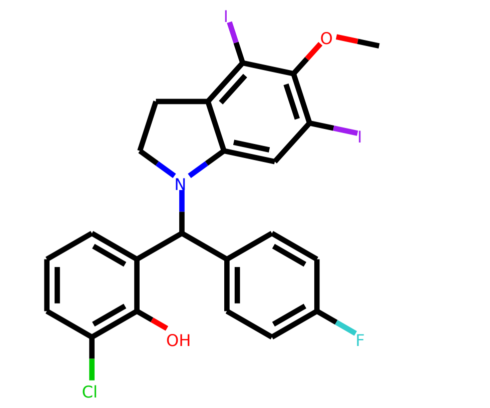
Osma-1490
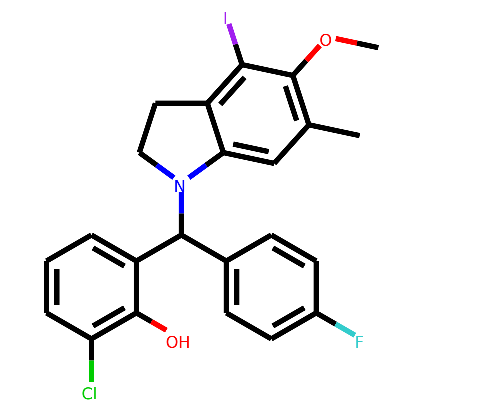
Osma-1491
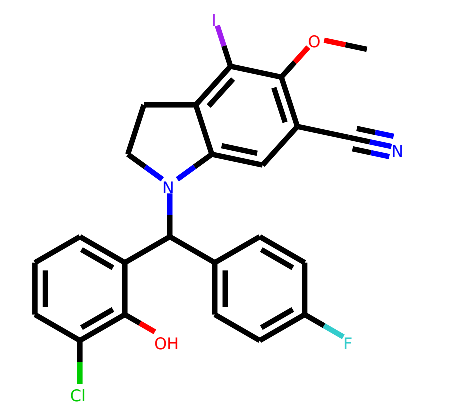
Osma-1492
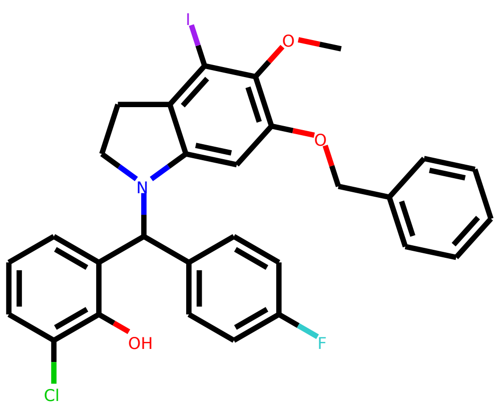
Osma-1493
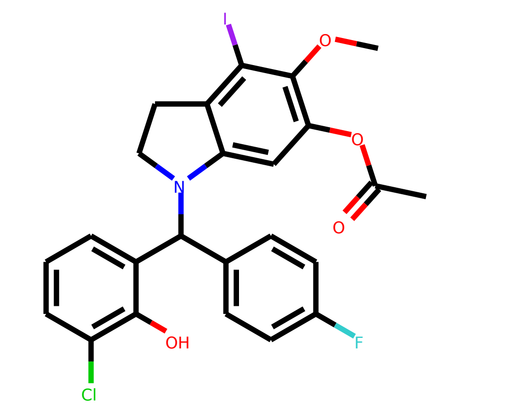
Osma-1494
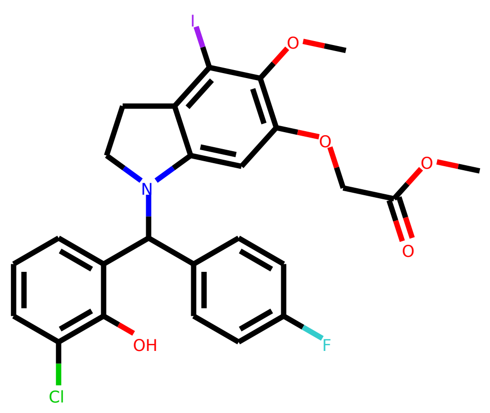
Osma-1495
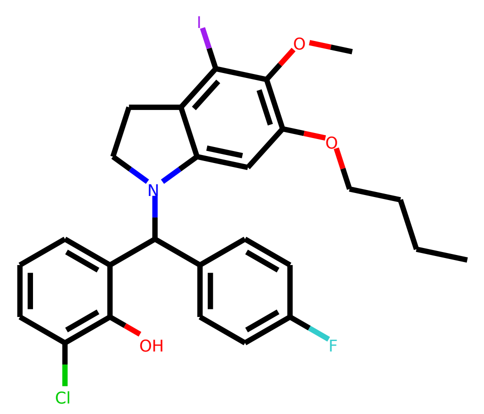
Osma-1496
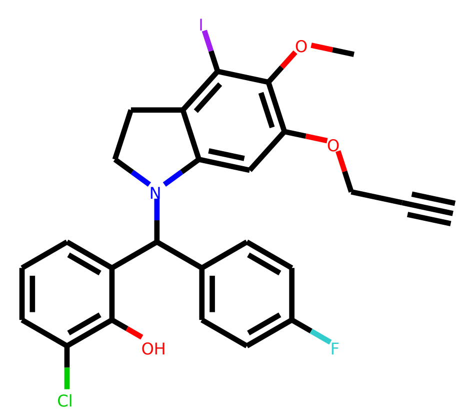
Osma-1497
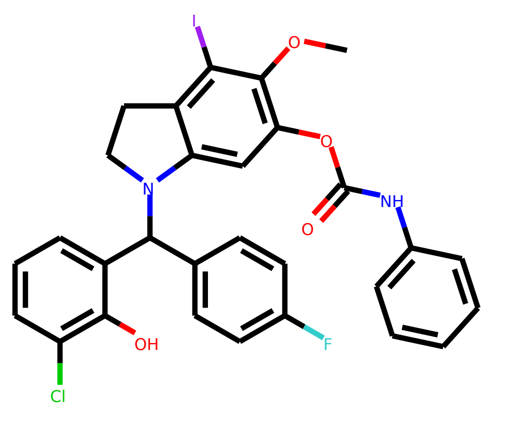
Osma-1498
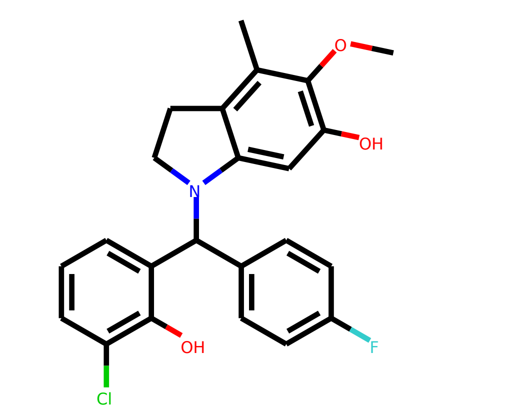
Osma-1499
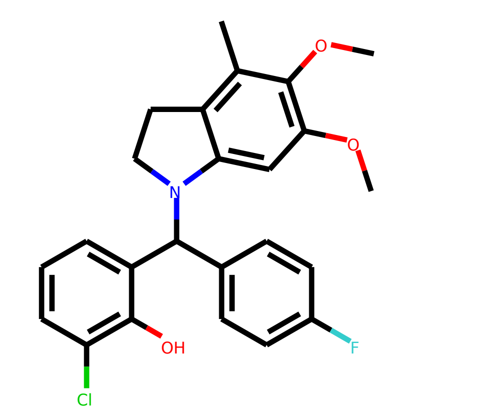
Osma-1500
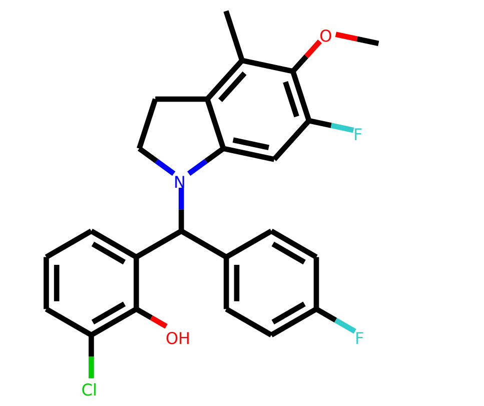
Osma-1501
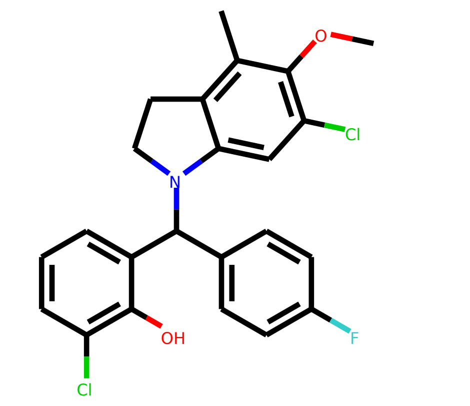
Osma-1502
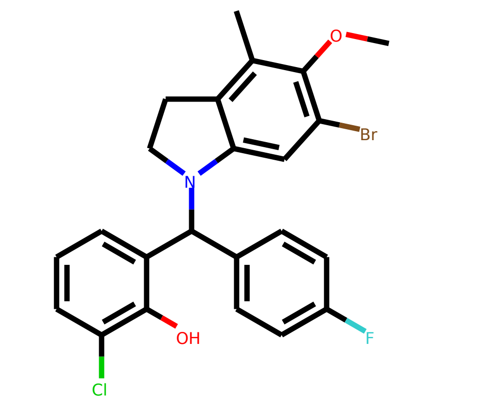
Osma-1503
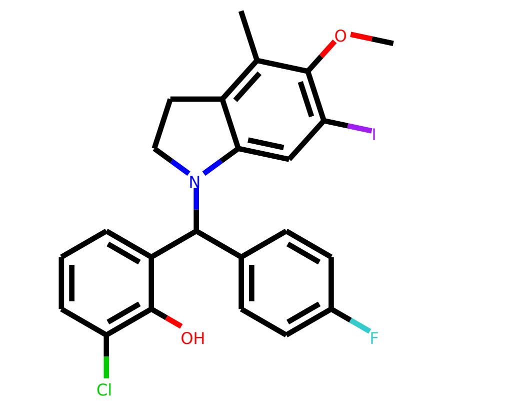
Osma-1504
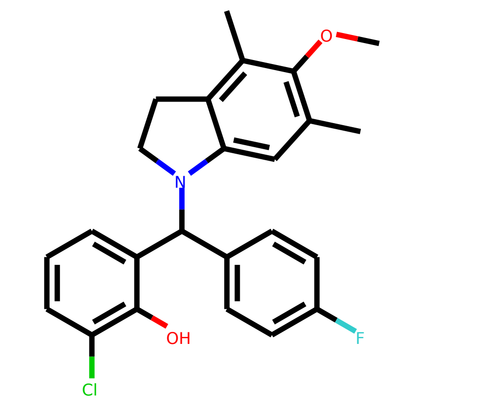
Osma-1505

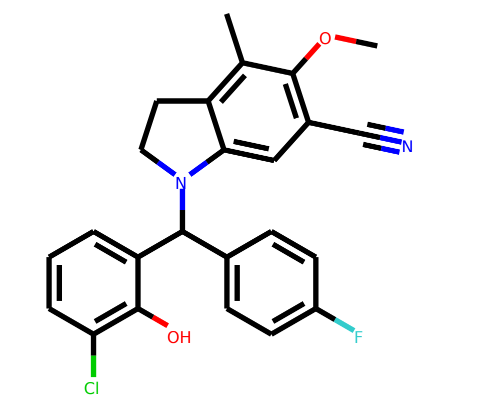
Osma-1506
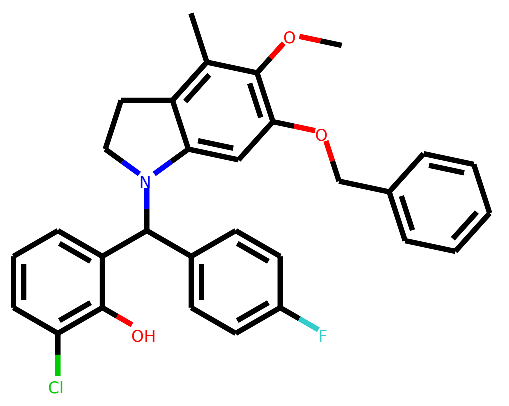
Osma-1507
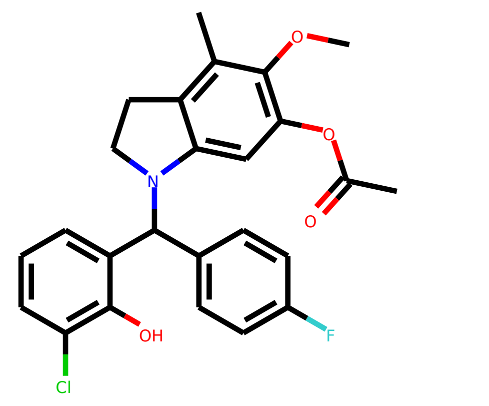
Osma-1508
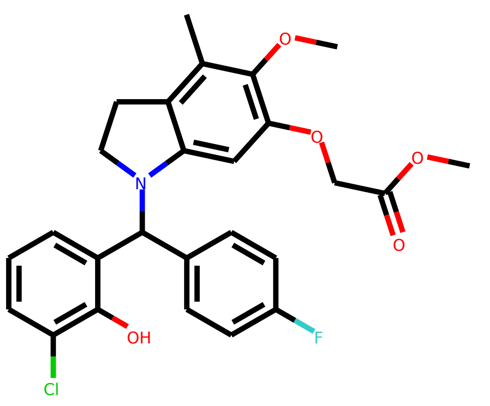
Osma-1509
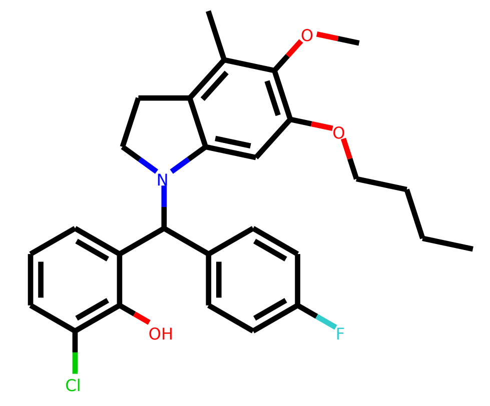
Osma-1510
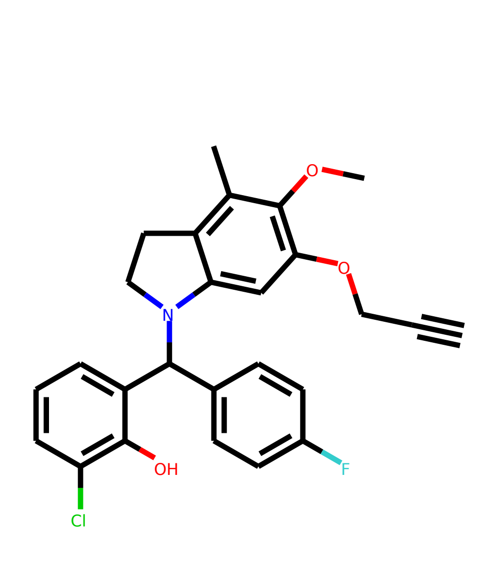
Osma-1511
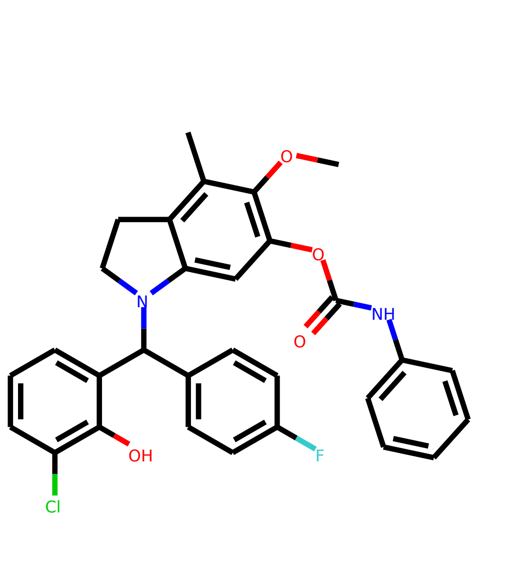
Osma-1512
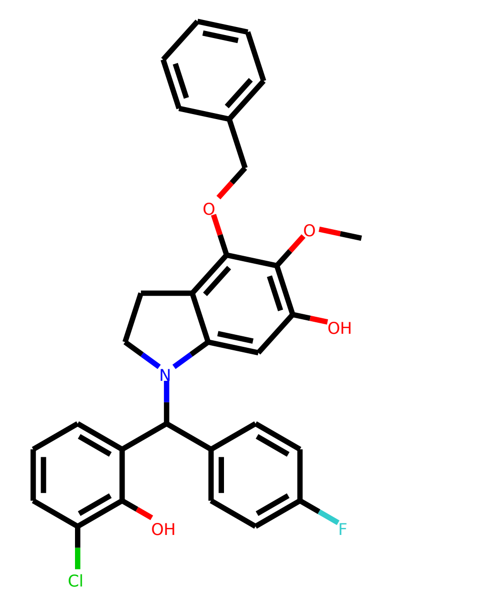
Osma-1513
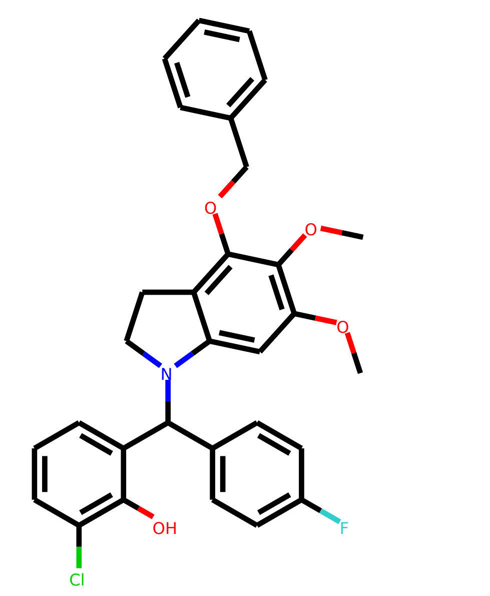
Osma-1514
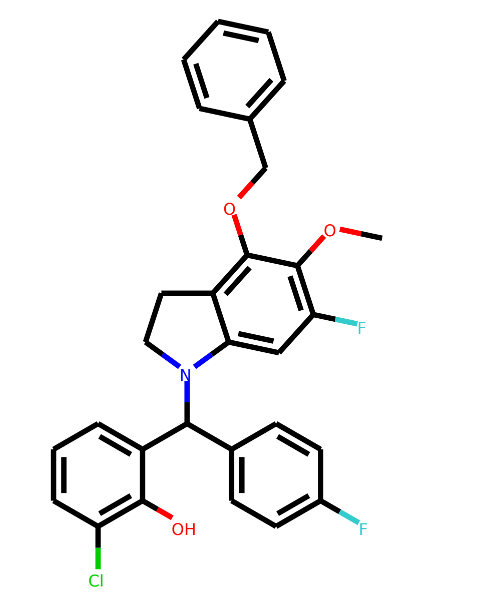
Osma-1515
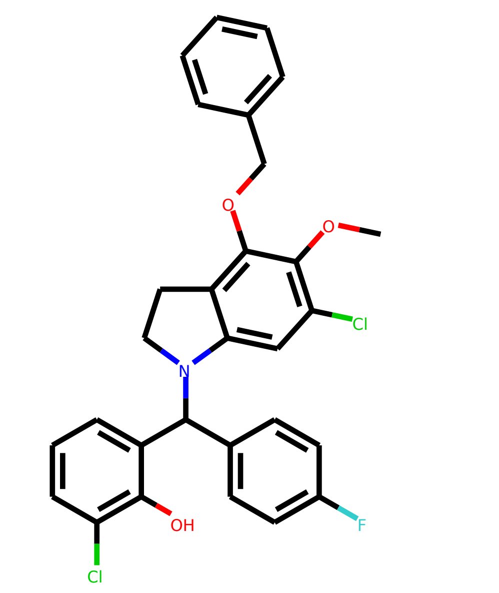
Osma-1516
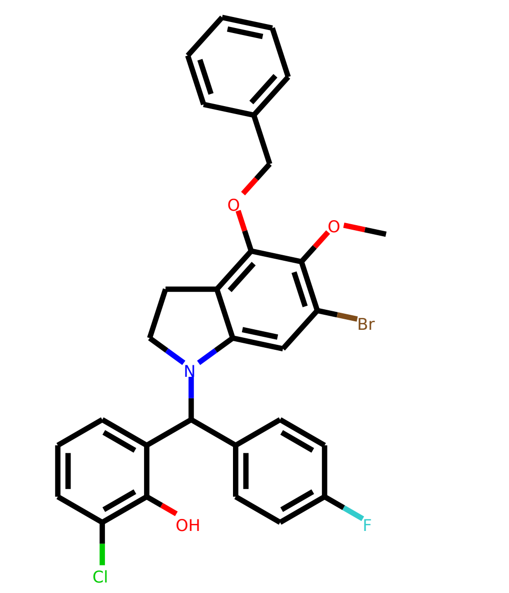
Osma-1517
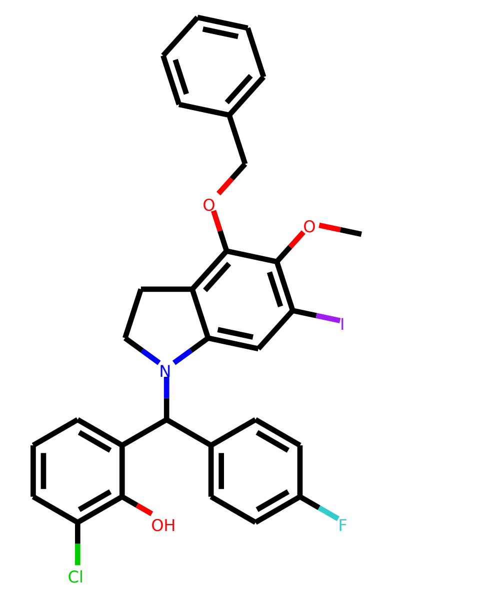
Osma-1518
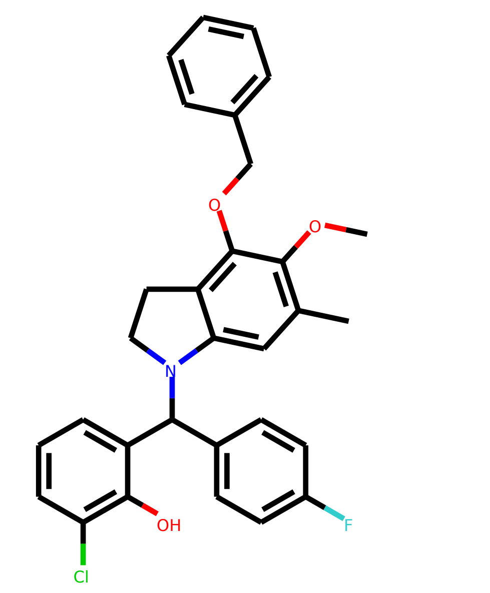
Osma-1519
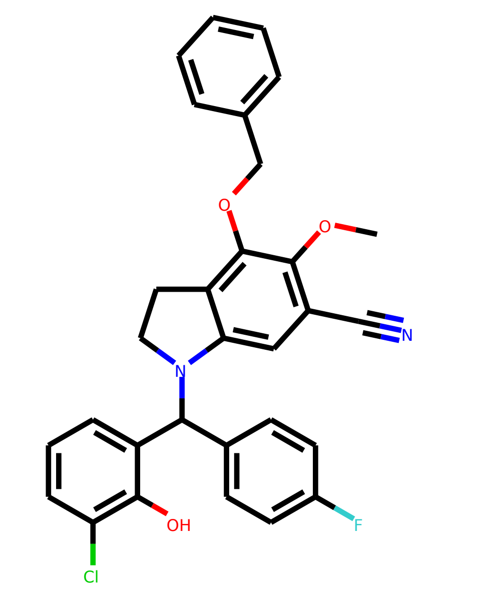
Osma-1520
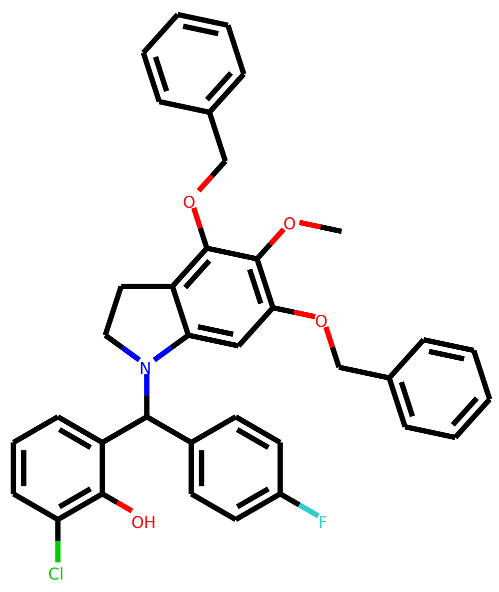
Osma-1521
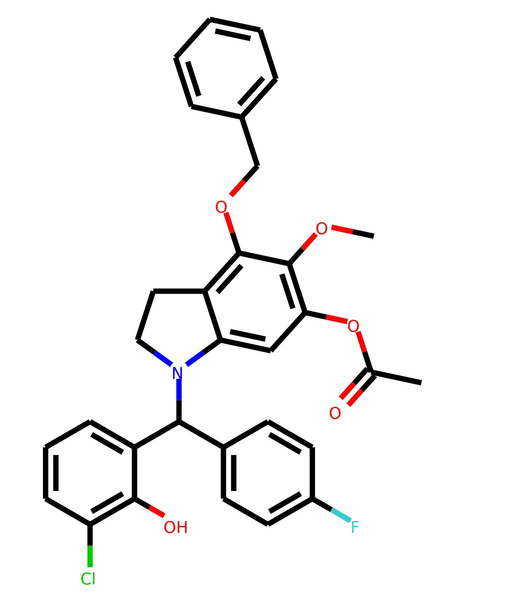
Osma-1522
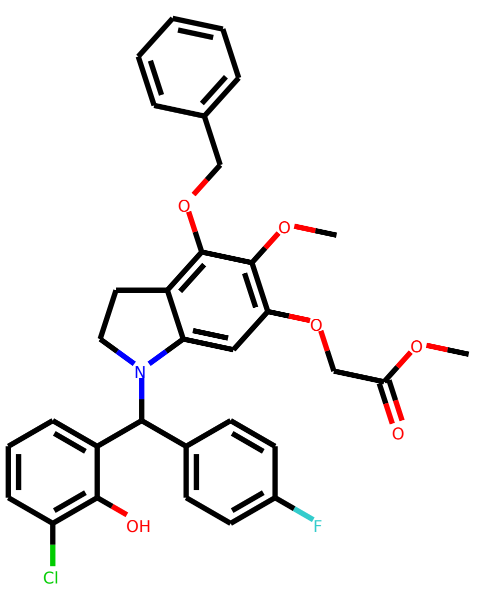
Osma-1523
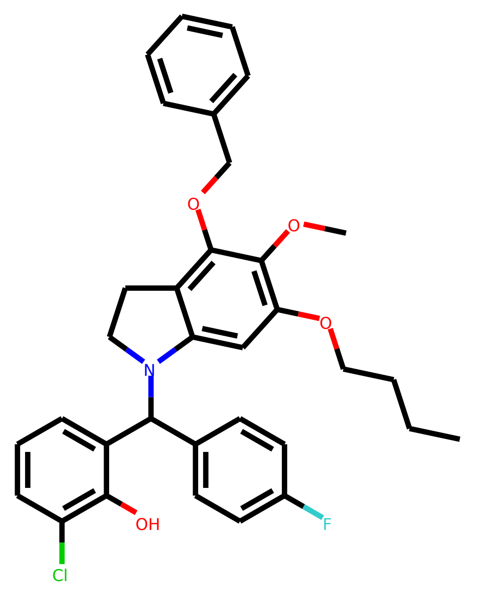
Osma-1524
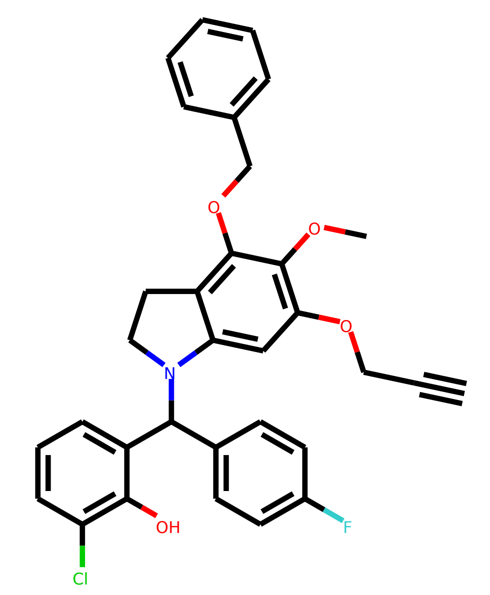
Osma-1525
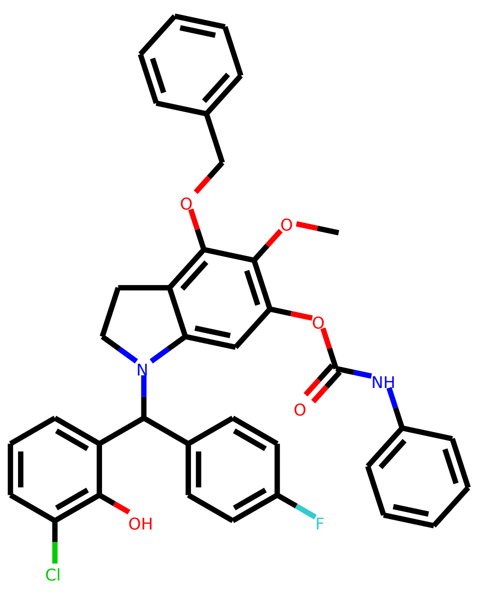
Osma-1526
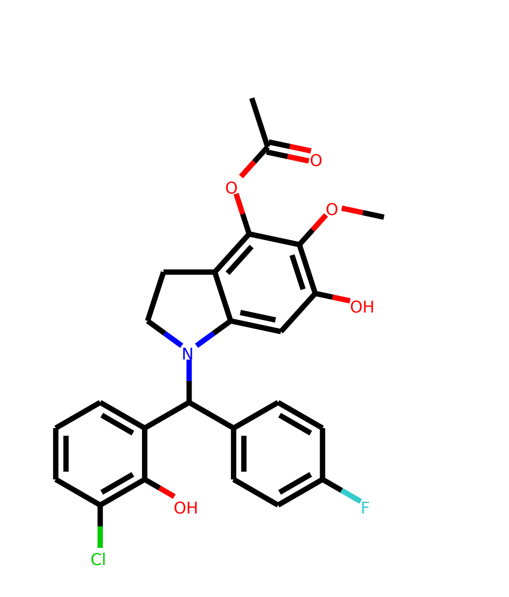
Osma-1527
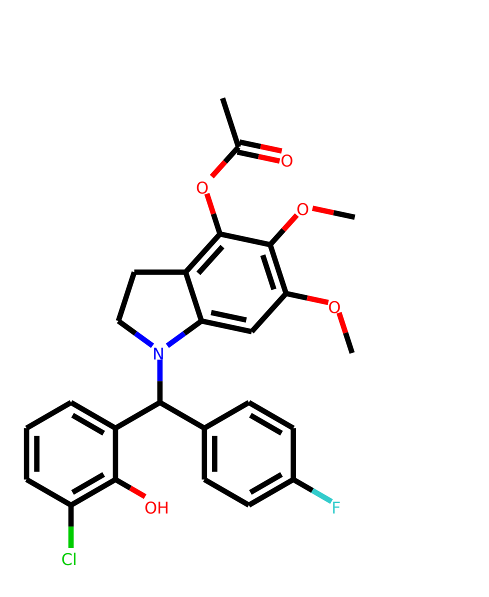
Osma-1528
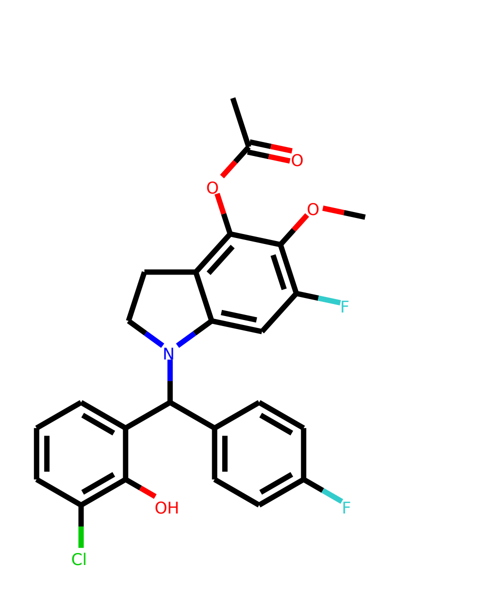
Osma-1529
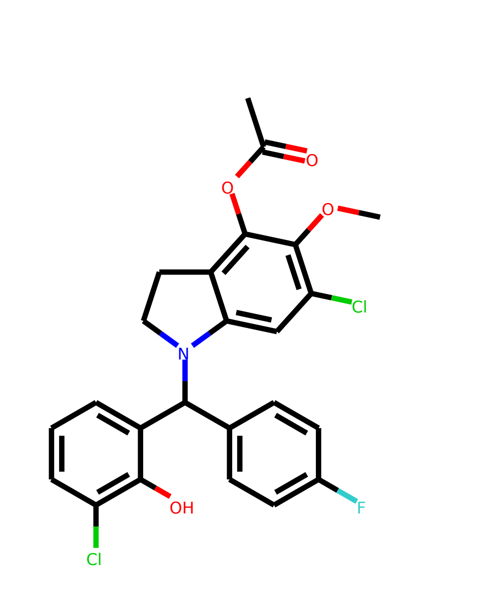
Osma-1530
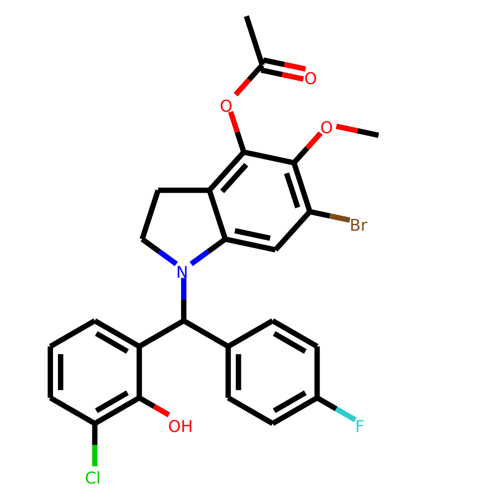
Osma-1531
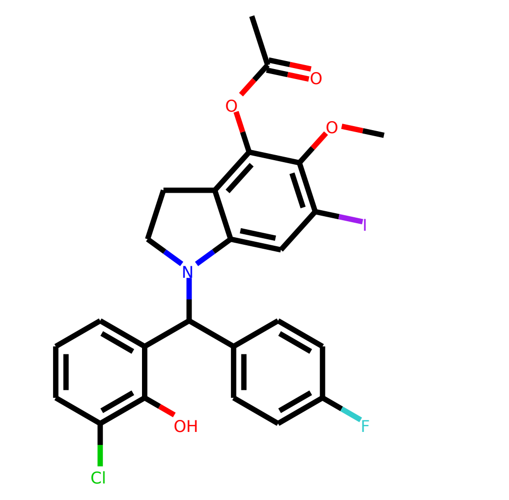
Osma-1532
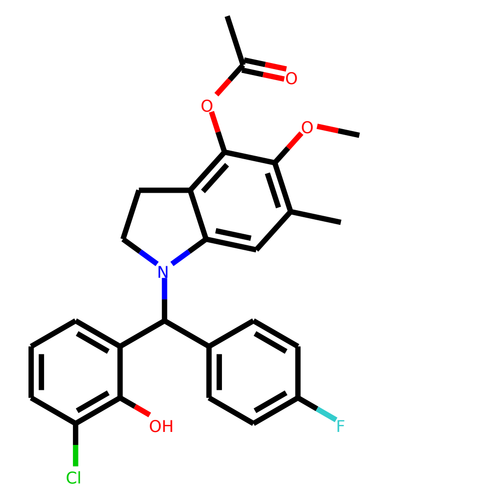
Osma-1533
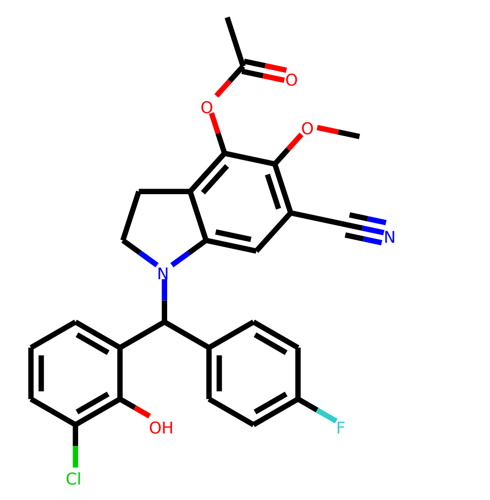
Osma-1534
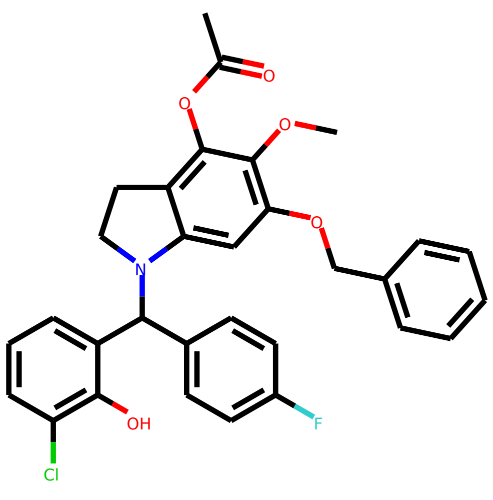
Osma-1535
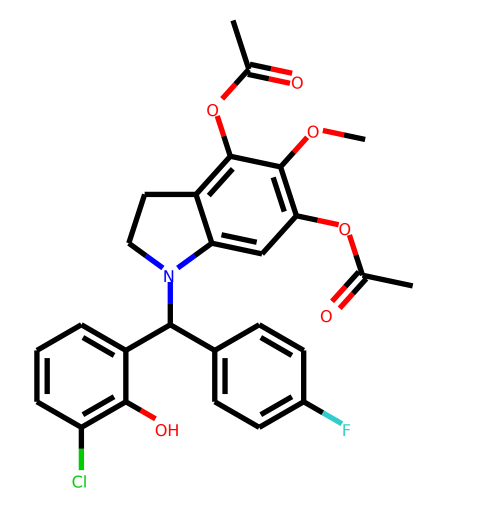
Osma-1536
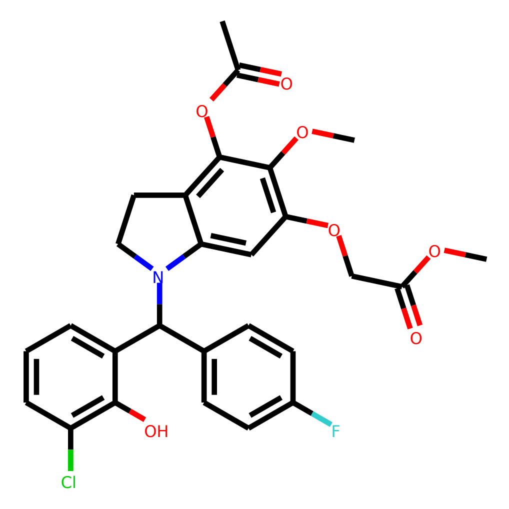
Osma-1537
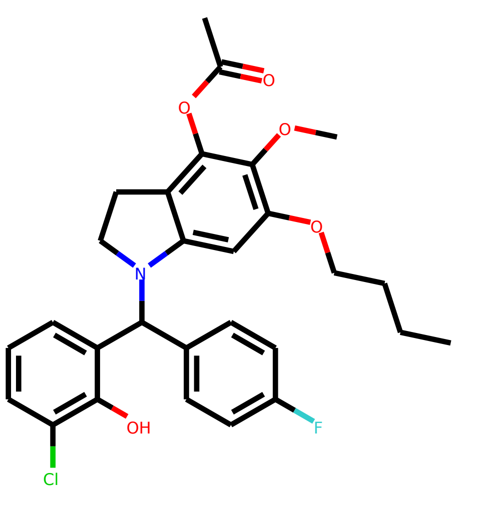
Osma-1538
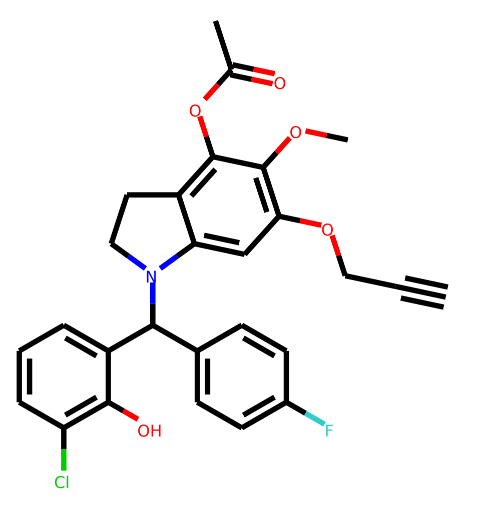
Osma-1539
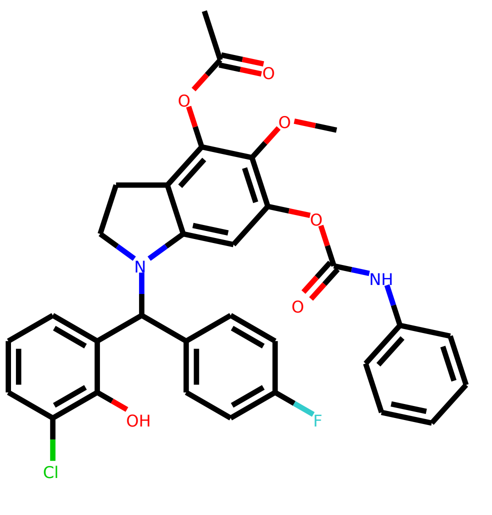
Osma-1540

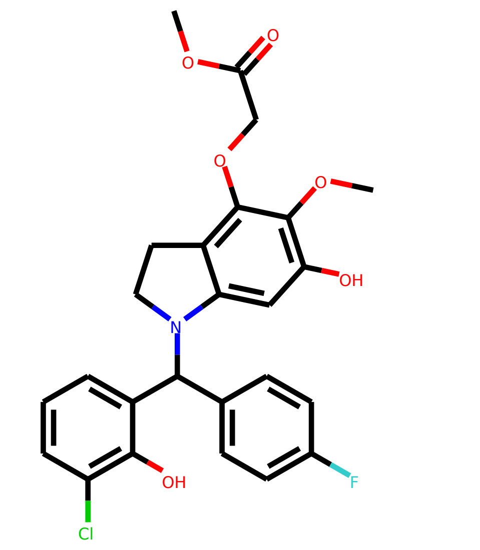
Osma-1541
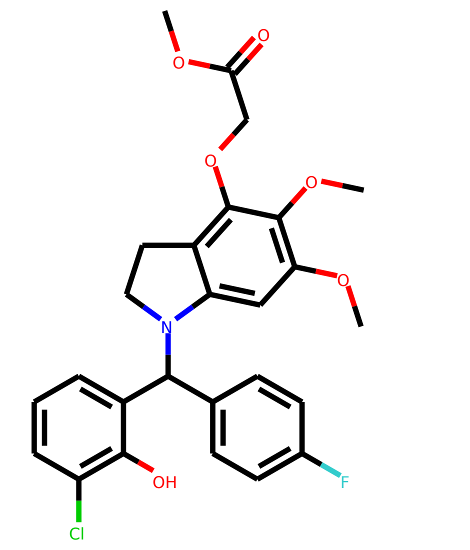
Osma-1542
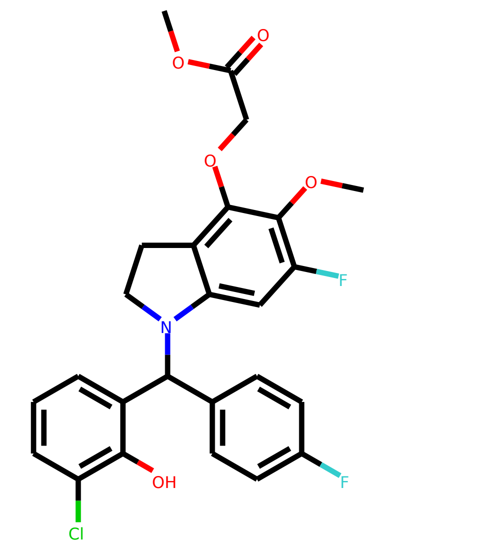
Osma-1543
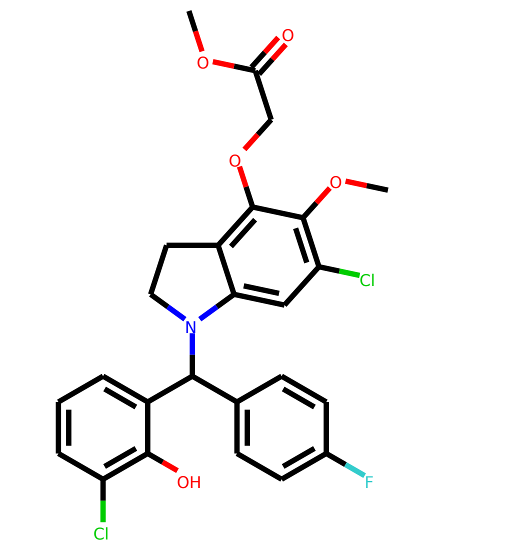
Osma-1544
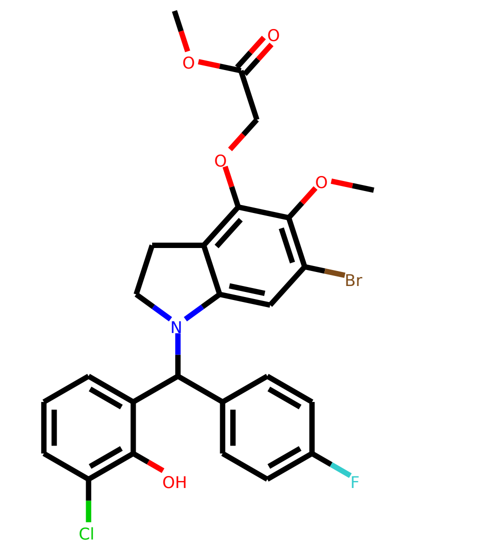
Osma-1545
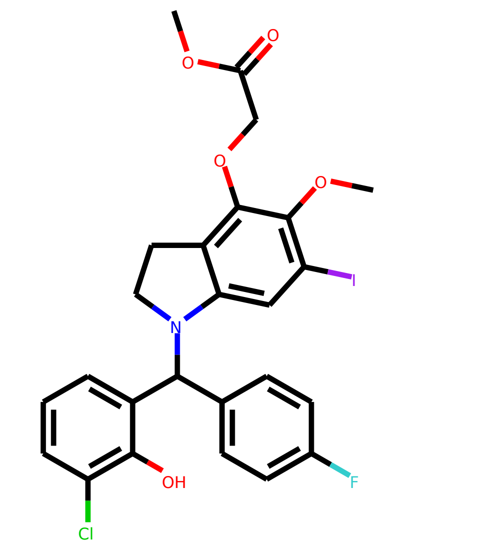
Osma-1546
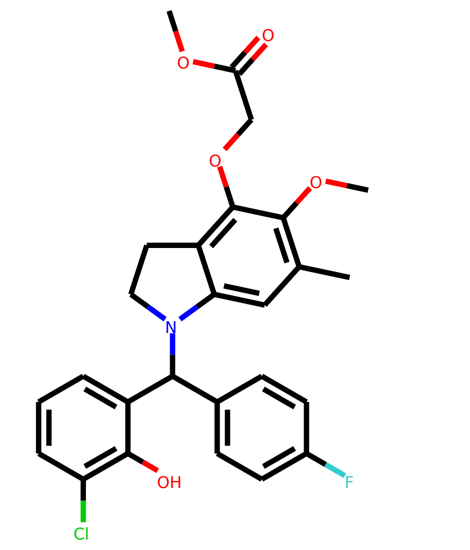
Osma-1547
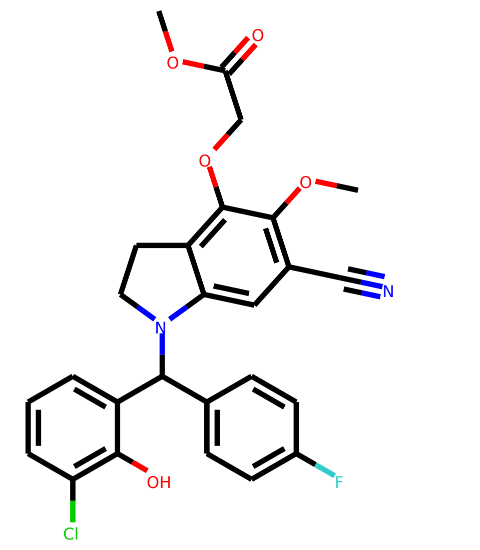
Osma-1548
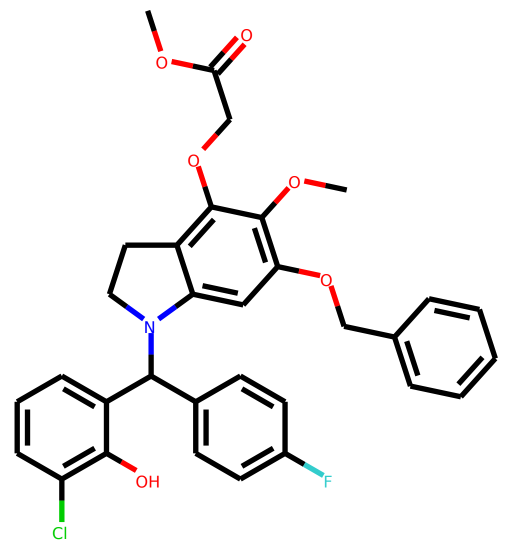
Osma-1549
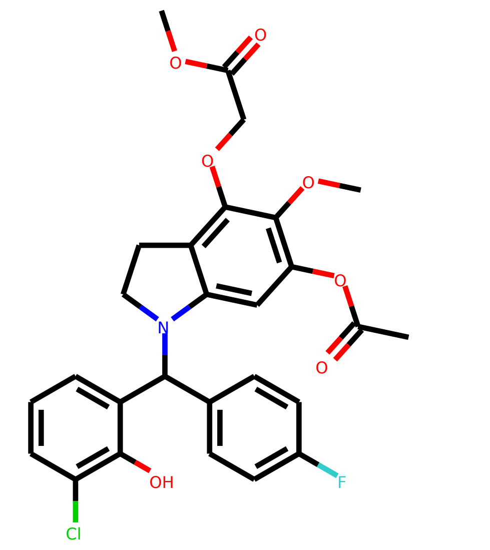
Osma-1550
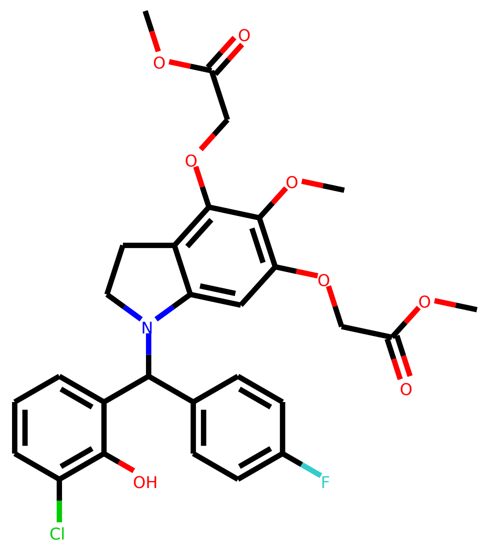
Osma-1551
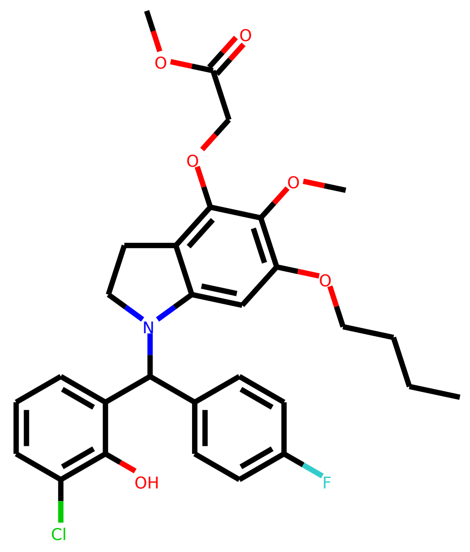
Osma-1552
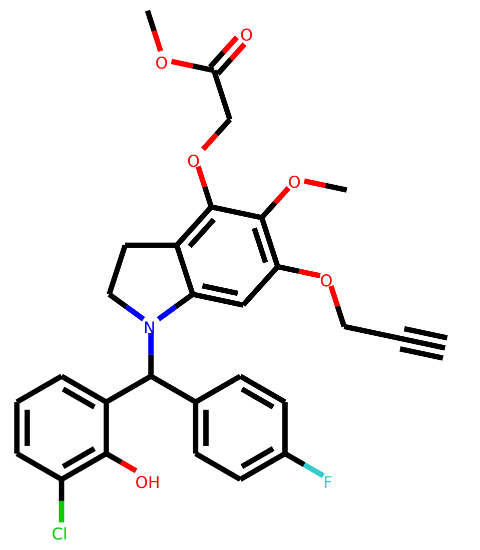
Osma-1553
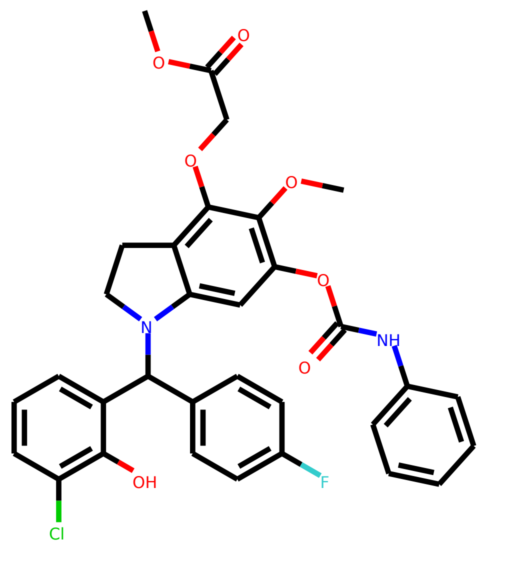
Osma-1554
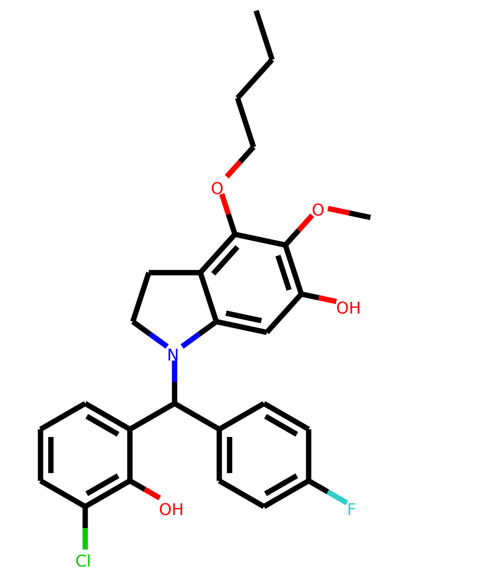
Osma-1555
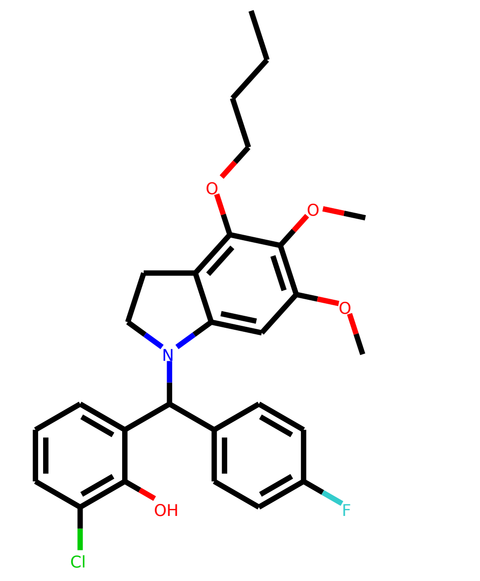
Osma-1556
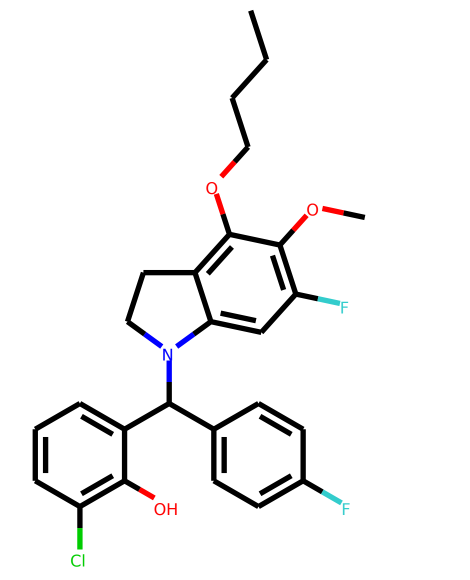
Osma-1557
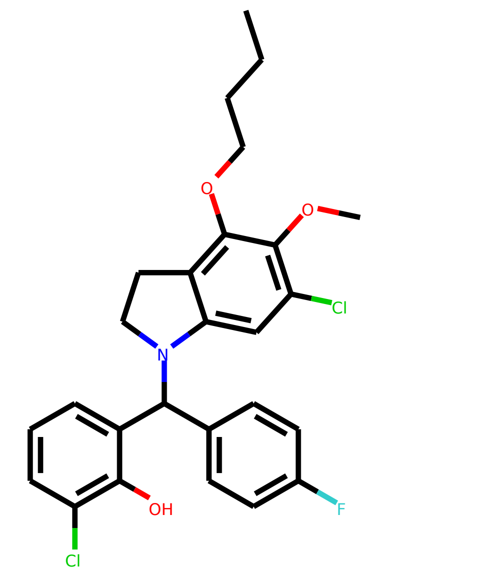
Osma-1558
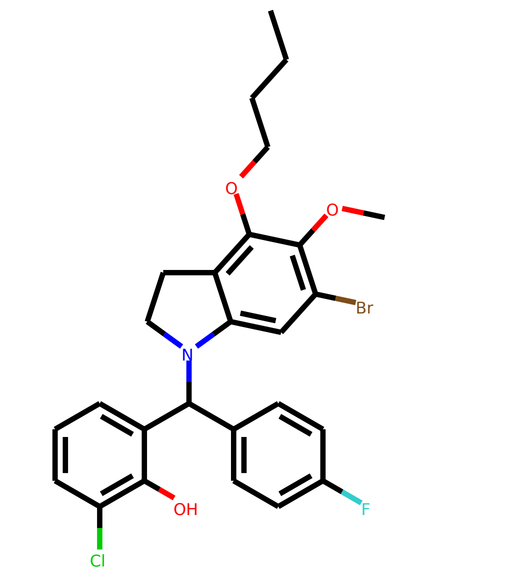
Osma-1559
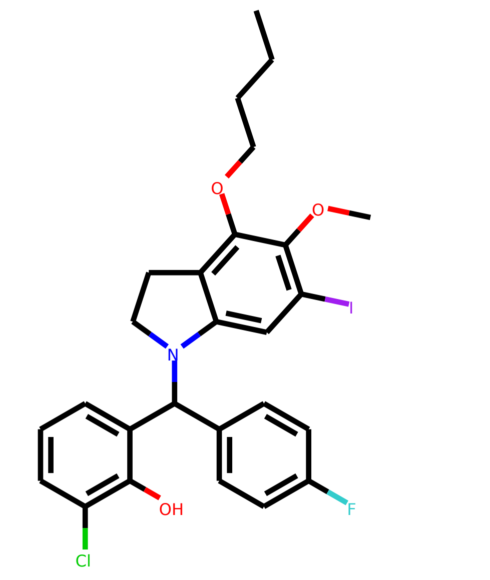
Osma-1560
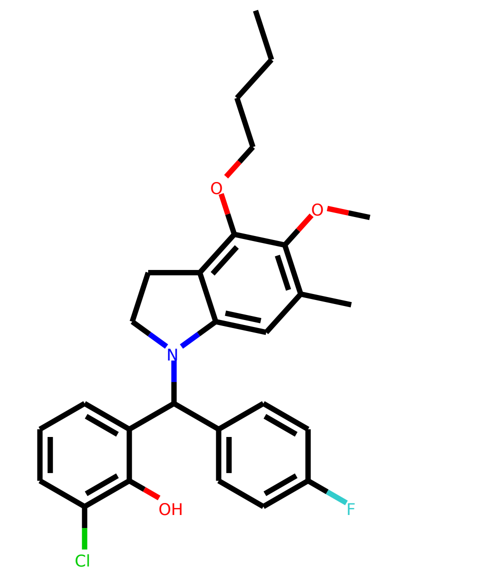
Osma-1561
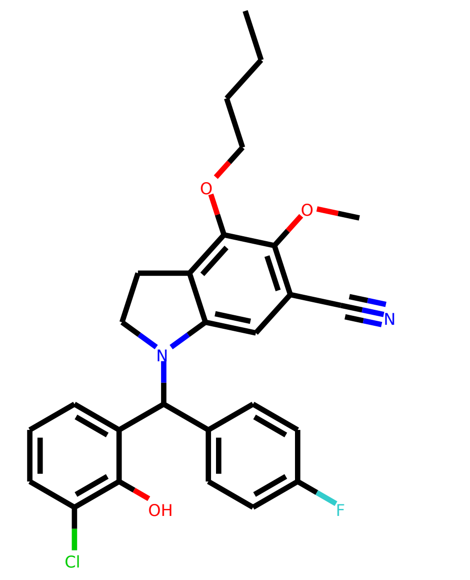
Osma-1562
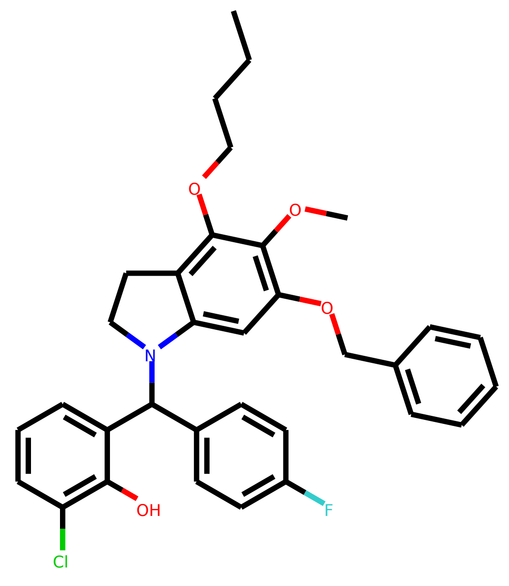
Osma-1563
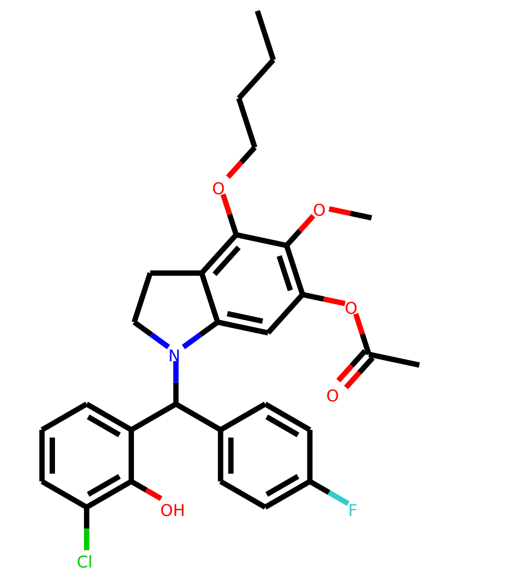
Osma-1564
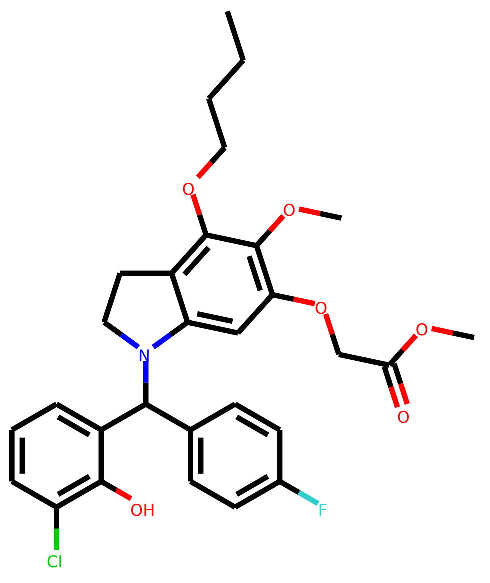
Osma-1565
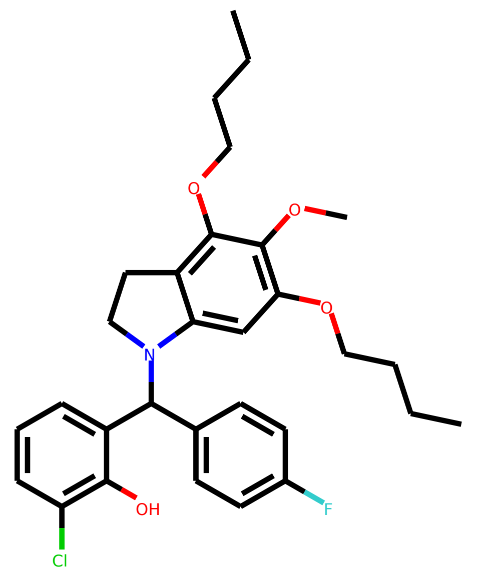
Osma-1566
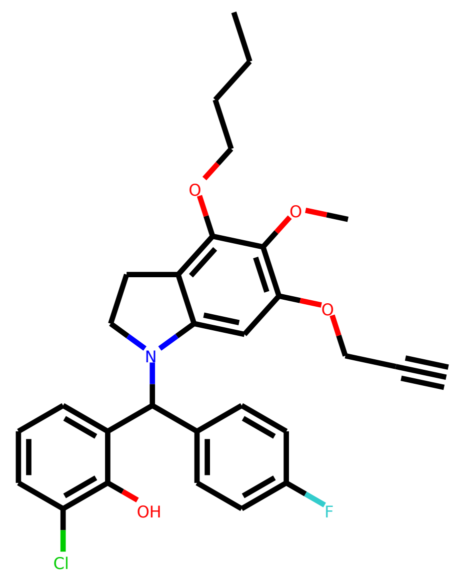
Osma-1567
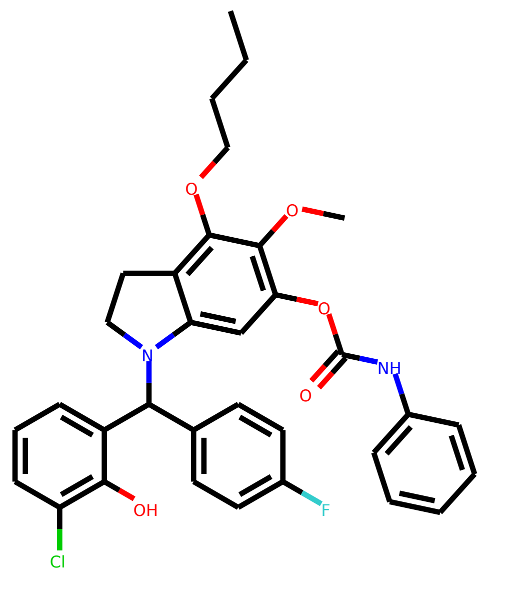
Osma-1568
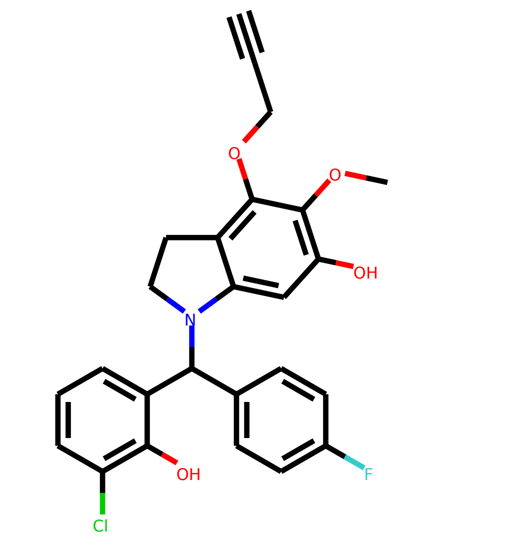
Osma-1569
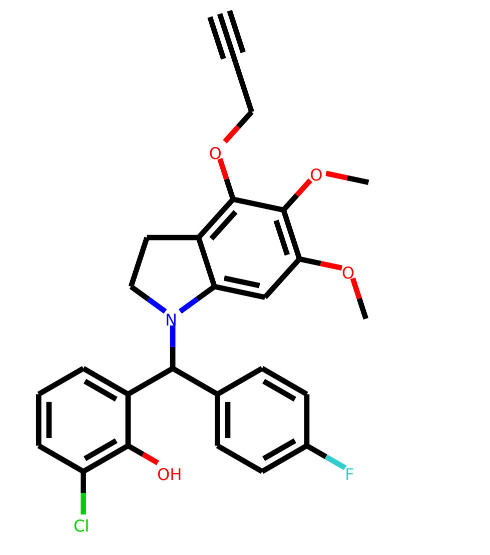
Osma-1570
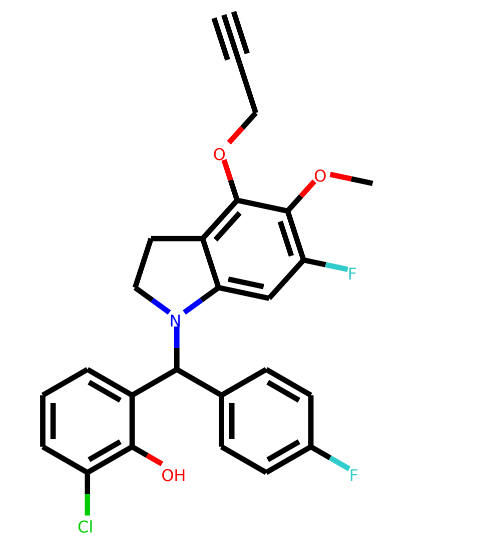
Osma-1571
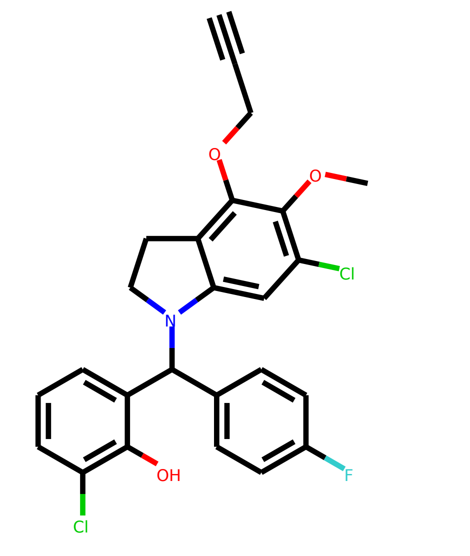
Osma-1572
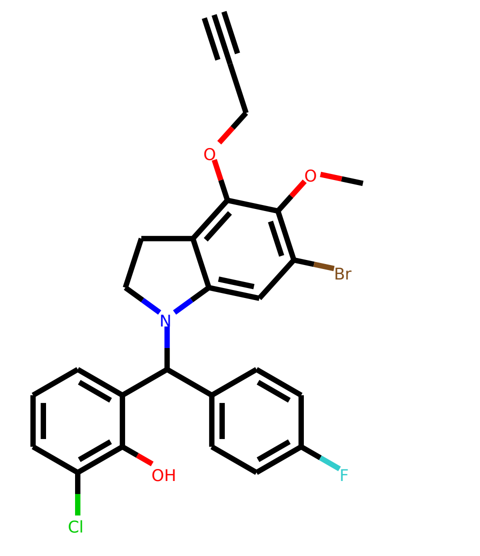
Osma-1573
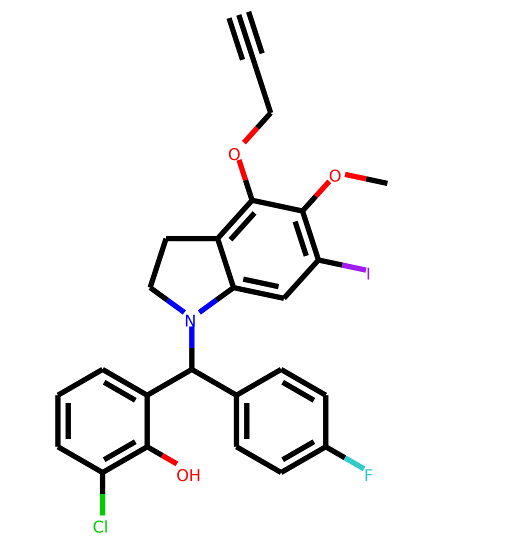
Osma-1574
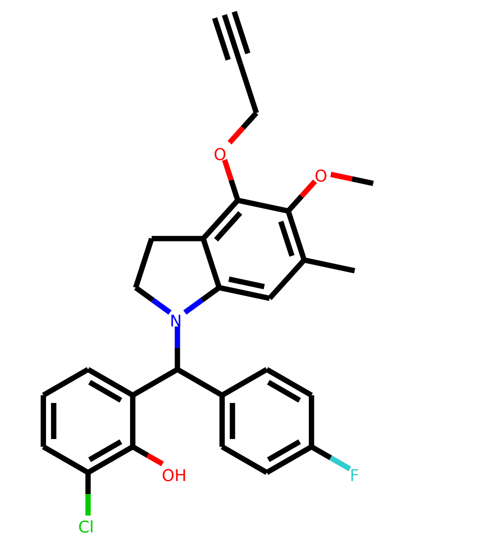
Osma-1575

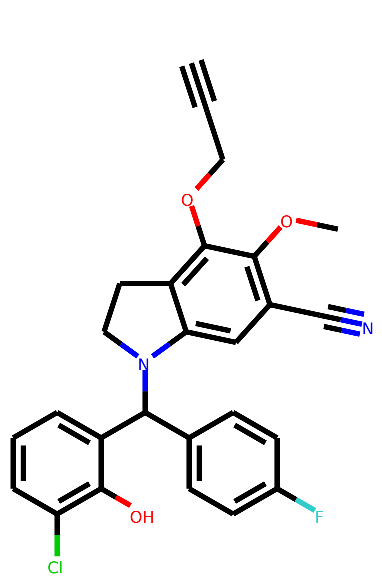
Osma-1576

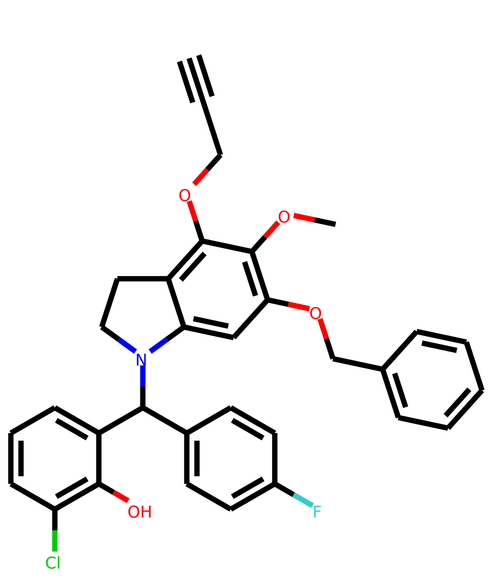
Osma-1577

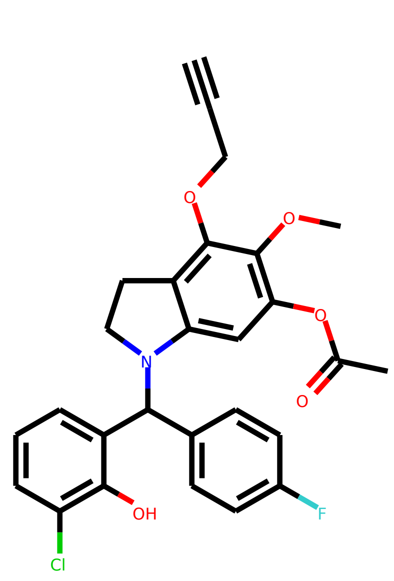
Osma-1578

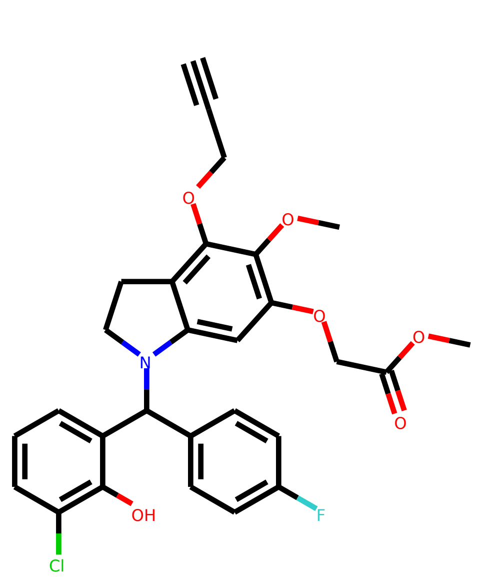
Osma-1579

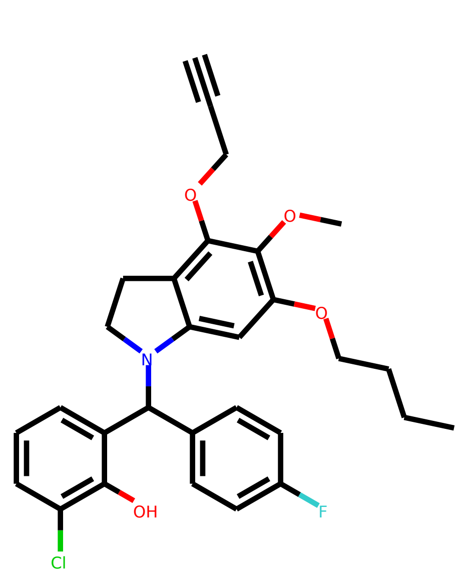
Osma-1580

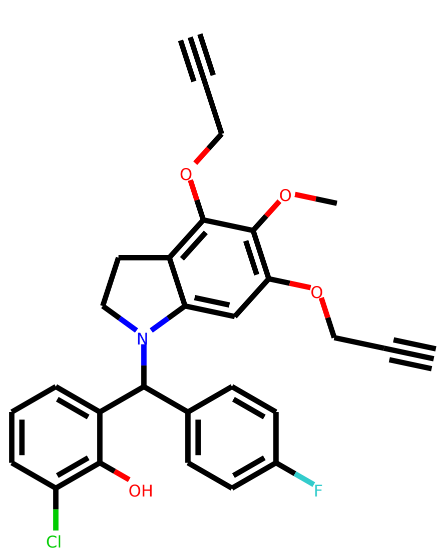
Osma-1581

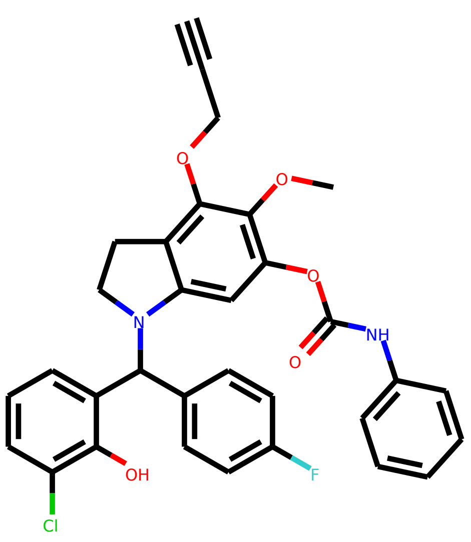
Osma-1582

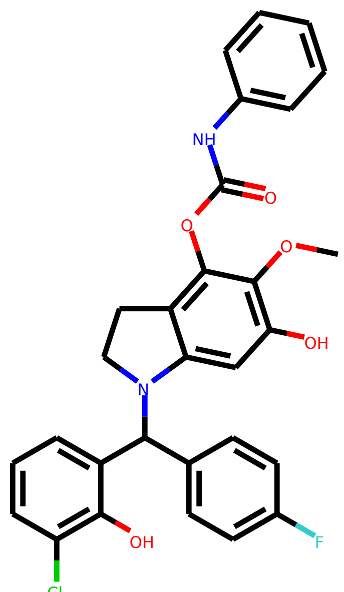
Osma-1583

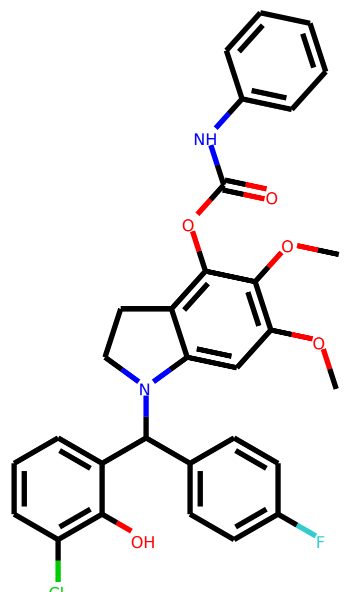
Osma-1584

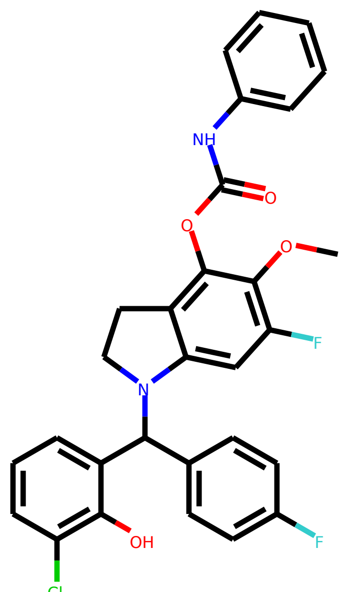
Osma-1585

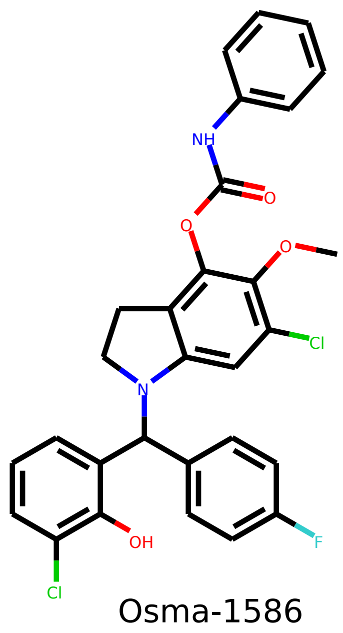
Osma-1586

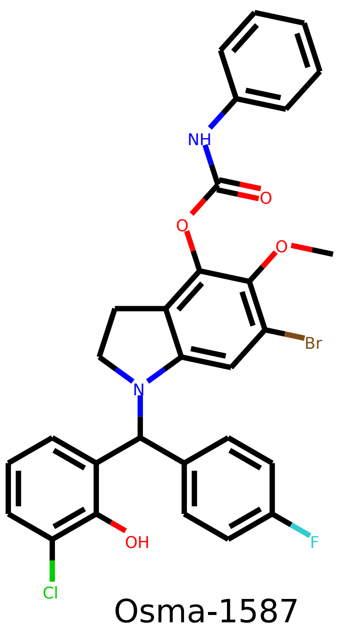
Osma-1587

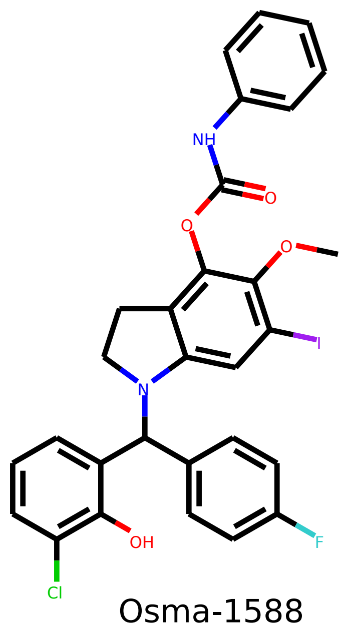
Osma-1588

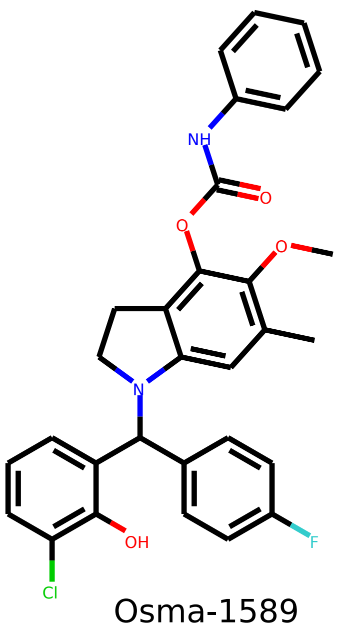
Osma-1589

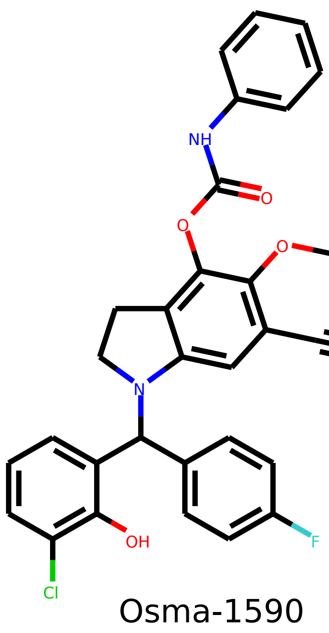
Osma-1590

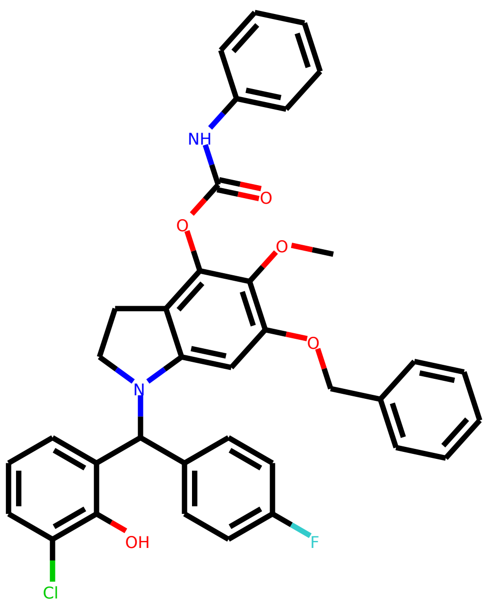
Osma-1591

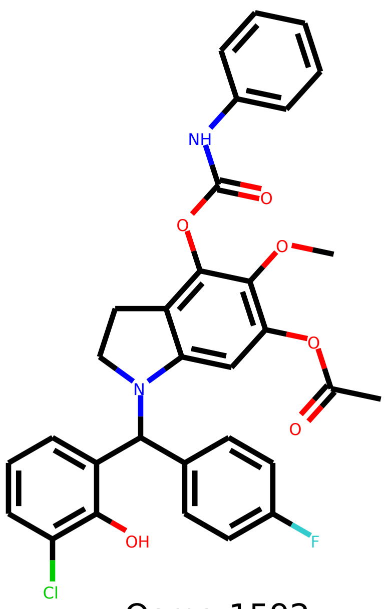
Osma-1592

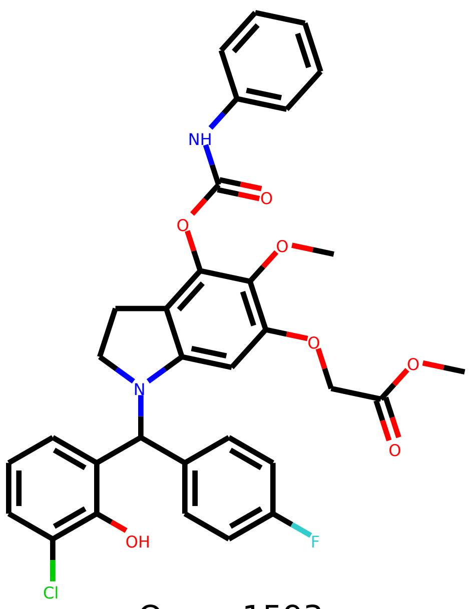
Osma-1593

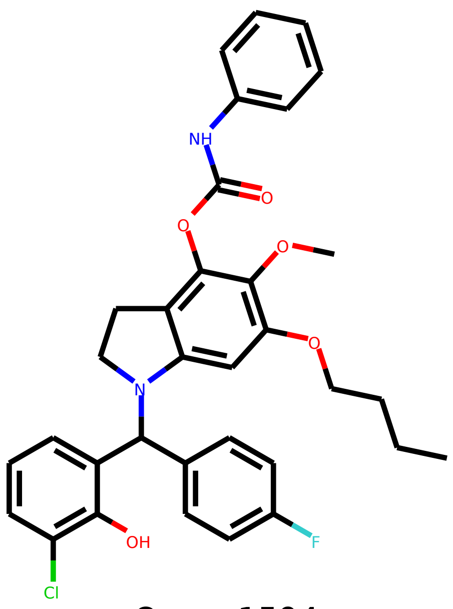
Osma-1594

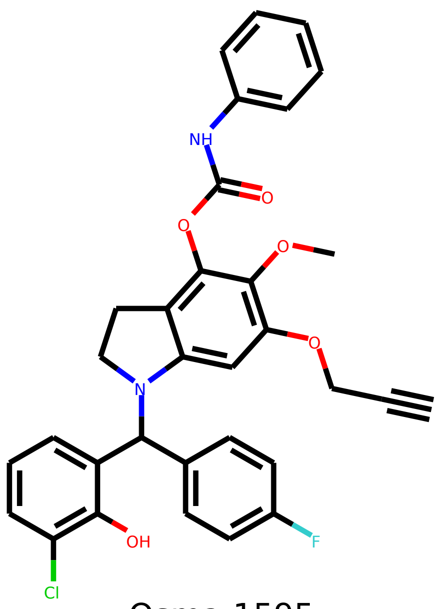
Osma-1595

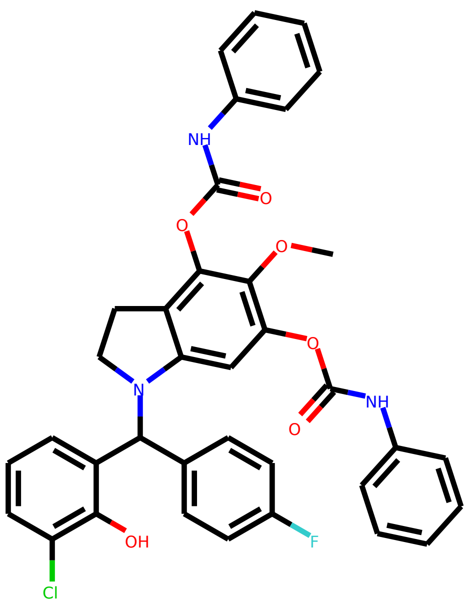
Osma-1596

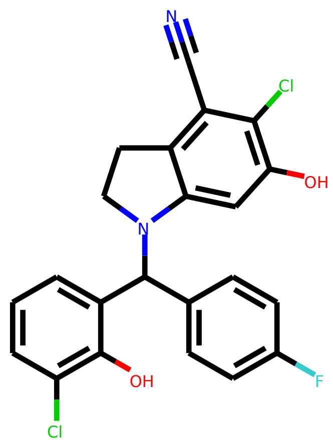
Osma-1597

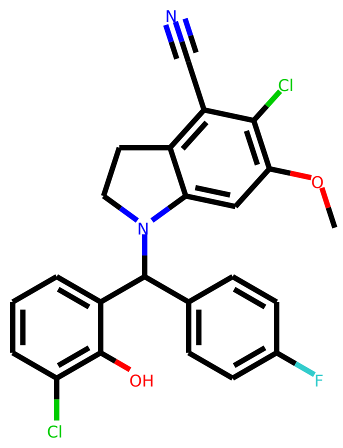
Osma-1598

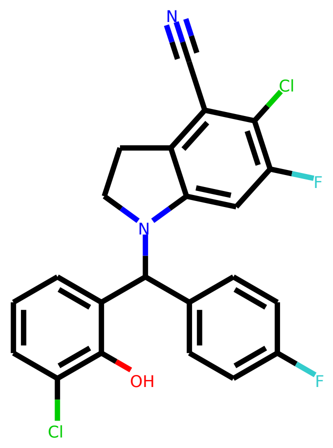
Osma-1599

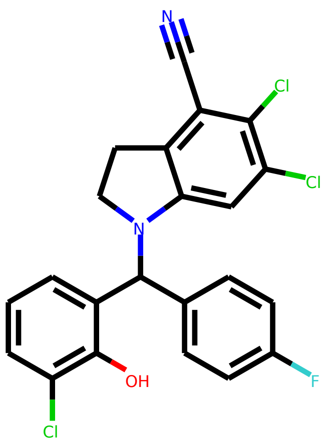
Osma-1600

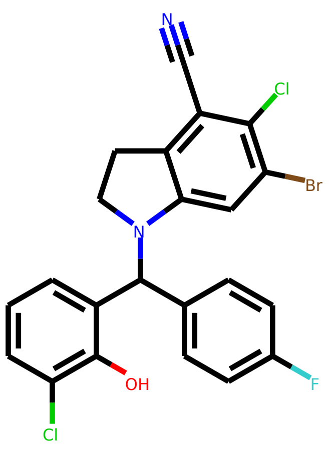
Osma-1601

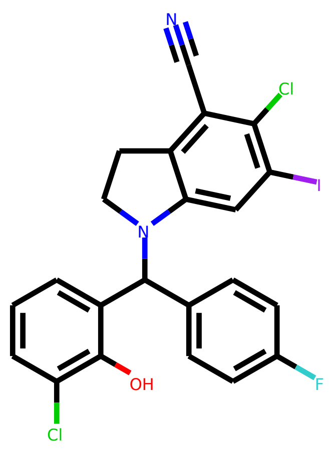
Osma-1602

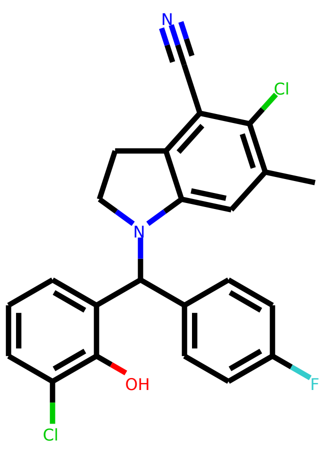
Osma-1603

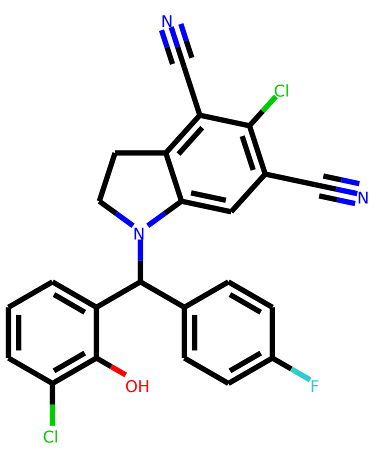
Osma-1604

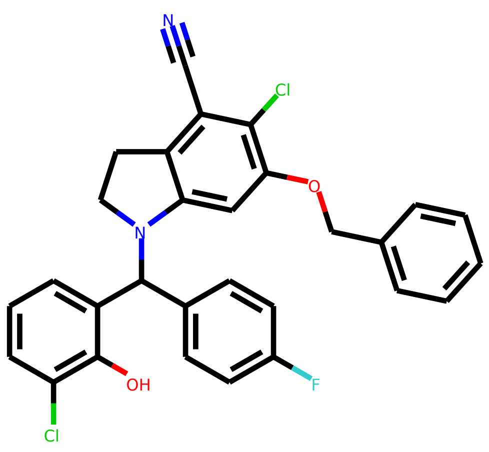
Osma-1605

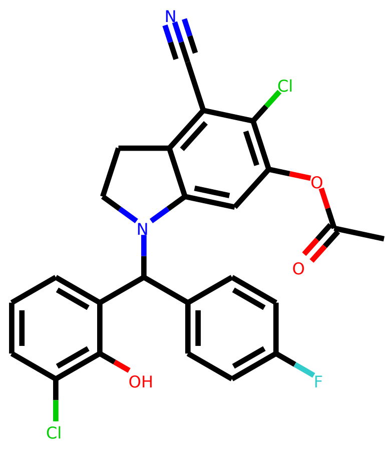
Osma-1606

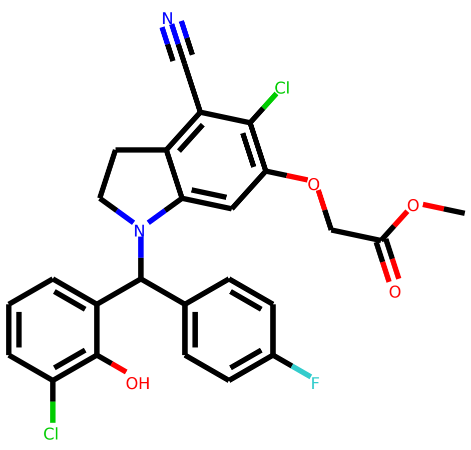
Osma-1607

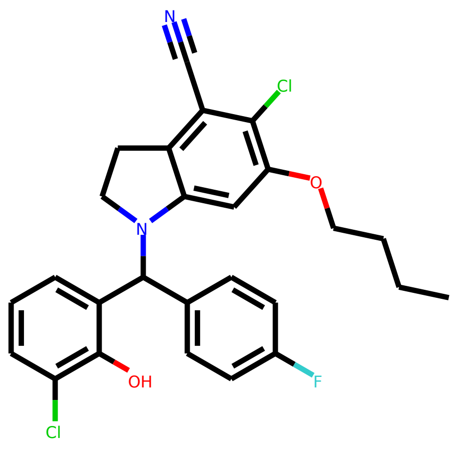
Osma-1608

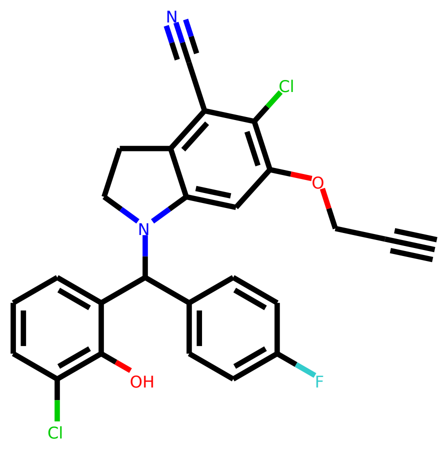
Osma-1609

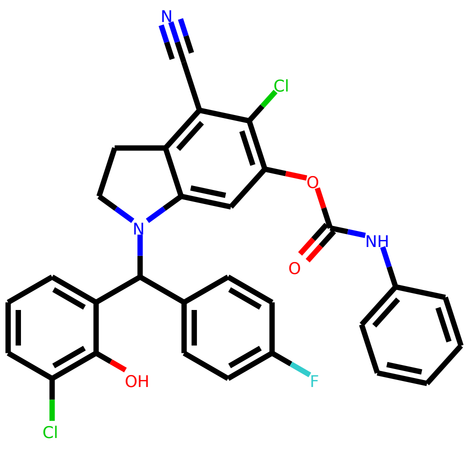
Osma-1610

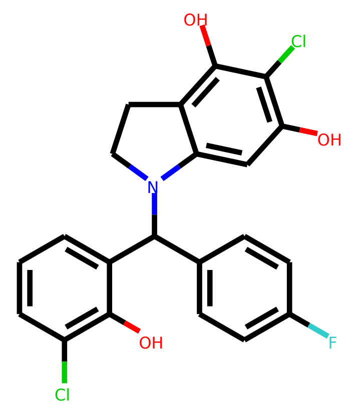

Osma-1611

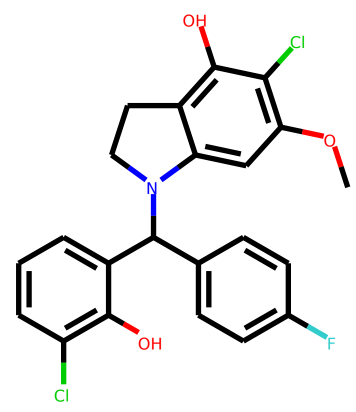

Osma-1612

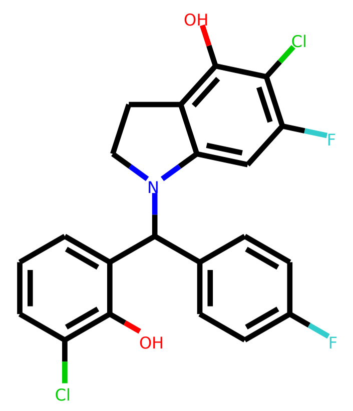

Osma-1613

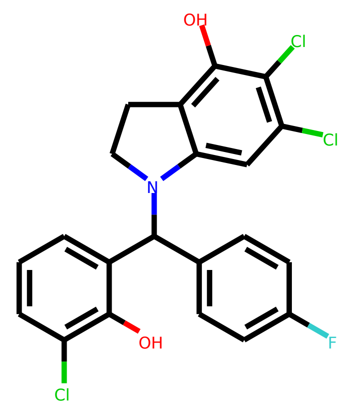

Osma-1614

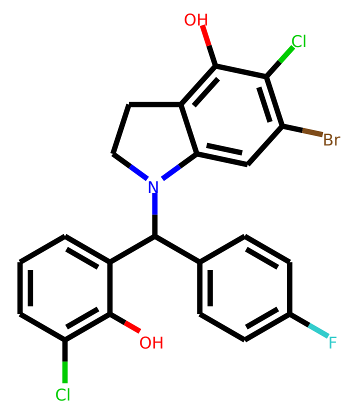

Osma-1615

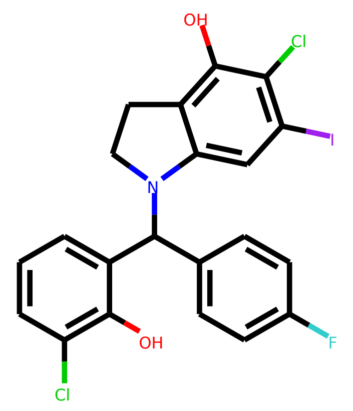

Osma-1616

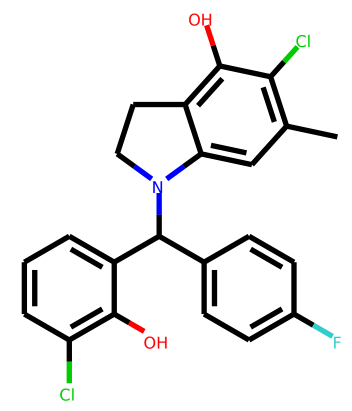

Osma-1617

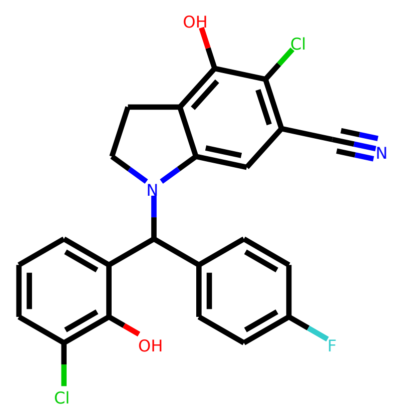

Osma-1618

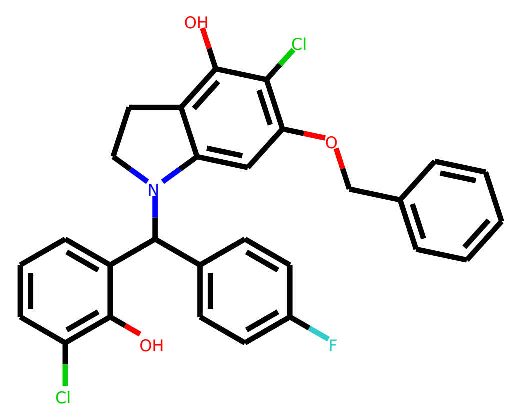

Osma-1619

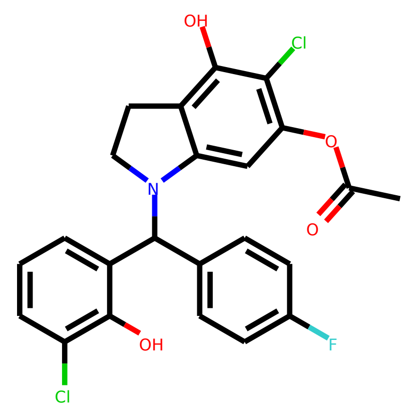

Osma-1620

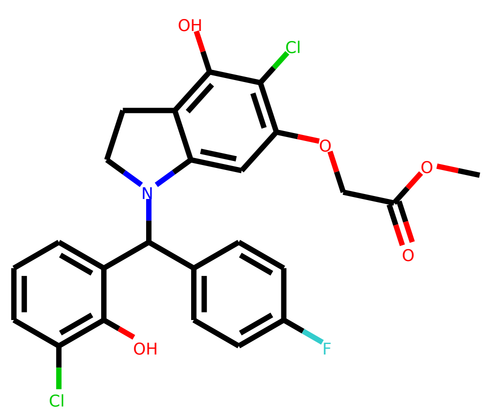

Osma-1621

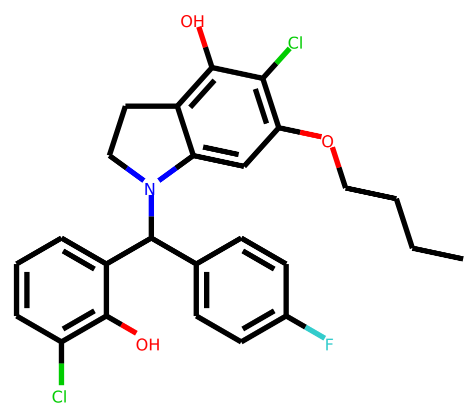

Osma-1622

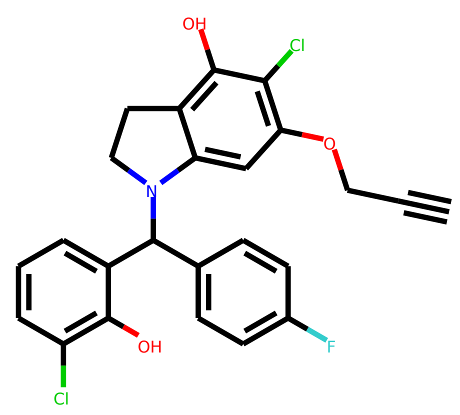

Osma-1623

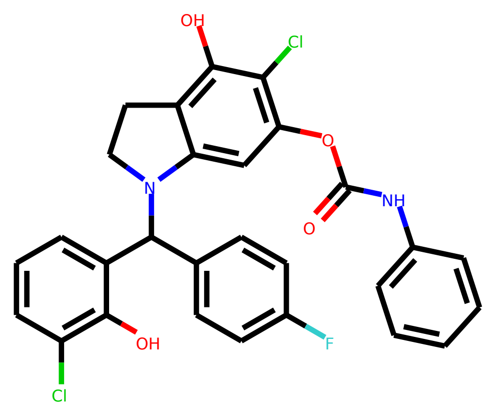

Osma-1624

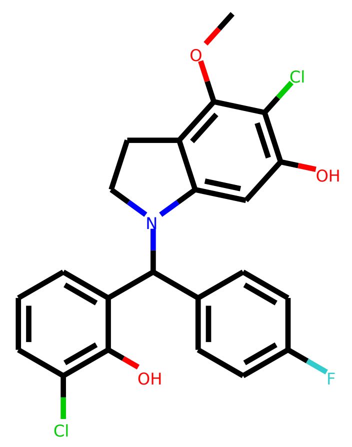

Osma-1625

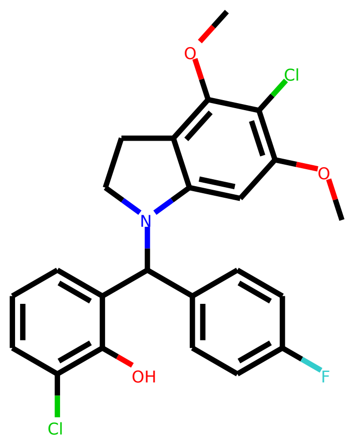

Osma-1626

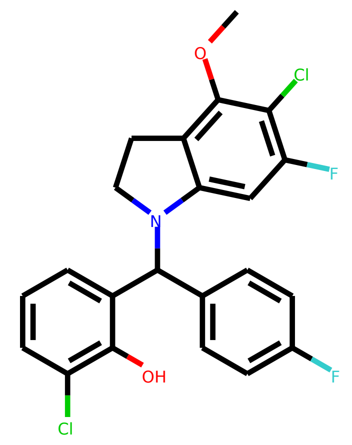

Osma-1627

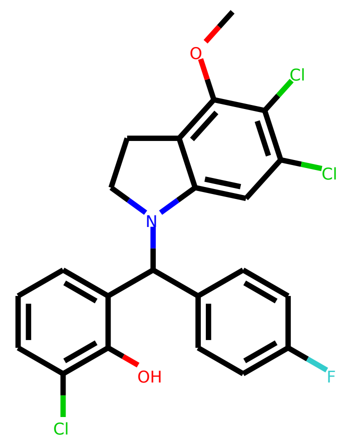

Osma-1628

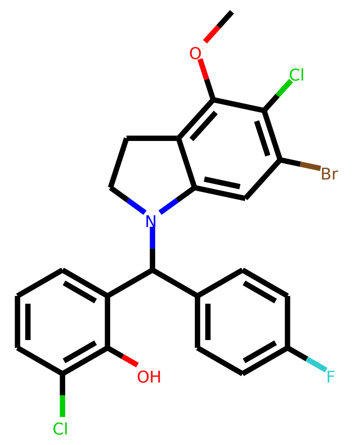

Osma-1629

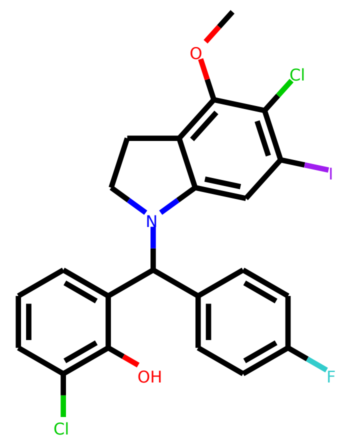

Osma-1630

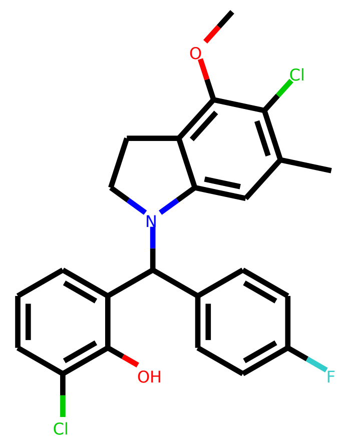

Osma-1631

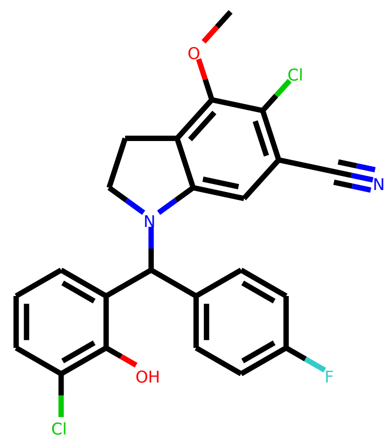

Osma-1632

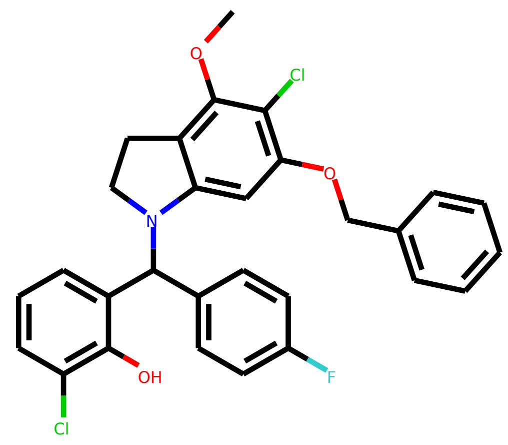

Osma-1633

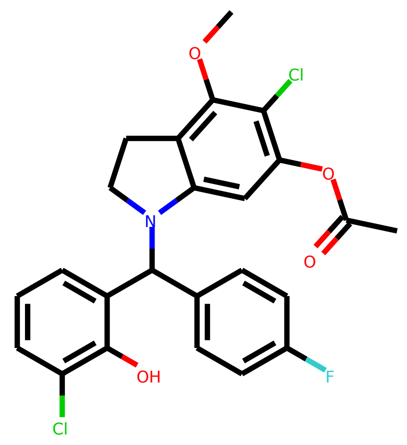

Osma-1634

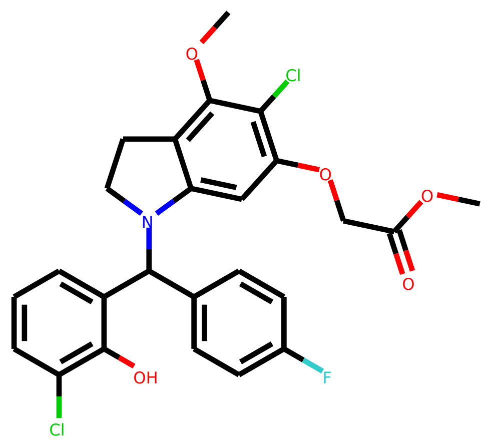

Osma-1635

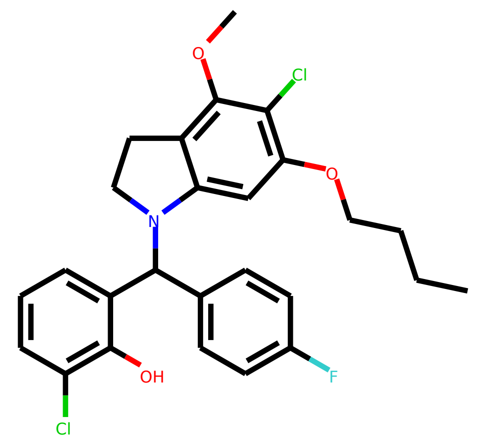

Osma-1636

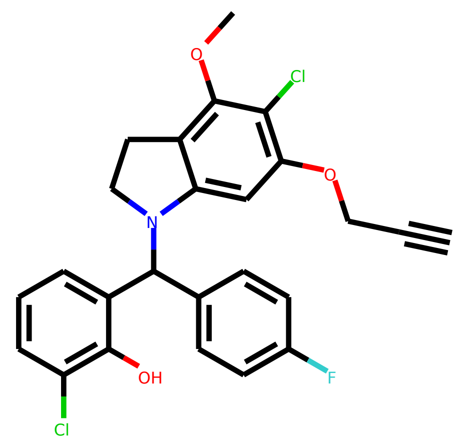

Osma-1637

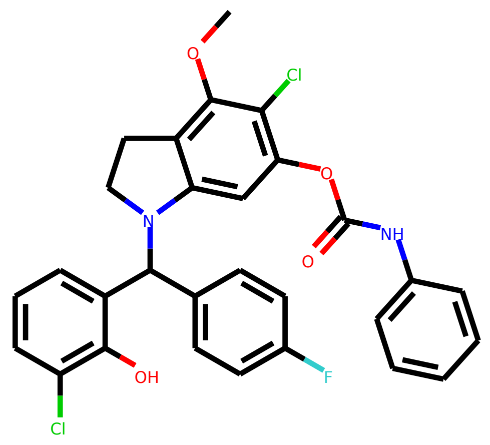

Osma-1638

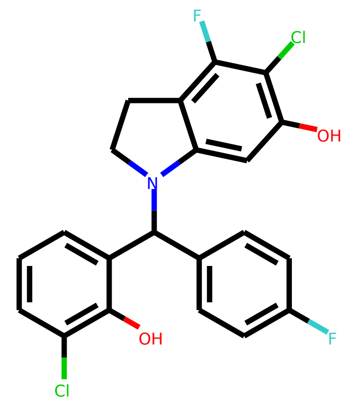

Osma-1639

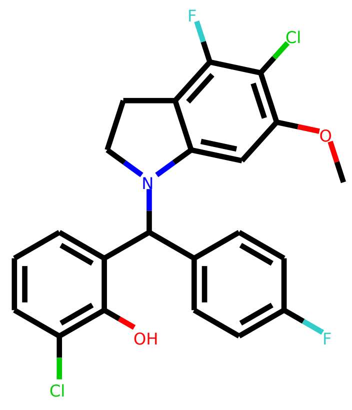

Osma-1640

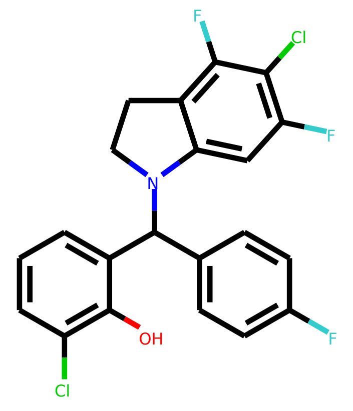

Osma-1641

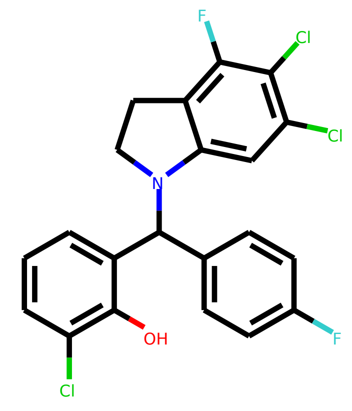

Osma-1642

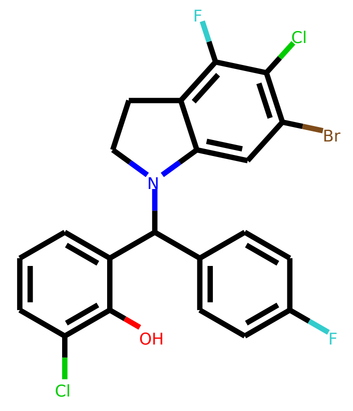

Osma-1643

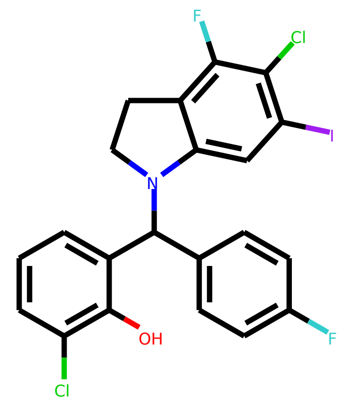

Osma-1644

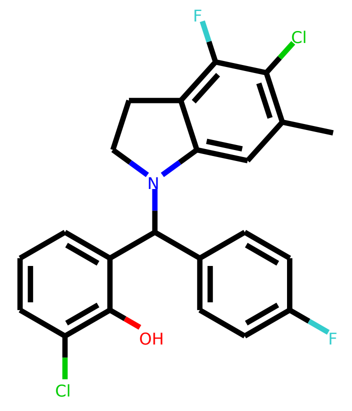

Osma-1645

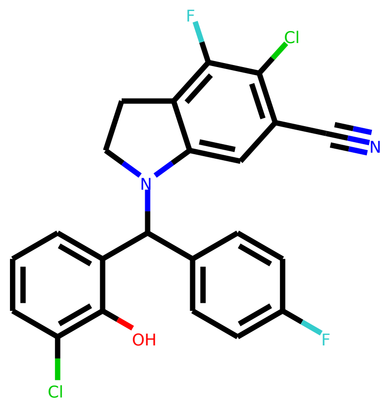

Osma-1646

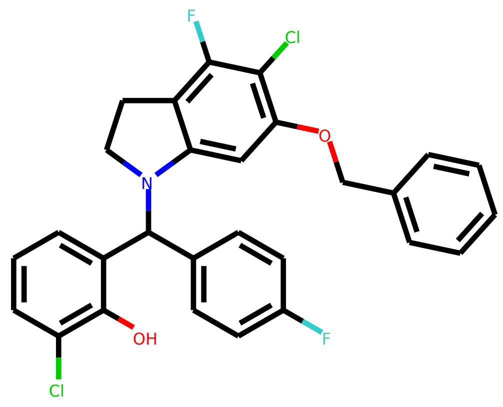

Osma-1647

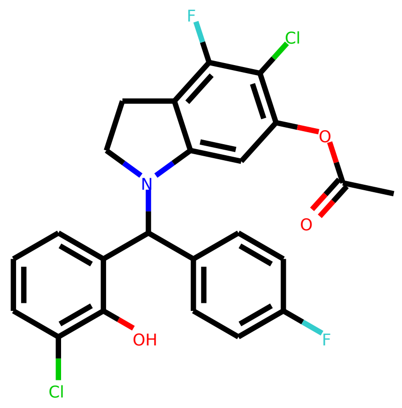

Osma-1648

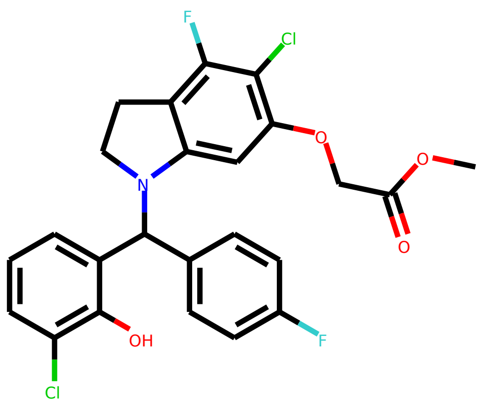

Osma-1649

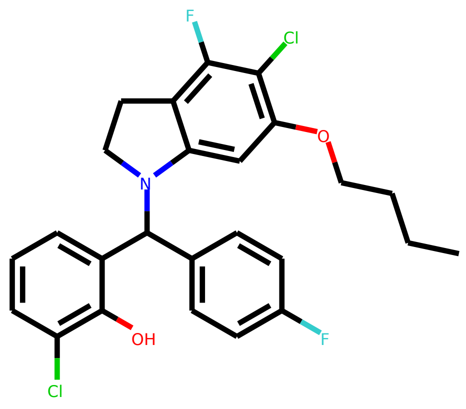

Osma-1650

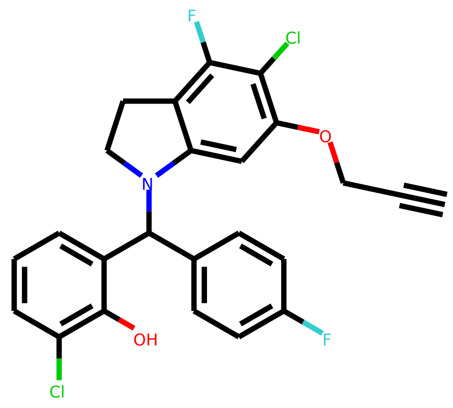

Osma-1651

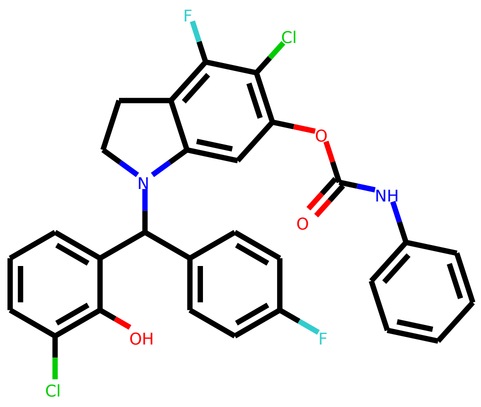

Osma-1652

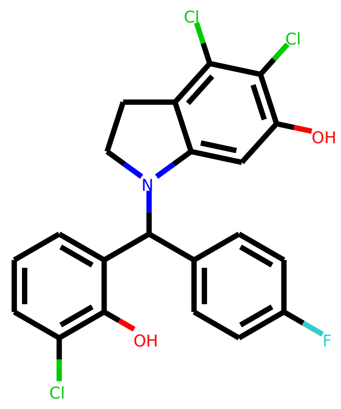

Osma-1653

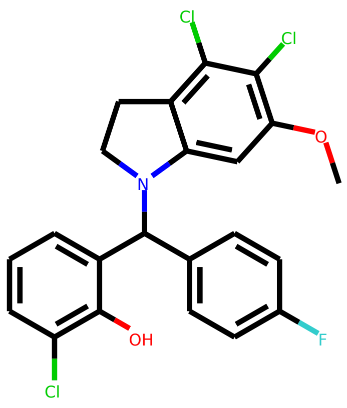

Osma-1654

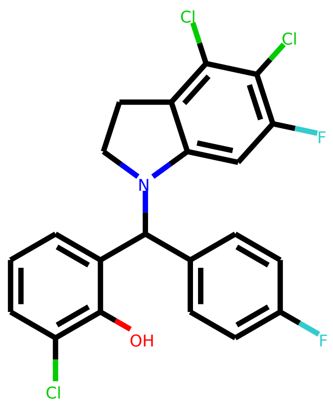

Osma-1655

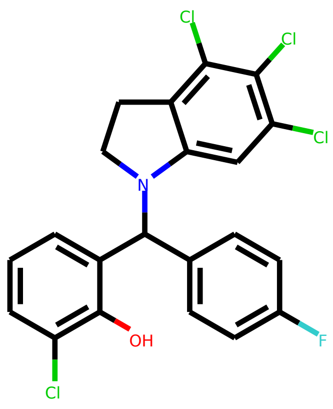

Osma-1656

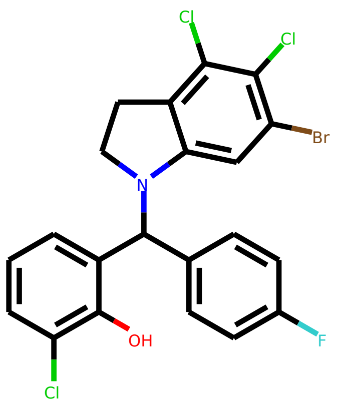

Osma-1657

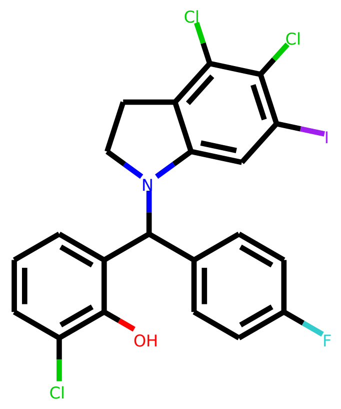

Osma-1658

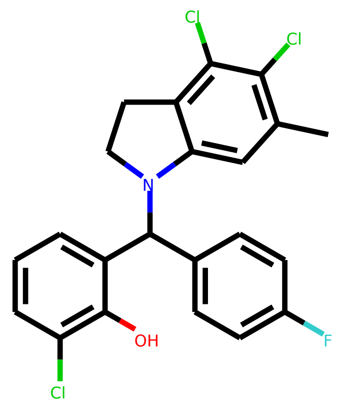

Osma-1659

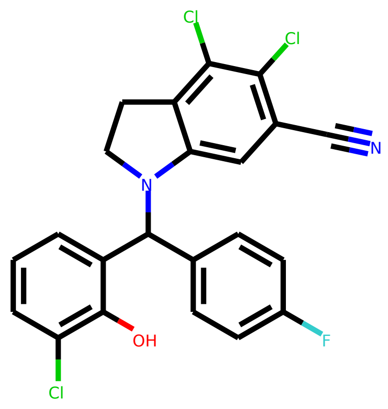

Osma-1660

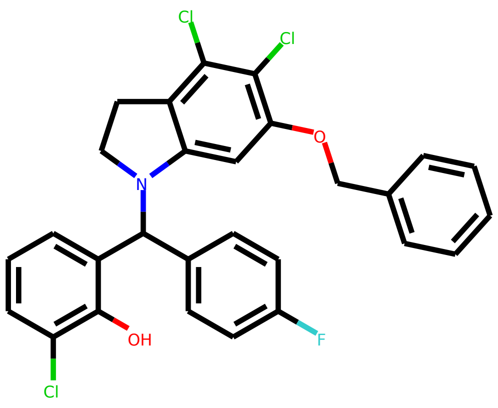

Osma-1661

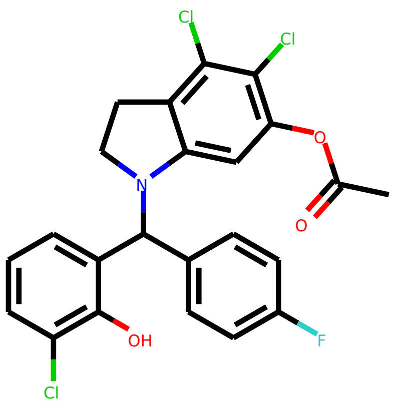

Osma-1662

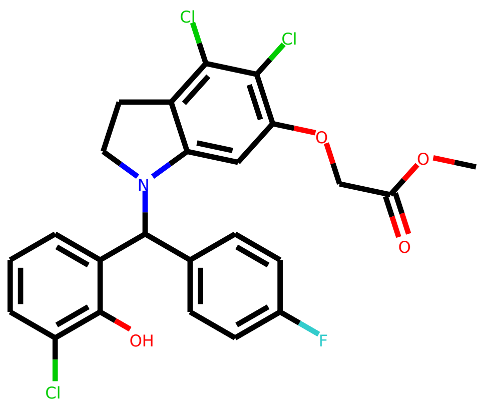

Osma-1663

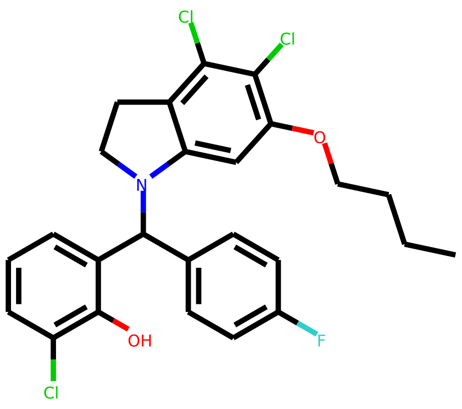

Osma-1664

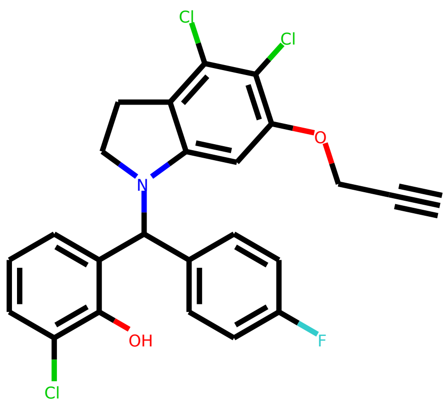

Osma-1665

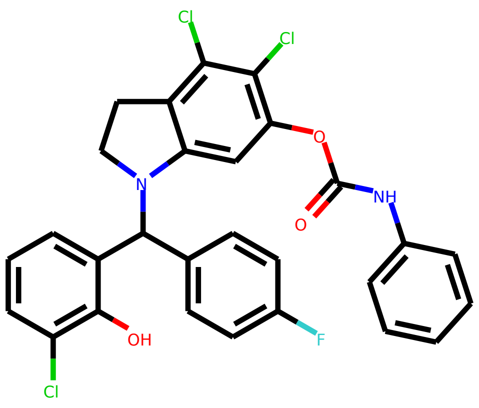

Osma-1666

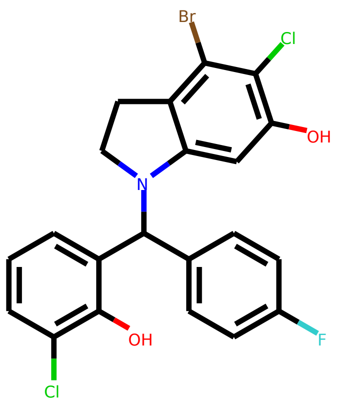

Osma-1667

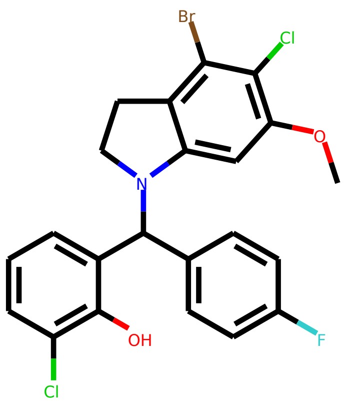

Osma-1668

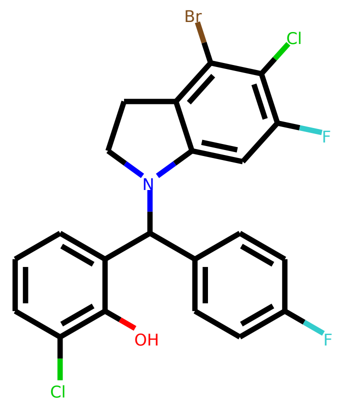

Osma-1669

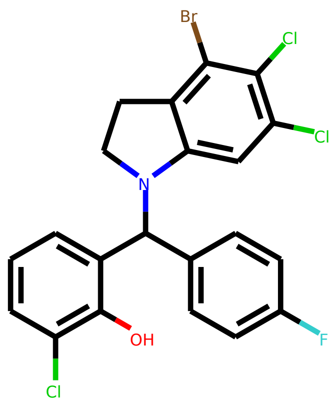

Osma-1670

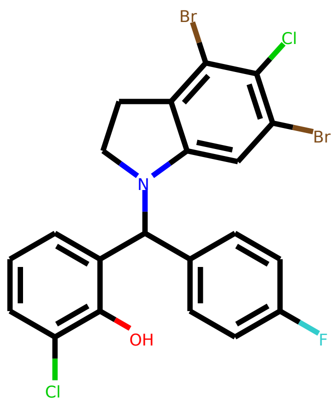

Osma-1671

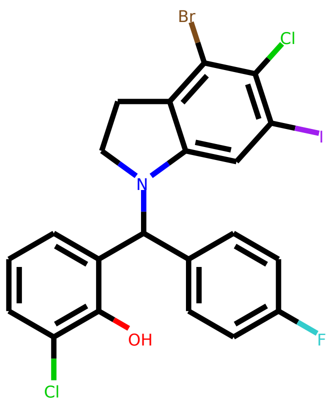

Osma-1672

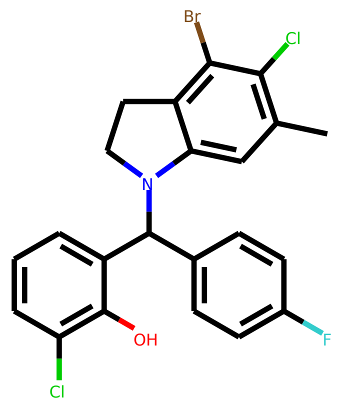

Osma-1673

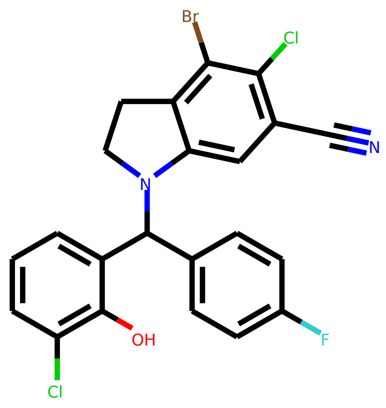

Osma-1674

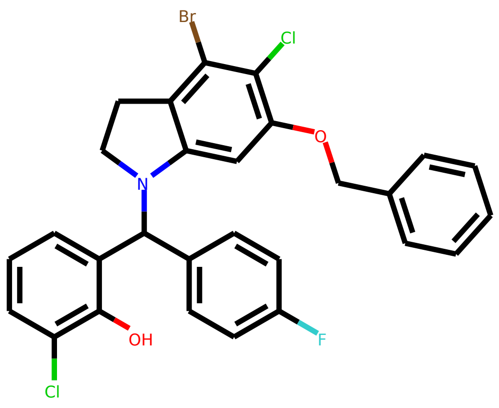

Osma-1675

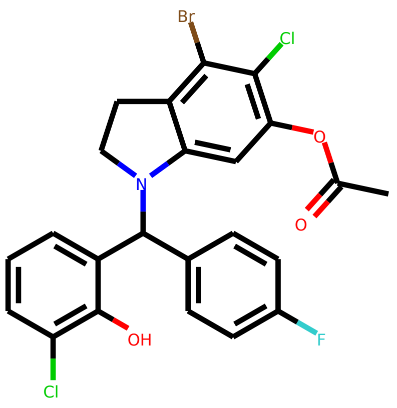

Osma-1676

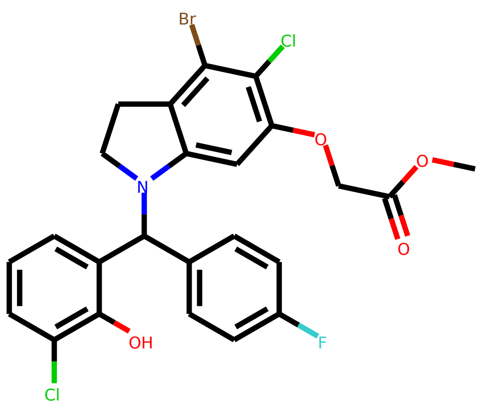

Osma-1677

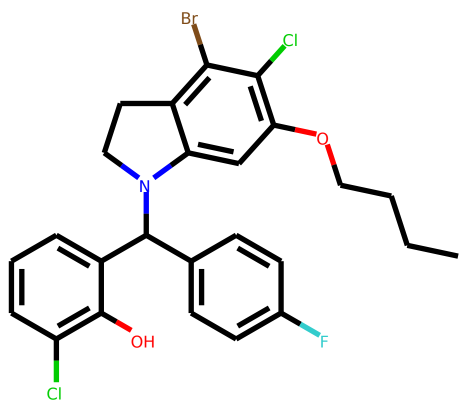

Osma-1678

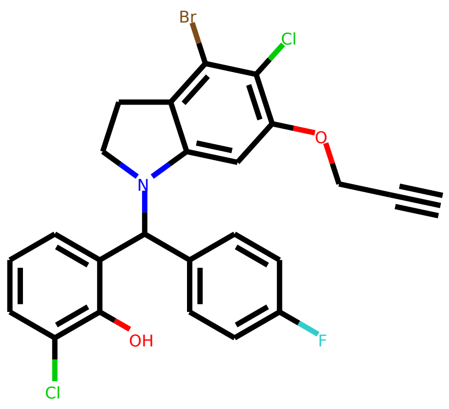

Osma-1679

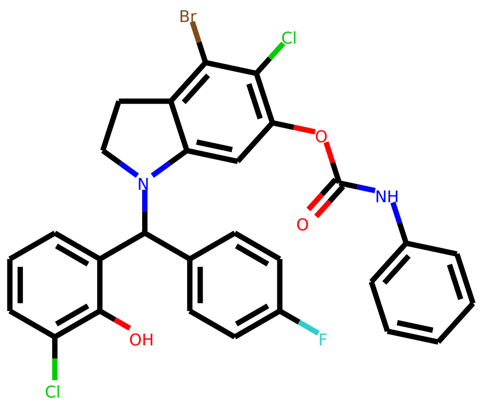

Osma-1680

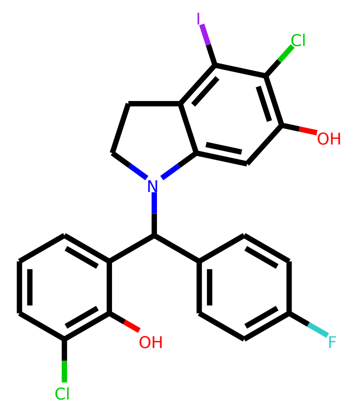

Osma-1681

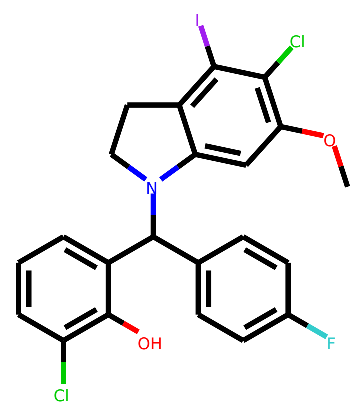

Osma-1682

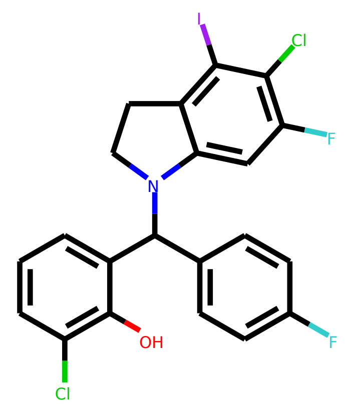

Osma-1683

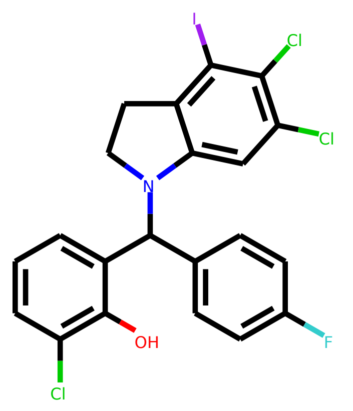

Osma-1684

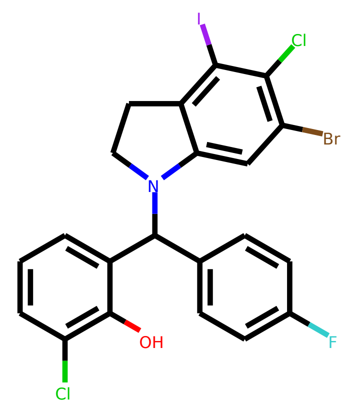

Osma-1685

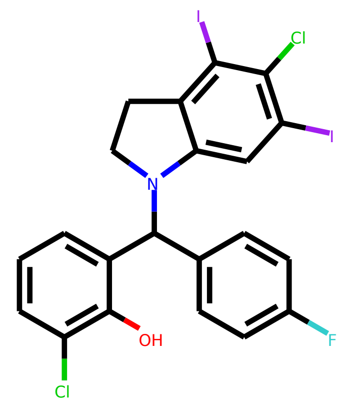

Osma-1686

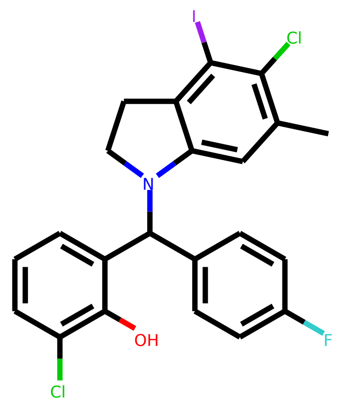

Osma-1687

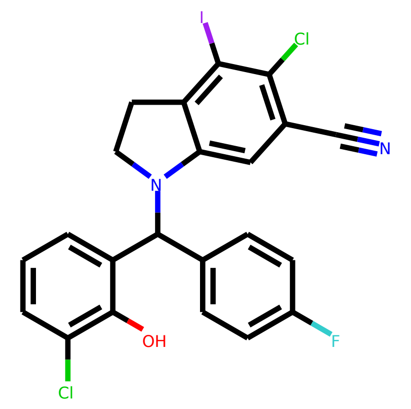

Osma-1688

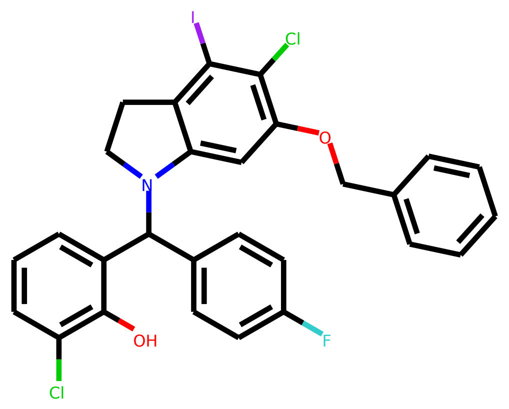

Osma-1689

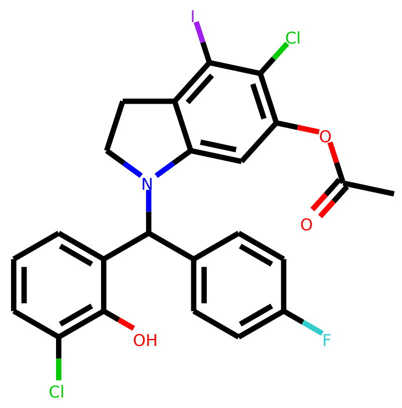

Osma-1690

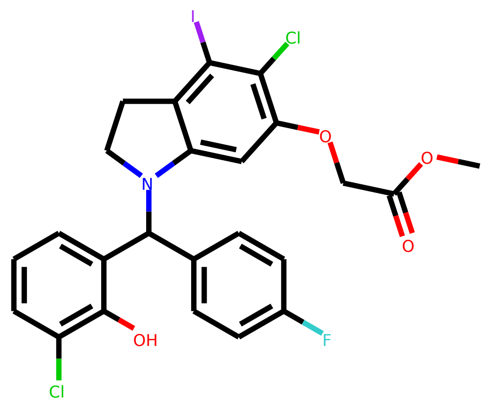

Osma-1691

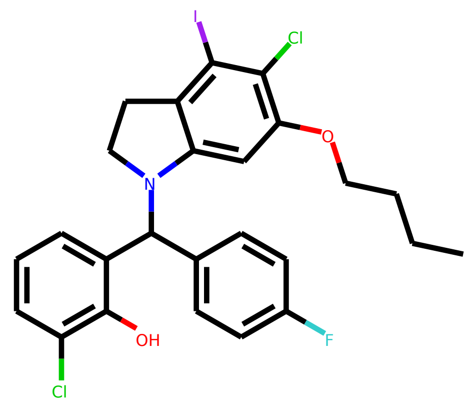

Osma-1692

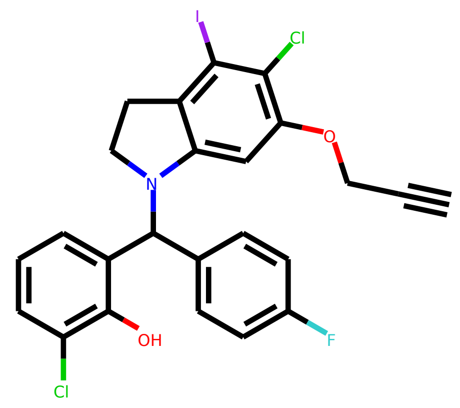

Osma-1693

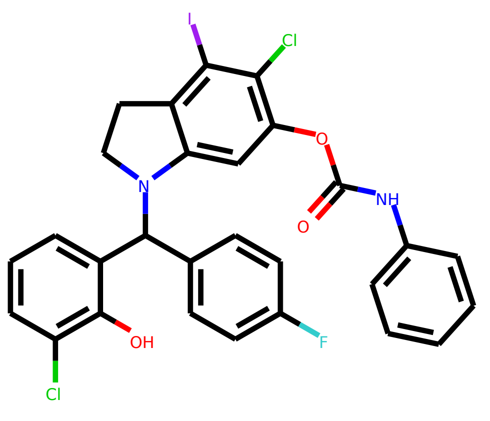

Osma-1694

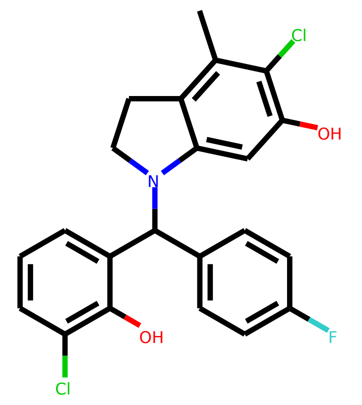

Osma-1695

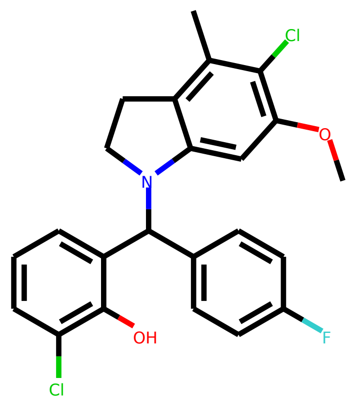

Osma-1696

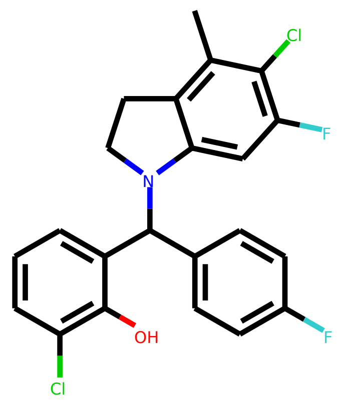

Osma-1697

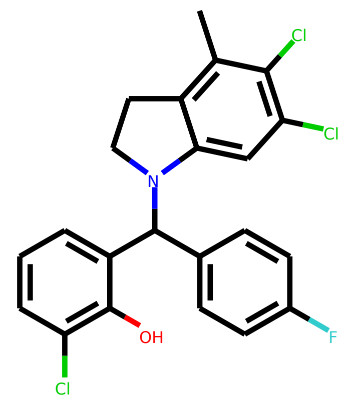

Osma-1698

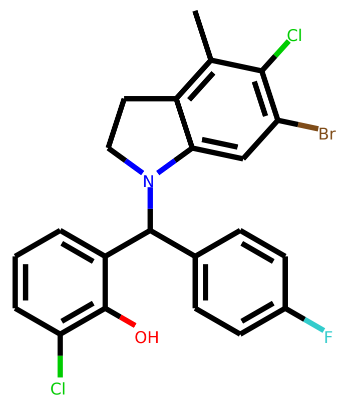

Osma-1699

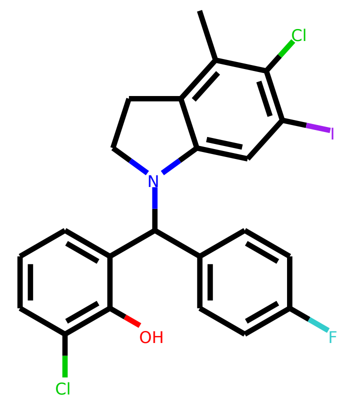

Osma-1700

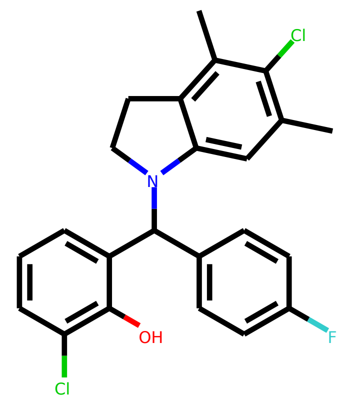

Osma-1701

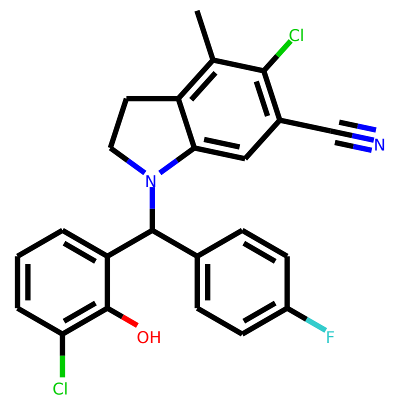

Osma-1702

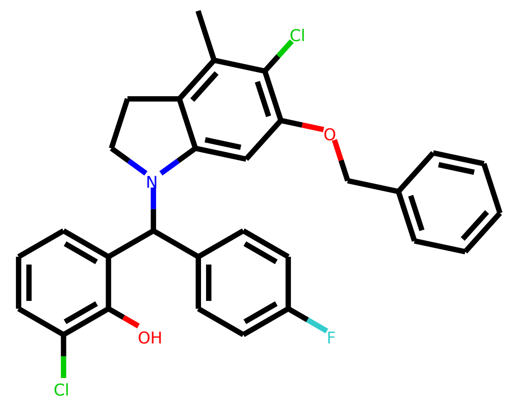

Osma-1703

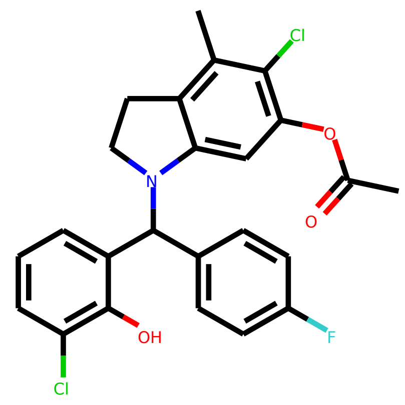

Osma-1704

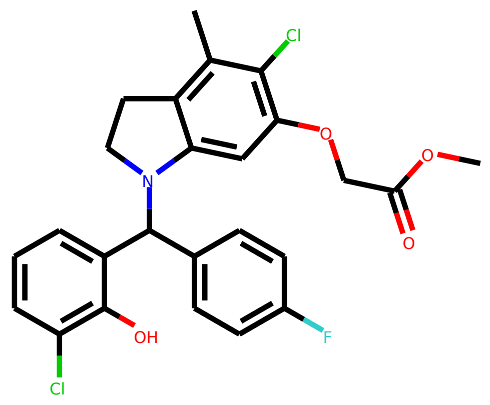

Osma-1705

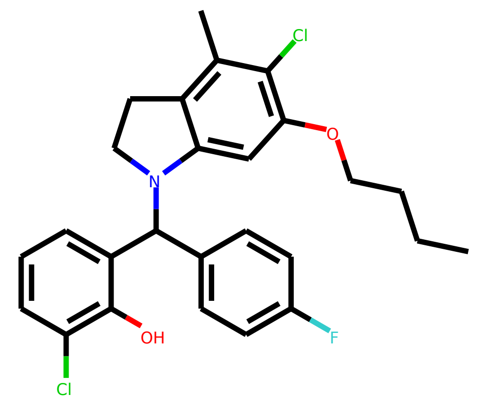

Osma-1706

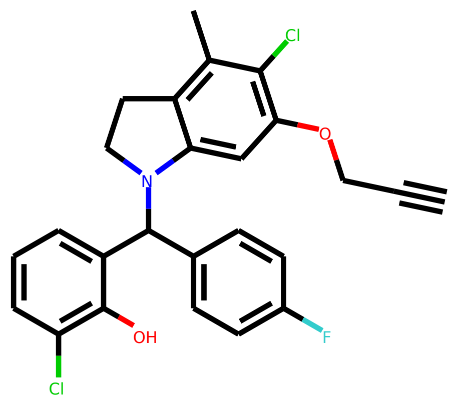

Osma-1707

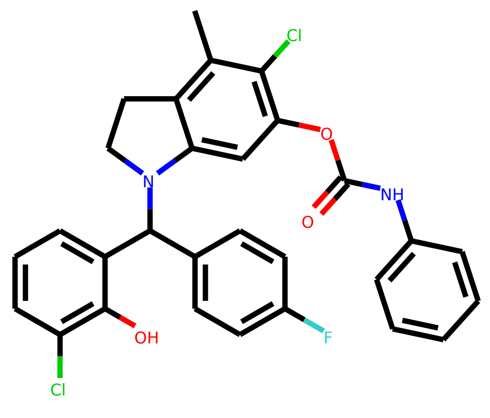

Osma-1708

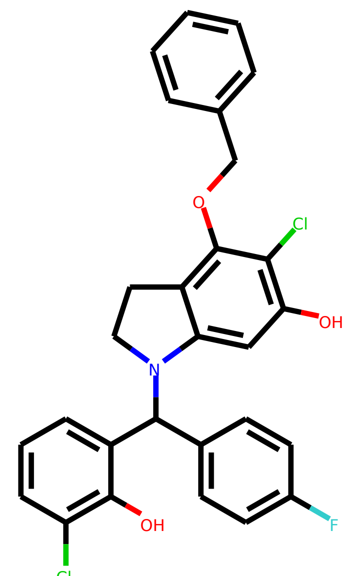

Osma-1709

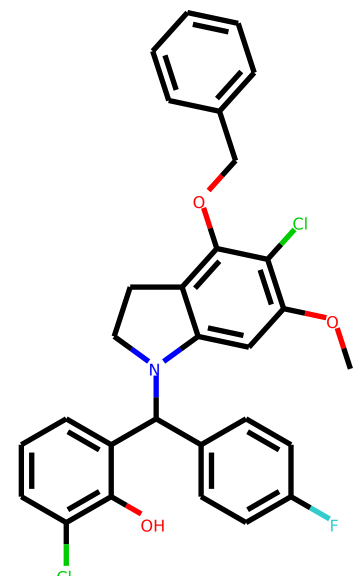

Osma-1710

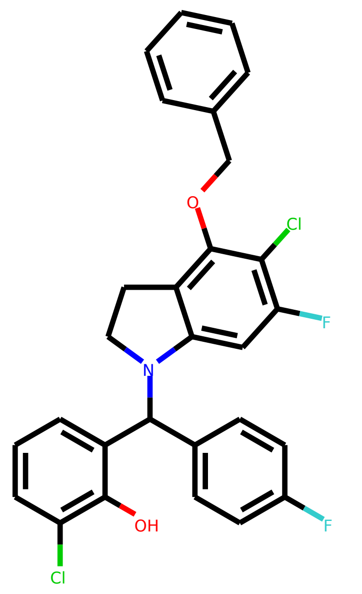

Osma-1711

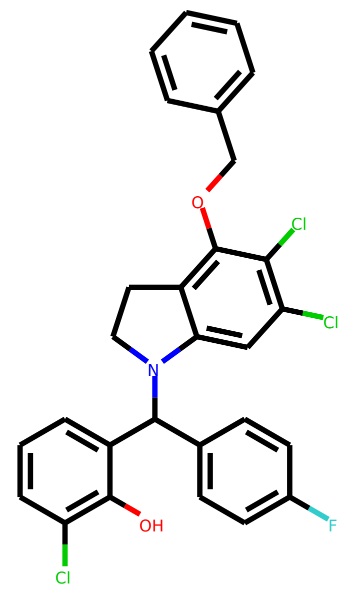

Osma-1712

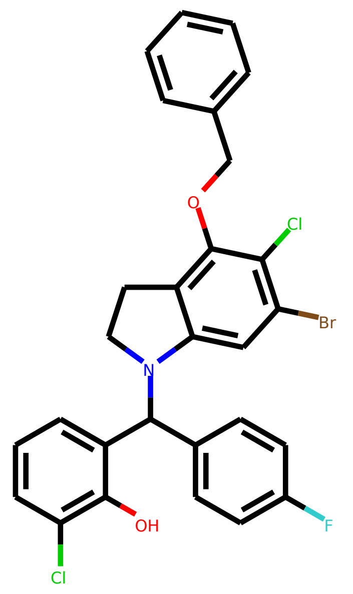

Osma-1713

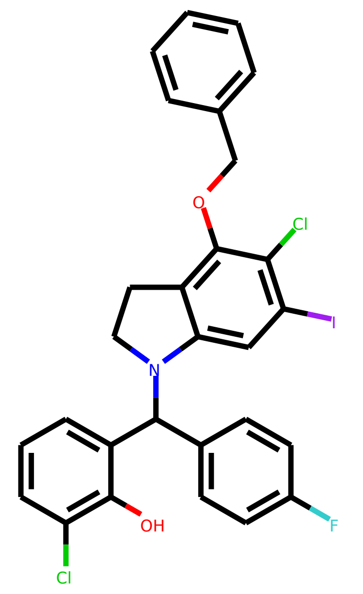

Osma-1714

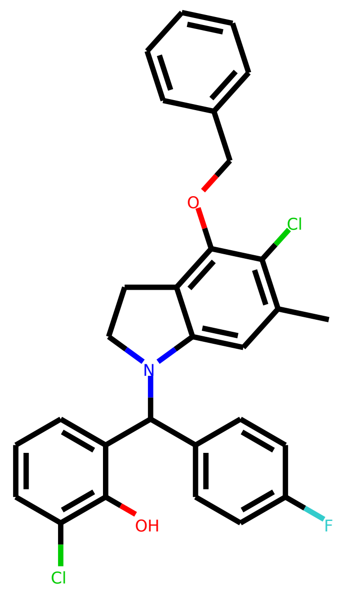

Osma-1715

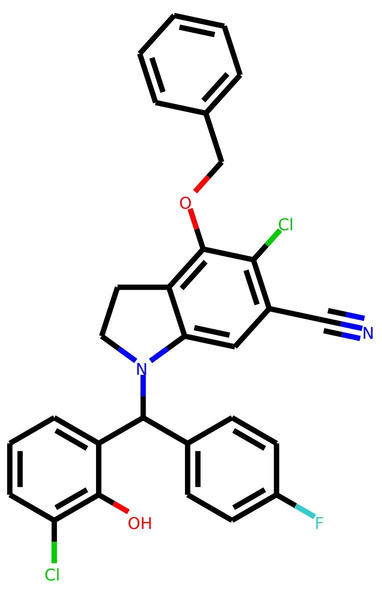
Osma-1716

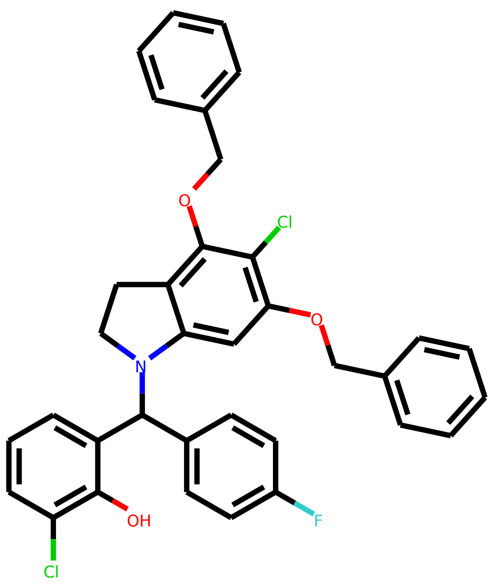
Osma-1717

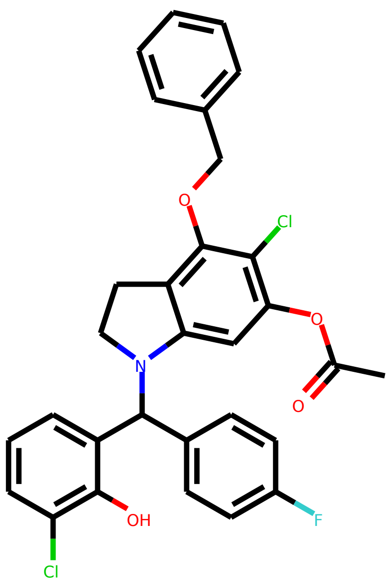
Osma-1718

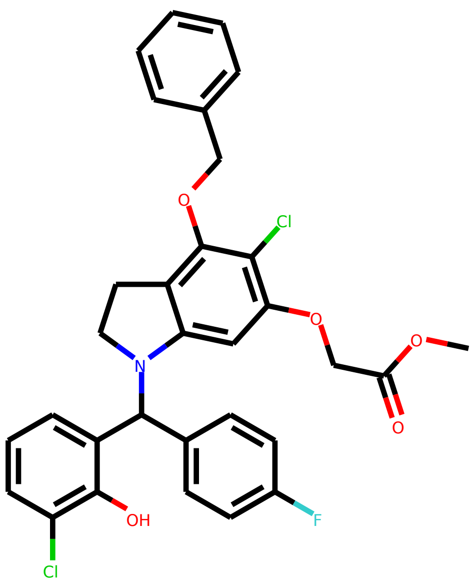
Osma-1719

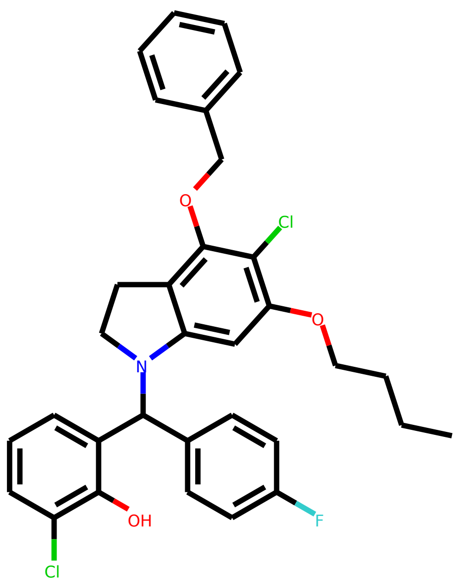
Osma-1720

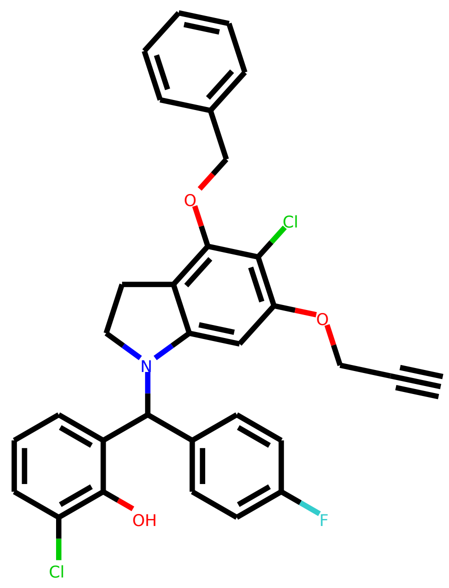
Osma-1721

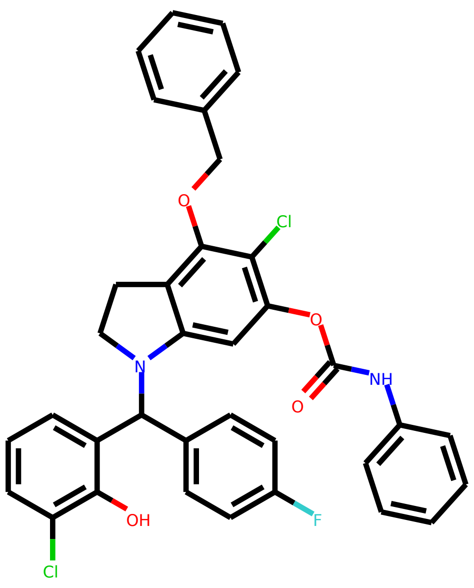
Osma-1722

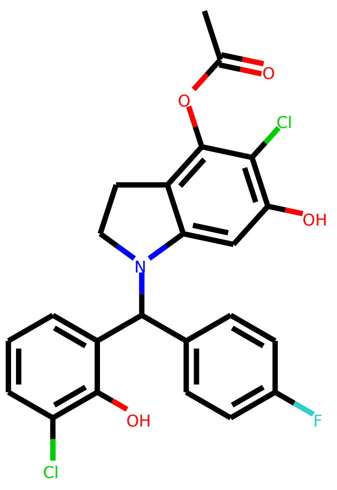
Osma-1723

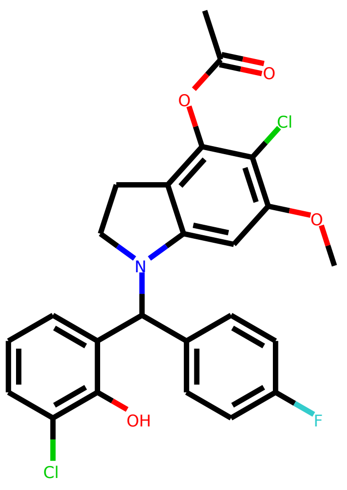
Osma-1724

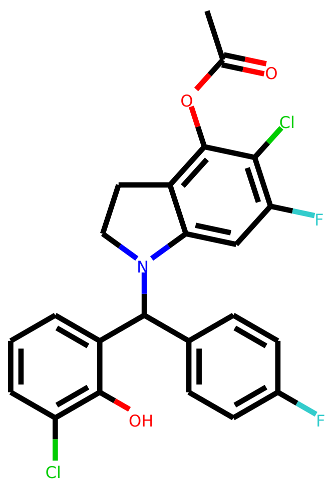
Osma-1725

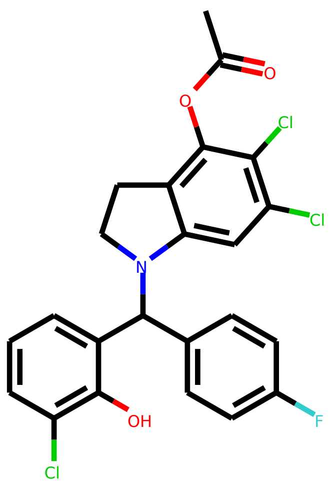
Osma-1726

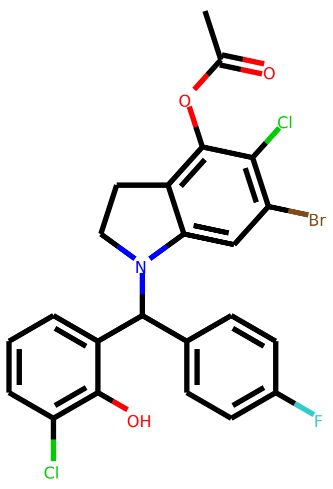
Osma-1727

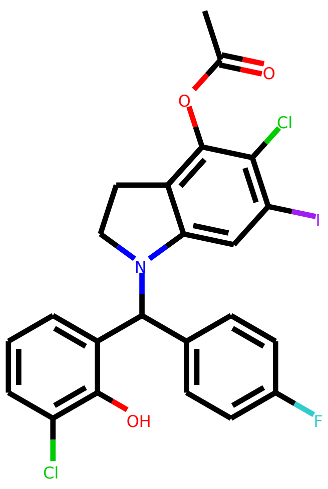
Osma-1728

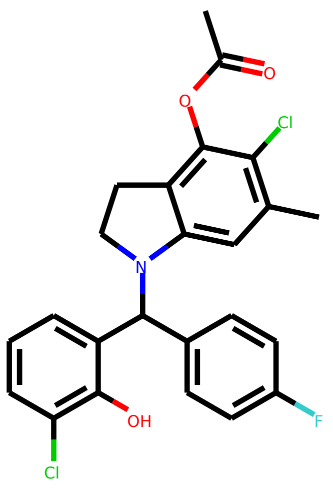
Osma-1729

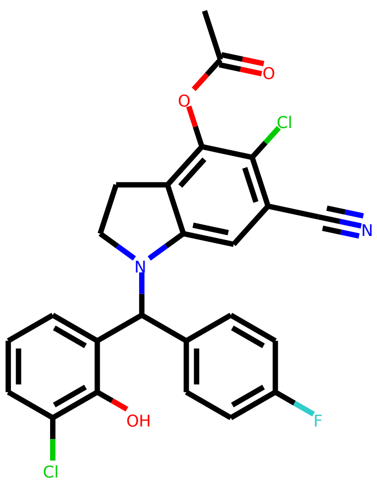
Osma-1730

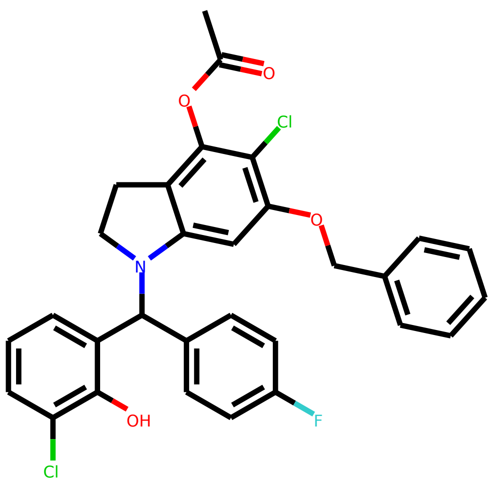
Osma-1731

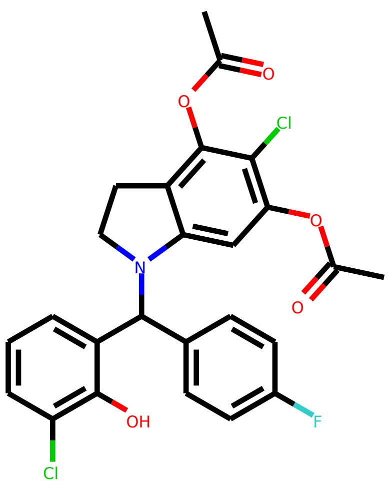
Osma-1732

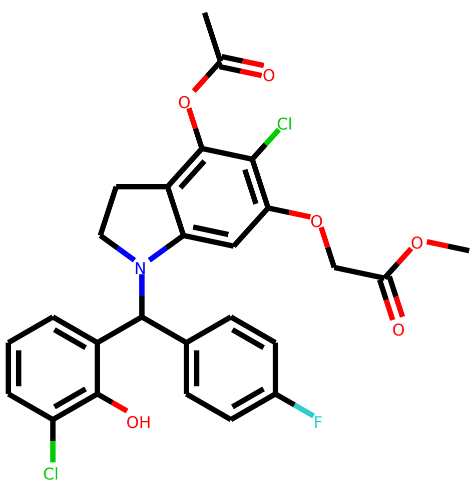
Osma-1733

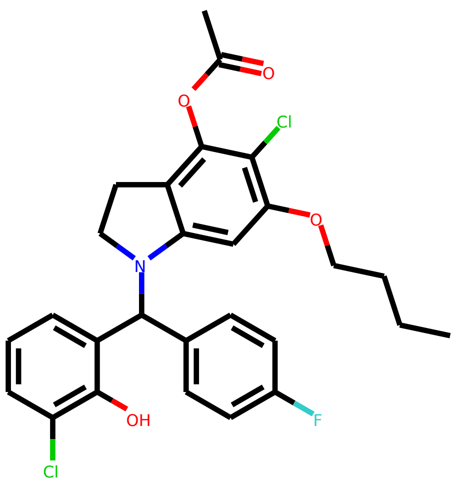
Osma-1734

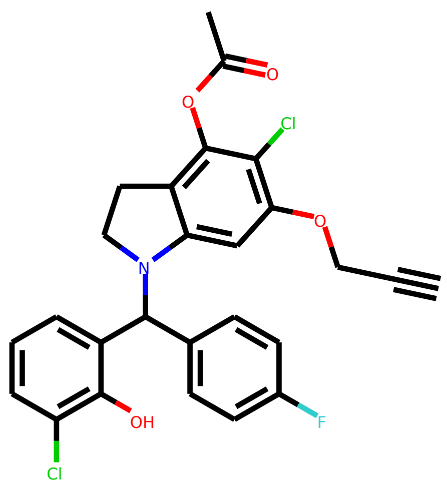
Osma-1735

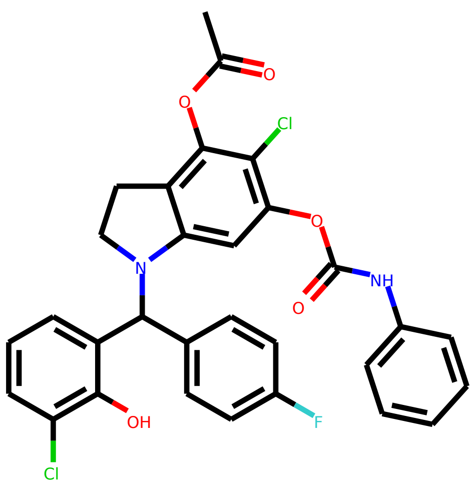
Osma-1736

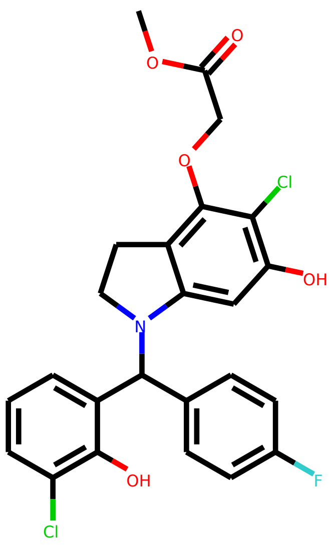
Osma-1737

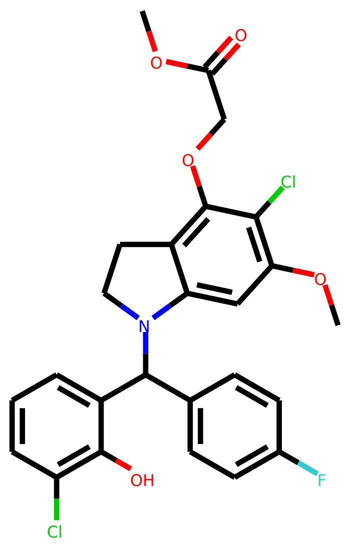
Osma-1738

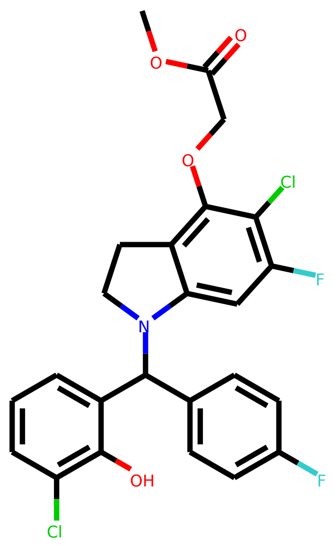
Osma-1739

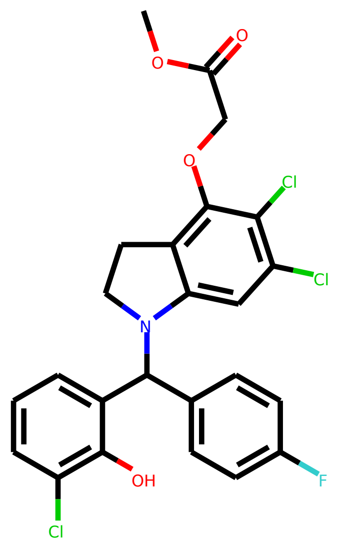
Osma-1740

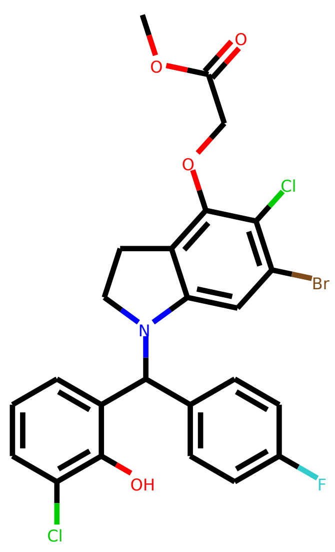
Osma-1741

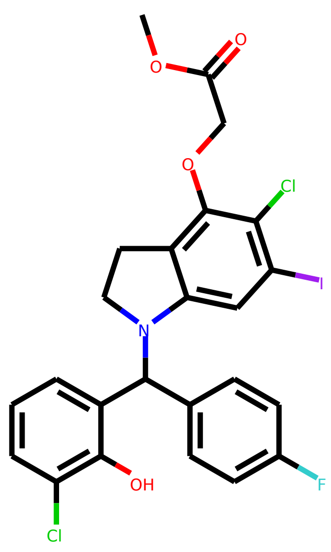
Osma-1742

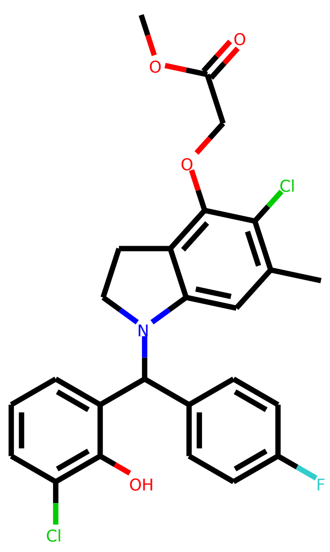
Osma-1743

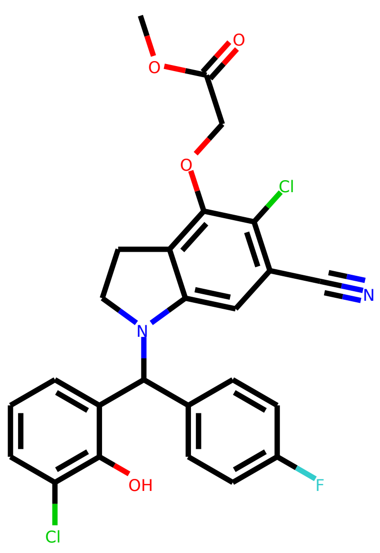
Osma-1744

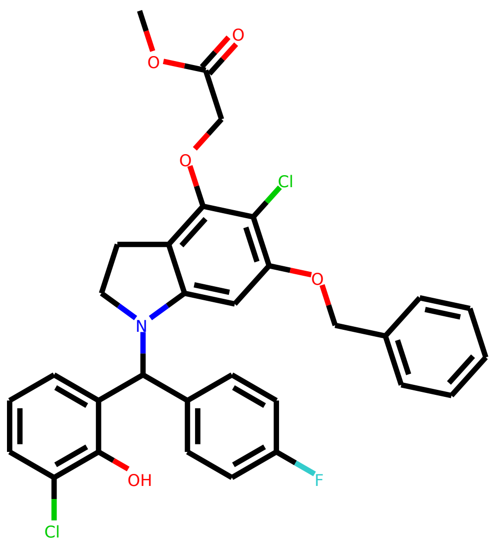
Osma-1745

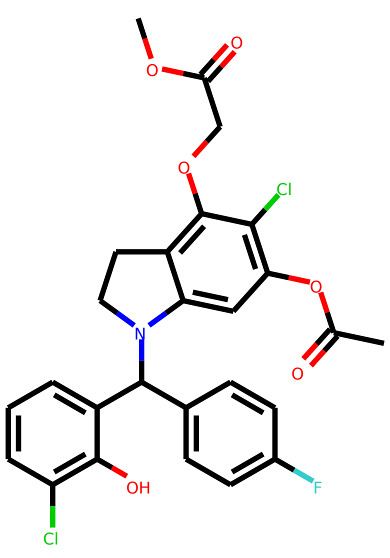
Osma-1746

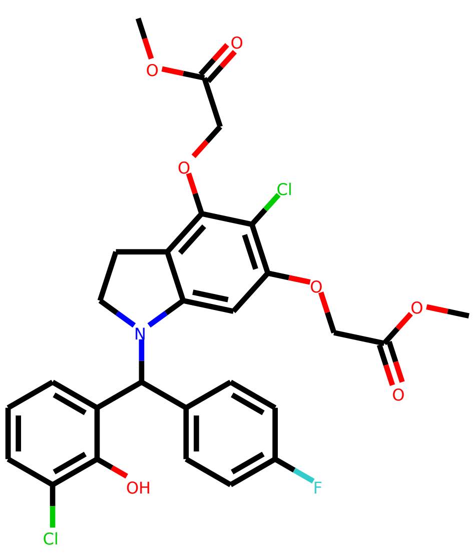
Osma-1747

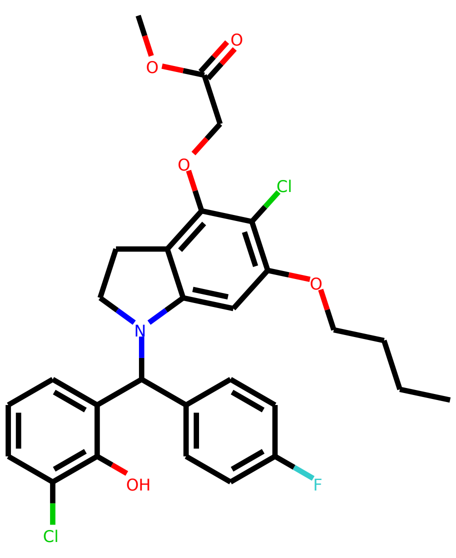
Osma-1748

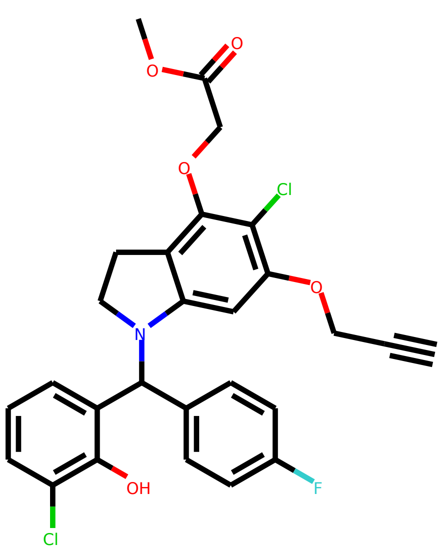
Osma-1749

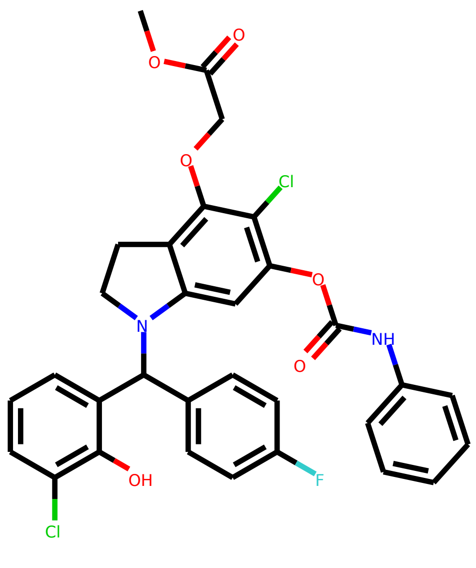
Osma-1750

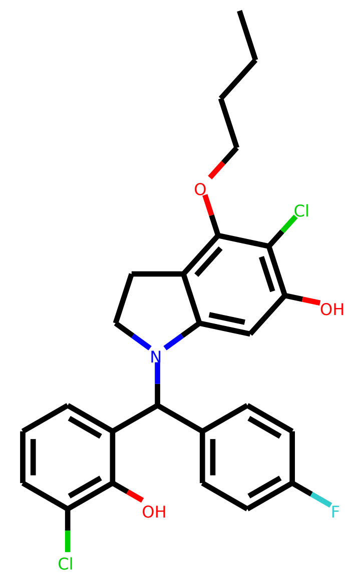
Osma-1751

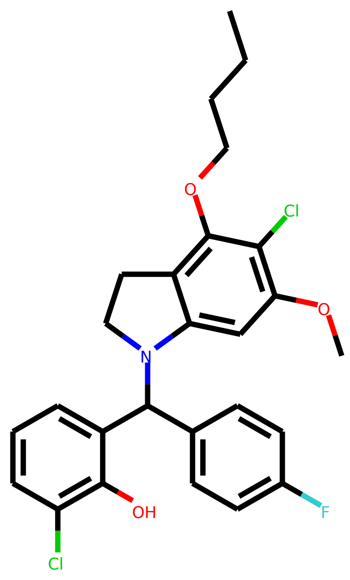
Osma-1752

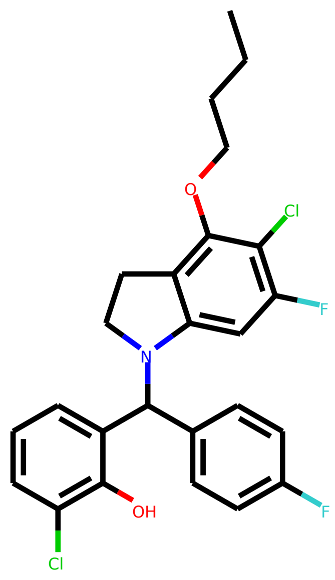
Osma-1753

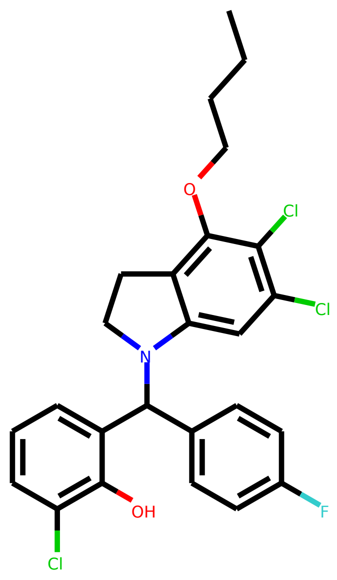
Osma-1754

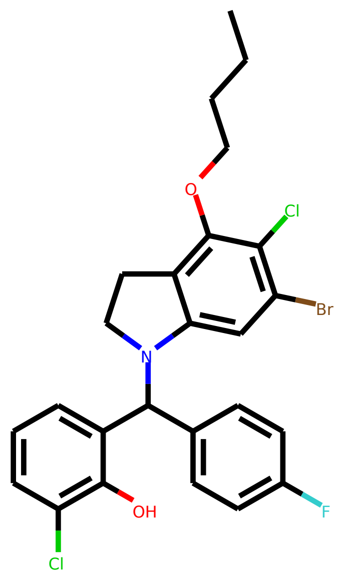
Osma-1755

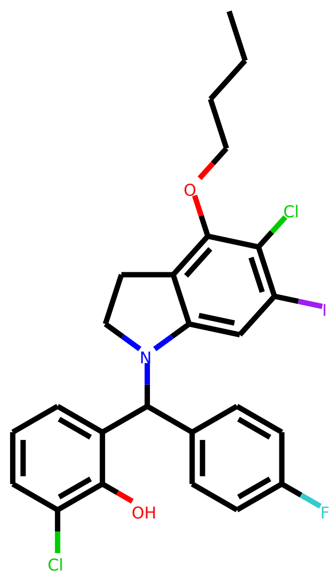
Osma-1756

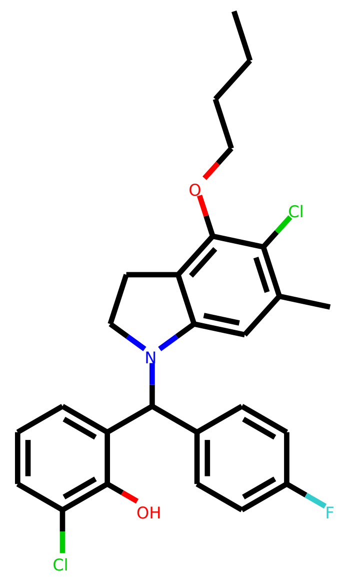
Osma-1757

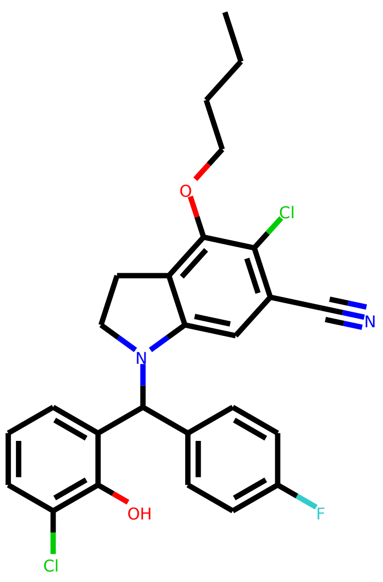
Osma-1758

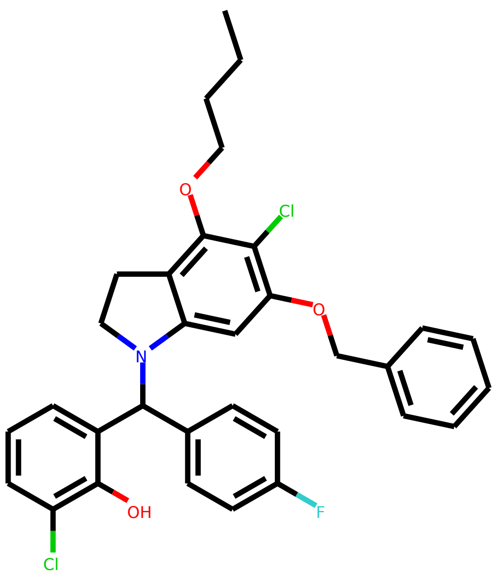
Osma-1759

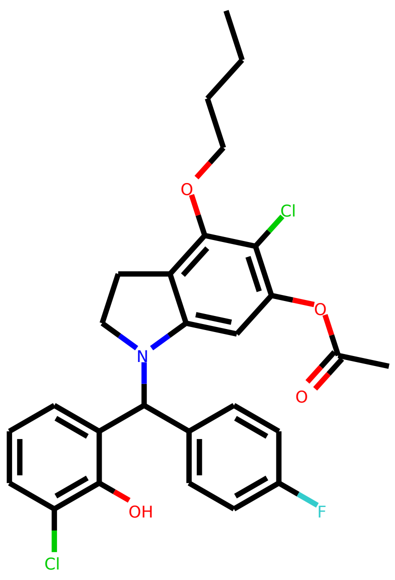
Osma-1760

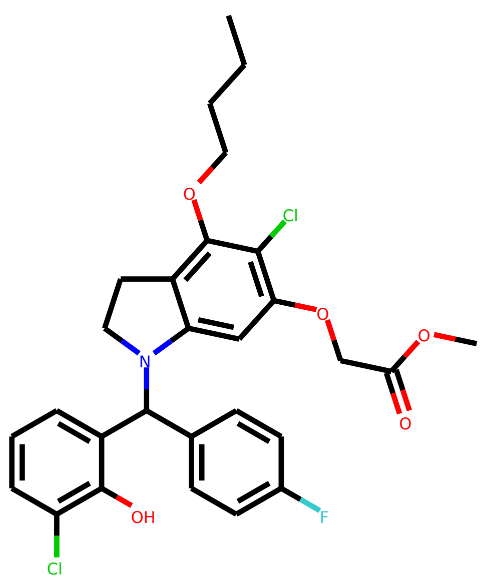
Osma-1761

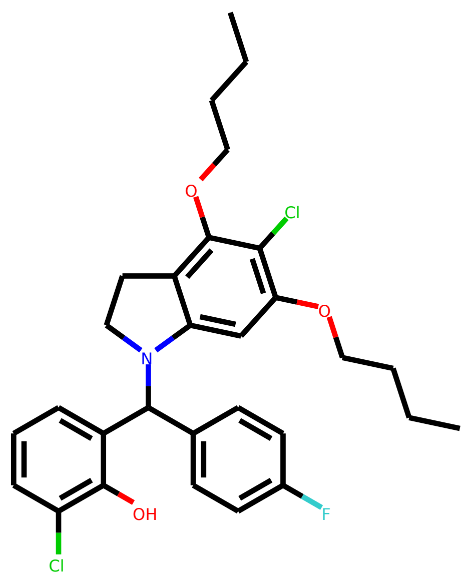
Osma-1762

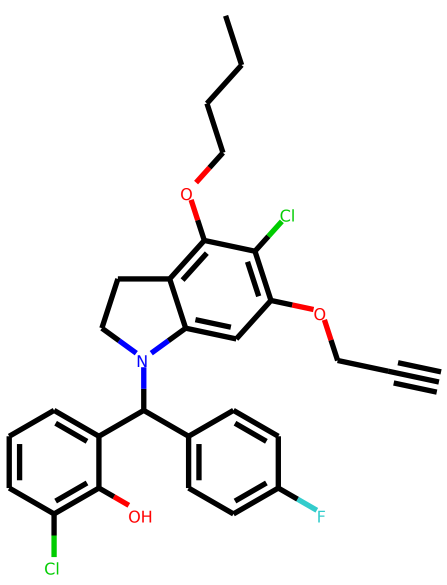
Osma-1763

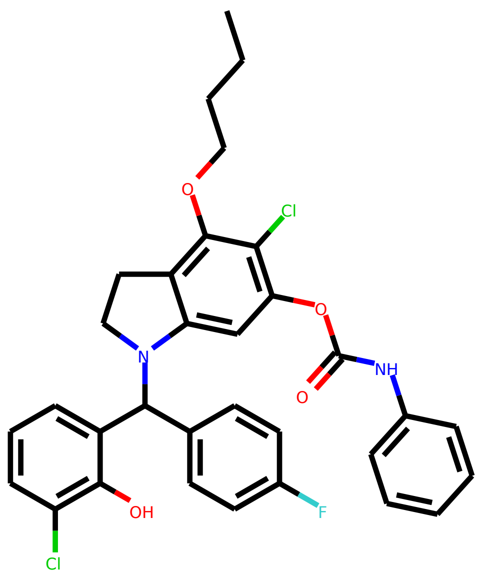
Osma-1764

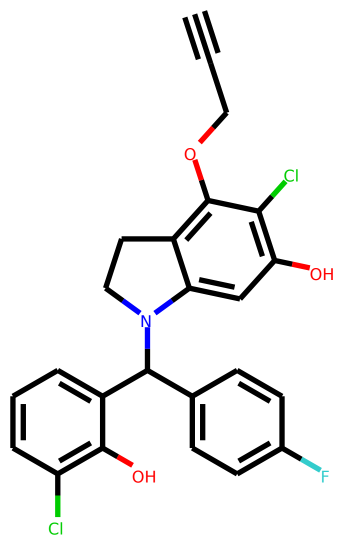
Osma-1765

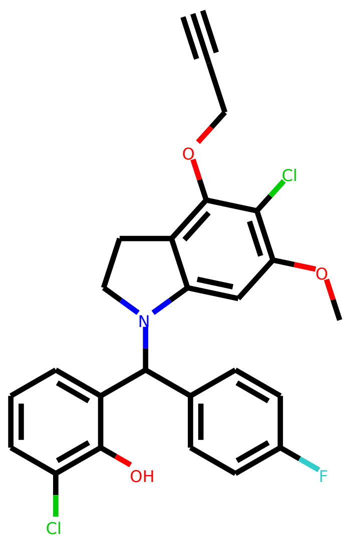
Osma-1766

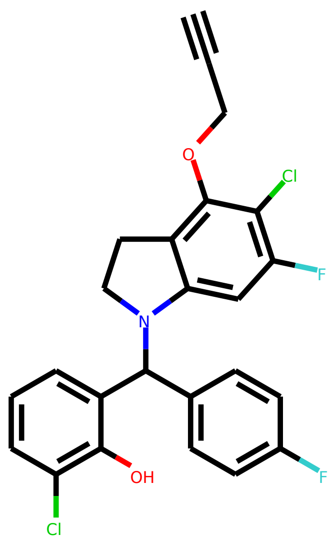
Osma-1767

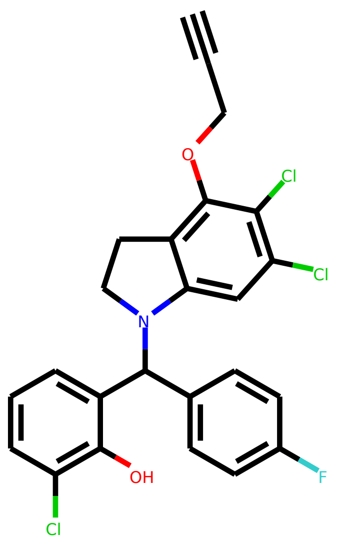
Osma-1768

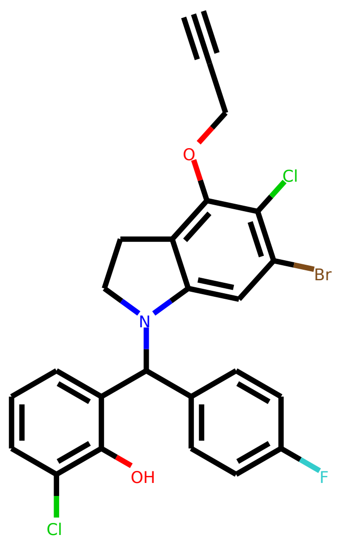
Osma-1769

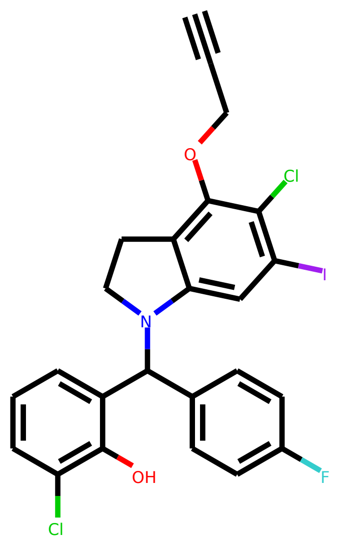
Osma-1770

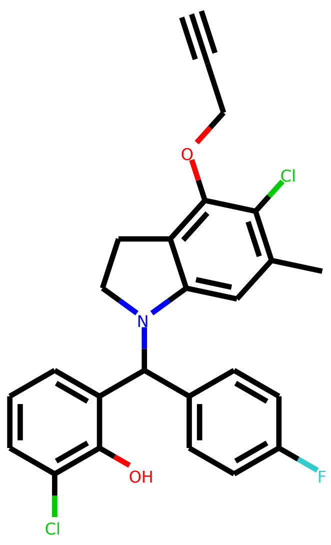
Osma-1771

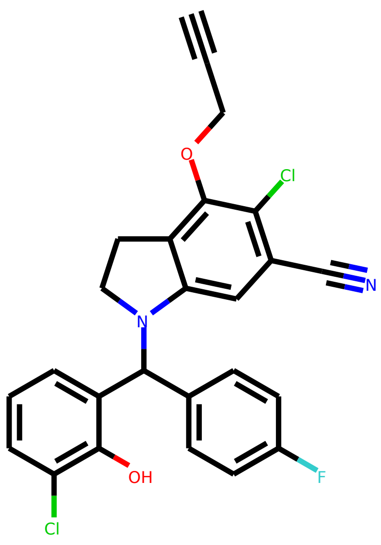
Osma-1772

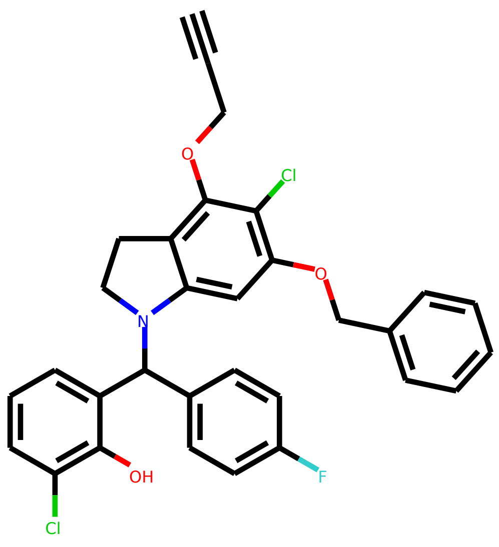
Osma-1773

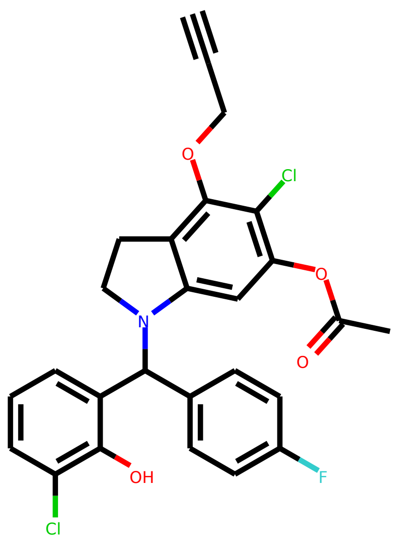
Osma-1774

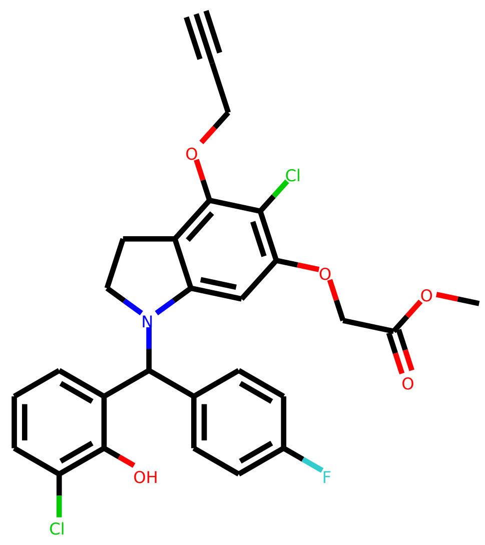
Osma-1775

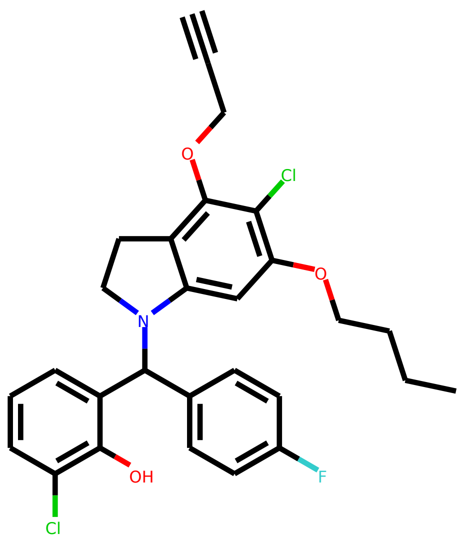
Osma-1776

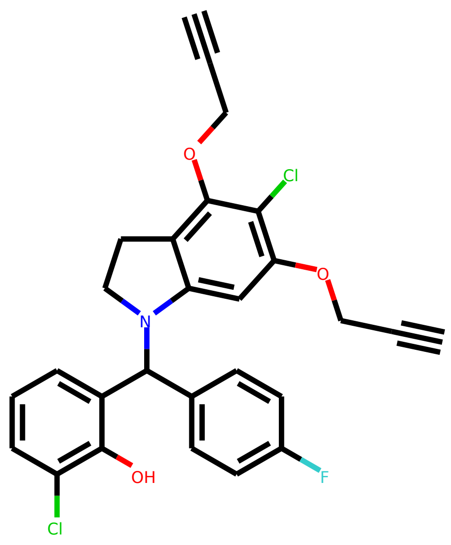
Osma-1777

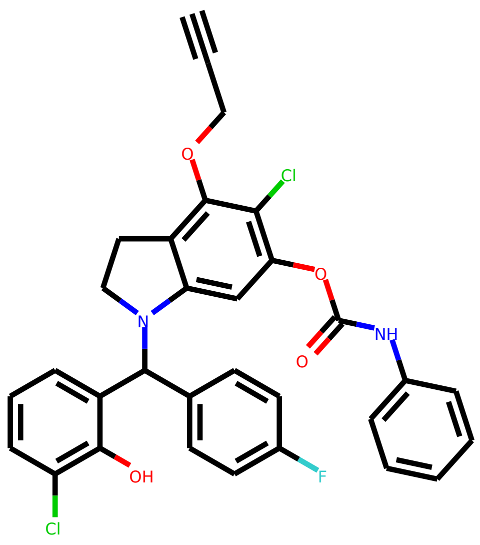
Osma-1778

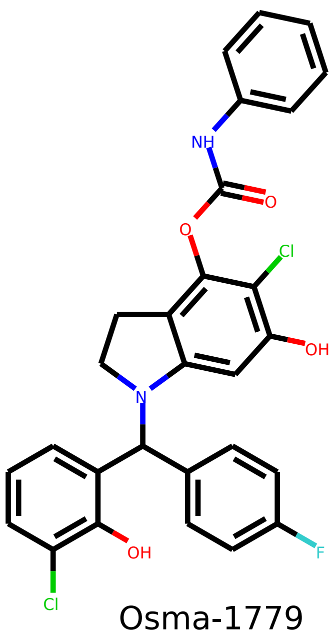
Osma-1779

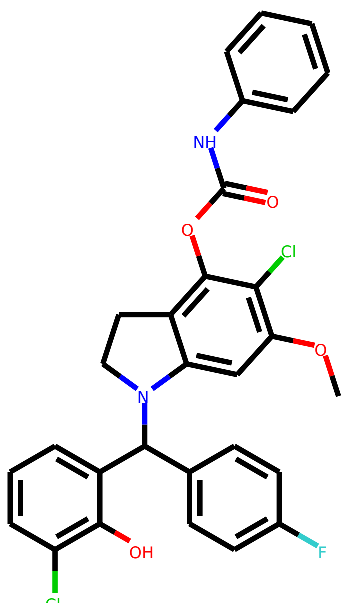
Osma-1780

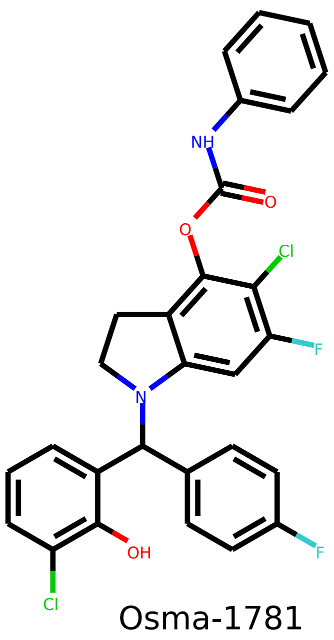
Osma-1781

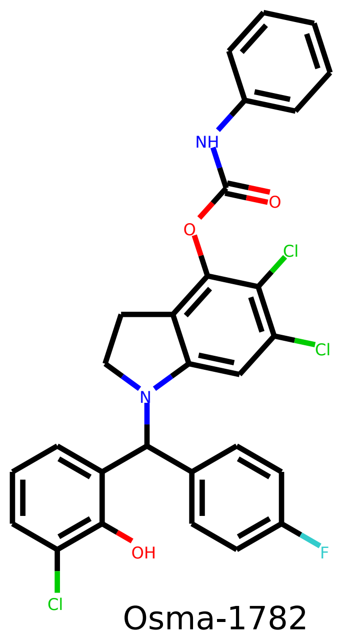
Osma-1782

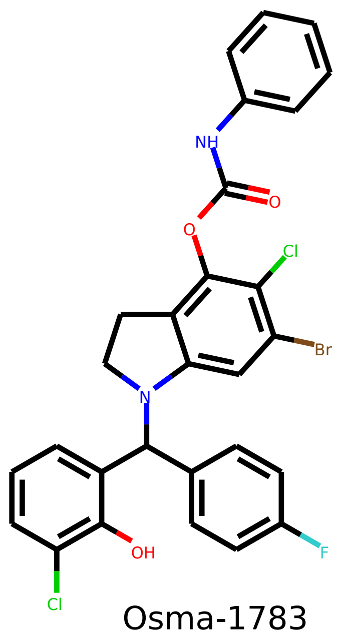
Osma-1783

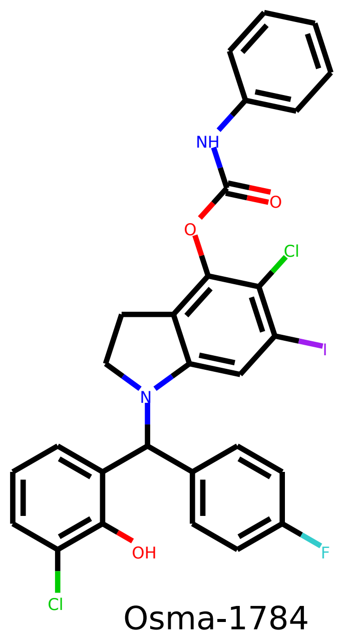
Osma-1784

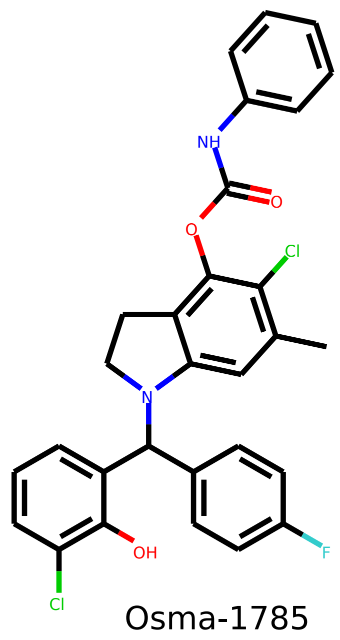
Osma-1785

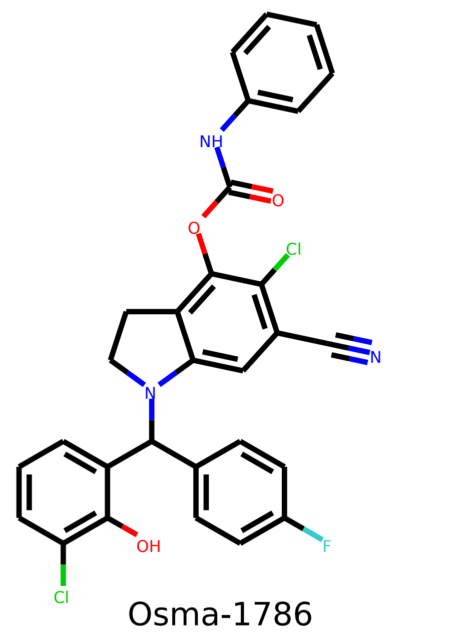
Osma-1786

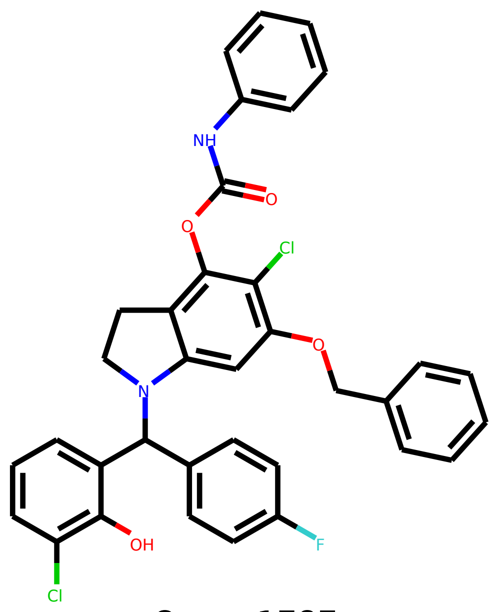
Osma-1787

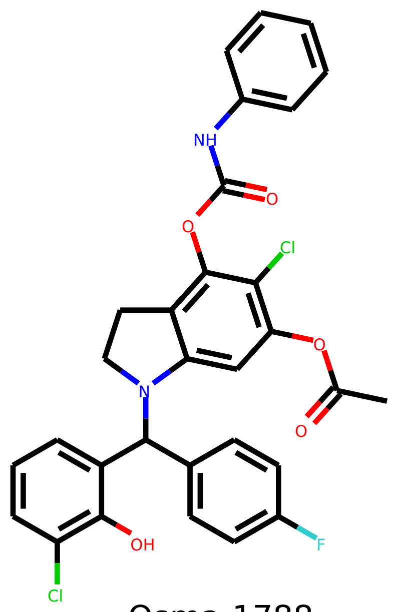
Osma-1788

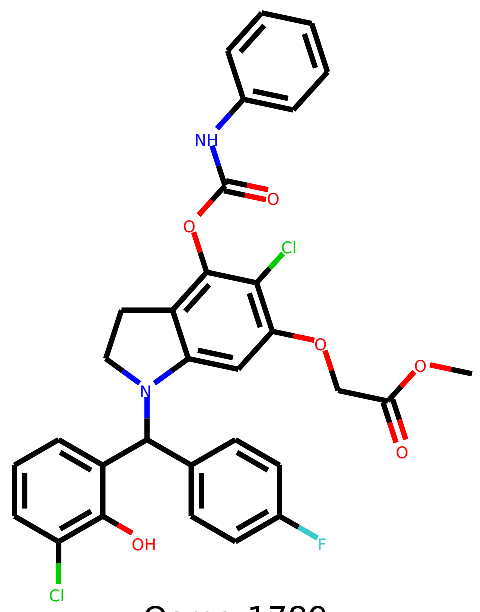
Osma-1789

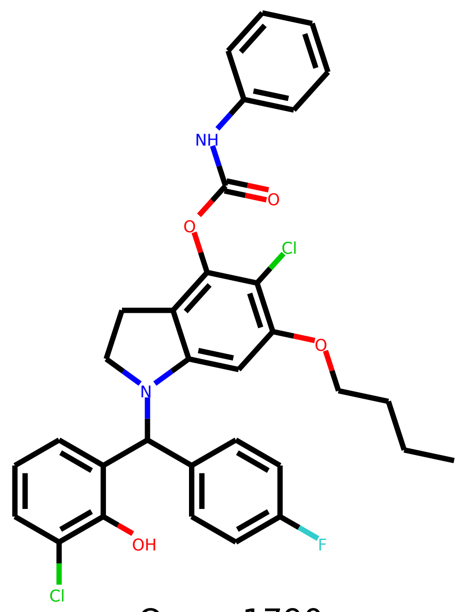
Osma-1790

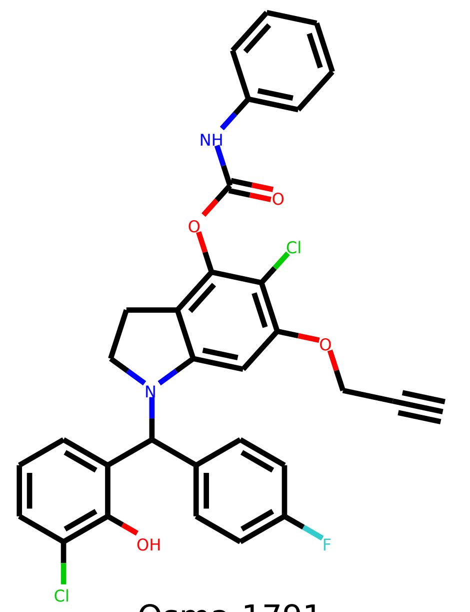
Osma-1791

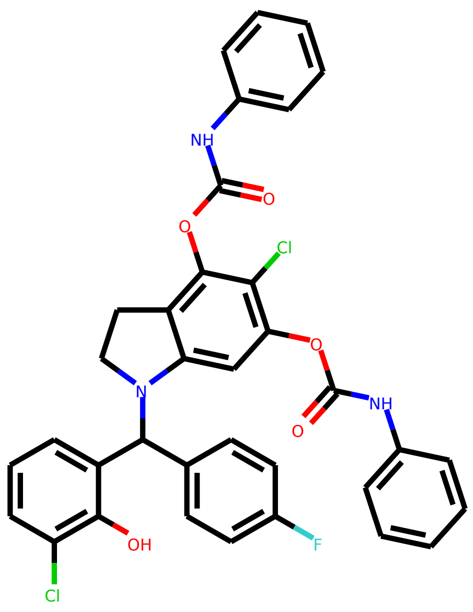
Osma-1792

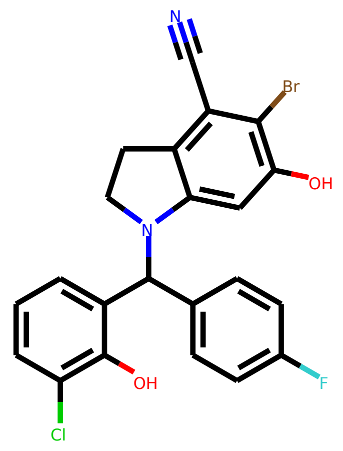
Osma-1793

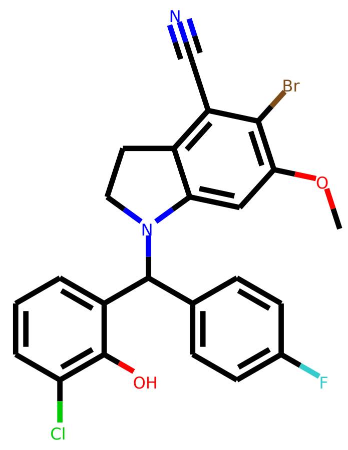
Osma-1794

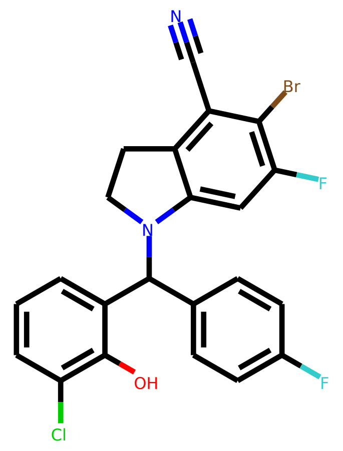
Osma-1795

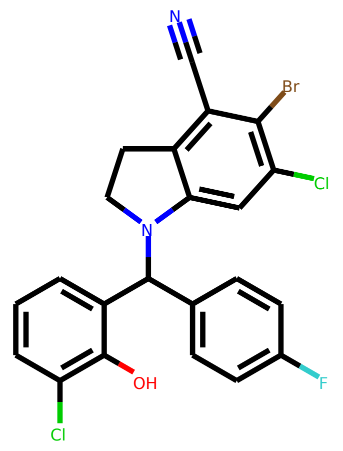
Osma-1796

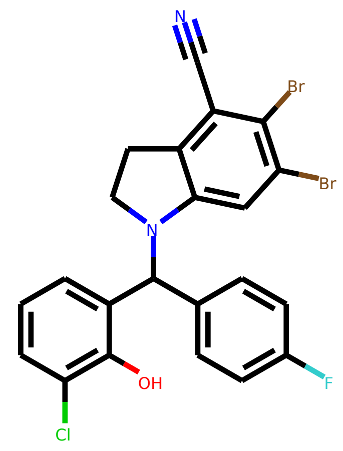
Osma-1797

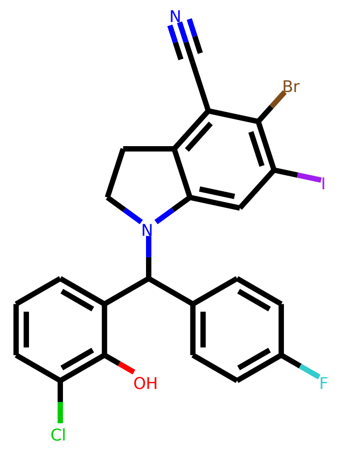
Osma-1798

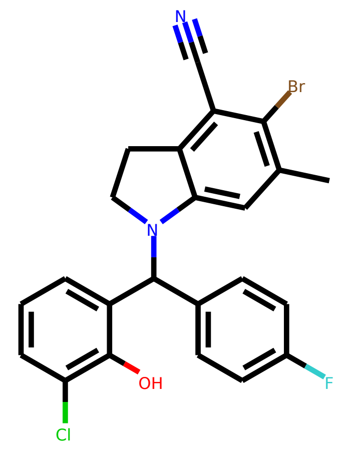
Osma-1799

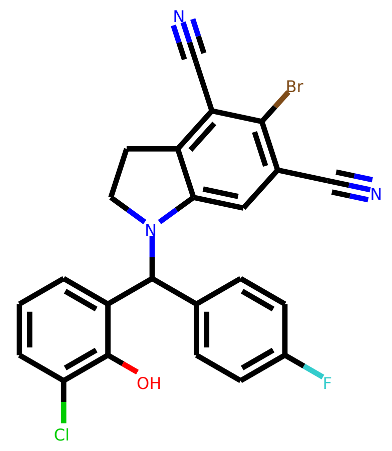
Osma-1800

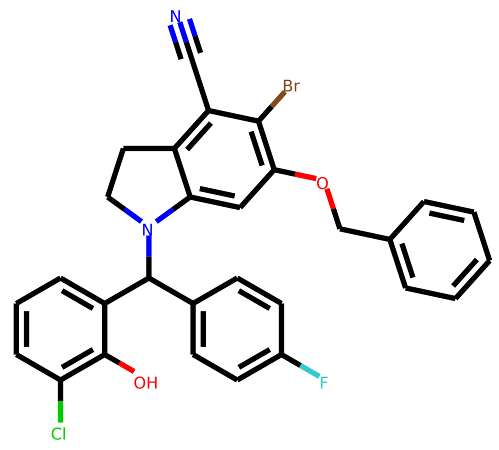
Osma-1801

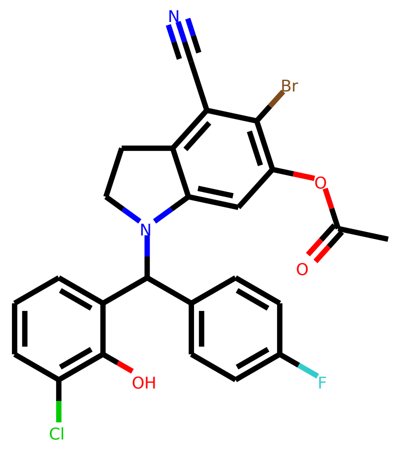
Osma-1802

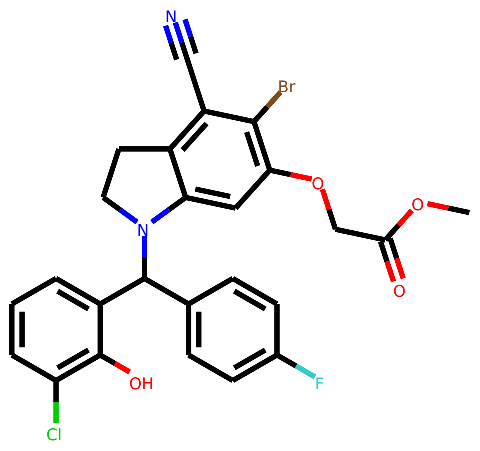
Osma-1803

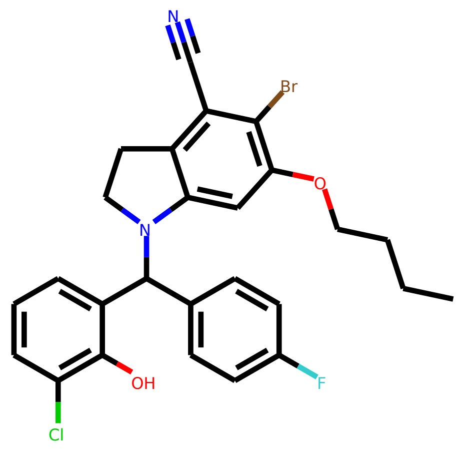
Osma-1804

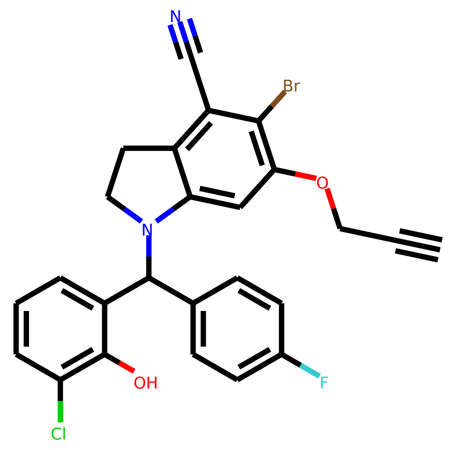
Osma-1805

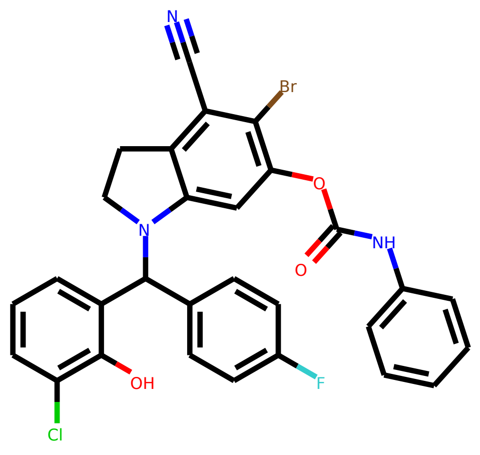
Osma-1806

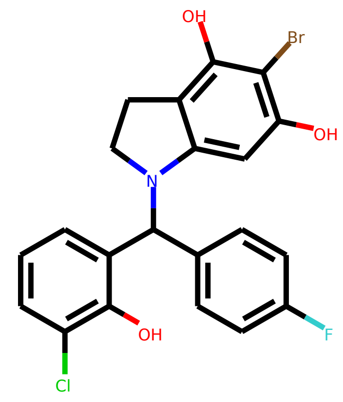
Osma-1807

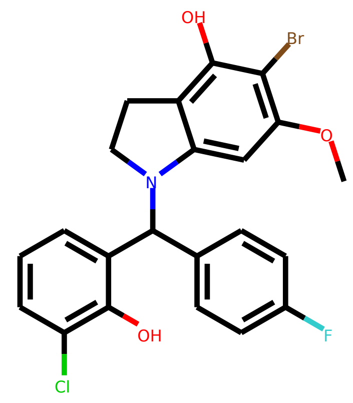
Osma-1808

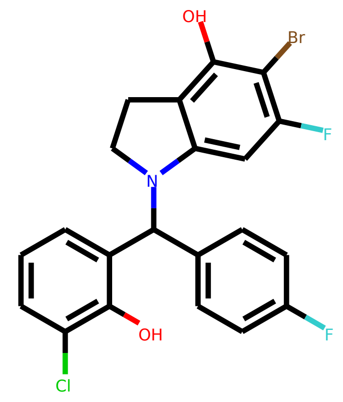
Osma-1809

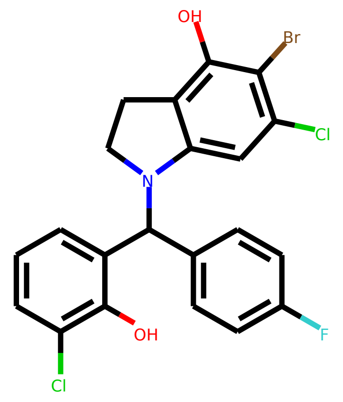
Osma-1810

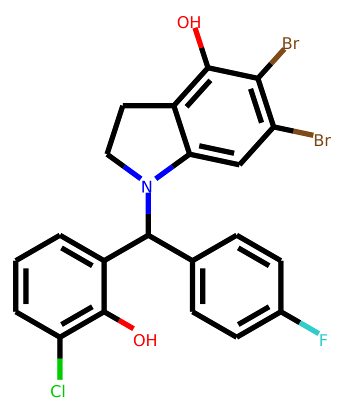
Osma-1811

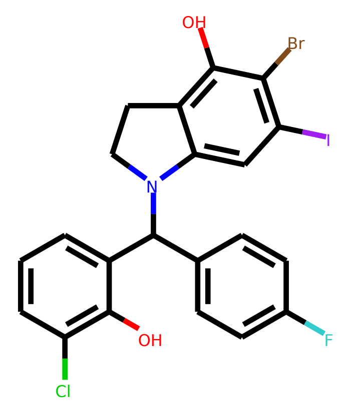
Osma-1812

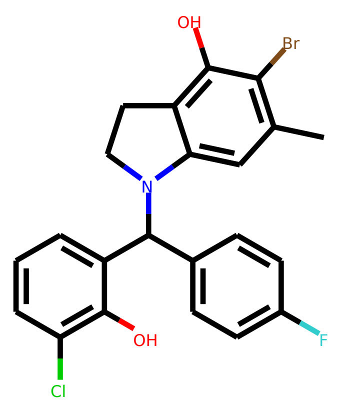
Osma-1813

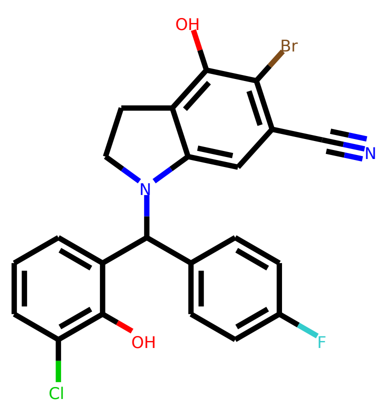
Osma-1814

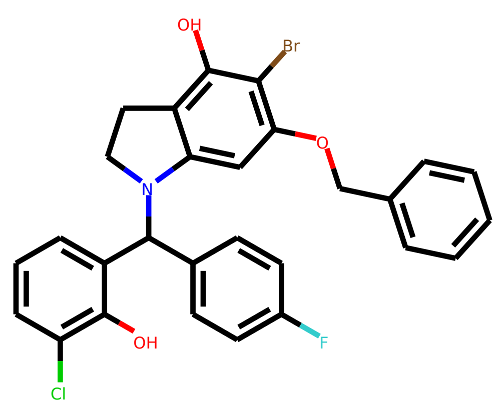
Osma-1815

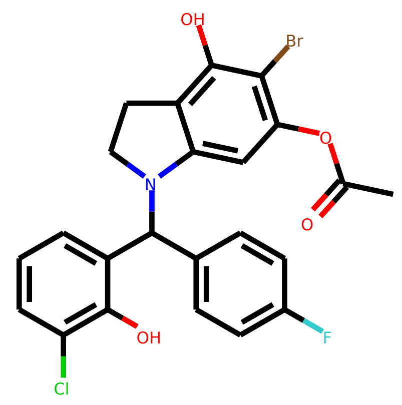
Osma-1816

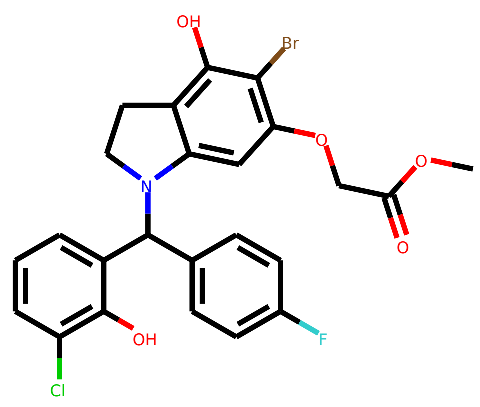
Osma-1817

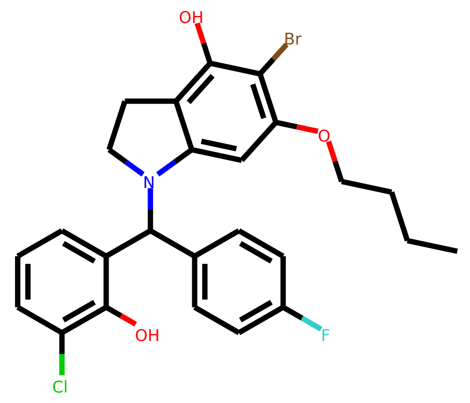
Osma-1818

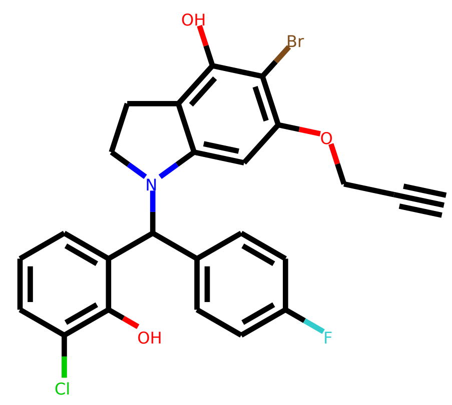
Osma-1819

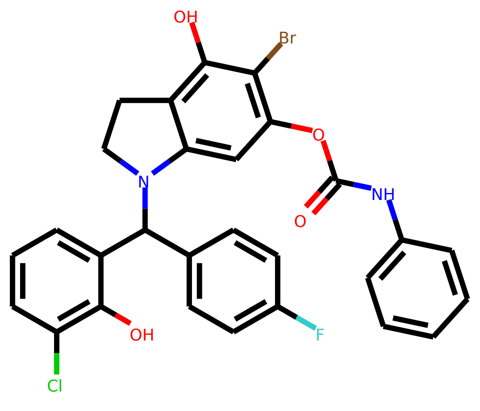
Osma-1820

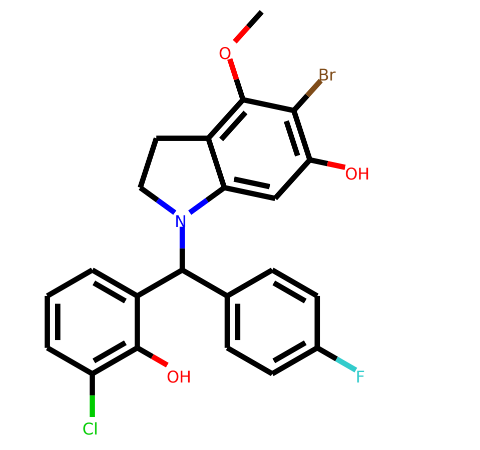
Osma-1821
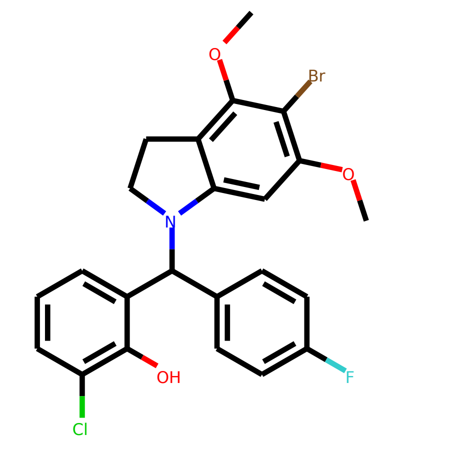
Osma-1822
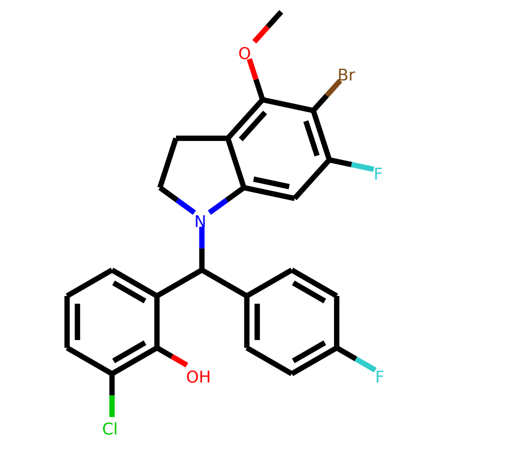
Osma-1823
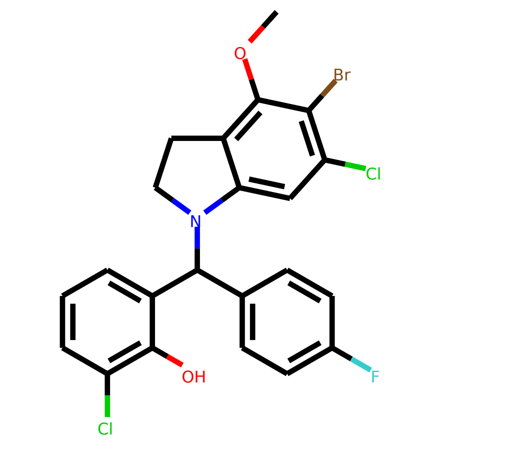
Osma-1824
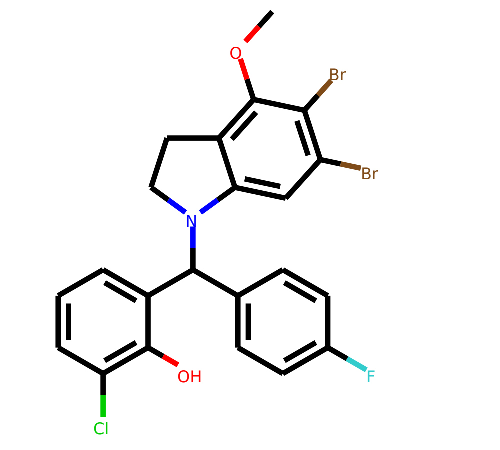
Osma-1825
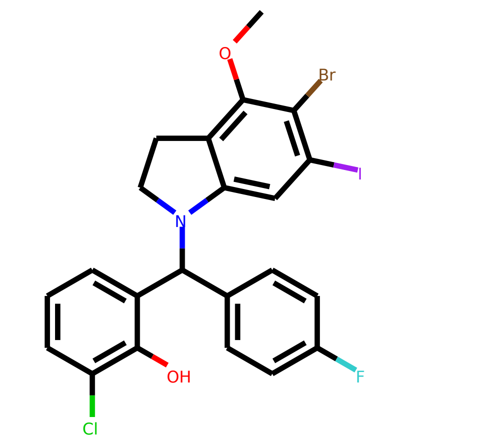
Osma-1826
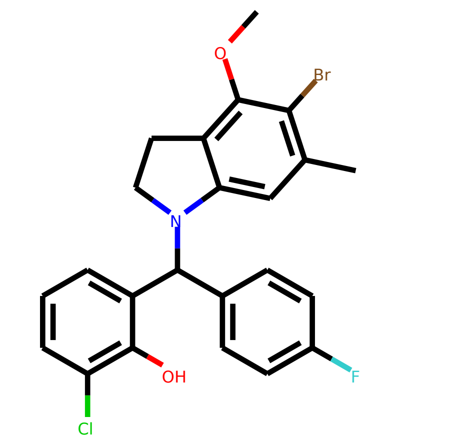
Osma-1827
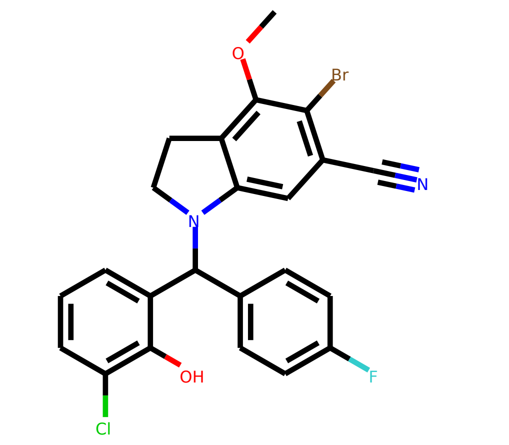
Osma-1828
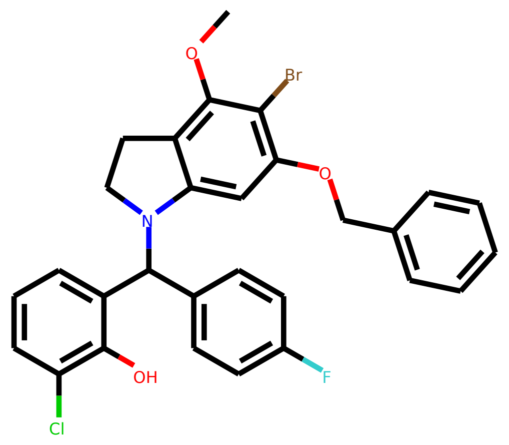
Osma-1829
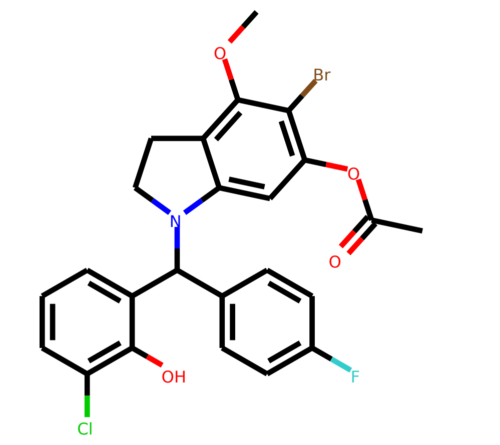
Osma-1830
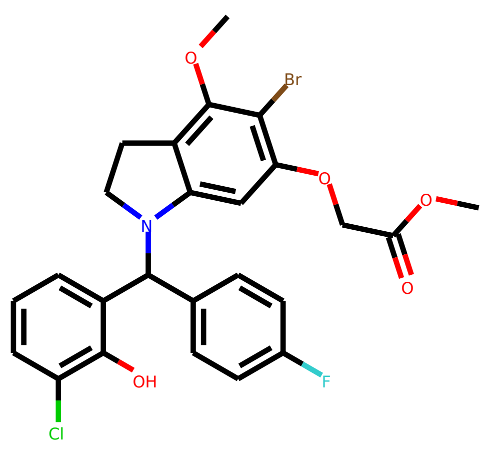
Osma-1831
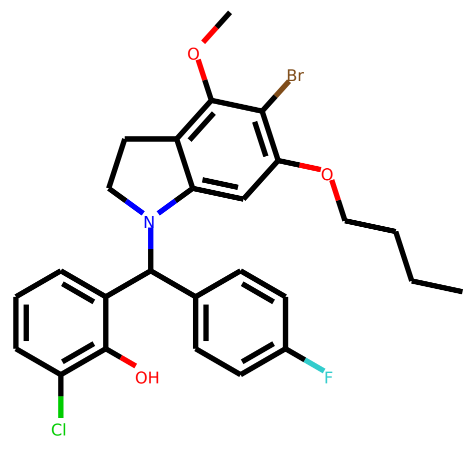
Osma-1832
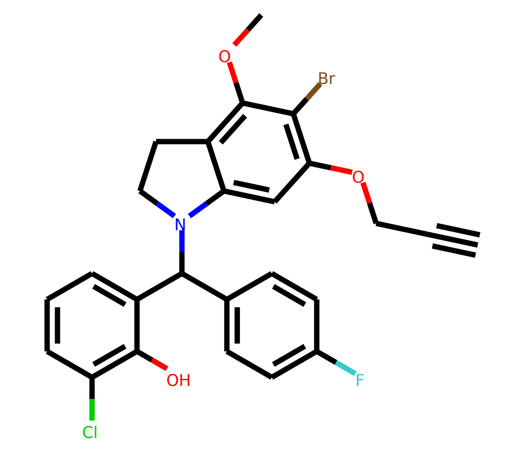
Osma-1833
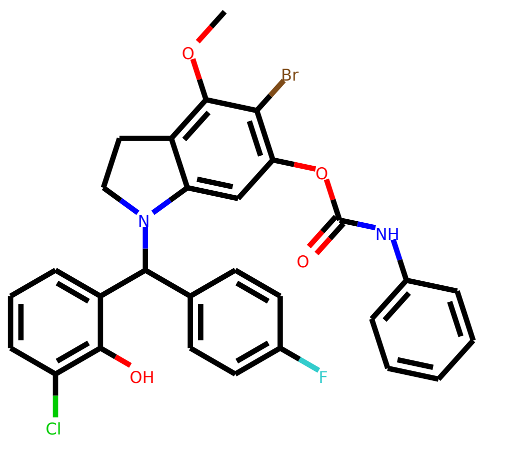
Osma-1834
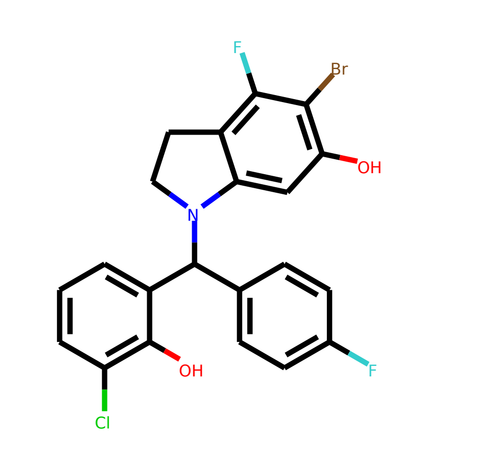
Osma-1835
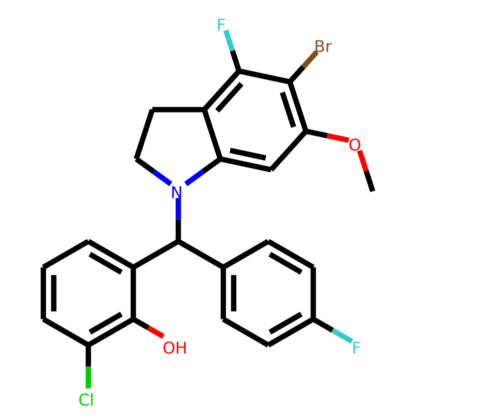
Osma-1836
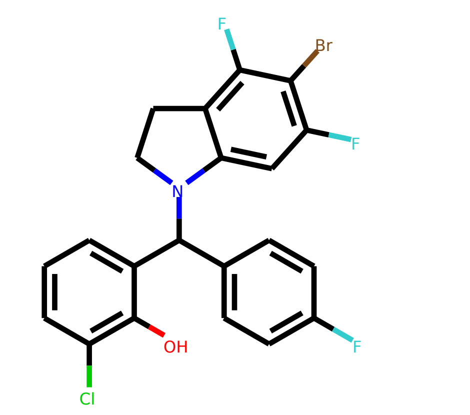
Osma-1837
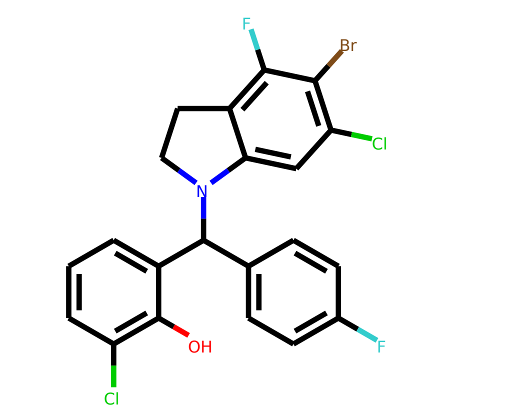
Osma-1838
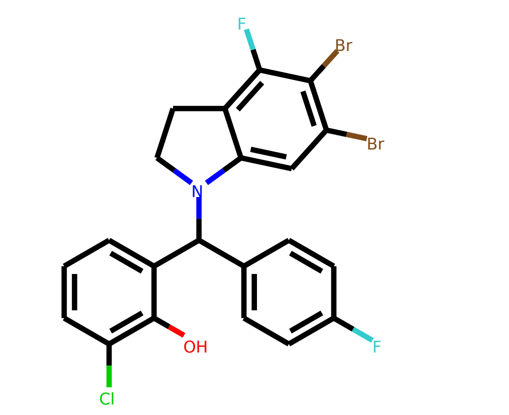
Osma-1839
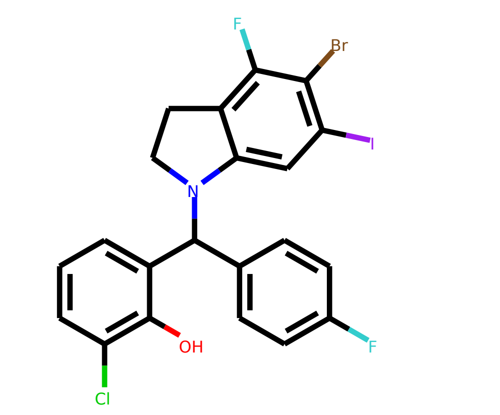
Osma-1840
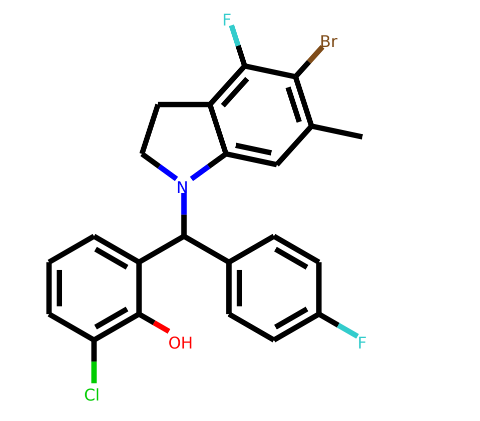
Osma-1841
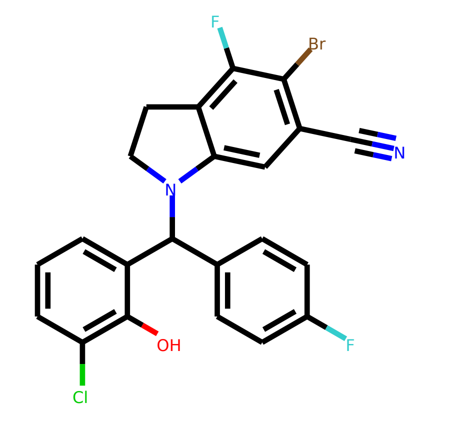
Osma-1842
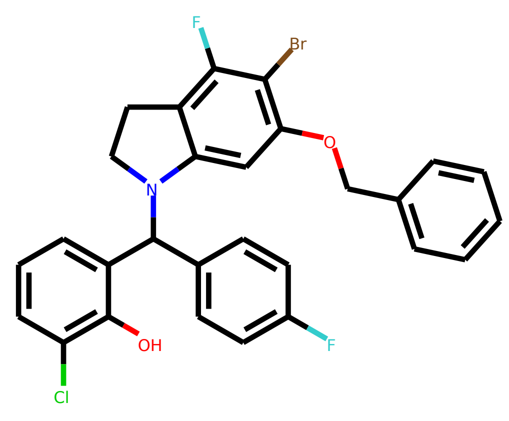
Osma-1843
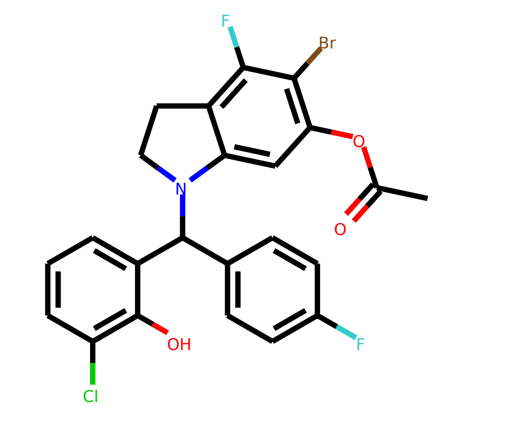
Osma-1844
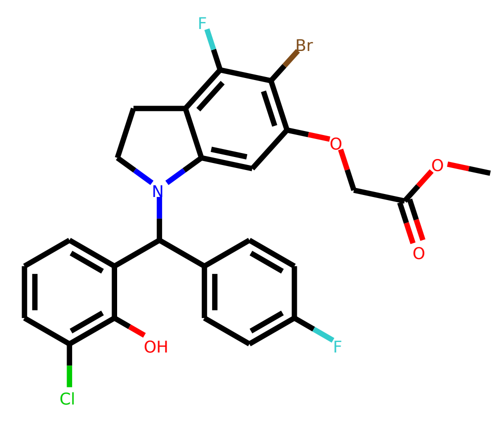
Osma-1845
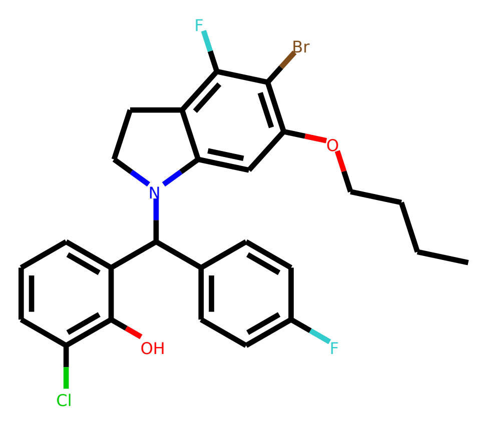
Osma-1846
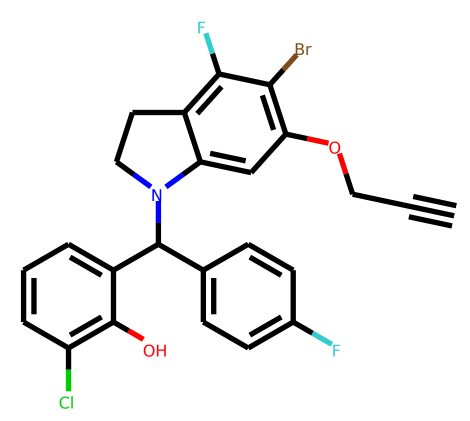
Osma-1847
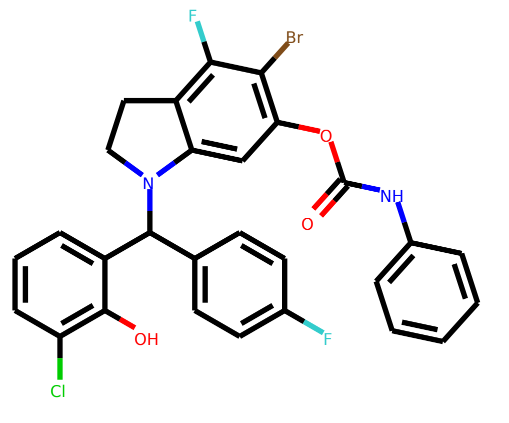
Osma-1848
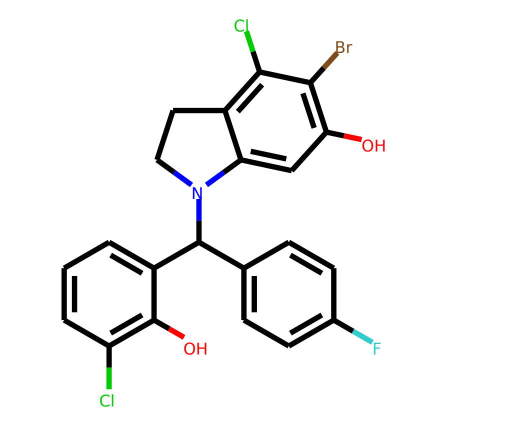
Osma-1849
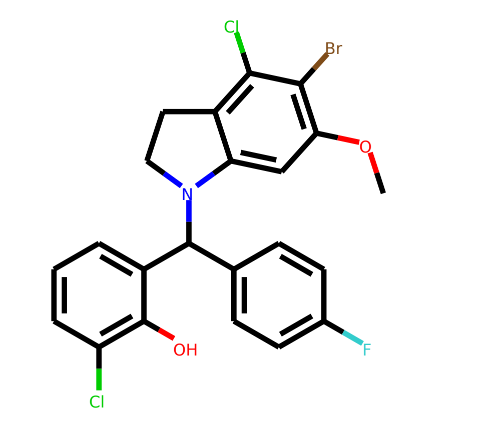
Osma-1850
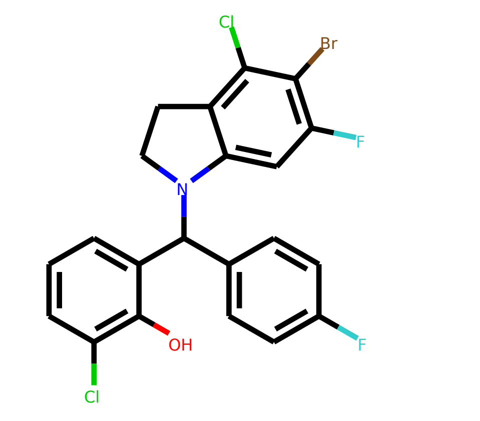
Osma-1851
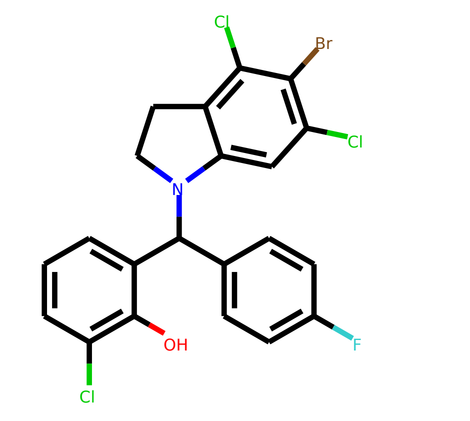
Osma-1852
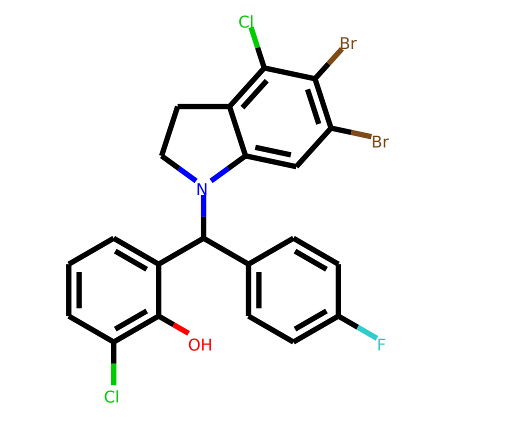
Osma-1853
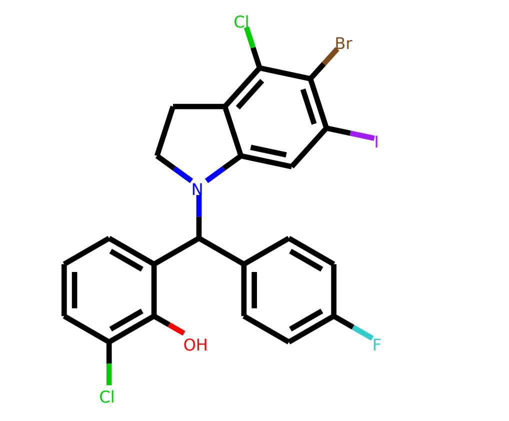
Osma-1854
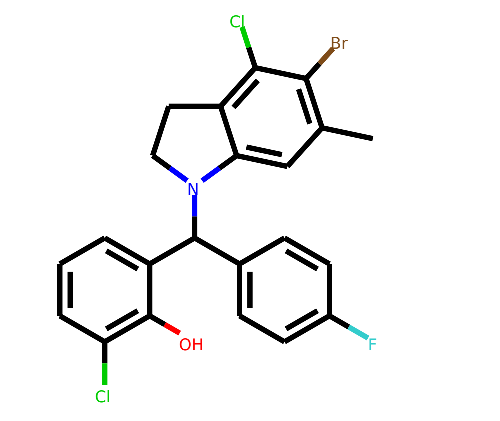
Osma-1855

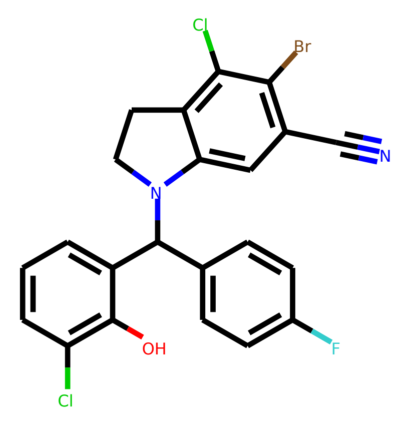

Osma-1856

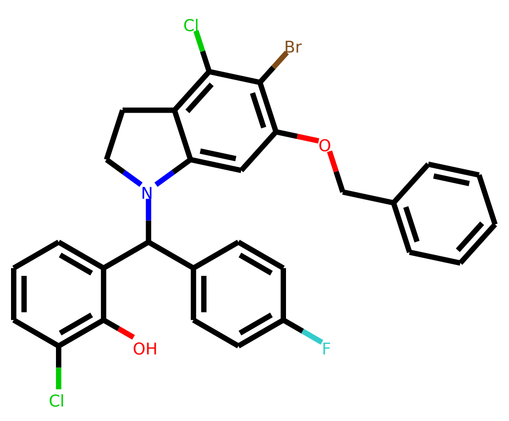

Osma-1857

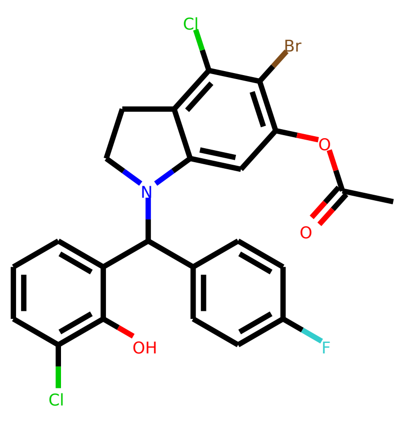

Osma-1858

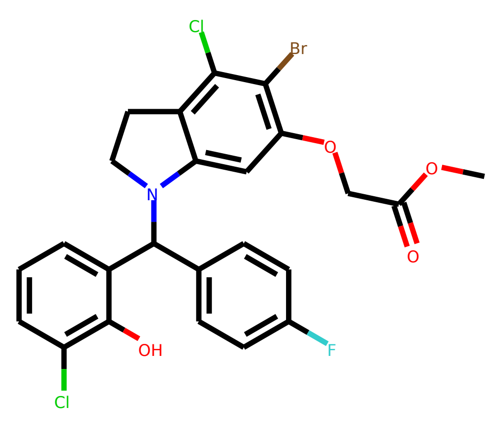

Osma-1859

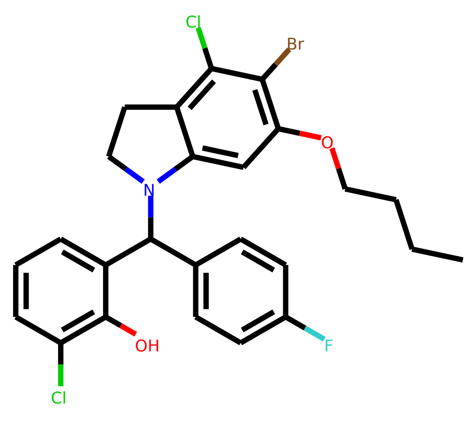

Osma-1860

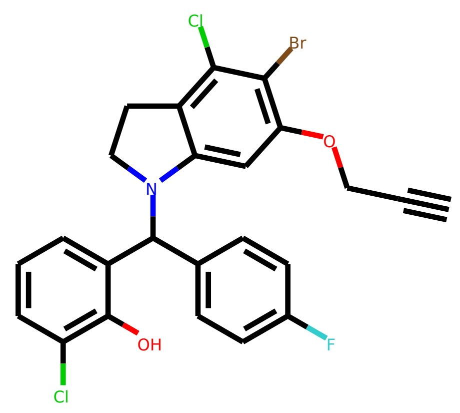

Osma-1861

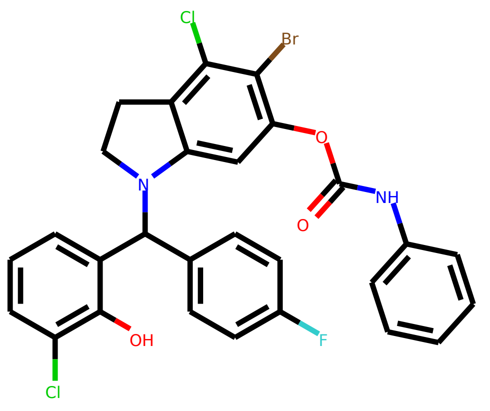

Osma-1862

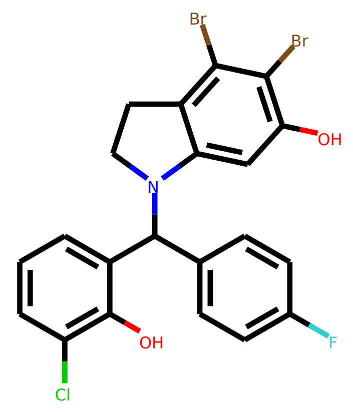

Osma-1863

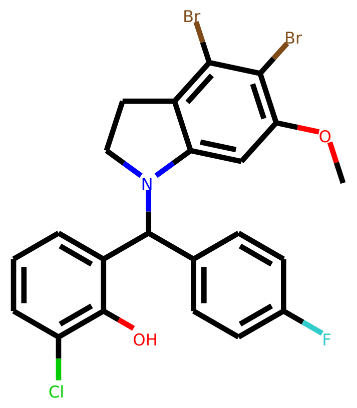

Osma-1864

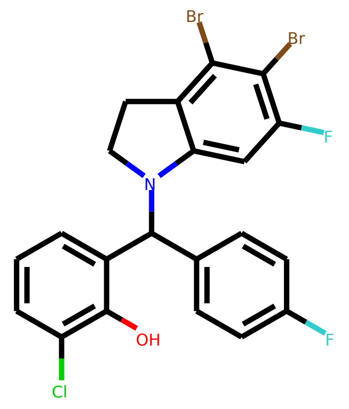

Osma-1865

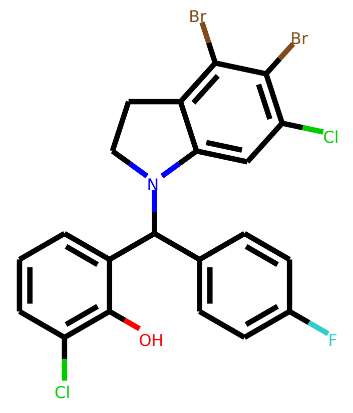

Osma-1866

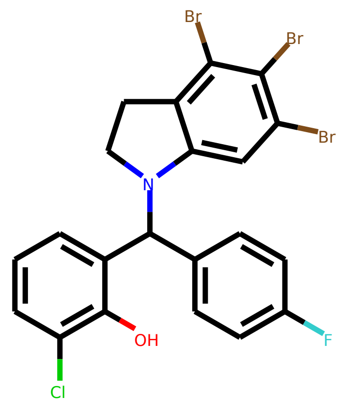

Osma-1867

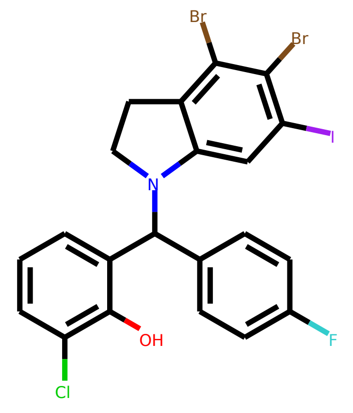

Osma-1868

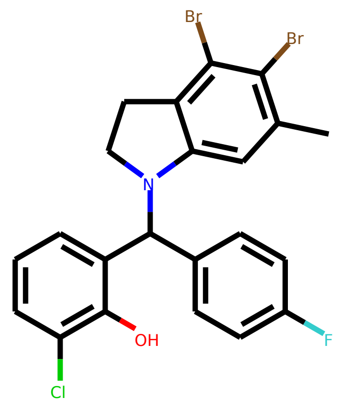

Osma-1869

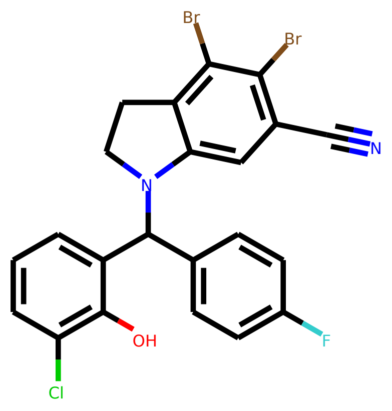

Osma-1870

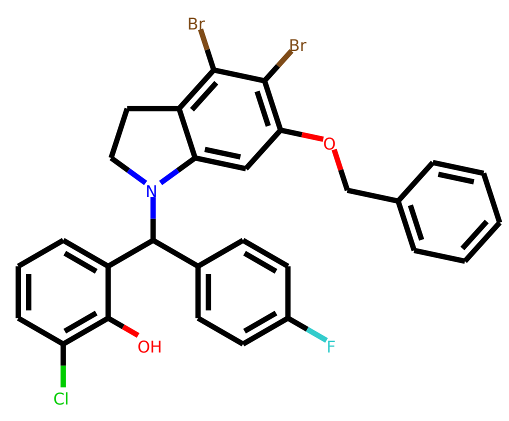

Osma-1871

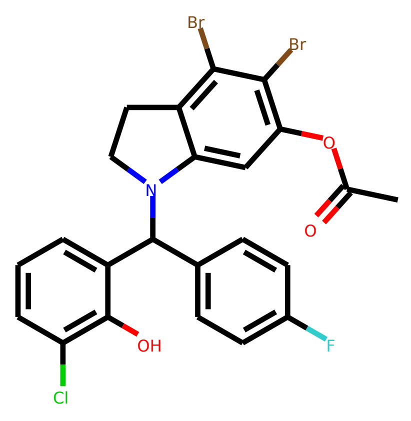

Osma-1872

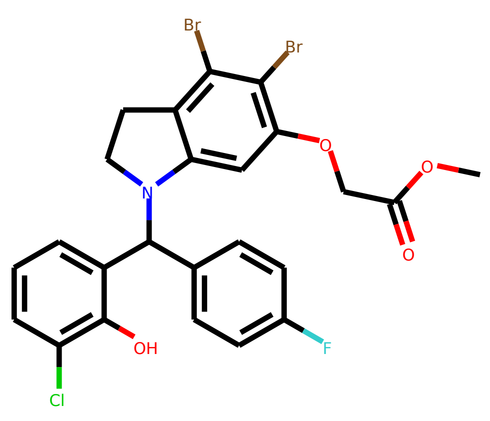

Osma-1873

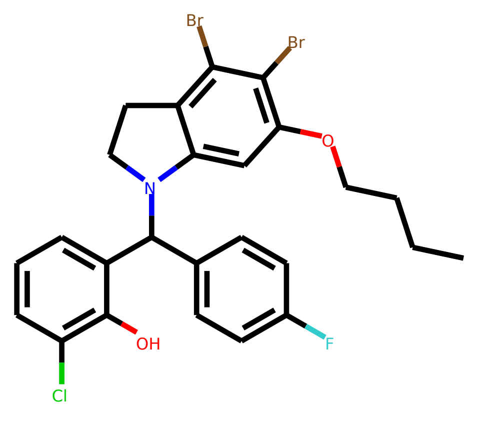

Osma-1874

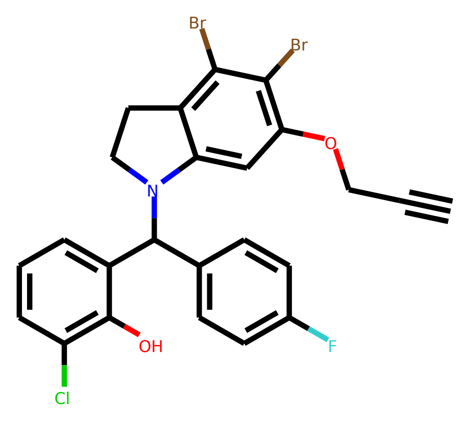

Osma-1875

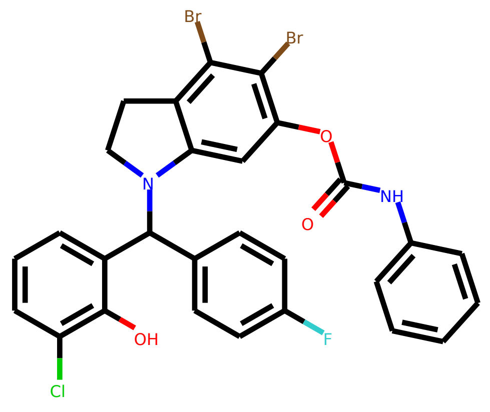

Osma-1876

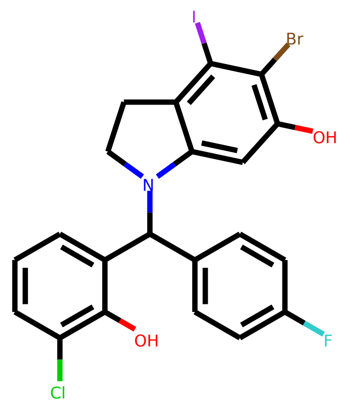

Osma-1877

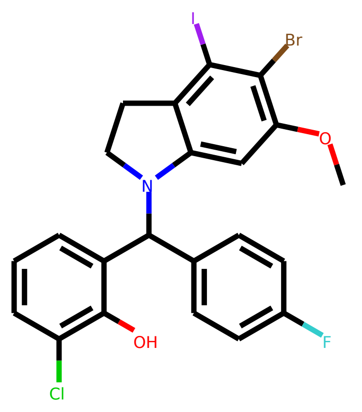

Osma-1878

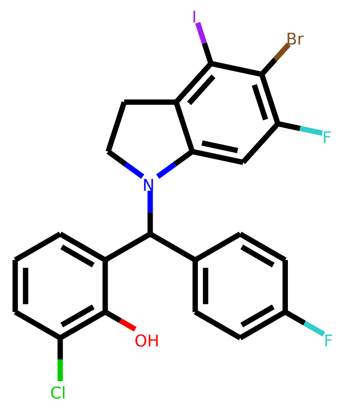

Osma-1879

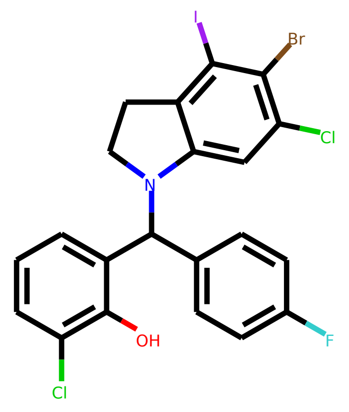

Osma-1880

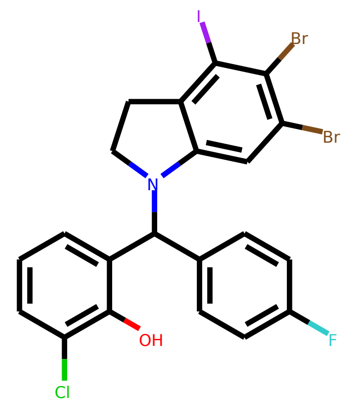

Osma-1881

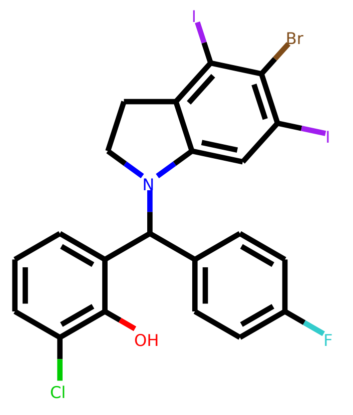

Osma-1882

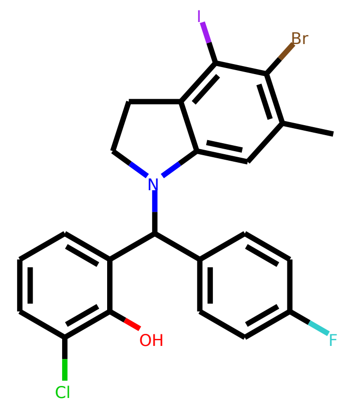

Osma-1883

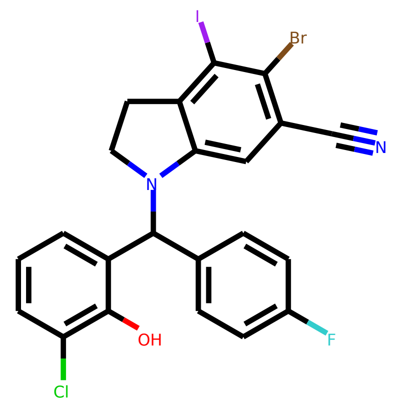

Osma-1884

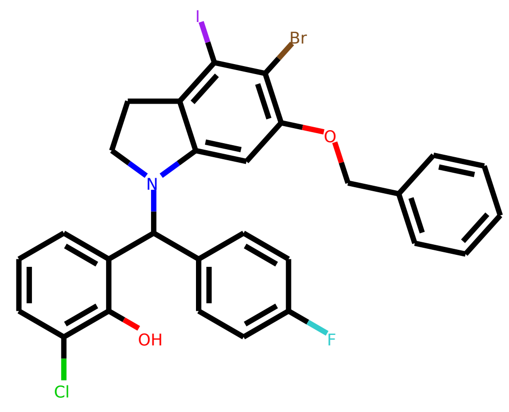

Osma-1885

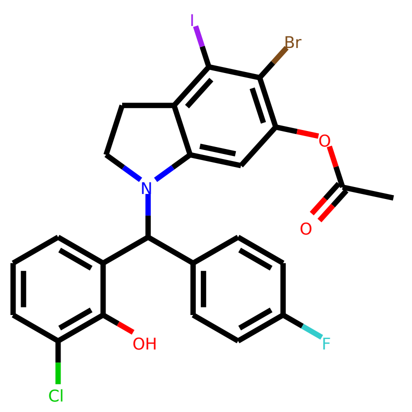

Osma-1886

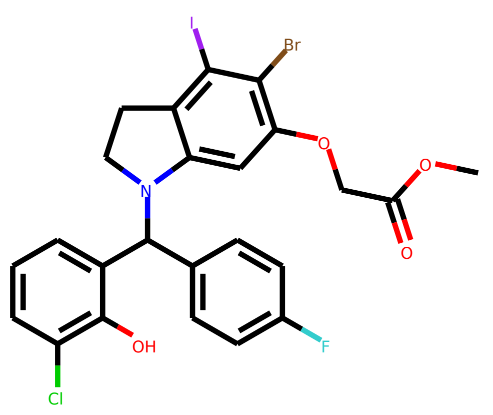

Osma-1887

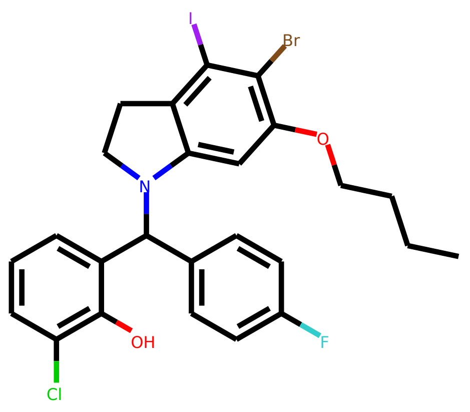

Osma-1888

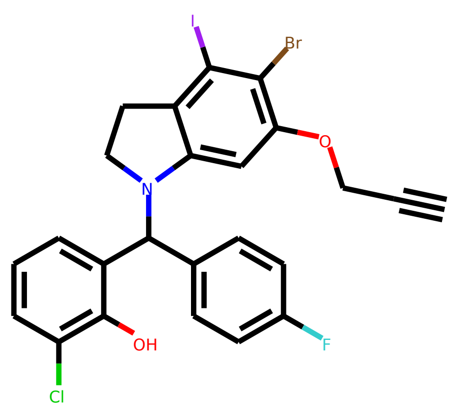

Osma-1889

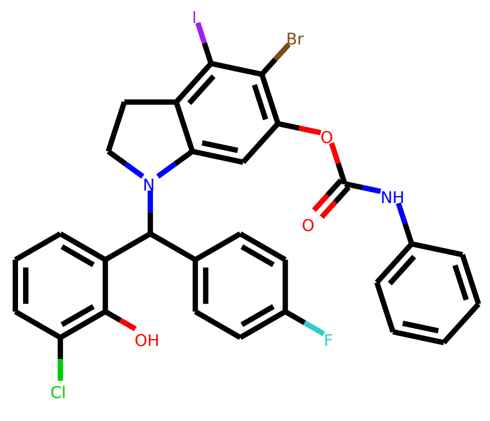

Osma-1890

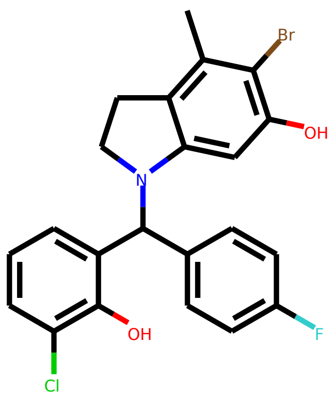
Osma-1891

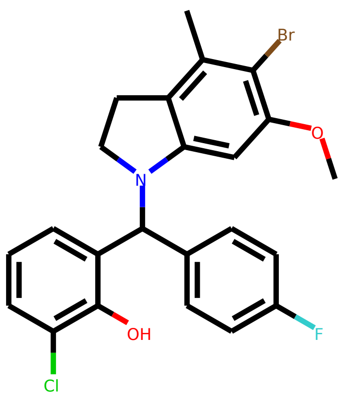
Osma-1892

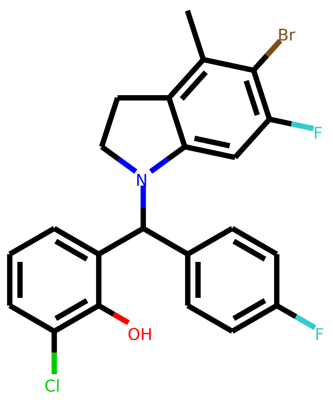
Osma-1893

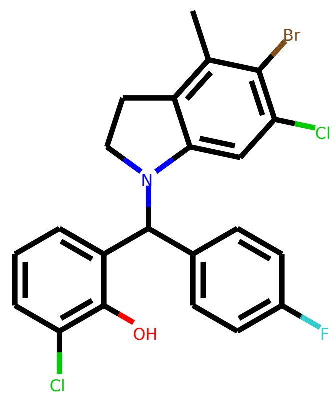
Osma-1894

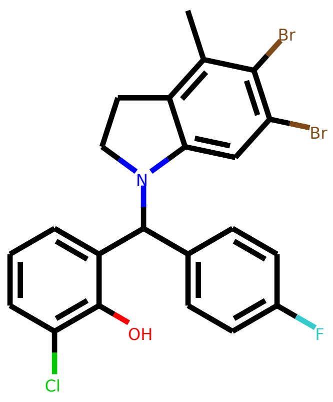
Osma-1895

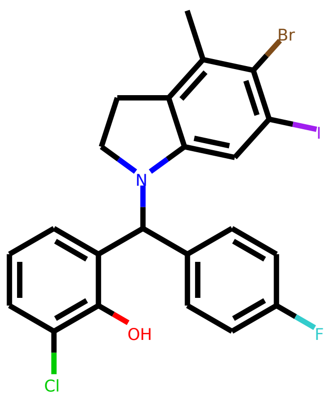
Osma-1896

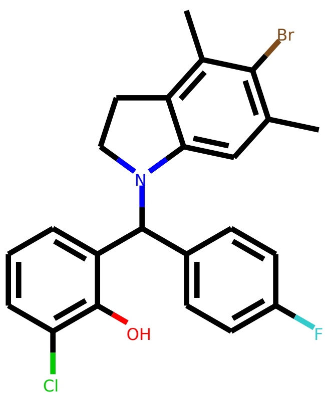
Osma-1897

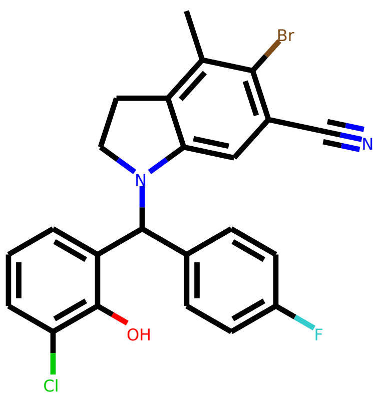
Osma-1898

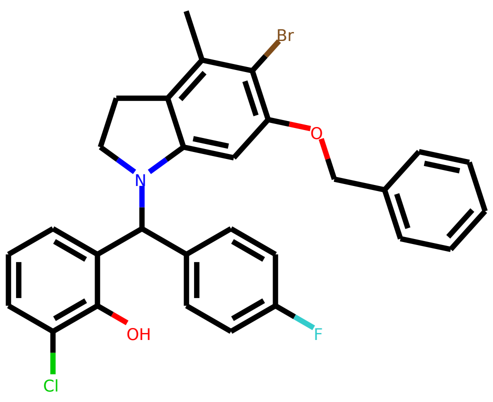
Osma-1899

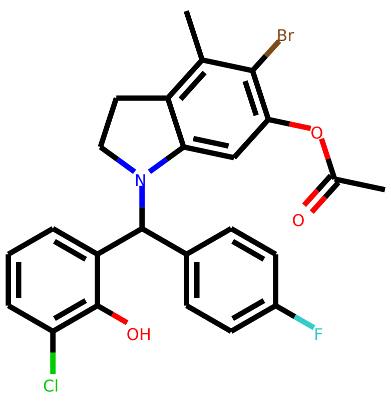
Osma-1900

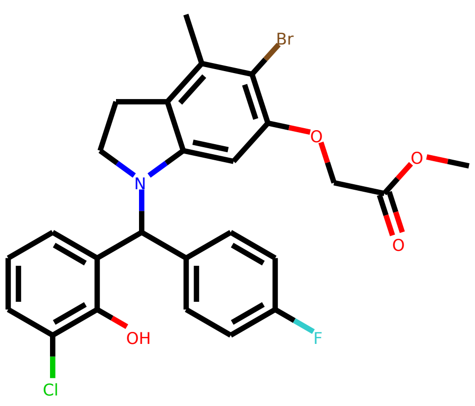
Osma-1901

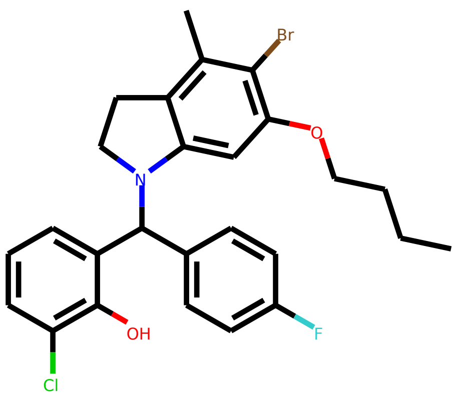
Osma-1902

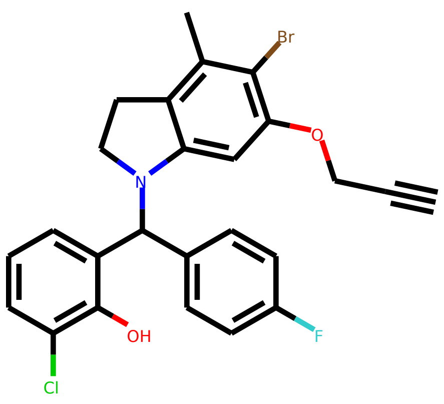
Osma-1903

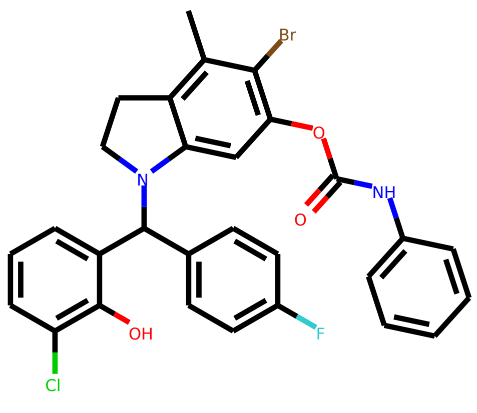
Osma-1904

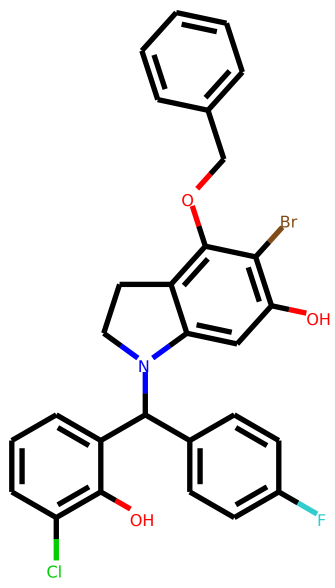
Osma-1905

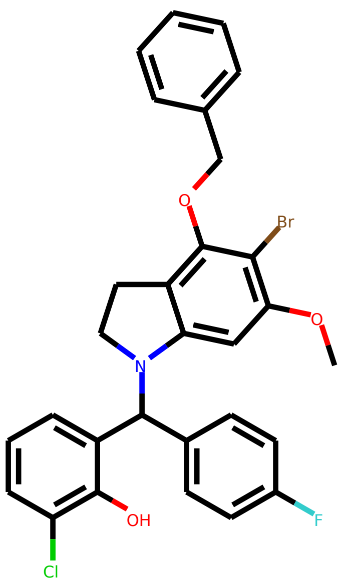
Osma-1906

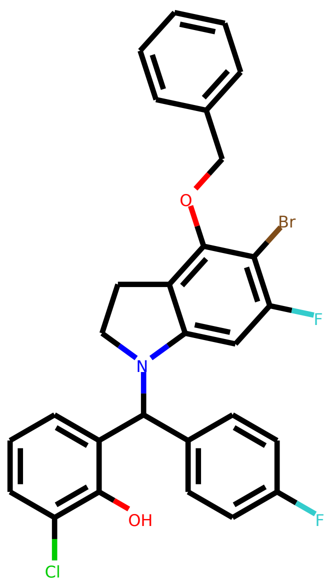
Osma-1907

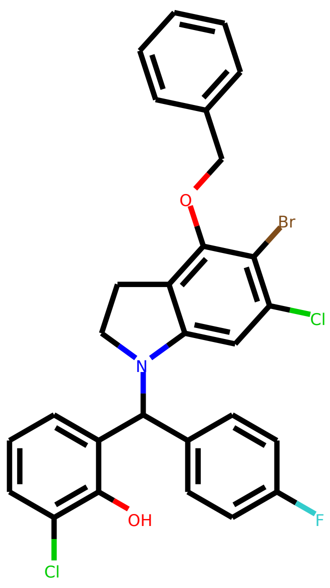
Osma-1908

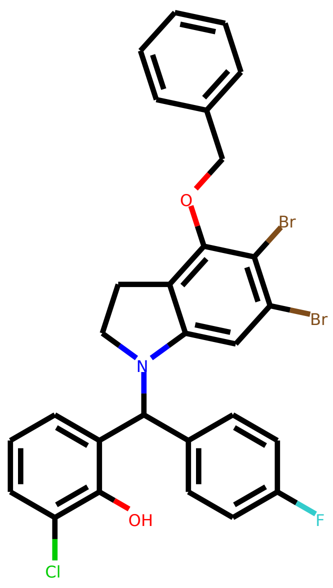
Osma-1909

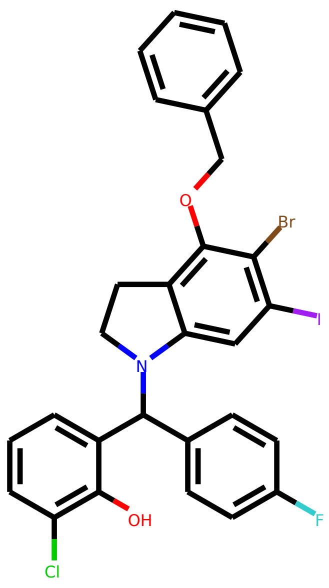
Osma-1910

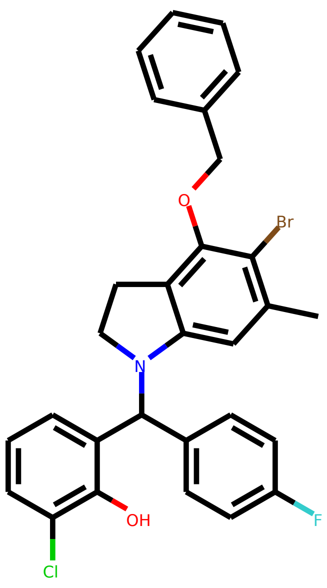
Osma-1911

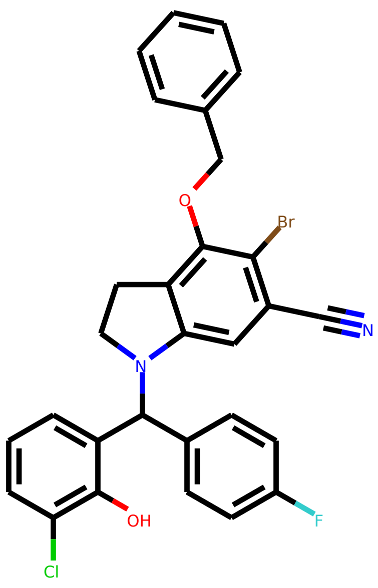
Osma-1912

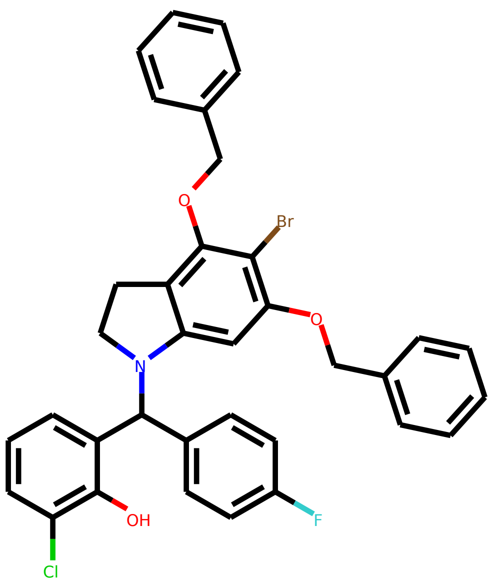
Osma-1913

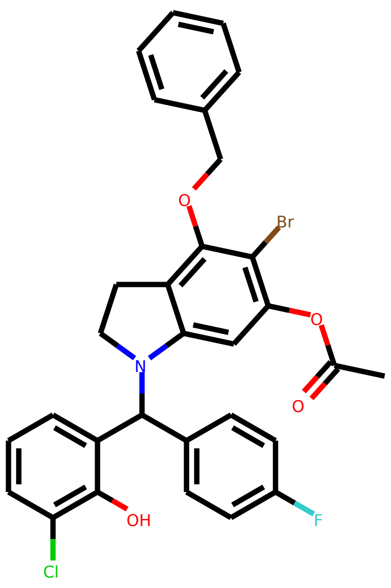
Osma-1914

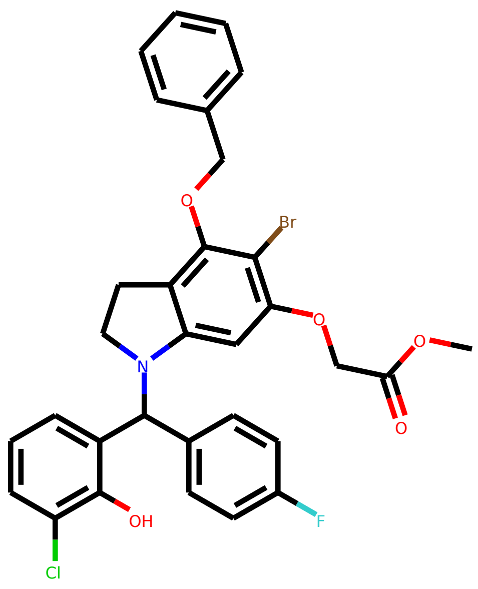
Osma-1915

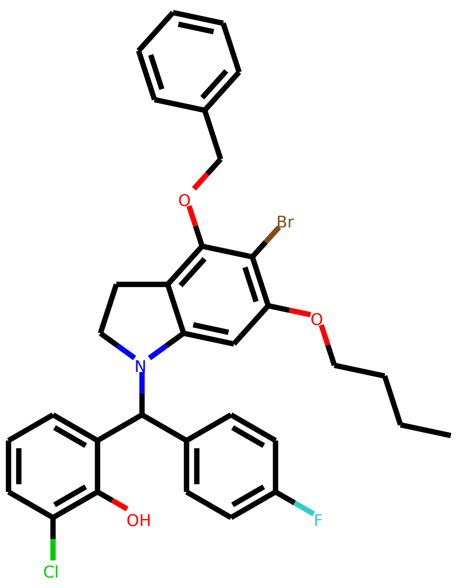
Osma-1916

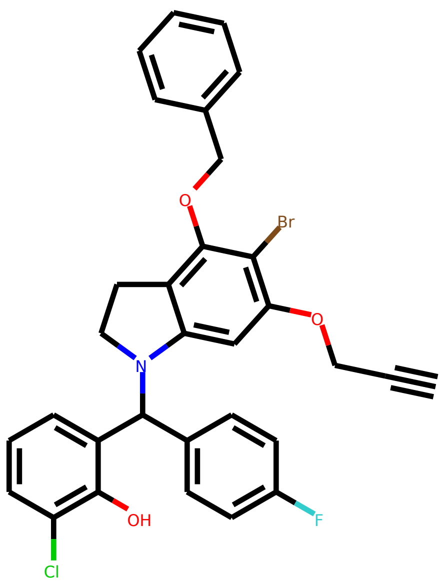
Osma-1917

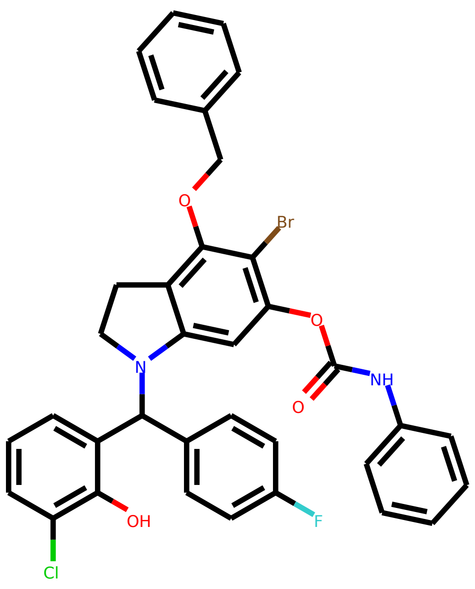
Osma-1918

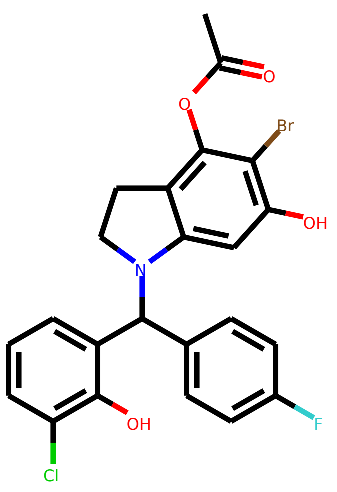
Osma-1919

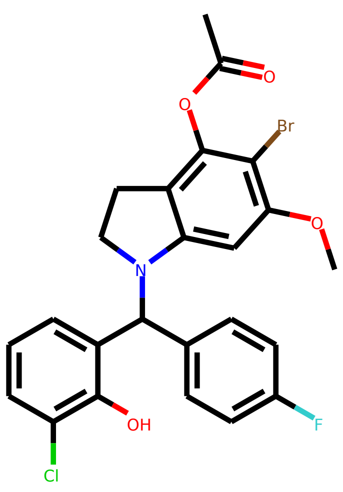
Osma-1920

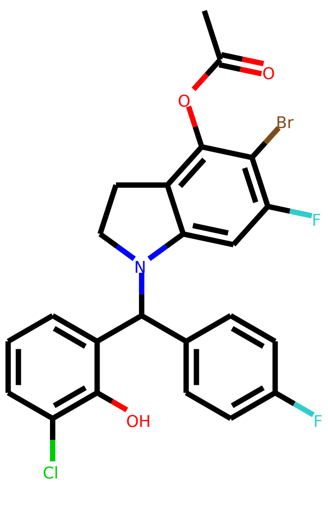
Osma-1921

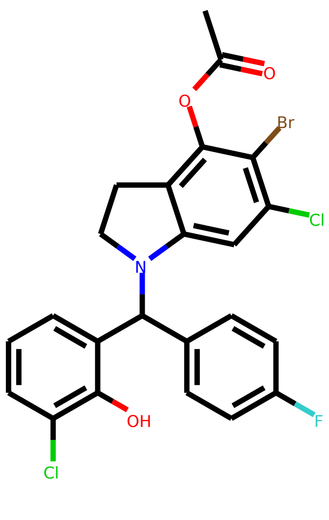
Osma-1922

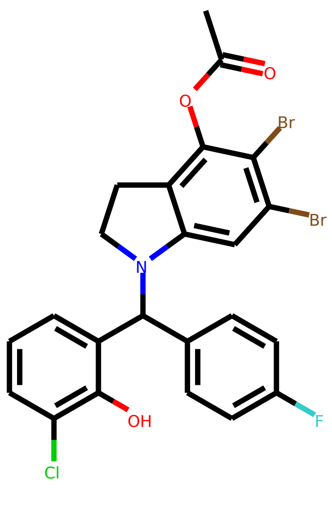
Osma-1923

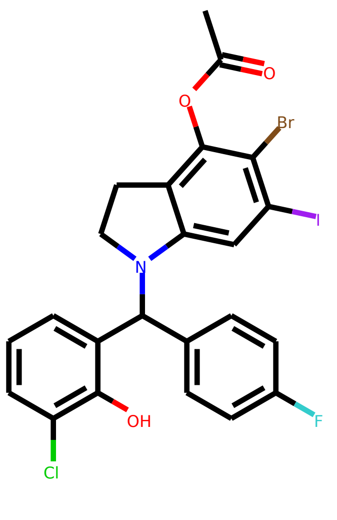
Osma-1924

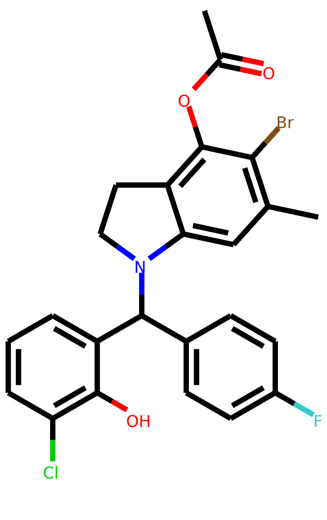
Osma-1925

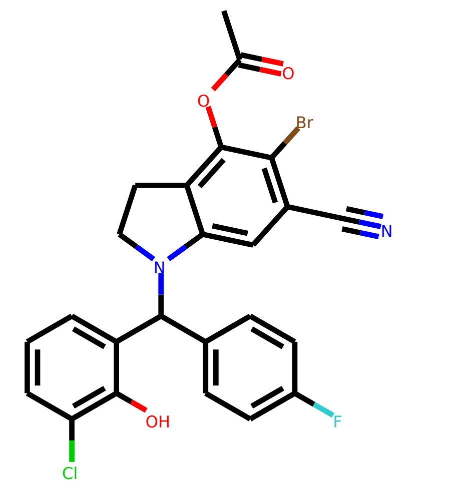
Osma-1926

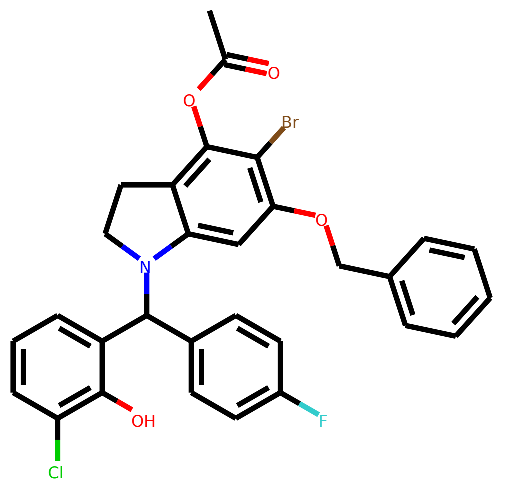
Osma-1927

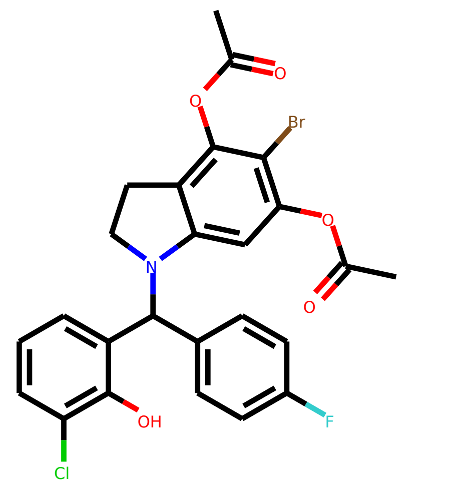
Osma-1928

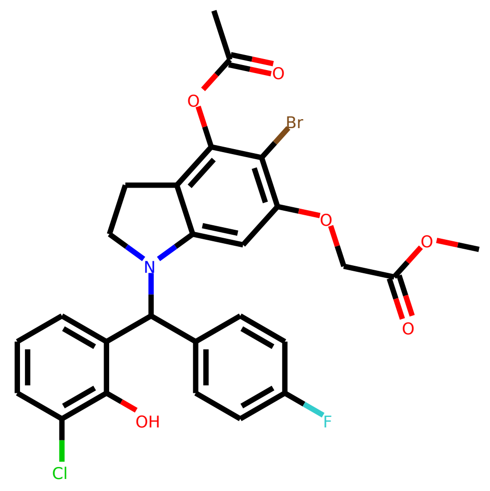
Osma-1929

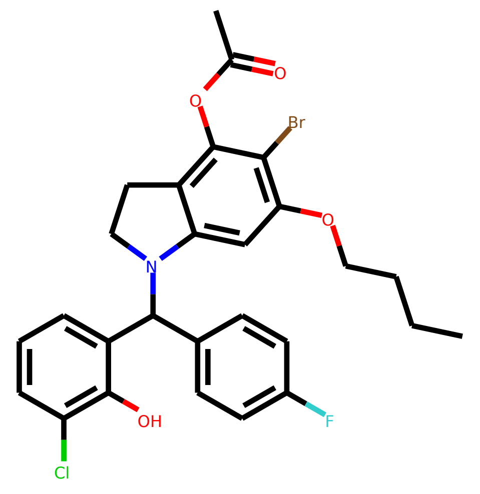
Osma-1930

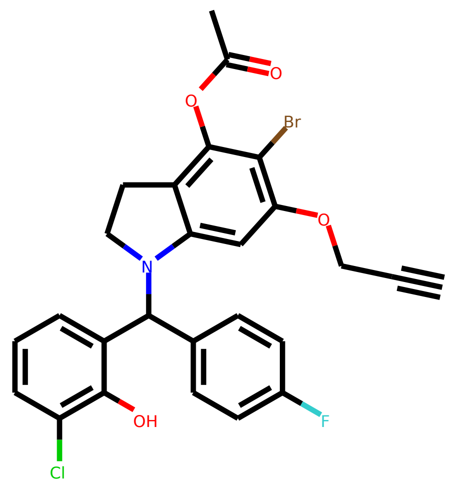
Osma-1931

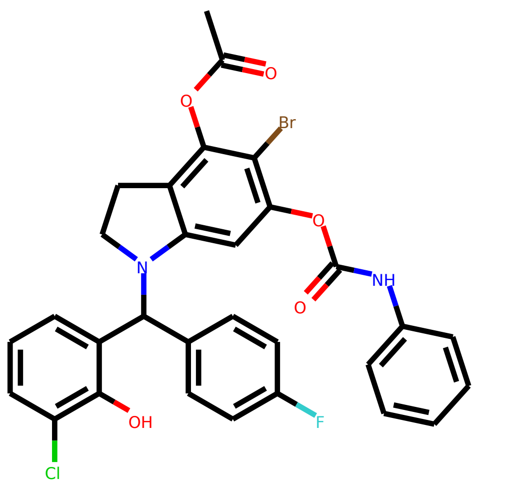
Osma-1932

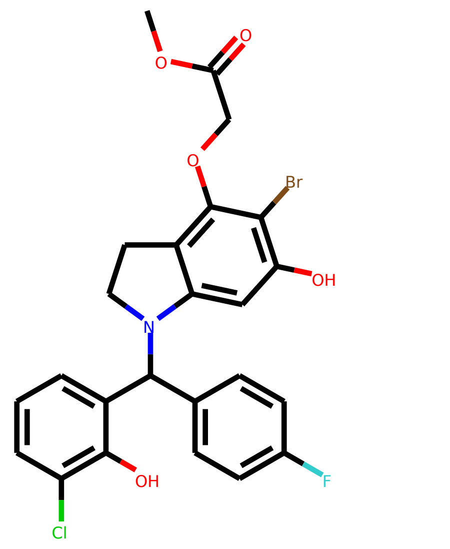
Osma-1933

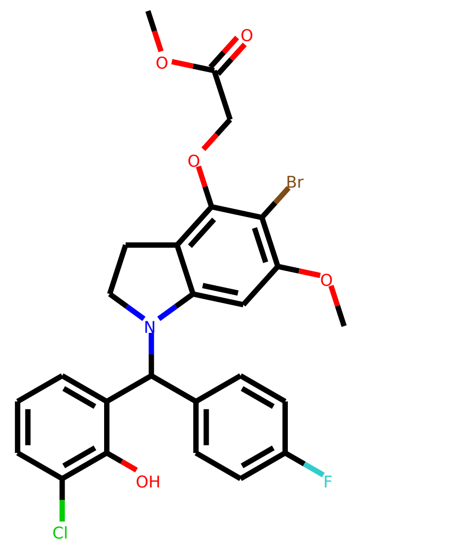
Osma-1934

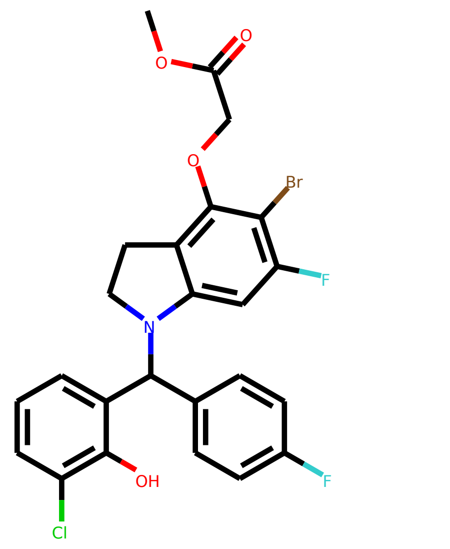
Osma-1935

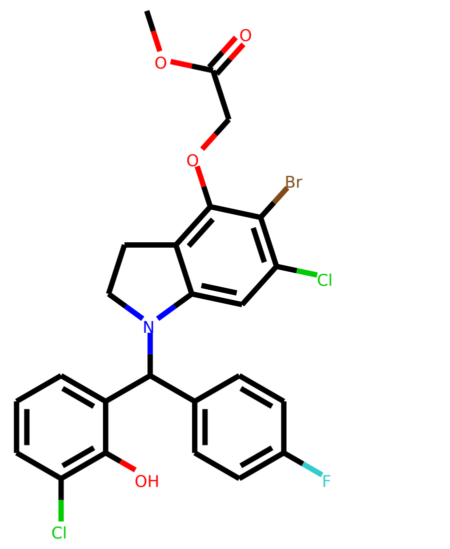
Osma-1936

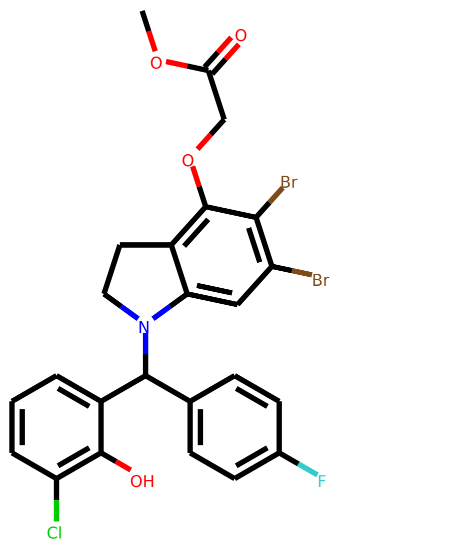
Osma-1937

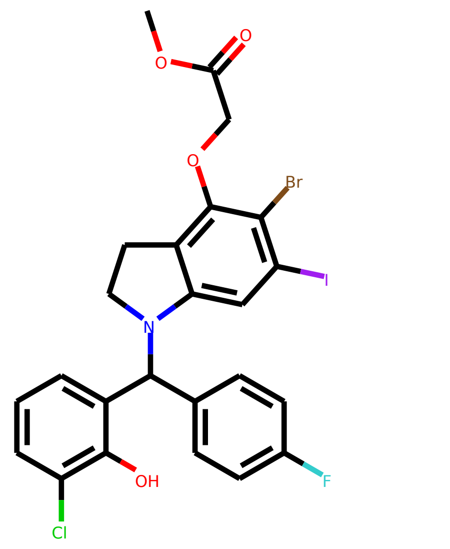
Osma-1938

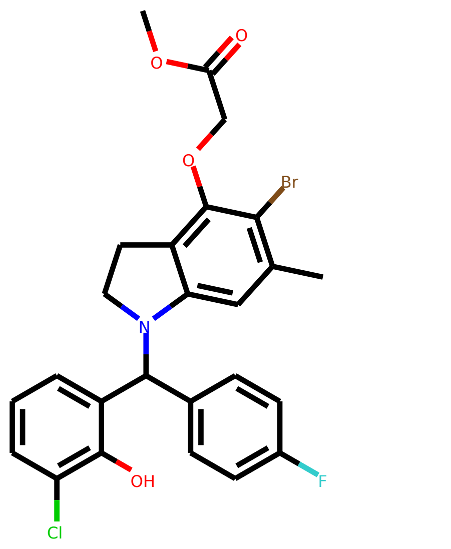
Osma-1939

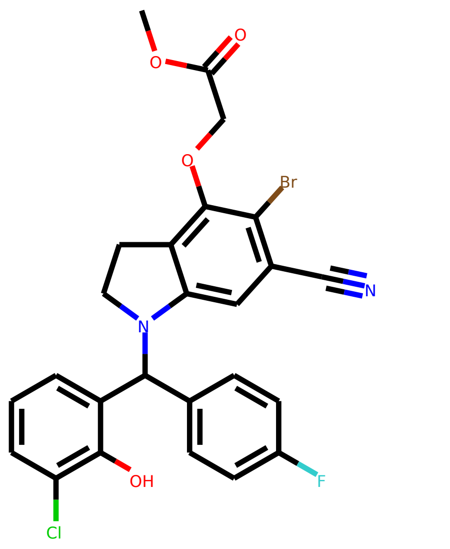
Osma-1940

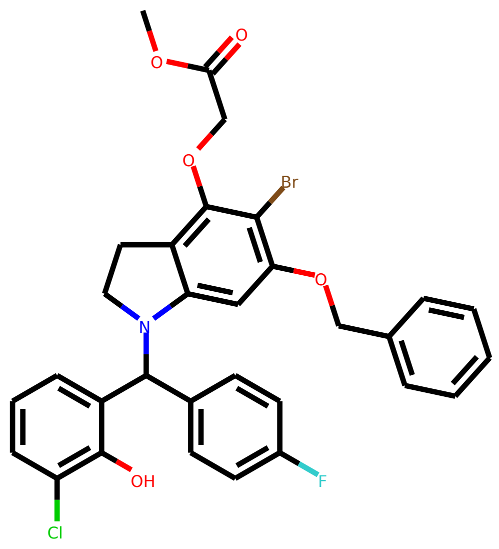
Osma-1941

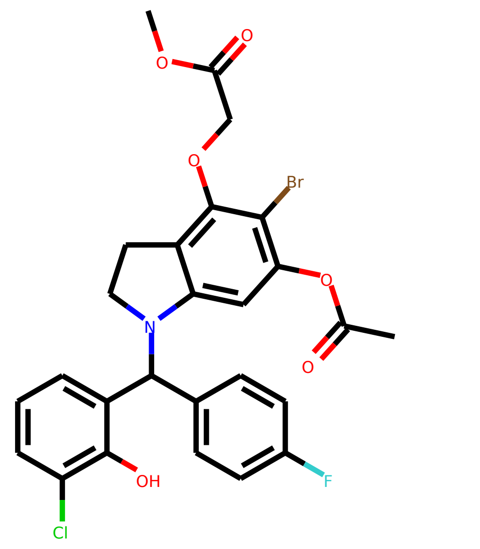
Osma-1942

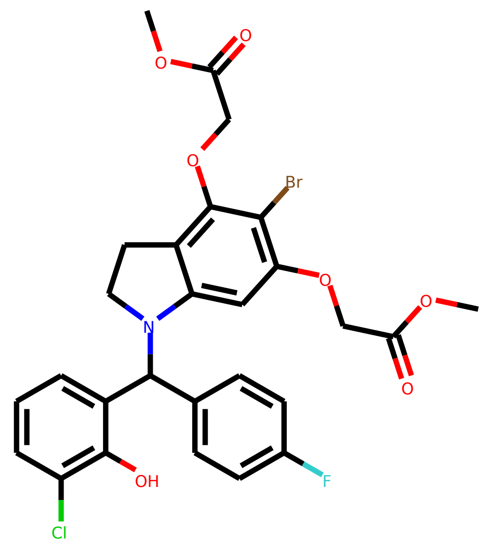
Osma-1943

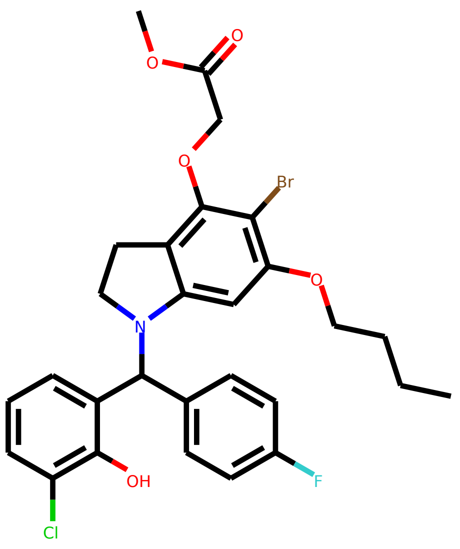
Osma-1944

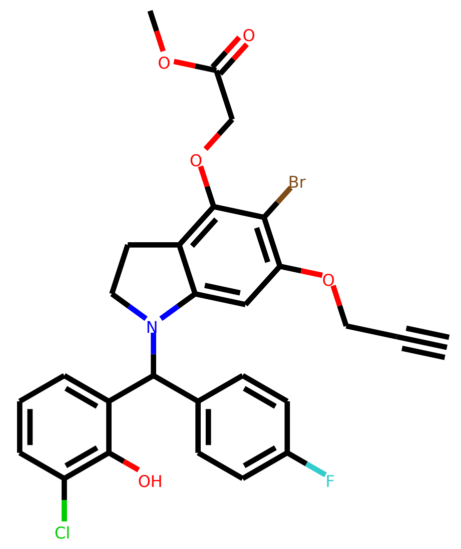
Osma-1945

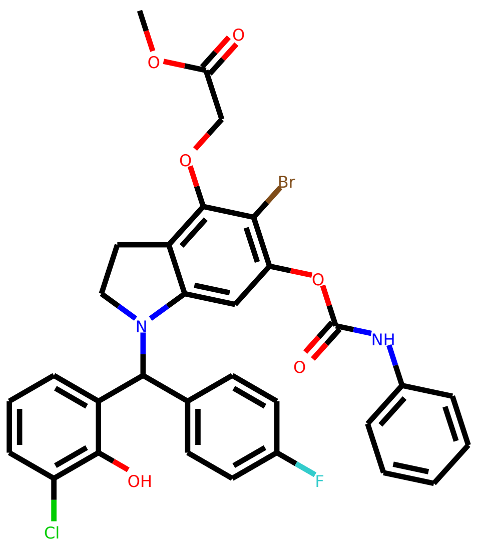
Osma-1946

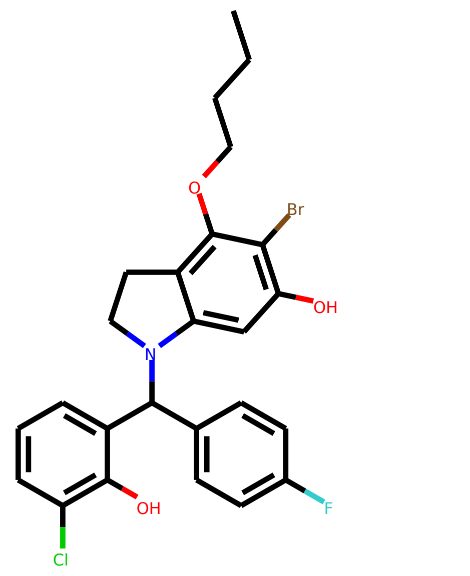
Osma-1947

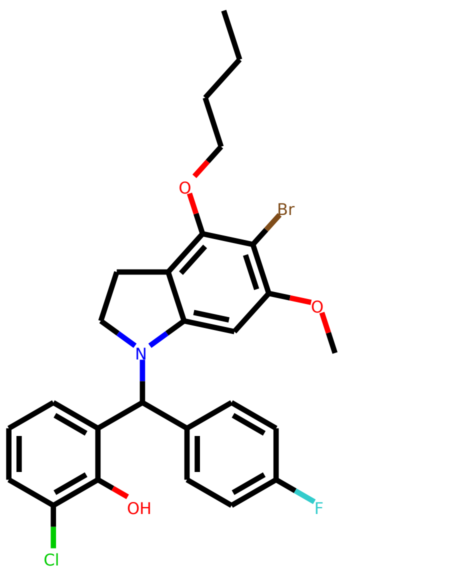
Osma-1948

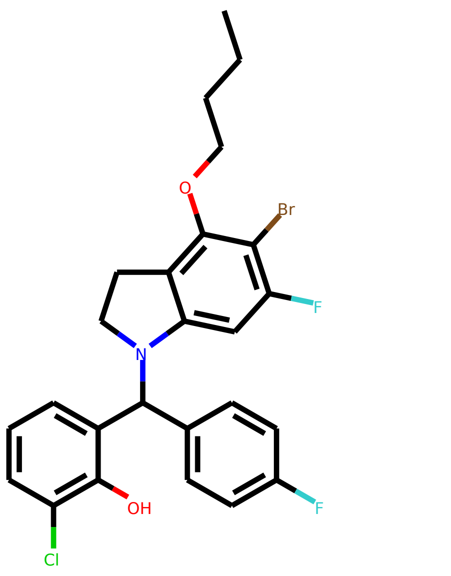
Osma-1949

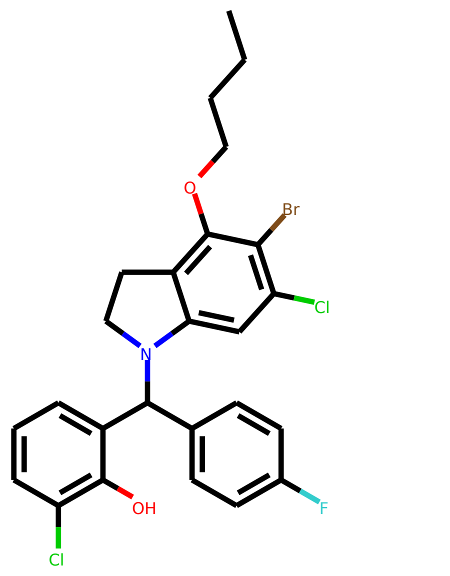
Osma-1950

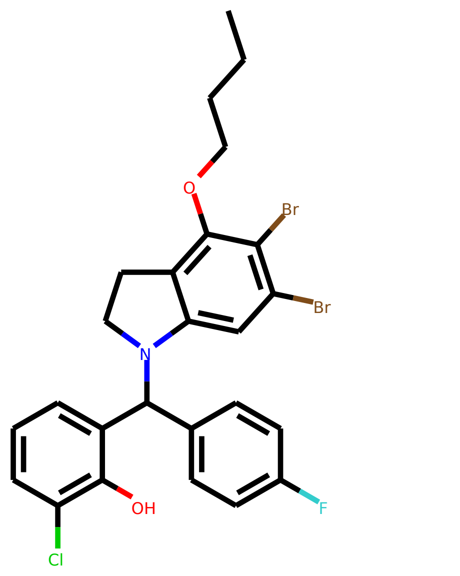
Osma-1951

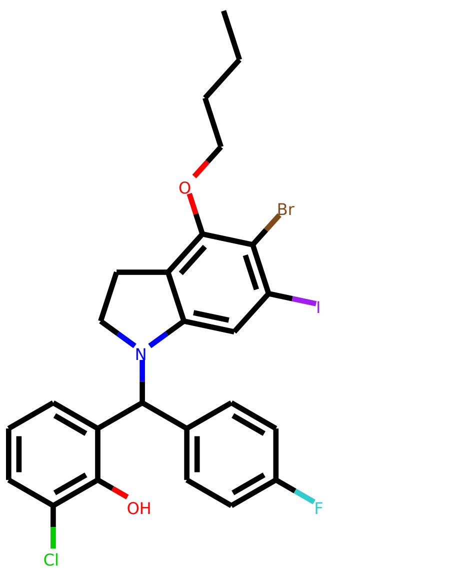
Osma-1952

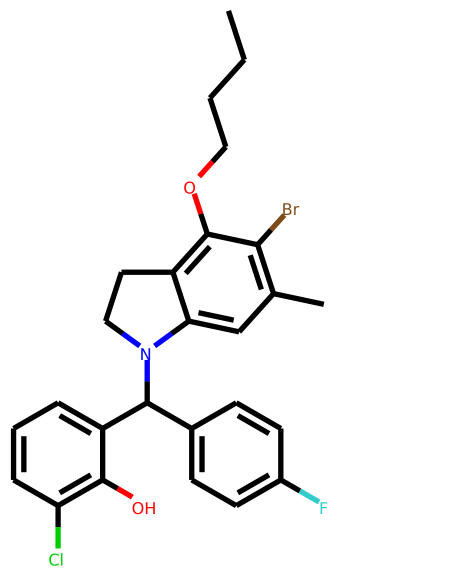
Osma-1953

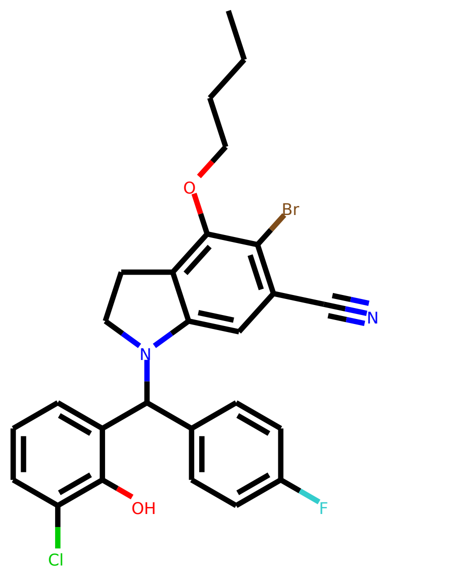
Osma-1954

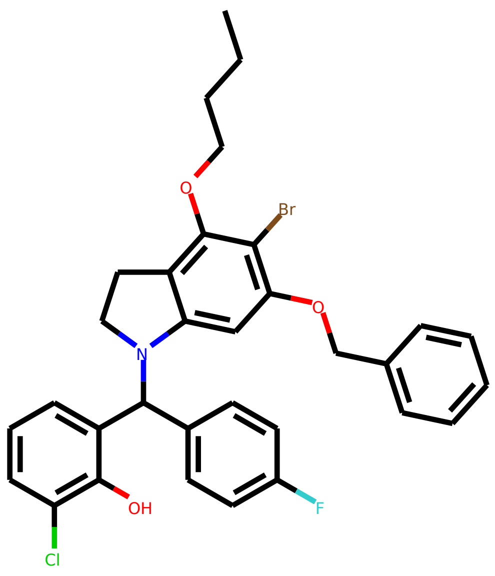
Osma-1955

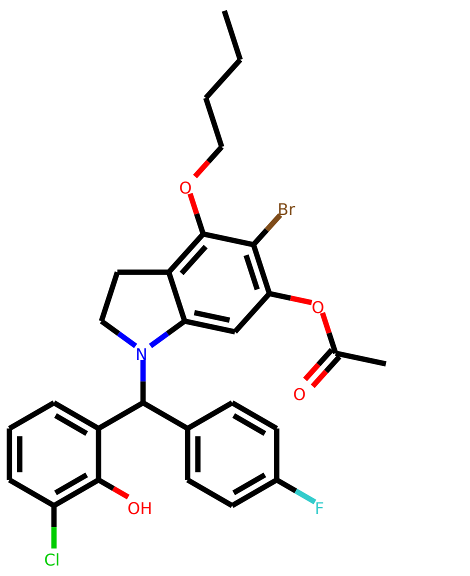
Osma-1956

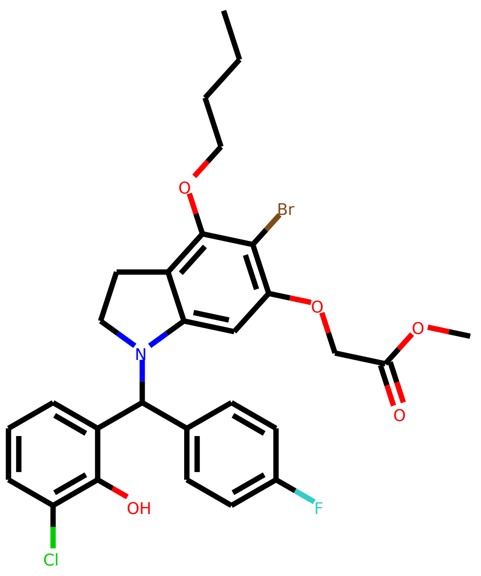
Osma-1957

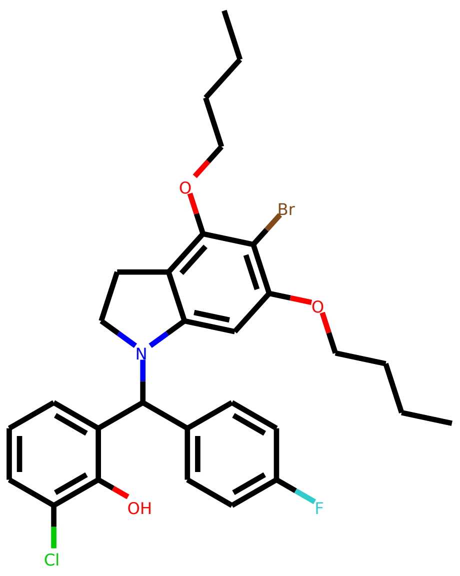
Osma-1958

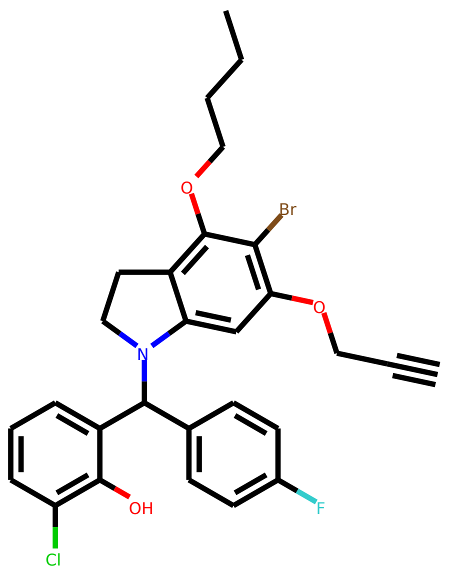
Osma-1959

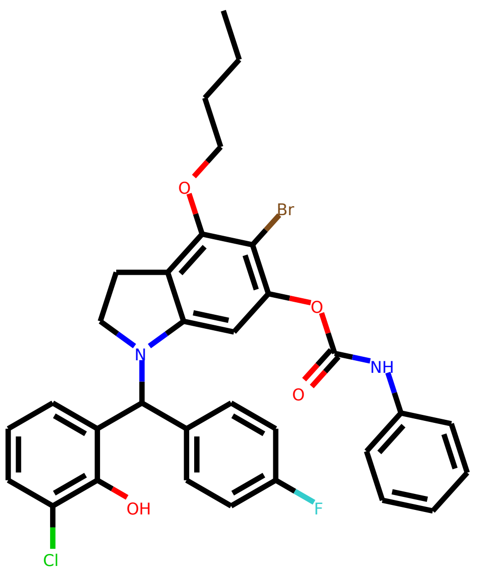
Osma-1960

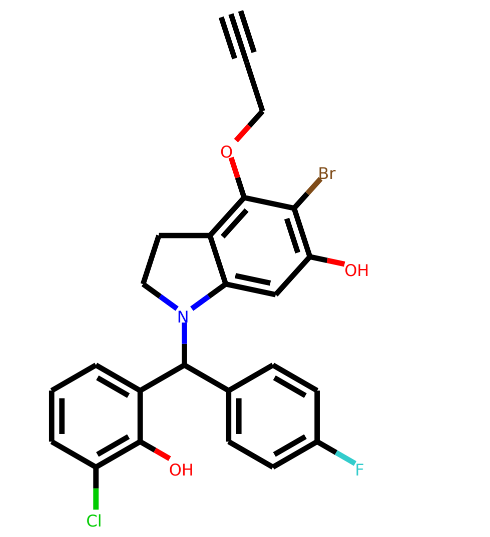

Osma-1961

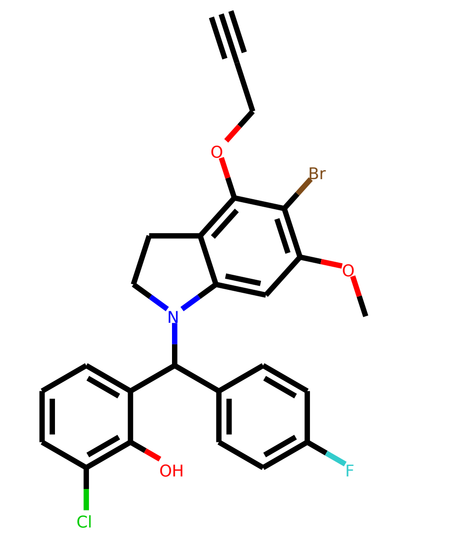

Osma-1962

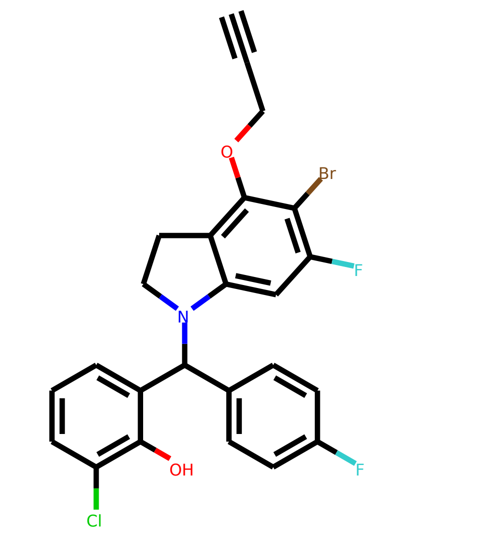

Osma-1963

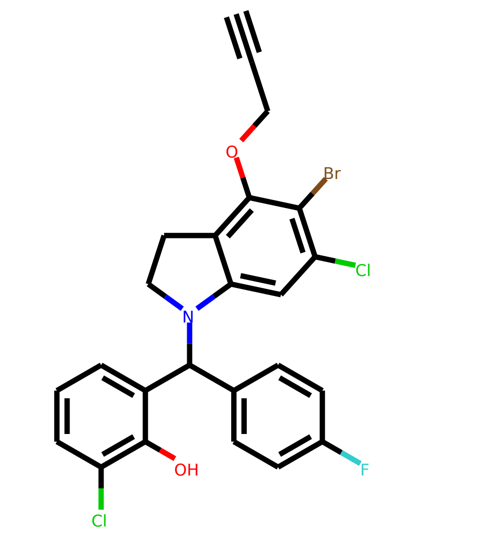

Osma-1964

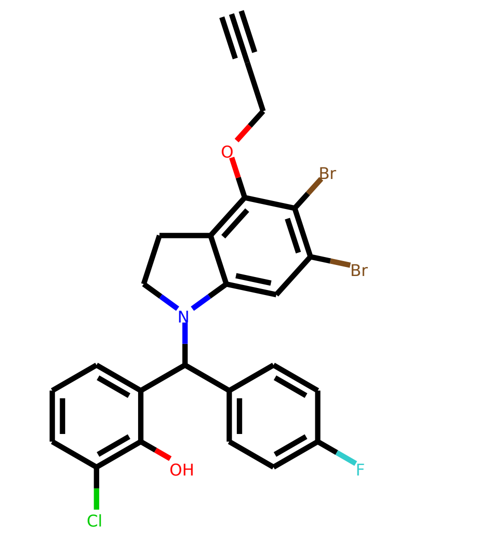

Osma-1965

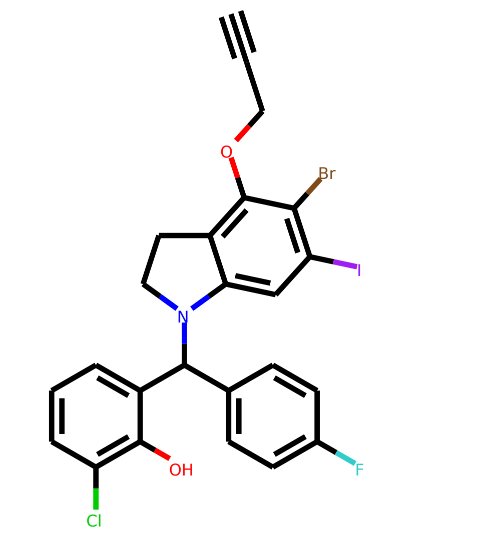

Osma-1966

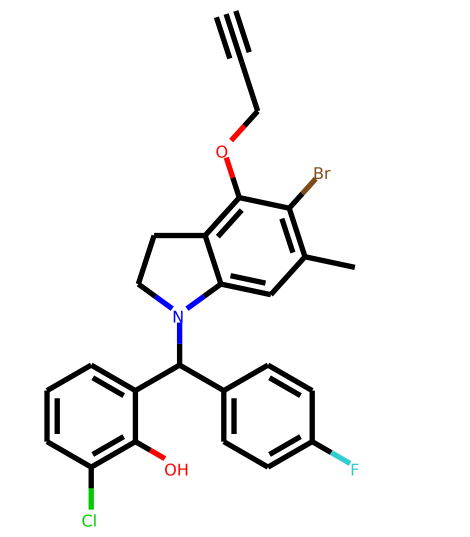

Osma-1967

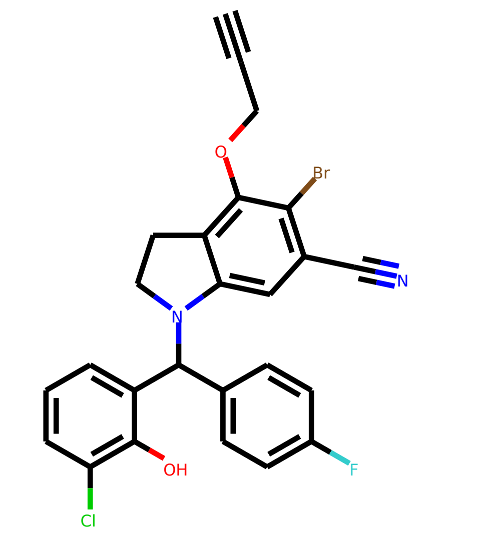

Osma-1968

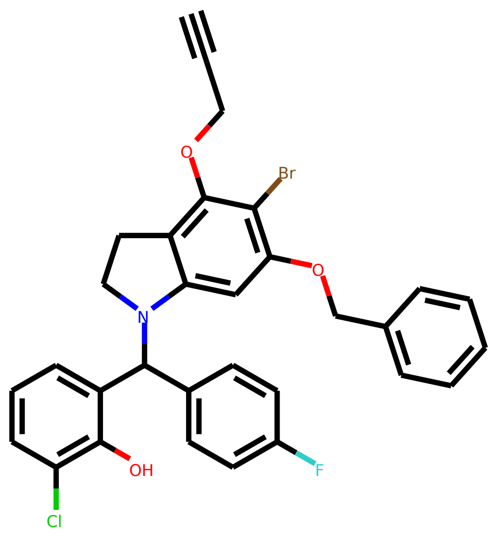

Osma-1969

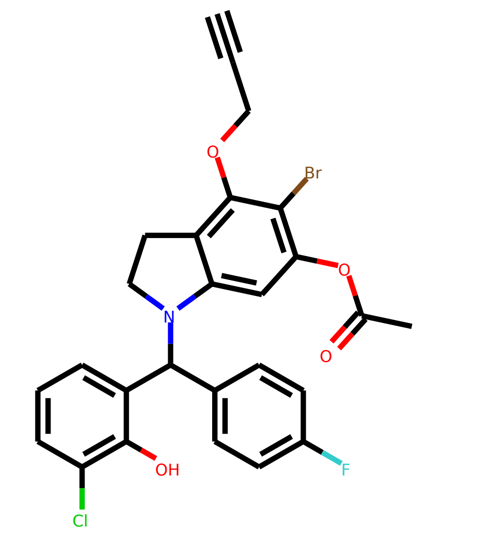

Osma-1970

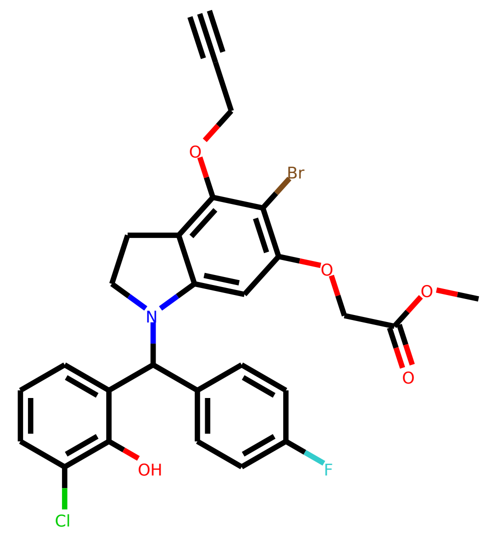

Osma-1971

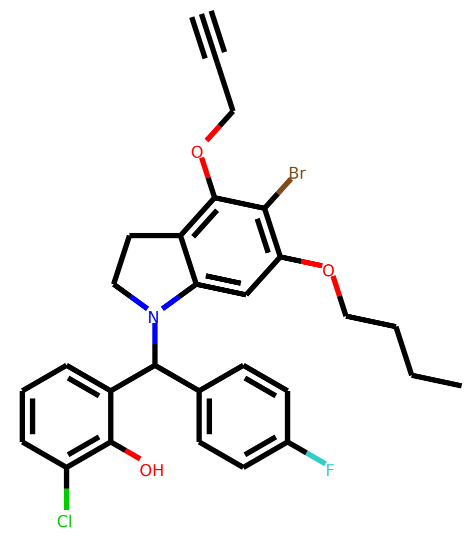

Osma-1972

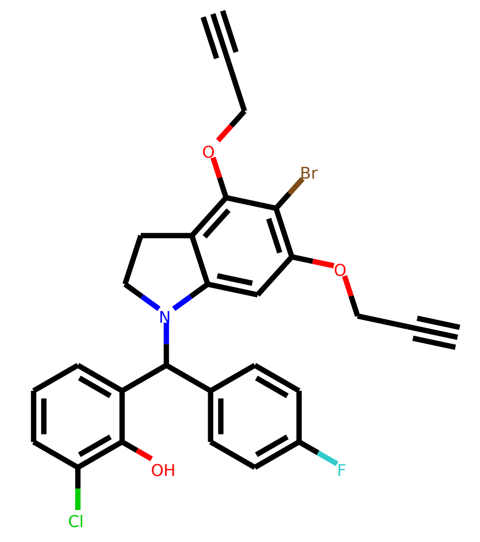

Osma-1973

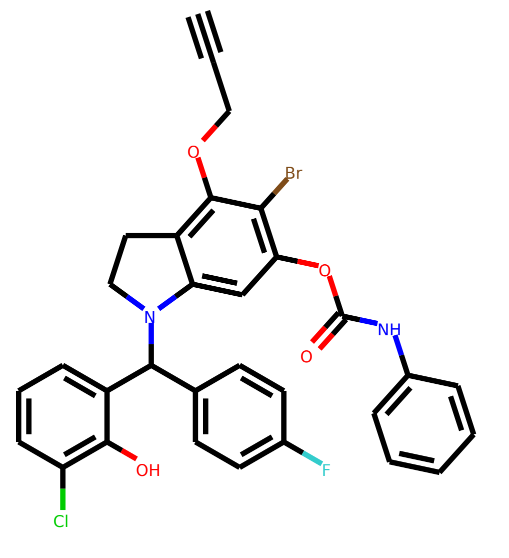

Osma-1974

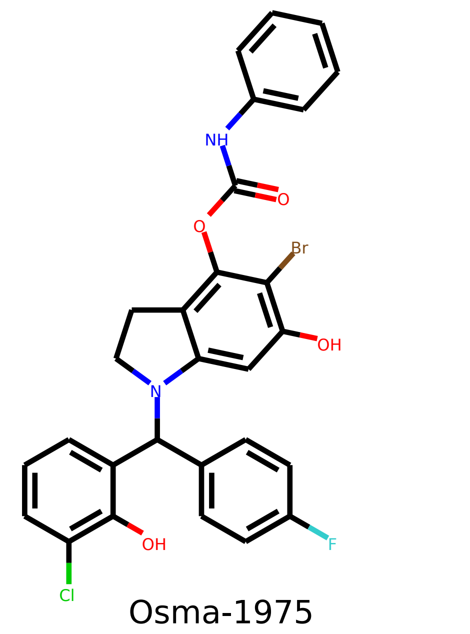

Osma-1975

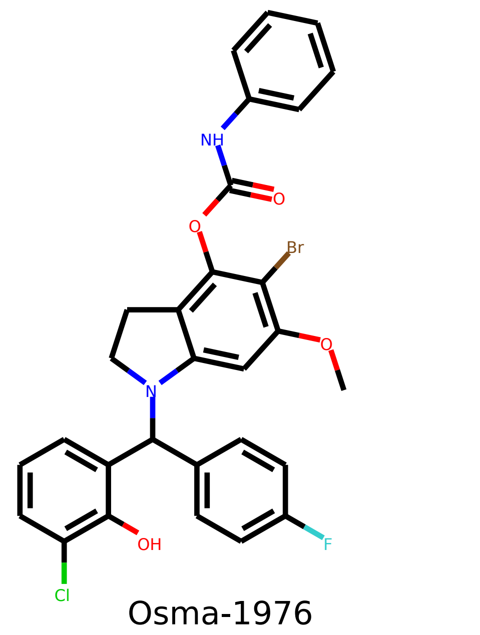

Osma-1976

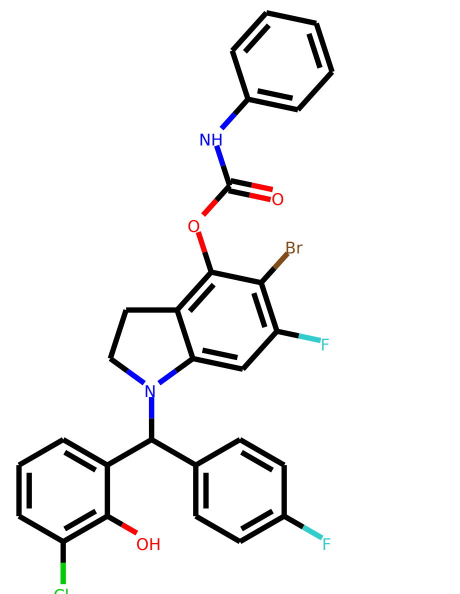

Osma-1977

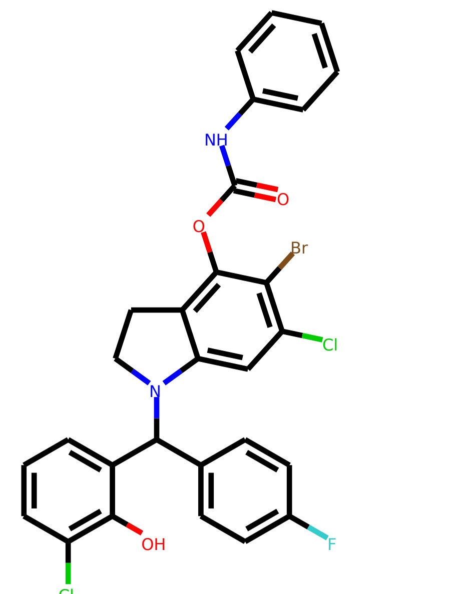

Osma-1978

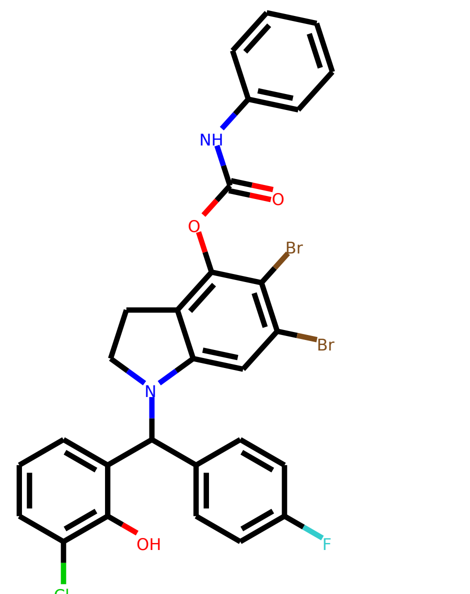

Osma-1979

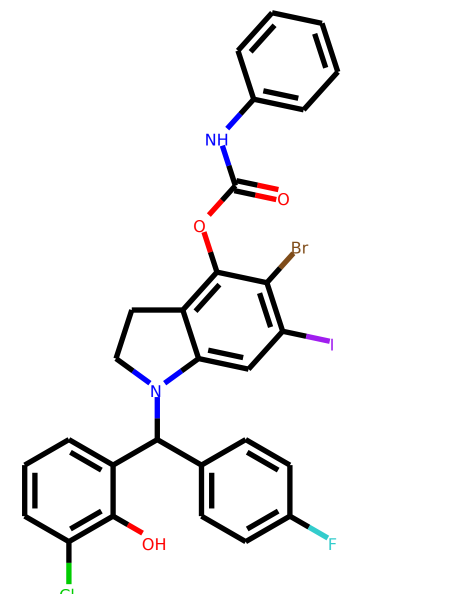

Osma-1980

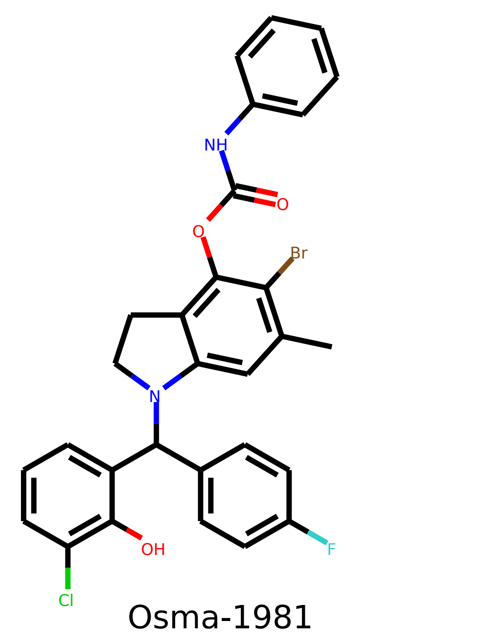

Osma-1981

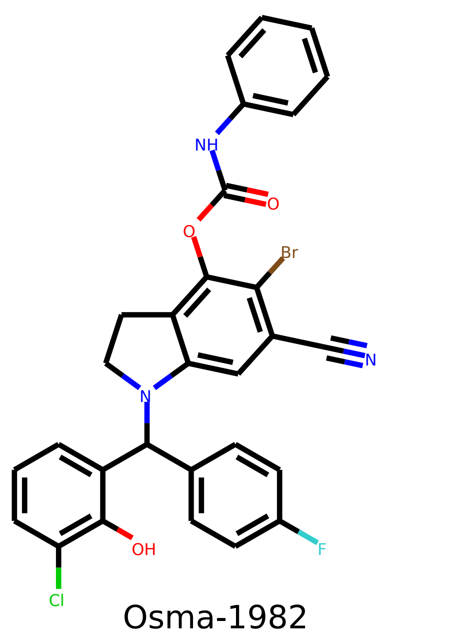

Osma-1982

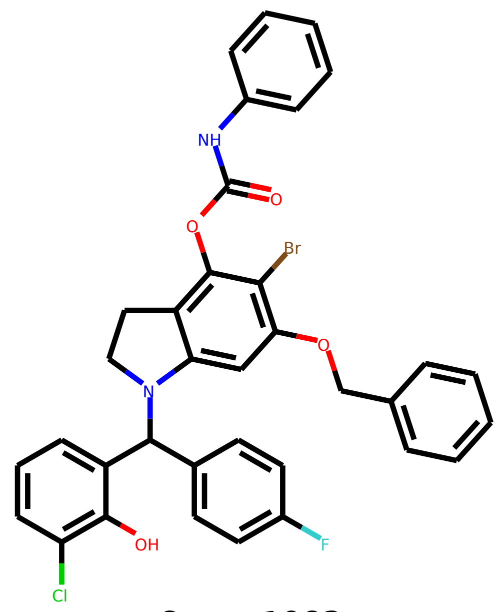

Osma-1983

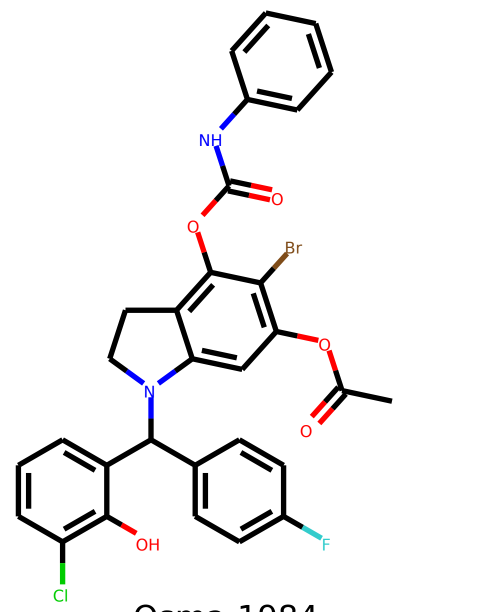

Osma-1984

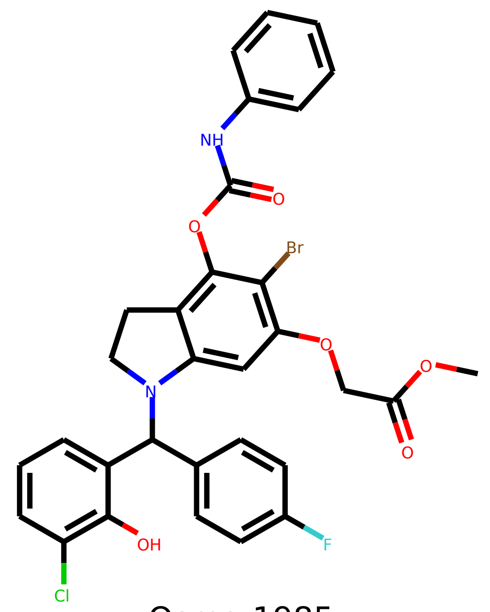

Osma-1985

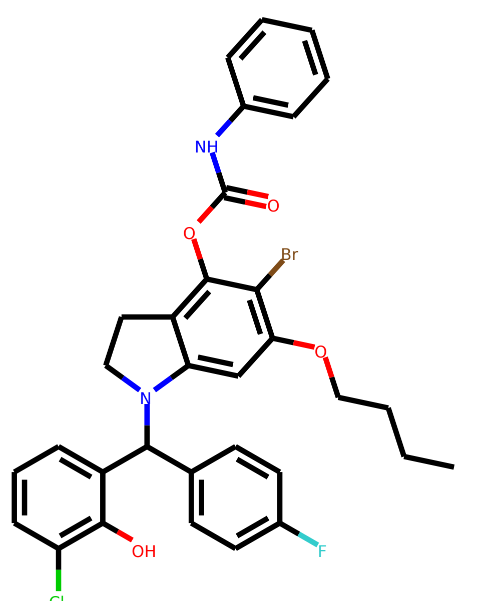

Osma-1986

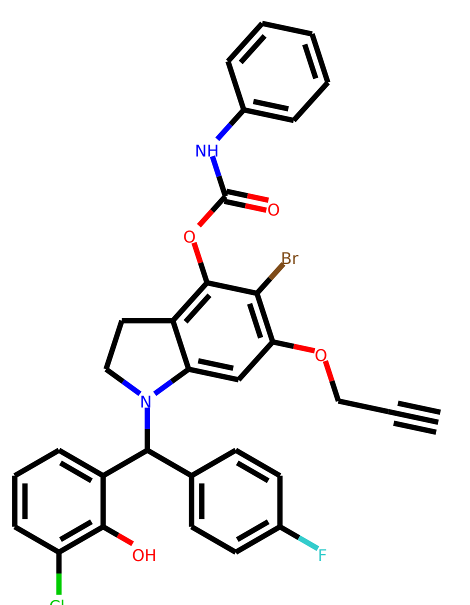

Osma-1987

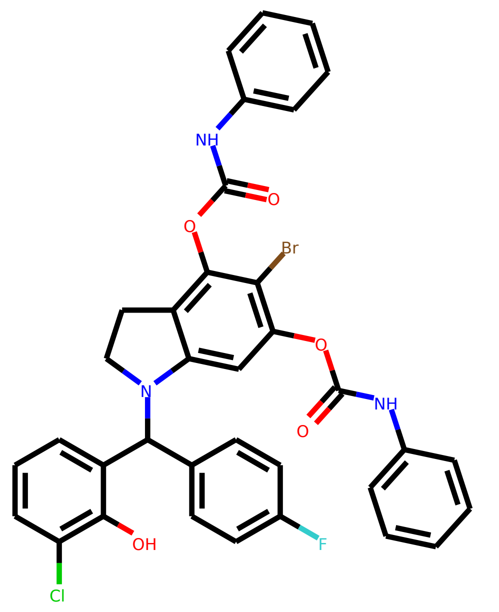

Osma-1988

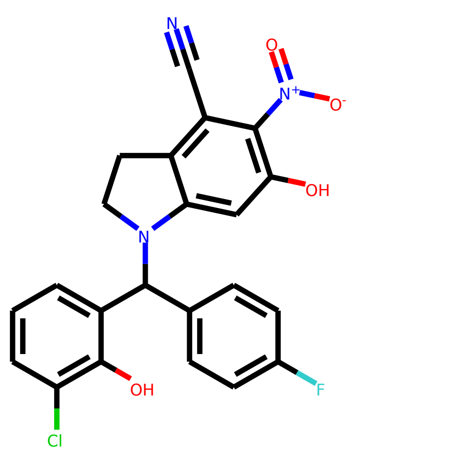

Osma-1989

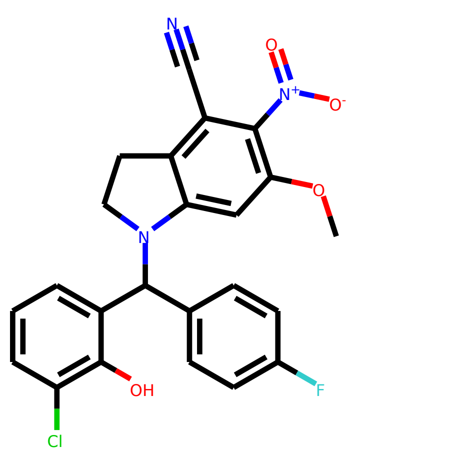

Osma-1990

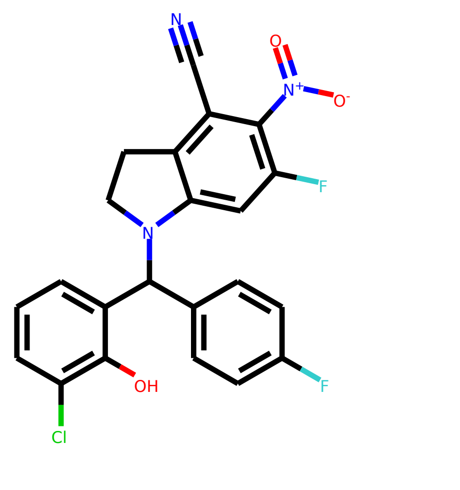

Osma-1991

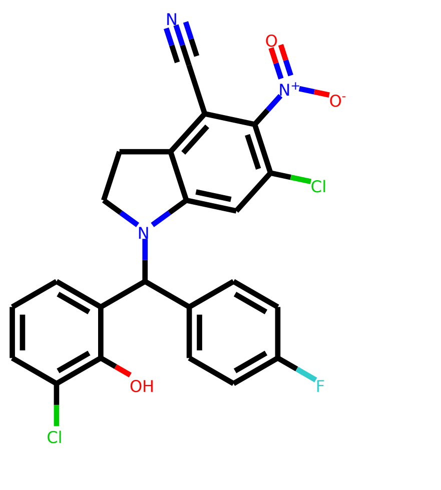

Osma-1992

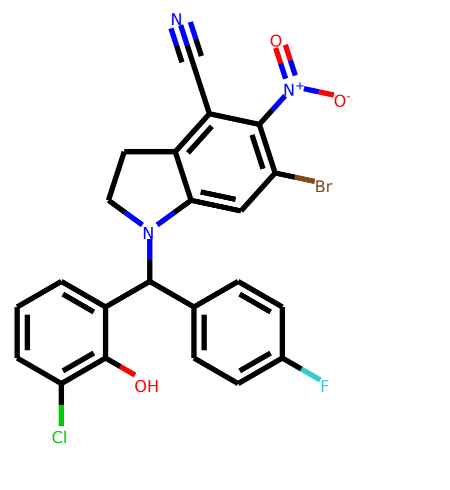

Osma-1993

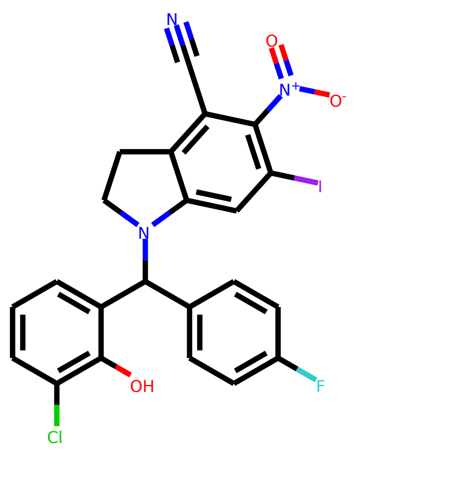

Osma-1994

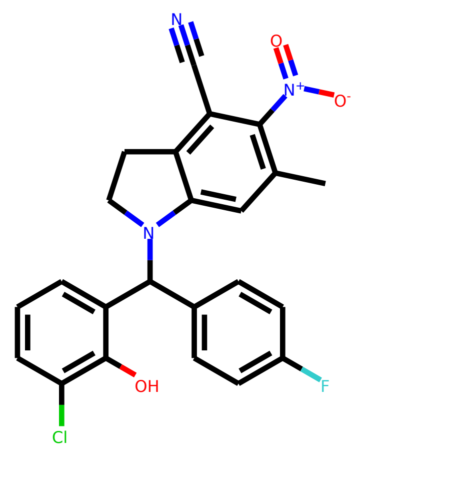

Osma-1995

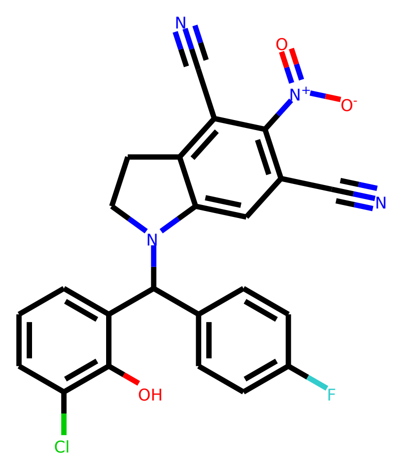
Osma-1996

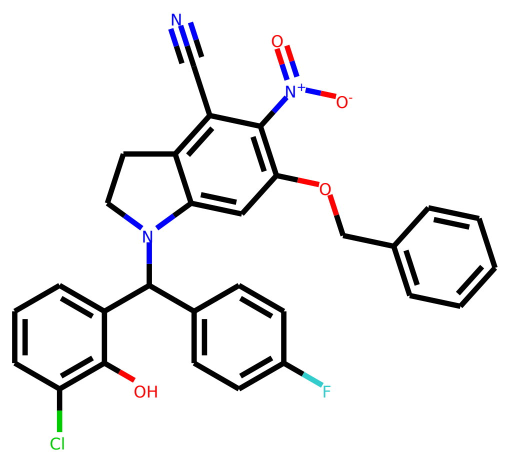
Osma-1997

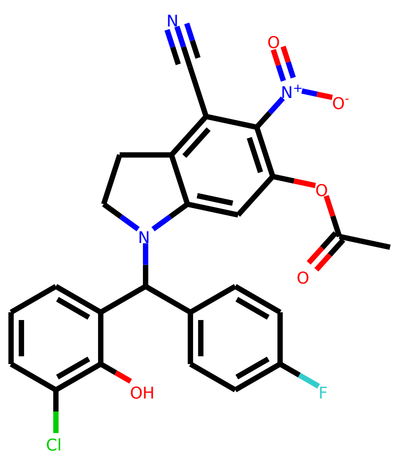
Osma-1998

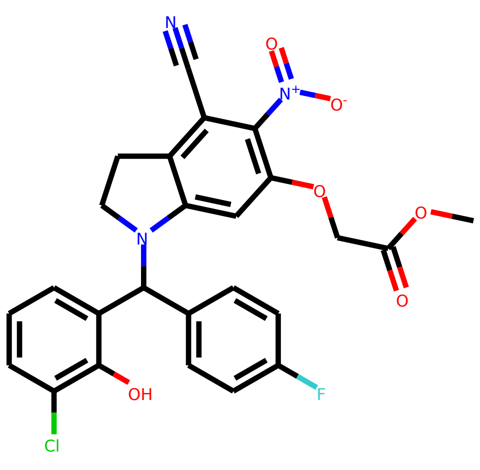
Osma-1999

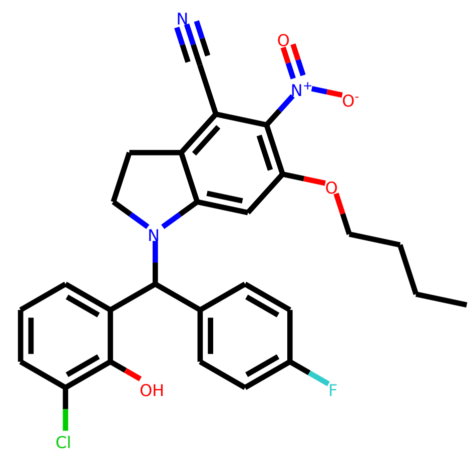
Osma-2000

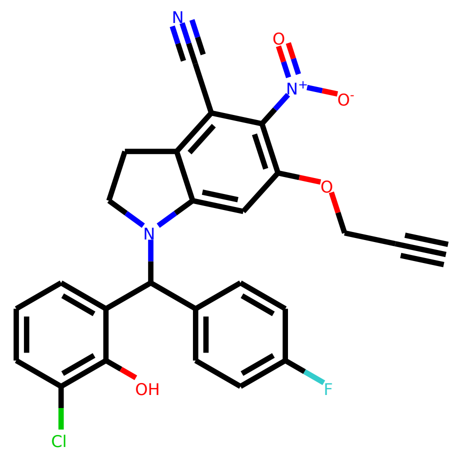
Osma-2001

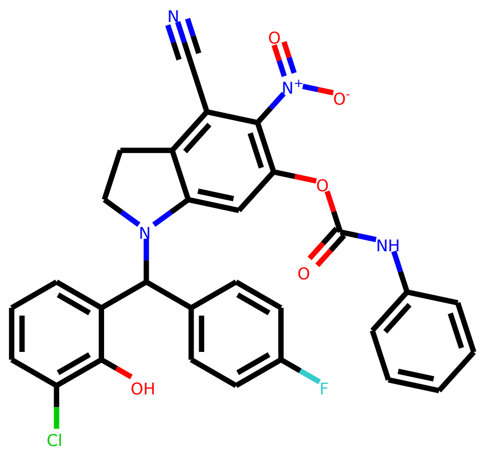
Osma-2002

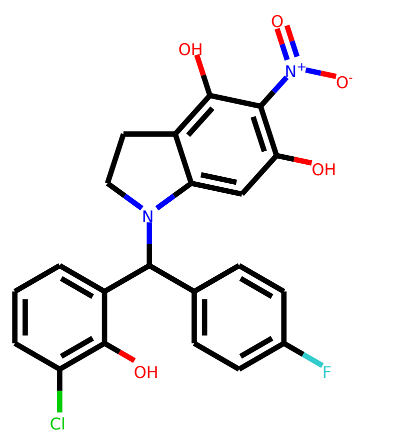
Osma-2003

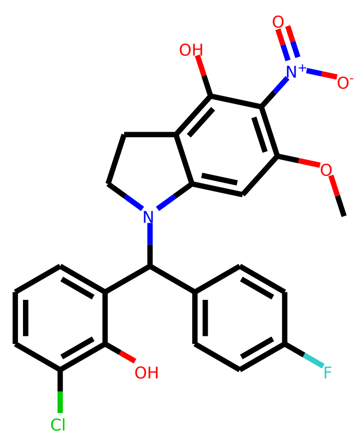
Osma-2004

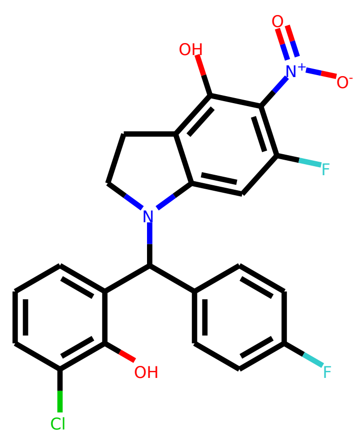
Osma-2005

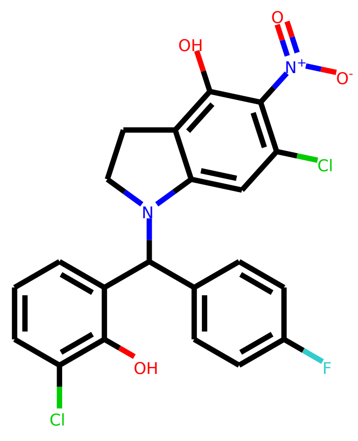
Osma-2006

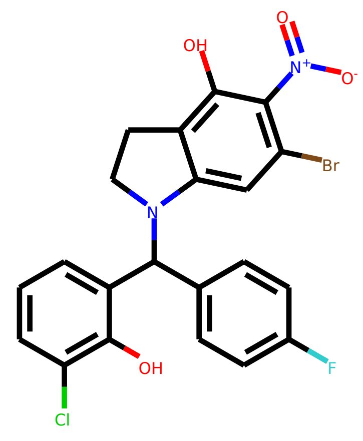
Osma-2007

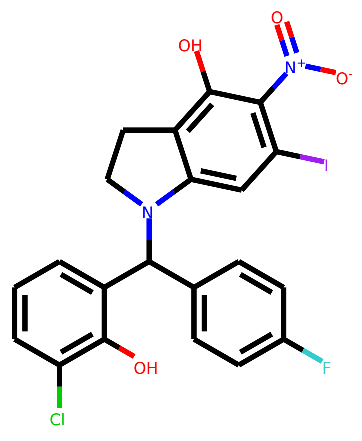
Osma-2008

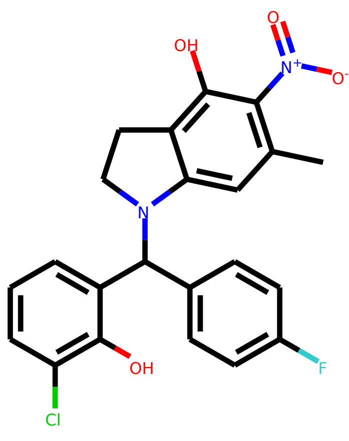
Osma-2009

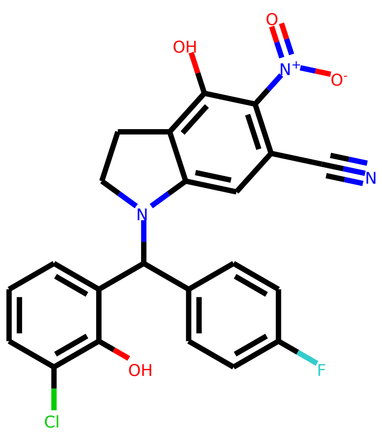
Osma-2010

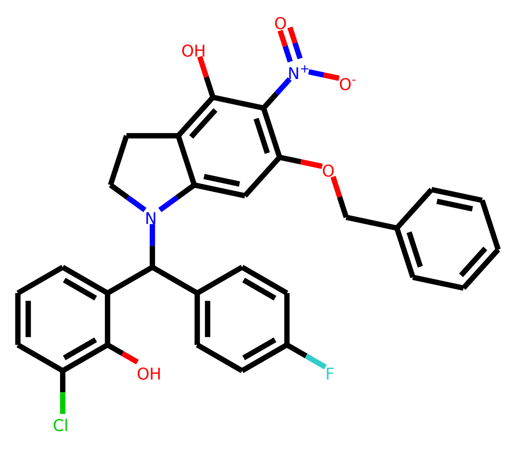
Osma-2011

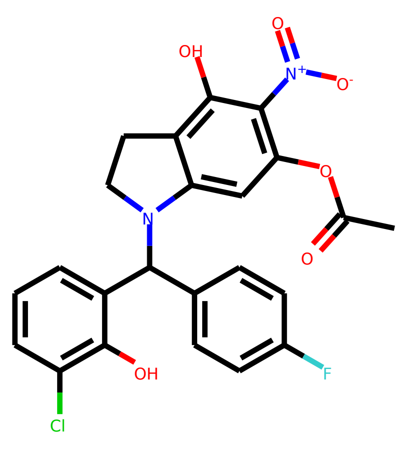
Osma-2012

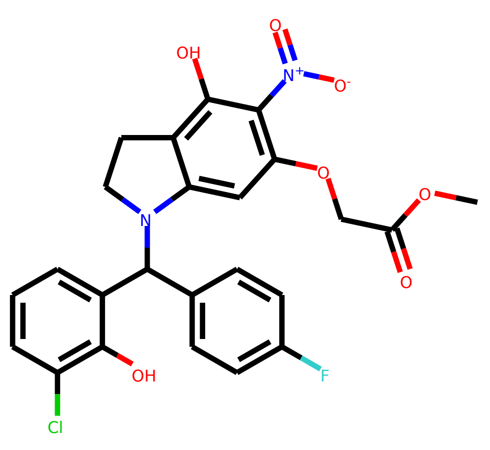
Osma-2013

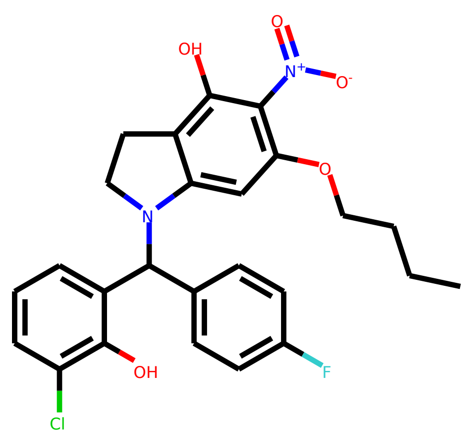
Osma-2014

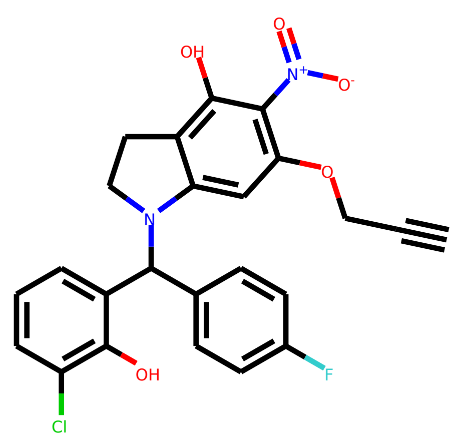
Osma-2015

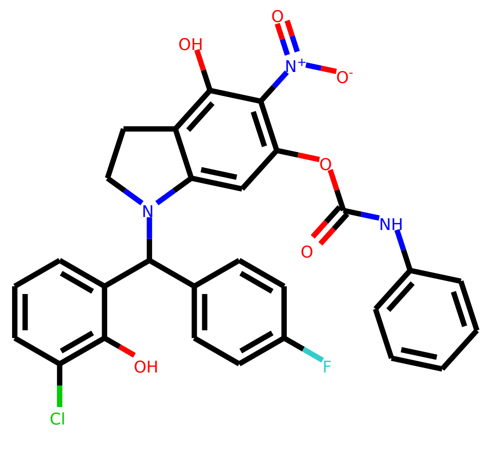
Osma-2016

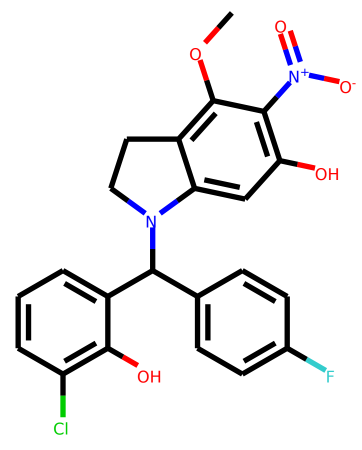
Osma-2017

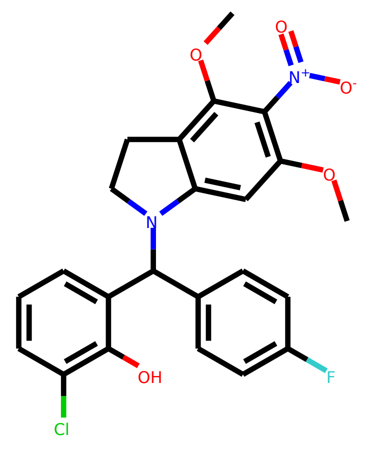
Osma-2018

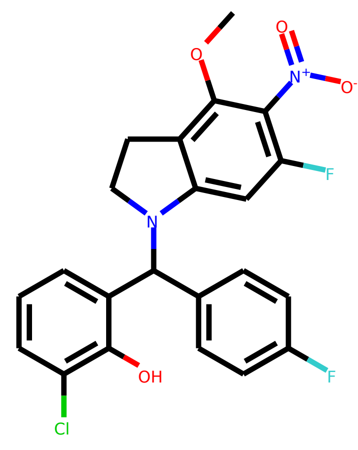
Osma-2019

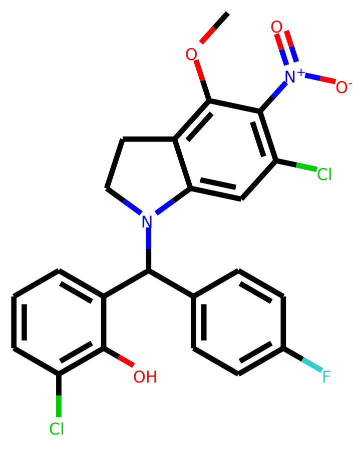
Osma-2020

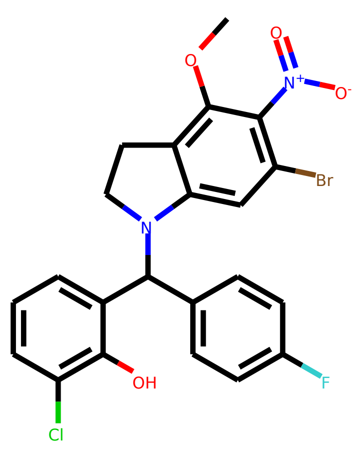
Osma-2021

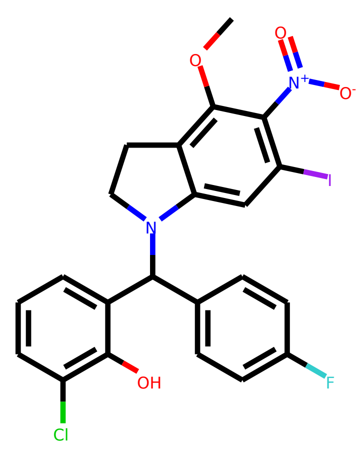
Osma-2022

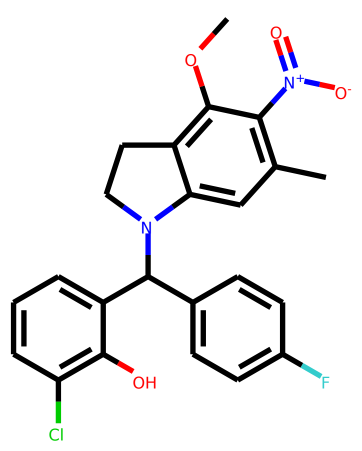
Osma-2023

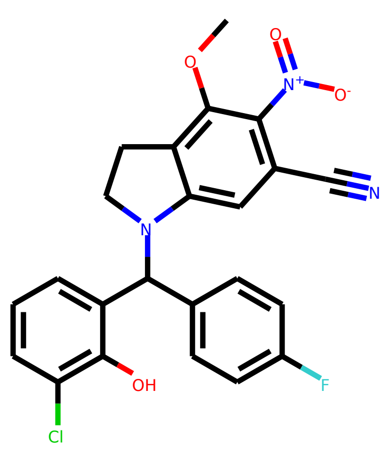
Osma-2024

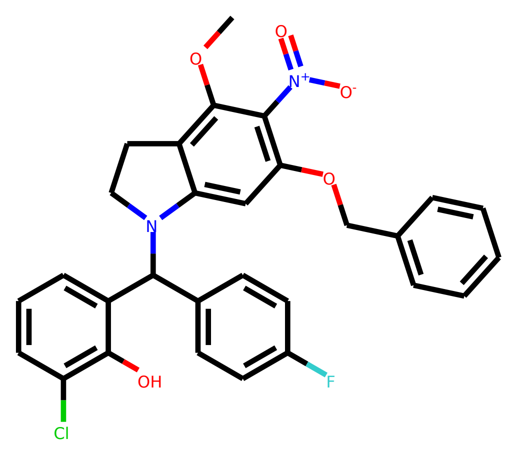
Osma-2025

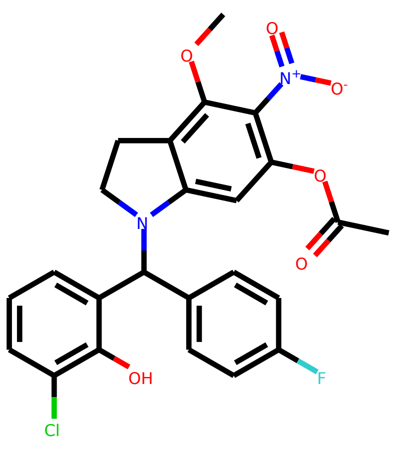
Osma-2026

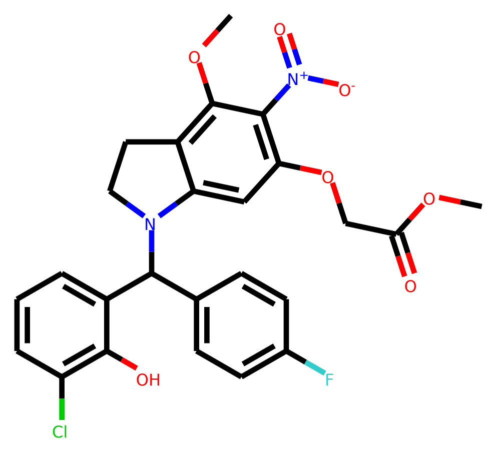
Osma-2027

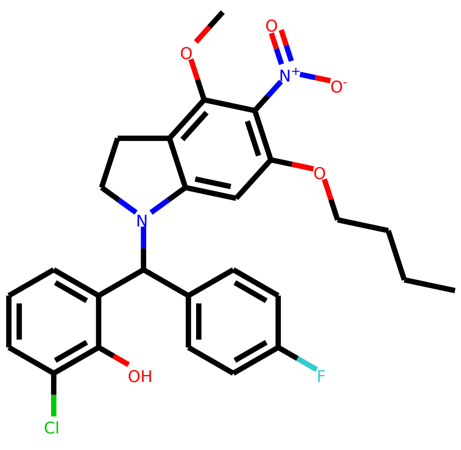
Osma-2028

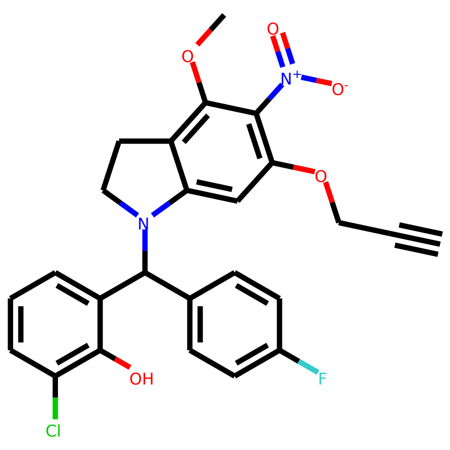
Osma-2029

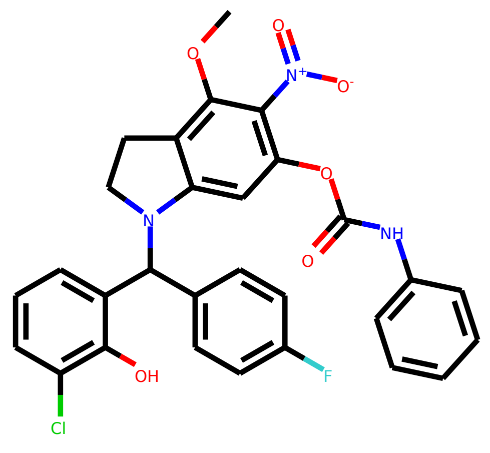
Osma-2030

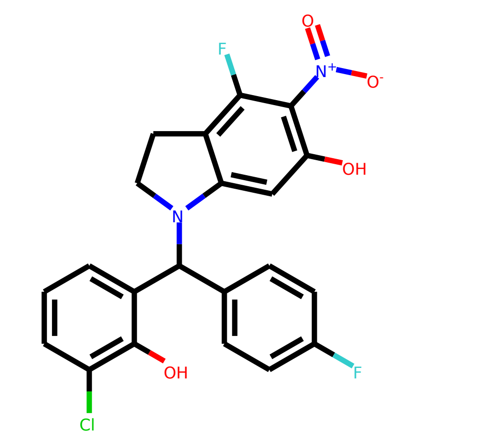

Osma-2031

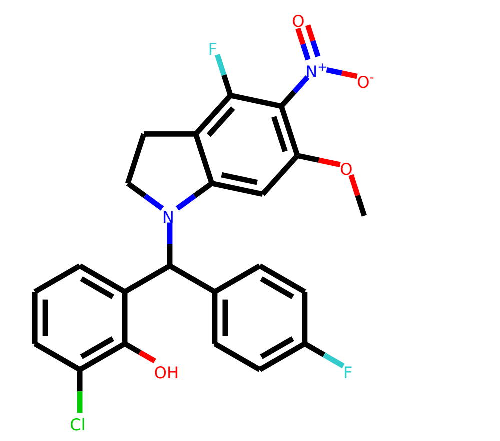

Osma-2032

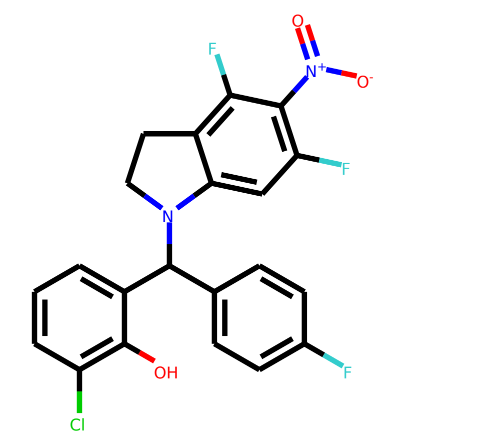

Osma-2033

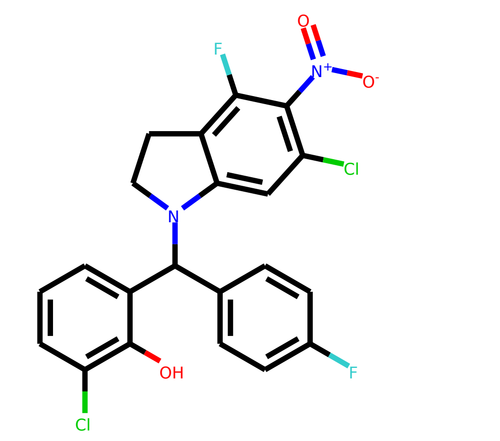

Osma-2034

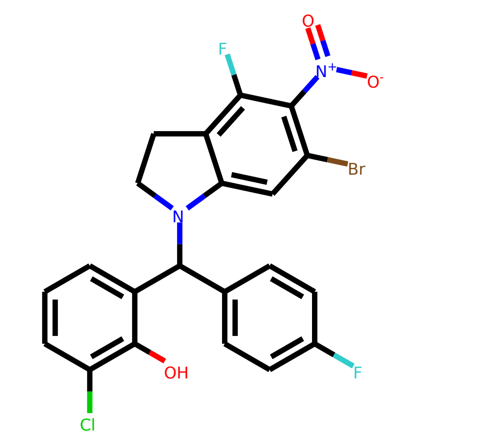

Osma-2035

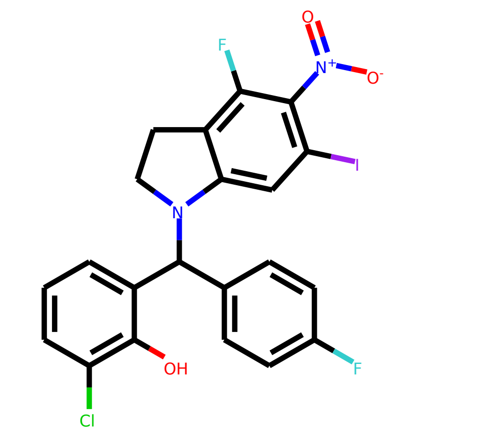

Osma-2036

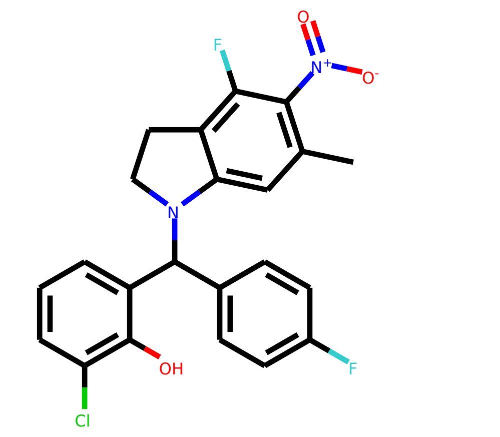

Osma-2037

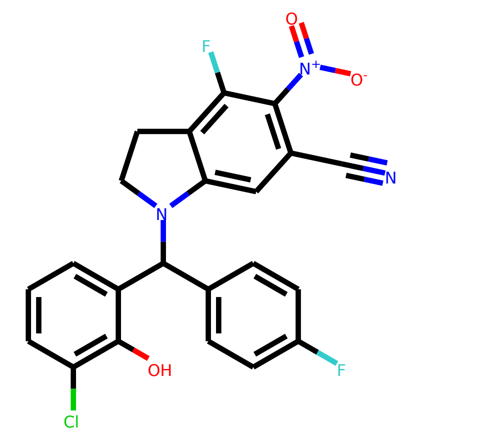

Osma-2038

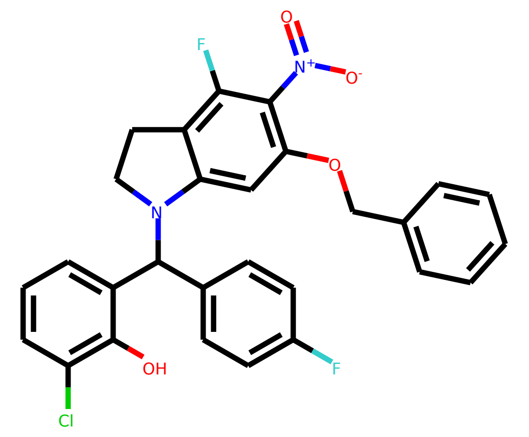

Osma-2039

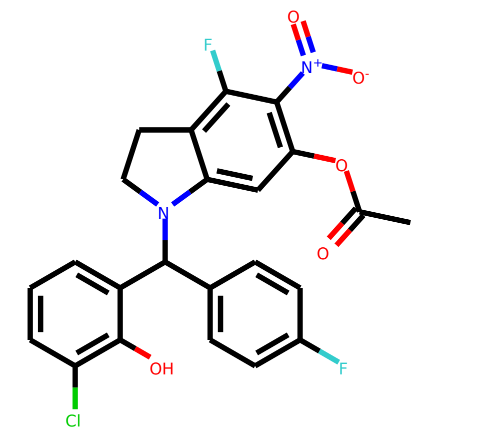

Osma-2040

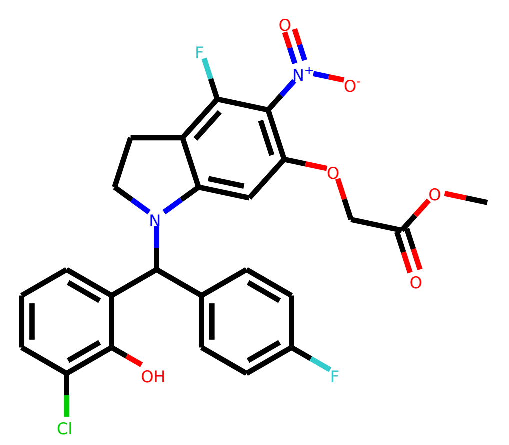

Osma-2041

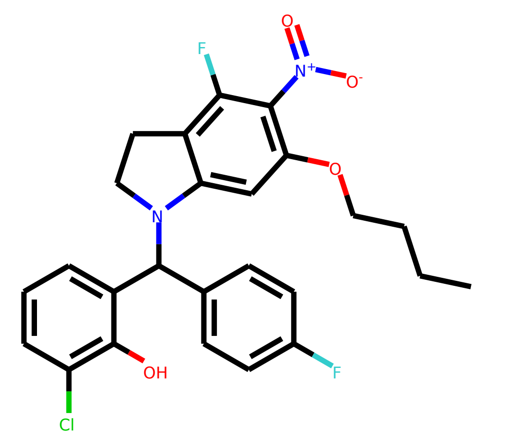

Osma-2042

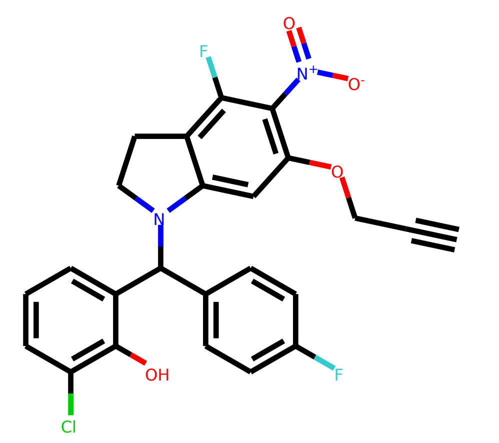

Osma-2043

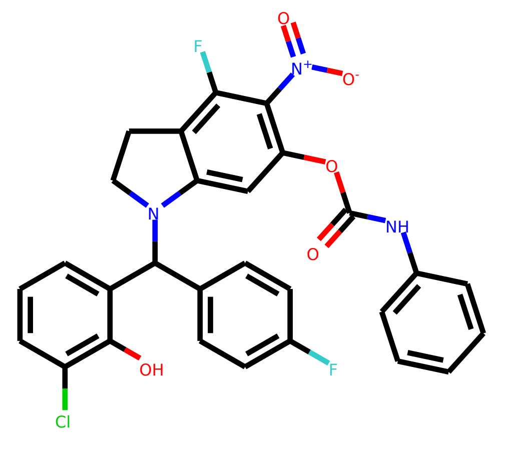

Osma-2044

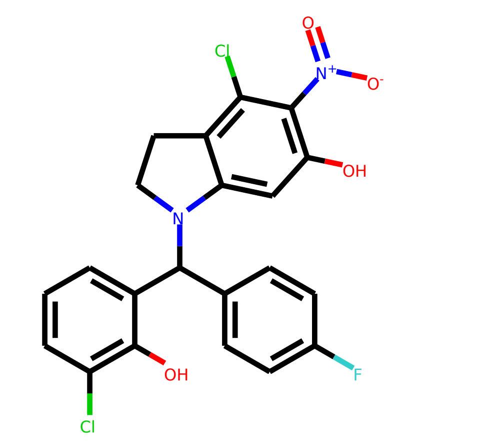

Osma-2045

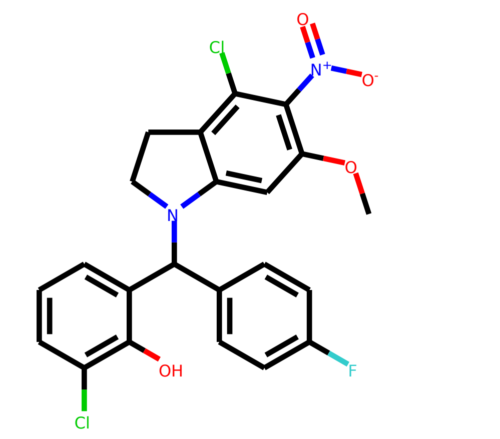

Osma-2046

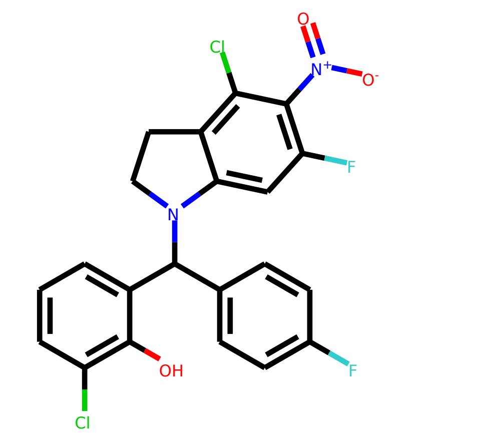

Osma-2047

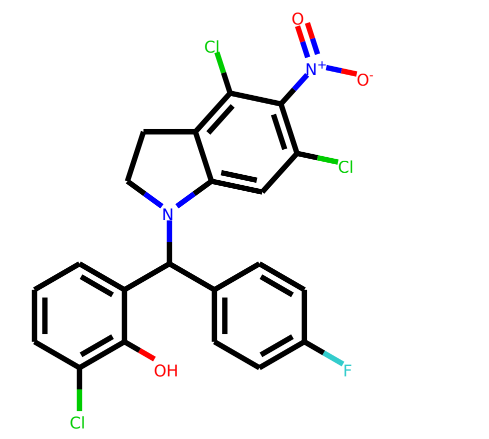

Osma-2048

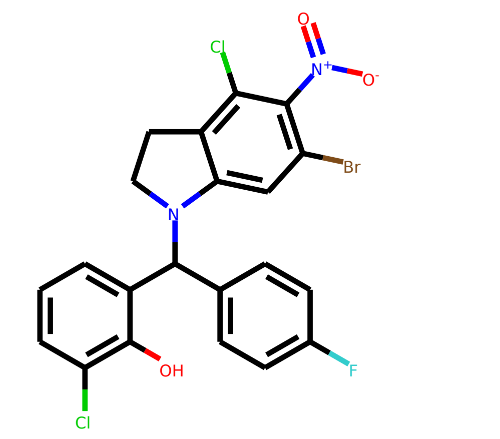

Osma-2049

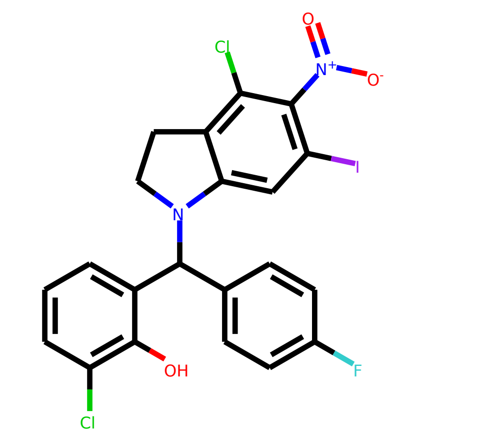

Osma-2050

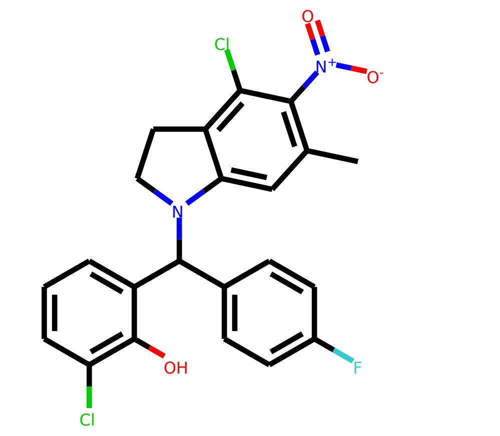

Osma-2051

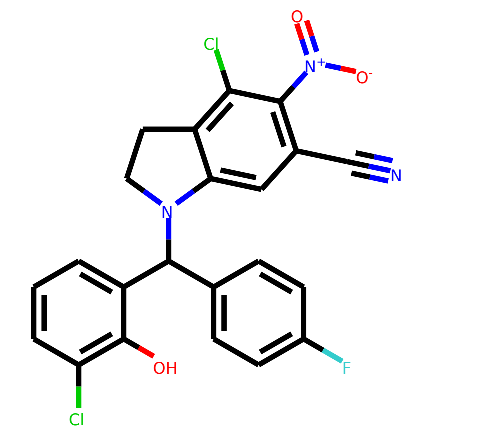

Osma-2052

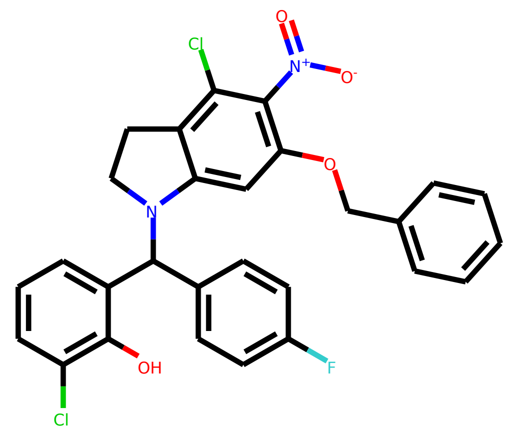

Osma-2053

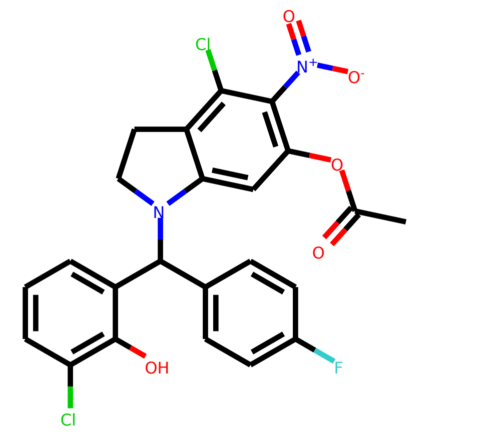

Osma-2054

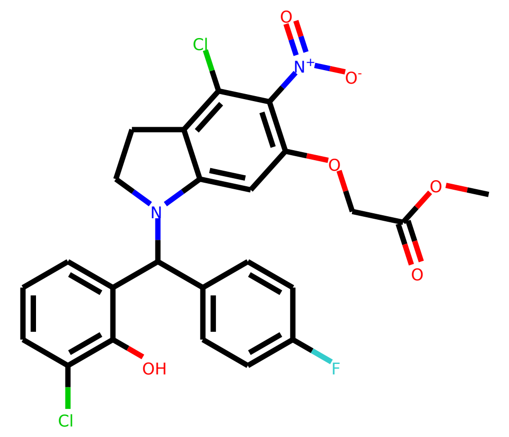

Osma-2055

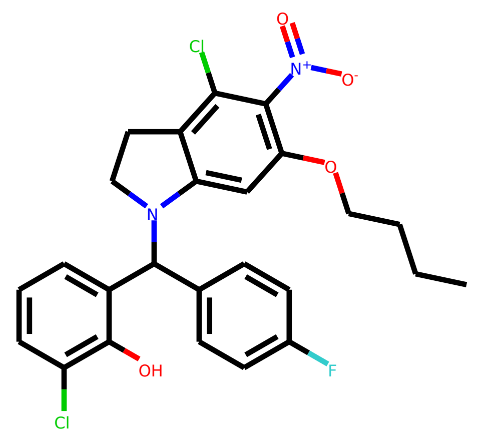

Osma-2056

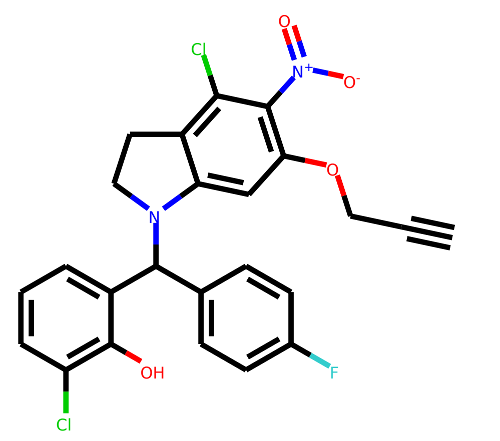

Osma-2057

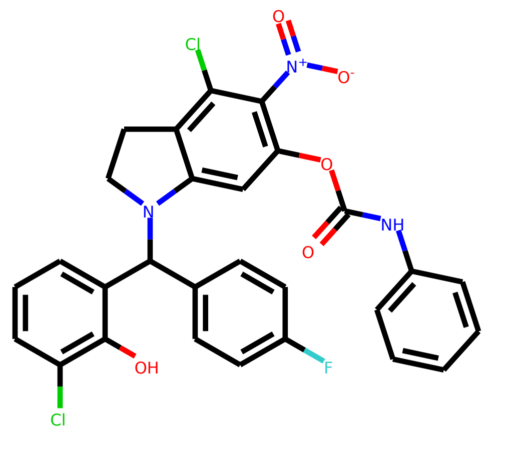

Osma-2058

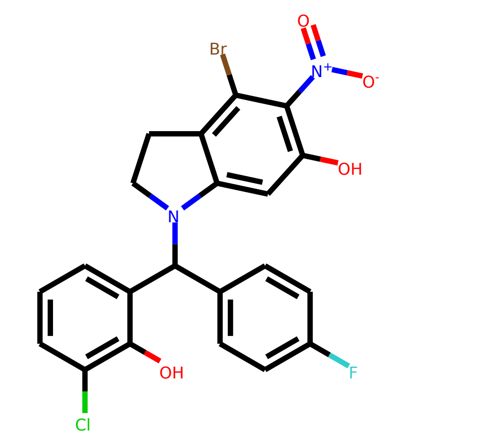

Osma-2059

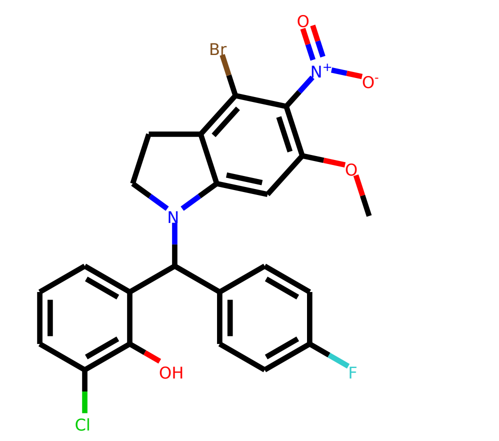

Osma-2060

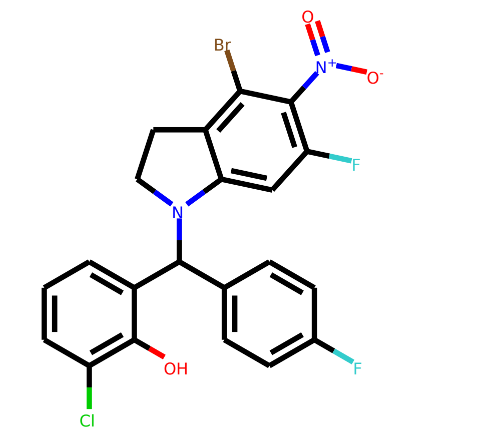

Osma-2061

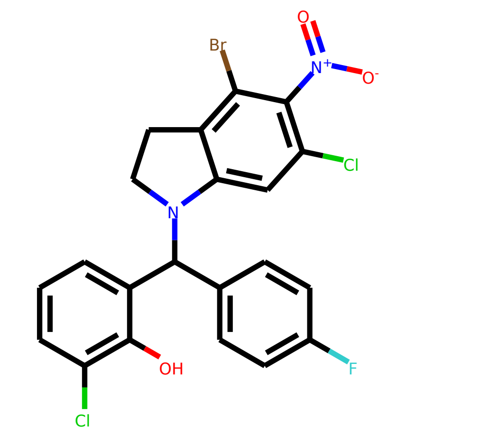

Osma-2062

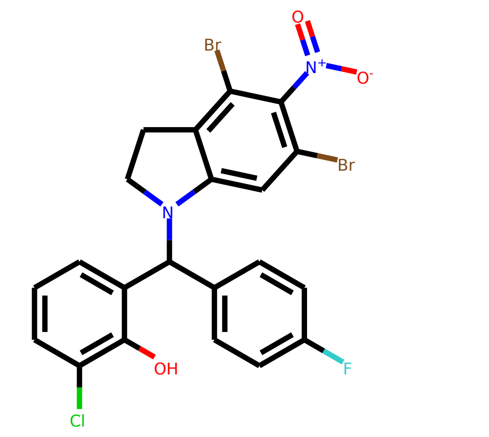

Osma-2063

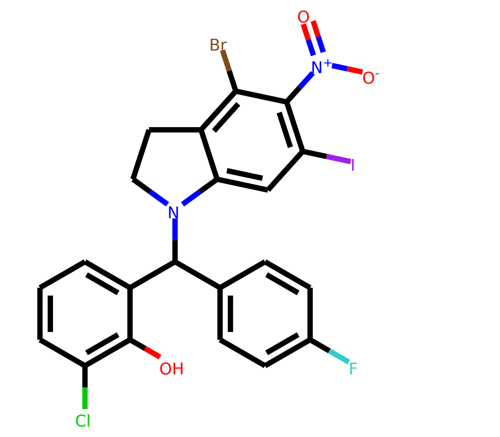

Osma-2064

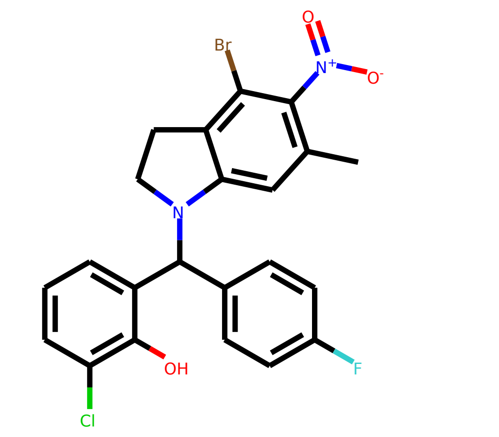

Osma-2065

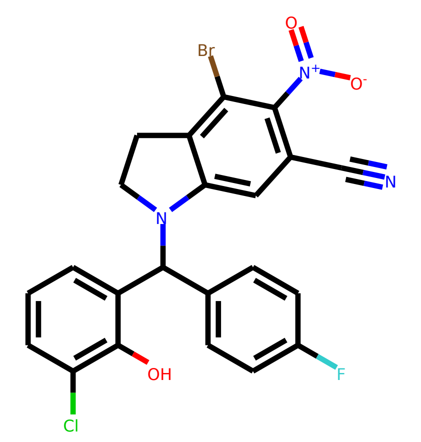
Osma-2066
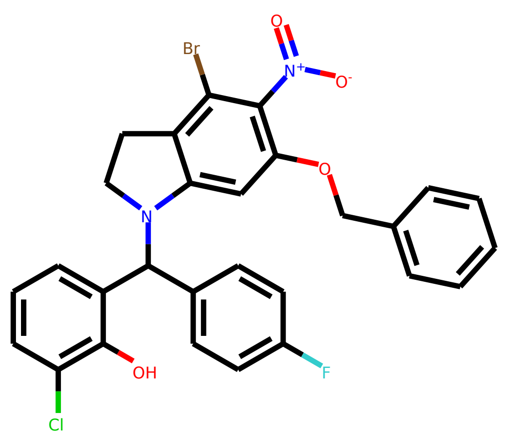
Osma-2067
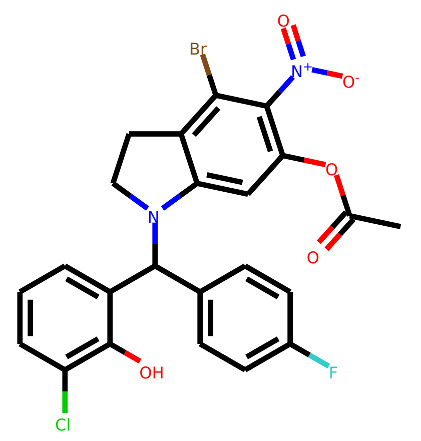
Osma-2068
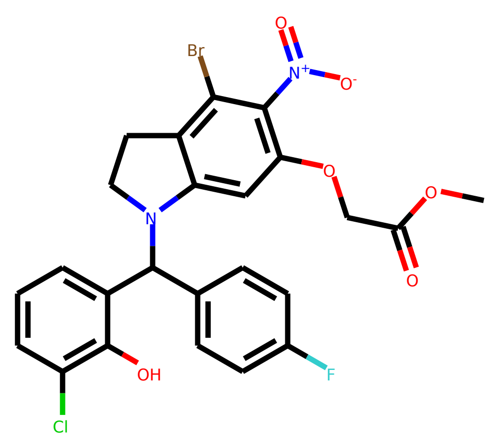
Osma-2069
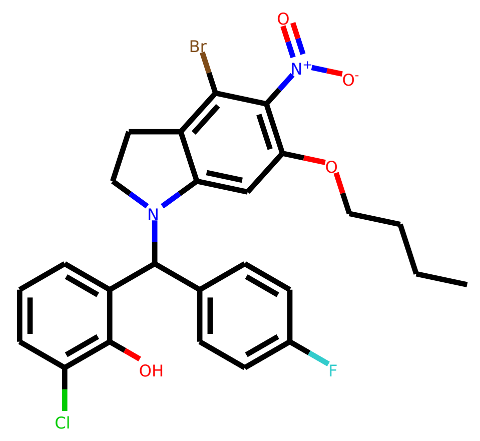
Osma-2070

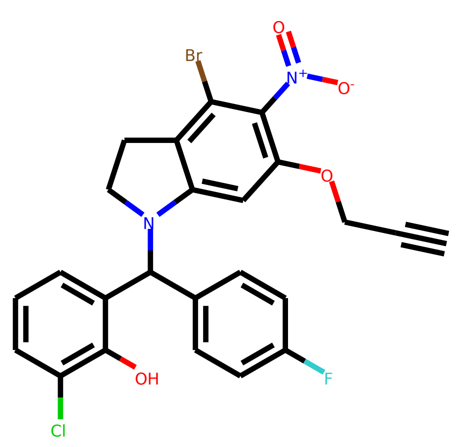
Osma-2071
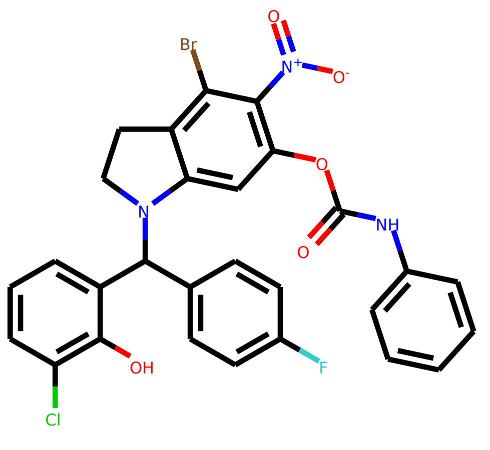
Osma-2072
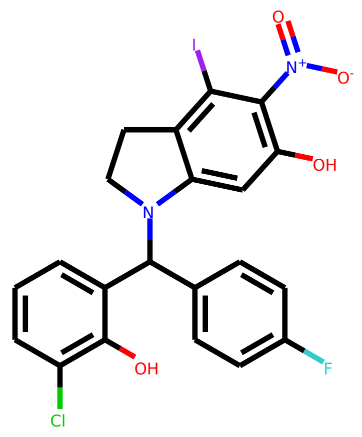
Osma-2073
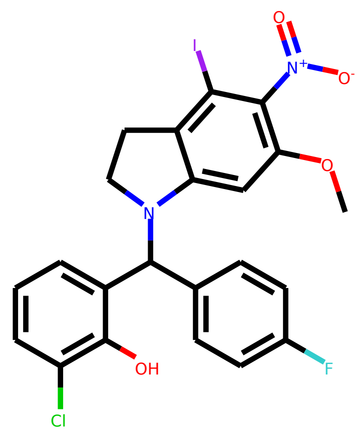
Osma-2074
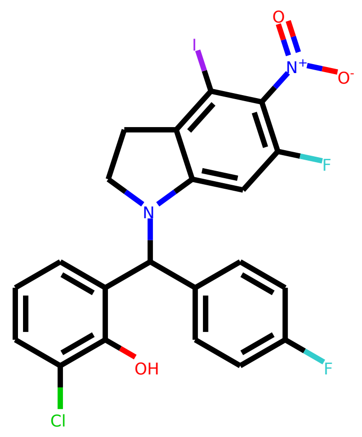
Osma-2075

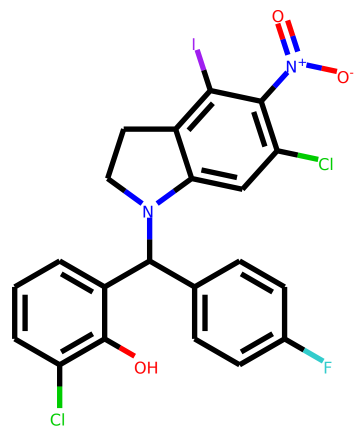
Osma-2076
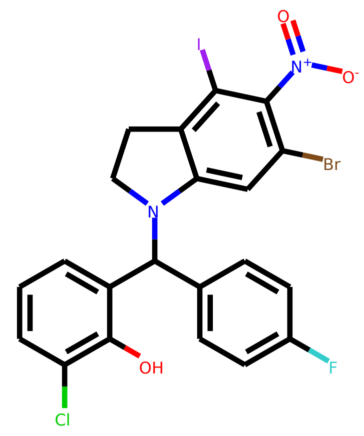
Osma-2077
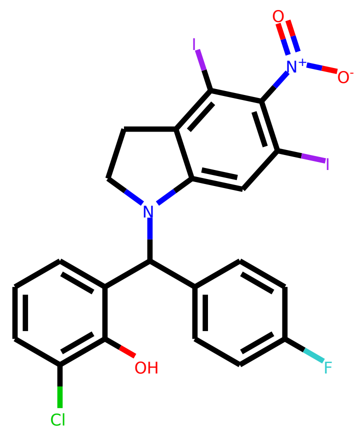
Osma-2078
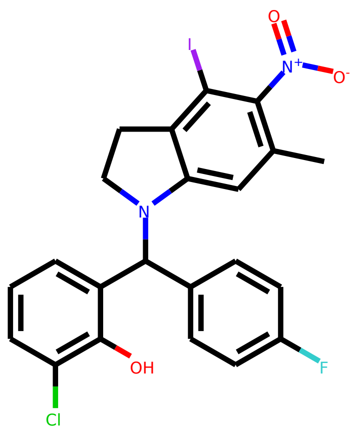
Osma-2079
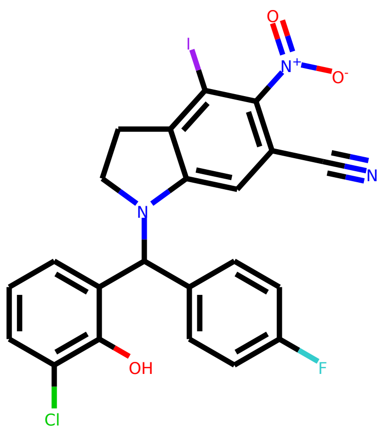
Osma-2080

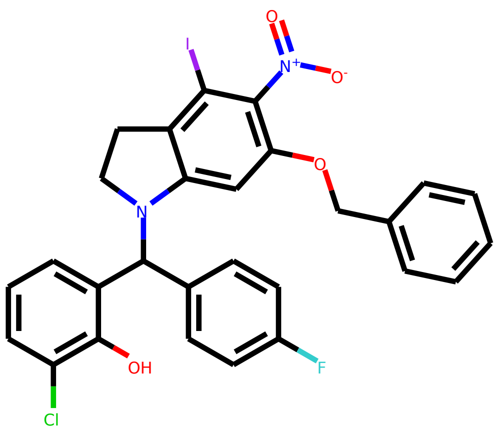
Osma-2081
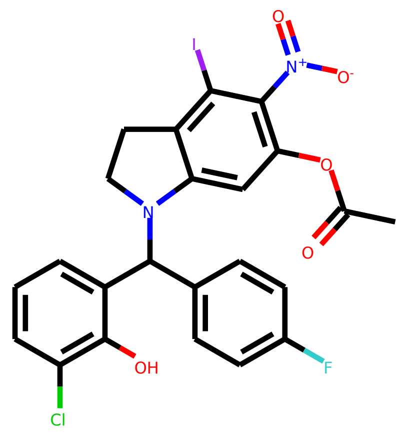
Osma-2082
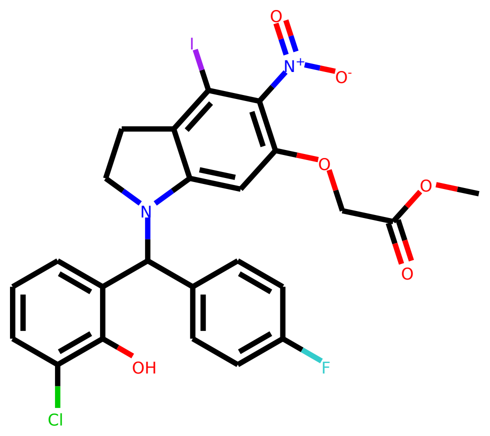
Osma-2083
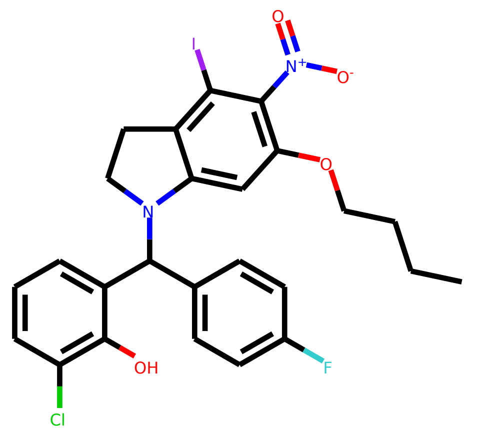
Osma-2084
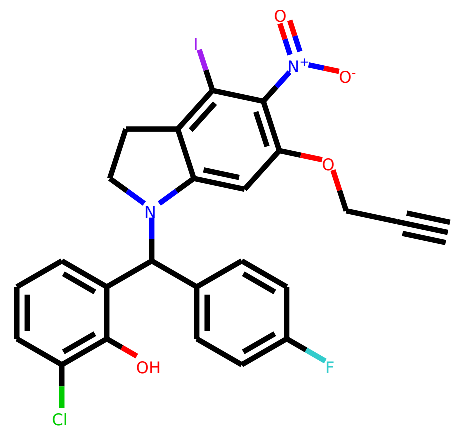
Osma-2085

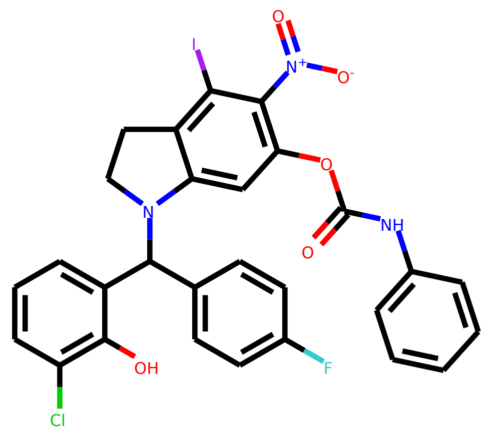
Osma-2086
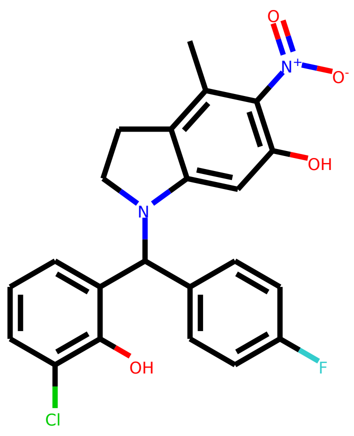
Osma-2087
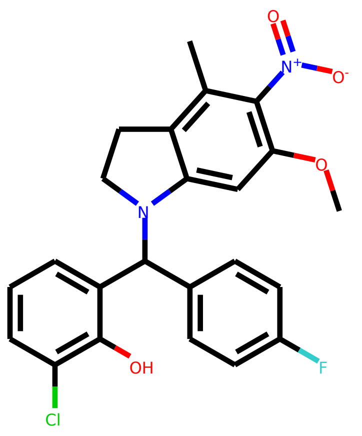
Osma-2088
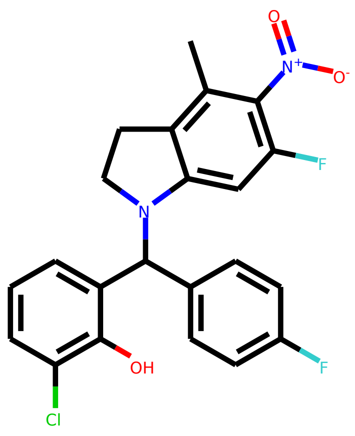
Osma-2089
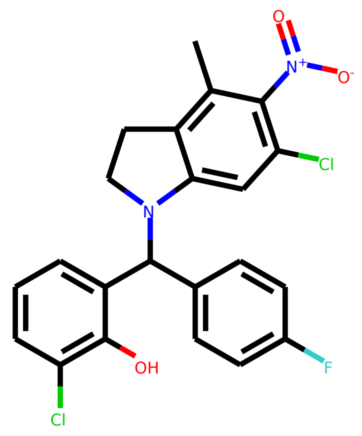
Osma-2090

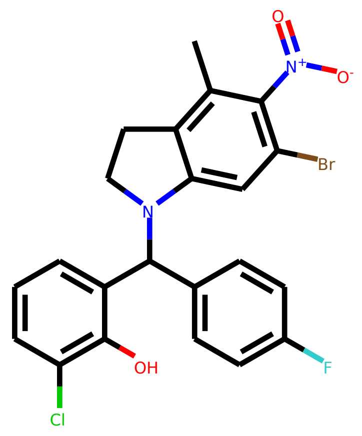
Osma-2091
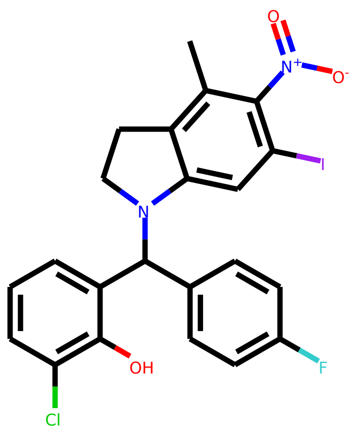
Osma-2092
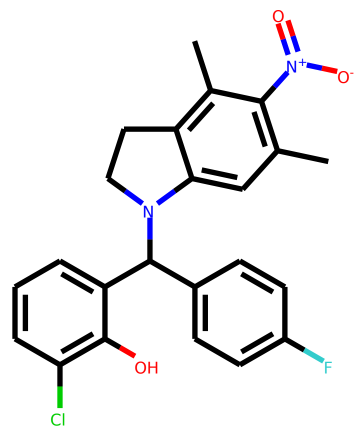
Osma-2093
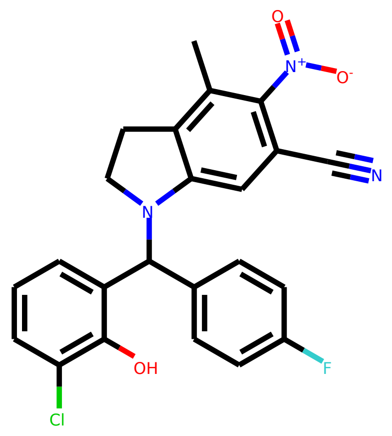
Osma-2094
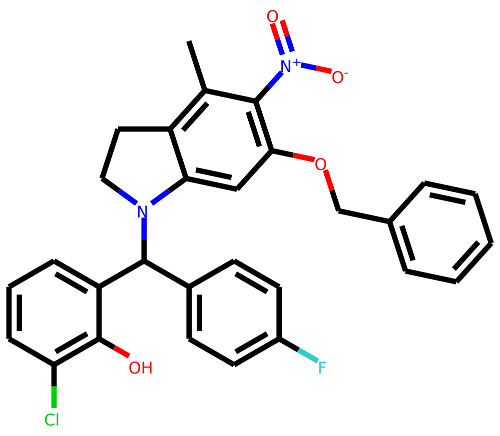
Osma-2095

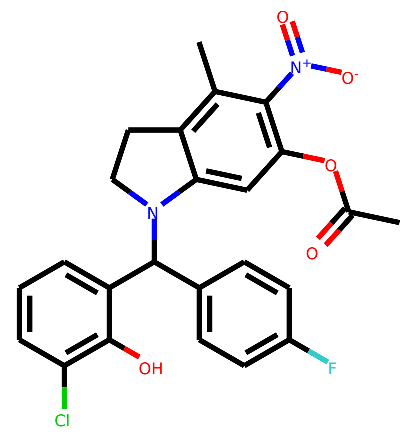
Osma-2096
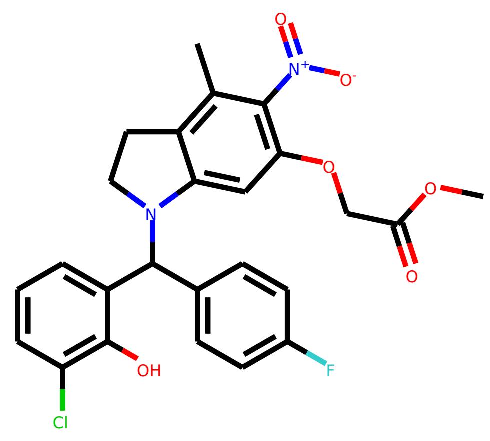
Osma-2097
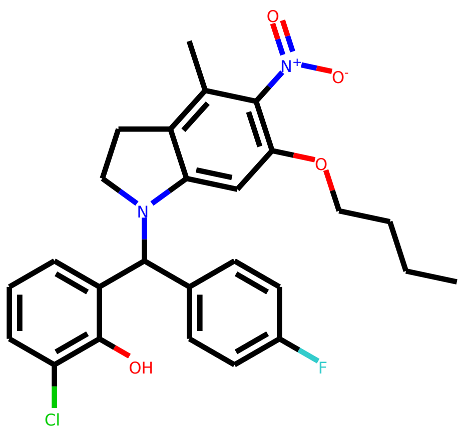
Osma-2098
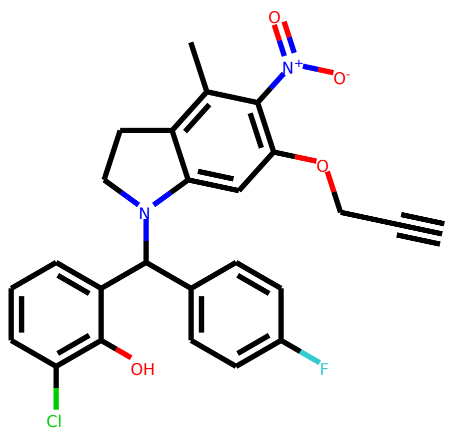
Osma-2099
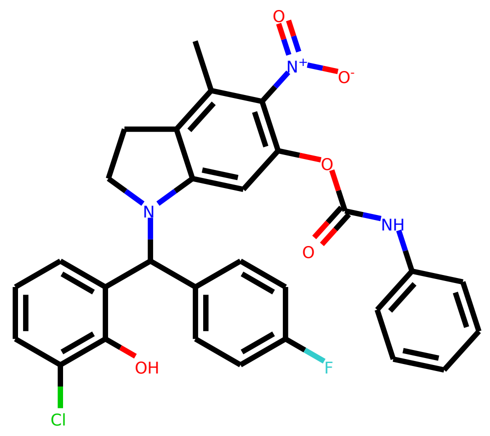
Osma-2100

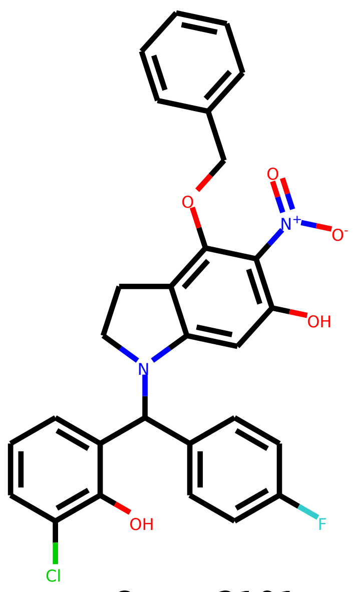
Osma-2101
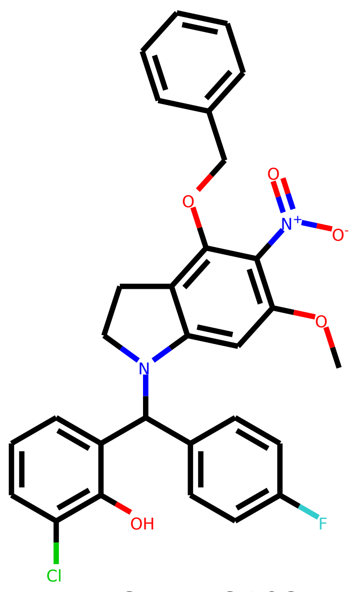
Osma-2102
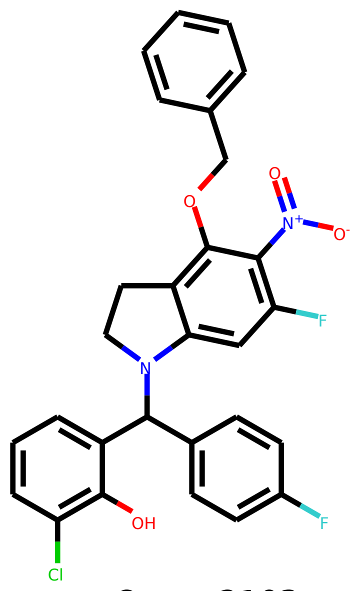
Osma-2103
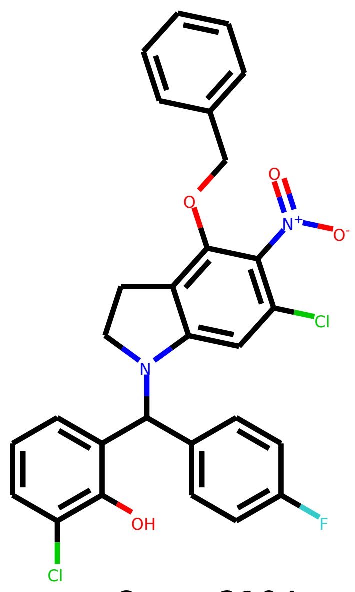
Osma-2104
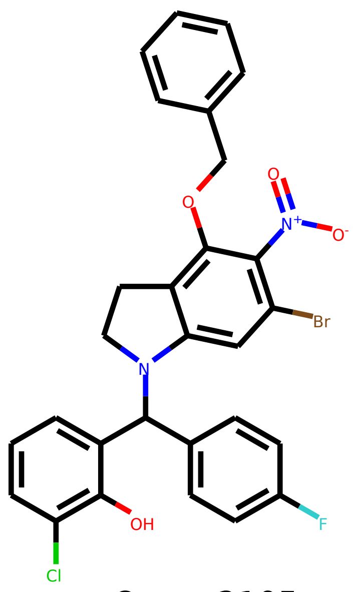
Osma-2105
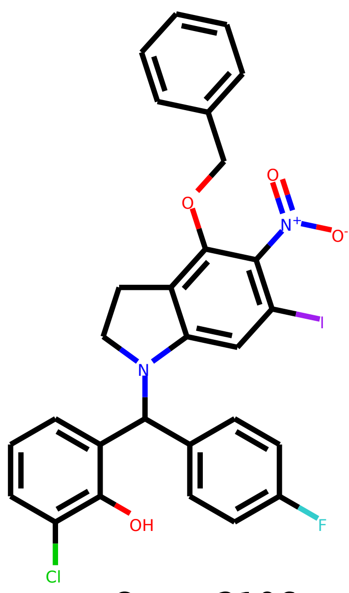
Osma-2106
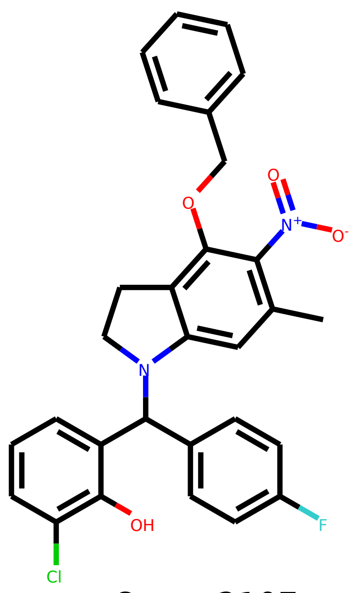
Osma-2107
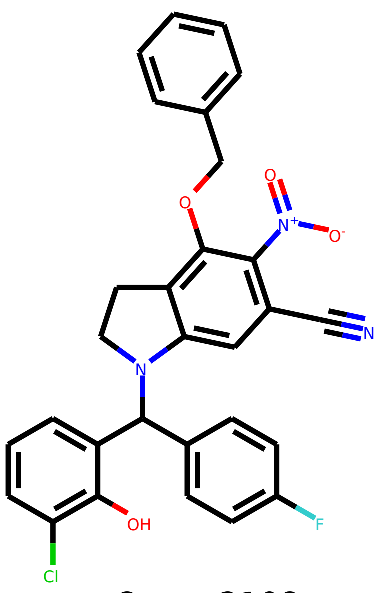
Osma-2108
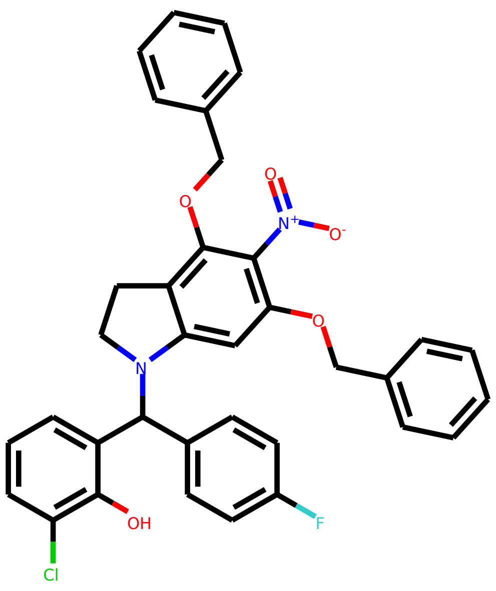
Osma-2109
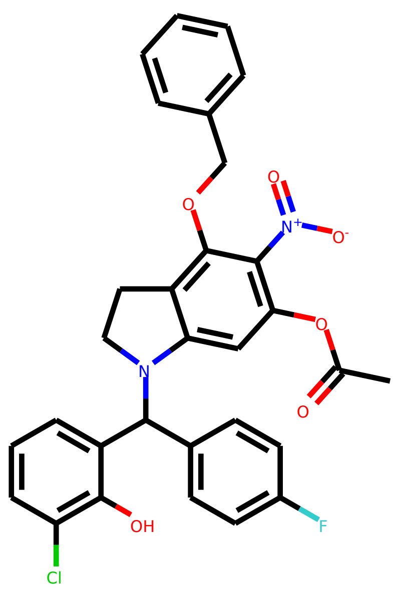
Osma-2110
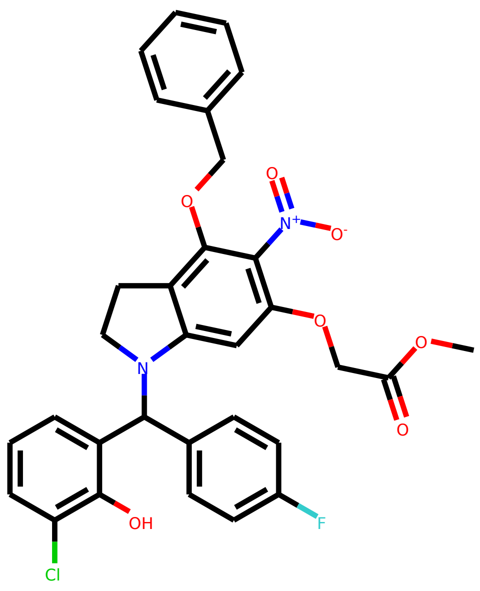
Osma-2111
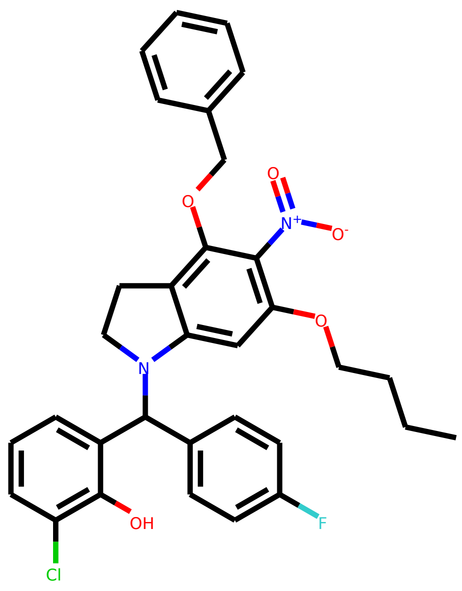
Osma-2112
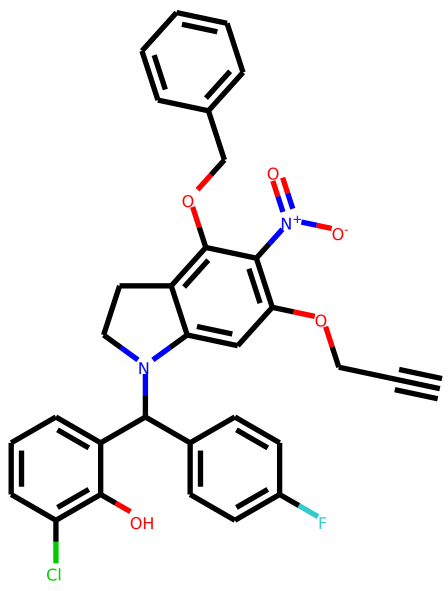
Osma-2113
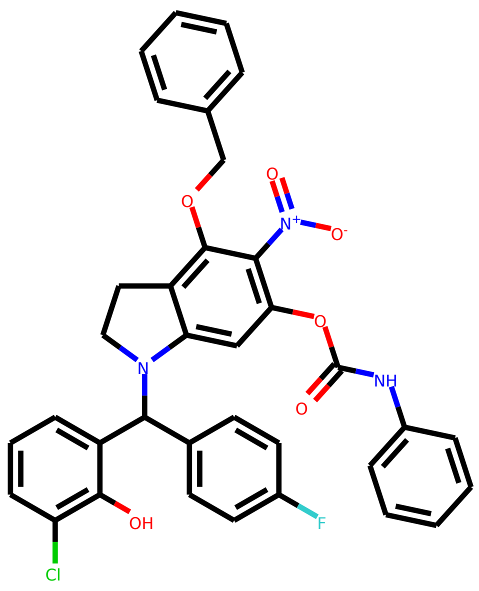
Osma-2114
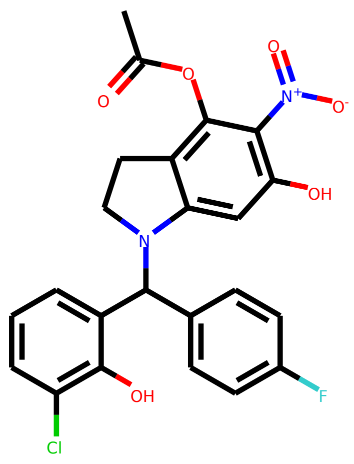
Osma-2115
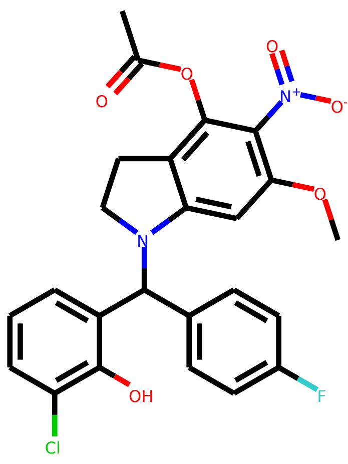
Osma-2116
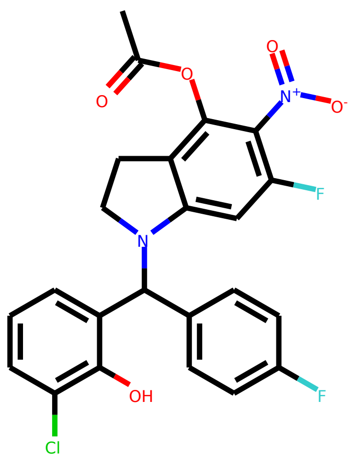
Osma-2117
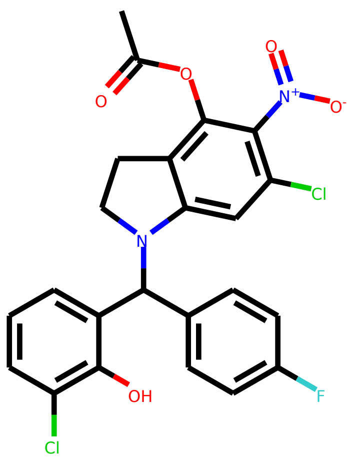
Osma-2118
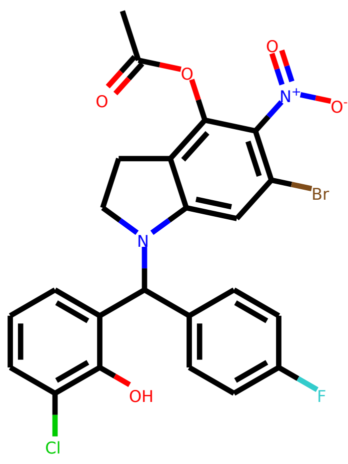
Osma-2119
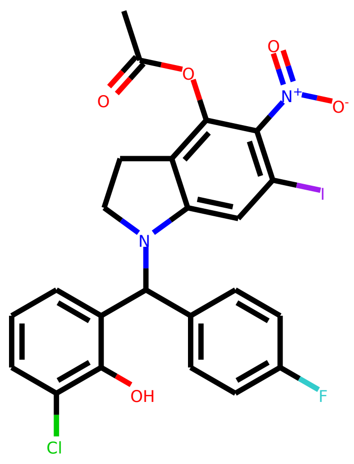
Osma-2120
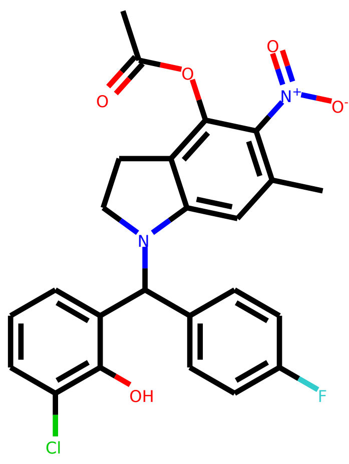
Osma-2121
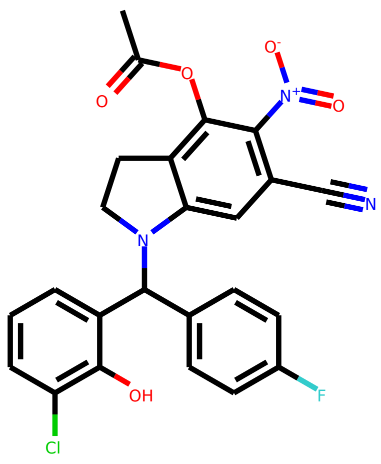
Osma-2122
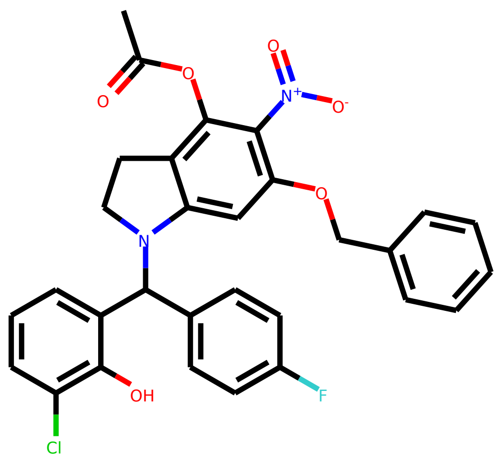
Osma-2123
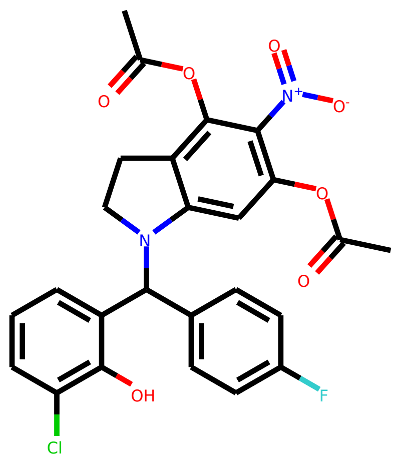
Osma-2124
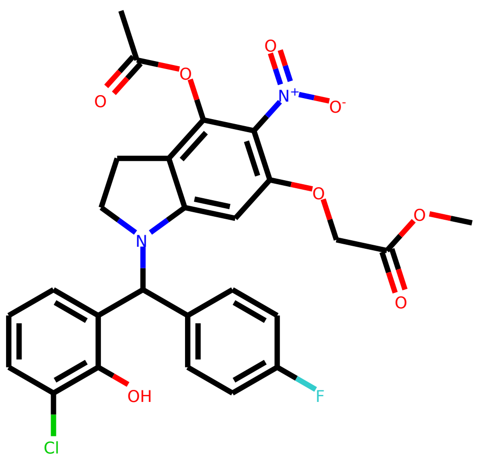
Osma-2125
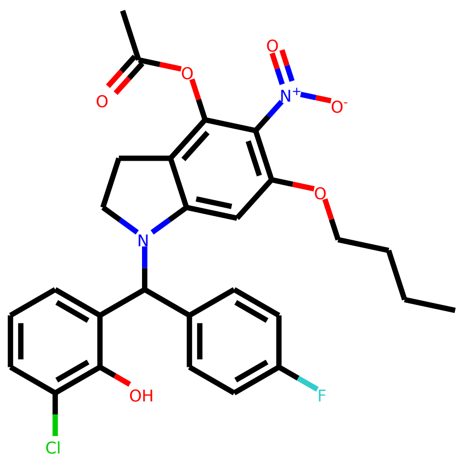
Osma-2126
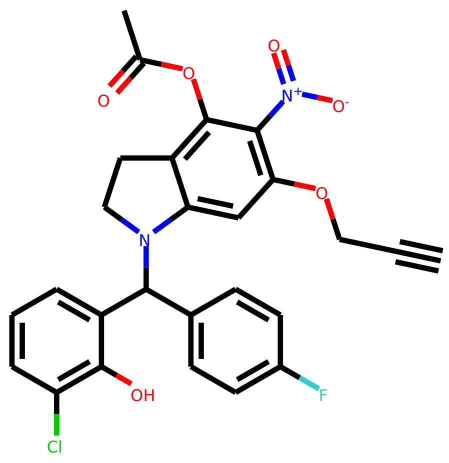
Osma-2127
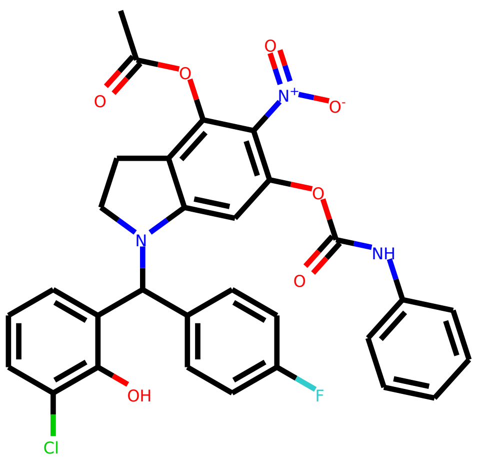
Osma-2128
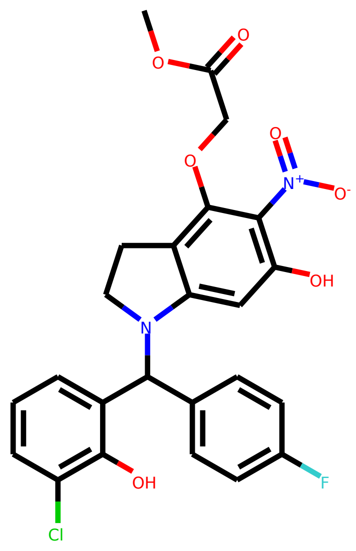
Osma-2129
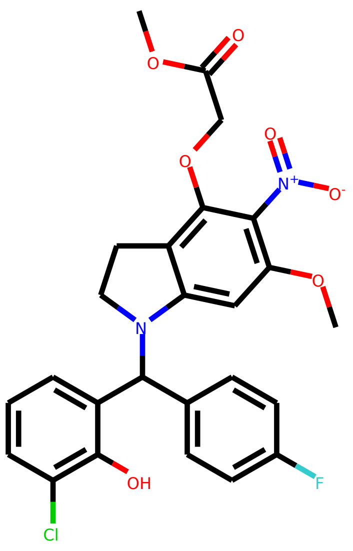
Osma-2130
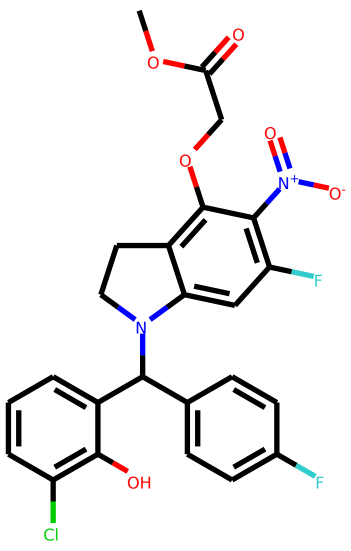
Osma-2131
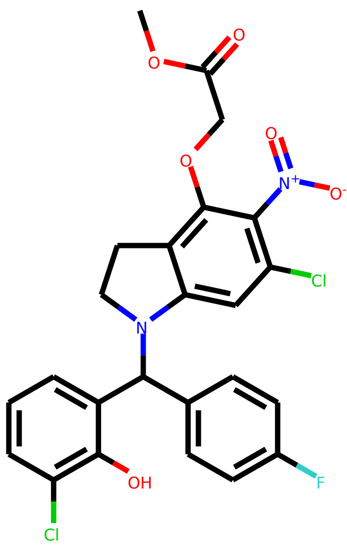
Osma-2132
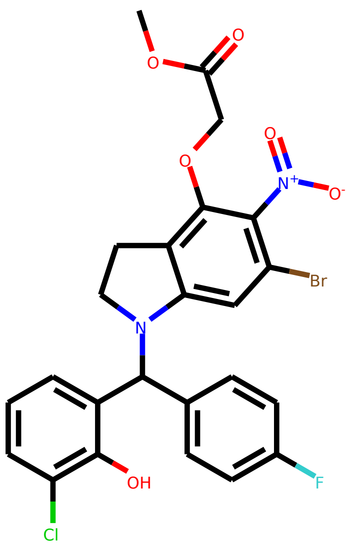
Osma-2133
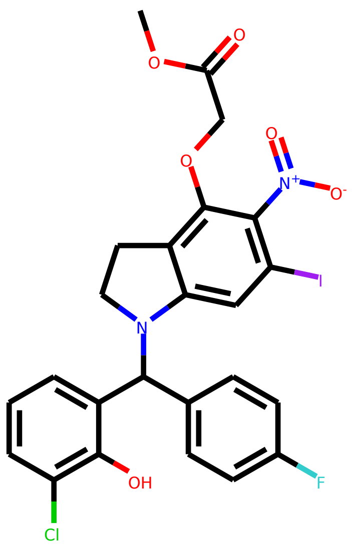
Osma-2134
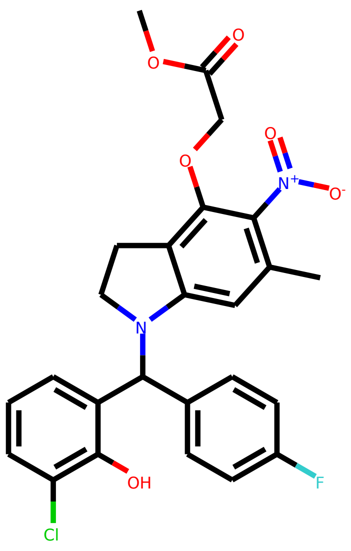
Osma-2135

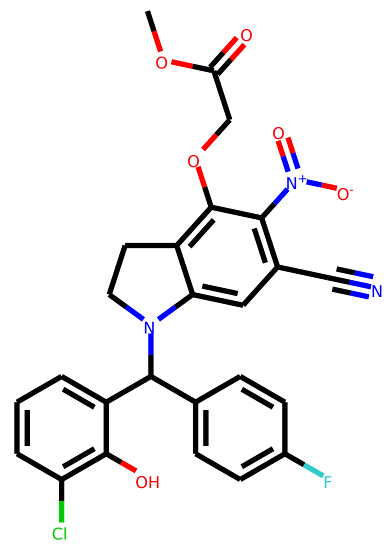
Osma-2136
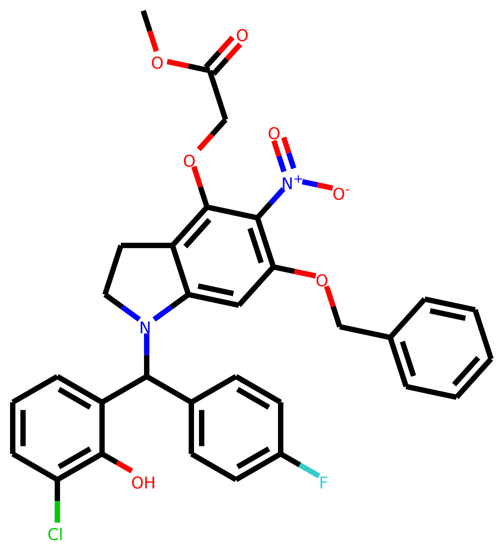
Osma-2137
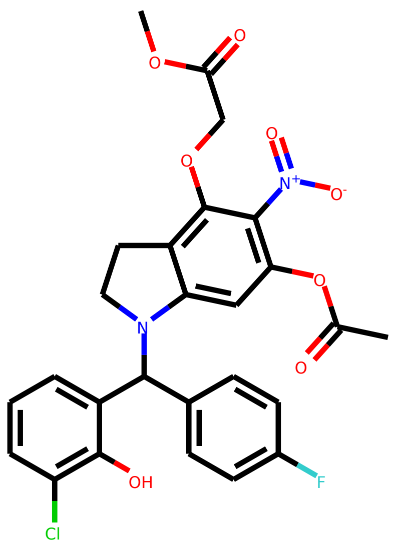
Osma-2138
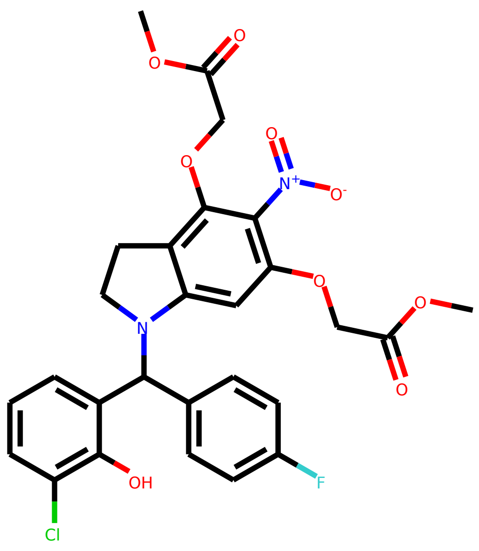
Osma-2139
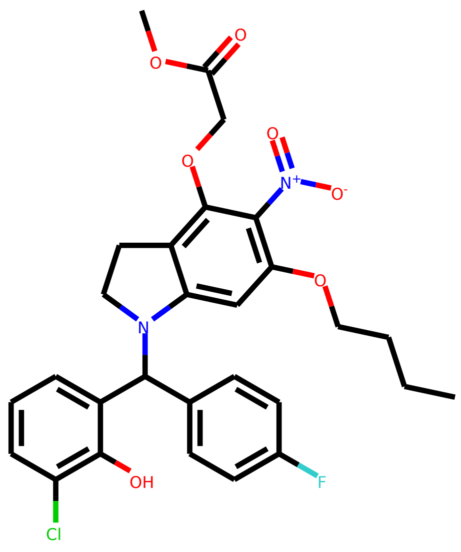
Osma-2140
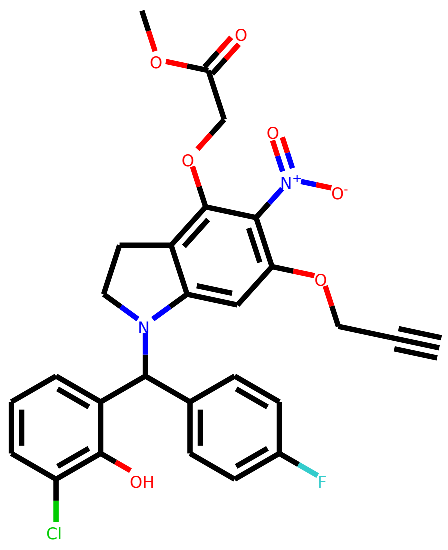
Osma-2141
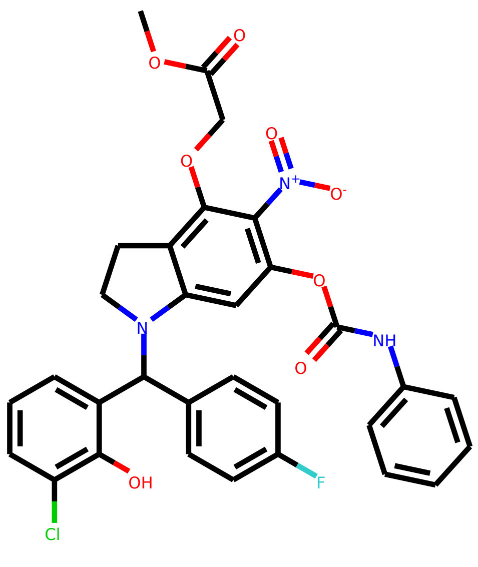
Osma-2142
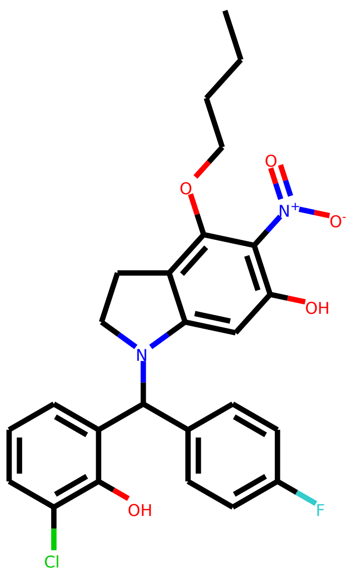
Osma-2143
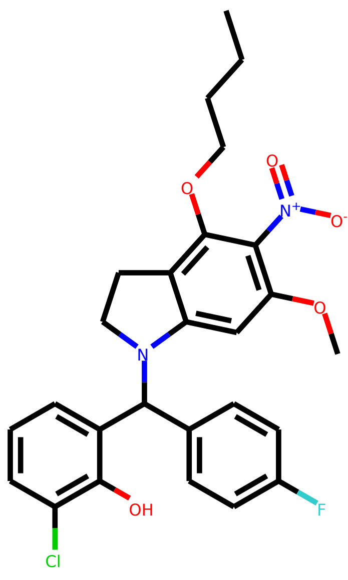
Osma-2144
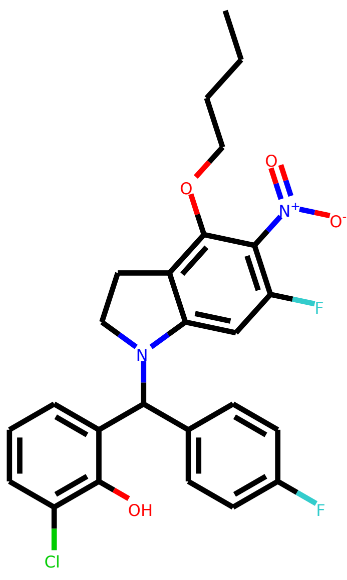
Osma-2145
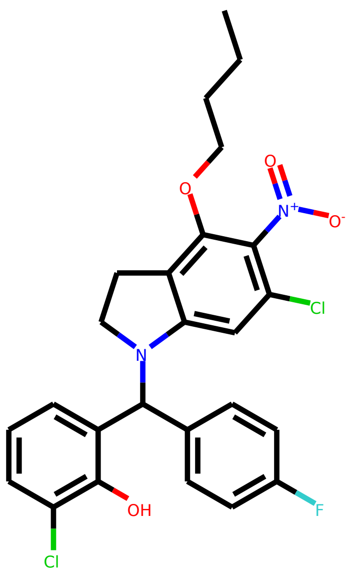
Osma-2146
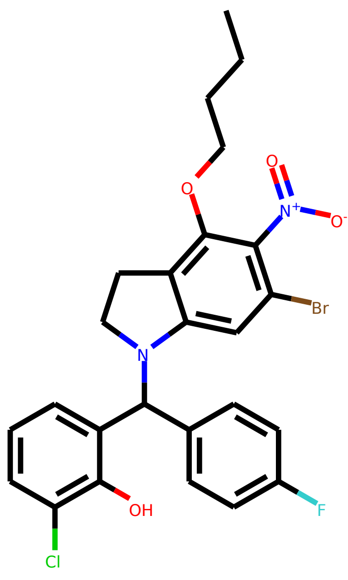
Osma-2147
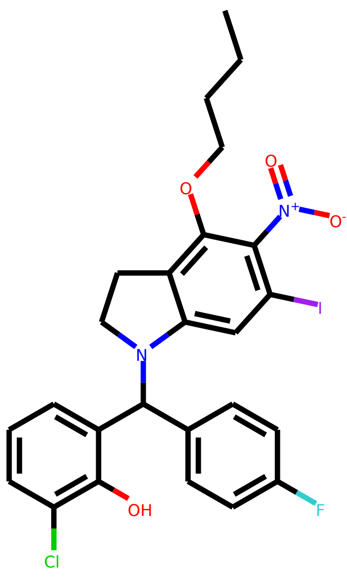
Osma-2148
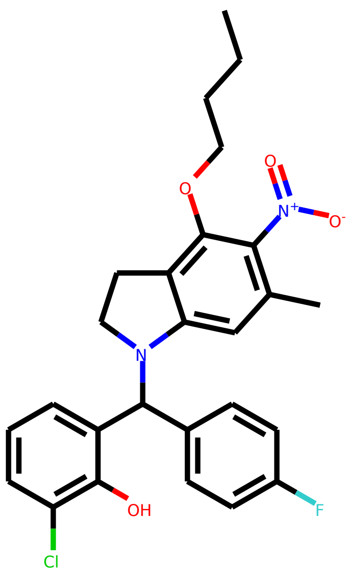
Osma-2149
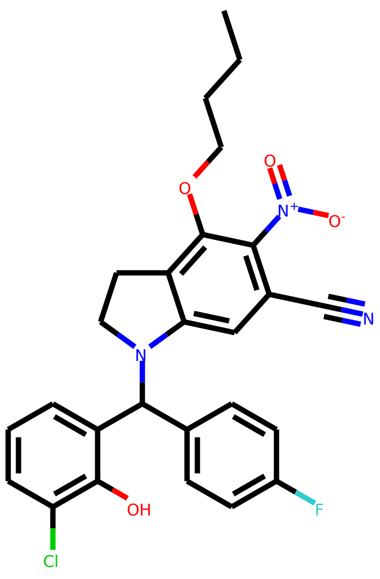
Osma-2150
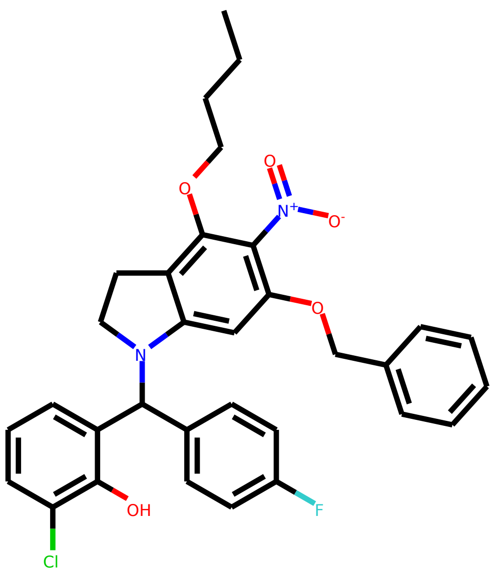
Osma-2151
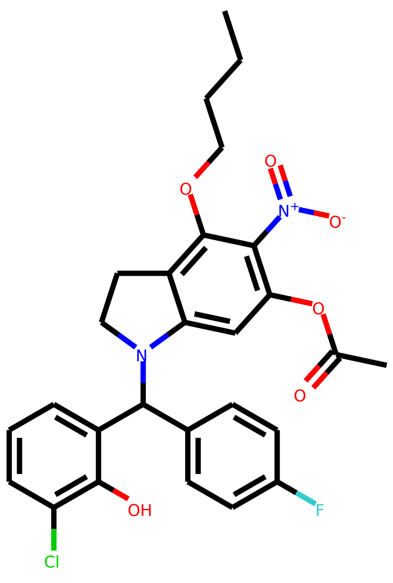
Osma-2152
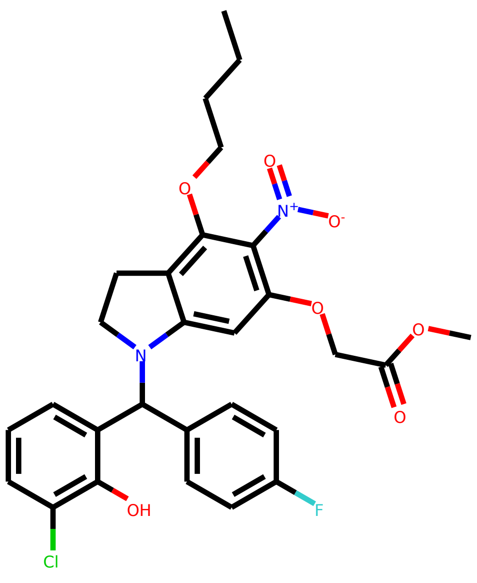
Osma-2153
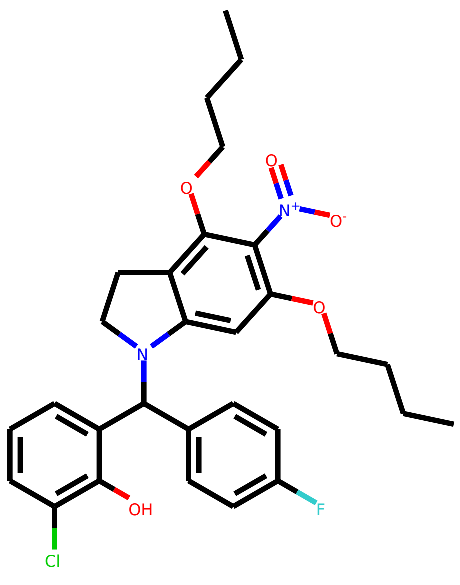
Osma-2154
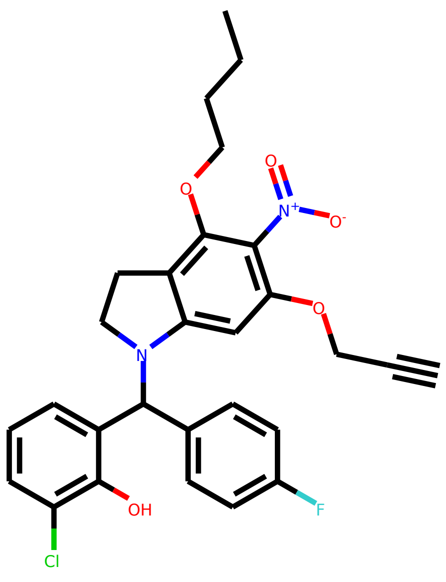
Osma-2155
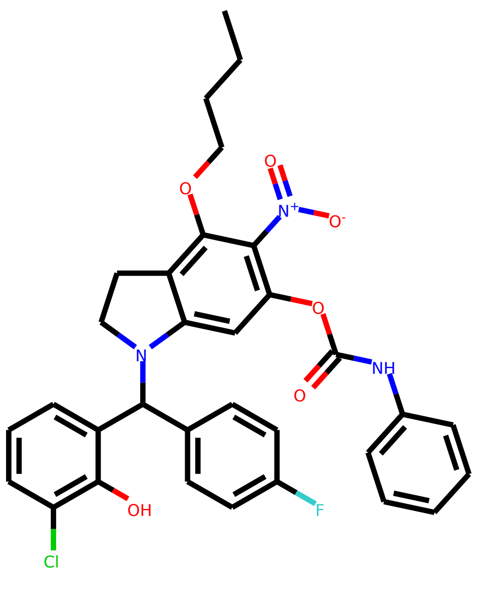
Osma-2156
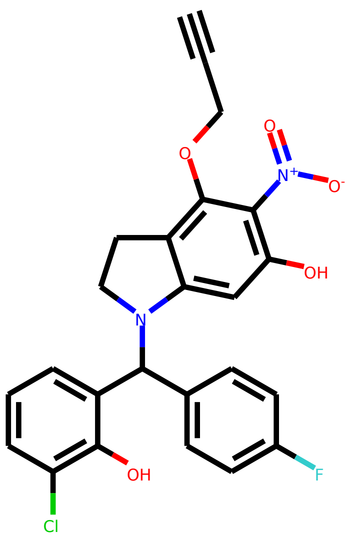
Osma-2157
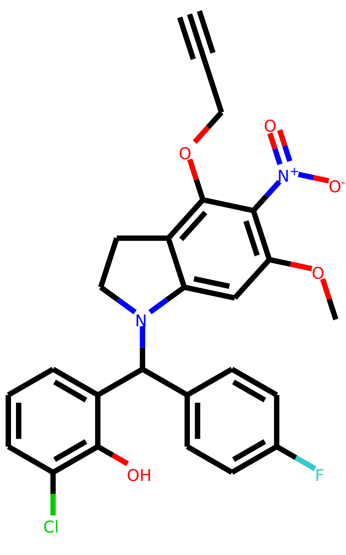
Osma-2158
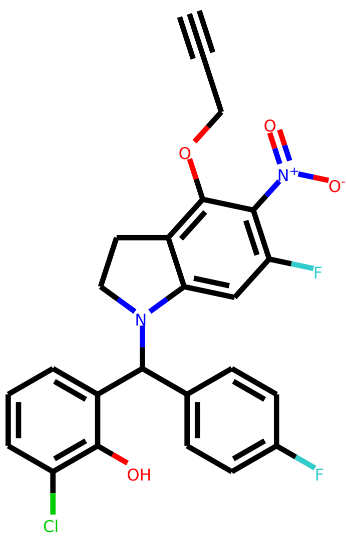
Osma-2159
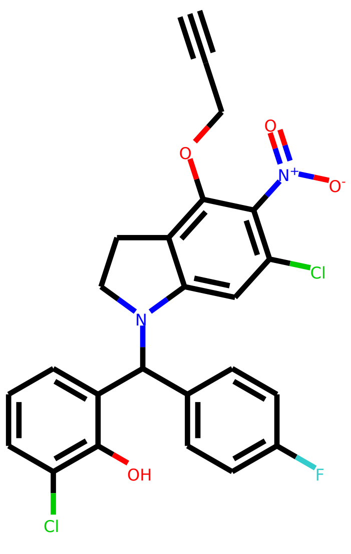
Osma-2160
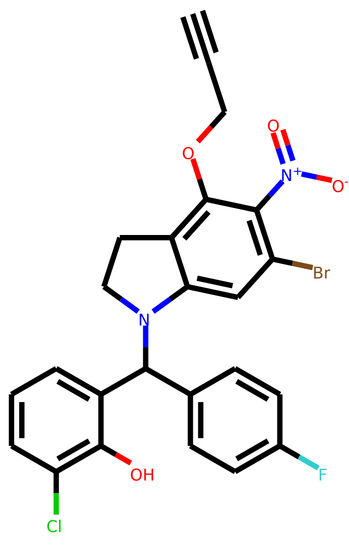
Osma-2161
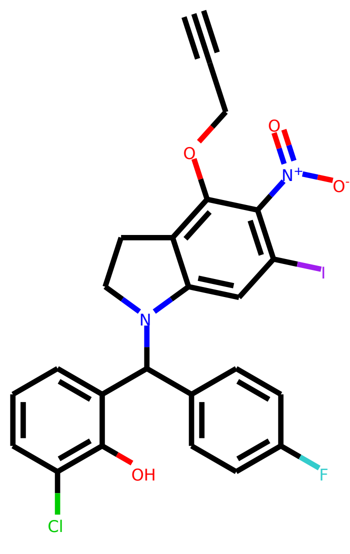
Osma-2162
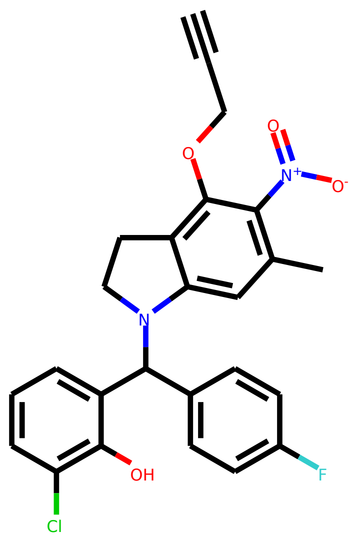
Osma-2163
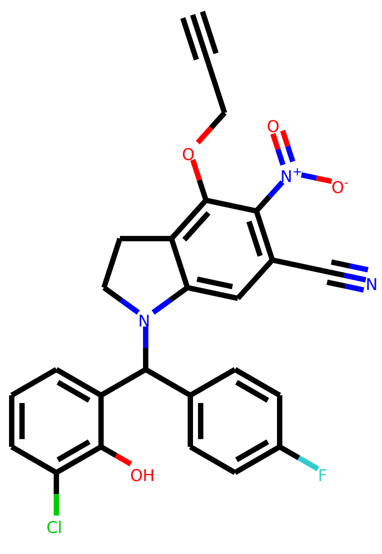
Osma-2164
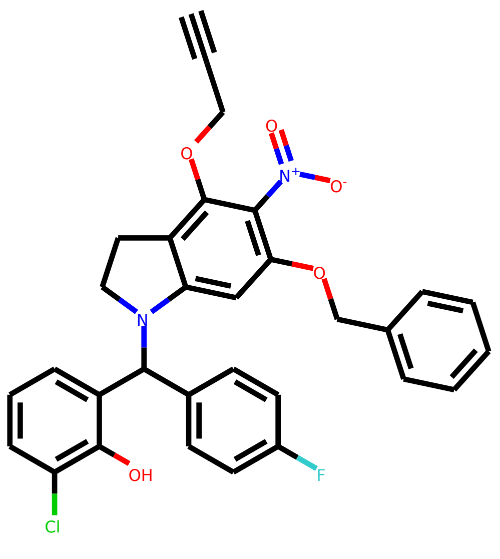
Osma-2165
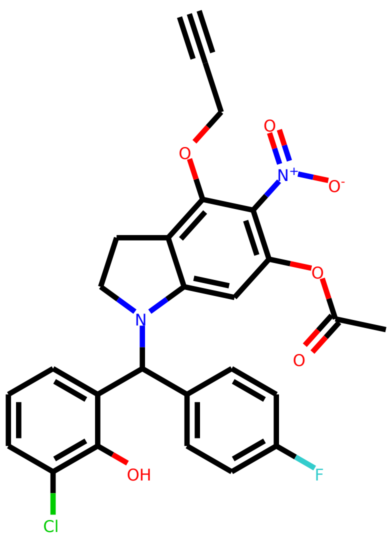
Osma-2166
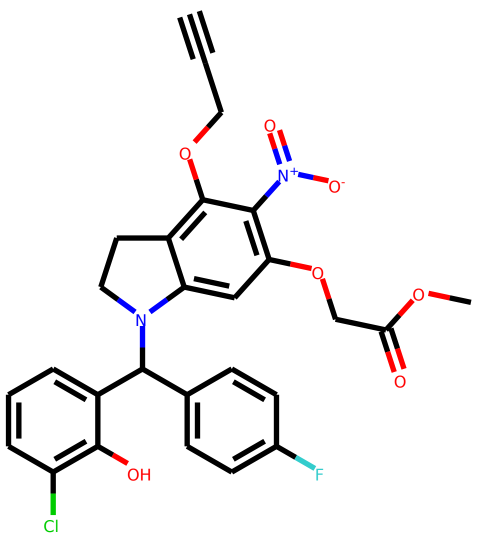
Osma-2167
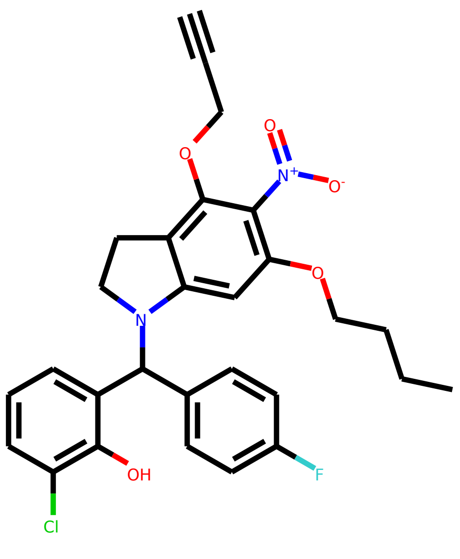
Osma-2168
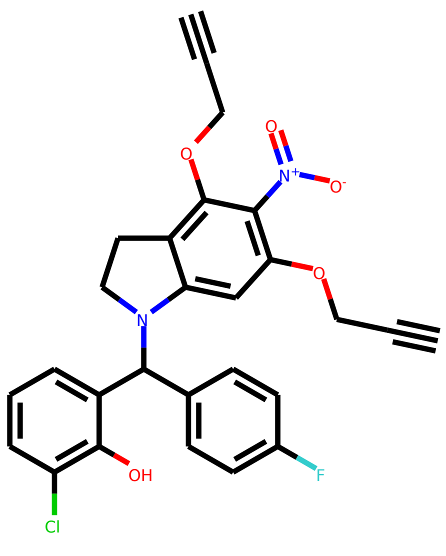
Osma-2169
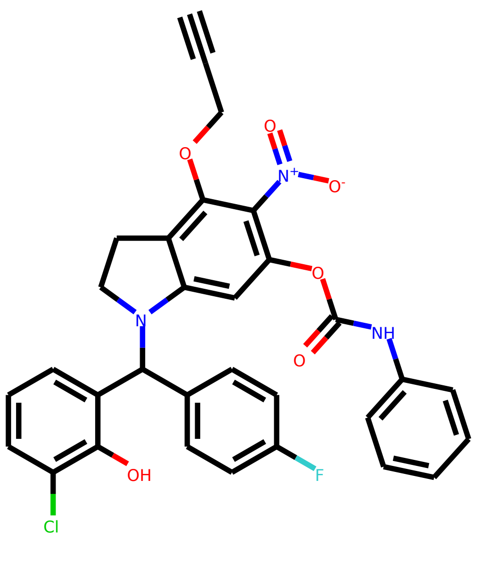
Osma-2170

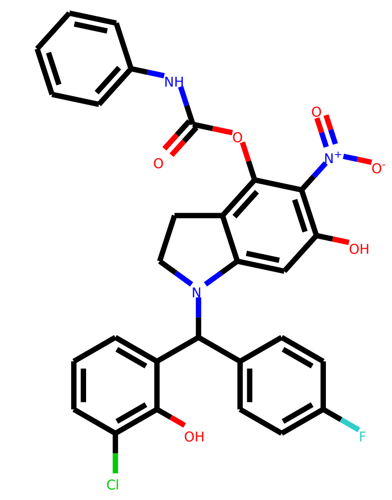
Osma-2171
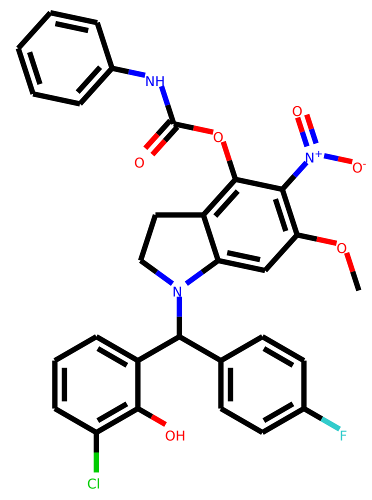
Osma-2172
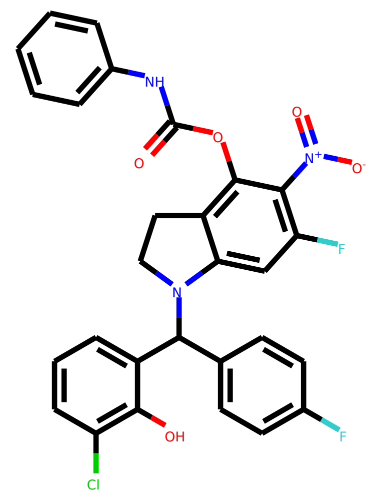
Osma-2173
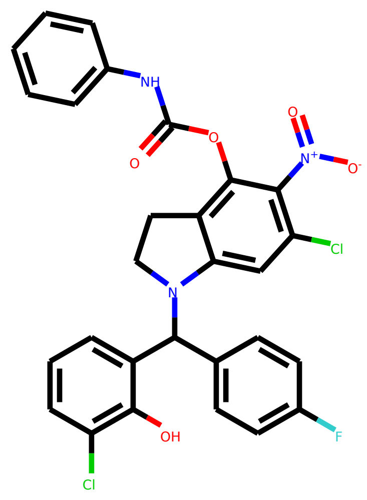
Osma-2174
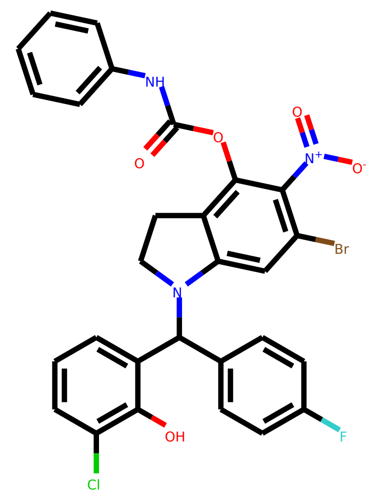
Osma-2175
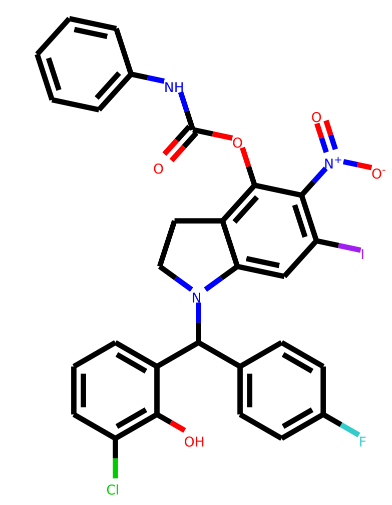
Osma-2176
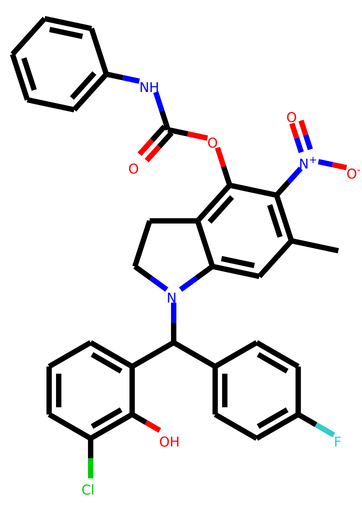
Osma-2177
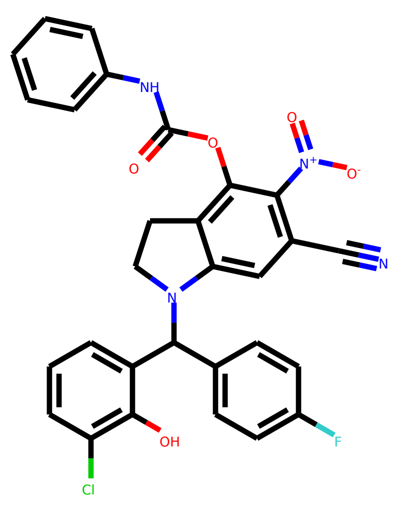
Osma-2178
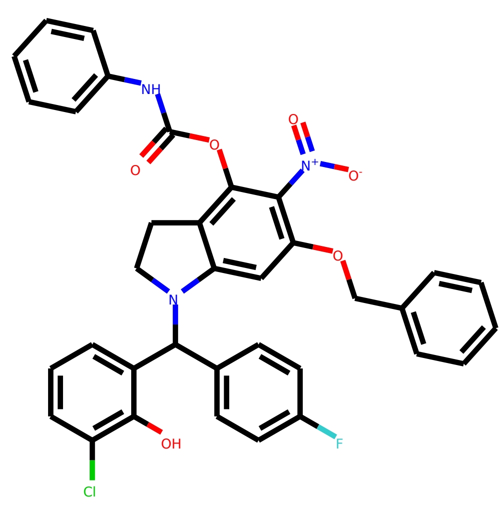
Osma-2179
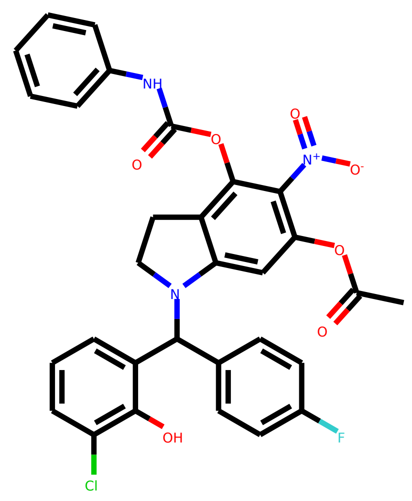
Osma-2180
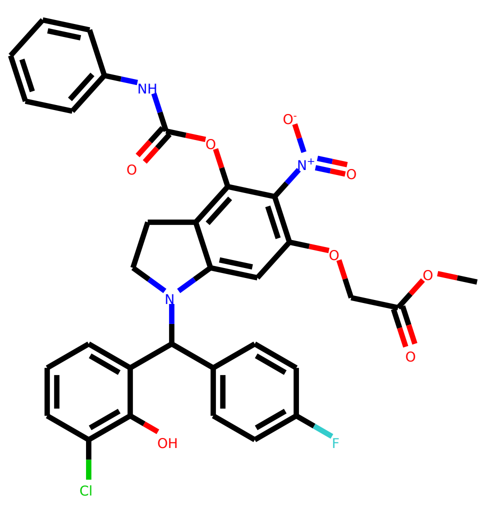
Osma-2181
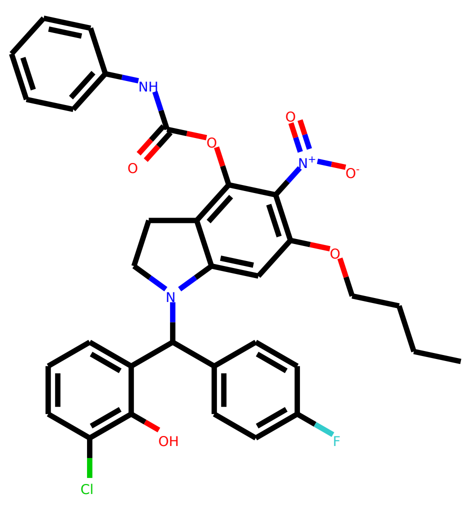
Osma-2182
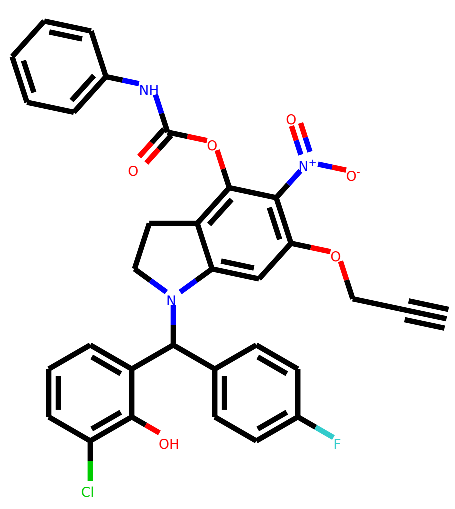
Osma-2183
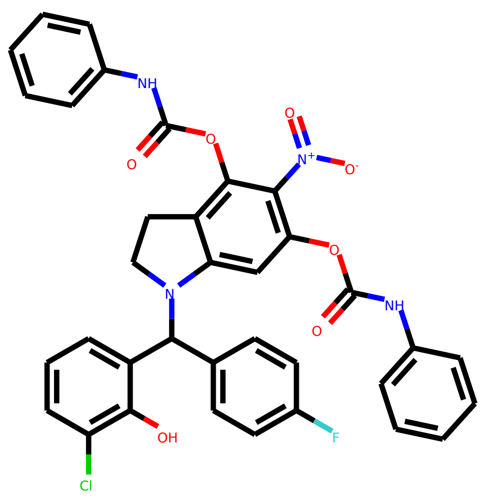
Osma-2184
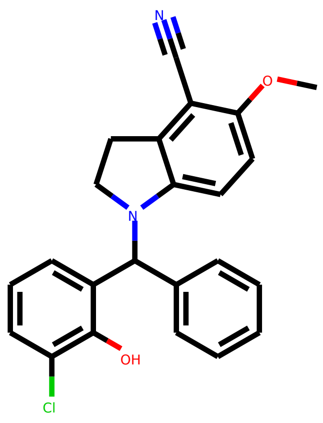
Osma-2185
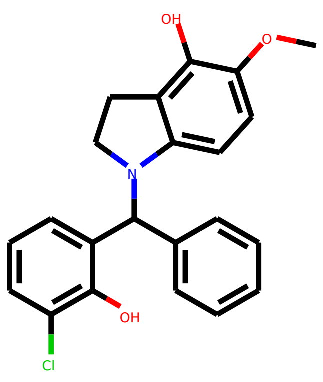
Osma-2186
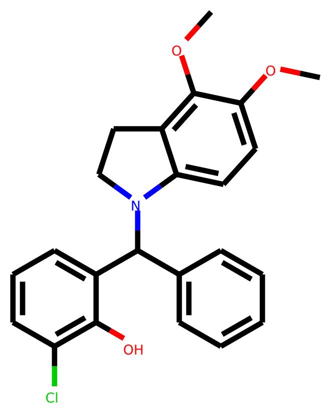
Osma-2187
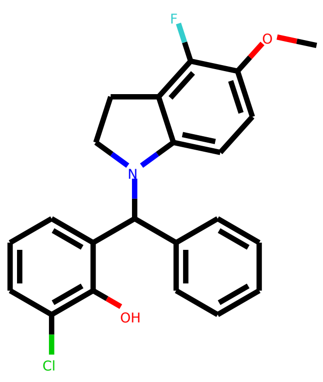
Osma-2188
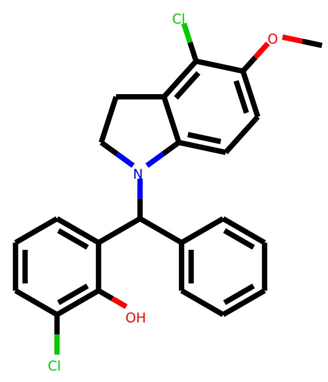
Osma-2189
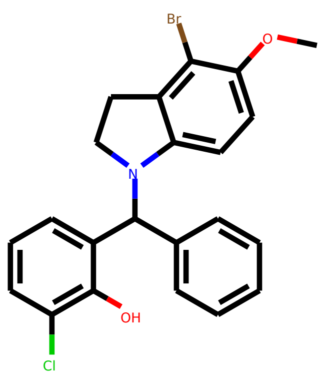
Osma-2190
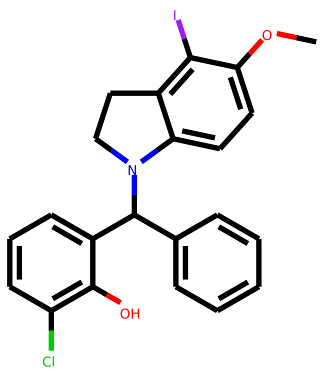
Osma-2191
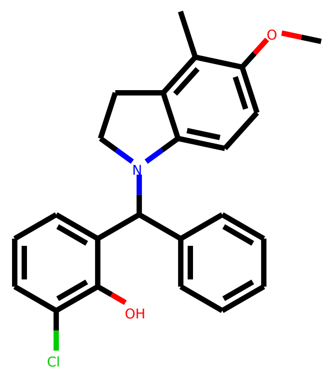
Osma-2192
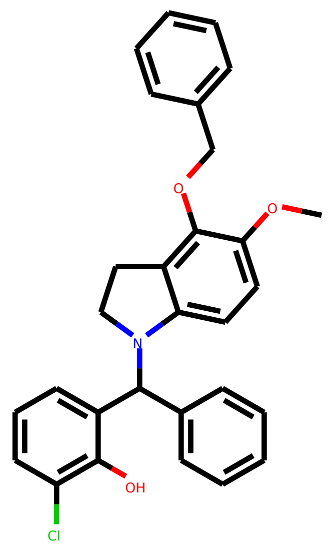
Osma-2193
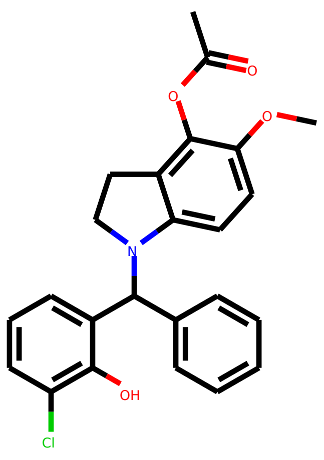
Osma-2194
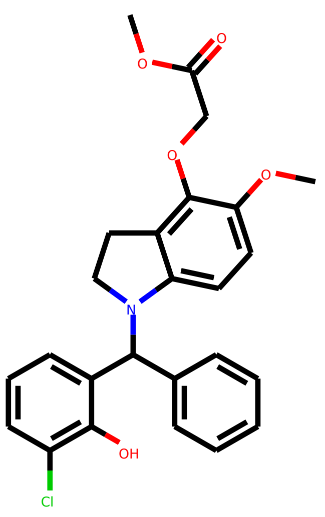
Osma-2195
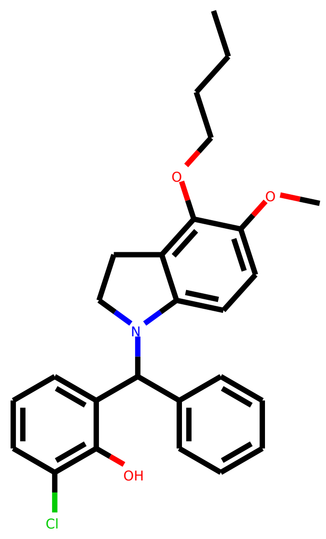
Osma-2196
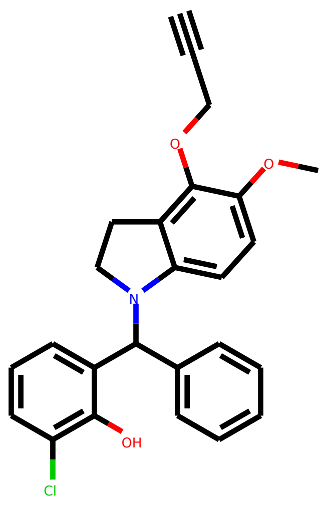
Osma-2197
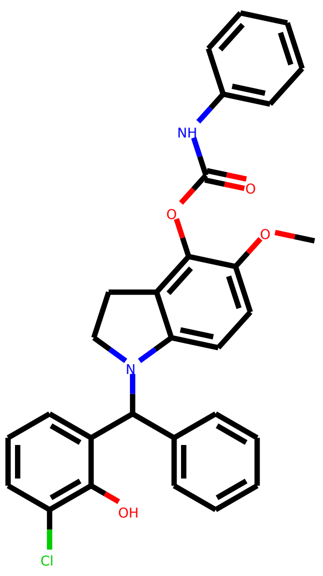
Osma-2198
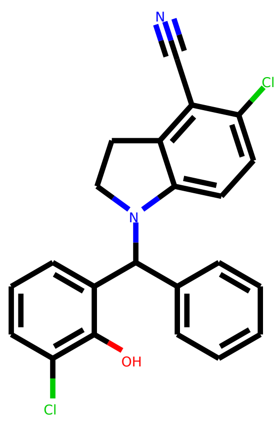
Osma-2199
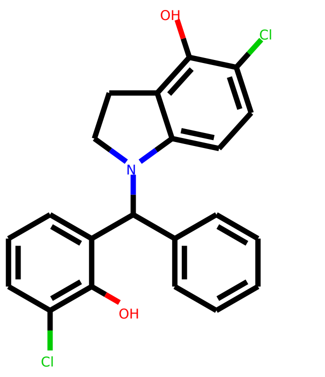
Osma-2200
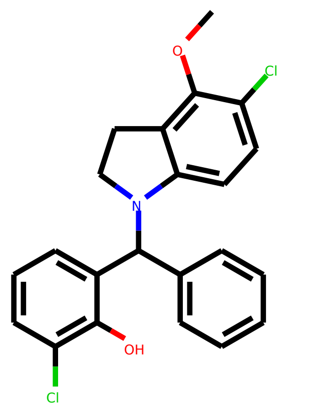
Osma-2201
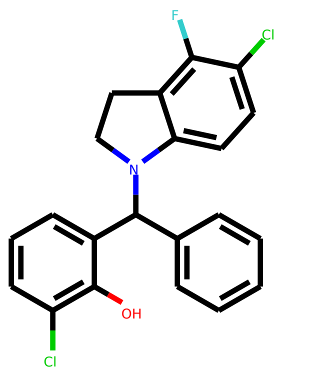
Osma-2202
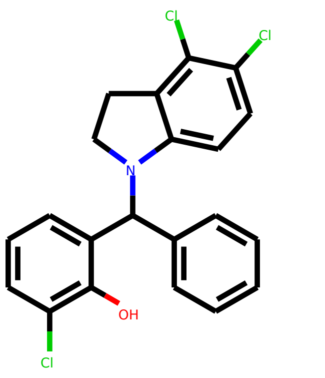
Osma-2203
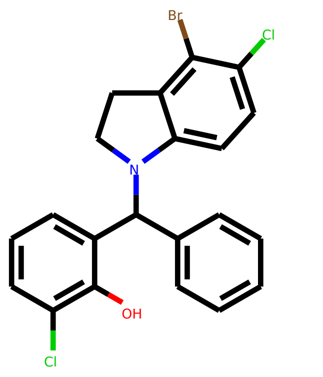
Osma-2204
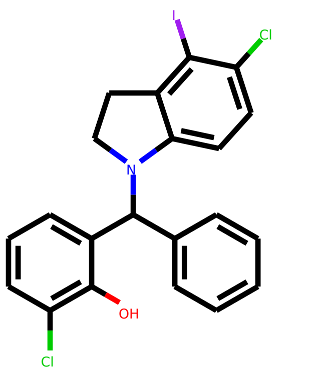
Osma-2205

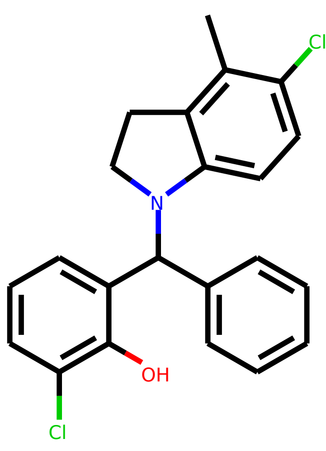
Osma-2206

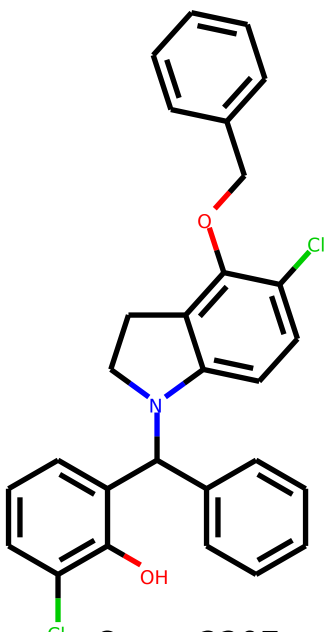
Osma-2207

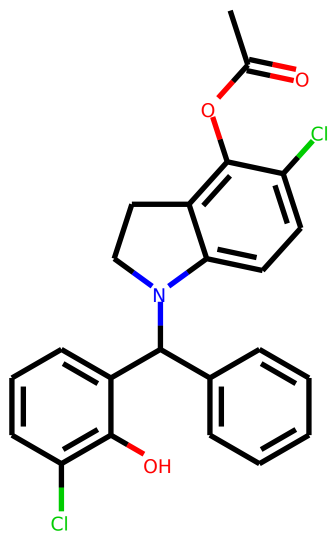
Osma-2208

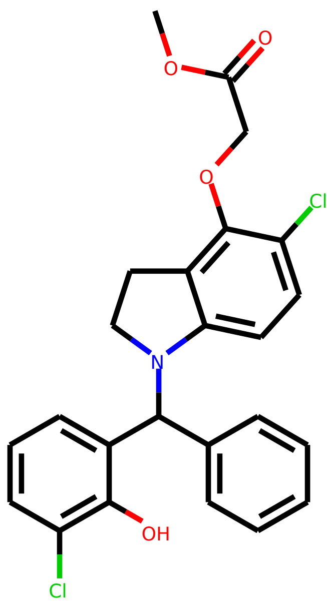
Osma-2209

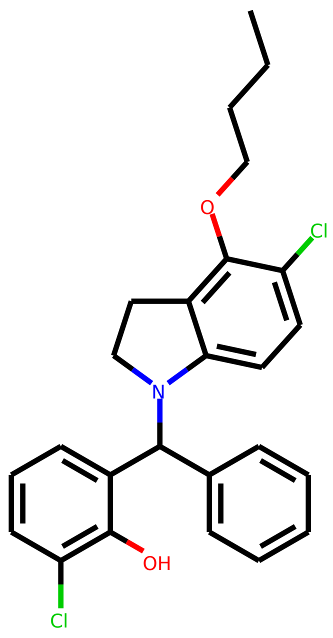
Osma-2210

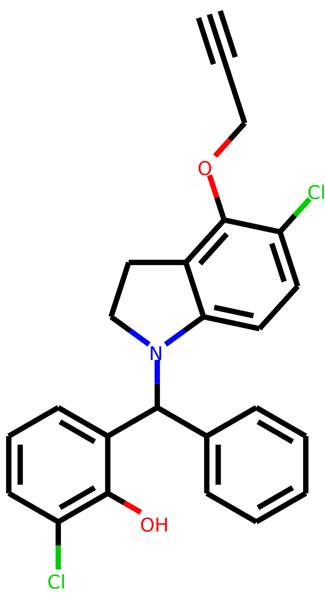
Osma-2211

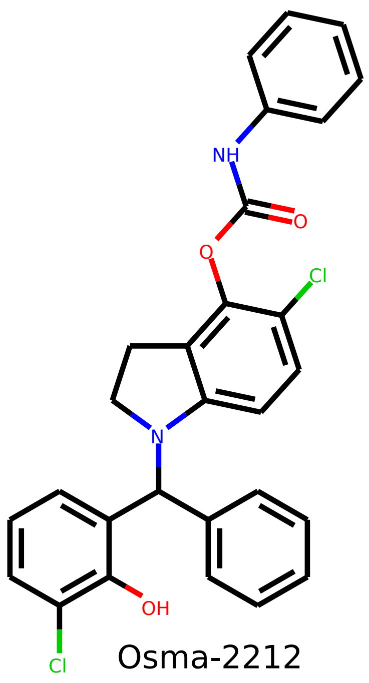
Osma-2212

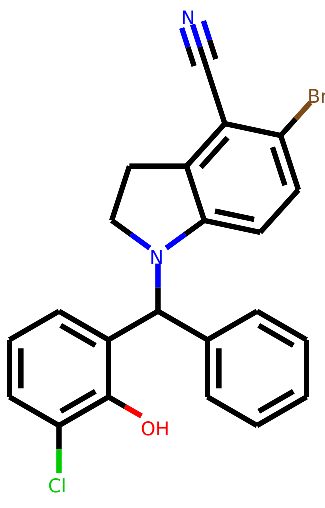
Osma-2213

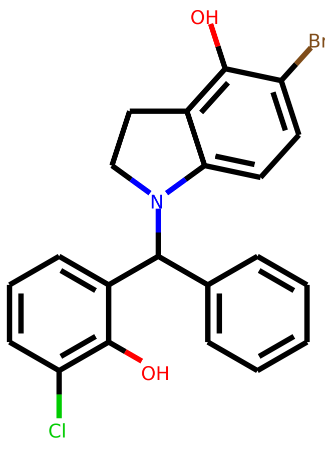
Osma-2214

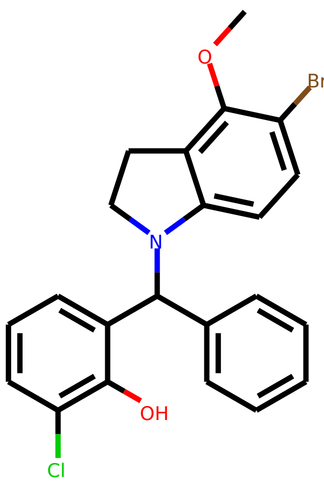
Osma-2215

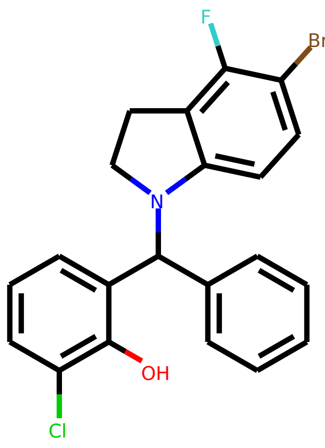
Osma-2216

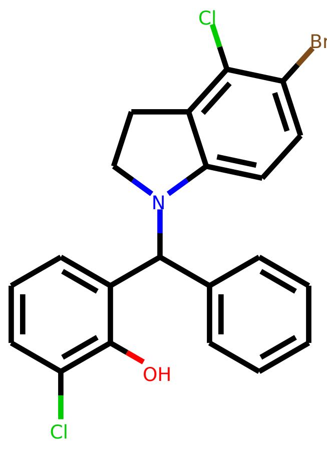
Osma-2217

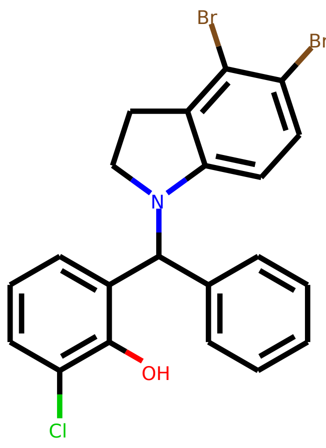
Osma-2218

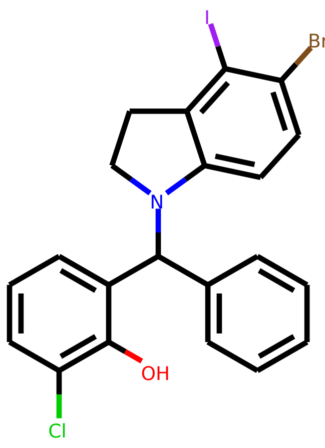
Osma-2219

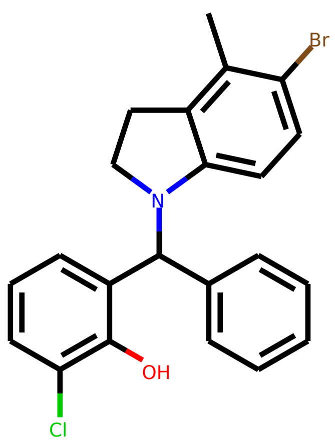
Osma-2220

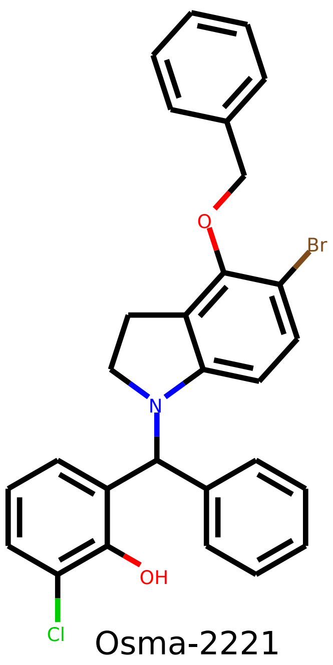
Osma-2221

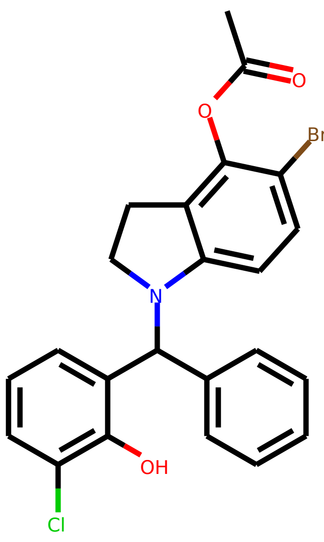
Osma-2222

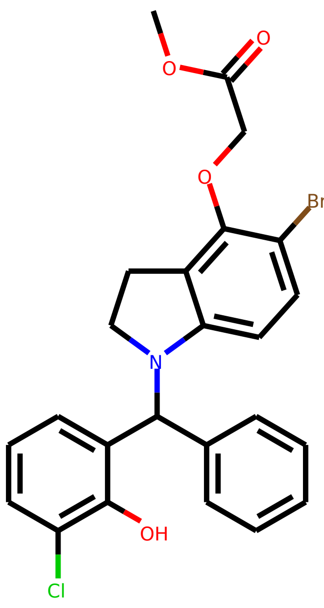
Osma-2223

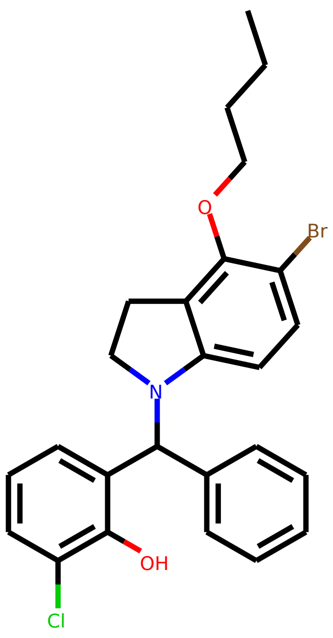
Osma-2224

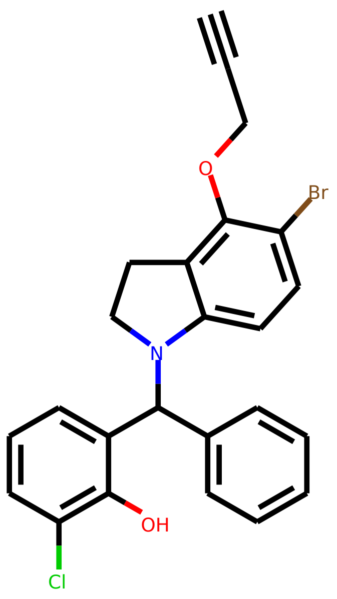
Osma-2225

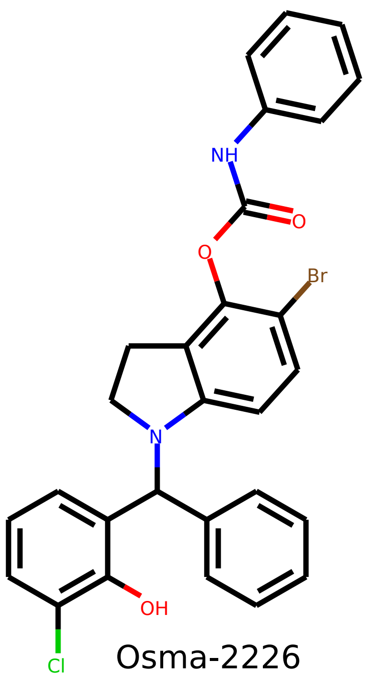
Osma-2226

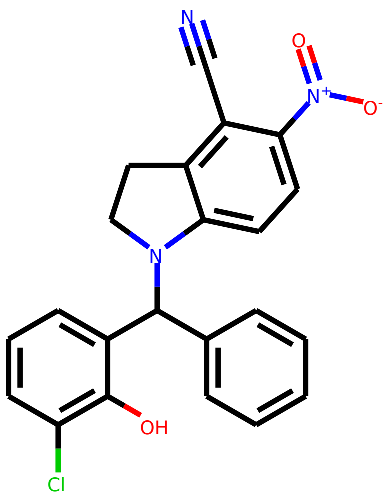
Osma-2227

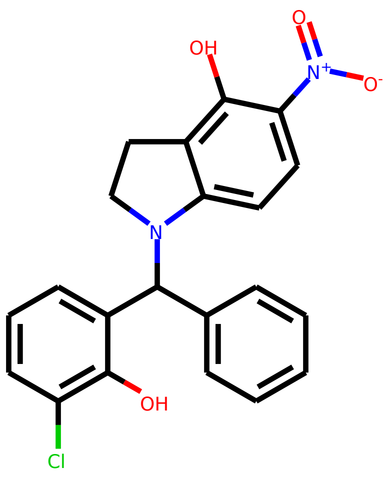
Osma-2228

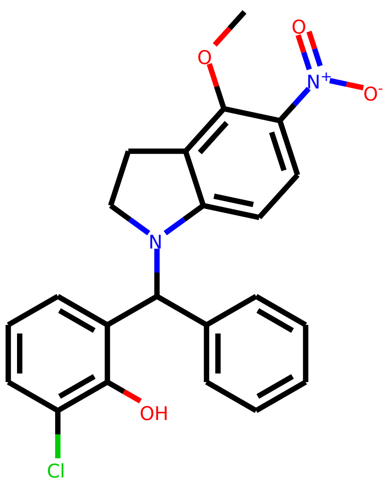
Osma-2229

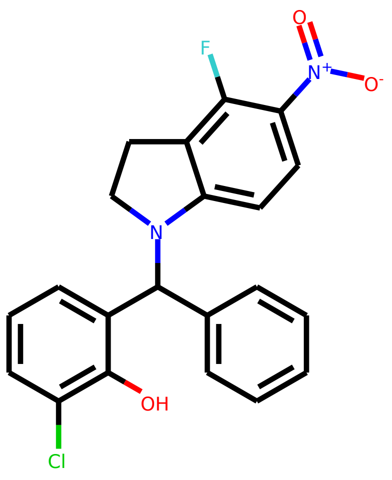
Osma-2230

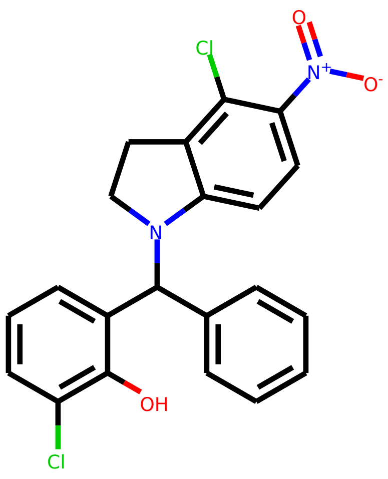
Osma-2231

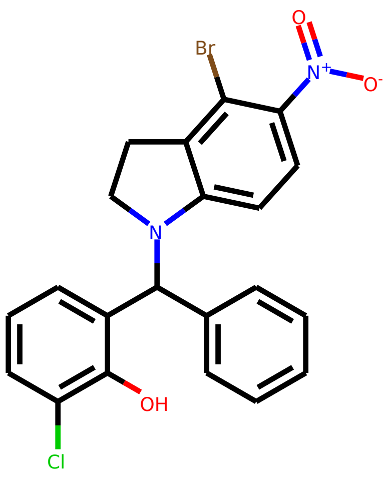
Osma-2232

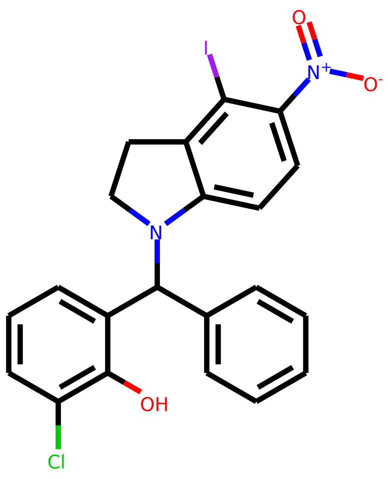
Osma-2233

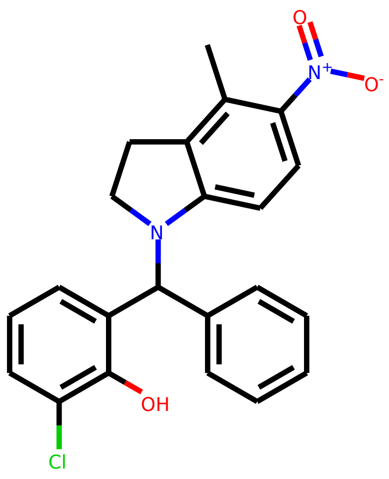
Osma-2234

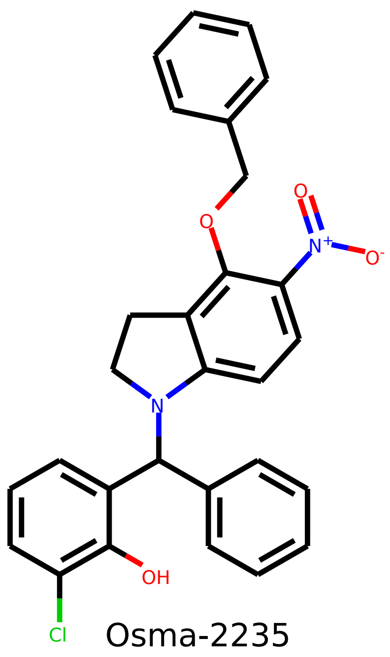
Osma-2235

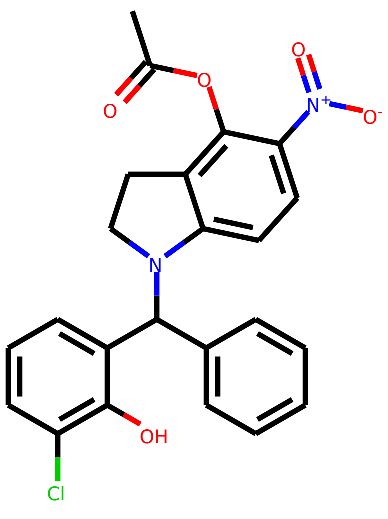
Osma-2236

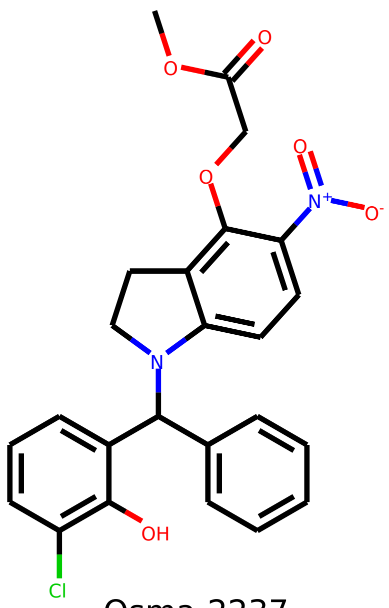
Osma-2237

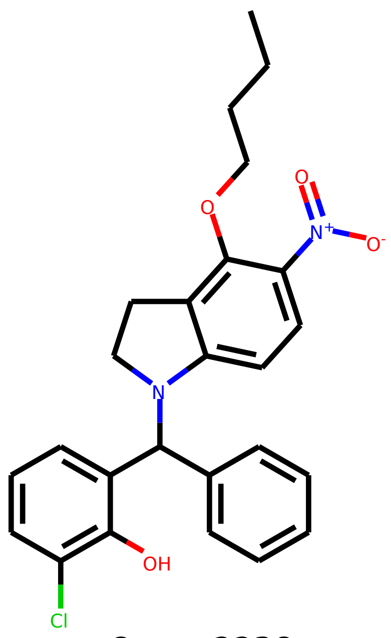
Osma-2238

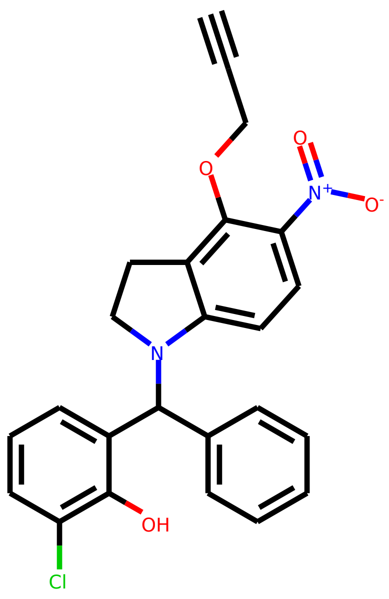
Osma-2239

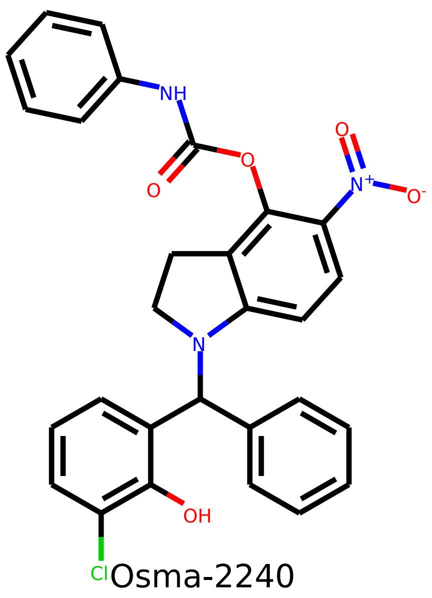
Osma-2240

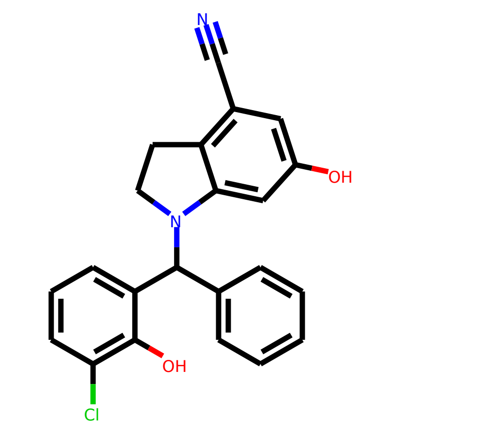
Osma-2241
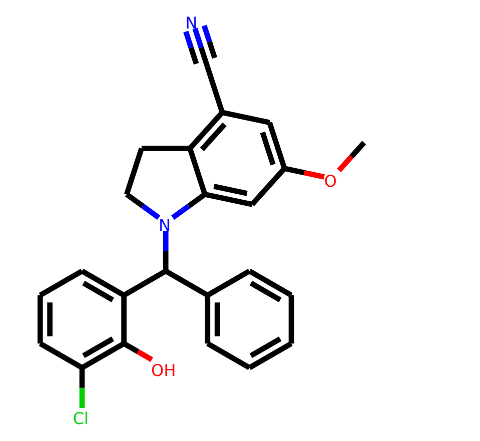
Osma-2242
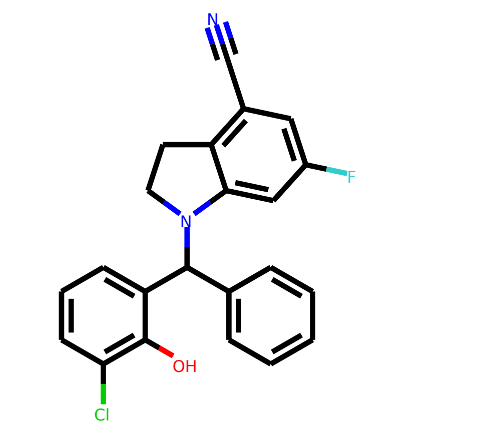
Osma-2243
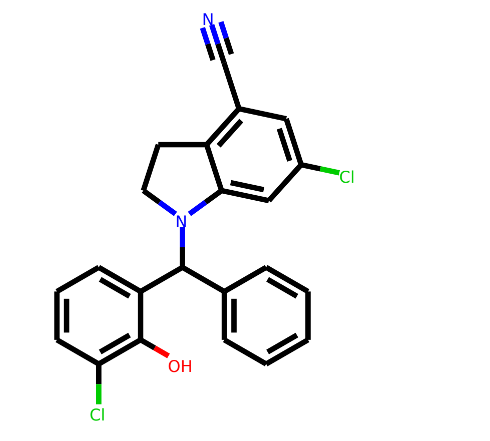
Osma-2244
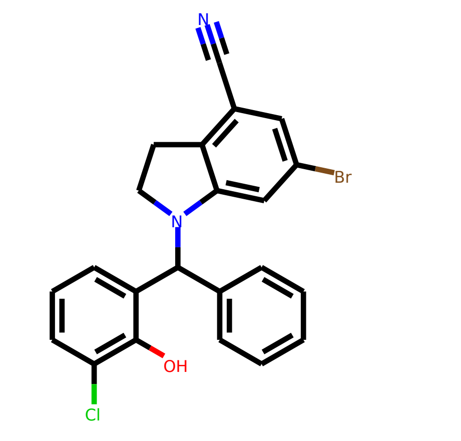
Osma-2245
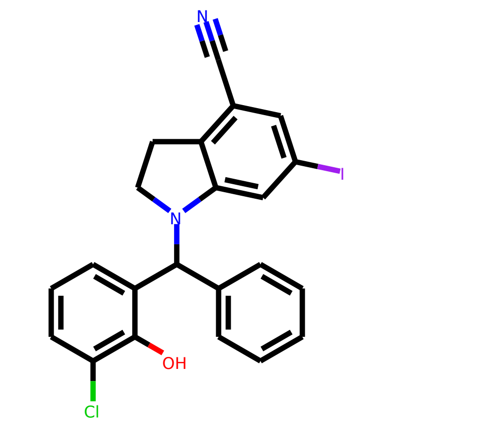
Osma-2246
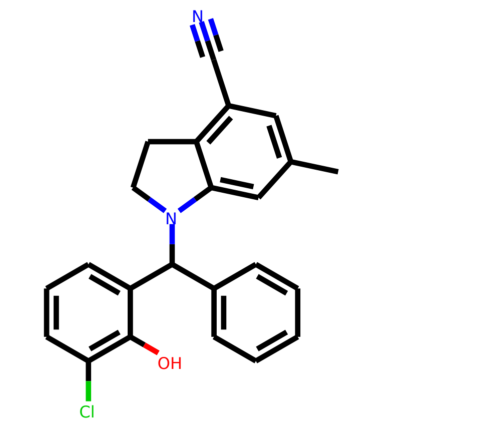
Osma-2247
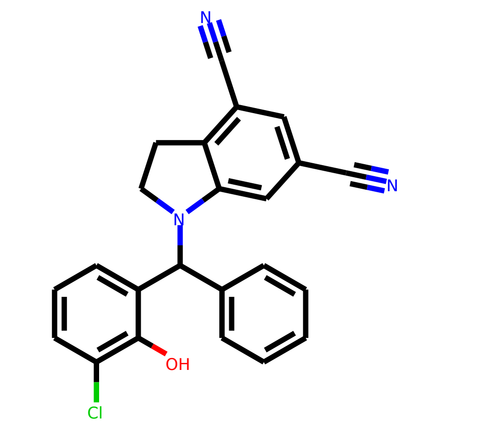
Osma-2248
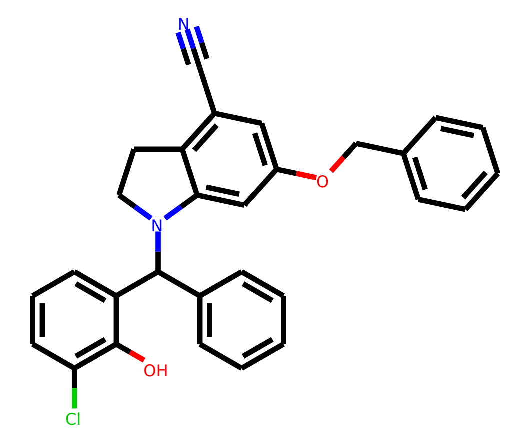
Osma-2249
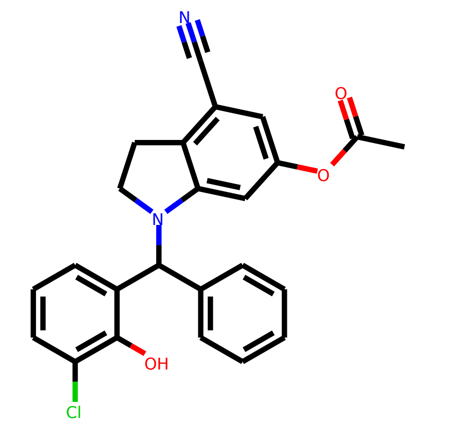
Osma-2250
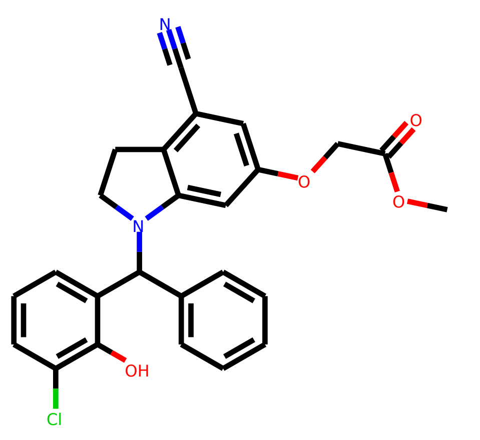
Osma-2251
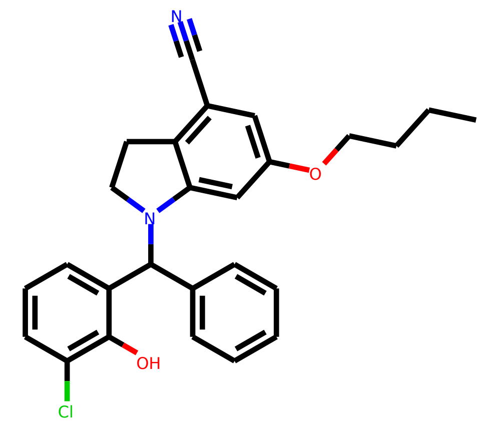
Osma-2252
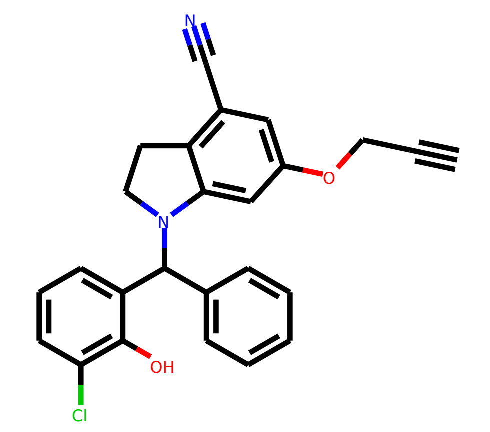
Osma-2253
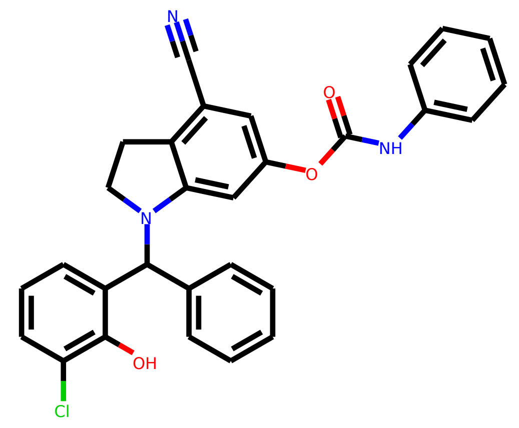
Osma-2254
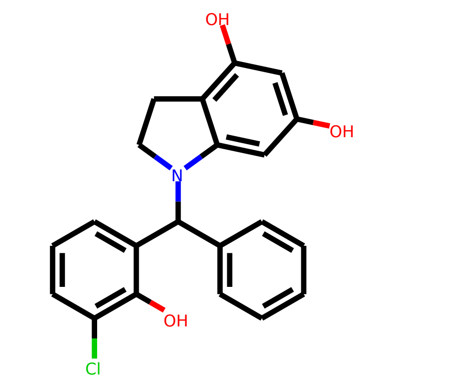
Osma-2255
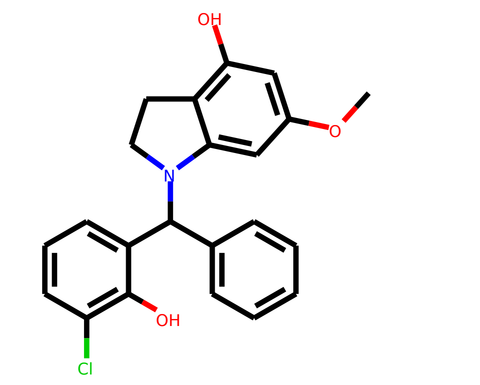
Osma-2256
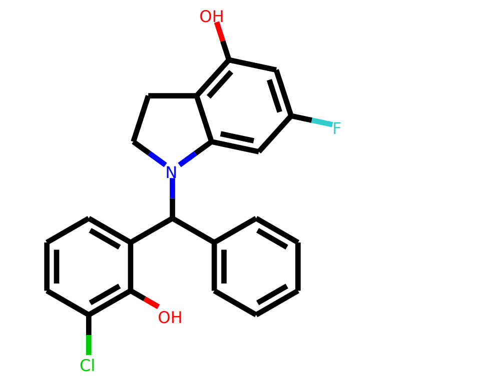
Osma-2257
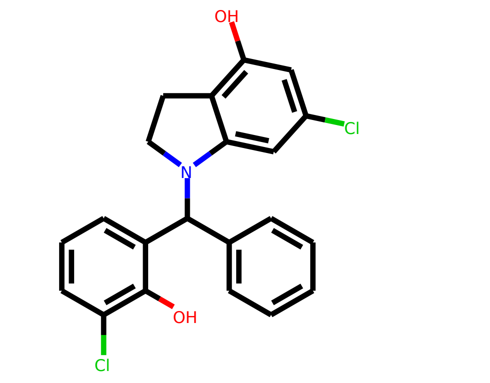
Osma-2258
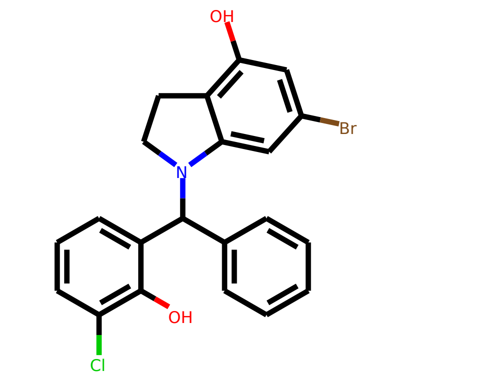
Osma-2259
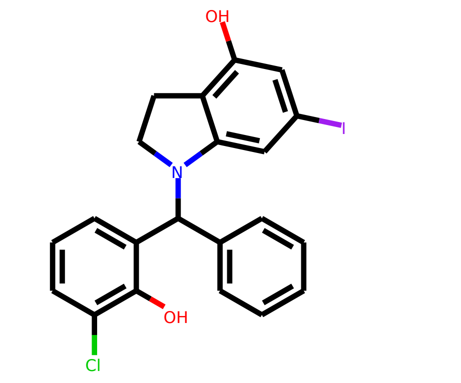
Osma-2260
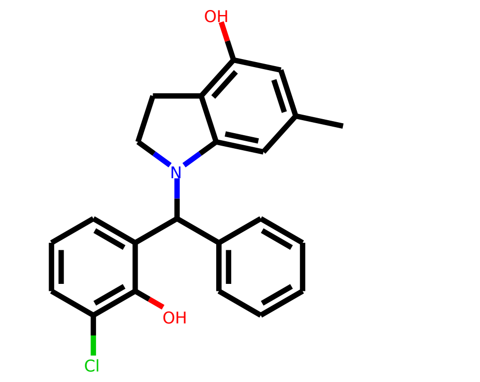
Osma-2261
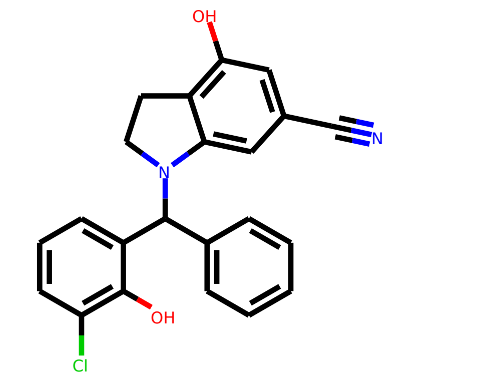
Osma-2262
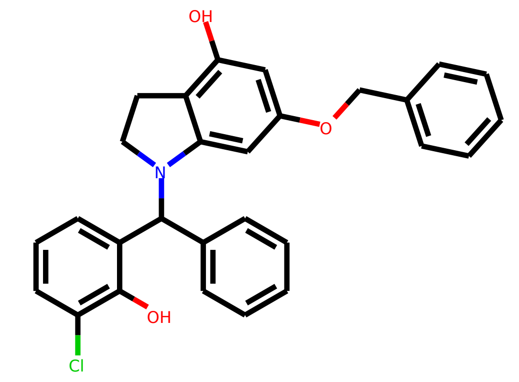
Osma-2263
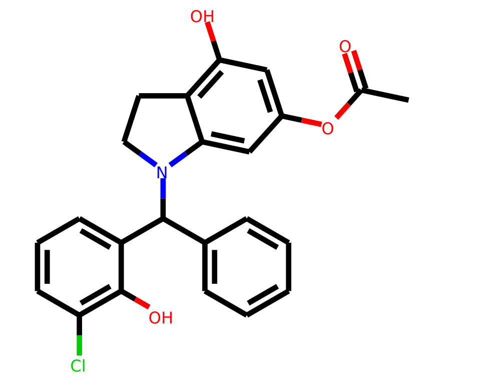
Osma-2264
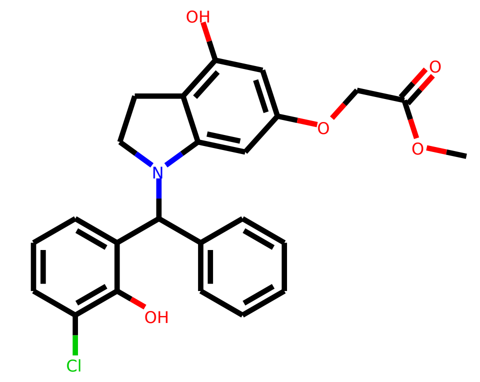
Osma-2265
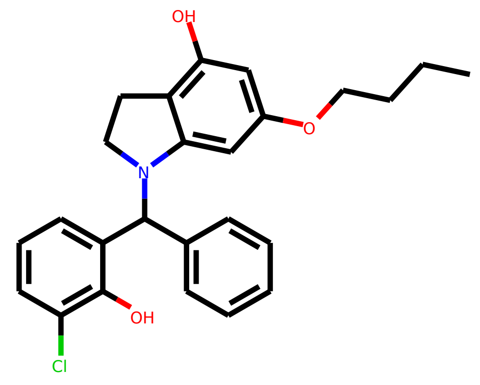
Osma-2266
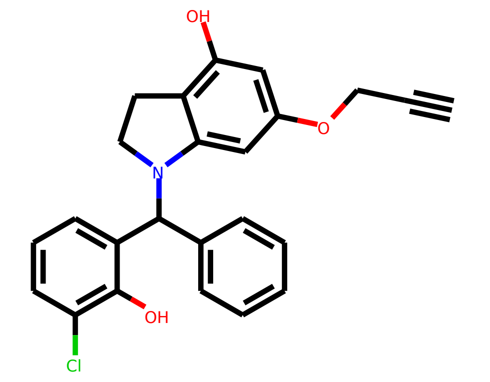
Osma-2267
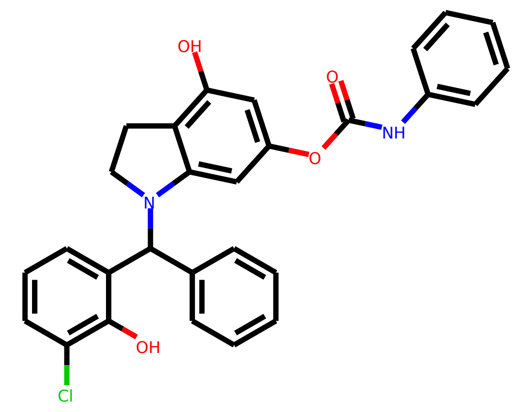
Osma-2268
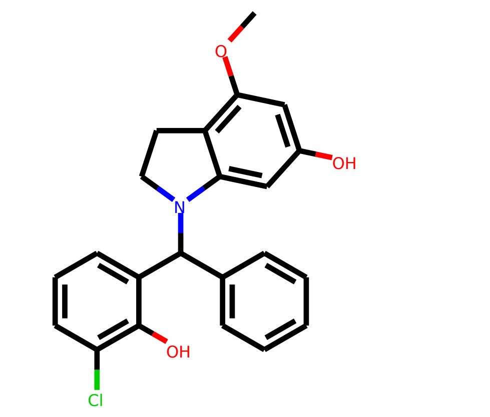
Osma-2269
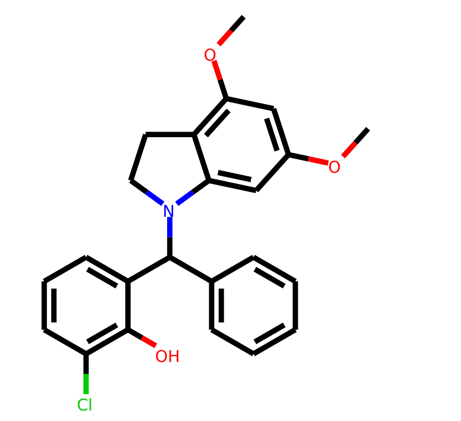
Osma-2270
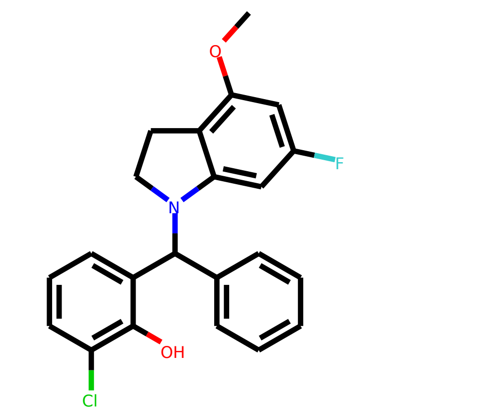
Osma-2271
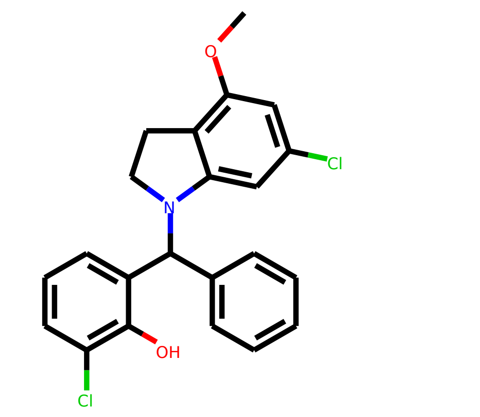
Osma-2272
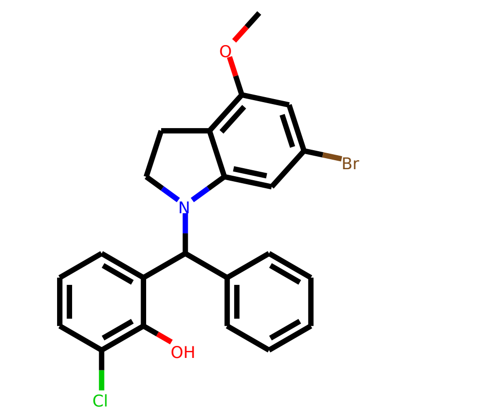
Osma-2273
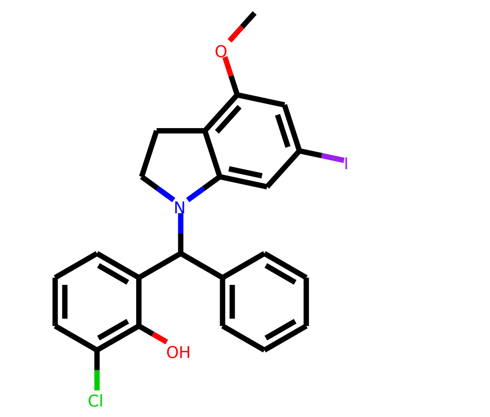
Osma-2274
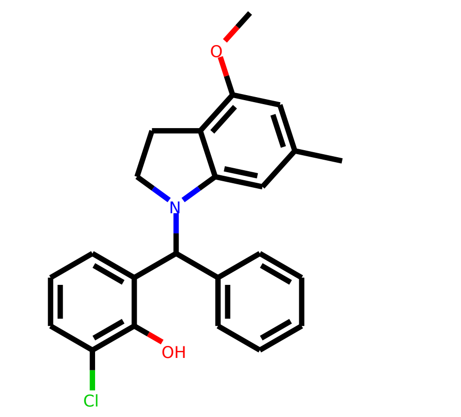
Osma-2275

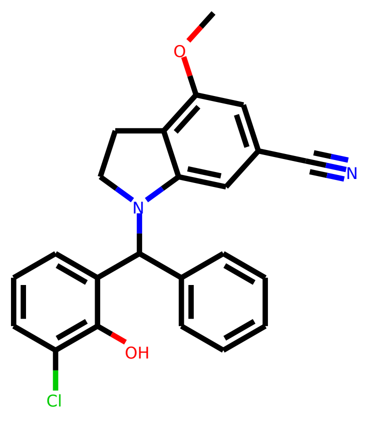

Osma-2276

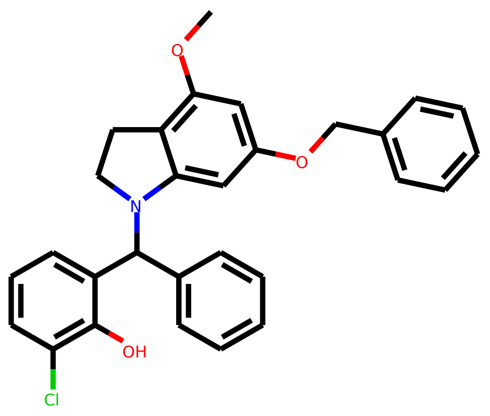

Osma-2277

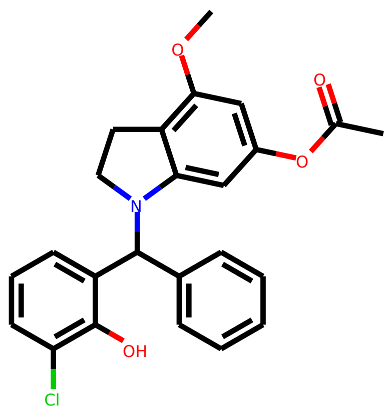

Osma-2278

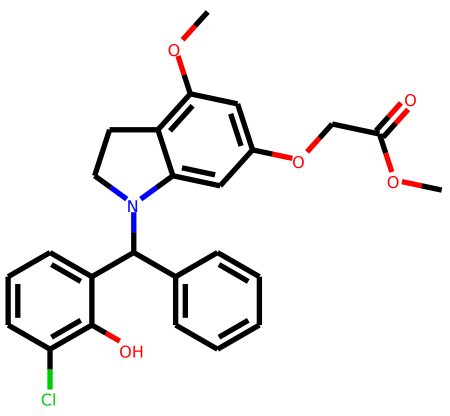

Osma-2279

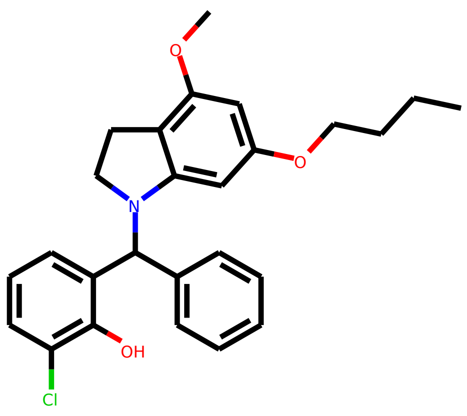

Osma-2280

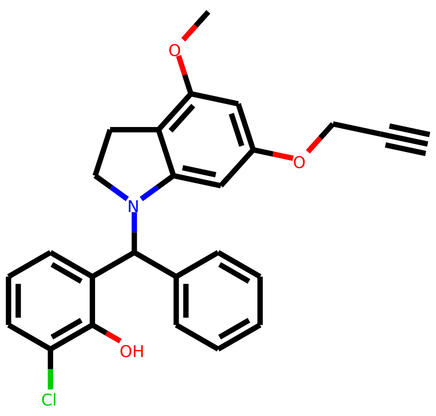

Osma-2281

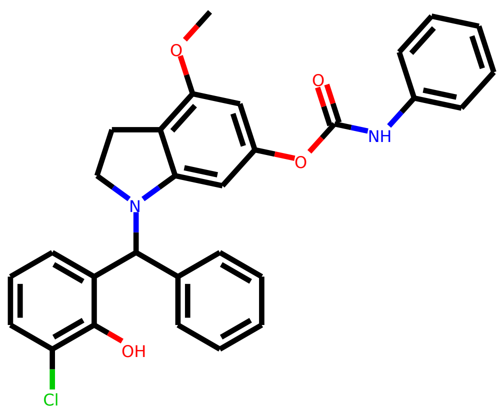

Osma-2282

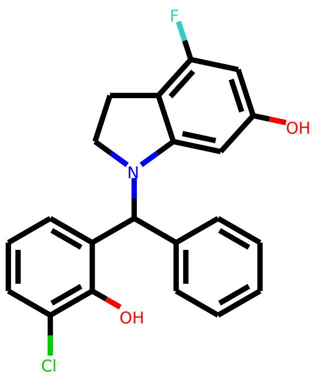

Osma-2283

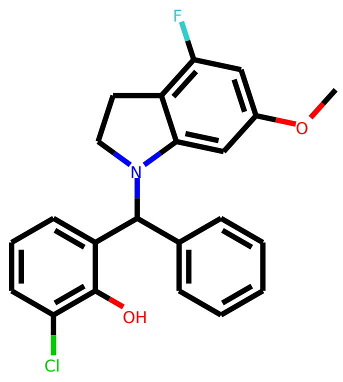

Osma-2284

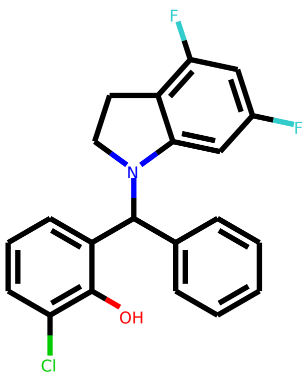

Osma-2285

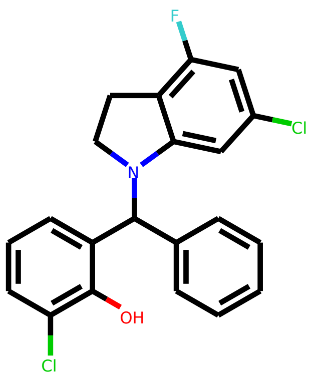

Osma-2286

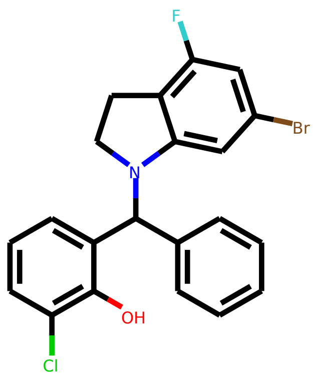

Osma-2287

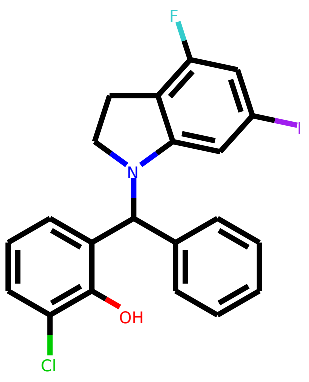

Osma-2288

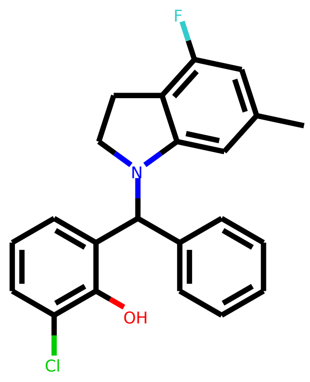

Osma-2289

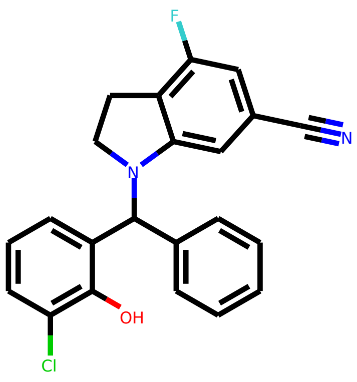

Osma-2290

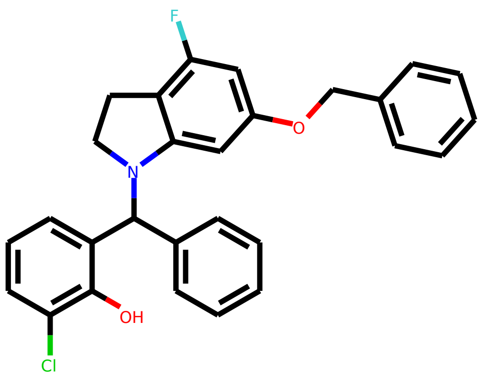

Osma-2291

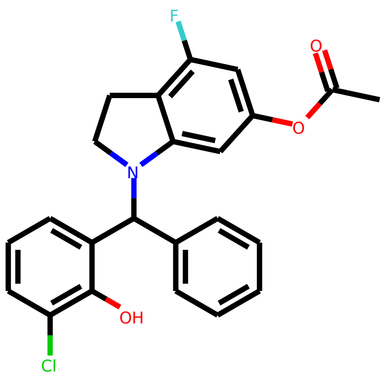

Osma-2292

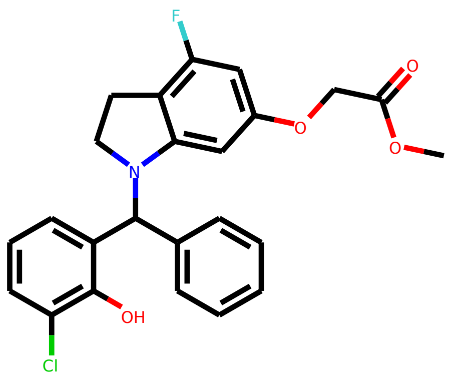

Osma-2293

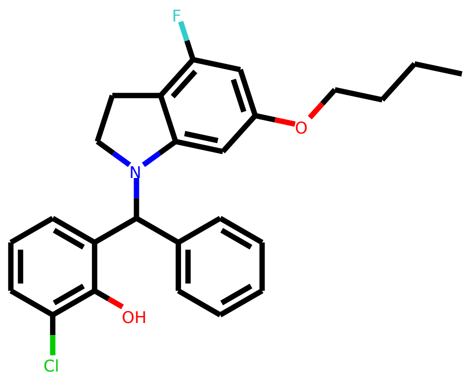

Osma-2294

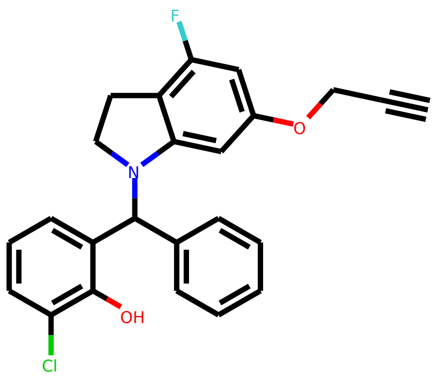

Osma-2295

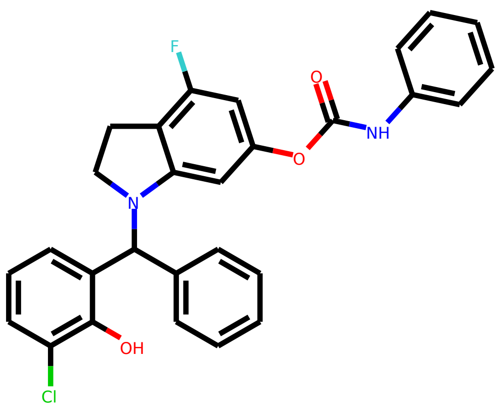

Osma-2296

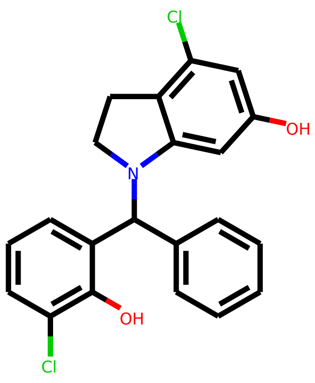

Osma-2297

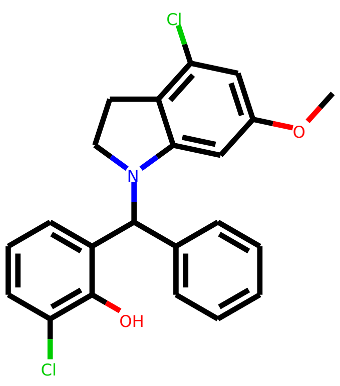

Osma-2298

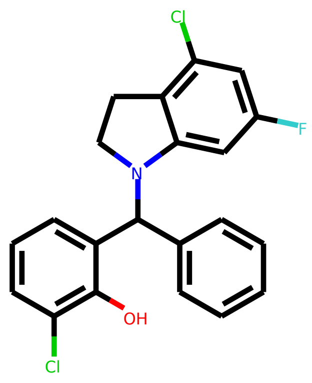

Osma-2299

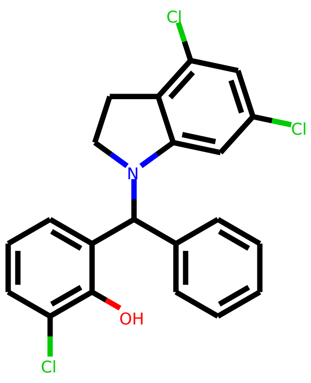

Osma-2300

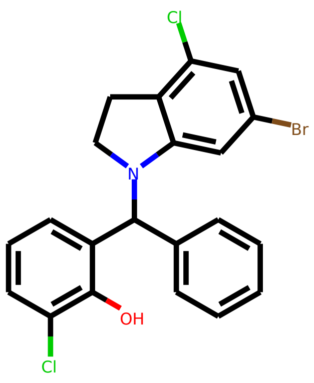

Osma-2301

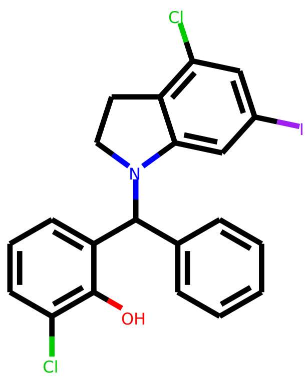

Osma-2302

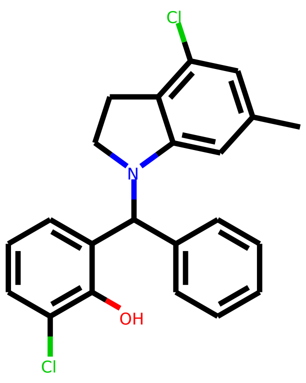

Osma-2303

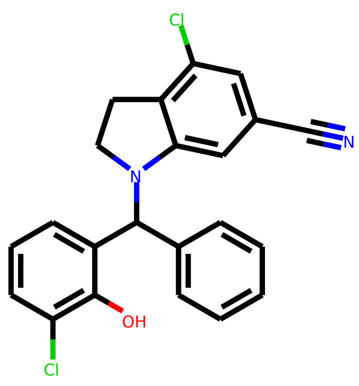

Osma-2304

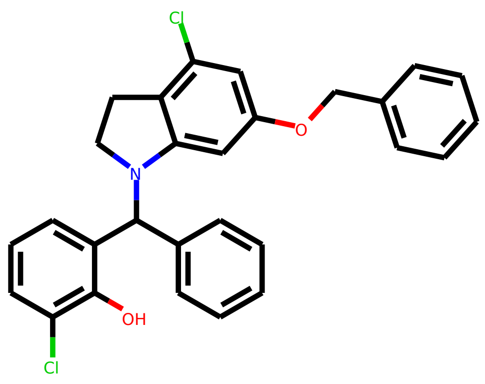

Osma-2305

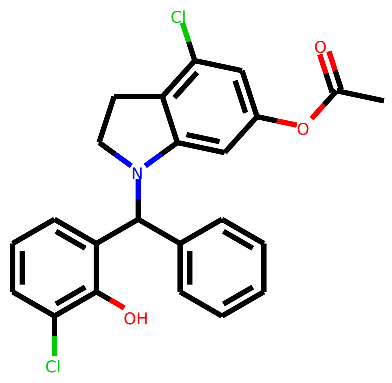

Osma-2306

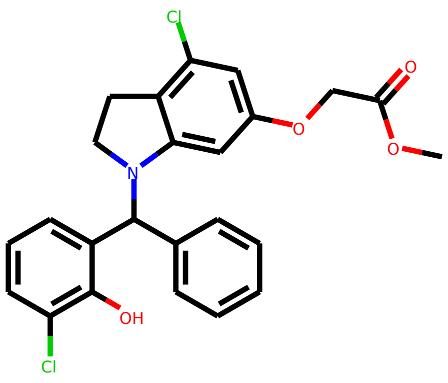

Osma-2307

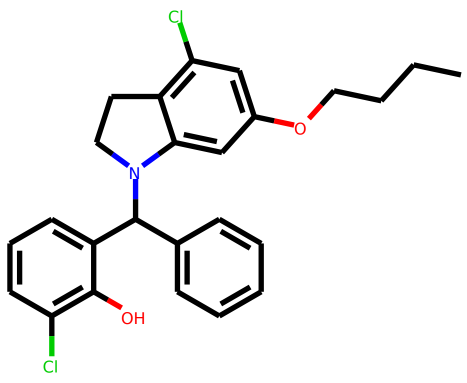

Osma-2308

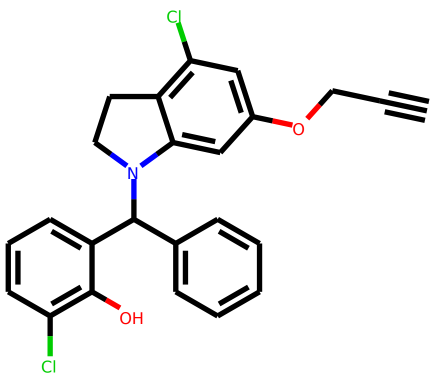

Osma-2309

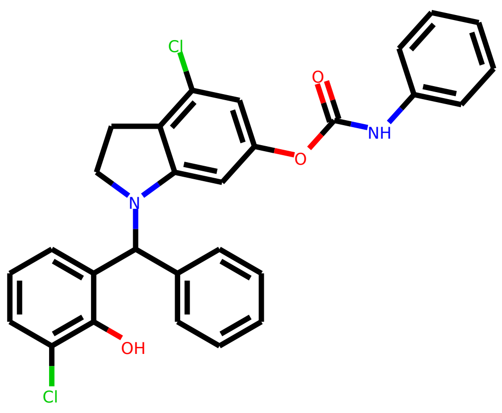

Osma-2310

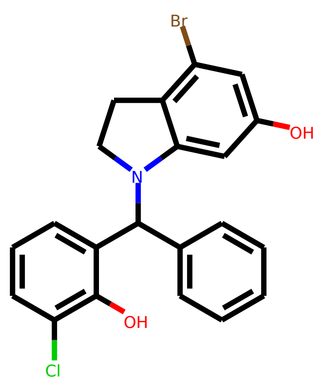
Osma-2311

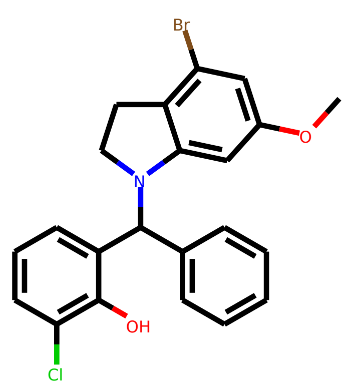
Osma-2312

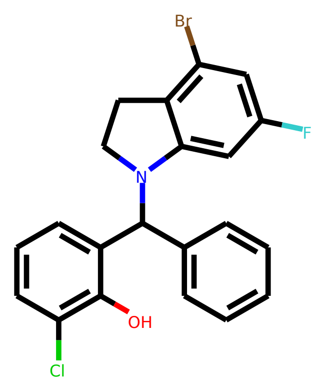
Osma-2313

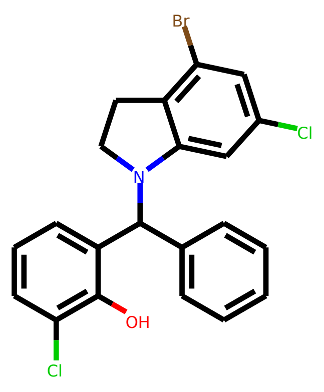
Osma-2314

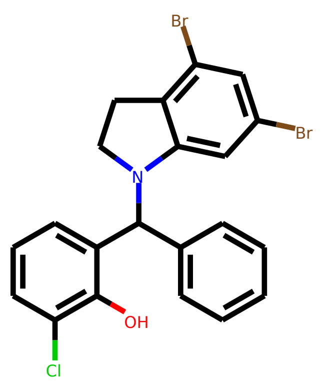
Osma-2315

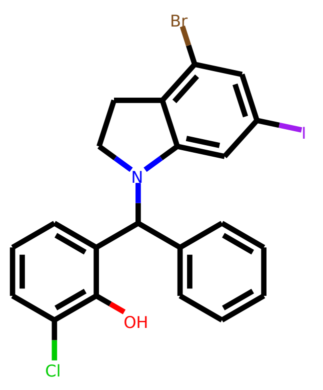
Osma-2316

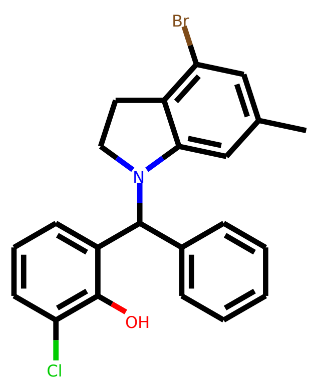
Osma-2317

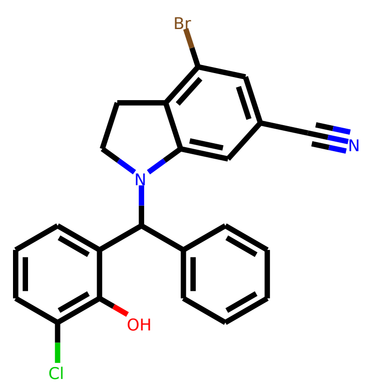
Osma-2318

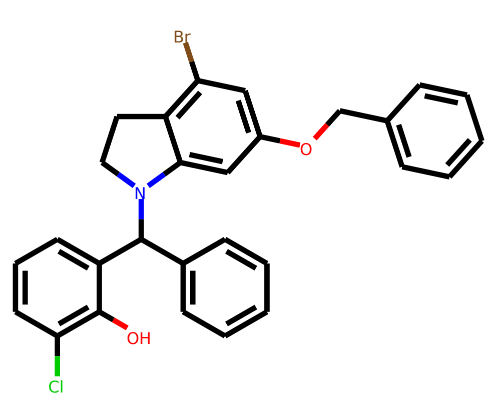
Osma-2319

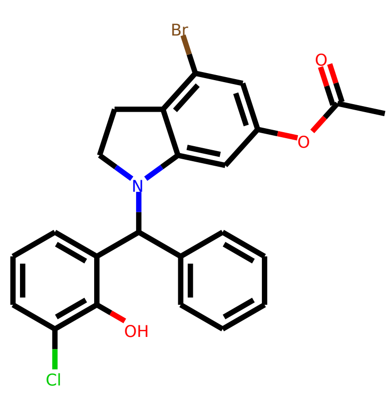
Osma-2320

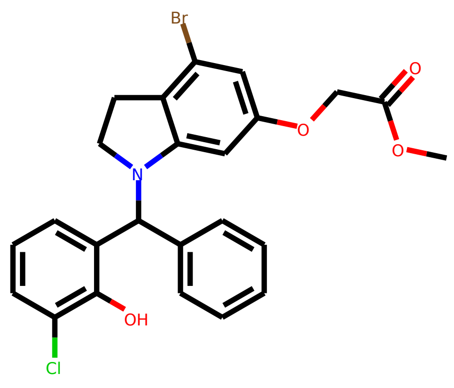
Osma-2321

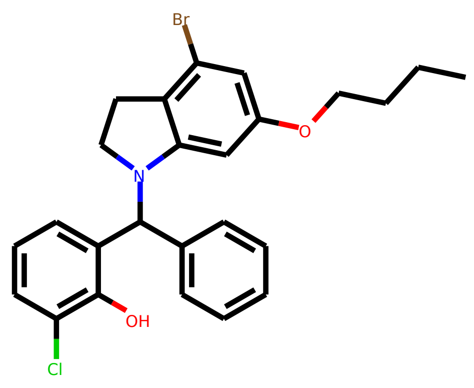
Osma-2322

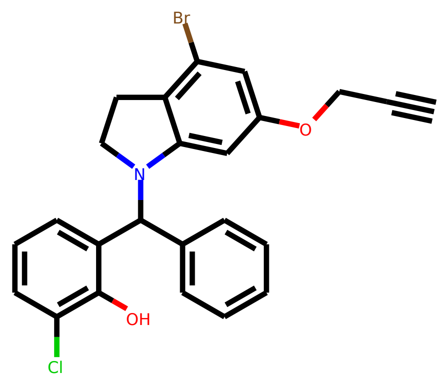
Osma-2323

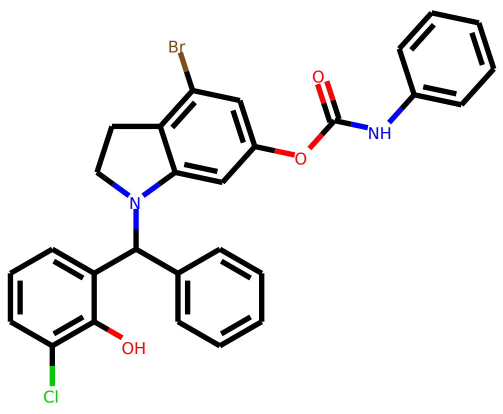
Osma-2324

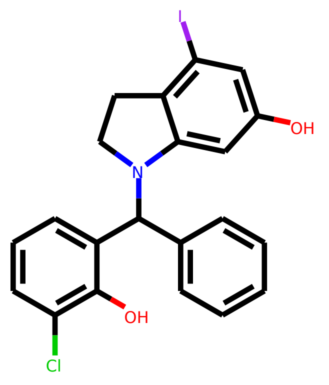
Osma-2325

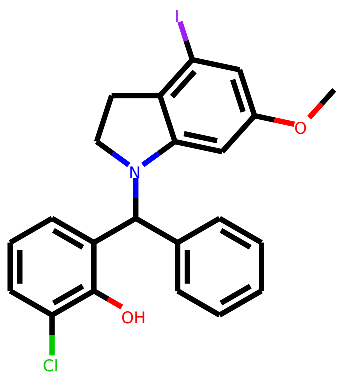
Osma-2326

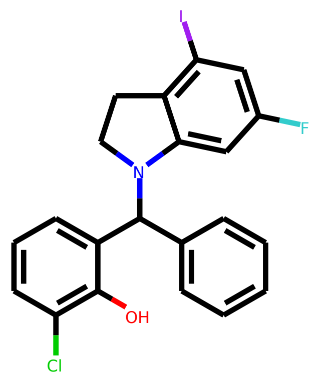
Osma-2327

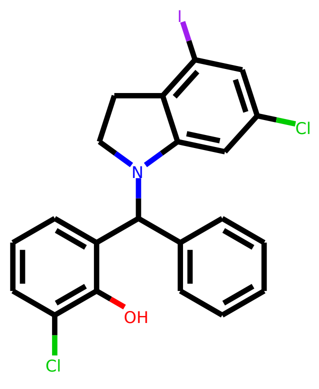
Osma-2328

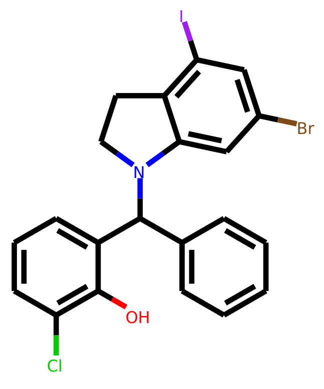
Osma-2329

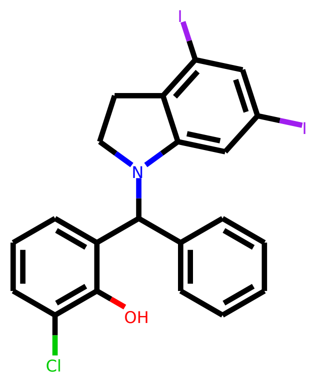
Osma-2330

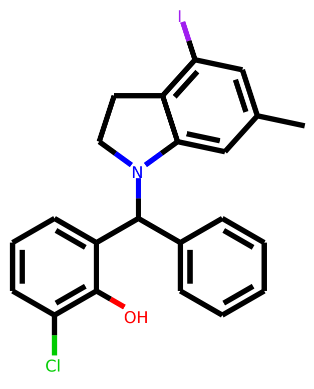
Osma-2331

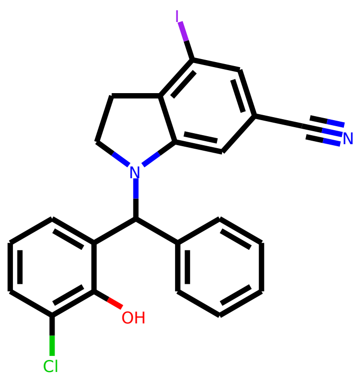
Osma-2332

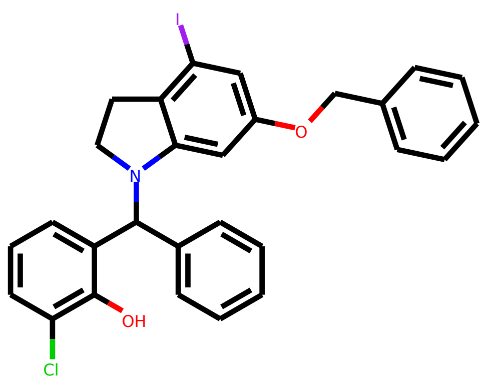
Osma-2333

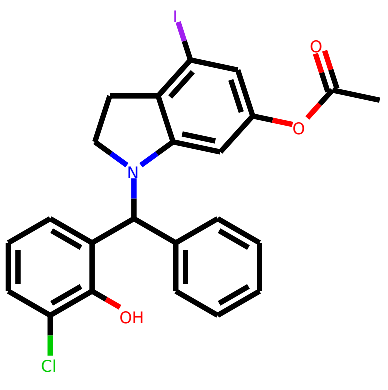
Osma-2334

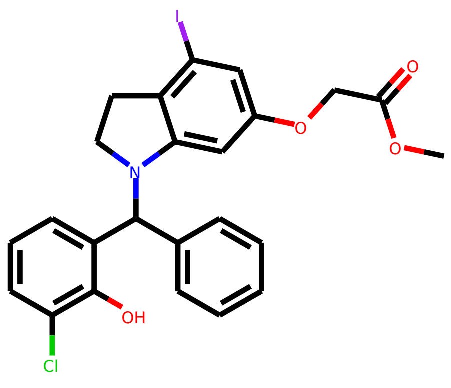
Osma-2335

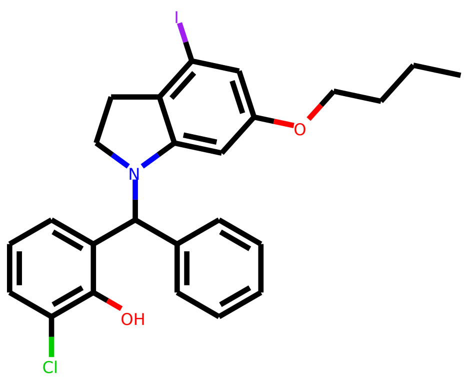
Osma-2336

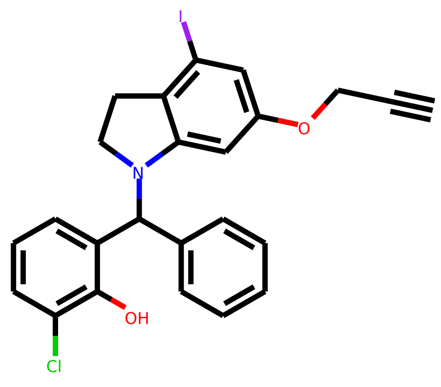
Osma-2337

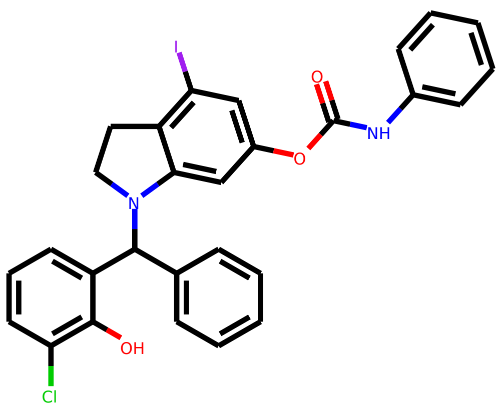
Osma-2338

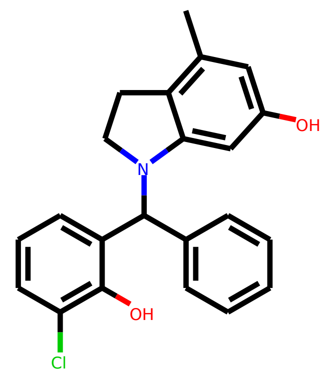
Osma-2339

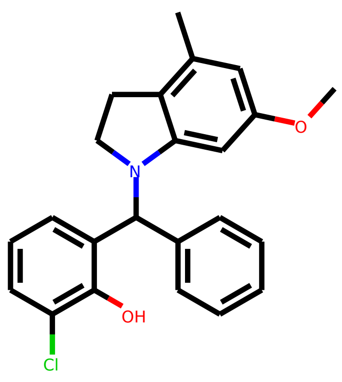
Osma-2340

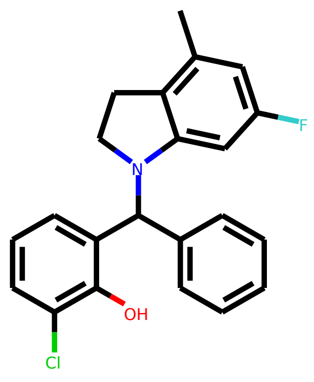
Osma-2341

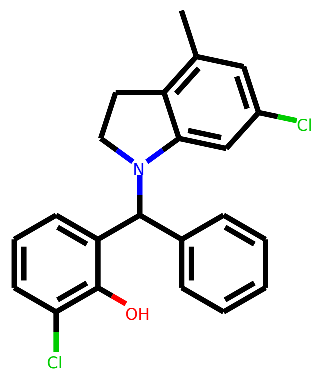
Osma-2342

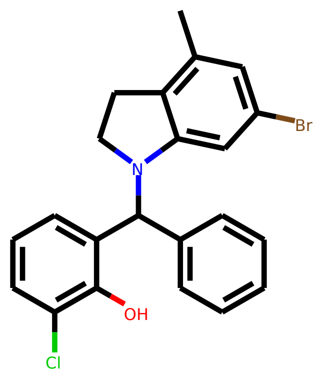
Osma-2343

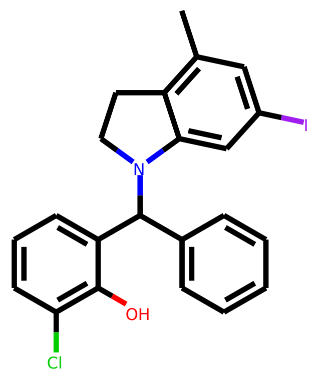
Osma-2344

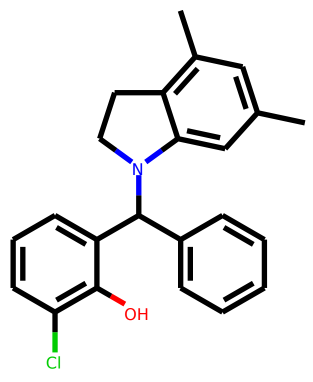
Osma-2345

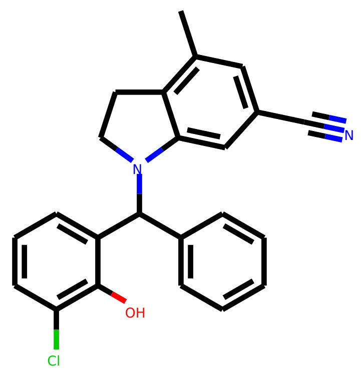
Osma-2346

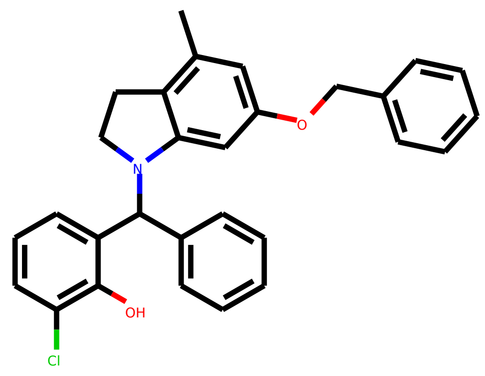
Osma-2347

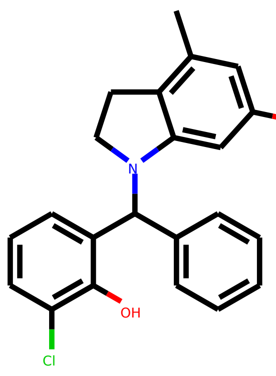
Osma-2348

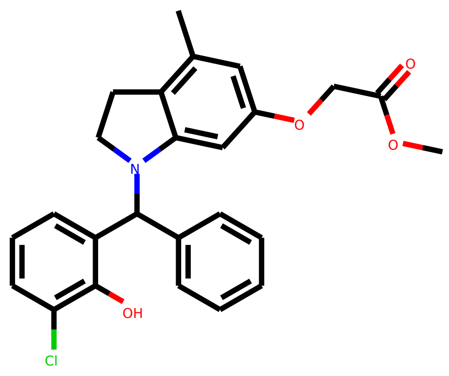
Osma-2349

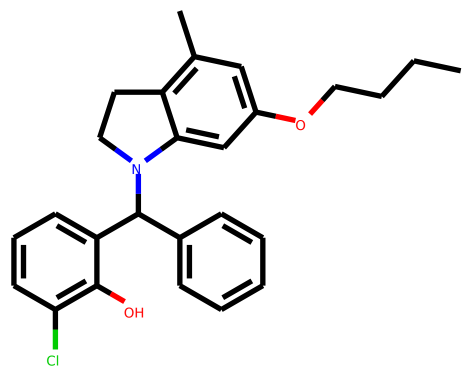
Osma-2350

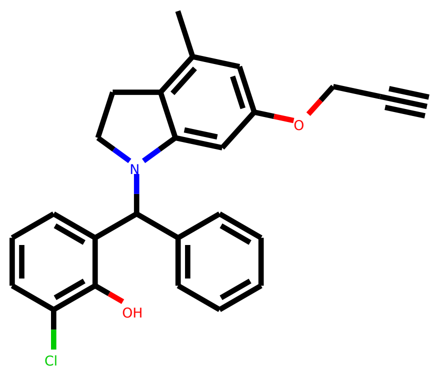
Osma-2351

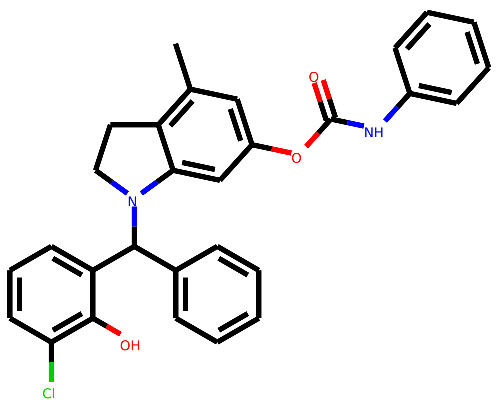
Osma-2352

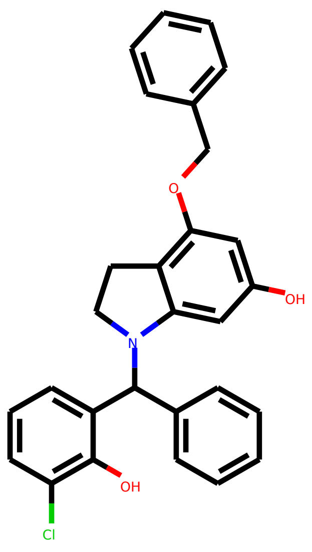
Osma-2353

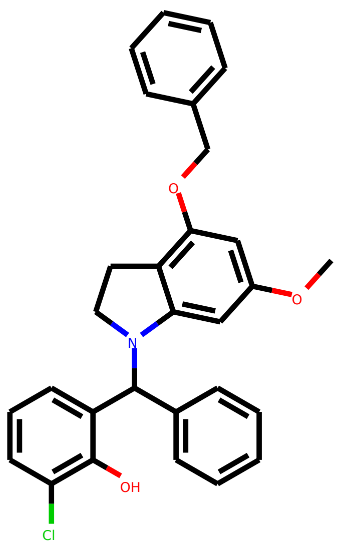
Osma-2354

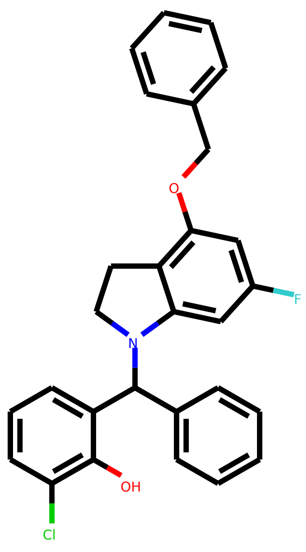
Osma-2355

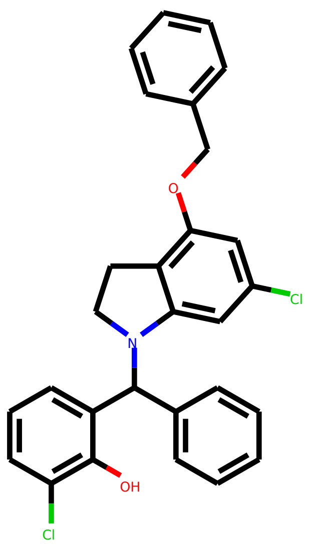
Osma-2356

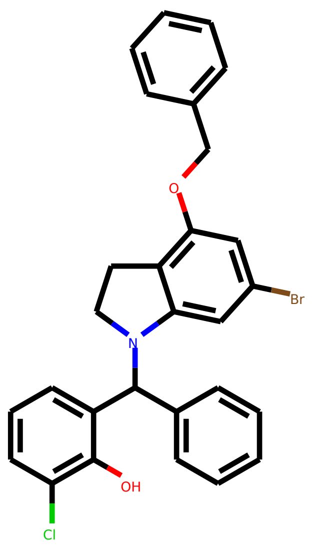
Osma-2357

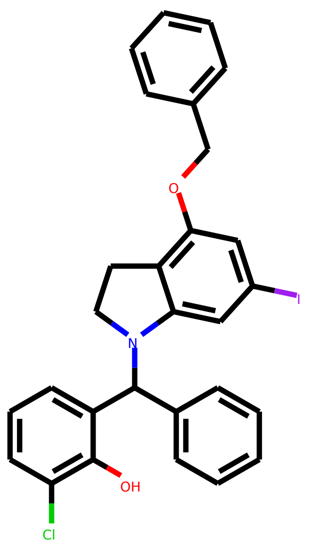
Osma-2358

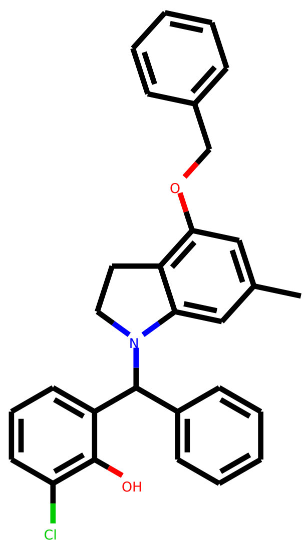
Osma-2359

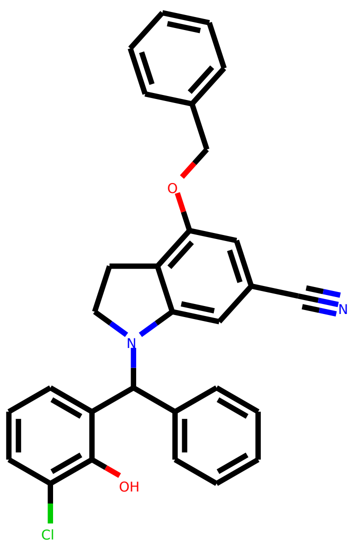
Osma-2360

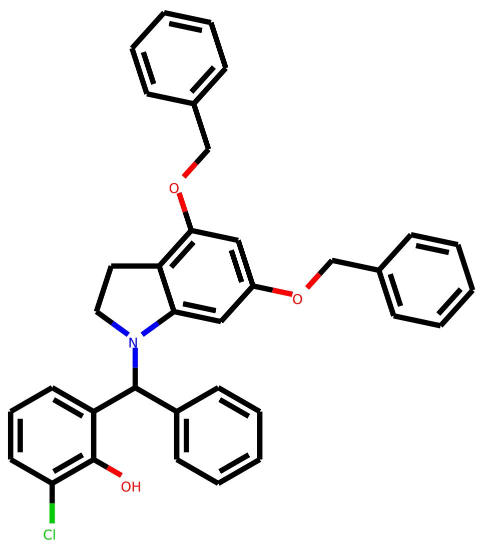
Osma-2361

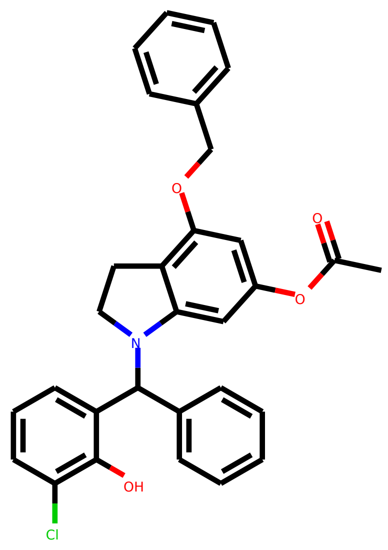
Osma-2362

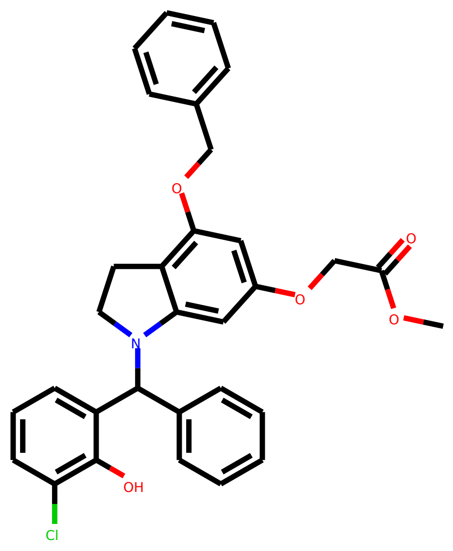
Osma-2363

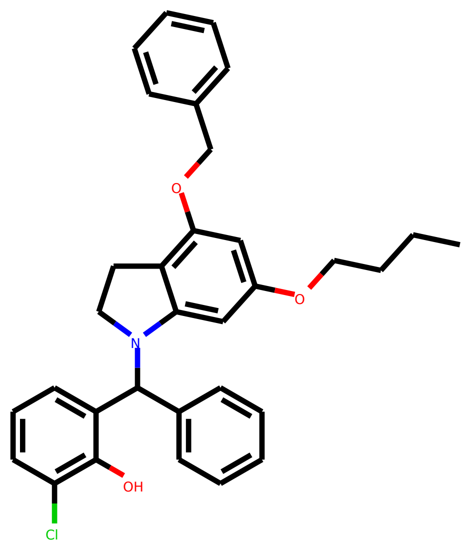
Osma-2364

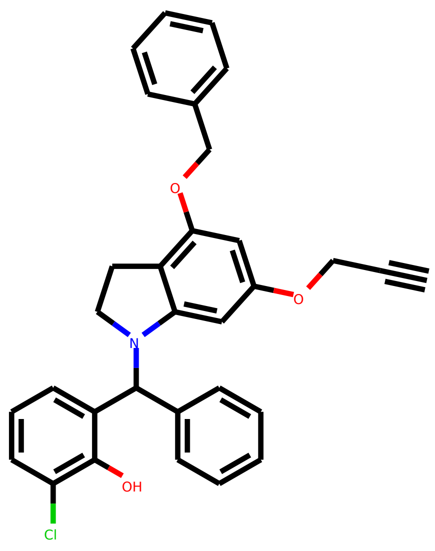
Osma-2365

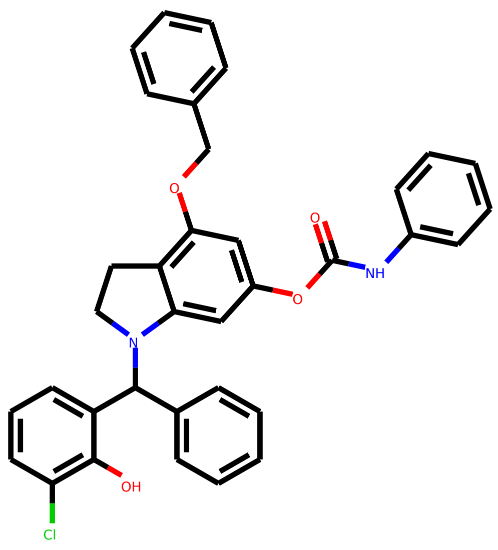
Osma-2366

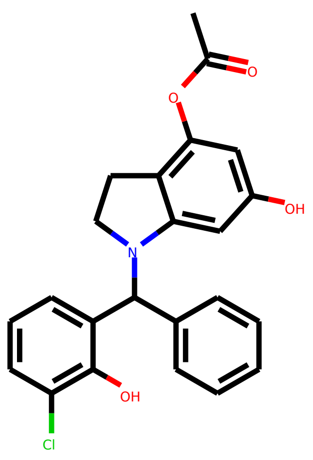
Osma-2367

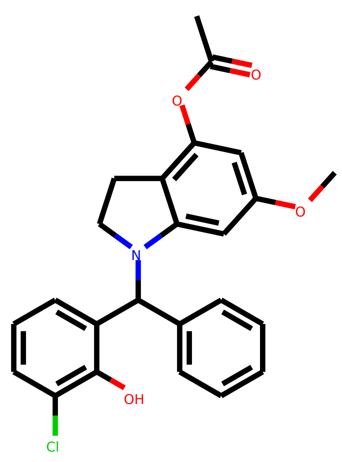
Osma-2368

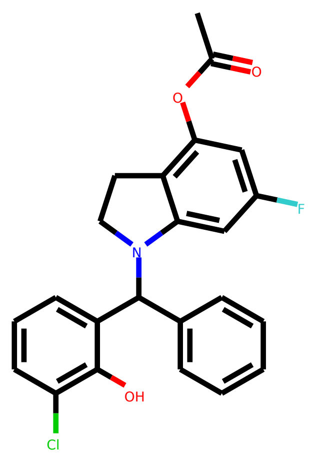
Osma-2369

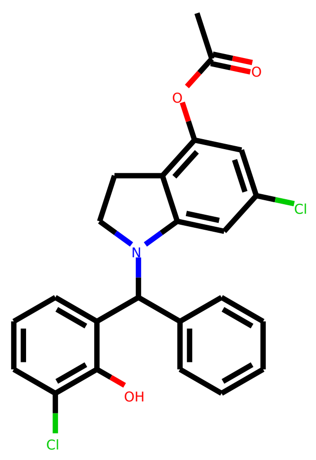
Osma-2370

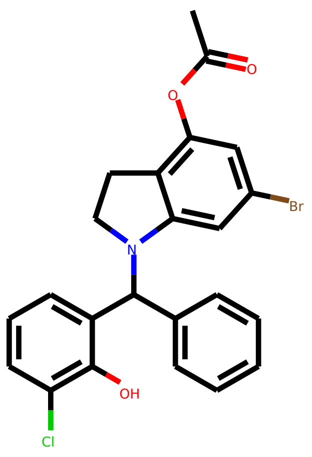
Osma-2371

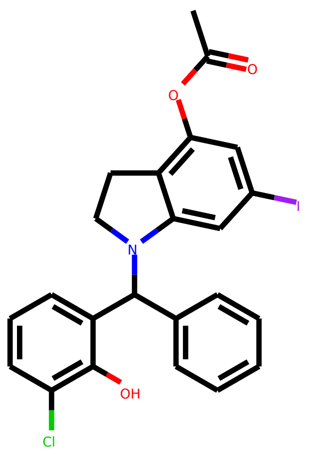
Osma-2372

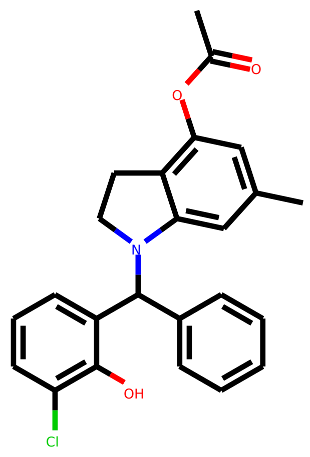
Osma-2373

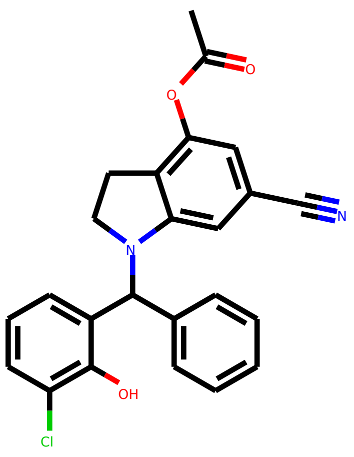
Osma-2374

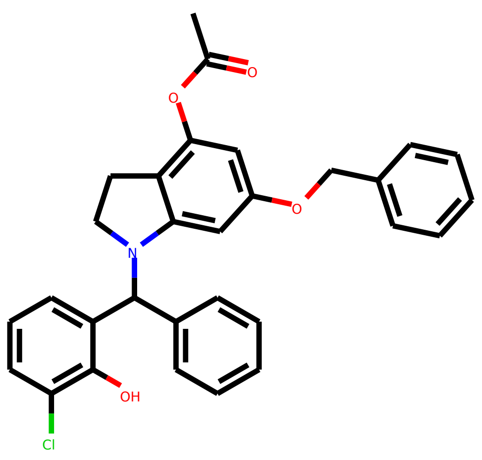
Osma-2375

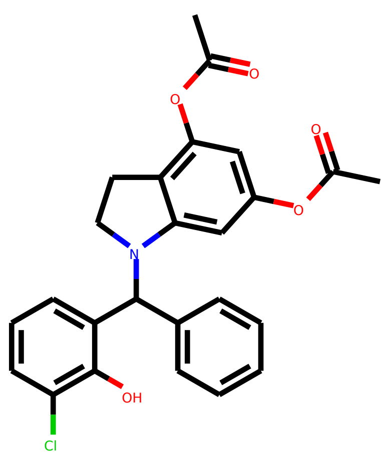
Osma-2376

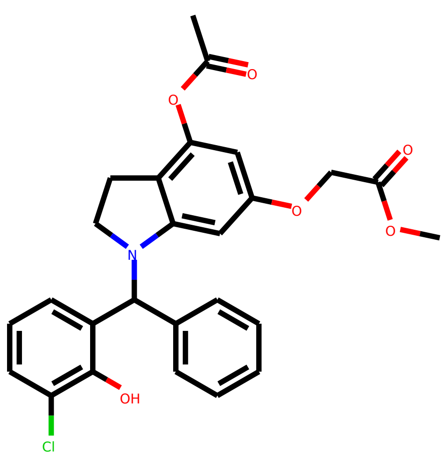
Osma-2377

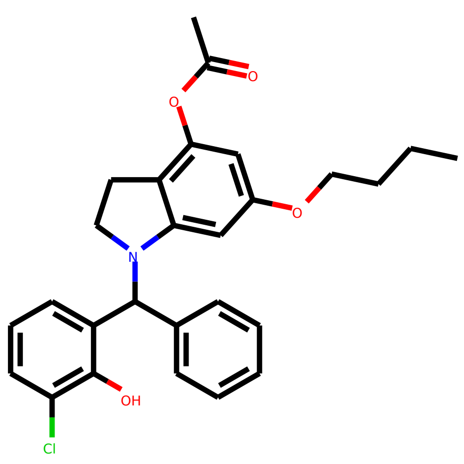
Osma-2378

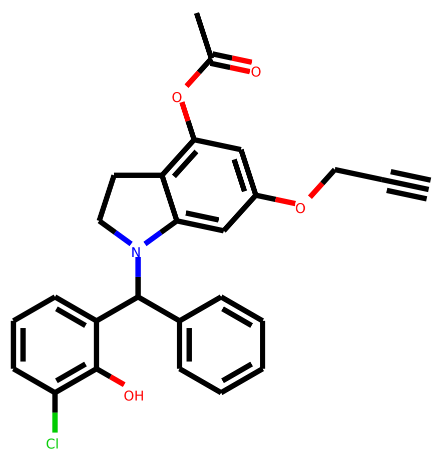
Osma-2379

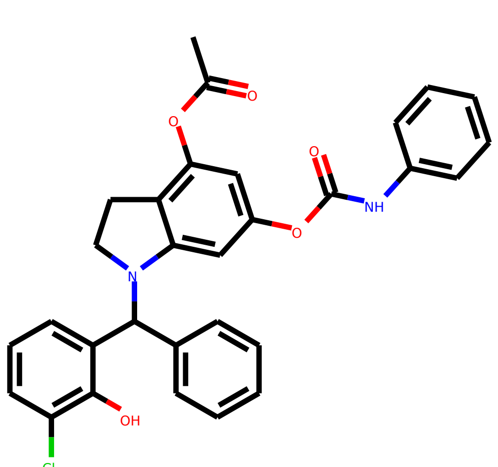
Osma-2380

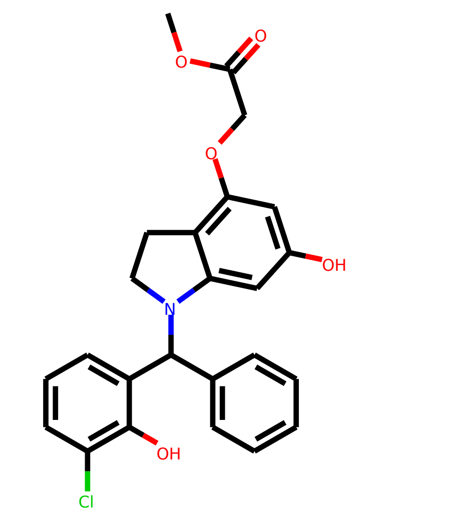

Osma-2381

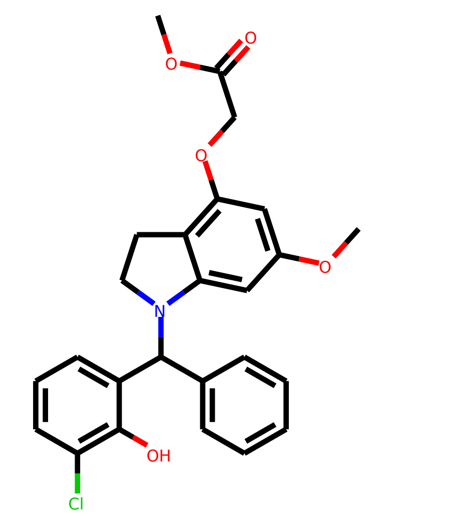

Osma-2382

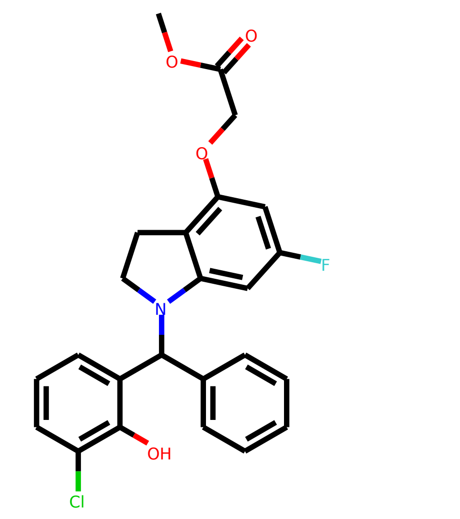

Osma-2383

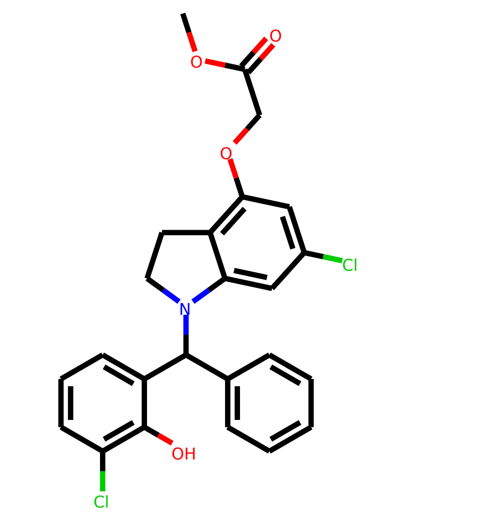

Osma-2384

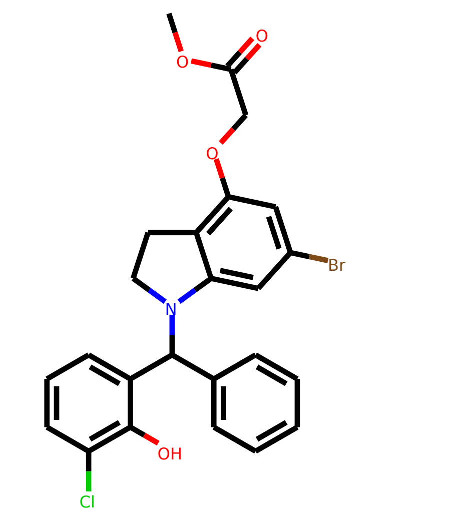

Osma-2385

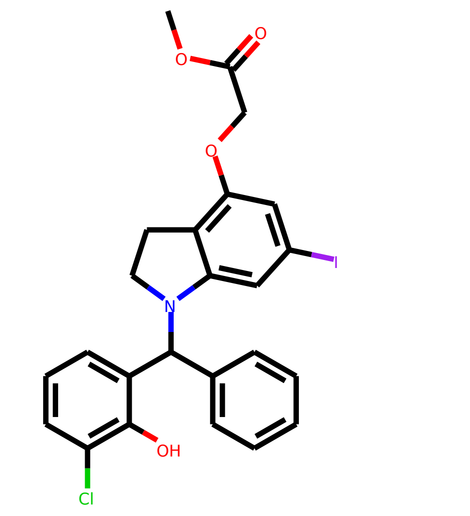

Osma-2386

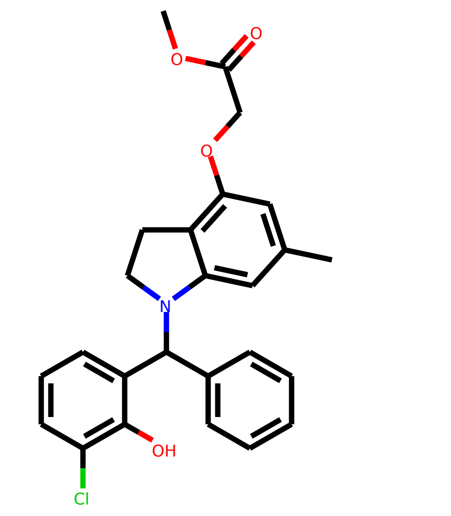

Osma-2387

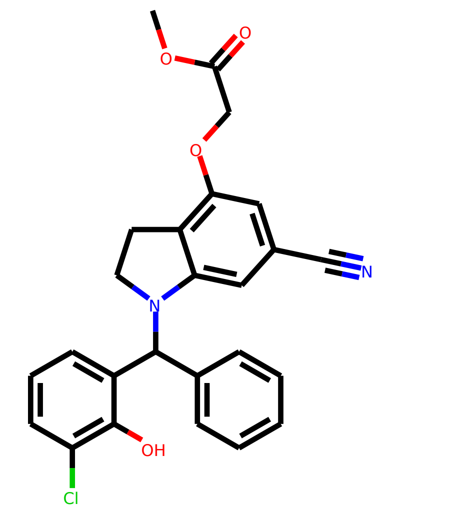

Osma-2388

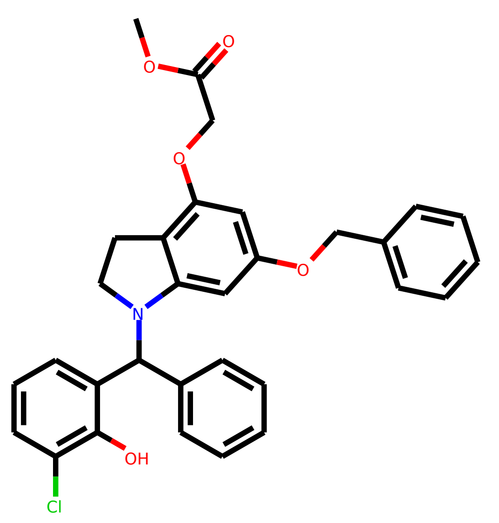

Osma-2389

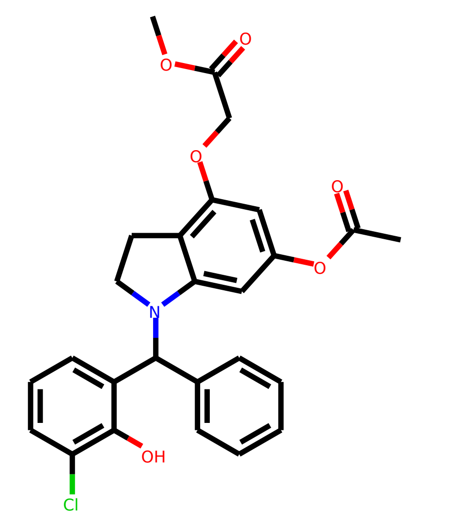

Osma-2390

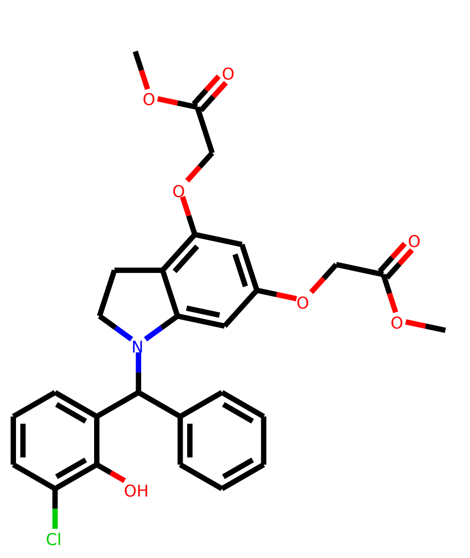

Osma-2391

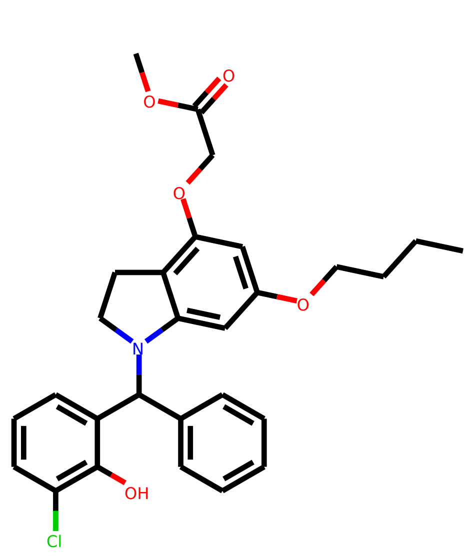

Osma-2392

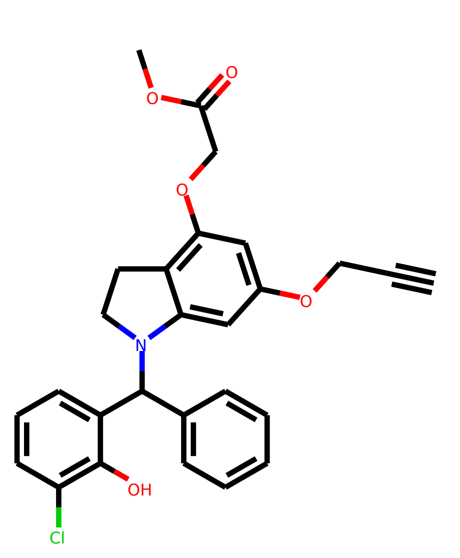

Osma-2393

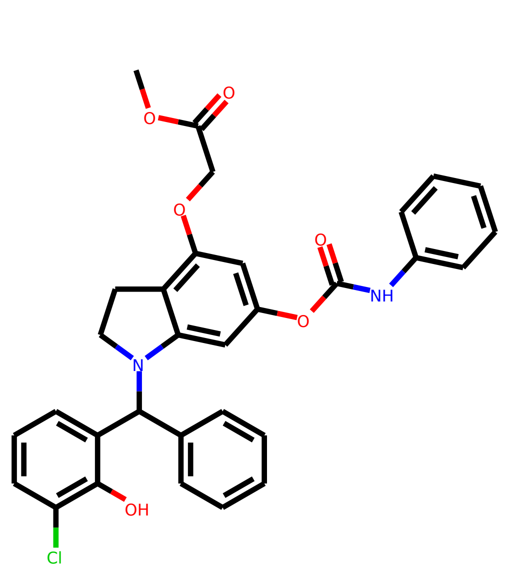

Osma-2394

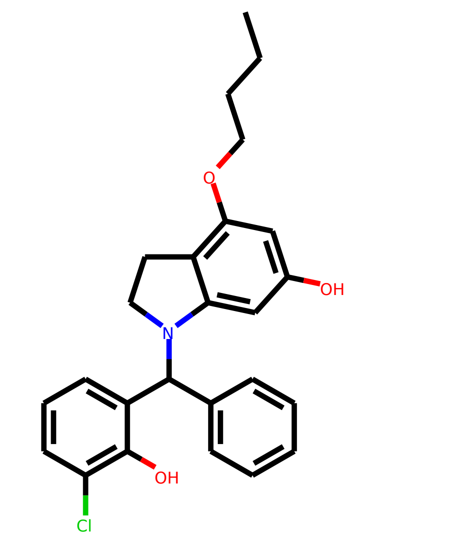

Osma-2395

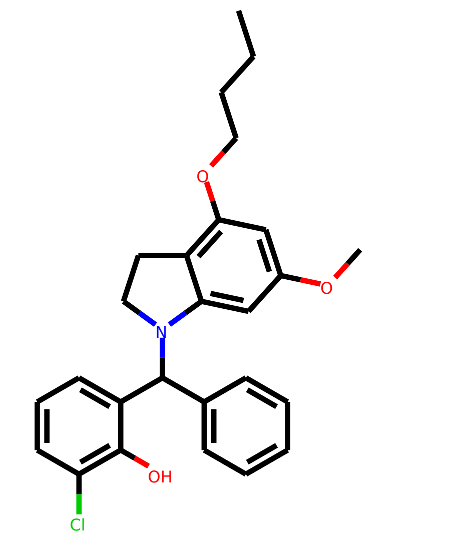

Osma-2396

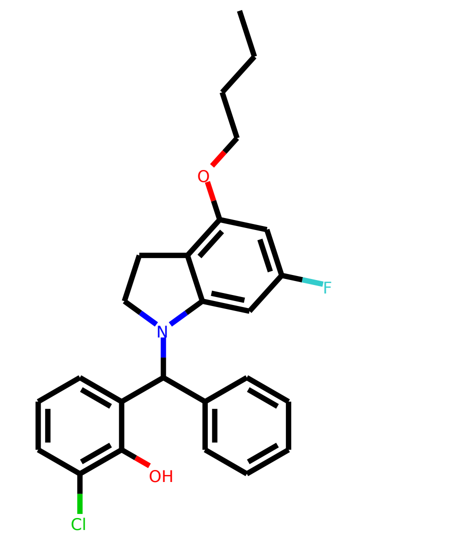

Osma-2397

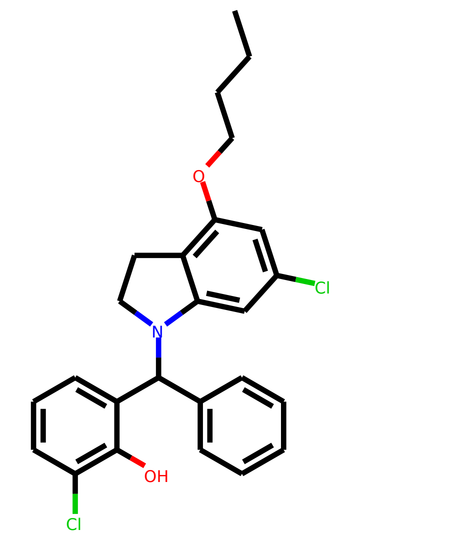

Osma-2398

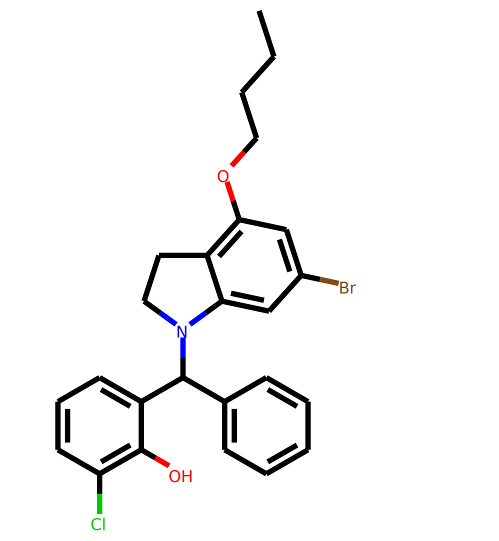

Osma-2399

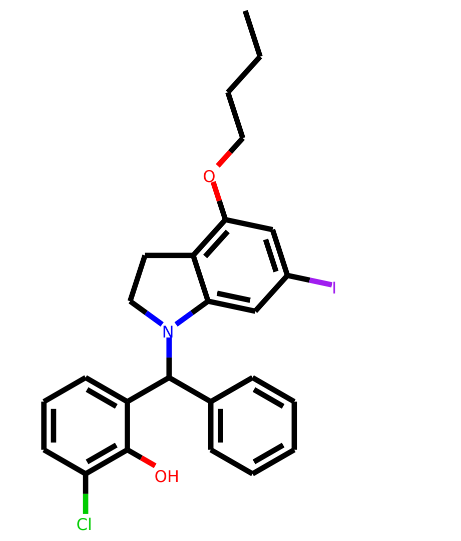

Osma-2400

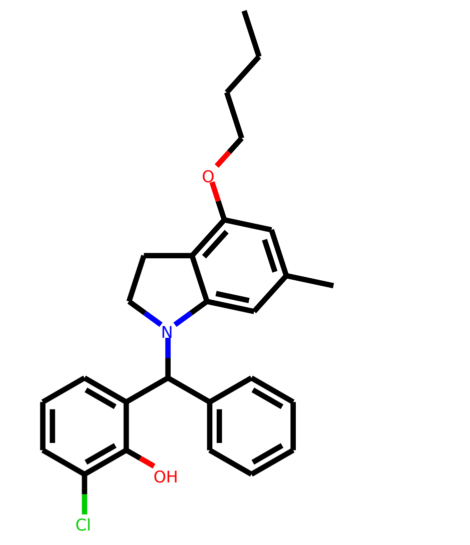

Osma-2401

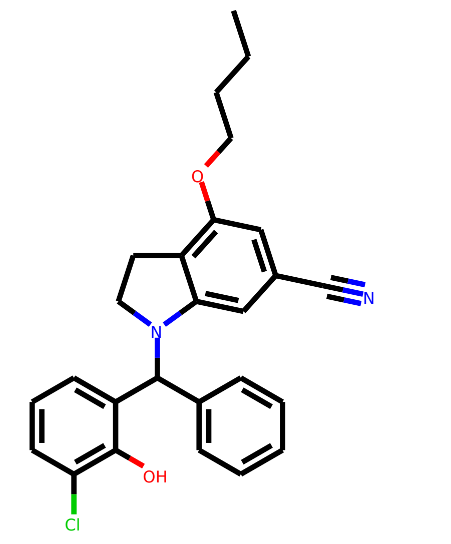

Osma-2402

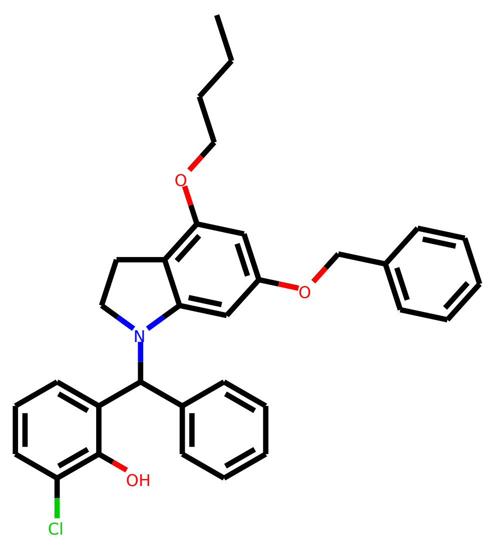

Osma-2403

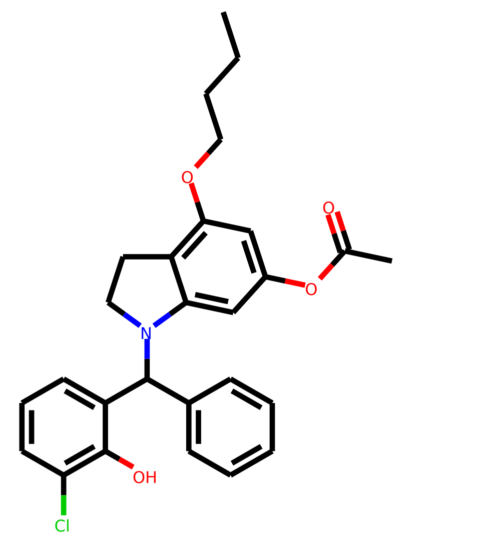

Osma-2404

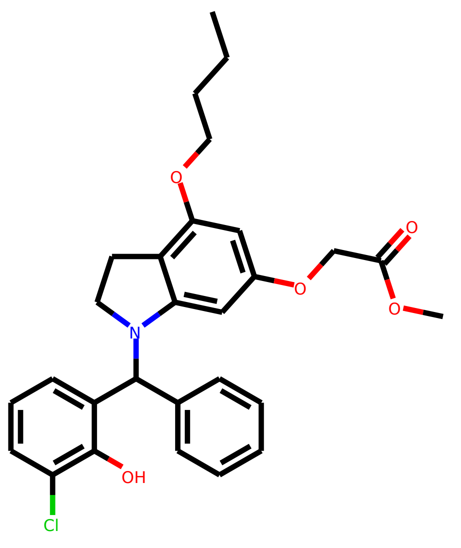

Osma-2405

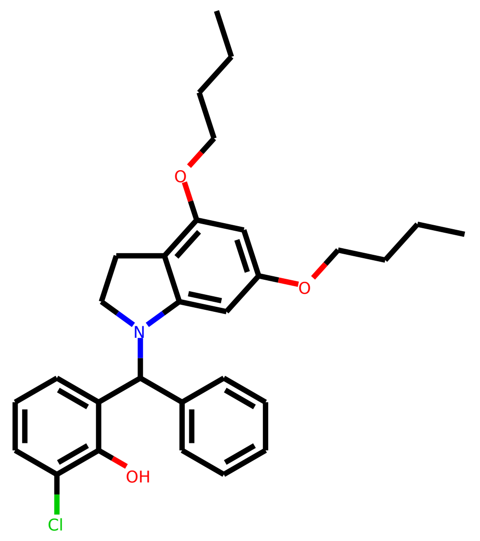

Osma-2406

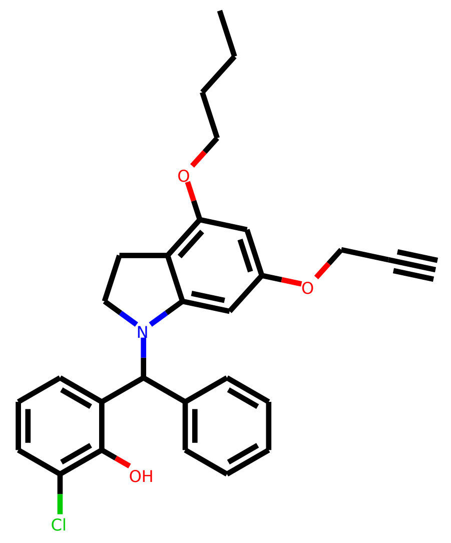

Osma-2407

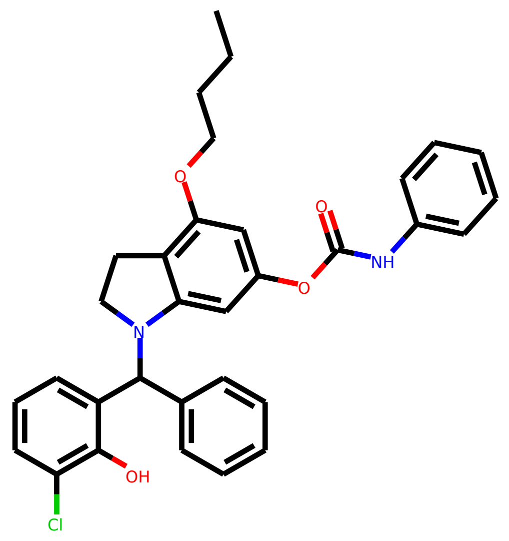

Osma-2408

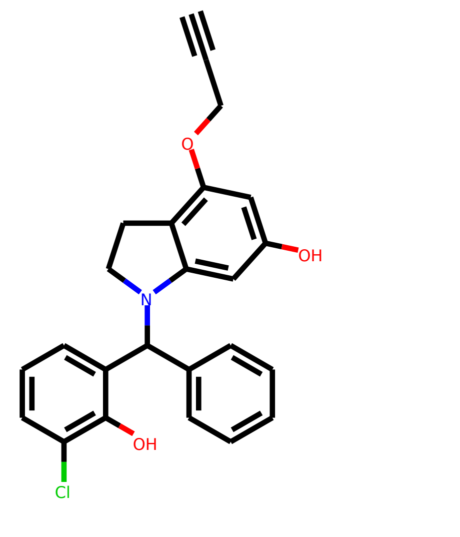

Osma-2409

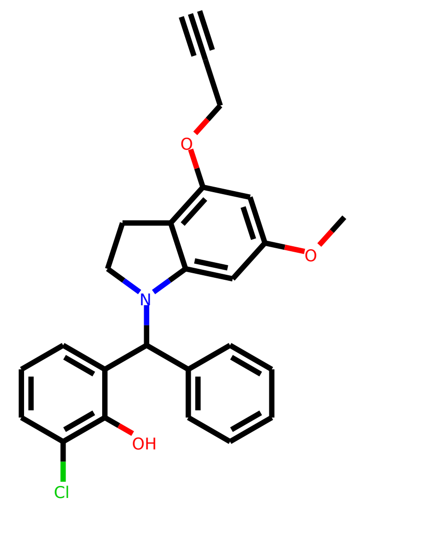

Osma-2410

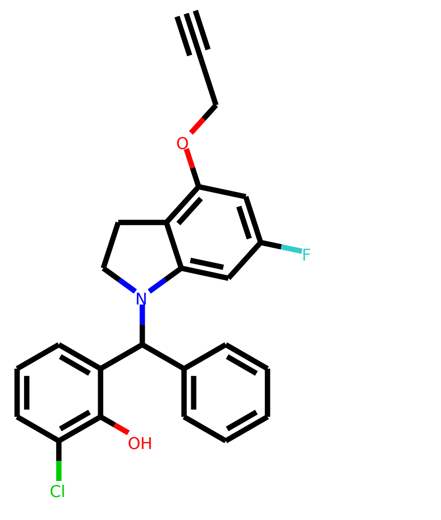

Osma-2411

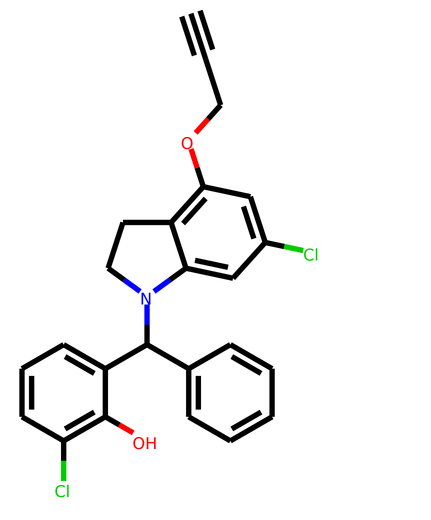

Osma-2412

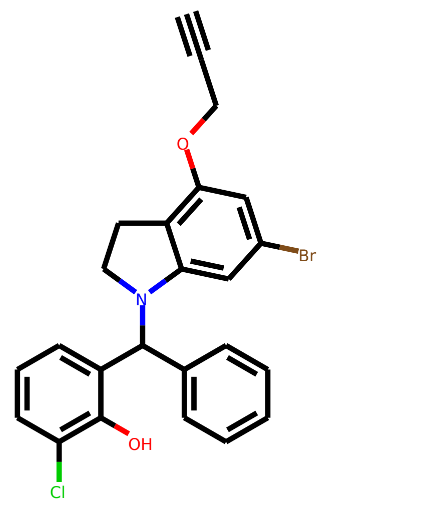

Osma-2413

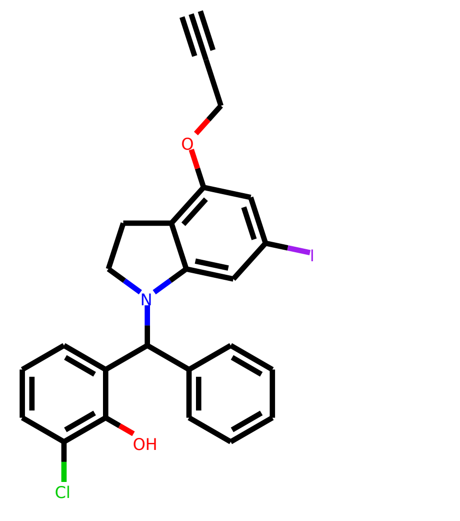

Osma-2414

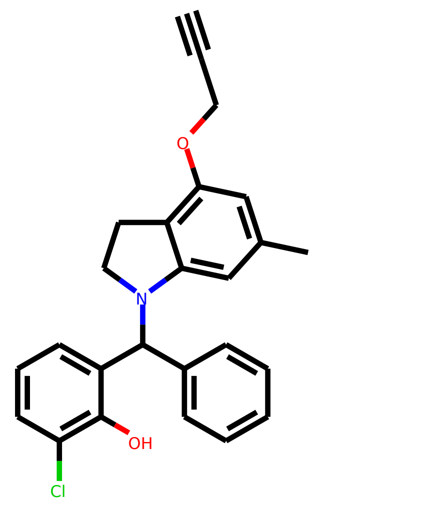

Osma-2415

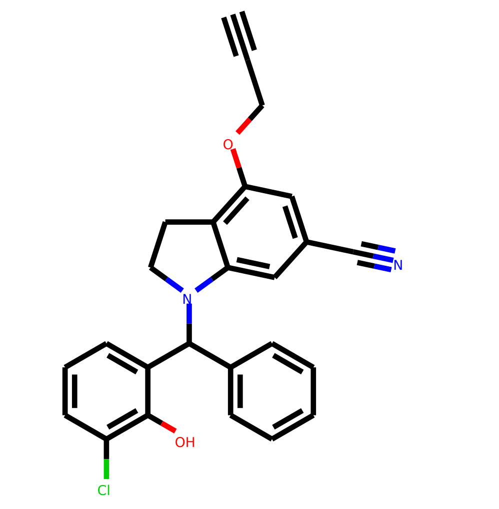
Osma-2416
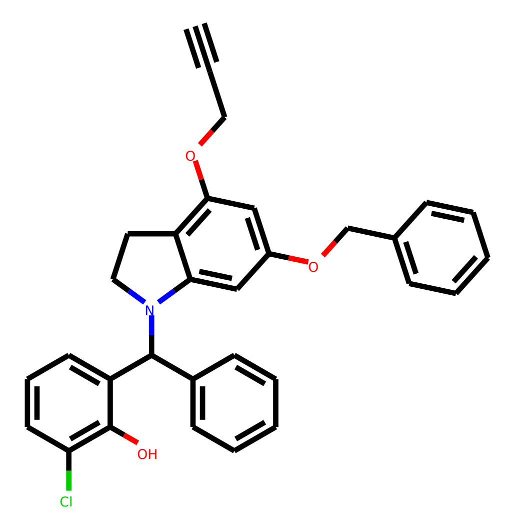
Osma-2417
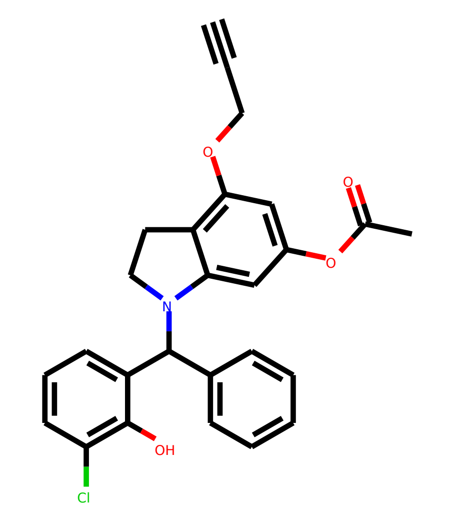
Osma-2418
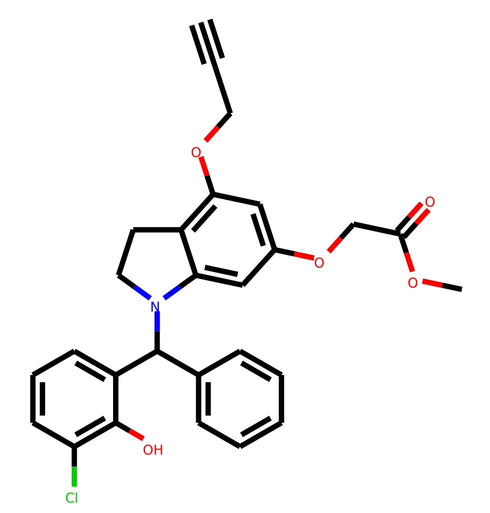
Osma-2419
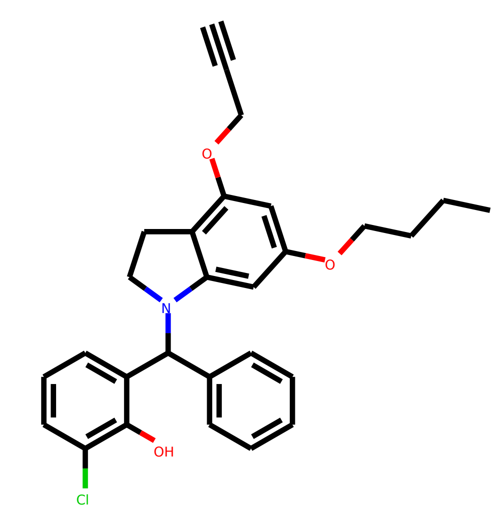
Osma-2420
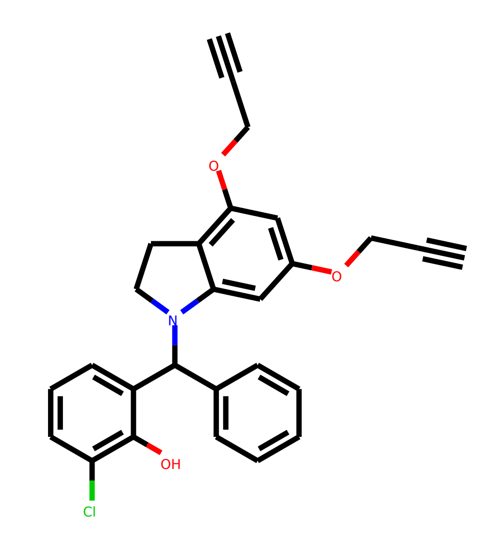
Osma-2421
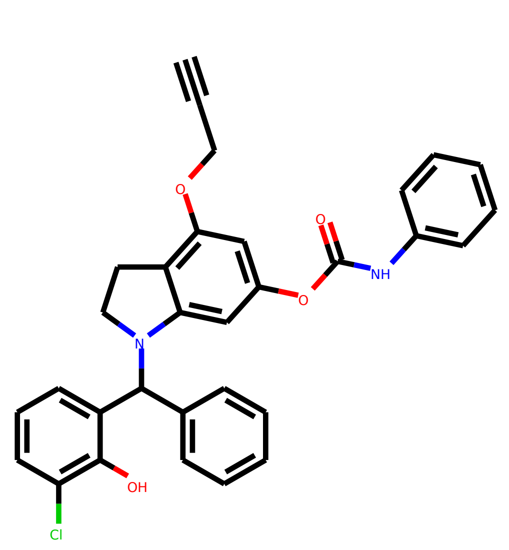
Osma-2422
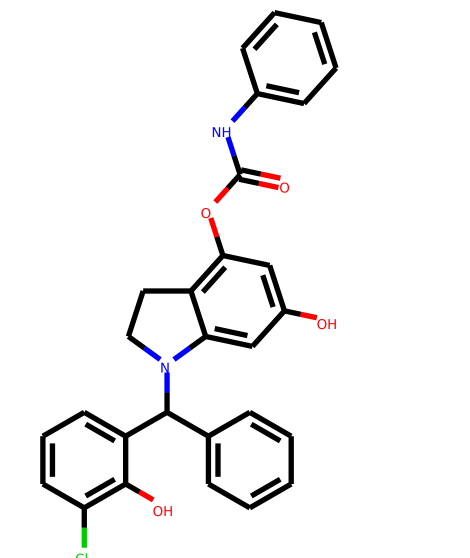
Osma-2423
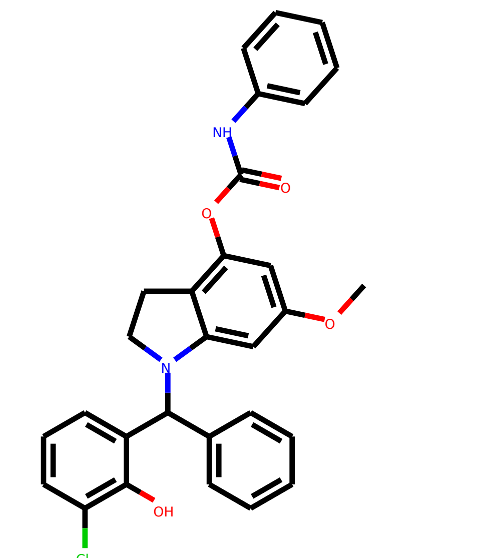
Osma-2424
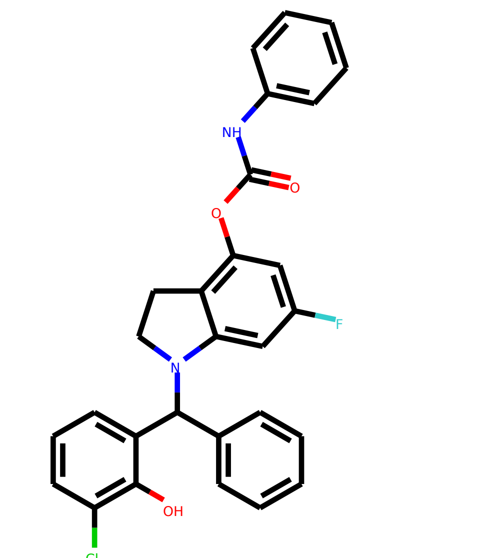
Osma-2425
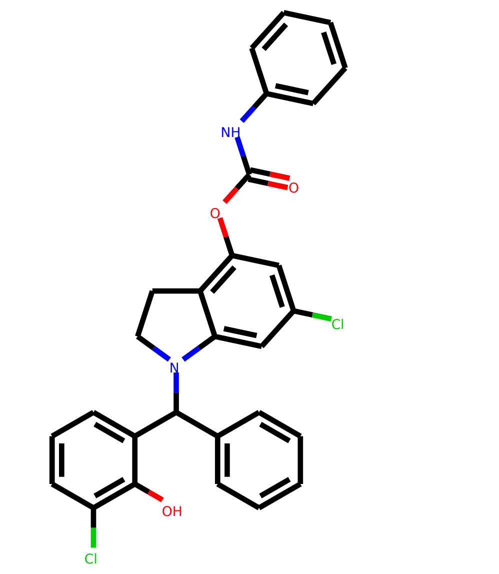
Osma-2426
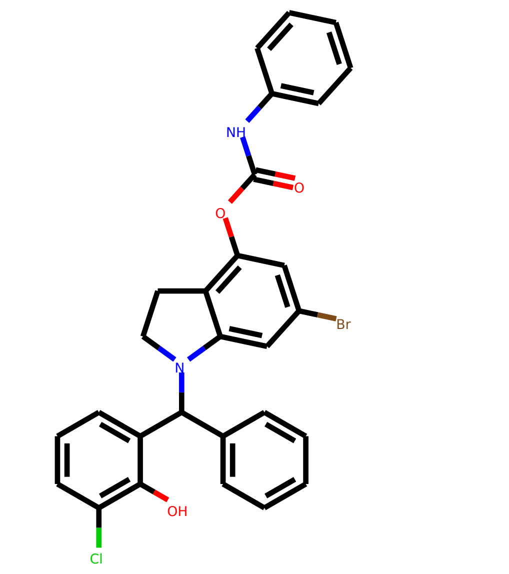
Osma-2427
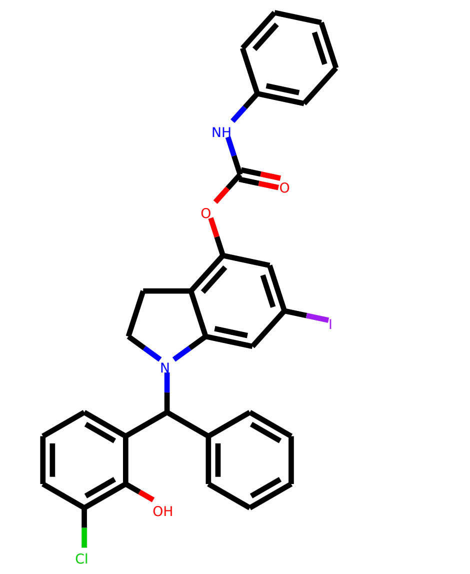
Osma-2428
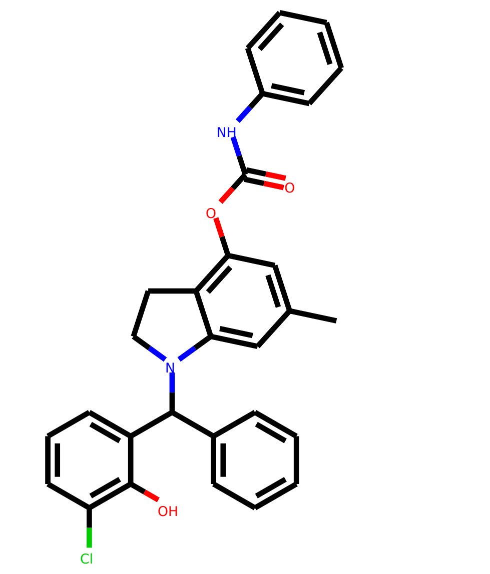
Osma-2429
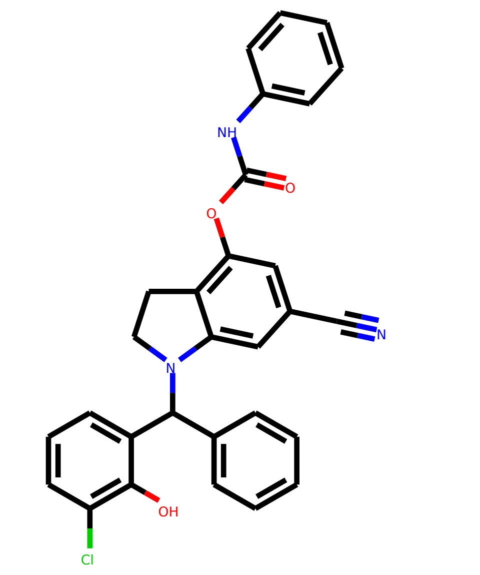
Osma-2430
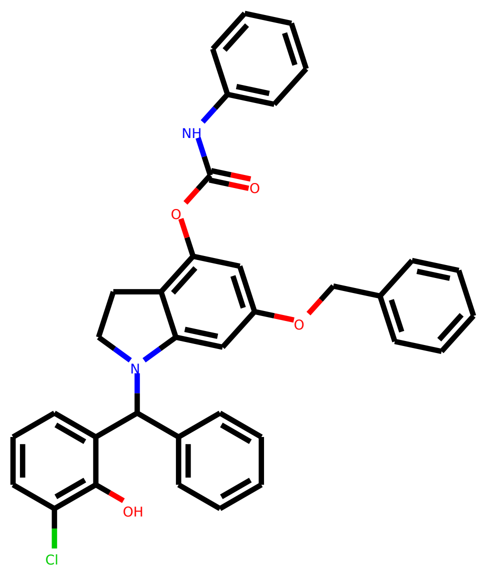
Osma-2431
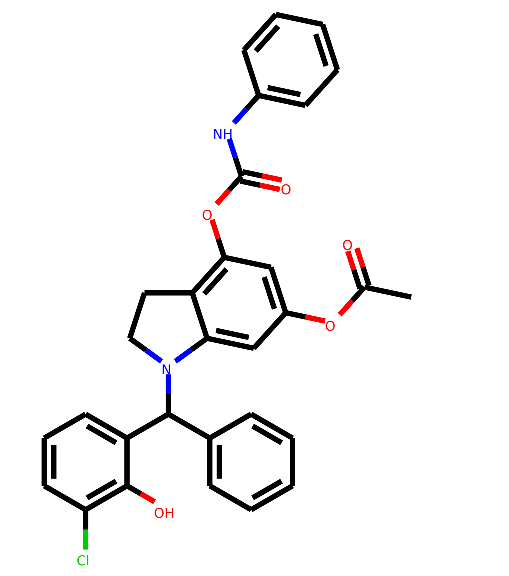
Osma-2432
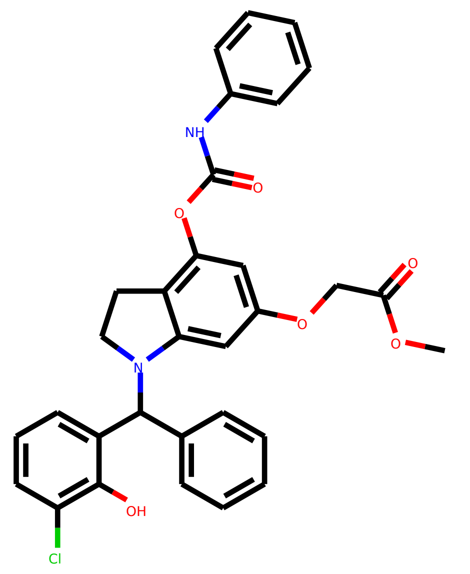
Osma-2433
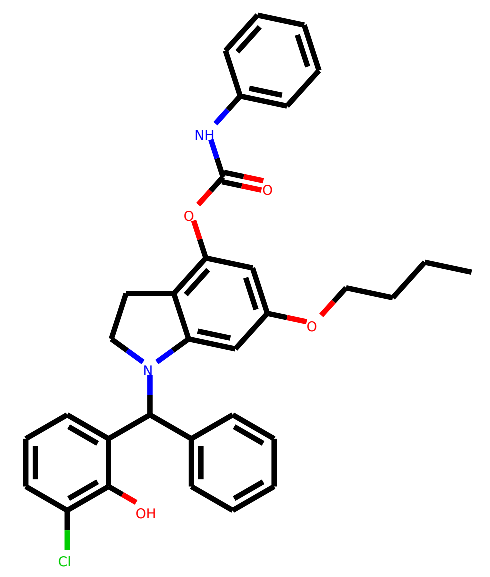
Osma-2434
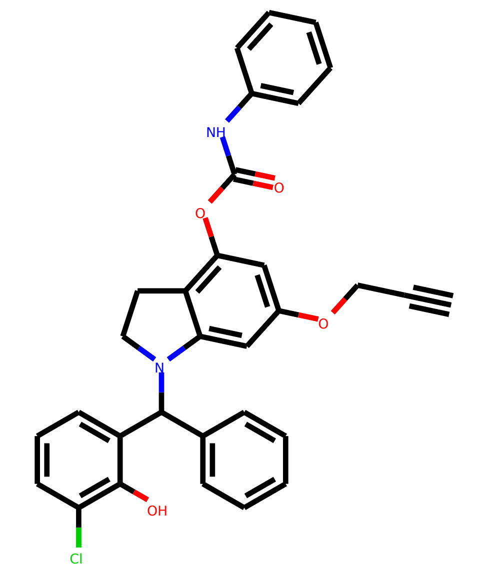
Osma-2435
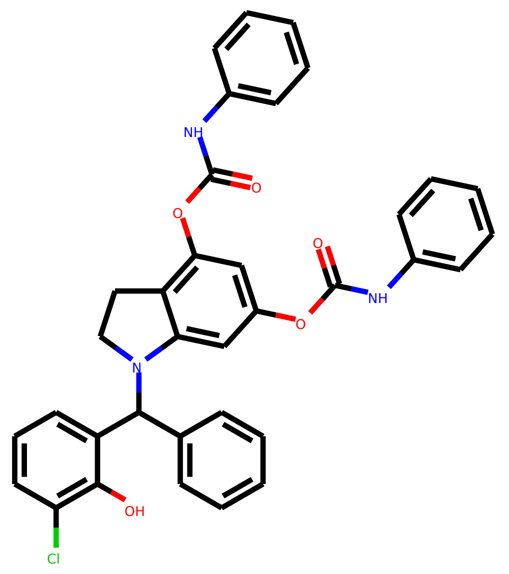
Osma-2436
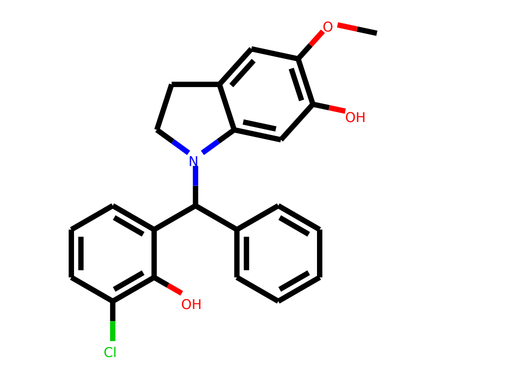
Osma-2437
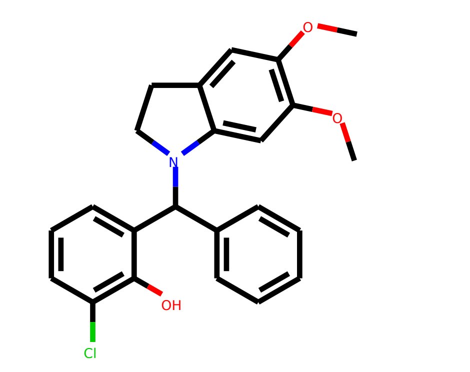
Osma-2438
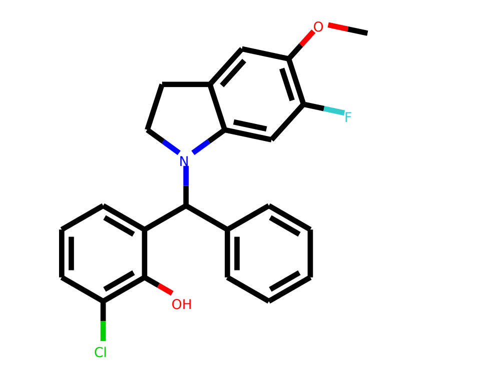
Osma-2439
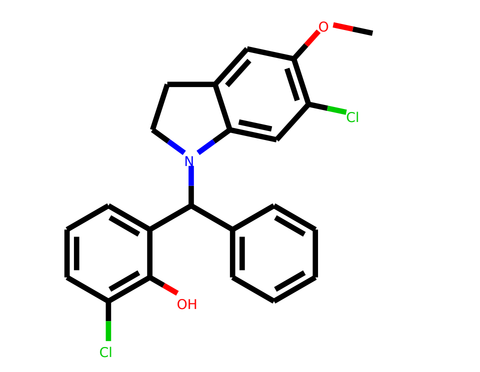
Osma-2440
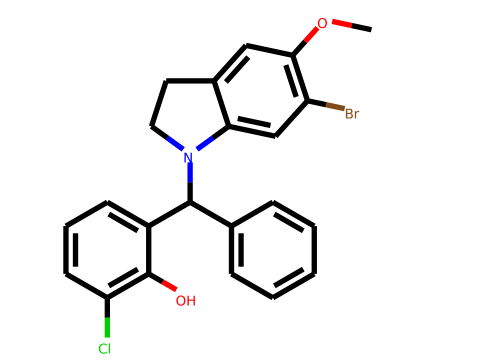
Osma-2441
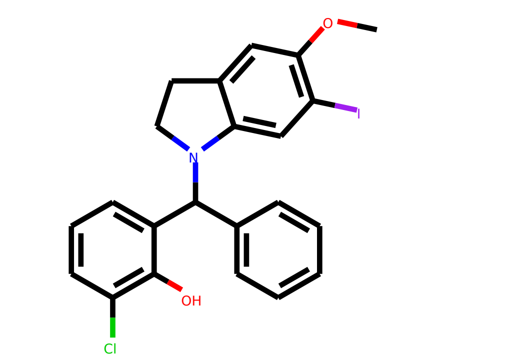
Osma-2442
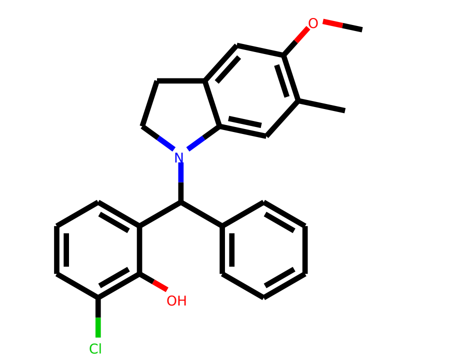
Osma-2443
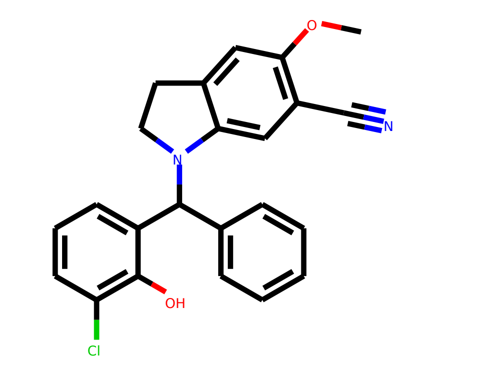
Osma-2444
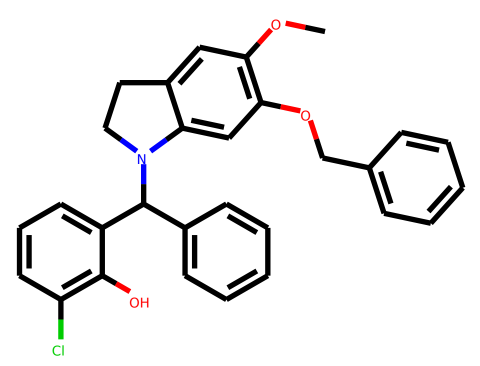
Osma-2445
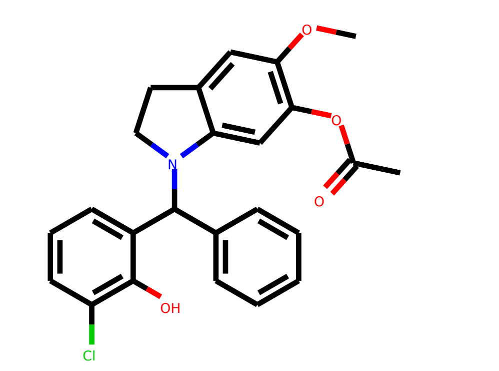
Osma-2446
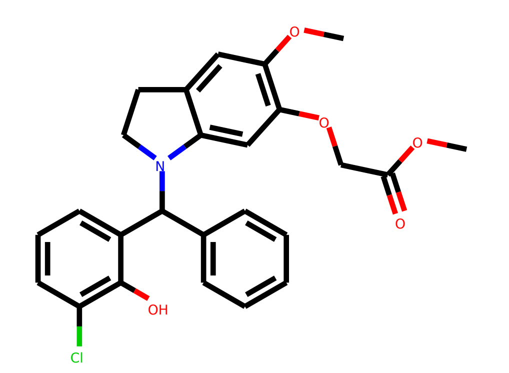
Osma-2447
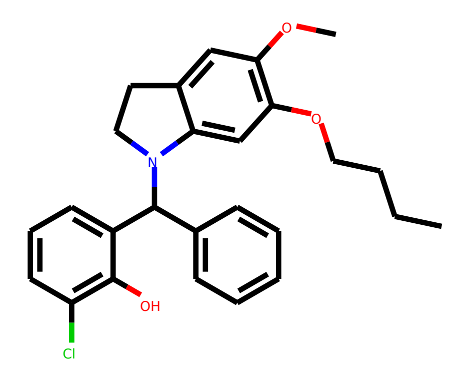
Osma-2448
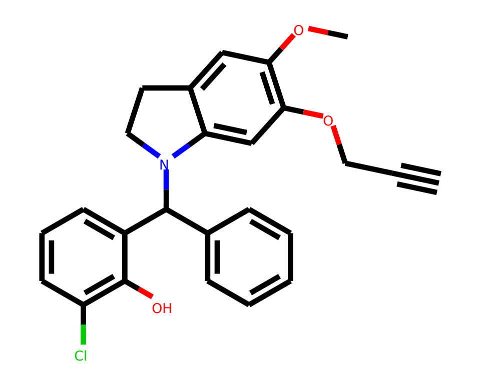
Osma-2449
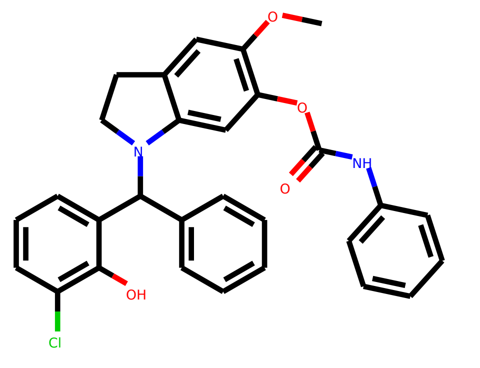
Osma-2450

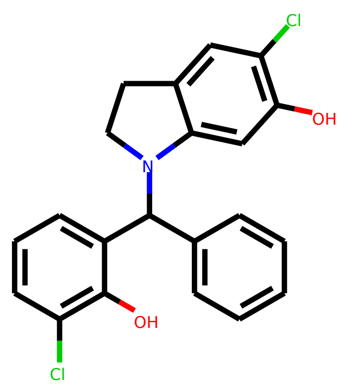
Osma-2451

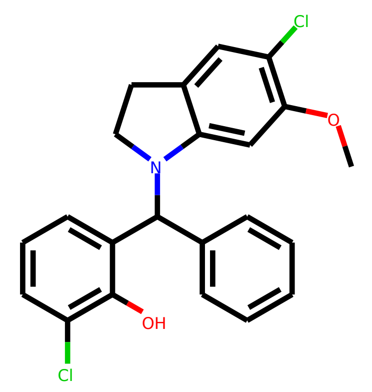
Osma-2452

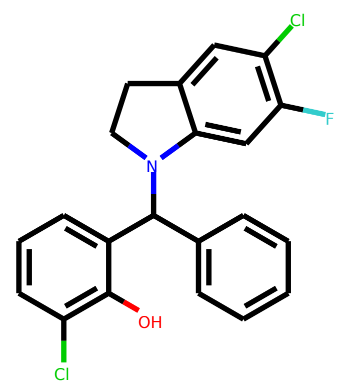
Osma-2453

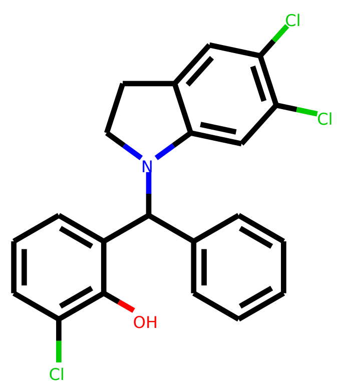
Osma-2454

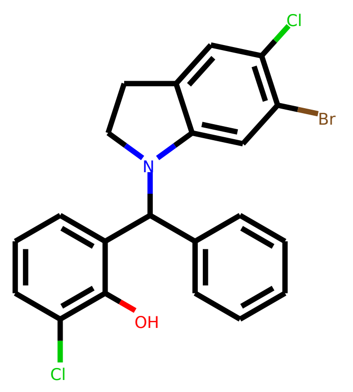
Osma-2455

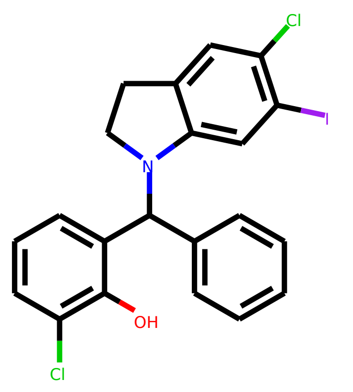
Osma-2456

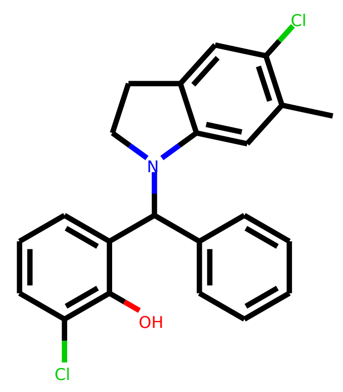
Osma-2457

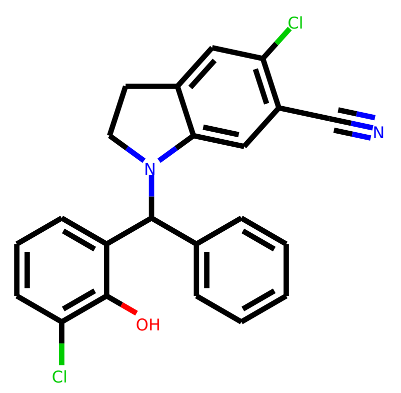
Osma-2458

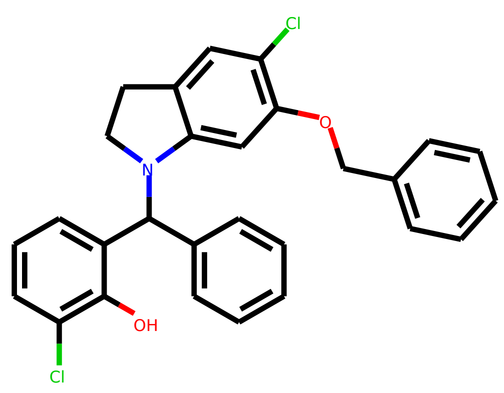
Osma-2459

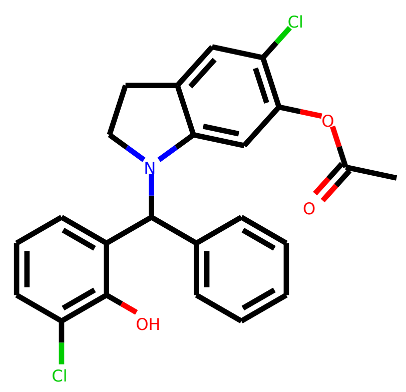
Osma-2460

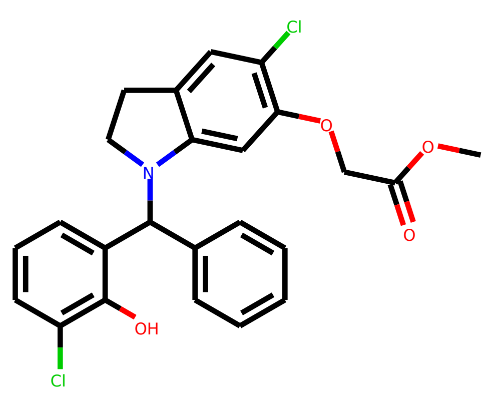
Osma-2461

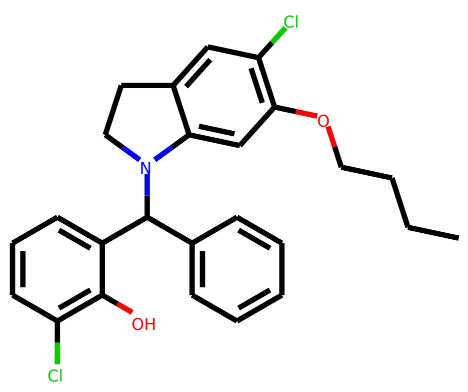
Osma-2462

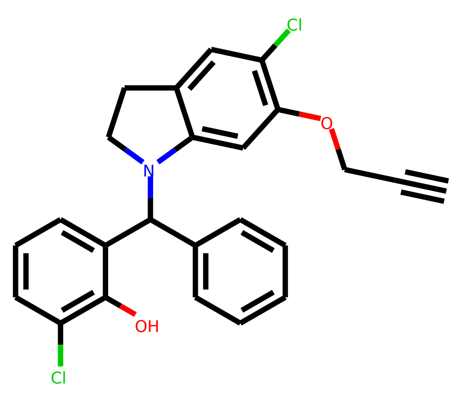
Osma-2463

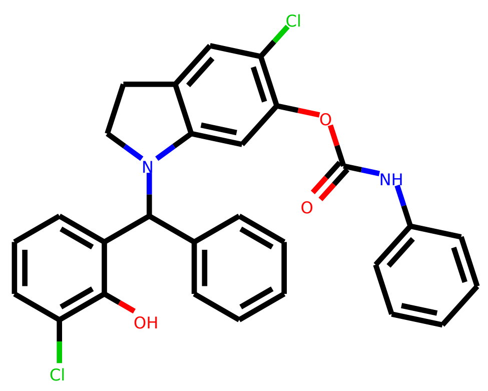
Osma-2464

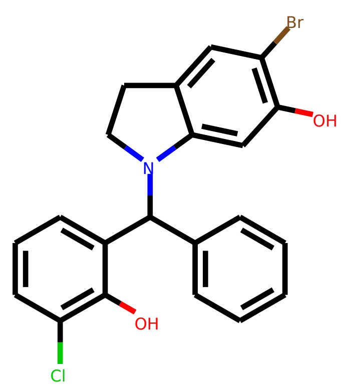
Osma-2465

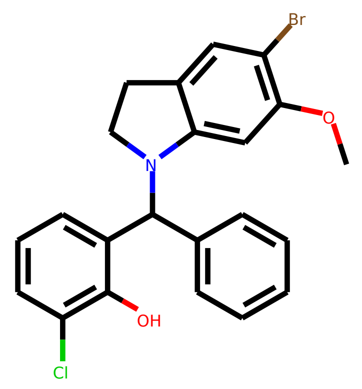
Osma-2466

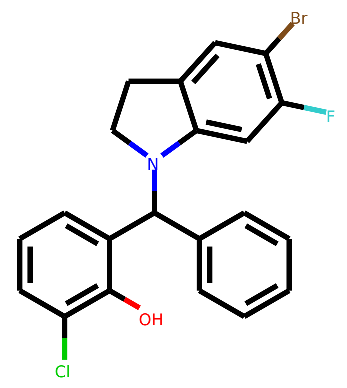
Osma-2467

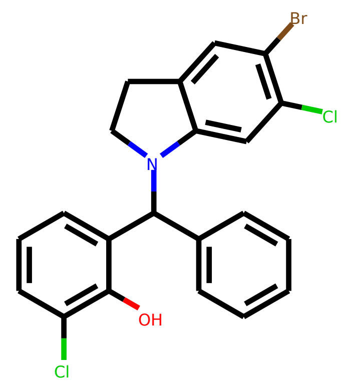
Osma-2468

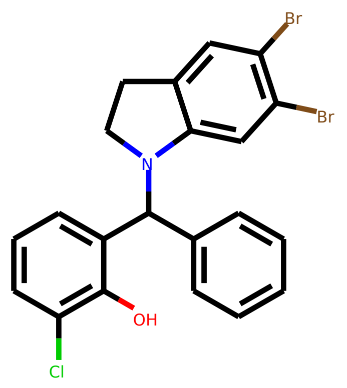
Osma-2469

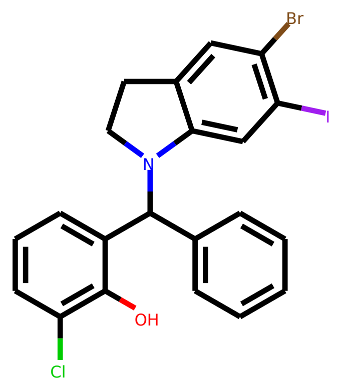
Osma-2470

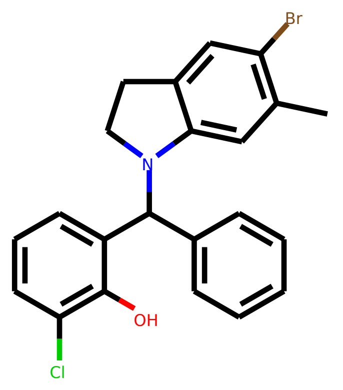
Osma-2471

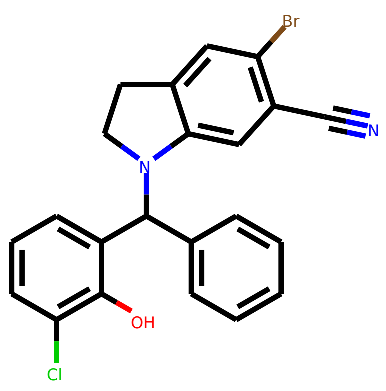
Osma-2472

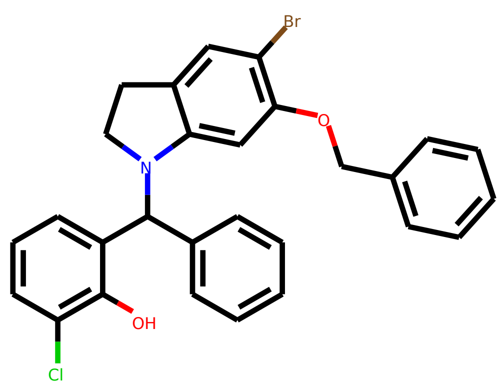
Osma-2473

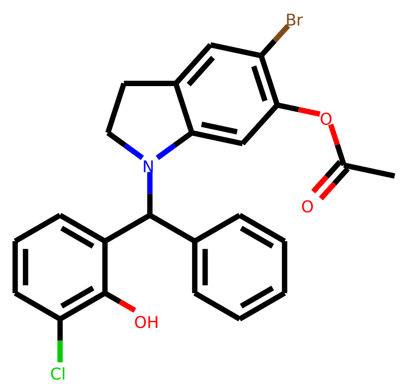
Osma-2474

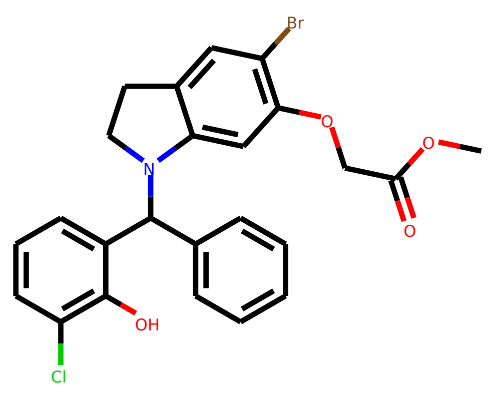
Osma-2475

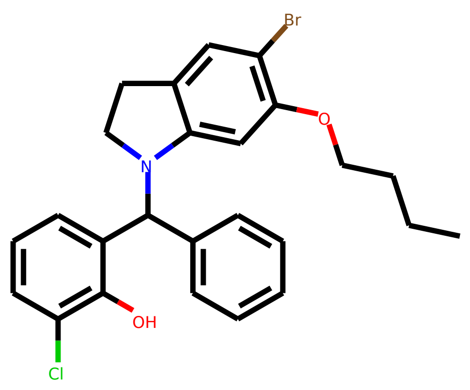
Osma-2476

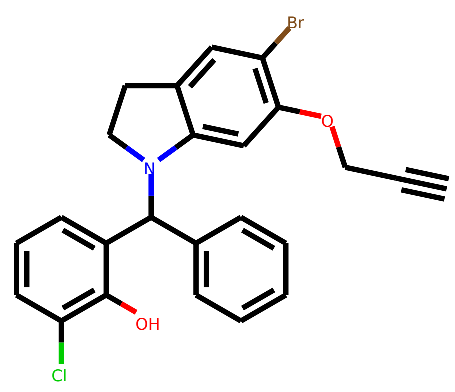
Osma-2477

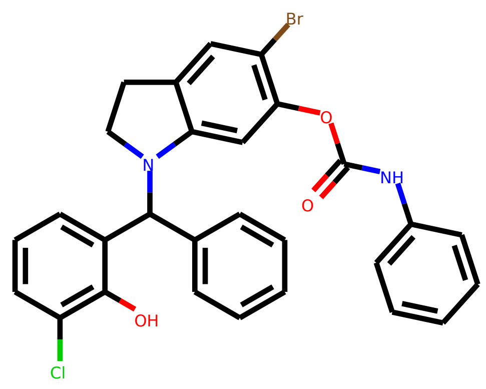
Osma-2478

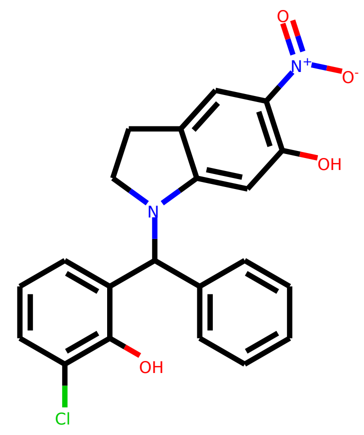
Osma-2479

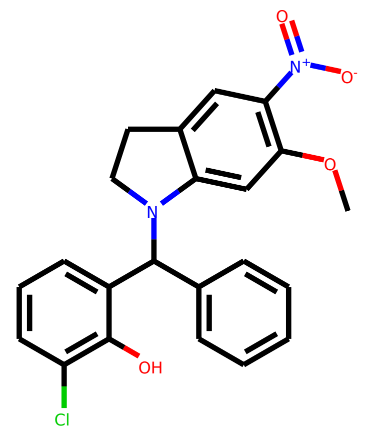
Osma-2480

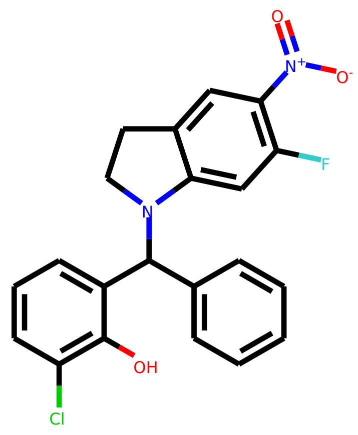
Osma-2481

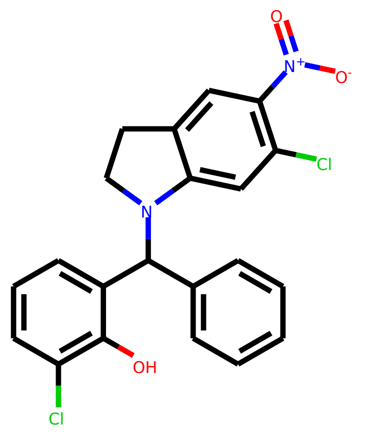
Osma-2482

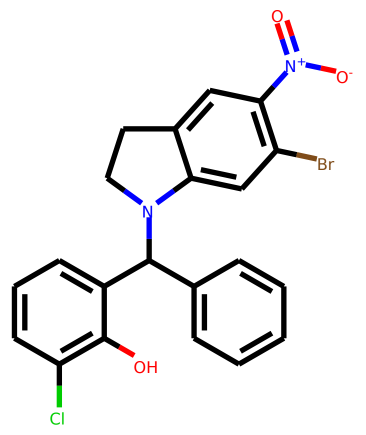
Osma-2483

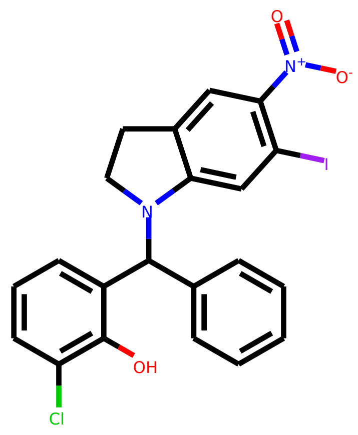
Osma-2484

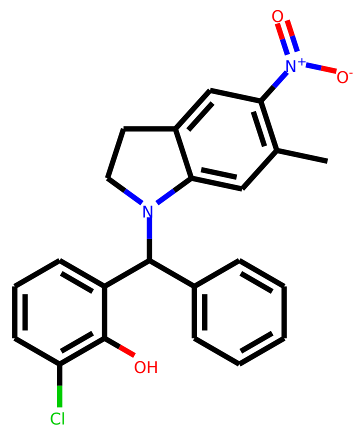
Osma-2485

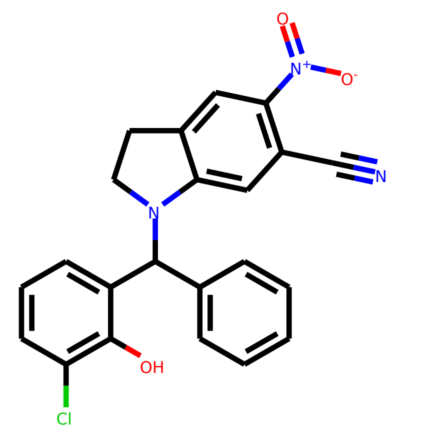
Osma-2486
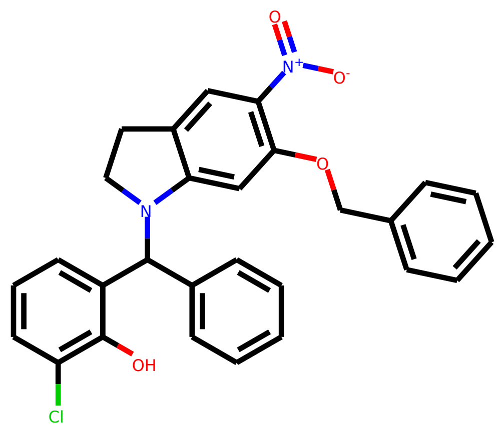
Osma-2487
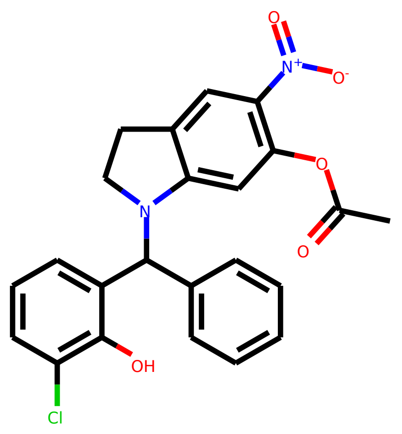
Osma-2488
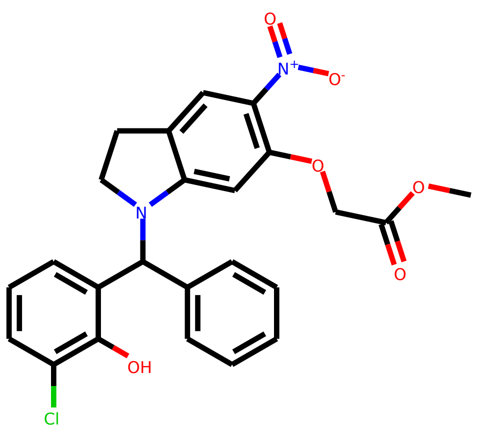
Osma-2489
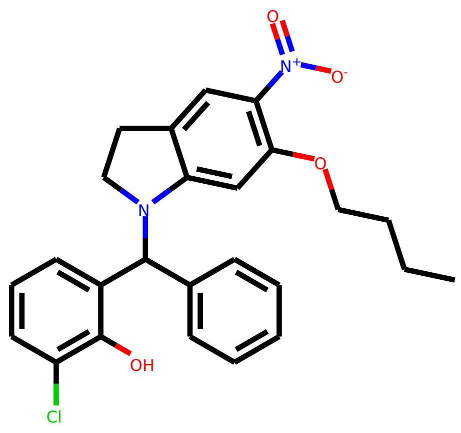
Osma-2490
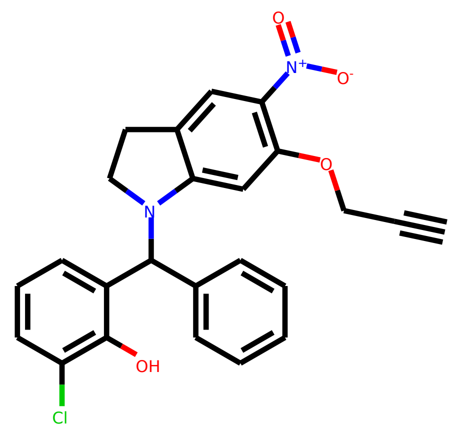
Osma-2491
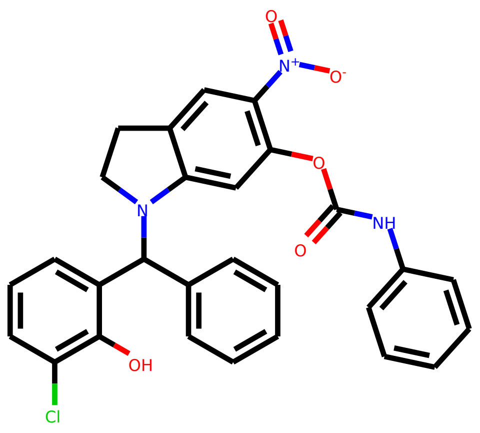
Osma-2492
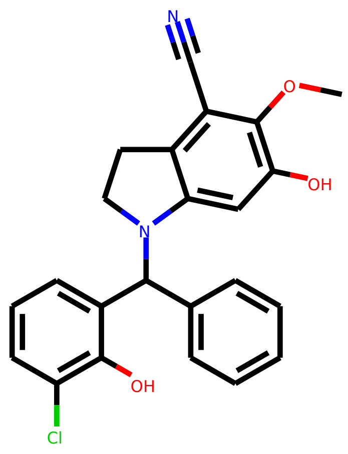
Osma-2493
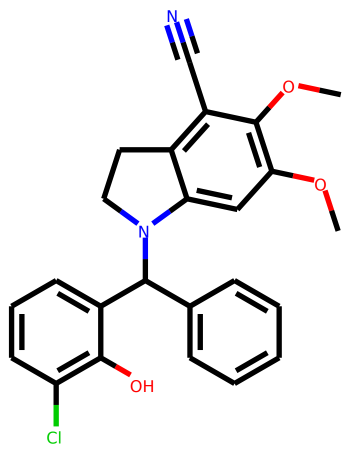
Osma-2494
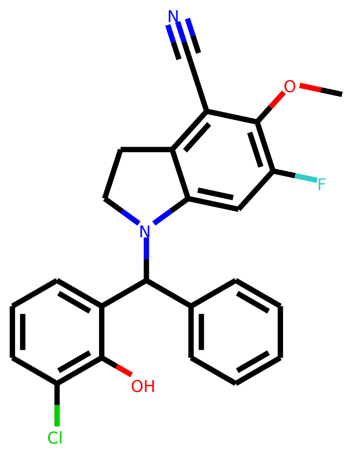
Osma-2495
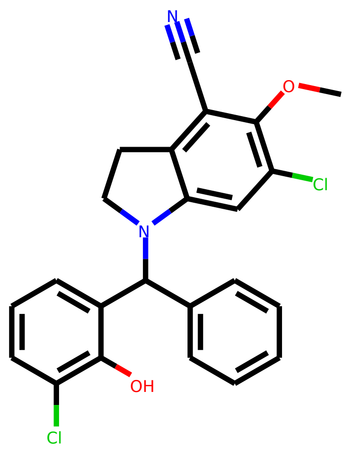
Osma-2496
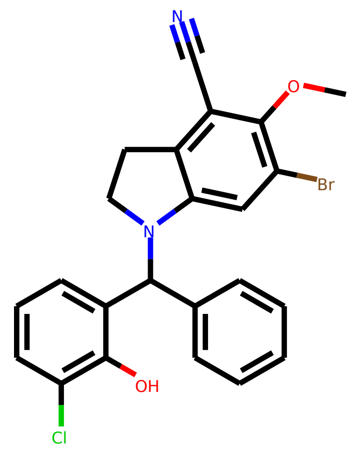
Osma-2497
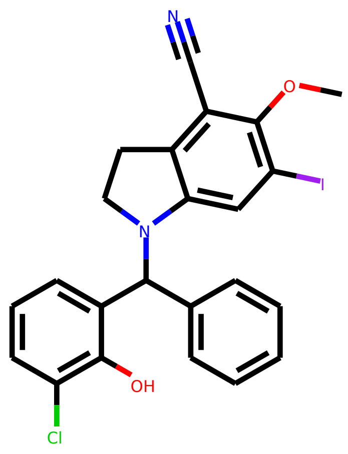
Osma-2498
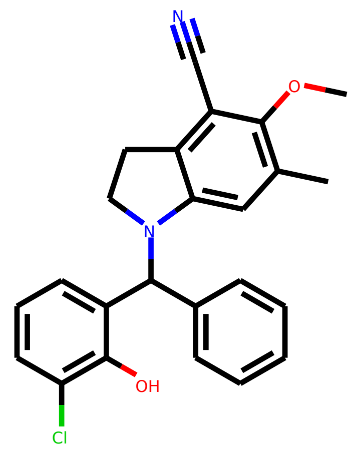
Osma-2499
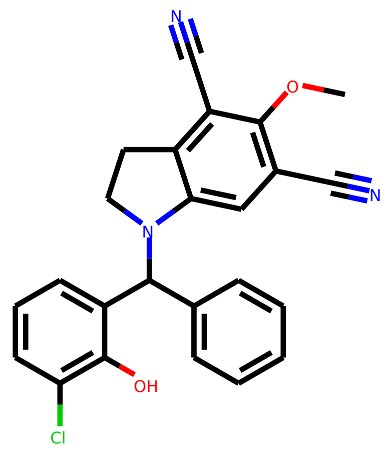
Osma-2500
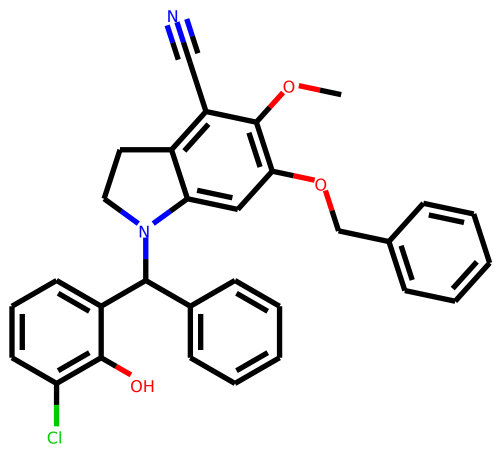
Osma-2501
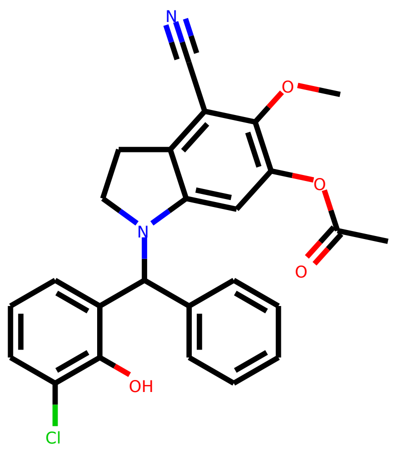
Osma-2502
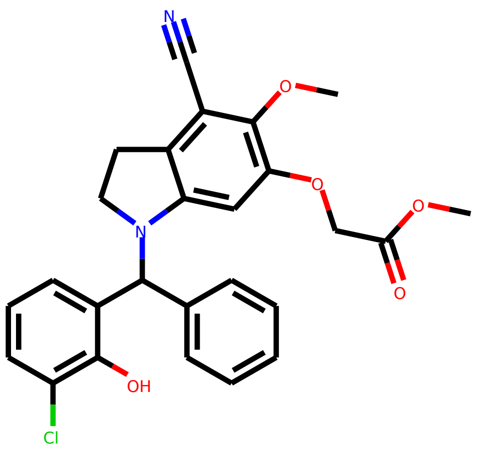
Osma-2503
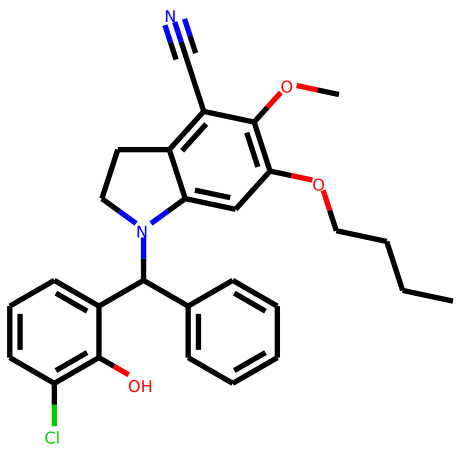
Osma-2504
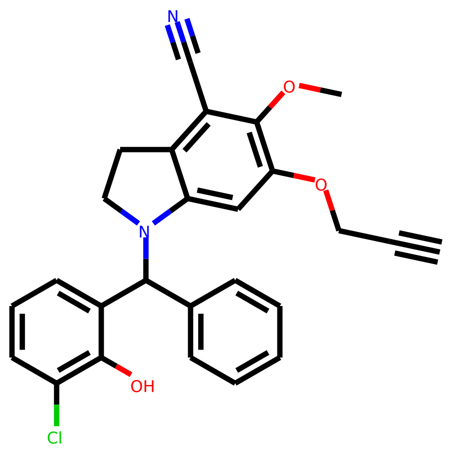
Osma-2505
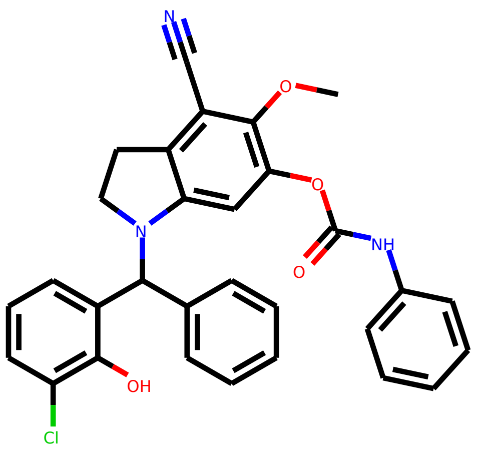
Osma-2506
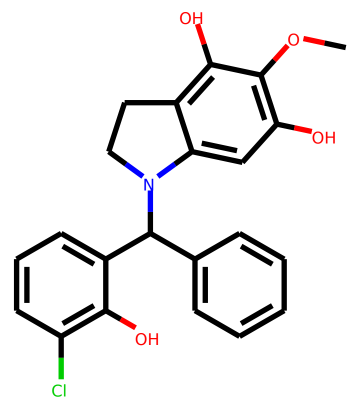
Osma-2507
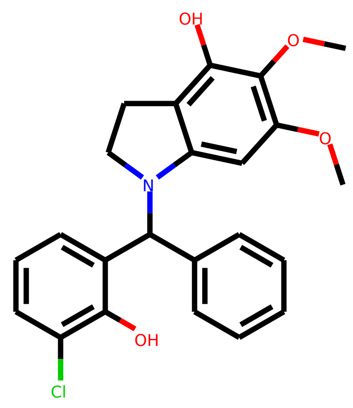
Osma-2508
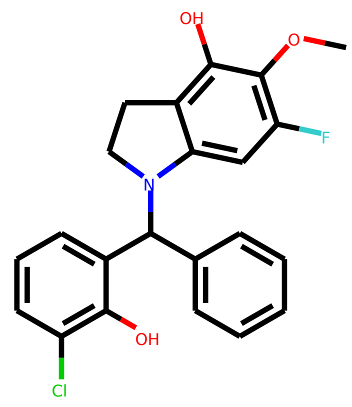
Osma-2509
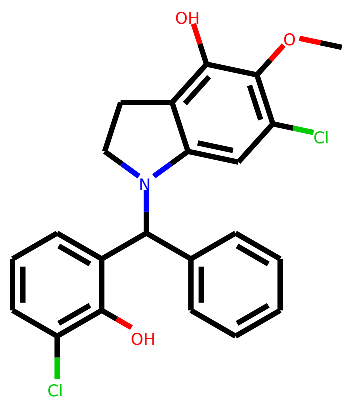
Osma-2510
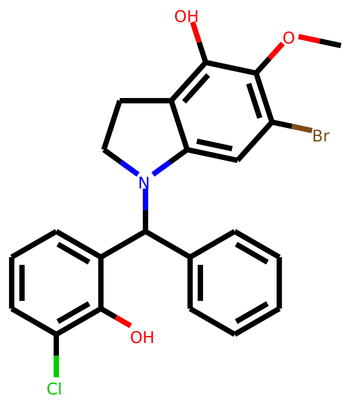
Osma-2511
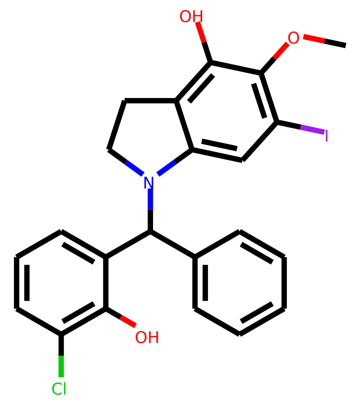
Osma-2512
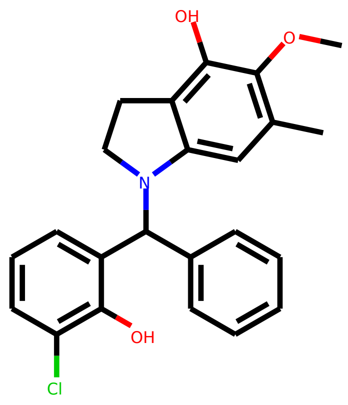
Osma-2513
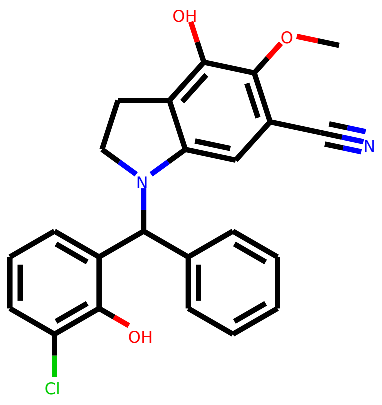
Osma-2514
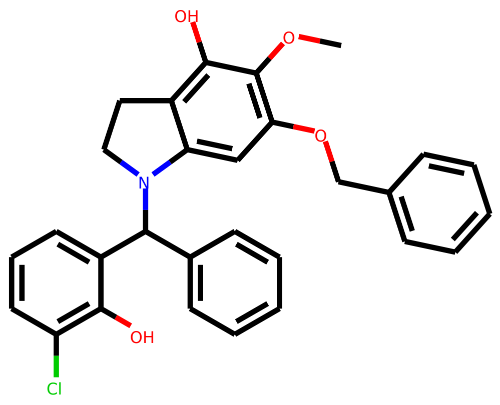
Osma-2515
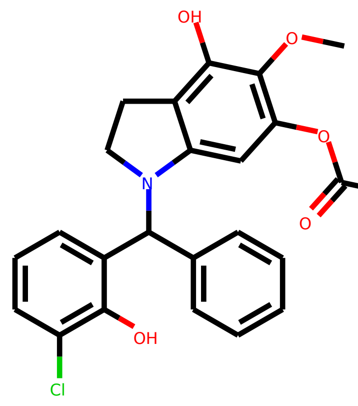
Osma-2516
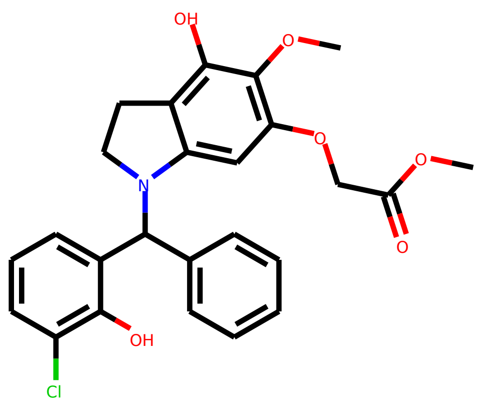
Osma-2517
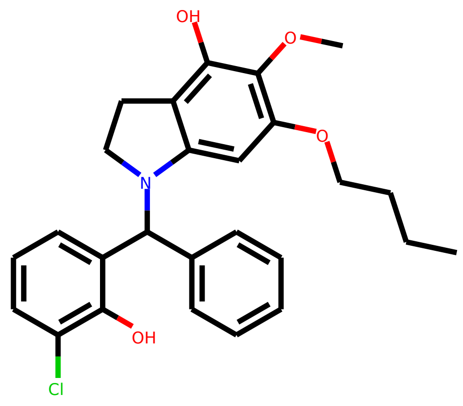
Osma-2518
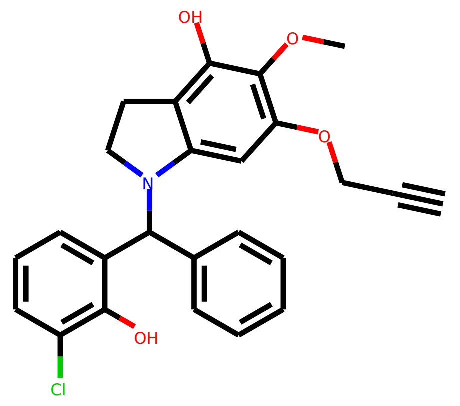
Osma-2519
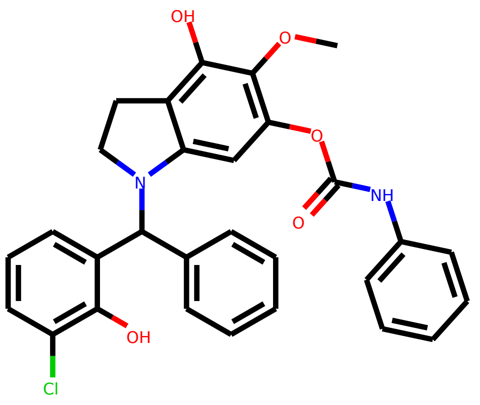
Osma-2520

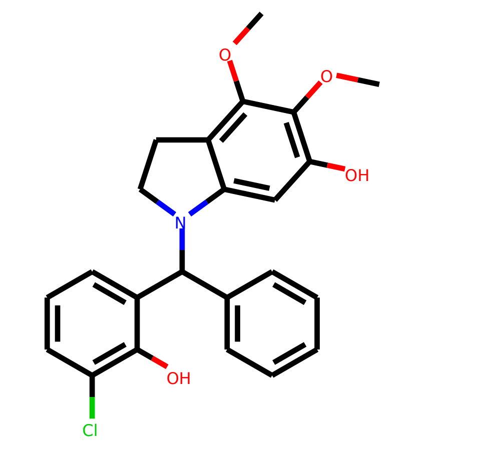

Osma-2521

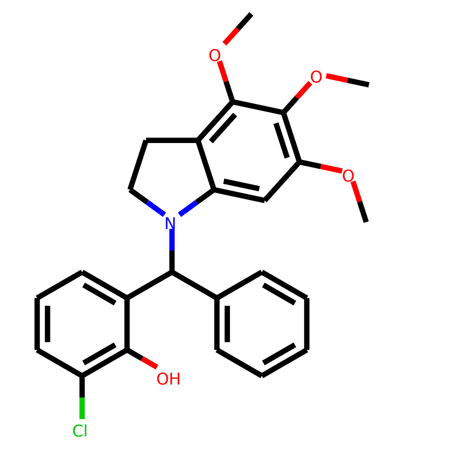

Osma-2522

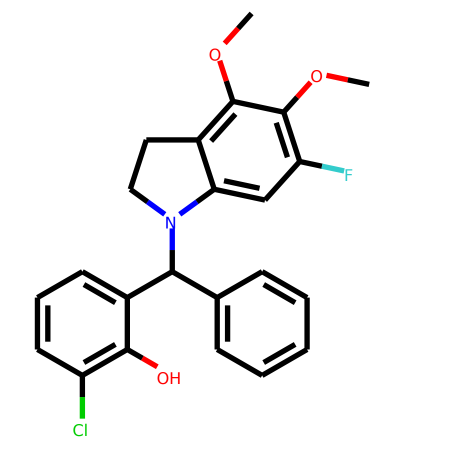

Osma-2523

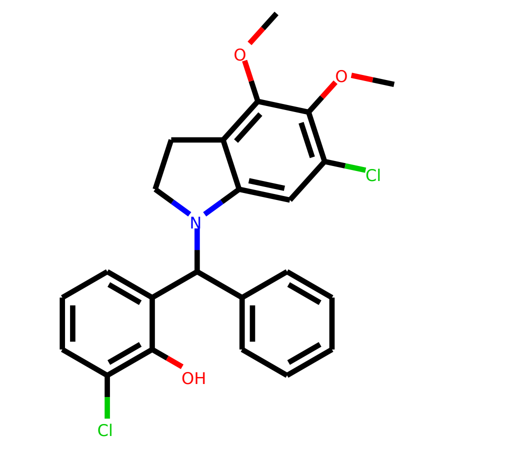

Osma-2524

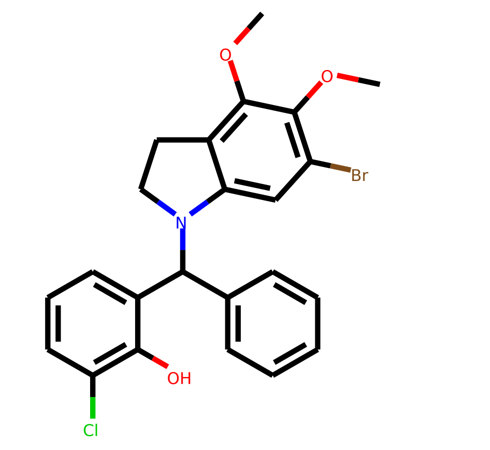

Osma-2525

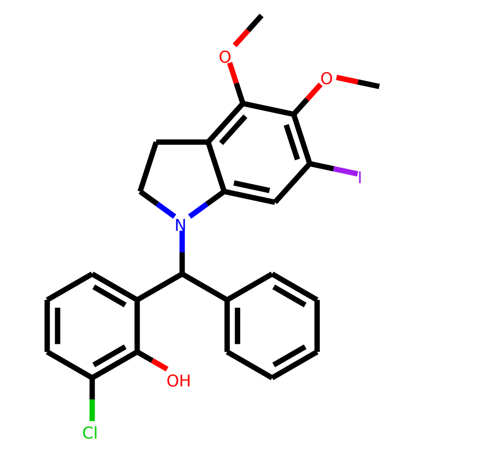

Osma-2526

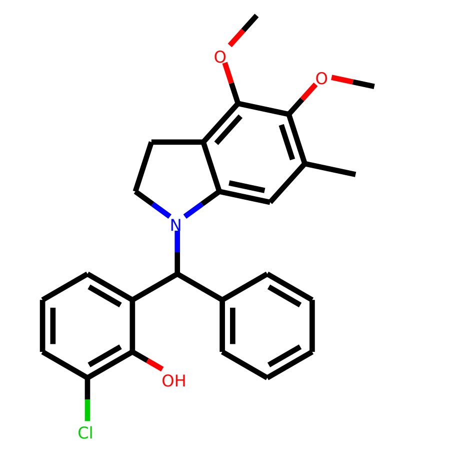

Osma-2527

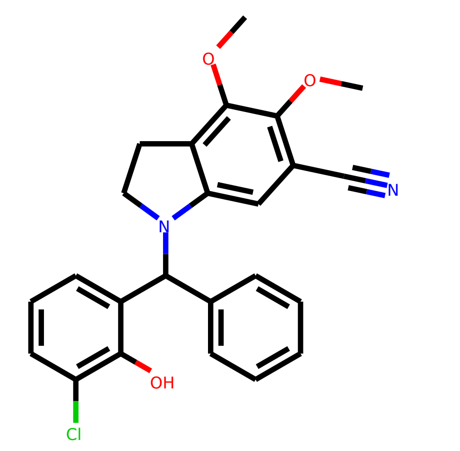

Osma-2528

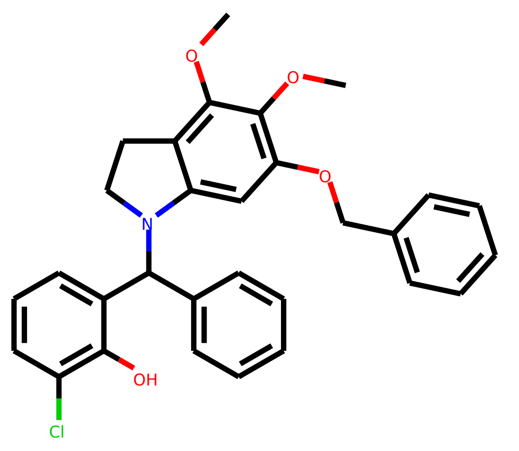

Osma-2529

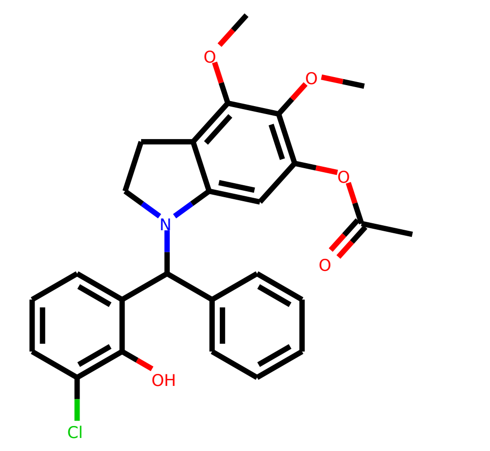

Osma-2530

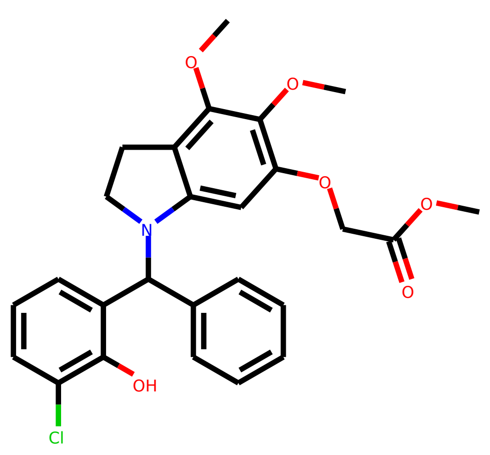

Osma-2531

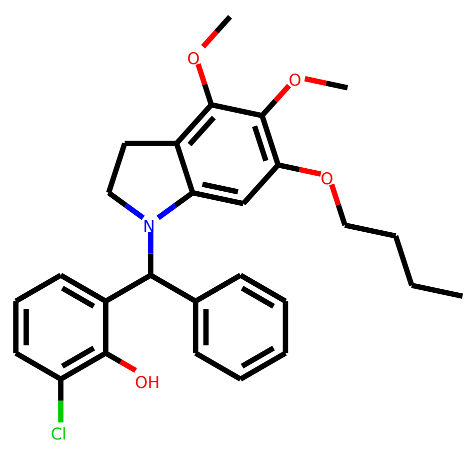

Osma-2532

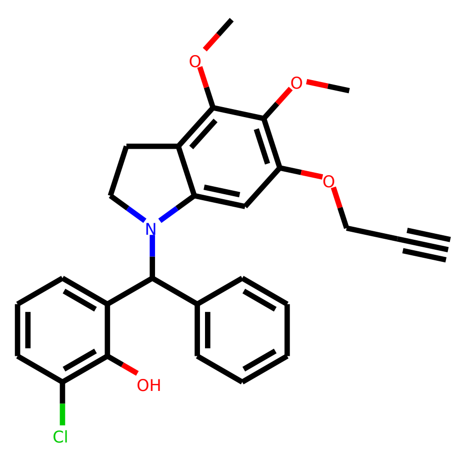

Osma-2533

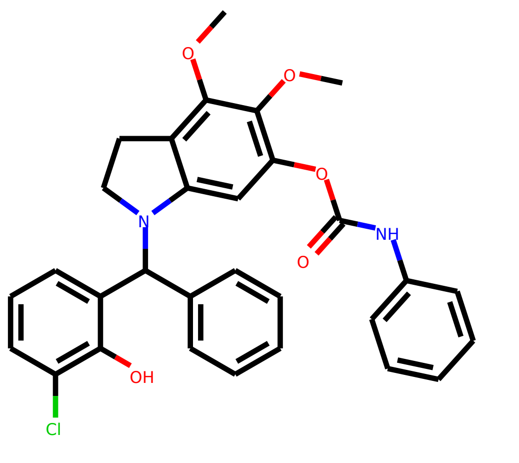

Osma-2534

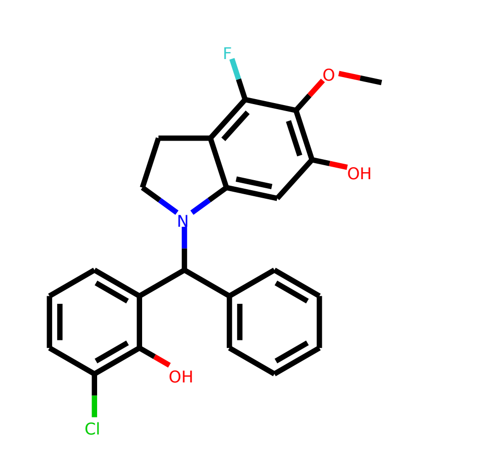

Osma-2535

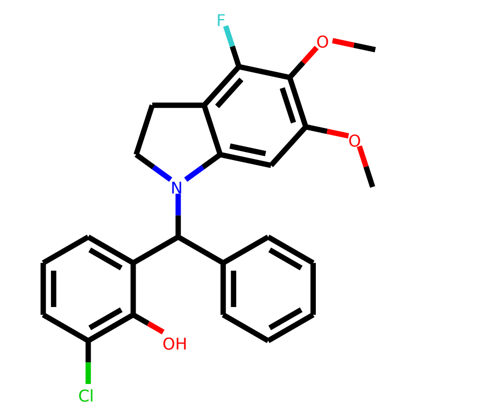

Osma-2536

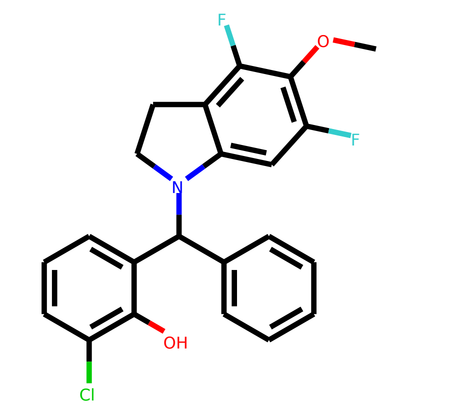

Osma-2537

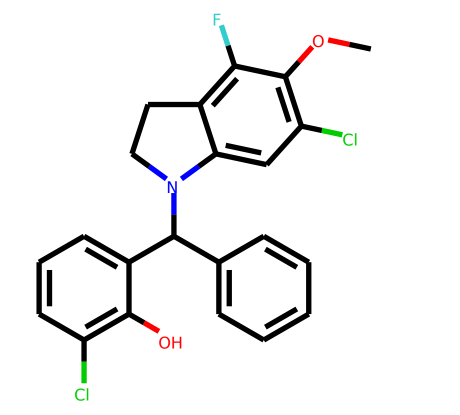

Osma-2538

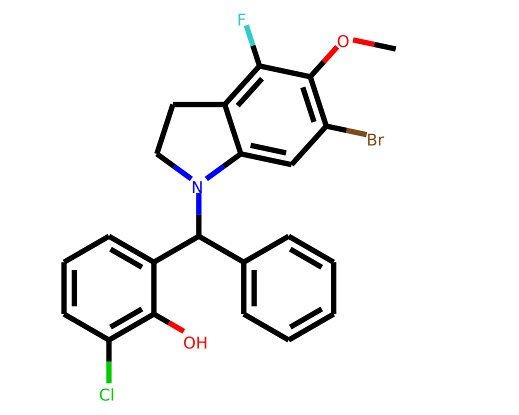

Osma-2539

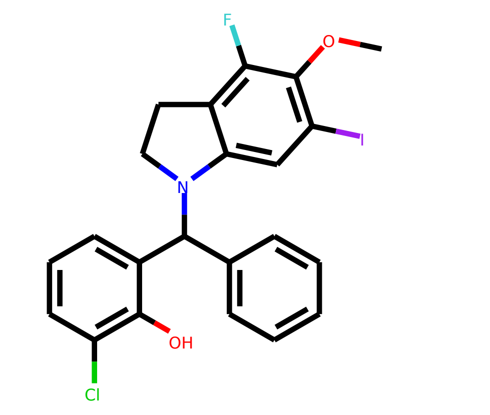

Osma-2540

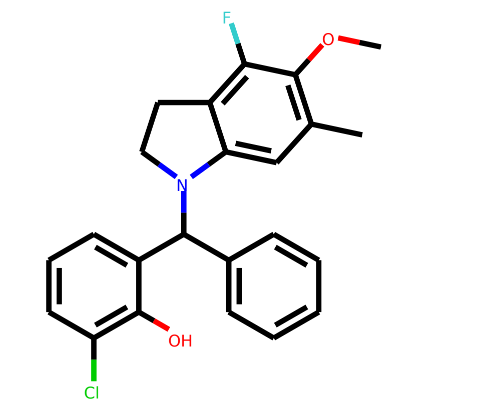

Osma-2541

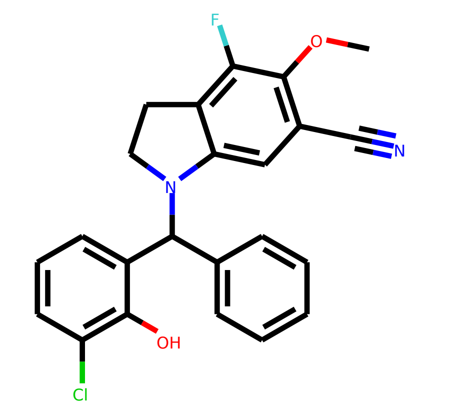

Osma-2542

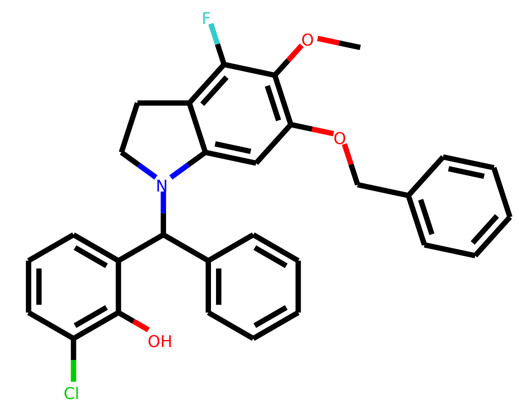

Osma-2543

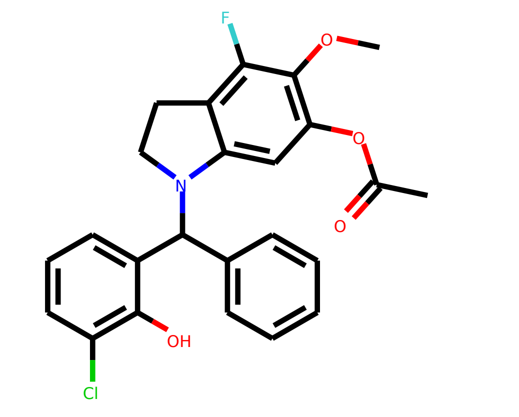

Osma-2544

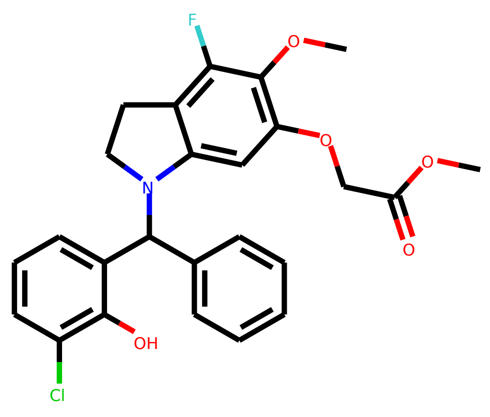

Osma-2545

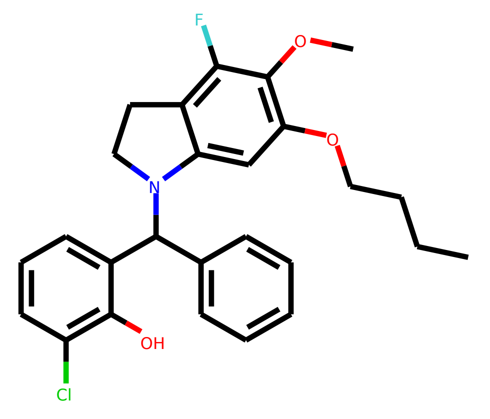

Osma-2546

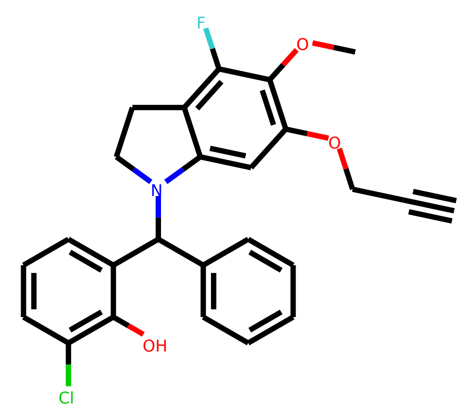

Osma-2547

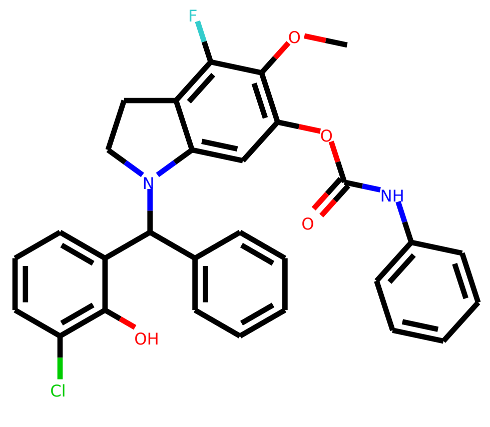

Osma-2548

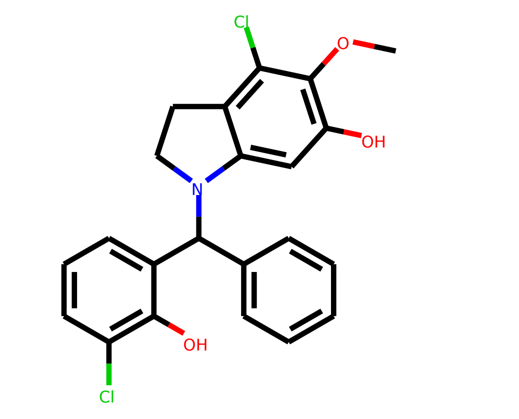

Osma-2549

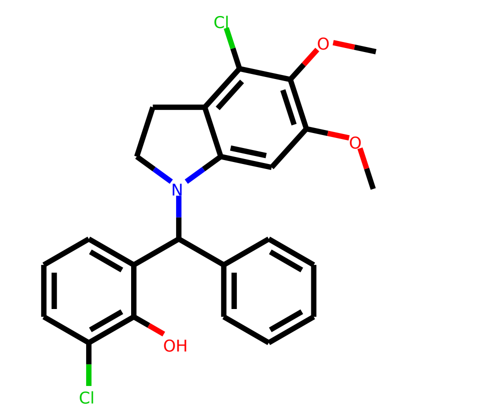

Osma-2550

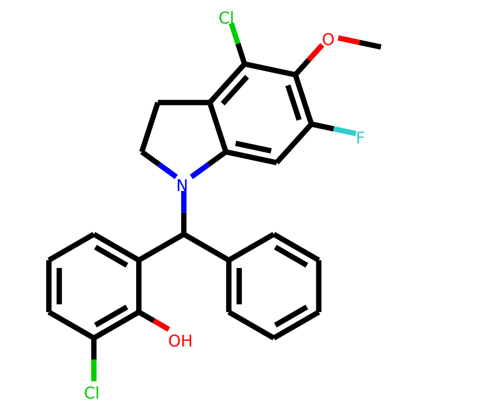

Osma-2551

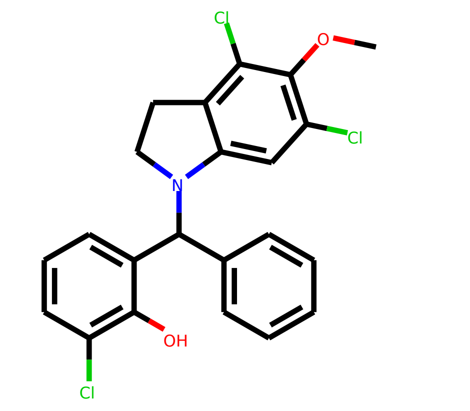

Osma-2552

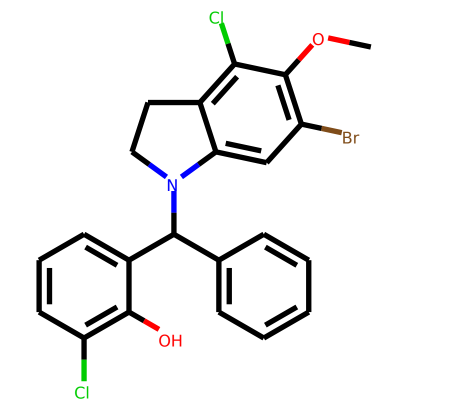

Osma-2553

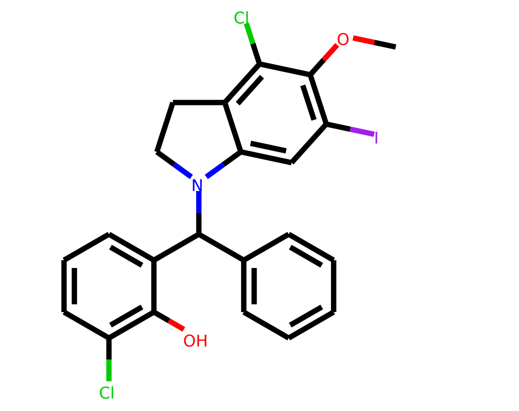

Osma-2554

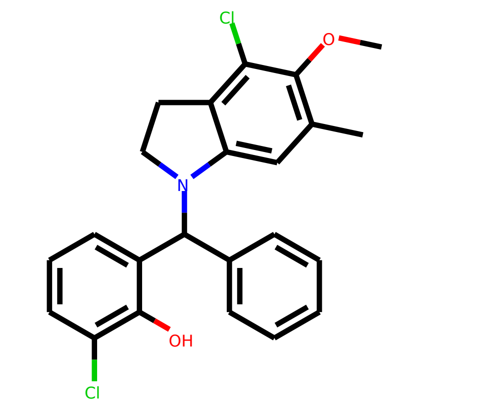

Osma-2555

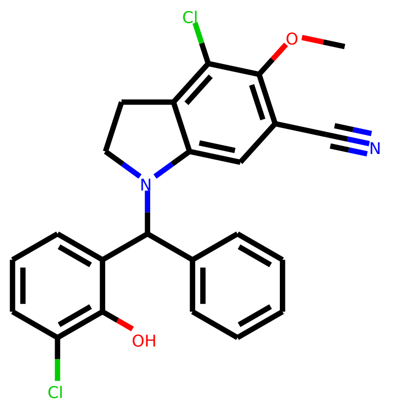

Osma-2556

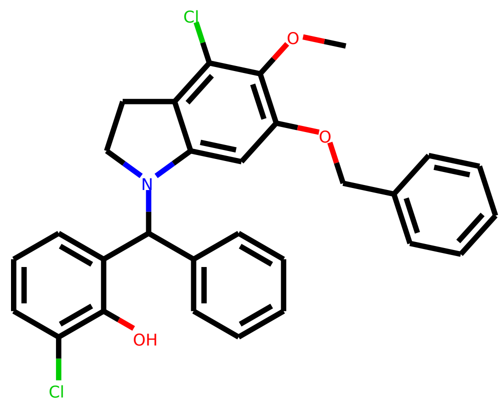

Osma-2557

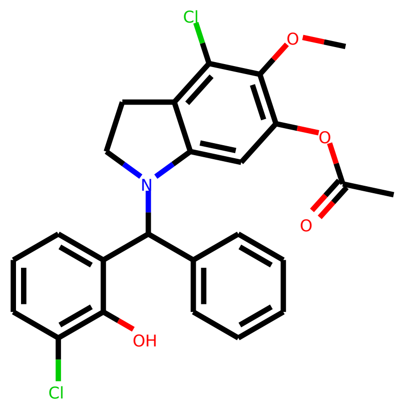

Osma-2558

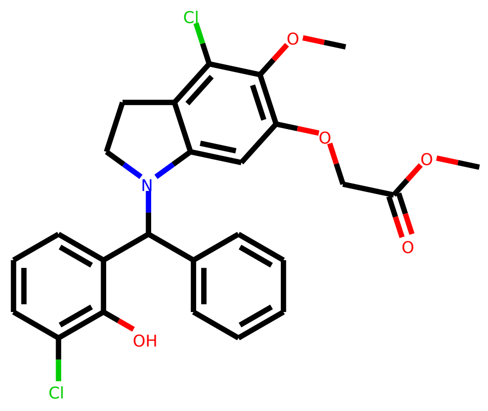

Osma-2559

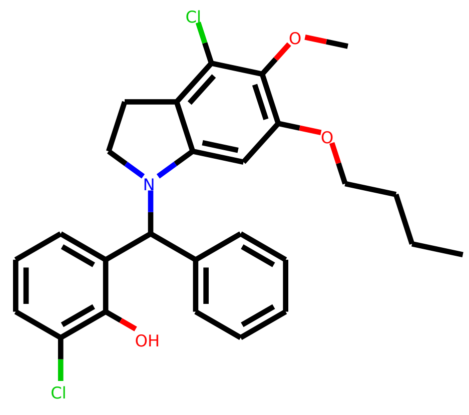

Osma-2560

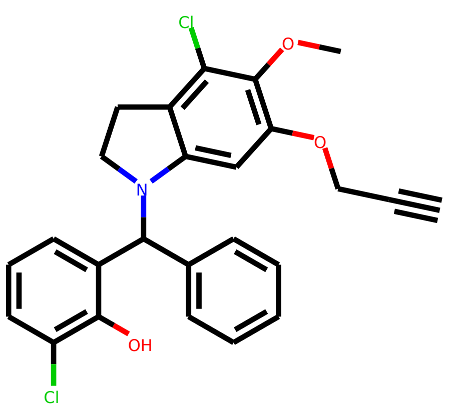

Osma-2561

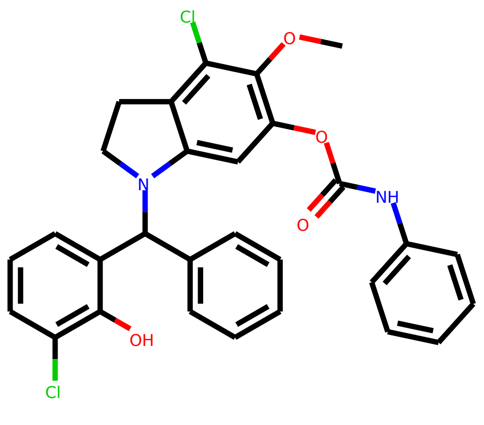

Osma-2562

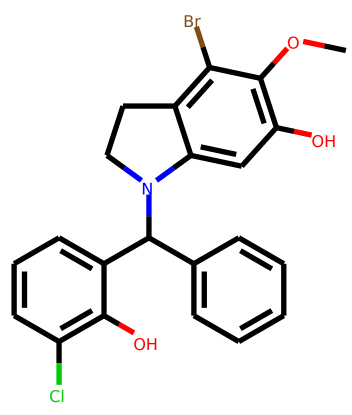

Osma-2563

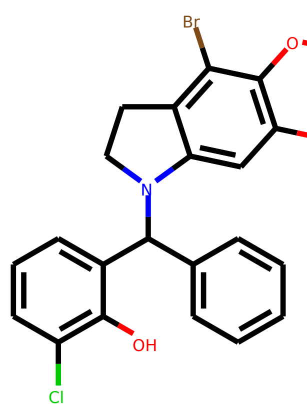

Osma-2564

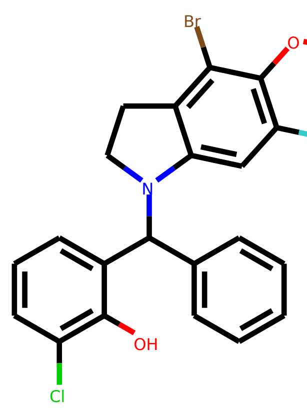

Osma-2565

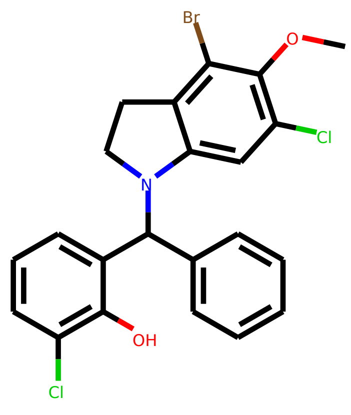

Osma-2566

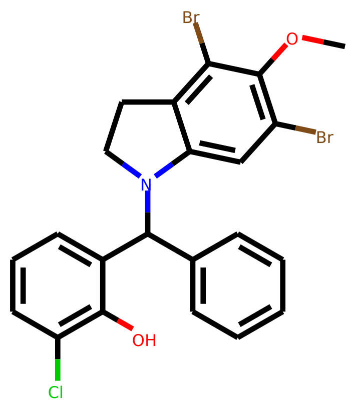

Osma-2567

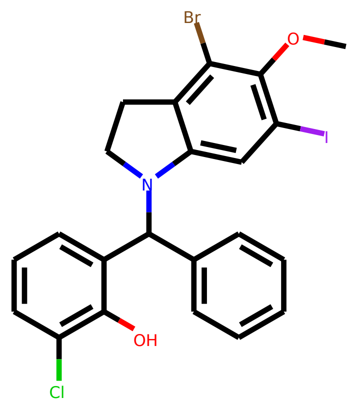

Osma-2568

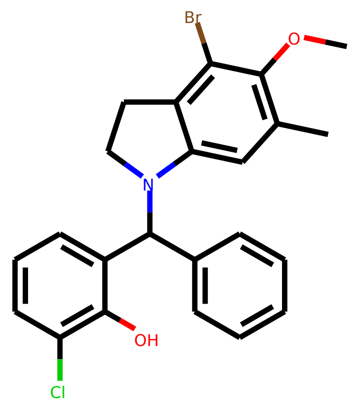

Osma-2569

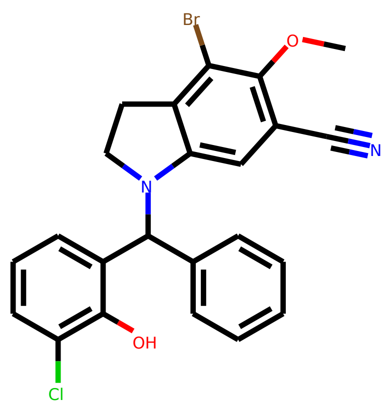

Osma-2570

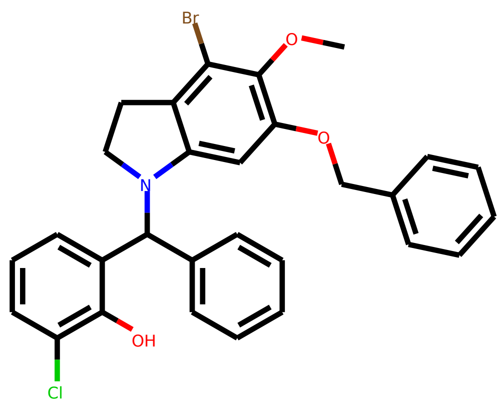

Osma-2571

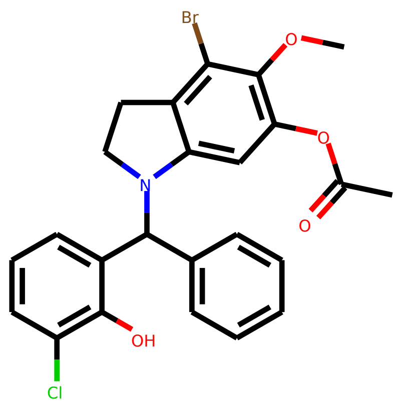

Osma-2572

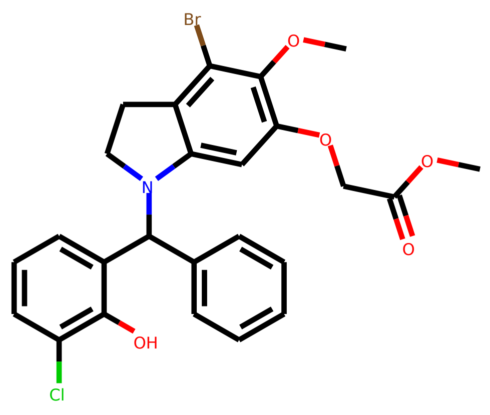

Osma-2573

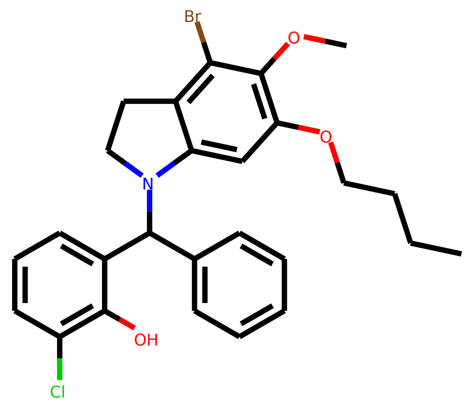

Osma-2574

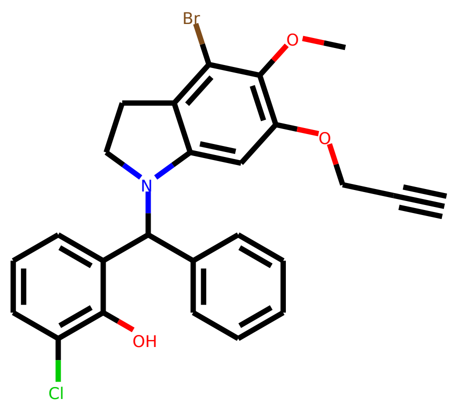

Osma-2575

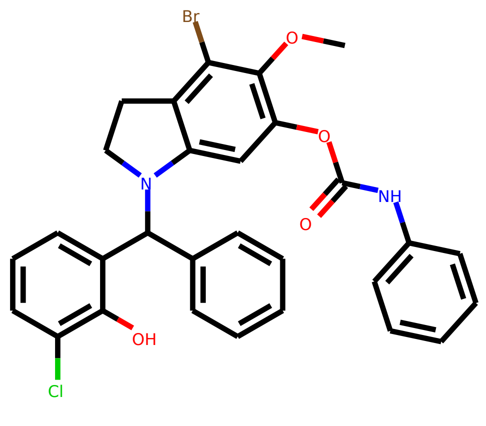

Osma-2576

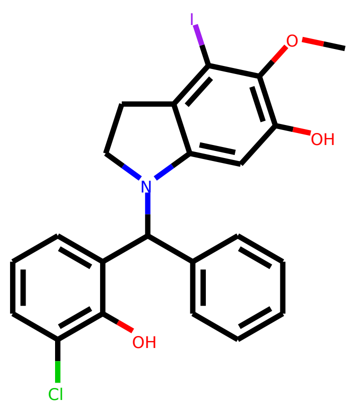

Osma-2577

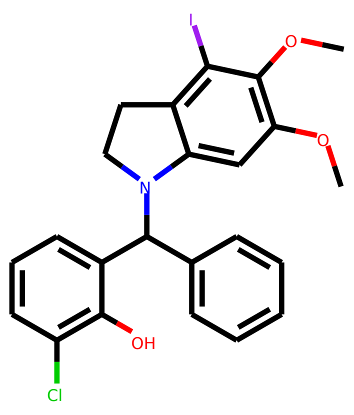

Osma-2578

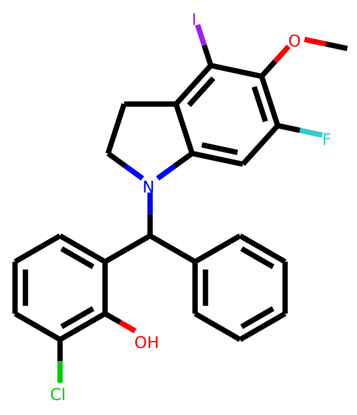

Osma-2579

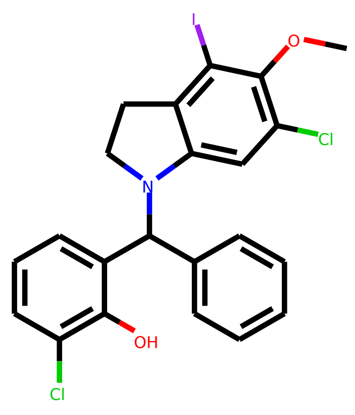

Osma-2580

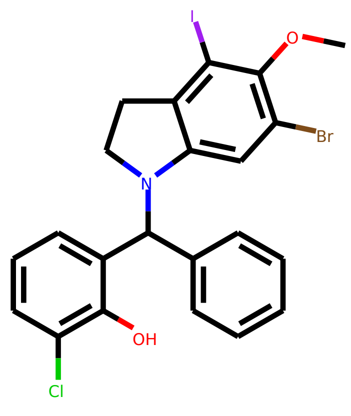

Osma-2581

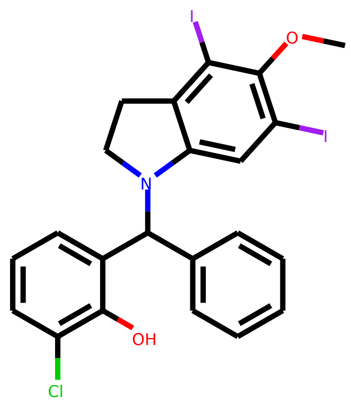

Osma-2582

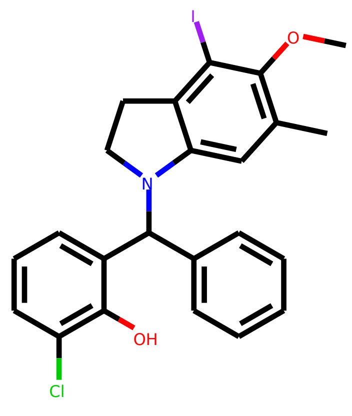

Osma-2583

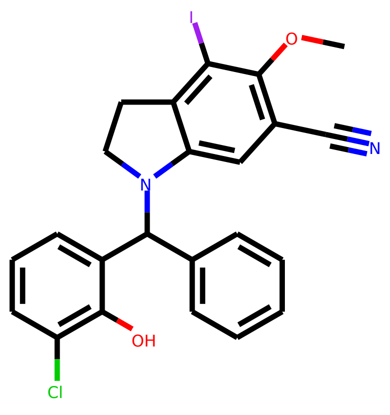

Osma-2584

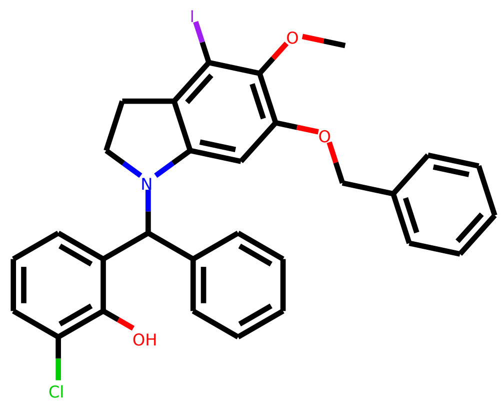

Osma-2585

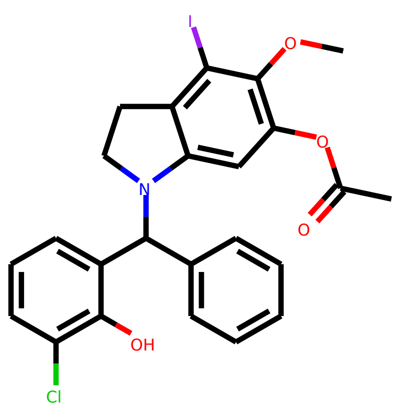

Osma-2586

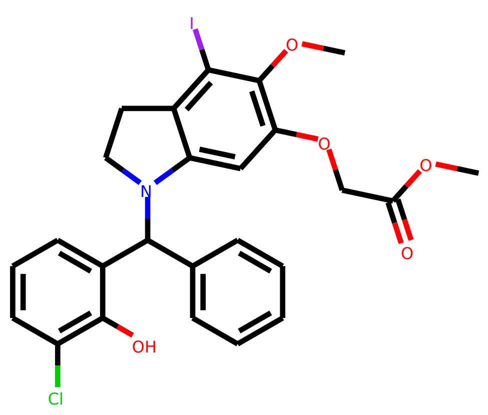

Osma-2587

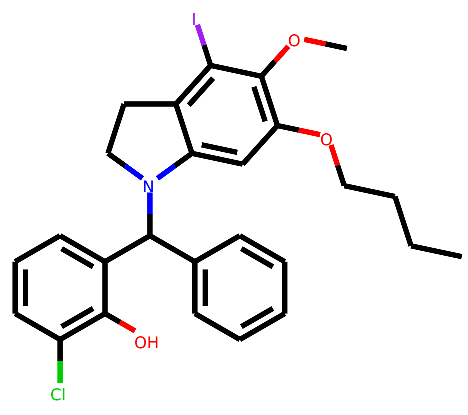

Osma-2588

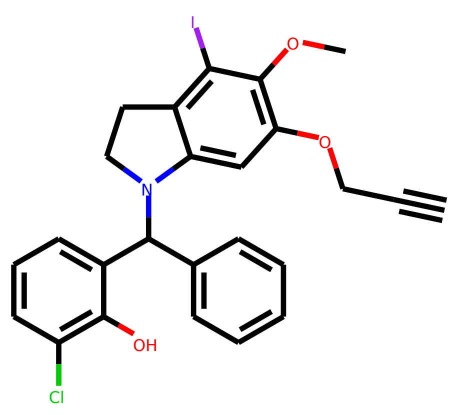

Osma-2589

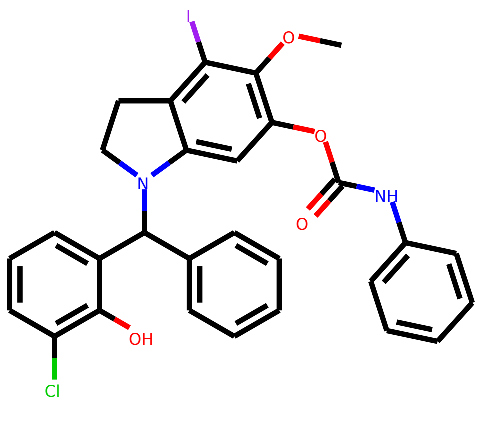

Osma-2590

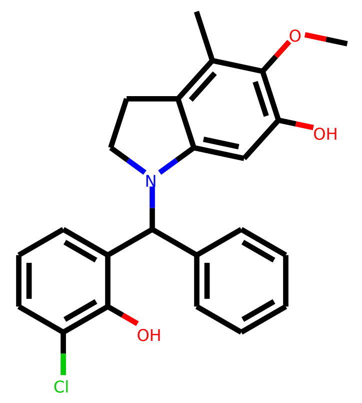
Osma-2591
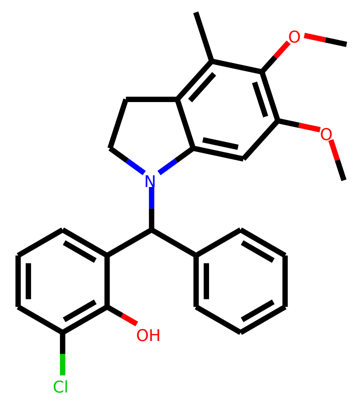
Osma-2592
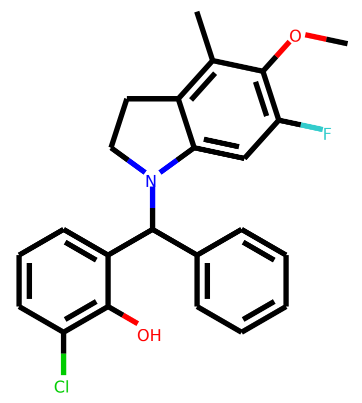
Osma-2593
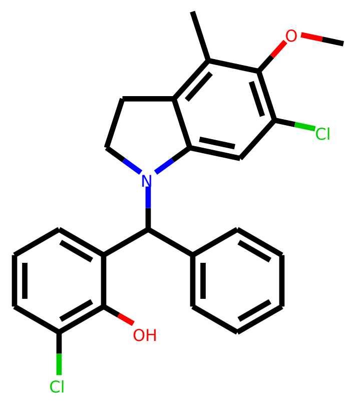
Osma-2594
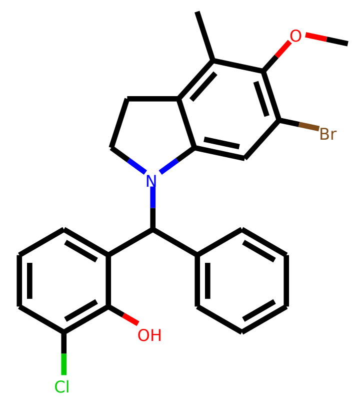
Osma-2595
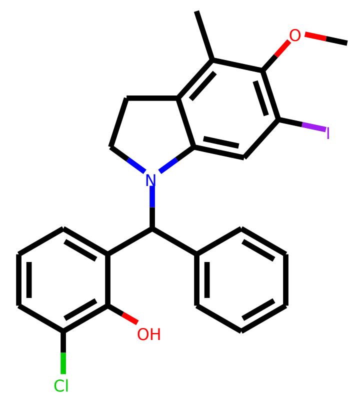
Osma-2596
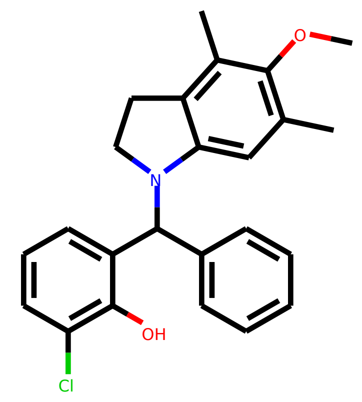
Osma-2597
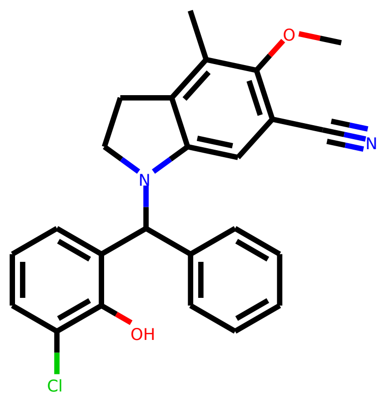
Osma-2598
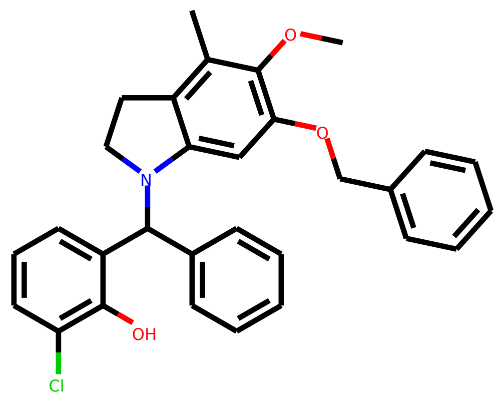
Osma-2599
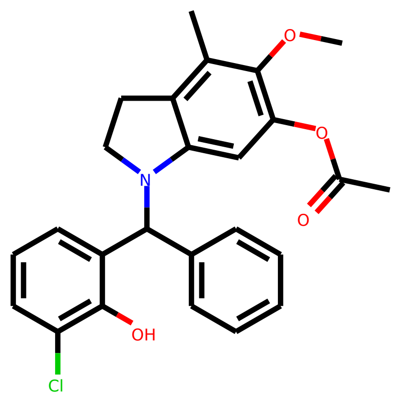
Osma-2600
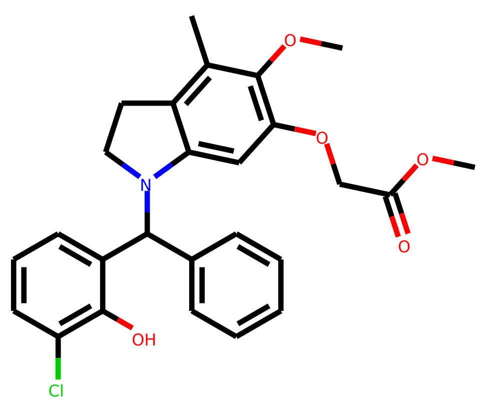
Osma-2601
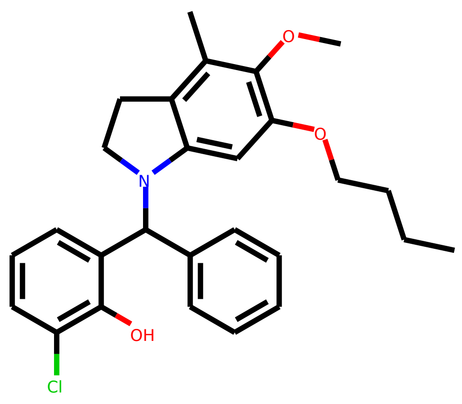
Osma-2602
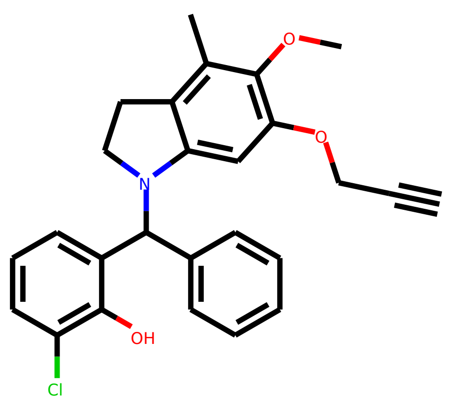
Osma-2603
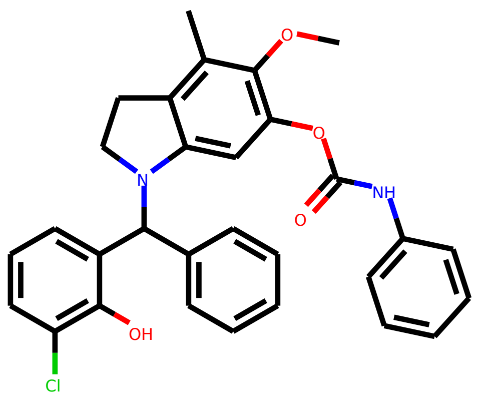
Osma-2604
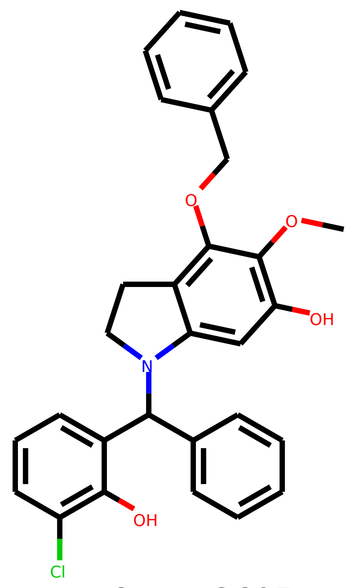
Osma-2605
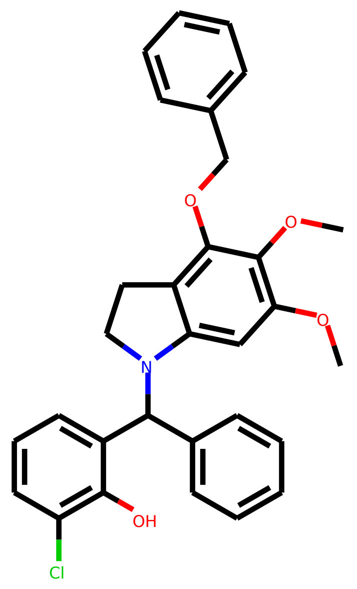
Osma-2606
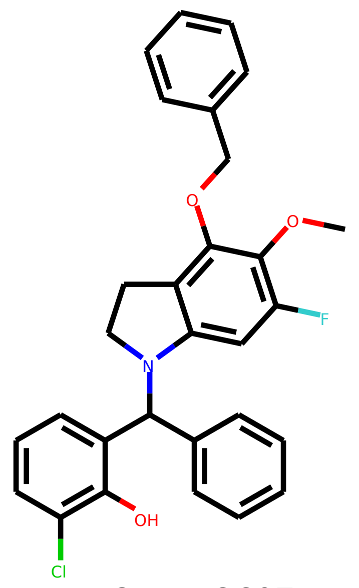
Osma-2607
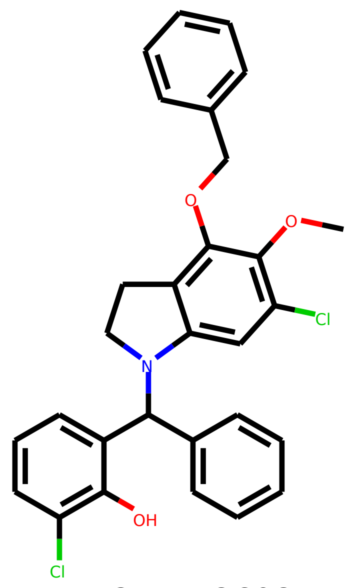
Osma-2608
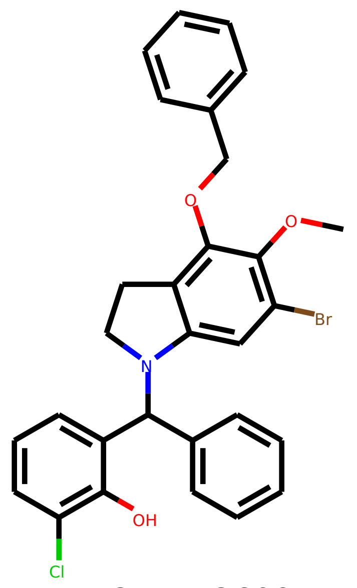
Osma-2609
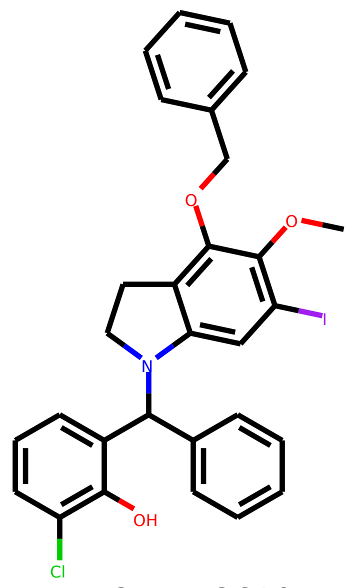
Osma-2610
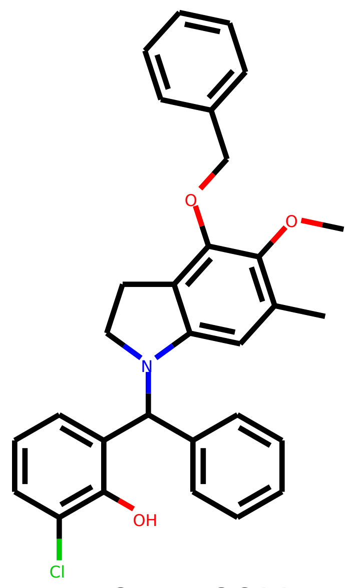
Osma-2611
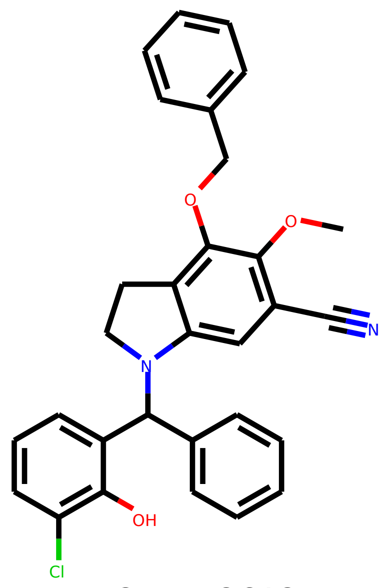
Osma-2612
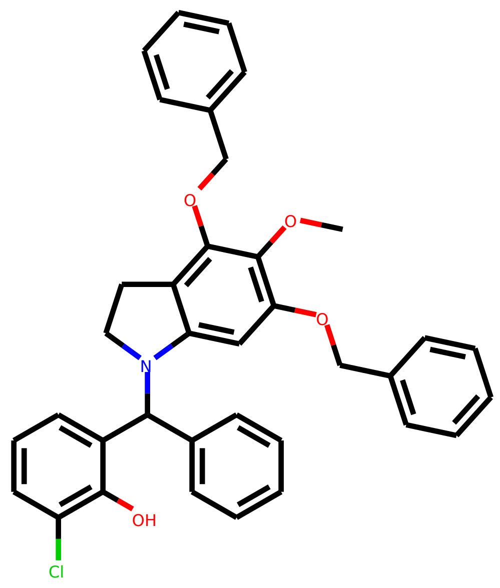
Osma-2613
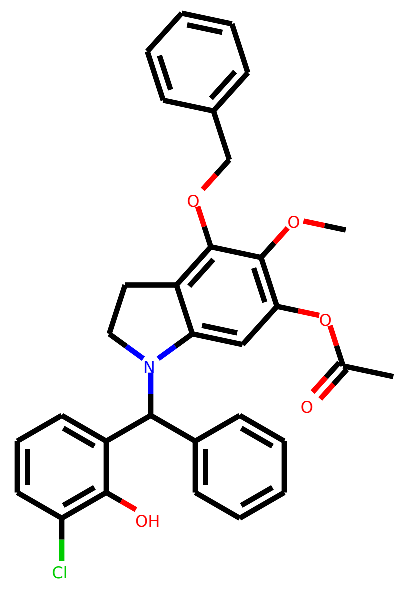
Osma-2614
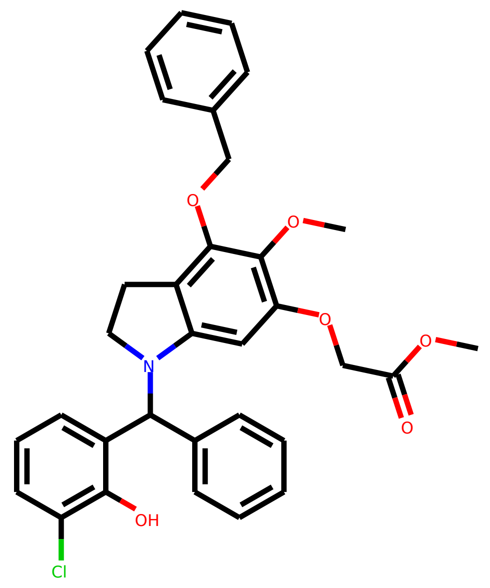
Osma-2615
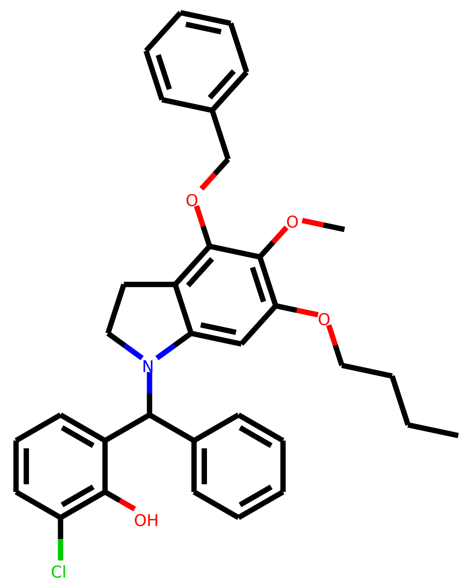
Osma-2616
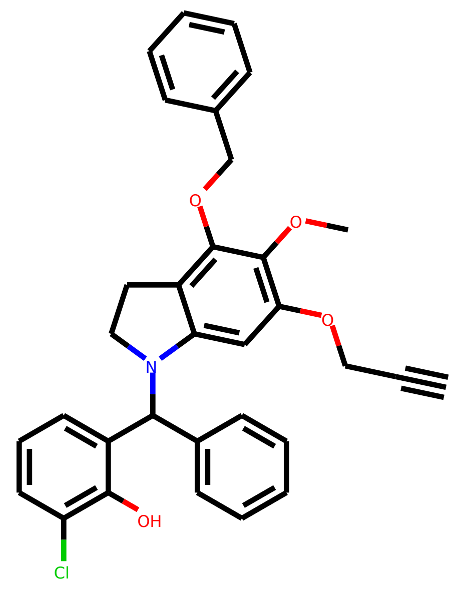
Osma-2617
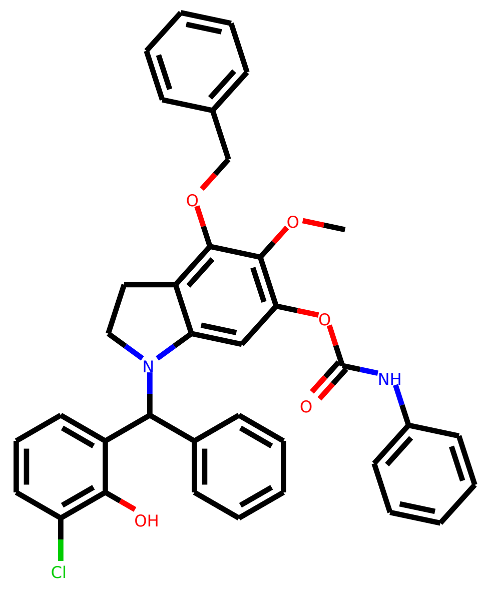
Osma-2618
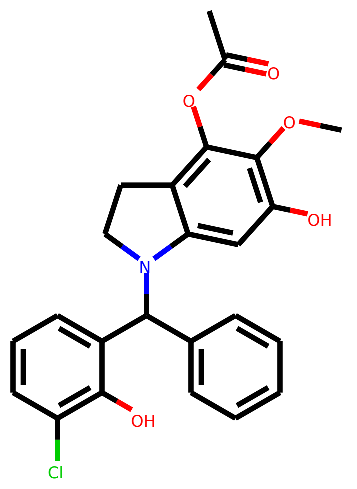
Osma-2619
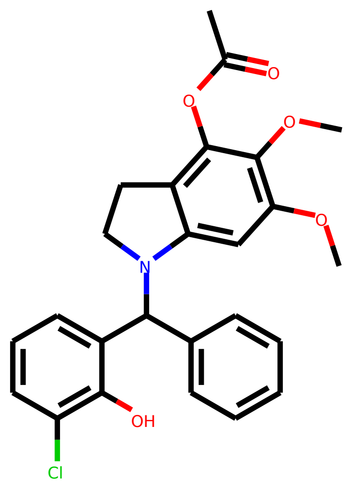
Osma-2620
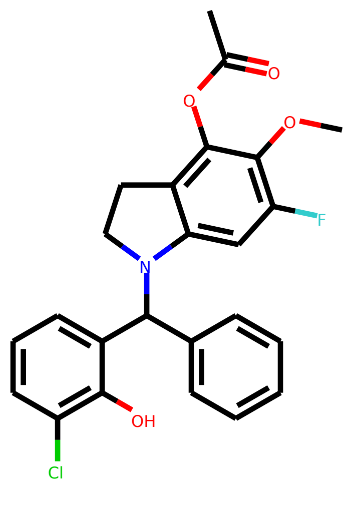
Osma-2621
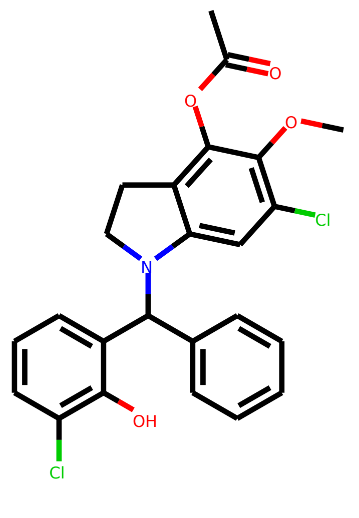
Osma-2622
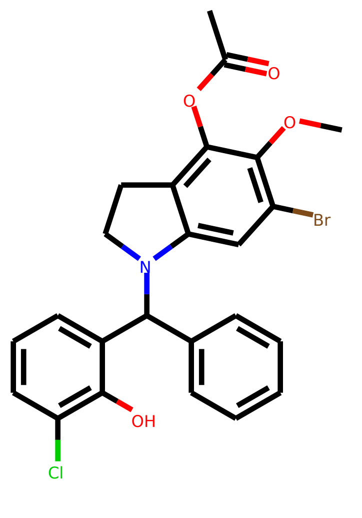
Osma-2623
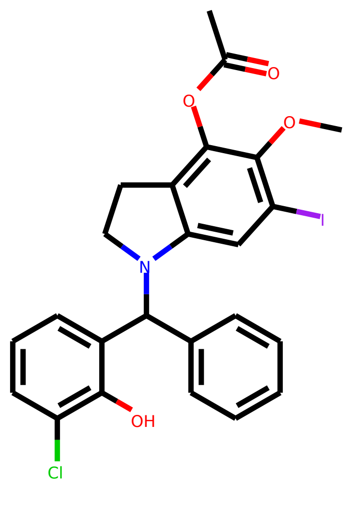
Osma-2624
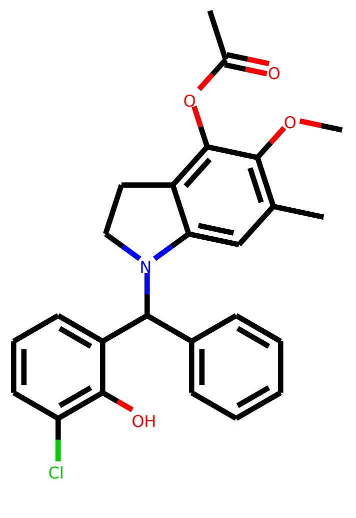
Osma-2625

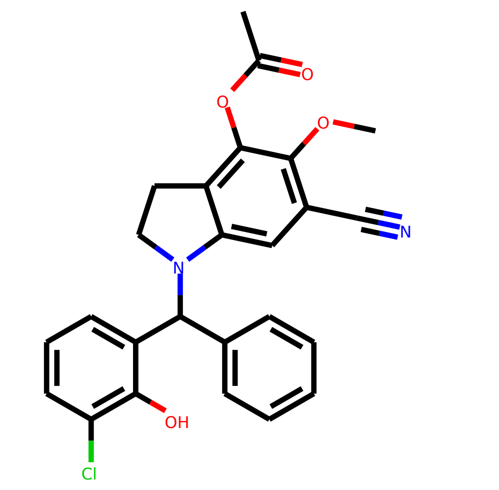
Osma-2626
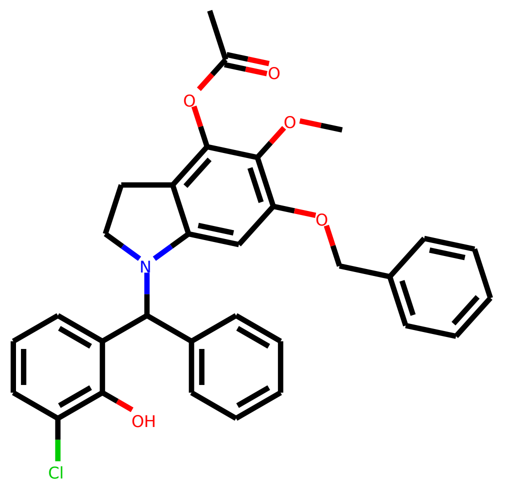
Osma-2627
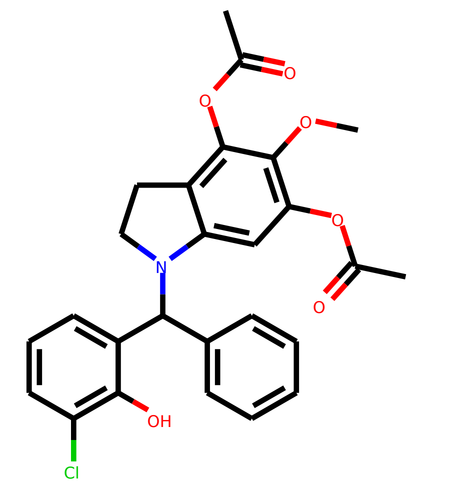
Osma-2628
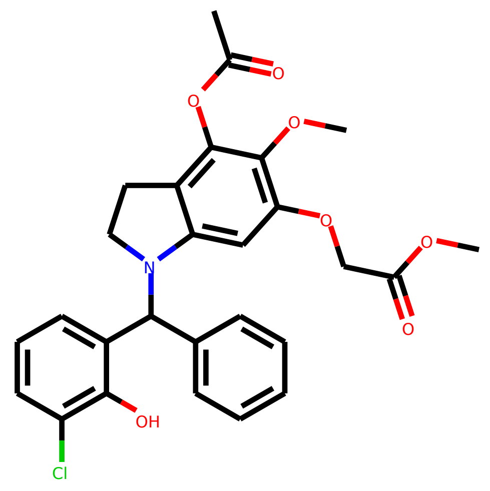
Osma-2629
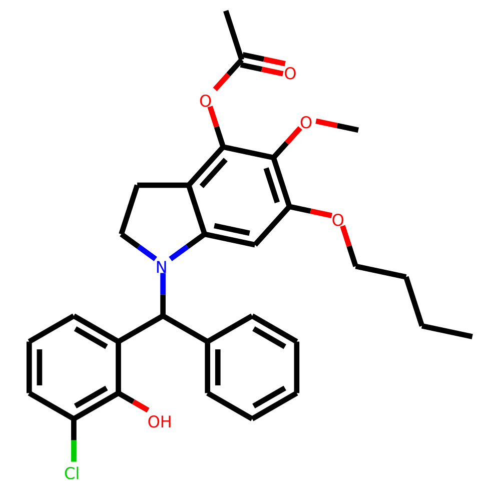
Osma-2630
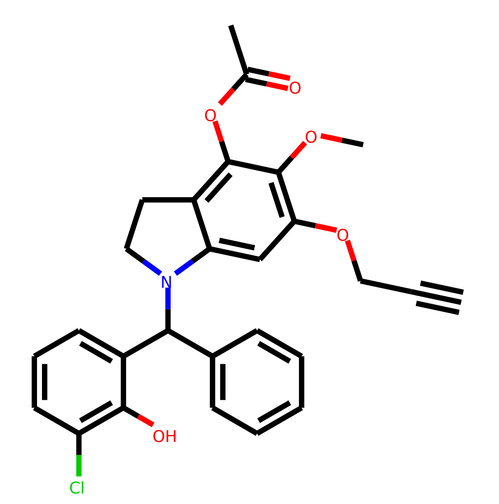
Osma-2631
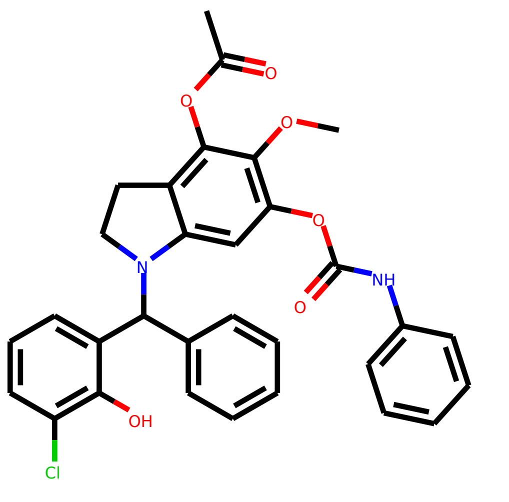
Osma-2632
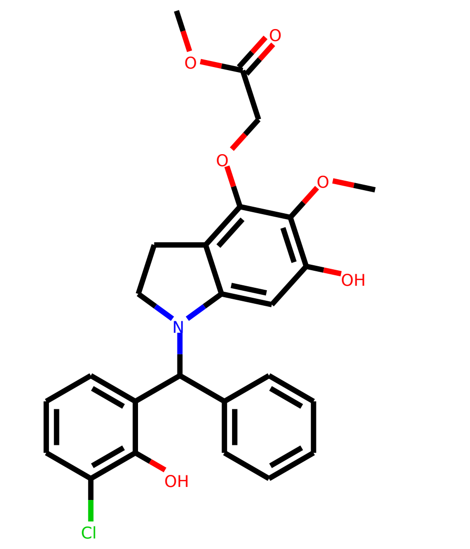
Osma-2633
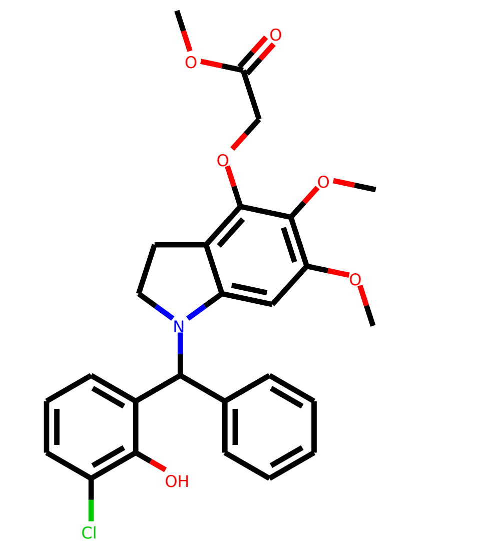
Osma-2634
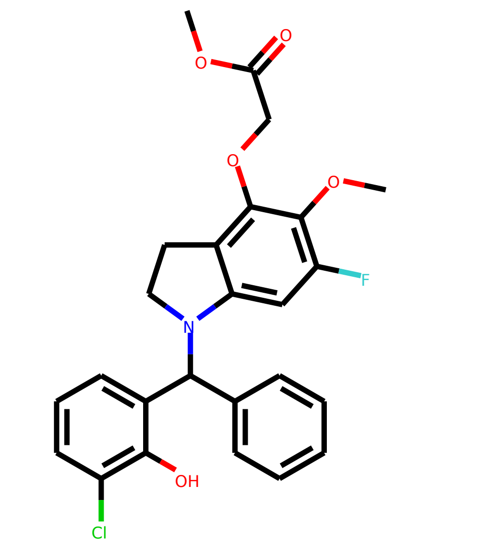
Osma-2635
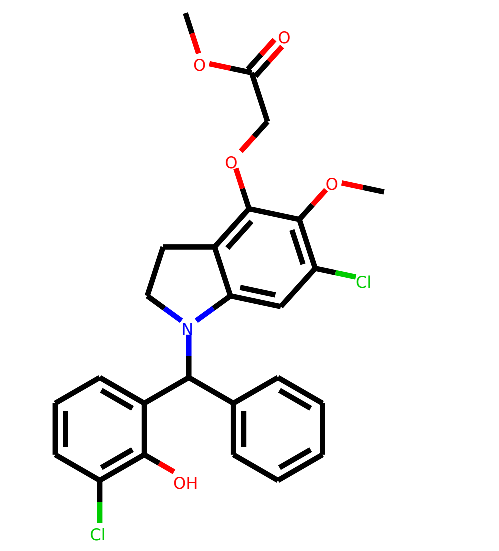
Osma-2636
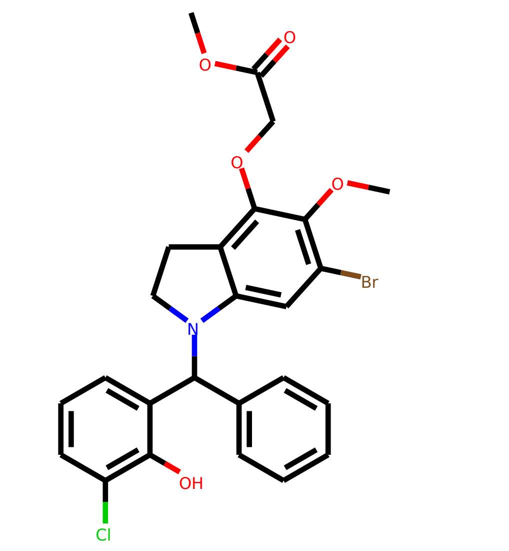
Osma-2637
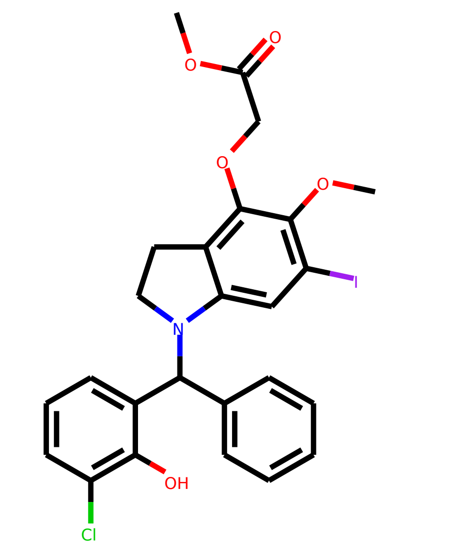
Osma-2638
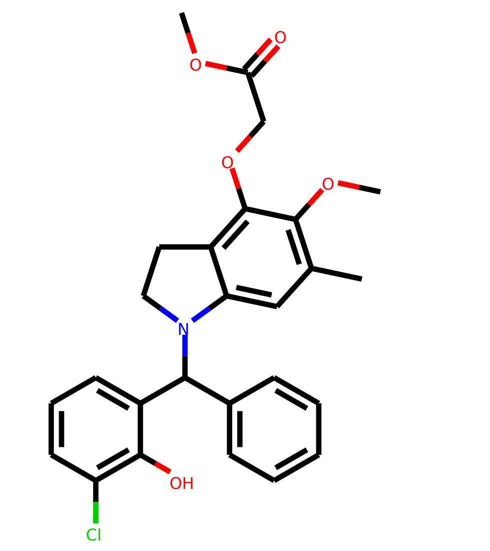
Osma-2639
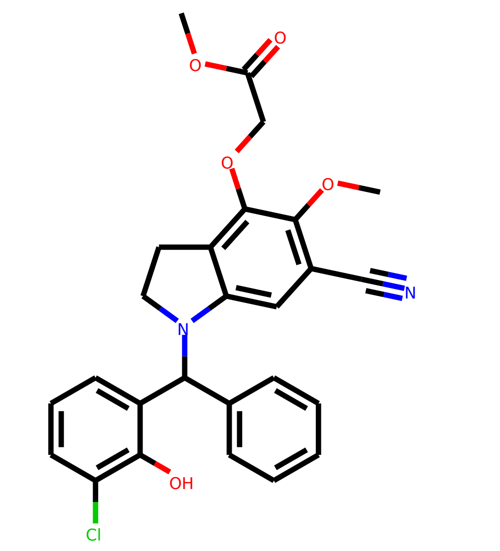
Osma-2640
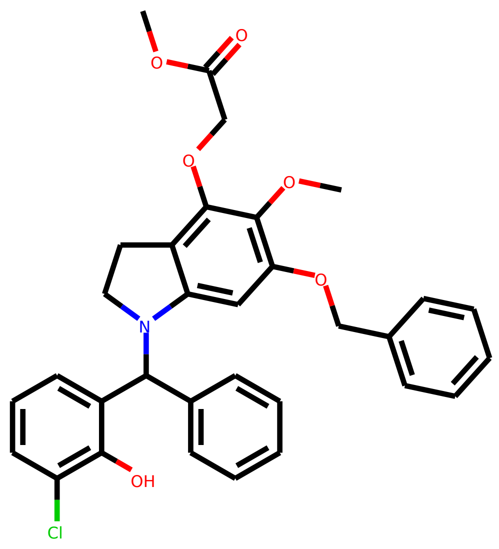
Osma-2641
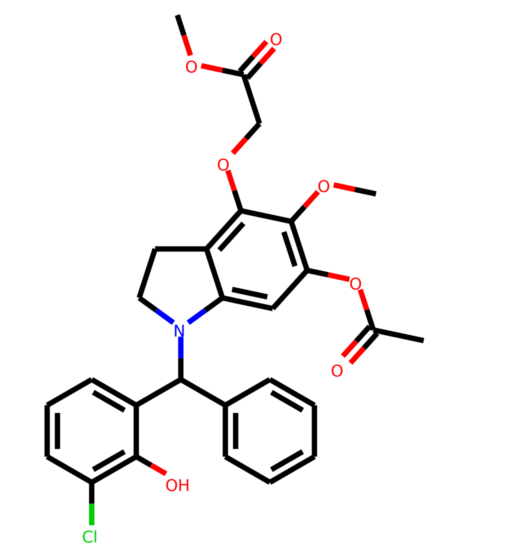
Osma-2642
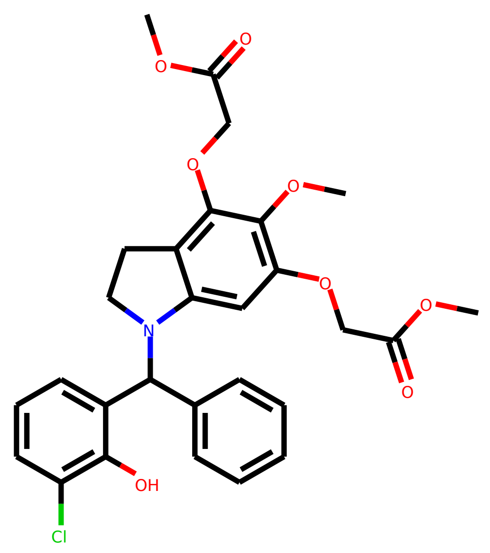
Osma-2643
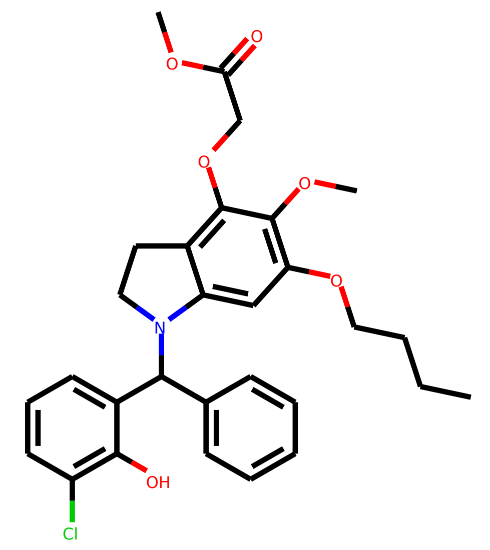
Osma-2644
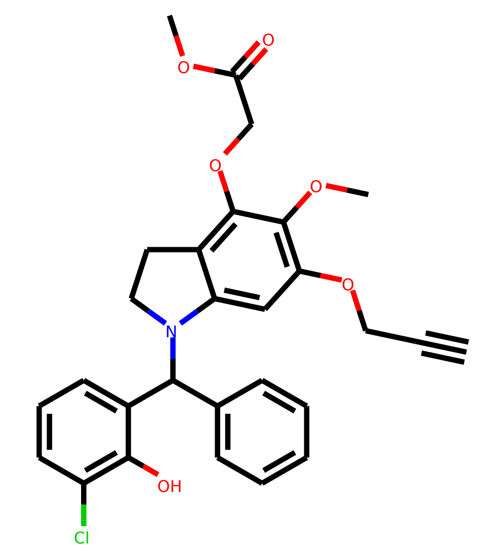
Osma-2645
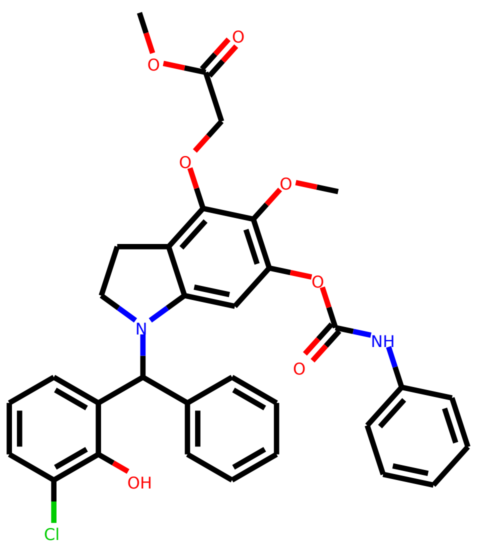
Osma-2646
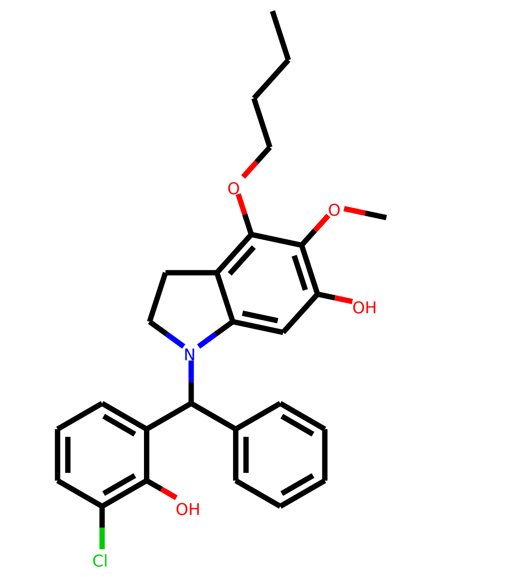
Osma-2647
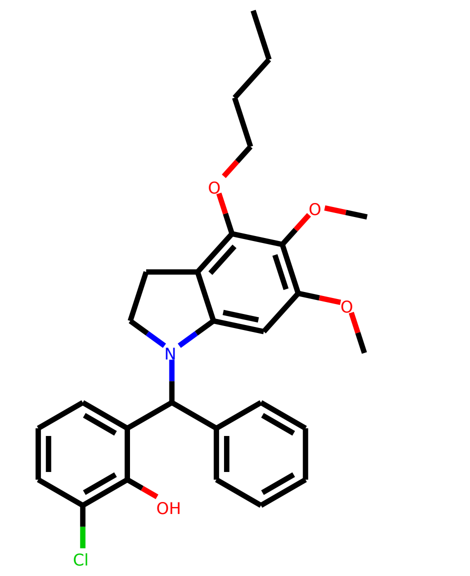
Osma-2648
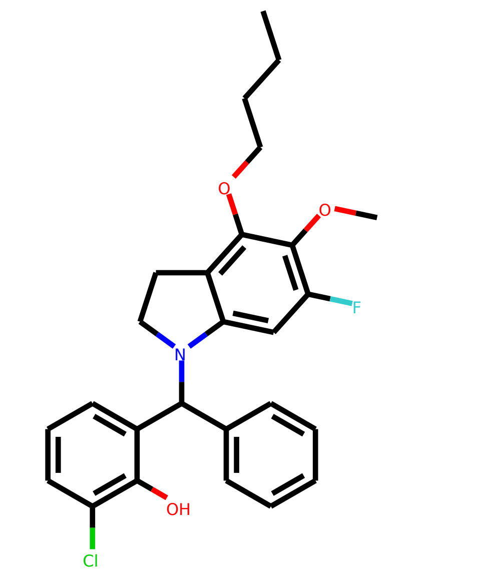
Osma-2649
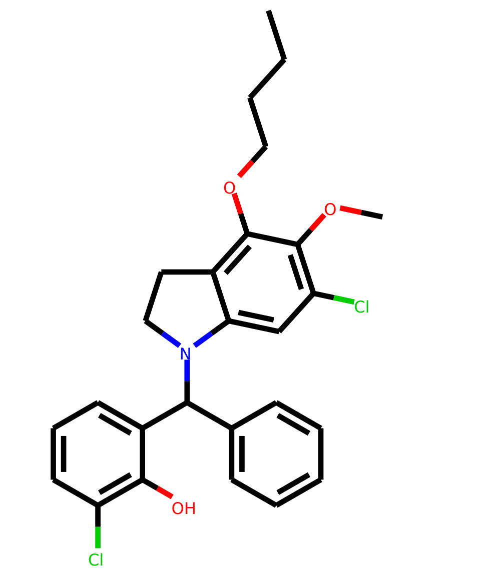
Osma-2650
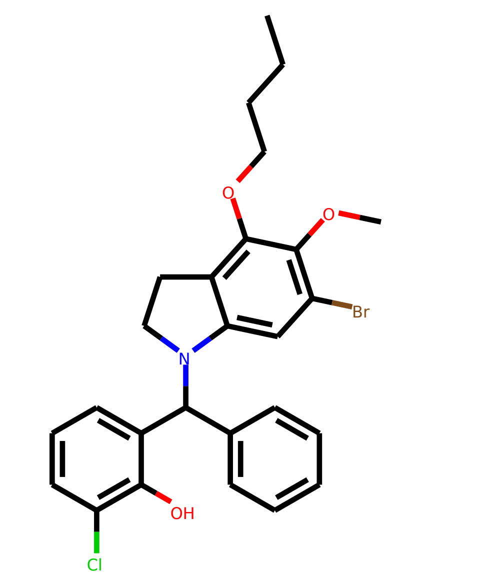
Osma-2651
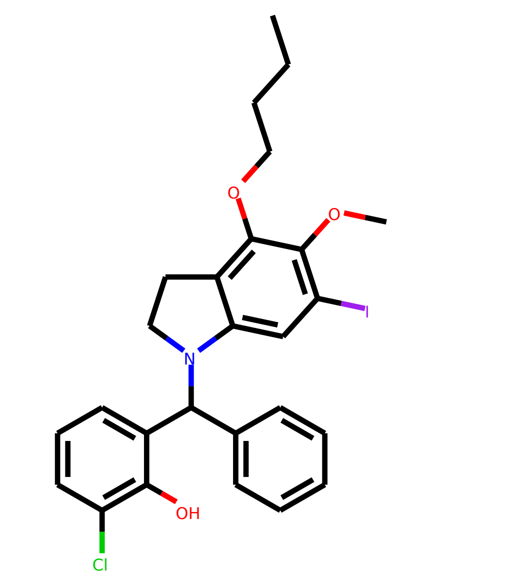
Osma-2652
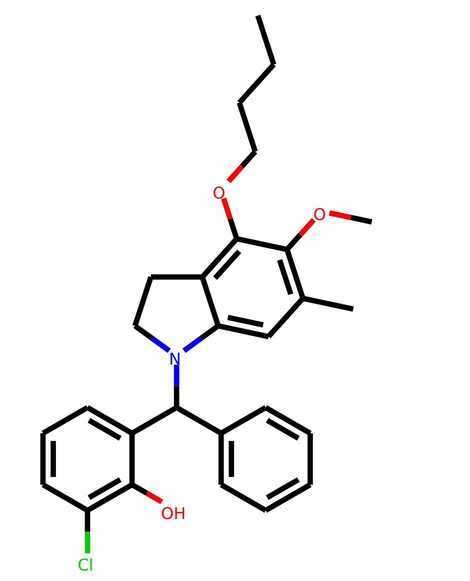
Osma-2653
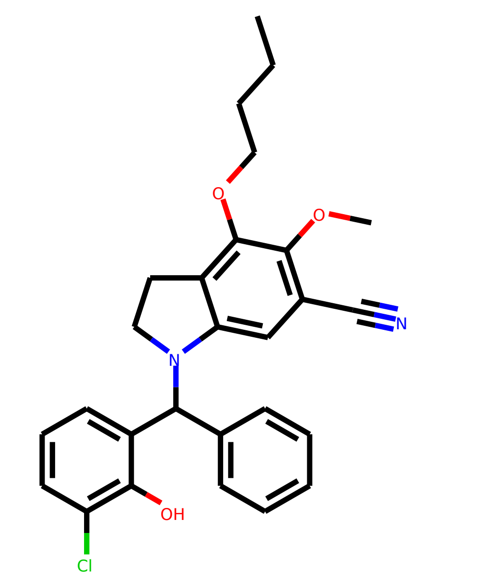
Osma-2654
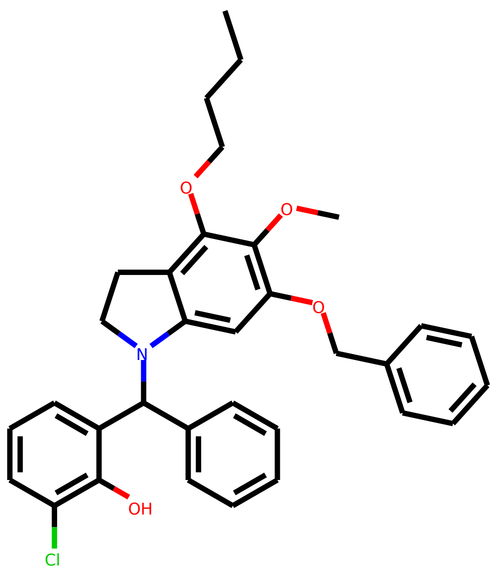
Osma-2655
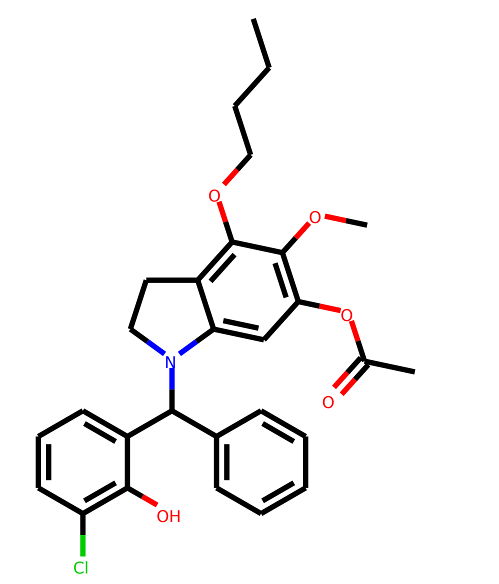
Osma-2656
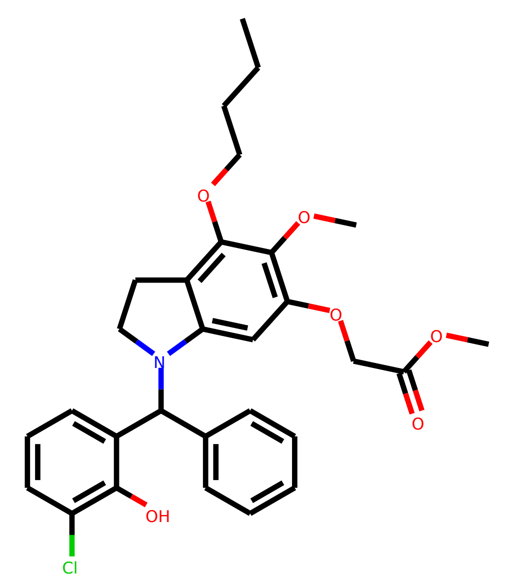
Osma-2657
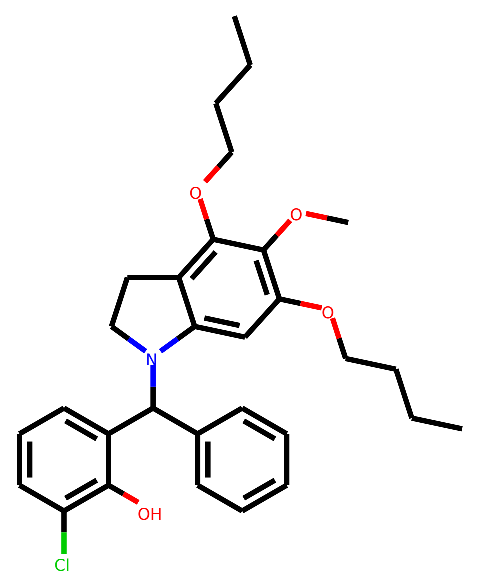
Osma-2658
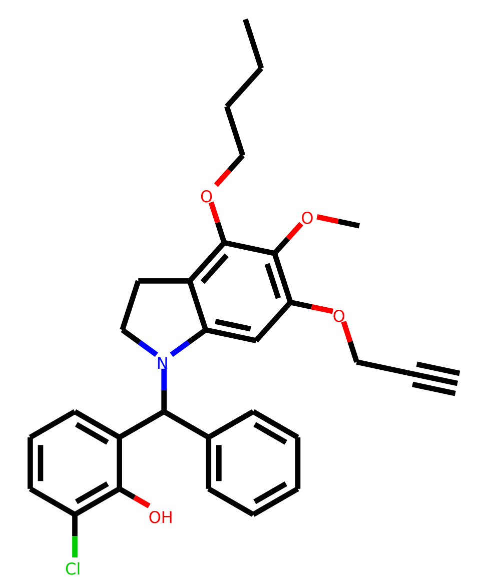
Osma-2659
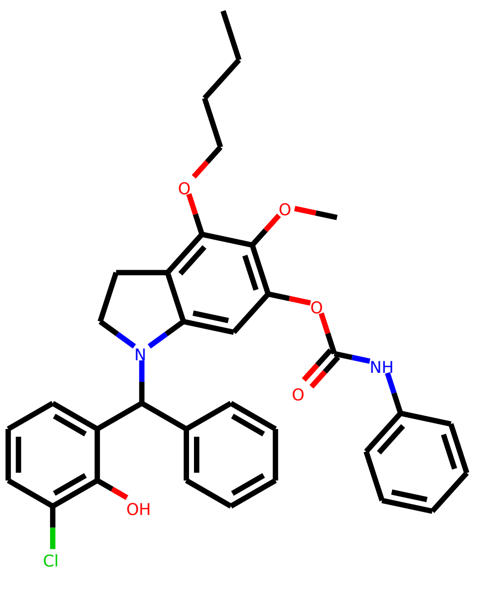
Osma-2660

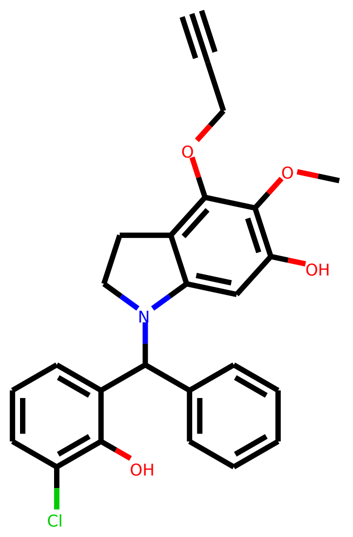
Osma-2661

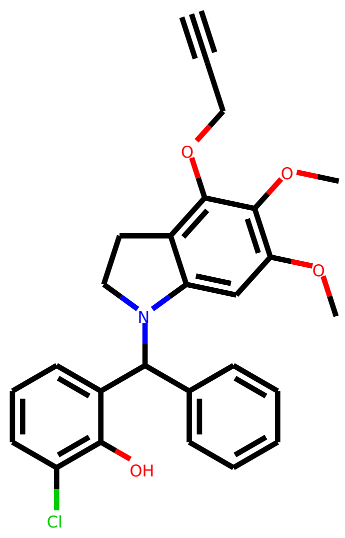
Osma-2662

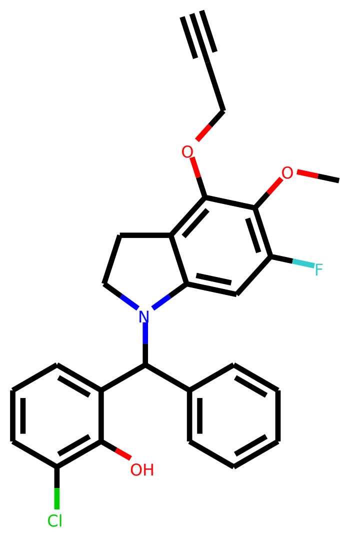
Osma-2663

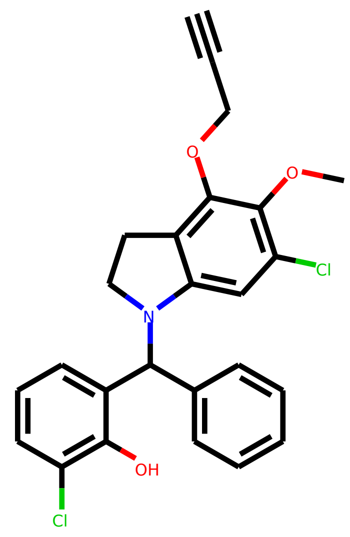
Osma-2664

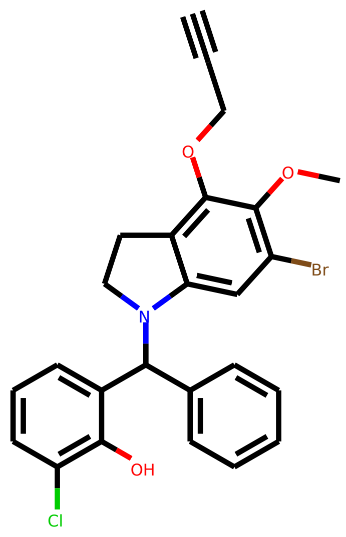
Osma-2665

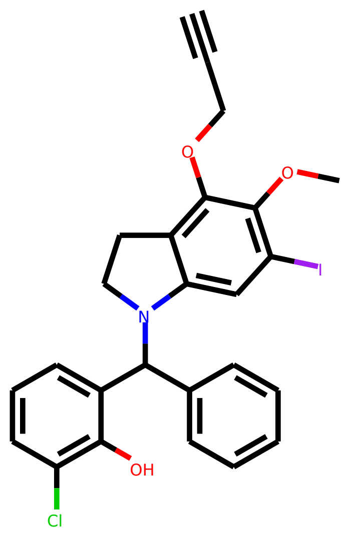
Osma-2666

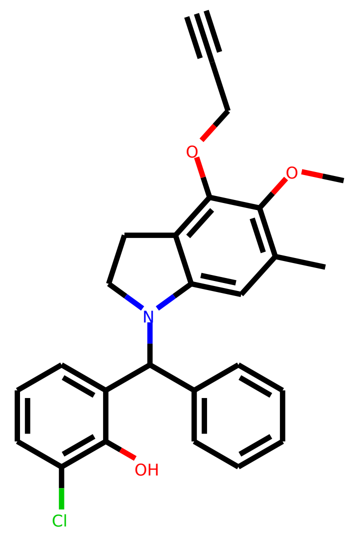
Osma-2667

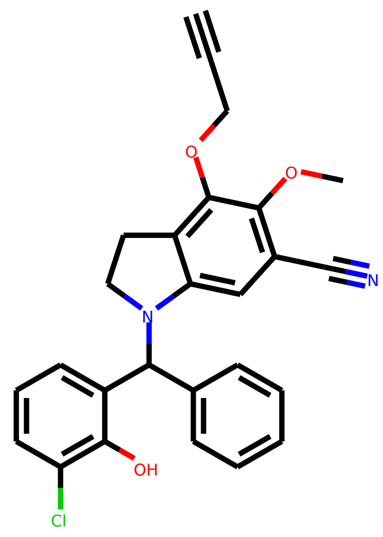
Osma-2668

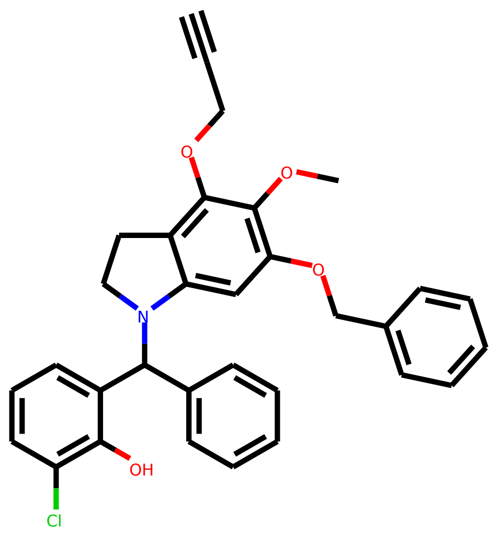
Osma-2669

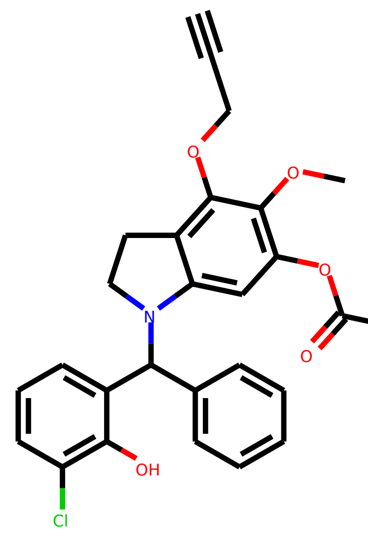
Osma-2670

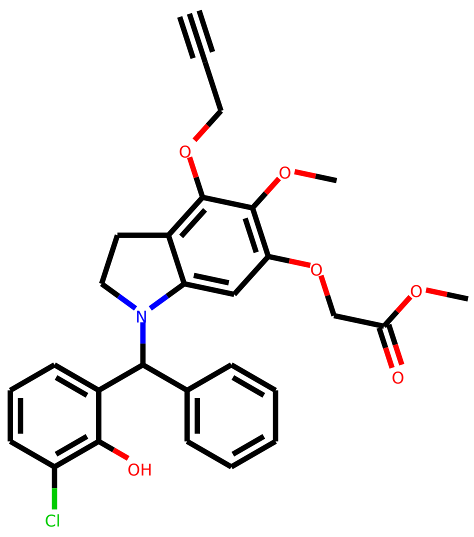
Osma-2671

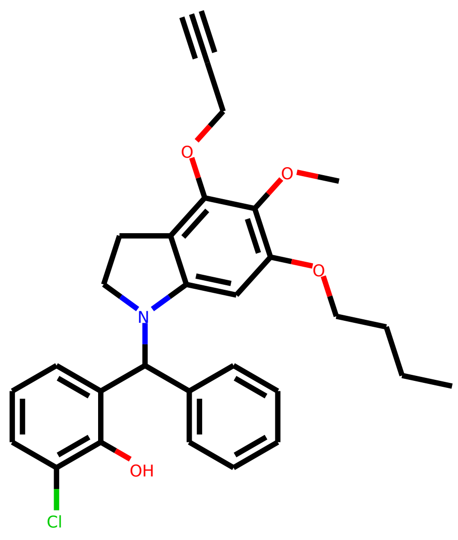
Osma-2672

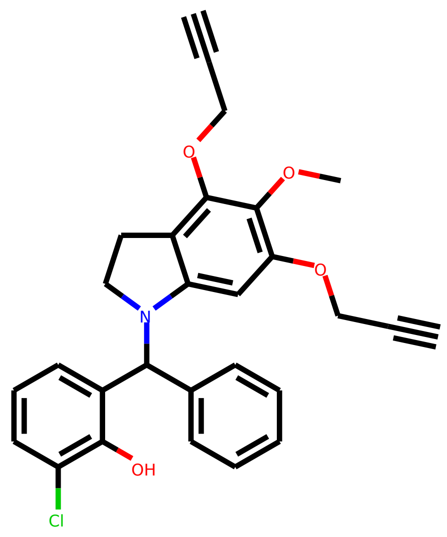
Osma-2673

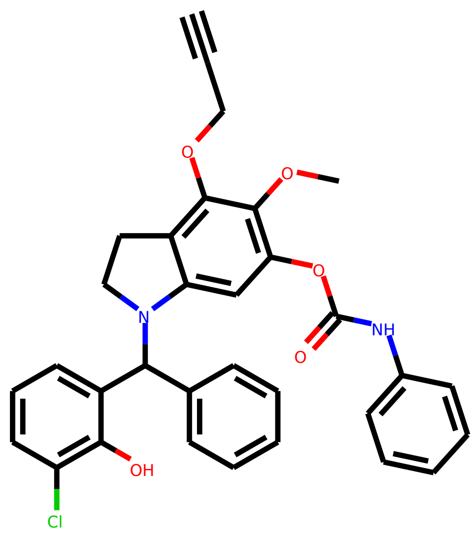
Osma-2674

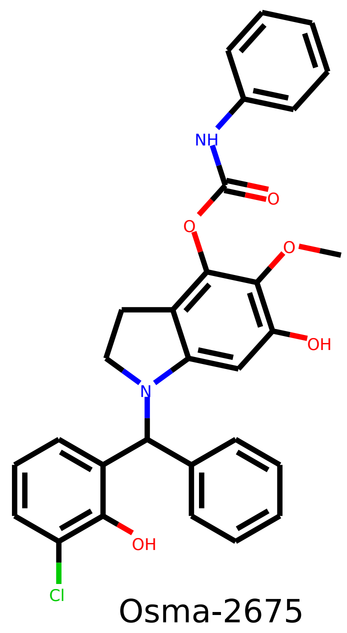
Osma-2675

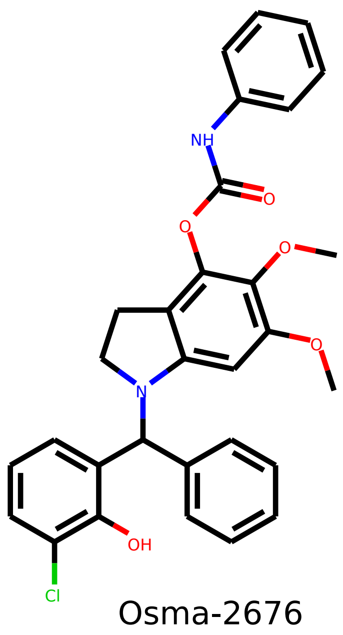
Osma-2676

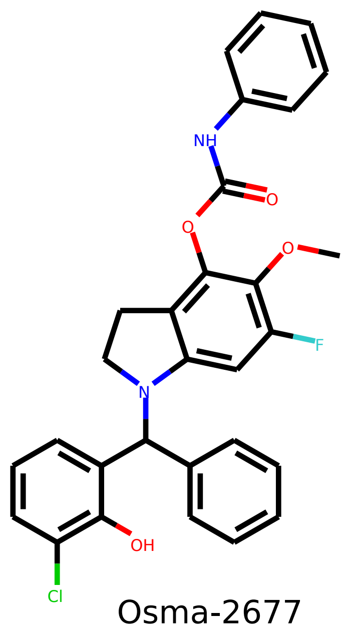
Osma-2677

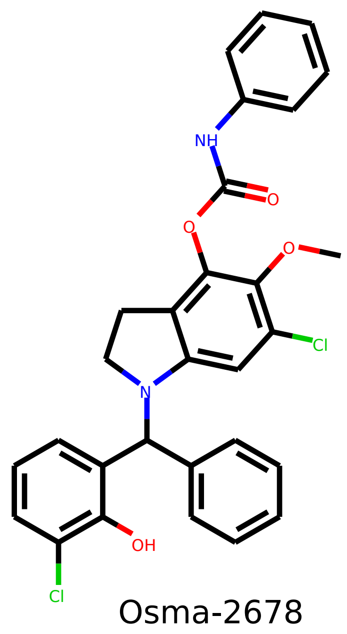
Osma-2678

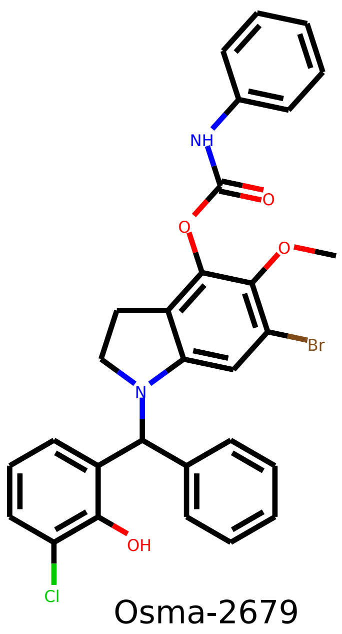
Osma-2679

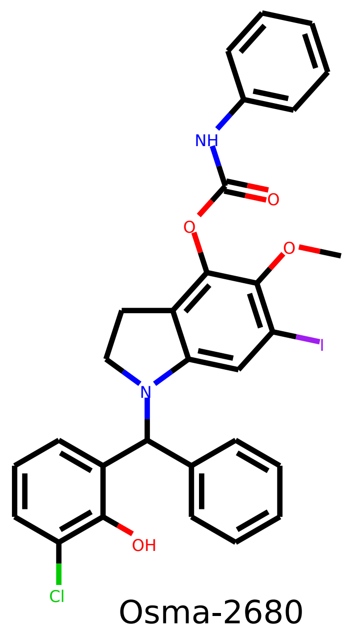
Osma-2680

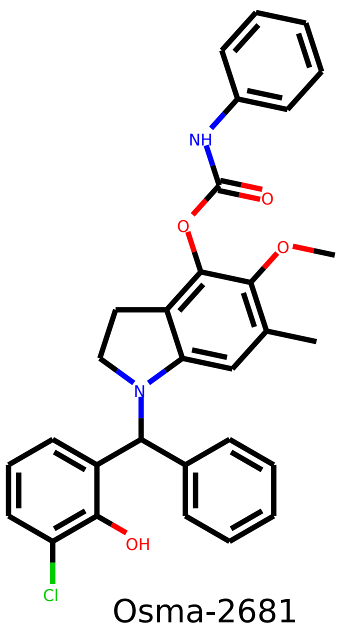
Osma-2681

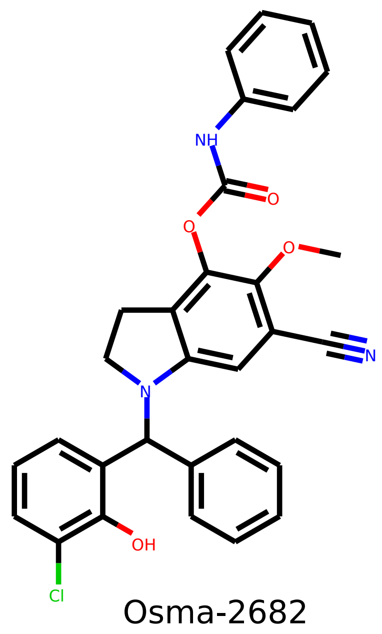
Osma-2682

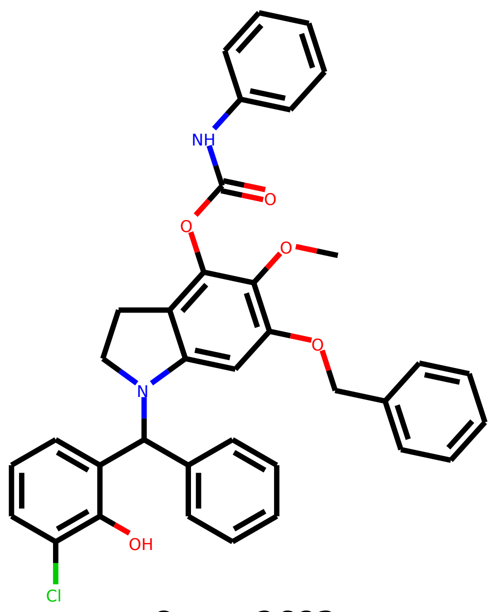
Osma-2683

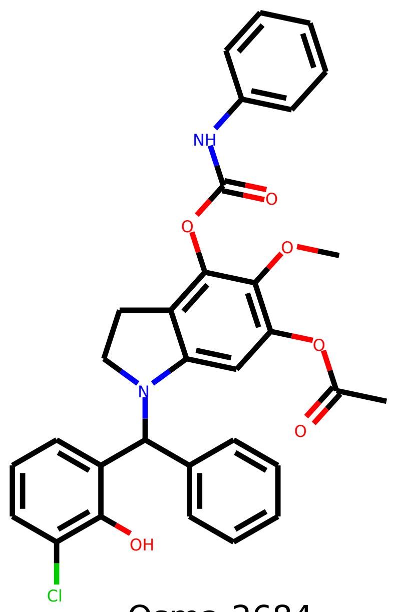
Osma-2684

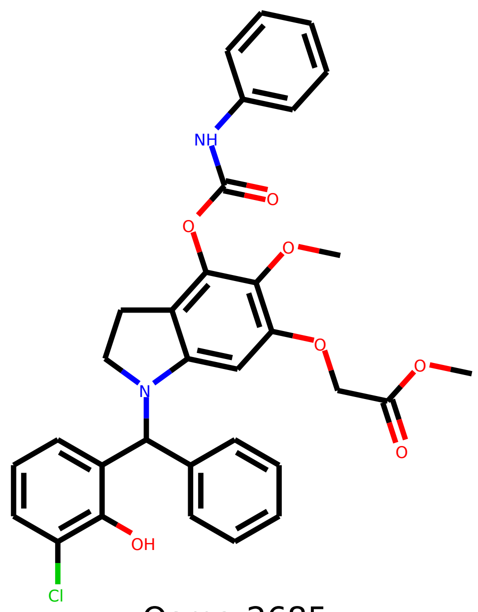
Osma-2685

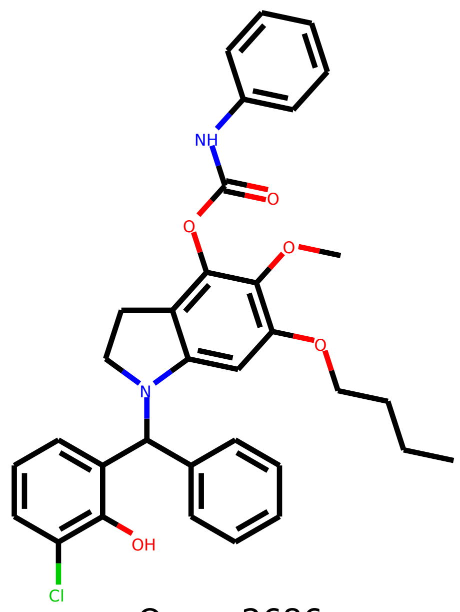
Osma-2686

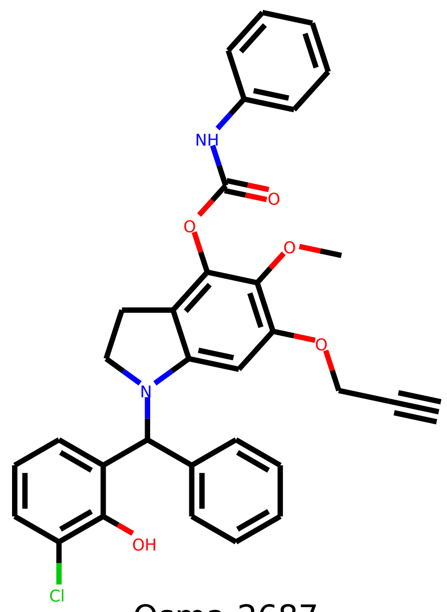
Osma-2687

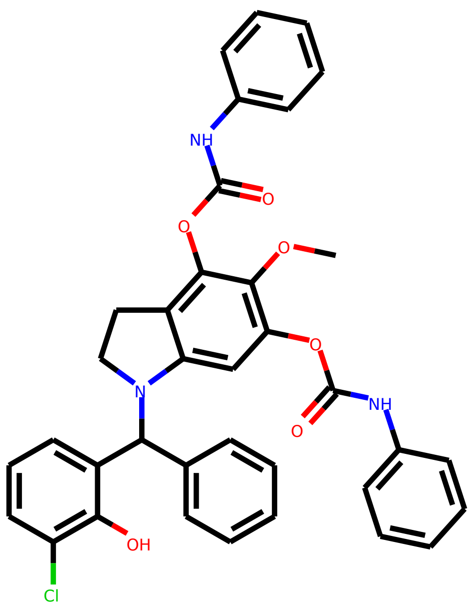
Osma-2688

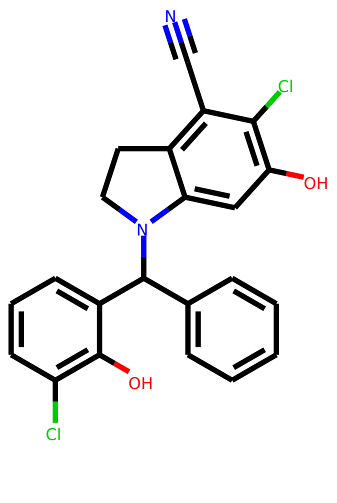
Osma-2689

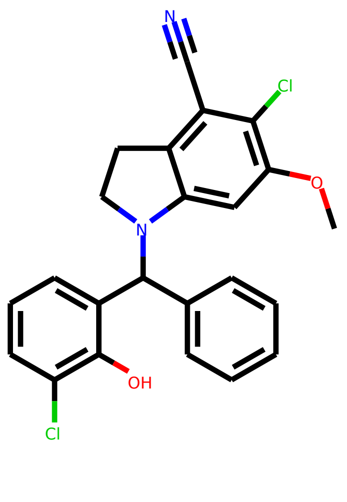
Osma-2690

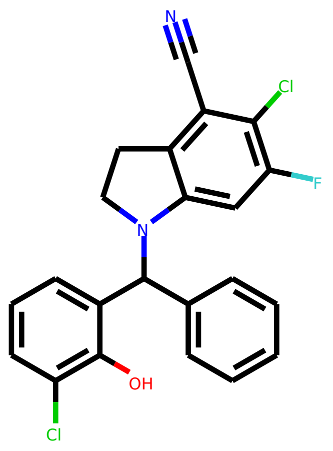
Osma-2691

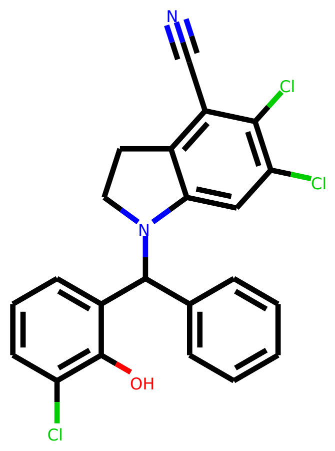
Osma-2692

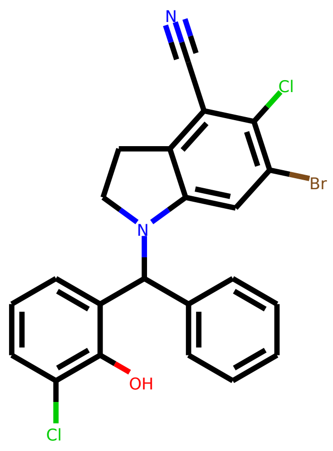
Osma-2693

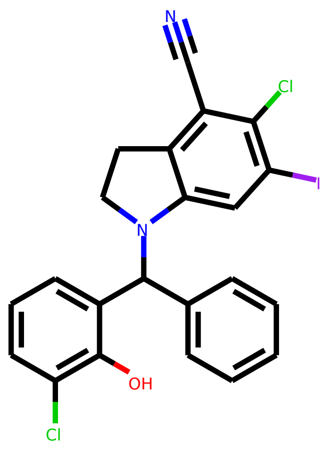
Osma-2694

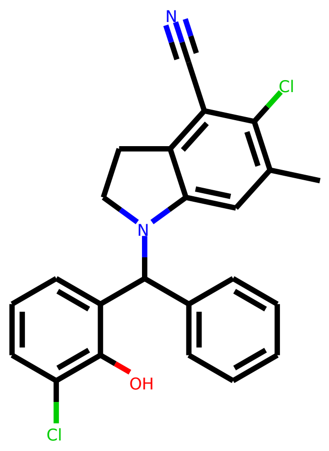
Osma-2695

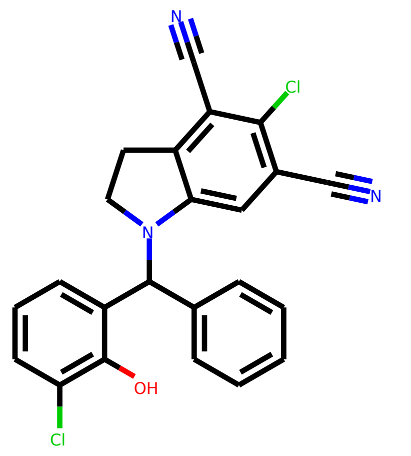
Osma-2696
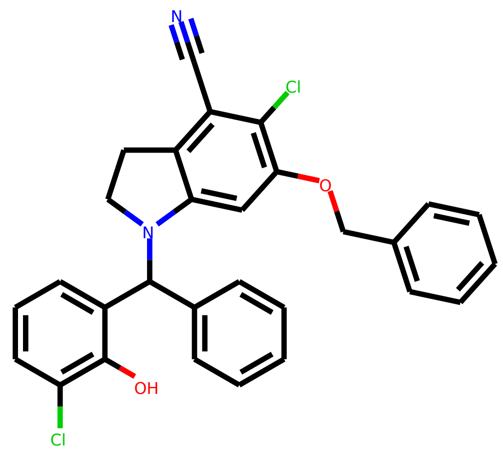
Osma-2697
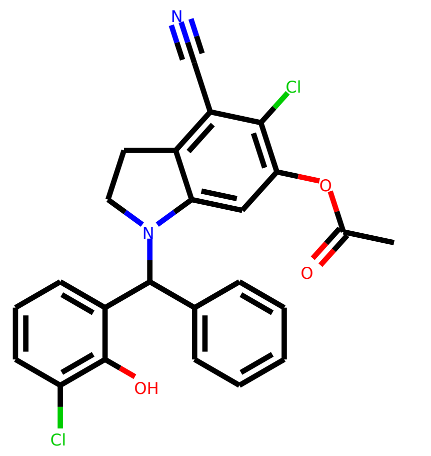
Osma-2698
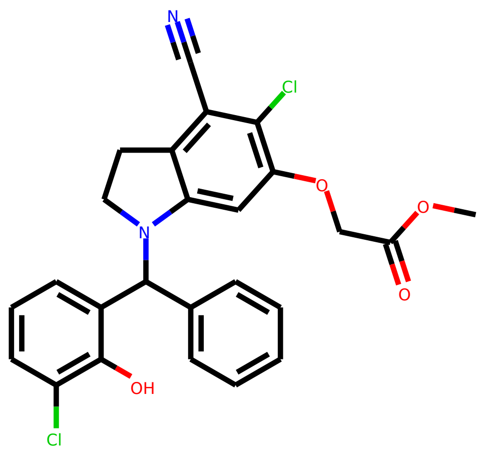
Osma-2699
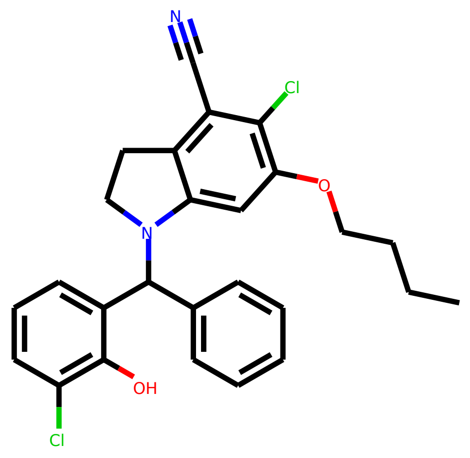
Osma-2700
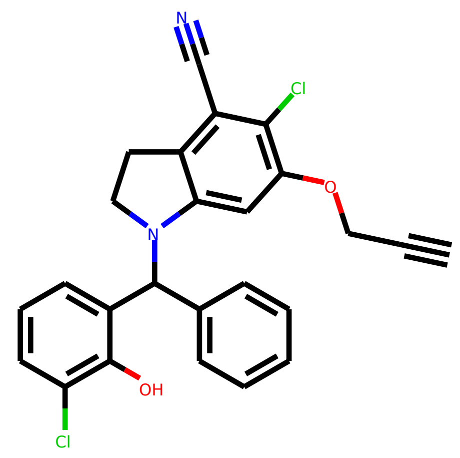
Osma-2701
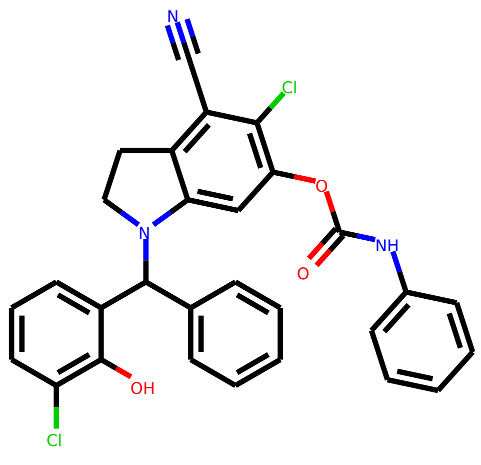
Osma-2702
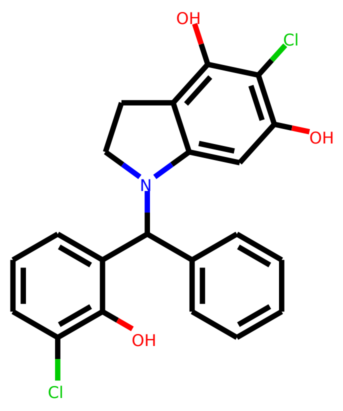
Osma-2703
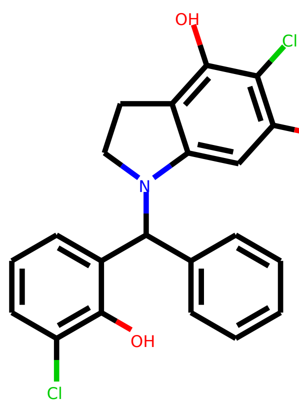
Osma-2704
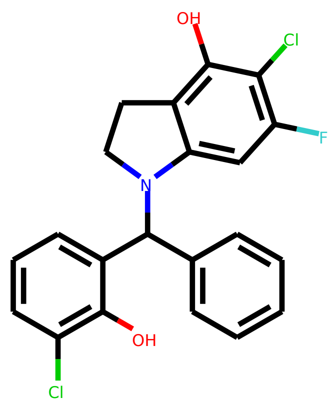
Osma-2705
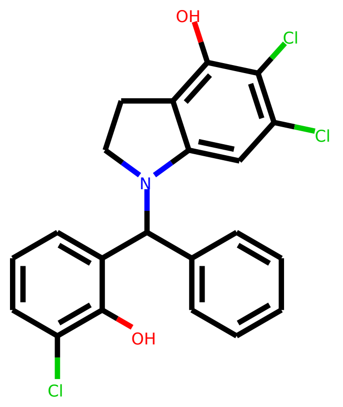
Osma-2706
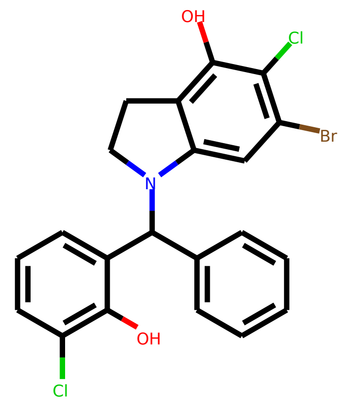
Osma-2707
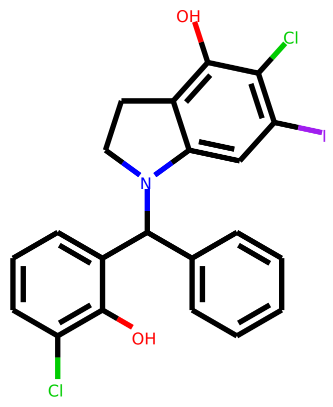
Osma-2708
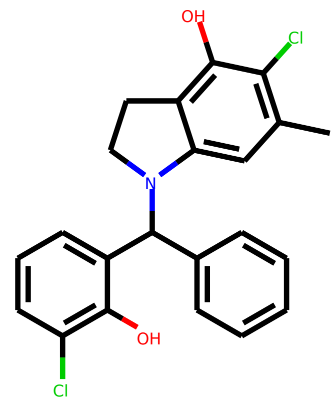
Osma-2709
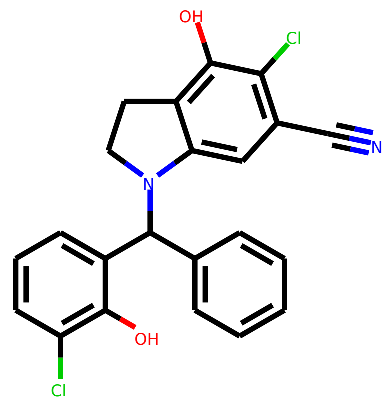
Osma-2710
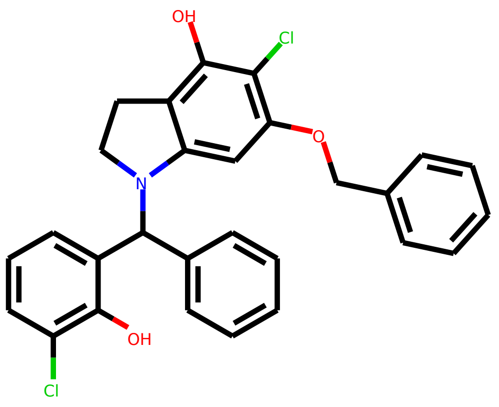
Osma-2711
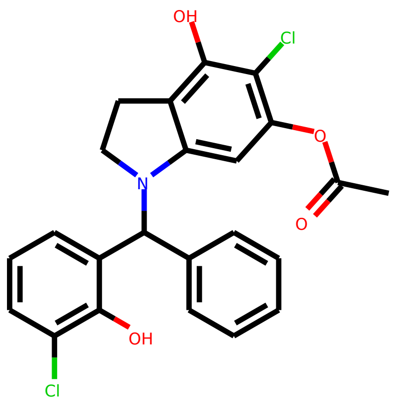
Osma-2712
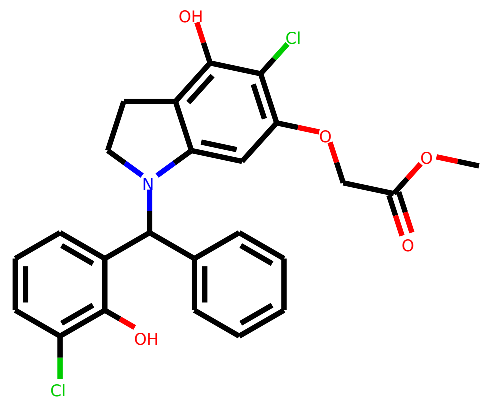
Osma-2713
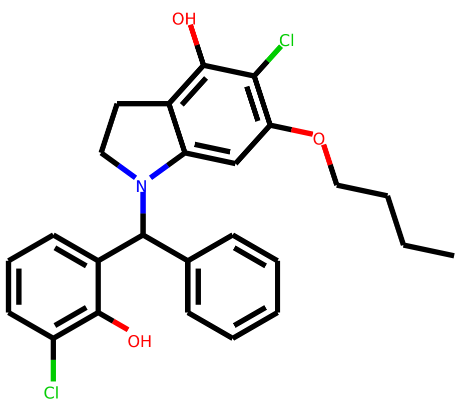
Osma-2714
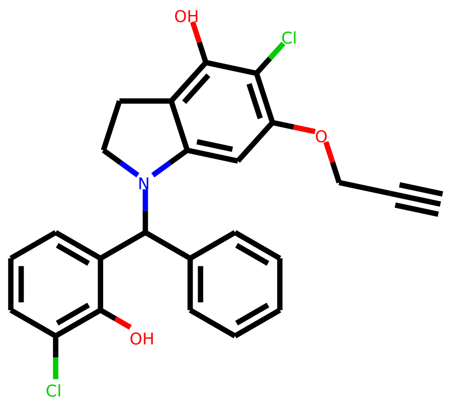
Osma-2715
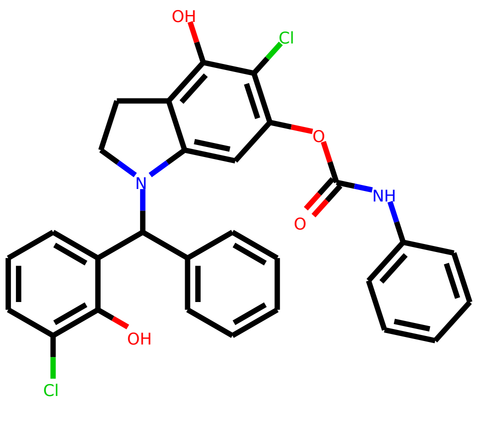
Osma-2716
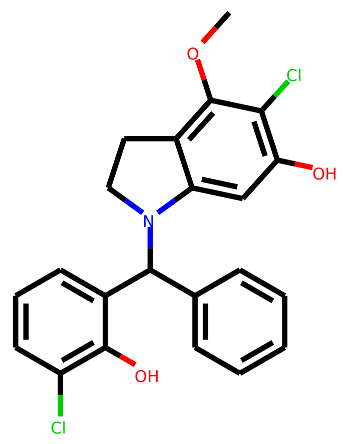
Osma-2717
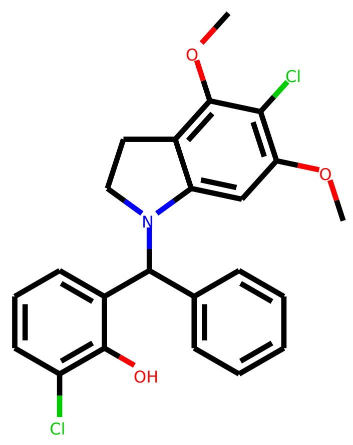
Osma-2718
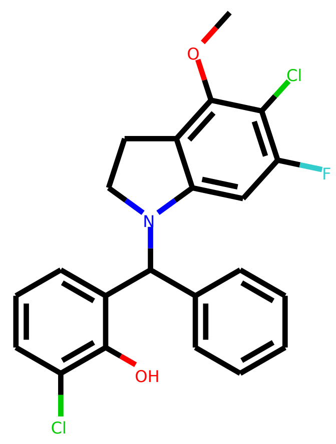
Osma-2719
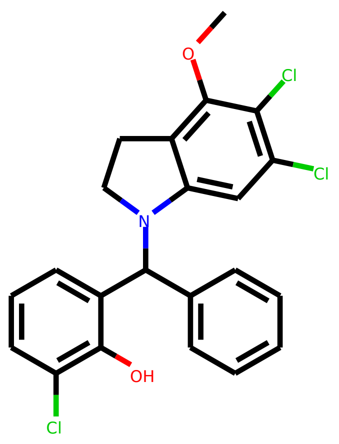
Osma-2720
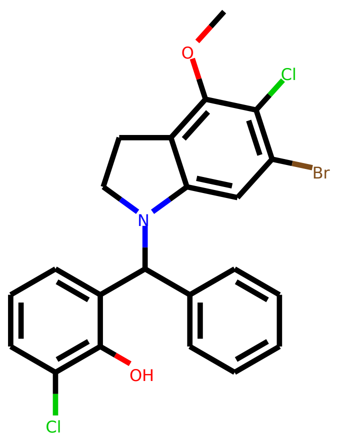
Osma-2721
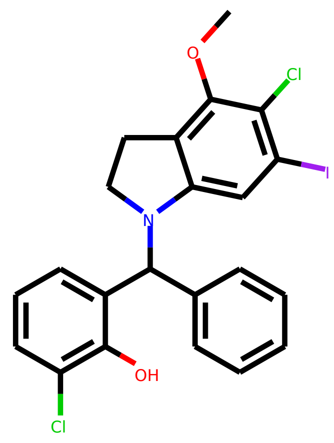
Osma-2722
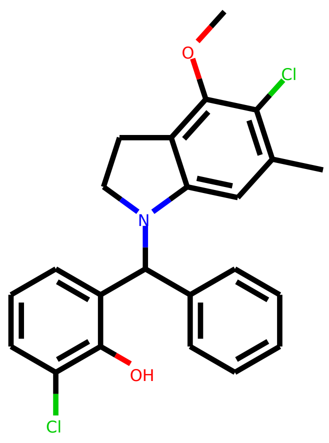
Osma-2723
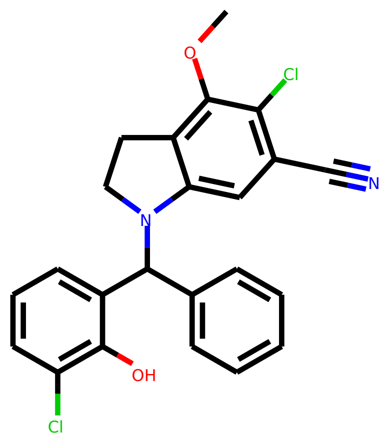
Osma-2724
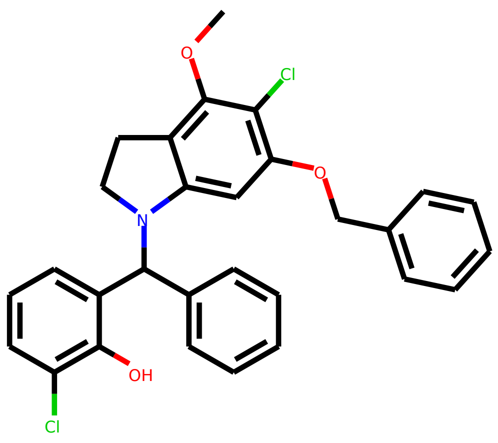
Osma-2725
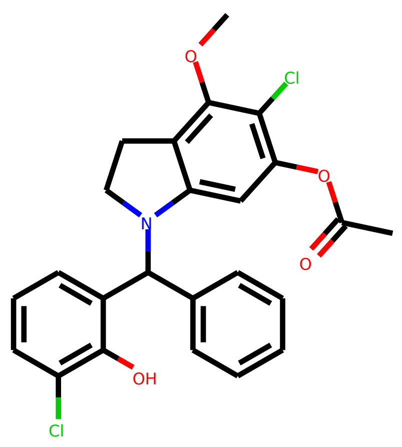
Osma-2726
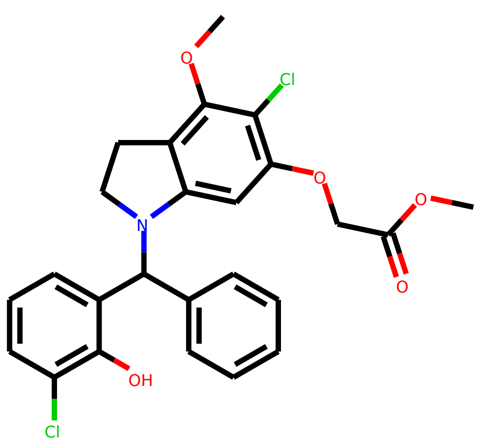
Osma-2727
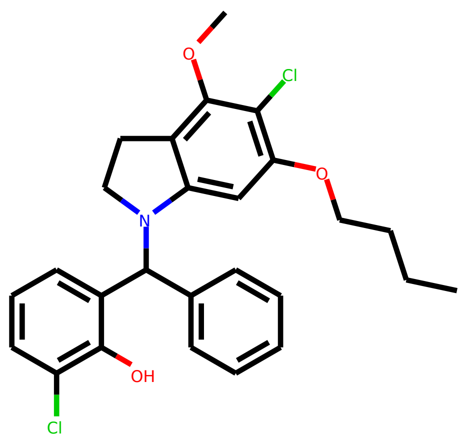
Osma-2728
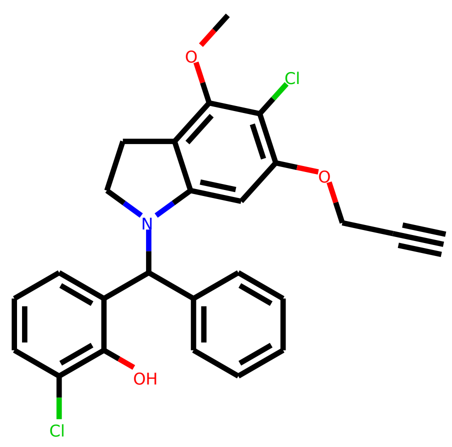
Osma-2729
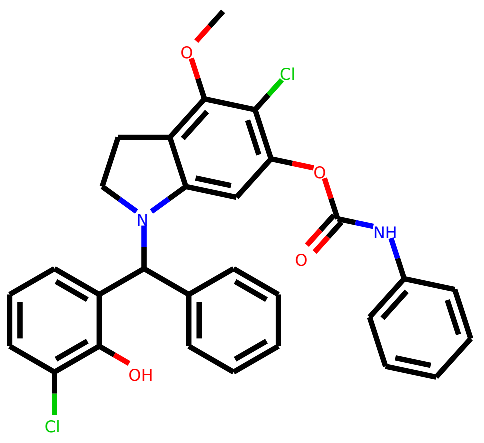
Osma-2730

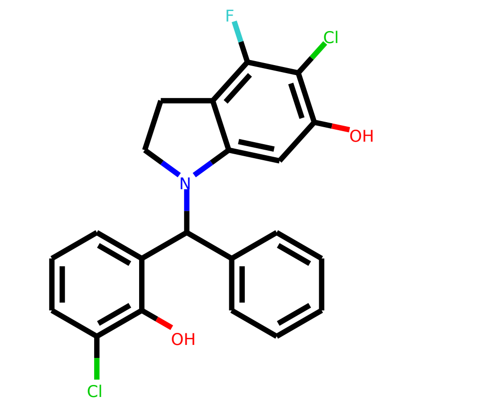

Osma-2731

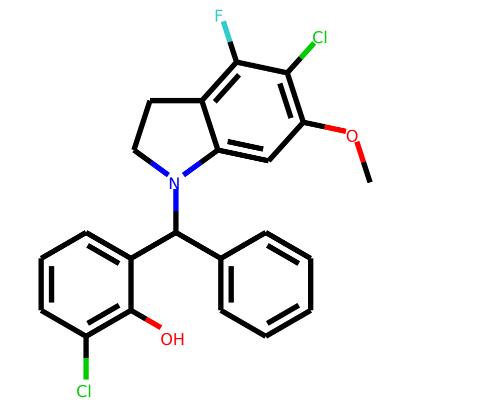

Osma-2732

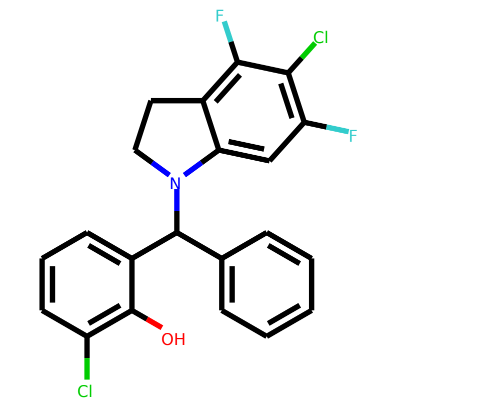

Osma-2733

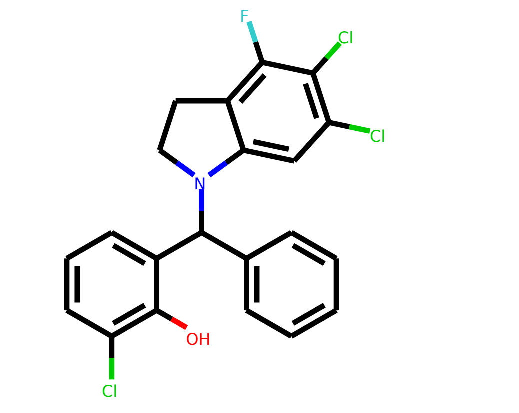

Osma-2734

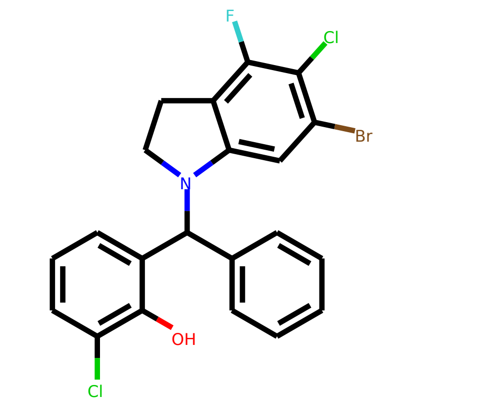

Osma-2735

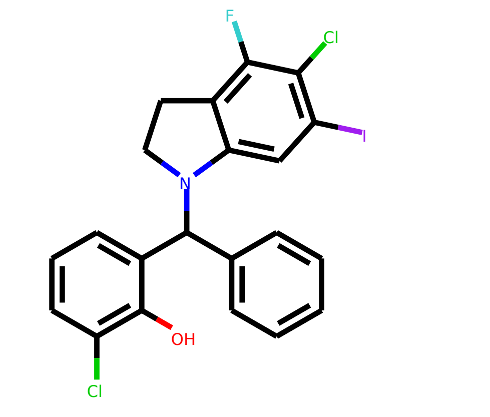

Osma-2736

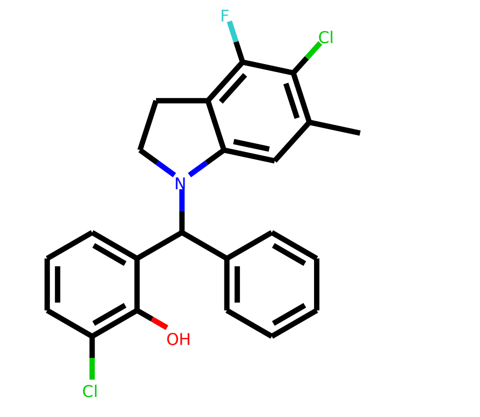

Osma-2737

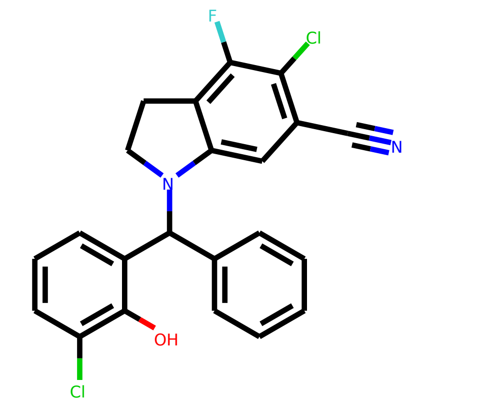

Osma-2738

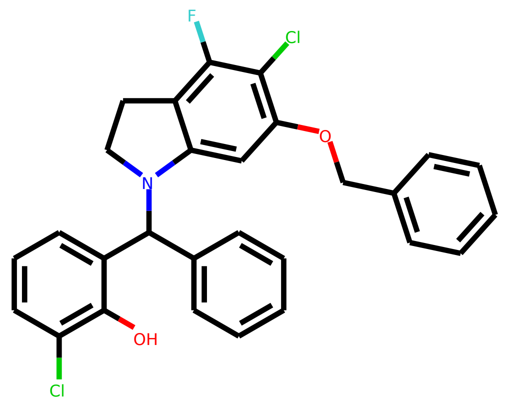

Osma-2739

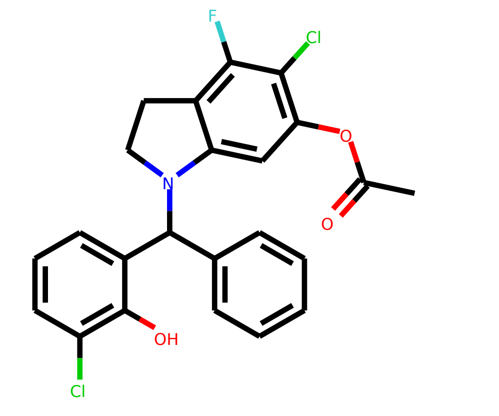

Osma-2740

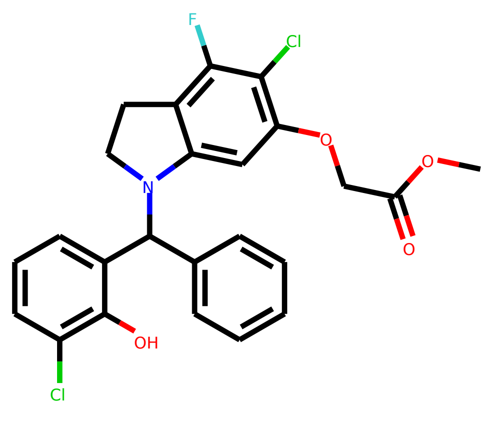

Osma-2741

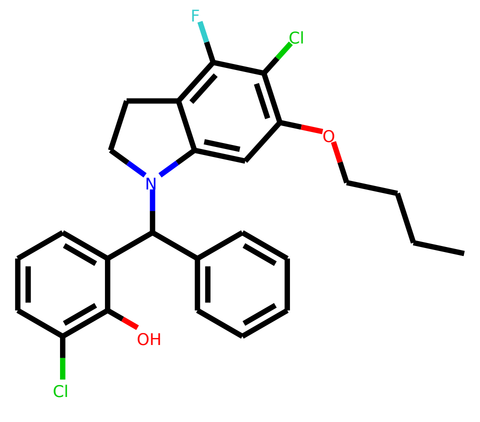

Osma-2742

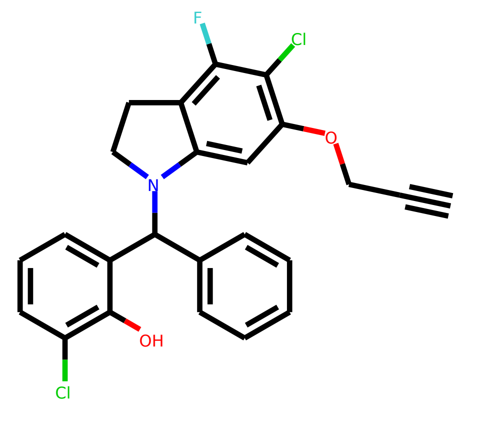

Osma-2743

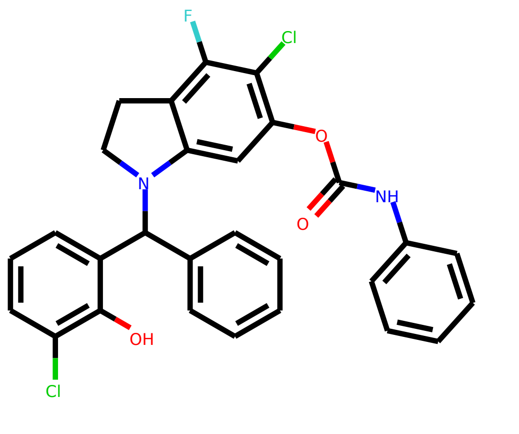

Osma-2744

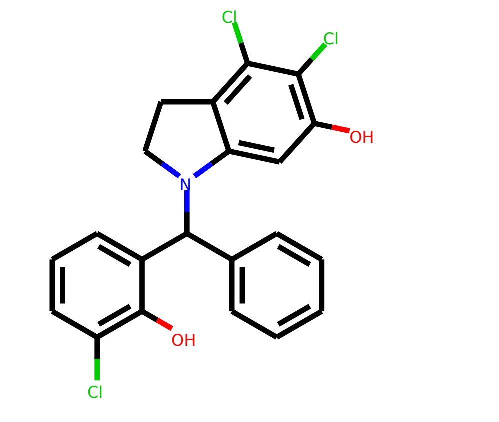

Osma-2745

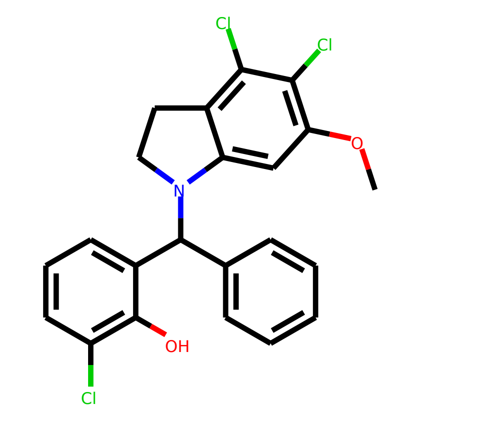

Osma-2746

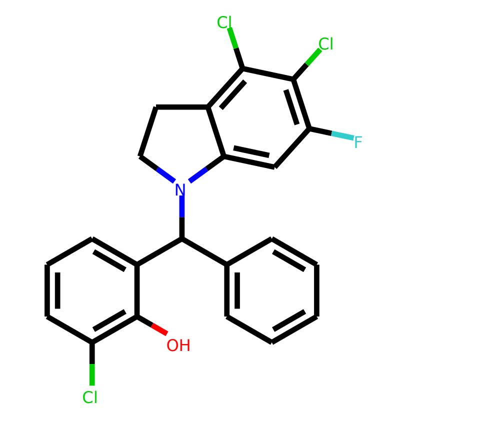

Osma-2747

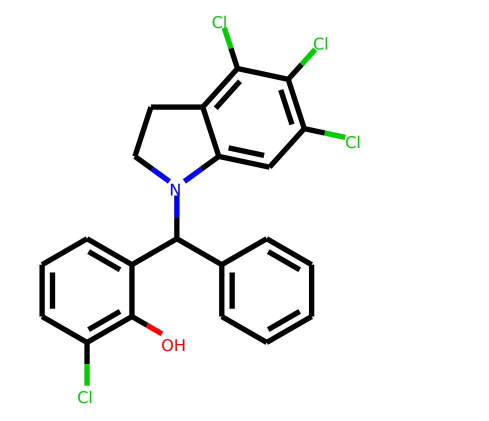

Osma-2748

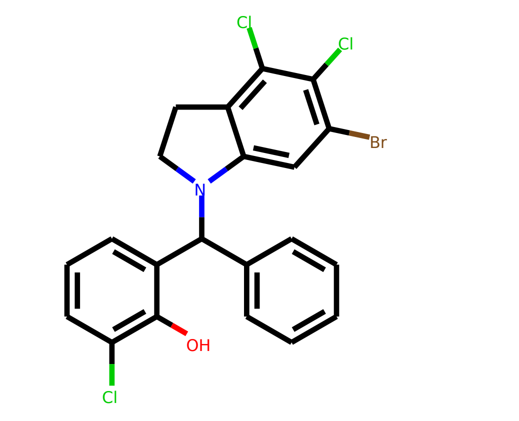

Osma-2749

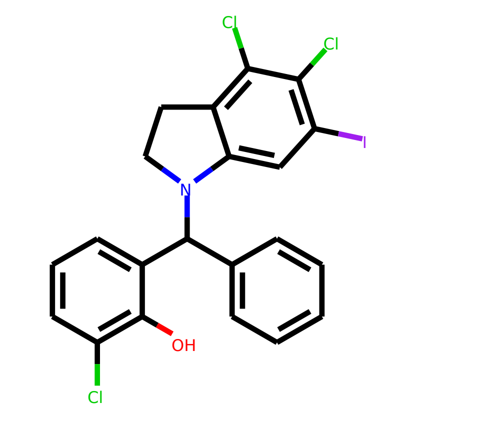

Osma-2750

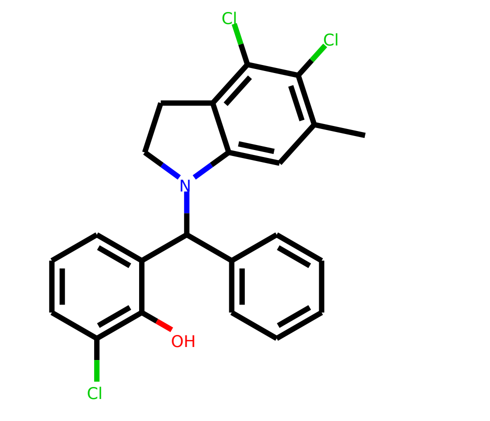

Osma-2751

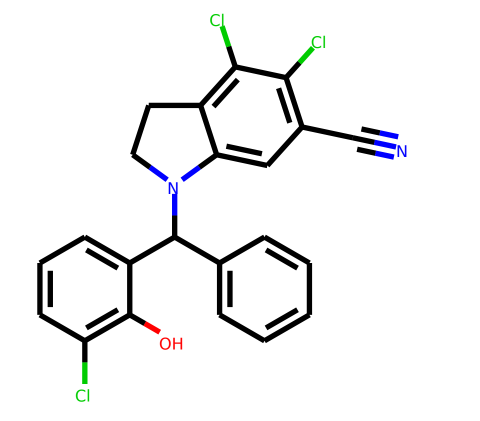

Osma-2752

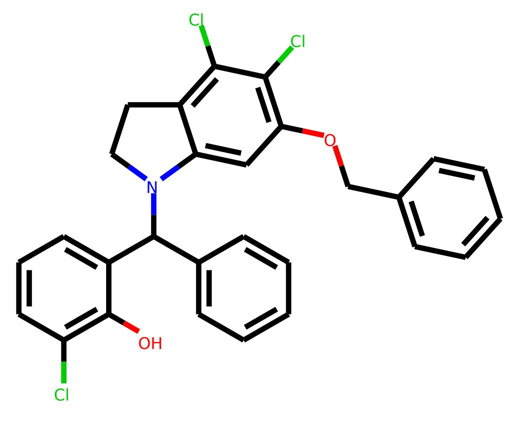

Osma-2753

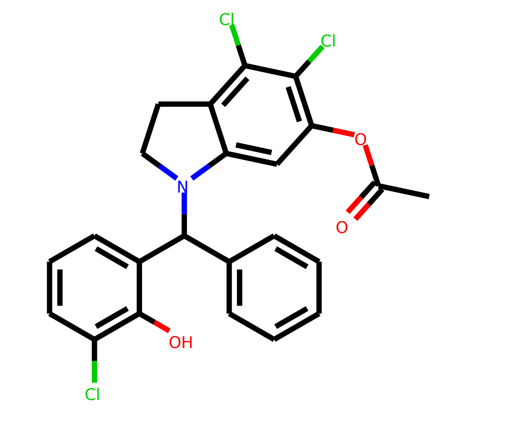

Osma-2754

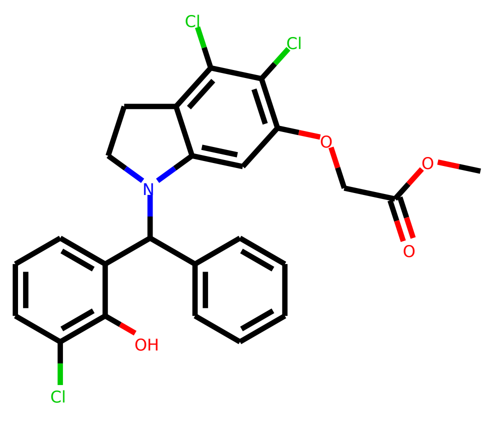

Osma-2755

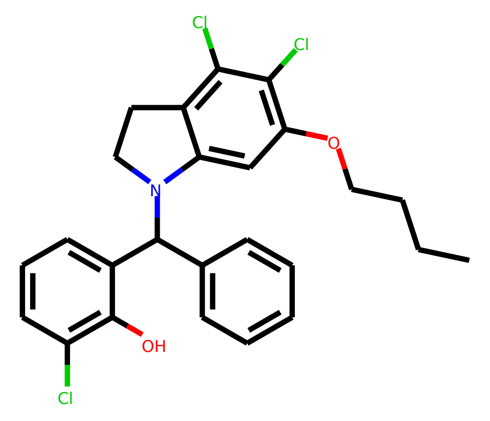

Osma-2756

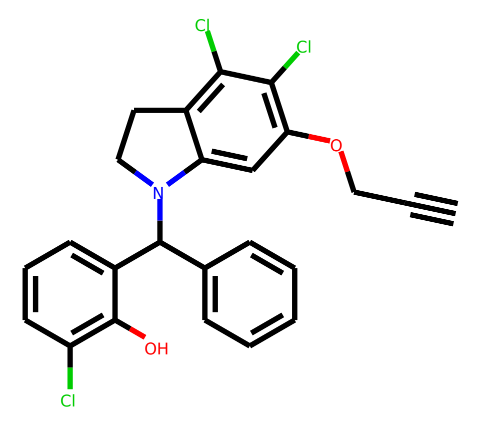

Osma-2757

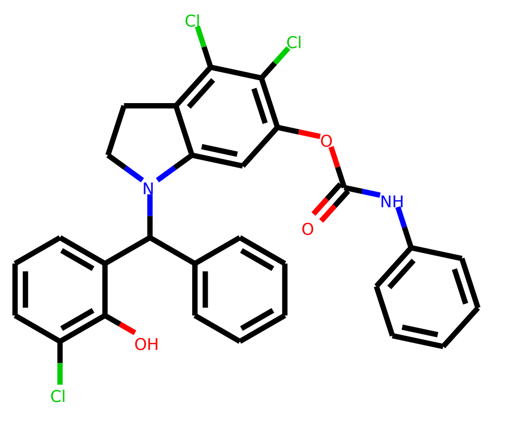

Osma-2758

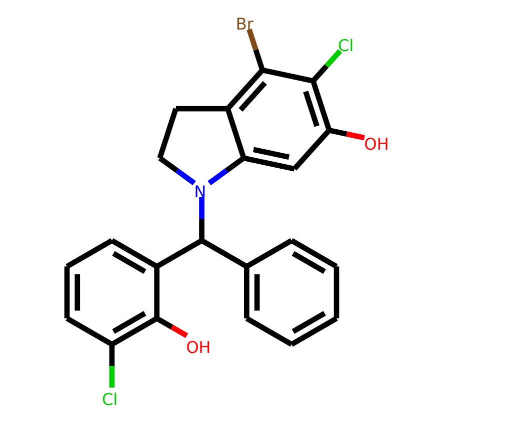

Osma-2759

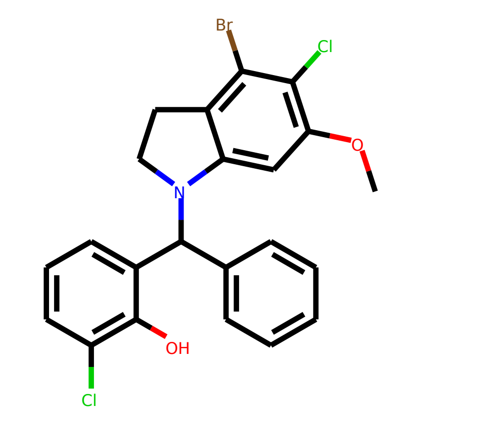

Osma-2760

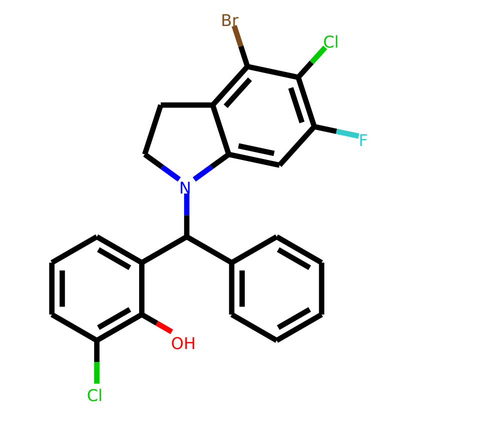

Osma-2761

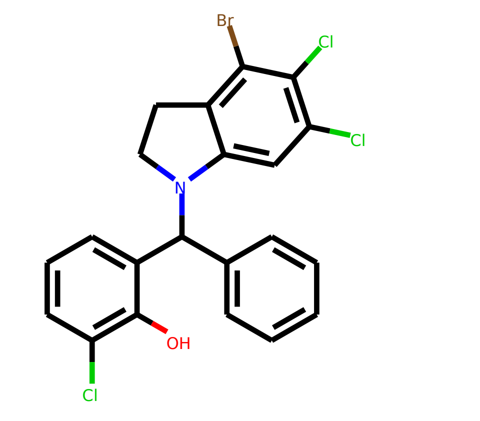

Osma-2762

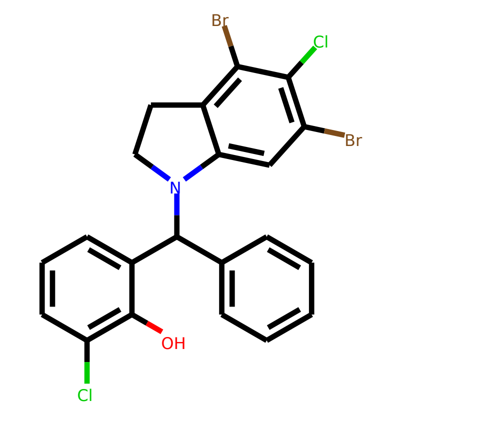

Osma-2763

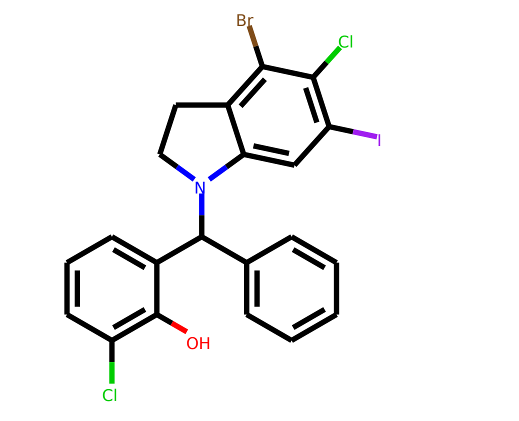

Osma-2764

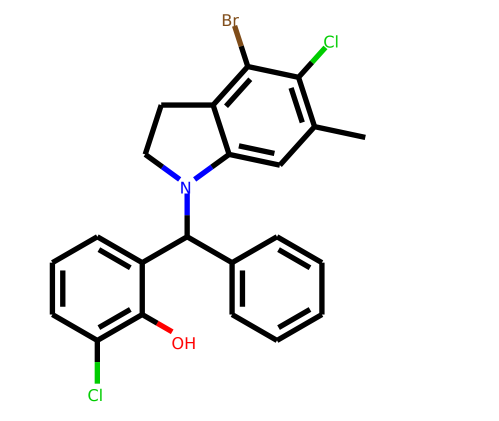

Osma-2765

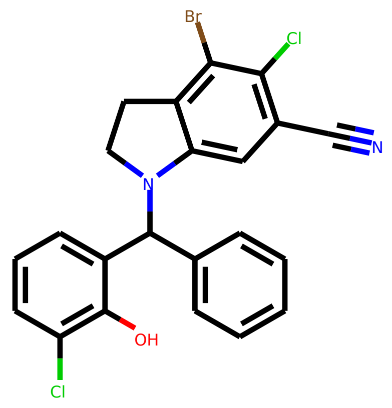

Osma-2766

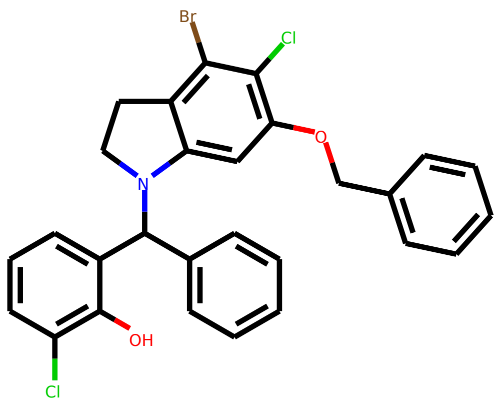

Osma-2767

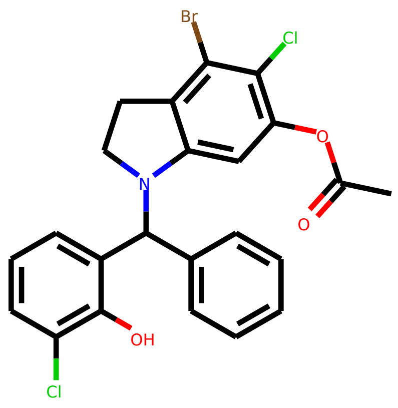

Osma-2768

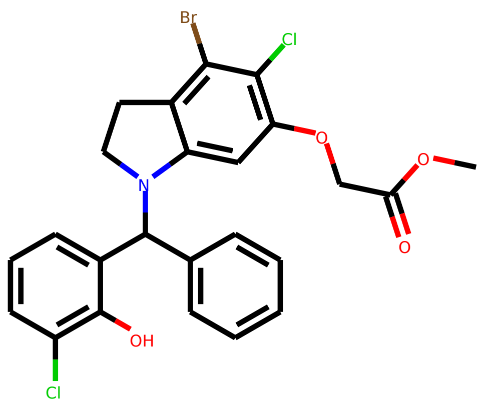

Osma-2769

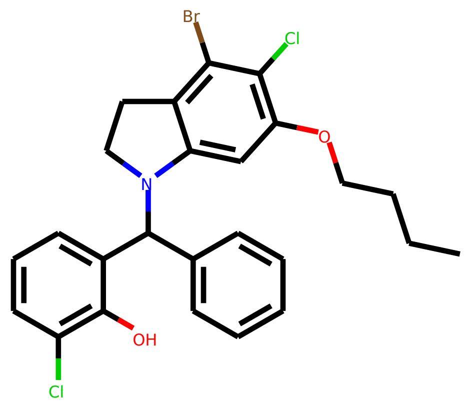

Osma-2770

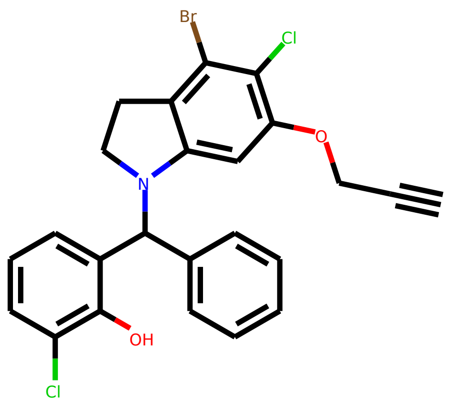

Osma-2771

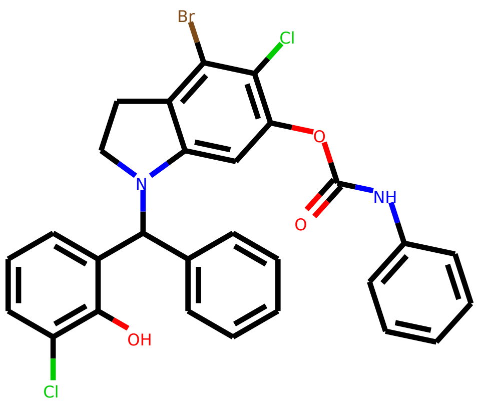

Osma-2772

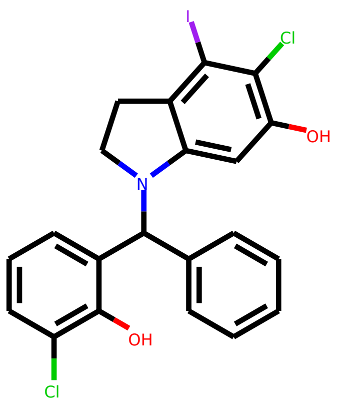

Osma-2773

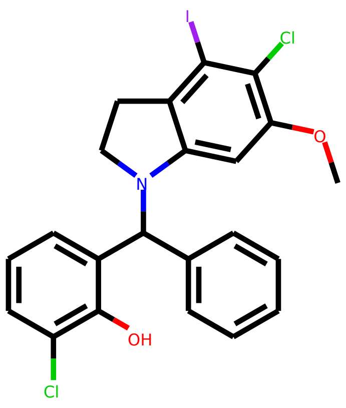

Osma-2774

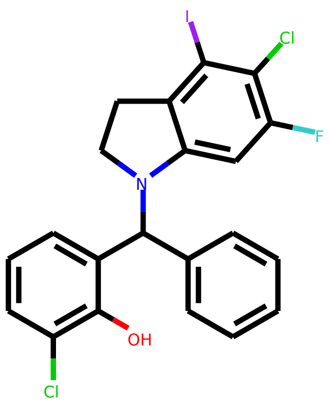

Osma-2775

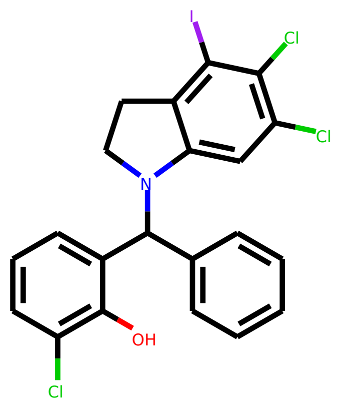

Osma-2776

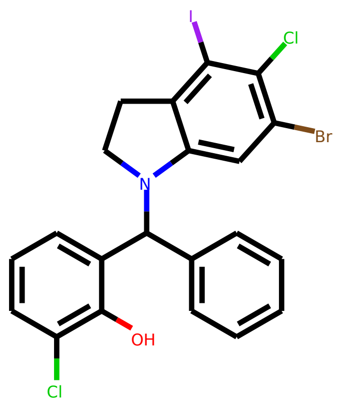

Osma-2777

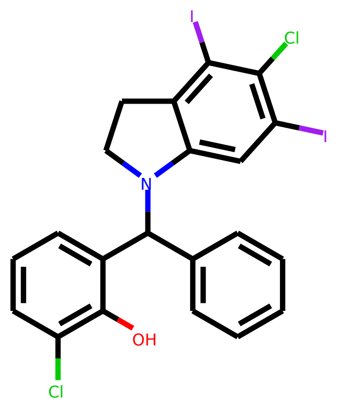

Osma-2778

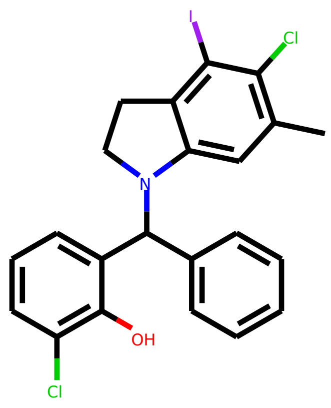

Osma-2779

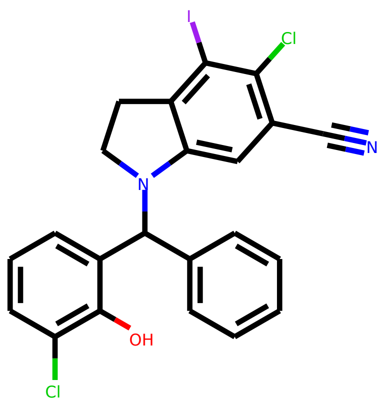

Osma-2780

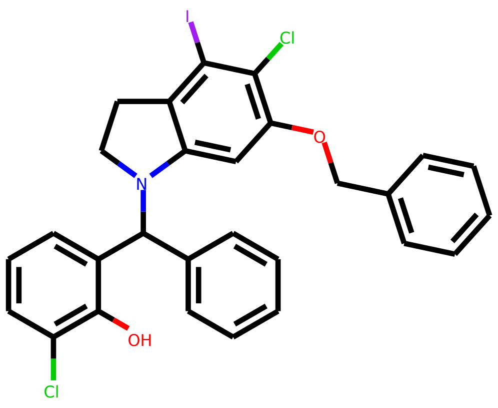

Osma-2781

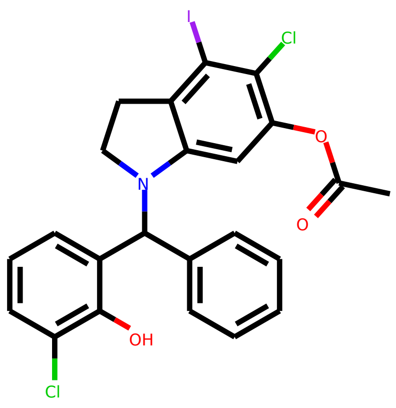

Osma-2782

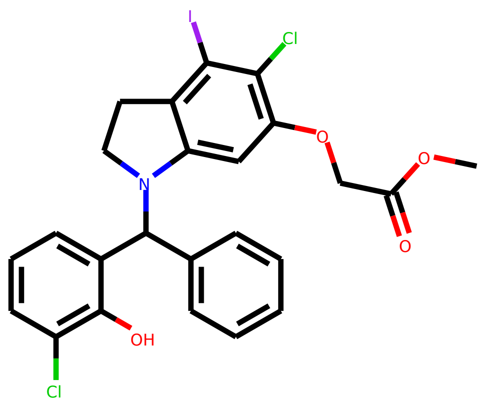

Osma-2783

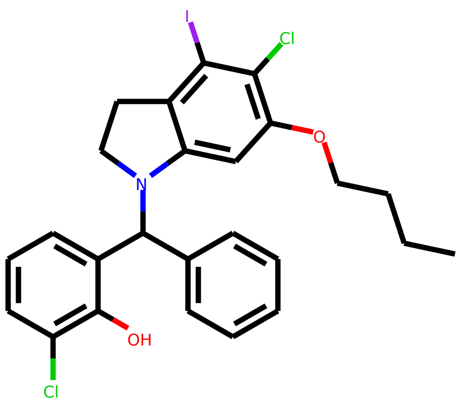

Osma-2784

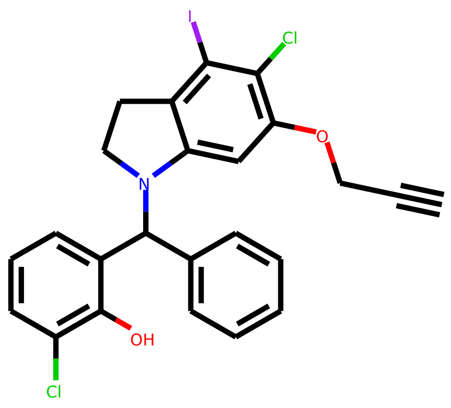

Osma-2785

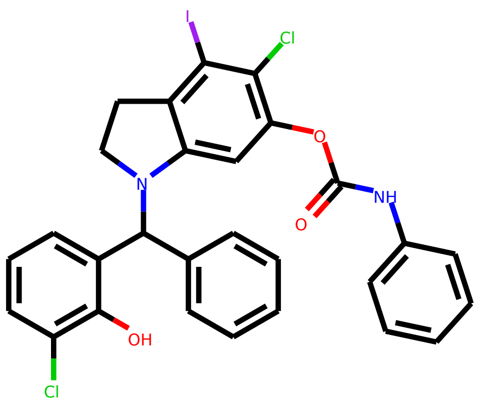

Osma-2786

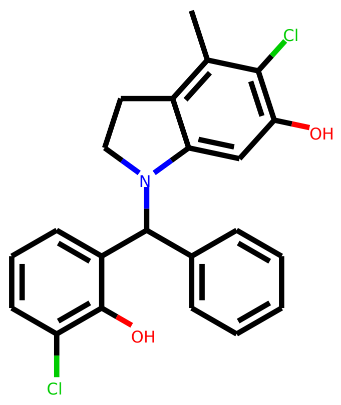

Osma-2787

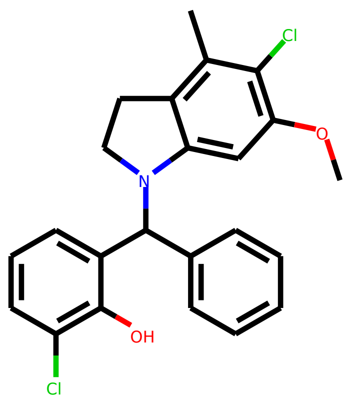

Osma-2788

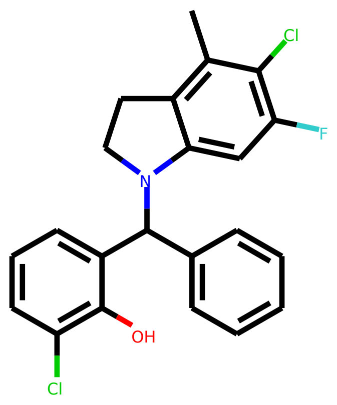

Osma-2789

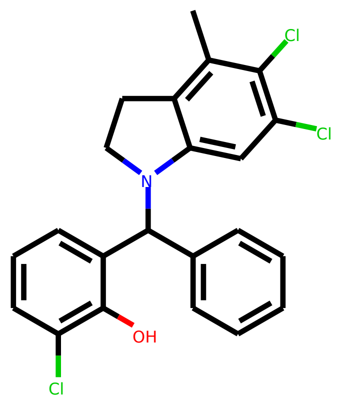

Osma-2790

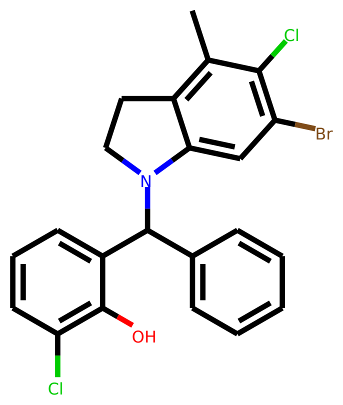

Osma-2791

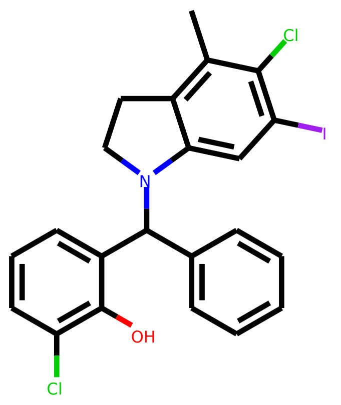

Osma-2792

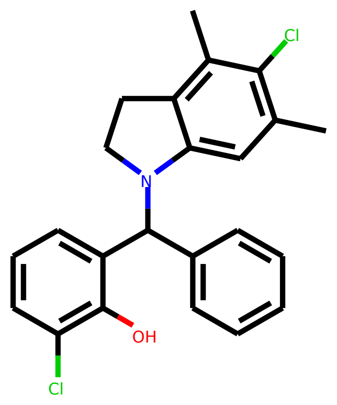

Osma-2793

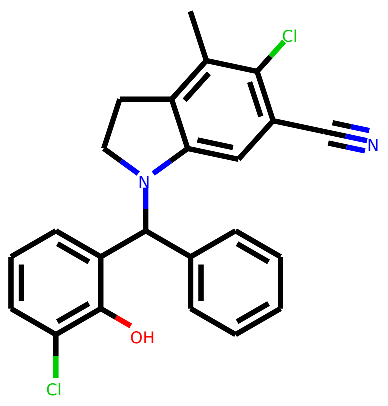

Osma-2794

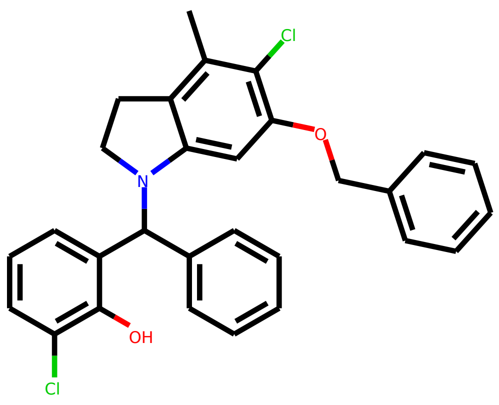

Osma-2795

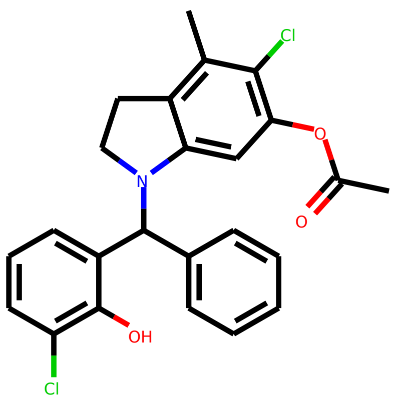

Osma-2796

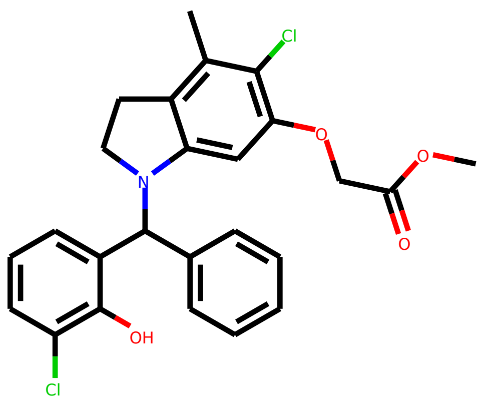

Osma-2797

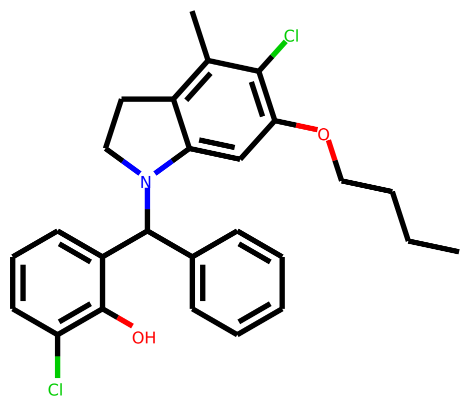

Osma-2798

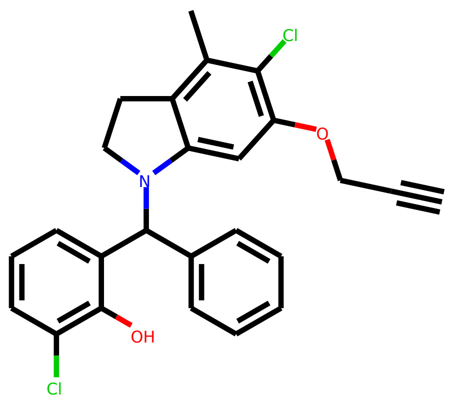

Osma-2799

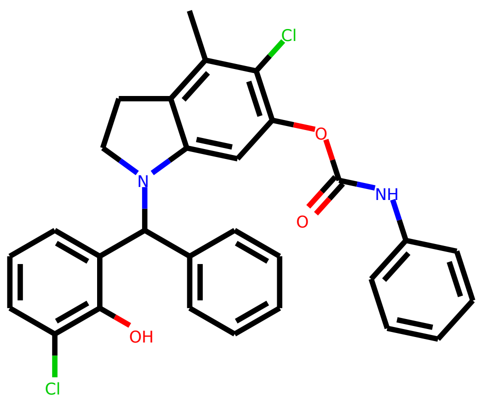

Osma-2800

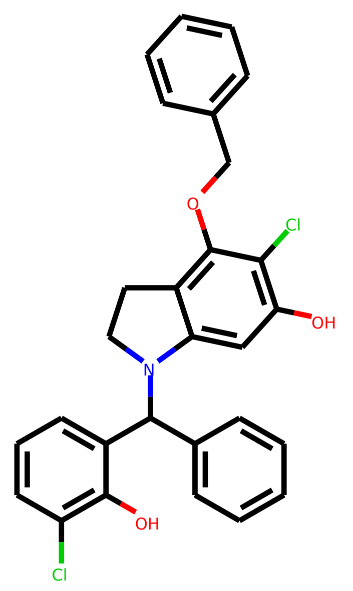
Osma-2801
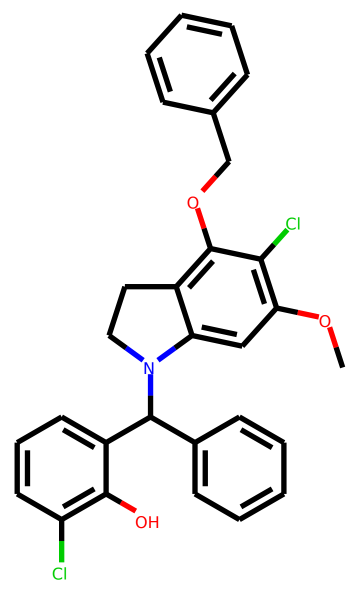
Osma-2802
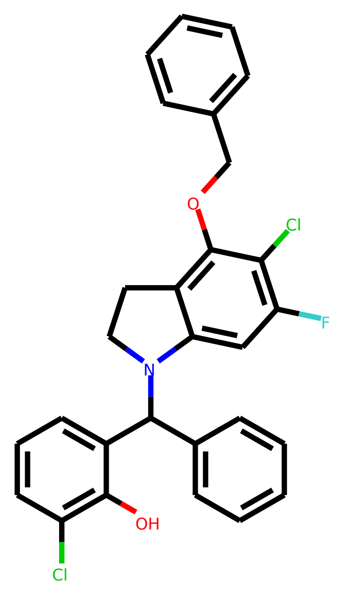
Osma-2803
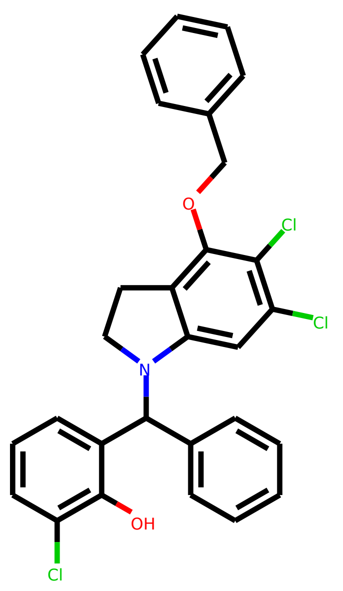
Osma-2804
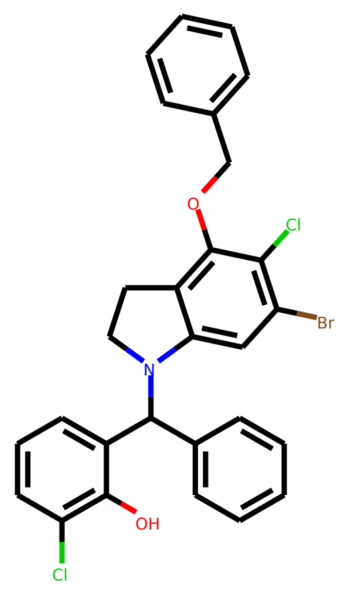
Osma-2805
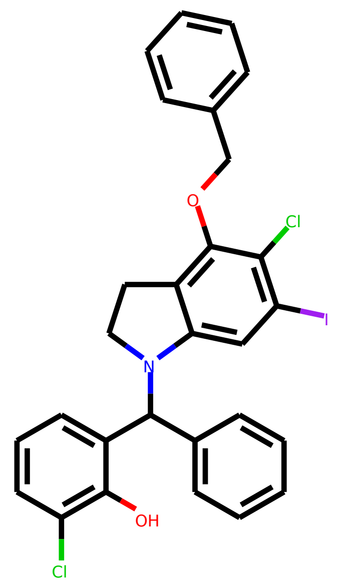
Osma-2806
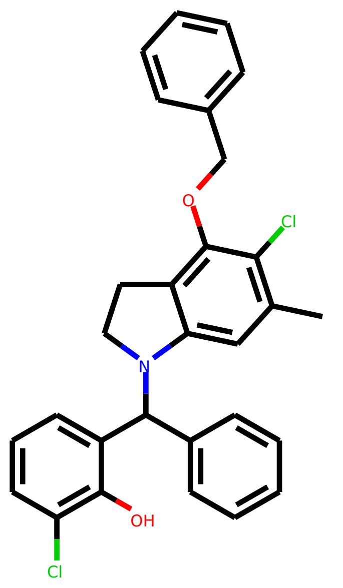
Osma-2807
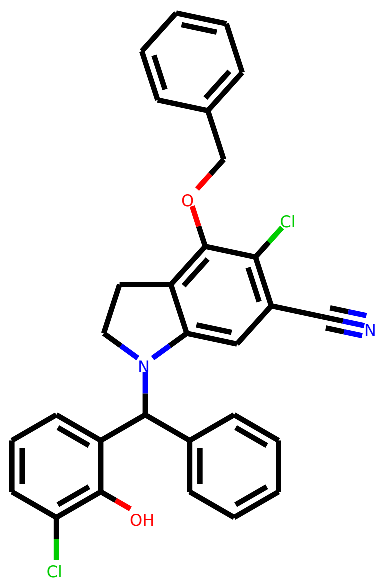
Osma-2808
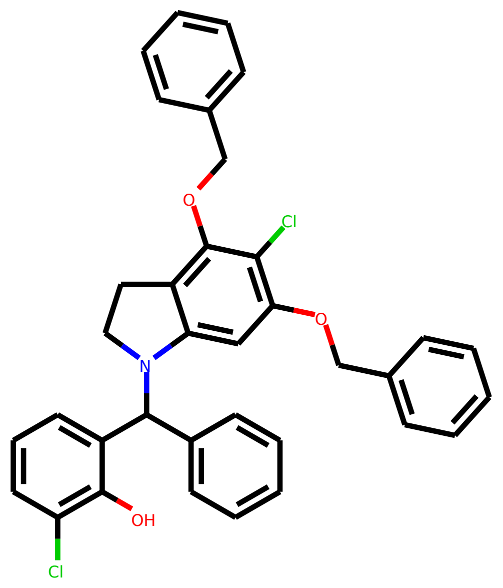
Osma-2809
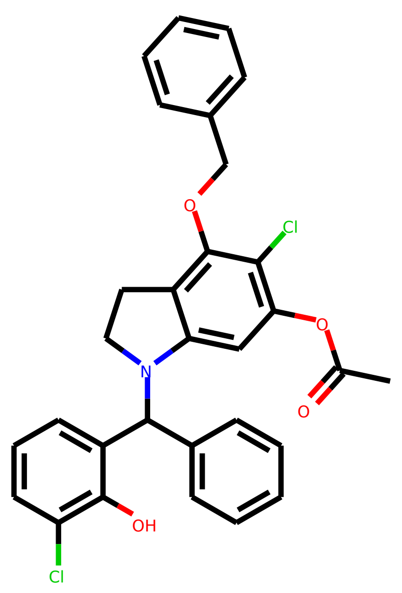
Osma-2810
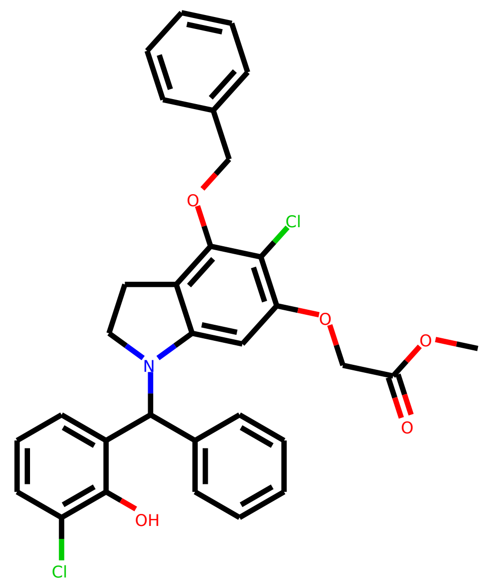
Osma-2811
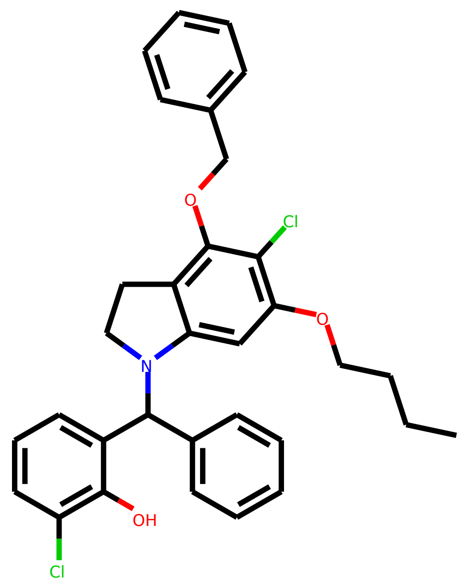
Osma-2812
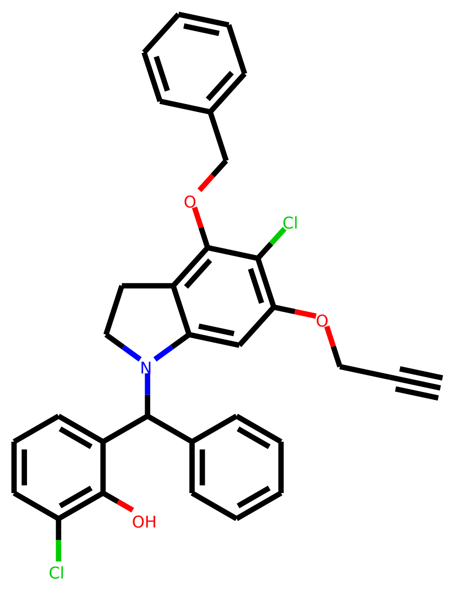
Osma-2813
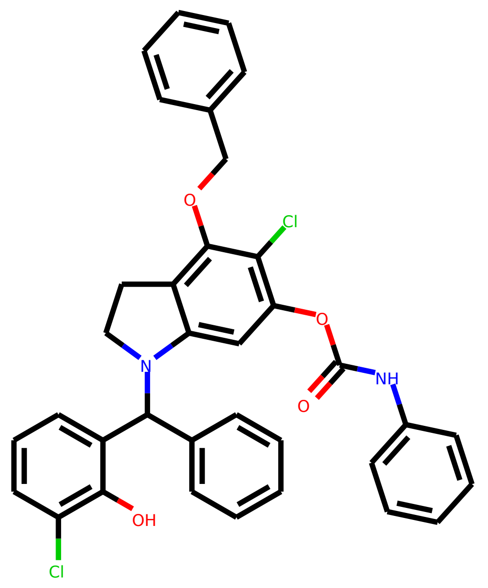
Osma-2814
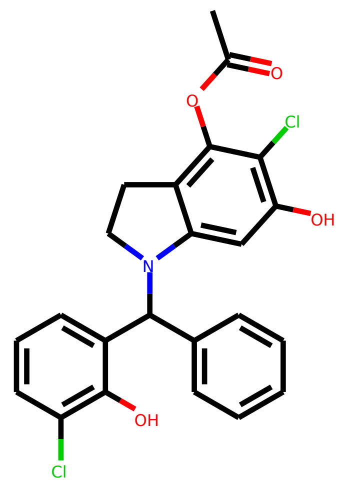
Osma-2815
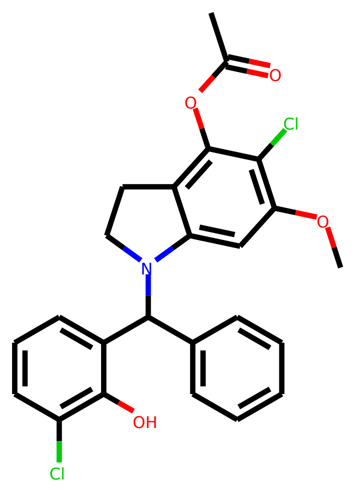
Osma-2816
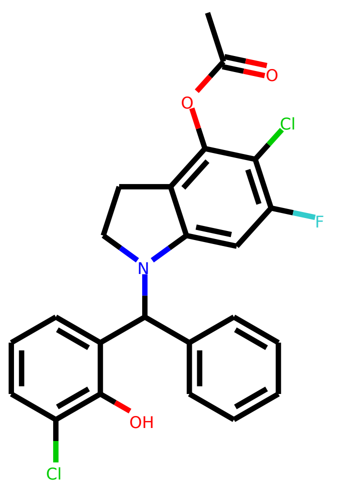
Osma-2817
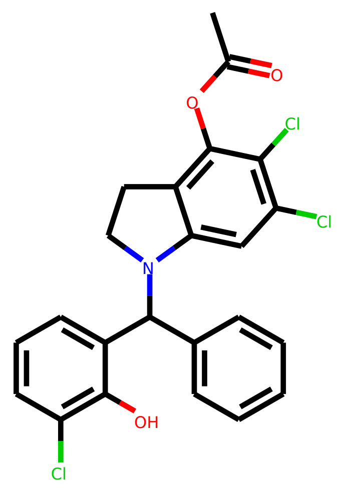
Osma-2818
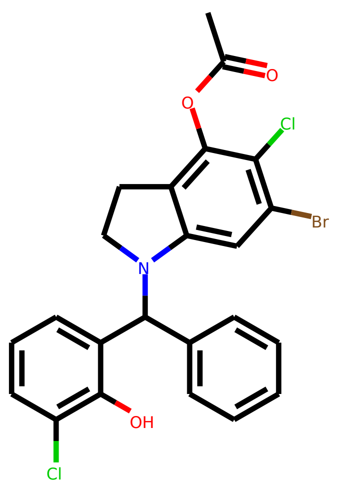
Osma-2819
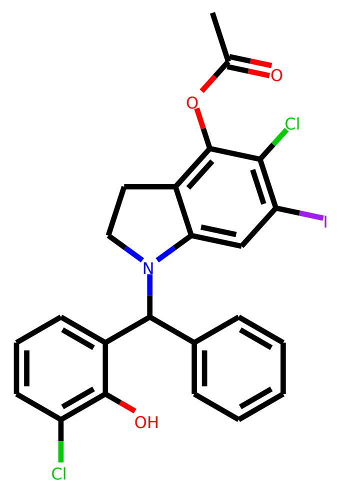
Osma-2820
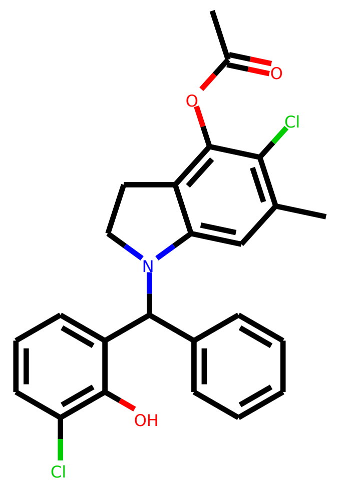
Osma-2821
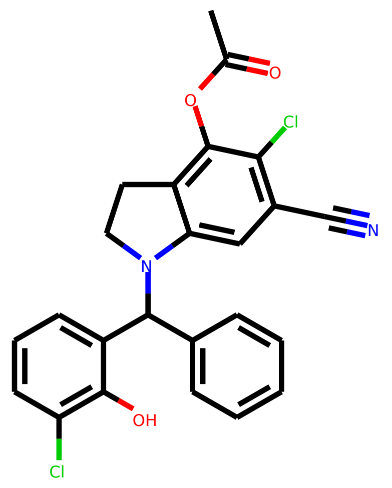
Osma-2822
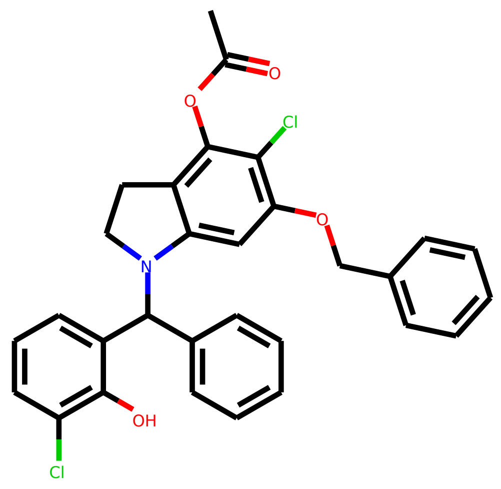
Osma-2823
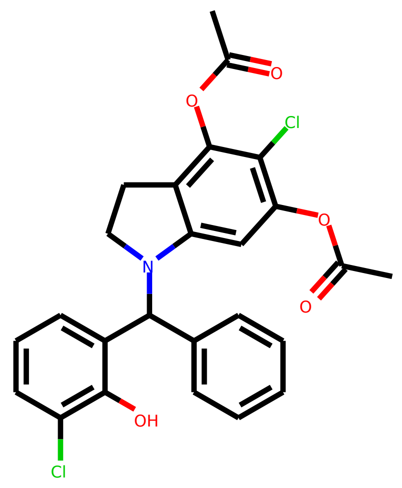
Osma-2824
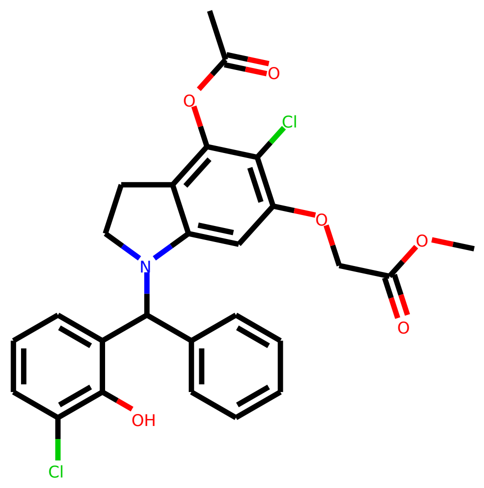
Osma-2825
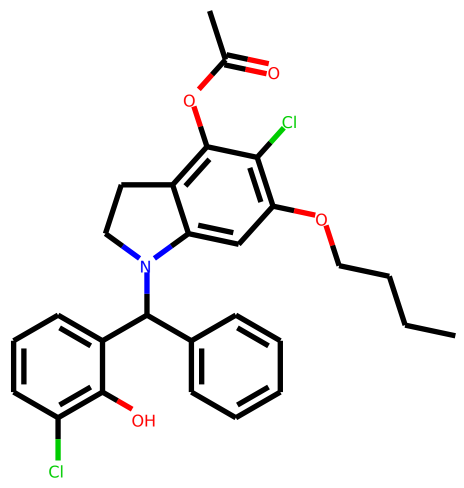
Osma-2826
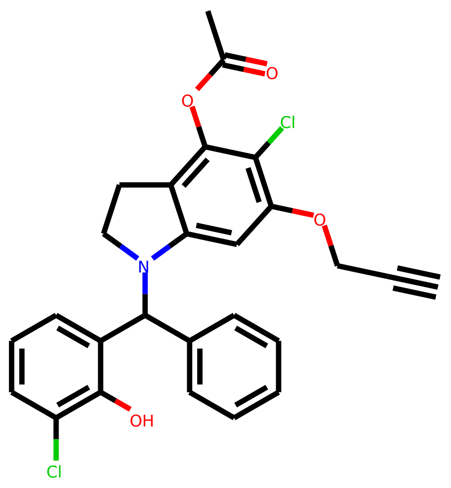
Osma-2827
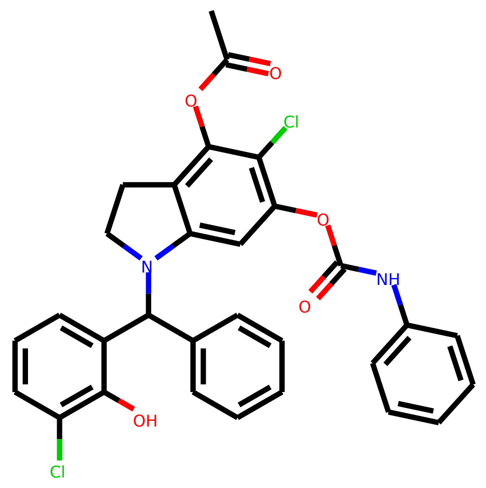
Osma-2828
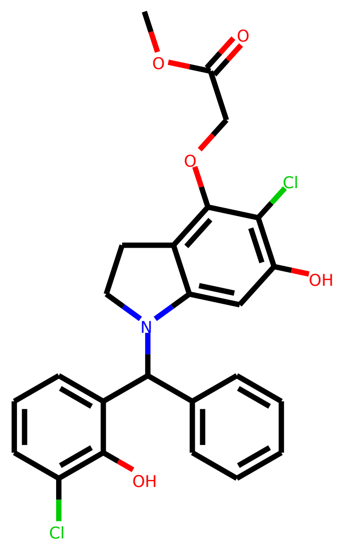
Osma-2829
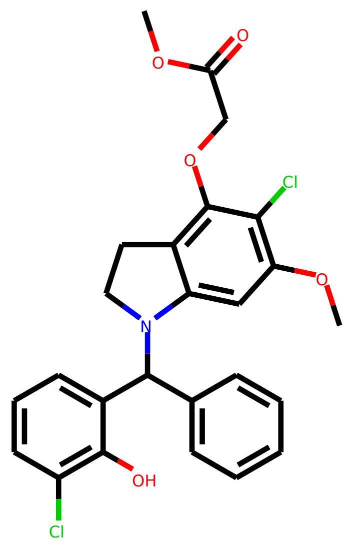
Osma-2830
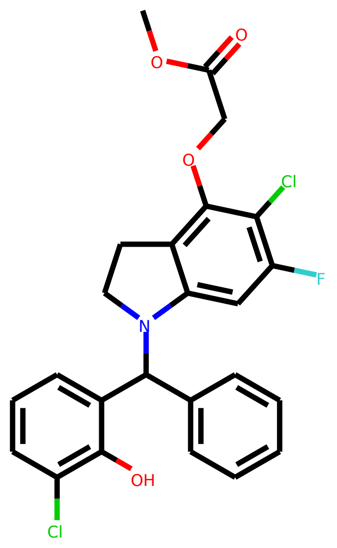
Osma-2831
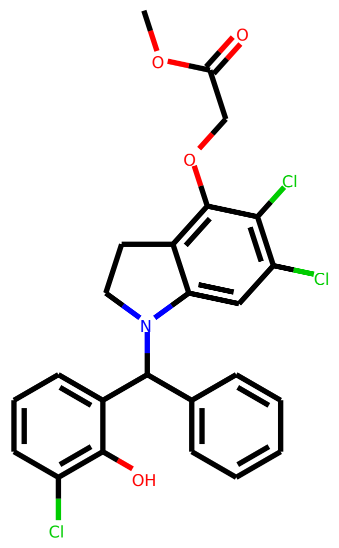
Osma-2832
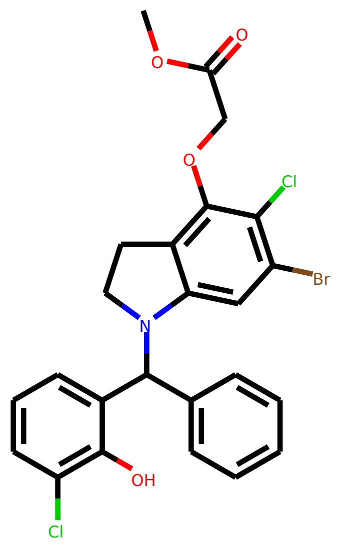
Osma-2833
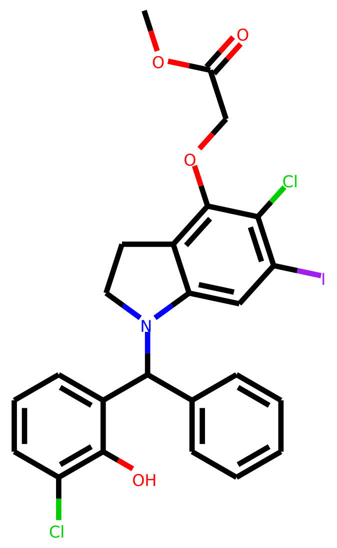
Osma-2834
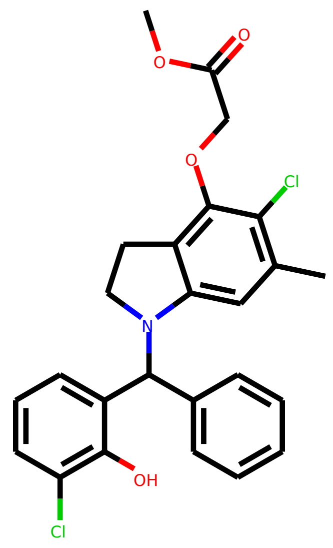
Osma-2835

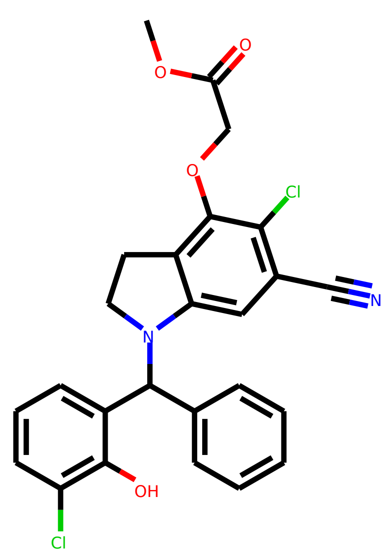
Osma-2836

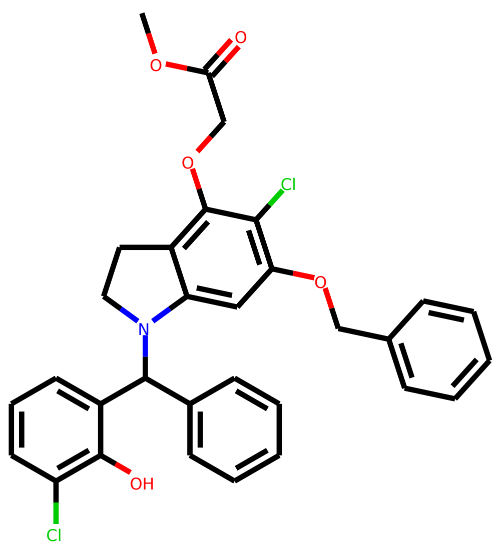
Osma-2837

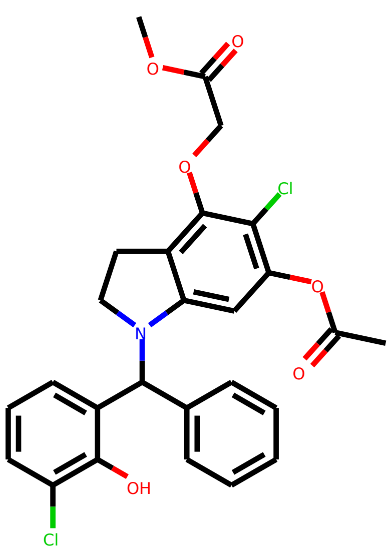
Osma-2838

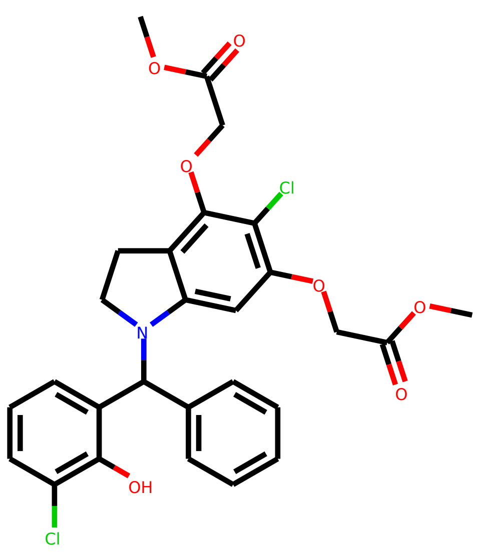
Osma-2839

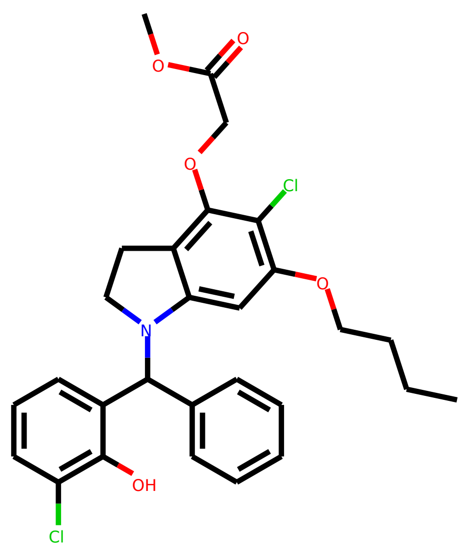
Osma-2840

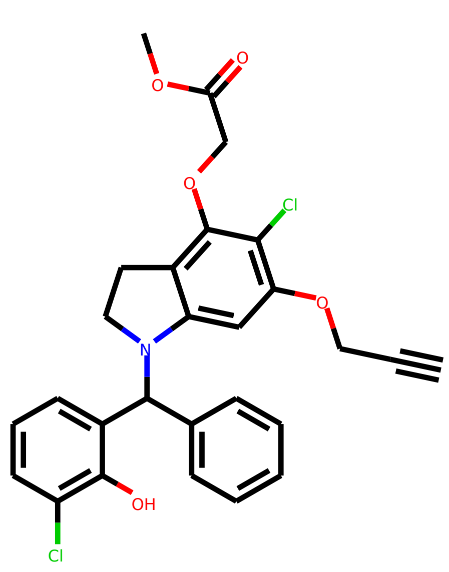
Osma-2841

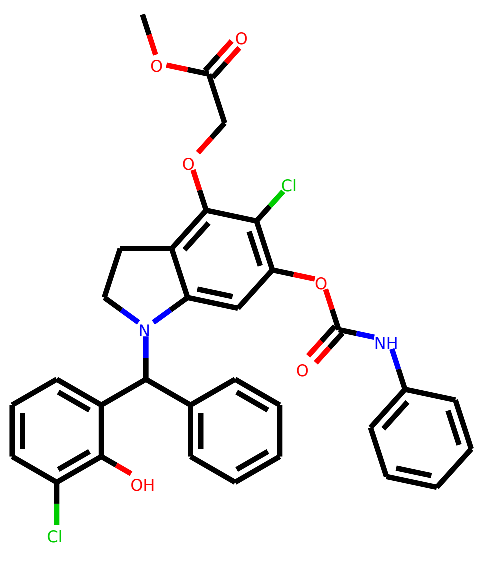
Osma-2842

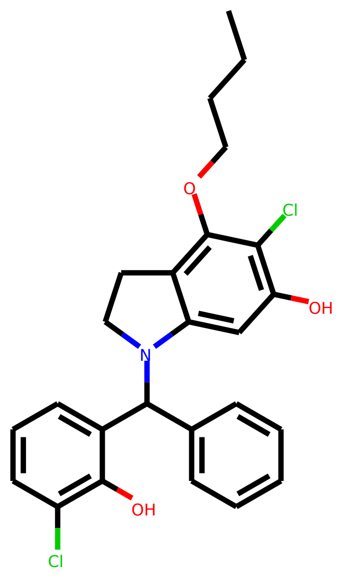
Osma-2843

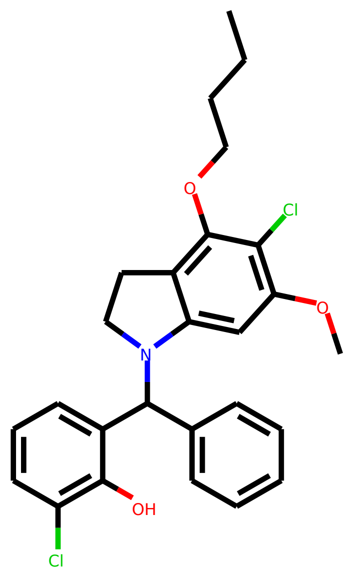
Osma-2844

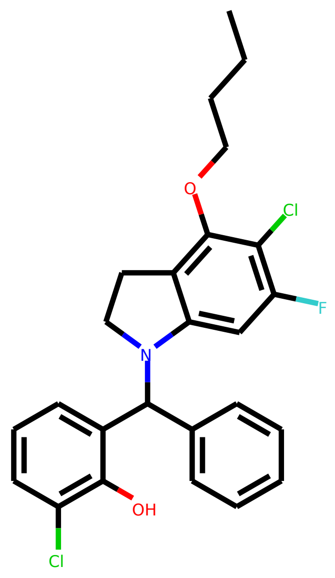
Osma-2845

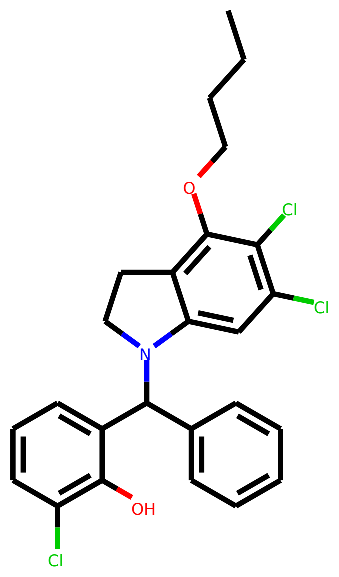
Osma-2846

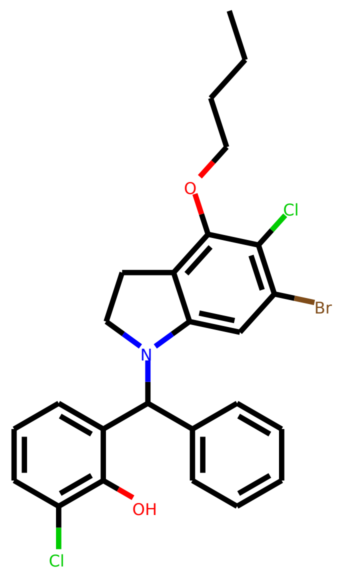
Osma-2847

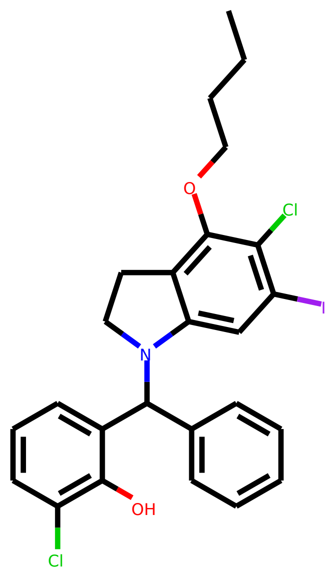
Osma-2848

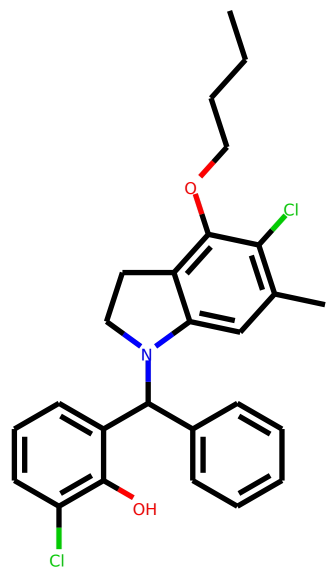
Osma-2849

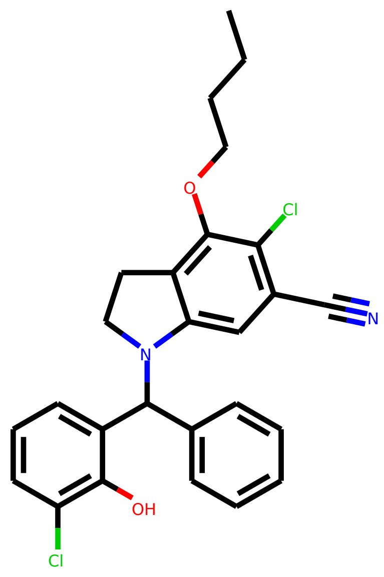
Osma-2850

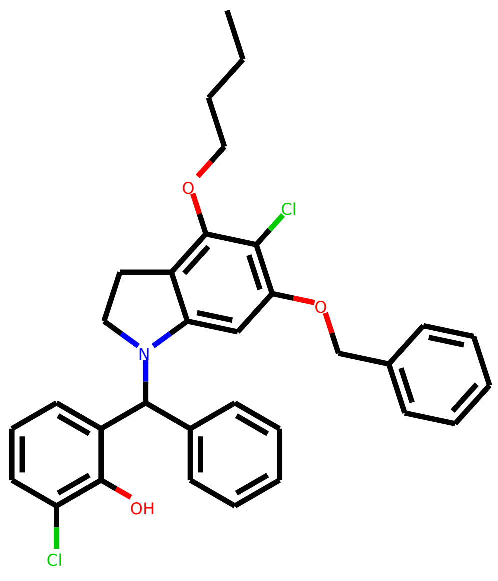
Osma-2851

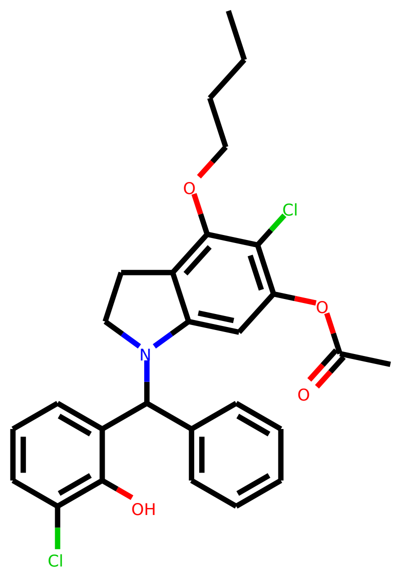
Osma-2852

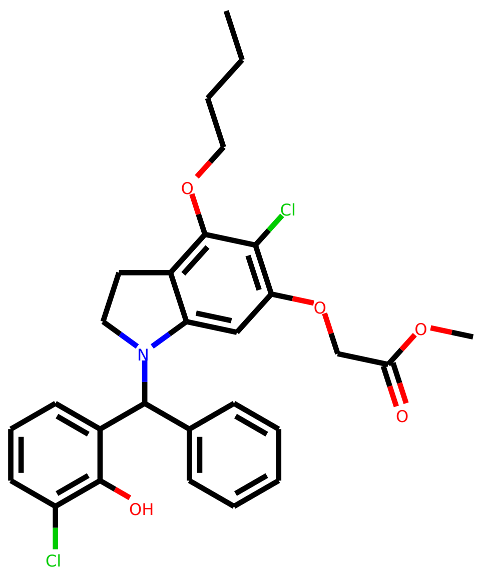
Osma-2853

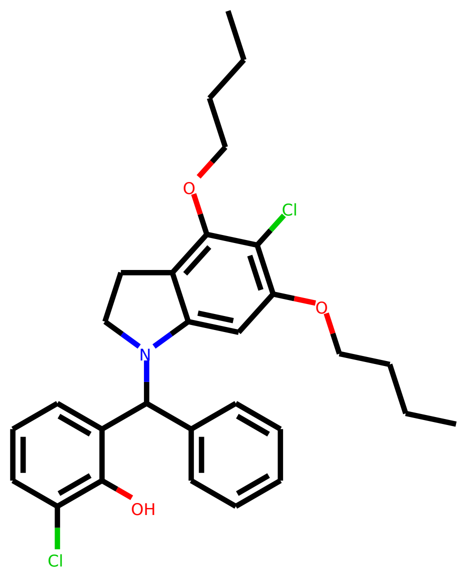
Osma-2854

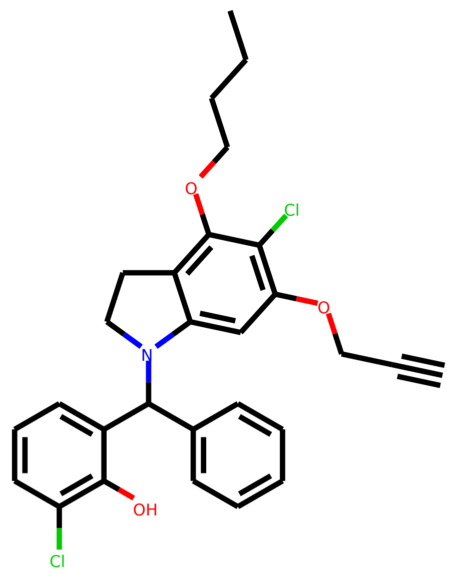
Osma-2855

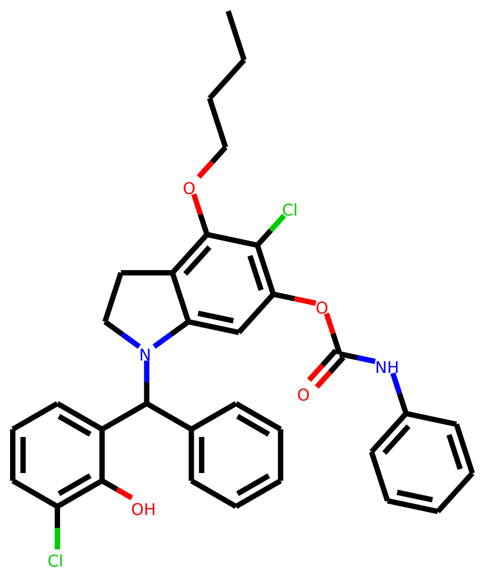
Osma-2856

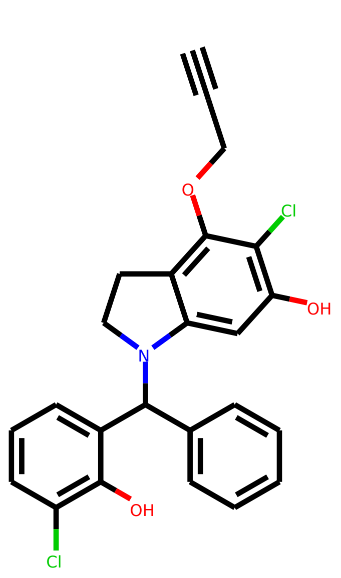
Osma-2857

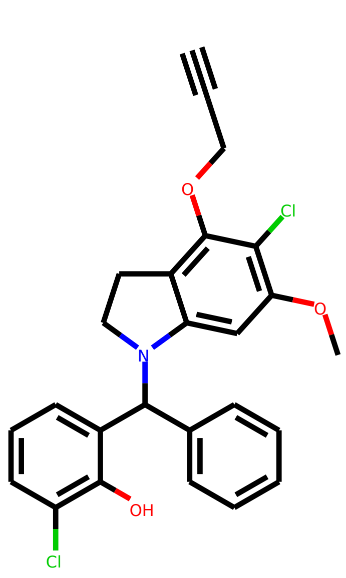
Osma-2858

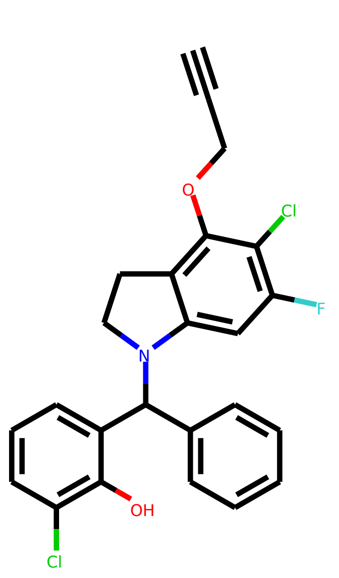
Osma-2859

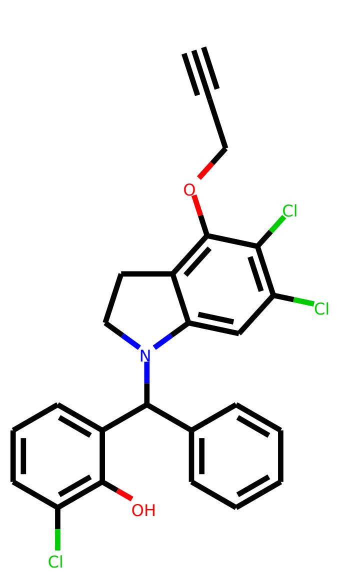
Osma-2860

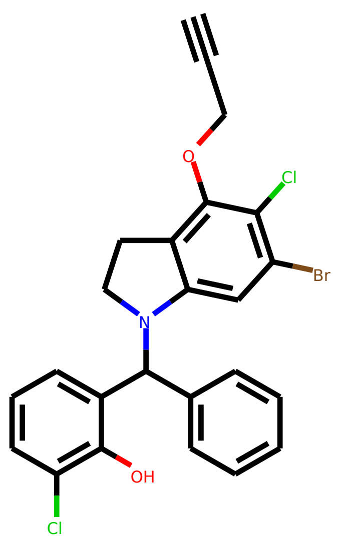
Osma-2861

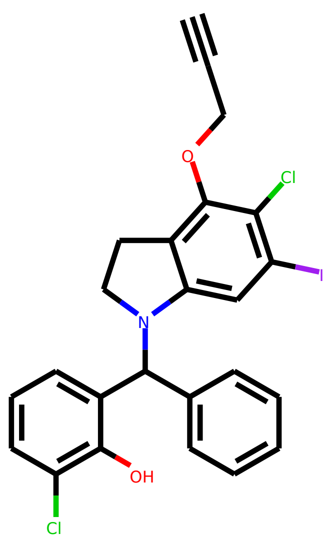
Osma-2862

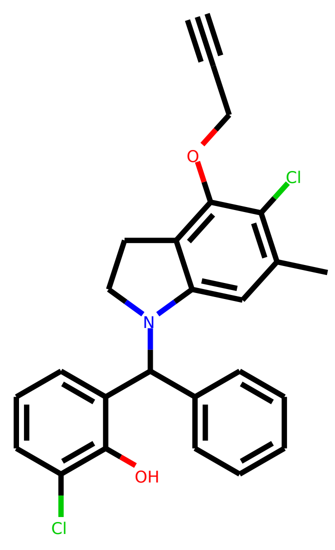
Osma-2863

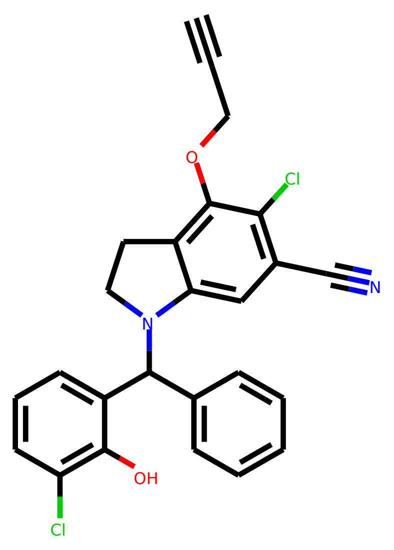
Osma-2864

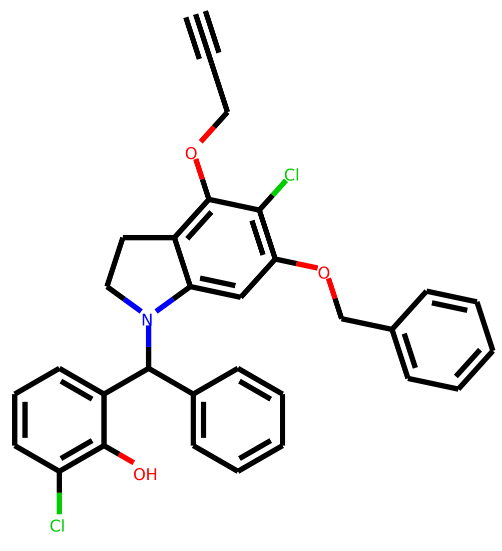
Osma-2865

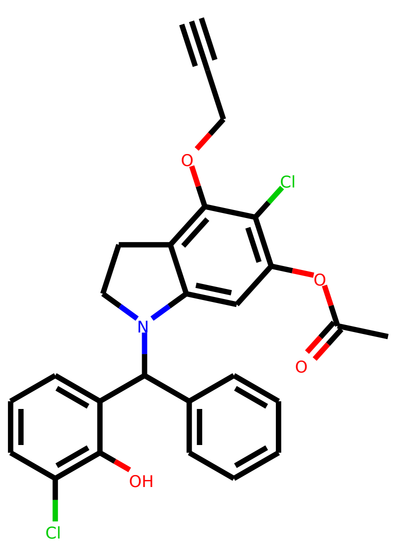
Osma-2866

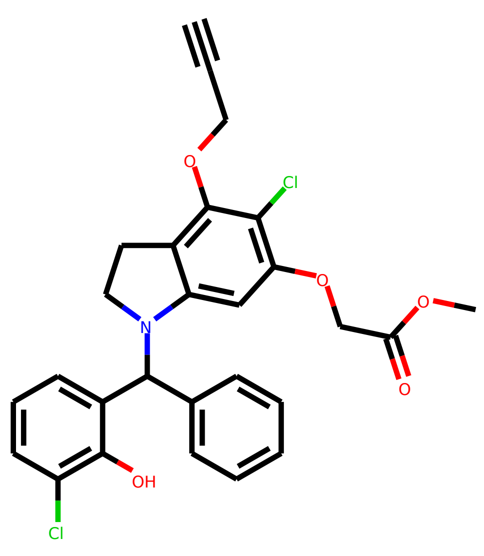
Osma-2867

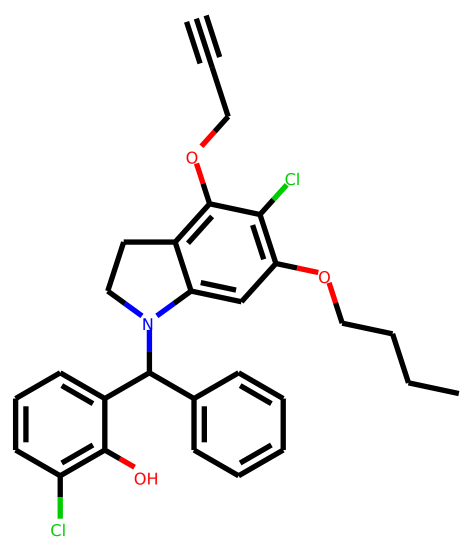
Osma-2868

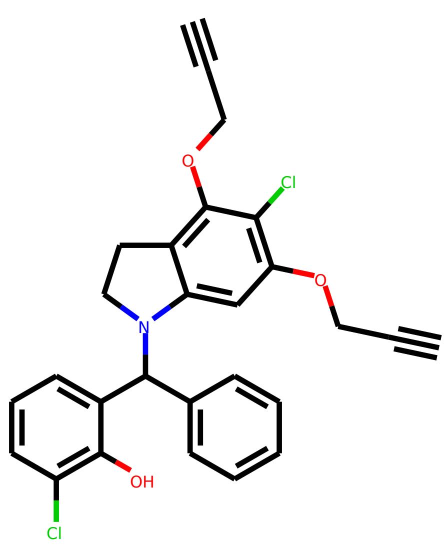
Osma-2869

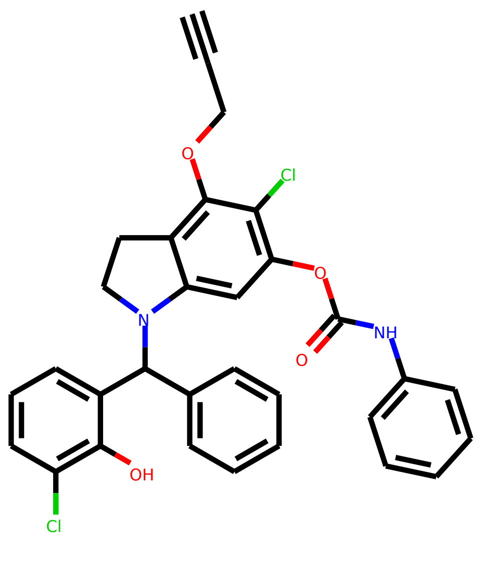
Osma-2870

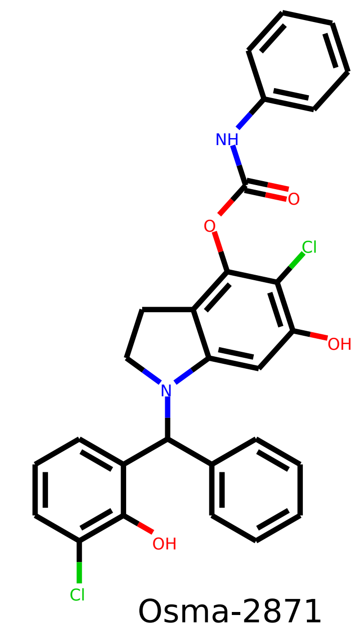
Osma-2871

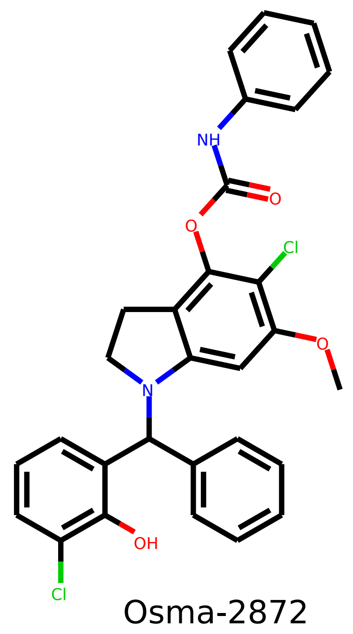
Osma-2872

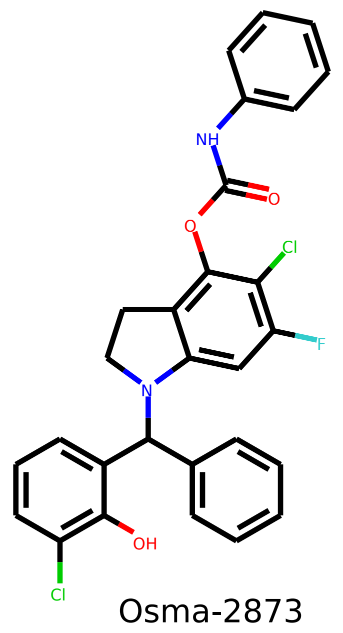
Osma-2873

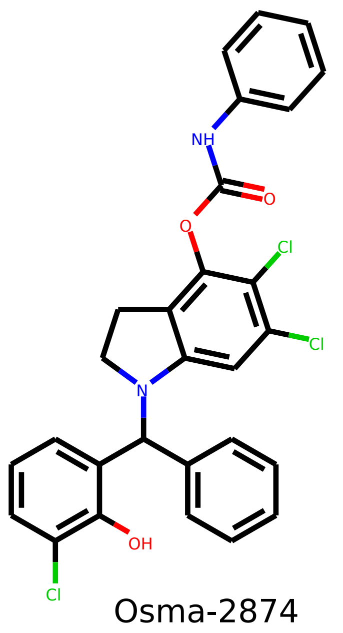
Osma-2874

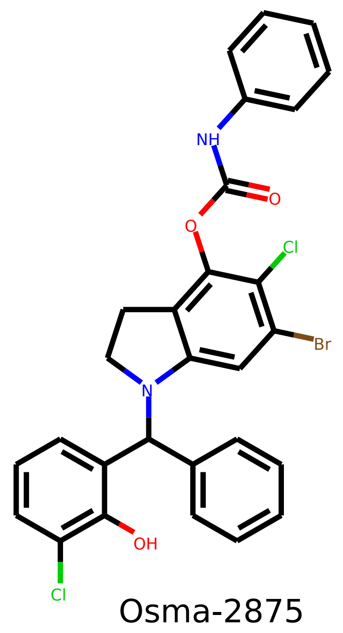
Osma-2875

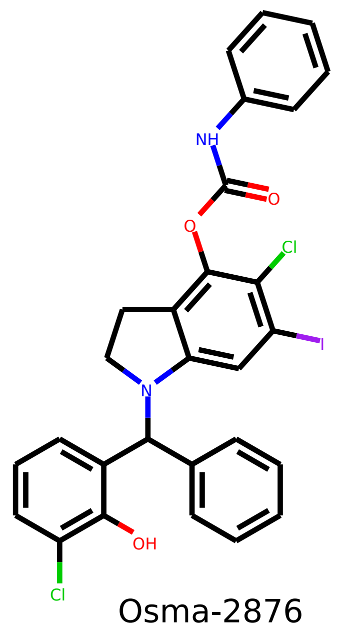
Osma-2876

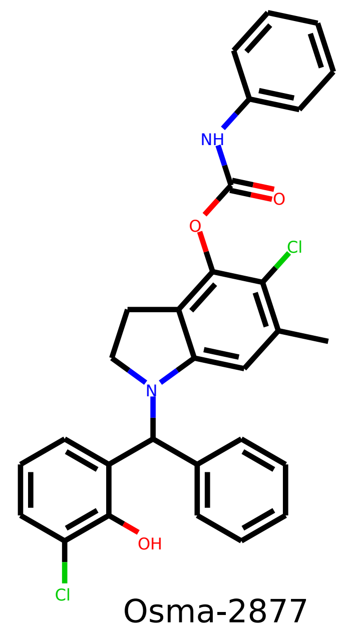
Osma-2877

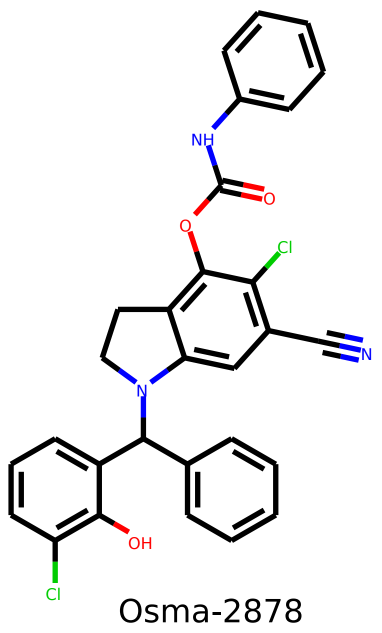
Osma-2878

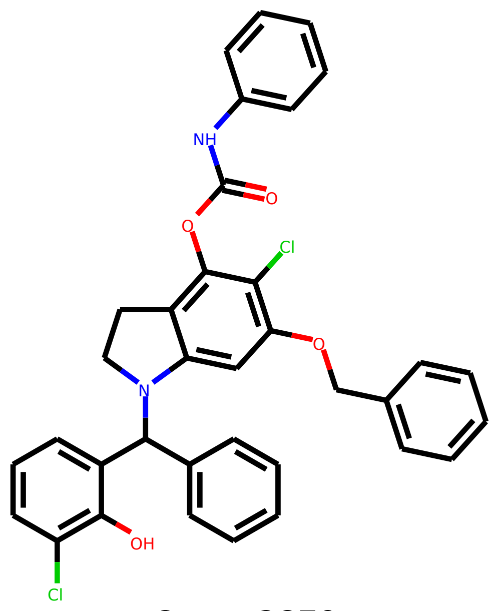
Osma-2879

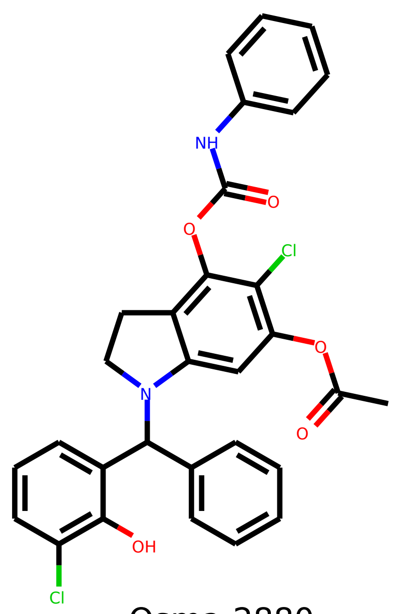
Osma-2880

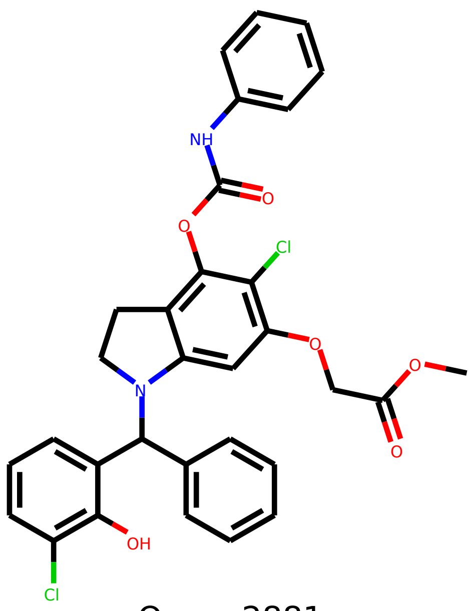
Osma-2881

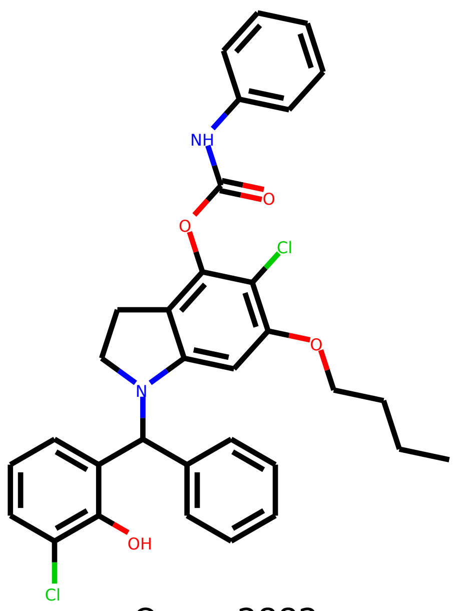
Osma-2882

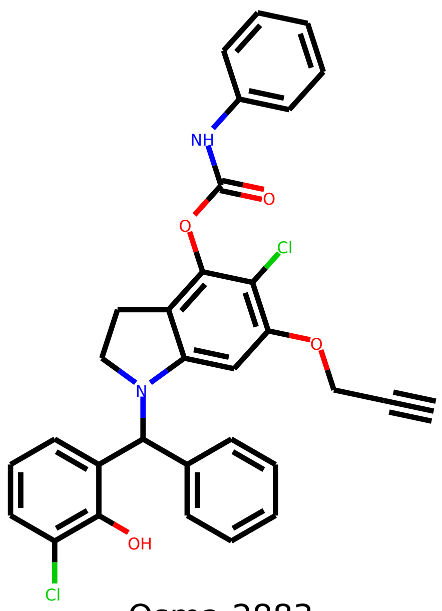
Osma-2883

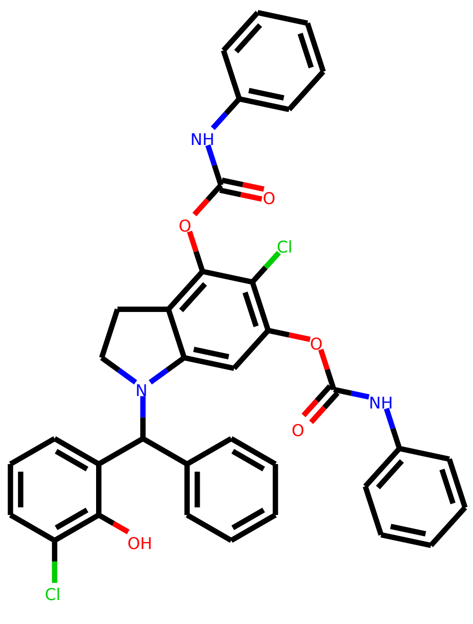
Osma-2884

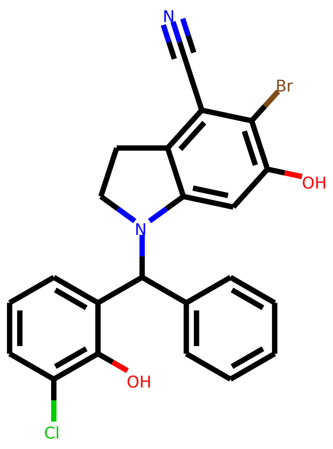
Osma-2885

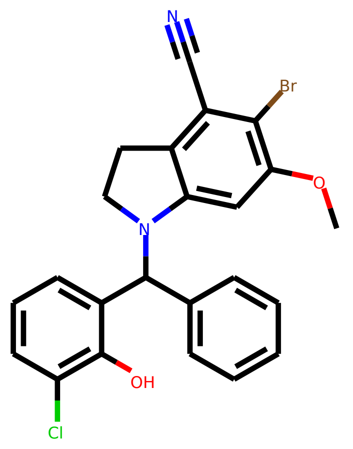
Osma-2886

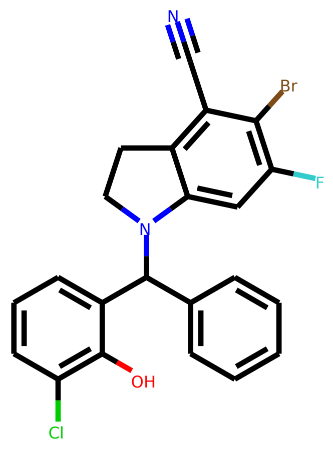
Osma-2887

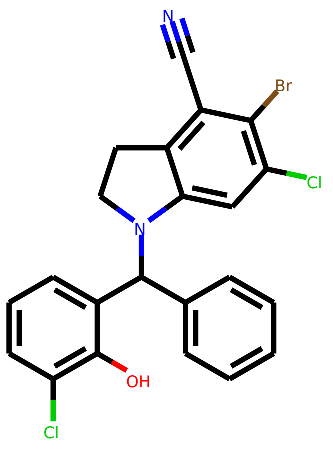
Osma-2888

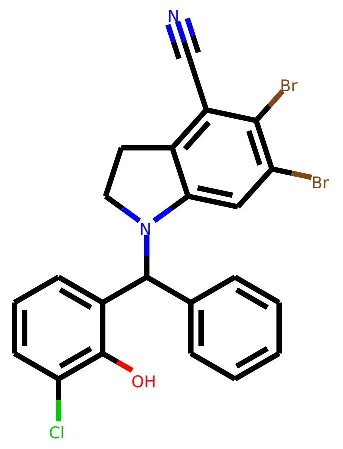
Osma-2889

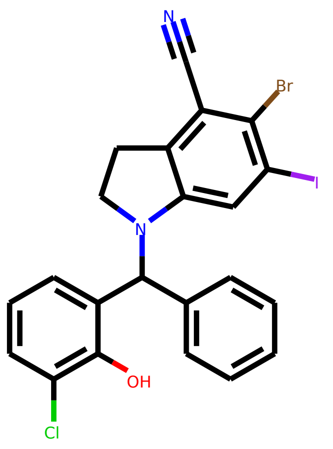
Osma-2890

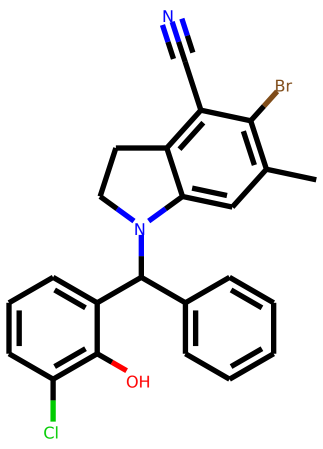
Osma-2891

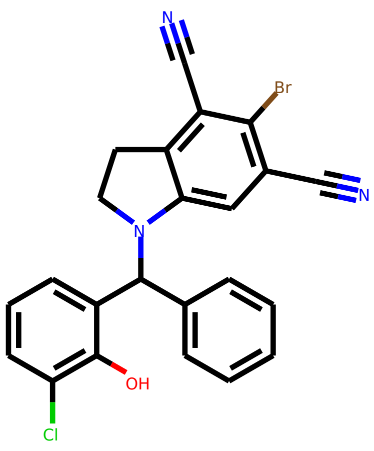
Osma-2892

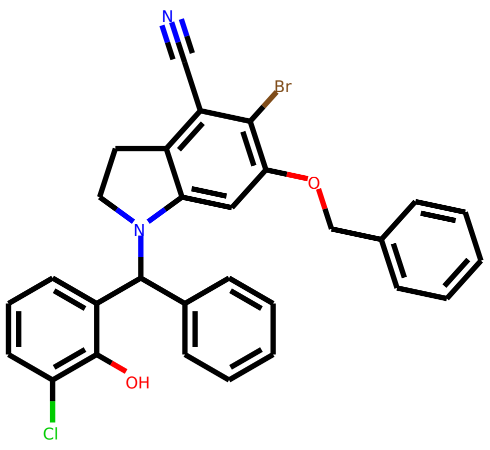
Osma-2893

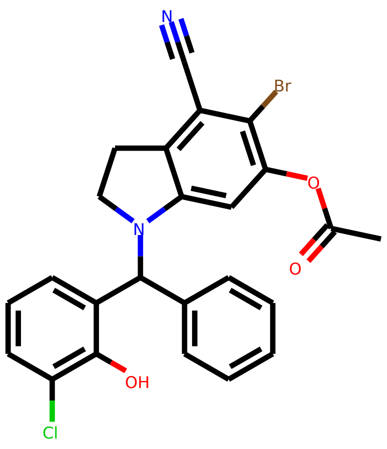
Osma-2894

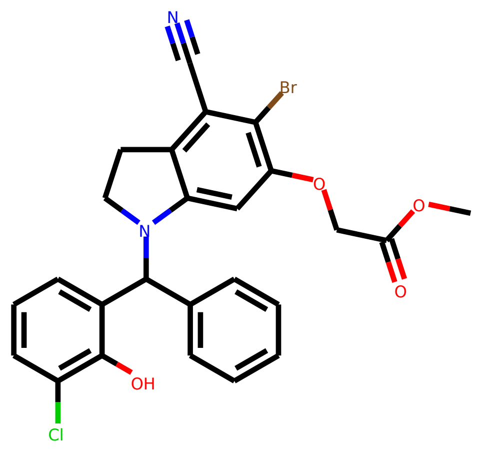
Osma-2895

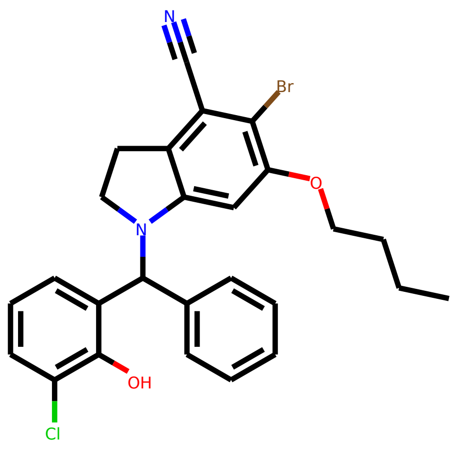
Osma-2896

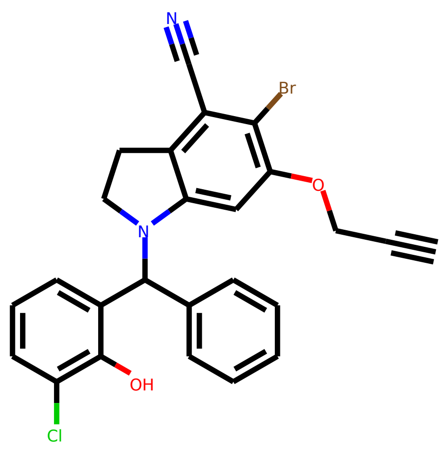
Osma-2897

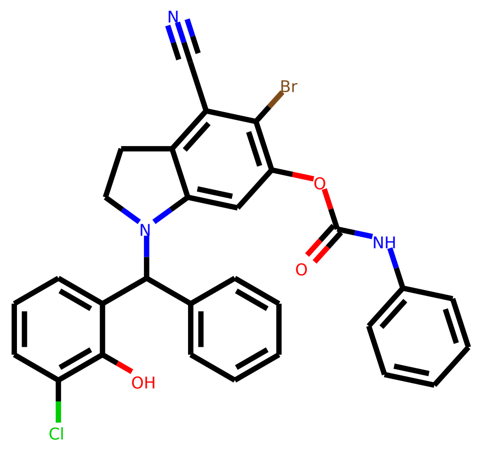
Osma-2898

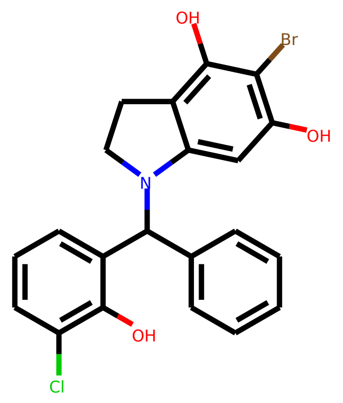
Osma-2899

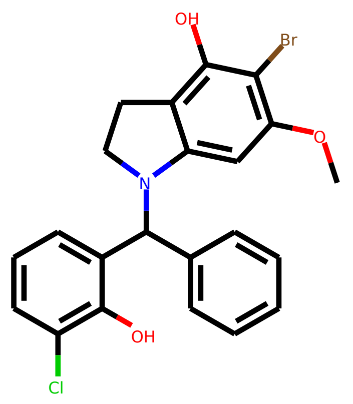
Osma-2900

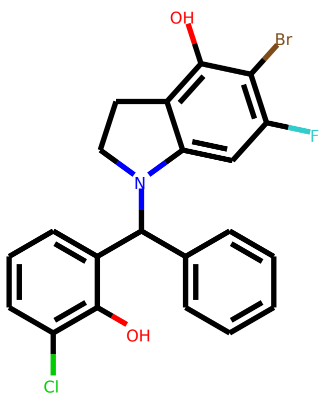
Osma-2901

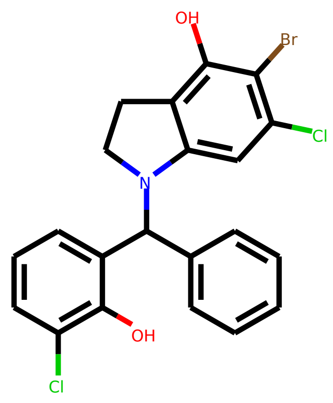
Osma-2902

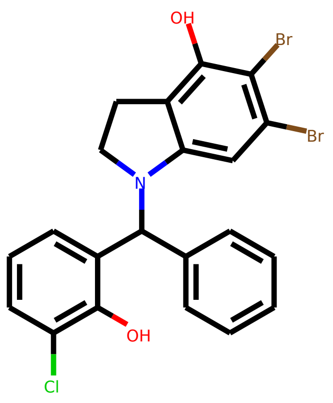
Osma-2903

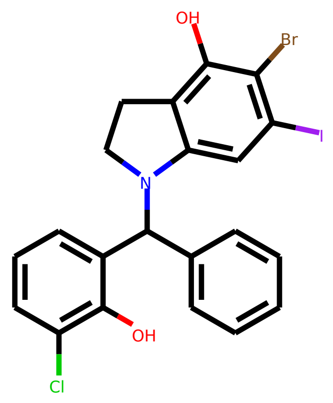
Osma-2904

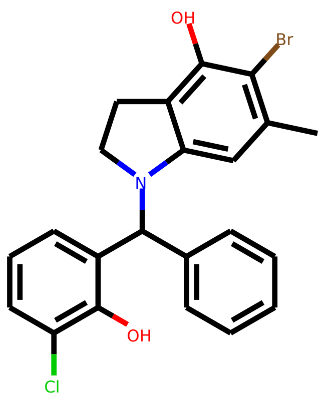
Osma-2905

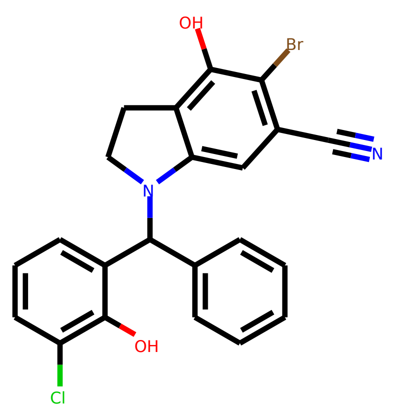

Osma-2906

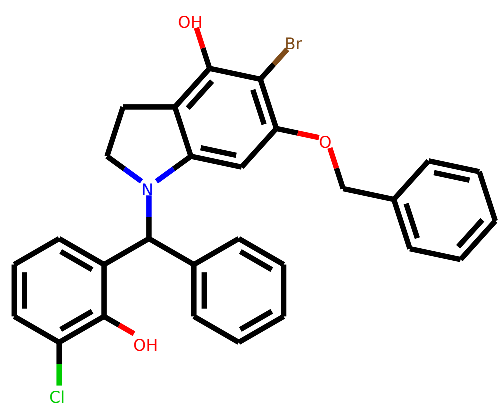

Osma-2907

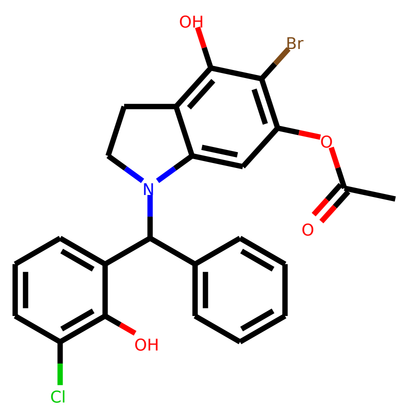

Osma-2908

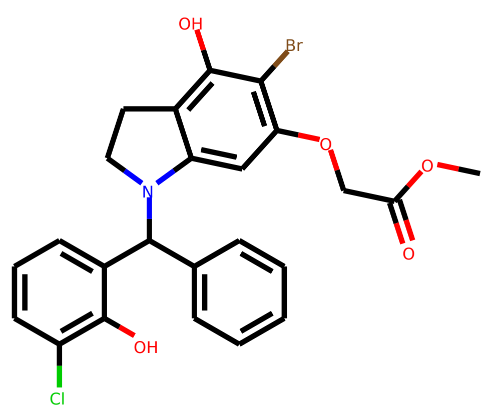

Osma-2909

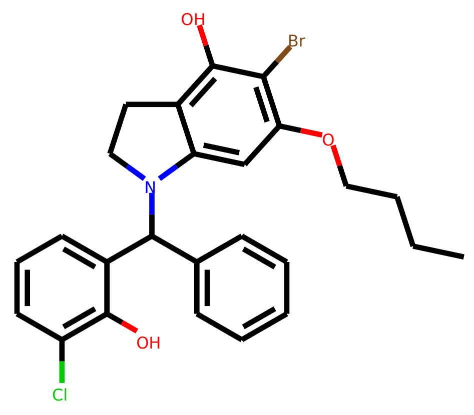

Osma-2910

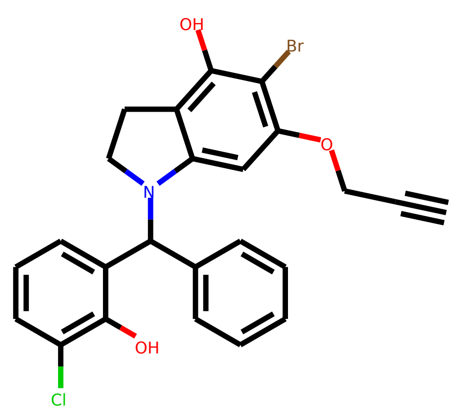

Osma-2911

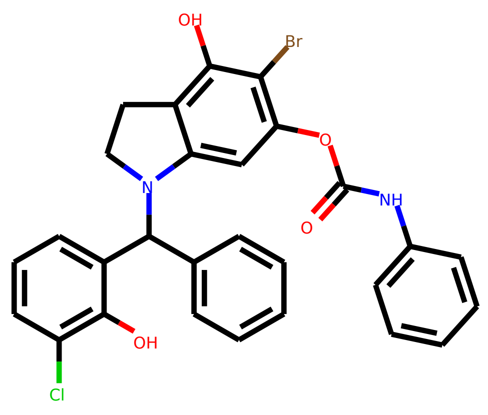

Osma-2912

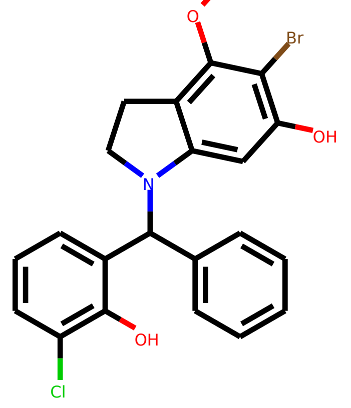

Osma-2913

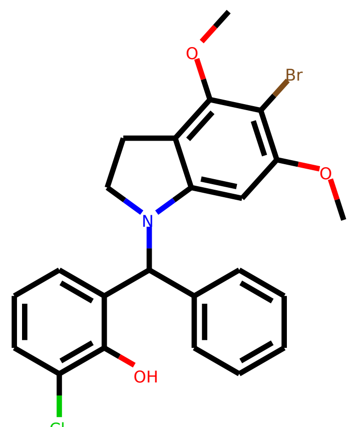

Osma-2914

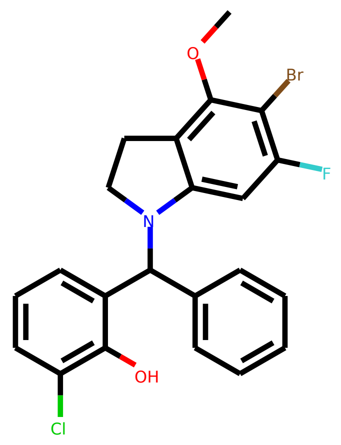

Osma-2915

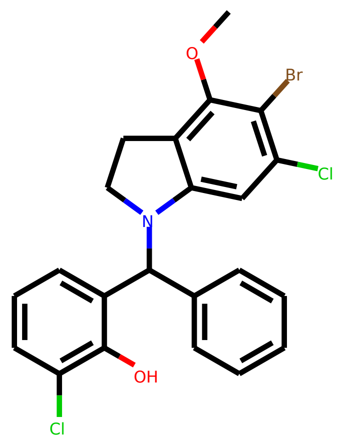

Osma-2916

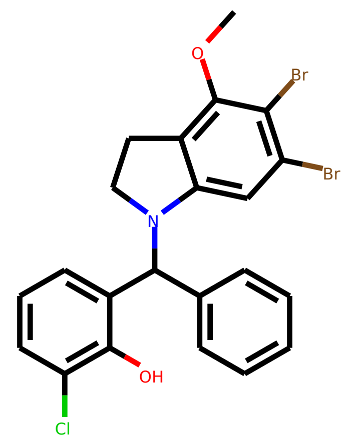

Osma-2917

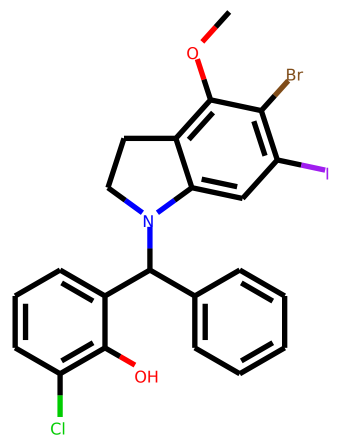

Osma-2918

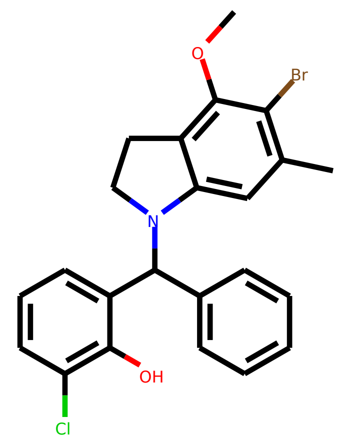

Osma-2919

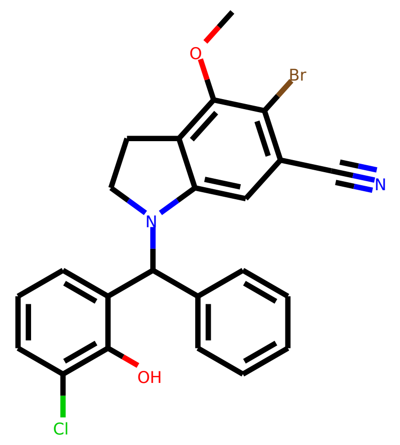

Osma-2920

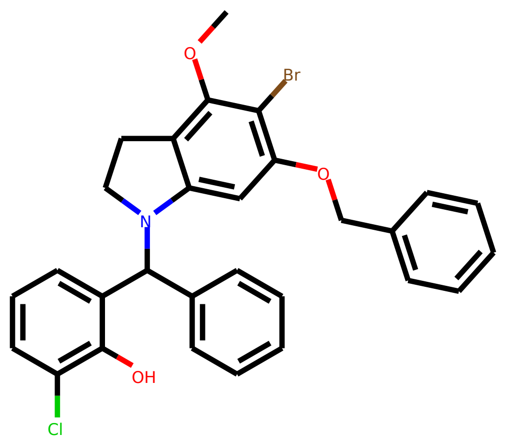

Osma-2921

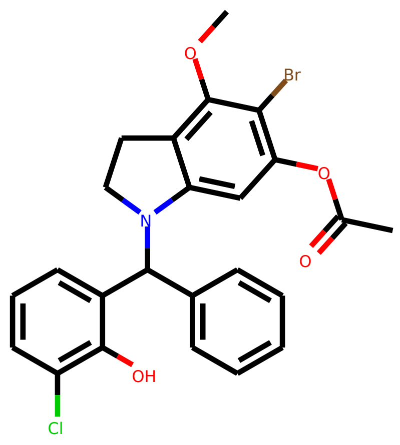

Osma-2922

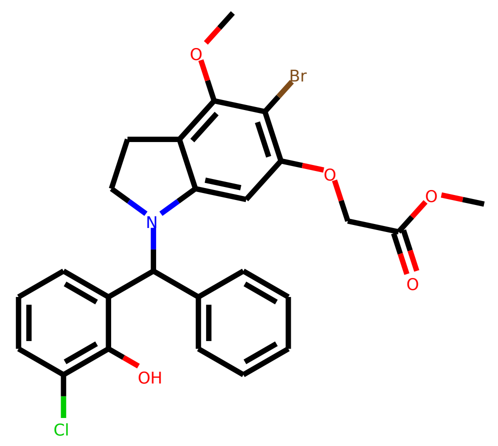

Osma-2923

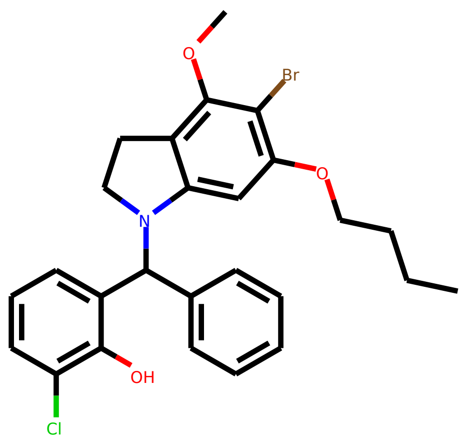

Osma-2924

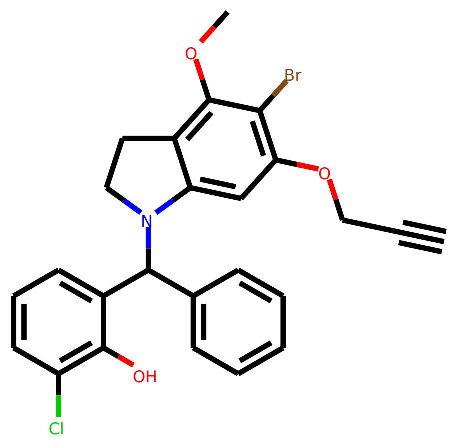

Osma-2925

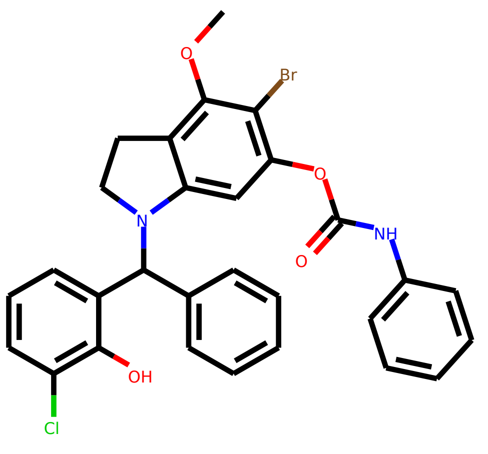

Osma-2926

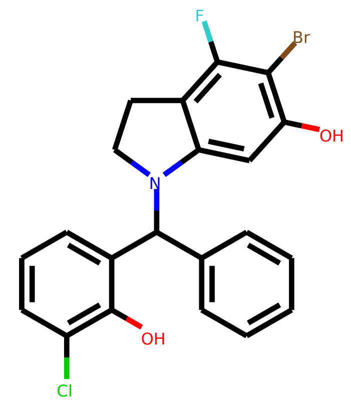

Osma-2927

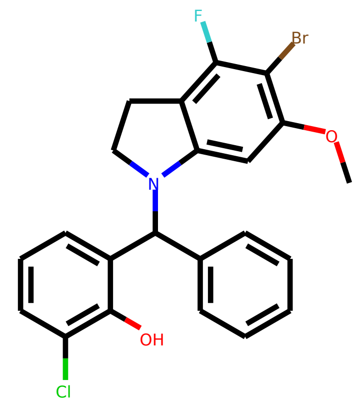

Osma-2928

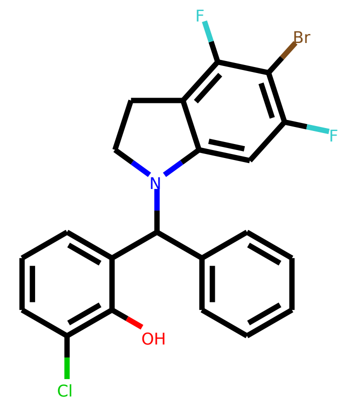

Osma-2929

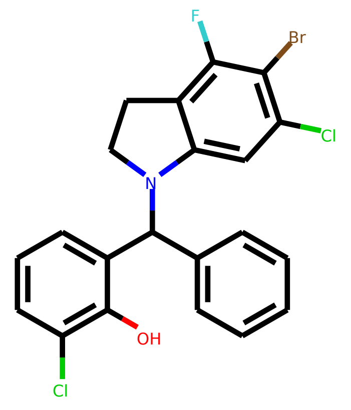

Osma-2930

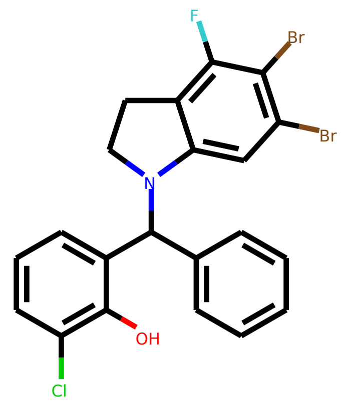

Osma-2931

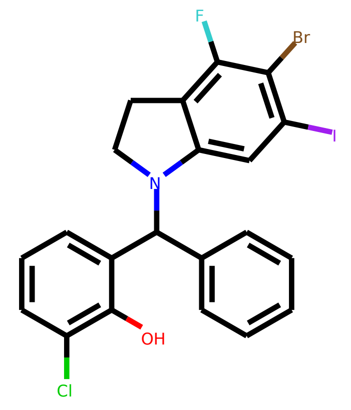

Osma-2932

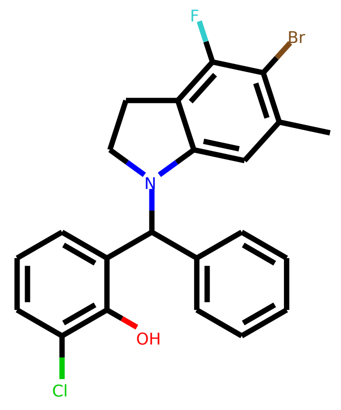

Osma-2933

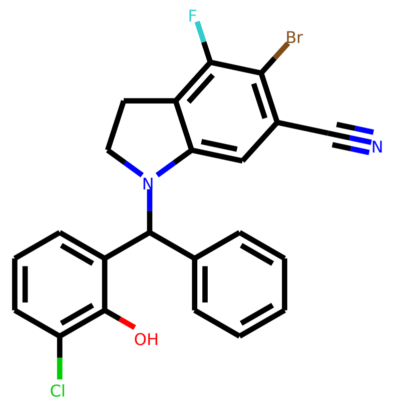

Osma-2934

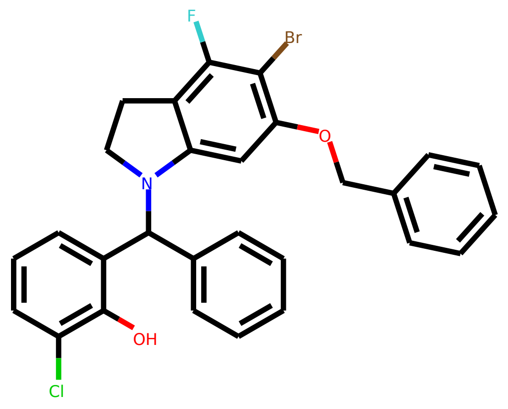

Osma-2935

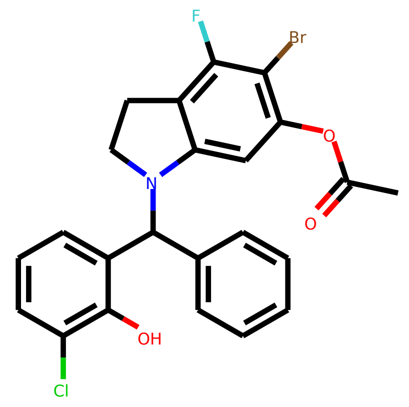

Osma-2936

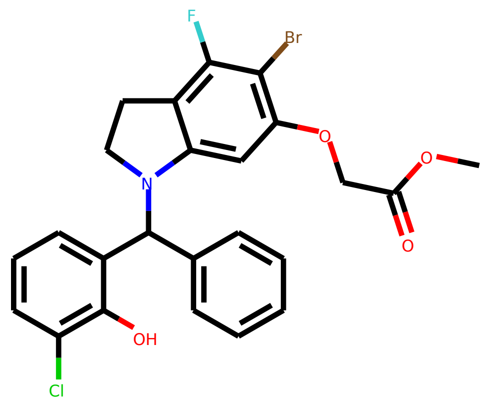

Osma-2937

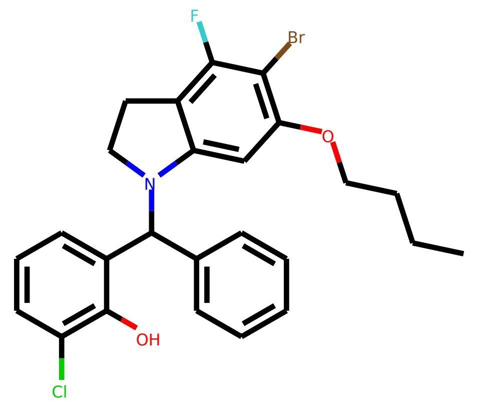

Osma-2938

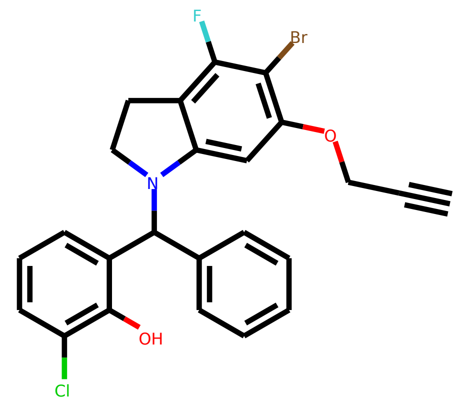

Osma-2939

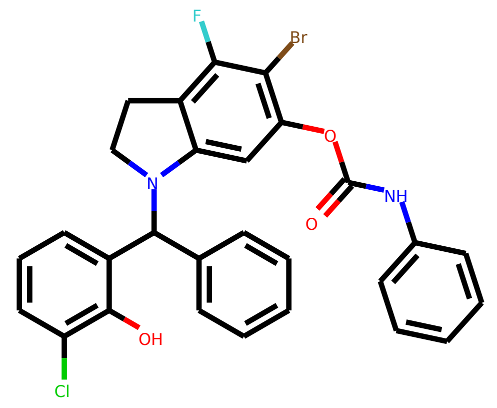

Osma-2940

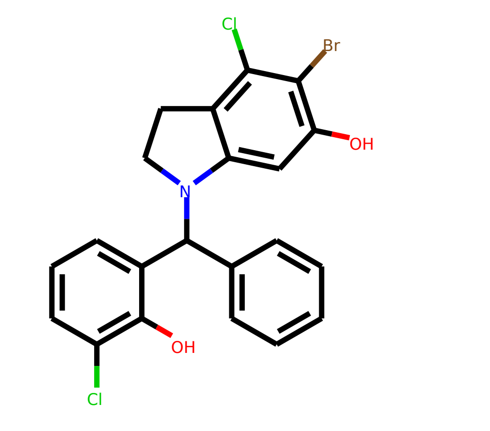
Osma-2941
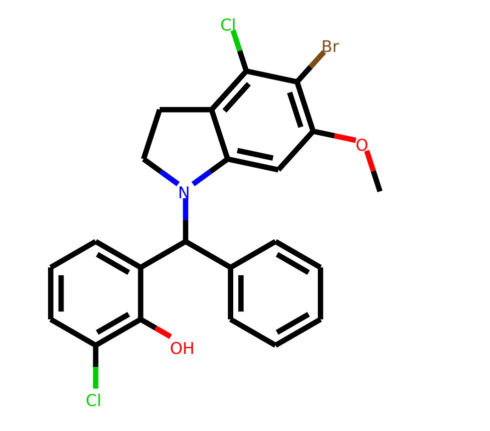
Osma-2942
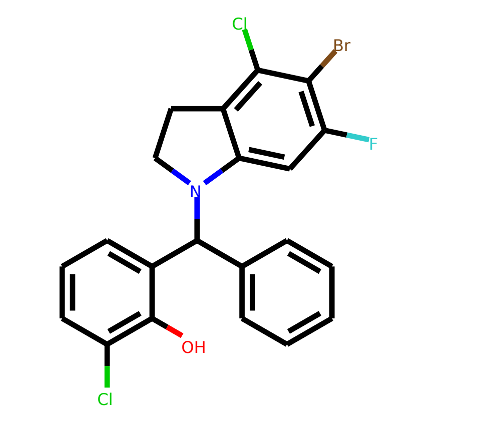
Osma-2943
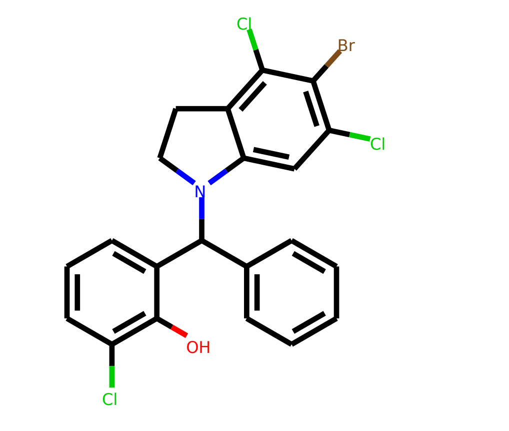
Osma-2944
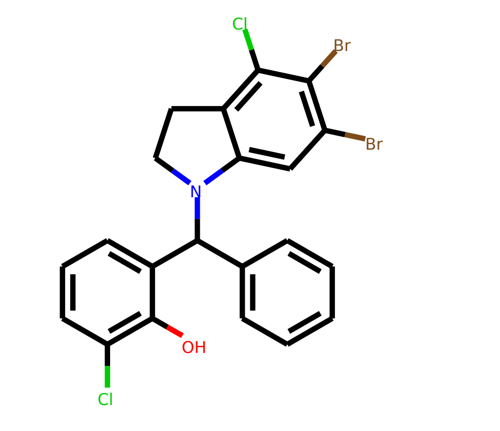
Osma-2945
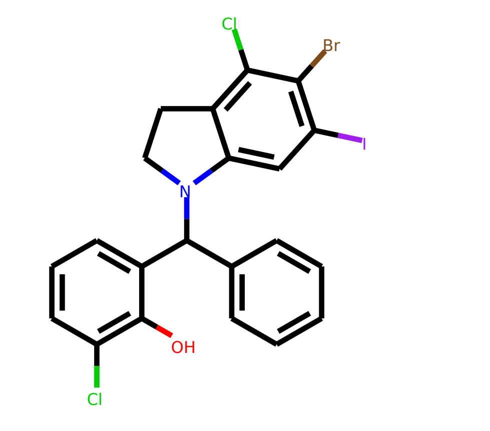
Osma-2946
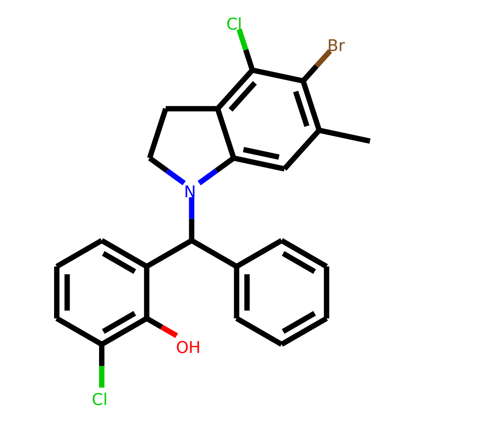
Osma-2947
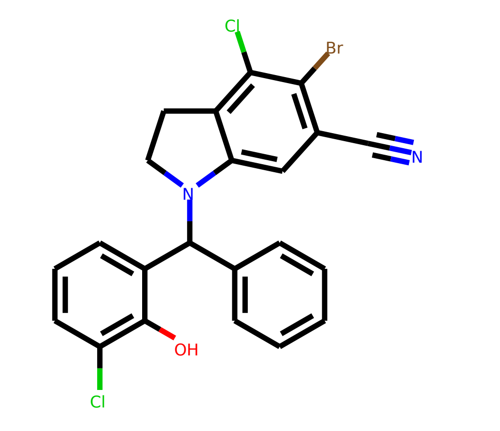
Osma-2948
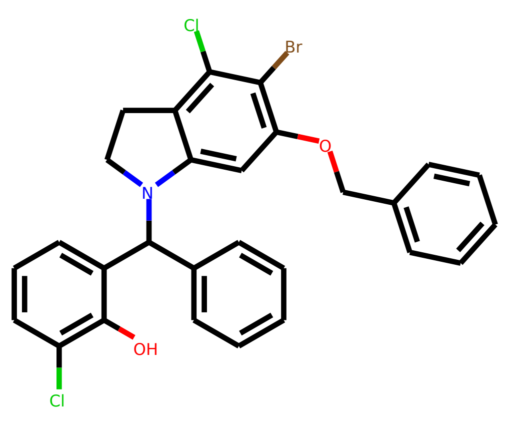
Osma-2949
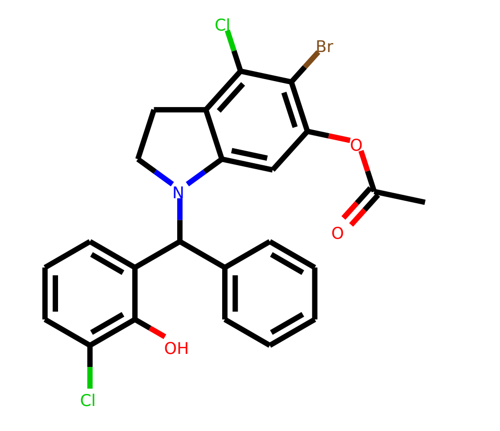
Osma-2950
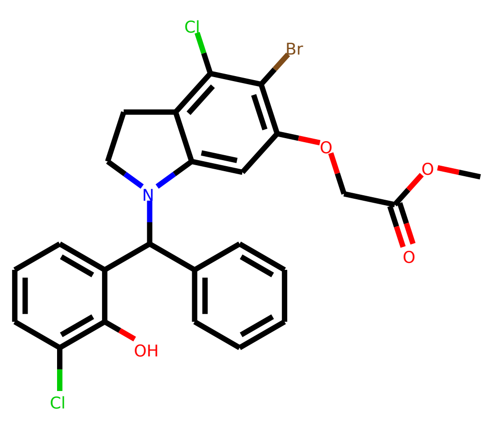
Osma-2951
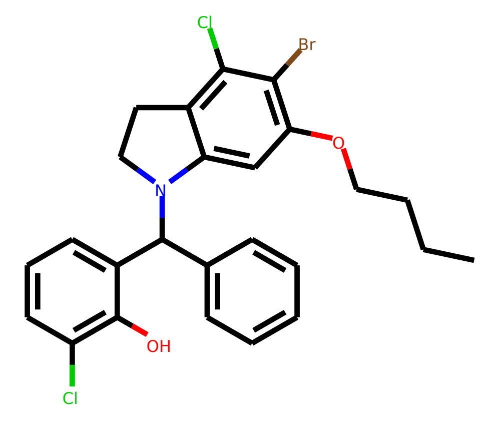
Osma-2952
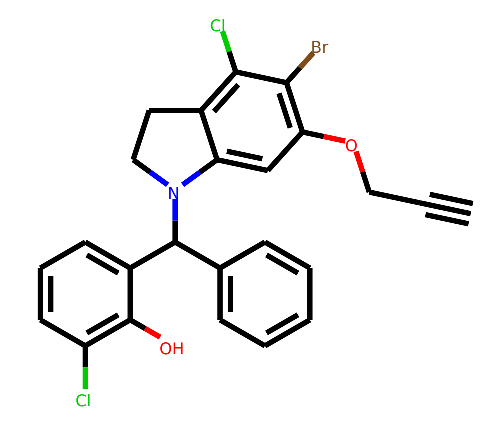
Osma-2953
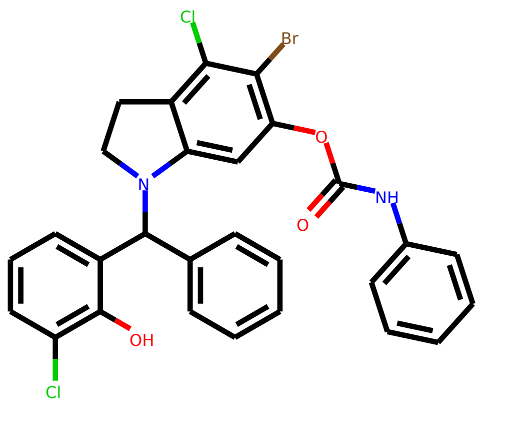
Osma-2954
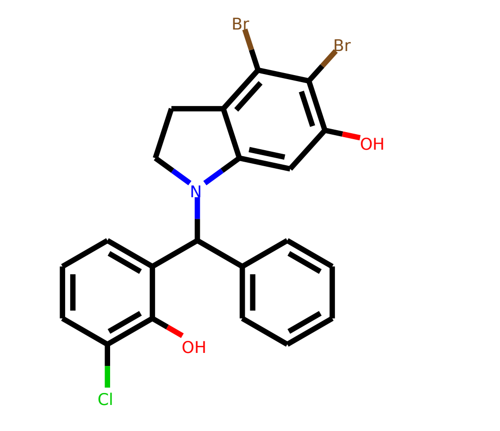
Osma-2955
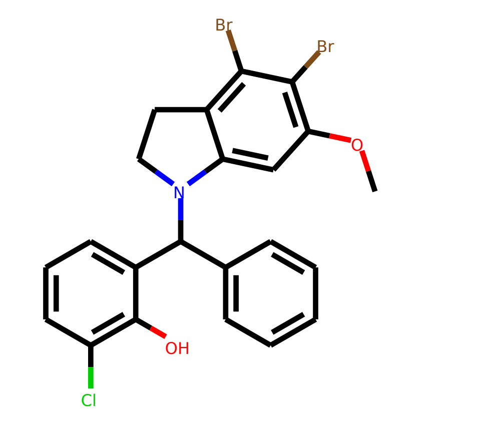
Osma-2956
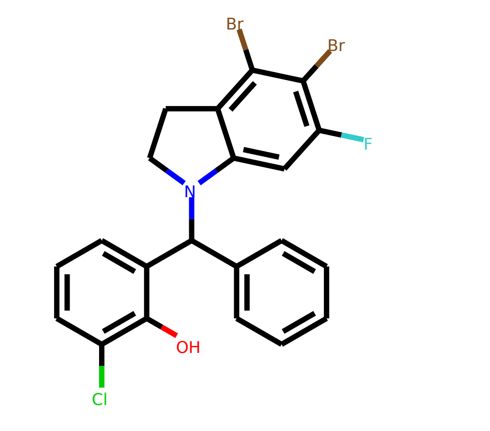
Osma-2957
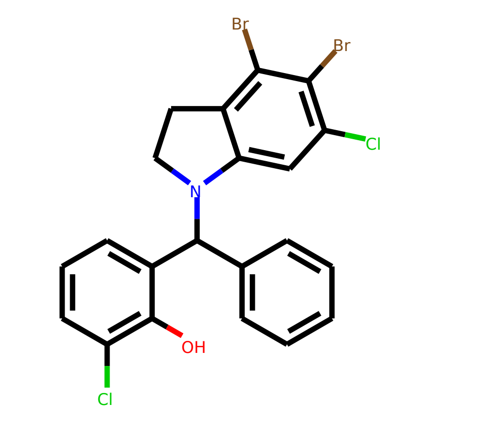
Osma-2958
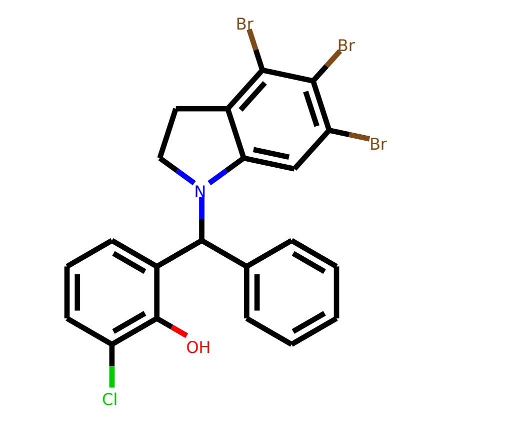
Osma-2959
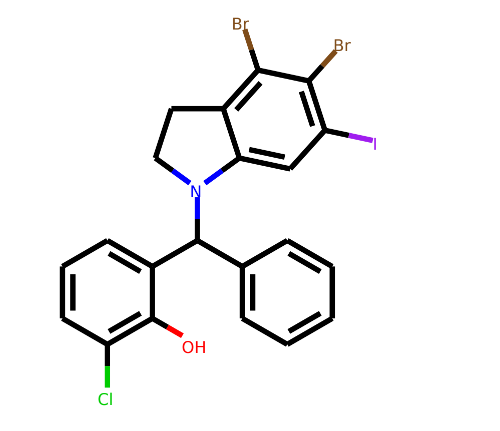
Osma-2960
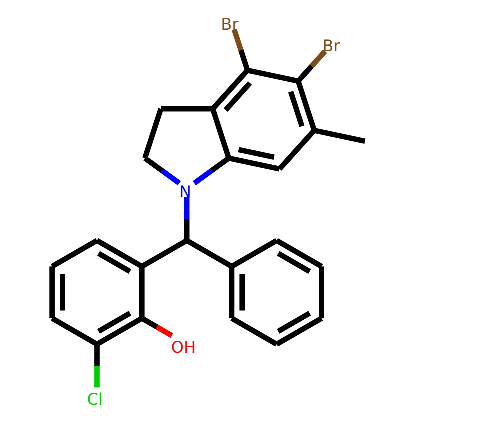
Osma-2961
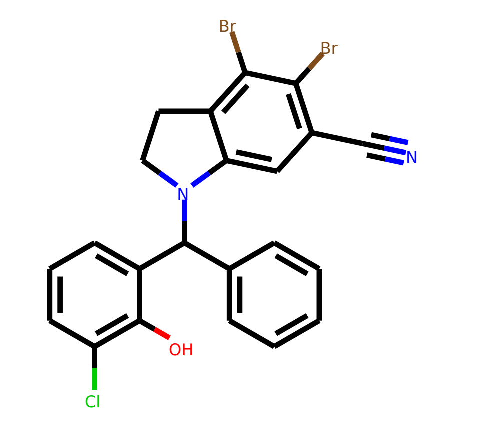
Osma-2962
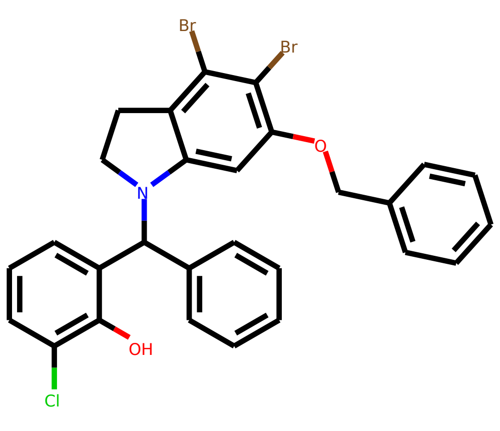
Osma-2963
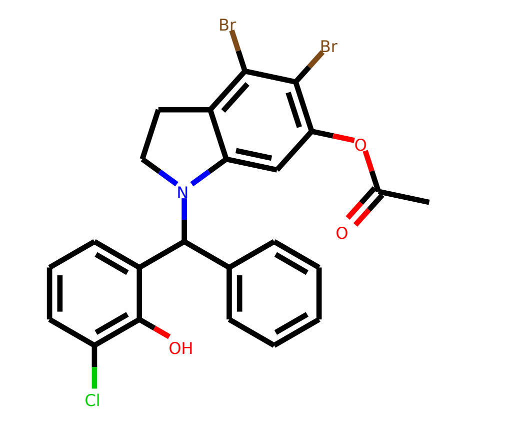
Osma-2964
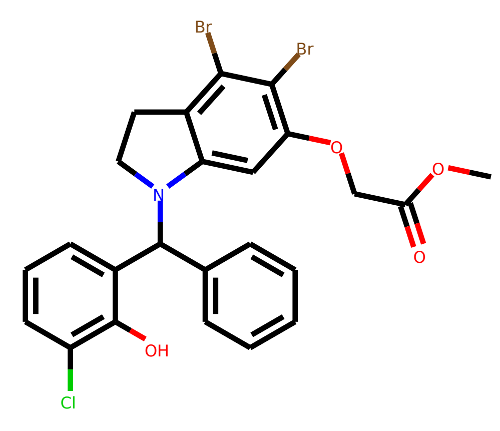
Osma-2965
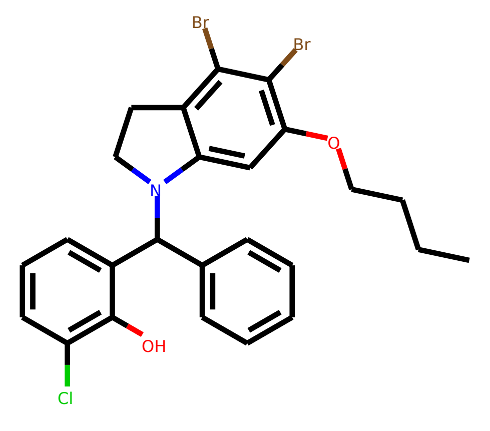
Osma-2966
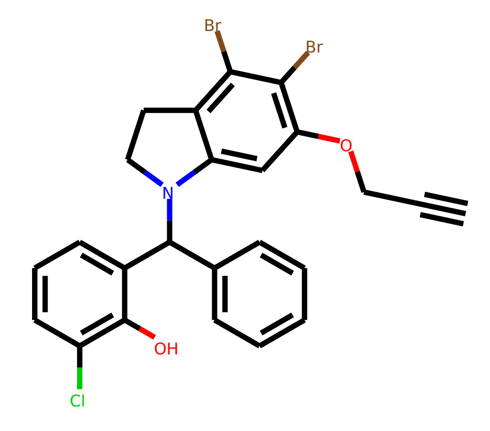
Osma-2967
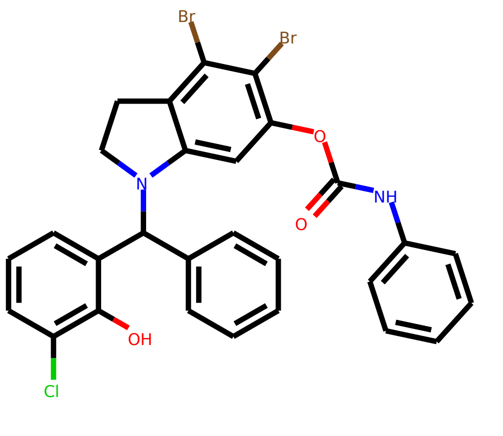
Osma-2968
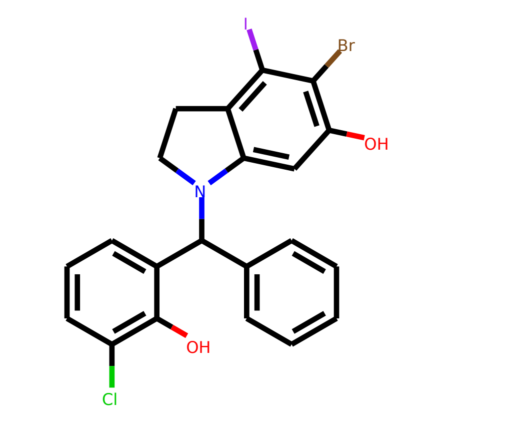
Osma-2969
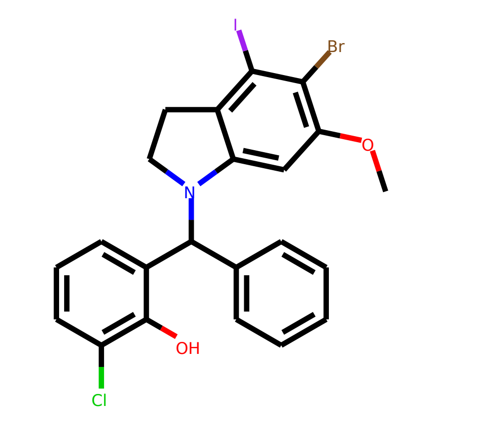
Osma-2970
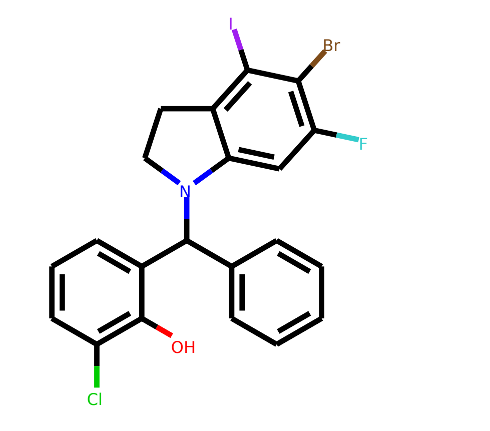
Osma-2971
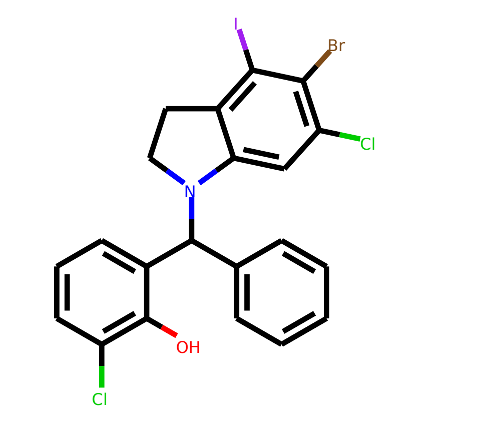
Osma-2972
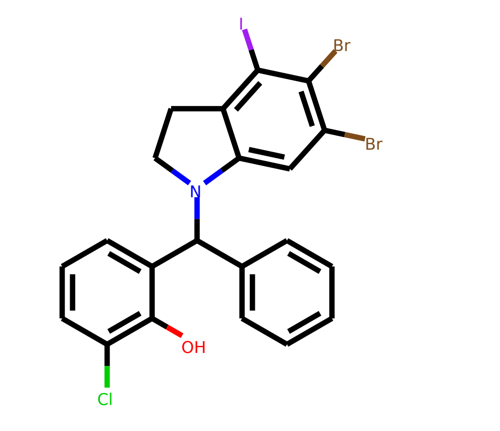
Osma-2973
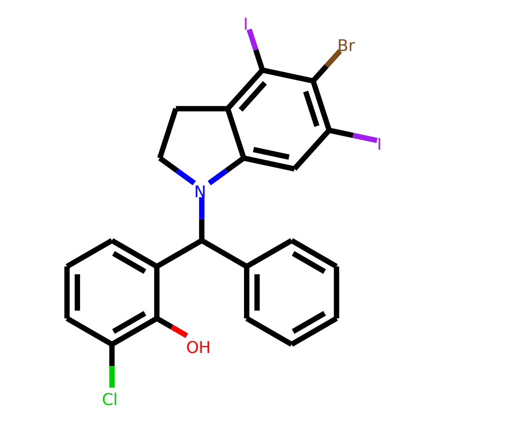
Osma-2974
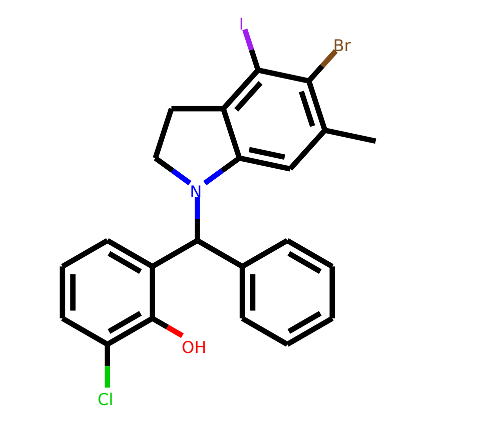
Osma-2975

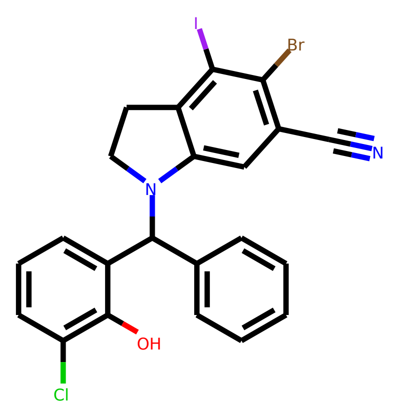
Osma-2976

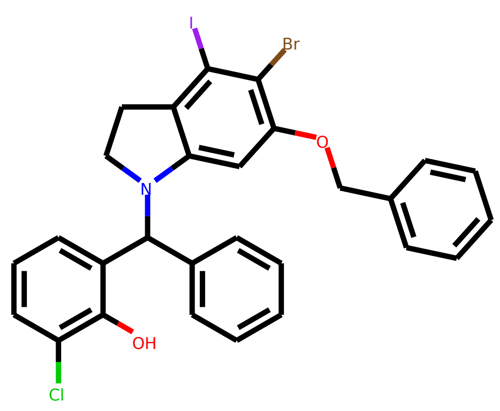
Osma-2977

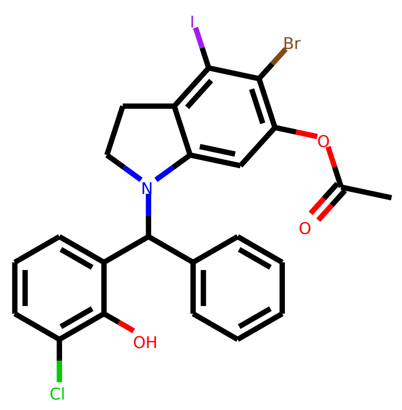
Osma-2978

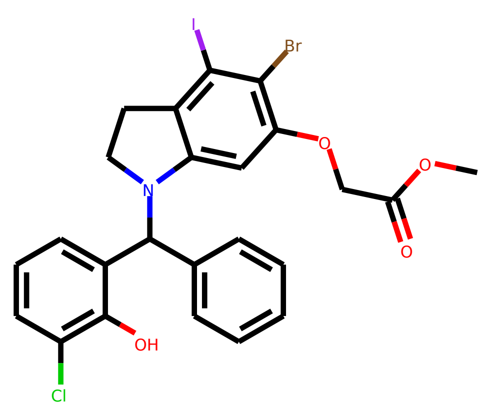
Osma-2979

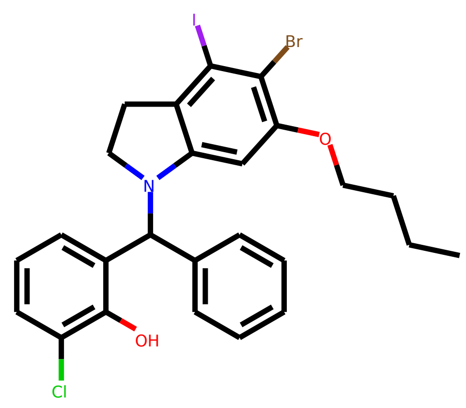
Osma-2980

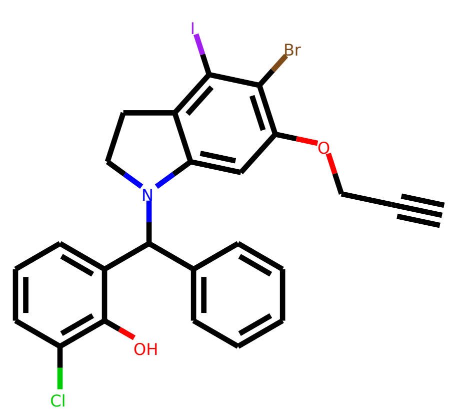
Osma-2981

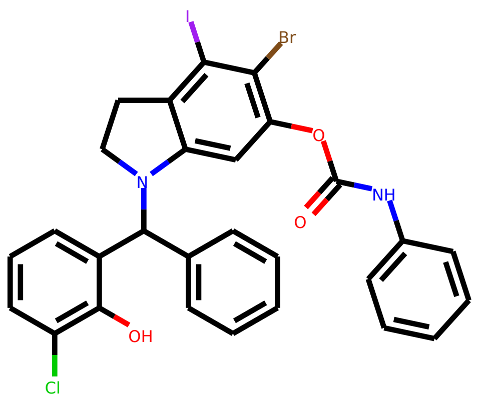
Osma-2982

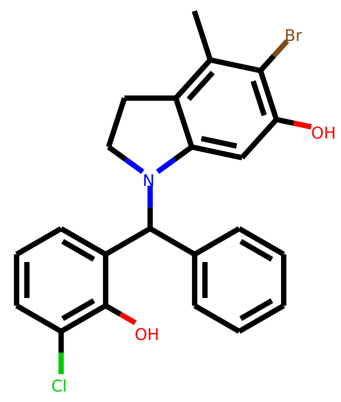
Osma-2983

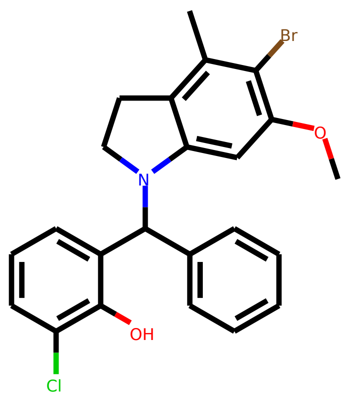
Osma-2984

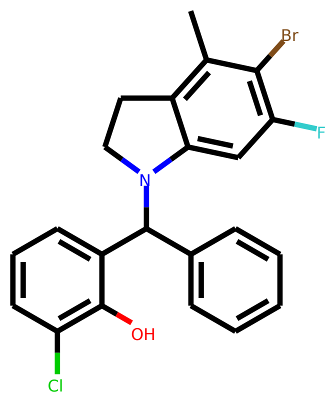
Osma-2985

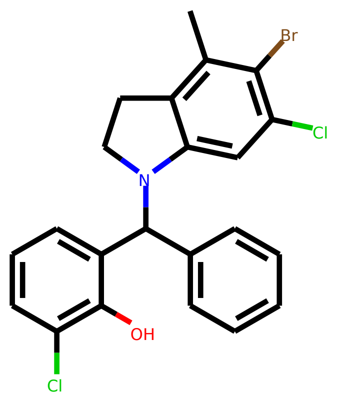
Osma-2986

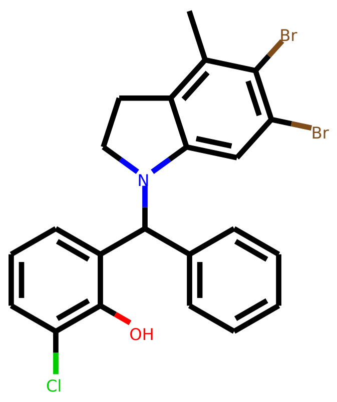
Osma-2987

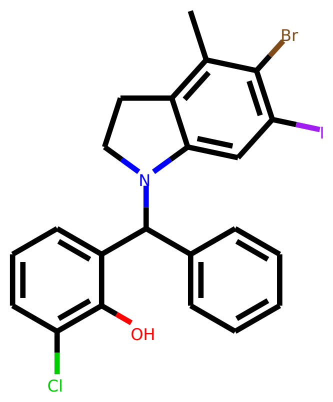
Osma-2988

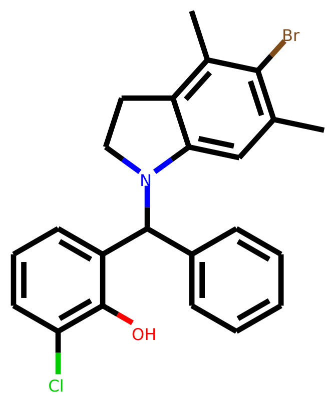
Osma-2989

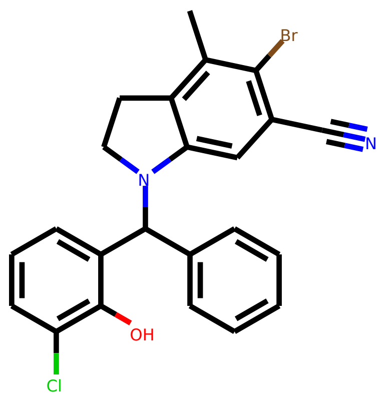
Osma-2990

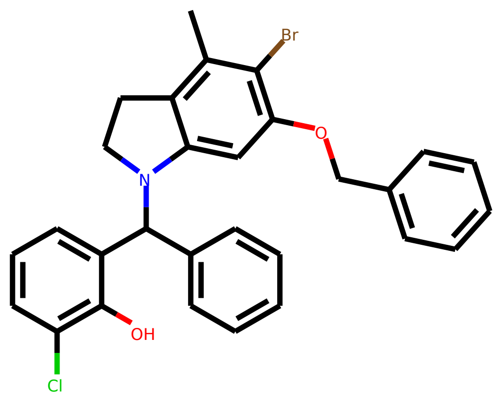
Osma-2991

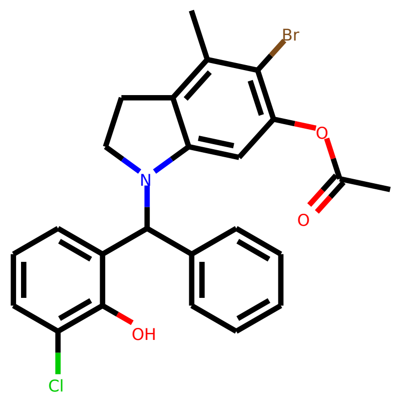
Osma-2992

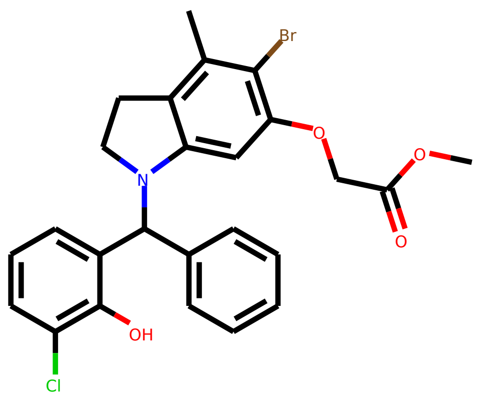
Osma-2993

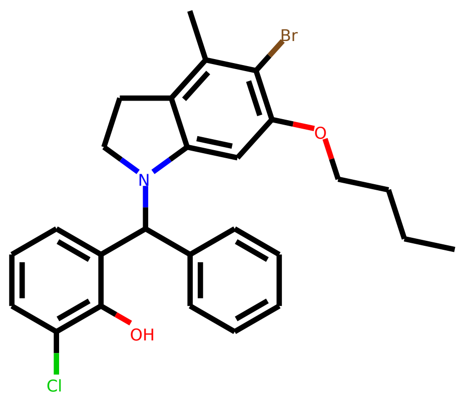
Osma-2994

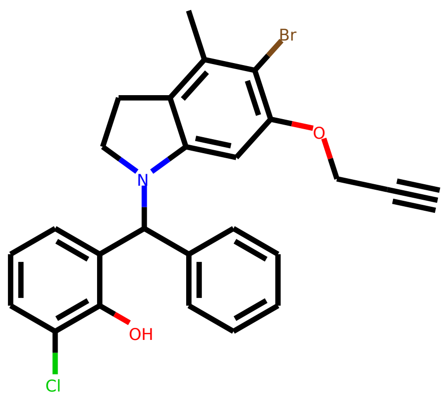
Osma-2995

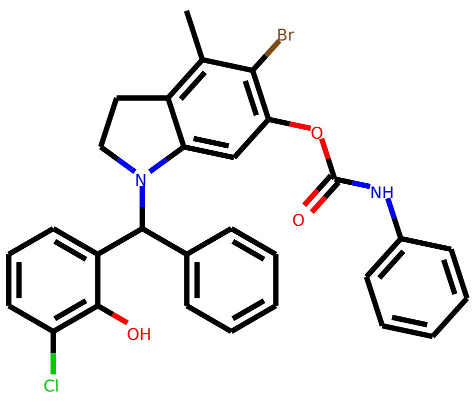
Osma-2996

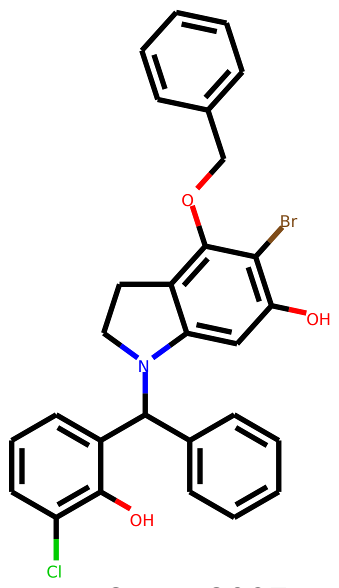
Osma-2997

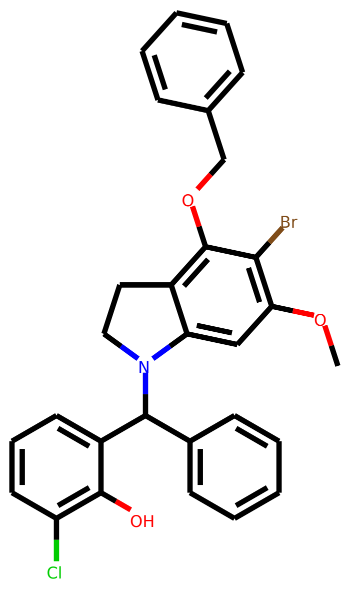
Osma-2998

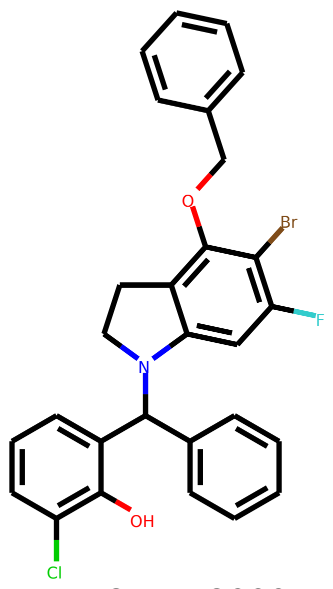
Osma-2999

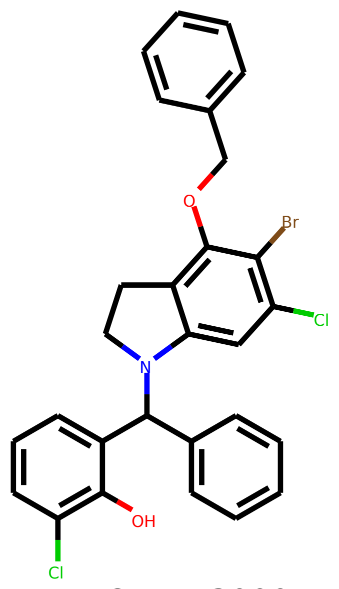
Osma-3000

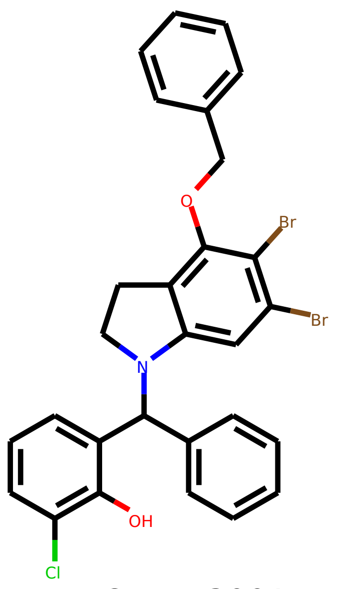
Osma-3001

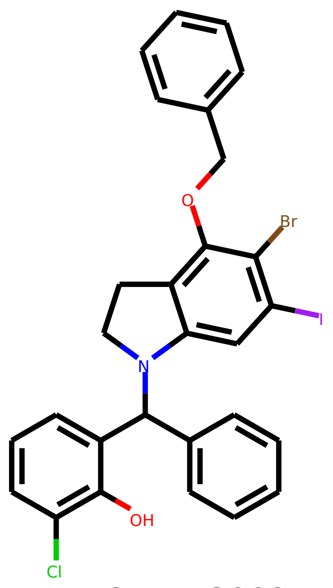
Osma-3002

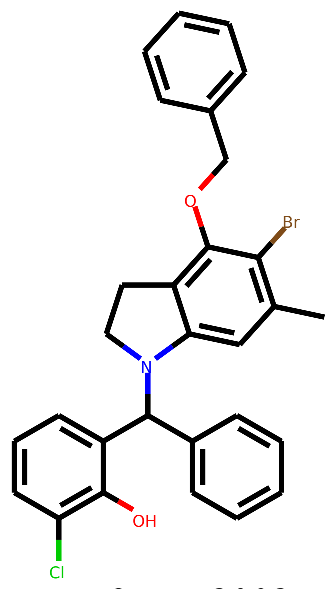
Osma-3003

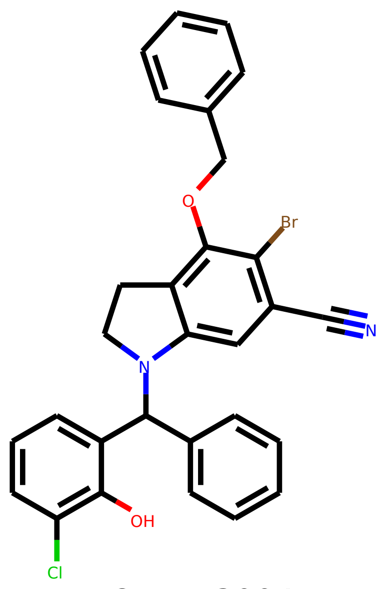
Osma-3004

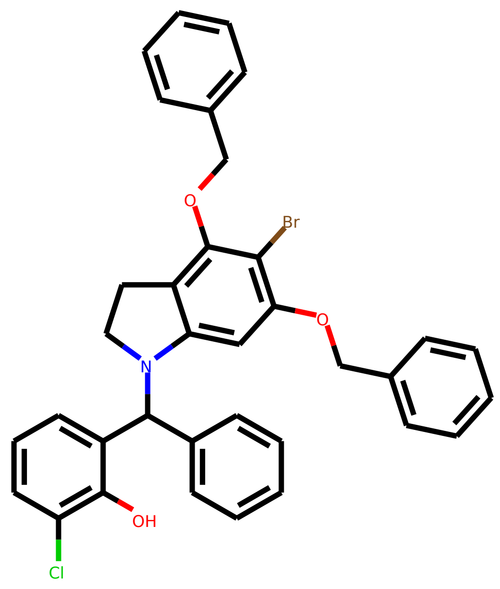
Osma-3005

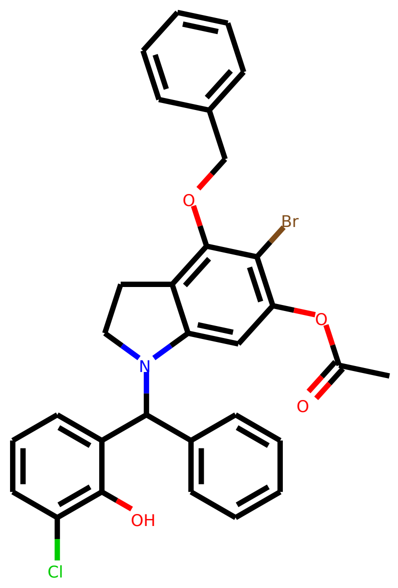
Osma-3006

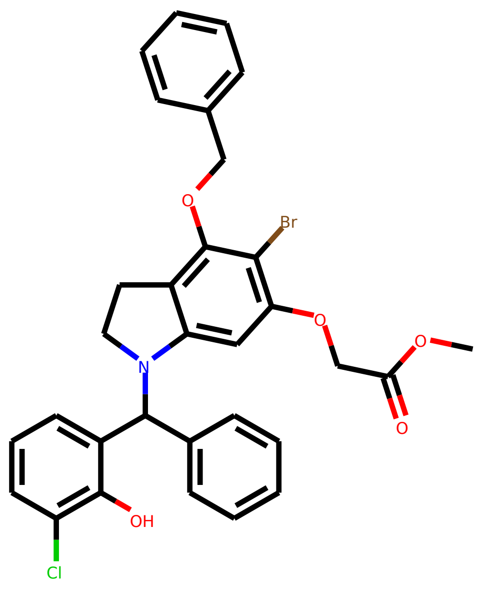
Osma-3007

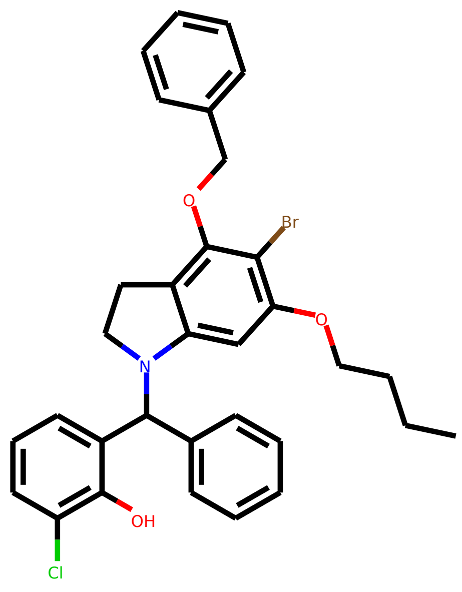
Osma-3008

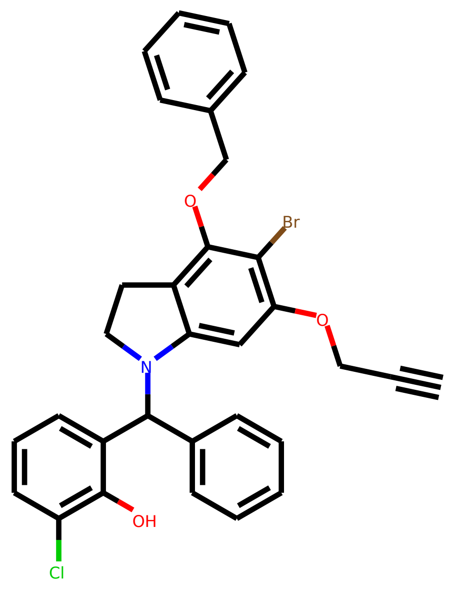
Osma-3009

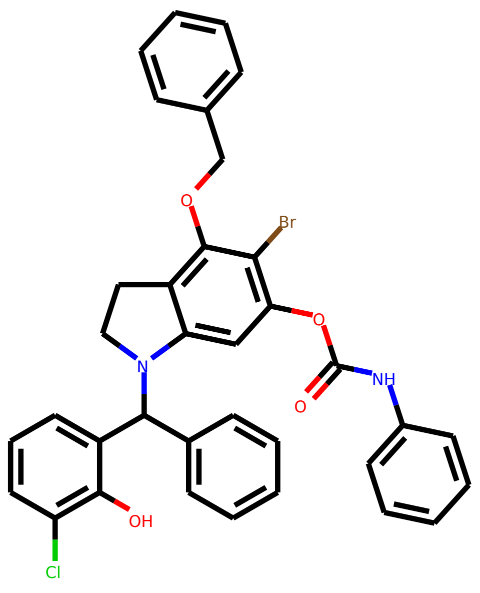
Osma-3010

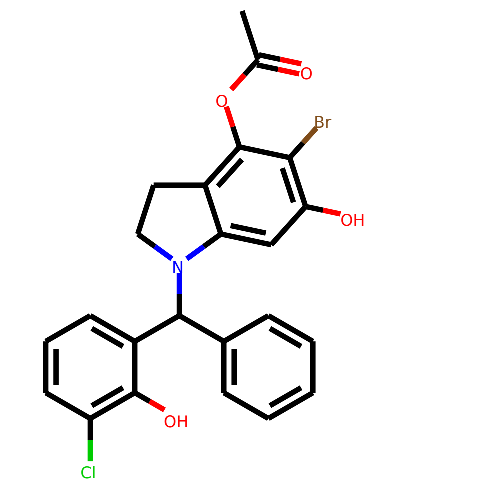
Osma-3011

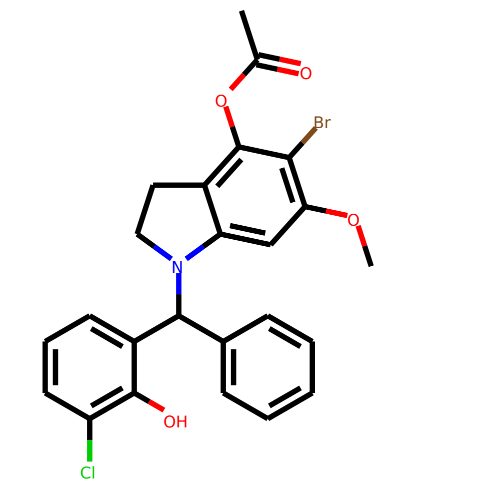
Osma-3012

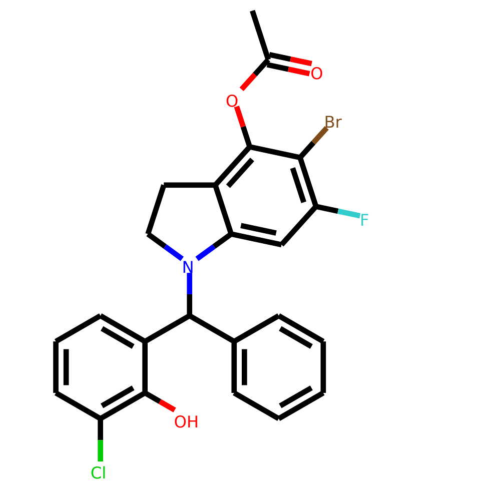
Osma-3013

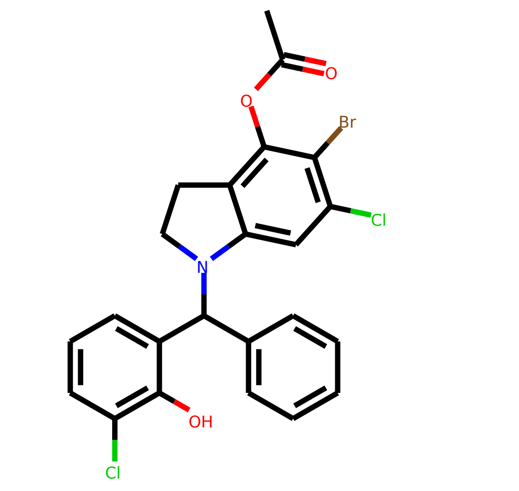
Osma-3014

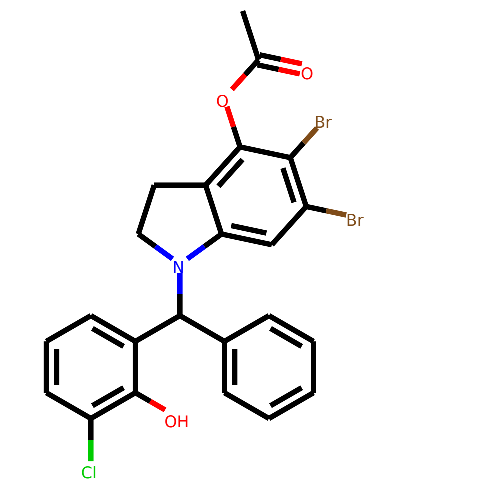
Osma-3015

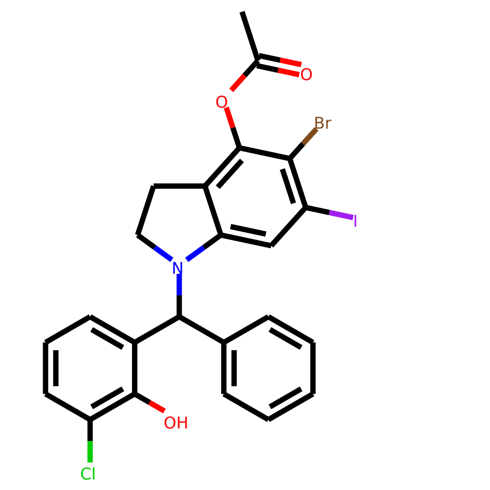
Osma-3016

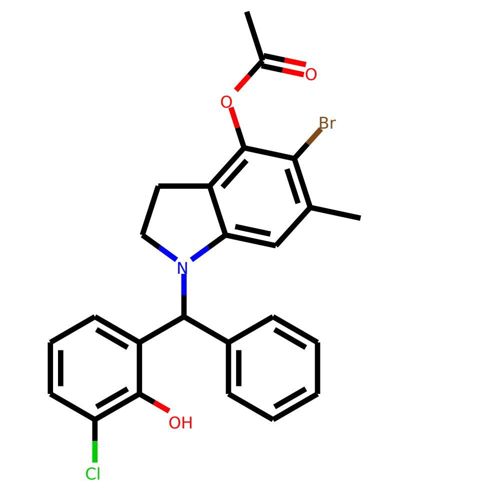
Osma-3017

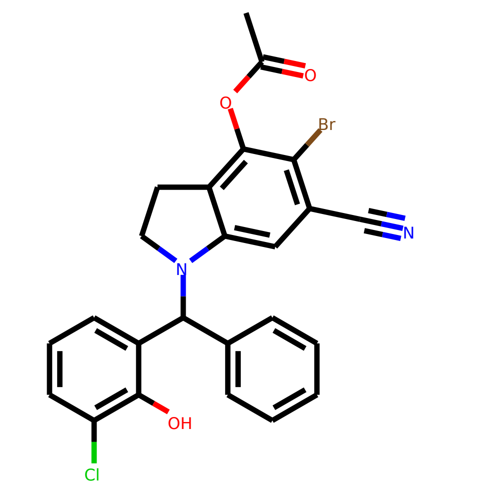
Osma-3018

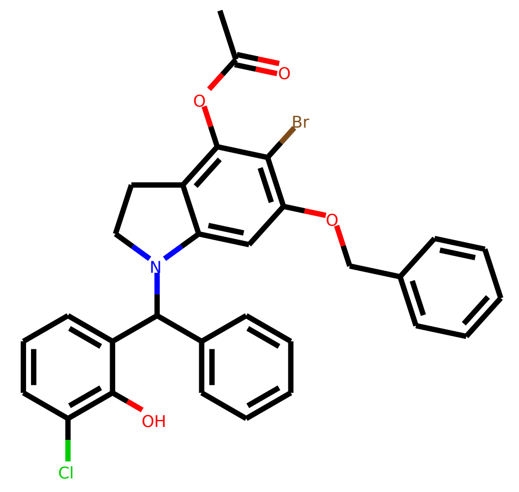
Osma-3019

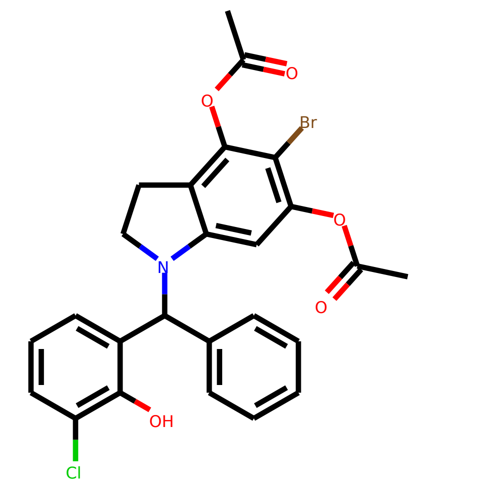
Osma-3020

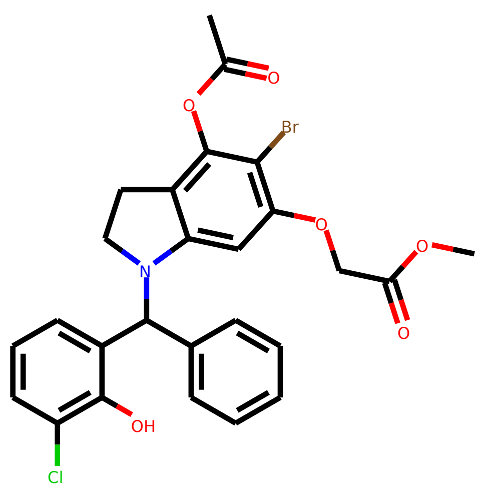
Osma-3021

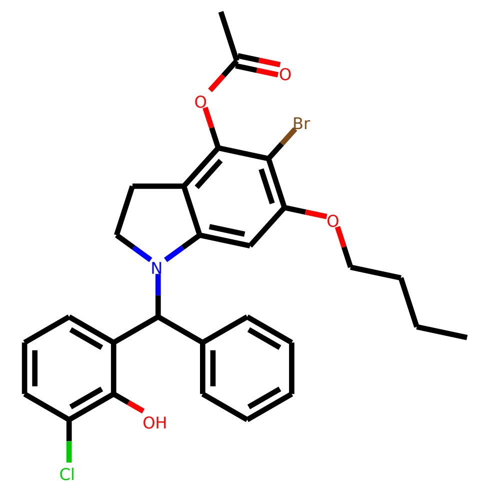
Osma-3022

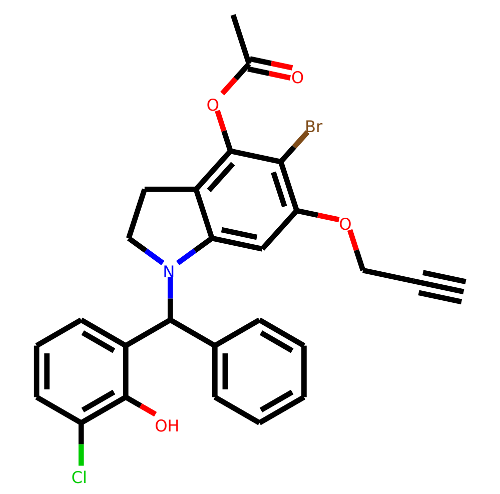
Osma-3023

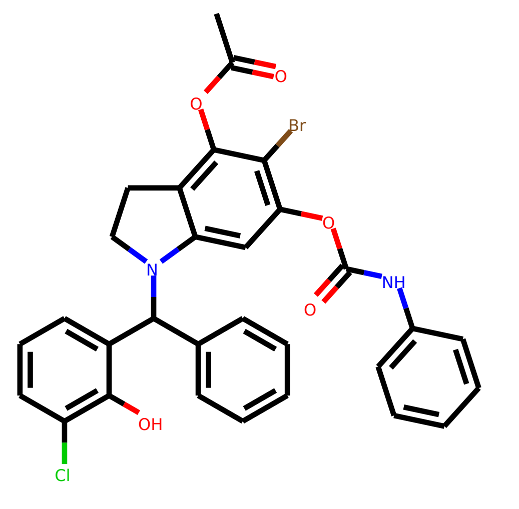
Osma-3024

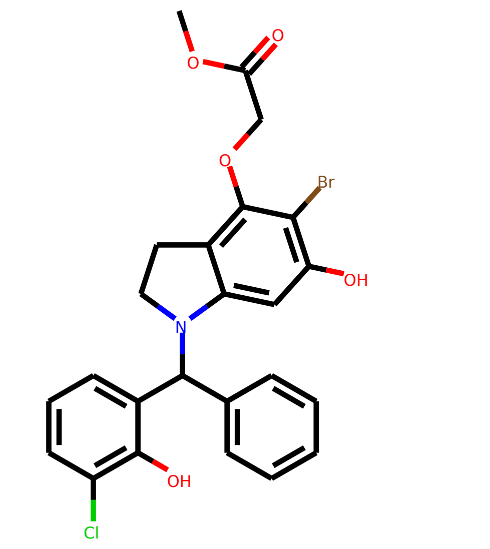
Osma-3025

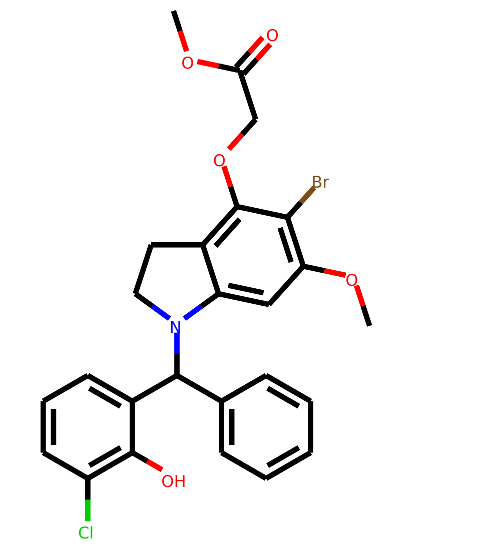
Osma-3026

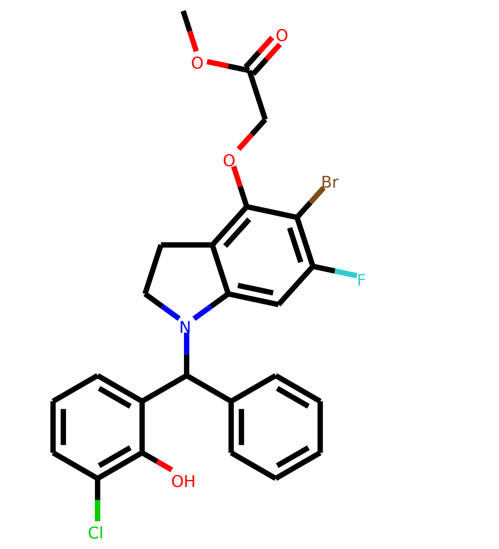
Osma-3027

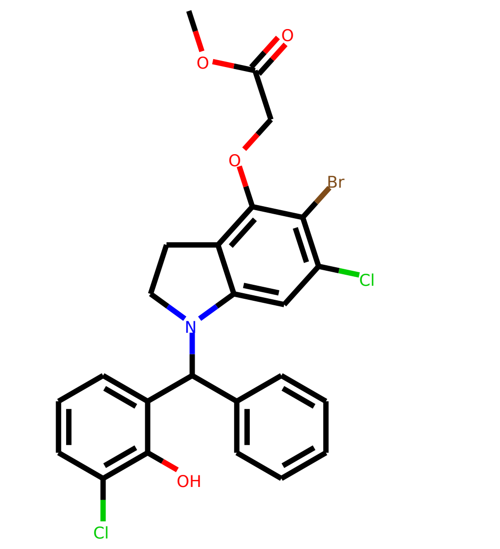
Osma-3028

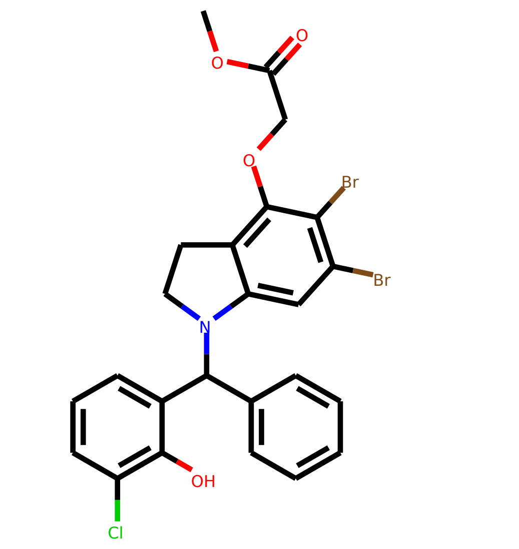
Osma-3029

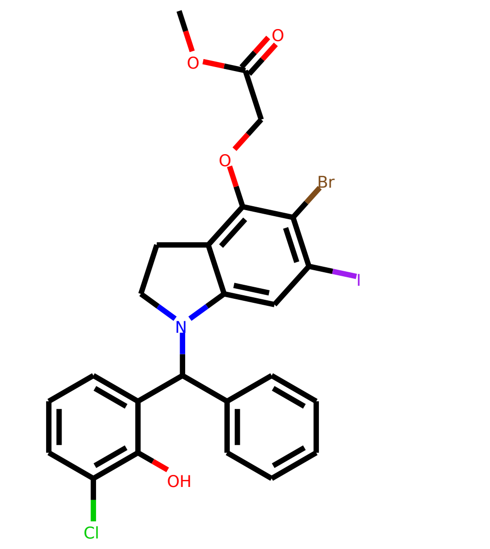
Osma-3030

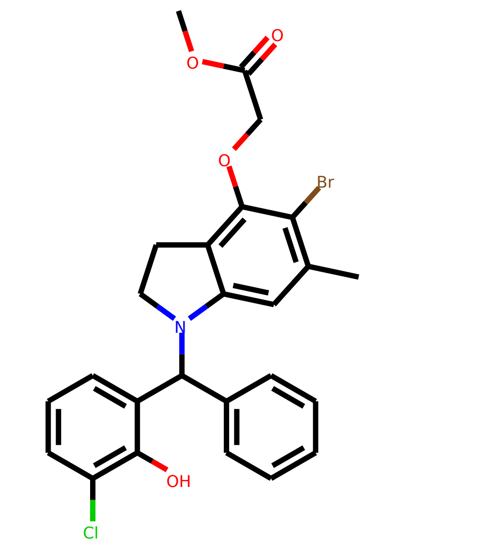
Osma-3031

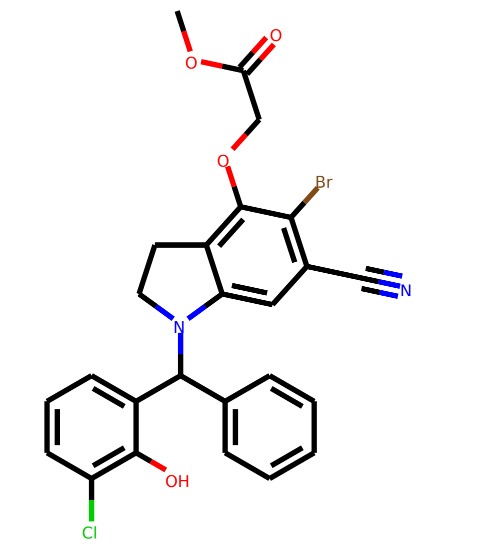
Osma-3032

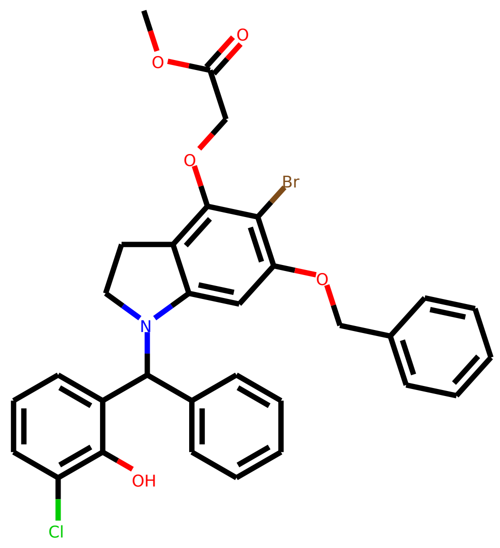
Osma-3033

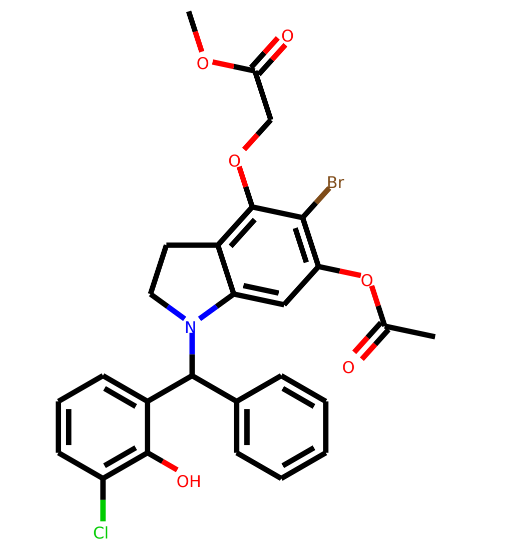
Osma-3034

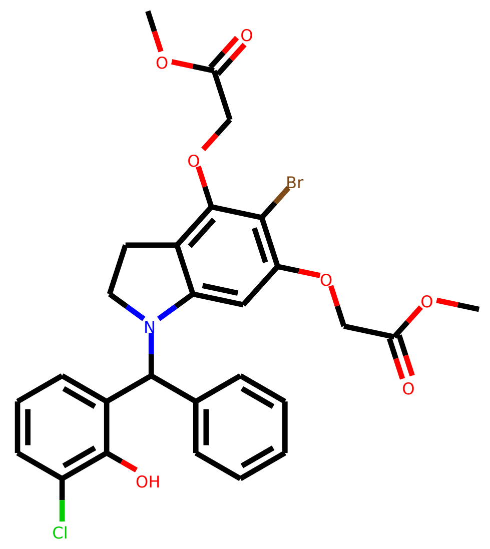
Osma-3035

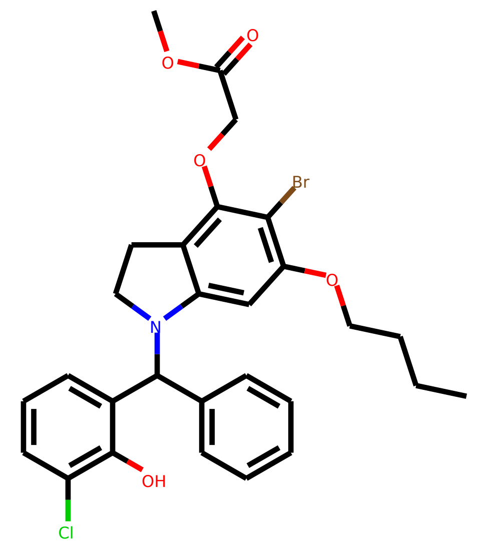
Osma-3036

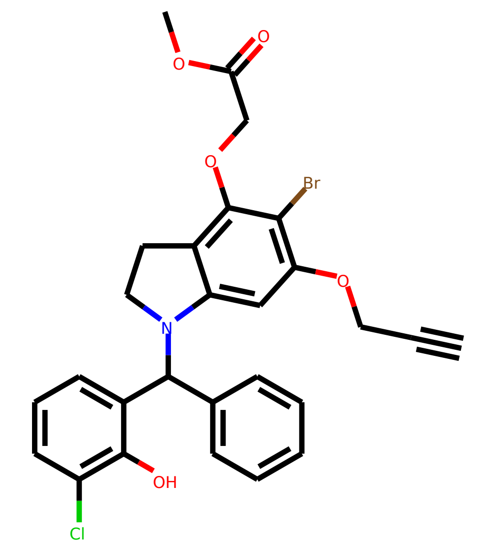
Osma-3037

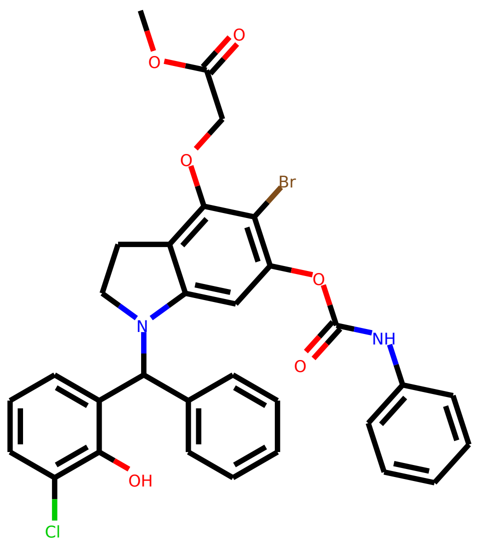
Osma-3038

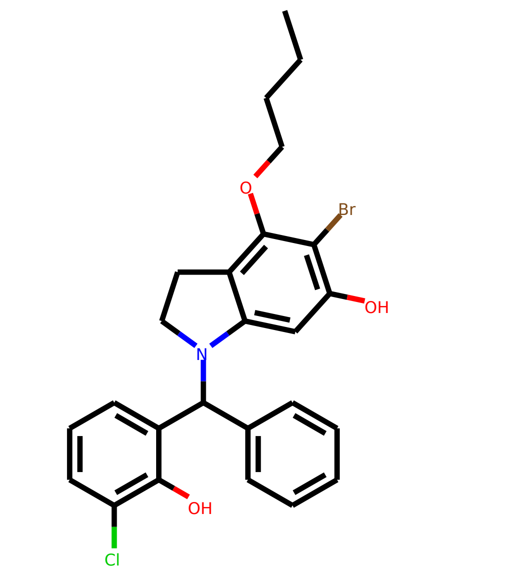
Osma-3039

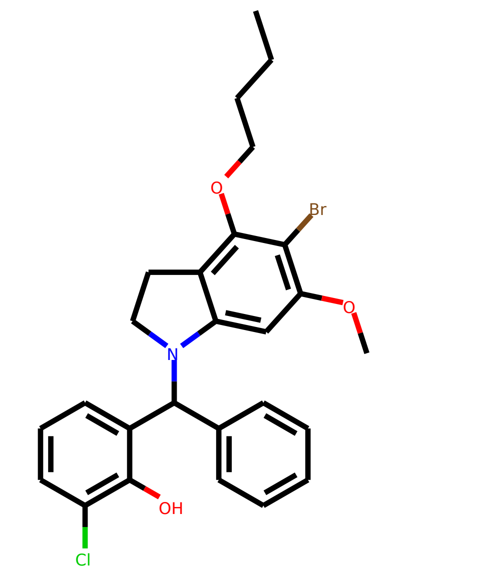
Osma-3040

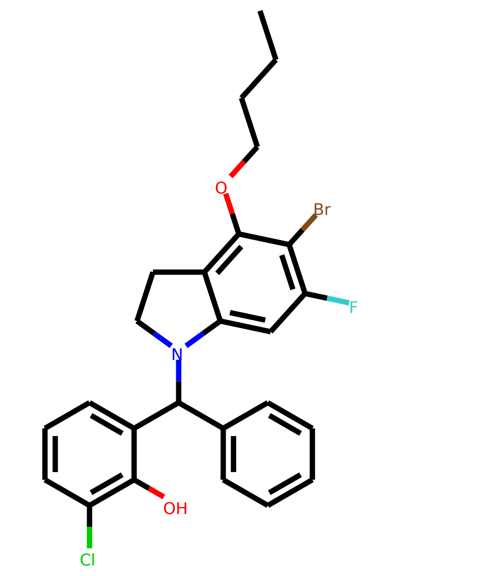
Osma-3041

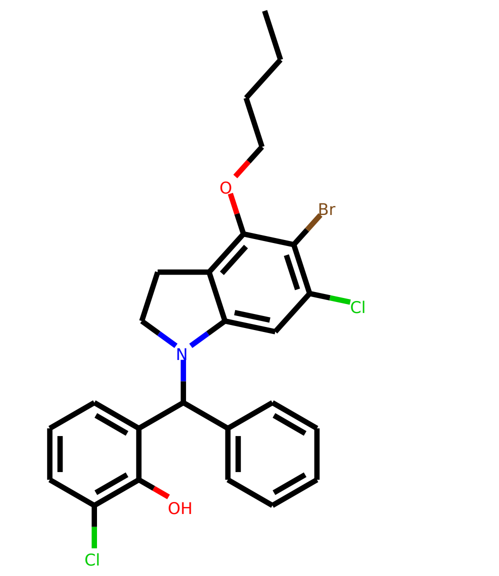
Osma-3042

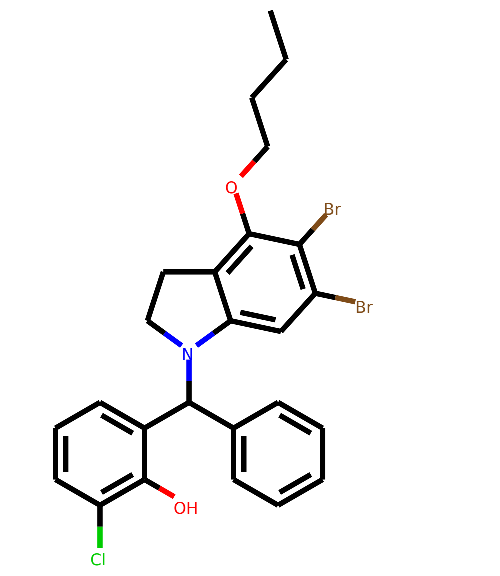
Osma-3043

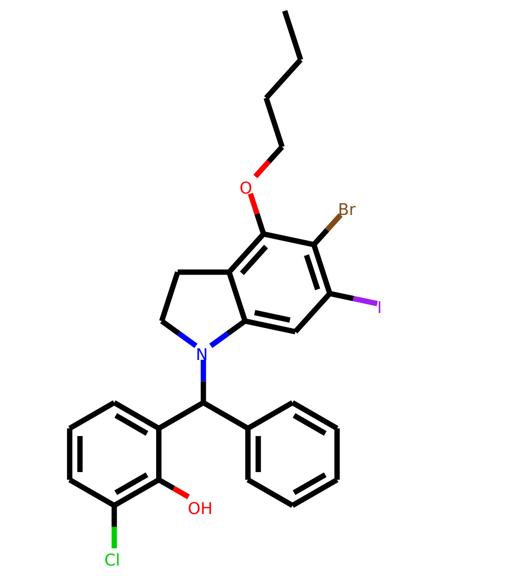
Osma-3044

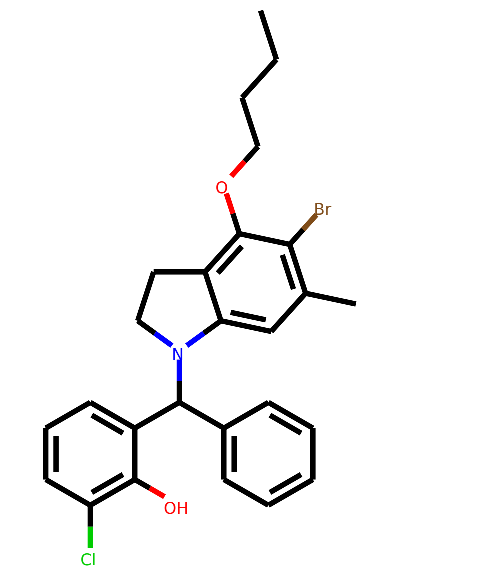
Osma-3045

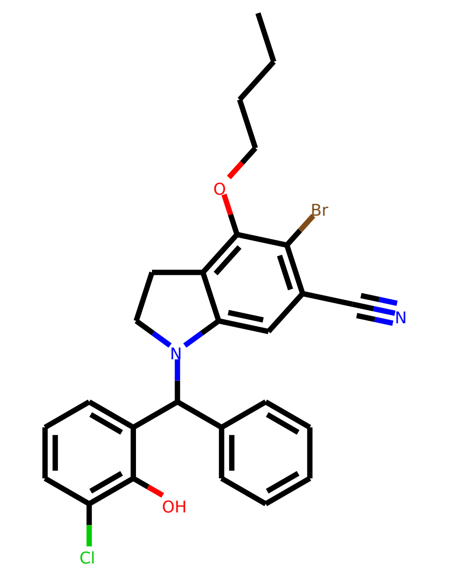

Osma-3046

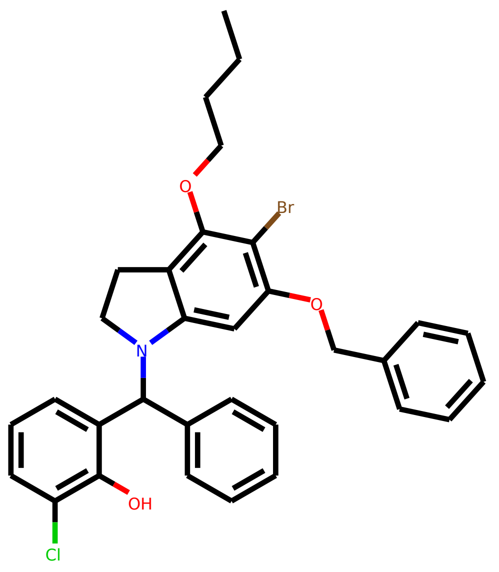

Osma-3047

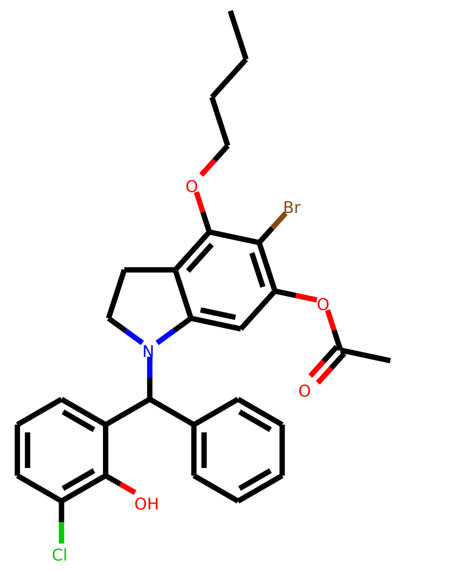

Osma-3048

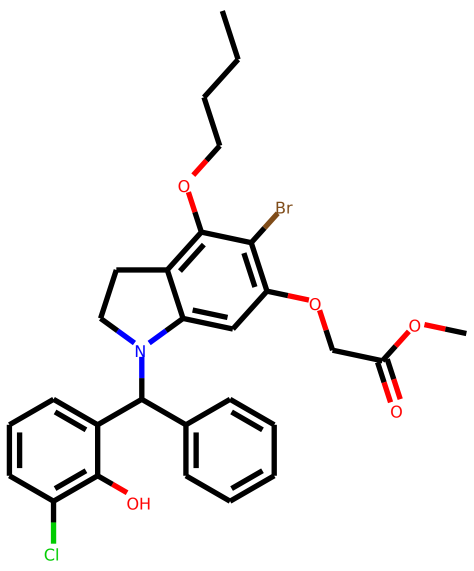

Osma-3049

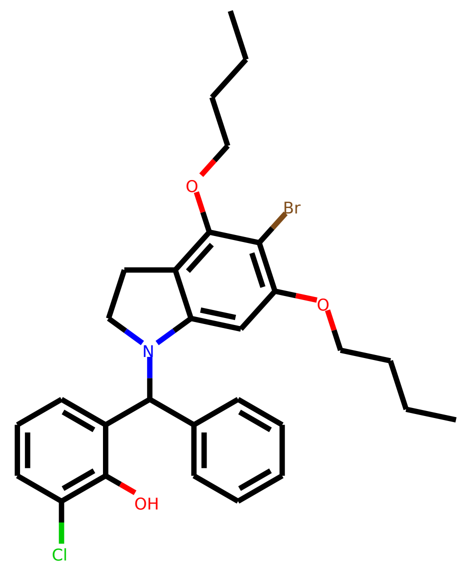

Osma-3050

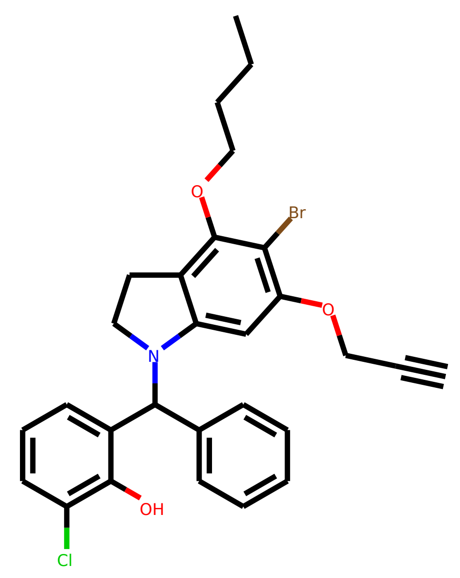

Osma-3051

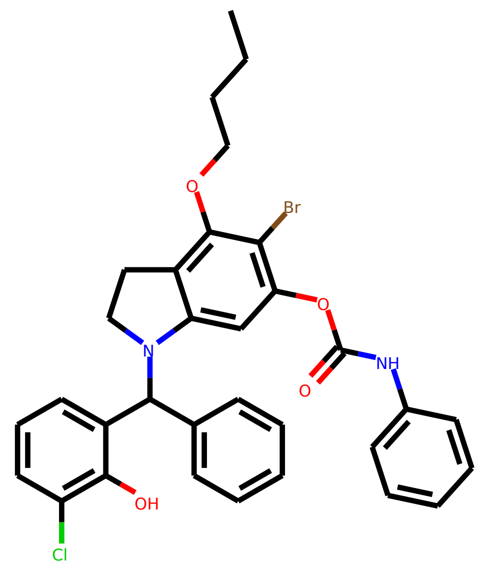

Osma-3052

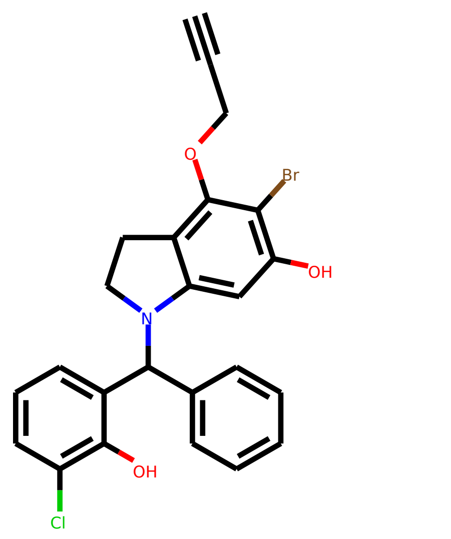

Osma-3053

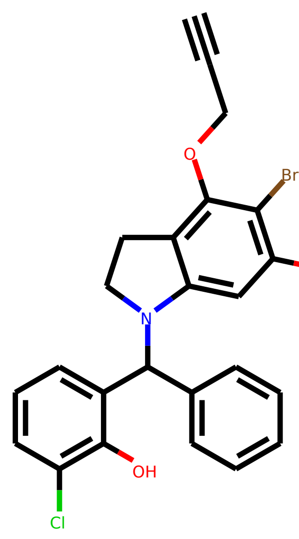

Osma-3054

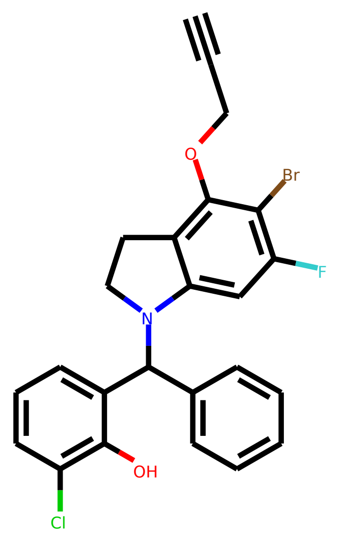

Osma-3055

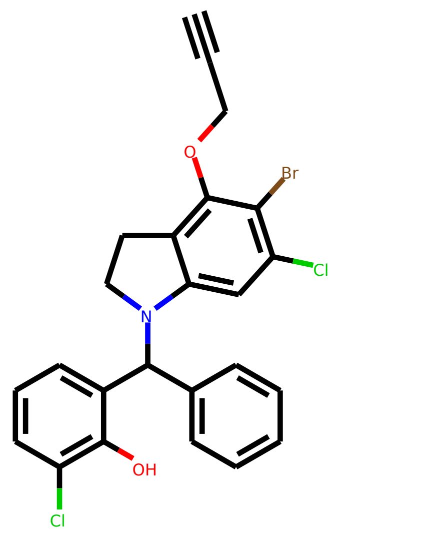

Osma-3056

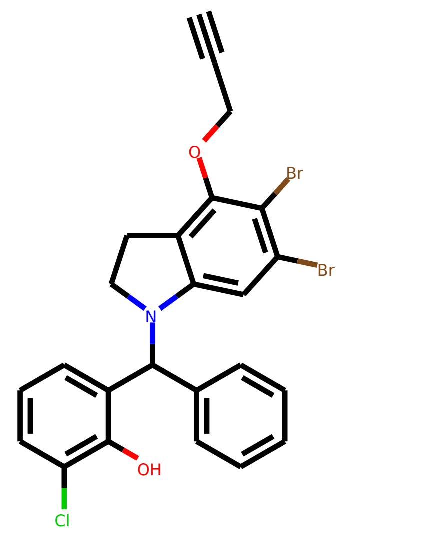

Osma-3057

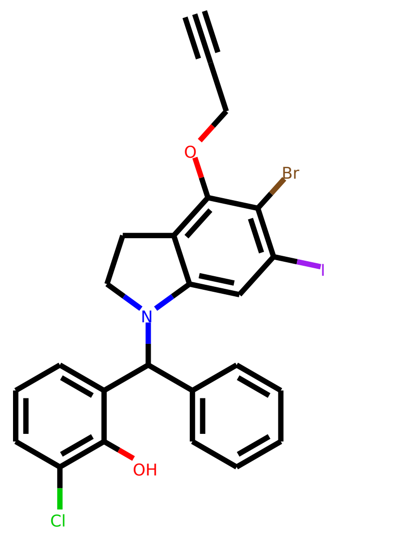

Osma-3058

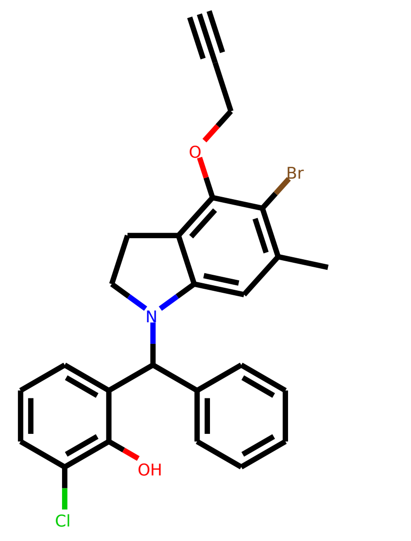

Osma-3059

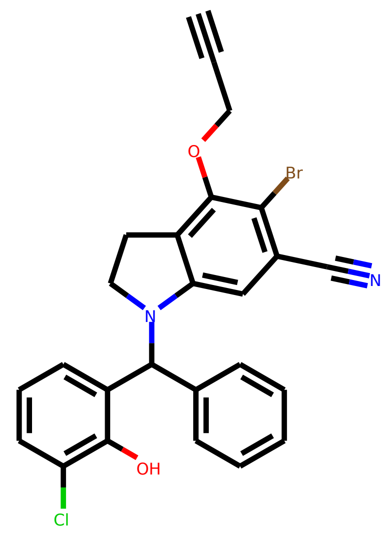

Osma-3060

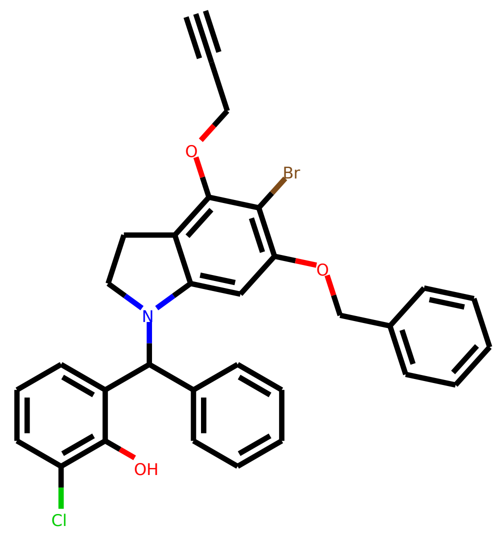

Osma-3061

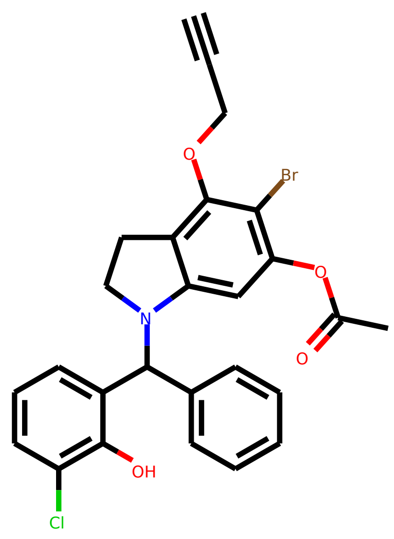

Osma-3062

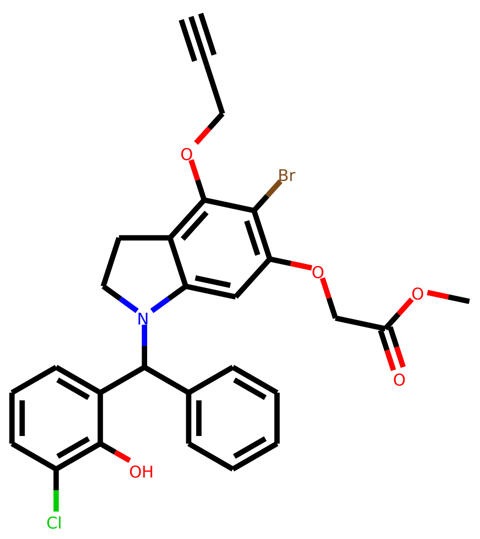

Osma-3063

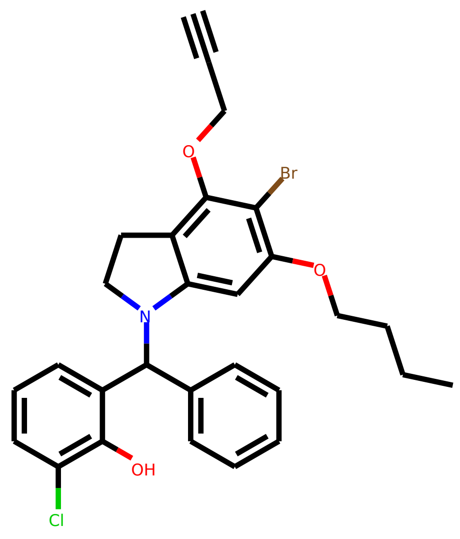

Osma-3064

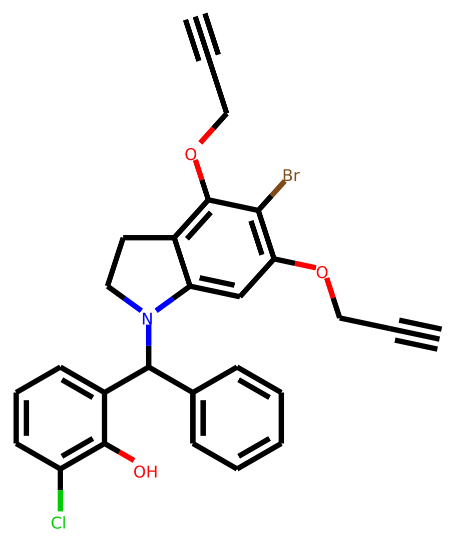

Osma-3065

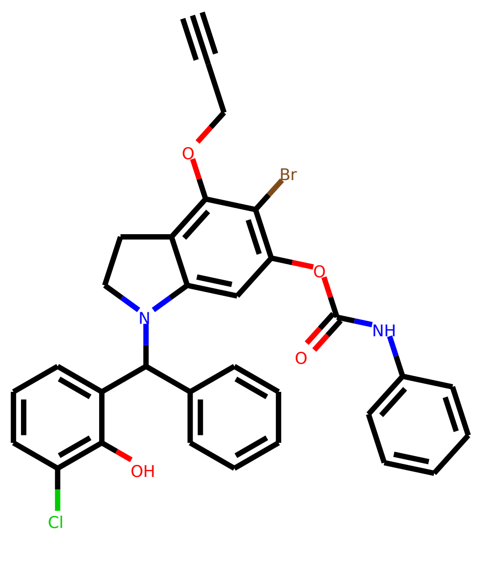

Osma-3066

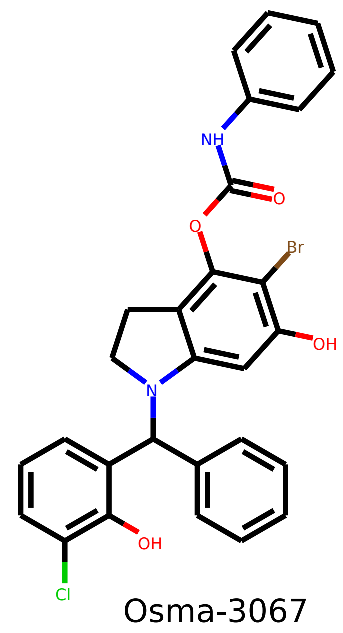

Osma-3067

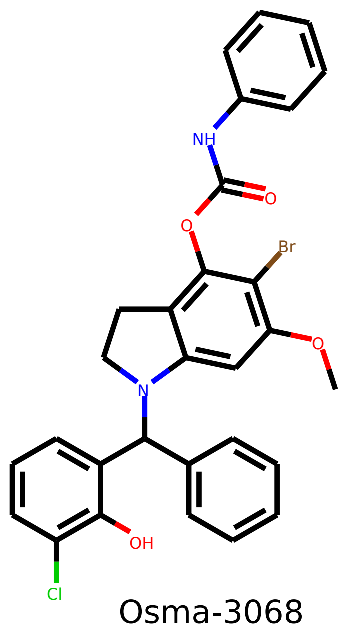

Osma-3068

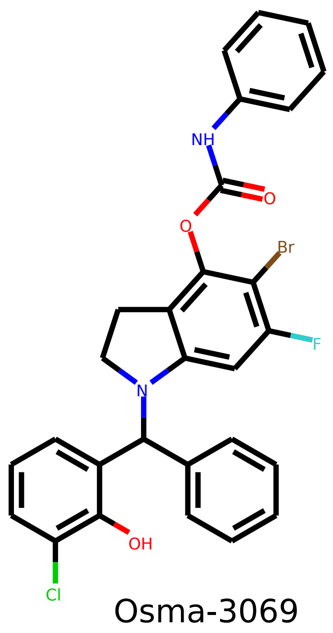

Osma-3069

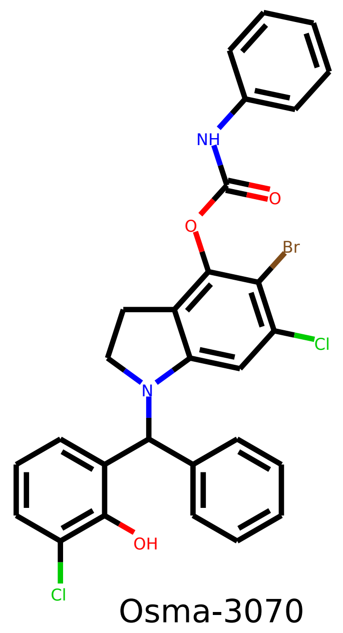

Osma-3070

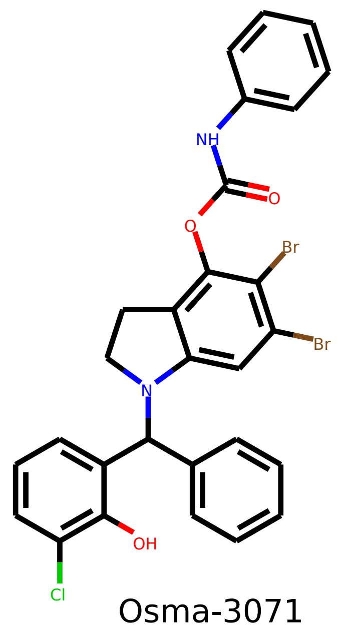

Osma-3071

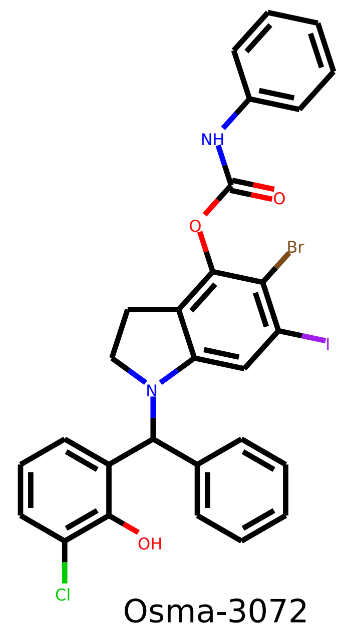

Osma-3072

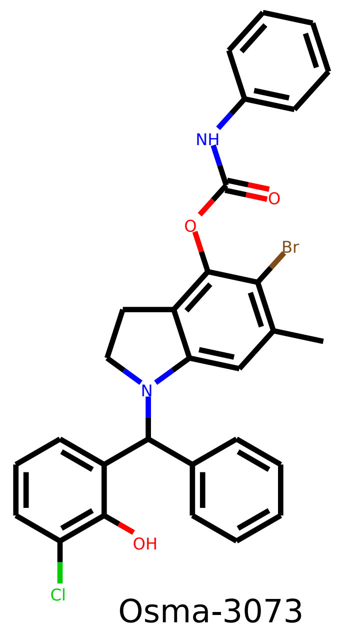

Osma-3073

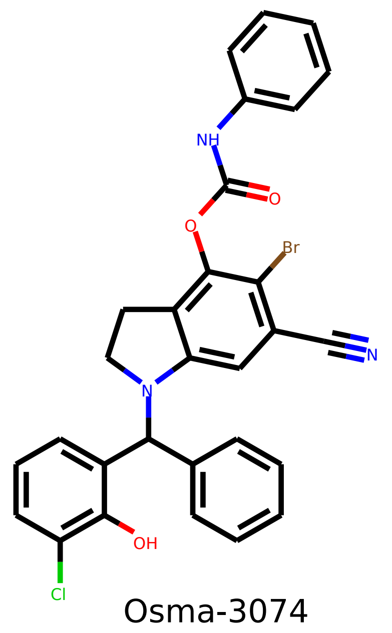

Osma-3074

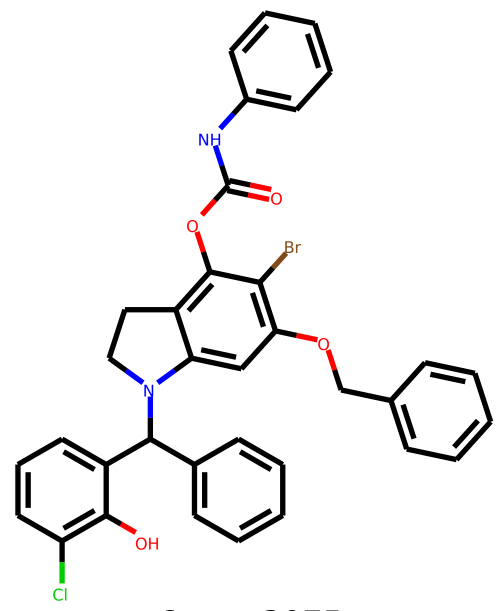

Osma-3075

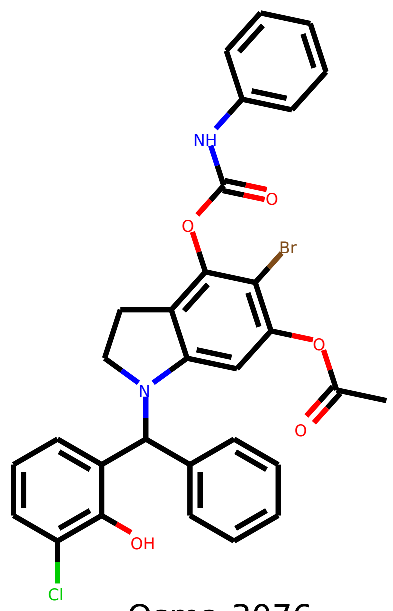

Osma-3076

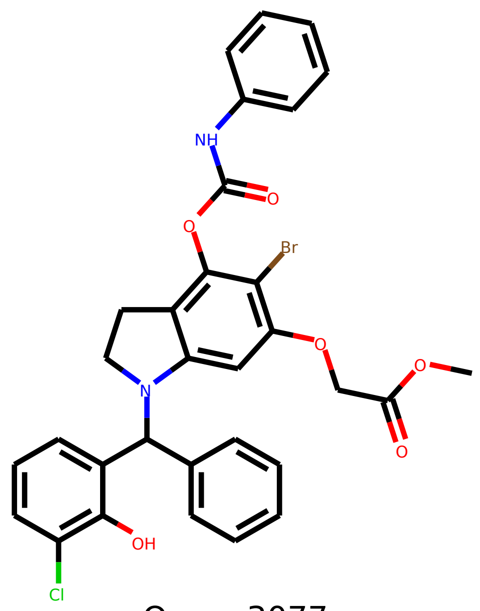

Osma-3077

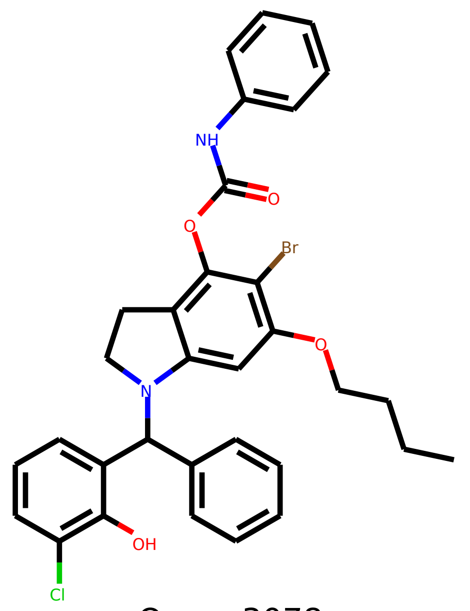

Osma-3078

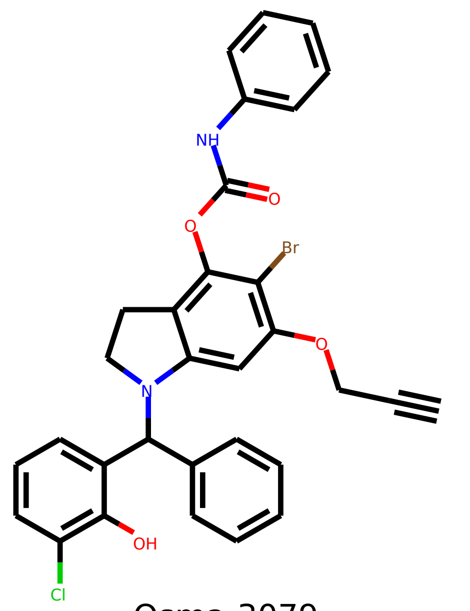

Osma-3079

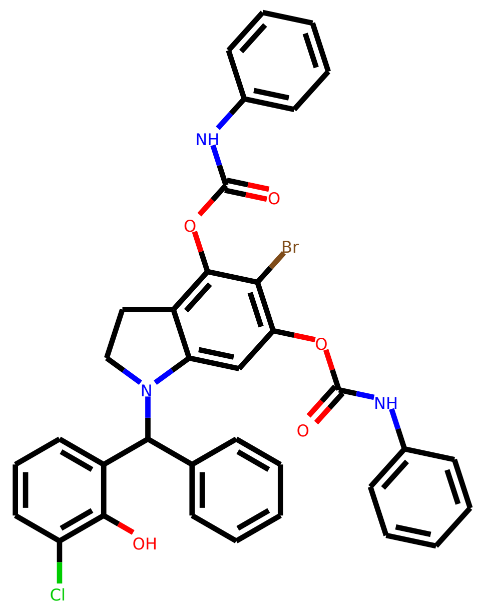

Osma-3080

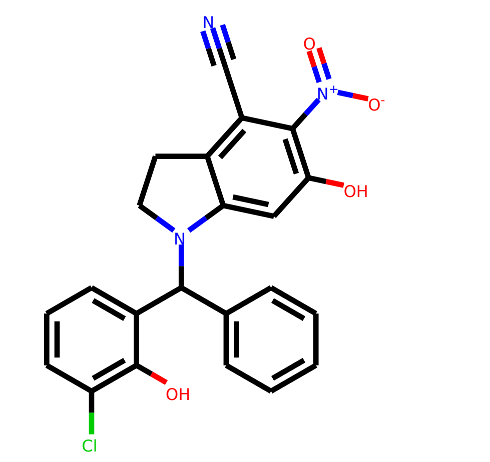
Osma-3081

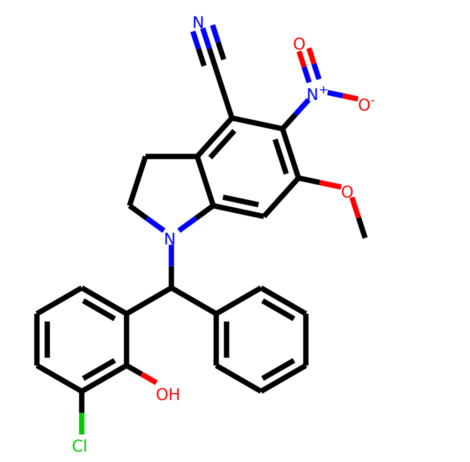
Osma-3082

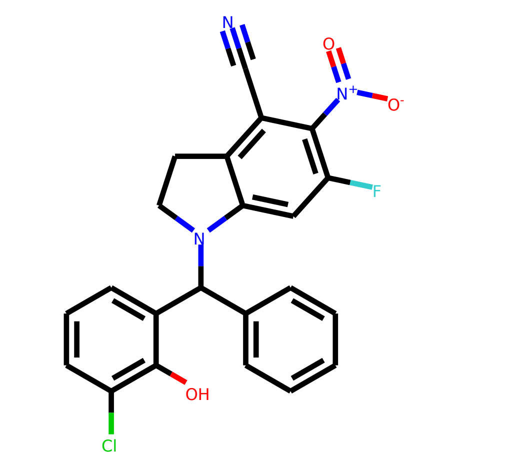
Osma-3083

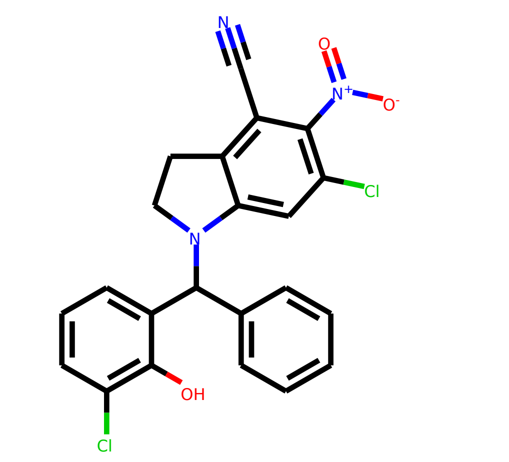
Osma-3084

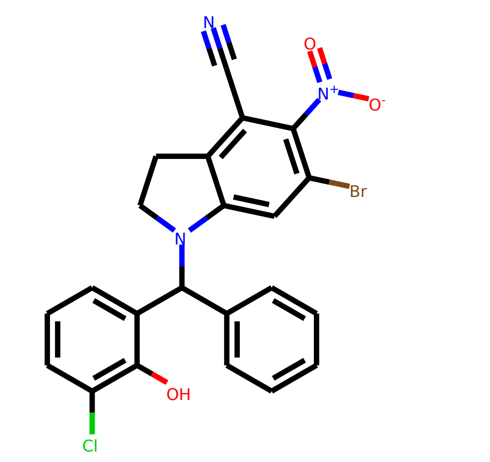
Osma-3085

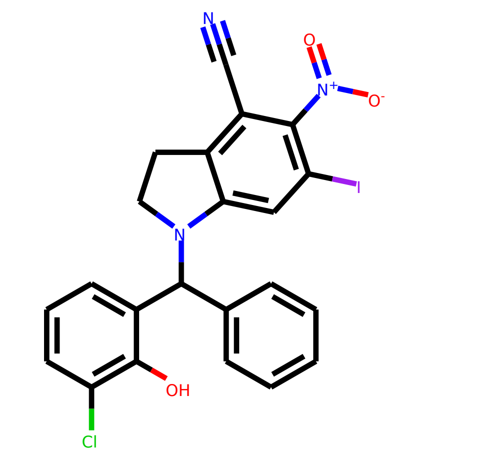
Osma-3086

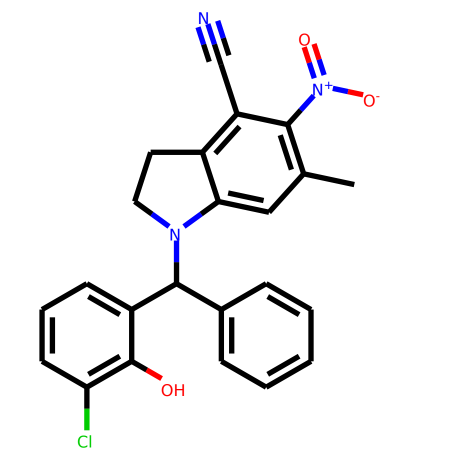
Osma-3087

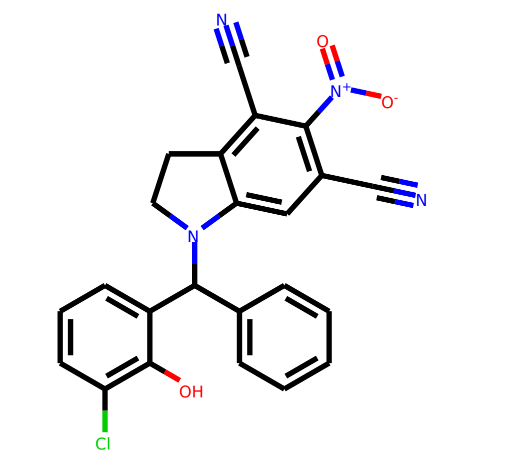
Osma-3088

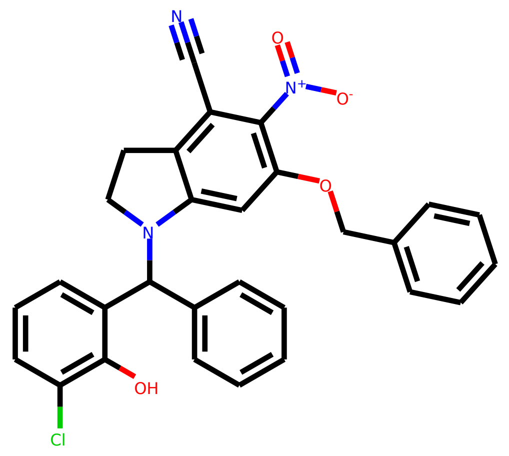
Osma-3089

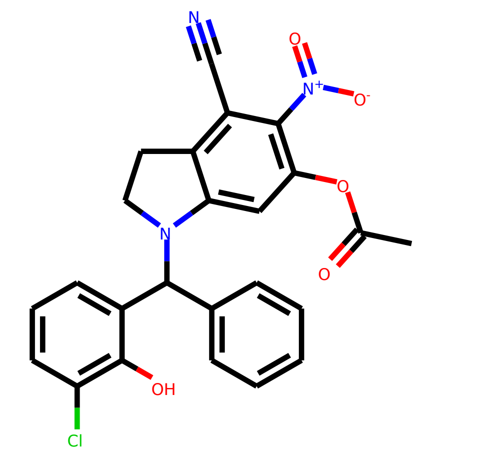
Osma-3090

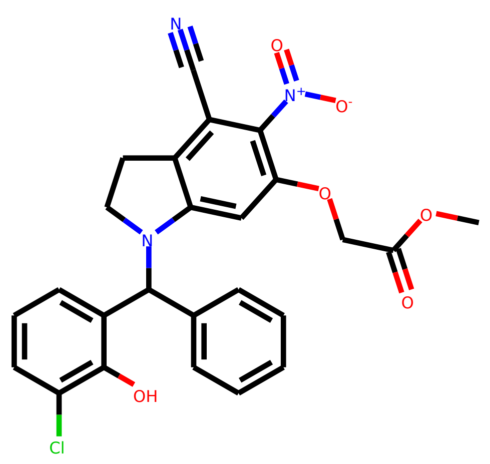
Osma-3091

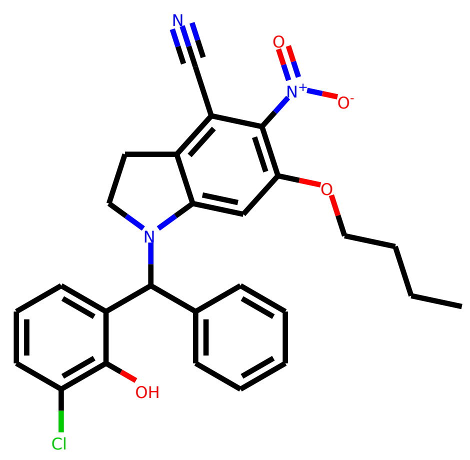
Osma-3092

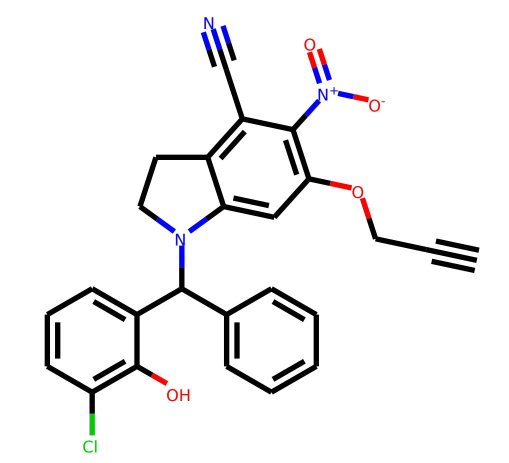
Osma-3093

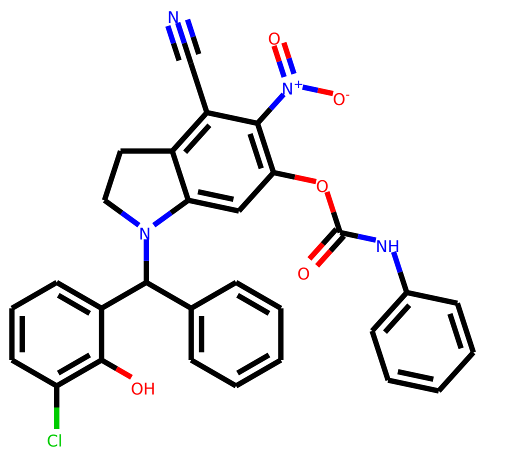
Osma-3094

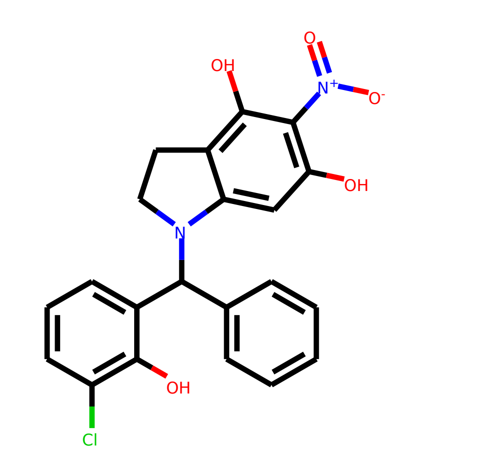
Osma-3095

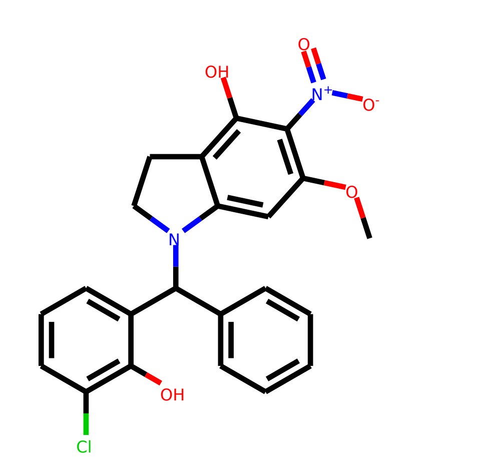
Osma-3096

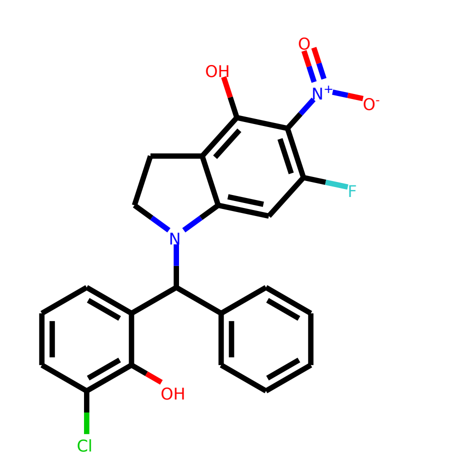
Osma-3097

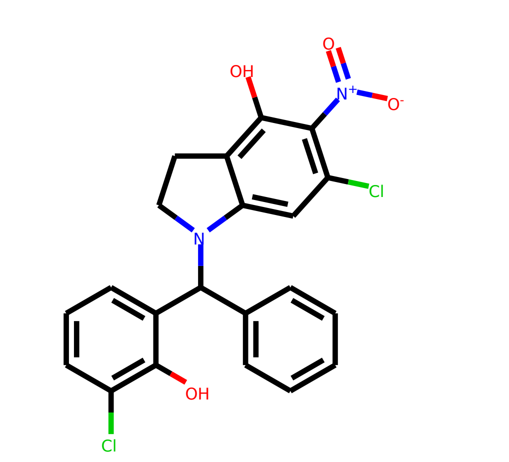
Osma-3098

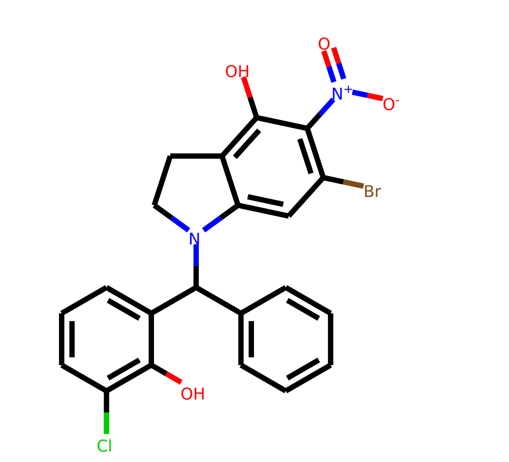
Osma-3099

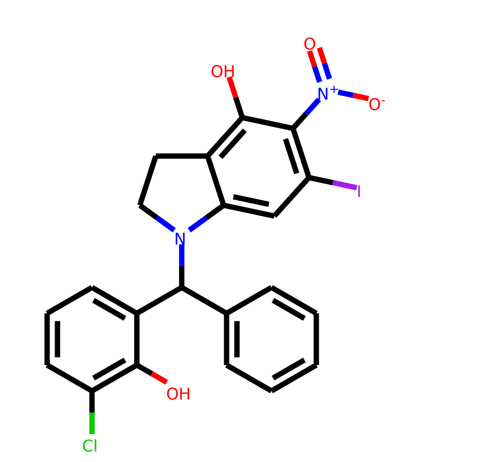
Osma-3100

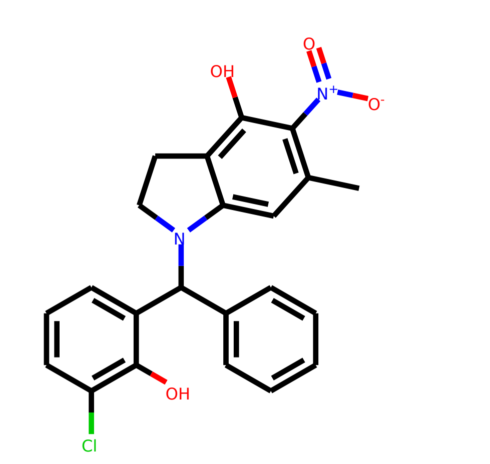
Osma-3101

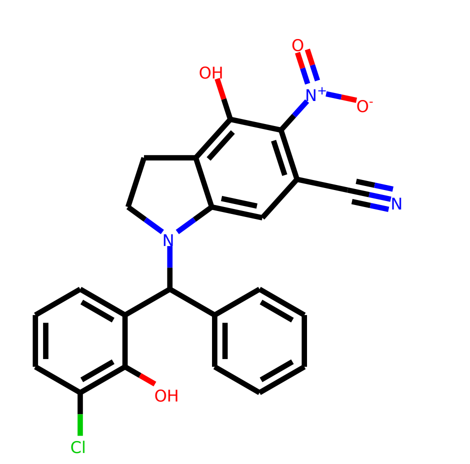
Osma-3102

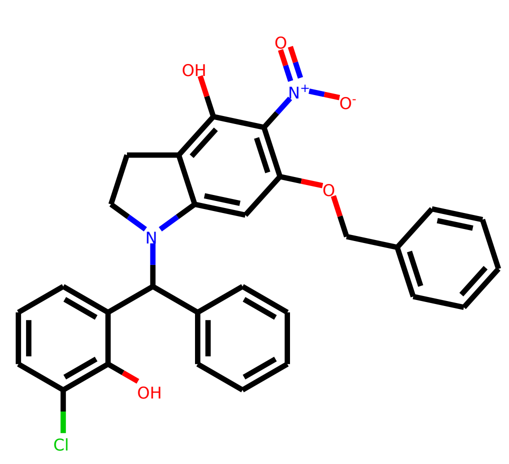
Osma-3103

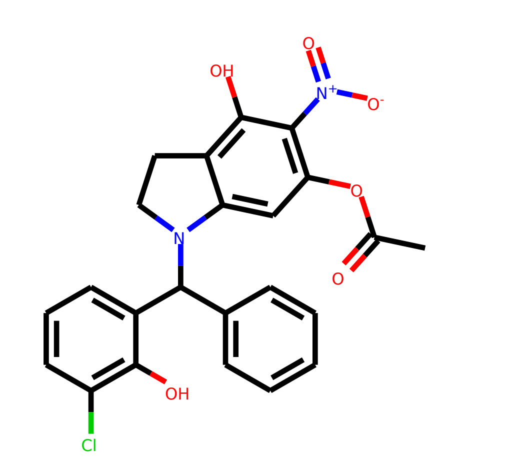
Osma-3104

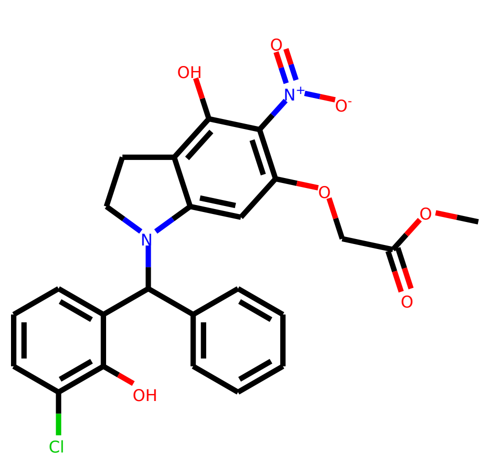
Osma-3105

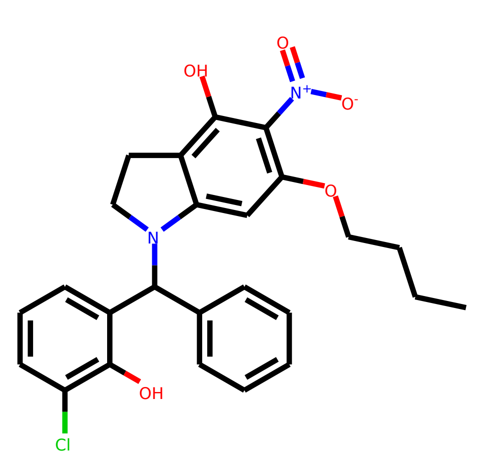
Osma-3106

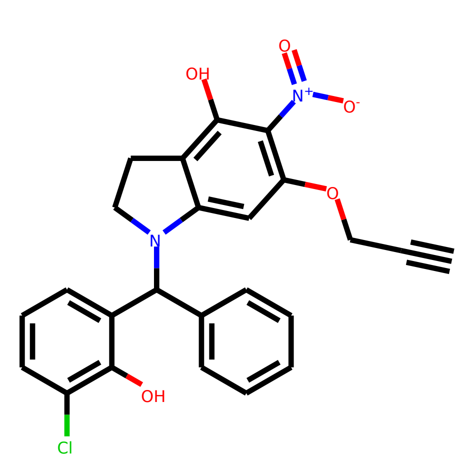
Osma-3107

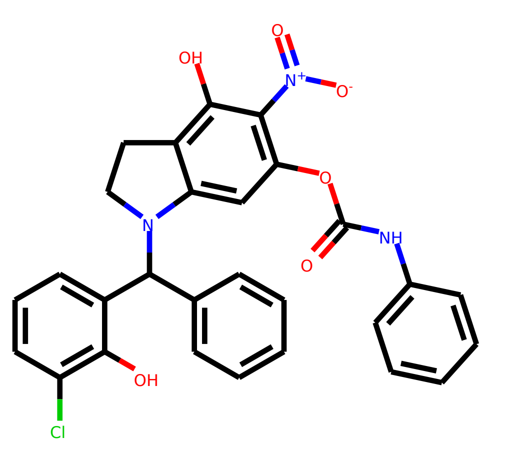
Osma-3108

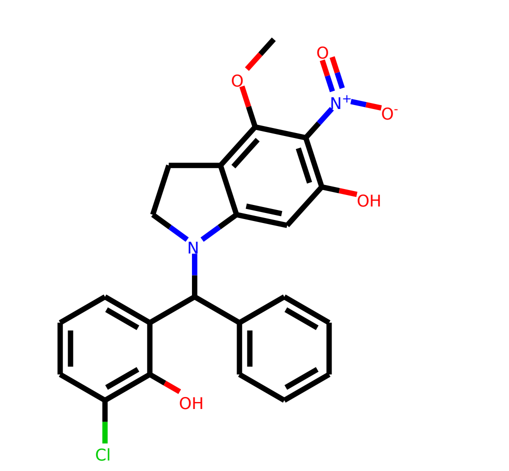
Osma-3109

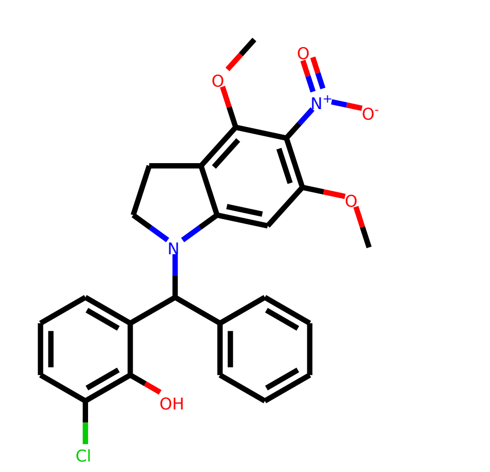
Osma-3110

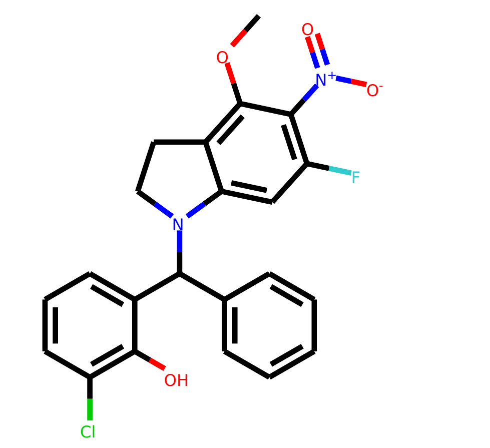
Osma-3111

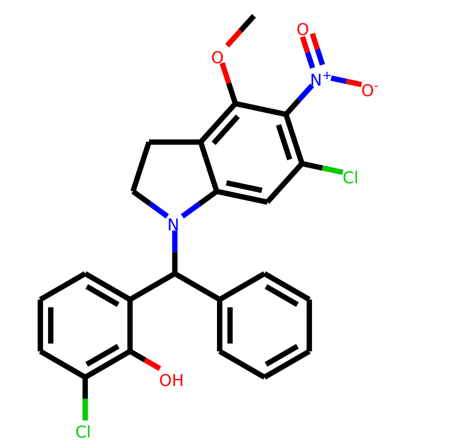
Osma-3112

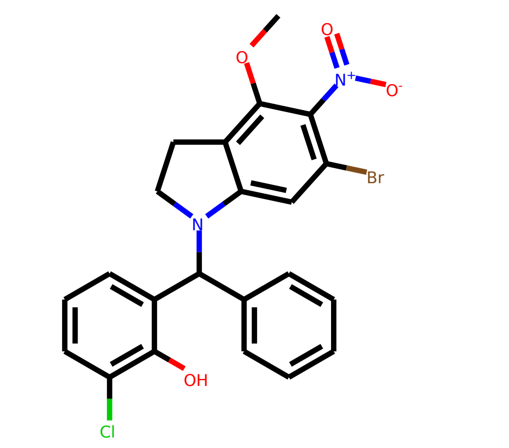
Osma-3113

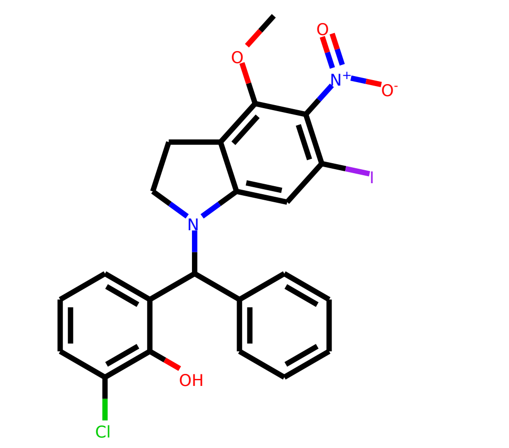
Osma-3114

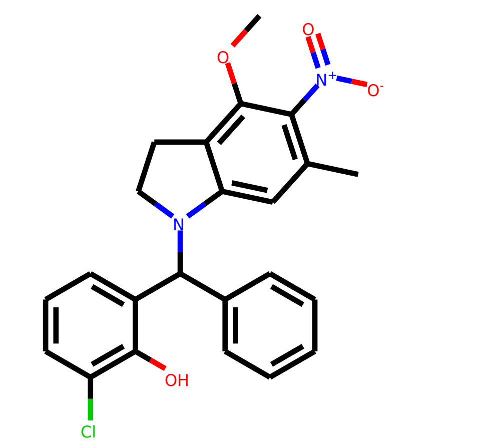
Osma-3115

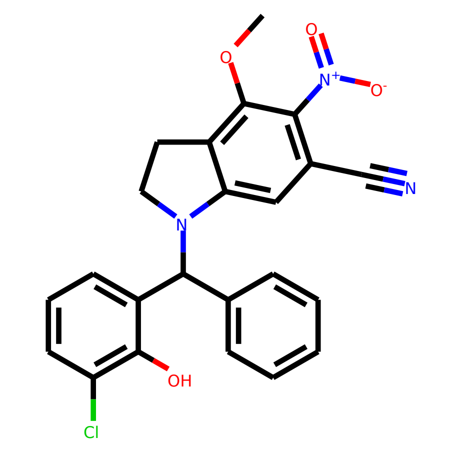
Osma-3116

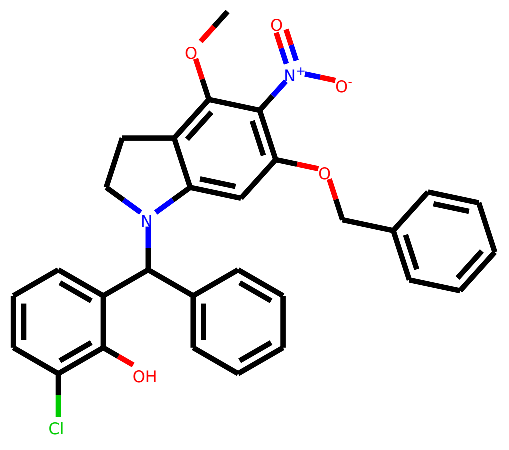
Osma-3117

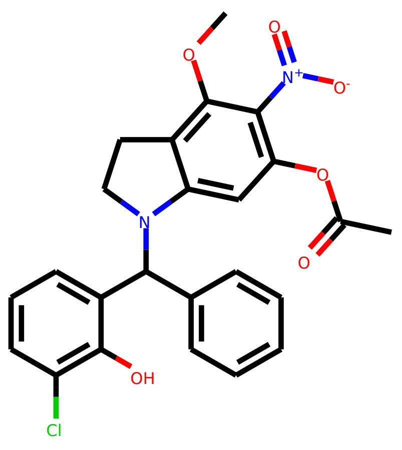
Osma-3118

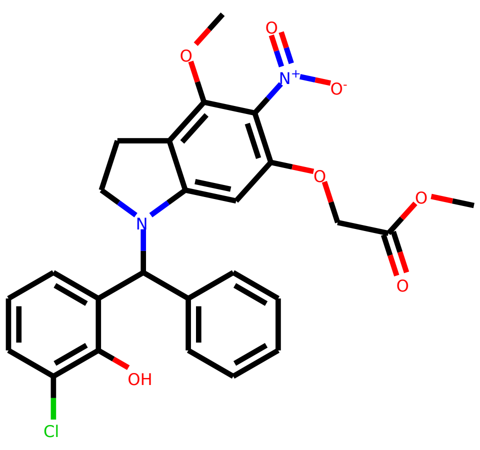
Osma-3119

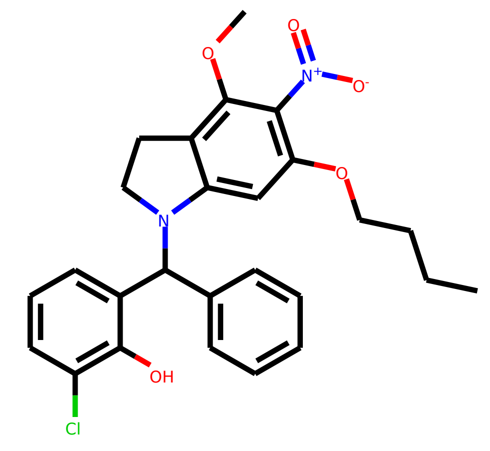
Osma-3120

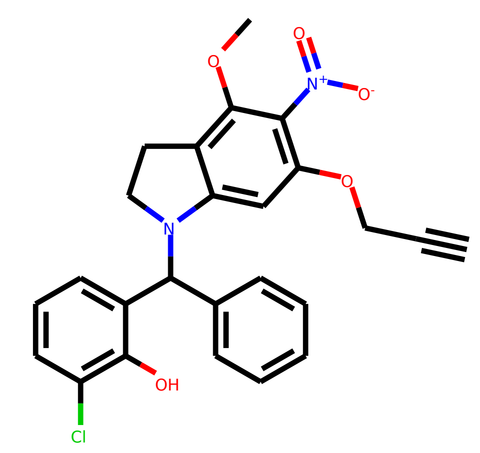
Osma-3121

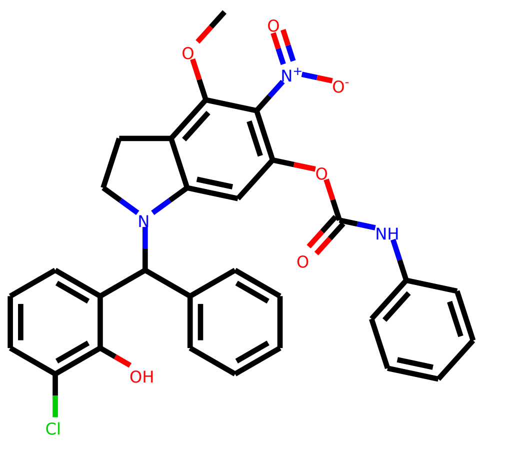
Osma-3122

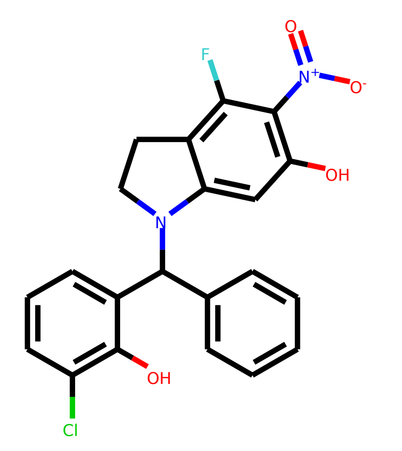
Osma-3123

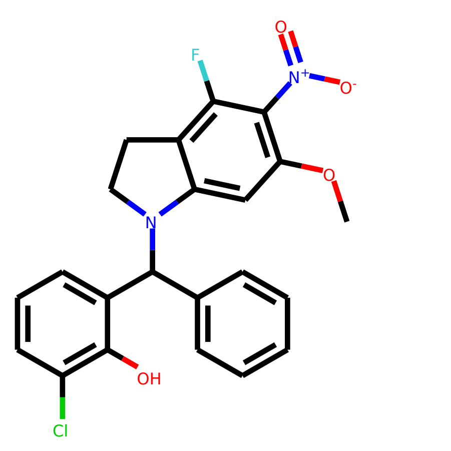
Osma-3124

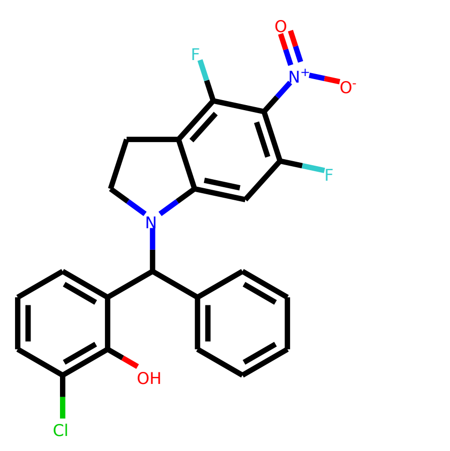
Osma-3125

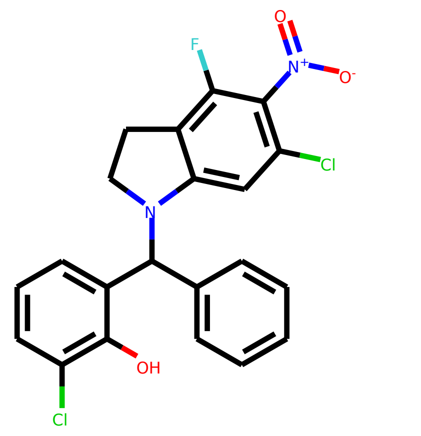
Osma-3126

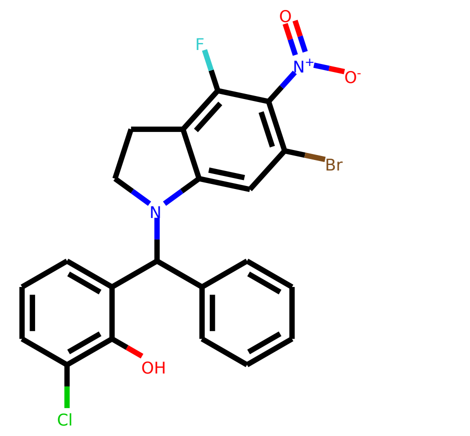
Osma-3127

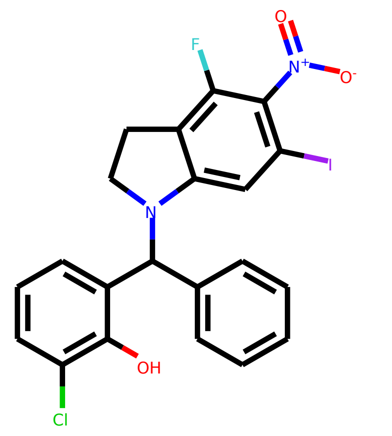
Osma-3128

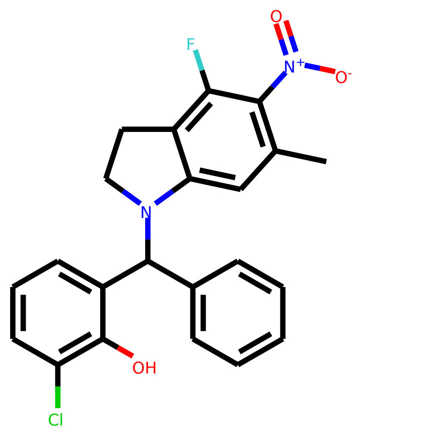
Osma-3129

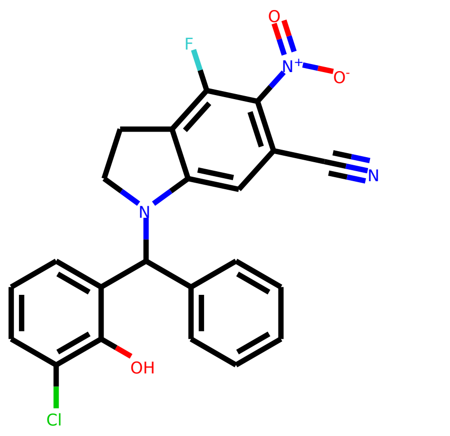
Osma-3130

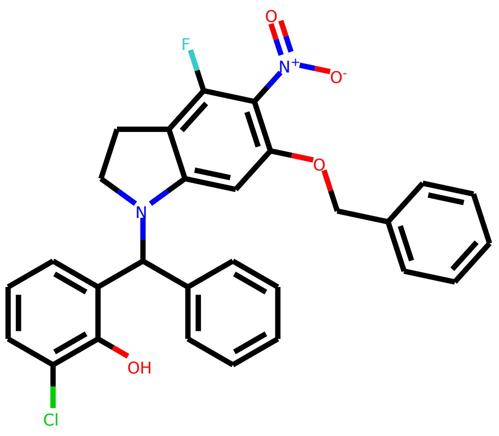
Osma-3131

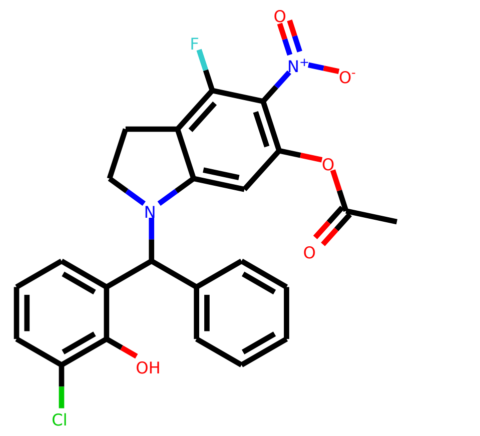
Osma-3132

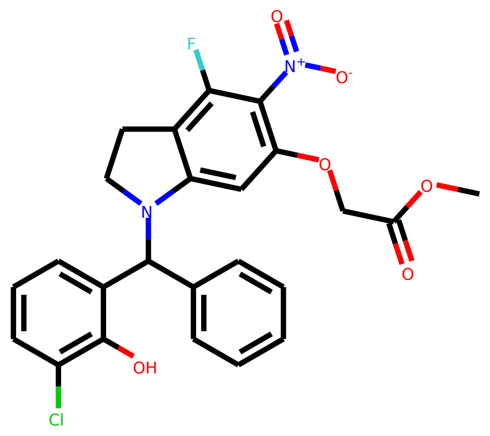
Osma-3133

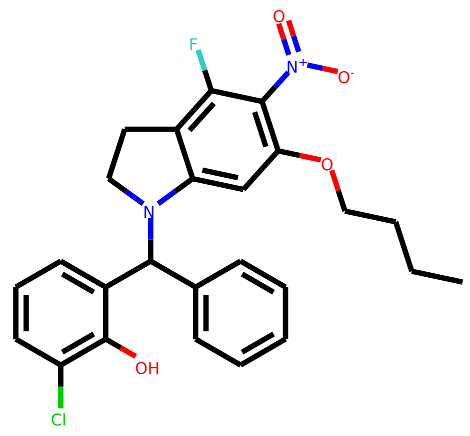
Osma-3134

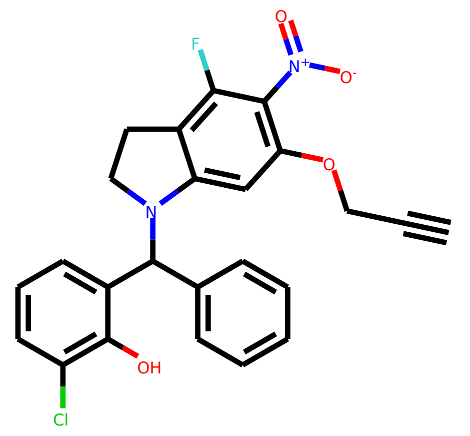
Osma-3135

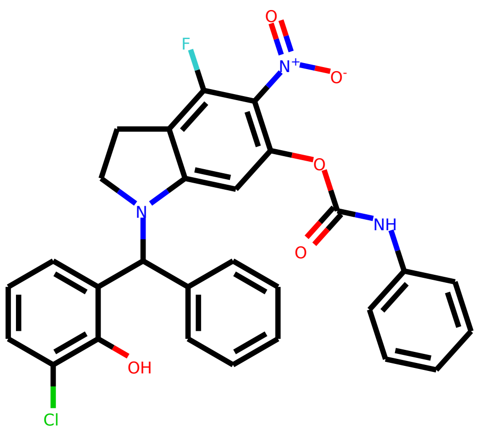
Osma-3136

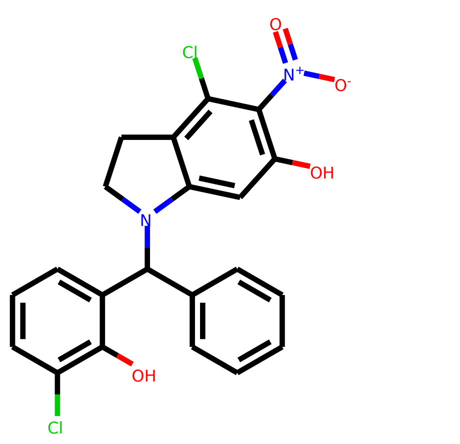
Osma-3137

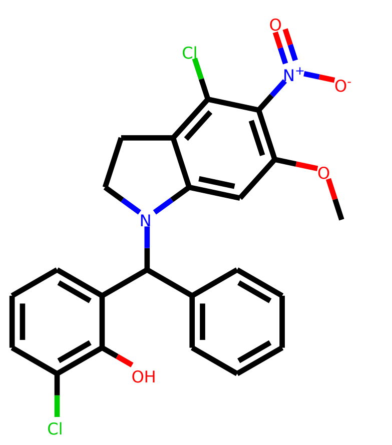
Osma-3138

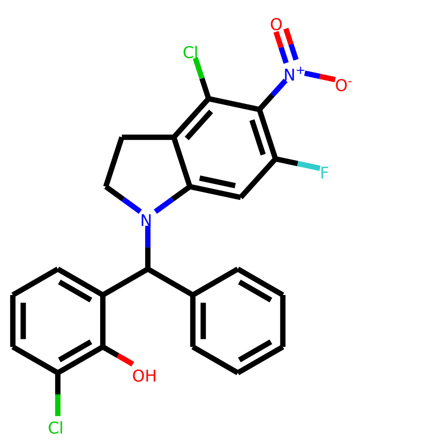
Osma-3139

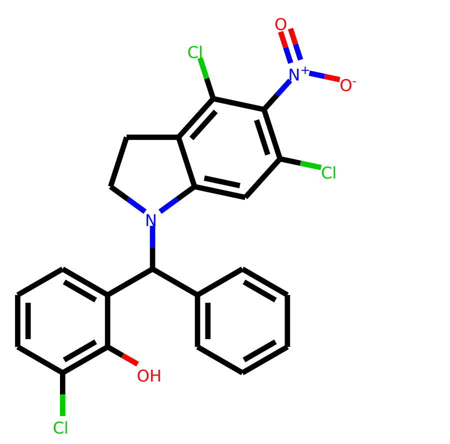
Osma-3140

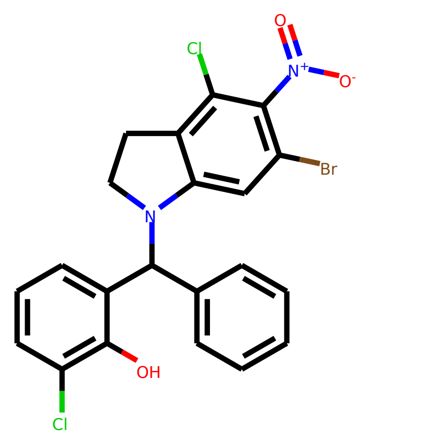
Osma-3141

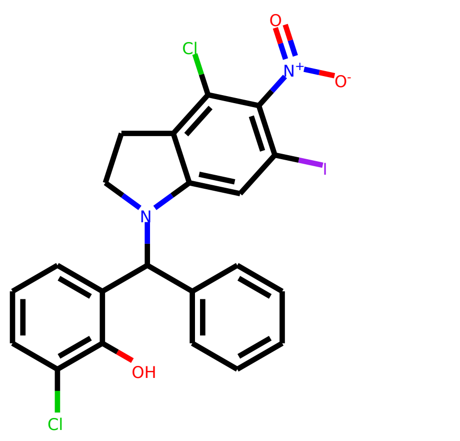
Osma-3142

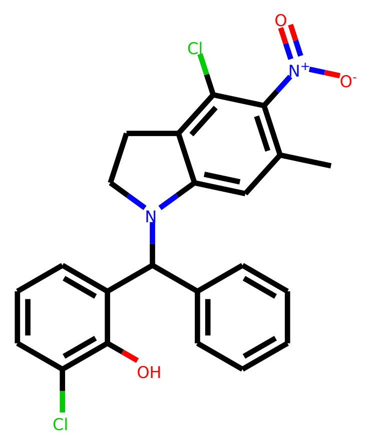
Osma-3143

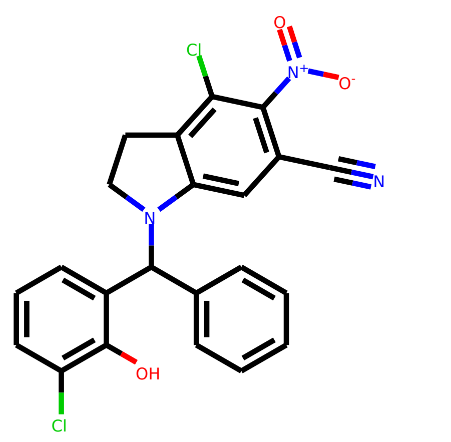
Osma-3144

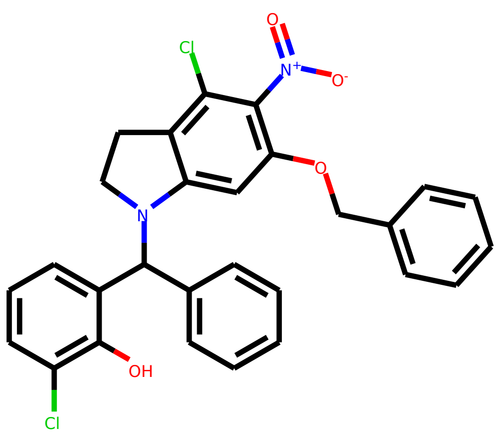
Osma-3145

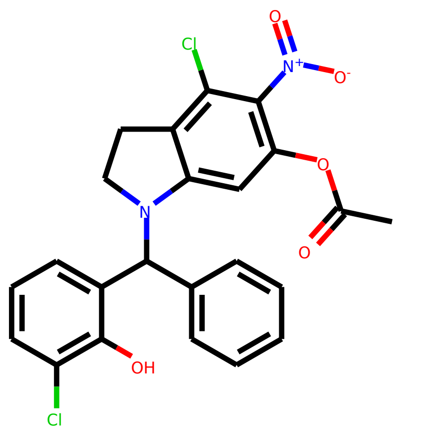
Osma-3146

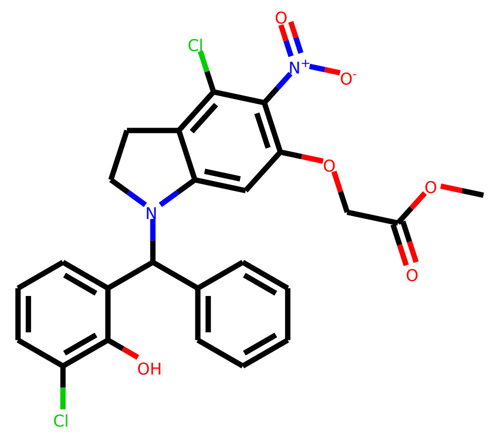
Osma-3147

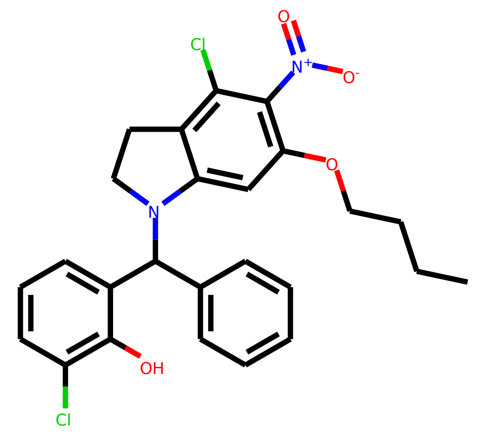
Osma-3148

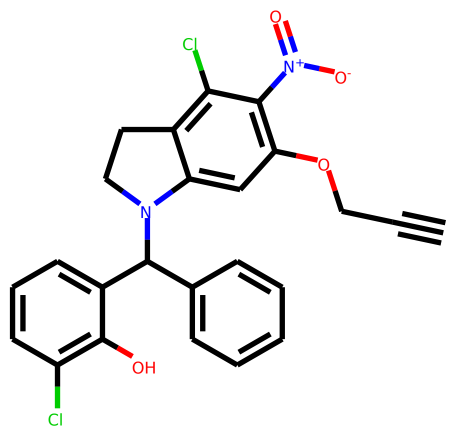
Osma-3149

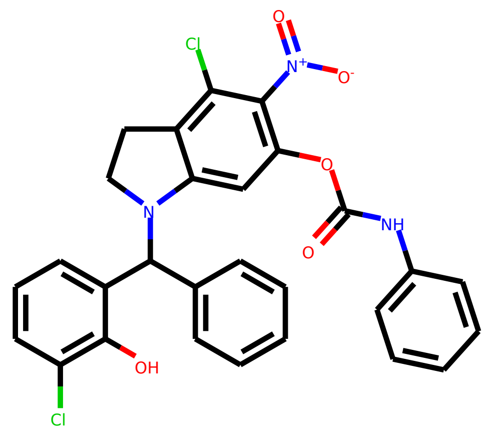
Osma-3150

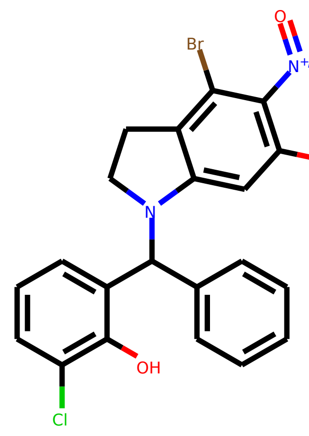

Osma-3151

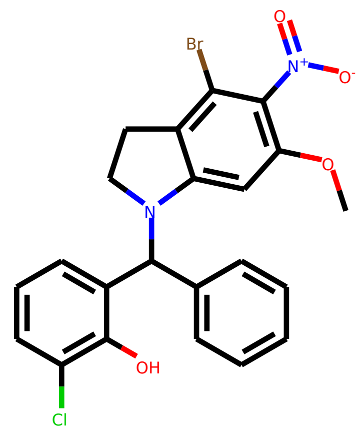

Osma-3152

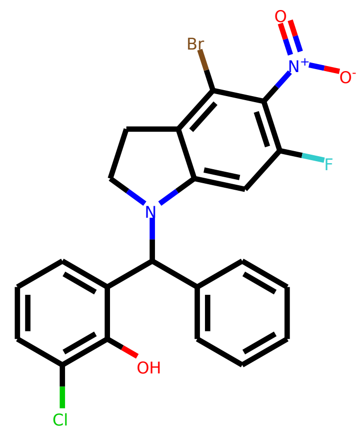

Osma-3153

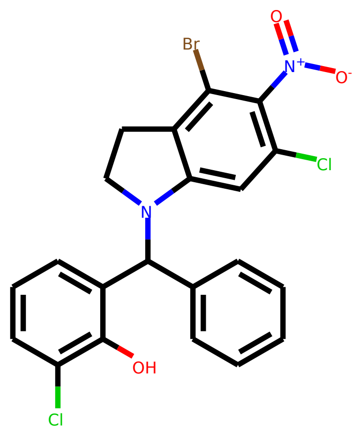

Osma-3154

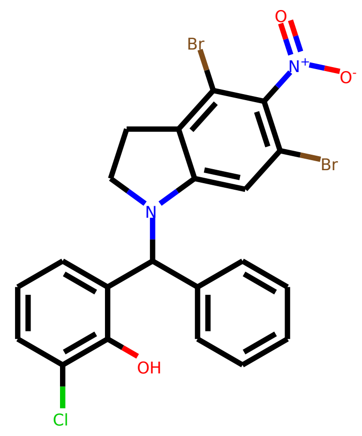

Osma-3155

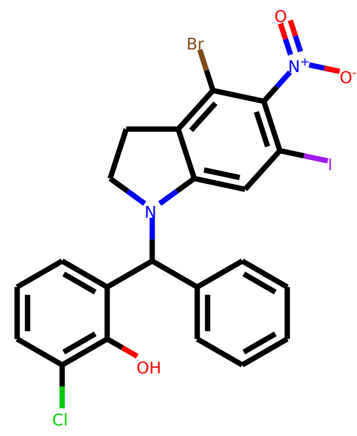

Osma-3156

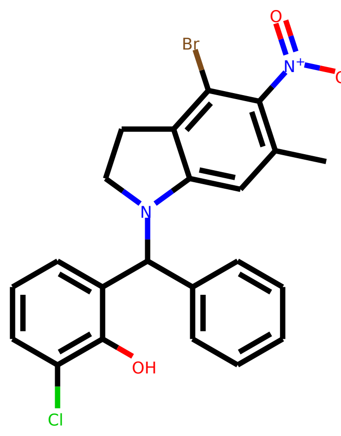

Osma-3157

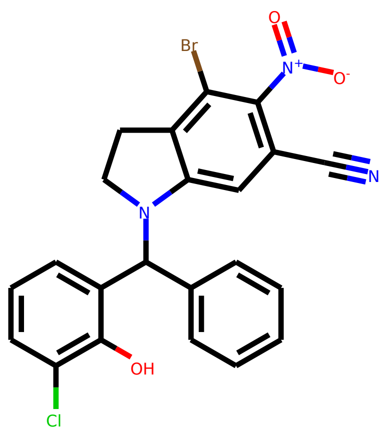

Osma-3158

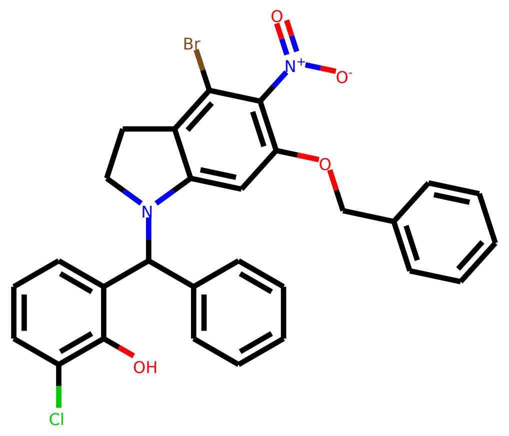

Osma-3159

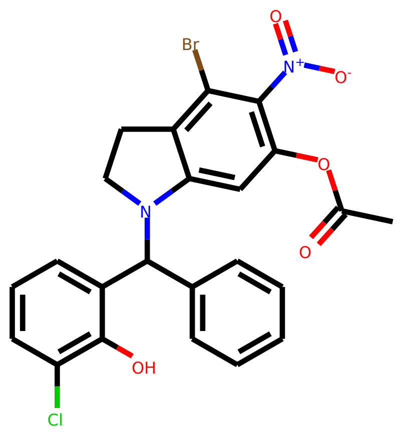

Osma-3160

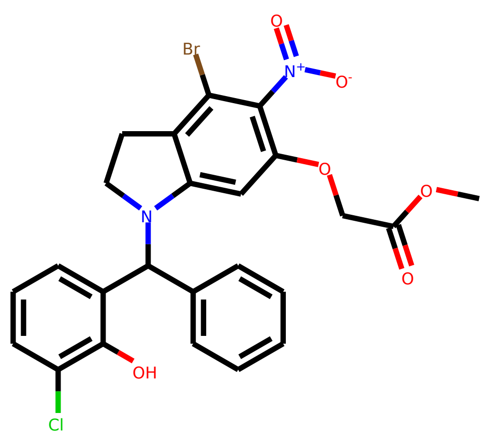

Osma-3161

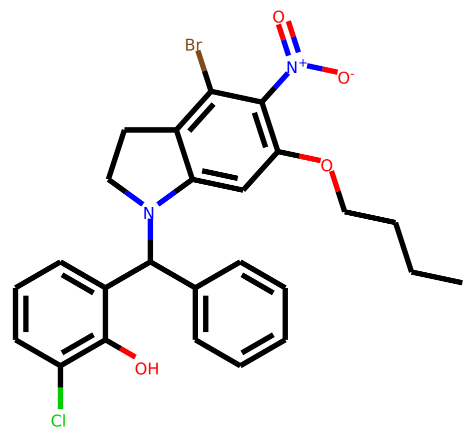

Osma-3162

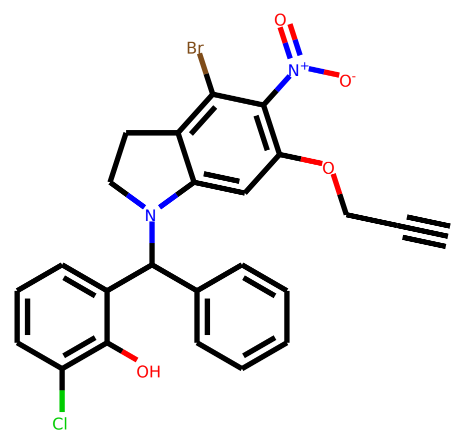

Osma-3163

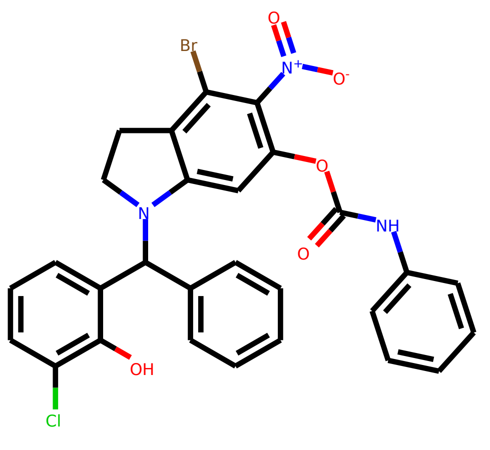

Osma-3164

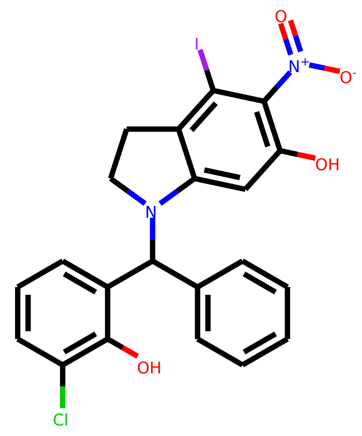

Osma-3165

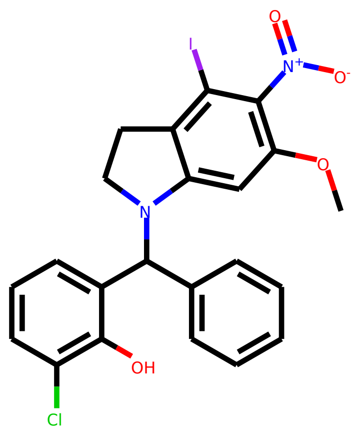

Osma-3166

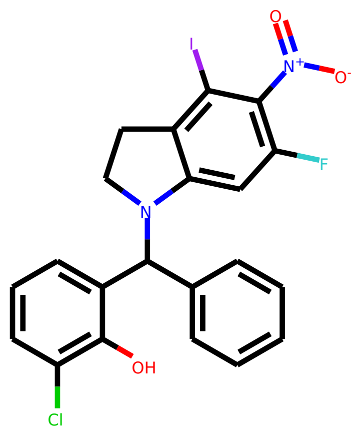

Osma-3167

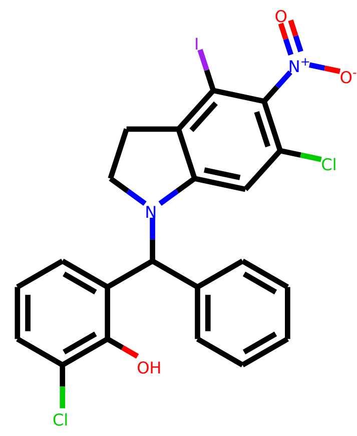

Osma-3168

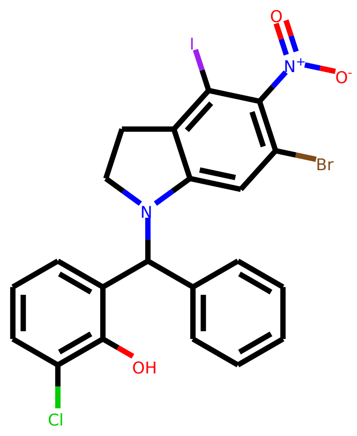

Osma-3169

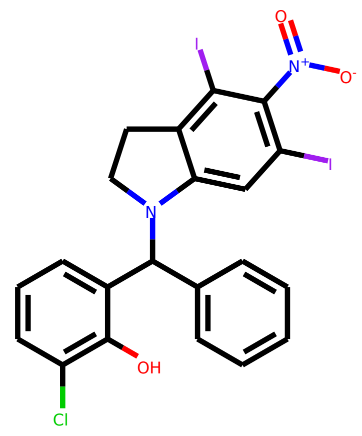

Osma-3170

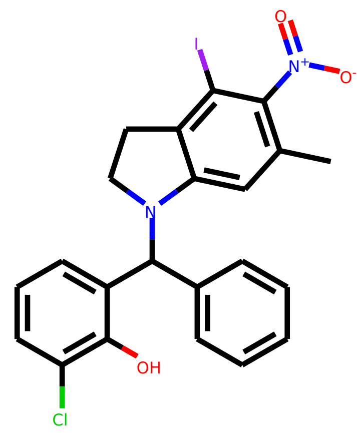

Osma-3171

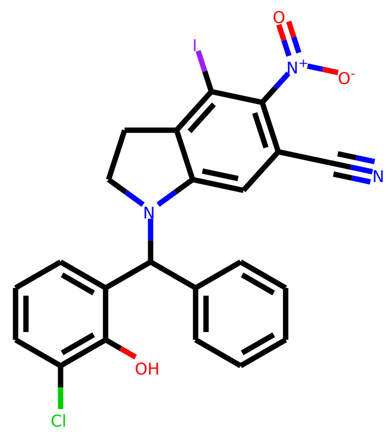

Osma-3172

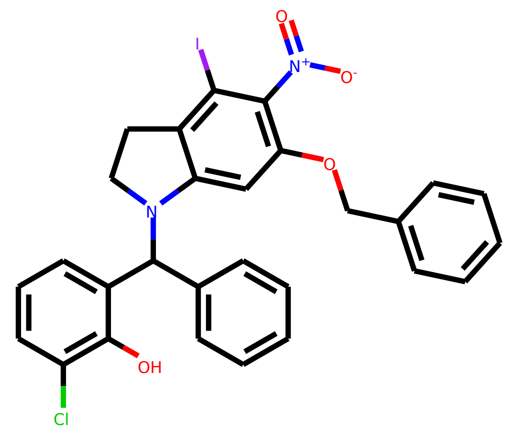

Osma-3173

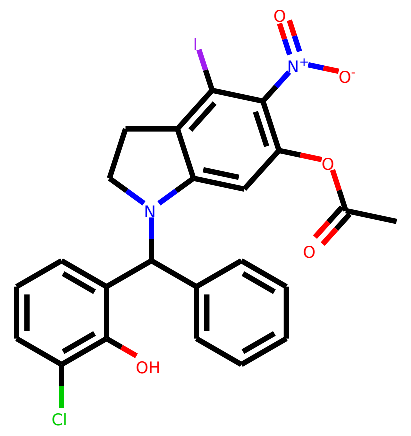

Osma-3174

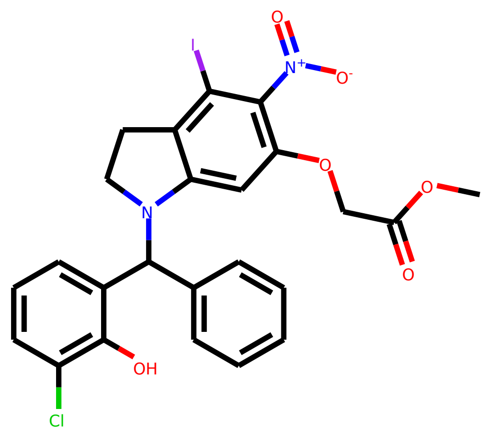

Osma-3175

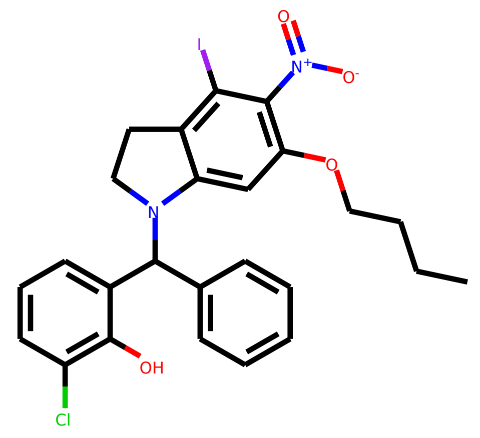

Osma-3176

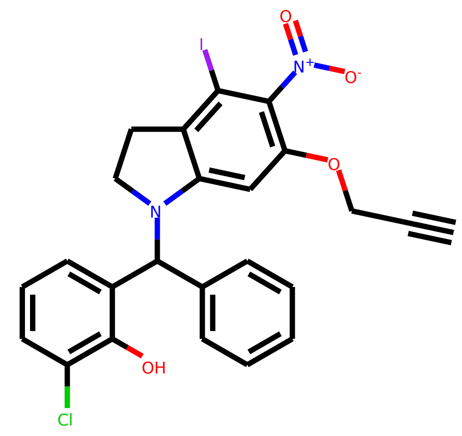

Osma-3177

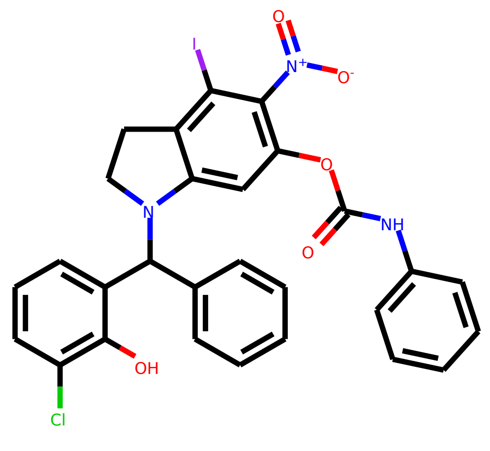

Osma-3178

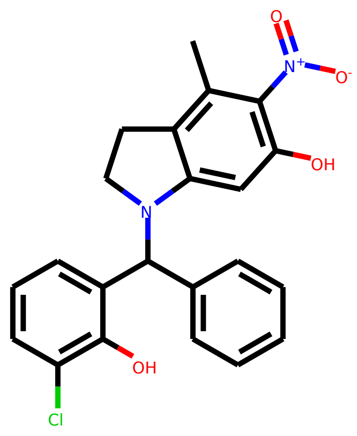

Osma-3179

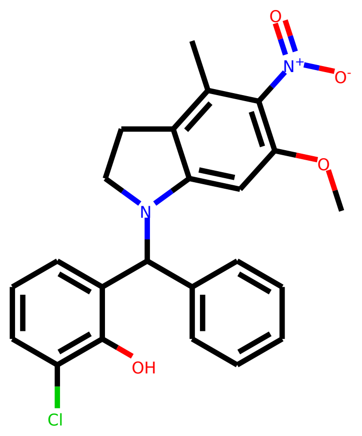

Osma-3180

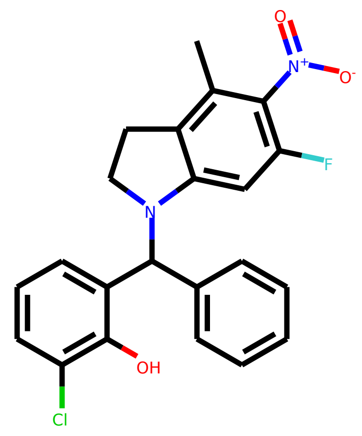

Osma-3181

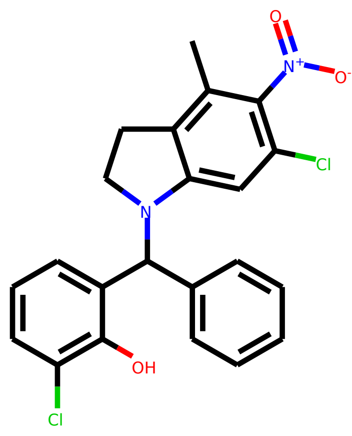

Osma-3182

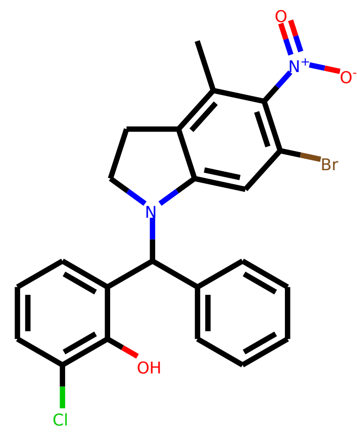

Osma-3183

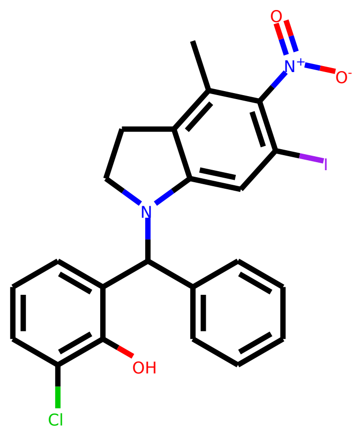

Osma-3184

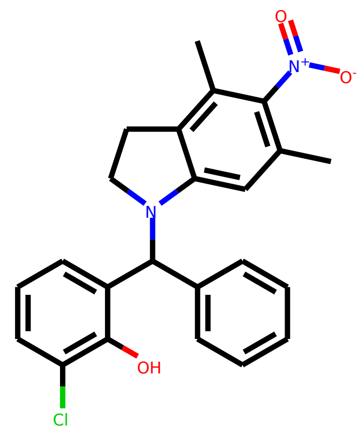

Osma-3185

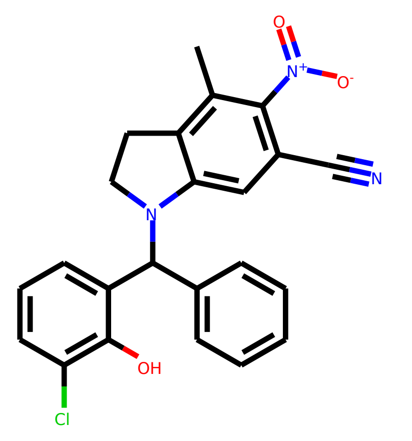
Osma-3186
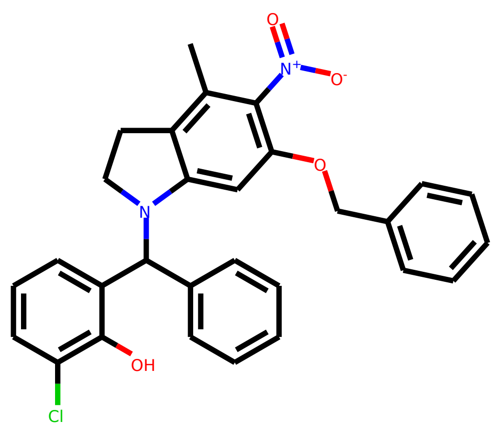
Osma-3187
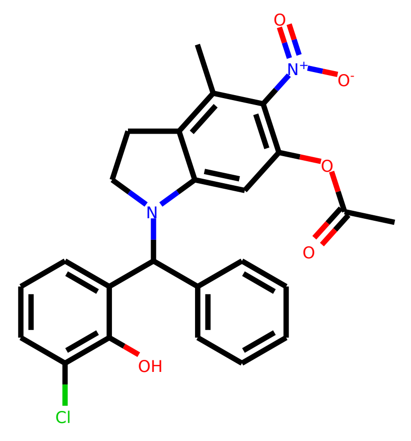
Osma-3188
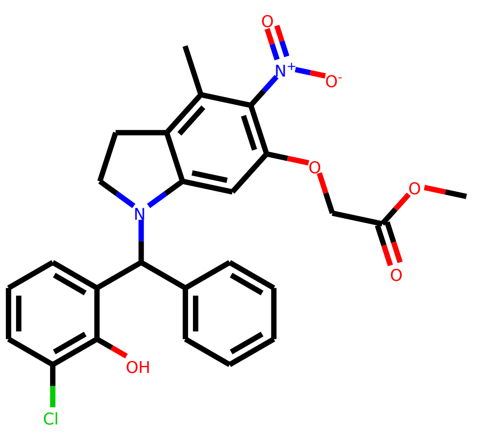
Osma-3189
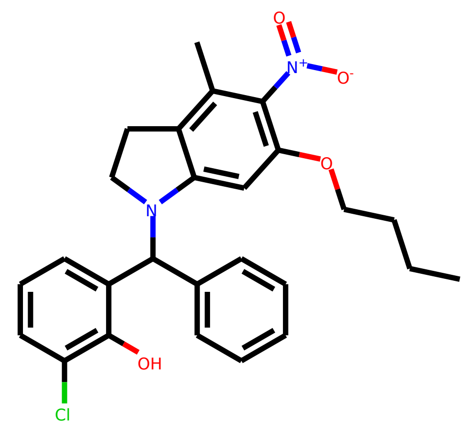
Osma-3190
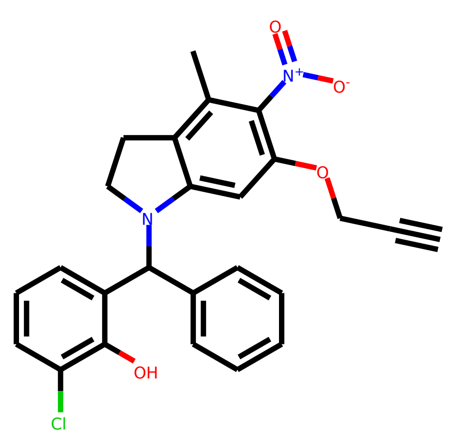
Osma-3191
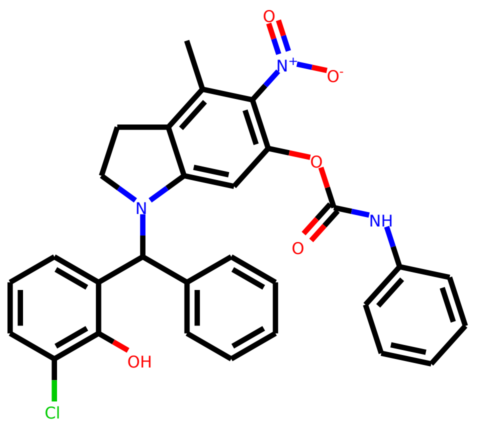
Osma-3192
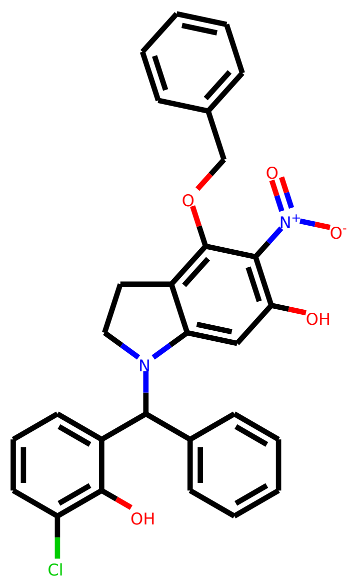
Osma-3193
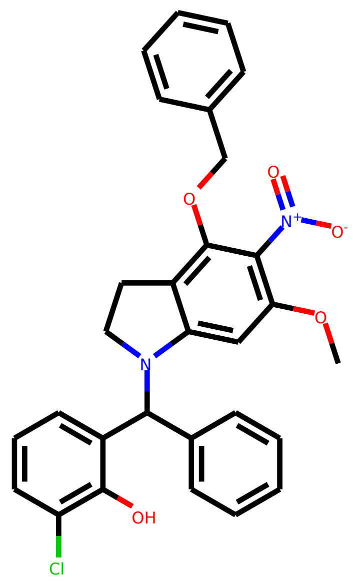
Osma-3194
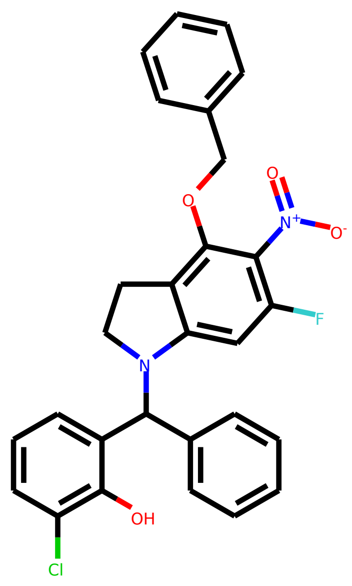
Osma-3195
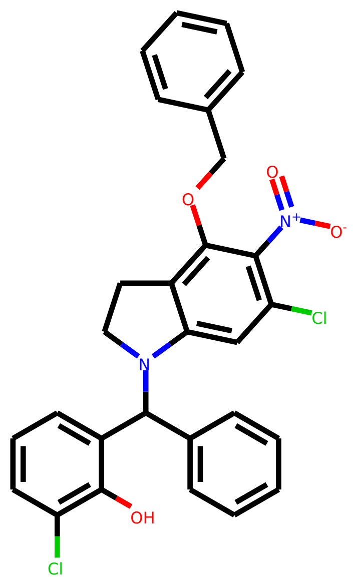
Osma-3196
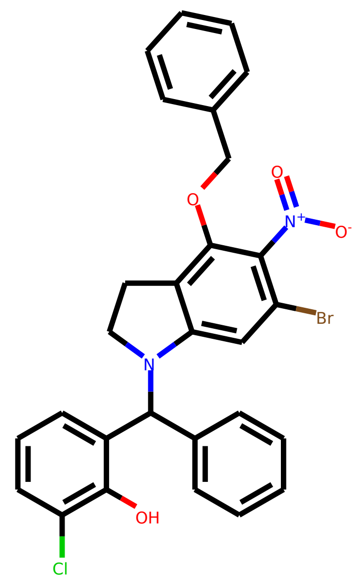
Osma-3197
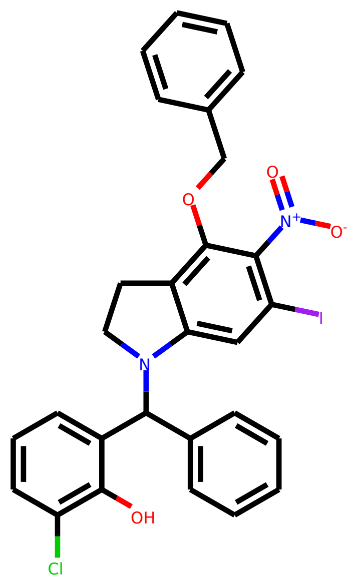
Osma-3198
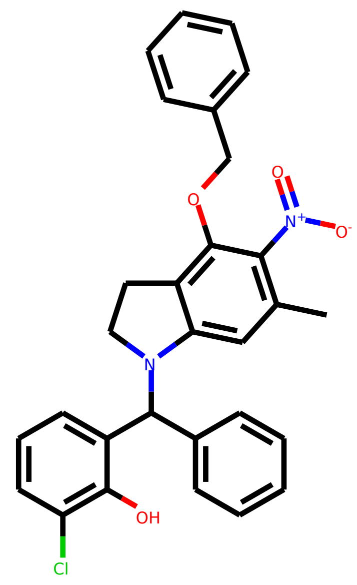
Osma-3199
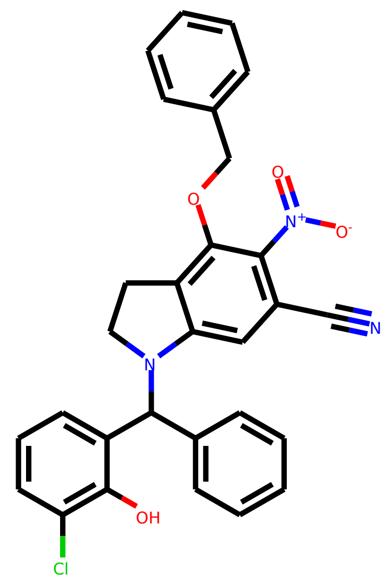
Osma-3200
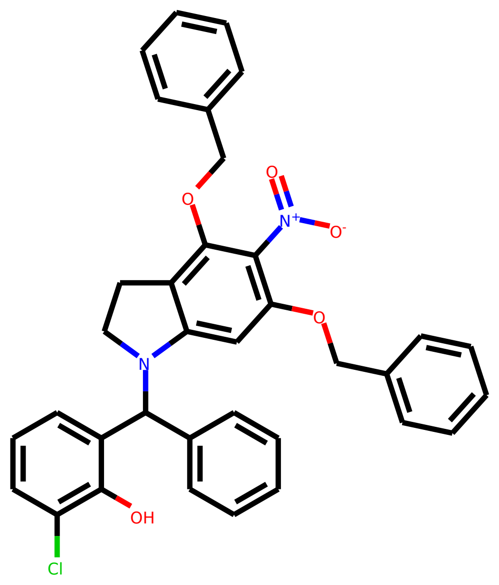
Osma-3201
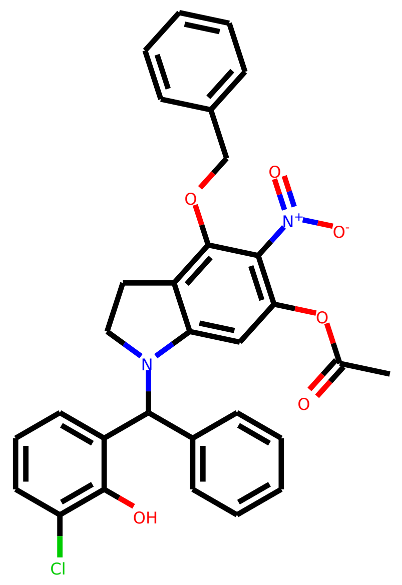
Osma-3202
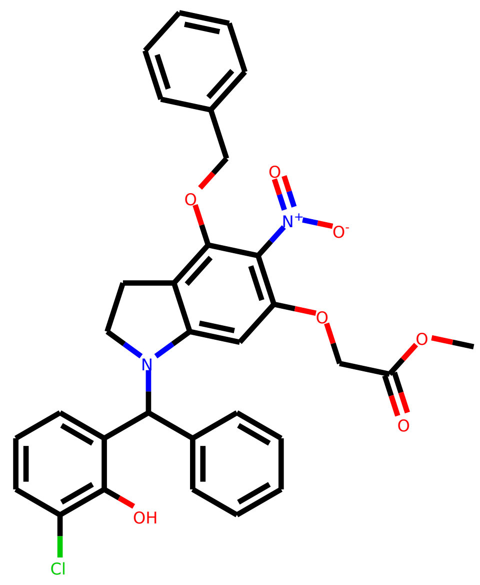
Osma-3203
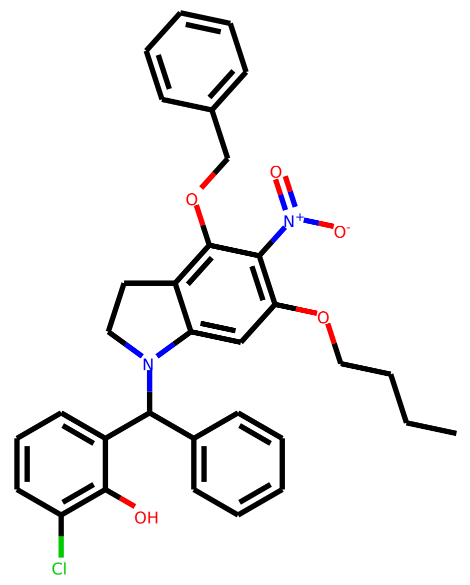
Osma-3204
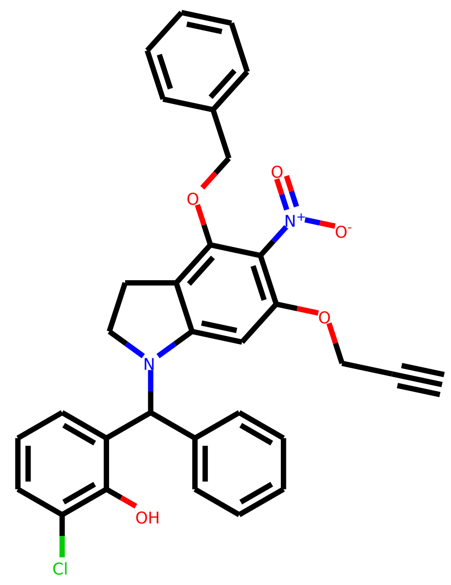
Osma-3205
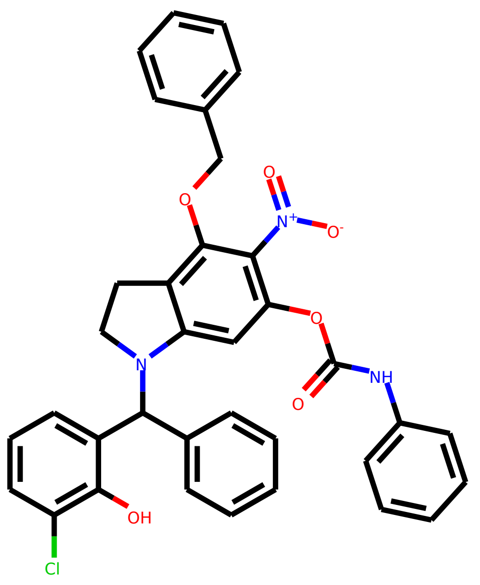
Osma-3206
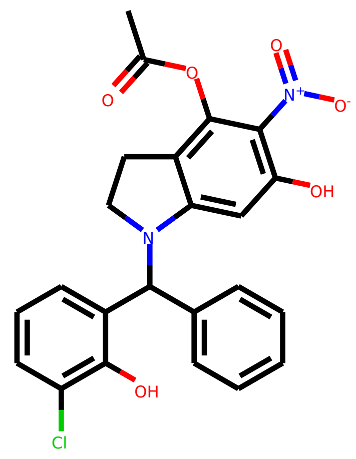
Osma-3207
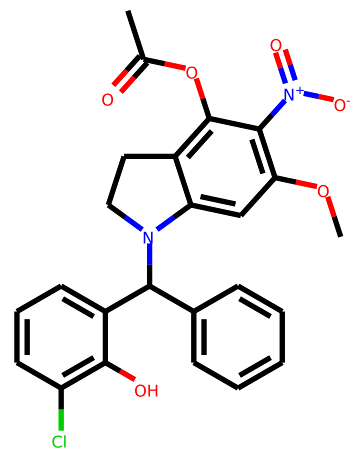
Osma-3208
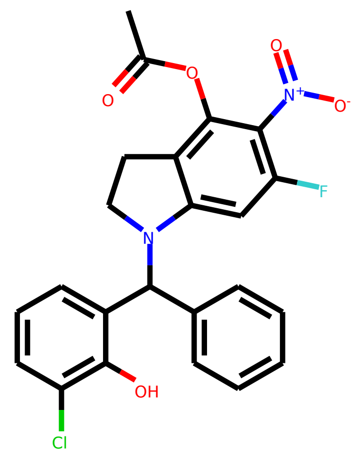
Osma-3209
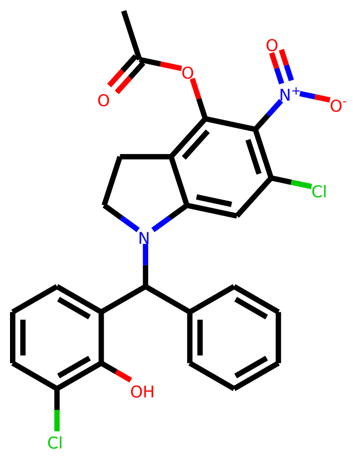
Osma-3210
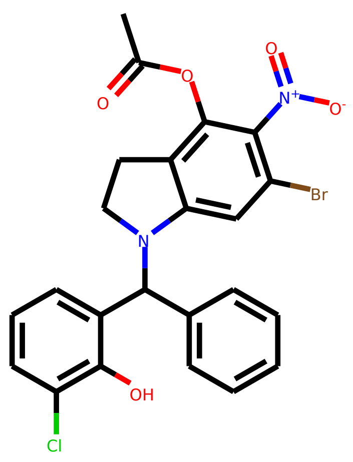
Osma-3211
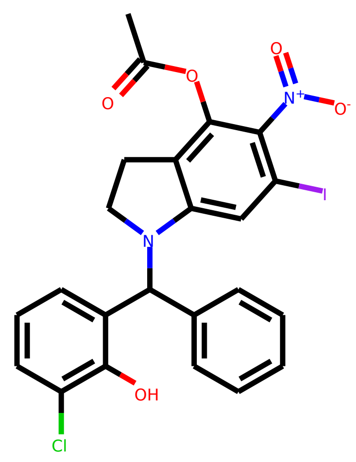
Osma-3212
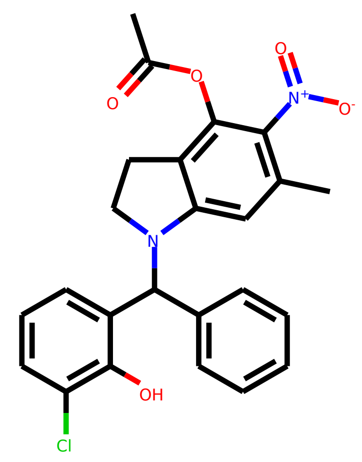
Osma-3213
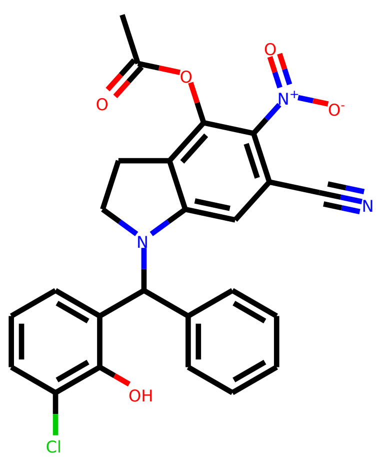
Osma-3214
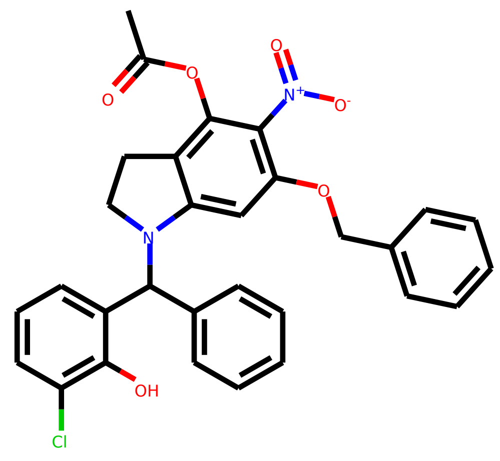
Osma-3215
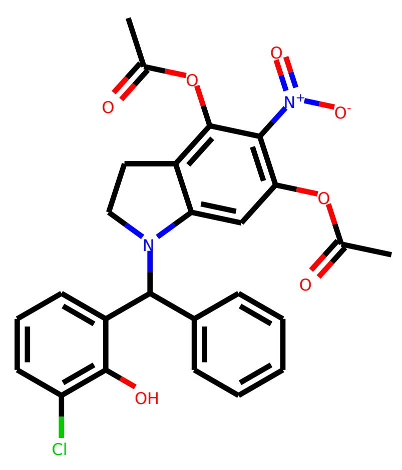
Osma-3216
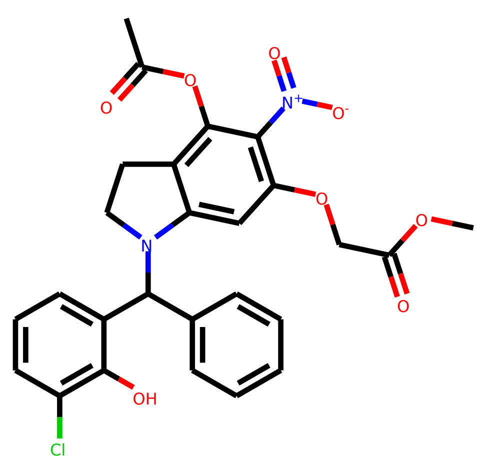
Osma-3217
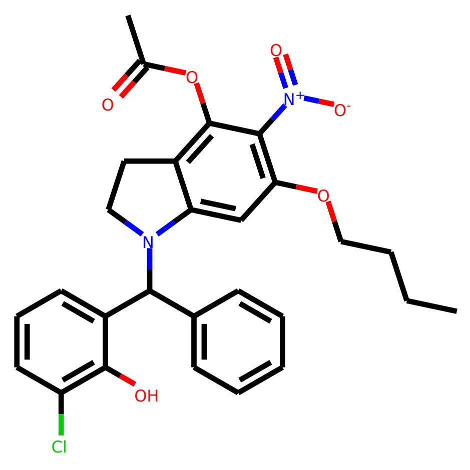
Osma-3218
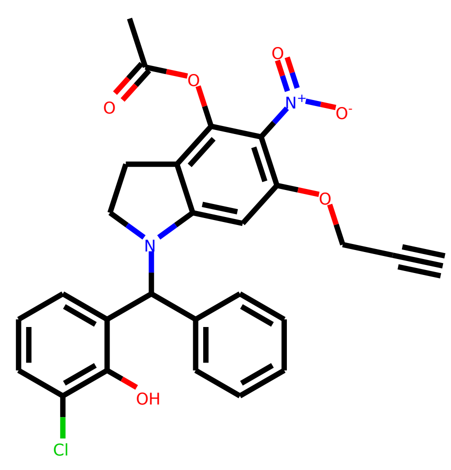
Osma-3219
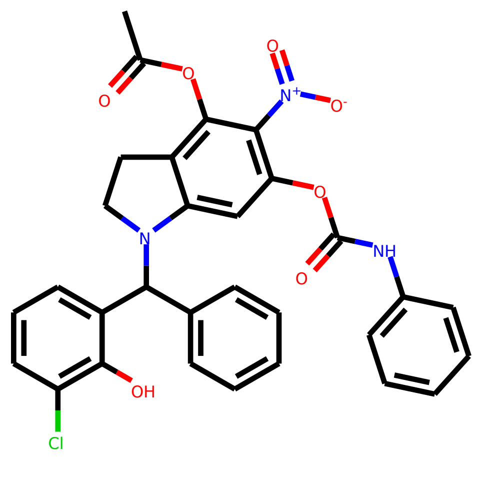
Osma-3220

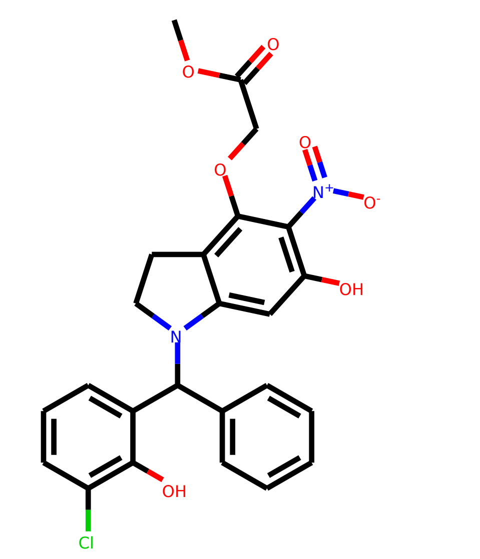
Osma-3221
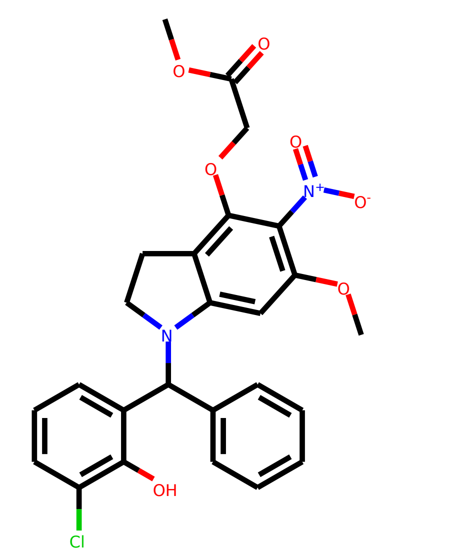
Osma-3222
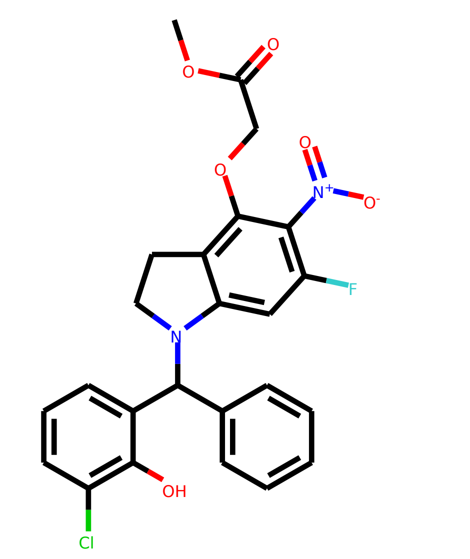
Osma-3223
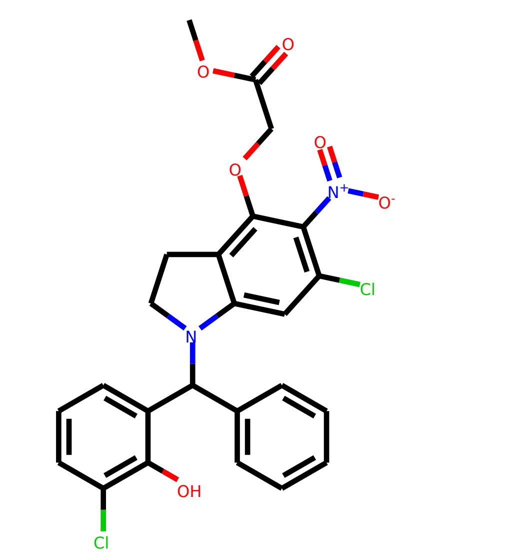
Osma-3224
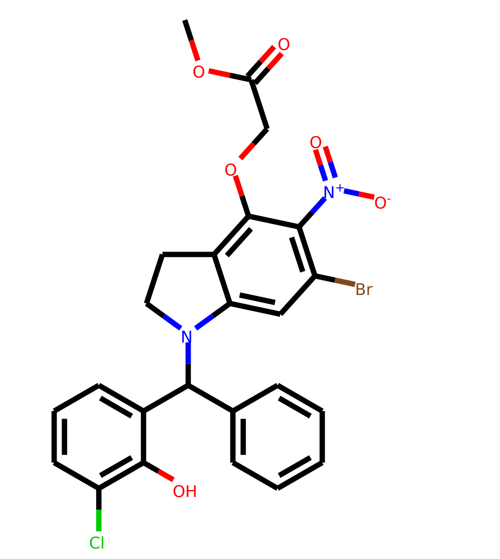
Osma-3225
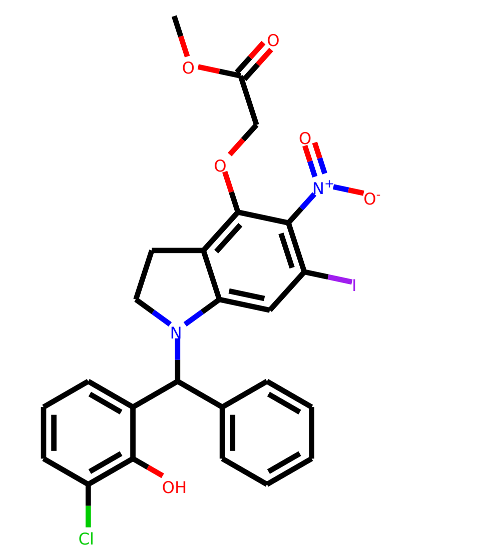
Osma-3226
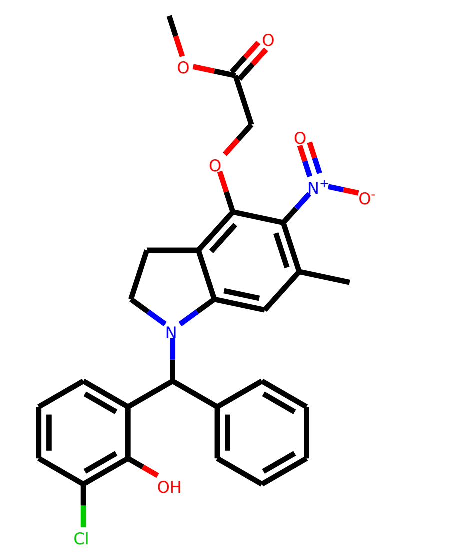
Osma-3227
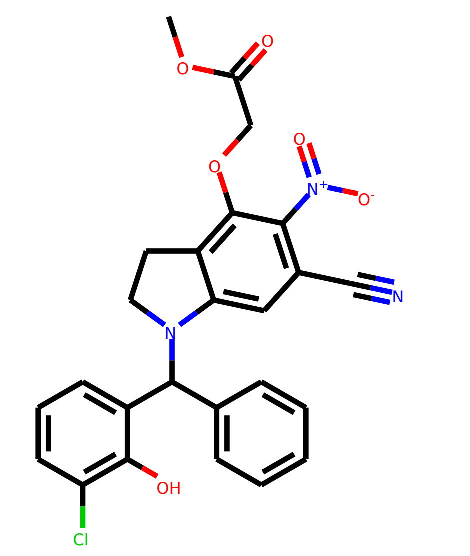
Osma-3228
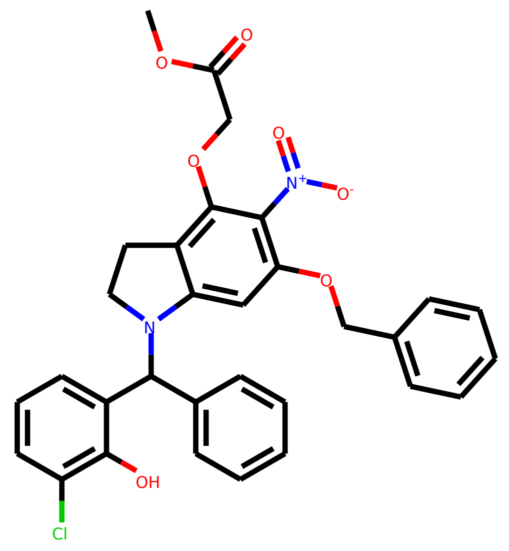
Osma-3229
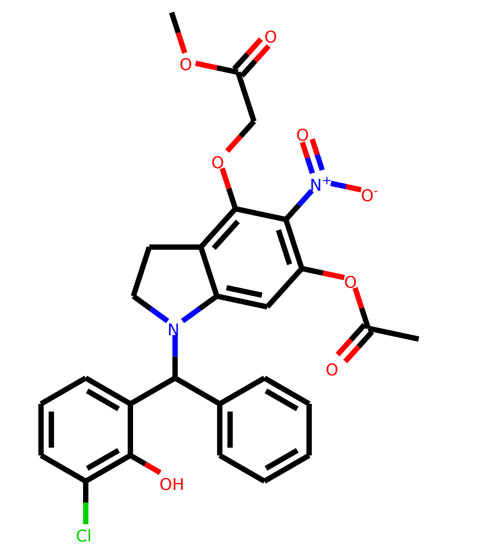
Osma-3230
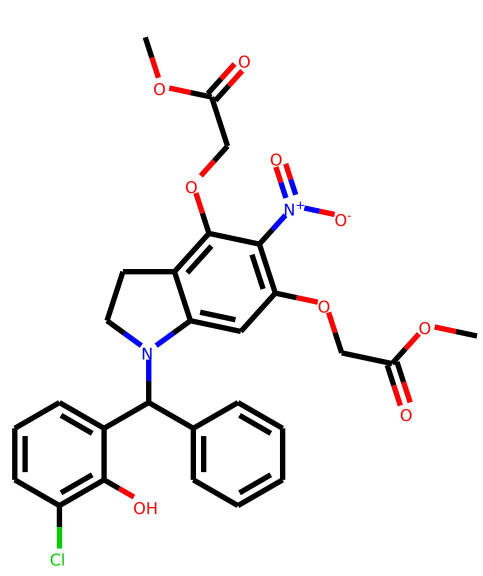
Osma-3231
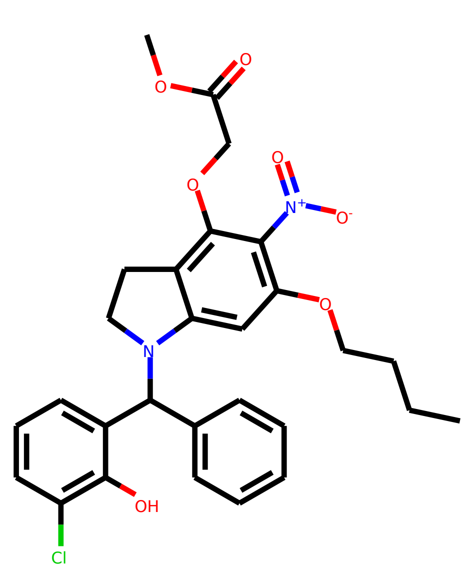
Osma-3232
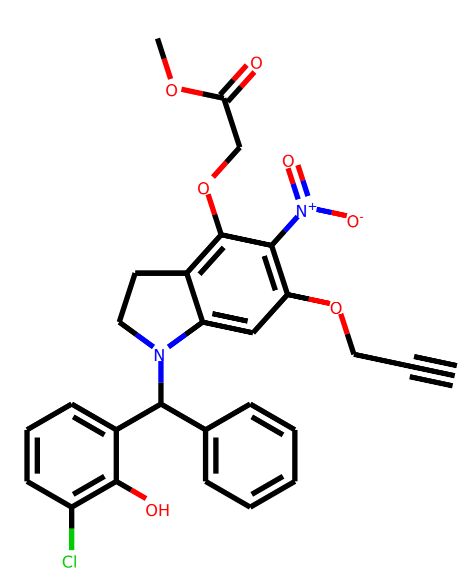
Osma-3233
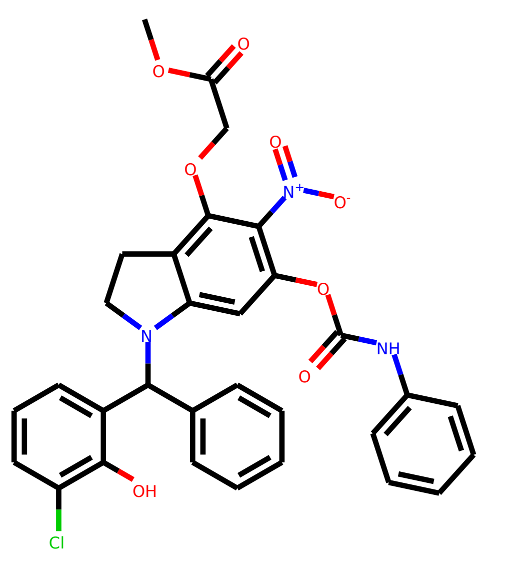
Osma-3234
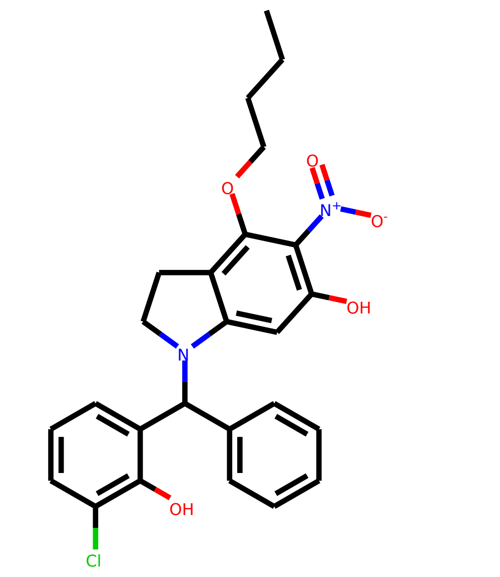
Osma-3235
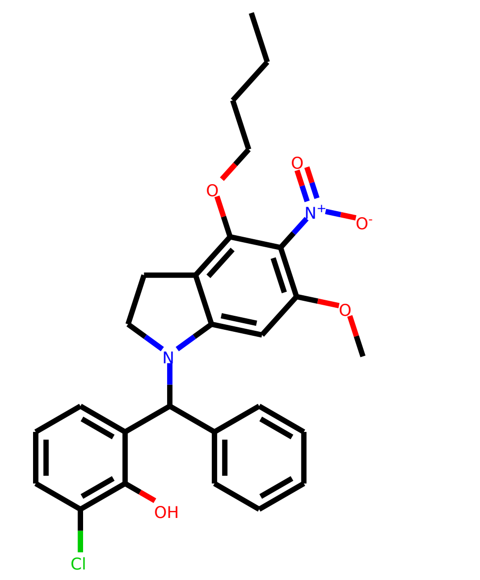
Osma-3236
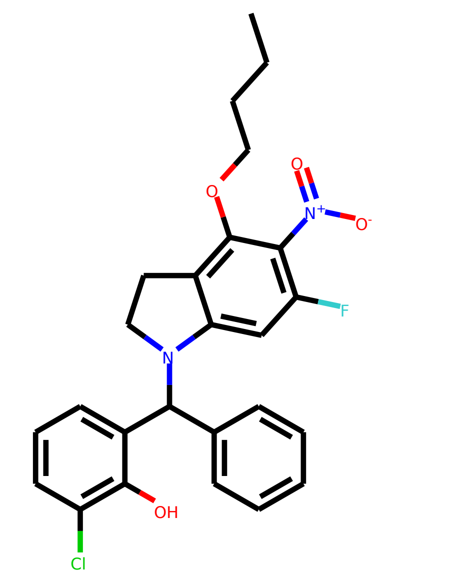
Osma-3237
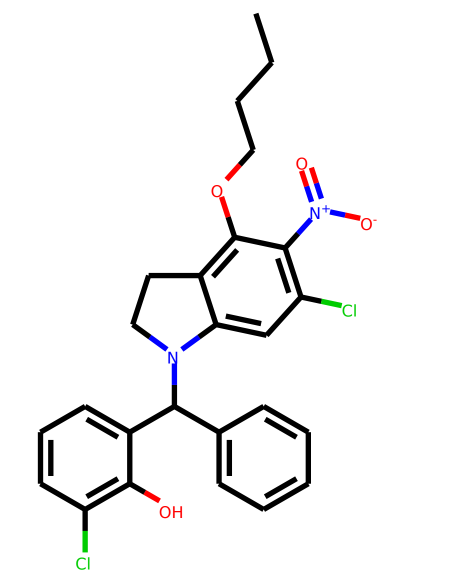
Osma-3238
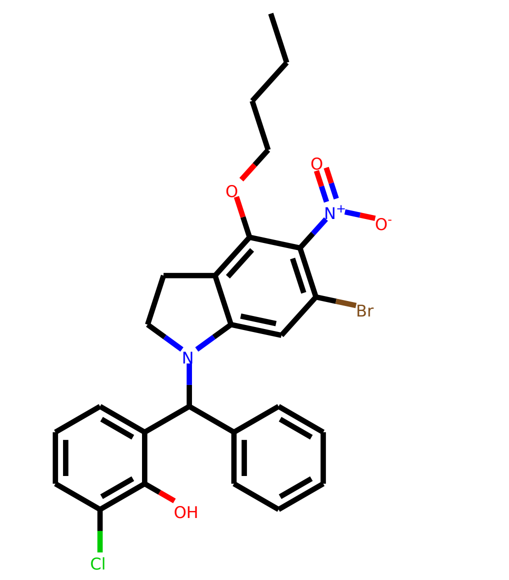
Osma-3239
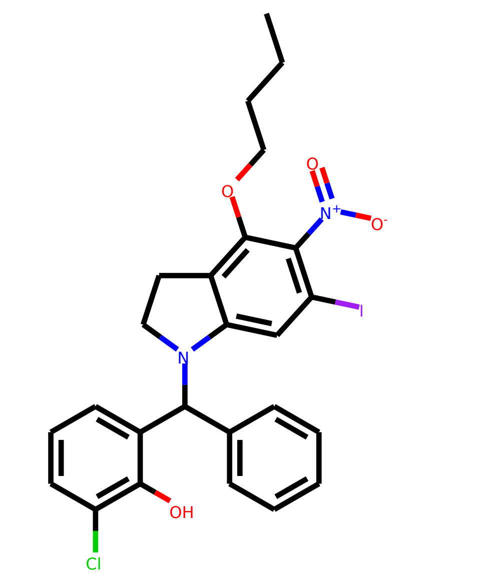
Osma-3240
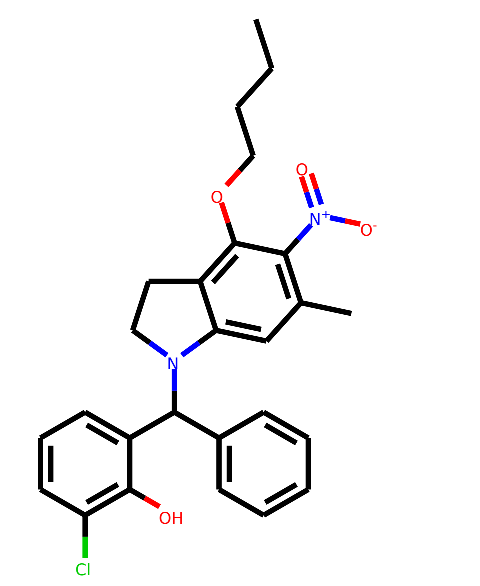
Osma-3241
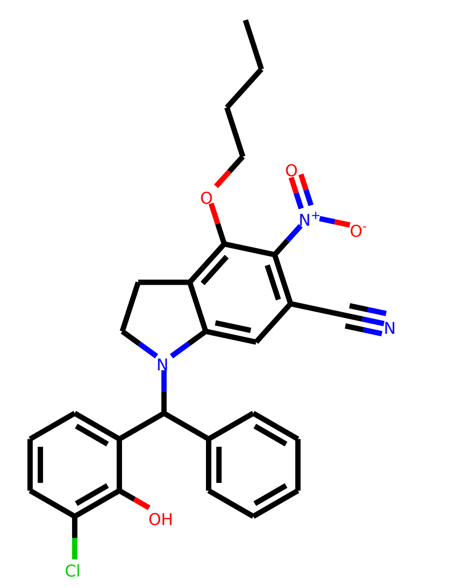
Osma-3242
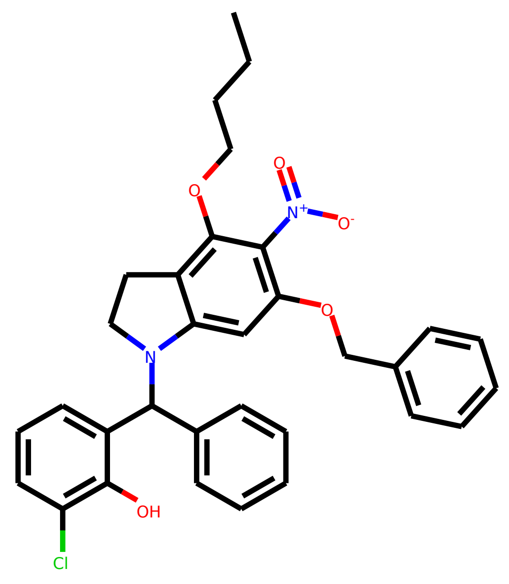
Osma-3243
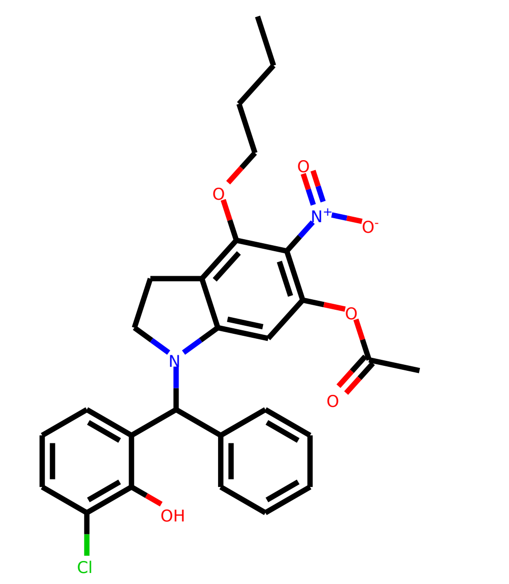
Osma-3244
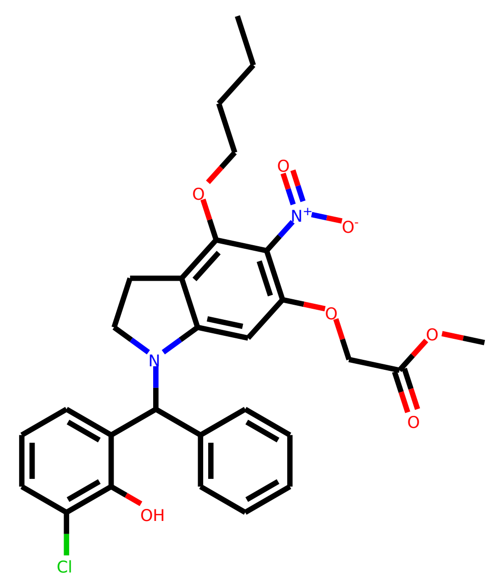
Osma-3245
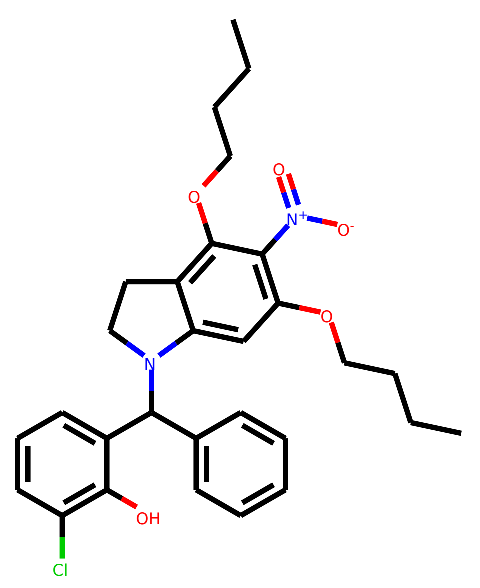
Osma-3246
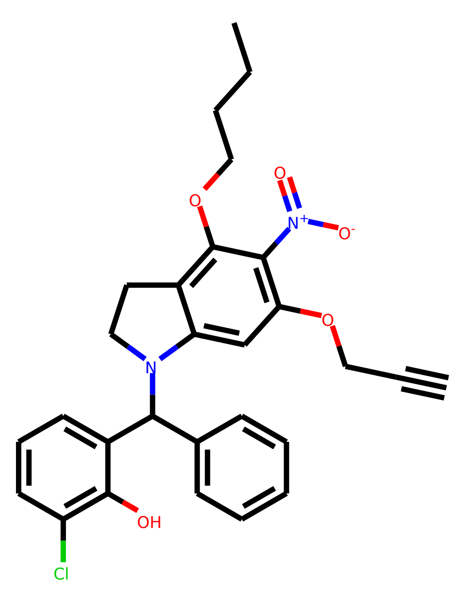
Osma-3247
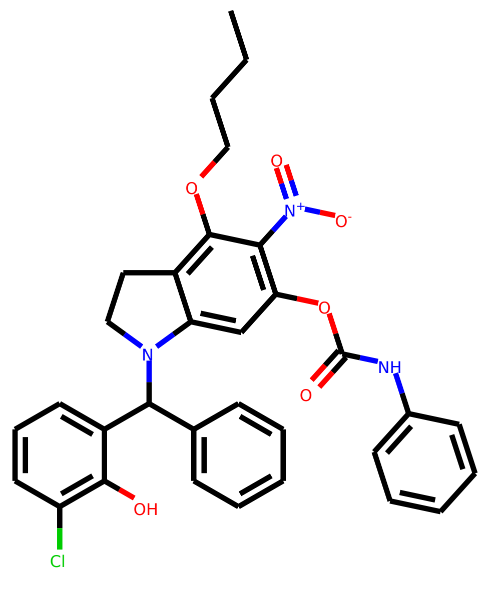
Osma-3248
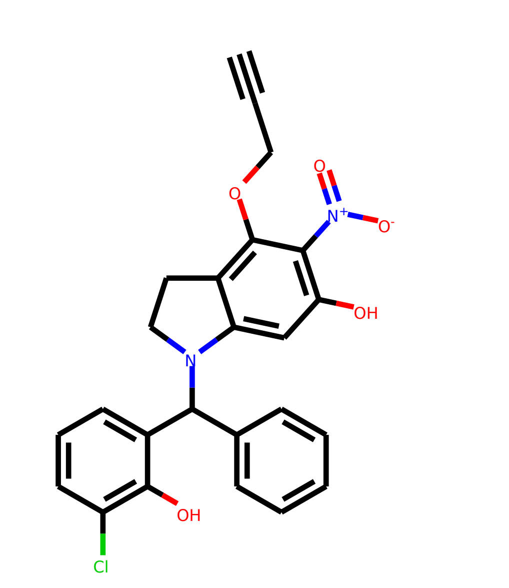
Osma-3249
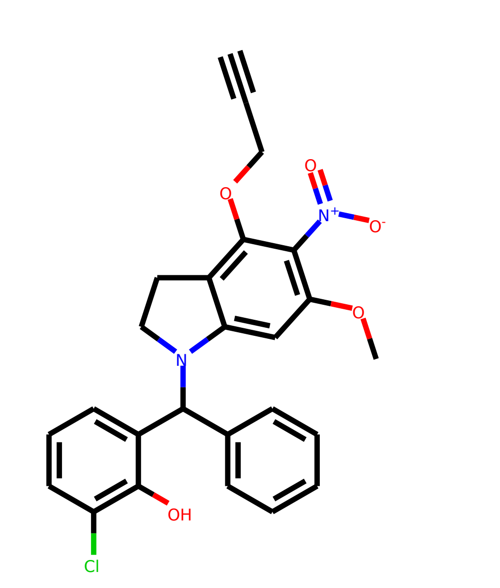
Osma-3250
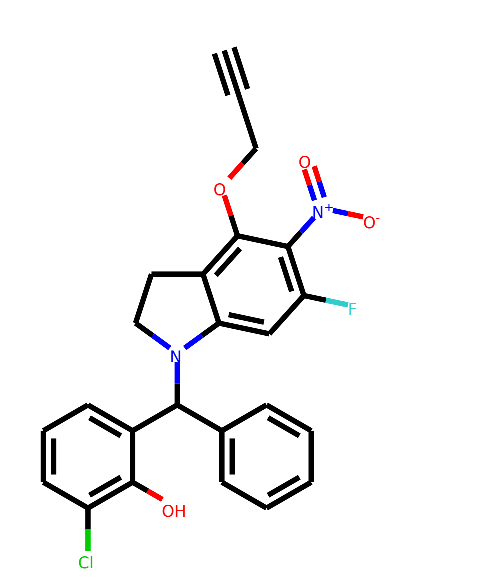
Osma-3251
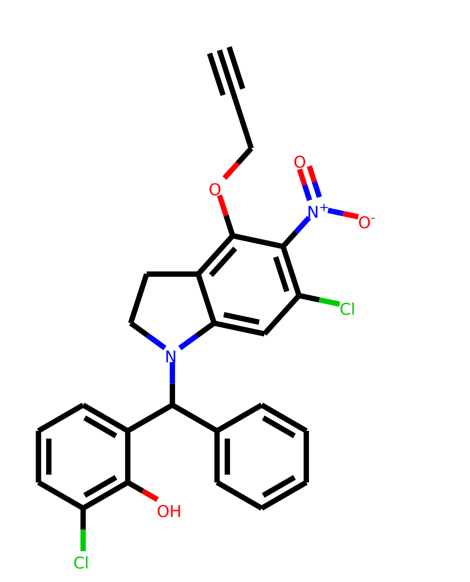
Osma-3252
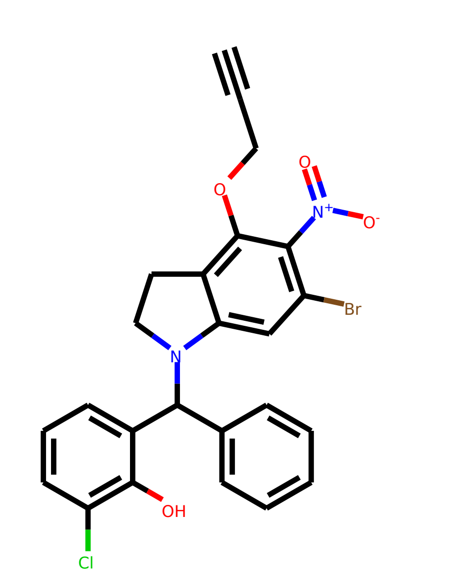
Osma-3253
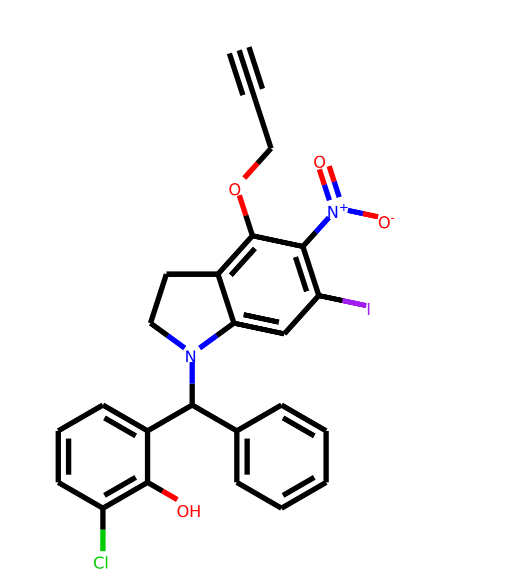
Osma-3254
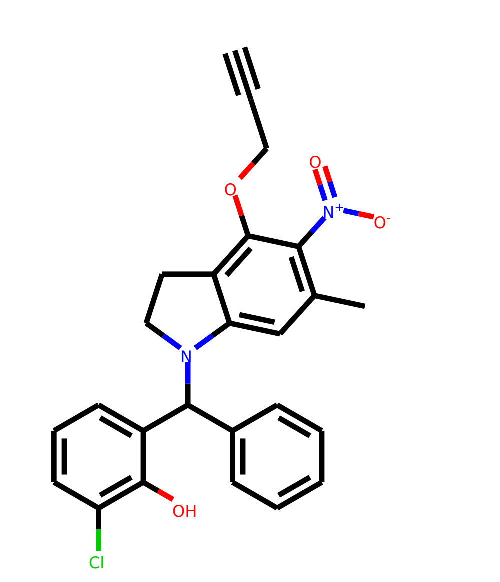
Osma-3255

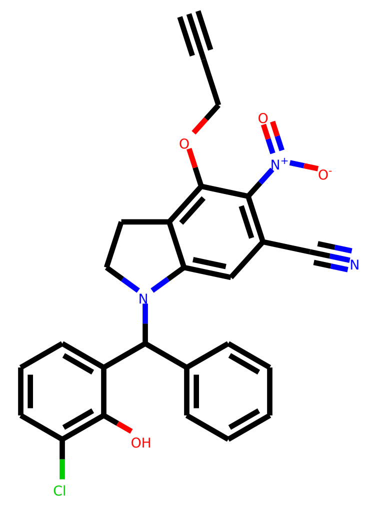

Osma-3256

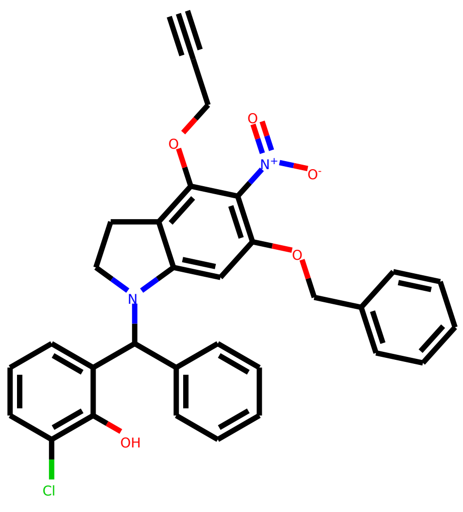

Osma-3257

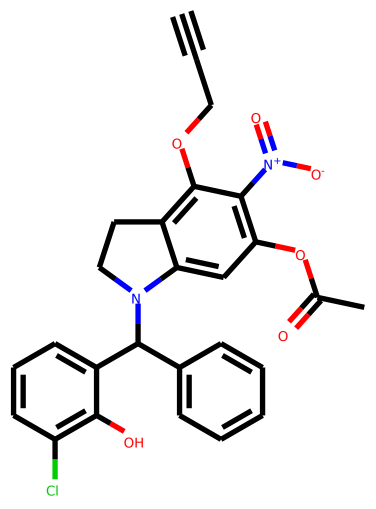

Osma-3258

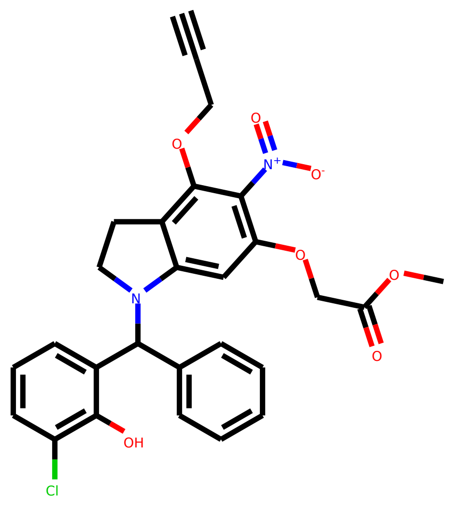

Osma-3259

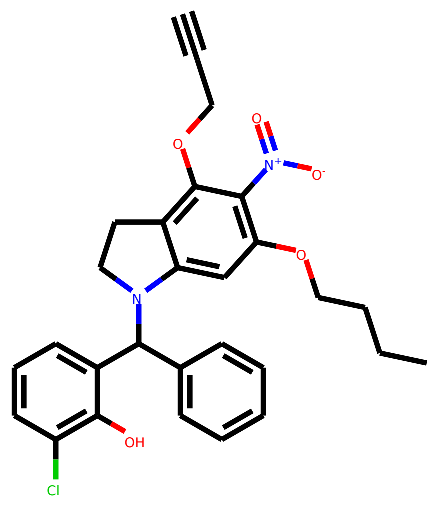

Osma-3260

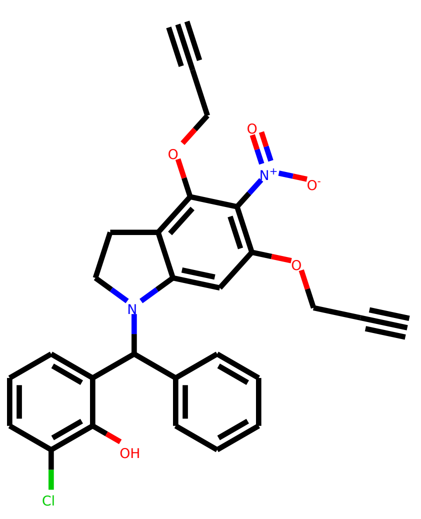

Osma-3261

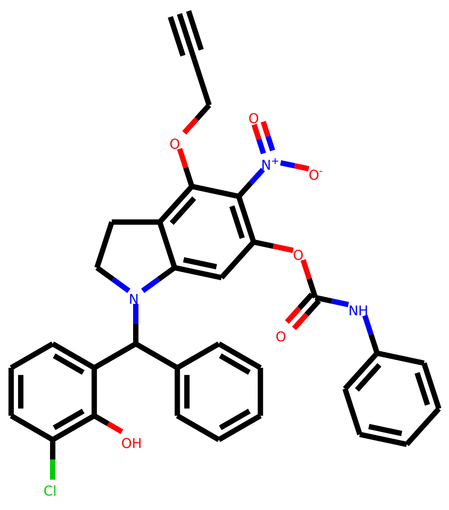

Osma-3262

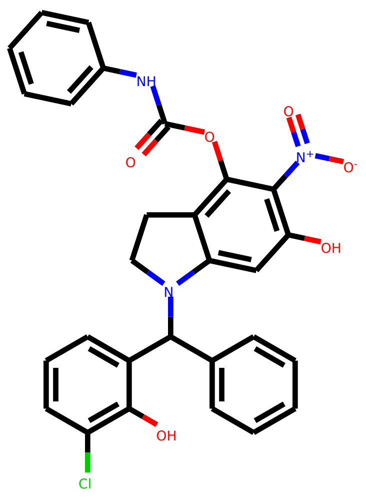

Osma-3263

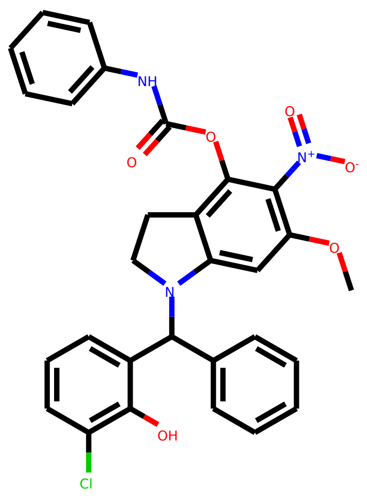

Osma-3264

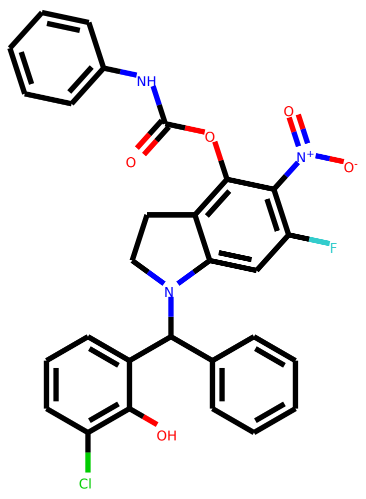

Osma-3265

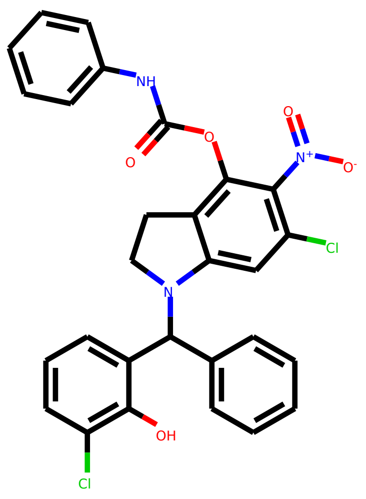

Osma-3266

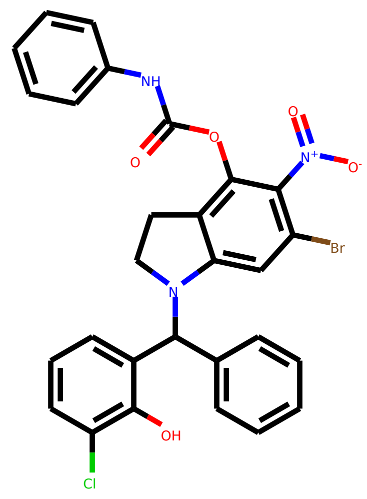

Osma-3267

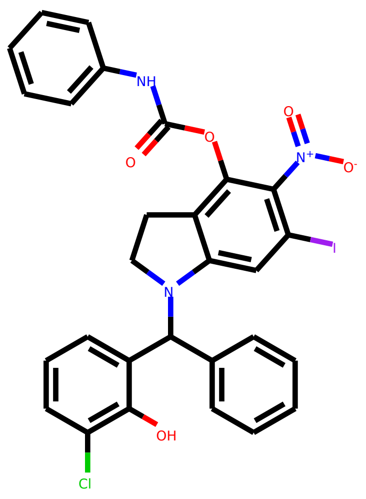

Osma-3268

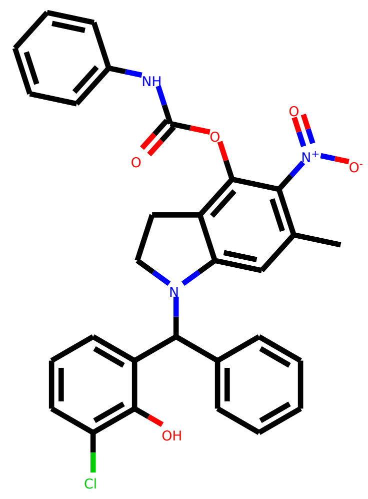

Osma-3269

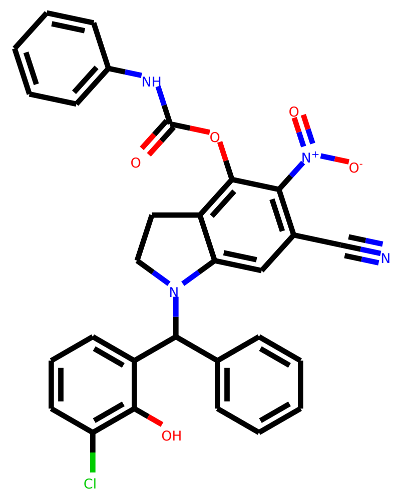

Osma-3270

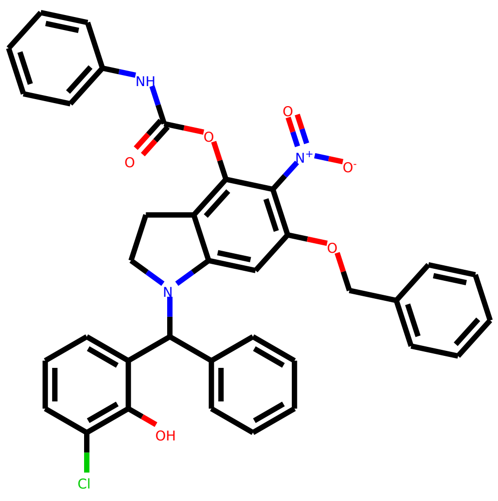

Osma-3271

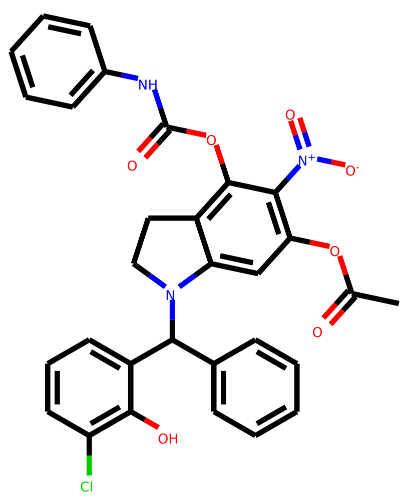

Osma-3272

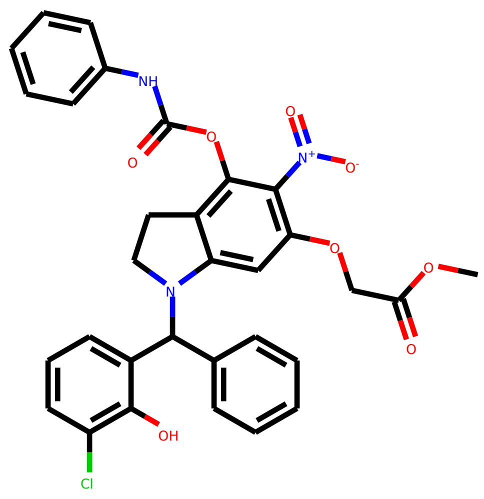

Osma-3273

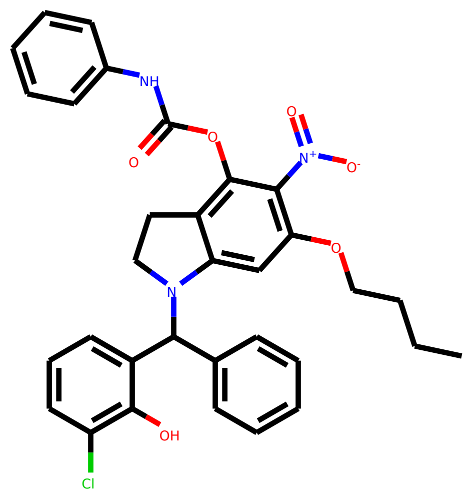

Osma-3274

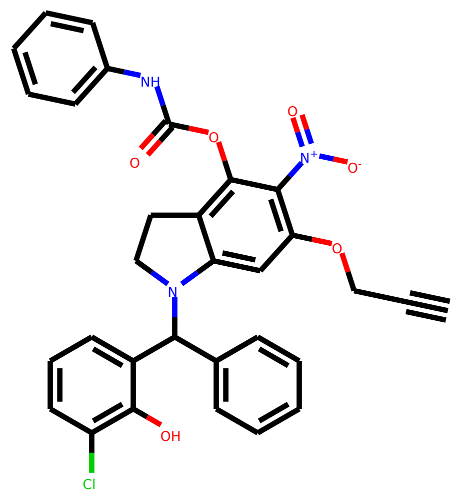

Osma-3275

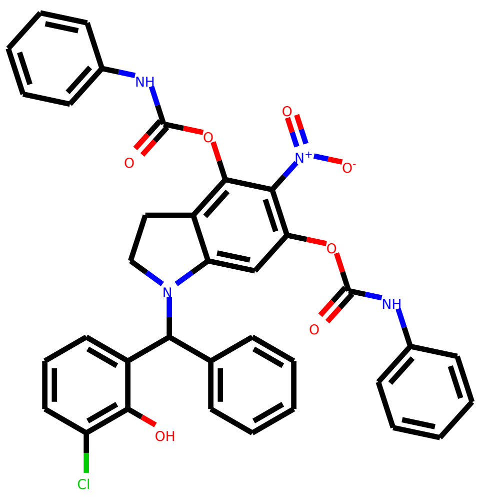

Osma-3276
